# Computing exponentially faster: implementing a non-deterministic universal Turing machine using DNA

**DOI:** 10.1098/rsif.2016.0990

**Published:** 2017-03-01

**Authors:** Andrew Currin, Konstantin Korovin, Maria Ababi, Katherine Roper, Douglas B. Kell, Philip J. Day, Ross D. King

**Affiliations:** 1 SYNBIOCHEM, Manchester Institute of Biotechnology, University of Manchester, Manchester, UK; 2 School of Chemistry, University of Manchester, Manchester, UK; 3 School of Computer Science, University of Manchester, Manchester, UK; 4 Manchester Institute of Biotechnology, University of Manchester, Manchester, UK; 5 Faculty of Biology, Medicine and Health, University of Manchester, Manchester, UK

**Keywords:** non-deterministic universal Turing machine, DNA computing, complexity theory

## Abstract

The theory of computer science is based around universal Turing machines (UTMs): abstract machines able to execute all possible algorithms. Modern digital computers are physical embodiments of classical UTMs. For the most important class of problem in computer science, non-deterministic polynomial complete problems, non-deterministic UTMs (NUTMs) are theoretically exponentially faster than both classical UTMs and quantum mechanical UTMs (QUTMs). However, no attempt has previously been made to build an NUTM, and their construction has been regarded as impossible. Here, we demonstrate the first physical design of an NUTM. This design is based on Thue string rewriting systems, and thereby avoids the limitations of most previous DNA computing schemes: all the computation is local (simple edits to strings) so there is no need for communication, and there is no need to order operations. The design exploits DNA's ability to replicate to execute an exponential number of computational paths in P time. Each Thue rewriting step is embodied in a DNA edit implemented using a novel combination of polymerase chain reactions and site-directed mutagenesis. We demonstrate that the design works using both computational modelling and *in vitro* molecular biology experimentation: the design is thermodynamically favourable, microprogramming can be used to encode arbitrary Thue rules, all classes of Thue rule can be implemented, and non-deterministic rule implementation. In an NUTM, the resource limitation is space, which contrasts with classical UTMs and QUTMs where it is time. This fundamental difference enables an NUTM to trade space for time, which is significant for both theoretical computer science and physics. It is also of practical importance, for to quote Richard Feynman ‘there's plenty of room at the bottom’. This means that a desktop DNA NUTM could potentially utilize more processors than all the electronic computers in the world combined, and thereby outperform the world's current fastest supercomputer, while consuming a tiny fraction of its energy.

## 1. Introduction

Universal Turing machines (UTMs) form the theoretical foundation of computer science [1–13]: the *Church–Turing thesis* states that UTMs exactly define the concept of an algorithm-effective calculability. UTMs also play a fundamental role in science: the *Church–Turing principle* states that they are sufficient to simulate perfectly all physically realizable systems [5,6,8].

UTMs are an abstract mathematical concept, but the language that describes them begs a physical interpretation. Digital electronic computers physically embody UTMs, but differ from them in that they have bounded memory, may only run for a bounded amount of time, make errors, etc. [9]. This tension between physical and abstract machines is at the heart of computer science.

**Figure 1:**
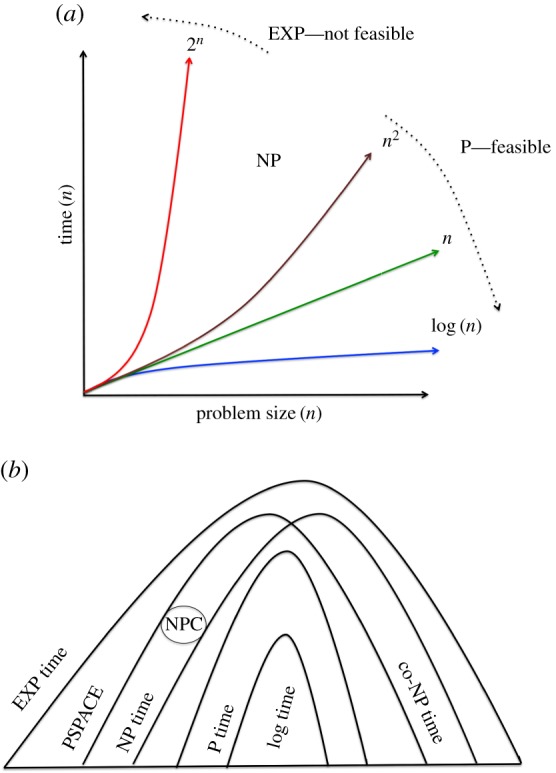
Computational complexity. (*a*) The feasibility thesis asserts that there is a fundamental qualitative difference between algorithms that run in polynomial time (P time) (e.g. schoolbook multiplication), and algorithms that run in exponential time (EXP time) (e.g. position evaluation in a generalized game) [2,11–18]. As problem size increases P time algorithms can still feasibly (efficiently) be executed on a physical computer, whereas EXP time algorithms cannot. The feasibility thesis also asserts that NP algorithms cannot feasibly be executed, but this is less clear as this assumes P ≠ NP. (*b*) Complexity classes are related through the subset relationship: log time ⊆ P time ⊆ NP ⊆ PSPACE ⊆ EXP time [2,11–18]. Little is known of the exact details of these relationships, e.g. does P = NP? (Online version in colour.)

The theory of computability investigates which problems a UTM can solve using unbounded space and time [1–4,11,12]. The related theory of computational complexity investigates how much time and space are needed to solve particular problem classes [2,4,11–18]. The complexity of an algorithm is its asymptotic worst-case use of a resource (space, time) as a function of the size of its input. A major conclusion of complexity theory is the ‘feasibility thesis’: that a natural problem has an efficient algorithm if and only if it has a polynomial-time (P) algorithm [2,11,12,15] (figure 1*a*). A function *f*: *I* → *I* is in the class P if there is an algorithm computing *f* and positive constants *A*, *k*, such that for every *n* and every |*x*| ≤ *n* the algorithm computes *f*(*x*) in at most *An^k^* steps.

The most significant concept in complexity theory is the class of non-deterministic polynomial time (NP) problems. Informally, this is the class of decision problems where the *solution* can be verified in P time, i.e. membership propositions have short efficiently verifiable proofs [2,11,12,14–17]. More formally, a decision problem *C* is in the class NP if there is a function *V_c_* ∉ P and a constant *k* such that

— If *x* ∈ *C* then ∃*y* with |*y*| ≤ |*x*|*^k^* and *V_c_*(*x, y*) = 1
— If *x* ∉ *C* then ∀*y* we have *V_c_*(*x, y*) = 0

A sequence *y* that ‘convinces’ *V_c_* that *x* ∈ *C* is often called a ‘witness’ or ‘certificate’ [17]. Many of the most important practical computational problems belong to the NP class, e.g. graph isomorphism, Boolean satisfiability, travelling salesman, graph colouring, etc. NP complete problems are the most difficult in NP, and all NP problems can be reduced to them in P time [2,4,11–18]. This means that if one can solve any type of NP complete problem in P time then one can solve all NP problems in P time.

To make these abstract concepts more concrete, consider the NP problems of prime factorization and 3SAT. In prime factorization, the problem is to determine the unique list (bag) of prime factors that when multiplied together produce a given integer. This is an NP problem because it is possible to verify in P time that the given prime factors actually multiply together to give the integer, but there is no known P time algorithm to find these prime factors. (The multiplication of two numbers is quadratic—O(*b*^2^), where *b* is the number of bits in the number, using schoolbook long multiplication—assuming a standard position based representation of numbers.) Integer factorization is important because its asymmetric difficulty is at the heart of the best-known public-key encryption method of RSA [19], and because it is the most celebrated problem where quantum mechanical UTMs (QUTMs) outperform classical UTMs, i.e. there is a P time QUTM algorithm to find prime factors. However, integer factoring is a special NP problem in a number of ways, for example every problem has a single unique solution, and so the problem is not thought to be NP complete [11].

The standard NP complete problem is 3SAT. In 3SAT, the problem is to find an assignment of Boolean variables to satisfy an expression of the following form: (X_1_∨X_2_∨X_3_) ∧ (∼X_1_∨X_4_∨∼X_5_) ∧ (∼X_2_∨X_3_∨X_6_)…. Satisfying such an equation means finding a way to assign the value either True (T) or False (F) to each of the Boolean variables X*_n_*, e.g. X_1_ = T, so as to make the overall expression True. It can easily be seen that verifying a solution is P time—just fill in the values evaluate the expression. However, there is no known P time algorithm to find solutions. 3SAT is NP complete because it has been proved possible to transform any NP problem into a 3SAT problem in P time [2,11–13]. This implies that if one could solve arbitrary 3SAT problems in P time then one could solve any NP problem in P time.

The NP class is commonly believed to be a strict superset of P, i.e. P ≠ NP; as it would seem generally harder to find a solution to a problem than to verify a correct solution (figure 1*b*). However, this has never been proved, and the P = NP question is the arguably the most important open problem in mathematics [11,15,18]. The problem is also of immense practical importance, for if P = NP it would essentially solve mathematics and transform science/engineering, but also have devastating consequences for activities that depend on cryptography for security, such as the banking system, the Internet, etc. [13,15,18].

It is important to distinguish the mathematical problem of the truth or falsehood of the proposition ‘P = NP’, and the practical problem of solving NP problems in P time [9]. A mathematical problem is constrained by a given set of axioms and proof methods, whereas all possible physical means may be used to solve a practical problem. In this paper, we do not address the P = NP mathematical problem, but instead present the physical design for a computer that has an exponential speedup over conventional computers on NP complete problems.

## 2. Design of a non-deterministic universal Turing machine

The state of a UTM is defined by a tuple of symbols [1,3]. In a classical (deterministic) UTM, computation is a 1 : 1 relation that takes an input state to an output state, with computing halting if an accepting state is reached. A non-deterministic UTM (NUTM) differs from a UTM in that from one input state there may be multiple output states, i.e. computing is a 1 : *n* relation [2,3]. A now old-fashioned, but insightful, way to define the NP class is through the use of NUTMs: the NP class is the set of problems that a NUTM can solve in P time [2]: 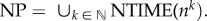


The customary interpretation of how a NUTM solves a NP problem in P time is through *serendipity* [7,10,11]: in each state it correctly guesses which of the output states to choose so as to reach the accepting state most quickly. Clearly, this interpretation precludes the possibility of a physical NUTM, and one reads that they are ‘magical’ [7], ‘hypothetical’ [10], ‘fictitious’ [11], etc. Our alternative *replicative* interpretation is that an NUTM is a UTM that can reproduce itself, and thereby follow all computational paths in parallel, with the computation ending when one path reaches the accepting state. Such a machine is physically implementable.

The theory of computational complexity treats time and space as fundamentally different: space is reusable while time is not. The resource limitation in a physical NUTM is space. The speed of an NUTM's computation increases exponentially, while the amount of space available is polynomially bound—the light-cone is cubic, and the bound (holographic) on the maximum amount of information in a volume of space is quadratic [13,20]. Computation in a physical NUTM therefore resembles an explosion. In contrast with an NUTM, the resource limitation for physical classical and QUTMs is time. This difference enables an NUTM to trade space for time.

When trading space for time it makes sense to use as small processors as possible: molecules. However, although molecules are very small (Avogadro's number is approximately 6 × 10^23^) they are still of finite size, and this restricts the size of NP problem that a molecular NUTM could practically solve before running out of space—the Earth has approximately 10^49^ atoms, and the observable Universe only approximately 10^80^. (This implies that what protects cryptographic systems from being broken is not just a lack of time, as is generally argued [11,13,18,19], but also a lack of space.) Despite a physical NUTM's restriction to using a polynomial amount of space, space is currently used very inefficiently in existing computers. It is therefore rational to expect that a molecular NUTM, through trading space for time, could outperform the world's current fastest supercomputer, while consuming a tiny fraction of its energy.

**Figure 2.**
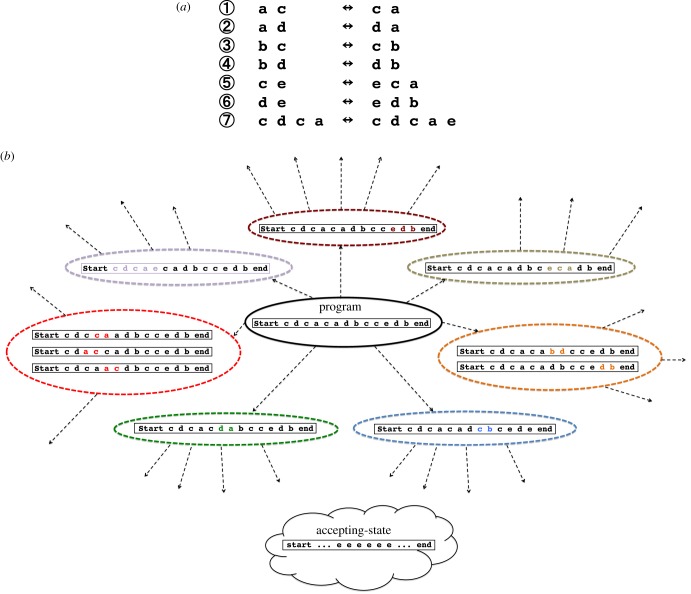
A universal Thue system. (*a*) A set of universal Thue rules: rules 1–4 require symbol transposition; rule 7 requires symbol insertion (forward) and deletion (reverse); and rules 5 and 6 require transposition, insertion (forward) and deletion (reverse). (*b*) Part of trace of the execution of the universal Thue system (an NUTM): the tree of all its possible computations. The root of the tree is the initial program. The child nodes of the root are the subsequent Thue sequences generated from the initial program by application of one of the seven Thue rules: note that the antecedent of a rule (e.g. **ca**—the reverse of rule 1) may occur multiple times. Thue rules are recursively applied until the accepting state is produced, thus execution of a program generates a potentially exponential number of states in P time.

We use a Thue rewriting system to implement an NUTM. Thue systems are a model of computation with equivalent power to Turing machines [2,3,21–25]. Formally, a Thue system is the presentation of a monoid [20]. Informally, a Thue system is a set of rules of the form ***w*** ↔ ***u***, where ***w***, ***u*** are strings in a finite alphabet of symbols. A string, for example, ***v w v'*** can be rewritten by the rule above to give ***v u v'***. The application of a Thue rule to a string therefore produces a new string—equivalent to change of state in a UTM (figure 2*a*). The starting state (program) is a specific string as is the accepting state. The execution of a Thue program consists of repeated application of Thue rewrite rules until an accepting state is produced (figure 2*b*). It is possible to translate any Turing machine into a Thue system, and vice versa [3,21]. We implement the Thue system shown in (figure 2*a*), which is universal, i.e. it has undecidable (word) problems [6,21–25].

Thue systems are similar to the more biologically familiar L-systems [26]. The main differences are that in an L-system there are no symbol deletions, and multiple rules are applied simultaneously. These differences have important theoretical and practical implications. Applying multiple rules simultaneously is difficult to implement in molecular systems.

It is important to note two key features of Thue systems. The first is that the order and position of application of Thue rules is naturally non-deterministic: multiple Thue rules may be applied to a string, and individual Thue rules may be applied to multiple positions in a string (figure 2*b*). The second feature is that all the computation is local: all that is needed to implement a universal Thue system is the ability to recognize a small number of local sub-strings and to locally edit these sub-strings, there is therefore no need for communication. These two features enable the practical exponential increase in speed in our NUTM design.

To solve any NP problem (e.g. integer factorization or 3SAT problem) using our NUTM, one would first translate the problem into an initial string of Thue symbols (the program), then determine the Thue string(s) that signals the accepting state—such a translation is always possible as the Thue system is universal. The program will then execute to produce all possible computational paths from the program, until an accepting state is found.

## 3. DNA computing

We use DNA computing to implement a Thue NUTM. Like other forms of molecular computing, DNA computing trades space for time: ‘there's plenty of room at the bottom’ [27]. The goal of building a molecular scale UTM has been pursued for over 50 years [27], with the most common molecule suggested being DNA [27–33], but protein has also been proposed [34]. DNA is an excellent medium for information processing and storage [32,33]. It is very stable, as the sequencing of ancient DNA demonstrates. It can also reliably be copied, and many genes have remained virtually unchanged for billions of years. These properties give DNA computing potential advantages in speed, energy efficiency and information storage over electronics [27,28,32,33,35]: the number of operations for a desktop DNA computer could plausibly be approximately 10^20^ s (approx. 10^3^ times faster than the fastest current supercomputer); it could execute approximately 2 × 10^19^ operations per joule (approx. 10^9^ more than current supercomputers); and utilize memory with an information density of approximately 1 bit per nm^3^ (approx. 10^8^ more dense than current memory). These advantages mean that it is feasible that a DNA NUTM based computer could potentially utilize more processors than all the electronic computers in the world combined, and so outperform all standard computers on significant practical problems [36].

The foundational work on DNA computing was that of Leonard Adleman [28]. He demonstrated the solution of a seven-point Hamiltonian path (an NP-complete problem) by generating a set of random DNA sequences (possible solutions), and selecting a solution from the set. This generate-and-test approach is useful for certain types of combinatorial problems, but is not well suited for general-purpose computation, as typically the (hardware) encoding of the symbols needs to redesigned for each new problem. By contrast, in a UTM only the software needs to be changed for a new problem, and the hardware stays fixed. No working molecular UTM yet exists, but several designs have been proposed. The most similar design to the one presented here is that of Khodor & Gifford [29] who proposed the use of site-directed mutagenesis (SDM) to implement a classical UTM. The authors presented an abstract proof that a classical UTM could be encoded using strings of DNA, and that SDM could be used to change state. However, the coding scheme in the proof is thermodynamically unrealistic, and they only physically implemented a simple counting scheme.

The use of Thue systems overcomes many problems with existing DNA computing designs. As Thue systems are non-deterministic there is no need for a specific order of operations, which is typically very difficult with molecular systems. The necessary ordering of operations is essential in most previous DNA computing designs, e.g. in direct implementations of UTMs [29]. Another key design advantage of Thue systems is that all the computation is local, a simple edit of a string, which means that there is no need for communication and the basic computational step takes a constant time. This contrasts with most previous DNA computing designs, where there is the requirement for unique molecules to find each other in solution, which takes time proportional to volume.

Most significantly our work is an advance on all previous other work in that we present the first NUTM design. This is important because NP complete problems are the most important class of problem in computer science, and on these problems NUTMs are theoretically exponentially faster than both classical UTMs, and QUTMs.

## 4. Implementation of a DNA non-deterministic universal Turing machine

**Figure 3.**
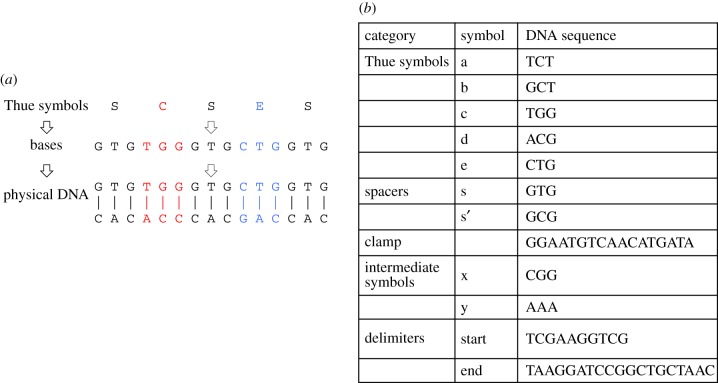
DNA computing. (*a*) Three levels of symbol exist in our NUTM: physical DNA, bases, and Thue symbols. Note that molecular structure of single-stranded DNA is asymmetric: one end is termed 3′, the other 5′ (these terms refer to the connectivity of the ribose sugars). When double-stranded DNA is formed the single strands bind together in an anti-parallel manner. DNA polymerases (enzymes for copying DNA) can only copy DNA in the 5′ to 3′ direction. (*b*) We encode the five Thue system symbols a, b, c, d, e using triplets of DNA. This length was found to provide the best balance between symbol specificity and the ability to mismatch—intriguingly the ‘genetic code’ (cypher) is also based on triplets. The use of triplets helps ensure that when performing PCRs with an annealing temperature in the range of 50–60°C only the desired target sequences are amplified. A spacer symbol s (or s') occurs between each Thue symbols to help enforce the specificity of desired mismatches. We require the marker symbol *clamp* for rule recognition. We use the intermediate symbols x and y to help ensure that unwanted cross-hybridization of symbols does not occur. Finally, the symbols *start* and *end* delimit the program. Physically, these delimiters are used as recognition site primers for PCRs.

**Figure 4.**
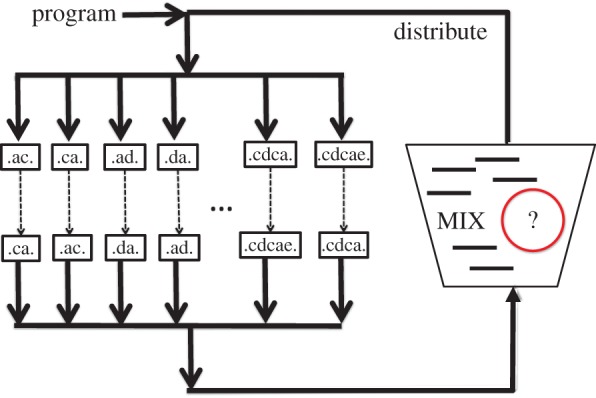
Implementation of a DNA NUTM. The physical NUTM consists of 14 paralleling executing processors (one each for both direction of the seven Thue rules) and a mixing vessel. Each processor executes a microprogram that implements a Thue rule in one direction. The resulting transformed DNA sequences are added back to the mixing vessel, mixed, and then distributed to the processors to continue the computation. In this way, all possible combinations of computational steps are executed. The mixing vessel also contains a detection method for recognizing accepting states. Such a system could be wholly automated, and work in continuous cycle.

In our NUTM starting states (programs) and accepting states (read-outs) are sequences of DNA that encode strings of Thue symbols (figure 3). The physical architecture of the computer, with a mixing chamber, and editing chambers for each different Thue rule/direction, ensures that every Thue rule is applied to every NUTM state (figure 4). To physically implement each Thue rewrite rule we have developed a novel combination of polymerase chain reactions (PCRs) to copy state, and SDM to change state [29]. This approach ensures that all possible applications of a Thue rule are made. In all stages in the process, well-formed strings can be recognized by the presence of appropriate sequences indicating the beginning and end of the well-formed string (see electronic supplementary material)*.*

The mechanism of the NUTM depends on the specificity of molecular binding—as do living organisms. The Boltzmann distribution determines the frequency of molecular states of differing energies (*E*): higher energy states are exponentially less likely to occur than lower energy ones. The energy of DNA binding depends on sequence similarity [37,38], so the probability of undesirable DNA bindings can be made exponentially less likely through sequence design—although this is constrained by upper limits on the temperature of DNA duplexes, etc.

To write programs (initial states), we use DNA synthesis technology. Accepting states are specific sequences of DNA that contain identifying certificates, and corresponding answers to the computation. We require that the accepting states be recognized from among a potential exponential number of other states generated by the NUTM (figure 2*b*). This is feasible thanks to the Boltzmann distribution of binding energies, and because PCRs enable an exponential amount of complementary sequences to be produced in linear time. In our development work, we read out accepting states directly using DNA sequencing. Other techniques are applicable, for example labelled complementary sequence to first identify the certificate, then sequencing to determine the result of the computation.

It is helpful to divide the task of applying a single Thue rule/direction into two steps: recognition and rewriting. This separates the more thermodynamically challenging step of recognition, from the technically more complex step of rewriting. In rule recognition, all antecedent strings of a given Thue rule are identified from among arbitrary complex strings of Thue symbols, and marked with a ‘clamp’ sequence. This clamp sequence is designed to be distinct from any DNA sequence encoded by Thue symbols, and thereby provide the specificity of binding required for rewriting. To insert the clamp sequence, we use DNA oligonucleotide primers: these have at their 3′ terminus a recognition sequence (the reverse complement of the antecedent of the rewrite rule), and at their 5′ end the clamp sequence. The PCR products from these primers encode the clamp sequence adjacent to the target symbol sequence. This type of insertion procedure is a well-established SDM technique [39,40].

**Figure 5.**
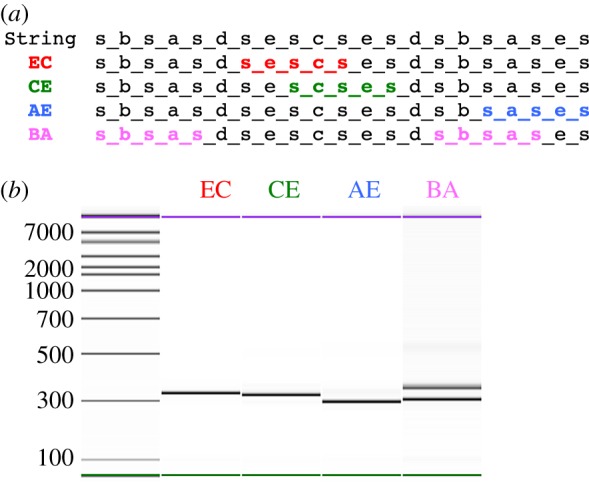
Thue rule recognition. (*a*) Sequence design of a DNA template encoding a string of 10 symbols separated by spacers. The symbol combinations **ec** (red), **ce** (green) and **ae** (blue) occurred once only within the string, whereas **ba** (pink) occurred twice. The complementary DNA primers consisted of a clamp sequence followed by the symbol combination and flanking spacers. (*b*) Capillary electrophoresis analysis (measurement of molecular weight—Bioanalyser 2100, Agilent Technologies) of PCR products for Thue rule recognition. Using the string template DNA described in (*a*), PCRs were carried out to insert a clamp sequence prior to each Thue rule symbol combination. For primers targeting the symbols **ec**, **ce** and **ae**, only one PCR product is created, while for **ba**, two products occur.

We have established *in vitro* that this recognition procedure works reliably. We have shown that we can recognize specific symbol string combinations and insert clamp sequences adjacent to them (**ec**, **ce**, **ae**, **ba**) in a Thue program (DNA template) containing multiple symbol combinations (figure 5). For the cases of **ec**, **ce**, **ae**, as expected, only one molecular weight (MW) band was produced. Sequencing demonstrated that the correct rule antecedent strings were identified, i.e. with the clamp sequence inserted at their 5′ ends. For the **ba** symbol string, which occurs twice in the Thue program, as expected, we detected two different MW bands, and sequencing revealed that both possible rule antecedent strings were correctly identified (figure 5). We have thus demonstrated non-deterministic rule recognition.

It would have been prohibitively expensive and time-consuming to physically demonstrate recognition against a background of all possible mismatching strings. We therefore applied computational modelling to demonstrate the specificity of recognition. The Gibbs free energy (*G*) of the hybridization of DNA sequences to each other can be modelled with good accuracy [37]. To calculate these estimates, we used the UNAFold software [38]. For each rewrite rule plus clamp we computationally verified that the binding that perfectly matches the rule antecedent sequence is energetically favourable (lower Δ*G*) compared with binding with any other possible string of Thue mismatching symbols (see electronic supplementary material). This modelling approach is conservative as it is not generally the case for a Thue program that all Thue symbol strings may be produced, and because PCR depends on 3′ binding, so the contribution of the 5′ clamp is relatively unimportant.

We use SDM to make the changes of state required to implement Thue rewriting rules. As it is difficult to directly implement the complex DNA editing required for the universal Thue rules, we decomposed the rules into basic operations that can be directly implemented (see the electronic supplementary material). These basic operations can then be arranged in different ways (‘microprograms’) to execute arbitrary complex Thue rules, and hence a variety of representations of an NUTM. The microprograms use a combination of symmetric and asymmetric PCRs to support the repeated targeting of multiple positions simultaneously [41,42]. In physical terms, the basic operations are DNA hybridizations, where the new sequence (encoded by a DNA primer) mismatches and binds with an existing template, with the products of primer extension encoding the new sequence. Note that this current string edit design process differs from an ideal Thue implementation in that the PCR processing is not purely local.

**Figure 6.**
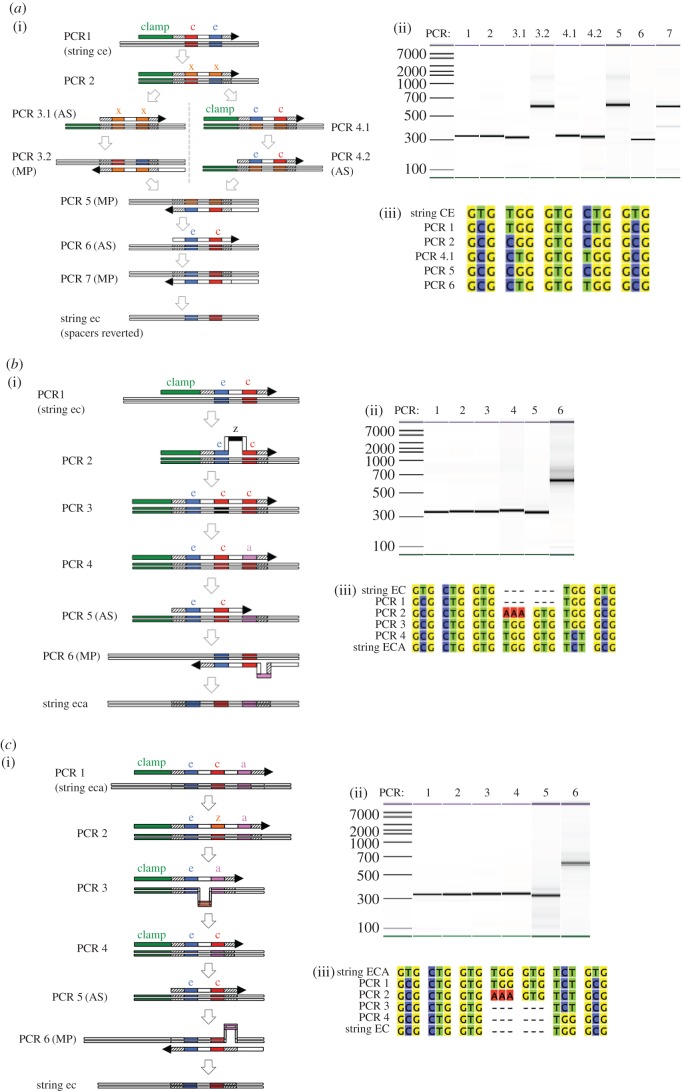
Thue rule implementation. (*a*) Microprogram for swapping **ce** → **ec**. (i) The clamp sequence is first inserted and the outer **s** symbols changed to **s**' (PCR1). This clamp and spacer sequence is then bound by the primer in PCR2, which replaces the **ce** symbols with an **xx** sequence. Asymmetric PCR (AS) is then used to remove the clamp and create a megaprimer, which is used to replace **ce** with **xx** in the string (megaprimer PCR annotated ‘MP’, PCR 3.1 and 3.2). In parallel, the **xx** symbols are changed to **ec** by symmetric PCR (PCR 4.1), and then a megaprimer produced removing the clamp (PCR 4.2). This megaprimer is then employed to replace the sequence **xx** with **ec** in the string (PCR 5). Finally, the **s**' symbols are returned to **s** to complete the microprogram (PCR 6 and 7). (ii) Capillary electrophoresis analysis of the PCR products from each PCR step. (iii) Sequence alignment of DNA sequencing of the key steps in the microprogram. (*b*) Microprogram for the insertion **ec** → **eca**. (i) Following the recognition of **ec** the clamp is inserted and outer spacers changed (**s** to **s'**, PCR 1). A non-coding symbol **z** is then inserted, which exploits the strong binding for the existing **e** and **c** with the modified **s**' spacers (PCR 2), which promotes a loop to occur during DNA hybridization. This symbol is then edited to **c** (PCR 3), and the other **c** changed to **a** (PCR 4). Asymmetric PCR is then used to generate a megaprimer for the **eca** sequence (PCR 5), which is then used to insert this new sequence into the tape (PCR 6). (ii) Capillary electrophoresis analysis of the PCR products from each PCR step. (iii) Sequence alignment of DNA sequencing of the key steps in the microprogram. (c) Microprogram for the deletion **eca** → **ec**. (i) The clamp and altered spacers **s**' are inserted upon recognition of **eca** in PCR 1. The middle symbol (**c**) is edited to a non-coding **z** (PCR 2) before deletion of this symbol by recognition of **ea** in the subsequent step (PCR 3). As with the insertion microprogram, the strength of DNA hybridization between the clamp, **s'e** and **as**' promotes the PCR primer to loop over the **z** symbol to delete it. Following deletion the symbol sequence **ec** is created (PCR 4), and the clamp removed by asymmetric PCR (PCR 5). Finally, the megaprimer is used to delete the original symbol **a** from the string. (ii) Capillary electrophoresis analysis of the PCR products from the deletion microprogram. (iii) Alignment of sequencing data from the key PCR steps.

All the microprograms follow a similar schema: a series of mismatching symmetric and asymmetric PCR operations that implement the designed DNA sequence changes. Each PCR operation generates a specific intermediate sequence that is either used as a template or megaprimer for subsequent operations. In all the microprograms, the first operation is insertion of the clamp. The second operation is change of the spacer sequence from **s** to **s**', which serves to further mark the position of rewriting and strengthen the binding of mismatching primers. (In our current *in vitro* implementation clamp, insertion and spacer change are combined.) DNA edits (insertions/deletions/swaps) are first made using symmetric PCRs to generate double-stranded DNA products (using the corresponding *end* (reverse) primer)—the edits being made to the truncated clamped sequence. Asymmetric PCRs are then used to generate megaprimers (single-stranded DNA product (figure 6)) that retain the required sequence changes, but have the clamp removed. Finally, the megaprimers are used to introduce the edits into the full-length DNA sequence, using the megaprimer and corresponding *start* (forward) primer.

There are three types of Thue rewriting edits: transpositions, insertions and deletions (figure 2*a*). To demonstrate that our SDM method is sufficient to implement transpositions, we used as examples the microprograms: **ce** → **ec** (figure 6*a*), and **ec** → **ce** (see the electronic supplementary material); for both microprograms we show the *in vitro* PCR steps, and the experimental evidence for the correct transformations. The universal Thue rules 1–4 require such transpositions (figure 2*a*).

To demonstrate insertions we used the microprogram **ec** → **eca** (figure 6*b*), and for deletions the microprogram **cea** → **ce** (figure 6*c*). The universal Thue rule 7 requires such insertions and deletions (figure 2*a*). Insertion/deletion edits require that the hybridized DNA ‘loops’, either in the template (for deletion), or the primer (for insertion). In all the microprograms, the most difficult case occurs when there are repeats of the Thue symbol to be swapped/inserted/deleted, as the primer and template often hybridize in an incorrect conformation. To overcome this a non-coding symbol (**x** or **z**) is inserted first and is then changed to its new symbol combination.

The most complex universal Thue rules are 5 and 6, as these involve transpositions, insertions and deletions (figure 2*a*). To demonstrate that this form of universal rule can be implemented using our methodology we used as an example rule 5: **ce** ↔ **eca** (see the electronic supplementary material). This rule can be implemented by integrating and adapting the above microprograms: **ce** → **ec** → **eca**; **eca** → **cea** → **ce** into a single workflow. Taken together, these results demonstrate that all the Thue rules required for an NUTM can be physically implemented using DNA mutagenesis.

## 5. Discussion

Our design for an NUTM physically embodies an abstract NUTM. We have demonstrated that the design works using both computational modelling, and *in vitro* molecular biology experimentation. We have shown the use of microprogramming to encode arbitrary Thue rules, shown that all classes of Thue rule can be implemented (reversible symbol transpositions, insertions and deletions), and validated non-deterministic rule implementation. However, we acknowledge that further experimentation is required to complete the physical construction of a fully working NUTM. Indeed, we are unaware of any fully working molecular implementation of a UTM, far less an NUTM. The key point about implementing a UTM compared with special purpose hardware is that special purpose hardware typically needs to be redesigned for each new problem. By contrast, in a UTM only the software needs to be changed for a new problem, and the hardware stays fixed. The situation for molecular UTMs is currently similar to that of QUTMs where hardware prototypes have executed significant computation, but no full physical implementation of a QUTM exists.

Perhaps the greatest challenge in developing a working NUTM is control of ‘noise’. Noise was a serious problem in the early days of electronic computers [43]; however, the problem has now essentially been solved. Noise is also the most serious hindrance to the physical implementation of QUTMs, and may actually make QUTMs physically impossible [44]. By contrast, in an NUTM, well-understood classical approaches can be employed to deal with noise. These classical methods enable unreliable components to be combined together to form extremely reliable overall systems.

Several promising approaches to noise reduction are available for NUTMs:

— *The use of error-correcting codes*. Such codes are used ubiquitously in electronic computers, and are also essential for QUTMs. Classical error-correcting code methods can be directly ported to NUTMs.
— *The repetition of computations*. The most basic way to reduce noise is to repeat computations, either spatially or temporally. The use of a polynomial number of repetitions does not affect the fundamental speed advantage of NUTMs over classical UTMs or QUTMs.
— *Kinetic proofreading*. This utilizes irreversible reactions to enable enzymes to discriminate between two possible reaction pathways (to correct or incorrect products) with an accuracy greater than expected based on the difference in the activation energy between these two pathways [45].
— *The use of restriction endonucleases and/or CRISPR/CAS9*. These DNA modification technologies (RNA/proteins) are essentially little nano-machines that are able to cut DNA at specific sequences. Restriction enzymes were the technology that first ignited the biotechnology industry. CRISPR/CAS9 is a recently discovered programmable technology for cutting DNA at specific places. The ability to cut DNA sequences is useful for NUTM error correction as it enables the removal of non-grammatical (e.g. no Thue symbol) sequences, i.e. sequences that have been produced by noisy computations. Cutting a DNA strand stops an NUTM process from executing as the resulting parts no longer has both *start* and *end* symbols. Similarly, if constraints on the correct solution are known, these imply constraints in the pattern of Thue symbol, and these patterns can be cut and the processes removed.
— *The use of labels.* This approach is complementary to the use of restriction enzymes and/or CRISPR. It uses special molecules with complementary sequences of patterns of Thue sequences of interest, and a label that enables the identified DNA strands to be fished out of the pool (figure 4). This approach can be used to both remove unwanted sequences, and identify desired sequences.
— *Checking certificates*. When a NP problem is putatively solved by an NUTM, the answer can be efficiently checked using an electronic computer in P time. This means that an NUTM is required to succeed only with a small probability of success.

To compete effectively with existing electronic computer hardware, one of the most successful technologies in history, will require fabrication of a NUTM with approximately 10^12^ processors executing in parallel—an order of magnitude more processors than in all the computers in the rest of the world combined. This would require the following plausible developments:

— Implementation of Thue rewriting rules using femtograms of DNA.
— Implementation of the accurate recognition of NUTM accepting states: precision of 1 − 1 × 10^−12^ and a recall of 1 − 1 × 10^−12^.
— Implementation of Thue rewriting rule error correction methods.
— A fluidic system capable of implementing multiple cycles of a multiple Thue rule system with at least 25 rules.
— An NUTM programming language that compiles down to Thue systems.

The molecular technology of CRISPR/CAS9 has the potential to rapidly advance the engineering of NUTMs. If the CRISPR/CAS9 system could be modified to edit programmed sequences of DNA, rather than just cut them this would be close to ideal for implementing Thue rule NUTMs. A large amount of research is being undertaken to achieve this redesign of CRISPR/CAS9, so it is not unreasonable to expect rapid progress in this area.

A major motivation for this work is to engineer a general-purpose way of controlling cells. The natural way cells are controlled is a very complex combination of DNA, RNA, protein and small-molecule interactions (supplemented by epigenetics, etc.) with multilevel control implemented through specific chemical interactions. This makes cells very difficult to reprogramme. Synthetic biology has sought to control cells through the design of simple switches, circuits, etc. and has some notable successes (e.g. [46]). However, we argue that a more radical and general approach is required: a DNA NUTM. This would in principle enable arbitrary biological processes to be programmed and executed. The NUTM could receive biological signals from the environment through interaction with transcription factors, etc. It could also use as effectors RNA/proteins generated using special sequences and RNA polymerase, etc. Our current *in vitro* implementation of an NUTM is not directly suitable for this. However, it would seem possible to implement the core ideas in a biological substrate. One way to do this would be to use plasmids as programs, and employ rolling circle amplification.

Computation in a deterministic UTM is in principle reversible, i.e. there is no lower bound on the amount of energy required per operation [47]. It is unclear whether NUTM computation is reversible in P time. This question is of importance in relation to power constraints in NUTMs, and to the P = NP question.

Given the prospect of engineering an NUTM it is natural to consider whether machines can be physically engineered for other complexity classes. A problem is a member of the class co-NP if and only if its complement is in the complexity class NP (figure 1*b*). The definition of NP uses an existential mode of computation: if any branch of the computation tree leads to an accepting state, then the whole computation accepts. The definition of co-NP uses a universal mode of computation: if all branches of the computation tree lead to an accepting state then the whole computation accepts. It would therefore be straightforward to adapt our NUTM design to compute co-NP problems: all accepting states are removed from the mixing vessel.

It would also be straightforward to add randomization to a physical NUTM (through the use of thermal noise). The class BPP (bounded-error probabilistic polynomial-time) is the class of decision problems where there exists a P time randomized algorithm [13]. Although the relationship between BPP and NP is unknown, it would seem computationally useful to generate an exponential number of randomized UTMs in P time, for example for simulations.

The complexity class PSPACE consists of those problems that can be solved by a Turing machine (deterministic or non-deterministic) using a polynomial amount of space (figure 1*b*). It is a superset of NP, but it is not known if this relation is strict i.e. if NP ≠ PSPACE. In an NUTM, all the computation is in a sense local: forks with no communication between computational paths. We hypothesize that a requirement for local computation is a fundamental definition of the NP class. By contrast, a physical PSPACE computer would seem to require highly efficient communication between computational paths, which seems challenging. We therefore conjecture that it is physically impossible to build a computer that can efficiently solve PSPACE complete problems.

Most effort on non-standard computation has focused on developing QUTMs [5,13,47]. Steady progress is being made in theory and implementation, but no QUTM currently exists. Although abstract QUTMs have not been proven to outperform classical UTMs, they are thought to be faster for certain problems [5,13,46]. The best evidence for this is Shor's integer factoring algorithm, which is exponentially faster than the current best classical algorithm [48]. While integer factoring is in NP, it is not thought to be NP complete [11], and it is generally believed that the class of problems solvable in P time by a QUTM (BQP) is not a superset of NP [13].

NUTMs and QUTMs both utilize exponential parallelism, but their advantages and disadvantages seem distinct. NUTMs utilize general parallelism, but this takes up physical space. In a QUTM, the parallelism is restricted, but does not occupy physical space (at least in our Universe). In principle therefore, it would seem to be possible to engineer an NUTM capable of utilizing an exponential number of QCs in P time.

Advocates of the many-worlds interpretation of quantum mechanics argue that QUTMs work through exploitation of the hypothesized parallel universes [8,13,49]. Intriguingly, if the multiverse were an NUTM this would explain the profligacy of worlds.

## Supplementary Material

King_information.pdf
