# Supplementary material for: Computing exponentially faster: implementing a non-deterministic universal Turing machine using DNA
Source: J R Soc Interface. 2017 Mar 1;14(128):20160990. doi: 10.1098/rsif.2016.0990 (PMC5378132; doi:10.1098/rsif.2016.0990)
Supplement: King_information.pdf [file rsif20160990supp1.pdf]

Supplementary Information

1. Thue Rule Recognition and Implementation

Supplementary Figure 1: Thue rule implementation.

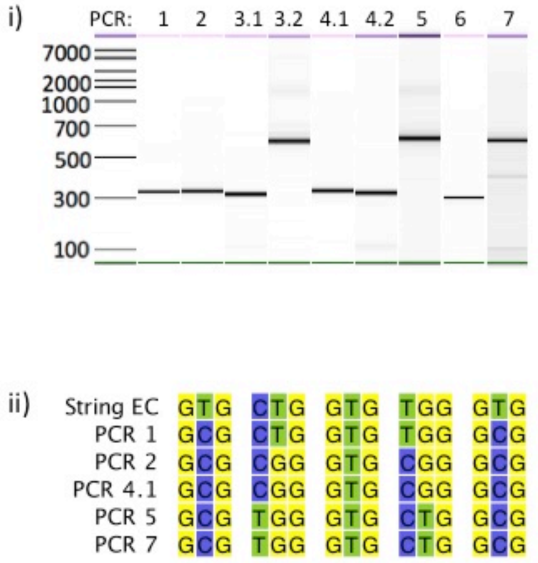

a)
Microprogram and results for swapping **ce** → **ec**. The microprogram is the same as shown in Fig. 5a, except the symbol sequences are transposed. i) Capillary electrophoresis analysis. ii) sequencing data alignment of each step.

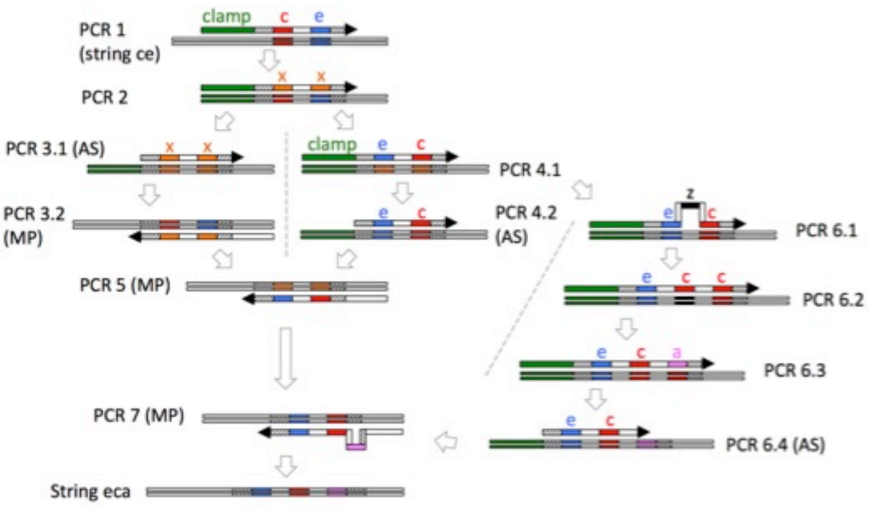

b)

The full microprogram for the Thue rule **ce → eca**. This combines the transposition **ce → ec** and the insertion **ec → eca**. To improve the overall efficiency of the PCRs *in vitro* we utilised DNA templates from earlier in the scheme (PCR 4.1) to implement the insertion rule in parallel with the transposition.

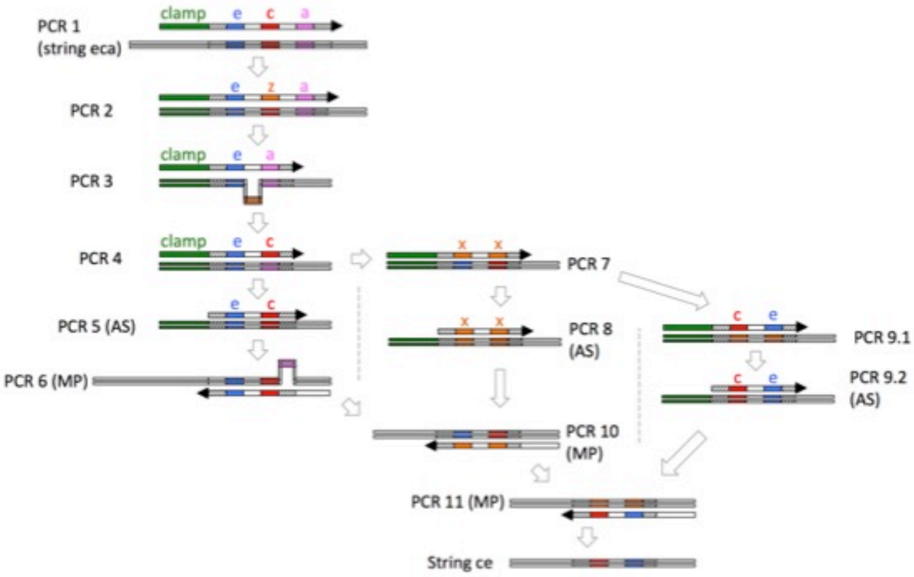

c)
The full microprogram for the Thue rule **eca → ce**. This combines the transposition **ec → ce** and the deletion **eca → ec**. The rest of the microprogram mirrors that of the inverse Thue rule **ce → eca.**

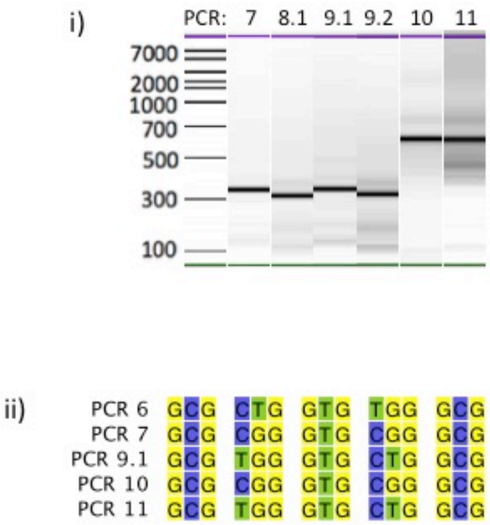

d)

Completion of Thue rule microprogram for **eca** → **ce**. Show is the additional swap microprogram **ec** ↔ **ce** that is performed following the deletion microprogram (Fig. 5c). i) Capillary electrophoresis analysis. ii) sequencing alignment of the PCR steps.

Materials and methods

The template DNA sequence, symbols, clamp and spacers were designed using GeneGenie [1] to ensure minimal erroneous hybridisations between the symbols and non-coding DNA template. Additional calculations of DNA hydridisation kinetics were performed using MFold [2], DINAMelt [3], and UNAFold [4] software with parameters matching the PCR conditions (50mM NaCl, 2mM $MgCl_2$, 500nM primers). All oligonucleotides were synthesised by Integrated DNA Technologies.

DNA templates (initial states) were synthesised by gene synthesis using the SpeedyGenes method [5]. PCR amplification was performed in both symmetric and asymmetric conditions, producing double- and single-stranded DNA products, respectively. For symmetric PCR the reaction contained 500nM of each forward and reverse primer together with 0.5ng DNA template, 0.2mM dNTP mix (New England Biolabs), ThermoPol reaction buffer and 1 unit Deep vent (exo-) DNA polymerase (New England Biolabs). The PCR had an initial denaturation at 95°C for 2 min, then 25 cycles of 95°C for 30 s, 60°C (or other Tm) for 40 s and 72°C for 1 min. PCR products were then purified using a PCR purification kit (Qiagen), following the manufacturer's protocol. These PCR products then served as the template for subsequent PCR steps in the microprogram using the same conditions.

For asymmetric PCR the concentration of primers was altered, with the 25nM forward (limiting) primer and 500nM reverse (excess) primer. All other buffer constituents and PCR conditions were the same as for symmetric PCR. Following purification, the ssDNA product was then used as a megaprimer in the subsequent PCR. This reaction contained 25-50% purified megaprimer and 250nM corresponding forward primer (all other components remained the same). The PCR contained an initial denaturation at 95°C for 2 min, followed by 25 cycles of 95°C for 30 s, 65°C (or other Tm) for 40 s and 72°C for 1 min. Samples were analysed by capillary electrophoresis using a 2100 Bioanalyzer instrument using the DNA 7500 kit (Agilent), following the manufacturer's instructions.

Purified PCR products were subject to Sanger sequencing (Eurofins) using the same primers as used in the PCR. When required, these products were also ligated into a linearised plasmid (pET16b, Novagen) using the In-Fusion cloning kit (Clontech), following the manufacturer's protocol. Samples were then transformed into *E. coli* 5α competent cells (New England Biolabs), streaked onto LB agar (100μg ml^{-1} ampicillin) and incubated overnight at 37°C, following the manufacturer's instructions. Individual colonies were then picked, incubated at 37°C at 200 rpm overnight and the plasmid extracted using a spin miniprep kit (Qiagen), following the manufacturer's protocol. Plasmids were then subject to DNA sequencing, as above.

1. Swainston N, Currin A, Day PJ, Kell DB. GeneGenie: optimized oligomer design for directed evolution. Nucleic Acids Res. 2014 Jul 1;42(W1):W395–400.
2. Zuker M. Mfold web server for nucleic acid folding and hybridization prediction. Nucleic Acids Res. 2003 Jul 1;31(13):3406–15.
3. Markham NR, Zuker M. DINAMelt web server for nucleic acid melting prediction. Nucleic Acids Res. 2005 Jul 1;33(suppl 2):W577–81.
4. Markham, N. R. & Zuker, M. UNAFold: software for nucleic acid folding and hybridization. In Keith, J. M., editor, 4 of 8 Bioinformatics, Volume II. Structure, Functions and Applications, number 453 in *Methods in Molecular Biology*, chapter 1, pages 3-31. Humana Press, Totowa, NJ. (2008).

5. Currin A, Swainston N, Day PJ, Kell DB. SpeedyGenes: an improved gene synthesis method for the efficient production of error-corrected, synthetic protein libraries for directed evolution. Protein Eng Des Sel. 2014 Sep 1;27(9):273–80.

2. Thue Rule Microprogramming Formalization.

Symbols:

H,T = Head, Tail the strings before and after a rewrite (H starts with start, T ends with end).

R = Thue rule head

s,t = {a,b,c,d,e} standard symbols, those in bold are activated (this involves changing spacer symbols).

x,y = special symbols, those in bold are primed

m = clamp
S = start
E = end

Operations:

1.

a)		m R + E	primers
		H R T	template
	→	m R T	Insertion of clamp at rule head
b)		R + E	primers
	+	m R T	template
	→	R T	Removal of clamp at rule head

2.

a)		m $\mathbf{s}_1$ $\mathbf{s}_2$ + E	primers
		m s_1 s_2 T	template
	→	m $\mathbf{s}_1$ $\mathbf{s}_2$ T	activate pair clamp
b)		S + $\mathbf{s}_1$ $\mathbf{s}_2$ T	primers
		H s_1 s_2 T	template
	→	H $\mathbf{s}_1$ $\mathbf{s}_2$ T	activate pair unification

3.

a)		m s_1 s_2 + E	primers
		m $\mathbf{s}_1$ $\mathbf{s}_2$ T	template
	→	m s_1 s_2 T	deactivate pair clamp
b)		S + s_1 s_2 T	primers
		H $\mathbf{s}_1$ $\mathbf{s}_2$ T	template
	→	H s_1 $\mathbf{s}_2$ T	deactivate pair unification

4.

a)		$m\ \mathbf{s_1}\ s_2\ \mathbf{s_3}$	$+\ E$	primers
	$+$	$m\ s_1\ s_2\ s_3\ T$		template
	$\rightarrow$	$m\ \mathbf{s_1}\ s_2\ \mathbf{s_3}\ T$		activate triplet clamp
b)	$+$	$S + \mathbf{s_1}\ s_2\ \mathbf{s_3}\ T$		primers
		$H\ s_1\ s_2\ s_3\ T$		template
	$\rightarrow$	$H\ \mathbf{s_1}\ s_2\ \mathbf{s_3}\ T$		activate triplet unification

5.

a)		$m\ s_1\ s_2\ s_3$	$+\ E$	primers
		$m\ \mathbf{s_1}\ s_2\ \mathbf{s_3}\ T$		template
	$\rightarrow$	$m\ s_1\ s_2\ s_3\ T$		deactivate triplet clamp
b)		$S + s_1\ s_2\ s_3\ T$		primers
		$H\ \mathbf{s_1}\ s_2\ \mathbf{s_3}\ T$		template
	$\rightarrow$	$H\ s_1\ s_2\ s_3\ T$		deactivate triplet unification

6.

a)		$m\ \mathbf{t_1}\ \mathbf{s_2}$	$+\ E$	primers
		$m\ \mathbf{s_1}\ \mathbf{s_2}\ T$		template
	$\rightarrow$	$m\ \mathbf{t_1}\ \mathbf{s_2}\ T$		change symbol clamp
b)		$S + \mathbf{t_1}\ \mathbf{s_2}\ T$		primers
		$H\ \mathbf{s_1}\ \mathbf{s_2}\ T$		template
	$\rightarrow$	$H\ \mathbf{t_1}\ \mathbf{s_2}\ T$		change symbol unification

7.

a)		$m\ \mathbf{s_1}\ \mathbf{t_2}$	$+\ E$	primers
	$+$	$m\ \mathbf{s_1}\ \mathbf{s_2}\ T$		template
	$\rightarrow$	$m\ \mathbf{s_1}\ \mathbf{t_2}\ T$		change symbol clamp
b)		$S + \mathbf{s_1}\ \mathbf{t_2}\ T$		primers
		$H\ \mathbf{s_1}\ \mathbf{s_2}\ T$		template
	$\rightarrow$	$H\ \mathbf{s_1}\ \mathbf{t_2}\ T$		change symbol unification

8.

a)		$m\ \mathbf{x_1}\ \mathbf{x_2}$	$+\ E$	primers
	$+$	$m\ \mathbf{s_1}\ \mathbf{s_2}\ T$		template
	$\rightarrow$	$m\ \mathbf{x_1}\ \mathbf{x_2}\ T$		change pair to xx clamp
b)		$S + \mathbf{x_1}\ \mathbf{x_2}\ T$		primers
		$H\ \mathbf{s_1}\ \mathbf{s_2}\ T$		template
	$\rightarrow$	$H\ \mathbf{x_1}\ \mathbf{x_2}\ T$		change pair to xx unification

9.

a)		$m\ \mathbf{s_1}\ \mathbf{s_2}$	$+\ E$	primers
	$+$	$m\ \mathbf{x_1}\ \mathbf{x_2}\ T$		template
	$\rightarrow$	$m\ \mathbf{s_1}\ \mathbf{s_2}\ T$		change pair from xx clamp

b)		$S + \mathbf{s}_1 \mathbf{s}_2 T$	primers
		$H \mathbf{x}_1 \mathbf{x}_2 T$	template
	→	$H \mathbf{s}_1 \mathbf{s}_2 T$	change pair from xx unification

10.

a)		$m \mathbf{t}_1 s_2 \mathbf{s}_3 + E$	primers
		$m \mathbf{s}_1 s_2 \mathbf{s}_3 T$	template
	→	$m \mathbf{t}_1 s_2 \mathbf{s}_3 T$	change triplet symbol clamp
b)		$S + \mathbf{t}_1 s_2 \mathbf{s}_3 T$	primers
		$H \mathbf{s}_1 s_2 \mathbf{s}_3 T$	template
	→	$H \mathbf{t}_1 s_2 \mathbf{s}_3 T$	change triplet symbol unification

11.

a)		$m \mathbf{s}_1 s_2 \mathbf{t}_3 + E$	primers
		$m \mathbf{s}_1 s_2 \mathbf{s}_3 T$	template
	→	$m \mathbf{s}_1 s_2 \mathbf{t}_3 T$	change triplet symbol clamp
b)		$S + \mathbf{s}_1 s_2 \mathbf{t}_3 T$	
		$H \mathbf{s}_1 s_2 \mathbf{s}_3 T$	
	→	$H \mathbf{s}_1 s_2 \mathbf{t}_3 T$	change triplet symbol unification

12.

a)		$m \mathbf{x}_1 x_2 \mathbf{s}_3 + E$	primers
	+	$m \mathbf{s}_1 s_2 \mathbf{s}_3 T$	template
	→	$m \mathbf{x}_1 x_2 \mathbf{s}_3 T$	change triplet to xx clamp
b)		$S + \mathbf{x}_1 x_2 \mathbf{s}_3 T$	primers
		$H \mathbf{s}_1 s_2 \mathbf{s}_3 T$	template
	→	$H \mathbf{x}_1 x_2 \mathbf{s}_3 T$	change triplet to xx unification

13.

a)		$m \mathbf{s}_1 x_2 \mathbf{x}_3 + E$	primers
	+	$m \mathbf{s}_1 s_2 \mathbf{s}_3 T$	template
	→	$m \mathbf{s}_1 x_2 \mathbf{x}_3 T$	change triplet to xx clamp
b)		$S + \mathbf{s}_1 x_2 \mathbf{x}_3 T$	
		$H \mathbf{s}_1 s_2 \mathbf{s}_3 T$	
	→	$H \mathbf{s}_1 x_2 \mathbf{x}_3 T$	change triplet to xx unification

14.

a)		$m \mathbf{s}_1 s_2 \mathbf{s}_3 + E$	primers
	+	$m \mathbf{x}_1 x_2 \mathbf{s}_3 T$	template
	→	$m \mathbf{s}_1 s_2 \mathbf{s}_3 T$	change triplet from xx clamp
b)		$S + \mathbf{s}_1 s_2 \mathbf{s}_3 T$	
		$H \mathbf{x}_1 x_2 \mathbf{s}_3 T$	

→	H $\mathbf{s}_1\, s_2\, \mathbf{s}_3$ T	change triplet from xx unification
15.		
a)	m $\mathbf{s}_1\, s_2\, \mathbf{s}_3$ + E	primers
	m $\mathbf{s}_1\, x_2\, \mathbf{x}_3$ T	template
→	m $\mathbf{s}_1\, s_2\, \mathbf{s}_3$ T	change triplet from xx clamp
b)	S + $\mathbf{s}_1\, s_2\, \mathbf{s}_3$ T	primers
	H $\mathbf{s}_1\, x_2\, \mathbf{x}_3$ T	template
→	H $\mathbf{s}_1\, s_2\, \mathbf{s}_3$ T	change triplet from xx unification
16.		
a)	m $\mathbf{s}_1\, y_2\, \mathbf{s}_3$ + E	primers
	m $\mathbf{s}_1\, s_2\, \mathbf{s}_3$ T	template
→	m $\mathbf{s}_1\, y_2\, \mathbf{s}_3$ T	change middle to y clamp
b)	S + $\mathbf{s}_1\, y_2\, \mathbf{s}_3$ T	primers
	H $\mathbf{s}_1\, s_2\, \mathbf{s}_3$ T	template
→	H $\mathbf{s}_1\, y_2\, \mathbf{s}_3$ T	change middle to y unification
17.		
a)	m $\mathbf{s}_1\, s_2\, \mathbf{s}_3$ + E	primers
	m $\mathbf{s}_1\, y_2\, \mathbf{s}_3$ T	template
→	m $\mathbf{s}_1\, s_2\, \mathbf{s}_3$ T	change middle from y clamp
b)	S + $\mathbf{s}_1\, s_2\, \mathbf{s}_3$ T	primers
	H $\mathbf{s}_1\, y_2\, \mathbf{s}_3$ T	template
→	H $\mathbf{s}_1\, s_2\, \mathbf{s}_3$ T	change middle from y unification
18.		
a)	m $\mathbf{s}_1\, y\, \mathbf{s}_2$ + E	primers
	m $\mathbf{s}_1\, \mathbf{s}_2$ T	template
→	m $\mathbf{s}_1\, y\, \mathbf{s}_2$ T	Insertion of y in middle clamp
b)	S + $\mathbf{s}_1\, s_2\, \mathbf{s}_3$ T	primers
	H $\mathbf{s}_1\, \mathbf{s}_2$	template
→	H $\mathbf{s}_1\, s_2\, \mathbf{s}_3$ T	Insertion of s in middle unification
19.		
a)	m $\mathbf{s}_1\, \mathbf{s}_2$ + E	primers
	m $\mathbf{s}_1\, y\, \mathbf{s}_2$ T	template
→	m $\mathbf{s}_1\, y\, \mathbf{s}_2$ T	Deletion of y in middle clamp
b)	S + $\mathbf{s}_1\, \mathbf{s}_2$ T	primers
	H $\mathbf{s}_1\, s_2\, \mathbf{s}_3$ T	template
→	H $\mathbf{s}_1\, \mathbf{s}_2$ T	Deletion of s in middle unification

%%%%%%%%%%%%%%%%%%%%
Swap ce->ec:

Step	In State1	In State2	Operation Type	Out State
1)	HceT	-	1.a	mceT
2)	mceT	-	2.a	m**ce**T
3)	m**ce**T	-	8.a	m**xx**T
4)	m**xx**T	-	9.a	m**ec**T
5)	m**ec**T	-	3.a	mecT
6)	m**ce**T	-	1.b	**ce**T
7)	m**xx**T	-	1.b	**xx**T
8)	m**ec**T	-	1.b	**ec**T
9)	mecT	-	1.b	ecT
10)	HceT	**ce**T	2.b	H**ce**T
11)	H**ce**T	**xx**T	8.b	H**xx**T
12)	H**xx**T	**ec**T	9.b	H**ec**T
13)	H**ec**T	ecT	3.b	HecT

%%%%%%%%%%%%%%%%%%%%

Insertion ec->eca:

Step	In State1	In State2	Operation Type	Out State
1)	HecT	-	1.a	mecT
2)	mecT	-	2.a	m**ec**T
3)	m**ec**T	-	18.a	m**eyc**T
4)	m**eyc**T	-	17.a	m**ecc**T
5)	m**ecc**T	-	11.a	m**eca**T
6)	m**eca**T	-	5.a	mecaT
7)	m**ec**T	-	1.b	**ec**T
8)	m**eca**T	-	1.b	**eca**T
9)	mecaT	-	1.b	ecaT
10)	HecT	**ec**T	2.b	H**ec**T
11)	H**ec**T	**eca**T	18.b	H**eca**T
12)	H**eca**T	ecaT	5.b	HecaT

%%%%%%%%%%%%%%%%%%%%

Deletion eca->ec:

Step	In State1	In State2	Operation Type	Out State
1)	HecaT	-	1.a	mecaT
2)	mecaT	-	4.a	m**eca**T

3)	mecaT	-	16.a	meyaT
4)	meyaT	-	19.a	meaT
5)	meaT	-	7.a	mecT
6)	mecT	-	3.a	mecT
7)	mecaT	-	1.b	ecaT
8)	mecT	-	1.b	ecT
9)	mecT	-	1.b	ecT
10)	HecaT	ecaT	4.b	HecaT
11)	HecaT	ecT	19.b	HecT
12)	HecT	ecT	3.b	HecT

%%%%%%%%%%%%%%%%%%%%

Rule ce->eca

Step	In State1	In State2	Operation Type	Out State
1)	HceT	-	1.a	mceT
2)	mceT	-	2.a	mceT
3)	mceT	-	8.a	mxxT
4)	mxxT	-	9.a	mecT
5)	mecT	-	18.a	meycT
6)	meycT	-	17.a	meccT
7)	meccT	-	11.a	mecaT
8)	mecaT	-	5.a	mecaT
9)	mceT	-	1.b	ceT
10)	mxxT	-	1.b	xxT
11)	mecT	-	1.b	ecT
12)	mecaT	-	1.b	ecaT
13)	mecaT	-	1.b	ecaT
14)	HceT	ceT	2.b	HceT
15)	HceT	xxT	8.b	HxxT
16)	HxxT	ecT	9.b	HecT
17)	HecT	ecaT	18.b	HecaT
18)	HecaT	ecaT	5.b	HecaT

%%%%%%%%%%%%%%%%%%%%

Rule eca->ce

Step	In State1	In State2	Operation Type	Out State
1)	HecaT	-	1.a	mecaT
2)	mecaT	-	4.a	mecaT
3)	mecaT	-	16.a	meyaT
4)	meyaT	-	19.a	meaT
5)	meaT	-	7.a	mecT
6)	mecT	-	8.a	mxxT

7)	m**xx**T	-	9.a	m**ce**T
8)	m**ce**T	-	3.a	mceT
9)	mec**a**T	-	1.b	**eca**T
10)	me**c**T	-	1.b	**ec**T
11)	m**xx**T	-	1.b	**xx**T
12)	m**ce**T	-	1.b	**ce**T
13)	mceT	-	1.b	ceT
14)	HecaT	**eca**T	4.b	Hec**a**T
15)	Hec**a**T	**ec**T	19.b	He**c**T
16)	He**c**T	**xx**T	8.b	H**xx**T
17)	H**xx**T	**ce**T	9.b	H**ce**T
18)	H**ce**T	ceT	3.b	HceT

3. DNA Binding Estimates.

Rule Recognition Description

The rule recognition data set contains hybridisation data between the antecedent of each rewrite rule extended with the clamp and target sequences consisting of all relevant combinations of Thue symbols. For each rule antecedent we generated two sets of target sequences: matching and mismatching. The aim is to demonstrate that matching sequences are energetically more favourable compared to mismatching sequences.

Let us describe the data in more detail using the following example. Consider a rule ac->ca and its antecedent sequence extended with the clamp: clamp_s_a_s_c_s, where s is the spacer symbol.

The matching set consists of all sequences of the form s_X_s_Y_s_Z_s_a_s_c_s where X,Y,Z are ranging over all Thue symbols. Let us note that the number of nucleobases in s_X_s_Y_s_Z_s it greater than the number of nucliobases in the clamp and therefore s_X_s_Y_s_Z_s_a_s_c_s covers all possible matching cases.

The mismatching set consists of all sequences of the form s_X_s_Y_s_Z_s_U_s_c_s and s_X_s_Y_s_Z_s_a_s_V_s, which do not match s_a_s_c_s, where X,Y,Z,U,V are ranging over all symbols. Informally, these target sequences represent all symbol combinations which cover the clamp and all possible one symbol mismatches of the rule antecedent. These sequences represent all mismatches closest to the prefect match.

The matching data for the rule antecedent "ac" is contained in the file s_a_s_c_s_matches_dG.data and the mismatching data is contained in the file s_a_s_c_s_mismatches_dG.data. The data is sorted in increasing order with respect to dG. The data set contains dG for matching and mismatching sequences for all rule antecedents of our universal Thue rewrite system.

The data is summarised below which contains maxima and minima of matching and mismatching energies for all rule antecedents. In all cases we can observe dG of matching sequences is lower than of mismatching sequences and therefore matching sequences are energetically more favorable than mismatching sequences.

The hybridisation energies where computed using the UNAFold v3.9 system [1] with parameters:
0.002M [Mg++]
0.05M [Na+]
0.0000005M <total strand concentration>

[1] Markham, N. R. & Zuker, M. (2008) UNAFold: software for nucleic acid folding and hybridization. In Keith, J. M., editor, 4 of 8 Bioinformatics, Volume II. Structure, Functions and Applications, number 453 in Methods in Molecular Biology, chapter 1, pages 3-31. Humana Press, Totowa, NJ.

```
***************************************************************************************

Rule Recognition Summary

========================================
s_a_s_c_s_matches_dG.data
```

```
     1 >s_a_s_c_s_d_s_a_s_c_s  GTGTCTGTGTGGGTGACGGTGTCTGTGTGGGTG;  >clamp_s_a_s_c_s:compl:rev
CACCCACACAGACACTATCATGTTGACATTCC; dG = -17.4 (min dG)
   125 >s_d_s_e_s_d_s_a_s_c_s  GTGACGGTGCTGGTGACGGTGTCTGTGTGGGTG;  >clamp_s_a_s_c_s:compl:rev
CACCCACACAGACACTATCATGTTGACATTCC; dG = -17.1 (max dG)

s_a_s_c_s_mismatches_dG.data
     1 >s_b_s_b_s_c_s_d_s_c_s  GTGGCTGTGGCTGTGTGGGTGACGGTGTGGGTG;  >clamp_s_a_s_c_s:compl:rev
CACCCACACAGACACTATCATGTTGACATTCC; dG = -16.0 (min dG)
   896 >s_e_s_e_s_e_s_a_s_b_s  GTGCTGGTGCTGGTGCTGGTGTCTGTGGCTGTG;  >clamp_s_a_s_c_s:compl:rev
CACCCACACAGACACTATCATGTTGACATTCC; dG = -9.9 (max dG)

========================================
s_a_s_d_s_matches_dG.data
     1 >s_a_s_d_s_d_s_a_s_d_s  GTGTCTGTGACGGTGACGGTGTCTGTGACGGTG;  >clamp_s_a_s_d_s:compl:rev
CACCGTCACAGACACTATCATGTTGACATTCC; dG = -17.6 (min dG)
   125 >s_d_s_e_s_d_s_a_s_d_s  GTGACGGTGCTGGTGACGGTGTCTGTGACGGTG;  >clamp_s_a_s_d_s:compl:rev
CACCGTCACAGACACTATCATGTTGACATTCC; dG = -17.2 (max dG)

s_a_s_d_s_mismatches_dG.data
     1 >s_a_s_a_s_b_s_d_s_d_s  GTGTCTGTGTCTGTGGCTGTGACGGTGACGGTG;  >clamp_s_a_s_d_s:compl:rev
CACCGTCACAGACACTATCATGTTGACATTCC; dG = -16.2 (min dG)
   896 >s_e_s_e_s_e_s_a_s_a_s  GTGCTGGTGCTGGTGCTGGTGTCTGTGTCTGTG;  >clamp_s_a_s_d_s:compl:rev
CACCGTCACAGACACTATCATGTTGACATTCC; dG = -9.4 (max dG)

========================================
s_b_s_c_s_matches_dG.data
     1 >s_b_s_b_s_c_s_b_s_c_s  GTGGCTGTGGCTGTGTGGGTGGCTGTGTGGGTG;  >clamp_s_b_s_c_s:compl:rev
CACCCACACAGCCACTATCATGTTGACATTCC; dG = -18.8 (min dG)
   125 >s_d_s_e_s_d_s_b_s_c_s  GTGACGGTGCTGGTGACGGTGGCTGTGTGGGTG;  >clamp_s_b_s_c_s:compl:rev
CACCCACACAGCCACTATCATGTTGACATTCC; dG = -18.4 (max dG)

s_b_s_c_s_mismatches_dG.data
     1 >s_b_s_a_s_c_s_d_s_c_s  GTGGCTGTGTCTGTGTGGGTGACGGTGTGGGTG;  >clamp_s_b_s_c_s:compl:rev
CACCCACACAGCCACTATCATGTTGACATTCC; dG = -13.6 (min dG)
   896 >s_e_s_e_s_e_s_d_s_c_s  GTGCTGGTGCTGGTGCTGGTGACGGTGTGGGTG;  >clamp_s_b_s_c_s:compl:rev
CACCCACACAGCCACTATCATGTTGACATTCC; dG = -10.1 (max dG)

========================================
s_b_s_d_s_matches_dG.data
     1 >s_b_s_b_s_d_s_b_s_d_s  GTGGCTGTGGCTGTGACGGTGGCTGTGACGGTG;  >clamp_s_b_s_d_s:compl:rev
CACCGTCACAGCCACTATCATGTTGACATTCC; dG = -19.0 (min dG)
   125 >s_d_s_e_s_d_s_b_s_d_s  GTGACGGTGCTGGTGACGGTGGCTGTGACGGTG;  >clamp_s_b_s_d_s:compl:rev
CACCGTCACAGCCACTATCATGTTGACATTCC; dG = -18.6 (max dG)

s_b_s_d_s_mismatches_dG.data
     1 >s_b_s_b_s_a_s_d_s_d_s  GTGGCTGTGGCTGTGTCTGTGACGGTGACGGTG;  >clamp_s_b_s_d_s:compl:rev
CACCGTCACAGCCACTATCATGTTGACATTCC; dG = -14.2 (min dG)
   896 >s_e_s_e_s_e_s_e_s_d_s  GTGCTGGTGCTGGTGCTGGTGCTGGTGACGGTG;  >clamp_s_b_s_d_s:compl:rev
CACCGTCACAGCCACTATCATGTTGACATTCC; dG = -10.3 (max dG)

========================================
s_c_s_a_s_matches_dG.data
     1 >s_a_s_c_s_a_s_c_s_a_s  GTGTCTGTGTGGGTGTCTGTGTGGGTGTCTGTG;  >clamp_s_c_s_a_s:compl:rev
CACAGACACCCACACTATCATGTTGACATTCC; dG = -17.4 (min dG)
   125 >s_d_s_e_s_d_s_c_s_a_s  GTGACGGTGCTGGTGACGGTGTGGGTGTCTGTG;  >clamp_s_c_s_a_s:compl:rev
CACAGACACCCACACTATCATGTTGACATTCC; dG = -17.1 (max dG)

s_c_s_a_s_mismatches_dG.data
     1 >s_b_s_c_s_b_s_d_s_a_s  GTGGCTGTGTGGGTGGCTGTGACGGTGTCTGTG;  >clamp_s_c_s_a_s:compl:rev
CACAGACACCCACACTATCATGTTGACATTCC; dG = -16.0 (min dG)
   896 >s_e_s_e_s_e_s_b_s_a_s  GTGCTGGTGCTGGTGCTGGTGGCTGTGTCTGTG;  >clamp_s_c_s_a_s:compl:rev
CACAGACACCCACACTATCATGTTGACATTCC; dG = -10.0 (max dG)

========================================
s_c_s_b_s_matches_dG.data
     1 >s_c_s_b_s_d_s_c_s_b_s  GTGTGGGTGGCTGTGACGGTGTGGGTGGCTGTG;  >clamp_s_c_s_b_s:compl:rev
CACAGCCACCCACACTATCATGTTGACATTCC; dG = -18.8 (min dG)
   125 >s_d_s_e_s_d_s_c_s_b_s  GTGACGGTGCTGGTGACGGTGTGGGTGGCTGTG;  >clamp_s_c_s_b_s:compl:rev
CACAGCCACCCACACTATCATGTTGACATTCC; dG = -18.4 (max dG)

s_c_s_b_s_mismatches_dG.data
     1 >s_b_s_c_s_a_s_d_s_b_s  GTGGCTGTGTGGGTGTCTGTGACGGTGGCTGTG;  >clamp_s_c_s_b_s:compl:rev
CACAGCCACCCACACTATCATGTTGACATTCC; dG = -13.6 (min dG)
```

```
   896 >s_e_s_e_s_e_s_c_s_c_s  GTGCTGGTGCTGGTGCTGGTGTGGGTGTGGGTG;  >clamp_s_c_s_b_s:compl:rev
CACAGCCACCCACACTATCATGTTGACATTCC; dG = -10.5 (max dG)

========================================
s_c_s_d_s_c_s_a_s_e_s_matches_dG.data
     1 >s_a_s_a_s_a_s_c_s_d_s_c_s_a_s_e_s
GTGTCTGTGTCTGTGTCTGTGTGGGTGACGGTGTGGGTGTCTGTGCTGGTG;    >clamp_s_c_s_d_s_c_s_a_s_e_s:compl:rev
CACCAGCACAGACACCCACACCGTCACCCACACTATCATGTTGACATTCC; dG = -42.6 (min dG)
   125 >s_d_s_e_s_d_s_c_s_d_s_c_s_a_s_e_s
GTGACGGTGCTGGTGACGGTGTGGGTGACGGTGTGGGTGTCTGTGCTGGTG;    >clamp_s_c_s_d_s_c_s_a_s_e_s:compl:rev
CACCAGCACAGACACCCACACCGTCACCCACACTATCATGTTGACATTCC; dG = -42.5 (max dG)

s_c_s_d_s_c_s_a_s_e_s_mismatches_dG.data
     1 >s_c_s_a_s_a_s_c_s_d_s_c_s_b_s_e_s
GTGTGGGTGTCTGTGTCTGTGTGGGTGACGGTGTGGGTGGCTGTGCTGGTG;    >clamp_s_c_s_d_s_c_s_a_s_e_s:compl:rev
CACCAGCACAGACACCCACACCGTCACCCACACTATCATGTTGACATTCC; dG = -40.6 (min dG)
  2500 >s_b_s_e_s_e_s_c_s_a_s_c_s_a_s_e_s
GTGGCTGTGCTGGTGCTGGTGTGGGTGTCTGTGTGGGTGTCTGTGCTGGTG;    >clamp_s_c_s_d_s_c_s_a_s_e_s:compl:rev
CACCAGCACAGACACCCACACCGTCACCCACACTATCATGTTGACATTCC; dG = -33.1 (max dG)

========================================
s_c_s_d_s_c_s_a_s_matches_dG.data
     1 >s_a_s_a_s_a_s_c_s_d_s_c_s_a_s          GTGTCTGTGTCTGTGTCTGTGTGGGTGACGGTGTGGGTGTCTGTG;
>clamp_s_c_s_d_s_c_s_a_s:compl:rev  CACAGACACCCACACCGTCACCCACACTATCATGTTGACATTCC;  dG  =  -34.0
(min dG)
   125 >s_d_s_e_s_d_s_c_s_d_s_c_s_a_s          GTGACGGTGCTGGTGACGGTGTGGGTGACGGTGTGGGTGTCTGTG;
>clamp_s_c_s_d_s_c_s_a_s:compl:rev  CACAGACACCCACACCGTCACCCACACTATCATGTTGACATTCC;  dG  =  -33.9
(max dG)

s_c_s_d_s_c_s_a_s_mismatches_dG.data
     1 >s_c_s_d_s_c_s_b_s_d_s_c_s_a_s          GTGTGGGTGACGGTGTGGGTGGCTGTGACGGTGTGGGTGTCTGTG;
>clamp_s_c_s_d_s_c_s_a_s:compl:rev  CACAGACACCCACACCGTCACCCACACTATCATGTTGACATTCC;  dG  =  -32.3
(min dG)
  1994 >s_a_s_b_s_a_s_c_s_a_s_c_s_a_s          GTGTCTGTGGCTGTGTCTGTGTGGGTGTCTGTGTGGGTGTCTGTG;
>clamp_s_c_s_d_s_c_s_a_s:compl:rev  CACAGACACCCACACCGTCACCCACACTATCATGTTGACATTCC;  dG  =  -24.5
(max dG)

========================================
s_c_s_e_s_matches_dG.data
     1 >s_c_s_e_s_d_s_c_s_e_s  GTGTGGGTGCTGGTGACGGTGTGGGTGCTGGTG;  >clamp_s_c_s_e_s:compl:rev
CACCAGCACCCACACTATCATGTTGACATTCC; dG = -18.8 (min dG)
   125 >s_d_s_e_s_d_s_c_s_e_s  GTGACGGTGCTGGTGACGGTGTGGGTGCTGGTG;  >clamp_s_c_s_e_s:compl:rev
CACCAGCACCCACACTATCATGTTGACATTCC; dG = -18.4 (max dG)

s_c_s_e_s_mismatches_dG.data
     1 >s_a_s_a_s_c_s_c_s_d_s  GTGTCTGTGTCTGTGTGGGTGTGGGTGACGGTG;  >clamp_s_c_s_e_s:compl:rev
CACCAGCACCCACACTATCATGTTGACATTCC; dG = -13.8 (min dG)
   896 >s_e_s_d_s_d_s_c_s_a_s  GTGCTGGTGACGGTGACGGTGTGGGTGTCTGTG;  >clamp_s_c_s_e_s:compl:rev
CACCAGCACCCACACTATCATGTTGACATTCC; dG = -10.7 (max dG)

========================================
s_d_s_a_s_matches_dG.data
     1 >s_e_s_d_s_a_s_d_s_a_s  GTGCTGGTGACGGTGTCTGTGACGGTGTCTGTG;  >clamp_s_d_s_a_s:compl:rev
CACAGACACCGTCACTATCATGTTGACATTCC; dG = -18.1 (min dG)
   125 >s_d_s_e_s_d_s_d_s_a_s  GTGACGGTGCTGGTGACGGTGACGGTGTCTGTG;  >clamp_s_d_s_a_s:compl:rev
CACAGACACCGTCACTATCATGTTGACATTCC; dG = -17.2 (max dG)

s_d_s_a_s_mismatches_dG.data
     1 >s_a_s_d_s_b_s_d_s_e_s  GTGTCTGTGACGGTGGCTGTGACGGTGCTGGTG;  >clamp_s_d_s_a_s:compl:rev
CACAGACACCGTCACTATCATGTTGACATTCC; dG = -16.2 (min dG)
   896 >s_a_s_a_s_a_s_a_s_a_s  GTGTCTGTGTCTGTGTCTGTGTCTGTGTCTGTG;  >clamp_s_d_s_a_s:compl:rev
CACAGACACCGTCACTATCATGTTGACATTCC; dG = -8.8 (max dG)

========================================
s_d_s_b_s_matches_dG.data
     1 >s_a_s_d_s_b_s_d_s_b_s  GTGTCTGTGACGGTGGCTGTGACGGTGGCTGTG;  >clamp_s_d_s_b_s:compl:rev
CACAGCCACCGTCACTATCATGTTGACATTCC; dG = -19.5 (min dG)
   125 >s_d_s_e_s_d_s_d_s_b_s  GTGACGGTGCTGGTGACGGTGACGGTGGCTGTG;  >clamp_s_d_s_b_s:compl:rev
CACAGCCACCGTCACTATCATGTTGACATTCC; dG = -18.6 (max dG)

s_d_s_b_s_mismatches_dG.data
     1 >s_a_s_d_s_a_s_d_s_e_s  GTGTCTGTGACGGTGTCTGTGACGGTGCTGGTG;  >clamp_s_d_s_b_s:compl:rev
CACAGCCACCGTCACTATCATGTTGACATTCC; dG = -13.8 (min dG)
```

896 >s_e_s_e_s_e_s_a_s_b_s GTGCTGGTGCTGGTGCTGGTGTCTGTGGCTGTG; >clamp_s_d_s_b_s:compl:rev CACAGCCACCGTCACTATCATGTTGACATTCC; dG = -9.9 (max dG)

==
s_d_s_e_s_matches_dG.data
1 >s_a_s_d_s_e_s_d_s_e_s GTGTCTGTGACGGTGCTGGTGACGGTGCTGGTG; >clamp_s_d_s_e_s:compl:rev CACCAGCACCGTCACTATCATGTTGACATTCC; dG = -19.5 (min dG)
125 >s_d_s_e_s_c_s_d_s_e_s GTGACGGTGCTGGTGTGGGTGACGGTGCTGGTG; >clamp_s_d_s_e_s:compl:rev CACCAGCACCGTCACTATCATGTTGACATTCC; dG = -18.6 (max dG)

s_d_s_e_s_mismatches_dG.data
1 >s_a_s_d_s_c_s_d_s_d_s GTGTCTGTGACGGTGTGGGTGACGGTGACGGTG; >clamp_s_d_s_e_s:compl:rev CACCAGCACCGTCACTATCATGTTGACATTCC; dG = -13.9 (min dG)
896 >s_a_s_c_s_b_s_a_s_e_s GTGTCTGTGTGGGTGGCTGTGTCTGTGCTGGTG; >clamp_s_d_s_e_s:compl:rev CACCAGCACCGTCACTATCATGTTGACATTCC; dG = -9.9 (max dG)

==
s_e_s_c_s_a_s_matches_dG.data
1 >s_a_s_a_s_a_s_e_s_c_s_a_s GTGTCTGTGTCTGTGTCTGTGCTGGTGTGGGTGTCTGTG; >clamp_s_e_s_c_s_a_s:compl:rev CACAGACACCCACACCAGCACTATCATGTTGACATTCC; dG = -25.7 (min dG)
125 >s_d_s_e_s_d_s_e_s_c_s_a_s GTGACGGTGCTGGTGACGGTGCTGGTGTGGGTGTCTGTG; >clamp_s_e_s_c_s_a_s:compl:rev CACAGACACCCACACCAGCACTATCATGTTGACATTCC; dG = -25.6 (max dG)

s_e_s_c_s_a_s_mismatches_dG.data
1 >s_e_s_c_s_b_s_d_s_c_s_a_s GTGCTGGTGTGGGTGGCTGTGACGGTGTGGGTGTCTGTG; >clamp_s_e_s_c_s_a_s:compl:rev CACAGACACCCACACCAGCACTATCATGTTGACATTCC; dG = -24.0 (min dG)
1483 >s_d_s_b_s_c_s_e_s_b_s_a_s GTGACGGTGGCTGTGTGGGTGCTGGTGGCTGTGTCTGTG; >clamp_s_e_s_c_s_a_s:compl:rev CACAGACACCCACACCAGCACTATCATGTTGACATTCC; dG = -16.5 (max dG)

==
s_e_s_d_s_b_s_matches_dG.data
1 >s_a_s_a_s_a_s_e_s_d_s_b_s GTGTCTGTGTCTGTGTCTGTGCTGGTGACGGTGGCTGTG; >clamp_s_e_s_d_s_b_s:compl:rev CACAGCCACCGTCACCAGCACTATCATGTTGACATTCC; dG = -27.2 (min dG)
125 >s_d_s_e_s_d_s_e_s_d_s_b_s GTGACGGTGCTGGTGACGGTGCTGGTGACGGTGGCTGTG; >clamp_s_e_s_d_s_b_s:compl:rev CACAGCCACCGTCACCAGCACTATCATGTTGACATTCC; dG = -27.2 (max dG)

s_e_s_d_s_b_s_mismatches_dG.data
1 >s_d_s_c_s_d_s_b_s_d_s_b_s GTGACGGTGTGGGTGACGGTGGCTGTGACGGTGGCTGTG; >clamp_s_e_s_d_s_b_s:compl:rev CACAGCCACCGTCACCAGCACTATCATGTTGACATTCC; dG = -22.5 (min dG)
1483 >s_d_s_a_s_e_s_e_s_a_s_b_s GTGACGGTGTCTGTGCTGGTGCTGGTGTCTGTGGCTGTG; >clamp_s_e_s_d_s_b_s:compl:rev CACAGCCACCGTCACCAGCACTATCATGTTGACATTCC; dG = -17.7 (max dG)

****

AC Matches

1 >s_a_s_c_s_d_s_a_s_c_s GTGTCTGTGTGGGTGACGGTGTCTGTGTGGGTG; >clamp_s_a_s_c_s:compl:rev CACCCACACAGACACTATCATGTTGACATTCC; dG = -17.4
2 >s_b_s_a_s_c_s_a_s_c_s GTGGCTGTGTCTGTGTGGGTGTCTGTGTGGGTG; >clamp_s_a_s_c_s:compl:rev CACCCACACAGACACTATCATGTTGACATTCC; dG = -17.4
3 >s_a_s_a_s_c_s_a_s_c_s GTGTCTGTGTCTGTGTGGGTGTCTGTGTGGGTG; >clamp_s_a_s_c_s:compl:rev CACCCACACAGACACTATCATGTTGACATTCC; dG = -17.4
4 >s_e_s_a_s_c_s_a_s_c_s GTGCTGGTGTCTGTGTGGGTGTCTGTGTGGGTG; >clamp_s_a_s_c_s:compl:rev CACCCACACAGACACTATCATGTTGACATTCC; dG = -17.3
5 >s_c_s_a_s_c_s_a_s_c_s GTGTGGGTGTCTGTGTGGGTGTCTGTGTGGGTG; >clamp_s_a_s_c_s:compl:rev CACCCACACAGACACTATCATGTTGACATTCC; dG = -17.3
6 >s_d_s_a_s_c_s_a_s_c_s GTGACGGTGTCTGTGTGGGTGTCTGTGTGGGTG; >clamp_s_a_s_c_s:compl:rev CACCCACACAGACACTATCATGTTGACATTCC; dG = -17.3
7 >s_a_s_a_s_a_s_a_s_c_s GTGTCTGTGTCTGTGTCTGTGTCTGTGTGGGTG; >clamp_s_a_s_c_s:compl:rev CACCCACACAGACACTATCATGTTGACATTCC; dG = -17.1
8 >s_a_s_b_s_e_s_a_s_c_s GTGTCTGTGGCTGTGCTGGTGTCTGTGTGGGTG; >clamp_s_a_s_c_s:compl:rev CACCCACACAGACACTATCATGTTGACATTCC; dG = -17.1
9 >s_d_s_e_s_e_s_a_s_c_s GTGACGGTGCTGGTGCTGGTGTCTGTGTGGGTG; >clamp_s_a_s_c_s:compl:rev CACCCACACAGACACTATCATGTTGACATTCC; dG = -17.1
10 >s_e_s_a_s_a_s_a_s_c_s GTGCTGGTGTCTGTGTCTGTGTCTGTGTGGGTG; >clamp_s_a_s_c_s:compl:rev CACCCACACAGACACTATCATGTTGACATTCC; dG = -17.1
11 >s_e_s_a_s_b_s_a_s_c_s GTGCTGGTGTCTGTGGCTGTGTCTGTGTGGGTG; >clamp_s_a_s_c_s:compl:rev CACCCACACAGACACTATCATGTTGACATTCC; dG = -17.1
12 >s_e_s_a_s_d_s_a_s_c_s GTGCTGGTGTCTGTGACGGTGTCTGTGTGGGTG; >clamp_s_a_s_c_s:compl:rev CACCCACACAGACACTATCATGTTGACATTCC; dG = -17.1
13 >s_e_s_a_s_e_s_a_s_c_s GTGCTGGTGTCTGTGCTGGTGTCTGTGTGGGTG; >clamp_s_a_s_c_s:compl:rev CACCCACACAGACACTATCATGTTGACATTCC; dG = -17.1

14 >s_e_s_b_s_a_s_a_s_c_s GTGCTGGTGGCTGTGTCTGTGTCTGTGTGGGTG; >clamp_s_a_s_c_s:compl:rev CACCCACACAGACACTATCATGTTGACATTCC; dG = -17.1
15 >s_e_s_b_s_b_s_a_s_c_s GTGCTGGTGGCTGTGGCTGTGTCTGTGTGGGTG; >clamp_s_a_s_c_s:compl:rev CACCCACACAGACACTATCATGTTGACATTCC; dG = -17.1
16 >s_e_s_b_s_c_s_a_s_c_s GTGCTGGTGGCTGTGTGGGTGTCTGTGTGGGTG; >clamp_s_a_s_c_s:compl:rev CACCCACACAGACACTATCATGTTGACATTCC; dG = -17.1
17 >s_e_s_b_s_d_s_a_s_c_s GTGCTGGTGGCTGTGACGGTGTCTGTGTGGGTG; >clamp_s_a_s_c_s:compl:rev CACCCACACAGACACTATCATGTTGACATTCC; dG = -17.1
18 >s_a_s_c_s_a_s_a_s_c_s GTGTCTGTGTGGGTGTCTGTGTCTGTGTGGGTG; >clamp_s_a_s_c_s:compl:rev CACCCACACAGACACTATCATGTTGACATTCC; dG = -17.1
19 >s_e_s_b_s_e_s_a_s_c_s GTGCTGGTGGCTGTGCTGGTGTCTGTGTGGGTG; >clamp_s_a_s_c_s:compl:rev CACCCACACAGACACTATCATGTTGACATTCC; dG = -17.1
20 >s_e_s_c_s_a_s_a_s_c_s GTGCTGGTGTGGGTGTCTGTGTCTGTGTGGGTG; >clamp_s_a_s_c_s:compl:rev CACCCACACAGACACTATCATGTTGACATTCC; dG = -17.1
21 >s_e_s_c_s_b_s_a_s_c_s GTGCTGGTGTGGGTGGCTGTGTCTGTGTGGGTG; >clamp_s_a_s_c_s:compl:rev CACCCACACAGACACTATCATGTTGACATTCC; dG = -17.1
22 >s_e_s_c_s_c_s_a_s_c_s GTGCTGGTGTGGGTGTGGGTGTCTGTGTGGGTG; >clamp_s_a_s_c_s:compl:rev CACCCACACAGACACTATCATGTTGACATTCC; dG = -17.1
23 >s_e_s_c_s_d_s_a_s_c_s GTGCTGGTGTGGGTGACGGTGTCTGTGTGGGTG; >clamp_s_a_s_c_s:compl:rev CACCCACACAGACACTATCATGTTGACATTCC; dG = -17.1
24 >s_e_s_c_s_e_s_a_s_c_s GTGCTGGTGTGGGTGCTGGTGTCTGTGTGGGTG; >clamp_s_a_s_c_s:compl:rev CACCCACACAGACACTATCATGTTGACATTCC; dG = -17.1
25 >s_e_s_d_s_a_s_a_s_c_s GTGCTGGTGACGGTGTCTGTGTCTGTGTGGGTG; >clamp_s_a_s_c_s:compl:rev CACCCACACAGACACTATCATGTTGACATTCC; dG = -17.1
26 >s_e_s_d_s_b_s_a_s_c_s GTGCTGGTGACGGTGGCTGTGTCTGTGTGGGTG; >clamp_s_a_s_c_s:compl:rev CACCCACACAGACACTATCATGTTGACATTCC; dG = -17.1
27 >s_e_s_d_s_c_s_a_s_c_s GTGCTGGTGACGGTGTGGGTGTCTGTGTGGGTG; >clamp_s_a_s_c_s:compl:rev CACCCACACAGACACTATCATGTTGACATTCC; dG = -17.1
28 >s_e_s_d_s_d_s_a_s_c_s GTGCTGGTGACGGTGACGGTGTCTGTGTGGGTG; >clamp_s_a_s_c_s:compl:rev CACCCACACAGACACTATCATGTTGACATTCC; dG = -17.1
29 >s_a_s_c_s_b_s_a_s_c_s GTGTCTGTGTGGGTGGCTGTGTCTGTGTGGGTG; >clamp_s_a_s_c_s:compl:rev CACCCACACAGACACTATCATGTTGACATTCC; dG = -17.1
30 >s_e_s_d_s_e_s_a_s_c_s GTGCTGGTGACGGTGCTGGTGTCTGTGTGGGTG; >clamp_s_a_s_c_s:compl:rev CACCCACACAGACACTATCATGTTGACATTCC; dG = -17.1
31 >s_e_s_e_s_a_s_a_s_c_s GTGCTGGTGCTGGTGTCTGTGTCTGTGTGGGTG; >clamp_s_a_s_c_s:compl:rev CACCCACACAGACACTATCATGTTGACATTCC; dG = -17.1
32 >s_e_s_e_s_b_s_a_s_c_s GTGCTGGTGCTGGTGGCTGTGTCTGTGTGGGTG; >clamp_s_a_s_c_s:compl:rev CACCCACACAGACACTATCATGTTGACATTCC; dG = -17.1
33 >s_e_s_e_s_c_s_a_s_c_s GTGCTGGTGCTGGTGTGGGTGTCTGTGTGGGTG; >clamp_s_a_s_c_s:compl:rev CACCCACACAGACACTATCATGTTGACATTCC; dG = -17.1
34 >s_e_s_e_s_d_s_a_s_c_s GTGCTGGTGCTGGTGACGGTGTCTGTGTGGGTG; >clamp_s_a_s_c_s:compl:rev CACCCACACAGACACTATCATGTTGACATTCC; dG = -17.1
35 >s_e_s_e_s_e_s_a_s_c_s GTGCTGGTGCTGGTGCTGGTGTCTGTGTGGGTG; >clamp_s_a_s_c_s:compl:rev CACCCACACAGACACTATCATGTTGACATTCC; dG = -17.1
36 >s_a_s_c_s_c_s_a_s_c_s GTGTCTGTGTGGGTGTGGGTGTCTGTGTGGGTG; >clamp_s_a_s_c_s:compl:rev CACCCACACAGACACTATCATGTTGACATTCC; dG = -17.1
37 >s_a_s_c_s_e_s_a_s_c_s GTGTCTGTGTGGGTGCTGGTGTCTGTGTGGGTG; >clamp_s_a_s_c_s:compl:rev CACCCACACAGACACTATCATGTTGACATTCC; dG = -17.1
38 >s_a_s_d_s_a_s_a_s_c_s GTGTCTGTGACGGTGTCTGTGTCTGTGTGGGTG; >clamp_s_a_s_c_s:compl:rev CACCCACACAGACACTATCATGTTGACATTCC; dG = -17.1
39 >s_a_s_d_s_b_s_a_s_c_s GTGTCTGTGACGGTGGCTGTGTCTGTGTGGGTG; >clamp_s_a_s_c_s:compl:rev CACCCACACAGACACTATCATGTTGACATTCC; dG = -17.1
40 >s_a_s_d_s_c_s_a_s_c_s GTGTCTGTGACGGTGTGGGTGTCTGTGTGGGTG; >clamp_s_a_s_c_s:compl:rev CACCCACACAGACACTATCATGTTGACATTCC; dG = -17.1
41 >s_a_s_d_s_d_s_a_s_c_s GTGTCTGTGACGGTGACGGTGTCTGTGTGGGTG; >clamp_s_a_s_c_s:compl:rev CACCCACACAGACACTATCATGTTGACATTCC; dG = -17.1
42 >s_a_s_a_s_b_s_a_s_c_s GTGTCTGTGTCTGTGGCTGTGTCTGTGTGGGTG; >clamp_s_a_s_c_s:compl:rev CACCCACACAGACACTATCATGTTGACATTCC; dG = -17.1
43 >s_a_s_d_s_e_s_a_s_c_s GTGTCTGTGACGGTGCTGGTGTCTGTGTGGGTG; >clamp_s_a_s_c_s:compl:rev CACCCACACAGACACTATCATGTTGACATTCC; dG = -17.1
44 >s_a_s_e_s_a_s_a_s_c_s GTGTCTGTGCTGGTGTCTGTGTCTGTGTGGGTG; >clamp_s_a_s_c_s:compl:rev CACCCACACAGACACTATCATGTTGACATTCC; dG = -17.1
45 >s_a_s_e_s_b_s_a_s_c_s GTGTCTGTGCTGGTGGCTGTGTCTGTGTGGGTG; >clamp_s_a_s_c_s:compl:rev CACCCACACAGACACTATCATGTTGACATTCC; dG = -17.1
46 >s_a_s_e_s_c_s_a_s_c_s GTGTCTGTGCTGGTGTGGGTGTCTGTGTGGGTG; >clamp_s_a_s_c_s:compl:rev CACCCACACAGACACTATCATGTTGACATTCC; dG = -17.1
47 >s_a_s_e_s_d_s_a_s_c_s GTGTCTGTGCTGGTGACGGTGTCTGTGTGGGTG; >clamp_s_a_s_c_s:compl:rev CACCCACACAGACACTATCATGTTGACATTCC; dG = -17.1
48 >s_a_s_e_s_e_s_a_s_c_s GTGTCTGTGCTGGTGCTGGTGTCTGTGTGGGTG; >clamp_s_a_s_c_s:compl:rev CACCCACACAGACACTATCATGTTGACATTCC; dG = -17.1
49 >s_b_s_a_s_a_s_a_s_c_s GTGGCTGTGTCTGTGTCTGTGTCTGTGTGGGTG; >clamp_s_a_s_c_s:compl:rev CACCCACACAGACACTATCATGTTGACATTCC; dG = -17.1
50 >s_b_s_a_s_b_s_a_s_c_s GTGGCTGTGTCTGTGGCTGTGTCTGTGTGGGTG; >clamp_s_a_s_c_s:compl:rev CACCCACACAGACACTATCATGTTGACATTCC; dG = -17.1

51 >s_b_s_a_s_d_s_a_s_c_s GTGGCTGTGTCTGTGACGGTGTCTGTGTGGGTG; >clamp_s_a_s_c_s:compl:rev
CACCCACACAGACACTATCATGTTGACATTCC; dG = -17.1
52 >s_b_s_a_s_e_s_a_s_c_s GTGGCTGTGTCTGTGCTGGTGTCTGTGTGGGTG; >clamp_s_a_s_c_s:compl:rev
CACCCACACAGACACTATCATGTTGACATTCC; dG = -17.1
53 >s_b_s_b_s_a_s_a_s_c_s GTGGCTGTGGCTGTGTCTGTGTCTGTGTGGGTG; >clamp_s_a_s_c_s:compl:rev
CACCCACACAGACACTATCATGTTGACATTCC; dG = -17.1
54 >s_b_s_b_s_b_s_a_s_c_s GTGGCTGTGGCTGTGGCTGTGTCTGTGTGGGTG; >clamp_s_a_s_c_s:compl:rev
CACCCACACAGACACTATCATGTTGACATTCC; dG = -17.1
55 >s_b_s_b_s_c_s_a_s_c_s GTGGCTGTGGCTGTGTGGGTGTCTGTGTGGGTG; >clamp_s_a_s_c_s:compl:rev
CACCCACACAGACACTATCATGTTGACATTCC; dG = -17.1
56 >s_b_s_b_s_d_s_a_s_c_s GTGGCTGTGGCTGTGACGGTGTCTGTGTGGGTG; >clamp_s_a_s_c_s:compl:rev
CACCCACACAGACACTATCATGTTGACATTCC; dG = -17.1
57 >s_b_s_b_s_e_s_a_s_c_s GTGGCTGTGGCTGTGCTGGTGTCTGTGTGGGTG; >clamp_s_a_s_c_s:compl:rev
CACCCACACAGACACTATCATGTTGACATTCC; dG = -17.1
58 >s_b_s_c_s_a_s_a_s_c_s GTGGCTGTGTGGGTGTCTGTGTCTGTGTGGGTG; >clamp_s_a_s_c_s:compl:rev
CACCCACACAGACACTATCATGTTGACATTCC; dG = -17.1
59 >s_b_s_c_s_b_s_a_s_c_s GTGGCTGTGTGGGTGGCTGTGTCTGTGTGGGTG; >clamp_s_a_s_c_s:compl:rev
CACCCACACAGACACTATCATGTTGACATTCC; dG = -17.1
60 >s_b_s_c_s_c_s_a_s_c_s GTGGCTGTGTGGGTGTGGGTGTCTGTGTGGGTG; >clamp_s_a_s_c_s:compl:rev
CACCCACACAGACACTATCATGTTGACATTCC; dG = -17.1
61 >s_b_s_c_s_d_s_a_s_c_s GTGGCTGTGTGGGTGACGGTGTCTGTGTGGGTG; >clamp_s_a_s_c_s:compl:rev
CACCCACACAGACACTATCATGTTGACATTCC; dG = -17.1
62 >s_a_s_a_s_d_s_a_s_c_s GTGTCTGTGTCTGTGACGGTGTCTGTGTGGGTG; >clamp_s_a_s_c_s:compl:rev
CACCCACACAGACACTATCATGTTGACATTCC; dG = -17.1
63 >s_b_s_c_s_e_s_a_s_c_s GTGGCTGTGTGGGTGCTGGTGTCTGTGTGGGTG; >clamp_s_a_s_c_s:compl:rev
CACCCACACAGACACTATCATGTTGACATTCC; dG = -17.1
64 >s_b_s_d_s_a_s_a_s_c_s GTGGCTGTGACGGTGTCTGTGTCTGTGTGGGTG; >clamp_s_a_s_c_s:compl:rev
CACCCACACAGACACTATCATGTTGACATTCC; dG = -17.1
65 >s_b_s_d_s_b_s_a_s_c_s GTGGCTGTGACGGTGGCTGTGTCTGTGTGGGTG; >clamp_s_a_s_c_s:compl:rev
CACCCACACAGACACTATCATGTTGACATTCC; dG = -17.1
66 >s_b_s_d_s_c_s_a_s_c_s GTGGCTGTGACGGTGTGGGTGTCTGTGTGGGTG; >clamp_s_a_s_c_s:compl:rev
CACCCACACAGACACTATCATGTTGACATTCC; dG = -17.1
67 >s_b_s_d_s_d_s_a_s_c_s GTGGCTGTGACGGTGACGGTGTCTGTGTGGGTG; >clamp_s_a_s_c_s:compl:rev
CACCCACACAGACACTATCATGTTGACATTCC; dG = -17.1
68 >s_b_s_d_s_e_s_a_s_c_s GTGGCTGTGACGGTGCTGGTGTCTGTGTGGGTG; >clamp_s_a_s_c_s:compl:rev
CACCCACACAGACACTATCATGTTGACATTCC; dG = -17.1
69 >s_b_s_e_s_a_s_a_s_c_s GTGGCTGTGCTGGTGTCTGTGTCTGTGTGGGTG; >clamp_s_a_s_c_s:compl:rev
CACCCACACAGACACTATCATGTTGACATTCC; dG = -17.1
70 >s_b_s_e_s_b_s_a_s_c_s GTGGCTGTGCTGGTGGCTGTGTCTGTGTGGGTG; >clamp_s_a_s_c_s:compl:rev
CACCCACACAGACACTATCATGTTGACATTCC; dG = -17.1
71 >s_b_s_e_s_c_s_a_s_c_s GTGGCTGTGCTGGTGTGGGTGTCTGTGTGGGTG; >clamp_s_a_s_c_s:compl:rev
CACCCACACAGACACTATCATGTTGACATTCC; dG = -17.1
72 >s_b_s_e_s_d_s_a_s_c_s GTGGCTGTGCTGGTGACGGTGTCTGTGTGGGTG; >clamp_s_a_s_c_s:compl:rev
CACCCACACAGACACTATCATGTTGACATTCC; dG = -17.1
73 >s_a_s_a_s_e_s_a_s_c_s GTGTCTGTGTCTGTGCTGGTGTCTGTGTGGGTG; >clamp_s_a_s_c_s:compl:rev
CACCCACACAGACACTATCATGTTGACATTCC; dG = -17.1
74 >s_b_s_e_s_e_s_a_s_c_s GTGGCTGTGCTGGTGCTGGTGTCTGTGTGGGTG; >clamp_s_a_s_c_s:compl:rev
CACCCACACAGACACTATCATGTTGACATTCC; dG = -17.1
75 >s_c_s_a_s_a_s_a_s_c_s GTGTGGGTGTCTGTGTCTGTGTCTGTGTGGGTG; >clamp_s_a_s_c_s:compl:rev
CACCCACACAGACACTATCATGTTGACATTCC; dG = -17.1
76 >s_c_s_a_s_b_s_a_s_c_s GTGTGGGTGTCTGTGGCTGTGTCTGTGTGGGTG; >clamp_s_a_s_c_s:compl:rev
CACCCACACAGACACTATCATGTTGACATTCC; dG = -17.1
77 >s_c_s_a_s_d_s_a_s_c_s GTGTGGGTGTCTGTGACGGTGTCTGTGTGGGTG; >clamp_s_a_s_c_s:compl:rev
CACCCACACAGACACTATCATGTTGACATTCC; dG = -17.1
78 >s_c_s_a_s_e_s_a_s_c_s GTGTGGGTGTCTGTGCTGGTGTCTGTGTGGGTG; >clamp_s_a_s_c_s:compl:rev
CACCCACACAGACACTATCATGTTGACATTCC; dG = -17.1
79 >s_c_s_b_s_a_s_a_s_c_s GTGTGGGTGGCTGTGTCTGTGTCTGTGTGGGTG; >clamp_s_a_s_c_s:compl:rev
CACCCACACAGACACTATCATGTTGACATTCC; dG = -17.1
80 >s_c_s_b_s_b_s_a_s_c_s GTGTGGGTGGCTGTGGCTGTGTCTGTGTGGGTG; >clamp_s_a_s_c_s:compl:rev
CACCCACACAGACACTATCATGTTGACATTCC; dG = -17.1
81 >s_c_s_b_s_c_s_a_s_c_s GTGTGGGTGGCTGTGTGGGTGTCTGTGTGGGTG; >clamp_s_a_s_c_s:compl:rev
CACCCACACAGACACTATCATGTTGACATTCC; dG = -17.1
82 >s_c_s_b_s_d_s_a_s_c_s GTGTGGGTGGCTGTGACGGTGTCTGTGTGGGTG; >clamp_s_a_s_c_s:compl:rev
CACCCACACAGACACTATCATGTTGACATTCC; dG = -17.1
83 >s_a_s_b_s_a_s_a_s_c_s GTGTCTGTGGCTGTGTCTGTGTCTGTGTGGGTG; >clamp_s_a_s_c_s:compl:rev
CACCCACACAGACACTATCATGTTGACATTCC; dG = -17.1
84 >s_c_s_b_s_e_s_a_s_c_s GTGTGGGTGGCTGTGCTGGTGTCTGTGTGGGTG; >clamp_s_a_s_c_s:compl:rev
CACCCACACAGACACTATCATGTTGACATTCC; dG = -17.1
85 >s_c_s_c_s_a_s_a_s_c_s GTGTGGGTGTGGGTGTCTGTGTCTGTGTGGGTG; >clamp_s_a_s_c_s:compl:rev
CACCCACACAGACACTATCATGTTGACATTCC; dG = -17.1
86 >s_c_s_c_s_b_s_a_s_c_s GTGTGGGTGTGGGTGGCTGTGTCTGTGTGGGTG; >clamp_s_a_s_c_s:compl:rev
CACCCACACAGACACTATCATGTTGACATTCC; dG = -17.1
87 >s_c_s_c_s_c_s_a_s_c_s GTGTGGGTGTGGGTGTGGGTGTCTGTGTGGGTG; >clamp_s_a_s_c_s:compl:rev
CACCCACACAGACACTATCATGTTGACATTCC; dG = -17.1

88 >s_c_s_c_s_d_s_a_s_c_s GTGTGGGTGTGGGTGACGGTGTCTGTGTGGGTG; >clamp_s_a_s_c_s:compl:rev CACCCACACAGACACTATCATGTTGACATTCC; dG = -17.1
89 >s_c_s_c_s_e_s_a_s_c_s GTGTGGGTGTGGGTGCTGGTGTCTGTGTGGGTG; >clamp_s_a_s_c_s:compl:rev CACCCACACAGACACTATCATGTTGACATTCC; dG = -17.1
90 >s_c_s_d_s_a_s_a_s_c_s GTGTGGGTGACGGTGTCTGTGTCTGTGTGGGTG; >clamp_s_a_s_c_s:compl:rev CACCCACACAGACACTATCATGTTGACATTCC; dG = -17.1
91 >s_c_s_d_s_b_s_a_s_c_s GTGTGGGTGACGGTGGCTGTGTCTGTGTGGGTG; >clamp_s_a_s_c_s:compl:rev CACCCACACAGACACTATCATGTTGACATTCC; dG = -17.1
92 >s_c_s_d_s_c_s_a_s_c_s GTGTGGGTGACGGTGTGGGTGTCTGTGTGGGTG; >clamp_s_a_s_c_s:compl:rev CACCCACACAGACACTATCATGTTGACATTCC; dG = -17.1
93 >s_c_s_d_s_d_s_a_s_c_s GTGTGGGTGACGGTGACGGTGTCTGTGTGGGTG; >clamp_s_a_s_c_s:compl:rev CACCCACACAGACACTATCATGTTGACATTCC; dG = -17.1
94 >s_a_s_b_s_b_s_a_s_c_s GTGTCTGTGGCTGTGGCTGTGTCTGTGTGGGTG; >clamp_s_a_s_c_s:compl:rev CACCCACACAGACACTATCATGTTGACATTCC; dG = -17.1
95 >s_c_s_d_s_e_s_a_s_c_s GTGTGGGTGACGGTGCTGGTGTCTGTGTGGGTG; >clamp_s_a_s_c_s:compl:rev CACCCACACAGACACTATCATGTTGACATTCC; dG = -17.1
96 >s_c_s_e_s_a_s_a_s_c_s GTGTGGGTGCTGGTGTCTGTGTCTGTGTGGGTG; >clamp_s_a_s_c_s:compl:rev CACCCACACAGACACTATCATGTTGACATTCC; dG = -17.1
97 >s_c_s_e_s_b_s_a_s_c_s GTGTGGGTGCTGGTGGCTGTGTCTGTGTGGGTG; >clamp_s_a_s_c_s:compl:rev CACCCACACAGACACTATCATGTTGACATTCC; dG = -17.1
98 >s_c_s_e_s_c_s_a_s_c_s GTGTGGGTGCTGGTGTGGGTGTCTGTGTGGGTG; >clamp_s_a_s_c_s:compl:rev CACCCACACAGACACTATCATGTTGACATTCC; dG = -17.1
99 >s_c_s_e_s_d_s_a_s_c_s GTGTGGGTGCTGGTGACGGTGTCTGTGTGGGTG; >clamp_s_a_s_c_s:compl:rev CACCCACACAGACACTATCATGTTGACATTCC; dG = -17.1
100 >s_c_s_e_s_e_s_a_s_c_s GTGTGGGTGCTGGTGCTGGTGTCTGTGTGGGTG; >clamp_s_a_s_c_s:compl:rev CACCCACACAGACACTATCATGTTGACATTCC; dG = -17.1
101 >s_d_s_a_s_a_s_a_s_c_s GTGACGGTGTCTGTGTCTGTGTCTGTGTGGGTG; >clamp_s_a_s_c_s:compl:rev CACCCACACAGACACTATCATGTTGACATTCC; dG = -17.1
102 >s_d_s_a_s_b_s_a_s_c_s GTGACGGTGTCTGTGGCTGTGTCTGTGTGGGTG; >clamp_s_a_s_c_s:compl:rev CACCCACACAGACACTATCATGTTGACATTCC; dG = -17.1
103 >s_d_s_a_s_d_s_a_s_c_s GTGACGGTGTCTGTGACGGTGTCTGTGTGGGTG; >clamp_s_a_s_c_s:compl:rev CACCCACACAGACACTATCATGTTGACATTCC; dG = -17.1
104 >s_a_s_b_s_c_s_a_s_c_s GTGTCTGTGGCTGTGTGGGTGTCTGTGTGGGTG; >clamp_s_a_s_c_s:compl:rev CACCCACACAGACACTATCATGTTGACATTCC; dG = -17.1
105 >s_d_s_a_s_e_s_a_s_c_s GTGACGGTGTCTGTGCTGGTGTCTGTGTGGGTG; >clamp_s_a_s_c_s:compl:rev CACCCACACAGACACTATCATGTTGACATTCC; dG = -17.1
106 >s_d_s_b_s_a_s_a_s_c_s GTGACGGTGGCTGTGTCTGTGTCTGTGTGGGTG; >clamp_s_a_s_c_s:compl:rev CACCCACACAGACACTATCATGTTGACATTCC; dG = -17.1
107 >s_d_s_b_s_b_s_a_s_c_s GTGACGGTGGCTGTGGCTGTGTCTGTGTGGGTG; >clamp_s_a_s_c_s:compl:rev CACCCACACAGACACTATCATGTTGACATTCC; dG = -17.1
108 >s_d_s_b_s_c_s_a_s_c_s GTGACGGTGGCTGTGTGGGTGTCTGTGTGGGTG; >clamp_s_a_s_c_s:compl:rev CACCCACACAGACACTATCATGTTGACATTCC; dG = -17.1
109 >s_d_s_b_s_d_s_a_s_c_s GTGACGGTGGCTGTGACGGTGTCTGTGTGGGTG; >clamp_s_a_s_c_s:compl:rev CACCCACACAGACACTATCATGTTGACATTCC; dG = -17.1
110 >s_d_s_b_s_e_s_a_s_c_s GTGACGGTGGCTGTGCTGGTGTCTGTGTGGGTG; >clamp_s_a_s_c_s:compl:rev CACCCACACAGACACTATCATGTTGACATTCC; dG = -17.1
111 >s_d_s_c_s_a_s_a_s_c_s GTGACGGTGTGGGTGTCTGTGTCTGTGTGGGTG; >clamp_s_a_s_c_s:compl:rev CACCCACACAGACACTATCATGTTGACATTCC; dG = -17.1
112 >s_d_s_c_s_b_s_a_s_c_s GTGACGGTGTGGGTGGCTGTGTCTGTGTGGGTG; >clamp_s_a_s_c_s:compl:rev CACCCACACAGACACTATCATGTTGACATTCC; dG = -17.1
113 >s_d_s_c_s_c_s_a_s_c_s GTGACGGTGTGGGTGTGGGTGTCTGTGTGGGTG; >clamp_s_a_s_c_s:compl:rev CACCCACACAGACACTATCATGTTGACATTCC; dG = -17.1
114 >s_d_s_c_s_d_s_a_s_c_s GTGACGGTGTGGGTGACGGTGTCTGTGTGGGTG; >clamp_s_a_s_c_s:compl:rev CACCCACACAGACACTATCATGTTGACATTCC; dG = -17.1
115 >s_a_s_b_s_d_s_a_s_c_s GTGTCTGTGGCTGTGACGGTGTCTGTGTGGGTG; >clamp_s_a_s_c_s:compl:rev CACCCACACAGACACTATCATGTTGACATTCC; dG = -17.1
116 >s_d_s_c_s_e_s_a_s_c_s GTGACGGTGTGGGTGCTGGTGTCTGTGTGGGTG; >clamp_s_a_s_c_s:compl:rev CACCCACACAGACACTATCATGTTGACATTCC; dG = -17.1
117 >s_d_s_d_s_a_s_a_s_c_s GTGACGGTGACGGTGTCTGTGTCTGTGTGGGTG; >clamp_s_a_s_c_s:compl:rev CACCCACACAGACACTATCATGTTGACATTCC; dG = -17.1
118 >s_d_s_d_s_b_s_a_s_c_s GTGACGGTGACGGTGGCTGTGTCTGTGTGGGTG; >clamp_s_a_s_c_s:compl:rev CACCCACACAGACACTATCATGTTGACATTCC; dG = -17.1
119 >s_d_s_d_s_c_s_a_s_c_s GTGACGGTGACGGTGTGGGTGTCTGTGTGGGTG; >clamp_s_a_s_c_s:compl:rev CACCCACACAGACACTATCATGTTGACATTCC; dG = -17.1
120 >s_d_s_d_s_d_s_a_s_c_s GTGACGGTGACGGTGACGGTGTCTGTGTGGGTG; >clamp_s_a_s_c_s:compl:rev CACCCACACAGACACTATCATGTTGACATTCC; dG = -17.1
121 >s_d_s_d_s_e_s_a_s_c_s GTGACGGTGACGGTGCTGGTGTCTGTGTGGGTG; >clamp_s_a_s_c_s:compl:rev CACCCACACAGACACTATCATGTTGACATTCC; dG = -17.1
122 >s_d_s_e_s_a_s_a_s_c_s GTGACGGTGCTGGTGTCTGTGTCTGTGTGGGTG; >clamp_s_a_s_c_s:compl:rev CACCCACACAGACACTATCATGTTGACATTCC; dG = -17.1
123 >s_d_s_e_s_b_s_a_s_c_s GTGACGGTGCTGGTGGCTGTGTCTGTGTGGGTG; >clamp_s_a_s_c_s:compl:rev CACCCACACAGACACTATCATGTTGACATTCC; dG = -17.1
124 >s_d_s_e_s_c_s_a_s_c_s GTGACGGTGCTGGTGTGGGTGTCTGTGTGGGTG; >clamp_s_a_s_c_s:compl:rev CACCCACACAGACACTATCATGTTGACATTCC; dG = -17.1

```
   125 >s_d_s_e_s_d_s_a_s_c_s  GTGACGGTGCTGGTGACGGTGTCTGTGTGGGTG;  >clamp_s_a_s_c_s:compl:rev
CACCCACACAGACACTATCATGTTGACATTCC; dG = -17.1

*************************************************************************************

AC Mismatches

     1 >s_b_s_b_s_c_s_d_s_c_s  GTGGCTGTGGCTGTGTGGGTGACGGTGTGGGTG;  >clamp_s_a_s_c_s:compl:rev
CACCCACACAGACACTATCATGTTGACATTCC; dG = -16.0
     2 >s_a_s_b_s_c_s_d_s_c_s  GTGTCTGTGGCTGTGTGGGTGACGGTGTGGGTG;  >clamp_s_a_s_c_s:compl:rev
CACCCACACAGACACTATCATGTTGACATTCC; dG = -16.0
     3 >s_c_s_b_s_c_s_d_s_c_s  GTGTGGGTGGCTGTGTGGGTGACGGTGTGGGTG;  >clamp_s_a_s_c_s:compl:rev
CACCCACACAGACACTATCATGTTGACATTCC; dG = -15.9
     4 >s_d_s_b_s_c_s_d_s_c_s  GTGACGGTGGCTGTGTGGGTGACGGTGTGGGTG;  >clamp_s_a_s_c_s:compl:rev
CACCCACACAGACACTATCATGTTGACATTCC; dG = -15.9
     5 >s_e_s_b_s_c_s_d_s_c_s  GTGCTGGTGGCTGTGTGGGTGACGGTGTGGGTG;  >clamp_s_a_s_c_s:compl:rev
CACCCACACAGACACTATCATGTTGACATTCC; dG = -15.9
     6 >s_b_s_b_s_c_s_b_s_c_s  GTGGCTGTGGCTGTGTGGGTGGCTGTGTGGGTG;  >clamp_s_a_s_c_s:compl:rev
CACCCACACAGACACTATCATGTTGACATTCC; dG = -15.5
     7 >s_b_s_c_s_d_s_a_s_e_s  GTGGCTGTGTGGGTGACGGTGTCTGTGCTGGTG;  >clamp_s_a_s_c_s:compl:rev
CACCCACACAGACACTATCATGTTGACATTCC; dG = -15.5
     8 >s_b_s_c_s_d_s_a_s_d_s  GTGGCTGTGTGGGTGACGGTGTCTGTGACGGTG;  >clamp_s_a_s_c_s:compl:rev
CACCCACACAGACACTATCATGTTGACATTCC; dG = -15.5
     9 >s_b_s_c_s_d_s_a_s_b_s  GTGGCTGTGTGGGTGACGGTGTCTGTGGCTGTG;  >clamp_s_a_s_c_s:compl:rev
CACCCACACAGACACTATCATGTTGACATTCC; dG = -15.5
    10 >s_b_s_c_s_d_s_a_s_a_s  GTGGCTGTGTGGGTGACGGTGTCTGTGTCTGTG;  >clamp_s_a_s_c_s:compl:rev
CACCCACACAGACACTATCATGTTGACATTCC; dG = -15.5
    11 >s_b_s_c_s_d_s_e_s_c_s  GTGGCTGTGTGGGTGACGGTGCTGGTGTGGGTG;  >clamp_s_a_s_c_s:compl:rev
CACCCACACAGACACTATCATGTTGACATTCC; dG = -15.5
    12 >s_b_s_c_s_d_s_d_s_c_s  GTGGCTGTGTGGGTGACGGTGACGGTGTGGGTG;  >clamp_s_a_s_c_s:compl:rev
CACCCACACAGACACTATCATGTTGACATTCC; dG = -15.5
    13 >s_b_s_c_s_d_s_c_s_c_s  GTGGCTGTGTGGGTGACGGTGTGGGTGTGGGTG;  >clamp_s_a_s_c_s:compl:rev
CACCCACACAGACACTATCATGTTGACATTCC; dG = -15.5
    14 >s_b_s_c_s_d_s_b_s_c_s  GTGGCTGTGTGGGTGACGGTGGCTGTGTGGGTG;  >clamp_s_a_s_c_s:compl:rev
CACCCACACAGACACTATCATGTTGACATTCC; dG = -15.5
    15 >s_a_s_b_s_c_s_b_s_c_s  GTGTCTGTGGCTGTGTGGGTGGCTGTGTGGGTG;  >clamp_s_a_s_c_s:compl:rev
CACCCACACAGACACTATCATGTTGACATTCC; dG = -15.5
    16 >s_a_s_e_s_b_s_c_s_c_s  GTGTCTGTGCTGGTGGCTGTGTGGGTGTGGGTG;  >clamp_s_a_s_c_s:compl:rev
CACCCACACAGACACTATCATGTTGACATTCC; dG = -15.4
    17 >s_a_s_a_s_b_s_c_s_c_s  GTGTCTGTGTCTGTGGCTGTGTGGGTGTGGGTG;  >clamp_s_a_s_c_s:compl:rev
CACCCACACAGACACTATCATGTTGACATTCC; dG = -15.4
    18 >s_b_s_a_s_b_s_c_s_c_s  GTGGCTGTGTCTGTGGCTGTGTGGGTGTGGGTG;  >clamp_s_a_s_c_s:compl:rev
CACCCACACAGACACTATCATGTTGACATTCC; dG = -15.4
    19 >s_b_s_b_s_b_s_c_s_c_s  GTGGCTGTGGCTGTGGCTGTGTGGGTGTGGGTG;  >clamp_s_a_s_c_s:compl:rev
CACCCACACAGACACTATCATGTTGACATTCC; dG = -15.4
    20 >s_b_s_b_s_c_s_a_s_e_s  GTGGCTGTGGCTGTGTGGGTGTCTGTGCTGGTG;  >clamp_s_a_s_c_s:compl:rev
CACCCACACAGACACTATCATGTTGACATTCC; dG = -15.4
    21 >s_b_s_b_s_c_s_a_s_d_s  GTGGCTGTGGCTGTGTGGGTGTCTGTGACGGTG;  >clamp_s_a_s_c_s:compl:rev
CACCCACACAGACACTATCATGTTGACATTCC; dG = -15.4
    22 >s_b_s_b_s_c_s_a_s_b_s  GTGGCTGTGGCTGTGTGGGTGTCTGTGGCTGTG;  >clamp_s_a_s_c_s:compl:rev
CACCCACACAGACACTATCATGTTGACATTCC; dG = -15.4
    23 >s_b_s_b_s_c_s_a_s_a_s  GTGGCTGTGGCTGTGTGGGTGTCTGTGTCTGTG;  >clamp_s_a_s_c_s:compl:rev
CACCCACACAGACACTATCATGTTGACATTCC; dG = -15.4
    24 >s_b_s_b_s_c_s_c_s_c_s  GTGGCTGTGGCTGTGTGGGTGTGGGTGTGGGTG;  >clamp_s_a_s_c_s:compl:rev
CACCCACACAGACACTATCATGTTGACATTCC; dG = -15.4
    25 >s_b_s_c_s_b_s_c_s_c_s  GTGGCTGTGTGGGTGGCTGTGTGGGTGTGGGTG;  >clamp_s_a_s_c_s:compl:rev
CACCCACACAGACACTATCATGTTGACATTCC; dG = -15.4
    26 >s_b_s_d_s_b_s_c_s_c_s  GTGGCTGTGACGGTGGCTGTGTGGGTGTGGGTG;  >clamp_s_a_s_c_s:compl:rev
CACCCACACAGACACTATCATGTTGACATTCC; dG = -15.4
    27 >s_b_s_e_s_b_s_c_s_c_s  GTGGCTGTGCTGGTGGCTGTGTGGGTGTGGGTG;  >clamp_s_a_s_c_s:compl:rev
CACCCACACAGACACTATCATGTTGACATTCC; dG = -15.4
    28 >s_c_s_a_s_b_s_c_s_c_s  GTGTGGGTGTCTGTGGCTGTGTGGGTGTGGGTG;  >clamp_s_a_s_c_s:compl:rev
CACCCACACAGACACTATCATGTTGACATTCC; dG = -15.4
    29 >s_c_s_b_s_b_s_c_s_c_s  GTGTGGGTGGCTGTGGCTGTGTGGGTGTGGGTG;  >clamp_s_a_s_c_s:compl:rev
CACCCACACAGACACTATCATGTTGACATTCC; dG = -15.4
    30 >s_c_s_b_s_c_s_a_s_e_s  GTGTGGGTGGCTGTGTGGGTGTCTGTGCTGGTG;  >clamp_s_a_s_c_s:compl:rev
CACCCACACAGACACTATCATGTTGACATTCC; dG = -15.4
    31 >s_c_s_b_s_c_s_a_s_d_s  GTGTGGGTGGCTGTGTGGGTGTCTGTGACGGTG;  >clamp_s_a_s_c_s:compl:rev
CACCCACACAGACACTATCATGTTGACATTCC; dG = -15.4
    32 >s_c_s_b_s_c_s_a_s_b_s  GTGTGGGTGGCTGTGTGGGTGTCTGTGGCTGTG;  >clamp_s_a_s_c_s:compl:rev
CACCCACACAGACACTATCATGTTGACATTCC; dG = -15.4
    33 >s_c_s_b_s_c_s_a_s_a_s  GTGTGGGTGGCTGTGTGGGTGTCTGTGTCTGTG;  >clamp_s_a_s_c_s:compl:rev
CACCCACACAGACACTATCATGTTGACATTCC; dG = -15.4
```

34 >s_c_s_b_s_c_s_c_s_c_s GTGTGGGTGGCTGTGTGGGTGTGGGTGTGGGTG; >clamp_s_a_s_c_s:compl:rev CACCCACACAGACACTATCATGTTGACATTCC; dG = -15.4

35 >s_c_s_b_s_c_s_b_s_c_s GTGTGGGTGGCTGTGTGGGTGGCTGTGTGGGTG; >clamp_s_a_s_c_s:compl:rev CACCCACACAGACACTATCATGTTGACATTCC; dG = -15.4

36 >s_c_s_c_s_b_s_c_s_c_s GTGTGGGTGTGGGTGGCTGTGTGGGTGTGGGTG; >clamp_s_a_s_c_s:compl:rev CACCCACACAGACACTATCATGTTGACATTCC; dG = -15.4

37 >s_a_s_b_s_b_s_c_s_c_s GTGTCTGTGGCTGTGGCTGTGTGGGTGTGGGTG; >clamp_s_a_s_c_s:compl:rev CACCCACACAGACACTATCATGTTGACATTCC; dG = -15.4

38 >s_c_s_d_s_b_s_c_s_c_s GTGTGGGTGACGGTGGCTGTGTGGGTGTGGGTG; >clamp_s_a_s_c_s:compl:rev CACCCACACAGACACTATCATGTTGACATTCC; dG = -15.4

39 >s_a_s_b_s_c_s_a_s_e_s GTGTCTGTGGCTGTGTGGGTGTCTGTGCTGGTG; >clamp_s_a_s_c_s:compl:rev CACCCACACAGACACTATCATGTTGACATTCC; dG = -15.4

40 >s_a_s_b_s_c_s_a_s_d_s GTGTCTGTGGCTGTGTGGGTGTCTGTGACGGTG; >clamp_s_a_s_c_s:compl:rev CACCCACACAGACACTATCATGTTGACATTCC; dG = -15.4

41 >s_a_s_b_s_c_s_a_s_b_s GTGTCTGTGGCTGTGTGGGTGTCTGTGGCTGTG; >clamp_s_a_s_c_s:compl:rev CACCCACACAGACACTATCATGTTGACATTCC; dG = -15.4

42 >s_c_s_e_s_b_s_c_s_c_s GTGTGGGTGCTGGTGGCTGTGTGGGTGTGGGTG; >clamp_s_a_s_c_s:compl:rev CACCCACACAGACACTATCATGTTGACATTCC; dG = -15.4

43 >s_a_s_b_s_c_s_a_s_a_s GTGTCTGTGGCTGTGTGGGTGTCTGTGTCTGTG; >clamp_s_a_s_c_s:compl:rev CACCCACACAGACACTATCATGTTGACATTCC; dG = -15.4

44 >s_a_s_b_s_c_s_c_s_c_s GTGTCTGTGGCTGTGTGGGTGTGGGTGTGGGTG; >clamp_s_a_s_c_s:compl:rev CACCCACACAGACACTATCATGTTGACATTCC; dG = -15.4

45 >s_d_s_a_s_b_s_c_s_c_s GTGACGGTGTCTGTGGCTGTGTGGGTGTGGGTG; >clamp_s_a_s_c_s:compl:rev CACCCACACAGACACTATCATGTTGACATTCC; dG = -15.4

46 >s_d_s_b_s_b_s_c_s_c_s GTGACGGTGGCTGTGGCTGTGTGGGTGTGGGTG; >clamp_s_a_s_c_s:compl:rev CACCCACACAGACACTATCATGTTGACATTCC; dG = -15.4

47 >s_d_s_b_s_c_s_a_s_e_s GTGACGGTGGCTGTGTGGGTGTCTGTGCTGGTG; >clamp_s_a_s_c_s:compl:rev CACCCACACAGACACTATCATGTTGACATTCC; dG = -15.4

48 >s_d_s_b_s_c_s_a_s_d_s GTGACGGTGGCTGTGTGGGTGTCTGTGACGGTG; >clamp_s_a_s_c_s:compl:rev CACCCACACAGACACTATCATGTTGACATTCC; dG = -15.4

49 >s_d_s_b_s_c_s_a_s_b_s GTGACGGTGGCTGTGTGGGTGTCTGTGGCTGTG; >clamp_s_a_s_c_s:compl:rev CACCCACACAGACACTATCATGTTGACATTCC; dG = -15.4

50 >s_d_s_b_s_c_s_a_s_a_s GTGACGGTGGCTGTGTGGGTGTCTGTGTCTGTG; >clamp_s_a_s_c_s:compl:rev CACCCACACAGACACTATCATGTTGACATTCC; dG = -15.4

51 >s_d_s_b_s_c_s_c_s_c_s GTGACGGTGGCTGTGTGGGTGTGGGTGTGGGTG; >clamp_s_a_s_c_s:compl:rev CACCCACACAGACACTATCATGTTGACATTCC; dG = -15.4

52 >s_d_s_b_s_c_s_b_s_c_s GTGACGGTGGCTGTGTGGGTGGCTGTGTGGGTG; >clamp_s_a_s_c_s:compl:rev CACCCACACAGACACTATCATGTTGACATTCC; dG = -15.4

53 >s_d_s_c_s_b_s_c_s_c_s GTGACGGTGTGGGTGGCTGTGTGGGTGTGGGTG; >clamp_s_a_s_c_s:compl:rev CACCCACACAGACACTATCATGTTGACATTCC; dG = -15.4

54 >s_d_s_d_s_b_s_c_s_c_s GTGACGGTGACGGTGGCTGTGTGGGTGTGGGTG; >clamp_s_a_s_c_s:compl:rev CACCCACACAGACACTATCATGTTGACATTCC; dG = -15.4

55 >s_d_s_e_s_b_s_c_s_c_s GTGACGGTGCTGGTGGCTGTGTGGGTGTGGGTG; >clamp_s_a_s_c_s:compl:rev CACCCACACAGACACTATCATGTTGACATTCC; dG = -15.4

56 >s_e_s_a_s_b_s_c_s_c_s GTGCTGGTGTCTGTGGCTGTGTGGGTGTGGGTG; >clamp_s_a_s_c_s:compl:rev CACCCACACAGACACTATCATGTTGACATTCC; dG = -15.4

57 >s_e_s_b_s_b_s_c_s_c_s GTGCTGGTGGCTGTGGCTGTGTGGGTGTGGGTG; >clamp_s_a_s_c_s:compl:rev CACCCACACAGACACTATCATGTTGACATTCC; dG = -15.4

58 >s_e_s_b_s_c_s_a_s_e_s GTGCTGGTGGCTGTGTGGGTGTCTGTGCTGGTG; >clamp_s_a_s_c_s:compl:rev CACCCACACAGACACTATCATGTTGACATTCC; dG = -15.4

59 >s_e_s_b_s_c_s_a_s_d_s GTGCTGGTGGCTGTGTGGGTGTCTGTGACGGTG; >clamp_s_a_s_c_s:compl:rev CACCCACACAGACACTATCATGTTGACATTCC; dG = -15.4

60 >s_e_s_b_s_c_s_a_s_b_s GTGCTGGTGGCTGTGTGGGTGTCTGTGGCTGTG; >clamp_s_a_s_c_s:compl:rev CACCCACACAGACACTATCATGTTGACATTCC; dG = -15.4

61 >s_e_s_b_s_c_s_a_s_a_s GTGCTGGTGGCTGTGTGGGTGTCTGTGTCTGTG; >clamp_s_a_s_c_s:compl:rev CACCCACACAGACACTATCATGTTGACATTCC; dG = -15.4

62 >s_e_s_b_s_c_s_c_s_c_s GTGCTGGTGGCTGTGTGGGTGTGGGTGTGGGTG; >clamp_s_a_s_c_s:compl:rev CACCCACACAGACACTATCATGTTGACATTCC; dG = -15.4

63 >s_e_s_b_s_c_s_b_s_c_s GTGCTGGTGGCTGTGTGGGTGGCTGTGTGGGTG; >clamp_s_a_s_c_s:compl:rev CACCCACACAGACACTATCATGTTGACATTCC; dG = -15.4

64 >s_e_s_c_s_b_s_c_s_c_s GTGCTGGTGTGGGTGGCTGTGTGGGTGTGGGTG; >clamp_s_a_s_c_s:compl:rev CACCCACACAGACACTATCATGTTGACATTCC; dG = -15.4

65 >s_e_s_d_s_b_s_c_s_c_s GTGCTGGTGACGGTGGCTGTGTGGGTGTGGGTG; >clamp_s_a_s_c_s:compl:rev CACCCACACAGACACTATCATGTTGACATTCC; dG = -15.4

66 >s_a_s_d_s_b_s_c_s_c_s GTGTCTGTGACGGTGGCTGTGTGGGTGTGGGTG; >clamp_s_a_s_c_s:compl:rev CACCCACACAGACACTATCATGTTGACATTCC; dG = -15.4

67 >s_e_s_e_s_b_s_c_s_c_s GTGCTGGTGCTGGTGGCTGTGTGGGTGTGGGTG; >clamp_s_a_s_c_s:compl:rev CACCCACACAGACACTATCATGTTGACATTCC; dG = -15.4

68 >s_b_s_b_s_c_s_e_s_c_s GTGGCTGTGGCTGTGTGGGTGCTGGTGTGGGTG; >clamp_s_a_s_c_s:compl:rev CACCCACACAGACACTATCATGTTGACATTCC; dG = -15.3

69 >s_a_s_b_s_c_s_e_s_c_s GTGTCTGTGGCTGTGTGGGTGCTGGTGTGGGTG; >clamp_s_a_s_c_s:compl:rev CACCCACACAGACACTATCATGTTGACATTCC; dG = -15.3

70 >s_c_s_b_s_c_s_e_s_c_s GTGTGGGTGGCTGTGTGGGTGCTGGTGTGGGTG; >clamp_s_a_s_c_s:compl:rev CACCCACACAGACACTATCATGTTGACATTCC; dG = -15.2

71 >s_d_s_b_s_c_s_e_s_c_s GTGACGGTGGCTGTGTGGGTGCTGGTGTGGGTG; >clamp_s_a_s_c_s:compl:rev
CACCCACACAGACACTATCATGTTGACATTCC; dG = -15.2
72 >s_e_s_b_s_c_s_e_s_c_s GTGCTGGTGGCTGTGTGGGTGCTGGTGTGGGTG; >clamp_s_a_s_c_s:compl:rev
CACCCACACAGACACTATCATGTTGACATTCC; dG = -15.2
73 >s_a_s_d_s_d_s_b_s_c_s GTGTCTGTGACGGTGACGGTGGCTGTGTGGGTG; >clamp_s_a_s_c_s:compl:rev
CACCCACACAGACACTATCATGTTGACATTCC; dG = -15.1
74 >s_a_s_d_s_e_s_b_s_c_s GTGTCTGTGACGGTGCTGGTGGCTGTGTGGGTG; >clamp_s_a_s_c_s:compl:rev
CACCCACACAGACACTATCATGTTGACATTCC; dG = -15.1
75 >s_a_s_e_s_a_s_b_s_c_s GTGTCTGTGCTGGTGTCTGTGGCTGTGTGGGTG; >clamp_s_a_s_c_s:compl:rev
CACCCACACAGACACTATCATGTTGACATTCC; dG = -15.1
76 >s_a_s_e_s_b_s_b_s_c_s GTGTCTGTGCTGGTGGCTGTGGCTGTGTGGGTG; >clamp_s_a_s_c_s:compl:rev
CACCCACACAGACACTATCATGTTGACATTCC; dG = -15.1
77 >s_a_s_e_s_c_s_b_s_c_s GTGTCTGTGCTGGTGTGGGTGGCTGTGTGGGTG; >clamp_s_a_s_c_s:compl:rev
CACCCACACAGACACTATCATGTTGACATTCC; dG = -15.1
78 >s_a_s_e_s_d_s_b_s_c_s GTGTCTGTGCTGGTGACGGTGGCTGTGTGGGTG; >clamp_s_a_s_c_s:compl:rev
CACCCACACAGACACTATCATGTTGACATTCC; dG = -15.1
79 >s_a_s_e_s_e_s_b_s_c_s GTGTCTGTGCTGGTGCTGGTGGCTGTGTGGGTG; >clamp_s_a_s_c_s:compl:rev
CACCCACACAGACACTATCATGTTGACATTCC; dG = -15.1
80 >s_a_s_a_s_b_s_b_s_c_s GTGTCTGTGTCTGTGGCTGTGGCTGTGTGGGTG; >clamp_s_a_s_c_s:compl:rev
CACCCACACAGACACTATCATGTTGACATTCC; dG = -15.1
81 >s_b_s_a_s_a_s_b_s_c_s GTGGCTGTGTCTGTGTCTGTGGCTGTGTGGGTG; >clamp_s_a_s_c_s:compl:rev
CACCCACACAGACACTATCATGTTGACATTCC; dG = -15.1
82 >s_b_s_a_s_b_s_b_s_c_s GTGGCTGTGTCTGTGGCTGTGGCTGTGTGGGTG; >clamp_s_a_s_c_s:compl:rev
CACCCACACAGACACTATCATGTTGACATTCC; dG = -15.1
83 >s_b_s_a_s_d_s_b_s_c_s GTGGCTGTGTCTGTGACGGTGGCTGTGTGGGTG; >clamp_s_a_s_c_s:compl:rev
CACCCACACAGACACTATCATGTTGACATTCC; dG = -15.1
84 >s_b_s_a_s_e_s_b_s_c_s GTGGCTGTGTCTGTGCTGGTGGCTGTGTGGGTG; >clamp_s_a_s_c_s:compl:rev
CACCCACACAGACACTATCATGTTGACATTCC; dG = -15.1
85 >s_b_s_b_s_a_s_b_s_c_s GTGGCTGTGGCTGTGTCTGTGGCTGTGTGGGTG; >clamp_s_a_s_c_s:compl:rev
CACCCACACAGACACTATCATGTTGACATTCC; dG = -15.1
86 >s_b_s_b_s_b_s_b_s_c_s GTGGCTGTGGCTGTGGCTGTGGCTGTGTGGGTG; >clamp_s_a_s_c_s:compl:rev
CACCCACACAGACACTATCATGTTGACATTCC; dG = -15.1
87 >s_b_s_b_s_d_s_b_s_c_s GTGGCTGTGGCTGTGACGGTGGCTGTGTGGGTG; >clamp_s_a_s_c_s:compl:rev
CACCCACACAGACACTATCATGTTGACATTCC; dG = -15.1
88 >s_b_s_b_s_e_s_b_s_c_s GTGGCTGTGGCTGTGCTGGTGGCTGTGTGGGTG; >clamp_s_a_s_c_s:compl:rev
CACCCACACAGACACTATCATGTTGACATTCC; dG = -15.1
89 >s_b_s_c_s_a_s_b_s_c_s GTGGCTGTGTGGGTGTCTGTGGCTGTGTGGGTG; >clamp_s_a_s_c_s:compl:rev
CACCCACACAGACACTATCATGTTGACATTCC; dG = -15.1
90 >s_a_s_a_s_d_s_b_s_c_s GTGTCTGTGTCTGTGACGGTGGCTGTGTGGGTG; >clamp_s_a_s_c_s:compl:rev
CACCCACACAGACACTATCATGTTGACATTCC; dG = -15.1
91 >s_b_s_c_s_b_s_b_s_c_s GTGGCTGTGTGGGTGGCTGTGGCTGTGTGGGTG; >clamp_s_a_s_c_s:compl:rev
CACCCACACAGACACTATCATGTTGACATTCC; dG = -15.1
92 >s_b_s_c_s_c_s_b_s_c_s GTGGCTGTGTGGGTGTGGGTGGCTGTGTGGGTG; >clamp_s_a_s_c_s:compl:rev
CACCCACACAGACACTATCATGTTGACATTCC; dG = -15.1
93 >s_b_s_c_s_e_s_b_s_c_s GTGGCTGTGTGGGTGCTGGTGGCTGTGTGGGTG; >clamp_s_a_s_c_s:compl:rev
CACCCACACAGACACTATCATGTTGACATTCC; dG = -15.1
94 >s_b_s_d_s_a_s_b_s_c_s GTGGCTGTGACGGTGTCTGTGGCTGTGTGGGTG; >clamp_s_a_s_c_s:compl:rev
CACCCACACAGACACTATCATGTTGACATTCC; dG = -15.1
95 >s_b_s_d_s_b_s_b_s_c_s GTGGCTGTGACGGTGGCTGTGGCTGTGTGGGTG; >clamp_s_a_s_c_s:compl:rev
CACCCACACAGACACTATCATGTTGACATTCC; dG = -15.1
96 >s_b_s_d_s_c_s_b_s_c_s GTGGCTGTGACGGTGTGGGTGGCTGTGTGGGTG; >clamp_s_a_s_c_s:compl:rev
CACCCACACAGACACTATCATGTTGACATTCC; dG = -15.1
97 >s_b_s_d_s_d_s_b_s_c_s GTGGCTGTGACGGTGACGGTGGCTGTGTGGGTG; >clamp_s_a_s_c_s:compl:rev
CACCCACACAGACACTATCATGTTGACATTCC; dG = -15.1
98 >s_b_s_d_s_e_s_b_s_c_s GTGGCTGTGACGGTGCTGGTGGCTGTGTGGGTG; >clamp_s_a_s_c_s:compl:rev
CACCCACACAGACACTATCATGTTGACATTCC; dG = -15.1
99 >s_b_s_e_s_a_s_b_s_c_s GTGGCTGTGCTGGTGTCTGTGGCTGTGTGGGTG; >clamp_s_a_s_c_s:compl:rev
CACCCACACAGACACTATCATGTTGACATTCC; dG = -15.1
100 >s_a_s_a_s_e_s_b_s_c_s GTGTCTGTGTCTGTGCTGGTGGCTGTGTGGGTG; >clamp_s_a_s_c_s:compl:rev
CACCCACACAGACACTATCATGTTGACATTCC; dG = -15.1
101 >s_b_s_e_s_b_s_b_s_c_s GTGGCTGTGCTGGTGGCTGTGGCTGTGTGGGTG; >clamp_s_a_s_c_s:compl:rev
CACCCACACAGACACTATCATGTTGACATTCC; dG = -15.1
102 >s_b_s_e_s_c_s_b_s_c_s GTGGCTGTGCTGGTGTGGGTGGCTGTGTGGGTG; >clamp_s_a_s_c_s:compl:rev
CACCCACACAGACACTATCATGTTGACATTCC; dG = -15.1
103 >s_b_s_e_s_d_s_b_s_c_s GTGGCTGTGCTGGTGACGGTGGCTGTGTGGGTG; >clamp_s_a_s_c_s:compl:rev
CACCCACACAGACACTATCATGTTGACATTCC; dG = -15.1
104 >s_b_s_e_s_e_s_b_s_c_s GTGGCTGTGCTGGTGCTGGTGGCTGTGTGGGTG; >clamp_s_a_s_c_s:compl:rev
CACCCACACAGACACTATCATGTTGACATTCC; dG = -15.1
105 >s_c_s_a_s_a_s_b_s_c_s GTGTGGGTGTCTGTGTCTGTGGCTGTGTGGGTG; >clamp_s_a_s_c_s:compl:rev
CACCCACACAGACACTATCATGTTGACATTCC; dG = -15.1
106 >s_c_s_a_s_b_s_b_s_c_s GTGTGGGTGTCTGTGGCTGTGGCTGTGTGGGTG; >clamp_s_a_s_c_s:compl:rev
CACCCACACAGACACTATCATGTTGACATTCC; dG = -15.1
107 >s_c_s_a_s_d_s_b_s_c_s GTGTGGGTGTCTGTGACGGTGGCTGTGTGGGTG; >clamp_s_a_s_c_s:compl:rev
CACCCACACAGACACTATCATGTTGACATTCC; dG = -15.1

108 >s_c_s_a_s_e_s_b_s_c_s GTGTGGGTGTCTGTGCTGGTGGCTGTGTGGGTG; >clamp_s_a_s_c_s:compl:rev CACCCACACAGACACTATCATGTTGACATTCC; dG = -15.1
109 >s_c_s_b_s_a_s_b_s_c_s GTGTGGGTGGCTGTGTCTGTGGCTGTGTGGGTG; >clamp_s_a_s_c_s:compl:rev CACCCACACAGACACTATCATGTTGACATTCC; dG = -15.1
110 >s_a_s_b_s_a_s_b_s_c_s GTGTCTGTGGCTGTGTCTGTGGCTGTGTGGGTG; >clamp_s_a_s_c_s:compl:rev CACCCACACAGACACTATCATGTTGACATTCC; dG = -15.1
111 >s_c_s_b_s_b_s_b_s_c_s GTGTGGGTGGCTGTGGCTGTGGCTGTGTGGGTG; >clamp_s_a_s_c_s:compl:rev CACCCACACAGACACTATCATGTTGACATTCC; dG = -15.1
112 >s_c_s_b_s_d_s_b_s_c_s GTGTGGGTGGCTGTGACGGTGGCTGTGTGGGTG; >clamp_s_a_s_c_s:compl:rev CACCCACACAGACACTATCATGTTGACATTCC; dG = -15.1
113 >s_c_s_b_s_e_s_b_s_c_s GTGTGGGTGGCTGTGCTGGTGGCTGTGTGGGTG; >clamp_s_a_s_c_s:compl:rev CACCCACACAGACACTATCATGTTGACATTCC; dG = -15.1
114 >s_c_s_c_s_a_s_b_s_c_s GTGTGGGTGTGGGTGTCTGTGGCTGTGTGGGTG; >clamp_s_a_s_c_s:compl:rev CACCCACACAGACACTATCATGTTGACATTCC; dG = -15.1
115 >s_c_s_c_s_b_s_b_s_c_s GTGTGGGTGTGGGTGGCTGTGGCTGTGTGGGTG; >clamp_s_a_s_c_s:compl:rev CACCCACACAGACACTATCATGTTGACATTCC; dG = -15.1
116 >s_c_s_c_s_c_s_b_s_c_s GTGTGGGTGTGGGTGTGGGTGGCTGTGTGGGTG; >clamp_s_a_s_c_s:compl:rev CACCCACACAGACACTATCATGTTGACATTCC; dG = -15.1
117 >s_c_s_c_s_d_s_b_s_c_s GTGTGGGTGTGGGTGACGGTGGCTGTGTGGGTG; >clamp_s_a_s_c_s:compl:rev CACCCACACAGACACTATCATGTTGACATTCC; dG = -15.1
118 >s_c_s_c_s_e_s_b_s_c_s GTGTGGGTGTGGGTGCTGGTGGCTGTGTGGGTG; >clamp_s_a_s_c_s:compl:rev CACCCACACAGACACTATCATGTTGACATTCC; dG = -15.1
119 >s_c_s_d_s_a_s_b_s_c_s GTGTGGGTGACGGTGTCTGTGGCTGTGTGGGTG; >clamp_s_a_s_c_s:compl:rev CACCCACACAGACACTATCATGTTGACATTCC; dG = -15.1
120 >s_c_s_d_s_b_s_b_s_c_s GTGTGGGTGACGGTGGCTGTGGCTGTGTGGGTG; >clamp_s_a_s_c_s:compl:rev CACCCACACAGACACTATCATGTTGACATTCC; dG = -15.1
121 >s_a_s_b_s_b_s_b_s_c_s GTGTCTGTGGCTGTGGCTGTGGCTGTGTGGGTG; >clamp_s_a_s_c_s:compl:rev CACCCACACAGACACTATCATGTTGACATTCC; dG = -15.1
122 >s_c_s_d_s_c_s_b_s_c_s GTGTGGGTGACGGTGTGGGTGGCTGTGTGGGTG; >clamp_s_a_s_c_s:compl:rev CACCCACACAGACACTATCATGTTGACATTCC; dG = -15.1
123 >s_c_s_d_s_d_s_b_s_c_s GTGTGGGTGACGGTGACGGTGGCTGTGTGGGTG; >clamp_s_a_s_c_s:compl:rev CACCCACACAGACACTATCATGTTGACATTCC; dG = -15.1
124 >s_c_s_d_s_e_s_b_s_c_s GTGTGGGTGACGGTGCTGGTGGCTGTGTGGGTG; >clamp_s_a_s_c_s:compl:rev CACCCACACAGACACTATCATGTTGACATTCC; dG = -15.1
125 >s_c_s_e_s_a_s_b_s_c_s GTGTGGGTGCTGGTGTCTGTGGCTGTGTGGGTG; >clamp_s_a_s_c_s:compl:rev CACCCACACAGACACTATCATGTTGACATTCC; dG = -15.1
126 >s_c_s_e_s_b_s_b_s_c_s GTGTGGGTGCTGGTGGCTGTGGCTGTGTGGGTG; >clamp_s_a_s_c_s:compl:rev CACCCACACAGACACTATCATGTTGACATTCC; dG = -15.1
127 >s_c_s_e_s_c_s_b_s_c_s GTGTGGGTGCTGGTGTGGGTGGCTGTGTGGGTG; >clamp_s_a_s_c_s:compl:rev CACCCACACAGACACTATCATGTTGACATTCC; dG = -15.1
128 >s_c_s_e_s_d_s_b_s_c_s GTGTGGGTGCTGGTGACGGTGGCTGTGTGGGTG; >clamp_s_a_s_c_s:compl:rev CACCCACACAGACACTATCATGTTGACATTCC; dG = -15.1
129 >s_c_s_e_s_e_s_b_s_c_s GTGTGGGTGCTGGTGCTGGTGGCTGTGTGGGTG; >clamp_s_a_s_c_s:compl:rev CACCCACACAGACACTATCATGTTGACATTCC; dG = -15.1
130 >s_d_s_a_s_a_s_b_s_c_s GTGACGGTGTCTGTGTCTGTGGCTGTGTGGGTG; >clamp_s_a_s_c_s:compl:rev CACCCACACAGACACTATCATGTTGACATTCC; dG = -15.1
131 >s_d_s_a_s_b_s_b_s_c_s GTGACGGTGTCTGTGGCTGTGGCTGTGTGGGTG; >clamp_s_a_s_c_s:compl:rev CACCCACACAGACACTATCATGTTGACATTCC; dG = -15.1
132 >s_d_s_a_s_d_s_b_s_c_s GTGACGGTGTCTGTGACGGTGGCTGTGTGGGTG; >clamp_s_a_s_c_s:compl:rev CACCCACACAGACACTATCATGTTGACATTCC; dG = -15.1
133 >s_d_s_a_s_e_s_b_s_c_s GTGACGGTGTCTGTGCTGGTGGCTGTGTGGGTG; >clamp_s_a_s_c_s:compl:rev CACCCACACAGACACTATCATGTTGACATTCC; dG = -15.1
134 >s_d_s_b_s_a_s_b_s_c_s GTGACGGTGGCTGTGTCTGTGGCTGTGTGGGTG; >clamp_s_a_s_c_s:compl:rev CACCCACACAGACACTATCATGTTGACATTCC; dG = -15.1
135 >s_d_s_b_s_b_s_b_s_c_s GTGACGGTGGCTGTGGCTGTGGCTGTGTGGGTG; >clamp_s_a_s_c_s:compl:rev CACCCACACAGACACTATCATGTTGACATTCC; dG = -15.1
136 >s_d_s_b_s_d_s_b_s_c_s GTGACGGTGGCTGTGACGGTGGCTGTGTGGGTG; >clamp_s_a_s_c_s:compl:rev CACCCACACAGACACTATCATGTTGACATTCC; dG = -15.1
137 >s_d_s_b_s_e_s_b_s_c_s GTGACGGTGGCTGTGCTGGTGGCTGTGTGGGTG; >clamp_s_a_s_c_s:compl:rev CACCCACACAGACACTATCATGTTGACATTCC; dG = -15.1
138 >s_d_s_c_s_a_s_b_s_c_s GTGACGGTGTGGGTGTCTGTGGCTGTGTGGGTG; >clamp_s_a_s_c_s:compl:rev CACCCACACAGACACTATCATGTTGACATTCC; dG = -15.1
139 >s_d_s_c_s_b_s_b_s_c_s GTGACGGTGTGGGTGGCTGTGGCTGTGTGGGTG; >clamp_s_a_s_c_s:compl:rev CACCCACACAGACACTATCATGTTGACATTCC; dG = -15.1
140 >s_d_s_c_s_c_s_b_s_c_s GTGACGGTGTGGGTGTGGGTGGCTGTGTGGGTG; >clamp_s_a_s_c_s:compl:rev CACCCACACAGACACTATCATGTTGACATTCC; dG = -15.1
141 >s_a_s_b_s_d_s_b_s_c_s GTGTCTGTGGCTGTGACGGTGGCTGTGTGGGTG; >clamp_s_a_s_c_s:compl:rev CACCCACACAGACACTATCATGTTGACATTCC; dG = -15.1
142 >s_d_s_c_s_d_s_b_s_c_s GTGACGGTGTGGGTGACGGTGGCTGTGTGGGTG; >clamp_s_a_s_c_s:compl:rev CACCCACACAGACACTATCATGTTGACATTCC; dG = -15.1
143 >s_d_s_c_s_e_s_b_s_c_s GTGACGGTGTGGGTGCTGGTGGCTGTGTGGGTG; >clamp_s_a_s_c_s:compl:rev CACCCACACAGACACTATCATGTTGACATTCC; dG = -15.1
144 >s_d_s_d_s_a_s_b_s_c_s GTGACGGTGACGGTGTCTGTGGCTGTGTGGGTG; >clamp_s_a_s_c_s:compl:rev CACCCACACAGACACTATCATGTTGACATTCC; dG = -15.1

145 >s_d_s_d_s_b_s_b_s_c_s GTGACGGTGACGGTGGCTGTGGCTGTGTGGGTG; >clamp_s_a_s_c_s:compl:rev
CACCCACACAGACACTATCATGTTGACATTCC; dG = -15.1
146 >s_d_s_d_s_c_s_b_s_c_s GTGACGGTGACGGTGTGGGTGGCTGTGTGGGTG; >clamp_s_a_s_c_s:compl:rev
CACCCACACAGACACTATCATGTTGACATTCC; dG = -15.1
147 >s_d_s_d_s_d_s_b_s_c_s GTGACGGTGACGGTGACGGTGGCTGTGTGGGTG; >clamp_s_a_s_c_s:compl:rev
CACCCACACAGACACTATCATGTTGACATTCC; dG = -15.1
148 >s_d_s_d_s_e_s_b_s_c_s GTGACGGTGACGGTGCTGGTGGCTGTGTGGGTG; >clamp_s_a_s_c_s:compl:rev
CACCCACACAGACACTATCATGTTGACATTCC; dG = -15.1
149 >s_d_s_e_s_a_s_b_s_c_s GTGACGGTGCTGGTGTCTGTGGCTGTGTGGGTG; >clamp_s_a_s_c_s:compl:rev
CACCCACACAGACACTATCATGTTGACATTCC; dG = -15.1
150 >s_d_s_e_s_b_s_b_s_c_s GTGACGGTGCTGGTGGCTGTGGCTGTGTGGGTG; >clamp_s_a_s_c_s:compl:rev
CACCCACACAGACACTATCATGTTGACATTCC; dG = -15.1
151 >s_d_s_e_s_c_s_b_s_c_s GTGACGGTGCTGGTGTGGGTGGCTGTGTGGGTG; >clamp_s_a_s_c_s:compl:rev
CACCCACACAGACACTATCATGTTGACATTCC; dG = -15.1
152 >s_a_s_a_s_a_s_b_s_c_s GTGTCTGTGTCTGTGTCTGTGGCTGTGTGGGTG; >clamp_s_a_s_c_s:compl:rev
CACCCACACAGACACTATCATGTTGACATTCC; dG = -15.1
153 >s_a_s_b_s_e_s_b_s_c_s GTGTCTGTGGCTGTGCTGGTGGCTGTGTGGGTG; >clamp_s_a_s_c_s:compl:rev
CACCCACACAGACACTATCATGTTGACATTCC; dG = -15.1
154 >s_d_s_e_s_d_s_b_s_c_s GTGACGGTGCTGGTGACGGTGGCTGTGTGGGTG; >clamp_s_a_s_c_s:compl:rev
CACCCACACAGACACTATCATGTTGACATTCC; dG = -15.1
155 >s_d_s_e_s_e_s_b_s_c_s GTGACGGTGCTGGTGCTGGTGGCTGTGTGGGTG; >clamp_s_a_s_c_s:compl:rev
CACCCACACAGACACTATCATGTTGACATTCC; dG = -15.1
156 >s_e_s_a_s_a_s_b_s_c_s GTGCTGGTGTCTGTGTCTGTGGCTGTGTGGGTG; >clamp_s_a_s_c_s:compl:rev
CACCCACACAGACACTATCATGTTGACATTCC; dG = -15.1
157 >s_e_s_a_s_b_s_b_s_c_s GTGCTGGTGTCTGTGGCTGTGGCTGTGTGGGTG; >clamp_s_a_s_c_s:compl:rev
CACCCACACAGACACTATCATGTTGACATTCC; dG = -15.1
158 >s_e_s_a_s_d_s_b_s_c_s GTGCTGGTGTCTGTGACGGTGGCTGTGTGGGTG; >clamp_s_a_s_c_s:compl:rev
CACCCACACAGACACTATCATGTTGACATTCC; dG = -15.1
159 >s_e_s_a_s_e_s_b_s_c_s GTGCTGGTGTCTGTGCTGGTGGCTGTGTGGGTG; >clamp_s_a_s_c_s:compl:rev
CACCCACACAGACACTATCATGTTGACATTCC; dG = -15.1
160 >s_e_s_b_s_a_s_b_s_c_s GTGCTGGTGGCTGTGTCTGTGGCTGTGTGGGTG; >clamp_s_a_s_c_s:compl:rev
CACCCACACAGACACTATCATGTTGACATTCC; dG = -15.1
161 >s_e_s_b_s_b_s_b_s_c_s GTGCTGGTGGCTGTGGCTGTGGCTGTGTGGGTG; >clamp_s_a_s_c_s:compl:rev
CACCCACACAGACACTATCATGTTGACATTCC; dG = -15.1
162 >s_a_s_d_s_a_s_b_s_c_s GTGTCTGTGACGGTGTCTGTGGCTGTGTGGGTG; >clamp_s_a_s_c_s:compl:rev
CACCCACACAGACACTATCATGTTGACATTCC; dG = -15.1
163 >s_e_s_b_s_d_s_b_s_c_s GTGCTGGTGGCTGTGACGGTGGCTGTGTGGGTG; >clamp_s_a_s_c_s:compl:rev
CACCCACACAGACACTATCATGTTGACATTCC; dG = -15.1
164 >s_e_s_b_s_e_s_b_s_c_s GTGCTGGTGGCTGTGCTGGTGGCTGTGTGGGTG; >clamp_s_a_s_c_s:compl:rev
CACCCACACAGACACTATCATGTTGACATTCC; dG = -15.1
165 >s_e_s_c_s_a_s_b_s_c_s GTGCTGGTGTGGGTGTCTGTGGCTGTGTGGGTG; >clamp_s_a_s_c_s:compl:rev
CACCCACACAGACACTATCATGTTGACATTCC; dG = -15.1
166 >s_e_s_c_s_b_s_b_s_c_s GTGCTGGTGTGGGTGGCTGTGGCTGTGTGGGTG; >clamp_s_a_s_c_s:compl:rev
CACCCACACAGACACTATCATGTTGACATTCC; dG = -15.1
167 >s_e_s_c_s_c_s_b_s_c_s GTGCTGGTGTGGGTGTGGGTGGCTGTGTGGGTG; >clamp_s_a_s_c_s:compl:rev
CACCCACACAGACACTATCATGTTGACATTCC; dG = -15.1
168 >s_e_s_c_s_d_s_b_s_c_s GTGCTGGTGTGGGTGACGGTGGCTGTGTGGGTG; >clamp_s_a_s_c_s:compl:rev
CACCCACACAGACACTATCATGTTGACATTCC; dG = -15.1
169 >s_e_s_c_s_e_s_b_s_c_s GTGCTGGTGTGGGTGCTGGTGGCTGTGTGGGTG; >clamp_s_a_s_c_s:compl:rev
CACCCACACAGACACTATCATGTTGACATTCC; dG = -15.1
170 >s_e_s_d_s_a_s_b_s_c_s GTGCTGGTGACGGTGTCTGTGGCTGTGTGGGTG; >clamp_s_a_s_c_s:compl:rev
CACCCACACAGACACTATCATGTTGACATTCC; dG = -15.1
171 >s_e_s_d_s_b_s_b_s_c_s GTGCTGGTGACGGTGGCTGTGGCTGTGTGGGTG; >clamp_s_a_s_c_s:compl:rev
CACCCACACAGACACTATCATGTTGACATTCC; dG = -15.1
172 >s_e_s_d_s_c_s_b_s_c_s GTGCTGGTGACGGTGTGGGTGGCTGTGTGGGTG; >clamp_s_a_s_c_s:compl:rev
CACCCACACAGACACTATCATGTTGACATTCC; dG = -15.1
173 >s_e_s_d_s_d_s_b_s_c_s GTGCTGGTGACGGTGACGGTGGCTGTGTGGGTG; >clamp_s_a_s_c_s:compl:rev
CACCCACACAGACACTATCATGTTGACATTCC; dG = -15.1
174 >s_a_s_d_s_b_s_b_s_c_s GTGTCTGTGACGGTGGCTGTGGCTGTGTGGGTG; >clamp_s_a_s_c_s:compl:rev
CACCCACACAGACACTATCATGTTGACATTCC; dG = -15.1
175 >s_e_s_d_s_e_s_b_s_c_s GTGCTGGTGACGGTGCTGGTGGCTGTGTGGGTG; >clamp_s_a_s_c_s:compl:rev
CACCCACACAGACACTATCATGTTGACATTCC; dG = -15.1
176 >s_e_s_e_s_a_s_b_s_c_s GTGCTGGTGCTGGTGTCTGTGGCTGTGTGGGTG; >clamp_s_a_s_c_s:compl:rev
CACCCACACAGACACTATCATGTTGACATTCC; dG = -15.1
177 >s_e_s_e_s_b_s_b_s_c_s GTGCTGGTGCTGGTGGCTGTGGCTGTGTGGGTG; >clamp_s_a_s_c_s:compl:rev
CACCCACACAGACACTATCATGTTGACATTCC; dG = -15.1
178 >s_e_s_e_s_c_s_b_s_c_s GTGCTGGTGCTGGTGTGGGTGGCTGTGTGGGTG; >clamp_s_a_s_c_s:compl:rev
CACCCACACAGACACTATCATGTTGACATTCC; dG = -15.1
179 >s_e_s_e_s_d_s_b_s_c_s GTGCTGGTGCTGGTGACGGTGGCTGTGTGGGTG; >clamp_s_a_s_c_s:compl:rev
CACCCACACAGACACTATCATGTTGACATTCC; dG = -15.1
180 >s_e_s_e_s_e_s_b_s_c_s GTGCTGGTGCTGGTGCTGGTGGCTGTGTGGGTG; >clamp_s_a_s_c_s:compl:rev
CACCCACACAGACACTATCATGTTGACATTCC; dG = -15.1
181 >s_a_s_d_s_c_s_b_s_c_s GTGTCTGTGACGGTGTGGGTGGCTGTGTGGGTG; >clamp_s_a_s_c_s:compl:rev
CACCCACACAGACACTATCATGTTGACATTCC; dG = -15.1

182 >s_b_s_c_s_b_s_a_s_e_s GTGGCTGTGTGGGTGGCTGTGTCTGTGCTGGTG; >clamp_s_a_s_c_s:compl:rev CACCCACACAGACACTATCATGTTGACATTCC; dG = -15.0
183 >s_b_s_c_s_b_s_a_s_d_s GTGGCTGTGTGGGTGGCTGTGTCTGTGACGGTG; >clamp_s_a_s_c_s:compl:rev CACCCACACAGACACTATCATGTTGACATTCC; dG = -15.0
184 >s_b_s_c_s_b_s_a_s_b_s GTGGCTGTGTGGGTGGCTGTGTCTGTGGCTGTG; >clamp_s_a_s_c_s:compl:rev CACCCACACAGACACTATCATGTTGACATTCC; dG = -15.0
185 >s_b_s_c_s_b_s_a_s_a_s GTGGCTGTGTGGGTGGCTGTGTCTGTGTCTGTG; >clamp_s_a_s_c_s:compl:rev CACCCACACAGACACTATCATGTTGACATTCC; dG = -15.0
186 >s_b_s_c_s_b_s_e_s_c_s GTGGCTGTGTGGGTGGCTGTGCTGGTGTGGGTG; >clamp_s_a_s_c_s:compl:rev CACCCACACAGACACTATCATGTTGACATTCC; dG = -15.0
187 >s_b_s_c_s_b_s_d_s_c_s GTGGCTGTGTGGGTGGCTGTGACGGTGTGGGTG; >clamp_s_a_s_c_s:compl:rev CACCCACACAGACACTATCATGTTGACATTCC; dG = -15.0
188 >s_b_s_c_s_a_s_a_s_e_s GTGGCTGTGTGGGTGTCTGTGTCTGTGCTGGTG; >clamp_s_a_s_c_s:compl:rev CACCCACACAGACACTATCATGTTGACATTCC; dG = -14.9
189 >s_b_s_c_s_a_s_a_s_d_s GTGGCTGTGTGGGTGTCTGTGTCTGTGACGGTG; >clamp_s_a_s_c_s:compl:rev CACCCACACAGACACTATCATGTTGACATTCC; dG = -14.9
190 >s_b_s_c_s_a_s_a_s_b_s GTGGCTGTGTGGGTGTCTGTGTCTGTGGCTGTG; >clamp_s_a_s_c_s:compl:rev CACCCACACAGACACTATCATGTTGACATTCC; dG = -14.9
191 >s_b_s_c_s_a_s_a_s_a_s GTGGCTGTGTGGGTGTCTGTGTCTGTGTCTGTG; >clamp_s_a_s_c_s:compl:rev CACCCACACAGACACTATCATGTTGACATTCC; dG = -14.9
192 >s_b_s_c_s_a_s_e_s_c_s GTGGCTGTGTGGGTGTCTGTGCTGGTGTGGGTG; >clamp_s_a_s_c_s:compl:rev CACCCACACAGACACTATCATGTTGACATTCC; dG = -14.9
193 >s_b_s_c_s_a_s_d_s_c_s GTGGCTGTGTGGGTGTCTGTGACGGTGTGGGTG; >clamp_s_a_s_c_s:compl:rev CACCCACACAGACACTATCATGTTGACATTCC; dG = -14.9
194 >s_b_s_c_s_c_s_a_s_e_s GTGGCTGTGTGGGTGTGGGTGTCTGTGCTGGTG; >clamp_s_a_s_c_s:compl:rev CACCCACACAGACACTATCATGTTGACATTCC; dG = -14.9
195 >s_b_s_c_s_c_s_a_s_d_s GTGGCTGTGTGGGTGTGGGTGTCTGTGACGGTG; >clamp_s_a_s_c_s:compl:rev CACCCACACAGACACTATCATGTTGACATTCC; dG = -14.9
196 >s_b_s_c_s_c_s_a_s_b_s GTGGCTGTGTGGGTGTGGGTGTCTGTGGCTGTG; >clamp_s_a_s_c_s:compl:rev CACCCACACAGACACTATCATGTTGACATTCC; dG = -14.9
197 >s_b_s_c_s_c_s_a_s_a_s GTGGCTGTGTGGGTGTGGGTGTCTGTGTCTGTG; >clamp_s_a_s_c_s:compl:rev CACCCACACAGACACTATCATGTTGACATTCC; dG = -14.9
198 >s_b_s_c_s_c_s_e_s_c_s GTGGCTGTGTGGGTGTGGGTGCTGGTGTGGGTG; >clamp_s_a_s_c_s:compl:rev CACCCACACAGACACTATCATGTTGACATTCC; dG = -14.9
199 >s_b_s_c_s_c_s_d_s_c_s GTGGCTGTGTGGGTGTGGGTGACGGTGTGGGTG; >clamp_s_a_s_c_s:compl:rev CACCCACACAGACACTATCATGTTGACATTCC; dG = -14.9
200 >s_b_s_c_s_c_s_c_s_c_s GTGGCTGTGTGGGTGTGGGTGTGGGTGTGGGTG; >clamp_s_a_s_c_s:compl:rev CACCCACACAGACACTATCATGTTGACATTCC; dG = -14.9
201 >s_b_s_c_s_e_s_a_s_e_s GTGGCTGTGTGGGTGCTGGTGTCTGTGCTGGTG; >clamp_s_a_s_c_s:compl:rev CACCCACACAGACACTATCATGTTGACATTCC; dG = -14.8
202 >s_b_s_c_s_e_s_a_s_d_s GTGGCTGTGTGGGTGCTGGTGTCTGTGACGGTG; >clamp_s_a_s_c_s:compl:rev CACCCACACAGACACTATCATGTTGACATTCC; dG = -14.8
203 >s_b_s_c_s_e_s_a_s_b_s GTGGCTGTGTGGGTGCTGGTGTCTGTGGCTGTG; >clamp_s_a_s_c_s:compl:rev CACCCACACAGACACTATCATGTTGACATTCC; dG = -14.8
204 >s_b_s_c_s_e_s_a_s_a_s GTGGCTGTGTGGGTGCTGGTGTCTGTGTCTGTG; >clamp_s_a_s_c_s:compl:rev CACCCACACAGACACTATCATGTTGACATTCC; dG = -14.8
205 >s_b_s_c_s_e_s_e_s_c_s GTGGCTGTGTGGGTGCTGGTGCTGGTGTGGGTG; >clamp_s_a_s_c_s:compl:rev CACCCACACAGACACTATCATGTTGACATTCC; dG = -14.8
206 >s_b_s_c_s_e_s_d_s_c_s GTGGCTGTGTGGGTGCTGGTGACGGTGTGGGTG; >clamp_s_a_s_c_s:compl:rev CACCCACACAGACACTATCATGTTGACATTCC; dG = -14.8
207 >s_b_s_c_s_e_s_c_s_c_s GTGGCTGTGTGGGTGCTGGTGTGGGTGTGGGTG; >clamp_s_a_s_c_s:compl:rev CACCCACACAGACACTATCATGTTGACATTCC; dG = -14.8
208 >s_c_s_c_s_c_s_d_s_c_s GTGTGGGTGTGGGTGTGGGTGACGGTGTGGGTG; >clamp_s_a_s_c_s:compl:rev CACCCACACAGACACTATCATGTTGACATTCC; dG = -12.3
209 >s_d_s_c_s_c_s_d_s_c_s GTGACGGTGTGGGTGTGGGTGACGGTGTGGGTG; >clamp_s_a_s_c_s:compl:rev CACCCACACAGACACTATCATGTTGACATTCC; dG = -12.3
210 >s_e_s_c_s_c_s_d_s_c_s GTGCTGGTGTGGGTGTGGGTGACGGTGTGGGTG; >clamp_s_a_s_c_s:compl:rev CACCCACACAGACACTATCATGTTGACATTCC; dG = -12.3
211 >s_a_s_e_s_c_s_d_s_c_s GTGTCTGTGCTGGTGTGGGTGACGGTGTGGGTG; >clamp_s_a_s_c_s:compl:rev CACCCACACAGACACTATCATGTTGACATTCC; dG = -12.0
212 >s_a_s_d_s_c_s_d_s_c_s GTGTCTGTGACGGTGTGGGTGACGGTGTGGGTG; >clamp_s_a_s_c_s:compl:rev CACCCACACAGACACTATCATGTTGACATTCC; dG = -12.0
213 >s_b_s_a_s_d_s_c_s_c_s GTGGCTGTGTCTGTGACGGTGTGGGTGTGGGTG; >clamp_s_a_s_c_s:compl:rev CACCCACACAGACACTATCATGTTGACATTCC; dG = -11.9
214 >s_b_s_a_s_e_s_c_s_c_s GTGGCTGTGTCTGTGCTGGTGTGGGTGTGGGTG; >clamp_s_a_s_c_s:compl:rev CACCCACACAGACACTATCATGTTGACATTCC; dG = -11.9
215 >s_a_s_a_s_d_s_c_s_c_s GTGTCTGTGTCTGTGACGGTGTGGGTGTGGGTG; >clamp_s_a_s_c_s:compl:rev CACCCACACAGACACTATCATGTTGACATTCC; dG = -11.9
216 >s_a_s_a_s_e_s_c_s_c_s GTGTCTGTGTCTGTGCTGGTGTGGGTGTGGGTG; >clamp_s_a_s_c_s:compl:rev CACCCACACAGACACTATCATGTTGACATTCC; dG = -11.9
217 >s_c_s_a_s_d_s_c_s_c_s GTGTGGGTGTCTGTGACGGTGTGGGTGTGGGTG; >clamp_s_a_s_c_s:compl:rev CACCCACACAGACACTATCATGTTGACATTCC; dG = -11.9
218 >s_c_s_a_s_e_s_c_s_c_s GTGTGGGTGTCTGTGCTGGTGTGGGTGTGGGTG; >clamp_s_a_s_c_s:compl:rev CACCCACACAGACACTATCATGTTGACATTCC; dG = -11.9

219 >s_d_s_a_s_d_s_c_s_c_s GTGACGGTGTCTGTGACGGTGTGGGTGTGGGTG; >clamp_s_a_s_c_s:compl:rev
CACCCACACAGACACTATCATGTTGACATTCC; dG = -11.9
220 >s_d_s_a_s_e_s_c_s_c_s GTGACGGTGTCTGTGCTGGTGTGGGTGTGGGTG; >clamp_s_a_s_c_s:compl:rev
CACCCACACAGACACTATCATGTTGACATTCC; dG = -11.9
221 >s_e_s_a_s_d_s_c_s_c_s GTGCTGGTGTCTGTGACGGTGTGGGTGTGGGTG; >clamp_s_a_s_c_s:compl:rev
CACCCACACAGACACTATCATGTTGACATTCC; dG = -11.9
222 >s_e_s_a_s_e_s_c_s_c_s GTGCTGGTGTCTGTGCTGGTGTGGGTGTGGGTG; >clamp_s_a_s_c_s:compl:rev
CACCCACACAGACACTATCATGTTGACATTCC; dG = -11.9
223 >s_c_s_c_s_d_s_a_s_e_s GTGTGGGTGTGGGTGACGGTGTCTGTGCTGGTG; >clamp_s_a_s_c_s:compl:rev
CACCCACACAGACACTATCATGTTGACATTCC; dG = -11.8
224 >s_c_s_c_s_d_s_a_s_d_s GTGTGGGTGTGGGTGACGGTGTCTGTGACGGTG; >clamp_s_a_s_c_s:compl:rev
CACCCACACAGACACTATCATGTTGACATTCC; dG = -11.8
225 >s_c_s_c_s_d_s_a_s_b_s GTGTGGGTGTGGGTGACGGTGTCTGTGGCTGTG; >clamp_s_a_s_c_s:compl:rev
CACCCACACAGACACTATCATGTTGACATTCC; dG = -11.8
226 >s_c_s_c_s_d_s_a_s_a_s GTGTGGGTGTGGGTGACGGTGTCTGTGTCTGTG; >clamp_s_a_s_c_s:compl:rev
CACCCACACAGACACTATCATGTTGACATTCC; dG = -11.8
227 >s_c_s_c_s_d_s_e_s_c_s GTGTGGGTGTGGGTGACGGTGCTGGTGTGGGTG; >clamp_s_a_s_c_s:compl:rev
CACCCACACAGACACTATCATGTTGACATTCC; dG = -11.8
228 >s_c_s_c_s_d_s_d_s_c_s GTGTGGGTGTGGGTGACGGTGACGGTGTGGGTG; >clamp_s_a_s_c_s:compl:rev
CACCCACACAGACACTATCATGTTGACATTCC; dG = -11.8
229 >s_c_s_c_s_d_s_c_s_c_s GTGTGGGTGTGGGTGACGGTGTGGGTGTGGGTG; >clamp_s_a_s_c_s:compl:rev
CACCCACACAGACACTATCATGTTGACATTCC; dG = -11.8
230 >s_a_s_e_s_c_s_c_s_c_s GTGTCTGTGCTGGTGTGGGTGTGGGTGTGGGTG; >clamp_s_a_s_c_s:compl:rev
CACCCACACAGACACTATCATGTTGACATTCC; dG = -11.7
231 >s_b_s_a_s_a_s_e_s_c_s GTGGCTGTGTCTGTGTCTGTGCTGGTGTGGGTG; >clamp_s_a_s_c_s:compl:rev
CACCCACACAGACACTATCATGTTGACATTCC; dG = -11.7
232 >s_b_s_a_s_a_s_d_s_c_s GTGGCTGTGTCTGTGTCTGTGACGGTGTGGGTG; >clamp_s_a_s_c_s:compl:rev
CACCCACACAGACACTATCATGTTGACATTCC; dG = -11.7
233 >s_b_s_a_s_d_s_d_s_c_s GTGGCTGTGTCTGTGACGGTGACGGTGTGGGTG; >clamp_s_a_s_c_s:compl:rev
CACCCACACAGACACTATCATGTTGACATTCC; dG = -11.7
234 >s_b_s_b_s_a_s_e_s_c_s GTGGCTGTGGCTGTGTCTGTGCTGGTGTGGGTG; >clamp_s_a_s_c_s:compl:rev
CACCCACACAGACACTATCATGTTGACATTCC; dG = -11.7
235 >s_b_s_b_s_a_s_d_s_c_s GTGGCTGTGGCTGTGTCTGTGACGGTGTGGGTG; >clamp_s_a_s_c_s:compl:rev
CACCCACACAGACACTATCATGTTGACATTCC; dG = -11.7
236 >s_a_s_a_s_d_s_d_s_c_s GTGTCTGTGTCTGTGACGGTGACGGTGTGGGTG; >clamp_s_a_s_c_s:compl:rev
CACCCACACAGACACTATCATGTTGACATTCC; dG = -11.7
237 >s_b_s_d_s_c_s_c_s_c_s GTGGCTGTGACGGTGTGGGTGTGGGTGTGGGTG; >clamp_s_a_s_c_s:compl:rev
CACCCACACAGACACTATCATGTTGACATTCC; dG = -11.7
238 >s_b_s_e_s_c_s_c_s_c_s GTGGCTGTGCTGGTGTGGGTGTGGGTGTGGGTG; >clamp_s_a_s_c_s:compl:rev
CACCCACACAGACACTATCATGTTGACATTCC; dG = -11.7
239 >s_c_s_a_s_a_s_e_s_c_s GTGTGGGTGTCTGTGTCTGTGCTGGTGTGGGTG; >clamp_s_a_s_c_s:compl:rev
CACCCACACAGACACTATCATGTTGACATTCC; dG = -11.7
240 >s_c_s_a_s_a_s_d_s_c_s GTGTGGGTGTCTGTGTCTGTGACGGTGTGGGTG; >clamp_s_a_s_c_s:compl:rev
CACCCACACAGACACTATCATGTTGACATTCC; dG = -11.7
241 >s_c_s_a_s_d_s_d_s_c_s GTGTGGGTGTCTGTGACGGTGACGGTGTGGGTG; >clamp_s_a_s_c_s:compl:rev
CACCCACACAGACACTATCATGTTGACATTCC; dG = -11.7
242 >s_a_s_b_s_a_s_e_s_c_s GTGTCTGTGGCTGTGTCTGTGCTGGTGTGGGTG; >clamp_s_a_s_c_s:compl:rev
CACCCACACAGACACTATCATGTTGACATTCC; dG = -11.7
243 >s_a_s_b_s_a_s_d_s_c_s GTGTCTGTGGCTGTGTCTGTGACGGTGTGGGTG; >clamp_s_a_s_c_s:compl:rev
CACCCACACAGACACTATCATGTTGACATTCC; dG = -11.7
244 >s_c_s_b_s_a_s_e_s_c_s GTGTGGGTGGCTGTGTCTGTGCTGGTGTGGGTG; >clamp_s_a_s_c_s:compl:rev
CACCCACACAGACACTATCATGTTGACATTCC; dG = -11.7
245 >s_c_s_b_s_a_s_d_s_c_s GTGTGGGTGGCTGTGTCTGTGACGGTGTGGGTG; >clamp_s_a_s_c_s:compl:rev
CACCCACACAGACACTATCATGTTGACATTCC; dG = -11.7
246 >s_c_s_c_s_c_s_a_s_e_s GTGTGGGTGTGGGTGTGGGTGTCTGTGCTGGTG; >clamp_s_a_s_c_s:compl:rev
CACCCACACAGACACTATCATGTTGACATTCC; dG = -11.7
247 >s_c_s_c_s_c_s_a_s_d_s GTGTGGGTGTGGGTGTGGGTGTCTGTGACGGTG; >clamp_s_a_s_c_s:compl:rev
CACCCACACAGACACTATCATGTTGACATTCC; dG = -11.7
248 >s_c_s_c_s_c_s_a_s_b_s GTGTGGGTGTGGGTGTGGGTGTCTGTGGCTGTG; >clamp_s_a_s_c_s:compl:rev
CACCCACACAGACACTATCATGTTGACATTCC; dG = -11.7
249 >s_c_s_c_s_c_s_a_s_a_s GTGTGGGTGTGGGTGTGGGTGTCTGTGTCTGTG; >clamp_s_a_s_c_s:compl:rev
CACCCACACAGACACTATCATGTTGACATTCC; dG = -11.7
250 >s_c_s_c_s_c_s_c_s_c_s GTGTGGGTGTGGGTGTGGGTGTGGGTGTGGGTG; >clamp_s_a_s_c_s:compl:rev
CACCCACACAGACACTATCATGTTGACATTCC; dG = -11.7
251 >s_c_s_d_s_c_s_c_s_c_s GTGTGGGTGACGGTGTGGGTGTGGGTGTGGGTG; >clamp_s_a_s_c_s:compl:rev
CACCCACACAGACACTATCATGTTGACATTCC; dG = -11.7
252 >s_a_s_a_s_a_s_e_s_c_s GTGTCTGTGTCTGTGTCTGTGCTGGTGTGGGTG; >clamp_s_a_s_c_s:compl:rev
CACCCACACAGACACTATCATGTTGACATTCC; dG = -11.7
253 >s_c_s_e_s_c_s_c_s_c_s GTGTGGGTGCTGGTGTGGGTGTGGGTGTGGGTG; >clamp_s_a_s_c_s:compl:rev
CACCCACACAGACACTATCATGTTGACATTCC; dG = -11.7
254 >s_d_s_a_s_a_s_e_s_c_s GTGACGGTGTCTGTGTCTGTGCTGGTGTGGGTG; >clamp_s_a_s_c_s:compl:rev
CACCCACACAGACACTATCATGTTGACATTCC; dG = -11.7
255 >s_d_s_a_s_a_s_d_s_c_s GTGACGGTGTCTGTGTCTGTGACGGTGTGGGTG; >clamp_s_a_s_c_s:compl:rev
CACCCACACAGACACTATCATGTTGACATTCC; dG = -11.7

256 >s_d_s_a_s_d_s_d_s_c_s GTGACGGTGTCTGTGACGGTGACGGTGTGGGTG; >clamp_s_a_s_c_s:compl:rev CACCCACACAGACACTATCATGTTGACATTCC; dG = -11.7
257 >s_d_s_b_s_a_s_e_s_c_s GTGACGGTGGCTGTGTCTGTGCTGGTGTGGGTG; >clamp_s_a_s_c_s:compl:rev CACCCACACAGACACTATCATGTTGACATTCC; dG = -11.7
258 >s_d_s_b_s_a_s_d_s_c_s GTGACGGTGGCTGTGTCTGTGACGGTGTGGGTG; >clamp_s_a_s_c_s:compl:rev CACCCACACAGACACTATCATGTTGACATTCC; dG = -11.7
259 >s_a_s_a_s_a_s_d_s_c_s GTGTCTGTGTCTGTGTCTGTGACGGTGTGGGTG; >clamp_s_a_s_c_s:compl:rev CACCCACACAGACACTATCATGTTGACATTCC; dG = -11.7
260 >s_d_s_c_s_c_s_a_s_e_s GTGACGGTGTGGGTGTGGGTGTCTGTGCTGGTG; >clamp_s_a_s_c_s:compl:rev CACCCACACAGACACTATCATGTTGACATTCC; dG = -11.7
261 >s_d_s_c_s_c_s_a_s_d_s GTGACGGTGTGGGTGTGGGTGTCTGTGACGGTG; >clamp_s_a_s_c_s:compl:rev CACCCACACAGACACTATCATGTTGACATTCC; dG = -11.7
262 >s_d_s_c_s_c_s_a_s_b_s GTGACGGTGTGGGTGTGGGTGTCTGTGGCTGTG; >clamp_s_a_s_c_s:compl:rev CACCCACACAGACACTATCATGTTGACATTCC; dG = -11.7
263 >s_d_s_c_s_c_s_a_s_a_s GTGACGGTGTGGGTGTGGGTGTCTGTGTCTGTG; >clamp_s_a_s_c_s:compl:rev CACCCACACAGACACTATCATGTTGACATTCC; dG = -11.7
264 >s_d_s_c_s_c_s_c_s_c_s GTGACGGTGTGGGTGTGGGTGTGGGTGTGGGTG; >clamp_s_a_s_c_s:compl:rev CACCCACACAGACACTATCATGTTGACATTCC; dG = -11.7
265 >s_d_s_d_s_c_s_c_s_c_s GTGACGGTGACGGTGTGGGTGTGGGTGTGGGTG; >clamp_s_a_s_c_s:compl:rev CACCCACACAGACACTATCATGTTGACATTCC; dG = -11.7
266 >s_d_s_e_s_c_s_c_s_c_s GTGACGGTGCTGGTGTGGGTGTGGGTGTGGGTG; >clamp_s_a_s_c_s:compl:rev CACCCACACAGACACTATCATGTTGACATTCC; dG = -11.7
267 >s_e_s_a_s_a_s_e_s_c_s GTGCTGGTGTCTGTGTCTGTGCTGGTGTGGGTG; >clamp_s_a_s_c_s:compl:rev CACCCACACAGACACTATCATGTTGACATTCC; dG = -11.7
268 >s_e_s_a_s_a_s_d_s_c_s GTGCTGGTGTCTGTGTCTGTGACGGTGTGGGTG; >clamp_s_a_s_c_s:compl:rev CACCCACACAGACACTATCATGTTGACATTCC; dG = -11.7
269 >s_e_s_a_s_d_s_d_s_c_s GTGCTGGTGTCTGTGACGGTGACGGTGTGGGTG; >clamp_s_a_s_c_s:compl:rev CACCCACACAGACACTATCATGTTGACATTCC; dG = -11.7
270 >s_e_s_b_s_a_s_e_s_c_s GTGCTGGTGGCTGTGTCTGTGCTGGTGTGGGTG; >clamp_s_a_s_c_s:compl:rev CACCCACACAGACACTATCATGTTGACATTCC; dG = -11.7
271 >s_e_s_b_s_a_s_d_s_c_s GTGCTGGTGGCTGTGTCTGTGACGGTGTGGGTG; >clamp_s_a_s_c_s:compl:rev CACCCACACAGACACTATCATGTTGACATTCC; dG = -11.7
272 >s_e_s_c_s_c_s_a_s_e_s GTGCTGGTGTGGGTGTGGGTGTCTGTGCTGGTG; >clamp_s_a_s_c_s:compl:rev CACCCACACAGACACTATCATGTTGACATTCC; dG = -11.7
273 >s_e_s_c_s_c_s_a_s_d_s GTGCTGGTGTGGGTGTGGGTGTCTGTGACGGTG; >clamp_s_a_s_c_s:compl:rev CACCCACACAGACACTATCATGTTGACATTCC; dG = -11.7
274 >s_e_s_c_s_c_s_a_s_b_s GTGCTGGTGTGGGTGTGGGTGTCTGTGGCTGTG; >clamp_s_a_s_c_s:compl:rev CACCCACACAGACACTATCATGTTGACATTCC; dG = -11.7
275 >s_e_s_c_s_c_s_a_s_a_s GTGCTGGTGTGGGTGTGGGTGTCTGTGTCTGTG; >clamp_s_a_s_c_s:compl:rev CACCCACACAGACACTATCATGTTGACATTCC; dG = -11.7
276 >s_e_s_c_s_c_s_c_s_c_s GTGCTGGTGTGGGTGTGGGTGTGGGTGTGGGTG; >clamp_s_a_s_c_s:compl:rev CACCCACACAGACACTATCATGTTGACATTCC; dG = -11.7
277 >s_e_s_d_s_c_s_c_s_c_s GTGCTGGTGACGGTGTGGGTGTGGGTGTGGGTG; >clamp_s_a_s_c_s:compl:rev CACCCACACAGACACTATCATGTTGACATTCC; dG = -11.7
278 >s_e_s_e_s_c_s_c_s_c_s GTGCTGGTGCTGGTGTGGGTGTGGGTGTGGGTG; >clamp_s_a_s_c_s:compl:rev CACCCACACAGACACTATCATGTTGACATTCC; dG = -11.7
279 >s_a_s_d_s_c_s_c_s_c_s GTGTCTGTGACGGTGTGGGTGTGGGTGTGGGTG; >clamp_s_a_s_c_s:compl:rev CACCCACACAGACACTATCATGTTGACATTCC; dG = -11.7
280 >s_a_s_e_s_a_s_e_s_c_s GTGTCTGTGCTGGTGTCTGTGCTGGTGTGGGTG; >clamp_s_a_s_c_s:compl:rev CACCCACACAGACACTATCATGTTGACATTCC; dG = -11.6
281 >s_a_s_e_s_a_s_d_s_c_s GTGTCTGTGCTGGTGTCTGTGACGGTGTGGGTG; >clamp_s_a_s_c_s:compl:rev CACCCACACAGACACTATCATGTTGACATTCC; dG = -11.6
282 >s_b_s_a_s_e_s_d_s_c_s GTGGCTGTGTCTGTGCTGGTGACGGTGTGGGTG; >clamp_s_a_s_c_s:compl:rev CACCCACACAGACACTATCATGTTGACATTCC; dG = -11.6
283 >s_b_s_d_s_a_s_e_s_c_s GTGGCTGTGACGGTGTCTGTGCTGGTGTGGGTG; >clamp_s_a_s_c_s:compl:rev CACCCACACAGACACTATCATGTTGACATTCC; dG = -11.6
284 >s_b_s_d_s_a_s_d_s_c_s GTGGCTGTGACGGTGTCTGTGACGGTGTGGGTG; >clamp_s_a_s_c_s:compl:rev CACCCACACAGACACTATCATGTTGACATTCC; dG = -11.6
285 >s_a_s_a_s_e_s_d_s_c_s GTGTCTGTGTCTGTGCTGGTGACGGTGTGGGTG; >clamp_s_a_s_c_s:compl:rev CACCCACACAGACACTATCATGTTGACATTCC; dG = -11.6
286 >s_b_s_e_s_a_s_e_s_c_s GTGGCTGTGCTGGTGTCTGTGCTGGTGTGGGTG; >clamp_s_a_s_c_s:compl:rev CACCCACACAGACACTATCATGTTGACATTCC; dG = -11.6
287 >s_b_s_e_s_a_s_d_s_c_s GTGGCTGTGCTGGTGTCTGTGACGGTGTGGGTG; >clamp_s_a_s_c_s:compl:rev CACCCACACAGACACTATCATGTTGACATTCC; dG = -11.6
288 >s_c_s_a_s_e_s_d_s_c_s GTGTGGGTGTCTGTGCTGGTGACGGTGTGGGTG; >clamp_s_a_s_c_s:compl:rev CACCCACACAGACACTATCATGTTGACATTCC; dG = -11.6
289 >s_c_s_c_s_a_s_e_s_c_s GTGTGGGTGTGGGTGTCTGTGCTGGTGTGGGTG; >clamp_s_a_s_c_s:compl:rev CACCCACACAGACACTATCATGTTGACATTCC; dG = -11.6
290 >s_c_s_c_s_a_s_d_s_c_s GTGTGGGTGTGGGTGTCTGTGACGGTGTGGGTG; >clamp_s_a_s_c_s:compl:rev CACCCACACAGACACTATCATGTTGACATTCC; dG = -11.6
291 >s_c_s_c_s_c_s_e_s_c_s GTGTGGGTGTGGGTGTGGGTGCTGGTGTGGGTG; >clamp_s_a_s_c_s:compl:rev CACCCACACAGACACTATCATGTTGACATTCC; dG = -11.6
292 >s_c_s_d_s_a_s_e_s_c_s GTGTGGGTGACGGTGTCTGTGCTGGTGTGGGTG; >clamp_s_a_s_c_s:compl:rev CACCCACACAGACACTATCATGTTGACATTCC; dG = -11.6

293 >s_c_s_d_s_a_s_d_s_c_s GTGTGGGTGACGGTGTCTGTGACGGTGTGGGTG; >clamp_s_a_s_c_s:compl:rev CACCCACACAGACACTATCATGTTGACATTCC; dG = -11.6
294 >s_c_s_e_s_a_s_e_s_c_s GTGTGGGTGCTGGTGTCTGTGCTGGTGTGGGTG; >clamp_s_a_s_c_s:compl:rev CACCCACACAGACACTATCATGTTGACATTCC; dG = -11.6
295 >s_c_s_e_s_a_s_d_s_c_s GTGTGGGTGCTGGTGTCTGTGACGGTGTGGGTG; >clamp_s_a_s_c_s:compl:rev CACCCACACAGACACTATCATGTTGACATTCC; dG = -11.6
296 >s_d_s_a_s_e_s_d_s_c_s GTGACGGTGTCTGTGCTGGTGACGGTGTGGGTG; >clamp_s_a_s_c_s:compl:rev CACCCACACAGACACTATCATGTTGACATTCC; dG = -11.6
297 >s_d_s_c_s_a_s_e_s_c_s GTGACGGTGTGGGTGTCTGTGCTGGTGTGGGTG; >clamp_s_a_s_c_s:compl:rev CACCCACACAGACACTATCATGTTGACATTCC; dG = -11.6
298 >s_d_s_c_s_a_s_d_s_c_s GTGACGGTGTGGGTGTCTGTGACGGTGTGGGTG; >clamp_s_a_s_c_s:compl:rev CACCCACACAGACACTATCATGTTGACATTCC; dG = -11.6
299 >s_d_s_c_s_c_s_e_s_c_s GTGACGGTGTGGGTGTGGGTGCTGGTGTGGGTG; >clamp_s_a_s_c_s:compl:rev CACCCACACAGACACTATCATGTTGACATTCC; dG = -11.6
300 >s_d_s_d_s_a_s_e_s_c_s GTGACGGTGACGGTGTCTGTGCTGGTGTGGGTG; >clamp_s_a_s_c_s:compl:rev CACCCACACAGACACTATCATGTTGACATTCC; dG = -11.6
301 >s_d_s_d_s_a_s_d_s_c_s GTGACGGTGACGGTGTCTGTGACGGTGTGGGTG; >clamp_s_a_s_c_s:compl:rev CACCCACACAGACACTATCATGTTGACATTCC; dG = -11.6
302 >s_d_s_e_s_a_s_e_s_c_s GTGACGGTGCTGGTGTCTGTGCTGGTGTGGGTG; >clamp_s_a_s_c_s:compl:rev CACCCACACAGACACTATCATGTTGACATTCC; dG = -11.6
303 >s_d_s_e_s_a_s_d_s_c_s GTGACGGTGCTGGTGTCTGTGACGGTGTGGGTG; >clamp_s_a_s_c_s:compl:rev CACCCACACAGACACTATCATGTTGACATTCC; dG = -11.6
304 >s_e_s_a_s_e_s_d_s_c_s GTGCTGGTGTCTGTGCTGGTGACGGTGTGGGTG; >clamp_s_a_s_c_s:compl:rev CACCCACACAGACACTATCATGTTGACATTCC; dG = -11.6
305 >s_a_s_d_s_a_s_e_s_c_s GTGTCTGTGACGGTGTCTGTGCTGGTGTGGGTG; >clamp_s_a_s_c_s:compl:rev CACCCACACAGACACTATCATGTTGACATTCC; dG = -11.6
306 >s_a_s_d_s_a_s_d_s_c_s GTGTCTGTGACGGTGTCTGTGACGGTGTGGGTG; >clamp_s_a_s_c_s:compl:rev CACCCACACAGACACTATCATGTTGACATTCC; dG = -11.6
307 >s_e_s_c_s_a_s_e_s_c_s GTGCTGGTGTGGGTGTCTGTGCTGGTGTGGGTG; >clamp_s_a_s_c_s:compl:rev CACCCACACAGACACTATCATGTTGACATTCC; dG = -11.6
308 >s_e_s_c_s_a_s_d_s_c_s GTGCTGGTGTGGGTGTCTGTGACGGTGTGGGTG; >clamp_s_a_s_c_s:compl:rev CACCCACACAGACACTATCATGTTGACATTCC; dG = -11.6
309 >s_e_s_c_s_c_s_e_s_c_s GTGCTGGTGTGGGTGTGGGTGCTGGTGTGGGTG; >clamp_s_a_s_c_s:compl:rev CACCCACACAGACACTATCATGTTGACATTCC; dG = -11.6
310 >s_e_s_d_s_a_s_e_s_c_s GTGCTGGTGACGGTGTCTGTGCTGGTGTGGGTG; >clamp_s_a_s_c_s:compl:rev CACCCACACAGACACTATCATGTTGACATTCC; dG = -11.6
311 >s_e_s_d_s_a_s_d_s_c_s GTGCTGGTGACGGTGTCTGTGACGGTGTGGGTG; >clamp_s_a_s_c_s:compl:rev CACCCACACAGACACTATCATGTTGACATTCC; dG = -11.6
312 >s_e_s_e_s_a_s_e_s_c_s GTGCTGGTGCTGGTGTCTGTGCTGGTGTGGGTG; >clamp_s_a_s_c_s:compl:rev CACCCACACAGACACTATCATGTTGACATTCC; dG = -11.6
313 >s_e_s_e_s_a_s_d_s_c_s GTGCTGGTGCTGGTGTCTGTGACGGTGTGGGTG; >clamp_s_a_s_c_s:compl:rev CACCCACACAGACACTATCATGTTGACATTCC; dG = -11.6
314 >s_a_s_d_s_d_s_c_s_c_s GTGTCTGTGACGGTGACGGTGTGGGTGTGGGTG; >clamp_s_a_s_c_s:compl:rev CACCCACACAGACACTATCATGTTGACATTCC; dG = -11.4
315 >s_a_s_d_s_e_s_c_s_c_s GTGTCTGTGACGGTGCTGGTGTGGGTGTGGGTG; >clamp_s_a_s_c_s:compl:rev CACCCACACAGACACTATCATGTTGACATTCC; dG = -11.4
316 >s_a_s_e_s_c_s_a_s_e_s GTGTCTGTGCTGGTGTGGGTGTCTGTGCTGGTG; >clamp_s_a_s_c_s:compl:rev CACCCACACAGACACTATCATGTTGACATTCC; dG = -11.4
317 >s_a_s_e_s_c_s_a_s_d_s GTGTCTGTGCTGGTGTGGGTGTCTGTGACGGTG; >clamp_s_a_s_c_s:compl:rev CACCCACACAGACACTATCATGTTGACATTCC; dG = -11.4
318 >s_a_s_e_s_c_s_a_s_b_s GTGTCTGTGCTGGTGTGGGTGTCTGTGGCTGTG; >clamp_s_a_s_c_s:compl:rev CACCCACACAGACACTATCATGTTGACATTCC; dG = -11.4
319 >s_a_s_e_s_c_s_a_s_a_s GTGTCTGTGCTGGTGTGGGTGTCTGTGTCTGTG; >clamp_s_a_s_c_s:compl:rev CACCCACACAGACACTATCATGTTGACATTCC; dG = -11.4
320 >s_a_s_e_s_d_s_c_s_c_s GTGTCTGTGCTGGTGACGGTGTGGGTGTGGGTG; >clamp_s_a_s_c_s:compl:rev CACCCACACAGACACTATCATGTTGACATTCC; dG = -11.4
321 >s_a_s_e_s_e_s_c_s_c_s GTGTCTGTGCTGGTGCTGGTGTGGGTGTGGGTG; >clamp_s_a_s_c_s:compl:rev CACCCACACAGACACTATCATGTTGACATTCC; dG = -11.4
322 >s_b_s_b_s_d_s_c_s_c_s GTGGCTGTGGCTGTGACGGTGTGGGTGTGGGTG; >clamp_s_a_s_c_s:compl:rev CACCCACACAGACACTATCATGTTGACATTCC; dG = -11.4
323 >s_b_s_b_s_e_s_c_s_c_s GTGGCTGTGGCTGTGCTGGTGTGGGTGTGGGTG; >clamp_s_a_s_c_s:compl:rev CACCCACACAGACACTATCATGTTGACATTCC; dG = -11.4
324 >s_b_s_d_s_d_s_c_s_c_s GTGGCTGTGACGGTGACGGTGTGGGTGTGGGTG; >clamp_s_a_s_c_s:compl:rev CACCCACACAGACACTATCATGTTGACATTCC; dG = -11.4
325 >s_b_s_d_s_e_s_c_s_c_s GTGGCTGTGACGGTGCTGGTGTGGGTGTGGGTG; >clamp_s_a_s_c_s:compl:rev CACCCACACAGACACTATCATGTTGACATTCC; dG = -11.4
326 >s_b_s_e_s_d_s_c_s_c_s GTGGCTGTGCTGGTGACGGTGTGGGTGTGGGTG; >clamp_s_a_s_c_s:compl:rev CACCCACACAGACACTATCATGTTGACATTCC; dG = -11.4
327 >s_b_s_e_s_e_s_c_s_c_s GTGGCTGTGCTGGTGCTGGTGTGGGTGTGGGTG; >clamp_s_a_s_c_s:compl:rev CACCCACACAGACACTATCATGTTGACATTCC; dG = -11.4
328 >s_c_s_b_s_d_s_c_s_c_s GTGTGGGTGGCTGTGACGGTGTGGGTGTGGGTG; >clamp_s_a_s_c_s:compl:rev CACCCACACAGACACTATCATGTTGACATTCC; dG = -11.4
329 >s_c_s_b_s_e_s_c_s_c_s GTGTGGGTGGCTGTGCTGGTGTGGGTGTGGGTG; >clamp_s_a_s_c_s:compl:rev CACCCACACAGACACTATCATGTTGACATTCC; dG = -11.4

330 >s_c_s_c_s_e_s_c_s_c_s GTGTGGGTGTGGGTGCTGGTGTGGGTGTGGGTG; >clamp_s_a_s_c_s:compl:rev
CACCCACACAGACACTATCATGTTGACATTCC; dG = -11.4
331 >s_c_s_d_s_d_s_c_s_c_s GTGTGGGTGACGGTGACGGTGTGGGTGTGGGTG; >clamp_s_a_s_c_s:compl:rev
CACCCACACAGACACTATCATGTTGACATTCC; dG = -11.4
332 >s_c_s_d_s_e_s_c_s_c_s GTGTGGGTGACGGTGCTGGTGTGGGTGTGGGTG; >clamp_s_a_s_c_s:compl:rev
CACCCACACAGACACTATCATGTTGACATTCC; dG = -11.4
333 >s_c_s_e_s_d_s_c_s_c_s GTGTGGGTGCTGGTGACGGTGTGGGTGTGGGTG; >clamp_s_a_s_c_s:compl:rev
CACCCACACAGACACTATCATGTTGACATTCC; dG = -11.4
334 >s_c_s_e_s_e_s_c_s_c_s GTGTGGGTGCTGGTGCTGGTGTGGGTGTGGGTG; >clamp_s_a_s_c_s:compl:rev
CACCCACACAGACACTATCATGTTGACATTCC; dG = -11.4
335 >s_d_s_b_s_d_s_c_s_c_s GTGACGGTGGCTGTGACGGTGTGGGTGTGGGTG; >clamp_s_a_s_c_s:compl:rev
CACCCACACAGACACTATCATGTTGACATTCC; dG = -11.4
336 >s_d_s_b_s_e_s_c_s_c_s GTGACGGTGGCTGTGCTGGTGTGGGTGTGGGTG; >clamp_s_a_s_c_s:compl:rev
CACCCACACAGACACTATCATGTTGACATTCC; dG = -11.4
337 >s_a_s_b_s_d_s_c_s_c_s GTGTCTGTGGCTGTGACGGTGTGGGTGTGGGTG; >clamp_s_a_s_c_s:compl:rev
CACCCACACAGACACTATCATGTTGACATTCC; dG = -11.4
338 >s_d_s_c_s_d_s_c_s_c_s GTGACGGTGTGGGTGACGGTGTGGGTGTGGGTG; >clamp_s_a_s_c_s:compl:rev
CACCCACACAGACACTATCATGTTGACATTCC; dG = -11.4
339 >s_d_s_c_s_e_s_c_s_c_s GTGACGGTGTGGGTGCTGGTGTGGGTGTGGGTG; >clamp_s_a_s_c_s:compl:rev
CACCCACACAGACACTATCATGTTGACATTCC; dG = -11.4
340 >s_d_s_d_s_d_s_c_s_c_s GTGACGGTGACGGTGACGGTGTGGGTGTGGGTG; >clamp_s_a_s_c_s:compl:rev
CACCCACACAGACACTATCATGTTGACATTCC; dG = -11.4
341 >s_d_s_d_s_e_s_c_s_c_s GTGACGGTGACGGTGCTGGTGTGGGTGTGGGTG; >clamp_s_a_s_c_s:compl:rev
CACCCACACAGACACTATCATGTTGACATTCC; dG = -11.4
342 >s_a_s_b_s_e_s_c_s_c_s GTGTCTGTGGCTGTGCTGGTGTGGGTGTGGGTG; >clamp_s_a_s_c_s:compl:rev
CACCCACACAGACACTATCATGTTGACATTCC; dG = -11.4
343 >s_d_s_e_s_d_s_c_s_c_s GTGACGGTGCTGGTGACGGTGTGGGTGTGGGTG; >clamp_s_a_s_c_s:compl:rev
CACCCACACAGACACTATCATGTTGACATTCC; dG = -11.4
344 >s_d_s_e_s_e_s_c_s_c_s GTGACGGTGCTGGTGCTGGTGTGGGTGTGGGTG; >clamp_s_a_s_c_s:compl:rev
CACCCACACAGACACTATCATGTTGACATTCC; dG = -11.4
345 >s_e_s_b_s_d_s_c_s_c_s GTGCTGGTGGCTGTGACGGTGTGGGTGTGGGTG; >clamp_s_a_s_c_s:compl:rev
CACCCACACAGACACTATCATGTTGACATTCC; dG = -11.4
346 >s_e_s_b_s_e_s_c_s_c_s GTGCTGGTGGCTGTGCTGGTGTGGGTGTGGGTG; >clamp_s_a_s_c_s:compl:rev
CACCCACACAGACACTATCATGTTGACATTCC; dG = -11.4
347 >s_e_s_c_s_d_s_c_s_c_s GTGCTGGTGTGGGTGACGGTGTGGGTGTGGGTG; >clamp_s_a_s_c_s:compl:rev
CACCCACACAGACACTATCATGTTGACATTCC; dG = -11.4
348 >s_e_s_c_s_e_s_c_s_c_s GTGCTGGTGTGGGTGCTGGTGTGGGTGTGGGTG; >clamp_s_a_s_c_s:compl:rev
CACCCACACAGACACTATCATGTTGACATTCC; dG = -11.4
349 >s_e_s_d_s_d_s_c_s_c_s GTGCTGGTGACGGTGACGGTGTGGGTGTGGGTG; >clamp_s_a_s_c_s:compl:rev
CACCCACACAGACACTATCATGTTGACATTCC; dG = -11.4
350 >s_e_s_d_s_e_s_c_s_c_s GTGCTGGTGACGGTGCTGGTGTGGGTGTGGGTG; >clamp_s_a_s_c_s:compl:rev
CACCCACACAGACACTATCATGTTGACATTCC; dG = -11.4
351 >s_a_s_d_s_c_s_a_s_e_s GTGTCTGTGACGGTGTGGGTGTCTGTGCTGGTG; >clamp_s_a_s_c_s:compl:rev
CACCCACACAGACACTATCATGTTGACATTCC; dG = -11.4
352 >s_a_s_d_s_c_s_a_s_d_s GTGTCTGTGACGGTGTGGGTGTCTGTGACGGTG; >clamp_s_a_s_c_s:compl:rev
CACCCACACAGACACTATCATGTTGACATTCC; dG = -11.4
353 >s_a_s_d_s_c_s_a_s_b_s GTGTCTGTGACGGTGTGGGTGTCTGTGGCTGTG; >clamp_s_a_s_c_s:compl:rev
CACCCACACAGACACTATCATGTTGACATTCC; dG = -11.4
354 >s_e_s_e_s_d_s_c_s_c_s GTGCTGGTGCTGGTGACGGTGTGGGTGTGGGTG; >clamp_s_a_s_c_s:compl:rev
CACCCACACAGACACTATCATGTTGACATTCC; dG = -11.4
355 >s_a_s_d_s_c_s_a_s_a_s GTGTCTGTGACGGTGTGGGTGTCTGTGTCTGTG; >clamp_s_a_s_c_s:compl:rev
CACCCACACAGACACTATCATGTTGACATTCC; dG = -11.4
356 >s_e_s_e_s_e_s_c_s_c_s GTGCTGGTGCTGGTGCTGGTGTGGGTGTGGGTG; >clamp_s_a_s_c_s:compl:rev
CACCCACACAGACACTATCATGTTGACATTCC; dG = -11.4
357 >s_a_s_e_s_c_s_e_s_c_s GTGTCTGTGCTGGTGTGGGTGCTGGTGTGGGTG; >clamp_s_a_s_c_s:compl:rev
CACCCACACAGACACTATCATGTTGACATTCC; dG = -11.3
358 >s_c_s_c_s_b_s_a_s_e_s GTGTGGGTGTGGGTGGCTGTGTCTGTGCTGGTG; >clamp_s_a_s_c_s:compl:rev
CACCCACACAGACACTATCATGTTGACATTCC; dG = -11.3
359 >s_c_s_c_s_b_s_a_s_d_s GTGTGGGTGTGGGTGGCTGTGTCTGTGACGGTG; >clamp_s_a_s_c_s:compl:rev
CACCCACACAGACACTATCATGTTGACATTCC; dG = -11.3
360 >s_c_s_c_s_b_s_a_s_b_s GTGTGGGTGTGGGTGGCTGTGTCTGTGGCTGTG; >clamp_s_a_s_c_s:compl:rev
CACCCACACAGACACTATCATGTTGACATTCC; dG = -11.3
361 >s_c_s_c_s_b_s_a_s_a_s GTGTGGGTGTGGGTGGCTGTGTCTGTGTCTGTG; >clamp_s_a_s_c_s:compl:rev
CACCCACACAGACACTATCATGTTGACATTCC; dG = -11.3
362 >s_c_s_c_s_b_s_e_s_c_s GTGTGGGTGTGGGTGGCTGTGCTGGTGTGGGTG; >clamp_s_a_s_c_s:compl:rev
CACCCACACAGACACTATCATGTTGACATTCC; dG = -11.3
363 >s_c_s_c_s_b_s_d_s_c_s GTGTGGGTGTGGGTGGCTGTGACGGTGTGGGTG; >clamp_s_a_s_c_s:compl:rev
CACCCACACAGACACTATCATGTTGACATTCC; dG = -11.3
364 >s_a_s_d_s_c_s_e_s_c_s GTGTCTGTGACGGTGTGGGTGCTGGTGTGGGTG; >clamp_s_a_s_c_s:compl:rev
CACCCACACAGACACTATCATGTTGACATTCC; dG = -11.3
365 >s_c_s_c_s_a_s_a_s_e_s GTGTGGGTGTGGGTGTCTGTGTCTGTGCTGGTG; >clamp_s_a_s_c_s:compl:rev
CACCCACACAGACACTATCATGTTGACATTCC; dG = -11.2
366 >s_c_s_c_s_a_s_a_s_d_s GTGTGGGTGTGGGTGTCTGTGTCTGTGACGGTG; >clamp_s_a_s_c_s:compl:rev
CACCCACACAGACACTATCATGTTGACATTCC; dG = -11.2

367 >s_c_s_c_s_a_s_a_s_b_s GTGTGGGTGTGGGTGTCTGTGTCTGTGGCTGTG; >clamp_s_a_s_c_s:compl:rev
CACCCACACAGACACTATCATGTTGACATTCC; dG = -11.2
368 >s_c_s_c_s_a_s_a_s_a_s GTGTGGGTGTGGGTGTCTGTGTCTGTGTCTGTG; >clamp_s_a_s_c_s:compl:rev
CACCCACACAGACACTATCATGTTGACATTCC; dG = -11.2
369 >s_a_s_d_s_d_s_a_s_e_s GTGTCTGTGACGGTGACGGTGTCTGTGCTGGTG; >clamp_s_a_s_c_s:compl:rev
CACCCACACAGACACTATCATGTTGACATTCC; dG = -11.2
370 >s_a_s_d_s_d_s_a_s_d_s GTGTCTGTGACGGTGACGGTGTCTGTGACGGTG; >clamp_s_a_s_c_s:compl:rev
CACCCACACAGACACTATCATGTTGACATTCC; dG = -11.2
371 >s_a_s_d_s_d_s_a_s_b_s GTGTCTGTGACGGTGACGGTGTCTGTGGCTGTG; >clamp_s_a_s_c_s:compl:rev
CACCCACACAGACACTATCATGTTGACATTCC; dG = -11.2
372 >s_a_s_d_s_d_s_a_s_a_s GTGTCTGTGACGGTGACGGTGTCTGTGTCTGTG; >clamp_s_a_s_c_s:compl:rev
CACCCACACAGACACTATCATGTTGACATTCC; dG = -11.2
373 >s_a_s_d_s_d_s_e_s_c_s GTGTCTGTGACGGTGACGGTGCTGGTGTGGGTG; >clamp_s_a_s_c_s:compl:rev
CACCCACACAGACACTATCATGTTGACATTCC; dG = -11.2
374 >s_a_s_d_s_d_s_d_s_c_s GTGTCTGTGACGGTGACGGTGACGGTGTGGGTG; >clamp_s_a_s_c_s:compl:rev
CACCCACACAGACACTATCATGTTGACATTCC; dG = -11.2
375 >s_a_s_e_s_d_s_a_s_e_s GTGTCTGTGCTGGTGACGGTGTCTGTGCTGGTG; >clamp_s_a_s_c_s:compl:rev
CACCCACACAGACACTATCATGTTGACATTCC; dG = -11.1
376 >s_a_s_e_s_d_s_a_s_d_s GTGTCTGTGCTGGTGACGGTGTCTGTGACGGTG; >clamp_s_a_s_c_s:compl:rev
CACCCACACAGACACTATCATGTTGACATTCC; dG = -11.1
377 >s_a_s_e_s_d_s_a_s_b_s GTGTCTGTGCTGGTGACGGTGTCTGTGGCTGTG; >clamp_s_a_s_c_s:compl:rev
CACCCACACAGACACTATCATGTTGACATTCC; dG = -11.1
378 >s_a_s_e_s_d_s_a_s_a_s GTGTCTGTGCTGGTGACGGTGTCTGTGTCTGTG; >clamp_s_a_s_c_s:compl:rev
CACCCACACAGACACTATCATGTTGACATTCC; dG = -11.1
379 >s_a_s_e_s_d_s_e_s_c_s GTGTCTGTGCTGGTGACGGTGCTGGTGTGGGTG; >clamp_s_a_s_c_s:compl:rev
CACCCACACAGACACTATCATGTTGACATTCC; dG = -11.1
380 >s_a_s_e_s_d_s_d_s_c_s GTGTCTGTGCTGGTGACGGTGACGGTGTGGGTG; >clamp_s_a_s_c_s:compl:rev
CACCCACACAGACACTATCATGTTGACATTCC; dG = -11.1
381 >s_a_s_a_s_d_s_a_s_e_s GTGTCTGTGTCTGTGACGGTGTCTGTGCTGGTG; >clamp_s_a_s_c_s:compl:rev
CACCCACACAGACACTATCATGTTGACATTCC; dG = -11.1
382 >s_b_s_a_s_d_s_a_s_e_s GTGGCTGTGTCTGTGACGGTGTCTGTGCTGGTG; >clamp_s_a_s_c_s:compl:rev
CACCCACACAGACACTATCATGTTGACATTCC; dG = -11.1
383 >s_b_s_a_s_d_s_a_s_d_s GTGGCTGTGTCTGTGACGGTGTCTGTGACGGTG; >clamp_s_a_s_c_s:compl:rev
CACCCACACAGACACTATCATGTTGACATTCC; dG = -11.1
384 >s_b_s_a_s_d_s_a_s_b_s GTGGCTGTGTCTGTGACGGTGTCTGTGGCTGTG; >clamp_s_a_s_c_s:compl:rev
CACCCACACAGACACTATCATGTTGACATTCC; dG = -11.1
385 >s_b_s_a_s_d_s_a_s_a_s GTGGCTGTGTCTGTGACGGTGTCTGTGTCTGTG; >clamp_s_a_s_c_s:compl:rev
CACCCACACAGACACTATCATGTTGACATTCC; dG = -11.1
386 >s_a_s_a_s_d_s_a_s_d_s GTGTCTGTGTCTGTGACGGTGTCTGTGACGGTG; >clamp_s_a_s_c_s:compl:rev
CACCCACACAGACACTATCATGTTGACATTCC; dG = -11.1
387 >s_a_s_a_s_d_s_a_s_b_s GTGTCTGTGTCTGTGACGGTGTCTGTGGCTGTG; >clamp_s_a_s_c_s:compl:rev
CACCCACACAGACACTATCATGTTGACATTCC; dG = -11.1
388 >s_a_s_a_s_d_s_a_s_a_s GTGTCTGTGTCTGTGACGGTGTCTGTGTCTGTG; >clamp_s_a_s_c_s:compl:rev
CACCCACACAGACACTATCATGTTGACATTCC; dG = -11.1
389 >s_c_s_a_s_d_s_a_s_e_s GTGTGGGTGTCTGTGACGGTGTCTGTGCTGGTG; >clamp_s_a_s_c_s:compl:rev
CACCCACACAGACACTATCATGTTGACATTCC; dG = -11.1
390 >s_c_s_a_s_d_s_a_s_d_s GTGTGGGTGTCTGTGACGGTGTCTGTGACGGTG; >clamp_s_a_s_c_s:compl:rev
CACCCACACAGACACTATCATGTTGACATTCC; dG = -11.1
391 >s_c_s_a_s_d_s_a_s_b_s GTGTGGGTGTCTGTGACGGTGTCTGTGGCTGTG; >clamp_s_a_s_c_s:compl:rev
CACCCACACAGACACTATCATGTTGACATTCC; dG = -11.1
392 >s_c_s_a_s_d_s_a_s_a_s GTGTGGGTGTCTGTGACGGTGTCTGTGTCTGTG; >clamp_s_a_s_c_s:compl:rev
CACCCACACAGACACTATCATGTTGACATTCC; dG = -11.1
393 >s_c_s_c_s_e_s_a_s_e_s GTGTGGGTGTGGGTGCTGGTGTCTGTGCTGGTG; >clamp_s_a_s_c_s:compl:rev
CACCCACACAGACACTATCATGTTGACATTCC; dG = -11.1
394 >s_c_s_c_s_e_s_a_s_d_s GTGTGGGTGTGGGTGCTGGTGTCTGTGACGGTG; >clamp_s_a_s_c_s:compl:rev
CACCCACACAGACACTATCATGTTGACATTCC; dG = -11.1
395 >s_c_s_c_s_e_s_a_s_b_s GTGTGGGTGTGGGTGCTGGTGTCTGTGGCTGTG; >clamp_s_a_s_c_s:compl:rev
CACCCACACAGACACTATCATGTTGACATTCC; dG = -11.1
396 >s_c_s_c_s_e_s_a_s_a_s GTGTGGGTGTGGGTGCTGGTGTCTGTGTCTGTG; >clamp_s_a_s_c_s:compl:rev
CACCCACACAGACACTATCATGTTGACATTCC; dG = -11.1
397 >s_c_s_c_s_e_s_e_s_c_s GTGTGGGTGTGGGTGCTGGTGCTGGTGTGGGTG; >clamp_s_a_s_c_s:compl:rev
CACCCACACAGACACTATCATGTTGACATTCC; dG = -11.1
398 >s_c_s_c_s_e_s_d_s_c_s GTGTGGGTGTGGGTGCTGGTGACGGTGTGGGTG; >clamp_s_a_s_c_s:compl:rev
CACCCACACAGACACTATCATGTTGACATTCC; dG = -11.1
399 >s_d_s_a_s_d_s_a_s_e_s GTGACGGTGTCTGTGACGGTGTCTGTGCTGGTG; >clamp_s_a_s_c_s:compl:rev
CACCCACACAGACACTATCATGTTGACATTCC; dG = -11.1
400 >s_d_s_a_s_d_s_a_s_d_s GTGACGGTGTCTGTGACGGTGTCTGTGACGGTG; >clamp_s_a_s_c_s:compl:rev
CACCCACACAGACACTATCATGTTGACATTCC; dG = -11.1
401 >s_d_s_a_s_d_s_a_s_b_s GTGACGGTGTCTGTGACGGTGTCTGTGGCTGTG; >clamp_s_a_s_c_s:compl:rev
CACCCACACAGACACTATCATGTTGACATTCC; dG = -11.1
402 >s_d_s_a_s_d_s_a_s_a_s GTGACGGTGTCTGTGACGGTGTCTGTGTCTGTG; >clamp_s_a_s_c_s:compl:rev
CACCCACACAGACACTATCATGTTGACATTCC; dG = -11.1
403 >s_e_s_a_s_d_s_a_s_e_s GTGCTGGTGTCTGTGACGGTGTCTGTGCTGGTG; >clamp_s_a_s_c_s:compl:rev
CACCCACACAGACACTATCATGTTGACATTCC; dG = -11.1

404 >s_e_s_a_s_d_s_a_s_d_s GTGCTGGTGTCTGTGACGGTGTCTGTGACGGTG; >clamp_s_a_s_c_s:compl:rev CACCCACACAGACACTATCATGTTGACATTCC; dG = -11.1
405 >s_e_s_a_s_d_s_a_s_b_s GTGCTGGTGTCTGTGACGGTGTCTGTGGCTGTG; >clamp_s_a_s_c_s:compl:rev CACCCACACAGACACTATCATGTTGACATTCC; dG = -11.1
406 >s_e_s_a_s_d_s_a_s_a_s GTGCTGGTGTCTGTGACGGTGTCTGTGTCTGTG; >clamp_s_a_s_c_s:compl:rev CACCCACACAGACACTATCATGTTGACATTCC; dG = -11.1
407 >s_b_s_a_s_d_s_e_s_c_s GTGGCTGTGTCTGTGACGGTGCTGGTGTGGGTG; >clamp_s_a_s_c_s:compl:rev CACCCACACAGACACTATCATGTTGACATTCC; dG = -11.0
408 >s_b_s_a_s_e_s_a_s_e_s GTGGCTGTGTCTGTGCTGGTGTCTGTGCTGGTG; >clamp_s_a_s_c_s:compl:rev CACCCACACAGACACTATCATGTTGACATTCC; dG = -11.0
409 >s_b_s_a_s_e_s_a_s_d_s GTGGCTGTGTCTGTGCTGGTGTCTGTGACGGTG; >clamp_s_a_s_c_s:compl:rev CACCCACACAGACACTATCATGTTGACATTCC; dG = -11.0
410 >s_b_s_a_s_e_s_a_s_b_s GTGGCTGTGTCTGTGCTGGTGTCTGTGGCTGTG; >clamp_s_a_s_c_s:compl:rev CACCCACACAGACACTATCATGTTGACATTCC; dG = -11.0
411 >s_b_s_a_s_e_s_a_s_a_s GTGGCTGTGTCTGTGCTGGTGTCTGTGTCTGTG; >clamp_s_a_s_c_s:compl:rev CACCCACACAGACACTATCATGTTGACATTCC; dG = -11.0
412 >s_a_s_a_s_d_s_e_s_c_s GTGTCTGTGTCTGTGACGGTGCTGGTGTGGGTG; >clamp_s_a_s_c_s:compl:rev CACCCACACAGACACTATCATGTTGACATTCC; dG = -11.0
413 >s_a_s_a_s_e_s_a_s_e_s GTGTCTGTGTCTGTGCTGGTGTCTGTGCTGGTG; >clamp_s_a_s_c_s:compl:rev CACCCACACAGACACTATCATGTTGACATTCC; dG = -11.0
414 >s_a_s_a_s_e_s_a_s_d_s GTGTCTGTGTCTGTGCTGGTGTCTGTGACGGTG; >clamp_s_a_s_c_s:compl:rev CACCCACACAGACACTATCATGTTGACATTCC; dG = -11.0
415 >s_a_s_a_s_e_s_a_s_b_s GTGTCTGTGTCTGTGCTGGTGTCTGTGGCTGTG; >clamp_s_a_s_c_s:compl:rev CACCCACACAGACACTATCATGTTGACATTCC; dG = -11.0
416 >s_a_s_a_s_e_s_a_s_a_s GTGTCTGTGTCTGTGCTGGTGTCTGTGTCTGTG; >clamp_s_a_s_c_s:compl:rev CACCCACACAGACACTATCATGTTGACATTCC; dG = -11.0
417 >s_c_s_a_s_d_s_e_s_c_s GTGTGGGTGTCTGTGACGGTGCTGGTGTGGGTG; >clamp_s_a_s_c_s:compl:rev CACCCACACAGACACTATCATGTTGACATTCC; dG = -11.0
418 >s_c_s_a_s_e_s_a_s_e_s GTGTGGGTGTCTGTGCTGGTGTCTGTGCTGGTG; >clamp_s_a_s_c_s:compl:rev CACCCACACAGACACTATCATGTTGACATTCC; dG = -11.0
419 >s_c_s_a_s_e_s_a_s_d_s GTGTGGGTGTCTGTGCTGGTGTCTGTGACGGTG; >clamp_s_a_s_c_s:compl:rev CACCCACACAGACACTATCATGTTGACATTCC; dG = -11.0
420 >s_c_s_a_s_e_s_a_s_b_s GTGTGGGTGTCTGTGCTGGTGTCTGTGGCTGTG; >clamp_s_a_s_c_s:compl:rev CACCCACACAGACACTATCATGTTGACATTCC; dG = -11.0
421 >s_c_s_a_s_e_s_a_s_a_s GTGTGGGTGTCTGTGCTGGTGTCTGTGTCTGTG; >clamp_s_a_s_c_s:compl:rev CACCCACACAGACACTATCATGTTGACATTCC; dG = -11.0
422 >s_d_s_a_s_d_s_e_s_c_s GTGACGGTGTCTGTGACGGTGCTGGTGTGGGTG; >clamp_s_a_s_c_s:compl:rev CACCCACACAGACACTATCATGTTGACATTCC; dG = -11.0
423 >s_d_s_a_s_e_s_a_s_e_s GTGACGGTGTCTGTGCTGGTGTCTGTGCTGGTG; >clamp_s_a_s_c_s:compl:rev CACCCACACAGACACTATCATGTTGACATTCC; dG = -11.0
424 >s_d_s_a_s_e_s_a_s_d_s GTGACGGTGTCTGTGCTGGTGTCTGTGACGGTG; >clamp_s_a_s_c_s:compl:rev CACCCACACAGACACTATCATGTTGACATTCC; dG = -11.0
425 >s_d_s_a_s_e_s_a_s_b_s GTGACGGTGTCTGTGCTGGTGTCTGTGGCTGTG; >clamp_s_a_s_c_s:compl:rev CACCCACACAGACACTATCATGTTGACATTCC; dG = -11.0
426 >s_d_s_a_s_e_s_a_s_a_s GTGACGGTGTCTGTGCTGGTGTCTGTGTCTGTG; >clamp_s_a_s_c_s:compl:rev CACCCACACAGACACTATCATGTTGACATTCC; dG = -11.0
427 >s_e_s_a_s_d_s_e_s_c_s GTGCTGGTGTCTGTGACGGTGCTGGTGTGGGTG; >clamp_s_a_s_c_s:compl:rev CACCCACACAGACACTATCATGTTGACATTCC; dG = -11.0
428 >s_e_s_a_s_e_s_a_s_e_s GTGCTGGTGTCTGTGCTGGTGTCTGTGCTGGTG; >clamp_s_a_s_c_s:compl:rev CACCCACACAGACACTATCATGTTGACATTCC; dG = -11.0
429 >s_e_s_a_s_e_s_a_s_d_s GTGCTGGTGTCTGTGCTGGTGTCTGTGACGGTG; >clamp_s_a_s_c_s:compl:rev CACCCACACAGACACTATCATGTTGACATTCC; dG = -11.0
430 >s_e_s_a_s_e_s_a_s_b_s GTGCTGGTGTCTGTGCTGGTGTCTGTGGCTGTG; >clamp_s_a_s_c_s:compl:rev CACCCACACAGACACTATCATGTTGACATTCC; dG = -11.0
431 >s_e_s_a_s_e_s_a_s_a_s GTGCTGGTGTCTGTGCTGGTGTCTGTGTCTGTG; >clamp_s_a_s_c_s:compl:rev CACCCACACAGACACTATCATGTTGACATTCC; dG = -11.0
432 >s_a_s_e_s_a_s_a_s_d_s GTGTCTGTGCTGGTGTCTGTGTCTGTGACGGTG; >clamp_s_a_s_c_s:compl:rev CACCCACACAGACACTATCATGTTGACATTCC; dG = -10.9
433 >s_a_s_e_s_b_s_a_s_d_s GTGTCTGTGCTGGTGGCTGTGTCTGTGACGGTG; >clamp_s_a_s_c_s:compl:rev CACCCACACAGACACTATCATGTTGACATTCC; dG = -10.9
434 >s_b_s_a_s_a_s_a_s_d_s GTGGCTGTGTCTGTGTCTGTGTCTGTGACGGTG; >clamp_s_a_s_c_s:compl:rev CACCCACACAGACACTATCATGTTGACATTCC; dG = -10.9
435 >s_b_s_a_s_b_s_a_s_d_s GTGGCTGTGTCTGTGGCTGTGTCTGTGACGGTG; >clamp_s_a_s_c_s:compl:rev CACCCACACAGACACTATCATGTTGACATTCC; dG = -10.9
436 >s_b_s_a_s_e_s_e_s_c_s GTGGCTGTGTCTGTGCTGGTGCTGGTGTGGGTG; >clamp_s_a_s_c_s:compl:rev CACCCACACAGACACTATCATGTTGACATTCC; dG = -10.9
437 >s_b_s_b_s_a_s_a_s_d_s GTGGCTGTGGCTGTGTCTGTGTCTGTGACGGTG; >clamp_s_a_s_c_s:compl:rev CACCCACACAGACACTATCATGTTGACATTCC; dG = -10.9
438 >s_b_s_b_s_b_s_a_s_d_s GTGGCTGTGGCTGTGGCTGTGTCTGTGACGGTG; >clamp_s_a_s_c_s:compl:rev CACCCACACAGACACTATCATGTTGACATTCC; dG = -10.9
439 >s_a_s_a_s_a_s_a_s_d_s GTGTCTGTGTCTGTGTCTGTGTCTGTGACGGTG; >clamp_s_a_s_c_s:compl:rev CACCCACACAGACACTATCATGTTGACATTCC; dG = -10.9
440 >s_b_s_d_s_a_s_a_s_d_s GTGGCTGTGACGGTGTCTGTGTCTGTGACGGTG; >clamp_s_a_s_c_s:compl:rev CACCCACACAGACACTATCATGTTGACATTCC; dG = -10.9

441 >s_b_s_d_s_b_s_a_s_d_s GTGGCTGTGACGGTGGCTGTGTCTGTGACGGTG; >clamp_s_a_s_c_s:compl:rev CACCCACACAGACACTATCATGTTGACATTCC; dG = -10.9
442 >s_b_s_d_s_c_s_d_s_c_s GTGGCTGTGACGGTGTGGGTGACGGTGTGGGTG; >clamp_s_a_s_c_s:compl:rev CACCCACACAGACACTATCATGTTGACATTCC; dG = -10.9
443 >s_a_s_a_s_e_s_e_s_c_s GTGTCTGTGTCTGTGCTGGTGCTGGTGTGGGTG; >clamp_s_a_s_c_s:compl:rev CACCCACACAGACACTATCATGTTGACATTCC; dG = -10.9
444 >s_b_s_e_s_a_s_a_s_d_s GTGGCTGTGCTGGTGTCTGTGTCTGTGACGGTG; >clamp_s_a_s_c_s:compl:rev CACCCACACAGACACTATCATGTTGACATTCC; dG = -10.9
445 >s_b_s_e_s_b_s_a_s_d_s GTGGCTGTGCTGGTGGCTGTGTCTGTGACGGTG; >clamp_s_a_s_c_s:compl:rev CACCCACACAGACACTATCATGTTGACATTCC; dG = -10.9
446 >s_b_s_e_s_c_s_d_s_c_s GTGGCTGTGCTGGTGTGGGTGACGGTGTGGGTG; >clamp_s_a_s_c_s:compl:rev CACCCACACAGACACTATCATGTTGACATTCC; dG = -10.9
447 >s_a_s_b_s_a_s_a_s_d_s GTGTCTGTGGCTGTGTCTGTGTCTGTGACGGTG; >clamp_s_a_s_c_s:compl:rev CACCCACACAGACACTATCATGTTGACATTCC; dG = -10.9
448 >s_c_s_a_s_a_s_a_s_d_s GTGTGGGTGTCTGTGTCTGTGTCTGTGACGGTG; >clamp_s_a_s_c_s:compl:rev CACCCACACAGACACTATCATGTTGACATTCC; dG = -10.9
449 >s_c_s_a_s_b_s_a_s_d_s GTGTGGGTGTCTGTGGCTGTGTCTGTGACGGTG; >clamp_s_a_s_c_s:compl:rev CACCCACACAGACACTATCATGTTGACATTCC; dG = -10.9
450 >s_c_s_a_s_e_s_e_s_c_s GTGTGGGTGTCTGTGCTGGTGCTGGTGTGGGTG; >clamp_s_a_s_c_s:compl:rev CACCCACACAGACACTATCATGTTGACATTCC; dG = -10.9
451 >s_c_s_b_s_a_s_a_s_d_s GTGTGGGTGGCTGTGTCTGTGTCTGTGACGGTG; >clamp_s_a_s_c_s:compl:rev CACCCACACAGACACTATCATGTTGACATTCC; dG = -10.9
452 >s_c_s_b_s_b_s_a_s_d_s GTGTGGGTGGCTGTGGCTGTGTCTGTGACGGTG; >clamp_s_a_s_c_s:compl:rev CACCCACACAGACACTATCATGTTGACATTCC; dG = -10.9
453 >s_a_s_b_s_b_s_a_s_d_s GTGTCTGTGGCTGTGGCTGTGTCTGTGACGGTG; >clamp_s_a_s_c_s:compl:rev CACCCACACAGACACTATCATGTTGACATTCC; dG = -10.9
454 >s_c_s_d_s_a_s_a_s_d_s GTGTGGGTGACGGTGTCTGTGTCTGTGACGGTG; >clamp_s_a_s_c_s:compl:rev CACCCACACAGACACTATCATGTTGACATTCC; dG = -10.9
455 >s_c_s_d_s_b_s_a_s_d_s GTGTGGGTGACGGTGGCTGTGTCTGTGACGGTG; >clamp_s_a_s_c_s:compl:rev CACCCACACAGACACTATCATGTTGACATTCC; dG = -10.9
456 >s_c_s_d_s_c_s_d_s_c_s GTGTGGGTGACGGTGTGGGTGACGGTGTGGGTG; >clamp_s_a_s_c_s:compl:rev CACCCACACAGACACTATCATGTTGACATTCC; dG = -10.9
457 >s_c_s_e_s_a_s_a_s_d_s GTGTGGGTGCTGGTGTCTGTGTCTGTGACGGTG; >clamp_s_a_s_c_s:compl:rev CACCCACACAGACACTATCATGTTGACATTCC; dG = -10.9
458 >s_c_s_e_s_b_s_a_s_d_s GTGTGGGTGCTGGTGGCTGTGTCTGTGACGGTG; >clamp_s_a_s_c_s:compl:rev CACCCACACAGACACTATCATGTTGACATTCC; dG = -10.9
459 >s_c_s_e_s_c_s_d_s_c_s GTGTGGGTGCTGGTGTGGGTGACGGTGTGGGTG; >clamp_s_a_s_c_s:compl:rev CACCCACACAGACACTATCATGTTGACATTCC; dG = -10.9
460 >s_d_s_a_s_a_s_a_s_d_s GTGACGGTGTCTGTGTCTGTGTCTGTGACGGTG; >clamp_s_a_s_c_s:compl:rev CACCCACACAGACACTATCATGTTGACATTCC; dG = -10.9
461 >s_d_s_a_s_b_s_a_s_d_s GTGACGGTGTCTGTGGCTGTGTCTGTGACGGTG; >clamp_s_a_s_c_s:compl:rev CACCCACACAGACACTATCATGTTGACATTCC; dG = -10.9
462 >s_d_s_a_s_e_s_e_s_c_s GTGACGGTGTCTGTGCTGGTGCTGGTGTGGGTG; >clamp_s_a_s_c_s:compl:rev CACCCACACAGACACTATCATGTTGACATTCC; dG = -10.9
463 >s_d_s_b_s_a_s_a_s_d_s GTGACGGTGGCTGTGTCTGTGTCTGTGACGGTG; >clamp_s_a_s_c_s:compl:rev CACCCACACAGACACTATCATGTTGACATTCC; dG = -10.9
464 >s_d_s_b_s_b_s_a_s_d_s GTGACGGTGGCTGTGGCTGTGTCTGTGACGGTG; >clamp_s_a_s_c_s:compl:rev CACCCACACAGACACTATCATGTTGACATTCC; dG = -10.9
465 >s_d_s_c_s_a_s_a_s_d_s GTGACGGTGTGGGTGTCTGTGTCTGTGACGGTG; >clamp_s_a_s_c_s:compl:rev CACCCACACAGACACTATCATGTTGACATTCC; dG = -10.9
466 >s_d_s_c_s_b_s_a_s_d_s GTGACGGTGTGGGTGGCTGTGTCTGTGACGGTG; >clamp_s_a_s_c_s:compl:rev CACCCACACAGACACTATCATGTTGACATTCC; dG = -10.9
467 >s_d_s_c_s_d_s_a_s_e_s GTGACGGTGTGGGTGACGGTGTCTGTGCTGGTG; >clamp_s_a_s_c_s:compl:rev CACCCACACAGACACTATCATGTTGACATTCC; dG = -10.9
468 >s_d_s_c_s_d_s_a_s_d_s GTGACGGTGTGGGTGACGGTGTCTGTGACGGTG; >clamp_s_a_s_c_s:compl:rev CACCCACACAGACACTATCATGTTGACATTCC; dG = -10.9
469 >s_d_s_c_s_d_s_a_s_b_s GTGACGGTGTGGGTGACGGTGTCTGTGGCTGTG; >clamp_s_a_s_c_s:compl:rev CACCCACACAGACACTATCATGTTGACATTCC; dG = -10.9
470 >s_d_s_c_s_d_s_a_s_a_s GTGACGGTGTGGGTGACGGTGTCTGTGTCTGTG; >clamp_s_a_s_c_s:compl:rev CACCCACACAGACACTATCATGTTGACATTCC; dG = -10.9
471 >s_d_s_c_s_d_s_e_s_c_s GTGACGGTGTGGGTGACGGTGCTGGTGTGGGTG; >clamp_s_a_s_c_s:compl:rev CACCCACACAGACACTATCATGTTGACATTCC; dG = -10.9
472 >s_d_s_c_s_d_s_d_s_c_s GTGACGGTGTGGGTGACGGTGACGGTGTGGGTG; >clamp_s_a_s_c_s:compl:rev CACCCACACAGACACTATCATGTTGACATTCC; dG = -10.9
473 >s_d_s_d_s_a_s_a_s_d_s GTGACGGTGACGGTGTCTGTGTCTGTGACGGTG; >clamp_s_a_s_c_s:compl:rev CACCCACACAGACACTATCATGTTGACATTCC; dG = -10.9
474 >s_d_s_d_s_b_s_a_s_d_s GTGACGGTGACGGTGGCTGTGTCTGTGACGGTG; >clamp_s_a_s_c_s:compl:rev CACCCACACAGACACTATCATGTTGACATTCC; dG = -10.9
475 >s_d_s_d_s_c_s_d_s_c_s GTGACGGTGACGGTGTGGGTGACGGTGTGGGTG; >clamp_s_a_s_c_s:compl:rev CACCCACACAGACACTATCATGTTGACATTCC; dG = -10.9
476 >s_d_s_e_s_a_s_a_s_d_s GTGACGGTGCTGGTGTCTGTGTCTGTGACGGTG; >clamp_s_a_s_c_s:compl:rev CACCCACACAGACACTATCATGTTGACATTCC; dG = -10.9
477 >s_d_s_e_s_b_s_a_s_d_s GTGACGGTGCTGGTGGCTGTGTCTGTGACGGTG; >clamp_s_a_s_c_s:compl:rev CACCCACACAGACACTATCATGTTGACATTCC; dG = -10.9

478 >s_d_s_e_s_c_s_d_s_c_s GTGACGGTGCTGGTGTGGGTGACGGTGTGGGTG; >clamp_s_a_s_c_s:compl:rev CACCCACACAGACACTATCATGTTGACATTCC; dG = -10.9
479 >s_e_s_a_s_a_s_a_s_d_s GTGCTGGTGTCTGTGTCTGTGTCTGTGACGGTG; >clamp_s_a_s_c_s:compl:rev CACCCACACAGACACTATCATGTTGACATTCC; dG = -10.9
480 >s_e_s_a_s_b_s_a_s_d_s GTGCTGGTGTCTGTGGCTGTGTCTGTGACGGTG; >clamp_s_a_s_c_s:compl:rev CACCCACACAGACACTATCATGTTGACATTCC; dG = -10.9
481 >s_a_s_d_s_a_s_a_s_d_s GTGTCTGTGACGGTGTCTGTGTCTGTGACGGTG; >clamp_s_a_s_c_s:compl:rev CACCCACACAGACACTATCATGTTGACATTCC; dG = -10.9
482 >s_e_s_a_s_e_s_e_s_c_s GTGCTGGTGTCTGTGCTGGTGCTGGTGTGGGTG; >clamp_s_a_s_c_s:compl:rev CACCCACACAGACACTATCATGTTGACATTCC; dG = -10.9
483 >s_e_s_b_s_a_s_a_s_d_s GTGCTGGTGGCTGTGTCTGTGTCTGTGACGGTG; >clamp_s_a_s_c_s:compl:rev CACCCACACAGACACTATCATGTTGACATTCC; dG = -10.9
484 >s_e_s_b_s_b_s_a_s_d_s GTGCTGGTGGCTGTGGCTGTGTCTGTGACGGTG; >clamp_s_a_s_c_s:compl:rev CACCCACACAGACACTATCATGTTGACATTCC; dG = -10.9
485 >s_e_s_c_s_a_s_a_s_d_s GTGCTGGTGTGGGTGTCTGTGTCTGTGACGGTG; >clamp_s_a_s_c_s:compl:rev CACCCACACAGACACTATCATGTTGACATTCC; dG = -10.9
486 >s_e_s_c_s_b_s_a_s_d_s GTGCTGGTGTGGGTGGCTGTGTCTGTGACGGTG; >clamp_s_a_s_c_s:compl:rev CACCCACACAGACACTATCATGTTGACATTCC; dG = -10.9
487 >s_a_s_d_s_b_s_a_s_d_s GTGTCTGTGACGGTGGCTGTGTCTGTGACGGTG; >clamp_s_a_s_c_s:compl:rev CACCCACACAGACACTATCATGTTGACATTCC; dG = -10.9
488 >s_e_s_c_s_d_s_a_s_e_s GTGCTGGTGTGGGTGACGGTGTCTGTGCTGGTG; >clamp_s_a_s_c_s:compl:rev CACCCACACAGACACTATCATGTTGACATTCC; dG = -10.9
489 >s_e_s_c_s_d_s_a_s_d_s GTGCTGGTGTGGGTGACGGTGTCTGTGACGGTG; >clamp_s_a_s_c_s:compl:rev CACCCACACAGACACTATCATGTTGACATTCC; dG = -10.9
490 >s_e_s_c_s_d_s_a_s_b_s GTGCTGGTGTGGGTGACGGTGTCTGTGGCTGTG; >clamp_s_a_s_c_s:compl:rev CACCCACACAGACACTATCATGTTGACATTCC; dG = -10.9
491 >s_e_s_c_s_d_s_a_s_a_s GTGCTGGTGTGGGTGACGGTGTCTGTGTCTGTG; >clamp_s_a_s_c_s:compl:rev CACCCACACAGACACTATCATGTTGACATTCC; dG = -10.9
492 >s_e_s_c_s_d_s_e_s_c_s GTGCTGGTGTGGGTGACGGTGCTGGTGTGGGTG; >clamp_s_a_s_c_s:compl:rev CACCCACACAGACACTATCATGTTGACATTCC; dG = -10.9
493 >s_e_s_c_s_d_s_d_s_c_s GTGCTGGTGTGGGTGACGGTGACGGTGTGGGTG; >clamp_s_a_s_c_s:compl:rev CACCCACACAGACACTATCATGTTGACATTCC; dG = -10.9
494 >s_e_s_d_s_a_s_a_s_d_s GTGCTGGTGACGGTGTCTGTGTCTGTGACGGTG; >clamp_s_a_s_c_s:compl:rev CACCCACACAGACACTATCATGTTGACATTCC; dG = -10.9
495 >s_e_s_d_s_b_s_a_s_d_s GTGCTGGTGACGGTGGCTGTGTCTGTGACGGTG; >clamp_s_a_s_c_s:compl:rev CACCCACACAGACACTATCATGTTGACATTCC; dG = -10.9
496 >s_e_s_d_s_c_s_d_s_c_s GTGCTGGTGACGGTGTGGGTGACGGTGTGGGTG; >clamp_s_a_s_c_s:compl:rev CACCCACACAGACACTATCATGTTGACATTCC; dG = -10.9
497 >s_e_s_e_s_a_s_a_s_d_s GTGCTGGTGCTGGTGTCTGTGTCTGTGACGGTG; >clamp_s_a_s_c_s:compl:rev CACCCACACAGACACTATCATGTTGACATTCC; dG = -10.9
498 >s_e_s_e_s_b_s_a_s_d_s GTGCTGGTGCTGGTGGCTGTGTCTGTGACGGTG; >clamp_s_a_s_c_s:compl:rev CACCCACACAGACACTATCATGTTGACATTCC; dG = -10.9
499 >s_e_s_e_s_c_s_d_s_c_s GTGCTGGTGCTGGTGTGGGTGACGGTGTGGGTG; >clamp_s_a_s_c_s:compl:rev CACCCACACAGACACTATCATGTTGACATTCC; dG = -10.9
500 >s_a_s_a_s_b_s_a_s_d_s GTGTCTGTGTCTGTGGCTGTGTCTGTGACGGTG; >clamp_s_a_s_c_s:compl:rev CACCCACACAGACACTATCATGTTGACATTCC; dG = -10.9
501 >s_a_s_a_s_a_s_a_s_e_s GTGTCTGTGTCTGTGTCTGTGTCTGTGCTGGTG; >clamp_s_a_s_c_s:compl:rev CACCCACACAGACACTATCATGTTGACATTCC; dG = -10.8
502 >s_a_s_d_s_e_s_a_s_d_s GTGTCTGTGACGGTGCTGGTGTCTGTGACGGTG; >clamp_s_a_s_c_s:compl:rev CACCCACACAGACACTATCATGTTGACATTCC; dG = -10.8
503 >s_a_s_e_s_a_s_a_s_e_s GTGTCTGTGCTGGTGTCTGTGTCTGTGCTGGTG; >clamp_s_a_s_c_s:compl:rev CACCCACACAGACACTATCATGTTGACATTCC; dG = -10.8
504 >s_a_s_e_s_b_s_a_s_e_s GTGTCTGTGCTGGTGGCTGTGTCTGTGCTGGTG; >clamp_s_a_s_c_s:compl:rev CACCCACACAGACACTATCATGTTGACATTCC; dG = -10.8
505 >s_a_s_e_s_e_s_a_s_d_s GTGTCTGTGCTGGTGCTGGTGTCTGTGACGGTG; >clamp_s_a_s_c_s:compl:rev CACCCACACAGACACTATCATGTTGACATTCC; dG = -10.8
506 >s_b_s_a_s_a_s_a_s_e_s GTGGCTGTGTCTGTGTCTGTGTCTGTGCTGGTG; >clamp_s_a_s_c_s:compl:rev CACCCACACAGACACTATCATGTTGACATTCC; dG = -10.8
507 >s_b_s_a_s_b_s_a_s_e_s GTGGCTGTGTCTGTGGCTGTGTCTGTGCTGGTG; >clamp_s_a_s_c_s:compl:rev CACCCACACAGACACTATCATGTTGACATTCC; dG = -10.8
508 >s_b_s_b_s_a_s_a_s_e_s GTGGCTGTGGCTGTGTCTGTGTCTGTGCTGGTG; >clamp_s_a_s_c_s:compl:rev CACCCACACAGACACTATCATGTTGACATTCC; dG = -10.8
509 >s_b_s_b_s_b_s_a_s_e_s GTGGCTGTGGCTGTGGCTGTGTCTGTGCTGGTG; >clamp_s_a_s_c_s:compl:rev CACCCACACAGACACTATCATGTTGACATTCC; dG = -10.8
510 >s_b_s_b_s_d_s_a_s_d_s GTGGCTGTGGCTGTGACGGTGTCTGTGACGGTG; >clamp_s_a_s_c_s:compl:rev CACCCACACAGACACTATCATGTTGACATTCC; dG = -10.8
511 >s_b_s_b_s_e_s_a_s_d_s GTGGCTGTGGCTGTGCTGGTGTCTGTGACGGTG; >clamp_s_a_s_c_s:compl:rev CACCCACACAGACACTATCATGTTGACATTCC; dG = -10.8
512 >s_b_s_d_s_a_s_a_s_e_s GTGGCTGTGACGGTGTCTGTGTCTGTGCTGGTG; >clamp_s_a_s_c_s:compl:rev CACCCACACAGACACTATCATGTTGACATTCC; dG = -10.8
513 >s_b_s_d_s_b_s_a_s_e_s GTGGCTGTGACGGTGGCTGTGTCTGTGCTGGTG; >clamp_s_a_s_c_s:compl:rev CACCCACACAGACACTATCATGTTGACATTCC; dG = -10.8
514 >s_b_s_d_s_c_s_a_s_d_s GTGGCTGTGACGGTGTGGGTGTCTGTGACGGTG; >clamp_s_a_s_c_s:compl:rev CACCCACACAGACACTATCATGTTGACATTCC; dG = -10.8

515 >s_b_s_d_s_d_s_a_s_d_s GTGGCTGTGACGGTGACGGTGTCTGTGACGGTG; >clamp_s_a_s_c_s:compl:rev
CACCCACACAGACACTATCATGTTGACATTCC; dG = -10.8
516 >s_b_s_d_s_e_s_a_s_d_s GTGGCTGTGACGGTGCTGGTGTCTGTGACGGTG; >clamp_s_a_s_c_s:compl:rev
CACCCACACAGACACTATCATGTTGACATTCC; dG = -10.8
517 >s_b_s_e_s_a_s_a_s_e_s GTGGCTGTGCTGGTGTCTGTGTCTGTGCTGGTG; >clamp_s_a_s_c_s:compl:rev
CACCCACACAGACACTATCATGTTGACATTCC; dG = -10.8
518 >s_b_s_e_s_b_s_a_s_e_s GTGGCTGTGCTGGTGGCTGTGTCTGTGCTGGTG; >clamp_s_a_s_c_s:compl:rev
CACCCACACAGACACTATCATGTTGACATTCC; dG = -10.8
519 >s_b_s_e_s_c_s_a_s_d_s GTGGCTGTGCTGGTGTGGGTGTCTGTGACGGTG; >clamp_s_a_s_c_s:compl:rev
CACCCACACAGACACTATCATGTTGACATTCC; dG = -10.8
520 >s_a_s_b_s_a_s_a_s_e_s GTGTCTGTGGCTGTGTCTGTGTCTGTGCTGGTG; >clamp_s_a_s_c_s:compl:rev
CACCCACACAGACACTATCATGTTGACATTCC; dG = -10.8
521 >s_b_s_e_s_d_s_a_s_d_s GTGGCTGTGCTGGTGACGGTGTCTGTGACGGTG; >clamp_s_a_s_c_s:compl:rev
CACCCACACAGACACTATCATGTTGACATTCC; dG = -10.8
522 >s_b_s_e_s_e_s_a_s_d_s GTGGCTGTGCTGGTGCTGGTGTCTGTGACGGTG; >clamp_s_a_s_c_s:compl:rev
CACCCACACAGACACTATCATGTTGACATTCC; dG = -10.8
523 >s_c_s_a_s_a_s_a_s_e_s GTGTGGGTGTCTGTGTCTGTGTCTGTGCTGGTG; >clamp_s_a_s_c_s:compl:rev
CACCCACACAGACACTATCATGTTGACATTCC; dG = -10.8
524 >s_c_s_a_s_b_s_a_s_e_s GTGTGGGTGTCTGTGGCTGTGTCTGTGCTGGTG; >clamp_s_a_s_c_s:compl:rev
CACCCACACAGACACTATCATGTTGACATTCC; dG = -10.8
525 >s_c_s_b_s_a_s_a_s_e_s GTGTGGGTGGCTGTGTCTGTGTCTGTGCTGGTG; >clamp_s_a_s_c_s:compl:rev
CACCCACACAGACACTATCATGTTGACATTCC; dG = -10.8
526 >s_c_s_b_s_b_s_a_s_e_s GTGTGGGTGGCTGTGGCTGTGTCTGTGCTGGTG; >clamp_s_a_s_c_s:compl:rev
CACCCACACAGACACTATCATGTTGACATTCC; dG = -10.8
527 >s_a_s_b_s_b_s_a_s_e_s GTGTCTGTGGCTGTGGCTGTGTCTGTGCTGGTG; >clamp_s_a_s_c_s:compl:rev
CACCCACACAGACACTATCATGTTGACATTCC; dG = -10.8
528 >s_c_s_b_s_d_s_a_s_d_s GTGTGGGTGGCTGTGACGGTGTCTGTGACGGTG; >clamp_s_a_s_c_s:compl:rev
CACCCACACAGACACTATCATGTTGACATTCC; dG = -10.8
529 >s_c_s_b_s_e_s_a_s_d_s GTGTGGGTGGCTGTGCTGGTGTCTGTGACGGTG; >clamp_s_a_s_c_s:compl:rev
CACCCACACAGACACTATCATGTTGACATTCC; dG = -10.8
530 >s_c_s_d_s_a_s_a_s_e_s GTGTGGGTGACGGTGTCTGTGTCTGTGCTGGTG; >clamp_s_a_s_c_s:compl:rev
CACCCACACAGACACTATCATGTTGACATTCC; dG = -10.8
531 >s_c_s_d_s_b_s_a_s_e_s GTGTGGGTGACGGTGGCTGTGTCTGTGCTGGTG; >clamp_s_a_s_c_s:compl:rev
CACCCACACAGACACTATCATGTTGACATTCC; dG = -10.8
532 >s_c_s_d_s_c_s_a_s_d_s GTGTGGGTGACGGTGTGGGTGTCTGTGACGGTG; >clamp_s_a_s_c_s:compl:rev
CACCCACACAGACACTATCATGTTGACATTCC; dG = -10.8
533 >s_c_s_d_s_d_s_a_s_d_s GTGTGGGTGACGGTGACGGTGTCTGTGACGGTG; >clamp_s_a_s_c_s:compl:rev
CACCCACACAGACACTATCATGTTGACATTCC; dG = -10.8
534 >s_c_s_d_s_e_s_a_s_d_s GTGTGGGTGACGGTGCTGGTGTCTGTGACGGTG; >clamp_s_a_s_c_s:compl:rev
CACCCACACAGACACTATCATGTTGACATTCC; dG = -10.8
535 >s_c_s_e_s_a_s_a_s_e_s GTGTGGGTGCTGGTGTCTGTGTCTGTGCTGGTG; >clamp_s_a_s_c_s:compl:rev
CACCCACACAGACACTATCATGTTGACATTCC; dG = -10.8
536 >s_c_s_e_s_b_s_a_s_e_s GTGTGGGTGCTGGTGGCTGTGTCTGTGCTGGTG; >clamp_s_a_s_c_s:compl:rev
CACCCACACAGACACTATCATGTTGACATTCC; dG = -10.8
537 >s_c_s_e_s_c_s_a_s_d_s GTGTGGGTGCTGGTGTGGGTGTCTGTGACGGTG; >clamp_s_a_s_c_s:compl:rev
CACCCACACAGACACTATCATGTTGACATTCC; dG = -10.8
538 >s_c_s_e_s_d_s_a_s_d_s GTGTGGGTGCTGGTGACGGTGTCTGTGACGGTG; >clamp_s_a_s_c_s:compl:rev
CACCCACACAGACACTATCATGTTGACATTCC; dG = -10.8
539 >s_c_s_e_s_e_s_a_s_d_s GTGTGGGTGCTGGTGCTGGTGTCTGTGACGGTG; >clamp_s_a_s_c_s:compl:rev
CACCCACACAGACACTATCATGTTGACATTCC; dG = -10.8
540 >s_d_s_a_s_a_s_a_s_e_s GTGACGGTGTCTGTGTCTGTGTCTGTGCTGGTG; >clamp_s_a_s_c_s:compl:rev
CACCCACACAGACACTATCATGTTGACATTCC; dG = -10.8
541 >s_d_s_a_s_b_s_a_s_e_s GTGACGGTGTCTGTGGCTGTGTCTGTGCTGGTG; >clamp_s_a_s_c_s:compl:rev
CACCCACACAGACACTATCATGTTGACATTCC; dG = -10.8
542 >s_d_s_b_s_a_s_a_s_e_s GTGACGGTGGCTGTGTCTGTGTCTGTGCTGGTG; >clamp_s_a_s_c_s:compl:rev
CACCCACACAGACACTATCATGTTGACATTCC; dG = -10.8
543 >s_a_s_b_s_d_s_a_s_d_s GTGTCTGTGGCTGTGACGGTGTCTGTGACGGTG; >clamp_s_a_s_c_s:compl:rev
CACCCACACAGACACTATCATGTTGACATTCC; dG = -10.8
544 >s_d_s_b_s_b_s_a_s_e_s GTGACGGTGGCTGTGGCTGTGTCTGTGCTGGTG; >clamp_s_a_s_c_s:compl:rev
CACCCACACAGACACTATCATGTTGACATTCC; dG = -10.8
545 >s_d_s_b_s_d_s_a_s_d_s GTGACGGTGGCTGTGACGGTGTCTGTGACGGTG; >clamp_s_a_s_c_s:compl:rev
CACCCACACAGACACTATCATGTTGACATTCC; dG = -10.8
546 >s_d_s_b_s_e_s_a_s_d_s GTGACGGTGGCTGTGCTGGTGTCTGTGACGGTG; >clamp_s_a_s_c_s:compl:rev
CACCCACACAGACACTATCATGTTGACATTCC; dG = -10.8
547 >s_d_s_c_s_a_s_a_s_e_s GTGACGGTGTGGGTGTCTGTGTCTGTGCTGGTG; >clamp_s_a_s_c_s:compl:rev
CACCCACACAGACACTATCATGTTGACATTCC; dG = -10.8
548 >s_d_s_c_s_b_s_a_s_e_s GTGACGGTGTGGGTGGCTGTGTCTGTGCTGGTG; >clamp_s_a_s_c_s:compl:rev
CACCCACACAGACACTATCATGTTGACATTCC; dG = -10.8
549 >s_d_s_c_s_e_s_a_s_d_s GTGACGGTGTGGGTGCTGGTGTCTGTGACGGTG; >clamp_s_a_s_c_s:compl:rev
CACCCACACAGACACTATCATGTTGACATTCC; dG = -10.8
550 >s_d_s_d_s_a_s_a_s_e_s GTGACGGTGACGGTGTCTGTGTCTGTGCTGGTG; >clamp_s_a_s_c_s:compl:rev
CACCCACACAGACACTATCATGTTGACATTCC; dG = -10.8
551 >s_d_s_d_s_b_s_a_s_e_s GTGACGGTGACGGTGGCTGTGTCTGTGCTGGTG; >clamp_s_a_s_c_s:compl:rev
CACCCACACAGACACTATCATGTTGACATTCC; dG = -10.8

552 >s_a_s_b_s_e_s_a_s_d_s GTGTCTGTGGCTGTGCTGGTGTCTGTGACGGTG; >clamp_s_a_s_c_s:compl:rev CACCCACACAGACACTATCATGTTGACATTCC; dG = -10.8
553 >s_d_s_d_s_c_s_a_s_d_s GTGACGGTGACGGTGTGGGTGTCTGTGACGGTG; >clamp_s_a_s_c_s:compl:rev CACCCACACAGACACTATCATGTTGACATTCC; dG = -10.8
554 >s_d_s_d_s_d_s_a_s_d_s GTGACGGTGACGGTGACGGTGTCTGTGACGGTG; >clamp_s_a_s_c_s:compl:rev CACCCACACAGACACTATCATGTTGACATTCC; dG = -10.8
555 >s_d_s_d_s_e_s_a_s_d_s GTGACGGTGACGGTGCTGGTGTCTGTGACGGTG; >clamp_s_a_s_c_s:compl:rev CACCCACACAGACACTATCATGTTGACATTCC; dG = -10.8
556 >s_d_s_e_s_a_s_a_s_e_s GTGACGGTGCTGGTGTCTGTGTCTGTGCTGGTG; >clamp_s_a_s_c_s:compl:rev CACCCACACAGACACTATCATGTTGACATTCC; dG = -10.8
557 >s_d_s_e_s_b_s_a_s_e_s GTGACGGTGCTGGTGGCTGTGTCTGTGCTGGTG; >clamp_s_a_s_c_s:compl:rev CACCCACACAGACACTATCATGTTGACATTCC; dG = -10.8
558 >s_d_s_e_s_c_s_a_s_d_s GTGACGGTGCTGGTGTGGGTGTCTGTGACGGTG; >clamp_s_a_s_c_s:compl:rev CACCCACACAGACACTATCATGTTGACATTCC; dG = -10.8
559 >s_d_s_e_s_d_s_a_s_d_s GTGACGGTGCTGGTGACGGTGTCTGTGACGGTG; >clamp_s_a_s_c_s:compl:rev CACCCACACAGACACTATCATGTTGACATTCC; dG = -10.8
560 >s_d_s_e_s_e_s_a_s_d_s GTGACGGTGCTGGTGCTGGTGTCTGTGACGGTG; >clamp_s_a_s_c_s:compl:rev CACCCACACAGACACTATCATGTTGACATTCC; dG = -10.8
561 >s_a_s_d_s_a_s_a_s_e_s GTGTCTGTGACGGTGTCTGTGTCTGTGCTGGTG; >clamp_s_a_s_c_s:compl:rev CACCCACACAGACACTATCATGTTGACATTCC; dG = -10.8
562 >s_e_s_a_s_a_s_a_s_e_s GTGCTGGTGTCTGTGTCTGTGTCTGTGCTGGTG; >clamp_s_a_s_c_s:compl:rev CACCCACACAGACACTATCATGTTGACATTCC; dG = -10.8
563 >s_e_s_a_s_b_s_a_s_e_s GTGCTGGTGTCTGTGGCTGTGTCTGTGCTGGTG; >clamp_s_a_s_c_s:compl:rev CACCCACACAGACACTATCATGTTGACATTCC; dG = -10.8
564 >s_e_s_b_s_a_s_a_s_e_s GTGCTGGTGGCTGTGTCTGTGTCTGTGCTGGTG; >clamp_s_a_s_c_s:compl:rev CACCCACACAGACACTATCATGTTGACATTCC; dG = -10.8
565 >s_e_s_b_s_b_s_a_s_e_s GTGCTGGTGGCTGTGGCTGTGTCTGTGCTGGTG; >clamp_s_a_s_c_s:compl:rev CACCCACACAGACACTATCATGTTGACATTCC; dG = -10.8
566 >s_e_s_b_s_d_s_a_s_d_s GTGCTGGTGGCTGTGACGGTGTCTGTGACGGTG; >clamp_s_a_s_c_s:compl:rev CACCCACACAGACACTATCATGTTGACATTCC; dG = -10.8
567 >s_e_s_b_s_e_s_a_s_d_s GTGCTGGTGGCTGTGCTGGTGTCTGTGACGGTG; >clamp_s_a_s_c_s:compl:rev CACCCACACAGACACTATCATGTTGACATTCC; dG = -10.8
568 >s_a_s_d_s_b_s_a_s_e_s GTGTCTGTGACGGTGGCTGTGTCTGTGCTGGTG; >clamp_s_a_s_c_s:compl:rev CACCCACACAGACACTATCATGTTGACATTCC; dG = -10.8
569 >s_e_s_c_s_a_s_a_s_e_s GTGCTGGTGTGGGTGTCTGTGTCTGTGCTGGTG; >clamp_s_a_s_c_s:compl:rev CACCCACACAGACACTATCATGTTGACATTCC; dG = -10.8
570 >s_e_s_c_s_b_s_a_s_e_s GTGCTGGTGTGGGTGGCTGTGTCTGTGCTGGTG; >clamp_s_a_s_c_s:compl:rev CACCCACACAGACACTATCATGTTGACATTCC; dG = -10.8
571 >s_a_s_a_s_b_s_a_s_e_s GTGTCTGTGTCTGTGGCTGTGTCTGTGCTGGTG; >clamp_s_a_s_c_s:compl:rev CACCCACACAGACACTATCATGTTGACATTCC; dG = -10.8
572 >s_e_s_c_s_e_s_a_s_d_s GTGCTGGTGTGGGTGCTGGTGTCTGTGACGGTG; >clamp_s_a_s_c_s:compl:rev CACCCACACAGACACTATCATGTTGACATTCC; dG = -10.8
573 >s_e_s_d_s_a_s_a_s_e_s GTGCTGGTGACGGTGTCTGTGTCTGTGCTGGTG; >clamp_s_a_s_c_s:compl:rev CACCCACACAGACACTATCATGTTGACATTCC; dG = -10.8
574 >s_e_s_d_s_b_s_a_s_e_s GTGCTGGTGACGGTGGCTGTGTCTGTGCTGGTG; >clamp_s_a_s_c_s:compl:rev CACCCACACAGACACTATCATGTTGACATTCC; dG = -10.8
575 >s_e_s_d_s_c_s_a_s_d_s GTGCTGGTGACGGTGTGGGTGTCTGTGACGGTG; >clamp_s_a_s_c_s:compl:rev CACCCACACAGACACTATCATGTTGACATTCC; dG = -10.8
576 >s_e_s_d_s_d_s_a_s_d_s GTGCTGGTGACGGTGACGGTGTCTGTGACGGTG; >clamp_s_a_s_c_s:compl:rev CACCCACACAGACACTATCATGTTGACATTCC; dG = -10.8
577 >s_e_s_d_s_e_s_a_s_d_s GTGCTGGTGACGGTGCTGGTGTCTGTGACGGTG; >clamp_s_a_s_c_s:compl:rev CACCCACACAGACACTATCATGTTGACATTCC; dG = -10.8
578 >s_e_s_e_s_a_s_a_s_e_s GTGCTGGTGCTGGTGTCTGTGTCTGTGCTGGTG; >clamp_s_a_s_c_s:compl:rev CACCCACACAGACACTATCATGTTGACATTCC; dG = -10.8
579 >s_e_s_e_s_b_s_a_s_e_s GTGCTGGTGCTGGTGGCTGTGTCTGTGCTGGTG; >clamp_s_a_s_c_s:compl:rev CACCCACACAGACACTATCATGTTGACATTCC; dG = -10.8
580 >s_e_s_e_s_c_s_a_s_d_s GTGCTGGTGCTGGTGTGGGTGTCTGTGACGGTG; >clamp_s_a_s_c_s:compl:rev CACCCACACAGACACTATCATGTTGACATTCC; dG = -10.8
581 >s_e_s_e_s_d_s_a_s_d_s GTGCTGGTGCTGGTGACGGTGTCTGTGACGGTG; >clamp_s_a_s_c_s:compl:rev CACCCACACAGACACTATCATGTTGACATTCC; dG = -10.8
582 >s_e_s_e_s_e_s_a_s_d_s GTGCTGGTGCTGGTGCTGGTGTCTGTGACGGTG; >clamp_s_a_s_c_s:compl:rev CACCCACACAGACACTATCATGTTGACATTCC; dG = -10.8
583 >s_a_s_d_s_e_s_a_s_e_s GTGTCTGTGACGGTGCTGGTGTCTGTGCTGGTG; >clamp_s_a_s_c_s:compl:rev CACCCACACAGACACTATCATGTTGACATTCC; dG = -10.7
584 >s_a_s_e_s_e_s_a_s_e_s GTGTCTGTGCTGGTGCTGGTGTCTGTGCTGGTG; >clamp_s_a_s_c_s:compl:rev CACCCACACAGACACTATCATGTTGACATTCC; dG = -10.7
585 >s_b_s_b_s_d_s_a_s_e_s GTGGCTGTGGCTGTGACGGTGTCTGTGCTGGTG; >clamp_s_a_s_c_s:compl:rev CACCCACACAGACACTATCATGTTGACATTCC; dG = -10.7
586 >s_b_s_b_s_e_s_a_s_e_s GTGGCTGTGGCTGTGCTGGTGTCTGTGCTGGTG; >clamp_s_a_s_c_s:compl:rev CACCCACACAGACACTATCATGTTGACATTCC; dG = -10.7
587 >s_b_s_d_s_c_s_a_s_e_s GTGGCTGTGACGGTGTGGGTGTCTGTGCTGGTG; >clamp_s_a_s_c_s:compl:rev CACCCACACAGACACTATCATGTTGACATTCC; dG = -10.7
588 >s_b_s_d_s_d_s_a_s_e_s GTGGCTGTGACGGTGACGGTGTCTGTGCTGGTG; >clamp_s_a_s_c_s:compl:rev CACCCACACAGACACTATCATGTTGACATTCC; dG = -10.7

589 >s_b_s_d_s_e_s_a_s_e_s GTGGCTGTGACGGTGCTGGTGTCTGTGCTGGTG; >clamp_s_a_s_c_s:compl:rev CACCCACACAGACACTATCATGTTGACATTCC; dG = -10.7
590 >s_b_s_e_s_c_s_a_s_e_s GTGGCTGTGCTGGTGTGGGTGTCTGTGCTGGTG; >clamp_s_a_s_c_s:compl:rev CACCCACACAGACACTATCATGTTGACATTCC; dG = -10.7
591 >s_b_s_e_s_d_s_a_s_e_s GTGGCTGTGCTGGTGACGGTGTCTGTGCTGGTG; >clamp_s_a_s_c_s:compl:rev CACCCACACAGACACTATCATGTTGACATTCC; dG = -10.7
592 >s_b_s_e_s_e_s_a_s_e_s GTGGCTGTGCTGGTGCTGGTGTCTGTGCTGGTG; >clamp_s_a_s_c_s:compl:rev CACCCACACAGACACTATCATGTTGACATTCC; dG = -10.7
593 >s_c_s_b_s_d_s_a_s_e_s GTGTGGGTGGCTGTGACGGTGTCTGTGCTGGTG; >clamp_s_a_s_c_s:compl:rev CACCCACACAGACACTATCATGTTGACATTCC; dG = -10.7
594 >s_c_s_b_s_e_s_a_s_e_s GTGTGGGTGGCTGTGCTGGTGTCTGTGCTGGTG; >clamp_s_a_s_c_s:compl:rev CACCCACACAGACACTATCATGTTGACATTCC; dG = -10.7
595 >s_c_s_d_s_c_s_a_s_e_s GTGTGGGTGACGGTGTGGGTGTCTGTGCTGGTG; >clamp_s_a_s_c_s:compl:rev CACCCACACAGACACTATCATGTTGACATTCC; dG = -10.7
596 >s_c_s_d_s_d_s_a_s_e_s GTGTGGGTGACGGTGACGGTGTCTGTGCTGGTG; >clamp_s_a_s_c_s:compl:rev CACCCACACAGACACTATCATGTTGACATTCC; dG = -10.7
597 >s_c_s_d_s_e_s_a_s_e_s GTGTGGGTGACGGTGCTGGTGTCTGTGCTGGTG; >clamp_s_a_s_c_s:compl:rev CACCCACACAGACACTATCATGTTGACATTCC; dG = -10.7
598 >s_c_s_e_s_c_s_a_s_e_s GTGTGGGTGCTGGTGTGGGTGTCTGTGCTGGTG; >clamp_s_a_s_c_s:compl:rev CACCCACACAGACACTATCATGTTGACATTCC; dG = -10.7
599 >s_c_s_e_s_d_s_a_s_e_s GTGTGGGTGCTGGTGACGGTGTCTGTGCTGGTG; >clamp_s_a_s_c_s:compl:rev CACCCACACAGACACTATCATGTTGACATTCC; dG = -10.7
600 >s_c_s_e_s_e_s_a_s_e_s GTGTGGGTGCTGGTGCTGGTGTCTGTGCTGGTG; >clamp_s_a_s_c_s:compl:rev CACCCACACAGACACTATCATGTTGACATTCC; dG = -10.7
601 >s_a_s_b_s_d_s_a_s_e_s GTGTCTGTGGCTGTGACGGTGTCTGTGCTGGTG; >clamp_s_a_s_c_s:compl:rev CACCCACACAGACACTATCATGTTGACATTCC; dG = -10.7
602 >s_d_s_b_s_d_s_a_s_e_s GTGACGGTGGCTGTGACGGTGTCTGTGCTGGTG; >clamp_s_a_s_c_s:compl:rev CACCCACACAGACACTATCATGTTGACATTCC; dG = -10.7
603 >s_d_s_b_s_e_s_a_s_e_s GTGACGGTGGCTGTGCTGGTGTCTGTGCTGGTG; >clamp_s_a_s_c_s:compl:rev CACCCACACAGACACTATCATGTTGACATTCC; dG = -10.7
604 >s_d_s_c_s_e_s_a_s_e_s GTGACGGTGTGGGTGCTGGTGTCTGTGCTGGTG; >clamp_s_a_s_c_s:compl:rev CACCCACACAGACACTATCATGTTGACATTCC; dG = -10.7
605 >s_a_s_b_s_e_s_a_s_e_s GTGTCTGTGGCTGTGCTGGTGTCTGTGCTGGTG; >clamp_s_a_s_c_s:compl:rev CACCCACACAGACACTATCATGTTGACATTCC; dG = -10.7
606 >s_d_s_d_s_c_s_a_s_e_s GTGACGGTGACGGTGTGGGTGTCTGTGCTGGTG; >clamp_s_a_s_c_s:compl:rev CACCCACACAGACACTATCATGTTGACATTCC; dG = -10.7
607 >s_d_s_d_s_d_s_a_s_e_s GTGACGGTGACGGTGACGGTGTCTGTGCTGGTG; >clamp_s_a_s_c_s:compl:rev CACCCACACAGACACTATCATGTTGACATTCC; dG = -10.7
608 >s_d_s_d_s_e_s_a_s_e_s GTGACGGTGACGGTGCTGGTGTCTGTGCTGGTG; >clamp_s_a_s_c_s:compl:rev CACCCACACAGACACTATCATGTTGACATTCC; dG = -10.7
609 >s_d_s_e_s_c_s_a_s_e_s GTGACGGTGCTGGTGTGGGTGTCTGTGCTGGTG; >clamp_s_a_s_c_s:compl:rev CACCCACACAGACACTATCATGTTGACATTCC; dG = -10.7
610 >s_d_s_e_s_d_s_a_s_e_s GTGACGGTGCTGGTGACGGTGTCTGTGCTGGTG; >clamp_s_a_s_c_s:compl:rev CACCCACACAGACACTATCATGTTGACATTCC; dG = -10.7
611 >s_d_s_e_s_e_s_a_s_e_s GTGACGGTGCTGGTGCTGGTGTCTGTGCTGGTG; >clamp_s_a_s_c_s:compl:rev CACCCACACAGACACTATCATGTTGACATTCC; dG = -10.7
612 >s_e_s_b_s_d_s_a_s_e_s GTGCTGGTGGCTGTGACGGTGTCTGTGCTGGTG; >clamp_s_a_s_c_s:compl:rev CACCCACACAGACACTATCATGTTGACATTCC; dG = -10.7
613 >s_e_s_b_s_e_s_a_s_e_s GTGCTGGTGGCTGTGCTGGTGTCTGTGCTGGTG; >clamp_s_a_s_c_s:compl:rev CACCCACACAGACACTATCATGTTGACATTCC; dG = -10.7
614 >s_a_s_d_s_b_s_a_s_b_s GTGTCTGTGACGGTGGCTGTGTCTGTGGCTGTG; >clamp_s_a_s_c_s:compl:rev CACCCACACAGACACTATCATGTTGACATTCC; dG = -10.7
615 >s_a_s_d_s_b_s_a_s_a_s GTGTCTGTGACGGTGGCTGTGTCTGTGTCTGTG; >clamp_s_a_s_c_s:compl:rev CACCCACACAGACACTATCATGTTGACATTCC; dG = -10.7
616 >s_e_s_c_s_e_s_a_s_e_s GTGCTGGTGTGGGTGCTGGTGTCTGTGCTGGTG; >clamp_s_a_s_c_s:compl:rev CACCCACACAGACACTATCATGTTGACATTCC; dG = -10.7
617 >s_a_s_d_s_b_s_e_s_c_s GTGTCTGTGACGGTGGCTGTGCTGGTGTGGGTG; >clamp_s_a_s_c_s:compl:rev CACCCACACAGACACTATCATGTTGACATTCC; dG = -10.7
618 >s_a_s_d_s_b_s_d_s_c_s GTGTCTGTGACGGTGGCTGTGACGGTGTGGGTG; >clamp_s_a_s_c_s:compl:rev CACCCACACAGACACTATCATGTTGACATTCC; dG = -10.7
619 >s_e_s_d_s_c_s_a_s_e_s GTGCTGGTGACGGTGTGGGTGTCTGTGCTGGTG; >clamp_s_a_s_c_s:compl:rev CACCCACACAGACACTATCATGTTGACATTCC; dG = -10.7
620 >s_e_s_d_s_d_s_a_s_e_s GTGCTGGTGACGGTGACGGTGTCTGTGCTGGTG; >clamp_s_a_s_c_s:compl:rev CACCCACACAGACACTATCATGTTGACATTCC; dG = -10.7
621 >s_e_s_d_s_e_s_a_s_e_s GTGCTGGTGACGGTGCTGGTGTCTGTGCTGGTG; >clamp_s_a_s_c_s:compl:rev CACCCACACAGACACTATCATGTTGACATTCC; dG = -10.7
622 >s_e_s_e_s_c_s_a_s_e_s GTGCTGGTGCTGGTGTGGGTGTCTGTGCTGGTG; >clamp_s_a_s_c_s:compl:rev CACCCACACAGACACTATCATGTTGACATTCC; dG = -10.7
623 >s_e_s_e_s_d_s_a_s_e_s GTGCTGGTGCTGGTGACGGTGTCTGTGCTGGTG; >clamp_s_a_s_c_s:compl:rev CACCCACACAGACACTATCATGTTGACATTCC; dG = -10.7
624 >s_e_s_e_s_e_s_a_s_e_s GTGCTGGTGCTGGTGCTGGTGTCTGTGCTGGTG; >clamp_s_a_s_c_s:compl:rev CACCCACACAGACACTATCATGTTGACATTCC; dG = -10.7
625 >s_a_s_e_s_b_s_a_s_b_s GTGTCTGTGCTGGTGGCTGTGTCTGTGGCTGTG; >clamp_s_a_s_c_s:compl:rev CACCCACACAGACACTATCATGTTGACATTCC; dG = -10.6

626 >s_a_s_e_s_b_s_a_s_a_s GTGTCTGTGCTGGTGGCTGTGTCTGTGTCTGTG; >clamp_s_a_s_c_s:compl:rev CACCCACACAGACACTATCATGTTGACATTCC; dG = -10.6
627 >s_a_s_e_s_b_s_e_s_c_s GTGTCTGTGCTGGTGGCTGTGCTGGTGTGGGTG; >clamp_s_a_s_c_s:compl:rev CACCCACACAGACACTATCATGTTGACATTCC; dG = -10.6
628 >s_a_s_e_s_b_s_d_s_c_s GTGTCTGTGCTGGTGGCTGTGACGGTGTGGGTG; >clamp_s_a_s_c_s:compl:rev CACCCACACAGACACTATCATGTTGACATTCC; dG = -10.6
629 >s_a_s_d_s_a_s_a_s_b_s GTGTCTGTGACGGTGTCTGTGTCTGTGGCTGTG; >clamp_s_a_s_c_s:compl:rev CACCCACACAGACACTATCATGTTGACATTCC; dG = -10.6
630 >s_a_s_d_s_a_s_a_s_a_s GTGTCTGTGACGGTGTCTGTGTCTGTGTCTGTG; >clamp_s_a_s_c_s:compl:rev CACCCACACAGACACTATCATGTTGACATTCC; dG = -10.6
631 >s_a_s_d_s_e_s_a_s_b_s GTGTCTGTGACGGTGCTGGTGTCTGTGGCTGTG; >clamp_s_a_s_c_s:compl:rev CACCCACACAGACACTATCATGTTGACATTCC; dG = -10.5
632 >s_a_s_d_s_e_s_a_s_a_s GTGTCTGTGACGGTGCTGGTGTCTGTGTCTGTG; >clamp_s_a_s_c_s:compl:rev CACCCACACAGACACTATCATGTTGACATTCC; dG = -10.5
633 >s_a_s_d_s_e_s_e_s_c_s GTGTCTGTGACGGTGCTGGTGCTGGTGTGGGTG; >clamp_s_a_s_c_s:compl:rev CACCCACACAGACACTATCATGTTGACATTCC; dG = -10.5
634 >s_a_s_d_s_e_s_d_s_c_s GTGTCTGTGACGGTGCTGGTGACGGTGTGGGTG; >clamp_s_a_s_c_s:compl:rev CACCCACACAGACACTATCATGTTGACATTCC; dG = -10.5
635 >s_a_s_e_s_a_s_a_s_b_s GTGTCTGTGCTGGTGTCTGTGTCTGTGGCTGTG; >clamp_s_a_s_c_s:compl:rev CACCCACACAGACACTATCATGTTGACATTCC; dG = -10.5
636 >s_a_s_e_s_a_s_a_s_a_s GTGTCTGTGCTGGTGTCTGTGTCTGTGTCTGTG; >clamp_s_a_s_c_s:compl:rev CACCCACACAGACACTATCATGTTGACATTCC; dG = -10.5
637 >s_a_s_a_s_b_s_e_s_c_s GTGTCTGTGTCTGTGGCTGTGCTGGTGTGGGTG; >clamp_s_a_s_c_s:compl:rev CACCCACACAGACACTATCATGTTGACATTCC; dG = -10.5
638 >s_a_s_a_s_b_s_d_s_c_s GTGTCTGTGTCTGTGGCTGTGACGGTGTGGGTG; >clamp_s_a_s_c_s:compl:rev CACCCACACAGACACTATCATGTTGACATTCC; dG = -10.5
639 >s_b_s_a_s_b_s_e_s_c_s GTGGCTGTGTCTGTGGCTGTGCTGGTGTGGGTG; >clamp_s_a_s_c_s:compl:rev CACCCACACAGACACTATCATGTTGACATTCC; dG = -10.5
640 >s_b_s_a_s_b_s_d_s_c_s GTGGCTGTGTCTGTGGCTGTGACGGTGTGGGTG; >clamp_s_a_s_c_s:compl:rev CACCCACACAGACACTATCATGTTGACATTCC; dG = -10.5
641 >s_c_s_d_s_a_s_a_s_b_s GTGTGGGTGACGGTGTCTGTGTCTGTGGCTGTG; >clamp_s_a_s_c_s:compl:rev CACCCACACAGACACTATCATGTTGACATTCC; dG = -10.5
642 >s_c_s_d_s_a_s_a_s_a_s GTGTGGGTGACGGTGTCTGTGTCTGTGTCTGTG; >clamp_s_a_s_c_s:compl:rev CACCCACACAGACACTATCATGTTGACATTCC; dG = -10.5
643 >s_c_s_d_s_b_s_a_s_b_s GTGTGGGTGACGGTGGCTGTGTCTGTGGCTGTG; >clamp_s_a_s_c_s:compl:rev CACCCACACAGACACTATCATGTTGACATTCC; dG = -10.5
644 >s_c_s_d_s_b_s_a_s_a_s GTGTGGGTGACGGTGGCTGTGTCTGTGTCTGTG; >clamp_s_a_s_c_s:compl:rev CACCCACACAGACACTATCATGTTGACATTCC; dG = -10.5
645 >s_c_s_d_s_b_s_e_s_c_s GTGTGGGTGACGGTGGCTGTGCTGGTGTGGGTG; >clamp_s_a_s_c_s:compl:rev CACCCACACAGACACTATCATGTTGACATTCC; dG = -10.5
646 >s_c_s_d_s_b_s_d_s_c_s GTGTGGGTGACGGTGGCTGTGACGGTGTGGGTG; >clamp_s_a_s_c_s:compl:rev CACCCACACAGACACTATCATGTTGACATTCC; dG = -10.5
647 >s_c_s_d_s_c_s_a_s_b_s GTGTGGGTGACGGTGTGGGTGTCTGTGGCTGTG; >clamp_s_a_s_c_s:compl:rev CACCCACACAGACACTATCATGTTGACATTCC; dG = -10.5
648 >s_c_s_d_s_c_s_a_s_a_s GTGTGGGTGACGGTGTGGGTGTCTGTGTCTGTG; >clamp_s_a_s_c_s:compl:rev CACCCACACAGACACTATCATGTTGACATTCC; dG = -10.5
649 >s_c_s_d_s_c_s_e_s_c_s GTGTGGGTGACGGTGTGGGTGCTGGTGTGGGTG; >clamp_s_a_s_c_s:compl:rev CACCCACACAGACACTATCATGTTGACATTCC; dG = -10.5
650 >s_c_s_d_s_d_s_a_s_b_s GTGTGGGTGACGGTGACGGTGTCTGTGGCTGTG; >clamp_s_a_s_c_s:compl:rev CACCCACACAGACACTATCATGTTGACATTCC; dG = -10.5
651 >s_c_s_d_s_d_s_a_s_a_s GTGTGGGTGACGGTGACGGTGTCTGTGTCTGTG; >clamp_s_a_s_c_s:compl:rev CACCCACACAGACACTATCATGTTGACATTCC; dG = -10.5
652 >s_c_s_d_s_d_s_e_s_c_s GTGTGGGTGACGGTGACGGTGCTGGTGTGGGTG; >clamp_s_a_s_c_s:compl:rev CACCCACACAGACACTATCATGTTGACATTCC; dG = -10.5
653 >s_c_s_d_s_d_s_d_s_c_s GTGTGGGTGACGGTGACGGTGACGGTGTGGGTG; >clamp_s_a_s_c_s:compl:rev CACCCACACAGACACTATCATGTTGACATTCC; dG = -10.5
654 >s_c_s_d_s_e_s_a_s_b_s GTGTGGGTGACGGTGCTGGTGTCTGTGGCTGTG; >clamp_s_a_s_c_s:compl:rev CACCCACACAGACACTATCATGTTGACATTCC; dG = -10.5
655 >s_c_s_d_s_e_s_a_s_a_s GTGTGGGTGACGGTGCTGGTGTCTGTGTCTGTG; >clamp_s_a_s_c_s:compl:rev CACCCACACAGACACTATCATGTTGACATTCC; dG = -10.5
656 >s_c_s_d_s_e_s_e_s_c_s GTGTGGGTGACGGTGCTGGTGCTGGTGTGGGTG; >clamp_s_a_s_c_s:compl:rev CACCCACACAGACACTATCATGTTGACATTCC; dG = -10.5
657 >s_c_s_d_s_e_s_d_s_c_s GTGTGGGTGACGGTGCTGGTGACGGTGTGGGTG; >clamp_s_a_s_c_s:compl:rev CACCCACACAGACACTATCATGTTGACATTCC; dG = -10.5
658 >s_d_s_c_s_b_s_a_s_b_s GTGACGGTGTGGGTGGCTGTGTCTGTGGCTGTG; >clamp_s_a_s_c_s:compl:rev CACCCACACAGACACTATCATGTTGACATTCC; dG = -10.5
659 >s_d_s_c_s_b_s_a_s_a_s GTGACGGTGTGGGTGGCTGTGTCTGTGTCTGTG; >clamp_s_a_s_c_s:compl:rev CACCCACACAGACACTATCATGTTGACATTCC; dG = -10.5
660 >s_d_s_c_s_b_s_e_s_c_s GTGACGGTGTGGGTGGCTGTGCTGGTGTGGGTG; >clamp_s_a_s_c_s:compl:rev CACCCACACAGACACTATCATGTTGACATTCC; dG = -10.5
661 >s_d_s_c_s_b_s_d_s_c_s GTGACGGTGTGGGTGGCTGTGACGGTGTGGGTG; >clamp_s_a_s_c_s:compl:rev CACCCACACAGACACTATCATGTTGACATTCC; dG = -10.5
662 >s_e_s_c_s_b_s_a_s_b_s GTGCTGGTGTGGGTGGCTGTGTCTGTGGCTGTG; >clamp_s_a_s_c_s:compl:rev CACCCACACAGACACTATCATGTTGACATTCC; dG = -10.5

663 >s_e_s_c_s_b_s_a_s_a_s GTGCTGGTGTGGGTGGCTGTGTCTGTGTCTGTG; >clamp_s_a_s_c_s:compl:rev
CACCCACACAGACACTATCATGTTGACATTCC; dG = -10.5

664 >s_e_s_c_s_b_s_e_s_c_s GTGCTGGTGTGGGTGGCTGTGCTGGTGTGGGTG; >clamp_s_a_s_c_s:compl:rev
CACCCACACAGACACTATCATGTTGACATTCC; dG = -10.5

665 >s_e_s_c_s_b_s_d_s_c_s GTGCTGGTGTGGGTGGCTGTGACGGTGTGGGTG; >clamp_s_a_s_c_s:compl:rev
CACCCACACAGACACTATCATGTTGACATTCC; dG = -10.5

666 >s_a_s_a_s_b_s_a_s_a_s GTGTCTGTGTCTGTGGCTGTGTCTGTGTCTGTG; >clamp_s_a_s_c_s:compl:rev
CACCCACACAGACACTATCATGTTGACATTCC; dG = -10.4

667 >s_a_s_e_s_e_s_a_s_b_s GTGTCTGTGCTGGTGCTGGTGTCTGTGGCTGTG; >clamp_s_a_s_c_s:compl:rev
CACCCACACAGACACTATCATGTTGACATTCC; dG = -10.4

668 >s_a_s_e_s_e_s_a_s_a_s GTGTCTGTGCTGGTGCTGGTGTCTGTGTCTGTG; >clamp_s_a_s_c_s:compl:rev
CACCCACACAGACACTATCATGTTGACATTCC; dG = -10.4

669 >s_a_s_e_s_e_s_e_s_c_s GTGTCTGTGCTGGTGCTGGTGCTGGTGTGGGTG; >clamp_s_a_s_c_s:compl:rev
CACCCACACAGACACTATCATGTTGACATTCC; dG = -10.4

670 >s_a_s_e_s_e_s_d_s_c_s GTGTCTGTGCTGGTGCTGGTGACGGTGTGGGTG; >clamp_s_a_s_c_s:compl:rev
CACCCACACAGACACTATCATGTTGACATTCC; dG = -10.4

671 >s_b_s_a_s_a_s_a_s_b_s GTGGCTGTGTCTGTGTCTGTGTCTGTGGCTGTG; >clamp_s_a_s_c_s:compl:rev
CACCCACACAGACACTATCATGTTGACATTCC; dG = -10.4

672 >s_b_s_a_s_a_s_a_s_a_s GTGGCTGTGTCTGTGTCTGTGTCTGTGTCTGTG; >clamp_s_a_s_c_s:compl:rev
CACCCACACAGACACTATCATGTTGACATTCC; dG = -10.4

673 >s_b_s_a_s_b_s_a_s_a_s GTGGCTGTGTCTGTGGCTGTGTCTGTGTCTGTG; >clamp_s_a_s_c_s:compl:rev
CACCCACACAGACACTATCATGTTGACATTCC; dG = -10.4

674 >s_b_s_b_s_a_s_a_s_b_s GTGGCTGTGGCTGTGTCTGTGTCTGTGGCTGTG; >clamp_s_a_s_c_s:compl:rev
CACCCACACAGACACTATCATGTTGACATTCC; dG = -10.4

675 >s_b_s_b_s_a_s_a_s_a_s GTGGCTGTGGCTGTGTCTGTGTCTGTGTCTGTG; >clamp_s_a_s_c_s:compl:rev
CACCCACACAGACACTATCATGTTGACATTCC; dG = -10.4

676 >s_b_s_b_s_b_s_a_s_a_s GTGGCTGTGGCTGTGGCTGTGTCTGTGTCTGTG; >clamp_s_a_s_c_s:compl:rev
CACCCACACAGACACTATCATGTTGACATTCC; dG = -10.4

677 >s_b_s_d_s_a_s_a_s_a_s GTGGCTGTGACGGTGTCTGTGTCTGTGTCTGTG; >clamp_s_a_s_c_s:compl:rev
CACCCACACAGACACTATCATGTTGACATTCC; dG = -10.4

678 >s_b_s_d_s_b_s_a_s_a_s GTGGCTGTGACGGTGGCTGTGTCTGTGTCTGTG; >clamp_s_a_s_c_s:compl:rev
CACCCACACAGACACTATCATGTTGACATTCC; dG = -10.4

679 >s_b_s_d_s_c_s_a_s_b_s GTGGCTGTGACGGTGTGGGTGTCTGTGGCTGTG; >clamp_s_a_s_c_s:compl:rev
CACCCACACAGACACTATCATGTTGACATTCC; dG = -10.4

680 >s_b_s_d_s_c_s_a_s_a_s GTGGCTGTGACGGTGTGGGTGTCTGTGTCTGTG; >clamp_s_a_s_c_s:compl:rev
CACCCACACAGACACTATCATGTTGACATTCC; dG = -10.4

681 >s_a_s_a_s_a_s_a_s_b_s GTGTCTGTGTCTGTGTCTGTGTCTGTGGCTGTG; >clamp_s_a_s_c_s:compl:rev
CACCCACACAGACACTATCATGTTGACATTCC; dG = -10.4

682 >s_b_s_e_s_a_s_a_s_a_s GTGGCTGTGCTGGTGTCTGTGTCTGTGTCTGTG; >clamp_s_a_s_c_s:compl:rev
CACCCACACAGACACTATCATGTTGACATTCC; dG = -10.4

683 >s_b_s_e_s_b_s_a_s_a_s GTGGCTGTGCTGGTGGCTGTGTCTGTGTCTGTG; >clamp_s_a_s_c_s:compl:rev
CACCCACACAGACACTATCATGTTGACATTCC; dG = -10.4

684 >s_b_s_e_s_c_s_a_s_b_s GTGGCTGTGCTGGTGTGGGTGTCTGTGGCTGTG; >clamp_s_a_s_c_s:compl:rev
CACCCACACAGACACTATCATGTTGACATTCC; dG = -10.4

685 >s_b_s_e_s_c_s_a_s_a_s GTGGCTGTGCTGGTGTGGGTGTCTGTGTCTGTG; >clamp_s_a_s_c_s:compl:rev
CACCCACACAGACACTATCATGTTGACATTCC; dG = -10.4

686 >s_c_s_a_s_a_s_a_s_b_s GTGTGGGTGTCTGTGTCTGTGTCTGTGGCTGTG; >clamp_s_a_s_c_s:compl:rev
CACCCACACAGACACTATCATGTTGACATTCC; dG = -10.4

687 >s_c_s_a_s_a_s_a_s_a_s GTGTGGGTGTCTGTGTCTGTGTCTGTGTCTGTG; >clamp_s_a_s_c_s:compl:rev
CACCCACACAGACACTATCATGTTGACATTCC; dG = -10.4

688 >s_a_s_b_s_a_s_a_s_b_s GTGTCTGTGGCTGTGTCTGTGTCTGTGGCTGTG; >clamp_s_a_s_c_s:compl:rev
CACCCACACAGACACTATCATGTTGACATTCC; dG = -10.4

689 >s_c_s_a_s_b_s_a_s_a_s GTGTGGGTGTCTGTGGCTGTGTCTGTGTCTGTG; >clamp_s_a_s_c_s:compl:rev
CACCCACACAGACACTATCATGTTGACATTCC; dG = -10.4

690 >s_c_s_a_s_b_s_e_s_c_s GTGTGGGTGTCTGTGGCTGTGCTGGTGTGGGTG; >clamp_s_a_s_c_s:compl:rev
CACCCACACAGACACTATCATGTTGACATTCC; dG = -10.4

691 >s_c_s_a_s_b_s_d_s_c_s GTGTGGGTGTCTGTGGCTGTGACGGTGTGGGTG; >clamp_s_a_s_c_s:compl:rev
CACCCACACAGACACTATCATGTTGACATTCC; dG = -10.4

692 >s_a_s_b_s_a_s_a_s_a_s GTGTCTGTGGCTGTGTCTGTGTCTGTGTCTGTG; >clamp_s_a_s_c_s:compl:rev
CACCCACACAGACACTATCATGTTGACATTCC; dG = -10.4

693 >s_c_s_b_s_a_s_a_s_b_s GTGTGGGTGGCTGTGTCTGTGTCTGTGGCTGTG; >clamp_s_a_s_c_s:compl:rev
CACCCACACAGACACTATCATGTTGACATTCC; dG = -10.4

694 >s_c_s_b_s_a_s_a_s_a_s GTGTGGGTGGCTGTGTCTGTGTCTGTGTCTGTG; >clamp_s_a_s_c_s:compl:rev
CACCCACACAGACACTATCATGTTGACATTCC; dG = -10.4

695 >s_c_s_b_s_b_s_a_s_a_s GTGTGGGTGGCTGTGGCTGTGTCTGTGTCTGTG; >clamp_s_a_s_c_s:compl:rev
CACCCACACAGACACTATCATGTTGACATTCC; dG = -10.4

696 >s_a_s_a_s_a_s_a_s_a_s GTGTCTGTGTCTGTGTCTGTGTCTGTGTCTGTG; >clamp_s_a_s_c_s:compl:rev
CACCCACACAGACACTATCATGTTGACATTCC; dG = -10.4

697 >s_a_s_b_s_b_s_a_s_a_s GTGTCTGTGGCTGTGGCTGTGTCTGTGTCTGTG; >clamp_s_a_s_c_s:compl:rev
CACCCACACAGACACTATCATGTTGACATTCC; dG = -10.4

698 >s_c_s_e_s_a_s_a_s_a_s GTGTGGGTGCTGGTGTCTGTGTCTGTGTCTGTG; >clamp_s_a_s_c_s:compl:rev
CACCCACACAGACACTATCATGTTGACATTCC; dG = -10.4

699 >s_c_s_e_s_b_s_a_s_a_s GTGTGGGTGCTGGTGGCTGTGTCTGTGTCTGTG; >clamp_s_a_s_c_s:compl:rev
CACCCACACAGACACTATCATGTTGACATTCC; dG = -10.4

700 >s_c_s_e_s_c_s_a_s_b_s GTGTGGGTGCTGGTGTGGGTGTCTGTGGCTGTG; >clamp_s_a_s_c_s:compl:rev
CACCCACACAGACACTATCATGTTGACATTCC; dG = -10.4
701 >s_c_s_e_s_c_s_a_s_a_s GTGTGGGTGCTGGTGTGGGTGTCTGTGTCTGTG; >clamp_s_a_s_c_s:compl:rev
CACCCACACAGACACTATCATGTTGACATTCC; dG = -10.4
702 >s_d_s_a_s_a_s_a_s_b_s GTGACGGTGTCTGTGTCTGTGTCTGTGGCTGTG; >clamp_s_a_s_c_s:compl:rev
CACCCACACAGACACTATCATGTTGACATTCC; dG = -10.4
703 >s_d_s_a_s_a_s_a_s_a_s GTGACGGTGTCTGTGTCTGTGTCTGTGTCTGTG; >clamp_s_a_s_c_s:compl:rev
CACCCACACAGACACTATCATGTTGACATTCC; dG = -10.4
704 >s_d_s_a_s_b_s_a_s_a_s GTGACGGTGTCTGTGGCTGTGTCTGTGTCTGTG; >clamp_s_a_s_c_s:compl:rev
CACCCACACAGACACTATCATGTTGACATTCC; dG = -10.4
705 >s_d_s_a_s_b_s_e_s_c_s GTGACGGTGTCTGTGGCTGTGCTGGTGTGGGTG; >clamp_s_a_s_c_s:compl:rev
CACCCACACAGACACTATCATGTTGACATTCC; dG = -10.4
706 >s_d_s_a_s_b_s_d_s_c_s GTGACGGTGTCTGTGGCTGTGACGGTGTGGGTG; >clamp_s_a_s_c_s:compl:rev
CACCCACACAGACACTATCATGTTGACATTCC; dG = -10.4
707 >s_d_s_b_s_a_s_a_s_b_s GTGACGGTGGCTGTGTCTGTGTCTGTGGCTGTG; >clamp_s_a_s_c_s:compl:rev
CACCCACACAGACACTATCATGTTGACATTCC; dG = -10.4
708 >s_d_s_b_s_a_s_a_s_a_s GTGACGGTGGCTGTGTCTGTGTCTGTGTCTGTG; >clamp_s_a_s_c_s:compl:rev
CACCCACACAGACACTATCATGTTGACATTCC; dG = -10.4
709 >s_d_s_b_s_b_s_a_s_a_s GTGACGGTGGCTGTGGCTGTGTCTGTGTCTGTG; >clamp_s_a_s_c_s:compl:rev
CACCCACACAGACACTATCATGTTGACATTCC; dG = -10.4
710 >s_d_s_c_s_a_s_a_s_b_s GTGACGGTGTGGGTGTCTGTGTCTGTGGCTGTG; >clamp_s_a_s_c_s:compl:rev
CACCCACACAGACACTATCATGTTGACATTCC; dG = -10.4
711 >s_d_s_c_s_a_s_a_s_a_s GTGACGGTGTGGGTGTCTGTGTCTGTGTCTGTG; >clamp_s_a_s_c_s:compl:rev
CACCCACACAGACACTATCATGTTGACATTCC; dG = -10.4
712 >s_d_s_d_s_a_s_a_s_a_s GTGACGGTGACGGTGTCTGTGTCTGTGTCTGTG; >clamp_s_a_s_c_s:compl:rev
CACCCACACAGACACTATCATGTTGACATTCC; dG = -10.4
713 >s_d_s_d_s_b_s_a_s_a_s GTGACGGTGACGGTGGCTGTGTCTGTGTCTGTG; >clamp_s_a_s_c_s:compl:rev
CACCCACACAGACACTATCATGTTGACATTCC; dG = -10.4
714 >s_d_s_d_s_c_s_a_s_b_s GTGACGGTGACGGTGTGGGTGTCTGTGGCTGTG; >clamp_s_a_s_c_s:compl:rev
CACCCACACAGACACTATCATGTTGACATTCC; dG = -10.4
715 >s_d_s_d_s_c_s_a_s_a_s GTGACGGTGACGGTGTGGGTGTCTGTGTCTGTG; >clamp_s_a_s_c_s:compl:rev
CACCCACACAGACACTATCATGTTGACATTCC; dG = -10.4
716 >s_d_s_e_s_a_s_a_s_a_s GTGACGGTGCTGGTGTCTGTGTCTGTGTCTGTG; >clamp_s_a_s_c_s:compl:rev
CACCCACACAGACACTATCATGTTGACATTCC; dG = -10.4
717 >s_d_s_e_s_b_s_a_s_a_s GTGACGGTGCTGGTGGCTGTGTCTGTGTCTGTG; >clamp_s_a_s_c_s:compl:rev
CACCCACACAGACACTATCATGTTGACATTCC; dG = -10.4
718 >s_d_s_e_s_c_s_a_s_b_s GTGACGGTGCTGGTGTGGGTGTCTGTGGCTGTG; >clamp_s_a_s_c_s:compl:rev
CACCCACACAGACACTATCATGTTGACATTCC; dG = -10.4
719 >s_d_s_e_s_c_s_a_s_a_s GTGACGGTGCTGGTGTGGGTGTCTGTGTCTGTG; >clamp_s_a_s_c_s:compl:rev
CACCCACACAGACACTATCATGTTGACATTCC; dG = -10.4
720 >s_e_s_a_s_a_s_a_s_b_s GTGCTGGTGTCTGTGTCTGTGTCTGTGGCTGTG; >clamp_s_a_s_c_s:compl:rev
CACCCACACAGACACTATCATGTTGACATTCC; dG = -10.4
721 >s_e_s_a_s_a_s_a_s_a_s GTGCTGGTGTCTGTGTCTGTGTCTGTGTCTGTG; >clamp_s_a_s_c_s:compl:rev
CACCCACACAGACACTATCATGTTGACATTCC; dG = -10.4
722 >s_e_s_a_s_b_s_a_s_a_s GTGCTGGTGTCTGTGGCTGTGTCTGTGTCTGTG; >clamp_s_a_s_c_s:compl:rev
CACCCACACAGACACTATCATGTTGACATTCC; dG = -10.4
723 >s_e_s_a_s_b_s_e_s_c_s GTGCTGGTGTCTGTGGCTGTGCTGGTGTGGGTG; >clamp_s_a_s_c_s:compl:rev
CACCCACACAGACACTATCATGTTGACATTCC; dG = -10.4
724 >s_e_s_a_s_b_s_d_s_c_s GTGCTGGTGTCTGTGGCTGTGACGGTGTGGGTG; >clamp_s_a_s_c_s:compl:rev
CACCCACACAGACACTATCATGTTGACATTCC; dG = -10.4
725 >s_e_s_b_s_a_s_a_s_b_s GTGCTGGTGGCTGTGTCTGTGTCTGTGGCTGTG; >clamp_s_a_s_c_s:compl:rev
CACCCACACAGACACTATCATGTTGACATTCC; dG = -10.4
726 >s_e_s_b_s_a_s_a_s_a_s GTGCTGGTGGCTGTGTCTGTGTCTGTGTCTGTG; >clamp_s_a_s_c_s:compl:rev
CACCCACACAGACACTATCATGTTGACATTCC; dG = -10.4
727 >s_e_s_b_s_b_s_a_s_a_s GTGCTGGTGGCTGTGGCTGTGTCTGTGTCTGTG; >clamp_s_a_s_c_s:compl:rev
CACCCACACAGACACTATCATGTTGACATTCC; dG = -10.4
728 >s_e_s_c_s_a_s_a_s_b_s GTGCTGGTGTGGGTGTCTGTGTCTGTGGCTGTG; >clamp_s_a_s_c_s:compl:rev
CACCCACACAGACACTATCATGTTGACATTCC; dG = -10.4
729 >s_e_s_c_s_a_s_a_s_a_s GTGCTGGTGTGGGTGTCTGTGTCTGTGTCTGTG; >clamp_s_a_s_c_s:compl:rev
CACCCACACAGACACTATCATGTTGACATTCC; dG = -10.4
730 >s_e_s_d_s_a_s_a_s_a_s GTGCTGGTGACGGTGTCTGTGTCTGTGTCTGTG; >clamp_s_a_s_c_s:compl:rev
CACCCACACAGACACTATCATGTTGACATTCC; dG = -10.4
731 >s_e_s_d_s_b_s_a_s_a_s GTGCTGGTGACGGTGGCTGTGTCTGTGTCTGTG; >clamp_s_a_s_c_s:compl:rev
CACCCACACAGACACTATCATGTTGACATTCC; dG = -10.4
732 >s_e_s_d_s_c_s_a_s_b_s GTGCTGGTGACGGTGTGGGTGTCTGTGGCTGTG; >clamp_s_a_s_c_s:compl:rev
CACCCACACAGACACTATCATGTTGACATTCC; dG = -10.4
733 >s_e_s_d_s_c_s_a_s_a_s GTGCTGGTGACGGTGTGGGTGTCTGTGTCTGTG; >clamp_s_a_s_c_s:compl:rev
CACCCACACAGACACTATCATGTTGACATTCC; dG = -10.4
734 >s_e_s_e_s_a_s_a_s_a_s GTGCTGGTGCTGGTGTCTGTGTCTGTGTCTGTG; >clamp_s_a_s_c_s:compl:rev
CACCCACACAGACACTATCATGTTGACATTCC; dG = -10.4
735 >s_e_s_e_s_b_s_a_s_a_s GTGCTGGTGCTGGTGGCTGTGTCTGTGTCTGTG; >clamp_s_a_s_c_s:compl:rev
CACCCACACAGACACTATCATGTTGACATTCC; dG = -10.4
736 >s_e_s_e_s_c_s_a_s_b_s GTGCTGGTGCTGGTGTGGGTGTCTGTGGCTGTG; >clamp_s_a_s_c_s:compl:rev
CACCCACACAGACACTATCATGTTGACATTCC; dG = -10.4

737 >s_e_s_e_s_c_s_a_s_a_s GTGCTGGTGCTGGTGTGGGTGTCTGTGTCTGTG; >clamp_s_a_s_c_s:compl:rev CACCCACACAGACACTATCATGTTGACATTCC; dG = -10.4
738 >s_b_s_b_s_d_s_a_s_a_s GTGGCTGTGGCTGTGACGGTGTCTGTGTCTGTG; >clamp_s_a_s_c_s:compl:rev CACCCACACAGACACTATCATGTTGACATTCC; dG = -10.3
739 >s_b_s_b_s_e_s_a_s_a_s GTGGCTGTGGCTGTGCTGGTGTCTGTGTCTGTG; >clamp_s_a_s_c_s:compl:rev CACCCACACAGACACTATCATGTTGACATTCC; dG = -10.3
740 >s_b_s_d_s_a_s_a_s_b_s GTGGCTGTGACGGTGTCTGTGTCTGTGGCTGTG; >clamp_s_a_s_c_s:compl:rev CACCCACACAGACACTATCATGTTGACATTCC; dG = -10.3
741 >s_b_s_d_s_d_s_a_s_a_s GTGGCTGTGACGGTGACGGTGTCTGTGTCTGTG; >clamp_s_a_s_c_s:compl:rev CACCCACACAGACACTATCATGTTGACATTCC; dG = -10.3
742 >s_b_s_d_s_e_s_a_s_a_s GTGGCTGTGACGGTGCTGGTGTCTGTGTCTGTG; >clamp_s_a_s_c_s:compl:rev CACCCACACAGACACTATCATGTTGACATTCC; dG = -10.3
743 >s_b_s_e_s_a_s_a_s_b_s GTGGCTGTGCTGGTGTCTGTGTCTGTGGCTGTG; >clamp_s_a_s_c_s:compl:rev CACCCACACAGACACTATCATGTTGACATTCC; dG = -10.3
744 >s_b_s_e_s_d_s_a_s_a_s GTGGCTGTGCTGGTGACGGTGTCTGTGTCTGTG; >clamp_s_a_s_c_s:compl:rev CACCCACACAGACACTATCATGTTGACATTCC; dG = -10.3
745 >s_b_s_e_s_e_s_a_s_a_s GTGGCTGTGCTGGTGCTGGTGTCTGTGTCTGTG; >clamp_s_a_s_c_s:compl:rev CACCCACACAGACACTATCATGTTGACATTCC; dG = -10.3
746 >s_c_s_b_s_d_s_a_s_a_s GTGTGGGTGGCTGTGACGGTGTCTGTGTCTGTG; >clamp_s_a_s_c_s:compl:rev CACCCACACAGACACTATCATGTTGACATTCC; dG = -10.3
747 >s_c_s_b_s_e_s_a_s_a_s GTGTGGGTGGCTGTGCTGGTGTCTGTGTCTGTG; >clamp_s_a_s_c_s:compl:rev CACCCACACAGACACTATCATGTTGACATTCC; dG = -10.3
748 >s_c_s_e_s_a_s_a_s_b_s GTGTGGGTGCTGGTGTCTGTGTCTGTGGCTGTG; >clamp_s_a_s_c_s:compl:rev CACCCACACAGACACTATCATGTTGACATTCC; dG = -10.3
749 >s_c_s_e_s_d_s_a_s_a_s GTGTGGGTGCTGGTGACGGTGTCTGTGTCTGTG; >clamp_s_a_s_c_s:compl:rev CACCCACACAGACACTATCATGTTGACATTCC; dG = -10.3
750 >s_c_s_e_s_e_s_a_s_a_s GTGTGGGTGCTGGTGCTGGTGTCTGTGTCTGTG; >clamp_s_a_s_c_s:compl:rev CACCCACACAGACACTATCATGTTGACATTCC; dG = -10.3
751 >s_a_s_b_s_d_s_a_s_a_s GTGTCTGTGGCTGTGACGGTGTCTGTGTCTGTG; >clamp_s_a_s_c_s:compl:rev CACCCACACAGACACTATCATGTTGACATTCC; dG = -10.3
752 >s_d_s_b_s_d_s_a_s_a_s GTGACGGTGGCTGTGACGGTGTCTGTGTCTGTG; >clamp_s_a_s_c_s:compl:rev CACCCACACAGACACTATCATGTTGACATTCC; dG = -10.3
753 >s_d_s_b_s_e_s_a_s_a_s GTGACGGTGGCTGTGCTGGTGTCTGTGTCTGTG; >clamp_s_a_s_c_s:compl:rev CACCCACACAGACACTATCATGTTGACATTCC; dG = -10.3
754 >s_d_s_c_s_e_s_a_s_a_s GTGACGGTGTGGGTGCTGGTGTCTGTGTCTGTG; >clamp_s_a_s_c_s:compl:rev CACCCACACAGACACTATCATGTTGACATTCC; dG = -10.3
755 >s_d_s_d_s_a_s_a_s_b_s GTGACGGTGACGGTGTCTGTGTCTGTGGCTGTG; >clamp_s_a_s_c_s:compl:rev CACCCACACAGACACTATCATGTTGACATTCC; dG = -10.3
756 >s_d_s_d_s_d_s_a_s_a_s GTGACGGTGACGGTGACGGTGTCTGTGTCTGTG; >clamp_s_a_s_c_s:compl:rev CACCCACACAGACACTATCATGTTGACATTCC; dG = -10.3
757 >s_a_s_b_s_e_s_a_s_a_s GTGTCTGTGGCTGTGCTGGTGTCTGTGTCTGTG; >clamp_s_a_s_c_s:compl:rev CACCCACACAGACACTATCATGTTGACATTCC; dG = -10.3
758 >s_d_s_d_s_e_s_a_s_a_s GTGACGGTGACGGTGCTGGTGTCTGTGTCTGTG; >clamp_s_a_s_c_s:compl:rev CACCCACACAGACACTATCATGTTGACATTCC; dG = -10.3
759 >s_d_s_e_s_a_s_a_s_b_s GTGACGGTGCTGGTGTCTGTGTCTGTGGCTGTG; >clamp_s_a_s_c_s:compl:rev CACCCACACAGACACTATCATGTTGACATTCC; dG = -10.3
760 >s_d_s_e_s_d_s_a_s_a_s GTGACGGTGCTGGTGACGGTGTCTGTGTCTGTG; >clamp_s_a_s_c_s:compl:rev CACCCACACAGACACTATCATGTTGACATTCC; dG = -10.3
761 >s_d_s_e_s_e_s_a_s_a_s GTGACGGTGCTGGTGCTGGTGTCTGTGTCTGTG; >clamp_s_a_s_c_s:compl:rev CACCCACACAGACACTATCATGTTGACATTCC; dG = -10.3
762 >s_e_s_b_s_d_s_a_s_a_s GTGCTGGTGGCTGTGACGGTGTCTGTGTCTGTG; >clamp_s_a_s_c_s:compl:rev CACCCACACAGACACTATCATGTTGACATTCC; dG = -10.3
763 >s_e_s_b_s_e_s_a_s_a_s GTGCTGGTGGCTGTGCTGGTGTCTGTGTCTGTG; >clamp_s_a_s_c_s:compl:rev CACCCACACAGACACTATCATGTTGACATTCC; dG = -10.3
764 >s_e_s_c_s_e_s_a_s_a_s GTGCTGGTGTGGGTGCTGGTGTCTGTGTCTGTG; >clamp_s_a_s_c_s:compl:rev CACCCACACAGACACTATCATGTTGACATTCC; dG = -10.3
765 >s_e_s_d_s_a_s_a_s_b_s GTGCTGGTGACGGTGTCTGTGTCTGTGGCTGTG; >clamp_s_a_s_c_s:compl:rev CACCCACACAGACACTATCATGTTGACATTCC; dG = -10.3
766 >s_e_s_d_s_d_s_a_s_a_s GTGCTGGTGACGGTGACGGTGTCTGTGTCTGTG; >clamp_s_a_s_c_s:compl:rev CACCCACACAGACACTATCATGTTGACATTCC; dG = -10.3
767 >s_e_s_d_s_e_s_a_s_a_s GTGCTGGTGACGGTGCTGGTGTCTGTGTCTGTG; >clamp_s_a_s_c_s:compl:rev CACCCACACAGACACTATCATGTTGACATTCC; dG = -10.3
768 >s_e_s_e_s_a_s_a_s_b_s GTGCTGGTGCTGGTGTCTGTGTCTGTGGCTGTG; >clamp_s_a_s_c_s:compl:rev CACCCACACAGACACTATCATGTTGACATTCC; dG = -10.3
769 >s_e_s_e_s_d_s_a_s_a_s GTGCTGGTGCTGGTGACGGTGTCTGTGTCTGTG; >clamp_s_a_s_c_s:compl:rev CACCCACACAGACACTATCATGTTGACATTCC; dG = -10.3
770 >s_e_s_e_s_e_s_a_s_a_s GTGCTGGTGCTGGTGCTGGTGTCTGTGTCTGTG; >clamp_s_a_s_c_s:compl:rev CACCCACACAGACACTATCATGTTGACATTCC; dG = -10.3
771 >s_b_s_d_s_c_s_e_s_c_s GTGGCTGTGACGGTGTGGGTGCTGGTGTGGGTG; >clamp_s_a_s_c_s:compl:rev CACCCACACAGACACTATCATGTTGACATTCC; dG = -10.2
772 >s_b_s_e_s_c_s_e_s_c_s GTGGCTGTGCTGGTGTGGGTGCTGGTGTGGGTG; >clamp_s_a_s_c_s:compl:rev CACCCACACAGACACTATCATGTTGACATTCC; dG = -10.2
773 >s_c_s_e_s_c_s_e_s_c_s GTGTGGGTGCTGGTGTGGGTGCTGGTGTGGGTG; >clamp_s_a_s_c_s:compl:rev CACCCACACAGACACTATCATGTTGACATTCC; dG = -10.2

774 >s_d_s_c_s_e_s_a_s_b_s GTGACGGTGTGGGTGCTGGTGTCTGTGGCTGTG; >clamp_s_a_s_c_s:compl:rev
CACCCACACAGACACTATCATGTTGACATTCC; dG = -10.2
775 >s_d_s_c_s_e_s_e_s_c_s GTGACGGTGTGGGTGCTGGTGCTGGTGTGGGTG; >clamp_s_a_s_c_s:compl:rev
CACCCACACAGACACTATCATGTTGACATTCC; dG = -10.2
776 >s_d_s_c_s_e_s_d_s_c_s GTGACGGTGTGGGTGCTGGTGACGGTGTGGGTG; >clamp_s_a_s_c_s:compl:rev
CACCCACACAGACACTATCATGTTGACATTCC; dG = -10.2
777 >s_d_s_d_s_c_s_e_s_c_s GTGACGGTGACGGTGTGGGTGCTGGTGTGGGTG; >clamp_s_a_s_c_s:compl:rev
CACCCACACAGACACTATCATGTTGACATTCC; dG = -10.2
778 >s_d_s_e_s_c_s_e_s_c_s GTGACGGTGCTGGTGTGGGTGCTGGTGTGGGTG; >clamp_s_a_s_c_s:compl:rev
CACCCACACAGACACTATCATGTTGACATTCC; dG = -10.2
779 >s_e_s_c_s_e_s_a_s_b_s GTGCTGGTGTGGGTGCTGGTGTCTGTGGCTGTG; >clamp_s_a_s_c_s:compl:rev
CACCCACACAGACACTATCATGTTGACATTCC; dG = -10.2
780 >s_e_s_c_s_e_s_e_s_c_s GTGCTGGTGTGGGTGCTGGTGCTGGTGTGGGTG; >clamp_s_a_s_c_s:compl:rev
CACCCACACAGACACTATCATGTTGACATTCC; dG = -10.2
781 >s_e_s_c_s_e_s_d_s_c_s GTGCTGGTGTGGGTGCTGGTGACGGTGTGGGTG; >clamp_s_a_s_c_s:compl:rev
CACCCACACAGACACTATCATGTTGACATTCC; dG = -10.2
782 >s_e_s_d_s_c_s_e_s_c_s GTGCTGGTGACGGTGTGGGTGCTGGTGTGGGTG; >clamp_s_a_s_c_s:compl:rev
CACCCACACAGACACTATCATGTTGACATTCC; dG = -10.2
783 >s_e_s_e_s_c_s_e_s_c_s GTGCTGGTGCTGGTGTGGGTGCTGGTGTGGGTG; >clamp_s_a_s_c_s:compl:rev
CACCCACACAGACACTATCATGTTGACATTCC; dG = -10.2
784 >s_b_s_b_s_b_s_e_s_c_s GTGGCTGTGGCTGTGGCTGTGCTGGTGTGGGTG; >clamp_s_a_s_c_s:compl:rev
CACCCACACAGACACTATCATGTTGACATTCC; dG = -10.1
785 >s_b_s_b_s_b_s_d_s_c_s GTGGCTGTGGCTGTGGCTGTGACGGTGTGGGTG; >clamp_s_a_s_c_s:compl:rev
CACCCACACAGACACTATCATGTTGACATTCC; dG = -10.1
786 >s_b_s_b_s_d_s_e_s_c_s GTGGCTGTGGCTGTGACGGTGCTGGTGTGGGTG; >clamp_s_a_s_c_s:compl:rev
CACCCACACAGACACTATCATGTTGACATTCC; dG = -10.1
787 >s_b_s_b_s_d_s_d_s_c_s GTGGCTGTGGCTGTGACGGTGACGGTGTGGGTG; >clamp_s_a_s_c_s:compl:rev
CACCCACACAGACACTATCATGTTGACATTCC; dG = -10.1
788 >s_b_s_b_s_e_s_e_s_c_s GTGGCTGTGGCTGTGCTGGTGCTGGTGTGGGTG; >clamp_s_a_s_c_s:compl:rev
CACCCACACAGACACTATCATGTTGACATTCC; dG = -10.1
789 >s_b_s_b_s_e_s_d_s_c_s GTGGCTGTGGCTGTGCTGGTGACGGTGTGGGTG; >clamp_s_a_s_c_s:compl:rev
CACCCACACAGACACTATCATGTTGACATTCC; dG = -10.1
790 >s_b_s_d_s_b_s_e_s_c_s GTGGCTGTGACGGTGGCTGTGCTGGTGTGGGTG; >clamp_s_a_s_c_s:compl:rev
CACCCACACAGACACTATCATGTTGACATTCC; dG = -10.1
791 >s_b_s_d_s_b_s_d_s_c_s GTGGCTGTGACGGTGGCTGTGACGGTGTGGGTG; >clamp_s_a_s_c_s:compl:rev
CACCCACACAGACACTATCATGTTGACATTCC; dG = -10.1
792 >s_b_s_d_s_d_s_e_s_c_s GTGGCTGTGACGGTGACGGTGCTGGTGTGGGTG; >clamp_s_a_s_c_s:compl:rev
CACCCACACAGACACTATCATGTTGACATTCC; dG = -10.1
793 >s_b_s_d_s_d_s_d_s_c_s GTGGCTGTGACGGTGACGGTGACGGTGTGGGTG; >clamp_s_a_s_c_s:compl:rev
CACCCACACAGACACTATCATGTTGACATTCC; dG = -10.1
794 >s_b_s_d_s_e_s_e_s_c_s GTGGCTGTGACGGTGCTGGTGCTGGTGTGGGTG; >clamp_s_a_s_c_s:compl:rev
CACCCACACAGACACTATCATGTTGACATTCC; dG = -10.1
795 >s_b_s_d_s_e_s_d_s_c_s GTGGCTGTGACGGTGCTGGTGACGGTGTGGGTG; >clamp_s_a_s_c_s:compl:rev
CACCCACACAGACACTATCATGTTGACATTCC; dG = -10.1
796 >s_b_s_e_s_b_s_e_s_c_s GTGGCTGTGCTGGTGGCTGTGCTGGTGTGGGTG; >clamp_s_a_s_c_s:compl:rev
CACCCACACAGACACTATCATGTTGACATTCC; dG = -10.1
797 >s_b_s_e_s_b_s_d_s_c_s GTGGCTGTGCTGGTGGCTGTGACGGTGTGGGTG; >clamp_s_a_s_c_s:compl:rev
CACCCACACAGACACTATCATGTTGACATTCC; dG = -10.1
798 >s_b_s_e_s_d_s_e_s_c_s GTGGCTGTGCTGGTGACGGTGCTGGTGTGGGTG; >clamp_s_a_s_c_s:compl:rev
CACCCACACAGACACTATCATGTTGACATTCC; dG = -10.1
799 >s_b_s_e_s_d_s_d_s_c_s GTGGCTGTGCTGGTGACGGTGACGGTGTGGGTG; >clamp_s_a_s_c_s:compl:rev
CACCCACACAGACACTATCATGTTGACATTCC; dG = -10.1
800 >s_b_s_e_s_e_s_e_s_c_s GTGGCTGTGCTGGTGCTGGTGCTGGTGTGGGTG; >clamp_s_a_s_c_s:compl:rev
CACCCACACAGACACTATCATGTTGACATTCC; dG = -10.1
801 >s_b_s_e_s_e_s_d_s_c_s GTGGCTGTGCTGGTGCTGGTGACGGTGTGGGTG; >clamp_s_a_s_c_s:compl:rev
CACCCACACAGACACTATCATGTTGACATTCC; dG = -10.1
802 >s_c_s_b_s_b_s_e_s_c_s GTGTGGGTGGCTGTGGCTGTGCTGGTGTGGGTG; >clamp_s_a_s_c_s:compl:rev
CACCCACACAGACACTATCATGTTGACATTCC; dG = -10.1
803 >s_c_s_b_s_b_s_d_s_c_s GTGTGGGTGGCTGTGGCTGTGACGGTGTGGGTG; >clamp_s_a_s_c_s:compl:rev
CACCCACACAGACACTATCATGTTGACATTCC; dG = -10.1
804 >s_c_s_b_s_d_s_e_s_c_s GTGTGGGTGGCTGTGACGGTGCTGGTGTGGGTG; >clamp_s_a_s_c_s:compl:rev
CACCCACACAGACACTATCATGTTGACATTCC; dG = -10.1
805 >s_c_s_b_s_d_s_d_s_c_s GTGTGGGTGGCTGTGACGGTGACGGTGTGGGTG; >clamp_s_a_s_c_s:compl:rev
CACCCACACAGACACTATCATGTTGACATTCC; dG = -10.1
806 >s_c_s_b_s_e_s_e_s_c_s GTGTGGGTGGCTGTGCTGGTGCTGGTGTGGGTG; >clamp_s_a_s_c_s:compl:rev
CACCCACACAGACACTATCATGTTGACATTCC; dG = -10.1
807 >s_c_s_b_s_e_s_d_s_c_s GTGTGGGTGGCTGTGCTGGTGACGGTGTGGGTG; >clamp_s_a_s_c_s:compl:rev
CACCCACACAGACACTATCATGTTGACATTCC; dG = -10.1
808 >s_a_s_b_s_b_s_e_s_c_s GTGTCTGTGGCTGTGGCTGTGCTGGTGTGGGTG; >clamp_s_a_s_c_s:compl:rev
CACCCACACAGACACTATCATGTTGACATTCC; dG = -10.1
809 >s_a_s_b_s_b_s_d_s_c_s GTGTCTGTGGCTGTGGCTGTGACGGTGTGGGTG; >clamp_s_a_s_c_s:compl:rev
CACCCACACAGACACTATCATGTTGACATTCC; dG = -10.1
810 >s_c_s_e_s_b_s_e_s_c_s GTGTGGGTGCTGGTGGCTGTGCTGGTGTGGGTG; >clamp_s_a_s_c_s:compl:rev
CACCCACACAGACACTATCATGTTGACATTCC; dG = -10.1

811 >s_c_s_e_s_b_s_d_s_c_s GTGTGGGTGCTGGTGGCTGTGACGGTGTGGGTG; >clamp_s_a_s_c_s:compl:rev CACCCACACAGACACTATCATGTTGACATTCC; dG = -10.1
812 >s_c_s_e_s_d_s_e_s_c_s GTGTGGGTGCTGGTGACGGTGCTGGTGTGGGTG; >clamp_s_a_s_c_s:compl:rev CACCCACACAGACACTATCATGTTGACATTCC; dG = -10.1
813 >s_c_s_e_s_d_s_d_s_c_s GTGTGGGTGCTGGTGACGGTGACGGTGTGGGTG; >clamp_s_a_s_c_s:compl:rev CACCCACACAGACACTATCATGTTGACATTCC; dG = -10.1
814 >s_c_s_e_s_e_s_e_s_c_s GTGTGGGTGCTGGTGCTGGTGCTGGTGTGGGTG; >clamp_s_a_s_c_s:compl:rev CACCCACACAGACACTATCATGTTGACATTCC; dG = -10.1
815 >s_c_s_e_s_e_s_d_s_c_s GTGTGGGTGCTGGTGCTGGTGACGGTGTGGGTG; >clamp_s_a_s_c_s:compl:rev CACCCACACAGACACTATCATGTTGACATTCC; dG = -10.1
816 >s_d_s_b_s_b_s_e_s_c_s GTGACGGTGGCTGTGGCTGTGCTGGTGTGGGTG; >clamp_s_a_s_c_s:compl:rev CACCCACACAGACACTATCATGTTGACATTCC; dG = -10.1
817 >s_d_s_b_s_b_s_d_s_c_s GTGACGGTGGCTGTGGCTGTGACGGTGTGGGTG; >clamp_s_a_s_c_s:compl:rev CACCCACACAGACACTATCATGTTGACATTCC; dG = -10.1
818 >s_d_s_b_s_d_s_e_s_c_s GTGACGGTGGCTGTGACGGTGCTGGTGTGGGTG; >clamp_s_a_s_c_s:compl:rev CACCCACACAGACACTATCATGTTGACATTCC; dG = -10.1
819 >s_d_s_b_s_d_s_d_s_c_s GTGACGGTGGCTGTGACGGTGACGGTGTGGGTG; >clamp_s_a_s_c_s:compl:rev CACCCACACAGACACTATCATGTTGACATTCC; dG = -10.1
820 >s_d_s_b_s_e_s_e_s_c_s GTGACGGTGGCTGTGCTGGTGCTGGTGTGGGTG; >clamp_s_a_s_c_s:compl:rev CACCCACACAGACACTATCATGTTGACATTCC; dG = -10.1
821 >s_a_s_b_s_d_s_e_s_c_s GTGTCTGTGGCTGTGACGGTGCTGGTGTGGGTG; >clamp_s_a_s_c_s:compl:rev CACCCACACAGACACTATCATGTTGACATTCC; dG = -10.1
822 >s_d_s_b_s_e_s_d_s_c_s GTGACGGTGGCTGTGCTGGTGACGGTGTGGGTG; >clamp_s_a_s_c_s:compl:rev CACCCACACAGACACTATCATGTTGACATTCC; dG = -10.1
823 >s_a_s_b_s_d_s_d_s_c_s GTGTCTGTGGCTGTGACGGTGACGGTGTGGGTG; >clamp_s_a_s_c_s:compl:rev CACCCACACAGACACTATCATGTTGACATTCC; dG = -10.1
824 >s_d_s_d_s_b_s_e_s_c_s GTGACGGTGACGGTGGCTGTGCTGGTGTGGGTG; >clamp_s_a_s_c_s:compl:rev CACCCACACAGACACTATCATGTTGACATTCC; dG = -10.1
825 >s_d_s_d_s_b_s_d_s_c_s GTGACGGTGACGGTGGCTGTGACGGTGTGGGTG; >clamp_s_a_s_c_s:compl:rev CACCCACACAGACACTATCATGTTGACATTCC; dG = -10.1
826 >s_d_s_d_s_d_s_e_s_c_s GTGACGGTGACGGTGACGGTGCTGGTGTGGGTG; >clamp_s_a_s_c_s:compl:rev CACCCACACAGACACTATCATGTTGACATTCC; dG = -10.1
827 >s_d_s_d_s_d_s_d_s_c_s GTGACGGTGACGGTGACGGTGACGGTGTGGGTG; >clamp_s_a_s_c_s:compl:rev CACCCACACAGACACTATCATGTTGACATTCC; dG = -10.1
828 >s_d_s_d_s_e_s_e_s_c_s GTGACGGTGACGGTGCTGGTGCTGGTGTGGGTG; >clamp_s_a_s_c_s:compl:rev CACCCACACAGACACTATCATGTTGACATTCC; dG = -10.1
829 >s_d_s_d_s_e_s_d_s_c_s GTGACGGTGACGGTGCTGGTGACGGTGTGGGTG; >clamp_s_a_s_c_s:compl:rev CACCCACACAGACACTATCATGTTGACATTCC; dG = -10.1
830 >s_a_s_b_s_e_s_e_s_c_s GTGTCTGTGGCTGTGCTGGTGCTGGTGTGGGTG; >clamp_s_a_s_c_s:compl:rev CACCCACACAGACACTATCATGTTGACATTCC; dG = -10.1
831 >s_a_s_b_s_e_s_d_s_c_s GTGTCTGTGGCTGTGCTGGTGACGGTGTGGGTG; >clamp_s_a_s_c_s:compl:rev CACCCACACAGACACTATCATGTTGACATTCC; dG = -10.1
832 >s_d_s_e_s_b_s_e_s_c_s GTGACGGTGCTGGTGGCTGTGCTGGTGTGGGTG; >clamp_s_a_s_c_s:compl:rev CACCCACACAGACACTATCATGTTGACATTCC; dG = -10.1
833 >s_d_s_e_s_b_s_d_s_c_s GTGACGGTGCTGGTGGCTGTGACGGTGTGGGTG; >clamp_s_a_s_c_s:compl:rev CACCCACACAGACACTATCATGTTGACATTCC; dG = -10.1
834 >s_d_s_e_s_d_s_e_s_c_s GTGACGGTGCTGGTGACGGTGCTGGTGTGGGTG; >clamp_s_a_s_c_s:compl:rev CACCCACACAGACACTATCATGTTGACATTCC; dG = -10.1
835 >s_d_s_e_s_d_s_d_s_c_s GTGACGGTGCTGGTGACGGTGACGGTGTGGGTG; >clamp_s_a_s_c_s:compl:rev CACCCACACAGACACTATCATGTTGACATTCC; dG = -10.1
836 >s_d_s_e_s_e_s_e_s_c_s GTGACGGTGCTGGTGCTGGTGCTGGTGTGGGTG; >clamp_s_a_s_c_s:compl:rev CACCCACACAGACACTATCATGTTGACATTCC; dG = -10.1
837 >s_d_s_e_s_e_s_d_s_c_s GTGACGGTGCTGGTGCTGGTGACGGTGTGGGTG; >clamp_s_a_s_c_s:compl:rev CACCCACACAGACACTATCATGTTGACATTCC; dG = -10.1
838 >s_e_s_b_s_b_s_e_s_c_s GTGCTGGTGGCTGTGGCTGTGCTGGTGTGGGTG; >clamp_s_a_s_c_s:compl:rev CACCCACACAGACACTATCATGTTGACATTCC; dG = -10.1
839 >s_e_s_b_s_b_s_d_s_c_s GTGCTGGTGGCTGTGGCTGTGACGGTGTGGGTG; >clamp_s_a_s_c_s:compl:rev CACCCACACAGACACTATCATGTTGACATTCC; dG = -10.1
840 >s_e_s_b_s_d_s_e_s_c_s GTGCTGGTGGCTGTGACGGTGCTGGTGTGGGTG; >clamp_s_a_s_c_s:compl:rev CACCCACACAGACACTATCATGTTGACATTCC; dG = -10.1
841 >s_e_s_b_s_d_s_d_s_c_s GTGCTGGTGGCTGTGACGGTGACGGTGTGGGTG; >clamp_s_a_s_c_s:compl:rev CACCCACACAGACACTATCATGTTGACATTCC; dG = -10.1
842 >s_e_s_b_s_e_s_e_s_c_s GTGCTGGTGGCTGTGCTGGTGCTGGTGTGGGTG; >clamp_s_a_s_c_s:compl:rev CACCCACACAGACACTATCATGTTGACATTCC; dG = -10.1
843 >s_e_s_b_s_e_s_d_s_c_s GTGCTGGTGGCTGTGCTGGTGACGGTGTGGGTG; >clamp_s_a_s_c_s:compl:rev CACCCACACAGACACTATCATGTTGACATTCC; dG = -10.1
844 >s_e_s_d_s_b_s_e_s_c_s GTGCTGGTGACGGTGGCTGTGCTGGTGTGGGTG; >clamp_s_a_s_c_s:compl:rev CACCCACACAGACACTATCATGTTGACATTCC; dG = -10.1
845 >s_e_s_d_s_b_s_d_s_c_s GTGCTGGTGACGGTGGCTGTGACGGTGTGGGTG; >clamp_s_a_s_c_s:compl:rev CACCCACACAGACACTATCATGTTGACATTCC; dG = -10.1
846 >s_e_s_d_s_d_s_e_s_c_s GTGCTGGTGACGGTGACGGTGCTGGTGTGGGTG; >clamp_s_a_s_c_s:compl:rev CACCCACACAGACACTATCATGTTGACATTCC; dG = -10.1
847 >s_e_s_d_s_d_s_d_s_c_s GTGCTGGTGACGGTGACGGTGACGGTGTGGGTG; >clamp_s_a_s_c_s:compl:rev CACCCACACAGACACTATCATGTTGACATTCC; dG = -10.1

848 >s_e_s_d_s_e_s_e_s_c_s GTGCTGGTGACGGTGCTGGTGCTGGTGTGGGTG; >clamp_s_a_s_c_s:compl:rev
CACCCACACAGACACTATCATGTTGACATTCC; dG = -10.1
849 >s_e_s_d_s_e_s_d_s_c_s GTGCTGGTGACGGTGCTGGTGACGGTGTGGGTG; >clamp_s_a_s_c_s:compl:rev
CACCCACACAGACACTATCATGTTGACATTCC; dG = -10.1
850 >s_e_s_e_s_b_s_e_s_c_s GTGCTGGTGCTGGTGGCTGTGCTGGTGTGGGTG; >clamp_s_a_s_c_s:compl:rev
CACCCACACAGACACTATCATGTTGACATTCC; dG = -10.1
851 >s_e_s_e_s_b_s_d_s_c_s GTGCTGGTGCTGGTGGCTGTGACGGTGTGGGTG; >clamp_s_a_s_c_s:compl:rev
CACCCACACAGACACTATCATGTTGACATTCC; dG = -10.1
852 >s_e_s_e_s_d_s_e_s_c_s GTGCTGGTGCTGGTGACGGTGCTGGTGTGGGTG; >clamp_s_a_s_c_s:compl:rev
CACCCACACAGACACTATCATGTTGACATTCC; dG = -10.1
853 >s_e_s_e_s_d_s_d_s_c_s GTGCTGGTGCTGGTGACGGTGACGGTGTGGGTG; >clamp_s_a_s_c_s:compl:rev
CACCCACACAGACACTATCATGTTGACATTCC; dG = -10.1
854 >s_e_s_e_s_e_s_e_s_c_s GTGCTGGTGCTGGTGCTGGTGCTGGTGTGGGTG; >clamp_s_a_s_c_s:compl:rev
CACCCACACAGACACTATCATGTTGACATTCC; dG = -10.1
855 >s_e_s_e_s_e_s_d_s_c_s GTGCTGGTGCTGGTGCTGGTGACGGTGTGGGTG; >clamp_s_a_s_c_s:compl:rev
CACCCACACAGACACTATCATGTTGACATTCC; dG = -10.1
856 >s_a_s_a_s_b_s_a_s_b_s GTGTCTGTGTCTGTGGCTGTGTCTGTGGCTGTG; >clamp_s_a_s_c_s:compl:rev
CACCCACACAGACACTATCATGTTGACATTCC; dG = -10.0
857 >s_b_s_a_s_b_s_a_s_b_s GTGGCTGTGTCTGTGGCTGTGTCTGTGGCTGTG; >clamp_s_a_s_c_s:compl:rev
CACCCACACAGACACTATCATGTTGACATTCC; dG = -10.0
858 >s_b_s_b_s_b_s_a_s_b_s GTGGCTGTGGCTGTGGCTGTGTCTGTGGCTGTG; >clamp_s_a_s_c_s:compl:rev
CACCCACACAGACACTATCATGTTGACATTCC; dG = -10.0
859 >s_b_s_d_s_b_s_a_s_b_s GTGGCTGTGACGGTGGCTGTGTCTGTGGCTGTG; >clamp_s_a_s_c_s:compl:rev
CACCCACACAGACACTATCATGTTGACATTCC; dG = -10.0
860 >s_b_s_e_s_b_s_a_s_b_s GTGGCTGTGCTGGTGGCTGTGTCTGTGGCTGTG; >clamp_s_a_s_c_s:compl:rev
CACCCACACAGACACTATCATGTTGACATTCC; dG = -10.0
861 >s_c_s_a_s_b_s_a_s_b_s GTGTGGGTGTCTGTGGCTGTGTCTGTGGCTGTG; >clamp_s_a_s_c_s:compl:rev
CACCCACACAGACACTATCATGTTGACATTCC; dG = -10.0
862 >s_c_s_b_s_b_s_a_s_b_s GTGTGGGTGGCTGTGGCTGTGTCTGTGGCTGTG; >clamp_s_a_s_c_s:compl:rev
CACCCACACAGACACTATCATGTTGACATTCC; dG = -10.0
863 >s_c_s_b_s_d_s_a_s_b_s GTGTGGGTGGCTGTGACGGTGTCTGTGGCTGTG; >clamp_s_a_s_c_s:compl:rev
CACCCACACAGACACTATCATGTTGACATTCC; dG = -10.0
864 >s_c_s_b_s_e_s_a_s_b_s GTGTGGGTGGCTGTGCTGGTGTCTGTGGCTGTG; >clamp_s_a_s_c_s:compl:rev
CACCCACACAGACACTATCATGTTGACATTCC; dG = -10.0
865 >s_a_s_b_s_b_s_a_s_b_s GTGTCTGTGGCTGTGGCTGTGTCTGTGGCTGTG; >clamp_s_a_s_c_s:compl:rev
CACCCACACAGACACTATCATGTTGACATTCC; dG = -10.0
866 >s_c_s_e_s_b_s_a_s_b_s GTGTGGGTGCTGGTGGCTGTGTCTGTGGCTGTG; >clamp_s_a_s_c_s:compl:rev
CACCCACACAGACACTATCATGTTGACATTCC; dG = -10.0
867 >s_d_s_a_s_b_s_a_s_b_s GTGACGGTGTCTGTGGCTGTGTCTGTGGCTGTG; >clamp_s_a_s_c_s:compl:rev
CACCCACACAGACACTATCATGTTGACATTCC; dG = -10.0
868 >s_d_s_b_s_b_s_a_s_b_s GTGACGGTGGCTGTGGCTGTGTCTGTGGCTGTG; >clamp_s_a_s_c_s:compl:rev
CACCCACACAGACACTATCATGTTGACATTCC; dG = -10.0
869 >s_d_s_d_s_b_s_a_s_b_s GTGACGGTGACGGTGGCTGTGTCTGTGGCTGTG; >clamp_s_a_s_c_s:compl:rev
CACCCACACAGACACTATCATGTTGACATTCC; dG = -10.0
870 >s_d_s_e_s_b_s_a_s_b_s GTGACGGTGCTGGTGGCTGTGTCTGTGGCTGTG; >clamp_s_a_s_c_s:compl:rev
CACCCACACAGACACTATCATGTTGACATTCC; dG = -10.0
871 >s_e_s_a_s_b_s_a_s_b_s GTGCTGGTGTCTGTGGCTGTGTCTGTGGCTGTG; >clamp_s_a_s_c_s:compl:rev
CACCCACACAGACACTATCATGTTGACATTCC; dG = -10.0
872 >s_e_s_b_s_b_s_a_s_b_s GTGCTGGTGGCTGTGGCTGTGTCTGTGGCTGTG; >clamp_s_a_s_c_s:compl:rev
CACCCACACAGACACTATCATGTTGACATTCC; dG = -10.0
873 >s_e_s_d_s_b_s_a_s_b_s GTGCTGGTGACGGTGGCTGTGTCTGTGGCTGTG; >clamp_s_a_s_c_s:compl:rev
CACCCACACAGACACTATCATGTTGACATTCC; dG = -10.0
874 >s_e_s_e_s_b_s_a_s_b_s GTGCTGGTGCTGGTGGCTGTGTCTGTGGCTGTG; >clamp_s_a_s_c_s:compl:rev
CACCCACACAGACACTATCATGTTGACATTCC; dG = -10.0
875 >s_b_s_b_s_d_s_a_s_b_s GTGGCTGTGGCTGTGACGGTGTCTGTGGCTGTG; >clamp_s_a_s_c_s:compl:rev
CACCCACACAGACACTATCATGTTGACATTCC; dG = -9.9
876 >s_b_s_b_s_e_s_a_s_b_s GTGGCTGTGGCTGTGCTGGTGTCTGTGGCTGTG; >clamp_s_a_s_c_s:compl:rev
CACCCACACAGACACTATCATGTTGACATTCC; dG = -9.9
877 >s_b_s_d_s_d_s_a_s_b_s GTGGCTGTGACGGTGACGGTGTCTGTGGCTGTG; >clamp_s_a_s_c_s:compl:rev
CACCCACACAGACACTATCATGTTGACATTCC; dG = -9.9
878 >s_b_s_d_s_e_s_a_s_b_s GTGGCTGTGACGGTGCTGGTGTCTGTGGCTGTG; >clamp_s_a_s_c_s:compl:rev
CACCCACACAGACACTATCATGTTGACATTCC; dG = -9.9
879 >s_b_s_e_s_d_s_a_s_b_s GTGGCTGTGCTGGTGACGGTGTCTGTGGCTGTG; >clamp_s_a_s_c_s:compl:rev
CACCCACACAGACACTATCATGTTGACATTCC; dG = -9.9
880 >s_b_s_e_s_e_s_a_s_b_s GTGGCTGTGCTGGTGCTGGTGTCTGTGGCTGTG; >clamp_s_a_s_c_s:compl:rev
CACCCACACAGACACTATCATGTTGACATTCC; dG = -9.9
881 >s_c_s_e_s_d_s_a_s_b_s GTGTGGGTGCTGGTGACGGTGTCTGTGGCTGTG; >clamp_s_a_s_c_s:compl:rev
CACCCACACAGACACTATCATGTTGACATTCC; dG = -9.9
882 >s_c_s_e_s_e_s_a_s_b_s GTGTGGGTGCTGGTGCTGGTGTCTGTGGCTGTG; >clamp_s_a_s_c_s:compl:rev
CACCCACACAGACACTATCATGTTGACATTCC; dG = -9.9
883 >s_a_s_b_s_d_s_a_s_b_s GTGTCTGTGGCTGTGACGGTGTCTGTGGCTGTG; >clamp_s_a_s_c_s:compl:rev
CACCCACACAGACACTATCATGTTGACATTCC; dG = -9.9
884 >s_d_s_b_s_d_s_a_s_b_s GTGACGGTGGCTGTGACGGTGTCTGTGGCTGTG; >clamp_s_a_s_c_s:compl:rev
CACCCACACAGACACTATCATGTTGACATTCC; dG = -9.9

885 >s_d_s_b_s_e_s_a_s_b_s GTGACGGTGGCTGTGCTGGTGTCTGTGGCTGTG; >clamp_s_a_s_c_s:compl:rev CACCCACACAGACACTATCATGTTGACATTCC; dG = -9.9
886 >s_a_s_b_s_e_s_a_s_b_s GTGTCTGTGGCTGTGCTGGTGTCTGTGGCTGTG; >clamp_s_a_s_c_s:compl:rev CACCCACACAGACACTATCATGTTGACATTCC; dG = -9.9
887 >s_d_s_d_s_d_s_a_s_b_s GTGACGGTGACGGTGACGGTGTCTGTGGCTGTG; >clamp_s_a_s_c_s:compl:rev CACCCACACAGACACTATCATGTTGACATTCC; dG = -9.9
888 >s_d_s_d_s_e_s_a_s_b_s GTGACGGTGACGGTGCTGGTGTCTGTGGCTGTG; >clamp_s_a_s_c_s:compl:rev CACCCACACAGACACTATCATGTTGACATTCC; dG = -9.9
889 >s_d_s_e_s_d_s_a_s_b_s GTGACGGTGCTGGTGACGGTGTCTGTGGCTGTG; >clamp_s_a_s_c_s:compl:rev CACCCACACAGACACTATCATGTTGACATTCC; dG = -9.9
890 >s_d_s_e_s_e_s_a_s_b_s GTGACGGTGCTGGTGCTGGTGTCTGTGGCTGTG; >clamp_s_a_s_c_s:compl:rev CACCCACACAGACACTATCATGTTGACATTCC; dG = -9.9
891 >s_e_s_b_s_d_s_a_s_b_s GTGCTGGTGGCTGTGACGGTGTCTGTGGCTGTG; >clamp_s_a_s_c_s:compl:rev CACCCACACAGACACTATCATGTTGACATTCC; dG = -9.9
892 >s_e_s_b_s_e_s_a_s_b_s GTGCTGGTGGCTGTGCTGGTGTCTGTGGCTGTG; >clamp_s_a_s_c_s:compl:rev CACCCACACAGACACTATCATGTTGACATTCC; dG = -9.9
893 >s_e_s_d_s_d_s_a_s_b_s GTGCTGGTGACGGTGACGGTGTCTGTGGCTGTG; >clamp_s_a_s_c_s:compl:rev CACCCACACAGACACTATCATGTTGACATTCC; dG = -9.9
894 >s_e_s_d_s_e_s_a_s_b_s GTGCTGGTGACGGTGCTGGTGTCTGTGGCTGTG; >clamp_s_a_s_c_s:compl:rev CACCCACACAGACACTATCATGTTGACATTCC; dG = -9.9
895 >s_e_s_e_s_d_s_a_s_b_s GTGCTGGTGCTGGTGACGGTGTCTGTGGCTGTG; >clamp_s_a_s_c_s:compl:rev CACCCACACAGACACTATCATGTTGACATTCC; dG = -9.9
896 >s_e_s_e_s_e_s_a_s_b_s GTGCTGGTGCTGGTGCTGGTGTCTGTGGCTGTG; >clamp_s_a_s_c_s:compl:rev CACCCACACAGACACTATCATGTTGACATTCC; dG = -9.9

****

AD Matches

1 >s_a_s_d_s_d_s_a_s_d_s GTGTCTGTGACGGTGACGGTGTCTGTGACGGTG; >clamp_s_a_s_d_s:compl:rev CACCGTCACAGACACTATCATGTTGACATTCC; dG = -17.6
2 >s_e_s_a_s_d_s_a_s_d_s GTGCTGGTGTCTGTGACGGTGTCTGTGACGGTG; >clamp_s_a_s_d_s:compl:rev CACCGTCACAGACACTATCATGTTGACATTCC; dG = -17.5
3 >s_b_s_a_s_d_s_a_s_d_s GTGGCTGTGTCTGTGACGGTGTCTGTGACGGTG; >clamp_s_a_s_d_s:compl:rev CACCGTCACAGACACTATCATGTTGACATTCC; dG = -17.5
4 >s_a_s_a_s_d_s_a_s_d_s GTGTCTGTGTCTGTGACGGTGTCTGTGACGGTG; >clamp_s_a_s_d_s:compl:rev CACCGTCACAGACACTATCATGTTGACATTCC; dG = -17.5
5 >s_c_s_a_s_d_s_a_s_d_s GTGTGGGTGTCTGTGACGGTGTCTGTGACGGTG; >clamp_s_a_s_d_s:compl:rev CACCGTCACAGACACTATCATGTTGACATTCC; dG = -17.5
6 >s_d_s_a_s_d_s_a_s_d_s GTGACGGTGTCTGTGACGGTGTCTGTGACGGTG; >clamp_s_a_s_d_s:compl:rev CACCGTCACAGACACTATCATGTTGACATTCC; dG = -17.5
7 >s_a_s_a_s_a_s_a_s_d_s GTGTCTGTGTCTGTGTCTGTGTCTGTGACGGTG; >clamp_s_a_s_d_s:compl:rev CACCGTCACAGACACTATCATGTTGACATTCC; dG = -17.3
8 >s_e_s_a_s_a_s_a_s_d_s GTGCTGGTGTCTGTGTCTGTGTCTGTGACGGTG; >clamp_s_a_s_d_s:compl:rev CACCGTCACAGACACTATCATGTTGACATTCC; dG = -17.3
9 >s_e_s_a_s_b_s_a_s_d_s GTGCTGGTGTCTGTGGCTGTGTCTGTGACGGTG; >clamp_s_a_s_d_s:compl:rev CACCGTCACAGACACTATCATGTTGACATTCC; dG = -17.3
10 >s_e_s_b_s_a_s_a_s_d_s GTGCTGGTGGCTGTGTCTGTGTCTGTGACGGTG; >clamp_s_a_s_d_s:compl:rev CACCGTCACAGACACTATCATGTTGACATTCC; dG = -17.3
11 >s_e_s_b_s_b_s_a_s_d_s GTGCTGGTGGCTGTGGCTGTGTCTGTGACGGTG; >clamp_s_a_s_d_s:compl:rev CACCGTCACAGACACTATCATGTTGACATTCC; dG = -17.3
12 >s_a_s_c_s_a_s_a_s_d_s GTGTCTGTGTGGGTGTCTGTGTCTGTGACGGTG; >clamp_s_a_s_d_s:compl:rev CACCGTCACAGACACTATCATGTTGACATTCC; dG = -17.3
13 >s_e_s_c_s_a_s_a_s_d_s GTGCTGGTGTGGGTGTCTGTGTCTGTGACGGTG; >clamp_s_a_s_d_s:compl:rev CACCGTCACAGACACTATCATGTTGACATTCC; dG = -17.3
14 >s_e_s_c_s_b_s_a_s_d_s GTGCTGGTGTGGGTGGCTGTGTCTGTGACGGTG; >clamp_s_a_s_d_s:compl:rev CACCGTCACAGACACTATCATGTTGACATTCC; dG = -17.3
15 >s_e_s_d_s_a_s_a_s_d_s GTGCTGGTGACGGTGTCTGTGTCTGTGACGGTG; >clamp_s_a_s_d_s:compl:rev CACCGTCACAGACACTATCATGTTGACATTCC; dG = -17.3
16 >s_e_s_d_s_b_s_a_s_d_s GTGCTGGTGACGGTGGCTGTGTCTGTGACGGTG; >clamp_s_a_s_d_s:compl:rev CACCGTCACAGACACTATCATGTTGACATTCC; dG = -17.3
17 >s_a_s_c_s_b_s_a_s_d_s GTGTCTGTGTGGGTGGCTGTGTCTGTGACGGTG; >clamp_s_a_s_d_s:compl:rev CACCGTCACAGACACTATCATGTTGACATTCC; dG = -17.3
18 >s_e_s_e_s_a_s_a_s_d_s GTGCTGGTGCTGGTGTCTGTGTCTGTGACGGTG; >clamp_s_a_s_d_s:compl:rev CACCGTCACAGACACTATCATGTTGACATTCC; dG = -17.3
19 >s_e_s_e_s_b_s_a_s_d_s GTGCTGGTGCTGGTGGCTGTGTCTGTGACGGTG; >clamp_s_a_s_d_s:compl:rev CACCGTCACAGACACTATCATGTTGACATTCC; dG = -17.3
20 >s_a_s_d_s_a_s_a_s_d_s GTGTCTGTGACGGTGTCTGTGTCTGTGACGGTG; >clamp_s_a_s_d_s:compl:rev CACCGTCACAGACACTATCATGTTGACATTCC; dG = -17.3
21 >s_a_s_d_s_b_s_a_s_d_s GTGTCTGTGACGGTGGCTGTGTCTGTGACGGTG; >clamp_s_a_s_d_s:compl:rev CACCGTCACAGACACTATCATGTTGACATTCC; dG = -17.3
22 >s_a_s_a_s_b_s_a_s_d_s GTGTCTGTGTCTGTGGCTGTGTCTGTGACGGTG; >clamp_s_a_s_d_s:compl:rev CACCGTCACAGACACTATCATGTTGACATTCC; dG = -17.3

23 >s_a_s_e_s_a_s_a_s_d_s GTGTCTGTGCTGGTGTCTGTGTCTGTGACGGTG; >clamp_s_a_s_d_s:compl:rev
CACCGTCACAGACACTATCATGTTGACATTCC; dG = -17.3

24 >s_a_s_e_s_b_s_a_s_d_s GTGTCTGTGCTGGTGGCTGTGTCTGTGACGGTG; >clamp_s_a_s_d_s:compl:rev
CACCGTCACAGACACTATCATGTTGACATTCC; dG = -17.3

25 >s_b_s_a_s_a_s_a_s_d_s GTGGCTGTGTCTGTGTCTGTGTCTGTGACGGTG; >clamp_s_a_s_d_s:compl:rev
CACCGTCACAGACACTATCATGTTGACATTCC; dG = -17.3

26 >s_b_s_a_s_b_s_a_s_d_s GTGGCTGTGTCTGTGGCTGTGTCTGTGACGGTG; >clamp_s_a_s_d_s:compl:rev
CACCGTCACAGACACTATCATGTTGACATTCC; dG = -17.3

27 >s_b_s_b_s_a_s_a_s_d_s GTGGCTGTGGCTGTGTCTGTGTCTGTGACGGTG; >clamp_s_a_s_d_s:compl:rev
CACCGTCACAGACACTATCATGTTGACATTCC; dG = -17.3

28 >s_b_s_b_s_b_s_a_s_d_s GTGGCTGTGGCTGTGGCTGTGTCTGTGACGGTG; >clamp_s_a_s_d_s:compl:rev
CACCGTCACAGACACTATCATGTTGACATTCC; dG = -17.3

29 >s_b_s_c_s_a_s_a_s_d_s GTGGCTGTGTGGGTGTCTGTGTCTGTGACGGTG; >clamp_s_a_s_d_s:compl:rev
CACCGTCACAGACACTATCATGTTGACATTCC; dG = -17.3

30 >s_b_s_c_s_b_s_a_s_d_s GTGGCTGTGTGGGTGGCTGTGTCTGTGACGGTG; >clamp_s_a_s_d_s:compl:rev
CACCGTCACAGACACTATCATGTTGACATTCC; dG = -17.3

31 >s_b_s_d_s_a_s_a_s_d_s GTGGCTGTGACGGTGTCTGTGTCTGTGACGGTG; >clamp_s_a_s_d_s:compl:rev
CACCGTCACAGACACTATCATGTTGACATTCC; dG = -17.3

32 >s_b_s_d_s_b_s_a_s_d_s GTGGCTGTGACGGTGGCTGTGTCTGTGACGGTG; >clamp_s_a_s_d_s:compl:rev
CACCGTCACAGACACTATCATGTTGACATTCC; dG = -17.3

33 >s_b_s_e_s_a_s_a_s_d_s GTGGCTGTGCTGGTGTCTGTGTCTGTGACGGTG; >clamp_s_a_s_d_s:compl:rev
CACCGTCACAGACACTATCATGTTGACATTCC; dG = -17.3

34 >s_b_s_e_s_b_s_a_s_d_s GTGGCTGTGCTGGTGGCTGTGTCTGTGACGGTG; >clamp_s_a_s_d_s:compl:rev
CACCGTCACAGACACTATCATGTTGACATTCC; dG = -17.3

35 >s_c_s_a_s_a_s_a_s_d_s GTGTGGGTGTCTGTGTCTGTGTCTGTGACGGTG; >clamp_s_a_s_d_s:compl:rev
CACCGTCACAGACACTATCATGTTGACATTCC; dG = -17.3

36 >s_c_s_a_s_b_s_a_s_d_s GTGTGGGTGTCTGTGGCTGTGTCTGTGACGGTG; >clamp_s_a_s_d_s:compl:rev
CACCGTCACAGACACTATCATGTTGACATTCC; dG = -17.3

37 >s_c_s_b_s_a_s_a_s_d_s GTGTGGGTGGCTGTGTCTGTGTCTGTGACGGTG; >clamp_s_a_s_d_s:compl:rev
CACCGTCACAGACACTATCATGTTGACATTCC; dG = -17.3

38 >s_c_s_b_s_b_s_a_s_d_s GTGTGGGTGGCTGTGGCTGTGTCTGTGACGGTG; >clamp_s_a_s_d_s:compl:rev
CACCGTCACAGACACTATCATGTTGACATTCC; dG = -17.3

39 >s_a_s_b_s_a_s_a_s_d_s GTGTCTGTGGCTGTGTCTGTGTCTGTGACGGTG; >clamp_s_a_s_d_s:compl:rev
CACCGTCACAGACACTATCATGTTGACATTCC; dG = -17.3

40 >s_c_s_c_s_a_s_a_s_d_s GTGTGGGTGTGGGTGTCTGTGTCTGTGACGGTG; >clamp_s_a_s_d_s:compl:rev
CACCGTCACAGACACTATCATGTTGACATTCC; dG = -17.3

41 >s_c_s_c_s_b_s_a_s_d_s GTGTGGGTGTGGGTGGCTGTGTCTGTGACGGTG; >clamp_s_a_s_d_s:compl:rev
CACCGTCACAGACACTATCATGTTGACATTCC; dG = -17.3

42 >s_c_s_d_s_a_s_a_s_d_s GTGTGGGTGACGGTGTCTGTGTCTGTGACGGTG; >clamp_s_a_s_d_s:compl:rev
CACCGTCACAGACACTATCATGTTGACATTCC; dG = -17.3

43 >s_c_s_d_s_b_s_a_s_d_s GTGTGGGTGACGGTGGCTGTGTCTGTGACGGTG; >clamp_s_a_s_d_s:compl:rev
CACCGTCACAGACACTATCATGTTGACATTCC; dG = -17.3

44 >s_a_s_b_s_b_s_a_s_d_s GTGTCTGTGGCTGTGGCTGTGTCTGTGACGGTG; >clamp_s_a_s_d_s:compl:rev
CACCGTCACAGACACTATCATGTTGACATTCC; dG = -17.3

45 >s_c_s_e_s_a_s_a_s_d_s GTGTGGGTGCTGGTGTCTGTGTCTGTGACGGTG; >clamp_s_a_s_d_s:compl:rev
CACCGTCACAGACACTATCATGTTGACATTCC; dG = -17.3

46 >s_c_s_e_s_b_s_a_s_d_s GTGTGGGTGCTGGTGGCTGTGTCTGTGACGGTG; >clamp_s_a_s_d_s:compl:rev
CACCGTCACAGACACTATCATGTTGACATTCC; dG = -17.3

47 >s_d_s_a_s_a_s_a_s_d_s GTGACGGTGTCTGTGTCTGTGTCTGTGACGGTG; >clamp_s_a_s_d_s:compl:rev
CACCGTCACAGACACTATCATGTTGACATTCC; dG = -17.3

48 >s_d_s_a_s_b_s_a_s_d_s GTGACGGTGTCTGTGGCTGTGTCTGTGACGGTG; >clamp_s_a_s_d_s:compl:rev
CACCGTCACAGACACTATCATGTTGACATTCC; dG = -17.3

49 >s_d_s_b_s_a_s_a_s_d_s GTGACGGTGGCTGTGTCTGTGTCTGTGACGGTG; >clamp_s_a_s_d_s:compl:rev
CACCGTCACAGACACTATCATGTTGACATTCC; dG = -17.3

50 >s_d_s_b_s_b_s_a_s_d_s GTGACGGTGGCTGTGGCTGTGTCTGTGACGGTG; >clamp_s_a_s_d_s:compl:rev
CACCGTCACAGACACTATCATGTTGACATTCC; dG = -17.3

51 >s_d_s_c_s_a_s_a_s_d_s GTGACGGTGTGGGTGTCTGTGTCTGTGACGGTG; >clamp_s_a_s_d_s:compl:rev
CACCGTCACAGACACTATCATGTTGACATTCC; dG = -17.3

52 >s_d_s_c_s_b_s_a_s_d_s GTGACGGTGTGGGTGGCTGTGTCTGTGACGGTG; >clamp_s_a_s_d_s:compl:rev
CACCGTCACAGACACTATCATGTTGACATTCC; dG = -17.3

53 >s_d_s_d_s_a_s_a_s_d_s GTGACGGTGACGGTGTCTGTGTCTGTGACGGTG; >clamp_s_a_s_d_s:compl:rev
CACCGTCACAGACACTATCATGTTGACATTCC; dG = -17.3

54 >s_d_s_d_s_b_s_a_s_d_s GTGACGGTGACGGTGGCTGTGTCTGTGACGGTG; >clamp_s_a_s_d_s:compl:rev
CACCGTCACAGACACTATCATGTTGACATTCC; dG = -17.3

55 >s_d_s_e_s_a_s_a_s_d_s GTGACGGTGCTGGTGTCTGTGTCTGTGACGGTG; >clamp_s_a_s_d_s:compl:rev
CACCGTCACAGACACTATCATGTTGACATTCC; dG = -17.3

56 >s_d_s_e_s_b_s_a_s_d_s GTGACGGTGCTGGTGGCTGTGTCTGTGACGGTG; >clamp_s_a_s_d_s:compl:rev
CACCGTCACAGACACTATCATGTTGACATTCC; dG = -17.3

57 >s_a_s_b_s_e_s_a_s_d_s GTGTCTGTGGCTGTGCTGGTGTCTGTGACGGTG; >clamp_s_a_s_d_s:compl:rev
CACCGTCACAGACACTATCATGTTGACATTCC; dG = -17.2

58 >s_d_s_e_s_e_s_a_s_d_s GTGACGGTGCTGGTGCTGGTGTCTGTGACGGTG; >clamp_s_a_s_d_s:compl:rev
CACCGTCACAGACACTATCATGTTGACATTCC; dG = -17.2

59 >s_e_s_a_s_c_s_a_s_d_s GTGCTGGTGTCTGTGTGGGTGTCTGTGACGGTG; >clamp_s_a_s_d_s:compl:rev
CACCGTCACAGACACTATCATGTTGACATTCC; dG = -17.2

60 >s_e_s_a_s_e_s_a_s_d_s GTGCTGGTGTCTGTGCTGGTGTCTGTGACGGTG; >clamp_s_a_s_d_s:compl:rev CACCGTCACAGACACTATCATGTTGACATTCC; dG = -17.2
61 >s_e_s_b_s_c_s_a_s_d_s GTGCTGGTGGCTGTGTGGGTGTCTGTGACGGTG; >clamp_s_a_s_d_s:compl:rev CACCGTCACAGACACTATCATGTTGACATTCC; dG = -17.2
62 >s_e_s_b_s_d_s_a_s_d_s GTGCTGGTGGCTGTGACGGTGTCTGTGACGGTG; >clamp_s_a_s_d_s:compl:rev CACCGTCACAGACACTATCATGTTGACATTCC; dG = -17.2
63 >s_e_s_b_s_e_s_a_s_d_s GTGCTGGTGGCTGTGCTGGTGTCTGTGACGGTG; >clamp_s_a_s_d_s:compl:rev CACCGTCACAGACACTATCATGTTGACATTCC; dG = -17.2
64 >s_e_s_c_s_c_s_a_s_d_s GTGCTGGTGTGGGTGTGGGTGTCTGTGACGGTG; >clamp_s_a_s_d_s:compl:rev CACCGTCACAGACACTATCATGTTGACATTCC; dG = -17.2
65 >s_e_s_c_s_d_s_a_s_d_s GTGCTGGTGTGGGTGACGGTGTCTGTGACGGTG; >clamp_s_a_s_d_s:compl:rev CACCGTCACAGACACTATCATGTTGACATTCC; dG = -17.2
66 >s_e_s_c_s_e_s_a_s_d_s GTGCTGGTGTGGGTGCTGGTGTCTGTGACGGTG; >clamp_s_a_s_d_s:compl:rev CACCGTCACAGACACTATCATGTTGACATTCC; dG = -17.2
67 >s_e_s_d_s_c_s_a_s_d_s GTGCTGGTGACGGTGTGGGTGTCTGTGACGGTG; >clamp_s_a_s_d_s:compl:rev CACCGTCACAGACACTATCATGTTGACATTCC; dG = -17.2
68 >s_e_s_d_s_d_s_a_s_d_s GTGCTGGTGACGGTGACGGTGTCTGTGACGGTG; >clamp_s_a_s_d_s:compl:rev CACCGTCACAGACACTATCATGTTGACATTCC; dG = -17.2
69 >s_e_s_d_s_e_s_a_s_d_s GTGCTGGTGACGGTGCTGGTGTCTGTGACGGTG; >clamp_s_a_s_d_s:compl:rev CACCGTCACAGACACTATCATGTTGACATTCC; dG = -17.2
70 >s_e_s_e_s_c_s_a_s_d_s GTGCTGGTGCTGGTGTGGGTGTCTGTGACGGTG; >clamp_s_a_s_d_s:compl:rev CACCGTCACAGACACTATCATGTTGACATTCC; dG = -17.2
71 >s_e_s_e_s_d_s_a_s_d_s GTGCTGGTGCTGGTGACGGTGTCTGTGACGGTG; >clamp_s_a_s_d_s:compl:rev CACCGTCACAGACACTATCATGTTGACATTCC; dG = -17.2
72 >s_e_s_e_s_e_s_a_s_d_s GTGCTGGTGCTGGTGCTGGTGTCTGTGACGGTG; >clamp_s_a_s_d_s:compl:rev CACCGTCACAGACACTATCATGTTGACATTCC; dG = -17.2
73 >s_a_s_c_s_c_s_a_s_d_s GTGTCTGTGTGGGTGTGGGTGTCTGTGACGGTG; >clamp_s_a_s_d_s:compl:rev CACCGTCACAGACACTATCATGTTGACATTCC; dG = -17.2
74 >s_a_s_c_s_d_s_a_s_d_s GTGTCTGTGTGGGTGACGGTGTCTGTGACGGTG; >clamp_s_a_s_d_s:compl:rev CACCGTCACAGACACTATCATGTTGACATTCC; dG = -17.2
75 >s_a_s_c_s_e_s_a_s_d_s GTGTCTGTGTGGGTGCTGGTGTCTGTGACGGTG; >clamp_s_a_s_d_s:compl:rev CACCGTCACAGACACTATCATGTTGACATTCC; dG = -17.2
76 >s_a_s_d_s_c_s_a_s_d_s GTGTCTGTGACGGTGTGGGTGTCTGTGACGGTG; >clamp_s_a_s_d_s:compl:rev CACCGTCACAGACACTATCATGTTGACATTCC; dG = -17.2
77 >s_a_s_d_s_e_s_a_s_d_s GTGTCTGTGACGGTGCTGGTGTCTGTGACGGTG; >clamp_s_a_s_d_s:compl:rev CACCGTCACAGACACTATCATGTTGACATTCC; dG = -17.2
78 >s_a_s_e_s_c_s_a_s_d_s GTGTCTGTGCTGGTGTGGGTGTCTGTGACGGTG; >clamp_s_a_s_d_s:compl:rev CACCGTCACAGACACTATCATGTTGACATTCC; dG = -17.2
79 >s_a_s_e_s_d_s_a_s_d_s GTGTCTGTGCTGGTGACGGTGTCTGTGACGGTG; >clamp_s_a_s_d_s:compl:rev CACCGTCACAGACACTATCATGTTGACATTCC; dG = -17.2
80 >s_a_s_e_s_e_s_a_s_d_s GTGTCTGTGCTGGTGCTGGTGTCTGTGACGGTG; >clamp_s_a_s_d_s:compl:rev CACCGTCACAGACACTATCATGTTGACATTCC; dG = -17.2
81 >s_b_s_a_s_c_s_a_s_d_s GTGGCTGTGTCTGTGTGGGTGTCTGTGACGGTG; >clamp_s_a_s_d_s:compl:rev CACCGTCACAGACACTATCATGTTGACATTCC; dG = -17.2
82 >s_a_s_a_s_c_s_a_s_d_s GTGTCTGTGTCTGTGTGGGTGTCTGTGACGGTG; >clamp_s_a_s_d_s:compl:rev CACCGTCACAGACACTATCATGTTGACATTCC; dG = -17.2
83 >s_b_s_a_s_e_s_a_s_d_s GTGGCTGTGTCTGTGCTGGTGTCTGTGACGGTG; >clamp_s_a_s_d_s:compl:rev CACCGTCACAGACACTATCATGTTGACATTCC; dG = -17.2
84 >s_b_s_b_s_c_s_a_s_d_s GTGGCTGTGGCTGTGTGGGTGTCTGTGACGGTG; >clamp_s_a_s_d_s:compl:rev CACCGTCACAGACACTATCATGTTGACATTCC; dG = -17.2
85 >s_b_s_b_s_d_s_a_s_d_s GTGGCTGTGGCTGTGACGGTGTCTGTGACGGTG; >clamp_s_a_s_d_s:compl:rev CACCGTCACAGACACTATCATGTTGACATTCC; dG = -17.2
86 >s_b_s_b_s_e_s_a_s_d_s GTGGCTGTGGCTGTGCTGGTGTCTGTGACGGTG; >clamp_s_a_s_d_s:compl:rev CACCGTCACAGACACTATCATGTTGACATTCC; dG = -17.2
87 >s_b_s_c_s_c_s_a_s_d_s GTGGCTGTGTGGGTGTGGGTGTCTGTGACGGTG; >clamp_s_a_s_d_s:compl:rev CACCGTCACAGACACTATCATGTTGACATTCC; dG = -17.2
88 >s_b_s_c_s_d_s_a_s_d_s GTGGCTGTGTGGGTGACGGTGTCTGTGACGGTG; >clamp_s_a_s_d_s:compl:rev CACCGTCACAGACACTATCATGTTGACATTCC; dG = -17.2
89 >s_b_s_c_s_e_s_a_s_d_s GTGGCTGTGTGGGTGCTGGTGTCTGTGACGGTG; >clamp_s_a_s_d_s:compl:rev CACCGTCACAGACACTATCATGTTGACATTCC; dG = -17.2
90 >s_b_s_d_s_c_s_a_s_d_s GTGGCTGTGACGGTGTGGGTGTCTGTGACGGTG; >clamp_s_a_s_d_s:compl:rev CACCGTCACAGACACTATCATGTTGACATTCC; dG = -17.2
91 >s_b_s_d_s_d_s_a_s_d_s GTGGCTGTGACGGTGACGGTGTCTGTGACGGTG; >clamp_s_a_s_d_s:compl:rev CACCGTCACAGACACTATCATGTTGACATTCC; dG = -17.2
92 >s_b_s_d_s_e_s_a_s_d_s GTGGCTGTGACGGTGCTGGTGTCTGTGACGGTG; >clamp_s_a_s_d_s:compl:rev CACCGTCACAGACACTATCATGTTGACATTCC; dG = -17.2
93 >s_b_s_e_s_c_s_a_s_d_s GTGGCTGTGCTGGTGTGGGTGTCTGTGACGGTG; >clamp_s_a_s_d_s:compl:rev CACCGTCACAGACACTATCATGTTGACATTCC; dG = -17.2
94 >s_b_s_e_s_d_s_a_s_d_s GTGGCTGTGCTGGTGACGGTGTCTGTGACGGTG; >clamp_s_a_s_d_s:compl:rev CACCGTCACAGACACTATCATGTTGACATTCC; dG = -17.2
95 >s_a_s_a_s_e_s_a_s_d_s GTGTCTGTGTCTGTGCTGGTGTCTGTGACGGTG; >clamp_s_a_s_d_s:compl:rev CACCGTCACAGACACTATCATGTTGACATTCC; dG = -17.2
96 >s_b_s_e_s_e_s_a_s_d_s GTGGCTGTGCTGGTGCTGGTGTCTGTGACGGTG; >clamp_s_a_s_d_s:compl:rev CACCGTCACAGACACTATCATGTTGACATTCC; dG = -17.2

97 >s_c_s_a_s_c_s_a_s_d_s GTGTGGGTGTCTGTGTGGGTGTCTGTGACGGTG; >clamp_s_a_s_d_s:compl:rev
CACCGTCACAGACACTATCATGTTGACATTCC; dG = -17.2
98 >s_c_s_a_s_e_s_a_s_d_s GTGTGGGTGTCTGTGCTGGTGTCTGTGACGGTG; >clamp_s_a_s_d_s:compl:rev
CACCGTCACAGACACTATCATGTTGACATTCC; dG = -17.2
99 >s_c_s_b_s_c_s_a_s_d_s GTGTGGGTGGCTGTGTGGGTGTCTGTGACGGTG; >clamp_s_a_s_d_s:compl:rev
CACCGTCACAGACACTATCATGTTGACATTCC; dG = -17.2
100 >s_c_s_b_s_d_s_a_s_d_s GTGTGGGTGGCTGTGACGGTGTCTGTGACGGTG; >clamp_s_a_s_d_s:compl:rev
CACCGTCACAGACACTATCATGTTGACATTCC; dG = -17.2
101 >s_c_s_b_s_e_s_a_s_d_s GTGTGGGTGGCTGTGCTGGTGTCTGTGACGGTG; >clamp_s_a_s_d_s:compl:rev
CACCGTCACAGACACTATCATGTTGACATTCC; dG = -17.2
102 >s_c_s_c_s_c_s_a_s_d_s GTGTGGGTGTGGGTGTGGGTGTCTGTGACGGTG; >clamp_s_a_s_d_s:compl:rev
CACCGTCACAGACACTATCATGTTGACATTCC; dG = -17.2
103 >s_c_s_c_s_d_s_a_s_d_s GTGTGGGTGTGGGTGACGGTGTCTGTGACGGTG; >clamp_s_a_s_d_s:compl:rev
CACCGTCACAGACACTATCATGTTGACATTCC; dG = -17.2
104 >s_c_s_c_s_e_s_a_s_d_s GTGTGGGTGTGGGTGCTGGTGTCTGTGACGGTG; >clamp_s_a_s_d_s:compl:rev
CACCGTCACAGACACTATCATGTTGACATTCC; dG = -17.2
105 >s_c_s_d_s_c_s_a_s_d_s GTGTGGGTGACGGTGTGGGTGTCTGTGACGGTG; >clamp_s_a_s_d_s:compl:rev
CACCGTCACAGACACTATCATGTTGACATTCC; dG = -17.2
106 >s_c_s_d_s_d_s_a_s_d_s GTGTGGGTGACGGTGACGGTGTCTGTGACGGTG; >clamp_s_a_s_d_s:compl:rev
CACCGTCACAGACACTATCATGTTGACATTCC; dG = -17.2
107 >s_c_s_d_s_e_s_a_s_d_s GTGTGGGTGACGGTGCTGGTGTCTGTGACGGTG; >clamp_s_a_s_d_s:compl:rev
CACCGTCACAGACACTATCATGTTGACATTCC; dG = -17.2
108 >s_c_s_e_s_c_s_a_s_d_s GTGTGGGTGCTGGTGTGGGTGTCTGTGACGGTG; >clamp_s_a_s_d_s:compl:rev
CACCGTCACAGACACTATCATGTTGACATTCC; dG = -17.2
109 >s_c_s_e_s_d_s_a_s_d_s GTGTGGGTGCTGGTGACGGTGTCTGTGACGGTG; >clamp_s_a_s_d_s:compl:rev
CACCGTCACAGACACTATCATGTTGACATTCC; dG = -17.2
110 >s_c_s_e_s_e_s_a_s_d_s GTGTGGGTGCTGGTGCTGGTGTCTGTGACGGTG; >clamp_s_a_s_d_s:compl:rev
CACCGTCACAGACACTATCATGTTGACATTCC; dG = -17.2
111 >s_d_s_a_s_c_s_a_s_d_s GTGACGGTGTCTGTGTGGGTGTCTGTGACGGTG; >clamp_s_a_s_d_s:compl:rev
CACCGTCACAGACACTATCATGTTGACATTCC; dG = -17.2
112 >s_a_s_b_s_c_s_a_s_d_s GTGTCTGTGGCTGTGTGGGTGTCTGTGACGGTG; >clamp_s_a_s_d_s:compl:rev
CACCGTCACAGACACTATCATGTTGACATTCC; dG = -17.2
113 >s_d_s_a_s_e_s_a_s_d_s GTGACGGTGTCTGTGCTGGTGTCTGTGACGGTG; >clamp_s_a_s_d_s:compl:rev
CACCGTCACAGACACTATCATGTTGACATTCC; dG = -17.2
114 >s_d_s_b_s_c_s_a_s_d_s GTGACGGTGGCTGTGTGGGTGTCTGTGACGGTG; >clamp_s_a_s_d_s:compl:rev
CACCGTCACAGACACTATCATGTTGACATTCC; dG = -17.2
115 >s_d_s_b_s_d_s_a_s_d_s GTGACGGTGGCTGTGACGGTGTCTGTGACGGTG; >clamp_s_a_s_d_s:compl:rev
CACCGTCACAGACACTATCATGTTGACATTCC; dG = -17.2
116 >s_d_s_b_s_e_s_a_s_d_s GTGACGGTGGCTGTGCTGGTGTCTGTGACGGTG; >clamp_s_a_s_d_s:compl:rev
CACCGTCACAGACACTATCATGTTGACATTCC; dG = -17.2
117 >s_d_s_c_s_c_s_a_s_d_s GTGACGGTGTGGGTGTGGGTGTCTGTGACGGTG; >clamp_s_a_s_d_s:compl:rev
CACCGTCACAGACACTATCATGTTGACATTCC; dG = -17.2
118 >s_d_s_c_s_d_s_a_s_d_s GTGACGGTGTGGGTGACGGTGTCTGTGACGGTG; >clamp_s_a_s_d_s:compl:rev
CACCGTCACAGACACTATCATGTTGACATTCC; dG = -17.2
119 >s_a_s_b_s_d_s_a_s_d_s GTGTCTGTGGCTGTGACGGTGTCTGTGACGGTG; >clamp_s_a_s_d_s:compl:rev
CACCGTCACAGACACTATCATGTTGACATTCC; dG = -17.2
120 >s_d_s_c_s_e_s_a_s_d_s GTGACGGTGTGGGTGCTGGTGTCTGTGACGGTG; >clamp_s_a_s_d_s:compl:rev
CACCGTCACAGACACTATCATGTTGACATTCC; dG = -17.2
121 >s_d_s_d_s_c_s_a_s_d_s GTGACGGTGACGGTGTGGGTGTCTGTGACGGTG; >clamp_s_a_s_d_s:compl:rev
CACCGTCACAGACACTATCATGTTGACATTCC; dG = -17.2
122 >s_d_s_d_s_d_s_a_s_d_s GTGACGGTGACGGTGACGGTGTCTGTGACGGTG; >clamp_s_a_s_d_s:compl:rev
CACCGTCACAGACACTATCATGTTGACATTCC; dG = -17.2
123 >s_d_s_d_s_e_s_a_s_d_s GTGACGGTGACGGTGCTGGTGTCTGTGACGGTG; >clamp_s_a_s_d_s:compl:rev
CACCGTCACAGACACTATCATGTTGACATTCC; dG = -17.2
124 >s_d_s_e_s_c_s_a_s_d_s GTGACGGTGCTGGTGTGGGTGTCTGTGACGGTG; >clamp_s_a_s_d_s:compl:rev
CACCGTCACAGACACTATCATGTTGACATTCC; dG = -17.2
125 >s_d_s_e_s_d_s_a_s_d_s GTGACGGTGCTGGTGACGGTGTCTGTGACGGTG; >clamp_s_a_s_d_s:compl:rev
CACCGTCACAGACACTATCATGTTGACATTCC; dG = -17.2

****

AD Mismatches

1 >s_a_s_a_s_b_s_d_s_d_s GTGTCTGTGTCTGTGGCTGTGACGGTGACGGTG; >clamp_s_a_s_d_s:compl:rev
CACCGTCACAGACACTATCATGTTGACATTCC; dG = -16.2
2 >s_b_s_a_s_b_s_d_s_d_s GTGGCTGTGTCTGTGGCTGTGACGGTGACGGTG; >clamp_s_a_s_d_s:compl:rev
CACCGTCACAGACACTATCATGTTGACATTCC; dG = -16.2
3 >s_b_s_b_s_b_s_d_s_d_s GTGGCTGTGGCTGTGGCTGTGACGGTGACGGTG; >clamp_s_a_s_d_s:compl:rev
CACCGTCACAGACACTATCATGTTGACATTCC; dG = -16.2
4 >s_b_s_b_s_d_s_d_s_d_s GTGGCTGTGGCTGTGACGGTGACGGTGACGGTG; >clamp_s_a_s_d_s:compl:rev
CACCGTCACAGACACTATCATGTTGACATTCC; dG = -16.2

5 >s_c_s_a_s_b_s_d_s_d_s GTGTGGGTGTCTGTGGCTGTGACGGTGACGGTG; >clamp_s_a_s_d_s:compl:rev
CACCGTCACAGACACTATCATGTTGACATTCC; dG = -16.2
6 >s_c_s_b_s_b_s_d_s_d_s GTGTGGGTGGCTGTGGCTGTGACGGTGACGGTG; >clamp_s_a_s_d_s:compl:rev
CACCGTCACAGACACTATCATGTTGACATTCC; dG = -16.2
7 >s_a_s_b_s_b_s_d_s_d_s GTGTCTGTGGCTGTGGCTGTGACGGTGACGGTG; >clamp_s_a_s_d_s:compl:rev
CACCGTCACAGACACTATCATGTTGACATTCC; dG = -16.2
8 >s_d_s_a_s_b_s_d_s_d_s GTGACGGTGTCTGTGGCTGTGACGGTGACGGTG; >clamp_s_a_s_d_s:compl:rev
CACCGTCACAGACACTATCATGTTGACATTCC; dG = -16.2
9 >s_d_s_b_s_b_s_d_s_d_s GTGACGGTGGCTGTGGCTGTGACGGTGACGGTG; >clamp_s_a_s_d_s:compl:rev
CACCGTCACAGACACTATCATGTTGACATTCC; dG = -16.2
10 >s_a_s_b_s_d_s_d_s_d_s GTGTCTGTGGCTGTGACGGTGACGGTGACGGTG; >clamp_s_a_s_d_s:compl:rev
CACCGTCACAGACACTATCATGTTGACATTCC; dG = -16.2
11 >s_e_s_a_s_b_s_d_s_d_s GTGCTGGTGTCTGTGGCTGTGACGGTGACGGTG; >clamp_s_a_s_d_s:compl:rev
CACCGTCACAGACACTATCATGTTGACATTCC; dG = -16.2
12 >s_e_s_b_s_b_s_d_s_d_s GTGCTGGTGGCTGTGGCTGTGACGGTGACGGTG; >clamp_s_a_s_d_s:compl:rev
CACCGTCACAGACACTATCATGTTGACATTCC; dG = -16.2
13 >s_a_s_e_s_b_s_d_s_d_s GTGTCTGTGCTGGTGGCTGTGACGGTGACGGTG; >clamp_s_a_s_d_s:compl:rev
CACCGTCACAGACACTATCATGTTGACATTCC; dG = -16.1
14 >s_b_s_c_s_b_s_d_s_d_s GTGGCTGTGTGGGTGGCTGTGACGGTGACGGTG; >clamp_s_a_s_d_s:compl:rev
CACCGTCACAGACACTATCATGTTGACATTCC; dG = -16.1
15 >s_b_s_d_s_b_s_d_s_d_s GTGGCTGTGACGGTGGCTGTGACGGTGACGGTG; >clamp_s_a_s_d_s:compl:rev
CACCGTCACAGACACTATCATGTTGACATTCC; dG = -16.1
16 >s_b_s_e_s_b_s_d_s_d_s GTGGCTGTGCTGGTGGCTGTGACGGTGACGGTG; >clamp_s_a_s_d_s:compl:rev
CACCGTCACAGACACTATCATGTTGACATTCC; dG = -16.1
17 >s_c_s_b_s_d_s_d_s_d_s GTGTGGGTGGCTGTGACGGTGACGGTGACGGTG; >clamp_s_a_s_d_s:compl:rev
CACCGTCACAGACACTATCATGTTGACATTCC; dG = -16.1
18 >s_c_s_c_s_b_s_d_s_d_s GTGTGGGTGTGGGTGGCTGTGACGGTGACGGTG; >clamp_s_a_s_d_s:compl:rev
CACCGTCACAGACACTATCATGTTGACATTCC; dG = -16.1
19 >s_c_s_d_s_b_s_d_s_d_s GTGTGGGTGACGGTGGCTGTGACGGTGACGGTG; >clamp_s_a_s_d_s:compl:rev
CACCGTCACAGACACTATCATGTTGACATTCC; dG = -16.1
20 >s_c_s_e_s_b_s_d_s_d_s GTGTGGGTGCTGGTGGCTGTGACGGTGACGGTG; >clamp_s_a_s_d_s:compl:rev
CACCGTCACAGACACTATCATGTTGACATTCC; dG = -16.1
21 >s_d_s_b_s_d_s_d_s_d_s GTGACGGTGGCTGTGACGGTGACGGTGACGGTG; >clamp_s_a_s_d_s:compl:rev
CACCGTCACAGACACTATCATGTTGACATTCC; dG = -16.1
22 >s_d_s_c_s_b_s_d_s_d_s GTGACGGTGTGGGTGGCTGTGACGGTGACGGTG; >clamp_s_a_s_d_s:compl:rev
CACCGTCACAGACACTATCATGTTGACATTCC; dG = -16.1
23 >s_d_s_d_s_b_s_d_s_d_s GTGACGGTGACGGTGGCTGTGACGGTGACGGTG; >clamp_s_a_s_d_s:compl:rev
CACCGTCACAGACACTATCATGTTGACATTCC; dG = -16.1
24 >s_d_s_e_s_b_s_d_s_d_s GTGACGGTGCTGGTGGCTGTGACGGTGACGGTG; >clamp_s_a_s_d_s:compl:rev
CACCGTCACAGACACTATCATGTTGACATTCC; dG = -16.1
25 >s_e_s_b_s_d_s_d_s_d_s GTGCTGGTGGCTGTGACGGTGACGGTGACGGTG; >clamp_s_a_s_d_s:compl:rev
CACCGTCACAGACACTATCATGTTGACATTCC; dG = -16.1
26 >s_e_s_c_s_b_s_d_s_d_s GTGCTGGTGTGGGTGGCTGTGACGGTGACGGTG; >clamp_s_a_s_d_s:compl:rev
CACCGTCACAGACACTATCATGTTGACATTCC; dG = -16.1
27 >s_a_s_c_s_b_s_d_s_d_s GTGTCTGTGTGGGTGGCTGTGACGGTGACGGTG; >clamp_s_a_s_d_s:compl:rev
CACCGTCACAGACACTATCATGTTGACATTCC; dG = -16.1
28 >s_e_s_d_s_b_s_d_s_d_s GTGCTGGTGACGGTGGCTGTGACGGTGACGGTG; >clamp_s_a_s_d_s:compl:rev
CACCGTCACAGACACTATCATGTTGACATTCC; dG = -16.1
29 >s_e_s_e_s_b_s_d_s_d_s GTGCTGGTGCTGGTGGCTGTGACGGTGACGGTG; >clamp_s_a_s_d_s:compl:rev
CACCGTCACAGACACTATCATGTTGACATTCC; dG = -16.1
30 >s_b_s_b_s_d_s_b_s_d_s GTGGCTGTGGCTGTGACGGTGGCTGTGACGGTG; >clamp_s_a_s_d_s:compl:rev
CACCGTCACAGACACTATCATGTTGACATTCC; dG = -15.7
31 >s_a_s_b_s_d_s_b_s_d_s GTGTCTGTGGCTGTGACGGTGGCTGTGACGGTG; >clamp_s_a_s_d_s:compl:rev
CACCGTCACAGACACTATCATGTTGACATTCC; dG = -15.7
32 >s_b_s_b_s_d_s_a_s_e_s GTGGCTGTGGCTGTGACGGTGTCTGTGCTGGTG; >clamp_s_a_s_d_s:compl:rev
CACCGTCACAGACACTATCATGTTGACATTCC; dG = -15.6
33 >s_b_s_b_s_d_s_a_s_c_s GTGGCTGTGGCTGTGACGGTGTCTGTGTGGGTG; >clamp_s_a_s_d_s:compl:rev
CACCGTCACAGACACTATCATGTTGACATTCC; dG = -15.6
34 >s_b_s_b_s_d_s_a_s_b_s GTGGCTGTGGCTGTGACGGTGTCTGTGGCTGTG; >clamp_s_a_s_d_s:compl:rev
CACCGTCACAGACACTATCATGTTGACATTCC; dG = -15.6
35 >s_b_s_b_s_d_s_a_s_a_s GTGGCTGTGGCTGTGACGGTGTCTGTGTCTGTG; >clamp_s_a_s_d_s:compl:rev
CACCGTCACAGACACTATCATGTTGACATTCC; dG = -15.6
36 >s_b_s_b_s_d_s_c_s_d_s GTGGCTGTGGCTGTGACGGTGTGGGTGACGGTG; >clamp_s_a_s_d_s:compl:rev
CACCGTCACAGACACTATCATGTTGACATTCC; dG = -15.6
37 >s_b_s_d_s_d_s_a_s_e_s GTGGCTGTGACGGTGACGGTGTCTGTGCTGGTG; >clamp_s_a_s_d_s:compl:rev
CACCGTCACAGACACTATCATGTTGACATTCC; dG = -15.6
38 >s_b_s_d_s_d_s_a_s_c_s GTGGCTGTGACGGTGACGGTGTCTGTGTGGGTG; >clamp_s_a_s_d_s:compl:rev
CACCGTCACAGACACTATCATGTTGACATTCC; dG = -15.6
39 >s_b_s_d_s_d_s_a_s_b_s GTGGCTGTGACGGTGACGGTGTCTGTGGCTGTG; >clamp_s_a_s_d_s:compl:rev
CACCGTCACAGACACTATCATGTTGACATTCC; dG = -15.6
40 >s_b_s_d_s_d_s_a_s_a_s GTGGCTGTGACGGTGACGGTGTCTGTGTCTGTG; >clamp_s_a_s_d_s:compl:rev
CACCGTCACAGACACTATCATGTTGACATTCC; dG = -15.6
41 >s_b_s_d_s_d_s_e_s_d_s GTGGCTGTGACGGTGACGGTGCTGGTGACGGTG; >clamp_s_a_s_d_s:compl:rev
CACCGTCACAGACACTATCATGTTGACATTCC; dG = -15.6

42 >s_b_s_d_s_d_s_d_s GTGGCTGTGACGGTGACGGTGACGGTGACGGTG; >clamp_s_a_s_d_s:compl:rev CACCGTCACAGACACTATCATGTTGACATTCC; dG = -15.6

43 >s_b_s_d_s_d_s_c_s_d_s GTGGCTGTGACGGTGACGGTGTGGGTGACGGTG; >clamp_s_a_s_d_s:compl:rev CACCGTCACAGACACTATCATGTTGACATTCC; dG = -15.6

44 >s_b_s_d_s_d_s_b_s_d_s GTGGCTGTGACGGTGACGGTGGCTGTGACGGTG; >clamp_s_a_s_d_s:compl:rev CACCGTCACAGACACTATCATGTTGACATTCC; dG = -15.6

45 >s_c_s_b_s_d_s_b_s_d_s GTGTGGGTGGCTGTGACGGTGGCTGTGACGGTG; >clamp_s_a_s_d_s:compl:rev CACCGTCACAGACACTATCATGTTGACATTCC; dG = -15.6

46 >s_a_s_b_s_d_s_a_s_e_s GTGTCTGTGGCTGTGACGGTGTCTGTGCTGGTG; >clamp_s_a_s_d_s:compl:rev CACCGTCACAGACACTATCATGTTGACATTCC; dG = -15.6

47 >s_a_s_b_s_d_s_a_s_c_s GTGTCTGTGGCTGTGACGGTGTCTGTGTGGGTG; >clamp_s_a_s_d_s:compl:rev CACCGTCACAGACACTATCATGTTGACATTCC; dG = -15.6

48 >s_a_s_b_s_d_s_a_s_b_s GTGTCTGTGGCTGTGACGGTGTCTGTGGCTGTG; >clamp_s_a_s_d_s:compl:rev CACCGTCACAGACACTATCATGTTGACATTCC; dG = -15.6

49 >s_a_s_b_s_d_s_a_s_a_s GTGTCTGTGGCTGTGACGGTGTCTGTGTCTGTG; >clamp_s_a_s_d_s:compl:rev CACCGTCACAGACACTATCATGTTGACATTCC; dG = -15.6

50 >s_d_s_b_s_d_s_b_s_d_s GTGACGGTGGCTGTGACGGTGGCTGTGACGGTG; >clamp_s_a_s_d_s:compl:rev CACCGTCACAGACACTATCATGTTGACATTCC; dG = -15.6

51 >s_a_s_b_s_d_s_c_s_d_s GTGTCTGTGGCTGTGACGGTGTGGGTGACGGTG; >clamp_s_a_s_d_s:compl:rev CACCGTCACAGACACTATCATGTTGACATTCC; dG = -15.6

52 >s_e_s_b_s_d_s_b_s_d_s GTGCTGGTGGCTGTGACGGTGGCTGTGACGGTG; >clamp_s_a_s_d_s:compl:rev CACCGTCACAGACACTATCATGTTGACATTCC; dG = -15.6

53 >s_b_s_b_s_d_s_e_s_d_s GTGGCTGTGGCTGTGACGGTGCTGGTGACGGTG; >clamp_s_a_s_d_s:compl:rev CACCGTCACAGACACTATCATGTTGACATTCC; dG = -15.5

54 >s_c_s_b_s_d_s_a_s_e_s GTGTGGGTGGCTGTGACGGTGTCTGTGCTGGTG; >clamp_s_a_s_d_s:compl:rev CACCGTCACAGACACTATCATGTTGACATTCC; dG = -15.5

55 >s_c_s_b_s_d_s_a_s_c_s GTGTGGGTGGCTGTGACGGTGTCTGTGTGGGTG; >clamp_s_a_s_d_s:compl:rev CACCGTCACAGACACTATCATGTTGACATTCC; dG = -15.5

56 >s_c_s_b_s_d_s_a_s_b_s GTGTGGGTGGCTGTGACGGTGTCTGTGGCTGTG; >clamp_s_a_s_d_s:compl:rev CACCGTCACAGACACTATCATGTTGACATTCC; dG = -15.5

57 >s_c_s_b_s_d_s_a_s_a_s GTGTGGGTGGCTGTGACGGTGTCTGTGTCTGTG; >clamp_s_a_s_d_s:compl:rev CACCGTCACAGACACTATCATGTTGACATTCC; dG = -15.5

58 >s_c_s_b_s_d_s_c_s_d_s GTGTGGGTGGCTGTGACGGTGTGGGTGACGGTG; >clamp_s_a_s_d_s:compl:rev CACCGTCACAGACACTATCATGTTGACATTCC; dG = -15.5

59 >s_d_s_b_s_d_s_a_s_e_s GTGACGGTGGCTGTGACGGTGTCTGTGCTGGTG; >clamp_s_a_s_d_s:compl:rev CACCGTCACAGACACTATCATGTTGACATTCC; dG = -15.5

60 >s_d_s_b_s_d_s_a_s_c_s GTGACGGTGGCTGTGACGGTGTCTGTGTGGGTG; >clamp_s_a_s_d_s:compl:rev CACCGTCACAGACACTATCATGTTGACATTCC; dG = -15.5

61 >s_d_s_b_s_d_s_a_s_b_s GTGACGGTGGCTGTGACGGTGTCTGTGGCTGTG; >clamp_s_a_s_d_s:compl:rev CACCGTCACAGACACTATCATGTTGACATTCC; dG = -15.5

62 >s_d_s_b_s_d_s_a_s_a_s GTGACGGTGGCTGTGACGGTGTCTGTGTCTGTG; >clamp_s_a_s_d_s:compl:rev CACCGTCACAGACACTATCATGTTGACATTCC; dG = -15.5

63 >s_d_s_b_s_d_s_c_s_d_s GTGACGGTGGCTGTGACGGTGTGGGTGACGGTG; >clamp_s_a_s_d_s:compl:rev CACCGTCACAGACACTATCATGTTGACATTCC; dG = -15.5

64 >s_a_s_b_s_d_s_e_s_d_s GTGTCTGTGGCTGTGACGGTGCTGGTGACGGTG; >clamp_s_a_s_d_s:compl:rev CACCGTCACAGACACTATCATGTTGACATTCC; dG = -15.5

65 >s_e_s_b_s_d_s_a_s_e_s GTGCTGGTGGCTGTGACGGTGTCTGTGCTGGTG; >clamp_s_a_s_d_s:compl:rev CACCGTCACAGACACTATCATGTTGACATTCC; dG = -15.5

66 >s_e_s_b_s_d_s_a_s_c_s GTGCTGGTGGCTGTGACGGTGTCTGTGTGGGTG; >clamp_s_a_s_d_s:compl:rev CACCGTCACAGACACTATCATGTTGACATTCC; dG = -15.5

67 >s_e_s_b_s_d_s_a_s_b_s GTGCTGGTGGCTGTGACGGTGTCTGTGGCTGTG; >clamp_s_a_s_d_s:compl:rev CACCGTCACAGACACTATCATGTTGACATTCC; dG = -15.5

68 >s_e_s_b_s_d_s_a_s_a_s GTGCTGGTGGCTGTGACGGTGTCTGTGTCTGTG; >clamp_s_a_s_d_s:compl:rev CACCGTCACAGACACTATCATGTTGACATTCC; dG = -15.5

69 >s_e_s_b_s_d_s_c_s_d_s GTGCTGGTGGCTGTGACGGTGTGGGTGACGGTG; >clamp_s_a_s_d_s:compl:rev CACCGTCACAGACACTATCATGTTGACATTCC; dG = -15.5

70 >s_c_s_b_s_d_s_e_s_d_s GTGTGGGTGGCTGTGACGGTGCTGGTGACGGTG; >clamp_s_a_s_d_s:compl:rev CACCGTCACAGACACTATCATGTTGACATTCC; dG = -15.4

71 >s_d_s_b_s_d_s_e_s_d_s GTGACGGTGGCTGTGACGGTGCTGGTGACGGTG; >clamp_s_a_s_d_s:compl:rev CACCGTCACAGACACTATCATGTTGACATTCC; dG = -15.4

72 >s_e_s_b_s_d_s_e_s_d_s GTGCTGGTGGCTGTGACGGTGCTGGTGACGGTG; >clamp_s_a_s_d_s:compl:rev CACCGTCACAGACACTATCATGTTGACATTCC; dG = -15.4

73 >s_a_s_c_s_d_s_b_s_d_s GTGTCTGTGTGGGTGACGGTGGCTGTGACGGTG; >clamp_s_a_s_d_s:compl:rev CACCGTCACAGACACTATCATGTTGACATTCC; dG = -15.3

74 >s_a_s_c_s_e_s_b_s_d_s GTGTCTGTGTGGGTGCTGGTGGCTGTGACGGTG; >clamp_s_a_s_d_s:compl:rev CACCGTCACAGACACTATCATGTTGACATTCC; dG = -15.3

75 >s_a_s_e_s_a_s_b_s_d_s GTGTCTGTGCTGGTGTCTGTGGCTGTGACGGTG; >clamp_s_a_s_d_s:compl:rev CACCGTCACAGACACTATCATGTTGACATTCC; dG = -15.3

76 >s_a_s_e_s_b_s_b_s_d_s GTGTCTGTGCTGGTGGCTGTGGCTGTGACGGTG; >clamp_s_a_s_d_s:compl:rev CACCGTCACAGACACTATCATGTTGACATTCC; dG = -15.3

77 >s_a_s_e_s_c_s_b_s_d_s GTGTCTGTGCTGGTGTGGGTGGCTGTGACGGTG; >clamp_s_a_s_d_s:compl:rev CACCGTCACAGACACTATCATGTTGACATTCC; dG = -15.3

78 >s_a_s_e_s_d_s_b_s_d_s GTGTCTGTGCTGGTGACGGTGGCTGTGACGGTG; >clamp_s_a_s_d_s:compl:rev CACCGTCACAGACACTATCATGTTGACATTCC; dG = -15.3

79 >s_a_s_e_s_e_s_b_s_d_s GTGTCTGTGCTGGTGCTGGTGGCTGTGACGGTG; >clamp_s_a_s_d_s:compl:rev
CACCGTCACAGACACTATCATGTTGACATTCC; dG = -15.3

80 >s_a_s_a_s_b_s_b_s_d_s GTGTCTGTGTCTGTGGCTGTGGCTGTGACGGTG; >clamp_s_a_s_d_s:compl:rev
CACCGTCACAGACACTATCATGTTGACATTCC; dG = -15.3

81 >s_b_s_a_s_a_s_b_s_d_s GTGGCTGTGTCTGTGTCTGTGGCTGTGACGGTG; >clamp_s_a_s_d_s:compl:rev
CACCGTCACAGACACTATCATGTTGACATTCC; dG = -15.3

82 >s_b_s_a_s_b_s_b_s_d_s GTGGCTGTGTCTGTGGCTGTGGCTGTGACGGTG; >clamp_s_a_s_d_s:compl:rev
CACCGTCACAGACACTATCATGTTGACATTCC; dG = -15.3

83 >s_b_s_a_s_c_s_b_s_d_s GTGGCTGTGTCTGTGTGGGTGGCTGTGACGGTG; >clamp_s_a_s_d_s:compl:rev
CACCGTCACAGACACTATCATGTTGACATTCC; dG = -15.3

84 >s_b_s_a_s_e_s_b_s_d_s GTGGCTGTGTCTGTGCTGGTGGCTGTGACGGTG; >clamp_s_a_s_d_s:compl:rev
CACCGTCACAGACACTATCATGTTGACATTCC; dG = -15.3

85 >s_b_s_b_s_a_s_b_s_d_s GTGGCTGTGGCTGTGTCTGTGGCTGTGACGGTG; >clamp_s_a_s_d_s:compl:rev
CACCGTCACAGACACTATCATGTTGACATTCC; dG = -15.3

86 >s_b_s_b_s_b_s_b_s_d_s GTGGCTGTGGCTGTGGCTGTGGCTGTGACGGTG; >clamp_s_a_s_d_s:compl:rev
CACCGTCACAGACACTATCATGTTGACATTCC; dG = -15.3

87 >s_b_s_b_s_c_s_b_s_d_s GTGGCTGTGGCTGTGTGGGTGGCTGTGACGGTG; >clamp_s_a_s_d_s:compl:rev
CACCGTCACAGACACTATCATGTTGACATTCC; dG = -15.3

88 >s_b_s_b_s_e_s_b_s_d_s GTGGCTGTGGCTGTGCTGGTGGCTGTGACGGTG; >clamp_s_a_s_d_s:compl:rev
CACCGTCACAGACACTATCATGTTGACATTCC; dG = -15.3

89 >s_b_s_c_s_a_s_b_s_d_s GTGGCTGTGTGGGTGTCTGTGGCTGTGACGGTG; >clamp_s_a_s_d_s:compl:rev
CACCGTCACAGACACTATCATGTTGACATTCC; dG = -15.3

90 >s_a_s_a_s_c_s_b_s_d_s GTGTCTGTGTCTGTGTGGGTGGCTGTGACGGTG; >clamp_s_a_s_d_s:compl:rev
CACCGTCACAGACACTATCATGTTGACATTCC; dG = -15.3

91 >s_b_s_c_s_b_s_b_s_d_s GTGGCTGTGTGGGTGGCTGTGGCTGTGACGGTG; >clamp_s_a_s_d_s:compl:rev
CACCGTCACAGACACTATCATGTTGACATTCC; dG = -15.3

92 >s_b_s_c_s_c_s_b_s_d_s GTGGCTGTGTGGGTGTGGGTGGCTGTGACGGTG; >clamp_s_a_s_d_s:compl:rev
CACCGTCACAGACACTATCATGTTGACATTCC; dG = -15.3

93 >s_b_s_c_s_d_s_b_s_d_s GTGGCTGTGTGGGTGACGGTGGCTGTGACGGTG; >clamp_s_a_s_d_s:compl:rev
CACCGTCACAGACACTATCATGTTGACATTCC; dG = -15.3

94 >s_b_s_c_s_e_s_b_s_d_s GTGGCTGTGTGGGTGCTGGTGGCTGTGACGGTG; >clamp_s_a_s_d_s:compl:rev
CACCGTCACAGACACTATCATGTTGACATTCC; dG = -15.3

95 >s_b_s_d_s_a_s_b_s_d_s GTGGCTGTGACGGTGTCTGTGGCTGTGACGGTG; >clamp_s_a_s_d_s:compl:rev
CACCGTCACAGACACTATCATGTTGACATTCC; dG = -15.3

96 >s_b_s_d_s_b_s_b_s_d_s GTGGCTGTGACGGTGGCTGTGGCTGTGACGGTG; >clamp_s_a_s_d_s:compl:rev
CACCGTCACAGACACTATCATGTTGACATTCC; dG = -15.3

97 >s_b_s_d_s_c_s_b_s_d_s GTGGCTGTGACGGTGTGGGTGGCTGTGACGGTG; >clamp_s_a_s_d_s:compl:rev
CACCGTCACAGACACTATCATGTTGACATTCC; dG = -15.3

98 >s_b_s_d_s_e_s_b_s_d_s GTGGCTGTGACGGTGCTGGTGGCTGTGACGGTG; >clamp_s_a_s_d_s:compl:rev
CACCGTCACAGACACTATCATGTTGACATTCC; dG = -15.3

99 >s_b_s_e_s_a_s_b_s_d_s GTGGCTGTGCTGGTGTCTGTGGCTGTGACGGTG; >clamp_s_a_s_d_s:compl:rev
CACCGTCACAGACACTATCATGTTGACATTCC; dG = -15.3

100 >s_a_s_a_s_e_s_b_s_d_s GTGTCTGTGTCTGTGCTGGTGGCTGTGACGGTG; >clamp_s_a_s_d_s:compl:rev
CACCGTCACAGACACTATCATGTTGACATTCC; dG = -15.3

101 >s_b_s_e_s_b_s_b_s_d_s GTGGCTGTGCTGGTGGCTGTGGCTGTGACGGTG; >clamp_s_a_s_d_s:compl:rev
CACCGTCACAGACACTATCATGTTGACATTCC; dG = -15.3

102 >s_b_s_e_s_c_s_b_s_d_s GTGGCTGTGCTGGTGTGGGTGGCTGTGACGGTG; >clamp_s_a_s_d_s:compl:rev
CACCGTCACAGACACTATCATGTTGACATTCC; dG = -15.3

103 >s_b_s_e_s_d_s_b_s_d_s GTGGCTGTGCTGGTGACGGTGGCTGTGACGGTG; >clamp_s_a_s_d_s:compl:rev
CACCGTCACAGACACTATCATGTTGACATTCC; dG = -15.3

104 >s_b_s_e_s_e_s_b_s_d_s GTGGCTGTGCTGGTGCTGGTGGCTGTGACGGTG; >clamp_s_a_s_d_s:compl:rev
CACCGTCACAGACACTATCATGTTGACATTCC; dG = -15.3

105 >s_c_s_a_s_a_s_b_s_d_s GTGTGGGTGTCTGTGTCTGTGGCTGTGACGGTG; >clamp_s_a_s_d_s:compl:rev
CACCGTCACAGACACTATCATGTTGACATTCC; dG = -15.3

106 >s_c_s_a_s_b_s_b_s_d_s GTGTGGGTGTCTGTGGCTGTGGCTGTGACGGTG; >clamp_s_a_s_d_s:compl:rev
CACCGTCACAGACACTATCATGTTGACATTCC; dG = -15.3

107 >s_c_s_a_s_c_s_b_s_d_s GTGTGGGTGTCTGTGTGGGTGGCTGTGACGGTG; >clamp_s_a_s_d_s:compl:rev
CACCGTCACAGACACTATCATGTTGACATTCC; dG = -15.3

108 >s_c_s_a_s_e_s_b_s_d_s GTGTGGGTGTCTGTGCTGGTGGCTGTGACGGTG; >clamp_s_a_s_d_s:compl:rev
CACCGTCACAGACACTATCATGTTGACATTCC; dG = -15.3

109 >s_c_s_b_s_a_s_b_s_d_s GTGTGGGTGGCTGTGTCTGTGGCTGTGACGGTG; >clamp_s_a_s_d_s:compl:rev
CACCGTCACAGACACTATCATGTTGACATTCC; dG = -15.3

110 >s_a_s_b_s_a_s_b_s_d_s GTGTCTGTGGCTGTGTCTGTGGCTGTGACGGTG; >clamp_s_a_s_d_s:compl:rev
CACCGTCACAGACACTATCATGTTGACATTCC; dG = -15.3

111 >s_c_s_b_s_b_s_b_s_d_s GTGTGGGTGGCTGTGGCTGTGGCTGTGACGGTG; >clamp_s_a_s_d_s:compl:rev
CACCGTCACAGACACTATCATGTTGACATTCC; dG = -15.3

112 >s_c_s_b_s_c_s_b_s_d_s GTGTGGGTGGCTGTGTGGGTGGCTGTGACGGTG; >clamp_s_a_s_d_s:compl:rev
CACCGTCACAGACACTATCATGTTGACATTCC; dG = -15.3

113 >s_c_s_b_s_e_s_b_s_d_s GTGTGGGTGGCTGTGCTGGTGGCTGTGACGGTG; >clamp_s_a_s_d_s:compl:rev
CACCGTCACAGACACTATCATGTTGACATTCC; dG = -15.3

114 >s_c_s_c_s_a_s_b_s_d_s GTGTGGGTGTGGGTGTCTGTGGCTGTGACGGTG; >clamp_s_a_s_d_s:compl:rev
CACCGTCACAGACACTATCATGTTGACATTCC; dG = -15.3

115 >s_c_s_c_s_b_s_b_s_d_s GTGTGGGTGTGGGTGGCTGTGGCTGTGACGGTG; >clamp_s_a_s_d_s:compl:rev
CACCGTCACAGACACTATCATGTTGACATTCC; dG = -15.3

116 >s_c_s_c_s_c_s_b_s_d_s GTGTGGGTGTGGGTGTGGGTGGCTGTGACGGTG; >clamp_s_a_s_d_s:compl:rev
CACCGTCACAGACACTATCATGTTGACATTCC; dG = -15.3
117 >s_c_s_c_s_d_s_b_s_d_s GTGTGGGTGTGGGTGACGGTGGCTGTGACGGTG; >clamp_s_a_s_d_s:compl:rev
CACCGTCACAGACACTATCATGTTGACATTCC; dG = -15.3
118 >s_c_s_c_s_e_s_b_s_d_s GTGTGGGTGTGGGTGCTGGTGGCTGTGACGGTG; >clamp_s_a_s_d_s:compl:rev
CACCGTCACAGACACTATCATGTTGACATTCC; dG = -15.3
119 >s_c_s_d_s_a_s_b_s_d_s GTGTGGGTGACGGTGTCTGTGGCTGTGACGGTG; >clamp_s_a_s_d_s:compl:rev
CACCGTCACAGACACTATCATGTTGACATTCC; dG = -15.3
120 >s_c_s_d_s_b_s_b_s_d_s GTGTGGGTGACGGTGGCTGTGGCTGTGACGGTG; >clamp_s_a_s_d_s:compl:rev
CACCGTCACAGACACTATCATGTTGACATTCC; dG = -15.3
121 >s_a_s_b_s_b_s_b_s_d_s GTGTCTGTGGCTGTGGCTGTGGCTGTGACGGTG; >clamp_s_a_s_d_s:compl:rev
CACCGTCACAGACACTATCATGTTGACATTCC; dG = -15.3
122 >s_c_s_d_s_c_s_b_s_d_s GTGTGGGTGACGGTGTGGGTGGCTGTGACGGTG; >clamp_s_a_s_d_s:compl:rev
CACCGTCACAGACACTATCATGTTGACATTCC; dG = -15.3
123 >s_c_s_d_s_d_s_b_s_d_s GTGTGGGTGACGGTGACGGTGGCTGTGACGGTG; >clamp_s_a_s_d_s:compl:rev
CACCGTCACAGACACTATCATGTTGACATTCC; dG = -15.3
124 >s_c_s_d_s_e_s_b_s_d_s GTGTGGGTGACGGTGCTGGTGGCTGTGACGGTG; >clamp_s_a_s_d_s:compl:rev
CACCGTCACAGACACTATCATGTTGACATTCC; dG = -15.3
125 >s_c_s_e_s_a_s_b_s_d_s GTGTGGGTGCTGGTGTCTGTGGCTGTGACGGTG; >clamp_s_a_s_d_s:compl:rev
CACCGTCACAGACACTATCATGTTGACATTCC; dG = -15.3
126 >s_c_s_e_s_b_s_b_s_d_s GTGTGGGTGCTGGTGGCTGTGGCTGTGACGGTG; >clamp_s_a_s_d_s:compl:rev
CACCGTCACAGACACTATCATGTTGACATTCC; dG = -15.3
127 >s_c_s_e_s_c_s_b_s_d_s GTGTGGGTGCTGGTGTGGGTGGCTGTGACGGTG; >clamp_s_a_s_d_s:compl:rev
CACCGTCACAGACACTATCATGTTGACATTCC; dG = -15.3
128 >s_c_s_e_s_d_s_b_s_d_s GTGTGGGTGCTGGTGACGGTGGCTGTGACGGTG; >clamp_s_a_s_d_s:compl:rev
CACCGTCACAGACACTATCATGTTGACATTCC; dG = -15.3
129 >s_c_s_e_s_e_s_b_s_d_s GTGTGGGTGCTGGTGCTGGTGGCTGTGACGGTG; >clamp_s_a_s_d_s:compl:rev
CACCGTCACAGACACTATCATGTTGACATTCC; dG = -15.3
130 >s_d_s_a_s_a_s_b_s_d_s GTGACGGTGTCTGTGTCTGTGGCTGTGACGGTG; >clamp_s_a_s_d_s:compl:rev
CACCGTCACAGACACTATCATGTTGACATTCC; dG = -15.3
131 >s_d_s_a_s_b_s_b_s_d_s GTGACGGTGTCTGTGGCTGTGGCTGTGACGGTG; >clamp_s_a_s_d_s:compl:rev
CACCGTCACAGACACTATCATGTTGACATTCC; dG = -15.3
132 >s_a_s_b_s_c_s_b_s_d_s GTGTCTGTGGCTGTGTGGGTGGCTGTGACGGTG; >clamp_s_a_s_d_s:compl:rev
CACCGTCACAGACACTATCATGTTGACATTCC; dG = -15.3
133 >s_d_s_a_s_c_s_b_s_d_s GTGACGGTGTCTGTGTGGGTGGCTGTGACGGTG; >clamp_s_a_s_d_s:compl:rev
CACCGTCACAGACACTATCATGTTGACATTCC; dG = -15.3
134 >s_d_s_a_s_e_s_b_s_d_s GTGACGGTGTCTGTGCTGGTGGCTGTGACGGTG; >clamp_s_a_s_d_s:compl:rev
CACCGTCACAGACACTATCATGTTGACATTCC; dG = -15.3
135 >s_d_s_b_s_a_s_b_s_d_s GTGACGGTGGCTGTGTCTGTGGCTGTGACGGTG; >clamp_s_a_s_d_s:compl:rev
CACCGTCACAGACACTATCATGTTGACATTCC; dG = -15.3
136 >s_d_s_b_s_b_s_b_s_d_s GTGACGGTGGCTGTGGCTGTGGCTGTGACGGTG; >clamp_s_a_s_d_s:compl:rev
CACCGTCACAGACACTATCATGTTGACATTCC; dG = -15.3
137 >s_d_s_b_s_c_s_b_s_d_s GTGACGGTGGCTGTGTGGGTGGCTGTGACGGTG; >clamp_s_a_s_d_s:compl:rev
CACCGTCACAGACACTATCATGTTGACATTCC; dG = -15.3
138 >s_d_s_b_s_e_s_b_s_d_s GTGACGGTGGCTGTGCTGGTGGCTGTGACGGTG; >clamp_s_a_s_d_s:compl:rev
CACCGTCACAGACACTATCATGTTGACATTCC; dG = -15.3
139 >s_d_s_c_s_a_s_b_s_d_s GTGACGGTGTGGGTGTCTGTGGCTGTGACGGTG; >clamp_s_a_s_d_s:compl:rev
CACCGTCACAGACACTATCATGTTGACATTCC; dG = -15.3
140 >s_d_s_c_s_b_s_b_s_d_s GTGACGGTGTGGGTGGCTGTGGCTGTGACGGTG; >clamp_s_a_s_d_s:compl:rev
CACCGTCACAGACACTATCATGTTGACATTCC; dG = -15.3
141 >s_d_s_c_s_c_s_b_s_d_s GTGACGGTGTGGGTGTGGGTGGCTGTGACGGTG; >clamp_s_a_s_d_s:compl:rev
CACCGTCACAGACACTATCATGTTGACATTCC; dG = -15.3
142 >s_d_s_c_s_d_s_b_s_d_s GTGACGGTGTGGGTGACGGTGGCTGTGACGGTG; >clamp_s_a_s_d_s:compl:rev
CACCGTCACAGACACTATCATGTTGACATTCC; dG = -15.3
143 >s_d_s_c_s_e_s_b_s_d_s GTGACGGTGTGGGTGCTGGTGGCTGTGACGGTG; >clamp_s_a_s_d_s:compl:rev
CACCGTCACAGACACTATCATGTTGACATTCC; dG = -15.3
144 >s_d_s_d_s_a_s_b_s_d_s GTGACGGTGACGGTGTCTGTGGCTGTGACGGTG; >clamp_s_a_s_d_s:compl:rev
CACCGTCACAGACACTATCATGTTGACATTCC; dG = -15.3
145 >s_d_s_d_s_b_s_b_s_d_s GTGACGGTGACGGTGGCTGTGGCTGTGACGGTG; >clamp_s_a_s_d_s:compl:rev
CACCGTCACAGACACTATCATGTTGACATTCC; dG = -15.3
146 >s_d_s_d_s_c_s_b_s_d_s GTGACGGTGACGGTGTGGGTGGCTGTGACGGTG; >clamp_s_a_s_d_s:compl:rev
CACCGTCACAGACACTATCATGTTGACATTCC; dG = -15.3
147 >s_d_s_d_s_d_s_b_s_d_s GTGACGGTGACGGTGACGGTGGCTGTGACGGTG; >clamp_s_a_s_d_s:compl:rev
CACCGTCACAGACACTATCATGTTGACATTCC; dG = -15.3
148 >s_d_s_d_s_e_s_b_s_d_s GTGACGGTGACGGTGCTGGTGGCTGTGACGGTG; >clamp_s_a_s_d_s:compl:rev
CACCGTCACAGACACTATCATGTTGACATTCC; dG = -15.3
149 >s_d_s_e_s_a_s_b_s_d_s GTGACGGTGCTGGTGTCTGTGGCTGTGACGGTG; >clamp_s_a_s_d_s:compl:rev
CACCGTCACAGACACTATCATGTTGACATTCC; dG = -15.3
150 >s_d_s_e_s_b_s_b_s_d_s GTGACGGTGCTGGTGGCTGTGGCTGTGACGGTG; >clamp_s_a_s_d_s:compl:rev
CACCGTCACAGACACTATCATGTTGACATTCC; dG = -15.3
151 >s_d_s_e_s_c_s_b_s_d_s GTGACGGTGCTGGTGTGGGTGGCTGTGACGGTG; >clamp_s_a_s_d_s:compl:rev
CACCGTCACAGACACTATCATGTTGACATTCC; dG = -15.3
152 >s_a_s_a_s_a_s_b_s_d_s GTGTCTGTGTCTGTGTCTGTGGCTGTGACGGTG; >clamp_s_a_s_d_s:compl:rev
CACCGTCACAGACACTATCATGTTGACATTCC; dG = -15.3

153 >s_a_s_b_s_e_s_b_s_d_s GTGTCTGTGGCTGTGCTGGTGGCTGTGACGGTG; >clamp_s_a_s_d_s:compl:rev CACCGTCACAGACACTATCATGTTGACATTCC; dG = -15.3
154 >s_d_s_e_s_d_s_b_s_d_s GTGACGGTGCTGGTGACGGTGGCTGTGACGGTG; >clamp_s_a_s_d_s:compl:rev CACCGTCACAGACACTATCATGTTGACATTCC; dG = -15.3
155 >s_d_s_e_s_e_s_b_s_d_s GTGACGGTGCTGGTGCTGGTGGCTGTGACGGTG; >clamp_s_a_s_d_s:compl:rev CACCGTCACAGACACTATCATGTTGACATTCC; dG = -15.3
156 >s_e_s_a_s_a_s_b_s_d_s GTGCTGGTGTCTGTGTCTGTGGCTGTGACGGTG; >clamp_s_a_s_d_s:compl:rev CACCGTCACAGACACTATCATGTTGACATTCC; dG = -15.3
157 >s_e_s_a_s_b_s_b_s_d_s GTGCTGGTGTCTGTGGCTGTGGCTGTGACGGTG; >clamp_s_a_s_d_s:compl:rev CACCGTCACAGACACTATCATGTTGACATTCC; dG = -15.3
158 >s_e_s_a_s_c_s_b_s_d_s GTGCTGGTGTCTGTGTGGGTGGCTGTGACGGTG; >clamp_s_a_s_d_s:compl:rev CACCGTCACAGACACTATCATGTTGACATTCC; dG = -15.3
159 >s_e_s_a_s_e_s_b_s_d_s GTGCTGGTGTCTGTGCTGGTGGCTGTGACGGTG; >clamp_s_a_s_d_s:compl:rev CACCGTCACAGACACTATCATGTTGACATTCC; dG = -15.3
160 >s_e_s_b_s_a_s_b_s_d_s GTGCTGGTGGCTGTGTCTGTGGCTGTGACGGTG; >clamp_s_a_s_d_s:compl:rev CACCGTCACAGACACTATCATGTTGACATTCC; dG = -15.3
161 >s_e_s_b_s_b_s_b_s_d_s GTGCTGGTGGCTGTGGCTGTGGCTGTGACGGTG; >clamp_s_a_s_d_s:compl:rev CACCGTCACAGACACTATCATGTTGACATTCC; dG = -15.3
162 >s_e_s_b_s_c_s_b_s_d_s GTGCTGGTGGCTGTGTGGGTGGCTGTGACGGTG; >clamp_s_a_s_d_s:compl:rev CACCGTCACAGACACTATCATGTTGACATTCC; dG = -15.3
163 >s_a_s_c_s_a_s_b_s_d_s GTGTCTGTGTGGGTGTCTGTGGCTGTGACGGTG; >clamp_s_a_s_d_s:compl:rev CACCGTCACAGACACTATCATGTTGACATTCC; dG = -15.3
164 >s_e_s_b_s_e_s_b_s_d_s GTGCTGGTGGCTGTGCTGGTGGCTGTGACGGTG; >clamp_s_a_s_d_s:compl:rev CACCGTCACAGACACTATCATGTTGACATTCC; dG = -15.3
165 >s_e_s_c_s_a_s_b_s_d_s GTGCTGGTGTGGGTGTCTGTGGCTGTGACGGTG; >clamp_s_a_s_d_s:compl:rev CACCGTCACAGACACTATCATGTTGACATTCC; dG = -15.3
166 >s_e_s_c_s_b_s_b_s_d_s GTGCTGGTGTGGGTGGCTGTGGCTGTGACGGTG; >clamp_s_a_s_d_s:compl:rev CACCGTCACAGACACTATCATGTTGACATTCC; dG = -15.3
167 >s_e_s_c_s_c_s_b_s_d_s GTGCTGGTGTGGGTGTGGGTGGCTGTGACGGTG; >clamp_s_a_s_d_s:compl:rev CACCGTCACAGACACTATCATGTTGACATTCC; dG = -15.3
168 >s_e_s_c_s_d_s_b_s_d_s GTGCTGGTGTGGGTGACGGTGGCTGTGACGGTG; >clamp_s_a_s_d_s:compl:rev CACCGTCACAGACACTATCATGTTGACATTCC; dG = -15.3
169 >s_e_s_c_s_e_s_b_s_d_s GTGCTGGTGTGGGTGCTGGTGGCTGTGACGGTG; >clamp_s_a_s_d_s:compl:rev CACCGTCACAGACACTATCATGTTGACATTCC; dG = -15.3
170 >s_e_s_d_s_a_s_b_s_d_s GTGCTGGTGACGGTGTCTGTGGCTGTGACGGTG; >clamp_s_a_s_d_s:compl:rev CACCGTCACAGACACTATCATGTTGACATTCC; dG = -15.3
171 >s_e_s_d_s_b_s_b_s_d_s GTGCTGGTGACGGTGGCTGTGGCTGTGACGGTG; >clamp_s_a_s_d_s:compl:rev CACCGTCACAGACACTATCATGTTGACATTCC; dG = -15.3
172 >s_e_s_d_s_c_s_b_s_d_s GTGCTGGTGACGGTGTGGGTGGCTGTGACGGTG; >clamp_s_a_s_d_s:compl:rev CACCGTCACAGACACTATCATGTTGACATTCC; dG = -15.3
173 >s_e_s_d_s_d_s_b_s_d_s GTGCTGGTGACGGTGACGGTGGCTGTGACGGTG; >clamp_s_a_s_d_s:compl:rev CACCGTCACAGACACTATCATGTTGACATTCC; dG = -15.3
174 >s_a_s_c_s_b_s_b_s_d_s GTGTCTGTGTGGGTGGCTGTGGCTGTGACGGTG; >clamp_s_a_s_d_s:compl:rev CACCGTCACAGACACTATCATGTTGACATTCC; dG = -15.3
175 >s_e_s_d_s_e_s_b_s_d_s GTGCTGGTGACGGTGCTGGTGGCTGTGACGGTG; >clamp_s_a_s_d_s:compl:rev CACCGTCACAGACACTATCATGTTGACATTCC; dG = -15.3
176 >s_e_s_e_s_a_s_b_s_d_s GTGCTGGTGCTGGTGTCTGTGGCTGTGACGGTG; >clamp_s_a_s_d_s:compl:rev CACCGTCACAGACACTATCATGTTGACATTCC; dG = -15.3
177 >s_e_s_e_s_b_s_b_s_d_s GTGCTGGTGCTGGTGGCTGTGGCTGTGACGGTG; >clamp_s_a_s_d_s:compl:rev CACCGTCACAGACACTATCATGTTGACATTCC; dG = -15.3
178 >s_e_s_e_s_c_s_b_s_d_s GTGCTGGTGCTGGTGTGGGTGGCTGTGACGGTG; >clamp_s_a_s_d_s:compl:rev CACCGTCACAGACACTATCATGTTGACATTCC; dG = -15.3
179 >s_e_s_e_s_d_s_b_s_d_s GTGCTGGTGCTGGTGACGGTGGCTGTGACGGTG; >clamp_s_a_s_d_s:compl:rev CACCGTCACAGACACTATCATGTTGACATTCC; dG = -15.3
180 >s_e_s_e_s_e_s_b_s_d_s GTGCTGGTGCTGGTGCTGGTGGCTGTGACGGTG; >clamp_s_a_s_d_s:compl:rev CACCGTCACAGACACTATCATGTTGACATTCC; dG = -15.3
181 >s_a_s_c_s_c_s_b_s_d_s GTGTCTGTGTGGGTGTGGGTGGCTGTGACGGTG; >clamp_s_a_s_d_s:compl:rev CACCGTCACAGACACTATCATGTTGACATTCC; dG = -15.3
182 >s_b_s_d_s_b_s_a_s_e_s GTGGCTGTGACGGTGGCTGTGTCTGTGCTGGTG; >clamp_s_a_s_d_s:compl:rev CACCGTCACAGACACTATCATGTTGACATTCC; dG = -15.2
183 >s_b_s_d_s_b_s_a_s_c_s GTGGCTGTGACGGTGGCTGTGTCTGTGTGGGTG; >clamp_s_a_s_d_s:compl:rev CACCGTCACAGACACTATCATGTTGACATTCC; dG = -15.2
184 >s_b_s_d_s_b_s_a_s_b_s GTGGCTGTGACGGTGGCTGTGTCTGTGGCTGTG; >clamp_s_a_s_d_s:compl:rev CACCGTCACAGACACTATCATGTTGACATTCC; dG = -15.2
185 >s_b_s_d_s_b_s_a_s_a_s GTGGCTGTGACGGTGGCTGTGTCTGTGTCTGTG; >clamp_s_a_s_d_s:compl:rev CACCGTCACAGACACTATCATGTTGACATTCC; dG = -15.2
186 >s_b_s_d_s_b_s_e_s_d_s GTGGCTGTGACGGTGGCTGTGCTGGTGACGGTG; >clamp_s_a_s_d_s:compl:rev CACCGTCACAGACACTATCATGTTGACATTCC; dG = -15.2
187 >s_b_s_d_s_b_s_c_s_d_s GTGGCTGTGACGGTGGCTGTGTGGGTGACGGTG; >clamp_s_a_s_d_s:compl:rev CACCGTCACAGACACTATCATGTTGACATTCC; dG = -15.2
188 >s_b_s_d_s_a_s_a_s_e_s GTGGCTGTGACGGTGTCTGTGTCTGTGCTGGTG; >clamp_s_a_s_d_s:compl:rev CACCGTCACAGACACTATCATGTTGACATTCC; dG = -15.1
189 >s_b_s_d_s_a_s_a_s_c_s GTGGCTGTGACGGTGTCTGTGTCTGTGTGGGTG; >clamp_s_a_s_d_s:compl:rev CACCGTCACAGACACTATCATGTTGACATTCC; dG = -15.1

190 >s_b_s_d_s_a_s_a_s_b_s GTGGCTGTGACGGTGTCTGTGTCTGTGGCTGTG; >clamp_s_a_s_d_s:compl:rev
CACCGTCACAGACACTATCATGTTGACATTCC; dG = -15.1
191 >s_b_s_d_s_a_s_a_s_a_s GTGGCTGTGACGGTGTCTGTGTCTGTGTCTGTG; >clamp_s_a_s_d_s:compl:rev
CACCGTCACAGACACTATCATGTTGACATTCC; dG = -15.1
192 >s_b_s_d_s_a_s_e_s_d_s GTGGCTGTGACGGTGTCTGTGCTGGTGACGGTG; >clamp_s_a_s_d_s:compl:rev
CACCGTCACAGACACTATCATGTTGACATTCC; dG = -15.1
193 >s_b_s_d_s_a_s_c_s_d_s GTGGCTGTGACGGTGTCTGTGTGGGTGACGGTG; >clamp_s_a_s_d_s:compl:rev
CACCGTCACAGACACTATCATGTTGACATTCC; dG = -15.1
194 >s_b_s_d_s_c_s_a_s_e_s GTGGCTGTGACGGTGTGGGTGTCTGTGCTGGTG; >clamp_s_a_s_d_s:compl:rev
CACCGTCACAGACACTATCATGTTGACATTCC; dG = -15.1
195 >s_b_s_d_s_c_s_a_s_c_s GTGGCTGTGACGGTGTGGGTGTCTGTGTGGGTG; >clamp_s_a_s_d_s:compl:rev
CACCGTCACAGACACTATCATGTTGACATTCC; dG = -15.1
196 >s_b_s_d_s_c_s_a_s_b_s GTGGCTGTGACGGTGTGGGTGTCTGTGGCTGTG; >clamp_s_a_s_d_s:compl:rev
CACCGTCACAGACACTATCATGTTGACATTCC; dG = -15.1
197 >s_b_s_d_s_c_s_a_s_a_s GTGGCTGTGACGGTGTGGGTGTCTGTGTCTGTG; >clamp_s_a_s_d_s:compl:rev
CACCGTCACAGACACTATCATGTTGACATTCC; dG = -15.1
198 >s_b_s_d_s_c_s_e_s_d_s GTGGCTGTGACGGTGTGGGTGCTGGTGACGGTG; >clamp_s_a_s_d_s:compl:rev
CACCGTCACAGACACTATCATGTTGACATTCC; dG = -15.1
199 >s_b_s_d_s_c_s_d_s_d_s GTGGCTGTGACGGTGTGGGTGACGGTGACGGTG; >clamp_s_a_s_d_s:compl:rev
CACCGTCACAGACACTATCATGTTGACATTCC; dG = -15.1
200 >s_b_s_d_s_c_s_c_s_d_s GTGGCTGTGACGGTGTGGGTGTGGGTGACGGTG; >clamp_s_a_s_d_s:compl:rev
CACCGTCACAGACACTATCATGTTGACATTCC; dG = -15.1
201 >s_b_s_d_s_e_s_a_s_e_s GTGGCTGTGACGGTGCTGGTGTCTGTGCTGGTG; >clamp_s_a_s_d_s:compl:rev
CACCGTCACAGACACTATCATGTTGACATTCC; dG = -14.9
202 >s_b_s_d_s_e_s_a_s_c_s GTGGCTGTGACGGTGCTGGTGTCTGTGTGGGTG; >clamp_s_a_s_d_s:compl:rev
CACCGTCACAGACACTATCATGTTGACATTCC; dG = -14.9
203 >s_b_s_d_s_e_s_a_s_b_s GTGGCTGTGACGGTGCTGGTGTCTGTGGCTGTG; >clamp_s_a_s_d_s:compl:rev
CACCGTCACAGACACTATCATGTTGACATTCC; dG = -14.9
204 >s_b_s_d_s_e_s_a_s_a_s GTGGCTGTGACGGTGCTGGTGTCTGTGTCTGTG; >clamp_s_a_s_d_s:compl:rev
CACCGTCACAGACACTATCATGTTGACATTCC; dG = -14.9
205 >s_b_s_d_s_e_s_e_s_d_s GTGGCTGTGACGGTGCTGGTGCTGGTGACGGTG; >clamp_s_a_s_d_s:compl:rev
CACCGTCACAGACACTATCATGTTGACATTCC; dG = -14.9
206 >s_b_s_d_s_e_s_d_s_d_s GTGGCTGTGACGGTGCTGGTGACGGTGACGGTG; >clamp_s_a_s_d_s:compl:rev
CACCGTCACAGACACTATCATGTTGACATTCC; dG = -14.9
207 >s_b_s_d_s_e_s_c_s_d_s GTGGCTGTGACGGTGCTGGTGTGGGTGACGGTG; >clamp_s_a_s_d_s:compl:rev
CACCGTCACAGACACTATCATGTTGACATTCC; dG = -14.9
208 >s_b_s_a_s_c_s_d_s_d_s GTGGCTGTGTCTGTGTGGGTGACGGTGACGGTG; >clamp_s_a_s_d_s:compl:rev
CACCGTCACAGACACTATCATGTTGACATTCC; dG = -12.8
209 >s_b_s_a_s_e_s_d_s_d_s GTGGCTGTGTCTGTGCTGGTGACGGTGACGGTG; >clamp_s_a_s_d_s:compl:rev
CACCGTCACAGACACTATCATGTTGACATTCC; dG = -12.8
210 >s_a_s_a_s_c_s_d_s_d_s GTGTCTGTGTCTGTGTGGGTGACGGTGACGGTG; >clamp_s_a_s_d_s:compl:rev
CACCGTCACAGACACTATCATGTTGACATTCC; dG = -12.8
211 >s_a_s_a_s_e_s_d_s_d_s GTGTCTGTGTCTGTGCTGGTGACGGTGACGGTG; >clamp_s_a_s_d_s:compl:rev
CACCGTCACAGACACTATCATGTTGACATTCC; dG = -12.8
212 >s_c_s_a_s_c_s_d_s_d_s GTGTGGGTGTCTGTGTGGGTGACGGTGACGGTG; >clamp_s_a_s_d_s:compl:rev
CACCGTCACAGACACTATCATGTTGACATTCC; dG = -12.8
213 >s_c_s_a_s_e_s_d_s_d_s GTGTGGGTGTCTGTGCTGGTGACGGTGACGGTG; >clamp_s_a_s_d_s:compl:rev
CACCGTCACAGACACTATCATGTTGACATTCC; dG = -12.8
214 >s_d_s_a_s_c_s_d_s_d_s GTGACGGTGTCTGTGTGGGTGACGGTGACGGTG; >clamp_s_a_s_d_s:compl:rev
CACCGTCACAGACACTATCATGTTGACATTCC; dG = -12.8
215 >s_d_s_a_s_e_s_d_s_d_s GTGACGGTGTCTGTGCTGGTGACGGTGACGGTG; >clamp_s_a_s_d_s:compl:rev
CACCGTCACAGACACTATCATGTTGACATTCC; dG = -12.8
216 >s_e_s_a_s_c_s_d_s_d_s GTGCTGGTGTCTGTGTGGGTGACGGTGACGGTG; >clamp_s_a_s_d_s:compl:rev
CACCGTCACAGACACTATCATGTTGACATTCC; dG = -12.8
217 >s_e_s_a_s_e_s_d_s_d_s GTGCTGGTGTCTGTGCTGGTGACGGTGACGGTG; >clamp_s_a_s_d_s:compl:rev
CACCGTCACAGACACTATCATGTTGACATTCC; dG = -12.8
218 >s_b_s_b_s_c_s_d_s_d_s GTGGCTGTGGCTGTGTGGGTGACGGTGACGGTG; >clamp_s_a_s_d_s:compl:rev
CACCGTCACAGACACTATCATGTTGACATTCC; dG = -12.5
219 >s_b_s_c_s_d_s_d_s_d_s GTGGCTGTGTGGGTGACGGTGACGGTGACGGTG; >clamp_s_a_s_d_s:compl:rev
CACCGTCACAGACACTATCATGTTGACATTCC; dG = -12.5
220 >s_c_s_b_s_c_s_d_s_d_s GTGTGGGTGGCTGTGTGGGTGACGGTGACGGTG; >clamp_s_a_s_d_s:compl:rev
CACCGTCACAGACACTATCATGTTGACATTCC; dG = -12.5
221 >s_a_s_b_s_c_s_d_s_d_s GTGTCTGTGGCTGTGTGGGTGACGGTGACGGTG; >clamp_s_a_s_d_s:compl:rev
CACCGTCACAGACACTATCATGTTGACATTCC; dG = -12.5
222 >s_d_s_b_s_c_s_d_s_d_s GTGACGGTGGCTGTGTGGGTGACGGTGACGGTG; >clamp_s_a_s_d_s:compl:rev
CACCGTCACAGACACTATCATGTTGACATTCC; dG = -12.5
223 >s_e_s_b_s_c_s_d_s_d_s GTGCTGGTGGCTGTGTGGGTGACGGTGACGGTG; >clamp_s_a_s_d_s:compl:rev
CACCGTCACAGACACTATCATGTTGACATTCC; dG = -12.5
224 >s_a_s_c_s_d_s_d_s_d_s GTGTCTGTGTGGGTGACGGTGACGGTGACGGTG; >clamp_s_a_s_d_s:compl:rev
CACCGTCACAGACACTATCATGTTGACATTCC; dG = -12.5
225 >s_a_s_e_s_c_s_d_s_d_s GTGTCTGTGCTGGTGTGGGTGACGGTGACGGTG; >clamp_s_a_s_d_s:compl:rev
CACCGTCACAGACACTATCATGTTGACATTCC; dG = -12.4
226 >s_b_s_c_s_c_s_d_s_d_s GTGGCTGTGTGGGTGTGGGTGACGGTGACGGTG; >clamp_s_a_s_d_s:compl:rev
CACCGTCACAGACACTATCATGTTGACATTCC; dG = -12.4

227 >s_b_s_e_s_c_s_d_s_d_s GTGGCTGTGCTGGTGTGGGTGACGGTGACGGTG; >clamp_s_a_s_d_s:compl:rev
CACCGTCACAGACACTATCATGTTGACATTCC; dG = -12.4
228 >s_c_s_c_s_c_s_d_s_d_s GTGTGGGTGTGGGTGTGGGTGACGGTGACGGTG; >clamp_s_a_s_d_s:compl:rev
CACCGTCACAGACACTATCATGTTGACATTCC; dG = -12.4
229 >s_c_s_c_s_d_s_d_s_d_s GTGTGGGTGTGGGTGACGGTGACGGTGACGGTG; >clamp_s_a_s_d_s:compl:rev
CACCGTCACAGACACTATCATGTTGACATTCC; dG = -12.4
230 >s_c_s_d_s_c_s_d_s_d_s GTGTGGGTGACGGTGTGGGTGACGGTGACGGTG; >clamp_s_a_s_d_s:compl:rev
CACCGTCACAGACACTATCATGTTGACATTCC; dG = -12.4
231 >s_c_s_e_s_c_s_d_s_d_s GTGTGGGTGCTGGTGTGGGTGACGGTGACGGTG; >clamp_s_a_s_d_s:compl:rev
CACCGTCACAGACACTATCATGTTGACATTCC; dG = -12.4
232 >s_d_s_c_s_c_s_d_s_d_s GTGACGGTGTGGGTGTGGGTGACGGTGACGGTG; >clamp_s_a_s_d_s:compl:rev
CACCGTCACAGACACTATCATGTTGACATTCC; dG = -12.4
233 >s_d_s_c_s_d_s_d_s_d_s GTGACGGTGTGGGTGACGGTGACGGTGACGGTG; >clamp_s_a_s_d_s:compl:rev
CACCGTCACAGACACTATCATGTTGACATTCC; dG = -12.4
234 >s_d_s_d_s_c_s_d_s_d_s GTGACGGTGACGGTGTGGGTGACGGTGACGGTG; >clamp_s_a_s_d_s:compl:rev
CACCGTCACAGACACTATCATGTTGACATTCC; dG = -12.4
235 >s_d_s_e_s_c_s_d_s_d_s GTGACGGTGCTGGTGTGGGTGACGGTGACGGTG; >clamp_s_a_s_d_s:compl:rev
CACCGTCACAGACACTATCATGTTGACATTCC; dG = -12.4
236 >s_e_s_c_s_c_s_d_s_d_s GTGCTGGTGTGGGTGTGGGTGACGGTGACGGTG; >clamp_s_a_s_d_s:compl:rev
CACCGTCACAGACACTATCATGTTGACATTCC; dG = -12.4
237 >s_e_s_c_s_d_s_d_s_d_s GTGCTGGTGTGGGTGACGGTGACGGTGACGGTG; >clamp_s_a_s_d_s:compl:rev
CACCGTCACAGACACTATCATGTTGACATTCC; dG = -12.4
238 >s_e_s_d_s_c_s_d_s_d_s GTGCTGGTGACGGTGTGGGTGACGGTGACGGTG; >clamp_s_a_s_d_s:compl:rev
CACCGTCACAGACACTATCATGTTGACATTCC; dG = -12.4
239 >s_e_s_e_s_c_s_d_s_d_s GTGCTGGTGCTGGTGTGGGTGACGGTGACGGTG; >clamp_s_a_s_d_s:compl:rev
CACCGTCACAGACACTATCATGTTGACATTCC; dG = -12.4
240 >s_a_s_c_s_c_s_d_s_d_s GTGTCTGTGTGGGTGTGGGTGACGGTGACGGTG; >clamp_s_a_s_d_s:compl:rev
CACCGTCACAGACACTATCATGTTGACATTCC; dG = -12.4
241 >s_a_s_e_s_d_s_d_s_d_s GTGTCTGTGCTGGTGACGGTGACGGTGACGGTG; >clamp_s_a_s_d_s:compl:rev
CACCGTCACAGACACTATCATGTTGACATTCC; dG = -12.3
242 >s_b_s_a_s_a_s_e_s_d_s GTGGCTGTGTCTGTGTCTGTGCTGGTGACGGTG; >clamp_s_a_s_d_s:compl:rev
CACCGTCACAGACACTATCATGTTGACATTCC; dG = -12.0
243 >s_b_s_a_s_a_s_c_s_d_s GTGGCTGTGTCTGTGTCTGTGTGGGTGACGGTG; >clamp_s_a_s_d_s:compl:rev
CACCGTCACAGACACTATCATGTTGACATTCC; dG = -12.0
244 >s_b_s_b_s_a_s_e_s_d_s GTGGCTGTGGCTGTGTCTGTGCTGGTGACGGTG; >clamp_s_a_s_d_s:compl:rev
CACCGTCACAGACACTATCATGTTGACATTCC; dG = -12.0
245 >s_b_s_b_s_a_s_c_s_d_s GTGGCTGTGGCTGTGTCTGTGTGGGTGACGGTG; >clamp_s_a_s_d_s:compl:rev
CACCGTCACAGACACTATCATGTTGACATTCC; dG = -12.0
246 >s_c_s_a_s_a_s_e_s_d_s GTGTGGGTGTCTGTGTCTGTGCTGGTGACGGTG; >clamp_s_a_s_d_s:compl:rev
CACCGTCACAGACACTATCATGTTGACATTCC; dG = -12.0
247 >s_c_s_a_s_a_s_c_s_d_s GTGTGGGTGTCTGTGTCTGTGTGGGTGACGGTG; >clamp_s_a_s_d_s:compl:rev
CACCGTCACAGACACTATCATGTTGACATTCC; dG = -12.0
248 >s_a_s_b_s_a_s_e_s_d_s GTGTCTGTGGCTGTGTCTGTGCTGGTGACGGTG; >clamp_s_a_s_d_s:compl:rev
CACCGTCACAGACACTATCATGTTGACATTCC; dG = -12.0
249 >s_a_s_b_s_a_s_c_s_d_s GTGTCTGTGGCTGTGTCTGTGTGGGTGACGGTG; >clamp_s_a_s_d_s:compl:rev
CACCGTCACAGACACTATCATGTTGACATTCC; dG = -12.0
250 >s_c_s_b_s_a_s_e_s_d_s GTGTGGGTGGCTGTGTCTGTGCTGGTGACGGTG; >clamp_s_a_s_d_s:compl:rev
CACCGTCACAGACACTATCATGTTGACATTCC; dG = -12.0
251 >s_c_s_b_s_a_s_c_s_d_s GTGTGGGTGGCTGTGTCTGTGTGGGTGACGGTG; >clamp_s_a_s_d_s:compl:rev
CACCGTCACAGACACTATCATGTTGACATTCC; dG = -12.0
252 >s_c_s_d_s_d_s_a_s_e_s GTGTGGGTGACGGTGACGGTGTCTGTGCTGGTG; >clamp_s_a_s_d_s:compl:rev
CACCGTCACAGACACTATCATGTTGACATTCC; dG = -12.0
253 >s_c_s_d_s_d_s_a_s_c_s GTGTGGGTGACGGTGACGGTGTCTGTGTGGGTG; >clamp_s_a_s_d_s:compl:rev
CACCGTCACAGACACTATCATGTTGACATTCC; dG = -12.0
254 >s_c_s_d_s_d_s_a_s_b_s GTGTGGGTGACGGTGACGGTGTCTGTGGCTGTG; >clamp_s_a_s_d_s:compl:rev
CACCGTCACAGACACTATCATGTTGACATTCC; dG = -12.0
255 >s_c_s_d_s_d_s_a_s_a_s GTGTGGGTGACGGTGACGGTGTCTGTGTCTGTG; >clamp_s_a_s_d_s:compl:rev
CACCGTCACAGACACTATCATGTTGACATTCC; dG = -12.0
256 >s_c_s_d_s_d_s_e_s_d_s GTGTGGGTGACGGTGACGGTGCTGGTGACGGTG; >clamp_s_a_s_d_s:compl:rev
CACCGTCACAGACACTATCATGTTGACATTCC; dG = -12.0
257 >s_c_s_d_s_d_s_d_s_d_s GTGTGGGTGACGGTGACGGTGACGGTGACGGTG; >clamp_s_a_s_d_s:compl:rev
CACCGTCACAGACACTATCATGTTGACATTCC; dG = -12.0
258 >s_c_s_d_s_d_s_c_s_d_s GTGTGGGTGACGGTGACGGTGTGGGTGACGGTG; >clamp_s_a_s_d_s:compl:rev
CACCGTCACAGACACTATCATGTTGACATTCC; dG = -12.0
259 >s_a_s_a_s_a_s_e_s_d_s GTGTCTGTGTCTGTGTCTGTGCTGGTGACGGTG; >clamp_s_a_s_d_s:compl:rev
CACCGTCACAGACACTATCATGTTGACATTCC; dG = -12.0
260 >s_d_s_a_s_a_s_e_s_d_s GTGACGGTGTCTGTGTCTGTGCTGGTGACGGTG; >clamp_s_a_s_d_s:compl:rev
CACCGTCACAGACACTATCATGTTGACATTCC; dG = -12.0
261 >s_d_s_a_s_a_s_c_s_d_s GTGACGGTGTCTGTGTCTGTGTGGGTGACGGTG; >clamp_s_a_s_d_s:compl:rev
CACCGTCACAGACACTATCATGTTGACATTCC; dG = -12.0
262 >s_d_s_b_s_a_s_e_s_d_s GTGACGGTGGCTGTGTCTGTGCTGGTGACGGTG; >clamp_s_a_s_d_s:compl:rev
CACCGTCACAGACACTATCATGTTGACATTCC; dG = -12.0
263 >s_d_s_b_s_a_s_c_s_d_s GTGACGGTGGCTGTGTCTGTGTGGGTGACGGTG; >clamp_s_a_s_d_s:compl:rev
CACCGTCACAGACACTATCATGTTGACATTCC; dG = -12.0

264 >s_a_s_a_s_a_s_c_s_d_s GTGTCTGTGTCTGTGTCTGTGTGGGTGACGGTG; >clamp_s_a_s_d_s:compl:rev CACCGTCACAGACACTATCATGTTGACATTCC; dG = -12.0
265 >s_e_s_a_s_a_s_e_s_d_s GTGCTGGTGTCTGTGTCTGTGCTGGTGACGGTG; >clamp_s_a_s_d_s:compl:rev CACCGTCACAGACACTATCATGTTGACATTCC; dG = -12.0
266 >s_e_s_a_s_a_s_c_s_d_s GTGCTGGTGTCTGTGTCTGTGTGGGTGACGGTG; >clamp_s_a_s_d_s:compl:rev CACCGTCACAGACACTATCATGTTGACATTCC; dG = -12.0
267 >s_e_s_b_s_a_s_e_s_d_s GTGCTGGTGGCTGTGTCTGTGCTGGTGACGGTG; >clamp_s_a_s_d_s:compl:rev CACCGTCACAGACACTATCATGTTGACATTCC; dG = -12.0
268 >s_e_s_b_s_a_s_c_s_d_s GTGCTGGTGGCTGTGTCTGTGTGGGTGACGGTG; >clamp_s_a_s_d_s:compl:rev CACCGTCACAGACACTATCATGTTGACATTCC; dG = -12.0
269 >s_a_s_c_s_d_s_c_s_d_s GTGTCTGTGTGGGTGACGGTGTGGGTGACGGTG; >clamp_s_a_s_d_s:compl:rev CACCGTCACAGACACTATCATGTTGACATTCC; dG = -11.9
270 >s_a_s_e_s_a_s_e_s_d_s GTGTCTGTGCTGGTGTCTGTGCTGGTGACGGTG; >clamp_s_a_s_d_s:compl:rev CACCGTCACAGACACTATCATGTTGACATTCC; dG = -11.9
271 >s_a_s_e_s_a_s_c_s_d_s GTGTCTGTGCTGGTGTCTGTGTGGGTGACGGTG; >clamp_s_a_s_d_s:compl:rev CACCGTCACAGACACTATCATGTTGACATTCC; dG = -11.9
272 >s_b_s_c_s_a_s_e_s_d_s GTGGCTGTGTGGGTGTCTGTGCTGGTGACGGTG; >clamp_s_a_s_d_s:compl:rev CACCGTCACAGACACTATCATGTTGACATTCC; dG = -11.9
273 >s_b_s_c_s_a_s_c_s_d_s GTGGCTGTGTGGGTGTCTGTGTGGGTGACGGTG; >clamp_s_a_s_d_s:compl:rev CACCGTCACAGACACTATCATGTTGACATTCC; dG = -11.9
274 >s_b_s_c_s_d_s_a_s_e_s GTGGCTGTGTGGGTGACGGTGTCTGTGCTGGTG; >clamp_s_a_s_d_s:compl:rev CACCGTCACAGACACTATCATGTTGACATTCC; dG = -11.9
275 >s_b_s_c_s_d_s_a_s_c_s GTGGCTGTGTGGGTGACGGTGTCTGTGTGGGTG; >clamp_s_a_s_d_s:compl:rev CACCGTCACAGACACTATCATGTTGACATTCC; dG = -11.9
276 >s_b_s_c_s_d_s_a_s_b_s GTGGCTGTGTGGGTGACGGTGTCTGTGGCTGTG; >clamp_s_a_s_d_s:compl:rev CACCGTCACAGACACTATCATGTTGACATTCC; dG = -11.9
277 >s_b_s_c_s_d_s_a_s_a_s GTGGCTGTGTGGGTGACGGTGTCTGTGTCTGTG; >clamp_s_a_s_d_s:compl:rev CACCGTCACAGACACTATCATGTTGACATTCC; dG = -11.9
278 >s_b_s_c_s_d_s_c_s_d_s GTGGCTGTGTGGGTGACGGTGTGGGTGACGGTG; >clamp_s_a_s_d_s:compl:rev CACCGTCACAGACACTATCATGTTGACATTCC; dG = -11.9
279 >s_b_s_e_s_a_s_e_s_d_s GTGGCTGTGCTGGTGTCTGTGCTGGTGACGGTG; >clamp_s_a_s_d_s:compl:rev CACCGTCACAGACACTATCATGTTGACATTCC; dG = -11.9
280 >s_b_s_e_s_a_s_c_s_d_s GTGGCTGTGCTGGTGTCTGTGTGGGTGACGGTG; >clamp_s_a_s_d_s:compl:rev CACCGTCACAGACACTATCATGTTGACATTCC; dG = -11.9
281 >s_c_s_c_s_a_s_e_s_d_s GTGTGGGTGTGGGTGTCTGTGCTGGTGACGGTG; >clamp_s_a_s_d_s:compl:rev CACCGTCACAGACACTATCATGTTGACATTCC; dG = -11.9
282 >s_c_s_c_s_a_s_c_s_d_s GTGTGGGTGTGGGTGTCTGTGTGGGTGACGGTG; >clamp_s_a_s_d_s:compl:rev CACCGTCACAGACACTATCATGTTGACATTCC; dG = -11.9
283 >s_c_s_c_s_d_s_a_s_e_s GTGTGGGTGTGGGTGACGGTGTCTGTGCTGGTG; >clamp_s_a_s_d_s:compl:rev CACCGTCACAGACACTATCATGTTGACATTCC; dG = -11.9
284 >s_c_s_c_s_d_s_a_s_c_s GTGTGGGTGTGGGTGACGGTGTCTGTGTGGGTG; >clamp_s_a_s_d_s:compl:rev CACCGTCACAGACACTATCATGTTGACATTCC; dG = -11.9
285 >s_c_s_c_s_d_s_a_s_b_s GTGTGGGTGTGGGTGACGGTGTCTGTGGCTGTG; >clamp_s_a_s_d_s:compl:rev CACCGTCACAGACACTATCATGTTGACATTCC; dG = -11.9
286 >s_c_s_c_s_d_s_a_s_a_s GTGTGGGTGTGGGTGACGGTGTCTGTGTCTGTG; >clamp_s_a_s_d_s:compl:rev CACCGTCACAGACACTATCATGTTGACATTCC; dG = -11.9
287 >s_c_s_c_s_d_s_c_s_d_s GTGTGGGTGTGGGTGACGGTGTGGGTGACGGTG; >clamp_s_a_s_d_s:compl:rev CACCGTCACAGACACTATCATGTTGACATTCC; dG = -11.9
288 >s_c_s_d_s_a_s_e_s_d_s GTGTGGGTGACGGTGTCTGTGCTGGTGACGGTG; >clamp_s_a_s_d_s:compl:rev CACCGTCACAGACACTATCATGTTGACATTCC; dG = -11.9
289 >s_c_s_d_s_a_s_c_s_d_s GTGTGGGTGACGGTGTCTGTGTGGGTGACGGTG; >clamp_s_a_s_d_s:compl:rev CACCGTCACAGACACTATCATGTTGACATTCC; dG = -11.9
290 >s_c_s_e_s_a_s_e_s_d_s GTGTGGGTGCTGGTGTCTGTGCTGGTGACGGTG; >clamp_s_a_s_d_s:compl:rev CACCGTCACAGACACTATCATGTTGACATTCC; dG = -11.9
291 >s_c_s_e_s_a_s_c_s_d_s GTGTGGGTGCTGGTGTCTGTGTGGGTGACGGTG; >clamp_s_a_s_d_s:compl:rev CACCGTCACAGACACTATCATGTTGACATTCC; dG = -11.9
292 >s_d_s_c_s_a_s_e_s_d_s GTGACGGTGTGGGTGTCTGTGCTGGTGACGGTG; >clamp_s_a_s_d_s:compl:rev CACCGTCACAGACACTATCATGTTGACATTCC; dG = -11.9
293 >s_d_s_c_s_a_s_c_s_d_s GTGACGGTGTGGGTGTCTGTGTGGGTGACGGTG; >clamp_s_a_s_d_s:compl:rev CACCGTCACAGACACTATCATGTTGACATTCC; dG = -11.9
294 >s_d_s_c_s_d_s_a_s_e_s GTGACGGTGTGGGTGACGGTGTCTGTGCTGGTG; >clamp_s_a_s_d_s:compl:rev CACCGTCACAGACACTATCATGTTGACATTCC; dG = -11.9
295 >s_d_s_c_s_d_s_a_s_c_s GTGACGGTGTGGGTGACGGTGTCTGTGTGGGTG; >clamp_s_a_s_d_s:compl:rev CACCGTCACAGACACTATCATGTTGACATTCC; dG = -11.9
296 >s_d_s_c_s_d_s_a_s_b_s GTGACGGTGTGGGTGACGGTGTCTGTGGCTGTG; >clamp_s_a_s_d_s:compl:rev CACCGTCACAGACACTATCATGTTGACATTCC; dG = -11.9
297 >s_d_s_c_s_d_s_a_s_a_s GTGACGGTGTGGGTGACGGTGTCTGTGTCTGTG; >clamp_s_a_s_d_s:compl:rev CACCGTCACAGACACTATCATGTTGACATTCC; dG = -11.9
298 >s_d_s_c_s_d_s_c_s_d_s GTGACGGTGTGGGTGACGGTGTGGGTGACGGTG; >clamp_s_a_s_d_s:compl:rev CACCGTCACAGACACTATCATGTTGACATTCC; dG = -11.9
299 >s_d_s_d_s_a_s_e_s_d_s GTGACGGTGACGGTGTCTGTGCTGGTGACGGTG; >clamp_s_a_s_d_s:compl:rev CACCGTCACAGACACTATCATGTTGACATTCC; dG = -11.9
300 >s_d_s_d_s_a_s_c_s_d_s GTGACGGTGACGGTGTCTGTGTGGGTGACGGTG; >clamp_s_a_s_d_s:compl:rev CACCGTCACAGACACTATCATGTTGACATTCC; dG = -11.9

301 >s_d_s_e_s_a_s_e_s_d_s GTGACGGTGCTGGTGTCTGTGCTGGTGACGGTG; >clamp_s_a_s_d_s:compl:rev CACCGTCACAGACACTATCATGTTGACATTCC; dG = -11.9
302 >s_d_s_e_s_a_s_c_s_d_s GTGACGGTGCTGGTGTCTGTGTGGGTGACGGTG; >clamp_s_a_s_d_s:compl:rev CACCGTCACAGACACTATCATGTTGACATTCC; dG = -11.9
303 >s_a_s_c_s_a_s_e_s_d_s GTGTCTGTGTGGGTGTCTGTGCTGGTGACGGTG; >clamp_s_a_s_d_s:compl:rev CACCGTCACAGACACTATCATGTTGACATTCC; dG = -11.9
304 >s_a_s_c_s_a_s_c_s_d_s GTGTCTGTGTGGGTGTCTGTGTGGGTGACGGTG; >clamp_s_a_s_d_s:compl:rev CACCGTCACAGACACTATCATGTTGACATTCC; dG = -11.9
305 >s_e_s_c_s_a_s_e_s_d_s GTGCTGGTGTGGGTGTCTGTGCTGGTGACGGTG; >clamp_s_a_s_d_s:compl:rev CACCGTCACAGACACTATCATGTTGACATTCC; dG = -11.9
306 >s_e_s_c_s_a_s_c_s_d_s GTGCTGGTGTGGGTGTCTGTGTGGGTGACGGTG; >clamp_s_a_s_d_s:compl:rev CACCGTCACAGACACTATCATGTTGACATTCC; dG = -11.9
307 >s_e_s_c_s_d_s_a_s_e_s GTGCTGGTGTGGGTGACGGTGTCTGTGCTGGTG; >clamp_s_a_s_d_s:compl:rev CACCGTCACAGACACTATCATGTTGACATTCC; dG = -11.9
308 >s_e_s_c_s_d_s_a_s_c_s GTGCTGGTGTGGGTGACGGTGTCTGTGTGGGTG; >clamp_s_a_s_d_s:compl:rev CACCGTCACAGACACTATCATGTTGACATTCC; dG = -11.9
309 >s_e_s_c_s_d_s_a_s_b_s GTGCTGGTGTGGGTGACGGTGTCTGTGGCTGTG; >clamp_s_a_s_d_s:compl:rev CACCGTCACAGACACTATCATGTTGACATTCC; dG = -11.9
310 >s_e_s_c_s_d_s_a_s_a_s GTGCTGGTGTGGGTGACGGTGTCTGTGTCTGTG; >clamp_s_a_s_d_s:compl:rev CACCGTCACAGACACTATCATGTTGACATTCC; dG = -11.9
311 >s_e_s_c_s_d_s_c_s_d_s GTGCTGGTGTGGGTGACGGTGTGGGTGACGGTG; >clamp_s_a_s_d_s:compl:rev CACCGTCACAGACACTATCATGTTGACATTCC; dG = -11.9
312 >s_e_s_d_s_a_s_e_s_d_s GTGCTGGTGACGGTGTCTGTGCTGGTGACGGTG; >clamp_s_a_s_d_s:compl:rev CACCGTCACAGACACTATCATGTTGACATTCC; dG = -11.9
313 >s_e_s_d_s_a_s_c_s_d_s GTGCTGGTGACGGTGTCTGTGTGGGTGACGGTG; >clamp_s_a_s_d_s:compl:rev CACCGTCACAGACACTATCATGTTGACATTCC; dG = -11.9
314 >s_e_s_e_s_a_s_e_s_d_s GTGCTGGTGCTGGTGTCTGTGCTGGTGACGGTG; >clamp_s_a_s_d_s:compl:rev CACCGTCACAGACACTATCATGTTGACATTCC; dG = -11.9
315 >s_e_s_e_s_a_s_c_s_d_s GTGCTGGTGCTGGTGTCTGTGTGGGTGACGGTG; >clamp_s_a_s_d_s:compl:rev CACCGTCACAGACACTATCATGTTGACATTCC; dG = -11.9
316 >s_a_s_c_s_d_s_a_s_e_s GTGTCTGTGTGGGTGACGGTGTCTGTGCTGGTG; >clamp_s_a_s_d_s:compl:rev CACCGTCACAGACACTATCATGTTGACATTCC; dG = -11.9
317 >s_a_s_c_s_d_s_a_s_c_s GTGTCTGTGTGGGTGACGGTGTCTGTGTGGGTG; >clamp_s_a_s_d_s:compl:rev CACCGTCACAGACACTATCATGTTGACATTCC; dG = -11.9
318 >s_a_s_c_s_d_s_a_s_b_s GTGTCTGTGTGGGTGACGGTGTCTGTGGCTGTG; >clamp_s_a_s_d_s:compl:rev CACCGTCACAGACACTATCATGTTGACATTCC; dG = -11.9
319 >s_a_s_c_s_d_s_a_s_a_s GTGTCTGTGTGGGTGACGGTGTCTGTGTCTGTG; >clamp_s_a_s_d_s:compl:rev CACCGTCACAGACACTATCATGTTGACATTCC; dG = -11.9
320 >s_b_s_c_s_d_s_e_s_d_s GTGGCTGTGTGGGTGACGGTGCTGGTGACGGTG; >clamp_s_a_s_d_s:compl:rev CACCGTCACAGACACTATCATGTTGACATTCC; dG = -11.8
321 >s_a_s_c_s_d_s_e_s_d_s GTGTCTGTGTGGGTGACGGTGCTGGTGACGGTG; >clamp_s_a_s_d_s:compl:rev CACCGTCACAGACACTATCATGTTGACATTCC; dG = -11.8
322 >s_a_s_e_s_b_s_c_s_d_s GTGTCTGTGCTGGTGGCTGTGTGGGTGACGGTG; >clamp_s_a_s_d_s:compl:rev CACCGTCACAGACACTATCATGTTGACATTCC; dG = -11.7
323 >s_a_s_e_s_d_s_a_s_e_s GTGTCTGTGCTGGTGACGGTGTCTGTGCTGGTG; >clamp_s_a_s_d_s:compl:rev CACCGTCACAGACACTATCATGTTGACATTCC; dG = -11.7
324 >s_a_s_e_s_d_s_a_s_c_s GTGTCTGTGCTGGTGACGGTGTCTGTGTGGGTG; >clamp_s_a_s_d_s:compl:rev CACCGTCACAGACACTATCATGTTGACATTCC; dG = -11.7
325 >s_a_s_e_s_d_s_a_s_b_s GTGTCTGTGCTGGTGACGGTGTCTGTGGCTGTG; >clamp_s_a_s_d_s:compl:rev CACCGTCACAGACACTATCATGTTGACATTCC; dG = -11.7
326 >s_a_s_e_s_d_s_a_s_a_s GTGTCTGTGCTGGTGACGGTGTCTGTGTCTGTG; >clamp_s_a_s_d_s:compl:rev CACCGTCACAGACACTATCATGTTGACATTCC; dG = -11.7
327 >s_a_s_e_s_d_s_c_s_d_s GTGTCTGTGCTGGTGACGGTGTGGGTGACGGTG; >clamp_s_a_s_d_s:compl:rev CACCGTCACAGACACTATCATGTTGACATTCC; dG = -11.7
328 >s_a_s_a_s_b_s_c_s_d_s GTGTCTGTGTCTGTGGCTGTGTGGGTGACGGTG; >clamp_s_a_s_d_s:compl:rev CACCGTCACAGACACTATCATGTTGACATTCC; dG = -11.7
329 >s_b_s_a_s_b_s_c_s_d_s GTGGCTGTGTCTGTGGCTGTGTGGGTGACGGTG; >clamp_s_a_s_d_s:compl:rev CACCGTCACAGACACTATCATGTTGACATTCC; dG = -11.7
330 >s_b_s_b_s_b_s_c_s_d_s GTGGCTGTGGCTGTGGCTGTGTGGGTGACGGTG; >clamp_s_a_s_d_s:compl:rev CACCGTCACAGACACTATCATGTTGACATTCC; dG = -11.7
331 >s_b_s_c_s_b_s_c_s_d_s GTGGCTGTGTGGGTGGCTGTGTGGGTGACGGTG; >clamp_s_a_s_d_s:compl:rev CACCGTCACAGACACTATCATGTTGACATTCC; dG = -11.7
332 >s_b_s_e_s_b_s_c_s_d_s GTGGCTGTGCTGGTGGCTGTGTGGGTGACGGTG; >clamp_s_a_s_d_s:compl:rev CACCGTCACAGACACTATCATGTTGACATTCC; dG = -11.7
333 >s_c_s_a_s_b_s_c_s_d_s GTGTGGGTGTCTGTGGCTGTGTGGGTGACGGTG; >clamp_s_a_s_d_s:compl:rev CACCGTCACAGACACTATCATGTTGACATTCC; dG = -11.7
334 >s_c_s_b_s_b_s_c_s_d_s GTGTGGGTGGCTGTGGCTGTGTGGGTGACGGTG; >clamp_s_a_s_d_s:compl:rev CACCGTCACAGACACTATCATGTTGACATTCC; dG = -11.7
335 >s_c_s_c_s_b_s_c_s_d_s GTGTGGGTGTGGGTGGCTGTGTGGGTGACGGTG; >clamp_s_a_s_d_s:compl:rev CACCGTCACAGACACTATCATGTTGACATTCC; dG = -11.7
336 >s_c_s_c_s_d_s_e_s_d_s GTGTGGGTGTGGGTGACGGTGCTGGTGACGGTG; >clamp_s_a_s_d_s:compl:rev CACCGTCACAGACACTATCATGTTGACATTCC; dG = -11.7
337 >s_a_s_b_s_b_s_c_s_d_s GTGTCTGTGGCTGTGGCTGTGTGGGTGACGGTG; >clamp_s_a_s_d_s:compl:rev CACCGTCACAGACACTATCATGTTGACATTCC; dG = -11.7

338 >s_c_s_d_s_b_s_c_s_d_s GTGTGGGTGACGGTGGCTGTGTGGGTGACGGTG; >clamp_s_a_s_d_s:compl:rev CACCGTCACAGACACTATCATGTTGACATTCC; dG = -11.7
339 >s_c_s_e_s_b_s_c_s_d_s GTGTGGGTGCTGGTGGCTGTGTGGGTGACGGTG; >clamp_s_a_s_d_s:compl:rev CACCGTCACAGACACTATCATGTTGACATTCC; dG = -11.7
340 >s_d_s_a_s_b_s_c_s_d_s GTGACGGTGTCTGTGGCTGTGTGGGTGACGGTG; >clamp_s_a_s_d_s:compl:rev CACCGTCACAGACACTATCATGTTGACATTCC; dG = -11.7
341 >s_d_s_b_s_b_s_c_s_d_s GTGACGGTGGCTGTGGCTGTGTGGGTGACGGTG; >clamp_s_a_s_d_s:compl:rev CACCGTCACAGACACTATCATGTTGACATTCC; dG = -11.7
342 >s_d_s_c_s_b_s_c_s_d_s GTGACGGTGTGGGTGGCTGTGTGGGTGACGGTG; >clamp_s_a_s_d_s:compl:rev CACCGTCACAGACACTATCATGTTGACATTCC; dG = -11.7
343 >s_d_s_c_s_d_s_e_s_d_s GTGACGGTGTGGGTGACGGTGCTGGTGACGGTG; >clamp_s_a_s_d_s:compl:rev CACCGTCACAGACACTATCATGTTGACATTCC; dG = -11.7
344 >s_d_s_d_s_b_s_c_s_d_s GTGACGGTGACGGTGGCTGTGTGGGTGACGGTG; >clamp_s_a_s_d_s:compl:rev CACCGTCACAGACACTATCATGTTGACATTCC; dG = -11.7
345 >s_d_s_e_s_b_s_c_s_d_s GTGACGGTGCTGGTGGCTGTGTGGGTGACGGTG; >clamp_s_a_s_d_s:compl:rev CACCGTCACAGACACTATCATGTTGACATTCC; dG = -11.7
346 >s_e_s_a_s_b_s_c_s_d_s GTGCTGGTGTCTGTGGCTGTGTGGGTGACGGTG; >clamp_s_a_s_d_s:compl:rev CACCGTCACAGACACTATCATGTTGACATTCC; dG = -11.7
347 >s_e_s_b_s_b_s_c_s_d_s GTGCTGGTGGCTGTGGCTGTGTGGGTGACGGTG; >clamp_s_a_s_d_s:compl:rev CACCGTCACAGACACTATCATGTTGACATTCC; dG = -11.7
348 >s_e_s_c_s_b_s_c_s_d_s GTGCTGGTGTGGGTGGCTGTGTGGGTGACGGTG; >clamp_s_a_s_d_s:compl:rev CACCGTCACAGACACTATCATGTTGACATTCC; dG = -11.7
349 >s_e_s_c_s_d_s_e_s_d_s GTGCTGGTGTGGGTGACGGTGCTGGTGACGGTG; >clamp_s_a_s_d_s:compl:rev CACCGTCACAGACACTATCATGTTGACATTCC; dG = -11.7
350 >s_e_s_d_s_b_s_c_s_d_s GTGCTGGTGACGGTGGCTGTGTGGGTGACGGTG; >clamp_s_a_s_d_s:compl:rev CACCGTCACAGACACTATCATGTTGACATTCC; dG = -11.7
351 >s_a_s_c_s_b_s_c_s_d_s GTGTCTGTGTGGGTGGCTGTGTGGGTGACGGTG; >clamp_s_a_s_d_s:compl:rev CACCGTCACAGACACTATCATGTTGACATTCC; dG = -11.7
352 >s_e_s_e_s_b_s_c_s_d_s GTGCTGGTGCTGGTGGCTGTGTGGGTGACGGTG; >clamp_s_a_s_d_s:compl:rev CACCGTCACAGACACTATCATGTTGACATTCC; dG = -11.7
353 >s_a_s_c_s_e_s_c_s_d_s GTGTCTGTGTGGGTGCTGGTGTGGGTGACGGTG; >clamp_s_a_s_d_s:compl:rev CACCGTCACAGACACTATCATGTTGACATTCC; dG = -11.6
354 >s_a_s_e_s_c_s_c_s_d_s GTGTCTGTGCTGGTGTGGGTGTGGGTGACGGTG; >clamp_s_a_s_d_s:compl:rev CACCGTCACAGACACTATCATGTTGACATTCC; dG = -11.6
355 >s_a_s_e_s_d_s_e_s_d_s GTGTCTGTGCTGGTGACGGTGCTGGTGACGGTG; >clamp_s_a_s_d_s:compl:rev CACCGTCACAGACACTATCATGTTGACATTCC; dG = -11.6
356 >s_a_s_e_s_e_s_c_s_d_s GTGTCTGTGCTGGTGCTGGTGTGGGTGACGGTG; >clamp_s_a_s_d_s:compl:rev CACCGTCACAGACACTATCATGTTGACATTCC; dG = -11.6
357 >s_b_s_a_s_c_s_c_s_d_s GTGGCTGTGTCTGTGTGGGTGTGGGTGACGGTG; >clamp_s_a_s_d_s:compl:rev CACCGTCACAGACACTATCATGTTGACATTCC; dG = -11.6
358 >s_b_s_a_s_e_s_c_s_d_s GTGGCTGTGTCTGTGCTGGTGTGGGTGACGGTG; >clamp_s_a_s_d_s:compl:rev CACCGTCACAGACACTATCATGTTGACATTCC; dG = -11.6
359 >s_b_s_b_s_c_s_c_s_d_s GTGGCTGTGGCTGTGTGGGTGTGGGTGACGGTG; >clamp_s_a_s_d_s:compl:rev CACCGTCACAGACACTATCATGTTGACATTCC; dG = -11.6
360 >s_b_s_b_s_e_s_c_s_d_s GTGGCTGTGGCTGTGCTGGTGTGGGTGACGGTG; >clamp_s_a_s_d_s:compl:rev CACCGTCACAGACACTATCATGTTGACATTCC; dG = -11.6
361 >s_a_s_a_s_c_s_c_s_d_s GTGTCTGTGTCTGTGTGGGTGTGGGTGACGGTG; >clamp_s_a_s_d_s:compl:rev CACCGTCACAGACACTATCATGTTGACATTCC; dG = -11.6
362 >s_b_s_c_s_c_s_c_s_d_s GTGGCTGTGTGGGTGTGGGTGTGGGTGACGGTG; >clamp_s_a_s_d_s:compl:rev CACCGTCACAGACACTATCATGTTGACATTCC; dG = -11.6
363 >s_b_s_c_s_e_s_c_s_d_s GTGGCTGTGTGGGTGCTGGTGTGGGTGACGGTG; >clamp_s_a_s_d_s:compl:rev CACCGTCACAGACACTATCATGTTGACATTCC; dG = -11.6
364 >s_a_s_a_s_e_s_c_s_d_s GTGTCTGTGTCTGTGCTGGTGTGGGTGACGGTG; >clamp_s_a_s_d_s:compl:rev CACCGTCACAGACACTATCATGTTGACATTCC; dG = -11.6
365 >s_b_s_e_s_c_s_c_s_d_s GTGGCTGTGCTGGTGTGGGTGTGGGTGACGGTG; >clamp_s_a_s_d_s:compl:rev CACCGTCACAGACACTATCATGTTGACATTCC; dG = -11.6
366 >s_b_s_e_s_d_s_c_s_d_s GTGGCTGTGCTGGTGACGGTGTGGGTGACGGTG; >clamp_s_a_s_d_s:compl:rev CACCGTCACAGACACTATCATGTTGACATTCC; dG = -11.6
367 >s_b_s_e_s_e_s_c_s_d_s GTGGCTGTGCTGGTGCTGGTGTGGGTGACGGTG; >clamp_s_a_s_d_s:compl:rev CACCGTCACAGACACTATCATGTTGACATTCC; dG = -11.6
368 >s_c_s_a_s_c_s_c_s_d_s GTGTGGGTGTCTGTGTGGGTGTGGGTGACGGTG; >clamp_s_a_s_d_s:compl:rev CACCGTCACAGACACTATCATGTTGACATTCC; dG = -11.6
369 >s_c_s_a_s_e_s_c_s_d_s GTGTGGGTGTCTGTGCTGGTGTGGGTGACGGTG; >clamp_s_a_s_d_s:compl:rev CACCGTCACAGACACTATCATGTTGACATTCC; dG = -11.6
370 >s_c_s_b_s_c_s_c_s_d_s GTGTGGGTGGCTGTGTGGGTGTGGGTGACGGTG; >clamp_s_a_s_d_s:compl:rev CACCGTCACAGACACTATCATGTTGACATTCC; dG = -11.6
371 >s_c_s_b_s_e_s_c_s_d_s GTGTGGGTGGCTGTGCTGGTGTGGGTGACGGTG; >clamp_s_a_s_d_s:compl:rev CACCGTCACAGACACTATCATGTTGACATTCC; dG = -11.6
372 >s_c_s_c_s_c_s_c_s_d_s GTGTGGGTGTGGGTGTGGGTGTGGGTGACGGTG; >clamp_s_a_s_d_s:compl:rev CACCGTCACAGACACTATCATGTTGACATTCC; dG = -11.6
373 >s_c_s_c_s_e_s_c_s_d_s GTGTGGGTGTGGGTGCTGGTGTGGGTGACGGTG; >clamp_s_a_s_d_s:compl:rev CACCGTCACAGACACTATCATGTTGACATTCC; dG = -11.6
374 >s_c_s_d_s_c_s_c_s_d_s GTGTGGGTGACGGTGTGGGTGTGGGTGACGGTG; >clamp_s_a_s_d_s:compl:rev CACCGTCACAGACACTATCATGTTGACATTCC; dG = -11.6

375 >s_c_s_d_s_e_s_c_s_d_s GTGTGGGTGACGGTGCTGGTGTGGGTGACGGTG; >clamp_s_a_s_d_s:compl:rev CACCGTCACAGACACTATCATGTTGACATTCC; dG = -11.6
376 >s_c_s_e_s_c_s_c_s_d_s GTGTGGGTGCTGGTGTGGGTGTGGGTGACGGTG; >clamp_s_a_s_d_s:compl:rev CACCGTCACAGACACTATCATGTTGACATTCC; dG = -11.6
377 >s_c_s_e_s_d_s_c_s_d_s GTGTGGGTGCTGGTGACGGTGTGGGTGACGGTG; >clamp_s_a_s_d_s:compl:rev CACCGTCACAGACACTATCATGTTGACATTCC; dG = -11.6
378 >s_c_s_e_s_e_s_c_s_d_s GTGTGGGTGCTGGTGCTGGTGTGGGTGACGGTG; >clamp_s_a_s_d_s:compl:rev CACCGTCACAGACACTATCATGTTGACATTCC; dG = -11.6
379 >s_a_s_b_s_c_s_c_s_d_s GTGTCTGTGGCTGTGTGGGTGTGGGTGACGGTG; >clamp_s_a_s_d_s:compl:rev CACCGTCACAGACACTATCATGTTGACATTCC; dG = -11.6
380 >s_d_s_a_s_c_s_c_s_d_s GTGACGGTGTCTGTGTGGGTGTGGGTGACGGTG; >clamp_s_a_s_d_s:compl:rev CACCGTCACAGACACTATCATGTTGACATTCC; dG = -11.6
381 >s_d_s_a_s_e_s_c_s_d_s GTGACGGTGTCTGTGCTGGTGTGGGTGACGGTG; >clamp_s_a_s_d_s:compl:rev CACCGTCACAGACACTATCATGTTGACATTCC; dG = -11.6
382 >s_d_s_b_s_c_s_c_s_d_s GTGACGGTGGCTGTGTGGGTGTGGGTGACGGTG; >clamp_s_a_s_d_s:compl:rev CACCGTCACAGACACTATCATGTTGACATTCC; dG = -11.6
383 >s_d_s_b_s_e_s_c_s_d_s GTGACGGTGGCTGTGCTGGTGTGGGTGACGGTG; >clamp_s_a_s_d_s:compl:rev CACCGTCACAGACACTATCATGTTGACATTCC; dG = -11.6
384 >s_d_s_c_s_c_s_c_s_d_s GTGACGGTGTGGGTGTGGGTGTGGGTGACGGTG; >clamp_s_a_s_d_s:compl:rev CACCGTCACAGACACTATCATGTTGACATTCC; dG = -11.6
385 >s_d_s_c_s_e_s_c_s_d_s GTGACGGTGTGGGTGCTGGTGTGGGTGACGGTG; >clamp_s_a_s_d_s:compl:rev CACCGTCACAGACACTATCATGTTGACATTCC; dG = -11.6
386 >s_d_s_d_s_c_s_c_s_d_s GTGACGGTGACGGTGTGGGTGTGGGTGACGGTG; >clamp_s_a_s_d_s:compl:rev CACCGTCACAGACACTATCATGTTGACATTCC; dG = -11.6
387 >s_d_s_d_s_d_s_c_s_d_s GTGACGGTGACGGTGACGGTGTGGGTGACGGTG; >clamp_s_a_s_d_s:compl:rev CACCGTCACAGACACTATCATGTTGACATTCC; dG = -11.6
388 >s_d_s_d_s_e_s_c_s_d_s GTGACGGTGACGGTGCTGGTGTGGGTGACGGTG; >clamp_s_a_s_d_s:compl:rev CACCGTCACAGACACTATCATGTTGACATTCC; dG = -11.6
389 >s_a_s_b_s_e_s_c_s_d_s GTGTCTGTGGCTGTGCTGGTGTGGGTGACGGTG; >clamp_s_a_s_d_s:compl:rev CACCGTCACAGACACTATCATGTTGACATTCC; dG = -11.6
390 >s_d_s_e_s_c_s_c_s_d_s GTGACGGTGCTGGTGTGGGTGTGGGTGACGGTG; >clamp_s_a_s_d_s:compl:rev CACCGTCACAGACACTATCATGTTGACATTCC; dG = -11.6
391 >s_d_s_e_s_d_s_c_s_d_s GTGACGGTGCTGGTGACGGTGTGGGTGACGGTG; >clamp_s_a_s_d_s:compl:rev CACCGTCACAGACACTATCATGTTGACATTCC; dG = -11.6
392 >s_d_s_e_s_e_s_c_s_d_s GTGACGGTGCTGGTGCTGGTGTGGGTGACGGTG; >clamp_s_a_s_d_s:compl:rev CACCGTCACAGACACTATCATGTTGACATTCC; dG = -11.6
393 >s_e_s_a_s_c_s_c_s_d_s GTGCTGGTGTCTGTGTGGGTGTGGGTGACGGTG; >clamp_s_a_s_d_s:compl:rev CACCGTCACAGACACTATCATGTTGACATTCC; dG = -11.6
394 >s_e_s_a_s_e_s_c_s_d_s GTGCTGGTGTCTGTGCTGGTGTGGGTGACGGTG; >clamp_s_a_s_d_s:compl:rev CACCGTCACAGACACTATCATGTTGACATTCC; dG = -11.6
395 >s_e_s_b_s_c_s_c_s_d_s GTGCTGGTGGCTGTGTGGGTGTGGGTGACGGTG; >clamp_s_a_s_d_s:compl:rev CACCGTCACAGACACTATCATGTTGACATTCC; dG = -11.6
396 >s_e_s_b_s_e_s_c_s_d_s GTGCTGGTGGCTGTGCTGGTGTGGGTGACGGTG; >clamp_s_a_s_d_s:compl:rev CACCGTCACAGACACTATCATGTTGACATTCC; dG = -11.6
397 >s_e_s_c_s_c_s_c_s_d_s GTGCTGGTGTGGGTGTGGGTGTGGGTGACGGTG; >clamp_s_a_s_d_s:compl:rev CACCGTCACAGACACTATCATGTTGACATTCC; dG = -11.6
398 >s_e_s_c_s_e_s_c_s_d_s GTGCTGGTGTGGGTGCTGGTGTGGGTGACGGTG; >clamp_s_a_s_d_s:compl:rev CACCGTCACAGACACTATCATGTTGACATTCC; dG = -11.6
399 >s_e_s_d_s_c_s_c_s_d_s GTGCTGGTGACGGTGTGGGTGTGGGTGACGGTG; >clamp_s_a_s_d_s:compl:rev CACCGTCACAGACACTATCATGTTGACATTCC; dG = -11.6
400 >s_e_s_d_s_d_s_c_s_d_s GTGCTGGTGACGGTGACGGTGTGGGTGACGGTG; >clamp_s_a_s_d_s:compl:rev CACCGTCACAGACACTATCATGTTGACATTCC; dG = -11.6
401 >s_e_s_d_s_e_s_c_s_d_s GTGCTGGTGACGGTGCTGGTGTGGGTGACGGTG; >clamp_s_a_s_d_s:compl:rev CACCGTCACAGACACTATCATGTTGACATTCC; dG = -11.6
402 >s_e_s_e_s_c_s_c_s_d_s GTGCTGGTGCTGGTGTGGGTGTGGGTGACGGTG; >clamp_s_a_s_d_s:compl:rev CACCGTCACAGACACTATCATGTTGACATTCC; dG = -11.6
403 >s_e_s_e_s_d_s_c_s_d_s GTGCTGGTGCTGGTGACGGTGTGGGTGACGGTG; >clamp_s_a_s_d_s:compl:rev CACCGTCACAGACACTATCATGTTGACATTCC; dG = -11.6
404 >s_e_s_e_s_e_s_c_s_d_s GTGCTGGTGCTGGTGCTGGTGTGGGTGACGGTG; >clamp_s_a_s_d_s:compl:rev CACCGTCACAGACACTATCATGTTGACATTCC; dG = -11.6
405 >s_a_s_c_s_c_s_c_s_d_s GTGTCTGTGTGGGTGTGGGTGTGGGTGACGGTG; >clamp_s_a_s_d_s:compl:rev CACCGTCACAGACACTATCATGTTGACATTCC; dG = -11.6
406 >s_c_s_d_s_b_s_a_s_e_s GTGTGGGTGACGGTGGCTGTGTCTGTGCTGGTG; >clamp_s_a_s_d_s:compl:rev CACCGTCACAGACACTATCATGTTGACATTCC; dG = -11.5
407 >s_c_s_d_s_b_s_a_s_c_s GTGTGGGTGACGGTGGCTGTGTCTGTGTGGGTG; >clamp_s_a_s_d_s:compl:rev CACCGTCACAGACACTATCATGTTGACATTCC; dG = -11.5
408 >s_c_s_d_s_b_s_a_s_b_s GTGTGGGTGACGGTGGCTGTGTCTGTGGCTGTG; >clamp_s_a_s_d_s:compl:rev CACCGTCACAGACACTATCATGTTGACATTCC; dG = -11.5
409 >s_c_s_d_s_b_s_a_s_a_s GTGTGGGTGACGGTGGCTGTGTCTGTGTCTGTG; >clamp_s_a_s_d_s:compl:rev CACCGTCACAGACACTATCATGTTGACATTCC; dG = -11.5
410 >s_c_s_d_s_b_s_e_s_d_s GTGTGGGTGACGGTGGCTGTGCTGGTGACGGTG; >clamp_s_a_s_d_s:compl:rev CACCGTCACAGACACTATCATGTTGACATTCC; dG = -11.5
411 >s_c_s_d_s_a_s_a_s_e_s GTGTGGGTGACGGTGTCTGTGTCTGTGCTGGTG; >clamp_s_a_s_d_s:compl:rev CACCGTCACAGACACTATCATGTTGACATTCC; dG = -11.4

412 >s_c_s_d_s_a_s_a_s_c_s GTGTGGGTGACGGTGTCTGTGTCTGTGTGGGTG; >clamp_s_a_s_d_s:compl:rev CACCGTCACAGACACTATCATGTTGACATTCC; dG = -11.4
413 >s_c_s_d_s_a_s_a_s_b_s GTGTGGGTGACGGTGTCTGTGTCTGTGGCTGTG; >clamp_s_a_s_d_s:compl:rev CACCGTCACAGACACTATCATGTTGACATTCC; dG = -11.4
414 >s_c_s_d_s_a_s_a_s_a_s GTGTGGGTGACGGTGTCTGTGTCTGTGTCTGTG; >clamp_s_a_s_d_s:compl:rev CACCGTCACAGACACTATCATGTTGACATTCC; dG = -11.4
415 >s_c_s_d_s_c_s_a_s_e_s GTGTGGGTGACGGTGTGGGTGTCTGTGCTGGTG; >clamp_s_a_s_d_s:compl:rev CACCGTCACAGACACTATCATGTTGACATTCC; dG = -11.4
416 >s_c_s_d_s_c_s_a_s_c_s GTGTGGGTGACGGTGTGGGTGTCTGTGTGGGTG; >clamp_s_a_s_d_s:compl:rev CACCGTCACAGACACTATCATGTTGACATTCC; dG = -11.4
417 >s_c_s_d_s_c_s_a_s_b_s GTGTGGGTGACGGTGTGGGTGTCTGTGGCTGTG; >clamp_s_a_s_d_s:compl:rev CACCGTCACAGACACTATCATGTTGACATTCC; dG = -11.4
418 >s_c_s_d_s_c_s_a_s_a_s GTGTGGGTGACGGTGTGGGTGTCTGTGTCTGTG; >clamp_s_a_s_d_s:compl:rev CACCGTCACAGACACTATCATGTTGACATTCC; dG = -11.4
419 >s_c_s_d_s_c_s_e_s_d_s GTGTGGGTGACGGTGTGGGTGCTGGTGACGGTG; >clamp_s_a_s_d_s:compl:rev CACCGTCACAGACACTATCATGTTGACATTCC; dG = -11.4
420 >s_a_s_a_s_c_s_a_s_e_s GTGTCTGTGTCTGTGTGGGTGTCTGTGCTGGTG; >clamp_s_a_s_d_s:compl:rev CACCGTCACAGACACTATCATGTTGACATTCC; dG = -11.3
421 >s_b_s_a_s_c_s_a_s_e_s GTGGCTGTGTCTGTGTGGGTGTCTGTGCTGGTG; >clamp_s_a_s_d_s:compl:rev CACCGTCACAGACACTATCATGTTGACATTCC; dG = -11.3
422 >s_b_s_a_s_c_s_a_s_c_s GTGGCTGTGTCTGTGTGGGTGTCTGTGTGGGTG; >clamp_s_a_s_d_s:compl:rev CACCGTCACAGACACTATCATGTTGACATTCC; dG = -11.3
423 >s_b_s_a_s_c_s_a_s_b_s GTGGCTGTGTCTGTGTGGGTGTCTGTGGCTGTG; >clamp_s_a_s_d_s:compl:rev CACCGTCACAGACACTATCATGTTGACATTCC; dG = -11.3
424 >s_b_s_a_s_c_s_a_s_a_s GTGGCTGTGTCTGTGTGGGTGTCTGTGTCTGTG; >clamp_s_a_s_d_s:compl:rev CACCGTCACAGACACTATCATGTTGACATTCC; dG = -11.3
425 >s_a_s_a_s_c_s_a_s_c_s GTGTCTGTGTCTGTGTGGGTGTCTGTGTGGGTG; >clamp_s_a_s_d_s:compl:rev CACCGTCACAGACACTATCATGTTGACATTCC; dG = -11.3
426 >s_a_s_a_s_c_s_a_s_b_s GTGTCTGTGTCTGTGTGGGTGTCTGTGGCTGTG; >clamp_s_a_s_d_s:compl:rev CACCGTCACAGACACTATCATGTTGACATTCC; dG = -11.3
427 >s_a_s_a_s_c_s_a_s_a_s GTGTCTGTGTCTGTGTGGGTGTCTGTGTCTGTG; >clamp_s_a_s_d_s:compl:rev CACCGTCACAGACACTATCATGTTGACATTCC; dG = -11.3
428 >s_c_s_a_s_c_s_a_s_e_s GTGTGGGTGTCTGTGTGGGTGTCTGTGCTGGTG; >clamp_s_a_s_d_s:compl:rev CACCGTCACAGACACTATCATGTTGACATTCC; dG = -11.3
429 >s_c_s_a_s_c_s_a_s_c_s GTGTGGGTGTCTGTGTGGGTGTCTGTGTGGGTG; >clamp_s_a_s_d_s:compl:rev CACCGTCACAGACACTATCATGTTGACATTCC; dG = -11.3
430 >s_c_s_a_s_c_s_a_s_b_s GTGTGGGTGTCTGTGTGGGTGTCTGTGGCTGTG; >clamp_s_a_s_d_s:compl:rev CACCGTCACAGACACTATCATGTTGACATTCC; dG = -11.3
431 >s_c_s_a_s_c_s_a_s_a_s GTGTGGGTGTCTGTGTGGGTGTCTGTGTCTGTG; >clamp_s_a_s_d_s:compl:rev CACCGTCACAGACACTATCATGTTGACATTCC; dG = -11.3
432 >s_c_s_d_s_e_s_a_s_e_s GTGTGGGTGACGGTGCTGGTGTCTGTGCTGGTG; >clamp_s_a_s_d_s:compl:rev CACCGTCACAGACACTATCATGTTGACATTCC; dG = -11.3
433 >s_c_s_d_s_e_s_a_s_c_s GTGTGGGTGACGGTGCTGGTGTCTGTGTGGGTG; >clamp_s_a_s_d_s:compl:rev CACCGTCACAGACACTATCATGTTGACATTCC; dG = -11.3
434 >s_c_s_d_s_e_s_a_s_b_s GTGTGGGTGACGGTGCTGGTGTCTGTGGCTGTG; >clamp_s_a_s_d_s:compl:rev CACCGTCACAGACACTATCATGTTGACATTCC; dG = -11.3
435 >s_c_s_d_s_e_s_a_s_a_s GTGTGGGTGACGGTGCTGGTGTCTGTGTCTGTG; >clamp_s_a_s_d_s:compl:rev CACCGTCACAGACACTATCATGTTGACATTCC; dG = -11.3
436 >s_c_s_d_s_e_s_e_s_d_s GTGTGGGTGACGGTGCTGGTGCTGGTGACGGTG; >clamp_s_a_s_d_s:compl:rev CACCGTCACAGACACTATCATGTTGACATTCC; dG = -11.3
437 >s_c_s_d_s_e_s_d_s_d_s GTGTGGGTGACGGTGCTGGTGACGGTGACGGTG; >clamp_s_a_s_d_s:compl:rev CACCGTCACAGACACTATCATGTTGACATTCC; dG = -11.3
438 >s_d_s_a_s_c_s_a_s_e_s GTGACGGTGTCTGTGTGGGTGTCTGTGCTGGTG; >clamp_s_a_s_d_s:compl:rev CACCGTCACAGACACTATCATGTTGACATTCC; dG = -11.3
439 >s_d_s_a_s_c_s_a_s_c_s GTGACGGTGTCTGTGTGGGTGTCTGTGTGGGTG; >clamp_s_a_s_d_s:compl:rev CACCGTCACAGACACTATCATGTTGACATTCC; dG = -11.3
440 >s_d_s_a_s_c_s_a_s_b_s GTGACGGTGTCTGTGTGGGTGTCTGTGGCTGTG; >clamp_s_a_s_d_s:compl:rev CACCGTCACAGACACTATCATGTTGACATTCC; dG = -11.3
441 >s_d_s_a_s_c_s_a_s_a_s GTGACGGTGTCTGTGTGGGTGTCTGTGTCTGTG; >clamp_s_a_s_d_s:compl:rev CACCGTCACAGACACTATCATGTTGACATTCC; dG = -11.3
442 >s_e_s_a_s_c_s_a_s_e_s GTGCTGGTGTCTGTGTGGGTGTCTGTGCTGGTG; >clamp_s_a_s_d_s:compl:rev CACCGTCACAGACACTATCATGTTGACATTCC; dG = -11.3
443 >s_e_s_a_s_c_s_a_s_c_s GTGCTGGTGTCTGTGTGGGTGTCTGTGTGGGTG; >clamp_s_a_s_d_s:compl:rev CACCGTCACAGACACTATCATGTTGACATTCC; dG = -11.3
444 >s_e_s_a_s_c_s_a_s_b_s GTGCTGGTGTCTGTGTGGGTGTCTGTGGCTGTG; >clamp_s_a_s_d_s:compl:rev CACCGTCACAGACACTATCATGTTGACATTCC; dG = -11.3
445 >s_e_s_a_s_c_s_a_s_a_s GTGCTGGTGTCTGTGTGGGTGTCTGTGTCTGTG; >clamp_s_a_s_d_s:compl:rev CACCGTCACAGACACTATCATGTTGACATTCC; dG = -11.3
446 >s_b_s_a_s_c_s_e_s_d_s GTGGCTGTGTCTGTGTGGGTGCTGGTGACGGTG; >clamp_s_a_s_d_s:compl:rev CACCGTCACAGACACTATCATGTTGACATTCC; dG = -11.2
447 >s_a_s_a_s_c_s_e_s_d_s GTGTCTGTGTCTGTGTGGGTGCTGGTGACGGTG; >clamp_s_a_s_d_s:compl:rev CACCGTCACAGACACTATCATGTTGACATTCC; dG = -11.2
448 >s_c_s_a_s_c_s_e_s_d_s GTGTGGGTGTCTGTGTGGGTGCTGGTGACGGTG; >clamp_s_a_s_d_s:compl:rev CACCGTCACAGACACTATCATGTTGACATTCC; dG = -11.2

449 >s_d_s_a_s_c_s_e_s_d_s GTGACGGTGTCTGTGTGGGTGCTGGTGACGGTG; >clamp_s_a_s_d_s:compl:rev CACCGTCACAGACACTATCATGTTGACATTCC; dG = -11.2
450 >s_e_s_a_s_c_s_e_s_d_s GTGCTGGTGTCTGTGTGGGTGCTGGTGACGGTG; >clamp_s_a_s_d_s:compl:rev CACCGTCACAGACACTATCATGTTGACATTCC; dG = -11.2
451 >s_a_s_c_s_e_s_d_s_d_s GTGTCTGTGTGGGTGCTGGTGACGGTGACGGTG; >clamp_s_a_s_d_s:compl:rev CACCGTCACAGACACTATCATGTTGACATTCC; dG = -11.1
452 >s_a_s_e_s_a_s_a_s_c_s GTGTCTGTGCTGGTGTCTGTGTCTGTGTGGGTG; >clamp_s_a_s_d_s:compl:rev CACCGTCACAGACACTATCATGTTGACATTCC; dG = -11.1
453 >s_a_s_e_s_b_s_a_s_c_s GTGTCTGTGCTGGTGGCTGTGTCTGTGTGGGTG; >clamp_s_a_s_d_s:compl:rev CACCGTCACAGACACTATCATGTTGACATTCC; dG = -11.1
454 >s_a_s_e_s_e_s_d_s_d_s GTGTCTGTGCTGGTGCTGGTGACGGTGACGGTG; >clamp_s_a_s_d_s:compl:rev CACCGTCACAGACACTATCATGTTGACATTCC; dG = -11.1
455 >s_b_s_a_s_a_s_a_s_c_s GTGGCTGTGTCTGTGTCTGTGTCTGTGTGGGTG; >clamp_s_a_s_d_s:compl:rev CACCGTCACAGACACTATCATGTTGACATTCC; dG = -11.1
456 >s_b_s_a_s_b_s_a_s_c_s GTGGCTGTGTCTGTGGCTGTGTCTGTGTGGGTG; >clamp_s_a_s_d_s:compl:rev CACCGTCACAGACACTATCATGTTGACATTCC; dG = -11.1
457 >s_b_s_b_s_a_s_a_s_c_s GTGGCTGTGGCTGTGTCTGTGTCTGTGTGGGTG; >clamp_s_a_s_d_s:compl:rev CACCGTCACAGACACTATCATGTTGACATTCC; dG = -11.1
458 >s_b_s_b_s_b_s_a_s_c_s GTGGCTGTGGCTGTGGCTGTGTCTGTGTGGGTG; >clamp_s_a_s_d_s:compl:rev CACCGTCACAGACACTATCATGTTGACATTCC; dG = -11.1
459 >s_a_s_a_s_a_s_a_s_c_s GTGTCTGTGTCTGTGTCTGTGTCTGTGTGGGTG; >clamp_s_a_s_d_s:compl:rev CACCGTCACAGACACTATCATGTTGACATTCC; dG = -11.1
460 >s_b_s_b_s_e_s_d_s_d_s GTGGCTGTGGCTGTGCTGGTGACGGTGACGGTG; >clamp_s_a_s_d_s:compl:rev CACCGTCACAGACACTATCATGTTGACATTCC; dG = -11.1
461 >s_b_s_c_s_a_s_a_s_c_s GTGGCTGTGTGGGTGTCTGTGTCTGTGTGGGTG; >clamp_s_a_s_d_s:compl:rev CACCGTCACAGACACTATCATGTTGACATTCC; dG = -11.1
462 >s_b_s_c_s_b_s_a_s_c_s GTGGCTGTGTGGGTGGCTGTGTCTGTGTGGGTG; >clamp_s_a_s_d_s:compl:rev CACCGTCACAGACACTATCATGTTGACATTCC; dG = -11.1
463 >s_b_s_c_s_e_s_d_s_d_s GTGGCTGTGTGGGTGCTGGTGACGGTGACGGTG; >clamp_s_a_s_d_s:compl:rev CACCGTCACAGACACTATCATGTTGACATTCC; dG = -11.1
464 >s_b_s_e_s_a_s_a_s_c_s GTGGCTGTGCTGGTGTCTGTGTCTGTGTGGGTG; >clamp_s_a_s_d_s:compl:rev CACCGTCACAGACACTATCATGTTGACATTCC; dG = -11.1
465 >s_b_s_e_s_b_s_a_s_c_s GTGGCTGTGCTGGTGGCTGTGTCTGTGTGGGTG; >clamp_s_a_s_d_s:compl:rev CACCGTCACAGACACTATCATGTTGACATTCC; dG = -11.1
466 >s_b_s_e_s_d_s_d_s_d_s GTGGCTGTGCTGGTGACGGTGACGGTGACGGTG; >clamp_s_a_s_d_s:compl:rev CACCGTCACAGACACTATCATGTTGACATTCC; dG = -11.1
467 >s_a_s_b_s_a_s_a_s_c_s GTGTCTGTGGCTGTGTCTGTGTCTGTGTGGGTG; >clamp_s_a_s_d_s:compl:rev CACCGTCACAGACACTATCATGTTGACATTCC; dG = -11.1
468 >s_b_s_e_s_e_s_d_s_d_s GTGGCTGTGCTGGTGCTGGTGACGGTGACGGTG; >clamp_s_a_s_d_s:compl:rev CACCGTCACAGACACTATCATGTTGACATTCC; dG = -11.1
469 >s_c_s_a_s_a_s_a_s_c_s GTGTGGGTGTCTGTGTCTGTGTCTGTGTGGGTG; >clamp_s_a_s_d_s:compl:rev CACCGTCACAGACACTATCATGTTGACATTCC; dG = -11.1
470 >s_c_s_a_s_b_s_a_s_c_s GTGTGGGTGTCTGTGGCTGTGTCTGTGTGGGTG; >clamp_s_a_s_d_s:compl:rev CACCGTCACAGACACTATCATGTTGACATTCC; dG = -11.1
471 >s_c_s_b_s_a_s_a_s_c_s GTGTGGGTGGCTGTGTCTGTGTCTGTGTGGGTG; >clamp_s_a_s_d_s:compl:rev CACCGTCACAGACACTATCATGTTGACATTCC; dG = -11.1
472 >s_c_s_b_s_b_s_a_s_c_s GTGTGGGTGGCTGTGGCTGTGTCTGTGTGGGTG; >clamp_s_a_s_d_s:compl:rev CACCGTCACAGACACTATCATGTTGACATTCC; dG = -11.1
473 >s_a_s_b_s_b_s_a_s_c_s GTGTCTGTGGCTGTGGCTGTGTCTGTGTGGGTG; >clamp_s_a_s_d_s:compl:rev CACCGTCACAGACACTATCATGTTGACATTCC; dG = -11.1
474 >s_c_s_b_s_e_s_d_s_d_s GTGTGGGTGGCTGTGCTGGTGACGGTGACGGTG; >clamp_s_a_s_d_s:compl:rev CACCGTCACAGACACTATCATGTTGACATTCC; dG = -11.1
475 >s_c_s_c_s_a_s_a_s_c_s GTGTGGGTGTGGGTGTCTGTGTCTGTGTGGGTG; >clamp_s_a_s_d_s:compl:rev CACCGTCACAGACACTATCATGTTGACATTCC; dG = -11.1
476 >s_c_s_c_s_b_s_a_s_c_s GTGTGGGTGTGGGTGGCTGTGTCTGTGTGGGTG; >clamp_s_a_s_d_s:compl:rev CACCGTCACAGACACTATCATGTTGACATTCC; dG = -11.1
477 >s_c_s_c_s_e_s_d_s_d_s GTGTGGGTGTGGGTGCTGGTGACGGTGACGGTG; >clamp_s_a_s_d_s:compl:rev CACCGTCACAGACACTATCATGTTGACATTCC; dG = -11.1
478 >s_c_s_e_s_a_s_a_s_c_s GTGTGGGTGCTGGTGTCTGTGTCTGTGTGGGTG; >clamp_s_a_s_d_s:compl:rev CACCGTCACAGACACTATCATGTTGACATTCC; dG = -11.1
479 >s_c_s_e_s_b_s_a_s_c_s GTGTGGGTGCTGGTGGCTGTGTCTGTGTGGGTG; >clamp_s_a_s_d_s:compl:rev CACCGTCACAGACACTATCATGTTGACATTCC; dG = -11.1
480 >s_c_s_e_s_d_s_d_s_d_s GTGTGGGTGCTGGTGACGGTGACGGTGACGGTG; >clamp_s_a_s_d_s:compl:rev CACCGTCACAGACACTATCATGTTGACATTCC; dG = -11.1
481 >s_c_s_e_s_e_s_d_s_d_s GTGTGGGTGCTGGTGCTGGTGACGGTGACGGTG; >clamp_s_a_s_d_s:compl:rev CACCGTCACAGACACTATCATGTTGACATTCC; dG = -11.1
482 >s_d_s_a_s_a_s_a_s_c_s GTGACGGTGTCTGTGTCTGTGTCTGTGTGGGTG; >clamp_s_a_s_d_s:compl:rev CACCGTCACAGACACTATCATGTTGACATTCC; dG = -11.1
483 >s_d_s_a_s_b_s_a_s_c_s GTGACGGTGTCTGTGGCTGTGTCTGTGTGGGTG; >clamp_s_a_s_d_s:compl:rev CACCGTCACAGACACTATCATGTTGACATTCC; dG = -11.1
484 >s_d_s_b_s_a_s_a_s_c_s GTGACGGTGGCTGTGTCTGTGTCTGTGTGGGTG; >clamp_s_a_s_d_s:compl:rev CACCGTCACAGACACTATCATGTTGACATTCC; dG = -11.1
485 >s_d_s_b_s_b_s_a_s_c_s GTGACGGTGGCTGTGGCTGTGTCTGTGTGGGTG; >clamp_s_a_s_d_s:compl:rev CACCGTCACAGACACTATCATGTTGACATTCC; dG = -11.1

486 >s_d_s_b_s_e_s_d_s_d_s GTGACGGTGGCTGTGCTGGTGACGGTGACGGTG; >clamp_s_a_s_d_s:compl:rev CACCGTCACAGACACTATCATGTTGACATTCC; dG = -11.1
487 >s_d_s_c_s_a_s_a_s_c_s GTGACGGTGTGGGTGTCTGTGTCTGTGTGGGTG; >clamp_s_a_s_d_s:compl:rev CACCGTCACAGACACTATCATGTTGACATTCC; dG = -11.1
488 >s_d_s_c_s_b_s_a_s_c_s GTGACGGTGTGGGTGGCTGTGTCTGTGTGGGTG; >clamp_s_a_s_d_s:compl:rev CACCGTCACAGACACTATCATGTTGACATTCC; dG = -11.1
489 >s_d_s_c_s_e_s_d_s_d_s GTGACGGTGTGGGTGCTGGTGACGGTGACGGTG; >clamp_s_a_s_d_s:compl:rev CACCGTCACAGACACTATCATGTTGACATTCC; dG = -11.1
490 >s_d_s_d_s_a_s_a_s_c_s GTGACGGTGACGGTGTCTGTGTCTGTGTGGGTG; >clamp_s_a_s_d_s:compl:rev CACCGTCACAGACACTATCATGTTGACATTCC; dG = -11.1
491 >s_d_s_d_s_b_s_a_s_c_s GTGACGGTGACGGTGGCTGTGTCTGTGTGGGTG; >clamp_s_a_s_d_s:compl:rev CACCGTCACAGACACTATCATGTTGACATTCC; dG = -11.1
492 >s_d_s_d_s_d_s_a_s_e_s GTGACGGTGACGGTGACGGTGTCTGTGCTGGTG; >clamp_s_a_s_d_s:compl:rev CACCGTCACAGACACTATCATGTTGACATTCC; dG = -11.1
493 >s_d_s_d_s_d_s_a_s_c_s GTGACGGTGACGGTGACGGTGTCTGTGTGGGTG; >clamp_s_a_s_d_s:compl:rev CACCGTCACAGACACTATCATGTTGACATTCC; dG = -11.1
494 >s_d_s_d_s_d_s_a_s_b_s GTGACGGTGACGGTGACGGTGTCTGTGGCTGTG; >clamp_s_a_s_d_s:compl:rev CACCGTCACAGACACTATCATGTTGACATTCC; dG = -11.1
495 >s_d_s_d_s_d_s_a_s_a_s GTGACGGTGACGGTGACGGTGTCTGTGTCTGTG; >clamp_s_a_s_d_s:compl:rev CACCGTCACAGACACTATCATGTTGACATTCC; dG = -11.1
496 >s_d_s_d_s_d_s_e_s_d_s GTGACGGTGACGGTGACGGTGCTGGTGACGGTG; >clamp_s_a_s_d_s:compl:rev CACCGTCACAGACACTATCATGTTGACATTCC; dG = -11.1
497 >s_d_s_d_s_d_s_d_s_d_s GTGACGGTGACGGTGACGGTGACGGTGACGGTG; >clamp_s_a_s_d_s:compl:rev CACCGTCACAGACACTATCATGTTGACATTCC; dG = -11.1
498 >s_d_s_d_s_e_s_d_s_d_s GTGACGGTGACGGTGCTGGTGACGGTGACGGTG; >clamp_s_a_s_d_s:compl:rev CACCGTCACAGACACTATCATGTTGACATTCC; dG = -11.1
499 >s_d_s_e_s_a_s_a_s_c_s GTGACGGTGCTGGTGTCTGTGTCTGTGTGGGTG; >clamp_s_a_s_d_s:compl:rev CACCGTCACAGACACTATCATGTTGACATTCC; dG = -11.1
500 >s_d_s_e_s_b_s_a_s_c_s GTGACGGTGCTGGTGGCTGTGTCTGTGTGGGTG; >clamp_s_a_s_d_s:compl:rev CACCGTCACAGACACTATCATGTTGACATTCC; dG = -11.1
501 >s_a_s_b_s_e_s_d_s_d_s GTGTCTGTGGCTGTGCTGGTGACGGTGACGGTG; >clamp_s_a_s_d_s:compl:rev CACCGTCACAGACACTATCATGTTGACATTCC; dG = -11.1
502 >s_d_s_e_s_d_s_d_s_d_s GTGACGGTGCTGGTGACGGTGACGGTGACGGTG; >clamp_s_a_s_d_s:compl:rev CACCGTCACAGACACTATCATGTTGACATTCC; dG = -11.1
503 >s_d_s_e_s_e_s_d_s_d_s GTGACGGTGCTGGTGCTGGTGACGGTGACGGTG; >clamp_s_a_s_d_s:compl:rev CACCGTCACAGACACTATCATGTTGACATTCC; dG = -11.1
504 >s_e_s_a_s_a_s_a_s_c_s GTGCTGGTGTCTGTGTCTGTGTCTGTGTGGGTG; >clamp_s_a_s_d_s:compl:rev CACCGTCACAGACACTATCATGTTGACATTCC; dG = -11.1
505 >s_e_s_a_s_b_s_a_s_c_s GTGCTGGTGTCTGTGGCTGTGTCTGTGTGGGTG; >clamp_s_a_s_d_s:compl:rev CACCGTCACAGACACTATCATGTTGACATTCC; dG = -11.1
506 >s_a_s_c_s_a_s_a_s_c_s GTGTCTGTGTGGGTGTCTGTGTCTGTGTGGGTG; >clamp_s_a_s_d_s:compl:rev CACCGTCACAGACACTATCATGTTGACATTCC; dG = -11.1
507 >s_e_s_b_s_a_s_a_s_c_s GTGCTGGTGGCTGTGTCTGTGTCTGTGTGGGTG; >clamp_s_a_s_d_s:compl:rev CACCGTCACAGACACTATCATGTTGACATTCC; dG = -11.1
508 >s_e_s_b_s_b_s_a_s_c_s GTGCTGGTGGCTGTGGCTGTGTCTGTGTGGGTG; >clamp_s_a_s_d_s:compl:rev CACCGTCACAGACACTATCATGTTGACATTCC; dG = -11.1
509 >s_e_s_b_s_e_s_d_s_d_s GTGCTGGTGGCTGTGCTGGTGACGGTGACGGTG; >clamp_s_a_s_d_s:compl:rev CACCGTCACAGACACTATCATGTTGACATTCC; dG = -11.1
510 >s_e_s_c_s_a_s_a_s_c_s GTGCTGGTGTGGGTGTCTGTGTCTGTGTGGGTG; >clamp_s_a_s_d_s:compl:rev CACCGTCACAGACACTATCATGTTGACATTCC; dG = -11.1
511 >s_e_s_c_s_b_s_a_s_c_s GTGCTGGTGTGGGTGGCTGTGTCTGTGTGGGTG; >clamp_s_a_s_d_s:compl:rev CACCGTCACAGACACTATCATGTTGACATTCC; dG = -11.1
512 >s_a_s_c_s_b_s_a_s_c_s GTGTCTGTGTGGGTGGCTGTGTCTGTGTGGGTG; >clamp_s_a_s_d_s:compl:rev CACCGTCACAGACACTATCATGTTGACATTCC; dG = -11.1
513 >s_e_s_c_s_e_s_d_s_d_s GTGCTGGTGTGGGTGCTGGTGACGGTGACGGTG; >clamp_s_a_s_d_s:compl:rev CACCGTCACAGACACTATCATGTTGACATTCC; dG = -11.1
514 >s_e_s_d_s_a_s_a_s_c_s GTGCTGGTGACGGTGTCTGTGTCTGTGTGGGTG; >clamp_s_a_s_d_s:compl:rev CACCGTCACAGACACTATCATGTTGACATTCC; dG = -11.1
515 >s_e_s_d_s_b_s_a_s_c_s GTGCTGGTGACGGTGGCTGTGTCTGTGTGGGTG; >clamp_s_a_s_d_s:compl:rev CACCGTCACAGACACTATCATGTTGACATTCC; dG = -11.1
516 >s_e_s_d_s_d_s_a_s_e_s GTGCTGGTGACGGTGACGGTGTCTGTGCTGGTG; >clamp_s_a_s_d_s:compl:rev CACCGTCACAGACACTATCATGTTGACATTCC; dG = -11.1
517 >s_e_s_d_s_d_s_a_s_c_s GTGCTGGTGACGGTGACGGTGTCTGTGTGGGTG; >clamp_s_a_s_d_s:compl:rev CACCGTCACAGACACTATCATGTTGACATTCC; dG = -11.1
518 >s_e_s_d_s_d_s_a_s_b_s GTGCTGGTGACGGTGACGGTGTCTGTGGCTGTG; >clamp_s_a_s_d_s:compl:rev CACCGTCACAGACACTATCATGTTGACATTCC; dG = -11.1
519 >s_e_s_d_s_d_s_a_s_a_s GTGCTGGTGACGGTGACGGTGTCTGTGTCTGTG; >clamp_s_a_s_d_s:compl:rev CACCGTCACAGACACTATCATGTTGACATTCC; dG = -11.1
520 >s_e_s_d_s_d_s_e_s_d_s GTGCTGGTGACGGTGACGGTGCTGGTGACGGTG; >clamp_s_a_s_d_s:compl:rev CACCGTCACAGACACTATCATGTTGACATTCC; dG = -11.1
521 >s_e_s_d_s_d_s_d_s_d_s GTGCTGGTGACGGTGACGGTGACGGTGACGGTG; >clamp_s_a_s_d_s:compl:rev CACCGTCACAGACACTATCATGTTGACATTCC; dG = -11.1
522 >s_e_s_d_s_e_s_d_s_d_s GTGCTGGTGACGGTGCTGGTGACGGTGACGGTG; >clamp_s_a_s_d_s:compl:rev CACCGTCACAGACACTATCATGTTGACATTCC; dG = -11.1

523 >s_e_s_e_s_a_s_a_s_c_s GTGCTGGTGCTGGTGTCTGTGTCTGTGTGGGTG; >clamp_s_a_s_d_s:compl:rev CACCGTCACAGACACTATCATGTTGACATTCC; dG = -11.1
524 >s_e_s_e_s_b_s_a_s_c_s GTGCTGGTGCTGGTGGCTGTGTCTGTGTGGGTG; >clamp_s_a_s_d_s:compl:rev CACCGTCACAGACACTATCATGTTGACATTCC; dG = -11.1
525 >s_e_s_e_s_d_s_d_s_d_s GTGCTGGTGCTGGTGACGGTGACGGTGACGGTG; >clamp_s_a_s_d_s:compl:rev CACCGTCACAGACACTATCATGTTGACATTCC; dG = -11.1
526 >s_e_s_e_s_e_s_d_s_d_s GTGCTGGTGCTGGTGCTGGTGACGGTGACGGTG; >clamp_s_a_s_d_s:compl:rev CACCGTCACAGACACTATCATGTTGACATTCC; dG = -11.1
527 >s_a_s_a_s_b_s_a_s_c_s GTGTCTGTGTCTGTGGCTGTGTCTGTGTGGGTG; >clamp_s_a_s_d_s:compl:rev CACCGTCACAGACACTATCATGTTGACATTCC; dG = -11.1
528 >s_a_s_c_s_e_s_a_s_c_s GTGTCTGTGTGGGTGCTGGTGTCTGTGTGGGTG; >clamp_s_a_s_d_s:compl:rev CACCGTCACAGACACTATCATGTTGACATTCC; dG = -11.0
529 >s_a_s_e_s_c_s_a_s_c_s GTGTCTGTGCTGGTGTGGGTGTCTGTGTGGGTG; >clamp_s_a_s_d_s:compl:rev CACCGTCACAGACACTATCATGTTGACATTCC; dG = -11.0
530 >s_a_s_e_s_e_s_a_s_c_s GTGTCTGTGCTGGTGCTGGTGTCTGTGTGGGTG; >clamp_s_a_s_d_s:compl:rev CACCGTCACAGACACTATCATGTTGACATTCC; dG = -11.0
531 >s_b_s_a_s_e_s_a_s_c_s GTGGCTGTGTCTGTGCTGGTGTCTGTGTGGGTG; >clamp_s_a_s_d_s:compl:rev CACCGTCACAGACACTATCATGTTGACATTCC; dG = -11.0
532 >s_b_s_b_s_c_s_a_s_c_s GTGGCTGTGGCTGTGTGGGTGTCTGTGTGGGTG; >clamp_s_a_s_d_s:compl:rev CACCGTCACAGACACTATCATGTTGACATTCC; dG = -11.0
533 >s_b_s_b_s_e_s_a_s_c_s GTGGCTGTGGCTGTGCTGGTGTCTGTGTGGGTG; >clamp_s_a_s_d_s:compl:rev CACCGTCACAGACACTATCATGTTGACATTCC; dG = -11.0
534 >s_b_s_c_s_c_s_a_s_c_s GTGGCTGTGTGGGTGTGGGTGTCTGTGTGGGTG; >clamp_s_a_s_d_s:compl:rev CACCGTCACAGACACTATCATGTTGACATTCC; dG = -11.0
535 >s_a_s_a_s_e_s_a_s_c_s GTGTCTGTGTCTGTGCTGGTGTCTGTGTGGGTG; >clamp_s_a_s_d_s:compl:rev CACCGTCACAGACACTATCATGTTGACATTCC; dG = -11.0
536 >s_b_s_c_s_e_s_a_s_c_s GTGGCTGTGTGGGTGCTGGTGTCTGTGTGGGTG; >clamp_s_a_s_d_s:compl:rev CACCGTCACAGACACTATCATGTTGACATTCC; dG = -11.0
537 >s_b_s_e_s_c_s_a_s_c_s GTGGCTGTGCTGGTGTGGGTGTCTGTGTGGGTG; >clamp_s_a_s_d_s:compl:rev CACCGTCACAGACACTATCATGTTGACATTCC; dG = -11.0
538 >s_b_s_e_s_d_s_a_s_c_s GTGGCTGTGCTGGTGACGGTGTCTGTGTGGGTG; >clamp_s_a_s_d_s:compl:rev CACCGTCACAGACACTATCATGTTGACATTCC; dG = -11.0
539 >s_b_s_e_s_e_s_a_s_c_s GTGGCTGTGCTGGTGCTGGTGTCTGTGTGGGTG; >clamp_s_a_s_d_s:compl:rev CACCGTCACAGACACTATCATGTTGACATTCC; dG = -11.0
540 >s_c_s_a_s_e_s_a_s_c_s GTGTGGGTGTCTGTGCTGGTGTCTGTGTGGGTG; >clamp_s_a_s_d_s:compl:rev CACCGTCACAGACACTATCATGTTGACATTCC; dG = -11.0
541 >s_c_s_b_s_c_s_a_s_c_s GTGTGGGTGGCTGTGTGGGTGTCTGTGTGGGTG; >clamp_s_a_s_d_s:compl:rev CACCGTCACAGACACTATCATGTTGACATTCC; dG = -11.0
542 >s_c_s_b_s_e_s_a_s_c_s GTGTGGGTGGCTGTGCTGGTGTCTGTGTGGGTG; >clamp_s_a_s_d_s:compl:rev CACCGTCACAGACACTATCATGTTGACATTCC; dG = -11.0
543 >s_c_s_c_s_c_s_a_s_c_s GTGTGGGTGTGGGTGTGGGTGTCTGTGTGGGTG; >clamp_s_a_s_d_s:compl:rev CACCGTCACAGACACTATCATGTTGACATTCC; dG = -11.0
544 >s_c_s_c_s_e_s_a_s_c_s GTGTGGGTGTGGGTGCTGGTGTCTGTGTGGGTG; >clamp_s_a_s_d_s:compl:rev CACCGTCACAGACACTATCATGTTGACATTCC; dG = -11.0
545 >s_a_s_b_s_c_s_a_s_c_s GTGTCTGTGGCTGTGTGGGTGTCTGTGTGGGTG; >clamp_s_a_s_d_s:compl:rev CACCGTCACAGACACTATCATGTTGACATTCC; dG = -11.0
546 >s_c_s_e_s_c_s_a_s_c_s GTGTGGGTGCTGGTGTGGGTGTCTGTGTGGGTG; >clamp_s_a_s_d_s:compl:rev CACCGTCACAGACACTATCATGTTGACATTCC; dG = -11.0
547 >s_c_s_e_s_d_s_a_s_c_s GTGTGGGTGCTGGTGACGGTGTCTGTGTGGGTG; >clamp_s_a_s_d_s:compl:rev CACCGTCACAGACACTATCATGTTGACATTCC; dG = -11.0
548 >s_c_s_e_s_e_s_a_s_c_s GTGTGGGTGCTGGTGCTGGTGTCTGTGTGGGTG; >clamp_s_a_s_d_s:compl:rev CACCGTCACAGACACTATCATGTTGACATTCC; dG = -11.0
549 >s_d_s_a_s_e_s_a_s_c_s GTGACGGTGTCTGTGCTGGTGTCTGTGTGGGTG; >clamp_s_a_s_d_s:compl:rev CACCGTCACAGACACTATCATGTTGACATTCC; dG = -11.0
550 >s_d_s_b_s_c_s_a_s_c_s GTGACGGTGGCTGTGTGGGTGTCTGTGTGGGTG; >clamp_s_a_s_d_s:compl:rev CACCGTCACAGACACTATCATGTTGACATTCC; dG = -11.0
551 >s_d_s_b_s_e_s_a_s_c_s GTGACGGTGGCTGTGCTGGTGTCTGTGTGGGTG; >clamp_s_a_s_d_s:compl:rev CACCGTCACAGACACTATCATGTTGACATTCC; dG = -11.0
552 >s_d_s_c_s_c_s_a_s_c_s GTGACGGTGTGGGTGTGGGTGTCTGTGTGGGTG; >clamp_s_a_s_d_s:compl:rev CACCGTCACAGACACTATCATGTTGACATTCC; dG = -11.0
553 >s_d_s_c_s_e_s_a_s_c_s GTGACGGTGTGGGTGCTGGTGTCTGTGTGGGTG; >clamp_s_a_s_d_s:compl:rev CACCGTCACAGACACTATCATGTTGACATTCC; dG = -11.0
554 >s_a_s_b_s_e_s_a_s_c_s GTGTCTGTGGCTGTGCTGGTGTCTGTGTGGGTG; >clamp_s_a_s_d_s:compl:rev CACCGTCACAGACACTATCATGTTGACATTCC; dG = -11.0
555 >s_d_s_d_s_c_s_a_s_c_s GTGACGGTGACGGTGTGGGTGTCTGTGTGGGTG; >clamp_s_a_s_d_s:compl:rev CACCGTCACAGACACTATCATGTTGACATTCC; dG = -11.0
556 >s_d_s_d_s_e_s_a_s_c_s GTGACGGTGACGGTGCTGGTGTCTGTGTGGGTG; >clamp_s_a_s_d_s:compl:rev CACCGTCACAGACACTATCATGTTGACATTCC; dG = -11.0
557 >s_d_s_e_s_c_s_a_s_c_s GTGACGGTGCTGGTGTGGGTGTCTGTGTGGGTG; >clamp_s_a_s_d_s:compl:rev CACCGTCACAGACACTATCATGTTGACATTCC; dG = -11.0
558 >s_d_s_e_s_d_s_a_s_c_s GTGACGGTGCTGGTGACGGTGTCTGTGTGGGTG; >clamp_s_a_s_d_s:compl:rev CACCGTCACAGACACTATCATGTTGACATTCC; dG = -11.0
559 >s_d_s_e_s_e_s_a_s_c_s GTGACGGTGCTGGTGCTGGTGTCTGTGTGGGTG; >clamp_s_a_s_d_s:compl:rev CACCGTCACAGACACTATCATGTTGACATTCC; dG = -11.0

560 >s_e_s_a_s_e_s_a_s_c_s GTGCTGGTGTCTGTGCTGGTGTCTGTGTGGGTG; >clamp_s_a_s_d_s:compl:rev
CACCGTCACAGACACTATCATGTTGACATTCC; dG = -11.0
561 >s_e_s_b_s_c_s_a_s_c_s GTGCTGGTGGCTGTGTGGGTGTCTGTGTGGGTG; >clamp_s_a_s_d_s:compl:rev
CACCGTCACAGACACTATCATGTTGACATTCC; dG = -11.0
562 >s_e_s_b_s_e_s_a_s_c_s GTGCTGGTGGCTGTGCTGGTGTCTGTGTGGGTG; >clamp_s_a_s_d_s:compl:rev
CACCGTCACAGACACTATCATGTTGACATTCC; dG = -11.0
563 >s_e_s_c_s_c_s_a_s_c_s GTGCTGGTGTGGGTGTGGGTGTCTGTGTGGGTG; >clamp_s_a_s_d_s:compl:rev
CACCGTCACAGACACTATCATGTTGACATTCC; dG = -11.0
564 >s_e_s_c_s_e_s_a_s_c_s GTGCTGGTGTGGGTGCTGGTGTCTGTGTGGGTG; >clamp_s_a_s_d_s:compl:rev
CACCGTCACAGACACTATCATGTTGACATTCC; dG = -11.0
565 >s_e_s_d_s_c_s_a_s_c_s GTGCTGGTGACGGTGTGGGTGTCTGTGTGGGTG; >clamp_s_a_s_d_s:compl:rev
CACCGTCACAGACACTATCATGTTGACATTCC; dG = -11.0
566 >s_e_s_d_s_e_s_a_s_c_s GTGCTGGTGACGGTGCTGGTGTCTGTGTGGGTG; >clamp_s_a_s_d_s:compl:rev
CACCGTCACAGACACTATCATGTTGACATTCC; dG = -11.0
567 >s_a_s_c_s_c_s_a_s_c_s GTGTCTGTGTGGGTGTGGGTGTCTGTGTGGGTG; >clamp_s_a_s_d_s:compl:rev
CACCGTCACAGACACTATCATGTTGACATTCC; dG = -11.0
568 >s_e_s_e_s_c_s_a_s_c_s GTGCTGGTGCTGGTGTGGGTGTCTGTGTGGGTG; >clamp_s_a_s_d_s:compl:rev
CACCGTCACAGACACTATCATGTTGACATTCC; dG = -11.0
569 >s_e_s_e_s_d_s_a_s_c_s GTGCTGGTGCTGGTGACGGTGTCTGTGTGGGTG; >clamp_s_a_s_d_s:compl:rev
CACCGTCACAGACACTATCATGTTGACATTCC; dG = -11.0
570 >s_e_s_e_s_e_s_a_s_c_s GTGCTGGTGCTGGTGCTGGTGTCTGTGTGGGTG; >clamp_s_a_s_d_s:compl:rev
CACCGTCACAGACACTATCATGTTGACATTCC; dG = -11.0
571 >s_a_s_c_s_b_s_a_s_e_s GTGTCTGTGTGGGTGGCTGTGTCTGTGCTGGTG; >clamp_s_a_s_d_s:compl:rev
CACCGTCACAGACACTATCATGTTGACATTCC; dG = -10.9
572 >s_a_s_c_s_b_s_a_s_b_s GTGTCTGTGTGGGTGGCTGTGTCTGTGGCTGTG; >clamp_s_a_s_d_s:compl:rev
CACCGTCACAGACACTATCATGTTGACATTCC; dG = -10.9
573 >s_a_s_c_s_b_s_a_s_a_s GTGTCTGTGTGGGTGGCTGTGTCTGTGTCTGTG; >clamp_s_a_s_d_s:compl:rev
CACCGTCACAGACACTATCATGTTGACATTCC; dG = -10.9
574 >s_a_s_c_s_b_s_e_s_d_s GTGTCTGTGTGGGTGGCTGTGCTGGTGACGGTG; >clamp_s_a_s_d_s:compl:rev
CACCGTCACAGACACTATCATGTTGACATTCC; dG = -10.9
575 >s_a_s_a_s_b_s_e_s_d_s GTGTCTGTGTCTGTGGCTGTGCTGGTGACGGTG; >clamp_s_a_s_d_s:compl:rev
CACCGTCACAGACACTATCATGTTGACATTCC; dG = -10.8
576 >s_b_s_a_s_b_s_e_s_d_s GTGGCTGTGTCTGTGGCTGTGCTGGTGACGGTG; >clamp_s_a_s_d_s:compl:rev
CACCGTCACAGACACTATCATGTTGACATTCC; dG = -10.8
577 >s_b_s_a_s_e_s_e_s_d_s GTGGCTGTGTCTGTGCTGGTGCTGGTGACGGTG; >clamp_s_a_s_d_s:compl:rev
CACCGTCACAGACACTATCATGTTGACATTCC; dG = -10.8
578 >s_a_s_a_s_e_s_e_s_d_s GTGTCTGTGTCTGTGCTGGTGCTGGTGACGGTG; >clamp_s_a_s_d_s:compl:rev
CACCGTCACAGACACTATCATGTTGACATTCC; dG = -10.8
579 >s_c_s_a_s_b_s_e_s_d_s GTGTGGGTGTCTGTGGCTGTGCTGGTGACGGTG; >clamp_s_a_s_d_s:compl:rev
CACCGTCACAGACACTATCATGTTGACATTCC; dG = -10.8
580 >s_c_s_a_s_e_s_e_s_d_s GTGTGGGTGTCTGTGCTGGTGCTGGTGACGGTG; >clamp_s_a_s_d_s:compl:rev
CACCGTCACAGACACTATCATGTTGACATTCC; dG = -10.8
581 >s_d_s_a_s_b_s_e_s_d_s GTGACGGTGTCTGTGGCTGTGCTGGTGACGGTG; >clamp_s_a_s_d_s:compl:rev
CACCGTCACAGACACTATCATGTTGACATTCC; dG = -10.8
582 >s_d_s_a_s_e_s_e_s_d_s GTGACGGTGTCTGTGCTGGTGCTGGTGACGGTG; >clamp_s_a_s_d_s:compl:rev
CACCGTCACAGACACTATCATGTTGACATTCC; dG = -10.8
583 >s_a_s_c_s_a_s_a_s_e_s GTGTCTGTGTGGGTGTCTGTGTCTGTGCTGGTG; >clamp_s_a_s_d_s:compl:rev
CACCGTCACAGACACTATCATGTTGACATTCC; dG = -10.8
584 >s_e_s_a_s_b_s_e_s_d_s GTGCTGGTGTCTGTGGCTGTGCTGGTGACGGTG; >clamp_s_a_s_d_s:compl:rev
CACCGTCACAGACACTATCATGTTGACATTCC; dG = -10.8
585 >s_a_s_c_s_a_s_a_s_b_s GTGTCTGTGTGGGTGTCTGTGTCTGTGGCTGTG; >clamp_s_a_s_d_s:compl:rev
CACCGTCACAGACACTATCATGTTGACATTCC; dG = -10.8
586 >s_e_s_a_s_e_s_e_s_d_s GTGCTGGTGTCTGTGCTGGTGCTGGTGACGGTG; >clamp_s_a_s_d_s:compl:rev
CACCGTCACAGACACTATCATGTTGACATTCC; dG = -10.8
587 >s_a_s_c_s_a_s_a_s_a_s GTGTCTGTGTGGGTGTCTGTGTCTGTGTCTGTG; >clamp_s_a_s_d_s:compl:rev
CACCGTCACAGACACTATCATGTTGACATTCC; dG = -10.8
588 >s_a_s_c_s_c_s_a_s_e_s GTGTCTGTGTGGGTGTGGGTGTCTGTGCTGGTG; >clamp_s_a_s_d_s:compl:rev
CACCGTCACAGACACTATCATGTTGACATTCC; dG = -10.8
589 >s_a_s_c_s_c_s_a_s_b_s GTGTCTGTGTGGGTGTGGGTGTCTGTGGCTGTG; >clamp_s_a_s_d_s:compl:rev
CACCGTCACAGACACTATCATGTTGACATTCC; dG = -10.8
590 >s_a_s_c_s_c_s_a_s_a_s GTGTCTGTGTGGGTGTGGGTGTCTGTGTCTGTG; >clamp_s_a_s_d_s:compl:rev
CACCGTCACAGACACTATCATGTTGACATTCC; dG = -10.8
591 >s_a_s_c_s_c_s_e_s_d_s GTGTCTGTGTGGGTGTGGGTGCTGGTGACGGTG; >clamp_s_a_s_d_s:compl:rev
CACCGTCACAGACACTATCATGTTGACATTCC; dG = -10.8
592 >s_a_s_c_s_e_s_a_s_e_s GTGTCTGTGTGGGTGCTGGTGTCTGTGCTGGTG; >clamp_s_a_s_d_s:compl:rev
CACCGTCACAGACACTATCATGTTGACATTCC; dG = -10.7
593 >s_a_s_c_s_e_s_a_s_b_s GTGTCTGTGTGGGTGCTGGTGTCTGTGGCTGTG; >clamp_s_a_s_d_s:compl:rev
CACCGTCACAGACACTATCATGTTGACATTCC; dG = -10.7
594 >s_a_s_c_s_e_s_a_s_a_s GTGTCTGTGTGGGTGCTGGTGTCTGTGTCTGTG; >clamp_s_a_s_d_s:compl:rev
CACCGTCACAGACACTATCATGTTGACATTCC; dG = -10.7
595 >s_a_s_c_s_e_s_e_s_d_s GTGTCTGTGTGGGTGCTGGTGCTGGTGACGGTG; >clamp_s_a_s_d_s:compl:rev
CACCGTCACAGACACTATCATGTTGACATTCC; dG = -10.7
596 >s_d_s_d_s_a_s_a_s_e_s GTGACGGTGACGGTGTCTGTGTCTGTGCTGGTG; >clamp_s_a_s_d_s:compl:rev
CACCGTCACAGACACTATCATGTTGACATTCC; dG = -10.6

597 >s_d_s_d_s_a_s_a_s_b_s GTGACGGTGACGGTGTCTGTGTCTGTGGCTGTG; >clamp_s_a_s_d_s:compl:rev
CACCGTCACAGACACTATCATGTTGACATTCC; dG = -10.6
598 >s_d_s_d_s_a_s_a_s_a_s GTGACGGTGACGGTGTCTGTGTCTGTGTCTGTG; >clamp_s_a_s_d_s:compl:rev
CACCGTCACAGACACTATCATGTTGACATTCC; dG = -10.6
599 >s_d_s_d_s_b_s_a_s_e_s GTGACGGTGACGGTGGCTGTGTCTGTGCTGGTG; >clamp_s_a_s_d_s:compl:rev
CACCGTCACAGACACTATCATGTTGACATTCC; dG = -10.6
600 >s_d_s_d_s_b_s_a_s_b_s GTGACGGTGACGGTGGCTGTGTCTGTGGCTGTG; >clamp_s_a_s_d_s:compl:rev
CACCGTCACAGACACTATCATGTTGACATTCC; dG = -10.6
601 >s_d_s_d_s_b_s_a_s_a_s GTGACGGTGACGGTGGCTGTGTCTGTGTCTGTG; >clamp_s_a_s_d_s:compl:rev
CACCGTCACAGACACTATCATGTTGACATTCC; dG = -10.6
602 >s_d_s_d_s_b_s_e_s_d_s GTGACGGTGACGGTGGCTGTGCTGGTGACGGTG; >clamp_s_a_s_d_s:compl:rev
CACCGTCACAGACACTATCATGTTGACATTCC; dG = -10.6
603 >s_d_s_d_s_c_s_a_s_e_s GTGACGGTGACGGTGTGGGTGTCTGTGCTGGTG; >clamp_s_a_s_d_s:compl:rev
CACCGTCACAGACACTATCATGTTGACATTCC; dG = -10.6
604 >s_d_s_d_s_c_s_a_s_b_s GTGACGGTGACGGTGTGGGTGTCTGTGGCTGTG; >clamp_s_a_s_d_s:compl:rev
CACCGTCACAGACACTATCATGTTGACATTCC; dG = -10.6
605 >s_d_s_d_s_c_s_a_s_a_s GTGACGGTGACGGTGTGGGTGTCTGTGTCTGTG; >clamp_s_a_s_d_s:compl:rev
CACCGTCACAGACACTATCATGTTGACATTCC; dG = -10.6
606 >s_d_s_d_s_c_s_e_s_d_s GTGACGGTGACGGTGTGGGTGCTGGTGACGGTG; >clamp_s_a_s_d_s:compl:rev
CACCGTCACAGACACTATCATGTTGACATTCC; dG = -10.6
607 >s_d_s_d_s_e_s_a_s_e_s GTGACGGTGACGGTGCTGGTGTCTGTGCTGGTG; >clamp_s_a_s_d_s:compl:rev
CACCGTCACAGACACTATCATGTTGACATTCC; dG = -10.6
608 >s_d_s_d_s_e_s_a_s_b_s GTGACGGTGACGGTGCTGGTGTCTGTGGCTGTG; >clamp_s_a_s_d_s:compl:rev
CACCGTCACAGACACTATCATGTTGACATTCC; dG = -10.6
609 >s_d_s_d_s_e_s_a_s_a_s GTGACGGTGACGGTGCTGGTGTCTGTGTCTGTG; >clamp_s_a_s_d_s:compl:rev
CACCGTCACAGACACTATCATGTTGACATTCC; dG = -10.6
610 >s_d_s_d_s_e_s_e_s_d_s GTGACGGTGACGGTGCTGGTGCTGGTGACGGTG; >clamp_s_a_s_d_s:compl:rev
CACCGTCACAGACACTATCATGTTGACATTCC; dG = -10.6
611 >s_e_s_d_s_b_s_a_s_e_s GTGCTGGTGACGGTGGCTGTGTCTGTGCTGGTG; >clamp_s_a_s_d_s:compl:rev
CACCGTCACAGACACTATCATGTTGACATTCC; dG = -10.6
612 >s_e_s_d_s_b_s_a_s_b_s GTGCTGGTGACGGTGGCTGTGTCTGTGGCTGTG; >clamp_s_a_s_d_s:compl:rev
CACCGTCACAGACACTATCATGTTGACATTCC; dG = -10.6
613 >s_e_s_d_s_b_s_a_s_a_s GTGCTGGTGACGGTGGCTGTGTCTGTGTCTGTG; >clamp_s_a_s_d_s:compl:rev
CACCGTCACAGACACTATCATGTTGACATTCC; dG = -10.6
614 >s_e_s_d_s_b_s_e_s_d_s GTGCTGGTGACGGTGGCTGTGCTGGTGACGGTG; >clamp_s_a_s_d_s:compl:rev
CACCGTCACAGACACTATCATGTTGACATTCC; dG = -10.6
615 >s_b_s_a_s_e_s_a_s_e_s GTGGCTGTGTCTGTGCTGGTGTCTGTGCTGGTG; >clamp_s_a_s_d_s:compl:rev
CACCGTCACAGACACTATCATGTTGACATTCC; dG = -10.5
616 >s_b_s_a_s_e_s_a_s_b_s GTGGCTGTGTCTGTGCTGGTGTCTGTGGCTGTG; >clamp_s_a_s_d_s:compl:rev
CACCGTCACAGACACTATCATGTTGACATTCC; dG = -10.5
617 >s_b_s_a_s_e_s_a_s_a_s GTGGCTGTGTCTGTGCTGGTGTCTGTGTCTGTG; >clamp_s_a_s_d_s:compl:rev
CACCGTCACAGACACTATCATGTTGACATTCC; dG = -10.5
618 >s_a_s_a_s_e_s_a_s_e_s GTGTCTGTGTCTGTGCTGGTGTCTGTGCTGGTG; >clamp_s_a_s_d_s:compl:rev
CACCGTCACAGACACTATCATGTTGACATTCC; dG = -10.5
619 >s_a_s_a_s_e_s_a_s_b_s GTGTCTGTGTCTGTGCTGGTGTCTGTGGCTGTG; >clamp_s_a_s_d_s:compl:rev
CACCGTCACAGACACTATCATGTTGACATTCC; dG = -10.5
620 >s_a_s_a_s_e_s_a_s_a_s GTGTCTGTGTCTGTGCTGGTGTCTGTGTCTGTG; >clamp_s_a_s_d_s:compl:rev
CACCGTCACAGACACTATCATGTTGACATTCC; dG = -10.5
621 >s_b_s_e_s_d_s_a_s_e_s GTGGCTGTGCTGGTGACGGTGTCTGTGCTGGTG; >clamp_s_a_s_d_s:compl:rev
CACCGTCACAGACACTATCATGTTGACATTCC; dG = -10.5
622 >s_b_s_e_s_d_s_a_s_b_s GTGGCTGTGCTGGTGACGGTGTCTGTGGCTGTG; >clamp_s_a_s_d_s:compl:rev
CACCGTCACAGACACTATCATGTTGACATTCC; dG = -10.5
623 >s_b_s_e_s_d_s_a_s_a_s GTGGCTGTGCTGGTGACGGTGTCTGTGTCTGTG; >clamp_s_a_s_d_s:compl:rev
CACCGTCACAGACACTATCATGTTGACATTCC; dG = -10.5
624 >s_c_s_a_s_e_s_a_s_e_s GTGTGGGTGTCTGTGCTGGTGTCTGTGCTGGTG; >clamp_s_a_s_d_s:compl:rev
CACCGTCACAGACACTATCATGTTGACATTCC; dG = -10.5
625 >s_c_s_a_s_e_s_a_s_b_s GTGTGGGTGTCTGTGCTGGTGTCTGTGGCTGTG; >clamp_s_a_s_d_s:compl:rev
CACCGTCACAGACACTATCATGTTGACATTCC; dG = -10.5
626 >s_c_s_a_s_e_s_a_s_a_s GTGTGGGTGTCTGTGCTGGTGTCTGTGTCTGTG; >clamp_s_a_s_d_s:compl:rev
CACCGTCACAGACACTATCATGTTGACATTCC; dG = -10.5
627 >s_c_s_e_s_d_s_a_s_e_s GTGTGGGTGCTGGTGACGGTGTCTGTGCTGGTG; >clamp_s_a_s_d_s:compl:rev
CACCGTCACAGACACTATCATGTTGACATTCC; dG = -10.5
628 >s_c_s_e_s_d_s_a_s_b_s GTGTGGGTGCTGGTGACGGTGTCTGTGGCTGTG; >clamp_s_a_s_d_s:compl:rev
CACCGTCACAGACACTATCATGTTGACATTCC; dG = -10.5
629 >s_c_s_e_s_d_s_a_s_a_s GTGTGGGTGCTGGTGACGGTGTCTGTGTCTGTG; >clamp_s_a_s_d_s:compl:rev
CACCGTCACAGACACTATCATGTTGACATTCC; dG = -10.5
630 >s_d_s_a_s_e_s_a_s_e_s GTGACGGTGTCTGTGCTGGTGTCTGTGCTGGTG; >clamp_s_a_s_d_s:compl:rev
CACCGTCACAGACACTATCATGTTGACATTCC; dG = -10.5
631 >s_d_s_a_s_e_s_a_s_b_s GTGACGGTGTCTGTGCTGGTGTCTGTGGCTGTG; >clamp_s_a_s_d_s:compl:rev
CACCGTCACAGACACTATCATGTTGACATTCC; dG = -10.5
632 >s_d_s_a_s_e_s_a_s_a_s GTGACGGTGTCTGTGCTGGTGTCTGTGTCTGTG; >clamp_s_a_s_d_s:compl:rev
CACCGTCACAGACACTATCATGTTGACATTCC; dG = -10.5
633 >s_d_s_e_s_d_s_a_s_e_s GTGACGGTGCTGGTGACGGTGTCTGTGCTGGTG; >clamp_s_a_s_d_s:compl:rev
CACCGTCACAGACACTATCATGTTGACATTCC; dG = -10.5

634 >s_d_s_e_s_d_s_a_s_b_s GTGACGGTGCTGGTGACGGTGTCTGTGGCTGTG; >clamp_s_a_s_d_s:compl:rev CACCGTCACAGACACTATCATGTTGACATTCC; dG = -10.5
635 >s_d_s_e_s_d_s_a_s_a_s GTGACGGTGCTGGTGACGGTGTCTGTGTCTGTG; >clamp_s_a_s_d_s:compl:rev CACCGTCACAGACACTATCATGTTGACATTCC; dG = -10.5
636 >s_e_s_a_s_e_s_a_s_e_s GTGCTGGTGTCTGTGCTGGTGTCTGTGCTGGTG; >clamp_s_a_s_d_s:compl:rev CACCGTCACAGACACTATCATGTTGACATTCC; dG = -10.5
637 >s_e_s_a_s_e_s_a_s_b_s GTGCTGGTGTCTGTGCTGGTGTCTGTGGCTGTG; >clamp_s_a_s_d_s:compl:rev CACCGTCACAGACACTATCATGTTGACATTCC; dG = -10.5
638 >s_e_s_a_s_e_s_a_s_a_s GTGCTGGTGTCTGTGCTGGTGTCTGTGTCTGTG; >clamp_s_a_s_d_s:compl:rev CACCGTCACAGACACTATCATGTTGACATTCC; dG = -10.5
639 >s_e_s_d_s_a_s_a_s_e_s GTGCTGGTGACGGTGTCTGTGTCTGTGCTGGTG; >clamp_s_a_s_d_s:compl:rev CACCGTCACAGACACTATCATGTTGACATTCC; dG = -10.5
640 >s_e_s_d_s_a_s_a_s_b_s GTGCTGGTGACGGTGTCTGTGTCTGTGGCTGTG; >clamp_s_a_s_d_s:compl:rev CACCGTCACAGACACTATCATGTTGACATTCC; dG = -10.5
641 >s_e_s_d_s_a_s_a_s_a_s GTGCTGGTGACGGTGTCTGTGTCTGTGTCTGTG; >clamp_s_a_s_d_s:compl:rev CACCGTCACAGACACTATCATGTTGACATTCC; dG = -10.5
642 >s_e_s_d_s_c_s_a_s_e_s GTGCTGGTGACGGTGTGGGTGTCTGTGCTGGTG; >clamp_s_a_s_d_s:compl:rev CACCGTCACAGACACTATCATGTTGACATTCC; dG = -10.5
643 >s_e_s_d_s_c_s_a_s_b_s GTGCTGGTGACGGTGTGGGTGTCTGTGGCTGTG; >clamp_s_a_s_d_s:compl:rev CACCGTCACAGACACTATCATGTTGACATTCC; dG = -10.5
644 >s_e_s_d_s_c_s_a_s_a_s GTGCTGGTGACGGTGTGGGTGTCTGTGTCTGTG; >clamp_s_a_s_d_s:compl:rev CACCGTCACAGACACTATCATGTTGACATTCC; dG = -10.5
645 >s_e_s_d_s_c_s_e_s_d_s GTGCTGGTGACGGTGTGGGTGCTGGTGACGGTG; >clamp_s_a_s_d_s:compl:rev CACCGTCACAGACACTATCATGTTGACATTCC; dG = -10.5
646 >s_e_s_e_s_d_s_a_s_e_s GTGCTGGTGCTGGTGACGGTGTCTGTGCTGGTG; >clamp_s_a_s_d_s:compl:rev CACCGTCACAGACACTATCATGTTGACATTCC; dG = -10.5
647 >s_e_s_e_s_d_s_a_s_b_s GTGCTGGTGCTGGTGACGGTGTCTGTGGCTGTG; >clamp_s_a_s_d_s:compl:rev CACCGTCACAGACACTATCATGTTGACATTCC; dG = -10.5
648 >s_e_s_e_s_d_s_a_s_a_s GTGCTGGTGCTGGTGACGGTGTCTGTGTCTGTG; >clamp_s_a_s_d_s:compl:rev CACCGTCACAGACACTATCATGTTGACATTCC; dG = -10.5
649 >s_b_s_e_s_d_s_e_s_d_s GTGGCTGTGCTGGTGACGGTGCTGGTGACGGTG; >clamp_s_a_s_d_s:compl:rev CACCGTCACAGACACTATCATGTTGACATTCC; dG = -10.4
650 >s_c_s_e_s_d_s_e_s_d_s GTGTGGGTGCTGGTGACGGTGCTGGTGACGGTG; >clamp_s_a_s_d_s:compl:rev CACCGTCACAGACACTATCATGTTGACATTCC; dG = -10.4
651 >s_d_s_e_s_d_s_e_s_d_s GTGACGGTGCTGGTGACGGTGCTGGTGACGGTG; >clamp_s_a_s_d_s:compl:rev CACCGTCACAGACACTATCATGTTGACATTCC; dG = -10.4
652 >s_e_s_d_s_e_s_a_s_e_s GTGCTGGTGACGGTGCTGGTGTCTGTGCTGGTG; >clamp_s_a_s_d_s:compl:rev CACCGTCACAGACACTATCATGTTGACATTCC; dG = -10.4
653 >s_e_s_d_s_e_s_a_s_b_s GTGCTGGTGACGGTGCTGGTGTCTGTGGCTGTG; >clamp_s_a_s_d_s:compl:rev CACCGTCACAGACACTATCATGTTGACATTCC; dG = -10.4
654 >s_e_s_d_s_e_s_a_s_a_s GTGCTGGTGACGGTGCTGGTGTCTGTGTCTGTG; >clamp_s_a_s_d_s:compl:rev CACCGTCACAGACACTATCATGTTGACATTCC; dG = -10.4
655 >s_e_s_d_s_e_s_e_s_d_s GTGCTGGTGACGGTGCTGGTGCTGGTGACGGTG; >clamp_s_a_s_d_s:compl:rev CACCGTCACAGACACTATCATGTTGACATTCC; dG = -10.4
656 >s_e_s_e_s_d_s_e_s_d_s GTGCTGGTGCTGGTGACGGTGCTGGTGACGGTG; >clamp_s_a_s_d_s:compl:rev CACCGTCACAGACACTATCATGTTGACATTCC; dG = -10.4
657 >s_a_s_a_s_b_s_a_s_b_s GTGTCTGTGTCTGTGGCTGTGTCTGTGGCTGTG; >clamp_s_a_s_d_s:compl:rev CACCGTCACAGACACTATCATGTTGACATTCC; dG = -10.3
658 >s_a_s_a_s_b_s_a_s_a_s GTGTCTGTGTCTGTGGCTGTGTCTGTGTCTGTG; >clamp_s_a_s_d_s:compl:rev CACCGTCACAGACACTATCATGTTGACATTCC; dG = -10.3
659 >s_a_s_e_s_b_s_e_s_d_s GTGTCTGTGCTGGTGGCTGTGCTGGTGACGGTG; >clamp_s_a_s_d_s:compl:rev CACCGTCACAGACACTATCATGTTGACATTCC; dG = -10.3
660 >s_a_s_e_s_c_s_e_s_d_s GTGTCTGTGCTGGTGTGGGTGCTGGTGACGGTG; >clamp_s_a_s_d_s:compl:rev CACCGTCACAGACACTATCATGTTGACATTCC; dG = -10.3
661 >s_a_s_e_s_e_s_e_s_d_s GTGTCTGTGCTGGTGCTGGTGCTGGTGACGGTG; >clamp_s_a_s_d_s:compl:rev CACCGTCACAGACACTATCATGTTGACATTCC; dG = -10.3
662 >s_b_s_a_s_b_s_a_s_e_s GTGGCTGTGTCTGTGGCTGTGTCTGTGCTGGTG; >clamp_s_a_s_d_s:compl:rev CACCGTCACAGACACTATCATGTTGACATTCC; dG = -10.3
663 >s_b_s_a_s_b_s_a_s_b_s GTGGCTGTGTCTGTGGCTGTGTCTGTGGCTGTG; >clamp_s_a_s_d_s:compl:rev CACCGTCACAGACACTATCATGTTGACATTCC; dG = -10.3
664 >s_b_s_a_s_b_s_a_s_a_s GTGGCTGTGTCTGTGGCTGTGTCTGTGTCTGTG; >clamp_s_a_s_d_s:compl:rev CACCGTCACAGACACTATCATGTTGACATTCC; dG = -10.3
665 >s_b_s_b_s_b_s_e_s_d_s GTGGCTGTGGCTGTGGCTGTGCTGGTGACGGTG; >clamp_s_a_s_d_s:compl:rev CACCGTCACAGACACTATCATGTTGACATTCC; dG = -10.3
666 >s_b_s_b_s_c_s_e_s_d_s GTGGCTGTGGCTGTGTGGGTGCTGGTGACGGTG; >clamp_s_a_s_d_s:compl:rev CACCGTCACAGACACTATCATGTTGACATTCC; dG = -10.3
667 >s_b_s_b_s_e_s_e_s_d_s GTGGCTGTGGCTGTGCTGGTGCTGGTGACGGTG; >clamp_s_a_s_d_s:compl:rev CACCGTCACAGACACTATCATGTTGACATTCC; dG = -10.3
668 >s_b_s_c_s_b_s_e_s_d_s GTGGCTGTGTGGGTGGCTGTGCTGGTGACGGTG; >clamp_s_a_s_d_s:compl:rev CACCGTCACAGACACTATCATGTTGACATTCC; dG = -10.3
669 >s_b_s_c_s_c_s_e_s_d_s GTGGCTGTGTGGGTGTGGGTGCTGGTGACGGTG; >clamp_s_a_s_d_s:compl:rev CACCGTCACAGACACTATCATGTTGACATTCC; dG = -10.3
670 >s_b_s_c_s_e_s_e_s_d_s GTGGCTGTGTGGGTGCTGGTGCTGGTGACGGTG; >clamp_s_a_s_d_s:compl:rev CACCGTCACAGACACTATCATGTTGACATTCC; dG = -10.3

671 >s_b_s_e_s_b_s_e_s_d_s GTGGCTGTGCTGGTGGCTGTGCTGGTGACGGTG; >clamp_s_a_s_d_s:compl:rev CACCGTCACAGACACTATCATGTTGACATTCC; dG = -10.3
672 >s_b_s_e_s_c_s_e_s_d_s GTGGCTGTGCTGGTGTGGGTGCTGGTGACGGTG; >clamp_s_a_s_d_s:compl:rev CACCGTCACAGACACTATCATGTTGACATTCC; dG = -10.3
673 >s_b_s_e_s_e_s_e_s_d_s GTGGCTGTGCTGGTGCTGGTGCTGGTGACGGTG; >clamp_s_a_s_d_s:compl:rev CACCGTCACAGACACTATCATGTTGACATTCC; dG = -10.3
674 >s_c_s_b_s_b_s_e_s_d_s GTGTGGGTGGCTGTGGCTGTGCTGGTGACGGTG; >clamp_s_a_s_d_s:compl:rev CACCGTCACAGACACTATCATGTTGACATTCC; dG = -10.3
675 >s_c_s_b_s_c_s_e_s_d_s GTGTGGGTGGCTGTGTGGGTGCTGGTGACGGTG; >clamp_s_a_s_d_s:compl:rev CACCGTCACAGACACTATCATGTTGACATTCC; dG = -10.3
676 >s_c_s_b_s_e_s_e_s_d_s GTGTGGGTGGCTGTGCTGGTGCTGGTGACGGTG; >clamp_s_a_s_d_s:compl:rev CACCGTCACAGACACTATCATGTTGACATTCC; dG = -10.3
677 >s_c_s_c_s_b_s_e_s_d_s GTGTGGGTGTGGGTGGCTGTGCTGGTGACGGTG; >clamp_s_a_s_d_s:compl:rev CACCGTCACAGACACTATCATGTTGACATTCC; dG = -10.3
678 >s_c_s_c_s_c_s_e_s_d_s GTGTGGGTGTGGGTGTGGGTGCTGGTGACGGTG; >clamp_s_a_s_d_s:compl:rev CACCGTCACAGACACTATCATGTTGACATTCC; dG = -10.3
679 >s_a_s_b_s_b_s_e_s_d_s GTGTCTGTGGCTGTGGCTGTGCTGGTGACGGTG; >clamp_s_a_s_d_s:compl:rev CACCGTCACAGACACTATCATGTTGACATTCC; dG = -10.3
680 >s_c_s_c_s_e_s_e_s_d_s GTGTGGGTGTGGGTGCTGGTGCTGGTGACGGTG; >clamp_s_a_s_d_s:compl:rev CACCGTCACAGACACTATCATGTTGACATTCC; dG = -10.3
681 >s_c_s_e_s_b_s_e_s_d_s GTGTGGGTGCTGGTGGCTGTGCTGGTGACGGTG; >clamp_s_a_s_d_s:compl:rev CACCGTCACAGACACTATCATGTTGACATTCC; dG = -10.3
682 >s_c_s_e_s_c_s_e_s_d_s GTGTGGGTGCTGGTGTGGGTGCTGGTGACGGTG; >clamp_s_a_s_d_s:compl:rev CACCGTCACAGACACTATCATGTTGACATTCC; dG = -10.3
683 >s_a_s_b_s_c_s_e_s_d_s GTGTCTGTGGCTGTGTGGGTGCTGGTGACGGTG; >clamp_s_a_s_d_s:compl:rev CACCGTCACAGACACTATCATGTTGACATTCC; dG = -10.3
684 >s_c_s_e_s_e_s_e_s_d_s GTGTGGGTGCTGGTGCTGGTGCTGGTGACGGTG; >clamp_s_a_s_d_s:compl:rev CACCGTCACAGACACTATCATGTTGACATTCC; dG = -10.3
685 >s_d_s_b_s_b_s_e_s_d_s GTGACGGTGGCTGTGGCTGTGCTGGTGACGGTG; >clamp_s_a_s_d_s:compl:rev CACCGTCACAGACACTATCATGTTGACATTCC; dG = -10.3
686 >s_d_s_b_s_c_s_e_s_d_s GTGACGGTGGCTGTGTGGGTGCTGGTGACGGTG; >clamp_s_a_s_d_s:compl:rev CACCGTCACAGACACTATCATGTTGACATTCC; dG = -10.3
687 >s_d_s_b_s_e_s_e_s_d_s GTGACGGTGGCTGTGCTGGTGCTGGTGACGGTG; >clamp_s_a_s_d_s:compl:rev CACCGTCACAGACACTATCATGTTGACATTCC; dG = -10.3
688 >s_d_s_c_s_b_s_e_s_d_s GTGACGGTGTGGGTGGCTGTGCTGGTGACGGTG; >clamp_s_a_s_d_s:compl:rev CACCGTCACAGACACTATCATGTTGACATTCC; dG = -10.3
689 >s_d_s_c_s_c_s_e_s_d_s GTGACGGTGTGGGTGTGGGTGCTGGTGACGGTG; >clamp_s_a_s_d_s:compl:rev CACCGTCACAGACACTATCATGTTGACATTCC; dG = -10.3
690 >s_d_s_c_s_e_s_e_s_d_s GTGACGGTGTGGGTGCTGGTGCTGGTGACGGTG; >clamp_s_a_s_d_s:compl:rev CACCGTCACAGACACTATCATGTTGACATTCC; dG = -10.3
691 >s_a_s_b_s_e_s_e_s_d_s GTGTCTGTGGCTGTGCTGGTGCTGGTGACGGTG; >clamp_s_a_s_d_s:compl:rev CACCGTCACAGACACTATCATGTTGACATTCC; dG = -10.3
692 >s_d_s_e_s_b_s_e_s_d_s GTGACGGTGCTGGTGGCTGTGCTGGTGACGGTG; >clamp_s_a_s_d_s:compl:rev CACCGTCACAGACACTATCATGTTGACATTCC; dG = -10.3
693 >s_d_s_e_s_c_s_e_s_d_s GTGACGGTGCTGGTGTGGGTGCTGGTGACGGTG; >clamp_s_a_s_d_s:compl:rev CACCGTCACAGACACTATCATGTTGACATTCC; dG = -10.3
694 >s_d_s_e_s_e_s_e_s_d_s GTGACGGTGCTGGTGCTGGTGCTGGTGACGGTG; >clamp_s_a_s_d_s:compl:rev CACCGTCACAGACACTATCATGTTGACATTCC; dG = -10.3
695 >s_e_s_b_s_b_s_e_s_d_s GTGCTGGTGGCTGTGGCTGTGCTGGTGACGGTG; >clamp_s_a_s_d_s:compl:rev CACCGTCACAGACACTATCATGTTGACATTCC; dG = -10.3
696 >s_e_s_b_s_c_s_e_s_d_s GTGCTGGTGGCTGTGTGGGTGCTGGTGACGGTG; >clamp_s_a_s_d_s:compl:rev CACCGTCACAGACACTATCATGTTGACATTCC; dG = -10.3
697 >s_e_s_b_s_e_s_e_s_d_s GTGCTGGTGGCTGTGCTGGTGCTGGTGACGGTG; >clamp_s_a_s_d_s:compl:rev CACCGTCACAGACACTATCATGTTGACATTCC; dG = -10.3
698 >s_e_s_c_s_b_s_e_s_d_s GTGCTGGTGTGGGTGGCTGTGCTGGTGACGGTG; >clamp_s_a_s_d_s:compl:rev CACCGTCACAGACACTATCATGTTGACATTCC; dG = -10.3
699 >s_e_s_c_s_c_s_e_s_d_s GTGCTGGTGTGGGTGTGGGTGCTGGTGACGGTG; >clamp_s_a_s_d_s:compl:rev CACCGTCACAGACACTATCATGTTGACATTCC; dG = -10.3
700 >s_a_s_a_s_b_s_a_s_e_s GTGTCTGTGTCTGTGGCTGTGTCTGTGCTGGTG; >clamp_s_a_s_d_s:compl:rev CACCGTCACAGACACTATCATGTTGACATTCC; dG = -10.3
701 >s_e_s_c_s_e_s_e_s_d_s GTGCTGGTGTGGGTGCTGGTGCTGGTGACGGTG; >clamp_s_a_s_d_s:compl:rev CACCGTCACAGACACTATCATGTTGACATTCC; dG = -10.3
702 >s_e_s_e_s_b_s_e_s_d_s GTGCTGGTGCTGGTGGCTGTGCTGGTGACGGTG; >clamp_s_a_s_d_s:compl:rev CACCGTCACAGACACTATCATGTTGACATTCC; dG = -10.3
703 >s_e_s_e_s_c_s_e_s_d_s GTGCTGGTGCTGGTGTGGGTGCTGGTGACGGTG; >clamp_s_a_s_d_s:compl:rev CACCGTCACAGACACTATCATGTTGACATTCC; dG = -10.3
704 >s_e_s_e_s_e_s_e_s_d_s GTGCTGGTGCTGGTGCTGGTGCTGGTGACGGTG; >clamp_s_a_s_d_s:compl:rev CACCGTCACAGACACTATCATGTTGACATTCC; dG = -10.3
705 >s_a_s_a_s_a_s_a_s_e_s GTGTCTGTGTCTGTGTCTGTGTCTGTGCTGGTG; >clamp_s_a_s_d_s:compl:rev CACCGTCACAGACACTATCATGTTGACATTCC; dG = -10.2
706 >s_a_s_e_s_a_s_a_s_e_s GTGTCTGTGCTGGTGTCTGTGTCTGTGCTGGTG; >clamp_s_a_s_d_s:compl:rev CACCGTCACAGACACTATCATGTTGACATTCC; dG = -10.2
707 >s_a_s_e_s_b_s_a_s_e_s GTGTCTGTGCTGGTGGCTGTGTCTGTGCTGGTG; >clamp_s_a_s_d_s:compl:rev CACCGTCACAGACACTATCATGTTGACATTCC; dG = -10.2

708 >s_a_s_e_s_c_s_a_s_e_s GTGTCTGTGCTGGTGTGGGTGTCTGTGCTGGTG; >clamp_s_a_s_d_s:compl:rev
CACCGTCACAGACACTATCATGTTGACATTCC; dG = -10.2
709 >s_a_s_e_s_e_s_a_s_e_s GTGTCTGTGCTGGTGCTGGTGTCTGTGCTGGTG; >clamp_s_a_s_d_s:compl:rev
CACCGTCACAGACACTATCATGTTGACATTCC; dG = -10.2
710 >s_b_s_a_s_a_s_a_s_e_s GTGGCTGTGTCTGTGTCTGTGTCTGTGCTGGTG; >clamp_s_a_s_d_s:compl:rev
CACCGTCACAGACACTATCATGTTGACATTCC; dG = -10.2
711 >s_b_s_b_s_a_s_a_s_e_s GTGGCTGTGGCTGTGTCTGTGTCTGTGCTGGTG; >clamp_s_a_s_d_s:compl:rev
CACCGTCACAGACACTATCATGTTGACATTCC; dG = -10.2
712 >s_b_s_b_s_b_s_a_s_e_s GTGGCTGTGGCTGTGGCTGTGTCTGTGCTGGTG; >clamp_s_a_s_d_s:compl:rev
CACCGTCACAGACACTATCATGTTGACATTCC; dG = -10.2
713 >s_b_s_b_s_c_s_a_s_e_s GTGGCTGTGGCTGTGTGGGTGTCTGTGCTGGTG; >clamp_s_a_s_d_s:compl:rev
CACCGTCACAGACACTATCATGTTGACATTCC; dG = -10.2
714 >s_b_s_b_s_e_s_a_s_e_s GTGGCTGTGGCTGTGCTGGTGTCTGTGCTGGTG; >clamp_s_a_s_d_s:compl:rev
CACCGTCACAGACACTATCATGTTGACATTCC; dG = -10.2
715 >s_b_s_c_s_a_s_a_s_e_s GTGGCTGTGTGGGTGTCTGTGTCTGTGCTGGTG; >clamp_s_a_s_d_s:compl:rev
CACCGTCACAGACACTATCATGTTGACATTCC; dG = -10.2
716 >s_b_s_c_s_b_s_a_s_e_s GTGGCTGTGTGGGTGGCTGTGTCTGTGCTGGTG; >clamp_s_a_s_d_s:compl:rev
CACCGTCACAGACACTATCATGTTGACATTCC; dG = -10.2
717 >s_b_s_c_s_c_s_a_s_e_s GTGGCTGTGTGGGTGTGGGTGTCTGTGCTGGTG; >clamp_s_a_s_d_s:compl:rev
CACCGTCACAGACACTATCATGTTGACATTCC; dG = -10.2
718 >s_b_s_c_s_e_s_a_s_e_s GTGGCTGTGTGGGTGCTGGTGTCTGTGCTGGTG; >clamp_s_a_s_d_s:compl:rev
CACCGTCACAGACACTATCATGTTGACATTCC; dG = -10.2
719 >s_b_s_e_s_a_s_a_s_e_s GTGGCTGTGCTGGTGTCTGTGTCTGTGCTGGTG; >clamp_s_a_s_d_s:compl:rev
CACCGTCACAGACACTATCATGTTGACATTCC; dG = -10.2
720 >s_b_s_e_s_b_s_a_s_e_s GTGGCTGTGCTGGTGGCTGTGTCTGTGCTGGTG; >clamp_s_a_s_d_s:compl:rev
CACCGTCACAGACACTATCATGTTGACATTCC; dG = -10.2
721 >s_b_s_e_s_c_s_a_s_e_s GTGGCTGTGCTGGTGTGGGTGTCTGTGCTGGTG; >clamp_s_a_s_d_s:compl:rev
CACCGTCACAGACACTATCATGTTGACATTCC; dG = -10.2
722 >s_a_s_b_s_a_s_a_s_e_s GTGTCTGTGGCTGTGTCTGTGTCTGTGCTGGTG; >clamp_s_a_s_d_s:compl:rev
CACCGTCACAGACACTATCATGTTGACATTCC; dG = -10.2
723 >s_b_s_e_s_e_s_a_s_e_s GTGGCTGTGCTGGTGCTGGTGTCTGTGCTGGTG; >clamp_s_a_s_d_s:compl:rev
CACCGTCACAGACACTATCATGTTGACATTCC; dG = -10.2
724 >s_c_s_a_s_a_s_a_s_e_s GTGTGGGTGTCTGTGTCTGTGTCTGTGCTGGTG; >clamp_s_a_s_d_s:compl:rev
CACCGTCACAGACACTATCATGTTGACATTCC; dG = -10.2
725 >s_c_s_a_s_b_s_a_s_e_s GTGTGGGTGTCTGTGGCTGTGTCTGTGCTGGTG; >clamp_s_a_s_d_s:compl:rev
CACCGTCACAGACACTATCATGTTGACATTCC; dG = -10.2
726 >s_c_s_a_s_b_s_a_s_b_s GTGTGGGTGTCTGTGGCTGTGTCTGTGGCTGTG; >clamp_s_a_s_d_s:compl:rev
CACCGTCACAGACACTATCATGTTGACATTCC; dG = -10.2
727 >s_c_s_a_s_b_s_a_s_a_s GTGTGGGTGTCTGTGGCTGTGTCTGTGTCTGTG; >clamp_s_a_s_d_s:compl:rev
CACCGTCACAGACACTATCATGTTGACATTCC; dG = -10.2
728 >s_c_s_b_s_a_s_a_s_e_s GTGTGGGTGGCTGTGTCTGTGTCTGTGCTGGTG; >clamp_s_a_s_d_s:compl:rev
CACCGTCACAGACACTATCATGTTGACATTCC; dG = -10.2
729 >s_c_s_b_s_b_s_a_s_e_s GTGTGGGTGGCTGTGGCTGTGTCTGTGCTGGTG; >clamp_s_a_s_d_s:compl:rev
CACCGTCACAGACACTATCATGTTGACATTCC; dG = -10.2
730 >s_c_s_b_s_c_s_a_s_e_s GTGTGGGTGGCTGTGTGGGTGTCTGTGCTGGTG; >clamp_s_a_s_d_s:compl:rev
CACCGTCACAGACACTATCATGTTGACATTCC; dG = -10.2
731 >s_a_s_b_s_b_s_a_s_e_s GTGTCTGTGGCTGTGGCTGTGTCTGTGCTGGTG; >clamp_s_a_s_d_s:compl:rev
CACCGTCACAGACACTATCATGTTGACATTCC; dG = -10.2
732 >s_c_s_b_s_e_s_a_s_e_s GTGTGGGTGGCTGTGCTGGTGTCTGTGCTGGTG; >clamp_s_a_s_d_s:compl:rev
CACCGTCACAGACACTATCATGTTGACATTCC; dG = -10.2
733 >s_c_s_c_s_a_s_a_s_e_s GTGTGGGTGTGGGTGTCTGTGTCTGTGCTGGTG; >clamp_s_a_s_d_s:compl:rev
CACCGTCACAGACACTATCATGTTGACATTCC; dG = -10.2
734 >s_c_s_c_s_b_s_a_s_e_s GTGTGGGTGTGGGTGGCTGTGTCTGTGCTGGTG; >clamp_s_a_s_d_s:compl:rev
CACCGTCACAGACACTATCATGTTGACATTCC; dG = -10.2
735 >s_c_s_c_s_c_s_a_s_e_s GTGTGGGTGTGGGTGTGGGTGTCTGTGCTGGTG; >clamp_s_a_s_d_s:compl:rev
CACCGTCACAGACACTATCATGTTGACATTCC; dG = -10.2
736 >s_c_s_c_s_e_s_a_s_e_s GTGTGGGTGTGGGTGCTGGTGTCTGTGCTGGTG; >clamp_s_a_s_d_s:compl:rev
CACCGTCACAGACACTATCATGTTGACATTCC; dG = -10.2
737 >s_a_s_b_s_c_s_a_s_e_s GTGTCTGTGGCTGTGTGGGTGTCTGTGCTGGTG; >clamp_s_a_s_d_s:compl:rev
CACCGTCACAGACACTATCATGTTGACATTCC; dG = -10.2
738 >s_c_s_e_s_a_s_a_s_e_s GTGTGGGTGCTGGTGTCTGTGTCTGTGCTGGTG; >clamp_s_a_s_d_s:compl:rev
CACCGTCACAGACACTATCATGTTGACATTCC; dG = -10.2
739 >s_c_s_e_s_b_s_a_s_e_s GTGTGGGTGCTGGTGGCTGTGTCTGTGCTGGTG; >clamp_s_a_s_d_s:compl:rev
CACCGTCACAGACACTATCATGTTGACATTCC; dG = -10.2
740 >s_c_s_e_s_c_s_a_s_e_s GTGTGGGTGCTGGTGTGGGTGTCTGTGCTGGTG; >clamp_s_a_s_d_s:compl:rev
CACCGTCACAGACACTATCATGTTGACATTCC; dG = -10.2
741 >s_c_s_e_s_e_s_a_s_e_s GTGTGGGTGCTGGTGCTGGTGTCTGTGCTGGTG; >clamp_s_a_s_d_s:compl:rev
CACCGTCACAGACACTATCATGTTGACATTCC; dG = -10.2
742 >s_d_s_a_s_a_s_a_s_e_s GTGACGGTGTCTGTGTCTGTGTCTGTGCTGGTG; >clamp_s_a_s_d_s:compl:rev
CACCGTCACAGACACTATCATGTTGACATTCC; dG = -10.2
743 >s_d_s_a_s_b_s_a_s_e_s GTGACGGTGTCTGTGGCTGTGTCTGTGCTGGTG; >clamp_s_a_s_d_s:compl:rev
CACCGTCACAGACACTATCATGTTGACATTCC; dG = -10.2
744 >s_d_s_a_s_b_s_a_s_b_s GTGACGGTGTCTGTGGCTGTGTCTGTGGCTGTG; >clamp_s_a_s_d_s:compl:rev
CACCGTCACAGACACTATCATGTTGACATTCC; dG = -10.2

745 >s_d_s_a_s_b_s_a_s_a_s GTGACGGTGTCTGTGGCTGTGTCTGTGTCTGTG; >clamp_s_a_s_d_s:compl:rev
CACCGTCACAGACACTATCATGTTGACATTCC; dG = -10.2
746 >s_d_s_b_s_a_s_a_s_e_s GTGACGGTGGCTGTGTCTGTGTCTGTGCTGGTG; >clamp_s_a_s_d_s:compl:rev
CACCGTCACAGACACTATCATGTTGACATTCC; dG = -10.2
747 >s_d_s_b_s_a_s_a_s_b_s GTGACGGTGGCTGTGTCTGTGTCTGTGGCTGTG; >clamp_s_a_s_d_s:compl:rev
CACCGTCACAGACACTATCATGTTGACATTCC; dG = -10.2
748 >s_d_s_b_s_a_s_a_s_a_s GTGACGGTGGCTGTGTCTGTGTCTGTGTCTGTG; >clamp_s_a_s_d_s:compl:rev
CACCGTCACAGACACTATCATGTTGACATTCC; dG = -10.2
749 >s_d_s_b_s_b_s_a_s_e_s GTGACGGTGGCTGTGGCTGTGTCTGTGCTGGTG; >clamp_s_a_s_d_s:compl:rev
CACCGTCACAGACACTATCATGTTGACATTCC; dG = -10.2
750 >s_d_s_b_s_b_s_a_s_b_s GTGACGGTGGCTGTGGCTGTGTCTGTGGCTGTG; >clamp_s_a_s_d_s:compl:rev
CACCGTCACAGACACTATCATGTTGACATTCC; dG = -10.2
751 >s_d_s_b_s_b_s_a_s_a_s GTGACGGTGGCTGTGGCTGTGTCTGTGTCTGTG; >clamp_s_a_s_d_s:compl:rev
CACCGTCACAGACACTATCATGTTGACATTCC; dG = -10.2
752 >s_d_s_b_s_c_s_a_s_e_s GTGACGGTGGCTGTGTGGGTGTCTGTGCTGGTG; >clamp_s_a_s_d_s:compl:rev
CACCGTCACAGACACTATCATGTTGACATTCC; dG = -10.2
753 >s_d_s_b_s_c_s_a_s_b_s GTGACGGTGGCTGTGTGGGTGTCTGTGGCTGTG; >clamp_s_a_s_d_s:compl:rev
CACCGTCACAGACACTATCATGTTGACATTCC; dG = -10.2
754 >s_d_s_b_s_c_s_a_s_a_s GTGACGGTGGCTGTGTGGGTGTCTGTGTCTGTG; >clamp_s_a_s_d_s:compl:rev
CACCGTCACAGACACTATCATGTTGACATTCC; dG = -10.2
755 >s_d_s_b_s_e_s_a_s_e_s GTGACGGTGGCTGTGCTGGTGTCTGTGCTGGTG; >clamp_s_a_s_d_s:compl:rev
CACCGTCACAGACACTATCATGTTGACATTCC; dG = -10.2
756 >s_d_s_b_s_e_s_a_s_b_s GTGACGGTGGCTGTGCTGGTGTCTGTGGCTGTG; >clamp_s_a_s_d_s:compl:rev
CACCGTCACAGACACTATCATGTTGACATTCC; dG = -10.2
757 >s_d_s_b_s_e_s_a_s_a_s GTGACGGTGGCTGTGCTGGTGTCTGTGTCTGTG; >clamp_s_a_s_d_s:compl:rev
CACCGTCACAGACACTATCATGTTGACATTCC; dG = -10.2
758 >s_d_s_c_s_a_s_a_s_e_s GTGACGGTGTGGGTGTCTGTGTCTGTGCTGGTG; >clamp_s_a_s_d_s:compl:rev
CACCGTCACAGACACTATCATGTTGACATTCC; dG = -10.2
759 >s_d_s_c_s_b_s_a_s_e_s GTGACGGTGTGGGTGGCTGTGTCTGTGCTGGTG; >clamp_s_a_s_d_s:compl:rev
CACCGTCACAGACACTATCATGTTGACATTCC; dG = -10.2
760 >s_d_s_c_s_c_s_a_s_e_s GTGACGGTGTGGGTGTGGGTGTCTGTGCTGGTG; >clamp_s_a_s_d_s:compl:rev
CACCGTCACAGACACTATCATGTTGACATTCC; dG = -10.2
761 >s_d_s_c_s_e_s_a_s_e_s GTGACGGTGTGGGTGCTGGTGTCTGTGCTGGTG; >clamp_s_a_s_d_s:compl:rev
CACCGTCACAGACACTATCATGTTGACATTCC; dG = -10.2
762 >s_a_s_b_s_e_s_a_s_e_s GTGTCTGTGGCTGTGCTGGTGTCTGTGCTGGTG; >clamp_s_a_s_d_s:compl:rev
CACCGTCACAGACACTATCATGTTGACATTCC; dG = -10.2
763 >s_d_s_e_s_a_s_a_s_e_s GTGACGGTGCTGGTGTCTGTGTCTGTGCTGGTG; >clamp_s_a_s_d_s:compl:rev
CACCGTCACAGACACTATCATGTTGACATTCC; dG = -10.2
764 >s_d_s_e_s_b_s_a_s_e_s GTGACGGTGCTGGTGGCTGTGTCTGTGCTGGTG; >clamp_s_a_s_d_s:compl:rev
CACCGTCACAGACACTATCATGTTGACATTCC; dG = -10.2
765 >s_d_s_e_s_c_s_a_s_e_s GTGACGGTGCTGGTGTGGGTGTCTGTGCTGGTG; >clamp_s_a_s_d_s:compl:rev
CACCGTCACAGACACTATCATGTTGACATTCC; dG = -10.2
766 >s_d_s_e_s_e_s_a_s_e_s GTGACGGTGCTGGTGCTGGTGTCTGTGCTGGTG; >clamp_s_a_s_d_s:compl:rev
CACCGTCACAGACACTATCATGTTGACATTCC; dG = -10.2
767 >s_e_s_a_s_a_s_a_s_e_s GTGCTGGTGTCTGTGTCTGTGTCTGTGCTGGTG; >clamp_s_a_s_d_s:compl:rev
CACCGTCACAGACACTATCATGTTGACATTCC; dG = -10.2
768 >s_e_s_a_s_b_s_a_s_e_s GTGCTGGTGTCTGTGGCTGTGTCTGTGCTGGTG; >clamp_s_a_s_d_s:compl:rev
CACCGTCACAGACACTATCATGTTGACATTCC; dG = -10.2
769 >s_e_s_a_s_b_s_a_s_b_s GTGCTGGTGTCTGTGGCTGTGTCTGTGGCTGTG; >clamp_s_a_s_d_s:compl:rev
CACCGTCACAGACACTATCATGTTGACATTCC; dG = -10.2
770 >s_e_s_a_s_b_s_a_s_a_s GTGCTGGTGTCTGTGGCTGTGTCTGTGTCTGTG; >clamp_s_a_s_d_s:compl:rev
CACCGTCACAGACACTATCATGTTGACATTCC; dG = -10.2
771 >s_e_s_b_s_a_s_a_s_e_s GTGCTGGTGGCTGTGTCTGTGTCTGTGCTGGTG; >clamp_s_a_s_d_s:compl:rev
CACCGTCACAGACACTATCATGTTGACATTCC; dG = -10.2
772 >s_e_s_b_s_b_s_a_s_e_s GTGCTGGTGGCTGTGGCTGTGTCTGTGCTGGTG; >clamp_s_a_s_d_s:compl:rev
CACCGTCACAGACACTATCATGTTGACATTCC; dG = -10.2
773 >s_e_s_b_s_c_s_a_s_e_s GTGCTGGTGGCTGTGTGGGTGTCTGTGCTGGTG; >clamp_s_a_s_d_s:compl:rev
CACCGTCACAGACACTATCATGTTGACATTCC; dG = -10.2
774 >s_e_s_b_s_e_s_a_s_e_s GTGCTGGTGGCTGTGCTGGTGTCTGTGCTGGTG; >clamp_s_a_s_d_s:compl:rev
CACCGTCACAGACACTATCATGTTGACATTCC; dG = -10.2
775 >s_e_s_c_s_a_s_a_s_e_s GTGCTGGTGTGGGTGTCTGTGTCTGTGCTGGTG; >clamp_s_a_s_d_s:compl:rev
CACCGTCACAGACACTATCATGTTGACATTCC; dG = -10.2
776 >s_e_s_c_s_b_s_a_s_e_s GTGCTGGTGTGGGTGGCTGTGTCTGTGCTGGTG; >clamp_s_a_s_d_s:compl:rev
CACCGTCACAGACACTATCATGTTGACATTCC; dG = -10.2
777 >s_e_s_c_s_c_s_a_s_e_s GTGCTGGTGTGGGTGTGGGTGTCTGTGCTGGTG; >clamp_s_a_s_d_s:compl:rev
CACCGTCACAGACACTATCATGTTGACATTCC; dG = -10.2
778 >s_e_s_c_s_e_s_a_s_e_s GTGCTGGTGTGGGTGCTGGTGTCTGTGCTGGTG; >clamp_s_a_s_d_s:compl:rev
CACCGTCACAGACACTATCATGTTGACATTCC; dG = -10.2
779 >s_e_s_e_s_a_s_a_s_e_s GTGCTGGTGCTGGTGTCTGTGTCTGTGCTGGTG; >clamp_s_a_s_d_s:compl:rev
CACCGTCACAGACACTATCATGTTGACATTCC; dG = -10.2
780 >s_e_s_e_s_b_s_a_s_e_s GTGCTGGTGCTGGTGGCTGTGTCTGTGCTGGTG; >clamp_s_a_s_d_s:compl:rev
CACCGTCACAGACACTATCATGTTGACATTCC; dG = -10.2
781 >s_e_s_e_s_c_s_a_s_e_s GTGCTGGTGCTGGTGTGGGTGTCTGTGCTGGTG; >clamp_s_a_s_d_s:compl:rev
CACCGTCACAGACACTATCATGTTGACATTCC; dG = -10.2

782 >s_e_s_e_s_e_s_a_s_e_s GTGCTGGTGCTGGTGCTGGTGTCTGTGCTGGTG; >clamp_s_a_s_d_s:compl:rev
CACCGTCACAGACACTATCATGTTGACATTCC; dG = -10.2
783 >s_a_s_e_s_b_s_a_s_b_s GTGTCTGTGCTGGTGGCTGTGTCTGTGGCTGTG; >clamp_s_a_s_d_s:compl:rev
CACCGTCACAGACACTATCATGTTGACATTCC; dG = -10.1
784 >s_a_s_e_s_b_s_a_s_a_s GTGTCTGTGCTGGTGGCTGTGTCTGTGTCTGTG; >clamp_s_a_s_d_s:compl:rev
CACCGTCACAGACACTATCATGTTGACATTCC; dG = -10.1
785 >s_d_s_a_s_a_s_a_s_b_s GTGACGGTGTCTGTGTCTGTGTCTGTGGCTGTG; >clamp_s_a_s_d_s:compl:rev
CACCGTCACAGACACTATCATGTTGACATTCC; dG = -10.1
786 >s_d_s_a_s_a_s_a_s_a_s GTGACGGTGTCTGTGTCTGTGTCTGTGTCTGTG; >clamp_s_a_s_d_s:compl:rev
CACCGTCACAGACACTATCATGTTGACATTCC; dG = -10.1
787 >s_d_s_c_s_a_s_a_s_b_s GTGACGGTGTGGGTGTCTGTGTCTGTGGCTGTG; >clamp_s_a_s_d_s:compl:rev
CACCGTCACAGACACTATCATGTTGACATTCC; dG = -10.1
788 >s_d_s_c_s_a_s_a_s_a_s GTGACGGTGTGGGTGTCTGTGTCTGTGTCTGTG; >clamp_s_a_s_d_s:compl:rev
CACCGTCACAGACACTATCATGTTGACATTCC; dG = -10.1
789 >s_d_s_c_s_b_s_a_s_b_s GTGACGGTGTGGGTGGCTGTGTCTGTGGCTGTG; >clamp_s_a_s_d_s:compl:rev
CACCGTCACAGACACTATCATGTTGACATTCC; dG = -10.1
790 >s_d_s_c_s_b_s_a_s_a_s GTGACGGTGTGGGTGGCTGTGTCTGTGTCTGTG; >clamp_s_a_s_d_s:compl:rev
CACCGTCACAGACACTATCATGTTGACATTCC; dG = -10.1
791 >s_d_s_c_s_c_s_a_s_b_s GTGACGGTGTGGGTGTGGGTGTCTGTGGCTGTG; >clamp_s_a_s_d_s:compl:rev
CACCGTCACAGACACTATCATGTTGACATTCC; dG = -10.1
792 >s_d_s_c_s_c_s_a_s_a_s GTGACGGTGTGGGTGTGGGTGTCTGTGTCTGTG; >clamp_s_a_s_d_s:compl:rev
CACCGTCACAGACACTATCATGTTGACATTCC; dG = -10.1
793 >s_d_s_c_s_e_s_a_s_b_s GTGACGGTGTGGGTGCTGGTGTCTGTGGCTGTG; >clamp_s_a_s_d_s:compl:rev
CACCGTCACAGACACTATCATGTTGACATTCC; dG = -10.1
794 >s_d_s_c_s_e_s_a_s_a_s GTGACGGTGTGGGTGCTGGTGTCTGTGTCTGTG; >clamp_s_a_s_d_s:compl:rev
CACCGTCACAGACACTATCATGTTGACATTCC; dG = -10.1
795 >s_a_s_e_s_a_s_a_s_b_s GTGTCTGTGCTGGTGTCTGTGTCTGTGGCTGTG; >clamp_s_a_s_d_s:compl:rev
CACCGTCACAGACACTATCATGTTGACATTCC; dG = -10.0
796 >s_a_s_e_s_a_s_a_s_a_s GTGTCTGTGCTGGTGTCTGTGTCTGTGTCTGTG; >clamp_s_a_s_d_s:compl:rev
CACCGTCACAGACACTATCATGTTGACATTCC; dG = -10.0
797 >s_a_s_e_s_c_s_a_s_b_s GTGTCTGTGCTGGTGTGGGTGTCTGTGGCTGTG; >clamp_s_a_s_d_s:compl:rev
CACCGTCACAGACACTATCATGTTGACATTCC; dG = -10.0
798 >s_a_s_e_s_c_s_a_s_a_s GTGTCTGTGCTGGTGTGGGTGTCTGTGTCTGTG; >clamp_s_a_s_d_s:compl:rev
CACCGTCACAGACACTATCATGTTGACATTCC; dG = -10.0
799 >s_a_s_e_s_e_s_a_s_b_s GTGTCTGTGCTGGTGCTGGTGTCTGTGGCTGTG; >clamp_s_a_s_d_s:compl:rev
CACCGTCACAGACACTATCATGTTGACATTCC; dG = -10.0
800 >s_b_s_a_s_a_s_a_s_b_s GTGGCTGTGTCTGTGTCTGTGTCTGTGGCTGTG; >clamp_s_a_s_d_s:compl:rev
CACCGTCACAGACACTATCATGTTGACATTCC; dG = -10.0
801 >s_b_s_b_s_a_s_a_s_b_s GTGGCTGTGGCTGTGTCTGTGTCTGTGGCTGTG; >clamp_s_a_s_d_s:compl:rev
CACCGTCACAGACACTATCATGTTGACATTCC; dG = -10.0
802 >s_b_s_b_s_b_s_a_s_b_s GTGGCTGTGGCTGTGGCTGTGTCTGTGGCTGTG; >clamp_s_a_s_d_s:compl:rev
CACCGTCACAGACACTATCATGTTGACATTCC; dG = -10.0
803 >s_b_s_b_s_c_s_a_s_b_s GTGGCTGTGGCTGTGTGGGTGTCTGTGGCTGTG; >clamp_s_a_s_d_s:compl:rev
CACCGTCACAGACACTATCATGTTGACATTCC; dG = -10.0
804 >s_b_s_b_s_e_s_a_s_b_s GTGGCTGTGGCTGTGCTGGTGTCTGTGGCTGTG; >clamp_s_a_s_d_s:compl:rev
CACCGTCACAGACACTATCATGTTGACATTCC; dG = -10.0
805 >s_b_s_c_s_a_s_a_s_b_s GTGGCTGTGTGGGTGTCTGTGTCTGTGGCTGTG; >clamp_s_a_s_d_s:compl:rev
CACCGTCACAGACACTATCATGTTGACATTCC; dG = -10.0
806 >s_b_s_c_s_b_s_a_s_b_s GTGGCTGTGTGGGTGGCTGTGTCTGTGGCTGTG; >clamp_s_a_s_d_s:compl:rev
CACCGTCACAGACACTATCATGTTGACATTCC; dG = -10.0
807 >s_b_s_c_s_c_s_a_s_b_s GTGGCTGTGTGGGTGTGGGTGTCTGTGGCTGTG; >clamp_s_a_s_d_s:compl:rev
CACCGTCACAGACACTATCATGTTGACATTCC; dG = -10.0
808 >s_b_s_c_s_e_s_a_s_b_s GTGGCTGTGTGGGTGCTGGTGTCTGTGGCTGTG; >clamp_s_a_s_d_s:compl:rev
CACCGTCACAGACACTATCATGTTGACATTCC; dG = -10.0
809 >s_b_s_e_s_a_s_a_s_b_s GTGGCTGTGCTGGTGTCTGTGTCTGTGGCTGTG; >clamp_s_a_s_d_s:compl:rev
CACCGTCACAGACACTATCATGTTGACATTCC; dG = -10.0
810 >s_a_s_a_s_a_s_a_s_b_s GTGTCTGTGTCTGTGTCTGTGTCTGTGGCTGTG; >clamp_s_a_s_d_s:compl:rev
CACCGTCACAGACACTATCATGTTGACATTCC; dG = -10.0
811 >s_b_s_e_s_b_s_a_s_b_s GTGGCTGTGCTGGTGGCTGTGTCTGTGGCTGTG; >clamp_s_a_s_d_s:compl:rev
CACCGTCACAGACACTATCATGTTGACATTCC; dG = -10.0
812 >s_b_s_e_s_c_s_a_s_b_s GTGGCTGTGCTGGTGTGGGTGTCTGTGGCTGTG; >clamp_s_a_s_d_s:compl:rev
CACCGTCACAGACACTATCATGTTGACATTCC; dG = -10.0
813 >s_b_s_e_s_e_s_a_s_b_s GTGGCTGTGCTGGTGCTGGTGTCTGTGGCTGTG; >clamp_s_a_s_d_s:compl:rev
CACCGTCACAGACACTATCATGTTGACATTCC; dG = -10.0
814 >s_c_s_a_s_a_s_a_s_b_s GTGTGGGTGTCTGTGTCTGTGTCTGTGGCTGTG; >clamp_s_a_s_d_s:compl:rev
CACCGTCACAGACACTATCATGTTGACATTCC; dG = -10.0
815 >s_a_s_b_s_a_s_a_s_b_s GTGTCTGTGGCTGTGTCTGTGTCTGTGGCTGTG; >clamp_s_a_s_d_s:compl:rev
CACCGTCACAGACACTATCATGTTGACATTCC; dG = -10.0
816 >s_c_s_b_s_a_s_a_s_b_s GTGTGGGTGGCTGTGTCTGTGTCTGTGGCTGTG; >clamp_s_a_s_d_s:compl:rev
CACCGTCACAGACACTATCATGTTGACATTCC; dG = -10.0
817 >s_c_s_b_s_b_s_a_s_b_s GTGTGGGTGGCTGTGGCTGTGTCTGTGGCTGTG; >clamp_s_a_s_d_s:compl:rev
CACCGTCACAGACACTATCATGTTGACATTCC; dG = -10.0
818 >s_c_s_b_s_c_s_a_s_b_s GTGTGGGTGGCTGTGTGGGTGTCTGTGGCTGTG; >clamp_s_a_s_d_s:compl:rev
CACCGTCACAGACACTATCATGTTGACATTCC; dG = -10.0

819 >s_c_s_b_s_e_s_a_s_b_s GTGTGGGTGGCTGTGCTGGTGTCTGTGGCTGTG; >clamp_s_a_s_d_s:compl:rev CACCGTCACAGACACTATCATGTTGACATTCC; dG = -10.0
820 >s_c_s_c_s_a_s_a_s_b_s GTGTGGGTGTGGGTGTCTGTGTCTGTGGCTGTG; >clamp_s_a_s_d_s:compl:rev CACCGTCACAGACACTATCATGTTGACATTCC; dG = -10.0
821 >s_a_s_b_s_b_s_a_s_b_s GTGTCTGTGGCTGTGGCTGTGTCTGTGGCTGTG; >clamp_s_a_s_d_s:compl:rev CACCGTCACAGACACTATCATGTTGACATTCC; dG = -10.0
822 >s_c_s_c_s_b_s_a_s_b_s GTGTGGGTGTGGGTGGCTGTGTCTGTGGCTGTG; >clamp_s_a_s_d_s:compl:rev CACCGTCACAGACACTATCATGTTGACATTCC; dG = -10.0
823 >s_c_s_c_s_c_s_a_s_b_s GTGTGGGTGTGGGTGTGGGTGTCTGTGGCTGTG; >clamp_s_a_s_d_s:compl:rev CACCGTCACAGACACTATCATGTTGACATTCC; dG = -10.0
824 >s_c_s_c_s_e_s_a_s_b_s GTGTGGGTGTGGGTGCTGGTGTCTGTGGCTGTG; >clamp_s_a_s_d_s:compl:rev CACCGTCACAGACACTATCATGTTGACATTCC; dG = -10.0
825 >s_c_s_e_s_a_s_a_s_b_s GTGTGGGTGCTGGTGTCTGTGTCTGTGGCTGTG; >clamp_s_a_s_d_s:compl:rev CACCGTCACAGACACTATCATGTTGACATTCC; dG = -10.0
826 >s_a_s_b_s_c_s_a_s_b_s GTGTCTGTGGCTGTGTGGGTGTCTGTGGCTGTG; >clamp_s_a_s_d_s:compl:rev CACCGTCACAGACACTATCATGTTGACATTCC; dG = -10.0
827 >s_c_s_e_s_b_s_a_s_b_s GTGTGGGTGCTGGTGGCTGTGTCTGTGGCTGTG; >clamp_s_a_s_d_s:compl:rev CACCGTCACAGACACTATCATGTTGACATTCC; dG = -10.0
828 >s_c_s_e_s_c_s_a_s_b_s GTGTGGGTGCTGGTGTGGGTGTCTGTGGCTGTG; >clamp_s_a_s_d_s:compl:rev CACCGTCACAGACACTATCATGTTGACATTCC; dG = -10.0
829 >s_c_s_e_s_e_s_a_s_b_s GTGTGGGTGCTGGTGCTGGTGTCTGTGGCTGTG; >clamp_s_a_s_d_s:compl:rev CACCGTCACAGACACTATCATGTTGACATTCC; dG = -10.0
830 >s_a_s_b_s_e_s_a_s_b_s GTGTCTGTGGCTGTGCTGGTGTCTGTGGCTGTG; >clamp_s_a_s_d_s:compl:rev CACCGTCACAGACACTATCATGTTGACATTCC; dG = -10.0
831 >s_d_s_e_s_a_s_a_s_b_s GTGACGGTGCTGGTGTCTGTGTCTGTGGCTGTG; >clamp_s_a_s_d_s:compl:rev CACCGTCACAGACACTATCATGTTGACATTCC; dG = -10.0
832 >s_d_s_e_s_a_s_a_s_a_s GTGACGGTGCTGGTGTCTGTGTCTGTGTCTGTG; >clamp_s_a_s_d_s:compl:rev CACCGTCACAGACACTATCATGTTGACATTCC; dG = -10.0
833 >s_d_s_e_s_b_s_a_s_b_s GTGACGGTGCTGGTGGCTGTGTCTGTGGCTGTG; >clamp_s_a_s_d_s:compl:rev CACCGTCACAGACACTATCATGTTGACATTCC; dG = -10.0
834 >s_d_s_e_s_b_s_a_s_a_s GTGACGGTGCTGGTGGCTGTGTCTGTGTCTGTG; >clamp_s_a_s_d_s:compl:rev CACCGTCACAGACACTATCATGTTGACATTCC; dG = -10.0
835 >s_d_s_e_s_c_s_a_s_b_s GTGACGGTGCTGGTGTGGGTGTCTGTGGCTGTG; >clamp_s_a_s_d_s:compl:rev CACCGTCACAGACACTATCATGTTGACATTCC; dG = -10.0
836 >s_d_s_e_s_c_s_a_s_a_s GTGACGGTGCTGGTGTGGGTGTCTGTGTCTGTG; >clamp_s_a_s_d_s:compl:rev CACCGTCACAGACACTATCATGTTGACATTCC; dG = -10.0
837 >s_d_s_e_s_e_s_a_s_b_s GTGACGGTGCTGGTGCTGGTGTCTGTGGCTGTG; >clamp_s_a_s_d_s:compl:rev CACCGTCACAGACACTATCATGTTGACATTCC; dG = -10.0
838 >s_d_s_e_s_e_s_a_s_a_s GTGACGGTGCTGGTGCTGGTGTCTGTGTCTGTG; >clamp_s_a_s_d_s:compl:rev CACCGTCACAGACACTATCATGTTGACATTCC; dG = -10.0
839 >s_e_s_a_s_a_s_a_s_b_s GTGCTGGTGTCTGTGTCTGTGTCTGTGGCTGTG; >clamp_s_a_s_d_s:compl:rev CACCGTCACAGACACTATCATGTTGACATTCC; dG = -10.0
840 >s_e_s_b_s_a_s_a_s_b_s GTGCTGGTGGCTGTGTCTGTGTCTGTGGCTGTG; >clamp_s_a_s_d_s:compl:rev CACCGTCACAGACACTATCATGTTGACATTCC; dG = -10.0
841 >s_e_s_b_s_b_s_a_s_b_s GTGCTGGTGGCTGTGGCTGTGTCTGTGGCTGTG; >clamp_s_a_s_d_s:compl:rev CACCGTCACAGACACTATCATGTTGACATTCC; dG = -10.0
842 >s_e_s_b_s_c_s_a_s_b_s GTGCTGGTGGCTGTGTGGGTGTCTGTGGCTGTG; >clamp_s_a_s_d_s:compl:rev CACCGTCACAGACACTATCATGTTGACATTCC; dG = -10.0
843 >s_e_s_b_s_e_s_a_s_b_s GTGCTGGTGGCTGTGCTGGTGTCTGTGGCTGTG; >clamp_s_a_s_d_s:compl:rev CACCGTCACAGACACTATCATGTTGACATTCC; dG = -10.0
844 >s_e_s_c_s_a_s_a_s_b_s GTGCTGGTGTGGGTGTCTGTGTCTGTGGCTGTG; >clamp_s_a_s_d_s:compl:rev CACCGTCACAGACACTATCATGTTGACATTCC; dG = -10.0
845 >s_e_s_c_s_b_s_a_s_b_s GTGCTGGTGTGGGTGGCTGTGTCTGTGGCTGTG; >clamp_s_a_s_d_s:compl:rev CACCGTCACAGACACTATCATGTTGACATTCC; dG = -10.0
846 >s_e_s_c_s_c_s_a_s_b_s GTGCTGGTGTGGGTGTGGGTGTCTGTGGCTGTG; >clamp_s_a_s_d_s:compl:rev CACCGTCACAGACACTATCATGTTGACATTCC; dG = -10.0
847 >s_e_s_c_s_e_s_a_s_b_s GTGCTGGTGTGGGTGCTGGTGTCTGTGGCTGTG; >clamp_s_a_s_d_s:compl:rev CACCGTCACAGACACTATCATGTTGACATTCC; dG = -10.0
848 >s_e_s_e_s_a_s_a_s_b_s GTGCTGGTGCTGGTGTCTGTGTCTGTGGCTGTG; >clamp_s_a_s_d_s:compl:rev CACCGTCACAGACACTATCATGTTGACATTCC; dG = -10.0
849 >s_e_s_e_s_b_s_a_s_b_s GTGCTGGTGCTGGTGGCTGTGTCTGTGGCTGTG; >clamp_s_a_s_d_s:compl:rev CACCGTCACAGACACTATCATGTTGACATTCC; dG = -10.0
850 >s_e_s_e_s_c_s_a_s_b_s GTGCTGGTGCTGGTGTGGGTGTCTGTGGCTGTG; >clamp_s_a_s_d_s:compl:rev CACCGTCACAGACACTATCATGTTGACATTCC; dG = -10.0
851 >s_e_s_e_s_e_s_a_s_b_s GTGCTGGTGCTGGTGCTGGTGTCTGTGGCTGTG; >clamp_s_a_s_d_s:compl:rev CACCGTCACAGACACTATCATGTTGACATTCC; dG = -10.0
852 >s_a_s_e_s_e_s_a_s_a_s GTGTCTGTGCTGGTGCTGGTGTCTGTGTCTGTG; >clamp_s_a_s_d_s:compl:rev CACCGTCACAGACACTATCATGTTGACATTCC; dG = -9.9
853 >s_a_s_b_s_b_s_a_s_a_s GTGTCTGTGGCTGTGGCTGTGTCTGTGTCTGTG; >clamp_s_a_s_d_s:compl:rev CACCGTCACAGACACTATCATGTTGACATTCC; dG = -9.9
854 >s_a_s_b_s_a_s_a_s_a_s GTGTCTGTGGCTGTGTCTGTGTCTGTGTCTGTG; >clamp_s_a_s_d_s:compl:rev CACCGTCACAGACACTATCATGTTGACATTCC; dG = -9.8
855 >s_a_s_b_s_c_s_a_s_a_s GTGTCTGTGGCTGTGTGGGTGTCTGTGTCTGTG; >clamp_s_a_s_d_s:compl:rev CACCGTCACAGACACTATCATGTTGACATTCC; dG = -9.8

856 >s_a_s_b_s_e_s_a_s_a_s GTGTCTGTGGCTGTGCTGGTGTCTGTGTCTGTG; >clamp_s_a_s_d_s:compl:rev
CACCGTCACAGACACTATCATGTTGACATTCC; dG = -9.6
857 >s_b_s_a_s_a_s_a_s_a_s GTGGCTGTGTCTGTGTCTGTGTCTGTGTCTGTG; >clamp_s_a_s_d_s:compl:rev
CACCGTCACAGACACTATCATGTTGACATTCC; dG = -9.5
858 >s_b_s_b_s_a_s_a_s_a_s GTGGCTGTGGCTGTGTCTGTGTCTGTGTCTGTG; >clamp_s_a_s_d_s:compl:rev
CACCGTCACAGACACTATCATGTTGACATTCC; dG = -9.5
859 >s_b_s_b_s_b_s_a_s_a_s GTGGCTGTGGCTGTGGCTGTGTCTGTGTCTGTG; >clamp_s_a_s_d_s:compl:rev
CACCGTCACAGACACTATCATGTTGACATTCC; dG = -9.5
860 >s_b_s_c_s_a_s_a_s_a_s GTGGCTGTGTGGGTGTCTGTGTCTGTGTCTGTG; >clamp_s_a_s_d_s:compl:rev
CACCGTCACAGACACTATCATGTTGACATTCC; dG = -9.5
861 >s_b_s_c_s_b_s_a_s_a_s GTGGCTGTGTGGGTGGCTGTGTCTGTGTCTGTG; >clamp_s_a_s_d_s:compl:rev
CACCGTCACAGACACTATCATGTTGACATTCC; dG = -9.5
862 >s_b_s_e_s_a_s_a_s_a_s GTGGCTGTGCTGGTGTCTGTGTCTGTGTCTGTG; >clamp_s_a_s_d_s:compl:rev
CACCGTCACAGACACTATCATGTTGACATTCC; dG = -9.5
863 >s_b_s_e_s_b_s_a_s_a_s GTGGCTGTGCTGGTGGCTGTGTCTGTGTCTGTG; >clamp_s_a_s_d_s:compl:rev
CACCGTCACAGACACTATCATGTTGACATTCC; dG = -9.5
864 >s_c_s_a_s_a_s_a_s_a_s GTGTGGGTGTCTGTGTCTGTGTCTGTGTCTGTG; >clamp_s_a_s_d_s:compl:rev
CACCGTCACAGACACTATCATGTTGACATTCC; dG = -9.5
865 >s_c_s_b_s_a_s_a_s_a_s GTGTGGGTGGCTGTGTCTGTGTCTGTGTCTGTG; >clamp_s_a_s_d_s:compl:rev
CACCGTCACAGACACTATCATGTTGACATTCC; dG = -9.5
866 >s_c_s_b_s_b_s_a_s_a_s GTGTGGGTGGCTGTGGCTGTGTCTGTGTCTGTG; >clamp_s_a_s_d_s:compl:rev
CACCGTCACAGACACTATCATGTTGACATTCC; dG = -9.5
867 >s_a_s_a_s_a_s_a_s_a_s GTGTCTGTGTCTGTGTCTGTGTCTGTGTCTGTG; >clamp_s_a_s_d_s:compl:rev
CACCGTCACAGACACTATCATGTTGACATTCC; dG = -9.5
868 >s_c_s_c_s_a_s_a_s_a_s GTGTGGGTGTGGGTGTCTGTGTCTGTGTCTGTG; >clamp_s_a_s_d_s:compl:rev
CACCGTCACAGACACTATCATGTTGACATTCC; dG = -9.5
869 >s_c_s_c_s_b_s_a_s_a_s GTGTGGGTGTGGGTGGCTGTGTCTGTGTCTGTG; >clamp_s_a_s_d_s:compl:rev
CACCGTCACAGACACTATCATGTTGACATTCC; dG = -9.5
870 >s_c_s_e_s_a_s_a_s_a_s GTGTGGGTGCTGGTGTCTGTGTCTGTGTCTGTG; >clamp_s_a_s_d_s:compl:rev
CACCGTCACAGACACTATCATGTTGACATTCC; dG = -9.5
871 >s_c_s_e_s_b_s_a_s_a_s GTGTGGGTGCTGGTGGCTGTGTCTGTGTCTGTG; >clamp_s_a_s_d_s:compl:rev
CACCGTCACAGACACTATCATGTTGACATTCC; dG = -9.5
872 >s_e_s_a_s_a_s_a_s_a_s GTGCTGGTGTCTGTGTCTGTGTCTGTGTCTGTG; >clamp_s_a_s_d_s:compl:rev
CACCGTCACAGACACTATCATGTTGACATTCC; dG = -9.5
873 >s_e_s_b_s_a_s_a_s_a_s GTGCTGGTGGCTGTGTCTGTGTCTGTGTCTGTG; >clamp_s_a_s_d_s:compl:rev
CACCGTCACAGACACTATCATGTTGACATTCC; dG = -9.5
874 >s_e_s_b_s_b_s_a_s_a_s GTGCTGGTGGCTGTGGCTGTGTCTGTGTCTGTG; >clamp_s_a_s_d_s:compl:rev
CACCGTCACAGACACTATCATGTTGACATTCC; dG = -9.5
875 >s_e_s_c_s_a_s_a_s_a_s GTGCTGGTGTGGGTGTCTGTGTCTGTGTCTGTG; >clamp_s_a_s_d_s:compl:rev
CACCGTCACAGACACTATCATGTTGACATTCC; dG = -9.5
876 >s_e_s_c_s_b_s_a_s_a_s GTGCTGGTGTGGGTGGCTGTGTCTGTGTCTGTG; >clamp_s_a_s_d_s:compl:rev
CACCGTCACAGACACTATCATGTTGACATTCC; dG = -9.5
877 >s_e_s_e_s_a_s_a_s_a_s GTGCTGGTGCTGGTGTCTGTGTCTGTGTCTGTG; >clamp_s_a_s_d_s:compl:rev
CACCGTCACAGACACTATCATGTTGACATTCC; dG = -9.5
878 >s_e_s_e_s_b_s_a_s_a_s GTGCTGGTGCTGGTGGCTGTGTCTGTGTCTGTG; >clamp_s_a_s_d_s:compl:rev
CACCGTCACAGACACTATCATGTTGACATTCC; dG = -9.5
879 >s_b_s_b_s_c_s_a_s_a_s GTGGCTGTGGCTGTGTGGGTGTCTGTGTCTGTG; >clamp_s_a_s_d_s:compl:rev
CACCGTCACAGACACTATCATGTTGACATTCC; dG = -9.4
880 >s_b_s_b_s_e_s_a_s_a_s GTGGCTGTGGCTGTGCTGGTGTCTGTGTCTGTG; >clamp_s_a_s_d_s:compl:rev
CACCGTCACAGACACTATCATGTTGACATTCC; dG = -9.4
881 >s_b_s_c_s_c_s_a_s_a_s GTGGCTGTGTGGGTGTGGGTGTCTGTGTCTGTG; >clamp_s_a_s_d_s:compl:rev
CACCGTCACAGACACTATCATGTTGACATTCC; dG = -9.4
882 >s_b_s_c_s_e_s_a_s_a_s GTGGCTGTGTGGGTGCTGGTGTCTGTGTCTGTG; >clamp_s_a_s_d_s:compl:rev
CACCGTCACAGACACTATCATGTTGACATTCC; dG = -9.4
883 >s_b_s_e_s_c_s_a_s_a_s GTGGCTGTGCTGGTGTGGGTGTCTGTGTCTGTG; >clamp_s_a_s_d_s:compl:rev
CACCGTCACAGACACTATCATGTTGACATTCC; dG = -9.4
884 >s_b_s_e_s_e_s_a_s_a_s GTGGCTGTGCTGGTGCTGGTGTCTGTGTCTGTG; >clamp_s_a_s_d_s:compl:rev
CACCGTCACAGACACTATCATGTTGACATTCC; dG = -9.4
885 >s_c_s_b_s_c_s_a_s_a_s GTGTGGGTGGCTGTGTGGGTGTCTGTGTCTGTG; >clamp_s_a_s_d_s:compl:rev
CACCGTCACAGACACTATCATGTTGACATTCC; dG = -9.4
886 >s_c_s_b_s_e_s_a_s_a_s GTGTGGGTGGCTGTGCTGGTGTCTGTGTCTGTG; >clamp_s_a_s_d_s:compl:rev
CACCGTCACAGACACTATCATGTTGACATTCC; dG = -9.4
887 >s_c_s_c_s_c_s_a_s_a_s GTGTGGGTGTGGGTGTGGGTGTCTGTGTCTGTG; >clamp_s_a_s_d_s:compl:rev
CACCGTCACAGACACTATCATGTTGACATTCC; dG = -9.4
888 >s_c_s_c_s_e_s_a_s_a_s GTGTGGGTGTGGGTGCTGGTGTCTGTGTCTGTG; >clamp_s_a_s_d_s:compl:rev
CACCGTCACAGACACTATCATGTTGACATTCC; dG = -9.4
889 >s_c_s_e_s_c_s_a_s_a_s GTGTGGGTGCTGGTGTGGGTGTCTGTGTCTGTG; >clamp_s_a_s_d_s:compl:rev
CACCGTCACAGACACTATCATGTTGACATTCC; dG = -9.4
890 >s_c_s_e_s_e_s_a_s_a_s GTGTGGGTGCTGGTGCTGGTGTCTGTGTCTGTG; >clamp_s_a_s_d_s:compl:rev
CACCGTCACAGACACTATCATGTTGACATTCC; dG = -9.4
891 >s_e_s_b_s_c_s_a_s_a_s GTGCTGGTGGCTGTGTGGGTGTCTGTGTCTGTG; >clamp_s_a_s_d_s:compl:rev
CACCGTCACAGACACTATCATGTTGACATTCC; dG = -9.4
892 >s_e_s_b_s_e_s_a_s_a_s GTGCTGGTGGCTGTGCTGGTGTCTGTGTCTGTG; >clamp_s_a_s_d_s:compl:rev
CACCGTCACAGACACTATCATGTTGACATTCC; dG = -9.4

893 >s_e_s_c_s_c_s_a_s_a_s GTGCTGGTGTGGGTGTGGGTGTCTGTGTCTGTG; >clamp_s_a_s_d_s:compl:rev
CACCGTCACAGACACTATCATGTTGACATTCC; dG = -9.4
894 >s_e_s_c_s_e_s_a_s_a_s GTGCTGGTGTGGGTGCTGGTGTCTGTGTCTGTG; >clamp_s_a_s_d_s:compl:rev
CACCGTCACAGACACTATCATGTTGACATTCC; dG = -9.4
895 >s_e_s_e_s_c_s_a_s_a_s GTGCTGGTGCTGGTGTGGGTGTCTGTGTCTGTG; >clamp_s_a_s_d_s:compl:rev
CACCGTCACAGACACTATCATGTTGACATTCC; dG = -9.4
896 >s_e_s_e_s_e_s_a_s_a_s GTGCTGGTGCTGGTGCTGGTGTCTGTGTCTGTG; >clamp_s_a_s_d_s:compl:rev
CACCGTCACAGACACTATCATGTTGACATTCC; dG = -9.4

****

BC Matches

1 >s_b_s_b_s_c_s_b_s_c_s GTGGCTGTGGCTGTGTGGGTGGCTGTGTGGGTG; >clamp_s_b_s_c_s:compl:rev
CACCCACACAGCCACTATCATGTTGACATTCC; dG = -18.8
2 >s_b_s_c_s_d_s_b_s_c_s GTGGCTGTGTGGGTGACGGTGGCTGTGTGGGTG; >clamp_s_b_s_c_s:compl:rev
CACCCACACAGCCACTATCATGTTGACATTCC; dG = -18.8
3 >s_a_s_b_s_c_s_b_s_c_s GTGTCTGTGGCTGTGTGGGTGGCTGTGTGGGTG; >clamp_s_b_s_c_s:compl:rev
CACCCACACAGCCACTATCATGTTGACATTCC; dG = -18.8
4 >s_e_s_b_s_c_s_b_s_c_s GTGCTGGTGGCTGTGTGGGTGGCTGTGTGGGTG; >clamp_s_b_s_c_s:compl:rev
CACCCACACAGCCACTATCATGTTGACATTCC; dG = -18.7
5 >s_c_s_b_s_c_s_b_s_c_s GTGTGGGTGGCTGTGTGGGTGGCTGTGTGGGTG; >clamp_s_b_s_c_s:compl:rev
CACCCACACAGCCACTATCATGTTGACATTCC; dG = -18.7
6 >s_d_s_b_s_c_s_b_s_c_s GTGACGGTGGCTGTGTGGGTGGCTGTGTGGGTG; >clamp_s_b_s_c_s:compl:rev
CACCCACACAGCCACTATCATGTTGACATTCC; dG = -18.7
7 >s_a_s_a_s_a_s_b_s_c_s GTGTCTGTGTCTGTGTCTGTGGCTGTGTGGGTG; >clamp_s_b_s_c_s:compl:rev
CACCCACACAGCCACTATCATGTTGACATTCC; dG = -18.4
8 >s_a_s_b_s_e_s_b_s_c_s GTGTCTGTGGCTGTGCTGGTGGCTGTGTGGGTG; >clamp_s_b_s_c_s:compl:rev
CACCCACACAGCCACTATCATGTTGACATTCC; dG = -18.4
9 >s_d_s_e_s_e_s_b_s_c_s GTGACGGTGCTGGTGCTGGTGGCTGTGTGGGTG; >clamp_s_b_s_c_s:compl:rev
CACCCACACAGCCACTATCATGTTGACATTCC; dG = -18.4
10 >s_e_s_a_s_a_s_b_s_c_s GTGCTGGTGTCTGTGTCTGTGGCTGTGTGGGTG; >clamp_s_b_s_c_s:compl:rev
CACCCACACAGCCACTATCATGTTGACATTCC; dG = -18.4
11 >s_e_s_a_s_b_s_b_s_c_s GTGCTGGTGTCTGTGGCTGTGGCTGTGTGGGTG; >clamp_s_b_s_c_s:compl:rev
CACCCACACAGCCACTATCATGTTGACATTCC; dG = -18.4
12 >s_e_s_a_s_c_s_b_s_c_s GTGCTGGTGTCTGTGTGGGTGGCTGTGTGGGTG; >clamp_s_b_s_c_s:compl:rev
CACCCACACAGCCACTATCATGTTGACATTCC; dG = -18.4
13 >s_e_s_a_s_d_s_b_s_c_s GTGCTGGTGTCTGTGACGGTGGCTGTGTGGGTG; >clamp_s_b_s_c_s:compl:rev
CACCCACACAGCCACTATCATGTTGACATTCC; dG = -18.4
14 >s_e_s_a_s_e_s_b_s_c_s GTGCTGGTGTCTGTGCTGGTGGCTGTGTGGGTG; >clamp_s_b_s_c_s:compl:rev
CACCCACACAGCCACTATCATGTTGACATTCC; dG = -18.4
15 >s_e_s_b_s_a_s_b_s_c_s GTGCTGGTGGCTGTGTCTGTGGCTGTGTGGGTG; >clamp_s_b_s_c_s:compl:rev
CACCCACACAGCCACTATCATGTTGACATTCC; dG = -18.4
16 >s_e_s_b_s_b_s_b_s_c_s GTGCTGGTGGCTGTGGCTGTGGCTGTGTGGGTG; >clamp_s_b_s_c_s:compl:rev
CACCCACACAGCCACTATCATGTTGACATTCC; dG = -18.4
17 >s_e_s_b_s_d_s_b_s_c_s GTGCTGGTGGCTGTGACGGTGGCTGTGTGGGTG; >clamp_s_b_s_c_s:compl:rev
CACCCACACAGCCACTATCATGTTGACATTCC; dG = -18.4
18 >s_a_s_c_s_a_s_b_s_c_s GTGTCTGTGTGGGTGTCTGTGGCTGTGTGGGTG; >clamp_s_b_s_c_s:compl:rev
CACCCACACAGCCACTATCATGTTGACATTCC; dG = -18.4
19 >s_e_s_b_s_e_s_b_s_c_s GTGCTGGTGGCTGTGCTGGTGGCTGTGTGGGTG; >clamp_s_b_s_c_s:compl:rev
CACCCACACAGCCACTATCATGTTGACATTCC; dG = -18.4
20 >s_e_s_c_s_a_s_b_s_c_s GTGCTGGTGTGGGTGTCTGTGGCTGTGTGGGTG; >clamp_s_b_s_c_s:compl:rev
CACCCACACAGCCACTATCATGTTGACATTCC; dG = -18.4
21 >s_e_s_c_s_b_s_b_s_c_s GTGCTGGTGTGGGTGGCTGTGGCTGTGTGGGTG; >clamp_s_b_s_c_s:compl:rev
CACCCACACAGCCACTATCATGTTGACATTCC; dG = -18.4
22 >s_e_s_c_s_c_s_b_s_c_s GTGCTGGTGTGGGTGTGGGTGGCTGTGTGGGTG; >clamp_s_b_s_c_s:compl:rev
CACCCACACAGCCACTATCATGTTGACATTCC; dG = -18.4
23 >s_e_s_c_s_d_s_b_s_c_s GTGCTGGTGTGGGTGACGGTGGCTGTGTGGGTG; >clamp_s_b_s_c_s:compl:rev
CACCCACACAGCCACTATCATGTTGACATTCC; dG = -18.4
24 >s_e_s_c_s_e_s_b_s_c_s GTGCTGGTGTGGGTGCTGGTGGCTGTGTGGGTG; >clamp_s_b_s_c_s:compl:rev
CACCCACACAGCCACTATCATGTTGACATTCC; dG = -18.4
25 >s_e_s_d_s_a_s_b_s_c_s GTGCTGGTGACGGTGTCTGTGGCTGTGTGGGTG; >clamp_s_b_s_c_s:compl:rev
CACCCACACAGCCACTATCATGTTGACATTCC; dG = -18.4
26 >s_e_s_d_s_b_s_b_s_c_s GTGCTGGTGACGGTGGCTGTGGCTGTGTGGGTG; >clamp_s_b_s_c_s:compl:rev
CACCCACACAGCCACTATCATGTTGACATTCC; dG = -18.4
27 >s_e_s_d_s_c_s_b_s_c_s GTGCTGGTGACGGTGTGGGTGGCTGTGTGGGTG; >clamp_s_b_s_c_s:compl:rev
CACCCACACAGCCACTATCATGTTGACATTCC; dG = -18.4
28 >s_e_s_d_s_d_s_b_s_c_s GTGCTGGTGACGGTGACGGTGGCTGTGTGGGTG; >clamp_s_b_s_c_s:compl:rev
CACCCACACAGCCACTATCATGTTGACATTCC; dG = -18.4
29 >s_a_s_c_s_b_s_b_s_c_s GTGTCTGTGTGGGTGGCTGTGGCTGTGTGGGTG; >clamp_s_b_s_c_s:compl:rev
CACCCACACAGCCACTATCATGTTGACATTCC; dG = -18.4

30 >s_e_s_d_s_e_s_b_s_c_s GTGCTGGTGACGGTGCTGGTGGCTGTGTGGGTG; >clamp_s_b_s_c_s:compl:rev CACCCACACAGCCACTATCATGTTGACATTCC; dG = -18.4
31 >s_e_s_e_s_a_s_b_s_c_s GTGCTGGTGCTGGTGTCTGTGGCTGTGTGGGTG; >clamp_s_b_s_c_s:compl:rev CACCCACACAGCCACTATCATGTTGACATTCC; dG = -18.4
32 >s_e_s_e_s_b_s_b_s_c_s GTGCTGGTGCTGGTGGCTGTGGCTGTGTGGGTG; >clamp_s_b_s_c_s:compl:rev CACCCACACAGCCACTATCATGTTGACATTCC; dG = -18.4
33 >s_e_s_e_s_c_s_b_s_c_s GTGCTGGTGCTGGTGTGGGTGGCTGTGTGGGTG; >clamp_s_b_s_c_s:compl:rev CACCCACACAGCCACTATCATGTTGACATTCC; dG = -18.4
34 >s_e_s_e_s_d_s_b_s_c_s GTGCTGGTGCTGGTGACGGTGGCTGTGTGGGTG; >clamp_s_b_s_c_s:compl:rev CACCCACACAGCCACTATCATGTTGACATTCC; dG = -18.4
35 >s_e_s_e_s_e_s_b_s_c_s GTGCTGGTGCTGGTGCTGGTGGCTGTGTGGGTG; >clamp_s_b_s_c_s:compl:rev CACCCACACAGCCACTATCATGTTGACATTCC; dG = -18.4
36 >s_a_s_c_s_c_s_b_s_c_s GTGTCTGTGTGGGTGTGGGTGGCTGTGTGGGTG; >clamp_s_b_s_c_s:compl:rev CACCCACACAGCCACTATCATGTTGACATTCC; dG = -18.4
37 >s_a_s_c_s_d_s_b_s_c_s GTGTCTGTGTGGGTGACGGTGGCTGTGTGGGTG; >clamp_s_b_s_c_s:compl:rev CACCCACACAGCCACTATCATGTTGACATTCC; dG = -18.4
38 >s_a_s_c_s_e_s_b_s_c_s GTGTCTGTGTGGGTGCTGGTGGCTGTGTGGGTG; >clamp_s_b_s_c_s:compl:rev CACCCACACAGCCACTATCATGTTGACATTCC; dG = -18.4
39 >s_a_s_d_s_a_s_b_s_c_s GTGTCTGTGACGGTGTCTGTGGCTGTGTGGGTG; >clamp_s_b_s_c_s:compl:rev CACCCACACAGCCACTATCATGTTGACATTCC; dG = -18.4
40 >s_a_s_d_s_b_s_b_s_c_s GTGTCTGTGACGGTGGCTGTGGCTGTGTGGGTG; >clamp_s_b_s_c_s:compl:rev CACCCACACAGCCACTATCATGTTGACATTCC; dG = -18.4
41 >s_a_s_d_s_c_s_b_s_c_s GTGTCTGTGACGGTGTGGGTGGCTGTGTGGGTG; >clamp_s_b_s_c_s:compl:rev CACCCACACAGCCACTATCATGTTGACATTCC; dG = -18.4
42 >s_a_s_d_s_d_s_b_s_c_s GTGTCTGTGACGGTGACGGTGGCTGTGTGGGTG; >clamp_s_b_s_c_s:compl:rev CACCCACACAGCCACTATCATGTTGACATTCC; dG = -18.4
43 >s_a_s_a_s_b_s_b_s_c_s GTGTCTGTGTCTGTGGCTGTGGCTGTGTGGGTG; >clamp_s_b_s_c_s:compl:rev CACCCACACAGCCACTATCATGTTGACATTCC; dG = -18.4
44 >s_a_s_d_s_e_s_b_s_c_s GTGTCTGTGACGGTGCTGGTGGCTGTGTGGGTG; >clamp_s_b_s_c_s:compl:rev CACCCACACAGCCACTATCATGTTGACATTCC; dG = -18.4
45 >s_a_s_e_s_a_s_b_s_c_s GTGTCTGTGCTGGTGTCTGTGGCTGTGTGGGTG; >clamp_s_b_s_c_s:compl:rev CACCCACACAGCCACTATCATGTTGACATTCC; dG = -18.4
46 >s_a_s_e_s_b_s_b_s_c_s GTGTCTGTGCTGGTGGCTGTGGCTGTGTGGGTG; >clamp_s_b_s_c_s:compl:rev CACCCACACAGCCACTATCATGTTGACATTCC; dG = -18.4
47 >s_a_s_e_s_c_s_b_s_c_s GTGTCTGTGCTGGTGTGGGTGGCTGTGTGGGTG; >clamp_s_b_s_c_s:compl:rev CACCCACACAGCCACTATCATGTTGACATTCC; dG = -18.4
48 >s_a_s_e_s_d_s_b_s_c_s GTGTCTGTGCTGGTGACGGTGGCTGTGTGGGTG; >clamp_s_b_s_c_s:compl:rev CACCCACACAGCCACTATCATGTTGACATTCC; dG = -18.4
49 >s_a_s_e_s_e_s_b_s_c_s GTGTCTGTGCTGGTGCTGGTGGCTGTGTGGGTG; >clamp_s_b_s_c_s:compl:rev CACCCACACAGCCACTATCATGTTGACATTCC; dG = -18.4
50 >s_b_s_a_s_a_s_b_s_c_s GTGGCTGTGTCTGTGTCTGTGGCTGTGTGGGTG; >clamp_s_b_s_c_s:compl:rev CACCCACACAGCCACTATCATGTTGACATTCC; dG = -18.4
51 >s_b_s_a_s_b_s_b_s_c_s GTGGCTGTGTCTGTGGCTGTGGCTGTGTGGGTG; >clamp_s_b_s_c_s:compl:rev CACCCACACAGCCACTATCATGTTGACATTCC; dG = -18.4
52 >s_b_s_a_s_c_s_b_s_c_s GTGGCTGTGTCTGTGTGGGTGGCTGTGTGGGTG; >clamp_s_b_s_c_s:compl:rev CACCCACACAGCCACTATCATGTTGACATTCC; dG = -18.4
53 >s_b_s_a_s_d_s_b_s_c_s GTGGCTGTGTCTGTGACGGTGGCTGTGTGGGTG; >clamp_s_b_s_c_s:compl:rev CACCCACACAGCCACTATCATGTTGACATTCC; dG = -18.4
54 >s_a_s_a_s_c_s_b_s_c_s GTGTCTGTGTCTGTGTGGGTGGCTGTGTGGGTG; >clamp_s_b_s_c_s:compl:rev CACCCACACAGCCACTATCATGTTGACATTCC; dG = -18.4
55 >s_b_s_a_s_e_s_b_s_c_s GTGGCTGTGTCTGTGCTGGTGGCTGTGTGGGTG; >clamp_s_b_s_c_s:compl:rev CACCCACACAGCCACTATCATGTTGACATTCC; dG = -18.4
56 >s_b_s_b_s_a_s_b_s_c_s GTGGCTGTGGCTGTGTCTGTGGCTGTGTGGGTG; >clamp_s_b_s_c_s:compl:rev CACCCACACAGCCACTATCATGTTGACATTCC; dG = -18.4
57 >s_b_s_b_s_b_s_b_s_c_s GTGGCTGTGGCTGTGGCTGTGGCTGTGTGGGTG; >clamp_s_b_s_c_s:compl:rev CACCCACACAGCCACTATCATGTTGACATTCC; dG = -18.4
58 >s_b_s_b_s_d_s_b_s_c_s GTGGCTGTGGCTGTGACGGTGGCTGTGTGGGTG; >clamp_s_b_s_c_s:compl:rev CACCCACACAGCCACTATCATGTTGACATTCC; dG = -18.4
59 >s_b_s_b_s_e_s_b_s_c_s GTGGCTGTGGCTGTGCTGGTGGCTGTGTGGGTG; >clamp_s_b_s_c_s:compl:rev CACCCACACAGCCACTATCATGTTGACATTCC; dG = -18.4
60 >s_b_s_c_s_a_s_b_s_c_s GTGGCTGTGTGGGTGTCTGTGGCTGTGTGGGTG; >clamp_s_b_s_c_s:compl:rev CACCCACACAGCCACTATCATGTTGACATTCC; dG = -18.4
61 >s_b_s_c_s_b_s_b_s_c_s GTGGCTGTGTGGGTGGCTGTGGCTGTGTGGGTG; >clamp_s_b_s_c_s:compl:rev CACCCACACAGCCACTATCATGTTGACATTCC; dG = -18.4
62 >s_b_s_c_s_c_s_b_s_c_s GTGGCTGTGTGGGTGTGGGTGGCTGTGTGGGTG; >clamp_s_b_s_c_s:compl:rev CACCCACACAGCCACTATCATGTTGACATTCC; dG = -18.4
63 >s_a_s_a_s_d_s_b_s_c_s GTGTCTGTGTCTGTGACGGTGGCTGTGTGGGTG; >clamp_s_b_s_c_s:compl:rev CACCCACACAGCCACTATCATGTTGACATTCC; dG = -18.4
64 >s_b_s_c_s_e_s_b_s_c_s GTGGCTGTGTGGGTGCTGGTGGCTGTGTGGGTG; >clamp_s_b_s_c_s:compl:rev CACCCACACAGCCACTATCATGTTGACATTCC; dG = -18.4
65 >s_b_s_d_s_a_s_b_s_c_s GTGGCTGTGACGGTGTCTGTGGCTGTGTGGGTG; >clamp_s_b_s_c_s:compl:rev CACCCACACAGCCACTATCATGTTGACATTCC; dG = -18.4
66 >s_b_s_d_s_b_s_b_s_c_s GTGGCTGTGACGGTGGCTGTGGCTGTGTGGGTG; >clamp_s_b_s_c_s:compl:rev CACCCACACAGCCACTATCATGTTGACATTCC; dG = -18.4

67 >s_b_s_d_s_c_s_b_s_c_s GTGGCTGTGACGGTGTGGGTGGCTGTGTGGGTG; >clamp_s_b_s_c_s:compl:rev CACCCACACAGCCACTATCATGTTGACATTCC; dG = -18.4
68 >s_b_s_d_s_d_s_b_s_c_s GTGGCTGTGACGGTGACGGTGGCTGTGTGGGTG; >clamp_s_b_s_c_s:compl:rev CACCCACACAGCCACTATCATGTTGACATTCC; dG = -18.4
69 >s_b_s_d_s_e_s_b_s_c_s GTGGCTGTGACGGTGCTGGTGGCTGTGTGGGTG; >clamp_s_b_s_c_s:compl:rev CACCCACACAGCCACTATCATGTTGACATTCC; dG = -18.4
70 >s_b_s_e_s_a_s_b_s_c_s GTGGCTGTGCTGGTGTCTGTGGCTGTGTGGGTG; >clamp_s_b_s_c_s:compl:rev CACCCACACAGCCACTATCATGTTGACATTCC; dG = -18.4
71 >s_b_s_e_s_b_s_b_s_c_s GTGGCTGTGCTGGTGGCTGTGGCTGTGTGGGTG; >clamp_s_b_s_c_s:compl:rev CACCCACACAGCCACTATCATGTTGACATTCC; dG = -18.4
72 >s_b_s_e_s_c_s_b_s_c_s GTGGCTGTGCTGGTGTGGGTGGCTGTGTGGGTG; >clamp_s_b_s_c_s:compl:rev CACCCACACAGCCACTATCATGTTGACATTCC; dG = -18.4
73 >s_b_s_e_s_d_s_b_s_c_s GTGGCTGTGCTGGTGACGGTGGCTGTGTGGGTG; >clamp_s_b_s_c_s:compl:rev CACCCACACAGCCACTATCATGTTGACATTCC; dG = -18.4
74 >s_a_s_a_s_e_s_b_s_c_s GTGTCTGTGTCTGTGCTGGTGGCTGTGTGGGTG; >clamp_s_b_s_c_s:compl:rev CACCCACACAGCCACTATCATGTTGACATTCC; dG = -18.4
75 >s_b_s_e_s_e_s_b_s_c_s GTGGCTGTGCTGGTGCTGGTGGCTGTGTGGGTG; >clamp_s_b_s_c_s:compl:rev CACCCACACAGCCACTATCATGTTGACATTCC; dG = -18.4
76 >s_c_s_a_s_a_s_b_s_c_s GTGTGGGTGTCTGTGTCTGTGGCTGTGTGGGTG; >clamp_s_b_s_c_s:compl:rev CACCCACACAGCCACTATCATGTTGACATTCC; dG = -18.4
77 >s_c_s_a_s_b_s_b_s_c_s GTGTGGGTGTCTGTGGCTGTGGCTGTGTGGGTG; >clamp_s_b_s_c_s:compl:rev CACCCACACAGCCACTATCATGTTGACATTCC; dG = -18.4
78 >s_c_s_a_s_c_s_b_s_c_s GTGTGGGTGTCTGTGTGGGTGGCTGTGTGGGTG; >clamp_s_b_s_c_s:compl:rev CACCCACACAGCCACTATCATGTTGACATTCC; dG = -18.4
79 >s_c_s_a_s_d_s_b_s_c_s GTGTGGGTGTCTGTGACGGTGGCTGTGTGGGTG; >clamp_s_b_s_c_s:compl:rev CACCCACACAGCCACTATCATGTTGACATTCC; dG = -18.4
80 >s_c_s_a_s_e_s_b_s_c_s GTGTGGGTGTCTGTGCTGGTGGCTGTGTGGGTG; >clamp_s_b_s_c_s:compl:rev CACCCACACAGCCACTATCATGTTGACATTCC; dG = -18.4
81 >s_c_s_b_s_a_s_b_s_c_s GTGTGGGTGGCTGTGTCTGTGGCTGTGTGGGTG; >clamp_s_b_s_c_s:compl:rev CACCCACACAGCCACTATCATGTTGACATTCC; dG = -18.4
82 >s_c_s_b_s_b_s_b_s_c_s GTGTGGGTGGCTGTGGCTGTGGCTGTGTGGGTG; >clamp_s_b_s_c_s:compl:rev CACCCACACAGCCACTATCATGTTGACATTCC; dG = -18.4
83 >s_c_s_b_s_d_s_b_s_c_s GTGTGGGTGGCTGTGACGGTGGCTGTGTGGGTG; >clamp_s_b_s_c_s:compl:rev CACCCACACAGCCACTATCATGTTGACATTCC; dG = -18.4
84 >s_a_s_b_s_a_s_b_s_c_s GTGTCTGTGGCTGTGTCTGTGGCTGTGTGGGTG; >clamp_s_b_s_c_s:compl:rev CACCCACACAGCCACTATCATGTTGACATTCC; dG = -18.4
85 >s_c_s_b_s_e_s_b_s_c_s GTGTGGGTGGCTGTGCTGGTGGCTGTGTGGGTG; >clamp_s_b_s_c_s:compl:rev CACCCACACAGCCACTATCATGTTGACATTCC; dG = -18.4
86 >s_c_s_c_s_a_s_b_s_c_s GTGTGGGTGTGGGTGTCTGTGGCTGTGTGGGTG; >clamp_s_b_s_c_s:compl:rev CACCCACACAGCCACTATCATGTTGACATTCC; dG = -18.4
87 >s_c_s_c_s_b_s_b_s_c_s GTGTGGGTGTGGGTGGCTGTGGCTGTGTGGGTG; >clamp_s_b_s_c_s:compl:rev CACCCACACAGCCACTATCATGTTGACATTCC; dG = -18.4
88 >s_c_s_c_s_c_s_b_s_c_s GTGTGGGTGTGGGTGTGGGTGGCTGTGTGGGTG; >clamp_s_b_s_c_s:compl:rev CACCCACACAGCCACTATCATGTTGACATTCC; dG = -18.4
89 >s_c_s_c_s_d_s_b_s_c_s GTGTGGGTGTGGGTGACGGTGGCTGTGTGGGTG; >clamp_s_b_s_c_s:compl:rev CACCCACACAGCCACTATCATGTTGACATTCC; dG = -18.4
90 >s_c_s_c_s_e_s_b_s_c_s GTGTGGGTGTGGGTGCTGGTGGCTGTGTGGGTG; >clamp_s_b_s_c_s:compl:rev CACCCACACAGCCACTATCATGTTGACATTCC; dG = -18.4
91 >s_c_s_d_s_a_s_b_s_c_s GTGTGGGTGACGGTGTCTGTGGCTGTGTGGGTG; >clamp_s_b_s_c_s:compl:rev CACCCACACAGCCACTATCATGTTGACATTCC; dG = -18.4
92 >s_c_s_d_s_b_s_b_s_c_s GTGTGGGTGACGGTGGCTGTGGCTGTGTGGGTG; >clamp_s_b_s_c_s:compl:rev CACCCACACAGCCACTATCATGTTGACATTCC; dG = -18.4
93 >s_c_s_d_s_c_s_b_s_c_s GTGTGGGTGACGGTGTGGGTGGCTGTGTGGGTG; >clamp_s_b_s_c_s:compl:rev CACCCACACAGCCACTATCATGTTGACATTCC; dG = -18.4
94 >s_c_s_d_s_d_s_b_s_c_s GTGTGGGTGACGGTGACGGTGGCTGTGTGGGTG; >clamp_s_b_s_c_s:compl:rev CACCCACACAGCCACTATCATGTTGACATTCC; dG = -18.4
95 >s_a_s_b_s_b_s_b_s_c_s GTGTCTGTGGCTGTGGCTGTGGCTGTGTGGGTG; >clamp_s_b_s_c_s:compl:rev CACCCACACAGCCACTATCATGTTGACATTCC; dG = -18.4
96 >s_c_s_d_s_e_s_b_s_c_s GTGTGGGTGACGGTGCTGGTGGCTGTGTGGGTG; >clamp_s_b_s_c_s:compl:rev CACCCACACAGCCACTATCATGTTGACATTCC; dG = -18.4
97 >s_c_s_e_s_a_s_b_s_c_s GTGTGGGTGCTGGTGTCTGTGGCTGTGTGGGTG; >clamp_s_b_s_c_s:compl:rev CACCCACACAGCCACTATCATGTTGACATTCC; dG = -18.4
98 >s_c_s_e_s_b_s_b_s_c_s GTGTGGGTGCTGGTGGCTGTGGCTGTGTGGGTG; >clamp_s_b_s_c_s:compl:rev CACCCACACAGCCACTATCATGTTGACATTCC; dG = -18.4
99 >s_c_s_e_s_c_s_b_s_c_s GTGTGGGTGCTGGTGTGGGTGGCTGTGTGGGTG; >clamp_s_b_s_c_s:compl:rev CACCCACACAGCCACTATCATGTTGACATTCC; dG = -18.4
100 >s_c_s_e_s_d_s_b_s_c_s GTGTGGGTGCTGGTGACGGTGGCTGTGTGGGTG; >clamp_s_b_s_c_s:compl:rev CACCCACACAGCCACTATCATGTTGACATTCC; dG = -18.4
101 >s_c_s_e_s_e_s_b_s_c_s GTGTGGGTGCTGGTGCTGGTGGCTGTGTGGGTG; >clamp_s_b_s_c_s:compl:rev CACCCACACAGCCACTATCATGTTGACATTCC; dG = -18.4
102 >s_d_s_a_s_a_s_b_s_c_s GTGACGGTGTCTGTGTCTGTGGCTGTGTGGGTG; >clamp_s_b_s_c_s:compl:rev CACCCACACAGCCACTATCATGTTGACATTCC; dG = -18.4
103 >s_d_s_a_s_b_s_b_s_c_s GTGACGGTGTCTGTGGCTGTGGCTGTGTGGGTG; >clamp_s_b_s_c_s:compl:rev CACCCACACAGCCACTATCATGTTGACATTCC; dG = -18.4

104 >s_d_s_a_s_c_s_b_s_c_s GTGACGGTGTCTGTGTGGGTGGCTGTGTGGGTG; >clamp_s_b_s_c_s:compl:rev CACCCACACAGCCACTATCATGTTGACATTCC; dG = -18.4
105 >s_d_s_a_s_d_s_b_s_c_s GTGACGGTGTCTGTGACGGTGGCTGTGTGGGTG; >clamp_s_b_s_c_s:compl:rev CACCCACACAGCCACTATCATGTTGACATTCC; dG = -18.4
106 >s_d_s_a_s_e_s_b_s_c_s GTGACGGTGTCTGTGCTGGTGGCTGTGTGGGTG; >clamp_s_b_s_c_s:compl:rev CACCCACACAGCCACTATCATGTTGACATTCC; dG = -18.4
107 >s_d_s_b_s_a_s_b_s_c_s GTGACGGTGGCTGTGTCTGTGGCTGTGTGGGTG; >clamp_s_b_s_c_s:compl:rev CACCCACACAGCCACTATCATGTTGACATTCC; dG = -18.4
108 >s_d_s_b_s_b_s_b_s_c_s GTGACGGTGGCTGTGGCTGTGGCTGTGTGGGTG; >clamp_s_b_s_c_s:compl:rev CACCCACACAGCCACTATCATGTTGACATTCC; dG = -18.4
109 >s_d_s_b_s_d_s_b_s_c_s GTGACGGTGGCTGTGACGGTGGCTGTGTGGGTG; >clamp_s_b_s_c_s:compl:rev CACCCACACAGCCACTATCATGTTGACATTCC; dG = -18.4
110 >s_d_s_b_s_e_s_b_s_c_s GTGACGGTGGCTGTGCTGGTGGCTGTGTGGGTG; >clamp_s_b_s_c_s:compl:rev CACCCACACAGCCACTATCATGTTGACATTCC; dG = -18.4
111 >s_d_s_c_s_a_s_b_s_c_s GTGACGGTGTGGGTGTCTGTGGCTGTGTGGGTG; >clamp_s_b_s_c_s:compl:rev CACCCACACAGCCACTATCATGTTGACATTCC; dG = -18.4
112 >s_d_s_c_s_b_s_b_s_c_s GTGACGGTGTGGGTGGCTGTGGCTGTGTGGGTG; >clamp_s_b_s_c_s:compl:rev CACCCACACAGCCACTATCATGTTGACATTCC; dG = -18.4
113 >s_d_s_c_s_c_s_b_s_c_s GTGACGGTGTGGGTGTGGGTGGCTGTGTGGGTG; >clamp_s_b_s_c_s:compl:rev CACCCACACAGCCACTATCATGTTGACATTCC; dG = -18.4
114 >s_d_s_c_s_d_s_b_s_c_s GTGACGGTGTGGGTGACGGTGGCTGTGTGGGTG; >clamp_s_b_s_c_s:compl:rev CACCCACACAGCCACTATCATGTTGACATTCC; dG = -18.4
115 >s_a_s_b_s_d_s_b_s_c_s GTGTCTGTGGCTGTGACGGTGGCTGTGTGGGTG; >clamp_s_b_s_c_s:compl:rev CACCCACACAGCCACTATCATGTTGACATTCC; dG = -18.4
116 >s_d_s_c_s_e_s_b_s_c_s GTGACGGTGTGGGTGCTGGTGGCTGTGTGGGTG; >clamp_s_b_s_c_s:compl:rev CACCCACACAGCCACTATCATGTTGACATTCC; dG = -18.4
117 >s_d_s_d_s_a_s_b_s_c_s GTGACGGTGACGGTGTCTGTGGCTGTGTGGGTG; >clamp_s_b_s_c_s:compl:rev CACCCACACAGCCACTATCATGTTGACATTCC; dG = -18.4
118 >s_d_s_d_s_b_s_b_s_c_s GTGACGGTGACGGTGGCTGTGGCTGTGTGGGTG; >clamp_s_b_s_c_s:compl:rev CACCCACACAGCCACTATCATGTTGACATTCC; dG = -18.4
119 >s_d_s_d_s_c_s_b_s_c_s GTGACGGTGACGGTGTGGGTGGCTGTGTGGGTG; >clamp_s_b_s_c_s:compl:rev CACCCACACAGCCACTATCATGTTGACATTCC; dG = -18.4
120 >s_d_s_d_s_d_s_b_s_c_s GTGACGGTGACGGTGACGGTGGCTGTGTGGGTG; >clamp_s_b_s_c_s:compl:rev CACCCACACAGCCACTATCATGTTGACATTCC; dG = -18.4
121 >s_d_s_d_s_e_s_b_s_c_s GTGACGGTGACGGTGCTGGTGGCTGTGTGGGTG; >clamp_s_b_s_c_s:compl:rev CACCCACACAGCCACTATCATGTTGACATTCC; dG = -18.4
122 >s_d_s_e_s_a_s_b_s_c_s GTGACGGTGCTGGTGTCTGTGGCTGTGTGGGTG; >clamp_s_b_s_c_s:compl:rev CACCCACACAGCCACTATCATGTTGACATTCC; dG = -18.4
123 >s_d_s_e_s_b_s_b_s_c_s GTGACGGTGCTGGTGGCTGTGGCTGTGTGGGTG; >clamp_s_b_s_c_s:compl:rev CACCCACACAGCCACTATCATGTTGACATTCC; dG = -18.4
124 >s_d_s_e_s_c_s_b_s_c_s GTGACGGTGCTGGTGTGGGTGGCTGTGTGGGTG; >clamp_s_b_s_c_s:compl:rev CACCCACACAGCCACTATCATGTTGACATTCC; dG = -18.4
125 >s_d_s_e_s_d_s_b_s_c_s GTGACGGTGCTGGTGACGGTGGCTGTGTGGGTG; >clamp_s_b_s_c_s:compl:rev CACCCACACAGCCACTATCATGTTGACATTCC; dG = -18.4

****

BC Mismatches

1 >s_b_s_a_s_c_s_d_s_c_s GTGGCTGTGTCTGTGTGGGTGACGGTGTGGGTG; >clamp_s_b_s_c_s:compl:rev CACCCACACAGCCACTATCATGTTGACATTCC; dG = -13.6
2 >s_a_s_a_s_c_s_d_s_c_s GTGTCTGTGTCTGTGTGGGTGACGGTGTGGGTG; >clamp_s_b_s_c_s:compl:rev CACCCACACAGCCACTATCATGTTGACATTCC; dG = -13.6
3 >s_c_s_a_s_c_s_d_s_c_s GTGTGGGTGTCTGTGTGGGTGACGGTGTGGGTG; >clamp_s_b_s_c_s:compl:rev CACCCACACAGCCACTATCATGTTGACATTCC; dG = -13.6
4 >s_d_s_a_s_c_s_d_s_c_s GTGACGGTGTCTGTGTGGGTGACGGTGTGGGTG; >clamp_s_b_s_c_s:compl:rev CACCCACACAGCCACTATCATGTTGACATTCC; dG = -13.5
5 >s_e_s_a_s_c_s_d_s_c_s GTGCTGGTGTCTGTGTGGGTGACGGTGTGGGTG; >clamp_s_b_s_c_s:compl:rev CACCCACACAGCCACTATCATGTTGACATTCC; dG = -13.5
6 >s_b_s_b_s_a_s_c_s_c_s GTGGCTGTGGCTGTGTCTGTGTGGGTGTGGGTG; >clamp_s_b_s_c_s:compl:rev CACCCACACAGCCACTATCATGTTGACATTCC; dG = -13.4
7 >s_c_s_b_s_a_s_c_s_c_s GTGTGGGTGGCTGTGTCTGTGTGGGTGTGGGTG; >clamp_s_b_s_c_s:compl:rev CACCCACACAGCCACTATCATGTTGACATTCC; dG = -13.4
8 >s_a_s_b_s_a_s_c_s_c_s GTGTCTGTGGCTGTGTCTGTGTGGGTGTGGGTG; >clamp_s_b_s_c_s:compl:rev CACCCACACAGCCACTATCATGTTGACATTCC; dG = -13.4
9 >s_d_s_b_s_a_s_c_s_c_s GTGACGGTGGCTGTGTCTGTGTGGGTGTGGGTG; >clamp_s_b_s_c_s:compl:rev CACCCACACAGCCACTATCATGTTGACATTCC; dG = -13.4
10 >s_e_s_b_s_a_s_c_s_c_s GTGCTGGTGGCTGTGTCTGTGTGGGTGTGGGTG; >clamp_s_b_s_c_s:compl:rev CACCCACACAGCCACTATCATGTTGACATTCC; dG = -13.4
11 >s_b_s_b_s_d_s_c_s_c_s GTGGCTGTGGCTGTGACGGTGTGGGTGTGGGTG; >clamp_s_b_s_c_s:compl:rev CACCCACACAGCCACTATCATGTTGACATTCC; dG = -13.3
12 >s_b_s_b_s_e_s_c_s_c_s GTGGCTGTGGCTGTGCTGGTGTGGGTGTGGGTG; >clamp_s_b_s_c_s:compl:rev CACCCACACAGCCACTATCATGTTGACATTCC; dG = -13.3

13 >s_b_s_d_s_c_s_d_s_c_s GTGGCTGTGACGGTGTGGGTGACGGTGTGGGTG; >clamp_s_b_s_c_s:compl:rev
CACCCACACAGCCACTATCATGTTGACATTCC; dG = -13.3
14 >s_b_s_e_s_c_s_d_s_c_s GTGGCTGTGCTGGTGTGGGTGACGGTGTGGGTG; >clamp_s_b_s_c_s:compl:rev
CACCCACACAGCCACTATCATGTTGACATTCC; dG = -13.3
15 >s_a_s_b_s_d_s_c_s_c_s GTGTCTGTGGCTGTGACGGTGTGGGTGTGGGTG; >clamp_s_b_s_c_s:compl:rev
CACCCACACAGCCACTATCATGTTGACATTCC; dG = -13.3
16 >s_a_s_b_s_e_s_c_s_c_s GTGTCTGTGGCTGTGCTGGTGTGGGTGTGGGTG; >clamp_s_b_s_c_s:compl:rev
CACCCACACAGCCACTATCATGTTGACATTCC; dG = -13.3
17 >s_a_s_a_s_b_s_a_s_c_s GTGTCTGTGTCTGTGGCTGTGTCTGTGTGGGTG; >clamp_s_b_s_c_s:compl:rev
CACCCACACAGCCACTATCATGTTGACATTCC; dG = -13.2
18 >s_b_s_a_s_b_s_a_s_c_s GTGGCTGTGTCTGTGGCTGTGTCTGTGTGGGTG; >clamp_s_b_s_c_s:compl:rev
CACCCACACAGCCACTATCATGTTGACATTCC; dG = -13.2
19 >s_b_s_b_s_b_s_a_s_c_s GTGGCTGTGGCTGTGGCTGTGTCTGTGTGGGTG; >clamp_s_b_s_c_s:compl:rev
CACCCACACAGCCACTATCATGTTGACATTCC; dG = -13.2
20 >s_c_s_a_s_b_s_a_s_c_s GTGTGGGTGTCTGTGGCTGTGTCTGTGTGGGTG; >clamp_s_b_s_c_s:compl:rev
CACCCACACAGCCACTATCATGTTGACATTCC; dG = -13.2
21 >s_c_s_b_s_b_s_a_s_c_s GTGTGGGTGGCTGTGGCTGTGTCTGTGTGGGTG; >clamp_s_b_s_c_s:compl:rev
CACCCACACAGCCACTATCATGTTGACATTCC; dG = -13.2
22 >s_c_s_b_s_d_s_c_s_c_s GTGTGGGTGGCTGTGACGGTGTGGGTGTGGGTG; >clamp_s_b_s_c_s:compl:rev
CACCCACACAGCCACTATCATGTTGACATTCC; dG = -13.2
23 >s_c_s_b_s_e_s_c_s_c_s GTGTGGGTGGCTGTGCTGGTGTGGGTGTGGGTG; >clamp_s_b_s_c_s:compl:rev
CACCCACACAGCCACTATCATGTTGACATTCC; dG = -13.2
24 >s_d_s_a_s_b_s_a_s_c_s GTGACGGTGTCTGTGGCTGTGTCTGTGTGGGTG; >clamp_s_b_s_c_s:compl:rev
CACCCACACAGCCACTATCATGTTGACATTCC; dG = -13.2
25 >s_a_s_b_s_b_s_a_s_c_s GTGTCTGTGGCTGTGGCTGTGTCTGTGTGGGTG; >clamp_s_b_s_c_s:compl:rev
CACCCACACAGCCACTATCATGTTGACATTCC; dG = -13.2
26 >s_d_s_b_s_b_s_a_s_c_s GTGACGGTGGCTGTGGCTGTGTCTGTGTGGGTG; >clamp_s_b_s_c_s:compl:rev
CACCCACACAGCCACTATCATGTTGACATTCC; dG = -13.2
27 >s_d_s_b_s_d_s_c_s_c_s GTGACGGTGGCTGTGACGGTGTGGGTGTGGGTG; >clamp_s_b_s_c_s:compl:rev
CACCCACACAGCCACTATCATGTTGACATTCC; dG = -13.2
28 >s_d_s_b_s_e_s_c_s_c_s GTGACGGTGGCTGTGCTGGTGTGGGTGTGGGTG; >clamp_s_b_s_c_s:compl:rev
CACCCACACAGCCACTATCATGTTGACATTCC; dG = -13.2
29 >s_e_s_a_s_b_s_a_s_c_s GTGCTGGTGTCTGTGGCTGTGTCTGTGTGGGTG; >clamp_s_b_s_c_s:compl:rev
CACCCACACAGCCACTATCATGTTGACATTCC; dG = -13.2
30 >s_e_s_b_s_b_s_a_s_c_s GTGCTGGTGGCTGTGGCTGTGTCTGTGTGGGTG; >clamp_s_b_s_c_s:compl:rev
CACCCACACAGCCACTATCATGTTGACATTCC; dG = -13.2
31 >s_e_s_b_s_d_s_c_s_c_s GTGCTGGTGGCTGTGACGGTGTGGGTGTGGGTG; >clamp_s_b_s_c_s:compl:rev
CACCCACACAGCCACTATCATGTTGACATTCC; dG = -13.2
32 >s_e_s_b_s_e_s_c_s_c_s GTGCTGGTGGCTGTGCTGGTGTGGGTGTGGGTG; >clamp_s_b_s_c_s:compl:rev
CACCCACACAGCCACTATCATGTTGACATTCC; dG = -13.2
33 >s_a_s_c_s_d_s_c_s_c_s GTGTCTGTGTGGGTGACGGTGTGGGTGTGGGTG; >clamp_s_b_s_c_s:compl:rev
CACCCACACAGCCACTATCATGTTGACATTCC; dG = -13.1
34 >s_a_s_c_s_d_s_a_s_c_s GTGTCTGTGTGGGTGACGGTGTCTGTGTGGGTG; >clamp_s_b_s_c_s:compl:rev
CACCCACACAGCCACTATCATGTTGACATTCC; dG = -13.1
35 >s_a_s_d_s_b_s_a_s_c_s GTGTCTGTGACGGTGGCTGTGTCTGTGTGGGTG; >clamp_s_b_s_c_s:compl:rev
CACCCACACAGCCACTATCATGTTGACATTCC; dG = -13.1
36 >s_a_s_a_s_c_s_b_s_e_s GTGTCTGTGTCTGTGTGGGTGGCTGTGCTGGTG; >clamp_s_b_s_c_s:compl:rev
CACCCACACAGCCACTATCATGTTGACATTCC; dG = -13.1
37 >s_a_s_e_s_b_s_a_s_c_s GTGTCTGTGCTGGTGGCTGTGTCTGTGTGGGTG; >clamp_s_b_s_c_s:compl:rev
CACCCACACAGCCACTATCATGTTGACATTCC; dG = -13.1
38 >s_a_s_a_s_c_s_b_s_d_s GTGTCTGTGTCTGTGTGGGTGGCTGTGACGGTG; >clamp_s_b_s_c_s:compl:rev
CACCCACACAGCCACTATCATGTTGACATTCC; dG = -13.1
39 >s_a_s_a_s_c_s_b_s_b_s GTGTCTGTGTCTGTGTGGGTGGCTGTGGCTGTG; >clamp_s_b_s_c_s:compl:rev
CACCCACACAGCCACTATCATGTTGACATTCC; dG = -13.1
40 >s_a_s_a_s_c_s_b_s_a_s GTGTCTGTGTCTGTGTGGGTGGCTGTGTCTGTG; >clamp_s_b_s_c_s:compl:rev
CACCCACACAGCCACTATCATGTTGACATTCC; dG = -13.1
41 >s_b_s_a_s_c_s_b_s_e_s GTGGCTGTGTCTGTGTGGGTGGCTGTGCTGGTG; >clamp_s_b_s_c_s:compl:rev
CACCCACACAGCCACTATCATGTTGACATTCC; dG = -13.1
42 >s_b_s_a_s_c_s_b_s_d_s GTGGCTGTGTCTGTGTGGGTGGCTGTGACGGTG; >clamp_s_b_s_c_s:compl:rev
CACCCACACAGCCACTATCATGTTGACATTCC; dG = -13.1
43 >s_b_s_a_s_c_s_b_s_b_s GTGGCTGTGTCTGTGTGGGTGGCTGTGGCTGTG; >clamp_s_b_s_c_s:compl:rev
CACCCACACAGCCACTATCATGTTGACATTCC; dG = -13.1
44 >s_b_s_a_s_c_s_b_s_a_s GTGGCTGTGTCTGTGTGGGTGGCTGTGTCTGTG; >clamp_s_b_s_c_s:compl:rev
CACCCACACAGCCACTATCATGTTGACATTCC; dG = -13.1
45 >s_b_s_d_s_b_s_a_s_c_s GTGGCTGTGACGGTGGCTGTGTCTGTGTGGGTG; >clamp_s_b_s_c_s:compl:rev
CACCCACACAGCCACTATCATGTTGACATTCC; dG = -13.1
46 >s_b_s_e_s_b_s_a_s_c_s GTGGCTGTGCTGGTGGCTGTGTCTGTGTGGGTG; >clamp_s_b_s_c_s:compl:rev
CACCCACACAGCCACTATCATGTTGACATTCC; dG = -13.1
47 >s_c_s_a_s_c_s_b_s_e_s GTGTGGGTGTCTGTGTGGGTGGCTGTGCTGGTG; >clamp_s_b_s_c_s:compl:rev
CACCCACACAGCCACTATCATGTTGACATTCC; dG = -13.1
48 >s_c_s_a_s_c_s_b_s_d_s GTGTGGGTGTCTGTGTGGGTGGCTGTGACGGTG; >clamp_s_b_s_c_s:compl:rev
CACCCACACAGCCACTATCATGTTGACATTCC; dG = -13.1
49 >s_c_s_a_s_c_s_b_s_b_s GTGTGGGTGTCTGTGTGGGTGGCTGTGGCTGTG; >clamp_s_b_s_c_s:compl:rev
CACCCACACAGCCACTATCATGTTGACATTCC; dG = -13.1

50 >s_c_s_a_s_c_s_b_s_a_s GTGTGGGTGTCTGTGTGGGTGGCTGTGTCTGTG; >clamp_s_b_s_c_s:compl:rev
CACCCACACAGCCACTATCATGTTGACATTCC; dG = -13.1
51 >s_c_s_c_s_b_s_a_s_c_s GTGTGGGTGTGGGTGGCTGTGTCTGTGTGGGTG; >clamp_s_b_s_c_s:compl:rev
CACCCACACAGCCACTATCATGTTGACATTCC; dG = -13.1
52 >s_c_s_d_s_b_s_a_s_c_s GTGTGGGTGACGGTGGCTGTGTCTGTGTGGGTG; >clamp_s_b_s_c_s:compl:rev
CACCCACACAGCCACTATCATGTTGACATTCC; dG = -13.1
53 >s_c_s_e_s_b_s_a_s_c_s GTGTGGGTGCTGGTGGCTGTGTCTGTGTGGGTG; >clamp_s_b_s_c_s:compl:rev
CACCCACACAGCCACTATCATGTTGACATTCC; dG = -13.1
54 >s_d_s_a_s_c_s_b_s_e_s GTGACGGTGTCTGTGTGGGTGGCTGTGCTGGTG; >clamp_s_b_s_c_s:compl:rev
CACCCACACAGCCACTATCATGTTGACATTCC; dG = -13.1
55 >s_d_s_a_s_c_s_b_s_d_s GTGACGGTGTCTGTGTGGGTGGCTGTGACGGTG; >clamp_s_b_s_c_s:compl:rev
CACCCACACAGCCACTATCATGTTGACATTCC; dG = -13.1
56 >s_d_s_a_s_c_s_b_s_b_s GTGACGGTGTCTGTGTGGGTGGCTGTGGCTGTG; >clamp_s_b_s_c_s:compl:rev
CACCCACACAGCCACTATCATGTTGACATTCC; dG = -13.1
57 >s_d_s_a_s_c_s_b_s_a_s GTGACGGTGTCTGTGTGGGTGGCTGTGTCTGTG; >clamp_s_b_s_c_s:compl:rev
CACCCACACAGCCACTATCATGTTGACATTCC; dG = -13.1
58 >s_d_s_c_s_b_s_a_s_c_s GTGACGGTGTGGGTGGCTGTGTCTGTGTGGGTG; >clamp_s_b_s_c_s:compl:rev
CACCCACACAGCCACTATCATGTTGACATTCC; dG = -13.1
59 >s_d_s_d_s_b_s_a_s_c_s GTGACGGTGACGGTGGCTGTGTCTGTGTGGGTG; >clamp_s_b_s_c_s:compl:rev
CACCCACACAGCCACTATCATGTTGACATTCC; dG = -13.1
60 >s_d_s_e_s_b_s_a_s_c_s GTGACGGTGCTGGTGGCTGTGTCTGTGTGGGTG; >clamp_s_b_s_c_s:compl:rev
CACCCACACAGCCACTATCATGTTGACATTCC; dG = -13.1
61 >s_e_s_a_s_c_s_b_s_e_s GTGCTGGTGTCTGTGTGGGTGGCTGTGCTGGTG; >clamp_s_b_s_c_s:compl:rev
CACCCACACAGCCACTATCATGTTGACATTCC; dG = -13.1
62 >s_e_s_a_s_c_s_b_s_d_s GTGCTGGTGTCTGTGTGGGTGGCTGTGACGGTG; >clamp_s_b_s_c_s:compl:rev
CACCCACACAGCCACTATCATGTTGACATTCC; dG = -13.1
63 >s_e_s_a_s_c_s_b_s_b_s GTGCTGGTGTCTGTGTGGGTGGCTGTGGCTGTG; >clamp_s_b_s_c_s:compl:rev
CACCCACACAGCCACTATCATGTTGACATTCC; dG = -13.1
64 >s_e_s_a_s_c_s_b_s_a_s GTGCTGGTGTCTGTGTGGGTGGCTGTGTCTGTG; >clamp_s_b_s_c_s:compl:rev
CACCCACACAGCCACTATCATGTTGACATTCC; dG = -13.1
65 >s_e_s_c_s_b_s_a_s_c_s GTGCTGGTGTGGGTGGCTGTGTCTGTGTGGGTG; >clamp_s_b_s_c_s:compl:rev
CACCCACACAGCCACTATCATGTTGACATTCC; dG = -13.1
66 >s_e_s_d_s_b_s_a_s_c_s GTGCTGGTGACGGTGGCTGTGTCTGTGTGGGTG; >clamp_s_b_s_c_s:compl:rev
CACCCACACAGCCACTATCATGTTGACATTCC; dG = -13.1
67 >s_a_s_c_s_b_s_a_s_c_s GTGTCTGTGTGGGTGGCTGTGTCTGTGTGGGTG; >clamp_s_b_s_c_s:compl:rev
CACCCACACAGCCACTATCATGTTGACATTCC; dG = -13.1
68 >s_e_s_e_s_b_s_a_s_c_s GTGCTGGTGCTGGTGGCTGTGTCTGTGTGGGTG; >clamp_s_b_s_c_s:compl:rev
CACCCACACAGCCACTATCATGTTGACATTCC; dG = -13.1
69 >s_a_s_c_s_d_s_b_s_e_s GTGTCTGTGTGGGTGACGGTGGCTGTGCTGGTG; >clamp_s_b_s_c_s:compl:rev
CACCCACACAGCCACTATCATGTTGACATTCC; dG = -13.1
70 >s_a_s_c_s_d_s_b_s_d_s GTGTCTGTGTGGGTGACGGTGGCTGTGACGGTG; >clamp_s_b_s_c_s:compl:rev
CACCCACACAGCCACTATCATGTTGACATTCC; dG = -13.1
71 >s_a_s_c_s_d_s_b_s_b_s GTGTCTGTGTGGGTGACGGTGGCTGTGGCTGTG; >clamp_s_b_s_c_s:compl:rev
CACCCACACAGCCACTATCATGTTGACATTCC; dG = -13.1
72 >s_a_s_c_s_d_s_b_s_a_s GTGTCTGTGTGGGTGACGGTGGCTGTGTCTGTG; >clamp_s_b_s_c_s:compl:rev
CACCCACACAGCCACTATCATGTTGACATTCC; dG = -13.1
73 >s_a_s_c_s_d_s_e_s_c_s GTGTCTGTGTGGGTGACGGTGCTGGTGTGGGTG; >clamp_s_b_s_c_s:compl:rev
CACCCACACAGCCACTATCATGTTGACATTCC; dG = -13.1
74 >s_a_s_c_s_d_s_d_s_c_s GTGTCTGTGTGGGTGACGGTGACGGTGTGGGTG; >clamp_s_b_s_c_s:compl:rev
CACCCACACAGCCACTATCATGTTGACATTCC; dG = -13.1
75 >s_a_s_d_s_a_s_c_s_c_s GTGTCTGTGACGGTGTCTGTGTGGGTGTGGGTG; >clamp_s_b_s_c_s:compl:rev
CACCCACACAGCCACTATCATGTTGACATTCC; dG = -13.0
76 >s_a_s_a_s_b_s_e_s_c_s GTGTCTGTGTCTGTGGCTGTGCTGGTGTGGGTG; >clamp_s_b_s_c_s:compl:rev
CACCCACACAGCCACTATCATGTTGACATTCC; dG = -13.0
77 >s_a_s_a_s_b_s_d_s_c_s GTGTCTGTGTCTGTGGCTGTGACGGTGTGGGTG; >clamp_s_b_s_c_s:compl:rev
CACCCACACAGCCACTATCATGTTGACATTCC; dG = -13.0
78 >s_a_s_e_s_a_s_c_s_c_s GTGTCTGTGCTGGTGTCTGTGTGGGTGTGGGTG; >clamp_s_b_s_c_s:compl:rev
CACCCACACAGCCACTATCATGTTGACATTCC; dG = -13.0
79 >s_b_s_a_s_a_s_c_s_c_s GTGGCTGTGTCTGTGTCTGTGTGGGTGTGGGTG; >clamp_s_b_s_c_s:compl:rev
CACCCACACAGCCACTATCATGTTGACATTCC; dG = -13.0
80 >s_b_s_a_s_b_s_e_s_c_s GTGGCTGTGTCTGTGGCTGTGCTGGTGTGGGTG; >clamp_s_b_s_c_s:compl:rev
CACCCACACAGCCACTATCATGTTGACATTCC; dG = -13.0
81 >s_b_s_a_s_b_s_d_s_c_s GTGGCTGTGTCTGTGGCTGTGACGGTGTGGGTG; >clamp_s_b_s_c_s:compl:rev
CACCCACACAGCCACTATCATGTTGACATTCC; dG = -13.0
82 >s_b_s_a_s_c_s_c_s_c_s GTGGCTGTGTCTGTGTGGGTGTGGGTGTGGGTG; >clamp_s_b_s_c_s:compl:rev
CACCCACACAGCCACTATCATGTTGACATTCC; dG = -13.0
83 >s_b_s_a_s_c_s_a_s_c_s GTGGCTGTGTCTGTGTGGGTGTCTGTGTGGGTG; >clamp_s_b_s_c_s:compl:rev
CACCCACACAGCCACTATCATGTTGACATTCC; dG = -13.0
84 >s_a_s_a_s_c_s_c_s_c_s GTGTCTGTGTCTGTGTGGGTGTGGGTGTGGGTG; >clamp_s_b_s_c_s:compl:rev
CACCCACACAGCCACTATCATGTTGACATTCC; dG = -13.0
85 >s_a_s_a_s_c_s_a_s_c_s GTGTCTGTGTCTGTGTGGGTGTCTGTGTGGGTG; >clamp_s_b_s_c_s:compl:rev
CACCCACACAGCCACTATCATGTTGACATTCC; dG = -13.0
86 >s_b_s_b_s_b_s_e_s_c_s GTGGCTGTGGCTGTGGCTGTGCTGGTGTGGGTG; >clamp_s_b_s_c_s:compl:rev
CACCCACACAGCCACTATCATGTTGACATTCC; dG = -13.0

87 >s_b_s_b_s_b_s_d_s_c_s GTGGCTGTGGCTGTGGCTGTGACGGTGTGGGTG; >clamp_s_b_s_c_s:compl:rev
CACCCACACAGCCACTATCATGTTGACATTCC; dG = -13.0

88 >s_b_s_b_s_d_s_d_s_c_s GTGGCTGTGGCTGTGACGGTGACGGTGTGGGTG; >clamp_s_b_s_c_s:compl:rev
CACCCACACAGCCACTATCATGTTGACATTCC; dG = -13.0

89 >s_b_s_d_s_a_s_c_s_c_s GTGGCTGTGACGGTGTCTGTGTGGGTGTGGGTG; >clamp_s_b_s_c_s:compl:rev
CACCCACACAGCCACTATCATGTTGACATTCC; dG = -13.0

90 >s_b_s_e_s_a_s_c_s_c_s GTGGCTGTGCTGGTGTCTGTGTGGGTGTGGGTG; >clamp_s_b_s_c_s:compl:rev
CACCCACACAGCCACTATCATGTTGACATTCC; dG = -13.0

91 >s_c_s_a_s_a_s_c_s_c_s GTGTGGGTGTCTGTGTCTGTGTGGGTGTGGGTG; >clamp_s_b_s_c_s:compl:rev
CACCCACACAGCCACTATCATGTTGACATTCC; dG = -13.0

92 >s_c_s_a_s_b_s_e_s_c_s GTGTGGGTGTCTGTGGCTGTGCTGGTGTGGGTG; >clamp_s_b_s_c_s:compl:rev
CACCCACACAGCCACTATCATGTTGACATTCC; dG = -13.0

93 >s_c_s_a_s_b_s_d_s_c_s GTGTGGGTGTCTGTGGCTGTGACGGTGTGGGTG; >clamp_s_b_s_c_s:compl:rev
CACCCACACAGCCACTATCATGTTGACATTCC; dG = -13.0

94 >s_c_s_a_s_c_s_c_s_c_s GTGTGGGTGTCTGTGTGGGTGTGGGTGTGGGTG; >clamp_s_b_s_c_s:compl:rev
CACCCACACAGCCACTATCATGTTGACATTCC; dG = -13.0

95 >s_c_s_a_s_c_s_a_s_c_s GTGTGGGTGTCTGTGTGGGTGTCTGTGTGGGTG; >clamp_s_b_s_c_s:compl:rev
CACCCACACAGCCACTATCATGTTGACATTCC; dG = -13.0

96 >s_c_s_b_s_b_s_e_s_c_s GTGTGGGTGGCTGTGGCTGTGCTGGTGTGGGTG; >clamp_s_b_s_c_s:compl:rev
CACCCACACAGCCACTATCATGTTGACATTCC; dG = -13.0

97 >s_c_s_b_s_b_s_d_s_c_s GTGTGGGTGGCTGTGGCTGTGACGGTGTGGGTG; >clamp_s_b_s_c_s:compl:rev
CACCCACACAGCCACTATCATGTTGACATTCC; dG = -13.0

98 >s_c_s_b_s_d_s_d_s_c_s GTGTGGGTGGCTGTGACGGTGACGGTGTGGGTG; >clamp_s_b_s_c_s:compl:rev
CACCCACACAGCCACTATCATGTTGACATTCC; dG = -13.0

99 >s_c_s_c_s_a_s_c_s_c_s GTGTGGGTGTGGGTGTCTGTGTGGGTGTGGGTG; >clamp_s_b_s_c_s:compl:rev
CACCCACACAGCCACTATCATGTTGACATTCC; dG = -13.0

100 >s_c_s_d_s_a_s_c_s_c_s GTGTGGGTGACGGTGTCTGTGTGGGTGTGGGTG; >clamp_s_b_s_c_s:compl:rev
CACCCACACAGCCACTATCATGTTGACATTCC; dG = -13.0

101 >s_c_s_e_s_a_s_c_s_c_s GTGTGGGTGCTGGTGTCTGTGTGGGTGTGGGTG; >clamp_s_b_s_c_s:compl:rev
CACCCACACAGCCACTATCATGTTGACATTCC; dG = -13.0

102 >s_a_s_b_s_b_s_e_s_c_s GTGTCTGTGGCTGTGGCTGTGCTGGTGTGGGTG; >clamp_s_b_s_c_s:compl:rev
CACCCACACAGCCACTATCATGTTGACATTCC; dG = -13.0

103 >s_d_s_a_s_a_s_c_s_c_s GTGACGGTGTCTGTGTCTGTGTGGGTGTGGGTG; >clamp_s_b_s_c_s:compl:rev
CACCCACACAGCCACTATCATGTTGACATTCC; dG = -13.0

104 >s_a_s_b_s_b_s_d_s_c_s GTGTCTGTGGCTGTGGCTGTGACGGTGTGGGTG; >clamp_s_b_s_c_s:compl:rev
CACCCACACAGCCACTATCATGTTGACATTCC; dG = -13.0

105 >s_d_s_a_s_b_s_e_s_c_s GTGACGGTGTCTGTGGCTGTGCTGGTGTGGGTG; >clamp_s_b_s_c_s:compl:rev
CACCCACACAGCCACTATCATGTTGACATTCC; dG = -13.0

106 >s_d_s_a_s_b_s_d_s_c_s GTGACGGTGTCTGTGGCTGTGACGGTGTGGGTG; >clamp_s_b_s_c_s:compl:rev
CACCCACACAGCCACTATCATGTTGACATTCC; dG = -13.0

107 >s_d_s_a_s_c_s_c_s_c_s GTGACGGTGTCTGTGTGGGTGTGGGTGTGGGTG; >clamp_s_b_s_c_s:compl:rev
CACCCACACAGCCACTATCATGTTGACATTCC; dG = -13.0

108 >s_d_s_a_s_c_s_a_s_c_s GTGACGGTGTCTGTGTGGGTGTCTGTGTGGGTG; >clamp_s_b_s_c_s:compl:rev
CACCCACACAGCCACTATCATGTTGACATTCC; dG = -13.0

109 >s_d_s_b_s_b_s_e_s_c_s GTGACGGTGGCTGTGGCTGTGCTGGTGTGGGTG; >clamp_s_b_s_c_s:compl:rev
CACCCACACAGCCACTATCATGTTGACATTCC; dG = -13.0

110 >s_d_s_b_s_b_s_d_s_c_s GTGACGGTGGCTGTGGCTGTGACGGTGTGGGTG; >clamp_s_b_s_c_s:compl:rev
CACCCACACAGCCACTATCATGTTGACATTCC; dG = -13.0

111 >s_d_s_b_s_d_s_d_s_c_s GTGACGGTGGCTGTGACGGTGACGGTGTGGGTG; >clamp_s_b_s_c_s:compl:rev
CACCCACACAGCCACTATCATGTTGACATTCC; dG = -13.0

112 >s_d_s_c_s_a_s_c_s_c_s GTGACGGTGTGGGTGTCTGTGTGGGTGTGGGTG; >clamp_s_b_s_c_s:compl:rev
CACCCACACAGCCACTATCATGTTGACATTCC; dG = -13.0

113 >s_a_s_b_s_d_s_d_s_c_s GTGTCTGTGGCTGTGACGGTGACGGTGTGGGTG; >clamp_s_b_s_c_s:compl:rev
CACCCACACAGCCACTATCATGTTGACATTCC; dG = -13.0

114 >s_d_s_d_s_a_s_c_s_c_s GTGACGGTGACGGTGTCTGTGTGGGTGTGGGTG; >clamp_s_b_s_c_s:compl:rev
CACCCACACAGCCACTATCATGTTGACATTCC; dG = -13.0

115 >s_d_s_e_s_a_s_c_s_c_s GTGACGGTGCTGGTGTCTGTGTGGGTGTGGGTG; >clamp_s_b_s_c_s:compl:rev
CACCCACACAGCCACTATCATGTTGACATTCC; dG = -13.0

116 >s_a_s_a_s_a_s_c_s_c_s GTGTCTGTGTCTGTGTCTGTGTGGGTGTGGGTG; >clamp_s_b_s_c_s:compl:rev
CACCCACACAGCCACTATCATGTTGACATTCC; dG = -13.0

117 >s_e_s_a_s_a_s_c_s_c_s GTGCTGGTGTCTGTGTCTGTGTGGGTGTGGGTG; >clamp_s_b_s_c_s:compl:rev
CACCCACACAGCCACTATCATGTTGACATTCC; dG = -13.0

118 >s_e_s_a_s_b_s_e_s_c_s GTGCTGGTGTCTGTGGCTGTGCTGGTGTGGGTG; >clamp_s_b_s_c_s:compl:rev
CACCCACACAGCCACTATCATGTTGACATTCC; dG = -13.0

119 >s_e_s_a_s_b_s_d_s_c_s GTGCTGGTGTCTGTGGCTGTGACGGTGTGGGTG; >clamp_s_b_s_c_s:compl:rev
CACCCACACAGCCACTATCATGTTGACATTCC; dG = -13.0

120 >s_e_s_a_s_c_s_c_s_c_s GTGCTGGTGTCTGTGTGGGTGTGGGTGTGGGTG; >clamp_s_b_s_c_s:compl:rev
CACCCACACAGCCACTATCATGTTGACATTCC; dG = -13.0

121 >s_e_s_a_s_c_s_a_s_c_s GTGCTGGTGTCTGTGTGGGTGTCTGTGTGGGTG; >clamp_s_b_s_c_s:compl:rev
CACCCACACAGCCACTATCATGTTGACATTCC; dG = -13.0

122 >s_e_s_b_s_b_s_e_s_c_s GTGCTGGTGGCTGTGGCTGTGCTGGTGTGGGTG; >clamp_s_b_s_c_s:compl:rev
CACCCACACAGCCACTATCATGTTGACATTCC; dG = -13.0

123 >s_e_s_b_s_b_s_d_s_c_s GTGCTGGTGGCTGTGGCTGTGACGGTGTGGGTG; >clamp_s_b_s_c_s:compl:rev
CACCCACACAGCCACTATCATGTTGACATTCC; dG = -13.0

124 >s_e_s_b_s_d_s_d_s_c_s GTGCTGGTGGCTGTGACGGTGACGGTGTGGGTG; >clamp_s_b_s_c_s:compl:rev
CACCCACACAGCCACTATCATGTTGACATTCC; dG = -13.0
125 >s_a_s_c_s_a_s_c_s_c_s GTGTCTGTGTGGGTGTCTGTGTGGGTGTGGGTG; >clamp_s_b_s_c_s:compl:rev
CACCCACACAGCCACTATCATGTTGACATTCC; dG = -13.0
126 >s_e_s_c_s_a_s_c_s_c_s GTGCTGGTGTGGGTGTCTGTGTGGGTGTGGGTG; >clamp_s_b_s_c_s:compl:rev
CACCCACACAGCCACTATCATGTTGACATTCC; dG = -13.0
127 >s_e_s_d_s_a_s_c_s_c_s GTGCTGGTGACGGTGTCTGTGTGGGTGTGGGTG; >clamp_s_b_s_c_s:compl:rev
CACCCACACAGCCACTATCATGTTGACATTCC; dG = -13.0
128 >s_e_s_e_s_a_s_c_s_c_s GTGCTGGTGCTGGTGTCTGTGTGGGTGTGGGTG; >clamp_s_b_s_c_s:compl:rev
CACCCACACAGCCACTATCATGTTGACATTCC; dG = -13.0
129 >s_a_s_d_s_b_s_e_s_c_s GTGTCTGTGACGGTGGCTGTGCTGGTGTGGGTG; >clamp_s_b_s_c_s:compl:rev
CACCCACACAGCCACTATCATGTTGACATTCC; dG = -12.9
130 >s_a_s_d_s_b_s_d_s_c_s GTGTCTGTGACGGTGGCTGTGACGGTGTGGGTG; >clamp_s_b_s_c_s:compl:rev
CACCCACACAGCCACTATCATGTTGACATTCC; dG = -12.9
131 >s_a_s_e_s_b_s_e_s_c_s GTGTCTGTGCTGGTGGCTGTGCTGGTGTGGGTG; >clamp_s_b_s_c_s:compl:rev
CACCCACACAGCCACTATCATGTTGACATTCC; dG = -12.9
132 >s_a_s_e_s_b_s_d_s_c_s GTGTCTGTGCTGGTGGCTGTGACGGTGTGGGTG; >clamp_s_b_s_c_s:compl:rev
CACCCACACAGCCACTATCATGTTGACATTCC; dG = -12.9
133 >s_a_s_a_s_c_s_e_s_c_s GTGTCTGTGTCTGTGTGGGTGCTGGTGTGGGTG; >clamp_s_b_s_c_s:compl:rev
CACCCACACAGCCACTATCATGTTGACATTCC; dG = -12.9
134 >s_b_s_a_s_c_s_e_s_c_s GTGGCTGTGTCTGTGTGGGTGCTGGTGTGGGTG; >clamp_s_b_s_c_s:compl:rev
CACCCACACAGCCACTATCATGTTGACATTCC; dG = -12.9
135 >s_b_s_b_s_e_s_d_s_c_s GTGGCTGTGGCTGTGCTGGTGACGGTGTGGGTG; >clamp_s_b_s_c_s:compl:rev
CACCCACACAGCCACTATCATGTTGACATTCC; dG = -12.9
136 >s_b_s_d_s_b_s_e_s_c_s GTGGCTGTGACGGTGGCTGTGCTGGTGTGGGTG; >clamp_s_b_s_c_s:compl:rev
CACCCACACAGCCACTATCATGTTGACATTCC; dG = -12.9
137 >s_b_s_d_s_b_s_d_s_c_s GTGGCTGTGACGGTGGCTGTGACGGTGTGGGTG; >clamp_s_b_s_c_s:compl:rev
CACCCACACAGCCACTATCATGTTGACATTCC; dG = -12.9
138 >s_b_s_e_s_b_s_e_s_c_s GTGGCTGTGCTGGTGGCTGTGCTGGTGTGGGTG; >clamp_s_b_s_c_s:compl:rev
CACCCACACAGCCACTATCATGTTGACATTCC; dG = -12.9
139 >s_b_s_e_s_b_s_d_s_c_s GTGGCTGTGCTGGTGGCTGTGACGGTGTGGGTG; >clamp_s_b_s_c_s:compl:rev
CACCCACACAGCCACTATCATGTTGACATTCC; dG = -12.9
140 >s_c_s_a_s_c_s_e_s_c_s GTGTGGGTGTCTGTGTGGGTGCTGGTGTGGGTG; >clamp_s_b_s_c_s:compl:rev
CACCCACACAGCCACTATCATGTTGACATTCC; dG = -12.9
141 >s_c_s_b_s_e_s_d_s_c_s GTGTGGGTGGCTGTGCTGGTGACGGTGTGGGTG; >clamp_s_b_s_c_s:compl:rev
CACCCACACAGCCACTATCATGTTGACATTCC; dG = -12.9
142 >s_c_s_c_s_b_s_e_s_c_s GTGTGGGTGTGGGTGGCTGTGCTGGTGTGGGTG; >clamp_s_b_s_c_s:compl:rev
CACCCACACAGCCACTATCATGTTGACATTCC; dG = -12.9
143 >s_c_s_c_s_b_s_d_s_c_s GTGTGGGTGTGGGTGGCTGTGACGGTGTGGGTG; >clamp_s_b_s_c_s:compl:rev
CACCCACACAGCCACTATCATGTTGACATTCC; dG = -12.9
144 >s_c_s_d_s_b_s_e_s_c_s GTGTGGGTGACGGTGGCTGTGCTGGTGTGGGTG; >clamp_s_b_s_c_s:compl:rev
CACCCACACAGCCACTATCATGTTGACATTCC; dG = -12.9
145 >s_c_s_d_s_b_s_d_s_c_s GTGTGGGTGACGGTGGCTGTGACGGTGTGGGTG; >clamp_s_b_s_c_s:compl:rev
CACCCACACAGCCACTATCATGTTGACATTCC; dG = -12.9
146 >s_c_s_e_s_b_s_e_s_c_s GTGTGGGTGCTGGTGGCTGTGCTGGTGTGGGTG; >clamp_s_b_s_c_s:compl:rev
CACCCACACAGCCACTATCATGTTGACATTCC; dG = -12.9
147 >s_c_s_e_s_b_s_d_s_c_s GTGTGGGTGCTGGTGGCTGTGACGGTGTGGGTG; >clamp_s_b_s_c_s:compl:rev
CACCCACACAGCCACTATCATGTTGACATTCC; dG = -12.9
148 >s_d_s_a_s_c_s_e_s_c_s GTGACGGTGTCTGTGTGGGTGCTGGTGTGGGTG; >clamp_s_b_s_c_s:compl:rev
CACCCACACAGCCACTATCATGTTGACATTCC; dG = -12.9
149 >s_d_s_b_s_e_s_d_s_c_s GTGACGGTGGCTGTGCTGGTGACGGTGTGGGTG; >clamp_s_b_s_c_s:compl:rev
CACCCACACAGCCACTATCATGTTGACATTCC; dG = -12.9
150 >s_d_s_c_s_b_s_e_s_c_s GTGACGGTGTGGGTGGCTGTGCTGGTGTGGGTG; >clamp_s_b_s_c_s:compl:rev
CACCCACACAGCCACTATCATGTTGACATTCC; dG = -12.9
151 >s_d_s_c_s_b_s_d_s_c_s GTGACGGTGTGGGTGGCTGTGACGGTGTGGGTG; >clamp_s_b_s_c_s:compl:rev
CACCCACACAGCCACTATCATGTTGACATTCC; dG = -12.9
152 >s_d_s_d_s_b_s_e_s_c_s GTGACGGTGACGGTGGCTGTGCTGGTGTGGGTG; >clamp_s_b_s_c_s:compl:rev
CACCCACACAGCCACTATCATGTTGACATTCC; dG = -12.9
153 >s_d_s_d_s_b_s_d_s_c_s GTGACGGTGACGGTGGCTGTGACGGTGTGGGTG; >clamp_s_b_s_c_s:compl:rev
CACCCACACAGCCACTATCATGTTGACATTCC; dG = -12.9
154 >s_a_s_b_s_e_s_d_s_c_s GTGTCTGTGGCTGTGCTGGTGACGGTGTGGGTG; >clamp_s_b_s_c_s:compl:rev
CACCCACACAGCCACTATCATGTTGACATTCC; dG = -12.9
155 >s_d_s_e_s_b_s_e_s_c_s GTGACGGTGCTGGTGGCTGTGCTGGTGTGGGTG; >clamp_s_b_s_c_s:compl:rev
CACCCACACAGCCACTATCATGTTGACATTCC; dG = -12.9
156 >s_d_s_e_s_b_s_d_s_c_s GTGACGGTGCTGGTGGCTGTGACGGTGTGGGTG; >clamp_s_b_s_c_s:compl:rev
CACCCACACAGCCACTATCATGTTGACATTCC; dG = -12.9
157 >s_e_s_a_s_c_s_e_s_c_s GTGCTGGTGTCTGTGTGGGTGCTGGTGTGGGTG; >clamp_s_b_s_c_s:compl:rev
CACCCACACAGCCACTATCATGTTGACATTCC; dG = -12.9
158 >s_e_s_b_s_e_s_d_s_c_s GTGCTGGTGGCTGTGCTGGTGACGGTGTGGGTG; >clamp_s_b_s_c_s:compl:rev
CACCCACACAGCCACTATCATGTTGACATTCC; dG = -12.9
159 >s_e_s_c_s_b_s_e_s_c_s GTGCTGGTGTGGGTGGCTGTGCTGGTGTGGGTG; >clamp_s_b_s_c_s:compl:rev
CACCCACACAGCCACTATCATGTTGACATTCC; dG = -12.9
160 >s_e_s_c_s_b_s_d_s_c_s GTGCTGGTGTGGGTGGCTGTGACGGTGTGGGTG; >clamp_s_b_s_c_s:compl:rev
CACCCACACAGCCACTATCATGTTGACATTCC; dG = -12.9

161 >s_a_s_c_s_b_s_e_s_c_s GTGTCTGTGTGGGTGGCTGTGCTGGTGTGGGTG; >clamp_s_b_s_c_s:compl:rev CACCCACACAGCCACTATCATGTTGACATTCC; dG = -12.9

162 >s_e_s_d_s_b_s_e_s_c_s GTGCTGGTGACGGTGGCTGTGCTGGTGTGGGTG; >clamp_s_b_s_c_s:compl:rev CACCCACACAGCCACTATCATGTTGACATTCC; dG = -12.9

163 >s_e_s_d_s_b_s_d_s_c_s GTGCTGGTGACGGTGGCTGTGACGGTGTGGGTG; >clamp_s_b_s_c_s:compl:rev CACCCACACAGCCACTATCATGTTGACATTCC; dG = -12.9

164 >s_a_s_c_s_b_s_d_s_c_s GTGTCTGTGTGGGTGGCTGTGACGGTGTGGGTG; >clamp_s_b_s_c_s:compl:rev CACCCACACAGCCACTATCATGTTGACATTCC; dG = -12.9

165 >s_e_s_e_s_b_s_e_s_c_s GTGCTGGTGCTGGTGGCTGTGCTGGTGTGGGTG; >clamp_s_b_s_c_s:compl:rev CACCCACACAGCCACTATCATGTTGACATTCC; dG = -12.9

166 >s_e_s_e_s_b_s_d_s_c_s GTGCTGGTGCTGGTGGCTGTGACGGTGTGGGTG; >clamp_s_b_s_c_s:compl:rev CACCCACACAGCCACTATCATGTTGACATTCC; dG = -12.9

167 >s_a_s_d_s_a_s_a_s_c_s GTGTCTGTGACGGTGTCTGTGTCTGTGTGGGTG; >clamp_s_b_s_c_s:compl:rev CACCCACACAGCCACTATCATGTTGACATTCC; dG = -12.8

168 >s_a_s_e_s_a_s_a_s_c_s GTGTCTGTGCTGGTGTCTGTGTCTGTGTGGGTG; >clamp_s_b_s_c_s:compl:rev CACCCACACAGCCACTATCATGTTGACATTCC; dG = -12.8

169 >s_b_s_a_s_a_s_a_s_c_s GTGGCTGTGTCTGTGTCTGTGTCTGTGTGGGTG; >clamp_s_b_s_c_s:compl:rev CACCCACACAGCCACTATCATGTTGACATTCC; dG = -12.8

170 >s_b_s_b_s_a_s_a_s_c_s GTGGCTGTGGCTGTGTCTGTGTCTGTGTGGGTG; >clamp_s_b_s_c_s:compl:rev CACCCACACAGCCACTATCATGTTGACATTCC; dG = -12.8

171 >s_b_s_d_s_a_s_a_s_c_s GTGGCTGTGACGGTGTCTGTGTCTGTGTGGGTG; >clamp_s_b_s_c_s:compl:rev CACCCACACAGCCACTATCATGTTGACATTCC; dG = -12.8

172 >s_b_s_d_s_c_s_b_s_e_s GTGGCTGTGACGGTGTGGGTGGCTGTGCTGGTG; >clamp_s_b_s_c_s:compl:rev CACCCACACAGCCACTATCATGTTGACATTCC; dG = -12.8

173 >s_b_s_d_s_c_s_b_s_d_s GTGGCTGTGACGGTGTGGGTGGCTGTGACGGTG; >clamp_s_b_s_c_s:compl:rev CACCCACACAGCCACTATCATGTTGACATTCC; dG = -12.8

174 >s_b_s_d_s_c_s_b_s_b_s GTGGCTGTGACGGTGTGGGTGGCTGTGGCTGTG; >clamp_s_b_s_c_s:compl:rev CACCCACACAGCCACTATCATGTTGACATTCC; dG = -12.8

175 >s_b_s_d_s_c_s_b_s_a_s GTGGCTGTGACGGTGTGGGTGGCTGTGTCTGTG; >clamp_s_b_s_c_s:compl:rev CACCCACACAGCCACTATCATGTTGACATTCC; dG = -12.8

176 >s_b_s_e_s_a_s_a_s_c_s GTGGCTGTGCTGGTGTCTGTGTCTGTGTGGGTG; >clamp_s_b_s_c_s:compl:rev CACCCACACAGCCACTATCATGTTGACATTCC; dG = -12.8

177 >s_b_s_e_s_c_s_b_s_e_s GTGGCTGTGCTGGTGTGGGTGGCTGTGCTGGTG; >clamp_s_b_s_c_s:compl:rev CACCCACACAGCCACTATCATGTTGACATTCC; dG = -12.8

178 >s_b_s_e_s_c_s_b_s_d_s GTGGCTGTGCTGGTGTGGGTGGCTGTGACGGTG; >clamp_s_b_s_c_s:compl:rev CACCCACACAGCCACTATCATGTTGACATTCC; dG = -12.8

179 >s_b_s_e_s_c_s_b_s_b_s GTGGCTGTGCTGGTGTGGGTGGCTGTGGCTGTG; >clamp_s_b_s_c_s:compl:rev CACCCACACAGCCACTATCATGTTGACATTCC; dG = -12.8

180 >s_b_s_e_s_c_s_b_s_a_s GTGGCTGTGCTGGTGTGGGTGGCTGTGTCTGTG; >clamp_s_b_s_c_s:compl:rev CACCCACACAGCCACTATCATGTTGACATTCC; dG = -12.8

181 >s_c_s_a_s_a_s_a_s_c_s GTGTGGGTGTCTGTGTCTGTGTCTGTGTGGGTG; >clamp_s_b_s_c_s:compl:rev CACCCACACAGCCACTATCATGTTGACATTCC; dG = -12.8

182 >s_c_s_b_s_a_s_a_s_c_s GTGTGGGTGGCTGTGTCTGTGTCTGTGTGGGTG; >clamp_s_b_s_c_s:compl:rev CACCCACACAGCCACTATCATGTTGACATTCC; dG = -12.8

183 >s_c_s_c_s_a_s_a_s_c_s GTGTGGGTGTGGGTGTCTGTGTCTGTGTGGGTG; >clamp_s_b_s_c_s:compl:rev CACCCACACAGCCACTATCATGTTGACATTCC; dG = -12.8

184 >s_c_s_d_s_a_s_a_s_c_s GTGTGGGTGACGGTGTCTGTGTCTGTGTGGGTG; >clamp_s_b_s_c_s:compl:rev CACCCACACAGCCACTATCATGTTGACATTCC; dG = -12.8

185 >s_a_s_b_s_a_s_a_s_c_s GTGTCTGTGGCTGTGTCTGTGTCTGTGTGGGTG; >clamp_s_b_s_c_s:compl:rev CACCCACACAGCCACTATCATGTTGACATTCC; dG = -12.8

186 >s_c_s_e_s_a_s_a_s_c_s GTGTGGGTGCTGGTGTCTGTGTCTGTGTGGGTG; >clamp_s_b_s_c_s:compl:rev CACCCACACAGCCACTATCATGTTGACATTCC; dG = -12.8

187 >s_d_s_a_s_a_s_a_s_c_s GTGACGGTGTCTGTGTCTGTGTCTGTGTGGGTG; >clamp_s_b_s_c_s:compl:rev CACCCACACAGCCACTATCATGTTGACATTCC; dG = -12.8

188 >s_d_s_b_s_a_s_a_s_c_s GTGACGGTGGCTGTGTCTGTGTCTGTGTGGGTG; >clamp_s_b_s_c_s:compl:rev CACCCACACAGCCACTATCATGTTGACATTCC; dG = -12.8

189 >s_d_s_c_s_a_s_a_s_c_s GTGACGGTGTGGGTGTCTGTGTCTGTGTGGGTG; >clamp_s_b_s_c_s:compl:rev CACCCACACAGCCACTATCATGTTGACATTCC; dG = -12.8

190 >s_d_s_d_s_a_s_a_s_c_s GTGACGGTGACGGTGTCTGTGTCTGTGTGGGTG; >clamp_s_b_s_c_s:compl:rev CACCCACACAGCCACTATCATGTTGACATTCC; dG = -12.8

191 >s_d_s_e_s_a_s_a_s_c_s GTGACGGTGCTGGTGTCTGTGTCTGTGTGGGTG; >clamp_s_b_s_c_s:compl:rev CACCCACACAGCCACTATCATGTTGACATTCC; dG = -12.8

192 >s_e_s_a_s_a_s_a_s_c_s GTGCTGGTGTCTGTGTCTGTGTCTGTGTGGGTG; >clamp_s_b_s_c_s:compl:rev CACCCACACAGCCACTATCATGTTGACATTCC; dG = -12.8

193 >s_e_s_b_s_a_s_a_s_c_s GTGCTGGTGGCTGTGTCTGTGTCTGTGTGGGTG; >clamp_s_b_s_c_s:compl:rev CACCCACACAGCCACTATCATGTTGACATTCC; dG = -12.8

194 >s_a_s_c_s_a_s_a_s_c_s GTGTCTGTGTGGGTGTCTGTGTCTGTGTGGGTG; >clamp_s_b_s_c_s:compl:rev CACCCACACAGCCACTATCATGTTGACATTCC; dG = -12.8

195 >s_e_s_c_s_a_s_a_s_c_s GTGCTGGTGTGGGTGTCTGTGTCTGTGTGGGTG; >clamp_s_b_s_c_s:compl:rev CACCCACACAGCCACTATCATGTTGACATTCC; dG = -12.8

196 >s_a_s_a_s_a_s_a_s_c_s GTGTCTGTGTCTGTGTCTGTGTCTGTGTGGGTG; >clamp_s_b_s_c_s:compl:rev CACCCACACAGCCACTATCATGTTGACATTCC; dG = -12.8

197 >s_e_s_d_s_a_s_a_s_c_s GTGCTGGTGACGGTGTCTGTGTCTGTGTGGGTG; >clamp_s_b_s_c_s:compl:rev CACCCACACAGCCACTATCATGTTGACATTCC; dG = -12.8

198 >s_e_s_e_s_a_s_a_s_c_s GTGCTGGTGCTGGTGTCTGTGTCTGTGTGGGTG; >clamp_s_b_s_c_s:compl:rev CACCCACACAGCCACTATCATGTTGACATTCC; dG = -12.8
199 >s_a_s_c_s_e_s_a_s_c_s GTGTCTGTGTGGGTGCTGGTGTCTGTGTGGGTG; >clamp_s_b_s_c_s:compl:rev CACCCACACAGCCACTATCATGTTGACATTCC; dG = -12.7
200 >s_a_s_d_s_c_s_a_s_c_s GTGTCTGTGACGGTGTGGGTGTCTGTGTGGGTG; >clamp_s_b_s_c_s:compl:rev CACCCACACAGCCACTATCATGTTGACATTCC; dG = -12.7
201 >s_a_s_d_s_d_s_a_s_c_s GTGTCTGTGACGGTGACGGTGTCTGTGTGGGTG; >clamp_s_b_s_c_s:compl:rev CACCCACACAGCCACTATCATGTTGACATTCC; dG = -12.7
202 >s_a_s_d_s_e_s_a_s_c_s GTGTCTGTGACGGTGCTGGTGTCTGTGTGGGTG; >clamp_s_b_s_c_s:compl:rev CACCCACACAGCCACTATCATGTTGACATTCC; dG = -12.7
203 >s_a_s_e_s_c_s_a_s_c_s GTGTCTGTGCTGGTGTGGGTGTCTGTGTGGGTG; >clamp_s_b_s_c_s:compl:rev CACCCACACAGCCACTATCATGTTGACATTCC; dG = -12.7
204 >s_a_s_e_s_d_s_a_s_c_s GTGTCTGTGCTGGTGACGGTGTCTGTGTGGGTG; >clamp_s_b_s_c_s:compl:rev CACCCACACAGCCACTATCATGTTGACATTCC; dG = -12.7
205 >s_a_s_e_s_e_s_a_s_c_s GTGTCTGTGCTGGTGCTGGTGTCTGTGTGGGTG; >clamp_s_b_s_c_s:compl:rev CACCCACACAGCCACTATCATGTTGACATTCC; dG = -12.7
206 >s_b_s_a_s_d_s_a_s_c_s GTGGCTGTGTCTGTGACGGTGTCTGTGTGGGTG; >clamp_s_b_s_c_s:compl:rev CACCCACACAGCCACTATCATGTTGACATTCC; dG = -12.7
207 >s_b_s_a_s_e_s_a_s_c_s GTGGCTGTGTCTGTGCTGGTGTCTGTGTGGGTG; >clamp_s_b_s_c_s:compl:rev CACCCACACAGCCACTATCATGTTGACATTCC; dG = -12.7
208 >s_b_s_b_s_d_s_a_s_c_s GTGGCTGTGGCTGTGACGGTGTCTGTGTGGGTG; >clamp_s_b_s_c_s:compl:rev CACCCACACAGCCACTATCATGTTGACATTCC; dG = -12.7
209 >s_b_s_b_s_e_s_a_s_c_s GTGGCTGTGGCTGTGCTGGTGTCTGTGTGGGTG; >clamp_s_b_s_c_s:compl:rev CACCCACACAGCCACTATCATGTTGACATTCC; dG = -12.7
210 >s_b_s_d_s_c_s_c_s_c_s GTGGCTGTGACGGTGTGGGTGTGGGTGTGGGTG; >clamp_s_b_s_c_s:compl:rev CACCCACACAGCCACTATCATGTTGACATTCC; dG = -12.7
211 >s_b_s_d_s_c_s_a_s_c_s GTGGCTGTGACGGTGTGGGTGTCTGTGTGGGTG; >clamp_s_b_s_c_s:compl:rev CACCCACACAGCCACTATCATGTTGACATTCC; dG = -12.7
212 >s_b_s_d_s_d_s_a_s_c_s GTGGCTGTGACGGTGACGGTGTCTGTGTGGGTG; >clamp_s_b_s_c_s:compl:rev CACCCACACAGCCACTATCATGTTGACATTCC; dG = -12.7
213 >s_b_s_d_s_e_s_a_s_c_s GTGGCTGTGACGGTGCTGGTGTCTGTGTGGGTG; >clamp_s_b_s_c_s:compl:rev CACCCACACAGCCACTATCATGTTGACATTCC; dG = -12.7
214 >s_a_s_a_s_d_s_a_s_c_s GTGTCTGTGTCTGTGACGGTGTCTGTGTGGGTG; >clamp_s_b_s_c_s:compl:rev CACCCACACAGCCACTATCATGTTGACATTCC; dG = -12.7
215 >s_b_s_e_s_c_s_c_s_c_s GTGGCTGTGCTGGTGTGGGTGTGGGTGTGGGTG; >clamp_s_b_s_c_s:compl:rev CACCCACACAGCCACTATCATGTTGACATTCC; dG = -12.7
216 >s_b_s_e_s_c_s_a_s_c_s GTGGCTGTGCTGGTGTGGGTGTCTGTGTGGGTG; >clamp_s_b_s_c_s:compl:rev CACCCACACAGCCACTATCATGTTGACATTCC; dG = -12.7
217 >s_b_s_e_s_d_s_a_s_c_s GTGGCTGTGCTGGTGACGGTGTCTGTGTGGGTG; >clamp_s_b_s_c_s:compl:rev CACCCACACAGCCACTATCATGTTGACATTCC; dG = -12.7
218 >s_b_s_e_s_e_s_a_s_c_s GTGGCTGTGCTGGTGCTGGTGTCTGTGTGGGTG; >clamp_s_b_s_c_s:compl:rev CACCCACACAGCCACTATCATGTTGACATTCC; dG = -12.7
219 >s_c_s_a_s_d_s_a_s_c_s GTGTGGGTGTCTGTGACGGTGTCTGTGTGGGTG; >clamp_s_b_s_c_s:compl:rev CACCCACACAGCCACTATCATGTTGACATTCC; dG = -12.7
220 >s_c_s_a_s_e_s_a_s_c_s GTGTGGGTGTCTGTGCTGGTGTCTGTGTGGGTG; >clamp_s_b_s_c_s:compl:rev CACCCACACAGCCACTATCATGTTGACATTCC; dG = -12.7
221 >s_a_s_a_s_e_s_a_s_c_s GTGTCTGTGTCTGTGCTGGTGTCTGTGTGGGTG; >clamp_s_b_s_c_s:compl:rev CACCCACACAGCCACTATCATGTTGACATTCC; dG = -12.7
222 >s_c_s_b_s_d_s_a_s_c_s GTGTGGGTGGCTGTGACGGTGTCTGTGTGGGTG; >clamp_s_b_s_c_s:compl:rev CACCCACACAGCCACTATCATGTTGACATTCC; dG = -12.7
223 >s_c_s_b_s_e_s_a_s_c_s GTGTGGGTGGCTGTGCTGGTGTCTGTGTGGGTG; >clamp_s_b_s_c_s:compl:rev CACCCACACAGCCACTATCATGTTGACATTCC; dG = -12.7
224 >s_c_s_c_s_c_s_a_s_c_s GTGTGGGTGTGGGTGTGGGTGTCTGTGTGGGTG; >clamp_s_b_s_c_s:compl:rev CACCCACACAGCCACTATCATGTTGACATTCC; dG = -12.7
225 >s_c_s_c_s_d_s_a_s_c_s GTGTGGGTGTGGGTGACGGTGTCTGTGTGGGTG; >clamp_s_b_s_c_s:compl:rev CACCCACACAGCCACTATCATGTTGACATTCC; dG = -12.7
226 >s_c_s_c_s_e_s_a_s_c_s GTGTGGGTGTGGGTGCTGGTGTCTGTGTGGGTG; >clamp_s_b_s_c_s:compl:rev CACCCACACAGCCACTATCATGTTGACATTCC; dG = -12.7
227 >s_c_s_d_s_c_s_a_s_c_s GTGTGGGTGACGGTGTGGGTGTCTGTGTGGGTG; >clamp_s_b_s_c_s:compl:rev CACCCACACAGCCACTATCATGTTGACATTCC; dG = -12.7
228 >s_c_s_d_s_d_s_a_s_c_s GTGTGGGTGACGGTGACGGTGTCTGTGTGGGTG; >clamp_s_b_s_c_s:compl:rev CACCCACACAGCCACTATCATGTTGACATTCC; dG = -12.7
229 >s_c_s_d_s_e_s_a_s_c_s GTGTGGGTGACGGTGCTGGTGTCTGTGTGGGTG; >clamp_s_b_s_c_s:compl:rev CACCCACACAGCCACTATCATGTTGACATTCC; dG = -12.7
230 >s_c_s_e_s_c_s_a_s_c_s GTGTGGGTGCTGGTGTGGGTGTCTGTGTGGGTG; >clamp_s_b_s_c_s:compl:rev CACCCACACAGCCACTATCATGTTGACATTCC; dG = -12.7
231 >s_c_s_e_s_d_s_a_s_c_s GTGTGGGTGCTGGTGACGGTGTCTGTGTGGGTG; >clamp_s_b_s_c_s:compl:rev CACCCACACAGCCACTATCATGTTGACATTCC; dG = -12.7
232 >s_c_s_e_s_e_s_a_s_c_s GTGTGGGTGCTGGTGCTGGTGTCTGTGTGGGTG; >clamp_s_b_s_c_s:compl:rev CACCCACACAGCCACTATCATGTTGACATTCC; dG = -12.7
233 >s_d_s_a_s_d_s_a_s_c_s GTGACGGTGTCTGTGACGGTGTCTGTGTGGGTG; >clamp_s_b_s_c_s:compl:rev CACCCACACAGCCACTATCATGTTGACATTCC; dG = -12.7
234 >s_d_s_a_s_e_s_a_s_c_s GTGACGGTGTCTGTGCTGGTGTCTGTGTGGGTG; >clamp_s_b_s_c_s:compl:rev CACCCACACAGCCACTATCATGTTGACATTCC; dG = -12.7

235 >s_d_s_b_s_d_s_a_s_c_s GTGACGGTGGCTGTGACGGTGTCTGTGTGGGTG; >clamp_s_b_s_c_s:compl:rev
CACCCACACAGCCACTATCATGTTGACATTCC; dG = -12.7
236 >s_d_s_b_s_e_s_a_s_c_s GTGACGGTGGCTGTGCTGGTGTCTGTGTGGGTG; >clamp_s_b_s_c_s:compl:rev
CACCCACACAGCCACTATCATGTTGACATTCC; dG = -12.7
237 >s_d_s_c_s_c_s_a_s_c_s GTGACGGTGTGGGTGTGGGTGTCTGTGTGGGTG; >clamp_s_b_s_c_s:compl:rev
CACCCACACAGCCACTATCATGTTGACATTCC; dG = -12.7
238 >s_a_s_b_s_d_s_a_s_c_s GTGTCTGTGGCTGTGACGGTGTCTGTGTGGGTG; >clamp_s_b_s_c_s:compl:rev
CACCCACACAGCCACTATCATGTTGACATTCC; dG = -12.7
239 >s_d_s_c_s_d_s_a_s_c_s GTGACGGTGTGGGTGACGGTGTCTGTGTGGGTG; >clamp_s_b_s_c_s:compl:rev
CACCCACACAGCCACTATCATGTTGACATTCC; dG = -12.7
240 >s_d_s_c_s_e_s_a_s_c_s GTGACGGTGTGGGTGCTGGTGTCTGTGTGGGTG; >clamp_s_b_s_c_s:compl:rev
CACCCACACAGCCACTATCATGTTGACATTCC; dG = -12.7
241 >s_d_s_d_s_c_s_a_s_c_s GTGACGGTGACGGTGTGGGTGTCTGTGTGGGTG; >clamp_s_b_s_c_s:compl:rev
CACCCACACAGCCACTATCATGTTGACATTCC; dG = -12.7
242 >s_d_s_d_s_d_s_a_s_c_s GTGACGGTGACGGTGACGGTGTCTGTGTGGGTG; >clamp_s_b_s_c_s:compl:rev
CACCCACACAGCCACTATCATGTTGACATTCC; dG = -12.7
243 >s_d_s_d_s_e_s_a_s_c_s GTGACGGTGACGGTGCTGGTGTCTGTGTGGGTG; >clamp_s_b_s_c_s:compl:rev
CACCCACACAGCCACTATCATGTTGACATTCC; dG = -12.7
244 >s_d_s_e_s_c_s_a_s_c_s GTGACGGTGCTGGTGTGGGTGTCTGTGTGGGTG; >clamp_s_b_s_c_s:compl:rev
CACCCACACAGCCACTATCATGTTGACATTCC; dG = -12.7
245 >s_a_s_b_s_e_s_a_s_c_s GTGTCTGTGGCTGTGCTGGTGTCTGTGTGGGTG; >clamp_s_b_s_c_s:compl:rev
CACCCACACAGCCACTATCATGTTGACATTCC; dG = -12.7
246 >s_d_s_e_s_d_s_a_s_c_s GTGACGGTGCTGGTGACGGTGTCTGTGTGGGTG; >clamp_s_b_s_c_s:compl:rev
CACCCACACAGCCACTATCATGTTGACATTCC; dG = -12.7
247 >s_d_s_e_s_e_s_a_s_c_s GTGACGGTGCTGGTGCTGGTGTCTGTGTGGGTG; >clamp_s_b_s_c_s:compl:rev
CACCCACACAGCCACTATCATGTTGACATTCC; dG = -12.7
248 >s_e_s_a_s_d_s_a_s_c_s GTGCTGGTGTCTGTGACGGTGTCTGTGTGGGTG; >clamp_s_b_s_c_s:compl:rev
CACCCACACAGCCACTATCATGTTGACATTCC; dG = -12.7
249 >s_e_s_a_s_e_s_a_s_c_s GTGCTGGTGTCTGTGCTGGTGTCTGTGTGGGTG; >clamp_s_b_s_c_s:compl:rev
CACCCACACAGCCACTATCATGTTGACATTCC; dG = -12.7
250 >s_e_s_b_s_d_s_a_s_c_s GTGCTGGTGGCTGTGACGGTGTCTGTGTGGGTG; >clamp_s_b_s_c_s:compl:rev
CACCCACACAGCCACTATCATGTTGACATTCC; dG = -12.7
251 >s_e_s_b_s_e_s_a_s_c_s GTGCTGGTGGCTGTGCTGGTGTCTGTGTGGGTG; >clamp_s_b_s_c_s:compl:rev
CACCCACACAGCCACTATCATGTTGACATTCC; dG = -12.7
252 >s_e_s_c_s_c_s_a_s_c_s GTGCTGGTGTGGGTGTGGGTGTCTGTGTGGGTG; >clamp_s_b_s_c_s:compl:rev
CACCCACACAGCCACTATCATGTTGACATTCC; dG = -12.7
253 >s_e_s_c_s_d_s_a_s_c_s GTGCTGGTGTGGGTGACGGTGTCTGTGTGGGTG; >clamp_s_b_s_c_s:compl:rev
CACCCACACAGCCACTATCATGTTGACATTCC; dG = -12.7
254 >s_e_s_c_s_e_s_a_s_c_s GTGCTGGTGTGGGTGCTGGTGTCTGTGTGGGTG; >clamp_s_b_s_c_s:compl:rev
CACCCACACAGCCACTATCATGTTGACATTCC; dG = -12.7
255 >s_e_s_d_s_c_s_a_s_c_s GTGCTGGTGACGGTGTGGGTGTCTGTGTGGGTG; >clamp_s_b_s_c_s:compl:rev
CACCCACACAGCCACTATCATGTTGACATTCC; dG = -12.7
256 >s_e_s_d_s_d_s_a_s_c_s GTGCTGGTGACGGTGACGGTGTCTGTGTGGGTG; >clamp_s_b_s_c_s:compl:rev
CACCCACACAGCCACTATCATGTTGACATTCC; dG = -12.7
257 >s_e_s_d_s_e_s_a_s_c_s GTGCTGGTGACGGTGCTGGTGTCTGTGTGGGTG; >clamp_s_b_s_c_s:compl:rev
CACCCACACAGCCACTATCATGTTGACATTCC; dG = -12.7
258 >s_e_s_e_s_c_s_a_s_c_s GTGCTGGTGCTGGTGTGGGTGTCTGTGTGGGTG; >clamp_s_b_s_c_s:compl:rev
CACCCACACAGCCACTATCATGTTGACATTCC; dG = -12.7
259 >s_e_s_e_s_d_s_a_s_c_s GTGCTGGTGCTGGTGACGGTGTCTGTGTGGGTG; >clamp_s_b_s_c_s:compl:rev
CACCCACACAGCCACTATCATGTTGACATTCC; dG = -12.7
260 >s_e_s_e_s_e_s_a_s_c_s GTGCTGGTGCTGGTGCTGGTGTCTGTGTGGGTG; >clamp_s_b_s_c_s:compl:rev
CACCCACACAGCCACTATCATGTTGACATTCC; dG = -12.7
261 >s_a_s_c_s_c_s_a_s_c_s GTGTCTGTGTGGGTGTGGGTGTCTGTGTGGGTG; >clamp_s_b_s_c_s:compl:rev
CACCCACACAGCCACTATCATGTTGACATTCC; dG = -12.7
262 >s_b_s_b_s_d_s_b_s_e_s GTGGCTGTGGCTGTGACGGTGGCTGTGCTGGTG; >clamp_s_b_s_c_s:compl:rev
CACCCACACAGCCACTATCATGTTGACATTCC; dG = -12.6
263 >s_b_s_b_s_d_s_b_s_d_s GTGGCTGTGGCTGTGACGGTGGCTGTGACGGTG; >clamp_s_b_s_c_s:compl:rev
CACCCACACAGCCACTATCATGTTGACATTCC; dG = -12.6
264 >s_b_s_b_s_d_s_b_s_b_s GTGGCTGTGGCTGTGACGGTGGCTGTGGCTGTG; >clamp_s_b_s_c_s:compl:rev
CACCCACACAGCCACTATCATGTTGACATTCC; dG = -12.6
265 >s_b_s_b_s_d_s_b_s_a_s GTGGCTGTGGCTGTGACGGTGGCTGTGTCTGTG; >clamp_s_b_s_c_s:compl:rev
CACCCACACAGCCACTATCATGTTGACATTCC; dG = -12.6
266 >s_b_s_d_s_c_s_e_s_c_s GTGGCTGTGACGGTGTGGGTGCTGGTGTGGGTG; >clamp_s_b_s_c_s:compl:rev
CACCCACACAGCCACTATCATGTTGACATTCC; dG = -12.6
267 >s_b_s_e_s_c_s_e_s_c_s GTGGCTGTGCTGGTGTGGGTGCTGGTGTGGGTG; >clamp_s_b_s_c_s:compl:rev
CACCCACACAGCCACTATCATGTTGACATTCC; dG = -12.6
268 >s_a_s_b_s_d_s_b_s_e_s GTGTCTGTGGCTGTGACGGTGGCTGTGCTGGTG; >clamp_s_b_s_c_s:compl:rev
CACCCACACAGCCACTATCATGTTGACATTCC; dG = -12.6
269 >s_a_s_b_s_d_s_b_s_d_s GTGTCTGTGGCTGTGACGGTGGCTGTGACGGTG; >clamp_s_b_s_c_s:compl:rev
CACCCACACAGCCACTATCATGTTGACATTCC; dG = -12.6
270 >s_a_s_b_s_d_s_b_s_b_s GTGTCTGTGGCTGTGACGGTGGCTGTGGCTGTG; >clamp_s_b_s_c_s:compl:rev
CACCCACACAGCCACTATCATGTTGACATTCC; dG = -12.6
271 >s_a_s_b_s_d_s_b_s_a_s GTGTCTGTGGCTGTGACGGTGGCTGTGTCTGTG; >clamp_s_b_s_c_s:compl:rev
CACCCACACAGCCACTATCATGTTGACATTCC; dG = -12.6

272 >s_a_s_c_s_b_s_b_s_e_s GTGTCTGTGTGGGTGGCTGTGGCTGTGCTGGTG; >clamp_s_b_s_c_s:compl:rev
CACCCACACAGCCACTATCATGTTGACATTCC; dG = -12.6
273 >s_a_s_c_s_b_s_b_s_d_s GTGTCTGTGTGGGTGGCTGTGGCTGTGACGGTG; >clamp_s_b_s_c_s:compl:rev
CACCCACACAGCCACTATCATGTTGACATTCC; dG = -12.6
274 >s_a_s_c_s_b_s_b_s_b_s GTGTCTGTGTGGGTGGCTGTGGCTGTGGCTGTG; >clamp_s_b_s_c_s:compl:rev
CACCCACACAGCCACTATCATGTTGACATTCC; dG = -12.6
275 >s_a_s_c_s_b_s_b_s_a_s GTGTCTGTGTGGGTGGCTGTGGCTGTGTCTGTG; >clamp_s_b_s_c_s:compl:rev
CACCCACACAGCCACTATCATGTTGACATTCC; dG = -12.6
276 >s_b_s_b_s_e_s_b_s_e_s GTGGCTGTGGCTGTGCTGGTGGCTGTGCTGGTG; >clamp_s_b_s_c_s:compl:rev
CACCCACACAGCCACTATCATGTTGACATTCC; dG = -12.5
277 >s_b_s_b_s_e_s_b_s_d_s GTGGCTGTGGCTGTGCTGGTGGCTGTGACGGTG; >clamp_s_b_s_c_s:compl:rev
CACCCACACAGCCACTATCATGTTGACATTCC; dG = -12.5
278 >s_b_s_b_s_e_s_b_s_b_s GTGGCTGTGGCTGTGCTGGTGGCTGTGGCTGTG; >clamp_s_b_s_c_s:compl:rev
CACCCACACAGCCACTATCATGTTGACATTCC; dG = -12.5
279 >s_b_s_b_s_e_s_b_s_a_s GTGGCTGTGGCTGTGCTGGTGGCTGTGTCTGTG; >clamp_s_b_s_c_s:compl:rev
CACCCACACAGCCACTATCATGTTGACATTCC; dG = -12.5
280 >s_b_s_d_s_d_s_b_s_e_s GTGGCTGTGACGGTGACGGTGGCTGTGCTGGTG; >clamp_s_b_s_c_s:compl:rev
CACCCACACAGCCACTATCATGTTGACATTCC; dG = -12.5
281 >s_b_s_d_s_d_s_b_s_d_s GTGGCTGTGACGGTGACGGTGGCTGTGACGGTG; >clamp_s_b_s_c_s:compl:rev
CACCCACACAGCCACTATCATGTTGACATTCC; dG = -12.5
282 >s_b_s_d_s_d_s_b_s_b_s GTGGCTGTGACGGTGACGGTGGCTGTGGCTGTG; >clamp_s_b_s_c_s:compl:rev
CACCCACACAGCCACTATCATGTTGACATTCC; dG = -12.5
283 >s_b_s_d_s_d_s_b_s_a_s GTGGCTGTGACGGTGACGGTGGCTGTGTCTGTG; >clamp_s_b_s_c_s:compl:rev
CACCCACACAGCCACTATCATGTTGACATTCC; dG = -12.5
284 >s_b_s_d_s_d_s_e_s_c_s GTGGCTGTGACGGTGACGGTGCTGGTGTGGGTG; >clamp_s_b_s_c_s:compl:rev
CACCCACACAGCCACTATCATGTTGACATTCC; dG = -12.5
285 >s_b_s_d_s_d_s_d_s_c_s GTGGCTGTGACGGTGACGGTGACGGTGTGGGTG; >clamp_s_b_s_c_s:compl:rev
CACCCACACAGCCACTATCATGTTGACATTCC; dG = -12.5
286 >s_b_s_d_s_d_s_c_s_c_s GTGGCTGTGACGGTGACGGTGTGGGTGTGGGTG; >clamp_s_b_s_c_s:compl:rev
CACCCACACAGCCACTATCATGTTGACATTCC; dG = -12.5
287 >s_c_s_b_s_d_s_b_s_e_s GTGTGGGTGGCTGTGACGGTGGCTGTGCTGGTG; >clamp_s_b_s_c_s:compl:rev
CACCCACACAGCCACTATCATGTTGACATTCC; dG = -12.5
288 >s_c_s_b_s_d_s_b_s_d_s GTGTGGGTGGCTGTGACGGTGGCTGTGACGGTG; >clamp_s_b_s_c_s:compl:rev
CACCCACACAGCCACTATCATGTTGACATTCC; dG = -12.5
289 >s_c_s_b_s_d_s_b_s_b_s GTGTGGGTGGCTGTGACGGTGGCTGTGGCTGTG; >clamp_s_b_s_c_s:compl:rev
CACCCACACAGCCACTATCATGTTGACATTCC; dG = -12.5
290 >s_c_s_b_s_d_s_b_s_a_s GTGTGGGTGGCTGTGACGGTGGCTGTGTCTGTG; >clamp_s_b_s_c_s:compl:rev
CACCCACACAGCCACTATCATGTTGACATTCC; dG = -12.5
291 >s_d_s_b_s_d_s_b_s_e_s GTGACGGTGGCTGTGACGGTGGCTGTGCTGGTG; >clamp_s_b_s_c_s:compl:rev
CACCCACACAGCCACTATCATGTTGACATTCC; dG = -12.5
292 >s_d_s_b_s_d_s_b_s_d_s GTGACGGTGGCTGTGACGGTGGCTGTGACGGTG; >clamp_s_b_s_c_s:compl:rev
CACCCACACAGCCACTATCATGTTGACATTCC; dG = -12.5
293 >s_d_s_b_s_d_s_b_s_b_s GTGACGGTGGCTGTGACGGTGGCTGTGGCTGTG; >clamp_s_b_s_c_s:compl:rev
CACCCACACAGCCACTATCATGTTGACATTCC; dG = -12.5
294 >s_d_s_b_s_d_s_b_s_a_s GTGACGGTGGCTGTGACGGTGGCTGTGTCTGTG; >clamp_s_b_s_c_s:compl:rev
CACCCACACAGCCACTATCATGTTGACATTCC; dG = -12.5
295 >s_a_s_b_s_e_s_b_s_e_s GTGTCTGTGGCTGTGCTGGTGGCTGTGCTGGTG; >clamp_s_b_s_c_s:compl:rev
CACCCACACAGCCACTATCATGTTGACATTCC; dG = -12.5
296 >s_a_s_b_s_e_s_b_s_d_s GTGTCTGTGGCTGTGCTGGTGGCTGTGACGGTG; >clamp_s_b_s_c_s:compl:rev
CACCCACACAGCCACTATCATGTTGACATTCC; dG = -12.5
297 >s_a_s_b_s_e_s_b_s_b_s GTGTCTGTGGCTGTGCTGGTGGCTGTGGCTGTG; >clamp_s_b_s_c_s:compl:rev
CACCCACACAGCCACTATCATGTTGACATTCC; dG = -12.5
298 >s_a_s_b_s_e_s_b_s_a_s GTGTCTGTGGCTGTGCTGGTGGCTGTGTCTGTG; >clamp_s_b_s_c_s:compl:rev
CACCCACACAGCCACTATCATGTTGACATTCC; dG = -12.5
299 >s_a_s_c_s_a_s_b_s_e_s GTGTCTGTGTGGGTGTCTGTGGCTGTGCTGGTG; >clamp_s_b_s_c_s:compl:rev
CACCCACACAGCCACTATCATGTTGACATTCC; dG = -12.5
300 >s_a_s_c_s_a_s_b_s_d_s GTGTCTGTGTGGGTGTCTGTGGCTGTGACGGTG; >clamp_s_b_s_c_s:compl:rev
CACCCACACAGCCACTATCATGTTGACATTCC; dG = -12.5
301 >s_a_s_c_s_a_s_b_s_b_s GTGTCTGTGTGGGTGTCTGTGGCTGTGGCTGTG; >clamp_s_b_s_c_s:compl:rev
CACCCACACAGCCACTATCATGTTGACATTCC; dG = -12.5
302 >s_a_s_c_s_a_s_b_s_a_s GTGTCTGTGTGGGTGTCTGTGGCTGTGTCTGTG; >clamp_s_b_s_c_s:compl:rev
CACCCACACAGCCACTATCATGTTGACATTCC; dG = -12.5
303 >s_a_s_c_s_a_s_e_s_c_s GTGTCTGTGTGGGTGTCTGTGCTGGTGTGGGTG; >clamp_s_b_s_c_s:compl:rev
CACCCACACAGCCACTATCATGTTGACATTCC; dG = -12.5
304 >s_a_s_c_s_a_s_d_s_c_s GTGTCTGTGTGGGTGTCTGTGACGGTGTGGGTG; >clamp_s_b_s_c_s:compl:rev
CACCCACACAGCCACTATCATGTTGACATTCC; dG = -12.5
305 >s_e_s_b_s_d_s_b_s_e_s GTGCTGGTGGCTGTGACGGTGGCTGTGCTGGTG; >clamp_s_b_s_c_s:compl:rev
CACCCACACAGCCACTATCATGTTGACATTCC; dG = -12.5
306 >s_e_s_b_s_d_s_b_s_d_s GTGCTGGTGGCTGTGACGGTGGCTGTGACGGTG; >clamp_s_b_s_c_s:compl:rev
CACCCACACAGCCACTATCATGTTGACATTCC; dG = -12.5
307 >s_e_s_b_s_d_s_b_s_b_s GTGCTGGTGGCTGTGACGGTGGCTGTGGCTGTG; >clamp_s_b_s_c_s:compl:rev
CACCCACACAGCCACTATCATGTTGACATTCC; dG = -12.5
308 >s_e_s_b_s_d_s_b_s_a_s GTGCTGGTGGCTGTGACGGTGGCTGTGTCTGTG; >clamp_s_b_s_c_s:compl:rev
CACCCACACAGCCACTATCATGTTGACATTCC; dG = -12.5

309 >s_a_s_c_s_c_s_b_s_e_s GTGTCTGTGTGGGTGTGGGTGGCTGTGCTGGTG; >clamp_s_b_s_c_s:compl:rev
CACCCACACAGCCACTATCATGTTGACATTCC; dG = -12.5
310 >s_a_s_c_s_c_s_b_s_d_s GTGTCTGTGTGGGTGTGGGTGGCTGTGACGGTG; >clamp_s_b_s_c_s:compl:rev
CACCCACACAGCCACTATCATGTTGACATTCC; dG = -12.5
311 >s_a_s_c_s_c_s_b_s_b_s GTGTCTGTGTGGGTGTGGGTGGCTGTGGCTGTG; >clamp_s_b_s_c_s:compl:rev
CACCCACACAGCCACTATCATGTTGACATTCC; dG = -12.5
312 >s_a_s_c_s_c_s_b_s_a_s GTGTCTGTGTGGGTGTGGGTGGCTGTGTCTGTG; >clamp_s_b_s_c_s:compl:rev
CACCCACACAGCCACTATCATGTTGACATTCC; dG = -12.5
313 >s_a_s_c_s_c_s_e_s_c_s GTGTCTGTGTGGGTGTGGGTGCTGGTGTGGGTG; >clamp_s_b_s_c_s:compl:rev
CACCCACACAGCCACTATCATGTTGACATTCC; dG = -12.5
314 >s_a_s_c_s_c_s_d_s_c_s GTGTCTGTGTGGGTGTGGGTGACGGTGTGGGTG; >clamp_s_b_s_c_s:compl:rev
CACCCACACAGCCACTATCATGTTGACATTCC; dG = -12.5
315 >s_a_s_c_s_c_s_c_s_c_s GTGTCTGTGTGGGTGTGGGTGTGGGTGTGGGTG; >clamp_s_b_s_c_s:compl:rev
CACCCACACAGCCACTATCATGTTGACATTCC; dG = -12.5
316 >s_a_s_c_s_e_s_b_s_e_s GTGTCTGTGTGGGTGCTGGTGGCTGTGCTGGTG; >clamp_s_b_s_c_s:compl:rev
CACCCACACAGCCACTATCATGTTGACATTCC; dG = -12.4
317 >s_a_s_c_s_e_s_b_s_d_s GTGTCTGTGTGGGTGCTGGTGGCTGTGACGGTG; >clamp_s_b_s_c_s:compl:rev
CACCCACACAGCCACTATCATGTTGACATTCC; dG = -12.4
318 >s_a_s_c_s_e_s_b_s_b_s GTGTCTGTGTGGGTGCTGGTGGCTGTGGCTGTG; >clamp_s_b_s_c_s:compl:rev
CACCCACACAGCCACTATCATGTTGACATTCC; dG = -12.4
319 >s_a_s_c_s_e_s_b_s_a_s GTGTCTGTGTGGGTGCTGGTGGCTGTGTCTGTG; >clamp_s_b_s_c_s:compl:rev
CACCCACACAGCCACTATCATGTTGACATTCC; dG = -12.4
320 >s_a_s_c_s_e_s_e_s_c_s GTGTCTGTGTGGGTGCTGGTGCTGGTGTGGGTG; >clamp_s_b_s_c_s:compl:rev
CACCCACACAGCCACTATCATGTTGACATTCC; dG = -12.4
321 >s_a_s_c_s_e_s_d_s_c_s GTGTCTGTGTGGGTGCTGGTGACGGTGTGGGTG; >clamp_s_b_s_c_s:compl:rev
CACCCACACAGCCACTATCATGTTGACATTCC; dG = -12.4
322 >s_a_s_c_s_e_s_c_s_c_s GTGTCTGTGTGGGTGCTGGTGTGGGTGTGGGTG; >clamp_s_b_s_c_s:compl:rev
CACCCACACAGCCACTATCATGTTGACATTCC; dG = -12.4
323 >s_b_s_b_s_d_s_e_s_c_s GTGGCTGTGGCTGTGACGGTGCTGGTGTGGGTG; >clamp_s_b_s_c_s:compl:rev
CACCCACACAGCCACTATCATGTTGACATTCC; dG = -12.4
324 >s_b_s_e_s_d_s_b_s_e_s GTGGCTGTGCTGGTGACGGTGGCTGTGCTGGTG; >clamp_s_b_s_c_s:compl:rev
CACCCACACAGCCACTATCATGTTGACATTCC; dG = -12.4
325 >s_b_s_e_s_d_s_b_s_d_s GTGGCTGTGCTGGTGACGGTGGCTGTGACGGTG; >clamp_s_b_s_c_s:compl:rev
CACCCACACAGCCACTATCATGTTGACATTCC; dG = -12.4
326 >s_b_s_e_s_d_s_b_s_b_s GTGGCTGTGCTGGTGACGGTGGCTGTGGCTGTG; >clamp_s_b_s_c_s:compl:rev
CACCCACACAGCCACTATCATGTTGACATTCC; dG = -12.4
327 >s_b_s_e_s_d_s_b_s_a_s GTGGCTGTGCTGGTGACGGTGGCTGTGTCTGTG; >clamp_s_b_s_c_s:compl:rev
CACCCACACAGCCACTATCATGTTGACATTCC; dG = -12.4
328 >s_b_s_e_s_d_s_e_s_c_s GTGGCTGTGCTGGTGACGGTGCTGGTGTGGGTG; >clamp_s_b_s_c_s:compl:rev
CACCCACACAGCCACTATCATGTTGACATTCC; dG = -12.4
329 >s_b_s_e_s_d_s_d_s_c_s GTGGCTGTGCTGGTGACGGTGACGGTGTGGGTG; >clamp_s_b_s_c_s:compl:rev
CACCCACACAGCCACTATCATGTTGACATTCC; dG = -12.4
330 >s_b_s_e_s_d_s_c_s_c_s GTGGCTGTGCTGGTGACGGTGTGGGTGTGGGTG; >clamp_s_b_s_c_s:compl:rev
CACCCACACAGCCACTATCATGTTGACATTCC; dG = -12.4
331 >s_c_s_b_s_e_s_b_s_e_s GTGTGGGTGGCTGTGCTGGTGGCTGTGCTGGTG; >clamp_s_b_s_c_s:compl:rev
CACCCACACAGCCACTATCATGTTGACATTCC; dG = -12.4
332 >s_c_s_b_s_e_s_b_s_d_s GTGTGGGTGGCTGTGCTGGTGGCTGTGACGGTG; >clamp_s_b_s_c_s:compl:rev
CACCCACACAGCCACTATCATGTTGACATTCC; dG = -12.4
333 >s_c_s_b_s_e_s_b_s_b_s GTGTGGGTGGCTGTGCTGGTGGCTGTGGCTGTG; >clamp_s_b_s_c_s:compl:rev
CACCCACACAGCCACTATCATGTTGACATTCC; dG = -12.4
334 >s_c_s_b_s_e_s_b_s_a_s GTGTGGGTGGCTGTGCTGGTGGCTGTGTCTGTG; >clamp_s_b_s_c_s:compl:rev
CACCCACACAGCCACTATCATGTTGACATTCC; dG = -12.4
335 >s_d_s_b_s_e_s_b_s_e_s GTGACGGTGGCTGTGCTGGTGGCTGTGCTGGTG; >clamp_s_b_s_c_s:compl:rev
CACCCACACAGCCACTATCATGTTGACATTCC; dG = -12.4
336 >s_d_s_b_s_e_s_b_s_d_s GTGACGGTGGCTGTGCTGGTGGCTGTGACGGTG; >clamp_s_b_s_c_s:compl:rev
CACCCACACAGCCACTATCATGTTGACATTCC; dG = -12.4
337 >s_d_s_b_s_e_s_b_s_b_s GTGACGGTGGCTGTGCTGGTGGCTGTGGCTGTG; >clamp_s_b_s_c_s:compl:rev
CACCCACACAGCCACTATCATGTTGACATTCC; dG = -12.4
338 >s_d_s_b_s_e_s_b_s_a_s GTGACGGTGGCTGTGCTGGTGGCTGTGTCTGTG; >clamp_s_b_s_c_s:compl:rev
CACCCACACAGCCACTATCATGTTGACATTCC; dG = -12.4
339 >s_a_s_b_s_d_s_e_s_c_s GTGTCTGTGGCTGTGACGGTGCTGGTGTGGGTG; >clamp_s_b_s_c_s:compl:rev
CACCCACACAGCCACTATCATGTTGACATTCC; dG = -12.4
340 >s_e_s_b_s_e_s_b_s_e_s GTGCTGGTGGCTGTGCTGGTGGCTGTGCTGGTG; >clamp_s_b_s_c_s:compl:rev
CACCCACACAGCCACTATCATGTTGACATTCC; dG = -12.4
341 >s_e_s_b_s_e_s_b_s_d_s GTGCTGGTGGCTGTGCTGGTGGCTGTGACGGTG; >clamp_s_b_s_c_s:compl:rev
CACCCACACAGCCACTATCATGTTGACATTCC; dG = -12.4
342 >s_e_s_b_s_e_s_b_s_b_s GTGCTGGTGGCTGTGCTGGTGGCTGTGGCTGTG; >clamp_s_b_s_c_s:compl:rev
CACCCACACAGCCACTATCATGTTGACATTCC; dG = -12.4
343 >s_e_s_b_s_e_s_b_s_a_s GTGCTGGTGGCTGTGCTGGTGGCTGTGTCTGTG; >clamp_s_b_s_c_s:compl:rev
CACCCACACAGCCACTATCATGTTGACATTCC; dG = -12.4
344 >s_b_s_b_s_e_s_e_s_c_s GTGGCTGTGGCTGTGCTGGTGCTGGTGTGGGTG; >clamp_s_b_s_c_s:compl:rev
CACCCACACAGCCACTATCATGTTGACATTCC; dG = -12.3
345 >s_c_s_b_s_d_s_e_s_c_s GTGTGGGTGGCTGTGACGGTGCTGGTGTGGGTG; >clamp_s_b_s_c_s:compl:rev
CACCCACACAGCCACTATCATGTTGACATTCC; dG = -12.3

346 >s_d_s_b_s_d_s_e_s_c_s GTGACGGTGGCTGTGACGGTGCTGGTGTGGGTG; >clamp_s_b_s_c_s:compl:rev CACCCACACAGCCACTATCATGTTGACATTCC; dG = -12.3

347 >s_a_s_b_s_e_s_e_s_c_s GTGTCTGTGGCTGTGCTGGTGCTGGTGTGGGTG; >clamp_s_b_s_c_s:compl:rev CACCCACACAGCCACTATCATGTTGACATTCC; dG = -12.3

348 >s_e_s_b_s_d_s_e_s_c_s GTGCTGGTGGCTGTGACGGTGCTGGTGTGGGTG; >clamp_s_b_s_c_s:compl:rev CACCCACACAGCCACTATCATGTTGACATTCC; dG = -12.3

349 >s_a_s_a_s_b_s_b_s_d_s GTGTCTGTGTCTGTGGCTGTGGCTGTGACGGTG; >clamp_s_b_s_c_s:compl:rev CACCCACACAGCCACTATCATGTTGACATTCC; dG = -12.2

350 >s_a_s_d_s_a_s_b_s_d_s GTGTCTGTGACGGTGTCTGTGGCTGTGACGGTG; >clamp_s_b_s_c_s:compl:rev CACCCACACAGCCACTATCATGTTGACATTCC; dG = -12.2

351 >s_a_s_d_s_b_s_b_s_d_s GTGTCTGTGACGGTGGCTGTGGCTGTGACGGTG; >clamp_s_b_s_c_s:compl:rev CACCCACACAGCCACTATCATGTTGACATTCC; dG = -12.2

352 >s_a_s_d_s_c_s_b_s_d_s GTGTCTGTGACGGTGTGGGTGGCTGTGACGGTG; >clamp_s_b_s_c_s:compl:rev CACCCACACAGCCACTATCATGTTGACATTCC; dG = -12.2

353 >s_a_s_d_s_d_s_b_s_d_s GTGTCTGTGACGGTGACGGTGGCTGTGACGGTG; >clamp_s_b_s_c_s:compl:rev CACCCACACAGCCACTATCATGTTGACATTCC; dG = -12.2

354 >s_a_s_d_s_e_s_b_s_d_s GTGTCTGTGACGGTGCTGGTGGCTGTGACGGTG; >clamp_s_b_s_c_s:compl:rev CACCCACACAGCCACTATCATGTTGACATTCC; dG = -12.2

355 >s_a_s_e_s_a_s_b_s_d_s GTGTCTGTGCTGGTGTCTGTGGCTGTGACGGTG; >clamp_s_b_s_c_s:compl:rev CACCCACACAGCCACTATCATGTTGACATTCC; dG = -12.2

356 >s_a_s_e_s_b_s_b_s_d_s GTGTCTGTGCTGGTGGCTGTGGCTGTGACGGTG; >clamp_s_b_s_c_s:compl:rev CACCCACACAGCCACTATCATGTTGACATTCC; dG = -12.2

357 >s_a_s_e_s_c_s_b_s_d_s GTGTCTGTGCTGGTGTGGGTGGCTGTGACGGTG; >clamp_s_b_s_c_s:compl:rev CACCCACACAGCCACTATCATGTTGACATTCC; dG = -12.2

358 >s_a_s_e_s_d_s_b_s_d_s GTGTCTGTGCTGGTGACGGTGGCTGTGACGGTG; >clamp_s_b_s_c_s:compl:rev CACCCACACAGCCACTATCATGTTGACATTCC; dG = -12.2

359 >s_a_s_e_s_e_s_b_s_d_s GTGTCTGTGCTGGTGCTGGTGGCTGTGACGGTG; >clamp_s_b_s_c_s:compl:rev CACCCACACAGCCACTATCATGTTGACATTCC; dG = -12.2

360 >s_b_s_a_s_a_s_b_s_d_s GTGGCTGTGTCTGTGTCTGTGGCTGTGACGGTG; >clamp_s_b_s_c_s:compl:rev CACCCACACAGCCACTATCATGTTGACATTCC; dG = -12.2

361 >s_b_s_a_s_b_s_b_s_d_s GTGGCTGTGTCTGTGGCTGTGGCTGTGACGGTG; >clamp_s_b_s_c_s:compl:rev CACCCACACAGCCACTATCATGTTGACATTCC; dG = -12.2

362 >s_a_s_a_s_a_s_b_s_d_s GTGTCTGTGTCTGTGTCTGTGGCTGTGACGGTG; >clamp_s_b_s_c_s:compl:rev CACCCACACAGCCACTATCATGTTGACATTCC; dG = -12.2

363 >s_b_s_a_s_d_s_b_s_d_s GTGGCTGTGTCTGTGACGGTGGCTGTGACGGTG; >clamp_s_b_s_c_s:compl:rev CACCCACACAGCCACTATCATGTTGACATTCC; dG = -12.2

364 >s_b_s_a_s_e_s_b_s_d_s GTGGCTGTGTCTGTGCTGGTGGCTGTGACGGTG; >clamp_s_b_s_c_s:compl:rev CACCCACACAGCCACTATCATGTTGACATTCC; dG = -12.2

365 >s_b_s_b_s_a_s_b_s_d_s GTGGCTGTGGCTGTGTCTGTGGCTGTGACGGTG; >clamp_s_b_s_c_s:compl:rev CACCCACACAGCCACTATCATGTTGACATTCC; dG = -12.2

366 >s_b_s_b_s_b_s_b_s_d_s GTGGCTGTGGCTGTGGCTGTGGCTGTGACGGTG; >clamp_s_b_s_c_s:compl:rev CACCCACACAGCCACTATCATGTTGACATTCC; dG = -12.2

367 >s_a_s_a_s_d_s_b_s_d_s GTGTCTGTGTCTGTGACGGTGGCTGTGACGGTG; >clamp_s_b_s_c_s:compl:rev CACCCACACAGCCACTATCATGTTGACATTCC; dG = -12.2

368 >s_b_s_d_s_a_s_b_s_d_s GTGGCTGTGACGGTGTCTGTGGCTGTGACGGTG; >clamp_s_b_s_c_s:compl:rev CACCCACACAGCCACTATCATGTTGACATTCC; dG = -12.2

369 >s_b_s_d_s_b_s_b_s_d_s GTGGCTGTGACGGTGGCTGTGGCTGTGACGGTG; >clamp_s_b_s_c_s:compl:rev CACCCACACAGCCACTATCATGTTGACATTCC; dG = -12.2

370 >s_b_s_d_s_e_s_b_s_d_s GTGGCTGTGACGGTGCTGGTGGCTGTGACGGTG; >clamp_s_b_s_c_s:compl:rev CACCCACACAGCCACTATCATGTTGACATTCC; dG = -12.2

371 >s_b_s_e_s_a_s_b_s_d_s GTGGCTGTGCTGGTGTCTGTGGCTGTGACGGTG; >clamp_s_b_s_c_s:compl:rev CACCCACACAGCCACTATCATGTTGACATTCC; dG = -12.2

372 >s_b_s_e_s_b_s_b_s_d_s GTGGCTGTGCTGGTGGCTGTGGCTGTGACGGTG; >clamp_s_b_s_c_s:compl:rev CACCCACACAGCCACTATCATGTTGACATTCC; dG = -12.2

373 >s_b_s_e_s_e_s_b_s_d_s GTGGCTGTGCTGGTGCTGGTGGCTGTGACGGTG; >clamp_s_b_s_c_s:compl:rev CACCCACACAGCCACTATCATGTTGACATTCC; dG = -12.2

374 >s_a_s_a_s_e_s_b_s_d_s GTGTCTGTGTCTGTGCTGGTGGCTGTGACGGTG; >clamp_s_b_s_c_s:compl:rev CACCCACACAGCCACTATCATGTTGACATTCC; dG = -12.2

375 >s_c_s_a_s_a_s_b_s_d_s GTGTGGGTGTCTGTGTCTGTGGCTGTGACGGTG; >clamp_s_b_s_c_s:compl:rev CACCCACACAGCCACTATCATGTTGACATTCC; dG = -12.2

376 >s_c_s_a_s_b_s_b_s_d_s GTGTGGGTGTCTGTGGCTGTGGCTGTGACGGTG; >clamp_s_b_s_c_s:compl:rev CACCCACACAGCCACTATCATGTTGACATTCC; dG = -12.2

377 >s_c_s_a_s_d_s_b_s_d_s GTGTGGGTGTCTGTGACGGTGGCTGTGACGGTG; >clamp_s_b_s_c_s:compl:rev CACCCACACAGCCACTATCATGTTGACATTCC; dG = -12.2

378 >s_c_s_a_s_e_s_b_s_d_s GTGTGGGTGTCTGTGCTGGTGGCTGTGACGGTG; >clamp_s_b_s_c_s:compl:rev CACCCACACAGCCACTATCATGTTGACATTCC; dG = -12.2

379 >s_c_s_b_s_a_s_b_s_d_s GTGTGGGTGGCTGTGTCTGTGGCTGTGACGGTG; >clamp_s_b_s_c_s:compl:rev CACCCACACAGCCACTATCATGTTGACATTCC; dG = -12.2

380 >s_c_s_b_s_b_s_b_s_d_s GTGTGGGTGGCTGTGGCTGTGGCTGTGACGGTG; >clamp_s_b_s_c_s:compl:rev CACCCACACAGCCACTATCATGTTGACATTCC; dG = -12.2

381 >s_c_s_b_s_e_s_e_s_c_s GTGTGGGTGGCTGTGCTGGTGCTGGTGTGGGTG; >clamp_s_b_s_c_s:compl:rev CACCCACACAGCCACTATCATGTTGACATTCC; dG = -12.2

382 >s_c_s_c_s_a_s_b_s_d_s GTGTGGGTGTGGGTGTCTGTGGCTGTGACGGTG; >clamp_s_b_s_c_s:compl:rev CACCCACACAGCCACTATCATGTTGACATTCC; dG = -12.2

383 >s_a_s_b_s_a_s_b_s_d_s GTGTCTGTGGCTGTGTCTGTGGCTGTGACGGTG; >clamp_s_b_s_c_s:compl:rev
CACCCACACAGCCACTATCATGTTGACATTCC; dG = -12.2
384 >s_c_s_c_s_b_s_b_s_d_s GTGTGGGTGTGGGTGGCTGTGGCTGTGACGGTG; >clamp_s_b_s_c_s:compl:rev
CACCCACACAGCCACTATCATGTTGACATTCC; dG = -12.2
385 >s_c_s_c_s_c_s_b_s_d_s GTGTGGGTGTGGGTGTGGGTGGCTGTGACGGTG; >clamp_s_b_s_c_s:compl:rev
CACCCACACAGCCACTATCATGTTGACATTCC; dG = -12.2
386 >s_c_s_c_s_d_s_b_s_d_s GTGTGGGTGTGGGTGACGGTGGCTGTGACGGTG; >clamp_s_b_s_c_s:compl:rev
CACCCACACAGCCACTATCATGTTGACATTCC; dG = -12.2
387 >s_c_s_c_s_e_s_b_s_d_s GTGTGGGTGTGGGTGCTGGTGGCTGTGACGGTG; >clamp_s_b_s_c_s:compl:rev
CACCCACACAGCCACTATCATGTTGACATTCC; dG = -12.2
388 >s_c_s_d_s_a_s_b_s_d_s GTGTGGGTGACGGTGTCTGTGGCTGTGACGGTG; >clamp_s_b_s_c_s:compl:rev
CACCCACACAGCCACTATCATGTTGACATTCC; dG = -12.2
389 >s_c_s_d_s_b_s_b_s_d_s GTGTGGGTGACGGTGGCTGTGGCTGTGACGGTG; >clamp_s_b_s_c_s:compl:rev
CACCCACACAGCCACTATCATGTTGACATTCC; dG = -12.2
390 >s_c_s_d_s_c_s_b_s_d_s GTGTGGGTGACGGTGTGGGTGGCTGTGACGGTG; >clamp_s_b_s_c_s:compl:rev
CACCCACACAGCCACTATCATGTTGACATTCC; dG = -12.2
391 >s_c_s_d_s_d_s_b_s_d_s GTGTGGGTGACGGTGACGGTGGCTGTGACGGTG; >clamp_s_b_s_c_s:compl:rev
CACCCACACAGCCACTATCATGTTGACATTCC; dG = -12.2
392 >s_c_s_d_s_e_s_b_s_d_s GTGTGGGTGACGGTGCTGGTGGCTGTGACGGTG; >clamp_s_b_s_c_s:compl:rev
CACCCACACAGCCACTATCATGTTGACATTCC; dG = -12.2
393 >s_c_s_e_s_a_s_b_s_d_s GTGTGGGTGCTGGTGTCTGTGGCTGTGACGGTG; >clamp_s_b_s_c_s:compl:rev
CACCCACACAGCCACTATCATGTTGACATTCC; dG = -12.2
394 >s_a_s_b_s_b_s_b_s_d_s GTGTCTGTGGCTGTGGCTGTGGCTGTGACGGTG; >clamp_s_b_s_c_s:compl:rev
CACCCACACAGCCACTATCATGTTGACATTCC; dG = -12.2
395 >s_c_s_e_s_b_s_b_s_d_s GTGTGGGTGCTGGTGGCTGTGGCTGTGACGGTG; >clamp_s_b_s_c_s:compl:rev
CACCCACACAGCCACTATCATGTTGACATTCC; dG = -12.2
396 >s_c_s_e_s_c_s_b_s_d_s GTGTGGGTGCTGGTGTGGGTGGCTGTGACGGTG; >clamp_s_b_s_c_s:compl:rev
CACCCACACAGCCACTATCATGTTGACATTCC; dG = -12.2
397 >s_c_s_e_s_d_s_b_s_d_s GTGTGGGTGCTGGTGACGGTGGCTGTGACGGTG; >clamp_s_b_s_c_s:compl:rev
CACCCACACAGCCACTATCATGTTGACATTCC; dG = -12.2
398 >s_c_s_e_s_e_s_b_s_d_s GTGTGGGTGCTGGTGCTGGTGGCTGTGACGGTG; >clamp_s_b_s_c_s:compl:rev
CACCCACACAGCCACTATCATGTTGACATTCC; dG = -12.2
399 >s_d_s_a_s_a_s_b_s_d_s GTGACGGTGTCTGTGTCTGTGGCTGTGACGGTG; >clamp_s_b_s_c_s:compl:rev
CACCCACACAGCCACTATCATGTTGACATTCC; dG = -12.2
400 >s_d_s_a_s_b_s_b_s_d_s GTGACGGTGTCTGTGGCTGTGGCTGTGACGGTG; >clamp_s_b_s_c_s:compl:rev
CACCCACACAGCCACTATCATGTTGACATTCC; dG = -12.2
401 >s_d_s_a_s_d_s_b_s_d_s GTGACGGTGTCTGTGACGGTGGCTGTGACGGTG; >clamp_s_b_s_c_s:compl:rev
CACCCACACAGCCACTATCATGTTGACATTCC; dG = -12.2
402 >s_d_s_a_s_e_s_b_s_d_s GTGACGGTGTCTGTGCTGGTGGCTGTGACGGTG; >clamp_s_b_s_c_s:compl:rev
CACCCACACAGCCACTATCATGTTGACATTCC; dG = -12.2
403 >s_d_s_b_s_a_s_b_s_d_s GTGACGGTGGCTGTGTCTGTGGCTGTGACGGTG; >clamp_s_b_s_c_s:compl:rev
CACCCACACAGCCACTATCATGTTGACATTCC; dG = -12.2
404 >s_d_s_b_s_b_s_b_s_d_s GTGACGGTGGCTGTGGCTGTGGCTGTGACGGTG; >clamp_s_b_s_c_s:compl:rev
CACCCACACAGCCACTATCATGTTGACATTCC; dG = -12.2
405 >s_d_s_b_s_e_s_e_s_c_s GTGACGGTGGCTGTGCTGGTGCTGGTGTGGGTG; >clamp_s_b_s_c_s:compl:rev
CACCCACACAGCCACTATCATGTTGACATTCC; dG = -12.2
406 >s_d_s_c_s_a_s_b_s_d_s GTGACGGTGTGGGTGTCTGTGGCTGTGACGGTG; >clamp_s_b_s_c_s:compl:rev
CACCCACACAGCCACTATCATGTTGACATTCC; dG = -12.2
407 >s_d_s_c_s_b_s_b_s_d_s GTGACGGTGTGGGTGGCTGTGGCTGTGACGGTG; >clamp_s_b_s_c_s:compl:rev
CACCCACACAGCCACTATCATGTTGACATTCC; dG = -12.2
408 >s_d_s_c_s_c_s_b_s_d_s GTGACGGTGTGGGTGTGGGTGGCTGTGACGGTG; >clamp_s_b_s_c_s:compl:rev
CACCCACACAGCCACTATCATGTTGACATTCC; dG = -12.2
409 >s_d_s_c_s_d_s_b_s_d_s GTGACGGTGTGGGTGACGGTGGCTGTGACGGTG; >clamp_s_b_s_c_s:compl:rev
CACCCACACAGCCACTATCATGTTGACATTCC; dG = -12.2
410 >s_d_s_c_s_e_s_b_s_d_s GTGACGGTGTGGGTGCTGGTGGCTGTGACGGTG; >clamp_s_b_s_c_s:compl:rev
CACCCACACAGCCACTATCATGTTGACATTCC; dG = -12.2
411 >s_d_s_d_s_a_s_b_s_d_s GTGACGGTGACGGTGTCTGTGGCTGTGACGGTG; >clamp_s_b_s_c_s:compl:rev
CACCCACACAGCCACTATCATGTTGACATTCC; dG = -12.2
412 >s_d_s_d_s_b_s_b_s_d_s GTGACGGTGACGGTGGCTGTGGCTGTGACGGTG; >clamp_s_b_s_c_s:compl:rev
CACCCACACAGCCACTATCATGTTGACATTCC; dG = -12.2
413 >s_d_s_d_s_c_s_b_s_d_s GTGACGGTGACGGTGTGGGTGGCTGTGACGGTG; >clamp_s_b_s_c_s:compl:rev
CACCCACACAGCCACTATCATGTTGACATTCC; dG = -12.2
414 >s_d_s_d_s_d_s_b_s_d_s GTGACGGTGACGGTGACGGTGGCTGTGACGGTG; >clamp_s_b_s_c_s:compl:rev
CACCCACACAGCCACTATCATGTTGACATTCC; dG = -12.2
415 >s_d_s_d_s_e_s_b_s_d_s GTGACGGTGACGGTGCTGGTGGCTGTGACGGTG; >clamp_s_b_s_c_s:compl:rev
CACCCACACAGCCACTATCATGTTGACATTCC; dG = -12.2
416 >s_d_s_e_s_a_s_b_s_d_s GTGACGGTGCTGGTGTCTGTGGCTGTGACGGTG; >clamp_s_b_s_c_s:compl:rev
CACCCACACAGCCACTATCATGTTGACATTCC; dG = -12.2
417 >s_d_s_e_s_b_s_b_s_d_s GTGACGGTGCTGGTGGCTGTGGCTGTGACGGTG; >clamp_s_b_s_c_s:compl:rev
CACCCACACAGCCACTATCATGTTGACATTCC; dG = -12.2
418 >s_d_s_e_s_c_s_b_s_d_s GTGACGGTGCTGGTGTGGGTGGCTGTGACGGTG; >clamp_s_b_s_c_s:compl:rev
CACCCACACAGCCACTATCATGTTGACATTCC; dG = -12.2
419 >s_d_s_e_s_d_s_b_s_d_s GTGACGGTGCTGGTGACGGTGGCTGTGACGGTG; >clamp_s_b_s_c_s:compl:rev
CACCCACACAGCCACTATCATGTTGACATTCC; dG = -12.2

420 >s_d_s_e_s_e_s_b_s_d_s GTGACGGTGCTGGTGCTGGTGGCTGTGACGGTG; >clamp_s_b_s_c_s:compl:rev CACCCACACAGCCACTATCATGTTGACATTCC; dG = -12.2
421 >s_e_s_a_s_a_s_b_s_d_s GTGCTGGTGTCTGTGTCTGTGGCTGTGACGGTG; >clamp_s_b_s_c_s:compl:rev CACCCACACAGCCACTATCATGTTGACATTCC; dG = -12.2
422 >s_e_s_a_s_b_s_b_s_d_s GTGCTGGTGTCTGTGGCTGTGGCTGTGACGGTG; >clamp_s_b_s_c_s:compl:rev CACCCACACAGCCACTATCATGTTGACATTCC; dG = -12.2
423 >s_e_s_a_s_d_s_b_s_d_s GTGCTGGTGTCTGTGACGGTGGCTGTGACGGTG; >clamp_s_b_s_c_s:compl:rev CACCCACACAGCCACTATCATGTTGACATTCC; dG = -12.2
424 >s_e_s_a_s_e_s_b_s_d_s GTGCTGGTGTCTGTGCTGGTGGCTGTGACGGTG; >clamp_s_b_s_c_s:compl:rev CACCCACACAGCCACTATCATGTTGACATTCC; dG = -12.2
425 >s_e_s_b_s_a_s_b_s_d_s GTGCTGGTGGCTGTGTCTGTGGCTGTGACGGTG; >clamp_s_b_s_c_s:compl:rev CACCCACACAGCCACTATCATGTTGACATTCC; dG = -12.2
426 >s_e_s_b_s_b_s_b_s_d_s GTGCTGGTGGCTGTGGCTGTGGCTGTGACGGTG; >clamp_s_b_s_c_s:compl:rev CACCCACACAGCCACTATCATGTTGACATTCC; dG = -12.2
427 >s_e_s_b_s_e_s_e_s_c_s GTGCTGGTGGCTGTGCTGGTGCTGGTGTGGGTG; >clamp_s_b_s_c_s:compl:rev CACCCACACAGCCACTATCATGTTGACATTCC; dG = -12.2
428 >s_e_s_c_s_a_s_b_s_d_s GTGCTGGTGTGGGTGTCTGTGGCTGTGACGGTG; >clamp_s_b_s_c_s:compl:rev CACCCACACAGCCACTATCATGTTGACATTCC; dG = -12.2
429 >s_e_s_c_s_b_s_b_s_d_s GTGCTGGTGTGGGTGGCTGTGGCTGTGACGGTG; >clamp_s_b_s_c_s:compl:rev CACCCACACAGCCACTATCATGTTGACATTCC; dG = -12.2
430 >s_e_s_c_s_c_s_b_s_d_s GTGCTGGTGTGGGTGTGGGTGGCTGTGACGGTG; >clamp_s_b_s_c_s:compl:rev CACCCACACAGCCACTATCATGTTGACATTCC; dG = -12.2
431 >s_e_s_c_s_d_s_b_s_d_s GTGCTGGTGTGGGTGACGGTGGCTGTGACGGTG; >clamp_s_b_s_c_s:compl:rev CACCCACACAGCCACTATCATGTTGACATTCC; dG = -12.2
432 >s_e_s_c_s_e_s_b_s_d_s GTGCTGGTGTGGGTGCTGGTGGCTGTGACGGTG; >clamp_s_b_s_c_s:compl:rev CACCCACACAGCCACTATCATGTTGACATTCC; dG = -12.2
433 >s_e_s_d_s_a_s_b_s_d_s GTGCTGGTGACGGTGTCTGTGGCTGTGACGGTG; >clamp_s_b_s_c_s:compl:rev CACCCACACAGCCACTATCATGTTGACATTCC; dG = -12.2
434 >s_e_s_d_s_b_s_b_s_d_s GTGCTGGTGACGGTGGCTGTGGCTGTGACGGTG; >clamp_s_b_s_c_s:compl:rev CACCCACACAGCCACTATCATGTTGACATTCC; dG = -12.2
435 >s_e_s_d_s_c_s_b_s_d_s GTGCTGGTGACGGTGTGGGTGGCTGTGACGGTG; >clamp_s_b_s_c_s:compl:rev CACCCACACAGCCACTATCATGTTGACATTCC; dG = -12.2
436 >s_e_s_d_s_d_s_b_s_d_s GTGCTGGTGACGGTGACGGTGGCTGTGACGGTG; >clamp_s_b_s_c_s:compl:rev CACCCACACAGCCACTATCATGTTGACATTCC; dG = -12.2
437 >s_e_s_d_s_e_s_b_s_d_s GTGCTGGTGACGGTGCTGGTGGCTGTGACGGTG; >clamp_s_b_s_c_s:compl:rev CACCCACACAGCCACTATCATGTTGACATTCC; dG = -12.2
438 >s_e_s_e_s_a_s_b_s_d_s GTGCTGGTGCTGGTGTCTGTGGCTGTGACGGTG; >clamp_s_b_s_c_s:compl:rev CACCCACACAGCCACTATCATGTTGACATTCC; dG = -12.2
439 >s_e_s_e_s_b_s_b_s_d_s GTGCTGGTGCTGGTGGCTGTGGCTGTGACGGTG; >clamp_s_b_s_c_s:compl:rev CACCCACACAGCCACTATCATGTTGACATTCC; dG = -12.2
440 >s_e_s_e_s_c_s_b_s_d_s GTGCTGGTGCTGGTGTGGGTGGCTGTGACGGTG; >clamp_s_b_s_c_s:compl:rev CACCCACACAGCCACTATCATGTTGACATTCC; dG = -12.2
441 >s_e_s_e_s_d_s_b_s_d_s GTGCTGGTGCTGGTGACGGTGGCTGTGACGGTG; >clamp_s_b_s_c_s:compl:rev CACCCACACAGCCACTATCATGTTGACATTCC; dG = -12.2
442 >s_e_s_e_s_e_s_b_s_d_s GTGCTGGTGCTGGTGCTGGTGGCTGTGACGGTG; >clamp_s_b_s_c_s:compl:rev CACCCACACAGCCACTATCATGTTGACATTCC; dG = -12.2
443 >s_a_s_a_s_a_s_b_s_e_s GTGTCTGTGTCTGTGTCTGTGGCTGTGCTGGTG; >clamp_s_b_s_c_s:compl:rev CACCCACACAGCCACTATCATGTTGACATTCC; dG = -12.1
444 >s_a_s_d_s_a_s_b_s_e_s GTGTCTGTGACGGTGTCTGTGGCTGTGCTGGTG; >clamp_s_b_s_c_s:compl:rev CACCCACACAGCCACTATCATGTTGACATTCC; dG = -12.1
445 >s_a_s_d_s_b_s_b_s_e_s GTGTCTGTGACGGTGGCTGTGGCTGTGCTGGTG; >clamp_s_b_s_c_s:compl:rev CACCCACACAGCCACTATCATGTTGACATTCC; dG = -12.1
446 >s_a_s_d_s_c_s_b_s_e_s GTGTCTGTGACGGTGTGGGTGGCTGTGCTGGTG; >clamp_s_b_s_c_s:compl:rev CACCCACACAGCCACTATCATGTTGACATTCC; dG = -12.1
447 >s_a_s_d_s_d_s_b_s_e_s GTGTCTGTGACGGTGACGGTGGCTGTGCTGGTG; >clamp_s_b_s_c_s:compl:rev CACCCACACAGCCACTATCATGTTGACATTCC; dG = -12.1
448 >s_a_s_d_s_e_s_b_s_e_s GTGTCTGTGACGGTGCTGGTGGCTGTGCTGGTG; >clamp_s_b_s_c_s:compl:rev CACCCACACAGCCACTATCATGTTGACATTCC; dG = -12.1
449 >s_a_s_e_s_a_s_b_s_e_s GTGTCTGTGCTGGTGTCTGTGGCTGTGCTGGTG; >clamp_s_b_s_c_s:compl:rev CACCCACACAGCCACTATCATGTTGACATTCC; dG = -12.1
450 >s_a_s_e_s_b_s_b_s_e_s GTGTCTGTGCTGGTGGCTGTGGCTGTGCTGGTG; >clamp_s_b_s_c_s:compl:rev CACCCACACAGCCACTATCATGTTGACATTCC; dG = -12.1
451 >s_a_s_e_s_c_s_b_s_e_s GTGTCTGTGCTGGTGTGGGTGGCTGTGCTGGTG; >clamp_s_b_s_c_s:compl:rev CACCCACACAGCCACTATCATGTTGACATTCC; dG = -12.1
452 >s_a_s_e_s_d_s_b_s_e_s GTGTCTGTGCTGGTGACGGTGGCTGTGCTGGTG; >clamp_s_b_s_c_s:compl:rev CACCCACACAGCCACTATCATGTTGACATTCC; dG = -12.1
453 >s_a_s_e_s_e_s_b_s_e_s GTGTCTGTGCTGGTGCTGGTGGCTGTGCTGGTG; >clamp_s_b_s_c_s:compl:rev CACCCACACAGCCACTATCATGTTGACATTCC; dG = -12.1
454 >s_b_s_a_s_a_s_b_s_e_s GTGGCTGTGTCTGTGTCTGTGGCTGTGCTGGTG; >clamp_s_b_s_c_s:compl:rev CACCCACACAGCCACTATCATGTTGACATTCC; dG = -12.1
455 >s_b_s_a_s_b_s_b_s_e_s GTGGCTGTGTCTGTGGCTGTGGCTGTGCTGGTG; >clamp_s_b_s_c_s:compl:rev CACCCACACAGCCACTATCATGTTGACATTCC; dG = -12.1
456 >s_b_s_a_s_d_s_b_s_e_s GTGGCTGTGTCTGTGACGGTGGCTGTGCTGGTG; >clamp_s_b_s_c_s:compl:rev CACCCACACAGCCACTATCATGTTGACATTCC; dG = -12.1

457 >s_b_s_a_s_e_s_b_s_e_s GTGGCTGTGTCTGTGCTGGTGGCTGTGCTGGTG; >clamp_s_b_s_c_s:compl:rev CACCCACACAGCCACTATCATGTTGACATTCC; dG = -12.1
458 >s_b_s_b_s_a_s_b_s_e_s GTGGCTGTGGCTGTGTCTGTGGCTGTGCTGGTG; >clamp_s_b_s_c_s:compl:rev CACCCACACAGCCACTATCATGTTGACATTCC; dG = -12.1
459 >s_b_s_b_s_b_s_b_s_e_s GTGGCTGTGGCTGTGGCTGTGGCTGTGCTGGTG; >clamp_s_b_s_c_s:compl:rev CACCCACACAGCCACTATCATGTTGACATTCC; dG = -12.1
460 >s_a_s_a_s_d_s_b_s_e_s GTGTCTGTGTCTGTGACGGTGGCTGTGCTGGTG; >clamp_s_b_s_c_s:compl:rev CACCCACACAGCCACTATCATGTTGACATTCC; dG = -12.1
461 >s_b_s_d_s_a_s_b_s_e_s GTGGCTGTGACGGTGTCTGTGGCTGTGCTGGTG; >clamp_s_b_s_c_s:compl:rev CACCCACACAGCCACTATCATGTTGACATTCC; dG = -12.1
462 >s_b_s_d_s_b_s_b_s_e_s GTGGCTGTGACGGTGGCTGTGGCTGTGCTGGTG; >clamp_s_b_s_c_s:compl:rev CACCCACACAGCCACTATCATGTTGACATTCC; dG = -12.1
463 >s_b_s_d_s_e_s_b_s_e_s GTGGCTGTGACGGTGCTGGTGGCTGTGCTGGTG; >clamp_s_b_s_c_s:compl:rev CACCCACACAGCCACTATCATGTTGACATTCC; dG = -12.1
464 >s_b_s_e_s_a_s_b_s_e_s GTGGCTGTGCTGGTGTCTGTGGCTGTGCTGGTG; >clamp_s_b_s_c_s:compl:rev CACCCACACAGCCACTATCATGTTGACATTCC; dG = -12.1
465 >s_b_s_e_s_b_s_b_s_e_s GTGGCTGTGCTGGTGGCTGTGGCTGTGCTGGTG; >clamp_s_b_s_c_s:compl:rev CACCCACACAGCCACTATCATGTTGACATTCC; dG = -12.1
466 >s_a_s_a_s_e_s_b_s_e_s GTGTCTGTGTCTGTGCTGGTGGCTGTGCTGGTG; >clamp_s_b_s_c_s:compl:rev CACCCACACAGCCACTATCATGTTGACATTCC; dG = -12.1
467 >s_b_s_e_s_e_s_b_s_e_s GTGGCTGTGCTGGTGCTGGTGGCTGTGCTGGTG; >clamp_s_b_s_c_s:compl:rev CACCCACACAGCCACTATCATGTTGACATTCC; dG = -12.1
468 >s_c_s_a_s_a_s_b_s_e_s GTGTGGGTGTCTGTGTCTGTGGCTGTGCTGGTG; >clamp_s_b_s_c_s:compl:rev CACCCACACAGCCACTATCATGTTGACATTCC; dG = -12.1
469 >s_c_s_a_s_b_s_b_s_e_s GTGTGGGTGTCTGTGGCTGTGGCTGTGCTGGTG; >clamp_s_b_s_c_s:compl:rev CACCCACACAGCCACTATCATGTTGACATTCC; dG = -12.1
470 >s_c_s_a_s_d_s_b_s_e_s GTGTGGGTGTCTGTGACGGTGGCTGTGCTGGTG; >clamp_s_b_s_c_s:compl:rev CACCCACACAGCCACTATCATGTTGACATTCC; dG = -12.1
471 >s_c_s_a_s_e_s_b_s_e_s GTGTGGGTGTCTGTGCTGGTGGCTGTGCTGGTG; >clamp_s_b_s_c_s:compl:rev CACCCACACAGCCACTATCATGTTGACATTCC; dG = -12.1
472 >s_c_s_b_s_a_s_b_s_e_s GTGTGGGTGGCTGTGTCTGTGGCTGTGCTGGTG; >clamp_s_b_s_c_s:compl:rev CACCCACACAGCCACTATCATGTTGACATTCC; dG = -12.1
473 >s_c_s_b_s_b_s_b_s_e_s GTGTGGGTGGCTGTGGCTGTGGCTGTGCTGGTG; >clamp_s_b_s_c_s:compl:rev CACCCACACAGCCACTATCATGTTGACATTCC; dG = -12.1
474 >s_a_s_b_s_a_s_b_s_e_s GTGTCTGTGGCTGTGTCTGTGGCTGTGCTGGTG; >clamp_s_b_s_c_s:compl:rev CACCCACACAGCCACTATCATGTTGACATTCC; dG = -12.1
475 >s_c_s_c_s_a_s_b_s_e_s GTGTGGGTGTGGGTGTCTGTGGCTGTGCTGGTG; >clamp_s_b_s_c_s:compl:rev CACCCACACAGCCACTATCATGTTGACATTCC; dG = -12.1
476 >s_c_s_c_s_b_s_b_s_e_s GTGTGGGTGTGGGTGGCTGTGGCTGTGCTGGTG; >clamp_s_b_s_c_s:compl:rev CACCCACACAGCCACTATCATGTTGACATTCC; dG = -12.1
477 >s_c_s_c_s_c_s_b_s_e_s GTGTGGGTGTGGGTGTGGGTGGCTGTGCTGGTG; >clamp_s_b_s_c_s:compl:rev CACCCACACAGCCACTATCATGTTGACATTCC; dG = -12.1
478 >s_c_s_c_s_d_s_b_s_e_s GTGTGGGTGTGGGTGACGGTGGCTGTGCTGGTG; >clamp_s_b_s_c_s:compl:rev CACCCACACAGCCACTATCATGTTGACATTCC; dG = -12.1
479 >s_c_s_c_s_e_s_b_s_e_s GTGTGGGTGTGGGTGCTGGTGGCTGTGCTGGTG; >clamp_s_b_s_c_s:compl:rev CACCCACACAGCCACTATCATGTTGACATTCC; dG = -12.1
480 >s_c_s_d_s_a_s_b_s_e_s GTGTGGGTGACGGTGTCTGTGGCTGTGCTGGTG; >clamp_s_b_s_c_s:compl:rev CACCCACACAGCCACTATCATGTTGACATTCC; dG = -12.1
481 >s_c_s_d_s_b_s_b_s_e_s GTGTGGGTGACGGTGGCTGTGGCTGTGCTGGTG; >clamp_s_b_s_c_s:compl:rev CACCCACACAGCCACTATCATGTTGACATTCC; dG = -12.1
482 >s_c_s_d_s_c_s_b_s_e_s GTGTGGGTGACGGTGTGGGTGGCTGTGCTGGTG; >clamp_s_b_s_c_s:compl:rev CACCCACACAGCCACTATCATGTTGACATTCC; dG = -12.1
483 >s_c_s_d_s_d_s_b_s_e_s GTGTGGGTGACGGTGACGGTGGCTGTGCTGGTG; >clamp_s_b_s_c_s:compl:rev CACCCACACAGCCACTATCATGTTGACATTCC; dG = -12.1
484 >s_c_s_d_s_e_s_b_s_e_s GTGTGGGTGACGGTGCTGGTGGCTGTGCTGGTG; >clamp_s_b_s_c_s:compl:rev CACCCACACAGCCACTATCATGTTGACATTCC; dG = -12.1
485 >s_a_s_b_s_b_s_b_s_e_s GTGTCTGTGGCTGTGGCTGTGGCTGTGCTGGTG; >clamp_s_b_s_c_s:compl:rev CACCCACACAGCCACTATCATGTTGACATTCC; dG = -12.1
486 >s_c_s_e_s_a_s_b_s_e_s GTGTGGGTGCTGGTGTCTGTGGCTGTGCTGGTG; >clamp_s_b_s_c_s:compl:rev CACCCACACAGCCACTATCATGTTGACATTCC; dG = -12.1
487 >s_c_s_e_s_b_s_b_s_e_s GTGTGGGTGCTGGTGGCTGTGGCTGTGCTGGTG; >clamp_s_b_s_c_s:compl:rev CACCCACACAGCCACTATCATGTTGACATTCC; dG = -12.1
488 >s_c_s_e_s_c_s_b_s_e_s GTGTGGGTGCTGGTGTGGGTGGCTGTGCTGGTG; >clamp_s_b_s_c_s:compl:rev CACCCACACAGCCACTATCATGTTGACATTCC; dG = -12.1
489 >s_c_s_e_s_d_s_b_s_e_s GTGTGGGTGCTGGTGACGGTGGCTGTGCTGGTG; >clamp_s_b_s_c_s:compl:rev CACCCACACAGCCACTATCATGTTGACATTCC; dG = -12.1
490 >s_c_s_e_s_e_s_b_s_e_s GTGTGGGTGCTGGTGCTGGTGGCTGTGCTGGTG; >clamp_s_b_s_c_s:compl:rev CACCCACACAGCCACTATCATGTTGACATTCC; dG = -12.1
491 >s_d_s_a_s_a_s_b_s_e_s GTGACGGTGTCTGTGTCTGTGGCTGTGCTGGTG; >clamp_s_b_s_c_s:compl:rev CACCCACACAGCCACTATCATGTTGACATTCC; dG = -12.1
492 >s_d_s_a_s_b_s_b_s_e_s GTGACGGTGTCTGTGGCTGTGGCTGTGCTGGTG; >clamp_s_b_s_c_s:compl:rev CACCCACACAGCCACTATCATGTTGACATTCC; dG = -12.1
493 >s_d_s_a_s_d_s_b_s_e_s GTGACGGTGTCTGTGACGGTGGCTGTGCTGGTG; >clamp_s_b_s_c_s:compl:rev CACCCACACAGCCACTATCATGTTGACATTCC; dG = -12.1

494 >s_d_s_a_s_e_s_b_s_e_s GTGACGGTGTCTGTGCTGGTGGCTGTGCTGGTG; >clamp_s_b_s_c_s:compl:rev CACCCACACAGCCACTATCATGTTGACATTCC; dG = -12.1
495 >s_d_s_b_s_a_s_b_s_e_s GTGACGGTGGCTGTGTCTGTGGCTGTGCTGGTG; >clamp_s_b_s_c_s:compl:rev CACCCACACAGCCACTATCATGTTGACATTCC; dG = -12.1
496 >s_d_s_b_s_b_s_b_s_e_s GTGACGGTGGCTGTGGCTGTGGCTGTGCTGGTG; >clamp_s_b_s_c_s:compl:rev CACCCACACAGCCACTATCATGTTGACATTCC; dG = -12.1
497 >s_d_s_c_s_a_s_b_s_e_s GTGACGGTGTGGGTGTCTGTGGCTGTGCTGGTG; >clamp_s_b_s_c_s:compl:rev CACCCACACAGCCACTATCATGTTGACATTCC; dG = -12.1
498 >s_d_s_c_s_b_s_b_s_e_s GTGACGGTGTGGGTGGCTGTGGCTGTGCTGGTG; >clamp_s_b_s_c_s:compl:rev CACCCACACAGCCACTATCATGTTGACATTCC; dG = -12.1
499 >s_d_s_c_s_c_s_b_s_e_s GTGACGGTGTGGGTGTGGGTGGCTGTGCTGGTG; >clamp_s_b_s_c_s:compl:rev CACCCACACAGCCACTATCATGTTGACATTCC; dG = -12.1
500 >s_d_s_c_s_d_s_b_s_e_s GTGACGGTGTGGGTGACGGTGGCTGTGCTGGTG; >clamp_s_b_s_c_s:compl:rev CACCCACACAGCCACTATCATGTTGACATTCC; dG = -12.1
501 >s_d_s_c_s_e_s_b_s_e_s GTGACGGTGTGGGTGCTGGTGGCTGTGCTGGTG; >clamp_s_b_s_c_s:compl:rev CACCCACACAGCCACTATCATGTTGACATTCC; dG = -12.1
502 >s_d_s_d_s_a_s_b_s_e_s GTGACGGTGACGGTGTCTGTGGCTGTGCTGGTG; >clamp_s_b_s_c_s:compl:rev CACCCACACAGCCACTATCATGTTGACATTCC; dG = -12.1
503 >s_d_s_d_s_b_s_b_s_e_s GTGACGGTGACGGTGGCTGTGGCTGTGCTGGTG; >clamp_s_b_s_c_s:compl:rev CACCCACACAGCCACTATCATGTTGACATTCC; dG = -12.1
504 >s_d_s_d_s_c_s_b_s_e_s GTGACGGTGACGGTGTGGGTGGCTGTGCTGGTG; >clamp_s_b_s_c_s:compl:rev CACCCACACAGCCACTATCATGTTGACATTCC; dG = -12.1
505 >s_d_s_d_s_d_s_b_s_e_s GTGACGGTGACGGTGACGGTGGCTGTGCTGGTG; >clamp_s_b_s_c_s:compl:rev CACCCACACAGCCACTATCATGTTGACATTCC; dG = -12.1
506 >s_d_s_d_s_e_s_b_s_e_s GTGACGGTGACGGTGCTGGTGGCTGTGCTGGTG; >clamp_s_b_s_c_s:compl:rev CACCCACACAGCCACTATCATGTTGACATTCC; dG = -12.1
507 >s_d_s_e_s_a_s_b_s_e_s GTGACGGTGCTGGTGTCTGTGGCTGTGCTGGTG; >clamp_s_b_s_c_s:compl:rev CACCCACACAGCCACTATCATGTTGACATTCC; dG = -12.1
508 >s_d_s_e_s_b_s_b_s_e_s GTGACGGTGCTGGTGGCTGTGGCTGTGCTGGTG; >clamp_s_b_s_c_s:compl:rev CACCCACACAGCCACTATCATGTTGACATTCC; dG = -12.1
509 >s_d_s_e_s_c_s_b_s_e_s GTGACGGTGCTGGTGTGGGTGGCTGTGCTGGTG; >clamp_s_b_s_c_s:compl:rev CACCCACACAGCCACTATCATGTTGACATTCC; dG = -12.1
510 >s_d_s_e_s_d_s_b_s_e_s GTGACGGTGCTGGTGACGGTGGCTGTGCTGGTG; >clamp_s_b_s_c_s:compl:rev CACCCACACAGCCACTATCATGTTGACATTCC; dG = -12.1
511 >s_d_s_e_s_e_s_b_s_e_s GTGACGGTGCTGGTGCTGGTGGCTGTGCTGGTG; >clamp_s_b_s_c_s:compl:rev CACCCACACAGCCACTATCATGTTGACATTCC; dG = -12.1
512 >s_e_s_a_s_a_s_b_s_e_s GTGCTGGTGTCTGTGTCTGTGGCTGTGCTGGTG; >clamp_s_b_s_c_s:compl:rev CACCCACACAGCCACTATCATGTTGACATTCC; dG = -12.1
513 >s_e_s_a_s_b_s_b_s_e_s GTGCTGGTGTCTGTGGCTGTGGCTGTGCTGGTG; >clamp_s_b_s_c_s:compl:rev CACCCACACAGCCACTATCATGTTGACATTCC; dG = -12.1
514 >s_e_s_a_s_d_s_b_s_e_s GTGCTGGTGTCTGTGACGGTGGCTGTGCTGGTG; >clamp_s_b_s_c_s:compl:rev CACCCACACAGCCACTATCATGTTGACATTCC; dG = -12.1
515 >s_e_s_a_s_e_s_b_s_e_s GTGCTGGTGTCTGTGCTGGTGGCTGTGCTGGTG; >clamp_s_b_s_c_s:compl:rev CACCCACACAGCCACTATCATGTTGACATTCC; dG = -12.1
516 >s_e_s_b_s_a_s_b_s_e_s GTGCTGGTGGCTGTGTCTGTGGCTGTGCTGGTG; >clamp_s_b_s_c_s:compl:rev CACCCACACAGCCACTATCATGTTGACATTCC; dG = -12.1
517 >s_e_s_b_s_b_s_b_s_e_s GTGCTGGTGGCTGTGGCTGTGGCTGTGCTGGTG; >clamp_s_b_s_c_s:compl:rev CACCCACACAGCCACTATCATGTTGACATTCC; dG = -12.1
518 >s_e_s_c_s_a_s_b_s_e_s GTGCTGGTGTGGGTGTCTGTGGCTGTGCTGGTG; >clamp_s_b_s_c_s:compl:rev CACCCACACAGCCACTATCATGTTGACATTCC; dG = -12.1
519 >s_e_s_c_s_b_s_b_s_e_s GTGCTGGTGTGGGTGGCTGTGGCTGTGCTGGTG; >clamp_s_b_s_c_s:compl:rev CACCCACACAGCCACTATCATGTTGACATTCC; dG = -12.1
520 >s_e_s_c_s_c_s_b_s_e_s GTGCTGGTGTGGGTGTGGGTGGCTGTGCTGGTG; >clamp_s_b_s_c_s:compl:rev CACCCACACAGCCACTATCATGTTGACATTCC; dG = -12.1
521 >s_e_s_c_s_d_s_b_s_e_s GTGCTGGTGTGGGTGACGGTGGCTGTGCTGGTG; >clamp_s_b_s_c_s:compl:rev CACCCACACAGCCACTATCATGTTGACATTCC; dG = -12.1
522 >s_e_s_c_s_e_s_b_s_e_s GTGCTGGTGTGGGTGCTGGTGGCTGTGCTGGTG; >clamp_s_b_s_c_s:compl:rev CACCCACACAGCCACTATCATGTTGACATTCC; dG = -12.1
523 >s_e_s_d_s_a_s_b_s_e_s GTGCTGGTGACGGTGTCTGTGGCTGTGCTGGTG; >clamp_s_b_s_c_s:compl:rev CACCCACACAGCCACTATCATGTTGACATTCC; dG = -12.1
524 >s_e_s_d_s_b_s_b_s_e_s GTGCTGGTGACGGTGGCTGTGGCTGTGCTGGTG; >clamp_s_b_s_c_s:compl:rev CACCCACACAGCCACTATCATGTTGACATTCC; dG = -12.1
525 >s_e_s_d_s_c_s_b_s_e_s GTGCTGGTGACGGTGTGGGTGGCTGTGCTGGTG; >clamp_s_b_s_c_s:compl:rev CACCCACACAGCCACTATCATGTTGACATTCC; dG = -12.1
526 >s_e_s_d_s_d_s_b_s_e_s GTGCTGGTGACGGTGACGGTGGCTGTGCTGGTG; >clamp_s_b_s_c_s:compl:rev CACCCACACAGCCACTATCATGTTGACATTCC; dG = -12.1
527 >s_e_s_d_s_e_s_b_s_e_s GTGCTGGTGACGGTGCTGGTGGCTGTGCTGGTG; >clamp_s_b_s_c_s:compl:rev CACCCACACAGCCACTATCATGTTGACATTCC; dG = -12.1
528 >s_e_s_e_s_a_s_b_s_e_s GTGCTGGTGCTGGTGTCTGTGGCTGTGCTGGTG; >clamp_s_b_s_c_s:compl:rev CACCCACACAGCCACTATCATGTTGACATTCC; dG = -12.1
529 >s_e_s_e_s_b_s_b_s_e_s GTGCTGGTGCTGGTGGCTGTGGCTGTGCTGGTG; >clamp_s_b_s_c_s:compl:rev CACCCACACAGCCACTATCATGTTGACATTCC; dG = -12.1
530 >s_e_s_e_s_c_s_b_s_e_s GTGCTGGTGCTGGTGTGGGTGGCTGTGCTGGTG; >clamp_s_b_s_c_s:compl:rev CACCCACACAGCCACTATCATGTTGACATTCC; dG = -12.1

531 >s_e_s_e_s_d_s_b_s_e_s GTGCTGGTGCTGGTGACGGTGGCTGTGCTGGTG; >clamp_s_b_s_c_s:compl:rev
CACCCACACAGCCACTATCATGTTGACATTCC; dG = -12.1
532 >s_e_s_e_s_e_s_b_s_e_s GTGCTGGTGCTGGTGCTGGTGGCTGTGCTGGTG; >clamp_s_b_s_c_s:compl:rev
CACCCACACAGCCACTATCATGTTGACATTCC; dG = -12.1
533 >s_a_s_a_s_b_s_b_s_e_s GTGTCTGTGTCTGTGGCTGTGGCTGTGCTGGTG; >clamp_s_b_s_c_s:compl:rev
CACCCACACAGCCACTATCATGTTGACATTCC; dG = -12.1
534 >s_b_s_b_s_a_s_d_s_c_s GTGGCTGTGGCTGTGTCTGTGACGGTGTGGGTG; >clamp_s_b_s_c_s:compl:rev
CACCCACACAGCCACTATCATGTTGACATTCC; dG = -12.0
535 >s_b_s_d_s_a_s_b_s_b_s GTGGCTGTGACGGTGTCTGTGGCTGTGGCTGTG; >clamp_s_b_s_c_s:compl:rev
CACCCACACAGCCACTATCATGTTGACATTCC; dG = -12.0
536 >s_b_s_d_s_a_s_b_s_a_s GTGGCTGTGACGGTGTCTGTGGCTGTGTCTGTG; >clamp_s_b_s_c_s:compl:rev
CACCCACACAGCCACTATCATGTTGACATTCC; dG = -12.0
537 >s_b_s_d_s_a_s_e_s_c_s GTGGCTGTGACGGTGTCTGTGCTGGTGTGGGTG; >clamp_s_b_s_c_s:compl:rev
CACCCACACAGCCACTATCATGTTGACATTCC; dG = -12.0
538 >s_b_s_d_s_a_s_d_s_c_s GTGGCTGTGACGGTGTCTGTGACGGTGTGGGTG; >clamp_s_b_s_c_s:compl:rev
CACCCACACAGCCACTATCATGTTGACATTCC; dG = -12.0
539 >s_b_s_d_s_b_s_b_s_b_s GTGGCTGTGACGGTGGCTGTGGCTGTGGCTGTG; >clamp_s_b_s_c_s:compl:rev
CACCCACACAGCCACTATCATGTTGACATTCC; dG = -12.0
540 >s_b_s_d_s_b_s_b_s_a_s GTGGCTGTGACGGTGGCTGTGGCTGTGTCTGTG; >clamp_s_b_s_c_s:compl:rev
CACCCACACAGCCACTATCATGTTGACATTCC; dG = -12.0
541 >s_c_s_b_s_a_s_d_s_c_s GTGTGGGTGGCTGTGTCTGTGACGGTGTGGGTG; >clamp_s_b_s_c_s:compl:rev
CACCCACACAGCCACTATCATGTTGACATTCC; dG = -12.0
542 >s_c_s_c_s_c_s_d_s_c_s GTGTGGGTGTGGGTGTGGGTGACGGTGTGGGTG; >clamp_s_b_s_c_s:compl:rev
CACCCACACAGCCACTATCATGTTGACATTCC; dG = -12.0
543 >s_a_s_b_s_a_s_d_s_c_s GTGTCTGTGGCTGTGTCTGTGACGGTGTGGGTG; >clamp_s_b_s_c_s:compl:rev
CACCCACACAGCCACTATCATGTTGACATTCC; dG = -12.0
544 >s_c_s_d_s_c_s_d_s_c_s GTGTGGGTGACGGTGTGGGTGACGGTGTGGGTG; >clamp_s_b_s_c_s:compl:rev
CACCCACACAGCCACTATCATGTTGACATTCC; dG = -12.0
545 >s_c_s_e_s_c_s_d_s_c_s GTGTGGGTGCTGGTGTGGGTGACGGTGTGGGTG; >clamp_s_b_s_c_s:compl:rev
CACCCACACAGCCACTATCATGTTGACATTCC; dG = -12.0
546 >s_d_s_b_s_a_s_d_s_c_s GTGACGGTGGCTGTGTCTGTGACGGTGTGGGTG; >clamp_s_b_s_c_s:compl:rev
CACCCACACAGCCACTATCATGTTGACATTCC; dG = -12.0
547 >s_e_s_b_s_a_s_d_s_c_s GTGCTGGTGGCTGTGTCTGTGACGGTGTGGGTG; >clamp_s_b_s_c_s:compl:rev
CACCCACACAGCCACTATCATGTTGACATTCC; dG = -12.0
548 >s_b_s_e_s_a_s_b_s_b_s GTGGCTGTGCTGGTGTCTGTGGCTGTGGCTGTG; >clamp_s_b_s_c_s:compl:rev
CACCCACACAGCCACTATCATGTTGACATTCC; dG = -11.9
549 >s_b_s_e_s_a_s_b_s_a_s GTGGCTGTGCTGGTGTCTGTGGCTGTGTCTGTG; >clamp_s_b_s_c_s:compl:rev
CACCCACACAGCCACTATCATGTTGACATTCC; dG = -11.9
550 >s_b_s_e_s_a_s_e_s_c_s GTGGCTGTGCTGGTGTCTGTGCTGGTGTGGGTG; >clamp_s_b_s_c_s:compl:rev
CACCCACACAGCCACTATCATGTTGACATTCC; dG = -11.9
551 >s_b_s_e_s_a_s_d_s_c_s GTGGCTGTGCTGGTGTCTGTGACGGTGTGGGTG; >clamp_s_b_s_c_s:compl:rev
CACCCACACAGCCACTATCATGTTGACATTCC; dG = -11.9
552 >s_b_s_e_s_b_s_b_s_b_s GTGGCTGTGCTGGTGGCTGTGGCTGTGGCTGTG; >clamp_s_b_s_c_s:compl:rev
CACCCACACAGCCACTATCATGTTGACATTCC; dG = -11.9
553 >s_b_s_e_s_b_s_b_s_a_s GTGGCTGTGCTGGTGGCTGTGGCTGTGTCTGTG; >clamp_s_b_s_c_s:compl:rev
CACCCACACAGCCACTATCATGTTGACATTCC; dG = -11.9
554 >s_b_s_b_s_a_s_e_s_c_s GTGGCTGTGGCTGTGTCTGTGCTGGTGTGGGTG; >clamp_s_b_s_c_s:compl:rev
CACCCACACAGCCACTATCATGTTGACATTCC; dG = -11.8
555 >s_b_s_d_s_e_s_b_s_b_s GTGGCTGTGACGGTGCTGGTGGCTGTGGCTGTG; >clamp_s_b_s_c_s:compl:rev
CACCCACACAGCCACTATCATGTTGACATTCC; dG = -11.8
556 >s_b_s_d_s_e_s_b_s_a_s GTGGCTGTGACGGTGCTGGTGGCTGTGTCTGTG; >clamp_s_b_s_c_s:compl:rev
CACCCACACAGCCACTATCATGTTGACATTCC; dG = -11.8
557 >s_b_s_d_s_e_s_e_s_c_s GTGGCTGTGACGGTGCTGGTGCTGGTGTGGGTG; >clamp_s_b_s_c_s:compl:rev
CACCCACACAGCCACTATCATGTTGACATTCC; dG = -11.8
558 >s_b_s_d_s_e_s_d_s_c_s GTGGCTGTGACGGTGCTGGTGACGGTGTGGGTG; >clamp_s_b_s_c_s:compl:rev
CACCCACACAGCCACTATCATGTTGACATTCC; dG = -11.8
559 >s_b_s_d_s_e_s_c_s_c_s GTGGCTGTGACGGTGCTGGTGTGGGTGTGGGTG; >clamp_s_b_s_c_s:compl:rev
CACCCACACAGCCACTATCATGTTGACATTCC; dG = -11.8
560 >s_c_s_b_s_a_s_e_s_c_s GTGTGGGTGGCTGTGTCTGTGCTGGTGTGGGTG; >clamp_s_b_s_c_s:compl:rev
CACCCACACAGCCACTATCATGTTGACATTCC; dG = -11.8
561 >s_a_s_b_s_a_s_e_s_c_s GTGTCTGTGGCTGTGTCTGTGCTGGTGTGGGTG; >clamp_s_b_s_c_s:compl:rev
CACCCACACAGCCACTATCATGTTGACATTCC; dG = -11.8
562 >s_d_s_b_s_a_s_e_s_c_s GTGACGGTGGCTGTGTCTGTGCTGGTGTGGGTG; >clamp_s_b_s_c_s:compl:rev
CACCCACACAGCCACTATCATGTTGACATTCC; dG = -11.8
563 >s_e_s_b_s_a_s_e_s_c_s GTGCTGGTGGCTGTGTCTGTGCTGGTGTGGGTG; >clamp_s_b_s_c_s:compl:rev
CACCCACACAGCCACTATCATGTTGACATTCC; dG = -11.8
564 >s_a_s_d_s_a_s_b_s_a_s GTGTCTGTGACGGTGTCTGTGGCTGTGTCTGTG; >clamp_s_b_s_c_s:compl:rev
CACCCACACAGCCACTATCATGTTGACATTCC; dG = -11.7
565 >s_a_s_a_s_b_s_b_s_a_s GTGTCTGTGTCTGTGGCTGTGGCTGTGTCTGTG; >clamp_s_b_s_c_s:compl:rev
CACCCACACAGCCACTATCATGTTGACATTCC; dG = -11.7
566 >s_a_s_d_s_b_s_b_s_a_s GTGTCTGTGACGGTGGCTGTGGCTGTGTCTGTG; >clamp_s_b_s_c_s:compl:rev
CACCCACACAGCCACTATCATGTTGACATTCC; dG = -11.7
567 >s_a_s_d_s_c_s_b_s_a_s GTGTCTGTGACGGTGTGGGTGGCTGTGTCTGTG; >clamp_s_b_s_c_s:compl:rev
CACCCACACAGCCACTATCATGTTGACATTCC; dG = -11.7

568 >s_a_s_d_s_d_s_b_s_a_s GTGTCTGTGACGGTGACGGTGGCTGTGTCTGTG; >clamp_s_b_s_c_s:compl:rev
CACCCACACAGCCACTATCATGTTGACATTCC; dG = -11.7
569 >s_a_s_d_s_e_s_b_s_a_s GTGTCTGTGACGGTGCTGGTGGCTGTGTCTGTG; >clamp_s_b_s_c_s:compl:rev
CACCCACACAGCCACTATCATGTTGACATTCC; dG = -11.7
570 >s_a_s_e_s_a_s_b_s_a_s GTGTCTGTGCTGGTGTCTGTGGCTGTGTCTGTG; >clamp_s_b_s_c_s:compl:rev
CACCCACACAGCCACTATCATGTTGACATTCC; dG = -11.7
571 >s_a_s_e_s_b_s_b_s_a_s GTGTCTGTGCTGGTGGCTGTGGCTGTGTCTGTG; >clamp_s_b_s_c_s:compl:rev
CACCCACACAGCCACTATCATGTTGACATTCC; dG = -11.7
572 >s_a_s_e_s_c_s_b_s_a_s GTGTCTGTGCTGGTGTGGGTGGCTGTGTCTGTG; >clamp_s_b_s_c_s:compl:rev
CACCCACACAGCCACTATCATGTTGACATTCC; dG = -11.7
573 >s_a_s_e_s_d_s_b_s_a_s GTGTCTGTGCTGGTGACGGTGGCTGTGTCTGTG; >clamp_s_b_s_c_s:compl:rev
CACCCACACAGCCACTATCATGTTGACATTCC; dG = -11.7
574 >s_a_s_e_s_e_s_b_s_a_s GTGTCTGTGCTGGTGCTGGTGGCTGTGTCTGTG; >clamp_s_b_s_c_s:compl:rev
CACCCACACAGCCACTATCATGTTGACATTCC; dG = -11.7
575 >s_b_s_a_s_a_s_b_s_a_s GTGGCTGTGTCTGTGTCTGTGGCTGTGTCTGTG; >clamp_s_b_s_c_s:compl:rev
CACCCACACAGCCACTATCATGTTGACATTCC; dG = -11.7
576 >s_b_s_a_s_b_s_b_s_a_s GTGGCTGTGTCTGTGGCTGTGGCTGTGTCTGTG; >clamp_s_b_s_c_s:compl:rev
CACCCACACAGCCACTATCATGTTGACATTCC; dG = -11.7
577 >s_b_s_a_s_d_s_b_s_a_s GTGGCTGTGTCTGTGACGGTGGCTGTGTCTGTG; >clamp_s_b_s_c_s:compl:rev
CACCCACACAGCCACTATCATGTTGACATTCC; dG = -11.7
578 >s_b_s_a_s_e_s_b_s_a_s GTGGCTGTGTCTGTGCTGGTGGCTGTGTCTGTG; >clamp_s_b_s_c_s:compl:rev
CACCCACACAGCCACTATCATGTTGACATTCC; dG = -11.7
579 >s_b_s_b_s_a_s_b_s_b_s GTGGCTGTGGCTGTGTCTGTGGCTGTGGCTGTG; >clamp_s_b_s_c_s:compl:rev
CACCCACACAGCCACTATCATGTTGACATTCC; dG = -11.7
580 >s_b_s_b_s_a_s_b_s_a_s GTGGCTGTGGCTGTGTCTGTGGCTGTGTCTGTG; >clamp_s_b_s_c_s:compl:rev
CACCCACACAGCCACTATCATGTTGACATTCC; dG = -11.7
581 >s_b_s_b_s_b_s_b_s_a_s GTGGCTGTGGCTGTGGCTGTGGCTGTGTCTGTG; >clamp_s_b_s_c_s:compl:rev
CACCCACACAGCCACTATCATGTTGACATTCC; dG = -11.7
582 >s_a_s_a_s_d_s_b_s_a_s GTGTCTGTGTCTGTGACGGTGGCTGTGTCTGTG; >clamp_s_b_s_c_s:compl:rev
CACCCACACAGCCACTATCATGTTGACATTCC; dG = -11.7
583 >s_b_s_e_s_e_s_b_s_b_s GTGGCTGTGCTGGTGCTGGTGGCTGTGGCTGTG; >clamp_s_b_s_c_s:compl:rev
CACCCACACAGCCACTATCATGTTGACATTCC; dG = -11.7
584 >s_b_s_e_s_e_s_b_s_a_s GTGGCTGTGCTGGTGCTGGTGGCTGTGTCTGTG; >clamp_s_b_s_c_s:compl:rev
CACCCACACAGCCACTATCATGTTGACATTCC; dG = -11.7
585 >s_b_s_e_s_e_s_e_s_c_s GTGGCTGTGCTGGTGCTGGTGCTGGTGTGGGTG; >clamp_s_b_s_c_s:compl:rev
CACCCACACAGCCACTATCATGTTGACATTCC; dG = -11.7
586 >s_b_s_e_s_e_s_d_s_c_s GTGGCTGTGCTGGTGCTGGTGACGGTGTGGGTG; >clamp_s_b_s_c_s:compl:rev
CACCCACACAGCCACTATCATGTTGACATTCC; dG = -11.7
587 >s_b_s_e_s_e_s_c_s_c_s GTGGCTGTGCTGGTGCTGGTGTGGGTGTGGGTG; >clamp_s_b_s_c_s:compl:rev
CACCCACACAGCCACTATCATGTTGACATTCC; dG = -11.7
588 >s_c_s_a_s_a_s_b_s_a_s GTGTGGGTGTCTGTGTCTGTGGCTGTGTCTGTG; >clamp_s_b_s_c_s:compl:rev
CACCCACACAGCCACTATCATGTTGACATTCC; dG = -11.7
589 >s_c_s_a_s_b_s_b_s_a_s GTGTGGGTGTCTGTGGCTGTGGCTGTGTCTGTG; >clamp_s_b_s_c_s:compl:rev
CACCCACACAGCCACTATCATGTTGACATTCC; dG = -11.7
590 >s_a_s_a_s_e_s_b_s_a_s GTGTCTGTGTCTGTGCTGGTGGCTGTGTCTGTG; >clamp_s_b_s_c_s:compl:rev
CACCCACACAGCCACTATCATGTTGACATTCC; dG = -11.7
591 >s_c_s_a_s_d_s_b_s_a_s GTGTGGGTGTCTGTGACGGTGGCTGTGTCTGTG; >clamp_s_b_s_c_s:compl:rev
CACCCACACAGCCACTATCATGTTGACATTCC; dG = -11.7
592 >s_c_s_a_s_e_s_b_s_a_s GTGTGGGTGTCTGTGCTGGTGGCTGTGTCTGTG; >clamp_s_b_s_c_s:compl:rev
CACCCACACAGCCACTATCATGTTGACATTCC; dG = -11.7
593 >s_c_s_b_s_a_s_b_s_b_s GTGTGGGTGGCTGTGTCTGTGGCTGTGGCTGTG; >clamp_s_b_s_c_s:compl:rev
CACCCACACAGCCACTATCATGTTGACATTCC; dG = -11.7
594 >s_c_s_b_s_a_s_b_s_a_s GTGTGGGTGGCTGTGTCTGTGGCTGTGTCTGTG; >clamp_s_b_s_c_s:compl:rev
CACCCACACAGCCACTATCATGTTGACATTCC; dG = -11.7
595 >s_c_s_b_s_b_s_b_s_a_s GTGTGGGTGGCTGTGGCTGTGGCTGTGTCTGTG; >clamp_s_b_s_c_s:compl:rev
CACCCACACAGCCACTATCATGTTGACATTCC; dG = -11.7
596 >s_a_s_a_s_a_s_b_s_a_s GTGTCTGTGTCTGTGTCTGTGGCTGTGTCTGTG; >clamp_s_b_s_c_s:compl:rev
CACCCACACAGCCACTATCATGTTGACATTCC; dG = -11.7
597 >s_c_s_c_s_a_s_b_s_a_s GTGTGGGTGTGGGTGTCTGTGGCTGTGTCTGTG; >clamp_s_b_s_c_s:compl:rev
CACCCACACAGCCACTATCATGTTGACATTCC; dG = -11.7
598 >s_c_s_c_s_b_s_b_s_a_s GTGTGGGTGTGGGTGGCTGTGGCTGTGTCTGTG; >clamp_s_b_s_c_s:compl:rev
CACCCACACAGCCACTATCATGTTGACATTCC; dG = -11.7
599 >s_a_s_b_s_a_s_b_s_b_s GTGTCTGTGGCTGTGTCTGTGGCTGTGGCTGTG; >clamp_s_b_s_c_s:compl:rev
CACCCACACAGCCACTATCATGTTGACATTCC; dG = -11.7
600 >s_c_s_c_s_c_s_b_s_a_s GTGTGGGTGTGGGTGTGGGTGGCTGTGTCTGTG; >clamp_s_b_s_c_s:compl:rev
CACCCACACAGCCACTATCATGTTGACATTCC; dG = -11.7
601 >s_a_s_b_s_a_s_b_s_a_s GTGTCTGTGGCTGTGTCTGTGGCTGTGTCTGTG; >clamp_s_b_s_c_s:compl:rev
CACCCACACAGCCACTATCATGTTGACATTCC; dG = -11.7
602 >s_c_s_c_s_d_s_b_s_b_s GTGTGGGTGTGGGTGACGGTGGCTGTGGCTGTG; >clamp_s_b_s_c_s:compl:rev
CACCCACACAGCCACTATCATGTTGACATTCC; dG = -11.7
603 >s_c_s_c_s_d_s_b_s_a_s GTGTGGGTGTGGGTGACGGTGGCTGTGTCTGTG; >clamp_s_b_s_c_s:compl:rev
CACCCACACAGCCACTATCATGTTGACATTCC; dG = -11.7
604 >s_c_s_c_s_d_s_e_s_c_s GTGTGGGTGTGGGTGACGGTGCTGGTGTGGGTG; >clamp_s_b_s_c_s:compl:rev
CACCCACACAGCCACTATCATGTTGACATTCC; dG = -11.7

605 >s_c_s_c_s_d_s_d_s_c_s GTGTGGGTGTGGGTGACGGTGACGGTGTGGGTG; >clamp_s_b_s_c_s:compl:rev CACCCACACAGCCACTATCATGTTGACATTCC; dG = -11.7
606 >s_c_s_c_s_d_s_c_s_c_s GTGTGGGTGTGGGTGACGGTGTGGGTGTGGGTG; >clamp_s_b_s_c_s:compl:rev CACCCACACAGCCACTATCATGTTGACATTCC; dG = -11.7
607 >s_c_s_c_s_e_s_b_s_a_s GTGTGGGTGTGGGTGCTGGTGGCTGTGTCTGTG; >clamp_s_b_s_c_s:compl:rev CACCCACACAGCCACTATCATGTTGACATTCC; dG = -11.7
608 >s_c_s_d_s_a_s_b_s_a_s GTGTGGGTGACGGTGTCTGTGGCTGTGTCTGTG; >clamp_s_b_s_c_s:compl:rev CACCCACACAGCCACTATCATGTTGACATTCC; dG = -11.7
609 >s_c_s_d_s_b_s_b_s_a_s GTGTGGGTGACGGTGGCTGTGGCTGTGTCTGTG; >clamp_s_b_s_c_s:compl:rev CACCCACACAGCCACTATCATGTTGACATTCC; dG = -11.7
610 >s_c_s_d_s_c_s_b_s_a_s GTGTGGGTGACGGTGTGGGTGGCTGTGTCTGTG; >clamp_s_b_s_c_s:compl:rev CACCCACACAGCCACTATCATGTTGACATTCC; dG = -11.7
611 >s_c_s_d_s_d_s_b_s_a_s GTGTGGGTGACGGTGACGGTGGCTGTGTCTGTG; >clamp_s_b_s_c_s:compl:rev CACCCACACAGCCACTATCATGTTGACATTCC; dG = -11.7
612 >s_c_s_d_s_e_s_b_s_a_s GTGTGGGTGACGGTGCTGGTGGCTGTGTCTGTG; >clamp_s_b_s_c_s:compl:rev CACCCACACAGCCACTATCATGTTGACATTCC; dG = -11.7
613 >s_c_s_e_s_a_s_b_s_a_s GTGTGGGTGCTGGTGTCTGTGGCTGTGTCTGTG; >clamp_s_b_s_c_s:compl:rev CACCCACACAGCCACTATCATGTTGACATTCC; dG = -11.7
614 >s_c_s_e_s_b_s_b_s_a_s GTGTGGGTGCTGGTGGCTGTGGCTGTGTCTGTG; >clamp_s_b_s_c_s:compl:rev CACCCACACAGCCACTATCATGTTGACATTCC; dG = -11.7
615 >s_c_s_e_s_c_s_b_s_a_s GTGTGGGTGCTGGTGTGGGTGGCTGTGTCTGTG; >clamp_s_b_s_c_s:compl:rev CACCCACACAGCCACTATCATGTTGACATTCC; dG = -11.7
616 >s_a_s_b_s_b_s_b_s_a_s GTGTCTGTGGCTGTGGCTGTGGCTGTGTCTGTG; >clamp_s_b_s_c_s:compl:rev CACCCACACAGCCACTATCATGTTGACATTCC; dG = -11.7
617 >s_c_s_e_s_d_s_b_s_a_s GTGTGGGTGCTGGTGACGGTGGCTGTGTCTGTG; >clamp_s_b_s_c_s:compl:rev CACCCACACAGCCACTATCATGTTGACATTCC; dG = -11.7
618 >s_c_s_e_s_e_s_b_s_a_s GTGTGGGTGCTGGTGCTGGTGGCTGTGTCTGTG; >clamp_s_b_s_c_s:compl:rev CACCCACACAGCCACTATCATGTTGACATTCC; dG = -11.7
619 >s_d_s_a_s_a_s_b_s_a_s GTGACGGTGTCTGTGTCTGTGGCTGTGTCTGTG; >clamp_s_b_s_c_s:compl:rev CACCCACACAGCCACTATCATGTTGACATTCC; dG = -11.7
620 >s_d_s_a_s_b_s_b_s_a_s GTGACGGTGTCTGTGGCTGTGGCTGTGTCTGTG; >clamp_s_b_s_c_s:compl:rev CACCCACACAGCCACTATCATGTTGACATTCC; dG = -11.7
621 >s_d_s_a_s_d_s_b_s_a_s GTGACGGTGTCTGTGACGGTGGCTGTGTCTGTG; >clamp_s_b_s_c_s:compl:rev CACCCACACAGCCACTATCATGTTGACATTCC; dG = -11.7
622 >s_d_s_a_s_e_s_b_s_a_s GTGACGGTGTCTGTGCTGGTGGCTGTGTCTGTG; >clamp_s_b_s_c_s:compl:rev CACCCACACAGCCACTATCATGTTGACATTCC; dG = -11.7
623 >s_d_s_b_s_a_s_b_s_b_s GTGACGGTGGCTGTGTCTGTGGCTGTGGCTGTG; >clamp_s_b_s_c_s:compl:rev CACCCACACAGCCACTATCATGTTGACATTCC; dG = -11.7
624 >s_d_s_b_s_a_s_b_s_a_s GTGACGGTGGCTGTGTCTGTGGCTGTGTCTGTG; >clamp_s_b_s_c_s:compl:rev CACCCACACAGCCACTATCATGTTGACATTCC; dG = -11.7
625 >s_d_s_b_s_b_s_b_s_a_s GTGACGGTGGCTGTGGCTGTGGCTGTGTCTGTG; >clamp_s_b_s_c_s:compl:rev CACCCACACAGCCACTATCATGTTGACATTCC; dG = -11.7
626 >s_d_s_c_s_a_s_b_s_a_s GTGACGGTGTGGGTGTCTGTGGCTGTGTCTGTG; >clamp_s_b_s_c_s:compl:rev CACCCACACAGCCACTATCATGTTGACATTCC; dG = -11.7
627 >s_d_s_c_s_b_s_b_s_a_s GTGACGGTGTGGGTGGCTGTGGCTGTGTCTGTG; >clamp_s_b_s_c_s:compl:rev CACCCACACAGCCACTATCATGTTGACATTCC; dG = -11.7
628 >s_d_s_c_s_c_s_b_s_a_s GTGACGGTGTGGGTGTGGGTGGCTGTGTCTGTG; >clamp_s_b_s_c_s:compl:rev CACCCACACAGCCACTATCATGTTGACATTCC; dG = -11.7
629 >s_d_s_c_s_c_s_d_s_c_s GTGACGGTGTGGGTGTGGGTGACGGTGTGGGTG; >clamp_s_b_s_c_s:compl:rev CACCCACACAGCCACTATCATGTTGACATTCC; dG = -11.7
630 >s_d_s_c_s_d_s_b_s_a_s GTGACGGTGTGGGTGACGGTGGCTGTGTCTGTG; >clamp_s_b_s_c_s:compl:rev CACCCACACAGCCACTATCATGTTGACATTCC; dG = -11.7
631 >s_d_s_c_s_e_s_b_s_a_s GTGACGGTGTGGGTGCTGGTGGCTGTGTCTGTG; >clamp_s_b_s_c_s:compl:rev CACCCACACAGCCACTATCATGTTGACATTCC; dG = -11.7
632 >s_d_s_d_s_a_s_b_s_a_s GTGACGGTGACGGTGTCTGTGGCTGTGTCTGTG; >clamp_s_b_s_c_s:compl:rev CACCCACACAGCCACTATCATGTTGACATTCC; dG = -11.7
633 >s_d_s_d_s_b_s_b_s_a_s GTGACGGTGACGGTGGCTGTGGCTGTGTCTGTG; >clamp_s_b_s_c_s:compl:rev CACCCACACAGCCACTATCATGTTGACATTCC; dG = -11.7
634 >s_d_s_d_s_c_s_b_s_a_s GTGACGGTGACGGTGTGGGTGGCTGTGTCTGTG; >clamp_s_b_s_c_s:compl:rev CACCCACACAGCCACTATCATGTTGACATTCC; dG = -11.7
635 >s_d_s_d_s_d_s_b_s_a_s GTGACGGTGACGGTGACGGTGGCTGTGTCTGTG; >clamp_s_b_s_c_s:compl:rev CACCCACACAGCCACTATCATGTTGACATTCC; dG = -11.7
636 >s_d_s_d_s_e_s_b_s_a_s GTGACGGTGACGGTGCTGGTGGCTGTGTCTGTG; >clamp_s_b_s_c_s:compl:rev CACCCACACAGCCACTATCATGTTGACATTCC; dG = -11.7
637 >s_d_s_e_s_a_s_b_s_a_s GTGACGGTGCTGGTGTCTGTGGCTGTGTCTGTG; >clamp_s_b_s_c_s:compl:rev CACCCACACAGCCACTATCATGTTGACATTCC; dG = -11.7
638 >s_d_s_e_s_b_s_b_s_a_s GTGACGGTGCTGGTGGCTGTGGCTGTGTCTGTG; >clamp_s_b_s_c_s:compl:rev CACCCACACAGCCACTATCATGTTGACATTCC; dG = -11.7
639 >s_d_s_e_s_c_s_b_s_a_s GTGACGGTGCTGGTGTGGGTGGCTGTGTCTGTG; >clamp_s_b_s_c_s:compl:rev CACCCACACAGCCACTATCATGTTGACATTCC; dG = -11.7
640 >s_d_s_e_s_d_s_b_s_a_s GTGACGGTGCTGGTGACGGTGGCTGTGTCTGTG; >clamp_s_b_s_c_s:compl:rev CACCCACACAGCCACTATCATGTTGACATTCC; dG = -11.7
641 >s_d_s_e_s_e_s_b_s_a_s GTGACGGTGCTGGTGCTGGTGGCTGTGTCTGTG; >clamp_s_b_s_c_s:compl:rev CACCCACACAGCCACTATCATGTTGACATTCC; dG = -11.7

642 >s_e_s_a_s_a_s_b_s_a_s GTGCTGGTGTCTGTGTCTGTGGCTGTGTCTGTG; >clamp_s_b_s_c_s:compl:rev
CACCCACACAGCCACTATCATGTTGACATTCC; dG = -11.7
643 >s_e_s_a_s_b_s_b_s_a_s GTGCTGGTGTCTGTGGCTGTGGCTGTGTCTGTG; >clamp_s_b_s_c_s:compl:rev
CACCCACACAGCCACTATCATGTTGACATTCC; dG = -11.7
644 >s_e_s_a_s_d_s_b_s_a_s GTGCTGGTGTCTGTGACGGTGGCTGTGTCTGTG; >clamp_s_b_s_c_s:compl:rev
CACCCACACAGCCACTATCATGTTGACATTCC; dG = -11.7
645 >s_e_s_a_s_e_s_b_s_a_s GTGCTGGTGTCTGTGCTGGTGGCTGTGTCTGTG; >clamp_s_b_s_c_s:compl:rev
CACCCACACAGCCACTATCATGTTGACATTCC; dG = -11.7
646 >s_e_s_b_s_a_s_b_s_b_s GTGCTGGTGGCTGTGTCTGTGGCTGTGGCTGTG; >clamp_s_b_s_c_s:compl:rev
CACCCACACAGCCACTATCATGTTGACATTCC; dG = -11.7
647 >s_e_s_b_s_a_s_b_s_a_s GTGCTGGTGGCTGTGTCTGTGGCTGTGTCTGTG; >clamp_s_b_s_c_s:compl:rev
CACCCACACAGCCACTATCATGTTGACATTCC; dG = -11.7
648 >s_e_s_b_s_b_s_b_s_a_s GTGCTGGTGGCTGTGGCTGTGGCTGTGTCTGTG; >clamp_s_b_s_c_s:compl:rev
CACCCACACAGCCACTATCATGTTGACATTCC; dG = -11.7
649 >s_e_s_c_s_a_s_b_s_a_s GTGCTGGTGTGGGTGTCTGTGGCTGTGTCTGTG; >clamp_s_b_s_c_s:compl:rev
CACCCACACAGCCACTATCATGTTGACATTCC; dG = -11.7
650 >s_e_s_c_s_b_s_b_s_a_s GTGCTGGTGTGGGTGGCTGTGGCTGTGTCTGTG; >clamp_s_b_s_c_s:compl:rev
CACCCACACAGCCACTATCATGTTGACATTCC; dG = -11.7
651 >s_e_s_c_s_c_s_b_s_a_s GTGCTGGTGTGGGTGTGGGTGGCTGTGTCTGTG; >clamp_s_b_s_c_s:compl:rev
CACCCACACAGCCACTATCATGTTGACATTCC; dG = -11.7
652 >s_e_s_c_s_c_s_d_s_c_s GTGCTGGTGTGGGTGTGGGTGACGGTGTGGGTG; >clamp_s_b_s_c_s:compl:rev
CACCCACACAGCCACTATCATGTTGACATTCC; dG = -11.7
653 >s_e_s_c_s_d_s_b_s_a_s GTGCTGGTGTGGGTGACGGTGGCTGTGTCTGTG; >clamp_s_b_s_c_s:compl:rev
CACCCACACAGCCACTATCATGTTGACATTCC; dG = -11.7
654 >s_e_s_c_s_e_s_b_s_a_s GTGCTGGTGTGGGTGCTGGTGGCTGTGTCTGTG; >clamp_s_b_s_c_s:compl:rev
CACCCACACAGCCACTATCATGTTGACATTCC; dG = -11.7
655 >s_e_s_d_s_a_s_b_s_a_s GTGCTGGTGACGGTGTCTGTGGCTGTGTCTGTG; >clamp_s_b_s_c_s:compl:rev
CACCCACACAGCCACTATCATGTTGACATTCC; dG = -11.7
656 >s_e_s_d_s_b_s_b_s_a_s GTGCTGGTGACGGTGGCTGTGGCTGTGTCTGTG; >clamp_s_b_s_c_s:compl:rev
CACCCACACAGCCACTATCATGTTGACATTCC; dG = -11.7
657 >s_e_s_d_s_c_s_b_s_a_s GTGCTGGTGACGGTGTGGGTGGCTGTGTCTGTG; >clamp_s_b_s_c_s:compl:rev
CACCCACACAGCCACTATCATGTTGACATTCC; dG = -11.7
658 >s_e_s_d_s_d_s_b_s_a_s GTGCTGGTGACGGTGACGGTGGCTGTGTCTGTG; >clamp_s_b_s_c_s:compl:rev
CACCCACACAGCCACTATCATGTTGACATTCC; dG = -11.7
659 >s_e_s_d_s_e_s_b_s_a_s GTGCTGGTGACGGTGCTGGTGGCTGTGTCTGTG; >clamp_s_b_s_c_s:compl:rev
CACCCACACAGCCACTATCATGTTGACATTCC; dG = -11.7
660 >s_e_s_e_s_a_s_b_s_a_s GTGCTGGTGCTGGTGTCTGTGGCTGTGTCTGTG; >clamp_s_b_s_c_s:compl:rev
CACCCACACAGCCACTATCATGTTGACATTCC; dG = -11.7
661 >s_e_s_e_s_b_s_b_s_a_s GTGCTGGTGCTGGTGGCTGTGGCTGTGTCTGTG; >clamp_s_b_s_c_s:compl:rev
CACCCACACAGCCACTATCATGTTGACATTCC; dG = -11.7
662 >s_e_s_e_s_c_s_b_s_a_s GTGCTGGTGCTGGTGTGGGTGGCTGTGTCTGTG; >clamp_s_b_s_c_s:compl:rev
CACCCACACAGCCACTATCATGTTGACATTCC; dG = -11.7
663 >s_e_s_e_s_d_s_b_s_a_s GTGCTGGTGCTGGTGACGGTGGCTGTGTCTGTG; >clamp_s_b_s_c_s:compl:rev
CACCCACACAGCCACTATCATGTTGACATTCC; dG = -11.7
664 >s_e_s_e_s_e_s_b_s_a_s GTGCTGGTGCTGGTGCTGGTGGCTGTGTCTGTG; >clamp_s_b_s_c_s:compl:rev
CACCCACACAGCCACTATCATGTTGACATTCC; dG = -11.7
665 >s_b_s_a_s_d_s_c_s_c_s GTGGCTGTGTCTGTGACGGTGTGGGTGTGGGTG; >clamp_s_b_s_c_s:compl:rev
CACCCACACAGCCACTATCATGTTGACATTCC; dG = -11.6
666 >s_b_s_a_s_e_s_c_s_c_s GTGGCTGTGTCTGTGCTGGTGTGGGTGTGGGTG; >clamp_s_b_s_c_s:compl:rev
CACCCACACAGCCACTATCATGTTGACATTCC; dG = -11.6
667 >s_b_s_a_s_d_s_b_s_b_s GTGGCTGTGTCTGTGACGGTGGCTGTGGCTGTG; >clamp_s_b_s_c_s:compl:rev
CACCCACACAGCCACTATCATGTTGACATTCC; dG = -11.5
668 >s_b_s_a_s_d_s_e_s_c_s GTGGCTGTGTCTGTGACGGTGCTGGTGTGGGTG; >clamp_s_b_s_c_s:compl:rev
CACCCACACAGCCACTATCATGTTGACATTCC; dG = -11.5
669 >s_b_s_a_s_d_s_d_s_c_s GTGGCTGTGTCTGTGACGGTGACGGTGTGGGTG; >clamp_s_b_s_c_s:compl:rev
CACCCACACAGCCACTATCATGTTGACATTCC; dG = -11.5
670 >s_c_s_c_s_c_s_b_s_b_s GTGTGGGTGTGGGTGTGGGTGGCTGTGGCTGTG; >clamp_s_b_s_c_s:compl:rev
CACCCACACAGCCACTATCATGTTGACATTCC; dG = -11.5
671 >s_c_s_c_s_c_s_c_s_c_s GTGTGGGTGTGGGTGTGGGTGTGGGTGTGGGTG; >clamp_s_b_s_c_s:compl:rev
CACCCACACAGCCACTATCATGTTGACATTCC; dG = -11.5
672 >s_c_s_c_s_e_s_c_s_c_s GTGTGGGTGTGGGTGCTGGTGTGGGTGTGGGTG; >clamp_s_b_s_c_s:compl:rev
CACCCACACAGCCACTATCATGTTGACATTCC; dG = -11.5
673 >s_c_s_d_s_c_s_b_s_b_s GTGTGGGTGACGGTGTGGGTGGCTGTGGCTGTG; >clamp_s_b_s_c_s:compl:rev
CACCCACACAGCCACTATCATGTTGACATTCC; dG = -11.5
674 >s_c_s_d_s_c_s_c_s_c_s GTGTGGGTGACGGTGTGGGTGTGGGTGTGGGTG; >clamp_s_b_s_c_s:compl:rev
CACCCACACAGCCACTATCATGTTGACATTCC; dG = -11.5
675 >s_c_s_e_s_c_s_b_s_b_s GTGTGGGTGCTGGTGTGGGTGGCTGTGGCTGTG; >clamp_s_b_s_c_s:compl:rev
CACCCACACAGCCACTATCATGTTGACATTCC; dG = -11.5
676 >s_c_s_e_s_c_s_c_s_c_s GTGTGGGTGCTGGTGTGGGTGTGGGTGTGGGTG; >clamp_s_b_s_c_s:compl:rev
CACCCACACAGCCACTATCATGTTGACATTCC; dG = -11.5
677 >s_d_s_c_s_c_s_c_s_c_s GTGACGGTGTGGGTGTGGGTGTGGGTGTGGGTG; >clamp_s_b_s_c_s:compl:rev
CACCCACACAGCCACTATCATGTTGACATTCC; dG = -11.5
678 >s_d_s_c_s_d_s_c_s_c_s GTGACGGTGTGGGTGACGGTGTGGGTGTGGGTG; >clamp_s_b_s_c_s:compl:rev
CACCCACACAGCCACTATCATGTTGACATTCC; dG = -11.5

679 >s_d_s_c_s_e_s_c_s_c_s GTGACGGTGTGGGTGCTGGTGTGGGTGTGGGTG; >clamp_s_b_s_c_s:compl:rev
CACCCACACAGCCACTATCATGTTGACATTCC; dG = -11.5
680 >s_e_s_c_s_c_s_c_s_c_s GTGCTGGTGTGGGTGTGGGTGTGGGTGTGGGTG; >clamp_s_b_s_c_s:compl:rev
CACCCACACAGCCACTATCATGTTGACATTCC; dG = -11.5
681 >s_e_s_c_s_d_s_c_s_c_s GTGCTGGTGTGGGTGACGGTGTGGGTGTGGGTG; >clamp_s_b_s_c_s:compl:rev
CACCCACACAGCCACTATCATGTTGACATTCC; dG = -11.5
682 >s_e_s_c_s_e_s_c_s_c_s GTGCTGGTGTGGGTGCTGGTGTGGGTGTGGGTG; >clamp_s_b_s_c_s:compl:rev
CACCCACACAGCCACTATCATGTTGACATTCC; dG = -11.5
683 >s_a_s_a_s_b_s_b_s_b_s GTGTCTGTGTCTGTGGCTGTGGCTGTGGCTGTG; >clamp_s_b_s_c_s:compl:rev
CACCCACACAGCCACTATCATGTTGACATTCC; dG = -11.3
684 >s_a_s_d_s_a_s_b_s_b_s GTGTCTGTGACGGTGTCTGTGGCTGTGGCTGTG; >clamp_s_b_s_c_s:compl:rev
CACCCACACAGCCACTATCATGTTGACATTCC; dG = -11.3
685 >s_a_s_d_s_b_s_b_s_b_s GTGTCTGTGACGGTGGCTGTGGCTGTGGCTGTG; >clamp_s_b_s_c_s:compl:rev
CACCCACACAGCCACTATCATGTTGACATTCC; dG = -11.3
686 >s_a_s_d_s_c_s_b_s_b_s GTGTCTGTGACGGTGTGGGTGGCTGTGGCTGTG; >clamp_s_b_s_c_s:compl:rev
CACCCACACAGCCACTATCATGTTGACATTCC; dG = -11.3
687 >s_a_s_d_s_d_s_b_s_b_s GTGTCTGTGACGGTGACGGTGGCTGTGGCTGTG; >clamp_s_b_s_c_s:compl:rev
CACCCACACAGCCACTATCATGTTGACATTCC; dG = -11.3
688 >s_a_s_d_s_e_s_b_s_b_s GTGTCTGTGACGGTGCTGGTGGCTGTGGCTGTG; >clamp_s_b_s_c_s:compl:rev
CACCCACACAGCCACTATCATGTTGACATTCC; dG = -11.3
689 >s_a_s_e_s_a_s_b_s_b_s GTGTCTGTGCTGGTGTCTGTGGCTGTGGCTGTG; >clamp_s_b_s_c_s:compl:rev
CACCCACACAGCCACTATCATGTTGACATTCC; dG = -11.3
690 >s_a_s_e_s_b_s_b_s_b_s GTGTCTGTGCTGGTGGCTGTGGCTGTGGCTGTG; >clamp_s_b_s_c_s:compl:rev
CACCCACACAGCCACTATCATGTTGACATTCC; dG = -11.3
691 >s_a_s_e_s_c_s_b_s_b_s GTGTCTGTGCTGGTGTGGGTGGCTGTGGCTGTG; >clamp_s_b_s_c_s:compl:rev
CACCCACACAGCCACTATCATGTTGACATTCC; dG = -11.3
692 >s_a_s_e_s_d_s_b_s_b_s GTGTCTGTGCTGGTGACGGTGGCTGTGGCTGTG; >clamp_s_b_s_c_s:compl:rev
CACCCACACAGCCACTATCATGTTGACATTCC; dG = -11.3
693 >s_a_s_e_s_e_s_b_s_b_s GTGTCTGTGCTGGTGCTGGTGGCTGTGGCTGTG; >clamp_s_b_s_c_s:compl:rev
CACCCACACAGCCACTATCATGTTGACATTCC; dG = -11.3
694 >s_b_s_a_s_a_s_b_s_b_s GTGGCTGTGTCTGTGTCTGTGGCTGTGGCTGTG; >clamp_s_b_s_c_s:compl:rev
CACCCACACAGCCACTATCATGTTGACATTCC; dG = -11.3
695 >s_b_s_a_s_b_s_b_s_b_s GTGGCTGTGTCTGTGGCTGTGGCTGTGGCTGTG; >clamp_s_b_s_c_s:compl:rev
CACCCACACAGCCACTATCATGTTGACATTCC; dG = -11.3
696 >s_b_s_a_s_e_s_b_s_b_s GTGGCTGTGTCTGTGCTGGTGGCTGTGGCTGTG; >clamp_s_b_s_c_s:compl:rev
CACCCACACAGCCACTATCATGTTGACATTCC; dG = -11.3
697 >s_b_s_b_s_b_s_b_s_b_s GTGGCTGTGGCTGTGGCTGTGGCTGTGGCTGTG; >clamp_s_b_s_c_s:compl:rev
CACCCACACAGCCACTATCATGTTGACATTCC; dG = -11.3
698 >s_a_s_a_s_d_s_b_s_b_s GTGTCTGTGTCTGTGACGGTGGCTGTGGCTGTG; >clamp_s_b_s_c_s:compl:rev
CACCCACACAGCCACTATCATGTTGACATTCC; dG = -11.3
699 >s_a_s_a_s_a_s_b_s_b_s GTGTCTGTGTCTGTGTCTGTGGCTGTGGCTGTG; >clamp_s_b_s_c_s:compl:rev
CACCCACACAGCCACTATCATGTTGACATTCC; dG = -11.3
700 >s_c_s_a_s_a_s_b_s_b_s GTGTGGGTGTCTGTGTCTGTGGCTGTGGCTGTG; >clamp_s_b_s_c_s:compl:rev
CACCCACACAGCCACTATCATGTTGACATTCC; dG = -11.3
701 >s_a_s_a_s_e_s_b_s_b_s GTGTCTGTGTCTGTGCTGGTGGCTGTGGCTGTG; >clamp_s_b_s_c_s:compl:rev
CACCCACACAGCCACTATCATGTTGACATTCC; dG = -11.3
702 >s_c_s_a_s_b_s_b_s_b_s GTGTGGGTGTCTGTGGCTGTGGCTGTGGCTGTG; >clamp_s_b_s_c_s:compl:rev
CACCCACACAGCCACTATCATGTTGACATTCC; dG = -11.3
703 >s_c_s_a_s_d_s_b_s_b_s GTGTGGGTGTCTGTGACGGTGGCTGTGGCTGTG; >clamp_s_b_s_c_s:compl:rev
CACCCACACAGCCACTATCATGTTGACATTCC; dG = -11.3
704 >s_c_s_a_s_e_s_b_s_b_s GTGTGGGTGTCTGTGCTGGTGGCTGTGGCTGTG; >clamp_s_b_s_c_s:compl:rev
CACCCACACAGCCACTATCATGTTGACATTCC; dG = -11.3
705 >s_c_s_b_s_b_s_b_s_b_s GTGTGGGTGGCTGTGGCTGTGGCTGTGGCTGTG; >clamp_s_b_s_c_s:compl:rev
CACCCACACAGCCACTATCATGTTGACATTCC; dG = -11.3
706 >s_c_s_c_s_a_s_b_s_b_s GTGTGGGTGTGGGTGTCTGTGGCTGTGGCTGTG; >clamp_s_b_s_c_s:compl:rev
CACCCACACAGCCACTATCATGTTGACATTCC; dG = -11.3
707 >s_c_s_c_s_b_s_b_s_b_s GTGTGGGTGTGGGTGGCTGTGGCTGTGGCTGTG; >clamp_s_b_s_c_s:compl:rev
CACCCACACAGCCACTATCATGTTGACATTCC; dG = -11.3
708 >s_c_s_c_s_c_s_e_s_c_s GTGTGGGTGTGGGTGTGGGTGCTGGTGTGGGTG; >clamp_s_b_s_c_s:compl:rev
CACCCACACAGCCACTATCATGTTGACATTCC; dG = -11.3
709 >s_c_s_c_s_e_s_b_s_b_s GTGTGGGTGTGGGTGCTGGTGGCTGTGGCTGTG; >clamp_s_b_s_c_s:compl:rev
CACCCACACAGCCACTATCATGTTGACATTCC; dG = -11.3
710 >s_c_s_d_s_a_s_b_s_b_s GTGTGGGTGACGGTGTCTGTGGCTGTGGCTGTG; >clamp_s_b_s_c_s:compl:rev
CACCCACACAGCCACTATCATGTTGACATTCC; dG = -11.3
711 >s_c_s_d_s_b_s_b_s_b_s GTGTGGGTGACGGTGGCTGTGGCTGTGGCTGTG; >clamp_s_b_s_c_s:compl:rev
CACCCACACAGCCACTATCATGTTGACATTCC; dG = -11.3
712 >s_c_s_d_s_c_s_e_s_c_s GTGTGGGTGACGGTGTGGGTGCTGGTGTGGGTG; >clamp_s_b_s_c_s:compl:rev
CACCCACACAGCCACTATCATGTTGACATTCC; dG = -11.3
713 >s_c_s_d_s_d_s_b_s_b_s GTGTGGGTGACGGTGACGGTGGCTGTGGCTGTG; >clamp_s_b_s_c_s:compl:rev
CACCCACACAGCCACTATCATGTTGACATTCC; dG = -11.3
714 >s_c_s_d_s_e_s_b_s_b_s GTGTGGGTGACGGTGCTGGTGGCTGTGGCTGTG; >clamp_s_b_s_c_s:compl:rev
CACCCACACAGCCACTATCATGTTGACATTCC; dG = -11.3
715 >s_c_s_e_s_a_s_b_s_b_s GTGTGGGTGCTGGTGTCTGTGGCTGTGGCTGTG; >clamp_s_b_s_c_s:compl:rev
CACCCACACAGCCACTATCATGTTGACATTCC; dG = -11.3

716 >s_c_s_e_s_b_s_b_s_b_s GTGTGGGTGCTGGTGGCTGTGGCTGTGGCTGTG; >clamp_s_b_s_c_s:compl:rev
CACCCACACAGCCACTATCATGTTGACATTCC; dG = -11.3
717 >s_a_s_b_s_b_s_b_s_b_s GTGTCTGTGGCTGTGGCTGTGGCTGTGGCTGTG; >clamp_s_b_s_c_s:compl:rev
CACCCACACAGCCACTATCATGTTGACATTCC; dG = -11.3
718 >s_c_s_e_s_c_s_e_s_c_s GTGTGGGTGCTGGTGTGGGTGCTGGTGTGGGTG; >clamp_s_b_s_c_s:compl:rev
CACCCACACAGCCACTATCATGTTGACATTCC; dG = -11.3
719 >s_c_s_e_s_d_s_b_s_b_s GTGTGGGTGCTGGTGACGGTGGCTGTGGCTGTG; >clamp_s_b_s_c_s:compl:rev
CACCCACACAGCCACTATCATGTTGACATTCC; dG = -11.3
720 >s_c_s_e_s_e_s_b_s_b_s GTGTGGGTGCTGGTGCTGGTGGCTGTGGCTGTG; >clamp_s_b_s_c_s:compl:rev
CACCCACACAGCCACTATCATGTTGACATTCC; dG = -11.3
721 >s_d_s_a_s_a_s_b_s_b_s GTGACGGTGTCTGTGTCTGTGGCTGTGGCTGTG; >clamp_s_b_s_c_s:compl:rev
CACCCACACAGCCACTATCATGTTGACATTCC; dG = -11.3
722 >s_d_s_a_s_b_s_b_s_b_s GTGACGGTGTCTGTGGCTGTGGCTGTGGCTGTG; >clamp_s_b_s_c_s:compl:rev
CACCCACACAGCCACTATCATGTTGACATTCC; dG = -11.3
723 >s_d_s_a_s_d_s_b_s_b_s GTGACGGTGTCTGTGACGGTGGCTGTGGCTGTG; >clamp_s_b_s_c_s:compl:rev
CACCCACACAGCCACTATCATGTTGACATTCC; dG = -11.3
724 >s_d_s_a_s_e_s_b_s_b_s GTGACGGTGTCTGTGCTGGTGGCTGTGGCTGTG; >clamp_s_b_s_c_s:compl:rev
CACCCACACAGCCACTATCATGTTGACATTCC; dG = -11.3
725 >s_d_s_b_s_b_s_b_s_b_s GTGACGGTGGCTGTGGCTGTGGCTGTGGCTGTG; >clamp_s_b_s_c_s:compl:rev
CACCCACACAGCCACTATCATGTTGACATTCC; dG = -11.3
726 >s_d_s_c_s_a_s_b_s_b_s GTGACGGTGTGGGTGTCTGTGGCTGTGGCTGTG; >clamp_s_b_s_c_s:compl:rev
CACCCACACAGCCACTATCATGTTGACATTCC; dG = -11.3
727 >s_d_s_c_s_b_s_b_s_b_s GTGACGGTGTGGGTGGCTGTGGCTGTGGCTGTG; >clamp_s_b_s_c_s:compl:rev
CACCCACACAGCCACTATCATGTTGACATTCC; dG = -11.3
728 >s_d_s_c_s_c_s_b_s_b_s GTGACGGTGTGGGTGTGGGTGGCTGTGGCTGTG; >clamp_s_b_s_c_s:compl:rev
CACCCACACAGCCACTATCATGTTGACATTCC; dG = -11.3
729 >s_d_s_c_s_d_s_b_s_b_s GTGACGGTGTGGGTGACGGTGGCTGTGGCTGTG; >clamp_s_b_s_c_s:compl:rev
CACCCACACAGCCACTATCATGTTGACATTCC; dG = -11.3
730 >s_d_s_c_s_e_s_b_s_b_s GTGACGGTGTGGGTGCTGGTGGCTGTGGCTGTG; >clamp_s_b_s_c_s:compl:rev
CACCCACACAGCCACTATCATGTTGACATTCC; dG = -11.3
731 >s_d_s_d_s_a_s_b_s_b_s GTGACGGTGACGGTGTCTGTGGCTGTGGCTGTG; >clamp_s_b_s_c_s:compl:rev
CACCCACACAGCCACTATCATGTTGACATTCC; dG = -11.3
732 >s_d_s_d_s_b_s_b_s_b_s GTGACGGTGACGGTGGCTGTGGCTGTGGCTGTG; >clamp_s_b_s_c_s:compl:rev
CACCCACACAGCCACTATCATGTTGACATTCC; dG = -11.3
733 >s_d_s_d_s_c_s_b_s_b_s GTGACGGTGACGGTGTGGGTGGCTGTGGCTGTG; >clamp_s_b_s_c_s:compl:rev
CACCCACACAGCCACTATCATGTTGACATTCC; dG = -11.3
734 >s_d_s_d_s_d_s_b_s_b_s GTGACGGTGACGGTGACGGTGGCTGTGGCTGTG; >clamp_s_b_s_c_s:compl:rev
CACCCACACAGCCACTATCATGTTGACATTCC; dG = -11.3
735 >s_d_s_d_s_e_s_b_s_b_s GTGACGGTGACGGTGCTGGTGGCTGTGGCTGTG; >clamp_s_b_s_c_s:compl:rev
CACCCACACAGCCACTATCATGTTGACATTCC; dG = -11.3
736 >s_d_s_e_s_a_s_b_s_b_s GTGACGGTGCTGGTGTCTGTGGCTGTGGCTGTG; >clamp_s_b_s_c_s:compl:rev
CACCCACACAGCCACTATCATGTTGACATTCC; dG = -11.3
737 >s_d_s_e_s_b_s_b_s_b_s GTGACGGTGCTGGTGGCTGTGGCTGTGGCTGTG; >clamp_s_b_s_c_s:compl:rev
CACCCACACAGCCACTATCATGTTGACATTCC; dG = -11.3
738 >s_d_s_e_s_c_s_b_s_b_s GTGACGGTGCTGGTGTGGGTGGCTGTGGCTGTG; >clamp_s_b_s_c_s:compl:rev
CACCCACACAGCCACTATCATGTTGACATTCC; dG = -11.3
739 >s_d_s_e_s_d_s_b_s_b_s GTGACGGTGCTGGTGACGGTGGCTGTGGCTGTG; >clamp_s_b_s_c_s:compl:rev
CACCCACACAGCCACTATCATGTTGACATTCC; dG = -11.3
740 >s_d_s_e_s_e_s_b_s_b_s GTGACGGTGCTGGTGCTGGTGGCTGTGGCTGTG; >clamp_s_b_s_c_s:compl:rev
CACCCACACAGCCACTATCATGTTGACATTCC; dG = -11.3
741 >s_e_s_a_s_a_s_b_s_b_s GTGCTGGTGTCTGTGTCTGTGGCTGTGGCTGTG; >clamp_s_b_s_c_s:compl:rev
CACCCACACAGCCACTATCATGTTGACATTCC; dG = -11.3
742 >s_e_s_a_s_b_s_b_s_b_s GTGCTGGTGTCTGTGGCTGTGGCTGTGGCTGTG; >clamp_s_b_s_c_s:compl:rev
CACCCACACAGCCACTATCATGTTGACATTCC; dG = -11.3
743 >s_e_s_a_s_d_s_b_s_b_s GTGCTGGTGTCTGTGACGGTGGCTGTGGCTGTG; >clamp_s_b_s_c_s:compl:rev
CACCCACACAGCCACTATCATGTTGACATTCC; dG = -11.3
744 >s_e_s_a_s_e_s_b_s_b_s GTGCTGGTGTCTGTGCTGGTGGCTGTGGCTGTG; >clamp_s_b_s_c_s:compl:rev
CACCCACACAGCCACTATCATGTTGACATTCC; dG = -11.3
745 >s_e_s_b_s_b_s_b_s_b_s GTGCTGGTGGCTGTGGCTGTGGCTGTGGCTGTG; >clamp_s_b_s_c_s:compl:rev
CACCCACACAGCCACTATCATGTTGACATTCC; dG = -11.3
746 >s_e_s_c_s_a_s_b_s_b_s GTGCTGGTGTGGGTGTCTGTGGCTGTGGCTGTG; >clamp_s_b_s_c_s:compl:rev
CACCCACACAGCCACTATCATGTTGACATTCC; dG = -11.3
747 >s_e_s_c_s_b_s_b_s_b_s GTGCTGGTGTGGGTGGCTGTGGCTGTGGCTGTG; >clamp_s_b_s_c_s:compl:rev
CACCCACACAGCCACTATCATGTTGACATTCC; dG = -11.3
748 >s_e_s_c_s_c_s_b_s_b_s GTGCTGGTGTGGGTGTGGGTGGCTGTGGCTGTG; >clamp_s_b_s_c_s:compl:rev
CACCCACACAGCCACTATCATGTTGACATTCC; dG = -11.3
749 >s_e_s_c_s_d_s_b_s_b_s GTGCTGGTGTGGGTGACGGTGGCTGTGGCTGTG; >clamp_s_b_s_c_s:compl:rev
CACCCACACAGCCACTATCATGTTGACATTCC; dG = -11.3
750 >s_e_s_c_s_e_s_b_s_b_s GTGCTGGTGTGGGTGCTGGTGGCTGTGGCTGTG; >clamp_s_b_s_c_s:compl:rev
CACCCACACAGCCACTATCATGTTGACATTCC; dG = -11.3
751 >s_e_s_d_s_a_s_b_s_b_s GTGCTGGTGACGGTGTCTGTGGCTGTGGCTGTG; >clamp_s_b_s_c_s:compl:rev
CACCCACACAGCCACTATCATGTTGACATTCC; dG = -11.3
752 >s_e_s_d_s_b_s_b_s_b_s GTGCTGGTGACGGTGGCTGTGGCTGTGGCTGTG; >clamp_s_b_s_c_s:compl:rev
CACCCACACAGCCACTATCATGTTGACATTCC; dG = -11.3

753 >s_e_s_d_s_c_s_b_s_b_s GTGCTGGTGACGGTGTGGGTGGCTGTGGCTGTG; >clamp_s_b_s_c_s:compl:rev CACCCACACAGCCACTATCATGTTGACATTCC; dG = -11.3
754 >s_e_s_d_s_d_s_b_s_b_s GTGCTGGTGACGGTGACGGTGGCTGTGGCTGTG; >clamp_s_b_s_c_s:compl:rev CACCCACACAGCCACTATCATGTTGACATTCC; dG = -11.3
755 >s_e_s_d_s_e_s_b_s_b_s GTGCTGGTGACGGTGCTGGTGGCTGTGGCTGTG; >clamp_s_b_s_c_s:compl:rev CACCCACACAGCCACTATCATGTTGACATTCC; dG = -11.3
756 >s_e_s_e_s_a_s_b_s_b_s GTGCTGGTGCTGGTGTCTGTGGCTGTGGCTGTG; >clamp_s_b_s_c_s:compl:rev CACCCACACAGCCACTATCATGTTGACATTCC; dG = -11.3
757 >s_e_s_e_s_b_s_b_s_b_s GTGCTGGTGCTGGTGGCTGTGGCTGTGGCTGTG; >clamp_s_b_s_c_s:compl:rev CACCCACACAGCCACTATCATGTTGACATTCC; dG = -11.3
758 >s_e_s_e_s_c_s_b_s_b_s GTGCTGGTGCTGGTGTGGGTGGCTGTGGCTGTG; >clamp_s_b_s_c_s:compl:rev CACCCACACAGCCACTATCATGTTGACATTCC; dG = -11.3
759 >s_e_s_e_s_d_s_b_s_b_s GTGCTGGTGCTGGTGACGGTGGCTGTGGCTGTG; >clamp_s_b_s_c_s:compl:rev CACCCACACAGCCACTATCATGTTGACATTCC; dG = -11.3
760 >s_e_s_e_s_e_s_b_s_b_s GTGCTGGTGCTGGTGCTGGTGGCTGTGGCTGTG; >clamp_s_b_s_c_s:compl:rev CACCCACACAGCCACTATCATGTTGACATTCC; dG = -11.3
761 >s_a_s_d_s_c_s_e_s_c_s GTGTCTGTGACGGTGTGGGTGCTGGTGTGGGTG; >clamp_s_b_s_c_s:compl:rev CACCCACACAGCCACTATCATGTTGACATTCC; dG = -11.2
762 >s_a_s_d_s_c_s_d_s_c_s GTGTCTGTGACGGTGTGGGTGACGGTGTGGGTG; >clamp_s_b_s_c_s:compl:rev CACCCACACAGCCACTATCATGTTGACATTCC; dG = -11.2
763 >s_a_s_d_s_c_s_c_s_c_s GTGTCTGTGACGGTGTGGGTGTGGGTGTGGGTG; >clamp_s_b_s_c_s:compl:rev CACCCACACAGCCACTATCATGTTGACATTCC; dG = -11.2
764 >s_a_s_e_s_c_s_e_s_c_s GTGTCTGTGCTGGTGTGGGTGCTGGTGTGGGTG; >clamp_s_b_s_c_s:compl:rev CACCCACACAGCCACTATCATGTTGACATTCC; dG = -11.2
765 >s_a_s_e_s_c_s_d_s_c_s GTGTCTGTGCTGGTGTGGGTGACGGTGTGGGTG; >clamp_s_b_s_c_s:compl:rev CACCCACACAGCCACTATCATGTTGACATTCC; dG = -11.2
766 >s_a_s_e_s_c_s_c_s_c_s GTGTCTGTGCTGGTGTGGGTGTGGGTGTGGGTG; >clamp_s_b_s_c_s:compl:rev CACCCACACAGCCACTATCATGTTGACATTCC; dG = -11.2
767 >s_b_s_a_s_a_s_e_s_c_s GTGGCTGTGTCTGTGTCTGTGCTGGTGTGGGTG; >clamp_s_b_s_c_s:compl:rev CACCCACACAGCCACTATCATGTTGACATTCC; dG = -11.2
768 >s_b_s_a_s_a_s_d_s_c_s GTGGCTGTGTCTGTGTCTGTGACGGTGTGGGTG; >clamp_s_b_s_c_s:compl:rev CACCCACACAGCCACTATCATGTTGACATTCC; dG = -11.2
769 >s_b_s_a_s_e_s_e_s_c_s GTGGCTGTGTCTGTGCTGGTGCTGGTGTGGGTG; >clamp_s_b_s_c_s:compl:rev CACCCACACAGCCACTATCATGTTGACATTCC; dG = -11.2
770 >s_b_s_a_s_e_s_d_s_c_s GTGGCTGTGTCTGTGCTGGTGACGGTGTGGGTG; >clamp_s_b_s_c_s:compl:rev CACCCACACAGCCACTATCATGTTGACATTCC; dG = -11.2
771 >s_d_s_c_s_c_s_e_s_c_s GTGACGGTGTGGGTGTGGGTGCTGGTGTGGGTG; >clamp_s_b_s_c_s:compl:rev CACCCACACAGCCACTATCATGTTGACATTCC; dG = -11.2
772 >s_d_s_d_s_c_s_e_s_c_s GTGACGGTGACGGTGTGGGTGCTGGTGTGGGTG; >clamp_s_b_s_c_s:compl:rev CACCCACACAGCCACTATCATGTTGACATTCC; dG = -11.2
773 >s_d_s_d_s_c_s_d_s_c_s GTGACGGTGACGGTGTGGGTGACGGTGTGGGTG; >clamp_s_b_s_c_s:compl:rev CACCCACACAGCCACTATCATGTTGACATTCC; dG = -11.2
774 >s_d_s_d_s_c_s_c_s_c_s GTGACGGTGACGGTGTGGGTGTGGGTGTGGGTG; >clamp_s_b_s_c_s:compl:rev CACCCACACAGCCACTATCATGTTGACATTCC; dG = -11.2
775 >s_d_s_e_s_c_s_e_s_c_s GTGACGGTGCTGGTGTGGGTGCTGGTGTGGGTG; >clamp_s_b_s_c_s:compl:rev CACCCACACAGCCACTATCATGTTGACATTCC; dG = -11.2
776 >s_d_s_e_s_c_s_d_s_c_s GTGACGGTGCTGGTGTGGGTGACGGTGTGGGTG; >clamp_s_b_s_c_s:compl:rev CACCCACACAGCCACTATCATGTTGACATTCC; dG = -11.2
777 >s_d_s_e_s_c_s_c_s_c_s GTGACGGTGCTGGTGTGGGTGTGGGTGTGGGTG; >clamp_s_b_s_c_s:compl:rev CACCCACACAGCCACTATCATGTTGACATTCC; dG = -11.2
778 >s_e_s_c_s_c_s_e_s_c_s GTGCTGGTGTGGGTGTGGGTGCTGGTGTGGGTG; >clamp_s_b_s_c_s:compl:rev CACCCACACAGCCACTATCATGTTGACATTCC; dG = -11.2
779 >s_e_s_d_s_c_s_e_s_c_s GTGCTGGTGACGGTGTGGGTGCTGGTGTGGGTG; >clamp_s_b_s_c_s:compl:rev CACCCACACAGCCACTATCATGTTGACATTCC; dG = -11.2
780 >s_e_s_d_s_c_s_d_s_c_s GTGCTGGTGACGGTGTGGGTGACGGTGTGGGTG; >clamp_s_b_s_c_s:compl:rev CACCCACACAGCCACTATCATGTTGACATTCC; dG = -11.2
781 >s_e_s_d_s_c_s_c_s_c_s GTGCTGGTGACGGTGTGGGTGTGGGTGTGGGTG; >clamp_s_b_s_c_s:compl:rev CACCCACACAGCCACTATCATGTTGACATTCC; dG = -11.2
782 >s_e_s_e_s_c_s_e_s_c_s GTGCTGGTGCTGGTGTGGGTGCTGGTGTGGGTG; >clamp_s_b_s_c_s:compl:rev CACCCACACAGCCACTATCATGTTGACATTCC; dG = -11.2
783 >s_e_s_e_s_c_s_d_s_c_s GTGCTGGTGCTGGTGTGGGTGACGGTGTGGGTG; >clamp_s_b_s_c_s:compl:rev CACCCACACAGCCACTATCATGTTGACATTCC; dG = -11.2
784 >s_e_s_e_s_c_s_c_s_c_s GTGCTGGTGCTGGTGTGGGTGTGGGTGTGGGTG; >clamp_s_b_s_c_s:compl:rev CACCCACACAGCCACTATCATGTTGACATTCC; dG = -11.2
785 >s_c_s_c_s_a_s_e_s_c_s GTGTGGGTGTGGGTGTCTGTGCTGGTGTGGGTG; >clamp_s_b_s_c_s:compl:rev CACCCACACAGCCACTATCATGTTGACATTCC; dG = -11.1
786 >s_c_s_c_s_a_s_d_s_c_s GTGTGGGTGTGGGTGTCTGTGACGGTGTGGGTG; >clamp_s_b_s_c_s:compl:rev CACCCACACAGCCACTATCATGTTGACATTCC; dG = -11.1
787 >s_c_s_c_s_e_s_e_s_c_s GTGTGGGTGTGGGTGCTGGTGCTGGTGTGGGTG; >clamp_s_b_s_c_s:compl:rev CACCCACACAGCCACTATCATGTTGACATTCC; dG = -11.0
788 >s_c_s_c_s_e_s_d_s_c_s GTGTGGGTGTGGGTGCTGGTGACGGTGTGGGTG; >clamp_s_b_s_c_s:compl:rev CACCCACACAGCCACTATCATGTTGACATTCC; dG = -11.0
789 >s_a_s_d_s_d_s_c_s_c_s GTGTCTGTGACGGTGACGGTGTGGGTGTGGGTG; >clamp_s_b_s_c_s:compl:rev CACCCACACAGCCACTATCATGTTGACATTCC; dG = -10.9

790 >s_a_s_d_s_e_s_c_s_c_s GTGTCTGTGACGGTGCTGGTGTGGGTGTGGGTG; >clamp_s_b_s_c_s:compl:rev CACCCACACAGCCACTATCATGTTGACATTCC; dG = -10.9
791 >s_a_s_e_s_d_s_c_s_c_s GTGTCTGTGCTGGTGACGGTGTGGGTGTGGGTG; >clamp_s_b_s_c_s:compl:rev CACCCACACAGCCACTATCATGTTGACATTCC; dG = -10.9
792 >s_a_s_e_s_e_s_c_s_c_s GTGTCTGTGCTGGTGCTGGTGTGGGTGTGGGTG; >clamp_s_b_s_c_s:compl:rev CACCCACACAGCCACTATCATGTTGACATTCC; dG = -10.9
793 >s_a_s_a_s_d_s_c_s_c_s GTGTCTGTGTCTGTGACGGTGTGGGTGTGGGTG; >clamp_s_b_s_c_s:compl:rev CACCCACACAGCCACTATCATGTTGACATTCC; dG = -10.9
794 >s_c_s_a_s_d_s_c_s_c_s GTGTGGGTGTCTGTGACGGTGTGGGTGTGGGTG; >clamp_s_b_s_c_s:compl:rev CACCCACACAGCCACTATCATGTTGACATTCC; dG = -10.9
795 >s_c_s_a_s_e_s_c_s_c_s GTGTGGGTGTCTGTGCTGGTGTGGGTGTGGGTG; >clamp_s_b_s_c_s:compl:rev CACCCACACAGCCACTATCATGTTGACATTCC; dG = -10.9
796 >s_a_s_a_s_e_s_c_s_c_s GTGTCTGTGTCTGTGCTGGTGTGGGTGTGGGTG; >clamp_s_b_s_c_s:compl:rev CACCCACACAGCCACTATCATGTTGACATTCC; dG = -10.9
797 >s_c_s_d_s_d_s_c_s_c_s GTGTGGGTGACGGTGACGGTGTGGGTGTGGGTG; >clamp_s_b_s_c_s:compl:rev CACCCACACAGCCACTATCATGTTGACATTCC; dG = -10.9
798 >s_c_s_d_s_e_s_c_s_c_s GTGTGGGTGACGGTGCTGGTGTGGGTGTGGGTG; >clamp_s_b_s_c_s:compl:rev CACCCACACAGCCACTATCATGTTGACATTCC; dG = -10.9
799 >s_c_s_e_s_d_s_c_s_c_s GTGTGGGTGCTGGTGACGGTGTGGGTGTGGGTG; >clamp_s_b_s_c_s:compl:rev CACCCACACAGCCACTATCATGTTGACATTCC; dG = -10.9
800 >s_c_s_e_s_e_s_c_s_c_s GTGTGGGTGCTGGTGCTGGTGTGGGTGTGGGTG; >clamp_s_b_s_c_s:compl:rev CACCCACACAGCCACTATCATGTTGACATTCC; dG = -10.9
801 >s_d_s_a_s_d_s_c_s_c_s GTGACGGTGTCTGTGACGGTGTGGGTGTGGGTG; >clamp_s_b_s_c_s:compl:rev CACCCACACAGCCACTATCATGTTGACATTCC; dG = -10.9
802 >s_d_s_a_s_e_s_c_s_c_s GTGACGGTGTCTGTGCTGGTGTGGGTGTGGGTG; >clamp_s_b_s_c_s:compl:rev CACCCACACAGCCACTATCATGTTGACATTCC; dG = -10.9
803 >s_d_s_c_s_d_s_e_s_c_s GTGACGGTGTGGGTGACGGTGCTGGTGTGGGTG; >clamp_s_b_s_c_s:compl:rev CACCCACACAGCCACTATCATGTTGACATTCC; dG = -10.9
804 >s_d_s_c_s_d_s_d_s_c_s GTGACGGTGTGGGTGACGGTGACGGTGTGGGTG; >clamp_s_b_s_c_s:compl:rev CACCCACACAGCCACTATCATGTTGACATTCC; dG = -10.9
805 >s_d_s_d_s_d_s_c_s_c_s GTGACGGTGACGGTGACGGTGTGGGTGTGGGTG; >clamp_s_b_s_c_s:compl:rev CACCCACACAGCCACTATCATGTTGACATTCC; dG = -10.9
806 >s_d_s_d_s_e_s_c_s_c_s GTGACGGTGACGGTGCTGGTGTGGGTGTGGGTG; >clamp_s_b_s_c_s:compl:rev CACCCACACAGCCACTATCATGTTGACATTCC; dG = -10.9
807 >s_d_s_e_s_d_s_c_s_c_s GTGACGGTGCTGGTGACGGTGTGGGTGTGGGTG; >clamp_s_b_s_c_s:compl:rev CACCCACACAGCCACTATCATGTTGACATTCC; dG = -10.9
808 >s_d_s_e_s_e_s_c_s_c_s GTGACGGTGCTGGTGCTGGTGTGGGTGTGGGTG; >clamp_s_b_s_c_s:compl:rev CACCCACACAGCCACTATCATGTTGACATTCC; dG = -10.9
809 >s_e_s_a_s_d_s_c_s_c_s GTGCTGGTGTCTGTGACGGTGTGGGTGTGGGTG; >clamp_s_b_s_c_s:compl:rev CACCCACACAGCCACTATCATGTTGACATTCC; dG = -10.9
810 >s_e_s_a_s_e_s_c_s_c_s GTGCTGGTGTCTGTGCTGGTGTGGGTGTGGGTG; >clamp_s_b_s_c_s:compl:rev CACCCACACAGCCACTATCATGTTGACATTCC; dG = -10.9
811 >s_e_s_c_s_d_s_e_s_c_s GTGCTGGTGTGGGTGACGGTGCTGGTGTGGGTG; >clamp_s_b_s_c_s:compl:rev CACCCACACAGCCACTATCATGTTGACATTCC; dG = -10.9
812 >s_e_s_c_s_d_s_d_s_c_s GTGCTGGTGTGGGTGACGGTGACGGTGTGGGTG; >clamp_s_b_s_c_s:compl:rev CACCCACACAGCCACTATCATGTTGACATTCC; dG = -10.9
813 >s_e_s_d_s_d_s_c_s_c_s GTGCTGGTGACGGTGACGGTGTGGGTGTGGGTG; >clamp_s_b_s_c_s:compl:rev CACCCACACAGCCACTATCATGTTGACATTCC; dG = -10.9
814 >s_e_s_d_s_e_s_c_s_c_s GTGCTGGTGACGGTGCTGGTGTGGGTGTGGGTG; >clamp_s_b_s_c_s:compl:rev CACCCACACAGCCACTATCATGTTGACATTCC; dG = -10.9
815 >s_e_s_e_s_d_s_c_s_c_s GTGCTGGTGCTGGTGACGGTGTGGGTGTGGGTG; >clamp_s_b_s_c_s:compl:rev CACCCACACAGCCACTATCATGTTGACATTCC; dG = -10.9
816 >s_e_s_e_s_e_s_c_s_c_s GTGCTGGTGCTGGTGCTGGTGTGGGTGTGGGTG; >clamp_s_b_s_c_s:compl:rev CACCCACACAGCCACTATCATGTTGACATTCC; dG = -10.9
817 >s_c_s_d_s_a_s_e_s_c_s GTGTGGGTGACGGTGTCTGTGCTGGTGTGGGTG; >clamp_s_b_s_c_s:compl:rev CACCCACACAGCCACTATCATGTTGACATTCC; dG = -10.5
818 >s_c_s_d_s_a_s_d_s_c_s GTGTGGGTGACGGTGTCTGTGACGGTGTGGGTG; >clamp_s_b_s_c_s:compl:rev CACCCACACAGCCACTATCATGTTGACATTCC; dG = -10.5
819 >s_c_s_d_s_d_s_e_s_c_s GTGTGGGTGACGGTGACGGTGCTGGTGTGGGTG; >clamp_s_b_s_c_s:compl:rev CACCCACACAGCCACTATCATGTTGACATTCC; dG = -10.5
820 >s_c_s_d_s_d_s_d_s_c_s GTGTGGGTGACGGTGACGGTGACGGTGTGGGTG; >clamp_s_b_s_c_s:compl:rev CACCCACACAGCCACTATCATGTTGACATTCC; dG = -10.5
821 >s_c_s_d_s_e_s_e_s_c_s GTGTGGGTGACGGTGCTGGTGCTGGTGTGGGTG; >clamp_s_b_s_c_s:compl:rev CACCCACACAGCCACTATCATGTTGACATTCC; dG = -10.5
822 >s_c_s_d_s_e_s_d_s_c_s GTGTGGGTGACGGTGCTGGTGACGGTGTGGGTG; >clamp_s_b_s_c_s:compl:rev CACCCACACAGCCACTATCATGTTGACATTCC; dG = -10.5
823 >s_d_s_c_s_a_s_e_s_c_s GTGACGGTGTGGGTGTCTGTGCTGGTGTGGGTG; >clamp_s_b_s_c_s:compl:rev CACCCACACAGCCACTATCATGTTGACATTCC; dG = -10.4
824 >s_d_s_c_s_a_s_d_s_c_s GTGACGGTGTGGGTGTCTGTGACGGTGTGGGTG; >clamp_s_b_s_c_s:compl:rev CACCCACACAGCCACTATCATGTTGACATTCC; dG = -10.4
825 >s_e_s_c_s_a_s_e_s_c_s GTGCTGGTGTGGGTGTCTGTGCTGGTGTGGGTG; >clamp_s_b_s_c_s:compl:rev CACCCACACAGCCACTATCATGTTGACATTCC; dG = -10.4
826 >s_e_s_c_s_a_s_d_s_c_s GTGCTGGTGTGGGTGTCTGTGACGGTGTGGGTG; >clamp_s_b_s_c_s:compl:rev CACCCACACAGCCACTATCATGTTGACATTCC; dG = -10.4

827 >s_d_s_c_s_e_s_e_s_c_s GTGACGGTGTGGGTGCTGGTGCTGGTGTGGGTG; >clamp_s_b_s_c_s:compl:rev
CACCCACACAGCCACTATCATGTTGACATTCC; dG = -10.2

828 >s_d_s_c_s_e_s_d_s_c_s GTGACGGTGTGGGTGCTGGTGACGGTGTGGGTG; >clamp_s_b_s_c_s:compl:rev
CACCCACACAGCCACTATCATGTTGACATTCC; dG = -10.2

829 >s_e_s_c_s_e_s_e_s_c_s GTGCTGGTGTGGGTGCTGGTGCTGGTGTGGGTG; >clamp_s_b_s_c_s:compl:rev
CACCCACACAGCCACTATCATGTTGACATTCC; dG = -10.2

830 >s_e_s_c_s_e_s_d_s_c_s GTGCTGGTGTGGGTGCTGGTGACGGTGTGGGTG; >clamp_s_b_s_c_s:compl:rev
CACCCACACAGCCACTATCATGTTGACATTCC; dG = -10.2

831 >s_a_s_d_s_a_s_e_s_c_s GTGTCTGTGACGGTGTCTGTGCTGGTGTGGGTG; >clamp_s_b_s_c_s:compl:rev
CACCCACACAGCCACTATCATGTTGACATTCC; dG = -10.1

832 >s_a_s_d_s_a_s_d_s_c_s GTGTCTGTGACGGTGTCTGTGACGGTGTGGGTG; >clamp_s_b_s_c_s:compl:rev
CACCCACACAGCCACTATCATGTTGACATTCC; dG = -10.1

833 >s_a_s_d_s_d_s_e_s_c_s GTGTCTGTGACGGTGACGGTGCTGGTGTGGGTG; >clamp_s_b_s_c_s:compl:rev
CACCCACACAGCCACTATCATGTTGACATTCC; dG = -10.1

834 >s_a_s_d_s_d_s_d_s_c_s GTGTCTGTGACGGTGACGGTGACGGTGTGGGTG; >clamp_s_b_s_c_s:compl:rev
CACCCACACAGCCACTATCATGTTGACATTCC; dG = -10.1

835 >s_a_s_d_s_e_s_e_s_c_s GTGTCTGTGACGGTGCTGGTGCTGGTGTGGGTG; >clamp_s_b_s_c_s:compl:rev
CACCCACACAGCCACTATCATGTTGACATTCC; dG = -10.1

836 >s_a_s_d_s_e_s_d_s_c_s GTGTCTGTGACGGTGCTGGTGACGGTGTGGGTG; >clamp_s_b_s_c_s:compl:rev
CACCCACACAGCCACTATCATGTTGACATTCC; dG = -10.1

837 >s_a_s_e_s_a_s_e_s_c_s GTGTCTGTGCTGGTGTCTGTGCTGGTGTGGGTG; >clamp_s_b_s_c_s:compl:rev
CACCCACACAGCCACTATCATGTTGACATTCC; dG = -10.1

838 >s_a_s_e_s_a_s_d_s_c_s GTGTCTGTGCTGGTGTCTGTGACGGTGTGGGTG; >clamp_s_b_s_c_s:compl:rev
CACCCACACAGCCACTATCATGTTGACATTCC; dG = -10.1

839 >s_a_s_e_s_d_s_e_s_c_s GTGTCTGTGCTGGTGACGGTGCTGGTGTGGGTG; >clamp_s_b_s_c_s:compl:rev
CACCCACACAGCCACTATCATGTTGACATTCC; dG = -10.1

840 >s_a_s_e_s_d_s_d_s_c_s GTGTCTGTGCTGGTGACGGTGACGGTGTGGGTG; >clamp_s_b_s_c_s:compl:rev
CACCCACACAGCCACTATCATGTTGACATTCC; dG = -10.1

841 >s_a_s_e_s_e_s_e_s_c_s GTGTCTGTGCTGGTGCTGGTGCTGGTGTGGGTG; >clamp_s_b_s_c_s:compl:rev
CACCCACACAGCCACTATCATGTTGACATTCC; dG = -10.1

842 >s_a_s_e_s_e_s_d_s_c_s GTGTCTGTGCTGGTGCTGGTGACGGTGTGGGTG; >clamp_s_b_s_c_s:compl:rev
CACCCACACAGCCACTATCATGTTGACATTCC; dG = -10.1

843 >s_a_s_a_s_d_s_e_s_c_s GTGTCTGTGTCTGTGACGGTGCTGGTGTGGGTG; >clamp_s_b_s_c_s:compl:rev
CACCCACACAGCCACTATCATGTTGACATTCC; dG = -10.1

844 >s_a_s_a_s_d_s_d_s_c_s GTGTCTGTGTCTGTGACGGTGACGGTGTGGGTG; >clamp_s_b_s_c_s:compl:rev
CACCCACACAGCCACTATCATGTTGACATTCC; dG = -10.1

845 >s_c_s_a_s_a_s_e_s_c_s GTGTGGGTGTCTGTGTCTGTGCTGGTGTGGGTG; >clamp_s_b_s_c_s:compl:rev
CACCCACACAGCCACTATCATGTTGACATTCC; dG = -10.1

846 >s_c_s_a_s_a_s_d_s_c_s GTGTGGGTGTCTGTGTCTGTGACGGTGTGGGTG; >clamp_s_b_s_c_s:compl:rev
CACCCACACAGCCACTATCATGTTGACATTCC; dG = -10.1

847 >s_a_s_a_s_e_s_e_s_c_s GTGTCTGTGTCTGTGCTGGTGCTGGTGTGGGTG; >clamp_s_b_s_c_s:compl:rev
CACCCACACAGCCACTATCATGTTGACATTCC; dG = -10.1

848 >s_c_s_a_s_d_s_e_s_c_s GTGTGGGTGTCTGTGACGGTGCTGGTGTGGGTG; >clamp_s_b_s_c_s:compl:rev
CACCCACACAGCCACTATCATGTTGACATTCC; dG = -10.1

849 >s_c_s_a_s_d_s_d_s_c_s GTGTGGGTGTCTGTGACGGTGACGGTGTGGGTG; >clamp_s_b_s_c_s:compl:rev
CACCCACACAGCCACTATCATGTTGACATTCC; dG = -10.1

850 >s_a_s_a_s_e_s_d_s_c_s GTGTCTGTGTCTGTGCTGGTGACGGTGTGGGTG; >clamp_s_b_s_c_s:compl:rev
CACCCACACAGCCACTATCATGTTGACATTCC; dG = -10.1

851 >s_c_s_a_s_e_s_e_s_c_s GTGTGGGTGTCTGTGCTGGTGCTGGTGTGGGTG; >clamp_s_b_s_c_s:compl:rev
CACCCACACAGCCACTATCATGTTGACATTCC; dG = -10.1

852 >s_c_s_a_s_e_s_d_s_c_s GTGTGGGTGTCTGTGCTGGTGACGGTGTGGGTG; >clamp_s_b_s_c_s:compl:rev
CACCCACACAGCCACTATCATGTTGACATTCC; dG = -10.1

853 >s_c_s_e_s_a_s_e_s_c_s GTGTGGGTGCTGGTGTCTGTGCTGGTGTGGGTG; >clamp_s_b_s_c_s:compl:rev
CACCCACACAGCCACTATCATGTTGACATTCC; dG = -10.1

854 >s_c_s_e_s_a_s_d_s_c_s GTGTGGGTGCTGGTGTCTGTGACGGTGTGGGTG; >clamp_s_b_s_c_s:compl:rev
CACCCACACAGCCACTATCATGTTGACATTCC; dG = -10.1

855 >s_a_s_a_s_a_s_e_s_c_s GTGTCTGTGTCTGTGTCTGTGCTGGTGTGGGTG; >clamp_s_b_s_c_s:compl:rev
CACCCACACAGCCACTATCATGTTGACATTCC; dG = -10.1

856 >s_c_s_e_s_d_s_e_s_c_s GTGTGGGTGCTGGTGACGGTGCTGGTGTGGGTG; >clamp_s_b_s_c_s:compl:rev
CACCCACACAGCCACTATCATGTTGACATTCC; dG = -10.1

857 >s_c_s_e_s_d_s_d_s_c_s GTGTGGGTGCTGGTGACGGTGACGGTGTGGGTG; >clamp_s_b_s_c_s:compl:rev
CACCCACACAGCCACTATCATGTTGACATTCC; dG = -10.1

858 >s_c_s_e_s_e_s_e_s_c_s GTGTGGGTGCTGGTGCTGGTGCTGGTGTGGGTG; >clamp_s_b_s_c_s:compl:rev
CACCCACACAGCCACTATCATGTTGACATTCC; dG = -10.1

859 >s_c_s_e_s_e_s_d_s_c_s GTGTGGGTGCTGGTGCTGGTGACGGTGTGGGTG; >clamp_s_b_s_c_s:compl:rev
CACCCACACAGCCACTATCATGTTGACATTCC; dG = -10.1

860 >s_d_s_a_s_a_s_e_s_c_s GTGACGGTGTCTGTGTCTGTGCTGGTGTGGGTG; >clamp_s_b_s_c_s:compl:rev
CACCCACACAGCCACTATCATGTTGACATTCC; dG = -10.1

861 >s_d_s_a_s_a_s_d_s_c_s GTGACGGTGTCTGTGTCTGTGACGGTGTGGGTG; >clamp_s_b_s_c_s:compl:rev
CACCCACACAGCCACTATCATGTTGACATTCC; dG = -10.1

862 >s_d_s_a_s_d_s_e_s_c_s GTGACGGTGTCTGTGACGGTGCTGGTGTGGGTG; >clamp_s_b_s_c_s:compl:rev
CACCCACACAGCCACTATCATGTTGACATTCC; dG = -10.1

863 >s_d_s_a_s_d_s_d_s_c_s GTGACGGTGTCTGTGACGGTGACGGTGTGGGTG; >clamp_s_b_s_c_s:compl:rev
CACCCACACAGCCACTATCATGTTGACATTCC; dG = -10.1

864 >s_d_s_a_s_e_s_e_s_c_s GTGACGGTGTCTGTGCTGGTGCTGGTGTGGGTG; >clamp_s_b_s_c_s:compl:rev
CACCCACACAGCCACTATCATGTTGACATTCC; dG = -10.1
865 >s_d_s_a_s_e_s_d_s_c_s GTGACGGTGTCTGTGCTGGTGACGGTGTGGGTG; >clamp_s_b_s_c_s:compl:rev
CACCCACACAGCCACTATCATGTTGACATTCC; dG = -10.1
866 >s_a_s_a_s_a_s_d_s_c_s GTGTCTGTGTCTGTGTCTGTGACGGTGTGGGTG; >clamp_s_b_s_c_s:compl:rev
CACCCACACAGCCACTATCATGTTGACATTCC; dG = -10.1
867 >s_d_s_d_s_a_s_e_s_c_s GTGACGGTGACGGTGTCTGTGCTGGTGTGGGTG; >clamp_s_b_s_c_s:compl:rev
CACCCACACAGCCACTATCATGTTGACATTCC; dG = -10.1
868 >s_d_s_d_s_a_s_d_s_c_s GTGACGGTGACGGTGTCTGTGACGGTGTGGGTG; >clamp_s_b_s_c_s:compl:rev
CACCCACACAGCCACTATCATGTTGACATTCC; dG = -10.1
869 >s_d_s_d_s_d_s_e_s_c_s GTGACGGTGACGGTGACGGTGCTGGTGTGGGTG; >clamp_s_b_s_c_s:compl:rev
CACCCACACAGCCACTATCATGTTGACATTCC; dG = -10.1
870 >s_d_s_d_s_d_s_d_s_c_s GTGACGGTGACGGTGACGGTGACGGTGTGGGTG; >clamp_s_b_s_c_s:compl:rev
CACCCACACAGCCACTATCATGTTGACATTCC; dG = -10.1
871 >s_d_s_d_s_e_s_e_s_c_s GTGACGGTGACGGTGCTGGTGCTGGTGTGGGTG; >clamp_s_b_s_c_s:compl:rev
CACCCACACAGCCACTATCATGTTGACATTCC; dG = -10.1
872 >s_d_s_d_s_e_s_d_s_c_s GTGACGGTGACGGTGCTGGTGACGGTGTGGGTG; >clamp_s_b_s_c_s:compl:rev
CACCCACACAGCCACTATCATGTTGACATTCC; dG = -10.1
873 >s_d_s_e_s_a_s_e_s_c_s GTGACGGTGCTGGTGTCTGTGCTGGTGTGGGTG; >clamp_s_b_s_c_s:compl:rev
CACCCACACAGCCACTATCATGTTGACATTCC; dG = -10.1
874 >s_d_s_e_s_a_s_d_s_c_s GTGACGGTGCTGGTGTCTGTGACGGTGTGGGTG; >clamp_s_b_s_c_s:compl:rev
CACCCACACAGCCACTATCATGTTGACATTCC; dG = -10.1
875 >s_d_s_e_s_d_s_e_s_c_s GTGACGGTGCTGGTGACGGTGCTGGTGTGGGTG; >clamp_s_b_s_c_s:compl:rev
CACCCACACAGCCACTATCATGTTGACATTCC; dG = -10.1
876 >s_d_s_e_s_d_s_d_s_c_s GTGACGGTGCTGGTGACGGTGACGGTGTGGGTG; >clamp_s_b_s_c_s:compl:rev
CACCCACACAGCCACTATCATGTTGACATTCC; dG = -10.1
877 >s_d_s_e_s_e_s_e_s_c_s GTGACGGTGCTGGTGCTGGTGCTGGTGTGGGTG; >clamp_s_b_s_c_s:compl:rev
CACCCACACAGCCACTATCATGTTGACATTCC; dG = -10.1
878 >s_d_s_e_s_e_s_d_s_c_s GTGACGGTGCTGGTGCTGGTGACGGTGTGGGTG; >clamp_s_b_s_c_s:compl:rev
CACCCACACAGCCACTATCATGTTGACATTCC; dG = -10.1
879 >s_e_s_a_s_a_s_e_s_c_s GTGCTGGTGTCTGTGTCTGTGCTGGTGTGGGTG; >clamp_s_b_s_c_s:compl:rev
CACCCACACAGCCACTATCATGTTGACATTCC; dG = -10.1
880 >s_e_s_a_s_a_s_d_s_c_s GTGCTGGTGTCTGTGTCTGTGACGGTGTGGGTG; >clamp_s_b_s_c_s:compl:rev
CACCCACACAGCCACTATCATGTTGACATTCC; dG = -10.1
881 >s_e_s_a_s_d_s_e_s_c_s GTGCTGGTGTCTGTGACGGTGCTGGTGTGGGTG; >clamp_s_b_s_c_s:compl:rev
CACCCACACAGCCACTATCATGTTGACATTCC; dG = -10.1
882 >s_e_s_a_s_d_s_d_s_c_s GTGCTGGTGTCTGTGACGGTGACGGTGTGGGTG; >clamp_s_b_s_c_s:compl:rev
CACCCACACAGCCACTATCATGTTGACATTCC; dG = -10.1
883 >s_e_s_a_s_e_s_e_s_c_s GTGCTGGTGTCTGTGCTGGTGCTGGTGTGGGTG; >clamp_s_b_s_c_s:compl:rev
CACCCACACAGCCACTATCATGTTGACATTCC; dG = -10.1
884 >s_e_s_a_s_e_s_d_s_c_s GTGCTGGTGTCTGTGCTGGTGACGGTGTGGGTG; >clamp_s_b_s_c_s:compl:rev
CACCCACACAGCCACTATCATGTTGACATTCC; dG = -10.1
885 >s_e_s_d_s_a_s_e_s_c_s GTGCTGGTGACGGTGTCTGTGCTGGTGTGGGTG; >clamp_s_b_s_c_s:compl:rev
CACCCACACAGCCACTATCATGTTGACATTCC; dG = -10.1
886 >s_e_s_d_s_a_s_d_s_c_s GTGCTGGTGACGGTGTCTGTGACGGTGTGGGTG; >clamp_s_b_s_c_s:compl:rev
CACCCACACAGCCACTATCATGTTGACATTCC; dG = -10.1
887 >s_e_s_d_s_d_s_e_s_c_s GTGCTGGTGACGGTGACGGTGCTGGTGTGGGTG; >clamp_s_b_s_c_s:compl:rev
CACCCACACAGCCACTATCATGTTGACATTCC; dG = -10.1
888 >s_e_s_d_s_d_s_d_s_c_s GTGCTGGTGACGGTGACGGTGACGGTGTGGGTG; >clamp_s_b_s_c_s:compl:rev
CACCCACACAGCCACTATCATGTTGACATTCC; dG = -10.1
889 >s_e_s_d_s_e_s_e_s_c_s GTGCTGGTGACGGTGCTGGTGCTGGTGTGGGTG; >clamp_s_b_s_c_s:compl:rev
CACCCACACAGCCACTATCATGTTGACATTCC; dG = -10.1
890 >s_e_s_d_s_e_s_d_s_c_s GTGCTGGTGACGGTGCTGGTGACGGTGTGGGTG; >clamp_s_b_s_c_s:compl:rev
CACCCACACAGCCACTATCATGTTGACATTCC; dG = -10.1
891 >s_e_s_e_s_a_s_e_s_c_s GTGCTGGTGCTGGTGTCTGTGCTGGTGTGGGTG; >clamp_s_b_s_c_s:compl:rev
CACCCACACAGCCACTATCATGTTGACATTCC; dG = -10.1
892 >s_e_s_e_s_a_s_d_s_c_s GTGCTGGTGCTGGTGTCTGTGACGGTGTGGGTG; >clamp_s_b_s_c_s:compl:rev
CACCCACACAGCCACTATCATGTTGACATTCC; dG = -10.1
893 >s_e_s_e_s_d_s_e_s_c_s GTGCTGGTGCTGGTGACGGTGCTGGTGTGGGTG; >clamp_s_b_s_c_s:compl:rev
CACCCACACAGCCACTATCATGTTGACATTCC; dG = -10.1
894 >s_e_s_e_s_d_s_d_s_c_s GTGCTGGTGCTGGTGACGGTGACGGTGTGGGTG; >clamp_s_b_s_c_s:compl:rev
CACCCACACAGCCACTATCATGTTGACATTCC; dG = -10.1
895 >s_e_s_e_s_e_s_e_s_c_s GTGCTGGTGCTGGTGCTGGTGCTGGTGTGGGTG; >clamp_s_b_s_c_s:compl:rev
CACCCACACAGCCACTATCATGTTGACATTCC; dG = -10.1
896 >s_e_s_e_s_e_s_d_s_c_s GTGCTGGTGCTGGTGCTGGTGACGGTGTGGGTG; >clamp_s_b_s_c_s:compl:rev
CACCCACACAGCCACTATCATGTTGACATTCC; dG = -10.1

****

BD Matches

```
     1 >s_b_s_b_s_d_s_b_s_d_s  GTGGCTGTGGCTGTGACGGTGGCTGTGACGGTG;  >clamp_s_b_s_d_s:compl:rev
CACCGTCACAGCCACTATCATGTTGACATTCC; dG = -19.0
     2 >s_a_s_b_s_d_s_b_s_d_s  GTGTCTGTGGCTGTGACGGTGGCTGTGACGGTG;  >clamp_s_b_s_d_s:compl:rev
CACCGTCACAGCCACTATCATGTTGACATTCC; dG = -19.0
     3 >s_e_s_b_s_d_s_b_s_d_s  GTGCTGGTGGCTGTGACGGTGGCTGTGACGGTG;  >clamp_s_b_s_d_s:compl:rev
CACCGTCACAGCCACTATCATGTTGACATTCC; dG = -18.9
     4 >s_b_s_d_s_d_s_b_s_d_s  GTGGCTGTGACGGTGACGGTGGCTGTGACGGTG;  >clamp_s_b_s_d_s:compl:rev
CACCGTCACAGCCACTATCATGTTGACATTCC; dG = -18.9
     5 >s_c_s_b_s_d_s_b_s_d_s  GTGTGGGTGGCTGTGACGGTGGCTGTGACGGTG;  >clamp_s_b_s_d_s:compl:rev
CACCGTCACAGCCACTATCATGTTGACATTCC; dG = -18.9
     6 >s_d_s_b_s_d_s_b_s_d_s  GTGACGGTGGCTGTGACGGTGGCTGTGACGGTG;  >clamp_s_b_s_d_s:compl:rev
CACCGTCACAGCCACTATCATGTTGACATTCC; dG = -18.9
     7 >s_a_s_a_s_a_s_b_s_d_s  GTGTCTGTGTCTGTGTCTGTGGCTGTGACGGTG;  >clamp_s_b_s_d_s:compl:rev
CACCGTCACAGCCACTATCATGTTGACATTCC; dG = -18.6
     8 >s_a_s_b_s_e_s_b_s_d_s  GTGTCTGTGGCTGTGCTGGTGGCTGTGACGGTG;  >clamp_s_b_s_d_s:compl:rev
CACCGTCACAGCCACTATCATGTTGACATTCC; dG = -18.6
     9 >s_d_s_e_s_e_s_b_s_d_s  GTGACGGTGCTGGTGCTGGTGGCTGTGACGGTG;  >clamp_s_b_s_d_s:compl:rev
CACCGTCACAGCCACTATCATGTTGACATTCC; dG = -18.6
    10 >s_e_s_a_s_a_s_b_s_d_s  GTGCTGGTGTCTGTGTCTGTGGCTGTGACGGTG;  >clamp_s_b_s_d_s:compl:rev
CACCGTCACAGCCACTATCATGTTGACATTCC; dG = -18.6
    11 >s_e_s_a_s_b_s_b_s_d_s  GTGCTGGTGTCTGTGGCTGTGGCTGTGACGGTG;  >clamp_s_b_s_d_s:compl:rev
CACCGTCACAGCCACTATCATGTTGACATTCC; dG = -18.6
    12 >s_e_s_a_s_c_s_b_s_d_s  GTGCTGGTGTCTGTGTGGGTGGCTGTGACGGTG;  >clamp_s_b_s_d_s:compl:rev
CACCGTCACAGCCACTATCATGTTGACATTCC; dG = -18.6
    13 >s_e_s_a_s_d_s_b_s_d_s  GTGCTGGTGTCTGTGACGGTGGCTGTGACGGTG;  >clamp_s_b_s_d_s:compl:rev
CACCGTCACAGCCACTATCATGTTGACATTCC; dG = -18.6
    14 >s_e_s_a_s_e_s_b_s_d_s  GTGCTGGTGTCTGTGCTGGTGGCTGTGACGGTG;  >clamp_s_b_s_d_s:compl:rev
CACCGTCACAGCCACTATCATGTTGACATTCC; dG = -18.6
    15 >s_e_s_b_s_a_s_b_s_d_s  GTGCTGGTGGCTGTGTCTGTGGCTGTGACGGTG;  >clamp_s_b_s_d_s:compl:rev
CACCGTCACAGCCACTATCATGTTGACATTCC; dG = -18.6
    16 >s_e_s_b_s_b_s_b_s_d_s  GTGCTGGTGGCTGTGGCTGTGGCTGTGACGGTG;  >clamp_s_b_s_d_s:compl:rev
CACCGTCACAGCCACTATCATGTTGACATTCC; dG = -18.6
    17 >s_e_s_b_s_c_s_b_s_d_s  GTGCTGGTGGCTGTGTGGGTGGCTGTGACGGTG;  >clamp_s_b_s_d_s:compl:rev
CACCGTCACAGCCACTATCATGTTGACATTCC; dG = -18.6
    18 >s_a_s_c_s_a_s_b_s_d_s  GTGTCTGTGTGGGTGTCTGTGGCTGTGACGGTG;  >clamp_s_b_s_d_s:compl:rev
CACCGTCACAGCCACTATCATGTTGACATTCC; dG = -18.6
    19 >s_e_s_b_s_e_s_b_s_d_s  GTGCTGGTGGCTGTGCTGGTGGCTGTGACGGTG;  >clamp_s_b_s_d_s:compl:rev
CACCGTCACAGCCACTATCATGTTGACATTCC; dG = -18.6
    20 >s_e_s_c_s_a_s_b_s_d_s  GTGCTGGTGTGGGTGTCTGTGGCTGTGACGGTG;  >clamp_s_b_s_d_s:compl:rev
CACCGTCACAGCCACTATCATGTTGACATTCC; dG = -18.6
    21 >s_e_s_c_s_b_s_b_s_d_s  GTGCTGGTGTGGGTGGCTGTGGCTGTGACGGTG;  >clamp_s_b_s_d_s:compl:rev
CACCGTCACAGCCACTATCATGTTGACATTCC; dG = -18.6
    22 >s_e_s_c_s_c_s_b_s_d_s  GTGCTGGTGTGGGTGTGGGTGGCTGTGACGGTG;  >clamp_s_b_s_d_s:compl:rev
CACCGTCACAGCCACTATCATGTTGACATTCC; dG = -18.6
    23 >s_e_s_c_s_d_s_b_s_d_s  GTGCTGGTGTGGGTGACGGTGGCTGTGACGGTG;  >clamp_s_b_s_d_s:compl:rev
CACCGTCACAGCCACTATCATGTTGACATTCC; dG = -18.6
    24 >s_e_s_c_s_e_s_b_s_d_s  GTGCTGGTGTGGGTGCTGGTGGCTGTGACGGTG;  >clamp_s_b_s_d_s:compl:rev
CACCGTCACAGCCACTATCATGTTGACATTCC; dG = -18.6
    25 >s_e_s_d_s_a_s_b_s_d_s  GTGCTGGTGACGGTGTCTGTGGCTGTGACGGTG;  >clamp_s_b_s_d_s:compl:rev
CACCGTCACAGCCACTATCATGTTGACATTCC; dG = -18.6
    26 >s_e_s_d_s_b_s_b_s_d_s  GTGCTGGTGACGGTGGCTGTGGCTGTGACGGTG;  >clamp_s_b_s_d_s:compl:rev
CACCGTCACAGCCACTATCATGTTGACATTCC; dG = -18.6
    27 >s_e_s_d_s_c_s_b_s_d_s  GTGCTGGTGACGGTGTGGGTGGCTGTGACGGTG;  >clamp_s_b_s_d_s:compl:rev
CACCGTCACAGCCACTATCATGTTGACATTCC; dG = -18.6
    28 >s_e_s_d_s_d_s_b_s_d_s  GTGCTGGTGACGGTGACGGTGGCTGTGACGGTG;  >clamp_s_b_s_d_s:compl:rev
CACCGTCACAGCCACTATCATGTTGACATTCC; dG = -18.6
    29 >s_a_s_c_s_b_s_b_s_d_s  GTGTCTGTGTGGGTGGCTGTGGCTGTGACGGTG;  >clamp_s_b_s_d_s:compl:rev
CACCGTCACAGCCACTATCATGTTGACATTCC; dG = -18.6
    30 >s_e_s_d_s_e_s_b_s_d_s  GTGCTGGTGACGGTGCTGGTGGCTGTGACGGTG;  >clamp_s_b_s_d_s:compl:rev
CACCGTCACAGCCACTATCATGTTGACATTCC; dG = -18.6
    31 >s_e_s_e_s_a_s_b_s_d_s  GTGCTGGTGCTGGTGTCTGTGGCTGTGACGGTG;  >clamp_s_b_s_d_s:compl:rev
CACCGTCACAGCCACTATCATGTTGACATTCC; dG = -18.6
    32 >s_e_s_e_s_b_s_b_s_d_s  GTGCTGGTGCTGGTGGCTGTGGCTGTGACGGTG;  >clamp_s_b_s_d_s:compl:rev
CACCGTCACAGCCACTATCATGTTGACATTCC; dG = -18.6
    33 >s_e_s_e_s_c_s_b_s_d_s  GTGCTGGTGCTGGTGTGGGTGGCTGTGACGGTG;  >clamp_s_b_s_d_s:compl:rev
CACCGTCACAGCCACTATCATGTTGACATTCC; dG = -18.6
    34 >s_e_s_e_s_d_s_b_s_d_s  GTGCTGGTGCTGGTGACGGTGGCTGTGACGGTG;  >clamp_s_b_s_d_s:compl:rev
CACCGTCACAGCCACTATCATGTTGACATTCC; dG = -18.6
    35 >s_e_s_e_s_e_s_b_s_d_s  GTGCTGGTGCTGGTGCTGGTGGCTGTGACGGTG;  >clamp_s_b_s_d_s:compl:rev
CACCGTCACAGCCACTATCATGTTGACATTCC; dG = -18.6
    36 >s_a_s_c_s_c_s_b_s_d_s  GTGTCTGTGTGGGTGTGGGTGGCTGTGACGGTG;  >clamp_s_b_s_d_s:compl:rev
CACCGTCACAGCCACTATCATGTTGACATTCC; dG = -18.6
    37 >s_a_s_c_s_d_s_b_s_d_s  GTGTCTGTGTGGGTGACGGTGGCTGTGACGGTG;  >clamp_s_b_s_d_s:compl:rev
CACCGTCACAGCCACTATCATGTTGACATTCC; dG = -18.6
```

38 >s_a_s_c_s_e_s_b_s_d_s GTGTCTGTGTGGGTGCTGGTGGCTGTGACGGTG; >clamp_s_b_s_d_s:compl:rev
CACCGTCACAGCCACTATCATGTTGACATTCC; dG = -18.6
39 >s_a_s_d_s_a_s_b_s_d_s GTGTCTGTGACGGTGTCTGTGGCTGTGACGGTG; >clamp_s_b_s_d_s:compl:rev
CACCGTCACAGCCACTATCATGTTGACATTCC; dG = -18.6
40 >s_a_s_d_s_b_s_b_s_d_s GTGTCTGTGACGGTGGCTGTGGCTGTGACGGTG; >clamp_s_b_s_d_s:compl:rev
CACCGTCACAGCCACTATCATGTTGACATTCC; dG = -18.6
41 >s_a_s_d_s_c_s_b_s_d_s GTGTCTGTGACGGTGTGGGTGGCTGTGACGGTG; >clamp_s_b_s_d_s:compl:rev
CACCGTCACAGCCACTATCATGTTGACATTCC; dG = -18.6
42 >s_a_s_d_s_d_s_b_s_d_s GTGTCTGTGACGGTGACGGTGGCTGTGACGGTG; >clamp_s_b_s_d_s:compl:rev
CACCGTCACAGCCACTATCATGTTGACATTCC; dG = -18.6
43 >s_a_s_a_s_b_s_b_s_d_s GTGTCTGTGTCTGTGGCTGTGGCTGTGACGGTG; >clamp_s_b_s_d_s:compl:rev
CACCGTCACAGCCACTATCATGTTGACATTCC; dG = -18.6
44 >s_a_s_d_s_e_s_b_s_d_s GTGTCTGTGACGGTGCTGGTGGCTGTGACGGTG; >clamp_s_b_s_d_s:compl:rev
CACCGTCACAGCCACTATCATGTTGACATTCC; dG = -18.6
45 >s_a_s_e_s_a_s_b_s_d_s GTGTCTGTGCTGGTGTCTGTGGCTGTGACGGTG; >clamp_s_b_s_d_s:compl:rev
CACCGTCACAGCCACTATCATGTTGACATTCC; dG = -18.6
46 >s_a_s_e_s_b_s_b_s_d_s GTGTCTGTGCTGGTGGCTGTGGCTGTGACGGTG; >clamp_s_b_s_d_s:compl:rev
CACCGTCACAGCCACTATCATGTTGACATTCC; dG = -18.6
47 >s_a_s_e_s_c_s_b_s_d_s GTGTCTGTGCTGGTGTGGGTGGCTGTGACGGTG; >clamp_s_b_s_d_s:compl:rev
CACCGTCACAGCCACTATCATGTTGACATTCC; dG = -18.6
48 >s_a_s_e_s_d_s_b_s_d_s GTGTCTGTGCTGGTGACGGTGGCTGTGACGGTG; >clamp_s_b_s_d_s:compl:rev
CACCGTCACAGCCACTATCATGTTGACATTCC; dG = -18.6
49 >s_a_s_e_s_e_s_b_s_d_s GTGTCTGTGCTGGTGCTGGTGGCTGTGACGGTG; >clamp_s_b_s_d_s:compl:rev
CACCGTCACAGCCACTATCATGTTGACATTCC; dG = -18.6
50 >s_b_s_a_s_a_s_b_s_d_s GTGGCTGTGTCTGTGTCTGTGGCTGTGACGGTG; >clamp_s_b_s_d_s:compl:rev
CACCGTCACAGCCACTATCATGTTGACATTCC; dG = -18.6
51 >s_b_s_a_s_b_s_b_s_d_s GTGGCTGTGTCTGTGGCTGTGGCTGTGACGGTG; >clamp_s_b_s_d_s:compl:rev
CACCGTCACAGCCACTATCATGTTGACATTCC; dG = -18.6
52 >s_b_s_a_s_c_s_b_s_d_s GTGGCTGTGTCTGTGTGGGTGGCTGTGACGGTG; >clamp_s_b_s_d_s:compl:rev
CACCGTCACAGCCACTATCATGTTGACATTCC; dG = -18.6
53 >s_b_s_a_s_d_s_b_s_d_s GTGGCTGTGTCTGTGACGGTGGCTGTGACGGTG; >clamp_s_b_s_d_s:compl:rev
CACCGTCACAGCCACTATCATGTTGACATTCC; dG = -18.6
54 >s_a_s_a_s_c_s_b_s_d_s GTGTCTGTGTCTGTGTGGGTGGCTGTGACGGTG; >clamp_s_b_s_d_s:compl:rev
CACCGTCACAGCCACTATCATGTTGACATTCC; dG = -18.6
55 >s_b_s_a_s_e_s_b_s_d_s GTGGCTGTGTCTGTGCTGGTGGCTGTGACGGTG; >clamp_s_b_s_d_s:compl:rev
CACCGTCACAGCCACTATCATGTTGACATTCC; dG = -18.6
56 >s_b_s_b_s_a_s_b_s_d_s GTGGCTGTGGCTGTGTCTGTGGCTGTGACGGTG; >clamp_s_b_s_d_s:compl:rev
CACCGTCACAGCCACTATCATGTTGACATTCC; dG = -18.6
57 >s_b_s_b_s_b_s_b_s_d_s GTGGCTGTGGCTGTGGCTGTGGCTGTGACGGTG; >clamp_s_b_s_d_s:compl:rev
CACCGTCACAGCCACTATCATGTTGACATTCC; dG = -18.6
58 >s_b_s_b_s_c_s_b_s_d_s GTGGCTGTGGCTGTGTGGGTGGCTGTGACGGTG; >clamp_s_b_s_d_s:compl:rev
CACCGTCACAGCCACTATCATGTTGACATTCC; dG = -18.6
59 >s_b_s_b_s_e_s_b_s_d_s GTGGCTGTGGCTGTGCTGGTGGCTGTGACGGTG; >clamp_s_b_s_d_s:compl:rev
CACCGTCACAGCCACTATCATGTTGACATTCC; dG = -18.6
60 >s_b_s_c_s_a_s_b_s_d_s GTGGCTGTGTGGGTGTCTGTGGCTGTGACGGTG; >clamp_s_b_s_d_s:compl:rev
CACCGTCACAGCCACTATCATGTTGACATTCC; dG = -18.6
61 >s_b_s_c_s_b_s_b_s_d_s GTGGCTGTGTGGGTGGCTGTGGCTGTGACGGTG; >clamp_s_b_s_d_s:compl:rev
CACCGTCACAGCCACTATCATGTTGACATTCC; dG = -18.6
62 >s_b_s_c_s_c_s_b_s_d_s GTGGCTGTGTGGGTGTGGGTGGCTGTGACGGTG; >clamp_s_b_s_d_s:compl:rev
CACCGTCACAGCCACTATCATGTTGACATTCC; dG = -18.6
63 >s_b_s_c_s_d_s_b_s_d_s GTGGCTGTGTGGGTGACGGTGGCTGTGACGGTG; >clamp_s_b_s_d_s:compl:rev
CACCGTCACAGCCACTATCATGTTGACATTCC; dG = -18.6
64 >s_a_s_a_s_d_s_b_s_d_s GTGTCTGTGTCTGTGACGGTGGCTGTGACGGTG; >clamp_s_b_s_d_s:compl:rev
CACCGTCACAGCCACTATCATGTTGACATTCC; dG = -18.6
65 >s_b_s_c_s_e_s_b_s_d_s GTGGCTGTGTGGGTGCTGGTGGCTGTGACGGTG; >clamp_s_b_s_d_s:compl:rev
CACCGTCACAGCCACTATCATGTTGACATTCC; dG = -18.6
66 >s_b_s_d_s_a_s_b_s_d_s GTGGCTGTGACGGTGTCTGTGGCTGTGACGGTG; >clamp_s_b_s_d_s:compl:rev
CACCGTCACAGCCACTATCATGTTGACATTCC; dG = -18.6
67 >s_b_s_d_s_b_s_b_s_d_s GTGGCTGTGACGGTGGCTGTGGCTGTGACGGTG; >clamp_s_b_s_d_s:compl:rev
CACCGTCACAGCCACTATCATGTTGACATTCC; dG = -18.6
68 >s_b_s_d_s_c_s_b_s_d_s GTGGCTGTGACGGTGTGGGTGGCTGTGACGGTG; >clamp_s_b_s_d_s:compl:rev
CACCGTCACAGCCACTATCATGTTGACATTCC; dG = -18.6
69 >s_b_s_d_s_e_s_b_s_d_s GTGGCTGTGACGGTGCTGGTGGCTGTGACGGTG; >clamp_s_b_s_d_s:compl:rev
CACCGTCACAGCCACTATCATGTTGACATTCC; dG = -18.6
70 >s_b_s_e_s_a_s_b_s_d_s GTGGCTGTGCTGGTGTCTGTGGCTGTGACGGTG; >clamp_s_b_s_d_s:compl:rev
CACCGTCACAGCCACTATCATGTTGACATTCC; dG = -18.6
71 >s_b_s_e_s_b_s_b_s_d_s GTGGCTGTGCTGGTGGCTGTGGCTGTGACGGTG; >clamp_s_b_s_d_s:compl:rev
CACCGTCACAGCCACTATCATGTTGACATTCC; dG = -18.6
72 >s_b_s_e_s_c_s_b_s_d_s GTGGCTGTGCTGGTGTGGGTGGCTGTGACGGTG; >clamp_s_b_s_d_s:compl:rev
CACCGTCACAGCCACTATCATGTTGACATTCC; dG = -18.6
73 >s_b_s_e_s_d_s_b_s_d_s GTGGCTGTGCTGGTGACGGTGGCTGTGACGGTG; >clamp_s_b_s_d_s:compl:rev
CACCGTCACAGCCACTATCATGTTGACATTCC; dG = -18.6
74 >s_a_s_a_s_e_s_b_s_d_s GTGTCTGTGTCTGTGCTGGTGGCTGTGACGGTG; >clamp_s_b_s_d_s:compl:rev
CACCGTCACAGCCACTATCATGTTGACATTCC; dG = -18.6

75 >s_b_s_e_s_e_s_b_s_d_s GTGGCTGTGCTGGTGCTGGTGGCTGTGACGGTG; >clamp_s_b_s_d_s:compl:rev CACCGTCACAGCCACTATCATGTTGACATTCC; dG = -18.6

76 >s_c_s_a_s_a_s_b_s_d_s GTGTGGGTGTCTGTGTCTGTGGCTGTGACGGTG; >clamp_s_b_s_d_s:compl:rev CACCGTCACAGCCACTATCATGTTGACATTCC; dG = -18.6

77 >s_c_s_a_s_b_s_b_s_d_s GTGTGGGTGTCTGTGGCTGTGGCTGTGACGGTG; >clamp_s_b_s_d_s:compl:rev CACCGTCACAGCCACTATCATGTTGACATTCC; dG = -18.6

78 >s_c_s_a_s_c_s_b_s_d_s GTGTGGGTGTCTGTGTGGGTGGCTGTGACGGTG; >clamp_s_b_s_d_s:compl:rev CACCGTCACAGCCACTATCATGTTGACATTCC; dG = -18.6

79 >s_c_s_a_s_d_s_b_s_d_s GTGTGGGTGTCTGTGACGGTGGCTGTGACGGTG; >clamp_s_b_s_d_s:compl:rev CACCGTCACAGCCACTATCATGTTGACATTCC; dG = -18.6

80 >s_c_s_a_s_e_s_b_s_d_s GTGTGGGTGTCTGTGCTGGTGGCTGTGACGGTG; >clamp_s_b_s_d_s:compl:rev CACCGTCACAGCCACTATCATGTTGACATTCC; dG = -18.6

81 >s_c_s_b_s_a_s_b_s_d_s GTGTGGGTGGCTGTGTCTGTGGCTGTGACGGTG; >clamp_s_b_s_d_s:compl:rev CACCGTCACAGCCACTATCATGTTGACATTCC; dG = -18.6

82 >s_c_s_b_s_b_s_b_s_d_s GTGTGGGTGGCTGTGGCTGTGGCTGTGACGGTG; >clamp_s_b_s_d_s:compl:rev CACCGTCACAGCCACTATCATGTTGACATTCC; dG = -18.6

83 >s_c_s_b_s_c_s_b_s_d_s GTGTGGGTGGCTGTGTGGGTGGCTGTGACGGTG; >clamp_s_b_s_d_s:compl:rev CACCGTCACAGCCACTATCATGTTGACATTCC; dG = -18.6

84 >s_a_s_b_s_a_s_b_s_d_s GTGTCTGTGGCTGTGTCTGTGGCTGTGACGGTG; >clamp_s_b_s_d_s:compl:rev CACCGTCACAGCCACTATCATGTTGACATTCC; dG = -18.6

85 >s_c_s_b_s_e_s_b_s_d_s GTGTGGGTGGCTGTGCTGGTGGCTGTGACGGTG; >clamp_s_b_s_d_s:compl:rev CACCGTCACAGCCACTATCATGTTGACATTCC; dG = -18.6

86 >s_c_s_c_s_a_s_b_s_d_s GTGTGGGTGTGGGTGTCTGTGGCTGTGACGGTG; >clamp_s_b_s_d_s:compl:rev CACCGTCACAGCCACTATCATGTTGACATTCC; dG = -18.6

87 >s_c_s_c_s_b_s_b_s_d_s GTGTGGGTGTGGGTGGCTGTGGCTGTGACGGTG; >clamp_s_b_s_d_s:compl:rev CACCGTCACAGCCACTATCATGTTGACATTCC; dG = -18.6

88 >s_c_s_c_s_c_s_b_s_d_s GTGTGGGTGTGGGTGTGGGTGGCTGTGACGGTG; >clamp_s_b_s_d_s:compl:rev CACCGTCACAGCCACTATCATGTTGACATTCC; dG = -18.6

89 >s_c_s_c_s_d_s_b_s_d_s GTGTGGGTGTGGGTGACGGTGGCTGTGACGGTG; >clamp_s_b_s_d_s:compl:rev CACCGTCACAGCCACTATCATGTTGACATTCC; dG = -18.6

90 >s_c_s_c_s_e_s_b_s_d_s GTGTGGGTGTGGGTGCTGGTGGCTGTGACGGTG; >clamp_s_b_s_d_s:compl:rev CACCGTCACAGCCACTATCATGTTGACATTCC; dG = -18.6

91 >s_c_s_d_s_a_s_b_s_d_s GTGTGGGTGACGGTGTCTGTGGCTGTGACGGTG; >clamp_s_b_s_d_s:compl:rev CACCGTCACAGCCACTATCATGTTGACATTCC; dG = -18.6

92 >s_c_s_d_s_b_s_b_s_d_s GTGTGGGTGACGGTGGCTGTGGCTGTGACGGTG; >clamp_s_b_s_d_s:compl:rev CACCGTCACAGCCACTATCATGTTGACATTCC; dG = -18.6

93 >s_c_s_d_s_c_s_b_s_d_s GTGTGGGTGACGGTGTGGGTGGCTGTGACGGTG; >clamp_s_b_s_d_s:compl:rev CACCGTCACAGCCACTATCATGTTGACATTCC; dG = -18.6

94 >s_c_s_d_s_d_s_b_s_d_s GTGTGGGTGACGGTGACGGTGGCTGTGACGGTG; >clamp_s_b_s_d_s:compl:rev CACCGTCACAGCCACTATCATGTTGACATTCC; dG = -18.6

95 >s_a_s_b_s_b_s_b_s_d_s GTGTCTGTGGCTGTGGCTGTGGCTGTGACGGTG; >clamp_s_b_s_d_s:compl:rev CACCGTCACAGCCACTATCATGTTGACATTCC; dG = -18.6

96 >s_c_s_d_s_e_s_b_s_d_s GTGTGGGTGACGGTGCTGGTGGCTGTGACGGTG; >clamp_s_b_s_d_s:compl:rev CACCGTCACAGCCACTATCATGTTGACATTCC; dG = -18.6

97 >s_c_s_e_s_a_s_b_s_d_s GTGTGGGTGCTGGTGTCTGTGGCTGTGACGGTG; >clamp_s_b_s_d_s:compl:rev CACCGTCACAGCCACTATCATGTTGACATTCC; dG = -18.6

98 >s_c_s_e_s_b_s_b_s_d_s GTGTGGGTGCTGGTGGCTGTGGCTGTGACGGTG; >clamp_s_b_s_d_s:compl:rev CACCGTCACAGCCACTATCATGTTGACATTCC; dG = -18.6

99 >s_c_s_e_s_c_s_b_s_d_s GTGTGGGTGCTGGTGTGGGTGGCTGTGACGGTG; >clamp_s_b_s_d_s:compl:rev CACCGTCACAGCCACTATCATGTTGACATTCC; dG = -18.6

100 >s_c_s_e_s_d_s_b_s_d_s GTGTGGGTGCTGGTGACGGTGGCTGTGACGGTG; >clamp_s_b_s_d_s:compl:rev CACCGTCACAGCCACTATCATGTTGACATTCC; dG = -18.6

101 >s_c_s_e_s_e_s_b_s_d_s GTGTGGGTGCTGGTGCTGGTGGCTGTGACGGTG; >clamp_s_b_s_d_s:compl:rev CACCGTCACAGCCACTATCATGTTGACATTCC; dG = -18.6

102 >s_d_s_a_s_a_s_b_s_d_s GTGACGGTGTCTGTGTCTGTGGCTGTGACGGTG; >clamp_s_b_s_d_s:compl:rev CACCGTCACAGCCACTATCATGTTGACATTCC; dG = -18.6

103 >s_d_s_a_s_b_s_b_s_d_s GTGACGGTGTCTGTGGCTGTGGCTGTGACGGTG; >clamp_s_b_s_d_s:compl:rev CACCGTCACAGCCACTATCATGTTGACATTCC; dG = -18.6

104 >s_d_s_a_s_c_s_b_s_d_s GTGACGGTGTCTGTGTGGGTGGCTGTGACGGTG; >clamp_s_b_s_d_s:compl:rev CACCGTCACAGCCACTATCATGTTGACATTCC; dG = -18.6

105 >s_d_s_a_s_d_s_b_s_d_s GTGACGGTGTCTGTGACGGTGGCTGTGACGGTG; >clamp_s_b_s_d_s:compl:rev CACCGTCACAGCCACTATCATGTTGACATTCC; dG = -18.6

106 >s_a_s_b_s_c_s_b_s_d_s GTGTCTGTGGCTGTGTGGGTGGCTGTGACGGTG; >clamp_s_b_s_d_s:compl:rev CACCGTCACAGCCACTATCATGTTGACATTCC; dG = -18.6

107 >s_d_s_a_s_e_s_b_s_d_s GTGACGGTGTCTGTGCTGGTGGCTGTGACGGTG; >clamp_s_b_s_d_s:compl:rev CACCGTCACAGCCACTATCATGTTGACATTCC; dG = -18.6

108 >s_d_s_b_s_a_s_b_s_d_s GTGACGGTGGCTGTGTCTGTGGCTGTGACGGTG; >clamp_s_b_s_d_s:compl:rev CACCGTCACAGCCACTATCATGTTGACATTCC; dG = -18.6

109 >s_d_s_b_s_b_s_b_s_d_s GTGACGGTGGCTGTGGCTGTGGCTGTGACGGTG; >clamp_s_b_s_d_s:compl:rev CACCGTCACAGCCACTATCATGTTGACATTCC; dG = -18.6

110 >s_d_s_b_s_c_s_b_s_d_s GTGACGGTGGCTGTGTGGGTGGCTGTGACGGTG; >clamp_s_b_s_d_s:compl:rev CACCGTCACAGCCACTATCATGTTGACATTCC; dG = -18.6

111 >s_d_s_b_s_e_s_b_s_d_s GTGACGGTGGCTGTGCTGGTGGCTGTGACGGTG; >clamp_s_b_s_d_s:compl:rev CACCGTCACAGCCACTATCATGTTGACATTCC; dG = -18.6

112 >s_d_s_c_s_a_s_b_s_d_s GTGACGGTGTGGGTGTCTGTGGCTGTGACGGTG; >clamp_s_b_s_d_s:compl:rev CACCGTCACAGCCACTATCATGTTGACATTCC; dG = -18.6
113 >s_d_s_c_s_b_s_b_s_d_s GTGACGGTGTGGGTGGCTGTGGCTGTGACGGTG; >clamp_s_b_s_d_s:compl:rev CACCGTCACAGCCACTATCATGTTGACATTCC; dG = -18.6
114 >s_d_s_c_s_c_s_b_s_d_s GTGACGGTGTGGGTGTGGGTGGCTGTGACGGTG; >clamp_s_b_s_d_s:compl:rev CACCGTCACAGCCACTATCATGTTGACATTCC; dG = -18.6
115 >s_d_s_c_s_d_s_b_s_d_s GTGACGGTGTGGGTGACGGTGGCTGTGACGGTG; >clamp_s_b_s_d_s:compl:rev CACCGTCACAGCCACTATCATGTTGACATTCC; dG = -18.6
116 >s_d_s_c_s_e_s_b_s_d_s GTGACGGTGTGGGTGCTGGTGGCTGTGACGGTG; >clamp_s_b_s_d_s:compl:rev CACCGTCACAGCCACTATCATGTTGACATTCC; dG = -18.6
117 >s_d_s_d_s_a_s_b_s_d_s GTGACGGTGACGGTGTCTGTGGCTGTGACGGTG; >clamp_s_b_s_d_s:compl:rev CACCGTCACAGCCACTATCATGTTGACATTCC; dG = -18.6
118 >s_d_s_d_s_b_s_b_s_d_s GTGACGGTGACGGTGGCTGTGGCTGTGACGGTG; >clamp_s_b_s_d_s:compl:rev CACCGTCACAGCCACTATCATGTTGACATTCC; dG = -18.6
119 >s_d_s_d_s_c_s_b_s_d_s GTGACGGTGACGGTGTGGGTGGCTGTGACGGTG; >clamp_s_b_s_d_s:compl:rev CACCGTCACAGCCACTATCATGTTGACATTCC; dG = -18.6
120 >s_d_s_d_s_d_s_b_s_d_s GTGACGGTGACGGTGACGGTGGCTGTGACGGTG; >clamp_s_b_s_d_s:compl:rev CACCGTCACAGCCACTATCATGTTGACATTCC; dG = -18.6
121 >s_d_s_d_s_e_s_b_s_d_s GTGACGGTGACGGTGCTGGTGGCTGTGACGGTG; >clamp_s_b_s_d_s:compl:rev CACCGTCACAGCCACTATCATGTTGACATTCC; dG = -18.6
122 >s_d_s_e_s_a_s_b_s_d_s GTGACGGTGCTGGTGTCTGTGGCTGTGACGGTG; >clamp_s_b_s_d_s:compl:rev CACCGTCACAGCCACTATCATGTTGACATTCC; dG = -18.6
123 >s_d_s_e_s_b_s_b_s_d_s GTGACGGTGCTGGTGGCTGTGGCTGTGACGGTG; >clamp_s_b_s_d_s:compl:rev CACCGTCACAGCCACTATCATGTTGACATTCC; dG = -18.6
124 >s_d_s_e_s_c_s_b_s_d_s GTGACGGTGCTGGTGTGGGTGGCTGTGACGGTG; >clamp_s_b_s_d_s:compl:rev CACCGTCACAGCCACTATCATGTTGACATTCC; dG = -18.6
125 >s_d_s_e_s_d_s_b_s_d_s GTGACGGTGCTGGTGACGGTGGCTGTGACGGTG; >clamp_s_b_s_d_s:compl:rev CACCGTCACAGCCACTATCATGTTGACATTCC; dG = -18.6

****

BD Mismatches

1 >s_b_s_b_s_a_s_d_s_d_s GTGGCTGTGGCTGTGTCTGTGACGGTGACGGTG; >clamp_s_b_s_d_s:compl:rev CACCGTCACAGCCACTATCATGTTGACATTCC; dG = -14.2
2 >s_a_s_b_s_a_s_d_s_d_s GTGTCTGTGGCTGTGTCTGTGACGGTGACGGTG; >clamp_s_b_s_d_s:compl:rev CACCGTCACAGCCACTATCATGTTGACATTCC; dG = -14.2
3 >s_b_s_b_s_c_s_d_s_d_s GTGGCTGTGGCTGTGTGGGTGACGGTGACGGTG; >clamp_s_b_s_d_s:compl:rev CACCGTCACAGCCACTATCATGTTGACATTCC; dG = -14.1
4 >s_b_s_b_s_e_s_d_s_d_s GTGGCTGTGGCTGTGCTGGTGACGGTGACGGTG; >clamp_s_b_s_d_s:compl:rev CACCGTCACAGCCACTATCATGTTGACATTCC; dG = -14.1
5 >s_c_s_b_s_a_s_d_s_d_s GTGTGGGTGGCTGTGTCTGTGACGGTGACGGTG; >clamp_s_b_s_d_s:compl:rev CACCGTCACAGCCACTATCATGTTGACATTCC; dG = -14.1
6 >s_c_s_b_s_c_s_d_s_d_s GTGTGGGTGGCTGTGTGGGTGACGGTGACGGTG; >clamp_s_b_s_d_s:compl:rev CACCGTCACAGCCACTATCATGTTGACATTCC; dG = -14.1
7 >s_c_s_b_s_e_s_d_s_d_s GTGTGGGTGGCTGTGCTGGTGACGGTGACGGTG; >clamp_s_b_s_d_s:compl:rev CACCGTCACAGCCACTATCATGTTGACATTCC; dG = -14.1
8 >s_d_s_b_s_a_s_d_s_d_s GTGACGGTGGCTGTGTCTGTGACGGTGACGGTG; >clamp_s_b_s_d_s:compl:rev CACCGTCACAGCCACTATCATGTTGACATTCC; dG = -14.1
9 >s_d_s_b_s_c_s_d_s_d_s GTGACGGTGGCTGTGTGGGTGACGGTGACGGTG; >clamp_s_b_s_d_s:compl:rev CACCGTCACAGCCACTATCATGTTGACATTCC; dG = -14.1
10 >s_d_s_b_s_e_s_d_s_d_s GTGACGGTGGCTGTGCTGGTGACGGTGACGGTG; >clamp_s_b_s_d_s:compl:rev CACCGTCACAGCCACTATCATGTTGACATTCC; dG = -14.1
11 >s_a_s_b_s_c_s_d_s_d_s GTGTCTGTGGCTGTGTGGGTGACGGTGACGGTG; >clamp_s_b_s_d_s:compl:rev CACCGTCACAGCCACTATCATGTTGACATTCC; dG = -14.1
12 >s_a_s_b_s_e_s_d_s_d_s GTGTCTGTGGCTGTGCTGGTGACGGTGACGGTG; >clamp_s_b_s_d_s:compl:rev CACCGTCACAGCCACTATCATGTTGACATTCC; dG = -14.1
13 >s_e_s_b_s_a_s_d_s_d_s GTGCTGGTGGCTGTGTCTGTGACGGTGACGGTG; >clamp_s_b_s_d_s:compl:rev CACCGTCACAGCCACTATCATGTTGACATTCC; dG = -14.1
14 >s_e_s_b_s_c_s_d_s_d_s GTGCTGGTGGCTGTGTGGGTGACGGTGACGGTG; >clamp_s_b_s_d_s:compl:rev CACCGTCACAGCCACTATCATGTTGACATTCC; dG = -14.1
15 >s_e_s_b_s_e_s_d_s_d_s GTGCTGGTGGCTGTGCTGGTGACGGTGACGGTG; >clamp_s_b_s_d_s:compl:rev CACCGTCACAGCCACTATCATGTTGACATTCC; dG = -14.1
16 >s_b_s_a_s_a_s_d_s_d_s GTGGCTGTGTCTGTGTCTGTGACGGTGACGGTG; >clamp_s_b_s_d_s:compl:rev CACCGTCACAGCCACTATCATGTTGACATTCC; dG = -13.8
17 >s_b_s_a_s_d_s_d_s_d_s GTGGCTGTGTCTGTGACGGTGACGGTGACGGTG; >clamp_s_b_s_d_s:compl:rev CACCGTCACAGCCACTATCATGTTGACATTCC; dG = -13.8
18 >s_a_s_a_s_d_s_d_s_d_s GTGTCTGTGTCTGTGACGGTGACGGTGACGGTG; >clamp_s_b_s_d_s:compl:rev CACCGTCACAGCCACTATCATGTTGACATTCC; dG = -13.8
19 >s_c_s_a_s_a_s_d_s_d_s GTGTGGGTGTCTGTGTCTGTGACGGTGACGGTG; >clamp_s_b_s_d_s:compl:rev CACCGTCACAGCCACTATCATGTTGACATTCC; dG = -13.8

20 >s_d_s_a_s_a_s_d_s_d_s GTGACGGTGTCTGTGTCTGTGACGGTGACGGTG; >clamp_s_b_s_d_s:compl:rev
CACCGTCACAGCCACTATCATGTTGACATTCC; dG = -13.8
21 >s_a_s_a_s_a_s_d_s_d_s GTGTCTGTGTCTGTGTCTGTGACGGTGACGGTG; >clamp_s_b_s_d_s:compl:rev
CACCGTCACAGCCACTATCATGTTGACATTCC; dG = -13.8
22 >s_e_s_a_s_a_s_d_s_d_s GTGCTGGTGTCTGTGTCTGTGACGGTGACGGTG; >clamp_s_b_s_d_s:compl:rev
CACCGTCACAGCCACTATCATGTTGACATTCC; dG = -13.8
23 >s_a_s_d_s_a_s_d_s_d_s GTGTCTGTGACGGTGTCTGTGACGGTGACGGTG; >clamp_s_b_s_d_s:compl:rev
CACCGTCACAGCCACTATCATGTTGACATTCC; dG = -13.7
24 >s_a_s_e_s_a_s_d_s_d_s GTGTCTGTGCTGGTGTCTGTGACGGTGACGGTG; >clamp_s_b_s_d_s:compl:rev
CACCGTCACAGCCACTATCATGTTGACATTCC; dG = -13.7
25 >s_b_s_c_s_a_s_d_s_d_s GTGGCTGTGTGGGTGTCTGTGACGGTGACGGTG; >clamp_s_b_s_d_s:compl:rev
CACCGTCACAGCCACTATCATGTTGACATTCC; dG = -13.7
26 >s_b_s_e_s_a_s_d_s_d_s GTGGCTGTGCTGGTGTCTGTGACGGTGACGGTG; >clamp_s_b_s_d_s:compl:rev
CACCGTCACAGCCACTATCATGTTGACATTCC; dG = -13.7
27 >s_c_s_a_s_d_s_d_s_d_s GTGTGGGTGTCTGTGACGGTGACGGTGACGGTG; >clamp_s_b_s_d_s:compl:rev
CACCGTCACAGCCACTATCATGTTGACATTCC; dG = -13.7
28 >s_c_s_c_s_a_s_d_s_d_s GTGTGGGTGTGGGTGTCTGTGACGGTGACGGTG; >clamp_s_b_s_d_s:compl:rev
CACCGTCACAGCCACTATCATGTTGACATTCC; dG = -13.7
29 >s_c_s_d_s_a_s_d_s_d_s GTGTGGGTGACGGTGTCTGTGACGGTGACGGTG; >clamp_s_b_s_d_s:compl:rev
CACCGTCACAGCCACTATCATGTTGACATTCC; dG = -13.7
30 >s_c_s_e_s_a_s_d_s_d_s GTGTGGGTGCTGGTGTCTGTGACGGTGACGGTG; >clamp_s_b_s_d_s:compl:rev
CACCGTCACAGCCACTATCATGTTGACATTCC; dG = -13.7
31 >s_d_s_a_s_d_s_d_s_d_s GTGACGGTGTCTGTGACGGTGACGGTGACGGTG; >clamp_s_b_s_d_s:compl:rev
CACCGTCACAGCCACTATCATGTTGACATTCC; dG = -13.7
32 >s_d_s_c_s_a_s_d_s_d_s GTGACGGTGTGGGTGTCTGTGACGGTGACGGTG; >clamp_s_b_s_d_s:compl:rev
CACCGTCACAGCCACTATCATGTTGACATTCC; dG = -13.7
33 >s_d_s_d_s_a_s_d_s_d_s GTGACGGTGACGGTGTCTGTGACGGTGACGGTG; >clamp_s_b_s_d_s:compl:rev
CACCGTCACAGCCACTATCATGTTGACATTCC; dG = -13.7
34 >s_d_s_e_s_a_s_d_s_d_s GTGACGGTGCTGGTGTCTGTGACGGTGACGGTG; >clamp_s_b_s_d_s:compl:rev
CACCGTCACAGCCACTATCATGTTGACATTCC; dG = -13.7
35 >s_e_s_a_s_d_s_d_s_d_s GTGCTGGTGTCTGTGACGGTGACGGTGACGGTG; >clamp_s_b_s_d_s:compl:rev
CACCGTCACAGCCACTATCATGTTGACATTCC; dG = -13.7
36 >s_a_s_c_s_a_s_d_s_d_s GTGTCTGTGTGGGTGTCTGTGACGGTGACGGTG; >clamp_s_b_s_d_s:compl:rev
CACCGTCACAGCCACTATCATGTTGACATTCC; dG = -13.7
37 >s_e_s_c_s_a_s_d_s_d_s GTGCTGGTGTGGGTGTCTGTGACGGTGACGGTG; >clamp_s_b_s_d_s:compl:rev
CACCGTCACAGCCACTATCATGTTGACATTCC; dG = -13.7
38 >s_e_s_d_s_a_s_d_s_d_s GTGCTGGTGACGGTGTCTGTGACGGTGACGGTG; >clamp_s_b_s_d_s:compl:rev
CACCGTCACAGCCACTATCATGTTGACATTCC; dG = -13.7
39 >s_e_s_e_s_a_s_d_s_d_s GTGCTGGTGCTGGTGTCTGTGACGGTGACGGTG; >clamp_s_b_s_d_s:compl:rev
CACCGTCACAGCCACTATCATGTTGACATTCC; dG = -13.7
40 >s_b_s_c_s_d_s_d_s_d_s GTGGCTGTGTGGGTGACGGTGACGGTGACGGTG; >clamp_s_b_s_d_s:compl:rev
CACCGTCACAGCCACTATCATGTTGACATTCC; dG = -13.6
41 >s_b_s_e_s_d_s_d_s_d_s GTGGCTGTGCTGGTGACGGTGACGGTGACGGTG; >clamp_s_b_s_d_s:compl:rev
CACCGTCACAGCCACTATCATGTTGACATTCC; dG = -13.6
42 >s_a_s_d_s_b_s_e_s_d_s GTGTCTGTGACGGTGGCTGTGCTGGTGACGGTG; >clamp_s_b_s_d_s:compl:rev
CACCGTCACAGCCACTATCATGTTGACATTCC; dG = -13.3
43 >s_a_s_d_s_b_s_c_s_d_s GTGTCTGTGACGGTGGCTGTGTGGGTGACGGTG; >clamp_s_b_s_d_s:compl:rev
CACCGTCACAGCCACTATCATGTTGACATTCC; dG = -13.3
44 >s_a_s_d_s_b_s_a_s_d_s GTGTCTGTGACGGTGGCTGTGTCTGTGACGGTG; >clamp_s_b_s_d_s:compl:rev
CACCGTCACAGCCACTATCATGTTGACATTCC; dG = -13.3
45 >s_a_s_a_s_b_s_e_s_d_s GTGTCTGTGTCTGTGGCTGTGCTGGTGACGGTG; >clamp_s_b_s_d_s:compl:rev
CACCGTCACAGCCACTATCATGTTGACATTCC; dG = -13.3
46 >s_a_s_d_s_d_s_b_s_e_s GTGTCTGTGACGGTGACGGTGGCTGTGCTGGTG; >clamp_s_b_s_d_s:compl:rev
CACCGTCACAGCCACTATCATGTTGACATTCC; dG = -13.3
47 >s_a_s_d_s_d_s_b_s_c_s GTGTCTGTGACGGTGACGGTGGCTGTGTGGGTG; >clamp_s_b_s_d_s:compl:rev
CACCGTCACAGCCACTATCATGTTGACATTCC; dG = -13.3
48 >s_a_s_d_s_d_s_b_s_b_s GTGTCTGTGACGGTGACGGTGGCTGTGGCTGTG; >clamp_s_b_s_d_s:compl:rev
CACCGTCACAGCCACTATCATGTTGACATTCC; dG = -13.3
49 >s_a_s_d_s_d_s_b_s_a_s GTGTCTGTGACGGTGACGGTGGCTGTGTCTGTG; >clamp_s_b_s_d_s:compl:rev
CACCGTCACAGCCACTATCATGTTGACATTCC; dG = -13.3
50 >s_a_s_d_s_d_s_e_s_d_s GTGTCTGTGACGGTGACGGTGCTGGTGACGGTG; >clamp_s_b_s_d_s:compl:rev
CACCGTCACAGCCACTATCATGTTGACATTCC; dG = -13.3
51 >s_a_s_d_s_d_s_d_s_d_s GTGTCTGTGACGGTGACGGTGACGGTGACGGTG; >clamp_s_b_s_d_s:compl:rev
CACCGTCACAGCCACTATCATGTTGACATTCC; dG = -13.3
52 >s_a_s_d_s_d_s_c_s_d_s GTGTCTGTGACGGTGACGGTGTGGGTGACGGTG; >clamp_s_b_s_d_s:compl:rev
CACCGTCACAGCCACTATCATGTTGACATTCC; dG = -13.3
53 >s_a_s_a_s_b_s_c_s_d_s GTGTCTGTGTCTGTGGCTGTGTGGGTGACGGTG; >clamp_s_b_s_d_s:compl:rev
CACCGTCACAGCCACTATCATGTTGACATTCC; dG = -13.3
54 >s_a_s_d_s_d_s_a_s_d_s GTGTCTGTGACGGTGACGGTGTCTGTGACGGTG; >clamp_s_b_s_d_s:compl:rev
CACCGTCACAGCCACTATCATGTTGACATTCC; dG = -13.3
55 >s_a_s_a_s_b_s_a_s_d_s GTGTCTGTGTCTGTGGCTGTGTCTGTGACGGTG; >clamp_s_b_s_d_s:compl:rev
CACCGTCACAGCCACTATCATGTTGACATTCC; dG = -13.3
56 >s_a_s_e_s_b_s_e_s_d_s GTGTCTGTGCTGGTGGCTGTGCTGGTGACGGTG; >clamp_s_b_s_d_s:compl:rev
CACCGTCACAGCCACTATCATGTTGACATTCC; dG = -13.3

57 >s_a_s_e_s_b_s_c_s_d_s GTGTCTGTGCTGGTGGCTGTGTGGGTGACGGTG; >clamp_s_b_s_d_s:compl:rev
CACCGTCACAGCCACTATCATGTTGACATTCC; dG = -13.3
58 >s_a_s_e_s_b_s_a_s_d_s GTGTCTGTGCTGGTGGCTGTGTCTGTGACGGTG; >clamp_s_b_s_d_s:compl:rev
CACCGTCACAGCCACTATCATGTTGACATTCC; dG = -13.3
59 >s_b_s_a_s_b_s_e_s_d_s GTGGCTGTGTCTGTGGCTGTGCTGGTGACGGTG; >clamp_s_b_s_d_s:compl:rev
CACCGTCACAGCCACTATCATGTTGACATTCC; dG = -13.3
60 >s_b_s_a_s_b_s_c_s_d_s GTGGCTGTGTCTGTGGCTGTGTGGGTGACGGTG; >clamp_s_b_s_d_s:compl:rev
CACCGTCACAGCCACTATCATGTTGACATTCC; dG = -13.3
61 >s_b_s_a_s_b_s_a_s_d_s GTGGCTGTGTCTGTGGCTGTGTCTGTGACGGTG; >clamp_s_b_s_d_s:compl:rev
CACCGTCACAGCCACTATCATGTTGACATTCC; dG = -13.3
62 >s_b_s_a_s_d_s_b_s_e_s GTGGCTGTGTCTGTGACGGTGGCTGTGCTGGTG; >clamp_s_b_s_d_s:compl:rev
CACCGTCACAGCCACTATCATGTTGACATTCC; dG = -13.3
63 >s_b_s_a_s_d_s_b_s_c_s GTGGCTGTGTCTGTGACGGTGGCTGTGTGGGTG; >clamp_s_b_s_d_s:compl:rev
CACCGTCACAGCCACTATCATGTTGACATTCC; dG = -13.3
64 >s_b_s_a_s_d_s_b_s_b_s GTGGCTGTGTCTGTGACGGTGGCTGTGGCTGTG; >clamp_s_b_s_d_s:compl:rev
CACCGTCACAGCCACTATCATGTTGACATTCC; dG = -13.3
65 >s_b_s_a_s_d_s_b_s_a_s GTGGCTGTGTCTGTGACGGTGGCTGTGTCTGTG; >clamp_s_b_s_d_s:compl:rev
CACCGTCACAGCCACTATCATGTTGACATTCC; dG = -13.3
66 >s_b_s_b_s_b_s_e_s_d_s GTGGCTGTGGCTGTGGCTGTGCTGGTGACGGTG; >clamp_s_b_s_d_s:compl:rev
CACCGTCACAGCCACTATCATGTTGACATTCC; dG = -13.3
67 >s_a_s_a_s_d_s_b_s_e_s GTGTCTGTGTCTGTGACGGTGGCTGTGCTGGTG; >clamp_s_b_s_d_s:compl:rev
CACCGTCACAGCCACTATCATGTTGACATTCC; dG = -13.3
68 >s_b_s_b_s_b_s_c_s_d_s GTGGCTGTGGCTGTGGCTGTGTGGGTGACGGTG; >clamp_s_b_s_d_s:compl:rev
CACCGTCACAGCCACTATCATGTTGACATTCC; dG = -13.3
69 >s_b_s_b_s_b_s_a_s_d_s GTGGCTGTGGCTGTGGCTGTGTCTGTGACGGTG; >clamp_s_b_s_d_s:compl:rev
CACCGTCACAGCCACTATCATGTTGACATTCC; dG = -13.3
70 >s_a_s_a_s_d_s_b_s_c_s GTGTCTGTGTCTGTGACGGTGGCTGTGTGGGTG; >clamp_s_b_s_d_s:compl:rev
CACCGTCACAGCCACTATCATGTTGACATTCC; dG = -13.3
71 >s_a_s_a_s_d_s_b_s_b_s GTGTCTGTGTCTGTGACGGTGGCTGTGGCTGTG; >clamp_s_b_s_d_s:compl:rev
CACCGTCACAGCCACTATCATGTTGACATTCC; dG = -13.3
72 >s_a_s_a_s_d_s_b_s_a_s GTGTCTGTGTCTGTGACGGTGGCTGTGTCTGTG; >clamp_s_b_s_d_s:compl:rev
CACCGTCACAGCCACTATCATGTTGACATTCC; dG = -13.3
73 >s_b_s_c_s_b_s_e_s_d_s GTGGCTGTGTGGGTGGCTGTGCTGGTGACGGTG; >clamp_s_b_s_d_s:compl:rev
CACCGTCACAGCCACTATCATGTTGACATTCC; dG = -13.3
74 >s_b_s_c_s_b_s_c_s_d_s GTGGCTGTGTGGGTGGCTGTGTGGGTGACGGTG; >clamp_s_b_s_d_s:compl:rev
CACCGTCACAGCCACTATCATGTTGACATTCC; dG = -13.3
75 >s_b_s_c_s_b_s_a_s_d_s GTGGCTGTGTGGGTGGCTGTGTCTGTGACGGTG; >clamp_s_b_s_d_s:compl:rev
CACCGTCACAGCCACTATCATGTTGACATTCC; dG = -13.3
76 >s_b_s_e_s_b_s_e_s_d_s GTGGCTGTGCTGGTGGCTGTGCTGGTGACGGTG; >clamp_s_b_s_d_s:compl:rev
CACCGTCACAGCCACTATCATGTTGACATTCC; dG = -13.3
77 >s_b_s_e_s_b_s_c_s_d_s GTGGCTGTGCTGGTGGCTGTGTGGGTGACGGTG; >clamp_s_b_s_d_s:compl:rev
CACCGTCACAGCCACTATCATGTTGACATTCC; dG = -13.3
78 >s_b_s_e_s_b_s_a_s_d_s GTGGCTGTGCTGGTGGCTGTGTCTGTGACGGTG; >clamp_s_b_s_d_s:compl:rev
CACCGTCACAGCCACTATCATGTTGACATTCC; dG = -13.3
79 >s_c_s_a_s_b_s_e_s_d_s GTGTGGGTGTCTGTGGCTGTGCTGGTGACGGTG; >clamp_s_b_s_d_s:compl:rev
CACCGTCACAGCCACTATCATGTTGACATTCC; dG = -13.3
80 >s_c_s_a_s_b_s_c_s_d_s GTGTGGGTGTCTGTGGCTGTGTGGGTGACGGTG; >clamp_s_b_s_d_s:compl:rev
CACCGTCACAGCCACTATCATGTTGACATTCC; dG = -13.3
81 >s_c_s_a_s_b_s_a_s_d_s GTGTGGGTGTCTGTGGCTGTGTCTGTGACGGTG; >clamp_s_b_s_d_s:compl:rev
CACCGTCACAGCCACTATCATGTTGACATTCC; dG = -13.3
82 >s_c_s_a_s_d_s_b_s_e_s GTGTGGGTGTCTGTGACGGTGGCTGTGCTGGTG; >clamp_s_b_s_d_s:compl:rev
CACCGTCACAGCCACTATCATGTTGACATTCC; dG = -13.3
83 >s_c_s_a_s_d_s_b_s_c_s GTGTGGGTGTCTGTGACGGTGGCTGTGTGGGTG; >clamp_s_b_s_d_s:compl:rev
CACCGTCACAGCCACTATCATGTTGACATTCC; dG = -13.3
84 >s_c_s_a_s_d_s_b_s_b_s GTGTGGGTGTCTGTGACGGTGGCTGTGGCTGTG; >clamp_s_b_s_d_s:compl:rev
CACCGTCACAGCCACTATCATGTTGACATTCC; dG = -13.3
85 >s_c_s_a_s_d_s_b_s_a_s GTGTGGGTGTCTGTGACGGTGGCTGTGTCTGTG; >clamp_s_b_s_d_s:compl:rev
CACCGTCACAGCCACTATCATGTTGACATTCC; dG = -13.3
86 >s_c_s_b_s_b_s_e_s_d_s GTGTGGGTGGCTGTGGCTGTGCTGGTGACGGTG; >clamp_s_b_s_d_s:compl:rev
CACCGTCACAGCCACTATCATGTTGACATTCC; dG = -13.3
87 >s_c_s_b_s_b_s_c_s_d_s GTGTGGGTGGCTGTGGCTGTGTGGGTGACGGTG; >clamp_s_b_s_d_s:compl:rev
CACCGTCACAGCCACTATCATGTTGACATTCC; dG = -13.3
88 >s_c_s_b_s_b_s_a_s_d_s GTGTGGGTGGCTGTGGCTGTGTCTGTGACGGTG; >clamp_s_b_s_d_s:compl:rev
CACCGTCACAGCCACTATCATGTTGACATTCC; dG = -13.3
89 >s_c_s_c_s_b_s_e_s_d_s GTGTGGGTGTGGGTGGCTGTGCTGGTGACGGTG; >clamp_s_b_s_d_s:compl:rev
CACCGTCACAGCCACTATCATGTTGACATTCC; dG = -13.3
90 >s_c_s_c_s_b_s_c_s_d_s GTGTGGGTGTGGGTGGCTGTGTGGGTGACGGTG; >clamp_s_b_s_d_s:compl:rev
CACCGTCACAGCCACTATCATGTTGACATTCC; dG = -13.3
91 >s_c_s_c_s_b_s_a_s_d_s GTGTGGGTGTGGGTGGCTGTGTCTGTGACGGTG; >clamp_s_b_s_d_s:compl:rev
CACCGTCACAGCCACTATCATGTTGACATTCC; dG = -13.3
92 >s_c_s_d_s_b_s_e_s_d_s GTGTGGGTGACGGTGGCTGTGCTGGTGACGGTG; >clamp_s_b_s_d_s:compl:rev
CACCGTCACAGCCACTATCATGTTGACATTCC; dG = -13.3
93 >s_c_s_d_s_b_s_c_s_d_s GTGTGGGTGACGGTGGCTGTGTGGGTGACGGTG; >clamp_s_b_s_d_s:compl:rev
CACCGTCACAGCCACTATCATGTTGACATTCC; dG = -13.3

94 >s_c_s_d_s_b_s_a_s_d_s GTGTGGGTGACGGTGGCTGTGTCTGTGACGGTG; >clamp_s_b_s_d_s:compl:rev
CACCGTCACAGCCACTATCATGTTGACATTCC; dG = -13.3
95 >s_c_s_e_s_b_s_e_s_d_s GTGTGGGTGCTGGTGGCTGTGCTGGTGACGGTG; >clamp_s_b_s_d_s:compl:rev
CACCGTCACAGCCACTATCATGTTGACATTCC; dG = -13.3
96 >s_c_s_e_s_b_s_c_s_d_s GTGTGGGTGCTGGTGGCTGTGTGGGTGACGGTG; >clamp_s_b_s_d_s:compl:rev
CACCGTCACAGCCACTATCATGTTGACATTCC; dG = -13.3
97 >s_c_s_e_s_b_s_a_s_d_s GTGTGGGTGCTGGTGGCTGTGTCTGTGACGGTG; >clamp_s_b_s_d_s:compl:rev
CACCGTCACAGCCACTATCATGTTGACATTCC; dG = -13.3
98 >s_a_s_b_s_b_s_e_s_d_s GTGTCTGTGGCTGTGGCTGTGCTGGTGACGGTG; >clamp_s_b_s_d_s:compl:rev
CACCGTCACAGCCACTATCATGTTGACATTCC; dG = -13.3
99 >s_a_s_b_s_b_s_c_s_d_s GTGTCTGTGGCTGTGGCTGTGTGGGTGACGGTG; >clamp_s_b_s_d_s:compl:rev
CACCGTCACAGCCACTATCATGTTGACATTCC; dG = -13.3
100 >s_d_s_a_s_b_s_e_s_d_s GTGACGGTGTCTGTGGCTGTGCTGGTGACGGTG; >clamp_s_b_s_d_s:compl:rev
CACCGTCACAGCCACTATCATGTTGACATTCC; dG = -13.3
101 >s_d_s_a_s_b_s_c_s_d_s GTGACGGTGTCTGTGGCTGTGTGGGTGACGGTG; >clamp_s_b_s_d_s:compl:rev
CACCGTCACAGCCACTATCATGTTGACATTCC; dG = -13.3
102 >s_d_s_a_s_b_s_a_s_d_s GTGACGGTGTCTGTGGCTGTGTCTGTGACGGTG; >clamp_s_b_s_d_s:compl:rev
CACCGTCACAGCCACTATCATGTTGACATTCC; dG = -13.3
103 >s_a_s_b_s_b_s_a_s_d_s GTGTCTGTGGCTGTGGCTGTGTCTGTGACGGTG; >clamp_s_b_s_d_s:compl:rev
CACCGTCACAGCCACTATCATGTTGACATTCC; dG = -13.3
104 >s_d_s_b_s_b_s_e_s_d_s GTGACGGTGGCTGTGGCTGTGCTGGTGACGGTG; >clamp_s_b_s_d_s:compl:rev
CACCGTCACAGCCACTATCATGTTGACATTCC; dG = -13.3
105 >s_d_s_b_s_b_s_c_s_d_s GTGACGGTGGCTGTGGCTGTGTGGGTGACGGTG; >clamp_s_b_s_d_s:compl:rev
CACCGTCACAGCCACTATCATGTTGACATTCC; dG = -13.3
106 >s_d_s_b_s_b_s_a_s_d_s GTGACGGTGGCTGTGGCTGTGTCTGTGACGGTG; >clamp_s_b_s_d_s:compl:rev
CACCGTCACAGCCACTATCATGTTGACATTCC; dG = -13.3
107 >s_d_s_c_s_b_s_e_s_d_s GTGACGGTGTGGGTGGCTGTGCTGGTGACGGTG; >clamp_s_b_s_d_s:compl:rev
CACCGTCACAGCCACTATCATGTTGACATTCC; dG = -13.3
108 >s_d_s_c_s_b_s_c_s_d_s GTGACGGTGTGGGTGGCTGTGTGGGTGACGGTG; >clamp_s_b_s_d_s:compl:rev
CACCGTCACAGCCACTATCATGTTGACATTCC; dG = -13.3
109 >s_d_s_c_s_b_s_a_s_d_s GTGACGGTGTGGGTGGCTGTGTCTGTGACGGTG; >clamp_s_b_s_d_s:compl:rev
CACCGTCACAGCCACTATCATGTTGACATTCC; dG = -13.3
110 >s_d_s_d_s_b_s_e_s_d_s GTGACGGTGACGGTGGCTGTGCTGGTGACGGTG; >clamp_s_b_s_d_s:compl:rev
CACCGTCACAGCCACTATCATGTTGACATTCC; dG = -13.3
111 >s_d_s_d_s_b_s_c_s_d_s GTGACGGTGACGGTGGCTGTGTGGGTGACGGTG; >clamp_s_b_s_d_s:compl:rev
CACCGTCACAGCCACTATCATGTTGACATTCC; dG = -13.3
112 >s_d_s_d_s_b_s_a_s_d_s GTGACGGTGACGGTGGCTGTGTCTGTGACGGTG; >clamp_s_b_s_d_s:compl:rev
CACCGTCACAGCCACTATCATGTTGACATTCC; dG = -13.3
113 >s_d_s_e_s_b_s_e_s_d_s GTGACGGTGCTGGTGGCTGTGCTGGTGACGGTG; >clamp_s_b_s_d_s:compl:rev
CACCGTCACAGCCACTATCATGTTGACATTCC; dG = -13.3
114 >s_d_s_e_s_b_s_c_s_d_s GTGACGGTGCTGGTGGCTGTGTGGGTGACGGTG; >clamp_s_b_s_d_s:compl:rev
CACCGTCACAGCCACTATCATGTTGACATTCC; dG = -13.3
115 >s_d_s_e_s_b_s_a_s_d_s GTGACGGTGCTGGTGGCTGTGTCTGTGACGGTG; >clamp_s_b_s_d_s:compl:rev
CACCGTCACAGCCACTATCATGTTGACATTCC; dG = -13.3
116 >s_e_s_a_s_b_s_e_s_d_s GTGCTGGTGTCTGTGGCTGTGCTGGTGACGGTG; >clamp_s_b_s_d_s:compl:rev
CACCGTCACAGCCACTATCATGTTGACATTCC; dG = -13.3
117 >s_e_s_a_s_b_s_c_s_d_s GTGCTGGTGTCTGTGGCTGTGTGGGTGACGGTG; >clamp_s_b_s_d_s:compl:rev
CACCGTCACAGCCACTATCATGTTGACATTCC; dG = -13.3
118 >s_e_s_a_s_b_s_a_s_d_s GTGCTGGTGTCTGTGGCTGTGTCTGTGACGGTG; >clamp_s_b_s_d_s:compl:rev
CACCGTCACAGCCACTATCATGTTGACATTCC; dG = -13.3
119 >s_e_s_b_s_b_s_e_s_d_s GTGCTGGTGGCTGTGGCTGTGCTGGTGACGGTG; >clamp_s_b_s_d_s:compl:rev
CACCGTCACAGCCACTATCATGTTGACATTCC; dG = -13.3
120 >s_e_s_b_s_b_s_c_s_d_s GTGCTGGTGGCTGTGGCTGTGTGGGTGACGGTG; >clamp_s_b_s_d_s:compl:rev
CACCGTCACAGCCACTATCATGTTGACATTCC; dG = -13.3
121 >s_e_s_b_s_b_s_a_s_d_s GTGCTGGTGGCTGTGGCTGTGTCTGTGACGGTG; >clamp_s_b_s_d_s:compl:rev
CACCGTCACAGCCACTATCATGTTGACATTCC; dG = -13.3
122 >s_e_s_c_s_b_s_e_s_d_s GTGCTGGTGTGGGTGGCTGTGCTGGTGACGGTG; >clamp_s_b_s_d_s:compl:rev
CACCGTCACAGCCACTATCATGTTGACATTCC; dG = -13.3
123 >s_e_s_c_s_b_s_c_s_d_s GTGCTGGTGTGGGTGGCTGTGTGGGTGACGGTG; >clamp_s_b_s_d_s:compl:rev
CACCGTCACAGCCACTATCATGTTGACATTCC; dG = -13.3
124 >s_e_s_c_s_b_s_a_s_d_s GTGCTGGTGTGGGTGGCTGTGTCTGTGACGGTG; >clamp_s_b_s_d_s:compl:rev
CACCGTCACAGCCACTATCATGTTGACATTCC; dG = -13.3
125 >s_a_s_c_s_b_s_e_s_d_s GTGTCTGTGTGGGTGGCTGTGCTGGTGACGGTG; >clamp_s_b_s_d_s:compl:rev
CACCGTCACAGCCACTATCATGTTGACATTCC; dG = -13.3
126 >s_e_s_d_s_b_s_e_s_d_s GTGCTGGTGACGGTGGCTGTGCTGGTGACGGTG; >clamp_s_b_s_d_s:compl:rev
CACCGTCACAGCCACTATCATGTTGACATTCC; dG = -13.3
127 >s_e_s_d_s_b_s_c_s_d_s GTGCTGGTGACGGTGGCTGTGTGGGTGACGGTG; >clamp_s_b_s_d_s:compl:rev
CACCGTCACAGCCACTATCATGTTGACATTCC; dG = -13.3
128 >s_e_s_d_s_b_s_a_s_d_s GTGCTGGTGACGGTGGCTGTGTCTGTGACGGTG; >clamp_s_b_s_d_s:compl:rev
CACCGTCACAGCCACTATCATGTTGACATTCC; dG = -13.3
129 >s_a_s_c_s_b_s_c_s_d_s GTGTCTGTGTGGGTGGCTGTGTGGGTGACGGTG; >clamp_s_b_s_d_s:compl:rev
CACCGTCACAGCCACTATCATGTTGACATTCC; dG = -13.3
130 >s_a_s_c_s_b_s_a_s_d_s GTGTCTGTGTGGGTGGCTGTGTCTGTGACGGTG; >clamp_s_b_s_d_s:compl:rev
CACCGTCACAGCCACTATCATGTTGACATTCC; dG = -13.3

131 >s_e_s_e_s_b_s_e_s_d_s GTGCTGGTGCTGGTGGCTGTGCTGGTGACGGTG; >clamp_s_b_s_d_s:compl:rev CACCGTCACAGCCACTATCATGTTGACATTCC; dG = -13.3

132 >s_e_s_e_s_b_s_c_s_d_s GTGCTGGTGCTGGTGGCTGTGTGGGTGACGGTG; >clamp_s_b_s_d_s:compl:rev CACCGTCACAGCCACTATCATGTTGACATTCC; dG = -13.3

133 >s_e_s_e_s_b_s_a_s_d_s GTGCTGGTGCTGGTGGCTGTGTCTGTGACGGTG; >clamp_s_b_s_d_s:compl:rev CACCGTCACAGCCACTATCATGTTGACATTCC; dG = -13.3

134 >s_b_s_a_s_d_s_c_s_d_s GTGGCTGTGTCTGTGACGGTGTGGGTGACGGTG; >clamp_s_b_s_d_s:compl:rev CACCGTCACAGCCACTATCATGTTGACATTCC; dG = -13.2

135 >s_b_s_a_s_d_s_a_s_d_s GTGGCTGTGTCTGTGACGGTGTCTGTGACGGTG; >clamp_s_b_s_d_s:compl:rev CACCGTCACAGCCACTATCATGTTGACATTCC; dG = -13.2

136 >s_a_s_a_s_d_s_c_s_d_s GTGTCTGTGTCTGTGACGGTGTGGGTGACGGTG; >clamp_s_b_s_d_s:compl:rev CACCGTCACAGCCACTATCATGTTGACATTCC; dG = -13.2

137 >s_a_s_a_s_d_s_a_s_d_s GTGTCTGTGTCTGTGACGGTGTCTGTGACGGTG; >clamp_s_b_s_d_s:compl:rev CACCGTCACAGCCACTATCATGTTGACATTCC; dG = -13.2

138 >s_c_s_a_s_d_s_c_s_d_s GTGTGGGTGTCTGTGACGGTGTGGGTGACGGTG; >clamp_s_b_s_d_s:compl:rev CACCGTCACAGCCACTATCATGTTGACATTCC; dG = -13.2

139 >s_c_s_a_s_d_s_a_s_d_s GTGTGGGTGTCTGTGACGGTGTCTGTGACGGTG; >clamp_s_b_s_d_s:compl:rev CACCGTCACAGCCACTATCATGTTGACATTCC; dG = -13.2

140 >s_d_s_a_s_d_s_b_s_e_s GTGACGGTGTCTGTGACGGTGGCTGTGCTGGTG; >clamp_s_b_s_d_s:compl:rev CACCGTCACAGCCACTATCATGTTGACATTCC; dG = -13.2

141 >s_d_s_a_s_d_s_b_s_c_s GTGACGGTGTCTGTGACGGTGGCTGTGTGGGTG; >clamp_s_b_s_d_s:compl:rev CACCGTCACAGCCACTATCATGTTGACATTCC; dG = -13.2

142 >s_d_s_a_s_d_s_b_s_b_s GTGACGGTGTCTGTGACGGTGGCTGTGGCTGTG; >clamp_s_b_s_d_s:compl:rev CACCGTCACAGCCACTATCATGTTGACATTCC; dG = -13.2

143 >s_d_s_a_s_d_s_b_s_a_s GTGACGGTGTCTGTGACGGTGGCTGTGTCTGTG; >clamp_s_b_s_d_s:compl:rev CACCGTCACAGCCACTATCATGTTGACATTCC; dG = -13.2

144 >s_d_s_a_s_d_s_c_s_d_s GTGACGGTGTCTGTGACGGTGTGGGTGACGGTG; >clamp_s_b_s_d_s:compl:rev CACCGTCACAGCCACTATCATGTTGACATTCC; dG = -13.2

145 >s_d_s_a_s_d_s_a_s_d_s GTGACGGTGTCTGTGACGGTGTCTGTGACGGTG; >clamp_s_b_s_d_s:compl:rev CACCGTCACAGCCACTATCATGTTGACATTCC; dG = -13.2

146 >s_e_s_a_s_d_s_b_s_e_s GTGCTGGTGTCTGTGACGGTGGCTGTGCTGGTG; >clamp_s_b_s_d_s:compl:rev CACCGTCACAGCCACTATCATGTTGACATTCC; dG = -13.2

147 >s_e_s_a_s_d_s_b_s_c_s GTGCTGGTGTCTGTGACGGTGGCTGTGTGGGTG; >clamp_s_b_s_d_s:compl:rev CACCGTCACAGCCACTATCATGTTGACATTCC; dG = -13.2

148 >s_e_s_a_s_d_s_b_s_b_s GTGCTGGTGTCTGTGACGGTGGCTGTGGCTGTG; >clamp_s_b_s_d_s:compl:rev CACCGTCACAGCCACTATCATGTTGACATTCC; dG = -13.2

149 >s_e_s_a_s_d_s_b_s_a_s GTGCTGGTGTCTGTGACGGTGGCTGTGTCTGTG; >clamp_s_b_s_d_s:compl:rev CACCGTCACAGCCACTATCATGTTGACATTCC; dG = -13.2

150 >s_e_s_a_s_d_s_c_s_d_s GTGCTGGTGTCTGTGACGGTGTGGGTGACGGTG; >clamp_s_b_s_d_s:compl:rev CACCGTCACAGCCACTATCATGTTGACATTCC; dG = -13.2

151 >s_e_s_a_s_d_s_a_s_d_s GTGCTGGTGTCTGTGACGGTGTCTGTGACGGTG; >clamp_s_b_s_d_s:compl:rev CACCGTCACAGCCACTATCATGTTGACATTCC; dG = -13.2

152 >s_b_s_a_s_d_s_e_s_d_s GTGGCTGTGTCTGTGACGGTGCTGGTGACGGTG; >clamp_s_b_s_d_s:compl:rev CACCGTCACAGCCACTATCATGTTGACATTCC; dG = -13.1

153 >s_a_s_a_s_d_s_e_s_d_s GTGTCTGTGTCTGTGACGGTGCTGGTGACGGTG; >clamp_s_b_s_d_s:compl:rev CACCGTCACAGCCACTATCATGTTGACATTCC; dG = -13.1

154 >s_b_s_c_s_d_s_b_s_e_s GTGGCTGTGTGGGTGACGGTGGCTGTGCTGGTG; >clamp_s_b_s_d_s:compl:rev CACCGTCACAGCCACTATCATGTTGACATTCC; dG = -13.1

155 >s_b_s_c_s_d_s_b_s_c_s GTGGCTGTGTGGGTGACGGTGGCTGTGTGGGTG; >clamp_s_b_s_d_s:compl:rev CACCGTCACAGCCACTATCATGTTGACATTCC; dG = -13.1

156 >s_b_s_c_s_d_s_b_s_b_s GTGGCTGTGTGGGTGACGGTGGCTGTGGCTGTG; >clamp_s_b_s_d_s:compl:rev CACCGTCACAGCCACTATCATGTTGACATTCC; dG = -13.1

157 >s_b_s_c_s_d_s_b_s_a_s GTGGCTGTGTGGGTGACGGTGGCTGTGTCTGTG; >clamp_s_b_s_d_s:compl:rev CACCGTCACAGCCACTATCATGTTGACATTCC; dG = -13.1

158 >s_b_s_c_s_d_s_c_s_d_s GTGGCTGTGTGGGTGACGGTGTGGGTGACGGTG; >clamp_s_b_s_d_s:compl:rev CACCGTCACAGCCACTATCATGTTGACATTCC; dG = -13.1

159 >s_b_s_c_s_d_s_a_s_d_s GTGGCTGTGTGGGTGACGGTGTCTGTGACGGTG; >clamp_s_b_s_d_s:compl:rev CACCGTCACAGCCACTATCATGTTGACATTCC; dG = -13.1

160 >s_b_s_e_s_d_s_b_s_e_s GTGGCTGTGCTGGTGACGGTGGCTGTGCTGGTG; >clamp_s_b_s_d_s:compl:rev CACCGTCACAGCCACTATCATGTTGACATTCC; dG = -13.1

161 >s_b_s_e_s_d_s_b_s_c_s GTGGCTGTGCTGGTGACGGTGGCTGTGTGGGTG; >clamp_s_b_s_d_s:compl:rev CACCGTCACAGCCACTATCATGTTGACATTCC; dG = -13.1

162 >s_b_s_e_s_d_s_b_s_b_s GTGGCTGTGCTGGTGACGGTGGCTGTGGCTGTG; >clamp_s_b_s_d_s:compl:rev CACCGTCACAGCCACTATCATGTTGACATTCC; dG = -13.1

163 >s_b_s_e_s_d_s_b_s_a_s GTGGCTGTGCTGGTGACGGTGGCTGTGTCTGTG; >clamp_s_b_s_d_s:compl:rev CACCGTCACAGCCACTATCATGTTGACATTCC; dG = -13.1

164 >s_b_s_e_s_d_s_c_s_d_s GTGGCTGTGCTGGTGACGGTGTGGGTGACGGTG; >clamp_s_b_s_d_s:compl:rev CACCGTCACAGCCACTATCATGTTGACATTCC; dG = -13.1

165 >s_b_s_e_s_d_s_a_s_d_s GTGGCTGTGCTGGTGACGGTGTCTGTGACGGTG; >clamp_s_b_s_d_s:compl:rev CACCGTCACAGCCACTATCATGTTGACATTCC; dG = -13.1

166 >s_c_s_a_s_d_s_e_s_d_s GTGTGGGTGTCTGTGACGGTGCTGGTGACGGTG; >clamp_s_b_s_d_s:compl:rev CACCGTCACAGCCACTATCATGTTGACATTCC; dG = -13.0

167 >s_d_s_a_s_d_s_e_s_d_s GTGACGGTGTCTGTGACGGTGCTGGTGACGGTG; >clamp_s_b_s_d_s:compl:rev CACCGTCACAGCCACTATCATGTTGACATTCC; dG = -13.0

168 >s_e_s_a_s_d_s_e_s_d_s GTGCTGGTGTCTGTGACGGTGCTGGTGACGGTG; >clamp_s_b_s_d_s:compl:rev
CACCGTCACAGCCACTATCATGTTGACATTCC; dG = -13.0
169 >s_a_s_c_s_d_s_a_s_d_s GTGTCTGTGTGGGTGACGGTGTCTGTGACGGTG; >clamp_s_b_s_d_s:compl:rev
CACCGTCACAGCCACTATCATGTTGACATTCC; dG = -12.9
170 >s_a_s_c_s_e_s_a_s_d_s GTGTCTGTGTGGGTGCTGGTGTCTGTGACGGTG; >clamp_s_b_s_d_s:compl:rev
CACCGTCACAGCCACTATCATGTTGACATTCC; dG = -12.9
171 >s_a_s_d_s_a_s_a_s_d_s GTGTCTGTGACGGTGTCTGTGTCTGTGACGGTG; >clamp_s_b_s_d_s:compl:rev
CACCGTCACAGCCACTATCATGTTGACATTCC; dG = -12.9
172 >s_a_s_d_s_c_s_a_s_d_s GTGTCTGTGACGGTGTGGGTGTCTGTGACGGTG; >clamp_s_b_s_d_s:compl:rev
CACCGTCACAGCCACTATCATGTTGACATTCC; dG = -12.9
173 >s_a_s_d_s_e_s_a_s_d_s GTGTCTGTGACGGTGCTGGTGTCTGTGACGGTG; >clamp_s_b_s_d_s:compl:rev
CACCGTCACAGCCACTATCATGTTGACATTCC; dG = -12.9
174 >s_a_s_e_s_a_s_a_s_d_s GTGTCTGTGCTGGTGTCTGTGTCTGTGACGGTG; >clamp_s_b_s_d_s:compl:rev
CACCGTCACAGCCACTATCATGTTGACATTCC; dG = -12.9
175 >s_a_s_e_s_c_s_a_s_d_s GTGTCTGTGCTGGTGTGGGTGTCTGTGACGGTG; >clamp_s_b_s_d_s:compl:rev
CACCGTCACAGCCACTATCATGTTGACATTCC; dG = -12.9
176 >s_a_s_e_s_d_s_a_s_d_s GTGTCTGTGCTGGTGACGGTGTCTGTGACGGTG; >clamp_s_b_s_d_s:compl:rev
CACCGTCACAGCCACTATCATGTTGACATTCC; dG = -12.9
177 >s_a_s_e_s_e_s_a_s_d_s GTGTCTGTGCTGGTGCTGGTGTCTGTGACGGTG; >clamp_s_b_s_d_s:compl:rev
CACCGTCACAGCCACTATCATGTTGACATTCC; dG = -12.9
178 >s_b_s_a_s_a_s_a_s_d_s GTGGCTGTGTCTGTGTCTGTGTCTGTGACGGTG; >clamp_s_b_s_d_s:compl:rev
CACCGTCACAGCCACTATCATGTTGACATTCC; dG = -12.9
179 >s_b_s_a_s_c_s_a_s_d_s GTGGCTGTGTCTGTGTGGGTGTCTGTGACGGTG; >clamp_s_b_s_d_s:compl:rev
CACCGTCACAGCCACTATCATGTTGACATTCC; dG = -12.9
180 >s_b_s_a_s_e_s_a_s_d_s GTGGCTGTGTCTGTGCTGGTGTCTGTGACGGTG; >clamp_s_b_s_d_s:compl:rev
CACCGTCACAGCCACTATCATGTTGACATTCC; dG = -12.9
181 >s_a_s_a_s_c_s_a_s_d_s GTGTCTGTGTCTGTGTGGGTGTCTGTGACGGTG; >clamp_s_b_s_d_s:compl:rev
CACCGTCACAGCCACTATCATGTTGACATTCC; dG = -12.9
182 >s_b_s_b_s_a_s_a_s_d_s GTGGCTGTGGCTGTGTCTGTGTCTGTGACGGTG; >clamp_s_b_s_d_s:compl:rev
CACCGTCACAGCCACTATCATGTTGACATTCC; dG = -12.9
183 >s_b_s_b_s_c_s_a_s_d_s GTGGCTGTGGCTGTGTGGGTGTCTGTGACGGTG; >clamp_s_b_s_d_s:compl:rev
CACCGTCACAGCCACTATCATGTTGACATTCC; dG = -12.9
184 >s_b_s_b_s_e_s_a_s_d_s GTGGCTGTGGCTGTGCTGGTGTCTGTGACGGTG; >clamp_s_b_s_d_s:compl:rev
CACCGTCACAGCCACTATCATGTTGACATTCC; dG = -12.9
185 >s_b_s_c_s_a_s_a_s_d_s GTGGCTGTGTGGGTGTCTGTGTCTGTGACGGTG; >clamp_s_b_s_d_s:compl:rev
CACCGTCACAGCCACTATCATGTTGACATTCC; dG = -12.9
186 >s_b_s_c_s_c_s_a_s_d_s GTGGCTGTGTGGGTGTGGGTGTCTGTGACGGTG; >clamp_s_b_s_d_s:compl:rev
CACCGTCACAGCCACTATCATGTTGACATTCC; dG = -12.9
187 >s_b_s_c_s_d_s_e_s_d_s GTGGCTGTGTGGGTGACGGTGCTGGTGACGGTG; >clamp_s_b_s_d_s:compl:rev
CACCGTCACAGCCACTATCATGTTGACATTCC; dG = -12.9
188 >s_b_s_c_s_e_s_a_s_d_s GTGGCTGTGTGGGTGCTGGTGTCTGTGACGGTG; >clamp_s_b_s_d_s:compl:rev
CACCGTCACAGCCACTATCATGTTGACATTCC; dG = -12.9
189 >s_b_s_e_s_a_s_a_s_d_s GTGGCTGTGCTGGTGTCTGTGTCTGTGACGGTG; >clamp_s_b_s_d_s:compl:rev
CACCGTCACAGCCACTATCATGTTGACATTCC; dG = -12.9
190 >s_b_s_e_s_c_s_a_s_d_s GTGGCTGTGCTGGTGTGGGTGTCTGTGACGGTG; >clamp_s_b_s_d_s:compl:rev
CACCGTCACAGCCACTATCATGTTGACATTCC; dG = -12.9
191 >s_b_s_e_s_d_s_e_s_d_s GTGGCTGTGCTGGTGACGGTGCTGGTGACGGTG; >clamp_s_b_s_d_s:compl:rev
CACCGTCACAGCCACTATCATGTTGACATTCC; dG = -12.9
192 >s_b_s_e_s_e_s_a_s_d_s GTGGCTGTGCTGGTGCTGGTGTCTGTGACGGTG; >clamp_s_b_s_d_s:compl:rev
CACCGTCACAGCCACTATCATGTTGACATTCC; dG = -12.9
193 >s_c_s_a_s_a_s_a_s_d_s GTGTGGGTGTCTGTGTCTGTGTCTGTGACGGTG; >clamp_s_b_s_d_s:compl:rev
CACCGTCACAGCCACTATCATGTTGACATTCC; dG = -12.9
194 >s_c_s_a_s_c_s_a_s_d_s GTGTGGGTGTCTGTGTGGGTGTCTGTGACGGTG; >clamp_s_b_s_d_s:compl:rev
CACCGTCACAGCCACTATCATGTTGACATTCC; dG = -12.9
195 >s_c_s_a_s_e_s_a_s_d_s GTGTGGGTGTCTGTGCTGGTGTCTGTGACGGTG; >clamp_s_b_s_d_s:compl:rev
CACCGTCACAGCCACTATCATGTTGACATTCC; dG = -12.9
196 >s_c_s_b_s_a_s_a_s_d_s GTGTGGGTGGCTGTGTCTGTGTCTGTGACGGTG; >clamp_s_b_s_d_s:compl:rev
CACCGTCACAGCCACTATCATGTTGACATTCC; dG = -12.9
197 >s_a_s_a_s_e_s_a_s_d_s GTGTCTGTGTCTGTGCTGGTGTCTGTGACGGTG; >clamp_s_b_s_d_s:compl:rev
CACCGTCACAGCCACTATCATGTTGACATTCC; dG = -12.9
198 >s_c_s_b_s_c_s_a_s_d_s GTGTGGGTGGCTGTGTGGGTGTCTGTGACGGTG; >clamp_s_b_s_d_s:compl:rev
CACCGTCACAGCCACTATCATGTTGACATTCC; dG = -12.9
199 >s_c_s_b_s_e_s_a_s_d_s GTGTGGGTGGCTGTGCTGGTGTCTGTGACGGTG; >clamp_s_b_s_d_s:compl:rev
CACCGTCACAGCCACTATCATGTTGACATTCC; dG = -12.9
200 >s_c_s_c_s_a_s_a_s_d_s GTGTGGGTGTGGGTGTCTGTGTCTGTGACGGTG; >clamp_s_b_s_d_s:compl:rev
CACCGTCACAGCCACTATCATGTTGACATTCC; dG = -12.9
201 >s_c_s_c_s_c_s_a_s_d_s GTGTGGGTGTGGGTGTGGGTGTCTGTGACGGTG; >clamp_s_b_s_d_s:compl:rev
CACCGTCACAGCCACTATCATGTTGACATTCC; dG = -12.9
202 >s_c_s_c_s_d_s_a_s_d_s GTGTGGGTGTGGGTGACGGTGTCTGTGACGGTG; >clamp_s_b_s_d_s:compl:rev
CACCGTCACAGCCACTATCATGTTGACATTCC; dG = -12.9
203 >s_c_s_c_s_e_s_a_s_d_s GTGTGGGTGTGGGTGCTGGTGTCTGTGACGGTG; >clamp_s_b_s_d_s:compl:rev
CACCGTCACAGCCACTATCATGTTGACATTCC; dG = -12.9
204 >s_c_s_d_s_a_s_a_s_d_s GTGTGGGTGACGGTGTCTGTGTCTGTGACGGTG; >clamp_s_b_s_d_s:compl:rev
CACCGTCACAGCCACTATCATGTTGACATTCC; dG = -12.9

205 >s_c_s_d_s_c_s_a_s_d_s GTGTGGGTGACGGTGTGGGTGTCTGTGACGGTG; >clamp_s_b_s_d_s:compl:rev CACCGTCACAGCCACTATCATGTTGACATTCC; dG = -12.9
206 >s_a_s_b_s_a_s_a_s_d_s GTGTCTGTGGCTGTGTCTGTGTCTGTGACGGTG; >clamp_s_b_s_d_s:compl:rev CACCGTCACAGCCACTATCATGTTGACATTCC; dG = -12.9
207 >s_c_s_d_s_d_s_a_s_d_s GTGTGGGTGACGGTGACGGTGTCTGTGACGGTG; >clamp_s_b_s_d_s:compl:rev CACCGTCACAGCCACTATCATGTTGACATTCC; dG = -12.9
208 >s_c_s_d_s_e_s_a_s_d_s GTGTGGGTGACGGTGCTGGTGTCTGTGACGGTG; >clamp_s_b_s_d_s:compl:rev CACCGTCACAGCCACTATCATGTTGACATTCC; dG = -12.9
209 >s_c_s_e_s_a_s_a_s_d_s GTGTGGGTGCTGGTGTCTGTGTCTGTGACGGTG; >clamp_s_b_s_d_s:compl:rev CACCGTCACAGCCACTATCATGTTGACATTCC; dG = -12.9
210 >s_c_s_e_s_c_s_a_s_d_s GTGTGGGTGCTGGTGTGGGTGTCTGTGACGGTG; >clamp_s_b_s_d_s:compl:rev CACCGTCACAGCCACTATCATGTTGACATTCC; dG = -12.9
211 >s_c_s_e_s_d_s_a_s_d_s GTGTGGGTGCTGGTGACGGTGTCTGTGACGGTG; >clamp_s_b_s_d_s:compl:rev CACCGTCACAGCCACTATCATGTTGACATTCC; dG = -12.9
212 >s_c_s_e_s_e_s_a_s_d_s GTGTGGGTGCTGGTGCTGGTGTCTGTGACGGTG; >clamp_s_b_s_d_s:compl:rev CACCGTCACAGCCACTATCATGTTGACATTCC; dG = -12.9
213 >s_d_s_a_s_a_s_a_s_d_s GTGACGGTGTCTGTGTCTGTGTCTGTGACGGTG; >clamp_s_b_s_d_s:compl:rev CACCGTCACAGCCACTATCATGTTGACATTCC; dG = -12.9
214 >s_d_s_a_s_c_s_a_s_d_s GTGACGGTGTCTGTGTGGGTGTCTGTGACGGTG; >clamp_s_b_s_d_s:compl:rev CACCGTCACAGCCACTATCATGTTGACATTCC; dG = -12.9
215 >s_d_s_a_s_e_s_a_s_d_s GTGACGGTGTCTGTGCTGGTGTCTGTGACGGTG; >clamp_s_b_s_d_s:compl:rev CACCGTCACAGCCACTATCATGTTGACATTCC; dG = -12.9
216 >s_d_s_b_s_a_s_a_s_d_s GTGACGGTGGCTGTGTCTGTGTCTGTGACGGTG; >clamp_s_b_s_d_s:compl:rev CACCGTCACAGCCACTATCATGTTGACATTCC; dG = -12.9
217 >s_d_s_b_s_c_s_a_s_d_s GTGACGGTGGCTGTGTGGGTGTCTGTGACGGTG; >clamp_s_b_s_d_s:compl:rev CACCGTCACAGCCACTATCATGTTGACATTCC; dG = -12.9
218 >s_d_s_b_s_e_s_a_s_d_s GTGACGGTGGCTGTGCTGGTGTCTGTGACGGTG; >clamp_s_b_s_d_s:compl:rev CACCGTCACAGCCACTATCATGTTGACATTCC; dG = -12.9
219 >s_d_s_c_s_a_s_a_s_d_s GTGACGGTGTGGGTGTCTGTGTCTGTGACGGTG; >clamp_s_b_s_d_s:compl:rev CACCGTCACAGCCACTATCATGTTGACATTCC; dG = -12.9
220 >s_d_s_c_s_c_s_a_s_d_s GTGACGGTGTGGGTGTGGGTGTCTGTGACGGTG; >clamp_s_b_s_d_s:compl:rev CACCGTCACAGCCACTATCATGTTGACATTCC; dG = -12.9
221 >s_a_s_b_s_c_s_a_s_d_s GTGTCTGTGGCTGTGTGGGTGTCTGTGACGGTG; >clamp_s_b_s_d_s:compl:rev CACCGTCACAGCCACTATCATGTTGACATTCC; dG = -12.9
222 >s_d_s_c_s_d_s_a_s_d_s GTGACGGTGTGGGTGACGGTGTCTGTGACGGTG; >clamp_s_b_s_d_s:compl:rev CACCGTCACAGCCACTATCATGTTGACATTCC; dG = -12.9
223 >s_d_s_c_s_e_s_a_s_d_s GTGACGGTGTGGGTGCTGGTGTCTGTGACGGTG; >clamp_s_b_s_d_s:compl:rev CACCGTCACAGCCACTATCATGTTGACATTCC; dG = -12.9
224 >s_d_s_d_s_a_s_a_s_d_s GTGACGGTGACGGTGTCTGTGTCTGTGACGGTG; >clamp_s_b_s_d_s:compl:rev CACCGTCACAGCCACTATCATGTTGACATTCC; dG = -12.9
225 >s_d_s_d_s_c_s_a_s_d_s GTGACGGTGACGGTGTGGGTGTCTGTGACGGTG; >clamp_s_b_s_d_s:compl:rev CACCGTCACAGCCACTATCATGTTGACATTCC; dG = -12.9
226 >s_d_s_d_s_d_s_a_s_d_s GTGACGGTGACGGTGACGGTGTCTGTGACGGTG; >clamp_s_b_s_d_s:compl:rev CACCGTCACAGCCACTATCATGTTGACATTCC; dG = -12.9
227 >s_d_s_d_s_e_s_a_s_d_s GTGACGGTGACGGTGCTGGTGTCTGTGACGGTG; >clamp_s_b_s_d_s:compl:rev CACCGTCACAGCCACTATCATGTTGACATTCC; dG = -12.9
228 >s_d_s_e_s_a_s_a_s_d_s GTGACGGTGCTGGTGTCTGTGTCTGTGACGGTG; >clamp_s_b_s_d_s:compl:rev CACCGTCACAGCCACTATCATGTTGACATTCC; dG = -12.9
229 >s_d_s_e_s_c_s_a_s_d_s GTGACGGTGCTGGTGTGGGTGTCTGTGACGGTG; >clamp_s_b_s_d_s:compl:rev CACCGTCACAGCCACTATCATGTTGACATTCC; dG = -12.9
230 >s_a_s_b_s_e_s_a_s_d_s GTGTCTGTGGCTGTGCTGGTGTCTGTGACGGTG; >clamp_s_b_s_d_s:compl:rev CACCGTCACAGCCACTATCATGTTGACATTCC; dG = -12.9
231 >s_d_s_e_s_d_s_a_s_d_s GTGACGGTGCTGGTGACGGTGTCTGTGACGGTG; >clamp_s_b_s_d_s:compl:rev CACCGTCACAGCCACTATCATGTTGACATTCC; dG = -12.9
232 >s_d_s_e_s_e_s_a_s_d_s GTGACGGTGCTGGTGCTGGTGTCTGTGACGGTG; >clamp_s_b_s_d_s:compl:rev CACCGTCACAGCCACTATCATGTTGACATTCC; dG = -12.9
233 >s_e_s_a_s_a_s_a_s_d_s GTGCTGGTGTCTGTGTCTGTGTCTGTGACGGTG; >clamp_s_b_s_d_s:compl:rev CACCGTCACAGCCACTATCATGTTGACATTCC; dG = -12.9
234 >s_e_s_a_s_c_s_a_s_d_s GTGCTGGTGTCTGTGTGGGTGTCTGTGACGGTG; >clamp_s_b_s_d_s:compl:rev CACCGTCACAGCCACTATCATGTTGACATTCC; dG = -12.9
235 >s_e_s_a_s_e_s_a_s_d_s GTGCTGGTGTCTGTGCTGGTGTCTGTGACGGTG; >clamp_s_b_s_d_s:compl:rev CACCGTCACAGCCACTATCATGTTGACATTCC; dG = -12.9
236 >s_e_s_b_s_a_s_a_s_d_s GTGCTGGTGGCTGTGTCTGTGTCTGTGACGGTG; >clamp_s_b_s_d_s:compl:rev CACCGTCACAGCCACTATCATGTTGACATTCC; dG = -12.9
237 >s_e_s_b_s_c_s_a_s_d_s GTGCTGGTGGCTGTGTGGGTGTCTGTGACGGTG; >clamp_s_b_s_d_s:compl:rev CACCGTCACAGCCACTATCATGTTGACATTCC; dG = -12.9
238 >s_e_s_b_s_e_s_a_s_d_s GTGCTGGTGGCTGTGCTGGTGTCTGTGACGGTG; >clamp_s_b_s_d_s:compl:rev CACCGTCACAGCCACTATCATGTTGACATTCC; dG = -12.9
239 >s_a_s_c_s_a_s_a_s_d_s GTGTCTGTGTGGGTGTCTGTGTCTGTGACGGTG; >clamp_s_b_s_d_s:compl:rev CACCGTCACAGCCACTATCATGTTGACATTCC; dG = -12.9
240 >s_e_s_c_s_a_s_a_s_d_s GTGCTGGTGTGGGTGTCTGTGTCTGTGACGGTG; >clamp_s_b_s_d_s:compl:rev CACCGTCACAGCCACTATCATGTTGACATTCC; dG = -12.9
241 >s_a_s_a_s_a_s_a_s_d_s GTGTCTGTGTCTGTGTCTGTGTCTGTGACGGTG; >clamp_s_b_s_d_s:compl:rev CACCGTCACAGCCACTATCATGTTGACATTCC; dG = -12.9

242 >s_e_s_c_s_c_s_a_s_d_s GTGCTGGTGTGGGTGTGGGTGTCTGTGACGGTG; >clamp_s_b_s_d_s:compl:rev CACCGTCACAGCCACTATCATGTTGACATTCC; dG = -12.9
243 >s_e_s_c_s_d_s_a_s_d_s GTGCTGGTGTGGGTGACGGTGTCTGTGACGGTG; >clamp_s_b_s_d_s:compl:rev CACCGTCACAGCCACTATCATGTTGACATTCC; dG = -12.9
244 >s_e_s_c_s_e_s_a_s_d_s GTGCTGGTGTGGGTGCTGGTGTCTGTGACGGTG; >clamp_s_b_s_d_s:compl:rev CACCGTCACAGCCACTATCATGTTGACATTCC; dG = -12.9
245 >s_e_s_d_s_a_s_a_s_d_s GTGCTGGTGACGGTGTCTGTGTCTGTGACGGTG; >clamp_s_b_s_d_s:compl:rev CACCGTCACAGCCACTATCATGTTGACATTCC; dG = -12.9
246 >s_e_s_d_s_c_s_a_s_d_s GTGCTGGTGACGGTGTGGGTGTCTGTGACGGTG; >clamp_s_b_s_d_s:compl:rev CACCGTCACAGCCACTATCATGTTGACATTCC; dG = -12.9
247 >s_e_s_d_s_d_s_a_s_d_s GTGCTGGTGACGGTGACGGTGTCTGTGACGGTG; >clamp_s_b_s_d_s:compl:rev CACCGTCACAGCCACTATCATGTTGACATTCC; dG = -12.9
248 >s_e_s_d_s_e_s_a_s_d_s GTGCTGGTGACGGTGCTGGTGTCTGTGACGGTG; >clamp_s_b_s_d_s:compl:rev CACCGTCACAGCCACTATCATGTTGACATTCC; dG = -12.9
249 >s_e_s_e_s_a_s_a_s_d_s GTGCTGGTGCTGGTGTCTGTGTCTGTGACGGTG; >clamp_s_b_s_d_s:compl:rev CACCGTCACAGCCACTATCATGTTGACATTCC; dG = -12.9
250 >s_e_s_e_s_c_s_a_s_d_s GTGCTGGTGCTGGTGTGGGTGTCTGTGACGGTG; >clamp_s_b_s_d_s:compl:rev CACCGTCACAGCCACTATCATGTTGACATTCC; dG = -12.9
251 >s_e_s_e_s_d_s_a_s_d_s GTGCTGGTGCTGGTGACGGTGTCTGTGACGGTG; >clamp_s_b_s_d_s:compl:rev CACCGTCACAGCCACTATCATGTTGACATTCC; dG = -12.9
252 >s_e_s_e_s_e_s_a_s_d_s GTGCTGGTGCTGGTGCTGGTGTCTGTGACGGTG; >clamp_s_b_s_d_s:compl:rev CACCGTCACAGCCACTATCATGTTGACATTCC; dG = -12.9
253 >s_a_s_c_s_c_s_a_s_d_s GTGTCTGTGTGGGTGTGGGTGTCTGTGACGGTG; >clamp_s_b_s_d_s:compl:rev CACCGTCACAGCCACTATCATGTTGACATTCC; dG = -12.9
254 >s_a_s_d_s_b_s_b_s_e_s GTGTCTGTGACGGTGGCTGTGGCTGTGCTGGTG; >clamp_s_b_s_d_s:compl:rev CACCGTCACAGCCACTATCATGTTGACATTCC; dG = -12.8
255 >s_a_s_d_s_b_s_b_s_c_s GTGTCTGTGACGGTGGCTGTGGCTGTGTGGGTG; >clamp_s_b_s_d_s:compl:rev CACCGTCACAGCCACTATCATGTTGACATTCC; dG = -12.8
256 >s_a_s_d_s_b_s_b_s_b_s GTGTCTGTGACGGTGGCTGTGGCTGTGGCTGTG; >clamp_s_b_s_d_s:compl:rev CACCGTCACAGCCACTATCATGTTGACATTCC; dG = -12.8
257 >s_a_s_d_s_b_s_b_s_a_s GTGTCTGTGACGGTGGCTGTGGCTGTGTCTGTG; >clamp_s_b_s_d_s:compl:rev CACCGTCACAGCCACTATCATGTTGACATTCC; dG = -12.8
258 >s_b_s_b_s_c_s_b_s_e_s GTGGCTGTGGCTGTGTGGGTGGCTGTGCTGGTG; >clamp_s_b_s_d_s:compl:rev CACCGTCACAGCCACTATCATGTTGACATTCC; dG = -12.8
259 >s_b_s_b_s_c_s_b_s_c_s GTGGCTGTGGCTGTGTGGGTGGCTGTGTGGGTG; >clamp_s_b_s_d_s:compl:rev CACCGTCACAGCCACTATCATGTTGACATTCC; dG = -12.8
260 >s_b_s_b_s_c_s_b_s_b_s GTGGCTGTGGCTGTGTGGGTGGCTGTGGCTGTG; >clamp_s_b_s_d_s:compl:rev CACCGTCACAGCCACTATCATGTTGACATTCC; dG = -12.8
261 >s_b_s_b_s_c_s_b_s_a_s GTGGCTGTGGCTGTGTGGGTGGCTGTGTCTGTG; >clamp_s_b_s_d_s:compl:rev CACCGTCACAGCCACTATCATGTTGACATTCC; dG = -12.8
262 >s_a_s_b_s_c_s_b_s_e_s GTGTCTGTGGCTGTGTGGGTGGCTGTGCTGGTG; >clamp_s_b_s_d_s:compl:rev CACCGTCACAGCCACTATCATGTTGACATTCC; dG = -12.8
263 >s_a_s_b_s_c_s_b_s_c_s GTGTCTGTGGCTGTGTGGGTGGCTGTGTGGGTG; >clamp_s_b_s_d_s:compl:rev CACCGTCACAGCCACTATCATGTTGACATTCC; dG = -12.8
264 >s_a_s_b_s_c_s_b_s_b_s GTGTCTGTGGCTGTGTGGGTGGCTGTGGCTGTG; >clamp_s_b_s_d_s:compl:rev CACCGTCACAGCCACTATCATGTTGACATTCC; dG = -12.8
265 >s_a_s_b_s_c_s_b_s_a_s GTGTCTGTGGCTGTGTGGGTGGCTGTGTCTGTG; >clamp_s_b_s_d_s:compl:rev CACCGTCACAGCCACTATCATGTTGACATTCC; dG = -12.8
266 >s_a_s_d_s_a_s_b_s_e_s GTGTCTGTGACGGTGTCTGTGGCTGTGCTGGTG; >clamp_s_b_s_d_s:compl:rev CACCGTCACAGCCACTATCATGTTGACATTCC; dG = -12.7
267 >s_a_s_d_s_a_s_b_s_c_s GTGTCTGTGACGGTGTCTGTGGCTGTGTGGGTG; >clamp_s_b_s_d_s:compl:rev CACCGTCACAGCCACTATCATGTTGACATTCC; dG = -12.7
268 >s_a_s_d_s_a_s_b_s_b_s GTGTCTGTGACGGTGTCTGTGGCTGTGGCTGTG; >clamp_s_b_s_d_s:compl:rev CACCGTCACAGCCACTATCATGTTGACATTCC; dG = -12.7
269 >s_a_s_d_s_a_s_b_s_a_s GTGTCTGTGACGGTGTCTGTGGCTGTGTCTGTG; >clamp_s_b_s_d_s:compl:rev CACCGTCACAGCCACTATCATGTTGACATTCC; dG = -12.7
270 >s_a_s_d_s_a_s_e_s_d_s GTGTCTGTGACGGTGTCTGTGCTGGTGACGGTG; >clamp_s_b_s_d_s:compl:rev CACCGTCACAGCCACTATCATGTTGACATTCC; dG = -12.7
271 >s_a_s_d_s_a_s_c_s_d_s GTGTCTGTGACGGTGTCTGTGTGGGTGACGGTG; >clamp_s_b_s_d_s:compl:rev CACCGTCACAGCCACTATCATGTTGACATTCC; dG = -12.7
272 >s_a_s_d_s_c_s_b_s_e_s GTGTCTGTGACGGTGTGGGTGGCTGTGCTGGTG; >clamp_s_b_s_d_s:compl:rev CACCGTCACAGCCACTATCATGTTGACATTCC; dG = -12.7
273 >s_a_s_d_s_c_s_b_s_c_s GTGTCTGTGACGGTGTGGGTGGCTGTGTGGGTG; >clamp_s_b_s_d_s:compl:rev CACCGTCACAGCCACTATCATGTTGACATTCC; dG = -12.7
274 >s_a_s_d_s_c_s_b_s_b_s GTGTCTGTGACGGTGTGGGTGGCTGTGGCTGTG; >clamp_s_b_s_d_s:compl:rev CACCGTCACAGCCACTATCATGTTGACATTCC; dG = -12.7
275 >s_a_s_d_s_c_s_b_s_a_s GTGTCTGTGACGGTGTGGGTGGCTGTGTCTGTG; >clamp_s_b_s_d_s:compl:rev CACCGTCACAGCCACTATCATGTTGACATTCC; dG = -12.7
276 >s_a_s_d_s_c_s_e_s_d_s GTGTCTGTGACGGTGTGGGTGCTGGTGACGGTG; >clamp_s_b_s_d_s:compl:rev CACCGTCACAGCCACTATCATGTTGACATTCC; dG = -12.7
277 >s_a_s_d_s_c_s_d_s_d_s GTGTCTGTGACGGTGTGGGTGACGGTGACGGTG; >clamp_s_b_s_d_s:compl:rev CACCGTCACAGCCACTATCATGTTGACATTCC; dG = -12.7
278 >s_a_s_d_s_c_s_c_s_d_s GTGTCTGTGACGGTGTGGGTGTGGGTGACGGTG; >clamp_s_b_s_d_s:compl:rev CACCGTCACAGCCACTATCATGTTGACATTCC; dG = -12.7

279 >s_b_s_b_s_c_s_c_s_d_s GTGGCTGTGGCTGTGTGGGTGTGGGTGACGGTG; >clamp_s_b_s_d_s:compl:rev CACCGTCACAGCCACTATCATGTTGACATTCC; dG = -12.7
280 >s_c_s_b_s_c_s_b_s_e_s GTGTGGGTGGCTGTGTGGGTGGCTGTGCTGGTG; >clamp_s_b_s_d_s:compl:rev CACCGTCACAGCCACTATCATGTTGACATTCC; dG = -12.7
281 >s_c_s_b_s_c_s_b_s_c_s GTGTGGGTGGCTGTGTGGGTGGCTGTGTGGGTG; >clamp_s_b_s_d_s:compl:rev CACCGTCACAGCCACTATCATGTTGACATTCC; dG = -12.7
282 >s_c_s_b_s_c_s_b_s_b_s GTGTGGGTGGCTGTGTGGGTGGCTGTGGCTGTG; >clamp_s_b_s_d_s:compl:rev CACCGTCACAGCCACTATCATGTTGACATTCC; dG = -12.7
283 >s_c_s_b_s_c_s_b_s_a_s GTGTGGGTGGCTGTGTGGGTGGCTGTGTCTGTG; >clamp_s_b_s_d_s:compl:rev CACCGTCACAGCCACTATCATGTTGACATTCC; dG = -12.7
284 >s_d_s_b_s_c_s_b_s_e_s GTGACGGTGGCTGTGTGGGTGGCTGTGCTGGTG; >clamp_s_b_s_d_s:compl:rev CACCGTCACAGCCACTATCATGTTGACATTCC; dG = -12.7
285 >s_d_s_b_s_c_s_b_s_c_s GTGACGGTGGCTGTGTGGGTGGCTGTGTGGGTG; >clamp_s_b_s_d_s:compl:rev CACCGTCACAGCCACTATCATGTTGACATTCC; dG = -12.7
286 >s_d_s_b_s_c_s_b_s_b_s GTGACGGTGGCTGTGTGGGTGGCTGTGGCTGTG; >clamp_s_b_s_d_s:compl:rev CACCGTCACAGCCACTATCATGTTGACATTCC; dG = -12.7
287 >s_d_s_b_s_c_s_b_s_a_s GTGACGGTGGCTGTGTGGGTGGCTGTGTCTGTG; >clamp_s_b_s_d_s:compl:rev CACCGTCACAGCCACTATCATGTTGACATTCC; dG = -12.7
288 >s_a_s_b_s_c_s_c_s_d_s GTGTCTGTGGCTGTGTGGGTGTGGGTGACGGTG; >clamp_s_b_s_d_s:compl:rev CACCGTCACAGCCACTATCATGTTGACATTCC; dG = -12.7
289 >s_e_s_b_s_c_s_b_s_e_s GTGCTGGTGGCTGTGTGGGTGGCTGTGCTGGTG; >clamp_s_b_s_d_s:compl:rev CACCGTCACAGCCACTATCATGTTGACATTCC; dG = -12.7
290 >s_e_s_b_s_c_s_b_s_c_s GTGCTGGTGGCTGTGTGGGTGGCTGTGTGGGTG; >clamp_s_b_s_d_s:compl:rev CACCGTCACAGCCACTATCATGTTGACATTCC; dG = -12.7
291 >s_e_s_b_s_c_s_b_s_b_s GTGCTGGTGGCTGTGTGGGTGGCTGTGGCTGTG; >clamp_s_b_s_d_s:compl:rev CACCGTCACAGCCACTATCATGTTGACATTCC; dG = -12.7
292 >s_e_s_b_s_c_s_b_s_a_s GTGCTGGTGGCTGTGTGGGTGGCTGTGTCTGTG; >clamp_s_b_s_d_s:compl:rev CACCGTCACAGCCACTATCATGTTGACATTCC; dG = -12.7
293 >s_a_s_d_s_e_s_b_s_e_s GTGTCTGTGACGGTGCTGGTGGCTGTGCTGGTG; >clamp_s_b_s_d_s:compl:rev CACCGTCACAGCCACTATCATGTTGACATTCC; dG = -12.6
294 >s_a_s_d_s_e_s_b_s_c_s GTGTCTGTGACGGTGCTGGTGGCTGTGTGGGTG; >clamp_s_b_s_d_s:compl:rev CACCGTCACAGCCACTATCATGTTGACATTCC; dG = -12.6
295 >s_a_s_d_s_e_s_b_s_b_s GTGTCTGTGACGGTGCTGGTGGCTGTGGCTGTG; >clamp_s_b_s_d_s:compl:rev CACCGTCACAGCCACTATCATGTTGACATTCC; dG = -12.6
296 >s_a_s_d_s_e_s_b_s_a_s GTGTCTGTGACGGTGCTGGTGGCTGTGTCTGTG; >clamp_s_b_s_d_s:compl:rev CACCGTCACAGCCACTATCATGTTGACATTCC; dG = -12.6
297 >s_a_s_d_s_e_s_e_s_d_s GTGTCTGTGACGGTGCTGGTGCTGGTGACGGTG; >clamp_s_b_s_d_s:compl:rev CACCGTCACAGCCACTATCATGTTGACATTCC; dG = -12.6
298 >s_a_s_d_s_e_s_d_s_d_s GTGTCTGTGACGGTGCTGGTGACGGTGACGGTG; >clamp_s_b_s_d_s:compl:rev CACCGTCACAGCCACTATCATGTTGACATTCC; dG = -12.6
299 >s_a_s_d_s_e_s_c_s_d_s GTGTCTGTGACGGTGCTGGTGTGGGTGACGGTG; >clamp_s_b_s_d_s:compl:rev CACCGTCACAGCCACTATCATGTTGACATTCC; dG = -12.6
300 >s_b_s_b_s_c_s_e_s_d_s GTGGCTGTGGCTGTGTGGGTGCTGGTGACGGTG; >clamp_s_b_s_d_s:compl:rev CACCGTCACAGCCACTATCATGTTGACATTCC; dG = -12.6
301 >s_c_s_b_s_c_s_c_s_d_s GTGTGGGTGGCTGTGTGGGTGTGGGTGACGGTG; >clamp_s_b_s_d_s:compl:rev CACCGTCACAGCCACTATCATGTTGACATTCC; dG = -12.6
302 >s_d_s_b_s_c_s_c_s_d_s GTGACGGTGGCTGTGTGGGTGTGGGTGACGGTG; >clamp_s_b_s_d_s:compl:rev CACCGTCACAGCCACTATCATGTTGACATTCC; dG = -12.6
303 >s_a_s_b_s_c_s_e_s_d_s GTGTCTGTGGCTGTGTGGGTGCTGGTGACGGTG; >clamp_s_b_s_d_s:compl:rev CACCGTCACAGCCACTATCATGTTGACATTCC; dG = -12.6
304 >s_e_s_b_s_c_s_c_s_d_s GTGCTGGTGGCTGTGTGGGTGTGGGTGACGGTG; >clamp_s_b_s_d_s:compl:rev CACCGTCACAGCCACTATCATGTTGACATTCC; dG = -12.6
305 >s_b_s_a_s_c_s_d_s_d_s GTGGCTGTGTCTGTGTGGGTGACGGTGACGGTG; >clamp_s_b_s_d_s:compl:rev CACCGTCACAGCCACTATCATGTTGACATTCC; dG = -12.5
306 >s_b_s_a_s_e_s_d_s_d_s GTGGCTGTGTCTGTGCTGGTGACGGTGACGGTG; >clamp_s_b_s_d_s:compl:rev CACCGTCACAGCCACTATCATGTTGACATTCC; dG = -12.5
307 >s_b_s_c_s_c_s_d_s_d_s GTGGCTGTGTGGGTGTGGGTGACGGTGACGGTG; >clamp_s_b_s_d_s:compl:rev CACCGTCACAGCCACTATCATGTTGACATTCC; dG = -12.5
308 >s_b_s_c_s_e_s_d_s_d_s GTGGCTGTGTGGGTGCTGGTGACGGTGACGGTG; >clamp_s_b_s_d_s:compl:rev CACCGTCACAGCCACTATCATGTTGACATTCC; dG = -12.5
309 >s_b_s_e_s_c_s_d_s_d_s GTGGCTGTGCTGGTGTGGGTGACGGTGACGGTG; >clamp_s_b_s_d_s:compl:rev CACCGTCACAGCCACTATCATGTTGACATTCC; dG = -12.5
310 >s_b_s_e_s_e_s_d_s_d_s GTGGCTGTGCTGGTGCTGGTGACGGTGACGGTG; >clamp_s_b_s_d_s:compl:rev CACCGTCACAGCCACTATCATGTTGACATTCC; dG = -12.5
311 >s_c_s_b_s_c_s_e_s_d_s GTGTGGGTGGCTGTGTGGGTGCTGGTGACGGTG; >clamp_s_b_s_d_s:compl:rev CACCGTCACAGCCACTATCATGTTGACATTCC; dG = -12.5
312 >s_d_s_b_s_c_s_e_s_d_s GTGACGGTGGCTGTGTGGGTGCTGGTGACGGTG; >clamp_s_b_s_d_s:compl:rev CACCGTCACAGCCACTATCATGTTGACATTCC; dG = -12.5
313 >s_e_s_b_s_c_s_e_s_d_s GTGCTGGTGGCTGTGTGGGTGCTGGTGACGGTG; >clamp_s_b_s_d_s:compl:rev CACCGTCACAGCCACTATCATGTTGACATTCC; dG = -12.5
314 >s_a_s_a_s_b_s_b_s_c_s GTGTCTGTGTCTGTGGCTGTGGCTGTGTGGGTG; >clamp_s_b_s_d_s:compl:rev CACCGTCACAGCCACTATCATGTTGACATTCC; dG = -12.4
315 >s_a_s_c_s_e_s_b_s_c_s GTGTCTGTGTGGGTGCTGGTGGCTGTGTGGGTG; >clamp_s_b_s_d_s:compl:rev CACCGTCACAGCCACTATCATGTTGACATTCC; dG = -12.4

316 >s_a_s_e_s_a_s_b_s_c_s GTGTCTGTGCTGGTGTCTGTGGCTGTGTGGGTG; >clamp_s_b_s_d_s:compl:rev CACCGTCACAGCCACTATCATGTTGACATTCC; dG = -12.4
317 >s_a_s_e_s_b_s_b_s_c_s GTGTCTGTGCTGGTGGCTGTGGCTGTGTGGGTG; >clamp_s_b_s_d_s:compl:rev CACCGTCACAGCCACTATCATGTTGACATTCC; dG = -12.4
318 >s_a_s_e_s_c_s_b_s_c_s GTGTCTGTGCTGGTGTGGGTGGCTGTGTGGGTG; >clamp_s_b_s_d_s:compl:rev CACCGTCACAGCCACTATCATGTTGACATTCC; dG = -12.4
319 >s_a_s_a_s_c_s_b_s_c_s GTGTCTGTGTCTGTGTGGGTGGCTGTGTGGGTG; >clamp_s_b_s_d_s:compl:rev CACCGTCACAGCCACTATCATGTTGACATTCC; dG = -12.4
320 >s_a_s_e_s_d_s_b_s_c_s GTGTCTGTGCTGGTGACGGTGGCTGTGTGGGTG; >clamp_s_b_s_d_s:compl:rev CACCGTCACAGCCACTATCATGTTGACATTCC; dG = -12.4
321 >s_a_s_e_s_e_s_b_s_c_s GTGTCTGTGCTGGTGCTGGTGGCTGTGTGGGTG; >clamp_s_b_s_d_s:compl:rev CACCGTCACAGCCACTATCATGTTGACATTCC; dG = -12.4
322 >s_b_s_a_s_a_s_b_s_c_s GTGGCTGTGTCTGTGTCTGTGGCTGTGTGGGTG; >clamp_s_b_s_d_s:compl:rev CACCGTCACAGCCACTATCATGTTGACATTCC; dG = -12.4
323 >s_b_s_a_s_b_s_b_s_c_s GTGGCTGTGTCTGTGGCTGTGGCTGTGTGGGTG; >clamp_s_b_s_d_s:compl:rev CACCGTCACAGCCACTATCATGTTGACATTCC; dG = -12.4
324 >s_a_s_a_s_a_s_b_s_c_s GTGTCTGTGTCTGTGTCTGTGGCTGTGTGGGTG; >clamp_s_b_s_d_s:compl:rev CACCGTCACAGCCACTATCATGTTGACATTCC; dG = -12.4
325 >s_b_s_a_s_c_s_b_s_c_s GTGGCTGTGTCTGTGTGGGTGGCTGTGTGGGTG; >clamp_s_b_s_d_s:compl:rev CACCGTCACAGCCACTATCATGTTGACATTCC; dG = -12.4
326 >s_b_s_a_s_e_s_b_s_c_s GTGGCTGTGTCTGTGCTGGTGGCTGTGTGGGTG; >clamp_s_b_s_d_s:compl:rev CACCGTCACAGCCACTATCATGTTGACATTCC; dG = -12.4
327 >s_b_s_b_s_a_s_b_s_c_s GTGGCTGTGGCTGTGTCTGTGGCTGTGTGGGTG; >clamp_s_b_s_d_s:compl:rev CACCGTCACAGCCACTATCATGTTGACATTCC; dG = -12.4
328 >s_b_s_b_s_b_s_b_s_c_s GTGGCTGTGGCTGTGGCTGTGGCTGTGTGGGTG; >clamp_s_b_s_d_s:compl:rev CACCGTCACAGCCACTATCATGTTGACATTCC; dG = -12.4
329 >s_b_s_b_s_e_s_b_s_c_s GTGGCTGTGGCTGTGCTGGTGGCTGTGTGGGTG; >clamp_s_b_s_d_s:compl:rev CACCGTCACAGCCACTATCATGTTGACATTCC; dG = -12.4
330 >s_b_s_c_s_a_s_b_s_c_s GTGGCTGTGTGGGTGTCTGTGGCTGTGTGGGTG; >clamp_s_b_s_d_s:compl:rev CACCGTCACAGCCACTATCATGTTGACATTCC; dG = -12.4
331 >s_b_s_c_s_b_s_b_s_c_s GTGGCTGTGTGGGTGGCTGTGGCTGTGTGGGTG; >clamp_s_b_s_d_s:compl:rev CACCGTCACAGCCACTATCATGTTGACATTCC; dG = -12.4
332 >s_b_s_c_s_c_s_b_s_c_s GTGGCTGTGTGGGTGTGGGTGGCTGTGTGGGTG; >clamp_s_b_s_d_s:compl:rev CACCGTCACAGCCACTATCATGTTGACATTCC; dG = -12.4
333 >s_b_s_c_s_e_s_b_s_c_s GTGGCTGTGTGGGTGCTGGTGGCTGTGTGGGTG; >clamp_s_b_s_d_s:compl:rev CACCGTCACAGCCACTATCATGTTGACATTCC; dG = -12.4
334 >s_b_s_e_s_a_s_b_s_c_s GTGGCTGTGCTGGTGTCTGTGGCTGTGTGGGTG; >clamp_s_b_s_d_s:compl:rev CACCGTCACAGCCACTATCATGTTGACATTCC; dG = -12.4
335 >s_b_s_e_s_b_s_b_s_c_s GTGGCTGTGCTGGTGGCTGTGGCTGTGTGGGTG; >clamp_s_b_s_d_s:compl:rev CACCGTCACAGCCACTATCATGTTGACATTCC; dG = -12.4
336 >s_b_s_e_s_c_s_b_s_c_s GTGGCTGTGCTGGTGTGGGTGGCTGTGTGGGTG; >clamp_s_b_s_d_s:compl:rev CACCGTCACAGCCACTATCATGTTGACATTCC; dG = -12.4
337 >s_b_s_e_s_e_s_b_s_c_s GTGGCTGTGCTGGTGCTGGTGGCTGTGTGGGTG; >clamp_s_b_s_d_s:compl:rev CACCGTCACAGCCACTATCATGTTGACATTCC; dG = -12.4
338 >s_a_s_a_s_e_s_b_s_c_s GTGTCTGTGTCTGTGCTGGTGGCTGTGTGGGTG; >clamp_s_b_s_d_s:compl:rev CACCGTCACAGCCACTATCATGTTGACATTCC; dG = -12.4
339 >s_c_s_a_s_a_s_b_s_c_s GTGTGGGTGTCTGTGTCTGTGGCTGTGTGGGTG; >clamp_s_b_s_d_s:compl:rev CACCGTCACAGCCACTATCATGTTGACATTCC; dG = -12.4
340 >s_c_s_a_s_b_s_b_s_c_s GTGTGGGTGTCTGTGGCTGTGGCTGTGTGGGTG; >clamp_s_b_s_d_s:compl:rev CACCGTCACAGCCACTATCATGTTGACATTCC; dG = -12.4
341 >s_c_s_a_s_c_s_b_s_c_s GTGTGGGTGTCTGTGTGGGTGGCTGTGTGGGTG; >clamp_s_b_s_d_s:compl:rev CACCGTCACAGCCACTATCATGTTGACATTCC; dG = -12.4
342 >s_c_s_a_s_e_s_b_s_c_s GTGTGGGTGTCTGTGCTGGTGGCTGTGTGGGTG; >clamp_s_b_s_d_s:compl:rev CACCGTCACAGCCACTATCATGTTGACATTCC; dG = -12.4
343 >s_c_s_b_s_a_s_b_s_c_s GTGTGGGTGGCTGTGTCTGTGGCTGTGTGGGTG; >clamp_s_b_s_d_s:compl:rev CACCGTCACAGCCACTATCATGTTGACATTCC; dG = -12.4
344 >s_c_s_b_s_b_s_b_s_c_s GTGTGGGTGGCTGTGGCTGTGGCTGTGTGGGTG; >clamp_s_b_s_d_s:compl:rev CACCGTCACAGCCACTATCATGTTGACATTCC; dG = -12.4
345 >s_c_s_b_s_e_s_b_s_c_s GTGTGGGTGGCTGTGCTGGTGGCTGTGTGGGTG; >clamp_s_b_s_d_s:compl:rev CACCGTCACAGCCACTATCATGTTGACATTCC; dG = -12.4
346 >s_c_s_c_s_a_s_b_s_c_s GTGTGGGTGTGGGTGTCTGTGGCTGTGTGGGTG; >clamp_s_b_s_d_s:compl:rev CACCGTCACAGCCACTATCATGTTGACATTCC; dG = -12.4
347 >s_a_s_b_s_a_s_b_s_c_s GTGTCTGTGGCTGTGTCTGTGGCTGTGTGGGTG; >clamp_s_b_s_d_s:compl:rev CACCGTCACAGCCACTATCATGTTGACATTCC; dG = -12.4
348 >s_c_s_c_s_b_s_b_s_c_s GTGTGGGTGTGGGTGGCTGTGGCTGTGTGGGTG; >clamp_s_b_s_d_s:compl:rev CACCGTCACAGCCACTATCATGTTGACATTCC; dG = -12.4
349 >s_c_s_c_s_c_s_b_s_c_s GTGTGGGTGTGGGTGTGGGTGGCTGTGTGGGTG; >clamp_s_b_s_d_s:compl:rev CACCGTCACAGCCACTATCATGTTGACATTCC; dG = -12.4
350 >s_c_s_c_s_d_s_b_s_c_s GTGTGGGTGTGGGTGACGGTGGCTGTGTGGGTG; >clamp_s_b_s_d_s:compl:rev CACCGTCACAGCCACTATCATGTTGACATTCC; dG = -12.4
351 >s_c_s_c_s_e_s_b_s_c_s GTGTGGGTGTGGGTGCTGGTGGCTGTGTGGGTG; >clamp_s_b_s_d_s:compl:rev CACCGTCACAGCCACTATCATGTTGACATTCC; dG = -12.4
352 >s_c_s_d_s_a_s_b_s_c_s GTGTGGGTGACGGTGTCTGTGGCTGTGTGGGTG; >clamp_s_b_s_d_s:compl:rev CACCGTCACAGCCACTATCATGTTGACATTCC; dG = -12.4

353 >s_c_s_d_s_b_s_b_s_c_s GTGTGGGTGACGGTGGCTGTGGCTGTGTGGGTG; >clamp_s_b_s_d_s:compl:rev
CACCGTCACAGCCACTATCATGTTGACATTCC; dG = -12.4
354 >s_c_s_d_s_c_s_b_s_c_s GTGTGGGTGACGGTGTGGGTGGCTGTGTGGGTG; >clamp_s_b_s_d_s:compl:rev
CACCGTCACAGCCACTATCATGTTGACATTCC; dG = -12.4
355 >s_c_s_d_s_d_s_b_s_c_s GTGTGGGTGACGGTGACGGTGGCTGTGTGGGTG; >clamp_s_b_s_d_s:compl:rev
CACCGTCACAGCCACTATCATGTTGACATTCC; dG = -12.4
356 >s_c_s_d_s_e_s_b_s_c_s GTGTGGGTGACGGTGCTGGTGGCTGTGTGGGTG; >clamp_s_b_s_d_s:compl:rev
CACCGTCACAGCCACTATCATGTTGACATTCC; dG = -12.4
357 >s_c_s_e_s_a_s_b_s_c_s GTGTGGGTGCTGGTGTCTGTGGCTGTGTGGGTG; >clamp_s_b_s_d_s:compl:rev
CACCGTCACAGCCACTATCATGTTGACATTCC; dG = -12.4
358 >s_a_s_b_s_b_s_b_s_c_s GTGTCTGTGGCTGTGGCTGTGGCTGTGTGGGTG; >clamp_s_b_s_d_s:compl:rev
CACCGTCACAGCCACTATCATGTTGACATTCC; dG = -12.4
359 >s_c_s_e_s_b_s_b_s_c_s GTGTGGGTGCTGGTGGCTGTGGCTGTGTGGGTG; >clamp_s_b_s_d_s:compl:rev
CACCGTCACAGCCACTATCATGTTGACATTCC; dG = -12.4
360 >s_c_s_e_s_c_s_b_s_c_s GTGTGGGTGCTGGTGTGGGTGGCTGTGTGGGTG; >clamp_s_b_s_d_s:compl:rev
CACCGTCACAGCCACTATCATGTTGACATTCC; dG = -12.4
361 >s_c_s_e_s_d_s_b_s_c_s GTGTGGGTGCTGGTGACGGTGGCTGTGTGGGTG; >clamp_s_b_s_d_s:compl:rev
CACCGTCACAGCCACTATCATGTTGACATTCC; dG = -12.4
362 >s_c_s_e_s_e_s_b_s_c_s GTGTGGGTGCTGGTGCTGGTGGCTGTGTGGGTG; >clamp_s_b_s_d_s:compl:rev
CACCGTCACAGCCACTATCATGTTGACATTCC; dG = -12.4
363 >s_d_s_a_s_a_s_b_s_c_s GTGACGGTGTCTGTGTCTGTGGCTGTGTGGGTG; >clamp_s_b_s_d_s:compl:rev
CACCGTCACAGCCACTATCATGTTGACATTCC; dG = -12.4
364 >s_d_s_a_s_b_s_b_s_c_s GTGACGGTGTCTGTGGCTGTGGCTGTGTGGGTG; >clamp_s_b_s_d_s:compl:rev
CACCGTCACAGCCACTATCATGTTGACATTCC; dG = -12.4
365 >s_d_s_a_s_c_s_b_s_c_s GTGACGGTGTCTGTGTGGGTGGCTGTGTGGGTG; >clamp_s_b_s_d_s:compl:rev
CACCGTCACAGCCACTATCATGTTGACATTCC; dG = -12.4
366 >s_d_s_a_s_e_s_b_s_c_s GTGACGGTGTCTGTGCTGGTGGCTGTGTGGGTG; >clamp_s_b_s_d_s:compl:rev
CACCGTCACAGCCACTATCATGTTGACATTCC; dG = -12.4
367 >s_d_s_b_s_a_s_b_s_c_s GTGACGGTGGCTGTGTCTGTGGCTGTGTGGGTG; >clamp_s_b_s_d_s:compl:rev
CACCGTCACAGCCACTATCATGTTGACATTCC; dG = -12.4
368 >s_d_s_b_s_b_s_b_s_c_s GTGACGGTGGCTGTGGCTGTGGCTGTGTGGGTG; >clamp_s_b_s_d_s:compl:rev
CACCGTCACAGCCACTATCATGTTGACATTCC; dG = -12.4
369 >s_d_s_b_s_e_s_b_s_c_s GTGACGGTGGCTGTGCTGGTGGCTGTGTGGGTG; >clamp_s_b_s_d_s:compl:rev
CACCGTCACAGCCACTATCATGTTGACATTCC; dG = -12.4
370 >s_d_s_c_s_a_s_b_s_c_s GTGACGGTGTGGGTGTCTGTGGCTGTGTGGGTG; >clamp_s_b_s_d_s:compl:rev
CACCGTCACAGCCACTATCATGTTGACATTCC; dG = -12.4
371 >s_d_s_c_s_b_s_b_s_c_s GTGACGGTGTGGGTGGCTGTGGCTGTGTGGGTG; >clamp_s_b_s_d_s:compl:rev
CACCGTCACAGCCACTATCATGTTGACATTCC; dG = -12.4
372 >s_d_s_c_s_c_s_b_s_c_s GTGACGGTGTGGGTGTGGGTGGCTGTGTGGGTG; >clamp_s_b_s_d_s:compl:rev
CACCGTCACAGCCACTATCATGTTGACATTCC; dG = -12.4
373 >s_d_s_c_s_d_s_b_s_c_s GTGACGGTGTGGGTGACGGTGGCTGTGTGGGTG; >clamp_s_b_s_d_s:compl:rev
CACCGTCACAGCCACTATCATGTTGACATTCC; dG = -12.4
374 >s_d_s_c_s_e_s_b_s_c_s GTGACGGTGTGGGTGCTGGTGGCTGTGTGGGTG; >clamp_s_b_s_d_s:compl:rev
CACCGTCACAGCCACTATCATGTTGACATTCC; dG = -12.4
375 >s_d_s_d_s_a_s_b_s_c_s GTGACGGTGACGGTGTCTGTGGCTGTGTGGGTG; >clamp_s_b_s_d_s:compl:rev
CACCGTCACAGCCACTATCATGTTGACATTCC; dG = -12.4
376 >s_a_s_b_s_e_s_b_s_c_s GTGTCTGTGGCTGTGCTGGTGGCTGTGTGGGTG; >clamp_s_b_s_d_s:compl:rev
CACCGTCACAGCCACTATCATGTTGACATTCC; dG = -12.4
377 >s_d_s_d_s_b_s_b_s_c_s GTGACGGTGACGGTGGCTGTGGCTGTGTGGGTG; >clamp_s_b_s_d_s:compl:rev
CACCGTCACAGCCACTATCATGTTGACATTCC; dG = -12.4
378 >s_d_s_d_s_c_s_b_s_c_s GTGACGGTGACGGTGTGGGTGGCTGTGTGGGTG; >clamp_s_b_s_d_s:compl:rev
CACCGTCACAGCCACTATCATGTTGACATTCC; dG = -12.4
379 >s_d_s_d_s_d_s_b_s_c_s GTGACGGTGACGGTGACGGTGGCTGTGTGGGTG; >clamp_s_b_s_d_s:compl:rev
CACCGTCACAGCCACTATCATGTTGACATTCC; dG = -12.4
380 >s_d_s_d_s_e_s_b_s_c_s GTGACGGTGACGGTGCTGGTGGCTGTGTGGGTG; >clamp_s_b_s_d_s:compl:rev
CACCGTCACAGCCACTATCATGTTGACATTCC; dG = -12.4
381 >s_d_s_e_s_a_s_b_s_c_s GTGACGGTGCTGGTGTCTGTGGCTGTGTGGGTG; >clamp_s_b_s_d_s:compl:rev
CACCGTCACAGCCACTATCATGTTGACATTCC; dG = -12.4
382 >s_d_s_e_s_b_s_b_s_c_s GTGACGGTGCTGGTGGCTGTGGCTGTGTGGGTG; >clamp_s_b_s_d_s:compl:rev
CACCGTCACAGCCACTATCATGTTGACATTCC; dG = -12.4
383 >s_d_s_e_s_c_s_b_s_c_s GTGACGGTGCTGGTGTGGGTGGCTGTGTGGGTG; >clamp_s_b_s_d_s:compl:rev
CACCGTCACAGCCACTATCATGTTGACATTCC; dG = -12.4
384 >s_d_s_e_s_d_s_b_s_c_s GTGACGGTGCTGGTGACGGTGGCTGTGTGGGTG; >clamp_s_b_s_d_s:compl:rev
CACCGTCACAGCCACTATCATGTTGACATTCC; dG = -12.4
385 >s_d_s_e_s_e_s_b_s_c_s GTGACGGTGCTGGTGCTGGTGGCTGTGTGGGTG; >clamp_s_b_s_d_s:compl:rev
CACCGTCACAGCCACTATCATGTTGACATTCC; dG = -12.4
386 >s_e_s_a_s_a_s_b_s_c_s GTGCTGGTGTCTGTGTCTGTGGCTGTGTGGGTG; >clamp_s_b_s_d_s:compl:rev
CACCGTCACAGCCACTATCATGTTGACATTCC; dG = -12.4
387 >s_e_s_a_s_b_s_b_s_c_s GTGCTGGTGTCTGTGGCTGTGGCTGTGTGGGTG; >clamp_s_b_s_d_s:compl:rev
CACCGTCACAGCCACTATCATGTTGACATTCC; dG = -12.4
388 >s_a_s_c_s_a_s_b_s_c_s GTGTCTGTGTGGGTGTCTGTGGCTGTGTGGGTG; >clamp_s_b_s_d_s:compl:rev
CACCGTCACAGCCACTATCATGTTGACATTCC; dG = -12.4
389 >s_e_s_a_s_c_s_b_s_c_s GTGCTGGTGTCTGTGTGGGTGGCTGTGTGGGTG; >clamp_s_b_s_d_s:compl:rev
CACCGTCACAGCCACTATCATGTTGACATTCC; dG = -12.4

390 >s_e_s_a_s_e_s_b_s_c_s GTGCTGGTGTCTGTGCTGGTGGCTGTGTGGGTG; >clamp_s_b_s_d_s:compl:rev CACCGTCACAGCCACTATCATGTTGACATTCC; dG = -12.4
391 >s_e_s_b_s_a_s_b_s_c_s GTGCTGGTGGCTGTGTCTGTGGCTGTGTGGGTG; >clamp_s_b_s_d_s:compl:rev CACCGTCACAGCCACTATCATGTTGACATTCC; dG = -12.4
392 >s_e_s_b_s_b_s_b_s_c_s GTGCTGGTGGCTGTGGCTGTGGCTGTGTGGGTG; >clamp_s_b_s_d_s:compl:rev CACCGTCACAGCCACTATCATGTTGACATTCC; dG = -12.4
393 >s_e_s_b_s_e_s_b_s_c_s GTGCTGGTGGCTGTGCTGGTGGCTGTGTGGGTG; >clamp_s_b_s_d_s:compl:rev CACCGTCACAGCCACTATCATGTTGACATTCC; dG = -12.4
394 >s_e_s_c_s_a_s_b_s_c_s GTGCTGGTGTGGGTGTCTGTGGCTGTGTGGGTG; >clamp_s_b_s_d_s:compl:rev CACCGTCACAGCCACTATCATGTTGACATTCC; dG = -12.4
395 >s_e_s_c_s_b_s_b_s_c_s GTGCTGGTGTGGGTGGCTGTGGCTGTGTGGGTG; >clamp_s_b_s_d_s:compl:rev CACCGTCACAGCCACTATCATGTTGACATTCC; dG = -12.4
396 >s_e_s_c_s_c_s_b_s_c_s GTGCTGGTGTGGGTGTGGGTGGCTGTGTGGGTG; >clamp_s_b_s_d_s:compl:rev CACCGTCACAGCCACTATCATGTTGACATTCC; dG = -12.4
397 >s_a_s_c_s_b_s_b_s_c_s GTGTCTGTGTGGGTGGCTGTGGCTGTGTGGGTG; >clamp_s_b_s_d_s:compl:rev CACCGTCACAGCCACTATCATGTTGACATTCC; dG = -12.4
398 >s_e_s_c_s_d_s_b_s_c_s GTGCTGGTGTGGGTGACGGTGGCTGTGTGGGTG; >clamp_s_b_s_d_s:compl:rev CACCGTCACAGCCACTATCATGTTGACATTCC; dG = -12.4
399 >s_e_s_c_s_e_s_b_s_c_s GTGCTGGTGTGGGTGCTGGTGGCTGTGTGGGTG; >clamp_s_b_s_d_s:compl:rev CACCGTCACAGCCACTATCATGTTGACATTCC; dG = -12.4
400 >s_e_s_d_s_a_s_b_s_c_s GTGCTGGTGACGGTGTCTGTGGCTGTGTGGGTG; >clamp_s_b_s_d_s:compl:rev CACCGTCACAGCCACTATCATGTTGACATTCC; dG = -12.4
401 >s_e_s_d_s_b_s_b_s_c_s GTGCTGGTGACGGTGGCTGTGGCTGTGTGGGTG; >clamp_s_b_s_d_s:compl:rev CACCGTCACAGCCACTATCATGTTGACATTCC; dG = -12.4
402 >s_e_s_d_s_c_s_b_s_c_s GTGCTGGTGACGGTGTGGGTGGCTGTGTGGGTG; >clamp_s_b_s_d_s:compl:rev CACCGTCACAGCCACTATCATGTTGACATTCC; dG = -12.4
403 >s_e_s_d_s_d_s_b_s_c_s GTGCTGGTGACGGTGACGGTGGCTGTGTGGGTG; >clamp_s_b_s_d_s:compl:rev CACCGTCACAGCCACTATCATGTTGACATTCC; dG = -12.4
404 >s_e_s_d_s_e_s_b_s_c_s GTGCTGGTGACGGTGCTGGTGGCTGTGTGGGTG; >clamp_s_b_s_d_s:compl:rev CACCGTCACAGCCACTATCATGTTGACATTCC; dG = -12.4
405 >s_e_s_e_s_a_s_b_s_c_s GTGCTGGTGCTGGTGTCTGTGGCTGTGTGGGTG; >clamp_s_b_s_d_s:compl:rev CACCGTCACAGCCACTATCATGTTGACATTCC; dG = -12.4
406 >s_e_s_e_s_b_s_b_s_c_s GTGCTGGTGCTGGTGGCTGTGGCTGTGTGGGTG; >clamp_s_b_s_d_s:compl:rev CACCGTCACAGCCACTATCATGTTGACATTCC; dG = -12.4
407 >s_a_s_c_s_c_s_b_s_c_s GTGTCTGTGTGGGTGTGGGTGGCTGTGTGGGTG; >clamp_s_b_s_d_s:compl:rev CACCGTCACAGCCACTATCATGTTGACATTCC; dG = -12.4
408 >s_e_s_e_s_c_s_b_s_c_s GTGCTGGTGCTGGTGTGGGTGGCTGTGTGGGTG; >clamp_s_b_s_d_s:compl:rev CACCGTCACAGCCACTATCATGTTGACATTCC; dG = -12.4
409 >s_e_s_e_s_d_s_b_s_c_s GTGCTGGTGCTGGTGACGGTGGCTGTGTGGGTG; >clamp_s_b_s_d_s:compl:rev CACCGTCACAGCCACTATCATGTTGACATTCC; dG = -12.4
410 >s_e_s_e_s_e_s_b_s_c_s GTGCTGGTGCTGGTGCTGGTGGCTGTGTGGGTG; >clamp_s_b_s_d_s:compl:rev CACCGTCACAGCCACTATCATGTTGACATTCC; dG = -12.4
411 >s_a_s_c_s_d_s_b_s_c_s GTGTCTGTGTGGGTGACGGTGGCTGTGTGGGTG; >clamp_s_b_s_d_s:compl:rev CACCGTCACAGCCACTATCATGTTGACATTCC; dG = -12.4
412 >s_c_s_c_s_c_s_d_s_d_s GTGTGGGTGTGGGTGTGGGTGACGGTGACGGTG; >clamp_s_b_s_d_s:compl:rev CACCGTCACAGCCACTATCATGTTGACATTCC; dG = -12.3
413 >s_c_s_c_s_d_s_d_s_d_s GTGTGGGTGTGGGTGACGGTGACGGTGACGGTG; >clamp_s_b_s_d_s:compl:rev CACCGTCACAGCCACTATCATGTTGACATTCC; dG = -12.3
414 >s_d_s_c_s_c_s_d_s_d_s GTGACGGTGTGGGTGTGGGTGACGGTGACGGTG; >clamp_s_b_s_d_s:compl:rev CACCGTCACAGCCACTATCATGTTGACATTCC; dG = -12.3
415 >s_e_s_c_s_c_s_d_s_d_s GTGCTGGTGTGGGTGTGGGTGACGGTGACGGTG; >clamp_s_b_s_d_s:compl:rev CACCGTCACAGCCACTATCATGTTGACATTCC; dG = -12.3
416 >s_a_s_c_s_c_s_d_s_d_s GTGTCTGTGTGGGTGTGGGTGACGGTGACGGTG; >clamp_s_b_s_d_s:compl:rev CACCGTCACAGCCACTATCATGTTGACATTCC; dG = -12.3
417 >s_a_s_c_s_e_s_d_s_d_s GTGTCTGTGTGGGTGCTGGTGACGGTGACGGTG; >clamp_s_b_s_d_s:compl:rev CACCGTCACAGCCACTATCATGTTGACATTCC; dG = -12.2
418 >s_b_s_b_s_a_s_e_s_d_s GTGGCTGTGGCTGTGTCTGTGCTGGTGACGGTG; >clamp_s_b_s_d_s:compl:rev CACCGTCACAGCCACTATCATGTTGACATTCC; dG = -12.2
419 >s_b_s_b_s_a_s_c_s_d_s GTGGCTGTGGCTGTGTCTGTGTGGGTGACGGTG; >clamp_s_b_s_d_s:compl:rev CACCGTCACAGCCACTATCATGTTGACATTCC; dG = -12.2
420 >s_b_s_b_s_e_s_e_s_d_s GTGGCTGTGGCTGTGCTGGTGCTGGTGACGGTG; >clamp_s_b_s_d_s:compl:rev CACCGTCACAGCCACTATCATGTTGACATTCC; dG = -12.2
421 >s_b_s_b_s_e_s_c_s_d_s GTGGCTGTGGCTGTGCTGGTGTGGGTGACGGTG; >clamp_s_b_s_d_s:compl:rev CACCGTCACAGCCACTATCATGTTGACATTCC; dG = -12.2
422 >s_b_s_c_s_a_s_b_s_e_s GTGGCTGTGTGGGTGTCTGTGGCTGTGCTGGTG; >clamp_s_b_s_d_s:compl:rev CACCGTCACAGCCACTATCATGTTGACATTCC; dG = -12.2
423 >s_b_s_c_s_a_s_b_s_b_s GTGGCTGTGTGGGTGTCTGTGGCTGTGGCTGTG; >clamp_s_b_s_d_s:compl:rev CACCGTCACAGCCACTATCATGTTGACATTCC; dG = -12.2
424 >s_b_s_c_s_a_s_b_s_a_s GTGGCTGTGTGGGTGTCTGTGGCTGTGTCTGTG; >clamp_s_b_s_d_s:compl:rev CACCGTCACAGCCACTATCATGTTGACATTCC; dG = -12.2
425 >s_b_s_c_s_a_s_e_s_d_s GTGGCTGTGTGGGTGTCTGTGCTGGTGACGGTG; >clamp_s_b_s_d_s:compl:rev CACCGTCACAGCCACTATCATGTTGACATTCC; dG = -12.2
426 >s_b_s_c_s_a_s_c_s_d_s GTGGCTGTGTGGGTGTCTGTGTGGGTGACGGTG; >clamp_s_b_s_d_s:compl:rev CACCGTCACAGCCACTATCATGTTGACATTCC; dG = -12.2

427 >s_b_s_c_s_b_s_b_s_e_s GTGGCTGTGTGGGTGGCTGTGGCTGTGCTGGTG; >clamp_s_b_s_d_s:compl:rev CACCGTCACAGCCACTATCATGTTGACATTCC; dG = -12.2
428 >s_b_s_c_s_b_s_b_s_b_s GTGGCTGTGTGGGTGGCTGTGGCTGTGGCTGTG; >clamp_s_b_s_d_s:compl:rev CACCGTCACAGCCACTATCATGTTGACATTCC; dG = -12.2
429 >s_b_s_c_s_b_s_b_s_a_s GTGGCTGTGTGGGTGGCTGTGGCTGTGTCTGTG; >clamp_s_b_s_d_s:compl:rev CACCGTCACAGCCACTATCATGTTGACATTCC; dG = -12.2
430 >s_b_s_c_s_c_s_b_s_e_s GTGGCTGTGTGGGTGTGGGTGGCTGTGCTGGTG; >clamp_s_b_s_d_s:compl:rev CACCGTCACAGCCACTATCATGTTGACATTCC; dG = -12.2
431 >s_b_s_c_s_c_s_b_s_b_s GTGGCTGTGTGGGTGTGGGTGGCTGTGGCTGTG; >clamp_s_b_s_d_s:compl:rev CACCGTCACAGCCACTATCATGTTGACATTCC; dG = -12.2
432 >s_b_s_c_s_c_s_b_s_a_s GTGGCTGTGTGGGTGTGGGTGGCTGTGTCTGTG; >clamp_s_b_s_d_s:compl:rev CACCGTCACAGCCACTATCATGTTGACATTCC; dG = -12.2
433 >s_b_s_c_s_c_s_e_s_d_s GTGGCTGTGTGGGTGTGGGTGCTGGTGACGGTG; >clamp_s_b_s_d_s:compl:rev CACCGTCACAGCCACTATCATGTTGACATTCC; dG = -12.2
434 >s_b_s_c_s_c_s_c_s_d_s GTGGCTGTGTGGGTGTGGGTGTGGGTGACGGTG; >clamp_s_b_s_d_s:compl:rev CACCGTCACAGCCACTATCATGTTGACATTCC; dG = -12.2
435 >s_a_s_b_s_a_s_e_s_d_s GTGTCTGTGGCTGTGTCTGTGCTGGTGACGGTG; >clamp_s_b_s_d_s:compl:rev CACCGTCACAGCCACTATCATGTTGACATTCC; dG = -12.2
436 >s_c_s_c_s_e_s_d_s_d_s GTGTGGGTGTGGGTGCTGGTGACGGTGACGGTG; >clamp_s_b_s_d_s:compl:rev CACCGTCACAGCCACTATCATGTTGACATTCC; dG = -12.2
437 >s_a_s_b_s_a_s_c_s_d_s GTGTCTGTGGCTGTGTCTGTGTGGGTGACGGTG; >clamp_s_b_s_d_s:compl:rev CACCGTCACAGCCACTATCATGTTGACATTCC; dG = -12.2
438 >s_c_s_d_s_d_s_d_s_d_s GTGTGGGTGACGGTGACGGTGACGGTGACGGTG; >clamp_s_b_s_d_s:compl:rev CACCGTCACAGCCACTATCATGTTGACATTCC; dG = -12.2
439 >s_c_s_e_s_d_s_d_s_d_s GTGTGGGTGCTGGTGACGGTGACGGTGACGGTG; >clamp_s_b_s_d_s:compl:rev CACCGTCACAGCCACTATCATGTTGACATTCC; dG = -12.2
440 >s_d_s_c_s_d_s_d_s_d_s GTGACGGTGTGGGTGACGGTGACGGTGACGGTG; >clamp_s_b_s_d_s:compl:rev CACCGTCACAGCCACTATCATGTTGACATTCC; dG = -12.2
441 >s_d_s_c_s_e_s_d_s_d_s GTGACGGTGTGGGTGCTGGTGACGGTGACGGTG; >clamp_s_b_s_d_s:compl:rev CACCGTCACAGCCACTATCATGTTGACATTCC; dG = -12.2
442 >s_a_s_b_s_e_s_e_s_d_s GTGTCTGTGGCTGTGCTGGTGCTGGTGACGGTG; >clamp_s_b_s_d_s:compl:rev CACCGTCACAGCCACTATCATGTTGACATTCC; dG = -12.2
443 >s_a_s_b_s_e_s_c_s_d_s GTGTCTGTGGCTGTGCTGGTGTGGGTGACGGTG; >clamp_s_b_s_d_s:compl:rev CACCGTCACAGCCACTATCATGTTGACATTCC; dG = -12.2
444 >s_e_s_c_s_d_s_d_s_d_s GTGCTGGTGTGGGTGACGGTGACGGTGACGGTG; >clamp_s_b_s_d_s:compl:rev CACCGTCACAGCCACTATCATGTTGACATTCC; dG = -12.2
445 >s_e_s_c_s_e_s_d_s_d_s GTGCTGGTGTGGGTGCTGGTGACGGTGACGGTG; >clamp_s_b_s_d_s:compl:rev CACCGTCACAGCCACTATCATGTTGACATTCC; dG = -12.2
446 >s_a_s_c_s_d_s_d_s_d_s GTGTCTGTGTGGGTGACGGTGACGGTGACGGTG; >clamp_s_b_s_d_s:compl:rev CACCGTCACAGCCACTATCATGTTGACATTCC; dG = -12.2
447 >s_c_s_b_s_a_s_e_s_d_s GTGTGGGTGGCTGTGTCTGTGCTGGTGACGGTG; >clamp_s_b_s_d_s:compl:rev CACCGTCACAGCCACTATCATGTTGACATTCC; dG = -12.1
448 >s_c_s_b_s_a_s_c_s_d_s GTGTGGGTGGCTGTGTCTGTGTGGGTGACGGTG; >clamp_s_b_s_d_s:compl:rev CACCGTCACAGCCACTATCATGTTGACATTCC; dG = -12.1
449 >s_c_s_b_s_e_s_e_s_d_s GTGTGGGTGGCTGTGCTGGTGCTGGTGACGGTG; >clamp_s_b_s_d_s:compl:rev CACCGTCACAGCCACTATCATGTTGACATTCC; dG = -12.1
450 >s_c_s_b_s_e_s_c_s_d_s GTGTGGGTGGCTGTGCTGGTGTGGGTGACGGTG; >clamp_s_b_s_d_s:compl:rev CACCGTCACAGCCACTATCATGTTGACATTCC; dG = -12.1
451 >s_d_s_b_s_a_s_e_s_d_s GTGACGGTGGCTGTGTCTGTGCTGGTGACGGTG; >clamp_s_b_s_d_s:compl:rev CACCGTCACAGCCACTATCATGTTGACATTCC; dG = -12.1
452 >s_d_s_b_s_a_s_c_s_d_s GTGACGGTGGCTGTGTCTGTGTGGGTGACGGTG; >clamp_s_b_s_d_s:compl:rev CACCGTCACAGCCACTATCATGTTGACATTCC; dG = -12.1
453 >s_d_s_b_s_e_s_e_s_d_s GTGACGGTGGCTGTGCTGGTGCTGGTGACGGTG; >clamp_s_b_s_d_s:compl:rev CACCGTCACAGCCACTATCATGTTGACATTCC; dG = -12.1
454 >s_d_s_b_s_e_s_c_s_d_s GTGACGGTGGCTGTGCTGGTGTGGGTGACGGTG; >clamp_s_b_s_d_s:compl:rev CACCGTCACAGCCACTATCATGTTGACATTCC; dG = -12.1
455 >s_e_s_b_s_a_s_e_s_d_s GTGCTGGTGGCTGTGTCTGTGCTGGTGACGGTG; >clamp_s_b_s_d_s:compl:rev CACCGTCACAGCCACTATCATGTTGACATTCC; dG = -12.1
456 >s_e_s_b_s_a_s_c_s_d_s GTGCTGGTGGCTGTGTCTGTGTGGGTGACGGTG; >clamp_s_b_s_d_s:compl:rev CACCGTCACAGCCACTATCATGTTGACATTCC; dG = -12.1
457 >s_e_s_b_s_e_s_e_s_d_s GTGCTGGTGGCTGTGCTGGTGCTGGTGACGGTG; >clamp_s_b_s_d_s:compl:rev CACCGTCACAGCCACTATCATGTTGACATTCC; dG = -12.1
458 >s_e_s_b_s_e_s_c_s_d_s GTGCTGGTGGCTGTGCTGGTGTGGGTGACGGTG; >clamp_s_b_s_d_s:compl:rev CACCGTCACAGCCACTATCATGTTGACATTCC; dG = -12.1
459 >s_b_s_c_s_e_s_b_s_e_s GTGGCTGTGTGGGTGCTGGTGGCTGTGCTGGTG; >clamp_s_b_s_d_s:compl:rev CACCGTCACAGCCACTATCATGTTGACATTCC; dG = -12.0
460 >s_b_s_c_s_e_s_b_s_b_s GTGGCTGTGTGGGTGCTGGTGGCTGTGGCTGTG; >clamp_s_b_s_d_s:compl:rev CACCGTCACAGCCACTATCATGTTGACATTCC; dG = -12.0
461 >s_b_s_c_s_e_s_b_s_a_s GTGGCTGTGTGGGTGCTGGTGGCTGTGTCTGTG; >clamp_s_b_s_d_s:compl:rev CACCGTCACAGCCACTATCATGTTGACATTCC; dG = -12.0
462 >s_b_s_c_s_e_s_e_s_d_s GTGGCTGTGTGGGTGCTGGTGCTGGTGACGGTG; >clamp_s_b_s_d_s:compl:rev CACCGTCACAGCCACTATCATGTTGACATTCC; dG = -12.0
463 >s_b_s_c_s_e_s_c_s_d_s GTGGCTGTGTGGGTGCTGGTGTGGGTGACGGTG; >clamp_s_b_s_d_s:compl:rev CACCGTCACAGCCACTATCATGTTGACATTCC; dG = -12.0

464 >s_a_s_e_s_c_s_d_s_d_s GTGTCTGTGCTGGTGTGGGTGACGGTGACGGTG; >clamp_s_b_s_d_s:compl:rev CACCGTCACAGCCACTATCATGTTGACATTCC; dG = -11.9
465 >s_a_s_a_s_c_s_d_s_d_s GTGTCTGTGTCTGTGTGGGTGACGGTGACGGTG; >clamp_s_b_s_d_s:compl:rev CACCGTCACAGCCACTATCATGTTGACATTCC; dG = -11.9
466 >s_b_s_b_s_e_s_b_s_e_s GTGGCTGTGGCTGTGCTGGTGGCTGTGCTGGTG; >clamp_s_b_s_d_s:compl:rev CACCGTCACAGCCACTATCATGTTGACATTCC; dG = -11.9
467 >s_b_s_b_s_e_s_b_s_b_s GTGGCTGTGGCTGTGCTGGTGGCTGTGGCTGTG; >clamp_s_b_s_d_s:compl:rev CACCGTCACAGCCACTATCATGTTGACATTCC; dG = -11.9
468 >s_b_s_b_s_e_s_b_s_a_s GTGGCTGTGGCTGTGCTGGTGGCTGTGTCTGTG; >clamp_s_b_s_d_s:compl:rev CACCGTCACAGCCACTATCATGTTGACATTCC; dG = -11.9
469 >s_c_s_a_s_c_s_d_s_d_s GTGTGGGTGTCTGTGTGGGTGACGGTGACGGTG; >clamp_s_b_s_d_s:compl:rev CACCGTCACAGCCACTATCATGTTGACATTCC; dG = -11.9
470 >s_c_s_b_s_e_s_b_s_e_s GTGTGGGTGGCTGTGCTGGTGGCTGTGCTGGTG; >clamp_s_b_s_d_s:compl:rev CACCGTCACAGCCACTATCATGTTGACATTCC; dG = -11.9
471 >s_c_s_b_s_e_s_b_s_b_s GTGTGGGTGGCTGTGCTGGTGGCTGTGGCTGTG; >clamp_s_b_s_d_s:compl:rev CACCGTCACAGCCACTATCATGTTGACATTCC; dG = -11.9
472 >s_c_s_b_s_e_s_b_s_a_s GTGTGGGTGGCTGTGCTGGTGGCTGTGTCTGTG; >clamp_s_b_s_d_s:compl:rev CACCGTCACAGCCACTATCATGTTGACATTCC; dG = -11.9
473 >s_c_s_c_s_d_s_b_s_e_s GTGTGGGTGTGGGTGACGGTGGCTGTGCTGGTG; >clamp_s_b_s_d_s:compl:rev CACCGTCACAGCCACTATCATGTTGACATTCC; dG = -11.9
474 >s_c_s_c_s_d_s_b_s_b_s GTGTGGGTGTGGGTGACGGTGGCTGTGGCTGTG; >clamp_s_b_s_d_s:compl:rev CACCGTCACAGCCACTATCATGTTGACATTCC; dG = -11.9
475 >s_c_s_c_s_d_s_b_s_a_s GTGTGGGTGTGGGTGACGGTGGCTGTGTCTGTG; >clamp_s_b_s_d_s:compl:rev CACCGTCACAGCCACTATCATGTTGACATTCC; dG = -11.9
476 >s_c_s_d_s_c_s_d_s_d_s GTGTGGGTGACGGTGTGGGTGACGGTGACGGTG; >clamp_s_b_s_d_s:compl:rev CACCGTCACAGCCACTATCATGTTGACATTCC; dG = -11.9
477 >s_c_s_d_s_d_s_b_s_e_s GTGTGGGTGACGGTGACGGTGGCTGTGCTGGTG; >clamp_s_b_s_d_s:compl:rev CACCGTCACAGCCACTATCATGTTGACATTCC; dG = -11.9
478 >s_c_s_d_s_d_s_b_s_b_s GTGTGGGTGACGGTGACGGTGGCTGTGGCTGTG; >clamp_s_b_s_d_s:compl:rev CACCGTCACAGCCACTATCATGTTGACATTCC; dG = -11.9
479 >s_c_s_d_s_d_s_b_s_a_s GTGTGGGTGACGGTGACGGTGGCTGTGTCTGTG; >clamp_s_b_s_d_s:compl:rev CACCGTCACAGCCACTATCATGTTGACATTCC; dG = -11.9
480 >s_c_s_d_s_d_s_e_s_d_s GTGTGGGTGACGGTGACGGTGCTGGTGACGGTG; >clamp_s_b_s_d_s:compl:rev CACCGTCACAGCCACTATCATGTTGACATTCC; dG = -11.9
481 >s_c_s_d_s_d_s_c_s_d_s GTGTGGGTGACGGTGACGGTGTGGGTGACGGTG; >clamp_s_b_s_d_s:compl:rev CACCGTCACAGCCACTATCATGTTGACATTCC; dG = -11.9
482 >s_c_s_e_s_c_s_d_s_d_s GTGTGGGTGCTGGTGTGGGTGACGGTGACGGTG; >clamp_s_b_s_d_s:compl:rev CACCGTCACAGCCACTATCATGTTGACATTCC; dG = -11.9
483 >s_d_s_a_s_c_s_d_s_d_s GTGACGGTGTCTGTGTGGGTGACGGTGACGGTG; >clamp_s_b_s_d_s:compl:rev CACCGTCACAGCCACTATCATGTTGACATTCC; dG = -11.9
484 >s_d_s_b_s_e_s_b_s_e_s GTGACGGTGGCTGTGCTGGTGGCTGTGCTGGTG; >clamp_s_b_s_d_s:compl:rev CACCGTCACAGCCACTATCATGTTGACATTCC; dG = -11.9
485 >s_d_s_b_s_e_s_b_s_b_s GTGACGGTGGCTGTGCTGGTGGCTGTGGCTGTG; >clamp_s_b_s_d_s:compl:rev CACCGTCACAGCCACTATCATGTTGACATTCC; dG = -11.9
486 >s_d_s_b_s_e_s_b_s_a_s GTGACGGTGGCTGTGCTGGTGGCTGTGTCTGTG; >clamp_s_b_s_d_s:compl:rev CACCGTCACAGCCACTATCATGTTGACATTCC; dG = -11.9
487 >s_a_s_b_s_e_s_b_s_e_s GTGTCTGTGGCTGTGCTGGTGGCTGTGCTGGTG; >clamp_s_b_s_d_s:compl:rev CACCGTCACAGCCACTATCATGTTGACATTCC; dG = -11.9
488 >s_a_s_b_s_e_s_b_s_b_s GTGTCTGTGGCTGTGCTGGTGGCTGTGGCTGTG; >clamp_s_b_s_d_s:compl:rev CACCGTCACAGCCACTATCATGTTGACATTCC; dG = -11.9
489 >s_d_s_d_s_c_s_d_s_d_s GTGACGGTGACGGTGTGGGTGACGGTGACGGTG; >clamp_s_b_s_d_s:compl:rev CACCGTCACAGCCACTATCATGTTGACATTCC; dG = -11.9
490 >s_a_s_b_s_e_s_b_s_a_s GTGTCTGTGGCTGTGCTGGTGGCTGTGTCTGTG; >clamp_s_b_s_d_s:compl:rev CACCGTCACAGCCACTATCATGTTGACATTCC; dG = -11.9
491 >s_d_s_e_s_c_s_d_s_d_s GTGACGGTGCTGGTGTGGGTGACGGTGACGGTG; >clamp_s_b_s_d_s:compl:rev CACCGTCACAGCCACTATCATGTTGACATTCC; dG = -11.9
492 >s_e_s_a_s_c_s_d_s_d_s GTGCTGGTGTCTGTGTGGGTGACGGTGACGGTG; >clamp_s_b_s_d_s:compl:rev CACCGTCACAGCCACTATCATGTTGACATTCC; dG = -11.9
493 >s_e_s_b_s_e_s_b_s_e_s GTGCTGGTGGCTGTGCTGGTGGCTGTGCTGGTG; >clamp_s_b_s_d_s:compl:rev CACCGTCACAGCCACTATCATGTTGACATTCC; dG = -11.9
494 >s_e_s_b_s_e_s_b_s_b_s GTGCTGGTGGCTGTGCTGGTGGCTGTGGCTGTG; >clamp_s_b_s_d_s:compl:rev CACCGTCACAGCCACTATCATGTTGACATTCC; dG = -11.9
495 >s_e_s_b_s_e_s_b_s_a_s GTGCTGGTGGCTGTGCTGGTGGCTGTGTCTGTG; >clamp_s_b_s_d_s:compl:rev CACCGTCACAGCCACTATCATGTTGACATTCC; dG = -11.9
496 >s_e_s_d_s_c_s_d_s_d_s GTGCTGGTGACGGTGTGGGTGACGGTGACGGTG; >clamp_s_b_s_d_s:compl:rev CACCGTCACAGCCACTATCATGTTGACATTCC; dG = -11.9
497 >s_e_s_e_s_c_s_d_s_d_s GTGCTGGTGCTGGTGTGGGTGACGGTGACGGTG; >clamp_s_b_s_d_s:compl:rev CACCGTCACAGCCACTATCATGTTGACATTCC; dG = -11.9
498 >s_c_s_c_s_d_s_c_s_d_s GTGTGGGTGTGGGTGACGGTGTGGGTGACGGTG; >clamp_s_b_s_d_s:compl:rev CACCGTCACAGCCACTATCATGTTGACATTCC; dG = -11.8
499 >s_a_s_a_s_b_s_b_s_b_s GTGTCTGTGTCTGTGGCTGTGGCTGTGGCTGTG; >clamp_s_b_s_d_s:compl:rev CACCGTCACAGCCACTATCATGTTGACATTCC; dG = -11.7
500 >s_a_s_e_s_b_s_b_s_b_s GTGTCTGTGCTGGTGGCTGTGGCTGTGGCTGTG; >clamp_s_b_s_d_s:compl:rev CACCGTCACAGCCACTATCATGTTGACATTCC; dG = -11.7

501 >s_b_s_a_s_b_s_b_s_b_s GTGGCTGTGTCTGTGGCTGTGGCTGTGGCTGTG; >clamp_s_b_s_d_s:compl:rev CACCGTCACAGCCACTATCATGTTGACATTCC; dG = -11.7
502 >s_b_s_b_s_b_s_b_s_e_s GTGGCTGTGGCTGTGGCTGTGGCTGTGCTGGTG; >clamp_s_b_s_d_s:compl:rev CACCGTCACAGCCACTATCATGTTGACATTCC; dG = -11.7
503 >s_b_s_b_s_b_s_b_s_b_s GTGGCTGTGGCTGTGGCTGTGGCTGTGGCTGTG; >clamp_s_b_s_d_s:compl:rev CACCGTCACAGCCACTATCATGTTGACATTCC; dG = -11.7
504 >s_b_s_b_s_b_s_b_s_a_s GTGGCTGTGGCTGTGGCTGTGGCTGTGTCTGTG; >clamp_s_b_s_d_s:compl:rev CACCGTCACAGCCACTATCATGTTGACATTCC; dG = -11.7
505 >s_b_s_e_s_b_s_b_s_b_s GTGGCTGTGCTGGTGGCTGTGGCTGTGGCTGTG; >clamp_s_b_s_d_s:compl:rev CACCGTCACAGCCACTATCATGTTGACATTCC; dG = -11.7
506 >s_c_s_a_s_b_s_b_s_b_s GTGTGGGTGTCTGTGGCTGTGGCTGTGGCTGTG; >clamp_s_b_s_d_s:compl:rev CACCGTCACAGCCACTATCATGTTGACATTCC; dG = -11.7
507 >s_c_s_b_s_b_s_b_s_e_s GTGTGGGTGGCTGTGGCTGTGGCTGTGCTGGTG; >clamp_s_b_s_d_s:compl:rev CACCGTCACAGCCACTATCATGTTGACATTCC; dG = -11.7
508 >s_c_s_b_s_b_s_b_s_b_s GTGTGGGTGGCTGTGGCTGTGGCTGTGGCTGTG; >clamp_s_b_s_d_s:compl:rev CACCGTCACAGCCACTATCATGTTGACATTCC; dG = -11.7
509 >s_c_s_b_s_b_s_b_s_a_s GTGTGGGTGGCTGTGGCTGTGGCTGTGTCTGTG; >clamp_s_b_s_d_s:compl:rev CACCGTCACAGCCACTATCATGTTGACATTCC; dG = -11.7
510 >s_c_s_c_s_b_s_b_s_b_s GTGTGGGTGTGGGTGGCTGTGGCTGTGGCTGTG; >clamp_s_b_s_d_s:compl:rev CACCGTCACAGCCACTATCATGTTGACATTCC; dG = -11.7
511 >s_c_s_c_s_d_s_e_s_d_s GTGTGGGTGTGGGTGACGGTGCTGGTGACGGTG; >clamp_s_b_s_d_s:compl:rev CACCGTCACAGCCACTATCATGTTGACATTCC; dG = -11.7
512 >s_c_s_d_s_b_s_b_s_b_s GTGTGGGTGACGGTGGCTGTGGCTGTGGCTGTG; >clamp_s_b_s_d_s:compl:rev CACCGTCACAGCCACTATCATGTTGACATTCC; dG = -11.7
513 >s_a_s_b_s_b_s_b_s_e_s GTGTCTGTGGCTGTGGCTGTGGCTGTGCTGGTG; >clamp_s_b_s_d_s:compl:rev CACCGTCACAGCCACTATCATGTTGACATTCC; dG = -11.7
514 >s_c_s_e_s_b_s_b_s_b_s GTGTGGGTGCTGGTGGCTGTGGCTGTGGCTGTG; >clamp_s_b_s_d_s:compl:rev CACCGTCACAGCCACTATCATGTTGACATTCC; dG = -11.7
515 >s_a_s_b_s_b_s_b_s_b_s GTGTCTGTGGCTGTGGCTGTGGCTGTGGCTGTG; >clamp_s_b_s_d_s:compl:rev CACCGTCACAGCCACTATCATGTTGACATTCC; dG = -11.7
516 >s_c_s_e_s_d_s_b_s_e_s GTGTGGGTGCTGGTGACGGTGGCTGTGCTGGTG; >clamp_s_b_s_d_s:compl:rev CACCGTCACAGCCACTATCATGTTGACATTCC; dG = -11.7
517 >s_c_s_e_s_d_s_b_s_b_s GTGTGGGTGCTGGTGACGGTGGCTGTGGCTGTG; >clamp_s_b_s_d_s:compl:rev CACCGTCACAGCCACTATCATGTTGACATTCC; dG = -11.7
518 >s_a_s_b_s_b_s_b_s_a_s GTGTCTGTGGCTGTGGCTGTGGCTGTGTCTGTG; >clamp_s_b_s_d_s:compl:rev CACCGTCACAGCCACTATCATGTTGACATTCC; dG = -11.7
519 >s_c_s_e_s_d_s_b_s_a_s GTGTGGGTGCTGGTGACGGTGGCTGTGTCTGTG; >clamp_s_b_s_d_s:compl:rev CACCGTCACAGCCACTATCATGTTGACATTCC; dG = -11.7
520 >s_d_s_a_s_b_s_b_s_b_s GTGACGGTGTCTGTGGCTGTGGCTGTGGCTGTG; >clamp_s_b_s_d_s:compl:rev CACCGTCACAGCCACTATCATGTTGACATTCC; dG = -11.7
521 >s_d_s_b_s_b_s_b_s_e_s GTGACGGTGGCTGTGGCTGTGGCTGTGCTGGTG; >clamp_s_b_s_d_s:compl:rev CACCGTCACAGCCACTATCATGTTGACATTCC; dG = -11.7
522 >s_d_s_b_s_b_s_b_s_b_s GTGACGGTGGCTGTGGCTGTGGCTGTGGCTGTG; >clamp_s_b_s_d_s:compl:rev CACCGTCACAGCCACTATCATGTTGACATTCC; dG = -11.7
523 >s_d_s_b_s_b_s_b_s_a_s GTGACGGTGGCTGTGGCTGTGGCTGTGTCTGTG; >clamp_s_b_s_d_s:compl:rev CACCGTCACAGCCACTATCATGTTGACATTCC; dG = -11.7
524 >s_d_s_c_s_b_s_b_s_b_s GTGACGGTGTGGGTGGCTGTGGCTGTGGCTGTG; >clamp_s_b_s_d_s:compl:rev CACCGTCACAGCCACTATCATGTTGACATTCC; dG = -11.7
525 >s_d_s_d_s_b_s_b_s_b_s GTGACGGTGACGGTGGCTGTGGCTGTGGCTGTG; >clamp_s_b_s_d_s:compl:rev CACCGTCACAGCCACTATCATGTTGACATTCC; dG = -11.7
526 >s_d_s_e_s_b_s_b_s_b_s GTGACGGTGCTGGTGGCTGTGGCTGTGGCTGTG; >clamp_s_b_s_d_s:compl:rev CACCGTCACAGCCACTATCATGTTGACATTCC; dG = -11.7
527 >s_e_s_a_s_b_s_b_s_b_s GTGCTGGTGTCTGTGGCTGTGGCTGTGGCTGTG; >clamp_s_b_s_d_s:compl:rev CACCGTCACAGCCACTATCATGTTGACATTCC; dG = -11.7
528 >s_e_s_b_s_b_s_b_s_e_s GTGCTGGTGGCTGTGGCTGTGGCTGTGCTGGTG; >clamp_s_b_s_d_s:compl:rev CACCGTCACAGCCACTATCATGTTGACATTCC; dG = -11.7
529 >s_e_s_b_s_b_s_b_s_b_s GTGCTGGTGGCTGTGGCTGTGGCTGTGGCTGTG; >clamp_s_b_s_d_s:compl:rev CACCGTCACAGCCACTATCATGTTGACATTCC; dG = -11.7
530 >s_e_s_b_s_b_s_b_s_a_s GTGCTGGTGGCTGTGGCTGTGGCTGTGTCTGTG; >clamp_s_b_s_d_s:compl:rev CACCGTCACAGCCACTATCATGTTGACATTCC; dG = -11.7
531 >s_e_s_c_s_b_s_b_s_b_s GTGCTGGTGTGGGTGGCTGTGGCTGTGGCTGTG; >clamp_s_b_s_d_s:compl:rev CACCGTCACAGCCACTATCATGTTGACATTCC; dG = -11.7
532 >s_a_s_c_s_b_s_b_s_b_s GTGTCTGTGTGGGTGGCTGTGGCTGTGGCTGTG; >clamp_s_b_s_d_s:compl:rev CACCGTCACAGCCACTATCATGTTGACATTCC; dG = -11.7
533 >s_e_s_d_s_b_s_b_s_b_s GTGCTGGTGACGGTGGCTGTGGCTGTGGCTGTG; >clamp_s_b_s_d_s:compl:rev CACCGTCACAGCCACTATCATGTTGACATTCC; dG = -11.7
534 >s_e_s_e_s_b_s_b_s_b_s GTGCTGGTGCTGGTGGCTGTGGCTGTGGCTGTG; >clamp_s_b_s_d_s:compl:rev CACCGTCACAGCCACTATCATGTTGACATTCC; dG = -11.7
535 >s_a_s_a_s_a_s_b_s_e_s GTGTCTGTGTCTGTGTCTGTGGCTGTGCTGGTG; >clamp_s_b_s_d_s:compl:rev CACCGTCACAGCCACTATCATGTTGACATTCC; dG = -11.6
536 >s_a_s_a_s_b_s_b_s_a_s GTGTCTGTGTCTGTGGCTGTGGCTGTGTCTGTG; >clamp_s_b_s_d_s:compl:rev CACCGTCACAGCCACTATCATGTTGACATTCC; dG = -11.6
537 >s_a_s_e_s_a_s_b_s_e_s GTGTCTGTGCTGGTGTCTGTGGCTGTGCTGGTG; >clamp_s_b_s_d_s:compl:rev CACCGTCACAGCCACTATCATGTTGACATTCC; dG = -11.6

538 >s_a_s_e_s_b_s_b_s_e_s GTGTCTGTGCTGGTGGCTGTGGCTGTGCTGGTG; >clamp_s_b_s_d_s:compl:rev CACCGTCACAGCCACTATCATGTTGACATTCC; dG = -11.6
539 >s_a_s_e_s_b_s_b_s_a_s GTGTCTGTGCTGGTGGCTGTGGCTGTGTCTGTG; >clamp_s_b_s_d_s:compl:rev CACCGTCACAGCCACTATCATGTTGACATTCC; dG = -11.6
540 >s_b_s_a_s_a_s_b_s_e_s GTGGCTGTGTCTGTGTCTGTGGCTGTGCTGGTG; >clamp_s_b_s_d_s:compl:rev CACCGTCACAGCCACTATCATGTTGACATTCC; dG = -11.6
541 >s_b_s_a_s_b_s_b_s_e_s GTGGCTGTGTCTGTGGCTGTGGCTGTGCTGGTG; >clamp_s_b_s_d_s:compl:rev CACCGTCACAGCCACTATCATGTTGACATTCC; dG = -11.6
542 >s_b_s_a_s_b_s_b_s_a_s GTGGCTGTGTCTGTGGCTGTGGCTGTGTCTGTG; >clamp_s_b_s_d_s:compl:rev CACCGTCACAGCCACTATCATGTTGACATTCC; dG = -11.6
543 >s_b_s_b_s_a_s_b_s_e_s GTGGCTGTGGCTGTGTCTGTGGCTGTGCTGGTG; >clamp_s_b_s_d_s:compl:rev CACCGTCACAGCCACTATCATGTTGACATTCC; dG = -11.6
544 >s_b_s_e_s_a_s_b_s_e_s GTGGCTGTGCTGGTGTCTGTGGCTGTGCTGGTG; >clamp_s_b_s_d_s:compl:rev CACCGTCACAGCCACTATCATGTTGACATTCC; dG = -11.6
545 >s_b_s_e_s_b_s_b_s_e_s GTGGCTGTGCTGGTGGCTGTGGCTGTGCTGGTG; >clamp_s_b_s_d_s:compl:rev CACCGTCACAGCCACTATCATGTTGACATTCC; dG = -11.6
546 >s_b_s_e_s_b_s_b_s_a_s GTGGCTGTGCTGGTGGCTGTGGCTGTGTCTGTG; >clamp_s_b_s_d_s:compl:rev CACCGTCACAGCCACTATCATGTTGACATTCC; dG = -11.6
547 >s_c_s_a_s_a_s_b_s_e_s GTGTGGGTGTCTGTGTCTGTGGCTGTGCTGGTG; >clamp_s_b_s_d_s:compl:rev CACCGTCACAGCCACTATCATGTTGACATTCC; dG = -11.6
548 >s_c_s_a_s_b_s_b_s_e_s GTGTGGGTGTCTGTGGCTGTGGCTGTGCTGGTG; >clamp_s_b_s_d_s:compl:rev CACCGTCACAGCCACTATCATGTTGACATTCC; dG = -11.6
549 >s_c_s_a_s_b_s_b_s_a_s GTGTGGGTGTCTGTGGCTGTGGCTGTGTCTGTG; >clamp_s_b_s_d_s:compl:rev CACCGTCACAGCCACTATCATGTTGACATTCC; dG = -11.6
550 >s_c_s_b_s_a_s_b_s_e_s GTGTGGGTGGCTGTGTCTGTGGCTGTGCTGGTG; >clamp_s_b_s_d_s:compl:rev CACCGTCACAGCCACTATCATGTTGACATTCC; dG = -11.6
551 >s_a_s_b_s_a_s_b_s_e_s GTGTCTGTGGCTGTGTCTGTGGCTGTGCTGGTG; >clamp_s_b_s_d_s:compl:rev CACCGTCACAGCCACTATCATGTTGACATTCC; dG = -11.6
552 >s_c_s_c_s_a_s_b_s_e_s GTGTGGGTGTGGGTGTCTGTGGCTGTGCTGGTG; >clamp_s_b_s_d_s:compl:rev CACCGTCACAGCCACTATCATGTTGACATTCC; dG = -11.6
553 >s_c_s_c_s_b_s_b_s_e_s GTGTGGGTGTGGGTGGCTGTGGCTGTGCTGGTG; >clamp_s_b_s_d_s:compl:rev CACCGTCACAGCCACTATCATGTTGACATTCC; dG = -11.6
554 >s_c_s_c_s_b_s_b_s_a_s GTGTGGGTGTGGGTGGCTGTGGCTGTGTCTGTG; >clamp_s_b_s_d_s:compl:rev CACCGTCACAGCCACTATCATGTTGACATTCC; dG = -11.6
555 >s_c_s_d_s_a_s_b_s_e_s GTGTGGGTGACGGTGTCTGTGGCTGTGCTGGTG; >clamp_s_b_s_d_s:compl:rev CACCGTCACAGCCACTATCATGTTGACATTCC; dG = -11.6
556 >s_c_s_d_s_b_s_b_s_e_s GTGTGGGTGACGGTGGCTGTGGCTGTGCTGGTG; >clamp_s_b_s_d_s:compl:rev CACCGTCACAGCCACTATCATGTTGACATTCC; dG = -11.6
557 >s_c_s_d_s_b_s_b_s_a_s GTGTGGGTGACGGTGGCTGTGGCTGTGTCTGTG; >clamp_s_b_s_d_s:compl:rev CACCGTCACAGCCACTATCATGTTGACATTCC; dG = -11.6
558 >s_c_s_e_s_a_s_b_s_e_s GTGTGGGTGCTGGTGTCTGTGGCTGTGCTGGTG; >clamp_s_b_s_d_s:compl:rev CACCGTCACAGCCACTATCATGTTGACATTCC; dG = -11.6
559 >s_c_s_e_s_b_s_b_s_e_s GTGTGGGTGCTGGTGGCTGTGGCTGTGCTGGTG; >clamp_s_b_s_d_s:compl:rev CACCGTCACAGCCACTATCATGTTGACATTCC; dG = -11.6
560 >s_c_s_e_s_b_s_b_s_a_s GTGTGGGTGCTGGTGGCTGTGGCTGTGTCTGTG; >clamp_s_b_s_d_s:compl:rev CACCGTCACAGCCACTATCATGTTGACATTCC; dG = -11.6
561 >s_c_s_e_s_d_s_c_s_d_s GTGTGGGTGCTGGTGACGGTGTGGGTGACGGTG; >clamp_s_b_s_d_s:compl:rev CACCGTCACAGCCACTATCATGTTGACATTCC; dG = -11.6
562 >s_d_s_a_s_a_s_b_s_e_s GTGACGGTGTCTGTGTCTGTGGCTGTGCTGGTG; >clamp_s_b_s_d_s:compl:rev CACCGTCACAGCCACTATCATGTTGACATTCC; dG = -11.6
563 >s_d_s_a_s_b_s_b_s_e_s GTGACGGTGTCTGTGGCTGTGGCTGTGCTGGTG; >clamp_s_b_s_d_s:compl:rev CACCGTCACAGCCACTATCATGTTGACATTCC; dG = -11.6
564 >s_d_s_a_s_b_s_b_s_a_s GTGACGGTGTCTGTGGCTGTGGCTGTGTCTGTG; >clamp_s_b_s_d_s:compl:rev CACCGTCACAGCCACTATCATGTTGACATTCC; dG = -11.6
565 >s_d_s_b_s_a_s_b_s_e_s GTGACGGTGGCTGTGTCTGTGGCTGTGCTGGTG; >clamp_s_b_s_d_s:compl:rev CACCGTCACAGCCACTATCATGTTGACATTCC; dG = -11.6
566 >s_d_s_c_s_a_s_b_s_e_s GTGACGGTGTGGGTGTCTGTGGCTGTGCTGGTG; >clamp_s_b_s_d_s:compl:rev CACCGTCACAGCCACTATCATGTTGACATTCC; dG = -11.6
567 >s_d_s_c_s_b_s_b_s_e_s GTGACGGTGTGGGTGGCTGTGGCTGTGCTGGTG; >clamp_s_b_s_d_s:compl:rev CACCGTCACAGCCACTATCATGTTGACATTCC; dG = -11.6
568 >s_d_s_c_s_b_s_b_s_a_s GTGACGGTGTGGGTGGCTGTGGCTGTGTCTGTG; >clamp_s_b_s_d_s:compl:rev CACCGTCACAGCCACTATCATGTTGACATTCC; dG = -11.6
569 >s_d_s_d_s_a_s_b_s_e_s GTGACGGTGACGGTGTCTGTGGCTGTGCTGGTG; >clamp_s_b_s_d_s:compl:rev CACCGTCACAGCCACTATCATGTTGACATTCC; dG = -11.6
570 >s_d_s_d_s_b_s_b_s_e_s GTGACGGTGACGGTGGCTGTGGCTGTGCTGGTG; >clamp_s_b_s_d_s:compl:rev CACCGTCACAGCCACTATCATGTTGACATTCC; dG = -11.6
571 >s_d_s_d_s_b_s_b_s_a_s GTGACGGTGACGGTGGCTGTGGCTGTGTCTGTG; >clamp_s_b_s_d_s:compl:rev CACCGTCACAGCCACTATCATGTTGACATTCC; dG = -11.6
572 >s_d_s_e_s_a_s_b_s_e_s GTGACGGTGCTGGTGTCTGTGGCTGTGCTGGTG; >clamp_s_b_s_d_s:compl:rev CACCGTCACAGCCACTATCATGTTGACATTCC; dG = -11.6
573 >s_d_s_e_s_b_s_b_s_e_s GTGACGGTGCTGGTGGCTGTGGCTGTGCTGGTG; >clamp_s_b_s_d_s:compl:rev CACCGTCACAGCCACTATCATGTTGACATTCC; dG = -11.6
574 >s_d_s_e_s_b_s_b_s_a_s GTGACGGTGCTGGTGGCTGTGGCTGTGTCTGTG; >clamp_s_b_s_d_s:compl:rev CACCGTCACAGCCACTATCATGTTGACATTCC; dG = -11.6

575 >s_a_s_c_s_a_s_b_s_e_s GTGTCTGTGTGGGTGTCTGTGGCTGTGCTGGTG; >clamp_s_b_s_d_s:compl:rev
CACCGTCACAGCCACTATCATGTTGACATTCC; dG = -11.6
576 >s_e_s_a_s_a_s_b_s_e_s GTGCTGGTGTCTGTGTCTGTGGCTGTGCTGGTG; >clamp_s_b_s_d_s:compl:rev
CACCGTCACAGCCACTATCATGTTGACATTCC; dG = -11.6
577 >s_e_s_a_s_b_s_b_s_e_s GTGCTGGTGTCTGTGGCTGTGGCTGTGCTGGTG; >clamp_s_b_s_d_s:compl:rev
CACCGTCACAGCCACTATCATGTTGACATTCC; dG = -11.6
578 >s_e_s_a_s_b_s_b_s_a_s GTGCTGGTGTCTGTGGCTGTGGCTGTGTCTGTG; >clamp_s_b_s_d_s:compl:rev
CACCGTCACAGCCACTATCATGTTGACATTCC; dG = -11.6
579 >s_e_s_b_s_a_s_b_s_e_s GTGCTGGTGGCTGTGTCTGTGGCTGTGCTGGTG; >clamp_s_b_s_d_s:compl:rev
CACCGTCACAGCCACTATCATGTTGACATTCC; dG = -11.6
580 >s_e_s_c_s_a_s_b_s_e_s GTGCTGGTGTGGGTGTCTGTGGCTGTGCTGGTG; >clamp_s_b_s_d_s:compl:rev
CACCGTCACAGCCACTATCATGTTGACATTCC; dG = -11.6
581 >s_e_s_c_s_b_s_b_s_e_s GTGCTGGTGTGGGTGGCTGTGGCTGTGCTGGTG; >clamp_s_b_s_d_s:compl:rev
CACCGTCACAGCCACTATCATGTTGACATTCC; dG = -11.6
582 >s_a_s_c_s_b_s_b_s_e_s GTGTCTGTGTGGGTGGCTGTGGCTGTGCTGGTG; >clamp_s_b_s_d_s:compl:rev
CACCGTCACAGCCACTATCATGTTGACATTCC; dG = -11.6
583 >s_e_s_c_s_b_s_b_s_a_s GTGCTGGTGTGGGTGGCTGTGGCTGTGTCTGTG; >clamp_s_b_s_d_s:compl:rev
CACCGTCACAGCCACTATCATGTTGACATTCC; dG = -11.6
584 >s_e_s_d_s_a_s_b_s_e_s GTGCTGGTGACGGTGTCTGTGGCTGTGCTGGTG; >clamp_s_b_s_d_s:compl:rev
CACCGTCACAGCCACTATCATGTTGACATTCC; dG = -11.6
585 >s_a_s_c_s_b_s_b_s_a_s GTGTCTGTGTGGGTGGCTGTGGCTGTGTCTGTG; >clamp_s_b_s_d_s:compl:rev
CACCGTCACAGCCACTATCATGTTGACATTCC; dG = -11.6
586 >s_e_s_d_s_b_s_b_s_e_s GTGCTGGTGACGGTGGCTGTGGCTGTGCTGGTG; >clamp_s_b_s_d_s:compl:rev
CACCGTCACAGCCACTATCATGTTGACATTCC; dG = -11.6
587 >s_e_s_d_s_b_s_b_s_a_s GTGCTGGTGACGGTGGCTGTGGCTGTGTCTGTG; >clamp_s_b_s_d_s:compl:rev
CACCGTCACAGCCACTATCATGTTGACATTCC; dG = -11.6
588 >s_e_s_e_s_a_s_b_s_e_s GTGCTGGTGCTGGTGTCTGTGGCTGTGCTGGTG; >clamp_s_b_s_d_s:compl:rev
CACCGTCACAGCCACTATCATGTTGACATTCC; dG = -11.6
589 >s_e_s_e_s_b_s_b_s_e_s GTGCTGGTGCTGGTGGCTGTGGCTGTGCTGGTG; >clamp_s_b_s_d_s:compl:rev
CACCGTCACAGCCACTATCATGTTGACATTCC; dG = -11.6
590 >s_e_s_e_s_b_s_b_s_a_s GTGCTGGTGCTGGTGGCTGTGGCTGTGTCTGTG; >clamp_s_b_s_d_s:compl:rev
CACCGTCACAGCCACTATCATGTTGACATTCC; dG = -11.6
591 >s_a_s_a_s_b_s_b_s_e_s GTGTCTGTGTCTGTGGCTGTGGCTGTGCTGGTG; >clamp_s_b_s_d_s:compl:rev
CACCGTCACAGCCACTATCATGTTGACATTCC; dG = -11.6
592 >s_a_s_c_s_e_s_b_s_e_s GTGTCTGTGTGGGTGCTGGTGGCTGTGCTGGTG; >clamp_s_b_s_d_s:compl:rev
CACCGTCACAGCCACTATCATGTTGACATTCC; dG = -11.5
593 >s_a_s_a_s_c_s_b_s_e_s GTGTCTGTGTCTGTGTGGGTGGCTGTGCTGGTG; >clamp_s_b_s_d_s:compl:rev
CACCGTCACAGCCACTATCATGTTGACATTCC; dG = -11.5
594 >s_a_s_e_s_c_s_b_s_e_s GTGTCTGTGCTGGTGTGGGTGGCTGTGCTGGTG; >clamp_s_b_s_d_s:compl:rev
CACCGTCACAGCCACTATCATGTTGACATTCC; dG = -11.5
595 >s_a_s_e_s_c_s_c_s_d_s GTGTCTGTGCTGGTGTGGGTGTGGGTGACGGTG; >clamp_s_b_s_d_s:compl:rev
CACCGTCACAGCCACTATCATGTTGACATTCC; dG = -11.5
596 >s_a_s_e_s_d_s_b_s_e_s GTGTCTGTGCTGGTGACGGTGGCTGTGCTGGTG; >clamp_s_b_s_d_s:compl:rev
CACCGTCACAGCCACTATCATGTTGACATTCC; dG = -11.5
597 >s_a_s_e_s_e_s_b_s_e_s GTGTCTGTGCTGGTGCTGGTGGCTGTGCTGGTG; >clamp_s_b_s_d_s:compl:rev
CACCGTCACAGCCACTATCATGTTGACATTCC; dG = -11.5
598 >s_b_s_a_s_c_s_b_s_e_s GTGGCTGTGTCTGTGTGGGTGGCTGTGCTGGTG; >clamp_s_b_s_d_s:compl:rev
CACCGTCACAGCCACTATCATGTTGACATTCC; dG = -11.5
599 >s_b_s_a_s_c_s_c_s_d_s GTGGCTGTGTCTGTGTGGGTGTGGGTGACGGTG; >clamp_s_b_s_d_s:compl:rev
CACCGTCACAGCCACTATCATGTTGACATTCC; dG = -11.5
600 >s_b_s_a_s_e_s_b_s_e_s GTGGCTGTGTCTGTGCTGGTGGCTGTGCTGGTG; >clamp_s_b_s_d_s:compl:rev
CACCGTCACAGCCACTATCATGTTGACATTCC; dG = -11.5
601 >s_a_s_a_s_c_s_c_s_d_s GTGTCTGTGTCTGTGTGGGTGTGGGTGACGGTG; >clamp_s_b_s_d_s:compl:rev
CACCGTCACAGCCACTATCATGTTGACATTCC; dG = -11.5
602 >s_b_s_e_s_c_s_b_s_e_s GTGGCTGTGCTGGTGTGGGTGGCTGTGCTGGTG; >clamp_s_b_s_d_s:compl:rev
CACCGTCACAGCCACTATCATGTTGACATTCC; dG = -11.5
603 >s_b_s_e_s_c_s_c_s_d_s GTGGCTGTGCTGGTGTGGGTGTGGGTGACGGTG; >clamp_s_b_s_d_s:compl:rev
CACCGTCACAGCCACTATCATGTTGACATTCC; dG = -11.5
604 >s_a_s_a_s_e_s_b_s_e_s GTGTCTGTGTCTGTGCTGGTGGCTGTGCTGGTG; >clamp_s_b_s_d_s:compl:rev
CACCGTCACAGCCACTATCATGTTGACATTCC; dG = -11.5
605 >s_b_s_e_s_e_s_b_s_e_s GTGGCTGTGCTGGTGCTGGTGGCTGTGCTGGTG; >clamp_s_b_s_d_s:compl:rev
CACCGTCACAGCCACTATCATGTTGACATTCC; dG = -11.5
606 >s_c_s_a_s_c_s_b_s_e_s GTGTGGGTGTCTGTGTGGGTGGCTGTGCTGGTG; >clamp_s_b_s_d_s:compl:rev
CACCGTCACAGCCACTATCATGTTGACATTCC; dG = -11.5
607 >s_c_s_a_s_c_s_c_s_d_s GTGTGGGTGTCTGTGTGGGTGTGGGTGACGGTG; >clamp_s_b_s_d_s:compl:rev
CACCGTCACAGCCACTATCATGTTGACATTCC; dG = -11.5
608 >s_c_s_a_s_e_s_b_s_e_s GTGTGGGTGTCTGTGCTGGTGGCTGTGCTGGTG; >clamp_s_b_s_d_s:compl:rev
CACCGTCACAGCCACTATCATGTTGACATTCC; dG = -11.5
609 >s_c_s_c_s_c_s_b_s_e_s GTGTGGGTGTGGGTGTGGGTGGCTGTGCTGGTG; >clamp_s_b_s_d_s:compl:rev
CACCGTCACAGCCACTATCATGTTGACATTCC; dG = -11.5
610 >s_c_s_c_s_c_s_c_s_d_s GTGTGGGTGTGGGTGTGGGTGTGGGTGACGGTG; >clamp_s_b_s_d_s:compl:rev
CACCGTCACAGCCACTATCATGTTGACATTCC; dG = -11.5
611 >s_c_s_c_s_e_s_b_s_e_s GTGTGGGTGTGGGTGCTGGTGGCTGTGCTGGTG; >clamp_s_b_s_d_s:compl:rev
CACCGTCACAGCCACTATCATGTTGACATTCC; dG = -11.5

612 >s_c_s_d_s_c_s_b_s_e_s GTGTGGGTGACGGTGTGGGTGGCTGTGCTGGTG; >clamp_s_b_s_d_s:compl:rev CACCGTCACAGCCACTATCATGTTGACATTCC; dG = -11.5
613 >s_c_s_d_s_c_s_c_s_d_s GTGTGGGTGACGGTGTGGGTGTGGGTGACGGTG; >clamp_s_b_s_d_s:compl:rev CACCGTCACAGCCACTATCATGTTGACATTCC; dG = -11.5
614 >s_c_s_d_s_e_s_b_s_e_s GTGTGGGTGACGGTGCTGGTGGCTGTGCTGGTG; >clamp_s_b_s_d_s:compl:rev CACCGTCACAGCCACTATCATGTTGACATTCC; dG = -11.5
615 >s_c_s_e_s_c_s_b_s_e_s GTGTGGGTGCTGGTGTGGGTGGCTGTGCTGGTG; >clamp_s_b_s_d_s:compl:rev CACCGTCACAGCCACTATCATGTTGACATTCC; dG = -11.5
616 >s_c_s_e_s_c_s_c_s_d_s GTGTGGGTGCTGGTGTGGGTGTGGGTGACGGTG; >clamp_s_b_s_d_s:compl:rev CACCGTCACAGCCACTATCATGTTGACATTCC; dG = -11.5
617 >s_c_s_e_s_d_s_e_s_d_s GTGTGGGTGCTGGTGACGGTGCTGGTGACGGTG; >clamp_s_b_s_d_s:compl:rev CACCGTCACAGCCACTATCATGTTGACATTCC; dG = -11.5
618 >s_c_s_e_s_e_s_b_s_e_s GTGTGGGTGCTGGTGCTGGTGGCTGTGCTGGTG; >clamp_s_b_s_d_s:compl:rev CACCGTCACAGCCACTATCATGTTGACATTCC; dG = -11.5
619 >s_d_s_a_s_c_s_b_s_e_s GTGACGGTGTCTGTGTGGGTGGCTGTGCTGGTG; >clamp_s_b_s_d_s:compl:rev CACCGTCACAGCCACTATCATGTTGACATTCC; dG = -11.5
620 >s_d_s_a_s_c_s_c_s_d_s GTGACGGTGTCTGTGTGGGTGTGGGTGACGGTG; >clamp_s_b_s_d_s:compl:rev CACCGTCACAGCCACTATCATGTTGACATTCC; dG = -11.5
621 >s_d_s_a_s_e_s_b_s_e_s GTGACGGTGTCTGTGCTGGTGGCTGTGCTGGTG; >clamp_s_b_s_d_s:compl:rev CACCGTCACAGCCACTATCATGTTGACATTCC; dG = -11.5
622 >s_d_s_c_s_c_s_b_s_e_s GTGACGGTGTGGGTGTGGGTGGCTGTGCTGGTG; >clamp_s_b_s_d_s:compl:rev CACCGTCACAGCCACTATCATGTTGACATTCC; dG = -11.5
623 >s_d_s_c_s_c_s_c_s_d_s GTGACGGTGTGGGTGTGGGTGTGGGTGACGGTG; >clamp_s_b_s_d_s:compl:rev CACCGTCACAGCCACTATCATGTTGACATTCC; dG = -11.5
624 >s_d_s_c_s_d_s_b_s_e_s GTGACGGTGTGGGTGACGGTGGCTGTGCTGGTG; >clamp_s_b_s_d_s:compl:rev CACCGTCACAGCCACTATCATGTTGACATTCC; dG = -11.5
625 >s_d_s_c_s_e_s_b_s_e_s GTGACGGTGTGGGTGCTGGTGGCTGTGCTGGTG; >clamp_s_b_s_d_s:compl:rev CACCGTCACAGCCACTATCATGTTGACATTCC; dG = -11.5
626 >s_d_s_d_s_c_s_b_s_e_s GTGACGGTGACGGTGTGGGTGGCTGTGCTGGTG; >clamp_s_b_s_d_s:compl:rev CACCGTCACAGCCACTATCATGTTGACATTCC; dG = -11.5
627 >s_d_s_d_s_c_s_c_s_d_s GTGACGGTGACGGTGTGGGTGTGGGTGACGGTG; >clamp_s_b_s_d_s:compl:rev CACCGTCACAGCCACTATCATGTTGACATTCC; dG = -11.5
628 >s_d_s_d_s_d_s_b_s_e_s GTGACGGTGACGGTGACGGTGGCTGTGCTGGTG; >clamp_s_b_s_d_s:compl:rev CACCGTCACAGCCACTATCATGTTGACATTCC; dG = -11.5
629 >s_d_s_d_s_e_s_b_s_e_s GTGACGGTGACGGTGCTGGTGGCTGTGCTGGTG; >clamp_s_b_s_d_s:compl:rev CACCGTCACAGCCACTATCATGTTGACATTCC; dG = -11.5
630 >s_d_s_e_s_c_s_b_s_e_s GTGACGGTGCTGGTGTGGGTGGCTGTGCTGGTG; >clamp_s_b_s_d_s:compl:rev CACCGTCACAGCCACTATCATGTTGACATTCC; dG = -11.5
631 >s_d_s_e_s_c_s_c_s_d_s GTGACGGTGCTGGTGTGGGTGTGGGTGACGGTG; >clamp_s_b_s_d_s:compl:rev CACCGTCACAGCCACTATCATGTTGACATTCC; dG = -11.5
632 >s_d_s_e_s_d_s_b_s_e_s GTGACGGTGCTGGTGACGGTGGCTGTGCTGGTG; >clamp_s_b_s_d_s:compl:rev CACCGTCACAGCCACTATCATGTTGACATTCC; dG = -11.5
633 >s_d_s_e_s_e_s_b_s_e_s GTGACGGTGCTGGTGCTGGTGGCTGTGCTGGTG; >clamp_s_b_s_d_s:compl:rev CACCGTCACAGCCACTATCATGTTGACATTCC; dG = -11.5
634 >s_e_s_a_s_c_s_b_s_e_s GTGCTGGTGTCTGTGTGGGTGGCTGTGCTGGTG; >clamp_s_b_s_d_s:compl:rev CACCGTCACAGCCACTATCATGTTGACATTCC; dG = -11.5
635 >s_e_s_a_s_c_s_c_s_d_s GTGCTGGTGTCTGTGTGGGTGTGGGTGACGGTG; >clamp_s_b_s_d_s:compl:rev CACCGTCACAGCCACTATCATGTTGACATTCC; dG = -11.5
636 >s_e_s_a_s_e_s_b_s_e_s GTGCTGGTGTCTGTGCTGGTGGCTGTGCTGGTG; >clamp_s_b_s_d_s:compl:rev CACCGTCACAGCCACTATCATGTTGACATTCC; dG = -11.5
637 >s_e_s_c_s_c_s_b_s_e_s GTGCTGGTGTGGGTGTGGGTGGCTGTGCTGGTG; >clamp_s_b_s_d_s:compl:rev CACCGTCACAGCCACTATCATGTTGACATTCC; dG = -11.5
638 >s_e_s_c_s_c_s_c_s_d_s GTGCTGGTGTGGGTGTGGGTGTGGGTGACGGTG; >clamp_s_b_s_d_s:compl:rev CACCGTCACAGCCACTATCATGTTGACATTCC; dG = -11.5
639 >s_e_s_c_s_d_s_b_s_e_s GTGCTGGTGTGGGTGACGGTGGCTGTGCTGGTG; >clamp_s_b_s_d_s:compl:rev CACCGTCACAGCCACTATCATGTTGACATTCC; dG = -11.5
640 >s_e_s_c_s_e_s_b_s_e_s GTGCTGGTGTGGGTGCTGGTGGCTGTGCTGGTG; >clamp_s_b_s_d_s:compl:rev CACCGTCACAGCCACTATCATGTTGACATTCC; dG = -11.5
641 >s_e_s_d_s_c_s_b_s_e_s GTGCTGGTGACGGTGTGGGTGGCTGTGCTGGTG; >clamp_s_b_s_d_s:compl:rev CACCGTCACAGCCACTATCATGTTGACATTCC; dG = -11.5
642 >s_e_s_d_s_c_s_c_s_d_s GTGCTGGTGACGGTGTGGGTGTGGGTGACGGTG; >clamp_s_b_s_d_s:compl:rev CACCGTCACAGCCACTATCATGTTGACATTCC; dG = -11.5
643 >s_e_s_d_s_d_s_b_s_e_s GTGCTGGTGACGGTGACGGTGGCTGTGCTGGTG; >clamp_s_b_s_d_s:compl:rev CACCGTCACAGCCACTATCATGTTGACATTCC; dG = -11.5
644 >s_e_s_d_s_e_s_b_s_e_s GTGCTGGTGACGGTGCTGGTGGCTGTGCTGGTG; >clamp_s_b_s_d_s:compl:rev CACCGTCACAGCCACTATCATGTTGACATTCC; dG = -11.5
645 >s_a_s_c_s_c_s_b_s_e_s GTGTCTGTGTGGGTGTGGGTGGCTGTGCTGGTG; >clamp_s_b_s_d_s:compl:rev CACCGTCACAGCCACTATCATGTTGACATTCC; dG = -11.5
646 >s_e_s_e_s_c_s_b_s_e_s GTGCTGGTGCTGGTGTGGGTGGCTGTGCTGGTG; >clamp_s_b_s_d_s:compl:rev CACCGTCACAGCCACTATCATGTTGACATTCC; dG = -11.5
647 >s_e_s_e_s_c_s_c_s_d_s GTGCTGGTGCTGGTGTGGGTGTGGGTGACGGTG; >clamp_s_b_s_d_s:compl:rev CACCGTCACAGCCACTATCATGTTGACATTCC; dG = -11.5
648 >s_e_s_e_s_d_s_b_s_e_s GTGCTGGTGCTGGTGACGGTGGCTGTGCTGGTG; >clamp_s_b_s_d_s:compl:rev CACCGTCACAGCCACTATCATGTTGACATTCC; dG = -11.5

649 >s_e_s_e_s_e_s_b_s_e_s GTGCTGGTGCTGGTGCTGGTGGCTGTGCTGGTG; >clamp_s_b_s_d_s:compl:rev
CACCGTCACAGCCACTATCATGTTGACATTCC; dG = -11.5
650 >s_a_s_c_s_c_s_c_s_d_s GTGTCTGTGTGGGTGTGGGTGTGGGTGACGGTG; >clamp_s_b_s_d_s:compl:rev
CACCGTCACAGCCACTATCATGTTGACATTCC; dG = -11.5
651 >s_a_s_c_s_d_s_b_s_e_s GTGTCTGTGTGGGTGACGGTGGCTGTGCTGGTG; >clamp_s_b_s_d_s:compl:rev
CACCGTCACAGCCACTATCATGTTGACATTCC; dG = -11.5
652 >s_a_s_e_s_a_s_b_s_b_s GTGTCTGTGCTGGTGTCTGTGGCTGTGGCTGTG; >clamp_s_b_s_d_s:compl:rev
CACCGTCACAGCCACTATCATGTTGACATTCC; dG = -11.4
653 >s_a_s_e_s_c_s_e_s_d_s GTGTCTGTGCTGGTGTGGGTGCTGGTGACGGTG; >clamp_s_b_s_d_s:compl:rev
CACCGTCACAGCCACTATCATGTTGACATTCC; dG = -11.4
654 >s_b_s_a_s_a_s_b_s_b_s GTGGCTGTGTCTGTGTCTGTGGCTGTGGCTGTG; >clamp_s_b_s_d_s:compl:rev
CACCGTCACAGCCACTATCATGTTGACATTCC; dG = -11.4
655 >s_a_s_a_s_c_s_e_s_d_s GTGTCTGTGTCTGTGTGGGTGCTGGTGACGGTG; >clamp_s_b_s_d_s:compl:rev
CACCGTCACAGCCACTATCATGTTGACATTCC; dG = -11.4
656 >s_b_s_a_s_c_s_e_s_d_s GTGGCTGTGTCTGTGTGGGTGCTGGTGACGGTG; >clamp_s_b_s_d_s:compl:rev
CACCGTCACAGCCACTATCATGTTGACATTCC; dG = -11.4
657 >s_b_s_b_s_a_s_b_s_b_s GTGGCTGTGGCTGTGTCTGTGGCTGTGGCTGTG; >clamp_s_b_s_d_s:compl:rev
CACCGTCACAGCCACTATCATGTTGACATTCC; dG = -11.4
658 >s_b_s_e_s_a_s_b_s_b_s GTGGCTGTGCTGGTGTCTGTGGCTGTGGCTGTG; >clamp_s_b_s_d_s:compl:rev
CACCGTCACAGCCACTATCATGTTGACATTCC; dG = -11.4
659 >s_a_s_a_s_a_s_b_s_b_s GTGTCTGTGTCTGTGTCTGTGGCTGTGGCTGTG; >clamp_s_b_s_d_s:compl:rev
CACCGTCACAGCCACTATCATGTTGACATTCC; dG = -11.4
660 >s_b_s_e_s_c_s_e_s_d_s GTGGCTGTGCTGGTGTGGGTGCTGGTGACGGTG; >clamp_s_b_s_d_s:compl:rev
CACCGTCACAGCCACTATCATGTTGACATTCC; dG = -11.4
661 >s_c_s_a_s_a_s_b_s_b_s GTGTGGGTGTCTGTGTCTGTGGCTGTGGCTGTG; >clamp_s_b_s_d_s:compl:rev
CACCGTCACAGCCACTATCATGTTGACATTCC; dG = -11.4
662 >s_c_s_a_s_c_s_e_s_d_s GTGTGGGTGTCTGTGTGGGTGCTGGTGACGGTG; >clamp_s_b_s_d_s:compl:rev
CACCGTCACAGCCACTATCATGTTGACATTCC; dG = -11.4
663 >s_c_s_b_s_a_s_b_s_b_s GTGTGGGTGGCTGTGTCTGTGGCTGTGGCTGTG; >clamp_s_b_s_d_s:compl:rev
CACCGTCACAGCCACTATCATGTTGACATTCC; dG = -11.4
664 >s_c_s_c_s_a_s_b_s_b_s GTGTGGGTGTGGGTGTCTGTGGCTGTGGCTGTG; >clamp_s_b_s_d_s:compl:rev
CACCGTCACAGCCACTATCATGTTGACATTCC; dG = -11.4
665 >s_a_s_b_s_a_s_b_s_b_s GTGTCTGTGGCTGTGTCTGTGGCTGTGGCTGTG; >clamp_s_b_s_d_s:compl:rev
CACCGTCACAGCCACTATCATGTTGACATTCC; dG = -11.4
666 >s_c_s_c_s_c_s_e_s_d_s GTGTGGGTGTGGGTGTGGGTGCTGGTGACGGTG; >clamp_s_b_s_d_s:compl:rev
CACCGTCACAGCCACTATCATGTTGACATTCC; dG = -11.4
667 >s_c_s_d_s_a_s_b_s_b_s GTGTGGGTGACGGTGTCTGTGGCTGTGGCTGTG; >clamp_s_b_s_d_s:compl:rev
CACCGTCACAGCCACTATCATGTTGACATTCC; dG = -11.4
668 >s_c_s_d_s_c_s_e_s_d_s GTGTGGGTGACGGTGTGGGTGCTGGTGACGGTG; >clamp_s_b_s_d_s:compl:rev
CACCGTCACAGCCACTATCATGTTGACATTCC; dG = -11.4
669 >s_c_s_e_s_a_s_b_s_b_s GTGTGGGTGCTGGTGTCTGTGGCTGTGGCTGTG; >clamp_s_b_s_d_s:compl:rev
CACCGTCACAGCCACTATCATGTTGACATTCC; dG = -11.4
670 >s_c_s_e_s_c_s_e_s_d_s GTGTGGGTGCTGGTGTGGGTGCTGGTGACGGTG; >clamp_s_b_s_d_s:compl:rev
CACCGTCACAGCCACTATCATGTTGACATTCC; dG = -11.4
671 >s_d_s_a_s_a_s_b_s_b_s GTGACGGTGTCTGTGTCTGTGGCTGTGGCTGTG; >clamp_s_b_s_d_s:compl:rev
CACCGTCACAGCCACTATCATGTTGACATTCC; dG = -11.4
672 >s_d_s_a_s_c_s_e_s_d_s GTGACGGTGTCTGTGTGGGTGCTGGTGACGGTG; >clamp_s_b_s_d_s:compl:rev
CACCGTCACAGCCACTATCATGTTGACATTCC; dG = -11.4
673 >s_d_s_b_s_a_s_b_s_b_s GTGACGGTGGCTGTGTCTGTGGCTGTGGCTGTG; >clamp_s_b_s_d_s:compl:rev
CACCGTCACAGCCACTATCATGTTGACATTCC; dG = -11.4
674 >s_d_s_c_s_a_s_b_s_b_s GTGACGGTGTGGGTGTCTGTGGCTGTGGCTGTG; >clamp_s_b_s_d_s:compl:rev
CACCGTCACAGCCACTATCATGTTGACATTCC; dG = -11.4
675 >s_d_s_c_s_c_s_e_s_d_s GTGACGGTGTGGGTGTGGGTGCTGGTGACGGTG; >clamp_s_b_s_d_s:compl:rev
CACCGTCACAGCCACTATCATGTTGACATTCC; dG = -11.4
676 >s_d_s_c_s_d_s_b_s_b_s GTGACGGTGTGGGTGACGGTGGCTGTGGCTGTG; >clamp_s_b_s_d_s:compl:rev
CACCGTCACAGCCACTATCATGTTGACATTCC; dG = -11.4
677 >s_d_s_c_s_d_s_b_s_a_s GTGACGGTGTGGGTGACGGTGGCTGTGTCTGTG; >clamp_s_b_s_d_s:compl:rev
CACCGTCACAGCCACTATCATGTTGACATTCC; dG = -11.4
678 >s_d_s_d_s_a_s_b_s_b_s GTGACGGTGACGGTGTCTGTGGCTGTGGCTGTG; >clamp_s_b_s_d_s:compl:rev
CACCGTCACAGCCACTATCATGTTGACATTCC; dG = -11.4
679 >s_d_s_d_s_c_s_e_s_d_s GTGACGGTGACGGTGTGGGTGCTGGTGACGGTG; >clamp_s_b_s_d_s:compl:rev
CACCGTCACAGCCACTATCATGTTGACATTCC; dG = -11.4
680 >s_d_s_e_s_a_s_b_s_b_s GTGACGGTGCTGGTGTCTGTGGCTGTGGCTGTG; >clamp_s_b_s_d_s:compl:rev
CACCGTCACAGCCACTATCATGTTGACATTCC; dG = -11.4
681 >s_d_s_e_s_c_s_e_s_d_s GTGACGGTGCTGGTGTGGGTGCTGGTGACGGTG; >clamp_s_b_s_d_s:compl:rev
CACCGTCACAGCCACTATCATGTTGACATTCC; dG = -11.4
682 >s_e_s_a_s_a_s_b_s_b_s GTGCTGGTGTCTGTGTCTGTGGCTGTGGCTGTG; >clamp_s_b_s_d_s:compl:rev
CACCGTCACAGCCACTATCATGTTGACATTCC; dG = -11.4
683 >s_e_s_a_s_c_s_e_s_d_s GTGCTGGTGTCTGTGTGGGTGCTGGTGACGGTG; >clamp_s_b_s_d_s:compl:rev
CACCGTCACAGCCACTATCATGTTGACATTCC; dG = -11.4
684 >s_a_s_c_s_a_s_b_s_b_s GTGTCTGTGTGGGTGTCTGTGGCTGTGGCTGTG; >clamp_s_b_s_d_s:compl:rev
CACCGTCACAGCCACTATCATGTTGACATTCC; dG = -11.4
685 >s_e_s_b_s_a_s_b_s_b_s GTGCTGGTGGCTGTGTCTGTGGCTGTGGCTGTG; >clamp_s_b_s_d_s:compl:rev
CACCGTCACAGCCACTATCATGTTGACATTCC; dG = -11.4

686 >s_e_s_c_s_a_s_b_s_b_s GTGCTGGTGTGGGTGTCTGTGGCTGTGGCTGTG; >clamp_s_b_s_d_s:compl:rev
CACCGTCACAGCCACTATCATGTTGACATTCC; dG = -11.4
687 >s_e_s_c_s_c_s_e_s_d_s GTGCTGGTGTGGGTGTGGGTGCTGGTGACGGTG; >clamp_s_b_s_d_s:compl:rev
CACCGTCACAGCCACTATCATGTTGACATTCC; dG = -11.4
688 >s_e_s_c_s_d_s_b_s_b_s GTGCTGGTGTGGGTGACGGTGGCTGTGGCTGTG; >clamp_s_b_s_d_s:compl:rev
CACCGTCACAGCCACTATCATGTTGACATTCC; dG = -11.4
689 >s_e_s_c_s_d_s_b_s_a_s GTGCTGGTGTGGGTGACGGTGGCTGTGTCTGTG; >clamp_s_b_s_d_s:compl:rev
CACCGTCACAGCCACTATCATGTTGACATTCC; dG = -11.4
690 >s_e_s_d_s_a_s_b_s_b_s GTGCTGGTGACGGTGTCTGTGGCTGTGGCTGTG; >clamp_s_b_s_d_s:compl:rev
CACCGTCACAGCCACTATCATGTTGACATTCC; dG = -11.4
691 >s_e_s_d_s_c_s_e_s_d_s GTGCTGGTGACGGTGTGGGTGCTGGTGACGGTG; >clamp_s_b_s_d_s:compl:rev
CACCGTCACAGCCACTATCATGTTGACATTCC; dG = -11.4
692 >s_e_s_e_s_a_s_b_s_b_s GTGCTGGTGCTGGTGTCTGTGGCTGTGGCTGTG; >clamp_s_b_s_d_s:compl:rev
CACCGTCACAGCCACTATCATGTTGACATTCC; dG = -11.4
693 >s_e_s_e_s_c_s_e_s_d_s GTGCTGGTGCTGGTGTGGGTGCTGGTGACGGTG; >clamp_s_b_s_d_s:compl:rev
CACCGTCACAGCCACTATCATGTTGACATTCC; dG = -11.4
694 >s_a_s_c_s_c_s_e_s_d_s GTGTCTGTGTGGGTGTGGGTGCTGGTGACGGTG; >clamp_s_b_s_d_s:compl:rev
CACCGTCACAGCCACTATCATGTTGACATTCC; dG = -11.4
695 >s_a_s_c_s_d_s_b_s_b_s GTGTCTGTGTGGGTGACGGTGGCTGTGGCTGTG; >clamp_s_b_s_d_s:compl:rev
CACCGTCACAGCCACTATCATGTTGACATTCC; dG = -11.4
696 >s_a_s_c_s_d_s_b_s_a_s GTGTCTGTGTGGGTGACGGTGGCTGTGTCTGTG; >clamp_s_b_s_d_s:compl:rev
CACCGTCACAGCCACTATCATGTTGACATTCC; dG = -11.4
697 >s_a_s_c_s_d_s_c_s_d_s GTGTCTGTGTGGGTGACGGTGTGGGTGACGGTG; >clamp_s_b_s_d_s:compl:rev
CACCGTCACAGCCACTATCATGTTGACATTCC; dG = -11.3
698 >s_a_s_c_s_e_s_b_s_b_s GTGTCTGTGTGGGTGCTGGTGGCTGTGGCTGTG; >clamp_s_b_s_d_s:compl:rev
CACCGTCACAGCCACTATCATGTTGACATTCC; dG = -11.3
699 >s_a_s_e_s_c_s_b_s_b_s GTGTCTGTGCTGGTGTGGGTGGCTGTGGCTGTG; >clamp_s_b_s_d_s:compl:rev
CACCGTCACAGCCACTATCATGTTGACATTCC; dG = -11.3
700 >s_a_s_e_s_d_s_b_s_b_s GTGTCTGTGCTGGTGACGGTGGCTGTGGCTGTG; >clamp_s_b_s_d_s:compl:rev
CACCGTCACAGCCACTATCATGTTGACATTCC; dG = -11.3
701 >s_a_s_a_s_c_s_b_s_b_s GTGTCTGTGTCTGTGTGGGTGGCTGTGGCTGTG; >clamp_s_b_s_d_s:compl:rev
CACCGTCACAGCCACTATCATGTTGACATTCC; dG = -11.3
702 >s_a_s_e_s_e_s_b_s_b_s GTGTCTGTGCTGGTGCTGGTGGCTGTGGCTGTG; >clamp_s_b_s_d_s:compl:rev
CACCGTCACAGCCACTATCATGTTGACATTCC; dG = -11.3
703 >s_b_s_a_s_a_s_e_s_d_s GTGGCTGTGTCTGTGTCTGTGCTGGTGACGGTG; >clamp_s_b_s_d_s:compl:rev
CACCGTCACAGCCACTATCATGTTGACATTCC; dG = -11.3
704 >s_b_s_a_s_a_s_c_s_d_s GTGGCTGTGTCTGTGTCTGTGTGGGTGACGGTG; >clamp_s_b_s_d_s:compl:rev
CACCGTCACAGCCACTATCATGTTGACATTCC; dG = -11.3
705 >s_b_s_a_s_c_s_b_s_b_s GTGGCTGTGTCTGTGTGGGTGGCTGTGGCTGTG; >clamp_s_b_s_d_s:compl:rev
CACCGTCACAGCCACTATCATGTTGACATTCC; dG = -11.3
706 >s_b_s_a_s_e_s_b_s_b_s GTGGCTGTGTCTGTGCTGGTGGCTGTGGCTGTG; >clamp_s_b_s_d_s:compl:rev
CACCGTCACAGCCACTATCATGTTGACATTCC; dG = -11.3
707 >s_b_s_a_s_e_s_e_s_d_s GTGGCTGTGTCTGTGCTGGTGCTGGTGACGGTG; >clamp_s_b_s_d_s:compl:rev
CACCGTCACAGCCACTATCATGTTGACATTCC; dG = -11.3
708 >s_b_s_a_s_e_s_c_s_d_s GTGGCTGTGTCTGTGCTGGTGTGGGTGACGGTG; >clamp_s_b_s_d_s:compl:rev
CACCGTCACAGCCACTATCATGTTGACATTCC; dG = -11.3
709 >s_b_s_e_s_a_s_b_s_a_s GTGGCTGTGCTGGTGTCTGTGGCTGTGTCTGTG; >clamp_s_b_s_d_s:compl:rev
CACCGTCACAGCCACTATCATGTTGACATTCC; dG = -11.3
710 >s_b_s_e_s_a_s_e_s_d_s GTGGCTGTGCTGGTGTCTGTGCTGGTGACGGTG; >clamp_s_b_s_d_s:compl:rev
CACCGTCACAGCCACTATCATGTTGACATTCC; dG = -11.3
711 >s_b_s_e_s_a_s_c_s_d_s GTGGCTGTGCTGGTGTCTGTGTGGGTGACGGTG; >clamp_s_b_s_d_s:compl:rev
CACCGTCACAGCCACTATCATGTTGACATTCC; dG = -11.3
712 >s_b_s_e_s_c_s_b_s_b_s GTGGCTGTGCTGGTGTGGGTGGCTGTGGCTGTG; >clamp_s_b_s_d_s:compl:rev
CACCGTCACAGCCACTATCATGTTGACATTCC; dG = -11.3
713 >s_b_s_e_s_c_s_b_s_a_s GTGGCTGTGCTGGTGTGGGTGGCTGTGTCTGTG; >clamp_s_b_s_d_s:compl:rev
CACCGTCACAGCCACTATCATGTTGACATTCC; dG = -11.3
714 >s_b_s_e_s_e_s_b_s_b_s GTGGCTGTGCTGGTGCTGGTGGCTGTGGCTGTG; >clamp_s_b_s_d_s:compl:rev
CACCGTCACAGCCACTATCATGTTGACATTCC; dG = -11.3
715 >s_b_s_e_s_e_s_e_s_d_s GTGGCTGTGCTGGTGCTGGTGCTGGTGACGGTG; >clamp_s_b_s_d_s:compl:rev
CACCGTCACAGCCACTATCATGTTGACATTCC; dG = -11.3
716 >s_b_s_e_s_e_s_c_s_d_s GTGGCTGTGCTGGTGCTGGTGTGGGTGACGGTG; >clamp_s_b_s_d_s:compl:rev
CACCGTCACAGCCACTATCATGTTGACATTCC; dG = -11.3
717 >s_a_s_a_s_e_s_b_s_b_s GTGTCTGTGTCTGTGCTGGTGGCTGTGGCTGTG; >clamp_s_b_s_d_s:compl:rev
CACCGTCACAGCCACTATCATGTTGACATTCC; dG = -11.3
718 >s_c_s_a_s_c_s_b_s_b_s GTGTGGGTGTCTGTGTGGGTGGCTGTGGCTGTG; >clamp_s_b_s_d_s:compl:rev
CACCGTCACAGCCACTATCATGTTGACATTCC; dG = -11.3
719 >s_c_s_a_s_e_s_b_s_b_s GTGTGGGTGTCTGTGCTGGTGGCTGTGGCTGTG; >clamp_s_b_s_d_s:compl:rev
CACCGTCACAGCCACTATCATGTTGACATTCC; dG = -11.3
720 >s_c_s_c_s_c_s_b_s_b_s GTGTGGGTGTGGGTGTGGGTGGCTGTGGCTGTG; >clamp_s_b_s_d_s:compl:rev
CACCGTCACAGCCACTATCATGTTGACATTCC; dG = -11.3
721 >s_c_s_c_s_e_s_b_s_b_s GTGTGGGTGTGGGTGCTGGTGGCTGTGGCTGTG; >clamp_s_b_s_d_s:compl:rev
CACCGTCACAGCCACTATCATGTTGACATTCC; dG = -11.3
722 >s_c_s_d_s_a_s_b_s_a_s GTGTGGGTGACGGTGTCTGTGGCTGTGTCTGTG; >clamp_s_b_s_d_s:compl:rev
CACCGTCACAGCCACTATCATGTTGACATTCC; dG = -11.3

723 >s_c_s_d_s_a_s_e_s_d_s GTGTGGGTGACGGTGTCTGTGCTGGTGACGGTG; >clamp_s_b_s_d_s:compl:rev
CACCGTCACAGCCACTATCATGTTGACATTCC; dG = -11.3
724 >s_c_s_d_s_a_s_c_s_d_s GTGTGGGTGACGGTGTCTGTGTGGGTGACGGTG; >clamp_s_b_s_d_s:compl:rev
CACCGTCACAGCCACTATCATGTTGACATTCC; dG = -11.3
725 >s_c_s_d_s_c_s_b_s_b_s GTGTGGGTGACGGTGTGGGTGGCTGTGGCTGTG; >clamp_s_b_s_d_s:compl:rev
CACCGTCACAGCCACTATCATGTTGACATTCC; dG = -11.3
726 >s_c_s_d_s_c_s_b_s_a_s GTGTGGGTGACGGTGTGGGTGGCTGTGTCTGTG; >clamp_s_b_s_d_s:compl:rev
CACCGTCACAGCCACTATCATGTTGACATTCC; dG = -11.3
727 >s_c_s_d_s_e_s_b_s_b_s GTGTGGGTGACGGTGCTGGTGGCTGTGGCTGTG; >clamp_s_b_s_d_s:compl:rev
CACCGTCACAGCCACTATCATGTTGACATTCC; dG = -11.3
728 >s_c_s_e_s_c_s_b_s_b_s GTGTGGGTGCTGGTGTGGGTGGCTGTGGCTGTG; >clamp_s_b_s_d_s:compl:rev
CACCGTCACAGCCACTATCATGTTGACATTCC; dG = -11.3
729 >s_c_s_e_s_e_s_b_s_b_s GTGTGGGTGCTGGTGCTGGTGGCTGTGGCTGTG; >clamp_s_b_s_d_s:compl:rev
CACCGTCACAGCCACTATCATGTTGACATTCC; dG = -11.3
730 >s_d_s_a_s_c_s_b_s_b_s GTGACGGTGTCTGTGTGGGTGGCTGTGGCTGTG; >clamp_s_b_s_d_s:compl:rev
CACCGTCACAGCCACTATCATGTTGACATTCC; dG = -11.3
731 >s_d_s_a_s_e_s_b_s_b_s GTGACGGTGTCTGTGCTGGTGGCTGTGGCTGTG; >clamp_s_b_s_d_s:compl:rev
CACCGTCACAGCCACTATCATGTTGACATTCC; dG = -11.3
732 >s_d_s_c_s_c_s_b_s_b_s GTGACGGTGTGGGTGTGGGTGGCTGTGGCTGTG; >clamp_s_b_s_d_s:compl:rev
CACCGTCACAGCCACTATCATGTTGACATTCC; dG = -11.3
733 >s_d_s_c_s_d_s_c_s_d_s GTGACGGTGTGGGTGACGGTGTGGGTGACGGTG; >clamp_s_b_s_d_s:compl:rev
CACCGTCACAGCCACTATCATGTTGACATTCC; dG = -11.3
734 >s_d_s_c_s_e_s_b_s_b_s GTGACGGTGTGGGTGCTGGTGGCTGTGGCTGTG; >clamp_s_b_s_d_s:compl:rev
CACCGTCACAGCCACTATCATGTTGACATTCC; dG = -11.3
735 >s_d_s_d_s_c_s_b_s_b_s GTGACGGTGACGGTGTGGGTGGCTGTGGCTGTG; >clamp_s_b_s_d_s:compl:rev
CACCGTCACAGCCACTATCATGTTGACATTCC; dG = -11.3
736 >s_d_s_d_s_d_s_b_s_b_s GTGACGGTGACGGTGACGGTGGCTGTGGCTGTG; >clamp_s_b_s_d_s:compl:rev
CACCGTCACAGCCACTATCATGTTGACATTCC; dG = -11.3
737 >s_d_s_d_s_e_s_b_s_b_s GTGACGGTGACGGTGCTGGTGGCTGTGGCTGTG; >clamp_s_b_s_d_s:compl:rev
CACCGTCACAGCCACTATCATGTTGACATTCC; dG = -11.3
738 >s_d_s_e_s_c_s_b_s_b_s GTGACGGTGCTGGTGTGGGTGGCTGTGGCTGTG; >clamp_s_b_s_d_s:compl:rev
CACCGTCACAGCCACTATCATGTTGACATTCC; dG = -11.3
739 >s_d_s_e_s_d_s_b_s_b_s GTGACGGTGCTGGTGACGGTGGCTGTGGCTGTG; >clamp_s_b_s_d_s:compl:rev
CACCGTCACAGCCACTATCATGTTGACATTCC; dG = -11.3
740 >s_d_s_e_s_e_s_b_s_b_s GTGACGGTGCTGGTGCTGGTGGCTGTGGCTGTG; >clamp_s_b_s_d_s:compl:rev
CACCGTCACAGCCACTATCATGTTGACATTCC; dG = -11.3
741 >s_e_s_a_s_c_s_b_s_b_s GTGCTGGTGTCTGTGTGGGTGGCTGTGGCTGTG; >clamp_s_b_s_d_s:compl:rev
CACCGTCACAGCCACTATCATGTTGACATTCC; dG = -11.3
742 >s_e_s_a_s_e_s_b_s_b_s GTGCTGGTGTCTGTGCTGGTGGCTGTGGCTGTG; >clamp_s_b_s_d_s:compl:rev
CACCGTCACAGCCACTATCATGTTGACATTCC; dG = -11.3
743 >s_e_s_c_s_c_s_b_s_b_s GTGCTGGTGTGGGTGTGGGTGGCTGTGGCTGTG; >clamp_s_b_s_d_s:compl:rev
CACCGTCACAGCCACTATCATGTTGACATTCC; dG = -11.3
744 >s_e_s_c_s_d_s_c_s_d_s GTGCTGGTGTGGGTGACGGTGTGGGTGACGGTG; >clamp_s_b_s_d_s:compl:rev
CACCGTCACAGCCACTATCATGTTGACATTCC; dG = -11.3
745 >s_e_s_c_s_e_s_b_s_b_s GTGCTGGTGTGGGTGCTGGTGGCTGTGGCTGTG; >clamp_s_b_s_d_s:compl:rev
CACCGTCACAGCCACTATCATGTTGACATTCC; dG = -11.3
746 >s_e_s_d_s_c_s_b_s_b_s GTGCTGGTGACGGTGTGGGTGGCTGTGGCTGTG; >clamp_s_b_s_d_s:compl:rev
CACCGTCACAGCCACTATCATGTTGACATTCC; dG = -11.3
747 >s_e_s_d_s_d_s_b_s_b_s GTGCTGGTGACGGTGACGGTGGCTGTGGCTGTG; >clamp_s_b_s_d_s:compl:rev
CACCGTCACAGCCACTATCATGTTGACATTCC; dG = -11.3
748 >s_e_s_d_s_e_s_b_s_b_s GTGCTGGTGACGGTGCTGGTGGCTGTGGCTGTG; >clamp_s_b_s_d_s:compl:rev
CACCGTCACAGCCACTATCATGTTGACATTCC; dG = -11.3
749 >s_e_s_e_s_c_s_b_s_b_s GTGCTGGTGCTGGTGTGGGTGGCTGTGGCTGTG; >clamp_s_b_s_d_s:compl:rev
CACCGTCACAGCCACTATCATGTTGACATTCC; dG = -11.3
750 >s_a_s_c_s_c_s_b_s_b_s GTGTCTGTGTGGGTGTGGGTGGCTGTGGCTGTG; >clamp_s_b_s_d_s:compl:rev
CACCGTCACAGCCACTATCATGTTGACATTCC; dG = -11.3
751 >s_e_s_e_s_d_s_b_s_b_s GTGCTGGTGCTGGTGACGGTGGCTGTGGCTGTG; >clamp_s_b_s_d_s:compl:rev
CACCGTCACAGCCACTATCATGTTGACATTCC; dG = -11.3
752 >s_e_s_e_s_e_s_b_s_b_s GTGCTGGTGCTGGTGCTGGTGGCTGTGGCTGTG; >clamp_s_b_s_d_s:compl:rev
CACCGTCACAGCCACTATCATGTTGACATTCC; dG = -11.3
753 >s_b_s_e_s_e_s_b_s_a_s GTGGCTGTGCTGGTGCTGGTGGCTGTGTCTGTG; >clamp_s_b_s_d_s:compl:rev
CACCGTCACAGCCACTATCATGTTGACATTCC; dG = -11.2
754 >s_c_s_d_s_e_s_b_s_a_s GTGTGGGTGACGGTGCTGGTGGCTGTGTCTGTG; >clamp_s_b_s_d_s:compl:rev
CACCGTCACAGCCACTATCATGTTGACATTCC; dG = -11.2
755 >s_c_s_d_s_e_s_e_s_d_s GTGTGGGTGACGGTGCTGGTGCTGGTGACGGTG; >clamp_s_b_s_d_s:compl:rev
CACCGTCACAGCCACTATCATGTTGACATTCC; dG = -11.2
756 >s_c_s_d_s_e_s_d_s_d_s GTGTGGGTGACGGTGCTGGTGACGGTGACGGTG; >clamp_s_b_s_d_s:compl:rev
CACCGTCACAGCCACTATCATGTTGACATTCC; dG = -11.2
757 >s_c_s_d_s_e_s_c_s_d_s GTGTGGGTGACGGTGCTGGTGTGGGTGACGGTG; >clamp_s_b_s_d_s:compl:rev
CACCGTCACAGCCACTATCATGTTGACATTCC; dG = -11.2
758 >s_d_s_c_s_d_s_e_s_d_s GTGACGGTGTGGGTGACGGTGCTGGTGACGGTG; >clamp_s_b_s_d_s:compl:rev
CACCGTCACAGCCACTATCATGTTGACATTCC; dG = -11.2
759 >s_e_s_c_s_d_s_e_s_d_s GTGCTGGTGTGGGTGACGGTGCTGGTGACGGTG; >clamp_s_b_s_d_s:compl:rev
CACCGTCACAGCCACTATCATGTTGACATTCC; dG = -11.2

760 >s_a_s_c_s_d_s_e_s_d_s GTGTCTGTGTGGGTGACGGTGCTGGTGACGGTG; >clamp_s_b_s_d_s:compl:rev
CACCGTCACAGCCACTATCATGTTGACATTCC; dG = -11.2
761 >s_a_s_e_s_d_s_d_s_d_s GTGTCTGTGCTGGTGACGGTGACGGTGACGGTG; >clamp_s_b_s_d_s:compl:rev
CACCGTCACAGCCACTATCATGTTGACATTCC; dG = -11.1
762 >s_a_s_e_s_e_s_d_s_d_s GTGTCTGTGCTGGTGCTGGTGACGGTGACGGTG; >clamp_s_b_s_d_s:compl:rev
CACCGTCACAGCCACTATCATGTTGACATTCC; dG = -11.1
763 >s_b_s_b_s_a_s_b_s_a_s GTGGCTGTGGCTGTGTCTGTGGCTGTGTCTGTG; >clamp_s_b_s_d_s:compl:rev
CACCGTCACAGCCACTATCATGTTGACATTCC; dG = -11.1
764 >s_a_s_a_s_e_s_d_s_d_s GTGTCTGTGTCTGTGCTGGTGACGGTGACGGTG; >clamp_s_b_s_d_s:compl:rev
CACCGTCACAGCCACTATCATGTTGACATTCC; dG = -11.1
765 >s_c_s_a_s_e_s_d_s_d_s GTGTGGGTGTCTGTGCTGGTGACGGTGACGGTG; >clamp_s_b_s_d_s:compl:rev
CACCGTCACAGCCACTATCATGTTGACATTCC; dG = -11.1
766 >s_c_s_e_s_e_s_d_s_d_s GTGTGGGTGCTGGTGCTGGTGACGGTGACGGTG; >clamp_s_b_s_d_s:compl:rev
CACCGTCACAGCCACTATCATGTTGACATTCC; dG = -11.1
767 >s_d_s_a_s_e_s_d_s_d_s GTGACGGTGTCTGTGCTGGTGACGGTGACGGTG; >clamp_s_b_s_d_s:compl:rev
CACCGTCACAGCCACTATCATGTTGACATTCC; dG = -11.1
768 >s_d_s_d_s_d_s_b_s_a_s GTGACGGTGACGGTGACGGTGGCTGTGTCTGTG; >clamp_s_b_s_d_s:compl:rev
CACCGTCACAGCCACTATCATGTTGACATTCC; dG = -11.1
769 >s_d_s_d_s_d_s_e_s_d_s GTGACGGTGACGGTGACGGTGCTGGTGACGGTG; >clamp_s_b_s_d_s:compl:rev
CACCGTCACAGCCACTATCATGTTGACATTCC; dG = -11.1
770 >s_d_s_d_s_d_s_d_s_d_s GTGACGGTGACGGTGACGGTGACGGTGACGGTG; >clamp_s_b_s_d_s:compl:rev
CACCGTCACAGCCACTATCATGTTGACATTCC; dG = -11.1
771 >s_d_s_d_s_d_s_c_s_d_s GTGACGGTGACGGTGACGGTGTGGGTGACGGTG; >clamp_s_b_s_d_s:compl:rev
CACCGTCACAGCCACTATCATGTTGACATTCC; dG = -11.1
772 >s_d_s_d_s_e_s_d_s_d_s GTGACGGTGACGGTGCTGGTGACGGTGACGGTG; >clamp_s_b_s_d_s:compl:rev
CACCGTCACAGCCACTATCATGTTGACATTCC; dG = -11.1
773 >s_d_s_e_s_d_s_d_s_d_s GTGACGGTGCTGGTGACGGTGACGGTGACGGTG; >clamp_s_b_s_d_s:compl:rev
CACCGTCACAGCCACTATCATGTTGACATTCC; dG = -11.1
774 >s_d_s_e_s_e_s_d_s_d_s GTGACGGTGCTGGTGCTGGTGACGGTGACGGTG; >clamp_s_b_s_d_s:compl:rev
CACCGTCACAGCCACTATCATGTTGACATTCC; dG = -11.1
775 >s_e_s_a_s_e_s_d_s_d_s GTGCTGGTGTCTGTGCTGGTGACGGTGACGGTG; >clamp_s_b_s_d_s:compl:rev
CACCGTCACAGCCACTATCATGTTGACATTCC; dG = -11.1
776 >s_e_s_d_s_d_s_b_s_a_s GTGCTGGTGACGGTGACGGTGGCTGTGTCTGTG; >clamp_s_b_s_d_s:compl:rev
CACCGTCACAGCCACTATCATGTTGACATTCC; dG = -11.1
777 >s_e_s_d_s_d_s_e_s_d_s GTGCTGGTGACGGTGACGGTGCTGGTGACGGTG; >clamp_s_b_s_d_s:compl:rev
CACCGTCACAGCCACTATCATGTTGACATTCC; dG = -11.1
778 >s_e_s_d_s_d_s_d_s_d_s GTGCTGGTGACGGTGACGGTGACGGTGACGGTG; >clamp_s_b_s_d_s:compl:rev
CACCGTCACAGCCACTATCATGTTGACATTCC; dG = -11.1
779 >s_e_s_d_s_d_s_c_s_d_s GTGCTGGTGACGGTGACGGTGTGGGTGACGGTG; >clamp_s_b_s_d_s:compl:rev
CACCGTCACAGCCACTATCATGTTGACATTCC; dG = -11.1
780 >s_e_s_d_s_e_s_d_s_d_s GTGCTGGTGACGGTGCTGGTGACGGTGACGGTG; >clamp_s_b_s_d_s:compl:rev
CACCGTCACAGCCACTATCATGTTGACATTCC; dG = -11.1
781 >s_e_s_e_s_d_s_d_s_d_s GTGCTGGTGCTGGTGACGGTGACGGTGACGGTG; >clamp_s_b_s_d_s:compl:rev
CACCGTCACAGCCACTATCATGTTGACATTCC; dG = -11.1
782 >s_e_s_e_s_e_s_d_s_d_s GTGCTGGTGCTGGTGCTGGTGACGGTGACGGTG; >clamp_s_b_s_d_s:compl:rev
CACCGTCACAGCCACTATCATGTTGACATTCC; dG = -11.1
783 >s_a_s_c_s_e_s_c_s_d_s GTGTCTGTGTGGGTGCTGGTGTGGGTGACGGTG; >clamp_s_b_s_d_s:compl:rev
CACCGTCACAGCCACTATCATGTTGACATTCC; dG = -11.0
784 >s_a_s_e_s_a_s_c_s_d_s GTGTCTGTGCTGGTGTCTGTGTGGGTGACGGTG; >clamp_s_b_s_d_s:compl:rev
CACCGTCACAGCCACTATCATGTTGACATTCC; dG = -11.0
785 >s_a_s_e_s_d_s_c_s_d_s GTGTCTGTGCTGGTGACGGTGTGGGTGACGGTG; >clamp_s_b_s_d_s:compl:rev
CACCGTCACAGCCACTATCATGTTGACATTCC; dG = -11.0
786 >s_a_s_e_s_e_s_c_s_d_s GTGTCTGTGCTGGTGCTGGTGTGGGTGACGGTG; >clamp_s_b_s_d_s:compl:rev
CACCGTCACAGCCACTATCATGTTGACATTCC; dG = -11.0
787 >s_c_s_a_s_a_s_c_s_d_s GTGTGGGTGTCTGTGTCTGTGTGGGTGACGGTG; >clamp_s_b_s_d_s:compl:rev
CACCGTCACAGCCACTATCATGTTGACATTCC; dG = -11.0
788 >s_c_s_a_s_e_s_c_s_d_s GTGTGGGTGTCTGTGCTGGTGTGGGTGACGGTG; >clamp_s_b_s_d_s:compl:rev
CACCGTCACAGCCACTATCATGTTGACATTCC; dG = -11.0
789 >s_a_s_a_s_e_s_c_s_d_s GTGTCTGTGTCTGTGCTGGTGTGGGTGACGGTG; >clamp_s_b_s_d_s:compl:rev
CACCGTCACAGCCACTATCATGTTGACATTCC; dG = -11.0
790 >s_c_s_c_s_a_s_c_s_d_s GTGTGGGTGTGGGTGTCTGTGTGGGTGACGGTG; >clamp_s_b_s_d_s:compl:rev
CACCGTCACAGCCACTATCATGTTGACATTCC; dG = -11.0
791 >s_c_s_c_s_e_s_c_s_d_s GTGTGGGTGTGGGTGCTGGTGTGGGTGACGGTG; >clamp_s_b_s_d_s:compl:rev
CACCGTCACAGCCACTATCATGTTGACATTCC; dG = -11.0
792 >s_c_s_e_s_a_s_c_s_d_s GTGTGGGTGCTGGTGTCTGTGTGGGTGACGGTG; >clamp_s_b_s_d_s:compl:rev
CACCGTCACAGCCACTATCATGTTGACATTCC; dG = -11.0
793 >s_c_s_e_s_e_s_c_s_d_s GTGTGGGTGCTGGTGCTGGTGTGGGTGACGGTG; >clamp_s_b_s_d_s:compl:rev
CACCGTCACAGCCACTATCATGTTGACATTCC; dG = -11.0
794 >s_d_s_a_s_a_s_c_s_d_s GTGACGGTGTCTGTGTCTGTGTGGGTGACGGTG; >clamp_s_b_s_d_s:compl:rev
CACCGTCACAGCCACTATCATGTTGACATTCC; dG = -11.0
795 >s_d_s_a_s_e_s_c_s_d_s GTGACGGTGTCTGTGCTGGTGTGGGTGACGGTG; >clamp_s_b_s_d_s:compl:rev
CACCGTCACAGCCACTATCATGTTGACATTCC; dG = -11.0
796 >s_d_s_c_s_a_s_c_s_d_s GTGACGGTGTGGGTGTCTGTGTGGGTGACGGTG; >clamp_s_b_s_d_s:compl:rev
CACCGTCACAGCCACTATCATGTTGACATTCC; dG = -11.0

797 >s_d_s_c_s_e_s_c_s_d_s GTGACGGTGTGGGTGCTGGTGTGGGTGACGGTG; >clamp_s_b_s_d_s:compl:rev
CACCGTCACAGCCACTATCATGTTGACATTCC; dG = -11.0
798 >s_d_s_d_s_a_s_c_s_d_s GTGACGGTGACGGTGTCTGTGTGGGTGACGGTG; >clamp_s_b_s_d_s:compl:rev
CACCGTCACAGCCACTATCATGTTGACATTCC; dG = -11.0
799 >s_d_s_d_s_e_s_c_s_d_s GTGACGGTGACGGTGCTGGTGTGGGTGACGGTG; >clamp_s_b_s_d_s:compl:rev
CACCGTCACAGCCACTATCATGTTGACATTCC; dG = -11.0
800 >s_d_s_e_s_a_s_c_s_d_s GTGACGGTGCTGGTGTCTGTGTGGGTGACGGTG; >clamp_s_b_s_d_s:compl:rev
CACCGTCACAGCCACTATCATGTTGACATTCC; dG = -11.0
801 >s_a_s_a_s_a_s_c_s_d_s GTGTCTGTGTCTGTGTCTGTGTGGGTGACGGTG; >clamp_s_b_s_d_s:compl:rev
CACCGTCACAGCCACTATCATGTTGACATTCC; dG = -11.0
802 >s_d_s_e_s_d_s_c_s_d_s GTGACGGTGCTGGTGACGGTGTGGGTGACGGTG; >clamp_s_b_s_d_s:compl:rev
CACCGTCACAGCCACTATCATGTTGACATTCC; dG = -11.0
803 >s_d_s_e_s_e_s_c_s_d_s GTGACGGTGCTGGTGCTGGTGTGGGTGACGGTG; >clamp_s_b_s_d_s:compl:rev
CACCGTCACAGCCACTATCATGTTGACATTCC; dG = -11.0
804 >s_e_s_a_s_a_s_c_s_d_s GTGCTGGTGTCTGTGTCTGTGTGGGTGACGGTG; >clamp_s_b_s_d_s:compl:rev
CACCGTCACAGCCACTATCATGTTGACATTCC; dG = -11.0
805 >s_e_s_a_s_e_s_c_s_d_s GTGCTGGTGTCTGTGCTGGTGTGGGTGACGGTG; >clamp_s_b_s_d_s:compl:rev
CACCGTCACAGCCACTATCATGTTGACATTCC; dG = -11.0
806 >s_a_s_c_s_a_s_c_s_d_s GTGTCTGTGTGGGTGTCTGTGTGGGTGACGGTG; >clamp_s_b_s_d_s:compl:rev
CACCGTCACAGCCACTATCATGTTGACATTCC; dG = -11.0
807 >s_e_s_c_s_a_s_c_s_d_s GTGCTGGTGTGGGTGTCTGTGTGGGTGACGGTG; >clamp_s_b_s_d_s:compl:rev
CACCGTCACAGCCACTATCATGTTGACATTCC; dG = -11.0
808 >s_e_s_c_s_e_s_c_s_d_s GTGCTGGTGTGGGTGCTGGTGTGGGTGACGGTG; >clamp_s_b_s_d_s:compl:rev
CACCGTCACAGCCACTATCATGTTGACATTCC; dG = -11.0
809 >s_e_s_d_s_a_s_c_s_d_s GTGCTGGTGACGGTGTCTGTGTGGGTGACGGTG; >clamp_s_b_s_d_s:compl:rev
CACCGTCACAGCCACTATCATGTTGACATTCC; dG = -11.0
810 >s_e_s_d_s_e_s_c_s_d_s GTGCTGGTGACGGTGCTGGTGTGGGTGACGGTG; >clamp_s_b_s_d_s:compl:rev
CACCGTCACAGCCACTATCATGTTGACATTCC; dG = -11.0
811 >s_e_s_e_s_a_s_c_s_d_s GTGCTGGTGCTGGTGTCTGTGTGGGTGACGGTG; >clamp_s_b_s_d_s:compl:rev
CACCGTCACAGCCACTATCATGTTGACATTCC; dG = -11.0
812 >s_e_s_e_s_d_s_c_s_d_s GTGCTGGTGCTGGTGACGGTGTGGGTGACGGTG; >clamp_s_b_s_d_s:compl:rev
CACCGTCACAGCCACTATCATGTTGACATTCC; dG = -11.0
813 >s_e_s_e_s_e_s_c_s_d_s GTGCTGGTGCTGGTGCTGGTGTGGGTGACGGTG; >clamp_s_b_s_d_s:compl:rev
CACCGTCACAGCCACTATCATGTTGACATTCC; dG = -11.0
814 >s_a_s_c_s_e_s_b_s_a_s GTGTCTGTGTGGGTGCTGGTGGCTGTGTCTGTG; >clamp_s_b_s_d_s:compl:rev
CACCGTCACAGCCACTATCATGTTGACATTCC; dG = -10.8
815 >s_a_s_e_s_a_s_b_s_a_s GTGTCTGTGCTGGTGTCTGTGGCTGTGTCTGTG; >clamp_s_b_s_d_s:compl:rev
CACCGTCACAGCCACTATCATGTTGACATTCC; dG = -10.8
816 >s_a_s_e_s_c_s_b_s_a_s GTGTCTGTGCTGGTGTGGGTGGCTGTGTCTGTG; >clamp_s_b_s_d_s:compl:rev
CACCGTCACAGCCACTATCATGTTGACATTCC; dG = -10.8
817 >s_a_s_e_s_d_s_b_s_a_s GTGTCTGTGCTGGTGACGGTGGCTGTGTCTGTG; >clamp_s_b_s_d_s:compl:rev
CACCGTCACAGCCACTATCATGTTGACATTCC; dG = -10.8
818 >s_a_s_e_s_e_s_b_s_a_s GTGTCTGTGCTGGTGCTGGTGGCTGTGTCTGTG; >clamp_s_b_s_d_s:compl:rev
CACCGTCACAGCCACTATCATGTTGACATTCC; dG = -10.8
819 >s_a_s_a_s_c_s_b_s_a_s GTGTCTGTGTCTGTGTGGGTGGCTGTGTCTGTG; >clamp_s_b_s_d_s:compl:rev
CACCGTCACAGCCACTATCATGTTGACATTCC; dG = -10.8
820 >s_b_s_a_s_a_s_b_s_a_s GTGGCTGTGTCTGTGTCTGTGGCTGTGTCTGTG; >clamp_s_b_s_d_s:compl:rev
CACCGTCACAGCCACTATCATGTTGACATTCC; dG = -10.8
821 >s_b_s_a_s_c_s_b_s_a_s GTGGCTGTGTCTGTGTGGGTGGCTGTGTCTGTG; >clamp_s_b_s_d_s:compl:rev
CACCGTCACAGCCACTATCATGTTGACATTCC; dG = -10.8
822 >s_b_s_a_s_e_s_b_s_a_s GTGGCTGTGTCTGTGCTGGTGGCTGTGTCTGTG; >clamp_s_b_s_d_s:compl:rev
CACCGTCACAGCCACTATCATGTTGACATTCC; dG = -10.8
823 >s_c_s_a_s_a_s_b_s_a_s GTGTGGGTGTCTGTGTCTGTGGCTGTGTCTGTG; >clamp_s_b_s_d_s:compl:rev
CACCGTCACAGCCACTATCATGTTGACATTCC; dG = -10.8
824 >s_a_s_a_s_e_s_b_s_a_s GTGTCTGTGTCTGTGCTGGTGGCTGTGTCTGTG; >clamp_s_b_s_d_s:compl:rev
CACCGTCACAGCCACTATCATGTTGACATTCC; dG = -10.8
825 >s_c_s_a_s_c_s_b_s_a_s GTGTGGGTGTCTGTGTGGGTGGCTGTGTCTGTG; >clamp_s_b_s_d_s:compl:rev
CACCGTCACAGCCACTATCATGTTGACATTCC; dG = -10.8
826 >s_c_s_a_s_e_s_b_s_a_s GTGTGGGTGTCTGTGCTGGTGGCTGTGTCTGTG; >clamp_s_b_s_d_s:compl:rev
CACCGTCACAGCCACTATCATGTTGACATTCC; dG = -10.8
827 >s_c_s_b_s_a_s_b_s_a_s GTGTGGGTGGCTGTGTCTGTGGCTGTGTCTGTG; >clamp_s_b_s_d_s:compl:rev
CACCGTCACAGCCACTATCATGTTGACATTCC; dG = -10.8
828 >s_a_s_a_s_a_s_b_s_a_s GTGTCTGTGTCTGTGTCTGTGGCTGTGTCTGTG; >clamp_s_b_s_d_s:compl:rev
CACCGTCACAGCCACTATCATGTTGACATTCC; dG = -10.8
829 >s_c_s_c_s_a_s_b_s_a_s GTGTGGGTGTGGGTGTCTGTGGCTGTGTCTGTG; >clamp_s_b_s_d_s:compl:rev
CACCGTCACAGCCACTATCATGTTGACATTCC; dG = -10.8
830 >s_c_s_c_s_c_s_b_s_a_s GTGTGGGTGTGGGTGTGGGTGGCTGTGTCTGTG; >clamp_s_b_s_d_s:compl:rev
CACCGTCACAGCCACTATCATGTTGACATTCC; dG = -10.8
831 >s_a_s_b_s_a_s_b_s_a_s GTGTCTGTGGCTGTGTCTGTGGCTGTGTCTGTG; >clamp_s_b_s_d_s:compl:rev
CACCGTCACAGCCACTATCATGTTGACATTCC; dG = -10.8
832 >s_c_s_c_s_e_s_b_s_a_s GTGTGGGTGTGGGTGCTGGTGGCTGTGTCTGTG; >clamp_s_b_s_d_s:compl:rev
CACCGTCACAGCCACTATCATGTTGACATTCC; dG = -10.8
833 >s_c_s_e_s_a_s_b_s_a_s GTGTGGGTGCTGGTGTCTGTGGCTGTGTCTGTG; >clamp_s_b_s_d_s:compl:rev
CACCGTCACAGCCACTATCATGTTGACATTCC; dG = -10.8

834 >s_c_s_e_s_c_s_b_s_a_s GTGTGGGTGCTGGTGTGGGTGGCTGTGTCTGTG; >clamp_s_b_s_d_s:compl:rev CACCGTCACAGCCACTATCATGTTGACATTCC; dG = -10.8
835 >s_c_s_e_s_e_s_b_s_a_s GTGTGGGTGCTGGTGCTGGTGGCTGTGTCTGTG; >clamp_s_b_s_d_s:compl:rev CACCGTCACAGCCACTATCATGTTGACATTCC; dG = -10.8
836 >s_d_s_a_s_a_s_b_s_a_s GTGACGGTGTCTGTGTCTGTGGCTGTGTCTGTG; >clamp_s_b_s_d_s:compl:rev CACCGTCACAGCCACTATCATGTTGACATTCC; dG = -10.8
837 >s_d_s_a_s_c_s_b_s_a_s GTGACGGTGTCTGTGTGGGTGGCTGTGTCTGTG; >clamp_s_b_s_d_s:compl:rev CACCGTCACAGCCACTATCATGTTGACATTCC; dG = -10.8
838 >s_d_s_a_s_e_s_b_s_a_s GTGACGGTGTCTGTGCTGGTGGCTGTGTCTGTG; >clamp_s_b_s_d_s:compl:rev CACCGTCACAGCCACTATCATGTTGACATTCC; dG = -10.8
839 >s_d_s_b_s_a_s_b_s_a_s GTGACGGTGGCTGTGTCTGTGGCTGTGTCTGTG; >clamp_s_b_s_d_s:compl:rev CACCGTCACAGCCACTATCATGTTGACATTCC; dG = -10.8
840 >s_d_s_c_s_a_s_b_s_a_s GTGACGGTGTGGGTGTCTGTGGCTGTGTCTGTG; >clamp_s_b_s_d_s:compl:rev CACCGTCACAGCCACTATCATGTTGACATTCC; dG = -10.8
841 >s_d_s_c_s_c_s_b_s_a_s GTGACGGTGTGGGTGTGGGTGGCTGTGTCTGTG; >clamp_s_b_s_d_s:compl:rev CACCGTCACAGCCACTATCATGTTGACATTCC; dG = -10.8
842 >s_d_s_c_s_e_s_b_s_a_s GTGACGGTGTGGGTGCTGGTGGCTGTGTCTGTG; >clamp_s_b_s_d_s:compl:rev CACCGTCACAGCCACTATCATGTTGACATTCC; dG = -10.8
843 >s_d_s_d_s_a_s_b_s_a_s GTGACGGTGACGGTGTCTGTGGCTGTGTCTGTG; >clamp_s_b_s_d_s:compl:rev CACCGTCACAGCCACTATCATGTTGACATTCC; dG = -10.8
844 >s_d_s_d_s_c_s_b_s_a_s GTGACGGTGACGGTGTGGGTGGCTGTGTCTGTG; >clamp_s_b_s_d_s:compl:rev CACCGTCACAGCCACTATCATGTTGACATTCC; dG = -10.8
845 >s_d_s_d_s_e_s_b_s_a_s GTGACGGTGACGGTGCTGGTGGCTGTGTCTGTG; >clamp_s_b_s_d_s:compl:rev CACCGTCACAGCCACTATCATGTTGACATTCC; dG = -10.8
846 >s_d_s_e_s_a_s_b_s_a_s GTGACGGTGCTGGTGTCTGTGGCTGTGTCTGTG; >clamp_s_b_s_d_s:compl:rev CACCGTCACAGCCACTATCATGTTGACATTCC; dG = -10.8
847 >s_d_s_e_s_c_s_b_s_a_s GTGACGGTGCTGGTGTGGGTGGCTGTGTCTGTG; >clamp_s_b_s_d_s:compl:rev CACCGTCACAGCCACTATCATGTTGACATTCC; dG = -10.8
848 >s_d_s_e_s_d_s_b_s_a_s GTGACGGTGCTGGTGACGGTGGCTGTGTCTGTG; >clamp_s_b_s_d_s:compl:rev CACCGTCACAGCCACTATCATGTTGACATTCC; dG = -10.8
849 >s_d_s_e_s_e_s_b_s_a_s GTGACGGTGCTGGTGCTGGTGGCTGTGTCTGTG; >clamp_s_b_s_d_s:compl:rev CACCGTCACAGCCACTATCATGTTGACATTCC; dG = -10.8
850 >s_e_s_a_s_a_s_b_s_a_s GTGCTGGTGTCTGTGTCTGTGGCTGTGTCTGTG; >clamp_s_b_s_d_s:compl:rev CACCGTCACAGCCACTATCATGTTGACATTCC; dG = -10.8
851 >s_e_s_a_s_c_s_b_s_a_s GTGCTGGTGTCTGTGTGGGTGGCTGTGTCTGTG; >clamp_s_b_s_d_s:compl:rev CACCGTCACAGCCACTATCATGTTGACATTCC; dG = -10.8
852 >s_a_s_c_s_a_s_b_s_a_s GTGTCTGTGTGGGTGTCTGTGGCTGTGTCTGTG; >clamp_s_b_s_d_s:compl:rev CACCGTCACAGCCACTATCATGTTGACATTCC; dG = -10.8
853 >s_e_s_a_s_e_s_b_s_a_s GTGCTGGTGTCTGTGCTGGTGGCTGTGTCTGTG; >clamp_s_b_s_d_s:compl:rev CACCGTCACAGCCACTATCATGTTGACATTCC; dG = -10.8
854 >s_e_s_b_s_a_s_b_s_a_s GTGCTGGTGGCTGTGTCTGTGGCTGTGTCTGTG; >clamp_s_b_s_d_s:compl:rev CACCGTCACAGCCACTATCATGTTGACATTCC; dG = -10.8
855 >s_e_s_c_s_a_s_b_s_a_s GTGCTGGTGTGGGTGTCTGTGGCTGTGTCTGTG; >clamp_s_b_s_d_s:compl:rev CACCGTCACAGCCACTATCATGTTGACATTCC; dG = -10.8
856 >s_e_s_c_s_c_s_b_s_a_s GTGCTGGTGTGGGTGTGGGTGGCTGTGTCTGTG; >clamp_s_b_s_d_s:compl:rev CACCGTCACAGCCACTATCATGTTGACATTCC; dG = -10.8
857 >s_e_s_c_s_e_s_b_s_a_s GTGCTGGTGTGGGTGCTGGTGGCTGTGTCTGTG; >clamp_s_b_s_d_s:compl:rev CACCGTCACAGCCACTATCATGTTGACATTCC; dG = -10.8
858 >s_e_s_d_s_a_s_b_s_a_s GTGCTGGTGACGGTGTCTGTGGCTGTGTCTGTG; >clamp_s_b_s_d_s:compl:rev CACCGTCACAGCCACTATCATGTTGACATTCC; dG = -10.8
859 >s_e_s_d_s_c_s_b_s_a_s GTGCTGGTGACGGTGTGGGTGGCTGTGTCTGTG; >clamp_s_b_s_d_s:compl:rev CACCGTCACAGCCACTATCATGTTGACATTCC; dG = -10.8
860 >s_e_s_d_s_e_s_b_s_a_s GTGCTGGTGACGGTGCTGGTGGCTGTGTCTGTG; >clamp_s_b_s_d_s:compl:rev CACCGTCACAGCCACTATCATGTTGACATTCC; dG = -10.8
861 >s_e_s_e_s_a_s_b_s_a_s GTGCTGGTGCTGGTGTCTGTGGCTGTGTCTGTG; >clamp_s_b_s_d_s:compl:rev CACCGTCACAGCCACTATCATGTTGACATTCC; dG = -10.8
862 >s_e_s_e_s_c_s_b_s_a_s GTGCTGGTGCTGGTGTGGGTGGCTGTGTCTGTG; >clamp_s_b_s_d_s:compl:rev CACCGTCACAGCCACTATCATGTTGACATTCC; dG = -10.8
863 >s_e_s_e_s_d_s_b_s_a_s GTGCTGGTGCTGGTGACGGTGGCTGTGTCTGTG; >clamp_s_b_s_d_s:compl:rev CACCGTCACAGCCACTATCATGTTGACATTCC; dG = -10.8
864 >s_a_s_c_s_c_s_b_s_a_s GTGTCTGTGTGGGTGTGGGTGGCTGTGTCTGTG; >clamp_s_b_s_d_s:compl:rev CACCGTCACAGCCACTATCATGTTGACATTCC; dG = -10.8
865 >s_e_s_e_s_e_s_b_s_a_s GTGCTGGTGCTGGTGCTGGTGGCTGTGTCTGTG; >clamp_s_b_s_d_s:compl:rev CACCGTCACAGCCACTATCATGTTGACATTCC; dG = -10.8
866 >s_d_s_d_s_a_s_e_s_d_s GTGACGGTGACGGTGTCTGTGCTGGTGACGGTG; >clamp_s_b_s_d_s:compl:rev CACCGTCACAGCCACTATCATGTTGACATTCC; dG = -10.6
867 >s_d_s_d_s_e_s_e_s_d_s GTGACGGTGACGGTGCTGGTGCTGGTGACGGTG; >clamp_s_b_s_d_s:compl:rev CACCGTCACAGCCACTATCATGTTGACATTCC; dG = -10.6
868 >s_e_s_d_s_a_s_e_s_d_s GTGCTGGTGACGGTGTCTGTGCTGGTGACGGTG; >clamp_s_b_s_d_s:compl:rev CACCGTCACAGCCACTATCATGTTGACATTCC; dG = -10.5
869 >s_a_s_e_s_d_s_e_s_d_s GTGTCTGTGCTGGTGACGGTGCTGGTGACGGTG; >clamp_s_b_s_d_s:compl:rev CACCGTCACAGCCACTATCATGTTGACATTCC; dG = -10.4
870 >s_d_s_e_s_d_s_e_s_d_s GTGACGGTGCTGGTGACGGTGCTGGTGACGGTG; >clamp_s_b_s_d_s:compl:rev CACCGTCACAGCCACTATCATGTTGACATTCC; dG = -10.4

871 >s_e_s_d_s_e_s_e_s_d_s GTGCTGGTGACGGTGCTGGTGCTGGTGACGGTG; >clamp_s_b_s_d_s:compl:rev CACCGTCACAGCCACTATCATGTTGACATTCC; dG = -10.4
872 >s_e_s_e_s_d_s_e_s_d_s GTGCTGGTGCTGGTGACGGTGCTGGTGACGGTG; >clamp_s_b_s_d_s:compl:rev CACCGTCACAGCCACTATCATGTTGACATTCC; dG = -10.4
873 >s_a_s_c_s_e_s_e_s_d_s GTGTCTGTGTGGGTGCTGGTGCTGGTGACGGTG; >clamp_s_b_s_d_s:compl:rev CACCGTCACAGCCACTATCATGTTGACATTCC; dG = -10.3
874 >s_a_s_e_s_a_s_e_s_d_s GTGTCTGTGCTGGTGTCTGTGCTGGTGACGGTG; >clamp_s_b_s_d_s:compl:rev CACCGTCACAGCCACTATCATGTTGACATTCC; dG = -10.3
875 >s_a_s_e_s_e_s_e_s_d_s GTGTCTGTGCTGGTGCTGGTGCTGGTGACGGTG; >clamp_s_b_s_d_s:compl:rev CACCGTCACAGCCACTATCATGTTGACATTCC; dG = -10.3
876 >s_c_s_a_s_a_s_e_s_d_s GTGTGGGTGTCTGTGTCTGTGCTGGTGACGGTG; >clamp_s_b_s_d_s:compl:rev CACCGTCACAGCCACTATCATGTTGACATTCC; dG = -10.3
877 >s_a_s_a_s_e_s_e_s_d_s GTGTCTGTGTCTGTGCTGGTGCTGGTGACGGTG; >clamp_s_b_s_d_s:compl:rev CACCGTCACAGCCACTATCATGTTGACATTCC; dG = -10.3
878 >s_c_s_a_s_e_s_e_s_d_s GTGTGGGTGTCTGTGCTGGTGCTGGTGACGGTG; >clamp_s_b_s_d_s:compl:rev CACCGTCACAGCCACTATCATGTTGACATTCC; dG = -10.3
879 >s_c_s_c_s_a_s_e_s_d_s GTGTGGGTGTGGGTGTCTGTGCTGGTGACGGTG; >clamp_s_b_s_d_s:compl:rev CACCGTCACAGCCACTATCATGTTGACATTCC; dG = -10.3
880 >s_c_s_c_s_e_s_e_s_d_s GTGTGGGTGTGGGTGCTGGTGCTGGTGACGGTG; >clamp_s_b_s_d_s:compl:rev CACCGTCACAGCCACTATCATGTTGACATTCC; dG = -10.3
881 >s_c_s_e_s_a_s_e_s_d_s GTGTGGGTGCTGGTGTCTGTGCTGGTGACGGTG; >clamp_s_b_s_d_s:compl:rev CACCGTCACAGCCACTATCATGTTGACATTCC; dG = -10.3
882 >s_a_s_a_s_a_s_e_s_d_s GTGTCTGTGTCTGTGTCTGTGCTGGTGACGGTG; >clamp_s_b_s_d_s:compl:rev CACCGTCACAGCCACTATCATGTTGACATTCC; dG = -10.3
883 >s_c_s_e_s_e_s_e_s_d_s GTGTGGGTGCTGGTGCTGGTGCTGGTGACGGTG; >clamp_s_b_s_d_s:compl:rev CACCGTCACAGCCACTATCATGTTGACATTCC; dG = -10.3
884 >s_d_s_a_s_a_s_e_s_d_s GTGACGGTGTCTGTGTCTGTGCTGGTGACGGTG; >clamp_s_b_s_d_s:compl:rev CACCGTCACAGCCACTATCATGTTGACATTCC; dG = -10.3
885 >s_d_s_a_s_e_s_e_s_d_s GTGACGGTGTCTGTGCTGGTGCTGGTGACGGTG; >clamp_s_b_s_d_s:compl:rev CACCGTCACAGCCACTATCATGTTGACATTCC; dG = -10.3
886 >s_d_s_c_s_a_s_e_s_d_s GTGACGGTGTGGGTGTCTGTGCTGGTGACGGTG; >clamp_s_b_s_d_s:compl:rev CACCGTCACAGCCACTATCATGTTGACATTCC; dG = -10.3
887 >s_d_s_c_s_e_s_e_s_d_s GTGACGGTGTGGGTGCTGGTGCTGGTGACGGTG; >clamp_s_b_s_d_s:compl:rev CACCGTCACAGCCACTATCATGTTGACATTCC; dG = -10.3
888 >s_d_s_e_s_a_s_e_s_d_s GTGACGGTGCTGGTGTCTGTGCTGGTGACGGTG; >clamp_s_b_s_d_s:compl:rev CACCGTCACAGCCACTATCATGTTGACATTCC; dG = -10.3
889 >s_d_s_e_s_e_s_e_s_d_s GTGACGGTGCTGGTGCTGGTGCTGGTGACGGTG; >clamp_s_b_s_d_s:compl:rev CACCGTCACAGCCACTATCATGTTGACATTCC; dG = -10.3
890 >s_e_s_a_s_a_s_e_s_d_s GTGCTGGTGTCTGTGTCTGTGCTGGTGACGGTG; >clamp_s_b_s_d_s:compl:rev CACCGTCACAGCCACTATCATGTTGACATTCC; dG = -10.3
891 >s_e_s_a_s_e_s_e_s_d_s GTGCTGGTGTCTGTGCTGGTGCTGGTGACGGTG; >clamp_s_b_s_d_s:compl:rev CACCGTCACAGCCACTATCATGTTGACATTCC; dG = -10.3
892 >s_a_s_c_s_a_s_e_s_d_s GTGTCTGTGTGGGTGTCTGTGCTGGTGACGGTG; >clamp_s_b_s_d_s:compl:rev CACCGTCACAGCCACTATCATGTTGACATTCC; dG = -10.3
893 >s_e_s_c_s_a_s_e_s_d_s GTGCTGGTGTGGGTGTCTGTGCTGGTGACGGTG; >clamp_s_b_s_d_s:compl:rev CACCGTCACAGCCACTATCATGTTGACATTCC; dG = -10.3
894 >s_e_s_c_s_e_s_e_s_d_s GTGCTGGTGTGGGTGCTGGTGCTGGTGACGGTG; >clamp_s_b_s_d_s:compl:rev CACCGTCACAGCCACTATCATGTTGACATTCC; dG = -10.3
895 >s_e_s_e_s_a_s_e_s_d_s GTGCTGGTGCTGGTGTCTGTGCTGGTGACGGTG; >clamp_s_b_s_d_s:compl:rev CACCGTCACAGCCACTATCATGTTGACATTCC; dG = -10.3
896 >s_e_s_e_s_e_s_e_s_d_s GTGCTGGTGCTGGTGCTGGTGCTGGTGACGGTG; >clamp_s_b_s_d_s:compl:rev CACCGTCACAGCCACTATCATGTTGACATTCC; dG = -10.3

****

CA Matches

1 >s_a_s_c_s_a_s_c_s_a_s GTGTCTGTGTGGGTGTCTGTGTGGGTGTCTGTG; >clamp_s_c_s_a_s:compl:rev CACAGACACCCACACTATCATGTTGACATTCC; dG = -17.4
2 >s_b_s_c_s_a_s_c_s_a_s GTGGCTGTGTGGGTGTCTGTGTGGGTGTCTGTG; >clamp_s_c_s_a_s:compl:rev CACAGACACCCACACTATCATGTTGACATTCC; dG = -17.4
3 >s_c_s_a_s_d_s_c_s_a_s GTGTGGGTGTCTGTGACGGTGTGGGTGTCTGTG; >clamp_s_c_s_a_s:compl:rev CACAGACACCCACACTATCATGTTGACATTCC; dG = -17.4
4 >s_e_s_c_s_a_s_c_s_a_s GTGCTGGTGTGGGTGTCTGTGTGGGTGTCTGTG; >clamp_s_c_s_a_s:compl:rev CACAGACACCCACACTATCATGTTGACATTCC; dG = -17.3
5 >s_c_s_c_s_a_s_c_s_a_s GTGTGGGTGTGGGTGTCTGTGTGGGTGTCTGTG; >clamp_s_c_s_a_s:compl:rev CACAGACACCCACACTATCATGTTGACATTCC; dG = -17.3
6 >s_d_s_c_s_a_s_c_s_a_s GTGACGGTGTGGGTGTCTGTGTGGGTGTCTGTG; >clamp_s_c_s_a_s:compl:rev CACAGACACCCACACTATCATGTTGACATTCC; dG = -17.3
7 >s_a_s_a_s_a_s_c_s_a_s GTGTCTGTGTCTGTGTCTGTGTGGGTGTCTGTG; >clamp_s_c_s_a_s:compl:rev CACAGACACCCACACTATCATGTTGACATTCC; dG = -17.1

8 >s_a_s_b_s_e_s_c_s_a_s GTGTCTGTGGCTGTGCTGGTGTGGGTGTCTGTG; >clamp_s_c_s_a_s:compl:rev
CACAGACACCCACACTATCATGTTGACATTCC; dG = -17.1

9 >s_d_s_e_s_e_s_c_s_a_s GTGACGGTGCTGGTGCTGGTGTGGGTGTCTGTG; >clamp_s_c_s_a_s:compl:rev
CACAGACACCCACACTATCATGTTGACATTCC; dG = -17.1

10 >s_e_s_a_s_a_s_c_s_a_s GTGCTGGTGTCTGTGTCTGTGTGGGTGTCTGTG; >clamp_s_c_s_a_s:compl:rev
CACAGACACCCACACTATCATGTTGACATTCC; dG = -17.1

11 >s_e_s_a_s_b_s_c_s_a_s GTGCTGGTGTCTGTGGCTGTGTGGGTGTCTGTG; >clamp_s_c_s_a_s:compl:rev
CACAGACACCCACACTATCATGTTGACATTCC; dG = -17.1

12 >s_e_s_a_s_c_s_c_s_a_s GTGCTGGTGTCTGTGTGGGTGTGGGTGTCTGTG; >clamp_s_c_s_a_s:compl:rev
CACAGACACCCACACTATCATGTTGACATTCC; dG = -17.1

13 >s_e_s_a_s_d_s_c_s_a_s GTGCTGGTGTCTGTGACGGTGTGGGTGTCTGTG; >clamp_s_c_s_a_s:compl:rev
CACAGACACCCACACTATCATGTTGACATTCC; dG = -17.1

14 >s_e_s_a_s_e_s_c_s_a_s GTGCTGGTGTCTGTGCTGGTGTGGGTGTCTGTG; >clamp_s_c_s_a_s:compl:rev
CACAGACACCCACACTATCATGTTGACATTCC; dG = -17.1

15 >s_e_s_b_s_a_s_c_s_a_s GTGCTGGTGGCTGTGTCTGTGTGGGTGTCTGTG; >clamp_s_c_s_a_s:compl:rev
CACAGACACCCACACTATCATGTTGACATTCC; dG = -17.1

16 >s_e_s_b_s_b_s_c_s_a_s GTGCTGGTGGCTGTGGCTGTGTGGGTGTCTGTG; >clamp_s_c_s_a_s:compl:rev
CACAGACACCCACACTATCATGTTGACATTCC; dG = -17.1

17 >s_e_s_b_s_c_s_c_s_a_s GTGCTGGTGGCTGTGTGGGTGTGGGTGTCTGTG; >clamp_s_c_s_a_s:compl:rev
CACAGACACCCACACTATCATGTTGACATTCC; dG = -17.1

18 >s_e_s_b_s_d_s_c_s_a_s GTGCTGGTGGCTGTGACGGTGTGGGTGTCTGTG; >clamp_s_c_s_a_s:compl:rev
CACAGACACCCACACTATCATGTTGACATTCC; dG = -17.1

19 >s_e_s_b_s_e_s_c_s_a_s GTGCTGGTGGCTGTGCTGGTGTGGGTGTCTGTG; >clamp_s_c_s_a_s:compl:rev
CACAGACACCCACACTATCATGTTGACATTCC; dG = -17.1

20 >s_e_s_c_s_b_s_c_s_a_s GTGCTGGTGTGGGTGGCTGTGTGGGTGTCTGTG; >clamp_s_c_s_a_s:compl:rev
CACAGACACCCACACTATCATGTTGACATTCC; dG = -17.1

21 >s_e_s_c_s_c_s_c_s_a_s GTGCTGGTGTGGGTGTGGGTGTGGGTGTCTGTG; >clamp_s_c_s_a_s:compl:rev
CACAGACACCCACACTATCATGTTGACATTCC; dG = -17.1

22 >s_e_s_c_s_d_s_c_s_a_s GTGCTGGTGTGGGTGACGGTGTGGGTGTCTGTG; >clamp_s_c_s_a_s:compl:rev
CACAGACACCCACACTATCATGTTGACATTCC; dG = -17.1

23 >s_e_s_c_s_e_s_c_s_a_s GTGCTGGTGTGGGTGCTGGTGTGGGTGTCTGTG; >clamp_s_c_s_a_s:compl:rev
CACAGACACCCACACTATCATGTTGACATTCC; dG = -17.1

24 >s_e_s_d_s_a_s_c_s_a_s GTGCTGGTGACGGTGTCTGTGTGGGTGTCTGTG; >clamp_s_c_s_a_s:compl:rev
CACAGACACCCACACTATCATGTTGACATTCC; dG = -17.1

25 >s_e_s_d_s_b_s_c_s_a_s GTGCTGGTGACGGTGGCTGTGTGGGTGTCTGTG; >clamp_s_c_s_a_s:compl:rev
CACAGACACCCACACTATCATGTTGACATTCC; dG = -17.1

26 >s_e_s_d_s_c_s_c_s_a_s GTGCTGGTGACGGTGTGGGTGTGGGTGTCTGTG; >clamp_s_c_s_a_s:compl:rev
CACAGACACCCACACTATCATGTTGACATTCC; dG = -17.1

27 >s_e_s_d_s_d_s_c_s_a_s GTGCTGGTGACGGTGACGGTGTGGGTGTCTGTG; >clamp_s_c_s_a_s:compl:rev
CACAGACACCCACACTATCATGTTGACATTCC; dG = -17.1

28 >s_a_s_c_s_b_s_c_s_a_s GTGTCTGTGTGGGTGGCTGTGTGGGTGTCTGTG; >clamp_s_c_s_a_s:compl:rev
CACAGACACCCACACTATCATGTTGACATTCC; dG = -17.1

29 >s_e_s_d_s_e_s_c_s_a_s GTGCTGGTGACGGTGCTGGTGTGGGTGTCTGTG; >clamp_s_c_s_a_s:compl:rev
CACAGACACCCACACTATCATGTTGACATTCC; dG = -17.1

30 >s_e_s_e_s_a_s_c_s_a_s GTGCTGGTGCTGGTGTCTGTGTGGGTGTCTGTG; >clamp_s_c_s_a_s:compl:rev
CACAGACACCCACACTATCATGTTGACATTCC; dG = -17.1

31 >s_e_s_e_s_b_s_c_s_a_s GTGCTGGTGCTGGTGGCTGTGTGGGTGTCTGTG; >clamp_s_c_s_a_s:compl:rev
CACAGACACCCACACTATCATGTTGACATTCC; dG = -17.1

32 >s_e_s_e_s_c_s_c_s_a_s GTGCTGGTGCTGGTGTGGGTGTGGGTGTCTGTG; >clamp_s_c_s_a_s:compl:rev
CACAGACACCCACACTATCATGTTGACATTCC; dG = -17.1

33 >s_e_s_e_s_d_s_c_s_a_s GTGCTGGTGCTGGTGACGGTGTGGGTGTCTGTG; >clamp_s_c_s_a_s:compl:rev
CACAGACACCCACACTATCATGTTGACATTCC; dG = -17.1

34 >s_e_s_e_s_e_s_c_s_a_s GTGCTGGTGCTGGTGCTGGTGTGGGTGTCTGTG; >clamp_s_c_s_a_s:compl:rev
CACAGACACCCACACTATCATGTTGACATTCC; dG = -17.1

35 >s_a_s_c_s_c_s_c_s_a_s GTGTCTGTGTGGGTGTGGGTGTGGGTGTCTGTG; >clamp_s_c_s_a_s:compl:rev
CACAGACACCCACACTATCATGTTGACATTCC; dG = -17.1

36 >s_a_s_c_s_d_s_c_s_a_s GTGTCTGTGTGGGTGACGGTGTGGGTGTCTGTG; >clamp_s_c_s_a_s:compl:rev
CACAGACACCCACACTATCATGTTGACATTCC; dG = -17.1

37 >s_a_s_c_s_e_s_c_s_a_s GTGTCTGTGTGGGTGCTGGTGTGGGTGTCTGTG; >clamp_s_c_s_a_s:compl:rev
CACAGACACCCACACTATCATGTTGACATTCC; dG = -17.1

38 >s_a_s_d_s_a_s_c_s_a_s GTGTCTGTGACGGTGTCTGTGTGGGTGTCTGTG; >clamp_s_c_s_a_s:compl:rev
CACAGACACCCACACTATCATGTTGACATTCC; dG = -17.1

39 >s_a_s_d_s_b_s_c_s_a_s GTGTCTGTGACGGTGGCTGTGTGGGTGTCTGTG; >clamp_s_c_s_a_s:compl:rev
CACAGACACCCACACTATCATGTTGACATTCC; dG = -17.1

40 >s_a_s_d_s_c_s_c_s_a_s GTGTCTGTGACGGTGTGGGTGTGGGTGTCTGTG; >clamp_s_c_s_a_s:compl:rev
CACAGACACCCACACTATCATGTTGACATTCC; dG = -17.1

41 >s_a_s_d_s_d_s_c_s_a_s GTGTCTGTGACGGTGACGGTGTGGGTGTCTGTG; >clamp_s_c_s_a_s:compl:rev
CACAGACACCCACACTATCATGTTGACATTCC; dG = -17.1

42 >s_a_s_a_s_b_s_c_s_a_s GTGTCTGTGTCTGTGGCTGTGTGGGTGTCTGTG; >clamp_s_c_s_a_s:compl:rev
CACAGACACCCACACTATCATGTTGACATTCC; dG = -17.1

43 >s_a_s_d_s_e_s_c_s_a_s GTGTCTGTGACGGTGCTGGTGTGGGTGTCTGTG; >clamp_s_c_s_a_s:compl:rev
CACAGACACCCACACTATCATGTTGACATTCC; dG = -17.1

44 >s_a_s_e_s_a_s_c_s_a_s GTGTCTGTGCTGGTGTCTGTGTGGGTGTCTGTG; >clamp_s_c_s_a_s:compl:rev
CACAGACACCCACACTATCATGTTGACATTCC; dG = -17.1

45 >s_a_s_e_s_b_s_c_s_a_s GTGTCTGTGCTGGTGGCTGTGTGGGTGTCTGTG; >clamp_s_c_s_a_s:compl:rev CACAGACACCCACACTATCATGTTGACATTCC; dG = -17.1

46 >s_a_s_e_s_c_s_c_s_a_s GTGTCTGTGCTGGTGTGGGTGTGGGTGTCTGTG; >clamp_s_c_s_a_s:compl:rev CACAGACACCCACACTATCATGTTGACATTCC; dG = -17.1

47 >s_a_s_e_s_d_s_c_s_a_s GTGTCTGTGCTGGTGACGGTGTGGGTGTCTGTG; >clamp_s_c_s_a_s:compl:rev CACAGACACCCACACTATCATGTTGACATTCC; dG = -17.1

48 >s_a_s_e_s_e_s_c_s_a_s GTGTCTGTGCTGGTGCTGGTGTGGGTGTCTGTG; >clamp_s_c_s_a_s:compl:rev CACAGACACCCACACTATCATGTTGACATTCC; dG = -17.1

49 >s_b_s_a_s_a_s_c_s_a_s GTGGCTGTGTCTGTGTCTGTGTGGGTGTCTGTG; >clamp_s_c_s_a_s:compl:rev CACAGACACCCACACTATCATGTTGACATTCC; dG = -17.1

50 >s_b_s_a_s_b_s_c_s_a_s GTGGCTGTGTCTGTGGCTGTGTGGGTGTCTGTG; >clamp_s_c_s_a_s:compl:rev CACAGACACCCACACTATCATGTTGACATTCC; dG = -17.1

51 >s_b_s_a_s_c_s_c_s_a_s GTGGCTGTGTCTGTGTGGGTGTGGGTGTCTGTG; >clamp_s_c_s_a_s:compl:rev CACAGACACCCACACTATCATGTTGACATTCC; dG = -17.1

52 >s_b_s_a_s_d_s_c_s_a_s GTGGCTGTGTCTGTGACGGTGTGGGTGTCTGTG; >clamp_s_c_s_a_s:compl:rev CACAGACACCCACACTATCATGTTGACATTCC; dG = -17.1

53 >s_a_s_a_s_c_s_c_s_a_s GTGTCTGTGTCTGTGTGGGTGTGGGTGTCTGTG; >clamp_s_c_s_a_s:compl:rev CACAGACACCCACACTATCATGTTGACATTCC; dG = -17.1

54 >s_b_s_a_s_e_s_c_s_a_s GTGGCTGTGTCTGTGCTGGTGTGGGTGTCTGTG; >clamp_s_c_s_a_s:compl:rev CACAGACACCCACACTATCATGTTGACATTCC; dG = -17.1

55 >s_b_s_b_s_a_s_c_s_a_s GTGGCTGTGGCTGTGTCTGTGTGGGTGTCTGTG; >clamp_s_c_s_a_s:compl:rev CACAGACACCCACACTATCATGTTGACATTCC; dG = -17.1

56 >s_b_s_b_s_b_s_c_s_a_s GTGGCTGTGGCTGTGGCTGTGTGGGTGTCTGTG; >clamp_s_c_s_a_s:compl:rev CACAGACACCCACACTATCATGTTGACATTCC; dG = -17.1

57 >s_b_s_b_s_c_s_c_s_a_s GTGGCTGTGGCTGTGTGGGTGTGGGTGTCTGTG; >clamp_s_c_s_a_s:compl:rev CACAGACACCCACACTATCATGTTGACATTCC; dG = -17.1

58 >s_b_s_b_s_d_s_c_s_a_s GTGGCTGTGGCTGTGACGGTGTGGGTGTCTGTG; >clamp_s_c_s_a_s:compl:rev CACAGACACCCACACTATCATGTTGACATTCC; dG = -17.1

59 >s_b_s_b_s_e_s_c_s_a_s GTGGCTGTGGCTGTGCTGGTGTGGGTGTCTGTG; >clamp_s_c_s_a_s:compl:rev CACAGACACCCACACTATCATGTTGACATTCC; dG = -17.1

60 >s_b_s_c_s_b_s_c_s_a_s GTGGCTGTGTGGGTGGCTGTGTGGGTGTCTGTG; >clamp_s_c_s_a_s:compl:rev CACAGACACCCACACTATCATGTTGACATTCC; dG = -17.1

61 >s_b_s_c_s_c_s_c_s_a_s GTGGCTGTGTGGGTGTGGGTGTGGGTGTCTGTG; >clamp_s_c_s_a_s:compl:rev CACAGACACCCACACTATCATGTTGACATTCC; dG = -17.1

62 >s_b_s_c_s_d_s_c_s_a_s GTGGCTGTGTGGGTGACGGTGTGGGTGTCTGTG; >clamp_s_c_s_a_s:compl:rev CACAGACACCCACACTATCATGTTGACATTCC; dG = -17.1

63 >s_a_s_a_s_d_s_c_s_a_s GTGTCTGTGTCTGTGACGGTGTGGGTGTCTGTG; >clamp_s_c_s_a_s:compl:rev CACAGACACCCACACTATCATGTTGACATTCC; dG = -17.1

64 >s_b_s_c_s_e_s_c_s_a_s GTGGCTGTGTGGGTGCTGGTGTGGGTGTCTGTG; >clamp_s_c_s_a_s:compl:rev CACAGACACCCACACTATCATGTTGACATTCC; dG = -17.1

65 >s_b_s_d_s_a_s_c_s_a_s GTGGCTGTGACGGTGTCTGTGTGGGTGTCTGTG; >clamp_s_c_s_a_s:compl:rev CACAGACACCCACACTATCATGTTGACATTCC; dG = -17.1

66 >s_b_s_d_s_b_s_c_s_a_s GTGGCTGTGACGGTGGCTGTGTGGGTGTCTGTG; >clamp_s_c_s_a_s:compl:rev CACAGACACCCACACTATCATGTTGACATTCC; dG = -17.1

67 >s_b_s_d_s_c_s_c_s_a_s GTGGCTGTGACGGTGTGGGTGTGGGTGTCTGTG; >clamp_s_c_s_a_s:compl:rev CACAGACACCCACACTATCATGTTGACATTCC; dG = -17.1

68 >s_b_s_d_s_d_s_c_s_a_s GTGGCTGTGACGGTGACGGTGTGGGTGTCTGTG; >clamp_s_c_s_a_s:compl:rev CACAGACACCCACACTATCATGTTGACATTCC; dG = -17.1

69 >s_b_s_d_s_e_s_c_s_a_s GTGGCTGTGACGGTGCTGGTGTGGGTGTCTGTG; >clamp_s_c_s_a_s:compl:rev CACAGACACCCACACTATCATGTTGACATTCC; dG = -17.1

70 >s_b_s_e_s_a_s_c_s_a_s GTGGCTGTGCTGGTGTCTGTGTGGGTGTCTGTG; >clamp_s_c_s_a_s:compl:rev CACAGACACCCACACTATCATGTTGACATTCC; dG = -17.1

71 >s_b_s_e_s_b_s_c_s_a_s GTGGCTGTGCTGGTGGCTGTGTGGGTGTCTGTG; >clamp_s_c_s_a_s:compl:rev CACAGACACCCACACTATCATGTTGACATTCC; dG = -17.1

72 >s_b_s_e_s_c_s_c_s_a_s GTGGCTGTGCTGGTGTGGGTGTGGGTGTCTGTG; >clamp_s_c_s_a_s:compl:rev CACAGACACCCACACTATCATGTTGACATTCC; dG = -17.1

73 >s_b_s_e_s_d_s_c_s_a_s GTGGCTGTGCTGGTGACGGTGTGGGTGTCTGTG; >clamp_s_c_s_a_s:compl:rev CACAGACACCCACACTATCATGTTGACATTCC; dG = -17.1

74 >s_a_s_a_s_e_s_c_s_a_s GTGTCTGTGTCTGTGCTGGTGTGGGTGTCTGTG; >clamp_s_c_s_a_s:compl:rev CACAGACACCCACACTATCATGTTGACATTCC; dG = -17.1

75 >s_b_s_e_s_e_s_c_s_a_s GTGGCTGTGCTGGTGCTGGTGTGGGTGTCTGTG; >clamp_s_c_s_a_s:compl:rev CACAGACACCCACACTATCATGTTGACATTCC; dG = -17.1

76 >s_c_s_a_s_a_s_c_s_a_s GTGTGGGTGTCTGTGTCTGTGTGGGTGTCTGTG; >clamp_s_c_s_a_s:compl:rev CACAGACACCCACACTATCATGTTGACATTCC; dG = -17.1

77 >s_c_s_a_s_b_s_c_s_a_s GTGTGGGTGTCTGTGGCTGTGTGGGTGTCTGTG; >clamp_s_c_s_a_s:compl:rev CACAGACACCCACACTATCATGTTGACATTCC; dG = -17.1

78 >s_c_s_a_s_c_s_c_s_a_s GTGTGGGTGTCTGTGTGGGTGTGGGTGTCTGTG; >clamp_s_c_s_a_s:compl:rev CACAGACACCCACACTATCATGTTGACATTCC; dG = -17.1

79 >s_c_s_a_s_e_s_c_s_a_s GTGTGGGTGTCTGTGCTGGTGTGGGTGTCTGTG; >clamp_s_c_s_a_s:compl:rev CACAGACACCCACACTATCATGTTGACATTCC; dG = -17.1

80 >s_c_s_b_s_a_s_c_s_a_s GTGTGGGTGGCTGTGTCTGTGTGGGTGTCTGTG; >clamp_s_c_s_a_s:compl:rev CACAGACACCCACACTATCATGTTGACATTCC; dG = -17.1

81 >s_c_s_b_s_b_s_c_s_a_s GTGTGGGTGGCTGTGGCTGTGTGGGTGTCTGTG; >clamp_s_c_s_a_s:compl:rev CACAGACACCCACACTATCATGTTGACATTCC; dG = -17.1

82 >s_c_s_b_s_c_s_c_s_a_s GTGTGGGTGGCTGTGTGGGTGTGGGTGTCTGTG; >clamp_s_c_s_a_s:compl:rev CACAGACACCCACACTATCATGTTGACATTCC; dG = -17.1
83 >s_c_s_b_s_d_s_c_s_a_s GTGTGGGTGGCTGTGACGGTGTGGGTGTCTGTG; >clamp_s_c_s_a_s:compl:rev CACAGACACCCACACTATCATGTTGACATTCC; dG = -17.1
84 >s_a_s_b_s_a_s_c_s_a_s GTGTCTGTGGCTGTGTCTGTGTGGGTGTCTGTG; >clamp_s_c_s_a_s:compl:rev CACAGACACCCACACTATCATGTTGACATTCC; dG = -17.1
85 >s_c_s_b_s_e_s_c_s_a_s GTGTGGGTGGCTGTGCTGGTGTGGGTGTCTGTG; >clamp_s_c_s_a_s:compl:rev CACAGACACCCACACTATCATGTTGACATTCC; dG = -17.1
86 >s_c_s_c_s_b_s_c_s_a_s GTGTGGGTGTGGGTGGCTGTGTGGGTGTCTGTG; >clamp_s_c_s_a_s:compl:rev CACAGACACCCACACTATCATGTTGACATTCC; dG = -17.1
87 >s_c_s_c_s_c_s_c_s_a_s GTGTGGGTGTGGGTGTGGGTGTGGGTGTCTGTG; >clamp_s_c_s_a_s:compl:rev CACAGACACCCACACTATCATGTTGACATTCC; dG = -17.1
88 >s_c_s_c_s_d_s_c_s_a_s GTGTGGGTGTGGGTGACGGTGTGGGTGTCTGTG; >clamp_s_c_s_a_s:compl:rev CACAGACACCCACACTATCATGTTGACATTCC; dG = -17.1
89 >s_c_s_c_s_e_s_c_s_a_s GTGTGGGTGTGGGTGCTGGTGTGGGTGTCTGTG; >clamp_s_c_s_a_s:compl:rev CACAGACACCCACACTATCATGTTGACATTCC; dG = -17.1
90 >s_c_s_d_s_a_s_c_s_a_s GTGTGGGTGACGGTGTCTGTGTGGGTGTCTGTG; >clamp_s_c_s_a_s:compl:rev CACAGACACCCACACTATCATGTTGACATTCC; dG = -17.1
91 >s_c_s_d_s_b_s_c_s_a_s GTGTGGGTGACGGTGGCTGTGTGGGTGTCTGTG; >clamp_s_c_s_a_s:compl:rev CACAGACACCCACACTATCATGTTGACATTCC; dG = -17.1
92 >s_c_s_d_s_c_s_c_s_a_s GTGTGGGTGACGGTGTGGGTGTGGGTGTCTGTG; >clamp_s_c_s_a_s:compl:rev CACAGACACCCACACTATCATGTTGACATTCC; dG = -17.1
93 >s_c_s_d_s_d_s_c_s_a_s GTGTGGGTGACGGTGACGGTGTGGGTGTCTGTG; >clamp_s_c_s_a_s:compl:rev CACAGACACCCACACTATCATGTTGACATTCC; dG = -17.1
94 >s_a_s_b_s_b_s_c_s_a_s GTGTCTGTGGCTGTGGCTGTGTGGGTGTCTGTG; >clamp_s_c_s_a_s:compl:rev CACAGACACCCACACTATCATGTTGACATTCC; dG = -17.1
95 >s_c_s_d_s_e_s_c_s_a_s GTGTGGGTGACGGTGCTGGTGTGGGTGTCTGTG; >clamp_s_c_s_a_s:compl:rev CACAGACACCCACACTATCATGTTGACATTCC; dG = -17.1
96 >s_c_s_e_s_a_s_c_s_a_s GTGTGGGTGCTGGTGTCTGTGTGGGTGTCTGTG; >clamp_s_c_s_a_s:compl:rev CACAGACACCCACACTATCATGTTGACATTCC; dG = -17.1
97 >s_c_s_e_s_b_s_c_s_a_s GTGTGGGTGCTGGTGGCTGTGTGGGTGTCTGTG; >clamp_s_c_s_a_s:compl:rev CACAGACACCCACACTATCATGTTGACATTCC; dG = -17.1
98 >s_c_s_e_s_c_s_c_s_a_s GTGTGGGTGCTGGTGTGGGTGTGGGTGTCTGTG; >clamp_s_c_s_a_s:compl:rev CACAGACACCCACACTATCATGTTGACATTCC; dG = -17.1
99 >s_c_s_e_s_d_s_c_s_a_s GTGTGGGTGCTGGTGACGGTGTGGGTGTCTGTG; >clamp_s_c_s_a_s:compl:rev CACAGACACCCACACTATCATGTTGACATTCC; dG = -17.1
100 >s_c_s_e_s_e_s_c_s_a_s GTGTGGGTGCTGGTGCTGGTGTGGGTGTCTGTG; >clamp_s_c_s_a_s:compl:rev CACAGACACCCACACTATCATGTTGACATTCC; dG = -17.1
101 >s_d_s_a_s_a_s_c_s_a_s GTGACGGTGTCTGTGTCTGTGTGGGTGTCTGTG; >clamp_s_c_s_a_s:compl:rev CACAGACACCCACACTATCATGTTGACATTCC; dG = -17.1
102 >s_d_s_a_s_b_s_c_s_a_s GTGACGGTGTCTGTGGCTGTGTGGGTGTCTGTG; >clamp_s_c_s_a_s:compl:rev CACAGACACCCACACTATCATGTTGACATTCC; dG = -17.1
103 >s_d_s_a_s_c_s_c_s_a_s GTGACGGTGTCTGTGTGGGTGTGGGTGTCTGTG; >clamp_s_c_s_a_s:compl:rev CACAGACACCCACACTATCATGTTGACATTCC; dG = -17.1
104 >s_d_s_a_s_d_s_c_s_a_s GTGACGGTGTCTGTGACGGTGTGGGTGTCTGTG; >clamp_s_c_s_a_s:compl:rev CACAGACACCCACACTATCATGTTGACATTCC; dG = -17.1
105 >s_a_s_b_s_c_s_c_s_a_s GTGTCTGTGGCTGTGTGGGTGTGGGTGTCTGTG; >clamp_s_c_s_a_s:compl:rev CACAGACACCCACACTATCATGTTGACATTCC; dG = -17.1
106 >s_d_s_a_s_e_s_c_s_a_s GTGACGGTGTCTGTGCTGGTGTGGGTGTCTGTG; >clamp_s_c_s_a_s:compl:rev CACAGACACCCACACTATCATGTTGACATTCC; dG = -17.1
107 >s_d_s_b_s_a_s_c_s_a_s GTGACGGTGGCTGTGTCTGTGTGGGTGTCTGTG; >clamp_s_c_s_a_s:compl:rev CACAGACACCCACACTATCATGTTGACATTCC; dG = -17.1
108 >s_d_s_b_s_b_s_c_s_a_s GTGACGGTGGCTGTGGCTGTGTGGGTGTCTGTG; >clamp_s_c_s_a_s:compl:rev CACAGACACCCACACTATCATGTTGACATTCC; dG = -17.1
109 >s_d_s_b_s_c_s_c_s_a_s GTGACGGTGGCTGTGTGGGTGTGGGTGTCTGTG; >clamp_s_c_s_a_s:compl:rev CACAGACACCCACACTATCATGTTGACATTCC; dG = -17.1
110 >s_d_s_b_s_d_s_c_s_a_s GTGACGGTGGCTGTGACGGTGTGGGTGTCTGTG; >clamp_s_c_s_a_s:compl:rev CACAGACACCCACACTATCATGTTGACATTCC; dG = -17.1
111 >s_d_s_b_s_e_s_c_s_a_s GTGACGGTGGCTGTGCTGGTGTGGGTGTCTGTG; >clamp_s_c_s_a_s:compl:rev CACAGACACCCACACTATCATGTTGACATTCC; dG = -17.1
112 >s_d_s_c_s_b_s_c_s_a_s GTGACGGTGTGGGTGGCTGTGTGGGTGTCTGTG; >clamp_s_c_s_a_s:compl:rev CACAGACACCCACACTATCATGTTGACATTCC; dG = -17.1
113 >s_d_s_c_s_c_s_c_s_a_s GTGACGGTGTGGGTGTGGGTGTGGGTGTCTGTG; >clamp_s_c_s_a_s:compl:rev CACAGACACCCACACTATCATGTTGACATTCC; dG = -17.1
114 >s_d_s_c_s_d_s_c_s_a_s GTGACGGTGTGGGTGACGGTGTGGGTGTCTGTG; >clamp_s_c_s_a_s:compl:rev CACAGACACCCACACTATCATGTTGACATTCC; dG = -17.1
115 >s_a_s_b_s_d_s_c_s_a_s GTGTCTGTGGCTGTGACGGTGTGGGTGTCTGTG; >clamp_s_c_s_a_s:compl:rev CACAGACACCCACACTATCATGTTGACATTCC; dG = -17.1
116 >s_d_s_c_s_e_s_c_s_a_s GTGACGGTGTGGGTGCTGGTGTGGGTGTCTGTG; >clamp_s_c_s_a_s:compl:rev CACAGACACCCACACTATCATGTTGACATTCC; dG = -17.1
117 >s_d_s_d_s_a_s_c_s_a_s GTGACGGTGACGGTGTCTGTGTGGGTGTCTGTG; >clamp_s_c_s_a_s:compl:rev CACAGACACCCACACTATCATGTTGACATTCC; dG = -17.1
118 >s_d_s_d_s_b_s_c_s_a_s GTGACGGTGACGGTGGCTGTGTGGGTGTCTGTG; >clamp_s_c_s_a_s:compl:rev CACAGACACCCACACTATCATGTTGACATTCC; dG = -17.1

119 >s_d_s_d_s_c_s_c_s_a_s GTGACGGTGACGGTGTGGGTGTGGGTGTCTGTG; >clamp_s_c_s_a_s:compl:rev
CACAGACACCCACACTATCATGTTGACATTCC; dG = -17.1
120 >s_d_s_d_s_d_s_c_s_a_s GTGACGGTGACGGTGACGGTGTGGGTGTCTGTG; >clamp_s_c_s_a_s:compl:rev
CACAGACACCCACACTATCATGTTGACATTCC; dG = -17.1
121 >s_d_s_d_s_e_s_c_s_a_s GTGACGGTGACGGTGCTGGTGTGGGTGTCTGTG; >clamp_s_c_s_a_s:compl:rev
CACAGACACCCACACTATCATGTTGACATTCC; dG = -17.1
122 >s_d_s_e_s_a_s_c_s_a_s GTGACGGTGCTGGTGTCTGTGTGGGTGTCTGTG; >clamp_s_c_s_a_s:compl:rev
CACAGACACCCACACTATCATGTTGACATTCC; dG = -17.1
123 >s_d_s_e_s_b_s_c_s_a_s GTGACGGTGCTGGTGGCTGTGTGGGTGTCTGTG; >clamp_s_c_s_a_s:compl:rev
CACAGACACCCACACTATCATGTTGACATTCC; dG = -17.1
124 >s_d_s_e_s_c_s_c_s_a_s GTGACGGTGCTGGTGTGGGTGTGGGTGTCTGTG; >clamp_s_c_s_a_s:compl:rev
CACAGACACCCACACTATCATGTTGACATTCC; dG = -17.1
125 >s_d_s_e_s_d_s_c_s_a_s GTGACGGTGCTGGTGACGGTGTGGGTGTCTGTG; >clamp_s_c_s_a_s:compl:rev
CACAGACACCCACACTATCATGTTGACATTCC; dG = -17.1

****

CA Mismatches

1 >s_b_s_c_s_b_s_d_s_a_s GTGGCTGTGTGGGTGGCTGTGACGGTGTCTGTG; >clamp_s_c_s_a_s:compl:rev
CACAGACACCCACACTATCATGTTGACATTCC; dG = -16.0
2 >s_a_s_c_s_b_s_d_s_a_s GTGTCTGTGTGGGTGGCTGTGACGGTGTCTGTG; >clamp_s_c_s_a_s:compl:rev
CACAGACACCCACACTATCATGTTGACATTCC; dG = -16.0
3 >s_c_s_c_s_b_s_d_s_a_s GTGTGGGTGTGGGTGGCTGTGACGGTGTCTGTG; >clamp_s_c_s_a_s:compl:rev
CACAGACACCCACACTATCATGTTGACATTCC; dG = -15.9
4 >s_d_s_c_s_b_s_d_s_a_s GTGACGGTGTGGGTGGCTGTGACGGTGTCTGTG; >clamp_s_c_s_a_s:compl:rev
CACAGACACCCACACTATCATGTTGACATTCC; dG = -15.9
5 >s_e_s_c_s_b_s_d_s_a_s GTGCTGGTGTGGGTGGCTGTGACGGTGTCTGTG; >clamp_s_c_s_a_s:compl:rev
CACAGACACCCACACTATCATGTTGACATTCC; dG = -15.9
6 >s_b_s_c_s_b_s_b_s_a_s GTGGCTGTGTGGGTGGCTGTGGCTGTGTCTGTG; >clamp_s_c_s_a_s:compl:rev
CACAGACACCCACACTATCATGTTGACATTCC; dG = -15.5
7 >s_c_s_b_s_d_s_c_s_e_s GTGTGGGTGGCTGTGACGGTGTGGGTGCTGGTG; >clamp_s_c_s_a_s:compl:rev
CACAGACACCCACACTATCATGTTGACATTCC; dG = -15.5
8 >s_c_s_b_s_d_s_c_s_d_s GTGTGGGTGGCTGTGACGGTGTGGGTGACGGTG; >clamp_s_c_s_a_s:compl:rev
CACAGACACCCACACTATCATGTTGACATTCC; dG = -15.5
9 >s_c_s_b_s_d_s_c_s_c_s GTGTGGGTGGCTGTGACGGTGTGGGTGTGGGTG; >clamp_s_c_s_a_s:compl:rev
CACAGACACCCACACTATCATGTTGACATTCC; dG = -15.5
10 >s_c_s_b_s_d_s_c_s_b_s GTGTGGGTGGCTGTGACGGTGTGGGTGGCTGTG; >clamp_s_c_s_a_s:compl:rev
CACAGACACCCACACTATCATGTTGACATTCC; dG = -15.5
11 >s_c_s_b_s_d_s_e_s_a_s GTGTGGGTGGCTGTGACGGTGCTGGTGTCTGTG; >clamp_s_c_s_a_s:compl:rev
CACAGACACCCACACTATCATGTTGACATTCC; dG = -15.5
12 >s_c_s_b_s_d_s_d_s_a_s GTGTGGGTGGCTGTGACGGTGACGGTGTCTGTG; >clamp_s_c_s_a_s:compl:rev
CACAGACACCCACACTATCATGTTGACATTCC; dG = -15.5
13 >s_c_s_b_s_d_s_b_s_a_s GTGTGGGTGGCTGTGACGGTGGCTGTGTCTGTG; >clamp_s_c_s_a_s:compl:rev
CACAGACACCCACACTATCATGTTGACATTCC; dG = -15.5
14 >s_c_s_b_s_d_s_a_s_a_s GTGTGGGTGGCTGTGACGGTGTCTGTGTCTGTG; >clamp_s_c_s_a_s:compl:rev
CACAGACACCCACACTATCATGTTGACATTCC; dG = -15.5
15 >s_a_s_c_s_b_s_b_s_a_s GTGTCTGTGTGGGTGGCTGTGGCTGTGTCTGTG; >clamp_s_c_s_a_s:compl:rev
CACAGACACCCACACTATCATGTTGACATTCC; dG = -15.5
16 >s_a_s_d_s_c_s_b_s_a_s GTGTCTGTGACGGTGTGGGTGGCTGTGTCTGTG; >clamp_s_c_s_a_s:compl:rev
CACAGACACCCACACTATCATGTTGACATTCC; dG = -15.4
17 >s_a_s_e_s_c_s_b_s_a_s GTGTCTGTGCTGGTGTGGGTGGCTGTGTCTGTG; >clamp_s_c_s_a_s:compl:rev
CACAGACACCCACACTATCATGTTGACATTCC; dG = -15.4
18 >s_b_s_a_s_c_s_b_s_a_s GTGGCTGTGTCTGTGTGGGTGGCTGTGTCTGTG; >clamp_s_c_s_a_s:compl:rev
CACAGACACCCACACTATCATGTTGACATTCC; dG = -15.4
19 >s_a_s_a_s_c_s_b_s_a_s GTGTCTGTGTCTGTGTGGGTGGCTGTGTCTGTG; >clamp_s_c_s_a_s:compl:rev
CACAGACACCCACACTATCATGTTGACATTCC; dG = -15.4
20 >s_b_s_b_s_c_s_b_s_a_s GTGGCTGTGGCTGTGTGGGTGGCTGTGTCTGTG; >clamp_s_c_s_a_s:compl:rev
CACAGACACCCACACTATCATGTTGACATTCC; dG = -15.4
21 >s_b_s_c_s_b_s_c_s_e_s GTGGCTGTGTGGGTGGCTGTGTGGGTGCTGGTG; >clamp_s_c_s_a_s:compl:rev
CACAGACACCCACACTATCATGTTGACATTCC; dG = -15.4
22 >s_b_s_c_s_b_s_c_s_d_s GTGGCTGTGTGGGTGGCTGTGTGGGTGACGGTG; >clamp_s_c_s_a_s:compl:rev
CACAGACACCCACACTATCATGTTGACATTCC; dG = -15.4
23 >s_b_s_c_s_b_s_c_s_c_s GTGGCTGTGTGGGTGGCTGTGTGGGTGTGGGTG; >clamp_s_c_s_a_s:compl:rev
CACAGACACCCACACTATCATGTTGACATTCC; dG = -15.4
24 >s_b_s_c_s_b_s_c_s_b_s GTGGCTGTGTGGGTGGCTGTGTGGGTGGCTGTG; >clamp_s_c_s_a_s:compl:rev
CACAGACACCCACACTATCATGTTGACATTCC; dG = -15.4
25 >s_b_s_c_s_b_s_a_s_a_s GTGGCTGTGTGGGTGGCTGTGTCTGTGTCTGTG; >clamp_s_c_s_a_s:compl:rev
CACAGACACCCACACTATCATGTTGACATTCC; dG = -15.4
26 >s_b_s_c_s_c_s_b_s_a_s GTGGCTGTGTGGGTGTGGGTGGCTGTGTCTGTG; >clamp_s_c_s_a_s:compl:rev
CACAGACACCCACACTATCATGTTGACATTCC; dG = -15.4

27 >s_b_s_d_s_c_s_b_s_a_s GTGGCTGTGACGGTGTGGGTGGCTGTGTCTGTG; >clamp_s_c_s_a_s:compl:rev
CACAGACACCCACACTATCATGTTGACATTCC; dG = -15.4
28 >s_b_s_e_s_c_s_b_s_a_s GTGGCTGTGCTGGTGTGGGTGGCTGTGTCTGTG; >clamp_s_c_s_a_s:compl:rev
CACAGACACCCACACTATCATGTTGACATTCC; dG = -15.4
29 >s_c_s_b_s_c_s_b_s_a_s GTGTGGGTGGCTGTGTGGGTGGCTGTGTCTGTG; >clamp_s_c_s_a_s:compl:rev
CACAGACACCCACACTATCATGTTGACATTCC; dG = -15.4
30 >s_c_s_c_s_b_s_c_s_e_s GTGTGGGTGTGGGTGGCTGTGTGGGTGCTGGTG; >clamp_s_c_s_a_s:compl:rev
CACAGACACCCACACTATCATGTTGACATTCC; dG = -15.4
31 >s_c_s_c_s_b_s_c_s_d_s GTGTGGGTGTGGGTGGCTGTGTGGGTGACGGTG; >clamp_s_c_s_a_s:compl:rev
CACAGACACCCACACTATCATGTTGACATTCC; dG = -15.4
32 >s_c_s_c_s_b_s_c_s_c_s GTGTGGGTGTGGGTGGCTGTGTGGGTGTGGGTG; >clamp_s_c_s_a_s:compl:rev
CACAGACACCCACACTATCATGTTGACATTCC; dG = -15.4
33 >s_c_s_c_s_b_s_c_s_b_s GTGTGGGTGTGGGTGGCTGTGTGGGTGGCTGTG; >clamp_s_c_s_a_s:compl:rev
CACAGACACCCACACTATCATGTTGACATTCC; dG = -15.4
34 >s_c_s_c_s_b_s_b_s_a_s GTGTGGGTGTGGGTGGCTGTGGCTGTGTCTGTG; >clamp_s_c_s_a_s:compl:rev
CACAGACACCCACACTATCATGTTGACATTCC; dG = -15.4
35 >s_c_s_c_s_b_s_a_s_a_s GTGTGGGTGTGGGTGGCTGTGTCTGTGTCTGTG; >clamp_s_c_s_a_s:compl:rev
CACAGACACCCACACTATCATGTTGACATTCC; dG = -15.4
36 >s_c_s_c_s_c_s_b_s_a_s GTGTGGGTGTGGGTGTGGGTGGCTGTGTCTGTG; >clamp_s_c_s_a_s:compl:rev
CACAGACACCCACACTATCATGTTGACATTCC; dG = -15.4
37 >s_c_s_d_s_c_s_b_s_a_s GTGTGGGTGACGGTGTGGGTGGCTGTGTCTGTG; >clamp_s_c_s_a_s:compl:rev
CACAGACACCCACACTATCATGTTGACATTCC; dG = -15.4
38 >s_c_s_e_s_c_s_b_s_a_s GTGTGGGTGCTGGTGTGGGTGGCTGTGTCTGTG; >clamp_s_c_s_a_s:compl:rev
CACAGACACCCACACTATCATGTTGACATTCC; dG = -15.4
39 >s_d_s_a_s_c_s_b_s_a_s GTGACGGTGTCTGTGTGGGTGGCTGTGTCTGTG; >clamp_s_c_s_a_s:compl:rev
CACAGACACCCACACTATCATGTTGACATTCC; dG = -15.4
40 >s_d_s_b_s_c_s_b_s_a_s GTGACGGTGGCTGTGTGGGTGGCTGTGTCTGTG; >clamp_s_c_s_a_s:compl:rev
CACAGACACCCACACTATCATGTTGACATTCC; dG = -15.4
41 >s_d_s_c_s_b_s_c_s_e_s GTGACGGTGTGGGTGGCTGTGTGGGTGCTGGTG; >clamp_s_c_s_a_s:compl:rev
CACAGACACCCACACTATCATGTTGACATTCC; dG = -15.4
42 >s_d_s_c_s_b_s_c_s_d_s GTGACGGTGTGGGTGGCTGTGTGGGTGACGGTG; >clamp_s_c_s_a_s:compl:rev
CACAGACACCCACACTATCATGTTGACATTCC; dG = -15.4
43 >s_d_s_c_s_b_s_c_s_c_s GTGACGGTGTGGGTGGCTGTGTGGGTGTGGGTG; >clamp_s_c_s_a_s:compl:rev
CACAGACACCCACACTATCATGTTGACATTCC; dG = -15.4
44 >s_d_s_c_s_b_s_c_s_b_s GTGACGGTGTGGGTGGCTGTGTGGGTGGCTGTG; >clamp_s_c_s_a_s:compl:rev
CACAGACACCCACACTATCATGTTGACATTCC; dG = -15.4
45 >s_d_s_c_s_b_s_b_s_a_s GTGACGGTGTGGGTGGCTGTGGCTGTGTCTGTG; >clamp_s_c_s_a_s:compl:rev
CACAGACACCCACACTATCATGTTGACATTCC; dG = -15.4
46 >s_d_s_c_s_b_s_a_s_a_s GTGACGGTGTGGGTGGCTGTGTCTGTGTCTGTG; >clamp_s_c_s_a_s:compl:rev
CACAGACACCCACACTATCATGTTGACATTCC; dG = -15.4
47 >s_d_s_c_s_c_s_b_s_a_s GTGACGGTGTGGGTGTGGGTGGCTGTGTCTGTG; >clamp_s_c_s_a_s:compl:rev
CACAGACACCCACACTATCATGTTGACATTCC; dG = -15.4
48 >s_a_s_b_s_c_s_b_s_a_s GTGTCTGTGGCTGTGTGGGTGGCTGTGTCTGTG; >clamp_s_c_s_a_s:compl:rev
CACAGACACCCACACTATCATGTTGACATTCC; dG = -15.4
49 >s_d_s_d_s_c_s_b_s_a_s GTGACGGTGACGGTGTGGGTGGCTGTGTCTGTG; >clamp_s_c_s_a_s:compl:rev
CACAGACACCCACACTATCATGTTGACATTCC; dG = -15.4
50 >s_d_s_e_s_c_s_b_s_a_s GTGACGGTGCTGGTGTGGGTGGCTGTGTCTGTG; >clamp_s_c_s_a_s:compl:rev
CACAGACACCCACACTATCATGTTGACATTCC; dG = -15.4
51 >s_e_s_a_s_c_s_b_s_a_s GTGCTGGTGTCTGTGTGGGTGGCTGTGTCTGTG; >clamp_s_c_s_a_s:compl:rev
CACAGACACCCACACTATCATGTTGACATTCC; dG = -15.4
52 >s_e_s_b_s_c_s_b_s_a_s GTGCTGGTGGCTGTGTGGGTGGCTGTGTCTGTG; >clamp_s_c_s_a_s:compl:rev
CACAGACACCCACACTATCATGTTGACATTCC; dG = -15.4
53 >s_e_s_c_s_b_s_c_s_e_s GTGCTGGTGTGGGTGGCTGTGTGGGTGCTGGTG; >clamp_s_c_s_a_s:compl:rev
CACAGACACCCACACTATCATGTTGACATTCC; dG = -15.4
54 >s_e_s_c_s_b_s_c_s_d_s GTGCTGGTGTGGGTGGCTGTGTGGGTGACGGTG; >clamp_s_c_s_a_s:compl:rev
CACAGACACCCACACTATCATGTTGACATTCC; dG = -15.4
55 >s_a_s_c_s_b_s_c_s_e_s GTGTCTGTGTGGGTGGCTGTGTGGGTGCTGGTG; >clamp_s_c_s_a_s:compl:rev
CACAGACACCCACACTATCATGTTGACATTCC; dG = -15.4
56 >s_e_s_c_s_b_s_c_s_c_s GTGCTGGTGTGGGTGGCTGTGTGGGTGTGGGTG; >clamp_s_c_s_a_s:compl:rev
CACAGACACCCACACTATCATGTTGACATTCC; dG = -15.4
57 >s_e_s_c_s_b_s_c_s_b_s GTGCTGGTGTGGGTGGCTGTGTGGGTGGCTGTG; >clamp_s_c_s_a_s:compl:rev
CACAGACACCCACACTATCATGTTGACATTCC; dG = -15.4
58 >s_e_s_c_s_b_s_b_s_a_s GTGCTGGTGTGGGTGGCTGTGGCTGTGTCTGTG; >clamp_s_c_s_a_s:compl:rev
CACAGACACCCACACTATCATGTTGACATTCC; dG = -15.4
59 >s_e_s_c_s_b_s_a_s_a_s GTGCTGGTGTGGGTGGCTGTGTCTGTGTCTGTG; >clamp_s_c_s_a_s:compl:rev
CACAGACACCCACACTATCATGTTGACATTCC; dG = -15.4
60 >s_a_s_c_s_b_s_c_s_d_s GTGTCTGTGTGGGTGGCTGTGTGGGTGACGGTG; >clamp_s_c_s_a_s:compl:rev
CACAGACACCCACACTATCATGTTGACATTCC; dG = -15.4
61 >s_e_s_c_s_c_s_b_s_a_s GTGCTGGTGTGGGTGTGGGTGGCTGTGTCTGTG; >clamp_s_c_s_a_s:compl:rev
CACAGACACCCACACTATCATGTTGACATTCC; dG = -15.4
62 >s_a_s_c_s_b_s_c_s_c_s GTGTCTGTGTGGGTGGCTGTGTGGGTGTGGGTG; >clamp_s_c_s_a_s:compl:rev
CACAGACACCCACACTATCATGTTGACATTCC; dG = -15.4
63 >s_a_s_c_s_b_s_c_s_b_s GTGTCTGTGTGGGTGGCTGTGTGGGTGGCTGTG; >clamp_s_c_s_a_s:compl:rev
CACAGACACCCACACTATCATGTTGACATTCC; dG = -15.4

64 >s_e_s_d_s_c_s_b_s_a_s GTGCTGGTGACGGTGTGGGTGGCTGTGTCTGTG; >clamp_s_c_s_a_s:compl:rev CACAGACACCCACACTATCATGTTGACATTCC; dG = -15.4
65 >s_a_s_c_s_b_s_a_s_a_s GTGTCTGTGTGGGTGGCTGTGTCTGTGTCTGTG; >clamp_s_c_s_a_s:compl:rev CACAGACACCCACACTATCATGTTGACATTCC; dG = -15.4
66 >s_e_s_e_s_c_s_b_s_a_s GTGCTGGTGCTGGTGTGGGTGGCTGTGTCTGTG; >clamp_s_c_s_a_s:compl:rev CACAGACACCCACACTATCATGTTGACATTCC; dG = -15.4
67 >s_a_s_c_s_c_s_b_s_a_s GTGTCTGTGTGGGTGTGGGTGGCTGTGTCTGTG; >clamp_s_c_s_a_s:compl:rev CACAGACACCCACACTATCATGTTGACATTCC; dG = -15.4
68 >s_b_s_c_s_b_s_e_s_a_s GTGGCTGTGTGGGTGGCTGTGCTGGTGTCTGTG; >clamp_s_c_s_a_s:compl:rev CACAGACACCCACACTATCATGTTGACATTCC; dG = -15.3
69 >s_a_s_c_s_b_s_e_s_a_s GTGTCTGTGTGGGTGGCTGTGCTGGTGTCTGTG; >clamp_s_c_s_a_s:compl:rev CACAGACACCCACACTATCATGTTGACATTCC; dG = -15.3
70 >s_c_s_c_s_b_s_e_s_a_s GTGTGGGTGTGGGTGGCTGTGCTGGTGTCTGTG; >clamp_s_c_s_a_s:compl:rev CACAGACACCCACACTATCATGTTGACATTCC; dG = -15.2
71 >s_d_s_c_s_b_s_e_s_a_s GTGACGGTGTGGGTGGCTGTGCTGGTGTCTGTG; >clamp_s_c_s_a_s:compl:rev CACAGACACCCACACTATCATGTTGACATTCC; dG = -15.2
72 >s_e_s_c_s_b_s_e_s_a_s GTGCTGGTGTGGGTGGCTGTGCTGGTGTCTGTG; >clamp_s_c_s_a_s:compl:rev CACAGACACCCACACTATCATGTTGACATTCC; dG = -15.2
73 >s_a_s_c_s_e_s_c_s_b_s GTGTCTGTGTGGGTGCTGGTGTGGGTGGCTGTG; >clamp_s_c_s_a_s:compl:rev CACAGACACCCACACTATCATGTTGACATTCC; dG = -15.1
74 >s_a_s_d_s_a_s_c_s_b_s GTGTCTGTGACGGTGTCTGTGTGGGTGGCTGTG; >clamp_s_c_s_a_s:compl:rev CACAGACACCCACACTATCATGTTGACATTCC; dG = -15.1
75 >s_a_s_a_s_b_s_c_s_b_s GTGTCTGTGTCTGTGGCTGTGTGGGTGGCTGTG; >clamp_s_c_s_a_s:compl:rev CACAGACACCCACACTATCATGTTGACATTCC; dG = -15.1
76 >s_a_s_d_s_b_s_c_s_b_s GTGTCTGTGACGGTGGCTGTGTGGGTGGCTGTG; >clamp_s_c_s_a_s:compl:rev CACAGACACCCACACTATCATGTTGACATTCC; dG = -15.1
77 >s_a_s_d_s_c_s_c_s_b_s GTGTCTGTGACGGTGTGGGTGTGGGTGGCTGTG; >clamp_s_c_s_a_s:compl:rev CACAGACACCCACACTATCATGTTGACATTCC; dG = -15.1
78 >s_a_s_d_s_d_s_c_s_b_s GTGTCTGTGACGGTGACGGTGTGGGTGGCTGTG; >clamp_s_c_s_a_s:compl:rev CACAGACACCCACACTATCATGTTGACATTCC; dG = -15.1
79 >s_a_s_d_s_e_s_c_s_b_s GTGTCTGTGACGGTGCTGGTGTGGGTGGCTGTG; >clamp_s_c_s_a_s:compl:rev CACAGACACCCACACTATCATGTTGACATTCC; dG = -15.1
80 >s_a_s_e_s_a_s_c_s_b_s GTGTCTGTGCTGGTGTCTGTGTGGGTGGCTGTG; >clamp_s_c_s_a_s:compl:rev CACAGACACCCACACTATCATGTTGACATTCC; dG = -15.1
81 >s_a_s_e_s_b_s_c_s_b_s GTGTCTGTGCTGGTGGCTGTGTGGGTGGCTGTG; >clamp_s_c_s_a_s:compl:rev CACAGACACCCACACTATCATGTTGACATTCC; dG = -15.1
82 >s_a_s_e_s_c_s_c_s_b_s GTGTCTGTGCTGGTGTGGGTGTGGGTGGCTGTG; >clamp_s_c_s_a_s:compl:rev CACAGACACCCACACTATCATGTTGACATTCC; dG = -15.1
83 >s_a_s_e_s_d_s_c_s_b_s GTGTCTGTGCTGGTGACGGTGTGGGTGGCTGTG; >clamp_s_c_s_a_s:compl:rev CACAGACACCCACACTATCATGTTGACATTCC; dG = -15.1
84 >s_a_s_e_s_e_s_c_s_b_s GTGTCTGTGCTGGTGCTGGTGTGGGTGGCTGTG; >clamp_s_c_s_a_s:compl:rev CACAGACACCCACACTATCATGTTGACATTCC; dG = -15.1
85 >s_b_s_a_s_a_s_c_s_b_s GTGGCTGTGTCTGTGTCTGTGTGGGTGGCTGTG; >clamp_s_c_s_a_s:compl:rev CACAGACACCCACACTATCATGTTGACATTCC; dG = -15.1
86 >s_b_s_a_s_b_s_c_s_b_s GTGGCTGTGTCTGTGGCTGTGTGGGTGGCTGTG; >clamp_s_c_s_a_s:compl:rev CACAGACACCCACACTATCATGTTGACATTCC; dG = -15.1
87 >s_a_s_a_s_c_s_c_s_b_s GTGTCTGTGTCTGTGTGGGTGTGGGTGGCTGTG; >clamp_s_c_s_a_s:compl:rev CACAGACACCCACACTATCATGTTGACATTCC; dG = -15.1
88 >s_b_s_a_s_c_s_c_s_b_s GTGGCTGTGTCTGTGTGGGTGTGGGTGGCTGTG; >clamp_s_c_s_a_s:compl:rev CACAGACACCCACACTATCATGTTGACATTCC; dG = -15.1
89 >s_b_s_a_s_d_s_c_s_b_s GTGGCTGTGTCTGTGACGGTGTGGGTGGCTGTG; >clamp_s_c_s_a_s:compl:rev CACAGACACCCACACTATCATGTTGACATTCC; dG = -15.1
90 >s_b_s_a_s_e_s_c_s_b_s GTGGCTGTGTCTGTGCTGGTGTGGGTGGCTGTG; >clamp_s_c_s_a_s:compl:rev CACAGACACCCACACTATCATGTTGACATTCC; dG = -15.1
91 >s_b_s_b_s_a_s_c_s_b_s GTGGCTGTGGCTGTGTCTGTGTGGGTGGCTGTG; >clamp_s_c_s_a_s:compl:rev CACAGACACCCACACTATCATGTTGACATTCC; dG = -15.1
92 >s_b_s_b_s_b_s_c_s_b_s GTGGCTGTGGCTGTGGCTGTGTGGGTGGCTGTG; >clamp_s_c_s_a_s:compl:rev CACAGACACCCACACTATCATGTTGACATTCC; dG = -15.1
93 >s_b_s_b_s_c_s_c_s_b_s GTGGCTGTGGCTGTGTGGGTGTGGGTGGCTGTG; >clamp_s_c_s_a_s:compl:rev CACAGACACCCACACTATCATGTTGACATTCC; dG = -15.1
94 >s_b_s_b_s_d_s_c_s_b_s GTGGCTGTGGCTGTGACGGTGTGGGTGGCTGTG; >clamp_s_c_s_a_s:compl:rev CACAGACACCCACACTATCATGTTGACATTCC; dG = -15.1
95 >s_b_s_b_s_e_s_c_s_b_s GTGGCTGTGGCTGTGCTGGTGTGGGTGGCTGTG; >clamp_s_c_s_a_s:compl:rev CACAGACACCCACACTATCATGTTGACATTCC; dG = -15.1
96 >s_a_s_a_s_d_s_c_s_b_s GTGTCTGTGTCTGTGACGGTGTGGGTGGCTGTG; >clamp_s_c_s_a_s:compl:rev CACAGACACCCACACTATCATGTTGACATTCC; dG = -15.1
97 >s_b_s_c_s_c_s_c_s_b_s GTGGCTGTGTGGGTGTGGGTGTGGGTGGCTGTG; >clamp_s_c_s_a_s:compl:rev CACAGACACCCACACTATCATGTTGACATTCC; dG = -15.1
98 >s_b_s_c_s_d_s_c_s_b_s GTGGCTGTGTGGGTGACGGTGTGGGTGGCTGTG; >clamp_s_c_s_a_s:compl:rev CACAGACACCCACACTATCATGTTGACATTCC; dG = -15.1
99 >s_b_s_c_s_e_s_c_s_b_s GTGGCTGTGTGGGTGCTGGTGTGGGTGGCTGTG; >clamp_s_c_s_a_s:compl:rev CACAGACACCCACACTATCATGTTGACATTCC; dG = -15.1
100 >s_b_s_d_s_a_s_c_s_b_s GTGGCTGTGACGGTGTCTGTGTGGGTGGCTGTG; >clamp_s_c_s_a_s:compl:rev CACAGACACCCACACTATCATGTTGACATTCC; dG = -15.1

101 >s_b_s_d_s_b_s_c_s_b_s GTGGCTGTGACGGTGGCTGTGTGGGTGGCTGTG; >clamp_s_c_s_a_s:compl:rev
CACAGACACCCACACTATCATGTTGACATTCC; dG = -15.1
102 >s_b_s_d_s_c_s_c_s_b_s GTGGCTGTGACGGTGTGGGTGTGGGTGGCTGTG; >clamp_s_c_s_a_s:compl:rev
CACAGACACCCACACTATCATGTTGACATTCC; dG = -15.1
103 >s_b_s_d_s_d_s_c_s_b_s GTGGCTGTGACGGTGACGGTGTGGGTGGCTGTG; >clamp_s_c_s_a_s:compl:rev
CACAGACACCCACACTATCATGTTGACATTCC; dG = -15.1
104 >s_b_s_d_s_e_s_c_s_b_s GTGGCTGTGACGGTGCTGGTGTGGGTGGCTGTG; >clamp_s_c_s_a_s:compl:rev
CACAGACACCCACACTATCATGTTGACATTCC; dG = -15.1
105 >s_b_s_e_s_a_s_c_s_b_s GTGGCTGTGCTGGTGTCTGTGTGGGTGGCTGTG; >clamp_s_c_s_a_s:compl:rev
CACAGACACCCACACTATCATGTTGACATTCC; dG = -15.1
106 >s_b_s_e_s_b_s_c_s_b_s GTGGCTGTGCTGGTGGCTGTGTGGGTGGCTGTG; >clamp_s_c_s_a_s:compl:rev
CACAGACACCCACACTATCATGTTGACATTCC; dG = -15.1
107 >s_a_s_a_s_e_s_c_s_b_s GTGTCTGTGTCTGTGCTGGTGTGGGTGGCTGTG; >clamp_s_c_s_a_s:compl:rev
CACAGACACCCACACTATCATGTTGACATTCC; dG = -15.1
108 >s_b_s_e_s_c_s_c_s_b_s GTGGCTGTGCTGGTGTGGGTGTGGGTGGCTGTG; >clamp_s_c_s_a_s:compl:rev
CACAGACACCCACACTATCATGTTGACATTCC; dG = -15.1
109 >s_b_s_e_s_d_s_c_s_b_s GTGGCTGTGCTGGTGACGGTGTGGGTGGCTGTG; >clamp_s_c_s_a_s:compl:rev
CACAGACACCCACACTATCATGTTGACATTCC; dG = -15.1
110 >s_b_s_e_s_e_s_c_s_b_s GTGGCTGTGCTGGTGCTGGTGTGGGTGGCTGTG; >clamp_s_c_s_a_s:compl:rev
CACAGACACCCACACTATCATGTTGACATTCC; dG = -15.1
111 >s_c_s_b_s_a_s_c_s_b_s GTGTGGGTGGCTGTGTCTGTGTGGGTGGCTGTG; >clamp_s_c_s_a_s:compl:rev
CACAGACACCCACACTATCATGTTGACATTCC; dG = -15.1
112 >s_c_s_b_s_b_s_c_s_b_s GTGTGGGTGGCTGTGGCTGTGTGGGTGGCTGTG; >clamp_s_c_s_a_s:compl:rev
CACAGACACCCACACTATCATGTTGACATTCC; dG = -15.1
113 >s_c_s_b_s_c_s_c_s_b_s GTGTGGGTGGCTGTGTGGGTGTGGGTGGCTGTG; >clamp_s_c_s_a_s:compl:rev
CACAGACACCCACACTATCATGTTGACATTCC; dG = -15.1
114 >s_a_s_a_s_a_s_c_s_b_s GTGTCTGTGTCTGTGTCTGTGTGGGTGGCTGTG; >clamp_s_c_s_a_s:compl:rev
CACAGACACCCACACTATCATGTTGACATTCC; dG = -15.1
115 >s_c_s_b_s_e_s_c_s_b_s GTGTGGGTGGCTGTGCTGGTGTGGGTGGCTGTG; >clamp_s_c_s_a_s:compl:rev
CACAGACACCCACACTATCATGTTGACATTCC; dG = -15.1
116 >s_c_s_c_s_c_s_c_s_b_s GTGTGGGTGTGGGTGTGGGTGTGGGTGGCTGTG; >clamp_s_c_s_a_s:compl:rev
CACAGACACCCACACTATCATGTTGACATTCC; dG = -15.1
117 >s_a_s_b_s_a_s_c_s_b_s GTGTCTGTGGCTGTGTCTGTGTGGGTGGCTGTG; >clamp_s_c_s_a_s:compl:rev
CACAGACACCCACACTATCATGTTGACATTCC; dG = -15.1
118 >s_c_s_c_s_d_s_c_s_b_s GTGTGGGTGTGGGTGACGGTGTGGGTGGCTGTG; >clamp_s_c_s_a_s:compl:rev
CACAGACACCCACACTATCATGTTGACATTCC; dG = -15.1
119 >s_c_s_c_s_e_s_c_s_b_s GTGTGGGTGTGGGTGCTGGTGTGGGTGGCTGTG; >clamp_s_c_s_a_s:compl:rev
CACAGACACCCACACTATCATGTTGACATTCC; dG = -15.1
120 >s_c_s_d_s_a_s_c_s_b_s GTGTGGGTGACGGTGTCTGTGTGGGTGGCTGTG; >clamp_s_c_s_a_s:compl:rev
CACAGACACCCACACTATCATGTTGACATTCC; dG = -15.1
121 >s_c_s_d_s_b_s_c_s_b_s GTGTGGGTGACGGTGGCTGTGTGGGTGGCTGTG; >clamp_s_c_s_a_s:compl:rev
CACAGACACCCACACTATCATGTTGACATTCC; dG = -15.1
122 >s_c_s_d_s_c_s_c_s_b_s GTGTGGGTGACGGTGTGGGTGTGGGTGGCTGTG; >clamp_s_c_s_a_s:compl:rev
CACAGACACCCACACTATCATGTTGACATTCC; dG = -15.1
123 >s_c_s_d_s_d_s_c_s_b_s GTGTGGGTGACGGTGACGGTGTGGGTGGCTGTG; >clamp_s_c_s_a_s:compl:rev
CACAGACACCCACACTATCATGTTGACATTCC; dG = -15.1
124 >s_c_s_d_s_e_s_c_s_b_s GTGTGGGTGACGGTGCTGGTGTGGGTGGCTGTG; >clamp_s_c_s_a_s:compl:rev
CACAGACACCCACACTATCATGTTGACATTCC; dG = -15.1
125 >s_c_s_e_s_a_s_c_s_b_s GTGTGGGTGCTGGTGTCTGTGTGGGTGGCTGTG; >clamp_s_c_s_a_s:compl:rev
CACAGACACCCACACTATCATGTTGACATTCC; dG = -15.1
126 >s_c_s_e_s_b_s_c_s_b_s GTGTGGGTGCTGGTGGCTGTGTGGGTGGCTGTG; >clamp_s_c_s_a_s:compl:rev
CACAGACACCCACACTATCATGTTGACATTCC; dG = -15.1
127 >s_c_s_e_s_c_s_c_s_b_s GTGTGGGTGCTGGTGTGGGTGTGGGTGGCTGTG; >clamp_s_c_s_a_s:compl:rev
CACAGACACCCACACTATCATGTTGACATTCC; dG = -15.1
128 >s_a_s_b_s_b_s_c_s_b_s GTGTCTGTGGCTGTGGCTGTGTGGGTGGCTGTG; >clamp_s_c_s_a_s:compl:rev
CACAGACACCCACACTATCATGTTGACATTCC; dG = -15.1
129 >s_c_s_e_s_d_s_c_s_b_s GTGTGGGTGCTGGTGACGGTGTGGGTGGCTGTG; >clamp_s_c_s_a_s:compl:rev
CACAGACACCCACACTATCATGTTGACATTCC; dG = -15.1
130 >s_c_s_e_s_e_s_c_s_b_s GTGTGGGTGCTGGTGCTGGTGTGGGTGGCTGTG; >clamp_s_c_s_a_s:compl:rev
CACAGACACCCACACTATCATGTTGACATTCC; dG = -15.1
131 >s_d_s_a_s_a_s_c_s_b_s GTGACGGTGTCTGTGTCTGTGTGGGTGGCTGTG; >clamp_s_c_s_a_s:compl:rev
CACAGACACCCACACTATCATGTTGACATTCC; dG = -15.1
132 >s_d_s_a_s_b_s_c_s_b_s GTGACGGTGTCTGTGGCTGTGTGGGTGGCTGTG; >clamp_s_c_s_a_s:compl:rev
CACAGACACCCACACTATCATGTTGACATTCC; dG = -15.1
133 >s_d_s_a_s_c_s_c_s_b_s GTGACGGTGTCTGTGTGGGTGTGGGTGGCTGTG; >clamp_s_c_s_a_s:compl:rev
CACAGACACCCACACTATCATGTTGACATTCC; dG = -15.1
134 >s_d_s_a_s_d_s_c_s_b_s GTGACGGTGTCTGTGACGGTGTGGGTGGCTGTG; >clamp_s_c_s_a_s:compl:rev
CACAGACACCCACACTATCATGTTGACATTCC; dG = -15.1
135 >s_d_s_a_s_e_s_c_s_b_s GTGACGGTGTCTGTGCTGGTGTGGGTGGCTGTG; >clamp_s_c_s_a_s:compl:rev
CACAGACACCCACACTATCATGTTGACATTCC; dG = -15.1
136 >s_d_s_b_s_a_s_c_s_b_s GTGACGGTGGCTGTGTCTGTGTGGGTGGCTGTG; >clamp_s_c_s_a_s:compl:rev
CACAGACACCCACACTATCATGTTGACATTCC; dG = -15.1
137 >s_d_s_b_s_b_s_c_s_b_s GTGACGGTGGCTGTGGCTGTGTGGGTGGCTGTG; >clamp_s_c_s_a_s:compl:rev
CACAGACACCCACACTATCATGTTGACATTCC; dG = -15.1

138 >s_d_s_b_s_c_s_c_s_b_s GTGACGGTGGCTGTGTGGGTGTGGGTGGCTGTG; >clamp_s_c_s_a_s:compl:rev
CACAGACACCCACACTATCATGTTGACATTCC; dG = -15.1
139 >s_d_s_b_s_d_s_c_s_b_s GTGACGGTGGCTGTGACGGTGTGGGTGGCTGTG; >clamp_s_c_s_a_s:compl:rev
CACAGACACCCACACTATCATGTTGACATTCC; dG = -15.1
140 >s_a_s_b_s_c_s_c_s_b_s GTGTCTGTGGCTGTGTGGGTGTGGGTGGCTGTG; >clamp_s_c_s_a_s:compl:rev
CACAGACACCCACACTATCATGTTGACATTCC; dG = -15.1
141 >s_d_s_b_s_e_s_c_s_b_s GTGACGGTGGCTGTGCTGGTGTGGGTGGCTGTG; >clamp_s_c_s_a_s:compl:rev
CACAGACACCCACACTATCATGTTGACATTCC; dG = -15.1
142 >s_d_s_c_s_c_s_c_s_b_s GTGACGGTGTGGGTGTGGGTGTGGGTGGCTGTG; >clamp_s_c_s_a_s:compl:rev
CACAGACACCCACACTATCATGTTGACATTCC; dG = -15.1
143 >s_d_s_c_s_d_s_c_s_b_s GTGACGGTGTGGGTGACGGTGTGGGTGGCTGTG; >clamp_s_c_s_a_s:compl:rev
CACAGACACCCACACTATCATGTTGACATTCC; dG = -15.1
144 >s_d_s_c_s_e_s_c_s_b_s GTGACGGTGTGGGTGCTGGTGTGGGTGGCTGTG; >clamp_s_c_s_a_s:compl:rev
CACAGACACCCACACTATCATGTTGACATTCC; dG = -15.1
145 >s_d_s_d_s_a_s_c_s_b_s GTGACGGTGACGGTGTCTGTGTGGGTGGCTGTG; >clamp_s_c_s_a_s:compl:rev
CACAGACACCCACACTATCATGTTGACATTCC; dG = -15.1
146 >s_d_s_d_s_b_s_c_s_b_s GTGACGGTGACGGTGGCTGTGTGGGTGGCTGTG; >clamp_s_c_s_a_s:compl:rev
CACAGACACCCACACTATCATGTTGACATTCC; dG = -15.1
147 >s_d_s_d_s_c_s_c_s_b_s GTGACGGTGACGGTGTGGGTGTGGGTGGCTGTG; >clamp_s_c_s_a_s:compl:rev
CACAGACACCCACACTATCATGTTGACATTCC; dG = -15.1
148 >s_d_s_d_s_d_s_c_s_b_s GTGACGGTGACGGTGACGGTGTGGGTGGCTGTG; >clamp_s_c_s_a_s:compl:rev
CACAGACACCCACACTATCATGTTGACATTCC; dG = -15.1
149 >s_a_s_b_s_d_s_c_s_b_s GTGTCTGTGGCTGTGACGGTGTGGGTGGCTGTG; >clamp_s_c_s_a_s:compl:rev
CACAGACACCCACACTATCATGTTGACATTCC; dG = -15.1
150 >s_d_s_d_s_e_s_c_s_b_s GTGACGGTGACGGTGCTGGTGTGGGTGGCTGTG; >clamp_s_c_s_a_s:compl:rev
CACAGACACCCACACTATCATGTTGACATTCC; dG = -15.1
151 >s_d_s_e_s_a_s_c_s_b_s GTGACGGTGCTGGTGTCTGTGTGGGTGGCTGTG; >clamp_s_c_s_a_s:compl:rev
CACAGACACCCACACTATCATGTTGACATTCC; dG = -15.1
152 >s_d_s_e_s_b_s_c_s_b_s GTGACGGTGCTGGTGGCTGTGTGGGTGGCTGTG; >clamp_s_c_s_a_s:compl:rev
CACAGACACCCACACTATCATGTTGACATTCC; dG = -15.1
153 >s_d_s_e_s_c_s_c_s_b_s GTGACGGTGCTGGTGTGGGTGTGGGTGGCTGTG; >clamp_s_c_s_a_s:compl:rev
CACAGACACCCACACTATCATGTTGACATTCC; dG = -15.1
154 >s_d_s_e_s_d_s_c_s_b_s GTGACGGTGCTGGTGACGGTGTGGGTGGCTGTG; >clamp_s_c_s_a_s:compl:rev
CACAGACACCCACACTATCATGTTGACATTCC; dG = -15.1
155 >s_d_s_e_s_e_s_c_s_b_s GTGACGGTGCTGGTGCTGGTGTGGGTGGCTGTG; >clamp_s_c_s_a_s:compl:rev
CACAGACACCCACACTATCATGTTGACATTCC; dG = -15.1
156 >s_e_s_a_s_a_s_c_s_b_s GTGCTGGTGTCTGTGTCTGTGTGGGTGGCTGTG; >clamp_s_c_s_a_s:compl:rev
CACAGACACCCACACTATCATGTTGACATTCC; dG = -15.1
157 >s_e_s_a_s_b_s_c_s_b_s GTGCTGGTGTCTGTGGCTGTGTGGGTGGCTGTG; >clamp_s_c_s_a_s:compl:rev
CACAGACACCCACACTATCATGTTGACATTCC; dG = -15.1
158 >s_e_s_a_s_c_s_c_s_b_s GTGCTGGTGTCTGTGTGGGTGTGGGTGGCTGTG; >clamp_s_c_s_a_s:compl:rev
CACAGACACCCACACTATCATGTTGACATTCC; dG = -15.1
159 >s_e_s_a_s_d_s_c_s_b_s GTGCTGGTGTCTGTGACGGTGTGGGTGGCTGTG; >clamp_s_c_s_a_s:compl:rev
CACAGACACCCACACTATCATGTTGACATTCC; dG = -15.1
160 >s_a_s_b_s_e_s_c_s_b_s GTGTCTGTGGCTGTGCTGGTGTGGGTGGCTGTG; >clamp_s_c_s_a_s:compl:rev
CACAGACACCCACACTATCATGTTGACATTCC; dG = -15.1
161 >s_e_s_a_s_e_s_c_s_b_s GTGCTGGTGTCTGTGCTGGTGTGGGTGGCTGTG; >clamp_s_c_s_a_s:compl:rev
CACAGACACCCACACTATCATGTTGACATTCC; dG = -15.1
162 >s_e_s_b_s_a_s_c_s_b_s GTGCTGGTGGCTGTGTCTGTGTGGGTGGCTGTG; >clamp_s_c_s_a_s:compl:rev
CACAGACACCCACACTATCATGTTGACATTCC; dG = -15.1
163 >s_e_s_b_s_b_s_c_s_b_s GTGCTGGTGGCTGTGGCTGTGTGGGTGGCTGTG; >clamp_s_c_s_a_s:compl:rev
CACAGACACCCACACTATCATGTTGACATTCC; dG = -15.1
164 >s_e_s_b_s_c_s_c_s_b_s GTGCTGGTGGCTGTGTGGGTGTGGGTGGCTGTG; >clamp_s_c_s_a_s:compl:rev
CACAGACACCCACACTATCATGTTGACATTCC; dG = -15.1
165 >s_e_s_b_s_d_s_c_s_b_s GTGCTGGTGGCTGTGACGGTGTGGGTGGCTGTG; >clamp_s_c_s_a_s:compl:rev
CACAGACACCCACACTATCATGTTGACATTCC; dG = -15.1
166 >s_e_s_b_s_e_s_c_s_b_s GTGCTGGTGGCTGTGCTGGTGTGGGTGGCTGTG; >clamp_s_c_s_a_s:compl:rev
CACAGACACCCACACTATCATGTTGACATTCC; dG = -15.1
167 >s_e_s_c_s_c_s_c_s_b_s GTGCTGGTGTGGGTGTGGGTGTGGGTGGCTGTG; >clamp_s_c_s_a_s:compl:rev
CACAGACACCCACACTATCATGTTGACATTCC; dG = -15.1
168 >s_e_s_c_s_d_s_c_s_b_s GTGCTGGTGTGGGTGACGGTGTGGGTGGCTGTG; >clamp_s_c_s_a_s:compl:rev
CACAGACACCCACACTATCATGTTGACATTCC; dG = -15.1
169 >s_e_s_c_s_e_s_c_s_b_s GTGCTGGTGTGGGTGCTGGTGTGGGTGGCTGTG; >clamp_s_c_s_a_s:compl:rev
CACAGACACCCACACTATCATGTTGACATTCC; dG = -15.1
170 >s_e_s_d_s_a_s_c_s_b_s GTGCTGGTGACGGTGTCTGTGTGGGTGGCTGTG; >clamp_s_c_s_a_s:compl:rev
CACAGACACCCACACTATCATGTTGACATTCC; dG = -15.1
171 >s_e_s_d_s_b_s_c_s_b_s GTGCTGGTGACGGTGGCTGTGTGGGTGGCTGTG; >clamp_s_c_s_a_s:compl:rev
CACAGACACCCACACTATCATGTTGACATTCC; dG = -15.1
172 >s_e_s_d_s_c_s_c_s_b_s GTGCTGGTGACGGTGTGGGTGTGGGTGGCTGTG; >clamp_s_c_s_a_s:compl:rev
CACAGACACCCACACTATCATGTTGACATTCC; dG = -15.1
173 >s_e_s_d_s_d_s_c_s_b_s GTGCTGGTGACGGTGACGGTGTGGGTGGCTGTG; >clamp_s_c_s_a_s:compl:rev
CACAGACACCCACACTATCATGTTGACATTCC; dG = -15.1
174 >s_e_s_d_s_e_s_c_s_b_s GTGCTGGTGACGGTGCTGGTGTGGGTGGCTGTG; >clamp_s_c_s_a_s:compl:rev
CACAGACACCCACACTATCATGTTGACATTCC; dG = -15.1

175 >s_e_s_e_s_a_s_c_s_b_s GTGCTGGTGCTGGTGTCTGTGTGGGTGGCTGTG; >clamp_s_c_s_a_s:compl:rev CACAGACACCCACACTATCATGTTGACATTCC; dG = -15.1
176 >s_e_s_e_s_b_s_c_s_b_s GTGCTGGTGCTGGTGGCTGTGTGGGTGGCTGTG; >clamp_s_c_s_a_s:compl:rev CACAGACACCCACACTATCATGTTGACATTCC; dG = -15.1
177 >s_e_s_e_s_c_s_c_s_b_s GTGCTGGTGCTGGTGTGGGTGTGGGTGGCTGTG; >clamp_s_c_s_a_s:compl:rev CACAGACACCCACACTATCATGTTGACATTCC; dG = -15.1
178 >s_e_s_e_s_d_s_c_s_b_s GTGCTGGTGCTGGTGACGGTGTGGGTGGCTGTG; >clamp_s_c_s_a_s:compl:rev CACAGACACCCACACTATCATGTTGACATTCC; dG = -15.1
179 >s_e_s_e_s_e_s_c_s_b_s GTGCTGGTGCTGGTGCTGGTGTGGGTGGCTGTG; >clamp_s_c_s_a_s:compl:rev CACAGACACCCACACTATCATGTTGACATTCC; dG = -15.1
180 >s_a_s_c_s_c_s_c_s_b_s GTGTCTGTGTGGGTGTGGGTGTGGGTGGCTGTG; >clamp_s_c_s_a_s:compl:rev CACAGACACCCACACTATCATGTTGACATTCC; dG = -15.1
181 >s_a_s_c_s_d_s_c_s_b_s GTGTCTGTGTGGGTGACGGTGTGGGTGGCTGTG; >clamp_s_c_s_a_s:compl:rev CACAGACACCCACACTATCATGTTGACATTCC; dG = -15.1
182 >s_c_s_b_s_b_s_c_s_e_s GTGTGGGTGGCTGTGGCTGTGTGGGTGCTGGTG; >clamp_s_c_s_a_s:compl:rev CACAGACACCCACACTATCATGTTGACATTCC; dG = -15.0
183 >s_c_s_b_s_b_s_c_s_d_s GTGTGGGTGGCTGTGGCTGTGTGGGTGACGGTG; >clamp_s_c_s_a_s:compl:rev CACAGACACCCACACTATCATGTTGACATTCC; dG = -15.0
184 >s_c_s_b_s_b_s_c_s_c_s GTGTGGGTGGCTGTGGCTGTGTGGGTGTGGGTG; >clamp_s_c_s_a_s:compl:rev CACAGACACCCACACTATCATGTTGACATTCC; dG = -15.0
185 >s_c_s_b_s_b_s_e_s_a_s GTGTGGGTGGCTGTGGCTGTGCTGGTGTCTGTG; >clamp_s_c_s_a_s:compl:rev CACAGACACCCACACTATCATGTTGACATTCC; dG = -15.0
186 >s_c_s_b_s_b_s_d_s_a_s GTGTGGGTGGCTGTGGCTGTGACGGTGTCTGTG; >clamp_s_c_s_a_s:compl:rev CACAGACACCCACACTATCATGTTGACATTCC; dG = -15.0
187 >s_c_s_b_s_b_s_b_s_a_s GTGTGGGTGGCTGTGGCTGTGGCTGTGTCTGTG; >clamp_s_c_s_a_s:compl:rev CACAGACACCCACACTATCATGTTGACATTCC; dG = -15.0
188 >s_c_s_b_s_b_s_a_s_a_s GTGTGGGTGGCTGTGGCTGTGTCTGTGTCTGTG; >clamp_s_c_s_a_s:compl:rev CACAGACACCCACACTATCATGTTGACATTCC; dG = -15.0
189 >s_c_s_b_s_a_s_c_s_e_s GTGTGGGTGGCTGTGTCTGTGTGGGTGCTGGTG; >clamp_s_c_s_a_s:compl:rev CACAGACACCCACACTATCATGTTGACATTCC; dG = -14.9
190 >s_c_s_b_s_a_s_c_s_d_s GTGTGGGTGGCTGTGTCTGTGTGGGTGACGGTG; >clamp_s_c_s_a_s:compl:rev CACAGACACCCACACTATCATGTTGACATTCC; dG = -14.9
191 >s_c_s_b_s_a_s_c_s_c_s GTGTGGGTGGCTGTGTCTGTGTGGGTGTGGGTG; >clamp_s_c_s_a_s:compl:rev CACAGACACCCACACTATCATGTTGACATTCC; dG = -14.9
192 >s_c_s_b_s_a_s_e_s_a_s GTGTGGGTGGCTGTGTCTGTGCTGGTGTCTGTG; >clamp_s_c_s_a_s:compl:rev CACAGACACCCACACTATCATGTTGACATTCC; dG = -14.9
193 >s_c_s_b_s_a_s_d_s_a_s GTGTGGGTGGCTGTGTCTGTGACGGTGTCTGTG; >clamp_s_c_s_a_s:compl:rev CACAGACACCCACACTATCATGTTGACATTCC; dG = -14.9
194 >s_c_s_b_s_a_s_b_s_a_s GTGTGGGTGGCTGTGTCTGTGGCTGTGTCTGTG; >clamp_s_c_s_a_s:compl:rev CACAGACACCCACACTATCATGTTGACATTCC; dG = -14.9
195 >s_c_s_b_s_a_s_a_s_a_s GTGTGGGTGGCTGTGTCTGTGTCTGTGTCTGTG; >clamp_s_c_s_a_s:compl:rev CACAGACACCCACACTATCATGTTGACATTCC; dG = -14.9
196 >s_c_s_b_s_c_s_c_s_e_s GTGTGGGTGGCTGTGTGGGTGTGGGTGCTGGTG; >clamp_s_c_s_a_s:compl:rev CACAGACACCCACACTATCATGTTGACATTCC; dG = -14.9
197 >s_c_s_b_s_c_s_c_s_d_s GTGTGGGTGGCTGTGTGGGTGTGGGTGACGGTG; >clamp_s_c_s_a_s:compl:rev CACAGACACCCACACTATCATGTTGACATTCC; dG = -14.9
198 >s_c_s_b_s_c_s_c_s_c_s GTGTGGGTGGCTGTGTGGGTGTGGGTGTGGGTG; >clamp_s_c_s_a_s:compl:rev CACAGACACCCACACTATCATGTTGACATTCC; dG = -14.9
199 >s_c_s_b_s_c_s_e_s_a_s GTGTGGGTGGCTGTGTGGGTGCTGGTGTCTGTG; >clamp_s_c_s_a_s:compl:rev CACAGACACCCACACTATCATGTTGACATTCC; dG = -14.9
200 >s_c_s_b_s_c_s_d_s_a_s GTGTGGGTGGCTGTGTGGGTGACGGTGTCTGTG; >clamp_s_c_s_a_s:compl:rev CACAGACACCCACACTATCATGTTGACATTCC; dG = -14.9
201 >s_c_s_b_s_e_s_c_s_e_s GTGTGGGTGGCTGTGCTGGTGTGGGTGCTGGTG; >clamp_s_c_s_a_s:compl:rev CACAGACACCCACACTATCATGTTGACATTCC; dG = -14.8
202 >s_c_s_b_s_e_s_c_s_d_s GTGTGGGTGGCTGTGCTGGTGTGGGTGACGGTG; >clamp_s_c_s_a_s:compl:rev CACAGACACCCACACTATCATGTTGACATTCC; dG = -14.8
203 >s_c_s_b_s_e_s_c_s_c_s GTGTGGGTGGCTGTGCTGGTGTGGGTGTGGGTG; >clamp_s_c_s_a_s:compl:rev CACAGACACCCACACTATCATGTTGACATTCC; dG = -14.8
204 >s_c_s_b_s_e_s_e_s_a_s GTGTGGGTGGCTGTGCTGGTGCTGGTGTCTGTG; >clamp_s_c_s_a_s:compl:rev CACAGACACCCACACTATCATGTTGACATTCC; dG = -14.8
205 >s_c_s_b_s_e_s_d_s_a_s GTGTGGGTGGCTGTGCTGGTGACGGTGTCTGTG; >clamp_s_c_s_a_s:compl:rev CACAGACACCCACACTATCATGTTGACATTCC; dG = -14.8
206 >s_c_s_b_s_e_s_b_s_a_s GTGTGGGTGGCTGTGCTGGTGGCTGTGTCTGTG; >clamp_s_c_s_a_s:compl:rev CACAGACACCCACACTATCATGTTGACATTCC; dG = -14.8
207 >s_c_s_b_s_e_s_a_s_a_s GTGTGGGTGGCTGTGCTGGTGTCTGTGTCTGTG; >clamp_s_c_s_a_s:compl:rev CACAGACACCCACACTATCATGTTGACATTCC; dG = -14.8
208 >s_a_s_d_s_c_s_c_s_d_s GTGTCTGTGACGGTGTGGGTGTGGGTGACGGTG; >clamp_s_c_s_a_s:compl:rev CACAGACACCCACACTATCATGTTGACATTCC; dG = -12.3
209 >s_a_s_e_s_c_s_c_s_d_s GTGTCTGTGCTGGTGTGGGTGTGGGTGACGGTG; >clamp_s_c_s_a_s:compl:rev CACAGACACCCACACTATCATGTTGACATTCC; dG = -12.3
210 >s_a_s_a_s_c_s_c_s_d_s GTGTCTGTGTCTGTGTGGGTGTGGGTGACGGTG; >clamp_s_c_s_a_s:compl:rev CACAGACACCCACACTATCATGTTGACATTCC; dG = -12.3
211 >s_b_s_a_s_c_s_c_s_d_s GTGGCTGTGTCTGTGTGGGTGTGGGTGACGGTG; >clamp_s_c_s_a_s:compl:rev CACAGACACCCACACTATCATGTTGACATTCC; dG = -12.3

212 >s_b_s_b_s_c_s_c_s_d_s GTGGCTGTGGCTGTGTGGGTGTGGGTGACGGTG; >clamp_s_c_s_a_s:compl:rev CACAGACACCCACACTATCATGTTGACATTCC; dG = -12.3
213 >s_b_s_c_s_c_s_c_s_d_s GTGGCTGTGTGGGTGTGGGTGTGGGTGACGGTG; >clamp_s_c_s_a_s:compl:rev CACAGACACCCACACTATCATGTTGACATTCC; dG = -12.3
214 >s_b_s_c_s_c_s_d_s_a_s GTGGCTGTGTGGGTGTGGGTGACGGTGTCTGTG; >clamp_s_c_s_a_s:compl:rev CACAGACACCCACACTATCATGTTGACATTCC; dG = -12.3
215 >s_b_s_d_s_c_s_c_s_d_s GTGGCTGTGACGGTGTGGGTGTGGGTGACGGTG; >clamp_s_c_s_a_s:compl:rev CACAGACACCCACACTATCATGTTGACATTCC; dG = -12.3
216 >s_b_s_e_s_c_s_c_s_d_s GTGGCTGTGCTGGTGTGGGTGTGGGTGACGGTG; >clamp_s_c_s_a_s:compl:rev CACAGACACCCACACTATCATGTTGACATTCC; dG = -12.3
217 >s_c_s_c_s_c_s_c_s_d_s GTGTGGGTGTGGGTGTGGGTGTGGGTGACGGTG; >clamp_s_c_s_a_s:compl:rev CACAGACACCCACACTATCATGTTGACATTCC; dG = -12.3
218 >s_c_s_c_s_c_s_d_s_a_s GTGTGGGTGTGGGTGTGGGTGACGGTGTCTGTG; >clamp_s_c_s_a_s:compl:rev CACAGACACCCACACTATCATGTTGACATTCC; dG = -12.3
219 >s_c_s_d_s_c_s_c_s_d_s GTGTGGGTGACGGTGTGGGTGTGGGTGACGGTG; >clamp_s_c_s_a_s:compl:rev CACAGACACCCACACTATCATGTTGACATTCC; dG = -12.3
220 >s_c_s_e_s_c_s_c_s_d_s GTGTGGGTGCTGGTGTGGGTGTGGGTGACGGTG; >clamp_s_c_s_a_s:compl:rev CACAGACACCCACACTATCATGTTGACATTCC; dG = -12.3
221 >s_d_s_a_s_c_s_c_s_d_s GTGACGGTGTCTGTGTGGGTGTGGGTGACGGTG; >clamp_s_c_s_a_s:compl:rev CACAGACACCCACACTATCATGTTGACATTCC; dG = -12.3
222 >s_a_s_b_s_c_s_c_s_d_s GTGTCTGTGGCTGTGTGGGTGTGGGTGACGGTG; >clamp_s_c_s_a_s:compl:rev CACAGACACCCACACTATCATGTTGACATTCC; dG = -12.3
223 >s_d_s_b_s_c_s_c_s_d_s GTGACGGTGGCTGTGTGGGTGTGGGTGACGGTG; >clamp_s_c_s_a_s:compl:rev CACAGACACCCACACTATCATGTTGACATTCC; dG = -12.3
224 >s_d_s_c_s_c_s_c_s_d_s GTGACGGTGTGGGTGTGGGTGTGGGTGACGGTG; >clamp_s_c_s_a_s:compl:rev CACAGACACCCACACTATCATGTTGACATTCC; dG = -12.3
225 >s_d_s_c_s_c_s_d_s_a_s GTGACGGTGTGGGTGTGGGTGACGGTGTCTGTG; >clamp_s_c_s_a_s:compl:rev CACAGACACCCACACTATCATGTTGACATTCC; dG = -12.3
226 >s_d_s_d_s_c_s_c_s_d_s GTGACGGTGACGGTGTGGGTGTGGGTGACGGTG; >clamp_s_c_s_a_s:compl:rev CACAGACACCCACACTATCATGTTGACATTCC; dG = -12.3
227 >s_d_s_e_s_c_s_c_s_d_s GTGACGGTGCTGGTGTGGGTGTGGGTGACGGTG; >clamp_s_c_s_a_s:compl:rev CACAGACACCCACACTATCATGTTGACATTCC; dG = -12.3
228 >s_e_s_a_s_c_s_c_s_d_s GTGCTGGTGTCTGTGTGGGTGTGGGTGACGGTG; >clamp_s_c_s_a_s:compl:rev CACAGACACCCACACTATCATGTTGACATTCC; dG = -12.3
229 >s_e_s_b_s_c_s_c_s_d_s GTGCTGGTGGCTGTGTGGGTGTGGGTGACGGTG; >clamp_s_c_s_a_s:compl:rev CACAGACACCCACACTATCATGTTGACATTCC; dG = -12.3
230 >s_e_s_c_s_c_s_c_s_d_s GTGCTGGTGTGGGTGTGGGTGTGGGTGACGGTG; >clamp_s_c_s_a_s:compl:rev CACAGACACCCACACTATCATGTTGACATTCC; dG = -12.3
231 >s_e_s_c_s_c_s_d_s_a_s GTGCTGGTGTGGGTGTGGGTGACGGTGTCTGTG; >clamp_s_c_s_a_s:compl:rev CACAGACACCCACACTATCATGTTGACATTCC; dG = -12.3
232 >s_e_s_d_s_c_s_c_s_d_s GTGCTGGTGACGGTGTGGGTGTGGGTGACGGTG; >clamp_s_c_s_a_s:compl:rev CACAGACACCCACACTATCATGTTGACATTCC; dG = -12.3
233 >s_e_s_e_s_c_s_c_s_d_s GTGCTGGTGCTGGTGTGGGTGTGGGTGACGGTG; >clamp_s_c_s_a_s:compl:rev CACAGACACCCACACTATCATGTTGACATTCC; dG = -12.3
234 >s_a_s_c_s_c_s_c_s_d_s GTGTCTGTGTGGGTGTGGGTGTGGGTGACGGTG; >clamp_s_c_s_a_s:compl:rev CACAGACACCCACACTATCATGTTGACATTCC; dG = -12.3
235 >s_a_s_c_s_c_s_d_s_a_s GTGTCTGTGTGGGTGTGGGTGACGGTGTCTGTG; >clamp_s_c_s_a_s:compl:rev CACAGACACCCACACTATCATGTTGACATTCC; dG = -12.3
236 >s_a_s_a_s_b_s_c_s_c_s GTGTCTGTGTCTGTGGCTGTGTGGGTGTGGGTG; >clamp_s_c_s_a_s:compl:rev CACAGACACCCACACTATCATGTTGACATTCC; dG = -12.0
237 >s_a_s_d_s_a_s_c_s_c_s GTGTCTGTGACGGTGTCTGTGTGGGTGTGGGTG; >clamp_s_c_s_a_s:compl:rev CACAGACACCCACACTATCATGTTGACATTCC; dG = -12.0
238 >s_a_s_d_s_b_s_c_s_c_s GTGTCTGTGACGGTGGCTGTGTGGGTGTGGGTG; >clamp_s_c_s_a_s:compl:rev CACAGACACCCACACTATCATGTTGACATTCC; dG = -12.0
239 >s_a_s_e_s_a_s_c_s_c_s GTGTCTGTGCTGGTGTCTGTGTGGGTGTGGGTG; >clamp_s_c_s_a_s:compl:rev CACAGACACCCACACTATCATGTTGACATTCC; dG = -12.0
240 >s_a_s_e_s_b_s_c_s_c_s GTGTCTGTGCTGGTGGCTGTGTGGGTGTGGGTG; >clamp_s_c_s_a_s:compl:rev CACAGACACCCACACTATCATGTTGACATTCC; dG = -12.0
241 >s_a_s_a_s_c_s_c_s_e_s GTGTCTGTGTCTGTGTGGGTGTGGGTGCTGGTG; >clamp_s_c_s_a_s:compl:rev CACAGACACCCACACTATCATGTTGACATTCC; dG = -12.0
242 >s_a_s_a_s_c_s_c_s_c_s GTGTCTGTGTCTGTGTGGGTGTGGGTGTGGGTG; >clamp_s_c_s_a_s:compl:rev CACAGACACCCACACTATCATGTTGACATTCC; dG = -12.0
243 >s_b_s_a_s_a_s_c_s_c_s GTGGCTGTGTCTGTGTCTGTGTGGGTGTGGGTG; >clamp_s_c_s_a_s:compl:rev CACAGACACCCACACTATCATGTTGACATTCC; dG = -12.0
244 >s_b_s_a_s_b_s_c_s_c_s GTGGCTGTGTCTGTGGCTGTGTGGGTGTGGGTG; >clamp_s_c_s_a_s:compl:rev CACAGACACCCACACTATCATGTTGACATTCC; dG = -12.0
245 >s_b_s_a_s_c_s_c_s_e_s GTGGCTGTGTCTGTGTGGGTGTGGGTGCTGGTG; >clamp_s_c_s_a_s:compl:rev CACAGACACCCACACTATCATGTTGACATTCC; dG = -12.0
246 >s_b_s_a_s_c_s_c_s_c_s GTGGCTGTGTCTGTGTGGGTGTGGGTGTGGGTG; >clamp_s_c_s_a_s:compl:rev CACAGACACCCACACTATCATGTTGACATTCC; dG = -12.0
247 >s_b_s_b_s_a_s_c_s_c_s GTGGCTGTGGCTGTGTCTGTGTGGGTGTGGGTG; >clamp_s_c_s_a_s:compl:rev CACAGACACCCACACTATCATGTTGACATTCC; dG = -12.0
248 >s_b_s_b_s_b_s_c_s_c_s GTGGCTGTGGCTGTGGCTGTGTGGGTGTGGGTG; >clamp_s_c_s_a_s:compl:rev CACAGACACCCACACTATCATGTTGACATTCC; dG = -12.0

249 >s_b_s_b_s_c_s_c_s_e_s GTGGCTGTGGCTGTGTGGGTGTGGGTGCTGGTG; >clamp_s_c_s_a_s:compl:rev
CACAGACACCCACACTATCATGTTGACATTCC; dG = -12.0
250 >s_b_s_b_s_c_s_c_s_c_s GTGGCTGTGGCTGTGTGGGTGTGGGTGTGGGTG; >clamp_s_c_s_a_s:compl:rev
CACAGACACCCACACTATCATGTTGACATTCC; dG = -12.0
251 >s_b_s_c_s_c_s_c_s_e_s GTGGCTGTGTGGGTGTGGGTGTGGGTGCTGGTG; >clamp_s_c_s_a_s:compl:rev
CACAGACACCCACACTATCATGTTGACATTCC; dG = -12.0
252 >s_b_s_c_s_c_s_c_s_c_s GTGGCTGTGTGGGTGTGGGTGTGGGTGTGGGTG; >clamp_s_c_s_a_s:compl:rev
CACAGACACCCACACTATCATGTTGACATTCC; dG = -12.0
253 >s_b_s_c_s_c_s_e_s_a_s GTGGCTGTGTGGGTGTGGGTGCTGGTGTCTGTG; >clamp_s_c_s_a_s:compl:rev
CACAGACACCCACACTATCATGTTGACATTCC; dG = -12.0
254 >s_b_s_d_s_a_s_c_s_c_s GTGGCTGTGACGGTGTCTGTGTGGGTGTGGGTG; >clamp_s_c_s_a_s:compl:rev
CACAGACACCCACACTATCATGTTGACATTCC; dG = -12.0
255 >s_a_s_a_s_a_s_c_s_c_s GTGTCTGTGTCTGTGTCTGTGTGGGTGTGGGTG; >clamp_s_c_s_a_s:compl:rev
CACAGACACCCACACTATCATGTTGACATTCC; dG = -12.0
256 >s_b_s_d_s_b_s_c_s_c_s GTGGCTGTGACGGTGGCTGTGTGGGTGTGGGTG; >clamp_s_c_s_a_s:compl:rev
CACAGACACCCACACTATCATGTTGACATTCC; dG = -12.0
257 >s_b_s_e_s_a_s_c_s_c_s GTGGCTGTGCTGGTGTCTGTGTGGGTGTGGGTG; >clamp_s_c_s_a_s:compl:rev
CACAGACACCCACACTATCATGTTGACATTCC; dG = -12.0
258 >s_b_s_e_s_b_s_c_s_c_s GTGGCTGTGCTGGTGGCTGTGTGGGTGTGGGTG; >clamp_s_c_s_a_s:compl:rev
CACAGACACCCACACTATCATGTTGACATTCC; dG = -12.0
259 >s_a_s_b_s_a_s_c_s_c_s GTGTCTGTGGCTGTGTCTGTGTGGGTGTGGGTG; >clamp_s_c_s_a_s:compl:rev
CACAGACACCCACACTATCATGTTGACATTCC; dG = -12.0
260 >s_c_s_d_s_a_s_c_s_c_s GTGTGGGTGACGGTGTCTGTGTGGGTGTGGGTG; >clamp_s_c_s_a_s:compl:rev
CACAGACACCCACACTATCATGTTGACATTCC; dG = -12.0
261 >s_c_s_d_s_a_s_d_s_a_s GTGTGGGTGACGGTGTCTGTGACGGTGTCTGTG; >clamp_s_c_s_a_s:compl:rev
CACAGACACCCACACTATCATGTTGACATTCC; dG = -12.0
262 >s_c_s_d_s_b_s_c_s_c_s GTGTGGGTGACGGTGGCTGTGTGGGTGTGGGTG; >clamp_s_c_s_a_s:compl:rev
CACAGACACCCACACTATCATGTTGACATTCC; dG = -12.0
263 >s_c_s_e_s_a_s_c_s_c_s GTGTGGGTGCTGGTGTCTGTGTGGGTGTGGGTG; >clamp_s_c_s_a_s:compl:rev
CACAGACACCCACACTATCATGTTGACATTCC; dG = -12.0
264 >s_c_s_e_s_a_s_d_s_a_s GTGTGGGTGCTGGTGTCTGTGACGGTGTCTGTG; >clamp_s_c_s_a_s:compl:rev
CACAGACACCCACACTATCATGTTGACATTCC; dG = -12.0
265 >s_c_s_e_s_b_s_c_s_c_s GTGTGGGTGCTGGTGGCTGTGTGGGTGTGGGTG; >clamp_s_c_s_a_s:compl:rev
CACAGACACCCACACTATCATGTTGACATTCC; dG = -12.0
266 >s_a_s_b_s_b_s_c_s_c_s GTGTCTGTGGCTGTGGCTGTGTGGGTGTGGGTG; >clamp_s_c_s_a_s:compl:rev
CACAGACACCCACACTATCATGTTGACATTCC; dG = -12.0
267 >s_d_s_a_s_a_s_c_s_c_s GTGACGGTGTCTGTGTCTGTGTGGGTGTGGGTG; >clamp_s_c_s_a_s:compl:rev
CACAGACACCCACACTATCATGTTGACATTCC; dG = -12.0
268 >s_d_s_a_s_b_s_c_s_c_s GTGACGGTGTCTGTGGCTGTGTGGGTGTGGGTG; >clamp_s_c_s_a_s:compl:rev
CACAGACACCCACACTATCATGTTGACATTCC; dG = -12.0
269 >s_d_s_a_s_c_s_c_s_e_s GTGACGGTGTCTGTGTGGGTGTGGGTGCTGGTG; >clamp_s_c_s_a_s:compl:rev
CACAGACACCCACACTATCATGTTGACATTCC; dG = -12.0
270 >s_d_s_a_s_c_s_c_s_c_s GTGACGGTGTCTGTGTGGGTGTGGGTGTGGGTG; >clamp_s_c_s_a_s:compl:rev
CACAGACACCCACACTATCATGTTGACATTCC; dG = -12.0
271 >s_a_s_b_s_c_s_c_s_e_s GTGTCTGTGGCTGTGTGGGTGTGGGTGCTGGTG; >clamp_s_c_s_a_s:compl:rev
CACAGACACCCACACTATCATGTTGACATTCC; dG = -12.0
272 >s_d_s_b_s_a_s_c_s_c_s GTGACGGTGGCTGTGTCTGTGTGGGTGTGGGTG; >clamp_s_c_s_a_s:compl:rev
CACAGACACCCACACTATCATGTTGACATTCC; dG = -12.0
273 >s_d_s_b_s_b_s_c_s_c_s GTGACGGTGGCTGTGGCTGTGTGGGTGTGGGTG; >clamp_s_c_s_a_s:compl:rev
CACAGACACCCACACTATCATGTTGACATTCC; dG = -12.0
274 >s_d_s_b_s_c_s_c_s_e_s GTGACGGTGGCTGTGTGGGTGTGGGTGCTGGTG; >clamp_s_c_s_a_s:compl:rev
CACAGACACCCACACTATCATGTTGACATTCC; dG = -12.0
275 >s_a_s_b_s_c_s_c_s_c_s GTGTCTGTGGCTGTGTGGGTGTGGGTGTGGGTG; >clamp_s_c_s_a_s:compl:rev
CACAGACACCCACACTATCATGTTGACATTCC; dG = -12.0
276 >s_d_s_b_s_c_s_c_s_c_s GTGACGGTGGCTGTGTGGGTGTGGGTGTGGGTG; >clamp_s_c_s_a_s:compl:rev
CACAGACACCCACACTATCATGTTGACATTCC; dG = -12.0
277 >s_d_s_d_s_a_s_c_s_c_s GTGACGGTGACGGTGTCTGTGTGGGTGTGGGTG; >clamp_s_c_s_a_s:compl:rev
CACAGACACCCACACTATCATGTTGACATTCC; dG = -12.0
278 >s_d_s_d_s_b_s_c_s_c_s GTGACGGTGACGGTGGCTGTGTGGGTGTGGGTG; >clamp_s_c_s_a_s:compl:rev
CACAGACACCCACACTATCATGTTGACATTCC; dG = -12.0
279 >s_d_s_e_s_a_s_c_s_c_s GTGACGGTGCTGGTGTCTGTGTGGGTGTGGGTG; >clamp_s_c_s_a_s:compl:rev
CACAGACACCCACACTATCATGTTGACATTCC; dG = -12.0
280 >s_d_s_e_s_b_s_c_s_c_s GTGACGGTGCTGGTGGCTGTGTGGGTGTGGGTG; >clamp_s_c_s_a_s:compl:rev
CACAGACACCCACACTATCATGTTGACATTCC; dG = -12.0
281 >s_e_s_a_s_a_s_c_s_c_s GTGCTGGTGTCTGTGTCTGTGTGGGTGTGGGTG; >clamp_s_c_s_a_s:compl:rev
CACAGACACCCACACTATCATGTTGACATTCC; dG = -12.0
282 >s_e_s_a_s_b_s_c_s_c_s GTGCTGGTGTCTGTGGCTGTGTGGGTGTGGGTG; >clamp_s_c_s_a_s:compl:rev
CACAGACACCCACACTATCATGTTGACATTCC; dG = -12.0
283 >s_e_s_a_s_c_s_c_s_e_s GTGCTGGTGTCTGTGTGGGTGTGGGTGCTGGTG; >clamp_s_c_s_a_s:compl:rev
CACAGACACCCACACTATCATGTTGACATTCC; dG = -12.0
284 >s_e_s_a_s_c_s_c_s_c_s GTGCTGGTGTCTGTGTGGGTGTGGGTGTGGGTG; >clamp_s_c_s_a_s:compl:rev
CACAGACACCCACACTATCATGTTGACATTCC; dG = -12.0
285 >s_e_s_b_s_a_s_c_s_c_s GTGCTGGTGGCTGTGTCTGTGTGGGTGTGGGTG; >clamp_s_c_s_a_s:compl:rev
CACAGACACCCACACTATCATGTTGACATTCC; dG = -12.0

286 >s_e_s_b_s_b_s_c_s_c_s GTGCTGGTGGCTGTGGCTGTGTGGGTGTGGGTG; >clamp_s_c_s_a_s:compl:rev
CACAGACACCCACACTATCATGTTGACATTCC; dG = -12.0
287 >s_e_s_b_s_c_s_c_s_e_s GTGCTGGTGGCTGTGTGGGTGTGGGTGCTGGTG; >clamp_s_c_s_a_s:compl:rev
CACAGACACCCACACTATCATGTTGACATTCC; dG = -12.0
288 >s_e_s_b_s_c_s_c_s_c_s GTGCTGGTGGCTGTGTGGGTGTGGGTGTGGGTG; >clamp_s_c_s_a_s:compl:rev
CACAGACACCCACACTATCATGTTGACATTCC; dG = -12.0
289 >s_e_s_d_s_a_s_c_s_c_s GTGCTGGTGACGGTGTCTGTGTGGGTGTGGGTG; >clamp_s_c_s_a_s:compl:rev
CACAGACACCCACACTATCATGTTGACATTCC; dG = -12.0
290 >s_e_s_d_s_b_s_c_s_c_s GTGCTGGTGACGGTGGCTGTGTGGGTGTGGGTG; >clamp_s_c_s_a_s:compl:rev
CACAGACACCCACACTATCATGTTGACATTCC; dG = -12.0
291 >s_e_s_e_s_a_s_c_s_c_s GTGCTGGTGCTGGTGTCTGTGTGGGTGTGGGTG; >clamp_s_c_s_a_s:compl:rev
CACAGACACCCACACTATCATGTTGACATTCC; dG = -12.0
292 >s_e_s_e_s_b_s_c_s_c_s GTGCTGGTGCTGGTGGCTGTGTGGGTGTGGGTG; >clamp_s_c_s_a_s:compl:rev
CACAGACACCCACACTATCATGTTGACATTCC; dG = -12.0
293 >s_a_s_c_s_c_s_c_s_e_s GTGTCTGTGTGGGTGTGGGTGTGGGTGCTGGTG; >clamp_s_c_s_a_s:compl:rev
CACAGACACCCACACTATCATGTTGACATTCC; dG = -12.0
294 >s_a_s_c_s_c_s_c_s_c_s GTGTCTGTGTGGGTGTGGGTGTGGGTGTGGGTG; >clamp_s_c_s_a_s:compl:rev
CACAGACACCCACACTATCATGTTGACATTCC; dG = -12.0
295 >s_a_s_c_s_c_s_e_s_a_s GTGTCTGTGTGGGTGTGGGTGCTGGTGTCTGTG; >clamp_s_c_s_a_s:compl:rev
CACAGACACCCACACTATCATGTTGACATTCC; dG = -12.0
296 >s_a_s_c_s_d_s_a_s_a_s GTGTCTGTGTGGGTGACGGTGTCTGTGTCTGTG; >clamp_s_c_s_a_s:compl:rev
CACAGACACCCACACTATCATGTTGACATTCC; dG = -11.9
297 >s_a_s_c_s_e_s_c_s_c_s GTGTCTGTGTGGGTGCTGGTGTGGGTGTGGGTG; >clamp_s_c_s_a_s:compl:rev
CACAGACACCCACACTATCATGTTGACATTCC; dG = -11.9
298 >s_a_s_c_s_e_s_a_s_a_s GTGTCTGTGTGGGTGCTGGTGTCTGTGTCTGTG; >clamp_s_c_s_a_s:compl:rev
CACAGACACCCACACTATCATGTTGACATTCC; dG = -11.9
299 >s_a_s_d_s_c_s_c_s_e_s GTGTCTGTGACGGTGTGGGTGTGGGTGCTGGTG; >clamp_s_c_s_a_s:compl:rev
CACAGACACCCACACTATCATGTTGACATTCC; dG = -11.9
300 >s_a_s_d_s_c_s_c_s_c_s GTGTCTGTGACGGTGTGGGTGTGGGTGTGGGTG; >clamp_s_c_s_a_s:compl:rev
CACAGACACCCACACTATCATGTTGACATTCC; dG = -11.9
301 >s_a_s_d_s_d_s_c_s_c_s GTGTCTGTGACGGTGACGGTGTGGGTGTGGGTG; >clamp_s_c_s_a_s:compl:rev
CACAGACACCCACACTATCATGTTGACATTCC; dG = -11.9
302 >s_a_s_d_s_e_s_c_s_c_s GTGTCTGTGACGGTGCTGGTGTGGGTGTGGGTG; >clamp_s_c_s_a_s:compl:rev
CACAGACACCCACACTATCATGTTGACATTCC; dG = -11.9
303 >s_a_s_e_s_c_s_c_s_e_s GTGTCTGTGCTGGTGTGGGTGTGGGTGCTGGTG; >clamp_s_c_s_a_s:compl:rev
CACAGACACCCACACTATCATGTTGACATTCC; dG = -11.9
304 >s_a_s_e_s_c_s_c_s_c_s GTGTCTGTGCTGGTGTGGGTGTGGGTGTGGGTG; >clamp_s_c_s_a_s:compl:rev
CACAGACACCCACACTATCATGTTGACATTCC; dG = -11.9
305 >s_a_s_e_s_d_s_c_s_c_s GTGTCTGTGCTGGTGACGGTGTGGGTGTGGGTG; >clamp_s_c_s_a_s:compl:rev
CACAGACACCCACACTATCATGTTGACATTCC; dG = -11.9
306 >s_a_s_e_s_e_s_c_s_c_s GTGTCTGTGCTGGTGCTGGTGTGGGTGTGGGTG; >clamp_s_c_s_a_s:compl:rev
CACAGACACCCACACTATCATGTTGACATTCC; dG = -11.9
307 >s_b_s_a_s_d_s_c_s_c_s GTGGCTGTGTCTGTGACGGTGTGGGTGTGGGTG; >clamp_s_c_s_a_s:compl:rev
CACAGACACCCACACTATCATGTTGACATTCC; dG = -11.9
308 >s_b_s_a_s_e_s_c_s_c_s GTGGCTGTGTCTGTGCTGGTGTGGGTGTGGGTG; >clamp_s_c_s_a_s:compl:rev
CACAGACACCCACACTATCATGTTGACATTCC; dG = -11.9
309 >s_b_s_b_s_d_s_c_s_c_s GTGGCTGTGGCTGTGACGGTGTGGGTGTGGGTG; >clamp_s_c_s_a_s:compl:rev
CACAGACACCCACACTATCATGTTGACATTCC; dG = -11.9
310 >s_a_s_a_s_d_s_c_s_c_s GTGTCTGTGTCTGTGACGGTGTGGGTGTGGGTG; >clamp_s_c_s_a_s:compl:rev
CACAGACACCCACACTATCATGTTGACATTCC; dG = -11.9
311 >s_b_s_b_s_e_s_c_s_c_s GTGGCTGTGGCTGTGCTGGTGTGGGTGTGGGTG; >clamp_s_c_s_a_s:compl:rev
CACAGACACCCACACTATCATGTTGACATTCC; dG = -11.9
312 >s_b_s_c_s_d_s_c_s_c_s GTGGCTGTGTGGGTGACGGTGTGGGTGTGGGTG; >clamp_s_c_s_a_s:compl:rev
CACAGACACCCACACTATCATGTTGACATTCC; dG = -11.9
313 >s_b_s_c_s_d_s_a_s_a_s GTGGCTGTGTGGGTGACGGTGTCTGTGTCTGTG; >clamp_s_c_s_a_s:compl:rev
CACAGACACCCACACTATCATGTTGACATTCC; dG = -11.9
314 >s_b_s_c_s_e_s_c_s_c_s GTGGCTGTGTGGGTGCTGGTGTGGGTGTGGGTG; >clamp_s_c_s_a_s:compl:rev
CACAGACACCCACACTATCATGTTGACATTCC; dG = -11.9
315 >s_b_s_c_s_e_s_a_s_a_s GTGGCTGTGTGGGTGCTGGTGTCTGTGTCTGTG; >clamp_s_c_s_a_s:compl:rev
CACAGACACCCACACTATCATGTTGACATTCC; dG = -11.9
316 >s_b_s_d_s_c_s_c_s_e_s GTGGCTGTGACGGTGTGGGTGTGGGTGCTGGTG; >clamp_s_c_s_a_s:compl:rev
CACAGACACCCACACTATCATGTTGACATTCC; dG = -11.9
317 >s_b_s_d_s_c_s_c_s_c_s GTGGCTGTGACGGTGTGGGTGTGGGTGTGGGTG; >clamp_s_c_s_a_s:compl:rev
CACAGACACCCACACTATCATGTTGACATTCC; dG = -11.9
318 >s_b_s_d_s_d_s_c_s_c_s GTGGCTGTGACGGTGACGGTGTGGGTGTGGGTG; >clamp_s_c_s_a_s:compl:rev
CACAGACACCCACACTATCATGTTGACATTCC; dG = -11.9
319 >s_b_s_d_s_e_s_c_s_c_s GTGGCTGTGACGGTGCTGGTGTGGGTGTGGGTG; >clamp_s_c_s_a_s:compl:rev
CACAGACACCCACACTATCATGTTGACATTCC; dG = -11.9
320 >s_a_s_a_s_e_s_c_s_c_s GTGTCTGTGTCTGTGCTGGTGTGGGTGTGGGTG; >clamp_s_c_s_a_s:compl:rev
CACAGACACCCACACTATCATGTTGACATTCC; dG = -11.9
321 >s_b_s_e_s_c_s_c_s_e_s GTGGCTGTGCTGGTGTGGGTGTGGGTGCTGGTG; >clamp_s_c_s_a_s:compl:rev
CACAGACACCCACACTATCATGTTGACATTCC; dG = -11.9
322 >s_b_s_e_s_c_s_c_s_c_s GTGGCTGTGCTGGTGTGGGTGTGGGTGTGGGTG; >clamp_s_c_s_a_s:compl:rev
CACAGACACCCACACTATCATGTTGACATTCC; dG = -11.9

323 >s_b_s_e_s_d_s_c_s_c_s GTGGCTGTGCTGGTGACGGTGTGGGTGTGGGTG; >clamp_s_c_s_a_s:compl:rev CACAGACACCCACACTATCATGTTGACATTCC; dG = -11.9
324 >s_b_s_e_s_e_s_c_s_c_s GTGGCTGTGCTGGTGCTGGTGTGGGTGTGGGTG; >clamp_s_c_s_a_s:compl:rev CACAGACACCCACACTATCATGTTGACATTCC; dG = -11.9
325 >s_c_s_c_s_c_s_c_s_e_s GTGTGGGTGTGGGTGTGGGTGTGGGTGCTGGTG; >clamp_s_c_s_a_s:compl:rev CACAGACACCCACACTATCATGTTGACATTCC; dG = -11.9
326 >s_c_s_c_s_c_s_c_s_c_s GTGTGGGTGTGGGTGTGGGTGTGGGTGTGGGTG; >clamp_s_c_s_a_s:compl:rev CACAGACACCCACACTATCATGTTGACATTCC; dG = -11.9
327 >s_c_s_c_s_c_s_e_s_a_s GTGTGGGTGTGGGTGTGGGTGCTGGTGTCTGTG; >clamp_s_c_s_a_s:compl:rev CACAGACACCCACACTATCATGTTGACATTCC; dG = -11.9
328 >s_c_s_c_s_d_s_c_s_c_s GTGTGGGTGTGGGTGACGGTGTGGGTGTGGGTG; >clamp_s_c_s_a_s:compl:rev CACAGACACCCACACTATCATGTTGACATTCC; dG = -11.9
329 >s_c_s_c_s_d_s_a_s_a_s GTGTGGGTGTGGGTGACGGTGTCTGTGTCTGTG; >clamp_s_c_s_a_s:compl:rev CACAGACACCCACACTATCATGTTGACATTCC; dG = -11.9
330 >s_c_s_c_s_e_s_c_s_c_s GTGTGGGTGTGGGTGCTGGTGTGGGTGTGGGTG; >clamp_s_c_s_a_s:compl:rev CACAGACACCCACACTATCATGTTGACATTCC; dG = -11.9
331 >s_c_s_c_s_e_s_a_s_a_s GTGTGGGTGTGGGTGCTGGTGTCTGTGTCTGTG; >clamp_s_c_s_a_s:compl:rev CACAGACACCCACACTATCATGTTGACATTCC; dG = -11.9
332 >s_c_s_d_s_c_s_c_s_e_s GTGTGGGTGACGGTGTGGGTGTGGGTGCTGGTG; >clamp_s_c_s_a_s:compl:rev CACAGACACCCACACTATCATGTTGACATTCC; dG = -11.9
333 >s_c_s_d_s_c_s_c_s_c_s GTGTGGGTGACGGTGTGGGTGTGGGTGTGGGTG; >clamp_s_c_s_a_s:compl:rev CACAGACACCCACACTATCATGTTGACATTCC; dG = -11.9
334 >s_c_s_d_s_d_s_c_s_c_s GTGTGGGTGACGGTGACGGTGTGGGTGTGGGTG; >clamp_s_c_s_a_s:compl:rev CACAGACACCCACACTATCATGTTGACATTCC; dG = -11.9
335 >s_c_s_d_s_e_s_c_s_c_s GTGTGGGTGACGGTGCTGGTGTGGGTGTGGGTG; >clamp_s_c_s_a_s:compl:rev CACAGACACCCACACTATCATGTTGACATTCC; dG = -11.9
336 >s_c_s_e_s_c_s_c_s_e_s GTGTGGGTGCTGGTGTGGGTGTGGGTGCTGGTG; >clamp_s_c_s_a_s:compl:rev CACAGACACCCACACTATCATGTTGACATTCC; dG = -11.9
337 >s_c_s_e_s_c_s_c_s_c_s GTGTGGGTGCTGGTGTGGGTGTGGGTGTGGGTG; >clamp_s_c_s_a_s:compl:rev CACAGACACCCACACTATCATGTTGACATTCC; dG = -11.9
338 >s_c_s_e_s_d_s_c_s_c_s GTGTGGGTGCTGGTGACGGTGTGGGTGTGGGTG; >clamp_s_c_s_a_s:compl:rev CACAGACACCCACACTATCATGTTGACATTCC; dG = -11.9
339 >s_c_s_e_s_e_s_c_s_c_s GTGTGGGTGCTGGTGCTGGTGTGGGTGTGGGTG; >clamp_s_c_s_a_s:compl:rev CACAGACACCCACACTATCATGTTGACATTCC; dG = -11.9
340 >s_d_s_a_s_d_s_c_s_c_s GTGACGGTGTCTGTGACGGTGTGGGTGTGGGTG; >clamp_s_c_s_a_s:compl:rev CACAGACACCCACACTATCATGTTGACATTCC; dG = -11.9
341 >s_d_s_a_s_e_s_c_s_c_s GTGACGGTGTCTGTGCTGGTGTGGGTGTGGGTG; >clamp_s_c_s_a_s:compl:rev CACAGACACCCACACTATCATGTTGACATTCC; dG = -11.9
342 >s_d_s_b_s_d_s_c_s_c_s GTGACGGTGGCTGTGACGGTGTGGGTGTGGGTG; >clamp_s_c_s_a_s:compl:rev CACAGACACCCACACTATCATGTTGACATTCC; dG = -11.9
343 >s_d_s_b_s_e_s_c_s_c_s GTGACGGTGGCTGTGCTGGTGTGGGTGTGGGTG; >clamp_s_c_s_a_s:compl:rev CACAGACACCCACACTATCATGTTGACATTCC; dG = -11.9
344 >s_d_s_c_s_c_s_c_s_e_s GTGACGGTGTGGGTGTGGGTGTGGGTGCTGGTG; >clamp_s_c_s_a_s:compl:rev CACAGACACCCACACTATCATGTTGACATTCC; dG = -11.9
345 >s_d_s_c_s_c_s_c_s_c_s GTGACGGTGTGGGTGTGGGTGTGGGTGTGGGTG; >clamp_s_c_s_a_s:compl:rev CACAGACACCCACACTATCATGTTGACATTCC; dG = -11.9
346 >s_d_s_c_s_c_s_e_s_a_s GTGACGGTGTGGGTGTGGGTGCTGGTGTCTGTG; >clamp_s_c_s_a_s:compl:rev CACAGACACCCACACTATCATGTTGACATTCC; dG = -11.9
347 >s_d_s_c_s_d_s_c_s_c_s GTGACGGTGTGGGTGACGGTGTGGGTGTGGGTG; >clamp_s_c_s_a_s:compl:rev CACAGACACCCACACTATCATGTTGACATTCC; dG = -11.9
348 >s_d_s_c_s_d_s_a_s_a_s GTGACGGTGTGGGTGACGGTGTCTGTGTCTGTG; >clamp_s_c_s_a_s:compl:rev CACAGACACCCACACTATCATGTTGACATTCC; dG = -11.9
349 >s_d_s_c_s_e_s_c_s_c_s GTGACGGTGTGGGTGCTGGTGTGGGTGTGGGTG; >clamp_s_c_s_a_s:compl:rev CACAGACACCCACACTATCATGTTGACATTCC; dG = -11.9
350 >s_d_s_c_s_e_s_a_s_a_s GTGACGGTGTGGGTGCTGGTGTCTGTGTCTGTG; >clamp_s_c_s_a_s:compl:rev CACAGACACCCACACTATCATGTTGACATTCC; dG = -11.9
351 >s_d_s_d_s_c_s_c_s_e_s GTGACGGTGACGGTGTGGGTGTGGGTGCTGGTG; >clamp_s_c_s_a_s:compl:rev CACAGACACCCACACTATCATGTTGACATTCC; dG = -11.9
352 >s_a_s_b_s_d_s_c_s_c_s GTGTCTGTGGCTGTGACGGTGTGGGTGTGGGTG; >clamp_s_c_s_a_s:compl:rev CACAGACACCCACACTATCATGTTGACATTCC; dG = -11.9
353 >s_d_s_d_s_c_s_c_s_c_s GTGACGGTGACGGTGTGGGTGTGGGTGTGGGTG; >clamp_s_c_s_a_s:compl:rev CACAGACACCCACACTATCATGTTGACATTCC; dG = -11.9
354 >s_d_s_d_s_d_s_c_s_c_s GTGACGGTGACGGTGACGGTGTGGGTGTGGGTG; >clamp_s_c_s_a_s:compl:rev CACAGACACCCACACTATCATGTTGACATTCC; dG = -11.9
355 >s_d_s_d_s_e_s_c_s_c_s GTGACGGTGACGGTGCTGGTGTGGGTGTGGGTG; >clamp_s_c_s_a_s:compl:rev CACAGACACCCACACTATCATGTTGACATTCC; dG = -11.9
356 >s_d_s_e_s_c_s_c_s_e_s GTGACGGTGCTGGTGTGGGTGTGGGTGCTGGTG; >clamp_s_c_s_a_s:compl:rev CACAGACACCCACACTATCATGTTGACATTCC; dG = -11.9
357 >s_d_s_e_s_c_s_c_s_c_s GTGACGGTGCTGGTGTGGGTGTGGGTGTGGGTG; >clamp_s_c_s_a_s:compl:rev CACAGACACCCACACTATCATGTTGACATTCC; dG = -11.9
358 >s_d_s_e_s_d_s_c_s_c_s GTGACGGTGCTGGTGACGGTGTGGGTGTGGGTG; >clamp_s_c_s_a_s:compl:rev CACAGACACCCACACTATCATGTTGACATTCC; dG = -11.9
359 >s_d_s_e_s_e_s_c_s_c_s GTGACGGTGCTGGTGCTGGTGTGGGTGTGGGTG; >clamp_s_c_s_a_s:compl:rev CACAGACACCCACACTATCATGTTGACATTCC; dG = -11.9

360 >s_a_s_b_s_e_s_c_s_c_s GTGTCTGTGGCTGTGCTGGTGTGGGTGTGGGTG; >clamp_s_c_s_a_s:compl:rev
CACAGACACCCACACTATCATGTTGACATTCC; dG = -11.9
361 >s_e_s_a_s_d_s_c_s_c_s GTGCTGGTGTCTGTGACGGTGTGGGTGTGGGTG; >clamp_s_c_s_a_s:compl:rev
CACAGACACCCACACTATCATGTTGACATTCC; dG = -11.9
362 >s_e_s_a_s_e_s_c_s_c_s GTGCTGGTGTCTGTGCTGGTGTGGGTGTGGGTG; >clamp_s_c_s_a_s:compl:rev
CACAGACACCCACACTATCATGTTGACATTCC; dG = -11.9
363 >s_e_s_b_s_d_s_c_s_c_s GTGCTGGTGGCTGTGACGGTGTGGGTGTGGGTG; >clamp_s_c_s_a_s:compl:rev
CACAGACACCCACACTATCATGTTGACATTCC; dG = -11.9
364 >s_e_s_b_s_e_s_c_s_c_s GTGCTGGTGGCTGTGCTGGTGTGGGTGTGGGTG; >clamp_s_c_s_a_s:compl:rev
CACAGACACCCACACTATCATGTTGACATTCC; dG = -11.9
365 >s_e_s_c_s_c_s_c_s_e_s GTGCTGGTGTGGGTGTGGGTGTGGGTGCTGGTG; >clamp_s_c_s_a_s:compl:rev
CACAGACACCCACACTATCATGTTGACATTCC; dG = -11.9
366 >s_e_s_c_s_c_s_c_s_c_s GTGCTGGTGTGGGTGTGGGTGTGGGTGTGGGTG; >clamp_s_c_s_a_s:compl:rev
CACAGACACCCACACTATCATGTTGACATTCC; dG = -11.9
367 >s_e_s_c_s_c_s_e_s_a_s GTGCTGGTGTGGGTGTGGGTGCTGGTGTCTGTG; >clamp_s_c_s_a_s:compl:rev
CACAGACACCCACACTATCATGTTGACATTCC; dG = -11.9
368 >s_e_s_c_s_d_s_c_s_c_s GTGCTGGTGTGGGTGACGGTGTGGGTGTGGGTG; >clamp_s_c_s_a_s:compl:rev
CACAGACACCCACACTATCATGTTGACATTCC; dG = -11.9
369 >s_e_s_c_s_d_s_a_s_a_s GTGCTGGTGTGGGTGACGGTGTCTGTGTCTGTG; >clamp_s_c_s_a_s:compl:rev
CACAGACACCCACACTATCATGTTGACATTCC; dG = -11.9
370 >s_e_s_c_s_e_s_c_s_c_s GTGCTGGTGTGGGTGCTGGTGTGGGTGTGGGTG; >clamp_s_c_s_a_s:compl:rev
CACAGACACCCACACTATCATGTTGACATTCC; dG = -11.9
371 >s_e_s_c_s_e_s_a_s_a_s GTGCTGGTGTGGGTGCTGGTGTCTGTGTCTGTG; >clamp_s_c_s_a_s:compl:rev
CACAGACACCCACACTATCATGTTGACATTCC; dG = -11.9
372 >s_e_s_d_s_c_s_c_s_e_s GTGCTGGTGACGGTGTGGGTGTGGGTGCTGGTG; >clamp_s_c_s_a_s:compl:rev
CACAGACACCCACACTATCATGTTGACATTCC; dG = -11.9
373 >s_e_s_d_s_c_s_c_s_c_s GTGCTGGTGACGGTGTGGGTGTGGGTGTGGGTG; >clamp_s_c_s_a_s:compl:rev
CACAGACACCCACACTATCATGTTGACATTCC; dG = -11.9
374 >s_e_s_d_s_d_s_c_s_c_s GTGCTGGTGACGGTGACGGTGTGGGTGTGGGTG; >clamp_s_c_s_a_s:compl:rev
CACAGACACCCACACTATCATGTTGACATTCC; dG = -11.9
375 >s_e_s_d_s_e_s_c_s_c_s GTGCTGGTGACGGTGCTGGTGTGGGTGTGGGTG; >clamp_s_c_s_a_s:compl:rev
CACAGACACCCACACTATCATGTTGACATTCC; dG = -11.9
376 >s_e_s_e_s_c_s_c_s_e_s GTGCTGGTGCTGGTGTGGGTGTGGGTGCTGGTG; >clamp_s_c_s_a_s:compl:rev
CACAGACACCCACACTATCATGTTGACATTCC; dG = -11.9
377 >s_e_s_e_s_c_s_c_s_c_s GTGCTGGTGCTGGTGTGGGTGTGGGTGTGGGTG; >clamp_s_c_s_a_s:compl:rev
CACAGACACCCACACTATCATGTTGACATTCC; dG = -11.9
378 >s_e_s_e_s_d_s_c_s_c_s GTGCTGGTGCTGGTGACGGTGTGGGTGTGGGTG; >clamp_s_c_s_a_s:compl:rev
CACAGACACCCACACTATCATGTTGACATTCC; dG = -11.9
379 >s_e_s_e_s_e_s_c_s_c_s GTGCTGGTGCTGGTGCTGGTGTGGGTGTGGGTG; >clamp_s_c_s_a_s:compl:rev
CACAGACACCCACACTATCATGTTGACATTCC; dG = -11.9
380 >s_a_s_c_s_d_s_c_s_c_s GTGTCTGTGTGGGTGACGGTGTGGGTGTGGGTG; >clamp_s_c_s_a_s:compl:rev
CACAGACACCCACACTATCATGTTGACATTCC; dG = -11.9
381 >s_c_s_c_s_d_s_c_s_e_s GTGTGGGTGTGGGTGACGGTGTGGGTGCTGGTG; >clamp_s_c_s_a_s:compl:rev
CACAGACACCCACACTATCATGTTGACATTCC; dG = -11.8
382 >s_c_s_c_s_d_s_c_s_d_s GTGTGGGTGTGGGTGACGGTGTGGGTGACGGTG; >clamp_s_c_s_a_s:compl:rev
CACAGACACCCACACTATCATGTTGACATTCC; dG = -11.8
383 >s_c_s_c_s_d_s_e_s_a_s GTGTGGGTGTGGGTGACGGTGCTGGTGTCTGTG; >clamp_s_c_s_a_s:compl:rev
CACAGACACCCACACTATCATGTTGACATTCC; dG = -11.8
384 >s_c_s_c_s_d_s_d_s_a_s GTGTGGGTGTGGGTGACGGTGACGGTGTCTGTG; >clamp_s_c_s_a_s:compl:rev
CACAGACACCCACACTATCATGTTGACATTCC; dG = -11.8
385 >s_c_s_c_s_d_s_b_s_a_s GTGTGGGTGTGGGTGACGGTGGCTGTGTCTGTG; >clamp_s_c_s_a_s:compl:rev
CACAGACACCCACACTATCATGTTGACATTCC; dG = -11.8
386 >s_a_s_d_s_a_s_d_s_a_s GTGTCTGTGACGGTGTCTGTGACGGTGTCTGTG; >clamp_s_c_s_a_s:compl:rev
CACAGACACCCACACTATCATGTTGACATTCC; dG = -11.7
387 >s_b_s_a_s_c_s_e_s_a_s GTGGCTGTGTCTGTGTGGGTGCTGGTGTCTGTG; >clamp_s_c_s_a_s:compl:rev
CACAGACACCCACACTATCATGTTGACATTCC; dG = -11.7
388 >s_b_s_a_s_c_s_d_s_a_s GTGGCTGTGTCTGTGTGGGTGACGGTGTCTGTG; >clamp_s_c_s_a_s:compl:rev
CACAGACACCCACACTATCATGTTGACATTCC; dG = -11.7
389 >s_a_s_a_s_c_s_e_s_a_s GTGTCTGTGTCTGTGTGGGTGCTGGTGTCTGTG; >clamp_s_c_s_a_s:compl:rev
CACAGACACCCACACTATCATGTTGACATTCC; dG = -11.7
390 >s_a_s_a_s_c_s_d_s_a_s GTGTCTGTGTCTGTGTGGGTGACGGTGTCTGTG; >clamp_s_c_s_a_s:compl:rev
CACAGACACCCACACTATCATGTTGACATTCC; dG = -11.7
391 >s_b_s_b_s_c_s_e_s_a_s GTGGCTGTGGCTGTGTGGGTGCTGGTGTCTGTG; >clamp_s_c_s_a_s:compl:rev
CACAGACACCCACACTATCATGTTGACATTCC; dG = -11.7
392 >s_b_s_b_s_c_s_d_s_a_s GTGGCTGTGGCTGTGTGGGTGACGGTGTCTGTG; >clamp_s_c_s_a_s:compl:rev
CACAGACACCCACACTATCATGTTGACATTCC; dG = -11.7
393 >s_b_s_d_s_a_s_d_s_a_s GTGGCTGTGACGGTGTCTGTGACGGTGTCTGTG; >clamp_s_c_s_a_s:compl:rev
CACAGACACCCACACTATCATGTTGACATTCC; dG = -11.7
394 >s_d_s_a_s_c_s_e_s_a_s GTGACGGTGTCTGTGTGGGTGCTGGTGTCTGTG; >clamp_s_c_s_a_s:compl:rev
CACAGACACCCACACTATCATGTTGACATTCC; dG = -11.7
395 >s_d_s_a_s_c_s_d_s_a_s GTGACGGTGTCTGTGTGGGTGACGGTGTCTGTG; >clamp_s_c_s_a_s:compl:rev
CACAGACACCCACACTATCATGTTGACATTCC; dG = -11.7
396 >s_d_s_b_s_c_s_e_s_a_s GTGACGGTGGCTGTGTGGGTGCTGGTGTCTGTG; >clamp_s_c_s_a_s:compl:rev
CACAGACACCCACACTATCATGTTGACATTCC; dG = -11.7

397 >s_d_s_b_s_c_s_d_s_a_s GTGACGGTGGCTGTGTGGGTGACGGTGTCTGTG; >clamp_s_c_s_a_s:compl:rev CACAGACACCCACACTATCATGTTGACATTCC; dG = -11.7
398 >s_a_s_b_s_c_s_e_s_a_s GTGTCTGTGGCTGTGTGGGTGCTGGTGTCTGTG; >clamp_s_c_s_a_s:compl:rev CACAGACACCCACACTATCATGTTGACATTCC; dG = -11.7
399 >s_a_s_b_s_c_s_d_s_a_s GTGTCTGTGGCTGTGTGGGTGACGGTGTCTGTG; >clamp_s_c_s_a_s:compl:rev CACAGACACCCACACTATCATGTTGACATTCC; dG = -11.7
400 >s_d_s_d_s_a_s_d_s_a_s GTGACGGTGACGGTGTCTGTGACGGTGTCTGTG; >clamp_s_c_s_a_s:compl:rev CACAGACACCCACACTATCATGTTGACATTCC; dG = -11.7
401 >s_e_s_a_s_c_s_e_s_a_s GTGCTGGTGTCTGTGTGGGTGCTGGTGTCTGTG; >clamp_s_c_s_a_s:compl:rev CACAGACACCCACACTATCATGTTGACATTCC; dG = -11.7
402 >s_e_s_a_s_c_s_d_s_a_s GTGCTGGTGTCTGTGTGGGTGACGGTGTCTGTG; >clamp_s_c_s_a_s:compl:rev CACAGACACCCACACTATCATGTTGACATTCC; dG = -11.7
403 >s_e_s_b_s_c_s_e_s_a_s GTGCTGGTGGCTGTGTGGGTGCTGGTGTCTGTG; >clamp_s_c_s_a_s:compl:rev CACAGACACCCACACTATCATGTTGACATTCC; dG = -11.7
404 >s_e_s_b_s_c_s_d_s_a_s GTGCTGGTGGCTGTGTGGGTGACGGTGTCTGTG; >clamp_s_c_s_a_s:compl:rev CACAGACACCCACACTATCATGTTGACATTCC; dG = -11.7
405 >s_e_s_d_s_a_s_d_s_a_s GTGCTGGTGACGGTGTCTGTGACGGTGTCTGTG; >clamp_s_c_s_a_s:compl:rev CACAGACACCCACACTATCATGTTGACATTCC; dG = -11.7
406 >s_a_s_a_s_b_s_c_s_d_s GTGTCTGTGTCTGTGGCTGTGTGGGTGACGGTG; >clamp_s_c_s_a_s:compl:rev CACAGACACCCACACTATCATGTTGACATTCC; dG = -11.6
407 >s_a_s_c_s_d_s_b_s_a_s GTGTCTGTGTGGGTGACGGTGGCTGTGTCTGTG; >clamp_s_c_s_a_s:compl:rev CACAGACACCCACACTATCATGTTGACATTCC; dG = -11.6
408 >s_a_s_d_s_a_s_c_s_d_s GTGTCTGTGACGGTGTCTGTGTGGGTGACGGTG; >clamp_s_c_s_a_s:compl:rev CACAGACACCCACACTATCATGTTGACATTCC; dG = -11.6
409 >s_a_s_d_s_b_s_c_s_d_s GTGTCTGTGACGGTGGCTGTGTGGGTGACGGTG; >clamp_s_c_s_a_s:compl:rev CACAGACACCCACACTATCATGTTGACATTCC; dG = -11.6
410 >s_a_s_d_s_c_s_e_s_a_s GTGTCTGTGACGGTGTGGGTGCTGGTGTCTGTG; >clamp_s_c_s_a_s:compl:rev CACAGACACCCACACTATCATGTTGACATTCC; dG = -11.6
411 >s_a_s_d_s_c_s_d_s_a_s GTGTCTGTGACGGTGTGGGTGACGGTGTCTGTG; >clamp_s_c_s_a_s:compl:rev CACAGACACCCACACTATCATGTTGACATTCC; dG = -11.6
412 >s_a_s_e_s_a_s_c_s_d_s GTGTCTGTGCTGGTGTCTGTGTGGGTGACGGTG; >clamp_s_c_s_a_s:compl:rev CACAGACACCCACACTATCATGTTGACATTCC; dG = -11.6
413 >s_a_s_e_s_a_s_d_s_a_s GTGTCTGTGCTGGTGTCTGTGACGGTGTCTGTG; >clamp_s_c_s_a_s:compl:rev CACAGACACCCACACTATCATGTTGACATTCC; dG = -11.6
414 >s_a_s_e_s_b_s_c_s_d_s GTGTCTGTGCTGGTGGCTGTGTGGGTGACGGTG; >clamp_s_c_s_a_s:compl:rev CACAGACACCCACACTATCATGTTGACATTCC; dG = -11.6
415 >s_a_s_e_s_c_s_e_s_a_s GTGTCTGTGCTGGTGTGGGTGCTGGTGTCTGTG; >clamp_s_c_s_a_s:compl:rev CACAGACACCCACACTATCATGTTGACATTCC; dG = -11.6
416 >s_a_s_e_s_c_s_d_s_a_s GTGTCTGTGCTGGTGTGGGTGACGGTGTCTGTG; >clamp_s_c_s_a_s:compl:rev CACAGACACCCACACTATCATGTTGACATTCC; dG = -11.6
417 >s_b_s_a_s_a_s_c_s_d_s GTGGCTGTGTCTGTGTCTGTGTGGGTGACGGTG; >clamp_s_c_s_a_s:compl:rev CACAGACACCCACACTATCATGTTGACATTCC; dG = -11.6
418 >s_b_s_a_s_b_s_c_s_d_s GTGGCTGTGTCTGTGGCTGTGTGGGTGACGGTG; >clamp_s_c_s_a_s:compl:rev CACAGACACCCACACTATCATGTTGACATTCC; dG = -11.6
419 >s_a_s_a_s_a_s_c_s_d_s GTGTCTGTGTCTGTGTCTGTGTGGGTGACGGTG; >clamp_s_c_s_a_s:compl:rev CACAGACACCCACACTATCATGTTGACATTCC; dG = -11.6
420 >s_b_s_b_s_a_s_c_s_d_s GTGGCTGTGGCTGTGTCTGTGTGGGTGACGGTG; >clamp_s_c_s_a_s:compl:rev CACAGACACCCACACTATCATGTTGACATTCC; dG = -11.6
421 >s_b_s_b_s_b_s_c_s_d_s GTGGCTGTGGCTGTGGCTGTGTGGGTGACGGTG; >clamp_s_c_s_a_s:compl:rev CACAGACACCCACACTATCATGTTGACATTCC; dG = -11.6
422 >s_b_s_c_s_d_s_c_s_e_s GTGGCTGTGTGGGTGACGGTGTGGGTGCTGGTG; >clamp_s_c_s_a_s:compl:rev CACAGACACCCACACTATCATGTTGACATTCC; dG = -11.6
423 >s_b_s_c_s_d_s_c_s_d_s GTGGCTGTGTGGGTGACGGTGTGGGTGACGGTG; >clamp_s_c_s_a_s:compl:rev CACAGACACCCACACTATCATGTTGACATTCC; dG = -11.6
424 >s_b_s_c_s_d_s_e_s_a_s GTGGCTGTGTGGGTGACGGTGCTGGTGTCTGTG; >clamp_s_c_s_a_s:compl:rev CACAGACACCCACACTATCATGTTGACATTCC; dG = -11.6
425 >s_b_s_c_s_d_s_d_s_a_s GTGGCTGTGTGGGTGACGGTGACGGTGTCTGTG; >clamp_s_c_s_a_s:compl:rev CACAGACACCCACACTATCATGTTGACATTCC; dG = -11.6
426 >s_b_s_c_s_d_s_b_s_a_s GTGGCTGTGTGGGTGACGGTGGCTGTGTCTGTG; >clamp_s_c_s_a_s:compl:rev CACAGACACCCACACTATCATGTTGACATTCC; dG = -11.6
427 >s_b_s_d_s_a_s_c_s_d_s GTGGCTGTGACGGTGTCTGTGTGGGTGACGGTG; >clamp_s_c_s_a_s:compl:rev CACAGACACCCACACTATCATGTTGACATTCC; dG = -11.6
428 >s_b_s_d_s_b_s_c_s_d_s GTGGCTGTGACGGTGGCTGTGTGGGTGACGGTG; >clamp_s_c_s_a_s:compl:rev CACAGACACCCACACTATCATGTTGACATTCC; dG = -11.6
429 >s_b_s_d_s_c_s_e_s_a_s GTGGCTGTGACGGTGTGGGTGCTGGTGTCTGTG; >clamp_s_c_s_a_s:compl:rev CACAGACACCCACACTATCATGTTGACATTCC; dG = -11.6
430 >s_b_s_d_s_c_s_d_s_a_s GTGGCTGTGACGGTGTGGGTGACGGTGTCTGTG; >clamp_s_c_s_a_s:compl:rev CACAGACACCCACACTATCATGTTGACATTCC; dG = -11.6
431 >s_b_s_e_s_a_s_c_s_d_s GTGGCTGTGCTGGTGTCTGTGTGGGTGACGGTG; >clamp_s_c_s_a_s:compl:rev CACAGACACCCACACTATCATGTTGACATTCC; dG = -11.6
432 >s_b_s_e_s_a_s_d_s_a_s GTGGCTGTGCTGGTGTCTGTGACGGTGTCTGTG; >clamp_s_c_s_a_s:compl:rev CACAGACACCCACACTATCATGTTGACATTCC; dG = -11.6
433 >s_b_s_e_s_b_s_c_s_d_s GTGGCTGTGCTGGTGGCTGTGTGGGTGACGGTG; >clamp_s_c_s_a_s:compl:rev CACAGACACCCACACTATCATGTTGACATTCC; dG = -11.6

434 >s_b_s_e_s_c_s_e_s_a_s GTGGCTGTGCTGGTGTGGGTGCTGGTGTCTGTG; >clamp_s_c_s_a_s:compl:rev
CACAGACACCCACACTATCATGTTGACATTCC; dG = -11.6
435 >s_b_s_e_s_c_s_d_s_a_s GTGGCTGTGCTGGTGTGGGTGACGGTGTCTGTG; >clamp_s_c_s_a_s:compl:rev
CACAGACACCCACACTATCATGTTGACATTCC; dG = -11.6
436 >s_a_s_b_s_a_s_c_s_d_s GTGTCTGTGGCTGTGTCTGTGTGGGTGACGGTG; >clamp_s_c_s_a_s:compl:rev
CACAGACACCCACACTATCATGTTGACATTCC; dG = -11.6
437 >s_c_s_d_s_a_s_c_s_d_s GTGTGGGTGACGGTGTCTGTGTGGGTGACGGTG; >clamp_s_c_s_a_s:compl:rev
CACAGACACCCACACTATCATGTTGACATTCC; dG = -11.6
438 >s_c_s_d_s_b_s_c_s_d_s GTGTGGGTGACGGTGGCTGTGTGGGTGACGGTG; >clamp_s_c_s_a_s:compl:rev
CACAGACACCCACACTATCATGTTGACATTCC; dG = -11.6
439 >s_c_s_d_s_c_s_e_s_a_s GTGTGGGTGACGGTGTGGGTGCTGGTGTCTGTG; >clamp_s_c_s_a_s:compl:rev
CACAGACACCCACACTATCATGTTGACATTCC; dG = -11.6
440 >s_c_s_d_s_c_s_d_s_a_s GTGTGGGTGACGGTGTGGGTGACGGTGTCTGTG; >clamp_s_c_s_a_s:compl:rev
CACAGACACCCACACTATCATGTTGACATTCC; dG = -11.6
441 >s_c_s_e_s_a_s_c_s_d_s GTGTGGGTGCTGGTGTCTGTGTGGGTGACGGTG; >clamp_s_c_s_a_s:compl:rev
CACAGACACCCACACTATCATGTTGACATTCC; dG = -11.6
442 >s_a_s_b_s_b_s_c_s_d_s GTGTCTGTGGCTGTGGCTGTGTGGGTGACGGTG; >clamp_s_c_s_a_s:compl:rev
CACAGACACCCACACTATCATGTTGACATTCC; dG = -11.6
443 >s_c_s_e_s_b_s_c_s_d_s GTGTGGGTGCTGGTGGCTGTGTGGGTGACGGTG; >clamp_s_c_s_a_s:compl:rev
CACAGACACCCACACTATCATGTTGACATTCC; dG = -11.6
444 >s_c_s_e_s_c_s_e_s_a_s GTGTGGGTGCTGGTGTGGGTGCTGGTGTCTGTG; >clamp_s_c_s_a_s:compl:rev
CACAGACACCCACACTATCATGTTGACATTCC; dG = -11.6
445 >s_c_s_e_s_c_s_d_s_a_s GTGTGGGTGCTGGTGTGGGTGACGGTGTCTGTG; >clamp_s_c_s_a_s:compl:rev
CACAGACACCCACACTATCATGTTGACATTCC; dG = -11.6
446 >s_d_s_a_s_a_s_c_s_d_s GTGACGGTGTCTGTGTCTGTGTGGGTGACGGTG; >clamp_s_c_s_a_s:compl:rev
CACAGACACCCACACTATCATGTTGACATTCC; dG = -11.6
447 >s_d_s_a_s_b_s_c_s_d_s GTGACGGTGTCTGTGGCTGTGTGGGTGACGGTG; >clamp_s_c_s_a_s:compl:rev
CACAGACACCCACACTATCATGTTGACATTCC; dG = -11.6
448 >s_d_s_b_s_a_s_c_s_d_s GTGACGGTGGCTGTGTCTGTGTGGGTGACGGTG; >clamp_s_c_s_a_s:compl:rev
CACAGACACCCACACTATCATGTTGACATTCC; dG = -11.6
449 >s_d_s_b_s_b_s_c_s_d_s GTGACGGTGGCTGTGGCTGTGTGGGTGACGGTG; >clamp_s_c_s_a_s:compl:rev
CACAGACACCCACACTATCATGTTGACATTCC; dG = -11.6
450 >s_d_s_d_s_a_s_c_s_d_s GTGACGGTGACGGTGTCTGTGTGGGTGACGGTG; >clamp_s_c_s_a_s:compl:rev
CACAGACACCCACACTATCATGTTGACATTCC; dG = -11.6
451 >s_d_s_d_s_b_s_c_s_d_s GTGACGGTGACGGTGGCTGTGTGGGTGACGGTG; >clamp_s_c_s_a_s:compl:rev
CACAGACACCCACACTATCATGTTGACATTCC; dG = -11.6
452 >s_d_s_d_s_c_s_e_s_a_s GTGACGGTGACGGTGTGGGTGCTGGTGTCTGTG; >clamp_s_c_s_a_s:compl:rev
CACAGACACCCACACTATCATGTTGACATTCC; dG = -11.6
453 >s_d_s_d_s_c_s_d_s_a_s GTGACGGTGACGGTGTGGGTGACGGTGTCTGTG; >clamp_s_c_s_a_s:compl:rev
CACAGACACCCACACTATCATGTTGACATTCC; dG = -11.6
454 >s_d_s_e_s_a_s_c_s_d_s GTGACGGTGCTGGTGTCTGTGTGGGTGACGGTG; >clamp_s_c_s_a_s:compl:rev
CACAGACACCCACACTATCATGTTGACATTCC; dG = -11.6
455 >s_d_s_e_s_a_s_d_s_a_s GTGACGGTGCTGGTGTCTGTGACGGTGTCTGTG; >clamp_s_c_s_a_s:compl:rev
CACAGACACCCACACTATCATGTTGACATTCC; dG = -11.6
456 >s_d_s_e_s_b_s_c_s_d_s GTGACGGTGCTGGTGGCTGTGTGGGTGACGGTG; >clamp_s_c_s_a_s:compl:rev
CACAGACACCCACACTATCATGTTGACATTCC; dG = -11.6
457 >s_d_s_e_s_c_s_e_s_a_s GTGACGGTGCTGGTGTGGGTGCTGGTGTCTGTG; >clamp_s_c_s_a_s:compl:rev
CACAGACACCCACACTATCATGTTGACATTCC; dG = -11.6
458 >s_d_s_e_s_c_s_d_s_a_s GTGACGGTGCTGGTGTGGGTGACGGTGTCTGTG; >clamp_s_c_s_a_s:compl:rev
CACAGACACCCACACTATCATGTTGACATTCC; dG = -11.6
459 >s_e_s_a_s_a_s_c_s_d_s GTGCTGGTGTCTGTGTCTGTGTGGGTGACGGTG; >clamp_s_c_s_a_s:compl:rev
CACAGACACCCACACTATCATGTTGACATTCC; dG = -11.6
460 >s_e_s_a_s_b_s_c_s_d_s GTGCTGGTGTCTGTGGCTGTGTGGGTGACGGTG; >clamp_s_c_s_a_s:compl:rev
CACAGACACCCACACTATCATGTTGACATTCC; dG = -11.6
461 >s_e_s_b_s_a_s_c_s_d_s GTGCTGGTGGCTGTGTCTGTGTGGGTGACGGTG; >clamp_s_c_s_a_s:compl:rev
CACAGACACCCACACTATCATGTTGACATTCC; dG = -11.6
462 >s_e_s_b_s_b_s_c_s_d_s GTGCTGGTGGCTGTGGCTGTGTGGGTGACGGTG; >clamp_s_c_s_a_s:compl:rev
CACAGACACCCACACTATCATGTTGACATTCC; dG = -11.6
463 >s_e_s_d_s_a_s_c_s_d_s GTGCTGGTGACGGTGTCTGTGTGGGTGACGGTG; >clamp_s_c_s_a_s:compl:rev
CACAGACACCCACACTATCATGTTGACATTCC; dG = -11.6
464 >s_e_s_d_s_b_s_c_s_d_s GTGCTGGTGACGGTGGCTGTGTGGGTGACGGTG; >clamp_s_c_s_a_s:compl:rev
CACAGACACCCACACTATCATGTTGACATTCC; dG = -11.6
465 >s_e_s_d_s_c_s_e_s_a_s GTGCTGGTGACGGTGTGGGTGCTGGTGTCTGTG; >clamp_s_c_s_a_s:compl:rev
CACAGACACCCACACTATCATGTTGACATTCC; dG = -11.6
466 >s_e_s_d_s_c_s_d_s_a_s GTGCTGGTGACGGTGTGGGTGACGGTGTCTGTG; >clamp_s_c_s_a_s:compl:rev
CACAGACACCCACACTATCATGTTGACATTCC; dG = -11.6
467 >s_e_s_e_s_a_s_c_s_d_s GTGCTGGTGCTGGTGTCTGTGTGGGTGACGGTG; >clamp_s_c_s_a_s:compl:rev
CACAGACACCCACACTATCATGTTGACATTCC; dG = -11.6
468 >s_e_s_e_s_a_s_d_s_a_s GTGCTGGTGCTGGTGTCTGTGACGGTGTCTGTG; >clamp_s_c_s_a_s:compl:rev
CACAGACACCCACACTATCATGTTGACATTCC; dG = -11.6
469 >s_e_s_e_s_b_s_c_s_d_s GTGCTGGTGCTGGTGGCTGTGTGGGTGACGGTG; >clamp_s_c_s_a_s:compl:rev
CACAGACACCCACACTATCATGTTGACATTCC; dG = -11.6
470 >s_e_s_e_s_c_s_e_s_a_s GTGCTGGTGCTGGTGTGGGTGCTGGTGTCTGTG; >clamp_s_c_s_a_s:compl:rev
CACAGACACCCACACTATCATGTTGACATTCC; dG = -11.6

471 >s_e_s_e_s_c_s_d_s_a_s GTGCTGGTGCTGGTGTGGGTGACGGTGTCTGTG; >clamp_s_c_s_a_s:compl:rev
CACAGACACCCACACTATCATGTTGACATTCC; dG = -11.6
472 >s_a_s_c_s_d_s_c_s_e_s GTGTCTGTGTGGGTGACGGTGTGGGTGCTGGTG; >clamp_s_c_s_a_s:compl:rev
CACAGACACCCACACTATCATGTTGACATTCC; dG = -11.6
473 >s_a_s_c_s_d_s_c_s_d_s GTGTCTGTGTGGGTGACGGTGTGGGTGACGGTG; >clamp_s_c_s_a_s:compl:rev
CACAGACACCCACACTATCATGTTGACATTCC; dG = -11.6
474 >s_a_s_c_s_d_s_e_s_a_s GTGTCTGTGTGGGTGACGGTGCTGGTGTCTGTG; >clamp_s_c_s_a_s:compl:rev
CACAGACACCCACACTATCATGTTGACATTCC; dG = -11.6
475 >s_a_s_c_s_d_s_d_s_a_s GTGTCTGTGTGGGTGACGGTGACGGTGTCTGTG; >clamp_s_c_s_a_s:compl:rev
CACAGACACCCACACTATCATGTTGACATTCC; dG = -11.6
476 >s_a_s_c_s_e_s_c_s_d_s GTGTCTGTGTGGGTGCTGGTGTGGGTGACGGTG; >clamp_s_c_s_a_s:compl:rev
CACAGACACCCACACTATCATGTTGACATTCC; dG = -11.5
477 >s_a_s_d_s_d_s_c_s_d_s GTGTCTGTGACGGTGACGGTGTGGGTGACGGTG; >clamp_s_c_s_a_s:compl:rev
CACAGACACCCACACTATCATGTTGACATTCC; dG = -11.5
478 >s_a_s_d_s_e_s_c_s_d_s GTGTCTGTGACGGTGCTGGTGTGGGTGACGGTG; >clamp_s_c_s_a_s:compl:rev
CACAGACACCCACACTATCATGTTGACATTCC; dG = -11.5
479 >s_a_s_e_s_d_s_c_s_d_s GTGTCTGTGCTGGTGACGGTGTGGGTGACGGTG; >clamp_s_c_s_a_s:compl:rev
CACAGACACCCACACTATCATGTTGACATTCC; dG = -11.5
480 >s_a_s_e_s_e_s_c_s_d_s GTGTCTGTGCTGGTGCTGGTGTGGGTGACGGTG; >clamp_s_c_s_a_s:compl:rev
CACAGACACCCACACTATCATGTTGACATTCC; dG = -11.5
481 >s_b_s_a_s_d_s_c_s_d_s GTGGCTGTGTCTGTGACGGTGTGGGTGACGGTG; >clamp_s_c_s_a_s:compl:rev
CACAGACACCCACACTATCATGTTGACATTCC; dG = -11.5
482 >s_b_s_a_s_e_s_c_s_d_s GTGGCTGTGTCTGTGCTGGTGTGGGTGACGGTG; >clamp_s_c_s_a_s:compl:rev
CACAGACACCCACACTATCATGTTGACATTCC; dG = -11.5
483 >s_a_s_a_s_d_s_c_s_d_s GTGTCTGTGTCTGTGACGGTGTGGGTGACGGTG; >clamp_s_c_s_a_s:compl:rev
CACAGACACCCACACTATCATGTTGACATTCC; dG = -11.5
484 >s_b_s_b_s_d_s_c_s_d_s GTGGCTGTGGCTGTGACGGTGTGGGTGACGGTG; >clamp_s_c_s_a_s:compl:rev
CACAGACACCCACACTATCATGTTGACATTCC; dG = -11.5
485 >s_b_s_b_s_e_s_c_s_d_s GTGGCTGTGGCTGTGCTGGTGTGGGTGACGGTG; >clamp_s_c_s_a_s:compl:rev
CACAGACACCCACACTATCATGTTGACATTCC; dG = -11.5
486 >s_b_s_c_s_e_s_c_s_d_s GTGGCTGTGTGGGTGCTGGTGTGGGTGACGGTG; >clamp_s_c_s_a_s:compl:rev
CACAGACACCCACACTATCATGTTGACATTCC; dG = -11.5
487 >s_b_s_d_s_d_s_c_s_d_s GTGGCTGTGACGGTGACGGTGTGGGTGACGGTG; >clamp_s_c_s_a_s:compl:rev
CACAGACACCCACACTATCATGTTGACATTCC; dG = -11.5
488 >s_b_s_d_s_e_s_c_s_d_s GTGGCTGTGACGGTGCTGGTGTGGGTGACGGTG; >clamp_s_c_s_a_s:compl:rev
CACAGACACCCACACTATCATGTTGACATTCC; dG = -11.5
489 >s_a_s_a_s_e_s_c_s_d_s GTGTCTGTGTCTGTGCTGGTGTGGGTGACGGTG; >clamp_s_c_s_a_s:compl:rev
CACAGACACCCACACTATCATGTTGACATTCC; dG = -11.5
490 >s_b_s_e_s_d_s_c_s_d_s GTGGCTGTGCTGGTGACGGTGTGGGTGACGGTG; >clamp_s_c_s_a_s:compl:rev
CACAGACACCCACACTATCATGTTGACATTCC; dG = -11.5
491 >s_b_s_e_s_e_s_c_s_d_s GTGGCTGTGCTGGTGCTGGTGTGGGTGACGGTG; >clamp_s_c_s_a_s:compl:rev
CACAGACACCCACACTATCATGTTGACATTCC; dG = -11.5
492 >s_c_s_c_s_e_s_c_s_e_s GTGTGGGTGTGGGTGCTGGTGTGGGTGCTGGTG; >clamp_s_c_s_a_s:compl:rev
CACAGACACCCACACTATCATGTTGACATTCC; dG = -11.5
493 >s_c_s_c_s_e_s_c_s_d_s GTGTGGGTGTGGGTGCTGGTGTGGGTGACGGTG; >clamp_s_c_s_a_s:compl:rev
CACAGACACCCACACTATCATGTTGACATTCC; dG = -11.5
494 >s_c_s_c_s_e_s_e_s_a_s GTGTGGGTGTGGGTGCTGGTGCTGGTGTCTGTG; >clamp_s_c_s_a_s:compl:rev
CACAGACACCCACACTATCATGTTGACATTCC; dG = -11.5
495 >s_c_s_c_s_e_s_d_s_a_s GTGTGGGTGTGGGTGCTGGTGACGGTGTCTGTG; >clamp_s_c_s_a_s:compl:rev
CACAGACACCCACACTATCATGTTGACATTCC; dG = -11.5
496 >s_c_s_c_s_e_s_b_s_a_s GTGTGGGTGTGGGTGCTGGTGGCTGTGTCTGTG; >clamp_s_c_s_a_s:compl:rev
CACAGACACCCACACTATCATGTTGACATTCC; dG = -11.5
497 >s_c_s_d_s_a_s_b_s_a_s GTGTGGGTGACGGTGTCTGTGGCTGTGTCTGTG; >clamp_s_c_s_a_s:compl:rev
CACAGACACCCACACTATCATGTTGACATTCC; dG = -11.5
498 >s_c_s_d_s_d_s_c_s_d_s GTGTGGGTGACGGTGACGGTGTGGGTGACGGTG; >clamp_s_c_s_a_s:compl:rev
CACAGACACCCACACTATCATGTTGACATTCC; dG = -11.5
499 >s_c_s_d_s_e_s_c_s_d_s GTGTGGGTGACGGTGCTGGTGTGGGTGACGGTG; >clamp_s_c_s_a_s:compl:rev
CACAGACACCCACACTATCATGTTGACATTCC; dG = -11.5
500 >s_c_s_e_s_a_s_b_s_a_s GTGTGGGTGCTGGTGTCTGTGGCTGTGTCTGTG; >clamp_s_c_s_a_s:compl:rev
CACAGACACCCACACTATCATGTTGACATTCC; dG = -11.5
501 >s_c_s_e_s_d_s_c_s_d_s GTGTGGGTGCTGGTGACGGTGTGGGTGACGGTG; >clamp_s_c_s_a_s:compl:rev
CACAGACACCCACACTATCATGTTGACATTCC; dG = -11.5
502 >s_c_s_e_s_e_s_c_s_d_s GTGTGGGTGCTGGTGCTGGTGTGGGTGACGGTG; >clamp_s_c_s_a_s:compl:rev
CACAGACACCCACACTATCATGTTGACATTCC; dG = -11.5
503 >s_d_s_a_s_d_s_c_s_d_s GTGACGGTGTCTGTGACGGTGTGGGTGACGGTG; >clamp_s_c_s_a_s:compl:rev
CACAGACACCCACACTATCATGTTGACATTCC; dG = -11.5
504 >s_d_s_a_s_e_s_c_s_d_s GTGACGGTGTCTGTGCTGGTGTGGGTGACGGTG; >clamp_s_c_s_a_s:compl:rev
CACAGACACCCACACTATCATGTTGACATTCC; dG = -11.5
505 >s_d_s_b_s_d_s_c_s_d_s GTGACGGTGGCTGTGACGGTGTGGGTGACGGTG; >clamp_s_c_s_a_s:compl:rev
CACAGACACCCACACTATCATGTTGACATTCC; dG = -11.5
506 >s_d_s_b_s_e_s_c_s_d_s GTGACGGTGGCTGTGCTGGTGTGGGTGACGGTG; >clamp_s_c_s_a_s:compl:rev
CACAGACACCCACACTATCATGTTGACATTCC; dG = -11.5
507 >s_d_s_c_s_d_s_c_s_e_s GTGACGGTGTGGGTGACGGTGTGGGTGCTGGTG; >clamp_s_c_s_a_s:compl:rev
CACAGACACCCACACTATCATGTTGACATTCC; dG = -11.5

508 >s_d_s_c_s_d_s_c_s_d_s GTGACGGTGTGGGTGACGGTGTGGGTGACGGTG; >clamp_s_c_s_a_s:compl:rev CACAGACACCCACACTATCATGTTGACATTCC; dG = -11.5
509 >s_d_s_c_s_d_s_e_s_a_s GTGACGGTGTGGGTGACGGTGCTGGTGTCTGTG; >clamp_s_c_s_a_s:compl:rev CACAGACACCCACACTATCATGTTGACATTCC; dG = -11.5
510 >s_d_s_c_s_d_s_d_s_a_s GTGACGGTGTGGGTGACGGTGACGGTGTCTGTG; >clamp_s_c_s_a_s:compl:rev CACAGACACCCACACTATCATGTTGACATTCC; dG = -11.5
511 >s_d_s_c_s_d_s_b_s_a_s GTGACGGTGTGGGTGACGGTGGCTGTGTCTGTG; >clamp_s_c_s_a_s:compl:rev CACAGACACCCACACTATCATGTTGACATTCC; dG = -11.5
512 >s_d_s_c_s_e_s_c_s_d_s GTGACGGTGTGGGTGCTGGTGTGGGTGACGGTG; >clamp_s_c_s_a_s:compl:rev CACAGACACCCACACTATCATGTTGACATTCC; dG = -11.5
513 >s_a_s_b_s_d_s_c_s_d_s GTGTCTGTGGCTGTGACGGTGTGGGTGACGGTG; >clamp_s_c_s_a_s:compl:rev CACAGACACCCACACTATCATGTTGACATTCC; dG = -11.5
514 >s_d_s_d_s_d_s_c_s_d_s GTGACGGTGACGGTGACGGTGTGGGTGACGGTG; >clamp_s_c_s_a_s:compl:rev CACAGACACCCACACTATCATGTTGACATTCC; dG = -11.5
515 >s_d_s_d_s_e_s_c_s_d_s GTGACGGTGACGGTGCTGGTGTGGGTGACGGTG; >clamp_s_c_s_a_s:compl:rev CACAGACACCCACACTATCATGTTGACATTCC; dG = -11.5
516 >s_d_s_e_s_d_s_c_s_d_s GTGACGGTGCTGGTGACGGTGTGGGTGACGGTG; >clamp_s_c_s_a_s:compl:rev CACAGACACCCACACTATCATGTTGACATTCC; dG = -11.5
517 >s_d_s_e_s_e_s_c_s_d_s GTGACGGTGCTGGTGCTGGTGTGGGTGACGGTG; >clamp_s_c_s_a_s:compl:rev CACAGACACCCACACTATCATGTTGACATTCC; dG = -11.5
518 >s_a_s_b_s_e_s_c_s_d_s GTGTCTGTGGCTGTGCTGGTGTGGGTGACGGTG; >clamp_s_c_s_a_s:compl:rev CACAGACACCCACACTATCATGTTGACATTCC; dG = -11.5
519 >s_e_s_a_s_d_s_c_s_d_s GTGCTGGTGTCTGTGACGGTGTGGGTGACGGTG; >clamp_s_c_s_a_s:compl:rev CACAGACACCCACACTATCATGTTGACATTCC; dG = -11.5
520 >s_e_s_a_s_e_s_c_s_d_s GTGCTGGTGTCTGTGCTGGTGTGGGTGACGGTG; >clamp_s_c_s_a_s:compl:rev CACAGACACCCACACTATCATGTTGACATTCC; dG = -11.5
521 >s_e_s_b_s_d_s_c_s_d_s GTGCTGGTGGCTGTGACGGTGTGGGTGACGGTG; >clamp_s_c_s_a_s:compl:rev CACAGACACCCACACTATCATGTTGACATTCC; dG = -11.5
522 >s_e_s_b_s_e_s_c_s_d_s GTGCTGGTGGCTGTGCTGGTGTGGGTGACGGTG; >clamp_s_c_s_a_s:compl:rev CACAGACACCCACACTATCATGTTGACATTCC; dG = -11.5
523 >s_e_s_c_s_d_s_c_s_e_s GTGCTGGTGTGGGTGACGGTGTGGGTGCTGGTG; >clamp_s_c_s_a_s:compl:rev CACAGACACCCACACTATCATGTTGACATTCC; dG = -11.5
524 >s_e_s_c_s_d_s_c_s_d_s GTGCTGGTGTGGGTGACGGTGTGGGTGACGGTG; >clamp_s_c_s_a_s:compl:rev CACAGACACCCACACTATCATGTTGACATTCC; dG = -11.5
525 >s_e_s_c_s_d_s_e_s_a_s GTGCTGGTGTGGGTGACGGTGCTGGTGTCTGTG; >clamp_s_c_s_a_s:compl:rev CACAGACACCCACACTATCATGTTGACATTCC; dG = -11.5
526 >s_e_s_c_s_d_s_d_s_a_s GTGCTGGTGTGGGTGACGGTGACGGTGTCTGTG; >clamp_s_c_s_a_s:compl:rev CACAGACACCCACACTATCATGTTGACATTCC; dG = -11.5
527 >s_e_s_c_s_d_s_b_s_a_s GTGCTGGTGTGGGTGACGGTGGCTGTGTCTGTG; >clamp_s_c_s_a_s:compl:rev CACAGACACCCACACTATCATGTTGACATTCC; dG = -11.5
528 >s_e_s_c_s_e_s_c_s_d_s GTGCTGGTGTGGGTGCTGGTGTGGGTGACGGTG; >clamp_s_c_s_a_s:compl:rev CACAGACACCCACACTATCATGTTGACATTCC; dG = -11.5
529 >s_e_s_d_s_d_s_c_s_d_s GTGCTGGTGACGGTGACGGTGTGGGTGACGGTG; >clamp_s_c_s_a_s:compl:rev CACAGACACCCACACTATCATGTTGACATTCC; dG = -11.5
530 >s_e_s_d_s_e_s_c_s_d_s GTGCTGGTGACGGTGCTGGTGTGGGTGACGGTG; >clamp_s_c_s_a_s:compl:rev CACAGACACCCACACTATCATGTTGACATTCC; dG = -11.5
531 >s_e_s_e_s_d_s_c_s_d_s GTGCTGGTGCTGGTGACGGTGTGGGTGACGGTG; >clamp_s_c_s_a_s:compl:rev CACAGACACCCACACTATCATGTTGACATTCC; dG = -11.5
532 >s_e_s_e_s_e_s_c_s_d_s GTGCTGGTGCTGGTGCTGGTGTGGGTGACGGTG; >clamp_s_c_s_a_s:compl:rev CACAGACACCCACACTATCATGTTGACATTCC; dG = -11.5
533 >s_c_s_d_s_a_s_c_s_e_s GTGTGGGTGACGGTGTCTGTGTGGGTGCTGGTG; >clamp_s_c_s_a_s:compl:rev CACAGACACCCACACTATCATGTTGACATTCC; dG = -11.4
534 >s_c_s_d_s_a_s_a_s_a_s GTGTGGGTGACGGTGTCTGTGTCTGTGTCTGTG; >clamp_s_c_s_a_s:compl:rev CACAGACACCCACACTATCATGTTGACATTCC; dG = -11.4
535 >s_c_s_e_s_a_s_c_s_e_s GTGTGGGTGCTGGTGTCTGTGTGGGTGCTGGTG; >clamp_s_c_s_a_s:compl:rev CACAGACACCCACACTATCATGTTGACATTCC; dG = -11.4
536 >s_c_s_e_s_a_s_a_s_a_s GTGTGGGTGCTGGTGTCTGTGTCTGTGTCTGTG; >clamp_s_c_s_a_s:compl:rev CACAGACACCCACACTATCATGTTGACATTCC; dG = -11.4
537 >s_a_s_a_s_a_s_c_s_e_s GTGTCTGTGTCTGTGTCTGTGTGGGTGCTGGTG; >clamp_s_c_s_a_s:compl:rev CACAGACACCCACACTATCATGTTGACATTCC; dG = -11.3
538 >s_a_s_c_s_e_s_c_s_e_s GTGTCTGTGTGGGTGCTGGTGTGGGTGCTGGTG; >clamp_s_c_s_a_s:compl:rev CACAGACACCCACACTATCATGTTGACATTCC; dG = -11.3
539 >s_a_s_c_s_e_s_e_s_a_s GTGTCTGTGTGGGTGCTGGTGCTGGTGTCTGTG; >clamp_s_c_s_a_s:compl:rev CACAGACACCCACACTATCATGTTGACATTCC; dG = -11.3
540 >s_a_s_c_s_e_s_d_s_a_s GTGTCTGTGTGGGTGCTGGTGACGGTGTCTGTG; >clamp_s_c_s_a_s:compl:rev CACAGACACCCACACTATCATGTTGACATTCC; dG = -11.3
541 >s_a_s_c_s_e_s_b_s_a_s GTGTCTGTGTGGGTGCTGGTGGCTGTGTCTGTG; >clamp_s_c_s_a_s:compl:rev CACAGACACCCACACTATCATGTTGACATTCC; dG = -11.3
542 >s_a_s_d_s_a_s_c_s_e_s GTGTCTGTGACGGTGTCTGTGTGGGTGCTGGTG; >clamp_s_c_s_a_s:compl:rev CACAGACACCCACACTATCATGTTGACATTCC; dG = -11.3
543 >s_a_s_d_s_b_s_c_s_e_s GTGTCTGTGACGGTGGCTGTGTGGGTGCTGGTG; >clamp_s_c_s_a_s:compl:rev CACAGACACCCACACTATCATGTTGACATTCC; dG = -11.3
544 >s_a_s_e_s_a_s_c_s_e_s GTGTCTGTGCTGGTGTCTGTGTGGGTGCTGGTG; >clamp_s_c_s_a_s:compl:rev CACAGACACCCACACTATCATGTTGACATTCC; dG = -11.3

545 >s_a_s_e_s_b_s_c_s_e_s GTGTCTGTGCTGGTGGCTGTGTGGGTGCTGGTG; >clamp_s_c_s_a_s:compl:rev
CACAGACACCCACACTATCATGTTGACATTCC; dG = -11.3
546 >s_b_s_a_s_a_s_c_s_e_s GTGGCTGTGTCTGTGTCTGTGTGGGTGCTGGTG; >clamp_s_c_s_a_s:compl:rev
CACAGACACCCACACTATCATGTTGACATTCC; dG = -11.3
547 >s_b_s_a_s_b_s_c_s_e_s GTGGCTGTGTCTGTGGCTGTGTGGGTGCTGGTG; >clamp_s_c_s_a_s:compl:rev
CACAGACACCCACACTATCATGTTGACATTCC; dG = -11.3
548 >s_b_s_b_s_a_s_c_s_e_s GTGGCTGTGGCTGTGTCTGTGTGGGTGCTGGTG; >clamp_s_c_s_a_s:compl:rev
CACAGACACCCACACTATCATGTTGACATTCC; dG = -11.3
549 >s_b_s_b_s_b_s_c_s_e_s GTGGCTGTGGCTGTGGCTGTGTGGGTGCTGGTG; >clamp_s_c_s_a_s:compl:rev
CACAGACACCCACACTATCATGTTGACATTCC; dG = -11.3
550 >s_b_s_c_s_e_s_c_s_e_s GTGGCTGTGTGGGTGCTGGTGTGGGTGCTGGTG; >clamp_s_c_s_a_s:compl:rev
CACAGACACCCACACTATCATGTTGACATTCC; dG = -11.3
551 >s_b_s_c_s_e_s_e_s_a_s GTGGCTGTGTGGGTGCTGGTGCTGGTGTCTGTG; >clamp_s_c_s_a_s:compl:rev
CACAGACACCCACACTATCATGTTGACATTCC; dG = -11.3
552 >s_b_s_c_s_e_s_d_s_a_s GTGGCTGTGTGGGTGCTGGTGACGGTGTCTGTG; >clamp_s_c_s_a_s:compl:rev
CACAGACACCCACACTATCATGTTGACATTCC; dG = -11.3
553 >s_b_s_c_s_e_s_b_s_a_s GTGGCTGTGTGGGTGCTGGTGGCTGTGTCTGTG; >clamp_s_c_s_a_s:compl:rev
CACAGACACCCACACTATCATGTTGACATTCC; dG = -11.3
554 >s_b_s_d_s_a_s_c_s_e_s GTGGCTGTGACGGTGTCTGTGTGGGTGCTGGTG; >clamp_s_c_s_a_s:compl:rev
CACAGACACCCACACTATCATGTTGACATTCC; dG = -11.3
555 >s_b_s_d_s_b_s_c_s_e_s GTGGCTGTGACGGTGGCTGTGTGGGTGCTGGTG; >clamp_s_c_s_a_s:compl:rev
CACAGACACCCACACTATCATGTTGACATTCC; dG = -11.3
556 >s_b_s_e_s_a_s_c_s_e_s GTGGCTGTGCTGGTGTCTGTGTGGGTGCTGGTG; >clamp_s_c_s_a_s:compl:rev
CACAGACACCCACACTATCATGTTGACATTCC; dG = -11.3
557 >s_b_s_e_s_b_s_c_s_e_s GTGGCTGTGCTGGTGGCTGTGTGGGTGCTGGTG; >clamp_s_c_s_a_s:compl:rev
CACAGACACCCACACTATCATGTTGACATTCC; dG = -11.3
558 >s_a_s_b_s_a_s_c_s_e_s GTGTCTGTGGCTGTGTCTGTGTGGGTGCTGGTG; >clamp_s_c_s_a_s:compl:rev
CACAGACACCCACACTATCATGTTGACATTCC; dG = -11.3
559 >s_c_s_d_s_a_s_e_s_a_s GTGTGGGTGACGGTGTCTGTGCTGGTGTCTGTG; >clamp_s_c_s_a_s:compl:rev
CACAGACACCCACACTATCATGTTGACATTCC; dG = -11.3
560 >s_c_s_d_s_b_s_c_s_e_s GTGTGGGTGACGGTGGCTGTGTGGGTGCTGGTG; >clamp_s_c_s_a_s:compl:rev
CACAGACACCCACACTATCATGTTGACATTCC; dG = -11.3
561 >s_a_s_b_s_b_s_c_s_e_s GTGTCTGTGGCTGTGGCTGTGTGGGTGCTGGTG; >clamp_s_c_s_a_s:compl:rev
CACAGACACCCACACTATCATGTTGACATTCC; dG = -11.3
562 >s_c_s_e_s_a_s_e_s_a_s GTGTGGGTGCTGGTGTCTGTGCTGGTGTCTGTG; >clamp_s_c_s_a_s:compl:rev
CACAGACACCCACACTATCATGTTGACATTCC; dG = -11.3
563 >s_c_s_e_s_b_s_c_s_e_s GTGTGGGTGCTGGTGGCTGTGTGGGTGCTGGTG; >clamp_s_c_s_a_s:compl:rev
CACAGACACCCACACTATCATGTTGACATTCC; dG = -11.3
564 >s_d_s_a_s_a_s_c_s_e_s GTGACGGTGTCTGTGTCTGTGTGGGTGCTGGTG; >clamp_s_c_s_a_s:compl:rev
CACAGACACCCACACTATCATGTTGACATTCC; dG = -11.3
565 >s_d_s_a_s_b_s_c_s_e_s GTGACGGTGTCTGTGGCTGTGTGGGTGCTGGTG; >clamp_s_c_s_a_s:compl:rev
CACAGACACCCACACTATCATGTTGACATTCC; dG = -11.3
566 >s_d_s_b_s_a_s_c_s_e_s GTGACGGTGGCTGTGTCTGTGTGGGTGCTGGTG; >clamp_s_c_s_a_s:compl:rev
CACAGACACCCACACTATCATGTTGACATTCC; dG = -11.3
567 >s_d_s_b_s_b_s_c_s_e_s GTGACGGTGGCTGTGGCTGTGTGGGTGCTGGTG; >clamp_s_c_s_a_s:compl:rev
CACAGACACCCACACTATCATGTTGACATTCC; dG = -11.3
568 >s_d_s_d_s_a_s_c_s_e_s GTGACGGTGACGGTGTCTGTGTGGGTGCTGGTG; >clamp_s_c_s_a_s:compl:rev
CACAGACACCCACACTATCATGTTGACATTCC; dG = -11.3
569 >s_d_s_d_s_b_s_c_s_e_s GTGACGGTGACGGTGGCTGTGTGGGTGCTGGTG; >clamp_s_c_s_a_s:compl:rev
CACAGACACCCACACTATCATGTTGACATTCC; dG = -11.3
570 >s_d_s_e_s_a_s_c_s_e_s GTGACGGTGCTGGTGTCTGTGTGGGTGCTGGTG; >clamp_s_c_s_a_s:compl:rev
CACAGACACCCACACTATCATGTTGACATTCC; dG = -11.3
571 >s_d_s_e_s_b_s_c_s_e_s GTGACGGTGCTGGTGGCTGTGTGGGTGCTGGTG; >clamp_s_c_s_a_s:compl:rev
CACAGACACCCACACTATCATGTTGACATTCC; dG = -11.3
572 >s_e_s_a_s_a_s_c_s_e_s GTGCTGGTGTCTGTGTCTGTGTGGGTGCTGGTG; >clamp_s_c_s_a_s:compl:rev
CACAGACACCCACACTATCATGTTGACATTCC; dG = -11.3
573 >s_e_s_a_s_b_s_c_s_e_s GTGCTGGTGTCTGTGGCTGTGTGGGTGCTGGTG; >clamp_s_c_s_a_s:compl:rev
CACAGACACCCACACTATCATGTTGACATTCC; dG = -11.3
574 >s_e_s_b_s_a_s_c_s_e_s GTGCTGGTGGCTGTGTCTGTGTGGGTGCTGGTG; >clamp_s_c_s_a_s:compl:rev
CACAGACACCCACACTATCATGTTGACATTCC; dG = -11.3
575 >s_e_s_b_s_b_s_c_s_e_s GTGCTGGTGGCTGTGGCTGTGTGGGTGCTGGTG; >clamp_s_c_s_a_s:compl:rev
CACAGACACCCACACTATCATGTTGACATTCC; dG = -11.3
576 >s_e_s_d_s_a_s_c_s_e_s GTGCTGGTGACGGTGTCTGTGTGGGTGCTGGTG; >clamp_s_c_s_a_s:compl:rev
CACAGACACCCACACTATCATGTTGACATTCC; dG = -11.3
577 >s_e_s_d_s_b_s_c_s_e_s GTGCTGGTGACGGTGGCTGTGTGGGTGCTGGTG; >clamp_s_c_s_a_s:compl:rev
CACAGACACCCACACTATCATGTTGACATTCC; dG = -11.3
578 >s_e_s_e_s_a_s_c_s_e_s GTGCTGGTGCTGGTGTCTGTGTGGGTGCTGGTG; >clamp_s_c_s_a_s:compl:rev
CACAGACACCCACACTATCATGTTGACATTCC; dG = -11.3
579 >s_e_s_e_s_b_s_c_s_e_s GTGCTGGTGCTGGTGGCTGTGTGGGTGCTGGTG; >clamp_s_c_s_a_s:compl:rev
CACAGACACCCACACTATCATGTTGACATTCC; dG = -11.3
580 >s_a_s_a_s_b_s_c_s_e_s GTGTCTGTGTCTGTGGCTGTGTGGGTGCTGGTG; >clamp_s_c_s_a_s:compl:rev
CACAGACACCCACACTATCATGTTGACATTCC; dG = -11.3
581 >s_a_s_d_s_a_s_b_s_a_s GTGTCTGTGACGGTGTCTGTGGCTGTGTCTGTG; >clamp_s_c_s_a_s:compl:rev
CACAGACACCCACACTATCATGTTGACATTCC; dG = -11.2

582 >s_a_s_d_s_d_s_c_s_e_s GTGTCTGTGACGGTGACGGTGTGGGTGCTGGTG; >clamp_s_c_s_a_s:compl:rev CACAGACACCCACACTATCATGTTGACATTCC; dG = -11.2
583 >s_a_s_d_s_e_s_c_s_e_s GTGTCTGTGACGGTGCTGGTGTGGGTGCTGGTG; >clamp_s_c_s_a_s:compl:rev CACAGACACCCACACTATCATGTTGACATTCC; dG = -11.2
584 >s_a_s_e_s_d_s_c_s_e_s GTGTCTGTGCTGGTGACGGTGTGGGTGCTGGTG; >clamp_s_c_s_a_s:compl:rev CACAGACACCCACACTATCATGTTGACATTCC; dG = -11.2
585 >s_a_s_e_s_e_s_c_s_e_s GTGTCTGTGCTGGTGCTGGTGTGGGTGCTGGTG; >clamp_s_c_s_a_s:compl:rev CACAGACACCCACACTATCATGTTGACATTCC; dG = -11.2
586 >s_b_s_a_s_d_s_c_s_e_s GTGGCTGTGTCTGTGACGGTGTGGGTGCTGGTG; >clamp_s_c_s_a_s:compl:rev CACAGACACCCACACTATCATGTTGACATTCC; dG = -11.2
587 >s_b_s_a_s_e_s_c_s_e_s GTGGCTGTGTCTGTGCTGGTGTGGGTGCTGGTG; >clamp_s_c_s_a_s:compl:rev CACAGACACCCACACTATCATGTTGACATTCC; dG = -11.2
588 >s_a_s_a_s_d_s_c_s_e_s GTGTCTGTGTCTGTGACGGTGTGGGTGCTGGTG; >clamp_s_c_s_a_s:compl:rev CACAGACACCCACACTATCATGTTGACATTCC; dG = -11.2
589 >s_b_s_b_s_d_s_c_s_e_s GTGGCTGTGGCTGTGACGGTGTGGGTGCTGGTG; >clamp_s_c_s_a_s:compl:rev CACAGACACCCACACTATCATGTTGACATTCC; dG = -11.2
590 >s_b_s_b_s_e_s_c_s_e_s GTGGCTGTGGCTGTGCTGGTGTGGGTGCTGGTG; >clamp_s_c_s_a_s:compl:rev CACAGACACCCACACTATCATGTTGACATTCC; dG = -11.2
591 >s_b_s_d_s_a_s_b_s_a_s GTGGCTGTGACGGTGTCTGTGGCTGTGTCTGTG; >clamp_s_c_s_a_s:compl:rev CACAGACACCCACACTATCATGTTGACATTCC; dG = -11.2
592 >s_a_s_a_s_e_s_c_s_e_s GTGTCTGTGTCTGTGCTGGTGTGGGTGCTGGTG; >clamp_s_c_s_a_s:compl:rev CACAGACACCCACACTATCATGTTGACATTCC; dG = -11.2
593 >s_b_s_d_s_d_s_c_s_e_s GTGGCTGTGACGGTGACGGTGTGGGTGCTGGTG; >clamp_s_c_s_a_s:compl:rev CACAGACACCCACACTATCATGTTGACATTCC; dG = -11.2
594 >s_b_s_d_s_e_s_c_s_e_s GTGGCTGTGACGGTGCTGGTGTGGGTGCTGGTG; >clamp_s_c_s_a_s:compl:rev CACAGACACCCACACTATCATGTTGACATTCC; dG = -11.2
595 >s_b_s_e_s_d_s_c_s_e_s GTGGCTGTGCTGGTGACGGTGTGGGTGCTGGTG; >clamp_s_c_s_a_s:compl:rev CACAGACACCCACACTATCATGTTGACATTCC; dG = -11.2
596 >s_b_s_e_s_e_s_c_s_e_s GTGGCTGTGCTGGTGCTGGTGTGGGTGCTGGTG; >clamp_s_c_s_a_s:compl:rev CACAGACACCCACACTATCATGTTGACATTCC; dG = -11.2
597 >s_c_s_d_s_d_s_c_s_e_s GTGTGGGTGACGGTGACGGTGTGGGTGCTGGTG; >clamp_s_c_s_a_s:compl:rev CACAGACACCCACACTATCATGTTGACATTCC; dG = -11.2
598 >s_c_s_d_s_e_s_c_s_e_s GTGTGGGTGACGGTGCTGGTGTGGGTGCTGGTG; >clamp_s_c_s_a_s:compl:rev CACAGACACCCACACTATCATGTTGACATTCC; dG = -11.2
599 >s_c_s_e_s_d_s_c_s_e_s GTGTGGGTGCTGGTGACGGTGTGGGTGCTGGTG; >clamp_s_c_s_a_s:compl:rev CACAGACACCCACACTATCATGTTGACATTCC; dG = -11.2
600 >s_c_s_e_s_e_s_c_s_e_s GTGTGGGTGCTGGTGCTGGTGTGGGTGCTGGTG; >clamp_s_c_s_a_s:compl:rev CACAGACACCCACACTATCATGTTGACATTCC; dG = -11.2
601 >s_d_s_a_s_d_s_c_s_e_s GTGACGGTGTCTGTGACGGTGTGGGTGCTGGTG; >clamp_s_c_s_a_s:compl:rev CACAGACACCCACACTATCATGTTGACATTCC; dG = -11.2
602 >s_d_s_a_s_d_s_e_s_a_s GTGACGGTGTCTGTGACGGTGCTGGTGTCTGTG; >clamp_s_c_s_a_s:compl:rev CACAGACACCCACACTATCATGTTGACATTCC; dG = -11.2
603 >s_d_s_a_s_d_s_d_s_a_s GTGACGGTGTCTGTGACGGTGACGGTGTCTGTG; >clamp_s_c_s_a_s:compl:rev CACAGACACCCACACTATCATGTTGACATTCC; dG = -11.2
604 >s_d_s_a_s_d_s_b_s_a_s GTGACGGTGTCTGTGACGGTGGCTGTGTCTGTG; >clamp_s_c_s_a_s:compl:rev CACAGACACCCACACTATCATGTTGACATTCC; dG = -11.2
605 >s_d_s_a_s_d_s_a_s_a_s GTGACGGTGTCTGTGACGGTGTCTGTGTCTGTG; >clamp_s_c_s_a_s:compl:rev CACAGACACCCACACTATCATGTTGACATTCC; dG = -11.2
606 >s_d_s_a_s_e_s_c_s_e_s GTGACGGTGTCTGTGCTGGTGTGGGTGCTGGTG; >clamp_s_c_s_a_s:compl:rev CACAGACACCCACACTATCATGTTGACATTCC; dG = -11.2
607 >s_d_s_b_s_d_s_c_s_e_s GTGACGGTGGCTGTGACGGTGTGGGTGCTGGTG; >clamp_s_c_s_a_s:compl:rev CACAGACACCCACACTATCATGTTGACATTCC; dG = -11.2
608 >s_d_s_b_s_e_s_c_s_e_s GTGACGGTGGCTGTGCTGGTGTGGGTGCTGGTG; >clamp_s_c_s_a_s:compl:rev CACAGACACCCACACTATCATGTTGACATTCC; dG = -11.2
609 >s_d_s_c_s_e_s_c_s_e_s GTGACGGTGTGGGTGCTGGTGTGGGTGCTGGTG; >clamp_s_c_s_a_s:compl:rev CACAGACACCCACACTATCATGTTGACATTCC; dG = -11.2
610 >s_d_s_c_s_e_s_e_s_a_s GTGACGGTGTGGGTGCTGGTGCTGGTGTCTGTG; >clamp_s_c_s_a_s:compl:rev CACAGACACCCACACTATCATGTTGACATTCC; dG = -11.2
611 >s_d_s_c_s_e_s_d_s_a_s GTGACGGTGTGGGTGCTGGTGACGGTGTCTGTG; >clamp_s_c_s_a_s:compl:rev CACAGACACCCACACTATCATGTTGACATTCC; dG = -11.2
612 >s_a_s_b_s_d_s_c_s_e_s GTGTCTGTGGCTGTGACGGTGTGGGTGCTGGTG; >clamp_s_c_s_a_s:compl:rev CACAGACACCCACACTATCATGTTGACATTCC; dG = -11.2
613 >s_d_s_c_s_e_s_b_s_a_s GTGACGGTGTGGGTGCTGGTGGCTGTGTCTGTG; >clamp_s_c_s_a_s:compl:rev CACAGACACCCACACTATCATGTTGACATTCC; dG = -11.2
614 >s_d_s_d_s_a_s_b_s_a_s GTGACGGTGACGGTGTCTGTGGCTGTGTCTGTG; >clamp_s_c_s_a_s:compl:rev CACAGACACCCACACTATCATGTTGACATTCC; dG = -11.2
615 >s_d_s_d_s_d_s_c_s_e_s GTGACGGTGACGGTGACGGTGTGGGTGCTGGTG; >clamp_s_c_s_a_s:compl:rev CACAGACACCCACACTATCATGTTGACATTCC; dG = -11.2
616 >s_d_s_d_s_e_s_c_s_e_s GTGACGGTGACGGTGCTGGTGTGGGTGCTGGTG; >clamp_s_c_s_a_s:compl:rev CACAGACACCCACACTATCATGTTGACATTCC; dG = -11.2
617 >s_d_s_e_s_d_s_c_s_e_s GTGACGGTGCTGGTGACGGTGTGGGTGCTGGTG; >clamp_s_c_s_a_s:compl:rev CACAGACACCCACACTATCATGTTGACATTCC; dG = -11.2
618 >s_d_s_e_s_e_s_c_s_e_s GTGACGGTGCTGGTGCTGGTGTGGGTGCTGGTG; >clamp_s_c_s_a_s:compl:rev CACAGACACCCACACTATCATGTTGACATTCC; dG = -11.2

619 >s_a_s_b_s_e_s_c_s_e_s GTGTCTGTGGCTGTGCTGGTGTGGGTGCTGGTG; >clamp_s_c_s_a_s:compl:rev
CACAGACACCCACACTATCATGTTGACATTCC; dG = -11.2
620 >s_e_s_a_s_d_s_c_s_e_s GTGCTGGTGTCTGTGACGGTGTGGGTGCTGGTG; >clamp_s_c_s_a_s:compl:rev
CACAGACACCCACACTATCATGTTGACATTCC; dG = -11.2
621 >s_e_s_a_s_e_s_c_s_e_s GTGCTGGTGTCTGTGCTGGTGTGGGTGCTGGTG; >clamp_s_c_s_a_s:compl:rev
CACAGACACCCACACTATCATGTTGACATTCC; dG = -11.2
622 >s_e_s_b_s_d_s_c_s_e_s GTGCTGGTGGCTGTGACGGTGTGGGTGCTGGTG; >clamp_s_c_s_a_s:compl:rev
CACAGACACCCACACTATCATGTTGACATTCC; dG = -11.2
623 >s_e_s_b_s_e_s_c_s_e_s GTGCTGGTGGCTGTGCTGGTGTGGGTGCTGGTG; >clamp_s_c_s_a_s:compl:rev
CACAGACACCCACACTATCATGTTGACATTCC; dG = -11.2
624 >s_e_s_c_s_e_s_c_s_e_s GTGCTGGTGTGGGTGCTGGTGTGGGTGCTGGTG; >clamp_s_c_s_a_s:compl:rev
CACAGACACCCACACTATCATGTTGACATTCC; dG = -11.2
625 >s_e_s_c_s_e_s_e_s_a_s GTGCTGGTGTGGGTGCTGGTGCTGGTGTCTGTG; >clamp_s_c_s_a_s:compl:rev
CACAGACACCCACACTATCATGTTGACATTCC; dG = -11.2
626 >s_e_s_c_s_e_s_d_s_a_s GTGCTGGTGTGGGTGCTGGTGACGGTGTCTGTG; >clamp_s_c_s_a_s:compl:rev
CACAGACACCCACACTATCATGTTGACATTCC; dG = -11.2
627 >s_e_s_c_s_e_s_b_s_a_s GTGCTGGTGTGGGTGCTGGTGGCTGTGTCTGTG; >clamp_s_c_s_a_s:compl:rev
CACAGACACCCACACTATCATGTTGACATTCC; dG = -11.2
628 >s_e_s_d_s_a_s_b_s_a_s GTGCTGGTGACGGTGTCTGTGGCTGTGTCTGTG; >clamp_s_c_s_a_s:compl:rev
CACAGACACCCACACTATCATGTTGACATTCC; dG = -11.2
629 >s_e_s_d_s_d_s_c_s_e_s GTGCTGGTGACGGTGACGGTGTGGGTGCTGGTG; >clamp_s_c_s_a_s:compl:rev
CACAGACACCCACACTATCATGTTGACATTCC; dG = -11.2
630 >s_e_s_d_s_e_s_c_s_e_s GTGCTGGTGACGGTGCTGGTGTGGGTGCTGGTG; >clamp_s_c_s_a_s:compl:rev
CACAGACACCCACACTATCATGTTGACATTCC; dG = -11.2
631 >s_e_s_e_s_d_s_c_s_e_s GTGCTGGTGCTGGTGACGGTGTGGGTGCTGGTG; >clamp_s_c_s_a_s:compl:rev
CACAGACACCCACACTATCATGTTGACATTCC; dG = -11.2
632 >s_e_s_e_s_e_s_c_s_e_s GTGCTGGTGCTGGTGCTGGTGTGGGTGCTGGTG; >clamp_s_c_s_a_s:compl:rev
CACAGACACCCACACTATCATGTTGACATTCC; dG = -11.2
633 >s_a_s_d_s_a_s_a_s_a_s GTGTCTGTGACGGTGTCTGTGTCTGTGTCTGTG; >clamp_s_c_s_a_s:compl:rev
CACAGACACCCACACTATCATGTTGACATTCC; dG = -11.1
634 >s_a_s_d_s_d_s_a_s_a_s GTGTCTGTGACGGTGACGGTGTCTGTGTCTGTG; >clamp_s_c_s_a_s:compl:rev
CACAGACACCCACACTATCATGTTGACATTCC; dG = -11.1
635 >s_a_s_e_s_a_s_b_s_a_s GTGTCTGTGCTGGTGTCTGTGGCTGTGTCTGTG; >clamp_s_c_s_a_s:compl:rev
CACAGACACCCACACTATCATGTTGACATTCC; dG = -11.1
636 >s_a_s_e_s_d_s_a_s_a_s GTGTCTGTGCTGGTGACGGTGTCTGTGTCTGTG; >clamp_s_c_s_a_s:compl:rev
CACAGACACCCACACTATCATGTTGACATTCC; dG = -11.1
637 >s_b_s_a_s_d_s_a_s_a_s GTGGCTGTGTCTGTGACGGTGTCTGTGTCTGTG; >clamp_s_c_s_a_s:compl:rev
CACAGACACCCACACTATCATGTTGACATTCC; dG = -11.1
638 >s_b_s_b_s_d_s_a_s_a_s GTGGCTGTGGCTGTGACGGTGTCTGTGTCTGTG; >clamp_s_c_s_a_s:compl:rev
CACAGACACCCACACTATCATGTTGACATTCC; dG = -11.1
639 >s_b_s_d_s_a_s_a_s_a_s GTGGCTGTGACGGTGTCTGTGTCTGTGTCTGTG; >clamp_s_c_s_a_s:compl:rev
CACAGACACCCACACTATCATGTTGACATTCC; dG = -11.1
640 >s_a_s_a_s_d_s_a_s_a_s GTGTCTGTGTCTGTGACGGTGTCTGTGTCTGTG; >clamp_s_c_s_a_s:compl:rev
CACAGACACCCACACTATCATGTTGACATTCC; dG = -11.1
641 >s_b_s_d_s_d_s_a_s_a_s GTGGCTGTGACGGTGACGGTGTCTGTGTCTGTG; >clamp_s_c_s_a_s:compl:rev
CACAGACACCCACACTATCATGTTGACATTCC; dG = -11.1
642 >s_b_s_e_s_a_s_b_s_a_s GTGGCTGTGCTGGTGTCTGTGGCTGTGTCTGTG; >clamp_s_c_s_a_s:compl:rev
CACAGACACCCACACTATCATGTTGACATTCC; dG = -11.1
643 >s_b_s_e_s_d_s_a_s_a_s GTGGCTGTGCTGGTGACGGTGTCTGTGTCTGTG; >clamp_s_c_s_a_s:compl:rev
CACAGACACCCACACTATCATGTTGACATTCC; dG = -11.1
644 >s_c_s_d_s_d_s_a_s_a_s GTGTGGGTGACGGTGACGGTGTCTGTGTCTGTG; >clamp_s_c_s_a_s:compl:rev
CACAGACACCCACACTATCATGTTGACATTCC; dG = -11.1
645 >s_c_s_e_s_d_s_a_s_a_s GTGTGGGTGCTGGTGACGGTGTCTGTGTCTGTG; >clamp_s_c_s_a_s:compl:rev
CACAGACACCCACACTATCATGTTGACATTCC; dG = -11.1
646 >s_d_s_b_s_d_s_a_s_a_s GTGACGGTGGCTGTGACGGTGTCTGTGTCTGTG; >clamp_s_c_s_a_s:compl:rev
CACAGACACCCACACTATCATGTTGACATTCC; dG = -11.1
647 >s_d_s_d_s_a_s_a_s_a_s GTGACGGTGACGGTGTCTGTGTCTGTGTCTGTG; >clamp_s_c_s_a_s:compl:rev
CACAGACACCCACACTATCATGTTGACATTCC; dG = -11.1
648 >s_d_s_d_s_d_s_a_s_a_s GTGACGGTGACGGTGACGGTGTCTGTGTCTGTG; >clamp_s_c_s_a_s:compl:rev
CACAGACACCCACACTATCATGTTGACATTCC; dG = -11.1
649 >s_d_s_e_s_a_s_b_s_a_s GTGACGGTGCTGGTGTCTGTGGCTGTGTCTGTG; >clamp_s_c_s_a_s:compl:rev
CACAGACACCCACACTATCATGTTGACATTCC; dG = -11.1
650 >s_a_s_b_s_d_s_a_s_a_s GTGTCTGTGGCTGTGACGGTGTCTGTGTCTGTG; >clamp_s_c_s_a_s:compl:rev
CACAGACACCCACACTATCATGTTGACATTCC; dG = -11.1
651 >s_d_s_e_s_d_s_a_s_a_s GTGACGGTGCTGGTGACGGTGTCTGTGTCTGTG; >clamp_s_c_s_a_s:compl:rev
CACAGACACCCACACTATCATGTTGACATTCC; dG = -11.1
652 >s_e_s_a_s_d_s_e_s_a_s GTGCTGGTGTCTGTGACGGTGCTGGTGTCTGTG; >clamp_s_c_s_a_s:compl:rev
CACAGACACCCACACTATCATGTTGACATTCC; dG = -11.1
653 >s_e_s_a_s_d_s_d_s_a_s GTGCTGGTGTCTGTGACGGTGACGGTGTCTGTG; >clamp_s_c_s_a_s:compl:rev
CACAGACACCCACACTATCATGTTGACATTCC; dG = -11.1
654 >s_e_s_a_s_d_s_b_s_a_s GTGCTGGTGTCTGTGACGGTGGCTGTGTCTGTG; >clamp_s_c_s_a_s:compl:rev
CACAGACACCCACACTATCATGTTGACATTCC; dG = -11.1
655 >s_e_s_a_s_d_s_a_s_a_s GTGCTGGTGTCTGTGACGGTGTCTGTGTCTGTG; >clamp_s_c_s_a_s:compl:rev
CACAGACACCCACACTATCATGTTGACATTCC; dG = -11.1

656 >s_e_s_b_s_d_s_a_s_a_s GTGCTGGTGGCTGTGACGGTGTCTGTGTCTGTG; >clamp_s_c_s_a_s:compl:rev CACAGACACCCACACTATCATGTTGACATTCC; dG = -11.1
657 >s_e_s_d_s_a_s_a_s_a_s GTGCTGGTGACGGTGTCTGTGTCTGTGTCTGTG; >clamp_s_c_s_a_s:compl:rev CACAGACACCCACACTATCATGTTGACATTCC; dG = -11.1
658 >s_e_s_d_s_d_s_a_s_a_s GTGCTGGTGACGGTGACGGTGTCTGTGTCTGTG; >clamp_s_c_s_a_s:compl:rev CACAGACACCCACACTATCATGTTGACATTCC; dG = -11.1
659 >s_e_s_e_s_a_s_b_s_a_s GTGCTGGTGCTGGTGTCTGTGGCTGTGTCTGTG; >clamp_s_c_s_a_s:compl:rev CACAGACACCCACACTATCATGTTGACATTCC; dG = -11.1
660 >s_e_s_e_s_d_s_a_s_a_s GTGCTGGTGCTGGTGACGGTGTCTGTGTCTGTG; >clamp_s_c_s_a_s:compl:rev CACAGACACCCACACTATCATGTTGACATTCC; dG = -11.1
661 >s_a_s_d_s_a_s_e_s_a_s GTGTCTGTGACGGTGTCTGTGCTGGTGTCTGTG; >clamp_s_c_s_a_s:compl:rev CACAGACACCCACACTATCATGTTGACATTCC; dG = -11.0
662 >s_a_s_d_s_e_s_a_s_a_s GTGTCTGTGACGGTGCTGGTGTCTGTGTCTGTG; >clamp_s_c_s_a_s:compl:rev CACAGACACCCACACTATCATGTTGACATTCC; dG = -11.0
663 >s_a_s_e_s_a_s_a_s_a_s GTGTCTGTGCTGGTGTCTGTGTCTGTGTCTGTG; >clamp_s_c_s_a_s:compl:rev CACAGACACCCACACTATCATGTTGACATTCC; dG = -11.0
664 >s_a_s_e_s_e_s_a_s_a_s GTGTCTGTGCTGGTGCTGGTGTCTGTGTCTGTG; >clamp_s_c_s_a_s:compl:rev CACAGACACCCACACTATCATGTTGACATTCC; dG = -11.0
665 >s_b_s_a_s_e_s_a_s_a_s GTGGCTGTGTCTGTGCTGGTGTCTGTGTCTGTG; >clamp_s_c_s_a_s:compl:rev CACAGACACCCACACTATCATGTTGACATTCC; dG = -11.0
666 >s_b_s_b_s_e_s_a_s_a_s GTGGCTGTGGCTGTGCTGGTGTCTGTGTCTGTG; >clamp_s_c_s_a_s:compl:rev CACAGACACCCACACTATCATGTTGACATTCC; dG = -11.0
667 >s_b_s_d_s_a_s_e_s_a_s GTGGCTGTGACGGTGTCTGTGCTGGTGTCTGTG; >clamp_s_c_s_a_s:compl:rev CACAGACACCCACACTATCATGTTGACATTCC; dG = -11.0
668 >s_b_s_d_s_e_s_a_s_a_s GTGGCTGTGACGGTGCTGGTGTCTGTGTCTGTG; >clamp_s_c_s_a_s:compl:rev CACAGACACCCACACTATCATGTTGACATTCC; dG = -11.0
669 >s_b_s_e_s_a_s_a_s_a_s GTGGCTGTGCTGGTGTCTGTGTCTGTGTCTGTG; >clamp_s_c_s_a_s:compl:rev CACAGACACCCACACTATCATGTTGACATTCC; dG = -11.0
670 >s_b_s_e_s_e_s_a_s_a_s GTGGCTGTGCTGGTGCTGGTGTCTGTGTCTGTG; >clamp_s_c_s_a_s:compl:rev CACAGACACCCACACTATCATGTTGACATTCC; dG = -11.0
671 >s_a_s_a_s_e_s_a_s_a_s GTGTCTGTGTCTGTGCTGGTGTCTGTGTCTGTG; >clamp_s_c_s_a_s:compl:rev CACAGACACCCACACTATCATGTTGACATTCC; dG = -11.0
672 >s_c_s_d_s_b_s_e_s_a_s GTGTGGGTGACGGTGGCTGTGCTGGTGTCTGTG; >clamp_s_c_s_a_s:compl:rev CACAGACACCCACACTATCATGTTGACATTCC; dG = -11.0
673 >s_c_s_d_s_b_s_d_s_a_s GTGTGGGTGACGGTGGCTGTGACGGTGTCTGTG; >clamp_s_c_s_a_s:compl:rev CACAGACACCCACACTATCATGTTGACATTCC; dG = -11.0
674 >s_c_s_d_s_b_s_b_s_a_s GTGTGGGTGACGGTGGCTGTGGCTGTGTCTGTG; >clamp_s_c_s_a_s:compl:rev CACAGACACCCACACTATCATGTTGACATTCC; dG = -11.0
675 >s_c_s_d_s_b_s_a_s_a_s GTGTGGGTGACGGTGGCTGTGTCTGTGTCTGTG; >clamp_s_c_s_a_s:compl:rev CACAGACACCCACACTATCATGTTGACATTCC; dG = -11.0
676 >s_c_s_d_s_d_s_e_s_a_s GTGTGGGTGACGGTGACGGTGCTGGTGTCTGTG; >clamp_s_c_s_a_s:compl:rev CACAGACACCCACACTATCATGTTGACATTCC; dG = -11.0
677 >s_c_s_d_s_d_s_d_s_a_s GTGTGGGTGACGGTGACGGTGACGGTGTCTGTG; >clamp_s_c_s_a_s:compl:rev CACAGACACCCACACTATCATGTTGACATTCC; dG = -11.0
678 >s_c_s_d_s_d_s_b_s_a_s GTGTGGGTGACGGTGACGGTGGCTGTGTCTGTG; >clamp_s_c_s_a_s:compl:rev CACAGACACCCACACTATCATGTTGACATTCC; dG = -11.0
679 >s_c_s_d_s_e_s_e_s_a_s GTGTGGGTGACGGTGCTGGTGCTGGTGTCTGTG; >clamp_s_c_s_a_s:compl:rev CACAGACACCCACACTATCATGTTGACATTCC; dG = -11.0
680 >s_c_s_d_s_e_s_d_s_a_s GTGTGGGTGACGGTGCTGGTGACGGTGTCTGTG; >clamp_s_c_s_a_s:compl:rev CACAGACACCCACACTATCATGTTGACATTCC; dG = -11.0
681 >s_c_s_d_s_e_s_b_s_a_s GTGTGGGTGACGGTGCTGGTGGCTGTGTCTGTG; >clamp_s_c_s_a_s:compl:rev CACAGACACCCACACTATCATGTTGACATTCC; dG = -11.0
682 >s_c_s_d_s_e_s_a_s_a_s GTGTGGGTGACGGTGCTGGTGTCTGTGTCTGTG; >clamp_s_c_s_a_s:compl:rev CACAGACACCCACACTATCATGTTGACATTCC; dG = -11.0
683 >s_c_s_e_s_e_s_a_s_a_s GTGTGGGTGCTGGTGCTGGTGTCTGTGTCTGTG; >clamp_s_c_s_a_s:compl:rev CACAGACACCCACACTATCATGTTGACATTCC; dG = -11.0
684 >s_d_s_a_s_e_s_a_s_a_s GTGACGGTGTCTGTGCTGGTGTCTGTGTCTGTG; >clamp_s_c_s_a_s:compl:rev CACAGACACCCACACTATCATGTTGACATTCC; dG = -11.0
685 >s_d_s_b_s_e_s_a_s_a_s GTGACGGTGGCTGTGCTGGTGTCTGTGTCTGTG; >clamp_s_c_s_a_s:compl:rev CACAGACACCCACACTATCATGTTGACATTCC; dG = -11.0
686 >s_d_s_d_s_a_s_e_s_a_s GTGACGGTGACGGTGTCTGTGCTGGTGTCTGTG; >clamp_s_c_s_a_s:compl:rev CACAGACACCCACACTATCATGTTGACATTCC; dG = -11.0
687 >s_d_s_d_s_e_s_a_s_a_s GTGACGGTGACGGTGCTGGTGTCTGTGTCTGTG; >clamp_s_c_s_a_s:compl:rev CACAGACACCCACACTATCATGTTGACATTCC; dG = -11.0
688 >s_d_s_e_s_a_s_a_s_a_s GTGACGGTGCTGGTGTCTGTGTCTGTGTCTGTG; >clamp_s_c_s_a_s:compl:rev CACAGACACCCACACTATCATGTTGACATTCC; dG = -11.0
689 >s_d_s_e_s_e_s_a_s_a_s GTGACGGTGCTGGTGCTGGTGTCTGTGTCTGTG; >clamp_s_c_s_a_s:compl:rev CACAGACACCCACACTATCATGTTGACATTCC; dG = -11.0
690 >s_e_s_a_s_e_s_a_s_a_s GTGCTGGTGTCTGTGCTGGTGTCTGTGTCTGTG; >clamp_s_c_s_a_s:compl:rev CACAGACACCCACACTATCATGTTGACATTCC; dG = -11.0
691 >s_a_s_b_s_e_s_a_s_a_s GTGTCTGTGGCTGTGCTGGTGTCTGTGTCTGTG; >clamp_s_c_s_a_s:compl:rev CACAGACACCCACACTATCATGTTGACATTCC; dG = -11.0
692 >s_e_s_b_s_e_s_a_s_a_s GTGCTGGTGGCTGTGCTGGTGTCTGTGTCTGTG; >clamp_s_c_s_a_s:compl:rev CACAGACACCCACACTATCATGTTGACATTCC; dG = -11.0

693 >s_e_s_d_s_a_s_e_s_a_s GTGCTGGTGACGGTGTCTGTGCTGGTGTCTGTG; >clamp_s_c_s_a_s:compl:rev CACAGACACCCACACTATCATGTTGACATTCC; dG = -11.0
694 >s_e_s_d_s_e_s_a_s_a_s GTGCTGGTGACGGTGCTGGTGTCTGTGTCTGTG; >clamp_s_c_s_a_s:compl:rev CACAGACACCCACACTATCATGTTGACATTCC; dG = -11.0
695 >s_e_s_e_s_a_s_a_s_a_s GTGCTGGTGCTGGTGTCTGTGTCTGTGTCTGTG; >clamp_s_c_s_a_s:compl:rev CACAGACACCCACACTATCATGTTGACATTCC; dG = -11.0
696 >s_e_s_e_s_e_s_a_s_a_s GTGCTGGTGCTGGTGCTGGTGTCTGTGTCTGTG; >clamp_s_c_s_a_s:compl:rev CACAGACACCCACACTATCATGTTGACATTCC; dG = -11.0
697 >s_a_s_d_s_b_s_d_s_a_s GTGTCTGTGACGGTGGCTGTGACGGTGTCTGTG; >clamp_s_c_s_a_s:compl:rev CACAGACACCCACACTATCATGTTGACATTCC; dG = -10.9
698 >s_a_s_a_s_b_s_d_s_a_s GTGTCTGTGTCTGTGGCTGTGACGGTGTCTGTG; >clamp_s_c_s_a_s:compl:rev CACAGACACCCACACTATCATGTTGACATTCC; dG = -10.9
699 >s_a_s_e_s_a_s_e_s_a_s GTGTCTGTGCTGGTGTCTGTGCTGGTGTCTGTG; >clamp_s_c_s_a_s:compl:rev CACAGACACCCACACTATCATGTTGACATTCC; dG = -10.9
700 >s_a_s_e_s_b_s_d_s_a_s GTGTCTGTGCTGGTGGCTGTGACGGTGTCTGTG; >clamp_s_c_s_a_s:compl:rev CACAGACACCCACACTATCATGTTGACATTCC; dG = -10.9
701 >s_b_s_a_s_a_s_d_s_a_s GTGGCTGTGTCTGTGTCTGTGACGGTGTCTGTG; >clamp_s_c_s_a_s:compl:rev CACAGACACCCACACTATCATGTTGACATTCC; dG = -10.9
702 >s_b_s_a_s_b_s_d_s_a_s GTGGCTGTGTCTGTGGCTGTGACGGTGTCTGTG; >clamp_s_c_s_a_s:compl:rev CACAGACACCCACACTATCATGTTGACATTCC; dG = -10.9
703 >s_b_s_b_s_a_s_d_s_a_s GTGGCTGTGGCTGTGTCTGTGACGGTGTCTGTG; >clamp_s_c_s_a_s:compl:rev CACAGACACCCACACTATCATGTTGACATTCC; dG = -10.9
704 >s_b_s_b_s_b_s_d_s_a_s GTGGCTGTGGCTGTGGCTGTGACGGTGTCTGTG; >clamp_s_c_s_a_s:compl:rev CACAGACACCCACACTATCATGTTGACATTCC; dG = -10.9
705 >s_b_s_d_s_b_s_d_s_a_s GTGGCTGTGACGGTGGCTGTGACGGTGTCTGTG; >clamp_s_c_s_a_s:compl:rev CACAGACACCCACACTATCATGTTGACATTCC; dG = -10.9
706 >s_b_s_e_s_a_s_e_s_a_s GTGGCTGTGCTGGTGTCTGTGCTGGTGTCTGTG; >clamp_s_c_s_a_s:compl:rev CACAGACACCCACACTATCATGTTGACATTCC; dG = -10.9
707 >s_b_s_e_s_b_s_d_s_a_s GTGGCTGTGCTGGTGGCTGTGACGGTGTCTGTG; >clamp_s_c_s_a_s:compl:rev CACAGACACCCACACTATCATGTTGACATTCC; dG = -10.9
708 >s_a_s_b_s_a_s_d_s_a_s GTGTCTGTGGCTGTGTCTGTGACGGTGTCTGTG; >clamp_s_c_s_a_s:compl:rev CACAGACACCCACACTATCATGTTGACATTCC; dG = -10.9
709 >s_c_s_e_s_b_s_d_s_a_s GTGTGGGTGCTGGTGGCTGTGACGGTGTCTGTG; >clamp_s_c_s_a_s:compl:rev CACAGACACCCACACTATCATGTTGACATTCC; dG = -10.9
710 >s_d_s_a_s_a_s_d_s_a_s GTGACGGTGTCTGTGTCTGTGACGGTGTCTGTG; >clamp_s_c_s_a_s:compl:rev CACAGACACCCACACTATCATGTTGACATTCC; dG = -10.9
711 >s_a_s_b_s_b_s_d_s_a_s GTGTCTGTGGCTGTGGCTGTGACGGTGTCTGTG; >clamp_s_c_s_a_s:compl:rev CACAGACACCCACACTATCATGTTGACATTCC; dG = -10.9
712 >s_d_s_a_s_b_s_d_s_a_s GTGACGGTGTCTGTGGCTGTGACGGTGTCTGTG; >clamp_s_c_s_a_s:compl:rev CACAGACACCCACACTATCATGTTGACATTCC; dG = -10.9
713 >s_d_s_b_s_a_s_d_s_a_s GTGACGGTGGCTGTGTCTGTGACGGTGTCTGTG; >clamp_s_c_s_a_s:compl:rev CACAGACACCCACACTATCATGTTGACATTCC; dG = -10.9
714 >s_d_s_b_s_b_s_d_s_a_s GTGACGGTGGCTGTGGCTGTGACGGTGTCTGTG; >clamp_s_c_s_a_s:compl:rev CACAGACACCCACACTATCATGTTGACATTCC; dG = -10.9
715 >s_a_s_a_s_a_s_d_s_a_s GTGTCTGTGTCTGTGTCTGTGACGGTGTCTGTG; >clamp_s_c_s_a_s:compl:rev CACAGACACCCACACTATCATGTTGACATTCC; dG = -10.9
716 >s_d_s_d_s_b_s_d_s_a_s GTGACGGTGACGGTGGCTGTGACGGTGTCTGTG; >clamp_s_c_s_a_s:compl:rev CACAGACACCCACACTATCATGTTGACATTCC; dG = -10.9
717 >s_d_s_e_s_a_s_e_s_a_s GTGACGGTGCTGGTGTCTGTGCTGGTGTCTGTG; >clamp_s_c_s_a_s:compl:rev CACAGACACCCACACTATCATGTTGACATTCC; dG = -10.9
718 >s_d_s_e_s_b_s_d_s_a_s GTGACGGTGCTGGTGGCTGTGACGGTGTCTGTG; >clamp_s_c_s_a_s:compl:rev CACAGACACCCACACTATCATGTTGACATTCC; dG = -10.9
719 >s_e_s_a_s_a_s_d_s_a_s GTGCTGGTGTCTGTGTCTGTGACGGTGTCTGTG; >clamp_s_c_s_a_s:compl:rev CACAGACACCCACACTATCATGTTGACATTCC; dG = -10.9
720 >s_e_s_a_s_b_s_d_s_a_s GTGCTGGTGTCTGTGGCTGTGACGGTGTCTGTG; >clamp_s_c_s_a_s:compl:rev CACAGACACCCACACTATCATGTTGACATTCC; dG = -10.9
721 >s_e_s_b_s_a_s_d_s_a_s GTGCTGGTGGCTGTGTCTGTGACGGTGTCTGTG; >clamp_s_c_s_a_s:compl:rev CACAGACACCCACACTATCATGTTGACATTCC; dG = -10.9
722 >s_e_s_b_s_b_s_d_s_a_s GTGCTGGTGGCTGTGGCTGTGACGGTGTCTGTG; >clamp_s_c_s_a_s:compl:rev CACAGACACCCACACTATCATGTTGACATTCC; dG = -10.9
723 >s_e_s_d_s_b_s_d_s_a_s GTGCTGGTGACGGTGGCTGTGACGGTGTCTGTG; >clamp_s_c_s_a_s:compl:rev CACAGACACCCACACTATCATGTTGACATTCC; dG = -10.9
724 >s_e_s_e_s_a_s_e_s_a_s GTGCTGGTGCTGGTGTCTGTGCTGGTGTCTGTG; >clamp_s_c_s_a_s:compl:rev CACAGACACCCACACTATCATGTTGACATTCC; dG = -10.9
725 >s_e_s_e_s_b_s_d_s_a_s GTGCTGGTGCTGGTGGCTGTGACGGTGTCTGTG; >clamp_s_c_s_a_s:compl:rev CACAGACACCCACACTATCATGTTGACATTCC; dG = -10.9
726 >s_a_s_d_s_b_s_e_s_a_s GTGTCTGTGACGGTGGCTGTGCTGGTGTCTGTG; >clamp_s_c_s_a_s:compl:rev CACAGACACCCACACTATCATGTTGACATTCC; dG = -10.8
727 >s_a_s_a_s_b_s_e_s_a_s GTGTCTGTGTCTGTGGCTGTGCTGGTGTCTGTG; >clamp_s_c_s_a_s:compl:rev CACAGACACCCACACTATCATGTTGACATTCC; dG = -10.8
728 >s_a_s_d_s_d_s_d_s_a_s GTGTCTGTGACGGTGACGGTGACGGTGTCTGTG; >clamp_s_c_s_a_s:compl:rev CACAGACACCCACACTATCATGTTGACATTCC; dG = -10.8
729 >s_a_s_d_s_e_s_d_s_a_s GTGTCTGTGACGGTGCTGGTGACGGTGTCTGTG; >clamp_s_c_s_a_s:compl:rev CACAGACACCCACACTATCATGTTGACATTCC; dG = -10.8

730 >s_a_s_e_s_b_s_e_s_a_s GTGTCTGTGCTGGTGGCTGTGCTGGTGTCTGTG; >clamp_s_c_s_a_s:compl:rev
CACAGACACCCACACTATCATGTTGACATTCC; dG = -10.8
731 >s_a_s_e_s_d_s_d_s_a_s GTGTCTGTGCTGGTGACGGTGACGGTGTCTGTG; >clamp_s_c_s_a_s:compl:rev
CACAGACACCCACACTATCATGTTGACATTCC; dG = -10.8
732 >s_a_s_e_s_e_s_d_s_a_s GTGTCTGTGCTGGTGCTGGTGACGGTGTCTGTG; >clamp_s_c_s_a_s:compl:rev
CACAGACACCCACACTATCATGTTGACATTCC; dG = -10.8
733 >s_b_s_a_s_a_s_e_s_a_s GTGGCTGTGTCTGTGTCTGTGCTGGTGTCTGTG; >clamp_s_c_s_a_s:compl:rev
CACAGACACCCACACTATCATGTTGACATTCC; dG = -10.8
734 >s_b_s_a_s_b_s_e_s_a_s GTGGCTGTGTCTGTGGCTGTGCTGGTGTCTGTG; >clamp_s_c_s_a_s:compl:rev
CACAGACACCCACACTATCATGTTGACATTCC; dG = -10.8
735 >s_b_s_a_s_d_s_d_s_a_s GTGGCTGTGTCTGTGACGGTGACGGTGTCTGTG; >clamp_s_c_s_a_s:compl:rev
CACAGACACCCACACTATCATGTTGACATTCC; dG = -10.8
736 >s_b_s_a_s_e_s_d_s_a_s GTGGCTGTGTCTGTGCTGGTGACGGTGTCTGTG; >clamp_s_c_s_a_s:compl:rev
CACAGACACCCACACTATCATGTTGACATTCC; dG = -10.8
737 >s_b_s_b_s_a_s_e_s_a_s GTGGCTGTGGCTGTGTCTGTGCTGGTGTCTGTG; >clamp_s_c_s_a_s:compl:rev
CACAGACACCCACACTATCATGTTGACATTCC; dG = -10.8
738 >s_b_s_b_s_b_s_e_s_a_s GTGGCTGTGGCTGTGGCTGTGCTGGTGTCTGTG; >clamp_s_c_s_a_s:compl:rev
CACAGACACCCACACTATCATGTTGACATTCC; dG = -10.8
739 >s_b_s_b_s_d_s_d_s_a_s GTGGCTGTGGCTGTGACGGTGACGGTGTCTGTG; >clamp_s_c_s_a_s:compl:rev
CACAGACACCCACACTATCATGTTGACATTCC; dG = -10.8
740 >s_b_s_b_s_e_s_d_s_a_s GTGGCTGTGGCTGTGCTGGTGACGGTGTCTGTG; >clamp_s_c_s_a_s:compl:rev
CACAGACACCCACACTATCATGTTGACATTCC; dG = -10.8
741 >s_a_s_a_s_d_s_d_s_a_s GTGTCTGTGTCTGTGACGGTGACGGTGTCTGTG; >clamp_s_c_s_a_s:compl:rev
CACAGACACCCACACTATCATGTTGACATTCC; dG = -10.8
742 >s_b_s_d_s_b_s_e_s_a_s GTGGCTGTGACGGTGGCTGTGCTGGTGTCTGTG; >clamp_s_c_s_a_s:compl:rev
CACAGACACCCACACTATCATGTTGACATTCC; dG = -10.8
743 >s_b_s_d_s_d_s_d_s_a_s GTGGCTGTGACGGTGACGGTGACGGTGTCTGTG; >clamp_s_c_s_a_s:compl:rev
CACAGACACCCACACTATCATGTTGACATTCC; dG = -10.8
744 >s_b_s_d_s_e_s_d_s_a_s GTGGCTGTGACGGTGCTGGTGACGGTGTCTGTG; >clamp_s_c_s_a_s:compl:rev
CACAGACACCCACACTATCATGTTGACATTCC; dG = -10.8
745 >s_b_s_e_s_b_s_e_s_a_s GTGGCTGTGCTGGTGGCTGTGCTGGTGTCTGTG; >clamp_s_c_s_a_s:compl:rev
CACAGACACCCACACTATCATGTTGACATTCC; dG = -10.8
746 >s_b_s_e_s_d_s_d_s_a_s GTGGCTGTGCTGGTGACGGTGACGGTGTCTGTG; >clamp_s_c_s_a_s:compl:rev
CACAGACACCCACACTATCATGTTGACATTCC; dG = -10.8
747 >s_a_s_a_s_e_s_d_s_a_s GTGTCTGTGTCTGTGCTGGTGACGGTGTCTGTG; >clamp_s_c_s_a_s:compl:rev
CACAGACACCCACACTATCATGTTGACATTCC; dG = -10.8
748 >s_b_s_e_s_e_s_d_s_a_s GTGGCTGTGCTGGTGCTGGTGACGGTGTCTGTG; >clamp_s_c_s_a_s:compl:rev
CACAGACACCCACACTATCATGTTGACATTCC; dG = -10.8
749 >s_a_s_b_s_a_s_e_s_a_s GTGTCTGTGGCTGTGTCTGTGCTGGTGTCTGTG; >clamp_s_c_s_a_s:compl:rev
CACAGACACCCACACTATCATGTTGACATTCC; dG = -10.8
750 >s_c_s_e_s_b_s_e_s_a_s GTGTGGGTGCTGGTGGCTGTGCTGGTGTCTGTG; >clamp_s_c_s_a_s:compl:rev
CACAGACACCCACACTATCATGTTGACATTCC; dG = -10.8
751 >s_c_s_e_s_b_s_b_s_a_s GTGTGGGTGCTGGTGGCTGTGGCTGTGTCTGTG; >clamp_s_c_s_a_s:compl:rev
CACAGACACCCACACTATCATGTTGACATTCC; dG = -10.8
752 >s_c_s_e_s_b_s_a_s_a_s GTGTGGGTGCTGGTGGCTGTGTCTGTGTCTGTG; >clamp_s_c_s_a_s:compl:rev
CACAGACACCCACACTATCATGTTGACATTCC; dG = -10.8
753 >s_a_s_a_s_a_s_e_s_a_s GTGTCTGTGTCTGTGTCTGTGCTGGTGTCTGTG; >clamp_s_c_s_a_s:compl:rev
CACAGACACCCACACTATCATGTTGACATTCC; dG = -10.8
754 >s_c_s_e_s_d_s_e_s_a_s GTGTGGGTGCTGGTGACGGTGCTGGTGTCTGTG; >clamp_s_c_s_a_s:compl:rev
CACAGACACCCACACTATCATGTTGACATTCC; dG = -10.8
755 >s_c_s_e_s_d_s_d_s_a_s GTGTGGGTGCTGGTGACGGTGACGGTGTCTGTG; >clamp_s_c_s_a_s:compl:rev
CACAGACACCCACACTATCATGTTGACATTCC; dG = -10.8
756 >s_c_s_e_s_d_s_b_s_a_s GTGTGGGTGCTGGTGACGGTGGCTGTGTCTGTG; >clamp_s_c_s_a_s:compl:rev
CACAGACACCCACACTATCATGTTGACATTCC; dG = -10.8
757 >s_c_s_e_s_e_s_e_s_a_s GTGTGGGTGCTGGTGCTGGTGCTGGTGTCTGTG; >clamp_s_c_s_a_s:compl:rev
CACAGACACCCACACTATCATGTTGACATTCC; dG = -10.8
758 >s_a_s_b_s_b_s_e_s_a_s GTGTCTGTGGCTGTGGCTGTGCTGGTGTCTGTG; >clamp_s_c_s_a_s:compl:rev
CACAGACACCCACACTATCATGTTGACATTCC; dG = -10.8
759 >s_c_s_e_s_e_s_d_s_a_s GTGTGGGTGCTGGTGCTGGTGACGGTGTCTGTG; >clamp_s_c_s_a_s:compl:rev
CACAGACACCCACACTATCATGTTGACATTCC; dG = -10.8
760 >s_c_s_e_s_e_s_b_s_a_s GTGTGGGTGCTGGTGCTGGTGGCTGTGTCTGTG; >clamp_s_c_s_a_s:compl:rev
CACAGACACCCACACTATCATGTTGACATTCC; dG = -10.8
761 >s_d_s_a_s_a_s_e_s_a_s GTGACGGTGTCTGTGTCTGTGCTGGTGTCTGTG; >clamp_s_c_s_a_s:compl:rev
CACAGACACCCACACTATCATGTTGACATTCC; dG = -10.8
762 >s_d_s_a_s_b_s_e_s_a_s GTGACGGTGTCTGTGGCTGTGCTGGTGTCTGTG; >clamp_s_c_s_a_s:compl:rev
CACAGACACCCACACTATCATGTTGACATTCC; dG = -10.8
763 >s_d_s_a_s_e_s_d_s_a_s GTGACGGTGTCTGTGCTGGTGACGGTGTCTGTG; >clamp_s_c_s_a_s:compl:rev
CACAGACACCCACACTATCATGTTGACATTCC; dG = -10.8
764 >s_d_s_b_s_a_s_e_s_a_s GTGACGGTGGCTGTGTCTGTGCTGGTGTCTGTG; >clamp_s_c_s_a_s:compl:rev
CACAGACACCCACACTATCATGTTGACATTCC; dG = -10.8
765 >s_d_s_b_s_b_s_e_s_a_s GTGACGGTGGCTGTGGCTGTGCTGGTGTCTGTG; >clamp_s_c_s_a_s:compl:rev
CACAGACACCCACACTATCATGTTGACATTCC; dG = -10.8
766 >s_d_s_b_s_d_s_d_s_a_s GTGACGGTGGCTGTGACGGTGACGGTGTCTGTG; >clamp_s_c_s_a_s:compl:rev
CACAGACACCCACACTATCATGTTGACATTCC; dG = -10.8

767 >s_d_s_b_s_e_s_d_s_a_s GTGACGGTGGCTGTGCTGGTGACGGTGTCTGTG; >clamp_s_c_s_a_s:compl:rev CACAGACACCCACACTATCATGTTGACATTCC; dG = -10.8
768 >s_d_s_d_s_b_s_e_s_a_s GTGACGGTGACGGTGGCTGTGCTGGTGTCTGTG; >clamp_s_c_s_a_s:compl:rev CACAGACACCCACACTATCATGTTGACATTCC; dG = -10.8
769 >s_d_s_d_s_d_s_d_s_a_s GTGACGGTGACGGTGACGGTGACGGTGTCTGTG; >clamp_s_c_s_a_s:compl:rev CACAGACACCCACACTATCATGTTGACATTCC; dG = -10.8
770 >s_d_s_d_s_e_s_d_s_a_s GTGACGGTGACGGTGCTGGTGACGGTGTCTGTG; >clamp_s_c_s_a_s:compl:rev CACAGACACCCACACTATCATGTTGACATTCC; dG = -10.8
771 >s_a_s_b_s_d_s_d_s_a_s GTGTCTGTGGCTGTGACGGTGACGGTGTCTGTG; >clamp_s_c_s_a_s:compl:rev CACAGACACCCACACTATCATGTTGACATTCC; dG = -10.8
772 >s_d_s_e_s_b_s_e_s_a_s GTGACGGTGCTGGTGGCTGTGCTGGTGTCTGTG; >clamp_s_c_s_a_s:compl:rev CACAGACACCCACACTATCATGTTGACATTCC; dG = -10.8
773 >s_d_s_e_s_d_s_d_s_a_s GTGACGGTGCTGGTGACGGTGACGGTGTCTGTG; >clamp_s_c_s_a_s:compl:rev CACAGACACCCACACTATCATGTTGACATTCC; dG = -10.8
774 >s_d_s_e_s_e_s_d_s_a_s GTGACGGTGCTGGTGCTGGTGACGGTGTCTGTG; >clamp_s_c_s_a_s:compl:rev CACAGACACCCACACTATCATGTTGACATTCC; dG = -10.8
775 >s_e_s_a_s_a_s_e_s_a_s GTGCTGGTGTCTGTGTCTGTGCTGGTGTCTGTG; >clamp_s_c_s_a_s:compl:rev CACAGACACCCACACTATCATGTTGACATTCC; dG = -10.8
776 >s_e_s_a_s_b_s_e_s_a_s GTGCTGGTGTCTGTGGCTGTGCTGGTGTCTGTG; >clamp_s_c_s_a_s:compl:rev CACAGACACCCACACTATCATGTTGACATTCC; dG = -10.8
777 >s_e_s_a_s_e_s_d_s_a_s GTGCTGGTGTCTGTGCTGGTGACGGTGTCTGTG; >clamp_s_c_s_a_s:compl:rev CACAGACACCCACACTATCATGTTGACATTCC; dG = -10.8
778 >s_e_s_b_s_a_s_e_s_a_s GTGCTGGTGGCTGTGTCTGTGCTGGTGTCTGTG; >clamp_s_c_s_a_s:compl:rev CACAGACACCCACACTATCATGTTGACATTCC; dG = -10.8
779 >s_a_s_b_s_e_s_d_s_a_s GTGTCTGTGGCTGTGCTGGTGACGGTGTCTGTG; >clamp_s_c_s_a_s:compl:rev CACAGACACCCACACTATCATGTTGACATTCC; dG = -10.8
780 >s_e_s_b_s_b_s_e_s_a_s GTGCTGGTGGCTGTGGCTGTGCTGGTGTCTGTG; >clamp_s_c_s_a_s:compl:rev CACAGACACCCACACTATCATGTTGACATTCC; dG = -10.8
781 >s_e_s_b_s_d_s_d_s_a_s GTGCTGGTGGCTGTGACGGTGACGGTGTCTGTG; >clamp_s_c_s_a_s:compl:rev CACAGACACCCACACTATCATGTTGACATTCC; dG = -10.8
782 >s_e_s_b_s_e_s_d_s_a_s GTGCTGGTGGCTGTGCTGGTGACGGTGTCTGTG; >clamp_s_c_s_a_s:compl:rev CACAGACACCCACACTATCATGTTGACATTCC; dG = -10.8
783 >s_e_s_d_s_b_s_e_s_a_s GTGCTGGTGACGGTGGCTGTGCTGGTGTCTGTG; >clamp_s_c_s_a_s:compl:rev CACAGACACCCACACTATCATGTTGACATTCC; dG = -10.8
784 >s_e_s_d_s_d_s_d_s_a_s GTGCTGGTGACGGTGACGGTGACGGTGTCTGTG; >clamp_s_c_s_a_s:compl:rev CACAGACACCCACACTATCATGTTGACATTCC; dG = -10.8
785 >s_e_s_d_s_e_s_d_s_a_s GTGCTGGTGACGGTGCTGGTGACGGTGTCTGTG; >clamp_s_c_s_a_s:compl:rev CACAGACACCCACACTATCATGTTGACATTCC; dG = -10.8
786 >s_e_s_e_s_b_s_e_s_a_s GTGCTGGTGCTGGTGGCTGTGCTGGTGTCTGTG; >clamp_s_c_s_a_s:compl:rev CACAGACACCCACACTATCATGTTGACATTCC; dG = -10.8
787 >s_e_s_e_s_d_s_d_s_a_s GTGCTGGTGCTGGTGACGGTGACGGTGTCTGTG; >clamp_s_c_s_a_s:compl:rev CACAGACACCCACACTATCATGTTGACATTCC; dG = -10.8
788 >s_e_s_e_s_e_s_d_s_a_s GTGCTGGTGCTGGTGCTGGTGACGGTGTCTGTG; >clamp_s_c_s_a_s:compl:rev CACAGACACCCACACTATCATGTTGACATTCC; dG = -10.8
789 >s_a_s_d_s_d_s_e_s_a_s GTGTCTGTGACGGTGACGGTGCTGGTGTCTGTG; >clamp_s_c_s_a_s:compl:rev CACAGACACCCACACTATCATGTTGACATTCC; dG = -10.7
790 >s_a_s_d_s_e_s_e_s_a_s GTGTCTGTGACGGTGCTGGTGCTGGTGTCTGTG; >clamp_s_c_s_a_s:compl:rev CACAGACACCCACACTATCATGTTGACATTCC; dG = -10.7
791 >s_a_s_e_s_d_s_e_s_a_s GTGTCTGTGCTGGTGACGGTGCTGGTGTCTGTG; >clamp_s_c_s_a_s:compl:rev CACAGACACCCACACTATCATGTTGACATTCC; dG = -10.7
792 >s_a_s_e_s_e_s_e_s_a_s GTGTCTGTGCTGGTGCTGGTGCTGGTGTCTGTG; >clamp_s_c_s_a_s:compl:rev CACAGACACCCACACTATCATGTTGACATTCC; dG = -10.7
793 >s_b_s_a_s_d_s_e_s_a_s GTGGCTGTGTCTGTGACGGTGCTGGTGTCTGTG; >clamp_s_c_s_a_s:compl:rev CACAGACACCCACACTATCATGTTGACATTCC; dG = -10.7
794 >s_b_s_a_s_e_s_e_s_a_s GTGGCTGTGTCTGTGCTGGTGCTGGTGTCTGTG; >clamp_s_c_s_a_s:compl:rev CACAGACACCCACACTATCATGTTGACATTCC; dG = -10.7
795 >s_b_s_b_s_d_s_e_s_a_s GTGGCTGTGGCTGTGACGGTGCTGGTGTCTGTG; >clamp_s_c_s_a_s:compl:rev CACAGACACCCACACTATCATGTTGACATTCC; dG = -10.7
796 >s_b_s_b_s_e_s_e_s_a_s GTGGCTGTGGCTGTGCTGGTGCTGGTGTCTGTG; >clamp_s_c_s_a_s:compl:rev CACAGACACCCACACTATCATGTTGACATTCC; dG = -10.7
797 >s_a_s_a_s_d_s_e_s_a_s GTGTCTGTGTCTGTGACGGTGCTGGTGTCTGTG; >clamp_s_c_s_a_s:compl:rev CACAGACACCCACACTATCATGTTGACATTCC; dG = -10.7
798 >s_b_s_d_s_d_s_e_s_a_s GTGGCTGTGACGGTGACGGTGCTGGTGTCTGTG; >clamp_s_c_s_a_s:compl:rev CACAGACACCCACACTATCATGTTGACATTCC; dG = -10.7
799 >s_b_s_d_s_e_s_e_s_a_s GTGGCTGTGACGGTGCTGGTGCTGGTGTCTGTG; >clamp_s_c_s_a_s:compl:rev CACAGACACCCACACTATCATGTTGACATTCC; dG = -10.7
800 >s_a_s_a_s_e_s_e_s_a_s GTGTCTGTGTCTGTGCTGGTGCTGGTGTCTGTG; >clamp_s_c_s_a_s:compl:rev CACAGACACCCACACTATCATGTTGACATTCC; dG = -10.7
801 >s_b_s_e_s_d_s_e_s_a_s GTGGCTGTGCTGGTGACGGTGCTGGTGTCTGTG; >clamp_s_c_s_a_s:compl:rev CACAGACACCCACACTATCATGTTGACATTCC; dG = -10.7
802 >s_b_s_e_s_e_s_e_s_a_s GTGGCTGTGCTGGTGCTGGTGCTGGTGTCTGTG; >clamp_s_c_s_a_s:compl:rev CACAGACACCCACACTATCATGTTGACATTCC; dG = -10.7
803 >s_d_s_a_s_b_s_b_s_a_s GTGACGGTGTCTGTGGCTGTGGCTGTGTCTGTG; >clamp_s_c_s_a_s:compl:rev CACAGACACCCACACTATCATGTTGACATTCC; dG = -10.7

804 >s_d_s_a_s_b_s_a_s_a_s GTGACGGTGTCTGTGGCTGTGTCTGTGTCTGTG; >clamp_s_c_s_a_s:compl:rev CACAGACACCCACACTATCATGTTGACATTCC; dG = -10.7
805 >s_d_s_a_s_e_s_e_s_a_s GTGACGGTGTCTGTGCTGGTGCTGGTGTCTGTG; >clamp_s_c_s_a_s:compl:rev CACAGACACCCACACTATCATGTTGACATTCC; dG = -10.7
806 >s_d_s_b_s_d_s_e_s_a_s GTGACGGTGGCTGTGACGGTGCTGGTGTCTGTG; >clamp_s_c_s_a_s:compl:rev CACAGACACCCACACTATCATGTTGACATTCC; dG = -10.7
807 >s_d_s_b_s_e_s_e_s_a_s GTGACGGTGGCTGTGCTGGTGCTGGTGTCTGTG; >clamp_s_c_s_a_s:compl:rev CACAGACACCCACACTATCATGTTGACATTCC; dG = -10.7
808 >s_d_s_d_s_d_s_e_s_a_s GTGACGGTGACGGTGACGGTGCTGGTGTCTGTG; >clamp_s_c_s_a_s:compl:rev CACAGACACCCACACTATCATGTTGACATTCC; dG = -10.7
809 >s_d_s_d_s_e_s_e_s_a_s GTGACGGTGACGGTGCTGGTGCTGGTGTCTGTG; >clamp_s_c_s_a_s:compl:rev CACAGACACCCACACTATCATGTTGACATTCC; dG = -10.7
810 >s_a_s_b_s_d_s_e_s_a_s GTGTCTGTGGCTGTGACGGTGCTGGTGTCTGTG; >clamp_s_c_s_a_s:compl:rev CACAGACACCCACACTATCATGTTGACATTCC; dG = -10.7
811 >s_d_s_e_s_d_s_e_s_a_s GTGACGGTGCTGGTGACGGTGCTGGTGTCTGTG; >clamp_s_c_s_a_s:compl:rev CACAGACACCCACACTATCATGTTGACATTCC; dG = -10.7
812 >s_d_s_e_s_e_s_e_s_a_s GTGACGGTGCTGGTGCTGGTGCTGGTGTCTGTG; >clamp_s_c_s_a_s:compl:rev CACAGACACCCACACTATCATGTTGACATTCC; dG = -10.7
813 >s_e_s_a_s_e_s_e_s_a_s GTGCTGGTGTCTGTGCTGGTGCTGGTGTCTGTG; >clamp_s_c_s_a_s:compl:rev CACAGACACCCACACTATCATGTTGACATTCC; dG = -10.7
814 >s_a_s_b_s_e_s_e_s_a_s GTGTCTGTGGCTGTGCTGGTGCTGGTGTCTGTG; >clamp_s_c_s_a_s:compl:rev CACAGACACCCACACTATCATGTTGACATTCC; dG = -10.7
815 >s_e_s_b_s_d_s_e_s_a_s GTGCTGGTGGCTGTGACGGTGCTGGTGTCTGTG; >clamp_s_c_s_a_s:compl:rev CACAGACACCCACACTATCATGTTGACATTCC; dG = -10.7
816 >s_e_s_b_s_e_s_e_s_a_s GTGCTGGTGGCTGTGCTGGTGCTGGTGTCTGTG; >clamp_s_c_s_a_s:compl:rev CACAGACACCCACACTATCATGTTGACATTCC; dG = -10.7
817 >s_e_s_d_s_d_s_e_s_a_s GTGCTGGTGACGGTGACGGTGCTGGTGTCTGTG; >clamp_s_c_s_a_s:compl:rev CACAGACACCCACACTATCATGTTGACATTCC; dG = -10.7
818 >s_e_s_d_s_e_s_e_s_a_s GTGCTGGTGACGGTGCTGGTGCTGGTGTCTGTG; >clamp_s_c_s_a_s:compl:rev CACAGACACCCACACTATCATGTTGACATTCC; dG = -10.7
819 >s_e_s_e_s_d_s_e_s_a_s GTGCTGGTGCTGGTGACGGTGCTGGTGTCTGTG; >clamp_s_c_s_a_s:compl:rev CACAGACACCCACACTATCATGTTGACATTCC; dG = -10.7
820 >s_e_s_e_s_e_s_e_s_a_s GTGCTGGTGCTGGTGCTGGTGCTGGTGTCTGTG; >clamp_s_c_s_a_s:compl:rev CACAGACACCCACACTATCATGTTGACATTCC; dG = -10.7
821 >s_d_s_a_s_a_s_b_s_a_s GTGACGGTGTCTGTGTCTGTGGCTGTGTCTGTG; >clamp_s_c_s_a_s:compl:rev CACAGACACCCACACTATCATGTTGACATTCC; dG = -10.6
822 >s_d_s_a_s_a_s_a_s_a_s GTGACGGTGTCTGTGTCTGTGTCTGTGTCTGTG; >clamp_s_c_s_a_s:compl:rev CACAGACACCCACACTATCATGTTGACATTCC; dG = -10.6
823 >s_e_s_a_s_b_s_b_s_a_s GTGCTGGTGTCTGTGGCTGTGGCTGTGTCTGTG; >clamp_s_c_s_a_s:compl:rev CACAGACACCCACACTATCATGTTGACATTCC; dG = -10.6
824 >s_e_s_a_s_b_s_a_s_a_s GTGCTGGTGTCTGTGGCTGTGTCTGTGTCTGTG; >clamp_s_c_s_a_s:compl:rev CACAGACACCCACACTATCATGTTGACATTCC; dG = -10.6
825 >s_d_s_a_s_e_s_b_s_a_s GTGACGGTGTCTGTGCTGGTGGCTGTGTCTGTG; >clamp_s_c_s_a_s:compl:rev CACAGACACCCACACTATCATGTTGACATTCC; dG = -10.5
826 >s_e_s_a_s_a_s_b_s_a_s GTGCTGGTGTCTGTGTCTGTGGCTGTGTCTGTG; >clamp_s_c_s_a_s:compl:rev CACAGACACCCACACTATCATGTTGACATTCC; dG = -10.5
827 >s_e_s_a_s_a_s_a_s_a_s GTGCTGGTGTCTGTGTCTGTGTCTGTGTCTGTG; >clamp_s_c_s_a_s:compl:rev CACAGACACCCACACTATCATGTTGACATTCC; dG = -10.5
828 >s_b_s_a_s_d_s_b_s_a_s GTGGCTGTGTCTGTGACGGTGGCTGTGTCTGTG; >clamp_s_c_s_a_s:compl:rev CACAGACACCCACACTATCATGTTGACATTCC; dG = -10.4
829 >s_b_s_b_s_a_s_b_s_a_s GTGGCTGTGGCTGTGTCTGTGGCTGTGTCTGTG; >clamp_s_c_s_a_s:compl:rev CACAGACACCCACACTATCATGTTGACATTCC; dG = -10.4
830 >s_a_s_b_s_a_s_b_s_a_s GTGTCTGTGGCTGTGTCTGTGGCTGTGTCTGTG; >clamp_s_c_s_a_s:compl:rev CACAGACACCCACACTATCATGTTGACATTCC; dG = -10.4
831 >s_d_s_b_s_a_s_b_s_a_s GTGACGGTGGCTGTGTCTGTGGCTGTGTCTGTG; >clamp_s_c_s_a_s:compl:rev CACAGACACCCACACTATCATGTTGACATTCC; dG = -10.4
832 >s_e_s_a_s_e_s_b_s_a_s GTGCTGGTGTCTGTGCTGGTGGCTGTGTCTGTG; >clamp_s_c_s_a_s:compl:rev CACAGACACCCACACTATCATGTTGACATTCC; dG = -10.4
833 >s_e_s_b_s_a_s_b_s_a_s GTGCTGGTGGCTGTGTCTGTGGCTGTGTCTGTG; >clamp_s_c_s_a_s:compl:rev CACAGACACCCACACTATCATGTTGACATTCC; dG = -10.4
834 >s_a_s_d_s_b_s_a_s_a_s GTGTCTGTGACGGTGGCTGTGTCTGTGTCTGTG; >clamp_s_c_s_a_s:compl:rev CACAGACACCCACACTATCATGTTGACATTCC; dG = -10.3
835 >s_a_s_a_s_b_s_a_s_a_s GTGTCTGTGTCTGTGGCTGTGTCTGTGTCTGTG; >clamp_s_c_s_a_s:compl:rev CACAGACACCCACACTATCATGTTGACATTCC; dG = -10.3
836 >s_a_s_e_s_b_s_a_s_a_s GTGTCTGTGCTGGTGGCTGTGTCTGTGTCTGTG; >clamp_s_c_s_a_s:compl:rev CACAGACACCCACACTATCATGTTGACATTCC; dG = -10.3
837 >s_b_s_a_s_b_s_a_s_a_s GTGGCTGTGTCTGTGGCTGTGTCTGTGTCTGTG; >clamp_s_c_s_a_s:compl:rev CACAGACACCCACACTATCATGTTGACATTCC; dG = -10.3
838 >s_b_s_b_s_a_s_a_s_a_s GTGGCTGTGGCTGTGTCTGTGTCTGTGTCTGTG; >clamp_s_c_s_a_s:compl:rev CACAGACACCCACACTATCATGTTGACATTCC; dG = -10.3
839 >s_b_s_b_s_b_s_a_s_a_s GTGGCTGTGGCTGTGGCTGTGTCTGTGTCTGTG; >clamp_s_c_s_a_s:compl:rev CACAGACACCCACACTATCATGTTGACATTCC; dG = -10.3
840 >s_b_s_d_s_b_s_a_s_a_s GTGGCTGTGACGGTGGCTGTGTCTGTGTCTGTG; >clamp_s_c_s_a_s:compl:rev CACAGACACCCACACTATCATGTTGACATTCC; dG = -10.3

841 >s_b_s_e_s_b_s_a_s_a_s GTGGCTGTGCTGGTGGCTGTGTCTGTGTCTGTG; >clamp_s_c_s_a_s:compl:rev CACAGACACCCACACTATCATGTTGACATTCC; dG = -10.3
842 >s_a_s_b_s_a_s_a_s_a_s GTGTCTGTGGCTGTGTCTGTGTCTGTGTCTGTG; >clamp_s_c_s_a_s:compl:rev CACAGACACCCACACTATCATGTTGACATTCC; dG = -10.3
843 >s_a_s_b_s_b_s_a_s_a_s GTGTCTGTGGCTGTGGCTGTGTCTGTGTCTGTG; >clamp_s_c_s_a_s:compl:rev CACAGACACCCACACTATCATGTTGACATTCC; dG = -10.3
844 >s_d_s_b_s_a_s_a_s_a_s GTGACGGTGGCTGTGTCTGTGTCTGTGTCTGTG; >clamp_s_c_s_a_s:compl:rev CACAGACACCCACACTATCATGTTGACATTCC; dG = -10.3
845 >s_d_s_b_s_b_s_a_s_a_s GTGACGGTGGCTGTGGCTGTGTCTGTGTCTGTG; >clamp_s_c_s_a_s:compl:rev CACAGACACCCACACTATCATGTTGACATTCC; dG = -10.3
846 >s_d_s_d_s_b_s_a_s_a_s GTGACGGTGACGGTGGCTGTGTCTGTGTCTGTG; >clamp_s_c_s_a_s:compl:rev CACAGACACCCACACTATCATGTTGACATTCC; dG = -10.3
847 >s_d_s_e_s_b_s_a_s_a_s GTGACGGTGCTGGTGGCTGTGTCTGTGTCTGTG; >clamp_s_c_s_a_s:compl:rev CACAGACACCCACACTATCATGTTGACATTCC; dG = -10.3
848 >s_e_s_b_s_a_s_a_s_a_s GTGCTGGTGGCTGTGTCTGTGTCTGTGTCTGTG; >clamp_s_c_s_a_s:compl:rev CACAGACACCCACACTATCATGTTGACATTCC; dG = -10.3
849 >s_e_s_b_s_b_s_a_s_a_s GTGCTGGTGGCTGTGGCTGTGTCTGTGTCTGTG; >clamp_s_c_s_a_s:compl:rev CACAGACACCCACACTATCATGTTGACATTCC; dG = -10.3
850 >s_e_s_d_s_b_s_a_s_a_s GTGCTGGTGACGGTGGCTGTGTCTGTGTCTGTG; >clamp_s_c_s_a_s:compl:rev CACAGACACCCACACTATCATGTTGACATTCC; dG = -10.3
851 >s_e_s_e_s_b_s_a_s_a_s GTGCTGGTGCTGGTGGCTGTGTCTGTGTCTGTG; >clamp_s_c_s_a_s:compl:rev CACAGACACCCACACTATCATGTTGACATTCC; dG = -10.3
852 >s_b_s_a_s_a_s_b_s_a_s GTGGCTGTGTCTGTGTCTGTGGCTGTGTCTGTG; >clamp_s_c_s_a_s:compl:rev CACAGACACCCACACTATCATGTTGACATTCC; dG = -10.2
853 >s_a_s_a_s_d_s_b_s_a_s GTGTCTGTGTCTGTGACGGTGGCTGTGTCTGTG; >clamp_s_c_s_a_s:compl:rev CACAGACACCCACACTATCATGTTGACATTCC; dG = -10.2
854 >s_a_s_a_s_a_s_b_s_a_s GTGTCTGTGTCTGTGTCTGTGGCTGTGTCTGTG; >clamp_s_c_s_a_s:compl:rev CACAGACACCCACACTATCATGTTGACATTCC; dG = -10.2
855 >s_a_s_d_s_b_s_b_s_a_s GTGTCTGTGACGGTGGCTGTGGCTGTGTCTGTG; >clamp_s_c_s_a_s:compl:rev CACAGACACCCACACTATCATGTTGACATTCC; dG = -10.1
856 >s_a_s_a_s_b_s_b_s_a_s GTGTCTGTGTCTGTGGCTGTGGCTGTGTCTGTG; >clamp_s_c_s_a_s:compl:rev CACAGACACCCACACTATCATGTTGACATTCC; dG = -10.1
857 >s_a_s_e_s_b_s_b_s_a_s GTGTCTGTGCTGGTGGCTGTGGCTGTGTCTGTG; >clamp_s_c_s_a_s:compl:rev CACAGACACCCACACTATCATGTTGACATTCC; dG = -10.1
858 >s_b_s_a_s_a_s_a_s_a_s GTGGCTGTGTCTGTGTCTGTGTCTGTGTCTGTG; >clamp_s_c_s_a_s:compl:rev CACAGACACCCACACTATCATGTTGACATTCC; dG = -10.1
859 >s_b_s_a_s_b_s_b_s_a_s GTGGCTGTGTCTGTGGCTGTGGCTGTGTCTGTG; >clamp_s_c_s_a_s:compl:rev CACAGACACCCACACTATCATGTTGACATTCC; dG = -10.1
860 >s_b_s_b_s_b_s_b_s_a_s GTGGCTGTGGCTGTGGCTGTGGCTGTGTCTGTG; >clamp_s_c_s_a_s:compl:rev CACAGACACCCACACTATCATGTTGACATTCC; dG = -10.1
861 >s_b_s_d_s_b_s_b_s_a_s GTGGCTGTGACGGTGGCTGTGGCTGTGTCTGTG; >clamp_s_c_s_a_s:compl:rev CACAGACACCCACACTATCATGTTGACATTCC; dG = -10.1
862 >s_b_s_e_s_b_s_b_s_a_s GTGGCTGTGCTGGTGGCTGTGGCTGTGTCTGTG; >clamp_s_c_s_a_s:compl:rev CACAGACACCCACACTATCATGTTGACATTCC; dG = -10.1
863 >s_a_s_b_s_b_s_b_s_a_s GTGTCTGTGGCTGTGGCTGTGGCTGTGTCTGTG; >clamp_s_c_s_a_s:compl:rev CACAGACACCCACACTATCATGTTGACATTCC; dG = -10.1
864 >s_d_s_b_s_b_s_b_s_a_s GTGACGGTGGCTGTGGCTGTGGCTGTGTCTGTG; >clamp_s_c_s_a_s:compl:rev CACAGACACCCACACTATCATGTTGACATTCC; dG = -10.1
865 >s_d_s_d_s_b_s_b_s_a_s GTGACGGTGACGGTGGCTGTGGCTGTGTCTGTG; >clamp_s_c_s_a_s:compl:rev CACAGACACCCACACTATCATGTTGACATTCC; dG = -10.1
866 >s_d_s_e_s_b_s_b_s_a_s GTGACGGTGCTGGTGGCTGTGGCTGTGTCTGTG; >clamp_s_c_s_a_s:compl:rev CACAGACACCCACACTATCATGTTGACATTCC; dG = -10.1
867 >s_e_s_b_s_b_s_b_s_a_s GTGCTGGTGGCTGTGGCTGTGGCTGTGTCTGTG; >clamp_s_c_s_a_s:compl:rev CACAGACACCCACACTATCATGTTGACATTCC; dG = -10.1
868 >s_a_s_a_s_a_s_a_s_a_s GTGTCTGTGTCTGTGTCTGTGTCTGTGTCTGTG; >clamp_s_c_s_a_s:compl:rev CACAGACACCCACACTATCATGTTGACATTCC; dG = -10.1
869 >s_e_s_d_s_b_s_b_s_a_s GTGCTGGTGACGGTGGCTGTGGCTGTGTCTGTG; >clamp_s_c_s_a_s:compl:rev CACAGACACCCACACTATCATGTTGACATTCC; dG = -10.1
870 >s_e_s_e_s_b_s_b_s_a_s GTGCTGGTGCTGGTGGCTGTGGCTGTGTCTGTG; >clamp_s_c_s_a_s:compl:rev CACAGACACCCACACTATCATGTTGACATTCC; dG = -10.1
871 >s_a_s_d_s_d_s_b_s_a_s GTGTCTGTGACGGTGACGGTGGCTGTGTCTGTG; >clamp_s_c_s_a_s:compl:rev CACAGACACCCACACTATCATGTTGACATTCC; dG = -10.0
872 >s_a_s_d_s_e_s_b_s_a_s GTGTCTGTGACGGTGCTGGTGGCTGTGTCTGTG; >clamp_s_c_s_a_s:compl:rev CACAGACACCCACACTATCATGTTGACATTCC; dG = -10.0
873 >s_a_s_e_s_d_s_b_s_a_s GTGTCTGTGCTGGTGACGGTGGCTGTGTCTGTG; >clamp_s_c_s_a_s:compl:rev CACAGACACCCACACTATCATGTTGACATTCC; dG = -10.0
874 >s_a_s_e_s_e_s_b_s_a_s GTGTCTGTGCTGGTGCTGGTGGCTGTGTCTGTG; >clamp_s_c_s_a_s:compl:rev CACAGACACCCACACTATCATGTTGACATTCC; dG = -10.0
875 >s_b_s_a_s_e_s_b_s_a_s GTGGCTGTGTCTGTGCTGGTGGCTGTGTCTGTG; >clamp_s_c_s_a_s:compl:rev CACAGACACCCACACTATCATGTTGACATTCC; dG = -10.0
876 >s_b_s_b_s_d_s_b_s_a_s GTGGCTGTGGCTGTGACGGTGGCTGTGTCTGTG; >clamp_s_c_s_a_s:compl:rev CACAGACACCCACACTATCATGTTGACATTCC; dG = -10.0
877 >s_b_s_b_s_e_s_b_s_a_s GTGGCTGTGGCTGTGCTGGTGGCTGTGTCTGTG; >clamp_s_c_s_a_s:compl:rev CACAGACACCCACACTATCATGTTGACATTCC; dG = -10.0

878 >s_b_s_d_s_d_s_b_s_a_s GTGGCTGTGACGGTGACGGTGGCTGTGTCTGTG; >clamp_s_c_s_a_s:compl:rev CACAGACACCCACACTATCATGTTGACATTCC; dG = -10.0
879 >s_b_s_d_s_e_s_b_s_a_s GTGGCTGTGACGGTGCTGGTGGCTGTGTCTGTG; >clamp_s_c_s_a_s:compl:rev CACAGACACCCACACTATCATGTTGACATTCC; dG = -10.0
880 >s_b_s_e_s_d_s_b_s_a_s GTGGCTGTGCTGGTGACGGTGGCTGTGTCTGTG; >clamp_s_c_s_a_s:compl:rev CACAGACACCCACACTATCATGTTGACATTCC; dG = -10.0
881 >s_b_s_e_s_e_s_b_s_a_s GTGGCTGTGCTGGTGCTGGTGGCTGTGTCTGTG; >clamp_s_c_s_a_s:compl:rev CACAGACACCCACACTATCATGTTGACATTCC; dG = -10.0
882 >s_a_s_a_s_e_s_b_s_a_s GTGTCTGTGTCTGTGCTGGTGGCTGTGTCTGTG; >clamp_s_c_s_a_s:compl:rev CACAGACACCCACACTATCATGTTGACATTCC; dG = -10.0
883 >s_d_s_b_s_d_s_b_s_a_s GTGACGGTGGCTGTGACGGTGGCTGTGTCTGTG; >clamp_s_c_s_a_s:compl:rev CACAGACACCCACACTATCATGTTGACATTCC; dG = -10.0
884 >s_d_s_b_s_e_s_b_s_a_s GTGACGGTGGCTGTGCTGGTGGCTGTGTCTGTG; >clamp_s_c_s_a_s:compl:rev CACAGACACCCACACTATCATGTTGACATTCC; dG = -10.0
885 >s_d_s_d_s_d_s_b_s_a_s GTGACGGTGACGGTGACGGTGGCTGTGTCTGTG; >clamp_s_c_s_a_s:compl:rev CACAGACACCCACACTATCATGTTGACATTCC; dG = -10.0
886 >s_d_s_d_s_e_s_b_s_a_s GTGACGGTGACGGTGCTGGTGGCTGTGTCTGTG; >clamp_s_c_s_a_s:compl:rev CACAGACACCCACACTATCATGTTGACATTCC; dG = -10.0
887 >s_a_s_b_s_d_s_b_s_a_s GTGTCTGTGGCTGTGACGGTGGCTGTGTCTGTG; >clamp_s_c_s_a_s:compl:rev CACAGACACCCACACTATCATGTTGACATTCC; dG = -10.0
888 >s_d_s_e_s_d_s_b_s_a_s GTGACGGTGCTGGTGACGGTGGCTGTGTCTGTG; >clamp_s_c_s_a_s:compl:rev CACAGACACCCACACTATCATGTTGACATTCC; dG = -10.0
889 >s_d_s_e_s_e_s_b_s_a_s GTGACGGTGCTGGTGCTGGTGGCTGTGTCTGTG; >clamp_s_c_s_a_s:compl:rev CACAGACACCCACACTATCATGTTGACATTCC; dG = -10.0
890 >s_a_s_b_s_e_s_b_s_a_s GTGTCTGTGGCTGTGCTGGTGGCTGTGTCTGTG; >clamp_s_c_s_a_s:compl:rev CACAGACACCCACACTATCATGTTGACATTCC; dG = -10.0
891 >s_e_s_b_s_d_s_b_s_a_s GTGCTGGTGGCTGTGACGGTGGCTGTGTCTGTG; >clamp_s_c_s_a_s:compl:rev CACAGACACCCACACTATCATGTTGACATTCC; dG = -10.0
892 >s_e_s_b_s_e_s_b_s_a_s GTGCTGGTGGCTGTGCTGGTGGCTGTGTCTGTG; >clamp_s_c_s_a_s:compl:rev CACAGACACCCACACTATCATGTTGACATTCC; dG = -10.0
893 >s_e_s_d_s_d_s_b_s_a_s GTGCTGGTGACGGTGACGGTGGCTGTGTCTGTG; >clamp_s_c_s_a_s:compl:rev CACAGACACCCACACTATCATGTTGACATTCC; dG = -10.0
894 >s_e_s_d_s_e_s_b_s_a_s GTGCTGGTGACGGTGCTGGTGGCTGTGTCTGTG; >clamp_s_c_s_a_s:compl:rev CACAGACACCCACACTATCATGTTGACATTCC; dG = -10.0
895 >s_e_s_e_s_d_s_b_s_a_s GTGCTGGTGCTGGTGACGGTGGCTGTGTCTGTG; >clamp_s_c_s_a_s:compl:rev CACAGACACCCACACTATCATGTTGACATTCC; dG = -10.0
896 >s_e_s_e_s_e_s_b_s_a_s GTGCTGGTGCTGGTGCTGGTGGCTGTGTCTGTG; >clamp_s_c_s_a_s:compl:rev CACAGACACCCACACTATCATGTTGACATTCC; dG = -10.0

****

CB Matches

1 >s_c_s_b_s_d_s_c_s_b_s GTGTGGGTGGCTGTGACGGTGTGGGTGGCTGTG; >clamp_s_c_s_b_s:compl:rev CACAGCCACCCACACTATCATGTTGACATTCC; dG = -18.8
2 >s_e_s_c_s_b_s_c_s_b_s GTGCTGGTGTGGGTGGCTGTGTGGGTGGCTGTG; >clamp_s_c_s_b_s:compl:rev CACAGCCACCCACACTATCATGTTGACATTCC; dG = -18.7
3 >s_a_s_c_s_b_s_c_s_b_s GTGTCTGTGTGGGTGGCTGTGTGGGTGGCTGTG; >clamp_s_c_s_b_s:compl:rev CACAGCCACCCACACTATCATGTTGACATTCC; dG = -18.7
4 >s_b_s_c_s_b_s_c_s_b_s GTGGCTGTGTGGGTGGCTGTGTGGGTGGCTGTG; >clamp_s_c_s_b_s:compl:rev CACAGCCACCCACACTATCATGTTGACATTCC; dG = -18.7
5 >s_c_s_c_s_b_s_c_s_b_s GTGTGGGTGTGGGTGGCTGTGTGGGTGGCTGTG; >clamp_s_c_s_b_s:compl:rev CACAGCCACCCACACTATCATGTTGACATTCC; dG = -18.7
6 >s_d_s_c_s_b_s_c_s_b_s GTGACGGTGTGGGTGGCTGTGTGGGTGGCTGTG; >clamp_s_c_s_b_s:compl:rev CACAGCCACCCACACTATCATGTTGACATTCC; dG = -18.7
7 >s_a_s_a_s_a_s_c_s_b_s GTGTCTGTGTCTGTGTCTGTGTGGGTGGCTGTG; >clamp_s_c_s_b_s:compl:rev CACAGCCACCCACACTATCATGTTGACATTCC; dG = -18.4
8 >s_a_s_b_s_e_s_c_s_b_s GTGTCTGTGGCTGTGCTGGTGTGGGTGGCTGTG; >clamp_s_c_s_b_s:compl:rev CACAGCCACCCACACTATCATGTTGACATTCC; dG = -18.4
9 >s_d_s_e_s_e_s_c_s_b_s GTGACGGTGCTGGTGCTGGTGTGGGTGGCTGTG; >clamp_s_c_s_b_s:compl:rev CACAGCCACCCACACTATCATGTTGACATTCC; dG = -18.4
10 >s_e_s_a_s_a_s_c_s_b_s GTGCTGGTGTCTGTGTCTGTGTGGGTGGCTGTG; >clamp_s_c_s_b_s:compl:rev CACAGCCACCCACACTATCATGTTGACATTCC; dG = -18.4
11 >s_e_s_a_s_b_s_c_s_b_s GTGCTGGTGTCTGTGGCTGTGTGGGTGGCTGTG; >clamp_s_c_s_b_s:compl:rev CACAGCCACCCACACTATCATGTTGACATTCC; dG = -18.4
12 >s_e_s_a_s_c_s_c_s_b_s GTGCTGGTGTCTGTGTGGGTGTGGGTGGCTGTG; >clamp_s_c_s_b_s:compl:rev CACAGCCACCCACACTATCATGTTGACATTCC; dG = -18.4
13 >s_e_s_a_s_d_s_c_s_b_s GTGCTGGTGTCTGTGACGGTGTGGGTGGCTGTG; >clamp_s_c_s_b_s:compl:rev CACAGCCACCCACACTATCATGTTGACATTCC; dG = -18.4
14 >s_e_s_a_s_e_s_c_s_b_s GTGCTGGTGTCTGTGCTGGTGTGGGTGGCTGTG; >clamp_s_c_s_b_s:compl:rev CACAGCCACCCACACTATCATGTTGACATTCC; dG = -18.4

15 >s_e_s_b_s_a_s_c_s_b_s GTGCTGGTGGCTGTGTCTGTGTGGGTGGCTGTG; >clamp_s_c_s_b_s:compl:rev CACAGCCACCCACACTATCATGTTGACATTCC; dG = -18.4
16 >s_e_s_b_s_b_s_c_s_b_s GTGCTGGTGGCTGTGGCTGTGTGGGTGGCTGTG; >clamp_s_c_s_b_s:compl:rev CACAGCCACCCACACTATCATGTTGACATTCC; dG = -18.4
17 >s_e_s_b_s_c_s_c_s_b_s GTGCTGGTGGCTGTGTGGGTGTGGGTGGCTGTG; >clamp_s_c_s_b_s:compl:rev CACAGCCACCCACACTATCATGTTGACATTCC; dG = -18.4
18 >s_e_s_b_s_d_s_c_s_b_s GTGCTGGTGGCTGTGACGGTGTGGGTGGCTGTG; >clamp_s_c_s_b_s:compl:rev CACAGCCACCCACACTATCATGTTGACATTCC; dG = -18.4
19 >s_a_s_c_s_a_s_c_s_b_s GTGTCTGTGTGGGTGTCTGTGTGGGTGGCTGTG; >clamp_s_c_s_b_s:compl:rev CACAGCCACCCACACTATCATGTTGACATTCC; dG = -18.4
20 >s_e_s_b_s_e_s_c_s_b_s GTGCTGGTGGCTGTGCTGGTGTGGGTGGCTGTG; >clamp_s_c_s_b_s:compl:rev CACAGCCACCCACACTATCATGTTGACATTCC; dG = -18.4
21 >s_e_s_c_s_a_s_c_s_b_s GTGCTGGTGTGGGTGTCTGTGTGGGTGGCTGTG; >clamp_s_c_s_b_s:compl:rev CACAGCCACCCACACTATCATGTTGACATTCC; dG = -18.4
22 >s_e_s_c_s_c_s_c_s_b_s GTGCTGGTGTGGGTGTGGGTGTGGGTGGCTGTG; >clamp_s_c_s_b_s:compl:rev CACAGCCACCCACACTATCATGTTGACATTCC; dG = -18.4
23 >s_e_s_c_s_d_s_c_s_b_s GTGCTGGTGTGGGTGACGGTGTGGGTGGCTGTG; >clamp_s_c_s_b_s:compl:rev CACAGCCACCCACACTATCATGTTGACATTCC; dG = -18.4
24 >s_e_s_c_s_e_s_c_s_b_s GTGCTGGTGTGGGTGCTGGTGTGGGTGGCTGTG; >clamp_s_c_s_b_s:compl:rev CACAGCCACCCACACTATCATGTTGACATTCC; dG = -18.4
25 >s_e_s_d_s_a_s_c_s_b_s GTGCTGGTGACGGTGTCTGTGTGGGTGGCTGTG; >clamp_s_c_s_b_s:compl:rev CACAGCCACCCACACTATCATGTTGACATTCC; dG = -18.4
26 >s_e_s_d_s_b_s_c_s_b_s GTGCTGGTGACGGTGGCTGTGTGGGTGGCTGTG; >clamp_s_c_s_b_s:compl:rev CACAGCCACCCACACTATCATGTTGACATTCC; dG = -18.4
27 >s_e_s_d_s_c_s_c_s_b_s GTGCTGGTGACGGTGTGGGTGTGGGTGGCTGTG; >clamp_s_c_s_b_s:compl:rev CACAGCCACCCACACTATCATGTTGACATTCC; dG = -18.4
28 >s_e_s_d_s_d_s_c_s_b_s GTGCTGGTGACGGTGACGGTGTGGGTGGCTGTG; >clamp_s_c_s_b_s:compl:rev CACAGCCACCCACACTATCATGTTGACATTCC; dG = -18.4
29 >s_e_s_d_s_e_s_c_s_b_s GTGCTGGTGACGGTGCTGGTGTGGGTGGCTGTG; >clamp_s_c_s_b_s:compl:rev CACAGCCACCCACACTATCATGTTGACATTCC; dG = -18.4
30 >s_e_s_e_s_a_s_c_s_b_s GTGCTGGTGCTGGTGTCTGTGTGGGTGGCTGTG; >clamp_s_c_s_b_s:compl:rev CACAGCCACCCACACTATCATGTTGACATTCC; dG = -18.4
31 >s_e_s_e_s_b_s_c_s_b_s GTGCTGGTGCTGGTGGCTGTGTGGGTGGCTGTG; >clamp_s_c_s_b_s:compl:rev CACAGCCACCCACACTATCATGTTGACATTCC; dG = -18.4
32 >s_e_s_e_s_c_s_c_s_b_s GTGCTGGTGCTGGTGTGGGTGTGGGTGGCTGTG; >clamp_s_c_s_b_s:compl:rev CACAGCCACCCACACTATCATGTTGACATTCC; dG = -18.4
33 >s_e_s_e_s_d_s_c_s_b_s GTGCTGGTGCTGGTGACGGTGTGGGTGGCTGTG; >clamp_s_c_s_b_s:compl:rev CACAGCCACCCACACTATCATGTTGACATTCC; dG = -18.4
34 >s_e_s_e_s_e_s_c_s_b_s GTGCTGGTGCTGGTGCTGGTGTGGGTGGCTGTG; >clamp_s_c_s_b_s:compl:rev CACAGCCACCCACACTATCATGTTGACATTCC; dG = -18.4
35 >s_a_s_c_s_c_s_c_s_b_s GTGTCTGTGTGGGTGTGGGTGTGGGTGGCTGTG; >clamp_s_c_s_b_s:compl:rev CACAGCCACCCACACTATCATGTTGACATTCC; dG = -18.4
36 >s_a_s_c_s_d_s_c_s_b_s GTGTCTGTGTGGGTGACGGTGTGGGTGGCTGTG; >clamp_s_c_s_b_s:compl:rev CACAGCCACCCACACTATCATGTTGACATTCC; dG = -18.4
37 >s_a_s_c_s_e_s_c_s_b_s GTGTCTGTGTGGGTGCTGGTGTGGGTGGCTGTG; >clamp_s_c_s_b_s:compl:rev CACAGCCACCCACACTATCATGTTGACATTCC; dG = -18.4
38 >s_a_s_d_s_a_s_c_s_b_s GTGTCTGTGACGGTGTCTGTGTGGGTGGCTGTG; >clamp_s_c_s_b_s:compl:rev CACAGCCACCCACACTATCATGTTGACATTCC; dG = -18.4
39 >s_a_s_d_s_b_s_c_s_b_s GTGTCTGTGACGGTGGCTGTGTGGGTGGCTGTG; >clamp_s_c_s_b_s:compl:rev CACAGCCACCCACACTATCATGTTGACATTCC; dG = -18.4
40 >s_a_s_d_s_c_s_c_s_b_s GTGTCTGTGACGGTGTGGGTGTGGGTGGCTGTG; >clamp_s_c_s_b_s:compl:rev CACAGCCACCCACACTATCATGTTGACATTCC; dG = -18.4
41 >s_a_s_d_s_d_s_c_s_b_s GTGTCTGTGACGGTGACGGTGTGGGTGGCTGTG; >clamp_s_c_s_b_s:compl:rev CACAGCCACCCACACTATCATGTTGACATTCC; dG = -18.4
42 >s_a_s_a_s_b_s_c_s_b_s GTGTCTGTGTCTGTGGCTGTGTGGGTGGCTGTG; >clamp_s_c_s_b_s:compl:rev CACAGCCACCCACACTATCATGTTGACATTCC; dG = -18.4
43 >s_a_s_d_s_e_s_c_s_b_s GTGTCTGTGACGGTGCTGGTGTGGGTGGCTGTG; >clamp_s_c_s_b_s:compl:rev CACAGCCACCCACACTATCATGTTGACATTCC; dG = -18.4
44 >s_a_s_e_s_a_s_c_s_b_s GTGTCTGTGCTGGTGTCTGTGTGGGTGGCTGTG; >clamp_s_c_s_b_s:compl:rev CACAGCCACCCACACTATCATGTTGACATTCC; dG = -18.4
45 >s_a_s_e_s_b_s_c_s_b_s GTGTCTGTGCTGGTGGCTGTGTGGGTGGCTGTG; >clamp_s_c_s_b_s:compl:rev CACAGCCACCCACACTATCATGTTGACATTCC; dG = -18.4
46 >s_a_s_e_s_c_s_c_s_b_s GTGTCTGTGCTGGTGTGGGTGTGGGTGGCTGTG; >clamp_s_c_s_b_s:compl:rev CACAGCCACCCACACTATCATGTTGACATTCC; dG = -18.4
47 >s_a_s_e_s_d_s_c_s_b_s GTGTCTGTGCTGGTGACGGTGTGGGTGGCTGTG; >clamp_s_c_s_b_s:compl:rev CACAGCCACCCACACTATCATGTTGACATTCC; dG = -18.4
48 >s_a_s_e_s_e_s_c_s_b_s GTGTCTGTGCTGGTGCTGGTGTGGGTGGCTGTG; >clamp_s_c_s_b_s:compl:rev CACAGCCACCCACACTATCATGTTGACATTCC; dG = -18.4
49 >s_b_s_a_s_a_s_c_s_b_s GTGGCTGTGTCTGTGTCTGTGTGGGTGGCTGTG; >clamp_s_c_s_b_s:compl:rev CACAGCCACCCACACTATCATGTTGACATTCC; dG = -18.4
50 >s_b_s_a_s_b_s_c_s_b_s GTGGCTGTGTCTGTGGCTGTGTGGGTGGCTGTG; >clamp_s_c_s_b_s:compl:rev CACAGCCACCCACACTATCATGTTGACATTCC; dG = -18.4
51 >s_b_s_a_s_c_s_c_s_b_s GTGGCTGTGTCTGTGTGGGTGTGGGTGGCTGTG; >clamp_s_c_s_b_s:compl:rev CACAGCCACCCACACTATCATGTTGACATTCC; dG = -18.4

52 >s_b_s_a_s_d_s_c_s_b_s GTGGCTGTGTCTGTGACGGTGTGGGTGGCTGTG; >clamp_s_c_s_b_s:compl:rev
CACAGCCACCCACACTATCATGTTGACATTCC; dG = -18.4
53 >s_a_s_a_s_c_s_c_s_b_s GTGTCTGTGTCTGTGTGGGTGTGGGTGGCTGTG; >clamp_s_c_s_b_s:compl:rev
CACAGCCACCCACACTATCATGTTGACATTCC; dG = -18.4
54 >s_b_s_a_s_e_s_c_s_b_s GTGGCTGTGTCTGTGCTGGTGTGGGTGGCTGTG; >clamp_s_c_s_b_s:compl:rev
CACAGCCACCCACACTATCATGTTGACATTCC; dG = -18.4
55 >s_b_s_b_s_a_s_c_s_b_s GTGGCTGTGGCTGTGTCTGTGTGGGTGGCTGTG; >clamp_s_c_s_b_s:compl:rev
CACAGCCACCCACACTATCATGTTGACATTCC; dG = -18.4
56 >s_b_s_b_s_b_s_c_s_b_s GTGGCTGTGGCTGTGGCTGTGTGGGTGGCTGTG; >clamp_s_c_s_b_s:compl:rev
CACAGCCACCCACACTATCATGTTGACATTCC; dG = -18.4
57 >s_b_s_b_s_c_s_c_s_b_s GTGGCTGTGGCTGTGTGGGTGTGGGTGGCTGTG; >clamp_s_c_s_b_s:compl:rev
CACAGCCACCCACACTATCATGTTGACATTCC; dG = -18.4
58 >s_b_s_b_s_d_s_c_s_b_s GTGGCTGTGGCTGTGACGGTGTGGGTGGCTGTG; >clamp_s_c_s_b_s:compl:rev
CACAGCCACCCACACTATCATGTTGACATTCC; dG = -18.4
59 >s_b_s_b_s_e_s_c_s_b_s GTGGCTGTGGCTGTGCTGGTGTGGGTGGCTGTG; >clamp_s_c_s_b_s:compl:rev
CACAGCCACCCACACTATCATGTTGACATTCC; dG = -18.4
60 >s_b_s_c_s_a_s_c_s_b_s GTGGCTGTGTGGGTGTCTGTGTGGGTGGCTGTG; >clamp_s_c_s_b_s:compl:rev
CACAGCCACCCACACTATCATGTTGACATTCC; dG = -18.4
61 >s_b_s_c_s_c_s_c_s_b_s GTGGCTGTGTGGGTGTGGGTGTGGGTGGCTGTG; >clamp_s_c_s_b_s:compl:rev
CACAGCCACCCACACTATCATGTTGACATTCC; dG = -18.4
62 >s_b_s_c_s_d_s_c_s_b_s GTGGCTGTGTGGGTGACGGTGTGGGTGGCTGTG; >clamp_s_c_s_b_s:compl:rev
CACAGCCACCCACACTATCATGTTGACATTCC; dG = -18.4
63 >s_a_s_a_s_d_s_c_s_b_s GTGTCTGTGTCTGTGACGGTGTGGGTGGCTGTG; >clamp_s_c_s_b_s:compl:rev
CACAGCCACCCACACTATCATGTTGACATTCC; dG = -18.4
64 >s_b_s_c_s_e_s_c_s_b_s GTGGCTGTGTGGGTGCTGGTGTGGGTGGCTGTG; >clamp_s_c_s_b_s:compl:rev
CACAGCCACCCACACTATCATGTTGACATTCC; dG = -18.4
65 >s_b_s_d_s_a_s_c_s_b_s GTGGCTGTGACGGTGTCTGTGTGGGTGGCTGTG; >clamp_s_c_s_b_s:compl:rev
CACAGCCACCCACACTATCATGTTGACATTCC; dG = -18.4
66 >s_b_s_d_s_b_s_c_s_b_s GTGGCTGTGACGGTGGCTGTGTGGGTGGCTGTG; >clamp_s_c_s_b_s:compl:rev
CACAGCCACCCACACTATCATGTTGACATTCC; dG = -18.4
67 >s_b_s_d_s_c_s_c_s_b_s GTGGCTGTGACGGTGTGGGTGTGGGTGGCTGTG; >clamp_s_c_s_b_s:compl:rev
CACAGCCACCCACACTATCATGTTGACATTCC; dG = -18.4
68 >s_b_s_d_s_d_s_c_s_b_s GTGGCTGTGACGGTGACGGTGTGGGTGGCTGTG; >clamp_s_c_s_b_s:compl:rev
CACAGCCACCCACACTATCATGTTGACATTCC; dG = -18.4
69 >s_b_s_d_s_e_s_c_s_b_s GTGGCTGTGACGGTGCTGGTGTGGGTGGCTGTG; >clamp_s_c_s_b_s:compl:rev
CACAGCCACCCACACTATCATGTTGACATTCC; dG = -18.4
70 >s_b_s_e_s_a_s_c_s_b_s GTGGCTGTGCTGGTGTCTGTGTGGGTGGCTGTG; >clamp_s_c_s_b_s:compl:rev
CACAGCCACCCACACTATCATGTTGACATTCC; dG = -18.4
71 >s_b_s_e_s_b_s_c_s_b_s GTGGCTGTGCTGGTGGCTGTGTGGGTGGCTGTG; >clamp_s_c_s_b_s:compl:rev
CACAGCCACCCACACTATCATGTTGACATTCC; dG = -18.4
72 >s_b_s_e_s_c_s_c_s_b_s GTGGCTGTGCTGGTGTGGGTGTGGGTGGCTGTG; >clamp_s_c_s_b_s:compl:rev
CACAGCCACCCACACTATCATGTTGACATTCC; dG = -18.4
73 >s_b_s_e_s_d_s_c_s_b_s GTGGCTGTGCTGGTGACGGTGTGGGTGGCTGTG; >clamp_s_c_s_b_s:compl:rev
CACAGCCACCCACACTATCATGTTGACATTCC; dG = -18.4
74 >s_a_s_a_s_e_s_c_s_b_s GTGTCTGTGTCTGTGCTGGTGTGGGTGGCTGTG; >clamp_s_c_s_b_s:compl:rev
CACAGCCACCCACACTATCATGTTGACATTCC; dG = -18.4
75 >s_b_s_e_s_e_s_c_s_b_s GTGGCTGTGCTGGTGCTGGTGTGGGTGGCTGTG; >clamp_s_c_s_b_s:compl:rev
CACAGCCACCCACACTATCATGTTGACATTCC; dG = -18.4
76 >s_c_s_a_s_a_s_c_s_b_s GTGTGGGTGTCTGTGTCTGTGTGGGTGGCTGTG; >clamp_s_c_s_b_s:compl:rev
CACAGCCACCCACACTATCATGTTGACATTCC; dG = -18.4
77 >s_c_s_a_s_b_s_c_s_b_s GTGTGGGTGTCTGTGGCTGTGTGGGTGGCTGTG; >clamp_s_c_s_b_s:compl:rev
CACAGCCACCCACACTATCATGTTGACATTCC; dG = -18.4
78 >s_c_s_a_s_c_s_c_s_b_s GTGTGGGTGTCTGTGTGGGTGTGGGTGGCTGTG; >clamp_s_c_s_b_s:compl:rev
CACAGCCACCCACACTATCATGTTGACATTCC; dG = -18.4
79 >s_c_s_a_s_d_s_c_s_b_s GTGTGGGTGTCTGTGACGGTGTGGGTGGCTGTG; >clamp_s_c_s_b_s:compl:rev
CACAGCCACCCACACTATCATGTTGACATTCC; dG = -18.4
80 >s_c_s_a_s_e_s_c_s_b_s GTGTGGGTGTCTGTGCTGGTGTGGGTGGCTGTG; >clamp_s_c_s_b_s:compl:rev
CACAGCCACCCACACTATCATGTTGACATTCC; dG = -18.4
81 >s_c_s_b_s_a_s_c_s_b_s GTGTGGGTGGCTGTGTCTGTGTGGGTGGCTGTG; >clamp_s_c_s_b_s:compl:rev
CACAGCCACCCACACTATCATGTTGACATTCC; dG = -18.4
82 >s_c_s_b_s_b_s_c_s_b_s GTGTGGGTGGCTGTGGCTGTGTGGGTGGCTGTG; >clamp_s_c_s_b_s:compl:rev
CACAGCCACCCACACTATCATGTTGACATTCC; dG = -18.4
83 >s_c_s_b_s_c_s_c_s_b_s GTGTGGGTGGCTGTGTGGGTGTGGGTGGCTGTG; >clamp_s_c_s_b_s:compl:rev
CACAGCCACCCACACTATCATGTTGACATTCC; dG = -18.4
84 >s_a_s_b_s_a_s_c_s_b_s GTGTCTGTGGCTGTGTCTGTGTGGGTGGCTGTG; >clamp_s_c_s_b_s:compl:rev
CACAGCCACCCACACTATCATGTTGACATTCC; dG = -18.4
85 >s_c_s_b_s_e_s_c_s_b_s GTGTGGGTGGCTGTGCTGGTGTGGGTGGCTGTG; >clamp_s_c_s_b_s:compl:rev
CACAGCCACCCACACTATCATGTTGACATTCC; dG = -18.4
86 >s_c_s_c_s_a_s_c_s_b_s GTGTGGGTGTGGGTGTCTGTGTGGGTGGCTGTG; >clamp_s_c_s_b_s:compl:rev
CACAGCCACCCACACTATCATGTTGACATTCC; dG = -18.4
87 >s_c_s_c_s_c_s_c_s_b_s GTGTGGGTGTGGGTGTGGGTGTGGGTGGCTGTG; >clamp_s_c_s_b_s:compl:rev
CACAGCCACCCACACTATCATGTTGACATTCC; dG = -18.4
88 >s_c_s_c_s_d_s_c_s_b_s GTGTGGGTGTGGGTGACGGTGTGGGTGGCTGTG; >clamp_s_c_s_b_s:compl:rev
CACAGCCACCCACACTATCATGTTGACATTCC; dG = -18.4

89 >s_c_s_c_s_e_s_c_s_b_s GTGTGGGTGTGGGTGCTGGTGTGGGTGGCTGTG; >clamp_s_c_s_b_s:compl:rev
CACAGCCACCCACACTATCATGTTGACATTCC; dG = -18.4
90 >s_c_s_d_s_a_s_c_s_b_s GTGTGGGTGACGGTGTCTGTGTGGGTGGCTGTG; >clamp_s_c_s_b_s:compl:rev
CACAGCCACCCACACTATCATGTTGACATTCC; dG = -18.4
91 >s_c_s_d_s_b_s_c_s_b_s GTGTGGGTGACGGTGGCTGTGTGGGTGGCTGTG; >clamp_s_c_s_b_s:compl:rev
CACAGCCACCCACACTATCATGTTGACATTCC; dG = -18.4
92 >s_c_s_d_s_c_s_c_s_b_s GTGTGGGTGACGGTGTGGGTGTGGGTGGCTGTG; >clamp_s_c_s_b_s:compl:rev
CACAGCCACCCACACTATCATGTTGACATTCC; dG = -18.4
93 >s_c_s_d_s_d_s_c_s_b_s GTGTGGGTGACGGTGACGGTGTGGGTGGCTGTG; >clamp_s_c_s_b_s:compl:rev
CACAGCCACCCACACTATCATGTTGACATTCC; dG = -18.4
94 >s_a_s_b_s_b_s_c_s_b_s GTGTCTGTGGCTGTGGCTGTGTGGGTGGCTGTG; >clamp_s_c_s_b_s:compl:rev
CACAGCCACCCACACTATCATGTTGACATTCC; dG = -18.4
95 >s_c_s_d_s_e_s_c_s_b_s GTGTGGGTGACGGTGCTGGTGTGGGTGGCTGTG; >clamp_s_c_s_b_s:compl:rev
CACAGCCACCCACACTATCATGTTGACATTCC; dG = -18.4
96 >s_c_s_e_s_a_s_c_s_b_s GTGTGGGTGCTGGTGTCTGTGTGGGTGGCTGTG; >clamp_s_c_s_b_s:compl:rev
CACAGCCACCCACACTATCATGTTGACATTCC; dG = -18.4
97 >s_c_s_e_s_b_s_c_s_b_s GTGTGGGTGCTGGTGGCTGTGTGGGTGGCTGTG; >clamp_s_c_s_b_s:compl:rev
CACAGCCACCCACACTATCATGTTGACATTCC; dG = -18.4
98 >s_c_s_e_s_c_s_c_s_b_s GTGTGGGTGCTGGTGTGGGTGTGGGTGGCTGTG; >clamp_s_c_s_b_s:compl:rev
CACAGCCACCCACACTATCATGTTGACATTCC; dG = -18.4
99 >s_c_s_e_s_d_s_c_s_b_s GTGTGGGTGCTGGTGACGGTGTGGGTGGCTGTG; >clamp_s_c_s_b_s:compl:rev
CACAGCCACCCACACTATCATGTTGACATTCC; dG = -18.4
100 >s_c_s_e_s_e_s_c_s_b_s GTGTGGGTGCTGGTGCTGGTGTGGGTGGCTGTG; >clamp_s_c_s_b_s:compl:rev
CACAGCCACCCACACTATCATGTTGACATTCC; dG = -18.4
101 >s_d_s_a_s_a_s_c_s_b_s GTGACGGTGTCTGTGTCTGTGTGGGTGGCTGTG; >clamp_s_c_s_b_s:compl:rev
CACAGCCACCCACACTATCATGTTGACATTCC; dG = -18.4
102 >s_d_s_a_s_b_s_c_s_b_s GTGACGGTGTCTGTGGCTGTGTGGGTGGCTGTG; >clamp_s_c_s_b_s:compl:rev
CACAGCCACCCACACTATCATGTTGACATTCC; dG = -18.4
103 >s_d_s_a_s_c_s_c_s_b_s GTGACGGTGTCTGTGTGGGTGTGGGTGGCTGTG; >clamp_s_c_s_b_s:compl:rev
CACAGCCACCCACACTATCATGTTGACATTCC; dG = -18.4
104 >s_d_s_a_s_d_s_c_s_b_s GTGACGGTGTCTGTGACGGTGTGGGTGGCTGTG; >clamp_s_c_s_b_s:compl:rev
CACAGCCACCCACACTATCATGTTGACATTCC; dG = -18.4
105 >s_a_s_b_s_c_s_c_s_b_s GTGTCTGTGGCTGTGTGGGTGTGGGTGGCTGTG; >clamp_s_c_s_b_s:compl:rev
CACAGCCACCCACACTATCATGTTGACATTCC; dG = -18.4
106 >s_d_s_a_s_e_s_c_s_b_s GTGACGGTGTCTGTGCTGGTGTGGGTGGCTGTG; >clamp_s_c_s_b_s:compl:rev
CACAGCCACCCACACTATCATGTTGACATTCC; dG = -18.4
107 >s_d_s_b_s_a_s_c_s_b_s GTGACGGTGGCTGTGTCTGTGTGGGTGGCTGTG; >clamp_s_c_s_b_s:compl:rev
CACAGCCACCCACACTATCATGTTGACATTCC; dG = -18.4
108 >s_d_s_b_s_b_s_c_s_b_s GTGACGGTGGCTGTGGCTGTGTGGGTGGCTGTG; >clamp_s_c_s_b_s:compl:rev
CACAGCCACCCACACTATCATGTTGACATTCC; dG = -18.4
109 >s_d_s_b_s_c_s_c_s_b_s GTGACGGTGGCTGTGTGGGTGTGGGTGGCTGTG; >clamp_s_c_s_b_s:compl:rev
CACAGCCACCCACACTATCATGTTGACATTCC; dG = -18.4
110 >s_d_s_b_s_d_s_c_s_b_s GTGACGGTGGCTGTGACGGTGTGGGTGGCTGTG; >clamp_s_c_s_b_s:compl:rev
CACAGCCACCCACACTATCATGTTGACATTCC; dG = -18.4
111 >s_d_s_b_s_e_s_c_s_b_s GTGACGGTGGCTGTGCTGGTGTGGGTGGCTGTG; >clamp_s_c_s_b_s:compl:rev
CACAGCCACCCACACTATCATGTTGACATTCC; dG = -18.4
112 >s_d_s_c_s_a_s_c_s_b_s GTGACGGTGTGGGTGTCTGTGTGGGTGGCTGTG; >clamp_s_c_s_b_s:compl:rev
CACAGCCACCCACACTATCATGTTGACATTCC; dG = -18.4
113 >s_d_s_c_s_c_s_c_s_b_s GTGACGGTGTGGGTGTGGGTGTGGGTGGCTGTG; >clamp_s_c_s_b_s:compl:rev
CACAGCCACCCACACTATCATGTTGACATTCC; dG = -18.4
114 >s_d_s_c_s_d_s_c_s_b_s GTGACGGTGTGGGTGACGGTGTGGGTGGCTGTG; >clamp_s_c_s_b_s:compl:rev
CACAGCCACCCACACTATCATGTTGACATTCC; dG = -18.4
115 >s_a_s_b_s_d_s_c_s_b_s GTGTCTGTGGCTGTGACGGTGTGGGTGGCTGTG; >clamp_s_c_s_b_s:compl:rev
CACAGCCACCCACACTATCATGTTGACATTCC; dG = -18.4
116 >s_d_s_c_s_e_s_c_s_b_s GTGACGGTGTGGGTGCTGGTGTGGGTGGCTGTG; >clamp_s_c_s_b_s:compl:rev
CACAGCCACCCACACTATCATGTTGACATTCC; dG = -18.4
117 >s_d_s_d_s_a_s_c_s_b_s GTGACGGTGACGGTGTCTGTGTGGGTGGCTGTG; >clamp_s_c_s_b_s:compl:rev
CACAGCCACCCACACTATCATGTTGACATTCC; dG = -18.4
118 >s_d_s_d_s_b_s_c_s_b_s GTGACGGTGACGGTGGCTGTGTGGGTGGCTGTG; >clamp_s_c_s_b_s:compl:rev
CACAGCCACCCACACTATCATGTTGACATTCC; dG = -18.4
119 >s_d_s_d_s_c_s_c_s_b_s GTGACGGTGACGGTGTGGGTGTGGGTGGCTGTG; >clamp_s_c_s_b_s:compl:rev
CACAGCCACCCACACTATCATGTTGACATTCC; dG = -18.4
120 >s_d_s_d_s_d_s_c_s_b_s GTGACGGTGACGGTGACGGTGTGGGTGGCTGTG; >clamp_s_c_s_b_s:compl:rev
CACAGCCACCCACACTATCATGTTGACATTCC; dG = -18.4
121 >s_d_s_d_s_e_s_c_s_b_s GTGACGGTGACGGTGCTGGTGTGGGTGGCTGTG; >clamp_s_c_s_b_s:compl:rev
CACAGCCACCCACACTATCATGTTGACATTCC; dG = -18.4
122 >s_d_s_e_s_a_s_c_s_b_s GTGACGGTGCTGGTGTCTGTGTGGGTGGCTGTG; >clamp_s_c_s_b_s:compl:rev
CACAGCCACCCACACTATCATGTTGACATTCC; dG = -18.4
123 >s_d_s_e_s_b_s_c_s_b_s GTGACGGTGCTGGTGGCTGTGTGGGTGGCTGTG; >clamp_s_c_s_b_s:compl:rev
CACAGCCACCCACACTATCATGTTGACATTCC; dG = -18.4
124 >s_d_s_e_s_c_s_c_s_b_s GTGACGGTGCTGGTGTGGGTGTGGGTGGCTGTG; >clamp_s_c_s_b_s:compl:rev
CACAGCCACCCACACTATCATGTTGACATTCC; dG = -18.4
125 >s_d_s_e_s_d_s_c_s_b_s GTGACGGTGCTGGTGACGGTGTGGGTGGCTGTG; >clamp_s_c_s_b_s:compl:rev
CACAGCCACCCACACTATCATGTTGACATTCC; dG = -18.4

```
*********************************************************************************

CB Mismatches

     1 >s_b_s_c_s_a_s_d_s_b_s  GTGGCTGTGTGGGTGTCTGTGACGGTGGCTGTG;  >clamp_s_c_s_b_s:compl:rev
CACAGCCACCCACACTATCATGTTGACATTCC; dG = -13.6
     2 >s_a_s_c_s_a_s_d_s_b_s  GTGTCTGTGTGGGTGTCTGTGACGGTGGCTGTG;  >clamp_s_c_s_b_s:compl:rev
CACAGCCACCCACACTATCATGTTGACATTCC; dG = -13.6
     3 >s_c_s_c_s_a_s_d_s_b_s  GTGTGGGTGTGGGTGTCTGTGACGGTGGCTGTG;  >clamp_s_c_s_b_s:compl:rev
CACAGCCACCCACACTATCATGTTGACATTCC; dG = -13.5
     4 >s_d_s_c_s_a_s_d_s_b_s  GTGACGGTGTGGGTGTCTGTGACGGTGGCTGTG;  >clamp_s_c_s_b_s:compl:rev
CACAGCCACCCACACTATCATGTTGACATTCC; dG = -13.5
     5 >s_e_s_c_s_a_s_d_s_b_s  GTGCTGGTGTGGGTGTCTGTGACGGTGGCTGTG;  >clamp_s_c_s_b_s:compl:rev
CACAGCCACCCACACTATCATGTTGACATTCC; dG = -13.5
     6 >s_a_s_c_s_d_s_b_s_b_s  GTGTCTGTGTGGGTGACGGTGGCTGTGGCTGTG;  >clamp_s_c_s_b_s:compl:rev
CACAGCCACCCACACTATCATGTTGACATTCC; dG = -13.3
     7 >s_a_s_c_s_e_s_b_s_b_s  GTGTCTGTGTGGGTGCTGGTGGCTGTGGCTGTG;  >clamp_s_c_s_b_s:compl:rev
CACAGCCACCCACACTATCATGTTGACATTCC; dG = -13.3
     8 >s_b_s_c_s_a_s_b_s_b_s  GTGGCTGTGTGGGTGTCTGTGGCTGTGGCTGTG;  >clamp_s_c_s_b_s:compl:rev
CACAGCCACCCACACTATCATGTTGACATTCC; dG = -13.3
     9 >s_b_s_c_s_d_s_b_s_b_s  GTGGCTGTGTGGGTGACGGTGGCTGTGGCTGTG;  >clamp_s_c_s_b_s:compl:rev
CACAGCCACCCACACTATCATGTTGACATTCC; dG = -13.3
    10 >s_b_s_c_s_e_s_b_s_b_s  GTGGCTGTGTGGGTGCTGGTGGCTGTGGCTGTG;  >clamp_s_c_s_b_s:compl:rev
CACAGCCACCCACACTATCATGTTGACATTCC; dG = -13.3
    11 >s_c_s_a_s_b_s_d_s_b_s  GTGTGGGTGTCTGTGGCTGTGACGGTGGCTGTG;  >clamp_s_c_s_b_s:compl:rev
CACAGCCACCCACACTATCATGTTGACATTCC; dG = -13.3
    12 >s_c_s_c_s_a_s_b_s_b_s  GTGTGGGTGTGGGTGTCTGTGGCTGTGGCTGTG;  >clamp_s_c_s_b_s:compl:rev
CACAGCCACCCACACTATCATGTTGACATTCC; dG = -13.3
    13 >s_c_s_c_s_d_s_b_s_b_s  GTGTGGGTGTGGGTGACGGTGGCTGTGGCTGTG;  >clamp_s_c_s_b_s:compl:rev
CACAGCCACCCACACTATCATGTTGACATTCC; dG = -13.3
    14 >s_c_s_c_s_e_s_b_s_b_s  GTGTGGGTGTGGGTGCTGGTGGCTGTGGCTGTG;  >clamp_s_c_s_b_s:compl:rev
CACAGCCACCCACACTATCATGTTGACATTCC; dG = -13.3
    15 >s_c_s_d_s_b_s_d_s_b_s  GTGTGGGTGACGGTGGCTGTGACGGTGGCTGTG;  >clamp_s_c_s_b_s:compl:rev
CACAGCCACCCACACTATCATGTTGACATTCC; dG = -13.3
    16 >s_c_s_e_s_b_s_d_s_b_s  GTGTGGGTGCTGGTGGCTGTGACGGTGGCTGTG;  >clamp_s_c_s_b_s:compl:rev
CACAGCCACCCACACTATCATGTTGACATTCC; dG = -13.3
    17 >s_d_s_c_s_a_s_b_s_b_s  GTGACGGTGTGGGTGTCTGTGGCTGTGGCTGTG;  >clamp_s_c_s_b_s:compl:rev
CACAGCCACCCACACTATCATGTTGACATTCC; dG = -13.3
    18 >s_d_s_c_s_d_s_b_s_b_s  GTGACGGTGTGGGTGACGGTGGCTGTGGCTGTG;  >clamp_s_c_s_b_s:compl:rev
CACAGCCACCCACACTATCATGTTGACATTCC; dG = -13.3
    19 >s_d_s_c_s_e_s_b_s_b_s  GTGACGGTGTGGGTGCTGGTGGCTGTGGCTGTG;  >clamp_s_c_s_b_s:compl:rev
CACAGCCACCCACACTATCATGTTGACATTCC; dG = -13.3
    20 >s_e_s_c_s_a_s_b_s_b_s  GTGCTGGTGTGGGTGTCTGTGGCTGTGGCTGTG;  >clamp_s_c_s_b_s:compl:rev
CACAGCCACCCACACTATCATGTTGACATTCC; dG = -13.3
    21 >s_e_s_c_s_d_s_b_s_b_s  GTGCTGGTGTGGGTGACGGTGGCTGTGGCTGTG;  >clamp_s_c_s_b_s:compl:rev
CACAGCCACCCACACTATCATGTTGACATTCC; dG = -13.3
    22 >s_e_s_c_s_e_s_b_s_b_s  GTGCTGGTGTGGGTGCTGGTGGCTGTGGCTGTG;  >clamp_s_c_s_b_s:compl:rev
CACAGCCACCCACACTATCATGTTGACATTCC; dG = -13.3
    23 >s_a_s_c_s_a_s_b_s_b_s  GTGTCTGTGTGGGTGTCTGTGGCTGTGGCTGTG;  >clamp_s_c_s_b_s:compl:rev
CACAGCCACCCACACTATCATGTTGACATTCC; dG = -13.3
    24 >s_a_s_d_s_c_s_a_s_b_s  GTGTCTGTGACGGTGTGGGTGTCTGTGGCTGTG;  >clamp_s_c_s_b_s:compl:rev
CACAGCCACCCACACTATCATGTTGACATTCC; dG = -13.1
    25 >s_a_s_e_s_c_s_a_s_b_s  GTGTCTGTGCTGGTGTGGGTGTCTGTGGCTGTG;  >clamp_s_c_s_b_s:compl:rev
CACAGCCACCCACACTATCATGTTGACATTCC; dG = -13.1
    26 >s_b_s_a_s_c_s_a_s_b_s  GTGGCTGTGTCTGTGTGGGTGTCTGTGGCTGTG;  >clamp_s_c_s_b_s:compl:rev
CACAGCCACCCACACTATCATGTTGACATTCC; dG = -13.1
    27 >s_a_s_a_s_c_s_a_s_b_s  GTGTCTGTGTCTGTGTGGGTGTCTGTGGCTGTG;  >clamp_s_c_s_b_s:compl:rev
CACAGCCACCCACACTATCATGTTGACATTCC; dG = -13.1
    28 >s_b_s_b_s_c_s_a_s_b_s  GTGGCTGTGGCTGTGTGGGTGTCTGTGGCTGTG;  >clamp_s_c_s_b_s:compl:rev
CACAGCCACCCACACTATCATGTTGACATTCC; dG = -13.1
    29 >s_b_s_c_s_c_s_a_s_b_s  GTGGCTGTGTGGGTGTGGGTGTCTGTGGCTGTG;  >clamp_s_c_s_b_s:compl:rev
CACAGCCACCCACACTATCATGTTGACATTCC; dG = -13.1
    30 >s_b_s_d_s_c_s_a_s_b_s  GTGGCTGTGACGGTGTGGGTGTCTGTGGCTGTG;  >clamp_s_c_s_b_s:compl:rev
CACAGCCACCCACACTATCATGTTGACATTCC; dG = -13.1
    31 >s_b_s_e_s_c_s_a_s_b_s  GTGGCTGTGCTGGTGTGGGTGTCTGTGGCTGTG;  >clamp_s_c_s_b_s:compl:rev
CACAGCCACCCACACTATCATGTTGACATTCC; dG = -13.1
    32 >s_c_s_a_s_c_s_a_s_b_s  GTGTGGGTGTCTGTGTGGGTGTCTGTGGCTGTG;  >clamp_s_c_s_b_s:compl:rev
CACAGCCACCCACACTATCATGTTGACATTCC; dG = -13.1
    33 >s_c_s_a_s_d_s_c_s_e_s  GTGTGGGTGTCTGTGACGGTGTGGGTGCTGGTG;  >clamp_s_c_s_b_s:compl:rev
CACAGCCACCCACACTATCATGTTGACATTCC; dG = -13.1
```

34 >s_c_s_a_s_d_s_c_s_d_s GTGTGGGTGTCTGTGACGGTGTGGGTGACGGTG; >clamp_s_c_s_b_s:compl:rev CACAGCCACCCACACTATCATGTTGACATTCC; dG = -13.1
35 >s_c_s_a_s_d_s_c_s_c_s GTGTGGGTGTCTGTGACGGTGTGGGTGTGGGTG; >clamp_s_c_s_b_s:compl:rev CACAGCCACCCACACTATCATGTTGACATTCC; dG = -13.1
36 >s_c_s_a_s_d_s_c_s_a_s GTGTGGGTGTCTGTGACGGTGTGGGTGTCTGTG; >clamp_s_c_s_b_s:compl:rev CACAGCCACCCACACTATCATGTTGACATTCC; dG = -13.1
37 >s_c_s_a_s_d_s_e_s_b_s GTGTGGGTGTCTGTGACGGTGCTGGTGGCTGTG; >clamp_s_c_s_b_s:compl:rev CACAGCCACCCACACTATCATGTTGACATTCC; dG = -13.1
38 >s_c_s_a_s_d_s_d_s_b_s GTGTGGGTGTCTGTGACGGTGACGGTGGCTGTG; >clamp_s_c_s_b_s:compl:rev CACAGCCACCCACACTATCATGTTGACATTCC; dG = -13.1
39 >s_c_s_a_s_d_s_b_s_b_s GTGTGGGTGTCTGTGACGGTGGCTGTGGCTGTG; >clamp_s_c_s_b_s:compl:rev CACAGCCACCCACACTATCATGTTGACATTCC; dG = -13.1
40 >s_c_s_a_s_d_s_a_s_b_s GTGTGGGTGTCTGTGACGGTGTCTGTGGCTGTG; >clamp_s_c_s_b_s:compl:rev CACAGCCACCCACACTATCATGTTGACATTCC; dG = -13.1
41 >s_c_s_c_s_c_s_a_s_b_s GTGTGGGTGTGGGTGTGGGTGTCTGTGGCTGTG; >clamp_s_c_s_b_s:compl:rev CACAGCCACCCACACTATCATGTTGACATTCC; dG = -13.1
42 >s_c_s_d_s_c_s_a_s_b_s GTGTGGGTGACGGTGTGGGTGTCTGTGGCTGTG; >clamp_s_c_s_b_s:compl:rev CACAGCCACCCACACTATCATGTTGACATTCC; dG = -13.1
43 >s_c_s_e_s_c_s_a_s_b_s GTGTGGGTGCTGGTGTGGGTGTCTGTGGCTGTG; >clamp_s_c_s_b_s:compl:rev CACAGCCACCCACACTATCATGTTGACATTCC; dG = -13.1
44 >s_d_s_a_s_c_s_a_s_b_s GTGACGGTGTCTGTGTGGGTGTCTGTGGCTGTG; >clamp_s_c_s_b_s:compl:rev CACAGCCACCCACACTATCATGTTGACATTCC; dG = -13.1
45 >s_d_s_b_s_c_s_a_s_b_s GTGACGGTGGCTGTGTGGGTGTCTGTGGCTGTG; >clamp_s_c_s_b_s:compl:rev CACAGCCACCCACACTATCATGTTGACATTCC; dG = -13.1
46 >s_d_s_c_s_c_s_a_s_b_s GTGACGGTGTGGGTGTGGGTGTCTGTGGCTGTG; >clamp_s_c_s_b_s:compl:rev CACAGCCACCCACACTATCATGTTGACATTCC; dG = -13.1
47 >s_a_s_b_s_c_s_a_s_b_s GTGTCTGTGGCTGTGTGGGTGTCTGTGGCTGTG; >clamp_s_c_s_b_s:compl:rev CACAGCCACCCACACTATCATGTTGACATTCC; dG = -13.1
48 >s_d_s_d_s_c_s_a_s_b_s GTGACGGTGACGGTGTGGGTGTCTGTGGCTGTG; >clamp_s_c_s_b_s:compl:rev CACAGCCACCCACACTATCATGTTGACATTCC; dG = -13.1
49 >s_d_s_e_s_c_s_a_s_b_s GTGACGGTGCTGGTGTGGGTGTCTGTGGCTGTG; >clamp_s_c_s_b_s:compl:rev CACAGCCACCCACACTATCATGTTGACATTCC; dG = -13.1
50 >s_e_s_a_s_c_s_a_s_b_s GTGCTGGTGTCTGTGTGGGTGTCTGTGGCTGTG; >clamp_s_c_s_b_s:compl:rev CACAGCCACCCACACTATCATGTTGACATTCC; dG = -13.1
51 >s_e_s_b_s_c_s_a_s_b_s GTGCTGGTGGCTGTGTGGGTGTCTGTGGCTGTG; >clamp_s_c_s_b_s:compl:rev CACAGCCACCCACACTATCATGTTGACATTCC; dG = -13.1
52 >s_e_s_c_s_c_s_a_s_b_s GTGCTGGTGTGGGTGTGGGTGTCTGTGGCTGTG; >clamp_s_c_s_b_s:compl:rev CACAGCCACCCACACTATCATGTTGACATTCC; dG = -13.1
53 >s_e_s_d_s_c_s_a_s_b_s GTGCTGGTGACGGTGTGGGTGTCTGTGGCTGTG; >clamp_s_c_s_b_s:compl:rev CACAGCCACCCACACTATCATGTTGACATTCC; dG = -13.1
54 >s_e_s_e_s_c_s_a_s_b_s GTGCTGGTGCTGGTGTGGGTGTCTGTGGCTGTG; >clamp_s_c_s_b_s:compl:rev CACAGCCACCCACACTATCATGTTGACATTCC; dG = -13.1
55 >s_a_s_c_s_c_s_a_s_b_s GTGTCTGTGTGGGTGTGGGTGTCTGTGGCTGTG; >clamp_s_c_s_b_s:compl:rev CACAGCCACCCACACTATCATGTTGACATTCC; dG = -13.1
56 >s_a_s_d_s_b_s_d_s_b_s GTGTCTGTGACGGTGGCTGTGACGGTGGCTGTG; >clamp_s_c_s_b_s:compl:rev CACAGCCACCCACACTATCATGTTGACATTCC; dG = -13.0
57 >s_b_s_a_s_c_s_e_s_b_s GTGGCTGTGTCTGTGTGGGTGCTGGTGGCTGTG; >clamp_s_c_s_b_s:compl:rev CACAGCCACCCACACTATCATGTTGACATTCC; dG = -13.0
58 >s_b_s_a_s_c_s_d_s_b_s GTGGCTGTGTCTGTGTGGGTGACGGTGGCTGTG; >clamp_s_c_s_b_s:compl:rev CACAGCCACCCACACTATCATGTTGACATTCC; dG = -13.0
59 >s_a_s_a_s_c_s_e_s_b_s GTGTCTGTGTCTGTGTGGGTGCTGGTGGCTGTG; >clamp_s_c_s_b_s:compl:rev CACAGCCACCCACACTATCATGTTGACATTCC; dG = -13.0
60 >s_a_s_a_s_c_s_d_s_b_s GTGTCTGTGTCTGTGTGGGTGACGGTGGCTGTG; >clamp_s_c_s_b_s:compl:rev CACAGCCACCCACACTATCATGTTGACATTCC; dG = -13.0
61 >s_b_s_b_s_c_s_e_s_b_s GTGGCTGTGGCTGTGTGGGTGCTGGTGGCTGTG; >clamp_s_c_s_b_s:compl:rev CACAGCCACCCACACTATCATGTTGACATTCC; dG = -13.0
62 >s_b_s_b_s_c_s_d_s_b_s GTGGCTGTGGCTGTGTGGGTGACGGTGGCTGTG; >clamp_s_c_s_b_s:compl:rev CACAGCCACCCACACTATCATGTTGACATTCC; dG = -13.0
63 >s_b_s_c_s_a_s_c_s_e_s GTGGCTGTGTGGGTGTCTGTGTGGGTGCTGGTG; >clamp_s_c_s_b_s:compl:rev CACAGCCACCCACACTATCATGTTGACATTCC; dG = -13.0
64 >s_b_s_c_s_a_s_c_s_d_s GTGGCTGTGTGGGTGTCTGTGTGGGTGACGGTG; >clamp_s_c_s_b_s:compl:rev CACAGCCACCCACACTATCATGTTGACATTCC; dG = -13.0
65 >s_b_s_c_s_a_s_c_s_c_s GTGGCTGTGTGGGTGTCTGTGTGGGTGTGGGTG; >clamp_s_c_s_b_s:compl:rev CACAGCCACCCACACTATCATGTTGACATTCC; dG = -13.0
66 >s_b_s_c_s_a_s_c_s_a_s GTGGCTGTGTGGGTGTCTGTGTGGGTGTCTGTG; >clamp_s_c_s_b_s:compl:rev CACAGCCACCCACACTATCATGTTGACATTCC; dG = -13.0
67 >s_b_s_c_s_a_s_a_s_b_s GTGGCTGTGTGGGTGTCTGTGTCTGTGGCTGTG; >clamp_s_c_s_b_s:compl:rev CACAGCCACCCACACTATCATGTTGACATTCC; dG = -13.0
68 >s_b_s_d_s_b_s_d_s_b_s GTGGCTGTGACGGTGGCTGTGACGGTGGCTGTG; >clamp_s_c_s_b_s:compl:rev CACAGCCACCCACACTATCATGTTGACATTCC; dG = -13.0
69 >s_c_s_a_s_c_s_e_s_b_s GTGTGGGTGTCTGTGTGGGTGCTGGTGGCTGTG; >clamp_s_c_s_b_s:compl:rev CACAGCCACCCACACTATCATGTTGACATTCC; dG = -13.0
70 >s_c_s_a_s_c_s_d_s_b_s GTGTGGGTGTCTGTGTGGGTGACGGTGGCTGTG; >clamp_s_c_s_b_s:compl:rev CACAGCCACCCACACTATCATGTTGACATTCC; dG = -13.0

71 >s_c_s_c_s_a_s_c_s_e_s GTGTGGGTGTGGGTGTCTGTGTGGGTGCTGGTG; >clamp_s_c_s_b_s:compl:rev CACAGCCACCCACACTATCATGTTGACATTCC; dG = -13.0
72 >s_c_s_c_s_a_s_c_s_d_s GTGTGGGTGTGGGTGTCTGTGTGGGTGACGGTG; >clamp_s_c_s_b_s:compl:rev CACAGCCACCCACACTATCATGTTGACATTCC; dG = -13.0
73 >s_c_s_c_s_a_s_c_s_c_s GTGTGGGTGTGGGTGTCTGTGTGGGTGTGGGTG; >clamp_s_c_s_b_s:compl:rev CACAGCCACCCACACTATCATGTTGACATTCC; dG = -13.0
74 >s_c_s_c_s_a_s_c_s_a_s GTGTGGGTGTGGGTGTCTGTGTGGGTGTCTGTG; >clamp_s_c_s_b_s:compl:rev CACAGCCACCCACACTATCATGTTGACATTCC; dG = -13.0
75 >s_c_s_c_s_a_s_a_s_b_s GTGTGGGTGTGGGTGTCTGTGTCTGTGGCTGTG; >clamp_s_c_s_b_s:compl:rev CACAGCCACCCACACTATCATGTTGACATTCC; dG = -13.0
76 >s_d_s_a_s_c_s_e_s_b_s GTGACGGTGTCTGTGTGGGTGCTGGTGGCTGTG; >clamp_s_c_s_b_s:compl:rev CACAGCCACCCACACTATCATGTTGACATTCC; dG = -13.0
77 >s_d_s_a_s_c_s_d_s_b_s GTGACGGTGTCTGTGTGGGTGACGGTGGCTGTG; >clamp_s_c_s_b_s:compl:rev CACAGCCACCCACACTATCATGTTGACATTCC; dG = -13.0
78 >s_d_s_b_s_c_s_e_s_b_s GTGACGGTGGCTGTGTGGGTGCTGGTGGCTGTG; >clamp_s_c_s_b_s:compl:rev CACAGCCACCCACACTATCATGTTGACATTCC; dG = -13.0
79 >s_d_s_b_s_c_s_d_s_b_s GTGACGGTGGCTGTGTGGGTGACGGTGGCTGTG; >clamp_s_c_s_b_s:compl:rev CACAGCCACCCACACTATCATGTTGACATTCC; dG = -13.0
80 >s_a_s_b_s_c_s_e_s_b_s GTGTCTGTGGCTGTGTGGGTGCTGGTGGCTGTG; >clamp_s_c_s_b_s:compl:rev CACAGCCACCCACACTATCATGTTGACATTCC; dG = -13.0
81 >s_d_s_c_s_a_s_c_s_e_s GTGACGGTGTGGGTGTCTGTGTGGGTGCTGGTG; >clamp_s_c_s_b_s:compl:rev CACAGCCACCCACACTATCATGTTGACATTCC; dG = -13.0
82 >s_d_s_c_s_a_s_c_s_d_s GTGACGGTGTGGGTGTCTGTGTGGGTGACGGTG; >clamp_s_c_s_b_s:compl:rev CACAGCCACCCACACTATCATGTTGACATTCC; dG = -13.0
83 >s_d_s_c_s_a_s_c_s_c_s GTGACGGTGTGGGTGTCTGTGTGGGTGTGGGTG; >clamp_s_c_s_b_s:compl:rev CACAGCCACCCACACTATCATGTTGACATTCC; dG = -13.0
84 >s_d_s_c_s_a_s_c_s_a_s GTGACGGTGTGGGTGTCTGTGTGGGTGTCTGTG; >clamp_s_c_s_b_s:compl:rev CACAGCCACCCACACTATCATGTTGACATTCC; dG = -13.0
85 >s_d_s_c_s_a_s_a_s_b_s GTGACGGTGTGGGTGTCTGTGTCTGTGGCTGTG; >clamp_s_c_s_b_s:compl:rev CACAGCCACCCACACTATCATGTTGACATTCC; dG = -13.0
86 >s_a_s_b_s_c_s_d_s_b_s GTGTCTGTGGCTGTGTGGGTGACGGTGGCTGTG; >clamp_s_c_s_b_s:compl:rev CACAGCCACCCACACTATCATGTTGACATTCC; dG = -13.0
87 >s_d_s_d_s_b_s_d_s_b_s GTGACGGTGACGGTGGCTGTGACGGTGGCTGTG; >clamp_s_c_s_b_s:compl:rev CACAGCCACCCACACTATCATGTTGACATTCC; dG = -13.0
88 >s_e_s_a_s_c_s_e_s_b_s GTGCTGGTGTCTGTGTGGGTGCTGGTGGCTGTG; >clamp_s_c_s_b_s:compl:rev CACAGCCACCCACACTATCATGTTGACATTCC; dG = -13.0
89 >s_e_s_a_s_c_s_d_s_b_s GTGCTGGTGTCTGTGTGGGTGACGGTGGCTGTG; >clamp_s_c_s_b_s:compl:rev CACAGCCACCCACACTATCATGTTGACATTCC; dG = -13.0
90 >s_e_s_b_s_c_s_e_s_b_s GTGCTGGTGGCTGTGTGGGTGCTGGTGGCTGTG; >clamp_s_c_s_b_s:compl:rev CACAGCCACCCACACTATCATGTTGACATTCC; dG = -13.0
91 >s_e_s_b_s_c_s_d_s_b_s GTGCTGGTGGCTGTGTGGGTGACGGTGGCTGTG; >clamp_s_c_s_b_s:compl:rev CACAGCCACCCACACTATCATGTTGACATTCC; dG = -13.0
92 >s_e_s_c_s_a_s_c_s_e_s GTGCTGGTGTGGGTGTCTGTGTGGGTGCTGGTG; >clamp_s_c_s_b_s:compl:rev CACAGCCACCCACACTATCATGTTGACATTCC; dG = -13.0
93 >s_e_s_c_s_a_s_c_s_d_s GTGCTGGTGTGGGTGTCTGTGTGGGTGACGGTG; >clamp_s_c_s_b_s:compl:rev CACAGCCACCCACACTATCATGTTGACATTCC; dG = -13.0
94 >s_a_s_c_s_a_s_c_s_e_s GTGTCTGTGTGGGTGTCTGTGTGGGTGCTGGTG; >clamp_s_c_s_b_s:compl:rev CACAGCCACCCACACTATCATGTTGACATTCC; dG = -13.0
95 >s_e_s_c_s_a_s_c_s_c_s GTGCTGGTGTGGGTGTCTGTGTGGGTGTGGGTG; >clamp_s_c_s_b_s:compl:rev CACAGCCACCCACACTATCATGTTGACATTCC; dG = -13.0
96 >s_e_s_c_s_a_s_c_s_a_s GTGCTGGTGTGGGTGTCTGTGTGGGTGTCTGTG; >clamp_s_c_s_b_s:compl:rev CACAGCCACCCACACTATCATGTTGACATTCC; dG = -13.0
97 >s_e_s_c_s_a_s_a_s_b_s GTGCTGGTGTGGGTGTCTGTGTCTGTGGCTGTG; >clamp_s_c_s_b_s:compl:rev CACAGCCACCCACACTATCATGTTGACATTCC; dG = -13.0
98 >s_a_s_c_s_a_s_c_s_d_s GTGTCTGTGTGGGTGTCTGTGTGGGTGACGGTG; >clamp_s_c_s_b_s:compl:rev CACAGCCACCCACACTATCATGTTGACATTCC; dG = -13.0
99 >s_a_s_c_s_a_s_c_s_c_s GTGTCTGTGTGGGTGTCTGTGTGGGTGTGGGTG; >clamp_s_c_s_b_s:compl:rev CACAGCCACCCACACTATCATGTTGACATTCC; dG = -13.0
100 >s_a_s_c_s_a_s_c_s_a_s GTGTCTGTGTGGGTGTCTGTGTGGGTGTCTGTG; >clamp_s_c_s_b_s:compl:rev CACAGCCACCCACACTATCATGTTGACATTCC; dG = -13.0
101 >s_e_s_d_s_b_s_d_s_b_s GTGCTGGTGACGGTGGCTGTGACGGTGGCTGTG; >clamp_s_c_s_b_s:compl:rev CACAGCCACCCACACTATCATGTTGACATTCC; dG = -13.0
102 >s_a_s_c_s_a_s_a_s_b_s GTGTCTGTGTGGGTGTCTGTGTCTGTGGCTGTG; >clamp_s_c_s_b_s:compl:rev CACAGCCACCCACACTATCATGTTGACATTCC; dG = -13.0
103 >s_a_s_d_s_c_s_e_s_b_s GTGTCTGTGACGGTGTGGGTGCTGGTGGCTGTG; >clamp_s_c_s_b_s:compl:rev CACAGCCACCCACACTATCATGTTGACATTCC; dG = -12.9
104 >s_a_s_d_s_c_s_d_s_b_s GTGTCTGTGACGGTGTGGGTGACGGTGGCTGTG; >clamp_s_c_s_b_s:compl:rev CACAGCCACCCACACTATCATGTTGACATTCC; dG = -12.9
105 >s_a_s_e_s_b_s_d_s_b_s GTGTCTGTGCTGGTGGCTGTGACGGTGGCTGTG; >clamp_s_c_s_b_s:compl:rev CACAGCCACCCACACTATCATGTTGACATTCC; dG = -12.9
106 >s_a_s_e_s_c_s_e_s_b_s GTGTCTGTGCTGGTGTGGGTGCTGGTGGCTGTG; >clamp_s_c_s_b_s:compl:rev CACAGCCACCCACACTATCATGTTGACATTCC; dG = -12.9
107 >s_a_s_e_s_c_s_d_s_b_s GTGTCTGTGCTGGTGTGGGTGACGGTGGCTGTG; >clamp_s_c_s_b_s:compl:rev CACAGCCACCCACACTATCATGTTGACATTCC; dG = -12.9

108 >s_b_s_c_s_a_s_e_s_b_s GTGGCTGTGTGGGTGTCTGTGCTGGTGGCTGTG; >clamp_s_c_s_b_s:compl:rev
CACAGCCACCCACACTATCATGTTGACATTCC; dG = -12.9
109 >s_b_s_c_s_c_s_e_s_b_s GTGGCTGTGTGGGTGTGGGTGCTGGTGGCTGTG; >clamp_s_c_s_b_s:compl:rev
CACAGCCACCCACACTATCATGTTGACATTCC; dG = -12.9
110 >s_b_s_c_s_c_s_d_s_b_s GTGGCTGTGTGGGTGTGGGTGACGGTGGCTGTG; >clamp_s_c_s_b_s:compl:rev
CACAGCCACCCACACTATCATGTTGACATTCC; dG = -12.9
111 >s_b_s_d_s_c_s_e_s_b_s GTGGCTGTGACGGTGTGGGTGCTGGTGGCTGTG; >clamp_s_c_s_b_s:compl:rev
CACAGCCACCCACACTATCATGTTGACATTCC; dG = -12.9
112 >s_b_s_d_s_c_s_d_s_b_s GTGGCTGTGACGGTGTGGGTGACGGTGGCTGTG; >clamp_s_c_s_b_s:compl:rev
CACAGCCACCCACACTATCATGTTGACATTCC; dG = -12.9
113 >s_b_s_e_s_b_s_d_s_b_s GTGGCTGTGCTGGTGGCTGTGACGGTGGCTGTG; >clamp_s_c_s_b_s:compl:rev
CACAGCCACCCACACTATCATGTTGACATTCC; dG = -12.9
114 >s_b_s_e_s_c_s_e_s_b_s GTGGCTGTGCTGGTGTGGGTGCTGGTGGCTGTG; >clamp_s_c_s_b_s:compl:rev
CACAGCCACCCACACTATCATGTTGACATTCC; dG = -12.9
115 >s_b_s_e_s_c_s_d_s_b_s GTGGCTGTGCTGGTGTGGGTGACGGTGGCTGTG; >clamp_s_c_s_b_s:compl:rev
CACAGCCACCCACACTATCATGTTGACATTCC; dG = -12.9
116 >s_c_s_c_s_a_s_e_s_b_s GTGTGGGTGTGGGTGTCTGTGCTGGTGGCTGTG; >clamp_s_c_s_b_s:compl:rev
CACAGCCACCCACACTATCATGTTGACATTCC; dG = -12.9
117 >s_c_s_c_s_c_s_e_s_b_s GTGTGGGTGTGGGTGTGGGTGCTGGTGGCTGTG; >clamp_s_c_s_b_s:compl:rev
CACAGCCACCCACACTATCATGTTGACATTCC; dG = -12.9
118 >s_c_s_c_s_c_s_d_s_b_s GTGTGGGTGTGGGTGTGGGTGACGGTGGCTGTG; >clamp_s_c_s_b_s:compl:rev
CACAGCCACCCACACTATCATGTTGACATTCC; dG = -12.9
119 >s_c_s_d_s_c_s_e_s_b_s GTGTGGGTGACGGTGTGGGTGCTGGTGGCTGTG; >clamp_s_c_s_b_s:compl:rev
CACAGCCACCCACACTATCATGTTGACATTCC; dG = -12.9
120 >s_c_s_d_s_c_s_d_s_b_s GTGTGGGTGACGGTGTGGGTGACGGTGGCTGTG; >clamp_s_c_s_b_s:compl:rev
CACAGCCACCCACACTATCATGTTGACATTCC; dG = -12.9
121 >s_c_s_e_s_c_s_e_s_b_s GTGTGGGTGCTGGTGTGGGTGCTGGTGGCTGTG; >clamp_s_c_s_b_s:compl:rev
CACAGCCACCCACACTATCATGTTGACATTCC; dG = -12.9
122 >s_c_s_e_s_c_s_d_s_b_s GTGTGGGTGCTGGTGTGGGTGACGGTGGCTGTG; >clamp_s_c_s_b_s:compl:rev
CACAGCCACCCACACTATCATGTTGACATTCC; dG = -12.9
123 >s_d_s_c_s_a_s_e_s_b_s GTGACGGTGTGGGTGTCTGTGCTGGTGGCTGTG; >clamp_s_c_s_b_s:compl:rev
CACAGCCACCCACACTATCATGTTGACATTCC; dG = -12.9
124 >s_d_s_c_s_c_s_e_s_b_s GTGACGGTGTGGGTGTGGGTGCTGGTGGCTGTG; >clamp_s_c_s_b_s:compl:rev
CACAGCCACCCACACTATCATGTTGACATTCC; dG = -12.9
125 >s_d_s_c_s_c_s_d_s_b_s GTGACGGTGTGGGTGTGGGTGACGGTGGCTGTG; >clamp_s_c_s_b_s:compl:rev
CACAGCCACCCACACTATCATGTTGACATTCC; dG = -12.9
126 >s_d_s_d_s_c_s_e_s_b_s GTGACGGTGACGGTGTGGGTGCTGGTGGCTGTG; >clamp_s_c_s_b_s:compl:rev
CACAGCCACCCACACTATCATGTTGACATTCC; dG = -12.9
127 >s_d_s_d_s_c_s_d_s_b_s GTGACGGTGACGGTGTGGGTGACGGTGGCTGTG; >clamp_s_c_s_b_s:compl:rev
CACAGCCACCCACACTATCATGTTGACATTCC; dG = -12.9
128 >s_d_s_e_s_b_s_d_s_b_s GTGACGGTGCTGGTGGCTGTGACGGTGGCTGTG; >clamp_s_c_s_b_s:compl:rev
CACAGCCACCCACACTATCATGTTGACATTCC; dG = -12.9
129 >s_d_s_e_s_c_s_e_s_b_s GTGACGGTGCTGGTGTGGGTGCTGGTGGCTGTG; >clamp_s_c_s_b_s:compl:rev
CACAGCCACCCACACTATCATGTTGACATTCC; dG = -12.9
130 >s_d_s_e_s_c_s_d_s_b_s GTGACGGTGCTGGTGTGGGTGACGGTGGCTGTG; >clamp_s_c_s_b_s:compl:rev
CACAGCCACCCACACTATCATGTTGACATTCC; dG = -12.9
131 >s_e_s_c_s_a_s_e_s_b_s GTGCTGGTGTGGGTGTCTGTGCTGGTGGCTGTG; >clamp_s_c_s_b_s:compl:rev
CACAGCCACCCACACTATCATGTTGACATTCC; dG = -12.9
132 >s_e_s_c_s_c_s_e_s_b_s GTGCTGGTGTGGGTGTGGGTGCTGGTGGCTGTG; >clamp_s_c_s_b_s:compl:rev
CACAGCCACCCACACTATCATGTTGACATTCC; dG = -12.9
133 >s_e_s_c_s_c_s_d_s_b_s GTGCTGGTGTGGGTGTGGGTGACGGTGGCTGTG; >clamp_s_c_s_b_s:compl:rev
CACAGCCACCCACACTATCATGTTGACATTCC; dG = -12.9
134 >s_a_s_c_s_a_s_e_s_b_s GTGTCTGTGTGGGTGTCTGTGCTGGTGGCTGTG; >clamp_s_c_s_b_s:compl:rev
CACAGCCACCCACACTATCATGTTGACATTCC; dG = -12.9
135 >s_e_s_d_s_c_s_e_s_b_s GTGCTGGTGACGGTGTGGGTGCTGGTGGCTGTG; >clamp_s_c_s_b_s:compl:rev
CACAGCCACCCACACTATCATGTTGACATTCC; dG = -12.9
136 >s_e_s_d_s_c_s_d_s_b_s GTGCTGGTGACGGTGTGGGTGACGGTGGCTGTG; >clamp_s_c_s_b_s:compl:rev
CACAGCCACCCACACTATCATGTTGACATTCC; dG = -12.9
137 >s_e_s_e_s_b_s_d_s_b_s GTGCTGGTGCTGGTGGCTGTGACGGTGGCTGTG; >clamp_s_c_s_b_s:compl:rev
CACAGCCACCCACACTATCATGTTGACATTCC; dG = -12.9
138 >s_e_s_e_s_c_s_e_s_b_s GTGCTGGTGCTGGTGTGGGTGCTGGTGGCTGTG; >clamp_s_c_s_b_s:compl:rev
CACAGCCACCCACACTATCATGTTGACATTCC; dG = -12.9
139 >s_e_s_e_s_c_s_d_s_b_s GTGCTGGTGCTGGTGTGGGTGACGGTGGCTGTG; >clamp_s_c_s_b_s:compl:rev
CACAGCCACCCACACTATCATGTTGACATTCC; dG = -12.9
140 >s_a_s_c_s_c_s_e_s_b_s GTGTCTGTGTGGGTGTGGGTGCTGGTGGCTGTG; >clamp_s_c_s_b_s:compl:rev
CACAGCCACCCACACTATCATGTTGACATTCC; dG = -12.9
141 >s_a_s_c_s_c_s_d_s_b_s GTGTCTGTGTGGGTGTGGGTGACGGTGGCTGTG; >clamp_s_c_s_b_s:compl:rev
CACAGCCACCCACACTATCATGTTGACATTCC; dG = -12.9
142 >s_a_s_d_s_a_s_c_s_a_s GTGTCTGTGACGGTGTCTGTGTGGGTGTCTGTG; >clamp_s_c_s_b_s:compl:rev
CACAGCCACCCACACTATCATGTTGACATTCC; dG = -12.8
143 >s_a_s_a_s_b_s_c_s_a_s GTGTCTGTGTCTGTGGCTGTGTGGGTGTCTGTG; >clamp_s_c_s_b_s:compl:rev
CACAGCCACCCACACTATCATGTTGACATTCC; dG = -12.8
144 >s_a_s_d_s_b_s_c_s_a_s GTGTCTGTGACGGTGGCTGTGTGGGTGTCTGTG; >clamp_s_c_s_b_s:compl:rev
CACAGCCACCCACACTATCATGTTGACATTCC; dG = -12.8

145 >s_a_s_e_s_a_s_c_s_a_s GTGTCTGTGCTGGTGTCTGTGTGGGTGTCTGTG; >clamp_s_c_s_b_s:compl:rev CACAGCCACCCACACTATCATGTTGACATTCC; dG = -12.8
146 >s_a_s_e_s_b_s_c_s_a_s GTGTCTGTGCTGGTGGCTGTGTGGGTGTCTGTG; >clamp_s_c_s_b_s:compl:rev CACAGCCACCCACACTATCATGTTGACATTCC; dG = -12.8
147 >s_b_s_a_s_a_s_c_s_a_s GTGGCTGTGTCTGTGTCTGTGTGGGTGTCTGTG; >clamp_s_c_s_b_s:compl:rev CACAGCCACCCACACTATCATGTTGACATTCC; dG = -12.8
148 >s_b_s_a_s_b_s_c_s_a_s GTGGCTGTGTCTGTGGCTGTGTGGGTGTCTGTG; >clamp_s_c_s_b_s:compl:rev CACAGCCACCCACACTATCATGTTGACATTCC; dG = -12.8
149 >s_b_s_b_s_a_s_c_s_a_s GTGGCTGTGGCTGTGTCTGTGTGGGTGTCTGTG; >clamp_s_c_s_b_s:compl:rev CACAGCCACCCACACTATCATGTTGACATTCC; dG = -12.8
150 >s_b_s_b_s_b_s_c_s_a_s GTGGCTGTGGCTGTGGCTGTGTGGGTGTCTGTG; >clamp_s_c_s_b_s:compl:rev CACAGCCACCCACACTATCATGTTGACATTCC; dG = -12.8
151 >s_b_s_d_s_a_s_c_s_a_s GTGGCTGTGACGGTGTCTGTGTGGGTGTCTGTG; >clamp_s_c_s_b_s:compl:rev CACAGCCACCCACACTATCATGTTGACATTCC; dG = -12.8
152 >s_b_s_d_s_b_s_c_s_a_s GTGGCTGTGACGGTGGCTGTGTGGGTGTCTGTG; >clamp_s_c_s_b_s:compl:rev CACAGCCACCCACACTATCATGTTGACATTCC; dG = -12.8
153 >s_b_s_e_s_a_s_c_s_a_s GTGGCTGTGCTGGTGTCTGTGTGGGTGTCTGTG; >clamp_s_c_s_b_s:compl:rev CACAGCCACCCACACTATCATGTTGACATTCC; dG = -12.8
154 >s_b_s_e_s_b_s_c_s_a_s GTGGCTGTGCTGGTGGCTGTGTGGGTGTCTGTG; >clamp_s_c_s_b_s:compl:rev CACAGCCACCCACACTATCATGTTGACATTCC; dG = -12.8
155 >s_c_s_a_s_a_s_c_s_a_s GTGTGGGTGTCTGTGTCTGTGTGGGTGTCTGTG; >clamp_s_c_s_b_s:compl:rev CACAGCCACCCACACTATCATGTTGACATTCC; dG = -12.8
156 >s_c_s_a_s_b_s_c_s_a_s GTGTGGGTGTCTGTGGCTGTGTGGGTGTCTGTG; >clamp_s_c_s_b_s:compl:rev CACAGCCACCCACACTATCATGTTGACATTCC; dG = -12.8
157 >s_c_s_a_s_b_s_b_s_b_s GTGTGGGTGTCTGTGGCTGTGGCTGTGGCTGTG; >clamp_s_c_s_b_s:compl:rev CACAGCCACCCACACTATCATGTTGACATTCC; dG = -12.8
158 >s_a_s_a_s_a_s_c_s_a_s GTGTCTGTGTCTGTGTCTGTGTGGGTGTCTGTG; >clamp_s_c_s_b_s:compl:rev CACAGCCACCCACACTATCATGTTGACATTCC; dG = -12.8
159 >s_a_s_b_s_a_s_c_s_a_s GTGTCTGTGGCTGTGTCTGTGTGGGTGTCTGTG; >clamp_s_c_s_b_s:compl:rev CACAGCCACCCACACTATCATGTTGACATTCC; dG = -12.8
160 >s_c_s_d_s_a_s_c_s_a_s GTGTGGGTGACGGTGTCTGTGTGGGTGTCTGTG; >clamp_s_c_s_b_s:compl:rev CACAGCCACCCACACTATCATGTTGACATTCC; dG = -12.8
161 >s_c_s_d_s_b_s_c_s_a_s GTGTGGGTGACGGTGGCTGTGTGGGTGTCTGTG; >clamp_s_c_s_b_s:compl:rev CACAGCCACCCACACTATCATGTTGACATTCC; dG = -12.8
162 >s_c_s_d_s_b_s_b_s_b_s GTGTGGGTGACGGTGGCTGTGGCTGTGGCTGTG; >clamp_s_c_s_b_s:compl:rev CACAGCCACCCACACTATCATGTTGACATTCC; dG = -12.8
163 >s_c_s_e_s_a_s_c_s_a_s GTGTGGGTGCTGGTGTCTGTGTGGGTGTCTGTG; >clamp_s_c_s_b_s:compl:rev CACAGCCACCCACACTATCATGTTGACATTCC; dG = -12.8
164 >s_c_s_e_s_b_s_c_s_a_s GTGTGGGTGCTGGTGGCTGTGTGGGTGTCTGTG; >clamp_s_c_s_b_s:compl:rev CACAGCCACCCACACTATCATGTTGACATTCC; dG = -12.8
165 >s_c_s_e_s_b_s_b_s_b_s GTGTGGGTGCTGGTGGCTGTGGCTGTGGCTGTG; >clamp_s_c_s_b_s:compl:rev CACAGCCACCCACACTATCATGTTGACATTCC; dG = -12.8
166 >s_a_s_b_s_b_s_c_s_a_s GTGTCTGTGGCTGTGGCTGTGTGGGTGTCTGTG; >clamp_s_c_s_b_s:compl:rev CACAGCCACCCACACTATCATGTTGACATTCC; dG = -12.8
167 >s_d_s_a_s_a_s_c_s_a_s GTGACGGTGTCTGTGTCTGTGTGGGTGTCTGTG; >clamp_s_c_s_b_s:compl:rev CACAGCCACCCACACTATCATGTTGACATTCC; dG = -12.8
168 >s_d_s_a_s_b_s_c_s_a_s GTGACGGTGTCTGTGGCTGTGTGGGTGTCTGTG; >clamp_s_c_s_b_s:compl:rev CACAGCCACCCACACTATCATGTTGACATTCC; dG = -12.8
169 >s_d_s_b_s_a_s_c_s_a_s GTGACGGTGGCTGTGTCTGTGTGGGTGTCTGTG; >clamp_s_c_s_b_s:compl:rev CACAGCCACCCACACTATCATGTTGACATTCC; dG = -12.8
170 >s_d_s_b_s_b_s_c_s_a_s GTGACGGTGGCTGTGGCTGTGTGGGTGTCTGTG; >clamp_s_c_s_b_s:compl:rev CACAGCCACCCACACTATCATGTTGACATTCC; dG = -12.8
171 >s_d_s_d_s_a_s_c_s_a_s GTGACGGTGACGGTGTCTGTGTGGGTGTCTGTG; >clamp_s_c_s_b_s:compl:rev CACAGCCACCCACACTATCATGTTGACATTCC; dG = -12.8
172 >s_d_s_d_s_b_s_c_s_a_s GTGACGGTGACGGTGGCTGTGTGGGTGTCTGTG; >clamp_s_c_s_b_s:compl:rev CACAGCCACCCACACTATCATGTTGACATTCC; dG = -12.8
173 >s_d_s_e_s_a_s_c_s_a_s GTGACGGTGCTGGTGTCTGTGTGGGTGTCTGTG; >clamp_s_c_s_b_s:compl:rev CACAGCCACCCACACTATCATGTTGACATTCC; dG = -12.8
174 >s_d_s_e_s_b_s_c_s_a_s GTGACGGTGCTGGTGGCTGTGTGGGTGTCTGTG; >clamp_s_c_s_b_s:compl:rev CACAGCCACCCACACTATCATGTTGACATTCC; dG = -12.8
175 >s_e_s_a_s_a_s_c_s_a_s GTGCTGGTGTCTGTGTCTGTGTGGGTGTCTGTG; >clamp_s_c_s_b_s:compl:rev CACAGCCACCCACACTATCATGTTGACATTCC; dG = -12.8
176 >s_e_s_a_s_b_s_c_s_a_s GTGCTGGTGTCTGTGGCTGTGTGGGTGTCTGTG; >clamp_s_c_s_b_s:compl:rev CACAGCCACCCACACTATCATGTTGACATTCC; dG = -12.8
177 >s_e_s_b_s_a_s_c_s_a_s GTGCTGGTGGCTGTGTCTGTGTGGGTGTCTGTG; >clamp_s_c_s_b_s:compl:rev CACAGCCACCCACACTATCATGTTGACATTCC; dG = -12.8
178 >s_e_s_b_s_b_s_c_s_a_s GTGCTGGTGGCTGTGGCTGTGTGGGTGTCTGTG; >clamp_s_c_s_b_s:compl:rev CACAGCCACCCACACTATCATGTTGACATTCC; dG = -12.8
179 >s_e_s_d_s_a_s_c_s_a_s GTGCTGGTGACGGTGTCTGTGTGGGTGTCTGTG; >clamp_s_c_s_b_s:compl:rev CACAGCCACCCACACTATCATGTTGACATTCC; dG = -12.8
180 >s_e_s_d_s_b_s_c_s_a_s GTGCTGGTGACGGTGGCTGTGTGGGTGTCTGTG; >clamp_s_c_s_b_s:compl:rev CACAGCCACCCACACTATCATGTTGACATTCC; dG = -12.8
181 >s_e_s_e_s_a_s_c_s_a_s GTGCTGGTGCTGGTGTCTGTGTGGGTGTCTGTG; >clamp_s_c_s_b_s:compl:rev CACAGCCACCCACACTATCATGTTGACATTCC; dG = -12.8

182 >s_e_s_e_s_b_s_c_s_a_s GTGCTGGTGCTGGTGGCTGTGTGGGTGTCTGTG; >clamp_s_c_s_b_s:compl:rev
CACAGCCACCCACACTATCATGTTGACATTCC; dG = -12.8
183 >s_a_s_c_s_e_s_c_s_a_s GTGTCTGTGTGGGTGCTGGTGTGGGTGTCTGTG; >clamp_s_c_s_b_s:compl:rev
CACAGCCACCCACACTATCATGTTGACATTCC; dG = -12.7
184 >s_a_s_d_s_c_s_c_s_a_s GTGTCTGTGACGGTGTGGGTGTGGGTGTCTGTG; >clamp_s_c_s_b_s:compl:rev
CACAGCCACCCACACTATCATGTTGACATTCC; dG = -12.7
185 >s_a_s_d_s_d_s_c_s_a_s GTGTCTGTGACGGTGACGGTGTGGGTGTCTGTG; >clamp_s_c_s_b_s:compl:rev
CACAGCCACCCACACTATCATGTTGACATTCC; dG = -12.7
186 >s_a_s_d_s_e_s_c_s_a_s GTGTCTGTGACGGTGCTGGTGTGGGTGTCTGTG; >clamp_s_c_s_b_s:compl:rev
CACAGCCACCCACACTATCATGTTGACATTCC; dG = -12.7
187 >s_a_s_e_s_c_s_c_s_a_s GTGTCTGTGCTGGTGTGGGTGTGGGTGTCTGTG; >clamp_s_c_s_b_s:compl:rev
CACAGCCACCCACACTATCATGTTGACATTCC; dG = -12.7
188 >s_a_s_e_s_d_s_c_s_a_s GTGTCTGTGCTGGTGACGGTGTGGGTGTCTGTG; >clamp_s_c_s_b_s:compl:rev
CACAGCCACCCACACTATCATGTTGACATTCC; dG = -12.7
189 >s_a_s_e_s_e_s_c_s_a_s GTGTCTGTGCTGGTGCTGGTGTGGGTGTCTGTG; >clamp_s_c_s_b_s:compl:rev
CACAGCCACCCACACTATCATGTTGACATTCC; dG = -12.7
190 >s_a_s_a_s_c_s_c_s_a_s GTGTCTGTGTCTGTGTGGGTGTGGGTGTCTGTG; >clamp_s_c_s_b_s:compl:rev
CACAGCCACCCACACTATCATGTTGACATTCC; dG = -12.7
191 >s_b_s_a_s_c_s_c_s_a_s GTGGCTGTGTCTGTGTGGGTGTGGGTGTCTGTG; >clamp_s_c_s_b_s:compl:rev
CACAGCCACCCACACTATCATGTTGACATTCC; dG = -12.7
192 >s_b_s_a_s_d_s_c_s_a_s GTGGCTGTGTCTGTGACGGTGTGGGTGTCTGTG; >clamp_s_c_s_b_s:compl:rev
CACAGCCACCCACACTATCATGTTGACATTCC; dG = -12.7
193 >s_b_s_a_s_e_s_c_s_a_s GTGGCTGTGTCTGTGCTGGTGTGGGTGTCTGTG; >clamp_s_c_s_b_s:compl:rev
CACAGCCACCCACACTATCATGTTGACATTCC; dG = -12.7
194 >s_b_s_b_s_c_s_c_s_a_s GTGGCTGTGGCTGTGTGGGTGTGGGTGTCTGTG; >clamp_s_c_s_b_s:compl:rev
CACAGCCACCCACACTATCATGTTGACATTCC; dG = -12.7
195 >s_b_s_b_s_d_s_c_s_a_s GTGGCTGTGGCTGTGACGGTGTGGGTGTCTGTG; >clamp_s_c_s_b_s:compl:rev
CACAGCCACCCACACTATCATGTTGACATTCC; dG = -12.7
196 >s_b_s_b_s_e_s_c_s_a_s GTGGCTGTGGCTGTGCTGGTGTGGGTGTCTGTG; >clamp_s_c_s_b_s:compl:rev
CACAGCCACCCACACTATCATGTTGACATTCC; dG = -12.7
197 >s_a_s_a_s_d_s_c_s_a_s GTGTCTGTGTCTGTGACGGTGTGGGTGTCTGTG; >clamp_s_c_s_b_s:compl:rev
CACAGCCACCCACACTATCATGTTGACATTCC; dG = -12.7
198 >s_b_s_c_s_c_s_c_s_a_s GTGGCTGTGTGGGTGTGGGTGTGGGTGTCTGTG; >clamp_s_c_s_b_s:compl:rev
CACAGCCACCCACACTATCATGTTGACATTCC; dG = -12.7
199 >s_b_s_c_s_d_s_c_s_a_s GTGGCTGTGTGGGTGACGGTGTGGGTGTCTGTG; >clamp_s_c_s_b_s:compl:rev
CACAGCCACCCACACTATCATGTTGACATTCC; dG = -12.7
200 >s_b_s_c_s_e_s_c_s_a_s GTGGCTGTGTGGGTGCTGGTGTGGGTGTCTGTG; >clamp_s_c_s_b_s:compl:rev
CACAGCCACCCACACTATCATGTTGACATTCC; dG = -12.7
201 >s_b_s_d_s_c_s_c_s_a_s GTGGCTGTGACGGTGTGGGTGTGGGTGTCTGTG; >clamp_s_c_s_b_s:compl:rev
CACAGCCACCCACACTATCATGTTGACATTCC; dG = -12.7
202 >s_b_s_d_s_d_s_c_s_a_s GTGGCTGTGACGGTGACGGTGTGGGTGTCTGTG; >clamp_s_c_s_b_s:compl:rev
CACAGCCACCCACACTATCATGTTGACATTCC; dG = -12.7
203 >s_b_s_d_s_e_s_c_s_a_s GTGGCTGTGACGGTGCTGGTGTGGGTGTCTGTG; >clamp_s_c_s_b_s:compl:rev
CACAGCCACCCACACTATCATGTTGACATTCC; dG = -12.7
204 >s_a_s_a_s_e_s_c_s_a_s GTGTCTGTGTCTGTGCTGGTGTGGGTGTCTGTG; >clamp_s_c_s_b_s:compl:rev
CACAGCCACCCACACTATCATGTTGACATTCC; dG = -12.7
205 >s_b_s_e_s_c_s_c_s_a_s GTGGCTGTGCTGGTGTGGGTGTGGGTGTCTGTG; >clamp_s_c_s_b_s:compl:rev
CACAGCCACCCACACTATCATGTTGACATTCC; dG = -12.7
206 >s_b_s_e_s_d_s_c_s_a_s GTGGCTGTGCTGGTGACGGTGTGGGTGTCTGTG; >clamp_s_c_s_b_s:compl:rev
CACAGCCACCCACACTATCATGTTGACATTCC; dG = -12.7
207 >s_b_s_e_s_e_s_c_s_a_s GTGGCTGTGCTGGTGCTGGTGTGGGTGTCTGTG; >clamp_s_c_s_b_s:compl:rev
CACAGCCACCCACACTATCATGTTGACATTCC; dG = -12.7
208 >s_c_s_a_s_b_s_c_s_e_s GTGTGGGTGTCTGTGGCTGTGTGGGTGCTGGTG; >clamp_s_c_s_b_s:compl:rev
CACAGCCACCCACACTATCATGTTGACATTCC; dG = -12.7
209 >s_c_s_a_s_b_s_c_s_d_s GTGTGGGTGTCTGTGGCTGTGTGGGTGACGGTG; >clamp_s_c_s_b_s:compl:rev
CACAGCCACCCACACTATCATGTTGACATTCC; dG = -12.7
210 >s_c_s_a_s_b_s_c_s_c_s GTGTGGGTGTCTGTGGCTGTGTGGGTGTGGGTG; >clamp_s_c_s_b_s:compl:rev
CACAGCCACCCACACTATCATGTTGACATTCC; dG = -12.7
211 >s_c_s_a_s_b_s_a_s_b_s GTGTGGGTGTCTGTGGCTGTGTCTGTGGCTGTG; >clamp_s_c_s_b_s:compl:rev
CACAGCCACCCACACTATCATGTTGACATTCC; dG = -12.7
212 >s_c_s_a_s_c_s_c_s_a_s GTGTGGGTGTCTGTGTGGGTGTGGGTGTCTGTG; >clamp_s_c_s_b_s:compl:rev
CACAGCCACCCACACTATCATGTTGACATTCC; dG = -12.7
213 >s_c_s_a_s_e_s_c_s_a_s GTGTGGGTGTCTGTGCTGGTGTGGGTGTCTGTG; >clamp_s_c_s_b_s:compl:rev
CACAGCCACCCACACTATCATGTTGACATTCC; dG = -12.7
214 >s_c_s_c_s_c_s_c_s_a_s GTGTGGGTGTGGGTGTGGGTGTGGGTGTCTGTG; >clamp_s_c_s_b_s:compl:rev
CACAGCCACCCACACTATCATGTTGACATTCC; dG = -12.7
215 >s_c_s_c_s_d_s_c_s_a_s GTGTGGGTGTGGGTGACGGTGTGGGTGTCTGTG; >clamp_s_c_s_b_s:compl:rev
CACAGCCACCCACACTATCATGTTGACATTCC; dG = -12.7
216 >s_c_s_c_s_e_s_c_s_a_s GTGTGGGTGTGGGTGCTGGTGTGGGTGTCTGTG; >clamp_s_c_s_b_s:compl:rev
CACAGCCACCCACACTATCATGTTGACATTCC; dG = -12.7
217 >s_c_s_d_s_b_s_c_s_e_s GTGTGGGTGACGGTGGCTGTGTGGGTGCTGGTG; >clamp_s_c_s_b_s:compl:rev
CACAGCCACCCACACTATCATGTTGACATTCC; dG = -12.7
218 >s_c_s_d_s_b_s_c_s_d_s GTGTGGGTGACGGTGGCTGTGTGGGTGACGGTG; >clamp_s_c_s_b_s:compl:rev
CACAGCCACCCACACTATCATGTTGACATTCC; dG = -12.7

219 >s_c_s_d_s_b_s_c_s_c_s GTGTGGGTGACGGTGGCTGTGTGGGTGTGGGTG; >clamp_s_c_s_b_s:compl:rev
CACAGCCACCCACACTATCATGTTGACATTCC; dG = -12.7
220 >s_c_s_d_s_b_s_a_s_b_s GTGTGGGTGACGGTGGCTGTGTCTGTGGCTGTG; >clamp_s_c_s_b_s:compl:rev
CACAGCCACCCACACTATCATGTTGACATTCC; dG = -12.7
221 >s_c_s_d_s_c_s_c_s_a_s GTGTGGGTGACGGTGTGGGTGTGGGTGTCTGTG; >clamp_s_c_s_b_s:compl:rev
CACAGCCACCCACACTATCATGTTGACATTCC; dG = -12.7
222 >s_c_s_d_s_d_s_c_s_a_s GTGTGGGTGACGGTGACGGTGTGGGTGTCTGTG; >clamp_s_c_s_b_s:compl:rev
CACAGCCACCCACACTATCATGTTGACATTCC; dG = -12.7
223 >s_c_s_d_s_e_s_c_s_a_s GTGTGGGTGACGGTGCTGGTGTGGGTGTCTGTG; >clamp_s_c_s_b_s:compl:rev
CACAGCCACCCACACTATCATGTTGACATTCC; dG = -12.7
224 >s_c_s_e_s_b_s_c_s_e_s GTGTGGGTGCTGGTGGCTGTGTGGGTGCTGGTG; >clamp_s_c_s_b_s:compl:rev
CACAGCCACCCACACTATCATGTTGACATTCC; dG = -12.7
225 >s_c_s_e_s_b_s_c_s_d_s GTGTGGGTGCTGGTGGCTGTGTGGGTGACGGTG; >clamp_s_c_s_b_s:compl:rev
CACAGCCACCCACACTATCATGTTGACATTCC; dG = -12.7
226 >s_c_s_e_s_b_s_c_s_c_s GTGTGGGTGCTGGTGGCTGTGTGGGTGTGGGTG; >clamp_s_c_s_b_s:compl:rev
CACAGCCACCCACACTATCATGTTGACATTCC; dG = -12.7
227 >s_c_s_e_s_b_s_a_s_b_s GTGTGGGTGCTGGTGGCTGTGTCTGTGGCTGTG; >clamp_s_c_s_b_s:compl:rev
CACAGCCACCCACACTATCATGTTGACATTCC; dG = -12.7
228 >s_c_s_e_s_c_s_c_s_a_s GTGTGGGTGCTGGTGTGGGTGTGGGTGTCTGTG; >clamp_s_c_s_b_s:compl:rev
CACAGCCACCCACACTATCATGTTGACATTCC; dG = -12.7
229 >s_c_s_e_s_d_s_c_s_a_s GTGTGGGTGCTGGTGACGGTGTGGGTGTCTGTG; >clamp_s_c_s_b_s:compl:rev
CACAGCCACCCACACTATCATGTTGACATTCC; dG = -12.7
230 >s_c_s_e_s_e_s_c_s_a_s GTGTGGGTGCTGGTGCTGGTGTGGGTGTCTGTG; >clamp_s_c_s_b_s:compl:rev
CACAGCCACCCACACTATCATGTTGACATTCC; dG = -12.7
231 >s_d_s_a_s_c_s_c_s_a_s GTGACGGTGTCTGTGTGGGTGTGGGTGTCTGTG; >clamp_s_c_s_b_s:compl:rev
CACAGCCACCCACACTATCATGTTGACATTCC; dG = -12.7
232 >s_d_s_a_s_d_s_c_s_a_s GTGACGGTGTCTGTGACGGTGTGGGTGTCTGTG; >clamp_s_c_s_b_s:compl:rev
CACAGCCACCCACACTATCATGTTGACATTCC; dG = -12.7
233 >s_d_s_a_s_e_s_c_s_a_s GTGACGGTGTCTGTGCTGGTGTGGGTGTCTGTG; >clamp_s_c_s_b_s:compl:rev
CACAGCCACCCACACTATCATGTTGACATTCC; dG = -12.7
234 >s_d_s_b_s_c_s_c_s_a_s GTGACGGTGGCTGTGTGGGTGTGGGTGTCTGTG; >clamp_s_c_s_b_s:compl:rev
CACAGCCACCCACACTATCATGTTGACATTCC; dG = -12.7
235 >s_d_s_b_s_d_s_c_s_a_s GTGACGGTGGCTGTGACGGTGTGGGTGTCTGTG; >clamp_s_c_s_b_s:compl:rev
CACAGCCACCCACACTATCATGTTGACATTCC; dG = -12.7
236 >s_a_s_b_s_c_s_c_s_a_s GTGTCTGTGGCTGTGTGGGTGTGGGTGTCTGTG; >clamp_s_c_s_b_s:compl:rev
CACAGCCACCCACACTATCATGTTGACATTCC; dG = -12.7
237 >s_d_s_b_s_e_s_c_s_a_s GTGACGGTGGCTGTGCTGGTGTGGGTGTCTGTG; >clamp_s_c_s_b_s:compl:rev
CACAGCCACCCACACTATCATGTTGACATTCC; dG = -12.7
238 >s_d_s_c_s_c_s_c_s_a_s GTGACGGTGTGGGTGTGGGTGTGGGTGTCTGTG; >clamp_s_c_s_b_s:compl:rev
CACAGCCACCCACACTATCATGTTGACATTCC; dG = -12.7
239 >s_d_s_c_s_d_s_c_s_a_s GTGACGGTGTGGGTGACGGTGTGGGTGTCTGTG; >clamp_s_c_s_b_s:compl:rev
CACAGCCACCCACACTATCATGTTGACATTCC; dG = -12.7
240 >s_d_s_c_s_e_s_c_s_a_s GTGACGGTGTGGGTGCTGGTGTGGGTGTCTGTG; >clamp_s_c_s_b_s:compl:rev
CACAGCCACCCACACTATCATGTTGACATTCC; dG = -12.7
241 >s_d_s_d_s_c_s_c_s_a_s GTGACGGTGACGGTGTGGGTGTGGGTGTCTGTG; >clamp_s_c_s_b_s:compl:rev
CACAGCCACCCACACTATCATGTTGACATTCC; dG = -12.7
242 >s_d_s_d_s_d_s_c_s_a_s GTGACGGTGACGGTGACGGTGTGGGTGTCTGTG; >clamp_s_c_s_b_s:compl:rev
CACAGCCACCCACACTATCATGTTGACATTCC; dG = -12.7
243 >s_a_s_b_s_d_s_c_s_a_s GTGTCTGTGGCTGTGACGGTGTGGGTGTCTGTG; >clamp_s_c_s_b_s:compl:rev
CACAGCCACCCACACTATCATGTTGACATTCC; dG = -12.7
244 >s_d_s_d_s_e_s_c_s_a_s GTGACGGTGACGGTGCTGGTGTGGGTGTCTGTG; >clamp_s_c_s_b_s:compl:rev
CACAGCCACCCACACTATCATGTTGACATTCC; dG = -12.7
245 >s_d_s_e_s_c_s_c_s_a_s GTGACGGTGCTGGTGTGGGTGTGGGTGTCTGTG; >clamp_s_c_s_b_s:compl:rev
CACAGCCACCCACACTATCATGTTGACATTCC; dG = -12.7
246 >s_d_s_e_s_d_s_c_s_a_s GTGACGGTGCTGGTGACGGTGTGGGTGTCTGTG; >clamp_s_c_s_b_s:compl:rev
CACAGCCACCCACACTATCATGTTGACATTCC; dG = -12.7
247 >s_d_s_e_s_e_s_c_s_a_s GTGACGGTGCTGGTGCTGGTGTGGGTGTCTGTG; >clamp_s_c_s_b_s:compl:rev
CACAGCCACCCACACTATCATGTTGACATTCC; dG = -12.7
248 >s_e_s_a_s_c_s_c_s_a_s GTGCTGGTGTCTGTGTGGGTGTGGGTGTCTGTG; >clamp_s_c_s_b_s:compl:rev
CACAGCCACCCACACTATCATGTTGACATTCC; dG = -12.7
249 >s_e_s_a_s_d_s_c_s_a_s GTGCTGGTGTCTGTGACGGTGTGGGTGTCTGTG; >clamp_s_c_s_b_s:compl:rev
CACAGCCACCCACACTATCATGTTGACATTCC; dG = -12.7
250 >s_a_s_b_s_e_s_c_s_a_s GTGTCTGTGGCTGTGCTGGTGTGGGTGTCTGTG; >clamp_s_c_s_b_s:compl:rev
CACAGCCACCCACACTATCATGTTGACATTCC; dG = -12.7
251 >s_e_s_a_s_e_s_c_s_a_s GTGCTGGTGTCTGTGCTGGTGTGGGTGTCTGTG; >clamp_s_c_s_b_s:compl:rev
CACAGCCACCCACACTATCATGTTGACATTCC; dG = -12.7
252 >s_e_s_b_s_c_s_c_s_a_s GTGCTGGTGGCTGTGTGGGTGTGGGTGTCTGTG; >clamp_s_c_s_b_s:compl:rev
CACAGCCACCCACACTATCATGTTGACATTCC; dG = -12.7
253 >s_e_s_b_s_d_s_c_s_a_s GTGCTGGTGGCTGTGACGGTGTGGGTGTCTGTG; >clamp_s_c_s_b_s:compl:rev
CACAGCCACCCACACTATCATGTTGACATTCC; dG = -12.7
254 >s_e_s_b_s_e_s_c_s_a_s GTGCTGGTGGCTGTGCTGGTGTGGGTGTCTGTG; >clamp_s_c_s_b_s:compl:rev
CACAGCCACCCACACTATCATGTTGACATTCC; dG = -12.7
255 >s_e_s_c_s_c_s_c_s_a_s GTGCTGGTGTGGGTGTGGGTGTGGGTGTCTGTG; >clamp_s_c_s_b_s:compl:rev
CACAGCCACCCACACTATCATGTTGACATTCC; dG = -12.7

256 >s_e_s_c_s_d_s_c_s_a_s GTGCTGGTGTGGGTGACGGTGTGGGTGTCTGTG; >clamp_s_c_s_b_s:compl:rev
CACAGCCACCCACACTATCATGTTGACATTCC; dG = -12.7
257 >s_e_s_c_s_e_s_c_s_a_s GTGCTGGTGTGGGTGCTGGTGTGGGTGTCTGTG; >clamp_s_c_s_b_s:compl:rev
CACAGCCACCCACACTATCATGTTGACATTCC; dG = -12.7
258 >s_e_s_d_s_c_s_c_s_a_s GTGCTGGTGACGGTGTGGGTGTGGGTGTCTGTG; >clamp_s_c_s_b_s:compl:rev
CACAGCCACCCACACTATCATGTTGACATTCC; dG = -12.7
259 >s_e_s_d_s_d_s_c_s_a_s GTGCTGGTGACGGTGACGGTGTGGGTGTCTGTG; >clamp_s_c_s_b_s:compl:rev
CACAGCCACCCACACTATCATGTTGACATTCC; dG = -12.7
260 >s_e_s_d_s_e_s_c_s_a_s GTGCTGGTGACGGTGCTGGTGTGGGTGTCTGTG; >clamp_s_c_s_b_s:compl:rev
CACAGCCACCCACACTATCATGTTGACATTCC; dG = -12.7
261 >s_e_s_e_s_c_s_c_s_a_s GTGCTGGTGCTGGTGTGGGTGTGGGTGTCTGTG; >clamp_s_c_s_b_s:compl:rev
CACAGCCACCCACACTATCATGTTGACATTCC; dG = -12.7
262 >s_e_s_e_s_d_s_c_s_a_s GTGCTGGTGCTGGTGACGGTGTGGGTGTCTGTG; >clamp_s_c_s_b_s:compl:rev
CACAGCCACCCACACTATCATGTTGACATTCC; dG = -12.7
263 >s_e_s_e_s_e_s_c_s_a_s GTGCTGGTGCTGGTGCTGGTGTGGGTGTCTGTG; >clamp_s_c_s_b_s:compl:rev
CACAGCCACCCACACTATCATGTTGACATTCC; dG = -12.7
264 >s_a_s_c_s_c_s_c_s_a_s GTGTCTGTGTGGGTGTGGGTGTGGGTGTCTGTG; >clamp_s_c_s_b_s:compl:rev
CACAGCCACCCACACTATCATGTTGACATTCC; dG = -12.7
265 >s_a_s_c_s_d_s_c_s_a_s GTGTCTGTGTGGGTGACGGTGTGGGTGTCTGTG; >clamp_s_c_s_b_s:compl:rev
CACAGCCACCCACACTATCATGTTGACATTCC; dG = -12.7
266 >s_a_s_d_s_b_s_b_s_b_s GTGTCTGTGACGGTGGCTGTGGCTGTGGCTGTG; >clamp_s_c_s_b_s:compl:rev
CACAGCCACCCACACTATCATGTTGACATTCC; dG = -12.6
267 >s_b_s_a_s_d_s_b_s_b_s GTGGCTGTGTCTGTGACGGTGGCTGTGGCTGTG; >clamp_s_c_s_b_s:compl:rev
CACAGCCACCCACACTATCATGTTGACATTCC; dG = -12.6
268 >s_b_s_b_s_d_s_b_s_b_s GTGGCTGTGGCTGTGACGGTGGCTGTGGCTGTG; >clamp_s_c_s_b_s:compl:rev
CACAGCCACCCACACTATCATGTTGACATTCC; dG = -12.6
269 >s_a_s_a_s_d_s_b_s_b_s GTGTCTGTGTCTGTGACGGTGGCTGTGGCTGTG; >clamp_s_c_s_b_s:compl:rev
CACAGCCACCCACACTATCATGTTGACATTCC; dG = -12.6
270 >s_b_s_d_s_b_s_b_s_b_s GTGGCTGTGACGGTGGCTGTGGCTGTGGCTGTG; >clamp_s_c_s_b_s:compl:rev
CACAGCCACCCACACTATCATGTTGACATTCC; dG = -12.6
271 >s_c_s_a_s_b_s_e_s_b_s GTGTGGGTGTCTGTGGCTGTGCTGGTGGCTGTG; >clamp_s_c_s_b_s:compl:rev
CACAGCCACCCACACTATCATGTTGACATTCC; dG = -12.6
272 >s_c_s_d_s_b_s_e_s_b_s GTGTGGGTGACGGTGGCTGTGCTGGTGGCTGTG; >clamp_s_c_s_b_s:compl:rev
CACAGCCACCCACACTATCATGTTGACATTCC; dG = -12.6
273 >s_c_s_e_s_b_s_e_s_b_s GTGTGGGTGCTGGTGGCTGTGCTGGTGGCTGTG; >clamp_s_c_s_b_s:compl:rev
CACAGCCACCCACACTATCATGTTGACATTCC; dG = -12.6
274 >s_d_s_a_s_d_s_b_s_b_s GTGACGGTGTCTGTGACGGTGGCTGTGGCTGTG; >clamp_s_c_s_b_s:compl:rev
CACAGCCACCCACACTATCATGTTGACATTCC; dG = -12.6
275 >s_d_s_b_s_d_s_b_s_b_s GTGACGGTGGCTGTGACGGTGGCTGTGGCTGTG; >clamp_s_c_s_b_s:compl:rev
CACAGCCACCCACACTATCATGTTGACATTCC; dG = -12.6
276 >s_a_s_b_s_d_s_b_s_b_s GTGTCTGTGGCTGTGACGGTGGCTGTGGCTGTG; >clamp_s_c_s_b_s:compl:rev
CACAGCCACCCACACTATCATGTTGACATTCC; dG = -12.6
277 >s_e_s_a_s_d_s_b_s_b_s GTGCTGGTGTCTGTGACGGTGGCTGTGGCTGTG; >clamp_s_c_s_b_s:compl:rev
CACAGCCACCCACACTATCATGTTGACATTCC; dG = -12.6
278 >s_e_s_b_s_d_s_b_s_b_s GTGCTGGTGGCTGTGACGGTGGCTGTGGCTGTG; >clamp_s_c_s_b_s:compl:rev
CACAGCCACCCACACTATCATGTTGACATTCC; dG = -12.6
279 >s_a_s_d_s_b_s_c_s_e_s GTGTCTGTGACGGTGGCTGTGTGGGTGCTGGTG; >clamp_s_c_s_b_s:compl:rev
CACAGCCACCCACACTATCATGTTGACATTCC; dG = -12.5
280 >s_a_s_d_s_b_s_c_s_d_s GTGTCTGTGACGGTGGCTGTGTGGGTGACGGTG; >clamp_s_c_s_b_s:compl:rev
CACAGCCACCCACACTATCATGTTGACATTCC; dG = -12.5
281 >s_a_s_d_s_b_s_c_s_c_s GTGTCTGTGACGGTGGCTGTGTGGGTGTGGGTG; >clamp_s_c_s_b_s:compl:rev
CACAGCCACCCACACTATCATGTTGACATTCC; dG = -12.5
282 >s_a_s_d_s_b_s_a_s_b_s GTGTCTGTGACGGTGGCTGTGTCTGTGGCTGTG; >clamp_s_c_s_b_s:compl:rev
CACAGCCACCCACACTATCATGTTGACATTCC; dG = -12.5
283 >s_a_s_d_s_d_s_b_s_b_s GTGTCTGTGACGGTGACGGTGGCTGTGGCTGTG; >clamp_s_c_s_b_s:compl:rev
CACAGCCACCCACACTATCATGTTGACATTCC; dG = -12.5
284 >s_a_s_e_s_b_s_b_s_b_s GTGTCTGTGCTGGTGGCTGTGGCTGTGGCTGTG; >clamp_s_c_s_b_s:compl:rev
CACAGCCACCCACACTATCATGTTGACATTCC; dG = -12.5
285 >s_a_s_e_s_d_s_b_s_b_s GTGTCTGTGCTGGTGACGGTGGCTGTGGCTGTG; >clamp_s_c_s_b_s:compl:rev
CACAGCCACCCACACTATCATGTTGACATTCC; dG = -12.5
286 >s_b_s_a_s_e_s_b_s_b_s GTGGCTGTGTCTGTGCTGGTGGCTGTGGCTGTG; >clamp_s_c_s_b_s:compl:rev
CACAGCCACCCACACTATCATGTTGACATTCC; dG = -12.5
287 >s_b_s_b_s_e_s_b_s_b_s GTGGCTGTGGCTGTGCTGGTGGCTGTGGCTGTG; >clamp_s_c_s_b_s:compl:rev
CACAGCCACCCACACTATCATGTTGACATTCC; dG = -12.5
288 >s_b_s_d_s_b_s_c_s_e_s GTGGCTGTGACGGTGGCTGTGTGGGTGCTGGTG; >clamp_s_c_s_b_s:compl:rev
CACAGCCACCCACACTATCATGTTGACATTCC; dG = -12.5
289 >s_b_s_d_s_b_s_c_s_d_s GTGGCTGTGACGGTGGCTGTGTGGGTGACGGTG; >clamp_s_c_s_b_s:compl:rev
CACAGCCACCCACACTATCATGTTGACATTCC; dG = -12.5
290 >s_b_s_d_s_b_s_c_s_c_s GTGGCTGTGACGGTGGCTGTGTGGGTGTGGGTG; >clamp_s_c_s_b_s:compl:rev
CACAGCCACCCACACTATCATGTTGACATTCC; dG = -12.5
291 >s_b_s_d_s_b_s_a_s_b_s GTGGCTGTGACGGTGGCTGTGTCTGTGGCTGTG; >clamp_s_c_s_b_s:compl:rev
CACAGCCACCCACACTATCATGTTGACATTCC; dG = -12.5
292 >s_b_s_d_s_d_s_b_s_b_s GTGGCTGTGACGGTGACGGTGGCTGTGGCTGTG; >clamp_s_c_s_b_s:compl:rev
CACAGCCACCCACACTATCATGTTGACATTCC; dG = -12.5

293 >s_b_s_e_s_b_s_b_s_b_s GTGGCTGTGCTGGTGGCTGTGGCTGTGGCTGTG; >clamp_s_c_s_b_s:compl:rev
CACAGCCACCCACACTATCATGTTGACATTCC; dG = -12.5
294 >s_b_s_e_s_d_s_b_s_b_s GTGGCTGTGCTGGTGACGGTGGCTGTGGCTGTG; >clamp_s_c_s_b_s:compl:rev
CACAGCCACCCACACTATCATGTTGACATTCC; dG = -12.5
295 >s_c_s_a_s_a_s_c_s_e_s GTGTGGGTGTCTGTGTCTGTGTGGGTGCTGGTG; >clamp_s_c_s_b_s:compl:rev
CACAGCCACCCACACTATCATGTTGACATTCC; dG = -12.5
296 >s_c_s_a_s_a_s_c_s_d_s GTGTGGGTGTCTGTGTCTGTGTGGGTGACGGTG; >clamp_s_c_s_b_s:compl:rev
CACAGCCACCCACACTATCATGTTGACATTCC; dG = -12.5
297 >s_c_s_a_s_a_s_c_s_c_s GTGTGGGTGTCTGTGTCTGTGTGGGTGTGGGTG; >clamp_s_c_s_b_s:compl:rev
CACAGCCACCCACACTATCATGTTGACATTCC; dG = -12.5
298 >s_c_s_a_s_a_s_e_s_b_s GTGTGGGTGTCTGTGTCTGTGCTGGTGGCTGTG; >clamp_s_c_s_b_s:compl:rev
CACAGCCACCCACACTATCATGTTGACATTCC; dG = -12.5
299 >s_a_s_a_s_e_s_b_s_b_s GTGTCTGTGTCTGTGCTGGTGGCTGTGGCTGTG; >clamp_s_c_s_b_s:compl:rev
CACAGCCACCCACACTATCATGTTGACATTCC; dG = -12.5
300 >s_c_s_a_s_a_s_d_s_b_s GTGTGGGTGTCTGTGTCTGTGACGGTGGCTGTG; >clamp_s_c_s_b_s:compl:rev
CACAGCCACCCACACTATCATGTTGACATTCC; dG = -12.5
301 >s_c_s_a_s_a_s_b_s_b_s GTGTGGGTGTCTGTGTCTGTGGCTGTGGCTGTG; >clamp_s_c_s_b_s:compl:rev
CACAGCCACCCACACTATCATGTTGACATTCC; dG = -12.5
302 >s_c_s_a_s_a_s_a_s_b_s GTGTGGGTGTCTGTGTCTGTGTCTGTGGCTGTG; >clamp_s_c_s_b_s:compl:rev
CACAGCCACCCACACTATCATGTTGACATTCC; dG = -12.5
303 >s_c_s_a_s_c_s_c_s_e_s GTGTGGGTGTCTGTGTGGGTGTGGGTGCTGGTG; >clamp_s_c_s_b_s:compl:rev
CACAGCCACCCACACTATCATGTTGACATTCC; dG = -12.5
304 >s_c_s_a_s_c_s_c_s_d_s GTGTGGGTGTCTGTGTGGGTGTGGGTGACGGTG; >clamp_s_c_s_b_s:compl:rev
CACAGCCACCCACACTATCATGTTGACATTCC; dG = -12.5
305 >s_c_s_a_s_c_s_c_s_c_s GTGTGGGTGTCTGTGTGGGTGTGGGTGTGGGTG; >clamp_s_c_s_b_s:compl:rev
CACAGCCACCCACACTATCATGTTGACATTCC; dG = -12.5
306 >s_c_s_a_s_e_s_b_s_b_s GTGTGGGTGTCTGTGCTGGTGGCTGTGGCTGTG; >clamp_s_c_s_b_s:compl:rev
CACAGCCACCCACACTATCATGTTGACATTCC; dG = -12.5
307 >s_c_s_d_s_d_s_b_s_b_s GTGTGGGTGACGGTGACGGTGGCTGTGGCTGTG; >clamp_s_c_s_b_s:compl:rev
CACAGCCACCCACACTATCATGTTGACATTCC; dG = -12.5
308 >s_c_s_e_s_d_s_b_s_b_s GTGTGGGTGCTGGTGACGGTGGCTGTGGCTGTG; >clamp_s_c_s_b_s:compl:rev
CACAGCCACCCACACTATCATGTTGACATTCC; dG = -12.5
309 >s_d_s_a_s_e_s_b_s_b_s GTGACGGTGTCTGTGCTGGTGGCTGTGGCTGTG; >clamp_s_c_s_b_s:compl:rev
CACAGCCACCCACACTATCATGTTGACATTCC; dG = -12.5
310 >s_d_s_b_s_d_s_c_s_e_s GTGACGGTGGCTGTGACGGTGTGGGTGCTGGTG; >clamp_s_c_s_b_s:compl:rev
CACAGCCACCCACACTATCATGTTGACATTCC; dG = -12.5
311 >s_d_s_b_s_d_s_c_s_d_s GTGACGGTGGCTGTGACGGTGTGGGTGACGGTG; >clamp_s_c_s_b_s:compl:rev
CACAGCCACCCACACTATCATGTTGACATTCC; dG = -12.5
312 >s_d_s_b_s_d_s_c_s_c_s GTGACGGTGGCTGTGACGGTGTGGGTGTGGGTG; >clamp_s_c_s_b_s:compl:rev
CACAGCCACCCACACTATCATGTTGACATTCC; dG = -12.5
313 >s_d_s_b_s_d_s_e_s_b_s GTGACGGTGGCTGTGACGGTGCTGGTGGCTGTG; >clamp_s_c_s_b_s:compl:rev
CACAGCCACCCACACTATCATGTTGACATTCC; dG = -12.5
314 >s_d_s_b_s_d_s_d_s_b_s GTGACGGTGGCTGTGACGGTGACGGTGGCTGTG; >clamp_s_c_s_b_s:compl:rev
CACAGCCACCCACACTATCATGTTGACATTCC; dG = -12.5
315 >s_d_s_b_s_d_s_a_s_b_s GTGACGGTGGCTGTGACGGTGTCTGTGGCTGTG; >clamp_s_c_s_b_s:compl:rev
CACAGCCACCCACACTATCATGTTGACATTCC; dG = -12.5
316 >s_d_s_b_s_e_s_b_s_b_s GTGACGGTGGCTGTGCTGGTGGCTGTGGCTGTG; >clamp_s_c_s_b_s:compl:rev
CACAGCCACCCACACTATCATGTTGACATTCC; dG = -12.5
317 >s_d_s_d_s_b_s_b_s_b_s GTGACGGTGACGGTGGCTGTGGCTGTGGCTGTG; >clamp_s_c_s_b_s:compl:rev
CACAGCCACCCACACTATCATGTTGACATTCC; dG = -12.5
318 >s_d_s_d_s_d_s_b_s_b_s GTGACGGTGACGGTGACGGTGGCTGTGGCTGTG; >clamp_s_c_s_b_s:compl:rev
CACAGCCACCCACACTATCATGTTGACATTCC; dG = -12.5
319 >s_d_s_e_s_d_s_b_s_b_s GTGACGGTGCTGGTGACGGTGGCTGTGGCTGTG; >clamp_s_c_s_b_s:compl:rev
CACAGCCACCCACACTATCATGTTGACATTCC; dG = -12.5
320 >s_e_s_a_s_e_s_b_s_b_s GTGCTGGTGTCTGTGCTGGTGGCTGTGGCTGTG; >clamp_s_c_s_b_s:compl:rev
CACAGCCACCCACACTATCATGTTGACATTCC; dG = -12.5
321 >s_a_s_b_s_e_s_b_s_b_s GTGTCTGTGGCTGTGCTGGTGGCTGTGGCTGTG; >clamp_s_c_s_b_s:compl:rev
CACAGCCACCCACACTATCATGTTGACATTCC; dG = -12.5
322 >s_e_s_b_s_e_s_b_s_b_s GTGCTGGTGGCTGTGCTGGTGGCTGTGGCTGTG; >clamp_s_c_s_b_s:compl:rev
CACAGCCACCCACACTATCATGTTGACATTCC; dG = -12.5
323 >s_e_s_d_s_b_s_b_s_b_s GTGCTGGTGACGGTGGCTGTGGCTGTGGCTGTG; >clamp_s_c_s_b_s:compl:rev
CACAGCCACCCACACTATCATGTTGACATTCC; dG = -12.5
324 >s_e_s_d_s_d_s_b_s_b_s GTGCTGGTGACGGTGACGGTGGCTGTGGCTGTG; >clamp_s_c_s_b_s:compl:rev
CACAGCCACCCACACTATCATGTTGACATTCC; dG = -12.5
325 >s_e_s_e_s_d_s_b_s_b_s GTGCTGGTGCTGGTGACGGTGGCTGTGGCTGTG; >clamp_s_c_s_b_s:compl:rev
CACAGCCACCCACACTATCATGTTGACATTCC; dG = -12.5
326 >s_a_s_d_s_b_s_e_s_b_s GTGTCTGTGACGGTGGCTGTGCTGGTGGCTGTG; >clamp_s_c_s_b_s:compl:rev
CACAGCCACCCACACTATCATGTTGACATTCC; dG = -12.4
327 >s_a_s_d_s_e_s_b_s_b_s GTGTCTGTGACGGTGCTGGTGGCTGTGGCTGTG; >clamp_s_c_s_b_s:compl:rev
CACAGCCACCCACACTATCATGTTGACATTCC; dG = -12.4
328 >s_a_s_e_s_b_s_c_s_e_s GTGTCTGTGCTGGTGGCTGTGTGGGTGCTGGTG; >clamp_s_c_s_b_s:compl:rev
CACAGCCACCCACACTATCATGTTGACATTCC; dG = -12.4
329 >s_a_s_e_s_b_s_c_s_d_s GTGTCTGTGCTGGTGGCTGTGTGGGTGACGGTG; >clamp_s_c_s_b_s:compl:rev
CACAGCCACCCACACTATCATGTTGACATTCC; dG = -12.4

330 >s_a_s_e_s_b_s_c_s_c_s GTGTCTGTGCTGGTGGCTGTGTGGGTGTGGGTG; >clamp_s_c_s_b_s:compl:rev
CACAGCCACCCACACTATCATGTTGACATTCC; dG = -12.4
331 >s_a_s_e_s_b_s_a_s_b_s GTGTCTGTGCTGGTGGCTGTGTCTGTGGCTGTG; >clamp_s_c_s_b_s:compl:rev
CACAGCCACCCACACTATCATGTTGACATTCC; dG = -12.4
332 >s_a_s_e_s_e_s_b_s_b_s GTGTCTGTGCTGGTGCTGGTGGCTGTGGCTGTG; >clamp_s_c_s_b_s:compl:rev
CACAGCCACCCACACTATCATGTTGACATTCC; dG = -12.4
333 >s_b_s_d_s_b_s_e_s_b_s GTGGCTGTGACGGTGGCTGTGCTGGTGGCTGTG; >clamp_s_c_s_b_s:compl:rev
CACAGCCACCCACACTATCATGTTGACATTCC; dG = -12.4
334 >s_b_s_d_s_e_s_b_s_b_s GTGGCTGTGACGGTGCTGGTGGCTGTGGCTGTG; >clamp_s_c_s_b_s:compl:rev
CACAGCCACCCACACTATCATGTTGACATTCC; dG = -12.4
335 >s_b_s_e_s_b_s_c_s_e_s GTGGCTGTGCTGGTGGCTGTGTGGGTGCTGGTG; >clamp_s_c_s_b_s:compl:rev
CACAGCCACCCACACTATCATGTTGACATTCC; dG = -12.4
336 >s_b_s_e_s_b_s_c_s_d_s GTGGCTGTGCTGGTGGCTGTGTGGGTGACGGTG; >clamp_s_c_s_b_s:compl:rev
CACAGCCACCCACACTATCATGTTGACATTCC; dG = -12.4
337 >s_b_s_e_s_b_s_c_s_c_s GTGGCTGTGCTGGTGGCTGTGTGGGTGTGGGTG; >clamp_s_c_s_b_s:compl:rev
CACAGCCACCCACACTATCATGTTGACATTCC; dG = -12.4
338 >s_b_s_e_s_b_s_a_s_b_s GTGGCTGTGCTGGTGGCTGTGTCTGTGGCTGTG; >clamp_s_c_s_b_s:compl:rev
CACAGCCACCCACACTATCATGTTGACATTCC; dG = -12.4
339 >s_b_s_e_s_e_s_b_s_b_s GTGGCTGTGCTGGTGCTGGTGGCTGTGGCTGTG; >clamp_s_c_s_b_s:compl:rev
CACAGCCACCCACACTATCATGTTGACATTCC; dG = -12.4
340 >s_c_s_a_s_e_s_c_s_e_s GTGTGGGTGTCTGTGCTGGTGTGGGTGCTGGTG; >clamp_s_c_s_b_s:compl:rev
CACAGCCACCCACACTATCATGTTGACATTCC; dG = -12.4
341 >s_c_s_a_s_e_s_c_s_d_s GTGTGGGTGTCTGTGCTGGTGTGGGTGACGGTG; >clamp_s_c_s_b_s:compl:rev
CACAGCCACCCACACTATCATGTTGACATTCC; dG = -12.4
342 >s_c_s_a_s_e_s_c_s_c_s GTGTGGGTGTCTGTGCTGGTGTGGGTGTGGGTG; >clamp_s_c_s_b_s:compl:rev
CACAGCCACCCACACTATCATGTTGACATTCC; dG = -12.4
343 >s_c_s_a_s_e_s_e_s_b_s GTGTGGGTGTCTGTGCTGGTGCTGGTGGCTGTG; >clamp_s_c_s_b_s:compl:rev
CACAGCCACCCACACTATCATGTTGACATTCC; dG = -12.4
344 >s_c_s_a_s_e_s_d_s_b_s GTGTGGGTGTCTGTGCTGGTGACGGTGGCTGTG; >clamp_s_c_s_b_s:compl:rev
CACAGCCACCCACACTATCATGTTGACATTCC; dG = -12.4
345 >s_c_s_a_s_e_s_a_s_b_s GTGTGGGTGTCTGTGCTGGTGTCTGTGGCTGTG; >clamp_s_c_s_b_s:compl:rev
CACAGCCACCCACACTATCATGTTGACATTCC; dG = -12.4
346 >s_c_s_d_s_e_s_b_s_b_s GTGTGGGTGACGGTGCTGGTGGCTGTGGCTGTG; >clamp_s_c_s_b_s:compl:rev
CACAGCCACCCACACTATCATGTTGACATTCC; dG = -12.4
347 >s_c_s_e_s_e_s_b_s_b_s GTGTGGGTGCTGGTGCTGGTGGCTGTGGCTGTG; >clamp_s_c_s_b_s:compl:rev
CACAGCCACCCACACTATCATGTTGACATTCC; dG = -12.4
348 >s_d_s_d_s_b_s_c_s_e_s GTGACGGTGACGGTGGCTGTGTGGGTGCTGGTG; >clamp_s_c_s_b_s:compl:rev
CACAGCCACCCACACTATCATGTTGACATTCC; dG = -12.4
349 >s_d_s_d_s_b_s_c_s_d_s GTGACGGTGACGGTGGCTGTGTGGGTGACGGTG; >clamp_s_c_s_b_s:compl:rev
CACAGCCACCCACACTATCATGTTGACATTCC; dG = -12.4
350 >s_d_s_d_s_b_s_c_s_c_s GTGACGGTGACGGTGGCTGTGTGGGTGTGGGTG; >clamp_s_c_s_b_s:compl:rev
CACAGCCACCCACACTATCATGTTGACATTCC; dG = -12.4
351 >s_d_s_d_s_b_s_a_s_b_s GTGACGGTGACGGTGGCTGTGTCTGTGGCTGTG; >clamp_s_c_s_b_s:compl:rev
CACAGCCACCCACACTATCATGTTGACATTCC; dG = -12.4
352 >s_d_s_d_s_e_s_b_s_b_s GTGACGGTGACGGTGCTGGTGGCTGTGGCTGTG; >clamp_s_c_s_b_s:compl:rev
CACAGCCACCCACACTATCATGTTGACATTCC; dG = -12.4
353 >s_d_s_e_s_b_s_b_s_b_s GTGACGGTGCTGGTGGCTGTGGCTGTGGCTGTG; >clamp_s_c_s_b_s:compl:rev
CACAGCCACCCACACTATCATGTTGACATTCC; dG = -12.4
354 >s_d_s_e_s_e_s_b_s_b_s GTGACGGTGCTGGTGCTGGTGGCTGTGGCTGTG; >clamp_s_c_s_b_s:compl:rev
CACAGCCACCCACACTATCATGTTGACATTCC; dG = -12.4
355 >s_e_s_b_s_d_s_c_s_e_s GTGCTGGTGGCTGTGACGGTGTGGGTGCTGGTG; >clamp_s_c_s_b_s:compl:rev
CACAGCCACCCACACTATCATGTTGACATTCC; dG = -12.4
356 >s_e_s_b_s_d_s_c_s_d_s GTGCTGGTGGCTGTGACGGTGTGGGTGACGGTG; >clamp_s_c_s_b_s:compl:rev
CACAGCCACCCACACTATCATGTTGACATTCC; dG = -12.4
357 >s_e_s_b_s_d_s_c_s_c_s GTGCTGGTGGCTGTGACGGTGTGGGTGTGGGTG; >clamp_s_c_s_b_s:compl:rev
CACAGCCACCCACACTATCATGTTGACATTCC; dG = -12.4
358 >s_e_s_b_s_d_s_e_s_b_s GTGCTGGTGGCTGTGACGGTGCTGGTGGCTGTG; >clamp_s_c_s_b_s:compl:rev
CACAGCCACCCACACTATCATGTTGACATTCC; dG = -12.4
359 >s_e_s_b_s_d_s_d_s_b_s GTGCTGGTGGCTGTGACGGTGACGGTGGCTGTG; >clamp_s_c_s_b_s:compl:rev
CACAGCCACCCACACTATCATGTTGACATTCC; dG = -12.4
360 >s_e_s_b_s_d_s_a_s_b_s GTGCTGGTGGCTGTGACGGTGTCTGTGGCTGTG; >clamp_s_c_s_b_s:compl:rev
CACAGCCACCCACACTATCATGTTGACATTCC; dG = -12.4
361 >s_e_s_d_s_b_s_c_s_e_s GTGCTGGTGACGGTGGCTGTGTGGGTGCTGGTG; >clamp_s_c_s_b_s:compl:rev
CACAGCCACCCACACTATCATGTTGACATTCC; dG = -12.4
362 >s_e_s_d_s_b_s_c_s_d_s GTGCTGGTGACGGTGGCTGTGTGGGTGACGGTG; >clamp_s_c_s_b_s:compl:rev
CACAGCCACCCACACTATCATGTTGACATTCC; dG = -12.4
363 >s_e_s_d_s_b_s_c_s_c_s GTGCTGGTGACGGTGGCTGTGTGGGTGTGGGTG; >clamp_s_c_s_b_s:compl:rev
CACAGCCACCCACACTATCATGTTGACATTCC; dG = -12.4
364 >s_e_s_d_s_b_s_a_s_b_s GTGCTGGTGACGGTGGCTGTGTCTGTGGCTGTG; >clamp_s_c_s_b_s:compl:rev
CACAGCCACCCACACTATCATGTTGACATTCC; dG = -12.4
365 >s_e_s_d_s_e_s_b_s_b_s GTGCTGGTGACGGTGCTGGTGGCTGTGGCTGTG; >clamp_s_c_s_b_s:compl:rev
CACAGCCACCCACACTATCATGTTGACATTCC; dG = -12.4
366 >s_e_s_e_s_b_s_b_s_b_s GTGCTGGTGCTGGTGGCTGTGGCTGTGGCTGTG; >clamp_s_c_s_b_s:compl:rev
CACAGCCACCCACACTATCATGTTGACATTCC; dG = -12.4

367 >s_e_s_e_s_e_s_b_s_b_s GTGCTGGTGCTGGTGCTGGTGGCTGTGGCTGTG; >clamp_s_c_s_b_s:compl:rev CACAGCCACCCACACTATCATGTTGACATTCC; dG = -12.4
368 >s_a_s_e_s_b_s_e_s_b_s GTGTCTGTGCTGGTGGCTGTGCTGGTGGCTGTG; >clamp_s_c_s_b_s:compl:rev CACAGCCACCCACACTATCATGTTGACATTCC; dG = -12.3
369 >s_b_s_e_s_b_s_e_s_b_s GTGGCTGTGCTGGTGGCTGTGCTGGTGGCTGTG; >clamp_s_c_s_b_s:compl:rev CACAGCCACCCACACTATCATGTTGACATTCC; dG = -12.3
370 >s_d_s_d_s_b_s_e_s_b_s GTGACGGTGACGGTGGCTGTGCTGGTGGCTGTG; >clamp_s_c_s_b_s:compl:rev CACAGCCACCCACACTATCATGTTGACATTCC; dG = -12.3
371 >s_d_s_e_s_b_s_c_s_e_s GTGACGGTGCTGGTGGCTGTGTGGGTGCTGGTG; >clamp_s_c_s_b_s:compl:rev CACAGCCACCCACACTATCATGTTGACATTCC; dG = -12.3
372 >s_d_s_e_s_b_s_c_s_d_s GTGACGGTGCTGGTGGCTGTGTGGGTGACGGTG; >clamp_s_c_s_b_s:compl:rev CACAGCCACCCACACTATCATGTTGACATTCC; dG = -12.3
373 >s_d_s_e_s_b_s_c_s_c_s GTGACGGTGCTGGTGGCTGTGTGGGTGTGGGTG; >clamp_s_c_s_b_s:compl:rev CACAGCCACCCACACTATCATGTTGACATTCC; dG = -12.3
374 >s_d_s_e_s_b_s_a_s_b_s GTGACGGTGCTGGTGGCTGTGTCTGTGGCTGTG; >clamp_s_c_s_b_s:compl:rev CACAGCCACCCACACTATCATGTTGACATTCC; dG = -12.3
375 >s_e_s_d_s_b_s_e_s_b_s GTGCTGGTGACGGTGGCTGTGCTGGTGGCTGTG; >clamp_s_c_s_b_s:compl:rev CACAGCCACCCACACTATCATGTTGACATTCC; dG = -12.3
376 >s_e_s_e_s_b_s_c_s_e_s GTGCTGGTGCTGGTGGCTGTGTGGGTGCTGGTG; >clamp_s_c_s_b_s:compl:rev CACAGCCACCCACACTATCATGTTGACATTCC; dG = -12.3
377 >s_e_s_e_s_b_s_c_s_d_s GTGCTGGTGCTGGTGGCTGTGTGGGTGACGGTG; >clamp_s_c_s_b_s:compl:rev CACAGCCACCCACACTATCATGTTGACATTCC; dG = -12.3
378 >s_e_s_e_s_b_s_c_s_c_s GTGCTGGTGCTGGTGGCTGTGTGGGTGTGGGTG; >clamp_s_c_s_b_s:compl:rev CACAGCCACCCACACTATCATGTTGACATTCC; dG = -12.3
379 >s_e_s_e_s_b_s_a_s_b_s GTGCTGGTGCTGGTGGCTGTGTCTGTGGCTGTG; >clamp_s_c_s_b_s:compl:rev CACAGCCACCCACACTATCATGTTGACATTCC; dG = -12.3
380 >s_a_s_c_s_e_s_d_s_b_s GTGTCTGTGTGGGTGCTGGTGACGGTGGCTGTG; >clamp_s_c_s_b_s:compl:rev CACAGCCACCCACACTATCATGTTGACATTCC; dG = -12.2
381 >s_a_s_d_s_a_s_d_s_b_s GTGTCTGTGACGGTGTCTGTGACGGTGGCTGTG; >clamp_s_c_s_b_s:compl:rev CACAGCCACCCACACTATCATGTTGACATTCC; dG = -12.2
382 >s_a_s_d_s_d_s_d_s_b_s GTGTCTGTGACGGTGACGGTGACGGTGGCTGTG; >clamp_s_c_s_b_s:compl:rev CACAGCCACCCACACTATCATGTTGACATTCC; dG = -12.2
383 >s_a_s_a_s_b_s_d_s_b_s GTGTCTGTGTCTGTGGCTGTGACGGTGGCTGTG; >clamp_s_c_s_b_s:compl:rev CACAGCCACCCACACTATCATGTTGACATTCC; dG = -12.2
384 >s_a_s_d_s_e_s_d_s_b_s GTGTCTGTGACGGTGCTGGTGACGGTGGCTGTG; >clamp_s_c_s_b_s:compl:rev CACAGCCACCCACACTATCATGTTGACATTCC; dG = -12.2
385 >s_a_s_e_s_a_s_d_s_b_s GTGTCTGTGCTGGTGTCTGTGACGGTGGCTGTG; >clamp_s_c_s_b_s:compl:rev CACAGCCACCCACACTATCATGTTGACATTCC; dG = -12.2
386 >s_a_s_e_s_d_s_d_s_b_s GTGTCTGTGCTGGTGACGGTGACGGTGGCTGTG; >clamp_s_c_s_b_s:compl:rev CACAGCCACCCACACTATCATGTTGACATTCC; dG = -12.2
387 >s_a_s_e_s_e_s_d_s_b_s GTGTCTGTGCTGGTGCTGGTGACGGTGGCTGTG; >clamp_s_c_s_b_s:compl:rev CACAGCCACCCACACTATCATGTTGACATTCC; dG = -12.2
388 >s_b_s_a_s_a_s_d_s_b_s GTGGCTGTGTCTGTGTCTGTGACGGTGGCTGTG; >clamp_s_c_s_b_s:compl:rev CACAGCCACCCACACTATCATGTTGACATTCC; dG = -12.2
389 >s_b_s_a_s_b_s_d_s_b_s GTGGCTGTGTCTGTGGCTGTGACGGTGGCTGTG; >clamp_s_c_s_b_s:compl:rev CACAGCCACCCACACTATCATGTTGACATTCC; dG = -12.2
390 >s_b_s_a_s_d_s_d_s_b_s GTGGCTGTGTCTGTGACGGTGACGGTGGCTGTG; >clamp_s_c_s_b_s:compl:rev CACAGCCACCCACACTATCATGTTGACATTCC; dG = -12.2
391 >s_b_s_a_s_e_s_d_s_b_s GTGGCTGTGTCTGTGCTGGTGACGGTGGCTGTG; >clamp_s_c_s_b_s:compl:rev CACAGCCACCCACACTATCATGTTGACATTCC; dG = -12.2
392 >s_b_s_b_s_a_s_d_s_b_s GTGGCTGTGGCTGTGTCTGTGACGGTGGCTGTG; >clamp_s_c_s_b_s:compl:rev CACAGCCACCCACACTATCATGTTGACATTCC; dG = -12.2
393 >s_b_s_b_s_b_s_d_s_b_s GTGGCTGTGGCTGTGGCTGTGACGGTGGCTGTG; >clamp_s_c_s_b_s:compl:rev CACAGCCACCCACACTATCATGTTGACATTCC; dG = -12.2
394 >s_b_s_b_s_d_s_d_s_b_s GTGGCTGTGGCTGTGACGGTGACGGTGGCTGTG; >clamp_s_c_s_b_s:compl:rev CACAGCCACCCACACTATCATGTTGACATTCC; dG = -12.2
395 >s_b_s_b_s_e_s_d_s_b_s GTGGCTGTGGCTGTGCTGGTGACGGTGGCTGTG; >clamp_s_c_s_b_s:compl:rev CACAGCCACCCACACTATCATGTTGACATTCC; dG = -12.2
396 >s_b_s_c_s_d_s_d_s_b_s GTGGCTGTGTGGGTGACGGTGACGGTGGCTGTG; >clamp_s_c_s_b_s:compl:rev CACAGCCACCCACACTATCATGTTGACATTCC; dG = -12.2
397 >s_a_s_a_s_d_s_d_s_b_s GTGTCTGTGTCTGTGACGGTGACGGTGGCTGTG; >clamp_s_c_s_b_s:compl:rev CACAGCCACCCACACTATCATGTTGACATTCC; dG = -12.2
398 >s_b_s_c_s_e_s_d_s_b_s GTGGCTGTGTGGGTGCTGGTGACGGTGGCTGTG; >clamp_s_c_s_b_s:compl:rev CACAGCCACCCACACTATCATGTTGACATTCC; dG = -12.2
399 >s_b_s_d_s_a_s_d_s_b_s GTGGCTGTGACGGTGTCTGTGACGGTGGCTGTG; >clamp_s_c_s_b_s:compl:rev CACAGCCACCCACACTATCATGTTGACATTCC; dG = -12.2
400 >s_b_s_d_s_d_s_d_s_b_s GTGGCTGTGACGGTGACGGTGACGGTGGCTGTG; >clamp_s_c_s_b_s:compl:rev CACAGCCACCCACACTATCATGTTGACATTCC; dG = -12.2
401 >s_b_s_d_s_e_s_d_s_b_s GTGGCTGTGACGGTGCTGGTGACGGTGGCTGTG; >clamp_s_c_s_b_s:compl:rev CACAGCCACCCACACTATCATGTTGACATTCC; dG = -12.2
402 >s_b_s_e_s_a_s_d_s_b_s GTGGCTGTGCTGGTGTCTGTGACGGTGGCTGTG; >clamp_s_c_s_b_s:compl:rev CACAGCCACCCACACTATCATGTTGACATTCC; dG = -12.2
403 >s_b_s_e_s_d_s_d_s_b_s GTGGCTGTGCTGGTGACGGTGACGGTGGCTGTG; >clamp_s_c_s_b_s:compl:rev CACAGCCACCCACACTATCATGTTGACATTCC; dG = -12.2

404 >s_a_s_a_s_e_s_d_s_b_s GTGTCTGTGTCTGTGCTGGTGACGGTGGCTGTG; >clamp_s_c_s_b_s:compl:rev CACAGCCACCCACACTATCATGTTGACATTCC; dG = -12.2
405 >s_b_s_e_s_e_s_d_s_b_s GTGGCTGTGCTGGTGCTGGTGACGGTGGCTGTG; >clamp_s_c_s_b_s:compl:rev CACAGCCACCCACACTATCATGTTGACATTCC; dG = -12.2
406 >s_c_s_c_s_d_s_d_s_b_s GTGTGGGTGTGGGTGACGGTGACGGTGGCTGTG; >clamp_s_c_s_b_s:compl:rev CACAGCCACCCACACTATCATGTTGACATTCC; dG = -12.2
407 >s_c_s_c_s_e_s_d_s_b_s GTGTGGGTGTGGGTGCTGGTGACGGTGGCTGTG; >clamp_s_c_s_b_s:compl:rev CACAGCCACCCACACTATCATGTTGACATTCC; dG = -12.2
408 >s_a_s_b_s_a_s_d_s_b_s GTGTCTGTGGCTGTGTCTGTGACGGTGGCTGTG; >clamp_s_c_s_b_s:compl:rev CACAGCCACCCACACTATCATGTTGACATTCC; dG = -12.2
409 >s_c_s_d_s_a_s_d_s_b_s GTGTGGGTGACGGTGTCTGTGACGGTGGCTGTG; >clamp_s_c_s_b_s:compl:rev CACAGCCACCCACACTATCATGTTGACATTCC; dG = -12.2
410 >s_c_s_d_s_d_s_d_s_b_s GTGTGGGTGACGGTGACGGTGACGGTGGCTGTG; >clamp_s_c_s_b_s:compl:rev CACAGCCACCCACACTATCATGTTGACATTCC; dG = -12.2
411 >s_c_s_d_s_e_s_d_s_b_s GTGTGGGTGACGGTGCTGGTGACGGTGGCTGTG; >clamp_s_c_s_b_s:compl:rev CACAGCCACCCACACTATCATGTTGACATTCC; dG = -12.2
412 >s_c_s_e_s_a_s_d_s_b_s GTGTGGGTGCTGGTGTCTGTGACGGTGGCTGTG; >clamp_s_c_s_b_s:compl:rev CACAGCCACCCACACTATCATGTTGACATTCC; dG = -12.2
413 >s_c_s_e_s_d_s_d_s_b_s GTGTGGGTGCTGGTGACGGTGACGGTGGCTGTG; >clamp_s_c_s_b_s:compl:rev CACAGCCACCCACACTATCATGTTGACATTCC; dG = -12.2
414 >s_c_s_e_s_e_s_d_s_b_s GTGTGGGTGCTGGTGCTGGTGACGGTGGCTGTG; >clamp_s_c_s_b_s:compl:rev CACAGCCACCCACACTATCATGTTGACATTCC; dG = -12.2
415 >s_d_s_a_s_a_s_d_s_b_s GTGACGGTGTCTGTGTCTGTGACGGTGGCTGTG; >clamp_s_c_s_b_s:compl:rev CACAGCCACCCACACTATCATGTTGACATTCC; dG = -12.2
416 >s_a_s_b_s_b_s_d_s_b_s GTGTCTGTGGCTGTGGCTGTGACGGTGGCTGTG; >clamp_s_c_s_b_s:compl:rev CACAGCCACCCACACTATCATGTTGACATTCC; dG = -12.2
417 >s_d_s_a_s_b_s_d_s_b_s GTGACGGTGTCTGTGGCTGTGACGGTGGCTGTG; >clamp_s_c_s_b_s:compl:rev CACAGCCACCCACACTATCATGTTGACATTCC; dG = -12.2
418 >s_d_s_a_s_d_s_d_s_b_s GTGACGGTGTCTGTGACGGTGACGGTGGCTGTG; >clamp_s_c_s_b_s:compl:rev CACAGCCACCCACACTATCATGTTGACATTCC; dG = -12.2
419 >s_d_s_a_s_e_s_d_s_b_s GTGACGGTGTCTGTGCTGGTGACGGTGGCTGTG; >clamp_s_c_s_b_s:compl:rev CACAGCCACCCACACTATCATGTTGACATTCC; dG = -12.2
420 >s_d_s_b_s_a_s_d_s_b_s GTGACGGTGGCTGTGTCTGTGACGGTGGCTGTG; >clamp_s_c_s_b_s:compl:rev CACAGCCACCCACACTATCATGTTGACATTCC; dG = -12.2
421 >s_d_s_b_s_b_s_d_s_b_s GTGACGGTGGCTGTGGCTGTGACGGTGGCTGTG; >clamp_s_c_s_b_s:compl:rev CACAGCCACCCACACTATCATGTTGACATTCC; dG = -12.2
422 >s_d_s_b_s_e_s_d_s_b_s GTGACGGTGGCTGTGCTGGTGACGGTGGCTGTG; >clamp_s_c_s_b_s:compl:rev CACAGCCACCCACACTATCATGTTGACATTCC; dG = -12.2
423 >s_a_s_a_s_a_s_d_s_b_s GTGTCTGTGTCTGTGTCTGTGACGGTGGCTGTG; >clamp_s_c_s_b_s:compl:rev CACAGCCACCCACACTATCATGTTGACATTCC; dG = -12.2
424 >s_d_s_c_s_d_s_d_s_b_s GTGACGGTGTGGGTGACGGTGACGGTGGCTGTG; >clamp_s_c_s_b_s:compl:rev CACAGCCACCCACACTATCATGTTGACATTCC; dG = -12.2
425 >s_d_s_c_s_e_s_d_s_b_s GTGACGGTGTGGGTGCTGGTGACGGTGGCTGTG; >clamp_s_c_s_b_s:compl:rev CACAGCCACCCACACTATCATGTTGACATTCC; dG = -12.2
426 >s_d_s_d_s_a_s_d_s_b_s GTGACGGTGACGGTGTCTGTGACGGTGGCTGTG; >clamp_s_c_s_b_s:compl:rev CACAGCCACCCACACTATCATGTTGACATTCC; dG = -12.2
427 >s_d_s_d_s_d_s_d_s_b_s GTGACGGTGACGGTGACGGTGACGGTGGCTGTG; >clamp_s_c_s_b_s:compl:rev CACAGCCACCCACACTATCATGTTGACATTCC; dG = -12.2
428 >s_d_s_d_s_e_s_d_s_b_s GTGACGGTGACGGTGCTGGTGACGGTGGCTGTG; >clamp_s_c_s_b_s:compl:rev CACAGCCACCCACACTATCATGTTGACATTCC; dG = -12.2
429 >s_d_s_e_s_a_s_d_s_b_s GTGACGGTGCTGGTGTCTGTGACGGTGGCTGTG; >clamp_s_c_s_b_s:compl:rev CACAGCCACCCACACTATCATGTTGACATTCC; dG = -12.2
430 >s_a_s_b_s_d_s_d_s_b_s GTGTCTGTGGCTGTGACGGTGACGGTGGCTGTG; >clamp_s_c_s_b_s:compl:rev CACAGCCACCCACACTATCATGTTGACATTCC; dG = -12.2
431 >s_d_s_e_s_b_s_e_s_b_s GTGACGGTGCTGGTGGCTGTGCTGGTGGCTGTG; >clamp_s_c_s_b_s:compl:rev CACAGCCACCCACACTATCATGTTGACATTCC; dG = -12.2
432 >s_d_s_e_s_d_s_d_s_b_s GTGACGGTGCTGGTGACGGTGACGGTGGCTGTG; >clamp_s_c_s_b_s:compl:rev CACAGCCACCCACACTATCATGTTGACATTCC; dG = -12.2
433 >s_d_s_e_s_e_s_d_s_b_s GTGACGGTGCTGGTGCTGGTGACGGTGGCTGTG; >clamp_s_c_s_b_s:compl:rev CACAGCCACCCACACTATCATGTTGACATTCC; dG = -12.2
434 >s_e_s_a_s_a_s_d_s_b_s GTGCTGGTGTCTGTGTCTGTGACGGTGGCTGTG; >clamp_s_c_s_b_s:compl:rev CACAGCCACCCACACTATCATGTTGACATTCC; dG = -12.2
435 >s_e_s_a_s_b_s_d_s_b_s GTGCTGGTGTCTGTGGCTGTGACGGTGGCTGTG; >clamp_s_c_s_b_s:compl:rev CACAGCCACCCACACTATCATGTTGACATTCC; dG = -12.2
436 >s_e_s_a_s_d_s_d_s_b_s GTGCTGGTGTCTGTGACGGTGACGGTGGCTGTG; >clamp_s_c_s_b_s:compl:rev CACAGCCACCCACACTATCATGTTGACATTCC; dG = -12.2
437 >s_e_s_a_s_e_s_d_s_b_s GTGCTGGTGTCTGTGCTGGTGACGGTGGCTGTG; >clamp_s_c_s_b_s:compl:rev CACAGCCACCCACACTATCATGTTGACATTCC; dG = -12.2
438 >s_e_s_b_s_a_s_d_s_b_s GTGCTGGTGGCTGTGTCTGTGACGGTGGCTGTG; >clamp_s_c_s_b_s:compl:rev CACAGCCACCCACACTATCATGTTGACATTCC; dG = -12.2
439 >s_a_s_b_s_e_s_d_s_b_s GTGTCTGTGGCTGTGCTGGTGACGGTGGCTGTG; >clamp_s_c_s_b_s:compl:rev CACAGCCACCCACACTATCATGTTGACATTCC; dG = -12.2
440 >s_e_s_b_s_b_s_d_s_b_s GTGCTGGTGGCTGTGGCTGTGACGGTGGCTGTG; >clamp_s_c_s_b_s:compl:rev CACAGCCACCCACACTATCATGTTGACATTCC; dG = -12.2

441 >s_e_s_b_s_e_s_d_s_b_s GTGCTGGTGGCTGTGCTGGTGACGGTGGCTGTG; >clamp_s_c_s_b_s:compl:rev CACAGCCACCCACACTATCATGTTGACATTCC; dG = -12.2
442 >s_e_s_c_s_d_s_d_s_b_s GTGCTGGTGTGGGTGACGGTGACGGTGGCTGTG; >clamp_s_c_s_b_s:compl:rev CACAGCCACCCACACTATCATGTTGACATTCC; dG = -12.2
443 >s_e_s_c_s_e_s_d_s_b_s GTGCTGGTGTGGGTGCTGGTGACGGTGGCTGTG; >clamp_s_c_s_b_s:compl:rev CACAGCCACCCACACTATCATGTTGACATTCC; dG = -12.2
444 >s_e_s_d_s_a_s_d_s_b_s GTGCTGGTGACGGTGTCTGTGACGGTGGCTGTG; >clamp_s_c_s_b_s:compl:rev CACAGCCACCCACACTATCATGTTGACATTCC; dG = -12.2
445 >s_e_s_d_s_d_s_d_s_b_s GTGCTGGTGACGGTGACGGTGACGGTGGCTGTG; >clamp_s_c_s_b_s:compl:rev CACAGCCACCCACACTATCATGTTGACATTCC; dG = -12.2
446 >s_e_s_d_s_e_s_d_s_b_s GTGCTGGTGACGGTGCTGGTGACGGTGGCTGTG; >clamp_s_c_s_b_s:compl:rev CACAGCCACCCACACTATCATGTTGACATTCC; dG = -12.2
447 >s_e_s_e_s_a_s_d_s_b_s GTGCTGGTGCTGGTGTCTGTGACGGTGGCTGTG; >clamp_s_c_s_b_s:compl:rev CACAGCCACCCACACTATCATGTTGACATTCC; dG = -12.2
448 >s_e_s_e_s_b_s_e_s_b_s GTGCTGGTGCTGGTGGCTGTGCTGGTGGCTGTG; >clamp_s_c_s_b_s:compl:rev CACAGCCACCCACACTATCATGTTGACATTCC; dG = -12.2
449 >s_e_s_e_s_d_s_d_s_b_s GTGCTGGTGCTGGTGACGGTGACGGTGGCTGTG; >clamp_s_c_s_b_s:compl:rev CACAGCCACCCACACTATCATGTTGACATTCC; dG = -12.2
450 >s_e_s_e_s_e_s_d_s_b_s GTGCTGGTGCTGGTGCTGGTGACGGTGGCTGTG; >clamp_s_c_s_b_s:compl:rev CACAGCCACCCACACTATCATGTTGACATTCC; dG = -12.2
451 >s_a_s_c_s_d_s_d_s_b_s GTGTCTGTGTGGGTGACGGTGACGGTGGCTGTG; >clamp_s_c_s_b_s:compl:rev CACAGCCACCCACACTATCATGTTGACATTCC; dG = -12.2
452 >s_a_s_c_s_e_s_e_s_b_s GTGTCTGTGTGGGTGCTGGTGCTGGTGGCTGTG; >clamp_s_c_s_b_s:compl:rev CACAGCCACCCACACTATCATGTTGACATTCC; dG = -12.1
453 >s_a_s_d_s_a_s_e_s_b_s GTGTCTGTGACGGTGTCTGTGCTGGTGGCTGTG; >clamp_s_c_s_b_s:compl:rev CACAGCCACCCACACTATCATGTTGACATTCC; dG = -12.1
454 >s_a_s_a_s_b_s_e_s_b_s GTGTCTGTGTCTGTGGCTGTGCTGGTGGCTGTG; >clamp_s_c_s_b_s:compl:rev CACAGCCACCCACACTATCATGTTGACATTCC; dG = -12.1
455 >s_a_s_d_s_d_s_e_s_b_s GTGTCTGTGACGGTGACGGTGCTGGTGGCTGTG; >clamp_s_c_s_b_s:compl:rev CACAGCCACCCACACTATCATGTTGACATTCC; dG = -12.1
456 >s_a_s_d_s_e_s_e_s_b_s GTGTCTGTGACGGTGCTGGTGCTGGTGGCTGTG; >clamp_s_c_s_b_s:compl:rev CACAGCCACCCACACTATCATGTTGACATTCC; dG = -12.1
457 >s_a_s_e_s_a_s_e_s_b_s GTGTCTGTGCTGGTGTCTGTGCTGGTGGCTGTG; >clamp_s_c_s_b_s:compl:rev CACAGCCACCCACACTATCATGTTGACATTCC; dG = -12.1
458 >s_a_s_e_s_d_s_e_s_b_s GTGTCTGTGCTGGTGACGGTGCTGGTGGCTGTG; >clamp_s_c_s_b_s:compl:rev CACAGCCACCCACACTATCATGTTGACATTCC; dG = -12.1
459 >s_a_s_e_s_e_s_e_s_b_s GTGTCTGTGCTGGTGCTGGTGCTGGTGGCTGTG; >clamp_s_c_s_b_s:compl:rev CACAGCCACCCACACTATCATGTTGACATTCC; dG = -12.1
460 >s_b_s_a_s_a_s_e_s_b_s GTGGCTGTGTCTGTGTCTGTGCTGGTGGCTGTG; >clamp_s_c_s_b_s:compl:rev CACAGCCACCCACACTATCATGTTGACATTCC; dG = -12.1
461 >s_b_s_a_s_b_s_e_s_b_s GTGGCTGTGTCTGTGGCTGTGCTGGTGGCTGTG; >clamp_s_c_s_b_s:compl:rev CACAGCCACCCACACTATCATGTTGACATTCC; dG = -12.1
462 >s_b_s_a_s_d_s_e_s_b_s GTGGCTGTGTCTGTGACGGTGCTGGTGGCTGTG; >clamp_s_c_s_b_s:compl:rev CACAGCCACCCACACTATCATGTTGACATTCC; dG = -12.1
463 >s_b_s_a_s_e_s_e_s_b_s GTGGCTGTGTCTGTGCTGGTGCTGGTGGCTGTG; >clamp_s_c_s_b_s:compl:rev CACAGCCACCCACACTATCATGTTGACATTCC; dG = -12.1
464 >s_b_s_b_s_a_s_e_s_b_s GTGGCTGTGGCTGTGTCTGTGCTGGTGGCTGTG; >clamp_s_c_s_b_s:compl:rev CACAGCCACCCACACTATCATGTTGACATTCC; dG = -12.1
465 >s_b_s_b_s_b_s_e_s_b_s GTGGCTGTGGCTGTGGCTGTGCTGGTGGCTGTG; >clamp_s_c_s_b_s:compl:rev CACAGCCACCCACACTATCATGTTGACATTCC; dG = -12.1
466 >s_b_s_b_s_d_s_e_s_b_s GTGGCTGTGGCTGTGACGGTGCTGGTGGCTGTG; >clamp_s_c_s_b_s:compl:rev CACAGCCACCCACACTATCATGTTGACATTCC; dG = -12.1
467 >s_b_s_b_s_e_s_e_s_b_s GTGGCTGTGGCTGTGCTGGTGCTGGTGGCTGTG; >clamp_s_c_s_b_s:compl:rev CACAGCCACCCACACTATCATGTTGACATTCC; dG = -12.1
468 >s_a_s_a_s_d_s_e_s_b_s GTGTCTGTGTCTGTGACGGTGCTGGTGGCTGTG; >clamp_s_c_s_b_s:compl:rev CACAGCCACCCACACTATCATGTTGACATTCC; dG = -12.1
469 >s_b_s_c_s_d_s_e_s_b_s GTGGCTGTGTGGGTGACGGTGCTGGTGGCTGTG; >clamp_s_c_s_b_s:compl:rev CACAGCCACCCACACTATCATGTTGACATTCC; dG = -12.1
470 >s_b_s_c_s_e_s_e_s_b_s GTGGCTGTGTGGGTGCTGGTGCTGGTGGCTGTG; >clamp_s_c_s_b_s:compl:rev CACAGCCACCCACACTATCATGTTGACATTCC; dG = -12.1
471 >s_b_s_d_s_a_s_e_s_b_s GTGGCTGTGACGGTGTCTGTGCTGGTGGCTGTG; >clamp_s_c_s_b_s:compl:rev CACAGCCACCCACACTATCATGTTGACATTCC; dG = -12.1
472 >s_b_s_d_s_d_s_e_s_b_s GTGGCTGTGACGGTGACGGTGCTGGTGGCTGTG; >clamp_s_c_s_b_s:compl:rev CACAGCCACCCACACTATCATGTTGACATTCC; dG = -12.1
473 >s_b_s_d_s_e_s_e_s_b_s GTGGCTGTGACGGTGCTGGTGCTGGTGGCTGTG; >clamp_s_c_s_b_s:compl:rev CACAGCCACCCACACTATCATGTTGACATTCC; dG = -12.1
474 >s_b_s_e_s_a_s_e_s_b_s GTGGCTGTGCTGGTGTCTGTGCTGGTGGCTGTG; >clamp_s_c_s_b_s:compl:rev CACAGCCACCCACACTATCATGTTGACATTCC; dG = -12.1
475 >s_a_s_a_s_e_s_e_s_b_s GTGTCTGTGTCTGTGCTGGTGCTGGTGGCTGTG; >clamp_s_c_s_b_s:compl:rev CACAGCCACCCACACTATCATGTTGACATTCC; dG = -12.1
476 >s_b_s_e_s_d_s_e_s_b_s GTGGCTGTGCTGGTGACGGTGCTGGTGGCTGTG; >clamp_s_c_s_b_s:compl:rev CACAGCCACCCACACTATCATGTTGACATTCC; dG = -12.1
477 >s_b_s_e_s_e_s_e_s_b_s GTGGCTGTGCTGGTGCTGGTGCTGGTGGCTGTG; >clamp_s_c_s_b_s:compl:rev CACAGCCACCCACACTATCATGTTGACATTCC; dG = -12.1

478 >s_c_s_c_s_d_s_e_s_b_s GTGTGGGTGTGGGTGACGGTGCTGGTGGCTGTG; >clamp_s_c_s_b_s:compl:rev
CACAGCCACCCACACTATCATGTTGACATTCC; dG = -12.1
479 >s_a_s_b_s_a_s_e_s_b_s GTGTCTGTGGCTGTGTCTGTGCTGGTGGCTGTG; >clamp_s_c_s_b_s:compl:rev
CACAGCCACCCACACTATCATGTTGACATTCC; dG = -12.1
480 >s_c_s_c_s_e_s_e_s_b_s GTGTGGGTGTGGGTGCTGGTGCTGGTGGCTGTG; >clamp_s_c_s_b_s:compl:rev
CACAGCCACCCACACTATCATGTTGACATTCC; dG = -12.1
481 >s_c_s_d_s_a_s_e_s_b_s GTGTGGGTGACGGTGTCTGTGCTGGTGGCTGTG; >clamp_s_c_s_b_s:compl:rev
CACAGCCACCCACACTATCATGTTGACATTCC; dG = -12.1
482 >s_c_s_d_s_d_s_e_s_b_s GTGTGGGTGACGGTGACGGTGCTGGTGGCTGTG; >clamp_s_c_s_b_s:compl:rev
CACAGCCACCCACACTATCATGTTGACATTCC; dG = -12.1
483 >s_c_s_d_s_e_s_e_s_b_s GTGTGGGTGACGGTGCTGGTGCTGGTGGCTGTG; >clamp_s_c_s_b_s:compl:rev
CACAGCCACCCACACTATCATGTTGACATTCC; dG = -12.1
484 >s_c_s_e_s_a_s_e_s_b_s GTGTGGGTGCTGGTGTCTGTGCTGGTGGCTGTG; >clamp_s_c_s_b_s:compl:rev
CACAGCCACCCACACTATCATGTTGACATTCC; dG = -12.1
485 >s_a_s_a_s_a_s_e_s_b_s GTGTCTGTGTCTGTGTCTGTGCTGGTGGCTGTG; >clamp_s_c_s_b_s:compl:rev
CACAGCCACCCACACTATCATGTTGACATTCC; dG = -12.1
486 >s_c_s_e_s_d_s_e_s_b_s GTGTGGGTGCTGGTGACGGTGCTGGTGGCTGTG; >clamp_s_c_s_b_s:compl:rev
CACAGCCACCCACACTATCATGTTGACATTCC; dG = -12.1
487 >s_c_s_e_s_e_s_e_s_b_s GTGTGGGTGCTGGTGCTGGTGCTGGTGGCTGTG; >clamp_s_c_s_b_s:compl:rev
CACAGCCACCCACACTATCATGTTGACATTCC; dG = -12.1
488 >s_a_s_b_s_b_s_e_s_b_s GTGTCTGTGGCTGTGGCTGTGCTGGTGGCTGTG; >clamp_s_c_s_b_s:compl:rev
CACAGCCACCCACACTATCATGTTGACATTCC; dG = -12.1
489 >s_d_s_a_s_a_s_e_s_b_s GTGACGGTGTCTGTGTCTGTGCTGGTGGCTGTG; >clamp_s_c_s_b_s:compl:rev
CACAGCCACCCACACTATCATGTTGACATTCC; dG = -12.1
490 >s_d_s_a_s_b_s_e_s_b_s GTGACGGTGTCTGTGGCTGTGCTGGTGGCTGTG; >clamp_s_c_s_b_s:compl:rev
CACAGCCACCCACACTATCATGTTGACATTCC; dG = -12.1
491 >s_d_s_a_s_d_s_e_s_b_s GTGACGGTGTCTGTGACGGTGCTGGTGGCTGTG; >clamp_s_c_s_b_s:compl:rev
CACAGCCACCCACACTATCATGTTGACATTCC; dG = -12.1
492 >s_d_s_a_s_e_s_e_s_b_s GTGACGGTGTCTGTGCTGGTGCTGGTGGCTGTG; >clamp_s_c_s_b_s:compl:rev
CACAGCCACCCACACTATCATGTTGACATTCC; dG = -12.1
493 >s_d_s_b_s_a_s_e_s_b_s GTGACGGTGGCTGTGTCTGTGCTGGTGGCTGTG; >clamp_s_c_s_b_s:compl:rev
CACAGCCACCCACACTATCATGTTGACATTCC; dG = -12.1
494 >s_d_s_b_s_b_s_e_s_b_s GTGACGGTGGCTGTGGCTGTGCTGGTGGCTGTG; >clamp_s_c_s_b_s:compl:rev
CACAGCCACCCACACTATCATGTTGACATTCC; dG = -12.1
495 >s_d_s_b_s_e_s_e_s_b_s GTGACGGTGGCTGTGCTGGTGCTGGTGGCTGTG; >clamp_s_c_s_b_s:compl:rev
CACAGCCACCCACACTATCATGTTGACATTCC; dG = -12.1
496 >s_d_s_c_s_d_s_e_s_b_s GTGACGGTGTGGGTGACGGTGCTGGTGGCTGTG; >clamp_s_c_s_b_s:compl:rev
CACAGCCACCCACACTATCATGTTGACATTCC; dG = -12.1
497 >s_d_s_c_s_e_s_e_s_b_s GTGACGGTGTGGGTGCTGGTGCTGGTGGCTGTG; >clamp_s_c_s_b_s:compl:rev
CACAGCCACCCACACTATCATGTTGACATTCC; dG = -12.1
498 >s_d_s_d_s_a_s_e_s_b_s GTGACGGTGACGGTGTCTGTGCTGGTGGCTGTG; >clamp_s_c_s_b_s:compl:rev
CACAGCCACCCACACTATCATGTTGACATTCC; dG = -12.1
499 >s_d_s_d_s_d_s_e_s_b_s GTGACGGTGACGGTGACGGTGCTGGTGGCTGTG; >clamp_s_c_s_b_s:compl:rev
CACAGCCACCCACACTATCATGTTGACATTCC; dG = -12.1
500 >s_d_s_d_s_e_s_e_s_b_s GTGACGGTGACGGTGCTGGTGCTGGTGGCTGTG; >clamp_s_c_s_b_s:compl:rev
CACAGCCACCCACACTATCATGTTGACATTCC; dG = -12.1
501 >s_a_s_b_s_d_s_e_s_b_s GTGTCTGTGGCTGTGACGGTGCTGGTGGCTGTG; >clamp_s_c_s_b_s:compl:rev
CACAGCCACCCACACTATCATGTTGACATTCC; dG = -12.1
502 >s_d_s_e_s_a_s_e_s_b_s GTGACGGTGCTGGTGTCTGTGCTGGTGGCTGTG; >clamp_s_c_s_b_s:compl:rev
CACAGCCACCCACACTATCATGTTGACATTCC; dG = -12.1
503 >s_d_s_e_s_d_s_e_s_b_s GTGACGGTGCTGGTGACGGTGCTGGTGGCTGTG; >clamp_s_c_s_b_s:compl:rev
CACAGCCACCCACACTATCATGTTGACATTCC; dG = -12.1
504 >s_d_s_e_s_e_s_e_s_b_s GTGACGGTGCTGGTGCTGGTGCTGGTGGCTGTG; >clamp_s_c_s_b_s:compl:rev
CACAGCCACCCACACTATCATGTTGACATTCC; dG = -12.1
505 >s_e_s_a_s_a_s_e_s_b_s GTGCTGGTGTCTGTGTCTGTGCTGGTGGCTGTG; >clamp_s_c_s_b_s:compl:rev
CACAGCCACCCACACTATCATGTTGACATTCC; dG = -12.1
506 >s_e_s_a_s_b_s_e_s_b_s GTGCTGGTGTCTGTGGCTGTGCTGGTGGCTGTG; >clamp_s_c_s_b_s:compl:rev
CACAGCCACCCACACTATCATGTTGACATTCC; dG = -12.1
507 >s_e_s_a_s_d_s_e_s_b_s GTGCTGGTGTCTGTGACGGTGCTGGTGGCTGTG; >clamp_s_c_s_b_s:compl:rev
CACAGCCACCCACACTATCATGTTGACATTCC; dG = -12.1
508 >s_e_s_a_s_e_s_e_s_b_s GTGCTGGTGTCTGTGCTGGTGCTGGTGGCTGTG; >clamp_s_c_s_b_s:compl:rev
CACAGCCACCCACACTATCATGTTGACATTCC; dG = -12.1
509 >s_a_s_b_s_e_s_e_s_b_s GTGTCTGTGGCTGTGCTGGTGCTGGTGGCTGTG; >clamp_s_c_s_b_s:compl:rev
CACAGCCACCCACACTATCATGTTGACATTCC; dG = -12.1
510 >s_e_s_b_s_a_s_e_s_b_s GTGCTGGTGGCTGTGTCTGTGCTGGTGGCTGTG; >clamp_s_c_s_b_s:compl:rev
CACAGCCACCCACACTATCATGTTGACATTCC; dG = -12.1
511 >s_e_s_b_s_b_s_e_s_b_s GTGCTGGTGGCTGTGGCTGTGCTGGTGGCTGTG; >clamp_s_c_s_b_s:compl:rev
CACAGCCACCCACACTATCATGTTGACATTCC; dG = -12.1
512 >s_e_s_b_s_e_s_e_s_b_s GTGCTGGTGGCTGTGCTGGTGCTGGTGGCTGTG; >clamp_s_c_s_b_s:compl:rev
CACAGCCACCCACACTATCATGTTGACATTCC; dG = -12.1
513 >s_e_s_c_s_d_s_e_s_b_s GTGCTGGTGTGGGTGACGGTGCTGGTGGCTGTG; >clamp_s_c_s_b_s:compl:rev
CACAGCCACCCACACTATCATGTTGACATTCC; dG = -12.1
514 >s_e_s_c_s_e_s_e_s_b_s GTGCTGGTGTGGGTGCTGGTGCTGGTGGCTGTG; >clamp_s_c_s_b_s:compl:rev
CACAGCCACCCACACTATCATGTTGACATTCC; dG = -12.1

515 >s_e_s_d_s_a_s_e_s_b_s GTGCTGGTGACGGTGTCTGTGCTGGTGGCTGTG; >clamp_s_c_s_b_s:compl:rev CACAGCCACCCACACTATCATGTTGACATTCC; dG = -12.1
516 >s_e_s_d_s_d_s_e_s_b_s GTGCTGGTGACGGTGACGGTGCTGGTGGCTGTG; >clamp_s_c_s_b_s:compl:rev CACAGCCACCCACACTATCATGTTGACATTCC; dG = -12.1
517 >s_e_s_d_s_e_s_e_s_b_s GTGCTGGTGACGGTGCTGGTGCTGGTGGCTGTG; >clamp_s_c_s_b_s:compl:rev CACAGCCACCCACACTATCATGTTGACATTCC; dG = -12.1
518 >s_e_s_e_s_a_s_e_s_b_s GTGCTGGTGCTGGTGTCTGTGCTGGTGGCTGTG; >clamp_s_c_s_b_s:compl:rev CACAGCCACCCACACTATCATGTTGACATTCC; dG = -12.1
519 >s_e_s_e_s_d_s_e_s_b_s GTGCTGGTGCTGGTGACGGTGCTGGTGGCTGTG; >clamp_s_c_s_b_s:compl:rev CACAGCCACCCACACTATCATGTTGACATTCC; dG = -12.1
520 >s_e_s_e_s_e_s_e_s_b_s GTGCTGGTGCTGGTGCTGGTGCTGGTGGCTGTG; >clamp_s_c_s_b_s:compl:rev CACAGCCACCCACACTATCATGTTGACATTCC; dG = -12.1
521 >s_a_s_c_s_d_s_e_s_b_s GTGTCTGTGTGGGTGACGGTGCTGGTGGCTGTG; >clamp_s_c_s_b_s:compl:rev CACAGCCACCCACACTATCATGTTGACATTCC; dG = -12.1
522 >s_a_s_d_s_c_s_c_s_c_s GTGTCTGTGACGGTGTGGGTGTGGGTGTGGGTG; >clamp_s_c_s_b_s:compl:rev CACAGCCACCCACACTATCATGTTGACATTCC; dG = -12.0
523 >s_a_s_e_s_c_s_c_s_c_s GTGTCTGTGCTGGTGTGGGTGTGGGTGTGGGTG; >clamp_s_c_s_b_s:compl:rev CACAGCCACCCACACTATCATGTTGACATTCC; dG = -12.0
524 >s_a_s_a_s_c_s_c_s_c_s GTGTCTGTGTCTGTGTGGGTGTGGGTGTGGGTG; >clamp_s_c_s_b_s:compl:rev CACAGCCACCCACACTATCATGTTGACATTCC; dG = -12.0
525 >s_b_s_a_s_c_s_c_s_c_s GTGGCTGTGTCTGTGTGGGTGTGGGTGTGGGTG; >clamp_s_c_s_b_s:compl:rev CACAGCCACCCACACTATCATGTTGACATTCC; dG = -12.0
526 >s_b_s_b_s_c_s_c_s_c_s GTGGCTGTGGCTGTGTGGGTGTGGGTGTGGGTG; >clamp_s_c_s_b_s:compl:rev CACAGCCACCCACACTATCATGTTGACATTCC; dG = -12.0
527 >s_b_s_c_s_c_s_c_s_e_s GTGGCTGTGTGGGTGTGGGTGTGGGTGCTGGTG; >clamp_s_c_s_b_s:compl:rev CACAGCCACCCACACTATCATGTTGACATTCC; dG = -12.0
528 >s_b_s_c_s_c_s_c_s_d_s GTGGCTGTGTGGGTGTGGGTGTGGGTGACGGTG; >clamp_s_c_s_b_s:compl:rev CACAGCCACCCACACTATCATGTTGACATTCC; dG = -12.0
529 >s_b_s_c_s_c_s_c_s_c_s GTGGCTGTGTGGGTGTGGGTGTGGGTGTGGGTG; >clamp_s_c_s_b_s:compl:rev CACAGCCACCCACACTATCATGTTGACATTCC; dG = -12.0
530 >s_b_s_d_s_c_s_c_s_c_s GTGGCTGTGACGGTGTGGGTGTGGGTGTGGGTG; >clamp_s_c_s_b_s:compl:rev CACAGCCACCCACACTATCATGTTGACATTCC; dG = -12.0
531 >s_b_s_e_s_c_s_c_s_c_s GTGGCTGTGCTGGTGTGGGTGTGGGTGTGGGTG; >clamp_s_c_s_b_s:compl:rev CACAGCCACCCACACTATCATGTTGACATTCC; dG = -12.0
532 >s_c_s_c_s_c_s_c_s_e_s GTGTGGGTGTGGGTGTGGGTGTGGGTGCTGGTG; >clamp_s_c_s_b_s:compl:rev CACAGCCACCCACACTATCATGTTGACATTCC; dG = -12.0
533 >s_c_s_c_s_c_s_c_s_d_s GTGTGGGTGTGGGTGTGGGTGTGGGTGACGGTG; >clamp_s_c_s_b_s:compl:rev CACAGCCACCCACACTATCATGTTGACATTCC; dG = -12.0
534 >s_c_s_c_s_c_s_c_s_c_s GTGTGGGTGTGGGTGTGGGTGTGGGTGTGGGTG; >clamp_s_c_s_b_s:compl:rev CACAGCCACCCACACTATCATGTTGACATTCC; dG = -12.0
535 >s_c_s_d_s_c_s_c_s_c_s GTGTGGGTGACGGTGTGGGTGTGGGTGTGGGTG; >clamp_s_c_s_b_s:compl:rev CACAGCCACCCACACTATCATGTTGACATTCC; dG = -12.0
536 >s_c_s_e_s_c_s_c_s_c_s GTGTGGGTGCTGGTGTGGGTGTGGGTGTGGGTG; >clamp_s_c_s_b_s:compl:rev CACAGCCACCCACACTATCATGTTGACATTCC; dG = -12.0
537 >s_d_s_a_s_c_s_c_s_c_s GTGACGGTGTCTGTGTGGGTGTGGGTGTGGGTG; >clamp_s_c_s_b_s:compl:rev CACAGCCACCCACACTATCATGTTGACATTCC; dG = -12.0
538 >s_d_s_b_s_a_s_c_s_e_s GTGACGGTGGCTGTGTCTGTGTGGGTGCTGGTG; >clamp_s_c_s_b_s:compl:rev CACAGCCACCCACACTATCATGTTGACATTCC; dG = -12.0
539 >s_d_s_b_s_a_s_c_s_d_s GTGACGGTGGCTGTGTCTGTGTGGGTGACGGTG; >clamp_s_c_s_b_s:compl:rev CACAGCCACCCACACTATCATGTTGACATTCC; dG = -12.0
540 >s_d_s_b_s_a_s_c_s_c_s GTGACGGTGGCTGTGTCTGTGTGGGTGTGGGTG; >clamp_s_c_s_b_s:compl:rev CACAGCCACCCACACTATCATGTTGACATTCC; dG = -12.0
541 >s_d_s_b_s_a_s_b_s_b_s GTGACGGTGGCTGTGTCTGTGGCTGTGGCTGTG; >clamp_s_c_s_b_s:compl:rev CACAGCCACCCACACTATCATGTTGACATTCC; dG = -12.0
542 >s_d_s_b_s_a_s_a_s_b_s GTGACGGTGGCTGTGTCTGTGTCTGTGGCTGTG; >clamp_s_c_s_b_s:compl:rev CACAGCCACCCACACTATCATGTTGACATTCC; dG = -12.0
543 >s_d_s_b_s_b_s_c_s_e_s GTGACGGTGGCTGTGGCTGTGTGGGTGCTGGTG; >clamp_s_c_s_b_s:compl:rev CACAGCCACCCACACTATCATGTTGACATTCC; dG = -12.0
544 >s_d_s_b_s_b_s_c_s_d_s GTGACGGTGGCTGTGGCTGTGTGGGTGACGGTG; >clamp_s_c_s_b_s:compl:rev CACAGCCACCCACACTATCATGTTGACATTCC; dG = -12.0
545 >s_d_s_b_s_b_s_c_s_c_s GTGACGGTGGCTGTGGCTGTGTGGGTGTGGGTG; >clamp_s_c_s_b_s:compl:rev CACAGCCACCCACACTATCATGTTGACATTCC; dG = -12.0
546 >s_d_s_b_s_b_s_b_s_b_s GTGACGGTGGCTGTGGCTGTGGCTGTGGCTGTG; >clamp_s_c_s_b_s:compl:rev CACAGCCACCCACACTATCATGTTGACATTCC; dG = -12.0
547 >s_d_s_b_s_b_s_a_s_b_s GTGACGGTGGCTGTGGCTGTGTCTGTGGCTGTG; >clamp_s_c_s_b_s:compl:rev CACAGCCACCCACACTATCATGTTGACATTCC; dG = -12.0
548 >s_d_s_b_s_c_s_c_s_e_s GTGACGGTGGCTGTGTGGGTGTGGGTGCTGGTG; >clamp_s_c_s_b_s:compl:rev CACAGCCACCCACACTATCATGTTGACATTCC; dG = -12.0
549 >s_d_s_b_s_c_s_c_s_d_s GTGACGGTGGCTGTGTGGGTGTGGGTGACGGTG; >clamp_s_c_s_b_s:compl:rev CACAGCCACCCACACTATCATGTTGACATTCC; dG = -12.0
550 >s_a_s_b_s_c_s_c_s_c_s GTGTCTGTGGCTGTGTGGGTGTGGGTGTGGGTG; >clamp_s_c_s_b_s:compl:rev CACAGCCACCCACACTATCATGTTGACATTCC; dG = -12.0
551 >s_d_s_b_s_c_s_c_s_c_s GTGACGGTGGCTGTGTGGGTGTGGGTGTGGGTG; >clamp_s_c_s_b_s:compl:rev CACAGCCACCCACACTATCATGTTGACATTCC; dG = -12.0

552 >s_d_s_c_s_c_s_c_s_e_s GTGACGGTGTGGGTGTGGGTGTGGGTGCTGGTG; >clamp_s_c_s_b_s:compl:rev
CACAGCCACCCACACTATCATGTTGACATTCC; dG = -12.0
553 >s_d_s_c_s_c_s_c_s_d_s GTGACGGTGTGGGTGTGGGTGTGGGTGACGGTG; >clamp_s_c_s_b_s:compl:rev
CACAGCCACCCACACTATCATGTTGACATTCC; dG = -12.0
554 >s_d_s_c_s_c_s_c_s_c_s GTGACGGTGTGGGTGTGGGTGTGGGTGTGGGTG; >clamp_s_c_s_b_s:compl:rev
CACAGCCACCCACACTATCATGTTGACATTCC; dG = -12.0
555 >s_d_s_d_s_c_s_c_s_c_s GTGACGGTGACGGTGTGGGTGTGGGTGTGGGTG; >clamp_s_c_s_b_s:compl:rev
CACAGCCACCCACACTATCATGTTGACATTCC; dG = -12.0
556 >s_d_s_e_s_c_s_c_s_c_s GTGACGGTGCTGGTGTGGGTGTGGGTGTGGGTG; >clamp_s_c_s_b_s:compl:rev
CACAGCCACCCACACTATCATGTTGACATTCC; dG = -12.0
557 >s_e_s_a_s_c_s_c_s_c_s GTGCTGGTGTCTGTGTGGGTGTGGGTGTGGGTG; >clamp_s_c_s_b_s:compl:rev
CACAGCCACCCACACTATCATGTTGACATTCC; dG = -12.0
558 >s_e_s_b_s_c_s_c_s_c_s GTGCTGGTGGCTGTGTGGGTGTGGGTGTGGGTG; >clamp_s_c_s_b_s:compl:rev
CACAGCCACCCACACTATCATGTTGACATTCC; dG = -12.0
559 >s_e_s_c_s_c_s_c_s_e_s GTGCTGGTGTGGGTGTGGGTGTGGGTGCTGGTG; >clamp_s_c_s_b_s:compl:rev
CACAGCCACCCACACTATCATGTTGACATTCC; dG = -12.0
560 >s_e_s_c_s_c_s_c_s_d_s GTGCTGGTGTGGGTGTGGGTGTGGGTGACGGTG; >clamp_s_c_s_b_s:compl:rev
CACAGCCACCCACACTATCATGTTGACATTCC; dG = -12.0
561 >s_e_s_c_s_c_s_c_s_c_s GTGCTGGTGTGGGTGTGGGTGTGGGTGTGGGTG; >clamp_s_c_s_b_s:compl:rev
CACAGCCACCCACACTATCATGTTGACATTCC; dG = -12.0
562 >s_e_s_d_s_c_s_c_s_c_s GTGCTGGTGACGGTGTGGGTGTGGGTGTGGGTG; >clamp_s_c_s_b_s:compl:rev
CACAGCCACCCACACTATCATGTTGACATTCC; dG = -12.0
563 >s_a_s_c_s_c_s_c_s_e_s GTGTCTGTGTGGGTGTGGGTGTGGGTGCTGGTG; >clamp_s_c_s_b_s:compl:rev
CACAGCCACCCACACTATCATGTTGACATTCC; dG = -12.0
564 >s_e_s_e_s_c_s_c_s_c_s GTGCTGGTGCTGGTGTGGGTGTGGGTGTGGGTG; >clamp_s_c_s_b_s:compl:rev
CACAGCCACCCACACTATCATGTTGACATTCC; dG = -12.0
565 >s_a_s_c_s_c_s_c_s_d_s GTGTCTGTGTGGGTGTGGGTGTGGGTGACGGTG; >clamp_s_c_s_b_s:compl:rev
CACAGCCACCCACACTATCATGTTGACATTCC; dG = -12.0
566 >s_a_s_c_s_c_s_c_s_c_s GTGTCTGTGTGGGTGTGGGTGTGGGTGTGGGTG; >clamp_s_c_s_b_s:compl:rev
CACAGCCACCCACACTATCATGTTGACATTCC; dG = -12.0
567 >s_e_s_b_s_a_s_c_s_e_s GTGCTGGTGGCTGTGTCTGTGTGGGTGCTGGTG; >clamp_s_c_s_b_s:compl:rev
CACAGCCACCCACACTATCATGTTGACATTCC; dG = -11.9
568 >s_e_s_b_s_a_s_c_s_d_s GTGCTGGTGGCTGTGTCTGTGTGGGTGACGGTG; >clamp_s_c_s_b_s:compl:rev
CACAGCCACCCACACTATCATGTTGACATTCC; dG = -11.9
569 >s_e_s_b_s_a_s_c_s_c_s GTGCTGGTGGCTGTGTCTGTGTGGGTGTGGGTG; >clamp_s_c_s_b_s:compl:rev
CACAGCCACCCACACTATCATGTTGACATTCC; dG = -11.9
570 >s_e_s_b_s_a_s_b_s_b_s GTGCTGGTGGCTGTGTCTGTGGCTGTGGCTGTG; >clamp_s_c_s_b_s:compl:rev
CACAGCCACCCACACTATCATGTTGACATTCC; dG = -11.9
571 >s_e_s_b_s_a_s_a_s_b_s GTGCTGGTGGCTGTGTCTGTGTCTGTGGCTGTG; >clamp_s_c_s_b_s:compl:rev
CACAGCCACCCACACTATCATGTTGACATTCC; dG = -11.9
572 >s_e_s_b_s_b_s_c_s_e_s GTGCTGGTGGCTGTGGCTGTGTGGGTGCTGGTG; >clamp_s_c_s_b_s:compl:rev
CACAGCCACCCACACTATCATGTTGACATTCC; dG = -11.9
573 >s_e_s_b_s_b_s_c_s_d_s GTGCTGGTGGCTGTGGCTGTGTGGGTGACGGTG; >clamp_s_c_s_b_s:compl:rev
CACAGCCACCCACACTATCATGTTGACATTCC; dG = -11.9
574 >s_e_s_b_s_b_s_c_s_c_s GTGCTGGTGGCTGTGGCTGTGTGGGTGTGGGTG; >clamp_s_c_s_b_s:compl:rev
CACAGCCACCCACACTATCATGTTGACATTCC; dG = -11.9
575 >s_e_s_b_s_b_s_b_s_b_s GTGCTGGTGGCTGTGGCTGTGGCTGTGGCTGTG; >clamp_s_c_s_b_s:compl:rev
CACAGCCACCCACACTATCATGTTGACATTCC; dG = -11.9
576 >s_e_s_b_s_b_s_a_s_b_s GTGCTGGTGGCTGTGGCTGTGTCTGTGGCTGTG; >clamp_s_c_s_b_s:compl:rev
CACAGCCACCCACACTATCATGTTGACATTCC; dG = -11.9
577 >s_e_s_b_s_c_s_c_s_e_s GTGCTGGTGGCTGTGTGGGTGTGGGTGCTGGTG; >clamp_s_c_s_b_s:compl:rev
CACAGCCACCCACACTATCATGTTGACATTCC; dG = -11.9
578 >s_e_s_b_s_c_s_c_s_d_s GTGCTGGTGGCTGTGTGGGTGTGGGTGACGGTG; >clamp_s_c_s_b_s:compl:rev
CACAGCCACCCACACTATCATGTTGACATTCC; dG = -11.9
579 >s_a_s_c_s_d_s_a_s_b_s GTGTCTGTGTGGGTGACGGTGTCTGTGGCTGTG; >clamp_s_c_s_b_s:compl:rev
CACAGCCACCCACACTATCATGTTGACATTCC; dG = -11.8
580 >s_a_s_c_s_e_s_a_s_b_s GTGTCTGTGTGGGTGCTGGTGTCTGTGGCTGTG; >clamp_s_c_s_b_s:compl:rev
CACAGCCACCCACACTATCATGTTGACATTCC; dG = -11.8
581 >s_b_s_c_s_d_s_a_s_b_s GTGGCTGTGTGGGTGACGGTGTCTGTGGCTGTG; >clamp_s_c_s_b_s:compl:rev
CACAGCCACCCACACTATCATGTTGACATTCC; dG = -11.8
582 >s_b_s_c_s_e_s_a_s_b_s GTGGCTGTGTGGGTGCTGGTGTCTGTGGCTGTG; >clamp_s_c_s_b_s:compl:rev
CACAGCCACCCACACTATCATGTTGACATTCC; dG = -11.8
583 >s_c_s_c_s_d_s_a_s_b_s GTGTGGGTGTGGGTGACGGTGTCTGTGGCTGTG; >clamp_s_c_s_b_s:compl:rev
CACAGCCACCCACACTATCATGTTGACATTCC; dG = -11.8
584 >s_c_s_c_s_e_s_a_s_b_s GTGTGGGTGTGGGTGCTGGTGTCTGTGGCTGTG; >clamp_s_c_s_b_s:compl:rev
CACAGCCACCCACACTATCATGTTGACATTCC; dG = -11.8
585 >s_d_s_b_s_e_s_c_s_e_s GTGACGGTGGCTGTGCTGGTGTGGGTGCTGGTG; >clamp_s_c_s_b_s:compl:rev
CACAGCCACCCACACTATCATGTTGACATTCC; dG = -11.8
586 >s_d_s_b_s_e_s_c_s_d_s GTGACGGTGGCTGTGCTGGTGTGGGTGACGGTG; >clamp_s_c_s_b_s:compl:rev
CACAGCCACCCACACTATCATGTTGACATTCC; dG = -11.8
587 >s_d_s_b_s_e_s_c_s_c_s GTGACGGTGGCTGTGCTGGTGTGGGTGTGGGTG; >clamp_s_c_s_b_s:compl:rev
CACAGCCACCCACACTATCATGTTGACATTCC; dG = -11.8
588 >s_d_s_b_s_e_s_a_s_b_s GTGACGGTGGCTGTGCTGGTGTCTGTGGCTGTG; >clamp_s_c_s_b_s:compl:rev
CACAGCCACCCACACTATCATGTTGACATTCC; dG = -11.8

589 >s_d_s_c_s_d_s_a_s_b_s GTGACGGTGTGGGTGACGGTGTCTGTGGCTGTG; >clamp_s_c_s_b_s:compl:rev CACAGCCACCCACACTATCATGTTGACATTCC; dG = -11.8
590 >s_d_s_c_s_e_s_a_s_b_s GTGACGGTGTGGGTGCTGGTGTCTGTGGCTGTG; >clamp_s_c_s_b_s:compl:rev CACAGCCACCCACACTATCATGTTGACATTCC; dG = -11.8
591 >s_e_s_c_s_d_s_a_s_b_s GTGCTGGTGTGGGTGACGGTGTCTGTGGCTGTG; >clamp_s_c_s_b_s:compl:rev CACAGCCACCCACACTATCATGTTGACATTCC; dG = -11.8
592 >s_e_s_c_s_e_s_a_s_b_s GTGCTGGTGTGGGTGCTGGTGTCTGTGGCTGTG; >clamp_s_c_s_b_s:compl:rev CACAGCCACCCACACTATCATGTTGACATTCC; dG = -11.8
593 >s_a_s_a_s_b_s_b_s_b_s GTGTCTGTGTCTGTGGCTGTGGCTGTGGCTGTG; >clamp_s_c_s_b_s:compl:rev CACAGCCACCCACACTATCATGTTGACATTCC; dG = -11.7
594 >s_b_s_a_s_b_s_b_s_b_s GTGGCTGTGTCTGTGGCTGTGGCTGTGGCTGTG; >clamp_s_c_s_b_s:compl:rev CACAGCCACCCACACTATCATGTTGACATTCC; dG = -11.7
595 >s_b_s_b_s_b_s_c_s_e_s GTGGCTGTGGCTGTGGCTGTGTGGGTGCTGGTG; >clamp_s_c_s_b_s:compl:rev CACAGCCACCCACACTATCATGTTGACATTCC; dG = -11.7
596 >s_b_s_b_s_b_s_c_s_d_s GTGGCTGTGGCTGTGGCTGTGTGGGTGACGGTG; >clamp_s_c_s_b_s:compl:rev CACAGCCACCCACACTATCATGTTGACATTCC; dG = -11.7
597 >s_b_s_b_s_b_s_c_s_c_s GTGGCTGTGGCTGTGGCTGTGTGGGTGTGGGTG; >clamp_s_c_s_b_s:compl:rev CACAGCCACCCACACTATCATGTTGACATTCC; dG = -11.7
598 >s_b_s_b_s_b_s_b_s_b_s GTGGCTGTGGCTGTGGCTGTGGCTGTGGCTGTG; >clamp_s_c_s_b_s:compl:rev CACAGCCACCCACACTATCATGTTGACATTCC; dG = -11.7
599 >s_b_s_b_s_b_s_a_s_b_s GTGGCTGTGGCTGTGGCTGTGTCTGTGGCTGTG; >clamp_s_c_s_b_s:compl:rev CACAGCCACCCACACTATCATGTTGACATTCC; dG = -11.7
600 >s_b_s_b_s_d_s_c_s_e_s GTGGCTGTGGCTGTGACGGTGTGGGTGCTGGTG; >clamp_s_c_s_b_s:compl:rev CACAGCCACCCACACTATCATGTTGACATTCC; dG = -11.7
601 >s_b_s_b_s_d_s_c_s_d_s GTGGCTGTGGCTGTGACGGTGTGGGTGACGGTG; >clamp_s_c_s_b_s:compl:rev CACAGCCACCCACACTATCATGTTGACATTCC; dG = -11.7
602 >s_b_s_b_s_d_s_c_s_c_s GTGGCTGTGGCTGTGACGGTGTGGGTGTGGGTG; >clamp_s_c_s_b_s:compl:rev CACAGCCACCCACACTATCATGTTGACATTCC; dG = -11.7
603 >s_b_s_b_s_d_s_a_s_b_s GTGGCTGTGGCTGTGACGGTGTCTGTGGCTGTG; >clamp_s_c_s_b_s:compl:rev CACAGCCACCCACACTATCATGTTGACATTCC; dG = -11.7
604 >s_c_s_d_s_a_s_b_s_b_s GTGTGGGTGACGGTGTCTGTGGCTGTGGCTGTG; >clamp_s_c_s_b_s:compl:rev CACAGCCACCCACACTATCATGTTGACATTCC; dG = -11.7
605 >s_a_s_b_s_b_s_c_s_e_s GTGTCTGTGGCTGTGGCTGTGTGGGTGCTGGTG; >clamp_s_c_s_b_s:compl:rev CACAGCCACCCACACTATCATGTTGACATTCC; dG = -11.7
606 >s_a_s_b_s_b_s_c_s_d_s GTGTCTGTGGCTGTGGCTGTGTGGGTGACGGTG; >clamp_s_c_s_b_s:compl:rev CACAGCCACCCACACTATCATGTTGACATTCC; dG = -11.7
607 >s_c_s_e_s_a_s_b_s_b_s GTGTGGGTGCTGGTGTCTGTGGCTGTGGCTGTG; >clamp_s_c_s_b_s:compl:rev CACAGCCACCCACACTATCATGTTGACATTCC; dG = -11.7
608 >s_a_s_b_s_b_s_c_s_c_s GTGTCTGTGGCTGTGGCTGTGTGGGTGTGGGTG; >clamp_s_c_s_b_s:compl:rev CACAGCCACCCACACTATCATGTTGACATTCC; dG = -11.7
609 >s_d_s_a_s_b_s_b_s_b_s GTGACGGTGTCTGTGGCTGTGGCTGTGGCTGTG; >clamp_s_c_s_b_s:compl:rev CACAGCCACCCACACTATCATGTTGACATTCC; dG = -11.7
610 >s_a_s_b_s_b_s_b_s_b_s GTGTCTGTGGCTGTGGCTGTGGCTGTGGCTGTG; >clamp_s_c_s_b_s:compl:rev CACAGCCACCCACACTATCATGTTGACATTCC; dG = -11.7
611 >s_a_s_b_s_b_s_a_s_b_s GTGTCTGTGGCTGTGGCTGTGTCTGTGGCTGTG; >clamp_s_c_s_b_s:compl:rev CACAGCCACCCACACTATCATGTTGACATTCC; dG = -11.7
612 >s_e_s_a_s_b_s_b_s_b_s GTGCTGGTGTCTGTGGCTGTGGCTGTGGCTGTG; >clamp_s_c_s_b_s:compl:rev CACAGCCACCCACACTATCATGTTGACATTCC; dG = -11.7
613 >s_e_s_b_s_e_s_c_s_e_s GTGCTGGTGGCTGTGCTGGTGTGGGTGCTGGTG; >clamp_s_c_s_b_s:compl:rev CACAGCCACCCACACTATCATGTTGACATTCC; dG = -11.7
614 >s_e_s_b_s_e_s_c_s_d_s GTGCTGGTGGCTGTGCTGGTGTGGGTGACGGTG; >clamp_s_c_s_b_s:compl:rev CACAGCCACCCACACTATCATGTTGACATTCC; dG = -11.7
615 >s_e_s_b_s_e_s_c_s_c_s GTGCTGGTGGCTGTGCTGGTGTGGGTGTGGGTG; >clamp_s_c_s_b_s:compl:rev CACAGCCACCCACACTATCATGTTGACATTCC; dG = -11.7
616 >s_e_s_b_s_e_s_a_s_b_s GTGCTGGTGGCTGTGCTGGTGTCTGTGGCTGTG; >clamp_s_c_s_b_s:compl:rev CACAGCCACCCACACTATCATGTTGACATTCC; dG = -11.7
617 >s_a_s_d_s_c_s_c_s_e_s GTGTCTGTGACGGTGTGGGTGTGGGTGCTGGTG; >clamp_s_c_s_b_s:compl:rev CACAGCCACCCACACTATCATGTTGACATTCC; dG = -11.6
618 >s_a_s_e_s_c_s_c_s_e_s GTGTCTGTGCTGGTGTGGGTGTGGGTGCTGGTG; >clamp_s_c_s_b_s:compl:rev CACAGCCACCCACACTATCATGTTGACATTCC; dG = -11.6
619 >s_a_s_a_s_c_s_c_s_e_s GTGTCTGTGTCTGTGTGGGTGTGGGTGCTGGTG; >clamp_s_c_s_b_s:compl:rev CACAGCCACCCACACTATCATGTTGACATTCC; dG = -11.6
620 >s_b_s_a_s_a_s_b_s_b_s GTGGCTGTGTCTGTGTCTGTGGCTGTGGCTGTG; >clamp_s_c_s_b_s:compl:rev CACAGCCACCCACACTATCATGTTGACATTCC; dG = -11.6
621 >s_b_s_a_s_c_s_c_s_e_s GTGGCTGTGTCTGTGTGGGTGTGGGTGCTGGTG; >clamp_s_c_s_b_s:compl:rev CACAGCCACCCACACTATCATGTTGACATTCC; dG = -11.6
622 >s_b_s_b_s_a_s_b_s_b_s GTGGCTGTGGCTGTGTCTGTGGCTGTGGCTGTG; >clamp_s_c_s_b_s:compl:rev CACAGCCACCCACACTATCATGTTGACATTCC; dG = -11.6
623 >s_b_s_b_s_c_s_c_s_e_s GTGGCTGTGGCTGTGTGGGTGTGGGTGCTGGTG; >clamp_s_c_s_b_s:compl:rev CACAGCCACCCACACTATCATGTTGACATTCC; dG = -11.6
624 >s_b_s_d_s_c_s_c_s_e_s GTGGCTGTGACGGTGTGGGTGTGGGTGCTGGTG; >clamp_s_c_s_b_s:compl:rev CACAGCCACCCACACTATCATGTTGACATTCC; dG = -11.6
625 >s_b_s_e_s_c_s_c_s_e_s GTGGCTGTGCTGGTGTGGGTGTGGGTGCTGGTG; >clamp_s_c_s_b_s:compl:rev CACAGCCACCCACACTATCATGTTGACATTCC; dG = -11.6

626 >s_a_s_b_s_a_s_b_s_b_s GTGTCTGTGGCTGTGTCTGTGGCTGTGGCTGTG; >clamp_s_c_s_b_s:compl:rev
CACAGCCACCCACACTATCATGTTGACATTCC; dG = -11.6
627 >s_c_s_d_s_c_s_c_s_e_s GTGTGGGTGACGGTGTGGGTGTGGGTGCTGGTG; >clamp_s_c_s_b_s:compl:rev
CACAGCCACCCACACTATCATGTTGACATTCC; dG = -11.6
628 >s_c_s_e_s_c_s_c_s_e_s GTGTGGGTGCTGGTGTGGGTGTGGGTGCTGGTG; >clamp_s_c_s_b_s:compl:rev
CACAGCCACCCACACTATCATGTTGACATTCC; dG = -11.6
629 >s_d_s_a_s_a_s_b_s_b_s GTGACGGTGTCTGTGTCTGTGGCTGTGGCTGTG; >clamp_s_c_s_b_s:compl:rev
CACAGCCACCCACACTATCATGTTGACATTCC; dG = -11.6
630 >s_d_s_a_s_c_s_c_s_e_s GTGACGGTGTCTGTGTGGGTGTGGGTGCTGGTG; >clamp_s_c_s_b_s:compl:rev
CACAGCCACCCACACTATCATGTTGACATTCC; dG = -11.6
631 >s_a_s_b_s_c_s_c_s_e_s GTGTCTGTGGCTGTGTGGGTGTGGGTGCTGGTG; >clamp_s_c_s_b_s:compl:rev
CACAGCCACCCACACTATCATGTTGACATTCC; dG = -11.6
632 >s_d_s_d_s_c_s_c_s_e_s GTGACGGTGACGGTGTGGGTGTGGGTGCTGGTG; >clamp_s_c_s_b_s:compl:rev
CACAGCCACCCACACTATCATGTTGACATTCC; dG = -11.6
633 >s_d_s_e_s_c_s_c_s_e_s GTGACGGTGCTGGTGTGGGTGTGGGTGCTGGTG; >clamp_s_c_s_b_s:compl:rev
CACAGCCACCCACACTATCATGTTGACATTCC; dG = -11.6
634 >s_a_s_a_s_a_s_b_s_b_s GTGTCTGTGTCTGTGTCTGTGGCTGTGGCTGTG; >clamp_s_c_s_b_s:compl:rev
CACAGCCACCCACACTATCATGTTGACATTCC; dG = -11.6
635 >s_e_s_a_s_a_s_b_s_b_s GTGCTGGTGTCTGTGTCTGTGGCTGTGGCTGTG; >clamp_s_c_s_b_s:compl:rev
CACAGCCACCCACACTATCATGTTGACATTCC; dG = -11.6
636 >s_e_s_a_s_c_s_c_s_e_s GTGCTGGTGTCTGTGTGGGTGTGGGTGCTGGTG; >clamp_s_c_s_b_s:compl:rev
CACAGCCACCCACACTATCATGTTGACATTCC; dG = -11.6
637 >s_e_s_d_s_c_s_c_s_e_s GTGCTGGTGACGGTGTGGGTGTGGGTGCTGGTG; >clamp_s_c_s_b_s:compl:rev
CACAGCCACCCACACTATCATGTTGACATTCC; dG = -11.6
638 >s_e_s_e_s_c_s_c_s_e_s GTGCTGGTGCTGGTGTGGGTGTGGGTGCTGGTG; >clamp_s_c_s_b_s:compl:rev
CACAGCCACCCACACTATCATGTTGACATTCC; dG = -11.6
639 >s_a_s_a_s_b_s_c_s_d_s GTGTCTGTGTCTGTGGCTGTGTGGGTGACGGTG; >clamp_s_c_s_b_s:compl:rev
CACAGCCACCCACACTATCATGTTGACATTCC; dG = -11.5
640 >s_a_s_a_s_b_s_c_s_c_s GTGTCTGTGTCTGTGGCTGTGTGGGTGTGGGTG; >clamp_s_c_s_b_s:compl:rev
CACAGCCACCCACACTATCATGTTGACATTCC; dG = -11.5
641 >s_a_s_d_s_a_s_b_s_b_s GTGTCTGTGACGGTGTCTGTGGCTGTGGCTGTG; >clamp_s_c_s_b_s:compl:rev
CACAGCCACCCACACTATCATGTTGACATTCC; dG = -11.5
642 >s_a_s_e_s_a_s_b_s_b_s GTGTCTGTGCTGGTGTCTGTGGCTGTGGCTGTG; >clamp_s_c_s_b_s:compl:rev
CACAGCCACCCACACTATCATGTTGACATTCC; dG = -11.5
643 >s_a_s_a_s_b_s_a_s_b_s GTGTCTGTGTCTGTGGCTGTGTCTGTGGCTGTG; >clamp_s_c_s_b_s:compl:rev
CACAGCCACCCACACTATCATGTTGACATTCC; dG = -11.5
644 >s_b_s_a_s_b_s_c_s_e_s GTGGCTGTGTCTGTGGCTGTGTGGGTGCTGGTG; >clamp_s_c_s_b_s:compl:rev
CACAGCCACCCACACTATCATGTTGACATTCC; dG = -11.5
645 >s_b_s_a_s_b_s_c_s_d_s GTGGCTGTGTCTGTGGCTGTGTGGGTGACGGTG; >clamp_s_c_s_b_s:compl:rev
CACAGCCACCCACACTATCATGTTGACATTCC; dG = -11.5
646 >s_b_s_a_s_b_s_c_s_c_s GTGGCTGTGTCTGTGGCTGTGTGGGTGTGGGTG; >clamp_s_c_s_b_s:compl:rev
CACAGCCACCCACACTATCATGTTGACATTCC; dG = -11.5
647 >s_b_s_a_s_b_s_a_s_b_s GTGGCTGTGTCTGTGGCTGTGTCTGTGGCTGTG; >clamp_s_c_s_b_s:compl:rev
CACAGCCACCCACACTATCATGTTGACATTCC; dG = -11.5
648 >s_b_s_d_s_a_s_b_s_b_s GTGGCTGTGACGGTGTCTGTGGCTGTGGCTGTG; >clamp_s_c_s_b_s:compl:rev
CACAGCCACCCACACTATCATGTTGACATTCC; dG = -11.5
649 >s_b_s_e_s_a_s_b_s_b_s GTGGCTGTGCTGGTGTCTGTGGCTGTGGCTGTG; >clamp_s_c_s_b_s:compl:rev
CACAGCCACCCACACTATCATGTTGACATTCC; dG = -11.5
650 >s_a_s_b_s_d_s_c_s_e_s GTGTCTGTGGCTGTGACGGTGTGGGTGCTGGTG; >clamp_s_c_s_b_s:compl:rev
CACAGCCACCCACACTATCATGTTGACATTCC; dG = -11.5
651 >s_d_s_d_s_a_s_b_s_b_s GTGACGGTGACGGTGTCTGTGGCTGTGGCTGTG; >clamp_s_c_s_b_s:compl:rev
CACAGCCACCCACACTATCATGTTGACATTCC; dG = -11.5
652 >s_a_s_b_s_d_s_c_s_d_s GTGTCTGTGGCTGTGACGGTGTGGGTGACGGTG; >clamp_s_c_s_b_s:compl:rev
CACAGCCACCCACACTATCATGTTGACATTCC; dG = -11.5
653 >s_a_s_b_s_d_s_c_s_c_s GTGTCTGTGGCTGTGACGGTGTGGGTGTGGGTG; >clamp_s_c_s_b_s:compl:rev
CACAGCCACCCACACTATCATGTTGACATTCC; dG = -11.5
654 >s_d_s_e_s_a_s_b_s_b_s GTGACGGTGCTGGTGTCTGTGGCTGTGGCTGTG; >clamp_s_c_s_b_s:compl:rev
CACAGCCACCCACACTATCATGTTGACATTCC; dG = -11.5
655 >s_a_s_b_s_d_s_a_s_b_s GTGTCTGTGGCTGTGACGGTGTCTGTGGCTGTG; >clamp_s_c_s_b_s:compl:rev
CACAGCCACCCACACTATCATGTTGACATTCC; dG = -11.5
656 >s_e_s_d_s_a_s_b_s_b_s GTGCTGGTGACGGTGTCTGTGGCTGTGGCTGTG; >clamp_s_c_s_b_s:compl:rev
CACAGCCACCCACACTATCATGTTGACATTCC; dG = -11.5
657 >s_e_s_e_s_a_s_b_s_b_s GTGCTGGTGCTGGTGTCTGTGGCTGTGGCTGTG; >clamp_s_c_s_b_s:compl:rev
CACAGCCACCCACACTATCATGTTGACATTCC; dG = -11.5
658 >s_a_s_a_s_b_s_c_s_e_s GTGTCTGTGTCTGTGGCTGTGTGGGTGCTGGTG; >clamp_s_c_s_b_s:compl:rev
CACAGCCACCCACACTATCATGTTGACATTCC; dG = -11.5
659 >s_d_s_a_s_b_s_c_s_e_s GTGACGGTGTCTGTGGCTGTGTGGGTGCTGGTG; >clamp_s_c_s_b_s:compl:rev
CACAGCCACCCACACTATCATGTTGACATTCC; dG = -11.4
660 >s_d_s_a_s_b_s_c_s_d_s GTGACGGTGTCTGTGGCTGTGTGGGTGACGGTG; >clamp_s_c_s_b_s:compl:rev
CACAGCCACCCACACTATCATGTTGACATTCC; dG = -11.4
661 >s_d_s_a_s_b_s_c_s_c_s GTGACGGTGTCTGTGGCTGTGTGGGTGTGGGTG; >clamp_s_c_s_b_s:compl:rev
CACAGCCACCCACACTATCATGTTGACATTCC; dG = -11.4
662 >s_d_s_a_s_b_s_a_s_b_s GTGACGGTGTCTGTGGCTGTGTCTGTGGCTGTG; >clamp_s_c_s_b_s:compl:rev
CACAGCCACCCACACTATCATGTTGACATTCC; dG = -11.4

663 >s_e_s_a_s_b_s_c_s_e_s GTGCTGGTGTCTGTGGCTGTGTGGGTGCTGGTG; >clamp_s_c_s_b_s:compl:rev
CACAGCCACCCACACTATCATGTTGACATTCC; dG = -11.4
664 >s_e_s_a_s_b_s_c_s_d_s GTGCTGGTGTCTGTGGCTGTGTGGGTGACGGTG; >clamp_s_c_s_b_s:compl:rev
CACAGCCACCCACACTATCATGTTGACATTCC; dG = -11.4
665 >s_e_s_a_s_b_s_c_s_c_s GTGCTGGTGTCTGTGGCTGTGTGGGTGTGGGTG; >clamp_s_c_s_b_s:compl:rev
CACAGCCACCCACACTATCATGTTGACATTCC; dG = -11.4
666 >s_e_s_a_s_b_s_a_s_b_s GTGCTGGTGTCTGTGGCTGTGTCTGTGGCTGTG; >clamp_s_c_s_b_s:compl:rev
CACAGCCACCCACACTATCATGTTGACATTCC; dG = -11.4
667 >s_a_s_a_s_a_s_c_s_e_s GTGTCTGTGTCTGTGTCTGTGTGGGTGCTGGTG; >clamp_s_c_s_b_s:compl:rev
CACAGCCACCCACACTATCATGTTGACATTCC; dG = -11.3
668 >s_a_s_c_s_e_s_c_s_e_s GTGTCTGTGTGGGTGCTGGTGTGGGTGCTGGTG; >clamp_s_c_s_b_s:compl:rev
CACAGCCACCCACACTATCATGTTGACATTCC; dG = -11.3
669 >s_a_s_c_s_e_s_c_s_d_s GTGTCTGTGTGGGTGCTGGTGTGGGTGACGGTG; >clamp_s_c_s_b_s:compl:rev
CACAGCCACCCACACTATCATGTTGACATTCC; dG = -11.3
670 >s_a_s_c_s_e_s_c_s_c_s GTGTCTGTGTGGGTGCTGGTGTGGGTGTGGGTG; >clamp_s_c_s_b_s:compl:rev
CACAGCCACCCACACTATCATGTTGACATTCC; dG = -11.3
671 >s_a_s_d_s_a_s_c_s_e_s GTGTCTGTGACGGTGTCTGTGTGGGTGCTGGTG; >clamp_s_c_s_b_s:compl:rev
CACAGCCACCCACACTATCATGTTGACATTCC; dG = -11.3
672 >s_a_s_e_s_a_s_c_s_e_s GTGTCTGTGCTGGTGTCTGTGTGGGTGCTGGTG; >clamp_s_c_s_b_s:compl:rev
CACAGCCACCCACACTATCATGTTGACATTCC; dG = -11.3
673 >s_b_s_a_s_a_s_c_s_e_s GTGGCTGTGTCTGTGTCTGTGTGGGTGCTGGTG; >clamp_s_c_s_b_s:compl:rev
CACAGCCACCCACACTATCATGTTGACATTCC; dG = -11.3
674 >s_b_s_b_s_a_s_c_s_e_s GTGGCTGTGGCTGTGTCTGTGTGGGTGCTGGTG; >clamp_s_c_s_b_s:compl:rev
CACAGCCACCCACACTATCATGTTGACATTCC; dG = -11.3
675 >s_b_s_c_s_e_s_c_s_e_s GTGGCTGTGTGGGTGCTGGTGTGGGTGCTGGTG; >clamp_s_c_s_b_s:compl:rev
CACAGCCACCCACACTATCATGTTGACATTCC; dG = -11.3
676 >s_b_s_c_s_e_s_c_s_d_s GTGGCTGTGTGGGTGCTGGTGTGGGTGACGGTG; >clamp_s_c_s_b_s:compl:rev
CACAGCCACCCACACTATCATGTTGACATTCC; dG = -11.3
677 >s_b_s_c_s_e_s_c_s_c_s GTGGCTGTGTGGGTGCTGGTGTGGGTGTGGGTG; >clamp_s_c_s_b_s:compl:rev
CACAGCCACCCACACTATCATGTTGACATTCC; dG = -11.3
678 >s_b_s_d_s_a_s_c_s_e_s GTGGCTGTGACGGTGTCTGTGTGGGTGCTGGTG; >clamp_s_c_s_b_s:compl:rev
CACAGCCACCCACACTATCATGTTGACATTCC; dG = -11.3
679 >s_b_s_e_s_a_s_c_s_e_s GTGGCTGTGCTGGTGTCTGTGTGGGTGCTGGTG; >clamp_s_c_s_b_s:compl:rev
CACAGCCACCCACACTATCATGTTGACATTCC; dG = -11.3
680 >s_a_s_b_s_a_s_c_s_e_s GTGTCTGTGGCTGTGTCTGTGTGGGTGCTGGTG; >clamp_s_c_s_b_s:compl:rev
CACAGCCACCCACACTATCATGTTGACATTCC; dG = -11.3
681 >s_c_s_d_s_a_s_c_s_e_s GTGTGGGTGACGGTGTCTGTGTGGGTGCTGGTG; >clamp_s_c_s_b_s:compl:rev
CACAGCCACCCACACTATCATGTTGACATTCC; dG = -11.3
682 >s_c_s_e_s_a_s_c_s_e_s GTGTGGGTGCTGGTGTCTGTGTGGGTGCTGGTG; >clamp_s_c_s_b_s:compl:rev
CACAGCCACCCACACTATCATGTTGACATTCC; dG = -11.3
683 >s_d_s_a_s_a_s_c_s_e_s GTGACGGTGTCTGTGTCTGTGTGGGTGCTGGTG; >clamp_s_c_s_b_s:compl:rev
CACAGCCACCCACACTATCATGTTGACATTCC; dG = -11.3
684 >s_d_s_d_s_a_s_c_s_e_s GTGACGGTGACGGTGTCTGTGTGGGTGCTGGTG; >clamp_s_c_s_b_s:compl:rev
CACAGCCACCCACACTATCATGTTGACATTCC; dG = -11.3
685 >s_d_s_e_s_a_s_c_s_e_s GTGACGGTGCTGGTGTCTGTGTGGGTGCTGGTG; >clamp_s_c_s_b_s:compl:rev
CACAGCCACCCACACTATCATGTTGACATTCC; dG = -11.3
686 >s_e_s_a_s_a_s_c_s_e_s GTGCTGGTGTCTGTGTCTGTGTGGGTGCTGGTG; >clamp_s_c_s_b_s:compl:rev
CACAGCCACCCACACTATCATGTTGACATTCC; dG = -11.3
687 >s_e_s_d_s_a_s_c_s_e_s GTGCTGGTGACGGTGTCTGTGTGGGTGCTGGTG; >clamp_s_c_s_b_s:compl:rev
CACAGCCACCCACACTATCATGTTGACATTCC; dG = -11.3
688 >s_e_s_e_s_a_s_c_s_e_s GTGCTGGTGCTGGTGTCTGTGTGGGTGCTGGTG; >clamp_s_c_s_b_s:compl:rev
CACAGCCACCCACACTATCATGTTGACATTCC; dG = -11.3
689 >s_a_s_d_s_a_s_a_s_b_s GTGTCTGTGACGGTGTCTGTGTCTGTGGCTGTG; >clamp_s_c_s_b_s:compl:rev
CACAGCCACCCACACTATCATGTTGACATTCC; dG = -11.2
690 >s_a_s_d_s_d_s_c_s_e_s GTGTCTGTGACGGTGACGGTGTGGGTGCTGGTG; >clamp_s_c_s_b_s:compl:rev
CACAGCCACCCACACTATCATGTTGACATTCC; dG = -11.2
691 >s_a_s_d_s_d_s_a_s_b_s GTGTCTGTGACGGTGACGGTGTCTGTGGCTGTG; >clamp_s_c_s_b_s:compl:rev
CACAGCCACCCACACTATCATGTTGACATTCC; dG = -11.2
692 >s_a_s_d_s_e_s_c_s_e_s GTGTCTGTGACGGTGCTGGTGTGGGTGCTGGTG; >clamp_s_c_s_b_s:compl:rev
CACAGCCACCCACACTATCATGTTGACATTCC; dG = -11.2
693 >s_a_s_d_s_e_s_a_s_b_s GTGTCTGTGACGGTGCTGGTGTCTGTGGCTGTG; >clamp_s_c_s_b_s:compl:rev
CACAGCCACCCACACTATCATGTTGACATTCC; dG = -11.2
694 >s_a_s_e_s_a_s_a_s_b_s GTGTCTGTGCTGGTGTCTGTGTCTGTGGCTGTG; >clamp_s_c_s_b_s:compl:rev
CACAGCCACCCACACTATCATGTTGACATTCC; dG = -11.2
695 >s_a_s_e_s_d_s_c_s_e_s GTGTCTGTGCTGGTGACGGTGTGGGTGCTGGTG; >clamp_s_c_s_b_s:compl:rev
CACAGCCACCCACACTATCATGTTGACATTCC; dG = -11.2
696 >s_a_s_e_s_d_s_a_s_b_s GTGTCTGTGCTGGTGACGGTGTCTGTGGCTGTG; >clamp_s_c_s_b_s:compl:rev
CACAGCCACCCACACTATCATGTTGACATTCC; dG = -11.2
697 >s_a_s_e_s_e_s_c_s_e_s GTGTCTGTGCTGGTGCTGGTGTGGGTGCTGGTG; >clamp_s_c_s_b_s:compl:rev
CACAGCCACCCACACTATCATGTTGACATTCC; dG = -11.2
698 >s_a_s_e_s_e_s_a_s_b_s GTGTCTGTGCTGGTGCTGGTGTCTGTGGCTGTG; >clamp_s_c_s_b_s:compl:rev
CACAGCCACCCACACTATCATGTTGACATTCC; dG = -11.2
699 >s_b_s_a_s_a_s_a_s_b_s GTGGCTGTGTCTGTGTCTGTGTCTGTGGCTGTG; >clamp_s_c_s_b_s:compl:rev
CACAGCCACCCACACTATCATGTTGACATTCC; dG = -11.2

700 >s_b_s_a_s_d_s_c_s_e_s GTGGCTGTGTCTGTGACGGTGTGGGTGCTGGTG; >clamp_s_c_s_b_s:compl:rev
CACAGCCACCCACACTATCATGTTGACATTCC; dG = -11.2
701 >s_b_s_a_s_d_s_a_s_b_s GTGGCTGTGTCTGTGACGGTGTCTGTGGCTGTG; >clamp_s_c_s_b_s:compl:rev
CACAGCCACCCACACTATCATGTTGACATTCC; dG = -11.2
702 >s_b_s_a_s_e_s_c_s_e_s GTGGCTGTGTCTGTGCTGGTGTGGGTGCTGGTG; >clamp_s_c_s_b_s:compl:rev
CACAGCCACCCACACTATCATGTTGACATTCC; dG = -11.2
703 >s_b_s_a_s_e_s_a_s_b_s GTGGCTGTGTCTGTGCTGGTGTCTGTGGCTGTG; >clamp_s_c_s_b_s:compl:rev
CACAGCCACCCACACTATCATGTTGACATTCC; dG = -11.2
704 >s_b_s_b_s_a_s_a_s_b_s GTGGCTGTGGCTGTGTCTGTGTCTGTGGCTGTG; >clamp_s_c_s_b_s:compl:rev
CACAGCCACCCACACTATCATGTTGACATTCC; dG = -11.2
705 >s_a_s_a_s_d_s_c_s_e_s GTGTCTGTGTCTGTGACGGTGTGGGTGCTGGTG; >clamp_s_c_s_b_s:compl:rev
CACAGCCACCCACACTATCATGTTGACATTCC; dG = -11.2
706 >s_b_s_b_s_e_s_c_s_e_s GTGGCTGTGGCTGTGCTGGTGTGGGTGCTGGTG; >clamp_s_c_s_b_s:compl:rev
CACAGCCACCCACACTATCATGTTGACATTCC; dG = -11.2
707 >s_b_s_b_s_e_s_a_s_b_s GTGGCTGTGGCTGTGCTGGTGTCTGTGGCTGTG; >clamp_s_c_s_b_s:compl:rev
CACAGCCACCCACACTATCATGTTGACATTCC; dG = -11.2
708 >s_b_s_c_s_d_s_c_s_e_s GTGGCTGTGTGGGTGACGGTGTGGGTGCTGGTG; >clamp_s_c_s_b_s:compl:rev
CACAGCCACCCACACTATCATGTTGACATTCC; dG = -11.2
709 >s_b_s_d_s_a_s_a_s_b_s GTGGCTGTGACGGTGTCTGTGTCTGTGGCTGTG; >clamp_s_c_s_b_s:compl:rev
CACAGCCACCCACACTATCATGTTGACATTCC; dG = -11.2
710 >s_a_s_a_s_d_s_a_s_b_s GTGTCTGTGTCTGTGACGGTGTCTGTGGCTGTG; >clamp_s_c_s_b_s:compl:rev
CACAGCCACCCACACTATCATGTTGACATTCC; dG = -11.2
711 >s_a_s_a_s_e_s_c_s_e_s GTGTCTGTGTCTGTGCTGGTGTGGGTGCTGGTG; >clamp_s_c_s_b_s:compl:rev
CACAGCCACCCACACTATCATGTTGACATTCC; dG = -11.2
712 >s_b_s_d_s_d_s_c_s_e_s GTGGCTGTGACGGTGACGGTGTGGGTGCTGGTG; >clamp_s_c_s_b_s:compl:rev
CACAGCCACCCACACTATCATGTTGACATTCC; dG = -11.2
713 >s_b_s_d_s_d_s_a_s_b_s GTGGCTGTGACGGTGACGGTGTCTGTGGCTGTG; >clamp_s_c_s_b_s:compl:rev
CACAGCCACCCACACTATCATGTTGACATTCC; dG = -11.2
714 >s_b_s_d_s_e_s_c_s_e_s GTGGCTGTGACGGTGCTGGTGTGGGTGCTGGTG; >clamp_s_c_s_b_s:compl:rev
CACAGCCACCCACACTATCATGTTGACATTCC; dG = -11.2
715 >s_b_s_d_s_e_s_a_s_b_s GTGGCTGTGACGGTGCTGGTGTCTGTGGCTGTG; >clamp_s_c_s_b_s:compl:rev
CACAGCCACCCACACTATCATGTTGACATTCC; dG = -11.2
716 >s_b_s_e_s_a_s_a_s_b_s GTGGCTGTGCTGGTGTCTGTGTCTGTGGCTGTG; >clamp_s_c_s_b_s:compl:rev
CACAGCCACCCACACTATCATGTTGACATTCC; dG = -11.2
717 >s_b_s_e_s_d_s_c_s_e_s GTGGCTGTGCTGGTGACGGTGTGGGTGCTGGTG; >clamp_s_c_s_b_s:compl:rev
CACAGCCACCCACACTATCATGTTGACATTCC; dG = -11.2
718 >s_b_s_e_s_d_s_a_s_b_s GTGGCTGTGCTGGTGACGGTGTCTGTGGCTGTG; >clamp_s_c_s_b_s:compl:rev
CACAGCCACCCACACTATCATGTTGACATTCC; dG = -11.2
719 >s_b_s_e_s_e_s_c_s_e_s GTGGCTGTGCTGGTGCTGGTGTGGGTGCTGGTG; >clamp_s_c_s_b_s:compl:rev
CACAGCCACCCACACTATCATGTTGACATTCC; dG = -11.2
720 >s_b_s_e_s_e_s_a_s_b_s GTGGCTGTGCTGGTGCTGGTGTCTGTGGCTGTG; >clamp_s_c_s_b_s:compl:rev
CACAGCCACCCACACTATCATGTTGACATTCC; dG = -11.2
721 >s_a_s_a_s_e_s_a_s_b_s GTGTCTGTGTCTGTGCTGGTGTCTGTGGCTGTG; >clamp_s_c_s_b_s:compl:rev
CACAGCCACCCACACTATCATGTTGACATTCC; dG = -11.2
722 >s_c_s_c_s_d_s_c_s_e_s GTGTGGGTGTGGGTGACGGTGTGGGTGCTGGTG; >clamp_s_c_s_b_s:compl:rev
CACAGCCACCCACACTATCATGTTGACATTCC; dG = -11.2
723 >s_c_s_c_s_e_s_c_s_e_s GTGTGGGTGTGGGTGCTGGTGTGGGTGCTGGTG; >clamp_s_c_s_b_s:compl:rev
CACAGCCACCCACACTATCATGTTGACATTCC; dG = -11.2
724 >s_c_s_c_s_e_s_c_s_d_s GTGTGGGTGTGGGTGCTGGTGTGGGTGACGGTG; >clamp_s_c_s_b_s:compl:rev
CACAGCCACCCACACTATCATGTTGACATTCC; dG = -11.2
725 >s_c_s_c_s_e_s_c_s_c_s GTGTGGGTGTGGGTGCTGGTGTGGGTGTGGGTG; >clamp_s_c_s_b_s:compl:rev
CACAGCCACCCACACTATCATGTTGACATTCC; dG = -11.2
726 >s_c_s_d_s_a_s_a_s_b_s GTGTGGGTGACGGTGTCTGTGTCTGTGGCTGTG; >clamp_s_c_s_b_s:compl:rev
CACAGCCACCCACACTATCATGTTGACATTCC; dG = -11.2
727 >s_c_s_d_s_d_s_c_s_e_s GTGTGGGTGACGGTGACGGTGTGGGTGCTGGTG; >clamp_s_c_s_b_s:compl:rev
CACAGCCACCCACACTATCATGTTGACATTCC; dG = -11.2
728 >s_a_s_b_s_a_s_a_s_b_s GTGTCTGTGGCTGTGTCTGTGTCTGTGGCTGTG; >clamp_s_c_s_b_s:compl:rev
CACAGCCACCCACACTATCATGTTGACATTCC; dG = -11.2
729 >s_c_s_d_s_d_s_a_s_b_s GTGTGGGTGACGGTGACGGTGTCTGTGGCTGTG; >clamp_s_c_s_b_s:compl:rev
CACAGCCACCCACACTATCATGTTGACATTCC; dG = -11.2
730 >s_c_s_d_s_e_s_c_s_e_s GTGTGGGTGACGGTGCTGGTGTGGGTGCTGGTG; >clamp_s_c_s_b_s:compl:rev
CACAGCCACCCACACTATCATGTTGACATTCC; dG = -11.2
731 >s_c_s_d_s_e_s_a_s_b_s GTGTGGGTGACGGTGCTGGTGTCTGTGGCTGTG; >clamp_s_c_s_b_s:compl:rev
CACAGCCACCCACACTATCATGTTGACATTCC; dG = -11.2
732 >s_c_s_e_s_a_s_a_s_b_s GTGTGGGTGCTGGTGTCTGTGTCTGTGGCTGTG; >clamp_s_c_s_b_s:compl:rev
CACAGCCACCCACACTATCATGTTGACATTCC; dG = -11.2
733 >s_c_s_e_s_d_s_c_s_e_s GTGTGGGTGCTGGTGACGGTGTGGGTGCTGGTG; >clamp_s_c_s_b_s:compl:rev
CACAGCCACCCACACTATCATGTTGACATTCC; dG = -11.2
734 >s_c_s_e_s_d_s_a_s_b_s GTGTGGGTGCTGGTGACGGTGTCTGTGGCTGTG; >clamp_s_c_s_b_s:compl:rev
CACAGCCACCCACACTATCATGTTGACATTCC; dG = -11.2
735 >s_c_s_e_s_e_s_c_s_e_s GTGTGGGTGCTGGTGCTGGTGTGGGTGCTGGTG; >clamp_s_c_s_b_s:compl:rev
CACAGCCACCCACACTATCATGTTGACATTCC; dG = -11.2
736 >s_c_s_e_s_e_s_a_s_b_s GTGTGGGTGCTGGTGCTGGTGTCTGTGGCTGTG; >clamp_s_c_s_b_s:compl:rev
CACAGCCACCCACACTATCATGTTGACATTCC; dG = -11.2

737 >s_d_s_a_s_a_s_a_s_b_s GTGACGGTGTCTGTGTCTGTGTCTGTGGCTGTG; >clamp_s_c_s_b_s:compl:rev CACAGCCACCCACACTATCATGTTGACATTCC; dG = -11.2
738 >s_d_s_a_s_d_s_c_s_e_s GTGACGGTGTCTGTGACGGTGTGGGTGCTGGTG; >clamp_s_c_s_b_s:compl:rev CACAGCCACCCACACTATCATGTTGACATTCC; dG = -11.2
739 >s_d_s_a_s_d_s_a_s_b_s GTGACGGTGTCTGTGACGGTGTCTGTGGCTGTG; >clamp_s_c_s_b_s:compl:rev CACAGCCACCCACACTATCATGTTGACATTCC; dG = -11.2
740 >s_d_s_a_s_e_s_c_s_e_s GTGACGGTGTCTGTGCTGGTGTGGGTGCTGGTG; >clamp_s_c_s_b_s:compl:rev CACAGCCACCCACACTATCATGTTGACATTCC; dG = -11.2
741 >s_d_s_a_s_e_s_a_s_b_s GTGACGGTGTCTGTGCTGGTGTCTGTGGCTGTG; >clamp_s_c_s_b_s:compl:rev CACAGCCACCCACACTATCATGTTGACATTCC; dG = -11.2
742 >s_d_s_c_s_d_s_c_s_e_s GTGACGGTGTGGGTGACGGTGTGGGTGCTGGTG; >clamp_s_c_s_b_s:compl:rev CACAGCCACCCACACTATCATGTTGACATTCC; dG = -11.2
743 >s_d_s_c_s_e_s_c_s_e_s GTGACGGTGTGGGTGCTGGTGTGGGTGCTGGTG; >clamp_s_c_s_b_s:compl:rev CACAGCCACCCACACTATCATGTTGACATTCC; dG = -11.2
744 >s_d_s_c_s_e_s_c_s_d_s GTGACGGTGTGGGTGCTGGTGTGGGTGACGGTG; >clamp_s_c_s_b_s:compl:rev CACAGCCACCCACACTATCATGTTGACATTCC; dG = -11.2
745 >s_d_s_c_s_e_s_c_s_c_s GTGACGGTGTGGGTGCTGGTGTGGGTGTGGGTG; >clamp_s_c_s_b_s:compl:rev CACAGCCACCCACACTATCATGTTGACATTCC; dG = -11.2
746 >s_d_s_d_s_a_s_a_s_b_s GTGACGGTGACGGTGTCTGTGTCTGTGGCTGTG; >clamp_s_c_s_b_s:compl:rev CACAGCCACCCACACTATCATGTTGACATTCC; dG = -11.2
747 >s_d_s_d_s_d_s_c_s_e_s GTGACGGTGACGGTGACGGTGTGGGTGCTGGTG; >clamp_s_c_s_b_s:compl:rev CACAGCCACCCACACTATCATGTTGACATTCC; dG = -11.2
748 >s_d_s_d_s_d_s_a_s_b_s GTGACGGTGACGGTGACGGTGTCTGTGGCTGTG; >clamp_s_c_s_b_s:compl:rev CACAGCCACCCACACTATCATGTTGACATTCC; dG = -11.2
749 >s_d_s_d_s_e_s_c_s_e_s GTGACGGTGACGGTGCTGGTGTGGGTGCTGGTG; >clamp_s_c_s_b_s:compl:rev CACAGCCACCCACACTATCATGTTGACATTCC; dG = -11.2
750 >s_d_s_d_s_e_s_a_s_b_s GTGACGGTGACGGTGCTGGTGTCTGTGGCTGTG; >clamp_s_c_s_b_s:compl:rev CACAGCCACCCACACTATCATGTTGACATTCC; dG = -11.2
751 >s_d_s_e_s_a_s_a_s_b_s GTGACGGTGCTGGTGTCTGTGTCTGTGGCTGTG; >clamp_s_c_s_b_s:compl:rev CACAGCCACCCACACTATCATGTTGACATTCC; dG = -11.2
752 >s_d_s_e_s_d_s_c_s_e_s GTGACGGTGCTGGTGACGGTGTGGGTGCTGGTG; >clamp_s_c_s_b_s:compl:rev CACAGCCACCCACACTATCATGTTGACATTCC; dG = -11.2
753 >s_d_s_e_s_d_s_a_s_b_s GTGACGGTGCTGGTGACGGTGTCTGTGGCTGTG; >clamp_s_c_s_b_s:compl:rev CACAGCCACCCACACTATCATGTTGACATTCC; dG = -11.2
754 >s_d_s_e_s_e_s_c_s_e_s GTGACGGTGCTGGTGCTGGTGTGGGTGCTGGTG; >clamp_s_c_s_b_s:compl:rev CACAGCCACCCACACTATCATGTTGACATTCC; dG = -11.2
755 >s_d_s_e_s_e_s_a_s_b_s GTGACGGTGCTGGTGCTGGTGTCTGTGGCTGTG; >clamp_s_c_s_b_s:compl:rev CACAGCCACCCACACTATCATGTTGACATTCC; dG = -11.2
756 >s_a_s_b_s_e_s_c_s_e_s GTGTCTGTGGCTGTGCTGGTGTGGGTGCTGGTG; >clamp_s_c_s_b_s:compl:rev CACAGCCACCCACACTATCATGTTGACATTCC; dG = -11.2
757 >s_e_s_a_s_a_s_a_s_b_s GTGCTGGTGTCTGTGTCTGTGTCTGTGGCTGTG; >clamp_s_c_s_b_s:compl:rev CACAGCCACCCACACTATCATGTTGACATTCC; dG = -11.2
758 >s_e_s_a_s_d_s_c_s_e_s GTGCTGGTGTCTGTGACGGTGTGGGTGCTGGTG; >clamp_s_c_s_b_s:compl:rev CACAGCCACCCACACTATCATGTTGACATTCC; dG = -11.2
759 >s_e_s_a_s_d_s_a_s_b_s GTGCTGGTGTCTGTGACGGTGTCTGTGGCTGTG; >clamp_s_c_s_b_s:compl:rev CACAGCCACCCACACTATCATGTTGACATTCC; dG = -11.2
760 >s_e_s_a_s_e_s_c_s_e_s GTGCTGGTGTCTGTGCTGGTGTGGGTGCTGGTG; >clamp_s_c_s_b_s:compl:rev CACAGCCACCCACACTATCATGTTGACATTCC; dG = -11.2
761 >s_e_s_a_s_e_s_a_s_b_s GTGCTGGTGTCTGTGCTGGTGTCTGTGGCTGTG; >clamp_s_c_s_b_s:compl:rev CACAGCCACCCACACTATCATGTTGACATTCC; dG = -11.2
762 >s_a_s_b_s_e_s_a_s_b_s GTGTCTGTGGCTGTGCTGGTGTCTGTGGCTGTG; >clamp_s_c_s_b_s:compl:rev CACAGCCACCCACACTATCATGTTGACATTCC; dG = -11.2
763 >s_a_s_a_s_a_s_a_s_b_s GTGTCTGTGTCTGTGTCTGTGTCTGTGGCTGTG; >clamp_s_c_s_b_s:compl:rev CACAGCCACCCACACTATCATGTTGACATTCC; dG = -11.2
764 >s_e_s_c_s_d_s_c_s_e_s GTGCTGGTGTGGGTGACGGTGTGGGTGCTGGTG; >clamp_s_c_s_b_s:compl:rev CACAGCCACCCACACTATCATGTTGACATTCC; dG = -11.2
765 >s_e_s_c_s_e_s_c_s_e_s GTGCTGGTGTGGGTGCTGGTGTGGGTGCTGGTG; >clamp_s_c_s_b_s:compl:rev CACAGCCACCCACACTATCATGTTGACATTCC; dG = -11.2
766 >s_e_s_c_s_e_s_c_s_d_s GTGCTGGTGTGGGTGCTGGTGTGGGTGACGGTG; >clamp_s_c_s_b_s:compl:rev CACAGCCACCCACACTATCATGTTGACATTCC; dG = -11.2
767 >s_e_s_c_s_e_s_c_s_c_s GTGCTGGTGTGGGTGCTGGTGTGGGTGTGGGTG; >clamp_s_c_s_b_s:compl:rev CACAGCCACCCACACTATCATGTTGACATTCC; dG = -11.2
768 >s_e_s_d_s_a_s_a_s_b_s GTGCTGGTGACGGTGTCTGTGTCTGTGGCTGTG; >clamp_s_c_s_b_s:compl:rev CACAGCCACCCACACTATCATGTTGACATTCC; dG = -11.2
769 >s_e_s_d_s_d_s_c_s_e_s GTGCTGGTGACGGTGACGGTGTGGGTGCTGGTG; >clamp_s_c_s_b_s:compl:rev CACAGCCACCCACACTATCATGTTGACATTCC; dG = -11.2
770 >s_e_s_d_s_d_s_a_s_b_s GTGCTGGTGACGGTGACGGTGTCTGTGGCTGTG; >clamp_s_c_s_b_s:compl:rev CACAGCCACCCACACTATCATGTTGACATTCC; dG = -11.2
771 >s_e_s_d_s_e_s_c_s_e_s GTGCTGGTGACGGTGCTGGTGTGGGTGCTGGTG; >clamp_s_c_s_b_s:compl:rev CACAGCCACCCACACTATCATGTTGACATTCC; dG = -11.2
772 >s_e_s_d_s_e_s_a_s_b_s GTGCTGGTGACGGTGCTGGTGTCTGTGGCTGTG; >clamp_s_c_s_b_s:compl:rev CACAGCCACCCACACTATCATGTTGACATTCC; dG = -11.2
773 >s_e_s_e_s_a_s_a_s_b_s GTGCTGGTGCTGGTGTCTGTGTCTGTGGCTGTG; >clamp_s_c_s_b_s:compl:rev CACAGCCACCCACACTATCATGTTGACATTCC; dG = -11.2

774 >s_e_s_e_s_d_s_c_s_e_s GTGCTGGTGCTGGTGACGGTGTGGGTGCTGGTG; >clamp_s_c_s_b_s:compl:rev
CACAGCCACCCACACTATCATGTTGACATTCC; dG = -11.2
775 >s_e_s_e_s_d_s_a_s_b_s GTGCTGGTGCTGGTGACGGTGTCTGTGGCTGTG; >clamp_s_c_s_b_s:compl:rev
CACAGCCACCCACACTATCATGTTGACATTCC; dG = -11.2
776 >s_e_s_e_s_e_s_c_s_e_s GTGCTGGTGCTGGTGCTGGTGTGGGTGCTGGTG; >clamp_s_c_s_b_s:compl:rev
CACAGCCACCCACACTATCATGTTGACATTCC; dG = -11.2
777 >s_e_s_e_s_e_s_a_s_b_s GTGCTGGTGCTGGTGCTGGTGTCTGTGGCTGTG; >clamp_s_c_s_b_s:compl:rev
CACAGCCACCCACACTATCATGTTGACATTCC; dG = -11.2
778 >s_a_s_c_s_d_s_c_s_e_s GTGTCTGTGTGGGTGACGGTGTGGGTGCTGGTG; >clamp_s_c_s_b_s:compl:rev
CACAGCCACCCACACTATCATGTTGACATTCC; dG = -11.2
779 >s_a_s_d_s_a_s_c_s_d_s GTGTCTGTGACGGTGTCTGTGTGGGTGACGGTG; >clamp_s_c_s_b_s:compl:rev
CACAGCCACCCACACTATCATGTTGACATTCC; dG = -11.1
780 >s_a_s_d_s_c_s_c_s_d_s GTGTCTGTGACGGTGTGGGTGTGGGTGACGGTG; >clamp_s_c_s_b_s:compl:rev
CACAGCCACCCACACTATCATGTTGACATTCC; dG = -11.1
781 >s_a_s_d_s_d_s_c_s_d_s GTGTCTGTGACGGTGACGGTGTGGGTGACGGTG; >clamp_s_c_s_b_s:compl:rev
CACAGCCACCCACACTATCATGTTGACATTCC; dG = -11.1
782 >s_a_s_d_s_e_s_c_s_d_s GTGTCTGTGACGGTGCTGGTGTGGGTGACGGTG; >clamp_s_c_s_b_s:compl:rev
CACAGCCACCCACACTATCATGTTGACATTCC; dG = -11.1
783 >s_a_s_e_s_a_s_c_s_d_s GTGTCTGTGCTGGTGTCTGTGTGGGTGACGGTG; >clamp_s_c_s_b_s:compl:rev
CACAGCCACCCACACTATCATGTTGACATTCC; dG = -11.1
784 >s_a_s_e_s_c_s_c_s_d_s GTGTCTGTGCTGGTGTGGGTGTGGGTGACGGTG; >clamp_s_c_s_b_s:compl:rev
CACAGCCACCCACACTATCATGTTGACATTCC; dG = -11.1
785 >s_a_s_e_s_d_s_c_s_d_s GTGTCTGTGCTGGTGACGGTGTGGGTGACGGTG; >clamp_s_c_s_b_s:compl:rev
CACAGCCACCCACACTATCATGTTGACATTCC; dG = -11.1
786 >s_a_s_a_s_c_s_c_s_d_s GTGTCTGTGTCTGTGTGGGTGTGGGTGACGGTG; >clamp_s_c_s_b_s:compl:rev
CACAGCCACCCACACTATCATGTTGACATTCC; dG = -11.1
787 >s_a_s_e_s_e_s_c_s_d_s GTGTCTGTGCTGGTGCTGGTGTGGGTGACGGTG; >clamp_s_c_s_b_s:compl:rev
CACAGCCACCCACACTATCATGTTGACATTCC; dG = -11.1
788 >s_b_s_a_s_a_s_c_s_d_s GTGGCTGTGTCTGTGTCTGTGTGGGTGACGGTG; >clamp_s_c_s_b_s:compl:rev
CACAGCCACCCACACTATCATGTTGACATTCC; dG = -11.1
789 >s_a_s_a_s_a_s_c_s_d_s GTGTCTGTGTCTGTGTCTGTGTGGGTGACGGTG; >clamp_s_c_s_b_s:compl:rev
CACAGCCACCCACACTATCATGTTGACATTCC; dG = -11.1
790 >s_b_s_a_s_c_s_c_s_d_s GTGGCTGTGTCTGTGTGGGTGTGGGTGACGGTG; >clamp_s_c_s_b_s:compl:rev
CACAGCCACCCACACTATCATGTTGACATTCC; dG = -11.1
791 >s_b_s_a_s_d_s_c_s_d_s GTGGCTGTGTCTGTGACGGTGTGGGTGACGGTG; >clamp_s_c_s_b_s:compl:rev
CACAGCCACCCACACTATCATGTTGACATTCC; dG = -11.1
792 >s_b_s_a_s_e_s_c_s_d_s GTGGCTGTGTCTGTGCTGGTGTGGGTGACGGTG; >clamp_s_c_s_b_s:compl:rev
CACAGCCACCCACACTATCATGTTGACATTCC; dG = -11.1
793 >s_b_s_b_s_a_s_c_s_d_s GTGGCTGTGGCTGTGTCTGTGTGGGTGACGGTG; >clamp_s_c_s_b_s:compl:rev
CACAGCCACCCACACTATCATGTTGACATTCC; dG = -11.1
794 >s_b_s_b_s_a_s_c_s_c_s GTGGCTGTGGCTGTGTCTGTGTGGGTGTGGGTG; >clamp_s_c_s_b_s:compl:rev
CACAGCCACCCACACTATCATGTTGACATTCC; dG = -11.1
795 >s_b_s_b_s_c_s_c_s_d_s GTGGCTGTGGCTGTGTGGGTGTGGGTGACGGTG; >clamp_s_c_s_b_s:compl:rev
CACAGCCACCCACACTATCATGTTGACATTCC; dG = -11.1
796 >s_a_s_a_s_d_s_c_s_d_s GTGTCTGTGTCTGTGACGGTGTGGGTGACGGTG; >clamp_s_c_s_b_s:compl:rev
CACAGCCACCCACACTATCATGTTGACATTCC; dG = -11.1
797 >s_b_s_b_s_e_s_c_s_d_s GTGGCTGTGGCTGTGCTGGTGTGGGTGACGGTG; >clamp_s_c_s_b_s:compl:rev
CACAGCCACCCACACTATCATGTTGACATTCC; dG = -11.1
798 >s_b_s_c_s_d_s_c_s_d_s GTGGCTGTGTGGGTGACGGTGTGGGTGACGGTG; >clamp_s_c_s_b_s:compl:rev
CACAGCCACCCACACTATCATGTTGACATTCC; dG = -11.1
799 >s_b_s_c_s_d_s_c_s_c_s GTGGCTGTGTGGGTGACGGTGTGGGTGTGGGTG; >clamp_s_c_s_b_s:compl:rev
CACAGCCACCCACACTATCATGTTGACATTCC; dG = -11.1
800 >s_b_s_d_s_a_s_c_s_d_s GTGGCTGTGACGGTGTCTGTGTGGGTGACGGTG; >clamp_s_c_s_b_s:compl:rev
CACAGCCACCCACACTATCATGTTGACATTCC; dG = -11.1
801 >s_b_s_d_s_c_s_c_s_d_s GTGGCTGTGACGGTGTGGGTGTGGGTGACGGTG; >clamp_s_c_s_b_s:compl:rev
CACAGCCACCCACACTATCATGTTGACATTCC; dG = -11.1
802 >s_b_s_d_s_d_s_c_s_d_s GTGGCTGTGACGGTGACGGTGTGGGTGACGGTG; >clamp_s_c_s_b_s:compl:rev
CACAGCCACCCACACTATCATGTTGACATTCC; dG = -11.1
803 >s_b_s_d_s_e_s_c_s_d_s GTGGCTGTGACGGTGCTGGTGTGGGTGACGGTG; >clamp_s_c_s_b_s:compl:rev
CACAGCCACCCACACTATCATGTTGACATTCC; dG = -11.1
804 >s_a_s_a_s_e_s_c_s_d_s GTGTCTGTGTCTGTGCTGGTGTGGGTGACGGTG; >clamp_s_c_s_b_s:compl:rev
CACAGCCACCCACACTATCATGTTGACATTCC; dG = -11.1
805 >s_b_s_e_s_a_s_c_s_d_s GTGGCTGTGCTGGTGTCTGTGTGGGTGACGGTG; >clamp_s_c_s_b_s:compl:rev
CACAGCCACCCACACTATCATGTTGACATTCC; dG = -11.1
806 >s_b_s_e_s_c_s_c_s_d_s GTGGCTGTGCTGGTGTGGGTGTGGGTGACGGTG; >clamp_s_c_s_b_s:compl:rev
CACAGCCACCCACACTATCATGTTGACATTCC; dG = -11.1
807 >s_b_s_e_s_d_s_c_s_d_s GTGGCTGTGCTGGTGACGGTGTGGGTGACGGTG; >clamp_s_c_s_b_s:compl:rev
CACAGCCACCCACACTATCATGTTGACATTCC; dG = -11.1
808 >s_b_s_e_s_e_s_c_s_d_s GTGGCTGTGCTGGTGCTGGTGTGGGTGACGGTG; >clamp_s_c_s_b_s:compl:rev
CACAGCCACCCACACTATCATGTTGACATTCC; dG = -11.1
809 >s_a_s_b_s_a_s_c_s_d_s GTGTCTGTGGCTGTGTCTGTGTGGGTGACGGTG; >clamp_s_c_s_b_s:compl:rev
CACAGCCACCCACACTATCATGTTGACATTCC; dG = -11.1
810 >s_c_s_c_s_d_s_c_s_d_s GTGTGGGTGTGGGTGACGGTGTGGGTGACGGTG; >clamp_s_c_s_b_s:compl:rev
CACAGCCACCCACACTATCATGTTGACATTCC; dG = -11.1

811 >s_c_s_c_s_d_s_c_s_c_s GTGTGGGTGTGGGTGACGGTGTGGGTGTGGGTG; >clamp_s_c_s_b_s:compl:rev
CACAGCCACCCACACTATCATGTTGACATTCC; dG = -11.1
812 >s_c_s_d_s_a_s_c_s_d_s GTGTGGGTGACGGTGTCTGTGTGGGTGACGGTG; >clamp_s_c_s_b_s:compl:rev
CACAGCCACCCACACTATCATGTTGACATTCC; dG = -11.1
813 >s_c_s_d_s_c_s_c_s_d_s GTGTGGGTGACGGTGTGGGTGTGGGTGACGGTG; >clamp_s_c_s_b_s:compl:rev
CACAGCCACCCACACTATCATGTTGACATTCC; dG = -11.1
814 >s_c_s_d_s_d_s_c_s_d_s GTGTGGGTGACGGTGACGGTGTGGGTGACGGTG; >clamp_s_c_s_b_s:compl:rev
CACAGCCACCCACACTATCATGTTGACATTCC; dG = -11.1
815 >s_c_s_d_s_e_s_c_s_d_s GTGTGGGTGACGGTGCTGGTGTGGGTGACGGTG; >clamp_s_c_s_b_s:compl:rev
CACAGCCACCCACACTATCATGTTGACATTCC; dG = -11.1
816 >s_c_s_d_s_e_s_c_s_c_s GTGTGGGTGACGGTGCTGGTGTGGGTGTGGGTG; >clamp_s_c_s_b_s:compl:rev
CACAGCCACCCACACTATCATGTTGACATTCC; dG = -11.1
817 >s_c_s_e_s_a_s_c_s_d_s GTGTGGGTGCTGGTGTCTGTGTGGGTGACGGTG; >clamp_s_c_s_b_s:compl:rev
CACAGCCACCCACACTATCATGTTGACATTCC; dG = -11.1
818 >s_c_s_e_s_c_s_c_s_d_s GTGTGGGTGCTGGTGTGGGTGTGGGTGACGGTG; >clamp_s_c_s_b_s:compl:rev
CACAGCCACCCACACTATCATGTTGACATTCC; dG = -11.1
819 >s_c_s_e_s_d_s_c_s_d_s GTGTGGGTGCTGGTGACGGTGTGGGTGACGGTG; >clamp_s_c_s_b_s:compl:rev
CACAGCCACCCACACTATCATGTTGACATTCC; dG = -11.1
820 >s_c_s_e_s_e_s_c_s_d_s GTGTGGGTGCTGGTGCTGGTGTGGGTGACGGTG; >clamp_s_c_s_b_s:compl:rev
CACAGCCACCCACACTATCATGTTGACATTCC; dG = -11.1
821 >s_c_s_e_s_e_s_c_s_c_s GTGTGGGTGCTGGTGCTGGTGTGGGTGTGGGTG; >clamp_s_c_s_b_s:compl:rev
CACAGCCACCCACACTATCATGTTGACATTCC; dG = -11.1
822 >s_d_s_a_s_a_s_c_s_d_s GTGACGGTGTCTGTGTCTGTGTGGGTGACGGTG; >clamp_s_c_s_b_s:compl:rev
CACAGCCACCCACACTATCATGTTGACATTCC; dG = -11.1
823 >s_d_s_a_s_c_s_c_s_d_s GTGACGGTGTCTGTGTGGGTGTGGGTGACGGTG; >clamp_s_c_s_b_s:compl:rev
CACAGCCACCCACACTATCATGTTGACATTCC; dG = -11.1
824 >s_d_s_a_s_d_s_c_s_d_s GTGACGGTGTCTGTGACGGTGTGGGTGACGGTG; >clamp_s_c_s_b_s:compl:rev
CACAGCCACCCACACTATCATGTTGACATTCC; dG = -11.1
825 >s_d_s_a_s_e_s_c_s_d_s GTGACGGTGTCTGTGCTGGTGTGGGTGACGGTG; >clamp_s_c_s_b_s:compl:rev
CACAGCCACCCACACTATCATGTTGACATTCC; dG = -11.1
826 >s_a_s_b_s_c_s_c_s_d_s GTGTCTGTGGCTGTGTGGGTGTGGGTGACGGTG; >clamp_s_c_s_b_s:compl:rev
CACAGCCACCCACACTATCATGTTGACATTCC; dG = -11.1
827 >s_d_s_c_s_d_s_c_s_d_s GTGACGGTGTGGGTGACGGTGTGGGTGACGGTG; >clamp_s_c_s_b_s:compl:rev
CACAGCCACCCACACTATCATGTTGACATTCC; dG = -11.1
828 >s_d_s_c_s_d_s_c_s_c_s GTGACGGTGTGGGTGACGGTGTGGGTGTGGGTG; >clamp_s_c_s_b_s:compl:rev
CACAGCCACCCACACTATCATGTTGACATTCC; dG = -11.1
829 >s_d_s_d_s_a_s_c_s_d_s GTGACGGTGACGGTGTCTGTGTGGGTGACGGTG; >clamp_s_c_s_b_s:compl:rev
CACAGCCACCCACACTATCATGTTGACATTCC; dG = -11.1
830 >s_d_s_d_s_c_s_c_s_d_s GTGACGGTGACGGTGTGGGTGTGGGTGACGGTG; >clamp_s_c_s_b_s:compl:rev
CACAGCCACCCACACTATCATGTTGACATTCC; dG = -11.1
831 >s_d_s_d_s_d_s_c_s_d_s GTGACGGTGACGGTGACGGTGTGGGTGACGGTG; >clamp_s_c_s_b_s:compl:rev
CACAGCCACCCACACTATCATGTTGACATTCC; dG = -11.1
832 >s_d_s_d_s_e_s_c_s_d_s GTGACGGTGACGGTGCTGGTGTGGGTGACGGTG; >clamp_s_c_s_b_s:compl:rev
CACAGCCACCCACACTATCATGTTGACATTCC; dG = -11.1
833 >s_d_s_e_s_a_s_c_s_d_s GTGACGGTGCTGGTGTCTGTGTGGGTGACGGTG; >clamp_s_c_s_b_s:compl:rev
CACAGCCACCCACACTATCATGTTGACATTCC; dG = -11.1
834 >s_d_s_e_s_c_s_c_s_d_s GTGACGGTGCTGGTGTGGGTGTGGGTGACGGTG; >clamp_s_c_s_b_s:compl:rev
CACAGCCACCCACACTATCATGTTGACATTCC; dG = -11.1
835 >s_d_s_e_s_d_s_c_s_d_s GTGACGGTGCTGGTGACGGTGTGGGTGACGGTG; >clamp_s_c_s_b_s:compl:rev
CACAGCCACCCACACTATCATGTTGACATTCC; dG = -11.1
836 >s_d_s_e_s_e_s_c_s_d_s GTGACGGTGCTGGTGCTGGTGTGGGTGACGGTG; >clamp_s_c_s_b_s:compl:rev
CACAGCCACCCACACTATCATGTTGACATTCC; dG = -11.1
837 >s_e_s_a_s_a_s_c_s_d_s GTGCTGGTGTCTGTGTCTGTGTGGGTGACGGTG; >clamp_s_c_s_b_s:compl:rev
CACAGCCACCCACACTATCATGTTGACATTCC; dG = -11.1
838 >s_a_s_b_s_e_s_c_s_d_s GTGTCTGTGGCTGTGCTGGTGTGGGTGACGGTG; >clamp_s_c_s_b_s:compl:rev
CACAGCCACCCACACTATCATGTTGACATTCC; dG = -11.1
839 >s_e_s_a_s_c_s_c_s_d_s GTGCTGGTGTCTGTGTGGGTGTGGGTGACGGTG; >clamp_s_c_s_b_s:compl:rev
CACAGCCACCCACACTATCATGTTGACATTCC; dG = -11.1
840 >s_e_s_a_s_d_s_c_s_d_s GTGCTGGTGTCTGTGACGGTGTGGGTGACGGTG; >clamp_s_c_s_b_s:compl:rev
CACAGCCACCCACACTATCATGTTGACATTCC; dG = -11.1
841 >s_e_s_a_s_e_s_c_s_d_s GTGCTGGTGTCTGTGCTGGTGTGGGTGACGGTG; >clamp_s_c_s_b_s:compl:rev
CACAGCCACCCACACTATCATGTTGACATTCC; dG = -11.1
842 >s_e_s_c_s_d_s_c_s_d_s GTGCTGGTGTGGGTGACGGTGTGGGTGACGGTG; >clamp_s_c_s_b_s:compl:rev
CACAGCCACCCACACTATCATGTTGACATTCC; dG = -11.1
843 >s_e_s_c_s_d_s_c_s_c_s GTGCTGGTGTGGGTGACGGTGTGGGTGTGGGTG; >clamp_s_c_s_b_s:compl:rev
CACAGCCACCCACACTATCATGTTGACATTCC; dG = -11.1
844 >s_e_s_d_s_a_s_c_s_d_s GTGCTGGTGACGGTGTCTGTGTGGGTGACGGTG; >clamp_s_c_s_b_s:compl:rev
CACAGCCACCCACACTATCATGTTGACATTCC; dG = -11.1
845 >s_e_s_d_s_c_s_c_s_d_s GTGCTGGTGACGGTGTGGGTGTGGGTGACGGTG; >clamp_s_c_s_b_s:compl:rev
CACAGCCACCCACACTATCATGTTGACATTCC; dG = -11.1
846 >s_e_s_d_s_d_s_c_s_d_s GTGCTGGTGACGGTGACGGTGTGGGTGACGGTG; >clamp_s_c_s_b_s:compl:rev
CACAGCCACCCACACTATCATGTTGACATTCC; dG = -11.1
847 >s_e_s_d_s_e_s_c_s_d_s GTGCTGGTGACGGTGCTGGTGTGGGTGACGGTG; >clamp_s_c_s_b_s:compl:rev
CACAGCCACCCACACTATCATGTTGACATTCC; dG = -11.1

848 >s_e_s_e_s_a_s_c_s_d_s GTGCTGGTGCTGGTGTCTGTGTGGGTGACGGTG; >clamp_s_c_s_b_s:compl:rev
CACAGCCACCCACACTATCATGTTGACATTCC; dG = -11.1
849 >s_e_s_e_s_c_s_c_s_d_s GTGCTGGTGCTGGTGTGGGTGTGGGTGACGGTG; >clamp_s_c_s_b_s:compl:rev
CACAGCCACCCACACTATCATGTTGACATTCC; dG = -11.1
850 >s_e_s_e_s_d_s_c_s_d_s GTGCTGGTGCTGGTGACGGTGTGGGTGACGGTG; >clamp_s_c_s_b_s:compl:rev
CACAGCCACCCACACTATCATGTTGACATTCC; dG = -11.1
851 >s_e_s_e_s_e_s_c_s_d_s GTGCTGGTGCTGGTGCTGGTGTGGGTGACGGTG; >clamp_s_c_s_b_s:compl:rev
CACAGCCACCCACACTATCATGTTGACATTCC; dG = -11.1
852 >s_a_s_c_s_d_s_c_s_d_s GTGTCTGTGTGGGTGACGGTGTGGGTGACGGTG; >clamp_s_c_s_b_s:compl:rev
CACAGCCACCCACACTATCATGTTGACATTCC; dG = -11.1
853 >s_a_s_c_s_d_s_c_s_c_s GTGTCTGTGTGGGTGACGGTGTGGGTGTGGGTG; >clamp_s_c_s_b_s:compl:rev
CACAGCCACCCACACTATCATGTTGACATTCC; dG = -11.1
854 >s_b_s_b_s_e_s_c_s_c_s GTGGCTGTGGCTGTGCTGGTGTGGGTGTGGGTG; >clamp_s_c_s_b_s:compl:rev
CACAGCCACCCACACTATCATGTTGACATTCC; dG = -11.0
855 >s_a_s_b_s_a_s_c_s_c_s GTGTCTGTGGCTGTGTCTGTGTGGGTGTGGGTG; >clamp_s_c_s_b_s:compl:rev
CACAGCCACCCACACTATCATGTTGACATTCC; dG = -11.0
856 >s_c_s_e_s_a_s_c_s_c_s GTGTGGGTGCTGGTGTCTGTGTGGGTGTGGGTG; >clamp_s_c_s_b_s:compl:rev
CACAGCCACCCACACTATCATGTTGACATTCC; dG = -10.8
857 >s_c_s_e_s_d_s_c_s_c_s GTGTGGGTGCTGGTGACGGTGTGGGTGTGGGTG; >clamp_s_c_s_b_s:compl:rev
CACAGCCACCCACACTATCATGTTGACATTCC; dG = -10.8
858 >s_a_s_b_s_e_s_c_s_c_s GTGTCTGTGGCTGTGCTGGTGTGGGTGTGGGTG; >clamp_s_c_s_b_s:compl:rev
CACAGCCACCCACACTATCATGTTGACATTCC; dG = -10.8
859 >s_c_s_d_s_a_s_c_s_c_s GTGTGGGTGACGGTGTCTGTGTGGGTGTGGGTG; >clamp_s_c_s_b_s:compl:rev
CACAGCCACCCACACTATCATGTTGACATTCC; dG = -10.6
860 >s_c_s_d_s_d_s_c_s_c_s GTGTGGGTGACGGTGACGGTGTGGGTGTGGGTG; >clamp_s_c_s_b_s:compl:rev
CACAGCCACCCACACTATCATGTTGACATTCC; dG = -10.6
861 >s_a_s_d_s_a_s_c_s_c_s GTGTCTGTGACGGTGTCTGTGTGGGTGTGGGTG; >clamp_s_c_s_b_s:compl:rev
CACAGCCACCCACACTATCATGTTGACATTCC; dG = -10.5
862 >s_a_s_d_s_d_s_c_s_c_s GTGTCTGTGACGGTGACGGTGTGGGTGTGGGTG; >clamp_s_c_s_b_s:compl:rev
CACAGCCACCCACACTATCATGTTGACATTCC; dG = -10.5
863 >s_a_s_d_s_e_s_c_s_c_s GTGTCTGTGACGGTGCTGGTGTGGGTGTGGGTG; >clamp_s_c_s_b_s:compl:rev
CACAGCCACCCACACTATCATGTTGACATTCC; dG = -10.5
864 >s_a_s_e_s_a_s_c_s_c_s GTGTCTGTGCTGGTGTCTGTGTGGGTGTGGGTG; >clamp_s_c_s_b_s:compl:rev
CACAGCCACCCACACTATCATGTTGACATTCC; dG = -10.5
865 >s_a_s_e_s_d_s_c_s_c_s GTGTCTGTGCTGGTGACGGTGTGGGTGTGGGTG; >clamp_s_c_s_b_s:compl:rev
CACAGCCACCCACACTATCATGTTGACATTCC; dG = -10.5
866 >s_a_s_e_s_e_s_c_s_c_s GTGTCTGTGCTGGTGCTGGTGTGGGTGTGGGTG; >clamp_s_c_s_b_s:compl:rev
CACAGCCACCCACACTATCATGTTGACATTCC; dG = -10.5
867 >s_b_s_a_s_a_s_c_s_c_s GTGGCTGTGTCTGTGTCTGTGTGGGTGTGGGTG; >clamp_s_c_s_b_s:compl:rev
CACAGCCACCCACACTATCATGTTGACATTCC; dG = -10.5
868 >s_b_s_a_s_d_s_c_s_c_s GTGGCTGTGTCTGTGACGGTGTGGGTGTGGGTG; >clamp_s_c_s_b_s:compl:rev
CACAGCCACCCACACTATCATGTTGACATTCC; dG = -10.5
869 >s_b_s_a_s_e_s_c_s_c_s GTGGCTGTGTCTGTGCTGGTGTGGGTGTGGGTG; >clamp_s_c_s_b_s:compl:rev
CACAGCCACCCACACTATCATGTTGACATTCC; dG = -10.5
870 >s_a_s_a_s_d_s_c_s_c_s GTGTCTGTGTCTGTGACGGTGTGGGTGTGGGTG; >clamp_s_c_s_b_s:compl:rev
CACAGCCACCCACACTATCATGTTGACATTCC; dG = -10.5
871 >s_b_s_d_s_a_s_c_s_c_s GTGGCTGTGACGGTGTCTGTGTGGGTGTGGGTG; >clamp_s_c_s_b_s:compl:rev
CACAGCCACCCACACTATCATGTTGACATTCC; dG = -10.5
872 >s_a_s_a_s_a_s_c_s_c_s GTGTCTGTGTCTGTGTCTGTGTGGGTGTGGGTG; >clamp_s_c_s_b_s:compl:rev
CACAGCCACCCACACTATCATGTTGACATTCC; dG = -10.5
873 >s_b_s_d_s_d_s_c_s_c_s GTGGCTGTGACGGTGACGGTGTGGGTGTGGGTG; >clamp_s_c_s_b_s:compl:rev
CACAGCCACCCACACTATCATGTTGACATTCC; dG = -10.5
874 >s_b_s_d_s_e_s_c_s_c_s GTGGCTGTGACGGTGCTGGTGTGGGTGTGGGTG; >clamp_s_c_s_b_s:compl:rev
CACAGCCACCCACACTATCATGTTGACATTCC; dG = -10.5
875 >s_b_s_e_s_a_s_c_s_c_s GTGGCTGTGCTGGTGTCTGTGTGGGTGTGGGTG; >clamp_s_c_s_b_s:compl:rev
CACAGCCACCCACACTATCATGTTGACATTCC; dG = -10.5
876 >s_a_s_a_s_e_s_c_s_c_s GTGTCTGTGTCTGTGCTGGTGTGGGTGTGGGTG; >clamp_s_c_s_b_s:compl:rev
CACAGCCACCCACACTATCATGTTGACATTCC; dG = -10.5
877 >s_b_s_e_s_d_s_c_s_c_s GTGGCTGTGCTGGTGACGGTGTGGGTGTGGGTG; >clamp_s_c_s_b_s:compl:rev
CACAGCCACCCACACTATCATGTTGACATTCC; dG = -10.5
878 >s_b_s_e_s_e_s_c_s_c_s GTGGCTGTGCTGGTGCTGGTGTGGGTGTGGGTG; >clamp_s_c_s_b_s:compl:rev
CACAGCCACCCACACTATCATGTTGACATTCC; dG = -10.5
879 >s_d_s_a_s_a_s_c_s_c_s GTGACGGTGTCTGTGTCTGTGTGGGTGTGGGTG; >clamp_s_c_s_b_s:compl:rev
CACAGCCACCCACACTATCATGTTGACATTCC; dG = -10.5
880 >s_d_s_a_s_d_s_c_s_c_s GTGACGGTGTCTGTGACGGTGTGGGTGTGGGTG; >clamp_s_c_s_b_s:compl:rev
CACAGCCACCCACACTATCATGTTGACATTCC; dG = -10.5
881 >s_d_s_a_s_e_s_c_s_c_s GTGACGGTGTCTGTGCTGGTGTGGGTGTGGGTG; >clamp_s_c_s_b_s:compl:rev
CACAGCCACCCACACTATCATGTTGACATTCC; dG = -10.5
882 >s_d_s_d_s_a_s_c_s_c_s GTGACGGTGACGGTGTCTGTGTGGGTGTGGGTG; >clamp_s_c_s_b_s:compl:rev
CACAGCCACCCACACTATCATGTTGACATTCC; dG = -10.5
883 >s_d_s_d_s_d_s_c_s_c_s GTGACGGTGACGGTGACGGTGTGGGTGTGGGTG; >clamp_s_c_s_b_s:compl:rev
CACAGCCACCCACACTATCATGTTGACATTCC; dG = -10.5
884 >s_d_s_d_s_e_s_c_s_c_s GTGACGGTGACGGTGCTGGTGTGGGTGTGGGTG; >clamp_s_c_s_b_s:compl:rev
CACAGCCACCCACACTATCATGTTGACATTCC; dG = -10.5

885 >s_d_s_e_s_a_s_c_s_c_s GTGACGGTGCTGGTGTCTGTGTGGGTGTGGGTG; >clamp_s_c_s_b_s:compl:rev CACAGCCACCCACACTATCATGTTGACATTCC; dG = -10.5
886 >s_d_s_e_s_d_s_c_s_c_s GTGACGGTGCTGGTGACGGTGTGGGTGTGGGTG; >clamp_s_c_s_b_s:compl:rev CACAGCCACCCACACTATCATGTTGACATTCC; dG = -10.5
887 >s_d_s_e_s_e_s_c_s_c_s GTGACGGTGCTGGTGCTGGTGTGGGTGTGGGTG; >clamp_s_c_s_b_s:compl:rev CACAGCCACCCACACTATCATGTTGACATTCC; dG = -10.5
888 >s_e_s_a_s_a_s_c_s_c_s GTGCTGGTGTCTGTGTCTGTGTGGGTGTGGGTG; >clamp_s_c_s_b_s:compl:rev CACAGCCACCCACACTATCATGTTGACATTCC; dG = -10.5
889 >s_e_s_a_s_d_s_c_s_c_s GTGCTGGTGTCTGTGACGGTGTGGGTGTGGGTG; >clamp_s_c_s_b_s:compl:rev CACAGCCACCCACACTATCATGTTGACATTCC; dG = -10.5
890 >s_e_s_a_s_e_s_c_s_c_s GTGCTGGTGTCTGTGCTGGTGTGGGTGTGGGTG; >clamp_s_c_s_b_s:compl:rev CACAGCCACCCACACTATCATGTTGACATTCC; dG = -10.5
891 >s_e_s_d_s_a_s_c_s_c_s GTGCTGGTGACGGTGTCTGTGTGGGTGTGGGTG; >clamp_s_c_s_b_s:compl:rev CACAGCCACCCACACTATCATGTTGACATTCC; dG = -10.5
892 >s_e_s_d_s_d_s_c_s_c_s GTGCTGGTGACGGTGACGGTGTGGGTGTGGGTG; >clamp_s_c_s_b_s:compl:rev CACAGCCACCCACACTATCATGTTGACATTCC; dG = -10.5
893 >s_e_s_d_s_e_s_c_s_c_s GTGCTGGTGACGGTGCTGGTGTGGGTGTGGGTG; >clamp_s_c_s_b_s:compl:rev CACAGCCACCCACACTATCATGTTGACATTCC; dG = -10.5
894 >s_e_s_e_s_a_s_c_s_c_s GTGCTGGTGCTGGTGTCTGTGTGGGTGTGGGTG; >clamp_s_c_s_b_s:compl:rev CACAGCCACCCACACTATCATGTTGACATTCC; dG = -10.5
895 >s_e_s_e_s_d_s_c_s_c_s GTGCTGGTGCTGGTGACGGTGTGGGTGTGGGTG; >clamp_s_c_s_b_s:compl:rev CACAGCCACCCACACTATCATGTTGACATTCC; dG = -10.5
896 >s_e_s_e_s_e_s_c_s_c_s GTGCTGGTGCTGGTGCTGGTGTGGGTGTGGGTG; >clamp_s_c_s_b_s:compl:rev CACAGCCACCCACACTATCATGTTGACATTCC; dG = -10.5

****

CDCAE Matches

1 >s_a_s_a_s_a_s_c_s_d_s_c_s_a_s_e_s
GTGTCTGTGTCTGTGTCTGTGTGGGTGACGGTGTGGGTGTCTGTGCTGGTG; >clamp_s_c_s_d_s_c_s_a_s_e_s:compl:rev CACCAGCACAGACACCCACACCGTCACCCACACTATCATGTTGACATTCC; dG = -42.6
2 >s_e_s_a_s_a_s_c_s_d_s_c_s_a_s_e_s
GTGCTGGTGTCTGTGTCTGTGTGGGTGACGGTGTGGGTGTCTGTGCTGGTG; >clamp_s_c_s_d_s_c_s_a_s_e_s:compl:rev CACCAGCACAGACACCCACACCGTCACCCACACTATCATGTTGACATTCC; dG = -42.6
3 >s_e_s_a_s_b_s_c_s_d_s_c_s_a_s_e_s
GTGCTGGTGTCTGTGGCTGTGTGGGTGACGGTGTGGGTGTCTGTGCTGGTG; >clamp_s_c_s_d_s_c_s_a_s_e_s:compl:rev CACCAGCACAGACACCCACACCGTCACCCACACTATCATGTTGACATTCC; dG = -42.6
4 >s_e_s_b_s_a_s_c_s_d_s_c_s_a_s_e_s
GTGCTGGTGGCTGTGTCTGTGTGGGTGACGGTGTGGGTGTCTGTGCTGGTG; >clamp_s_c_s_d_s_c_s_a_s_e_s:compl:rev CACCAGCACAGACACCCACACCGTCACCCACACTATCATGTTGACATTCC; dG = -42.6
5 >s_e_s_b_s_b_s_c_s_d_s_c_s_a_s_e_s
GTGCTGGTGGCTGTGGCTGTGTGGGTGACGGTGTGGGTGTCTGTGCTGGTG; >clamp_s_c_s_d_s_c_s_a_s_e_s:compl:rev CACCAGCACAGACACCCACACCGTCACCCACACTATCATGTTGACATTCC; dG = -42.6
6 >s_a_s_c_s_a_s_c_s_d_s_c_s_a_s_e_s
GTGTCTGTGTGGGTGTCTGTGTGGGTGACGGTGTGGGTGTCTGTGCTGGTG; >clamp_s_c_s_d_s_c_s_a_s_e_s:compl:rev CACCAGCACAGACACCCACACCGTCACCCACACTATCATGTTGACATTCC; dG = -42.6
7 >s_e_s_c_s_a_s_c_s_d_s_c_s_a_s_e_s
GTGCTGGTGTGGGTGTCTGTGTGGGTGACGGTGTGGGTGTCTGTGCTGGTG; >clamp_s_c_s_d_s_c_s_a_s_e_s:compl:rev CACCAGCACAGACACCCACACCGTCACCCACACTATCATGTTGACATTCC; dG = -42.6
8 >s_e_s_c_s_b_s_c_s_d_s_c_s_a_s_e_s
GTGCTGGTGTGGGTGGCTGTGTGGGTGACGGTGTGGGTGTCTGTGCTGGTG; >clamp_s_c_s_d_s_c_s_a_s_e_s:compl:rev CACCAGCACAGACACCCACACCGTCACCCACACTATCATGTTGACATTCC; dG = -42.6
9 >s_e_s_d_s_a_s_c_s_d_s_c_s_a_s_e_s
GTGCTGGTGACGGTGTCTGTGTGGGTGACGGTGTGGGTGTCTGTGCTGGTG; >clamp_s_c_s_d_s_c_s_a_s_e_s:compl:rev CACCAGCACAGACACCCACACCGTCACCCACACTATCATGTTGACATTCC; dG = -42.6
10 >s_e_s_d_s_b_s_c_s_d_s_c_s_a_s_e_s
GTGCTGGTGACGGTGGCTGTGTGGGTGACGGTGTGGGTGTCTGTGCTGGTG; >clamp_s_c_s_d_s_c_s_a_s_e_s:compl:rev CACCAGCACAGACACCCACACCGTCACCCACACTATCATGTTGACATTCC; dG = -42.6
11 >s_a_s_c_s_b_s_c_s_d_s_c_s_a_s_e_s
GTGTCTGTGTGGGTGGCTGTGTGGGTGACGGTGTGGGTGTCTGTGCTGGTG; >clamp_s_c_s_d_s_c_s_a_s_e_s:compl:rev CACCAGCACAGACACCCACACCGTCACCCACACTATCATGTTGACATTCC; dG = -42.6
12 >s_e_s_e_s_a_s_c_s_d_s_c_s_a_s_e_s
GTGCTGGTGCTGGTGTCTGTGTGGGTGACGGTGTGGGTGTCTGTGCTGGTG; >clamp_s_c_s_d_s_c_s_a_s_e_s:compl:rev CACCAGCACAGACACCCACACCGTCACCCACACTATCATGTTGACATTCC; dG = -42.6
13 >s_e_s_e_s_b_s_c_s_d_s_c_s_a_s_e_s
GTGCTGGTGCTGGTGGCTGTGTGGGTGACGGTGTGGGTGTCTGTGCTGGTG; >clamp_s_c_s_d_s_c_s_a_s_e_s:compl:rev CACCAGCACAGACACCCACACCGTCACCCACACTATCATGTTGACATTCC; dG = -42.6
14 >s_a_s_d_s_a_s_c_s_d_s_c_s_a_s_e_s
GTGTCTGTGACGGTGTCTGTGTGGGTGACGGTGTGGGTGTCTGTGCTGGTG; >clamp_s_c_s_d_s_c_s_a_s_e_s:compl:rev CACCAGCACAGACACCCACACCGTCACCCACACTATCATGTTGACATTCC; dG = -42.6

15 >s_a_s_d_s_b_s_c_s_d_s_c_s_a_s_e_s
GTGTCTGTGACGGTGGCTGTGTGGGTGACGGTGTGGGTGTCTGTGCTGGTG; >clamp_s_c_s_d_s_c_s_a_s_e_s:compl:rev
CACCAGCACAGACACCCACACCGTCACCCACACTATCATGTTGACATTCC; dG = -42.6
16 >s_a_s_a_s_b_s_c_s_d_s_c_s_a_s_e_s
GTGTCTGTGTCTGTGGCTGTGTGGGTGACGGTGTGGGTGTCTGTGCTGGTG; >clamp_s_c_s_d_s_c_s_a_s_e_s:compl:rev
CACCAGCACAGACACCCACACCGTCACCCACACTATCATGTTGACATTCC; dG = -42.6
17 >s_a_s_e_s_a_s_c_s_d_s_c_s_a_s_e_s
GTGTCTGTGCTGGTGTCTGTGTGGGTGACGGTGTGGGTGTCTGTGCTGGTG; >clamp_s_c_s_d_s_c_s_a_s_e_s:compl:rev
CACCAGCACAGACACCCACACCGTCACCCACACTATCATGTTGACATTCC; dG = -42.6
18 >s_a_s_e_s_b_s_c_s_d_s_c_s_a_s_e_s
GTGTCTGTGCTGGTGGCTGTGTGGGTGACGGTGTGGGTGTCTGTGCTGGTG; >clamp_s_c_s_d_s_c_s_a_s_e_s:compl:rev
CACCAGCACAGACACCCACACCGTCACCCACACTATCATGTTGACATTCC; dG = -42.6
19 >s_b_s_a_s_a_s_c_s_d_s_c_s_a_s_e_s
GTGGCTGTGTCTGTGTCTGTGTGGGTGACGGTGTGGGTGTCTGTGCTGGTG; >clamp_s_c_s_d_s_c_s_a_s_e_s:compl:rev
CACCAGCACAGACACCCACACCGTCACCCACACTATCATGTTGACATTCC; dG = -42.6
20 >s_b_s_a_s_b_s_c_s_d_s_c_s_a_s_e_s
GTGGCTGTGTCTGTGGCTGTGTGGGTGACGGTGTGGGTGTCTGTGCTGGTG; >clamp_s_c_s_d_s_c_s_a_s_e_s:compl:rev
CACCAGCACAGACACCCACACCGTCACCCACACTATCATGTTGACATTCC; dG = -42.6
21 >s_b_s_b_s_a_s_c_s_d_s_c_s_a_s_e_s
GTGGCTGTGGCTGTGTCTGTGTGGGTGACGGTGTGGGTGTCTGTGCTGGTG; >clamp_s_c_s_d_s_c_s_a_s_e_s:compl:rev
CACCAGCACAGACACCCACACCGTCACCCACACTATCATGTTGACATTCC; dG = -42.6
22 >s_b_s_b_s_b_s_c_s_d_s_c_s_a_s_e_s
GTGGCTGTGGCTGTGGCTGTGTGGGTGACGGTGTGGGTGTCTGTGCTGGTG; >clamp_s_c_s_d_s_c_s_a_s_e_s:compl:rev
CACCAGCACAGACACCCACACCGTCACCCACACTATCATGTTGACATTCC; dG = -42.6
23 >s_b_s_c_s_a_s_c_s_d_s_c_s_a_s_e_s
GTGGCTGTGTGGGTGTCTGTGTGGGTGACGGTGTGGGTGTCTGTGCTGGTG; >clamp_s_c_s_d_s_c_s_a_s_e_s:compl:rev
CACCAGCACAGACACCCACACCGTCACCCACACTATCATGTTGACATTCC; dG = -42.6
24 >s_b_s_c_s_b_s_c_s_d_s_c_s_a_s_e_s
GTGGCTGTGTGGGTGGCTGTGTGGGTGACGGTGTGGGTGTCTGTGCTGGTG; >clamp_s_c_s_d_s_c_s_a_s_e_s:compl:rev
CACCAGCACAGACACCCACACCGTCACCCACACTATCATGTTGACATTCC; dG = -42.6
25 >s_b_s_d_s_a_s_c_s_d_s_c_s_a_s_e_s
GTGGCTGTGACGGTGTCTGTGTGGGTGACGGTGTGGGTGTCTGTGCTGGTG; >clamp_s_c_s_d_s_c_s_a_s_e_s:compl:rev
CACCAGCACAGACACCCACACCGTCACCCACACTATCATGTTGACATTCC; dG = -42.6
26 >s_b_s_d_s_b_s_c_s_d_s_c_s_a_s_e_s
GTGGCTGTGACGGTGGCTGTGTGGGTGACGGTGTGGGTGTCTGTGCTGGTG; >clamp_s_c_s_d_s_c_s_a_s_e_s:compl:rev
CACCAGCACAGACACCCACACCGTCACCCACACTATCATGTTGACATTCC; dG = -42.6
27 >s_b_s_e_s_a_s_c_s_d_s_c_s_a_s_e_s
GTGGCTGTGCTGGTGTCTGTGTGGGTGACGGTGTGGGTGTCTGTGCTGGTG; >clamp_s_c_s_d_s_c_s_a_s_e_s:compl:rev
CACCAGCACAGACACCCACACCGTCACCCACACTATCATGTTGACATTCC; dG = -42.6
28 >s_b_s_e_s_b_s_c_s_d_s_c_s_a_s_e_s
GTGGCTGTGCTGGTGGCTGTGTGGGTGACGGTGTGGGTGTCTGTGCTGGTG; >clamp_s_c_s_d_s_c_s_a_s_e_s:compl:rev
CACCAGCACAGACACCCACACCGTCACCCACACTATCATGTTGACATTCC; dG = -42.6
29 >s_c_s_a_s_a_s_c_s_d_s_c_s_a_s_e_s
GTGTGGGTGTCTGTGTCTGTGTGGGTGACGGTGTGGGTGTCTGTGCTGGTG; >clamp_s_c_s_d_s_c_s_a_s_e_s:compl:rev
CACCAGCACAGACACCCACACCGTCACCCACACTATCATGTTGACATTCC; dG = -42.6
30 >s_c_s_a_s_b_s_c_s_d_s_c_s_a_s_e_s
GTGTGGGTGTCTGTGGCTGTGTGGGTGACGGTGTGGGTGTCTGTGCTGGTG; >clamp_s_c_s_d_s_c_s_a_s_e_s:compl:rev
CACCAGCACAGACACCCACACCGTCACCCACACTATCATGTTGACATTCC; dG = -42.6
31 >s_c_s_b_s_a_s_c_s_d_s_c_s_a_s_e_s
GTGTGGGTGGCTGTGTCTGTGTGGGTGACGGTGTGGGTGTCTGTGCTGGTG; >clamp_s_c_s_d_s_c_s_a_s_e_s:compl:rev
CACCAGCACAGACACCCACACCGTCACCCACACTATCATGTTGACATTCC; dG = -42.6
32 >s_c_s_b_s_b_s_c_s_d_s_c_s_a_s_e_s
GTGTGGGTGGCTGTGGCTGTGTGGGTGACGGTGTGGGTGTCTGTGCTGGTG; >clamp_s_c_s_d_s_c_s_a_s_e_s:compl:rev
CACCAGCACAGACACCCACACCGTCACCCACACTATCATGTTGACATTCC; dG = -42.6
33 >s_a_s_b_s_a_s_c_s_d_s_c_s_a_s_e_s
GTGTCTGTGGCTGTGTCTGTGTGGGTGACGGTGTGGGTGTCTGTGCTGGTG; >clamp_s_c_s_d_s_c_s_a_s_e_s:compl:rev
CACCAGCACAGACACCCACACCGTCACCCACACTATCATGTTGACATTCC; dG = -42.6
34 >s_c_s_c_s_a_s_c_s_d_s_c_s_a_s_e_s
GTGTGGGTGTGGGTGTCTGTGTGGGTGACGGTGTGGGTGTCTGTGCTGGTG; >clamp_s_c_s_d_s_c_s_a_s_e_s:compl:rev
CACCAGCACAGACACCCACACCGTCACCCACACTATCATGTTGACATTCC; dG = -42.6
35 >s_c_s_c_s_b_s_c_s_d_s_c_s_a_s_e_s
GTGTGGGTGTGGGTGGCTGTGTGGGTGACGGTGTGGGTGTCTGTGCTGGTG; >clamp_s_c_s_d_s_c_s_a_s_e_s:compl:rev
CACCAGCACAGACACCCACACCGTCACCCACACTATCATGTTGACATTCC; dG = -42.6
36 >s_c_s_d_s_a_s_c_s_d_s_c_s_a_s_e_s
GTGTGGGTGACGGTGTCTGTGTGGGTGACGGTGTGGGTGTCTGTGCTGGTG; >clamp_s_c_s_d_s_c_s_a_s_e_s:compl:rev
CACCAGCACAGACACCCACACCGTCACCCACACTATCATGTTGACATTCC; dG = -42.6
37 >s_c_s_d_s_b_s_c_s_d_s_c_s_a_s_e_s
GTGTGGGTGACGGTGGCTGTGTGGGTGACGGTGTGGGTGTCTGTGCTGGTG; >clamp_s_c_s_d_s_c_s_a_s_e_s:compl:rev
CACCAGCACAGACACCCACACCGTCACCCACACTATCATGTTGACATTCC; dG = -42.6
38 >s_a_s_b_s_b_s_c_s_d_s_c_s_a_s_e_s
GTGTCTGTGGCTGTGGCTGTGTGGGTGACGGTGTGGGTGTCTGTGCTGGTG; >clamp_s_c_s_d_s_c_s_a_s_e_s:compl:rev
CACCAGCACAGACACCCACACCGTCACCCACACTATCATGTTGACATTCC; dG = -42.6

```
    39 >s_c_s_e_s_a_s_c_s_d_s_c_s_a_s_e_s
GTGTGGGTGCTGGTGTCTGTGTGGGTGACGGTGTGGGTGTCTGTGCTGGTG;    >clamp_s_c_s_d_s_c_s_a_s_e_s:compl:rev
CACCAGCACAGACACCCACACCGTCACCCACACTATCATGTTGACATTCC; dG = -42.6
    40 >s_c_s_e_s_b_s_c_s_d_s_c_s_a_s_e_s
GTGTGGGTGCTGGTGGCTGTGTGGGTGACGGTGTGGGTGTCTGTGCTGGTG;    >clamp_s_c_s_d_s_c_s_a_s_e_s:compl:rev
CACCAGCACAGACACCCACACCGTCACCCACACTATCATGTTGACATTCC; dG = -42.6
    41 >s_d_s_a_s_a_s_c_s_d_s_c_s_a_s_e_s
GTGACGGTGTCTGTGTCTGTGTGGGTGACGGTGTGGGTGTCTGTGCTGGTG;    >clamp_s_c_s_d_s_c_s_a_s_e_s:compl:rev
CACCAGCACAGACACCCACACCGTCACCCACACTATCATGTTGACATTCC; dG = -42.6
    42 >s_d_s_a_s_b_s_c_s_d_s_c_s_a_s_e_s
GTGACGGTGTCTGTGGCTGTGTGGGTGACGGTGTGGGTGTCTGTGCTGGTG;    >clamp_s_c_s_d_s_c_s_a_s_e_s:compl:rev
CACCAGCACAGACACCCACACCGTCACCCACACTATCATGTTGACATTCC; dG = -42.6
    43 >s_d_s_b_s_a_s_c_s_d_s_c_s_a_s_e_s
GTGACGGTGGCTGTGTCTGTGTGGGTGACGGTGTGGGTGTCTGTGCTGGTG;    >clamp_s_c_s_d_s_c_s_a_s_e_s:compl:rev
CACCAGCACAGACACCCACACCGTCACCCACACTATCATGTTGACATTCC; dG = -42.6
    44 >s_d_s_b_s_b_s_c_s_d_s_c_s_a_s_e_s
GTGACGGTGGCTGTGGCTGTGTGGGTGACGGTGTGGGTGTCTGTGCTGGTG;    >clamp_s_c_s_d_s_c_s_a_s_e_s:compl:rev
CACCAGCACAGACACCCACACCGTCACCCACACTATCATGTTGACATTCC; dG = -42.6
    45 >s_d_s_c_s_a_s_c_s_d_s_c_s_a_s_e_s
GTGACGGTGTGGGTGTCTGTGTGGGTGACGGTGTGGGTGTCTGTGCTGGTG;    >clamp_s_c_s_d_s_c_s_a_s_e_s:compl:rev
CACCAGCACAGACACCCACACCGTCACCCACACTATCATGTTGACATTCC; dG = -42.6
    46 >s_d_s_c_s_b_s_c_s_d_s_c_s_a_s_e_s
GTGACGGTGTGGGTGGCTGTGTGGGTGACGGTGTGGGTGTCTGTGCTGGTG;    >clamp_s_c_s_d_s_c_s_a_s_e_s:compl:rev
CACCAGCACAGACACCCACACCGTCACCCACACTATCATGTTGACATTCC; dG = -42.6
    47 >s_d_s_d_s_a_s_c_s_d_s_c_s_a_s_e_s
GTGACGGTGACGGTGTCTGTGTGGGTGACGGTGTGGGTGTCTGTGCTGGTG;    >clamp_s_c_s_d_s_c_s_a_s_e_s:compl:rev
CACCAGCACAGACACCCACACCGTCACCCACACTATCATGTTGACATTCC; dG = -42.6
    48 >s_d_s_d_s_b_s_c_s_d_s_c_s_a_s_e_s
GTGACGGTGACGGTGGCTGTGTGGGTGACGGTGTGGGTGTCTGTGCTGGTG;    >clamp_s_c_s_d_s_c_s_a_s_e_s:compl:rev
CACCAGCACAGACACCCACACCGTCACCCACACTATCATGTTGACATTCC; dG = -42.6
    49 >s_d_s_e_s_a_s_c_s_d_s_c_s_a_s_e_s
GTGACGGTGCTGGTGTCTGTGTGGGTGACGGTGTGGGTGTCTGTGCTGGTG;    >clamp_s_c_s_d_s_c_s_a_s_e_s:compl:rev
CACCAGCACAGACACCCACACCGTCACCCACACTATCATGTTGACATTCC; dG = -42.6
    50 >s_d_s_e_s_b_s_c_s_d_s_c_s_a_s_e_s
GTGACGGTGCTGGTGGCTGTGTGGGTGACGGTGTGGGTGTCTGTGCTGGTG;    >clamp_s_c_s_d_s_c_s_a_s_e_s:compl:rev
CACCAGCACAGACACCCACACCGTCACCCACACTATCATGTTGACATTCC; dG = -42.6
    51 >s_a_s_b_s_e_s_c_s_d_s_c_s_a_s_e_s
GTGTCTGTGGCTGTGCTGGTGTGGGTGACGGTGTGGGTGTCTGTGCTGGTG;    >clamp_s_c_s_d_s_c_s_a_s_e_s:compl:rev
CACCAGCACAGACACCCACACCGTCACCCACACTATCATGTTGACATTCC; dG = -42.5
    52 >s_d_s_e_s_e_s_c_s_d_s_c_s_a_s_e_s
GTGACGGTGCTGGTGCTGGTGTGGGTGACGGTGTGGGTGTCTGTGCTGGTG;    >clamp_s_c_s_d_s_c_s_a_s_e_s:compl:rev
CACCAGCACAGACACCCACACCGTCACCCACACTATCATGTTGACATTCC; dG = -42.5
    53 >s_e_s_a_s_c_s_c_s_d_s_c_s_a_s_e_s
GTGCTGGTGTCTGTGTGGGTGTGGGTGACGGTGTGGGTGTCTGTGCTGGTG;    >clamp_s_c_s_d_s_c_s_a_s_e_s:compl:rev
CACCAGCACAGACACCCACACCGTCACCCACACTATCATGTTGACATTCC; dG = -42.5
    54 >s_e_s_a_s_d_s_c_s_d_s_c_s_a_s_e_s
GTGCTGGTGTCTGTGACGGTGTGGGTGACGGTGTGGGTGTCTGTGCTGGTG;    >clamp_s_c_s_d_s_c_s_a_s_e_s:compl:rev
CACCAGCACAGACACCCACACCGTCACCCACACTATCATGTTGACATTCC; dG = -42.5
    55 >s_e_s_a_s_e_s_c_s_d_s_c_s_a_s_e_s
GTGCTGGTGTCTGTGCTGGTGTGGGTGACGGTGTGGGTGTCTGTGCTGGTG;    >clamp_s_c_s_d_s_c_s_a_s_e_s:compl:rev
CACCAGCACAGACACCCACACCGTCACCCACACTATCATGTTGACATTCC; dG = -42.5
    56 >s_e_s_b_s_c_s_c_s_d_s_c_s_a_s_e_s
GTGCTGGTGGCTGTGTGGGTGTGGGTGACGGTGTGGGTGTCTGTGCTGGTG;    >clamp_s_c_s_d_s_c_s_a_s_e_s:compl:rev
CACCAGCACAGACACCCACACCGTCACCCACACTATCATGTTGACATTCC; dG = -42.5
    57 >s_e_s_b_s_d_s_c_s_d_s_c_s_a_s_e_s
GTGCTGGTGGCTGTGACGGTGTGGGTGACGGTGTGGGTGTCTGTGCTGGTG;    >clamp_s_c_s_d_s_c_s_a_s_e_s:compl:rev
CACCAGCACAGACACCCACACCGTCACCCACACTATCATGTTGACATTCC; dG = -42.5
    58 >s_e_s_b_s_e_s_c_s_d_s_c_s_a_s_e_s
GTGCTGGTGGCTGTGCTGGTGTGGGTGACGGTGTGGGTGTCTGTGCTGGTG;    >clamp_s_c_s_d_s_c_s_a_s_e_s:compl:rev
CACCAGCACAGACACCCACACCGTCACCCACACTATCATGTTGACATTCC; dG = -42.5
    59 >s_e_s_c_s_c_s_c_s_d_s_c_s_a_s_e_s
GTGCTGGTGTGGGTGTGGGTGTGGGTGACGGTGTGGGTGTCTGTGCTGGTG;    >clamp_s_c_s_d_s_c_s_a_s_e_s:compl:rev
CACCAGCACAGACACCCACACCGTCACCCACACTATCATGTTGACATTCC; dG = -42.5
    60 >s_e_s_c_s_d_s_c_s_d_s_c_s_a_s_e_s
GTGCTGGTGTGGGTGACGGTGTGGGTGACGGTGTGGGTGTCTGTGCTGGTG;    >clamp_s_c_s_d_s_c_s_a_s_e_s:compl:rev
CACCAGCACAGACACCCACACCGTCACCCACACTATCATGTTGACATTCC; dG = -42.5
    61 >s_e_s_c_s_e_s_c_s_d_s_c_s_a_s_e_s
GTGCTGGTGTGGGTGCTGGTGTGGGTGACGGTGTGGGTGTCTGTGCTGGTG;    >clamp_s_c_s_d_s_c_s_a_s_e_s:compl:rev
CACCAGCACAGACACCCACACCGTCACCCACACTATCATGTTGACATTCC; dG = -42.5
    62 >s_e_s_d_s_c_s_c_s_d_s_c_s_a_s_e_s
GTGCTGGTGACGGTGTGGGTGTGGGTGACGGTGTGGGTGTCTGTGCTGGTG;    >clamp_s_c_s_d_s_c_s_a_s_e_s:compl:rev
CACCAGCACAGACACCCACACCGTCACCCACACTATCATGTTGACATTCC; dG = -42.5
```

63 >s_e_s_d_s_d_s_c_s_d_s_c_s_a_s_e_s
GTGCTGGTGACGGTGACGGTGTGGGTGACGGTGTGGGTGTCTGTGCTGGTG; >clamp_s_c_s_d_s_c_s_a_s_e_s:compl:rev
CACCAGCACAGACACCCACACCGTCACCCACACTATCATGTTGACATTCC; dG = -42.5
64 >s_e_s_d_s_e_s_c_s_d_s_c_s_a_s_e_s
GTGCTGGTGACGGTGCTGGTGTGGGTGACGGTGTGGGTGTCTGTGCTGGTG; >clamp_s_c_s_d_s_c_s_a_s_e_s:compl:rev
CACCAGCACAGACACCCACACCGTCACCCACACTATCATGTTGACATTCC; dG = -42.5
65 >s_e_s_e_s_c_s_c_s_d_s_c_s_a_s_e_s
GTGCTGGTGCTGGTGTGGGTGTGGGTGACGGTGTGGGTGTCTGTGCTGGTG; >clamp_s_c_s_d_s_c_s_a_s_e_s:compl:rev
CACCAGCACAGACACCCACACCGTCACCCACACTATCATGTTGACATTCC; dG = -42.5
66 >s_e_s_e_s_d_s_c_s_d_s_c_s_a_s_e_s
GTGCTGGTGCTGGTGACGGTGTGGGTGACGGTGTGGGTGTCTGTGCTGGTG; >clamp_s_c_s_d_s_c_s_a_s_e_s:compl:rev
CACCAGCACAGACACCCACACCGTCACCCACACTATCATGTTGACATTCC; dG = -42.5
67 >s_e_s_e_s_e_s_c_s_d_s_c_s_a_s_e_s
GTGCTGGTGCTGGTGCTGGTGTGGGTGACGGTGTGGGTGTCTGTGCTGGTG; >clamp_s_c_s_d_s_c_s_a_s_e_s:compl:rev
CACCAGCACAGACACCCACACCGTCACCCACACTATCATGTTGACATTCC; dG = -42.5
68 >s_a_s_c_s_c_s_c_s_d_s_c_s_a_s_e_s
GTGTCTGTGTGGGTGTGGGTGTGGGTGACGGTGTGGGTGTCTGTGCTGGTG; >clamp_s_c_s_d_s_c_s_a_s_e_s:compl:rev
CACCAGCACAGACACCCACACCGTCACCCACACTATCATGTTGACATTCC; dG = -42.5
69 >s_a_s_c_s_d_s_c_s_d_s_c_s_a_s_e_s
GTGTCTGTGTGGGTGACGGTGTGGGTGACGGTGTGGGTGTCTGTGCTGGTG; >clamp_s_c_s_d_s_c_s_a_s_e_s:compl:rev
CACCAGCACAGACACCCACACCGTCACCCACACTATCATGTTGACATTCC; dG = -42.5
70 >s_a_s_c_s_e_s_c_s_d_s_c_s_a_s_e_s
GTGTCTGTGTGGGTGCTGGTGTGGGTGACGGTGTGGGTGTCTGTGCTGGTG; >clamp_s_c_s_d_s_c_s_a_s_e_s:compl:rev
CACCAGCACAGACACCCACACCGTCACCCACACTATCATGTTGACATTCC; dG = -42.5
71 >s_a_s_d_s_c_s_c_s_d_s_c_s_a_s_e_s
GTGTCTGTGACGGTGTGGGTGTGGGTGACGGTGTGGGTGTCTGTGCTGGTG; >clamp_s_c_s_d_s_c_s_a_s_e_s:compl:rev
CACCAGCACAGACACCCACACCGTCACCCACACTATCATGTTGACATTCC; dG = -42.5
72 >s_a_s_d_s_d_s_c_s_d_s_c_s_a_s_e_s
GTGTCTGTGACGGTGACGGTGTGGGTGACGGTGTGGGTGTCTGTGCTGGTG; >clamp_s_c_s_d_s_c_s_a_s_e_s:compl:rev
CACCAGCACAGACACCCACACCGTCACCCACACTATCATGTTGACATTCC; dG = -42.5
73 >s_a_s_d_s_e_s_c_s_d_s_c_s_a_s_e_s
GTGTCTGTGACGGTGCTGGTGTGGGTGACGGTGTGGGTGTCTGTGCTGGTG; >clamp_s_c_s_d_s_c_s_a_s_e_s:compl:rev
CACCAGCACAGACACCCACACCGTCACCCACACTATCATGTTGACATTCC; dG = -42.5
74 >s_a_s_e_s_c_s_c_s_d_s_c_s_a_s_e_s
GTGTCTGTGCTGGTGTGGGTGTGGGTGACGGTGTGGGTGTCTGTGCTGGTG; >clamp_s_c_s_d_s_c_s_a_s_e_s:compl:rev
CACCAGCACAGACACCCACACCGTCACCCACACTATCATGTTGACATTCC; dG = -42.5
75 >s_a_s_e_s_d_s_c_s_d_s_c_s_a_s_e_s
GTGTCTGTGCTGGTGACGGTGTGGGTGACGGTGTGGGTGTCTGTGCTGGTG; >clamp_s_c_s_d_s_c_s_a_s_e_s:compl:rev
CACCAGCACAGACACCCACACCGTCACCCACACTATCATGTTGACATTCC; dG = -42.5
76 >s_a_s_e_s_e_s_c_s_d_s_c_s_a_s_e_s
GTGTCTGTGCTGGTGCTGGTGTGGGTGACGGTGTGGGTGTCTGTGCTGGTG; >clamp_s_c_s_d_s_c_s_a_s_e_s:compl:rev
CACCAGCACAGACACCCACACCGTCACCCACACTATCATGTTGACATTCC; dG = -42.5
77 >s_b_s_a_s_c_s_c_s_d_s_c_s_a_s_e_s
GTGGCTGTGTCTGTGTGGGTGTGGGTGACGGTGTGGGTGTCTGTGCTGGTG; >clamp_s_c_s_d_s_c_s_a_s_e_s:compl:rev
CACCAGCACAGACACCCACACCGTCACCCACACTATCATGTTGACATTCC; dG = -42.5
78 >s_b_s_a_s_d_s_c_s_d_s_c_s_a_s_e_s
GTGGCTGTGTCTGTGACGGTGTGGGTGACGGTGTGGGTGTCTGTGCTGGTG; >clamp_s_c_s_d_s_c_s_a_s_e_s:compl:rev
CACCAGCACAGACACCCACACCGTCACCCACACTATCATGTTGACATTCC; dG = -42.5
79 >s_a_s_a_s_c_s_c_s_d_s_c_s_a_s_e_s
GTGTCTGTGTCTGTGTGGGTGTGGGTGACGGTGTGGGTGTCTGTGCTGGTG; >clamp_s_c_s_d_s_c_s_a_s_e_s:compl:rev
CACCAGCACAGACACCCACACCGTCACCCACACTATCATGTTGACATTCC; dG = -42.5
80 >s_b_s_a_s_e_s_c_s_d_s_c_s_a_s_e_s
GTGGCTGTGTCTGTGCTGGTGTGGGTGACGGTGTGGGTGTCTGTGCTGGTG; >clamp_s_c_s_d_s_c_s_a_s_e_s:compl:rev
CACCAGCACAGACACCCACACCGTCACCCACACTATCATGTTGACATTCC; dG = -42.5
81 >s_b_s_b_s_c_s_c_s_d_s_c_s_a_s_e_s
GTGGCTGTGGCTGTGTGGGTGTGGGTGACGGTGTGGGTGTCTGTGCTGGTG; >clamp_s_c_s_d_s_c_s_a_s_e_s:compl:rev
CACCAGCACAGACACCCACACCGTCACCCACACTATCATGTTGACATTCC; dG = -42.5
82 >s_b_s_b_s_d_s_c_s_d_s_c_s_a_s_e_s
GTGGCTGTGGCTGTGACGGTGTGGGTGACGGTGTGGGTGTCTGTGCTGGTG; >clamp_s_c_s_d_s_c_s_a_s_e_s:compl:rev
CACCAGCACAGACACCCACACCGTCACCCACACTATCATGTTGACATTCC; dG = -42.5
83 >s_b_s_b_s_e_s_c_s_d_s_c_s_a_s_e_s
GTGGCTGTGGCTGTGCTGGTGTGGGTGACGGTGTGGGTGTCTGTGCTGGTG; >clamp_s_c_s_d_s_c_s_a_s_e_s:compl:rev
CACCAGCACAGACACCCACACCGTCACCCACACTATCATGTTGACATTCC; dG = -42.5
84 >s_b_s_c_s_c_s_c_s_d_s_c_s_a_s_e_s
GTGGCTGTGTGGGTGTGGGTGTGGGTGACGGTGTGGGTGTCTGTGCTGGTG; >clamp_s_c_s_d_s_c_s_a_s_e_s:compl:rev
CACCAGCACAGACACCCACACCGTCACCCACACTATCATGTTGACATTCC; dG = -42.5
85 >s_b_s_c_s_d_s_c_s_d_s_c_s_a_s_e_s
GTGGCTGTGTGGGTGACGGTGTGGGTGACGGTGTGGGTGTCTGTGCTGGTG; >clamp_s_c_s_d_s_c_s_a_s_e_s:compl:rev
CACCAGCACAGACACCCACACCGTCACCCACACTATCATGTTGACATTCC; dG = -42.5
86 >s_a_s_a_s_d_s_c_s_d_s_c_s_a_s_e_s
GTGTCTGTGTCTGTGACGGTGTGGGTGACGGTGTGGGTGTCTGTGCTGGTG; >clamp_s_c_s_d_s_c_s_a_s_e_s:compl:rev
CACCAGCACAGACACCCACACCGTCACCCACACTATCATGTTGACATTCC; dG = -42.5

```
    87 >s_b_s_c_s_e_s_c_s_d_s_c_s_a_s_e_s
GTGGCTGTGTGGGTGCTGGTGTGGGTGACGGTGTGGGTGTCTGTGCTGGTG;    >clamp_s_c_s_d_s_c_s_a_s_e_s:compl:rev
CACCAGCACAGACACCCACACCGTCACCCACACTATCATGTTGACATTCC; dG = -42.5
    88 >s_b_s_d_s_c_s_c_s_d_s_c_s_a_s_e_s
GTGGCTGTGACGGTGTGGGTGTGGGTGACGGTGTGGGTGTCTGTGCTGGTG;    >clamp_s_c_s_d_s_c_s_a_s_e_s:compl:rev
CACCAGCACAGACACCCACACCGTCACCCACACTATCATGTTGACATTCC; dG = -42.5
    89 >s_b_s_d_s_d_s_c_s_d_s_c_s_a_s_e_s
GTGGCTGTGACGGTGACGGTGTGGGTGACGGTGTGGGTGTCTGTGCTGGTG;    >clamp_s_c_s_d_s_c_s_a_s_e_s:compl:rev
CACCAGCACAGACACCCACACCGTCACCCACACTATCATGTTGACATTCC; dG = -42.5
    90 >s_b_s_d_s_e_s_c_s_d_s_c_s_a_s_e_s
GTGGCTGTGACGGTGCTGGTGTGGGTGACGGTGTGGGTGTCTGTGCTGGTG;    >clamp_s_c_s_d_s_c_s_a_s_e_s:compl:rev
CACCAGCACAGACACCCACACCGTCACCCACACTATCATGTTGACATTCC; dG = -42.5
    91 >s_b_s_e_s_c_s_c_s_d_s_c_s_a_s_e_s
GTGGCTGTGCTGGTGTGGGTGTGGGTGACGGTGTGGGTGTCTGTGCTGGTG;    >clamp_s_c_s_d_s_c_s_a_s_e_s:compl:rev
CACCAGCACAGACACCCACACCGTCACCCACACTATCATGTTGACATTCC; dG = -42.5
    92 >s_b_s_e_s_d_s_c_s_d_s_c_s_a_s_e_s
GTGGCTGTGCTGGTGACGGTGTGGGTGACGGTGTGGGTGTCTGTGCTGGTG;    >clamp_s_c_s_d_s_c_s_a_s_e_s:compl:rev
CACCAGCACAGACACCCACACCGTCACCCACACTATCATGTTGACATTCC; dG = -42.5
    93 >s_a_s_a_s_e_s_c_s_d_s_c_s_a_s_e_s
GTGTCTGTGTCTGTGCTGGTGTGGGTGACGGTGTGGGTGTCTGTGCTGGTG;    >clamp_s_c_s_d_s_c_s_a_s_e_s:compl:rev
CACCAGCACAGACACCCACACCGTCACCCACACTATCATGTTGACATTCC; dG = -42.5
    94 >s_b_s_e_s_e_s_c_s_d_s_c_s_a_s_e_s
GTGGCTGTGCTGGTGCTGGTGTGGGTGACGGTGTGGGTGTCTGTGCTGGTG;    >clamp_s_c_s_d_s_c_s_a_s_e_s:compl:rev
CACCAGCACAGACACCCACACCGTCACCCACACTATCATGTTGACATTCC; dG = -42.5
    95 >s_c_s_a_s_c_s_c_s_d_s_c_s_a_s_e_s
GTGTGGGTGTCTGTGTGGGTGTGGGTGACGGTGTGGGTGTCTGTGCTGGTG;    >clamp_s_c_s_d_s_c_s_a_s_e_s:compl:rev
CACCAGCACAGACACCCACACCGTCACCCACACTATCATGTTGACATTCC; dG = -42.5
    96 >s_c_s_a_s_d_s_c_s_d_s_c_s_a_s_e_s
GTGTGGGTGTCTGTGACGGTGTGGGTGACGGTGTGGGTGTCTGTGCTGGTG;    >clamp_s_c_s_d_s_c_s_a_s_e_s:compl:rev
CACCAGCACAGACACCCACACCGTCACCCACACTATCATGTTGACATTCC; dG = -42.5
    97 >s_c_s_a_s_e_s_c_s_d_s_c_s_a_s_e_s
GTGTGGGTGTCTGTGCTGGTGTGGGTGACGGTGTGGGTGTCTGTGCTGGTG;    >clamp_s_c_s_d_s_c_s_a_s_e_s:compl:rev
CACCAGCACAGACACCCACACCGTCACCCACACTATCATGTTGACATTCC; dG = -42.5
    98 >s_c_s_b_s_c_s_c_s_d_s_c_s_a_s_e_s
GTGTGGGTGGCTGTGTGGGTGTGGGTGACGGTGTGGGTGTCTGTGCTGGTG;    >clamp_s_c_s_d_s_c_s_a_s_e_s:compl:rev
CACCAGCACAGACACCCACACCGTCACCCACACTATCATGTTGACATTCC; dG = -42.5
    99 >s_c_s_b_s_d_s_c_s_d_s_c_s_a_s_e_s
GTGTGGGTGGCTGTGACGGTGTGGGTGACGGTGTGGGTGTCTGTGCTGGTG;    >clamp_s_c_s_d_s_c_s_a_s_e_s:compl:rev
CACCAGCACAGACACCCACACCGTCACCCACACTATCATGTTGACATTCC; dG = -42.5
   100 >s_c_s_b_s_e_s_c_s_d_s_c_s_a_s_e_s
GTGTGGGTGGCTGTGCTGGTGTGGGTGACGGTGTGGGTGTCTGTGCTGGTG;    >clamp_s_c_s_d_s_c_s_a_s_e_s:compl:rev
CACCAGCACAGACACCCACACCGTCACCCACACTATCATGTTGACATTCC; dG = -42.5
   101 >s_c_s_c_s_c_s_c_s_d_s_c_s_a_s_e_s
GTGTGGGTGTGGGTGTGGGTGTGGGTGACGGTGTGGGTGTCTGTGCTGGTG;    >clamp_s_c_s_d_s_c_s_a_s_e_s:compl:rev
CACCAGCACAGACACCCACACCGTCACCCACACTATCATGTTGACATTCC; dG = -42.5
   102 >s_c_s_c_s_d_s_c_s_d_s_c_s_a_s_e_s
GTGTGGGTGTGGGTGACGGTGTGGGTGACGGTGTGGGTGTCTGTGCTGGTG;    >clamp_s_c_s_d_s_c_s_a_s_e_s:compl:rev
CACCAGCACAGACACCCACACCGTCACCCACACTATCATGTTGACATTCC; dG = -42.5
   103 >s_c_s_c_s_e_s_c_s_d_s_c_s_a_s_e_s
GTGTGGGTGTGGGTGCTGGTGTGGGTGACGGTGTGGGTGTCTGTGCTGGTG;    >clamp_s_c_s_d_s_c_s_a_s_e_s:compl:rev
CACCAGCACAGACACCCACACCGTCACCCACACTATCATGTTGACATTCC; dG = -42.5
   104 >s_c_s_d_s_c_s_c_s_d_s_c_s_a_s_e_s
GTGTGGGTGACGGTGTGGGTGTGGGTGACGGTGTGGGTGTCTGTGCTGGTG;    >clamp_s_c_s_d_s_c_s_a_s_e_s:compl:rev
CACCAGCACAGACACCCACACCGTCACCCACACTATCATGTTGACATTCC; dG = -42.5
   105 >s_c_s_d_s_d_s_c_s_d_s_c_s_a_s_e_s
GTGTGGGTGACGGTGACGGTGTGGGTGACGGTGTGGGTGTCTGTGCTGGTG;    >clamp_s_c_s_d_s_c_s_a_s_e_s:compl:rev
CACCAGCACAGACACCCACACCGTCACCCACACTATCATGTTGACATTCC; dG = -42.5
   106 >s_c_s_d_s_e_s_c_s_d_s_c_s_a_s_e_s
GTGTGGGTGACGGTGCTGGTGTGGGTGACGGTGTGGGTGTCTGTGCTGGTG;    >clamp_s_c_s_d_s_c_s_a_s_e_s:compl:rev
CACCAGCACAGACACCCACACCGTCACCCACACTATCATGTTGACATTCC; dG = -42.5
   107 >s_c_s_e_s_c_s_c_s_d_s_c_s_a_s_e_s
GTGTGGGTGCTGGTGTGGGTGTGGGTGACGGTGTGGGTGTCTGTGCTGGTG;    >clamp_s_c_s_d_s_c_s_a_s_e_s:compl:rev
CACCAGCACAGACACCCACACCGTCACCCACACTATCATGTTGACATTCC; dG = -42.5
   108 >s_c_s_e_s_d_s_c_s_d_s_c_s_a_s_e_s
GTGTGGGTGCTGGTGACGGTGTGGGTGACGGTGTGGGTGTCTGTGCTGGTG;    >clamp_s_c_s_d_s_c_s_a_s_e_s:compl:rev
CACCAGCACAGACACCCACACCGTCACCCACACTATCATGTTGACATTCC; dG = -42.5
   109 >s_c_s_e_s_e_s_c_s_d_s_c_s_a_s_e_s
GTGTGGGTGCTGGTGCTGGTGTGGGTGACGGTGTGGGTGTCTGTGCTGGTG;    >clamp_s_c_s_d_s_c_s_a_s_e_s:compl:rev
CACCAGCACAGACACCCACACCGTCACCCACACTATCATGTTGACATTCC; dG = -42.5
   110 >s_d_s_a_s_c_s_c_s_d_s_c_s_a_s_e_s
GTGACGGTGTCTGTGTGGGTGTGGGTGACGGTGTGGGTGTCTGTGCTGGTG;    >clamp_s_c_s_d_s_c_s_a_s_e_s:compl:rev
CACCAGCACAGACACCCACACCGTCACCCACACTATCATGTTGACATTCC; dG = -42.5
```

111 >s_d_s_a_s_d_s_c_s_d_s_c_s_a_s_e_s
GTGACGGTGTCTGTGACGGTGTGGGTGACGGTGTGGGTGTCTGTGCTGGTG;   >clamp_s_c_s_d_s_c_s_a_s_e_s:compl:rev
CACCAGCACAGACACCCACACCGTCACCCACACTATCATGTTGACATTCC; dG = -42.5
112 >s_a_s_b_s_c_s_c_s_d_s_c_s_a_s_e_s
GTGTCTGTGGCTGTGTGGGTGTGGGTGACGGTGTGGGTGTCTGTGCTGGTG;   >clamp_s_c_s_d_s_c_s_a_s_e_s:compl:rev
CACCAGCACAGACACCCACACCGTCACCCACACTATCATGTTGACATTCC; dG = -42.5
113 >s_d_s_a_s_e_s_c_s_d_s_c_s_a_s_e_s
GTGACGGTGTCTGTGCTGGTGTGGGTGACGGTGTGGGTGTCTGTGCTGGTG;   >clamp_s_c_s_d_s_c_s_a_s_e_s:compl:rev
CACCAGCACAGACACCCACACCGTCACCCACACTATCATGTTGACATTCC; dG = -42.5
114 >s_d_s_b_s_c_s_c_s_d_s_c_s_a_s_e_s
GTGACGGTGGCTGTGTGGGTGTGGGTGACGGTGTGGGTGTCTGTGCTGGTG;   >clamp_s_c_s_d_s_c_s_a_s_e_s:compl:rev
CACCAGCACAGACACCCACACCGTCACCCACACTATCATGTTGACATTCC; dG = -42.5
115 >s_d_s_b_s_d_s_c_s_d_s_c_s_a_s_e_s
GTGACGGTGGCTGTGACGGTGTGGGTGACGGTGTGGGTGTCTGTGCTGGTG;   >clamp_s_c_s_d_s_c_s_a_s_e_s:compl:rev
CACCAGCACAGACACCCACACCGTCACCCACACTATCATGTTGACATTCC; dG = -42.5
116 >s_d_s_b_s_e_s_c_s_d_s_c_s_a_s_e_s
GTGACGGTGGCTGTGCTGGTGTGGGTGACGGTGTGGGTGTCTGTGCTGGTG;   >clamp_s_c_s_d_s_c_s_a_s_e_s:compl:rev
CACCAGCACAGACACCCACACCGTCACCCACACTATCATGTTGACATTCC; dG = -42.5
117 >s_d_s_c_s_c_s_c_s_d_s_c_s_a_s_e_s
GTGACGGTGTGGGTGTGGGTGTGGGTGACGGTGTGGGTGTCTGTGCTGGTG;   >clamp_s_c_s_d_s_c_s_a_s_e_s:compl:rev
CACCAGCACAGACACCCACACCGTCACCCACACTATCATGTTGACATTCC; dG = -42.5
118 >s_d_s_c_s_d_s_c_s_d_s_c_s_a_s_e_s
GTGACGGTGTGGGTGACGGTGTGGGTGACGGTGTGGGTGTCTGTGCTGGTG;   >clamp_s_c_s_d_s_c_s_a_s_e_s:compl:rev
CACCAGCACAGACACCCACACCGTCACCCACACTATCATGTTGACATTCC; dG = -42.5
119 >s_a_s_b_s_d_s_c_s_d_s_c_s_a_s_e_s
GTGTCTGTGGCTGTGACGGTGTGGGTGACGGTGTGGGTGTCTGTGCTGGTG;   >clamp_s_c_s_d_s_c_s_a_s_e_s:compl:rev
CACCAGCACAGACACCCACACCGTCACCCACACTATCATGTTGACATTCC; dG = -42.5
120 >s_d_s_c_s_e_s_c_s_d_s_c_s_a_s_e_s
GTGACGGTGTGGGTGCTGGTGTGGGTGACGGTGTGGGTGTCTGTGCTGGTG;   >clamp_s_c_s_d_s_c_s_a_s_e_s:compl:rev
CACCAGCACAGACACCCACACCGTCACCCACACTATCATGTTGACATTCC; dG = -42.5
121 >s_d_s_d_s_c_s_c_s_d_s_c_s_a_s_e_s
GTGACGGTGACGGTGTGGGTGTGGGTGACGGTGTGGGTGTCTGTGCTGGTG;   >clamp_s_c_s_d_s_c_s_a_s_e_s:compl:rev
CACCAGCACAGACACCCACACCGTCACCCACACTATCATGTTGACATTCC; dG = -42.5
122 >s_d_s_d_s_d_s_c_s_d_s_c_s_a_s_e_s
GTGACGGTGACGGTGACGGTGTGGGTGACGGTGTGGGTGTCTGTGCTGGTG;   >clamp_s_c_s_d_s_c_s_a_s_e_s:compl:rev
CACCAGCACAGACACCCACACCGTCACCCACACTATCATGTTGACATTCC; dG = -42.5
123 >s_d_s_d_s_e_s_c_s_d_s_c_s_a_s_e_s
GTGACGGTGACGGTGCTGGTGTGGGTGACGGTGTGGGTGTCTGTGCTGGTG;   >clamp_s_c_s_d_s_c_s_a_s_e_s:compl:rev
CACCAGCACAGACACCCACACCGTCACCCACACTATCATGTTGACATTCC; dG = -42.5
124 >s_d_s_e_s_c_s_c_s_d_s_c_s_a_s_e_s
GTGACGGTGCTGGTGTGGGTGTGGGTGACGGTGTGGGTGTCTGTGCTGGTG;   >clamp_s_c_s_d_s_c_s_a_s_e_s:compl:rev
CACCAGCACAGACACCCACACCGTCACCCACACTATCATGTTGACATTCC; dG = -42.5
125 >s_d_s_e_s_d_s_c_s_d_s_c_s_a_s_e_s
GTGACGGTGCTGGTGACGGTGTGGGTGACGGTGTGGGTGTCTGTGCTGGTG;   >clamp_s_c_s_d_s_c_s_a_s_e_s:compl:rev
CACCAGCACAGACACCCACACCGTCACCCACACTATCATGTTGACATTCC; dG = -42.5

****

CDCAE Mismatches

1 >s_c_s_a_s_a_s_c_s_d_s_c_s_b_s_e_s
GTGTGGGTGTCTGTGTCTGTGTGGGTGACGGTGTGGGTGGCTGTGCTGGTG;   >clamp_s_c_s_d_s_c_s_a_s_e_s:compl:rev
CACCAGCACAGACACCCACACCGTCACCCACACTATCATGTTGACATTCC; dG = -40.6
2 >s_c_s_a_s_b_s_c_s_d_s_c_s_b_s_e_s
GTGTGGGTGTCTGTGGCTGTGTGGGTGACGGTGTGGGTGGCTGTGCTGGTG;   >clamp_s_c_s_d_s_c_s_a_s_e_s:compl:rev
CACCAGCACAGACACCCACACCGTCACCCACACTATCATGTTGACATTCC; dG = -40.6
3 >s_c_s_a_s_c_s_c_s_d_s_c_s_b_s_e_s
GTGTGGGTGTCTGTGTGGGTGTGGGTGACGGTGTGGGTGGCTGTGCTGGTG;   >clamp_s_c_s_d_s_c_s_a_s_e_s:compl:rev
CACCAGCACAGACACCCACACCGTCACCCACACTATCATGTTGACATTCC; dG = -40.6
4 >s_c_s_a_s_d_s_c_s_d_s_c_s_b_s_e_s
GTGTGGGTGTCTGTGACGGTGTGGGTGACGGTGTGGGTGGCTGTGCTGGTG;   >clamp_s_c_s_d_s_c_s_a_s_e_s:compl:rev
CACCAGCACAGACACCCACACCGTCACCCACACTATCATGTTGACATTCC; dG = -40.6
5 >s_a_s_b_s_a_s_c_s_d_s_c_s_b_s_e_s
GTGTCTGTGGCTGTGTCTGTGTGGGTGACGGTGTGGGTGGCTGTGCTGGTG;   >clamp_s_c_s_d_s_c_s_a_s_e_s:compl:rev
CACCAGCACAGACACCCACACCGTCACCCACACTATCATGTTGACATTCC; dG = -40.6
6 >s_c_s_a_s_e_s_c_s_d_s_c_s_b_s_e_s
GTGTGGGTGTCTGTGCTGGTGTGGGTGACGGTGTGGGTGGCTGTGCTGGTG;   >clamp_s_c_s_d_s_c_s_a_s_e_s:compl:rev
CACCAGCACAGACACCCACACCGTCACCCACACTATCATGTTGACATTCC; dG = -40.6
7 >s_c_s_b_s_a_s_c_s_d_s_c_s_b_s_e_s
GTGTGGGTGGCTGTGTCTGTGTGGGTGACGGTGTGGGTGGCTGTGCTGGTG;   >clamp_s_c_s_d_s_c_s_a_s_e_s:compl:rev
CACCAGCACAGACACCCACACCGTCACCCACACTATCATGTTGACATTCC; dG = -40.6

```
     8  >s_c_s_b_s_b_s_c_s_d_s_c_s_b_s_e_s
GTGTGGGTGGCTGTGGCTGTGTGGGTGACGGTGTGGGTGGCTGTGCTGGTG;    >clamp_s_c_s_d_s_c_s_a_s_e_s:compl:rev
CACCAGCACAGACACCCACACCGTCACCCACACTATCATGTTGACATTCC; dG = -40.6
     9  >s_c_s_b_s_c_s_c_s_d_s_c_s_b_s_e_s
GTGTGGGTGGCTGTGTGGGTGTGGGTGACGGTGTGGGTGGCTGTGCTGGTG;    >clamp_s_c_s_d_s_c_s_a_s_e_s:compl:rev
CACCAGCACAGACACCCACACCGTCACCCACACTATCATGTTGACATTCC; dG = -40.6
    10  >s_c_s_b_s_d_s_c_s_d_s_c_s_b_s_e_s
GTGTGGGTGGCTGTGACGGTGTGGGTGACGGTGTGGGTGGCTGTGCTGGTG;    >clamp_s_c_s_d_s_c_s_a_s_e_s:compl:rev
CACCAGCACAGACACCCACACCGTCACCCACACTATCATGTTGACATTCC; dG = -40.6
    11  >s_c_s_b_s_e_s_c_s_d_s_c_s_b_s_e_s
GTGTGGGTGGCTGTGCTGGTGTGGGTGACGGTGTGGGTGGCTGTGCTGGTG;    >clamp_s_c_s_d_s_c_s_a_s_e_s:compl:rev
CACCAGCACAGACACCCACACCGTCACCCACACTATCATGTTGACATTCC; dG = -40.6
    12  >s_c_s_c_s_a_s_c_s_d_s_c_s_b_s_e_s
GTGTGGGTGTGGGTGTCTGTGTGGGTGACGGTGTGGGTGGCTGTGCTGGTG;    >clamp_s_c_s_d_s_c_s_a_s_e_s:compl:rev
CACCAGCACAGACACCCACACCGTCACCCACACTATCATGTTGACATTCC; dG = -40.6
    13  >s_c_s_c_s_b_s_c_s_d_s_c_s_b_s_e_s
GTGTGGGTGTGGGTGGCTGTGTGGGTGACGGTGTGGGTGGCTGTGCTGGTG;    >clamp_s_c_s_d_s_c_s_a_s_e_s:compl:rev
CACCAGCACAGACACCCACACCGTCACCCACACTATCATGTTGACATTCC; dG = -40.6
    14  >s_c_s_c_s_c_s_c_s_d_s_c_s_b_s_e_s
GTGTGGGTGTGGGTGTGGGTGTGGGTGACGGTGTGGGTGGCTGTGCTGGTG;    >clamp_s_c_s_d_s_c_s_a_s_e_s:compl:rev
CACCAGCACAGACACCCACACCGTCACCCACACTATCATGTTGACATTCC; dG = -40.6
    15  >s_c_s_c_s_d_s_c_s_d_s_c_s_b_s_e_s
GTGTGGGTGTGGGTGACGGTGTGGGTGACGGTGTGGGTGGCTGTGCTGGTG;    >clamp_s_c_s_d_s_c_s_a_s_e_s:compl:rev
CACCAGCACAGACACCCACACCGTCACCCACACTATCATGTTGACATTCC; dG = -40.6
    16  >s_a_s_b_s_b_s_c_s_d_s_c_s_b_s_e_s
GTGTCTGTGGCTGTGGCTGTGTGGGTGACGGTGTGGGTGGCTGTGCTGGTG;    >clamp_s_c_s_d_s_c_s_a_s_e_s:compl:rev
CACCAGCACAGACACCCACACCGTCACCCACACTATCATGTTGACATTCC; dG = -40.6
    17  >s_c_s_c_s_e_s_c_s_d_s_c_s_b_s_e_s
GTGTGGGTGTGGGTGCTGGTGTGGGTGACGGTGTGGGTGGCTGTGCTGGTG;    >clamp_s_c_s_d_s_c_s_a_s_e_s:compl:rev
CACCAGCACAGACACCCACACCGTCACCCACACTATCATGTTGACATTCC; dG = -40.6
    18  >s_c_s_d_s_a_s_c_s_d_s_c_s_b_s_e_s
GTGTGGGTGACGGTGTCTGTGTGGGTGACGGTGTGGGTGGCTGTGCTGGTG;    >clamp_s_c_s_d_s_c_s_a_s_e_s:compl:rev
CACCAGCACAGACACCCACACCGTCACCCACACTATCATGTTGACATTCC; dG = -40.6
    19  >s_c_s_d_s_b_s_c_s_d_s_c_s_b_s_e_s
GTGTGGGTGACGGTGGCTGTGTGGGTGACGGTGTGGGTGGCTGTGCTGGTG;    >clamp_s_c_s_d_s_c_s_a_s_e_s:compl:rev
CACCAGCACAGACACCCACACCGTCACCCACACTATCATGTTGACATTCC; dG = -40.6
    20  >s_c_s_d_s_c_s_c_s_d_s_c_s_b_s_e_s
GTGTGGGTGACGGTGTGGGTGTGGGTGACGGTGTGGGTGGCTGTGCTGGTG;    >clamp_s_c_s_d_s_c_s_a_s_e_s:compl:rev
CACCAGCACAGACACCCACACCGTCACCCACACTATCATGTTGACATTCC; dG = -40.6
    21  >s_c_s_d_s_d_s_c_s_d_s_c_s_b_s_e_s
GTGTGGGTGACGGTGACGGTGTGGGTGACGGTGTGGGTGGCTGTGCTGGTG;    >clamp_s_c_s_d_s_c_s_a_s_e_s:compl:rev
CACCAGCACAGACACCCACACCGTCACCCACACTATCATGTTGACATTCC; dG = -40.6
    22  >s_c_s_d_s_e_s_c_s_d_s_c_s_b_s_e_s
GTGTGGGTGACGGTGCTGGTGTGGGTGACGGTGTGGGTGGCTGTGCTGGTG;    >clamp_s_c_s_d_s_c_s_a_s_e_s:compl:rev
CACCAGCACAGACACCCACACCGTCACCCACACTATCATGTTGACATTCC; dG = -40.6
    23  >s_c_s_e_s_a_s_c_s_d_s_c_s_b_s_e_s
GTGTGGGTGCTGGTGTCTGTGTGGGTGACGGTGTGGGTGGCTGTGCTGGTG;    >clamp_s_c_s_d_s_c_s_a_s_e_s:compl:rev
CACCAGCACAGACACCCACACCGTCACCCACACTATCATGTTGACATTCC; dG = -40.6
    24  >s_c_s_e_s_b_s_c_s_d_s_c_s_b_s_e_s
GTGTGGGTGCTGGTGGCTGTGTGGGTGACGGTGTGGGTGGCTGTGCTGGTG;    >clamp_s_c_s_d_s_c_s_a_s_e_s:compl:rev
CACCAGCACAGACACCCACACCGTCACCCACACTATCATGTTGACATTCC; dG = -40.6
    25  >s_c_s_e_s_c_s_c_s_d_s_c_s_b_s_e_s
GTGTGGGTGCTGGTGTGGGTGTGGGTGACGGTGTGGGTGGCTGTGCTGGTG;    >clamp_s_c_s_d_s_c_s_a_s_e_s:compl:rev
CACCAGCACAGACACCCACACCGTCACCCACACTATCATGTTGACATTCC; dG = -40.6
    26  >s_c_s_e_s_d_s_c_s_d_s_c_s_b_s_e_s
GTGTGGGTGCTGGTGACGGTGTGGGTGACGGTGTGGGTGGCTGTGCTGGTG;    >clamp_s_c_s_d_s_c_s_a_s_e_s:compl:rev
CACCAGCACAGACACCCACACCGTCACCCACACTATCATGTTGACATTCC; dG = -40.6
    27  >s_a_s_b_s_c_s_c_s_d_s_c_s_b_s_e_s
GTGTCTGTGGCTGTGTGGGTGTGGGTGACGGTGTGGGTGGCTGTGCTGGTG;    >clamp_s_c_s_d_s_c_s_a_s_e_s:compl:rev
CACCAGCACAGACACCCACACCGTCACCCACACTATCATGTTGACATTCC; dG = -40.6
    28  >s_c_s_e_s_e_s_c_s_d_s_c_s_b_s_e_s
GTGTGGGTGCTGGTGCTGGTGTGGGTGACGGTGTGGGTGGCTGTGCTGGTG;    >clamp_s_c_s_d_s_c_s_a_s_e_s:compl:rev
CACCAGCACAGACACCCACACCGTCACCCACACTATCATGTTGACATTCC; dG = -40.6
    29  >s_d_s_a_s_a_s_c_s_d_s_c_s_b_s_e_s
GTGACGGTGTCTGTGTCTGTGTGGGTGACGGTGTGGGTGGCTGTGCTGGTG;    >clamp_s_c_s_d_s_c_s_a_s_e_s:compl:rev
CACCAGCACAGACACCCACACCGTCACCCACACTATCATGTTGACATTCC; dG = -40.6
    30  >s_d_s_a_s_b_s_c_s_d_s_c_s_b_s_e_s
GTGACGGTGTCTGTGGCTGTGTGGGTGACGGTGTGGGTGGCTGTGCTGGTG;    >clamp_s_c_s_d_s_c_s_a_s_e_s:compl:rev
CACCAGCACAGACACCCACACCGTCACCCACACTATCATGTTGACATTCC; dG = -40.6
    31  >s_d_s_a_s_c_s_c_s_d_s_c_s_b_s_e_s
GTGACGGTGTCTGTGTGGGTGTGGGTGACGGTGTGGGTGGCTGTGCTGGTG;    >clamp_s_c_s_d_s_c_s_a_s_e_s:compl:rev
CACCAGCACAGACACCCACACCGTCACCCACACTATCATGTTGACATTCC; dG = -40.6
```

32 >s_d_s_a_s_d_s_c_s_d_s_c_s_b_s_e_s
GTGACGGTGTCTGTGACGGTGTGGGTGACGGTGTGGGTGGCTGTGCTGGTG; >clamp_s_c_s_d_s_c_s_a_s_e_s:compl:rev
CACCAGCACAGACACCCACACCGTCACCCACACTATCATGTTGACATTCC; dG = -40.6
33 >s_d_s_a_s_e_s_c_s_d_s_c_s_b_s_e_s
GTGACGGTGTCTGTGCTGGTGTGGGTGACGGTGTGGGTGGCTGTGCTGGTG; >clamp_s_c_s_d_s_c_s_a_s_e_s:compl:rev
CACCAGCACAGACACCCACACCGTCACCCACACTATCATGTTGACATTCC; dG = -40.6
34 >s_d_s_b_s_a_s_c_s_d_s_c_s_b_s_e_s
GTGACGGTGGCTGTGTCTGTGTGGGTGACGGTGTGGGTGGCTGTGCTGGTG; >clamp_s_c_s_d_s_c_s_a_s_e_s:compl:rev
CACCAGCACAGACACCCACACCGTCACCCACACTATCATGTTGACATTCC; dG = -40.6
35 >s_d_s_b_s_b_s_c_s_d_s_c_s_b_s_e_s
GTGACGGTGGCTGTGGCTGTGTGGGTGACGGTGTGGGTGGCTGTGCTGGTG; >clamp_s_c_s_d_s_c_s_a_s_e_s:compl:rev
CACCAGCACAGACACCCACACCGTCACCCACACTATCATGTTGACATTCC; dG = -40.6
36 >s_d_s_b_s_c_s_c_s_d_s_c_s_b_s_e_s
GTGACGGTGGCTGTGTGGGTGTGGGTGACGGTGTGGGTGGCTGTGCTGGTG; >clamp_s_c_s_d_s_c_s_a_s_e_s:compl:rev
CACCAGCACAGACACCCACACCGTCACCCACACTATCATGTTGACATTCC; dG = -40.6
37 >s_d_s_b_s_d_s_c_s_d_s_c_s_b_s_e_s
GTGACGGTGGCTGTGACGGTGTGGGTGACGGTGTGGGTGGCTGTGCTGGTG; >clamp_s_c_s_d_s_c_s_a_s_e_s:compl:rev
CACCAGCACAGACACCCACACCGTCACCCACACTATCATGTTGACATTCC; dG = -40.6
38 >s_a_s_b_s_d_s_c_s_d_s_c_s_b_s_e_s
GTGTCTGTGGCTGTGACGGTGTGGGTGACGGTGTGGGTGGCTGTGCTGGTG; >clamp_s_c_s_d_s_c_s_a_s_e_s:compl:rev
CACCAGCACAGACACCCACACCGTCACCCACACTATCATGTTGACATTCC; dG = -40.6
39 >s_d_s_b_s_e_s_c_s_d_s_c_s_b_s_e_s
GTGACGGTGGCTGTGCTGGTGTGGGTGACGGTGTGGGTGGCTGTGCTGGTG; >clamp_s_c_s_d_s_c_s_a_s_e_s:compl:rev
CACCAGCACAGACACCCACACCGTCACCCACACTATCATGTTGACATTCC; dG = -40.6
40 >s_d_s_c_s_a_s_c_s_d_s_c_s_b_s_e_s
GTGACGGTGTGGGTGTCTGTGTGGGTGACGGTGTGGGTGGCTGTGCTGGTG; >clamp_s_c_s_d_s_c_s_a_s_e_s:compl:rev
CACCAGCACAGACACCCACACCGTCACCCACACTATCATGTTGACATTCC; dG = -40.6
41 >s_d_s_c_s_b_s_c_s_d_s_c_s_b_s_e_s
GTGACGGTGTGGGTGGCTGTGTGGGTGACGGTGTGGGTGGCTGTGCTGGTG; >clamp_s_c_s_d_s_c_s_a_s_e_s:compl:rev
CACCAGCACAGACACCCACACCGTCACCCACACTATCATGTTGACATTCC; dG = -40.6
42 >s_d_s_c_s_c_s_c_s_d_s_c_s_b_s_e_s
GTGACGGTGTGGGTGTGGGTGTGGGTGACGGTGTGGGTGGCTGTGCTGGTG; >clamp_s_c_s_d_s_c_s_a_s_e_s:compl:rev
CACCAGCACAGACACCCACACCGTCACCCACACTATCATGTTGACATTCC; dG = -40.6
43 >s_d_s_c_s_d_s_c_s_d_s_c_s_b_s_e_s
GTGACGGTGTGGGTGACGGTGTGGGTGACGGTGTGGGTGGCTGTGCTGGTG; >clamp_s_c_s_d_s_c_s_a_s_e_s:compl:rev
CACCAGCACAGACACCCACACCGTCACCCACACTATCATGTTGACATTCC; dG = -40.6
44 >s_d_s_c_s_e_s_c_s_d_s_c_s_b_s_e_s
GTGACGGTGTGGGTGCTGGTGTGGGTGACGGTGTGGGTGGCTGTGCTGGTG; >clamp_s_c_s_d_s_c_s_a_s_e_s:compl:rev
CACCAGCACAGACACCCACACCGTCACCCACACTATCATGTTGACATTCC; dG = -40.6
45 >s_d_s_d_s_a_s_c_s_d_s_c_s_b_s_e_s
GTGACGGTGACGGTGTCTGTGTGGGTGACGGTGTGGGTGGCTGTGCTGGTG; >clamp_s_c_s_d_s_c_s_a_s_e_s:compl:rev
CACCAGCACAGACACCCACACCGTCACCCACACTATCATGTTGACATTCC; dG = -40.6
46 >s_d_s_d_s_b_s_c_s_d_s_c_s_b_s_e_s
GTGACGGTGACGGTGGCTGTGTGGGTGACGGTGTGGGTGGCTGTGCTGGTG; >clamp_s_c_s_d_s_c_s_a_s_e_s:compl:rev
CACCAGCACAGACACCCACACCGTCACCCACACTATCATGTTGACATTCC; dG = -40.6
47 >s_d_s_d_s_c_s_c_s_d_s_c_s_b_s_e_s
GTGACGGTGACGGTGTGGGTGTGGGTGACGGTGTGGGTGGCTGTGCTGGTG; >clamp_s_c_s_d_s_c_s_a_s_e_s:compl:rev
CACCAGCACAGACACCCACACCGTCACCCACACTATCATGTTGACATTCC; dG = -40.6
48 >s_d_s_d_s_d_s_c_s_d_s_c_s_b_s_e_s
GTGACGGTGACGGTGACGGTGTGGGTGACGGTGTGGGTGGCTGTGCTGGTG; >clamp_s_c_s_d_s_c_s_a_s_e_s:compl:rev
CACCAGCACAGACACCCACACCGTCACCCACACTATCATGTTGACATTCC; dG = -40.6
49 >s_a_s_b_s_e_s_c_s_d_s_c_s_b_s_e_s
GTGTCTGTGGCTGTGCTGGTGTGGGTGACGGTGTGGGTGGCTGTGCTGGTG; >clamp_s_c_s_d_s_c_s_a_s_e_s:compl:rev
CACCAGCACAGACACCCACACCGTCACCCACACTATCATGTTGACATTCC; dG = -40.6
50 >s_d_s_d_s_e_s_c_s_d_s_c_s_b_s_e_s
GTGACGGTGACGGTGCTGGTGTGGGTGACGGTGTGGGTGGCTGTGCTGGTG; >clamp_s_c_s_d_s_c_s_a_s_e_s:compl:rev
CACCAGCACAGACACCCACACCGTCACCCACACTATCATGTTGACATTCC; dG = -40.6
51 >s_d_s_e_s_a_s_c_s_d_s_c_s_b_s_e_s
GTGACGGTGCTGGTGTCTGTGTGGGTGACGGTGTGGGTGGCTGTGCTGGTG; >clamp_s_c_s_d_s_c_s_a_s_e_s:compl:rev
CACCAGCACAGACACCCACACCGTCACCCACACTATCATGTTGACATTCC; dG = -40.6
52 >s_d_s_e_s_b_s_c_s_d_s_c_s_b_s_e_s
GTGACGGTGCTGGTGGCTGTGTGGGTGACGGTGTGGGTGGCTGTGCTGGTG; >clamp_s_c_s_d_s_c_s_a_s_e_s:compl:rev
CACCAGCACAGACACCCACACCGTCACCCACACTATCATGTTGACATTCC; dG = -40.6
53 >s_d_s_e_s_c_s_c_s_d_s_c_s_b_s_e_s
GTGACGGTGCTGGTGTGGGTGTGGGTGACGGTGTGGGTGGCTGTGCTGGTG; >clamp_s_c_s_d_s_c_s_a_s_e_s:compl:rev
CACCAGCACAGACACCCACACCGTCACCCACACTATCATGTTGACATTCC; dG = -40.6
54 >s_d_s_e_s_d_s_c_s_d_s_c_s_b_s_e_s
GTGACGGTGCTGGTGACGGTGTGGGTGACGGTGTGGGTGGCTGTGCTGGTG; >clamp_s_c_s_d_s_c_s_a_s_e_s:compl:rev
CACCAGCACAGACACCCACACCGTCACCCACACTATCATGTTGACATTCC; dG = -40.6
55 >s_d_s_e_s_e_s_c_s_d_s_c_s_b_s_e_s
GTGACGGTGCTGGTGCTGGTGTGGGTGACGGTGTGGGTGGCTGTGCTGGTG; >clamp_s_c_s_d_s_c_s_a_s_e_s:compl:rev
CACCAGCACAGACACCCACACCGTCACCCACACTATCATGTTGACATTCC; dG = -40.6

56 >s_e_s_a_s_a_s_c_s_d_s_c_s_b_s_e_s
GTGCTGGTGTCTGTGTCTGTGTGGGTGACGGTGTGGGTGGCTGTGCTGGTG; >clamp_s_c_s_d_s_c_s_a_s_e_s:compl:rev
CACCAGCACAGACACCCACACCGTCACCCACACTATCATGTTGACATTCC; dG = -40.6
57 >s_e_s_a_s_b_s_c_s_d_s_c_s_b_s_e_s
GTGCTGGTGTCTGTGGCTGTGTGGGTGACGGTGTGGGTGGCTGTGCTGGTG; >clamp_s_c_s_d_s_c_s_a_s_e_s:compl:rev
CACCAGCACAGACACCCACACCGTCACCCACACTATCATGTTGACATTCC; dG = -40.6
58 >s_e_s_a_s_c_s_c_s_d_s_c_s_b_s_e_s
GTGCTGGTGTCTGTGTGGGTGTGGGTGACGGTGTGGGTGGCTGTGCTGGTG; >clamp_s_c_s_d_s_c_s_a_s_e_s:compl:rev
CACCAGCACAGACACCCACACCGTCACCCACACTATCATGTTGACATTCC; dG = -40.6
59 >s_e_s_a_s_d_s_c_s_d_s_c_s_b_s_e_s
GTGCTGGTGTCTGTGACGGTGTGGGTGACGGTGTGGGTGGCTGTGCTGGTG; >clamp_s_c_s_d_s_c_s_a_s_e_s:compl:rev
CACCAGCACAGACACCCACACCGTCACCCACACTATCATGTTGACATTCC; dG = -40.6
60 >s_a_s_c_s_a_s_c_s_d_s_c_s_b_s_e_s
GTGTCTGTGTGGGTGTCTGTGTGGGTGACGGTGTGGGTGGCTGTGCTGGTG; >clamp_s_c_s_d_s_c_s_a_s_e_s:compl:rev
CACCAGCACAGACACCCACACCGTCACCCACACTATCATGTTGACATTCC; dG = -40.6
61 >s_e_s_a_s_e_s_c_s_d_s_c_s_b_s_e_s
GTGCTGGTGTCTGTGCTGGTGTGGGTGACGGTGTGGGTGGCTGTGCTGGTG; >clamp_s_c_s_d_s_c_s_a_s_e_s:compl:rev
CACCAGCACAGACACCCACACCGTCACCCACACTATCATGTTGACATTCC; dG = -40.6
62 >s_e_s_b_s_a_s_c_s_d_s_c_s_b_s_e_s
GTGCTGGTGGCTGTGTCTGTGTGGGTGACGGTGTGGGTGGCTGTGCTGGTG; >clamp_s_c_s_d_s_c_s_a_s_e_s:compl:rev
CACCAGCACAGACACCCACACCGTCACCCACACTATCATGTTGACATTCC; dG = -40.6
63 >s_e_s_b_s_b_s_c_s_d_s_c_s_b_s_e_s
GTGCTGGTGGCTGTGGCTGTGTGGGTGACGGTGTGGGTGGCTGTGCTGGTG; >clamp_s_c_s_d_s_c_s_a_s_e_s:compl:rev
CACCAGCACAGACACCCACACCGTCACCCACACTATCATGTTGACATTCC; dG = -40.6
64 >s_e_s_b_s_c_s_c_s_d_s_c_s_b_s_e_s
GTGCTGGTGGCTGTGTGGGTGTGGGTGACGGTGTGGGTGGCTGTGCTGGTG; >clamp_s_c_s_d_s_c_s_a_s_e_s:compl:rev
CACCAGCACAGACACCCACACCGTCACCCACACTATCATGTTGACATTCC; dG = -40.6
65 >s_e_s_b_s_d_s_c_s_d_s_c_s_b_s_e_s
GTGCTGGTGGCTGTGACGGTGTGGGTGACGGTGTGGGTGGCTGTGCTGGTG; >clamp_s_c_s_d_s_c_s_a_s_e_s:compl:rev
CACCAGCACAGACACCCACACCGTCACCCACACTATCATGTTGACATTCC; dG = -40.6
66 >s_e_s_b_s_e_s_c_s_d_s_c_s_b_s_e_s
GTGCTGGTGGCTGTGCTGGTGTGGGTGACGGTGTGGGTGGCTGTGCTGGTG; >clamp_s_c_s_d_s_c_s_a_s_e_s:compl:rev
CACCAGCACAGACACCCACACCGTCACCCACACTATCATGTTGACATTCC; dG = -40.6
67 >s_e_s_c_s_a_s_c_s_d_s_c_s_b_s_e_s
GTGCTGGTGTGGGTGTCTGTGTGGGTGACGGTGTGGGTGGCTGTGCTGGTG; >clamp_s_c_s_d_s_c_s_a_s_e_s:compl:rev
CACCAGCACAGACACCCACACCGTCACCCACACTATCATGTTGACATTCC; dG = -40.6
68 >s_e_s_c_s_b_s_c_s_d_s_c_s_b_s_e_s
GTGCTGGTGTGGGTGGCTGTGTGGGTGACGGTGTGGGTGGCTGTGCTGGTG; >clamp_s_c_s_d_s_c_s_a_s_e_s:compl:rev
CACCAGCACAGACACCCACACCGTCACCCACACTATCATGTTGACATTCC; dG = -40.6
69 >s_e_s_c_s_c_s_c_s_d_s_c_s_b_s_e_s
GTGCTGGTGTGGGTGTGGGTGTGGGTGACGGTGTGGGTGGCTGTGCTGGTG; >clamp_s_c_s_d_s_c_s_a_s_e_s:compl:rev
CACCAGCACAGACACCCACACCGTCACCCACACTATCATGTTGACATTCC; dG = -40.6
70 >s_e_s_c_s_d_s_c_s_d_s_c_s_b_s_e_s
GTGCTGGTGTGGGTGACGGTGTGGGTGACGGTGTGGGTGGCTGTGCTGGTG; >clamp_s_c_s_d_s_c_s_a_s_e_s:compl:rev
CACCAGCACAGACACCCACACCGTCACCCACACTATCATGTTGACATTCC; dG = -40.6
71 >s_a_s_c_s_b_s_c_s_d_s_c_s_b_s_e_s
GTGTCTGTGTGGGTGGCTGTGTGGGTGACGGTGTGGGTGGCTGTGCTGGTG; >clamp_s_c_s_d_s_c_s_a_s_e_s:compl:rev
CACCAGCACAGACACCCACACCGTCACCCACACTATCATGTTGACATTCC; dG = -40.6
72 >s_e_s_c_s_e_s_c_s_d_s_c_s_b_s_e_s
GTGCTGGTGTGGGTGCTGGTGTGGGTGACGGTGTGGGTGGCTGTGCTGGTG; >clamp_s_c_s_d_s_c_s_a_s_e_s:compl:rev
CACCAGCACAGACACCCACACCGTCACCCACACTATCATGTTGACATTCC; dG = -40.6
73 >s_e_s_d_s_a_s_c_s_d_s_c_s_b_s_e_s
GTGCTGGTGACGGTGTCTGTGTGGGTGACGGTGTGGGTGGCTGTGCTGGTG; >clamp_s_c_s_d_s_c_s_a_s_e_s:compl:rev
CACCAGCACAGACACCCACACCGTCACCCACACTATCATGTTGACATTCC; dG = -40.6
74 >s_e_s_d_s_b_s_c_s_d_s_c_s_b_s_e_s
GTGCTGGTGACGGTGGCTGTGTGGGTGACGGTGTGGGTGGCTGTGCTGGTG; >clamp_s_c_s_d_s_c_s_a_s_e_s:compl:rev
CACCAGCACAGACACCCACACCGTCACCCACACTATCATGTTGACATTCC; dG = -40.6
75 >s_e_s_d_s_c_s_c_s_d_s_c_s_b_s_e_s
GTGCTGGTGACGGTGTGGGTGTGGGTGACGGTGTGGGTGGCTGTGCTGGTG; >clamp_s_c_s_d_s_c_s_a_s_e_s:compl:rev
CACCAGCACAGACACCCACACCGTCACCCACACTATCATGTTGACATTCC; dG = -40.6
76 >s_e_s_d_s_d_s_c_s_d_s_c_s_b_s_e_s
GTGCTGGTGACGGTGACGGTGTGGGTGACGGTGTGGGTGGCTGTGCTGGTG; >clamp_s_c_s_d_s_c_s_a_s_e_s:compl:rev
CACCAGCACAGACACCCACACCGTCACCCACACTATCATGTTGACATTCC; dG = -40.6
77 >s_e_s_d_s_e_s_c_s_d_s_c_s_b_s_e_s
GTGCTGGTGACGGTGCTGGTGTGGGTGACGGTGTGGGTGGCTGTGCTGGTG; >clamp_s_c_s_d_s_c_s_a_s_e_s:compl:rev
CACCAGCACAGACACCCACACCGTCACCCACACTATCATGTTGACATTCC; dG = -40.6
78 >s_e_s_e_s_a_s_c_s_d_s_c_s_b_s_e_s
GTGCTGGTGCTGGTGTCTGTGTGGGTGACGGTGTGGGTGGCTGTGCTGGTG; >clamp_s_c_s_d_s_c_s_a_s_e_s:compl:rev
CACCAGCACAGACACCCACACCGTCACCCACACTATCATGTTGACATTCC; dG = -40.6
79 >s_e_s_e_s_b_s_c_s_d_s_c_s_b_s_e_s
GTGCTGGTGCTGGTGGCTGTGTGGGTGACGGTGTGGGTGGCTGTGCTGGTG; >clamp_s_c_s_d_s_c_s_a_s_e_s:compl:rev
CACCAGCACAGACACCCACACCGTCACCCACACTATCATGTTGACATTCC; dG = -40.6

80 >s_e_s_e_s_c_s_c_s_d_s_c_s_b_s_e_s
GTGCTGGTGCTGGTGTGGGTGTGGGTGACGGTGTGGGTGGCTGTGCTGGTG; >clamp_s_c_s_d_s_c_s_a_s_e_s:compl:rev
CACCAGCACAGACACCCACACCGTCACCCACACTATCATGTTGACATTCC; dG = -40.6
81 >s_e_s_e_s_d_s_c_s_d_s_c_s_b_s_e_s
GTGCTGGTGCTGGTGACGGTGTGGGTGACGGTGTGGGTGGCTGTGCTGGTG; >clamp_s_c_s_d_s_c_s_a_s_e_s:compl:rev
CACCAGCACAGACACCCACACCGTCACCCACACTATCATGTTGACATTCC; dG = -40.6
82 >s_a_s_c_s_c_s_c_s_d_s_c_s_b_s_e_s
GTGTCTGTGTGGGTGTGGGTGTGGGTGACGGTGTGGGTGGCTGTGCTGGTG; >clamp_s_c_s_d_s_c_s_a_s_e_s:compl:rev
CACCAGCACAGACACCCACACCGTCACCCACACTATCATGTTGACATTCC; dG = -40.6
83 >s_e_s_e_s_e_s_c_s_d_s_c_s_b_s_e_s
GTGCTGGTGCTGGTGCTGGTGTGGGTGACGGTGTGGGTGGCTGTGCTGGTG; >clamp_s_c_s_d_s_c_s_a_s_e_s:compl:rev
CACCAGCACAGACACCCACACCGTCACCCACACTATCATGTTGACATTCC; dG = -40.6
84 >s_a_s_c_s_d_s_c_s_d_s_c_s_b_s_e_s
GTGTCTGTGTGGGTGACGGTGTGGGTGACGGTGTGGGTGGCTGTGCTGGTG; >clamp_s_c_s_d_s_c_s_a_s_e_s:compl:rev
CACCAGCACAGACACCCACACCGTCACCCACACTATCATGTTGACATTCC; dG = -40.6
85 >s_a_s_a_s_b_s_c_s_d_s_c_s_b_s_e_s
GTGTCTGTGTCTGTGGCTGTGTGGGTGACGGTGTGGGTGGCTGTGCTGGTG; >clamp_s_c_s_d_s_c_s_a_s_e_s:compl:rev
CACCAGCACAGACACCCACACCGTCACCCACACTATCATGTTGACATTCC; dG = -40.6
86 >s_a_s_c_s_e_s_c_s_d_s_c_s_b_s_e_s
GTGTCTGTGTGGGTGCTGGTGTGGGTGACGGTGTGGGTGGCTGTGCTGGTG; >clamp_s_c_s_d_s_c_s_a_s_e_s:compl:rev
CACCAGCACAGACACCCACACCGTCACCCACACTATCATGTTGACATTCC; dG = -40.6
87 >s_a_s_d_s_a_s_c_s_d_s_c_s_b_s_e_s
GTGTCTGTGACGGTGTCTGTGTGGGTGACGGTGTGGGTGGCTGTGCTGGTG; >clamp_s_c_s_d_s_c_s_a_s_e_s:compl:rev
CACCAGCACAGACACCCACACCGTCACCCACACTATCATGTTGACATTCC; dG = -40.6
88 >s_a_s_d_s_b_s_c_s_d_s_c_s_b_s_e_s
GTGTCTGTGACGGTGGCTGTGTGGGTGACGGTGTGGGTGGCTGTGCTGGTG; >clamp_s_c_s_d_s_c_s_a_s_e_s:compl:rev
CACCAGCACAGACACCCACACCGTCACCCACACTATCATGTTGACATTCC; dG = -40.6
89 >s_a_s_d_s_c_s_c_s_d_s_c_s_b_s_e_s
GTGTCTGTGACGGTGTGGGTGTGGGTGACGGTGTGGGTGGCTGTGCTGGTG; >clamp_s_c_s_d_s_c_s_a_s_e_s:compl:rev
CACCAGCACAGACACCCACACCGTCACCCACACTATCATGTTGACATTCC; dG = -40.6
90 >s_a_s_d_s_d_s_c_s_d_s_c_s_b_s_e_s
GTGTCTGTGACGGTGACGGTGTGGGTGACGGTGTGGGTGGCTGTGCTGGTG; >clamp_s_c_s_d_s_c_s_a_s_e_s:compl:rev
CACCAGCACAGACACCCACACCGTCACCCACACTATCATGTTGACATTCC; dG = -40.6
91 >s_a_s_d_s_e_s_c_s_d_s_c_s_b_s_e_s
GTGTCTGTGACGGTGCTGGTGTGGGTGACGGTGTGGGTGGCTGTGCTGGTG; >clamp_s_c_s_d_s_c_s_a_s_e_s:compl:rev
CACCAGCACAGACACCCACACCGTCACCCACACTATCATGTTGACATTCC; dG = -40.6
92 >s_a_s_e_s_a_s_c_s_d_s_c_s_b_s_e_s
GTGTCTGTGCTGGTGTCTGTGTGGGTGACGGTGTGGGTGGCTGTGCTGGTG; >clamp_s_c_s_d_s_c_s_a_s_e_s:compl:rev
CACCAGCACAGACACCCACACCGTCACCCACACTATCATGTTGACATTCC; dG = -40.6
93 >s_a_s_e_s_b_s_c_s_d_s_c_s_b_s_e_s
GTGTCTGTGCTGGTGGCTGTGTGGGTGACGGTGTGGGTGGCTGTGCTGGTG; >clamp_s_c_s_d_s_c_s_a_s_e_s:compl:rev
CACCAGCACAGACACCCACACCGTCACCCACACTATCATGTTGACATTCC; dG = -40.6
94 >s_a_s_e_s_c_s_c_s_d_s_c_s_b_s_e_s
GTGTCTGTGCTGGTGTGGGTGTGGGTGACGGTGTGGGTGGCTGTGCTGGTG; >clamp_s_c_s_d_s_c_s_a_s_e_s:compl:rev
CACCAGCACAGACACCCACACCGTCACCCACACTATCATGTTGACATTCC; dG = -40.6
95 >s_a_s_e_s_d_s_c_s_d_s_c_s_b_s_e_s
GTGTCTGTGCTGGTGACGGTGTGGGTGACGGTGTGGGTGGCTGTGCTGGTG; >clamp_s_c_s_d_s_c_s_a_s_e_s:compl:rev
CACCAGCACAGACACCCACACCGTCACCCACACTATCATGTTGACATTCC; dG = -40.6
96 >s_a_s_a_s_c_s_c_s_d_s_c_s_b_s_e_s
GTGTCTGTGTCTGTGTGGGTGTGGGTGACGGTGTGGGTGGCTGTGCTGGTG; >clamp_s_c_s_d_s_c_s_a_s_e_s:compl:rev
CACCAGCACAGACACCCACACCGTCACCCACACTATCATGTTGACATTCC; dG = -40.6
97 >s_a_s_e_s_e_s_c_s_d_s_c_s_b_s_e_s
GTGTCTGTGCTGGTGCTGGTGTGGGTGACGGTGTGGGTGGCTGTGCTGGTG; >clamp_s_c_s_d_s_c_s_a_s_e_s:compl:rev
CACCAGCACAGACACCCACACCGTCACCCACACTATCATGTTGACATTCC; dG = -40.6
98 >s_b_s_a_s_a_s_c_s_d_s_c_s_b_s_e_s
GTGGCTGTGTCTGTGTCTGTGTGGGTGACGGTGTGGGTGGCTGTGCTGGTG; >clamp_s_c_s_d_s_c_s_a_s_e_s:compl:rev
CACCAGCACAGACACCCACACCGTCACCCACACTATCATGTTGACATTCC; dG = -40.6
99 >s_b_s_a_s_b_s_c_s_d_s_c_s_b_s_e_s
GTGGCTGTGTCTGTGGCTGTGTGGGTGACGGTGTGGGTGGCTGTGCTGGTG; >clamp_s_c_s_d_s_c_s_a_s_e_s:compl:rev
CACCAGCACAGACACCCACACCGTCACCCACACTATCATGTTGACATTCC; dG = -40.6
100 >s_b_s_a_s_c_s_c_s_d_s_c_s_b_s_e_s
GTGGCTGTGTCTGTGTGGGTGTGGGTGACGGTGTGGGTGGCTGTGCTGGTG; >clamp_s_c_s_d_s_c_s_a_s_e_s:compl:rev
CACCAGCACAGACACCCACACCGTCACCCACACTATCATGTTGACATTCC; dG = -40.6
101 >s_b_s_a_s_d_s_c_s_d_s_c_s_b_s_e_s
GTGGCTGTGTCTGTGACGGTGTGGGTGACGGTGTGGGTGGCTGTGCTGGTG; >clamp_s_c_s_d_s_c_s_a_s_e_s:compl:rev
CACCAGCACAGACACCCACACCGTCACCCACACTATCATGTTGACATTCC; dG = -40.6
102 >s_b_s_a_s_e_s_c_s_d_s_c_s_b_s_e_s
GTGGCTGTGTCTGTGCTGGTGTGGGTGACGGTGTGGGTGGCTGTGCTGGTG; >clamp_s_c_s_d_s_c_s_a_s_e_s:compl:rev
CACCAGCACAGACACCCACACCGTCACCCACACTATCATGTTGACATTCC; dG = -40.6
103 >s_b_s_b_s_a_s_c_s_d_s_c_s_b_s_e_s
GTGGCTGTGGCTGTGTCTGTGTGGGTGACGGTGTGGGTGGCTGTGCTGGTG; >clamp_s_c_s_d_s_c_s_a_s_e_s:compl:rev
CACCAGCACAGACACCCACACCGTCACCCACACTATCATGTTGACATTCC; dG = -40.6

104 >s_b_s_b_s_b_s_c_s_d_s_c_s_b_s_e_s
GTGGCTGTGGCTGTGGCTGTGTGGGTGACGGTGTGGGTGGCTGTGCTGGTG;   >clamp_s_c_s_d_s_c_s_a_s_e_s:compl:rev
CACCAGCACAGACACCCACACCGTCACCCACACTATCATGTTGACATTCC; dG = -40.6
105 >s_b_s_b_s_c_s_c_s_d_s_c_s_b_s_e_s
GTGGCTGTGGCTGTGTGGGTGTGGGTGACGGTGTGGGTGGCTGTGCTGGTG;   >clamp_s_c_s_d_s_c_s_a_s_e_s:compl:rev
CACCAGCACAGACACCCACACCGTCACCCACACTATCATGTTGACATTCC; dG = -40.6
106 >s_b_s_b_s_d_s_c_s_d_s_c_s_b_s_e_s
GTGGCTGTGGCTGTGACGGTGTGGGTGACGGTGTGGGTGGCTGTGCTGGTG;   >clamp_s_c_s_d_s_c_s_a_s_e_s:compl:rev
CACCAGCACAGACACCCACACCGTCACCCACACTATCATGTTGACATTCC; dG = -40.6
107 >s_a_s_a_s_d_s_c_s_d_s_c_s_b_s_e_s
GTGTCTGTGTCTGTGACGGTGTGGGTGACGGTGTGGGTGGCTGTGCTGGTG;   >clamp_s_c_s_d_s_c_s_a_s_e_s:compl:rev
CACCAGCACAGACACCCACACCGTCACCCACACTATCATGTTGACATTCC; dG = -40.6
108 >s_b_s_b_s_e_s_c_s_d_s_c_s_b_s_e_s
GTGGCTGTGGCTGTGCTGGTGTGGGTGACGGTGTGGGTGGCTGTGCTGGTG;   >clamp_s_c_s_d_s_c_s_a_s_e_s:compl:rev
CACCAGCACAGACACCCACACCGTCACCCACACTATCATGTTGACATTCC; dG = -40.6
109 >s_b_s_c_s_a_s_c_s_d_s_c_s_b_s_e_s
GTGGCTGTGTGGGTGTCTGTGTGGGTGACGGTGTGGGTGGCTGTGCTGGTG;   >clamp_s_c_s_d_s_c_s_a_s_e_s:compl:rev
CACCAGCACAGACACCCACACCGTCACCCACACTATCATGTTGACATTCC; dG = -40.6
110 >s_b_s_c_s_b_s_c_s_d_s_c_s_b_s_e_s
GTGGCTGTGTGGGTGGCTGTGTGGGTGACGGTGTGGGTGGCTGTGCTGGTG;   >clamp_s_c_s_d_s_c_s_a_s_e_s:compl:rev
CACCAGCACAGACACCCACACCGTCACCCACACTATCATGTTGACATTCC; dG = -40.6
111 >s_b_s_c_s_c_s_c_s_d_s_c_s_b_s_e_s
GTGGCTGTGTGGGTGTGGGTGTGGGTGACGGTGTGGGTGGCTGTGCTGGTG;   >clamp_s_c_s_d_s_c_s_a_s_e_s:compl:rev
CACCAGCACAGACACCCACACCGTCACCCACACTATCATGTTGACATTCC; dG = -40.6
112 >s_b_s_c_s_d_s_c_s_d_s_c_s_b_s_e_s
GTGGCTGTGTGGGTGACGGTGTGGGTGACGGTGTGGGTGGCTGTGCTGGTG;   >clamp_s_c_s_d_s_c_s_a_s_e_s:compl:rev
CACCAGCACAGACACCCACACCGTCACCCACACTATCATGTTGACATTCC; dG = -40.6
113 >s_b_s_c_s_e_s_c_s_d_s_c_s_b_s_e_s
GTGGCTGTGTGGGTGCTGGTGTGGGTGACGGTGTGGGTGGCTGTGCTGGTG;   >clamp_s_c_s_d_s_c_s_a_s_e_s:compl:rev
CACCAGCACAGACACCCACACCGTCACCCACACTATCATGTTGACATTCC; dG = -40.6
114 >s_a_s_a_s_a_s_c_s_d_s_c_s_b_s_e_s
GTGTCTGTGTCTGTGTCTGTGTGGGTGACGGTGTGGGTGGCTGTGCTGGTG;   >clamp_s_c_s_d_s_c_s_a_s_e_s:compl:rev
CACCAGCACAGACACCCACACCGTCACCCACACTATCATGTTGACATTCC; dG = -40.6
115 >s_b_s_d_s_a_s_c_s_d_s_c_s_b_s_e_s
GTGGCTGTGACGGTGTCTGTGTGGGTGACGGTGTGGGTGGCTGTGCTGGTG;   >clamp_s_c_s_d_s_c_s_a_s_e_s:compl:rev
CACCAGCACAGACACCCACACCGTCACCCACACTATCATGTTGACATTCC; dG = -40.6
116 >s_b_s_d_s_b_s_c_s_d_s_c_s_b_s_e_s
GTGGCTGTGACGGTGGCTGTGTGGGTGACGGTGTGGGTGGCTGTGCTGGTG;   >clamp_s_c_s_d_s_c_s_a_s_e_s:compl:rev
CACCAGCACAGACACCCACACCGTCACCCACACTATCATGTTGACATTCC; dG = -40.6
117 >s_b_s_d_s_c_s_c_s_d_s_c_s_b_s_e_s
GTGGCTGTGACGGTGTGGGTGTGGGTGACGGTGTGGGTGGCTGTGCTGGTG;   >clamp_s_c_s_d_s_c_s_a_s_e_s:compl:rev
CACCAGCACAGACACCCACACCGTCACCCACACTATCATGTTGACATTCC; dG = -40.6
118 >s_b_s_d_s_d_s_c_s_d_s_c_s_b_s_e_s
GTGGCTGTGACGGTGACGGTGTGGGTGACGGTGTGGGTGGCTGTGCTGGTG;   >clamp_s_c_s_d_s_c_s_a_s_e_s:compl:rev
CACCAGCACAGACACCCACACCGTCACCCACACTATCATGTTGACATTCC; dG = -40.6
119 >s_a_s_a_s_e_s_c_s_d_s_c_s_b_s_e_s
GTGTCTGTGTCTGTGCTGGTGTGGGTGACGGTGTGGGTGGCTGTGCTGGTG;   >clamp_s_c_s_d_s_c_s_a_s_e_s:compl:rev
CACCAGCACAGACACCCACACCGTCACCCACACTATCATGTTGACATTCC; dG = -40.6
120 >s_b_s_d_s_e_s_c_s_d_s_c_s_b_s_e_s
GTGGCTGTGACGGTGCTGGTGTGGGTGACGGTGTGGGTGGCTGTGCTGGTG;   >clamp_s_c_s_d_s_c_s_a_s_e_s:compl:rev
CACCAGCACAGACACCCACACCGTCACCCACACTATCATGTTGACATTCC; dG = -40.6
121 >s_b_s_e_s_a_s_c_s_d_s_c_s_b_s_e_s
GTGGCTGTGCTGGTGTCTGTGTGGGTGACGGTGTGGGTGGCTGTGCTGGTG;   >clamp_s_c_s_d_s_c_s_a_s_e_s:compl:rev
CACCAGCACAGACACCCACACCGTCACCCACACTATCATGTTGACATTCC; dG = -40.6
122 >s_b_s_e_s_b_s_c_s_d_s_c_s_b_s_e_s
GTGGCTGTGCTGGTGGCTGTGTGGGTGACGGTGTGGGTGGCTGTGCTGGTG;   >clamp_s_c_s_d_s_c_s_a_s_e_s:compl:rev
CACCAGCACAGACACCCACACCGTCACCCACACTATCATGTTGACATTCC; dG = -40.6
123 >s_b_s_e_s_c_s_c_s_d_s_c_s_b_s_e_s
GTGGCTGTGCTGGTGTGGGTGTGGGTGACGGTGTGGGTGGCTGTGCTGGTG;   >clamp_s_c_s_d_s_c_s_a_s_e_s:compl:rev
CACCAGCACAGACACCCACACCGTCACCCACACTATCATGTTGACATTCC; dG = -40.6
124 >s_b_s_e_s_d_s_c_s_d_s_c_s_b_s_e_s
GTGGCTGTGCTGGTGACGGTGTGGGTGACGGTGTGGGTGGCTGTGCTGGTG;   >clamp_s_c_s_d_s_c_s_a_s_e_s:compl:rev
CACCAGCACAGACACCCACACCGTCACCCACACTATCATGTTGACATTCC; dG = -40.6
125 >s_b_s_e_s_e_s_c_s_d_s_c_s_b_s_e_s
GTGGCTGTGCTGGTGCTGGTGTGGGTGACGGTGTGGGTGGCTGTGCTGGTG;   >clamp_s_c_s_d_s_c_s_a_s_e_s:compl:rev
CACCAGCACAGACACCCACACCGTCACCCACACTATCATGTTGACATTCC; dG = -40.6
126 >s_c_s_a_s_c_s_e_s_d_s_c_s_a_s_e_s
GTGTGGGTGTCTGTGTGGGTGCTGGTGACGGTGTGGGTGTCTGTGCTGGTG;   >clamp_s_c_s_d_s_c_s_a_s_e_s:compl:rev
CACCAGCACAGACACCCACACCGTCACCCACACTATCATGTTGACATTCC; dG = -37.3
127 >s_c_s_a_s_c_s_d_s_d_s_c_s_a_s_e_s
GTGTGGGTGTCTGTGTGGGTGACGGTGACGGTGTGGGTGTCTGTGCTGGTG;   >clamp_s_c_s_d_s_c_s_a_s_e_s:compl:rev
CACCAGCACAGACACCCACACCGTCACCCACACTATCATGTTGACATTCC; dG = -37.3

```
   128 >s_c_s_a_s_c_s_b_s_d_s_c_s_a_s_e_s
GTGTGGGTGTCTGTGTGGGTGGCTGTGACGGTGTGGGTGTCTGTGCTGGTG;    >clamp_s_c_s_d_s_c_s_a_s_e_s:compl:rev
CACCAGCACAGACACCCACACCGTCACCCACACTATCATGTTGACATTCC; dG = -37.3
   129 >s_c_s_a_s_c_s_a_s_d_s_c_s_a_s_e_s
GTGTGGGTGTCTGTGTGGGTGTCTGTGACGGTGTGGGTGTCTGTGCTGGTG;    >clamp_s_c_s_d_s_c_s_a_s_e_s:compl:rev
CACCAGCACAGACACCCACACCGTCACCCACACTATCATGTTGACATTCC; dG = -37.3
   130 >s_c_s_b_s_c_s_e_s_d_s_c_s_a_s_e_s
GTGTGGGTGGCTGTGTGGGTGCTGGTGACGGTGTGGGTGTCTGTGCTGGTG;    >clamp_s_c_s_d_s_c_s_a_s_e_s:compl:rev
CACCAGCACAGACACCCACACCGTCACCCACACTATCATGTTGACATTCC; dG = -37.3
   131 >s_c_s_b_s_c_s_d_s_d_s_c_s_a_s_e_s
GTGTGGGTGGCTGTGTGGGTGACGGTGACGGTGTGGGTGTCTGTGCTGGTG;    >clamp_s_c_s_d_s_c_s_a_s_e_s:compl:rev
CACCAGCACAGACACCCACACCGTCACCCACACTATCATGTTGACATTCC; dG = -37.3
   132 >s_c_s_b_s_c_s_b_s_d_s_c_s_a_s_e_s
GTGTGGGTGGCTGTGTGGGTGGCTGTGACGGTGTGGGTGTCTGTGCTGGTG;    >clamp_s_c_s_d_s_c_s_a_s_e_s:compl:rev
CACCAGCACAGACACCCACACCGTCACCCACACTATCATGTTGACATTCC; dG = -37.3
   133 >s_c_s_b_s_c_s_a_s_d_s_c_s_a_s_e_s
GTGTGGGTGGCTGTGTGGGTGTCTGTGACGGTGTGGGTGTCTGTGCTGGTG;    >clamp_s_c_s_d_s_c_s_a_s_e_s:compl:rev
CACCAGCACAGACACCCACACCGTCACCCACACTATCATGTTGACATTCC; dG = -37.3
   134 >s_c_s_c_s_d_s_c_s_a_s_c_s_a_s_e_s
GTGTGGGTGTGGGTGACGGTGTGGGTGTCTGTGTGGGTGTCTGTGCTGGTG;    >clamp_s_c_s_d_s_c_s_a_s_e_s:compl:rev
CACCAGCACAGACACCCACACCGTCACCCACACTATCATGTTGACATTCC; dG = -37.3
   135 >s_d_s_a_s_c_s_e_s_d_s_c_s_a_s_e_s
GTGACGGTGTCTGTGTGGGTGCTGGTGACGGTGTGGGTGTCTGTGCTGGTG;    >clamp_s_c_s_d_s_c_s_a_s_e_s:compl:rev
CACCAGCACAGACACCCACACCGTCACCCACACTATCATGTTGACATTCC; dG = -37.3
   136 >s_d_s_a_s_c_s_d_s_d_s_c_s_a_s_e_s
GTGACGGTGTCTGTGTGGGTGACGGTGACGGTGTGGGTGTCTGTGCTGGTG;    >clamp_s_c_s_d_s_c_s_a_s_e_s:compl:rev
CACCAGCACAGACACCCACACCGTCACCCACACTATCATGTTGACATTCC; dG = -37.3
   137 >s_d_s_a_s_c_s_b_s_d_s_c_s_a_s_e_s
GTGACGGTGTCTGTGTGGGTGGCTGTGACGGTGTGGGTGTCTGTGCTGGTG;    >clamp_s_c_s_d_s_c_s_a_s_e_s:compl:rev
CACCAGCACAGACACCCACACCGTCACCCACACTATCATGTTGACATTCC; dG = -37.3
   138 >s_d_s_a_s_c_s_a_s_d_s_c_s_a_s_e_s
GTGACGGTGTCTGTGTGGGTGTCTGTGACGGTGTGGGTGTCTGTGCTGGTG;    >clamp_s_c_s_d_s_c_s_a_s_e_s:compl:rev
CACCAGCACAGACACCCACACCGTCACCCACACTATCATGTTGACATTCC; dG = -37.3
   139 >s_a_s_b_s_c_s_e_s_d_s_c_s_a_s_e_s
GTGTCTGTGGCTGTGTGGGTGCTGGTGACGGTGTGGGTGTCTGTGCTGGTG;    >clamp_s_c_s_d_s_c_s_a_s_e_s:compl:rev
CACCAGCACAGACACCCACACCGTCACCCACACTATCATGTTGACATTCC; dG = -37.3
   140 >s_a_s_b_s_c_s_d_s_d_s_c_s_a_s_e_s
GTGTCTGTGGCTGTGTGGGTGACGGTGACGGTGTGGGTGTCTGTGCTGGTG;    >clamp_s_c_s_d_s_c_s_a_s_e_s:compl:rev
CACCAGCACAGACACCCACACCGTCACCCACACTATCATGTTGACATTCC; dG = -37.3
   141 >s_a_s_b_s_c_s_b_s_d_s_c_s_a_s_e_s
GTGTCTGTGGCTGTGTGGGTGGCTGTGACGGTGTGGGTGTCTGTGCTGGTG;    >clamp_s_c_s_d_s_c_s_a_s_e_s:compl:rev
CACCAGCACAGACACCCACACCGTCACCCACACTATCATGTTGACATTCC; dG = -37.3
   142 >s_a_s_b_s_c_s_a_s_d_s_c_s_a_s_e_s
GTGTCTGTGGCTGTGTGGGTGTCTGTGACGGTGTGGGTGTCTGTGCTGGTG;    >clamp_s_c_s_d_s_c_s_a_s_e_s:compl:rev
CACCAGCACAGACACCCACACCGTCACCCACACTATCATGTTGACATTCC; dG = -37.3
   143 >s_d_s_b_s_c_s_e_s_d_s_c_s_a_s_e_s
GTGACGGTGGCTGTGTGGGTGCTGGTGACGGTGTGGGTGTCTGTGCTGGTG;    >clamp_s_c_s_d_s_c_s_a_s_e_s:compl:rev
CACCAGCACAGACACCCACACCGTCACCCACACTATCATGTTGACATTCC; dG = -37.3
   144 >s_d_s_b_s_c_s_d_s_d_s_c_s_a_s_e_s
GTGACGGTGGCTGTGTGGGTGACGGTGACGGTGTGGGTGTCTGTGCTGGTG;    >clamp_s_c_s_d_s_c_s_a_s_e_s:compl:rev
CACCAGCACAGACACCCACACCGTCACCCACACTATCATGTTGACATTCC; dG = -37.3
   145 >s_d_s_b_s_c_s_b_s_d_s_c_s_a_s_e_s
GTGACGGTGGCTGTGTGGGTGGCTGTGACGGTGTGGGTGTCTGTGCTGGTG;    >clamp_s_c_s_d_s_c_s_a_s_e_s:compl:rev
CACCAGCACAGACACCCACACCGTCACCCACACTATCATGTTGACATTCC; dG = -37.3
   146 >s_d_s_b_s_c_s_a_s_d_s_c_s_a_s_e_s
GTGACGGTGGCTGTGTGGGTGTCTGTGACGGTGTGGGTGTCTGTGCTGGTG;    >clamp_s_c_s_d_s_c_s_a_s_e_s:compl:rev
CACCAGCACAGACACCCACACCGTCACCCACACTATCATGTTGACATTCC; dG = -37.3
   147 >s_d_s_c_s_d_s_c_s_a_s_c_s_a_s_e_s
GTGACGGTGTGGGTGACGGTGTGGGTGTCTGTGTGGGTGTCTGTGCTGGTG;    >clamp_s_c_s_d_s_c_s_a_s_e_s:compl:rev
CACCAGCACAGACACCCACACCGTCACCCACACTATCATGTTGACATTCC; dG = -37.3
   148 >s_e_s_a_s_c_s_e_s_d_s_c_s_a_s_e_s
GTGCTGGTGTCTGTGTGGGTGCTGGTGACGGTGTGGGTGTCTGTGCTGGTG;    >clamp_s_c_s_d_s_c_s_a_s_e_s:compl:rev
CACCAGCACAGACACCCACACCGTCACCCACACTATCATGTTGACATTCC; dG = -37.3
   149 >s_e_s_a_s_c_s_d_s_d_s_c_s_a_s_e_s
GTGCTGGTGTCTGTGTGGGTGACGGTGACGGTGTGGGTGTCTGTGCTGGTG;    >clamp_s_c_s_d_s_c_s_a_s_e_s:compl:rev
CACCAGCACAGACACCCACACCGTCACCCACACTATCATGTTGACATTCC; dG = -37.3
   150 >s_e_s_a_s_c_s_b_s_d_s_c_s_a_s_e_s
GTGCTGGTGTCTGTGTGGGTGGCTGTGACGGTGTGGGTGTCTGTGCTGGTG;    >clamp_s_c_s_d_s_c_s_a_s_e_s:compl:rev
CACCAGCACAGACACCCACACCGTCACCCACACTATCATGTTGACATTCC; dG = -37.3
   151 >s_e_s_a_s_c_s_a_s_d_s_c_s_a_s_e_s
GTGCTGGTGTCTGTGTGGGTGTCTGTGACGGTGTGGGTGTCTGTGCTGGTG;    >clamp_s_c_s_d_s_c_s_a_s_e_s:compl:rev
CACCAGCACAGACACCCACACCGTCACCCACACTATCATGTTGACATTCC; dG = -37.3
```

152 >s_e_s_b_s_c_s_e_s_d_s_c_s_a_s_e_s
GTGCTGGTGGCTGTGTGGGTGCTGGTGACGGTGTGGGTGTCTGTGCTGGTG; >clamp_s_c_s_d_s_c_s_a_s_e_s:compl:rev
CACCAGCACAGACACCCACACCGTCACCCACACTATCATGTTGACATTCC; dG = -37.3

153 >s_e_s_b_s_c_s_d_s_d_s_c_s_a_s_e_s
GTGCTGGTGGCTGTGTGGGTGACGGTGACGGTGTGGGTGTCTGTGCTGGTG; >clamp_s_c_s_d_s_c_s_a_s_e_s:compl:rev
CACCAGCACAGACACCCACACCGTCACCCACACTATCATGTTGACATTCC; dG = -37.3

154 >s_e_s_b_s_c_s_b_s_d_s_c_s_a_s_e_s
GTGCTGGTGGCTGTGTGGGTGGCTGTGACGGTGTGGGTGTCTGTGCTGGTG; >clamp_s_c_s_d_s_c_s_a_s_e_s:compl:rev
CACCAGCACAGACACCCACACCGTCACCCACACTATCATGTTGACATTCC; dG = -37.3

155 >s_e_s_b_s_c_s_a_s_d_s_c_s_a_s_e_s
GTGCTGGTGGCTGTGTGGGTGTCTGTGACGGTGTGGGTGTCTGTGCTGGTG; >clamp_s_c_s_d_s_c_s_a_s_e_s:compl:rev
CACCAGCACAGACACCCACACCGTCACCCACACTATCATGTTGACATTCC; dG = -37.3

156 >s_e_s_c_s_d_s_c_s_a_s_c_s_a_s_e_s
GTGCTGGTGTGGGTGACGGTGTGGGTGTCTGTGTGGGTGTCTGTGCTGGTG; >clamp_s_c_s_d_s_c_s_a_s_e_s:compl:rev
CACCAGCACAGACACCCACACCGTCACCCACACTATCATGTTGACATTCC; dG = -37.3

157 >s_a_s_c_s_d_s_c_s_a_s_c_s_a_s_e_s
GTGTCTGTGTGGGTGACGGTGTGGGTGTCTGTGTGGGTGTCTGTGCTGGTG; >clamp_s_c_s_d_s_c_s_a_s_e_s:compl:rev
CACCAGCACAGACACCCACACCGTCACCCACACTATCATGTTGACATTCC; dG = -37.3

158 >s_b_s_a_s_c_s_e_s_d_s_c_s_a_s_e_s
GTGGCTGTGTCTGTGTGGGTGCTGGTGACGGTGTGGGTGTCTGTGCTGGTG; >clamp_s_c_s_d_s_c_s_a_s_e_s:compl:rev
CACCAGCACAGACACCCACACCGTCACCCACACTATCATGTTGACATTCC; dG = -37.3

159 >s_b_s_a_s_c_s_d_s_d_s_c_s_a_s_e_s
GTGGCTGTGTCTGTGTGGGTGACGGTGACGGTGTGGGTGTCTGTGCTGGTG; >clamp_s_c_s_d_s_c_s_a_s_e_s:compl:rev
CACCAGCACAGACACCCACACCGTCACCCACACTATCATGTTGACATTCC; dG = -37.3

160 >s_b_s_a_s_c_s_b_s_d_s_c_s_a_s_e_s
GTGGCTGTGTCTGTGTGGGTGGCTGTGACGGTGTGGGTGTCTGTGCTGGTG; >clamp_s_c_s_d_s_c_s_a_s_e_s:compl:rev
CACCAGCACAGACACCCACACCGTCACCCACACTATCATGTTGACATTCC; dG = -37.3

161 >s_b_s_a_s_c_s_a_s_d_s_c_s_a_s_e_s
GTGGCTGTGTCTGTGTGGGTGTCTGTGACGGTGTGGGTGTCTGTGCTGGTG; >clamp_s_c_s_d_s_c_s_a_s_e_s:compl:rev
CACCAGCACAGACACCCACACCGTCACCCACACTATCATGTTGACATTCC; dG = -37.3

162 >s_a_s_a_s_c_s_e_s_d_s_c_s_a_s_e_s
GTGTCTGTGTCTGTGTGGGTGCTGGTGACGGTGTGGGTGTCTGTGCTGGTG; >clamp_s_c_s_d_s_c_s_a_s_e_s:compl:rev
CACCAGCACAGACACCCACACCGTCACCCACACTATCATGTTGACATTCC; dG = -37.3

163 >s_a_s_a_s_c_s_d_s_d_s_c_s_a_s_e_s
GTGTCTGTGTCTGTGTGGGTGACGGTGACGGTGTGGGTGTCTGTGCTGGTG; >clamp_s_c_s_d_s_c_s_a_s_e_s:compl:rev
CACCAGCACAGACACCCACACCGTCACCCACACTATCATGTTGACATTCC; dG = -37.3

164 >s_a_s_a_s_c_s_b_s_d_s_c_s_a_s_e_s
GTGTCTGTGTCTGTGTGGGTGGCTGTGACGGTGTGGGTGTCTGTGCTGGTG; >clamp_s_c_s_d_s_c_s_a_s_e_s:compl:rev
CACCAGCACAGACACCCACACCGTCACCCACACTATCATGTTGACATTCC; dG = -37.3

165 >s_a_s_a_s_c_s_a_s_d_s_c_s_a_s_e_s
GTGTCTGTGTCTGTGTGGGTGTCTGTGACGGTGTGGGTGTCTGTGCTGGTG; >clamp_s_c_s_d_s_c_s_a_s_e_s:compl:rev
CACCAGCACAGACACCCACACCGTCACCCACACTATCATGTTGACATTCC; dG = -37.3

166 >s_b_s_b_s_c_s_e_s_d_s_c_s_a_s_e_s
GTGGCTGTGGCTGTGTGGGTGCTGGTGACGGTGTGGGTGTCTGTGCTGGTG; >clamp_s_c_s_d_s_c_s_a_s_e_s:compl:rev
CACCAGCACAGACACCCACACCGTCACCCACACTATCATGTTGACATTCC; dG = -37.3

167 >s_b_s_b_s_c_s_d_s_d_s_c_s_a_s_e_s
GTGGCTGTGGCTGTGTGGGTGACGGTGACGGTGTGGGTGTCTGTGCTGGTG; >clamp_s_c_s_d_s_c_s_a_s_e_s:compl:rev
CACCAGCACAGACACCCACACCGTCACCCACACTATCATGTTGACATTCC; dG = -37.3

168 >s_b_s_b_s_c_s_b_s_d_s_c_s_a_s_e_s
GTGGCTGTGGCTGTGTGGGTGGCTGTGACGGTGTGGGTGTCTGTGCTGGTG; >clamp_s_c_s_d_s_c_s_a_s_e_s:compl:rev
CACCAGCACAGACACCCACACCGTCACCCACACTATCATGTTGACATTCC; dG = -37.3

169 >s_b_s_b_s_c_s_a_s_d_s_c_s_a_s_e_s
GTGGCTGTGGCTGTGTGGGTGTCTGTGACGGTGTGGGTGTCTGTGCTGGTG; >clamp_s_c_s_d_s_c_s_a_s_e_s:compl:rev
CACCAGCACAGACACCCACACCGTCACCCACACTATCATGTTGACATTCC; dG = -37.3

170 >s_b_s_c_s_d_s_c_s_a_s_c_s_a_s_e_s
GTGGCTGTGTGGGTGACGGTGTGGGTGTCTGTGTGGGTGTCTGTGCTGGTG; >clamp_s_c_s_d_s_c_s_a_s_e_s:compl:rev
CACCAGCACAGACACCCACACCGTCACCCACACTATCATGTTGACATTCC; dG = -37.3

171 >s_c_s_c_s_c_s_e_s_d_s_c_s_a_s_e_s
GTGTGGGTGTGGGTGTGGGTGCTGGTGACGGTGTGGGTGTCTGTGCTGGTG; >clamp_s_c_s_d_s_c_s_a_s_e_s:compl:rev
CACCAGCACAGACACCCACACCGTCACCCACACTATCATGTTGACATTCC; dG = -37.2

172 >s_c_s_c_s_c_s_d_s_d_s_c_s_a_s_e_s
GTGTGGGTGTGGGTGTGGGTGACGGTGACGGTGTGGGTGTCTGTGCTGGTG; >clamp_s_c_s_d_s_c_s_a_s_e_s:compl:rev
CACCAGCACAGACACCCACACCGTCACCCACACTATCATGTTGACATTCC; dG = -37.2

173 >s_c_s_c_s_c_s_b_s_d_s_c_s_a_s_e_s
GTGTGGGTGTGGGTGTGGGTGGCTGTGACGGTGTGGGTGTCTGTGCTGGTG; >clamp_s_c_s_d_s_c_s_a_s_e_s:compl:rev
CACCAGCACAGACACCCACACCGTCACCCACACTATCATGTTGACATTCC; dG = -37.2

174 >s_c_s_c_s_c_s_a_s_d_s_c_s_a_s_e_s
GTGTGGGTGTGGGTGTGGGTGTCTGTGACGGTGTGGGTGTCTGTGCTGGTG; >clamp_s_c_s_d_s_c_s_a_s_e_s:compl:rev
CACCAGCACAGACACCCACACCGTCACCCACACTATCATGTTGACATTCC; dG = -37.2

175 >s_c_s_d_s_c_s_e_s_d_s_c_s_a_s_e_s
GTGTGGGTGACGGTGTGGGTGCTGGTGACGGTGTGGGTGTCTGTGCTGGTG; >clamp_s_c_s_d_s_c_s_a_s_e_s:compl:rev
CACCAGCACAGACACCCACACCGTCACCCACACTATCATGTTGACATTCC; dG = -37.2

176 >s_c_s_d_s_c_s_d_s_d_s_c_s_a_s_e_s
GTGTGGGTGACGGTGTGGGTGACGGTGACGGTGTGGGTGTCTGTGCTGGTG; >clamp_s_c_s_d_s_c_s_a_s_e_s:compl:rev
CACCAGCACAGACACCCACACCGTCACCCACACTATCATGTTGACATTCC; dG = -37.2
177 >s_c_s_d_s_c_s_b_s_d_s_c_s_a_s_e_s
GTGTGGGTGACGGTGTGGGTGGCTGTGACGGTGTGGGTGTCTGTGCTGGTG; >clamp_s_c_s_d_s_c_s_a_s_e_s:compl:rev
CACCAGCACAGACACCCACACCGTCACCCACACTATCATGTTGACATTCC; dG = -37.2
178 >s_c_s_d_s_c_s_a_s_d_s_c_s_a_s_e_s
GTGTGGGTGACGGTGTGGGTGTCTGTGACGGTGTGGGTGTCTGTGCTGGTG; >clamp_s_c_s_d_s_c_s_a_s_e_s:compl:rev
CACCAGCACAGACACCCACACCGTCACCCACACTATCATGTTGACATTCC; dG = -37.2
179 >s_c_s_e_s_c_s_e_s_d_s_c_s_a_s_e_s
GTGTGGGTGCTGGTGTGGGTGCTGGTGACGGTGTGGGTGTCTGTGCTGGTG; >clamp_s_c_s_d_s_c_s_a_s_e_s:compl:rev
CACCAGCACAGACACCCACACCGTCACCCACACTATCATGTTGACATTCC; dG = -37.2
180 >s_c_s_e_s_c_s_d_s_d_s_c_s_a_s_e_s
GTGTGGGTGCTGGTGTGGGTGACGGTGACGGTGTGGGTGTCTGTGCTGGTG; >clamp_s_c_s_d_s_c_s_a_s_e_s:compl:rev
CACCAGCACAGACACCCACACCGTCACCCACACTATCATGTTGACATTCC; dG = -37.2
181 >s_c_s_e_s_c_s_b_s_d_s_c_s_a_s_e_s
GTGTGGGTGCTGGTGTGGGTGGCTGTGACGGTGTGGGTGTCTGTGCTGGTG; >clamp_s_c_s_d_s_c_s_a_s_e_s:compl:rev
CACCAGCACAGACACCCACACCGTCACCCACACTATCATGTTGACATTCC; dG = -37.2
182 >s_c_s_e_s_c_s_a_s_d_s_c_s_a_s_e_s
GTGTGGGTGCTGGTGTGGGTGTCTGTGACGGTGTGGGTGTCTGTGCTGGTG; >clamp_s_c_s_d_s_c_s_a_s_e_s:compl:rev
CACCAGCACAGACACCCACACCGTCACCCACACTATCATGTTGACATTCC; dG = -37.2
183 >s_d_s_c_s_c_s_e_s_d_s_c_s_a_s_e_s
GTGACGGTGTGGGTGTGGGTGCTGGTGACGGTGTGGGTGTCTGTGCTGGTG; >clamp_s_c_s_d_s_c_s_a_s_e_s:compl:rev
CACCAGCACAGACACCCACACCGTCACCCACACTATCATGTTGACATTCC; dG = -37.2
184 >s_d_s_c_s_c_s_d_s_d_s_c_s_a_s_e_s
GTGACGGTGTGGGTGTGGGTGACGGTGACGGTGTGGGTGTCTGTGCTGGTG; >clamp_s_c_s_d_s_c_s_a_s_e_s:compl:rev
CACCAGCACAGACACCCACACCGTCACCCACACTATCATGTTGACATTCC; dG = -37.2
185 >s_d_s_c_s_c_s_b_s_d_s_c_s_a_s_e_s
GTGACGGTGTGGGTGTGGGTGGCTGTGACGGTGTGGGTGTCTGTGCTGGTG; >clamp_s_c_s_d_s_c_s_a_s_e_s:compl:rev
CACCAGCACAGACACCCACACCGTCACCCACACTATCATGTTGACATTCC; dG = -37.2
186 >s_d_s_c_s_c_s_a_s_d_s_c_s_a_s_e_s
GTGACGGTGTGGGTGTGGGTGTCTGTGACGGTGTGGGTGTCTGTGCTGGTG; >clamp_s_c_s_d_s_c_s_a_s_e_s:compl:rev
CACCAGCACAGACACCCACACCGTCACCCACACTATCATGTTGACATTCC; dG = -37.2
187 >s_d_s_d_s_c_s_e_s_d_s_c_s_a_s_e_s
GTGACGGTGACGGTGTGGGTGCTGGTGACGGTGTGGGTGTCTGTGCTGGTG; >clamp_s_c_s_d_s_c_s_a_s_e_s:compl:rev
CACCAGCACAGACACCCACACCGTCACCCACACTATCATGTTGACATTCC; dG = -37.2
188 >s_d_s_d_s_c_s_d_s_d_s_c_s_a_s_e_s
GTGACGGTGACGGTGTGGGTGACGGTGACGGTGTGGGTGTCTGTGCTGGTG; >clamp_s_c_s_d_s_c_s_a_s_e_s:compl:rev
CACCAGCACAGACACCCACACCGTCACCCACACTATCATGTTGACATTCC; dG = -37.2
189 >s_d_s_d_s_c_s_b_s_d_s_c_s_a_s_e_s
GTGACGGTGACGGTGTGGGTGGCTGTGACGGTGTGGGTGTCTGTGCTGGTG; >clamp_s_c_s_d_s_c_s_a_s_e_s:compl:rev
CACCAGCACAGACACCCACACCGTCACCCACACTATCATGTTGACATTCC; dG = -37.2
190 >s_d_s_d_s_c_s_a_s_d_s_c_s_a_s_e_s
GTGACGGTGACGGTGTGGGTGTCTGTGACGGTGTGGGTGTCTGTGCTGGTG; >clamp_s_c_s_d_s_c_s_a_s_e_s:compl:rev
CACCAGCACAGACACCCACACCGTCACCCACACTATCATGTTGACATTCC; dG = -37.2
191 >s_d_s_e_s_c_s_e_s_d_s_c_s_a_s_e_s
GTGACGGTGCTGGTGTGGGTGCTGGTGACGGTGTGGGTGTCTGTGCTGGTG; >clamp_s_c_s_d_s_c_s_a_s_e_s:compl:rev
CACCAGCACAGACACCCACACCGTCACCCACACTATCATGTTGACATTCC; dG = -37.2
192 >s_d_s_e_s_c_s_d_s_d_s_c_s_a_s_e_s
GTGACGGTGCTGGTGTGGGTGACGGTGACGGTGTGGGTGTCTGTGCTGGTG; >clamp_s_c_s_d_s_c_s_a_s_e_s:compl:rev
CACCAGCACAGACACCCACACCGTCACCCACACTATCATGTTGACATTCC; dG = -37.2
193 >s_d_s_e_s_c_s_b_s_d_s_c_s_a_s_e_s
GTGACGGTGCTGGTGTGGGTGGCTGTGACGGTGTGGGTGTCTGTGCTGGTG; >clamp_s_c_s_d_s_c_s_a_s_e_s:compl:rev
CACCAGCACAGACACCCACACCGTCACCCACACTATCATGTTGACATTCC; dG = -37.2
194 >s_d_s_e_s_c_s_a_s_d_s_c_s_a_s_e_s
GTGACGGTGCTGGTGTGGGTGTCTGTGACGGTGTGGGTGTCTGTGCTGGTG; >clamp_s_c_s_d_s_c_s_a_s_e_s:compl:rev
CACCAGCACAGACACCCACACCGTCACCCACACTATCATGTTGACATTCC; dG = -37.2
195 >s_e_s_c_s_c_s_e_s_d_s_c_s_a_s_e_s
GTGCTGGTGTGGGTGTGGGTGCTGGTGACGGTGTGGGTGTCTGTGCTGGTG; >clamp_s_c_s_d_s_c_s_a_s_e_s:compl:rev
CACCAGCACAGACACCCACACCGTCACCCACACTATCATGTTGACATTCC; dG = -37.2
196 >s_e_s_c_s_c_s_d_s_d_s_c_s_a_s_e_s
GTGCTGGTGTGGGTGTGGGTGACGGTGACGGTGTGGGTGTCTGTGCTGGTG; >clamp_s_c_s_d_s_c_s_a_s_e_s:compl:rev
CACCAGCACAGACACCCACACCGTCACCCACACTATCATGTTGACATTCC; dG = -37.2
197 >s_e_s_c_s_c_s_b_s_d_s_c_s_a_s_e_s
GTGCTGGTGTGGGTGTGGGTGGCTGTGACGGTGTGGGTGTCTGTGCTGGTG; >clamp_s_c_s_d_s_c_s_a_s_e_s:compl:rev
CACCAGCACAGACACCCACACCGTCACCCACACTATCATGTTGACATTCC; dG = -37.2
198 >s_e_s_c_s_c_s_a_s_d_s_c_s_a_s_e_s
GTGCTGGTGTGGGTGTGGGTGTCTGTGACGGTGTGGGTGTCTGTGCTGGTG; >clamp_s_c_s_d_s_c_s_a_s_e_s:compl:rev
CACCAGCACAGACACCCACACCGTCACCCACACTATCATGTTGACATTCC; dG = -37.2
199 >s_e_s_d_s_c_s_e_s_d_s_c_s_a_s_e_s
GTGCTGGTGACGGTGTGGGTGCTGGTGACGGTGTGGGTGTCTGTGCTGGTG; >clamp_s_c_s_d_s_c_s_a_s_e_s:compl:rev
CACCAGCACAGACACCCACACCGTCACCCACACTATCATGTTGACATTCC; dG = -37.2

200 >s_e_s_d_s_c_s_d_s_d_s_c_s_a_s_e_s
GTGCTGGTGACGGTGTGGGTGACGGTGACGGTGTGGGTGTCTGTGCTGGTG; >clamp_s_c_s_d_s_c_s_a_s_e_s:compl:rev
CACCAGCACAGACACCCACACCGTCACCCACACTATCATGTTGACATTCC; dG = -37.2
201 >s_e_s_d_s_c_s_b_s_d_s_c_s_a_s_e_s
GTGCTGGTGACGGTGTGGGTGGCTGTGACGGTGTGGGTGTCTGTGCTGGTG; >clamp_s_c_s_d_s_c_s_a_s_e_s:compl:rev
CACCAGCACAGACACCCACACCGTCACCCACACTATCATGTTGACATTCC; dG = -37.2
202 >s_e_s_d_s_c_s_a_s_d_s_c_s_a_s_e_s
GTGCTGGTGACGGTGTGGGTGTCTGTGACGGTGTGGGTGTCTGTGCTGGTG; >clamp_s_c_s_d_s_c_s_a_s_e_s:compl:rev
CACCAGCACAGACACCCACACCGTCACCCACACTATCATGTTGACATTCC; dG = -37.2
203 >s_e_s_e_s_c_s_e_s_d_s_c_s_a_s_e_s
GTGCTGGTGCTGGTGTGGGTGCTGGTGACGGTGTGGGTGTCTGTGCTGGTG; >clamp_s_c_s_d_s_c_s_a_s_e_s:compl:rev
CACCAGCACAGACACCCACACCGTCACCCACACTATCATGTTGACATTCC; dG = -37.2
204 >s_e_s_e_s_c_s_d_s_d_s_c_s_a_s_e_s
GTGCTGGTGCTGGTGTGGGTGACGGTGACGGTGTGGGTGTCTGTGCTGGTG; >clamp_s_c_s_d_s_c_s_a_s_e_s:compl:rev
CACCAGCACAGACACCCACACCGTCACCCACACTATCATGTTGACATTCC; dG = -37.2
205 >s_e_s_e_s_c_s_b_s_d_s_c_s_a_s_e_s
GTGCTGGTGCTGGTGTGGGTGGCTGTGACGGTGTGGGTGTCTGTGCTGGTG; >clamp_s_c_s_d_s_c_s_a_s_e_s:compl:rev
CACCAGCACAGACACCCACACCGTCACCCACACTATCATGTTGACATTCC; dG = -37.2
206 >s_e_s_e_s_c_s_a_s_d_s_c_s_a_s_e_s
GTGCTGGTGCTGGTGTGGGTGTCTGTGACGGTGTGGGTGTCTGTGCTGGTG; >clamp_s_c_s_d_s_c_s_a_s_e_s:compl:rev
CACCAGCACAGACACCCACACCGTCACCCACACTATCATGTTGACATTCC; dG = -37.2
207 >s_a_s_c_s_c_s_e_s_d_s_c_s_a_s_e_s
GTGTCTGTGTGGGTGTGGGTGCTGGTGACGGTGTGGGTGTCTGTGCTGGTG; >clamp_s_c_s_d_s_c_s_a_s_e_s:compl:rev
CACCAGCACAGACACCCACACCGTCACCCACACTATCATGTTGACATTCC; dG = -37.2
208 >s_a_s_c_s_c_s_d_s_d_s_c_s_a_s_e_s
GTGTCTGTGTGGGTGTGGGTGACGGTGACGGTGTGGGTGTCTGTGCTGGTG; >clamp_s_c_s_d_s_c_s_a_s_e_s:compl:rev
CACCAGCACAGACACCCACACCGTCACCCACACTATCATGTTGACATTCC; dG = -37.2
209 >s_a_s_c_s_c_s_b_s_d_s_c_s_a_s_e_s
GTGTCTGTGTGGGTGTGGGTGGCTGTGACGGTGTGGGTGTCTGTGCTGGTG; >clamp_s_c_s_d_s_c_s_a_s_e_s:compl:rev
CACCAGCACAGACACCCACACCGTCACCCACACTATCATGTTGACATTCC; dG = -37.2
210 >s_a_s_c_s_c_s_a_s_d_s_c_s_a_s_e_s
GTGTCTGTGTGGGTGTGGGTGTCTGTGACGGTGTGGGTGTCTGTGCTGGTG; >clamp_s_c_s_d_s_c_s_a_s_e_s:compl:rev
CACCAGCACAGACACCCACACCGTCACCCACACTATCATGTTGACATTCC; dG = -37.2
211 >s_a_s_d_s_c_s_e_s_d_s_c_s_a_s_e_s
GTGTCTGTGACGGTGTGGGTGCTGGTGACGGTGTGGGTGTCTGTGCTGGTG; >clamp_s_c_s_d_s_c_s_a_s_e_s:compl:rev
CACCAGCACAGACACCCACACCGTCACCCACACTATCATGTTGACATTCC; dG = -37.2
212 >s_a_s_d_s_c_s_d_s_d_s_c_s_a_s_e_s
GTGTCTGTGACGGTGTGGGTGACGGTGACGGTGTGGGTGTCTGTGCTGGTG; >clamp_s_c_s_d_s_c_s_a_s_e_s:compl:rev
CACCAGCACAGACACCCACACCGTCACCCACACTATCATGTTGACATTCC; dG = -37.2
213 >s_a_s_d_s_c_s_b_s_d_s_c_s_a_s_e_s
GTGTCTGTGACGGTGTGGGTGGCTGTGACGGTGTGGGTGTCTGTGCTGGTG; >clamp_s_c_s_d_s_c_s_a_s_e_s:compl:rev
CACCAGCACAGACACCCACACCGTCACCCACACTATCATGTTGACATTCC; dG = -37.2
214 >s_a_s_d_s_c_s_a_s_d_s_c_s_a_s_e_s
GTGTCTGTGACGGTGTGGGTGTCTGTGACGGTGTGGGTGTCTGTGCTGGTG; >clamp_s_c_s_d_s_c_s_a_s_e_s:compl:rev
CACCAGCACAGACACCCACACCGTCACCCACACTATCATGTTGACATTCC; dG = -37.2
215 >s_a_s_e_s_c_s_e_s_d_s_c_s_a_s_e_s
GTGTCTGTGCTGGTGTGGGTGCTGGTGACGGTGTGGGTGTCTGTGCTGGTG; >clamp_s_c_s_d_s_c_s_a_s_e_s:compl:rev
CACCAGCACAGACACCCACACCGTCACCCACACTATCATGTTGACATTCC; dG = -37.2
216 >s_a_s_e_s_c_s_d_s_d_s_c_s_a_s_e_s
GTGTCTGTGCTGGTGTGGGTGACGGTGACGGTGTGGGTGTCTGTGCTGGTG; >clamp_s_c_s_d_s_c_s_a_s_e_s:compl:rev
CACCAGCACAGACACCCACACCGTCACCCACACTATCATGTTGACATTCC; dG = -37.2
217 >s_a_s_e_s_c_s_b_s_d_s_c_s_a_s_e_s
GTGTCTGTGCTGGTGTGGGTGGCTGTGACGGTGTGGGTGTCTGTGCTGGTG; >clamp_s_c_s_d_s_c_s_a_s_e_s:compl:rev
CACCAGCACAGACACCCACACCGTCACCCACACTATCATGTTGACATTCC; dG = -37.2
218 >s_a_s_e_s_c_s_a_s_d_s_c_s_a_s_e_s
GTGTCTGTGCTGGTGTGGGTGTCTGTGACGGTGTGGGTGTCTGTGCTGGTG; >clamp_s_c_s_d_s_c_s_a_s_e_s:compl:rev
CACCAGCACAGACACCCACACCGTCACCCACACTATCATGTTGACATTCC; dG = -37.2
219 >s_b_s_c_s_c_s_e_s_d_s_c_s_a_s_e_s
GTGGCTGTGTGGGTGTGGGTGCTGGTGACGGTGTGGGTGTCTGTGCTGGTG; >clamp_s_c_s_d_s_c_s_a_s_e_s:compl:rev
CACCAGCACAGACACCCACACCGTCACCCACACTATCATGTTGACATTCC; dG = -37.2
220 >s_b_s_c_s_c_s_d_s_d_s_c_s_a_s_e_s
GTGGCTGTGTGGGTGTGGGTGACGGTGACGGTGTGGGTGTCTGTGCTGGTG; >clamp_s_c_s_d_s_c_s_a_s_e_s:compl:rev
CACCAGCACAGACACCCACACCGTCACCCACACTATCATGTTGACATTCC; dG = -37.2
221 >s_b_s_c_s_c_s_b_s_d_s_c_s_a_s_e_s
GTGGCTGTGTGGGTGTGGGTGGCTGTGACGGTGTGGGTGTCTGTGCTGGTG; >clamp_s_c_s_d_s_c_s_a_s_e_s:compl:rev
CACCAGCACAGACACCCACACCGTCACCCACACTATCATGTTGACATTCC; dG = -37.2
222 >s_b_s_c_s_c_s_a_s_d_s_c_s_a_s_e_s
GTGGCTGTGTGGGTGTGGGTGTCTGTGACGGTGTGGGTGTCTGTGCTGGTG; >clamp_s_c_s_d_s_c_s_a_s_e_s:compl:rev
CACCAGCACAGACACCCACACCGTCACCCACACTATCATGTTGACATTCC; dG = -37.2
223 >s_b_s_d_s_c_s_e_s_d_s_c_s_a_s_e_s
GTGGCTGTGACGGTGTGGGTGCTGGTGACGGTGTGGGTGTCTGTGCTGGTG; >clamp_s_c_s_d_s_c_s_a_s_e_s:compl:rev
CACCAGCACAGACACCCACACCGTCACCCACACTATCATGTTGACATTCC; dG = -37.2

```
   224 >s_b_s_d_s_c_s_d_s_d_s_c_s_a_s_e_s
GTGGCTGTGACGGTGTGGGTGACGGTGACGGTGTGGGTGTCTGTGCTGGTG;    >clamp_s_c_s_d_s_c_s_a_s_e_s:compl:rev
CACCAGCACAGACACCCACACCGTCACCCACACTATCATGTTGACATTCC; dG = -37.2
   225 >s_b_s_d_s_c_s_b_s_d_s_c_s_a_s_e_s
GTGGCTGTGACGGTGTGGGTGGCTGTGACGGTGTGGGTGTCTGTGCTGGTG;    >clamp_s_c_s_d_s_c_s_a_s_e_s:compl:rev
CACCAGCACAGACACCCACACCGTCACCCACACTATCATGTTGACATTCC; dG = -37.2
   226 >s_b_s_d_s_c_s_a_s_d_s_c_s_a_s_e_s
GTGGCTGTGACGGTGTGGGTGTCTGTGACGGTGTGGGTGTCTGTGCTGGTG;    >clamp_s_c_s_d_s_c_s_a_s_e_s:compl:rev
CACCAGCACAGACACCCACACCGTCACCCACACTATCATGTTGACATTCC; dG = -37.2
   227 >s_b_s_e_s_c_s_e_s_d_s_c_s_a_s_e_s
GTGGCTGTGCTGGTGTGGGTGCTGGTGACGGTGTGGGTGTCTGTGCTGGTG;    >clamp_s_c_s_d_s_c_s_a_s_e_s:compl:rev
CACCAGCACAGACACCCACACCGTCACCCACACTATCATGTTGACATTCC; dG = -37.2
   228 >s_b_s_e_s_c_s_d_s_d_s_c_s_a_s_e_s
GTGGCTGTGCTGGTGTGGGTGACGGTGACGGTGTGGGTGTCTGTGCTGGTG;    >clamp_s_c_s_d_s_c_s_a_s_e_s:compl:rev
CACCAGCACAGACACCCACACCGTCACCCACACTATCATGTTGACATTCC; dG = -37.2
   229 >s_b_s_e_s_c_s_b_s_d_s_c_s_a_s_e_s
GTGGCTGTGCTGGTGTGGGTGGCTGTGACGGTGTGGGTGTCTGTGCTGGTG;    >clamp_s_c_s_d_s_c_s_a_s_e_s:compl:rev
CACCAGCACAGACACCCACACCGTCACCCACACTATCATGTTGACATTCC; dG = -37.2
   230 >s_b_s_e_s_c_s_a_s_d_s_c_s_a_s_e_s
GTGGCTGTGCTGGTGTGGGTGTCTGTGACGGTGTGGGTGTCTGTGCTGGTG;    >clamp_s_c_s_d_s_c_s_a_s_e_s:compl:rev
CACCAGCACAGACACCCACACCGTCACCCACACTATCATGTTGACATTCC; dG = -37.2
   231 >s_c_s_a_s_a_s_c_s_d_s_c_s_a_s_c_s
GTGTGGGTGTCTGTGTCTGTGTGGGTGACGGTGTGGGTGTCTGTGTGGGTG;    >clamp_s_c_s_d_s_c_s_a_s_e_s:compl:rev
CACCAGCACAGACACCCACACCGTCACCCACACTATCATGTTGACATTCC; dG = -37.1
   232 >s_a_s_b_s_a_s_c_s_d_s_c_s_a_s_c_s
GTGTCTGTGGCTGTGTCTGTGTGGGTGACGGTGTGGGTGTCTGTGTGGGTG;    >clamp_s_c_s_d_s_c_s_a_s_e_s:compl:rev
CACCAGCACAGACACCCACACCGTCACCCACACTATCATGTTGACATTCC; dG = -37.1
   233 >s_c_s_a_s_b_s_c_s_d_s_c_s_a_s_c_s
GTGTGGGTGTCTGTGGCTGTGTGGGTGACGGTGTGGGTGTCTGTGTGGGTG;    >clamp_s_c_s_d_s_c_s_a_s_e_s:compl:rev
CACCAGCACAGACACCCACACCGTCACCCACACTATCATGTTGACATTCC; dG = -37.1
   234 >s_c_s_b_s_a_s_c_s_d_s_c_s_a_s_c_s
GTGTGGGTGGCTGTGTCTGTGTGGGTGACGGTGTGGGTGTCTGTGTGGGTG;    >clamp_s_c_s_d_s_c_s_a_s_e_s:compl:rev
CACCAGCACAGACACCCACACCGTCACCCACACTATCATGTTGACATTCC; dG = -37.1
   235 >s_c_s_b_s_b_s_c_s_d_s_c_s_a_s_c_s
GTGTGGGTGGCTGTGGCTGTGTGGGTGACGGTGTGGGTGTCTGTGTGGGTG;    >clamp_s_c_s_d_s_c_s_a_s_e_s:compl:rev
CACCAGCACAGACACCCACACCGTCACCCACACTATCATGTTGACATTCC; dG = -37.1
   236 >s_c_s_c_s_a_s_c_s_d_s_c_s_a_s_c_s
GTGTGGGTGTGGGTGTCTGTGTGGGTGACGGTGTGGGTGTCTGTGTGGGTG;    >clamp_s_c_s_d_s_c_s_a_s_e_s:compl:rev
CACCAGCACAGACACCCACACCGTCACCCACACTATCATGTTGACATTCC; dG = -37.1
   237 >s_a_s_b_s_b_s_c_s_d_s_c_s_a_s_c_s
GTGTCTGTGGCTGTGGCTGTGTGGGTGACGGTGTGGGTGTCTGTGTGGGTG;    >clamp_s_c_s_d_s_c_s_a_s_e_s:compl:rev
CACCAGCACAGACACCCACACCGTCACCCACACTATCATGTTGACATTCC; dG = -37.1
   238 >s_c_s_c_s_b_s_c_s_d_s_c_s_a_s_c_s
GTGTGGGTGTGGGTGGCTGTGTGGGTGACGGTGTGGGTGTCTGTGTGGGTG;    >clamp_s_c_s_d_s_c_s_a_s_e_s:compl:rev
CACCAGCACAGACACCCACACCGTCACCCACACTATCATGTTGACATTCC; dG = -37.1
   239 >s_c_s_d_s_a_s_c_s_d_s_c_s_a_s_c_s
GTGTGGGTGACGGTGTCTGTGTGGGTGACGGTGTGGGTGTCTGTGTGGGTG;    >clamp_s_c_s_d_s_c_s_a_s_e_s:compl:rev
CACCAGCACAGACACCCACACCGTCACCCACACTATCATGTTGACATTCC; dG = -37.1
   240 >s_c_s_d_s_b_s_c_s_d_s_c_s_a_s_c_s
GTGTGGGTGACGGTGGCTGTGTGGGTGACGGTGTGGGTGTCTGTGTGGGTG;    >clamp_s_c_s_d_s_c_s_a_s_e_s:compl:rev
CACCAGCACAGACACCCACACCGTCACCCACACTATCATGTTGACATTCC; dG = -37.1
   241 >s_c_s_e_s_a_s_c_s_d_s_c_s_a_s_c_s
GTGTGGGTGCTGGTGTCTGTGTGGGTGACGGTGTGGGTGTCTGTGTGGGTG;    >clamp_s_c_s_d_s_c_s_a_s_e_s:compl:rev
CACCAGCACAGACACCCACACCGTCACCCACACTATCATGTTGACATTCC; dG = -37.1
   242 >s_c_s_e_s_b_s_c_s_d_s_c_s_a_s_c_s
GTGTGGGTGCTGGTGGCTGTGTGGGTGACGGTGTGGGTGTCTGTGTGGGTG;    >clamp_s_c_s_d_s_c_s_a_s_e_s:compl:rev
CACCAGCACAGACACCCACACCGTCACCCACACTATCATGTTGACATTCC; dG = -37.1
   243 >s_d_s_a_s_a_s_c_s_d_s_c_s_a_s_c_s
GTGACGGTGTCTGTGTCTGTGTGGGTGACGGTGTGGGTGTCTGTGTGGGTG;    >clamp_s_c_s_d_s_c_s_a_s_e_s:compl:rev
CACCAGCACAGACACCCACACCGTCACCCACACTATCATGTTGACATTCC; dG = -37.1
   244 >s_d_s_a_s_b_s_c_s_d_s_c_s_a_s_c_s
GTGACGGTGTCTGTGGCTGTGTGGGTGACGGTGTGGGTGTCTGTGTGGGTG;    >clamp_s_c_s_d_s_c_s_a_s_e_s:compl:rev
CACCAGCACAGACACCCACACCGTCACCCACACTATCATGTTGACATTCC; dG = -37.1
   245 >s_d_s_b_s_a_s_c_s_d_s_c_s_a_s_c_s
GTGACGGTGGCTGTGTCTGTGTGGGTGACGGTGTGGGTGTCTGTGTGGGTG;    >clamp_s_c_s_d_s_c_s_a_s_e_s:compl:rev
CACCAGCACAGACACCCACACCGTCACCCACACTATCATGTTGACATTCC; dG = -37.1
   246 >s_d_s_b_s_b_s_c_s_d_s_c_s_a_s_c_s
GTGACGGTGGCTGTGGCTGTGTGGGTGACGGTGTGGGTGTCTGTGTGGGTG;    >clamp_s_c_s_d_s_c_s_a_s_e_s:compl:rev
CACCAGCACAGACACCCACACCGTCACCCACACTATCATGTTGACATTCC; dG = -37.1
   247 >s_d_s_c_s_a_s_c_s_d_s_c_s_a_s_c_s
GTGACGGTGTGGGTGTCTGTGTGGGTGACGGTGTGGGTGTCTGTGTGGGTG;    >clamp_s_c_s_d_s_c_s_a_s_e_s:compl:rev
CACCAGCACAGACACCCACACCGTCACCCACACTATCATGTTGACATTCC; dG = -37.1
```

248 >s_d_s_c_s_b_s_c_s_d_s_c_s_a_s_c_s
GTGACGGTGTGGGTGGCTGTGTGGGTGACGGTGTGGGTGTCTGTGTGGGTG; >clamp_s_c_s_d_s_c_s_a_s_e_s:compl:rev
CACCAGCACAGACACCCACACCGTCACCCACACTATCATGTTGACATTCC; dG = -37.1
249 >s_d_s_d_s_a_s_c_s_d_s_c_s_a_s_c_s
GTGACGGTGACGGTGTCTGTGTGGGTGACGGTGTGGGTGTCTGTGTGGGTG; >clamp_s_c_s_d_s_c_s_a_s_e_s:compl:rev
CACCAGCACAGACACCCACACCGTCACCCACACTATCATGTTGACATTCC; dG = -37.1
250 >s_d_s_d_s_b_s_c_s_d_s_c_s_a_s_c_s
GTGACGGTGACGGTGGCTGTGTGGGTGACGGTGTGGGTGTCTGTGTGGGTG; >clamp_s_c_s_d_s_c_s_a_s_e_s:compl:rev
CACCAGCACAGACACCCACACCGTCACCCACACTATCATGTTGACATTCC; dG = -37.1
251 >s_d_s_e_s_a_s_c_s_d_s_c_s_a_s_c_s
GTGACGGTGCTGGTGTCTGTGTGGGTGACGGTGTGGGTGTCTGTGTGGGTG; >clamp_s_c_s_d_s_c_s_a_s_e_s:compl:rev
CACCAGCACAGACACCCACACCGTCACCCACACTATCATGTTGACATTCC; dG = -37.1
252 >s_d_s_e_s_b_s_c_s_d_s_c_s_a_s_c_s
GTGACGGTGCTGGTGGCTGTGTGGGTGACGGTGTGGGTGTCTGTGTGGGTG; >clamp_s_c_s_d_s_c_s_a_s_e_s:compl:rev
CACCAGCACAGACACCCACACCGTCACCCACACTATCATGTTGACATTCC; dG = -37.1
253 >s_a_s_a_s_a_s_c_s_d_s_c_s_a_s_c_s
GTGTCTGTGTCTGTGTCTGTGTGGGTGACGGTGTGGGTGTCTGTGTGGGTG; >clamp_s_c_s_d_s_c_s_a_s_e_s:compl:rev
CACCAGCACAGACACCCACACCGTCACCCACACTATCATGTTGACATTCC; dG = -37.1
254 >s_e_s_a_s_a_s_c_s_d_s_c_s_a_s_c_s
GTGCTGGTGTCTGTGTCTGTGTGGGTGACGGTGTGGGTGTCTGTGTGGGTG; >clamp_s_c_s_d_s_c_s_a_s_e_s:compl:rev
CACCAGCACAGACACCCACACCGTCACCCACACTATCATGTTGACATTCC; dG = -37.1
255 >s_a_s_c_s_a_s_c_s_d_s_c_s_a_s_c_s
GTGTCTGTGTGGGTGTCTGTGTGGGTGACGGTGTGGGTGTCTGTGTGGGTG; >clamp_s_c_s_d_s_c_s_a_s_e_s:compl:rev
CACCAGCACAGACACCCACACCGTCACCCACACTATCATGTTGACATTCC; dG = -37.1
256 >s_e_s_a_s_b_s_c_s_d_s_c_s_a_s_c_s
GTGCTGGTGTCTGTGGCTGTGTGGGTGACGGTGTGGGTGTCTGTGTGGGTG; >clamp_s_c_s_d_s_c_s_a_s_e_s:compl:rev
CACCAGCACAGACACCCACACCGTCACCCACACTATCATGTTGACATTCC; dG = -37.1
257 >s_e_s_b_s_a_s_c_s_d_s_c_s_a_s_c_s
GTGCTGGTGGCTGTGTCTGTGTGGGTGACGGTGTGGGTGTCTGTGTGGGTG; >clamp_s_c_s_d_s_c_s_a_s_e_s:compl:rev
CACCAGCACAGACACCCACACCGTCACCCACACTATCATGTTGACATTCC; dG = -37.1
258 >s_e_s_b_s_b_s_c_s_d_s_c_s_a_s_c_s
GTGCTGGTGGCTGTGGCTGTGTGGGTGACGGTGTGGGTGTCTGTGTGGGTG; >clamp_s_c_s_d_s_c_s_a_s_e_s:compl:rev
CACCAGCACAGACACCCACACCGTCACCCACACTATCATGTTGACATTCC; dG = -37.1
259 >s_a_s_a_s_b_s_c_s_d_s_c_s_a_s_c_s
GTGTCTGTGTCTGTGGCTGTGTGGGTGACGGTGTGGGTGTCTGTGTGGGTG; >clamp_s_c_s_d_s_c_s_a_s_e_s:compl:rev
CACCAGCACAGACACCCACACCGTCACCCACACTATCATGTTGACATTCC; dG = -37.1
260 >s_e_s_c_s_a_s_c_s_d_s_c_s_a_s_c_s
GTGCTGGTGTGGGTGTCTGTGTGGGTGACGGTGTGGGTGTCTGTGTGGGTG; >clamp_s_c_s_d_s_c_s_a_s_e_s:compl:rev
CACCAGCACAGACACCCACACCGTCACCCACACTATCATGTTGACATTCC; dG = -37.1
261 >s_a_s_c_s_b_s_c_s_d_s_c_s_a_s_c_s
GTGTCTGTGTGGGTGGCTGTGTGGGTGACGGTGTGGGTGTCTGTGTGGGTG; >clamp_s_c_s_d_s_c_s_a_s_e_s:compl:rev
CACCAGCACAGACACCCACACCGTCACCCACACTATCATGTTGACATTCC; dG = -37.1
262 >s_e_s_c_s_b_s_c_s_d_s_c_s_a_s_c_s
GTGCTGGTGTGGGTGGCTGTGTGGGTGACGGTGTGGGTGTCTGTGTGGGTG; >clamp_s_c_s_d_s_c_s_a_s_e_s:compl:rev
CACCAGCACAGACACCCACACCGTCACCCACACTATCATGTTGACATTCC; dG = -37.1
263 >s_e_s_d_s_a_s_c_s_d_s_c_s_a_s_c_s
GTGCTGGTGACGGTGTCTGTGTGGGTGACGGTGTGGGTGTCTGTGTGGGTG; >clamp_s_c_s_d_s_c_s_a_s_e_s:compl:rev
CACCAGCACAGACACCCACACCGTCACCCACACTATCATGTTGACATTCC; dG = -37.1
264 >s_e_s_d_s_b_s_c_s_d_s_c_s_a_s_c_s
GTGCTGGTGACGGTGGCTGTGTGGGTGACGGTGTGGGTGTCTGTGTGGGTG; >clamp_s_c_s_d_s_c_s_a_s_e_s:compl:rev
CACCAGCACAGACACCCACACCGTCACCCACACTATCATGTTGACATTCC; dG = -37.1
265 >s_e_s_e_s_a_s_c_s_d_s_c_s_a_s_c_s
GTGCTGGTGCTGGTGTCTGTGTGGGTGACGGTGTGGGTGTCTGTGTGGGTG; >clamp_s_c_s_d_s_c_s_a_s_e_s:compl:rev
CACCAGCACAGACACCCACACCGTCACCCACACTATCATGTTGACATTCC; dG = -37.1
266 >s_e_s_e_s_b_s_c_s_d_s_c_s_a_s_c_s
GTGCTGGTGCTGGTGGCTGTGTGGGTGACGGTGTGGGTGTCTGTGTGGGTG; >clamp_s_c_s_d_s_c_s_a_s_e_s:compl:rev
CACCAGCACAGACACCCACACCGTCACCCACACTATCATGTTGACATTCC; dG = -37.1
267 >s_a_s_d_s_a_s_c_s_d_s_c_s_a_s_c_s
GTGTCTGTGACGGTGTCTGTGTGGGTGACGGTGTGGGTGTCTGTGTGGGTG; >clamp_s_c_s_d_s_c_s_a_s_e_s:compl:rev
CACCAGCACAGACACCCACACCGTCACCCACACTATCATGTTGACATTCC; dG = -37.1
268 >s_a_s_d_s_b_s_c_s_d_s_c_s_a_s_c_s
GTGTCTGTGACGGTGGCTGTGTGGGTGACGGTGTGGGTGTCTGTGTGGGTG; >clamp_s_c_s_d_s_c_s_a_s_e_s:compl:rev
CACCAGCACAGACACCCACACCGTCACCCACACTATCATGTTGACATTCC; dG = -37.1
269 >s_a_s_e_s_a_s_c_s_d_s_c_s_a_s_c_s
GTGTCTGTGCTGGTGTCTGTGTGGGTGACGGTGTGGGTGTCTGTGTGGGTG; >clamp_s_c_s_d_s_c_s_a_s_e_s:compl:rev
CACCAGCACAGACACCCACACCGTCACCCACACTATCATGTTGACATTCC; dG = -37.1
270 >s_a_s_e_s_b_s_c_s_d_s_c_s_a_s_c_s
GTGTCTGTGCTGGTGGCTGTGTGGGTGACGGTGTGGGTGTCTGTGTGGGTG; >clamp_s_c_s_d_s_c_s_a_s_e_s:compl:rev
CACCAGCACAGACACCCACACCGTCACCCACACTATCATGTTGACATTCC; dG = -37.1
271 >s_b_s_a_s_a_s_c_s_d_s_c_s_a_s_c_s
GTGGCTGTGTCTGTGTCTGTGTGGGTGACGGTGTGGGTGTCTGTGTGGGTG; >clamp_s_c_s_d_s_c_s_a_s_e_s:compl:rev
CACCAGCACAGACACCCACACCGTCACCCACACTATCATGTTGACATTCC; dG = -37.1

272 >s_b_s_a_s_b_s_c_s_d_s_c_s_a_s_c_s
GTGGCTGTGTCTGTGGCTGTGTGGGTGACGGTGTGGGTGTCTGTGTGGGTG; >clamp_s_c_s_d_s_c_s_a_s_e_s:compl:rev
CACCAGCACAGACACCCACACCGTCACCCACACTATCATGTTGACATTCC; dG = -37.1
273 >s_b_s_b_s_a_s_c_s_d_s_c_s_a_s_c_s
GTGGCTGTGGCTGTGTCTGTGTGGGTGACGGTGTGGGTGTCTGTGTGGGTG; >clamp_s_c_s_d_s_c_s_a_s_e_s:compl:rev
CACCAGCACAGACACCCACACCGTCACCCACACTATCATGTTGACATTCC; dG = -37.1
274 >s_b_s_b_s_b_s_c_s_d_s_c_s_a_s_c_s
GTGGCTGTGGCTGTGGCTGTGTGGGTGACGGTGTGGGTGTCTGTGTGGGTG; >clamp_s_c_s_d_s_c_s_a_s_e_s:compl:rev
CACCAGCACAGACACCCACACCGTCACCCACACTATCATGTTGACATTCC; dG = -37.1
275 >s_b_s_c_s_a_s_c_s_d_s_c_s_a_s_c_s
GTGGCTGTGTGGGTGTCTGTGTGGGTGACGGTGTGGGTGTCTGTGTGGGTG; >clamp_s_c_s_d_s_c_s_a_s_e_s:compl:rev
CACCAGCACAGACACCCACACCGTCACCCACACTATCATGTTGACATTCC; dG = -37.1
276 >s_b_s_c_s_b_s_c_s_d_s_c_s_a_s_c_s
GTGGCTGTGTGGGTGGCTGTGTGGGTGACGGTGTGGGTGTCTGTGTGGGTG; >clamp_s_c_s_d_s_c_s_a_s_e_s:compl:rev
CACCAGCACAGACACCCACACCGTCACCCACACTATCATGTTGACATTCC; dG = -37.1
277 >s_b_s_d_s_a_s_c_s_d_s_c_s_a_s_c_s
GTGGCTGTGACGGTGTCTGTGTGGGTGACGGTGTGGGTGTCTGTGTGGGTG; >clamp_s_c_s_d_s_c_s_a_s_e_s:compl:rev
CACCAGCACAGACACCCACACCGTCACCCACACTATCATGTTGACATTCC; dG = -37.1
278 >s_b_s_d_s_b_s_c_s_d_s_c_s_a_s_c_s
GTGGCTGTGACGGTGGCTGTGTGGGTGACGGTGTGGGTGTCTGTGTGGGTG; >clamp_s_c_s_d_s_c_s_a_s_e_s:compl:rev
CACCAGCACAGACACCCACACCGTCACCCACACTATCATGTTGACATTCC; dG = -37.1
279 >s_b_s_e_s_a_s_c_s_d_s_c_s_a_s_c_s
GTGGCTGTGCTGGTGTCTGTGTGGGTGACGGTGTGGGTGTCTGTGTGGGTG; >clamp_s_c_s_d_s_c_s_a_s_e_s:compl:rev
CACCAGCACAGACACCCACACCGTCACCCACACTATCATGTTGACATTCC; dG = -37.1
280 >s_b_s_e_s_b_s_c_s_d_s_c_s_a_s_c_s
GTGGCTGTGCTGGTGGCTGTGTGGGTGACGGTGTGGGTGTCTGTGTGGGTG; >clamp_s_c_s_d_s_c_s_a_s_e_s:compl:rev
CACCAGCACAGACACCCACACCGTCACCCACACTATCATGTTGACATTCC; dG = -37.1
281 >s_c_s_a_s_c_s_c_s_d_s_c_s_a_s_c_s
GTGTGGGTGTCTGTGTGGGTGTGGGTGACGGTGTGGGTGTCTGTGTGGGTG; >clamp_s_c_s_d_s_c_s_a_s_e_s:compl:rev
CACCAGCACAGACACCCACACCGTCACCCACACTATCATGTTGACATTCC; dG = -37.0
282 >s_c_s_a_s_d_s_c_s_d_s_c_s_a_s_c_s
GTGTGGGTGTCTGTGACGGTGTGGGTGACGGTGTGGGTGTCTGTGTGGGTG; >clamp_s_c_s_d_s_c_s_a_s_e_s:compl:rev
CACCAGCACAGACACCCACACCGTCACCCACACTATCATGTTGACATTCC; dG = -37.0
283 >s_c_s_a_s_e_s_c_s_d_s_c_s_a_s_c_s
GTGTGGGTGTCTGTGCTGGTGTGGGTGACGGTGTGGGTGTCTGTGTGGGTG; >clamp_s_c_s_d_s_c_s_a_s_e_s:compl:rev
CACCAGCACAGACACCCACACCGTCACCCACACTATCATGTTGACATTCC; dG = -37.0
284 >s_c_s_b_s_c_s_c_s_d_s_c_s_a_s_c_s
GTGTGGGTGGCTGTGTGGGTGTGGGTGACGGTGTGGGTGTCTGTGTGGGTG; >clamp_s_c_s_d_s_c_s_a_s_e_s:compl:rev
CACCAGCACAGACACCCACACCGTCACCCACACTATCATGTTGACATTCC; dG = -37.0
285 >s_c_s_b_s_d_s_c_s_d_s_c_s_a_s_c_s
GTGTGGGTGGCTGTGACGGTGTGGGTGACGGTGTGGGTGTCTGTGTGGGTG; >clamp_s_c_s_d_s_c_s_a_s_e_s:compl:rev
CACCAGCACAGACACCCACACCGTCACCCACACTATCATGTTGACATTCC; dG = -37.0
286 >s_c_s_b_s_e_s_c_s_d_s_c_s_a_s_c_s
GTGTGGGTGGCTGTGCTGGTGTGGGTGACGGTGTGGGTGTCTGTGTGGGTG; >clamp_s_c_s_d_s_c_s_a_s_e_s:compl:rev
CACCAGCACAGACACCCACACCGTCACCCACACTATCATGTTGACATTCC; dG = -37.0
287 >s_c_s_c_s_c_s_c_s_d_s_c_s_a_s_c_s
GTGTGGGTGTGGGTGTGGGTGTGGGTGACGGTGTGGGTGTCTGTGTGGGTG; >clamp_s_c_s_d_s_c_s_a_s_e_s:compl:rev
CACCAGCACAGACACCCACACCGTCACCCACACTATCATGTTGACATTCC; dG = -37.0
288 >s_c_s_c_s_d_s_c_s_d_s_c_s_a_s_c_s
GTGTGGGTGTGGGTGACGGTGTGGGTGACGGTGTGGGTGTCTGTGTGGGTG; >clamp_s_c_s_d_s_c_s_a_s_e_s:compl:rev
CACCAGCACAGACACCCACACCGTCACCCACACTATCATGTTGACATTCC; dG = -37.0
289 >s_c_s_c_s_e_s_c_s_d_s_c_s_a_s_c_s
GTGTGGGTGTGGGTGCTGGTGTGGGTGACGGTGTGGGTGTCTGTGTGGGTG; >clamp_s_c_s_d_s_c_s_a_s_e_s:compl:rev
CACCAGCACAGACACCCACACCGTCACCCACACTATCATGTTGACATTCC; dG = -37.0
290 >s_c_s_d_s_c_s_c_s_d_s_c_s_a_s_c_s
GTGTGGGTGACGGTGTGGGTGTGGGTGACGGTGTGGGTGTCTGTGTGGGTG; >clamp_s_c_s_d_s_c_s_a_s_e_s:compl:rev
CACCAGCACAGACACCCACACCGTCACCCACACTATCATGTTGACATTCC; dG = -37.0
291 >s_c_s_d_s_d_s_c_s_d_s_c_s_a_s_c_s
GTGTGGGTGACGGTGACGGTGTGGGTGACGGTGTGGGTGTCTGTGTGGGTG; >clamp_s_c_s_d_s_c_s_a_s_e_s:compl:rev
CACCAGCACAGACACCCACACCGTCACCCACACTATCATGTTGACATTCC; dG = -37.0
292 >s_c_s_d_s_e_s_c_s_d_s_c_s_a_s_c_s
GTGTGGGTGACGGTGCTGGTGTGGGTGACGGTGTGGGTGTCTGTGTGGGTG; >clamp_s_c_s_d_s_c_s_a_s_e_s:compl:rev
CACCAGCACAGACACCCACACCGTCACCCACACTATCATGTTGACATTCC; dG = -37.0
293 >s_a_s_b_s_c_s_c_s_d_s_c_s_a_s_c_s
GTGTCTGTGGCTGTGTGGGTGTGGGTGACGGTGTGGGTGTCTGTGTGGGTG; >clamp_s_c_s_d_s_c_s_a_s_e_s:compl:rev
CACCAGCACAGACACCCACACCGTCACCCACACTATCATGTTGACATTCC; dG = -37.0
294 >s_c_s_e_s_c_s_c_s_d_s_c_s_a_s_c_s
GTGTGGGTGCTGGTGTGGGTGTGGGTGACGGTGTGGGTGTCTGTGTGGGTG; >clamp_s_c_s_d_s_c_s_a_s_e_s:compl:rev
CACCAGCACAGACACCCACACCGTCACCCACACTATCATGTTGACATTCC; dG = -37.0
295 >s_c_s_e_s_d_s_c_s_d_s_c_s_a_s_c_s
GTGTGGGTGCTGGTGACGGTGTGGGTGACGGTGTGGGTGTCTGTGTGGGTG; >clamp_s_c_s_d_s_c_s_a_s_e_s:compl:rev
CACCAGCACAGACACCCACACCGTCACCCACACTATCATGTTGACATTCC; dG = -37.0

296 >s_c_s_e_s_e_s_c_s_d_s_c_s_a_s_c_s
GTGTGGGTGCTGGTGCTGGTGTGGGTGACGGTGTGGGTGTCTGTGTGGGTG; >clamp_s_c_s_d_s_c_s_a_s_e_s:compl:rev
CACCAGCACAGACACCCACACCGTCACCCACACTATCATGTTGACATTCC; dG = -37.0
297 >s_d_s_a_s_c_s_c_s_d_s_c_s_a_s_c_s
GTGACGGTGTCTGTGTGGGTGTGGGTGACGGTGTGGGTGTCTGTGTGGGTG; >clamp_s_c_s_d_s_c_s_a_s_e_s:compl:rev
CACCAGCACAGACACCCACACCGTCACCCACACTATCATGTTGACATTCC; dG = -37.0
298 >s_d_s_a_s_d_s_c_s_d_s_c_s_a_s_c_s
GTGACGGTGTCTGTGACGGTGTGGGTGACGGTGTGGGTGTCTGTGTGGGTG; >clamp_s_c_s_d_s_c_s_a_s_e_s:compl:rev
CACCAGCACAGACACCCACACCGTCACCCACACTATCATGTTGACATTCC; dG = -37.0
299 >s_d_s_a_s_e_s_c_s_d_s_c_s_a_s_c_s
GTGACGGTGTCTGTGCTGGTGTGGGTGACGGTGTGGGTGTCTGTGTGGGTG; >clamp_s_c_s_d_s_c_s_a_s_e_s:compl:rev
CACCAGCACAGACACCCACACCGTCACCCACACTATCATGTTGACATTCC; dG = -37.0
300 >s_a_s_b_s_d_s_c_s_d_s_c_s_a_s_c_s
GTGTCTGTGGCTGTGACGGTGTGGGTGACGGTGTGGGTGTCTGTGTGGGTG; >clamp_s_c_s_d_s_c_s_a_s_e_s:compl:rev
CACCAGCACAGACACCCACACCGTCACCCACACTATCATGTTGACATTCC; dG = -37.0
301 >s_d_s_b_s_c_s_c_s_d_s_c_s_a_s_c_s
GTGACGGTGGCTGTGTGGGTGTGGGTGACGGTGTGGGTGTCTGTGTGGGTG; >clamp_s_c_s_d_s_c_s_a_s_e_s:compl:rev
CACCAGCACAGACACCCACACCGTCACCCACACTATCATGTTGACATTCC; dG = -37.0
302 >s_d_s_b_s_d_s_c_s_d_s_c_s_a_s_c_s
GTGACGGTGGCTGTGACGGTGTGGGTGACGGTGTGGGTGTCTGTGTGGGTG; >clamp_s_c_s_d_s_c_s_a_s_e_s:compl:rev
CACCAGCACAGACACCCACACCGTCACCCACACTATCATGTTGACATTCC; dG = -37.0
303 >s_d_s_b_s_e_s_c_s_d_s_c_s_a_s_c_s
GTGACGGTGGCTGTGCTGGTGTGGGTGACGGTGTGGGTGTCTGTGTGGGTG; >clamp_s_c_s_d_s_c_s_a_s_e_s:compl:rev
CACCAGCACAGACACCCACACCGTCACCCACACTATCATGTTGACATTCC; dG = -37.0
304 >s_d_s_c_s_c_s_c_s_d_s_c_s_a_s_c_s
GTGACGGTGTGGGTGTGGGTGTGGGTGACGGTGTGGGTGTCTGTGTGGGTG; >clamp_s_c_s_d_s_c_s_a_s_e_s:compl:rev
CACCAGCACAGACACCCACACCGTCACCCACACTATCATGTTGACATTCC; dG = -37.0
305 >s_d_s_c_s_d_s_c_s_d_s_c_s_a_s_c_s
GTGACGGTGTGGGTGACGGTGTGGGTGACGGTGTGGGTGTCTGTGTGGGTG; >clamp_s_c_s_d_s_c_s_a_s_e_s:compl:rev
CACCAGCACAGACACCCACACCGTCACCCACACTATCATGTTGACATTCC; dG = -37.0
306 >s_d_s_c_s_e_s_c_s_d_s_c_s_a_s_c_s
GTGACGGTGTGGGTGCTGGTGTGGGTGACGGTGTGGGTGTCTGTGTGGGTG; >clamp_s_c_s_d_s_c_s_a_s_e_s:compl:rev
CACCAGCACAGACACCCACACCGTCACCCACACTATCATGTTGACATTCC; dG = -37.0
307 >s_a_s_b_s_e_s_c_s_d_s_c_s_a_s_c_s
GTGTCTGTGGCTGTGCTGGTGTGGGTGACGGTGTGGGTGTCTGTGTGGGTG; >clamp_s_c_s_d_s_c_s_a_s_e_s:compl:rev
CACCAGCACAGACACCCACACCGTCACCCACACTATCATGTTGACATTCC; dG = -37.0
308 >s_d_s_d_s_c_s_c_s_d_s_c_s_a_s_c_s
GTGACGGTGACGGTGTGGGTGTGGGTGACGGTGTGGGTGTCTGTGTGGGTG; >clamp_s_c_s_d_s_c_s_a_s_e_s:compl:rev
CACCAGCACAGACACCCACACCGTCACCCACACTATCATGTTGACATTCC; dG = -37.0
309 >s_d_s_d_s_d_s_c_s_d_s_c_s_a_s_c_s
GTGACGGTGACGGTGACGGTGTGGGTGACGGTGTGGGTGTCTGTGTGGGTG; >clamp_s_c_s_d_s_c_s_a_s_e_s:compl:rev
CACCAGCACAGACACCCACACCGTCACCCACACTATCATGTTGACATTCC; dG = -37.0
310 >s_d_s_d_s_e_s_c_s_d_s_c_s_a_s_c_s
GTGACGGTGACGGTGCTGGTGTGGGTGACGGTGTGGGTGTCTGTGTGGGTG; >clamp_s_c_s_d_s_c_s_a_s_e_s:compl:rev
CACCAGCACAGACACCCACACCGTCACCCACACTATCATGTTGACATTCC; dG = -37.0
311 >s_d_s_e_s_c_s_c_s_d_s_c_s_a_s_c_s
GTGACGGTGCTGGTGTGGGTGTGGGTGACGGTGTGGGTGTCTGTGTGGGTG; >clamp_s_c_s_d_s_c_s_a_s_e_s:compl:rev
CACCAGCACAGACACCCACACCGTCACCCACACTATCATGTTGACATTCC; dG = -37.0
312 >s_d_s_e_s_d_s_c_s_d_s_c_s_a_s_c_s
GTGACGGTGCTGGTGACGGTGTGGGTGACGGTGTGGGTGTCTGTGTGGGTG; >clamp_s_c_s_d_s_c_s_a_s_e_s:compl:rev
CACCAGCACAGACACCCACACCGTCACCCACACTATCATGTTGACATTCC; dG = -37.0
313 >s_d_s_e_s_e_s_c_s_d_s_c_s_a_s_c_s
GTGACGGTGCTGGTGCTGGTGTGGGTGACGGTGTGGGTGTCTGTGTGGGTG; >clamp_s_c_s_d_s_c_s_a_s_e_s:compl:rev
CACCAGCACAGACACCCACACCGTCACCCACACTATCATGTTGACATTCC; dG = -37.0
314 >s_e_s_a_s_c_s_c_s_d_s_c_s_a_s_c_s
GTGCTGGTGTCTGTGTGGGTGTGGGTGACGGTGTGGGTGTCTGTGTGGGTG; >clamp_s_c_s_d_s_c_s_a_s_e_s:compl:rev
CACCAGCACAGACACCCACACCGTCACCCACACTATCATGTTGACATTCC; dG = -37.0
315 >s_e_s_a_s_d_s_c_s_d_s_c_s_a_s_c_s
GTGCTGGTGTCTGTGACGGTGTGGGTGACGGTGTGGGTGTCTGTGTGGGTG; >clamp_s_c_s_d_s_c_s_a_s_e_s:compl:rev
CACCAGCACAGACACCCACACCGTCACCCACACTATCATGTTGACATTCC; dG = -37.0
316 >s_e_s_a_s_e_s_c_s_d_s_c_s_a_s_c_s
GTGCTGGTGTCTGTGCTGGTGTGGGTGACGGTGTGGGTGTCTGTGTGGGTG; >clamp_s_c_s_d_s_c_s_a_s_e_s:compl:rev
CACCAGCACAGACACCCACACCGTCACCCACACTATCATGTTGACATTCC; dG = -37.0
317 >s_e_s_b_s_c_s_c_s_d_s_c_s_a_s_c_s
GTGCTGGTGGCTGTGTGGGTGTGGGTGACGGTGTGGGTGTCTGTGTGGGTG; >clamp_s_c_s_d_s_c_s_a_s_e_s:compl:rev
CACCAGCACAGACACCCACACCGTCACCCACACTATCATGTTGACATTCC; dG = -37.0
318 >s_e_s_b_s_d_s_c_s_d_s_c_s_a_s_c_s
GTGCTGGTGGCTGTGACGGTGTGGGTGACGGTGTGGGTGTCTGTGTGGGTG; >clamp_s_c_s_d_s_c_s_a_s_e_s:compl:rev
CACCAGCACAGACACCCACACCGTCACCCACACTATCATGTTGACATTCC; dG = -37.0
319 >s_e_s_b_s_e_s_c_s_d_s_c_s_a_s_c_s
GTGCTGGTGGCTGTGCTGGTGTGGGTGACGGTGTGGGTGTCTGTGTGGGTG; >clamp_s_c_s_d_s_c_s_a_s_e_s:compl:rev
CACCAGCACAGACACCCACACCGTCACCCACACTATCATGTTGACATTCC; dG = -37.0

320 >s_e_s_c_s_c_s_c_s_d_s_c_s_a_s_c_s
GTGCTGGTGTGGGTGTGGGTGTGGGTGACGGTGTGGGTGTCTGTGTGGGTG; >clamp_s_c_s_d_s_c_s_a_s_e_s:compl:rev
CACCAGCACAGACACCCACACCGTCACCCACACTATCATGTTGACATTCC; dG = -37.0
321 >s_e_s_c_s_d_s_c_s_d_s_c_s_a_s_c_s
GTGCTGGTGTGGGTGACGGTGTGGGTGACGGTGTGGGTGTCTGTGTGGGTG; >clamp_s_c_s_d_s_c_s_a_s_e_s:compl:rev
CACCAGCACAGACACCCACACCGTCACCCACACTATCATGTTGACATTCC; dG = -37.0
322 >s_e_s_c_s_e_s_c_s_d_s_c_s_a_s_c_s
GTGCTGGTGTGGGTGCTGGTGTGGGTGACGGTGTGGGTGTCTGTGTGGGTG; >clamp_s_c_s_d_s_c_s_a_s_e_s:compl:rev
CACCAGCACAGACACCCACACCGTCACCCACACTATCATGTTGACATTCC; dG = -37.0
323 >s_e_s_d_s_c_s_c_s_d_s_c_s_a_s_c_s
GTGCTGGTGACGGTGTGGGTGTGGGTGACGGTGTGGGTGTCTGTGTGGGTG; >clamp_s_c_s_d_s_c_s_a_s_e_s:compl:rev
CACCAGCACAGACACCCACACCGTCACCCACACTATCATGTTGACATTCC; dG = -37.0
324 >s_e_s_d_s_d_s_c_s_d_s_c_s_a_s_c_s
GTGCTGGTGACGGTGACGGTGTGGGTGACGGTGTGGGTGTCTGTGTGGGTG; >clamp_s_c_s_d_s_c_s_a_s_e_s:compl:rev
CACCAGCACAGACACCCACACCGTCACCCACACTATCATGTTGACATTCC; dG = -37.0
325 >s_e_s_d_s_e_s_c_s_d_s_c_s_a_s_c_s
GTGCTGGTGACGGTGCTGGTGTGGGTGACGGTGTGGGTGTCTGTGTGGGTG; >clamp_s_c_s_d_s_c_s_a_s_e_s:compl:rev
CACCAGCACAGACACCCACACCGTCACCCACACTATCATGTTGACATTCC; dG = -37.0
326 >s_a_s_c_s_c_s_c_s_d_s_c_s_a_s_c_s
GTGTCTGTGTGGGTGTGGGTGTGGGTGACGGTGTGGGTGTCTGTGTGGGTG; >clamp_s_c_s_d_s_c_s_a_s_e_s:compl:rev
CACCAGCACAGACACCCACACCGTCACCCACACTATCATGTTGACATTCC; dG = -37.0
327 >s_e_s_e_s_c_s_c_s_d_s_c_s_a_s_c_s
GTGCTGGTGCTGGTGTGGGTGTGGGTGACGGTGTGGGTGTCTGTGTGGGTG; >clamp_s_c_s_d_s_c_s_a_s_e_s:compl:rev
CACCAGCACAGACACCCACACCGTCACCCACACTATCATGTTGACATTCC; dG = -37.0
328 >s_e_s_e_s_d_s_c_s_d_s_c_s_a_s_c_s
GTGCTGGTGCTGGTGACGGTGTGGGTGACGGTGTGGGTGTCTGTGTGGGTG; >clamp_s_c_s_d_s_c_s_a_s_e_s:compl:rev
CACCAGCACAGACACCCACACCGTCACCCACACTATCATGTTGACATTCC; dG = -37.0
329 >s_e_s_e_s_e_s_c_s_d_s_c_s_a_s_c_s
GTGCTGGTGCTGGTGCTGGTGTGGGTGACGGTGTGGGTGTCTGTGTGGGTG; >clamp_s_c_s_d_s_c_s_a_s_e_s:compl:rev
CACCAGCACAGACACCCACACCGTCACCCACACTATCATGTTGACATTCC; dG = -37.0
330 >s_a_s_c_s_d_s_c_s_d_s_c_s_a_s_c_s
GTGTCTGTGTGGGTGACGGTGTGGGTGACGGTGTGGGTGTCTGTGTGGGTG; >clamp_s_c_s_d_s_c_s_a_s_e_s:compl:rev
CACCAGCACAGACACCCACACCGTCACCCACACTATCATGTTGACATTCC; dG = -37.0
331 >s_a_s_c_s_e_s_c_s_d_s_c_s_a_s_c_s
GTGTCTGTGTGGGTGCTGGTGTGGGTGACGGTGTGGGTGTCTGTGTGGGTG; >clamp_s_c_s_d_s_c_s_a_s_e_s:compl:rev
CACCAGCACAGACACCCACACCGTCACCCACACTATCATGTTGACATTCC; dG = -37.0
332 >s_a_s_d_s_c_s_c_s_d_s_c_s_a_s_c_s
GTGTCTGTGACGGTGTGGGTGTGGGTGACGGTGTGGGTGTCTGTGTGGGTG; >clamp_s_c_s_d_s_c_s_a_s_e_s:compl:rev
CACCAGCACAGACACCCACACCGTCACCCACACTATCATGTTGACATTCC; dG = -37.0
333 >s_a_s_d_s_d_s_c_s_d_s_c_s_a_s_c_s
GTGTCTGTGACGGTGACGGTGTGGGTGACGGTGTGGGTGTCTGTGTGGGTG; >clamp_s_c_s_d_s_c_s_a_s_e_s:compl:rev
CACCAGCACAGACACCCACACCGTCACCCACACTATCATGTTGACATTCC; dG = -37.0
334 >s_a_s_d_s_e_s_c_s_d_s_c_s_a_s_c_s
GTGTCTGTGACGGTGCTGGTGTGGGTGACGGTGTGGGTGTCTGTGTGGGTG; >clamp_s_c_s_d_s_c_s_a_s_e_s:compl:rev
CACCAGCACAGACACCCACACCGTCACCCACACTATCATGTTGACATTCC; dG = -37.0
335 >s_a_s_a_s_c_s_c_s_d_s_c_s_a_s_c_s
GTGTCTGTGTCTGTGTGGGTGTGGGTGACGGTGTGGGTGTCTGTGTGGGTG; >clamp_s_c_s_d_s_c_s_a_s_e_s:compl:rev
CACCAGCACAGACACCCACACCGTCACCCACACTATCATGTTGACATTCC; dG = -37.0
336 >s_a_s_e_s_c_s_c_s_d_s_c_s_a_s_c_s
GTGTCTGTGCTGGTGTGGGTGTGGGTGACGGTGTGGGTGTCTGTGTGGGTG; >clamp_s_c_s_d_s_c_s_a_s_e_s:compl:rev
CACCAGCACAGACACCCACACCGTCACCCACACTATCATGTTGACATTCC; dG = -37.0
337 >s_a_s_e_s_d_s_c_s_d_s_c_s_a_s_c_s
GTGTCTGTGCTGGTGACGGTGTGGGTGACGGTGTGGGTGTCTGTGTGGGTG; >clamp_s_c_s_d_s_c_s_a_s_e_s:compl:rev
CACCAGCACAGACACCCACACCGTCACCCACACTATCATGTTGACATTCC; dG = -37.0
338 >s_a_s_e_s_e_s_c_s_d_s_c_s_a_s_c_s
GTGTCTGTGCTGGTGCTGGTGTGGGTGACGGTGTGGGTGTCTGTGTGGGTG; >clamp_s_c_s_d_s_c_s_a_s_e_s:compl:rev
CACCAGCACAGACACCCACACCGTCACCCACACTATCATGTTGACATTCC; dG = -37.0
339 >s_b_s_a_s_c_s_c_s_d_s_c_s_a_s_c_s
GTGGCTGTGTCTGTGTGGGTGTGGGTGACGGTGTGGGTGTCTGTGTGGGTG; >clamp_s_c_s_d_s_c_s_a_s_e_s:compl:rev
CACCAGCACAGACACCCACACCGTCACCCACACTATCATGTTGACATTCC; dG = -37.0
340 >s_b_s_a_s_d_s_c_s_d_s_c_s_a_s_c_s
GTGGCTGTGTCTGTGACGGTGTGGGTGACGGTGTGGGTGTCTGTGTGGGTG; >clamp_s_c_s_d_s_c_s_a_s_e_s:compl:rev
CACCAGCACAGACACCCACACCGTCACCCACACTATCATGTTGACATTCC; dG = -37.0
341 >s_b_s_a_s_e_s_c_s_d_s_c_s_a_s_c_s
GTGGCTGTGTCTGTGCTGGTGTGGGTGACGGTGTGGGTGTCTGTGTGGGTG; >clamp_s_c_s_d_s_c_s_a_s_e_s:compl:rev
CACCAGCACAGACACCCACACCGTCACCCACACTATCATGTTGACATTCC; dG = -37.0
342 >s_a_s_a_s_d_s_c_s_d_s_c_s_a_s_c_s
GTGTCTGTGTCTGTGACGGTGTGGGTGACGGTGTGGGTGTCTGTGTGGGTG; >clamp_s_c_s_d_s_c_s_a_s_e_s:compl:rev
CACCAGCACAGACACCCACACCGTCACCCACACTATCATGTTGACATTCC; dG = -37.0
343 >s_b_s_b_s_c_s_c_s_d_s_c_s_a_s_c_s
GTGGCTGTGGCTGTGTGGGTGTGGGTGACGGTGTGGGTGTCTGTGTGGGTG; >clamp_s_c_s_d_s_c_s_a_s_e_s:compl:rev
CACCAGCACAGACACCCACACCGTCACCCACACTATCATGTTGACATTCC; dG = -37.0

344 >s_b_s_b_s_d_s_c_s_d_s_c_s_a_s_c_s
GTGGCTGTGGCTGTGACGGTGTGGGTGACGGTGTGGGTGTCTGTGTGGGTG; >clamp_s_c_s_d_s_c_s_a_s_e_s:compl:rev
CACCAGCACAGACACCCACACCGTCACCCACACTATCATGTTGACATTCC; dG = -37.0
345 >s_b_s_b_s_e_s_c_s_d_s_c_s_a_s_c_s
GTGGCTGTGGCTGTGCTGGTGTGGGTGACGGTGTGGGTGTCTGTGTGGGTG; >clamp_s_c_s_d_s_c_s_a_s_e_s:compl:rev
CACCAGCACAGACACCCACACCGTCACCCACACTATCATGTTGACATTCC; dG = -37.0
346 >s_b_s_c_s_c_s_c_s_d_s_c_s_a_s_c_s
GTGGCTGTGTGGGTGTGGGTGTGGGTGACGGTGTGGGTGTCTGTGTGGGTG; >clamp_s_c_s_d_s_c_s_a_s_e_s:compl:rev
CACCAGCACAGACACCCACACCGTCACCCACACTATCATGTTGACATTCC; dG = -37.0
347 >s_b_s_c_s_d_s_c_s_d_s_c_s_a_s_c_s
GTGGCTGTGTGGGTGACGGTGTGGGTGACGGTGTGGGTGTCTGTGTGGGTG; >clamp_s_c_s_d_s_c_s_a_s_e_s:compl:rev
CACCAGCACAGACACCCACACCGTCACCCACACTATCATGTTGACATTCC; dG = -37.0
348 >s_b_s_c_s_e_s_c_s_d_s_c_s_a_s_c_s
GTGGCTGTGTGGGTGCTGGTGTGGGTGACGGTGTGGGTGTCTGTGTGGGTG; >clamp_s_c_s_d_s_c_s_a_s_e_s:compl:rev
CACCAGCACAGACACCCACACCGTCACCCACACTATCATGTTGACATTCC; dG = -37.0
349 >s_a_s_a_s_e_s_c_s_d_s_c_s_a_s_c_s
GTGTCTGTGTCTGTGCTGGTGTGGGTGACGGTGTGGGTGTCTGTGTGGGTG; >clamp_s_c_s_d_s_c_s_a_s_e_s:compl:rev
CACCAGCACAGACACCCACACCGTCACCCACACTATCATGTTGACATTCC; dG = -37.0
350 >s_b_s_d_s_c_s_c_s_d_s_c_s_a_s_c_s
GTGGCTGTGACGGTGTGGGTGTGGGTGACGGTGTGGGTGTCTGTGTGGGTG; >clamp_s_c_s_d_s_c_s_a_s_e_s:compl:rev
CACCAGCACAGACACCCACACCGTCACCCACACTATCATGTTGACATTCC; dG = -37.0
351 >s_b_s_d_s_d_s_c_s_d_s_c_s_a_s_c_s
GTGGCTGTGACGGTGACGGTGTGGGTGACGGTGTGGGTGTCTGTGTGGGTG; >clamp_s_c_s_d_s_c_s_a_s_e_s:compl:rev
CACCAGCACAGACACCCACACCGTCACCCACACTATCATGTTGACATTCC; dG = -37.0
352 >s_b_s_d_s_e_s_c_s_d_s_c_s_a_s_c_s
GTGGCTGTGACGGTGCTGGTGTGGGTGACGGTGTGGGTGTCTGTGTGGGTG; >clamp_s_c_s_d_s_c_s_a_s_e_s:compl:rev
CACCAGCACAGACACCCACACCGTCACCCACACTATCATGTTGACATTCC; dG = -37.0
353 >s_b_s_e_s_c_s_c_s_d_s_c_s_a_s_c_s
GTGGCTGTGCTGGTGTGGGTGTGGGTGACGGTGTGGGTGTCTGTGTGGGTG; >clamp_s_c_s_d_s_c_s_a_s_e_s:compl:rev
CACCAGCACAGACACCCACACCGTCACCCACACTATCATGTTGACATTCC; dG = -37.0
354 >s_b_s_e_s_d_s_c_s_d_s_c_s_a_s_c_s
GTGGCTGTGCTGGTGACGGTGTGGGTGACGGTGTGGGTGTCTGTGTGGGTG; >clamp_s_c_s_d_s_c_s_a_s_e_s:compl:rev
CACCAGCACAGACACCCACACCGTCACCCACACTATCATGTTGACATTCC; dG = -37.0
355 >s_b_s_e_s_e_s_c_s_d_s_c_s_a_s_c_s
GTGGCTGTGCTGGTGCTGGTGTGGGTGACGGTGTGGGTGTCTGTGTGGGTG; >clamp_s_c_s_d_s_c_s_a_s_e_s:compl:rev
CACCAGCACAGACACCCACACCGTCACCCACACTATCATGTTGACATTCC; dG = -37.0
356 >s_c_s_a_s_a_s_c_s_d_s_c_s_c_s_e_s
GTGTGGGTGTCTGTGTCTGTGTGGGTGACGGTGTGGGTGTGGGTGCTGGTG; >clamp_s_c_s_d_s_c_s_a_s_e_s:compl:rev
CACCAGCACAGACACCCACACCGTCACCCACACTATCATGTTGACATTCC; dG = -36.9
357 >s_c_s_a_s_b_s_c_s_d_s_c_s_c_s_e_s
GTGTGGGTGTCTGTGGCTGTGTGGGTGACGGTGTGGGTGTGGGTGCTGGTG; >clamp_s_c_s_d_s_c_s_a_s_e_s:compl:rev
CACCAGCACAGACACCCACACCGTCACCCACACTATCATGTTGACATTCC; dG = -36.9
358 >s_c_s_a_s_c_s_c_s_d_s_c_s_c_s_e_s
GTGTGGGTGTCTGTGTGGGTGTGGGTGACGGTGTGGGTGTGGGTGCTGGTG; >clamp_s_c_s_d_s_c_s_a_s_e_s:compl:rev
CACCAGCACAGACACCCACACCGTCACCCACACTATCATGTTGACATTCC; dG = -36.9
359 >s_c_s_a_s_d_s_c_s_d_s_c_s_c_s_e_s
GTGTGGGTGTCTGTGACGGTGTGGGTGACGGTGTGGGTGTGGGTGCTGGTG; >clamp_s_c_s_d_s_c_s_a_s_e_s:compl:rev
CACCAGCACAGACACCCACACCGTCACCCACACTATCATGTTGACATTCC; dG = -36.9
360 >s_a_s_b_s_a_s_c_s_d_s_c_s_c_s_e_s
GTGTCTGTGGCTGTGTCTGTGTGGGTGACGGTGTGGGTGTGGGTGCTGGTG; >clamp_s_c_s_d_s_c_s_a_s_e_s:compl:rev
CACCAGCACAGACACCCACACCGTCACCCACACTATCATGTTGACATTCC; dG = -36.9
361 >s_c_s_a_s_e_s_c_s_d_s_c_s_c_s_e_s
GTGTGGGTGTCTGTGCTGGTGTGGGTGACGGTGTGGGTGTGGGTGCTGGTG; >clamp_s_c_s_d_s_c_s_a_s_e_s:compl:rev
CACCAGCACAGACACCCACACCGTCACCCACACTATCATGTTGACATTCC; dG = -36.9
362 >s_c_s_b_s_a_s_c_s_d_s_c_s_c_s_e_s
GTGTGGGTGGCTGTGTCTGTGTGGGTGACGGTGTGGGTGTGGGTGCTGGTG; >clamp_s_c_s_d_s_c_s_a_s_e_s:compl:rev
CACCAGCACAGACACCCACACCGTCACCCACACTATCATGTTGACATTCC; dG = -36.9
363 >s_c_s_b_s_b_s_c_s_d_s_c_s_c_s_e_s
GTGTGGGTGGCTGTGGCTGTGTGGGTGACGGTGTGGGTGTGGGTGCTGGTG; >clamp_s_c_s_d_s_c_s_a_s_e_s:compl:rev
CACCAGCACAGACACCCACACCGTCACCCACACTATCATGTTGACATTCC; dG = -36.9
364 >s_c_s_b_s_c_s_c_s_d_s_c_s_c_s_e_s
GTGTGGGTGGCTGTGTGGGTGTGGGTGACGGTGTGGGTGTGGGTGCTGGTG; >clamp_s_c_s_d_s_c_s_a_s_e_s:compl:rev
CACCAGCACAGACACCCACACCGTCACCCACACTATCATGTTGACATTCC; dG = -36.9
365 >s_c_s_b_s_d_s_c_s_d_s_c_s_c_s_e_s
GTGTGGGTGGCTGTGACGGTGTGGGTGACGGTGTGGGTGTGGGTGCTGGTG; >clamp_s_c_s_d_s_c_s_a_s_e_s:compl:rev
CACCAGCACAGACACCCACACCGTCACCCACACTATCATGTTGACATTCC; dG = -36.9
366 >s_c_s_b_s_e_s_c_s_d_s_c_s_c_s_e_s
GTGTGGGTGGCTGTGCTGGTGTGGGTGACGGTGTGGGTGTGGGTGCTGGTG; >clamp_s_c_s_d_s_c_s_a_s_e_s:compl:rev
CACCAGCACAGACACCCACACCGTCACCCACACTATCATGTTGACATTCC; dG = -36.9
367 >s_c_s_c_s_a_s_c_s_d_s_c_s_c_s_e_s
GTGTGGGTGTGGGTGTCTGTGTGGGTGACGGTGTGGGTGTGGGTGCTGGTG; >clamp_s_c_s_d_s_c_s_a_s_e_s:compl:rev
CACCAGCACAGACACCCACACCGTCACCCACACTATCATGTTGACATTCC; dG = -36.9

368 >s_c_s_c_s_b_s_c_s_d_s_c_s_c_s_e_s
GTGTGGGTGTGGGTGGCTGTGTGGGTGACGGTGTGGGTGTGGGTGCTGGTG; >clamp_s_c_s_d_s_c_s_a_s_e_s:compl:rev
CACCAGCACAGACACCCACACCGTCACCCACACTATCATGTTGACATTCC; dG = -36.9
369 >s_c_s_c_s_c_s_c_s_d_s_c_s_c_s_e_s
GTGTGGGTGTGGGTGTGGGTGTGGGTGACGGTGTGGGTGTGGGTGCTGGTG; >clamp_s_c_s_d_s_c_s_a_s_e_s:compl:rev
CACCAGCACAGACACCCACACCGTCACCCACACTATCATGTTGACATTCC; dG = -36.9
370 >s_c_s_c_s_d_s_c_s_d_s_c_s_c_s_e_s
GTGTGGGTGTGGGTGACGGTGTGGGTGACGGTGTGGGTGTGGGTGCTGGTG; >clamp_s_c_s_d_s_c_s_a_s_e_s:compl:rev
CACCAGCACAGACACCCACACCGTCACCCACACTATCATGTTGACATTCC; dG = -36.9
371 >s_a_s_b_s_b_s_c_s_d_s_c_s_c_s_e_s
GTGTCTGTGGCTGTGGCTGTGTGGGTGACGGTGTGGGTGTGGGTGCTGGTG; >clamp_s_c_s_d_s_c_s_a_s_e_s:compl:rev
CACCAGCACAGACACCCACACCGTCACCCACACTATCATGTTGACATTCC; dG = -36.9
372 >s_c_s_c_s_e_s_c_s_d_s_c_s_c_s_e_s
GTGTGGGTGTGGGTGCTGGTGTGGGTGACGGTGTGGGTGTGGGTGCTGGTG; >clamp_s_c_s_d_s_c_s_a_s_e_s:compl:rev
CACCAGCACAGACACCCACACCGTCACCCACACTATCATGTTGACATTCC; dG = -36.9
373 >s_c_s_d_s_a_s_c_s_d_s_c_s_c_s_e_s
GTGTGGGTGACGGTGTCTGTGTGGGTGACGGTGTGGGTGTGGGTGCTGGTG; >clamp_s_c_s_d_s_c_s_a_s_e_s:compl:rev
CACCAGCACAGACACCCACACCGTCACCCACACTATCATGTTGACATTCC; dG = -36.9
374 >s_c_s_d_s_b_s_c_s_d_s_c_s_c_s_e_s
GTGTGGGTGACGGTGGCTGTGTGGGTGACGGTGTGGGTGTGGGTGCTGGTG; >clamp_s_c_s_d_s_c_s_a_s_e_s:compl:rev
CACCAGCACAGACACCCACACCGTCACCCACACTATCATGTTGACATTCC; dG = -36.9
375 >s_c_s_d_s_c_s_c_s_d_s_c_s_c_s_e_s
GTGTGGGTGACGGTGTGGGTGTGGGTGACGGTGTGGGTGTGGGTGCTGGTG; >clamp_s_c_s_d_s_c_s_a_s_e_s:compl:rev
CACCAGCACAGACACCCACACCGTCACCCACACTATCATGTTGACATTCC; dG = -36.9
376 >s_c_s_d_s_d_s_c_s_d_s_c_s_c_s_e_s
GTGTGGGTGACGGTGACGGTGTGGGTGACGGTGTGGGTGTGGGTGCTGGTG; >clamp_s_c_s_d_s_c_s_a_s_e_s:compl:rev
CACCAGCACAGACACCCACACCGTCACCCACACTATCATGTTGACATTCC; dG = -36.9
377 >s_c_s_d_s_e_s_c_s_d_s_c_s_c_s_e_s
GTGTGGGTGACGGTGCTGGTGTGGGTGACGGTGTGGGTGTGGGTGCTGGTG; >clamp_s_c_s_d_s_c_s_a_s_e_s:compl:rev
CACCAGCACAGACACCCACACCGTCACCCACACTATCATGTTGACATTCC; dG = -36.9
378 >s_c_s_e_s_a_s_c_s_d_s_c_s_c_s_e_s
GTGTGGGTGCTGGTGTCTGTGTGGGTGACGGTGTGGGTGTGGGTGCTGGTG; >clamp_s_c_s_d_s_c_s_a_s_e_s:compl:rev
CACCAGCACAGACACCCACACCGTCACCCACACTATCATGTTGACATTCC; dG = -36.9
379 >s_c_s_e_s_b_s_c_s_d_s_c_s_c_s_e_s
GTGTGGGTGCTGGTGGCTGTGTGGGTGACGGTGTGGGTGTGGGTGCTGGTG; >clamp_s_c_s_d_s_c_s_a_s_e_s:compl:rev
CACCAGCACAGACACCCACACCGTCACCCACACTATCATGTTGACATTCC; dG = -36.9
380 >s_c_s_e_s_c_s_c_s_d_s_c_s_c_s_e_s
GTGTGGGTGCTGGTGTGGGTGTGGGTGACGGTGTGGGTGTGGGTGCTGGTG; >clamp_s_c_s_d_s_c_s_a_s_e_s:compl:rev
CACCAGCACAGACACCCACACCGTCACCCACACTATCATGTTGACATTCC; dG = -36.9
381 >s_c_s_e_s_d_s_c_s_d_s_c_s_c_s_e_s
GTGTGGGTGCTGGTGACGGTGTGGGTGACGGTGTGGGTGTGGGTGCTGGTG; >clamp_s_c_s_d_s_c_s_a_s_e_s:compl:rev
CACCAGCACAGACACCCACACCGTCACCCACACTATCATGTTGACATTCC; dG = -36.9
382 >s_a_s_b_s_c_s_c_s_d_s_c_s_c_s_e_s
GTGTCTGTGGCTGTGTGGGTGTGGGTGACGGTGTGGGTGTGGGTGCTGGTG; >clamp_s_c_s_d_s_c_s_a_s_e_s:compl:rev
CACCAGCACAGACACCCACACCGTCACCCACACTATCATGTTGACATTCC; dG = -36.9
383 >s_c_s_e_s_e_s_c_s_d_s_c_s_c_s_e_s
GTGTGGGTGCTGGTGCTGGTGTGGGTGACGGTGTGGGTGTGGGTGCTGGTG; >clamp_s_c_s_d_s_c_s_a_s_e_s:compl:rev
CACCAGCACAGACACCCACACCGTCACCCACACTATCATGTTGACATTCC; dG = -36.9
384 >s_d_s_a_s_a_s_c_s_d_s_c_s_c_s_e_s
GTGACGGTGTCTGTGTCTGTGTGGGTGACGGTGTGGGTGTGGGTGCTGGTG; >clamp_s_c_s_d_s_c_s_a_s_e_s:compl:rev
CACCAGCACAGACACCCACACCGTCACCCACACTATCATGTTGACATTCC; dG = -36.9
385 >s_d_s_a_s_b_s_c_s_d_s_c_s_c_s_e_s
GTGACGGTGTCTGTGGCTGTGTGGGTGACGGTGTGGGTGTGGGTGCTGGTG; >clamp_s_c_s_d_s_c_s_a_s_e_s:compl:rev
CACCAGCACAGACACCCACACCGTCACCCACACTATCATGTTGACATTCC; dG = -36.9
386 >s_d_s_a_s_c_s_c_s_d_s_c_s_c_s_e_s
GTGACGGTGTCTGTGTGGGTGTGGGTGACGGTGTGGGTGTGGGTGCTGGTG; >clamp_s_c_s_d_s_c_s_a_s_e_s:compl:rev
CACCAGCACAGACACCCACACCGTCACCCACACTATCATGTTGACATTCC; dG = -36.9
387 >s_d_s_a_s_d_s_c_s_d_s_c_s_c_s_e_s
GTGACGGTGTCTGTGACGGTGTGGGTGACGGTGTGGGTGTGGGTGCTGGTG; >clamp_s_c_s_d_s_c_s_a_s_e_s:compl:rev
CACCAGCACAGACACCCACACCGTCACCCACACTATCATGTTGACATTCC; dG = -36.9
388 >s_d_s_a_s_e_s_c_s_d_s_c_s_c_s_e_s
GTGACGGTGTCTGTGCTGGTGTGGGTGACGGTGTGGGTGTGGGTGCTGGTG; >clamp_s_c_s_d_s_c_s_a_s_e_s:compl:rev
CACCAGCACAGACACCCACACCGTCACCCACACTATCATGTTGACATTCC; dG = -36.9
389 >s_d_s_b_s_a_s_c_s_d_s_c_s_c_s_e_s
GTGACGGTGGCTGTGTCTGTGTGGGTGACGGTGTGGGTGTGGGTGCTGGTG; >clamp_s_c_s_d_s_c_s_a_s_e_s:compl:rev
CACCAGCACAGACACCCACACCGTCACCCACACTATCATGTTGACATTCC; dG = -36.9
390 >s_d_s_b_s_b_s_c_s_d_s_c_s_c_s_e_s
GTGACGGTGGCTGTGGCTGTGTGGGTGACGGTGTGGGTGTGGGTGCTGGTG; >clamp_s_c_s_d_s_c_s_a_s_e_s:compl:rev
CACCAGCACAGACACCCACACCGTCACCCACACTATCATGTTGACATTCC; dG = -36.9
391 >s_d_s_b_s_c_s_c_s_d_s_c_s_c_s_e_s
GTGACGGTGGCTGTGTGGGTGTGGGTGACGGTGTGGGTGTGGGTGCTGGTG; >clamp_s_c_s_d_s_c_s_a_s_e_s:compl:rev
CACCAGCACAGACACCCACACCGTCACCCACACTATCATGTTGACATTCC; dG = -36.9

```
   392 >s_d_s_b_s_d_s_c_s_d_s_c_s_c_s_e_s
GTGACGGTGGCTGTGACGGTGTGGGTGACGGTGTGGGTGTGGGTGCTGGTG;    >clamp_s_c_s_d_s_c_s_a_s_e_s:compl:rev
CACCAGCACAGACACCCACACCGTCACCCACACTATCATGTTGACATTCC; dG = -36.9
   393 >s_a_s_b_s_d_s_c_s_d_s_c_s_c_s_e_s
GTGTCTGTGGCTGTGACGGTGTGGGTGACGGTGTGGGTGTGGGTGCTGGTG;    >clamp_s_c_s_d_s_c_s_a_s_e_s:compl:rev
CACCAGCACAGACACCCACACCGTCACCCACACTATCATGTTGACATTCC; dG = -36.9
   394 >s_d_s_b_s_e_s_c_s_d_s_c_s_c_s_e_s
GTGACGGTGGCTGTGCTGGTGTGGGTGACGGTGTGGGTGTGGGTGCTGGTG;    >clamp_s_c_s_d_s_c_s_a_s_e_s:compl:rev
CACCAGCACAGACACCCACACCGTCACCCACACTATCATGTTGACATTCC; dG = -36.9
   395 >s_d_s_c_s_a_s_c_s_d_s_c_s_c_s_e_s
GTGACGGTGTGGGTGTCTGTGTGGGTGACGGTGTGGGTGTGGGTGCTGGTG;    >clamp_s_c_s_d_s_c_s_a_s_e_s:compl:rev
CACCAGCACAGACACCCACACCGTCACCCACACTATCATGTTGACATTCC; dG = -36.9
   396 >s_d_s_c_s_b_s_c_s_d_s_c_s_c_s_e_s
GTGACGGTGTGGGTGGCTGTGTGGGTGACGGTGTGGGTGTGGGTGCTGGTG;    >clamp_s_c_s_d_s_c_s_a_s_e_s:compl:rev
CACCAGCACAGACACCCACACCGTCACCCACACTATCATGTTGACATTCC; dG = -36.9
   397 >s_d_s_c_s_c_s_c_s_d_s_c_s_c_s_e_s
GTGACGGTGTGGGTGTGGGTGTGGGTGACGGTGTGGGTGTGGGTGCTGGTG;    >clamp_s_c_s_d_s_c_s_a_s_e_s:compl:rev
CACCAGCACAGACACCCACACCGTCACCCACACTATCATGTTGACATTCC; dG = -36.9
   398 >s_d_s_c_s_d_s_c_s_d_s_c_s_c_s_e_s
GTGACGGTGTGGGTGACGGTGTGGGTGACGGTGTGGGTGTGGGTGCTGGTG;    >clamp_s_c_s_d_s_c_s_a_s_e_s:compl:rev
CACCAGCACAGACACCCACACCGTCACCCACACTATCATGTTGACATTCC; dG = -36.9
   399 >s_d_s_c_s_e_s_c_s_d_s_c_s_c_s_e_s
GTGACGGTGTGGGTGCTGGTGTGGGTGACGGTGTGGGTGTGGGTGCTGGTG;    >clamp_s_c_s_d_s_c_s_a_s_e_s:compl:rev
CACCAGCACAGACACCCACACCGTCACCCACACTATCATGTTGACATTCC; dG = -36.9
   400 >s_d_s_d_s_a_s_c_s_d_s_c_s_c_s_e_s
GTGACGGTGACGGTGTCTGTGTGGGTGACGGTGTGGGTGTGGGTGCTGGTG;    >clamp_s_c_s_d_s_c_s_a_s_e_s:compl:rev
CACCAGCACAGACACCCACACCGTCACCCACACTATCATGTTGACATTCC; dG = -36.9
   401 >s_d_s_d_s_b_s_c_s_d_s_c_s_c_s_e_s
GTGACGGTGACGGTGGCTGTGTGGGTGACGGTGTGGGTGTGGGTGCTGGTG;    >clamp_s_c_s_d_s_c_s_a_s_e_s:compl:rev
CACCAGCACAGACACCCACACCGTCACCCACACTATCATGTTGACATTCC; dG = -36.9
   402 >s_d_s_d_s_c_s_c_s_d_s_c_s_c_s_e_s
GTGACGGTGACGGTGTGGGTGTGGGTGACGGTGTGGGTGTGGGTGCTGGTG;    >clamp_s_c_s_d_s_c_s_a_s_e_s:compl:rev
CACCAGCACAGACACCCACACCGTCACCCACACTATCATGTTGACATTCC; dG = -36.9
   403 >s_d_s_d_s_d_s_c_s_d_s_c_s_c_s_e_s
GTGACGGTGACGGTGACGGTGTGGGTGACGGTGTGGGTGTGGGTGCTGGTG;    >clamp_s_c_s_d_s_c_s_a_s_e_s:compl:rev
CACCAGCACAGACACCCACACCGTCACCCACACTATCATGTTGACATTCC; dG = -36.9
   404 >s_a_s_b_s_e_s_c_s_d_s_c_s_c_s_e_s
GTGTCTGTGGCTGTGCTGGTGTGGGTGACGGTGTGGGTGTGGGTGCTGGTG;    >clamp_s_c_s_d_s_c_s_a_s_e_s:compl:rev
CACCAGCACAGACACCCACACCGTCACCCACACTATCATGTTGACATTCC; dG = -36.9
   405 >s_d_s_d_s_e_s_c_s_d_s_c_s_c_s_e_s
GTGACGGTGACGGTGCTGGTGTGGGTGACGGTGTGGGTGTGGGTGCTGGTG;    >clamp_s_c_s_d_s_c_s_a_s_e_s:compl:rev
CACCAGCACAGACACCCACACCGTCACCCACACTATCATGTTGACATTCC; dG = -36.9
   406 >s_d_s_e_s_a_s_c_s_d_s_c_s_c_s_e_s
GTGACGGTGCTGGTGTCTGTGTGGGTGACGGTGTGGGTGTGGGTGCTGGTG;    >clamp_s_c_s_d_s_c_s_a_s_e_s:compl:rev
CACCAGCACAGACACCCACACCGTCACCCACACTATCATGTTGACATTCC; dG = -36.9
   407 >s_d_s_e_s_b_s_c_s_d_s_c_s_c_s_e_s
GTGACGGTGCTGGTGGCTGTGTGGGTGACGGTGTGGGTGTGGGTGCTGGTG;    >clamp_s_c_s_d_s_c_s_a_s_e_s:compl:rev
CACCAGCACAGACACCCACACCGTCACCCACACTATCATGTTGACATTCC; dG = -36.9
   408 >s_d_s_e_s_c_s_c_s_d_s_c_s_c_s_e_s
GTGACGGTGCTGGTGTGGGTGTGGGTGACGGTGTGGGTGTGGGTGCTGGTG;    >clamp_s_c_s_d_s_c_s_a_s_e_s:compl:rev
CACCAGCACAGACACCCACACCGTCACCCACACTATCATGTTGACATTCC; dG = -36.9
   409 >s_d_s_e_s_d_s_c_s_d_s_c_s_c_s_e_s
GTGACGGTGCTGGTGACGGTGTGGGTGACGGTGTGGGTGTGGGTGCTGGTG;    >clamp_s_c_s_d_s_c_s_a_s_e_s:compl:rev
CACCAGCACAGACACCCACACCGTCACCCACACTATCATGTTGACATTCC; dG = -36.9
   410 >s_d_s_e_s_e_s_c_s_d_s_c_s_c_s_e_s
GTGACGGTGCTGGTGCTGGTGTGGGTGACGGTGTGGGTGTGGGTGCTGGTG;    >clamp_s_c_s_d_s_c_s_a_s_e_s:compl:rev
CACCAGCACAGACACCCACACCGTCACCCACACTATCATGTTGACATTCC; dG = -36.9
   411 >s_e_s_a_s_a_s_c_s_d_s_c_s_c_s_e_s
GTGCTGGTGTCTGTGTCTGTGTGGGTGACGGTGTGGGTGTGGGTGCTGGTG;    >clamp_s_c_s_d_s_c_s_a_s_e_s:compl:rev
CACCAGCACAGACACCCACACCGTCACCCACACTATCATGTTGACATTCC; dG = -36.9
   412 >s_e_s_a_s_b_s_c_s_d_s_c_s_c_s_e_s
GTGCTGGTGTCTGTGGCTGTGTGGGTGACGGTGTGGGTGTGGGTGCTGGTG;    >clamp_s_c_s_d_s_c_s_a_s_e_s:compl:rev
CACCAGCACAGACACCCACACCGTCACCCACACTATCATGTTGACATTCC; dG = -36.9
   413 >s_e_s_a_s_c_s_c_s_d_s_c_s_c_s_e_s
GTGCTGGTGTCTGTGTGGGTGTGGGTGACGGTGTGGGTGTGGGTGCTGGTG;    >clamp_s_c_s_d_s_c_s_a_s_e_s:compl:rev
CACCAGCACAGACACCCACACCGTCACCCACACTATCATGTTGACATTCC; dG = -36.9
   414 >s_e_s_a_s_d_s_c_s_d_s_c_s_c_s_e_s
GTGCTGGTGTCTGTGACGGTGTGGGTGACGGTGTGGGTGTGGGTGCTGGTG;    >clamp_s_c_s_d_s_c_s_a_s_e_s:compl:rev
CACCAGCACAGACACCCACACCGTCACCCACACTATCATGTTGACATTCC; dG = -36.9
   415 >s_a_s_c_s_a_s_c_s_d_s_c_s_c_s_e_s
GTGTCTGTGTGGGTGTCTGTGTGGGTGACGGTGTGGGTGTGGGTGCTGGTG;    >clamp_s_c_s_d_s_c_s_a_s_e_s:compl:rev
CACCAGCACAGACACCCACACCGTCACCCACACTATCATGTTGACATTCC; dG = -36.9
```

416 >s_e_s_a_s_e_s_c_s_d_s_c_s_c_s_e_s
GTGCTGGTGTCTGTGCTGGTGTGGGTGACGGTGTGGGTGTGGGTGCTGGTG; >clamp_s_c_s_d_s_c_s_a_s_e_s:compl:rev
CACCAGCACAGACACCCACACCGTCACCCACACTATCATGTTGACATTCC; dG = -36.9
417 >s_e_s_b_s_a_s_c_s_d_s_c_s_c_s_e_s
GTGCTGGTGGCTGTGTCTGTGTGGGTGACGGTGTGGGTGTGGGTGCTGGTG; >clamp_s_c_s_d_s_c_s_a_s_e_s:compl:rev
CACCAGCACAGACACCCACACCGTCACCCACACTATCATGTTGACATTCC; dG = -36.9
418 >s_e_s_b_s_b_s_c_s_d_s_c_s_c_s_e_s
GTGCTGGTGGCTGTGGCTGTGTGGGTGACGGTGTGGGTGTGGGTGCTGGTG; >clamp_s_c_s_d_s_c_s_a_s_e_s:compl:rev
CACCAGCACAGACACCCACACCGTCACCCACACTATCATGTTGACATTCC; dG = -36.9
419 >s_e_s_b_s_c_s_c_s_d_s_c_s_c_s_e_s
GTGCTGGTGGCTGTGTGGGTGTGGGTGACGGTGTGGGTGTGGGTGCTGGTG; >clamp_s_c_s_d_s_c_s_a_s_e_s:compl:rev
CACCAGCACAGACACCCACACCGTCACCCACACTATCATGTTGACATTCC; dG = -36.9
420 >s_e_s_b_s_d_s_c_s_d_s_c_s_c_s_e_s
GTGCTGGTGGCTGTGACGGTGTGGGTGACGGTGTGGGTGTGGGTGCTGGTG; >clamp_s_c_s_d_s_c_s_a_s_e_s:compl:rev
CACCAGCACAGACACCCACACCGTCACCCACACTATCATGTTGACATTCC; dG = -36.9
421 >s_e_s_b_s_e_s_c_s_d_s_c_s_c_s_e_s
GTGCTGGTGGCTGTGCTGGTGTGGGTGACGGTGTGGGTGTGGGTGCTGGTG; >clamp_s_c_s_d_s_c_s_a_s_e_s:compl:rev
CACCAGCACAGACACCCACACCGTCACCCACACTATCATGTTGACATTCC; dG = -36.9
422 >s_e_s_c_s_a_s_c_s_d_s_c_s_c_s_e_s
GTGCTGGTGTGGGTGTCTGTGTGGGTGACGGTGTGGGTGTGGGTGCTGGTG; >clamp_s_c_s_d_s_c_s_a_s_e_s:compl:rev
CACCAGCACAGACACCCACACCGTCACCCACACTATCATGTTGACATTCC; dG = -36.9
423 >s_e_s_c_s_b_s_c_s_d_s_c_s_c_s_e_s
GTGCTGGTGTGGGTGGCTGTGTGGGTGACGGTGTGGGTGTGGGTGCTGGTG; >clamp_s_c_s_d_s_c_s_a_s_e_s:compl:rev
CACCAGCACAGACACCCACACCGTCACCCACACTATCATGTTGACATTCC; dG = -36.9
424 >s_e_s_c_s_c_s_c_s_d_s_c_s_c_s_e_s
GTGCTGGTGTGGGTGTGGGTGTGGGTGACGGTGTGGGTGTGGGTGCTGGTG; >clamp_s_c_s_d_s_c_s_a_s_e_s:compl:rev
CACCAGCACAGACACCCACACCGTCACCCACACTATCATGTTGACATTCC; dG = -36.9
425 >s_e_s_c_s_d_s_c_s_d_s_c_s_c_s_e_s
GTGCTGGTGTGGGTGACGGTGTGGGTGACGGTGTGGGTGTGGGTGCTGGTG; >clamp_s_c_s_d_s_c_s_a_s_e_s:compl:rev
CACCAGCACAGACACCCACACCGTCACCCACACTATCATGTTGACATTCC; dG = -36.9
426 >s_a_s_c_s_b_s_c_s_d_s_c_s_c_s_e_s
GTGTCTGTGTGGGTGGCTGTGTGGGTGACGGTGTGGGTGTGGGTGCTGGTG; >clamp_s_c_s_d_s_c_s_a_s_e_s:compl:rev
CACCAGCACAGACACCCACACCGTCACCCACACTATCATGTTGACATTCC; dG = -36.9
427 >s_e_s_c_s_e_s_c_s_d_s_c_s_c_s_e_s
GTGCTGGTGTGGGTGCTGGTGTGGGTGACGGTGTGGGTGTGGGTGCTGGTG; >clamp_s_c_s_d_s_c_s_a_s_e_s:compl:rev
CACCAGCACAGACACCCACACCGTCACCCACACTATCATGTTGACATTCC; dG = -36.9
428 >s_e_s_d_s_a_s_c_s_d_s_c_s_c_s_e_s
GTGCTGGTGACGGTGTCTGTGTGGGTGACGGTGTGGGTGTGGGTGCTGGTG; >clamp_s_c_s_d_s_c_s_a_s_e_s:compl:rev
CACCAGCACAGACACCCACACCGTCACCCACACTATCATGTTGACATTCC; dG = -36.9
429 >s_e_s_d_s_b_s_c_s_d_s_c_s_c_s_e_s
GTGCTGGTGACGGTGGCTGTGTGGGTGACGGTGTGGGTGTGGGTGCTGGTG; >clamp_s_c_s_d_s_c_s_a_s_e_s:compl:rev
CACCAGCACAGACACCCACACCGTCACCCACACTATCATGTTGACATTCC; dG = -36.9
430 >s_e_s_d_s_c_s_c_s_d_s_c_s_c_s_e_s
GTGCTGGTGACGGTGTGGGTGTGGGTGACGGTGTGGGTGTGGGTGCTGGTG; >clamp_s_c_s_d_s_c_s_a_s_e_s:compl:rev
CACCAGCACAGACACCCACACCGTCACCCACACTATCATGTTGACATTCC; dG = -36.9
431 >s_e_s_d_s_d_s_c_s_d_s_c_s_c_s_e_s
GTGCTGGTGACGGTGACGGTGTGGGTGACGGTGTGGGTGTGGGTGCTGGTG; >clamp_s_c_s_d_s_c_s_a_s_e_s:compl:rev
CACCAGCACAGACACCCACACCGTCACCCACACTATCATGTTGACATTCC; dG = -36.9
432 >s_e_s_d_s_e_s_c_s_d_s_c_s_c_s_e_s
GTGCTGGTGACGGTGCTGGTGTGGGTGACGGTGTGGGTGTGGGTGCTGGTG; >clamp_s_c_s_d_s_c_s_a_s_e_s:compl:rev
CACCAGCACAGACACCCACACCGTCACCCACACTATCATGTTGACATTCC; dG = -36.9
433 >s_e_s_e_s_a_s_c_s_d_s_c_s_c_s_e_s
GTGCTGGTGCTGGTGTCTGTGTGGGTGACGGTGTGGGTGTGGGTGCTGGTG; >clamp_s_c_s_d_s_c_s_a_s_e_s:compl:rev
CACCAGCACAGACACCCACACCGTCACCCACACTATCATGTTGACATTCC; dG = -36.9
434 >s_e_s_e_s_b_s_c_s_d_s_c_s_c_s_e_s
GTGCTGGTGCTGGTGGCTGTGTGGGTGACGGTGTGGGTGTGGGTGCTGGTG; >clamp_s_c_s_d_s_c_s_a_s_e_s:compl:rev
CACCAGCACAGACACCCACACCGTCACCCACACTATCATGTTGACATTCC; dG = -36.9
435 >s_e_s_e_s_c_s_c_s_d_s_c_s_c_s_e_s
GTGCTGGTGCTGGTGTGGGTGTGGGTGACGGTGTGGGTGTGGGTGCTGGTG; >clamp_s_c_s_d_s_c_s_a_s_e_s:compl:rev
CACCAGCACAGACACCCACACCGTCACCCACACTATCATGTTGACATTCC; dG = -36.9
436 >s_e_s_e_s_d_s_c_s_d_s_c_s_c_s_e_s
GTGCTGGTGCTGGTGACGGTGTGGGTGACGGTGTGGGTGTGGGTGCTGGTG; >clamp_s_c_s_d_s_c_s_a_s_e_s:compl:rev
CACCAGCACAGACACCCACACCGTCACCCACACTATCATGTTGACATTCC; dG = -36.9
437 >s_a_s_c_s_c_s_c_s_d_s_c_s_c_s_e_s
GTGTCTGTGTGGGTGTGGGTGTGGGTGACGGTGTGGGTGTGGGTGCTGGTG; >clamp_s_c_s_d_s_c_s_a_s_e_s:compl:rev
CACCAGCACAGACACCCACACCGTCACCCACACTATCATGTTGACATTCC; dG = -36.9
438 >s_e_s_e_s_e_s_c_s_d_s_c_s_c_s_e_s
GTGCTGGTGCTGGTGCTGGTGTGGGTGACGGTGTGGGTGTGGGTGCTGGTG; >clamp_s_c_s_d_s_c_s_a_s_e_s:compl:rev
CACCAGCACAGACACCCACACCGTCACCCACACTATCATGTTGACATTCC; dG = -36.9
439 >s_a_s_c_s_d_s_c_s_d_s_c_s_c_s_e_s
GTGTCTGTGTGGGTGACGGTGTGGGTGACGGTGTGGGTGTGGGTGCTGGTG; >clamp_s_c_s_d_s_c_s_a_s_e_s:compl:rev
CACCAGCACAGACACCCACACCGTCACCCACACTATCATGTTGACATTCC; dG = -36.9

```
   440 >s_a_s_a_s_b_s_c_s_d_s_c_s_c_s_e_s
GTGTCTGTGTCTGTGGCTGTGTGGGTGACGGTGTGGGTGTGGGTGCTGGTG;    >clamp_s_c_s_d_s_c_s_a_s_e_s:compl:rev
CACCAGCACAGACACCCACACCGTCACCCACACTATCATGTTGACATTCC; dG = -36.9
   441 >s_a_s_c_s_e_s_c_s_d_s_c_s_c_s_e_s
GTGTCTGTGTGGGTGCTGGTGTGGGTGACGGTGTGGGTGTGGGTGCTGGTG;    >clamp_s_c_s_d_s_c_s_a_s_e_s:compl:rev
CACCAGCACAGACACCCACACCGTCACCCACACTATCATGTTGACATTCC; dG = -36.9
   442 >s_a_s_d_s_a_s_c_s_d_s_c_s_c_s_e_s
GTGTCTGTGACGGTGTCTGTGTGGGTGACGGTGTGGGTGTGGGTGCTGGTG;    >clamp_s_c_s_d_s_c_s_a_s_e_s:compl:rev
CACCAGCACAGACACCCACACCGTCACCCACACTATCATGTTGACATTCC; dG = -36.9
   443 >s_a_s_d_s_b_s_c_s_d_s_c_s_c_s_e_s
GTGTCTGTGACGGTGGCTGTGTGGGTGACGGTGTGGGTGTGGGTGCTGGTG;    >clamp_s_c_s_d_s_c_s_a_s_e_s:compl:rev
CACCAGCACAGACACCCACACCGTCACCCACACTATCATGTTGACATTCC; dG = -36.9
   444 >s_a_s_d_s_c_s_c_s_d_s_c_s_c_s_e_s
GTGTCTGTGACGGTGTGGGTGTGGGTGACGGTGTGGGTGTGGGTGCTGGTG;    >clamp_s_c_s_d_s_c_s_a_s_e_s:compl:rev
CACCAGCACAGACACCCACACCGTCACCCACACTATCATGTTGACATTCC; dG = -36.9
   445 >s_a_s_d_s_d_s_c_s_d_s_c_s_c_s_e_s
GTGTCTGTGACGGTGACGGTGTGGGTGACGGTGTGGGTGTGGGTGCTGGTG;    >clamp_s_c_s_d_s_c_s_a_s_e_s:compl:rev
CACCAGCACAGACACCCACACCGTCACCCACACTATCATGTTGACATTCC; dG = -36.9
   446 >s_a_s_d_s_e_s_c_s_d_s_c_s_c_s_e_s
GTGTCTGTGACGGTGCTGGTGTGGGTGACGGTGTGGGTGTGGGTGCTGGTG;    >clamp_s_c_s_d_s_c_s_a_s_e_s:compl:rev
CACCAGCACAGACACCCACACCGTCACCCACACTATCATGTTGACATTCC; dG = -36.9
   447 >s_a_s_e_s_a_s_c_s_d_s_c_s_c_s_e_s
GTGTCTGTGCTGGTGTCTGTGTGGGTGACGGTGTGGGTGTGGGTGCTGGTG;    >clamp_s_c_s_d_s_c_s_a_s_e_s:compl:rev
CACCAGCACAGACACCCACACCGTCACCCACACTATCATGTTGACATTCC; dG = -36.9
   448 >s_a_s_e_s_b_s_c_s_d_s_c_s_c_s_e_s
GTGTCTGTGCTGGTGGCTGTGTGGGTGACGGTGTGGGTGTGGGTGCTGGTG;    >clamp_s_c_s_d_s_c_s_a_s_e_s:compl:rev
CACCAGCACAGACACCCACACCGTCACCCACACTATCATGTTGACATTCC; dG = -36.9
   449 >s_a_s_e_s_c_s_c_s_d_s_c_s_c_s_e_s
GTGTCTGTGCTGGTGTGGGTGTGGGTGACGGTGTGGGTGTGGGTGCTGGTG;    >clamp_s_c_s_d_s_c_s_a_s_e_s:compl:rev
CACCAGCACAGACACCCACACCGTCACCCACACTATCATGTTGACATTCC; dG = -36.9
   450 >s_a_s_e_s_d_s_c_s_d_s_c_s_c_s_e_s
GTGTCTGTGCTGGTGACGGTGTGGGTGACGGTGTGGGTGTGGGTGCTGGTG;    >clamp_s_c_s_d_s_c_s_a_s_e_s:compl:rev
CACCAGCACAGACACCCACACCGTCACCCACACTATCATGTTGACATTCC; dG = -36.9
   451 >s_a_s_a_s_c_s_c_s_d_s_c_s_c_s_e_s
GTGTCTGTGTCTGTGTGGGTGTGGGTGACGGTGTGGGTGTGGGTGCTGGTG;    >clamp_s_c_s_d_s_c_s_a_s_e_s:compl:rev
CACCAGCACAGACACCCACACCGTCACCCACACTATCATGTTGACATTCC; dG = -36.9
   452 >s_a_s_e_s_e_s_c_s_d_s_c_s_c_s_e_s
GTGTCTGTGCTGGTGCTGGTGTGGGTGACGGTGTGGGTGTGGGTGCTGGTG;    >clamp_s_c_s_d_s_c_s_a_s_e_s:compl:rev
CACCAGCACAGACACCCACACCGTCACCCACACTATCATGTTGACATTCC; dG = -36.9
   453 >s_b_s_a_s_a_s_c_s_d_s_c_s_c_s_e_s
GTGGCTGTGTCTGTGTCTGTGTGGGTGACGGTGTGGGTGTGGGTGCTGGTG;    >clamp_s_c_s_d_s_c_s_a_s_e_s:compl:rev
CACCAGCACAGACACCCACACCGTCACCCACACTATCATGTTGACATTCC; dG = -36.9
   454 >s_b_s_a_s_b_s_c_s_d_s_c_s_c_s_e_s
GTGGCTGTGTCTGTGGCTGTGTGGGTGACGGTGTGGGTGTGGGTGCTGGTG;    >clamp_s_c_s_d_s_c_s_a_s_e_s:compl:rev
CACCAGCACAGACACCCACACCGTCACCCACACTATCATGTTGACATTCC; dG = -36.9
   455 >s_b_s_a_s_c_s_c_s_d_s_c_s_c_s_e_s
GTGGCTGTGTCTGTGTGGGTGTGGGTGACGGTGTGGGTGTGGGTGCTGGTG;    >clamp_s_c_s_d_s_c_s_a_s_e_s:compl:rev
CACCAGCACAGACACCCACACCGTCACCCACACTATCATGTTGACATTCC; dG = -36.9
   456 >s_b_s_a_s_d_s_c_s_d_s_c_s_c_s_e_s
GTGGCTGTGTCTGTGACGGTGTGGGTGACGGTGTGGGTGTGGGTGCTGGTG;    >clamp_s_c_s_d_s_c_s_a_s_e_s:compl:rev
CACCAGCACAGACACCCACACCGTCACCCACACTATCATGTTGACATTCC; dG = -36.9
   457 >s_b_s_a_s_e_s_c_s_d_s_c_s_c_s_e_s
GTGGCTGTGTCTGTGCTGGTGTGGGTGACGGTGTGGGTGTGGGTGCTGGTG;    >clamp_s_c_s_d_s_c_s_a_s_e_s:compl:rev
CACCAGCACAGACACCCACACCGTCACCCACACTATCATGTTGACATTCC; dG = -36.9
   458 >s_b_s_b_s_a_s_c_s_d_s_c_s_c_s_e_s
GTGGCTGTGGCTGTGTCTGTGTGGGTGACGGTGTGGGTGTGGGTGCTGGTG;    >clamp_s_c_s_d_s_c_s_a_s_e_s:compl:rev
CACCAGCACAGACACCCACACCGTCACCCACACTATCATGTTGACATTCC; dG = -36.9
   459 >s_b_s_b_s_b_s_c_s_d_s_c_s_c_s_e_s
GTGGCTGTGGCTGTGGCTGTGTGGGTGACGGTGTGGGTGTGGGTGCTGGTG;    >clamp_s_c_s_d_s_c_s_a_s_e_s:compl:rev
CACCAGCACAGACACCCACACCGTCACCCACACTATCATGTTGACATTCC; dG = -36.9
   460 >s_b_s_b_s_c_s_c_s_d_s_c_s_c_s_e_s
GTGGCTGTGGCTGTGTGGGTGTGGGTGACGGTGTGGGTGTGGGTGCTGGTG;    >clamp_s_c_s_d_s_c_s_a_s_e_s:compl:rev
CACCAGCACAGACACCCACACCGTCACCCACACTATCATGTTGACATTCC; dG = -36.9
   461 >s_b_s_b_s_d_s_c_s_d_s_c_s_c_s_e_s
GTGGCTGTGGCTGTGACGGTGTGGGTGACGGTGTGGGTGTGGGTGCTGGTG;    >clamp_s_c_s_d_s_c_s_a_s_e_s:compl:rev
CACCAGCACAGACACCCACACCGTCACCCACACTATCATGTTGACATTCC; dG = -36.9
   462 >s_a_s_a_s_d_s_c_s_d_s_c_s_c_s_e_s
GTGTCTGTGTCTGTGACGGTGTGGGTGACGGTGTGGGTGTGGGTGCTGGTG;    >clamp_s_c_s_d_s_c_s_a_s_e_s:compl:rev
CACCAGCACAGACACCCACACCGTCACCCACACTATCATGTTGACATTCC; dG = -36.9
   463 >s_b_s_b_s_e_s_c_s_d_s_c_s_c_s_e_s
GTGGCTGTGGCTGTGCTGGTGTGGGTGACGGTGTGGGTGTGGGTGCTGGTG;    >clamp_s_c_s_d_s_c_s_a_s_e_s:compl:rev
CACCAGCACAGACACCCACACCGTCACCCACACTATCATGTTGACATTCC; dG = -36.9
```

464 >s_a_s_a_s_a_s_c_s_d_s_c_s_c_s_e_s
GTGTCTGTGTCTGTGTCTGTGTGGGTGACGGTGTGGGTGTGGGTGCTGGTG; >clamp_s_c_s_d_s_c_s_a_s_e_s:compl:rev
CACCAGCACAGACACCCACACCGTCACCCACACTATCATGTTGACATTCC; dG = -36.9
465 >s_b_s_c_s_a_s_c_s_d_s_c_s_c_s_e_s
GTGGCTGTGTGGGTGTCTGTGTGGGTGACGGTGTGGGTGTGGGTGCTGGTG; >clamp_s_c_s_d_s_c_s_a_s_e_s:compl:rev
CACCAGCACAGACACCCACACCGTCACCCACACTATCATGTTGACATTCC; dG = -36.9
466 >s_b_s_c_s_b_s_c_s_d_s_c_s_c_s_e_s
GTGGCTGTGTGGGTGGCTGTGTGGGTGACGGTGTGGGTGTGGGTGCTGGTG; >clamp_s_c_s_d_s_c_s_a_s_e_s:compl:rev
CACCAGCACAGACACCCACACCGTCACCCACACTATCATGTTGACATTCC; dG = -36.9
467 >s_b_s_c_s_c_s_c_s_d_s_c_s_c_s_e_s
GTGGCTGTGTGGGTGTGGGTGTGGGTGACGGTGTGGGTGTGGGTGCTGGTG; >clamp_s_c_s_d_s_c_s_a_s_e_s:compl:rev
CACCAGCACAGACACCCACACCGTCACCCACACTATCATGTTGACATTCC; dG = -36.9
468 >s_b_s_c_s_d_s_c_s_d_s_c_s_c_s_e_s
GTGGCTGTGTGGGTGACGGTGTGGGTGACGGTGTGGGTGTGGGTGCTGGTG; >clamp_s_c_s_d_s_c_s_a_s_e_s:compl:rev
CACCAGCACAGACACCCACACCGTCACCCACACTATCATGTTGACATTCC; dG = -36.9
469 >s_b_s_c_s_e_s_c_s_d_s_c_s_c_s_e_s
GTGGCTGTGTGGGTGCTGGTGTGGGTGACGGTGTGGGTGTGGGTGCTGGTG; >clamp_s_c_s_d_s_c_s_a_s_e_s:compl:rev
CACCAGCACAGACACCCACACCGTCACCCACACTATCATGTTGACATTCC; dG = -36.9
470 >s_b_s_d_s_a_s_c_s_d_s_c_s_c_s_e_s
GTGGCTGTGACGGTGTCTGTGTGGGTGACGGTGTGGGTGTGGGTGCTGGTG; >clamp_s_c_s_d_s_c_s_a_s_e_s:compl:rev
CACCAGCACAGACACCCACACCGTCACCCACACTATCATGTTGACATTCC; dG = -36.9
471 >s_b_s_d_s_b_s_c_s_d_s_c_s_c_s_e_s
GTGGCTGTGACGGTGGCTGTGTGGGTGACGGTGTGGGTGTGGGTGCTGGTG; >clamp_s_c_s_d_s_c_s_a_s_e_s:compl:rev
CACCAGCACAGACACCCACACCGTCACCCACACTATCATGTTGACATTCC; dG = -36.9
472 >s_b_s_d_s_c_s_c_s_d_s_c_s_c_s_e_s
GTGGCTGTGACGGTGTGGGTGTGGGTGACGGTGTGGGTGTGGGTGCTGGTG; >clamp_s_c_s_d_s_c_s_a_s_e_s:compl:rev
CACCAGCACAGACACCCACACCGTCACCCACACTATCATGTTGACATTCC; dG = -36.9
473 >s_b_s_d_s_d_s_c_s_d_s_c_s_c_s_e_s
GTGGCTGTGACGGTGACGGTGTGGGTGACGGTGTGGGTGTGGGTGCTGGTG; >clamp_s_c_s_d_s_c_s_a_s_e_s:compl:rev
CACCAGCACAGACACCCACACCGTCACCCACACTATCATGTTGACATTCC; dG = -36.9
474 >s_a_s_a_s_e_s_c_s_d_s_c_s_c_s_e_s
GTGTCTGTGTCTGTGCTGGTGTGGGTGACGGTGTGGGTGTGGGTGCTGGTG; >clamp_s_c_s_d_s_c_s_a_s_e_s:compl:rev
CACCAGCACAGACACCCACACCGTCACCCACACTATCATGTTGACATTCC; dG = -36.9
475 >s_b_s_d_s_e_s_c_s_d_s_c_s_c_s_e_s
GTGGCTGTGACGGTGCTGGTGTGGGTGACGGTGTGGGTGTGGGTGCTGGTG; >clamp_s_c_s_d_s_c_s_a_s_e_s:compl:rev
CACCAGCACAGACACCCACACCGTCACCCACACTATCATGTTGACATTCC; dG = -36.9
476 >s_b_s_e_s_a_s_c_s_d_s_c_s_c_s_e_s
GTGGCTGTGCTGGTGTCTGTGTGGGTGACGGTGTGGGTGTGGGTGCTGGTG; >clamp_s_c_s_d_s_c_s_a_s_e_s:compl:rev
CACCAGCACAGACACCCACACCGTCACCCACACTATCATGTTGACATTCC; dG = -36.9
477 >s_b_s_e_s_b_s_c_s_d_s_c_s_c_s_e_s
GTGGCTGTGCTGGTGGCTGTGTGGGTGACGGTGTGGGTGTGGGTGCTGGTG; >clamp_s_c_s_d_s_c_s_a_s_e_s:compl:rev
CACCAGCACAGACACCCACACCGTCACCCACACTATCATGTTGACATTCC; dG = -36.9
478 >s_b_s_e_s_c_s_c_s_d_s_c_s_c_s_e_s
GTGGCTGTGCTGGTGTGGGTGTGGGTGACGGTGTGGGTGTGGGTGCTGGTG; >clamp_s_c_s_d_s_c_s_a_s_e_s:compl:rev
CACCAGCACAGACACCCACACCGTCACCCACACTATCATGTTGACATTCC; dG = -36.9
479 >s_b_s_e_s_d_s_c_s_d_s_c_s_c_s_e_s
GTGGCTGTGCTGGTGACGGTGTGGGTGACGGTGTGGGTGTGGGTGCTGGTG; >clamp_s_c_s_d_s_c_s_a_s_e_s:compl:rev
CACCAGCACAGACACCCACACCGTCACCCACACTATCATGTTGACATTCC; dG = -36.9
480 >s_b_s_e_s_e_s_c_s_d_s_c_s_c_s_e_s
GTGGCTGTGCTGGTGCTGGTGTGGGTGACGGTGTGGGTGTGGGTGCTGGTG; >clamp_s_c_s_d_s_c_s_a_s_e_s:compl:rev
CACCAGCACAGACACCCACACCGTCACCCACACTATCATGTTGACATTCC; dG = -36.9
481 >s_c_s_d_s_c_s_c_s_e_s_c_s_a_s_e_s
GTGTGGGTGACGGTGTGGGTGTGGGTGCTGGTGTGGGTGTCTGTGCTGGTG; >clamp_s_c_s_d_s_c_s_a_s_e_s:compl:rev
CACCAGCACAGACACCCACACCGTCACCCACACTATCATGTTGACATTCC; dG = -36.7
482 >s_c_s_a_s_a_s_c_s_c_s_c_s_a_s_e_s
GTGTGGGTGTCTGTGTCTGTGTGGGTGTGGGTGTGGGTGTCTGTGCTGGTG; >clamp_s_c_s_d_s_c_s_a_s_e_s:compl:rev
CACCAGCACAGACACCCACACCGTCACCCACACTATCATGTTGACATTCC; dG = -36.4
483 >s_c_s_a_s_b_s_c_s_c_s_c_s_a_s_e_s
GTGTGGGTGTCTGTGGCTGTGTGGGTGTGGGTGTGGGTGTCTGTGCTGGTG; >clamp_s_c_s_d_s_c_s_a_s_e_s:compl:rev
CACCAGCACAGACACCCACACCGTCACCCACACTATCATGTTGACATTCC; dG = -36.4
484 >s_c_s_b_s_a_s_c_s_c_s_c_s_a_s_e_s
GTGTGGGTGGCTGTGTCTGTGTGGGTGTGGGTGTGGGTGTCTGTGCTGGTG; >clamp_s_c_s_d_s_c_s_a_s_e_s:compl:rev
CACCAGCACAGACACCCACACCGTCACCCACACTATCATGTTGACATTCC; dG = -36.4
485 >s_c_s_b_s_b_s_c_s_c_s_c_s_a_s_e_s
GTGTGGGTGGCTGTGGCTGTGTGGGTGTGGGTGTGGGTGTCTGTGCTGGTG; >clamp_s_c_s_d_s_c_s_a_s_e_s:compl:rev
CACCAGCACAGACACCCACACCGTCACCCACACTATCATGTTGACATTCC; dG = -36.4
486 >s_a_s_b_s_a_s_c_s_c_s_c_s_a_s_e_s
GTGTCTGTGGCTGTGTCTGTGTGGGTGTGGGTGTGGGTGTCTGTGCTGGTG; >clamp_s_c_s_d_s_c_s_a_s_e_s:compl:rev
CACCAGCACAGACACCCACACCGTCACCCACACTATCATGTTGACATTCC; dG = -36.4
487 >s_c_s_c_s_a_s_c_s_c_s_c_s_a_s_e_s
GTGTGGGTGTGGGTGTCTGTGTGGGTGTGGGTGTGGGTGTCTGTGCTGGTG; >clamp_s_c_s_d_s_c_s_a_s_e_s:compl:rev
CACCAGCACAGACACCCACACCGTCACCCACACTATCATGTTGACATTCC; dG = -36.4

```
   488 >s_c_s_c_s_b_s_c_s_c_s_c_s_a_s_e_s
GTGTGGGTGTGGGTGGCTGTGTGGGTGTGGGTGTGGGTGTCTGTGCTGGTG;    >clamp_s_c_s_d_s_c_s_a_s_e_s:compl:rev
CACCAGCACAGACACCCACACCGTCACCCACACTATCATGTTGACATTCC; dG = -36.4
   489 >s_c_s_d_s_a_s_c_s_c_s_c_s_a_s_e_s
GTGTGGGTGACGGTGTCTGTGTGGGTGTGGGTGTGGGTGTCTGTGCTGGTG;    >clamp_s_c_s_d_s_c_s_a_s_e_s:compl:rev
CACCAGCACAGACACCCACACCGTCACCCACACTATCATGTTGACATTCC; dG = -36.4
   490 >s_c_s_d_s_b_s_c_s_c_s_c_s_a_s_e_s
GTGTGGGTGACGGTGGCTGTGTGGGTGTGGGTGTGGGTGTCTGTGCTGGTG;    >clamp_s_c_s_d_s_c_s_a_s_e_s:compl:rev
CACCAGCACAGACACCCACACCGTCACCCACACTATCATGTTGACATTCC; dG = -36.4
   491 >s_a_s_b_s_b_s_c_s_c_s_c_s_a_s_e_s
GTGTCTGTGGCTGTGGCTGTGTGGGTGTGGGTGTGGGTGTCTGTGCTGGTG;    >clamp_s_c_s_d_s_c_s_a_s_e_s:compl:rev
CACCAGCACAGACACCCACACCGTCACCCACACTATCATGTTGACATTCC; dG = -36.4
   492 >s_a_s_a_s_a_s_c_s_c_s_c_s_a_s_e_s
GTGTCTGTGTCTGTGTCTGTGTGGGTGTGGGTGTGGGTGTCTGTGCTGGTG;    >clamp_s_c_s_d_s_c_s_a_s_e_s:compl:rev
CACCAGCACAGACACCCACACCGTCACCCACACTATCATGTTGACATTCC; dG = -36.4
   493 >s_c_s_e_s_a_s_c_s_c_s_c_s_a_s_e_s
GTGTGGGTGCTGGTGTCTGTGTGGGTGTGGGTGTGGGTGTCTGTGCTGGTG;    >clamp_s_c_s_d_s_c_s_a_s_e_s:compl:rev
CACCAGCACAGACACCCACACCGTCACCCACACTATCATGTTGACATTCC; dG = -36.4
   494 >s_c_s_e_s_b_s_c_s_c_s_c_s_a_s_e_s
GTGTGGGTGCTGGTGGCTGTGTGGGTGTGGGTGTGGGTGTCTGTGCTGGTG;    >clamp_s_c_s_d_s_c_s_a_s_e_s:compl:rev
CACCAGCACAGACACCCACACCGTCACCCACACTATCATGTTGACATTCC; dG = -36.4
   495 >s_d_s_a_s_a_s_c_s_c_s_c_s_a_s_e_s
GTGACGGTGTCTGTGTCTGTGTGGGTGTGGGTGTGGGTGTCTGTGCTGGTG;    >clamp_s_c_s_d_s_c_s_a_s_e_s:compl:rev
CACCAGCACAGACACCCACACCGTCACCCACACTATCATGTTGACATTCC; dG = -36.4
   496 >s_d_s_a_s_b_s_c_s_c_s_c_s_a_s_e_s
GTGACGGTGTCTGTGGCTGTGTGGGTGTGGGTGTGGGTGTCTGTGCTGGTG;    >clamp_s_c_s_d_s_c_s_a_s_e_s:compl:rev
CACCAGCACAGACACCCACACCGTCACCCACACTATCATGTTGACATTCC; dG = -36.4
   497 >s_d_s_b_s_a_s_c_s_c_s_c_s_a_s_e_s
GTGACGGTGGCTGTGTCTGTGTGGGTGTGGGTGTGGGTGTCTGTGCTGGTG;    >clamp_s_c_s_d_s_c_s_a_s_e_s:compl:rev
CACCAGCACAGACACCCACACCGTCACCCACACTATCATGTTGACATTCC; dG = -36.4
   498 >s_d_s_b_s_b_s_c_s_c_s_c_s_a_s_e_s
GTGACGGTGGCTGTGGCTGTGTGGGTGTGGGTGTGGGTGTCTGTGCTGGTG;    >clamp_s_c_s_d_s_c_s_a_s_e_s:compl:rev
CACCAGCACAGACACCCACACCGTCACCCACACTATCATGTTGACATTCC; dG = -36.4
   499 >s_d_s_c_s_a_s_c_s_c_s_c_s_a_s_e_s
GTGACGGTGTGGGTGTCTGTGTGGGTGTGGGTGTGGGTGTCTGTGCTGGTG;    >clamp_s_c_s_d_s_c_s_a_s_e_s:compl:rev
CACCAGCACAGACACCCACACCGTCACCCACACTATCATGTTGACATTCC; dG = -36.4
   500 >s_d_s_c_s_b_s_c_s_c_s_c_s_a_s_e_s
GTGACGGTGTGGGTGGCTGTGTGGGTGTGGGTGTGGGTGTCTGTGCTGGTG;    >clamp_s_c_s_d_s_c_s_a_s_e_s:compl:rev
CACCAGCACAGACACCCACACCGTCACCCACACTATCATGTTGACATTCC; dG = -36.4
   501 >s_d_s_d_s_a_s_c_s_c_s_c_s_a_s_e_s
GTGACGGTGACGGTGTCTGTGTGGGTGTGGGTGTGGGTGTCTGTGCTGGTG;    >clamp_s_c_s_d_s_c_s_a_s_e_s:compl:rev
CACCAGCACAGACACCCACACCGTCACCCACACTATCATGTTGACATTCC; dG = -36.4
   502 >s_d_s_d_s_b_s_c_s_c_s_c_s_a_s_e_s
GTGACGGTGACGGTGGCTGTGTGGGTGTGGGTGTGGGTGTCTGTGCTGGTG;    >clamp_s_c_s_d_s_c_s_a_s_e_s:compl:rev
CACCAGCACAGACACCCACACCGTCACCCACACTATCATGTTGACATTCC; dG = -36.4
   503 >s_d_s_e_s_a_s_c_s_c_s_c_s_a_s_e_s
GTGACGGTGCTGGTGTCTGTGTGGGTGTGGGTGTGGGTGTCTGTGCTGGTG;    >clamp_s_c_s_d_s_c_s_a_s_e_s:compl:rev
CACCAGCACAGACACCCACACCGTCACCCACACTATCATGTTGACATTCC; dG = -36.4
   504 >s_d_s_e_s_b_s_c_s_c_s_c_s_a_s_e_s
GTGACGGTGCTGGTGGCTGTGTGGGTGTGGGTGTGGGTGTCTGTGCTGGTG;    >clamp_s_c_s_d_s_c_s_a_s_e_s:compl:rev
CACCAGCACAGACACCCACACCGTCACCCACACTATCATGTTGACATTCC; dG = -36.4
   505 >s_e_s_a_s_a_s_c_s_c_s_c_s_a_s_e_s
GTGCTGGTGTCTGTGTCTGTGTGGGTGTGGGTGTGGGTGTCTGTGCTGGTG;    >clamp_s_c_s_d_s_c_s_a_s_e_s:compl:rev
CACCAGCACAGACACCCACACCGTCACCCACACTATCATGTTGACATTCC; dG = -36.4
   506 >s_e_s_a_s_b_s_c_s_c_s_c_s_a_s_e_s
GTGCTGGTGTCTGTGGCTGTGTGGGTGTGGGTGTGGGTGTCTGTGCTGGTG;    >clamp_s_c_s_d_s_c_s_a_s_e_s:compl:rev
CACCAGCACAGACACCCACACCGTCACCCACACTATCATGTTGACATTCC; dG = -36.4
   507 >s_e_s_b_s_a_s_c_s_c_s_c_s_a_s_e_s
GTGCTGGTGGCTGTGTCTGTGTGGGTGTGGGTGTGGGTGTCTGTGCTGGTG;    >clamp_s_c_s_d_s_c_s_a_s_e_s:compl:rev
CACCAGCACAGACACCCACACCGTCACCCACACTATCATGTTGACATTCC; dG = -36.4
   508 >s_e_s_b_s_b_s_c_s_c_s_c_s_a_s_e_s
GTGCTGGTGGCTGTGGCTGTGTGGGTGTGGGTGTGGGTGTCTGTGCTGGTG;    >clamp_s_c_s_d_s_c_s_a_s_e_s:compl:rev
CACCAGCACAGACACCCACACCGTCACCCACACTATCATGTTGACATTCC; dG = -36.4
   509 >s_a_s_c_s_a_s_c_s_c_s_c_s_a_s_e_s
GTGTCTGTGTGGGTGTCTGTGTGGGTGTGGGTGTGGGTGTCTGTGCTGGTG;    >clamp_s_c_s_d_s_c_s_a_s_e_s:compl:rev
CACCAGCACAGACACCCACACCGTCACCCACACTATCATGTTGACATTCC; dG = -36.4
   510 >s_e_s_c_s_a_s_c_s_c_s_c_s_a_s_e_s
GTGCTGGTGTGGGTGTCTGTGTGGGTGTGGGTGTGGGTGTCTGTGCTGGTG;    >clamp_s_c_s_d_s_c_s_a_s_e_s:compl:rev
CACCAGCACAGACACCCACACCGTCACCCACACTATCATGTTGACATTCC; dG = -36.4
   511 >s_e_s_c_s_b_s_c_s_c_s_c_s_a_s_e_s
GTGCTGGTGTGGGTGGCTGTGTGGGTGTGGGTGTGGGTGTCTGTGCTGGTG;    >clamp_s_c_s_d_s_c_s_a_s_e_s:compl:rev
CACCAGCACAGACACCCACACCGTCACCCACACTATCATGTTGACATTCC; dG = -36.4
```

512 >s_e_s_d_s_a_s_c_s_c_s_c_s_a_s_e_s
GTGCTGGTGACGGTGTCTGTGTGGGTGTGGGTGTGGGTGTCTGTGCTGGTG; >clamp_s_c_s_d_s_c_s_a_s_e_s:compl:rev
CACCAGCACAGACACCCACACCGTCACCCACACTATCATGTTGACATTCC; dG = -36.4
513 >s_e_s_d_s_b_s_c_s_c_s_c_s_a_s_e_s
GTGCTGGTGACGGTGGCTGTGTGGGTGTGGGTGTGGGTGTCTGTGCTGGTG; >clamp_s_c_s_d_s_c_s_a_s_e_s:compl:rev
CACCAGCACAGACACCCACACCGTCACCCACACTATCATGTTGACATTCC; dG = -36.4
514 >s_a_s_c_s_b_s_c_s_c_s_c_s_a_s_e_s
GTGTCTGTGTGGGTGGCTGTGTGGGTGTGGGTGTGGGTGTCTGTGCTGGTG; >clamp_s_c_s_d_s_c_s_a_s_e_s:compl:rev
CACCAGCACAGACACCCACACCGTCACCCACACTATCATGTTGACATTCC; dG = -36.4
515 >s_e_s_e_s_a_s_c_s_c_s_c_s_a_s_e_s
GTGCTGGTGCTGGTGTCTGTGTGGGTGTGGGTGTGGGTGTCTGTGCTGGTG; >clamp_s_c_s_d_s_c_s_a_s_e_s:compl:rev
CACCAGCACAGACACCCACACCGTCACCCACACTATCATGTTGACATTCC; dG = -36.4
516 >s_e_s_e_s_b_s_c_s_c_s_c_s_a_s_e_s
GTGCTGGTGCTGGTGGCTGTGTGGGTGTGGGTGTGGGTGTCTGTGCTGGTG; >clamp_s_c_s_d_s_c_s_a_s_e_s:compl:rev
CACCAGCACAGACACCCACACCGTCACCCACACTATCATGTTGACATTCC; dG = -36.4
517 >s_a_s_d_s_a_s_c_s_c_s_c_s_a_s_e_s
GTGTCTGTGACGGTGTCTGTGTGGGTGTGGGTGTGGGTGTCTGTGCTGGTG; >clamp_s_c_s_d_s_c_s_a_s_e_s:compl:rev
CACCAGCACAGACACCCACACCGTCACCCACACTATCATGTTGACATTCC; dG = -36.4
518 >s_a_s_d_s_b_s_c_s_c_s_c_s_a_s_e_s
GTGTCTGTGACGGTGGCTGTGTGGGTGTGGGTGTGGGTGTCTGTGCTGGTG; >clamp_s_c_s_d_s_c_s_a_s_e_s:compl:rev
CACCAGCACAGACACCCACACCGTCACCCACACTATCATGTTGACATTCC; dG = -36.4
519 >s_a_s_a_s_b_s_c_s_c_s_c_s_a_s_e_s
GTGTCTGTGTCTGTGGCTGTGTGGGTGTGGGTGTGGGTGTCTGTGCTGGTG; >clamp_s_c_s_d_s_c_s_a_s_e_s:compl:rev
CACCAGCACAGACACCCACACCGTCACCCACACTATCATGTTGACATTCC; dG = -36.4
520 >s_a_s_e_s_a_s_c_s_c_s_c_s_a_s_e_s
GTGTCTGTGCTGGTGTCTGTGTGGGTGTGGGTGTGGGTGTCTGTGCTGGTG; >clamp_s_c_s_d_s_c_s_a_s_e_s:compl:rev
CACCAGCACAGACACCCACACCGTCACCCACACTATCATGTTGACATTCC; dG = -36.4
521 >s_a_s_e_s_b_s_c_s_c_s_c_s_a_s_e_s
GTGTCTGTGCTGGTGGCTGTGTGGGTGTGGGTGTGGGTGTCTGTGCTGGTG; >clamp_s_c_s_d_s_c_s_a_s_e_s:compl:rev
CACCAGCACAGACACCCACACCGTCACCCACACTATCATGTTGACATTCC; dG = -36.4
522 >s_b_s_a_s_a_s_c_s_c_s_c_s_a_s_e_s
GTGGCTGTGTCTGTGTCTGTGTGGGTGTGGGTGTGGGTGTCTGTGCTGGTG; >clamp_s_c_s_d_s_c_s_a_s_e_s:compl:rev
CACCAGCACAGACACCCACACCGTCACCCACACTATCATGTTGACATTCC; dG = -36.4
523 >s_b_s_a_s_b_s_c_s_c_s_c_s_a_s_e_s
GTGGCTGTGTCTGTGGCTGTGTGGGTGTGGGTGTGGGTGTCTGTGCTGGTG; >clamp_s_c_s_d_s_c_s_a_s_e_s:compl:rev
CACCAGCACAGACACCCACACCGTCACCCACACTATCATGTTGACATTCC; dG = -36.4
524 >s_b_s_b_s_a_s_c_s_c_s_c_s_a_s_e_s
GTGGCTGTGGCTGTGTCTGTGTGGGTGTGGGTGTGGGTGTCTGTGCTGGTG; >clamp_s_c_s_d_s_c_s_a_s_e_s:compl:rev
CACCAGCACAGACACCCACACCGTCACCCACACTATCATGTTGACATTCC; dG = -36.4
525 >s_b_s_b_s_b_s_c_s_c_s_c_s_a_s_e_s
GTGGCTGTGGCTGTGGCTGTGTGGGTGTGGGTGTGGGTGTCTGTGCTGGTG; >clamp_s_c_s_d_s_c_s_a_s_e_s:compl:rev
CACCAGCACAGACACCCACACCGTCACCCACACTATCATGTTGACATTCC; dG = -36.4
526 >s_b_s_c_s_a_s_c_s_c_s_c_s_a_s_e_s
GTGGCTGTGTGGGTGTCTGTGTGGGTGTGGGTGTGGGTGTCTGTGCTGGTG; >clamp_s_c_s_d_s_c_s_a_s_e_s:compl:rev
CACCAGCACAGACACCCACACCGTCACCCACACTATCATGTTGACATTCC; dG = -36.4
527 >s_b_s_c_s_b_s_c_s_c_s_c_s_a_s_e_s
GTGGCTGTGTGGGTGGCTGTGTGGGTGTGGGTGTGGGTGTCTGTGCTGGTG; >clamp_s_c_s_d_s_c_s_a_s_e_s:compl:rev
CACCAGCACAGACACCCACACCGTCACCCACACTATCATGTTGACATTCC; dG = -36.4
528 >s_b_s_d_s_a_s_c_s_c_s_c_s_a_s_e_s
GTGGCTGTGACGGTGTCTGTGTGGGTGTGGGTGTGGGTGTCTGTGCTGGTG; >clamp_s_c_s_d_s_c_s_a_s_e_s:compl:rev
CACCAGCACAGACACCCACACCGTCACCCACACTATCATGTTGACATTCC; dG = -36.4
529 >s_b_s_d_s_b_s_c_s_c_s_c_s_a_s_e_s
GTGGCTGTGACGGTGGCTGTGTGGGTGTGGGTGTGGGTGTCTGTGCTGGTG; >clamp_s_c_s_d_s_c_s_a_s_e_s:compl:rev
CACCAGCACAGACACCCACACCGTCACCCACACTATCATGTTGACATTCC; dG = -36.4
530 >s_b_s_e_s_a_s_c_s_c_s_c_s_a_s_e_s
GTGGCTGTGCTGGTGTCTGTGTGGGTGTGGGTGTGGGTGTCTGTGCTGGTG; >clamp_s_c_s_d_s_c_s_a_s_e_s:compl:rev
CACCAGCACAGACACCCACACCGTCACCCACACTATCATGTTGACATTCC; dG = -36.4
531 >s_b_s_e_s_b_s_c_s_c_s_c_s_a_s_e_s
GTGGCTGTGCTGGTGGCTGTGTGGGTGTGGGTGTGGGTGTCTGTGCTGGTG; >clamp_s_c_s_d_s_c_s_a_s_e_s:compl:rev
CACCAGCACAGACACCCACACCGTCACCCACACTATCATGTTGACATTCC; dG = -36.4
532 >s_a_s_a_s_a_s_c_s_d_s_d_s_a_s_e_s
GTGTCTGTGTCTGTGTCTGTGTGGGTGACGGTGACGGTGTCTGTGCTGGTG; >clamp_s_c_s_d_s_c_s_a_s_e_s:compl:rev
CACCAGCACAGACACCCACACCGTCACCCACACTATCATGTTGACATTCC; dG = -36.3
533 >s_c_s_a_s_a_s_c_s_d_s_d_s_a_s_e_s
GTGTGGGTGTCTGTGTCTGTGTGGGTGACGGTGACGGTGTCTGTGCTGGTG; >clamp_s_c_s_d_s_c_s_a_s_e_s:compl:rev
CACCAGCACAGACACCCACACCGTCACCCACACTATCATGTTGACATTCC; dG = -36.3
534 >s_c_s_a_s_a_s_d_s_d_s_c_s_a_s_e_s
GTGTGGGTGTCTGTGTCTGTGACGGTGACGGTGTGGGTGTCTGTGCTGGTG; >clamp_s_c_s_d_s_c_s_a_s_e_s:compl:rev
CACCAGCACAGACACCCACACCGTCACCCACACTATCATGTTGACATTCC; dG = -36.3
535 >s_c_s_a_s_b_s_c_s_d_s_d_s_a_s_e_s
GTGTGGGTGTCTGTGGCTGTGTGGGTGACGGTGACGGTGTCTGTGCTGGTG; >clamp_s_c_s_d_s_c_s_a_s_e_s:compl:rev
CACCAGCACAGACACCCACACCGTCACCCACACTATCATGTTGACATTCC; dG = -36.3

536 >s_c_s_a_s_b_s_d_s_d_s_c_s_a_s_e_s
GTGTGGGTGTCTGTGGCTGTGACGGTGACGGTGTGGGTGTCTGTGCTGGTG; >clamp_s_c_s_d_s_c_s_a_s_e_s:compl:rev
CACCAGCACAGACACCCACACCGTCACCCACACTATCATGTTGACATTCC; dG = -36.3
537 >s_c_s_a_s_c_s_c_s_d_s_d_s_a_s_e_s
GTGTGGGTGTCTGTGTGGGTGTGGGTGACGGTGACGGTGTCTGTGCTGGTG; >clamp_s_c_s_d_s_c_s_a_s_e_s:compl:rev
CACCAGCACAGACACCCACACCGTCACCCACACTATCATGTTGACATTCC; dG = -36.3
538 >s_c_s_a_s_c_s_c_s_c_s_c_s_a_s_e_s
GTGTGGGTGTCTGTGTGGGTGTGGGTGTGGGTGTGGGTGTCTGTGCTGGTG; >clamp_s_c_s_d_s_c_s_a_s_e_s:compl:rev
CACCAGCACAGACACCCACACCGTCACCCACACTATCATGTTGACATTCC; dG = -36.3
539 >s_c_s_a_s_d_s_c_s_d_s_d_s_a_s_e_s
GTGTGGGTGTCTGTGACGGTGTGGGTGACGGTGACGGTGTCTGTGCTGGTG; >clamp_s_c_s_d_s_c_s_a_s_e_s:compl:rev
CACCAGCACAGACACCCACACCGTCACCCACACTATCATGTTGACATTCC; dG = -36.3
540 >s_c_s_a_s_d_s_c_s_c_s_c_s_a_s_e_s
GTGTGGGTGTCTGTGACGGTGTGGGTGTGGGTGTGGGTGTCTGTGCTGGTG; >clamp_s_c_s_d_s_c_s_a_s_e_s:compl:rev
CACCAGCACAGACACCCACACCGTCACCCACACTATCATGTTGACATTCC; dG = -36.3
541 >s_c_s_a_s_d_s_d_s_d_s_c_s_a_s_e_s
GTGTGGGTGTCTGTGACGGTGACGGTGACGGTGTGGGTGTCTGTGCTGGTG; >clamp_s_c_s_d_s_c_s_a_s_e_s:compl:rev
CACCAGCACAGACACCCACACCGTCACCCACACTATCATGTTGACATTCC; dG = -36.3
542 >s_c_s_a_s_e_s_c_s_d_s_d_s_a_s_e_s
GTGTGGGTGTCTGTGCTGGTGTGGGTGACGGTGACGGTGTCTGTGCTGGTG; >clamp_s_c_s_d_s_c_s_a_s_e_s:compl:rev
CACCAGCACAGACACCCACACCGTCACCCACACTATCATGTTGACATTCC; dG = -36.3
543 >s_c_s_a_s_e_s_c_s_c_s_c_s_a_s_e_s
GTGTGGGTGTCTGTGCTGGTGTGGGTGTGGGTGTGGGTGTCTGTGCTGGTG; >clamp_s_c_s_d_s_c_s_a_s_e_s:compl:rev
CACCAGCACAGACACCCACACCGTCACCCACACTATCATGTTGACATTCC; dG = -36.3
544 >s_c_s_a_s_e_s_d_s_d_s_c_s_a_s_e_s
GTGTGGGTGTCTGTGCTGGTGACGGTGACGGTGTGGGTGTCTGTGCTGGTG; >clamp_s_c_s_d_s_c_s_a_s_e_s:compl:rev
CACCAGCACAGACACCCACACCGTCACCCACACTATCATGTTGACATTCC; dG = -36.3
545 >s_a_s_b_s_a_s_c_s_d_s_d_s_a_s_e_s
GTGTCTGTGGCTGTGTCTGTGTGGGTGACGGTGACGGTGTCTGTGCTGGTG; >clamp_s_c_s_d_s_c_s_a_s_e_s:compl:rev
CACCAGCACAGACACCCACACCGTCACCCACACTATCATGTTGACATTCC; dG = -36.3
546 >s_c_s_b_s_a_s_c_s_d_s_d_s_a_s_e_s
GTGTGGGTGGCTGTGTCTGTGTGGGTGACGGTGACGGTGTCTGTGCTGGTG; >clamp_s_c_s_d_s_c_s_a_s_e_s:compl:rev
CACCAGCACAGACACCCACACCGTCACCCACACTATCATGTTGACATTCC; dG = -36.3
547 >s_c_s_b_s_a_s_d_s_d_s_c_s_a_s_e_s
GTGTGGGTGGCTGTGTCTGTGACGGTGACGGTGTGGGTGTCTGTGCTGGTG; >clamp_s_c_s_d_s_c_s_a_s_e_s:compl:rev
CACCAGCACAGACACCCACACCGTCACCCACACTATCATGTTGACATTCC; dG = -36.3
548 >s_c_s_b_s_b_s_c_s_d_s_d_s_a_s_e_s
GTGTGGGTGGCTGTGGCTGTGTGGGTGACGGTGACGGTGTCTGTGCTGGTG; >clamp_s_c_s_d_s_c_s_a_s_e_s:compl:rev
CACCAGCACAGACACCCACACCGTCACCCACACTATCATGTTGACATTCC; dG = -36.3
549 >s_c_s_b_s_b_s_d_s_d_s_c_s_a_s_e_s
GTGTGGGTGGCTGTGGCTGTGACGGTGACGGTGTGGGTGTCTGTGCTGGTG; >clamp_s_c_s_d_s_c_s_a_s_e_s:compl:rev
CACCAGCACAGACACCCACACCGTCACCCACACTATCATGTTGACATTCC; dG = -36.3
550 >s_c_s_b_s_c_s_c_s_d_s_d_s_a_s_e_s
GTGTGGGTGGCTGTGTGGGTGTGGGTGACGGTGACGGTGTCTGTGCTGGTG; >clamp_s_c_s_d_s_c_s_a_s_e_s:compl:rev
CACCAGCACAGACACCCACACCGTCACCCACACTATCATGTTGACATTCC; dG = -36.3
551 >s_c_s_b_s_c_s_c_s_c_s_c_s_a_s_e_s
GTGTGGGTGGCTGTGTGGGTGTGGGTGTGGGTGTGGGTGTCTGTGCTGGTG; >clamp_s_c_s_d_s_c_s_a_s_e_s:compl:rev
CACCAGCACAGACACCCACACCGTCACCCACACTATCATGTTGACATTCC; dG = -36.3
552 >s_c_s_b_s_d_s_c_s_d_s_d_s_a_s_e_s
GTGTGGGTGGCTGTGACGGTGTGGGTGACGGTGACGGTGTCTGTGCTGGTG; >clamp_s_c_s_d_s_c_s_a_s_e_s:compl:rev
CACCAGCACAGACACCCACACCGTCACCCACACTATCATGTTGACATTCC; dG = -36.3
553 >s_c_s_b_s_d_s_c_s_c_s_c_s_a_s_e_s
GTGTGGGTGGCTGTGACGGTGTGGGTGTGGGTGTGGGTGTCTGTGCTGGTG; >clamp_s_c_s_d_s_c_s_a_s_e_s:compl:rev
CACCAGCACAGACACCCACACCGTCACCCACACTATCATGTTGACATTCC; dG = -36.3
554 >s_c_s_b_s_d_s_d_s_d_s_c_s_a_s_e_s
GTGTGGGTGGCTGTGACGGTGACGGTGACGGTGTGGGTGTCTGTGCTGGTG; >clamp_s_c_s_d_s_c_s_a_s_e_s:compl:rev
CACCAGCACAGACACCCACACCGTCACCCACACTATCATGTTGACATTCC; dG = -36.3
555 >s_a_s_b_s_a_s_d_s_d_s_c_s_a_s_e_s
GTGTCTGTGGCTGTGTCTGTGACGGTGACGGTGTGGGTGTCTGTGCTGGTG; >clamp_s_c_s_d_s_c_s_a_s_e_s:compl:rev
CACCAGCACAGACACCCACACCGTCACCCACACTATCATGTTGACATTCC; dG = -36.3
556 >s_c_s_b_s_e_s_c_s_d_s_d_s_a_s_e_s
GTGTGGGTGGCTGTGCTGGTGTGGGTGACGGTGACGGTGTCTGTGCTGGTG; >clamp_s_c_s_d_s_c_s_a_s_e_s:compl:rev
CACCAGCACAGACACCCACACCGTCACCCACACTATCATGTTGACATTCC; dG = -36.3
557 >s_c_s_b_s_e_s_c_s_c_s_c_s_a_s_e_s
GTGTGGGTGGCTGTGCTGGTGTGGGTGTGGGTGTGGGTGTCTGTGCTGGTG; >clamp_s_c_s_d_s_c_s_a_s_e_s:compl:rev
CACCAGCACAGACACCCACACCGTCACCCACACTATCATGTTGACATTCC; dG = -36.3
558 >s_c_s_b_s_e_s_d_s_d_s_c_s_a_s_e_s
GTGTGGGTGGCTGTGCTGGTGACGGTGACGGTGTGGGTGTCTGTGCTGGTG; >clamp_s_c_s_d_s_c_s_a_s_e_s:compl:rev
CACCAGCACAGACACCCACACCGTCACCCACACTATCATGTTGACATTCC; dG = -36.3
559 >s_c_s_c_s_a_s_c_s_d_s_d_s_a_s_e_s
GTGTGGGTGTGGGTGTCTGTGTGGGTGACGGTGACGGTGTCTGTGCTGGTG; >clamp_s_c_s_d_s_c_s_a_s_e_s:compl:rev
CACCAGCACAGACACCCACACCGTCACCCACACTATCATGTTGACATTCC; dG = -36.3

```
   560 >s_c_s_c_s_a_s_d_s_d_s_c_s_a_s_e_s
GTGTGGGTGTGGGTGTCTGTGACGGTGACGGTGTGGGTGTCTGTGCTGGTG;    >clamp_s_c_s_d_s_c_s_a_s_e_s:compl:rev
CACCAGCACAGACACCCACACCGTCACCCACACTATCATGTTGACATTCC; dG = -36.3
   561 >s_c_s_c_s_b_s_c_s_d_s_d_s_a_s_e_s
GTGTGGGTGTGGGTGGCTGTGTGGGTGACGGTGACGGTGTCTGTGCTGGTG;    >clamp_s_c_s_d_s_c_s_a_s_e_s:compl:rev
CACCAGCACAGACACCCACACCGTCACCCACACTATCATGTTGACATTCC; dG = -36.3
   562 >s_c_s_c_s_b_s_d_s_d_s_c_s_a_s_e_s
GTGTGGGTGTGGGTGGCTGTGACGGTGACGGTGTGGGTGTCTGTGCTGGTG;    >clamp_s_c_s_d_s_c_s_a_s_e_s:compl:rev
CACCAGCACAGACACCCACACCGTCACCCACACTATCATGTTGACATTCC; dG = -36.3
   563 >s_c_s_c_s_c_s_c_s_d_s_d_s_a_s_e_s
GTGTGGGTGTGGGTGTGGGTGTGGGTGACGGTGACGGTGTCTGTGCTGGTG;    >clamp_s_c_s_d_s_c_s_a_s_e_s:compl:rev
CACCAGCACAGACACCCACACCGTCACCCACACTATCATGTTGACATTCC; dG = -36.3
   564 >s_c_s_c_s_c_s_c_s_c_s_c_s_a_s_e_s
GTGTGGGTGTGGGTGTGGGTGTGGGTGTGGGTGTGGGTGTCTGTGCTGGTG;    >clamp_s_c_s_d_s_c_s_a_s_e_s:compl:rev
CACCAGCACAGACACCCACACCGTCACCCACACTATCATGTTGACATTCC; dG = -36.3
   565 >s_c_s_c_s_d_s_c_s_d_s_d_s_a_s_e_s
GTGTGGGTGTGGGTGACGGTGTGGGTGACGGTGACGGTGTCTGTGCTGGTG;    >clamp_s_c_s_d_s_c_s_a_s_e_s:compl:rev
CACCAGCACAGACACCCACACCGTCACCCACACTATCATGTTGACATTCC; dG = -36.3
   566 >s_c_s_c_s_d_s_c_s_c_s_c_s_a_s_e_s
GTGTGGGTGTGGGTGACGGTGTGGGTGTGGGTGTGGGTGTCTGTGCTGGTG;    >clamp_s_c_s_d_s_c_s_a_s_e_s:compl:rev
CACCAGCACAGACACCCACACCGTCACCCACACTATCATGTTGACATTCC; dG = -36.3
   567 >s_c_s_c_s_d_s_d_s_d_s_c_s_a_s_e_s
GTGTGGGTGTGGGTGACGGTGACGGTGACGGTGTGGGTGTCTGTGCTGGTG;    >clamp_s_c_s_d_s_c_s_a_s_e_s:compl:rev
CACCAGCACAGACACCCACACCGTCACCCACACTATCATGTTGACATTCC; dG = -36.3
   568 >s_c_s_c_s_e_s_c_s_d_s_d_s_a_s_e_s
GTGTGGGTGTGGGTGCTGGTGTGGGTGACGGTGACGGTGTCTGTGCTGGTG;    >clamp_s_c_s_d_s_c_s_a_s_e_s:compl:rev
CACCAGCACAGACACCCACACCGTCACCCACACTATCATGTTGACATTCC; dG = -36.3
   569 >s_c_s_c_s_e_s_c_s_c_s_c_s_a_s_e_s
GTGTGGGTGTGGGTGCTGGTGTGGGTGTGGGTGTGGGTGTCTGTGCTGGTG;    >clamp_s_c_s_d_s_c_s_a_s_e_s:compl:rev
CACCAGCACAGACACCCACACCGTCACCCACACTATCATGTTGACATTCC; dG = -36.3
   570 >s_c_s_c_s_e_s_d_s_d_s_c_s_a_s_e_s
GTGTGGGTGTGGGTGCTGGTGACGGTGACGGTGTGGGTGTCTGTGCTGGTG;    >clamp_s_c_s_d_s_c_s_a_s_e_s:compl:rev
CACCAGCACAGACACCCACACCGTCACCCACACTATCATGTTGACATTCC; dG = -36.3
   571 >s_a_s_b_s_b_s_c_s_d_s_d_s_a_s_e_s
GTGTCTGTGGCTGTGGCTGTGTGGGTGACGGTGACGGTGTCTGTGCTGGTG;    >clamp_s_c_s_d_s_c_s_a_s_e_s:compl:rev
CACCAGCACAGACACCCACACCGTCACCCACACTATCATGTTGACATTCC; dG = -36.3
   572 >s_c_s_d_s_a_s_c_s_d_s_d_s_a_s_e_s
GTGTGGGTGACGGTGTCTGTGTGGGTGACGGTGACGGTGTCTGTGCTGGTG;    >clamp_s_c_s_d_s_c_s_a_s_e_s:compl:rev
CACCAGCACAGACACCCACACCGTCACCCACACTATCATGTTGACATTCC; dG = -36.3
   573 >s_c_s_d_s_a_s_d_s_d_s_c_s_a_s_e_s
GTGTGGGTGACGGTGTCTGTGACGGTGACGGTGTGGGTGTCTGTGCTGGTG;    >clamp_s_c_s_d_s_c_s_a_s_e_s:compl:rev
CACCAGCACAGACACCCACACCGTCACCCACACTATCATGTTGACATTCC; dG = -36.3
   574 >s_c_s_d_s_b_s_c_s_d_s_d_s_a_s_e_s
GTGTGGGTGACGGTGGCTGTGTGGGTGACGGTGACGGTGTCTGTGCTGGTG;    >clamp_s_c_s_d_s_c_s_a_s_e_s:compl:rev
CACCAGCACAGACACCCACACCGTCACCCACACTATCATGTTGACATTCC; dG = -36.3
   575 >s_c_s_d_s_b_s_d_s_d_s_c_s_a_s_e_s
GTGTGGGTGACGGTGGCTGTGACGGTGACGGTGTGGGTGTCTGTGCTGGTG;    >clamp_s_c_s_d_s_c_s_a_s_e_s:compl:rev
CACCAGCACAGACACCCACACCGTCACCCACACTATCATGTTGACATTCC; dG = -36.3
   576 >s_c_s_d_s_c_s_c_s_d_s_d_s_a_s_e_s
GTGTGGGTGACGGTGTGGGTGTGGGTGACGGTGACGGTGTCTGTGCTGGTG;    >clamp_s_c_s_d_s_c_s_a_s_e_s:compl:rev
CACCAGCACAGACACCCACACCGTCACCCACACTATCATGTTGACATTCC; dG = -36.3
   577 >s_c_s_d_s_c_s_c_s_c_s_c_s_a_s_e_s
GTGTGGGTGACGGTGTGGGTGTGGGTGTGGGTGTGGGTGTCTGTGCTGGTG;    >clamp_s_c_s_d_s_c_s_a_s_e_s:compl:rev
CACCAGCACAGACACCCACACCGTCACCCACACTATCATGTTGACATTCC; dG = -36.3
   578 >s_c_s_d_s_d_s_c_s_d_s_d_s_a_s_e_s
GTGTGGGTGACGGTGACGGTGTGGGTGACGGTGACGGTGTCTGTGCTGGTG;    >clamp_s_c_s_d_s_c_s_a_s_e_s:compl:rev
CACCAGCACAGACACCCACACCGTCACCCACACTATCATGTTGACATTCC; dG = -36.3
   579 >s_c_s_d_s_d_s_c_s_c_s_c_s_a_s_e_s
GTGTGGGTGACGGTGACGGTGTGGGTGTGGGTGTGGGTGTCTGTGCTGGTG;    >clamp_s_c_s_d_s_c_s_a_s_e_s:compl:rev
CACCAGCACAGACACCCACACCGTCACCCACACTATCATGTTGACATTCC; dG = -36.3
   580 >s_c_s_d_s_d_s_d_s_d_s_c_s_a_s_e_s
GTGTGGGTGACGGTGACGGTGACGGTGACGGTGTGGGTGTCTGTGCTGGTG;    >clamp_s_c_s_d_s_c_s_a_s_e_s:compl:rev
CACCAGCACAGACACCCACACCGTCACCCACACTATCATGTTGACATTCC; dG = -36.3
   581 >s_a_s_b_s_b_s_d_s_d_s_c_s_a_s_e_s
GTGTCTGTGGCTGTGGCTGTGACGGTGACGGTGTGGGTGTCTGTGCTGGTG;    >clamp_s_c_s_d_s_c_s_a_s_e_s:compl:rev
CACCAGCACAGACACCCACACCGTCACCCACACTATCATGTTGACATTCC; dG = -36.3
   582 >s_c_s_d_s_e_s_c_s_d_s_d_s_a_s_e_s
GTGTGGGTGACGGTGCTGGTGTGGGTGACGGTGACGGTGTCTGTGCTGGTG;    >clamp_s_c_s_d_s_c_s_a_s_e_s:compl:rev
CACCAGCACAGACACCCACACCGTCACCCACACTATCATGTTGACATTCC; dG = -36.3
   583 >s_c_s_d_s_e_s_c_s_c_s_c_s_a_s_e_s
GTGTGGGTGACGGTGCTGGTGTGGGTGTGGGTGTGGGTGTCTGTGCTGGTG;    >clamp_s_c_s_d_s_c_s_a_s_e_s:compl:rev
CACCAGCACAGACACCCACACCGTCACCCACACTATCATGTTGACATTCC; dG = -36.3
```

584 >s_c_s_d_s_e_s_d_s_d_s_c_s_a_s_e_s
GTGTGGGTGACGGTGCTGGTGACGGTGACGGTGTGGGTGTCTGTGCTGGTG; >clamp_s_c_s_d_s_c_s_a_s_e_s:compl:rev
CACCAGCACAGACACCCACACCGTCACCCACACTATCATGTTGACATTCC; dG = -36.3
585 >s_c_s_e_s_a_s_c_s_d_s_d_s_a_s_e_s
GTGTGGGTGCTGGTGTCTGTGTGGGTGACGGTGACGGTGTCTGTGCTGGTG; >clamp_s_c_s_d_s_c_s_a_s_e_s:compl:rev
CACCAGCACAGACACCCACACCGTCACCCACACTATCATGTTGACATTCC; dG = -36.3
586 >s_c_s_e_s_a_s_d_s_d_s_c_s_a_s_e_s
GTGTGGGTGCTGGTGTCTGTGACGGTGACGGTGTGGGTGTCTGTGCTGGTG; >clamp_s_c_s_d_s_c_s_a_s_e_s:compl:rev
CACCAGCACAGACACCCACACCGTCACCCACACTATCATGTTGACATTCC; dG = -36.3
587 >s_c_s_e_s_b_s_c_s_d_s_d_s_a_s_e_s
GTGTGGGTGCTGGTGGCTGTGTGGGTGACGGTGACGGTGTCTGTGCTGGTG; >clamp_s_c_s_d_s_c_s_a_s_e_s:compl:rev
CACCAGCACAGACACCCACACCGTCACCCACACTATCATGTTGACATTCC; dG = -36.3
588 >s_c_s_e_s_b_s_d_s_d_s_c_s_a_s_e_s
GTGTGGGTGCTGGTGGCTGTGACGGTGACGGTGTGGGTGTCTGTGCTGGTG; >clamp_s_c_s_d_s_c_s_a_s_e_s:compl:rev
CACCAGCACAGACACCCACACCGTCACCCACACTATCATGTTGACATTCC; dG = -36.3
589 >s_c_s_e_s_c_s_c_s_d_s_d_s_a_s_e_s
GTGTGGGTGCTGGTGTGGGTGTGGGTGACGGTGACGGTGTCTGTGCTGGTG; >clamp_s_c_s_d_s_c_s_a_s_e_s:compl:rev
CACCAGCACAGACACCCACACCGTCACCCACACTATCATGTTGACATTCC; dG = -36.3
590 >s_c_s_e_s_c_s_c_s_c_s_c_s_a_s_e_s
GTGTGGGTGCTGGTGTGGGTGTGGGTGTGGGTGTGGGTGTCTGTGCTGGTG; >clamp_s_c_s_d_s_c_s_a_s_e_s:compl:rev
CACCAGCACAGACACCCACACCGTCACCCACACTATCATGTTGACATTCC; dG = -36.3
591 >s_c_s_e_s_d_s_c_s_d_s_d_s_a_s_e_s
GTGTGGGTGCTGGTGACGGTGTGGGTGACGGTGACGGTGTCTGTGCTGGTG; >clamp_s_c_s_d_s_c_s_a_s_e_s:compl:rev
CACCAGCACAGACACCCACACCGTCACCCACACTATCATGTTGACATTCC; dG = -36.3
592 >s_c_s_e_s_d_s_c_s_c_s_c_s_a_s_e_s
GTGTGGGTGCTGGTGACGGTGTGGGTGTGGGTGTGGGTGTCTGTGCTGGTG; >clamp_s_c_s_d_s_c_s_a_s_e_s:compl:rev
CACCAGCACAGACACCCACACCGTCACCCACACTATCATGTTGACATTCC; dG = -36.3
593 >s_c_s_e_s_d_s_d_s_d_s_c_s_a_s_e_s
GTGTGGGTGCTGGTGACGGTGACGGTGACGGTGTGGGTGTCTGTGCTGGTG; >clamp_s_c_s_d_s_c_s_a_s_e_s:compl:rev
CACCAGCACAGACACCCACACCGTCACCCACACTATCATGTTGACATTCC; dG = -36.3
594 >s_c_s_e_s_e_s_c_s_d_s_d_s_a_s_e_s
GTGTGGGTGCTGGTGCTGGTGTGGGTGACGGTGACGGTGTCTGTGCTGGTG; >clamp_s_c_s_d_s_c_s_a_s_e_s:compl:rev
CACCAGCACAGACACCCACACCGTCACCCACACTATCATGTTGACATTCC; dG = -36.3
595 >s_c_s_e_s_e_s_c_s_c_s_c_s_a_s_e_s
GTGTGGGTGCTGGTGCTGGTGTGGGTGTGGGTGTGGGTGTCTGTGCTGGTG; >clamp_s_c_s_d_s_c_s_a_s_e_s:compl:rev
CACCAGCACAGACACCCACACCGTCACCCACACTATCATGTTGACATTCC; dG = -36.3
596 >s_c_s_e_s_e_s_d_s_d_s_c_s_a_s_e_s
GTGTGGGTGCTGGTGCTGGTGACGGTGACGGTGTGGGTGTCTGTGCTGGTG; >clamp_s_c_s_d_s_c_s_a_s_e_s:compl:rev
CACCAGCACAGACACCCACACCGTCACCCACACTATCATGTTGACATTCC; dG = -36.3
597 >s_a_s_b_s_c_s_c_s_d_s_d_s_a_s_e_s
GTGTCTGTGGCTGTGTGGGTGTGGGTGACGGTGACGGTGTCTGTGCTGGTG; >clamp_s_c_s_d_s_c_s_a_s_e_s:compl:rev
CACCAGCACAGACACCCACACCGTCACCCACACTATCATGTTGACATTCC; dG = -36.3
598 >s_d_s_a_s_a_s_c_s_d_s_d_s_a_s_e_s
GTGACGGTGTCTGTGTCTGTGTGGGTGACGGTGACGGTGTCTGTGCTGGTG; >clamp_s_c_s_d_s_c_s_a_s_e_s:compl:rev
CACCAGCACAGACACCCACACCGTCACCCACACTATCATGTTGACATTCC; dG = -36.3
599 >s_d_s_a_s_a_s_d_s_d_s_c_s_a_s_e_s
GTGACGGTGTCTGTGTCTGTGACGGTGACGGTGTGGGTGTCTGTGCTGGTG; >clamp_s_c_s_d_s_c_s_a_s_e_s:compl:rev
CACCAGCACAGACACCCACACCGTCACCCACACTATCATGTTGACATTCC; dG = -36.3
600 >s_d_s_a_s_b_s_c_s_d_s_d_s_a_s_e_s
GTGACGGTGTCTGTGGCTGTGTGGGTGACGGTGACGGTGTCTGTGCTGGTG; >clamp_s_c_s_d_s_c_s_a_s_e_s:compl:rev
CACCAGCACAGACACCCACACCGTCACCCACACTATCATGTTGACATTCC; dG = -36.3
601 >s_d_s_a_s_b_s_d_s_d_s_c_s_a_s_e_s
GTGACGGTGTCTGTGGCTGTGACGGTGACGGTGTGGGTGTCTGTGCTGGTG; >clamp_s_c_s_d_s_c_s_a_s_e_s:compl:rev
CACCAGCACAGACACCCACACCGTCACCCACACTATCATGTTGACATTCC; dG = -36.3
602 >s_a_s_b_s_c_s_c_s_c_s_c_s_a_s_e_s
GTGTCTGTGGCTGTGTGGGTGTGGGTGTGGGTGTGGGTGTCTGTGCTGGTG; >clamp_s_c_s_d_s_c_s_a_s_e_s:compl:rev
CACCAGCACAGACACCCACACCGTCACCCACACTATCATGTTGACATTCC; dG = -36.3
603 >s_d_s_a_s_c_s_c_s_d_s_d_s_a_s_e_s
GTGACGGTGTCTGTGTGGGTGTGGGTGACGGTGACGGTGTCTGTGCTGGTG; >clamp_s_c_s_d_s_c_s_a_s_e_s:compl:rev
CACCAGCACAGACACCCACACCGTCACCCACACTATCATGTTGACATTCC; dG = -36.3
604 >s_d_s_a_s_c_s_c_s_c_s_c_s_a_s_e_s
GTGACGGTGTCTGTGTGGGTGTGGGTGTGGGTGTGGGTGTCTGTGCTGGTG; >clamp_s_c_s_d_s_c_s_a_s_e_s:compl:rev
CACCAGCACAGACACCCACACCGTCACCCACACTATCATGTTGACATTCC; dG = -36.3
605 >s_d_s_a_s_d_s_c_s_d_s_d_s_a_s_e_s
GTGACGGTGTCTGTGACGGTGTGGGTGACGGTGACGGTGTCTGTGCTGGTG; >clamp_s_c_s_d_s_c_s_a_s_e_s:compl:rev
CACCAGCACAGACACCCACACCGTCACCCACACTATCATGTTGACATTCC; dG = -36.3
606 >s_d_s_a_s_d_s_c_s_c_s_c_s_a_s_e_s
GTGACGGTGTCTGTGACGGTGTGGGTGTGGGTGTGGGTGTCTGTGCTGGTG; >clamp_s_c_s_d_s_c_s_a_s_e_s:compl:rev
CACCAGCACAGACACCCACACCGTCACCCACACTATCATGTTGACATTCC; dG = -36.3
607 >s_d_s_a_s_d_s_d_s_d_s_c_s_a_s_e_s
GTGACGGTGTCTGTGACGGTGACGGTGACGGTGTGGGTGTCTGTGCTGGTG; >clamp_s_c_s_d_s_c_s_a_s_e_s:compl:rev
CACCAGCACAGACACCCACACCGTCACCCACACTATCATGTTGACATTCC; dG = -36.3

608 >s_d_s_a_s_e_s_c_s_d_s_d_s_a_s_e_s
GTGACGGTGTCTGTGCTGGTGTGGGTGACGGTGACGGTGTCTGTGCTGGTG; >clamp_s_c_s_d_s_c_s_a_s_e_s:compl:rev
CACCAGCACAGACACCCACACCGTCACCCACACTATCATGTTGACATTCC; dG = -36.3
609 >s_d_s_a_s_e_s_c_s_c_s_c_s_a_s_e_s
GTGACGGTGTCTGTGCTGGTGTGGGTGTGGGTGTGGGTGTCTGTGCTGGTG; >clamp_s_c_s_d_s_c_s_a_s_e_s:compl:rev
CACCAGCACAGACACCCACACCGTCACCCACACTATCATGTTGACATTCC; dG = -36.3
610 >s_d_s_a_s_e_s_d_s_d_s_c_s_a_s_e_s
GTGACGGTGTCTGTGCTGGTGACGGTGACGGTGTGGGTGTCTGTGCTGGTG; >clamp_s_c_s_d_s_c_s_a_s_e_s:compl:rev
CACCAGCACAGACACCCACACCGTCACCCACACTATCATGTTGACATTCC; dG = -36.3
611 >s_d_s_b_s_a_s_c_s_d_s_d_s_a_s_e_s
GTGACGGTGGCTGTGTCTGTGTGGGTGACGGTGACGGTGTCTGTGCTGGTG; >clamp_s_c_s_d_s_c_s_a_s_e_s:compl:rev
CACCAGCACAGACACCCACACCGTCACCCACACTATCATGTTGACATTCC; dG = -36.3
612 >s_d_s_b_s_a_s_d_s_d_s_c_s_a_s_e_s
GTGACGGTGGCTGTGTCTGTGACGGTGACGGTGTGGGTGTCTGTGCTGGTG; >clamp_s_c_s_d_s_c_s_a_s_e_s:compl:rev
CACCAGCACAGACACCCACACCGTCACCCACACTATCATGTTGACATTCC; dG = -36.3
613 >s_d_s_b_s_b_s_c_s_d_s_d_s_a_s_e_s
GTGACGGTGGCTGTGGCTGTGTGGGTGACGGTGACGGTGTCTGTGCTGGTG; >clamp_s_c_s_d_s_c_s_a_s_e_s:compl:rev
CACCAGCACAGACACCCACACCGTCACCCACACTATCATGTTGACATTCC; dG = -36.3
614 >s_d_s_b_s_b_s_d_s_d_s_c_s_a_s_e_s
GTGACGGTGGCTGTGGCTGTGACGGTGACGGTGTGGGTGTCTGTGCTGGTG; >clamp_s_c_s_d_s_c_s_a_s_e_s:compl:rev
CACCAGCACAGACACCCACACCGTCACCCACACTATCATGTTGACATTCC; dG = -36.3
615 >s_d_s_b_s_c_s_c_s_d_s_d_s_a_s_e_s
GTGACGGTGGCTGTGTGGGTGTGGGTGACGGTGACGGTGTCTGTGCTGGTG; >clamp_s_c_s_d_s_c_s_a_s_e_s:compl:rev
CACCAGCACAGACACCCACACCGTCACCCACACTATCATGTTGACATTCC; dG = -36.3
616 >s_d_s_b_s_c_s_c_s_c_s_c_s_a_s_e_s
GTGACGGTGGCTGTGTGGGTGTGGGTGTGGGTGTGGGTGTCTGTGCTGGTG; >clamp_s_c_s_d_s_c_s_a_s_e_s:compl:rev
CACCAGCACAGACACCCACACCGTCACCCACACTATCATGTTGACATTCC; dG = -36.3
617 >s_d_s_b_s_d_s_c_s_d_s_d_s_a_s_e_s
GTGACGGTGGCTGTGACGGTGTGGGTGACGGTGACGGTGTCTGTGCTGGTG; >clamp_s_c_s_d_s_c_s_a_s_e_s:compl:rev
CACCAGCACAGACACCCACACCGTCACCCACACTATCATGTTGACATTCC; dG = -36.3
618 >s_d_s_b_s_d_s_c_s_c_s_c_s_a_s_e_s
GTGACGGTGGCTGTGACGGTGTGGGTGTGGGTGTGGGTGTCTGTGCTGGTG; >clamp_s_c_s_d_s_c_s_a_s_e_s:compl:rev
CACCAGCACAGACACCCACACCGTCACCCACACTATCATGTTGACATTCC; dG = -36.3
619 >s_d_s_b_s_d_s_d_s_d_s_c_s_a_s_e_s
GTGACGGTGGCTGTGACGGTGACGGTGACGGTGTGGGTGTCTGTGCTGGTG; >clamp_s_c_s_d_s_c_s_a_s_e_s:compl:rev
CACCAGCACAGACACCCACACCGTCACCCACACTATCATGTTGACATTCC; dG = -36.3
620 >s_d_s_b_s_e_s_c_s_d_s_d_s_a_s_e_s
GTGACGGTGGCTGTGCTGGTGTGGGTGACGGTGACGGTGTCTGTGCTGGTG; >clamp_s_c_s_d_s_c_s_a_s_e_s:compl:rev
CACCAGCACAGACACCCACACCGTCACCCACACTATCATGTTGACATTCC; dG = -36.3
621 >s_d_s_b_s_e_s_c_s_c_s_c_s_a_s_e_s
GTGACGGTGGCTGTGCTGGTGTGGGTGTGGGTGTGGGTGTCTGTGCTGGTG; >clamp_s_c_s_d_s_c_s_a_s_e_s:compl:rev
CACCAGCACAGACACCCACACCGTCACCCACACTATCATGTTGACATTCC; dG = -36.3
622 >s_d_s_b_s_e_s_d_s_d_s_c_s_a_s_e_s
GTGACGGTGGCTGTGCTGGTGACGGTGACGGTGTGGGTGTCTGTGCTGGTG; >clamp_s_c_s_d_s_c_s_a_s_e_s:compl:rev
CACCAGCACAGACACCCACACCGTCACCCACACTATCATGTTGACATTCC; dG = -36.3
623 >s_a_s_b_s_d_s_c_s_d_s_d_s_a_s_e_s
GTGTCTGTGGCTGTGACGGTGTGGGTGACGGTGACGGTGTCTGTGCTGGTG; >clamp_s_c_s_d_s_c_s_a_s_e_s:compl:rev
CACCAGCACAGACACCCACACCGTCACCCACACTATCATGTTGACATTCC; dG = -36.3
624 >s_d_s_c_s_a_s_c_s_d_s_d_s_a_s_e_s
GTGACGGTGTGGGTGTCTGTGTGGGTGACGGTGACGGTGTCTGTGCTGGTG; >clamp_s_c_s_d_s_c_s_a_s_e_s:compl:rev
CACCAGCACAGACACCCACACCGTCACCCACACTATCATGTTGACATTCC; dG = -36.3
625 >s_d_s_c_s_a_s_d_s_d_s_c_s_a_s_e_s
GTGACGGTGTGGGTGTCTGTGACGGTGACGGTGTGGGTGTCTGTGCTGGTG; >clamp_s_c_s_d_s_c_s_a_s_e_s:compl:rev
CACCAGCACAGACACCCACACCGTCACCCACACTATCATGTTGACATTCC; dG = -36.3
626 >s_d_s_c_s_b_s_c_s_d_s_d_s_a_s_e_s
GTGACGGTGTGGGTGGCTGTGTGGGTGACGGTGACGGTGTCTGTGCTGGTG; >clamp_s_c_s_d_s_c_s_a_s_e_s:compl:rev
CACCAGCACAGACACCCACACCGTCACCCACACTATCATGTTGACATTCC; dG = -36.3
627 >s_d_s_c_s_b_s_d_s_d_s_c_s_a_s_e_s
GTGACGGTGTGGGTGGCTGTGACGGTGACGGTGTGGGTGTCTGTGCTGGTG; >clamp_s_c_s_d_s_c_s_a_s_e_s:compl:rev
CACCAGCACAGACACCCACACCGTCACCCACACTATCATGTTGACATTCC; dG = -36.3
628 >s_a_s_b_s_d_s_c_s_c_s_c_s_a_s_e_s
GTGTCTGTGGCTGTGACGGTGTGGGTGTGGGTGTGGGTGTCTGTGCTGGTG; >clamp_s_c_s_d_s_c_s_a_s_e_s:compl:rev
CACCAGCACAGACACCCACACCGTCACCCACACTATCATGTTGACATTCC; dG = -36.3
629 >s_d_s_c_s_c_s_c_s_d_s_d_s_a_s_e_s
GTGACGGTGTGGGTGTGGGTGTGGGTGACGGTGACGGTGTCTGTGCTGGTG; >clamp_s_c_s_d_s_c_s_a_s_e_s:compl:rev
CACCAGCACAGACACCCACACCGTCACCCACACTATCATGTTGACATTCC; dG = -36.3
630 >s_d_s_c_s_c_s_c_s_c_s_c_s_a_s_e_s
GTGACGGTGTGGGTGTGGGTGTGGGTGTGGGTGTGGGTGTCTGTGCTGGTG; >clamp_s_c_s_d_s_c_s_a_s_e_s:compl:rev
CACCAGCACAGACACCCACACCGTCACCCACACTATCATGTTGACATTCC; dG = -36.3
631 >s_d_s_c_s_d_s_c_s_d_s_d_s_a_s_e_s
GTGACGGTGTGGGTGACGGTGTGGGTGACGGTGACGGTGTCTGTGCTGGTG; >clamp_s_c_s_d_s_c_s_a_s_e_s:compl:rev
CACCAGCACAGACACCCACACCGTCACCCACACTATCATGTTGACATTCC; dG = -36.3

632 >s_d_s_c_s_d_s_c_s_c_s_c_s_a_s_e_s
GTGACGGTGTGGGTGACGGTGTGGGTGTGGGTGTGGGTGTCTGTGCTGGTG; >clamp_s_c_s_d_s_c_s_a_s_e_s:compl:rev
CACCAGCACAGACACCCACACCGTCACCCACACTATCATGTTGACATTCC; dG = -36.3
633 >s_d_s_c_s_d_s_d_s_d_s_c_s_a_s_e_s
GTGACGGTGTGGGTGACGGTGACGGTGACGGTGTGGGTGTCTGTGCTGGTG; >clamp_s_c_s_d_s_c_s_a_s_e_s:compl:rev
CACCAGCACAGACACCCACACCGTCACCCACACTATCATGTTGACATTCC; dG = -36.3
634 >s_a_s_b_s_d_s_d_s_d_s_c_s_a_s_e_s
GTGTCTGTGGCTGTGACGGTGACGGTGACGGTGTGGGTGTCTGTGCTGGTG; >clamp_s_c_s_d_s_c_s_a_s_e_s:compl:rev
CACCAGCACAGACACCCACACCGTCACCCACACTATCATGTTGACATTCC; dG = -36.3
635 >s_d_s_c_s_e_s_c_s_d_s_d_s_a_s_e_s
GTGACGGTGTGGGTGCTGGTGTGGGTGACGGTGACGGTGTCTGTGCTGGTG; >clamp_s_c_s_d_s_c_s_a_s_e_s:compl:rev
CACCAGCACAGACACCCACACCGTCACCCACACTATCATGTTGACATTCC; dG = -36.3
636 >s_d_s_c_s_e_s_c_s_c_s_c_s_a_s_e_s
GTGACGGTGTGGGTGCTGGTGTGGGTGTGGGTGTGGGTGTCTGTGCTGGTG; >clamp_s_c_s_d_s_c_s_a_s_e_s:compl:rev
CACCAGCACAGACACCCACACCGTCACCCACACTATCATGTTGACATTCC; dG = -36.3
637 >s_d_s_c_s_e_s_d_s_d_s_c_s_a_s_e_s
GTGACGGTGTGGGTGCTGGTGACGGTGACGGTGTGGGTGTCTGTGCTGGTG; >clamp_s_c_s_d_s_c_s_a_s_e_s:compl:rev
CACCAGCACAGACACCCACACCGTCACCCACACTATCATGTTGACATTCC; dG = -36.3
638 >s_a_s_a_s_a_s_d_s_d_s_c_s_a_s_e_s
GTGTCTGTGTCTGTGTCTGTGACGGTGACGGTGTGGGTGTCTGTGCTGGTG; >clamp_s_c_s_d_s_c_s_a_s_e_s:compl:rev
CACCAGCACAGACACCCACACCGTCACCCACACTATCATGTTGACATTCC; dG = -36.3
639 >s_d_s_d_s_a_s_c_s_d_s_d_s_a_s_e_s
GTGACGGTGACGGTGTCTGTGTGGGTGACGGTGACGGTGTCTGTGCTGGTG; >clamp_s_c_s_d_s_c_s_a_s_e_s:compl:rev
CACCAGCACAGACACCCACACCGTCACCCACACTATCATGTTGACATTCC; dG = -36.3
640 >s_d_s_d_s_a_s_d_s_d_s_c_s_a_s_e_s
GTGACGGTGACGGTGTCTGTGACGGTGACGGTGTGGGTGTCTGTGCTGGTG; >clamp_s_c_s_d_s_c_s_a_s_e_s:compl:rev
CACCAGCACAGACACCCACACCGTCACCCACACTATCATGTTGACATTCC; dG = -36.3
641 >s_d_s_d_s_b_s_c_s_d_s_d_s_a_s_e_s
GTGACGGTGACGGTGGCTGTGTGGGTGACGGTGACGGTGTCTGTGCTGGTG; >clamp_s_c_s_d_s_c_s_a_s_e_s:compl:rev
CACCAGCACAGACACCCACACCGTCACCCACACTATCATGTTGACATTCC; dG = -36.3
642 >s_d_s_d_s_b_s_d_s_d_s_c_s_a_s_e_s
GTGACGGTGACGGTGGCTGTGACGGTGACGGTGTGGGTGTCTGTGCTGGTG; >clamp_s_c_s_d_s_c_s_a_s_e_s:compl:rev
CACCAGCACAGACACCCACACCGTCACCCACACTATCATGTTGACATTCC; dG = -36.3
643 >s_d_s_d_s_c_s_c_s_d_s_d_s_a_s_e_s
GTGACGGTGACGGTGTGGGTGTGGGTGACGGTGACGGTGTCTGTGCTGGTG; >clamp_s_c_s_d_s_c_s_a_s_e_s:compl:rev
CACCAGCACAGACACCCACACCGTCACCCACACTATCATGTTGACATTCC; dG = -36.3
644 >s_d_s_d_s_c_s_c_s_c_s_c_s_a_s_e_s
GTGACGGTGACGGTGTGGGTGTGGGTGTGGGTGTGGGTGTCTGTGCTGGTG; >clamp_s_c_s_d_s_c_s_a_s_e_s:compl:rev
CACCAGCACAGACACCCACACCGTCACCCACACTATCATGTTGACATTCC; dG = -36.3
645 >s_d_s_d_s_d_s_c_s_d_s_d_s_a_s_e_s
GTGACGGTGACGGTGACGGTGTGGGTGACGGTGACGGTGTCTGTGCTGGTG; >clamp_s_c_s_d_s_c_s_a_s_e_s:compl:rev
CACCAGCACAGACACCCACACCGTCACCCACACTATCATGTTGACATTCC; dG = -36.3
646 >s_d_s_d_s_d_s_c_s_c_s_c_s_a_s_e_s
GTGACGGTGACGGTGACGGTGTGGGTGTGGGTGTGGGTGTCTGTGCTGGTG; >clamp_s_c_s_d_s_c_s_a_s_e_s:compl:rev
CACCAGCACAGACACCCACACCGTCACCCACACTATCATGTTGACATTCC; dG = -36.3
647 >s_d_s_d_s_d_s_d_s_d_s_c_s_a_s_e_s
GTGACGGTGACGGTGACGGTGACGGTGACGGTGTGGGTGTCTGTGCTGGTG; >clamp_s_c_s_d_s_c_s_a_s_e_s:compl:rev
CACCAGCACAGACACCCACACCGTCACCCACACTATCATGTTGACATTCC; dG = -36.3
648 >s_d_s_d_s_e_s_c_s_d_s_d_s_a_s_e_s
GTGACGGTGACGGTGCTGGTGTGGGTGACGGTGACGGTGTCTGTGCTGGTG; >clamp_s_c_s_d_s_c_s_a_s_e_s:compl:rev
CACCAGCACAGACACCCACACCGTCACCCACACTATCATGTTGACATTCC; dG = -36.3
649 >s_d_s_d_s_e_s_c_s_c_s_c_s_a_s_e_s
GTGACGGTGACGGTGCTGGTGTGGGTGTGGGTGTGGGTGTCTGTGCTGGTG; >clamp_s_c_s_d_s_c_s_a_s_e_s:compl:rev
CACCAGCACAGACACCCACACCGTCACCCACACTATCATGTTGACATTCC; dG = -36.3
650 >s_d_s_d_s_e_s_d_s_d_s_c_s_a_s_e_s
GTGACGGTGACGGTGCTGGTGACGGTGACGGTGTGGGTGTCTGTGCTGGTG; >clamp_s_c_s_d_s_c_s_a_s_e_s:compl:rev
CACCAGCACAGACACCCACACCGTCACCCACACTATCATGTTGACATTCC; dG = -36.3
651 >s_a_s_b_s_e_s_c_s_d_s_d_s_a_s_e_s
GTGTCTGTGGCTGTGCTGGTGTGGGTGACGGTGACGGTGTCTGTGCTGGTG; >clamp_s_c_s_d_s_c_s_a_s_e_s:compl:rev
CACCAGCACAGACACCCACACCGTCACCCACACTATCATGTTGACATTCC; dG = -36.3
652 >s_d_s_e_s_a_s_c_s_d_s_d_s_a_s_e_s
GTGACGGTGCTGGTGTCTGTGTGGGTGACGGTGACGGTGTCTGTGCTGGTG; >clamp_s_c_s_d_s_c_s_a_s_e_s:compl:rev
CACCAGCACAGACACCCACACCGTCACCCACACTATCATGTTGACATTCC; dG = -36.3
653 >s_d_s_e_s_a_s_d_s_d_s_c_s_a_s_e_s
GTGACGGTGCTGGTGTCTGTGACGGTGACGGTGTGGGTGTCTGTGCTGGTG; >clamp_s_c_s_d_s_c_s_a_s_e_s:compl:rev
CACCAGCACAGACACCCACACCGTCACCCACACTATCATGTTGACATTCC; dG = -36.3
654 >s_d_s_e_s_b_s_c_s_d_s_d_s_a_s_e_s
GTGACGGTGCTGGTGGCTGTGTGGGTGACGGTGACGGTGTCTGTGCTGGTG; >clamp_s_c_s_d_s_c_s_a_s_e_s:compl:rev
CACCAGCACAGACACCCACACCGTCACCCACACTATCATGTTGACATTCC; dG = -36.3
655 >s_d_s_e_s_b_s_d_s_d_s_c_s_a_s_e_s
GTGACGGTGCTGGTGGCTGTGACGGTGACGGTGTGGGTGTCTGTGCTGGTG; >clamp_s_c_s_d_s_c_s_a_s_e_s:compl:rev
CACCAGCACAGACACCCACACCGTCACCCACACTATCATGTTGACATTCC; dG = -36.3

656 >s_a_s_b_s_e_s_c_s_c_s_c_s_a_s_e_s
GTGTCTGTGGCTGTGCTGGTGTGGGTGTGGGTGTGGGTGTCTGTGCTGGTG; >clamp_s_c_s_d_s_c_s_a_s_e_s:compl:rev
CACCAGCACAGACACCCACACCGTCACCCACACTATCATGTTGACATTCC; dG = -36.3
657 >s_d_s_e_s_c_s_c_s_d_s_d_s_a_s_e_s
GTGACGGTGCTGGTGTGGGTGTGGGTGACGGTGACGGTGTCTGTGCTGGTG; >clamp_s_c_s_d_s_c_s_a_s_e_s:compl:rev
CACCAGCACAGACACCCACACCGTCACCCACACTATCATGTTGACATTCC; dG = -36.3
658 >s_d_s_e_s_c_s_c_s_c_s_c_s_a_s_e_s
GTGACGGTGCTGGTGTGGGTGTGGGTGTGGGTGTGGGTGTCTGTGCTGGTG; >clamp_s_c_s_d_s_c_s_a_s_e_s:compl:rev
CACCAGCACAGACACCCACACCGTCACCCACACTATCATGTTGACATTCC; dG = -36.3
659 >s_d_s_e_s_d_s_c_s_d_s_d_s_a_s_e_s
GTGACGGTGCTGGTGACGGTGTGGGTGACGGTGACGGTGTCTGTGCTGGTG; >clamp_s_c_s_d_s_c_s_a_s_e_s:compl:rev
CACCAGCACAGACACCCACACCGTCACCCACACTATCATGTTGACATTCC; dG = -36.3
660 >s_d_s_e_s_d_s_c_s_c_s_c_s_a_s_e_s
GTGACGGTGCTGGTGACGGTGTGGGTGTGGGTGTGGGTGTCTGTGCTGGTG; >clamp_s_c_s_d_s_c_s_a_s_e_s:compl:rev
CACCAGCACAGACACCCACACCGTCACCCACACTATCATGTTGACATTCC; dG = -36.3
661 >s_d_s_e_s_d_s_d_s_d_s_c_s_a_s_e_s
GTGACGGTGCTGGTGACGGTGACGGTGACGGTGTGGGTGTCTGTGCTGGTG; >clamp_s_c_s_d_s_c_s_a_s_e_s:compl:rev
CACCAGCACAGACACCCACACCGTCACCCACACTATCATGTTGACATTCC; dG = -36.3
662 >s_a_s_b_s_e_s_d_s_d_s_c_s_a_s_e_s
GTGTCTGTGGCTGTGCTGGTGACGGTGACGGTGTGGGTGTCTGTGCTGGTG; >clamp_s_c_s_d_s_c_s_a_s_e_s:compl:rev
CACCAGCACAGACACCCACACCGTCACCCACACTATCATGTTGACATTCC; dG = -36.3
663 >s_d_s_e_s_e_s_c_s_d_s_d_s_a_s_e_s
GTGACGGTGCTGGTGCTGGTGTGGGTGACGGTGACGGTGTCTGTGCTGGTG; >clamp_s_c_s_d_s_c_s_a_s_e_s:compl:rev
CACCAGCACAGACACCCACACCGTCACCCACACTATCATGTTGACATTCC; dG = -36.3
664 >s_d_s_e_s_e_s_c_s_c_s_c_s_a_s_e_s
GTGACGGTGCTGGTGCTGGTGTGGGTGTGGGTGTGGGTGTCTGTGCTGGTG; >clamp_s_c_s_d_s_c_s_a_s_e_s:compl:rev
CACCAGCACAGACACCCACACCGTCACCCACACTATCATGTTGACATTCC; dG = -36.3
665 >s_d_s_e_s_e_s_d_s_d_s_c_s_a_s_e_s
GTGACGGTGCTGGTGCTGGTGACGGTGACGGTGTGGGTGTCTGTGCTGGTG; >clamp_s_c_s_d_s_c_s_a_s_e_s:compl:rev
CACCAGCACAGACACCCACACCGTCACCCACACTATCATGTTGACATTCC; dG = -36.3
666 >s_e_s_a_s_a_s_c_s_d_s_d_s_a_s_e_s
GTGCTGGTGTCTGTGTCTGTGTGGGTGACGGTGACGGTGTCTGTGCTGGTG; >clamp_s_c_s_d_s_c_s_a_s_e_s:compl:rev
CACCAGCACAGACACCCACACCGTCACCCACACTATCATGTTGACATTCC; dG = -36.3
667 >s_e_s_a_s_a_s_d_s_d_s_c_s_a_s_e_s
GTGCTGGTGTCTGTGTCTGTGACGGTGACGGTGTGGGTGTCTGTGCTGGTG; >clamp_s_c_s_d_s_c_s_a_s_e_s:compl:rev
CACCAGCACAGACACCCACACCGTCACCCACACTATCATGTTGACATTCC; dG = -36.3
668 >s_e_s_a_s_b_s_c_s_d_s_d_s_a_s_e_s
GTGCTGGTGTCTGTGGCTGTGTGGGTGACGGTGACGGTGTCTGTGCTGGTG; >clamp_s_c_s_d_s_c_s_a_s_e_s:compl:rev
CACCAGCACAGACACCCACACCGTCACCCACACTATCATGTTGACATTCC; dG = -36.3
669 >s_e_s_a_s_b_s_d_s_d_s_c_s_a_s_e_s
GTGCTGGTGTCTGTGGCTGTGACGGTGACGGTGTGGGTGTCTGTGCTGGTG; >clamp_s_c_s_d_s_c_s_a_s_e_s:compl:rev
CACCAGCACAGACACCCACACCGTCACCCACACTATCATGTTGACATTCC; dG = -36.3
670 >s_e_s_a_s_c_s_c_s_d_s_d_s_a_s_e_s
GTGCTGGTGTCTGTGTGGGTGTGGGTGACGGTGACGGTGTCTGTGCTGGTG; >clamp_s_c_s_d_s_c_s_a_s_e_s:compl:rev
CACCAGCACAGACACCCACACCGTCACCCACACTATCATGTTGACATTCC; dG = -36.3
671 >s_e_s_a_s_c_s_c_s_c_s_c_s_a_s_e_s
GTGCTGGTGTCTGTGTGGGTGTGGGTGTGGGTGTGGGTGTCTGTGCTGGTG; >clamp_s_c_s_d_s_c_s_a_s_e_s:compl:rev
CACCAGCACAGACACCCACACCGTCACCCACACTATCATGTTGACATTCC; dG = -36.3
672 >s_e_s_a_s_d_s_c_s_d_s_d_s_a_s_e_s
GTGCTGGTGTCTGTGACGGTGTGGGTGACGGTGACGGTGTCTGTGCTGGTG; >clamp_s_c_s_d_s_c_s_a_s_e_s:compl:rev
CACCAGCACAGACACCCACACCGTCACCCACACTATCATGTTGACATTCC; dG = -36.3
673 >s_e_s_a_s_d_s_c_s_c_s_c_s_a_s_e_s
GTGCTGGTGTCTGTGACGGTGTGGGTGTGGGTGTGGGTGTCTGTGCTGGTG; >clamp_s_c_s_d_s_c_s_a_s_e_s:compl:rev
CACCAGCACAGACACCCACACCGTCACCCACACTATCATGTTGACATTCC; dG = -36.3
674 >s_e_s_a_s_d_s_d_s_d_s_c_s_a_s_e_s
GTGCTGGTGTCTGTGACGGTGACGGTGACGGTGTGGGTGTCTGTGCTGGTG; >clamp_s_c_s_d_s_c_s_a_s_e_s:compl:rev
CACCAGCACAGACACCCACACCGTCACCCACACTATCATGTTGACATTCC; dG = -36.3
675 >s_e_s_a_s_e_s_c_s_d_s_d_s_a_s_e_s
GTGCTGGTGTCTGTGCTGGTGTGGGTGACGGTGACGGTGTCTGTGCTGGTG; >clamp_s_c_s_d_s_c_s_a_s_e_s:compl:rev
CACCAGCACAGACACCCACACCGTCACCCACACTATCATGTTGACATTCC; dG = -36.3
676 >s_e_s_a_s_e_s_c_s_c_s_c_s_a_s_e_s
GTGCTGGTGTCTGTGCTGGTGTGGGTGTGGGTGTGGGTGTCTGTGCTGGTG; >clamp_s_c_s_d_s_c_s_a_s_e_s:compl:rev
CACCAGCACAGACACCCACACCGTCACCCACACTATCATGTTGACATTCC; dG = -36.3
677 >s_e_s_a_s_e_s_d_s_d_s_c_s_a_s_e_s
GTGCTGGTGTCTGTGCTGGTGACGGTGACGGTGTGGGTGTCTGTGCTGGTG; >clamp_s_c_s_d_s_c_s_a_s_e_s:compl:rev
CACCAGCACAGACACCCACACCGTCACCCACACTATCATGTTGACATTCC; dG = -36.3
678 >s_a_s_c_s_a_s_c_s_d_s_d_s_a_s_e_s
GTGTCTGTGTGGGTGTCTGTGTGGGTGACGGTGACGGTGTCTGTGCTGGTG; >clamp_s_c_s_d_s_c_s_a_s_e_s:compl:rev
CACCAGCACAGACACCCACACCGTCACCCACACTATCATGTTGACATTCC; dG = -36.3
679 >s_e_s_b_s_a_s_c_s_d_s_d_s_a_s_e_s
GTGCTGGTGGCTGTGTCTGTGTGGGTGACGGTGACGGTGTCTGTGCTGGTG; >clamp_s_c_s_d_s_c_s_a_s_e_s:compl:rev
CACCAGCACAGACACCCACACCGTCACCCACACTATCATGTTGACATTCC; dG = -36.3

680 >s_e_s_b_s_a_s_d_s_d_s_c_s_a_s_e_s
GTGCTGGTGGCTGTGTCTGTGACGGTGACGGTGTGGGTGTCTGTGCTGGTG; >clamp_s_c_s_d_s_c_s_a_s_e_s:compl:rev
CACCAGCACAGACACCCACACCGTCACCCACACTATCATGTTGACATTCC; dG = -36.3
681 >s_e_s_b_s_b_s_c_s_d_s_d_s_a_s_e_s
GTGCTGGTGGCTGTGGCTGTGTGGGTGACGGTGACGGTGTCTGTGCTGGTG; >clamp_s_c_s_d_s_c_s_a_s_e_s:compl:rev
CACCAGCACAGACACCCACACCGTCACCCACACTATCATGTTGACATTCC; dG = -36.3
682 >s_e_s_b_s_b_s_d_s_d_s_c_s_a_s_e_s
GTGCTGGTGGCTGTGGCTGTGACGGTGACGGTGTGGGTGTCTGTGCTGGTG; >clamp_s_c_s_d_s_c_s_a_s_e_s:compl:rev
CACCAGCACAGACACCCACACCGTCACCCACACTATCATGTTGACATTCC; dG = -36.3
683 >s_e_s_b_s_c_s_c_s_d_s_d_s_a_s_e_s
GTGCTGGTGGCTGTGTGGGTGTGGGTGACGGTGACGGTGTCTGTGCTGGTG; >clamp_s_c_s_d_s_c_s_a_s_e_s:compl:rev
CACCAGCACAGACACCCACACCGTCACCCACACTATCATGTTGACATTCC; dG = -36.3
684 >s_e_s_b_s_c_s_c_s_c_s_c_s_a_s_e_s
GTGCTGGTGGCTGTGTGGGTGTGGGTGTGGGTGTGGGTGTCTGTGCTGGTG; >clamp_s_c_s_d_s_c_s_a_s_e_s:compl:rev
CACCAGCACAGACACCCACACCGTCACCCACACTATCATGTTGACATTCC; dG = -36.3
685 >s_e_s_b_s_d_s_c_s_d_s_d_s_a_s_e_s
GTGCTGGTGGCTGTGACGGTGTGGGTGACGGTGACGGTGTCTGTGCTGGTG; >clamp_s_c_s_d_s_c_s_a_s_e_s:compl:rev
CACCAGCACAGACACCCACACCGTCACCCACACTATCATGTTGACATTCC; dG = -36.3
686 >s_e_s_b_s_d_s_c_s_c_s_c_s_a_s_e_s
GTGCTGGTGGCTGTGACGGTGTGGGTGTGGGTGTGGGTGTCTGTGCTGGTG; >clamp_s_c_s_d_s_c_s_a_s_e_s:compl:rev
CACCAGCACAGACACCCACACCGTCACCCACACTATCATGTTGACATTCC; dG = -36.3
687 >s_e_s_b_s_d_s_d_s_d_s_c_s_a_s_e_s
GTGCTGGTGGCTGTGACGGTGACGGTGACGGTGTGGGTGTCTGTGCTGGTG; >clamp_s_c_s_d_s_c_s_a_s_e_s:compl:rev
CACCAGCACAGACACCCACACCGTCACCCACACTATCATGTTGACATTCC; dG = -36.3
688 >s_a_s_c_s_a_s_d_s_d_s_c_s_a_s_e_s
GTGTCTGTGTGGGTGTCTGTGACGGTGACGGTGTGGGTGTCTGTGCTGGTG; >clamp_s_c_s_d_s_c_s_a_s_e_s:compl:rev
CACCAGCACAGACACCCACACCGTCACCCACACTATCATGTTGACATTCC; dG = -36.3
689 >s_e_s_b_s_e_s_c_s_d_s_d_s_a_s_e_s
GTGCTGGTGGCTGTGCTGGTGTGGGTGACGGTGACGGTGTCTGTGCTGGTG; >clamp_s_c_s_d_s_c_s_a_s_e_s:compl:rev
CACCAGCACAGACACCCACACCGTCACCCACACTATCATGTTGACATTCC; dG = -36.3
690 >s_e_s_b_s_e_s_c_s_c_s_c_s_a_s_e_s
GTGCTGGTGGCTGTGCTGGTGTGGGTGTGGGTGTGGGTGTCTGTGCTGGTG; >clamp_s_c_s_d_s_c_s_a_s_e_s:compl:rev
CACCAGCACAGACACCCACACCGTCACCCACACTATCATGTTGACATTCC; dG = -36.3
691 >s_e_s_b_s_e_s_d_s_d_s_c_s_a_s_e_s
GTGCTGGTGGCTGTGCTGGTGACGGTGACGGTGTGGGTGTCTGTGCTGGTG; >clamp_s_c_s_d_s_c_s_a_s_e_s:compl:rev
CACCAGCACAGACACCCACACCGTCACCCACACTATCATGTTGACATTCC; dG = -36.3
692 >s_e_s_c_s_a_s_c_s_d_s_d_s_a_s_e_s
GTGCTGGTGTGGGTGTCTGTGTGGGTGACGGTGACGGTGTCTGTGCTGGTG; >clamp_s_c_s_d_s_c_s_a_s_e_s:compl:rev
CACCAGCACAGACACCCACACCGTCACCCACACTATCATGTTGACATTCC; dG = -36.3
693 >s_e_s_c_s_a_s_d_s_d_s_c_s_a_s_e_s
GTGCTGGTGTGGGTGTCTGTGACGGTGACGGTGTGGGTGTCTGTGCTGGTG; >clamp_s_c_s_d_s_c_s_a_s_e_s:compl:rev
CACCAGCACAGACACCCACACCGTCACCCACACTATCATGTTGACATTCC; dG = -36.3
694 >s_e_s_c_s_b_s_c_s_d_s_d_s_a_s_e_s
GTGCTGGTGTGGGTGGCTGTGTGGGTGACGGTGACGGTGTCTGTGCTGGTG; >clamp_s_c_s_d_s_c_s_a_s_e_s:compl:rev
CACCAGCACAGACACCCACACCGTCACCCACACTATCATGTTGACATTCC; dG = -36.3
695 >s_e_s_c_s_b_s_d_s_d_s_c_s_a_s_e_s
GTGCTGGTGTGGGTGGCTGTGACGGTGACGGTGTGGGTGTCTGTGCTGGTG; >clamp_s_c_s_d_s_c_s_a_s_e_s:compl:rev
CACCAGCACAGACACCCACACCGTCACCCACACTATCATGTTGACATTCC; dG = -36.3
696 >s_e_s_c_s_c_s_c_s_d_s_d_s_a_s_e_s
GTGCTGGTGTGGGTGTGGGTGTGGGTGACGGTGACGGTGTCTGTGCTGGTG; >clamp_s_c_s_d_s_c_s_a_s_e_s:compl:rev
CACCAGCACAGACACCCACACCGTCACCCACACTATCATGTTGACATTCC; dG = -36.3
697 >s_e_s_c_s_c_s_c_s_c_s_c_s_a_s_e_s
GTGCTGGTGTGGGTGTGGGTGTGGGTGTGGGTGTGGGTGTCTGTGCTGGTG; >clamp_s_c_s_d_s_c_s_a_s_e_s:compl:rev
CACCAGCACAGACACCCACACCGTCACCCACACTATCATGTTGACATTCC; dG = -36.3
698 >s_e_s_c_s_d_s_c_s_d_s_d_s_a_s_e_s
GTGCTGGTGTGGGTGACGGTGTGGGTGACGGTGACGGTGTCTGTGCTGGTG; >clamp_s_c_s_d_s_c_s_a_s_e_s:compl:rev
CACCAGCACAGACACCCACACCGTCACCCACACTATCATGTTGACATTCC; dG = -36.3
699 >s_e_s_c_s_d_s_c_s_c_s_c_s_a_s_e_s
GTGCTGGTGTGGGTGACGGTGTGGGTGTGGGTGTGGGTGTCTGTGCTGGTG; >clamp_s_c_s_d_s_c_s_a_s_e_s:compl:rev
CACCAGCACAGACACCCACACCGTCACCCACACTATCATGTTGACATTCC; dG = -36.3
700 >s_e_s_c_s_d_s_d_s_d_s_c_s_a_s_e_s
GTGCTGGTGTGGGTGACGGTGACGGTGACGGTGTGGGTGTCTGTGCTGGTG; >clamp_s_c_s_d_s_c_s_a_s_e_s:compl:rev
CACCAGCACAGACACCCACACCGTCACCCACACTATCATGTTGACATTCC; dG = -36.3
701 >s_e_s_c_s_e_s_c_s_d_s_d_s_a_s_e_s
GTGCTGGTGTGGGTGCTGGTGTGGGTGACGGTGACGGTGTCTGTGCTGGTG; >clamp_s_c_s_d_s_c_s_a_s_e_s:compl:rev
CACCAGCACAGACACCCACACCGTCACCCACACTATCATGTTGACATTCC; dG = -36.3
702 >s_e_s_c_s_e_s_c_s_c_s_c_s_a_s_e_s
GTGCTGGTGTGGGTGCTGGTGTGGGTGTGGGTGTGGGTGTCTGTGCTGGTG; >clamp_s_c_s_d_s_c_s_a_s_e_s:compl:rev
CACCAGCACAGACACCCACACCGTCACCCACACTATCATGTTGACATTCC; dG = -36.3
703 >s_e_s_c_s_e_s_d_s_d_s_c_s_a_s_e_s
GTGCTGGTGTGGGTGCTGGTGACGGTGACGGTGTGGGTGTCTGTGCTGGTG; >clamp_s_c_s_d_s_c_s_a_s_e_s:compl:rev
CACCAGCACAGACACCCACACCGTCACCCACACTATCATGTTGACATTCC; dG = -36.3

704 >s_a_s_c_s_b_s_c_s_d_s_d_s_a_s_e_s
GTGTCTGTGTGGGTGGCTGTGTGGGTGACGGTGACGGTGTCTGTGCTGGTG; >clamp_s_c_s_d_s_c_s_a_s_e_s:compl:rev
CACCAGCACAGACACCCACACCGTCACCCACACTATCATGTTGACATTCC; dG = -36.3
705 >s_e_s_d_s_a_s_c_s_d_s_d_s_a_s_e_s
GTGCTGGTGACGGTGTCTGTGTGGGTGACGGTGACGGTGTCTGTGCTGGTG; >clamp_s_c_s_d_s_c_s_a_s_e_s:compl:rev
CACCAGCACAGACACCCACACCGTCACCCACACTATCATGTTGACATTCC; dG = -36.3
706 >s_e_s_d_s_a_s_d_s_d_s_c_s_a_s_e_s
GTGCTGGTGACGGTGTCTGTGACGGTGACGGTGTGGGTGTCTGTGCTGGTG; >clamp_s_c_s_d_s_c_s_a_s_e_s:compl:rev
CACCAGCACAGACACCCACACCGTCACCCACACTATCATGTTGACATTCC; dG = -36.3
707 >s_e_s_d_s_b_s_c_s_d_s_d_s_a_s_e_s
GTGCTGGTGACGGTGGCTGTGTGGGTGACGGTGACGGTGTCTGTGCTGGTG; >clamp_s_c_s_d_s_c_s_a_s_e_s:compl:rev
CACCAGCACAGACACCCACACCGTCACCCACACTATCATGTTGACATTCC; dG = -36.3
708 >s_e_s_d_s_b_s_d_s_d_s_c_s_a_s_e_s
GTGCTGGTGACGGTGGCTGTGACGGTGACGGTGTGGGTGTCTGTGCTGGTG; >clamp_s_c_s_d_s_c_s_a_s_e_s:compl:rev
CACCAGCACAGACACCCACACCGTCACCCACACTATCATGTTGACATTCC; dG = -36.3
709 >s_e_s_d_s_c_s_c_s_d_s_d_s_a_s_e_s
GTGCTGGTGACGGTGTGGGTGTGGGTGACGGTGACGGTGTCTGTGCTGGTG; >clamp_s_c_s_d_s_c_s_a_s_e_s:compl:rev
CACCAGCACAGACACCCACACCGTCACCCACACTATCATGTTGACATTCC; dG = -36.3
710 >s_e_s_d_s_c_s_c_s_c_s_c_s_a_s_e_s
GTGCTGGTGACGGTGTGGGTGTGGGTGTGGGTGTGGGTGTCTGTGCTGGTG; >clamp_s_c_s_d_s_c_s_a_s_e_s:compl:rev
CACCAGCACAGACACCCACACCGTCACCCACACTATCATGTTGACATTCC; dG = -36.3
711 >s_e_s_d_s_d_s_c_s_d_s_d_s_a_s_e_s
GTGCTGGTGACGGTGACGGTGTGGGTGACGGTGACGGTGTCTGTGCTGGTG; >clamp_s_c_s_d_s_c_s_a_s_e_s:compl:rev
CACCAGCACAGACACCCACACCGTCACCCACACTATCATGTTGACATTCC; dG = -36.3
712 >s_e_s_d_s_d_s_c_s_c_s_c_s_a_s_e_s
GTGCTGGTGACGGTGACGGTGTGGGTGTGGGTGTGGGTGTCTGTGCTGGTG; >clamp_s_c_s_d_s_c_s_a_s_e_s:compl:rev
CACCAGCACAGACACCCACACCGTCACCCACACTATCATGTTGACATTCC; dG = -36.3
713 >s_e_s_d_s_d_s_d_s_d_s_c_s_a_s_e_s
GTGCTGGTGACGGTGACGGTGACGGTGACGGTGTGGGTGTCTGTGCTGGTG; >clamp_s_c_s_d_s_c_s_a_s_e_s:compl:rev
CACCAGCACAGACACCCACACCGTCACCCACACTATCATGTTGACATTCC; dG = -36.3
714 >s_a_s_c_s_b_s_d_s_d_s_c_s_a_s_e_s
GTGTCTGTGTGGGTGGCTGTGACGGTGACGGTGTGGGTGTCTGTGCTGGTG; >clamp_s_c_s_d_s_c_s_a_s_e_s:compl:rev
CACCAGCACAGACACCCACACCGTCACCCACACTATCATGTTGACATTCC; dG = -36.3
715 >s_e_s_d_s_e_s_c_s_d_s_d_s_a_s_e_s
GTGCTGGTGACGGTGCTGGTGTGGGTGACGGTGACGGTGTCTGTGCTGGTG; >clamp_s_c_s_d_s_c_s_a_s_e_s:compl:rev
CACCAGCACAGACACCCACACCGTCACCCACACTATCATGTTGACATTCC; dG = -36.3
716 >s_e_s_d_s_e_s_c_s_c_s_c_s_a_s_e_s
GTGCTGGTGACGGTGCTGGTGTGGGTGTGGGTGTGGGTGTCTGTGCTGGTG; >clamp_s_c_s_d_s_c_s_a_s_e_s:compl:rev
CACCAGCACAGACACCCACACCGTCACCCACACTATCATGTTGACATTCC; dG = -36.3
717 >s_e_s_d_s_e_s_d_s_d_s_c_s_a_s_e_s
GTGCTGGTGACGGTGCTGGTGACGGTGACGGTGTGGGTGTCTGTGCTGGTG; >clamp_s_c_s_d_s_c_s_a_s_e_s:compl:rev
CACCAGCACAGACACCCACACCGTCACCCACACTATCATGTTGACATTCC; dG = -36.3
718 >s_e_s_e_s_a_s_c_s_d_s_d_s_a_s_e_s
GTGCTGGTGCTGGTGTCTGTGTGGGTGACGGTGACGGTGTCTGTGCTGGTG; >clamp_s_c_s_d_s_c_s_a_s_e_s:compl:rev
CACCAGCACAGACACCCACACCGTCACCCACACTATCATGTTGACATTCC; dG = -36.3
719 >s_e_s_e_s_a_s_d_s_d_s_c_s_a_s_e_s
GTGCTGGTGCTGGTGTCTGTGACGGTGACGGTGTGGGTGTCTGTGCTGGTG; >clamp_s_c_s_d_s_c_s_a_s_e_s:compl:rev
CACCAGCACAGACACCCACACCGTCACCCACACTATCATGTTGACATTCC; dG = -36.3
720 >s_e_s_e_s_b_s_c_s_d_s_d_s_a_s_e_s
GTGCTGGTGCTGGTGGCTGTGTGGGTGACGGTGACGGTGTCTGTGCTGGTG; >clamp_s_c_s_d_s_c_s_a_s_e_s:compl:rev
CACCAGCACAGACACCCACACCGTCACCCACACTATCATGTTGACATTCC; dG = -36.3
721 >s_e_s_e_s_b_s_d_s_d_s_c_s_a_s_e_s
GTGCTGGTGCTGGTGGCTGTGACGGTGACGGTGTGGGTGTCTGTGCTGGTG; >clamp_s_c_s_d_s_c_s_a_s_e_s:compl:rev
CACCAGCACAGACACCCACACCGTCACCCACACTATCATGTTGACATTCC; dG = -36.3
722 >s_e_s_e_s_c_s_c_s_d_s_d_s_a_s_e_s
GTGCTGGTGCTGGTGTGGGTGTGGGTGACGGTGACGGTGTCTGTGCTGGTG; >clamp_s_c_s_d_s_c_s_a_s_e_s:compl:rev
CACCAGCACAGACACCCACACCGTCACCCACACTATCATGTTGACATTCC; dG = -36.3
723 >s_e_s_e_s_c_s_c_s_c_s_c_s_a_s_e_s
GTGCTGGTGCTGGTGTGGGTGTGGGTGTGGGTGTGGGTGTCTGTGCTGGTG; >clamp_s_c_s_d_s_c_s_a_s_e_s:compl:rev
CACCAGCACAGACACCCACACCGTCACCCACACTATCATGTTGACATTCC; dG = -36.3
724 >s_e_s_e_s_d_s_c_s_d_s_d_s_a_s_e_s
GTGCTGGTGCTGGTGACGGTGTGGGTGACGGTGACGGTGTCTGTGCTGGTG; >clamp_s_c_s_d_s_c_s_a_s_e_s:compl:rev
CACCAGCACAGACACCCACACCGTCACCCACACTATCATGTTGACATTCC; dG = -36.3
725 >s_e_s_e_s_d_s_c_s_c_s_c_s_a_s_e_s
GTGCTGGTGCTGGTGACGGTGTGGGTGTGGGTGTGGGTGTCTGTGCTGGTG; >clamp_s_c_s_d_s_c_s_a_s_e_s:compl:rev
CACCAGCACAGACACCCACACCGTCACCCACACTATCATGTTGACATTCC; dG = -36.3
726 >s_e_s_e_s_d_s_d_s_d_s_c_s_a_s_e_s
GTGCTGGTGCTGGTGACGGTGACGGTGACGGTGTGGGTGTCTGTGCTGGTG; >clamp_s_c_s_d_s_c_s_a_s_e_s:compl:rev
CACCAGCACAGACACCCACACCGTCACCCACACTATCATGTTGACATTCC; dG = -36.3
727 >s_e_s_e_s_e_s_c_s_d_s_d_s_a_s_e_s
GTGCTGGTGCTGGTGCTGGTGTGGGTGACGGTGACGGTGTCTGTGCTGGTG; >clamp_s_c_s_d_s_c_s_a_s_e_s:compl:rev
CACCAGCACAGACACCCACACCGTCACCCACACTATCATGTTGACATTCC; dG = -36.3

728 >s_e_s_e_s_e_s_c_s_c_s_c_s_a_s_e_s
GTGCTGGTGCTGGTGCTGGTGTGGGTGTGGGTGTGGGTGTCTGTGCTGGTG; >clamp_s_c_s_d_s_c_s_a_s_e_s:compl:rev
CACCAGCACAGACACCCACACCGTCACCCACACTATCATGTTGACATTCC; dG = -36.3
729 >s_e_s_e_s_e_s_d_s_d_s_c_s_a_s_e_s
GTGCTGGTGCTGGTGCTGGTGACGGTGACGGTGTGGGTGTCTGTGCTGGTG; >clamp_s_c_s_d_s_c_s_a_s_e_s:compl:rev
CACCAGCACAGACACCCACACCGTCACCCACACTATCATGTTGACATTCC; dG = -36.3
730 >s_a_s_c_s_c_s_c_s_d_s_d_s_a_s_e_s
GTGTCTGTGTGGGTGTGGGTGTGGGTGACGGTGACGGTGTCTGTGCTGGTG; >clamp_s_c_s_d_s_c_s_a_s_e_s:compl:rev
CACCAGCACAGACACCCACACCGTCACCCACACTATCATGTTGACATTCC; dG = -36.3
731 >s_a_s_c_s_c_s_c_s_c_s_c_s_a_s_e_s
GTGTCTGTGTGGGTGTGGGTGTGGGTGTGGGTGTGGGTGTCTGTGCTGGTG; >clamp_s_c_s_d_s_c_s_a_s_e_s:compl:rev
CACCAGCACAGACACCCACACCGTCACCCACACTATCATGTTGACATTCC; dG = -36.3
732 >s_a_s_c_s_d_s_c_s_d_s_d_s_a_s_e_s
GTGTCTGTGTGGGTGACGGTGTGGGTGACGGTGACGGTGTCTGTGCTGGTG; >clamp_s_c_s_d_s_c_s_a_s_e_s:compl:rev
CACCAGCACAGACACCCACACCGTCACCCACACTATCATGTTGACATTCC; dG = -36.3
733 >s_a_s_c_s_d_s_c_s_c_s_c_s_a_s_e_s
GTGTCTGTGTGGGTGACGGTGTGGGTGTGGGTGTGGGTGTCTGTGCTGGTG; >clamp_s_c_s_d_s_c_s_a_s_e_s:compl:rev
CACCAGCACAGACACCCACACCGTCACCCACACTATCATGTTGACATTCC; dG = -36.3
734 >s_a_s_c_s_d_s_d_s_d_s_c_s_a_s_e_s
GTGTCTGTGTGGGTGACGGTGACGGTGACGGTGTGGGTGTCTGTGCTGGTG; >clamp_s_c_s_d_s_c_s_a_s_e_s:compl:rev
CACCAGCACAGACACCCACACCGTCACCCACACTATCATGTTGACATTCC; dG = -36.3
735 >s_a_s_c_s_e_s_c_s_d_s_d_s_a_s_e_s
GTGTCTGTGTGGGTGCTGGTGTGGGTGACGGTGACGGTGTCTGTGCTGGTG; >clamp_s_c_s_d_s_c_s_a_s_e_s:compl:rev
CACCAGCACAGACACCCACACCGTCACCCACACTATCATGTTGACATTCC; dG = -36.3
736 >s_a_s_c_s_e_s_c_s_c_s_c_s_a_s_e_s
GTGTCTGTGTGGGTGCTGGTGTGGGTGTGGGTGTGGGTGTCTGTGCTGGTG; >clamp_s_c_s_d_s_c_s_a_s_e_s:compl:rev
CACCAGCACAGACACCCACACCGTCACCCACACTATCATGTTGACATTCC; dG = -36.3
737 >s_a_s_c_s_e_s_d_s_d_s_c_s_a_s_e_s
GTGTCTGTGTGGGTGCTGGTGACGGTGACGGTGTGGGTGTCTGTGCTGGTG; >clamp_s_c_s_d_s_c_s_a_s_e_s:compl:rev
CACCAGCACAGACACCCACACCGTCACCCACACTATCATGTTGACATTCC; dG = -36.3
738 >s_a_s_a_s_b_s_c_s_d_s_d_s_a_s_e_s
GTGTCTGTGTCTGTGGCTGTGTGGGTGACGGTGACGGTGTCTGTGCTGGTG; >clamp_s_c_s_d_s_c_s_a_s_e_s:compl:rev
CACCAGCACAGACACCCACACCGTCACCCACACTATCATGTTGACATTCC; dG = -36.3
739 >s_a_s_d_s_a_s_c_s_d_s_d_s_a_s_e_s
GTGTCTGTGACGGTGTCTGTGTGGGTGACGGTGACGGTGTCTGTGCTGGTG; >clamp_s_c_s_d_s_c_s_a_s_e_s:compl:rev
CACCAGCACAGACACCCACACCGTCACCCACACTATCATGTTGACATTCC; dG = -36.3
740 >s_a_s_d_s_a_s_d_s_d_s_c_s_a_s_e_s
GTGTCTGTGACGGTGTCTGTGACGGTGACGGTGTGGGTGTCTGTGCTGGTG; >clamp_s_c_s_d_s_c_s_a_s_e_s:compl:rev
CACCAGCACAGACACCCACACCGTCACCCACACTATCATGTTGACATTCC; dG = -36.3
741 >s_a_s_d_s_b_s_c_s_d_s_d_s_a_s_e_s
GTGTCTGTGACGGTGGCTGTGTGGGTGACGGTGACGGTGTCTGTGCTGGTG; >clamp_s_c_s_d_s_c_s_a_s_e_s:compl:rev
CACCAGCACAGACACCCACACCGTCACCCACACTATCATGTTGACATTCC; dG = -36.3
742 >s_a_s_d_s_b_s_d_s_d_s_c_s_a_s_e_s
GTGTCTGTGACGGTGGCTGTGACGGTGACGGTGTGGGTGTCTGTGCTGGTG; >clamp_s_c_s_d_s_c_s_a_s_e_s:compl:rev
CACCAGCACAGACACCCACACCGTCACCCACACTATCATGTTGACATTCC; dG = -36.3
743 >s_a_s_d_s_c_s_c_s_d_s_d_s_a_s_e_s
GTGTCTGTGACGGTGTGGGTGTGGGTGACGGTGACGGTGTCTGTGCTGGTG; >clamp_s_c_s_d_s_c_s_a_s_e_s:compl:rev
CACCAGCACAGACACCCACACCGTCACCCACACTATCATGTTGACATTCC; dG = -36.3
744 >s_a_s_d_s_c_s_c_s_c_s_c_s_a_s_e_s
GTGTCTGTGACGGTGTGGGTGTGGGTGTGGGTGTGGGTGTCTGTGCTGGTG; >clamp_s_c_s_d_s_c_s_a_s_e_s:compl:rev
CACCAGCACAGACACCCACACCGTCACCCACACTATCATGTTGACATTCC; dG = -36.3
745 >s_a_s_d_s_d_s_c_s_d_s_d_s_a_s_e_s
GTGTCTGTGACGGTGACGGTGTGGGTGACGGTGACGGTGTCTGTGCTGGTG; >clamp_s_c_s_d_s_c_s_a_s_e_s:compl:rev
CACCAGCACAGACACCCACACCGTCACCCACACTATCATGTTGACATTCC; dG = -36.3
746 >s_a_s_d_s_d_s_c_s_c_s_c_s_a_s_e_s
GTGTCTGTGACGGTGACGGTGTGGGTGTGGGTGTGGGTGTCTGTGCTGGTG; >clamp_s_c_s_d_s_c_s_a_s_e_s:compl:rev
CACCAGCACAGACACCCACACCGTCACCCACACTATCATGTTGACATTCC; dG = -36.3
747 >s_a_s_d_s_d_s_d_s_d_s_c_s_a_s_e_s
GTGTCTGTGACGGTGACGGTGACGGTGACGGTGTGGGTGTCTGTGCTGGTG; >clamp_s_c_s_d_s_c_s_a_s_e_s:compl:rev
CACCAGCACAGACACCCACACCGTCACCCACACTATCATGTTGACATTCC; dG = -36.3
748 >s_a_s_a_s_b_s_d_s_d_s_c_s_a_s_e_s
GTGTCTGTGTCTGTGGCTGTGACGGTGACGGTGTGGGTGTCTGTGCTGGTG; >clamp_s_c_s_d_s_c_s_a_s_e_s:compl:rev
CACCAGCACAGACACCCACACCGTCACCCACACTATCATGTTGACATTCC; dG = -36.3
749 >s_a_s_d_s_e_s_c_s_d_s_d_s_a_s_e_s
GTGTCTGTGACGGTGCTGGTGTGGGTGACGGTGACGGTGTCTGTGCTGGTG; >clamp_s_c_s_d_s_c_s_a_s_e_s:compl:rev
CACCAGCACAGACACCCACACCGTCACCCACACTATCATGTTGACATTCC; dG = -36.3
750 >s_a_s_d_s_e_s_c_s_c_s_c_s_a_s_e_s
GTGTCTGTGACGGTGCTGGTGTGGGTGTGGGTGTGGGTGTCTGTGCTGGTG; >clamp_s_c_s_d_s_c_s_a_s_e_s:compl:rev
CACCAGCACAGACACCCACACCGTCACCCACACTATCATGTTGACATTCC; dG = -36.3
751 >s_a_s_d_s_e_s_d_s_d_s_c_s_a_s_e_s
GTGTCTGTGACGGTGCTGGTGACGGTGACGGTGTGGGTGTCTGTGCTGGTG; >clamp_s_c_s_d_s_c_s_a_s_e_s:compl:rev
CACCAGCACAGACACCCACACCGTCACCCACACTATCATGTTGACATTCC; dG = -36.3

752 >s_a_s_e_s_a_s_c_s_d_s_d_s_a_s_e_s
GTGTCTGTGCTGGTGTCTGTGTGGGTGACGGTGACGGTGTCTGTGCTGGTG; >clamp_s_c_s_d_s_c_s_a_s_e_s:compl:rev
CACCAGCACAGACACCCACACCGTCACCCACACTATCATGTTGACATTCC; dG = -36.3
753 >s_a_s_e_s_a_s_d_s_d_s_c_s_a_s_e_s
GTGTCTGTGCTGGTGTCTGTGACGGTGACGGTGTGGGTGTCTGTGCTGGTG; >clamp_s_c_s_d_s_c_s_a_s_e_s:compl:rev
CACCAGCACAGACACCCACACCGTCACCCACACTATCATGTTGACATTCC; dG = -36.3
754 >s_a_s_e_s_b_s_c_s_d_s_d_s_a_s_e_s
GTGTCTGTGCTGGTGGCTGTGTGGGTGACGGTGACGGTGTCTGTGCTGGTG; >clamp_s_c_s_d_s_c_s_a_s_e_s:compl:rev
CACCAGCACAGACACCCACACCGTCACCCACACTATCATGTTGACATTCC; dG = -36.3
755 >s_a_s_e_s_b_s_d_s_d_s_c_s_a_s_e_s
GTGTCTGTGCTGGTGGCTGTGACGGTGACGGTGTGGGTGTCTGTGCTGGTG; >clamp_s_c_s_d_s_c_s_a_s_e_s:compl:rev
CACCAGCACAGACACCCACACCGTCACCCACACTATCATGTTGACATTCC; dG = -36.3
756 >s_a_s_e_s_c_s_c_s_d_s_d_s_a_s_e_s
GTGTCTGTGCTGGTGTGGGTGTGGGTGACGGTGACGGTGTCTGTGCTGGTG; >clamp_s_c_s_d_s_c_s_a_s_e_s:compl:rev
CACCAGCACAGACACCCACACCGTCACCCACACTATCATGTTGACATTCC; dG = -36.3
757 >s_a_s_e_s_c_s_c_s_c_s_c_s_a_s_e_s
GTGTCTGTGCTGGTGTGGGTGTGGGTGTGGGTGTGGGTGTCTGTGCTGGTG; >clamp_s_c_s_d_s_c_s_a_s_e_s:compl:rev
CACCAGCACAGACACCCACACCGTCACCCACACTATCATGTTGACATTCC; dG = -36.3
758 >s_a_s_e_s_d_s_c_s_d_s_d_s_a_s_e_s
GTGTCTGTGCTGGTGACGGTGTGGGTGACGGTGACGGTGTCTGTGCTGGTG; >clamp_s_c_s_d_s_c_s_a_s_e_s:compl:rev
CACCAGCACAGACACCCACACCGTCACCCACACTATCATGTTGACATTCC; dG = -36.3
759 >s_a_s_e_s_d_s_c_s_c_s_c_s_a_s_e_s
GTGTCTGTGCTGGTGACGGTGTGGGTGTGGGTGTGGGTGTCTGTGCTGGTG; >clamp_s_c_s_d_s_c_s_a_s_e_s:compl:rev
CACCAGCACAGACACCCACACCGTCACCCACACTATCATGTTGACATTCC; dG = -36.3
760 >s_a_s_e_s_d_s_d_s_d_s_c_s_a_s_e_s
GTGTCTGTGCTGGTGACGGTGACGGTGACGGTGTGGGTGTCTGTGCTGGTG; >clamp_s_c_s_d_s_c_s_a_s_e_s:compl:rev
CACCAGCACAGACACCCACACCGTCACCCACACTATCATGTTGACATTCC; dG = -36.3
761 >s_a_s_e_s_e_s_c_s_d_s_d_s_a_s_e_s
GTGTCTGTGCTGGTGCTGGTGTGGGTGACGGTGACGGTGTCTGTGCTGGTG; >clamp_s_c_s_d_s_c_s_a_s_e_s:compl:rev
CACCAGCACAGACACCCACACCGTCACCCACACTATCATGTTGACATTCC; dG = -36.3
762 >s_a_s_e_s_e_s_c_s_c_s_c_s_a_s_e_s
GTGTCTGTGCTGGTGCTGGTGTGGGTGTGGGTGTGGGTGTCTGTGCTGGTG; >clamp_s_c_s_d_s_c_s_a_s_e_s:compl:rev
CACCAGCACAGACACCCACACCGTCACCCACACTATCATGTTGACATTCC; dG = -36.3
763 >s_a_s_e_s_e_s_d_s_d_s_c_s_a_s_e_s
GTGTCTGTGCTGGTGCTGGTGACGGTGACGGTGTGGGTGTCTGTGCTGGTG; >clamp_s_c_s_d_s_c_s_a_s_e_s:compl:rev
CACCAGCACAGACACCCACACCGTCACCCACACTATCATGTTGACATTCC; dG = -36.3
764 >s_a_s_a_s_c_s_c_s_d_s_d_s_a_s_e_s
GTGTCTGTGTCTGTGTGGGTGTGGGTGACGGTGACGGTGTCTGTGCTGGTG; >clamp_s_c_s_d_s_c_s_a_s_e_s:compl:rev
CACCAGCACAGACACCCACACCGTCACCCACACTATCATGTTGACATTCC; dG = -36.3
765 >s_b_s_a_s_a_s_c_s_d_s_d_s_a_s_e_s
GTGGCTGTGTCTGTGTCTGTGTGGGTGACGGTGACGGTGTCTGTGCTGGTG; >clamp_s_c_s_d_s_c_s_a_s_e_s:compl:rev
CACCAGCACAGACACCCACACCGTCACCCACACTATCATGTTGACATTCC; dG = -36.3
766 >s_b_s_a_s_a_s_d_s_d_s_c_s_a_s_e_s
GTGGCTGTGTCTGTGTCTGTGACGGTGACGGTGTGGGTGTCTGTGCTGGTG; >clamp_s_c_s_d_s_c_s_a_s_e_s:compl:rev
CACCAGCACAGACACCCACACCGTCACCCACACTATCATGTTGACATTCC; dG = -36.3
767 >s_b_s_a_s_b_s_c_s_d_s_d_s_a_s_e_s
GTGGCTGTGTCTGTGGCTGTGTGGGTGACGGTGACGGTGTCTGTGCTGGTG; >clamp_s_c_s_d_s_c_s_a_s_e_s:compl:rev
CACCAGCACAGACACCCACACCGTCACCCACACTATCATGTTGACATTCC; dG = -36.3
768 >s_b_s_a_s_b_s_d_s_d_s_c_s_a_s_e_s
GTGGCTGTGTCTGTGGCTGTGACGGTGACGGTGTGGGTGTCTGTGCTGGTG; >clamp_s_c_s_d_s_c_s_a_s_e_s:compl:rev
CACCAGCACAGACACCCACACCGTCACCCACACTATCATGTTGACATTCC; dG = -36.3
769 >s_a_s_a_s_c_s_c_s_c_s_c_s_a_s_e_s
GTGTCTGTGTCTGTGTGGGTGTGGGTGTGGGTGTGGGTGTCTGTGCTGGTG; >clamp_s_c_s_d_s_c_s_a_s_e_s:compl:rev
CACCAGCACAGACACCCACACCGTCACCCACACTATCATGTTGACATTCC; dG = -36.3
770 >s_b_s_a_s_c_s_c_s_d_s_d_s_a_s_e_s
GTGGCTGTGTCTGTGTGGGTGTGGGTGACGGTGACGGTGTCTGTGCTGGTG; >clamp_s_c_s_d_s_c_s_a_s_e_s:compl:rev
CACCAGCACAGACACCCACACCGTCACCCACACTATCATGTTGACATTCC; dG = -36.3
771 >s_b_s_a_s_c_s_c_s_c_s_c_s_a_s_e_s
GTGGCTGTGTCTGTGTGGGTGTGGGTGTGGGTGTGGGTGTCTGTGCTGGTG; >clamp_s_c_s_d_s_c_s_a_s_e_s:compl:rev
CACCAGCACAGACACCCACACCGTCACCCACACTATCATGTTGACATTCC; dG = -36.3
772 >s_b_s_a_s_d_s_c_s_d_s_d_s_a_s_e_s
GTGGCTGTGTCTGTGACGGTGTGGGTGACGGTGACGGTGTCTGTGCTGGTG; >clamp_s_c_s_d_s_c_s_a_s_e_s:compl:rev
CACCAGCACAGACACCCACACCGTCACCCACACTATCATGTTGACATTCC; dG = -36.3
773 >s_b_s_a_s_d_s_c_s_c_s_c_s_a_s_e_s
GTGGCTGTGTCTGTGACGGTGTGGGTGTGGGTGTGGGTGTCTGTGCTGGTG; >clamp_s_c_s_d_s_c_s_a_s_e_s:compl:rev
CACCAGCACAGACACCCACACCGTCACCCACACTATCATGTTGACATTCC; dG = -36.3
774 >s_b_s_a_s_d_s_d_s_d_s_c_s_a_s_e_s
GTGGCTGTGTCTGTGACGGTGACGGTGACGGTGTGGGTGTCTGTGCTGGTG; >clamp_s_c_s_d_s_c_s_a_s_e_s:compl:rev
CACCAGCACAGACACCCACACCGTCACCCACACTATCATGTTGACATTCC; dG = -36.3
775 >s_b_s_a_s_e_s_c_s_d_s_d_s_a_s_e_s
GTGGCTGTGTCTGTGCTGGTGTGGGTGACGGTGACGGTGTCTGTGCTGGTG; >clamp_s_c_s_d_s_c_s_a_s_e_s:compl:rev
CACCAGCACAGACACCCACACCGTCACCCACACTATCATGTTGACATTCC; dG = -36.3

776 >s_b_s_a_s_e_s_c_s_c_s_c_s_a_s_e_s
GTGGCTGTGTCTGTGCTGGTGTGGGTGTGGGTGTGGGTGTCTGTGCTGGTG; >clamp_s_c_s_d_s_c_s_a_s_e_s:compl:rev
CACCAGCACAGACACCCACACCGTCACCCACACTATCATGTTGACATTCC; dG = -36.3
777 >s_b_s_a_s_e_s_d_s_d_s_c_s_a_s_e_s
GTGGCTGTGTCTGTGCTGGTGACGGTGACGGTGTGGGTGTCTGTGCTGGTG; >clamp_s_c_s_d_s_c_s_a_s_e_s:compl:rev
CACCAGCACAGACACCCACACCGTCACCCACACTATCATGTTGACATTCC; dG = -36.3
778 >s_b_s_b_s_a_s_c_s_d_s_d_s_a_s_e_s
GTGGCTGTGGCTGTGTCTGTGTGGGTGACGGTGACGGTGTCTGTGCTGGTG; >clamp_s_c_s_d_s_c_s_a_s_e_s:compl:rev
CACCAGCACAGACACCCACACCGTCACCCACACTATCATGTTGACATTCC; dG = -36.3
779 >s_b_s_b_s_a_s_d_s_d_s_c_s_a_s_e_s
GTGGCTGTGGCTGTGTCTGTGACGGTGACGGTGTGGGTGTCTGTGCTGGTG; >clamp_s_c_s_d_s_c_s_a_s_e_s:compl:rev
CACCAGCACAGACACCCACACCGTCACCCACACTATCATGTTGACATTCC; dG = -36.3
780 >s_b_s_b_s_b_s_c_s_d_s_d_s_a_s_e_s
GTGGCTGTGGCTGTGGCTGTGTGGGTGACGGTGACGGTGTCTGTGCTGGTG; >clamp_s_c_s_d_s_c_s_a_s_e_s:compl:rev
CACCAGCACAGACACCCACACCGTCACCCACACTATCATGTTGACATTCC; dG = -36.3
781 >s_b_s_b_s_b_s_d_s_d_s_c_s_a_s_e_s
GTGGCTGTGGCTGTGGCTGTGACGGTGACGGTGTGGGTGTCTGTGCTGGTG; >clamp_s_c_s_d_s_c_s_a_s_e_s:compl:rev
CACCAGCACAGACACCCACACCGTCACCCACACTATCATGTTGACATTCC; dG = -36.3
782 >s_b_s_b_s_c_s_c_s_d_s_d_s_a_s_e_s
GTGGCTGTGGCTGTGTGGGTGTGGGTGACGGTGACGGTGTCTGTGCTGGTG; >clamp_s_c_s_d_s_c_s_a_s_e_s:compl:rev
CACCAGCACAGACACCCACACCGTCACCCACACTATCATGTTGACATTCC; dG = -36.3
783 >s_b_s_b_s_c_s_c_s_c_s_c_s_a_s_e_s
GTGGCTGTGGCTGTGTGGGTGTGGGTGTGGGTGTGGGTGTCTGTGCTGGTG; >clamp_s_c_s_d_s_c_s_a_s_e_s:compl:rev
CACCAGCACAGACACCCACACCGTCACCCACACTATCATGTTGACATTCC; dG = -36.3
784 >s_b_s_b_s_d_s_c_s_d_s_d_s_a_s_e_s
GTGGCTGTGGCTGTGACGGTGTGGGTGACGGTGACGGTGTCTGTGCTGGTG; >clamp_s_c_s_d_s_c_s_a_s_e_s:compl:rev
CACCAGCACAGACACCCACACCGTCACCCACACTATCATGTTGACATTCC; dG = -36.3
785 >s_b_s_b_s_d_s_c_s_c_s_c_s_a_s_e_s
GTGGCTGTGGCTGTGACGGTGTGGGTGTGGGTGTGGGTGTCTGTGCTGGTG; >clamp_s_c_s_d_s_c_s_a_s_e_s:compl:rev
CACCAGCACAGACACCCACACCGTCACCCACACTATCATGTTGACATTCC; dG = -36.3
786 >s_b_s_b_s_d_s_d_s_d_s_c_s_a_s_e_s
GTGGCTGTGGCTGTGACGGTGACGGTGACGGTGTGGGTGTCTGTGCTGGTG; >clamp_s_c_s_d_s_c_s_a_s_e_s:compl:rev
CACCAGCACAGACACCCACACCGTCACCCACACTATCATGTTGACATTCC; dG = -36.3
787 >s_b_s_b_s_e_s_c_s_d_s_d_s_a_s_e_s
GTGGCTGTGGCTGTGCTGGTGTGGGTGACGGTGACGGTGTCTGTGCTGGTG; >clamp_s_c_s_d_s_c_s_a_s_e_s:compl:rev
CACCAGCACAGACACCCACACCGTCACCCACACTATCATGTTGACATTCC; dG = -36.3
788 >s_b_s_b_s_e_s_c_s_c_s_c_s_a_s_e_s
GTGGCTGTGGCTGTGCTGGTGTGGGTGTGGGTGTGGGTGTCTGTGCTGGTG; >clamp_s_c_s_d_s_c_s_a_s_e_s:compl:rev
CACCAGCACAGACACCCACACCGTCACCCACACTATCATGTTGACATTCC; dG = -36.3
789 >s_b_s_b_s_e_s_d_s_d_s_c_s_a_s_e_s
GTGGCTGTGGCTGTGCTGGTGACGGTGACGGTGTGGGTGTCTGTGCTGGTG; >clamp_s_c_s_d_s_c_s_a_s_e_s:compl:rev
CACCAGCACAGACACCCACACCGTCACCCACACTATCATGTTGACATTCC; dG = -36.3
790 >s_a_s_a_s_d_s_c_s_d_s_d_s_a_s_e_s
GTGTCTGTGTCTGTGACGGTGTGGGTGACGGTGACGGTGTCTGTGCTGGTG; >clamp_s_c_s_d_s_c_s_a_s_e_s:compl:rev
CACCAGCACAGACACCCACACCGTCACCCACACTATCATGTTGACATTCC; dG = -36.3
791 >s_b_s_c_s_a_s_c_s_d_s_d_s_a_s_e_s
GTGGCTGTGTGGGTGTCTGTGTGGGTGACGGTGACGGTGTCTGTGCTGGTG; >clamp_s_c_s_d_s_c_s_a_s_e_s:compl:rev
CACCAGCACAGACACCCACACCGTCACCCACACTATCATGTTGACATTCC; dG = -36.3
792 >s_b_s_c_s_a_s_d_s_d_s_c_s_a_s_e_s
GTGGCTGTGTGGGTGTCTGTGACGGTGACGGTGTGGGTGTCTGTGCTGGTG; >clamp_s_c_s_d_s_c_s_a_s_e_s:compl:rev
CACCAGCACAGACACCCACACCGTCACCCACACTATCATGTTGACATTCC; dG = -36.3
793 >s_b_s_c_s_b_s_c_s_d_s_d_s_a_s_e_s
GTGGCTGTGTGGGTGGCTGTGTGGGTGACGGTGACGGTGTCTGTGCTGGTG; >clamp_s_c_s_d_s_c_s_a_s_e_s:compl:rev
CACCAGCACAGACACCCACACCGTCACCCACACTATCATGTTGACATTCC; dG = -36.3
794 >s_b_s_c_s_b_s_d_s_d_s_c_s_a_s_e_s
GTGGCTGTGTGGGTGGCTGTGACGGTGACGGTGTGGGTGTCTGTGCTGGTG; >clamp_s_c_s_d_s_c_s_a_s_e_s:compl:rev
CACCAGCACAGACACCCACACCGTCACCCACACTATCATGTTGACATTCC; dG = -36.3
795 >s_a_s_a_s_d_s_c_s_c_s_c_s_a_s_e_s
GTGTCTGTGTCTGTGACGGTGTGGGTGTGGGTGTGGGTGTCTGTGCTGGTG; >clamp_s_c_s_d_s_c_s_a_s_e_s:compl:rev
CACCAGCACAGACACCCACACCGTCACCCACACTATCATGTTGACATTCC; dG = -36.3
796 >s_b_s_c_s_c_s_c_s_d_s_d_s_a_s_e_s
GTGGCTGTGTGGGTGTGGGTGTGGGTGACGGTGACGGTGTCTGTGCTGGTG; >clamp_s_c_s_d_s_c_s_a_s_e_s:compl:rev
CACCAGCACAGACACCCACACCGTCACCCACACTATCATGTTGACATTCC; dG = -36.3
797 >s_b_s_c_s_c_s_c_s_c_s_c_s_a_s_e_s
GTGGCTGTGTGGGTGTGGGTGTGGGTGTGGGTGTGGGTGTCTGTGCTGGTG; >clamp_s_c_s_d_s_c_s_a_s_e_s:compl:rev
CACCAGCACAGACACCCACACCGTCACCCACACTATCATGTTGACATTCC; dG = -36.3
798 >s_b_s_c_s_d_s_c_s_d_s_d_s_a_s_e_s
GTGGCTGTGTGGGTGACGGTGTGGGTGACGGTGACGGTGTCTGTGCTGGTG; >clamp_s_c_s_d_s_c_s_a_s_e_s:compl:rev
CACCAGCACAGACACCCACACCGTCACCCACACTATCATGTTGACATTCC; dG = -36.3
799 >s_b_s_c_s_d_s_c_s_c_s_c_s_a_s_e_s
GTGGCTGTGTGGGTGACGGTGTGGGTGTGGGTGTGGGTGTCTGTGCTGGTG; >clamp_s_c_s_d_s_c_s_a_s_e_s:compl:rev
CACCAGCACAGACACCCACACCGTCACCCACACTATCATGTTGACATTCC; dG = -36.3

800 >s_b_s_c_s_d_s_d_s_d_s_c_s_a_s_e_s
GTGGCTGTGTGGGTGACGGTGACGGTGACGGTGTGGGTGTCTGTGCTGGTG; >clamp_s_c_s_d_s_c_s_a_s_e_s:compl:rev
CACCAGCACAGACACCCACACCGTCACCCACACTATCATGTTGACATTCC; dG = -36.3
801 >s_a_s_a_s_d_s_d_s_d_s_c_s_a_s_e_s
GTGTCTGTGTCTGTGACGGTGACGGTGACGGTGTGGGTGTCTGTGCTGGTG; >clamp_s_c_s_d_s_c_s_a_s_e_s:compl:rev
CACCAGCACAGACACCCACACCGTCACCCACACTATCATGTTGACATTCC; dG = -36.3
802 >s_b_s_c_s_e_s_c_s_d_s_d_s_a_s_e_s
GTGGCTGTGTGGGTGCTGGTGTGGGTGACGGTGACGGTGTCTGTGCTGGTG; >clamp_s_c_s_d_s_c_s_a_s_e_s:compl:rev
CACCAGCACAGACACCCACACCGTCACCCACACTATCATGTTGACATTCC; dG = -36.3
803 >s_b_s_c_s_e_s_c_s_c_s_c_s_a_s_e_s
GTGGCTGTGTGGGTGCTGGTGTGGGTGTGGGTGTGGGTGTCTGTGCTGGTG; >clamp_s_c_s_d_s_c_s_a_s_e_s:compl:rev
CACCAGCACAGACACCCACACCGTCACCCACACTATCATGTTGACATTCC; dG = -36.3
804 >s_b_s_c_s_e_s_d_s_d_s_c_s_a_s_e_s
GTGGCTGTGTGGGTGCTGGTGACGGTGACGGTGTGGGTGTCTGTGCTGGTG; >clamp_s_c_s_d_s_c_s_a_s_e_s:compl:rev
CACCAGCACAGACACCCACACCGTCACCCACACTATCATGTTGACATTCC; dG = -36.3
805 >s_b_s_d_s_a_s_c_s_d_s_d_s_a_s_e_s
GTGGCTGTGACGGTGTCTGTGTGGGTGACGGTGACGGTGTCTGTGCTGGTG; >clamp_s_c_s_d_s_c_s_a_s_e_s:compl:rev
CACCAGCACAGACACCCACACCGTCACCCACACTATCATGTTGACATTCC; dG = -36.3
806 >s_b_s_d_s_a_s_d_s_d_s_c_s_a_s_e_s
GTGGCTGTGACGGTGTCTGTGACGGTGACGGTGTGGGTGTCTGTGCTGGTG; >clamp_s_c_s_d_s_c_s_a_s_e_s:compl:rev
CACCAGCACAGACACCCACACCGTCACCCACACTATCATGTTGACATTCC; dG = -36.3
807 >s_b_s_d_s_b_s_c_s_d_s_d_s_a_s_e_s
GTGGCTGTGACGGTGGCTGTGTGGGTGACGGTGACGGTGTCTGTGCTGGTG; >clamp_s_c_s_d_s_c_s_a_s_e_s:compl:rev
CACCAGCACAGACACCCACACCGTCACCCACACTATCATGTTGACATTCC; dG = -36.3
808 >s_b_s_d_s_b_s_d_s_d_s_c_s_a_s_e_s
GTGGCTGTGACGGTGGCTGTGACGGTGACGGTGTGGGTGTCTGTGCTGGTG; >clamp_s_c_s_d_s_c_s_a_s_e_s:compl:rev
CACCAGCACAGACACCCACACCGTCACCCACACTATCATGTTGACATTCC; dG = -36.3
809 >s_b_s_d_s_c_s_c_s_d_s_d_s_a_s_e_s
GTGGCTGTGACGGTGTGGGTGTGGGTGACGGTGACGGTGTCTGTGCTGGTG; >clamp_s_c_s_d_s_c_s_a_s_e_s:compl:rev
CACCAGCACAGACACCCACACCGTCACCCACACTATCATGTTGACATTCC; dG = -36.3
810 >s_b_s_d_s_c_s_c_s_c_s_c_s_a_s_e_s
GTGGCTGTGACGGTGTGGGTGTGGGTGTGGGTGTGGGTGTCTGTGCTGGTG; >clamp_s_c_s_d_s_c_s_a_s_e_s:compl:rev
CACCAGCACAGACACCCACACCGTCACCCACACTATCATGTTGACATTCC; dG = -36.3
811 >s_b_s_d_s_d_s_c_s_d_s_d_s_a_s_e_s
GTGGCTGTGACGGTGACGGTGTGGGTGACGGTGACGGTGTCTGTGCTGGTG; >clamp_s_c_s_d_s_c_s_a_s_e_s:compl:rev
CACCAGCACAGACACCCACACCGTCACCCACACTATCATGTTGACATTCC; dG = -36.3
812 >s_b_s_d_s_d_s_c_s_c_s_c_s_a_s_e_s
GTGGCTGTGACGGTGACGGTGTGGGTGTGGGTGTGGGTGTCTGTGCTGGTG; >clamp_s_c_s_d_s_c_s_a_s_e_s:compl:rev
CACCAGCACAGACACCCACACCGTCACCCACACTATCATGTTGACATTCC; dG = -36.3
813 >s_b_s_d_s_d_s_d_s_d_s_c_s_a_s_e_s
GTGGCTGTGACGGTGACGGTGACGGTGACGGTGTGGGTGTCTGTGCTGGTG; >clamp_s_c_s_d_s_c_s_a_s_e_s:compl:rev
CACCAGCACAGACACCCACACCGTCACCCACACTATCATGTTGACATTCC; dG = -36.3
814 >s_b_s_d_s_e_s_c_s_d_s_d_s_a_s_e_s
GTGGCTGTGACGGTGCTGGTGTGGGTGACGGTGACGGTGTCTGTGCTGGTG; >clamp_s_c_s_d_s_c_s_a_s_e_s:compl:rev
CACCAGCACAGACACCCACACCGTCACCCACACTATCATGTTGACATTCC; dG = -36.3
815 >s_b_s_d_s_e_s_c_s_c_s_c_s_a_s_e_s
GTGGCTGTGACGGTGCTGGTGTGGGTGTGGGTGTGGGTGTCTGTGCTGGTG; >clamp_s_c_s_d_s_c_s_a_s_e_s:compl:rev
CACCAGCACAGACACCCACACCGTCACCCACACTATCATGTTGACATTCC; dG = -36.3
816 >s_b_s_d_s_e_s_d_s_d_s_c_s_a_s_e_s
GTGGCTGTGACGGTGCTGGTGACGGTGACGGTGTGGGTGTCTGTGCTGGTG; >clamp_s_c_s_d_s_c_s_a_s_e_s:compl:rev
CACCAGCACAGACACCCACACCGTCACCCACACTATCATGTTGACATTCC; dG = -36.3
817 >s_a_s_a_s_e_s_c_s_d_s_d_s_a_s_e_s
GTGTCTGTGTCTGTGCTGGTGTGGGTGACGGTGACGGTGTCTGTGCTGGTG; >clamp_s_c_s_d_s_c_s_a_s_e_s:compl:rev
CACCAGCACAGACACCCACACCGTCACCCACACTATCATGTTGACATTCC; dG = -36.3
818 >s_b_s_e_s_a_s_c_s_d_s_d_s_a_s_e_s
GTGGCTGTGCTGGTGTCTGTGTGGGTGACGGTGACGGTGTCTGTGCTGGTG; >clamp_s_c_s_d_s_c_s_a_s_e_s:compl:rev
CACCAGCACAGACACCCACACCGTCACCCACACTATCATGTTGACATTCC; dG = -36.3
819 >s_b_s_e_s_a_s_d_s_d_s_c_s_a_s_e_s
GTGGCTGTGCTGGTGTCTGTGACGGTGACGGTGTGGGTGTCTGTGCTGGTG; >clamp_s_c_s_d_s_c_s_a_s_e_s:compl:rev
CACCAGCACAGACACCCACACCGTCACCCACACTATCATGTTGACATTCC; dG = -36.3
820 >s_b_s_e_s_b_s_c_s_d_s_d_s_a_s_e_s
GTGGCTGTGCTGGTGGCTGTGTGGGTGACGGTGACGGTGTCTGTGCTGGTG; >clamp_s_c_s_d_s_c_s_a_s_e_s:compl:rev
CACCAGCACAGACACCCACACCGTCACCCACACTATCATGTTGACATTCC; dG = -36.3
821 >s_b_s_e_s_b_s_d_s_d_s_c_s_a_s_e_s
GTGGCTGTGCTGGTGGCTGTGACGGTGACGGTGTGGGTGTCTGTGCTGGTG; >clamp_s_c_s_d_s_c_s_a_s_e_s:compl:rev
CACCAGCACAGACACCCACACCGTCACCCACACTATCATGTTGACATTCC; dG = -36.3
822 >s_a_s_a_s_e_s_c_s_c_s_c_s_a_s_e_s
GTGTCTGTGTCTGTGCTGGTGTGGGTGTGGGTGTGGGTGTCTGTGCTGGTG; >clamp_s_c_s_d_s_c_s_a_s_e_s:compl:rev
CACCAGCACAGACACCCACACCGTCACCCACACTATCATGTTGACATTCC; dG = -36.3
823 >s_b_s_e_s_c_s_c_s_d_s_d_s_a_s_e_s
GTGGCTGTGCTGGTGTGGGTGTGGGTGACGGTGACGGTGTCTGTGCTGGTG; >clamp_s_c_s_d_s_c_s_a_s_e_s:compl:rev
CACCAGCACAGACACCCACACCGTCACCCACACTATCATGTTGACATTCC; dG = -36.3

824 >s_b_s_e_s_c_s_c_s_c_s_c_s_a_s_e_s
GTGGCTGTGCTGGTGTGGGTGTGGGTGTGGGTGTGGGTGTCTGTGCTGGTG; >clamp_s_c_s_d_s_c_s_a_s_e_s:compl:rev
CACCAGCACAGACACCCACACCGTCACCCACACTATCATGTTGACATTCC; dG = -36.3
825 >s_b_s_e_s_d_s_c_s_d_s_d_s_a_s_e_s
GTGGCTGTGCTGGTGACGGTGTGGGTGACGGTGACGGTGTCTGTGCTGGTG; >clamp_s_c_s_d_s_c_s_a_s_e_s:compl:rev
CACCAGCACAGACACCCACACCGTCACCCACACTATCATGTTGACATTCC; dG = -36.3
826 >s_b_s_e_s_d_s_c_s_c_s_c_s_a_s_e_s
GTGGCTGTGCTGGTGACGGTGTGGGTGTGGGTGTGGGTGTCTGTGCTGGTG; >clamp_s_c_s_d_s_c_s_a_s_e_s:compl:rev
CACCAGCACAGACACCCACACCGTCACCCACACTATCATGTTGACATTCC; dG = -36.3
827 >s_b_s_e_s_d_s_d_s_d_s_c_s_a_s_e_s
GTGGCTGTGCTGGTGACGGTGACGGTGACGGTGTGGGTGTCTGTGCTGGTG; >clamp_s_c_s_d_s_c_s_a_s_e_s:compl:rev
CACCAGCACAGACACCCACACCGTCACCCACACTATCATGTTGACATTCC; dG = -36.3
828 >s_a_s_a_s_e_s_d_s_d_s_c_s_a_s_e_s
GTGTCTGTGTCTGTGCTGGTGACGGTGACGGTGTGGGTGTCTGTGCTGGTG; >clamp_s_c_s_d_s_c_s_a_s_e_s:compl:rev
CACCAGCACAGACACCCACACCGTCACCCACACTATCATGTTGACATTCC; dG = -36.3
829 >s_b_s_e_s_e_s_c_s_d_s_d_s_a_s_e_s
GTGGCTGTGCTGGTGCTGGTGTGGGTGACGGTGACGGTGTCTGTGCTGGTG; >clamp_s_c_s_d_s_c_s_a_s_e_s:compl:rev
CACCAGCACAGACACCCACACCGTCACCCACACTATCATGTTGACATTCC; dG = -36.3
830 >s_b_s_e_s_e_s_c_s_c_s_c_s_a_s_e_s
GTGGCTGTGCTGGTGCTGGTGTGGGTGTGGGTGTGGGTGTCTGTGCTGGTG; >clamp_s_c_s_d_s_c_s_a_s_e_s:compl:rev
CACCAGCACAGACACCCACACCGTCACCCACACTATCATGTTGACATTCC; dG = -36.3
831 >s_b_s_e_s_e_s_d_s_d_s_c_s_a_s_e_s
GTGGCTGTGCTGGTGCTGGTGACGGTGACGGTGTGGGTGTCTGTGCTGGTG; >clamp_s_c_s_d_s_c_s_a_s_e_s:compl:rev
CACCAGCACAGACACCCACACCGTCACCCACACTATCATGTTGACATTCC; dG = -36.3
832 >s_a_s_a_s_a_s_c_s_d_s_c_s_a_s_d_s
GTGTCTGTGTCTGTGTCTGTGTGGGTGACGGTGTGGGTGTCTGTGACGGTG; >clamp_s_c_s_d_s_c_s_a_s_e_s:compl:rev
CACCAGCACAGACACCCACACCGTCACCCACACTATCATGTTGACATTCC; dG = -36.2
833 >s_c_s_a_s_a_s_c_s_d_s_c_s_a_s_d_s
GTGTGGGTGTCTGTGTCTGTGTGGGTGACGGTGTGGGTGTCTGTGACGGTG; >clamp_s_c_s_d_s_c_s_a_s_e_s:compl:rev
CACCAGCACAGACACCCACACCGTCACCCACACTATCATGTTGACATTCC; dG = -36.2
834 >s_c_s_a_s_a_s_c_s_d_s_e_s_a_s_e_s
GTGTGGGTGTCTGTGTCTGTGTGGGTGACGGTGCTGGTGTCTGTGCTGGTG; >clamp_s_c_s_d_s_c_s_a_s_e_s:compl:rev
CACCAGCACAGACACCCACACCGTCACCCACACTATCATGTTGACATTCC; dG = -36.2
835 >s_a_s_b_s_a_s_c_s_d_s_c_s_a_s_d_s
GTGTCTGTGGCTGTGTCTGTGTGGGTGACGGTGTGGGTGTCTGTGACGGTG; >clamp_s_c_s_d_s_c_s_a_s_e_s:compl:rev
CACCAGCACAGACACCCACACCGTCACCCACACTATCATGTTGACATTCC; dG = -36.2
836 >s_c_s_a_s_a_s_e_s_d_s_c_s_a_s_e_s
GTGTGGGTGTCTGTGTCTGTGCTGGTGACGGTGTGGGTGTCTGTGCTGGTG; >clamp_s_c_s_d_s_c_s_a_s_e_s:compl:rev
CACCAGCACAGACACCCACACCGTCACCCACACTATCATGTTGACATTCC; dG = -36.2
837 >s_c_s_a_s_b_s_c_s_d_s_c_s_a_s_d_s
GTGTGGGTGTCTGTGGCTGTGTGGGTGACGGTGTGGGTGTCTGTGACGGTG; >clamp_s_c_s_d_s_c_s_a_s_e_s:compl:rev
CACCAGCACAGACACCCACACCGTCACCCACACTATCATGTTGACATTCC; dG = -36.2
838 >s_c_s_a_s_b_s_c_s_d_s_e_s_a_s_e_s
GTGTGGGTGTCTGTGGCTGTGTGGGTGACGGTGCTGGTGTCTGTGCTGGTG; >clamp_s_c_s_d_s_c_s_a_s_e_s:compl:rev
CACCAGCACAGACACCCACACCGTCACCCACACTATCATGTTGACATTCC; dG = -36.2
839 >s_c_s_a_s_b_s_e_s_d_s_c_s_a_s_e_s
GTGTGGGTGTCTGTGGCTGTGCTGGTGACGGTGTGGGTGTCTGTGCTGGTG; >clamp_s_c_s_d_s_c_s_a_s_e_s:compl:rev
CACCAGCACAGACACCCACACCGTCACCCACACTATCATGTTGACATTCC; dG = -36.2
840 >s_c_s_a_s_c_s_c_s_d_s_c_s_a_s_d_s
GTGTGGGTGTCTGTGTGGGTGTGGGTGACGGTGTGGGTGTCTGTGACGGTG; >clamp_s_c_s_d_s_c_s_a_s_e_s:compl:rev
CACCAGCACAGACACCCACACCGTCACCCACACTATCATGTTGACATTCC; dG = -36.2
841 >s_c_s_a_s_c_s_c_s_d_s_e_s_a_s_e_s
GTGTGGGTGTCTGTGTGGGTGTGGGTGACGGTGCTGGTGTCTGTGCTGGTG; >clamp_s_c_s_d_s_c_s_a_s_e_s:compl:rev
CACCAGCACAGACACCCACACCGTCACCCACACTATCATGTTGACATTCC; dG = -36.2
842 >s_c_s_a_s_d_s_c_s_d_s_c_s_a_s_d_s
GTGTGGGTGTCTGTGACGGTGTGGGTGACGGTGTGGGTGTCTGTGACGGTG; >clamp_s_c_s_d_s_c_s_a_s_e_s:compl:rev
CACCAGCACAGACACCCACACCGTCACCCACACTATCATGTTGACATTCC; dG = -36.2
843 >s_c_s_a_s_d_s_c_s_d_s_e_s_a_s_e_s
GTGTGGGTGTCTGTGACGGTGTGGGTGACGGTGCTGGTGTCTGTGCTGGTG; >clamp_s_c_s_d_s_c_s_a_s_e_s:compl:rev
CACCAGCACAGACACCCACACCGTCACCCACACTATCATGTTGACATTCC; dG = -36.2
844 >s_c_s_a_s_d_s_e_s_d_s_c_s_a_s_e_s
GTGTGGGTGTCTGTGACGGTGCTGGTGACGGTGTGGGTGTCTGTGCTGGTG; >clamp_s_c_s_d_s_c_s_a_s_e_s:compl:rev
CACCAGCACAGACACCCACACCGTCACCCACACTATCATGTTGACATTCC; dG = -36.2
845 >s_c_s_a_s_e_s_c_s_d_s_c_s_a_s_d_s
GTGTGGGTGTCTGTGCTGGTGTGGGTGACGGTGTGGGTGTCTGTGACGGTG; >clamp_s_c_s_d_s_c_s_a_s_e_s:compl:rev
CACCAGCACAGACACCCACACCGTCACCCACACTATCATGTTGACATTCC; dG = -36.2
846 >s_c_s_a_s_e_s_c_s_d_s_e_s_a_s_e_s
GTGTGGGTGTCTGTGCTGGTGTGGGTGACGGTGCTGGTGTCTGTGCTGGTG; >clamp_s_c_s_d_s_c_s_a_s_e_s:compl:rev
CACCAGCACAGACACCCACACCGTCACCCACACTATCATGTTGACATTCC; dG = -36.2
847 >s_a_s_b_s_a_s_c_s_d_s_e_s_a_s_e_s
GTGTCTGTGGCTGTGTCTGTGTGGGTGACGGTGCTGGTGTCTGTGCTGGTG; >clamp_s_c_s_d_s_c_s_a_s_e_s:compl:rev
CACCAGCACAGACACCCACACCGTCACCCACACTATCATGTTGACATTCC; dG = -36.2

848 >s_c_s_a_s_e_s_e_s_d_s_c_s_a_s_e_s
GTGTGGGTGTCTGTGCTGGTGCTGGTGACGGTGTGGGTGTCTGTGCTGGTG; >clamp_s_c_s_d_s_c_s_a_s_e_s:compl:rev
CACCAGCACAGACACCCACACCGTCACCCACACTATCATGTTGACATTCC; dG = -36.2
849 >s_c_s_b_s_a_s_c_s_d_s_c_s_a_s_d_s
GTGTGGGTGGCTGTGTCTGTGTGGGTGACGGTGTGGGTGTCTGTGACGGTG; >clamp_s_c_s_d_s_c_s_a_s_e_s:compl:rev
CACCAGCACAGACACCCACACCGTCACCCACACTATCATGTTGACATTCC; dG = -36.2
850 >s_c_s_b_s_a_s_c_s_d_s_e_s_a_s_e_s
GTGTGGGTGGCTGTGTCTGTGTGGGTGACGGTGCTGGTGTCTGTGCTGGTG; >clamp_s_c_s_d_s_c_s_a_s_e_s:compl:rev
CACCAGCACAGACACCCACACCGTCACCCACACTATCATGTTGACATTCC; dG = -36.2
851 >s_c_s_b_s_a_s_e_s_d_s_c_s_a_s_e_s
GTGTGGGTGGCTGTGTCTGTGCTGGTGACGGTGTGGGTGTCTGTGCTGGTG; >clamp_s_c_s_d_s_c_s_a_s_e_s:compl:rev
CACCAGCACAGACACCCACACCGTCACCCACACTATCATGTTGACATTCC; dG = -36.2
852 >s_c_s_b_s_b_s_c_s_d_s_c_s_a_s_d_s
GTGTGGGTGGCTGTGGCTGTGTGGGTGACGGTGTGGGTGTCTGTGACGGTG; >clamp_s_c_s_d_s_c_s_a_s_e_s:compl:rev
CACCAGCACAGACACCCACACCGTCACCCACACTATCATGTTGACATTCC; dG = -36.2
853 >s_c_s_b_s_b_s_c_s_d_s_e_s_a_s_e_s
GTGTGGGTGGCTGTGGCTGTGTGGGTGACGGTGCTGGTGTCTGTGCTGGTG; >clamp_s_c_s_d_s_c_s_a_s_e_s:compl:rev
CACCAGCACAGACACCCACACCGTCACCCACACTATCATGTTGACATTCC; dG = -36.2
854 >s_c_s_b_s_b_s_e_s_d_s_c_s_a_s_e_s
GTGTGGGTGGCTGTGGCTGTGCTGGTGACGGTGTGGGTGTCTGTGCTGGTG; >clamp_s_c_s_d_s_c_s_a_s_e_s:compl:rev
CACCAGCACAGACACCCACACCGTCACCCACACTATCATGTTGACATTCC; dG = -36.2
855 >s_c_s_b_s_c_s_c_s_d_s_c_s_a_s_d_s
GTGTGGGTGGCTGTGTGGGTGTGGGTGACGGTGTGGGTGTCTGTGACGGTG; >clamp_s_c_s_d_s_c_s_a_s_e_s:compl:rev
CACCAGCACAGACACCCACACCGTCACCCACACTATCATGTTGACATTCC; dG = -36.2
856 >s_c_s_b_s_c_s_c_s_d_s_e_s_a_s_e_s
GTGTGGGTGGCTGTGTGGGTGTGGGTGACGGTGCTGGTGTCTGTGCTGGTG; >clamp_s_c_s_d_s_c_s_a_s_e_s:compl:rev
CACCAGCACAGACACCCACACCGTCACCCACACTATCATGTTGACATTCC; dG = -36.2
857 >s_c_s_b_s_d_s_c_s_d_s_c_s_a_s_d_s
GTGTGGGTGGCTGTGACGGTGTGGGTGACGGTGTGGGTGTCTGTGACGGTG; >clamp_s_c_s_d_s_c_s_a_s_e_s:compl:rev
CACCAGCACAGACACCCACACCGTCACCCACACTATCATGTTGACATTCC; dG = -36.2
858 >s_c_s_b_s_d_s_c_s_d_s_e_s_a_s_e_s
GTGTGGGTGGCTGTGACGGTGTGGGTGACGGTGCTGGTGTCTGTGCTGGTG; >clamp_s_c_s_d_s_c_s_a_s_e_s:compl:rev
CACCAGCACAGACACCCACACCGTCACCCACACTATCATGTTGACATTCC; dG = -36.2
859 >s_a_s_b_s_a_s_e_s_d_s_c_s_a_s_e_s
GTGTCTGTGGCTGTGTCTGTGCTGGTGACGGTGTGGGTGTCTGTGCTGGTG; >clamp_s_c_s_d_s_c_s_a_s_e_s:compl:rev
CACCAGCACAGACACCCACACCGTCACCCACACTATCATGTTGACATTCC; dG = -36.2
860 >s_c_s_b_s_d_s_e_s_d_s_c_s_a_s_e_s
GTGTGGGTGGCTGTGACGGTGCTGGTGACGGTGTGGGTGTCTGTGCTGGTG; >clamp_s_c_s_d_s_c_s_a_s_e_s:compl:rev
CACCAGCACAGACACCCACACCGTCACCCACACTATCATGTTGACATTCC; dG = -36.2
861 >s_c_s_b_s_e_s_c_s_d_s_c_s_a_s_d_s
GTGTGGGTGGCTGTGCTGGTGTGGGTGACGGTGTGGGTGTCTGTGACGGTG; >clamp_s_c_s_d_s_c_s_a_s_e_s:compl:rev
CACCAGCACAGACACCCACACCGTCACCCACACTATCATGTTGACATTCC; dG = -36.2
862 >s_c_s_b_s_e_s_c_s_d_s_e_s_a_s_e_s
GTGTGGGTGGCTGTGCTGGTGTGGGTGACGGTGCTGGTGTCTGTGCTGGTG; >clamp_s_c_s_d_s_c_s_a_s_e_s:compl:rev
CACCAGCACAGACACCCACACCGTCACCCACACTATCATGTTGACATTCC; dG = -36.2
863 >s_c_s_b_s_e_s_e_s_d_s_c_s_a_s_e_s
GTGTGGGTGGCTGTGCTGGTGCTGGTGACGGTGTGGGTGTCTGTGCTGGTG; >clamp_s_c_s_d_s_c_s_a_s_e_s:compl:rev
CACCAGCACAGACACCCACACCGTCACCCACACTATCATGTTGACATTCC; dG = -36.2
864 >s_c_s_c_s_a_s_c_s_d_s_c_s_a_s_d_s
GTGTGGGTGTGGGTGTCTGTGTGGGTGACGGTGTGGGTGTCTGTGACGGTG; >clamp_s_c_s_d_s_c_s_a_s_e_s:compl:rev
CACCAGCACAGACACCCACACCGTCACCCACACTATCATGTTGACATTCC; dG = -36.2
865 >s_c_s_c_s_a_s_c_s_d_s_e_s_a_s_e_s
GTGTGGGTGTGGGTGTCTGTGTGGGTGACGGTGCTGGTGTCTGTGCTGGTG; >clamp_s_c_s_d_s_c_s_a_s_e_s:compl:rev
CACCAGCACAGACACCCACACCGTCACCCACACTATCATGTTGACATTCC; dG = -36.2
866 >s_a_s_b_s_b_s_c_s_d_s_c_s_a_s_d_s
GTGTCTGTGGCTGTGGCTGTGTGGGTGACGGTGTGGGTGTCTGTGACGGTG; >clamp_s_c_s_d_s_c_s_a_s_e_s:compl:rev
CACCAGCACAGACACCCACACCGTCACCCACACTATCATGTTGACATTCC; dG = -36.2
867 >s_c_s_c_s_a_s_e_s_d_s_c_s_a_s_e_s
GTGTGGGTGTGGGTGTCTGTGCTGGTGACGGTGTGGGTGTCTGTGCTGGTG; >clamp_s_c_s_d_s_c_s_a_s_e_s:compl:rev
CACCAGCACAGACACCCACACCGTCACCCACACTATCATGTTGACATTCC; dG = -36.2
868 >s_c_s_c_s_b_s_c_s_d_s_c_s_a_s_d_s
GTGTGGGTGTGGGTGGCTGTGTGGGTGACGGTGTGGGTGTCTGTGACGGTG; >clamp_s_c_s_d_s_c_s_a_s_e_s:compl:rev
CACCAGCACAGACACCCACACCGTCACCCACACTATCATGTTGACATTCC; dG = -36.2
869 >s_c_s_c_s_b_s_c_s_d_s_e_s_a_s_e_s
GTGTGGGTGTGGGTGGCTGTGTGGGTGACGGTGCTGGTGTCTGTGCTGGTG; >clamp_s_c_s_d_s_c_s_a_s_e_s:compl:rev
CACCAGCACAGACACCCACACCGTCACCCACACTATCATGTTGACATTCC; dG = -36.2
870 >s_c_s_c_s_b_s_e_s_d_s_c_s_a_s_e_s
GTGTGGGTGTGGGTGGCTGTGCTGGTGACGGTGTGGGTGTCTGTGCTGGTG; >clamp_s_c_s_d_s_c_s_a_s_e_s:compl:rev
CACCAGCACAGACACCCACACCGTCACCCACACTATCATGTTGACATTCC; dG = -36.2
871 >s_c_s_c_s_c_s_c_s_d_s_c_s_a_s_d_s
GTGTGGGTGTGGGTGTGGGTGTGGGTGACGGTGTGGGTGTCTGTGACGGTG; >clamp_s_c_s_d_s_c_s_a_s_e_s:compl:rev
CACCAGCACAGACACCCACACCGTCACCCACACTATCATGTTGACATTCC; dG = -36.2

872 >s_c_s_c_s_c_s_c_s_d_s_e_s_a_s_e_s
GTGTGGGTGTGGGTGTGGGTGTGGGTGACGGTGCTGGTGTCTGTGCTGGTG; >clamp_s_c_s_d_s_c_s_a_s_e_s:compl:rev
CACCAGCACAGACACCCACACCGTCACCCACACTATCATGTTGACATTCC; dG = -36.2
873 >s_c_s_c_s_d_s_c_s_d_s_c_s_a_s_d_s
GTGTGGGTGTGGGTGACGGTGTGGGTGACGGTGTGGGTGTCTGTGACGGTG; >clamp_s_c_s_d_s_c_s_a_s_e_s:compl:rev
CACCAGCACAGACACCCACACCGTCACCCACACTATCATGTTGACATTCC; dG = -36.2
874 >s_c_s_c_s_d_s_c_s_d_s_e_s_a_s_e_s
GTGTGGGTGTGGGTGACGGTGTGGGTGACGGTGCTGGTGTCTGTGCTGGTG; >clamp_s_c_s_d_s_c_s_a_s_e_s:compl:rev
CACCAGCACAGACACCCACACCGTCACCCACACTATCATGTTGACATTCC; dG = -36.2
875 >s_c_s_c_s_d_s_e_s_d_s_c_s_a_s_e_s
GTGTGGGTGTGGGTGACGGTGCTGGTGACGGTGTGGGTGTCTGTGCTGGTG; >clamp_s_c_s_d_s_c_s_a_s_e_s:compl:rev
CACCAGCACAGACACCCACACCGTCACCCACACTATCATGTTGACATTCC; dG = -36.2
876 >s_c_s_c_s_e_s_c_s_d_s_c_s_a_s_d_s
GTGTGGGTGTGGGTGCTGGTGTGGGTGACGGTGTGGGTGTCTGTGACGGTG; >clamp_s_c_s_d_s_c_s_a_s_e_s:compl:rev
CACCAGCACAGACACCCACACCGTCACCCACACTATCATGTTGACATTCC; dG = -36.2
877 >s_c_s_c_s_e_s_c_s_d_s_e_s_a_s_e_s
GTGTGGGTGTGGGTGCTGGTGTGGGTGACGGTGCTGGTGTCTGTGCTGGTG; >clamp_s_c_s_d_s_c_s_a_s_e_s:compl:rev
CACCAGCACAGACACCCACACCGTCACCCACACTATCATGTTGACATTCC; dG = -36.2
878 >s_a_s_b_s_b_s_c_s_d_s_e_s_a_s_e_s
GTGTCTGTGGCTGTGGCTGTGTGGGTGACGGTGCTGGTGTCTGTGCTGGTG; >clamp_s_c_s_d_s_c_s_a_s_e_s:compl:rev
CACCAGCACAGACACCCACACCGTCACCCACACTATCATGTTGACATTCC; dG = -36.2
879 >s_c_s_c_s_e_s_e_s_d_s_c_s_a_s_e_s
GTGTGGGTGTGGGTGCTGGTGCTGGTGACGGTGTGGGTGTCTGTGCTGGTG; >clamp_s_c_s_d_s_c_s_a_s_e_s:compl:rev
CACCAGCACAGACACCCACACCGTCACCCACACTATCATGTTGACATTCC; dG = -36.2
880 >s_c_s_d_s_a_s_c_s_d_s_c_s_a_s_d_s
GTGTGGGTGACGGTGTCTGTGTGGGTGACGGTGTGGGTGTCTGTGACGGTG; >clamp_s_c_s_d_s_c_s_a_s_e_s:compl:rev
CACCAGCACAGACACCCACACCGTCACCCACACTATCATGTTGACATTCC; dG = -36.2
881 >s_c_s_d_s_a_s_c_s_d_s_e_s_a_s_e_s
GTGTGGGTGACGGTGTCTGTGTGGGTGACGGTGCTGGTGTCTGTGCTGGTG; >clamp_s_c_s_d_s_c_s_a_s_e_s:compl:rev
CACCAGCACAGACACCCACACCGTCACCCACACTATCATGTTGACATTCC; dG = -36.2
882 >s_c_s_d_s_a_s_e_s_d_s_c_s_a_s_e_s
GTGTGGGTGACGGTGTCTGTGCTGGTGACGGTGTGGGTGTCTGTGCTGGTG; >clamp_s_c_s_d_s_c_s_a_s_e_s:compl:rev
CACCAGCACAGACACCCACACCGTCACCCACACTATCATGTTGACATTCC; dG = -36.2
883 >s_c_s_d_s_b_s_c_s_d_s_c_s_a_s_d_s
GTGTGGGTGACGGTGGCTGTGTGGGTGACGGTGTGGGTGTCTGTGACGGTG; >clamp_s_c_s_d_s_c_s_a_s_e_s:compl:rev
CACCAGCACAGACACCCACACCGTCACCCACACTATCATGTTGACATTCC; dG = -36.2
884 >s_c_s_d_s_b_s_c_s_d_s_e_s_a_s_e_s
GTGTGGGTGACGGTGGCTGTGTGGGTGACGGTGCTGGTGTCTGTGCTGGTG; >clamp_s_c_s_d_s_c_s_a_s_e_s:compl:rev
CACCAGCACAGACACCCACACCGTCACCCACACTATCATGTTGACATTCC; dG = -36.2
885 >s_c_s_d_s_b_s_e_s_d_s_c_s_a_s_e_s
GTGTGGGTGACGGTGGCTGTGCTGGTGACGGTGTGGGTGTCTGTGCTGGTG; >clamp_s_c_s_d_s_c_s_a_s_e_s:compl:rev
CACCAGCACAGACACCCACACCGTCACCCACACTATCATGTTGACATTCC; dG = -36.2
886 >s_c_s_d_s_c_s_c_s_d_s_c_s_a_s_d_s
GTGTGGGTGACGGTGTGGGTGTGGGTGACGGTGTGGGTGTCTGTGACGGTG; >clamp_s_c_s_d_s_c_s_a_s_e_s:compl:rev
CACCAGCACAGACACCCACACCGTCACCCACACTATCATGTTGACATTCC; dG = -36.2
887 >s_c_s_d_s_c_s_c_s_d_s_e_s_a_s_e_s
GTGTGGGTGACGGTGTGGGTGTGGGTGACGGTGCTGGTGTCTGTGCTGGTG; >clamp_s_c_s_d_s_c_s_a_s_e_s:compl:rev
CACCAGCACAGACACCCACACCGTCACCCACACTATCATGTTGACATTCC; dG = -36.2
888 >s_c_s_d_s_d_s_c_s_d_s_c_s_a_s_d_s
GTGTGGGTGACGGTGACGGTGTGGGTGACGGTGTGGGTGTCTGTGACGGTG; >clamp_s_c_s_d_s_c_s_a_s_e_s:compl:rev
CACCAGCACAGACACCCACACCGTCACCCACACTATCATGTTGACATTCC; dG = -36.2
889 >s_c_s_d_s_d_s_c_s_d_s_e_s_a_s_e_s
GTGTGGGTGACGGTGACGGTGTGGGTGACGGTGCTGGTGTCTGTGCTGGTG; >clamp_s_c_s_d_s_c_s_a_s_e_s:compl:rev
CACCAGCACAGACACCCACACCGTCACCCACACTATCATGTTGACATTCC; dG = -36.2
890 >s_a_s_b_s_b_s_e_s_d_s_c_s_a_s_e_s
GTGTCTGTGGCTGTGGCTGTGCTGGTGACGGTGTGGGTGTCTGTGCTGGTG; >clamp_s_c_s_d_s_c_s_a_s_e_s:compl:rev
CACCAGCACAGACACCCACACCGTCACCCACACTATCATGTTGACATTCC; dG = -36.2
891 >s_c_s_d_s_d_s_e_s_d_s_c_s_a_s_e_s
GTGTGGGTGACGGTGACGGTGCTGGTGACGGTGTGGGTGTCTGTGCTGGTG; >clamp_s_c_s_d_s_c_s_a_s_e_s:compl:rev
CACCAGCACAGACACCCACACCGTCACCCACACTATCATGTTGACATTCC; dG = -36.2
892 >s_c_s_d_s_e_s_c_s_d_s_c_s_a_s_d_s
GTGTGGGTGACGGTGCTGGTGTGGGTGACGGTGTGGGTGTCTGTGACGGTG; >clamp_s_c_s_d_s_c_s_a_s_e_s:compl:rev
CACCAGCACAGACACCCACACCGTCACCCACACTATCATGTTGACATTCC; dG = -36.2
893 >s_c_s_d_s_e_s_c_s_d_s_e_s_a_s_e_s
GTGTGGGTGACGGTGCTGGTGTGGGTGACGGTGCTGGTGTCTGTGCTGGTG; >clamp_s_c_s_d_s_c_s_a_s_e_s:compl:rev
CACCAGCACAGACACCCACACCGTCACCCACACTATCATGTTGACATTCC; dG = -36.2
894 >s_c_s_d_s_e_s_e_s_d_s_c_s_a_s_e_s
GTGTGGGTGACGGTGCTGGTGCTGGTGACGGTGTGGGTGTCTGTGCTGGTG; >clamp_s_c_s_d_s_c_s_a_s_e_s:compl:rev
CACCAGCACAGACACCCACACCGTCACCCACACTATCATGTTGACATTCC; dG = -36.2
895 >s_c_s_e_s_a_s_c_s_d_s_c_s_a_s_d_s
GTGTGGGTGCTGGTGTCTGTGTGGGTGACGGTGTGGGTGTCTGTGACGGTG; >clamp_s_c_s_d_s_c_s_a_s_e_s:compl:rev
CACCAGCACAGACACCCACACCGTCACCCACACTATCATGTTGACATTCC; dG = -36.2

896 >s_c_s_e_s_a_s_c_s_d_s_e_s_a_s_e_s
GTGTGGGTGCTGGTGTCTGTGTGGGTGACGGTGCTGGTGTCTGTGCTGGTG; >clamp_s_c_s_d_s_c_s_a_s_e_s:compl:rev
CACCAGCACAGACACCCACACCGTCACCCACACTATCATGTTGACATTCC; dG = -36.2
897 >s_a_s_b_s_c_s_c_s_d_s_c_s_a_s_d_s
GTGTCTGTGGCTGTGTGGGTGTGGGTGACGGTGTGGGTGTCTGTGACGGTG; >clamp_s_c_s_d_s_c_s_a_s_e_s:compl:rev
CACCAGCACAGACACCCACACCGTCACCCACACTATCATGTTGACATTCC; dG = -36.2
898 >s_c_s_e_s_a_s_e_s_d_s_c_s_a_s_e_s
GTGTGGGTGCTGGTGTCTGTGCTGGTGACGGTGTGGGTGTCTGTGCTGGTG; >clamp_s_c_s_d_s_c_s_a_s_e_s:compl:rev
CACCAGCACAGACACCCACACCGTCACCCACACTATCATGTTGACATTCC; dG = -36.2
899 >s_c_s_e_s_b_s_c_s_d_s_c_s_a_s_d_s
GTGTGGGTGCTGGTGGCTGTGTGGGTGACGGTGTGGGTGTCTGTGACGGTG; >clamp_s_c_s_d_s_c_s_a_s_e_s:compl:rev
CACCAGCACAGACACCCACACCGTCACCCACACTATCATGTTGACATTCC; dG = -36.2
900 >s_c_s_e_s_b_s_c_s_d_s_e_s_a_s_e_s
GTGTGGGTGCTGGTGGCTGTGTGGGTGACGGTGCTGGTGTCTGTGCTGGTG; >clamp_s_c_s_d_s_c_s_a_s_e_s:compl:rev
CACCAGCACAGACACCCACACCGTCACCCACACTATCATGTTGACATTCC; dG = -36.2
901 >s_c_s_e_s_b_s_e_s_d_s_c_s_a_s_e_s
GTGTGGGTGCTGGTGGCTGTGCTGGTGACGGTGTGGGTGTCTGTGCTGGTG; >clamp_s_c_s_d_s_c_s_a_s_e_s:compl:rev
CACCAGCACAGACACCCACACCGTCACCCACACTATCATGTTGACATTCC; dG = -36.2
902 >s_c_s_e_s_c_s_c_s_d_s_c_s_a_s_d_s
GTGTGGGTGCTGGTGTGGGTGTGGGTGACGGTGTGGGTGTCTGTGACGGTG; >clamp_s_c_s_d_s_c_s_a_s_e_s:compl:rev
CACCAGCACAGACACCCACACCGTCACCCACACTATCATGTTGACATTCC; dG = -36.2
903 >s_c_s_e_s_c_s_c_s_d_s_e_s_a_s_e_s
GTGTGGGTGCTGGTGTGGGTGTGGGTGACGGTGCTGGTGTCTGTGCTGGTG; >clamp_s_c_s_d_s_c_s_a_s_e_s:compl:rev
CACCAGCACAGACACCCACACCGTCACCCACACTATCATGTTGACATTCC; dG = -36.2
904 >s_c_s_e_s_d_s_c_s_d_s_c_s_a_s_d_s
GTGTGGGTGCTGGTGACGGTGTGGGTGACGGTGTGGGTGTCTGTGACGGTG; >clamp_s_c_s_d_s_c_s_a_s_e_s:compl:rev
CACCAGCACAGACACCCACACCGTCACCCACACTATCATGTTGACATTCC; dG = -36.2
905 >s_c_s_e_s_d_s_c_s_d_s_e_s_a_s_e_s
GTGTGGGTGCTGGTGACGGTGTGGGTGACGGTGCTGGTGTCTGTGCTGGTG; >clamp_s_c_s_d_s_c_s_a_s_e_s:compl:rev
CACCAGCACAGACACCCACACCGTCACCCACACTATCATGTTGACATTCC; dG = -36.2
906 >s_c_s_e_s_d_s_e_s_d_s_c_s_a_s_e_s
GTGTGGGTGCTGGTGACGGTGCTGGTGACGGTGTGGGTGTCTGTGCTGGTG; >clamp_s_c_s_d_s_c_s_a_s_e_s:compl:rev
CACCAGCACAGACACCCACACCGTCACCCACACTATCATGTTGACATTCC; dG = -36.2
907 >s_c_s_e_s_e_s_c_s_d_s_c_s_a_s_d_s
GTGTGGGTGCTGGTGCTGGTGTGGGTGACGGTGTGGGTGTCTGTGACGGTG; >clamp_s_c_s_d_s_c_s_a_s_e_s:compl:rev
CACCAGCACAGACACCCACACCGTCACCCACACTATCATGTTGACATTCC; dG = -36.2
908 >s_c_s_e_s_e_s_c_s_d_s_e_s_a_s_e_s
GTGTGGGTGCTGGTGCTGGTGTGGGTGACGGTGCTGGTGTCTGTGCTGGTG; >clamp_s_c_s_d_s_c_s_a_s_e_s:compl:rev
CACCAGCACAGACACCCACACCGTCACCCACACTATCATGTTGACATTCC; dG = -36.2
909 >s_a_s_b_s_c_s_c_s_d_s_e_s_a_s_e_s
GTGTCTGTGGCTGTGTGGGTGTGGGTGACGGTGCTGGTGTCTGTGCTGGTG; >clamp_s_c_s_d_s_c_s_a_s_e_s:compl:rev
CACCAGCACAGACACCCACACCGTCACCCACACTATCATGTTGACATTCC; dG = -36.2
910 >s_c_s_e_s_e_s_e_s_d_s_c_s_a_s_e_s
GTGTGGGTGCTGGTGCTGGTGCTGGTGACGGTGTGGGTGTCTGTGCTGGTG; >clamp_s_c_s_d_s_c_s_a_s_e_s:compl:rev
CACCAGCACAGACACCCACACCGTCACCCACACTATCATGTTGACATTCC; dG = -36.2
911 >s_d_s_a_s_a_s_c_s_d_s_c_s_a_s_d_s
GTGACGGTGTCTGTGTCTGTGTGGGTGACGGTGTGGGTGTCTGTGACGGTG; >clamp_s_c_s_d_s_c_s_a_s_e_s:compl:rev
CACCAGCACAGACACCCACACCGTCACCCACACTATCATGTTGACATTCC; dG = -36.2
912 >s_d_s_a_s_a_s_c_s_d_s_e_s_a_s_e_s
GTGACGGTGTCTGTGTCTGTGTGGGTGACGGTGCTGGTGTCTGTGCTGGTG; >clamp_s_c_s_d_s_c_s_a_s_e_s:compl:rev
CACCAGCACAGACACCCACACCGTCACCCACACTATCATGTTGACATTCC; dG = -36.2
913 >s_d_s_a_s_a_s_e_s_d_s_c_s_a_s_e_s
GTGACGGTGTCTGTGTCTGTGCTGGTGACGGTGTGGGTGTCTGTGCTGGTG; >clamp_s_c_s_d_s_c_s_a_s_e_s:compl:rev
CACCAGCACAGACACCCACACCGTCACCCACACTATCATGTTGACATTCC; dG = -36.2
914 >s_d_s_a_s_b_s_c_s_d_s_c_s_a_s_d_s
GTGACGGTGTCTGTGGCTGTGTGGGTGACGGTGTGGGTGTCTGTGACGGTG; >clamp_s_c_s_d_s_c_s_a_s_e_s:compl:rev
CACCAGCACAGACACCCACACCGTCACCCACACTATCATGTTGACATTCC; dG = -36.2
915 >s_d_s_a_s_b_s_c_s_d_s_e_s_a_s_e_s
GTGACGGTGTCTGTGGCTGTGTGGGTGACGGTGCTGGTGTCTGTGCTGGTG; >clamp_s_c_s_d_s_c_s_a_s_e_s:compl:rev
CACCAGCACAGACACCCACACCGTCACCCACACTATCATGTTGACATTCC; dG = -36.2
916 >s_d_s_a_s_b_s_e_s_d_s_c_s_a_s_e_s
GTGACGGTGTCTGTGGCTGTGCTGGTGACGGTGTGGGTGTCTGTGCTGGTG; >clamp_s_c_s_d_s_c_s_a_s_e_s:compl:rev
CACCAGCACAGACACCCACACCGTCACCCACACTATCATGTTGACATTCC; dG = -36.2
917 >s_d_s_a_s_c_s_c_s_d_s_c_s_a_s_d_s
GTGACGGTGTCTGTGTGGGTGTGGGTGACGGTGTGGGTGTCTGTGACGGTG; >clamp_s_c_s_d_s_c_s_a_s_e_s:compl:rev
CACCAGCACAGACACCCACACCGTCACCCACACTATCATGTTGACATTCC; dG = -36.2
918 >s_d_s_a_s_c_s_c_s_d_s_e_s_a_s_e_s
GTGACGGTGTCTGTGTGGGTGTGGGTGACGGTGCTGGTGTCTGTGCTGGTG; >clamp_s_c_s_d_s_c_s_a_s_e_s:compl:rev
CACCAGCACAGACACCCACACCGTCACCCACACTATCATGTTGACATTCC; dG = -36.2
919 >s_d_s_a_s_d_s_c_s_d_s_c_s_a_s_d_s
GTGACGGTGTCTGTGACGGTGTGGGTGACGGTGTGGGTGTCTGTGACGGTG; >clamp_s_c_s_d_s_c_s_a_s_e_s:compl:rev
CACCAGCACAGACACCCACACCGTCACCCACACTATCATGTTGACATTCC; dG = -36.2

920 >s_d_s_a_s_d_s_c_s_d_s_e_s_a_s_e_s
GTGACGGTGTCTGTGACGGTGTGGGTGACGGTGCTGGTGTCTGTGCTGGTG; >clamp_s_c_s_d_s_c_s_a_s_e_s:compl:rev
CACCAGCACAGACACCCACACCGTCACCCACACTATCATGTTGACATTCC; dG = -36.2
921 >s_d_s_a_s_d_s_e_s_d_s_c_s_a_s_e_s
GTGACGGTGTCTGTGACGGTGCTGGTGACGGTGTGGGTGTCTGTGCTGGTG; >clamp_s_c_s_d_s_c_s_a_s_e_s:compl:rev
CACCAGCACAGACACCCACACCGTCACCCACACTATCATGTTGACATTCC; dG = -36.2
922 >s_d_s_a_s_e_s_c_s_d_s_c_s_a_s_d_s
GTGACGGTGTCTGTGCTGGTGTGGGTGACGGTGTGGGTGTCTGTGACGGTG; >clamp_s_c_s_d_s_c_s_a_s_e_s:compl:rev
CACCAGCACAGACACCCACACCGTCACCCACACTATCATGTTGACATTCC; dG = -36.2
923 >s_d_s_a_s_e_s_c_s_d_s_e_s_a_s_e_s
GTGACGGTGTCTGTGCTGGTGTGGGTGACGGTGCTGGTGTCTGTGCTGGTG; >clamp_s_c_s_d_s_c_s_a_s_e_s:compl:rev
CACCAGCACAGACACCCACACCGTCACCCACACTATCATGTTGACATTCC; dG = -36.2
924 >s_d_s_a_s_e_s_e_s_d_s_c_s_a_s_e_s
GTGACGGTGTCTGTGCTGGTGCTGGTGACGGTGTGGGTGTCTGTGCTGGTG; >clamp_s_c_s_d_s_c_s_a_s_e_s:compl:rev
CACCAGCACAGACACCCACACCGTCACCCACACTATCATGTTGACATTCC; dG = -36.2
925 >s_d_s_b_s_a_s_c_s_d_s_c_s_a_s_d_s
GTGACGGTGGCTGTGTCTGTGTGGGTGACGGTGTGGGTGTCTGTGACGGTG; >clamp_s_c_s_d_s_c_s_a_s_e_s:compl:rev
CACCAGCACAGACACCCACACCGTCACCCACACTATCATGTTGACATTCC; dG = -36.2
926 >s_d_s_b_s_a_s_c_s_d_s_e_s_a_s_e_s
GTGACGGTGGCTGTGTCTGTGTGGGTGACGGTGCTGGTGTCTGTGCTGGTG; >clamp_s_c_s_d_s_c_s_a_s_e_s:compl:rev
CACCAGCACAGACACCCACACCGTCACCCACACTATCATGTTGACATTCC; dG = -36.2
927 >s_a_s_b_s_d_s_c_s_d_s_c_s_a_s_d_s
GTGTCTGTGGCTGTGACGGTGTGGGTGACGGTGTGGGTGTCTGTGACGGTG; >clamp_s_c_s_d_s_c_s_a_s_e_s:compl:rev
CACCAGCACAGACACCCACACCGTCACCCACACTATCATGTTGACATTCC; dG = -36.2
928 >s_d_s_b_s_a_s_e_s_d_s_c_s_a_s_e_s
GTGACGGTGGCTGTGTCTGTGCTGGTGACGGTGTGGGTGTCTGTGCTGGTG; >clamp_s_c_s_d_s_c_s_a_s_e_s:compl:rev
CACCAGCACAGACACCCACACCGTCACCCACACTATCATGTTGACATTCC; dG = -36.2
929 >s_d_s_b_s_b_s_c_s_d_s_c_s_a_s_d_s
GTGACGGTGGCTGTGGCTGTGTGGGTGACGGTGTGGGTGTCTGTGACGGTG; >clamp_s_c_s_d_s_c_s_a_s_e_s:compl:rev
CACCAGCACAGACACCCACACCGTCACCCACACTATCATGTTGACATTCC; dG = -36.2
930 >s_d_s_b_s_b_s_c_s_d_s_e_s_a_s_e_s
GTGACGGTGGCTGTGGCTGTGTGGGTGACGGTGCTGGTGTCTGTGCTGGTG; >clamp_s_c_s_d_s_c_s_a_s_e_s:compl:rev
CACCAGCACAGACACCCACACCGTCACCCACACTATCATGTTGACATTCC; dG = -36.2
931 >s_d_s_b_s_b_s_e_s_d_s_c_s_a_s_e_s
GTGACGGTGGCTGTGGCTGTGCTGGTGACGGTGTGGGTGTCTGTGCTGGTG; >clamp_s_c_s_d_s_c_s_a_s_e_s:compl:rev
CACCAGCACAGACACCCACACCGTCACCCACACTATCATGTTGACATTCC; dG = -36.2
932 >s_d_s_b_s_c_s_c_s_d_s_c_s_a_s_d_s
GTGACGGTGGCTGTGTGGGTGTGGGTGACGGTGTGGGTGTCTGTGACGGTG; >clamp_s_c_s_d_s_c_s_a_s_e_s:compl:rev
CACCAGCACAGACACCCACACCGTCACCCACACTATCATGTTGACATTCC; dG = -36.2
933 >s_d_s_b_s_c_s_c_s_d_s_e_s_a_s_e_s
GTGACGGTGGCTGTGTGGGTGTGGGTGACGGTGCTGGTGTCTGTGCTGGTG; >clamp_s_c_s_d_s_c_s_a_s_e_s:compl:rev
CACCAGCACAGACACCCACACCGTCACCCACACTATCATGTTGACATTCC; dG = -36.2
934 >s_d_s_b_s_d_s_c_s_d_s_c_s_a_s_d_s
GTGACGGTGGCTGTGACGGTGTGGGTGACGGTGTGGGTGTCTGTGACGGTG; >clamp_s_c_s_d_s_c_s_a_s_e_s:compl:rev
CACCAGCACAGACACCCACACCGTCACCCACACTATCATGTTGACATTCC; dG = -36.2
935 >s_d_s_b_s_d_s_c_s_d_s_e_s_a_s_e_s
GTGACGGTGGCTGTGACGGTGTGGGTGACGGTGCTGGTGTCTGTGCTGGTG; >clamp_s_c_s_d_s_c_s_a_s_e_s:compl:rev
CACCAGCACAGACACCCACACCGTCACCCACACTATCATGTTGACATTCC; dG = -36.2
936 >s_d_s_b_s_d_s_e_s_d_s_c_s_a_s_e_s
GTGACGGTGGCTGTGACGGTGCTGGTGACGGTGTGGGTGTCTGTGCTGGTG; >clamp_s_c_s_d_s_c_s_a_s_e_s:compl:rev
CACCAGCACAGACACCCACACCGTCACCCACACTATCATGTTGACATTCC; dG = -36.2
937 >s_d_s_b_s_e_s_c_s_d_s_c_s_a_s_d_s
GTGACGGTGGCTGTGCTGGTGTGGGTGACGGTGTGGGTGTCTGTGACGGTG; >clamp_s_c_s_d_s_c_s_a_s_e_s:compl:rev
CACCAGCACAGACACCCACACCGTCACCCACACTATCATGTTGACATTCC; dG = -36.2
938 >s_d_s_b_s_e_s_c_s_d_s_e_s_a_s_e_s
GTGACGGTGGCTGTGCTGGTGTGGGTGACGGTGCTGGTGTCTGTGCTGGTG; >clamp_s_c_s_d_s_c_s_a_s_e_s:compl:rev
CACCAGCACAGACACCCACACCGTCACCCACACTATCATGTTGACATTCC; dG = -36.2
939 >s_a_s_b_s_d_s_c_s_d_s_e_s_a_s_e_s
GTGTCTGTGGCTGTGACGGTGTGGGTGACGGTGCTGGTGTCTGTGCTGGTG; >clamp_s_c_s_d_s_c_s_a_s_e_s:compl:rev
CACCAGCACAGACACCCACACCGTCACCCACACTATCATGTTGACATTCC; dG = -36.2
940 >s_d_s_b_s_e_s_e_s_d_s_c_s_a_s_e_s
GTGACGGTGGCTGTGCTGGTGCTGGTGACGGTGTGGGTGTCTGTGCTGGTG; >clamp_s_c_s_d_s_c_s_a_s_e_s:compl:rev
CACCAGCACAGACACCCACACCGTCACCCACACTATCATGTTGACATTCC; dG = -36.2
941 >s_a_s_a_s_a_s_e_s_d_s_c_s_a_s_e_s
GTGTCTGTGTCTGTGTCTGTGCTGGTGACGGTGTGGGTGTCTGTGCTGGTG; >clamp_s_c_s_d_s_c_s_a_s_e_s:compl:rev
CACCAGCACAGACACCCACACCGTCACCCACACTATCATGTTGACATTCC; dG = -36.2
942 >s_d_s_c_s_a_s_c_s_d_s_c_s_a_s_d_s
GTGACGGTGTGGGTGTCTGTGTGGGTGACGGTGTGGGTGTCTGTGACGGTG; >clamp_s_c_s_d_s_c_s_a_s_e_s:compl:rev
CACCAGCACAGACACCCACACCGTCACCCACACTATCATGTTGACATTCC; dG = -36.2
943 >s_d_s_c_s_a_s_c_s_d_s_e_s_a_s_e_s
GTGACGGTGTGGGTGTCTGTGTGGGTGACGGTGCTGGTGTCTGTGCTGGTG; >clamp_s_c_s_d_s_c_s_a_s_e_s:compl:rev
CACCAGCACAGACACCCACACCGTCACCCACACTATCATGTTGACATTCC; dG = -36.2

944 >s_d_s_c_s_a_s_e_s_d_s_c_s_a_s_e_s
GTGACGGTGTGGGTGTCTGTGCTGGTGACGGTGTGGGTGTCTGTGCTGGTG; >clamp_s_c_s_d_s_c_s_a_s_e_s:compl:rev
CACCAGCACAGACACCCACACCGTCACCCACACTATCATGTTGACATTCC; dG = -36.2
945 >s_d_s_c_s_b_s_c_s_d_s_c_s_a_s_d_s
GTGACGGTGTGGGTGGCTGTGTGGGTGACGGTGTGGGTGTCTGTGACGGTG; >clamp_s_c_s_d_s_c_s_a_s_e_s:compl:rev
CACCAGCACAGACACCCACACCGTCACCCACACTATCATGTTGACATTCC; dG = -36.2
946 >s_d_s_c_s_b_s_c_s_d_s_e_s_a_s_e_s
GTGACGGTGTGGGTGGCTGTGTGGGTGACGGTGCTGGTGTCTGTGCTGGTG; >clamp_s_c_s_d_s_c_s_a_s_e_s:compl:rev
CACCAGCACAGACACCCACACCGTCACCCACACTATCATGTTGACATTCC; dG = -36.2
947 >s_d_s_c_s_b_s_e_s_d_s_c_s_a_s_e_s
GTGACGGTGTGGGTGGCTGTGCTGGTGACGGTGTGGGTGTCTGTGCTGGTG; >clamp_s_c_s_d_s_c_s_a_s_e_s:compl:rev
CACCAGCACAGACACCCACACCGTCACCCACACTATCATGTTGACATTCC; dG = -36.2
948 >s_d_s_c_s_c_s_c_s_d_s_c_s_a_s_d_s
GTGACGGTGTGGGTGTGGGTGTGGGTGACGGTGTGGGTGTCTGTGACGGTG; >clamp_s_c_s_d_s_c_s_a_s_e_s:compl:rev
CACCAGCACAGACACCCACACCGTCACCCACACTATCATGTTGACATTCC; dG = -36.2
949 >s_d_s_c_s_c_s_c_s_d_s_e_s_a_s_e_s
GTGACGGTGTGGGTGTGGGTGTGGGTGACGGTGCTGGTGTCTGTGCTGGTG; >clamp_s_c_s_d_s_c_s_a_s_e_s:compl:rev
CACCAGCACAGACACCCACACCGTCACCCACACTATCATGTTGACATTCC; dG = -36.2
950 >s_d_s_c_s_d_s_c_s_d_s_c_s_a_s_d_s
GTGACGGTGTGGGTGACGGTGTGGGTGACGGTGTGGGTGTCTGTGACGGTG; >clamp_s_c_s_d_s_c_s_a_s_e_s:compl:rev
CACCAGCACAGACACCCACACCGTCACCCACACTATCATGTTGACATTCC; dG = -36.2
951 >s_d_s_c_s_d_s_c_s_d_s_e_s_a_s_e_s
GTGACGGTGTGGGTGACGGTGTGGGTGACGGTGCTGGTGTCTGTGCTGGTG; >clamp_s_c_s_d_s_c_s_a_s_e_s:compl:rev
CACCAGCACAGACACCCACACCGTCACCCACACTATCATGTTGACATTCC; dG = -36.2
952 >s_a_s_b_s_d_s_e_s_d_s_c_s_a_s_e_s
GTGTCTGTGGCTGTGACGGTGCTGGTGACGGTGTGGGTGTCTGTGCTGGTG; >clamp_s_c_s_d_s_c_s_a_s_e_s:compl:rev
CACCAGCACAGACACCCACACCGTCACCCACACTATCATGTTGACATTCC; dG = -36.2
953 >s_d_s_c_s_d_s_e_s_d_s_c_s_a_s_e_s
GTGACGGTGTGGGTGACGGTGCTGGTGACGGTGTGGGTGTCTGTGCTGGTG; >clamp_s_c_s_d_s_c_s_a_s_e_s:compl:rev
CACCAGCACAGACACCCACACCGTCACCCACACTATCATGTTGACATTCC; dG = -36.2
954 >s_d_s_c_s_e_s_c_s_d_s_c_s_a_s_d_s
GTGACGGTGTGGGTGCTGGTGTGGGTGACGGTGTGGGTGTCTGTGACGGTG; >clamp_s_c_s_d_s_c_s_a_s_e_s:compl:rev
CACCAGCACAGACACCCACACCGTCACCCACACTATCATGTTGACATTCC; dG = -36.2
955 >s_d_s_c_s_e_s_c_s_d_s_e_s_a_s_e_s
GTGACGGTGTGGGTGCTGGTGTGGGTGACGGTGCTGGTGTCTGTGCTGGTG; >clamp_s_c_s_d_s_c_s_a_s_e_s:compl:rev
CACCAGCACAGACACCCACACCGTCACCCACACTATCATGTTGACATTCC; dG = -36.2
956 >s_d_s_c_s_e_s_e_s_d_s_c_s_a_s_e_s
GTGACGGTGTGGGTGCTGGTGCTGGTGACGGTGTGGGTGTCTGTGCTGGTG; >clamp_s_c_s_d_s_c_s_a_s_e_s:compl:rev
CACCAGCACAGACACCCACACCGTCACCCACACTATCATGTTGACATTCC; dG = -36.2
957 >s_d_s_d_s_a_s_c_s_d_s_c_s_a_s_d_s
GTGACGGTGACGGTGTCTGTGTGGGTGACGGTGTGGGTGTCTGTGACGGTG; >clamp_s_c_s_d_s_c_s_a_s_e_s:compl:rev
CACCAGCACAGACACCCACACCGTCACCCACACTATCATGTTGACATTCC; dG = -36.2
958 >s_d_s_d_s_a_s_c_s_d_s_e_s_a_s_e_s
GTGACGGTGACGGTGTCTGTGTGGGTGACGGTGCTGGTGTCTGTGCTGGTG; >clamp_s_c_s_d_s_c_s_a_s_e_s:compl:rev
CACCAGCACAGACACCCACACCGTCACCCACACTATCATGTTGACATTCC; dG = -36.2
959 >s_a_s_b_s_e_s_c_s_d_s_c_s_a_s_d_s
GTGTCTGTGGCTGTGCTGGTGTGGGTGACGGTGTGGGTGTCTGTGACGGTG; >clamp_s_c_s_d_s_c_s_a_s_e_s:compl:rev
CACCAGCACAGACACCCACACCGTCACCCACACTATCATGTTGACATTCC; dG = -36.2
960 >s_d_s_d_s_a_s_e_s_d_s_c_s_a_s_e_s
GTGACGGTGACGGTGTCTGTGCTGGTGACGGTGTGGGTGTCTGTGCTGGTG; >clamp_s_c_s_d_s_c_s_a_s_e_s:compl:rev
CACCAGCACAGACACCCACACCGTCACCCACACTATCATGTTGACATTCC; dG = -36.2
961 >s_d_s_d_s_b_s_c_s_d_s_c_s_a_s_d_s
GTGACGGTGACGGTGGCTGTGTGGGTGACGGTGTGGGTGTCTGTGACGGTG; >clamp_s_c_s_d_s_c_s_a_s_e_s:compl:rev
CACCAGCACAGACACCCACACCGTCACCCACACTATCATGTTGACATTCC; dG = -36.2
962 >s_d_s_d_s_b_s_c_s_d_s_e_s_a_s_e_s
GTGACGGTGACGGTGGCTGTGTGGGTGACGGTGCTGGTGTCTGTGCTGGTG; >clamp_s_c_s_d_s_c_s_a_s_e_s:compl:rev
CACCAGCACAGACACCCACACCGTCACCCACACTATCATGTTGACATTCC; dG = -36.2
963 >s_d_s_d_s_b_s_e_s_d_s_c_s_a_s_e_s
GTGACGGTGACGGTGGCTGTGCTGGTGACGGTGTGGGTGTCTGTGCTGGTG; >clamp_s_c_s_d_s_c_s_a_s_e_s:compl:rev
CACCAGCACAGACACCCACACCGTCACCCACACTATCATGTTGACATTCC; dG = -36.2
964 >s_d_s_d_s_c_s_c_s_d_s_c_s_a_s_d_s
GTGACGGTGACGGTGTGGGTGTGGGTGACGGTGTGGGTGTCTGTGACGGTG; >clamp_s_c_s_d_s_c_s_a_s_e_s:compl:rev
CACCAGCACAGACACCCACACCGTCACCCACACTATCATGTTGACATTCC; dG = -36.2
965 >s_d_s_d_s_c_s_c_s_d_s_e_s_a_s_e_s
GTGACGGTGACGGTGTGGGTGTGGGTGACGGTGCTGGTGTCTGTGCTGGTG; >clamp_s_c_s_d_s_c_s_a_s_e_s:compl:rev
CACCAGCACAGACACCCACACCGTCACCCACACTATCATGTTGACATTCC; dG = -36.2
966 >s_d_s_d_s_d_s_c_s_d_s_c_s_a_s_d_s
GTGACGGTGACGGTGACGGTGTGGGTGACGGTGTGGGTGTCTGTGACGGTG; >clamp_s_c_s_d_s_c_s_a_s_e_s:compl:rev
CACCAGCACAGACACCCACACCGTCACCCACACTATCATGTTGACATTCC; dG = -36.2
967 >s_d_s_d_s_d_s_c_s_d_s_e_s_a_s_e_s
GTGACGGTGACGGTGACGGTGTGGGTGACGGTGCTGGTGTCTGTGCTGGTG; >clamp_s_c_s_d_s_c_s_a_s_e_s:compl:rev
CACCAGCACAGACACCCACACCGTCACCCACACTATCATGTTGACATTCC; dG = -36.2

968 >s_d_s_d_s_d_s_e_s_d_s_c_s_a_s_e_s
GTGACGGTGACGGTGACGGTGCTGGTGACGGTGTGGGTGTCTGTGCTGGTG; >clamp_s_c_s_d_s_c_s_a_s_e_s:compl:rev
CACCAGCACAGACACCCACACCGTCACCCACACTATCATGTTGACATTCC; dG = -36.2
969 >s_d_s_d_s_e_s_c_s_d_s_c_s_a_s_d_s
GTGACGGTGACGGTGCTGGTGTGGGTGACGGTGTGGGTGTCTGTGACGGTG; >clamp_s_c_s_d_s_c_s_a_s_e_s:compl:rev
CACCAGCACAGACACCCACACCGTCACCCACACTATCATGTTGACATTCC; dG = -36.2
970 >s_d_s_d_s_e_s_c_s_d_s_e_s_a_s_e_s
GTGACGGTGACGGTGCTGGTGTGGGTGACGGTGCTGGTGTCTGTGCTGGTG; >clamp_s_c_s_d_s_c_s_a_s_e_s:compl:rev
CACCAGCACAGACACCCACACCGTCACCCACACTATCATGTTGACATTCC; dG = -36.2
971 >s_a_s_b_s_e_s_c_s_d_s_e_s_a_s_e_s
GTGTCTGTGGCTGTGCTGGTGTGGGTGACGGTGCTGGTGTCTGTGCTGGTG; >clamp_s_c_s_d_s_c_s_a_s_e_s:compl:rev
CACCAGCACAGACACCCACACCGTCACCCACACTATCATGTTGACATTCC; dG = -36.2
972 >s_d_s_d_s_e_s_e_s_d_s_c_s_a_s_e_s
GTGACGGTGACGGTGCTGGTGCTGGTGACGGTGTGGGTGTCTGTGCTGGTG; >clamp_s_c_s_d_s_c_s_a_s_e_s:compl:rev
CACCAGCACAGACACCCACACCGTCACCCACACTATCATGTTGACATTCC; dG = -36.2
973 >s_d_s_e_s_a_s_c_s_d_s_c_s_a_s_d_s
GTGACGGTGCTGGTGTCTGTGTGGGTGACGGTGTGGGTGTCTGTGACGGTG; >clamp_s_c_s_d_s_c_s_a_s_e_s:compl:rev
CACCAGCACAGACACCCACACCGTCACCCACACTATCATGTTGACATTCC; dG = -36.2
974 >s_d_s_e_s_a_s_c_s_d_s_e_s_a_s_e_s
GTGACGGTGCTGGTGTCTGTGTGGGTGACGGTGCTGGTGTCTGTGCTGGTG; >clamp_s_c_s_d_s_c_s_a_s_e_s:compl:rev
CACCAGCACAGACACCCACACCGTCACCCACACTATCATGTTGACATTCC; dG = -36.2
975 >s_d_s_e_s_a_s_e_s_d_s_c_s_a_s_e_s
GTGACGGTGCTGGTGTCTGTGCTGGTGACGGTGTGGGTGTCTGTGCTGGTG; >clamp_s_c_s_d_s_c_s_a_s_e_s:compl:rev
CACCAGCACAGACACCCACACCGTCACCCACACTATCATGTTGACATTCC; dG = -36.2
976 >s_d_s_e_s_b_s_c_s_d_s_c_s_a_s_d_s
GTGACGGTGCTGGTGGCTGTGTGGGTGACGGTGTGGGTGTCTGTGACGGTG; >clamp_s_c_s_d_s_c_s_a_s_e_s:compl:rev
CACCAGCACAGACACCCACACCGTCACCCACACTATCATGTTGACATTCC; dG = -36.2
977 >s_d_s_e_s_b_s_c_s_d_s_e_s_a_s_e_s
GTGACGGTGCTGGTGGCTGTGTGGGTGACGGTGCTGGTGTCTGTGCTGGTG; >clamp_s_c_s_d_s_c_s_a_s_e_s:compl:rev
CACCAGCACAGACACCCACACCGTCACCCACACTATCATGTTGACATTCC; dG = -36.2
978 >s_d_s_e_s_b_s_e_s_d_s_c_s_a_s_e_s
GTGACGGTGCTGGTGGCTGTGCTGGTGACGGTGTGGGTGTCTGTGCTGGTG; >clamp_s_c_s_d_s_c_s_a_s_e_s:compl:rev
CACCAGCACAGACACCCACACCGTCACCCACACTATCATGTTGACATTCC; dG = -36.2
979 >s_d_s_e_s_c_s_c_s_d_s_c_s_a_s_d_s
GTGACGGTGCTGGTGTGGGTGTGGGTGACGGTGTGGGTGTCTGTGACGGTG; >clamp_s_c_s_d_s_c_s_a_s_e_s:compl:rev
CACCAGCACAGACACCCACACCGTCACCCACACTATCATGTTGACATTCC; dG = -36.2
980 >s_d_s_e_s_c_s_c_s_d_s_e_s_a_s_e_s
GTGACGGTGCTGGTGTGGGTGTGGGTGACGGTGCTGGTGTCTGTGCTGGTG; >clamp_s_c_s_d_s_c_s_a_s_e_s:compl:rev
CACCAGCACAGACACCCACACCGTCACCCACACTATCATGTTGACATTCC; dG = -36.2
981 >s_d_s_e_s_d_s_c_s_d_s_c_s_a_s_d_s
GTGACGGTGCTGGTGACGGTGTGGGTGACGGTGTGGGTGTCTGTGACGGTG; >clamp_s_c_s_d_s_c_s_a_s_e_s:compl:rev
CACCAGCACAGACACCCACACCGTCACCCACACTATCATGTTGACATTCC; dG = -36.2
982 >s_d_s_e_s_d_s_c_s_d_s_e_s_a_s_e_s
GTGACGGTGCTGGTGACGGTGTGGGTGACGGTGCTGGTGTCTGTGCTGGTG; >clamp_s_c_s_d_s_c_s_a_s_e_s:compl:rev
CACCAGCACAGACACCCACACCGTCACCCACACTATCATGTTGACATTCC; dG = -36.2
983 >s_a_s_b_s_e_s_e_s_d_s_c_s_a_s_e_s
GTGTCTGTGGCTGTGCTGGTGCTGGTGACGGTGTGGGTGTCTGTGCTGGTG; >clamp_s_c_s_d_s_c_s_a_s_e_s:compl:rev
CACCAGCACAGACACCCACACCGTCACCCACACTATCATGTTGACATTCC; dG = -36.2
984 >s_d_s_e_s_d_s_e_s_d_s_c_s_a_s_e_s
GTGACGGTGCTGGTGACGGTGCTGGTGACGGTGTGGGTGTCTGTGCTGGTG; >clamp_s_c_s_d_s_c_s_a_s_e_s:compl:rev
CACCAGCACAGACACCCACACCGTCACCCACACTATCATGTTGACATTCC; dG = -36.2
985 >s_d_s_e_s_e_s_c_s_d_s_c_s_a_s_d_s
GTGACGGTGCTGGTGCTGGTGTGGGTGACGGTGTGGGTGTCTGTGACGGTG; >clamp_s_c_s_d_s_c_s_a_s_e_s:compl:rev
CACCAGCACAGACACCCACACCGTCACCCACACTATCATGTTGACATTCC; dG = -36.2
986 >s_d_s_e_s_e_s_c_s_d_s_e_s_a_s_e_s
GTGACGGTGCTGGTGCTGGTGTGGGTGACGGTGCTGGTGTCTGTGCTGGTG; >clamp_s_c_s_d_s_c_s_a_s_e_s:compl:rev
CACCAGCACAGACACCCACACCGTCACCCACACTATCATGTTGACATTCC; dG = -36.2
987 >s_d_s_e_s_e_s_e_s_d_s_c_s_a_s_e_s
GTGACGGTGCTGGTGCTGGTGCTGGTGACGGTGTGGGTGTCTGTGCTGGTG; >clamp_s_c_s_d_s_c_s_a_s_e_s:compl:rev
CACCAGCACAGACACCCACACCGTCACCCACACTATCATGTTGACATTCC; dG = -36.2
988 >s_e_s_a_s_a_s_c_s_d_s_c_s_a_s_d_s
GTGCTGGTGTCTGTGTCTGTGTGGGTGACGGTGTGGGTGTCTGTGACGGTG; >clamp_s_c_s_d_s_c_s_a_s_e_s:compl:rev
CACCAGCACAGACACCCACACCGTCACCCACACTATCATGTTGACATTCC; dG = -36.2
989 >s_e_s_a_s_a_s_c_s_d_s_e_s_a_s_e_s
GTGCTGGTGTCTGTGTCTGTGTGGGTGACGGTGCTGGTGTCTGTGCTGGTG; >clamp_s_c_s_d_s_c_s_a_s_e_s:compl:rev
CACCAGCACAGACACCCACACCGTCACCCACACTATCATGTTGACATTCC; dG = -36.2
990 >s_a_s_c_s_a_s_c_s_d_s_c_s_a_s_d_s
GTGTCTGTGTGGGTGTCTGTGTGGGTGACGGTGTGGGTGTCTGTGACGGTG; >clamp_s_c_s_d_s_c_s_a_s_e_s:compl:rev
CACCAGCACAGACACCCACACCGTCACCCACACTATCATGTTGACATTCC; dG = -36.2
991 >s_e_s_a_s_a_s_e_s_d_s_c_s_a_s_e_s
GTGCTGGTGTCTGTGTCTGTGCTGGTGACGGTGTGGGTGTCTGTGCTGGTG; >clamp_s_c_s_d_s_c_s_a_s_e_s:compl:rev
CACCAGCACAGACACCCACACCGTCACCCACACTATCATGTTGACATTCC; dG = -36.2

```
   992 >s_e_s_a_s_b_s_c_s_d_s_c_s_a_s_d_s
GTGCTGGTGTCTGTGGCTGTGTGGGTGACGGTGTGGGTGTCTGTGACGGTG;    >clamp_s_c_s_d_s_c_s_a_s_e_s:compl:rev
CACCAGCACAGACACCCACACCGTCACCCACACTATCATGTTGACATTCC; dG = -36.2
   993 >s_e_s_a_s_b_s_c_s_d_s_e_s_a_s_e_s
GTGCTGGTGTCTGTGGCTGTGTGGGTGACGGTGCTGGTGTCTGTGCTGGTG;    >clamp_s_c_s_d_s_c_s_a_s_e_s:compl:rev
CACCAGCACAGACACCCACACCGTCACCCACACTATCATGTTGACATTCC; dG = -36.2
   994 >s_e_s_a_s_b_s_e_s_d_s_c_s_a_s_e_s
GTGCTGGTGTCTGTGGCTGTGCTGGTGACGGTGTGGGTGTCTGTGCTGGTG;    >clamp_s_c_s_d_s_c_s_a_s_e_s:compl:rev
CACCAGCACAGACACCCACACCGTCACCCACACTATCATGTTGACATTCC; dG = -36.2
   995 >s_e_s_a_s_c_s_c_s_d_s_c_s_a_s_d_s
GTGCTGGTGTCTGTGTGGGTGTGGGTGACGGTGTGGGTGTCTGTGACGGTG;    >clamp_s_c_s_d_s_c_s_a_s_e_s:compl:rev
CACCAGCACAGACACCCACACCGTCACCCACACTATCATGTTGACATTCC; dG = -36.2
   996 >s_e_s_a_s_c_s_c_s_d_s_e_s_a_s_e_s
GTGCTGGTGTCTGTGTGGGTGTGGGTGACGGTGCTGGTGTCTGTGCTGGTG;    >clamp_s_c_s_d_s_c_s_a_s_e_s:compl:rev
CACCAGCACAGACACCCACACCGTCACCCACACTATCATGTTGACATTCC; dG = -36.2
   997 >s_e_s_a_s_d_s_c_s_d_s_c_s_a_s_d_s
GTGCTGGTGTCTGTGACGGTGTGGGTGACGGTGTGGGTGTCTGTGACGGTG;    >clamp_s_c_s_d_s_c_s_a_s_e_s:compl:rev
CACCAGCACAGACACCCACACCGTCACCCACACTATCATGTTGACATTCC; dG = -36.2
   998 >s_e_s_a_s_d_s_c_s_d_s_e_s_a_s_e_s
GTGCTGGTGTCTGTGACGGTGTGGGTGACGGTGCTGGTGTCTGTGCTGGTG;    >clamp_s_c_s_d_s_c_s_a_s_e_s:compl:rev
CACCAGCACAGACACCCACACCGTCACCCACACTATCATGTTGACATTCC; dG = -36.2
   999 >s_e_s_a_s_d_s_e_s_d_s_c_s_a_s_e_s
GTGCTGGTGTCTGTGACGGTGCTGGTGACGGTGTGGGTGTCTGTGCTGGTG;    >clamp_s_c_s_d_s_c_s_a_s_e_s:compl:rev
CACCAGCACAGACACCCACACCGTCACCCACACTATCATGTTGACATTCC; dG = -36.2
  1000 >s_e_s_a_s_e_s_c_s_d_s_c_s_a_s_d_s
GTGCTGGTGTCTGTGCTGGTGTGGGTGACGGTGTGGGTGTCTGTGACGGTG;    >clamp_s_c_s_d_s_c_s_a_s_e_s:compl:rev
CACCAGCACAGACACCCACACCGTCACCCACACTATCATGTTGACATTCC; dG = -36.2
  1001 >s_e_s_a_s_e_s_c_s_d_s_e_s_a_s_e_s
GTGCTGGTGTCTGTGCTGGTGTGGGTGACGGTGCTGGTGTCTGTGCTGGTG;    >clamp_s_c_s_d_s_c_s_a_s_e_s:compl:rev
CACCAGCACAGACACCCACACCGTCACCCACACTATCATGTTGACATTCC; dG = -36.2
  1002 >s_a_s_c_s_a_s_c_s_d_s_e_s_a_s_e_s
GTGTCTGTGTGGGTGTCTGTGTGGGTGACGGTGCTGGTGTCTGTGCTGGTG;    >clamp_s_c_s_d_s_c_s_a_s_e_s:compl:rev
CACCAGCACAGACACCCACACCGTCACCCACACTATCATGTTGACATTCC; dG = -36.2
  1003 >s_e_s_a_s_e_s_e_s_d_s_c_s_a_s_e_s
GTGCTGGTGTCTGTGCTGGTGCTGGTGACGGTGTGGGTGTCTGTGCTGGTG;    >clamp_s_c_s_d_s_c_s_a_s_e_s:compl:rev
CACCAGCACAGACACCCACACCGTCACCCACACTATCATGTTGACATTCC; dG = -36.2
  1004 >s_a_s_a_s_b_s_c_s_d_s_c_s_a_s_d_s
GTGTCTGTGTCTGTGGCTGTGTGGGTGACGGTGTGGGTGTCTGTGACGGTG;    >clamp_s_c_s_d_s_c_s_a_s_e_s:compl:rev
CACCAGCACAGACACCCACACCGTCACCCACACTATCATGTTGACATTCC; dG = -36.2
  1005 >s_e_s_b_s_a_s_c_s_d_s_c_s_a_s_d_s
GTGCTGGTGGCTGTGTCTGTGTGGGTGACGGTGTGGGTGTCTGTGACGGTG;    >clamp_s_c_s_d_s_c_s_a_s_e_s:compl:rev
CACCAGCACAGACACCCACACCGTCACCCACACTATCATGTTGACATTCC; dG = -36.2
  1006 >s_e_s_b_s_a_s_c_s_d_s_e_s_a_s_e_s
GTGCTGGTGGCTGTGTCTGTGTGGGTGACGGTGCTGGTGTCTGTGCTGGTG;    >clamp_s_c_s_d_s_c_s_a_s_e_s:compl:rev
CACCAGCACAGACACCCACACCGTCACCCACACTATCATGTTGACATTCC; dG = -36.2
  1007 >s_e_s_b_s_a_s_e_s_d_s_c_s_a_s_e_s
GTGCTGGTGGCTGTGTCTGTGCTGGTGACGGTGTGGGTGTCTGTGCTGGTG;    >clamp_s_c_s_d_s_c_s_a_s_e_s:compl:rev
CACCAGCACAGACACCCACACCGTCACCCACACTATCATGTTGACATTCC; dG = -36.2
  1008 >s_e_s_b_s_b_s_c_s_d_s_c_s_a_s_d_s
GTGCTGGTGGCTGTGGCTGTGTGGGTGACGGTGTGGGTGTCTGTGACGGTG;    >clamp_s_c_s_d_s_c_s_a_s_e_s:compl:rev
CACCAGCACAGACACCCACACCGTCACCCACACTATCATGTTGACATTCC; dG = -36.2
  1009 >s_e_s_b_s_b_s_c_s_d_s_e_s_a_s_e_s
GTGCTGGTGGCTGTGGCTGTGTGGGTGACGGTGCTGGTGTCTGTGCTGGTG;    >clamp_s_c_s_d_s_c_s_a_s_e_s:compl:rev
CACCAGCACAGACACCCACACCGTCACCCACACTATCATGTTGACATTCC; dG = -36.2
  1010 >s_e_s_b_s_b_s_e_s_d_s_c_s_a_s_e_s
GTGCTGGTGGCTGTGGCTGTGCTGGTGACGGTGTGGGTGTCTGTGCTGGTG;    >clamp_s_c_s_d_s_c_s_a_s_e_s:compl:rev
CACCAGCACAGACACCCACACCGTCACCCACACTATCATGTTGACATTCC; dG = -36.2
  1011 >s_e_s_b_s_c_s_c_s_d_s_c_s_a_s_d_s
GTGCTGGTGGCTGTGTGGGTGTGGGTGACGGTGTGGGTGTCTGTGACGGTG;    >clamp_s_c_s_d_s_c_s_a_s_e_s:compl:rev
CACCAGCACAGACACCCACACCGTCACCCACACTATCATGTTGACATTCC; dG = -36.2
  1012 >s_e_s_b_s_c_s_c_s_d_s_e_s_a_s_e_s
GTGCTGGTGGCTGTGTGGGTGTGGGTGACGGTGCTGGTGTCTGTGCTGGTG;    >clamp_s_c_s_d_s_c_s_a_s_e_s:compl:rev
CACCAGCACAGACACCCACACCGTCACCCACACTATCATGTTGACATTCC; dG = -36.2
  1013 >s_e_s_b_s_d_s_c_s_d_s_c_s_a_s_d_s
GTGCTGGTGGCTGTGACGGTGTGGGTGACGGTGTGGGTGTCTGTGACGGTG;    >clamp_s_c_s_d_s_c_s_a_s_e_s:compl:rev
CACCAGCACAGACACCCACACCGTCACCCACACTATCATGTTGACATTCC; dG = -36.2
  1014 >s_e_s_b_s_d_s_c_s_d_s_e_s_a_s_e_s
GTGCTGGTGGCTGTGACGGTGTGGGTGACGGTGCTGGTGTCTGTGCTGGTG;    >clamp_s_c_s_d_s_c_s_a_s_e_s:compl:rev
CACCAGCACAGACACCCACACCGTCACCCACACTATCATGTTGACATTCC; dG = -36.2
  1015 >s_a_s_c_s_a_s_e_s_d_s_c_s_a_s_e_s
GTGTCTGTGTGGGTGTCTGTGCTGGTGACGGTGTGGGTGTCTGTGCTGGTG;    >clamp_s_c_s_d_s_c_s_a_s_e_s:compl:rev
CACCAGCACAGACACCCACACCGTCACCCACACTATCATGTTGACATTCC; dG = -36.2
```

```
  1016 >s_e_s_b_s_d_s_e_s_d_s_c_s_a_s_e_s
GTGCTGGTGGCTGTGACGGTGCTGGTGACGGTGTGGGTGTCTGTGCTGGTG;    >clamp_s_c_s_d_s_c_s_a_s_e_s:compl:rev
CACCAGCACAGACACCCACACCGTCACCCACACTATCATGTTGACATTCC; dG = -36.2
  1017 >s_e_s_b_s_e_s_c_s_d_s_c_s_a_s_d_s
GTGCTGGTGGCTGTGCTGGTGTGGGTGACGGTGTGGGTGTCTGTGACGGTG;    >clamp_s_c_s_d_s_c_s_a_s_e_s:compl:rev
CACCAGCACAGACACCCACACCGTCACCCACACTATCATGTTGACATTCC; dG = -36.2
  1018 >s_e_s_b_s_e_s_c_s_d_s_e_s_a_s_e_s
GTGCTGGTGGCTGTGCTGGTGTGGGTGACGGTGCTGGTGTCTGTGCTGGTG;    >clamp_s_c_s_d_s_c_s_a_s_e_s:compl:rev
CACCAGCACAGACACCCACACCGTCACCCACACTATCATGTTGACATTCC; dG = -36.2
  1019 >s_e_s_b_s_e_s_e_s_d_s_c_s_a_s_e_s
GTGCTGGTGGCTGTGCTGGTGCTGGTGACGGTGTGGGTGTCTGTGCTGGTG;    >clamp_s_c_s_d_s_c_s_a_s_e_s:compl:rev
CACCAGCACAGACACCCACACCGTCACCCACACTATCATGTTGACATTCC; dG = -36.2
  1020 >s_e_s_c_s_a_s_c_s_d_s_c_s_a_s_d_s
GTGCTGGTGTGGGTGTCTGTGTGGGTGACGGTGTGGGTGTCTGTGACGGTG;    >clamp_s_c_s_d_s_c_s_a_s_e_s:compl:rev
CACCAGCACAGACACCCACACCGTCACCCACACTATCATGTTGACATTCC; dG = -36.2
  1021 >s_e_s_c_s_a_s_c_s_d_s_e_s_a_s_e_s
GTGCTGGTGTGGGTGTCTGTGTGGGTGACGGTGCTGGTGTCTGTGCTGGTG;    >clamp_s_c_s_d_s_c_s_a_s_e_s:compl:rev
CACCAGCACAGACACCCACACCGTCACCCACACTATCATGTTGACATTCC; dG = -36.2
  1022 >s_a_s_c_s_b_s_c_s_d_s_c_s_a_s_d_s
GTGTCTGTGTGGGTGGCTGTGTGGGTGACGGTGTGGGTGTCTGTGACGGTG;    >clamp_s_c_s_d_s_c_s_a_s_e_s:compl:rev
CACCAGCACAGACACCCACACCGTCACCCACACTATCATGTTGACATTCC; dG = -36.2
  1023 >s_e_s_c_s_a_s_e_s_d_s_c_s_a_s_e_s
GTGCTGGTGTGGGTGTCTGTGCTGGTGACGGTGTGGGTGTCTGTGCTGGTG;    >clamp_s_c_s_d_s_c_s_a_s_e_s:compl:rev
CACCAGCACAGACACCCACACCGTCACCCACACTATCATGTTGACATTCC; dG = -36.2
  1024 >s_e_s_c_s_b_s_c_s_d_s_c_s_a_s_d_s
GTGCTGGTGTGGGTGGCTGTGTGGGTGACGGTGTGGGTGTCTGTGACGGTG;    >clamp_s_c_s_d_s_c_s_a_s_e_s:compl:rev
CACCAGCACAGACACCCACACCGTCACCCACACTATCATGTTGACATTCC; dG = -36.2
  1025 >s_e_s_c_s_b_s_c_s_d_s_e_s_a_s_e_s
GTGCTGGTGTGGGTGGCTGTGTGGGTGACGGTGCTGGTGTCTGTGCTGGTG;    >clamp_s_c_s_d_s_c_s_a_s_e_s:compl:rev
CACCAGCACAGACACCCACACCGTCACCCACACTATCATGTTGACATTCC; dG = -36.2
  1026 >s_e_s_c_s_b_s_e_s_d_s_c_s_a_s_e_s
GTGCTGGTGTGGGTGGCTGTGCTGGTGACGGTGTGGGTGTCTGTGCTGGTG;    >clamp_s_c_s_d_s_c_s_a_s_e_s:compl:rev
CACCAGCACAGACACCCACACCGTCACCCACACTATCATGTTGACATTCC; dG = -36.2
  1027 >s_e_s_c_s_c_s_c_s_d_s_c_s_a_s_d_s
GTGCTGGTGTGGGTGTGGGTGTGGGTGACGGTGTGGGTGTCTGTGACGGTG;    >clamp_s_c_s_d_s_c_s_a_s_e_s:compl:rev
CACCAGCACAGACACCCACACCGTCACCCACACTATCATGTTGACATTCC; dG = -36.2
  1028 >s_e_s_c_s_c_s_c_s_d_s_e_s_a_s_e_s
GTGCTGGTGTGGGTGTGGGTGTGGGTGACGGTGCTGGTGTCTGTGCTGGTG;    >clamp_s_c_s_d_s_c_s_a_s_e_s:compl:rev
CACCAGCACAGACACCCACACCGTCACCCACACTATCATGTTGACATTCC; dG = -36.2
  1029 >s_e_s_c_s_d_s_c_s_d_s_c_s_a_s_d_s
GTGCTGGTGTGGGTGACGGTGTGGGTGACGGTGTGGGTGTCTGTGACGGTG;    >clamp_s_c_s_d_s_c_s_a_s_e_s:compl:rev
CACCAGCACAGACACCCACACCGTCACCCACACTATCATGTTGACATTCC; dG = -36.2
  1030 >s_e_s_c_s_d_s_c_s_d_s_e_s_a_s_e_s
GTGCTGGTGTGGGTGACGGTGTGGGTGACGGTGCTGGTGTCTGTGCTGGTG;    >clamp_s_c_s_d_s_c_s_a_s_e_s:compl:rev
CACCAGCACAGACACCCACACCGTCACCCACACTATCATGTTGACATTCC; dG = -36.2
  1031 >s_e_s_c_s_d_s_e_s_d_s_c_s_a_s_e_s
GTGCTGGTGTGGGTGACGGTGCTGGTGACGGTGTGGGTGTCTGTGCTGGTG;    >clamp_s_c_s_d_s_c_s_a_s_e_s:compl:rev
CACCAGCACAGACACCCACACCGTCACCCACACTATCATGTTGACATTCC; dG = -36.2
  1032 >s_e_s_c_s_e_s_c_s_d_s_c_s_a_s_d_s
GTGCTGGTGTGGGTGCTGGTGTGGGTGACGGTGTGGGTGTCTGTGACGGTG;    >clamp_s_c_s_d_s_c_s_a_s_e_s:compl:rev
CACCAGCACAGACACCCACACCGTCACCCACACTATCATGTTGACATTCC; dG = -36.2
  1033 >s_e_s_c_s_e_s_c_s_d_s_e_s_a_s_e_s
GTGCTGGTGTGGGTGCTGGTGTGGGTGACGGTGCTGGTGTCTGTGCTGGTG;    >clamp_s_c_s_d_s_c_s_a_s_e_s:compl:rev
CACCAGCACAGACACCCACACCGTCACCCACACTATCATGTTGACATTCC; dG = -36.2
  1034 >s_a_s_c_s_b_s_c_s_d_s_e_s_a_s_e_s
GTGTCTGTGTGGGTGGCTGTGTGGGTGACGGTGCTGGTGTCTGTGCTGGTG;    >clamp_s_c_s_d_s_c_s_a_s_e_s:compl:rev
CACCAGCACAGACACCCACACCGTCACCCACACTATCATGTTGACATTCC; dG = -36.2
  1035 >s_e_s_c_s_e_s_e_s_d_s_c_s_a_s_e_s
GTGCTGGTGTGGGTGCTGGTGCTGGTGACGGTGTGGGTGTCTGTGCTGGTG;    >clamp_s_c_s_d_s_c_s_a_s_e_s:compl:rev
CACCAGCACAGACACCCACACCGTCACCCACACTATCATGTTGACATTCC; dG = -36.2
  1036 >s_e_s_d_s_a_s_c_s_d_s_c_s_a_s_d_s
GTGCTGGTGACGGTGTCTGTGTGGGTGACGGTGTGGGTGTCTGTGACGGTG;    >clamp_s_c_s_d_s_c_s_a_s_e_s:compl:rev
CACCAGCACAGACACCCACACCGTCACCCACACTATCATGTTGACATTCC; dG = -36.2
  1037 >s_e_s_d_s_a_s_c_s_d_s_e_s_a_s_e_s
GTGCTGGTGACGGTGTCTGTGTGGGTGACGGTGCTGGTGTCTGTGCTGGTG;    >clamp_s_c_s_d_s_c_s_a_s_e_s:compl:rev
CACCAGCACAGACACCCACACCGTCACCCACACTATCATGTTGACATTCC; dG = -36.2
  1038 >s_e_s_d_s_a_s_e_s_d_s_c_s_a_s_e_s
GTGCTGGTGACGGTGTCTGTGCTGGTGACGGTGTGGGTGTCTGTGCTGGTG;    >clamp_s_c_s_d_s_c_s_a_s_e_s:compl:rev
CACCAGCACAGACACCCACACCGTCACCCACACTATCATGTTGACATTCC; dG = -36.2
  1039 >s_e_s_d_s_b_s_c_s_d_s_c_s_a_s_d_s
GTGCTGGTGACGGTGGCTGTGTGGGTGACGGTGTGGGTGTCTGTGACGGTG;    >clamp_s_c_s_d_s_c_s_a_s_e_s:compl:rev
CACCAGCACAGACACCCACACCGTCACCCACACTATCATGTTGACATTCC; dG = -36.2
```

1040 >s_e_s_d_s_b_s_c_s_d_s_e_s_a_s_e_s
GTGCTGGTGACGGTGGCTGTGTGGGTGACGGTGCTGGTGTCTGTGCTGGTG;   >clamp_s_c_s_d_s_c_s_a_s_e_s:compl:rev
CACCAGCACAGACACCCACACCGTCACCCACACTATCATGTTGACATTCC; dG = -36.2
1041 >s_e_s_d_s_b_s_e_s_d_s_c_s_a_s_e_s
GTGCTGGTGACGGTGGCTGTGCTGGTGACGGTGTGGGTGTCTGTGCTGGTG;   >clamp_s_c_s_d_s_c_s_a_s_e_s:compl:rev
CACCAGCACAGACACCCACACCGTCACCCACACTATCATGTTGACATTCC; dG = -36.2
1042 >s_e_s_d_s_c_s_c_s_d_s_c_s_a_s_d_s
GTGCTGGTGACGGTGTGGGTGTGGGTGACGGTGTGGGTGTCTGTGACGGTG;   >clamp_s_c_s_d_s_c_s_a_s_e_s:compl:rev
CACCAGCACAGACACCCACACCGTCACCCACACTATCATGTTGACATTCC; dG = -36.2
1043 >s_e_s_d_s_c_s_c_s_d_s_e_s_a_s_e_s
GTGCTGGTGACGGTGTGGGTGTGGGTGACGGTGCTGGTGTCTGTGCTGGTG;   >clamp_s_c_s_d_s_c_s_a_s_e_s:compl:rev
CACCAGCACAGACACCCACACCGTCACCCACACTATCATGTTGACATTCC; dG = -36.2
1044 >s_e_s_d_s_d_s_c_s_d_s_c_s_a_s_d_s
GTGCTGGTGACGGTGACGGTGTGGGTGACGGTGTGGGTGTCTGTGACGGTG;   >clamp_s_c_s_d_s_c_s_a_s_e_s:compl:rev
CACCAGCACAGACACCCACACCGTCACCCACACTATCATGTTGACATTCC; dG = -36.2
1045 >s_e_s_d_s_d_s_c_s_d_s_e_s_a_s_e_s
GTGCTGGTGACGGTGACGGTGTGGGTGACGGTGCTGGTGTCTGTGCTGGTG;   >clamp_s_c_s_d_s_c_s_a_s_e_s:compl:rev
CACCAGCACAGACACCCACACCGTCACCCACACTATCATGTTGACATTCC; dG = -36.2
1046 >s_a_s_c_s_b_s_e_s_d_s_c_s_a_s_e_s
GTGTCTGTGTGGGTGGCTGTGCTGGTGACGGTGTGGGTGTCTGTGCTGGTG;   >clamp_s_c_s_d_s_c_s_a_s_e_s:compl:rev
CACCAGCACAGACACCCACACCGTCACCCACACTATCATGTTGACATTCC; dG = -36.2
1047 >s_e_s_d_s_d_s_e_s_d_s_c_s_a_s_e_s
GTGCTGGTGACGGTGACGGTGCTGGTGACGGTGTGGGTGTCTGTGCTGGTG;   >clamp_s_c_s_d_s_c_s_a_s_e_s:compl:rev
CACCAGCACAGACACCCACACCGTCACCCACACTATCATGTTGACATTCC; dG = -36.2
1048 >s_e_s_d_s_e_s_c_s_d_s_c_s_a_s_d_s
GTGCTGGTGACGGTGCTGGTGTGGGTGACGGTGTGGGTGTCTGTGACGGTG;   >clamp_s_c_s_d_s_c_s_a_s_e_s:compl:rev
CACCAGCACAGACACCCACACCGTCACCCACACTATCATGTTGACATTCC; dG = -36.2
1049 >s_e_s_d_s_e_s_c_s_d_s_e_s_a_s_e_s
GTGCTGGTGACGGTGCTGGTGTGGGTGACGGTGCTGGTGTCTGTGCTGGTG;   >clamp_s_c_s_d_s_c_s_a_s_e_s:compl:rev
CACCAGCACAGACACCCACACCGTCACCCACACTATCATGTTGACATTCC; dG = -36.2
1050 >s_e_s_d_s_e_s_e_s_d_s_c_s_a_s_e_s
GTGCTGGTGACGGTGCTGGTGCTGGTGACGGTGTGGGTGTCTGTGCTGGTG;   >clamp_s_c_s_d_s_c_s_a_s_e_s:compl:rev
CACCAGCACAGACACCCACACCGTCACCCACACTATCATGTTGACATTCC; dG = -36.2
1051 >s_e_s_e_s_a_s_c_s_d_s_c_s_a_s_d_s
GTGCTGGTGCTGGTGTCTGTGTGGGTGACGGTGTGGGTGTCTGTGACGGTG;   >clamp_s_c_s_d_s_c_s_a_s_e_s:compl:rev
CACCAGCACAGACACCCACACCGTCACCCACACTATCATGTTGACATTCC; dG = -36.2
1052 >s_e_s_e_s_a_s_c_s_d_s_e_s_a_s_e_s
GTGCTGGTGCTGGTGTCTGTGTGGGTGACGGTGCTGGTGTCTGTGCTGGTG;   >clamp_s_c_s_d_s_c_s_a_s_e_s:compl:rev
CACCAGCACAGACACCCACACCGTCACCCACACTATCATGTTGACATTCC; dG = -36.2
1053 >s_a_s_c_s_c_s_c_s_d_s_c_s_a_s_d_s
GTGTCTGTGTGGGTGTGGGTGTGGGTGACGGTGTGGGTGTCTGTGACGGTG;   >clamp_s_c_s_d_s_c_s_a_s_e_s:compl:rev
CACCAGCACAGACACCCACACCGTCACCCACACTATCATGTTGACATTCC; dG = -36.2
1054 >s_e_s_e_s_a_s_e_s_d_s_c_s_a_s_e_s
GTGCTGGTGCTGGTGTCTGTGCTGGTGACGGTGTGGGTGTCTGTGCTGGTG;   >clamp_s_c_s_d_s_c_s_a_s_e_s:compl:rev
CACCAGCACAGACACCCACACCGTCACCCACACTATCATGTTGACATTCC; dG = -36.2
1055 >s_e_s_e_s_b_s_c_s_d_s_c_s_a_s_d_s
GTGCTGGTGCTGGTGGCTGTGTGGGTGACGGTGTGGGTGTCTGTGACGGTG;   >clamp_s_c_s_d_s_c_s_a_s_e_s:compl:rev
CACCAGCACAGACACCCACACCGTCACCCACACTATCATGTTGACATTCC; dG = -36.2
1056 >s_e_s_e_s_b_s_c_s_d_s_e_s_a_s_e_s
GTGCTGGTGCTGGTGGCTGTGTGGGTGACGGTGCTGGTGTCTGTGCTGGTG;   >clamp_s_c_s_d_s_c_s_a_s_e_s:compl:rev
CACCAGCACAGACACCCACACCGTCACCCACACTATCATGTTGACATTCC; dG = -36.2
1057 >s_e_s_e_s_b_s_e_s_d_s_c_s_a_s_e_s
GTGCTGGTGCTGGTGGCTGTGCTGGTGACGGTGTGGGTGTCTGTGCTGGTG;   >clamp_s_c_s_d_s_c_s_a_s_e_s:compl:rev
CACCAGCACAGACACCCACACCGTCACCCACACTATCATGTTGACATTCC; dG = -36.2
1058 >s_e_s_e_s_c_s_c_s_d_s_c_s_a_s_d_s
GTGCTGGTGCTGGTGTGGGTGTGGGTGACGGTGTGGGTGTCTGTGACGGTG;   >clamp_s_c_s_d_s_c_s_a_s_e_s:compl:rev
CACCAGCACAGACACCCACACCGTCACCCACACTATCATGTTGACATTCC; dG = -36.2
1059 >s_e_s_e_s_c_s_c_s_d_s_e_s_a_s_e_s
GTGCTGGTGCTGGTGTGGGTGTGGGTGACGGTGCTGGTGTCTGTGCTGGTG;   >clamp_s_c_s_d_s_c_s_a_s_e_s:compl:rev
CACCAGCACAGACACCCACACCGTCACCCACACTATCATGTTGACATTCC; dG = -36.2
1060 >s_e_s_e_s_d_s_c_s_d_s_c_s_a_s_d_s
GTGCTGGTGCTGGTGACGGTGTGGGTGACGGTGTGGGTGTCTGTGACGGTG;   >clamp_s_c_s_d_s_c_s_a_s_e_s:compl:rev
CACCAGCACAGACACCCACACCGTCACCCACACTATCATGTTGACATTCC; dG = -36.2
1061 >s_e_s_e_s_d_s_c_s_d_s_e_s_a_s_e_s
GTGCTGGTGCTGGTGACGGTGTGGGTGACGGTGCTGGTGTCTGTGCTGGTG;   >clamp_s_c_s_d_s_c_s_a_s_e_s:compl:rev
CACCAGCACAGACACCCACACCGTCACCCACACTATCATGTTGACATTCC; dG = -36.2
1062 >s_e_s_e_s_d_s_e_s_d_s_c_s_a_s_e_s
GTGCTGGTGCTGGTGACGGTGCTGGTGACGGTGTGGGTGTCTGTGCTGGTG;   >clamp_s_c_s_d_s_c_s_a_s_e_s:compl:rev
CACCAGCACAGACACCCACACCGTCACCCACACTATCATGTTGACATTCC; dG = -36.2
1063 >s_e_s_e_s_e_s_c_s_d_s_c_s_a_s_d_s
GTGCTGGTGCTGGTGCTGGTGTGGGTGACGGTGTGGGTGTCTGTGACGGTG;   >clamp_s_c_s_d_s_c_s_a_s_e_s:compl:rev
CACCAGCACAGACACCCACACCGTCACCCACACTATCATGTTGACATTCC; dG = -36.2

1064 >s_e_s_e_s_e_s_c_s_d_s_e_s_a_s_e_s
GTGCTGGTGCTGGTGCTGGTGTGGGTGACGGTGCTGGTGTCTGTGCTGGTG;   >clamp_s_c_s_d_s_c_s_a_s_e_s:compl:rev
CACCAGCACAGACACCCACACCGTCACCCACACTATCATGTTGACATTCC; dG = -36.2
1065 >s_a_s_c_s_c_s_c_s_d_s_e_s_a_s_e_s
GTGTCTGTGTGGGTGTGGGTGTGGGTGACGGTGCTGGTGTCTGTGCTGGTG;   >clamp_s_c_s_d_s_c_s_a_s_e_s:compl:rev
CACCAGCACAGACACCCACACCGTCACCCACACTATCATGTTGACATTCC; dG = -36.2
1066 >s_e_s_e_s_e_s_e_s_d_s_c_s_a_s_e_s
GTGCTGGTGCTGGTGCTGGTGCTGGTGACGGTGTGGGTGTCTGTGCTGGTG;   >clamp_s_c_s_d_s_c_s_a_s_e_s:compl:rev
CACCAGCACAGACACCCACACCGTCACCCACACTATCATGTTGACATTCC; dG = -36.2
1067 >s_a_s_c_s_d_s_c_s_d_s_c_s_a_s_d_s
GTGTCTGTGTGGGTGACGGTGTGGGTGACGGTGTGGGTGTCTGTGACGGTG;   >clamp_s_c_s_d_s_c_s_a_s_e_s:compl:rev
CACCAGCACAGACACCCACACCGTCACCCACACTATCATGTTGACATTCC; dG = -36.2
1068 >s_a_s_c_s_d_s_c_s_d_s_e_s_a_s_e_s
GTGTCTGTGTGGGTGACGGTGTGGGTGACGGTGCTGGTGTCTGTGCTGGTG;   >clamp_s_c_s_d_s_c_s_a_s_e_s:compl:rev
CACCAGCACAGACACCCACACCGTCACCCACACTATCATGTTGACATTCC; dG = -36.2
1069 >s_a_s_c_s_d_s_e_s_d_s_c_s_a_s_e_s
GTGTCTGTGTGGGTGACGGTGCTGGTGACGGTGTGGGTGTCTGTGCTGGTG;   >clamp_s_c_s_d_s_c_s_a_s_e_s:compl:rev
CACCAGCACAGACACCCACACCGTCACCCACACTATCATGTTGACATTCC; dG = -36.2
1070 >s_a_s_c_s_e_s_c_s_d_s_c_s_a_s_d_s
GTGTCTGTGTGGGTGCTGGTGTGGGTGACGGTGTGGGTGTCTGTGACGGTG;   >clamp_s_c_s_d_s_c_s_a_s_e_s:compl:rev
CACCAGCACAGACACCCACACCGTCACCCACACTATCATGTTGACATTCC; dG = -36.2
1071 >s_a_s_c_s_e_s_c_s_d_s_e_s_a_s_e_s
GTGTCTGTGTGGGTGCTGGTGTGGGTGACGGTGCTGGTGTCTGTGCTGGTG;   >clamp_s_c_s_d_s_c_s_a_s_e_s:compl:rev
CACCAGCACAGACACCCACACCGTCACCCACACTATCATGTTGACATTCC; dG = -36.2
1072 >s_a_s_a_s_b_s_c_s_d_s_e_s_a_s_e_s
GTGTCTGTGTCTGTGGCTGTGTGGGTGACGGTGCTGGTGTCTGTGCTGGTG;   >clamp_s_c_s_d_s_c_s_a_s_e_s:compl:rev
CACCAGCACAGACACCCACACCGTCACCCACACTATCATGTTGACATTCC; dG = -36.2
1073 >s_a_s_c_s_e_s_e_s_d_s_c_s_a_s_e_s
GTGTCTGTGTGGGTGCTGGTGCTGGTGACGGTGTGGGTGTCTGTGCTGGTG;   >clamp_s_c_s_d_s_c_s_a_s_e_s:compl:rev
CACCAGCACAGACACCCACACCGTCACCCACACTATCATGTTGACATTCC; dG = -36.2
1074 >s_a_s_d_s_a_s_c_s_d_s_c_s_a_s_d_s
GTGTCTGTGACGGTGTCTGTGTGGGTGACGGTGTGGGTGTCTGTGACGGTG;   >clamp_s_c_s_d_s_c_s_a_s_e_s:compl:rev
CACCAGCACAGACACCCACACCGTCACCCACACTATCATGTTGACATTCC; dG = -36.2
1075 >s_a_s_d_s_a_s_c_s_d_s_e_s_a_s_e_s
GTGTCTGTGACGGTGTCTGTGTGGGTGACGGTGCTGGTGTCTGTGCTGGTG;   >clamp_s_c_s_d_s_c_s_a_s_e_s:compl:rev
CACCAGCACAGACACCCACACCGTCACCCACACTATCATGTTGACATTCC; dG = -36.2
1076 >s_a_s_d_s_a_s_e_s_d_s_c_s_a_s_e_s
GTGTCTGTGACGGTGTCTGTGCTGGTGACGGTGTGGGTGTCTGTGCTGGTG;   >clamp_s_c_s_d_s_c_s_a_s_e_s:compl:rev
CACCAGCACAGACACCCACACCGTCACCCACACTATCATGTTGACATTCC; dG = -36.2
1077 >s_a_s_d_s_b_s_c_s_d_s_c_s_a_s_d_s
GTGTCTGTGACGGTGGCTGTGTGGGTGACGGTGTGGGTGTCTGTGACGGTG;   >clamp_s_c_s_d_s_c_s_a_s_e_s:compl:rev
CACCAGCACAGACACCCACACCGTCACCCACACTATCATGTTGACATTCC; dG = -36.2
1078 >s_a_s_d_s_b_s_c_s_d_s_e_s_a_s_e_s
GTGTCTGTGACGGTGGCTGTGTGGGTGACGGTGCTGGTGTCTGTGCTGGTG;   >clamp_s_c_s_d_s_c_s_a_s_e_s:compl:rev
CACCAGCACAGACACCCACACCGTCACCCACACTATCATGTTGACATTCC; dG = -36.2
1079 >s_a_s_d_s_b_s_e_s_d_s_c_s_a_s_e_s
GTGTCTGTGACGGTGGCTGTGCTGGTGACGGTGTGGGTGTCTGTGCTGGTG;   >clamp_s_c_s_d_s_c_s_a_s_e_s:compl:rev
CACCAGCACAGACACCCACACCGTCACCCACACTATCATGTTGACATTCC; dG = -36.2
1080 >s_a_s_d_s_c_s_c_s_d_s_c_s_a_s_d_s
GTGTCTGTGACGGTGTGGGTGTGGGTGACGGTGTGGGTGTCTGTGACGGTG;   >clamp_s_c_s_d_s_c_s_a_s_e_s:compl:rev
CACCAGCACAGACACCCACACCGTCACCCACACTATCATGTTGACATTCC; dG = -36.2
1081 >s_a_s_d_s_c_s_c_s_d_s_e_s_a_s_e_s
GTGTCTGTGACGGTGTGGGTGTGGGTGACGGTGCTGGTGTCTGTGCTGGTG;   >clamp_s_c_s_d_s_c_s_a_s_e_s:compl:rev
CACCAGCACAGACACCCACACCGTCACCCACACTATCATGTTGACATTCC; dG = -36.2
1082 >s_a_s_d_s_d_s_c_s_d_s_c_s_a_s_d_s
GTGTCTGTGACGGTGACGGTGTGGGTGACGGTGTGGGTGTCTGTGACGGTG;   >clamp_s_c_s_d_s_c_s_a_s_e_s:compl:rev
CACCAGCACAGACACCCACACCGTCACCCACACTATCATGTTGACATTCC; dG = -36.2
1083 >s_a_s_d_s_d_s_c_s_d_s_e_s_a_s_e_s
GTGTCTGTGACGGTGACGGTGTGGGTGACGGTGCTGGTGTCTGTGCTGGTG;   >clamp_s_c_s_d_s_c_s_a_s_e_s:compl:rev
CACCAGCACAGACACCCACACCGTCACCCACACTATCATGTTGACATTCC; dG = -36.2
1084 >s_a_s_a_s_b_s_e_s_d_s_c_s_a_s_e_s
GTGTCTGTGTCTGTGGCTGTGCTGGTGACGGTGTGGGTGTCTGTGCTGGTG;   >clamp_s_c_s_d_s_c_s_a_s_e_s:compl:rev
CACCAGCACAGACACCCACACCGTCACCCACACTATCATGTTGACATTCC; dG = -36.2
1085 >s_a_s_d_s_d_s_e_s_d_s_c_s_a_s_e_s
GTGTCTGTGACGGTGACGGTGCTGGTGACGGTGTGGGTGTCTGTGCTGGTG;   >clamp_s_c_s_d_s_c_s_a_s_e_s:compl:rev
CACCAGCACAGACACCCACACCGTCACCCACACTATCATGTTGACATTCC; dG = -36.2
1086 >s_a_s_d_s_e_s_c_s_d_s_c_s_a_s_d_s
GTGTCTGTGACGGTGCTGGTGTGGGTGACGGTGTGGGTGTCTGTGACGGTG;   >clamp_s_c_s_d_s_c_s_a_s_e_s:compl:rev
CACCAGCACAGACACCCACACCGTCACCCACACTATCATGTTGACATTCC; dG = -36.2
1087 >s_a_s_d_s_e_s_c_s_d_s_e_s_a_s_e_s
GTGTCTGTGACGGTGCTGGTGTGGGTGACGGTGCTGGTGTCTGTGCTGGTG;   >clamp_s_c_s_d_s_c_s_a_s_e_s:compl:rev
CACCAGCACAGACACCCACACCGTCACCCACACTATCATGTTGACATTCC; dG = -36.2

1088 >s_a_s_d_s_e_s_e_s_d_s_c_s_a_s_e_s
GTGTCTGTGACGGTGCTGGTGCTGGTGACGGTGTGGGTGTCTGTGCTGGTG; >clamp_s_c_s_d_s_c_s_a_s_e_s:compl:rev
CACCAGCACAGACACCCACACCGTCACCCACACTATCATGTTGACATTCC; dG = -36.2
1089 >s_a_s_e_s_a_s_c_s_d_s_c_s_a_s_d_s
GTGTCTGTGCTGGTGTCTGTGTGGGTGACGGTGTGGGTGTCTGTGACGGTG; >clamp_s_c_s_d_s_c_s_a_s_e_s:compl:rev
CACCAGCACAGACACCCACACCGTCACCCACACTATCATGTTGACATTCC; dG = -36.2
1090 >s_a_s_e_s_a_s_c_s_d_s_e_s_a_s_e_s
GTGTCTGTGCTGGTGTCTGTGTGGGTGACGGTGCTGGTGTCTGTGCTGGTG; >clamp_s_c_s_d_s_c_s_a_s_e_s:compl:rev
CACCAGCACAGACACCCACACCGTCACCCACACTATCATGTTGACATTCC; dG = -36.2
1091 >s_a_s_a_s_c_s_c_s_d_s_c_s_a_s_d_s
GTGTCTGTGTCTGTGTGGGTGTGGGTGACGGTGTGGGTGTCTGTGACGGTG; >clamp_s_c_s_d_s_c_s_a_s_e_s:compl:rev
CACCAGCACAGACACCCACACCGTCACCCACACTATCATGTTGACATTCC; dG = -36.2
1092 >s_a_s_e_s_a_s_e_s_d_s_c_s_a_s_e_s
GTGTCTGTGCTGGTGTCTGTGCTGGTGACGGTGTGGGTGTCTGTGCTGGTG; >clamp_s_c_s_d_s_c_s_a_s_e_s:compl:rev
CACCAGCACAGACACCCACACCGTCACCCACACTATCATGTTGACATTCC; dG = -36.2
1093 >s_a_s_e_s_b_s_c_s_d_s_c_s_a_s_d_s
GTGTCTGTGCTGGTGGCTGTGTGGGTGACGGTGTGGGTGTCTGTGACGGTG; >clamp_s_c_s_d_s_c_s_a_s_e_s:compl:rev
CACCAGCACAGACACCCACACCGTCACCCACACTATCATGTTGACATTCC; dG = -36.2
1094 >s_a_s_e_s_b_s_c_s_d_s_e_s_a_s_e_s
GTGTCTGTGCTGGTGGCTGTGTGGGTGACGGTGCTGGTGTCTGTGCTGGTG; >clamp_s_c_s_d_s_c_s_a_s_e_s:compl:rev
CACCAGCACAGACACCCACACCGTCACCCACACTATCATGTTGACATTCC; dG = -36.2
1095 >s_a_s_e_s_b_s_e_s_d_s_c_s_a_s_e_s
GTGTCTGTGCTGGTGGCTGTGCTGGTGACGGTGTGGGTGTCTGTGCTGGTG; >clamp_s_c_s_d_s_c_s_a_s_e_s:compl:rev
CACCAGCACAGACACCCACACCGTCACCCACACTATCATGTTGACATTCC; dG = -36.2
1096 >s_a_s_e_s_c_s_c_s_d_s_c_s_a_s_d_s
GTGTCTGTGCTGGTGTGGGTGTGGGTGACGGTGTGGGTGTCTGTGACGGTG; >clamp_s_c_s_d_s_c_s_a_s_e_s:compl:rev
CACCAGCACAGACACCCACACCGTCACCCACACTATCATGTTGACATTCC; dG = -36.2
1097 >s_a_s_e_s_c_s_c_s_d_s_e_s_a_s_e_s
GTGTCTGTGCTGGTGTGGGTGTGGGTGACGGTGCTGGTGTCTGTGCTGGTG; >clamp_s_c_s_d_s_c_s_a_s_e_s:compl:rev
CACCAGCACAGACACCCACACCGTCACCCACACTATCATGTTGACATTCC; dG = -36.2
1098 >s_a_s_e_s_d_s_c_s_d_s_c_s_a_s_d_s
GTGTCTGTGCTGGTGACGGTGTGGGTGACGGTGTGGGTGTCTGTGACGGTG; >clamp_s_c_s_d_s_c_s_a_s_e_s:compl:rev
CACCAGCACAGACACCCACACCGTCACCCACACTATCATGTTGACATTCC; dG = -36.2
1099 >s_a_s_e_s_d_s_c_s_d_s_e_s_a_s_e_s
GTGTCTGTGCTGGTGACGGTGTGGGTGACGGTGCTGGTGTCTGTGCTGGTG; >clamp_s_c_s_d_s_c_s_a_s_e_s:compl:rev
CACCAGCACAGACACCCACACCGTCACCCACACTATCATGTTGACATTCC; dG = -36.2
1100 >s_a_s_e_s_d_s_e_s_d_s_c_s_a_s_e_s
GTGTCTGTGCTGGTGACGGTGCTGGTGACGGTGTGGGTGTCTGTGCTGGTG; >clamp_s_c_s_d_s_c_s_a_s_e_s:compl:rev
CACCAGCACAGACACCCACACCGTCACCCACACTATCATGTTGACATTCC; dG = -36.2
1101 >s_a_s_e_s_e_s_c_s_d_s_c_s_a_s_d_s
GTGTCTGTGCTGGTGCTGGTGTGGGTGACGGTGTGGGTGTCTGTGACGGTG; >clamp_s_c_s_d_s_c_s_a_s_e_s:compl:rev
CACCAGCACAGACACCCACACCGTCACCCACACTATCATGTTGACATTCC; dG = -36.2
1102 >s_a_s_e_s_e_s_c_s_d_s_e_s_a_s_e_s
GTGTCTGTGCTGGTGCTGGTGTGGGTGACGGTGCTGGTGTCTGTGCTGGTG; >clamp_s_c_s_d_s_c_s_a_s_e_s:compl:rev
CACCAGCACAGACACCCACACCGTCACCCACACTATCATGTTGACATTCC; dG = -36.2
1103 >s_a_s_a_s_c_s_c_s_d_s_e_s_a_s_e_s
GTGTCTGTGTCTGTGTGGGTGTGGGTGACGGTGCTGGTGTCTGTGCTGGTG; >clamp_s_c_s_d_s_c_s_a_s_e_s:compl:rev
CACCAGCACAGACACCCACACCGTCACCCACACTATCATGTTGACATTCC; dG = -36.2
1104 >s_a_s_e_s_e_s_e_s_d_s_c_s_a_s_e_s
GTGTCTGTGCTGGTGCTGGTGCTGGTGACGGTGTGGGTGTCTGTGCTGGTG; >clamp_s_c_s_d_s_c_s_a_s_e_s:compl:rev
CACCAGCACAGACACCCACACCGTCACCCACACTATCATGTTGACATTCC; dG = -36.2
1105 >s_b_s_a_s_a_s_c_s_d_s_c_s_a_s_d_s
GTGGCTGTGTCTGTGTCTGTGTGGGTGACGGTGTGGGTGTCTGTGACGGTG; >clamp_s_c_s_d_s_c_s_a_s_e_s:compl:rev
CACCAGCACAGACACCCACACCGTCACCCACACTATCATGTTGACATTCC; dG = -36.2
1106 >s_b_s_a_s_a_s_c_s_d_s_e_s_a_s_e_s
GTGGCTGTGTCTGTGTCTGTGTGGGTGACGGTGCTGGTGTCTGTGCTGGTG; >clamp_s_c_s_d_s_c_s_a_s_e_s:compl:rev
CACCAGCACAGACACCCACACCGTCACCCACACTATCATGTTGACATTCC; dG = -36.2
1107 >s_b_s_a_s_a_s_e_s_d_s_c_s_a_s_e_s
GTGGCTGTGTCTGTGTCTGTGCTGGTGACGGTGTGGGTGTCTGTGCTGGTG; >clamp_s_c_s_d_s_c_s_a_s_e_s:compl:rev
CACCAGCACAGACACCCACACCGTCACCCACACTATCATGTTGACATTCC; dG = -36.2
1108 >s_b_s_a_s_b_s_c_s_d_s_c_s_a_s_d_s
GTGGCTGTGTCTGTGGCTGTGTGGGTGACGGTGTGGGTGTCTGTGACGGTG; >clamp_s_c_s_d_s_c_s_a_s_e_s:compl:rev
CACCAGCACAGACACCCACACCGTCACCCACACTATCATGTTGACATTCC; dG = -36.2
1109 >s_b_s_a_s_b_s_c_s_d_s_e_s_a_s_e_s
GTGGCTGTGTCTGTGGCTGTGTGGGTGACGGTGCTGGTGTCTGTGCTGGTG; >clamp_s_c_s_d_s_c_s_a_s_e_s:compl:rev
CACCAGCACAGACACCCACACCGTCACCCACACTATCATGTTGACATTCC; dG = -36.2
1110 >s_b_s_a_s_b_s_e_s_d_s_c_s_a_s_e_s
GTGGCTGTGTCTGTGGCTGTGCTGGTGACGGTGTGGGTGTCTGTGCTGGTG; >clamp_s_c_s_d_s_c_s_a_s_e_s:compl:rev
CACCAGCACAGACACCCACACCGTCACCCACACTATCATGTTGACATTCC; dG = -36.2
1111 >s_b_s_a_s_c_s_c_s_d_s_c_s_a_s_d_s
GTGGCTGTGTCTGTGTGGGTGTGGGTGACGGTGTGGGTGTCTGTGACGGTG; >clamp_s_c_s_d_s_c_s_a_s_e_s:compl:rev
CACCAGCACAGACACCCACACCGTCACCCACACTATCATGTTGACATTCC; dG = -36.2

1112 >s_b_s_a_s_c_s_c_s_d_s_e_s_a_s_e_s
GTGGCTGTGTCTGTGTGGGTGTGGGTGACGGTGCTGGTGTCTGTGCTGGTG; >clamp_s_c_s_d_s_c_s_a_s_e_s:compl:rev
CACCAGCACAGACACCCACACCGTCACCCACACTATCATGTTGACATTCC; dG = -36.2
1113 >s_b_s_a_s_d_s_c_s_d_s_c_s_a_s_d_s
GTGGCTGTGTCTGTGACGGTGTGGGTGACGGTGTGGGTGTCTGTGACGGTG; >clamp_s_c_s_d_s_c_s_a_s_e_s:compl:rev
CACCAGCACAGACACCCACACCGTCACCCACACTATCATGTTGACATTCC; dG = -36.2
1114 >s_b_s_a_s_d_s_c_s_d_s_e_s_a_s_e_s
GTGGCTGTGTCTGTGACGGTGTGGGTGACGGTGCTGGTGTCTGTGCTGGTG; >clamp_s_c_s_d_s_c_s_a_s_e_s:compl:rev
CACCAGCACAGACACCCACACCGTCACCCACACTATCATGTTGACATTCC; dG = -36.2
1115 >s_b_s_a_s_d_s_e_s_d_s_c_s_a_s_e_s
GTGGCTGTGTCTGTGACGGTGCTGGTGACGGTGTGGGTGTCTGTGCTGGTG; >clamp_s_c_s_d_s_c_s_a_s_e_s:compl:rev
CACCAGCACAGACACCCACACCGTCACCCACACTATCATGTTGACATTCC; dG = -36.2
1116 >s_b_s_a_s_e_s_c_s_d_s_c_s_a_s_d_s
GTGGCTGTGTCTGTGCTGGTGTGGGTGACGGTGTGGGTGTCTGTGACGGTG; >clamp_s_c_s_d_s_c_s_a_s_e_s:compl:rev
CACCAGCACAGACACCCACACCGTCACCCACACTATCATGTTGACATTCC; dG = -36.2
1117 >s_b_s_a_s_e_s_c_s_d_s_e_s_a_s_e_s
GTGGCTGTGTCTGTGCTGGTGTGGGTGACGGTGCTGGTGTCTGTGCTGGTG; >clamp_s_c_s_d_s_c_s_a_s_e_s:compl:rev
CACCAGCACAGACACCCACACCGTCACCCACACTATCATGTTGACATTCC; dG = -36.2
1118 >s_b_s_a_s_e_s_e_s_d_s_c_s_a_s_e_s
GTGGCTGTGTCTGTGCTGGTGCTGGTGACGGTGTGGGTGTCTGTGCTGGTG; >clamp_s_c_s_d_s_c_s_a_s_e_s:compl:rev
CACCAGCACAGACACCCACACCGTCACCCACACTATCATGTTGACATTCC; dG = -36.2
1119 >s_b_s_b_s_a_s_c_s_d_s_c_s_a_s_d_s
GTGGCTGTGGCTGTGTCTGTGTGGGTGACGGTGTGGGTGTCTGTGACGGTG; >clamp_s_c_s_d_s_c_s_a_s_e_s:compl:rev
CACCAGCACAGACACCCACACCGTCACCCACACTATCATGTTGACATTCC; dG = -36.2
1120 >s_b_s_b_s_a_s_c_s_d_s_e_s_a_s_e_s
GTGGCTGTGGCTGTGTCTGTGTGGGTGACGGTGCTGGTGTCTGTGCTGGTG; >clamp_s_c_s_d_s_c_s_a_s_e_s:compl:rev
CACCAGCACAGACACCCACACCGTCACCCACACTATCATGTTGACATTCC; dG = -36.2
1121 >s_a_s_a_s_d_s_c_s_d_s_c_s_a_s_d_s
GTGTCTGTGTCTGTGACGGTGTGGGTGACGGTGTGGGTGTCTGTGACGGTG; >clamp_s_c_s_d_s_c_s_a_s_e_s:compl:rev
CACCAGCACAGACACCCACACCGTCACCCACACTATCATGTTGACATTCC; dG = -36.2
1122 >s_b_s_b_s_a_s_e_s_d_s_c_s_a_s_e_s
GTGGCTGTGGCTGTGTCTGTGCTGGTGACGGTGTGGGTGTCTGTGCTGGTG; >clamp_s_c_s_d_s_c_s_a_s_e_s:compl:rev
CACCAGCACAGACACCCACACCGTCACCCACACTATCATGTTGACATTCC; dG = -36.2
1123 >s_b_s_b_s_b_s_c_s_d_s_c_s_a_s_d_s
GTGGCTGTGGCTGTGGCTGTGTGGGTGACGGTGTGGGTGTCTGTGACGGTG; >clamp_s_c_s_d_s_c_s_a_s_e_s:compl:rev
CACCAGCACAGACACCCACACCGTCACCCACACTATCATGTTGACATTCC; dG = -36.2
1124 >s_b_s_b_s_b_s_c_s_d_s_e_s_a_s_e_s
GTGGCTGTGGCTGTGGCTGTGTGGGTGACGGTGCTGGTGTCTGTGCTGGTG; >clamp_s_c_s_d_s_c_s_a_s_e_s:compl:rev
CACCAGCACAGACACCCACACCGTCACCCACACTATCATGTTGACATTCC; dG = -36.2
1125 >s_b_s_b_s_b_s_e_s_d_s_c_s_a_s_e_s
GTGGCTGTGGCTGTGGCTGTGCTGGTGACGGTGTGGGTGTCTGTGCTGGTG; >clamp_s_c_s_d_s_c_s_a_s_e_s:compl:rev
CACCAGCACAGACACCCACACCGTCACCCACACTATCATGTTGACATTCC; dG = -36.2
1126 >s_b_s_b_s_c_s_c_s_d_s_c_s_a_s_d_s
GTGGCTGTGGCTGTGTGGGTGTGGGTGACGGTGTGGGTGTCTGTGACGGTG; >clamp_s_c_s_d_s_c_s_a_s_e_s:compl:rev
CACCAGCACAGACACCCACACCGTCACCCACACTATCATGTTGACATTCC; dG = -36.2
1127 >s_b_s_b_s_c_s_c_s_d_s_e_s_a_s_e_s
GTGGCTGTGGCTGTGTGGGTGTGGGTGACGGTGCTGGTGTCTGTGCTGGTG; >clamp_s_c_s_d_s_c_s_a_s_e_s:compl:rev
CACCAGCACAGACACCCACACCGTCACCCACACTATCATGTTGACATTCC; dG = -36.2
1128 >s_b_s_b_s_d_s_c_s_d_s_c_s_a_s_d_s
GTGGCTGTGGCTGTGACGGTGTGGGTGACGGTGTGGGTGTCTGTGACGGTG; >clamp_s_c_s_d_s_c_s_a_s_e_s:compl:rev
CACCAGCACAGACACCCACACCGTCACCCACACTATCATGTTGACATTCC; dG = -36.2
1129 >s_b_s_b_s_d_s_c_s_d_s_e_s_a_s_e_s
GTGGCTGTGGCTGTGACGGTGTGGGTGACGGTGCTGGTGTCTGTGCTGGTG; >clamp_s_c_s_d_s_c_s_a_s_e_s:compl:rev
CACCAGCACAGACACCCACACCGTCACCCACACTATCATGTTGACATTCC; dG = -36.2
1130 >s_b_s_b_s_d_s_e_s_d_s_c_s_a_s_e_s
GTGGCTGTGGCTGTGACGGTGCTGGTGACGGTGTGGGTGTCTGTGCTGGTG; >clamp_s_c_s_d_s_c_s_a_s_e_s:compl:rev
CACCAGCACAGACACCCACACCGTCACCCACACTATCATGTTGACATTCC; dG = -36.2
1131 >s_b_s_b_s_e_s_c_s_d_s_c_s_a_s_d_s
GTGGCTGTGGCTGTGCTGGTGTGGGTGACGGTGTGGGTGTCTGTGACGGTG; >clamp_s_c_s_d_s_c_s_a_s_e_s:compl:rev
CACCAGCACAGACACCCACACCGTCACCCACACTATCATGTTGACATTCC; dG = -36.2
1132 >s_b_s_b_s_e_s_c_s_d_s_e_s_a_s_e_s
GTGGCTGTGGCTGTGCTGGTGTGGGTGACGGTGCTGGTGTCTGTGCTGGTG; >clamp_s_c_s_d_s_c_s_a_s_e_s:compl:rev
CACCAGCACAGACACCCACACCGTCACCCACACTATCATGTTGACATTCC; dG = -36.2
1133 >s_a_s_a_s_d_s_c_s_d_s_e_s_a_s_e_s
GTGTCTGTGTCTGTGACGGTGTGGGTGACGGTGCTGGTGTCTGTGCTGGTG; >clamp_s_c_s_d_s_c_s_a_s_e_s:compl:rev
CACCAGCACAGACACCCACACCGTCACCCACACTATCATGTTGACATTCC; dG = -36.2
1134 >s_b_s_b_s_e_s_e_s_d_s_c_s_a_s_e_s
GTGGCTGTGGCTGTGCTGGTGCTGGTGACGGTGTGGGTGTCTGTGCTGGTG; >clamp_s_c_s_d_s_c_s_a_s_e_s:compl:rev
CACCAGCACAGACACCCACACCGTCACCCACACTATCATGTTGACATTCC; dG = -36.2
1135 >s_b_s_c_s_a_s_c_s_d_s_c_s_a_s_d_s
GTGGCTGTGTGGGTGTCTGTGTGGGTGACGGTGTGGGTGTCTGTGACGGTG; >clamp_s_c_s_d_s_c_s_a_s_e_s:compl:rev
CACCAGCACAGACACCCACACCGTCACCCACACTATCATGTTGACATTCC; dG = -36.2

1136 >s_b_s_c_s_a_s_c_s_d_s_e_s_a_s_e_s
GTGGCTGTGTGGGTGTCTGTGTGGGTGACGGTGCTGGTGTCTGTGCTGGTG; >clamp_s_c_s_d_s_c_s_a_s_e_s:compl:rev
CACCAGCACAGACACCCACACCGTCACCCACACTATCATGTTGACATTCC; dG = -36.2
1137 >s_b_s_c_s_a_s_e_s_d_s_c_s_a_s_e_s
GTGGCTGTGTGGGTGTCTGTGCTGGTGACGGTGTGGGTGTCTGTGCTGGTG; >clamp_s_c_s_d_s_c_s_a_s_e_s:compl:rev
CACCAGCACAGACACCCACACCGTCACCCACACTATCATGTTGACATTCC; dG = -36.2
1138 >s_b_s_c_s_b_s_c_s_d_s_c_s_a_s_d_s
GTGGCTGTGTGGGTGGCTGTGTGGGTGACGGTGTGGGTGTCTGTGACGGTG; >clamp_s_c_s_d_s_c_s_a_s_e_s:compl:rev
CACCAGCACAGACACCCACACCGTCACCCACACTATCATGTTGACATTCC; dG = -36.2
1139 >s_b_s_c_s_b_s_c_s_d_s_e_s_a_s_e_s
GTGGCTGTGTGGGTGGCTGTGTGGGTGACGGTGCTGGTGTCTGTGCTGGTG; >clamp_s_c_s_d_s_c_s_a_s_e_s:compl:rev
CACCAGCACAGACACCCACACCGTCACCCACACTATCATGTTGACATTCC; dG = -36.2
1140 >s_b_s_c_s_b_s_e_s_d_s_c_s_a_s_e_s
GTGGCTGTGTGGGTGGCTGTGCTGGTGACGGTGTGGGTGTCTGTGCTGGTG; >clamp_s_c_s_d_s_c_s_a_s_e_s:compl:rev
CACCAGCACAGACACCCACACCGTCACCCACACTATCATGTTGACATTCC; dG = -36.2
1141 >s_b_s_c_s_c_s_c_s_d_s_c_s_a_s_d_s
GTGGCTGTGTGGGTGTGGGTGTGGGTGACGGTGTGGGTGTCTGTGACGGTG; >clamp_s_c_s_d_s_c_s_a_s_e_s:compl:rev
CACCAGCACAGACACCCACACCGTCACCCACACTATCATGTTGACATTCC; dG = -36.2
1142 >s_b_s_c_s_c_s_c_s_d_s_e_s_a_s_e_s
GTGGCTGTGTGGGTGTGGGTGTGGGTGACGGTGCTGGTGTCTGTGCTGGTG; >clamp_s_c_s_d_s_c_s_a_s_e_s:compl:rev
CACCAGCACAGACACCCACACCGTCACCCACACTATCATGTTGACATTCC; dG = -36.2
1143 >s_b_s_c_s_d_s_c_s_d_s_c_s_a_s_d_s
GTGGCTGTGTGGGTGACGGTGTGGGTGACGGTGTGGGTGTCTGTGACGGTG; >clamp_s_c_s_d_s_c_s_a_s_e_s:compl:rev
CACCAGCACAGACACCCACACCGTCACCCACACTATCATGTTGACATTCC; dG = -36.2
1144 >s_b_s_c_s_d_s_c_s_d_s_e_s_a_s_e_s
GTGGCTGTGTGGGTGACGGTGTGGGTGACGGTGCTGGTGTCTGTGCTGGTG; >clamp_s_c_s_d_s_c_s_a_s_e_s:compl:rev
CACCAGCACAGACACCCACACCGTCACCCACACTATCATGTTGACATTCC; dG = -36.2
1145 >s_a_s_a_s_d_s_e_s_d_s_c_s_a_s_e_s
GTGTCTGTGTCTGTGACGGTGCTGGTGACGGTGTGGGTGTCTGTGCTGGTG; >clamp_s_c_s_d_s_c_s_a_s_e_s:compl:rev
CACCAGCACAGACACCCACACCGTCACCCACACTATCATGTTGACATTCC; dG = -36.2
1146 >s_b_s_c_s_d_s_e_s_d_s_c_s_a_s_e_s
GTGGCTGTGTGGGTGACGGTGCTGGTGACGGTGTGGGTGTCTGTGCTGGTG; >clamp_s_c_s_d_s_c_s_a_s_e_s:compl:rev
CACCAGCACAGACACCCACACCGTCACCCACACTATCATGTTGACATTCC; dG = -36.2
1147 >s_b_s_c_s_e_s_c_s_d_s_c_s_a_s_d_s
GTGGCTGTGTGGGTGCTGGTGTGGGTGACGGTGTGGGTGTCTGTGACGGTG; >clamp_s_c_s_d_s_c_s_a_s_e_s:compl:rev
CACCAGCACAGACACCCACACCGTCACCCACACTATCATGTTGACATTCC; dG = -36.2
1148 >s_b_s_c_s_e_s_c_s_d_s_e_s_a_s_e_s
GTGGCTGTGTGGGTGCTGGTGTGGGTGACGGTGCTGGTGTCTGTGCTGGTG; >clamp_s_c_s_d_s_c_s_a_s_e_s:compl:rev
CACCAGCACAGACACCCACACCGTCACCCACACTATCATGTTGACATTCC; dG = -36.2
1149 >s_b_s_c_s_e_s_e_s_d_s_c_s_a_s_e_s
GTGGCTGTGTGGGTGCTGGTGCTGGTGACGGTGTGGGTGTCTGTGCTGGTG; >clamp_s_c_s_d_s_c_s_a_s_e_s:compl:rev
CACCAGCACAGACACCCACACCGTCACCCACACTATCATGTTGACATTCC; dG = -36.2
1150 >s_b_s_d_s_a_s_c_s_d_s_c_s_a_s_d_s
GTGGCTGTGACGGTGTCTGTGTGGGTGACGGTGTGGGTGTCTGTGACGGTG; >clamp_s_c_s_d_s_c_s_a_s_e_s:compl:rev
CACCAGCACAGACACCCACACCGTCACCCACACTATCATGTTGACATTCC; dG = -36.2
1151 >s_b_s_d_s_a_s_c_s_d_s_e_s_a_s_e_s
GTGGCTGTGACGGTGTCTGTGTGGGTGACGGTGCTGGTGTCTGTGCTGGTG; >clamp_s_c_s_d_s_c_s_a_s_e_s:compl:rev
CACCAGCACAGACACCCACACCGTCACCCACACTATCATGTTGACATTCC; dG = -36.2
1152 >s_a_s_a_s_e_s_c_s_d_s_c_s_a_s_d_s
GTGTCTGTGTCTGTGCTGGTGTGGGTGACGGTGTGGGTGTCTGTGACGGTG; >clamp_s_c_s_d_s_c_s_a_s_e_s:compl:rev
CACCAGCACAGACACCCACACCGTCACCCACACTATCATGTTGACATTCC; dG = -36.2
1153 >s_b_s_d_s_a_s_e_s_d_s_c_s_a_s_e_s
GTGGCTGTGACGGTGTCTGTGCTGGTGACGGTGTGGGTGTCTGTGCTGGTG; >clamp_s_c_s_d_s_c_s_a_s_e_s:compl:rev
CACCAGCACAGACACCCACACCGTCACCCACACTATCATGTTGACATTCC; dG = -36.2
1154 >s_b_s_d_s_b_s_c_s_d_s_c_s_a_s_d_s
GTGGCTGTGACGGTGGCTGTGTGGGTGACGGTGTGGGTGTCTGTGACGGTG; >clamp_s_c_s_d_s_c_s_a_s_e_s:compl:rev
CACCAGCACAGACACCCACACCGTCACCCACACTATCATGTTGACATTCC; dG = -36.2
1155 >s_b_s_d_s_b_s_c_s_d_s_e_s_a_s_e_s
GTGGCTGTGACGGTGGCTGTGTGGGTGACGGTGCTGGTGTCTGTGCTGGTG; >clamp_s_c_s_d_s_c_s_a_s_e_s:compl:rev
CACCAGCACAGACACCCACACCGTCACCCACACTATCATGTTGACATTCC; dG = -36.2
1156 >s_b_s_d_s_b_s_e_s_d_s_c_s_a_s_e_s
GTGGCTGTGACGGTGGCTGTGCTGGTGACGGTGTGGGTGTCTGTGCTGGTG; >clamp_s_c_s_d_s_c_s_a_s_e_s:compl:rev
CACCAGCACAGACACCCACACCGTCACCCACACTATCATGTTGACATTCC; dG = -36.2
1157 >s_b_s_d_s_c_s_c_s_d_s_c_s_a_s_d_s
GTGGCTGTGACGGTGTGGGTGTGGGTGACGGTGTGGGTGTCTGTGACGGTG; >clamp_s_c_s_d_s_c_s_a_s_e_s:compl:rev
CACCAGCACAGACACCCACACCGTCACCCACACTATCATGTTGACATTCC; dG = -36.2
1158 >s_b_s_d_s_c_s_c_s_d_s_e_s_a_s_e_s
GTGGCTGTGACGGTGTGGGTGTGGGTGACGGTGCTGGTGTCTGTGCTGGTG; >clamp_s_c_s_d_s_c_s_a_s_e_s:compl:rev
CACCAGCACAGACACCCACACCGTCACCCACACTATCATGTTGACATTCC; dG = -36.2
1159 >s_b_s_d_s_d_s_c_s_d_s_c_s_a_s_d_s
GTGGCTGTGACGGTGACGGTGTGGGTGACGGTGTGGGTGTCTGTGACGGTG; >clamp_s_c_s_d_s_c_s_a_s_e_s:compl:rev
CACCAGCACAGACACCCACACCGTCACCCACACTATCATGTTGACATTCC; dG = -36.2

1160 >s_b_s_d_s_d_s_c_s_d_s_e_s_a_s_e_s
GTGGCTGTGACGGTGACGGTGTGGGTGACGGTGCTGGTGTCTGTGCTGGTG; >clamp_s_c_s_d_s_c_s_a_s_e_s:compl:rev
CACCAGCACAGACACCCACACCGTCACCCACACTATCATGTTGACATTCC; dG = -36.2
1161 >s_b_s_d_s_d_s_e_s_d_s_c_s_a_s_e_s
GTGGCTGTGACGGTGACGGTGCTGGTGACGGTGTGGGTGTCTGTGCTGGTG; >clamp_s_c_s_d_s_c_s_a_s_e_s:compl:rev
CACCAGCACAGACACCCACACCGTCACCCACACTATCATGTTGACATTCC; dG = -36.2
1162 >s_b_s_d_s_e_s_c_s_d_s_c_s_a_s_d_s
GTGGCTGTGACGGTGCTGGTGTGGGTGACGGTGTGGGTGTCTGTGACGGTG; >clamp_s_c_s_d_s_c_s_a_s_e_s:compl:rev
CACCAGCACAGACACCCACACCGTCACCCACACTATCATGTTGACATTCC; dG = -36.2
1163 >s_b_s_d_s_e_s_c_s_d_s_e_s_a_s_e_s
GTGGCTGTGACGGTGCTGGTGTGGGTGACGGTGCTGGTGTCTGTGCTGGTG; >clamp_s_c_s_d_s_c_s_a_s_e_s:compl:rev
CACCAGCACAGACACCCACACCGTCACCCACACTATCATGTTGACATTCC; dG = -36.2
1164 >s_a_s_a_s_e_s_c_s_d_s_e_s_a_s_e_s
GTGTCTGTGTCTGTGCTGGTGTGGGTGACGGTGCTGGTGTCTGTGCTGGTG; >clamp_s_c_s_d_s_c_s_a_s_e_s:compl:rev
CACCAGCACAGACACCCACACCGTCACCCACACTATCATGTTGACATTCC; dG = -36.2
1165 >s_b_s_d_s_e_s_e_s_d_s_c_s_a_s_e_s
GTGGCTGTGACGGTGCTGGTGCTGGTGACGGTGTGGGTGTCTGTGCTGGTG; >clamp_s_c_s_d_s_c_s_a_s_e_s:compl:rev
CACCAGCACAGACACCCACACCGTCACCCACACTATCATGTTGACATTCC; dG = -36.2
1166 >s_a_s_a_s_a_s_c_s_d_s_e_s_a_s_e_s
GTGTCTGTGTCTGTGTCTGTGTGGGTGACGGTGCTGGTGTCTGTGCTGGTG; >clamp_s_c_s_d_s_c_s_a_s_e_s:compl:rev
CACCAGCACAGACACCCACACCGTCACCCACACTATCATGTTGACATTCC; dG = -36.2
1167 >s_b_s_e_s_a_s_c_s_d_s_c_s_a_s_d_s
GTGGCTGTGCTGGTGTCTGTGTGGGTGACGGTGTGGGTGTCTGTGACGGTG; >clamp_s_c_s_d_s_c_s_a_s_e_s:compl:rev
CACCAGCACAGACACCCACACCGTCACCCACACTATCATGTTGACATTCC; dG = -36.2
1168 >s_b_s_e_s_a_s_c_s_d_s_e_s_a_s_e_s
GTGGCTGTGCTGGTGTCTGTGTGGGTGACGGTGCTGGTGTCTGTGCTGGTG; >clamp_s_c_s_d_s_c_s_a_s_e_s:compl:rev
CACCAGCACAGACACCCACACCGTCACCCACACTATCATGTTGACATTCC; dG = -36.2
1169 >s_b_s_e_s_a_s_e_s_d_s_c_s_a_s_e_s
GTGGCTGTGCTGGTGTCTGTGCTGGTGACGGTGTGGGTGTCTGTGCTGGTG; >clamp_s_c_s_d_s_c_s_a_s_e_s:compl:rev
CACCAGCACAGACACCCACACCGTCACCCACACTATCATGTTGACATTCC; dG = -36.2
1170 >s_b_s_e_s_b_s_c_s_d_s_c_s_a_s_d_s
GTGGCTGTGCTGGTGGCTGTGTGGGTGACGGTGTGGGTGTCTGTGACGGTG; >clamp_s_c_s_d_s_c_s_a_s_e_s:compl:rev
CACCAGCACAGACACCCACACCGTCACCCACACTATCATGTTGACATTCC; dG = -36.2
1171 >s_b_s_e_s_b_s_c_s_d_s_e_s_a_s_e_s
GTGGCTGTGCTGGTGGCTGTGTGGGTGACGGTGCTGGTGTCTGTGCTGGTG; >clamp_s_c_s_d_s_c_s_a_s_e_s:compl:rev
CACCAGCACAGACACCCACACCGTCACCCACACTATCATGTTGACATTCC; dG = -36.2
1172 >s_b_s_e_s_b_s_e_s_d_s_c_s_a_s_e_s
GTGGCTGTGCTGGTGGCTGTGCTGGTGACGGTGTGGGTGTCTGTGCTGGTG; >clamp_s_c_s_d_s_c_s_a_s_e_s:compl:rev
CACCAGCACAGACACCCACACCGTCACCCACACTATCATGTTGACATTCC; dG = -36.2
1173 >s_b_s_e_s_c_s_c_s_d_s_c_s_a_s_d_s
GTGGCTGTGCTGGTGTGGGTGTGGGTGACGGTGTGGGTGTCTGTGACGGTG; >clamp_s_c_s_d_s_c_s_a_s_e_s:compl:rev
CACCAGCACAGACACCCACACCGTCACCCACACTATCATGTTGACATTCC; dG = -36.2
1174 >s_b_s_e_s_c_s_c_s_d_s_e_s_a_s_e_s
GTGGCTGTGCTGGTGTGGGTGTGGGTGACGGTGCTGGTGTCTGTGCTGGTG; >clamp_s_c_s_d_s_c_s_a_s_e_s:compl:rev
CACCAGCACAGACACCCACACCGTCACCCACACTATCATGTTGACATTCC; dG = -36.2
1175 >s_b_s_e_s_d_s_c_s_d_s_c_s_a_s_d_s
GTGGCTGTGCTGGTGACGGTGTGGGTGACGGTGTGGGTGTCTGTGACGGTG; >clamp_s_c_s_d_s_c_s_a_s_e_s:compl:rev
CACCAGCACAGACACCCACACCGTCACCCACACTATCATGTTGACATTCC; dG = -36.2
1176 >s_b_s_e_s_d_s_c_s_d_s_e_s_a_s_e_s
GTGGCTGTGCTGGTGACGGTGTGGGTGACGGTGCTGGTGTCTGTGCTGGTG; >clamp_s_c_s_d_s_c_s_a_s_e_s:compl:rev
CACCAGCACAGACACCCACACCGTCACCCACACTATCATGTTGACATTCC; dG = -36.2
1177 >s_a_s_a_s_e_s_e_s_d_s_c_s_a_s_e_s
GTGTCTGTGTCTGTGCTGGTGCTGGTGACGGTGTGGGTGTCTGTGCTGGTG; >clamp_s_c_s_d_s_c_s_a_s_e_s:compl:rev
CACCAGCACAGACACCCACACCGTCACCCACACTATCATGTTGACATTCC; dG = -36.2
1178 >s_b_s_e_s_d_s_e_s_d_s_c_s_a_s_e_s
GTGGCTGTGCTGGTGACGGTGCTGGTGACGGTGTGGGTGTCTGTGCTGGTG; >clamp_s_c_s_d_s_c_s_a_s_e_s:compl:rev
CACCAGCACAGACACCCACACCGTCACCCACACTATCATGTTGACATTCC; dG = -36.2
1179 >s_b_s_e_s_e_s_c_s_d_s_c_s_a_s_d_s
GTGGCTGTGCTGGTGCTGGTGTGGGTGACGGTGTGGGTGTCTGTGACGGTG; >clamp_s_c_s_d_s_c_s_a_s_e_s:compl:rev
CACCAGCACAGACACCCACACCGTCACCCACACTATCATGTTGACATTCC; dG = -36.2
1180 >s_b_s_e_s_e_s_c_s_d_s_e_s_a_s_e_s
GTGGCTGTGCTGGTGCTGGTGTGGGTGACGGTGCTGGTGTCTGTGCTGGTG; >clamp_s_c_s_d_s_c_s_a_s_e_s:compl:rev
CACCAGCACAGACACCCACACCGTCACCCACACTATCATGTTGACATTCC; dG = -36.2
1181 >s_b_s_e_s_e_s_e_s_d_s_c_s_a_s_e_s
GTGGCTGTGCTGGTGCTGGTGCTGGTGACGGTGTGGGTGTCTGTGCTGGTG; >clamp_s_c_s_d_s_c_s_a_s_e_s:compl:rev
CACCAGCACAGACACCCACACCGTCACCCACACTATCATGTTGACATTCC; dG = -36.2
1182 >s_c_s_c_s_a_s_b_s_d_s_c_s_a_s_e_s
GTGTGGGTGTGGGTGTCTGTGGCTGTGACGGTGTGGGTGTCTGTGCTGGTG; >clamp_s_c_s_d_s_c_s_a_s_e_s:compl:rev
CACCAGCACAGACACCCACACCGTCACCCACACTATCATGTTGACATTCC; dG = -36.1
1183 >s_c_s_c_s_a_s_a_s_d_s_c_s_a_s_e_s
GTGTGGGTGTGGGTGTCTGTGTCTGTGACGGTGTGGGTGTCTGTGCTGGTG; >clamp_s_c_s_d_s_c_s_a_s_e_s:compl:rev
CACCAGCACAGACACCCACACCGTCACCCACACTATCATGTTGACATTCC; dG = -36.1

1184 >s_c_s_c_s_b_s_b_s_d_s_c_s_a_s_e_s
GTGTGGGTGTGGGTGGCTGTGGCTGTGACGGTGTGGGTGTCTGTGCTGGTG; >clamp_s_c_s_d_s_c_s_a_s_e_s:compl:rev
CACCAGCACAGACACCCACACCGTCACCCACACTATCATGTTGACATTCC; dG = -36.1
1185 >s_c_s_c_s_b_s_a_s_d_s_c_s_a_s_e_s
GTGTGGGTGTGGGTGGCTGTGTCTGTGACGGTGTGGGTGTCTGTGCTGGTG; >clamp_s_c_s_d_s_c_s_a_s_e_s:compl:rev
CACCAGCACAGACACCCACACCGTCACCCACACTATCATGTTGACATTCC; dG = -36.1
1186 >s_c_s_c_s_d_s_b_s_d_s_c_s_a_s_e_s
GTGTGGGTGTGGGTGACGGTGGCTGTGACGGTGTGGGTGTCTGTGCTGGTG; >clamp_s_c_s_d_s_c_s_a_s_e_s:compl:rev
CACCAGCACAGACACCCACACCGTCACCCACACTATCATGTTGACATTCC; dG = -36.1
1187 >s_c_s_c_s_d_s_a_s_d_s_c_s_a_s_e_s
GTGTGGGTGTGGGTGACGGTGTCTGTGACGGTGTGGGTGTCTGTGCTGGTG; >clamp_s_c_s_d_s_c_s_a_s_e_s:compl:rev
CACCAGCACAGACACCCACACCGTCACCCACACTATCATGTTGACATTCC; dG = -36.1
1188 >s_c_s_c_s_e_s_b_s_d_s_c_s_a_s_e_s
GTGTGGGTGTGGGTGCTGGTGGCTGTGACGGTGTGGGTGTCTGTGCTGGTG; >clamp_s_c_s_d_s_c_s_a_s_e_s:compl:rev
CACCAGCACAGACACCCACACCGTCACCCACACTATCATGTTGACATTCC; dG = -36.1
1189 >s_c_s_c_s_e_s_a_s_d_s_c_s_a_s_e_s
GTGTGGGTGTGGGTGCTGGTGTCTGTGACGGTGTGGGTGTCTGTGCTGGTG; >clamp_s_c_s_d_s_c_s_a_s_e_s:compl:rev
CACCAGCACAGACACCCACACCGTCACCCACACTATCATGTTGACATTCC; dG = -36.1
1190 >s_d_s_c_s_a_s_b_s_d_s_c_s_a_s_e_s
GTGACGGTGTGGGTGTCTGTGGCTGTGACGGTGTGGGTGTCTGTGCTGGTG; >clamp_s_c_s_d_s_c_s_a_s_e_s:compl:rev
CACCAGCACAGACACCCACACCGTCACCCACACTATCATGTTGACATTCC; dG = -36.1
1191 >s_d_s_c_s_a_s_a_s_d_s_c_s_a_s_e_s
GTGACGGTGTGGGTGTCTGTGTCTGTGACGGTGTGGGTGTCTGTGCTGGTG; >clamp_s_c_s_d_s_c_s_a_s_e_s:compl:rev
CACCAGCACAGACACCCACACCGTCACCCACACTATCATGTTGACATTCC; dG = -36.1
1192 >s_d_s_c_s_b_s_b_s_d_s_c_s_a_s_e_s
GTGACGGTGTGGGTGGCTGTGGCTGTGACGGTGTGGGTGTCTGTGCTGGTG; >clamp_s_c_s_d_s_c_s_a_s_e_s:compl:rev
CACCAGCACAGACACCCACACCGTCACCCACACTATCATGTTGACATTCC; dG = -36.1
1193 >s_d_s_c_s_b_s_a_s_d_s_c_s_a_s_e_s
GTGACGGTGTGGGTGGCTGTGTCTGTGACGGTGTGGGTGTCTGTGCTGGTG; >clamp_s_c_s_d_s_c_s_a_s_e_s:compl:rev
CACCAGCACAGACACCCACACCGTCACCCACACTATCATGTTGACATTCC; dG = -36.1
1194 >s_d_s_c_s_d_s_b_s_d_s_c_s_a_s_e_s
GTGACGGTGTGGGTGACGGTGGCTGTGACGGTGTGGGTGTCTGTGCTGGTG; >clamp_s_c_s_d_s_c_s_a_s_e_s:compl:rev
CACCAGCACAGACACCCACACCGTCACCCACACTATCATGTTGACATTCC; dG = -36.1
1195 >s_d_s_c_s_d_s_a_s_d_s_c_s_a_s_e_s
GTGACGGTGTGGGTGACGGTGTCTGTGACGGTGTGGGTGTCTGTGCTGGTG; >clamp_s_c_s_d_s_c_s_a_s_e_s:compl:rev
CACCAGCACAGACACCCACACCGTCACCCACACTATCATGTTGACATTCC; dG = -36.1
1196 >s_d_s_c_s_e_s_b_s_d_s_c_s_a_s_e_s
GTGACGGTGTGGGTGCTGGTGGCTGTGACGGTGTGGGTGTCTGTGCTGGTG; >clamp_s_c_s_d_s_c_s_a_s_e_s:compl:rev
CACCAGCACAGACACCCACACCGTCACCCACACTATCATGTTGACATTCC; dG = -36.1
1197 >s_d_s_c_s_e_s_a_s_d_s_c_s_a_s_e_s
GTGACGGTGTGGGTGCTGGTGTCTGTGACGGTGTGGGTGTCTGTGCTGGTG; >clamp_s_c_s_d_s_c_s_a_s_e_s:compl:rev
CACCAGCACAGACACCCACACCGTCACCCACACTATCATGTTGACATTCC; dG = -36.1
1198 >s_a_s_c_s_a_s_b_s_d_s_c_s_a_s_e_s
GTGTCTGTGTGGGTGTCTGTGGCTGTGACGGTGTGGGTGTCTGTGCTGGTG; >clamp_s_c_s_d_s_c_s_a_s_e_s:compl:rev
CACCAGCACAGACACCCACACCGTCACCCACACTATCATGTTGACATTCC; dG = -36.1
1199 >s_a_s_c_s_a_s_a_s_d_s_c_s_a_s_e_s
GTGTCTGTGTGGGTGTCTGTGTCTGTGACGGTGTGGGTGTCTGTGCTGGTG; >clamp_s_c_s_d_s_c_s_a_s_e_s:compl:rev
CACCAGCACAGACACCCACACCGTCACCCACACTATCATGTTGACATTCC; dG = -36.1
1200 >s_e_s_c_s_a_s_b_s_d_s_c_s_a_s_e_s
GTGCTGGTGTGGGTGTCTGTGGCTGTGACGGTGTGGGTGTCTGTGCTGGTG; >clamp_s_c_s_d_s_c_s_a_s_e_s:compl:rev
CACCAGCACAGACACCCACACCGTCACCCACACTATCATGTTGACATTCC; dG = -36.1
1201 >s_e_s_c_s_a_s_a_s_d_s_c_s_a_s_e_s
GTGCTGGTGTGGGTGTCTGTGTCTGTGACGGTGTGGGTGTCTGTGCTGGTG; >clamp_s_c_s_d_s_c_s_a_s_e_s:compl:rev
CACCAGCACAGACACCCACACCGTCACCCACACTATCATGTTGACATTCC; dG = -36.1
1202 >s_e_s_c_s_b_s_b_s_d_s_c_s_a_s_e_s
GTGCTGGTGTGGGTGGCTGTGGCTGTGACGGTGTGGGTGTCTGTGCTGGTG; >clamp_s_c_s_d_s_c_s_a_s_e_s:compl:rev
CACCAGCACAGACACCCACACCGTCACCCACACTATCATGTTGACATTCC; dG = -36.1
1203 >s_e_s_c_s_b_s_a_s_d_s_c_s_a_s_e_s
GTGCTGGTGTGGGTGGCTGTGTCTGTGACGGTGTGGGTGTCTGTGCTGGTG; >clamp_s_c_s_d_s_c_s_a_s_e_s:compl:rev
CACCAGCACAGACACCCACACCGTCACCCACACTATCATGTTGACATTCC; dG = -36.1
1204 >s_e_s_c_s_d_s_b_s_d_s_c_s_a_s_e_s
GTGCTGGTGTGGGTGACGGTGGCTGTGACGGTGTGGGTGTCTGTGCTGGTG; >clamp_s_c_s_d_s_c_s_a_s_e_s:compl:rev
CACCAGCACAGACACCCACACCGTCACCCACACTATCATGTTGACATTCC; dG = -36.1
1205 >s_e_s_c_s_d_s_a_s_d_s_c_s_a_s_e_s
GTGCTGGTGTGGGTGACGGTGTCTGTGACGGTGTGGGTGTCTGTGCTGGTG; >clamp_s_c_s_d_s_c_s_a_s_e_s:compl:rev
CACCAGCACAGACACCCACACCGTCACCCACACTATCATGTTGACATTCC; dG = -36.1
1206 >s_e_s_c_s_e_s_b_s_d_s_c_s_a_s_e_s
GTGCTGGTGTGGGTGCTGGTGGCTGTGACGGTGTGGGTGTCTGTGCTGGTG; >clamp_s_c_s_d_s_c_s_a_s_e_s:compl:rev
CACCAGCACAGACACCCACACCGTCACCCACACTATCATGTTGACATTCC; dG = -36.1
1207 >s_e_s_c_s_e_s_a_s_d_s_c_s_a_s_e_s
GTGCTGGTGTGGGTGCTGGTGTCTGTGACGGTGTGGGTGTCTGTGCTGGTG; >clamp_s_c_s_d_s_c_s_a_s_e_s:compl:rev
CACCAGCACAGACACCCACACCGTCACCCACACTATCATGTTGACATTCC; dG = -36.1

1208 >s_a_s_c_s_b_s_b_s_d_s_c_s_a_s_e_s
GTGTCTGTGTGGGTGGCTGTGGCTGTGACGGTGTGGGTGTCTGTGCTGGTG; >clamp_s_c_s_d_s_c_s_a_s_e_s:compl:rev
CACCAGCACAGACACCCACACCGTCACCCACACTATCATGTTGACATTCC; dG = -36.1
1209 >s_a_s_c_s_b_s_a_s_d_s_c_s_a_s_e_s
GTGTCTGTGTGGGTGGCTGTGTCTGTGACGGTGTGGGTGTCTGTGCTGGTG; >clamp_s_c_s_d_s_c_s_a_s_e_s:compl:rev
CACCAGCACAGACACCCACACCGTCACCCACACTATCATGTTGACATTCC; dG = -36.1
1210 >s_a_s_c_s_d_s_b_s_d_s_c_s_a_s_e_s
GTGTCTGTGTGGGTGACGGTGGCTGTGACGGTGTGGGTGTCTGTGCTGGTG; >clamp_s_c_s_d_s_c_s_a_s_e_s:compl:rev
CACCAGCACAGACACCCACACCGTCACCCACACTATCATGTTGACATTCC; dG = -36.1
1211 >s_a_s_c_s_d_s_a_s_d_s_c_s_a_s_e_s
GTGTCTGTGTGGGTGACGGTGTCTGTGACGGTGTGGGTGTCTGTGCTGGTG; >clamp_s_c_s_d_s_c_s_a_s_e_s:compl:rev
CACCAGCACAGACACCCACACCGTCACCCACACTATCATGTTGACATTCC; dG = -36.1
1212 >s_a_s_c_s_e_s_b_s_d_s_c_s_a_s_e_s
GTGTCTGTGTGGGTGCTGGTGGCTGTGACGGTGTGGGTGTCTGTGCTGGTG; >clamp_s_c_s_d_s_c_s_a_s_e_s:compl:rev
CACCAGCACAGACACCCACACCGTCACCCACACTATCATGTTGACATTCC; dG = -36.1
1213 >s_a_s_c_s_e_s_a_s_d_s_c_s_a_s_e_s
GTGTCTGTGTGGGTGCTGGTGTCTGTGACGGTGTGGGTGTCTGTGCTGGTG; >clamp_s_c_s_d_s_c_s_a_s_e_s:compl:rev
CACCAGCACAGACACCCACACCGTCACCCACACTATCATGTTGACATTCC; dG = -36.1
1214 >s_b_s_c_s_a_s_b_s_d_s_c_s_a_s_e_s
GTGGCTGTGTGGGTGTCTGTGGCTGTGACGGTGTGGGTGTCTGTGCTGGTG; >clamp_s_c_s_d_s_c_s_a_s_e_s:compl:rev
CACCAGCACAGACACCCACACCGTCACCCACACTATCATGTTGACATTCC; dG = -36.1
1215 >s_b_s_c_s_a_s_a_s_d_s_c_s_a_s_e_s
GTGGCTGTGTGGGTGTCTGTGTCTGTGACGGTGTGGGTGTCTGTGCTGGTG; >clamp_s_c_s_d_s_c_s_a_s_e_s:compl:rev
CACCAGCACAGACACCCACACCGTCACCCACACTATCATGTTGACATTCC; dG = -36.1
1216 >s_b_s_c_s_b_s_b_s_d_s_c_s_a_s_e_s
GTGGCTGTGTGGGTGGCTGTGGCTGTGACGGTGTGGGTGTCTGTGCTGGTG; >clamp_s_c_s_d_s_c_s_a_s_e_s:compl:rev
CACCAGCACAGACACCCACACCGTCACCCACACTATCATGTTGACATTCC; dG = -36.1
1217 >s_b_s_c_s_b_s_a_s_d_s_c_s_a_s_e_s
GTGGCTGTGTGGGTGGCTGTGTCTGTGACGGTGTGGGTGTCTGTGCTGGTG; >clamp_s_c_s_d_s_c_s_a_s_e_s:compl:rev
CACCAGCACAGACACCCACACCGTCACCCACACTATCATGTTGACATTCC; dG = -36.1
1218 >s_b_s_c_s_d_s_b_s_d_s_c_s_a_s_e_s
GTGGCTGTGTGGGTGACGGTGGCTGTGACGGTGTGGGTGTCTGTGCTGGTG; >clamp_s_c_s_d_s_c_s_a_s_e_s:compl:rev
CACCAGCACAGACACCCACACCGTCACCCACACTATCATGTTGACATTCC; dG = -36.1
1219 >s_b_s_c_s_d_s_a_s_d_s_c_s_a_s_e_s
GTGGCTGTGTGGGTGACGGTGTCTGTGACGGTGTGGGTGTCTGTGCTGGTG; >clamp_s_c_s_d_s_c_s_a_s_e_s:compl:rev
CACCAGCACAGACACCCACACCGTCACCCACACTATCATGTTGACATTCC; dG = -36.1
1220 >s_b_s_c_s_e_s_b_s_d_s_c_s_a_s_e_s
GTGGCTGTGTGGGTGCTGGTGGCTGTGACGGTGTGGGTGTCTGTGCTGGTG; >clamp_s_c_s_d_s_c_s_a_s_e_s:compl:rev
CACCAGCACAGACACCCACACCGTCACCCACACTATCATGTTGACATTCC; dG = -36.1
1221 >s_b_s_c_s_e_s_a_s_d_s_c_s_a_s_e_s
GTGGCTGTGTGGGTGCTGGTGTCTGTGACGGTGTGGGTGTCTGTGCTGGTG; >clamp_s_c_s_d_s_c_s_a_s_e_s:compl:rev
CACCAGCACAGACACCCACACCGTCACCCACACTATCATGTTGACATTCC; dG = -36.1
1222 >s_a_s_c_s_d_s_c_s_d_s_b_s_a_s_e_s
GTGTCTGTGTGGGTGACGGTGTGGGTGACGGTGGCTGTGTCTGTGCTGGTG; >clamp_s_c_s_d_s_c_s_a_s_e_s:compl:rev
CACCAGCACAGACACCCACACCGTCACCCACACTATCATGTTGACATTCC; dG = -36.0
1223 >s_a_s_c_s_d_s_c_s_d_s_a_s_a_s_e_s
GTGTCTGTGTGGGTGACGGTGTGGGTGACGGTGTCTGTGTCTGTGCTGGTG; >clamp_s_c_s_d_s_c_s_a_s_e_s:compl:rev
CACCAGCACAGACACCCACACCGTCACCCACACTATCATGTTGACATTCC; dG = -36.0
1224 >s_a_s_c_s_d_s_c_s_e_s_c_s_a_s_e_s
GTGTCTGTGTGGGTGACGGTGTGGGTGCTGGTGTGGGTGTCTGTGCTGGTG; >clamp_s_c_s_d_s_c_s_a_s_e_s:compl:rev
CACCAGCACAGACACCCACACCGTCACCCACACTATCATGTTGACATTCC; dG = -36.0
1225 >s_a_s_c_s_d_s_c_s_b_s_c_s_a_s_e_s
GTGTCTGTGTGGGTGACGGTGTGGGTGGCTGTGTGGGTGTCTGTGCTGGTG; >clamp_s_c_s_d_s_c_s_a_s_e_s:compl:rev
CACCAGCACAGACACCCACACCGTCACCCACACTATCATGTTGACATTCC; dG = -36.0
1226 >s_b_s_c_s_d_s_c_s_d_s_b_s_a_s_e_s
GTGGCTGTGTGGGTGACGGTGTGGGTGACGGTGGCTGTGTCTGTGCTGGTG; >clamp_s_c_s_d_s_c_s_a_s_e_s:compl:rev
CACCAGCACAGACACCCACACCGTCACCCACACTATCATGTTGACATTCC; dG = -36.0
1227 >s_b_s_c_s_d_s_c_s_d_s_a_s_a_s_e_s
GTGGCTGTGTGGGTGACGGTGTGGGTGACGGTGTCTGTGTCTGTGCTGGTG; >clamp_s_c_s_d_s_c_s_a_s_e_s:compl:rev
CACCAGCACAGACACCCACACCGTCACCCACACTATCATGTTGACATTCC; dG = -36.0
1228 >s_b_s_c_s_d_s_c_s_e_s_c_s_a_s_e_s
GTGGCTGTGTGGGTGACGGTGTGGGTGCTGGTGTGGGTGTCTGTGCTGGTG; >clamp_s_c_s_d_s_c_s_a_s_e_s:compl:rev
CACCAGCACAGACACCCACACCGTCACCCACACTATCATGTTGACATTCC; dG = -36.0
1229 >s_b_s_c_s_d_s_c_s_b_s_c_s_a_s_e_s
GTGGCTGTGTGGGTGACGGTGTGGGTGGCTGTGTGGGTGTCTGTGCTGGTG; >clamp_s_c_s_d_s_c_s_a_s_e_s:compl:rev
CACCAGCACAGACACCCACACCGTCACCCACACTATCATGTTGACATTCC; dG = -36.0
1230 >s_c_s_c_s_d_s_c_s_d_s_b_s_a_s_e_s
GTGTGGGTGTGGGTGACGGTGTGGGTGACGGTGGCTGTGTCTGTGCTGGTG; >clamp_s_c_s_d_s_c_s_a_s_e_s:compl:rev
CACCAGCACAGACACCCACACCGTCACCCACACTATCATGTTGACATTCC; dG = -35.9
1231 >s_c_s_c_s_d_s_c_s_d_s_a_s_a_s_e_s
GTGTGGGTGTGGGTGACGGTGTGGGTGACGGTGTCTGTGTCTGTGCTGGTG; >clamp_s_c_s_d_s_c_s_a_s_e_s:compl:rev
CACCAGCACAGACACCCACACCGTCACCCACACTATCATGTTGACATTCC; dG = -35.9

1232 >s_c_s_c_s_d_s_c_s_e_s_c_s_a_s_e_s
GTGTGGGTGTGGGTGACGGTGTGGGTGCTGGTGTGGGTGTCTGTGCTGGTG; >clamp_s_c_s_d_s_c_s_a_s_e_s:compl:rev
CACCAGCACAGACACCCACACCGTCACCCACACTATCATGTTGACATTCC; dG = -35.9
1233 >s_c_s_c_s_d_s_c_s_b_s_c_s_a_s_e_s
GTGTGGGTGTGGGTGACGGTGTGGGTGGCTGTGTGGGTGTCTGTGCTGGTG; >clamp_s_c_s_d_s_c_s_a_s_e_s:compl:rev
CACCAGCACAGACACCCACACCGTCACCCACACTATCATGTTGACATTCC; dG = -35.9
1234 >s_d_s_c_s_d_s_c_s_d_s_b_s_a_s_e_s
GTGACGGTGTGGGTGACGGTGTGGGTGACGGTGGCTGTGTCTGTGCTGGTG; >clamp_s_c_s_d_s_c_s_a_s_e_s:compl:rev
CACCAGCACAGACACCCACACCGTCACCCACACTATCATGTTGACATTCC; dG = -35.9
1235 >s_d_s_c_s_d_s_c_s_d_s_a_s_a_s_e_s
GTGACGGTGTGGGTGACGGTGTGGGTGACGGTGTCTGTGTCTGTGCTGGTG; >clamp_s_c_s_d_s_c_s_a_s_e_s:compl:rev
CACCAGCACAGACACCCACACCGTCACCCACACTATCATGTTGACATTCC; dG = -35.9
1236 >s_d_s_c_s_d_s_c_s_e_s_c_s_a_s_e_s
GTGACGGTGTGGGTGACGGTGTGGGTGCTGGTGTGGGTGTCTGTGCTGGTG; >clamp_s_c_s_d_s_c_s_a_s_e_s:compl:rev
CACCAGCACAGACACCCACACCGTCACCCACACTATCATGTTGACATTCC; dG = -35.9
1237 >s_d_s_c_s_d_s_c_s_b_s_c_s_a_s_e_s
GTGACGGTGTGGGTGACGGTGTGGGTGGCTGTGTGGGTGTCTGTGCTGGTG; >clamp_s_c_s_d_s_c_s_a_s_e_s:compl:rev
CACCAGCACAGACACCCACACCGTCACCCACACTATCATGTTGACATTCC; dG = -35.9
1238 >s_e_s_c_s_d_s_c_s_d_s_b_s_a_s_e_s
GTGCTGGTGTGGGTGACGGTGTGGGTGACGGTGGCTGTGTCTGTGCTGGTG; >clamp_s_c_s_d_s_c_s_a_s_e_s:compl:rev
CACCAGCACAGACACCCACACCGTCACCCACACTATCATGTTGACATTCC; dG = -35.9
1239 >s_e_s_c_s_d_s_c_s_d_s_a_s_a_s_e_s
GTGCTGGTGTGGGTGACGGTGTGGGTGACGGTGTCTGTGTCTGTGCTGGTG; >clamp_s_c_s_d_s_c_s_a_s_e_s:compl:rev
CACCAGCACAGACACCCACACCGTCACCCACACTATCATGTTGACATTCC; dG = -35.9
1240 >s_e_s_c_s_d_s_c_s_e_s_c_s_a_s_e_s
GTGCTGGTGTGGGTGACGGTGTGGGTGCTGGTGTGGGTGTCTGTGCTGGTG; >clamp_s_c_s_d_s_c_s_a_s_e_s:compl:rev
CACCAGCACAGACACCCACACCGTCACCCACACTATCATGTTGACATTCC; dG = -35.9
1241 >s_e_s_c_s_d_s_c_s_b_s_c_s_a_s_e_s
GTGCTGGTGTGGGTGACGGTGTGGGTGGCTGTGTGGGTGTCTGTGCTGGTG; >clamp_s_c_s_d_s_c_s_a_s_e_s:compl:rev
CACCAGCACAGACACCCACACCGTCACCCACACTATCATGTTGACATTCC; dG = -35.9
1242 >s_c_s_a_s_a_s_c_s_e_s_c_s_a_s_e_s
GTGTGGGTGTCTGTGTCTGTGTGGGTGCTGGTGTGGGTGTCTGTGCTGGTG; >clamp_s_c_s_d_s_c_s_a_s_e_s:compl:rev
CACCAGCACAGACACCCACACCGTCACCCACACTATCATGTTGACATTCC; dG = -35.5
1243 >s_c_s_a_s_a_s_b_s_d_s_c_s_a_s_e_s
GTGTGGGTGTCTGTGTCTGTGGCTGTGACGGTGTGGGTGTCTGTGCTGGTG; >clamp_s_c_s_d_s_c_s_a_s_e_s:compl:rev
CACCAGCACAGACACCCACACCGTCACCCACACTATCATGTTGACATTCC; dG = -35.5
1244 >s_c_s_a_s_b_s_c_s_e_s_c_s_a_s_e_s
GTGTGGGTGTCTGTGGCTGTGTGGGTGCTGGTGTGGGTGTCTGTGCTGGTG; >clamp_s_c_s_d_s_c_s_a_s_e_s:compl:rev
CACCAGCACAGACACCCACACCGTCACCCACACTATCATGTTGACATTCC; dG = -35.5
1245 >s_c_s_a_s_b_s_b_s_d_s_c_s_a_s_e_s
GTGTGGGTGTCTGTGGCTGTGGCTGTGACGGTGTGGGTGTCTGTGCTGGTG; >clamp_s_c_s_d_s_c_s_a_s_e_s:compl:rev
CACCAGCACAGACACCCACACCGTCACCCACACTATCATGTTGACATTCC; dG = -35.5
1246 >s_c_s_a_s_c_s_c_s_e_s_c_s_a_s_e_s
GTGTGGGTGTCTGTGTGGGTGTGGGTGCTGGTGTGGGTGTCTGTGCTGGTG; >clamp_s_c_s_d_s_c_s_a_s_e_s:compl:rev
CACCAGCACAGACACCCACACCGTCACCCACACTATCATGTTGACATTCC; dG = -35.5
1247 >s_c_s_a_s_d_s_c_s_e_s_c_s_a_s_e_s
GTGTGGGTGTCTGTGACGGTGTGGGTGCTGGTGTGGGTGTCTGTGCTGGTG; >clamp_s_c_s_d_s_c_s_a_s_e_s:compl:rev
CACCAGCACAGACACCCACACCGTCACCCACACTATCATGTTGACATTCC; dG = -35.5
1248 >s_c_s_a_s_d_s_b_s_d_s_c_s_a_s_e_s
GTGTGGGTGTCTGTGACGGTGGCTGTGACGGTGTGGGTGTCTGTGCTGGTG; >clamp_s_c_s_d_s_c_s_a_s_e_s:compl:rev
CACCAGCACAGACACCCACACCGTCACCCACACTATCATGTTGACATTCC; dG = -35.5
1249 >s_c_s_a_s_e_s_c_s_e_s_c_s_a_s_e_s
GTGTGGGTGTCTGTGCTGGTGTGGGTGCTGGTGTGGGTGTCTGTGCTGGTG; >clamp_s_c_s_d_s_c_s_a_s_e_s:compl:rev
CACCAGCACAGACACCCACACCGTCACCCACACTATCATGTTGACATTCC; dG = -35.5
1250 >s_c_s_a_s_e_s_b_s_d_s_c_s_a_s_e_s
GTGTGGGTGTCTGTGCTGGTGGCTGTGACGGTGTGGGTGTCTGTGCTGGTG; >clamp_s_c_s_d_s_c_s_a_s_e_s:compl:rev
CACCAGCACAGACACCCACACCGTCACCCACACTATCATGTTGACATTCC; dG = -35.5
1251 >s_c_s_b_s_a_s_c_s_e_s_c_s_a_s_e_s
GTGTGGGTGGCTGTGTCTGTGTGGGTGCTGGTGTGGGTGTCTGTGCTGGTG; >clamp_s_c_s_d_s_c_s_a_s_e_s:compl:rev
CACCAGCACAGACACCCACACCGTCACCCACACTATCATGTTGACATTCC; dG = -35.5
1252 >s_c_s_b_s_a_s_b_s_d_s_c_s_a_s_e_s
GTGTGGGTGGCTGTGTCTGTGGCTGTGACGGTGTGGGTGTCTGTGCTGGTG; >clamp_s_c_s_d_s_c_s_a_s_e_s:compl:rev
CACCAGCACAGACACCCACACCGTCACCCACACTATCATGTTGACATTCC; dG = -35.5
1253 >s_a_s_b_s_a_s_c_s_e_s_c_s_a_s_e_s
GTGTCTGTGGCTGTGTCTGTGTGGGTGCTGGTGTGGGTGTCTGTGCTGGTG; >clamp_s_c_s_d_s_c_s_a_s_e_s:compl:rev
CACCAGCACAGACACCCACACCGTCACCCACACTATCATGTTGACATTCC; dG = -35.5
1254 >s_c_s_b_s_b_s_c_s_e_s_c_s_a_s_e_s
GTGTGGGTGGCTGTGGCTGTGTGGGTGCTGGTGTGGGTGTCTGTGCTGGTG; >clamp_s_c_s_d_s_c_s_a_s_e_s:compl:rev
CACCAGCACAGACACCCACACCGTCACCCACACTATCATGTTGACATTCC; dG = -35.5
1255 >s_c_s_b_s_b_s_b_s_d_s_c_s_a_s_e_s
GTGTGGGTGGCTGTGGCTGTGGCTGTGACGGTGTGGGTGTCTGTGCTGGTG; >clamp_s_c_s_d_s_c_s_a_s_e_s:compl:rev
CACCAGCACAGACACCCACACCGTCACCCACACTATCATGTTGACATTCC; dG = -35.5

1256 >s_c_s_b_s_c_s_c_s_e_s_c_s_a_s_e_s
GTGTGGGTGGCTGTGTGGGTGTGGGTGCTGGTGTGGGTGTCTGTGCTGGTG; >clamp_s_c_s_d_s_c_s_a_s_e_s:compl:rev
CACCAGCACAGACACCCACACCGTCACCCACACTATCATGTTGACATTCC; dG = -35.5
1257 >s_c_s_b_s_d_s_c_s_e_s_c_s_a_s_e_s
GTGTGGGTGGCTGTGACGGTGTGGGTGCTGGTGTGGGTGTCTGTGCTGGTG; >clamp_s_c_s_d_s_c_s_a_s_e_s:compl:rev
CACCAGCACAGACACCCACACCGTCACCCACACTATCATGTTGACATTCC; dG = -35.5
1258 >s_c_s_b_s_d_s_b_s_d_s_c_s_a_s_e_s
GTGTGGGTGGCTGTGACGGTGGCTGTGACGGTGTGGGTGTCTGTGCTGGTG; >clamp_s_c_s_d_s_c_s_a_s_e_s:compl:rev
CACCAGCACAGACACCCACACCGTCACCCACACTATCATGTTGACATTCC; dG = -35.5
1259 >s_a_s_b_s_a_s_b_s_d_s_c_s_a_s_e_s
GTGTCTGTGGCTGTGTCTGTGGCTGTGACGGTGTGGGTGTCTGTGCTGGTG; >clamp_s_c_s_d_s_c_s_a_s_e_s:compl:rev
CACCAGCACAGACACCCACACCGTCACCCACACTATCATGTTGACATTCC; dG = -35.5
1260 >s_c_s_b_s_e_s_c_s_e_s_c_s_a_s_e_s
GTGTGGGTGGCTGTGCTGGTGTGGGTGCTGGTGTGGGTGTCTGTGCTGGTG; >clamp_s_c_s_d_s_c_s_a_s_e_s:compl:rev
CACCAGCACAGACACCCACACCGTCACCCACACTATCATGTTGACATTCC; dG = -35.5
1261 >s_c_s_b_s_e_s_b_s_d_s_c_s_a_s_e_s
GTGTGGGTGGCTGTGCTGGTGGCTGTGACGGTGTGGGTGTCTGTGCTGGTG; >clamp_s_c_s_d_s_c_s_a_s_e_s:compl:rev
CACCAGCACAGACACCCACACCGTCACCCACACTATCATGTTGACATTCC; dG = -35.5
1262 >s_c_s_c_s_a_s_c_s_e_s_c_s_a_s_e_s
GTGTGGGTGTGGGTGTCTGTGTGGGTGCTGGTGTGGGTGTCTGTGCTGGTG; >clamp_s_c_s_d_s_c_s_a_s_e_s:compl:rev
CACCAGCACAGACACCCACACCGTCACCCACACTATCATGTTGACATTCC; dG = -35.5
1263 >s_c_s_c_s_b_s_c_s_e_s_c_s_a_s_e_s
GTGTGGGTGTGGGTGGCTGTGTGGGTGCTGGTGTGGGTGTCTGTGCTGGTG; >clamp_s_c_s_d_s_c_s_a_s_e_s:compl:rev
CACCAGCACAGACACCCACACCGTCACCCACACTATCATGTTGACATTCC; dG = -35.5
1264 >s_c_s_c_s_c_s_c_s_e_s_c_s_a_s_e_s
GTGTGGGTGTGGGTGTGGGTGTGGGTGCTGGTGTGGGTGTCTGTGCTGGTG; >clamp_s_c_s_d_s_c_s_a_s_e_s:compl:rev
CACCAGCACAGACACCCACACCGTCACCCACACTATCATGTTGACATTCC; dG = -35.5
1265 >s_c_s_c_s_e_s_c_s_e_s_c_s_a_s_e_s
GTGTGGGTGTGGGTGCTGGTGTGGGTGCTGGTGTGGGTGTCTGTGCTGGTG; >clamp_s_c_s_d_s_c_s_a_s_e_s:compl:rev
CACCAGCACAGACACCCACACCGTCACCCACACTATCATGTTGACATTCC; dG = -35.5
1266 >s_a_s_a_s_a_s_c_s_e_s_c_s_a_s_e_s
GTGTCTGTGTCTGTGTCTGTGTGGGTGCTGGTGTGGGTGTCTGTGCTGGTG; >clamp_s_c_s_d_s_c_s_a_s_e_s:compl:rev
CACCAGCACAGACACCCACACCGTCACCCACACTATCATGTTGACATTCC; dG = -35.5
1267 >s_c_s_d_s_a_s_c_s_e_s_c_s_a_s_e_s
GTGTGGGTGACGGTGTCTGTGTGGGTGCTGGTGTGGGTGTCTGTGCTGGTG; >clamp_s_c_s_d_s_c_s_a_s_e_s:compl:rev
CACCAGCACAGACACCCACACCGTCACCCACACTATCATGTTGACATTCC; dG = -35.5
1268 >s_c_s_d_s_a_s_b_s_d_s_c_s_a_s_e_s
GTGTGGGTGACGGTGTCTGTGGCTGTGACGGTGTGGGTGTCTGTGCTGGTG; >clamp_s_c_s_d_s_c_s_a_s_e_s:compl:rev
CACCAGCACAGACACCCACACCGTCACCCACACTATCATGTTGACATTCC; dG = -35.5
1269 >s_a_s_b_s_b_s_c_s_e_s_c_s_a_s_e_s
GTGTCTGTGGCTGTGGCTGTGTGGGTGCTGGTGTGGGTGTCTGTGCTGGTG; >clamp_s_c_s_d_s_c_s_a_s_e_s:compl:rev
CACCAGCACAGACACCCACACCGTCACCCACACTATCATGTTGACATTCC; dG = -35.5
1270 >s_c_s_d_s_b_s_c_s_e_s_c_s_a_s_e_s
GTGTGGGTGACGGTGGCTGTGTGGGTGCTGGTGTGGGTGTCTGTGCTGGTG; >clamp_s_c_s_d_s_c_s_a_s_e_s:compl:rev
CACCAGCACAGACACCCACACCGTCACCCACACTATCATGTTGACATTCC; dG = -35.5
1271 >s_c_s_d_s_b_s_b_s_d_s_c_s_a_s_e_s
GTGTGGGTGACGGTGGCTGTGGCTGTGACGGTGTGGGTGTCTGTGCTGGTG; >clamp_s_c_s_d_s_c_s_a_s_e_s:compl:rev
CACCAGCACAGACACCCACACCGTCACCCACACTATCATGTTGACATTCC; dG = -35.5
1272 >s_c_s_d_s_d_s_c_s_e_s_c_s_a_s_e_s
GTGTGGGTGACGGTGACGGTGTGGGTGCTGGTGTGGGTGTCTGTGCTGGTG; >clamp_s_c_s_d_s_c_s_a_s_e_s:compl:rev
CACCAGCACAGACACCCACACCGTCACCCACACTATCATGTTGACATTCC; dG = -35.5
1273 >s_c_s_d_s_d_s_b_s_d_s_c_s_a_s_e_s
GTGTGGGTGACGGTGACGGTGGCTGTGACGGTGTGGGTGTCTGTGCTGGTG; >clamp_s_c_s_d_s_c_s_a_s_e_s:compl:rev
CACCAGCACAGACACCCACACCGTCACCCACACTATCATGTTGACATTCC; dG = -35.5
1274 >s_a_s_b_s_b_s_b_s_d_s_c_s_a_s_e_s
GTGTCTGTGGCTGTGGCTGTGGCTGTGACGGTGTGGGTGTCTGTGCTGGTG; >clamp_s_c_s_d_s_c_s_a_s_e_s:compl:rev
CACCAGCACAGACACCCACACCGTCACCCACACTATCATGTTGACATTCC; dG = -35.5
1275 >s_c_s_d_s_e_s_c_s_e_s_c_s_a_s_e_s
GTGTGGGTGACGGTGCTGGTGTGGGTGCTGGTGTGGGTGTCTGTGCTGGTG; >clamp_s_c_s_d_s_c_s_a_s_e_s:compl:rev
CACCAGCACAGACACCCACACCGTCACCCACACTATCATGTTGACATTCC; dG = -35.5
1276 >s_c_s_d_s_e_s_b_s_d_s_c_s_a_s_e_s
GTGTGGGTGACGGTGCTGGTGGCTGTGACGGTGTGGGTGTCTGTGCTGGTG; >clamp_s_c_s_d_s_c_s_a_s_e_s:compl:rev
CACCAGCACAGACACCCACACCGTCACCCACACTATCATGTTGACATTCC; dG = -35.5
1277 >s_c_s_e_s_a_s_c_s_e_s_c_s_a_s_e_s
GTGTGGGTGCTGGTGTCTGTGTGGGTGCTGGTGTGGGTGTCTGTGCTGGTG; >clamp_s_c_s_d_s_c_s_a_s_e_s:compl:rev
CACCAGCACAGACACCCACACCGTCACCCACACTATCATGTTGACATTCC; dG = -35.5
1278 >s_c_s_e_s_a_s_b_s_d_s_c_s_a_s_e_s
GTGTGGGTGCTGGTGTCTGTGGCTGTGACGGTGTGGGTGTCTGTGCTGGTG; >clamp_s_c_s_d_s_c_s_a_s_e_s:compl:rev
CACCAGCACAGACACCCACACCGTCACCCACACTATCATGTTGACATTCC; dG = -35.5
1279 >s_c_s_e_s_b_s_c_s_e_s_c_s_a_s_e_s
GTGTGGGTGCTGGTGGCTGTGTGGGTGCTGGTGTGGGTGTCTGTGCTGGTG; >clamp_s_c_s_d_s_c_s_a_s_e_s:compl:rev
CACCAGCACAGACACCCACACCGTCACCCACACTATCATGTTGACATTCC; dG = -35.5

1280 >s_c_s_e_s_b_s_b_s_d_s_c_s_a_s_e_s
GTGTGGGTGCTGGTGGCTGTGGCTGTGACGGTGTGGGTGTCTGTGCTGGTG; >clamp_s_c_s_d_s_c_s_a_s_e_s:compl:rev
CACCAGCACAGACACCCACACCGTCACCCACACTATCATGTTGACATTCC; dG = -35.5
1281 >s_c_s_e_s_c_s_c_s_e_s_c_s_a_s_e_s
GTGTGGGTGCTGGTGTGGGTGTGGGTGCTGGTGTGGGTGTCTGTGCTGGTG; >clamp_s_c_s_d_s_c_s_a_s_e_s:compl:rev
CACCAGCACAGACACCCACACCGTCACCCACACTATCATGTTGACATTCC; dG = -35.5
1282 >s_c_s_e_s_d_s_c_s_e_s_c_s_a_s_e_s
GTGTGGGTGCTGGTGACGGTGTGGGTGCTGGTGTGGGTGTCTGTGCTGGTG; >clamp_s_c_s_d_s_c_s_a_s_e_s:compl:rev
CACCAGCACAGACACCCACACCGTCACCCACACTATCATGTTGACATTCC; dG = -35.5
1283 >s_c_s_e_s_d_s_b_s_d_s_c_s_a_s_e_s
GTGTGGGTGCTGGTGACGGTGGCTGTGACGGTGTGGGTGTCTGTGCTGGTG; >clamp_s_c_s_d_s_c_s_a_s_e_s:compl:rev
CACCAGCACAGACACCCACACCGTCACCCACACTATCATGTTGACATTCC; dG = -35.5
1284 >s_c_s_e_s_e_s_c_s_e_s_c_s_a_s_e_s
GTGTGGGTGCTGGTGCTGGTGTGGGTGCTGGTGTGGGTGTCTGTGCTGGTG; >clamp_s_c_s_d_s_c_s_a_s_e_s:compl:rev
CACCAGCACAGACACCCACACCGTCACCCACACTATCATGTTGACATTCC; dG = -35.5
1285 >s_c_s_e_s_e_s_b_s_d_s_c_s_a_s_e_s
GTGTGGGTGCTGGTGCTGGTGGCTGTGACGGTGTGGGTGTCTGTGCTGGTG; >clamp_s_c_s_d_s_c_s_a_s_e_s:compl:rev
CACCAGCACAGACACCCACACCGTCACCCACACTATCATGTTGACATTCC; dG = -35.5
1286 >s_d_s_a_s_a_s_c_s_e_s_c_s_a_s_e_s
GTGACGGTGTCTGTGTCTGTGTGGGTGCTGGTGTGGGTGTCTGTGCTGGTG; >clamp_s_c_s_d_s_c_s_a_s_e_s:compl:rev
CACCAGCACAGACACCCACACCGTCACCCACACTATCATGTTGACATTCC; dG = -35.5
1287 >s_d_s_a_s_a_s_b_s_d_s_c_s_a_s_e_s
GTGACGGTGTCTGTGTCTGTGGCTGTGACGGTGTGGGTGTCTGTGCTGGTG; >clamp_s_c_s_d_s_c_s_a_s_e_s:compl:rev
CACCAGCACAGACACCCACACCGTCACCCACACTATCATGTTGACATTCC; dG = -35.5
1288 >s_a_s_b_s_c_s_c_s_e_s_c_s_a_s_e_s
GTGTCTGTGGCTGTGTGGGTGTGGGTGCTGGTGTGGGTGTCTGTGCTGGTG; >clamp_s_c_s_d_s_c_s_a_s_e_s:compl:rev
CACCAGCACAGACACCCACACCGTCACCCACACTATCATGTTGACATTCC; dG = -35.5
1289 >s_d_s_a_s_b_s_c_s_e_s_c_s_a_s_e_s
GTGACGGTGTCTGTGGCTGTGTGGGTGCTGGTGTGGGTGTCTGTGCTGGTG; >clamp_s_c_s_d_s_c_s_a_s_e_s:compl:rev
CACCAGCACAGACACCCACACCGTCACCCACACTATCATGTTGACATTCC; dG = -35.5
1290 >s_d_s_a_s_b_s_b_s_d_s_c_s_a_s_e_s
GTGACGGTGTCTGTGGCTGTGGCTGTGACGGTGTGGGTGTCTGTGCTGGTG; >clamp_s_c_s_d_s_c_s_a_s_e_s:compl:rev
CACCAGCACAGACACCCACACCGTCACCCACACTATCATGTTGACATTCC; dG = -35.5
1291 >s_d_s_a_s_c_s_c_s_e_s_c_s_a_s_e_s
GTGACGGTGTCTGTGTGGGTGTGGGTGCTGGTGTGGGTGTCTGTGCTGGTG; >clamp_s_c_s_d_s_c_s_a_s_e_s:compl:rev
CACCAGCACAGACACCCACACCGTCACCCACACTATCATGTTGACATTCC; dG = -35.5
1292 >s_d_s_a_s_d_s_c_s_e_s_c_s_a_s_e_s
GTGACGGTGTCTGTGACGGTGTGGGTGCTGGTGTGGGTGTCTGTGCTGGTG; >clamp_s_c_s_d_s_c_s_a_s_e_s:compl:rev
CACCAGCACAGACACCCACACCGTCACCCACACTATCATGTTGACATTCC; dG = -35.5
1293 >s_d_s_a_s_d_s_b_s_d_s_c_s_a_s_e_s
GTGACGGTGTCTGTGACGGTGGCTGTGACGGTGTGGGTGTCTGTGCTGGTG; >clamp_s_c_s_d_s_c_s_a_s_e_s:compl:rev
CACCAGCACAGACACCCACACCGTCACCCACACTATCATGTTGACATTCC; dG = -35.5
1294 >s_d_s_a_s_e_s_c_s_e_s_c_s_a_s_e_s
GTGACGGTGTCTGTGCTGGTGTGGGTGCTGGTGTGGGTGTCTGTGCTGGTG; >clamp_s_c_s_d_s_c_s_a_s_e_s:compl:rev
CACCAGCACAGACACCCACACCGTCACCCACACTATCATGTTGACATTCC; dG = -35.5
1295 >s_d_s_a_s_e_s_b_s_d_s_c_s_a_s_e_s
GTGACGGTGTCTGTGCTGGTGGCTGTGACGGTGTGGGTGTCTGTGCTGGTG; >clamp_s_c_s_d_s_c_s_a_s_e_s:compl:rev
CACCAGCACAGACACCCACACCGTCACCCACACTATCATGTTGACATTCC; dG = -35.5
1296 >s_d_s_b_s_a_s_c_s_e_s_c_s_a_s_e_s
GTGACGGTGGCTGTGTCTGTGTGGGTGCTGGTGTGGGTGTCTGTGCTGGTG; >clamp_s_c_s_d_s_c_s_a_s_e_s:compl:rev
CACCAGCACAGACACCCACACCGTCACCCACACTATCATGTTGACATTCC; dG = -35.5
1297 >s_d_s_b_s_a_s_b_s_d_s_c_s_a_s_e_s
GTGACGGTGGCTGTGTCTGTGGCTGTGACGGTGTGGGTGTCTGTGCTGGTG; >clamp_s_c_s_d_s_c_s_a_s_e_s:compl:rev
CACCAGCACAGACACCCACACCGTCACCCACACTATCATGTTGACATTCC; dG = -35.5
1298 >s_d_s_b_s_b_s_c_s_e_s_c_s_a_s_e_s
GTGACGGTGGCTGTGGCTGTGTGGGTGCTGGTGTGGGTGTCTGTGCTGGTG; >clamp_s_c_s_d_s_c_s_a_s_e_s:compl:rev
CACCAGCACAGACACCCACACCGTCACCCACACTATCATGTTGACATTCC; dG = -35.5
1299 >s_d_s_b_s_b_s_b_s_d_s_c_s_a_s_e_s
GTGACGGTGGCTGTGGCTGTGGCTGTGACGGTGTGGGTGTCTGTGCTGGTG; >clamp_s_c_s_d_s_c_s_a_s_e_s:compl:rev
CACCAGCACAGACACCCACACCGTCACCCACACTATCATGTTGACATTCC; dG = -35.5
1300 >s_d_s_b_s_c_s_c_s_e_s_c_s_a_s_e_s
GTGACGGTGGCTGTGTGGGTGTGGGTGCTGGTGTGGGTGTCTGTGCTGGTG; >clamp_s_c_s_d_s_c_s_a_s_e_s:compl:rev
CACCAGCACAGACACCCACACCGTCACCCACACTATCATGTTGACATTCC; dG = -35.5
1301 >s_d_s_b_s_d_s_c_s_e_s_c_s_a_s_e_s
GTGACGGTGGCTGTGACGGTGTGGGTGCTGGTGTGGGTGTCTGTGCTGGTG; >clamp_s_c_s_d_s_c_s_a_s_e_s:compl:rev
CACCAGCACAGACACCCACACCGTCACCCACACTATCATGTTGACATTCC; dG = -35.5
1302 >s_d_s_b_s_d_s_b_s_d_s_c_s_a_s_e_s
GTGACGGTGGCTGTGACGGTGGCTGTGACGGTGTGGGTGTCTGTGCTGGTG; >clamp_s_c_s_d_s_c_s_a_s_e_s:compl:rev
CACCAGCACAGACACCCACACCGTCACCCACACTATCATGTTGACATTCC; dG = -35.5
1303 >s_d_s_b_s_e_s_c_s_e_s_c_s_a_s_e_s
GTGACGGTGGCTGTGCTGGTGTGGGTGCTGGTGTGGGTGTCTGTGCTGGTG; >clamp_s_c_s_d_s_c_s_a_s_e_s:compl:rev
CACCAGCACAGACACCCACACCGTCACCCACACTATCATGTTGACATTCC; dG = -35.5

1304 >s_d_s_b_s_e_s_b_s_d_s_c_s_a_s_e_s
GTGACGGTGGCTGTGCTGGTGGCTGTGACGGTGTGGGTGTCTGTGCTGGTG; >clamp_s_c_s_d_s_c_s_a_s_e_s:compl:rev
CACCAGCACAGACACCCACACCGTCACCCACACTATCATGTTGACATTCC; dG = -35.5
1305 >s_d_s_c_s_a_s_c_s_e_s_c_s_a_s_e_s
GTGACGGTGTGGGTGTCTGTGTGGGTGCTGGTGTGGGTGTCTGTGCTGGTG; >clamp_s_c_s_d_s_c_s_a_s_e_s:compl:rev
CACCAGCACAGACACCCACACCGTCACCCACACTATCATGTTGACATTCC; dG = -35.5
1306 >s_a_s_b_s_d_s_c_s_e_s_c_s_a_s_e_s
GTGTCTGTGGCTGTGACGGTGTGGGTGCTGGTGTGGGTGTCTGTGCTGGTG; >clamp_s_c_s_d_s_c_s_a_s_e_s:compl:rev
CACCAGCACAGACACCCACACCGTCACCCACACTATCATGTTGACATTCC; dG = -35.5
1307 >s_d_s_c_s_b_s_c_s_e_s_c_s_a_s_e_s
GTGACGGTGTGGGTGGCTGTGTGGGTGCTGGTGTGGGTGTCTGTGCTGGTG; >clamp_s_c_s_d_s_c_s_a_s_e_s:compl:rev
CACCAGCACAGACACCCACACCGTCACCCACACTATCATGTTGACATTCC; dG = -35.5
1308 >s_d_s_c_s_c_s_c_s_e_s_c_s_a_s_e_s
GTGACGGTGTGGGTGTGGGTGTGGGTGCTGGTGTGGGTGTCTGTGCTGGTG; >clamp_s_c_s_d_s_c_s_a_s_e_s:compl:rev
CACCAGCACAGACACCCACACCGTCACCCACACTATCATGTTGACATTCC; dG = -35.5
1309 >s_a_s_b_s_d_s_b_s_d_s_c_s_a_s_e_s
GTGTCTGTGGCTGTGACGGTGGCTGTGACGGTGTGGGTGTCTGTGCTGGTG; >clamp_s_c_s_d_s_c_s_a_s_e_s:compl:rev
CACCAGCACAGACACCCACACCGTCACCCACACTATCATGTTGACATTCC; dG = -35.5
1310 >s_d_s_c_s_e_s_c_s_e_s_c_s_a_s_e_s
GTGACGGTGTGGGTGCTGGTGTGGGTGCTGGTGTGGGTGTCTGTGCTGGTG; >clamp_s_c_s_d_s_c_s_a_s_e_s:compl:rev
CACCAGCACAGACACCCACACCGTCACCCACACTATCATGTTGACATTCC; dG = -35.5
1311 >s_d_s_d_s_a_s_c_s_e_s_c_s_a_s_e_s
GTGACGGTGACGGTGTCTGTGTGGGTGCTGGTGTGGGTGTCTGTGCTGGTG; >clamp_s_c_s_d_s_c_s_a_s_e_s:compl:rev
CACCAGCACAGACACCCACACCGTCACCCACACTATCATGTTGACATTCC; dG = -35.5
1312 >s_d_s_d_s_a_s_b_s_d_s_c_s_a_s_e_s
GTGACGGTGACGGTGTCTGTGGCTGTGACGGTGTGGGTGTCTGTGCTGGTG; >clamp_s_c_s_d_s_c_s_a_s_e_s:compl:rev
CACCAGCACAGACACCCACACCGTCACCCACACTATCATGTTGACATTCC; dG = -35.5
1313 >s_d_s_d_s_b_s_c_s_e_s_c_s_a_s_e_s
GTGACGGTGACGGTGGCTGTGTGGGTGCTGGTGTGGGTGTCTGTGCTGGTG; >clamp_s_c_s_d_s_c_s_a_s_e_s:compl:rev
CACCAGCACAGACACCCACACCGTCACCCACACTATCATGTTGACATTCC; dG = -35.5
1314 >s_d_s_d_s_b_s_b_s_d_s_c_s_a_s_e_s
GTGACGGTGACGGTGGCTGTGGCTGTGACGGTGTGGGTGTCTGTGCTGGTG; >clamp_s_c_s_d_s_c_s_a_s_e_s:compl:rev
CACCAGCACAGACACCCACACCGTCACCCACACTATCATGTTGACATTCC; dG = -35.5
1315 >s_d_s_d_s_c_s_c_s_e_s_c_s_a_s_e_s
GTGACGGTGACGGTGTGGGTGTGGGTGCTGGTGTGGGTGTCTGTGCTGGTG; >clamp_s_c_s_d_s_c_s_a_s_e_s:compl:rev
CACCAGCACAGACACCCACACCGTCACCCACACTATCATGTTGACATTCC; dG = -35.5
1316 >s_d_s_d_s_d_s_c_s_e_s_c_s_a_s_e_s
GTGACGGTGACGGTGACGGTGTGGGTGCTGGTGTGGGTGTCTGTGCTGGTG; >clamp_s_c_s_d_s_c_s_a_s_e_s:compl:rev
CACCAGCACAGACACCCACACCGTCACCCACACTATCATGTTGACATTCC; dG = -35.5
1317 >s_d_s_d_s_d_s_b_s_d_s_c_s_a_s_e_s
GTGACGGTGACGGTGACGGTGGCTGTGACGGTGTGGGTGTCTGTGCTGGTG; >clamp_s_c_s_d_s_c_s_a_s_e_s:compl:rev
CACCAGCACAGACACCCACACCGTCACCCACACTATCATGTTGACATTCC; dG = -35.5
1318 >s_d_s_d_s_e_s_c_s_e_s_c_s_a_s_e_s
GTGACGGTGACGGTGCTGGTGTGGGTGCTGGTGTGGGTGTCTGTGCTGGTG; >clamp_s_c_s_d_s_c_s_a_s_e_s:compl:rev
CACCAGCACAGACACCCACACCGTCACCCACACTATCATGTTGACATTCC; dG = -35.5
1319 >s_d_s_d_s_e_s_b_s_d_s_c_s_a_s_e_s
GTGACGGTGACGGTGCTGGTGGCTGTGACGGTGTGGGTGTCTGTGCTGGTG; >clamp_s_c_s_d_s_c_s_a_s_e_s:compl:rev
CACCAGCACAGACACCCACACCGTCACCCACACTATCATGTTGACATTCC; dG = -35.5
1320 >s_a_s_a_s_a_s_b_s_d_s_c_s_a_s_e_s
GTGTCTGTGTCTGTGTCTGTGGCTGTGACGGTGTGGGTGTCTGTGCTGGTG; >clamp_s_c_s_d_s_c_s_a_s_e_s:compl:rev
CACCAGCACAGACACCCACACCGTCACCCACACTATCATGTTGACATTCC; dG = -35.5
1321 >s_d_s_e_s_a_s_c_s_e_s_c_s_a_s_e_s
GTGACGGTGCTGGTGTCTGTGTGGGTGCTGGTGTGGGTGTCTGTGCTGGTG; >clamp_s_c_s_d_s_c_s_a_s_e_s:compl:rev
CACCAGCACAGACACCCACACCGTCACCCACACTATCATGTTGACATTCC; dG = -35.5
1322 >s_d_s_e_s_a_s_b_s_d_s_c_s_a_s_e_s
GTGACGGTGCTGGTGTCTGTGGCTGTGACGGTGTGGGTGTCTGTGCTGGTG; >clamp_s_c_s_d_s_c_s_a_s_e_s:compl:rev
CACCAGCACAGACACCCACACCGTCACCCACACTATCATGTTGACATTCC; dG = -35.5
1323 >s_a_s_b_s_e_s_c_s_e_s_c_s_a_s_e_s
GTGTCTGTGGCTGTGCTGGTGTGGGTGCTGGTGTGGGTGTCTGTGCTGGTG; >clamp_s_c_s_d_s_c_s_a_s_e_s:compl:rev
CACCAGCACAGACACCCACACCGTCACCCACACTATCATGTTGACATTCC; dG = -35.5
1324 >s_d_s_e_s_b_s_c_s_e_s_c_s_a_s_e_s
GTGACGGTGCTGGTGGCTGTGTGGGTGCTGGTGTGGGTGTCTGTGCTGGTG; >clamp_s_c_s_d_s_c_s_a_s_e_s:compl:rev
CACCAGCACAGACACCCACACCGTCACCCACACTATCATGTTGACATTCC; dG = -35.5
1325 >s_d_s_e_s_b_s_b_s_d_s_c_s_a_s_e_s
GTGACGGTGCTGGTGGCTGTGGCTGTGACGGTGTGGGTGTCTGTGCTGGTG; >clamp_s_c_s_d_s_c_s_a_s_e_s:compl:rev
CACCAGCACAGACACCCACACCGTCACCCACACTATCATGTTGACATTCC; dG = -35.5
1326 >s_d_s_e_s_c_s_c_s_e_s_c_s_a_s_e_s
GTGACGGTGCTGGTGTGGGTGTGGGTGCTGGTGTGGGTGTCTGTGCTGGTG; >clamp_s_c_s_d_s_c_s_a_s_e_s:compl:rev
CACCAGCACAGACACCCACACCGTCACCCACACTATCATGTTGACATTCC; dG = -35.5
1327 >s_d_s_e_s_d_s_c_s_e_s_c_s_a_s_e_s
GTGACGGTGCTGGTGACGGTGTGGGTGCTGGTGTGGGTGTCTGTGCTGGTG; >clamp_s_c_s_d_s_c_s_a_s_e_s:compl:rev
CACCAGCACAGACACCCACACCGTCACCCACACTATCATGTTGACATTCC; dG = -35.5

1328 >s_d_s_e_s_d_s_b_s_d_s_c_s_a_s_e_s
GTGACGGTGCTGGTGACGGTGGCTGTGACGGTGTGGGTGTCTGTGCTGGTG; >clamp_s_c_s_d_s_c_s_a_s_e_s:compl:rev
CACCAGCACAGACACCCACACCGTCACCCACACTATCATGTTGACATTCC; dG = -35.5
1329 >s_a_s_b_s_e_s_b_s_d_s_c_s_a_s_e_s
GTGTCTGTGGCTGTGCTGGTGGCTGTGACGGTGTGGGTGTCTGTGCTGGTG; >clamp_s_c_s_d_s_c_s_a_s_e_s:compl:rev
CACCAGCACAGACACCCACACCGTCACCCACACTATCATGTTGACATTCC; dG = -35.5
1330 >s_d_s_e_s_e_s_c_s_e_s_c_s_a_s_e_s
GTGACGGTGCTGGTGCTGGTGTGGGTGCTGGTGTGGGTGTCTGTGCTGGTG; >clamp_s_c_s_d_s_c_s_a_s_e_s:compl:rev
CACCAGCACAGACACCCACACCGTCACCCACACTATCATGTTGACATTCC; dG = -35.5
1331 >s_d_s_e_s_e_s_b_s_d_s_c_s_a_s_e_s
GTGACGGTGCTGGTGCTGGTGGCTGTGACGGTGTGGGTGTCTGTGCTGGTG; >clamp_s_c_s_d_s_c_s_a_s_e_s:compl:rev
CACCAGCACAGACACCCACACCGTCACCCACACTATCATGTTGACATTCC; dG = -35.5
1332 >s_e_s_a_s_a_s_c_s_e_s_c_s_a_s_e_s
GTGCTGGTGTCTGTGTCTGTGTGGGTGCTGGTGTGGGTGTCTGTGCTGGTG; >clamp_s_c_s_d_s_c_s_a_s_e_s:compl:rev
CACCAGCACAGACACCCACACCGTCACCCACACTATCATGTTGACATTCC; dG = -35.5
1333 >s_e_s_a_s_a_s_b_s_d_s_c_s_a_s_e_s
GTGCTGGTGTCTGTGTCTGTGGCTGTGACGGTGTGGGTGTCTGTGCTGGTG; >clamp_s_c_s_d_s_c_s_a_s_e_s:compl:rev
CACCAGCACAGACACCCACACCGTCACCCACACTATCATGTTGACATTCC; dG = -35.5
1334 >s_e_s_a_s_b_s_c_s_e_s_c_s_a_s_e_s
GTGCTGGTGTCTGTGGCTGTGTGGGTGCTGGTGTGGGTGTCTGTGCTGGTG; >clamp_s_c_s_d_s_c_s_a_s_e_s:compl:rev
CACCAGCACAGACACCCACACCGTCACCCACACTATCATGTTGACATTCC; dG = -35.5
1335 >s_e_s_a_s_b_s_b_s_d_s_c_s_a_s_e_s
GTGCTGGTGTCTGTGGCTGTGGCTGTGACGGTGTGGGTGTCTGTGCTGGTG; >clamp_s_c_s_d_s_c_s_a_s_e_s:compl:rev
CACCAGCACAGACACCCACACCGTCACCCACACTATCATGTTGACATTCC; dG = -35.5
1336 >s_e_s_a_s_c_s_c_s_e_s_c_s_a_s_e_s
GTGCTGGTGTCTGTGTGGGTGTGGGTGCTGGTGTGGGTGTCTGTGCTGGTG; >clamp_s_c_s_d_s_c_s_a_s_e_s:compl:rev
CACCAGCACAGACACCCACACCGTCACCCACACTATCATGTTGACATTCC; dG = -35.5
1337 >s_e_s_a_s_d_s_c_s_e_s_c_s_a_s_e_s
GTGCTGGTGTCTGTGACGGTGTGGGTGCTGGTGTGGGTGTCTGTGCTGGTG; >clamp_s_c_s_d_s_c_s_a_s_e_s:compl:rev
CACCAGCACAGACACCCACACCGTCACCCACACTATCATGTTGACATTCC; dG = -35.5
1338 >s_e_s_a_s_d_s_b_s_d_s_c_s_a_s_e_s
GTGCTGGTGTCTGTGACGGTGGCTGTGACGGTGTGGGTGTCTGTGCTGGTG; >clamp_s_c_s_d_s_c_s_a_s_e_s:compl:rev
CACCAGCACAGACACCCACACCGTCACCCACACTATCATGTTGACATTCC; dG = -35.5
1339 >s_e_s_a_s_e_s_c_s_e_s_c_s_a_s_e_s
GTGCTGGTGTCTGTGCTGGTGTGGGTGCTGGTGTGGGTGTCTGTGCTGGTG; >clamp_s_c_s_d_s_c_s_a_s_e_s:compl:rev
CACCAGCACAGACACCCACACCGTCACCCACACTATCATGTTGACATTCC; dG = -35.5
1340 >s_e_s_a_s_e_s_b_s_d_s_c_s_a_s_e_s
GTGCTGGTGTCTGTGCTGGTGGCTGTGACGGTGTGGGTGTCTGTGCTGGTG; >clamp_s_c_s_d_s_c_s_a_s_e_s:compl:rev
CACCAGCACAGACACCCACACCGTCACCCACACTATCATGTTGACATTCC; dG = -35.5
1341 >s_e_s_b_s_a_s_c_s_e_s_c_s_a_s_e_s
GTGCTGGTGGCTGTGTCTGTGTGGGTGCTGGTGTGGGTGTCTGTGCTGGTG; >clamp_s_c_s_d_s_c_s_a_s_e_s:compl:rev
CACCAGCACAGACACCCACACCGTCACCCACACTATCATGTTGACATTCC; dG = -35.5
1342 >s_e_s_b_s_a_s_b_s_d_s_c_s_a_s_e_s
GTGCTGGTGGCTGTGTCTGTGGCTGTGACGGTGTGGGTGTCTGTGCTGGTG; >clamp_s_c_s_d_s_c_s_a_s_e_s:compl:rev
CACCAGCACAGACACCCACACCGTCACCCACACTATCATGTTGACATTCC; dG = -35.5
1343 >s_a_s_c_s_a_s_c_s_e_s_c_s_a_s_e_s
GTGTCTGTGTGGGTGTCTGTGTGGGTGCTGGTGTGGGTGTCTGTGCTGGTG; >clamp_s_c_s_d_s_c_s_a_s_e_s:compl:rev
CACCAGCACAGACACCCACACCGTCACCCACACTATCATGTTGACATTCC; dG = -35.5
1344 >s_e_s_b_s_b_s_c_s_e_s_c_s_a_s_e_s
GTGCTGGTGGCTGTGGCTGTGTGGGTGCTGGTGTGGGTGTCTGTGCTGGTG; >clamp_s_c_s_d_s_c_s_a_s_e_s:compl:rev
CACCAGCACAGACACCCACACCGTCACCCACACTATCATGTTGACATTCC; dG = -35.5
1345 >s_e_s_b_s_b_s_b_s_d_s_c_s_a_s_e_s
GTGCTGGTGGCTGTGGCTGTGGCTGTGACGGTGTGGGTGTCTGTGCTGGTG; >clamp_s_c_s_d_s_c_s_a_s_e_s:compl:rev
CACCAGCACAGACACCCACACCGTCACCCACACTATCATGTTGACATTCC; dG = -35.5
1346 >s_e_s_b_s_c_s_c_s_e_s_c_s_a_s_e_s
GTGCTGGTGGCTGTGTGGGTGTGGGTGCTGGTGTGGGTGTCTGTGCTGGTG; >clamp_s_c_s_d_s_c_s_a_s_e_s:compl:rev
CACCAGCACAGACACCCACACCGTCACCCACACTATCATGTTGACATTCC; dG = -35.5
1347 >s_e_s_b_s_d_s_c_s_e_s_c_s_a_s_e_s
GTGCTGGTGGCTGTGACGGTGTGGGTGCTGGTGTGGGTGTCTGTGCTGGTG; >clamp_s_c_s_d_s_c_s_a_s_e_s:compl:rev
CACCAGCACAGACACCCACACCGTCACCCACACTATCATGTTGACATTCC; dG = -35.5
1348 >s_e_s_b_s_d_s_b_s_d_s_c_s_a_s_e_s
GTGCTGGTGGCTGTGACGGTGGCTGTGACGGTGTGGGTGTCTGTGCTGGTG; >clamp_s_c_s_d_s_c_s_a_s_e_s:compl:rev
CACCAGCACAGACACCCACACCGTCACCCACACTATCATGTTGACATTCC; dG = -35.5
1349 >s_e_s_b_s_e_s_c_s_e_s_c_s_a_s_e_s
GTGCTGGTGGCTGTGCTGGTGTGGGTGCTGGTGTGGGTGTCTGTGCTGGTG; >clamp_s_c_s_d_s_c_s_a_s_e_s:compl:rev
CACCAGCACAGACACCCACACCGTCACCCACACTATCATGTTGACATTCC; dG = -35.5
1350 >s_e_s_b_s_e_s_b_s_d_s_c_s_a_s_e_s
GTGCTGGTGGCTGTGCTGGTGGCTGTGACGGTGTGGGTGTCTGTGCTGGTG; >clamp_s_c_s_d_s_c_s_a_s_e_s:compl:rev
CACCAGCACAGACACCCACACCGTCACCCACACTATCATGTTGACATTCC; dG = -35.5
1351 >s_e_s_c_s_a_s_c_s_e_s_c_s_a_s_e_s
GTGCTGGTGTGGGTGTCTGTGTGGGTGCTGGTGTGGGTGTCTGTGCTGGTG; >clamp_s_c_s_d_s_c_s_a_s_e_s:compl:rev
CACCAGCACAGACACCCACACCGTCACCCACACTATCATGTTGACATTCC; dG = -35.5

1352 >s_e_s_c_s_b_s_c_s_e_s_c_s_a_s_e_s
GTGCTGGTGTGGGTGGCTGTGTGGGTGCTGGTGTGGGTGTCTGTGCTGGTG; >clamp_s_c_s_d_s_c_s_a_s_e_s:compl:rev
CACCAGCACAGACACCCACACCGTCACCCACACTATCATGTTGACATTCC; dG = -35.5
1353 >s_e_s_c_s_c_s_c_s_e_s_c_s_a_s_e_s
GTGCTGGTGTGGGTGTGGGTGTGGGTGCTGGTGTGGGTGTCTGTGCTGGTG; >clamp_s_c_s_d_s_c_s_a_s_e_s:compl:rev
CACCAGCACAGACACCCACACCGTCACCCACACTATCATGTTGACATTCC; dG = -35.5
1354 >s_e_s_c_s_e_s_c_s_e_s_c_s_a_s_e_s
GTGCTGGTGTGGGTGCTGGTGTGGGTGCTGGTGTGGGTGTCTGTGCTGGTG; >clamp_s_c_s_d_s_c_s_a_s_e_s:compl:rev
CACCAGCACAGACACCCACACCGTCACCCACACTATCATGTTGACATTCC; dG = -35.5
1355 >s_e_s_d_s_a_s_c_s_e_s_c_s_a_s_e_s
GTGCTGGTGACGGTGTCTGTGTGGGTGCTGGTGTGGGTGTCTGTGCTGGTG; >clamp_s_c_s_d_s_c_s_a_s_e_s:compl:rev
CACCAGCACAGACACCCACACCGTCACCCACACTATCATGTTGACATTCC; dG = -35.5
1356 >s_e_s_d_s_a_s_b_s_d_s_c_s_a_s_e_s
GTGCTGGTGACGGTGTCTGTGGCTGTGACGGTGTGGGTGTCTGTGCTGGTG; >clamp_s_c_s_d_s_c_s_a_s_e_s:compl:rev
CACCAGCACAGACACCCACACCGTCACCCACACTATCATGTTGACATTCC; dG = -35.5
1357 >s_a_s_c_s_b_s_c_s_e_s_c_s_a_s_e_s
GTGTCTGTGTGGGTGGCTGTGTGGGTGCTGGTGTGGGTGTCTGTGCTGGTG; >clamp_s_c_s_d_s_c_s_a_s_e_s:compl:rev
CACCAGCACAGACACCCACACCGTCACCCACACTATCATGTTGACATTCC; dG = -35.5
1358 >s_e_s_d_s_b_s_c_s_e_s_c_s_a_s_e_s
GTGCTGGTGACGGTGGCTGTGTGGGTGCTGGTGTGGGTGTCTGTGCTGGTG; >clamp_s_c_s_d_s_c_s_a_s_e_s:compl:rev
CACCAGCACAGACACCCACACCGTCACCCACACTATCATGTTGACATTCC; dG = -35.5
1359 >s_e_s_d_s_b_s_b_s_d_s_c_s_a_s_e_s
GTGCTGGTGACGGTGGCTGTGGCTGTGACGGTGTGGGTGTCTGTGCTGGTG; >clamp_s_c_s_d_s_c_s_a_s_e_s:compl:rev
CACCAGCACAGACACCCACACCGTCACCCACACTATCATGTTGACATTCC; dG = -35.5
1360 >s_e_s_d_s_c_s_c_s_e_s_c_s_a_s_e_s
GTGCTGGTGACGGTGTGGGTGTGGGTGCTGGTGTGGGTGTCTGTGCTGGTG; >clamp_s_c_s_d_s_c_s_a_s_e_s:compl:rev
CACCAGCACAGACACCCACACCGTCACCCACACTATCATGTTGACATTCC; dG = -35.5
1361 >s_e_s_d_s_d_s_c_s_e_s_c_s_a_s_e_s
GTGCTGGTGACGGTGACGGTGTGGGTGCTGGTGTGGGTGTCTGTGCTGGTG; >clamp_s_c_s_d_s_c_s_a_s_e_s:compl:rev
CACCAGCACAGACACCCACACCGTCACCCACACTATCATGTTGACATTCC; dG = -35.5
1362 >s_e_s_d_s_d_s_b_s_d_s_c_s_a_s_e_s
GTGCTGGTGACGGTGACGGTGGCTGTGACGGTGTGGGTGTCTGTGCTGGTG; >clamp_s_c_s_d_s_c_s_a_s_e_s:compl:rev
CACCAGCACAGACACCCACACCGTCACCCACACTATCATGTTGACATTCC; dG = -35.5
1363 >s_e_s_d_s_e_s_c_s_e_s_c_s_a_s_e_s
GTGCTGGTGACGGTGCTGGTGTGGGTGCTGGTGTGGGTGTCTGTGCTGGTG; >clamp_s_c_s_d_s_c_s_a_s_e_s:compl:rev
CACCAGCACAGACACCCACACCGTCACCCACACTATCATGTTGACATTCC; dG = -35.5
1364 >s_e_s_d_s_e_s_b_s_d_s_c_s_a_s_e_s
GTGCTGGTGACGGTGCTGGTGGCTGTGACGGTGTGGGTGTCTGTGCTGGTG; >clamp_s_c_s_d_s_c_s_a_s_e_s:compl:rev
CACCAGCACAGACACCCACACCGTCACCCACACTATCATGTTGACATTCC; dG = -35.5
1365 >s_e_s_e_s_a_s_c_s_e_s_c_s_a_s_e_s
GTGCTGGTGCTGGTGTCTGTGTGGGTGCTGGTGTGGGTGTCTGTGCTGGTG; >clamp_s_c_s_d_s_c_s_a_s_e_s:compl:rev
CACCAGCACAGACACCCACACCGTCACCCACACTATCATGTTGACATTCC; dG = -35.5
1366 >s_e_s_e_s_a_s_b_s_d_s_c_s_a_s_e_s
GTGCTGGTGCTGGTGTCTGTGGCTGTGACGGTGTGGGTGTCTGTGCTGGTG; >clamp_s_c_s_d_s_c_s_a_s_e_s:compl:rev
CACCAGCACAGACACCCACACCGTCACCCACACTATCATGTTGACATTCC; dG = -35.5
1367 >s_e_s_e_s_b_s_c_s_e_s_c_s_a_s_e_s
GTGCTGGTGCTGGTGGCTGTGTGGGTGCTGGTGTGGGTGTCTGTGCTGGTG; >clamp_s_c_s_d_s_c_s_a_s_e_s:compl:rev
CACCAGCACAGACACCCACACCGTCACCCACACTATCATGTTGACATTCC; dG = -35.5
1368 >s_e_s_e_s_b_s_b_s_d_s_c_s_a_s_e_s
GTGCTGGTGCTGGTGGCTGTGGCTGTGACGGTGTGGGTGTCTGTGCTGGTG; >clamp_s_c_s_d_s_c_s_a_s_e_s:compl:rev
CACCAGCACAGACACCCACACCGTCACCCACACTATCATGTTGACATTCC; dG = -35.5
1369 >s_e_s_e_s_c_s_c_s_e_s_c_s_a_s_e_s
GTGCTGGTGCTGGTGTGGGTGTGGGTGCTGGTGTGGGTGTCTGTGCTGGTG; >clamp_s_c_s_d_s_c_s_a_s_e_s:compl:rev
CACCAGCACAGACACCCACACCGTCACCCACACTATCATGTTGACATTCC; dG = -35.5
1370 >s_e_s_e_s_d_s_c_s_e_s_c_s_a_s_e_s
GTGCTGGTGCTGGTGACGGTGTGGGTGCTGGTGTGGGTGTCTGTGCTGGTG; >clamp_s_c_s_d_s_c_s_a_s_e_s:compl:rev
CACCAGCACAGACACCCACACCGTCACCCACACTATCATGTTGACATTCC; dG = -35.5
1371 >s_e_s_e_s_d_s_b_s_d_s_c_s_a_s_e_s
GTGCTGGTGCTGGTGACGGTGGCTGTGACGGTGTGGGTGTCTGTGCTGGTG; >clamp_s_c_s_d_s_c_s_a_s_e_s:compl:rev
CACCAGCACAGACACCCACACCGTCACCCACACTATCATGTTGACATTCC; dG = -35.5
1372 >s_e_s_e_s_e_s_c_s_e_s_c_s_a_s_e_s
GTGCTGGTGCTGGTGCTGGTGTGGGTGCTGGTGTGGGTGTCTGTGCTGGTG; >clamp_s_c_s_d_s_c_s_a_s_e_s:compl:rev
CACCAGCACAGACACCCACACCGTCACCCACACTATCATGTTGACATTCC; dG = -35.5
1373 >s_e_s_e_s_e_s_b_s_d_s_c_s_a_s_e_s
GTGCTGGTGCTGGTGCTGGTGGCTGTGACGGTGTGGGTGTCTGTGCTGGTG; >clamp_s_c_s_d_s_c_s_a_s_e_s:compl:rev
CACCAGCACAGACACCCACACCGTCACCCACACTATCATGTTGACATTCC; dG = -35.5
1374 >s_a_s_c_s_c_s_c_s_e_s_c_s_a_s_e_s
GTGTCTGTGTGGGTGTGGGTGTGGGTGCTGGTGTGGGTGTCTGTGCTGGTG; >clamp_s_c_s_d_s_c_s_a_s_e_s:compl:rev
CACCAGCACAGACACCCACACCGTCACCCACACTATCATGTTGACATTCC; dG = -35.5
1375 >s_a_s_c_s_e_s_c_s_e_s_c_s_a_s_e_s
GTGTCTGTGTGGGTGCTGGTGTGGGTGCTGGTGTGGGTGTCTGTGCTGGTG; >clamp_s_c_s_d_s_c_s_a_s_e_s:compl:rev
CACCAGCACAGACACCCACACCGTCACCCACACTATCATGTTGACATTCC; dG = -35.5

1376 >s_a_s_d_s_a_s_c_s_e_s_c_s_a_s_e_s
GTGTCTGTGACGGTGTCTGTGTGGGTGCTGGTGTGGGTGTCTGTGCTGGTG; >clamp_s_c_s_d_s_c_s_a_s_e_s:compl:rev
CACCAGCACAGACACCCACACCGTCACCCACACTATCATGTTGACATTCC; dG = -35.5
1377 >s_a_s_d_s_a_s_b_s_d_s_c_s_a_s_e_s
GTGTCTGTGACGGTGTCTGTGGCTGTGACGGTGTGGGTGTCTGTGCTGGTG; >clamp_s_c_s_d_s_c_s_a_s_e_s:compl:rev
CACCAGCACAGACACCCACACCGTCACCCACACTATCATGTTGACATTCC; dG = -35.5
1378 >s_a_s_a_s_b_s_c_s_e_s_c_s_a_s_e_s
GTGTCTGTGTCTGTGGCTGTGTGGGTGCTGGTGTGGGTGTCTGTGCTGGTG; >clamp_s_c_s_d_s_c_s_a_s_e_s:compl:rev
CACCAGCACAGACACCCACACCGTCACCCACACTATCATGTTGACATTCC; dG = -35.5
1379 >s_a_s_d_s_b_s_c_s_e_s_c_s_a_s_e_s
GTGTCTGTGACGGTGGCTGTGTGGGTGCTGGTGTGGGTGTCTGTGCTGGTG; >clamp_s_c_s_d_s_c_s_a_s_e_s:compl:rev
CACCAGCACAGACACCCACACCGTCACCCACACTATCATGTTGACATTCC; dG = -35.5
1380 >s_a_s_d_s_b_s_b_s_d_s_c_s_a_s_e_s
GTGTCTGTGACGGTGGCTGTGGCTGTGACGGTGTGGGTGTCTGTGCTGGTG; >clamp_s_c_s_d_s_c_s_a_s_e_s:compl:rev
CACCAGCACAGACACCCACACCGTCACCCACACTATCATGTTGACATTCC; dG = -35.5
1381 >s_a_s_d_s_c_s_c_s_e_s_c_s_a_s_e_s
GTGTCTGTGACGGTGTGGGTGTGGGTGCTGGTGTGGGTGTCTGTGCTGGTG; >clamp_s_c_s_d_s_c_s_a_s_e_s:compl:rev
CACCAGCACAGACACCCACACCGTCACCCACACTATCATGTTGACATTCC; dG = -35.5
1382 >s_a_s_d_s_d_s_c_s_e_s_c_s_a_s_e_s
GTGTCTGTGACGGTGACGGTGTGGGTGCTGGTGTGGGTGTCTGTGCTGGTG; >clamp_s_c_s_d_s_c_s_a_s_e_s:compl:rev
CACCAGCACAGACACCCACACCGTCACCCACACTATCATGTTGACATTCC; dG = -35.5
1383 >s_a_s_d_s_d_s_b_s_d_s_c_s_a_s_e_s
GTGTCTGTGACGGTGACGGTGGCTGTGACGGTGTGGGTGTCTGTGCTGGTG; >clamp_s_c_s_d_s_c_s_a_s_e_s:compl:rev
CACCAGCACAGACACCCACACCGTCACCCACACTATCATGTTGACATTCC; dG = -35.5
1384 >s_a_s_a_s_b_s_b_s_d_s_c_s_a_s_e_s
GTGTCTGTGTCTGTGGCTGTGGCTGTGACGGTGTGGGTGTCTGTGCTGGTG; >clamp_s_c_s_d_s_c_s_a_s_e_s:compl:rev
CACCAGCACAGACACCCACACCGTCACCCACACTATCATGTTGACATTCC; dG = -35.5
1385 >s_a_s_d_s_e_s_c_s_e_s_c_s_a_s_e_s
GTGTCTGTGACGGTGCTGGTGTGGGTGCTGGTGTGGGTGTCTGTGCTGGTG; >clamp_s_c_s_d_s_c_s_a_s_e_s:compl:rev
CACCAGCACAGACACCCACACCGTCACCCACACTATCATGTTGACATTCC; dG = -35.5
1386 >s_a_s_d_s_e_s_b_s_d_s_c_s_a_s_e_s
GTGTCTGTGACGGTGCTGGTGGCTGTGACGGTGTGGGTGTCTGTGCTGGTG; >clamp_s_c_s_d_s_c_s_a_s_e_s:compl:rev
CACCAGCACAGACACCCACACCGTCACCCACACTATCATGTTGACATTCC; dG = -35.5
1387 >s_a_s_e_s_a_s_c_s_e_s_c_s_a_s_e_s
GTGTCTGTGCTGGTGTCTGTGTGGGTGCTGGTGTGGGTGTCTGTGCTGGTG; >clamp_s_c_s_d_s_c_s_a_s_e_s:compl:rev
CACCAGCACAGACACCCACACCGTCACCCACACTATCATGTTGACATTCC; dG = -35.5
1388 >s_a_s_e_s_a_s_b_s_d_s_c_s_a_s_e_s
GTGTCTGTGCTGGTGTCTGTGGCTGTGACGGTGTGGGTGTCTGTGCTGGTG; >clamp_s_c_s_d_s_c_s_a_s_e_s:compl:rev
CACCAGCACAGACACCCACACCGTCACCCACACTATCATGTTGACATTCC; dG = -35.5
1389 >s_a_s_e_s_b_s_c_s_e_s_c_s_a_s_e_s
GTGTCTGTGCTGGTGGCTGTGTGGGTGCTGGTGTGGGTGTCTGTGCTGGTG; >clamp_s_c_s_d_s_c_s_a_s_e_s:compl:rev
CACCAGCACAGACACCCACACCGTCACCCACACTATCATGTTGACATTCC; dG = -35.5
1390 >s_a_s_e_s_b_s_b_s_d_s_c_s_a_s_e_s
GTGTCTGTGCTGGTGGCTGTGGCTGTGACGGTGTGGGTGTCTGTGCTGGTG; >clamp_s_c_s_d_s_c_s_a_s_e_s:compl:rev
CACCAGCACAGACACCCACACCGTCACCCACACTATCATGTTGACATTCC; dG = -35.5
1391 >s_a_s_e_s_c_s_c_s_e_s_c_s_a_s_e_s
GTGTCTGTGCTGGTGTGGGTGTGGGTGCTGGTGTGGGTGTCTGTGCTGGTG; >clamp_s_c_s_d_s_c_s_a_s_e_s:compl:rev
CACCAGCACAGACACCCACACCGTCACCCACACTATCATGTTGACATTCC; dG = -35.5
1392 >s_a_s_e_s_d_s_c_s_e_s_c_s_a_s_e_s
GTGTCTGTGCTGGTGACGGTGTGGGTGCTGGTGTGGGTGTCTGTGCTGGTG; >clamp_s_c_s_d_s_c_s_a_s_e_s:compl:rev
CACCAGCACAGACACCCACACCGTCACCCACACTATCATGTTGACATTCC; dG = -35.5
1393 >s_a_s_e_s_d_s_b_s_d_s_c_s_a_s_e_s
GTGTCTGTGCTGGTGACGGTGGCTGTGACGGTGTGGGTGTCTGTGCTGGTG; >clamp_s_c_s_d_s_c_s_a_s_e_s:compl:rev
CACCAGCACAGACACCCACACCGTCACCCACACTATCATGTTGACATTCC; dG = -35.5
1394 >s_a_s_e_s_e_s_c_s_e_s_c_s_a_s_e_s
GTGTCTGTGCTGGTGCTGGTGTGGGTGCTGGTGTGGGTGTCTGTGCTGGTG; >clamp_s_c_s_d_s_c_s_a_s_e_s:compl:rev
CACCAGCACAGACACCCACACCGTCACCCACACTATCATGTTGACATTCC; dG = -35.5
1395 >s_a_s_e_s_e_s_b_s_d_s_c_s_a_s_e_s
GTGTCTGTGCTGGTGCTGGTGGCTGTGACGGTGTGGGTGTCTGTGCTGGTG; >clamp_s_c_s_d_s_c_s_a_s_e_s:compl:rev
CACCAGCACAGACACCCACACCGTCACCCACACTATCATGTTGACATTCC; dG = -35.5
1396 >s_b_s_a_s_a_s_c_s_e_s_c_s_a_s_e_s
GTGGCTGTGTCTGTGTCTGTGTGGGTGCTGGTGTGGGTGTCTGTGCTGGTG; >clamp_s_c_s_d_s_c_s_a_s_e_s:compl:rev
CACCAGCACAGACACCCACACCGTCACCCACACTATCATGTTGACATTCC; dG = -35.5
1397 >s_b_s_a_s_a_s_b_s_d_s_c_s_a_s_e_s
GTGGCTGTGTCTGTGTCTGTGGCTGTGACGGTGTGGGTGTCTGTGCTGGTG; >clamp_s_c_s_d_s_c_s_a_s_e_s:compl:rev
CACCAGCACAGACACCCACACCGTCACCCACACTATCATGTTGACATTCC; dG = -35.5
1398 >s_a_s_a_s_c_s_c_s_e_s_c_s_a_s_e_s
GTGTCTGTGTCTGTGTGGGTGTGGGTGCTGGTGTGGGTGTCTGTGCTGGTG; >clamp_s_c_s_d_s_c_s_a_s_e_s:compl:rev
CACCAGCACAGACACCCACACCGTCACCCACACTATCATGTTGACATTCC; dG = -35.5
1399 >s_b_s_a_s_b_s_c_s_e_s_c_s_a_s_e_s
GTGGCTGTGTCTGTGGCTGTGTGGGTGCTGGTGTGGGTGTCTGTGCTGGTG; >clamp_s_c_s_d_s_c_s_a_s_e_s:compl:rev
CACCAGCACAGACACCCACACCGTCACCCACACTATCATGTTGACATTCC; dG = -35.5

1400 >s_b_s_a_s_b_s_b_s_d_s_c_s_a_s_e_s
GTGGCTGTGTCTGTGGCTGTGGCTGTGACGGTGTGGGTGTCTGTGCTGGTG; >clamp_s_c_s_d_s_c_s_a_s_e_s:compl:rev
CACCAGCACAGACACCCACACCGTCACCCACACTATCATGTTGACATTCC; dG = -35.5
1401 >s_b_s_a_s_c_s_c_s_e_s_c_s_a_s_e_s
GTGGCTGTGTCTGTGTGGGTGTGGGTGCTGGTGTGGGTGTCTGTGCTGGTG; >clamp_s_c_s_d_s_c_s_a_s_e_s:compl:rev
CACCAGCACAGACACCCACACCGTCACCCACACTATCATGTTGACATTCC; dG = -35.5
1402 >s_b_s_a_s_d_s_c_s_e_s_c_s_a_s_e_s
GTGGCTGTGTCTGTGACGGTGTGGGTGCTGGTGTGGGTGTCTGTGCTGGTG; >clamp_s_c_s_d_s_c_s_a_s_e_s:compl:rev
CACCAGCACAGACACCCACACCGTCACCCACACTATCATGTTGACATTCC; dG = -35.5
1403 >s_b_s_a_s_d_s_b_s_d_s_c_s_a_s_e_s
GTGGCTGTGTCTGTGACGGTGGCTGTGACGGTGTGGGTGTCTGTGCTGGTG; >clamp_s_c_s_d_s_c_s_a_s_e_s:compl:rev
CACCAGCACAGACACCCACACCGTCACCCACACTATCATGTTGACATTCC; dG = -35.5
1404 >s_b_s_a_s_e_s_c_s_e_s_c_s_a_s_e_s
GTGGCTGTGTCTGTGCTGGTGTGGGTGCTGGTGTGGGTGTCTGTGCTGGTG; >clamp_s_c_s_d_s_c_s_a_s_e_s:compl:rev
CACCAGCACAGACACCCACACCGTCACCCACACTATCATGTTGACATTCC; dG = -35.5
1405 >s_b_s_a_s_e_s_b_s_d_s_c_s_a_s_e_s
GTGGCTGTGTCTGTGCTGGTGGCTGTGACGGTGTGGGTGTCTGTGCTGGTG; >clamp_s_c_s_d_s_c_s_a_s_e_s:compl:rev
CACCAGCACAGACACCCACACCGTCACCCACACTATCATGTTGACATTCC; dG = -35.5
1406 >s_b_s_b_s_a_s_c_s_e_s_c_s_a_s_e_s
GTGGCTGTGGCTGTGTCTGTGTGGGTGCTGGTGTGGGTGTCTGTGCTGGTG; >clamp_s_c_s_d_s_c_s_a_s_e_s:compl:rev
CACCAGCACAGACACCCACACCGTCACCCACACTATCATGTTGACATTCC; dG = -35.5
1407 >s_b_s_b_s_a_s_b_s_d_s_c_s_a_s_e_s
GTGGCTGTGGCTGTGTCTGTGGCTGTGACGGTGTGGGTGTCTGTGCTGGTG; >clamp_s_c_s_d_s_c_s_a_s_e_s:compl:rev
CACCAGCACAGACACCCACACCGTCACCCACACTATCATGTTGACATTCC; dG = -35.5
1408 >s_b_s_b_s_b_s_c_s_e_s_c_s_a_s_e_s
GTGGCTGTGGCTGTGGCTGTGTGGGTGCTGGTGTGGGTGTCTGTGCTGGTG; >clamp_s_c_s_d_s_c_s_a_s_e_s:compl:rev
CACCAGCACAGACACCCACACCGTCACCCACACTATCATGTTGACATTCC; dG = -35.5
1409 >s_b_s_b_s_b_s_b_s_d_s_c_s_a_s_e_s
GTGGCTGTGGCTGTGGCTGTGGCTGTGACGGTGTGGGTGTCTGTGCTGGTG; >clamp_s_c_s_d_s_c_s_a_s_e_s:compl:rev
CACCAGCACAGACACCCACACCGTCACCCACACTATCATGTTGACATTCC; dG = -35.5
1410 >s_b_s_b_s_c_s_c_s_e_s_c_s_a_s_e_s
GTGGCTGTGGCTGTGTGGGTGTGGGTGCTGGTGTGGGTGTCTGTGCTGGTG; >clamp_s_c_s_d_s_c_s_a_s_e_s:compl:rev
CACCAGCACAGACACCCACACCGTCACCCACACTATCATGTTGACATTCC; dG = -35.5
1411 >s_b_s_b_s_d_s_c_s_e_s_c_s_a_s_e_s
GTGGCTGTGGCTGTGACGGTGTGGGTGCTGGTGTGGGTGTCTGTGCTGGTG; >clamp_s_c_s_d_s_c_s_a_s_e_s:compl:rev
CACCAGCACAGACACCCACACCGTCACCCACACTATCATGTTGACATTCC; dG = -35.5
1412 >s_b_s_b_s_d_s_b_s_d_s_c_s_a_s_e_s
GTGGCTGTGGCTGTGACGGTGGCTGTGACGGTGTGGGTGTCTGTGCTGGTG; >clamp_s_c_s_d_s_c_s_a_s_e_s:compl:rev
CACCAGCACAGACACCCACACCGTCACCCACACTATCATGTTGACATTCC; dG = -35.5
1413 >s_b_s_b_s_e_s_c_s_e_s_c_s_a_s_e_s
GTGGCTGTGGCTGTGCTGGTGTGGGTGCTGGTGTGGGTGTCTGTGCTGGTG; >clamp_s_c_s_d_s_c_s_a_s_e_s:compl:rev
CACCAGCACAGACACCCACACCGTCACCCACACTATCATGTTGACATTCC; dG = -35.5
1414 >s_b_s_b_s_e_s_b_s_d_s_c_s_a_s_e_s
GTGGCTGTGGCTGTGCTGGTGGCTGTGACGGTGTGGGTGTCTGTGCTGGTG; >clamp_s_c_s_d_s_c_s_a_s_e_s:compl:rev
CACCAGCACAGACACCCACACCGTCACCCACACTATCATGTTGACATTCC; dG = -35.5
1415 >s_b_s_c_s_a_s_c_s_e_s_c_s_a_s_e_s
GTGGCTGTGTGGGTGTCTGTGTGGGTGCTGGTGTGGGTGTCTGTGCTGGTG; >clamp_s_c_s_d_s_c_s_a_s_e_s:compl:rev
CACCAGCACAGACACCCACACCGTCACCCACACTATCATGTTGACATTCC; dG = -35.5
1416 >s_a_s_a_s_d_s_c_s_e_s_c_s_a_s_e_s
GTGTCTGTGTCTGTGACGGTGTGGGTGCTGGTGTGGGTGTCTGTGCTGGTG; >clamp_s_c_s_d_s_c_s_a_s_e_s:compl:rev
CACCAGCACAGACACCCACACCGTCACCCACACTATCATGTTGACATTCC; dG = -35.5
1417 >s_b_s_c_s_b_s_c_s_e_s_c_s_a_s_e_s
GTGGCTGTGTGGGTGGCTGTGTGGGTGCTGGTGTGGGTGTCTGTGCTGGTG; >clamp_s_c_s_d_s_c_s_a_s_e_s:compl:rev
CACCAGCACAGACACCCACACCGTCACCCACACTATCATGTTGACATTCC; dG = -35.5
1418 >s_b_s_c_s_c_s_c_s_e_s_c_s_a_s_e_s
GTGGCTGTGTGGGTGTGGGTGTGGGTGCTGGTGTGGGTGTCTGTGCTGGTG; >clamp_s_c_s_d_s_c_s_a_s_e_s:compl:rev
CACCAGCACAGACACCCACACCGTCACCCACACTATCATGTTGACATTCC; dG = -35.5
1419 >s_a_s_a_s_d_s_b_s_d_s_c_s_a_s_e_s
GTGTCTGTGTCTGTGACGGTGGCTGTGACGGTGTGGGTGTCTGTGCTGGTG; >clamp_s_c_s_d_s_c_s_a_s_e_s:compl:rev
CACCAGCACAGACACCCACACCGTCACCCACACTATCATGTTGACATTCC; dG = -35.5
1420 >s_b_s_c_s_e_s_c_s_e_s_c_s_a_s_e_s
GTGGCTGTGTGGGTGCTGGTGTGGGTGCTGGTGTGGGTGTCTGTGCTGGTG; >clamp_s_c_s_d_s_c_s_a_s_e_s:compl:rev
CACCAGCACAGACACCCACACCGTCACCCACACTATCATGTTGACATTCC; dG = -35.5
1421 >s_b_s_d_s_a_s_c_s_e_s_c_s_a_s_e_s
GTGGCTGTGACGGTGTCTGTGTGGGTGCTGGTGTGGGTGTCTGTGCTGGTG; >clamp_s_c_s_d_s_c_s_a_s_e_s:compl:rev
CACCAGCACAGACACCCACACCGTCACCCACACTATCATGTTGACATTCC; dG = -35.5
1422 >s_b_s_d_s_a_s_b_s_d_s_c_s_a_s_e_s
GTGGCTGTGACGGTGTCTGTGGCTGTGACGGTGTGGGTGTCTGTGCTGGTG; >clamp_s_c_s_d_s_c_s_a_s_e_s:compl:rev
CACCAGCACAGACACCCACACCGTCACCCACACTATCATGTTGACATTCC; dG = -35.5
1423 >s_b_s_d_s_b_s_c_s_e_s_c_s_a_s_e_s
GTGGCTGTGACGGTGGCTGTGTGGGTGCTGGTGTGGGTGTCTGTGCTGGTG; >clamp_s_c_s_d_s_c_s_a_s_e_s:compl:rev
CACCAGCACAGACACCCACACCGTCACCCACACTATCATGTTGACATTCC; dG = -35.5

1424 >s_b_s_d_s_b_s_b_s_d_s_c_s_a_s_e_s
GTGGCTGTGACGGTGGCTGTGGCTGTGACGGTGTGGGTGTCTGTGCTGGTG; >clamp_s_c_s_d_s_c_s_a_s_e_s:compl:rev
CACCAGCACAGACACCCACACCGTCACCCACACTATCATGTTGACATTCC; dG = -35.5
1425 >s_b_s_d_s_c_s_c_s_e_s_c_s_a_s_e_s
GTGGCTGTGACGGTGTGGGTGTGGGTGCTGGTGTGGGTGTCTGTGCTGGTG; >clamp_s_c_s_d_s_c_s_a_s_e_s:compl:rev
CACCAGCACAGACACCCACACCGTCACCCACACTATCATGTTGACATTCC; dG = -35.5
1426 >s_b_s_d_s_d_s_c_s_e_s_c_s_a_s_e_s
GTGGCTGTGACGGTGACGGTGTGGGTGCTGGTGTGGGTGTCTGTGCTGGTG; >clamp_s_c_s_d_s_c_s_a_s_e_s:compl:rev
CACCAGCACAGACACCCACACCGTCACCCACACTATCATGTTGACATTCC; dG = -35.5
1427 >s_b_s_d_s_d_s_b_s_d_s_c_s_a_s_e_s
GTGGCTGTGACGGTGACGGTGGCTGTGACGGTGTGGGTGTCTGTGCTGGTG; >clamp_s_c_s_d_s_c_s_a_s_e_s:compl:rev
CACCAGCACAGACACCCACACCGTCACCCACACTATCATGTTGACATTCC; dG = -35.5
1428 >s_b_s_d_s_e_s_c_s_e_s_c_s_a_s_e_s
GTGGCTGTGACGGTGCTGGTGTGGGTGCTGGTGTGGGTGTCTGTGCTGGTG; >clamp_s_c_s_d_s_c_s_a_s_e_s:compl:rev
CACCAGCACAGACACCCACACCGTCACCCACACTATCATGTTGACATTCC; dG = -35.5
1429 >s_b_s_d_s_e_s_b_s_d_s_c_s_a_s_e_s
GTGGCTGTGACGGTGCTGGTGGCTGTGACGGTGTGGGTGTCTGTGCTGGTG; >clamp_s_c_s_d_s_c_s_a_s_e_s:compl:rev
CACCAGCACAGACACCCACACCGTCACCCACACTATCATGTTGACATTCC; dG = -35.5
1430 >s_b_s_e_s_a_s_c_s_e_s_c_s_a_s_e_s
GTGGCTGTGCTGGTGTCTGTGTGGGTGCTGGTGTGGGTGTCTGTGCTGGTG; >clamp_s_c_s_d_s_c_s_a_s_e_s:compl:rev
CACCAGCACAGACACCCACACCGTCACCCACACTATCATGTTGACATTCC; dG = -35.5
1431 >s_b_s_e_s_a_s_b_s_d_s_c_s_a_s_e_s
GTGGCTGTGCTGGTGTCTGTGGCTGTGACGGTGTGGGTGTCTGTGCTGGTG; >clamp_s_c_s_d_s_c_s_a_s_e_s:compl:rev
CACCAGCACAGACACCCACACCGTCACCCACACTATCATGTTGACATTCC; dG = -35.5
1432 >s_a_s_a_s_e_s_c_s_e_s_c_s_a_s_e_s
GTGTCTGTGTCTGTGCTGGTGTGGGTGCTGGTGTGGGTGTCTGTGCTGGTG; >clamp_s_c_s_d_s_c_s_a_s_e_s:compl:rev
CACCAGCACAGACACCCACACCGTCACCCACACTATCATGTTGACATTCC; dG = -35.5
1433 >s_b_s_e_s_b_s_c_s_e_s_c_s_a_s_e_s
GTGGCTGTGCTGGTGGCTGTGTGGGTGCTGGTGTGGGTGTCTGTGCTGGTG; >clamp_s_c_s_d_s_c_s_a_s_e_s:compl:rev
CACCAGCACAGACACCCACACCGTCACCCACACTATCATGTTGACATTCC; dG = -35.5
1434 >s_b_s_e_s_b_s_b_s_d_s_c_s_a_s_e_s
GTGGCTGTGCTGGTGGCTGTGGCTGTGACGGTGTGGGTGTCTGTGCTGGTG; >clamp_s_c_s_d_s_c_s_a_s_e_s:compl:rev
CACCAGCACAGACACCCACACCGTCACCCACACTATCATGTTGACATTCC; dG = -35.5
1435 >s_b_s_e_s_c_s_c_s_e_s_c_s_a_s_e_s
GTGGCTGTGCTGGTGTGGGTGTGGGTGCTGGTGTGGGTGTCTGTGCTGGTG; >clamp_s_c_s_d_s_c_s_a_s_e_s:compl:rev
CACCAGCACAGACACCCACACCGTCACCCACACTATCATGTTGACATTCC; dG = -35.5
1436 >s_b_s_e_s_d_s_c_s_e_s_c_s_a_s_e_s
GTGGCTGTGCTGGTGACGGTGTGGGTGCTGGTGTGGGTGTCTGTGCTGGTG; >clamp_s_c_s_d_s_c_s_a_s_e_s:compl:rev
CACCAGCACAGACACCCACACCGTCACCCACACTATCATGTTGACATTCC; dG = -35.5
1437 >s_b_s_e_s_d_s_b_s_d_s_c_s_a_s_e_s
GTGGCTGTGCTGGTGACGGTGGCTGTGACGGTGTGGGTGTCTGTGCTGGTG; >clamp_s_c_s_d_s_c_s_a_s_e_s:compl:rev
CACCAGCACAGACACCCACACCGTCACCCACACTATCATGTTGACATTCC; dG = -35.5
1438 >s_a_s_a_s_e_s_b_s_d_s_c_s_a_s_e_s
GTGTCTGTGTCTGTGCTGGTGGCTGTGACGGTGTGGGTGTCTGTGCTGGTG; >clamp_s_c_s_d_s_c_s_a_s_e_s:compl:rev
CACCAGCACAGACACCCACACCGTCACCCACACTATCATGTTGACATTCC; dG = -35.5
1439 >s_b_s_e_s_e_s_c_s_e_s_c_s_a_s_e_s
GTGGCTGTGCTGGTGCTGGTGTGGGTGCTGGTGTGGGTGTCTGTGCTGGTG; >clamp_s_c_s_d_s_c_s_a_s_e_s:compl:rev
CACCAGCACAGACACCCACACCGTCACCCACACTATCATGTTGACATTCC; dG = -35.5
1440 >s_b_s_e_s_e_s_b_s_d_s_c_s_a_s_e_s
GTGGCTGTGCTGGTGCTGGTGGCTGTGACGGTGTGGGTGTCTGTGCTGGTG; >clamp_s_c_s_d_s_c_s_a_s_e_s:compl:rev
CACCAGCACAGACACCCACACCGTCACCCACACTATCATGTTGACATTCC; dG = -35.5
1441 >s_a_s_a_s_e_s_a_s_d_s_c_s_a_s_e_s
GTGTCTGTGTCTGTGCTGGTGTCTGTGACGGTGTGGGTGTCTGTGCTGGTG; >clamp_s_c_s_d_s_c_s_a_s_e_s:compl:rev
CACCAGCACAGACACCCACACCGTCACCCACACTATCATGTTGACATTCC; dG = -35.3
1442 >s_b_s_e_s_e_s_a_s_d_s_c_s_a_s_e_s
GTGGCTGTGCTGGTGCTGGTGTCTGTGACGGTGTGGGTGTCTGTGCTGGTG; >clamp_s_c_s_d_s_c_s_a_s_e_s:compl:rev
CACCAGCACAGACACCCACACCGTCACCCACACTATCATGTTGACATTCC; dG = -35.3
1443 >s_c_s_a_s_a_s_c_s_d_s_a_s_a_s_e_s
GTGTGGGTGTCTGTGTCTGTGTGGGTGACGGTGTCTGTGTCTGTGCTGGTG; >clamp_s_c_s_d_s_c_s_a_s_e_s:compl:rev
CACCAGCACAGACACCCACACCGTCACCCACACTATCATGTTGACATTCC; dG = -35.3
1444 >s_c_s_a_s_a_s_c_s_b_s_c_s_a_s_e_s
GTGTGGGTGTCTGTGTCTGTGTGGGTGGCTGTGTGGGTGTCTGTGCTGGTG; >clamp_s_c_s_d_s_c_s_a_s_e_s:compl:rev
CACCAGCACAGACACCCACACCGTCACCCACACTATCATGTTGACATTCC; dG = -35.3
1445 >s_c_s_a_s_a_s_a_s_d_s_c_s_a_s_e_s
GTGTGGGTGTCTGTGTCTGTGTCTGTGACGGTGTGGGTGTCTGTGCTGGTG; >clamp_s_c_s_d_s_c_s_a_s_e_s:compl:rev
CACCAGCACAGACACCCACACCGTCACCCACACTATCATGTTGACATTCC; dG = -35.3
1446 >s_c_s_a_s_b_s_c_s_d_s_a_s_a_s_e_s
GTGTGGGTGTCTGTGGCTGTGTGGGTGACGGTGTCTGTGTCTGTGCTGGTG; >clamp_s_c_s_d_s_c_s_a_s_e_s:compl:rev
CACCAGCACAGACACCCACACCGTCACCCACACTATCATGTTGACATTCC; dG = -35.3
1447 >s_c_s_a_s_b_s_c_s_b_s_c_s_a_s_e_s
GTGTGGGTGTCTGTGGCTGTGTGGGTGGCTGTGTGGGTGTCTGTGCTGGTG; >clamp_s_c_s_d_s_c_s_a_s_e_s:compl:rev
CACCAGCACAGACACCCACACCGTCACCCACACTATCATGTTGACATTCC; dG = -35.3

1448 >s_c_s_a_s_b_s_a_s_d_s_c_s_a_s_e_s
GTGTGGGTGTCTGTGGCTGTGTCTGTGACGGTGTGGGTGTCTGTGCTGGTG; >clamp_s_c_s_d_s_c_s_a_s_e_s:compl:rev
CACCAGCACAGACACCCACACCGTCACCCACACTATCATGTTGACATTCC; dG = -35.3
1449 >s_c_s_a_s_c_s_c_s_d_s_a_s_a_s_e_s
GTGTGGGTGTCTGTGTGGGTGTGGGTGACGGTGTCTGTGTCTGTGCTGGTG; >clamp_s_c_s_d_s_c_s_a_s_e_s:compl:rev
CACCAGCACAGACACCCACACCGTCACCCACACTATCATGTTGACATTCC; dG = -35.3
1450 >s_c_s_a_s_c_s_c_s_b_s_c_s_a_s_e_s
GTGTGGGTGTCTGTGTGGGTGTGGGTGGCTGTGTGGGTGTCTGTGCTGGTG; >clamp_s_c_s_d_s_c_s_a_s_e_s:compl:rev
CACCAGCACAGACACCCACACCGTCACCCACACTATCATGTTGACATTCC; dG = -35.3
1451 >s_c_s_a_s_d_s_c_s_d_s_a_s_a_s_e_s
GTGTGGGTGTCTGTGACGGTGTGGGTGACGGTGTCTGTGTCTGTGCTGGTG; >clamp_s_c_s_d_s_c_s_a_s_e_s:compl:rev
CACCAGCACAGACACCCACACCGTCACCCACACTATCATGTTGACATTCC; dG = -35.3
1452 >s_c_s_a_s_d_s_c_s_b_s_c_s_a_s_e_s
GTGTGGGTGTCTGTGACGGTGTGGGTGGCTGTGTGGGTGTCTGTGCTGGTG; >clamp_s_c_s_d_s_c_s_a_s_e_s:compl:rev
CACCAGCACAGACACCCACACCGTCACCCACACTATCATGTTGACATTCC; dG = -35.3
1453 >s_c_s_a_s_d_s_a_s_d_s_c_s_a_s_e_s
GTGTGGGTGTCTGTGACGGTGTCTGTGACGGTGTGGGTGTCTGTGCTGGTG; >clamp_s_c_s_d_s_c_s_a_s_e_s:compl:rev
CACCAGCACAGACACCCACACCGTCACCCACACTATCATGTTGACATTCC; dG = -35.3
1454 >s_c_s_a_s_e_s_c_s_d_s_a_s_a_s_e_s
GTGTGGGTGTCTGTGCTGGTGTGGGTGACGGTGTCTGTGTCTGTGCTGGTG; >clamp_s_c_s_d_s_c_s_a_s_e_s:compl:rev
CACCAGCACAGACACCCACACCGTCACCCACACTATCATGTTGACATTCC; dG = -35.3
1455 >s_c_s_a_s_e_s_c_s_b_s_c_s_a_s_e_s
GTGTGGGTGTCTGTGCTGGTGTGGGTGGCTGTGTGGGTGTCTGTGCTGGTG; >clamp_s_c_s_d_s_c_s_a_s_e_s:compl:rev
CACCAGCACAGACACCCACACCGTCACCCACACTATCATGTTGACATTCC; dG = -35.3
1456 >s_c_s_a_s_e_s_a_s_d_s_c_s_a_s_e_s
GTGTGGGTGTCTGTGCTGGTGTCTGTGACGGTGTGGGTGTCTGTGCTGGTG; >clamp_s_c_s_d_s_c_s_a_s_e_s:compl:rev
CACCAGCACAGACACCCACACCGTCACCCACACTATCATGTTGACATTCC; dG = -35.3
1457 >s_c_s_b_s_a_s_c_s_d_s_a_s_a_s_e_s
GTGTGGGTGGCTGTGTCTGTGTGGGTGACGGTGTCTGTGTCTGTGCTGGTG; >clamp_s_c_s_d_s_c_s_a_s_e_s:compl:rev
CACCAGCACAGACACCCACACCGTCACCCACACTATCATGTTGACATTCC; dG = -35.3
1458 >s_c_s_b_s_a_s_c_s_b_s_c_s_a_s_e_s
GTGTGGGTGGCTGTGTCTGTGTGGGTGGCTGTGTGGGTGTCTGTGCTGGTG; >clamp_s_c_s_d_s_c_s_a_s_e_s:compl:rev
CACCAGCACAGACACCCACACCGTCACCCACACTATCATGTTGACATTCC; dG = -35.3
1459 >s_a_s_b_s_a_s_c_s_d_s_a_s_a_s_e_s
GTGTCTGTGGCTGTGTCTGTGTGGGTGACGGTGTCTGTGTCTGTGCTGGTG; >clamp_s_c_s_d_s_c_s_a_s_e_s:compl:rev
CACCAGCACAGACACCCACACCGTCACCCACACTATCATGTTGACATTCC; dG = -35.3
1460 >s_c_s_b_s_a_s_a_s_d_s_c_s_a_s_e_s
GTGTGGGTGGCTGTGTCTGTGTCTGTGACGGTGTGGGTGTCTGTGCTGGTG; >clamp_s_c_s_d_s_c_s_a_s_e_s:compl:rev
CACCAGCACAGACACCCACACCGTCACCCACACTATCATGTTGACATTCC; dG = -35.3
1461 >s_c_s_b_s_b_s_c_s_d_s_a_s_a_s_e_s
GTGTGGGTGGCTGTGGCTGTGTGGGTGACGGTGTCTGTGTCTGTGCTGGTG; >clamp_s_c_s_d_s_c_s_a_s_e_s:compl:rev
CACCAGCACAGACACCCACACCGTCACCCACACTATCATGTTGACATTCC; dG = -35.3
1462 >s_c_s_b_s_b_s_c_s_b_s_c_s_a_s_e_s
GTGTGGGTGGCTGTGGCTGTGTGGGTGGCTGTGTGGGTGTCTGTGCTGGTG; >clamp_s_c_s_d_s_c_s_a_s_e_s:compl:rev
CACCAGCACAGACACCCACACCGTCACCCACACTATCATGTTGACATTCC; dG = -35.3
1463 >s_c_s_b_s_b_s_a_s_d_s_c_s_a_s_e_s
GTGTGGGTGGCTGTGGCTGTGTCTGTGACGGTGTGGGTGTCTGTGCTGGTG; >clamp_s_c_s_d_s_c_s_a_s_e_s:compl:rev
CACCAGCACAGACACCCACACCGTCACCCACACTATCATGTTGACATTCC; dG = -35.3
1464 >s_a_s_b_s_a_s_c_s_b_s_c_s_a_s_e_s
GTGTCTGTGGCTGTGTCTGTGTGGGTGGCTGTGTGGGTGTCTGTGCTGGTG; >clamp_s_c_s_d_s_c_s_a_s_e_s:compl:rev
CACCAGCACAGACACCCACACCGTCACCCACACTATCATGTTGACATTCC; dG = -35.3
1465 >s_c_s_b_s_c_s_c_s_d_s_a_s_a_s_e_s
GTGTGGGTGGCTGTGTGGGTGTGGGTGACGGTGTCTGTGTCTGTGCTGGTG; >clamp_s_c_s_d_s_c_s_a_s_e_s:compl:rev
CACCAGCACAGACACCCACACCGTCACCCACACTATCATGTTGACATTCC; dG = -35.3
1466 >s_c_s_b_s_c_s_c_s_b_s_c_s_a_s_e_s
GTGTGGGTGGCTGTGTGGGTGTGGGTGGCTGTGTGGGTGTCTGTGCTGGTG; >clamp_s_c_s_d_s_c_s_a_s_e_s:compl:rev
CACCAGCACAGACACCCACACCGTCACCCACACTATCATGTTGACATTCC; dG = -35.3
1467 >s_c_s_b_s_d_s_c_s_d_s_a_s_a_s_e_s
GTGTGGGTGGCTGTGACGGTGTGGGTGACGGTGTCTGTGTCTGTGCTGGTG; >clamp_s_c_s_d_s_c_s_a_s_e_s:compl:rev
CACCAGCACAGACACCCACACCGTCACCCACACTATCATGTTGACATTCC; dG = -35.3
1468 >s_c_s_b_s_d_s_c_s_b_s_c_s_a_s_e_s
GTGTGGGTGGCTGTGACGGTGTGGGTGGCTGTGTGGGTGTCTGTGCTGGTG; >clamp_s_c_s_d_s_c_s_a_s_e_s:compl:rev
CACCAGCACAGACACCCACACCGTCACCCACACTATCATGTTGACATTCC; dG = -35.3
1469 >s_c_s_b_s_d_s_a_s_d_s_c_s_a_s_e_s
GTGTGGGTGGCTGTGACGGTGTCTGTGACGGTGTGGGTGTCTGTGCTGGTG; >clamp_s_c_s_d_s_c_s_a_s_e_s:compl:rev
CACCAGCACAGACACCCACACCGTCACCCACACTATCATGTTGACATTCC; dG = -35.3
1470 >s_c_s_b_s_e_s_c_s_d_s_a_s_a_s_e_s
GTGTGGGTGGCTGTGCTGGTGTGGGTGACGGTGTCTGTGTCTGTGCTGGTG; >clamp_s_c_s_d_s_c_s_a_s_e_s:compl:rev
CACCAGCACAGACACCCACACCGTCACCCACACTATCATGTTGACATTCC; dG = -35.3
1471 >s_c_s_b_s_e_s_c_s_b_s_c_s_a_s_e_s
GTGTGGGTGGCTGTGCTGGTGTGGGTGGCTGTGTGGGTGTCTGTGCTGGTG; >clamp_s_c_s_d_s_c_s_a_s_e_s:compl:rev
CACCAGCACAGACACCCACACCGTCACCCACACTATCATGTTGACATTCC; dG = -35.3

1472 >s_a_s_a_s_a_s_c_s_d_s_a_s_a_s_e_s
GTGTCTGTGTCTGTGTCTGTGTGGGTGACGGTGTCTGTGTCTGTGCTGGTG; >clamp_s_c_s_d_s_c_s_a_s_e_s:compl:rev
CACCAGCACAGACACCCACACCGTCACCCACACTATCATGTTGACATTCC; dG = -35.3
1473 >s_a_s_b_s_a_s_a_s_d_s_c_s_a_s_e_s
GTGTCTGTGGCTGTGTCTGTGTCTGTGACGGTGTGGGTGTCTGTGCTGGTG; >clamp_s_c_s_d_s_c_s_a_s_e_s:compl:rev
CACCAGCACAGACACCCACACCGTCACCCACACTATCATGTTGACATTCC; dG = -35.3
1474 >s_c_s_b_s_e_s_a_s_d_s_c_s_a_s_e_s
GTGTGGGTGGCTGTGCTGGTGTCTGTGACGGTGTGGGTGTCTGTGCTGGTG; >clamp_s_c_s_d_s_c_s_a_s_e_s:compl:rev
CACCAGCACAGACACCCACACCGTCACCCACACTATCATGTTGACATTCC; dG = -35.3
1475 >s_c_s_c_s_a_s_c_s_d_s_a_s_a_s_e_s
GTGTGGGTGTGGGTGTCTGTGTGGGTGACGGTGTCTGTGTCTGTGCTGGTG; >clamp_s_c_s_d_s_c_s_a_s_e_s:compl:rev
CACCAGCACAGACACCCACACCGTCACCCACACTATCATGTTGACATTCC; dG = -35.3
1476 >s_c_s_c_s_a_s_c_s_b_s_c_s_a_s_e_s
GTGTGGGTGTGGGTGTCTGTGTGGGTGGCTGTGTGGGTGTCTGTGCTGGTG; >clamp_s_c_s_d_s_c_s_a_s_e_s:compl:rev
CACCAGCACAGACACCCACACCGTCACCCACACTATCATGTTGACATTCC; dG = -35.3
1477 >s_c_s_c_s_b_s_c_s_d_s_a_s_a_s_e_s
GTGTGGGTGTGGGTGGCTGTGTGGGTGACGGTGTCTGTGTCTGTGCTGGTG; >clamp_s_c_s_d_s_c_s_a_s_e_s:compl:rev
CACCAGCACAGACACCCACACCGTCACCCACACTATCATGTTGACATTCC; dG = -35.3
1478 >s_c_s_c_s_b_s_c_s_b_s_c_s_a_s_e_s
GTGTGGGTGTGGGTGGCTGTGTGGGTGGCTGTGTGGGTGTCTGTGCTGGTG; >clamp_s_c_s_d_s_c_s_a_s_e_s:compl:rev
CACCAGCACAGACACCCACACCGTCACCCACACTATCATGTTGACATTCC; dG = -35.3
1479 >s_c_s_c_s_c_s_c_s_d_s_a_s_a_s_e_s
GTGTGGGTGTGGGTGTGGGTGTGGGTGACGGTGTCTGTGTCTGTGCTGGTG; >clamp_s_c_s_d_s_c_s_a_s_e_s:compl:rev
CACCAGCACAGACACCCACACCGTCACCCACACTATCATGTTGACATTCC; dG = -35.3
1480 >s_c_s_c_s_c_s_c_s_b_s_c_s_a_s_e_s
GTGTGGGTGTGGGTGTGGGTGTGGGTGGCTGTGTGGGTGTCTGTGCTGGTG; >clamp_s_c_s_d_s_c_s_a_s_e_s:compl:rev
CACCAGCACAGACACCCACACCGTCACCCACACTATCATGTTGACATTCC; dG = -35.3
1481 >s_c_s_c_s_e_s_c_s_d_s_a_s_a_s_e_s
GTGTGGGTGTGGGTGCTGGTGTGGGTGACGGTGTCTGTGTCTGTGCTGGTG; >clamp_s_c_s_d_s_c_s_a_s_e_s:compl:rev
CACCAGCACAGACACCCACACCGTCACCCACACTATCATGTTGACATTCC; dG = -35.3
1482 >s_c_s_c_s_e_s_c_s_b_s_c_s_a_s_e_s
GTGTGGGTGTGGGTGCTGGTGTGGGTGGCTGTGTGGGTGTCTGTGCTGGTG; >clamp_s_c_s_d_s_c_s_a_s_e_s:compl:rev
CACCAGCACAGACACCCACACCGTCACCCACACTATCATGTTGACATTCC; dG = -35.3
1483 >s_c_s_d_s_a_s_c_s_d_s_a_s_a_s_e_s
GTGTGGGTGACGGTGTCTGTGTGGGTGACGGTGTCTGTGTCTGTGCTGGTG; >clamp_s_c_s_d_s_c_s_a_s_e_s:compl:rev
CACCAGCACAGACACCCACACCGTCACCCACACTATCATGTTGACATTCC; dG = -35.3
1484 >s_c_s_d_s_a_s_c_s_b_s_c_s_a_s_e_s
GTGTGGGTGACGGTGTCTGTGTGGGTGGCTGTGTGGGTGTCTGTGCTGGTG; >clamp_s_c_s_d_s_c_s_a_s_e_s:compl:rev
CACCAGCACAGACACCCACACCGTCACCCACACTATCATGTTGACATTCC; dG = -35.3
1485 >s_a_s_b_s_b_s_c_s_d_s_a_s_a_s_e_s
GTGTCTGTGGCTGTGGCTGTGTGGGTGACGGTGTCTGTGTCTGTGCTGGTG; >clamp_s_c_s_d_s_c_s_a_s_e_s:compl:rev
CACCAGCACAGACACCCACACCGTCACCCACACTATCATGTTGACATTCC; dG = -35.3
1486 >s_c_s_d_s_a_s_a_s_d_s_c_s_a_s_e_s
GTGTGGGTGACGGTGTCTGTGTCTGTGACGGTGTGGGTGTCTGTGCTGGTG; >clamp_s_c_s_d_s_c_s_a_s_e_s:compl:rev
CACCAGCACAGACACCCACACCGTCACCCACACTATCATGTTGACATTCC; dG = -35.3
1487 >s_c_s_d_s_b_s_c_s_d_s_a_s_a_s_e_s
GTGTGGGTGACGGTGGCTGTGTGGGTGACGGTGTCTGTGTCTGTGCTGGTG; >clamp_s_c_s_d_s_c_s_a_s_e_s:compl:rev
CACCAGCACAGACACCCACACCGTCACCCACACTATCATGTTGACATTCC; dG = -35.3
1488 >s_c_s_d_s_b_s_c_s_b_s_c_s_a_s_e_s
GTGTGGGTGACGGTGGCTGTGTGGGTGGCTGTGTGGGTGTCTGTGCTGGTG; >clamp_s_c_s_d_s_c_s_a_s_e_s:compl:rev
CACCAGCACAGACACCCACACCGTCACCCACACTATCATGTTGACATTCC; dG = -35.3
1489 >s_c_s_d_s_b_s_a_s_d_s_c_s_a_s_e_s
GTGTGGGTGACGGTGGCTGTGTCTGTGACGGTGTGGGTGTCTGTGCTGGTG; >clamp_s_c_s_d_s_c_s_a_s_e_s:compl:rev
CACCAGCACAGACACCCACACCGTCACCCACACTATCATGTTGACATTCC; dG = -35.3
1490 >s_a_s_b_s_b_s_c_s_b_s_c_s_a_s_e_s
GTGTCTGTGGCTGTGGCTGTGTGGGTGGCTGTGTGGGTGTCTGTGCTGGTG; >clamp_s_c_s_d_s_c_s_a_s_e_s:compl:rev
CACCAGCACAGACACCCACACCGTCACCCACACTATCATGTTGACATTCC; dG = -35.3
1491 >s_c_s_d_s_c_s_c_s_d_s_b_s_a_s_e_s
GTGTGGGTGACGGTGTGGGTGTGGGTGACGGTGGCTGTGTCTGTGCTGGTG; >clamp_s_c_s_d_s_c_s_a_s_e_s:compl:rev
CACCAGCACAGACACCCACACCGTCACCCACACTATCATGTTGACATTCC; dG = -35.3
1492 >s_c_s_d_s_c_s_c_s_d_s_a_s_a_s_e_s
GTGTGGGTGACGGTGTGGGTGTGGGTGACGGTGTCTGTGTCTGTGCTGGTG; >clamp_s_c_s_d_s_c_s_a_s_e_s:compl:rev
CACCAGCACAGACACCCACACCGTCACCCACACTATCATGTTGACATTCC; dG = -35.3
1493 >s_c_s_d_s_c_s_c_s_b_s_c_s_a_s_e_s
GTGTGGGTGACGGTGTGGGTGTGGGTGGCTGTGTGGGTGTCTGTGCTGGTG; >clamp_s_c_s_d_s_c_s_a_s_e_s:compl:rev
CACCAGCACAGACACCCACACCGTCACCCACACTATCATGTTGACATTCC; dG = -35.3
1494 >s_c_s_d_s_c_s_c_s_a_s_c_s_a_s_e_s
GTGTGGGTGACGGTGTGGGTGTGGGTGTCTGTGTGGGTGTCTGTGCTGGTG; >clamp_s_c_s_d_s_c_s_a_s_e_s:compl:rev
CACCAGCACAGACACCCACACCGTCACCCACACTATCATGTTGACATTCC; dG = -35.3
1495 >s_c_s_d_s_d_s_c_s_d_s_a_s_a_s_e_s
GTGTGGGTGACGGTGACGGTGTGGGTGACGGTGTCTGTGTCTGTGCTGGTG; >clamp_s_c_s_d_s_c_s_a_s_e_s:compl:rev
CACCAGCACAGACACCCACACCGTCACCCACACTATCATGTTGACATTCC; dG = -35.3

1496 >s_c_s_d_s_d_s_c_s_b_s_c_s_a_s_e_s
GTGTGGGTGACGGTGACGGTGTGGGTGGCTGTGTGGGTGTCTGTGCTGGTG; >clamp_s_c_s_d_s_c_s_a_s_e_s:compl:rev
CACCAGCACAGACACCCACACCGTCACCCACACTATCATGTTGACATTCC; dG = -35.3
1497 >s_c_s_d_s_d_s_a_s_d_s_c_s_a_s_e_s
GTGTGGGTGACGGTGACGGTGTCTGTGACGGTGTGGGTGTCTGTGCTGGTG; >clamp_s_c_s_d_s_c_s_a_s_e_s:compl:rev
CACCAGCACAGACACCCACACCGTCACCCACACTATCATGTTGACATTCC; dG = -35.3
1498 >s_c_s_d_s_e_s_c_s_d_s_a_s_a_s_e_s
GTGTGGGTGACGGTGCTGGTGTGGGTGACGGTGTCTGTGTCTGTGCTGGTG; >clamp_s_c_s_d_s_c_s_a_s_e_s:compl:rev
CACCAGCACAGACACCCACACCGTCACCCACACTATCATGTTGACATTCC; dG = -35.3
1499 >s_c_s_d_s_e_s_c_s_b_s_c_s_a_s_e_s
GTGTGGGTGACGGTGCTGGTGTGGGTGGCTGTGTGGGTGTCTGTGCTGGTG; >clamp_s_c_s_d_s_c_s_a_s_e_s:compl:rev
CACCAGCACAGACACCCACACCGTCACCCACACTATCATGTTGACATTCC; dG = -35.3
1500 >s_a_s_b_s_b_s_a_s_d_s_c_s_a_s_e_s
GTGTCTGTGGCTGTGGCTGTGTCTGTGACGGTGTGGGTGTCTGTGCTGGTG; >clamp_s_c_s_d_s_c_s_a_s_e_s:compl:rev
CACCAGCACAGACACCCACACCGTCACCCACACTATCATGTTGACATTCC; dG = -35.3
1501 >s_c_s_d_s_e_s_a_s_d_s_c_s_a_s_e_s
GTGTGGGTGACGGTGCTGGTGTCTGTGACGGTGTGGGTGTCTGTGCTGGTG; >clamp_s_c_s_d_s_c_s_a_s_e_s:compl:rev
CACCAGCACAGACACCCACACCGTCACCCACACTATCATGTTGACATTCC; dG = -35.3
1502 >s_c_s_e_s_a_s_c_s_d_s_a_s_a_s_e_s
GTGTGGGTGCTGGTGTCTGTGTGGGTGACGGTGTCTGTGTCTGTGCTGGTG; >clamp_s_c_s_d_s_c_s_a_s_e_s:compl:rev
CACCAGCACAGACACCCACACCGTCACCCACACTATCATGTTGACATTCC; dG = -35.3
1503 >s_c_s_e_s_a_s_c_s_b_s_c_s_a_s_e_s
GTGTGGGTGCTGGTGTCTGTGTGGGTGGCTGTGTGGGTGTCTGTGCTGGTG; >clamp_s_c_s_d_s_c_s_a_s_e_s:compl:rev
CACCAGCACAGACACCCACACCGTCACCCACACTATCATGTTGACATTCC; dG = -35.3
1504 >s_c_s_e_s_a_s_a_s_d_s_c_s_a_s_e_s
GTGTGGGTGCTGGTGTCTGTGTCTGTGACGGTGTGGGTGTCTGTGCTGGTG; >clamp_s_c_s_d_s_c_s_a_s_e_s:compl:rev
CACCAGCACAGACACCCACACCGTCACCCACACTATCATGTTGACATTCC; dG = -35.3
1505 >s_c_s_e_s_b_s_c_s_d_s_a_s_a_s_e_s
GTGTGGGTGCTGGTGGCTGTGTGGGTGACGGTGTCTGTGTCTGTGCTGGTG; >clamp_s_c_s_d_s_c_s_a_s_e_s:compl:rev
CACCAGCACAGACACCCACACCGTCACCCACACTATCATGTTGACATTCC; dG = -35.3
1506 >s_c_s_e_s_b_s_c_s_b_s_c_s_a_s_e_s
GTGTGGGTGCTGGTGGCTGTGTGGGTGGCTGTGTGGGTGTCTGTGCTGGTG; >clamp_s_c_s_d_s_c_s_a_s_e_s:compl:rev
CACCAGCACAGACACCCACACCGTCACCCACACTATCATGTTGACATTCC; dG = -35.3
1507 >s_c_s_e_s_b_s_a_s_d_s_c_s_a_s_e_s
GTGTGGGTGCTGGTGGCTGTGTCTGTGACGGTGTGGGTGTCTGTGCTGGTG; >clamp_s_c_s_d_s_c_s_a_s_e_s:compl:rev
CACCAGCACAGACACCCACACCGTCACCCACACTATCATGTTGACATTCC; dG = -35.3
1508 >s_c_s_e_s_c_s_c_s_d_s_a_s_a_s_e_s
GTGTGGGTGCTGGTGTGGGTGTGGGTGACGGTGTCTGTGTCTGTGCTGGTG; >clamp_s_c_s_d_s_c_s_a_s_e_s:compl:rev
CACCAGCACAGACACCCACACCGTCACCCACACTATCATGTTGACATTCC; dG = -35.3
1509 >s_c_s_e_s_c_s_c_s_b_s_c_s_a_s_e_s
GTGTGGGTGCTGGTGTGGGTGTGGGTGGCTGTGTGGGTGTCTGTGCTGGTG; >clamp_s_c_s_d_s_c_s_a_s_e_s:compl:rev
CACCAGCACAGACACCCACACCGTCACCCACACTATCATGTTGACATTCC; dG = -35.3
1510 >s_c_s_e_s_d_s_c_s_d_s_a_s_a_s_e_s
GTGTGGGTGCTGGTGACGGTGTGGGTGACGGTGTCTGTGTCTGTGCTGGTG; >clamp_s_c_s_d_s_c_s_a_s_e_s:compl:rev
CACCAGCACAGACACCCACACCGTCACCCACACTATCATGTTGACATTCC; dG = -35.3
1511 >s_c_s_e_s_d_s_c_s_b_s_c_s_a_s_e_s
GTGTGGGTGCTGGTGACGGTGTGGGTGGCTGTGTGGGTGTCTGTGCTGGTG; >clamp_s_c_s_d_s_c_s_a_s_e_s:compl:rev
CACCAGCACAGACACCCACACCGTCACCCACACTATCATGTTGACATTCC; dG = -35.3
1512 >s_c_s_e_s_d_s_a_s_d_s_c_s_a_s_e_s
GTGTGGGTGCTGGTGACGGTGTCTGTGACGGTGTGGGTGTCTGTGCTGGTG; >clamp_s_c_s_d_s_c_s_a_s_e_s:compl:rev
CACCAGCACAGACACCCACACCGTCACCCACACTATCATGTTGACATTCC; dG = -35.3
1513 >s_c_s_e_s_e_s_c_s_d_s_a_s_a_s_e_s
GTGTGGGTGCTGGTGCTGGTGTGGGTGACGGTGTCTGTGTCTGTGCTGGTG; >clamp_s_c_s_d_s_c_s_a_s_e_s:compl:rev
CACCAGCACAGACACCCACACCGTCACCCACACTATCATGTTGACATTCC; dG = -35.3
1514 >s_c_s_e_s_e_s_c_s_b_s_c_s_a_s_e_s
GTGTGGGTGCTGGTGCTGGTGTGGGTGGCTGTGTGGGTGTCTGTGCTGGTG; >clamp_s_c_s_d_s_c_s_a_s_e_s:compl:rev
CACCAGCACAGACACCCACACCGTCACCCACACTATCATGTTGACATTCC; dG = -35.3
1515 >s_a_s_a_s_a_s_c_s_b_s_c_s_a_s_e_s
GTGTCTGTGTCTGTGTCTGTGTGGGTGGCTGTGTGGGTGTCTGTGCTGGTG; >clamp_s_c_s_d_s_c_s_a_s_e_s:compl:rev
CACCAGCACAGACACCCACACCGTCACCCACACTATCATGTTGACATTCC; dG = -35.3
1516 >s_c_s_e_s_e_s_a_s_d_s_c_s_a_s_e_s
GTGTGGGTGCTGGTGCTGGTGTCTGTGACGGTGTGGGTGTCTGTGCTGGTG; >clamp_s_c_s_d_s_c_s_a_s_e_s:compl:rev
CACCAGCACAGACACCCACACCGTCACCCACACTATCATGTTGACATTCC; dG = -35.3
1517 >s_d_s_a_s_a_s_c_s_d_s_a_s_a_s_e_s
GTGACGGTGTCTGTGTCTGTGTGGGTGACGGTGTCTGTGTCTGTGCTGGTG; >clamp_s_c_s_d_s_c_s_a_s_e_s:compl:rev
CACCAGCACAGACACCCACACCGTCACCCACACTATCATGTTGACATTCC; dG = -35.3
1518 >s_d_s_a_s_a_s_c_s_b_s_c_s_a_s_e_s
GTGACGGTGTCTGTGTCTGTGTGGGTGGCTGTGTGGGTGTCTGTGCTGGTG; >clamp_s_c_s_d_s_c_s_a_s_e_s:compl:rev
CACCAGCACAGACACCCACACCGTCACCCACACTATCATGTTGACATTCC; dG = -35.3
1519 >s_a_s_b_s_c_s_c_s_d_s_a_s_a_s_e_s
GTGTCTGTGGCTGTGTGGGTGTGGGTGACGGTGTCTGTGTCTGTGCTGGTG; >clamp_s_c_s_d_s_c_s_a_s_e_s:compl:rev
CACCAGCACAGACACCCACACCGTCACCCACACTATCATGTTGACATTCC; dG = -35.3

1520 >s_d_s_a_s_a_s_a_s_d_s_c_s_a_s_e_s
GTGACGGTGTCTGTGTCTGTGTCTGTGACGGTGTGGGTGTCTGTGCTGGTG; >clamp_s_c_s_d_s_c_s_a_s_e_s:compl:rev
CACCAGCACAGACACCCACACCGTCACCCACACTATCATGTTGACATTCC; dG = -35.3
1521 >s_d_s_a_s_b_s_c_s_d_s_a_s_a_s_e_s
GTGACGGTGTCTGTGGCTGTGTGGGTGACGGTGTCTGTGTCTGTGCTGGTG; >clamp_s_c_s_d_s_c_s_a_s_e_s:compl:rev
CACCAGCACAGACACCCACACCGTCACCCACACTATCATGTTGACATTCC; dG = -35.3
1522 >s_d_s_a_s_b_s_c_s_b_s_c_s_a_s_e_s
GTGACGGTGTCTGTGGCTGTGTGGGTGGCTGTGTGGGTGTCTGTGCTGGTG; >clamp_s_c_s_d_s_c_s_a_s_e_s:compl:rev
CACCAGCACAGACACCCACACCGTCACCCACACTATCATGTTGACATTCC; dG = -35.3
1523 >s_d_s_a_s_b_s_a_s_d_s_c_s_a_s_e_s
GTGACGGTGTCTGTGGCTGTGTCTGTGACGGTGTGGGTGTCTGTGCTGGTG; >clamp_s_c_s_d_s_c_s_a_s_e_s:compl:rev
CACCAGCACAGACACCCACACCGTCACCCACACTATCATGTTGACATTCC; dG = -35.3
1524 >s_a_s_b_s_c_s_c_s_b_s_c_s_a_s_e_s
GTGTCTGTGGCTGTGTGGGTGTGGGTGGCTGTGTGGGTGTCTGTGCTGGTG; >clamp_s_c_s_d_s_c_s_a_s_e_s:compl:rev
CACCAGCACAGACACCCACACCGTCACCCACACTATCATGTTGACATTCC; dG = -35.3
1525 >s_d_s_a_s_c_s_c_s_d_s_a_s_a_s_e_s
GTGACGGTGTCTGTGTGGGTGTGGGTGACGGTGTCTGTGTCTGTGCTGGTG; >clamp_s_c_s_d_s_c_s_a_s_e_s:compl:rev
CACCAGCACAGACACCCACACCGTCACCCACACTATCATGTTGACATTCC; dG = -35.3
1526 >s_d_s_a_s_c_s_c_s_b_s_c_s_a_s_e_s
GTGACGGTGTCTGTGTGGGTGTGGGTGGCTGTGTGGGTGTCTGTGCTGGTG; >clamp_s_c_s_d_s_c_s_a_s_e_s:compl:rev
CACCAGCACAGACACCCACACCGTCACCCACACTATCATGTTGACATTCC; dG = -35.3
1527 >s_d_s_a_s_d_s_c_s_d_s_a_s_a_s_e_s
GTGACGGTGTCTGTGACGGTGTGGGTGACGGTGTCTGTGTCTGTGCTGGTG; >clamp_s_c_s_d_s_c_s_a_s_e_s:compl:rev
CACCAGCACAGACACCCACACCGTCACCCACACTATCATGTTGACATTCC; dG = -35.3
1528 >s_d_s_a_s_d_s_c_s_b_s_c_s_a_s_e_s
GTGACGGTGTCTGTGACGGTGTGGGTGGCTGTGTGGGTGTCTGTGCTGGTG; >clamp_s_c_s_d_s_c_s_a_s_e_s:compl:rev
CACCAGCACAGACACCCACACCGTCACCCACACTATCATGTTGACATTCC; dG = -35.3
1529 >s_d_s_a_s_d_s_a_s_d_s_c_s_a_s_e_s
GTGACGGTGTCTGTGACGGTGTCTGTGACGGTGTGGGTGTCTGTGCTGGTG; >clamp_s_c_s_d_s_c_s_a_s_e_s:compl:rev
CACCAGCACAGACACCCACACCGTCACCCACACTATCATGTTGACATTCC; dG = -35.3
1530 >s_d_s_a_s_e_s_c_s_d_s_a_s_a_s_e_s
GTGACGGTGTCTGTGCTGGTGTGGGTGACGGTGTCTGTGTCTGTGCTGGTG; >clamp_s_c_s_d_s_c_s_a_s_e_s:compl:rev
CACCAGCACAGACACCCACACCGTCACCCACACTATCATGTTGACATTCC; dG = -35.3
1531 >s_d_s_a_s_e_s_c_s_b_s_c_s_a_s_e_s
GTGACGGTGTCTGTGCTGGTGTGGGTGGCTGTGTGGGTGTCTGTGCTGGTG; >clamp_s_c_s_d_s_c_s_a_s_e_s:compl:rev
CACCAGCACAGACACCCACACCGTCACCCACACTATCATGTTGACATTCC; dG = -35.3
1532 >s_d_s_a_s_e_s_a_s_d_s_c_s_a_s_e_s
GTGACGGTGTCTGTGCTGGTGTCTGTGACGGTGTGGGTGTCTGTGCTGGTG; >clamp_s_c_s_d_s_c_s_a_s_e_s:compl:rev
CACCAGCACAGACACCCACACCGTCACCCACACTATCATGTTGACATTCC; dG = -35.3
1533 >s_d_s_b_s_a_s_c_s_d_s_a_s_a_s_e_s
GTGACGGTGGCTGTGTCTGTGTGGGTGACGGTGTCTGTGTCTGTGCTGGTG; >clamp_s_c_s_d_s_c_s_a_s_e_s:compl:rev
CACCAGCACAGACACCCACACCGTCACCCACACTATCATGTTGACATTCC; dG = -35.3
1534 >s_d_s_b_s_a_s_c_s_b_s_c_s_a_s_e_s
GTGACGGTGGCTGTGTCTGTGTGGGTGGCTGTGTGGGTGTCTGTGCTGGTG; >clamp_s_c_s_d_s_c_s_a_s_e_s:compl:rev
CACCAGCACAGACACCCACACCGTCACCCACACTATCATGTTGACATTCC; dG = -35.3
1535 >s_d_s_b_s_a_s_a_s_d_s_c_s_a_s_e_s
GTGACGGTGGCTGTGTCTGTGTCTGTGACGGTGTGGGTGTCTGTGCTGGTG; >clamp_s_c_s_d_s_c_s_a_s_e_s:compl:rev
CACCAGCACAGACACCCACACCGTCACCCACACTATCATGTTGACATTCC; dG = -35.3
1536 >s_d_s_b_s_b_s_c_s_d_s_a_s_a_s_e_s
GTGACGGTGGCTGTGGCTGTGTGGGTGACGGTGTCTGTGTCTGTGCTGGTG; >clamp_s_c_s_d_s_c_s_a_s_e_s:compl:rev
CACCAGCACAGACACCCACACCGTCACCCACACTATCATGTTGACATTCC; dG = -35.3
1537 >s_d_s_b_s_b_s_c_s_b_s_c_s_a_s_e_s
GTGACGGTGGCTGTGGCTGTGTGGGTGGCTGTGTGGGTGTCTGTGCTGGTG; >clamp_s_c_s_d_s_c_s_a_s_e_s:compl:rev
CACCAGCACAGACACCCACACCGTCACCCACACTATCATGTTGACATTCC; dG = -35.3
1538 >s_d_s_b_s_b_s_a_s_d_s_c_s_a_s_e_s
GTGACGGTGGCTGTGGCTGTGTCTGTGACGGTGTGGGTGTCTGTGCTGGTG; >clamp_s_c_s_d_s_c_s_a_s_e_s:compl:rev
CACCAGCACAGACACCCACACCGTCACCCACACTATCATGTTGACATTCC; dG = -35.3
1539 >s_d_s_b_s_c_s_c_s_d_s_a_s_a_s_e_s
GTGACGGTGGCTGTGTGGGTGTGGGTGACGGTGTCTGTGTCTGTGCTGGTG; >clamp_s_c_s_d_s_c_s_a_s_e_s:compl:rev
CACCAGCACAGACACCCACACCGTCACCCACACTATCATGTTGACATTCC; dG = -35.3
1540 >s_d_s_b_s_c_s_c_s_b_s_c_s_a_s_e_s
GTGACGGTGGCTGTGTGGGTGTGGGTGGCTGTGTGGGTGTCTGTGCTGGTG; >clamp_s_c_s_d_s_c_s_a_s_e_s:compl:rev
CACCAGCACAGACACCCACACCGTCACCCACACTATCATGTTGACATTCC; dG = -35.3
1541 >s_d_s_b_s_d_s_c_s_d_s_a_s_a_s_e_s
GTGACGGTGGCTGTGACGGTGTGGGTGACGGTGTCTGTGTCTGTGCTGGTG; >clamp_s_c_s_d_s_c_s_a_s_e_s:compl:rev
CACCAGCACAGACACCCACACCGTCACCCACACTATCATGTTGACATTCC; dG = -35.3
1542 >s_d_s_b_s_d_s_c_s_b_s_c_s_a_s_e_s
GTGACGGTGGCTGTGACGGTGTGGGTGGCTGTGTGGGTGTCTGTGCTGGTG; >clamp_s_c_s_d_s_c_s_a_s_e_s:compl:rev
CACCAGCACAGACACCCACACCGTCACCCACACTATCATGTTGACATTCC; dG = -35.3
1543 >s_d_s_b_s_d_s_a_s_d_s_c_s_a_s_e_s
GTGACGGTGGCTGTGACGGTGTCTGTGACGGTGTGGGTGTCTGTGCTGGTG; >clamp_s_c_s_d_s_c_s_a_s_e_s:compl:rev
CACCAGCACAGACACCCACACCGTCACCCACACTATCATGTTGACATTCC; dG = -35.3

1544 >s_d_s_b_s_e_s_c_s_d_s_a_s_a_s_e_s
GTGACGGTGGCTGTGCTGGTGTGGGTGACGGTGTCTGTGTCTGTGCTGGTG; >clamp_s_c_s_d_s_c_s_a_s_e_s:compl:rev
CACCAGCACAGACACCCACACCGTCACCCACACTATCATGTTGACATTCC; dG = -35.3
1545 >s_d_s_b_s_e_s_c_s_b_s_c_s_a_s_e_s
GTGACGGTGGCTGTGCTGGTGTGGGTGGCTGTGTGGGTGTCTGTGCTGGTG; >clamp_s_c_s_d_s_c_s_a_s_e_s:compl:rev
CACCAGCACAGACACCCACACCGTCACCCACACTATCATGTTGACATTCC; dG = -35.3
1546 >s_d_s_b_s_e_s_a_s_d_s_c_s_a_s_e_s
GTGACGGTGGCTGTGCTGGTGTCTGTGACGGTGTGGGTGTCTGTGCTGGTG; >clamp_s_c_s_d_s_c_s_a_s_e_s:compl:rev
CACCAGCACAGACACCCACACCGTCACCCACACTATCATGTTGACATTCC; dG = -35.3
1547 >s_d_s_c_s_a_s_c_s_d_s_a_s_a_s_e_s
GTGACGGTGTGGGTGTCTGTGTGGGTGACGGTGTCTGTGTCTGTGCTGGTG; >clamp_s_c_s_d_s_c_s_a_s_e_s:compl:rev
CACCAGCACAGACACCCACACCGTCACCCACACTATCATGTTGACATTCC; dG = -35.3
1548 >s_d_s_c_s_a_s_c_s_b_s_c_s_a_s_e_s
GTGACGGTGTGGGTGTCTGTGTGGGTGGCTGTGTGGGTGTCTGTGCTGGTG; >clamp_s_c_s_d_s_c_s_a_s_e_s:compl:rev
CACCAGCACAGACACCCACACCGTCACCCACACTATCATGTTGACATTCC; dG = -35.3
1549 >s_a_s_b_s_d_s_c_s_d_s_a_s_a_s_e_s
GTGTCTGTGGCTGTGACGGTGTGGGTGACGGTGTCTGTGTCTGTGCTGGTG; >clamp_s_c_s_d_s_c_s_a_s_e_s:compl:rev
CACCAGCACAGACACCCACACCGTCACCCACACTATCATGTTGACATTCC; dG = -35.3
1550 >s_d_s_c_s_b_s_c_s_d_s_a_s_a_s_e_s
GTGACGGTGTGGGTGGCTGTGTGGGTGACGGTGTCTGTGTCTGTGCTGGTG; >clamp_s_c_s_d_s_c_s_a_s_e_s:compl:rev
CACCAGCACAGACACCCACACCGTCACCCACACTATCATGTTGACATTCC; dG = -35.3
1551 >s_d_s_c_s_b_s_c_s_b_s_c_s_a_s_e_s
GTGACGGTGTGGGTGGCTGTGTGGGTGGCTGTGTGGGTGTCTGTGCTGGTG; >clamp_s_c_s_d_s_c_s_a_s_e_s:compl:rev
CACCAGCACAGACACCCACACCGTCACCCACACTATCATGTTGACATTCC; dG = -35.3
1552 >s_a_s_b_s_d_s_c_s_b_s_c_s_a_s_e_s
GTGTCTGTGGCTGTGACGGTGTGGGTGGCTGTGTGGGTGTCTGTGCTGGTG; >clamp_s_c_s_d_s_c_s_a_s_e_s:compl:rev
CACCAGCACAGACACCCACACCGTCACCCACACTATCATGTTGACATTCC; dG = -35.3
1553 >s_d_s_c_s_c_s_c_s_d_s_a_s_a_s_e_s
GTGACGGTGTGGGTGTGGGTGTGGGTGACGGTGTCTGTGTCTGTGCTGGTG; >clamp_s_c_s_d_s_c_s_a_s_e_s:compl:rev
CACCAGCACAGACACCCACACCGTCACCCACACTATCATGTTGACATTCC; dG = -35.3
1554 >s_d_s_c_s_c_s_c_s_b_s_c_s_a_s_e_s
GTGACGGTGTGGGTGTGGGTGTGGGTGGCTGTGTGGGTGTCTGTGCTGGTG; >clamp_s_c_s_d_s_c_s_a_s_e_s:compl:rev
CACCAGCACAGACACCCACACCGTCACCCACACTATCATGTTGACATTCC; dG = -35.3
1555 >s_d_s_c_s_e_s_c_s_d_s_a_s_a_s_e_s
GTGACGGTGTGGGTGCTGGTGTGGGTGACGGTGTCTGTGTCTGTGCTGGTG; >clamp_s_c_s_d_s_c_s_a_s_e_s:compl:rev
CACCAGCACAGACACCCACACCGTCACCCACACTATCATGTTGACATTCC; dG = -35.3
1556 >s_d_s_c_s_e_s_c_s_b_s_c_s_a_s_e_s
GTGACGGTGTGGGTGCTGGTGTGGGTGGCTGTGTGGGTGTCTGTGCTGGTG; >clamp_s_c_s_d_s_c_s_a_s_e_s:compl:rev
CACCAGCACAGACACCCACACCGTCACCCACACTATCATGTTGACATTCC; dG = -35.3
1557 >s_a_s_b_s_d_s_a_s_d_s_c_s_a_s_e_s
GTGTCTGTGGCTGTGACGGTGTCTGTGACGGTGTGGGTGTCTGTGCTGGTG; >clamp_s_c_s_d_s_c_s_a_s_e_s:compl:rev
CACCAGCACAGACACCCACACCGTCACCCACACTATCATGTTGACATTCC; dG = -35.3
1558 >s_d_s_d_s_a_s_c_s_d_s_a_s_a_s_e_s
GTGACGGTGACGGTGTCTGTGTGGGTGACGGTGTCTGTGTCTGTGCTGGTG; >clamp_s_c_s_d_s_c_s_a_s_e_s:compl:rev
CACCAGCACAGACACCCACACCGTCACCCACACTATCATGTTGACATTCC; dG = -35.3
1559 >s_d_s_d_s_a_s_c_s_b_s_c_s_a_s_e_s
GTGACGGTGACGGTGTCTGTGTGGGTGGCTGTGTGGGTGTCTGTGCTGGTG; >clamp_s_c_s_d_s_c_s_a_s_e_s:compl:rev
CACCAGCACAGACACCCACACCGTCACCCACACTATCATGTTGACATTCC; dG = -35.3
1560 >s_d_s_d_s_a_s_a_s_d_s_c_s_a_s_e_s
GTGACGGTGACGGTGTCTGTGTCTGTGACGGTGTGGGTGTCTGTGCTGGTG; >clamp_s_c_s_d_s_c_s_a_s_e_s:compl:rev
CACCAGCACAGACACCCACACCGTCACCCACACTATCATGTTGACATTCC; dG = -35.3
1561 >s_d_s_d_s_b_s_c_s_d_s_a_s_a_s_e_s
GTGACGGTGACGGTGGCTGTGTGGGTGACGGTGTCTGTGTCTGTGCTGGTG; >clamp_s_c_s_d_s_c_s_a_s_e_s:compl:rev
CACCAGCACAGACACCCACACCGTCACCCACACTATCATGTTGACATTCC; dG = -35.3
1562 >s_d_s_d_s_b_s_c_s_b_s_c_s_a_s_e_s
GTGACGGTGACGGTGGCTGTGTGGGTGGCTGTGTGGGTGTCTGTGCTGGTG; >clamp_s_c_s_d_s_c_s_a_s_e_s:compl:rev
CACCAGCACAGACACCCACACCGTCACCCACACTATCATGTTGACATTCC; dG = -35.3
1563 >s_d_s_d_s_b_s_a_s_d_s_c_s_a_s_e_s
GTGACGGTGACGGTGGCTGTGTCTGTGACGGTGTGGGTGTCTGTGCTGGTG; >clamp_s_c_s_d_s_c_s_a_s_e_s:compl:rev
CACCAGCACAGACACCCACACCGTCACCCACACTATCATGTTGACATTCC; dG = -35.3
1564 >s_d_s_d_s_c_s_c_s_d_s_a_s_a_s_e_s
GTGACGGTGACGGTGTGGGTGTGGGTGACGGTGTCTGTGTCTGTGCTGGTG; >clamp_s_c_s_d_s_c_s_a_s_e_s:compl:rev
CACCAGCACAGACACCCACACCGTCACCCACACTATCATGTTGACATTCC; dG = -35.3
1565 >s_d_s_d_s_c_s_c_s_b_s_c_s_a_s_e_s
GTGACGGTGACGGTGTGGGTGTGGGTGGCTGTGTGGGTGTCTGTGCTGGTG; >clamp_s_c_s_d_s_c_s_a_s_e_s:compl:rev
CACCAGCACAGACACCCACACCGTCACCCACACTATCATGTTGACATTCC; dG = -35.3
1566 >s_d_s_d_s_d_s_c_s_d_s_a_s_a_s_e_s
GTGACGGTGACGGTGACGGTGTGGGTGACGGTGTCTGTGTCTGTGCTGGTG; >clamp_s_c_s_d_s_c_s_a_s_e_s:compl:rev
CACCAGCACAGACACCCACACCGTCACCCACACTATCATGTTGACATTCC; dG = -35.3
1567 >s_d_s_d_s_d_s_c_s_b_s_c_s_a_s_e_s
GTGACGGTGACGGTGACGGTGTGGGTGGCTGTGTGGGTGTCTGTGCTGGTG; >clamp_s_c_s_d_s_c_s_a_s_e_s:compl:rev
CACCAGCACAGACACCCACACCGTCACCCACACTATCATGTTGACATTCC; dG = -35.3

1568 >s_d_s_d_s_d_s_a_s_d_s_c_s_a_s_e_s
GTGACGGTGACGGTGACGGTGTCTGTGACGGTGTGGGTGTCTGTGCTGGTG;   >clamp_s_c_s_d_s_c_s_a_s_e_s:compl:rev
CACCAGCACAGACACCCACACCGTCACCCACACTATCATGTTGACATTCC; dG = -35.3
1569 >s_d_s_d_s_e_s_c_s_d_s_a_s_a_s_e_s
GTGACGGTGACGGTGCTGGTGTGGGTGACGGTGTCTGTGTCTGTGCTGGTG;   >clamp_s_c_s_d_s_c_s_a_s_e_s:compl:rev
CACCAGCACAGACACCCACACCGTCACCCACACTATCATGTTGACATTCC; dG = -35.3
1570 >s_d_s_d_s_e_s_c_s_b_s_c_s_a_s_e_s
GTGACGGTGACGGTGCTGGTGTGGGTGGCTGTGTGGGTGTCTGTGCTGGTG;   >clamp_s_c_s_d_s_c_s_a_s_e_s:compl:rev
CACCAGCACAGACACCCACACCGTCACCCACACTATCATGTTGACATTCC; dG = -35.3
1571 >s_d_s_d_s_e_s_a_s_d_s_c_s_a_s_e_s
GTGACGGTGACGGTGCTGGTGTCTGTGACGGTGTGGGTGTCTGTGCTGGTG;   >clamp_s_c_s_d_s_c_s_a_s_e_s:compl:rev
CACCAGCACAGACACCCACACCGTCACCCACACTATCATGTTGACATTCC; dG = -35.3
1572 >s_d_s_e_s_a_s_c_s_d_s_a_s_a_s_e_s
GTGACGGTGCTGGTGTCTGTGTGGGTGACGGTGTCTGTGTCTGTGCTGGTG;   >clamp_s_c_s_d_s_c_s_a_s_e_s:compl:rev
CACCAGCACAGACACCCACACCGTCACCCACACTATCATGTTGACATTCC; dG = -35.3
1573 >s_d_s_e_s_a_s_c_s_b_s_c_s_a_s_e_s
GTGACGGTGCTGGTGTCTGTGTGGGTGGCTGTGTGGGTGTCTGTGCTGGTG;   >clamp_s_c_s_d_s_c_s_a_s_e_s:compl:rev
CACCAGCACAGACACCCACACCGTCACCCACACTATCATGTTGACATTCC; dG = -35.3
1574 >s_a_s_b_s_e_s_c_s_d_s_a_s_a_s_e_s
GTGTCTGTGGCTGTGCTGGTGTGGGTGACGGTGTCTGTGTCTGTGCTGGTG;   >clamp_s_c_s_d_s_c_s_a_s_e_s:compl:rev
CACCAGCACAGACACCCACACCGTCACCCACACTATCATGTTGACATTCC; dG = -35.3
1575 >s_d_s_e_s_a_s_a_s_d_s_c_s_a_s_e_s
GTGACGGTGCTGGTGTCTGTGTCTGTGACGGTGTGGGTGTCTGTGCTGGTG;   >clamp_s_c_s_d_s_c_s_a_s_e_s:compl:rev
CACCAGCACAGACACCCACACCGTCACCCACACTATCATGTTGACATTCC; dG = -35.3
1576 >s_d_s_e_s_b_s_c_s_d_s_a_s_a_s_e_s
GTGACGGTGCTGGTGGCTGTGTGGGTGACGGTGTCTGTGTCTGTGCTGGTG;   >clamp_s_c_s_d_s_c_s_a_s_e_s:compl:rev
CACCAGCACAGACACCCACACCGTCACCCACACTATCATGTTGACATTCC; dG = -35.3
1577 >s_d_s_e_s_b_s_c_s_b_s_c_s_a_s_e_s
GTGACGGTGCTGGTGGCTGTGTGGGTGGCTGTGTGGGTGTCTGTGCTGGTG;   >clamp_s_c_s_d_s_c_s_a_s_e_s:compl:rev
CACCAGCACAGACACCCACACCGTCACCCACACTATCATGTTGACATTCC; dG = -35.3
1578 >s_d_s_e_s_b_s_a_s_d_s_c_s_a_s_e_s
GTGACGGTGCTGGTGGCTGTGTCTGTGACGGTGTGGGTGTCTGTGCTGGTG;   >clamp_s_c_s_d_s_c_s_a_s_e_s:compl:rev
CACCAGCACAGACACCCACACCGTCACCCACACTATCATGTTGACATTCC; dG = -35.3
1579 >s_a_s_b_s_e_s_c_s_b_s_c_s_a_s_e_s
GTGTCTGTGGCTGTGCTGGTGTGGGTGGCTGTGTGGGTGTCTGTGCTGGTG;   >clamp_s_c_s_d_s_c_s_a_s_e_s:compl:rev
CACCAGCACAGACACCCACACCGTCACCCACACTATCATGTTGACATTCC; dG = -35.3
1580 >s_d_s_e_s_c_s_c_s_d_s_a_s_a_s_e_s
GTGACGGTGCTGGTGTGGGTGTGGGTGACGGTGTCTGTGTCTGTGCTGGTG;   >clamp_s_c_s_d_s_c_s_a_s_e_s:compl:rev
CACCAGCACAGACACCCACACCGTCACCCACACTATCATGTTGACATTCC; dG = -35.3
1581 >s_d_s_e_s_c_s_c_s_b_s_c_s_a_s_e_s
GTGACGGTGCTGGTGTGGGTGTGGGTGGCTGTGTGGGTGTCTGTGCTGGTG;   >clamp_s_c_s_d_s_c_s_a_s_e_s:compl:rev
CACCAGCACAGACACCCACACCGTCACCCACACTATCATGTTGACATTCC; dG = -35.3
1582 >s_d_s_e_s_d_s_c_s_d_s_a_s_a_s_e_s
GTGACGGTGCTGGTGACGGTGTGGGTGACGGTGTCTGTGTCTGTGCTGGTG;   >clamp_s_c_s_d_s_c_s_a_s_e_s:compl:rev
CACCAGCACAGACACCCACACCGTCACCCACACTATCATGTTGACATTCC; dG = -35.3
1583 >s_d_s_e_s_d_s_c_s_b_s_c_s_a_s_e_s
GTGACGGTGCTGGTGACGGTGTGGGTGGCTGTGTGGGTGTCTGTGCTGGTG;   >clamp_s_c_s_d_s_c_s_a_s_e_s:compl:rev
CACCAGCACAGACACCCACACCGTCACCCACACTATCATGTTGACATTCC; dG = -35.3
1584 >s_d_s_e_s_d_s_a_s_d_s_c_s_a_s_e_s
GTGACGGTGCTGGTGACGGTGTCTGTGACGGTGTGGGTGTCTGTGCTGGTG;   >clamp_s_c_s_d_s_c_s_a_s_e_s:compl:rev
CACCAGCACAGACACCCACACCGTCACCCACACTATCATGTTGACATTCC; dG = -35.3
1585 >s_d_s_e_s_e_s_c_s_d_s_a_s_a_s_e_s
GTGACGGTGCTGGTGCTGGTGTGGGTGACGGTGTCTGTGTCTGTGCTGGTG;   >clamp_s_c_s_d_s_c_s_a_s_e_s:compl:rev
CACCAGCACAGACACCCACACCGTCACCCACACTATCATGTTGACATTCC; dG = -35.3
1586 >s_d_s_e_s_e_s_c_s_b_s_c_s_a_s_e_s
GTGACGGTGCTGGTGCTGGTGTGGGTGGCTGTGTGGGTGTCTGTGCTGGTG;   >clamp_s_c_s_d_s_c_s_a_s_e_s:compl:rev
CACCAGCACAGACACCCACACCGTCACCCACACTATCATGTTGACATTCC; dG = -35.3
1587 >s_a_s_a_s_a_s_a_s_d_s_c_s_a_s_e_s
GTGTCTGTGTCTGTGTCTGTGTCTGTGACGGTGTGGGTGTCTGTGCTGGTG;   >clamp_s_c_s_d_s_c_s_a_s_e_s:compl:rev
CACCAGCACAGACACCCACACCGTCACCCACACTATCATGTTGACATTCC; dG = -35.3
1588 >s_a_s_b_s_e_s_a_s_d_s_c_s_a_s_e_s
GTGTCTGTGGCTGTGCTGGTGTCTGTGACGGTGTGGGTGTCTGTGCTGGTG;   >clamp_s_c_s_d_s_c_s_a_s_e_s:compl:rev
CACCAGCACAGACACCCACACCGTCACCCACACTATCATGTTGACATTCC; dG = -35.3
1589 >s_d_s_e_s_e_s_a_s_d_s_c_s_a_s_e_s
GTGACGGTGCTGGTGCTGGTGTCTGTGACGGTGTGGGTGTCTGTGCTGGTG;   >clamp_s_c_s_d_s_c_s_a_s_e_s:compl:rev
CACCAGCACAGACACCCACACCGTCACCCACACTATCATGTTGACATTCC; dG = -35.3
1590 >s_e_s_a_s_a_s_c_s_d_s_a_s_a_s_e_s
GTGCTGGTGTCTGTGTCTGTGTGGGTGACGGTGTCTGTGTCTGTGCTGGTG;   >clamp_s_c_s_d_s_c_s_a_s_e_s:compl:rev
CACCAGCACAGACACCCACACCGTCACCCACACTATCATGTTGACATTCC; dG = -35.3
1591 >s_e_s_a_s_a_s_c_s_b_s_c_s_a_s_e_s
GTGCTGGTGTCTGTGTCTGTGTGGGTGGCTGTGTGGGTGTCTGTGCTGGTG;   >clamp_s_c_s_d_s_c_s_a_s_e_s:compl:rev
CACCAGCACAGACACCCACACCGTCACCCACACTATCATGTTGACATTCC; dG = -35.3

1592 >s_e_s_a_s_a_s_a_s_d_s_c_s_a_s_e_s
GTGCTGGTGTCTGTGTCTGTGTCTGTGACGGTGTGGGTGTCTGTGCTGGTG; >clamp_s_c_s_d_s_c_s_a_s_e_s:compl:rev
CACCAGCACAGACACCCACACCGTCACCCACACTATCATGTTGACATTCC; dG = -35.3
1593 >s_e_s_a_s_b_s_c_s_d_s_a_s_a_s_e_s
GTGCTGGTGTCTGTGGCTGTGTGGGTGACGGTGTCTGTGTCTGTGCTGGTG; >clamp_s_c_s_d_s_c_s_a_s_e_s:compl:rev
CACCAGCACAGACACCCACACCGTCACCCACACTATCATGTTGACATTCC; dG = -35.3
1594 >s_e_s_a_s_b_s_c_s_b_s_c_s_a_s_e_s
GTGCTGGTGTCTGTGGCTGTGTGGGTGGCTGTGTGGGTGTCTGTGCTGGTG; >clamp_s_c_s_d_s_c_s_a_s_e_s:compl:rev
CACCAGCACAGACACCCACACCGTCACCCACACTATCATGTTGACATTCC; dG = -35.3
1595 >s_e_s_a_s_b_s_a_s_d_s_c_s_a_s_e_s
GTGCTGGTGTCTGTGGCTGTGTCTGTGACGGTGTGGGTGTCTGTGCTGGTG; >clamp_s_c_s_d_s_c_s_a_s_e_s:compl:rev
CACCAGCACAGACACCCACACCGTCACCCACACTATCATGTTGACATTCC; dG = -35.3
1596 >s_e_s_a_s_c_s_c_s_d_s_a_s_a_s_e_s
GTGCTGGTGTCTGTGTGGGTGTGGGTGACGGTGTCTGTGTCTGTGCTGGTG; >clamp_s_c_s_d_s_c_s_a_s_e_s:compl:rev
CACCAGCACAGACACCCACACCGTCACCCACACTATCATGTTGACATTCC; dG = -35.3
1597 >s_e_s_a_s_c_s_c_s_b_s_c_s_a_s_e_s
GTGCTGGTGTCTGTGTGGGTGTGGGTGGCTGTGTGGGTGTCTGTGCTGGTG; >clamp_s_c_s_d_s_c_s_a_s_e_s:compl:rev
CACCAGCACAGACACCCACACCGTCACCCACACTATCATGTTGACATTCC; dG = -35.3
1598 >s_e_s_a_s_d_s_c_s_d_s_a_s_a_s_e_s
GTGCTGGTGTCTGTGACGGTGTGGGTGACGGTGTCTGTGTCTGTGCTGGTG; >clamp_s_c_s_d_s_c_s_a_s_e_s:compl:rev
CACCAGCACAGACACCCACACCGTCACCCACACTATCATGTTGACATTCC; dG = -35.3
1599 >s_e_s_a_s_d_s_c_s_b_s_c_s_a_s_e_s
GTGCTGGTGTCTGTGACGGTGTGGGTGGCTGTGTGGGTGTCTGTGCTGGTG; >clamp_s_c_s_d_s_c_s_a_s_e_s:compl:rev
CACCAGCACAGACACCCACACCGTCACCCACACTATCATGTTGACATTCC; dG = -35.3
1600 >s_e_s_a_s_d_s_a_s_d_s_c_s_a_s_e_s
GTGCTGGTGTCTGTGACGGTGTCTGTGACGGTGTGGGTGTCTGTGCTGGTG; >clamp_s_c_s_d_s_c_s_a_s_e_s:compl:rev
CACCAGCACAGACACCCACACCGTCACCCACACTATCATGTTGACATTCC; dG = -35.3
1601 >s_e_s_a_s_e_s_c_s_d_s_a_s_a_s_e_s
GTGCTGGTGTCTGTGCTGGTGTGGGTGACGGTGTCTGTGTCTGTGCTGGTG; >clamp_s_c_s_d_s_c_s_a_s_e_s:compl:rev
CACCAGCACAGACACCCACACCGTCACCCACACTATCATGTTGACATTCC; dG = -35.3
1602 >s_e_s_a_s_e_s_c_s_b_s_c_s_a_s_e_s
GTGCTGGTGTCTGTGCTGGTGTGGGTGGCTGTGTGGGTGTCTGTGCTGGTG; >clamp_s_c_s_d_s_c_s_a_s_e_s:compl:rev
CACCAGCACAGACACCCACACCGTCACCCACACTATCATGTTGACATTCC; dG = -35.3
1603 >s_e_s_a_s_e_s_a_s_d_s_c_s_a_s_e_s
GTGCTGGTGTCTGTGCTGGTGTCTGTGACGGTGTGGGTGTCTGTGCTGGTG; >clamp_s_c_s_d_s_c_s_a_s_e_s:compl:rev
CACCAGCACAGACACCCACACCGTCACCCACACTATCATGTTGACATTCC; dG = -35.3
1604 >s_e_s_b_s_a_s_c_s_d_s_a_s_a_s_e_s
GTGCTGGTGGCTGTGTCTGTGTGGGTGACGGTGTCTGTGTCTGTGCTGGTG; >clamp_s_c_s_d_s_c_s_a_s_e_s:compl:rev
CACCAGCACAGACACCCACACCGTCACCCACACTATCATGTTGACATTCC; dG = -35.3
1605 >s_e_s_b_s_a_s_c_s_b_s_c_s_a_s_e_s
GTGCTGGTGGCTGTGTCTGTGTGGGTGGCTGTGTGGGTGTCTGTGCTGGTG; >clamp_s_c_s_d_s_c_s_a_s_e_s:compl:rev
CACCAGCACAGACACCCACACCGTCACCCACACTATCATGTTGACATTCC; dG = -35.3
1606 >s_a_s_c_s_a_s_c_s_d_s_a_s_a_s_e_s
GTGTCTGTGTGGGTGTCTGTGTGGGTGACGGTGTCTGTGTCTGTGCTGGTG; >clamp_s_c_s_d_s_c_s_a_s_e_s:compl:rev
CACCAGCACAGACACCCACACCGTCACCCACACTATCATGTTGACATTCC; dG = -35.3
1607 >s_e_s_b_s_a_s_a_s_d_s_c_s_a_s_e_s
GTGCTGGTGGCTGTGTCTGTGTCTGTGACGGTGTGGGTGTCTGTGCTGGTG; >clamp_s_c_s_d_s_c_s_a_s_e_s:compl:rev
CACCAGCACAGACACCCACACCGTCACCCACACTATCATGTTGACATTCC; dG = -35.3
1608 >s_e_s_b_s_b_s_c_s_d_s_a_s_a_s_e_s
GTGCTGGTGGCTGTGGCTGTGTGGGTGACGGTGTCTGTGTCTGTGCTGGTG; >clamp_s_c_s_d_s_c_s_a_s_e_s:compl:rev
CACCAGCACAGACACCCACACCGTCACCCACACTATCATGTTGACATTCC; dG = -35.3
1609 >s_e_s_b_s_b_s_c_s_b_s_c_s_a_s_e_s
GTGCTGGTGGCTGTGGCTGTGTGGGTGGCTGTGTGGGTGTCTGTGCTGGTG; >clamp_s_c_s_d_s_c_s_a_s_e_s:compl:rev
CACCAGCACAGACACCCACACCGTCACCCACACTATCATGTTGACATTCC; dG = -35.3
1610 >s_e_s_b_s_b_s_a_s_d_s_c_s_a_s_e_s
GTGCTGGTGGCTGTGGCTGTGTCTGTGACGGTGTGGGTGTCTGTGCTGGTG; >clamp_s_c_s_d_s_c_s_a_s_e_s:compl:rev
CACCAGCACAGACACCCACACCGTCACCCACACTATCATGTTGACATTCC; dG = -35.3
1611 >s_a_s_c_s_a_s_c_s_b_s_c_s_a_s_e_s
GTGTCTGTGTGGGTGTCTGTGTGGGTGGCTGTGTGGGTGTCTGTGCTGGTG; >clamp_s_c_s_d_s_c_s_a_s_e_s:compl:rev
CACCAGCACAGACACCCACACCGTCACCCACACTATCATGTTGACATTCC; dG = -35.3
1612 >s_e_s_b_s_c_s_c_s_d_s_a_s_a_s_e_s
GTGCTGGTGGCTGTGTGGGTGTGGGTGACGGTGTCTGTGTCTGTGCTGGTG; >clamp_s_c_s_d_s_c_s_a_s_e_s:compl:rev
CACCAGCACAGACACCCACACCGTCACCCACACTATCATGTTGACATTCC; dG = -35.3
1613 >s_e_s_b_s_c_s_c_s_b_s_c_s_a_s_e_s
GTGCTGGTGGCTGTGTGGGTGTGGGTGGCTGTGTGGGTGTCTGTGCTGGTG; >clamp_s_c_s_d_s_c_s_a_s_e_s:compl:rev
CACCAGCACAGACACCCACACCGTCACCCACACTATCATGTTGACATTCC; dG = -35.3
1614 >s_e_s_b_s_d_s_c_s_d_s_a_s_a_s_e_s
GTGCTGGTGGCTGTGACGGTGTGGGTGACGGTGTCTGTGTCTGTGCTGGTG; >clamp_s_c_s_d_s_c_s_a_s_e_s:compl:rev
CACCAGCACAGACACCCACACCGTCACCCACACTATCATGTTGACATTCC; dG = -35.3
1615 >s_e_s_b_s_d_s_c_s_b_s_c_s_a_s_e_s
GTGCTGGTGGCTGTGACGGTGTGGGTGGCTGTGTGGGTGTCTGTGCTGGTG; >clamp_s_c_s_d_s_c_s_a_s_e_s:compl:rev
CACCAGCACAGACACCCACACCGTCACCCACACTATCATGTTGACATTCC; dG = -35.3

1616 >s_e_s_b_s_d_s_a_s_d_s_c_s_a_s_e_s
GTGCTGGTGGCTGTGACGGTGTCTGTGACGGTGTGGGTGTCTGTGCTGGTG; >clamp_s_c_s_d_s_c_s_a_s_e_s:compl:rev
CACCAGCACAGACACCCACACCGTCACCCACACTATCATGTTGACATTCC; dG = -35.3
1617 >s_e_s_b_s_e_s_c_s_d_s_a_s_a_s_e_s
GTGCTGGTGGCTGTGCTGGTGTGGGTGACGGTGTCTGTGTCTGTGCTGGTG; >clamp_s_c_s_d_s_c_s_a_s_e_s:compl:rev
CACCAGCACAGACACCCACACCGTCACCCACACTATCATGTTGACATTCC; dG = -35.3
1618 >s_e_s_b_s_e_s_c_s_b_s_c_s_a_s_e_s
GTGCTGGTGGCTGTGCTGGTGTGGGTGGCTGTGTGGGTGTCTGTGCTGGTG; >clamp_s_c_s_d_s_c_s_a_s_e_s:compl:rev
CACCAGCACAGACACCCACACCGTCACCCACACTATCATGTTGACATTCC; dG = -35.3
1619 >s_e_s_b_s_e_s_a_s_d_s_c_s_a_s_e_s
GTGCTGGTGGCTGTGCTGGTGTCTGTGACGGTGTGGGTGTCTGTGCTGGTG; >clamp_s_c_s_d_s_c_s_a_s_e_s:compl:rev
CACCAGCACAGACACCCACACCGTCACCCACACTATCATGTTGACATTCC; dG = -35.3
1620 >s_e_s_c_s_a_s_c_s_d_s_a_s_a_s_e_s
GTGCTGGTGTGGGTGTCTGTGTGGGTGACGGTGTCTGTGTCTGTGCTGGTG; >clamp_s_c_s_d_s_c_s_a_s_e_s:compl:rev
CACCAGCACAGACACCCACACCGTCACCCACACTATCATGTTGACATTCC; dG = -35.3
1621 >s_e_s_c_s_a_s_c_s_b_s_c_s_a_s_e_s
GTGCTGGTGTGGGTGTCTGTGTGGGTGGCTGTGTGGGTGTCTGTGCTGGTG; >clamp_s_c_s_d_s_c_s_a_s_e_s:compl:rev
CACCAGCACAGACACCCACACCGTCACCCACACTATCATGTTGACATTCC; dG = -35.3
1622 >s_e_s_c_s_b_s_c_s_d_s_a_s_a_s_e_s
GTGCTGGTGTGGGTGGCTGTGTGGGTGACGGTGTCTGTGTCTGTGCTGGTG; >clamp_s_c_s_d_s_c_s_a_s_e_s:compl:rev
CACCAGCACAGACACCCACACCGTCACCCACACTATCATGTTGACATTCC; dG = -35.3
1623 >s_e_s_c_s_b_s_c_s_b_s_c_s_a_s_e_s
GTGCTGGTGTGGGTGGCTGTGTGGGTGGCTGTGTGGGTGTCTGTGCTGGTG; >clamp_s_c_s_d_s_c_s_a_s_e_s:compl:rev
CACCAGCACAGACACCCACACCGTCACCCACACTATCATGTTGACATTCC; dG = -35.3
1624 >s_e_s_c_s_c_s_c_s_d_s_a_s_a_s_e_s
GTGCTGGTGTGGGTGTGGGTGTGGGTGACGGTGTCTGTGTCTGTGCTGGTG; >clamp_s_c_s_d_s_c_s_a_s_e_s:compl:rev
CACCAGCACAGACACCCACACCGTCACCCACACTATCATGTTGACATTCC; dG = -35.3
1625 >s_e_s_c_s_c_s_c_s_b_s_c_s_a_s_e_s
GTGCTGGTGTGGGTGTGGGTGTGGGTGGCTGTGTGGGTGTCTGTGCTGGTG; >clamp_s_c_s_d_s_c_s_a_s_e_s:compl:rev
CACCAGCACAGACACCCACACCGTCACCCACACTATCATGTTGACATTCC; dG = -35.3
1626 >s_e_s_c_s_e_s_c_s_d_s_a_s_a_s_e_s
GTGCTGGTGTGGGTGCTGGTGTGGGTGACGGTGTCTGTGTCTGTGCTGGTG; >clamp_s_c_s_d_s_c_s_a_s_e_s:compl:rev
CACCAGCACAGACACCCACACCGTCACCCACACTATCATGTTGACATTCC; dG = -35.3
1627 >s_e_s_c_s_e_s_c_s_b_s_c_s_a_s_e_s
GTGCTGGTGTGGGTGCTGGTGTGGGTGGCTGTGTGGGTGTCTGTGCTGGTG; >clamp_s_c_s_d_s_c_s_a_s_e_s:compl:rev
CACCAGCACAGACACCCACACCGTCACCCACACTATCATGTTGACATTCC; dG = -35.3
1628 >s_e_s_d_s_a_s_c_s_d_s_a_s_a_s_e_s
GTGCTGGTGACGGTGTCTGTGTGGGTGACGGTGTCTGTGTCTGTGCTGGTG; >clamp_s_c_s_d_s_c_s_a_s_e_s:compl:rev
CACCAGCACAGACACCCACACCGTCACCCACACTATCATGTTGACATTCC; dG = -35.3
1629 >s_e_s_d_s_a_s_c_s_b_s_c_s_a_s_e_s
GTGCTGGTGACGGTGTCTGTGTGGGTGGCTGTGTGGGTGTCTGTGCTGGTG; >clamp_s_c_s_d_s_c_s_a_s_e_s:compl:rev
CACCAGCACAGACACCCACACCGTCACCCACACTATCATGTTGACATTCC; dG = -35.3
1630 >s_a_s_c_s_b_s_c_s_d_s_a_s_a_s_e_s
GTGTCTGTGTGGGTGGCTGTGTGGGTGACGGTGTCTGTGTCTGTGCTGGTG; >clamp_s_c_s_d_s_c_s_a_s_e_s:compl:rev
CACCAGCACAGACACCCACACCGTCACCCACACTATCATGTTGACATTCC; dG = -35.3
1631 >s_e_s_d_s_a_s_a_s_d_s_c_s_a_s_e_s
GTGCTGGTGACGGTGTCTGTGTCTGTGACGGTGTGGGTGTCTGTGCTGGTG; >clamp_s_c_s_d_s_c_s_a_s_e_s:compl:rev
CACCAGCACAGACACCCACACCGTCACCCACACTATCATGTTGACATTCC; dG = -35.3
1632 >s_e_s_d_s_b_s_c_s_d_s_a_s_a_s_e_s
GTGCTGGTGACGGTGGCTGTGTGGGTGACGGTGTCTGTGTCTGTGCTGGTG; >clamp_s_c_s_d_s_c_s_a_s_e_s:compl:rev
CACCAGCACAGACACCCACACCGTCACCCACACTATCATGTTGACATTCC; dG = -35.3
1633 >s_e_s_d_s_b_s_c_s_b_s_c_s_a_s_e_s
GTGCTGGTGACGGTGGCTGTGTGGGTGGCTGTGTGGGTGTCTGTGCTGGTG; >clamp_s_c_s_d_s_c_s_a_s_e_s:compl:rev
CACCAGCACAGACACCCACACCGTCACCCACACTATCATGTTGACATTCC; dG = -35.3
1634 >s_e_s_d_s_b_s_a_s_d_s_c_s_a_s_e_s
GTGCTGGTGACGGTGGCTGTGTCTGTGACGGTGTGGGTGTCTGTGCTGGTG; >clamp_s_c_s_d_s_c_s_a_s_e_s:compl:rev
CACCAGCACAGACACCCACACCGTCACCCACACTATCATGTTGACATTCC; dG = -35.3
1635 >s_a_s_c_s_b_s_c_s_b_s_c_s_a_s_e_s
GTGTCTGTGTGGGTGGCTGTGTGGGTGGCTGTGTGGGTGTCTGTGCTGGTG; >clamp_s_c_s_d_s_c_s_a_s_e_s:compl:rev
CACCAGCACAGACACCCACACCGTCACCCACACTATCATGTTGACATTCC; dG = -35.3
1636 >s_e_s_d_s_c_s_c_s_d_s_a_s_a_s_e_s
GTGCTGGTGACGGTGTGGGTGTGGGTGACGGTGTCTGTGTCTGTGCTGGTG; >clamp_s_c_s_d_s_c_s_a_s_e_s:compl:rev
CACCAGCACAGACACCCACACCGTCACCCACACTATCATGTTGACATTCC; dG = -35.3
1637 >s_e_s_d_s_c_s_c_s_b_s_c_s_a_s_e_s
GTGCTGGTGACGGTGTGGGTGTGGGTGGCTGTGTGGGTGTCTGTGCTGGTG; >clamp_s_c_s_d_s_c_s_a_s_e_s:compl:rev
CACCAGCACAGACACCCACACCGTCACCCACACTATCATGTTGACATTCC; dG = -35.3
1638 >s_e_s_d_s_d_s_c_s_d_s_a_s_a_s_e_s
GTGCTGGTGACGGTGACGGTGTGGGTGACGGTGTCTGTGTCTGTGCTGGTG; >clamp_s_c_s_d_s_c_s_a_s_e_s:compl:rev
CACCAGCACAGACACCCACACCGTCACCCACACTATCATGTTGACATTCC; dG = -35.3
1639 >s_e_s_d_s_d_s_c_s_b_s_c_s_a_s_e_s
GTGCTGGTGACGGTGACGGTGTGGGTGGCTGTGTGGGTGTCTGTGCTGGTG; >clamp_s_c_s_d_s_c_s_a_s_e_s:compl:rev
CACCAGCACAGACACCCACACCGTCACCCACACTATCATGTTGACATTCC; dG = -35.3

1640 >s_e_s_d_s_d_s_a_s_d_s_c_s_a_s_e_s
GTGCTGGTGACGGTGACGGTGTCTGTGACGGTGTGGGTGTCTGTGCTGGTG;   >clamp_s_c_s_d_s_c_s_a_s_e_s:compl:rev
CACCAGCACAGACACCCACACCGTCACCCACACTATCATGTTGACATTCC; dG = -35.3
1641 >s_e_s_d_s_e_s_c_s_d_s_a_s_a_s_e_s
GTGCTGGTGACGGTGCTGGTGTGGGTGACGGTGTCTGTGTCTGTGCTGGTG;   >clamp_s_c_s_d_s_c_s_a_s_e_s:compl:rev
CACCAGCACAGACACCCACACCGTCACCCACACTATCATGTTGACATTCC; dG = -35.3
1642 >s_e_s_d_s_e_s_c_s_b_s_c_s_a_s_e_s
GTGCTGGTGACGGTGCTGGTGTGGGTGGCTGTGTGGGTGTCTGTGCTGGTG;   >clamp_s_c_s_d_s_c_s_a_s_e_s:compl:rev
CACCAGCACAGACACCCACACCGTCACCCACACTATCATGTTGACATTCC; dG = -35.3
1643 >s_e_s_d_s_e_s_a_s_d_s_c_s_a_s_e_s
GTGCTGGTGACGGTGCTGGTGTCTGTGACGGTGTGGGTGTCTGTGCTGGTG;   >clamp_s_c_s_d_s_c_s_a_s_e_s:compl:rev
CACCAGCACAGACACCCACACCGTCACCCACACTATCATGTTGACATTCC; dG = -35.3
1644 >s_e_s_e_s_a_s_c_s_d_s_a_s_a_s_e_s
GTGCTGGTGCTGGTGTCTGTGTGGGTGACGGTGTCTGTGTCTGTGCTGGTG;   >clamp_s_c_s_d_s_c_s_a_s_e_s:compl:rev
CACCAGCACAGACACCCACACCGTCACCCACACTATCATGTTGACATTCC; dG = -35.3
1645 >s_e_s_e_s_a_s_c_s_b_s_c_s_a_s_e_s
GTGCTGGTGCTGGTGTCTGTGTGGGTGGCTGTGTGGGTGTCTGTGCTGGTG;   >clamp_s_c_s_d_s_c_s_a_s_e_s:compl:rev
CACCAGCACAGACACCCACACCGTCACCCACACTATCATGTTGACATTCC; dG = -35.3
1646 >s_e_s_e_s_a_s_a_s_d_s_c_s_a_s_e_s
GTGCTGGTGCTGGTGTCTGTGTCTGTGACGGTGTGGGTGTCTGTGCTGGTG;   >clamp_s_c_s_d_s_c_s_a_s_e_s:compl:rev
CACCAGCACAGACACCCACACCGTCACCCACACTATCATGTTGACATTCC; dG = -35.3
1647 >s_e_s_e_s_b_s_c_s_d_s_a_s_a_s_e_s
GTGCTGGTGCTGGTGGCTGTGTGGGTGACGGTGTCTGTGTCTGTGCTGGTG;   >clamp_s_c_s_d_s_c_s_a_s_e_s:compl:rev
CACCAGCACAGACACCCACACCGTCACCCACACTATCATGTTGACATTCC; dG = -35.3
1648 >s_e_s_e_s_b_s_c_s_b_s_c_s_a_s_e_s
GTGCTGGTGCTGGTGGCTGTGTGGGTGGCTGTGTGGGTGTCTGTGCTGGTG;   >clamp_s_c_s_d_s_c_s_a_s_e_s:compl:rev
CACCAGCACAGACACCCACACCGTCACCCACACTATCATGTTGACATTCC; dG = -35.3
1649 >s_e_s_e_s_b_s_a_s_d_s_c_s_a_s_e_s
GTGCTGGTGCTGGTGGCTGTGTCTGTGACGGTGTGGGTGTCTGTGCTGGTG;   >clamp_s_c_s_d_s_c_s_a_s_e_s:compl:rev
CACCAGCACAGACACCCACACCGTCACCCACACTATCATGTTGACATTCC; dG = -35.3
1650 >s_e_s_e_s_c_s_c_s_d_s_a_s_a_s_e_s
GTGCTGGTGCTGGTGTGGGTGTGGGTGACGGTGTCTGTGTCTGTGCTGGTG;   >clamp_s_c_s_d_s_c_s_a_s_e_s:compl:rev
CACCAGCACAGACACCCACACCGTCACCCACACTATCATGTTGACATTCC; dG = -35.3
1651 >s_e_s_e_s_c_s_c_s_b_s_c_s_a_s_e_s
GTGCTGGTGCTGGTGTGGGTGTGGGTGGCTGTGTGGGTGTCTGTGCTGGTG;   >clamp_s_c_s_d_s_c_s_a_s_e_s:compl:rev
CACCAGCACAGACACCCACACCGTCACCCACACTATCATGTTGACATTCC; dG = -35.3
1652 >s_e_s_e_s_d_s_c_s_d_s_a_s_a_s_e_s
GTGCTGGTGCTGGTGACGGTGTGGGTGACGGTGTCTGTGTCTGTGCTGGTG;   >clamp_s_c_s_d_s_c_s_a_s_e_s:compl:rev
CACCAGCACAGACACCCACACCGTCACCCACACTATCATGTTGACATTCC; dG = -35.3
1653 >s_e_s_e_s_d_s_c_s_b_s_c_s_a_s_e_s
GTGCTGGTGCTGGTGACGGTGTGGGTGGCTGTGTGGGTGTCTGTGCTGGTG;   >clamp_s_c_s_d_s_c_s_a_s_e_s:compl:rev
CACCAGCACAGACACCCACACCGTCACCCACACTATCATGTTGACATTCC; dG = -35.3
1654 >s_e_s_e_s_d_s_a_s_d_s_c_s_a_s_e_s
GTGCTGGTGCTGGTGACGGTGTCTGTGACGGTGTGGGTGTCTGTGCTGGTG;   >clamp_s_c_s_d_s_c_s_a_s_e_s:compl:rev
CACCAGCACAGACACCCACACCGTCACCCACACTATCATGTTGACATTCC; dG = -35.3
1655 >s_e_s_e_s_e_s_c_s_d_s_a_s_a_s_e_s
GTGCTGGTGCTGGTGCTGGTGTGGGTGACGGTGTCTGTGTCTGTGCTGGTG;   >clamp_s_c_s_d_s_c_s_a_s_e_s:compl:rev
CACCAGCACAGACACCCACACCGTCACCCACACTATCATGTTGACATTCC; dG = -35.3
1656 >s_e_s_e_s_e_s_c_s_b_s_c_s_a_s_e_s
GTGCTGGTGCTGGTGCTGGTGTGGGTGGCTGTGTGGGTGTCTGTGCTGGTG;   >clamp_s_c_s_d_s_c_s_a_s_e_s:compl:rev
CACCAGCACAGACACCCACACCGTCACCCACACTATCATGTTGACATTCC; dG = -35.3
1657 >s_e_s_e_s_e_s_a_s_d_s_c_s_a_s_e_s
GTGCTGGTGCTGGTGCTGGTGTCTGTGACGGTGTGGGTGTCTGTGCTGGTG;   >clamp_s_c_s_d_s_c_s_a_s_e_s:compl:rev
CACCAGCACAGACACCCACACCGTCACCCACACTATCATGTTGACATTCC; dG = -35.3
1658 >s_a_s_c_s_c_s_c_s_d_s_a_s_a_s_e_s
GTGTCTGTGTGGGTGTGGGTGTGGGTGACGGTGTCTGTGTCTGTGCTGGTG;   >clamp_s_c_s_d_s_c_s_a_s_e_s:compl:rev
CACCAGCACAGACACCCACACCGTCACCCACACTATCATGTTGACATTCC; dG = -35.3
1659 >s_a_s_c_s_c_s_c_s_b_s_c_s_a_s_e_s
GTGTCTGTGTGGGTGTGGGTGTGGGTGGCTGTGTGGGTGTCTGTGCTGGTG;   >clamp_s_c_s_d_s_c_s_a_s_e_s:compl:rev
CACCAGCACAGACACCCACACCGTCACCCACACTATCATGTTGACATTCC; dG = -35.3
1660 >s_a_s_c_s_e_s_c_s_d_s_a_s_a_s_e_s
GTGTCTGTGTGGGTGCTGGTGTGGGTGACGGTGTCTGTGTCTGTGCTGGTG;   >clamp_s_c_s_d_s_c_s_a_s_e_s:compl:rev
CACCAGCACAGACACCCACACCGTCACCCACACTATCATGTTGACATTCC; dG = -35.3
1661 >s_a_s_c_s_e_s_c_s_b_s_c_s_a_s_e_s
GTGTCTGTGTGGGTGCTGGTGTGGGTGGCTGTGTGGGTGTCTGTGCTGGTG;   >clamp_s_c_s_d_s_c_s_a_s_e_s:compl:rev
CACCAGCACAGACACCCACACCGTCACCCACACTATCATGTTGACATTCC; dG = -35.3
1662 >s_a_s_d_s_a_s_c_s_d_s_a_s_a_s_e_s
GTGTCTGTGACGGTGTCTGTGTGGGTGACGGTGTCTGTGTCTGTGCTGGTG;   >clamp_s_c_s_d_s_c_s_a_s_e_s:compl:rev
CACCAGCACAGACACCCACACCGTCACCCACACTATCATGTTGACATTCC; dG = -35.3
1663 >s_a_s_d_s_a_s_c_s_b_s_c_s_a_s_e_s
GTGTCTGTGACGGTGTCTGTGTGGGTGGCTGTGTGGGTGTCTGTGCTGGTG;   >clamp_s_c_s_d_s_c_s_a_s_e_s:compl:rev
CACCAGCACAGACACCCACACCGTCACCCACACTATCATGTTGACATTCC; dG = -35.3

1664 >s_a_s_a_s_b_s_c_s_d_s_a_s_a_s_e_s
GTGTCTGTGTCTGTGGCTGTGTGGGTGACGGTGTCTGTGTCTGTGCTGGTG; >clamp_s_c_s_d_s_c_s_a_s_e_s:compl:rev
CACCAGCACAGACACCCACACCGTCACCCACACTATCATGTTGACATTCC; dG = -35.3
1665 >s_a_s_d_s_a_s_a_s_d_s_c_s_a_s_e_s
GTGTCTGTGACGGTGTCTGTGTCTGTGACGGTGTGGGTGTCTGTGCTGGTG; >clamp_s_c_s_d_s_c_s_a_s_e_s:compl:rev
CACCAGCACAGACACCCACACCGTCACCCACACTATCATGTTGACATTCC; dG = -35.3
1666 >s_a_s_d_s_b_s_c_s_d_s_a_s_a_s_e_s
GTGTCTGTGACGGTGGCTGTGTGGGTGACGGTGTCTGTGTCTGTGCTGGTG; >clamp_s_c_s_d_s_c_s_a_s_e_s:compl:rev
CACCAGCACAGACACCCACACCGTCACCCACACTATCATGTTGACATTCC; dG = -35.3
1667 >s_a_s_d_s_b_s_c_s_b_s_c_s_a_s_e_s
GTGTCTGTGACGGTGGCTGTGTGGGTGGCTGTGTGGGTGTCTGTGCTGGTG; >clamp_s_c_s_d_s_c_s_a_s_e_s:compl:rev
CACCAGCACAGACACCCACACCGTCACCCACACTATCATGTTGACATTCC; dG = -35.3
1668 >s_a_s_d_s_b_s_a_s_d_s_c_s_a_s_e_s
GTGTCTGTGACGGTGGCTGTGTCTGTGACGGTGTGGGTGTCTGTGCTGGTG; >clamp_s_c_s_d_s_c_s_a_s_e_s:compl:rev
CACCAGCACAGACACCCACACCGTCACCCACACTATCATGTTGACATTCC; dG = -35.3
1669 >s_a_s_a_s_b_s_c_s_b_s_c_s_a_s_e_s
GTGTCTGTGTCTGTGGCTGTGTGGGTGGCTGTGTGGGTGTCTGTGCTGGTG; >clamp_s_c_s_d_s_c_s_a_s_e_s:compl:rev
CACCAGCACAGACACCCACACCGTCACCCACACTATCATGTTGACATTCC; dG = -35.3
1670 >s_a_s_d_s_c_s_c_s_d_s_a_s_a_s_e_s
GTGTCTGTGACGGTGTGGGTGTGGGTGACGGTGTCTGTGTCTGTGCTGGTG; >clamp_s_c_s_d_s_c_s_a_s_e_s:compl:rev
CACCAGCACAGACACCCACACCGTCACCCACACTATCATGTTGACATTCC; dG = -35.3
1671 >s_a_s_d_s_c_s_c_s_b_s_c_s_a_s_e_s
GTGTCTGTGACGGTGTGGGTGTGGGTGGCTGTGTGGGTGTCTGTGCTGGTG; >clamp_s_c_s_d_s_c_s_a_s_e_s:compl:rev
CACCAGCACAGACACCCACACCGTCACCCACACTATCATGTTGACATTCC; dG = -35.3
1672 >s_a_s_d_s_d_s_c_s_d_s_a_s_a_s_e_s
GTGTCTGTGACGGTGACGGTGTGGGTGACGGTGTCTGTGTCTGTGCTGGTG; >clamp_s_c_s_d_s_c_s_a_s_e_s:compl:rev
CACCAGCACAGACACCCACACCGTCACCCACACTATCATGTTGACATTCC; dG = -35.3
1673 >s_a_s_d_s_d_s_c_s_b_s_c_s_a_s_e_s
GTGTCTGTGACGGTGACGGTGTGGGTGGCTGTGTGGGTGTCTGTGCTGGTG; >clamp_s_c_s_d_s_c_s_a_s_e_s:compl:rev
CACCAGCACAGACACCCACACCGTCACCCACACTATCATGTTGACATTCC; dG = -35.3
1674 >s_a_s_d_s_d_s_a_s_d_s_c_s_a_s_e_s
GTGTCTGTGACGGTGACGGTGTCTGTGACGGTGTGGGTGTCTGTGCTGGTG; >clamp_s_c_s_d_s_c_s_a_s_e_s:compl:rev
CACCAGCACAGACACCCACACCGTCACCCACACTATCATGTTGACATTCC; dG = -35.3
1675 >s_a_s_d_s_e_s_c_s_d_s_a_s_a_s_e_s
GTGTCTGTGACGGTGCTGGTGTGGGTGACGGTGTCTGTGTCTGTGCTGGTG; >clamp_s_c_s_d_s_c_s_a_s_e_s:compl:rev
CACCAGCACAGACACCCACACCGTCACCCACACTATCATGTTGACATTCC; dG = -35.3
1676 >s_a_s_d_s_e_s_c_s_b_s_c_s_a_s_e_s
GTGTCTGTGACGGTGCTGGTGTGGGTGGCTGTGTGGGTGTCTGTGCTGGTG; >clamp_s_c_s_d_s_c_s_a_s_e_s:compl:rev
CACCAGCACAGACACCCACACCGTCACCCACACTATCATGTTGACATTCC; dG = -35.3
1677 >s_a_s_a_s_b_s_a_s_d_s_c_s_a_s_e_s
GTGTCTGTGTCTGTGGCTGTGTCTGTGACGGTGTGGGTGTCTGTGCTGGTG; >clamp_s_c_s_d_s_c_s_a_s_e_s:compl:rev
CACCAGCACAGACACCCACACCGTCACCCACACTATCATGTTGACATTCC; dG = -35.3
1678 >s_a_s_d_s_e_s_a_s_d_s_c_s_a_s_e_s
GTGTCTGTGACGGTGCTGGTGTCTGTGACGGTGTGGGTGTCTGTGCTGGTG; >clamp_s_c_s_d_s_c_s_a_s_e_s:compl:rev
CACCAGCACAGACACCCACACCGTCACCCACACTATCATGTTGACATTCC; dG = -35.3
1679 >s_a_s_e_s_a_s_c_s_d_s_a_s_a_s_e_s
GTGTCTGTGCTGGTGTCTGTGTGGGTGACGGTGTCTGTGTCTGTGCTGGTG; >clamp_s_c_s_d_s_c_s_a_s_e_s:compl:rev
CACCAGCACAGACACCCACACCGTCACCCACACTATCATGTTGACATTCC; dG = -35.3
1680 >s_a_s_e_s_a_s_c_s_b_s_c_s_a_s_e_s
GTGTCTGTGCTGGTGTCTGTGTGGGTGGCTGTGTGGGTGTCTGTGCTGGTG; >clamp_s_c_s_d_s_c_s_a_s_e_s:compl:rev
CACCAGCACAGACACCCACACCGTCACCCACACTATCATGTTGACATTCC; dG = -35.3
1681 >s_a_s_e_s_a_s_a_s_d_s_c_s_a_s_e_s
GTGTCTGTGCTGGTGTCTGTGTCTGTGACGGTGTGGGTGTCTGTGCTGGTG; >clamp_s_c_s_d_s_c_s_a_s_e_s:compl:rev
CACCAGCACAGACACCCACACCGTCACCCACACTATCATGTTGACATTCC; dG = -35.3
1682 >s_a_s_e_s_b_s_c_s_d_s_a_s_a_s_e_s
GTGTCTGTGCTGGTGGCTGTGTGGGTGACGGTGTCTGTGTCTGTGCTGGTG; >clamp_s_c_s_d_s_c_s_a_s_e_s:compl:rev
CACCAGCACAGACACCCACACCGTCACCCACACTATCATGTTGACATTCC; dG = -35.3
1683 >s_a_s_e_s_b_s_c_s_b_s_c_s_a_s_e_s
GTGTCTGTGCTGGTGGCTGTGTGGGTGGCTGTGTGGGTGTCTGTGCTGGTG; >clamp_s_c_s_d_s_c_s_a_s_e_s:compl:rev
CACCAGCACAGACACCCACACCGTCACCCACACTATCATGTTGACATTCC; dG = -35.3
1684 >s_a_s_e_s_b_s_a_s_d_s_c_s_a_s_e_s
GTGTCTGTGCTGGTGGCTGTGTCTGTGACGGTGTGGGTGTCTGTGCTGGTG; >clamp_s_c_s_d_s_c_s_a_s_e_s:compl:rev
CACCAGCACAGACACCCACACCGTCACCCACACTATCATGTTGACATTCC; dG = -35.3
1685 >s_a_s_e_s_c_s_c_s_d_s_a_s_a_s_e_s
GTGTCTGTGCTGGTGTGGGTGTGGGTGACGGTGTCTGTGTCTGTGCTGGTG; >clamp_s_c_s_d_s_c_s_a_s_e_s:compl:rev
CACCAGCACAGACACCCACACCGTCACCCACACTATCATGTTGACATTCC; dG = -35.3
1686 >s_a_s_e_s_c_s_c_s_b_s_c_s_a_s_e_s
GTGTCTGTGCTGGTGTGGGTGTGGGTGGCTGTGTGGGTGTCTGTGCTGGTG; >clamp_s_c_s_d_s_c_s_a_s_e_s:compl:rev
CACCAGCACAGACACCCACACCGTCACCCACACTATCATGTTGACATTCC; dG = -35.3
1687 >s_a_s_e_s_d_s_c_s_d_s_a_s_a_s_e_s
GTGTCTGTGCTGGTGACGGTGTGGGTGACGGTGTCTGTGTCTGTGCTGGTG; >clamp_s_c_s_d_s_c_s_a_s_e_s:compl:rev
CACCAGCACAGACACCCACACCGTCACCCACACTATCATGTTGACATTCC; dG = -35.3

1688 >s_a_s_e_s_d_s_c_s_b_s_c_s_a_s_e_s
GTGTCTGTGCTGGTGACGGTGTGGGTGGCTGTGTGGGTGTCTGTGCTGGTG; >clamp_s_c_s_d_s_c_s_a_s_e_s:compl:rev
CACCAGCACAGACACCCACACCGTCACCCACACTATCATGTTGACATTCC; dG = -35.3
1689 >s_a_s_e_s_d_s_a_s_d_s_c_s_a_s_e_s
GTGTCTGTGCTGGTGACGGTGTCTGTGACGGTGTGGGTGTCTGTGCTGGTG; >clamp_s_c_s_d_s_c_s_a_s_e_s:compl:rev
CACCAGCACAGACACCCACACCGTCACCCACACTATCATGTTGACATTCC; dG = -35.3
1690 >s_a_s_e_s_e_s_c_s_d_s_a_s_a_s_e_s
GTGTCTGTGCTGGTGCTGGTGTGGGTGACGGTGTCTGTGTCTGTGCTGGTG; >clamp_s_c_s_d_s_c_s_a_s_e_s:compl:rev
CACCAGCACAGACACCCACACCGTCACCCACACTATCATGTTGACATTCC; dG = -35.3
1691 >s_a_s_e_s_e_s_c_s_b_s_c_s_a_s_e_s
GTGTCTGTGCTGGTGCTGGTGTGGGTGGCTGTGTGGGTGTCTGTGCTGGTG; >clamp_s_c_s_d_s_c_s_a_s_e_s:compl:rev
CACCAGCACAGACACCCACACCGTCACCCACACTATCATGTTGACATTCC; dG = -35.3
1692 >s_a_s_e_s_e_s_a_s_d_s_c_s_a_s_e_s
GTGTCTGTGCTGGTGCTGGTGTCTGTGACGGTGTGGGTGTCTGTGCTGGTG; >clamp_s_c_s_d_s_c_s_a_s_e_s:compl:rev
CACCAGCACAGACACCCACACCGTCACCCACACTATCATGTTGACATTCC; dG = -35.3
1693 >s_b_s_a_s_a_s_c_s_d_s_a_s_a_s_e_s
GTGGCTGTGTCTGTGTCTGTGTGGGTGACGGTGTCTGTGTCTGTGCTGGTG; >clamp_s_c_s_d_s_c_s_a_s_e_s:compl:rev
CACCAGCACAGACACCCACACCGTCACCCACACTATCATGTTGACATTCC; dG = -35.3
1694 >s_b_s_a_s_a_s_c_s_b_s_c_s_a_s_e_s
GTGGCTGTGTCTGTGTCTGTGTGGGTGGCTGTGTGGGTGTCTGTGCTGGTG; >clamp_s_c_s_d_s_c_s_a_s_e_s:compl:rev
CACCAGCACAGACACCCACACCGTCACCCACACTATCATGTTGACATTCC; dG = -35.3
1695 >s_a_s_a_s_c_s_c_s_d_s_a_s_a_s_e_s
GTGTCTGTGTCTGTGTGGGTGTGGGTGACGGTGTCTGTGTCTGTGCTGGTG; >clamp_s_c_s_d_s_c_s_a_s_e_s:compl:rev
CACCAGCACAGACACCCACACCGTCACCCACACTATCATGTTGACATTCC; dG = -35.3
1696 >s_b_s_a_s_a_s_a_s_d_s_c_s_a_s_e_s
GTGGCTGTGTCTGTGTCTGTGTCTGTGACGGTGTGGGTGTCTGTGCTGGTG; >clamp_s_c_s_d_s_c_s_a_s_e_s:compl:rev
CACCAGCACAGACACCCACACCGTCACCCACACTATCATGTTGACATTCC; dG = -35.3
1697 >s_b_s_a_s_b_s_c_s_d_s_a_s_a_s_e_s
GTGGCTGTGTCTGTGGCTGTGTGGGTGACGGTGTCTGTGTCTGTGCTGGTG; >clamp_s_c_s_d_s_c_s_a_s_e_s:compl:rev
CACCAGCACAGACACCCACACCGTCACCCACACTATCATGTTGACATTCC; dG = -35.3
1698 >s_b_s_a_s_b_s_c_s_b_s_c_s_a_s_e_s
GTGGCTGTGTCTGTGGCTGTGTGGGTGGCTGTGTGGGTGTCTGTGCTGGTG; >clamp_s_c_s_d_s_c_s_a_s_e_s:compl:rev
CACCAGCACAGACACCCACACCGTCACCCACACTATCATGTTGACATTCC; dG = -35.3
1699 >s_b_s_a_s_b_s_a_s_d_s_c_s_a_s_e_s
GTGGCTGTGTCTGTGGCTGTGTCTGTGACGGTGTGGGTGTCTGTGCTGGTG; >clamp_s_c_s_d_s_c_s_a_s_e_s:compl:rev
CACCAGCACAGACACCCACACCGTCACCCACACTATCATGTTGACATTCC; dG = -35.3
1700 >s_a_s_a_s_c_s_c_s_b_s_c_s_a_s_e_s
GTGTCTGTGTCTGTGTGGGTGTGGGTGGCTGTGTGGGTGTCTGTGCTGGTG; >clamp_s_c_s_d_s_c_s_a_s_e_s:compl:rev
CACCAGCACAGACACCCACACCGTCACCCACACTATCATGTTGACATTCC; dG = -35.3
1701 >s_b_s_a_s_c_s_c_s_d_s_a_s_a_s_e_s
GTGGCTGTGTCTGTGTGGGTGTGGGTGACGGTGTCTGTGTCTGTGCTGGTG; >clamp_s_c_s_d_s_c_s_a_s_e_s:compl:rev
CACCAGCACAGACACCCACACCGTCACCCACACTATCATGTTGACATTCC; dG = -35.3
1702 >s_b_s_a_s_c_s_c_s_b_s_c_s_a_s_e_s
GTGGCTGTGTCTGTGTGGGTGTGGGTGGCTGTGTGGGTGTCTGTGCTGGTG; >clamp_s_c_s_d_s_c_s_a_s_e_s:compl:rev
CACCAGCACAGACACCCACACCGTCACCCACACTATCATGTTGACATTCC; dG = -35.3
1703 >s_b_s_a_s_d_s_c_s_d_s_a_s_a_s_e_s
GTGGCTGTGTCTGTGACGGTGTGGGTGACGGTGTCTGTGTCTGTGCTGGTG; >clamp_s_c_s_d_s_c_s_a_s_e_s:compl:rev
CACCAGCACAGACACCCACACCGTCACCCACACTATCATGTTGACATTCC; dG = -35.3
1704 >s_b_s_a_s_d_s_c_s_b_s_c_s_a_s_e_s
GTGGCTGTGTCTGTGACGGTGTGGGTGGCTGTGTGGGTGTCTGTGCTGGTG; >clamp_s_c_s_d_s_c_s_a_s_e_s:compl:rev
CACCAGCACAGACACCCACACCGTCACCCACACTATCATGTTGACATTCC; dG = -35.3
1705 >s_b_s_a_s_d_s_a_s_d_s_c_s_a_s_e_s
GTGGCTGTGTCTGTGACGGTGTCTGTGACGGTGTGGGTGTCTGTGCTGGTG; >clamp_s_c_s_d_s_c_s_a_s_e_s:compl:rev
CACCAGCACAGACACCCACACCGTCACCCACACTATCATGTTGACATTCC; dG = -35.3
1706 >s_b_s_a_s_e_s_c_s_d_s_a_s_a_s_e_s
GTGGCTGTGTCTGTGCTGGTGTGGGTGACGGTGTCTGTGTCTGTGCTGGTG; >clamp_s_c_s_d_s_c_s_a_s_e_s:compl:rev
CACCAGCACAGACACCCACACCGTCACCCACACTATCATGTTGACATTCC; dG = -35.3
1707 >s_b_s_a_s_e_s_c_s_b_s_c_s_a_s_e_s
GTGGCTGTGTCTGTGCTGGTGTGGGTGGCTGTGTGGGTGTCTGTGCTGGTG; >clamp_s_c_s_d_s_c_s_a_s_e_s:compl:rev
CACCAGCACAGACACCCACACCGTCACCCACACTATCATGTTGACATTCC; dG = -35.3
1708 >s_b_s_a_s_e_s_a_s_d_s_c_s_a_s_e_s
GTGGCTGTGTCTGTGCTGGTGTCTGTGACGGTGTGGGTGTCTGTGCTGGTG; >clamp_s_c_s_d_s_c_s_a_s_e_s:compl:rev
CACCAGCACAGACACCCACACCGTCACCCACACTATCATGTTGACATTCC; dG = -35.3
1709 >s_b_s_b_s_a_s_c_s_d_s_a_s_a_s_e_s
GTGGCTGTGGCTGTGTCTGTGTGGGTGACGGTGTCTGTGTCTGTGCTGGTG; >clamp_s_c_s_d_s_c_s_a_s_e_s:compl:rev
CACCAGCACAGACACCCACACCGTCACCCACACTATCATGTTGACATTCC; dG = -35.3
1710 >s_b_s_b_s_a_s_c_s_b_s_c_s_a_s_e_s
GTGGCTGTGGCTGTGTCTGTGTGGGTGGCTGTGTGGGTGTCTGTGCTGGTG; >clamp_s_c_s_d_s_c_s_a_s_e_s:compl:rev
CACCAGCACAGACACCCACACCGTCACCCACACTATCATGTTGACATTCC; dG = -35.3
1711 >s_b_s_b_s_a_s_a_s_d_s_c_s_a_s_e_s
GTGGCTGTGGCTGTGTCTGTGTCTGTGACGGTGTGGGTGTCTGTGCTGGTG; >clamp_s_c_s_d_s_c_s_a_s_e_s:compl:rev
CACCAGCACAGACACCCACACCGTCACCCACACTATCATGTTGACATTCC; dG = -35.3

1712 >s_b_s_b_s_b_s_c_s_d_s_a_s_a_s_e_s
GTGGCTGTGGCTGTGGCTGTGTGGGTGACGGTGTCTGTGTCTGTGCTGGTG; >clamp_s_c_s_d_s_c_s_a_s_e_s:compl:rev
CACCAGCACAGACACCCACACCGTCACCCACACTATCATGTTGACATTCC; dG = -35.3
1713 >s_b_s_b_s_b_s_c_s_b_s_c_s_a_s_e_s
GTGGCTGTGGCTGTGGCTGTGTGGGTGGCTGTGTGGGTGTCTGTGCTGGTG; >clamp_s_c_s_d_s_c_s_a_s_e_s:compl:rev
CACCAGCACAGACACCCACACCGTCACCCACACTATCATGTTGACATTCC; dG = -35.3
1714 >s_b_s_b_s_b_s_a_s_d_s_c_s_a_s_e_s
GTGGCTGTGGCTGTGGCTGTGTCTGTGACGGTGTGGGTGTCTGTGCTGGTG; >clamp_s_c_s_d_s_c_s_a_s_e_s:compl:rev
CACCAGCACAGACACCCACACCGTCACCCACACTATCATGTTGACATTCC; dG = -35.3
1715 >s_b_s_b_s_c_s_c_s_d_s_a_s_a_s_e_s
GTGGCTGTGGCTGTGTGGGTGTGGGTGACGGTGTCTGTGTCTGTGCTGGTG; >clamp_s_c_s_d_s_c_s_a_s_e_s:compl:rev
CACCAGCACAGACACCCACACCGTCACCCACACTATCATGTTGACATTCC; dG = -35.3
1716 >s_b_s_b_s_c_s_c_s_b_s_c_s_a_s_e_s
GTGGCTGTGGCTGTGTGGGTGTGGGTGGCTGTGTGGGTGTCTGTGCTGGTG; >clamp_s_c_s_d_s_c_s_a_s_e_s:compl:rev
CACCAGCACAGACACCCACACCGTCACCCACACTATCATGTTGACATTCC; dG = -35.3
1717 >s_b_s_b_s_d_s_c_s_d_s_a_s_a_s_e_s
GTGGCTGTGGCTGTGACGGTGTGGGTGACGGTGTCTGTGTCTGTGCTGGTG; >clamp_s_c_s_d_s_c_s_a_s_e_s:compl:rev
CACCAGCACAGACACCCACACCGTCACCCACACTATCATGTTGACATTCC; dG = -35.3
1718 >s_b_s_b_s_d_s_c_s_b_s_c_s_a_s_e_s
GTGGCTGTGGCTGTGACGGTGTGGGTGGCTGTGTGGGTGTCTGTGCTGGTG; >clamp_s_c_s_d_s_c_s_a_s_e_s:compl:rev
CACCAGCACAGACACCCACACCGTCACCCACACTATCATGTTGACATTCC; dG = -35.3
1719 >s_b_s_b_s_d_s_a_s_d_s_c_s_a_s_e_s
GTGGCTGTGGCTGTGACGGTGTCTGTGACGGTGTGGGTGTCTGTGCTGGTG; >clamp_s_c_s_d_s_c_s_a_s_e_s:compl:rev
CACCAGCACAGACACCCACACCGTCACCCACACTATCATGTTGACATTCC; dG = -35.3
1720 >s_b_s_b_s_e_s_c_s_d_s_a_s_a_s_e_s
GTGGCTGTGGCTGTGCTGGTGTGGGTGACGGTGTCTGTGTCTGTGCTGGTG; >clamp_s_c_s_d_s_c_s_a_s_e_s:compl:rev
CACCAGCACAGACACCCACACCGTCACCCACACTATCATGTTGACATTCC; dG = -35.3
1721 >s_b_s_b_s_e_s_c_s_b_s_c_s_a_s_e_s
GTGGCTGTGGCTGTGCTGGTGTGGGTGGCTGTGTGGGTGTCTGTGCTGGTG; >clamp_s_c_s_d_s_c_s_a_s_e_s:compl:rev
CACCAGCACAGACACCCACACCGTCACCCACACTATCATGTTGACATTCC; dG = -35.3
1722 >s_b_s_b_s_e_s_a_s_d_s_c_s_a_s_e_s
GTGGCTGTGGCTGTGCTGGTGTCTGTGACGGTGTGGGTGTCTGTGCTGGTG; >clamp_s_c_s_d_s_c_s_a_s_e_s:compl:rev
CACCAGCACAGACACCCACACCGTCACCCACACTATCATGTTGACATTCC; dG = -35.3
1723 >s_b_s_c_s_a_s_c_s_d_s_a_s_a_s_e_s
GTGGCTGTGTGGGTGTCTGTGTGGGTGACGGTGTCTGTGTCTGTGCTGGTG; >clamp_s_c_s_d_s_c_s_a_s_e_s:compl:rev
CACCAGCACAGACACCCACACCGTCACCCACACTATCATGTTGACATTCC; dG = -35.3
1724 >s_b_s_c_s_a_s_c_s_b_s_c_s_a_s_e_s
GTGGCTGTGTGGGTGTCTGTGTGGGTGGCTGTGTGGGTGTCTGTGCTGGTG; >clamp_s_c_s_d_s_c_s_a_s_e_s:compl:rev
CACCAGCACAGACACCCACACCGTCACCCACACTATCATGTTGACATTCC; dG = -35.3
1725 >s_a_s_a_s_d_s_c_s_d_s_a_s_a_s_e_s
GTGTCTGTGTCTGTGACGGTGTGGGTGACGGTGTCTGTGTCTGTGCTGGTG; >clamp_s_c_s_d_s_c_s_a_s_e_s:compl:rev
CACCAGCACAGACACCCACACCGTCACCCACACTATCATGTTGACATTCC; dG = -35.3
1726 >s_b_s_c_s_b_s_c_s_d_s_a_s_a_s_e_s
GTGGCTGTGTGGGTGGCTGTGTGGGTGACGGTGTCTGTGTCTGTGCTGGTG; >clamp_s_c_s_d_s_c_s_a_s_e_s:compl:rev
CACCAGCACAGACACCCACACCGTCACCCACACTATCATGTTGACATTCC; dG = -35.3
1727 >s_b_s_c_s_b_s_c_s_b_s_c_s_a_s_e_s
GTGGCTGTGTGGGTGGCTGTGTGGGTGGCTGTGTGGGTGTCTGTGCTGGTG; >clamp_s_c_s_d_s_c_s_a_s_e_s:compl:rev
CACCAGCACAGACACCCACACCGTCACCCACACTATCATGTTGACATTCC; dG = -35.3
1728 >s_a_s_a_s_d_s_c_s_b_s_c_s_a_s_e_s
GTGTCTGTGTCTGTGACGGTGTGGGTGGCTGTGTGGGTGTCTGTGCTGGTG; >clamp_s_c_s_d_s_c_s_a_s_e_s:compl:rev
CACCAGCACAGACACCCACACCGTCACCCACACTATCATGTTGACATTCC; dG = -35.3
1729 >s_b_s_c_s_c_s_c_s_d_s_a_s_a_s_e_s
GTGGCTGTGTGGGTGTGGGTGTGGGTGACGGTGTCTGTGTCTGTGCTGGTG; >clamp_s_c_s_d_s_c_s_a_s_e_s:compl:rev
CACCAGCACAGACACCCACACCGTCACCCACACTATCATGTTGACATTCC; dG = -35.3
1730 >s_b_s_c_s_c_s_c_s_b_s_c_s_a_s_e_s
GTGGCTGTGTGGGTGTGGGTGTGGGTGGCTGTGTGGGTGTCTGTGCTGGTG; >clamp_s_c_s_d_s_c_s_a_s_e_s:compl:rev
CACCAGCACAGACACCCACACCGTCACCCACACTATCATGTTGACATTCC; dG = -35.3
1731 >s_b_s_c_s_e_s_c_s_d_s_a_s_a_s_e_s
GTGGCTGTGTGGGTGCTGGTGTGGGTGACGGTGTCTGTGTCTGTGCTGGTG; >clamp_s_c_s_d_s_c_s_a_s_e_s:compl:rev
CACCAGCACAGACACCCACACCGTCACCCACACTATCATGTTGACATTCC; dG = -35.3
1732 >s_b_s_c_s_e_s_c_s_b_s_c_s_a_s_e_s
GTGGCTGTGTGGGTGCTGGTGTGGGTGGCTGTGTGGGTGTCTGTGCTGGTG; >clamp_s_c_s_d_s_c_s_a_s_e_s:compl:rev
CACCAGCACAGACACCCACACCGTCACCCACACTATCATGTTGACATTCC; dG = -35.3
1733 >s_a_s_a_s_d_s_a_s_d_s_c_s_a_s_e_s
GTGTCTGTGTCTGTGACGGTGTCTGTGACGGTGTGGGTGTCTGTGCTGGTG; >clamp_s_c_s_d_s_c_s_a_s_e_s:compl:rev
CACCAGCACAGACACCCACACCGTCACCCACACTATCATGTTGACATTCC; dG = -35.3
1734 >s_b_s_d_s_a_s_c_s_d_s_a_s_a_s_e_s
GTGGCTGTGACGGTGTCTGTGTGGGTGACGGTGTCTGTGTCTGTGCTGGTG; >clamp_s_c_s_d_s_c_s_a_s_e_s:compl:rev
CACCAGCACAGACACCCACACCGTCACCCACACTATCATGTTGACATTCC; dG = -35.3
1735 >s_b_s_d_s_a_s_c_s_b_s_c_s_a_s_e_s
GTGGCTGTGACGGTGTCTGTGTGGGTGGCTGTGTGGGTGTCTGTGCTGGTG; >clamp_s_c_s_d_s_c_s_a_s_e_s:compl:rev
CACCAGCACAGACACCCACACCGTCACCCACACTATCATGTTGACATTCC; dG = -35.3

1736 >s_b_s_d_s_a_s_a_s_d_s_c_s_a_s_e_s
GTGGCTGTGACGGTGTCTGTGTCTGTGACGGTGTGGGTGTCTGTGCTGGTG; >clamp_s_c_s_d_s_c_s_a_s_e_s:compl:rev
CACCAGCACAGACACCCACACCGTCACCCACACTATCATGTTGACATTCC; dG = -35.3
1737 >s_b_s_d_s_b_s_c_s_d_s_a_s_a_s_e_s
GTGGCTGTGACGGTGGCTGTGTGGGTGACGGTGTCTGTGTCTGTGCTGGTG; >clamp_s_c_s_d_s_c_s_a_s_e_s:compl:rev
CACCAGCACAGACACCCACACCGTCACCCACACTATCATGTTGACATTCC; dG = -35.3
1738 >s_b_s_d_s_b_s_c_s_b_s_c_s_a_s_e_s
GTGGCTGTGACGGTGGCTGTGTGGGTGGCTGTGTGGGTGTCTGTGCTGGTG; >clamp_s_c_s_d_s_c_s_a_s_e_s:compl:rev
CACCAGCACAGACACCCACACCGTCACCCACACTATCATGTTGACATTCC; dG = -35.3
1739 >s_b_s_d_s_b_s_a_s_d_s_c_s_a_s_e_s
GTGGCTGTGACGGTGGCTGTGTCTGTGACGGTGTGGGTGTCTGTGCTGGTG; >clamp_s_c_s_d_s_c_s_a_s_e_s:compl:rev
CACCAGCACAGACACCCACACCGTCACCCACACTATCATGTTGACATTCC; dG = -35.3
1740 >s_b_s_d_s_c_s_c_s_d_s_a_s_a_s_e_s
GTGGCTGTGACGGTGTGGGTGTGGGTGACGGTGTCTGTGTCTGTGCTGGTG; >clamp_s_c_s_d_s_c_s_a_s_e_s:compl:rev
CACCAGCACAGACACCCACACCGTCACCCACACTATCATGTTGACATTCC; dG = -35.3
1741 >s_b_s_d_s_c_s_c_s_b_s_c_s_a_s_e_s
GTGGCTGTGACGGTGTGGGTGTGGGTGGCTGTGTGGGTGTCTGTGCTGGTG; >clamp_s_c_s_d_s_c_s_a_s_e_s:compl:rev
CACCAGCACAGACACCCACACCGTCACCCACACTATCATGTTGACATTCC; dG = -35.3
1742 >s_b_s_d_s_d_s_c_s_d_s_a_s_a_s_e_s
GTGGCTGTGACGGTGACGGTGTGGGTGACGGTGTCTGTGTCTGTGCTGGTG; >clamp_s_c_s_d_s_c_s_a_s_e_s:compl:rev
CACCAGCACAGACACCCACACCGTCACCCACACTATCATGTTGACATTCC; dG = -35.3
1743 >s_b_s_d_s_d_s_c_s_b_s_c_s_a_s_e_s
GTGGCTGTGACGGTGACGGTGTGGGTGGCTGTGTGGGTGTCTGTGCTGGTG; >clamp_s_c_s_d_s_c_s_a_s_e_s:compl:rev
CACCAGCACAGACACCCACACCGTCACCCACACTATCATGTTGACATTCC; dG = -35.3
1744 >s_b_s_d_s_d_s_a_s_d_s_c_s_a_s_e_s
GTGGCTGTGACGGTGACGGTGTCTGTGACGGTGTGGGTGTCTGTGCTGGTG; >clamp_s_c_s_d_s_c_s_a_s_e_s:compl:rev
CACCAGCACAGACACCCACACCGTCACCCACACTATCATGTTGACATTCC; dG = -35.3
1745 >s_b_s_d_s_e_s_c_s_d_s_a_s_a_s_e_s
GTGGCTGTGACGGTGCTGGTGTGGGTGACGGTGTCTGTGTCTGTGCTGGTG; >clamp_s_c_s_d_s_c_s_a_s_e_s:compl:rev
CACCAGCACAGACACCCACACCGTCACCCACACTATCATGTTGACATTCC; dG = -35.3
1746 >s_b_s_d_s_e_s_c_s_b_s_c_s_a_s_e_s
GTGGCTGTGACGGTGCTGGTGTGGGTGGCTGTGTGGGTGTCTGTGCTGGTG; >clamp_s_c_s_d_s_c_s_a_s_e_s:compl:rev
CACCAGCACAGACACCCACACCGTCACCCACACTATCATGTTGACATTCC; dG = -35.3
1747 >s_b_s_d_s_e_s_a_s_d_s_c_s_a_s_e_s
GTGGCTGTGACGGTGCTGGTGTCTGTGACGGTGTGGGTGTCTGTGCTGGTG; >clamp_s_c_s_d_s_c_s_a_s_e_s:compl:rev
CACCAGCACAGACACCCACACCGTCACCCACACTATCATGTTGACATTCC; dG = -35.3
1748 >s_b_s_e_s_a_s_c_s_d_s_a_s_a_s_e_s
GTGGCTGTGCTGGTGTCTGTGTGGGTGACGGTGTCTGTGTCTGTGCTGGTG; >clamp_s_c_s_d_s_c_s_a_s_e_s:compl:rev
CACCAGCACAGACACCCACACCGTCACCCACACTATCATGTTGACATTCC; dG = -35.3
1749 >s_b_s_e_s_a_s_c_s_b_s_c_s_a_s_e_s
GTGGCTGTGCTGGTGTCTGTGTGGGTGGCTGTGTGGGTGTCTGTGCTGGTG; >clamp_s_c_s_d_s_c_s_a_s_e_s:compl:rev
CACCAGCACAGACACCCACACCGTCACCCACACTATCATGTTGACATTCC; dG = -35.3
1750 >s_a_s_a_s_e_s_c_s_d_s_a_s_a_s_e_s
GTGTCTGTGTCTGTGCTGGTGTGGGTGACGGTGTCTGTGTCTGTGCTGGTG; >clamp_s_c_s_d_s_c_s_a_s_e_s:compl:rev
CACCAGCACAGACACCCACACCGTCACCCACACTATCATGTTGACATTCC; dG = -35.3
1751 >s_b_s_e_s_a_s_a_s_d_s_c_s_a_s_e_s
GTGGCTGTGCTGGTGTCTGTGTCTGTGACGGTGTGGGTGTCTGTGCTGGTG; >clamp_s_c_s_d_s_c_s_a_s_e_s:compl:rev
CACCAGCACAGACACCCACACCGTCACCCACACTATCATGTTGACATTCC; dG = -35.3
1752 >s_b_s_e_s_b_s_c_s_d_s_a_s_a_s_e_s
GTGGCTGTGCTGGTGGCTGTGTGGGTGACGGTGTCTGTGTCTGTGCTGGTG; >clamp_s_c_s_d_s_c_s_a_s_e_s:compl:rev
CACCAGCACAGACACCCACACCGTCACCCACACTATCATGTTGACATTCC; dG = -35.3
1753 >s_b_s_e_s_b_s_c_s_b_s_c_s_a_s_e_s
GTGGCTGTGCTGGTGGCTGTGTGGGTGGCTGTGTGGGTGTCTGTGCTGGTG; >clamp_s_c_s_d_s_c_s_a_s_e_s:compl:rev
CACCAGCACAGACACCCACACCGTCACCCACACTATCATGTTGACATTCC; dG = -35.3
1754 >s_b_s_e_s_b_s_a_s_d_s_c_s_a_s_e_s
GTGGCTGTGCTGGTGGCTGTGTCTGTGACGGTGTGGGTGTCTGTGCTGGTG; >clamp_s_c_s_d_s_c_s_a_s_e_s:compl:rev
CACCAGCACAGACACCCACACCGTCACCCACACTATCATGTTGACATTCC; dG = -35.3
1755 >s_a_s_a_s_e_s_c_s_b_s_c_s_a_s_e_s
GTGTCTGTGTCTGTGCTGGTGTGGGTGGCTGTGTGGGTGTCTGTGCTGGTG; >clamp_s_c_s_d_s_c_s_a_s_e_s:compl:rev
CACCAGCACAGACACCCACACCGTCACCCACACTATCATGTTGACATTCC; dG = -35.3
1756 >s_b_s_e_s_c_s_c_s_d_s_a_s_a_s_e_s
GTGGCTGTGCTGGTGTGGGTGTGGGTGACGGTGTCTGTGTCTGTGCTGGTG; >clamp_s_c_s_d_s_c_s_a_s_e_s:compl:rev
CACCAGCACAGACACCCACACCGTCACCCACACTATCATGTTGACATTCC; dG = -35.3
1757 >s_b_s_e_s_c_s_c_s_b_s_c_s_a_s_e_s
GTGGCTGTGCTGGTGTGGGTGTGGGTGGCTGTGTGGGTGTCTGTGCTGGTG; >clamp_s_c_s_d_s_c_s_a_s_e_s:compl:rev
CACCAGCACAGACACCCACACCGTCACCCACACTATCATGTTGACATTCC; dG = -35.3
1758 >s_b_s_e_s_d_s_c_s_d_s_a_s_a_s_e_s
GTGGCTGTGCTGGTGACGGTGTGGGTGACGGTGTCTGTGTCTGTGCTGGTG; >clamp_s_c_s_d_s_c_s_a_s_e_s:compl:rev
CACCAGCACAGACACCCACACCGTCACCCACACTATCATGTTGACATTCC; dG = -35.3
1759 >s_b_s_e_s_d_s_c_s_b_s_c_s_a_s_e_s
GTGGCTGTGCTGGTGACGGTGTGGGTGGCTGTGTGGGTGTCTGTGCTGGTG; >clamp_s_c_s_d_s_c_s_a_s_e_s:compl:rev
CACCAGCACAGACACCCACACCGTCACCCACACTATCATGTTGACATTCC; dG = -35.3

1760 >s_b_s_e_s_d_s_a_s_d_s_c_s_a_s_e_s
GTGGCTGTGCTGGTGACGGTGTCTGTGACGGTGTGGGTGTCTGTGCTGGTG; >clamp_s_c_s_d_s_c_s_a_s_e_s:compl:rev
CACCAGCACAGACACCCACACCGTCACCCACACTATCATGTTGACATTCC; dG = -35.3
1761 >s_b_s_e_s_e_s_c_s_d_s_a_s_a_s_e_s
GTGGCTGTGCTGGTGCTGGTGTGGGTGACGGTGTCTGTGTCTGTGCTGGTG; >clamp_s_c_s_d_s_c_s_a_s_e_s:compl:rev
CACCAGCACAGACACCCACACCGTCACCCACACTATCATGTTGACATTCC; dG = -35.3
1762 >s_b_s_e_s_e_s_c_s_b_s_c_s_a_s_e_s
GTGGCTGTGCTGGTGCTGGTGTGGGTGGCTGTGTGGGTGTCTGTGCTGGTG; >clamp_s_c_s_d_s_c_s_a_s_e_s:compl:rev
CACCAGCACAGACACCCACACCGTCACCCACACTATCATGTTGACATTCC; dG = -35.3
1763 >s_c_s_d_s_a_s_c_s_a_s_c_s_a_s_e_s
GTGTGGGTGACGGTGTCTGTGTGGGTGTCTGTGTGGGTGTCTGTGCTGGTG; >clamp_s_c_s_d_s_c_s_a_s_e_s:compl:rev
CACCAGCACAGACACCCACACCGTCACCCACACTATCATGTTGACATTCC; dG = -35.1
1764 >s_c_s_d_s_b_s_c_s_a_s_c_s_a_s_e_s
GTGTGGGTGACGGTGGCTGTGTGGGTGTCTGTGTGGGTGTCTGTGCTGGTG; >clamp_s_c_s_d_s_c_s_a_s_e_s:compl:rev
CACCAGCACAGACACCCACACCGTCACCCACACTATCATGTTGACATTCC; dG = -35.1
1765 >s_c_s_d_s_d_s_c_s_a_s_c_s_a_s_e_s
GTGTGGGTGACGGTGACGGTGTGGGTGTCTGTGTGGGTGTCTGTGCTGGTG; >clamp_s_c_s_d_s_c_s_a_s_e_s:compl:rev
CACCAGCACAGACACCCACACCGTCACCCACACTATCATGTTGACATTCC; dG = -35.1
1766 >s_c_s_d_s_e_s_c_s_a_s_c_s_a_s_e_s
GTGTGGGTGACGGTGCTGGTGTGGGTGTCTGTGTGGGTGTCTGTGCTGGTG; >clamp_s_c_s_d_s_c_s_a_s_e_s:compl:rev
CACCAGCACAGACACCCACACCGTCACCCACACTATCATGTTGACATTCC; dG = -35.1
1767 >s_c_s_a_s_a_s_c_s_d_s_c_s_a_s_b_s
GTGTGGGTGTCTGTGTCTGTGTGGGTGACGGTGTGGGTGTCTGTGGCTGTG; >clamp_s_c_s_d_s_c_s_a_s_e_s:compl:rev
CACCAGCACAGACACCCACACCGTCACCCACACTATCATGTTGACATTCC; dG = -35.0
1768 >s_c_s_a_s_b_s_c_s_d_s_c_s_a_s_b_s
GTGTGGGTGTCTGTGGCTGTGTGGGTGACGGTGTGGGTGTCTGTGGCTGTG; >clamp_s_c_s_d_s_c_s_a_s_e_s:compl:rev
CACCAGCACAGACACCCACACCGTCACCCACACTATCATGTTGACATTCC; dG = -35.0
1769 >s_a_s_b_s_a_s_c_s_d_s_c_s_a_s_b_s
GTGTCTGTGGCTGTGTCTGTGTGGGTGACGGTGTGGGTGTCTGTGGCTGTG; >clamp_s_c_s_d_s_c_s_a_s_e_s:compl:rev
CACCAGCACAGACACCCACACCGTCACCCACACTATCATGTTGACATTCC; dG = -35.0
1770 >s_c_s_b_s_a_s_c_s_d_s_c_s_a_s_b_s
GTGTGGGTGGCTGTGTCTGTGTGGGTGACGGTGTGGGTGTCTGTGGCTGTG; >clamp_s_c_s_d_s_c_s_a_s_e_s:compl:rev
CACCAGCACAGACACCCACACCGTCACCCACACTATCATGTTGACATTCC; dG = -35.0
1771 >s_c_s_b_s_b_s_c_s_d_s_c_s_a_s_b_s
GTGTGGGTGGCTGTGGCTGTGTGGGTGACGGTGTGGGTGTCTGTGGCTGTG; >clamp_s_c_s_d_s_c_s_a_s_e_s:compl:rev
CACCAGCACAGACACCCACACCGTCACCCACACTATCATGTTGACATTCC; dG = -35.0
1772 >s_c_s_c_s_a_s_c_s_d_s_c_s_a_s_b_s
GTGTGGGTGTGGGTGTCTGTGTGGGTGACGGTGTGGGTGTCTGTGGCTGTG; >clamp_s_c_s_d_s_c_s_a_s_e_s:compl:rev
CACCAGCACAGACACCCACACCGTCACCCACACTATCATGTTGACATTCC; dG = -35.0
1773 >s_c_s_c_s_b_s_c_s_d_s_c_s_a_s_b_s
GTGTGGGTGTGGGTGGCTGTGTGGGTGACGGTGTGGGTGTCTGTGGCTGTG; >clamp_s_c_s_d_s_c_s_a_s_e_s:compl:rev
CACCAGCACAGACACCCACACCGTCACCCACACTATCATGTTGACATTCC; dG = -35.0
1774 >s_a_s_b_s_b_s_c_s_d_s_c_s_a_s_b_s
GTGTCTGTGGCTGTGGCTGTGTGGGTGACGGTGTGGGTGTCTGTGGCTGTG; >clamp_s_c_s_d_s_c_s_a_s_e_s:compl:rev
CACCAGCACAGACACCCACACCGTCACCCACACTATCATGTTGACATTCC; dG = -35.0
1775 >s_c_s_d_s_a_s_c_s_d_s_c_s_a_s_b_s
GTGTGGGTGACGGTGTCTGTGTGGGTGACGGTGTGGGTGTCTGTGGCTGTG; >clamp_s_c_s_d_s_c_s_a_s_e_s:compl:rev
CACCAGCACAGACACCCACACCGTCACCCACACTATCATGTTGACATTCC; dG = -35.0
1776 >s_c_s_d_s_b_s_c_s_d_s_c_s_a_s_b_s
GTGTGGGTGACGGTGGCTGTGTGGGTGACGGTGTGGGTGTCTGTGGCTGTG; >clamp_s_c_s_d_s_c_s_a_s_e_s:compl:rev
CACCAGCACAGACACCCACACCGTCACCCACACTATCATGTTGACATTCC; dG = -35.0
1777 >s_c_s_e_s_a_s_c_s_d_s_c_s_a_s_b_s
GTGTGGGTGCTGGTGTCTGTGTGGGTGACGGTGTGGGTGTCTGTGGCTGTG; >clamp_s_c_s_d_s_c_s_a_s_e_s:compl:rev
CACCAGCACAGACACCCACACCGTCACCCACACTATCATGTTGACATTCC; dG = -35.0
1778 >s_c_s_e_s_b_s_c_s_d_s_c_s_a_s_b_s
GTGTGGGTGCTGGTGGCTGTGTGGGTGACGGTGTGGGTGTCTGTGGCTGTG; >clamp_s_c_s_d_s_c_s_a_s_e_s:compl:rev
CACCAGCACAGACACCCACACCGTCACCCACACTATCATGTTGACATTCC; dG = -35.0
1779 >s_d_s_a_s_a_s_c_s_d_s_c_s_a_s_b_s
GTGACGGTGTCTGTGTCTGTGTGGGTGACGGTGTGGGTGTCTGTGGCTGTG; >clamp_s_c_s_d_s_c_s_a_s_e_s:compl:rev
CACCAGCACAGACACCCACACCGTCACCCACACTATCATGTTGACATTCC; dG = -35.0
1780 >s_d_s_a_s_b_s_c_s_d_s_c_s_a_s_b_s
GTGACGGTGTCTGTGGCTGTGTGGGTGACGGTGTGGGTGTCTGTGGCTGTG; >clamp_s_c_s_d_s_c_s_a_s_e_s:compl:rev
CACCAGCACAGACACCCACACCGTCACCCACACTATCATGTTGACATTCC; dG = -35.0
1781 >s_d_s_b_s_a_s_c_s_d_s_c_s_a_s_b_s
GTGACGGTGGCTGTGTCTGTGTGGGTGACGGTGTGGGTGTCTGTGGCTGTG; >clamp_s_c_s_d_s_c_s_a_s_e_s:compl:rev
CACCAGCACAGACACCCACACCGTCACCCACACTATCATGTTGACATTCC; dG = -35.0
1782 >s_d_s_b_s_b_s_c_s_d_s_c_s_a_s_b_s
GTGACGGTGGCTGTGGCTGTGTGGGTGACGGTGTGGGTGTCTGTGGCTGTG; >clamp_s_c_s_d_s_c_s_a_s_e_s:compl:rev
CACCAGCACAGACACCCACACCGTCACCCACACTATCATGTTGACATTCC; dG = -35.0
1783 >s_d_s_c_s_a_s_c_s_d_s_c_s_a_s_b_s
GTGACGGTGTGGGTGTCTGTGTGGGTGACGGTGTGGGTGTCTGTGGCTGTG; >clamp_s_c_s_d_s_c_s_a_s_e_s:compl:rev
CACCAGCACAGACACCCACACCGTCACCCACACTATCATGTTGACATTCC; dG = -35.0

1784 >s_d_s_c_s_b_s_c_s_d_s_c_s_a_s_b_s
GTGACGGTGTGGGTGGCTGTGTGGGTGACGGTGTGGGTGTCTGTGGCTGTG;   >clamp_s_c_s_d_s_c_s_a_s_e_s:compl:rev
CACCAGCACAGACACCCACACCGTCACCCACACTATCATGTTGACATTCC; dG = -35.0
1785 >s_d_s_d_s_a_s_c_s_d_s_c_s_a_s_b_s
GTGACGGTGACGGTGTCTGTGTGGGTGACGGTGTGGGTGTCTGTGGCTGTG;   >clamp_s_c_s_d_s_c_s_a_s_e_s:compl:rev
CACCAGCACAGACACCCACACCGTCACCCACACTATCATGTTGACATTCC; dG = -35.0
1786 >s_d_s_d_s_b_s_c_s_d_s_c_s_a_s_b_s
GTGACGGTGACGGTGGCTGTGTGGGTGACGGTGTGGGTGTCTGTGGCTGTG;   >clamp_s_c_s_d_s_c_s_a_s_e_s:compl:rev
CACCAGCACAGACACCCACACCGTCACCCACACTATCATGTTGACATTCC; dG = -35.0
1787 >s_d_s_e_s_a_s_c_s_d_s_c_s_a_s_b_s
GTGACGGTGCTGGTGTCTGTGTGGGTGACGGTGTGGGTGTCTGTGGCTGTG;   >clamp_s_c_s_d_s_c_s_a_s_e_s:compl:rev
CACCAGCACAGACACCCACACCGTCACCCACACTATCATGTTGACATTCC; dG = -35.0
1788 >s_d_s_e_s_b_s_c_s_d_s_c_s_a_s_b_s
GTGACGGTGCTGGTGGCTGTGTGGGTGACGGTGTGGGTGTCTGTGGCTGTG;   >clamp_s_c_s_d_s_c_s_a_s_e_s:compl:rev
CACCAGCACAGACACCCACACCGTCACCCACACTATCATGTTGACATTCC; dG = -35.0
1789 >s_e_s_a_s_a_s_c_s_d_s_c_s_a_s_b_s
GTGCTGGTGTCTGTGTCTGTGTGGGTGACGGTGTGGGTGTCTGTGGCTGTG;   >clamp_s_c_s_d_s_c_s_a_s_e_s:compl:rev
CACCAGCACAGACACCCACACCGTCACCCACACTATCATGTTGACATTCC; dG = -35.0
1790 >s_e_s_a_s_b_s_c_s_d_s_c_s_a_s_b_s
GTGCTGGTGTCTGTGGCTGTGTGGGTGACGGTGTGGGTGTCTGTGGCTGTG;   >clamp_s_c_s_d_s_c_s_a_s_e_s:compl:rev
CACCAGCACAGACACCCACACCGTCACCCACACTATCATGTTGACATTCC; dG = -35.0
1791 >s_a_s_c_s_a_s_c_s_d_s_c_s_a_s_b_s
GTGTCTGTGTGGGTGTCTGTGTGGGTGACGGTGTGGGTGTCTGTGGCTGTG;   >clamp_s_c_s_d_s_c_s_a_s_e_s:compl:rev
CACCAGCACAGACACCCACACCGTCACCCACACTATCATGTTGACATTCC; dG = -35.0
1792 >s_e_s_b_s_a_s_c_s_d_s_c_s_a_s_b_s
GTGCTGGTGGCTGTGTCTGTGTGGGTGACGGTGTGGGTGTCTGTGGCTGTG;   >clamp_s_c_s_d_s_c_s_a_s_e_s:compl:rev
CACCAGCACAGACACCCACACCGTCACCCACACTATCATGTTGACATTCC; dG = -35.0
1793 >s_e_s_b_s_b_s_c_s_d_s_c_s_a_s_b_s
GTGCTGGTGGCTGTGGCTGTGTGGGTGACGGTGTGGGTGTCTGTGGCTGTG;   >clamp_s_c_s_d_s_c_s_a_s_e_s:compl:rev
CACCAGCACAGACACCCACACCGTCACCCACACTATCATGTTGACATTCC; dG = -35.0
1794 >s_e_s_c_s_a_s_c_s_d_s_c_s_a_s_b_s
GTGCTGGTGTGGGTGTCTGTGTGGGTGACGGTGTGGGTGTCTGTGGCTGTG;   >clamp_s_c_s_d_s_c_s_a_s_e_s:compl:rev
CACCAGCACAGACACCCACACCGTCACCCACACTATCATGTTGACATTCC; dG = -35.0
1795 >s_e_s_c_s_b_s_c_s_d_s_c_s_a_s_b_s
GTGCTGGTGTGGGTGGCTGTGTGGGTGACGGTGTGGGTGTCTGTGGCTGTG;   >clamp_s_c_s_d_s_c_s_a_s_e_s:compl:rev
CACCAGCACAGACACCCACACCGTCACCCACACTATCATGTTGACATTCC; dG = -35.0
1796 >s_a_s_c_s_b_s_c_s_d_s_c_s_a_s_b_s
GTGTCTGTGTGGGTGGCTGTGTGGGTGACGGTGTGGGTGTCTGTGGCTGTG;   >clamp_s_c_s_d_s_c_s_a_s_e_s:compl:rev
CACCAGCACAGACACCCACACCGTCACCCACACTATCATGTTGACATTCC; dG = -35.0
1797 >s_a_s_a_s_b_s_c_s_d_s_c_s_a_s_b_s
GTGTCTGTGTCTGTGGCTGTGTGGGTGACGGTGTGGGTGTCTGTGGCTGTG;   >clamp_s_c_s_d_s_c_s_a_s_e_s:compl:rev
CACCAGCACAGACACCCACACCGTCACCCACACTATCATGTTGACATTCC; dG = -35.0
1798 >s_e_s_d_s_a_s_c_s_d_s_c_s_a_s_b_s
GTGCTGGTGACGGTGTCTGTGTGGGTGACGGTGTGGGTGTCTGTGGCTGTG;   >clamp_s_c_s_d_s_c_s_a_s_e_s:compl:rev
CACCAGCACAGACACCCACACCGTCACCCACACTATCATGTTGACATTCC; dG = -35.0
1799 >s_e_s_d_s_b_s_c_s_d_s_c_s_a_s_b_s
GTGCTGGTGACGGTGGCTGTGTGGGTGACGGTGTGGGTGTCTGTGGCTGTG;   >clamp_s_c_s_d_s_c_s_a_s_e_s:compl:rev
CACCAGCACAGACACCCACACCGTCACCCACACTATCATGTTGACATTCC; dG = -35.0
1800 >s_e_s_e_s_a_s_c_s_d_s_c_s_a_s_b_s
GTGCTGGTGCTGGTGTCTGTGTGGGTGACGGTGTGGGTGTCTGTGGCTGTG;   >clamp_s_c_s_d_s_c_s_a_s_e_s:compl:rev
CACCAGCACAGACACCCACACCGTCACCCACACTATCATGTTGACATTCC; dG = -35.0
1801 >s_e_s_e_s_b_s_c_s_d_s_c_s_a_s_b_s
GTGCTGGTGCTGGTGGCTGTGTGGGTGACGGTGTGGGTGTCTGTGGCTGTG;   >clamp_s_c_s_d_s_c_s_a_s_e_s:compl:rev
CACCAGCACAGACACCCACACCGTCACCCACACTATCATGTTGACATTCC; dG = -35.0
1802 >s_a_s_a_s_a_s_c_s_d_s_c_s_a_s_b_s
GTGTCTGTGTCTGTGTCTGTGTGGGTGACGGTGTGGGTGTCTGTGGCTGTG;   >clamp_s_c_s_d_s_c_s_a_s_e_s:compl:rev
CACCAGCACAGACACCCACACCGTCACCCACACTATCATGTTGACATTCC; dG = -35.0
1803 >s_a_s_d_s_a_s_c_s_d_s_c_s_a_s_b_s
GTGTCTGTGACGGTGTCTGTGTGGGTGACGGTGTGGGTGTCTGTGGCTGTG;   >clamp_s_c_s_d_s_c_s_a_s_e_s:compl:rev
CACCAGCACAGACACCCACACCGTCACCCACACTATCATGTTGACATTCC; dG = -35.0
1804 >s_a_s_d_s_b_s_c_s_d_s_c_s_a_s_b_s
GTGTCTGTGACGGTGGCTGTGTGGGTGACGGTGTGGGTGTCTGTGGCTGTG;   >clamp_s_c_s_d_s_c_s_a_s_e_s:compl:rev
CACCAGCACAGACACCCACACCGTCACCCACACTATCATGTTGACATTCC; dG = -35.0
1805 >s_a_s_e_s_a_s_c_s_d_s_c_s_a_s_b_s
GTGTCTGTGCTGGTGTCTGTGTGGGTGACGGTGTGGGTGTCTGTGGCTGTG;   >clamp_s_c_s_d_s_c_s_a_s_e_s:compl:rev
CACCAGCACAGACACCCACACCGTCACCCACACTATCATGTTGACATTCC; dG = -35.0
1806 >s_a_s_e_s_b_s_c_s_d_s_c_s_a_s_b_s
GTGTCTGTGCTGGTGGCTGTGTGGGTGACGGTGTGGGTGTCTGTGGCTGTG;   >clamp_s_c_s_d_s_c_s_a_s_e_s:compl:rev
CACCAGCACAGACACCCACACCGTCACCCACACTATCATGTTGACATTCC; dG = -35.0
1807 >s_b_s_a_s_a_s_c_s_d_s_c_s_a_s_b_s
GTGGCTGTGTCTGTGTCTGTGTGGGTGACGGTGTGGGTGTCTGTGGCTGTG;   >clamp_s_c_s_d_s_c_s_a_s_e_s:compl:rev
CACCAGCACAGACACCCACACCGTCACCCACACTATCATGTTGACATTCC; dG = -35.0

1808 >s_b_s_a_s_b_s_c_s_d_s_c_s_a_s_b_s
GTGGCTGTGTCTGTGGCTGTGTGGGTGACGGTGTGGGTGTCTGTGGCTGTG; >clamp_s_c_s_d_s_c_s_a_s_e_s:compl:rev
CACCAGCACAGACACCCACACCGTCACCCACACTATCATGTTGACATTCC; dG = -35.0
1809 >s_b_s_b_s_a_s_c_s_d_s_c_s_a_s_b_s
GTGGCTGTGGCTGTGTCTGTGTGGGTGACGGTGTGGGTGTCTGTGGCTGTG; >clamp_s_c_s_d_s_c_s_a_s_e_s:compl:rev
CACCAGCACAGACACCCACACCGTCACCCACACTATCATGTTGACATTCC; dG = -35.0
1810 >s_b_s_b_s_b_s_c_s_d_s_c_s_a_s_b_s
GTGGCTGTGGCTGTGGCTGTGTGGGTGACGGTGTGGGTGTCTGTGGCTGTG; >clamp_s_c_s_d_s_c_s_a_s_e_s:compl:rev
CACCAGCACAGACACCCACACCGTCACCCACACTATCATGTTGACATTCC; dG = -35.0
1811 >s_b_s_c_s_a_s_c_s_d_s_c_s_a_s_b_s
GTGGCTGTGTGGGTGTCTGTGTGGGTGACGGTGTGGGTGTCTGTGGCTGTG; >clamp_s_c_s_d_s_c_s_a_s_e_s:compl:rev
CACCAGCACAGACACCCACACCGTCACCCACACTATCATGTTGACATTCC; dG = -35.0
1812 >s_b_s_c_s_b_s_c_s_d_s_c_s_a_s_b_s
GTGGCTGTGTGGGTGGCTGTGTGGGTGACGGTGTGGGTGTCTGTGGCTGTG; >clamp_s_c_s_d_s_c_s_a_s_e_s:compl:rev
CACCAGCACAGACACCCACACCGTCACCCACACTATCATGTTGACATTCC; dG = -35.0
1813 >s_b_s_d_s_a_s_c_s_d_s_c_s_a_s_b_s
GTGGCTGTGACGGTGTCTGTGTGGGTGACGGTGTGGGTGTCTGTGGCTGTG; >clamp_s_c_s_d_s_c_s_a_s_e_s:compl:rev
CACCAGCACAGACACCCACACCGTCACCCACACTATCATGTTGACATTCC; dG = -35.0
1814 >s_b_s_d_s_b_s_c_s_d_s_c_s_a_s_b_s
GTGGCTGTGACGGTGGCTGTGTGGGTGACGGTGTGGGTGTCTGTGGCTGTG; >clamp_s_c_s_d_s_c_s_a_s_e_s:compl:rev
CACCAGCACAGACACCCACACCGTCACCCACACTATCATGTTGACATTCC; dG = -35.0
1815 >s_b_s_e_s_a_s_c_s_d_s_c_s_a_s_b_s
GTGGCTGTGCTGGTGTCTGTGTGGGTGACGGTGTGGGTGTCTGTGGCTGTG; >clamp_s_c_s_d_s_c_s_a_s_e_s:compl:rev
CACCAGCACAGACACCCACACCGTCACCCACACTATCATGTTGACATTCC; dG = -35.0
1816 >s_b_s_e_s_b_s_c_s_d_s_c_s_a_s_b_s
GTGGCTGTGCTGGTGGCTGTGTGGGTGACGGTGTGGGTGTCTGTGGCTGTG; >clamp_s_c_s_d_s_c_s_a_s_e_s:compl:rev
CACCAGCACAGACACCCACACCGTCACCCACACTATCATGTTGACATTCC; dG = -35.0
1817 >s_c_s_a_s_a_s_c_s_d_s_c_s_a_s_a_s
GTGTGGGTGTCTGTGTCTGTGTGGGTGACGGTGTGGGTGTCTGTGTCTGTG; >clamp_s_c_s_d_s_c_s_a_s_e_s:compl:rev
CACCAGCACAGACACCCACACCGTCACCCACACTATCATGTTGACATTCC; dG = -34.9
1818 >s_c_s_a_s_b_s_c_s_d_s_c_s_a_s_a_s
GTGTGGGTGTCTGTGGCTGTGTGGGTGACGGTGTGGGTGTCTGTGTCTGTG; >clamp_s_c_s_d_s_c_s_a_s_e_s:compl:rev
CACCAGCACAGACACCCACACCGTCACCCACACTATCATGTTGACATTCC; dG = -34.9
1819 >s_a_s_b_s_a_s_c_s_d_s_c_s_a_s_a_s
GTGTCTGTGGCTGTGTCTGTGTGGGTGACGGTGTGGGTGTCTGTGTCTGTG; >clamp_s_c_s_d_s_c_s_a_s_e_s:compl:rev
CACCAGCACAGACACCCACACCGTCACCCACACTATCATGTTGACATTCC; dG = -34.9
1820 >s_c_s_a_s_c_s_c_s_d_s_c_s_a_s_b_s
GTGTGGGTGTCTGTGTGGGTGTGGGTGACGGTGTGGGTGTCTGTGGCTGTG; >clamp_s_c_s_d_s_c_s_a_s_e_s:compl:rev
CACCAGCACAGACACCCACACCGTCACCCACACTATCATGTTGACATTCC; dG = -34.9
1821 >s_c_s_a_s_c_s_c_s_d_s_c_s_a_s_a_s
GTGTGGGTGTCTGTGTGGGTGTGGGTGACGGTGTGGGTGTCTGTGTCTGTG; >clamp_s_c_s_d_s_c_s_a_s_e_s:compl:rev
CACCAGCACAGACACCCACACCGTCACCCACACTATCATGTTGACATTCC; dG = -34.9
1822 >s_c_s_a_s_d_s_c_s_d_s_c_s_a_s_b_s
GTGTGGGTGTCTGTGACGGTGTGGGTGACGGTGTGGGTGTCTGTGGCTGTG; >clamp_s_c_s_d_s_c_s_a_s_e_s:compl:rev
CACCAGCACAGACACCCACACCGTCACCCACACTATCATGTTGACATTCC; dG = -34.9
1823 >s_c_s_a_s_d_s_c_s_d_s_c_s_a_s_a_s
GTGTGGGTGTCTGTGACGGTGTGGGTGACGGTGTGGGTGTCTGTGTCTGTG; >clamp_s_c_s_d_s_c_s_a_s_e_s:compl:rev
CACCAGCACAGACACCCACACCGTCACCCACACTATCATGTTGACATTCC; dG = -34.9
1824 >s_c_s_a_s_e_s_c_s_d_s_c_s_a_s_b_s
GTGTGGGTGTCTGTGCTGGTGTGGGTGACGGTGTGGGTGTCTGTGGCTGTG; >clamp_s_c_s_d_s_c_s_a_s_e_s:compl:rev
CACCAGCACAGACACCCACACCGTCACCCACACTATCATGTTGACATTCC; dG = -34.9
1825 >s_c_s_a_s_e_s_c_s_d_s_c_s_a_s_a_s
GTGTGGGTGTCTGTGCTGGTGTGGGTGACGGTGTGGGTGTCTGTGTCTGTG; >clamp_s_c_s_d_s_c_s_a_s_e_s:compl:rev
CACCAGCACAGACACCCACACCGTCACCCACACTATCATGTTGACATTCC; dG = -34.9
1826 >s_c_s_b_s_a_s_c_s_d_s_c_s_a_s_a_s
GTGTGGGTGGCTGTGTCTGTGTGGGTGACGGTGTGGGTGTCTGTGTCTGTG; >clamp_s_c_s_d_s_c_s_a_s_e_s:compl:rev
CACCAGCACAGACACCCACACCGTCACCCACACTATCATGTTGACATTCC; dG = -34.9
1827 >s_c_s_b_s_b_s_c_s_d_s_c_s_a_s_a_s
GTGTGGGTGGCTGTGGCTGTGTGGGTGACGGTGTGGGTGTCTGTGTCTGTG; >clamp_s_c_s_d_s_c_s_a_s_e_s:compl:rev
CACCAGCACAGACACCCACACCGTCACCCACACTATCATGTTGACATTCC; dG = -34.9
1828 >s_c_s_b_s_c_s_c_s_d_s_c_s_a_s_b_s
GTGTGGGTGGCTGTGTGGGTGTGGGTGACGGTGTGGGTGTCTGTGGCTGTG; >clamp_s_c_s_d_s_c_s_a_s_e_s:compl:rev
CACCAGCACAGACACCCACACCGTCACCCACACTATCATGTTGACATTCC; dG = -34.9
1829 >s_c_s_b_s_c_s_c_s_d_s_c_s_a_s_a_s
GTGTGGGTGGCTGTGTGGGTGTGGGTGACGGTGTGGGTGTCTGTGTCTGTG; >clamp_s_c_s_d_s_c_s_a_s_e_s:compl:rev
CACCAGCACAGACACCCACACCGTCACCCACACTATCATGTTGACATTCC; dG = -34.9
1830 >s_c_s_b_s_d_s_c_s_d_s_c_s_a_s_b_s
GTGTGGGTGGCTGTGACGGTGTGGGTGACGGTGTGGGTGTCTGTGGCTGTG; >clamp_s_c_s_d_s_c_s_a_s_e_s:compl:rev
CACCAGCACAGACACCCACACCGTCACCCACACTATCATGTTGACATTCC; dG = -34.9
1831 >s_c_s_b_s_d_s_c_s_d_s_c_s_a_s_a_s
GTGTGGGTGGCTGTGACGGTGTGGGTGACGGTGTGGGTGTCTGTGTCTGTG; >clamp_s_c_s_d_s_c_s_a_s_e_s:compl:rev
CACCAGCACAGACACCCACACCGTCACCCACACTATCATGTTGACATTCC; dG = -34.9

1832 >s_c_s_b_s_e_s_c_s_d_s_c_s_a_s_b_s
GTGTGGGTGGCTGTGCTGGTGTGGGTGACGGTGTGGGTGTCTGTGGCTGTG; >clamp_s_c_s_d_s_c_s_a_s_e_s:compl:rev
CACCAGCACAGACACCCACACCGTCACCCACACTATCATGTTGACATTCC; dG = -34.9
1833 >s_c_s_b_s_e_s_c_s_d_s_c_s_a_s_a_s
GTGTGGGTGGCTGTGCTGGTGTGGGTGACGGTGTGGGTGTCTGTGTCTGTG; >clamp_s_c_s_d_s_c_s_a_s_e_s:compl:rev
CACCAGCACAGACACCCACACCGTCACCCACACTATCATGTTGACATTCC; dG = -34.9
1834 >s_c_s_c_s_a_s_c_s_d_s_c_s_a_s_a_s
GTGTGGGTGTGGGTGTCTGTGTGGGTGACGGTGTGGGTGTCTGTGTCTGTG; >clamp_s_c_s_d_s_c_s_a_s_e_s:compl:rev
CACCAGCACAGACACCCACACCGTCACCCACACTATCATGTTGACATTCC; dG = -34.9
1835 >s_c_s_c_s_b_s_c_s_d_s_c_s_a_s_a_s
GTGTGGGTGTGGGTGGCTGTGTGGGTGACGGTGTGGGTGTCTGTGTCTGTG; >clamp_s_c_s_d_s_c_s_a_s_e_s:compl:rev
CACCAGCACAGACACCCACACCGTCACCCACACTATCATGTTGACATTCC; dG = -34.9
1836 >s_a_s_b_s_b_s_c_s_d_s_c_s_a_s_a_s
GTGTCTGTGGCTGTGGCTGTGTGGGTGACGGTGTGGGTGTCTGTGTCTGTG; >clamp_s_c_s_d_s_c_s_a_s_e_s:compl:rev
CACCAGCACAGACACCCACACCGTCACCCACACTATCATGTTGACATTCC; dG = -34.9
1837 >s_c_s_c_s_c_s_c_s_d_s_c_s_a_s_b_s
GTGTGGGTGTGGGTGTGGGTGTGGGTGACGGTGTGGGTGTCTGTGGCTGTG; >clamp_s_c_s_d_s_c_s_a_s_e_s:compl:rev
CACCAGCACAGACACCCACACCGTCACCCACACTATCATGTTGACATTCC; dG = -34.9
1838 >s_c_s_c_s_c_s_c_s_d_s_c_s_a_s_a_s
GTGTGGGTGTGGGTGTGGGTGTGGGTGACGGTGTGGGTGTCTGTGTCTGTG; >clamp_s_c_s_d_s_c_s_a_s_e_s:compl:rev
CACCAGCACAGACACCCACACCGTCACCCACACTATCATGTTGACATTCC; dG = -34.9
1839 >s_c_s_c_s_d_s_c_s_d_s_c_s_a_s_b_s
GTGTGGGTGTGGGTGACGGTGTGGGTGACGGTGTGGGTGTCTGTGGCTGTG; >clamp_s_c_s_d_s_c_s_a_s_e_s:compl:rev
CACCAGCACAGACACCCACACCGTCACCCACACTATCATGTTGACATTCC; dG = -34.9
1840 >s_c_s_c_s_d_s_c_s_d_s_c_s_a_s_a_s
GTGTGGGTGTGGGTGACGGTGTGGGTGACGGTGTGGGTGTCTGTGTCTGTG; >clamp_s_c_s_d_s_c_s_a_s_e_s:compl:rev
CACCAGCACAGACACCCACACCGTCACCCACACTATCATGTTGACATTCC; dG = -34.9
1841 >s_c_s_c_s_e_s_c_s_d_s_c_s_a_s_b_s
GTGTGGGTGTGGGTGCTGGTGTGGGTGACGGTGTGGGTGTCTGTGGCTGTG; >clamp_s_c_s_d_s_c_s_a_s_e_s:compl:rev
CACCAGCACAGACACCCACACCGTCACCCACACTATCATGTTGACATTCC; dG = -34.9
1842 >s_c_s_c_s_e_s_c_s_d_s_c_s_a_s_a_s
GTGTGGGTGTGGGTGCTGGTGTGGGTGACGGTGTGGGTGTCTGTGTCTGTG; >clamp_s_c_s_d_s_c_s_a_s_e_s:compl:rev
CACCAGCACAGACACCCACACCGTCACCCACACTATCATGTTGACATTCC; dG = -34.9
1843 >s_c_s_d_s_a_s_c_s_d_s_c_s_a_s_a_s
GTGTGGGTGACGGTGTCTGTGTGGGTGACGGTGTGGGTGTCTGTGTCTGTG; >clamp_s_c_s_d_s_c_s_a_s_e_s:compl:rev
CACCAGCACAGACACCCACACCGTCACCCACACTATCATGTTGACATTCC; dG = -34.9
1844 >s_c_s_d_s_b_s_c_s_d_s_c_s_a_s_a_s
GTGTGGGTGACGGTGGCTGTGTGGGTGACGGTGTGGGTGTCTGTGTCTGTG; >clamp_s_c_s_d_s_c_s_a_s_e_s:compl:rev
CACCAGCACAGACACCCACACCGTCACCCACACTATCATGTTGACATTCC; dG = -34.9
1845 >s_c_s_d_s_c_s_c_s_d_s_c_s_a_s_b_s
GTGTGGGTGACGGTGTGGGTGTGGGTGACGGTGTGGGTGTCTGTGGCTGTG; >clamp_s_c_s_d_s_c_s_a_s_e_s:compl:rev
CACCAGCACAGACACCCACACCGTCACCCACACTATCATGTTGACATTCC; dG = -34.9
1846 >s_c_s_d_s_c_s_c_s_d_s_c_s_a_s_a_s
GTGTGGGTGACGGTGTGGGTGTGGGTGACGGTGTGGGTGTCTGTGTCTGTG; >clamp_s_c_s_d_s_c_s_a_s_e_s:compl:rev
CACCAGCACAGACACCCACACCGTCACCCACACTATCATGTTGACATTCC; dG = -34.9
1847 >s_c_s_d_s_d_s_c_s_d_s_c_s_a_s_b_s
GTGTGGGTGACGGTGACGGTGTGGGTGACGGTGTGGGTGTCTGTGGCTGTG; >clamp_s_c_s_d_s_c_s_a_s_e_s:compl:rev
CACCAGCACAGACACCCACACCGTCACCCACACTATCATGTTGACATTCC; dG = -34.9
1848 >s_c_s_d_s_d_s_c_s_d_s_c_s_a_s_a_s
GTGTGGGTGACGGTGACGGTGTGGGTGACGGTGTGGGTGTCTGTGTCTGTG; >clamp_s_c_s_d_s_c_s_a_s_e_s:compl:rev
CACCAGCACAGACACCCACACCGTCACCCACACTATCATGTTGACATTCC; dG = -34.9
1849 >s_c_s_d_s_e_s_c_s_d_s_c_s_a_s_b_s
GTGTGGGTGACGGTGCTGGTGTGGGTGACGGTGTGGGTGTCTGTGGCTGTG; >clamp_s_c_s_d_s_c_s_a_s_e_s:compl:rev
CACCAGCACAGACACCCACACCGTCACCCACACTATCATGTTGACATTCC; dG = -34.9
1850 >s_c_s_d_s_e_s_c_s_d_s_c_s_a_s_a_s
GTGTGGGTGACGGTGCTGGTGTGGGTGACGGTGTGGGTGTCTGTGTCTGTG; >clamp_s_c_s_d_s_c_s_a_s_e_s:compl:rev
CACCAGCACAGACACCCACACCGTCACCCACACTATCATGTTGACATTCC; dG = -34.9
1851 >s_c_s_e_s_a_s_c_s_d_s_c_s_a_s_a_s
GTGTGGGTGCTGGTGTCTGTGTGGGTGACGGTGTGGGTGTCTGTGTCTGTG; >clamp_s_c_s_d_s_c_s_a_s_e_s:compl:rev
CACCAGCACAGACACCCACACCGTCACCCACACTATCATGTTGACATTCC; dG = -34.9
1852 >s_c_s_e_s_b_s_c_s_d_s_c_s_a_s_a_s
GTGTGGGTGCTGGTGGCTGTGTGGGTGACGGTGTGGGTGTCTGTGTCTGTG; >clamp_s_c_s_d_s_c_s_a_s_e_s:compl:rev
CACCAGCACAGACACCCACACCGTCACCCACACTATCATGTTGACATTCC; dG = -34.9
1853 >s_a_s_b_s_c_s_c_s_d_s_c_s_a_s_b_s
GTGTCTGTGGCTGTGTGGGTGTGGGTGACGGTGTGGGTGTCTGTGGCTGTG; >clamp_s_c_s_d_s_c_s_a_s_e_s:compl:rev
CACCAGCACAGACACCCACACCGTCACCCACACTATCATGTTGACATTCC; dG = -34.9
1854 >s_a_s_b_s_c_s_c_s_d_s_c_s_a_s_a_s
GTGTCTGTGGCTGTGTGGGTGTGGGTGACGGTGTGGGTGTCTGTGTCTGTG; >clamp_s_c_s_d_s_c_s_a_s_e_s:compl:rev
CACCAGCACAGACACCCACACCGTCACCCACACTATCATGTTGACATTCC; dG = -34.9
1855 >s_c_s_e_s_c_s_c_s_d_s_c_s_a_s_b_s
GTGTGGGTGCTGGTGTGGGTGTGGGTGACGGTGTGGGTGTCTGTGGCTGTG; >clamp_s_c_s_d_s_c_s_a_s_e_s:compl:rev
CACCAGCACAGACACCCACACCGTCACCCACACTATCATGTTGACATTCC; dG = -34.9

1856 >s_c_s_e_s_c_s_c_s_d_s_c_s_a_s_a_s
GTGTGGGTGCTGGTGTGGGTGTGGGTGACGGTGTGGGTGTCTGTGTCTGTG; >clamp_s_c_s_d_s_c_s_a_s_e_s:compl:rev
CACCAGCACAGACACCCACACCGTCACCCACACTATCATGTTGACATTCC; dG = -34.9
1857 >s_c_s_e_s_d_s_c_s_d_s_c_s_a_s_b_s
GTGTGGGTGCTGGTGACGGTGTGGGTGACGGTGTGGGTGTCTGTGGCTGTG; >clamp_s_c_s_d_s_c_s_a_s_e_s:compl:rev
CACCAGCACAGACACCCACACCGTCACCCACACTATCATGTTGACATTCC; dG = -34.9
1858 >s_c_s_e_s_d_s_c_s_d_s_c_s_a_s_a_s
GTGTGGGTGCTGGTGACGGTGTGGGTGACGGTGTGGGTGTCTGTGTCTGTG; >clamp_s_c_s_d_s_c_s_a_s_e_s:compl:rev
CACCAGCACAGACACCCACACCGTCACCCACACTATCATGTTGACATTCC; dG = -34.9
1859 >s_c_s_e_s_e_s_c_s_d_s_c_s_a_s_b_s
GTGTGGGTGCTGGTGCTGGTGTGGGTGACGGTGTGGGTGTCTGTGGCTGTG; >clamp_s_c_s_d_s_c_s_a_s_e_s:compl:rev
CACCAGCACAGACACCCACACCGTCACCCACACTATCATGTTGACATTCC; dG = -34.9
1860 >s_c_s_e_s_e_s_c_s_d_s_c_s_a_s_a_s
GTGTGGGTGCTGGTGCTGGTGTGGGTGACGGTGTGGGTGTCTGTGTCTGTG; >clamp_s_c_s_d_s_c_s_a_s_e_s:compl:rev
CACCAGCACAGACACCCACACCGTCACCCACACTATCATGTTGACATTCC; dG = -34.9
1861 >s_d_s_a_s_a_s_c_s_d_s_c_s_a_s_a_s
GTGACGGTGTCTGTGTCTGTGTGGGTGACGGTGTGGGTGTCTGTGTCTGTG; >clamp_s_c_s_d_s_c_s_a_s_e_s:compl:rev
CACCAGCACAGACACCCACACCGTCACCCACACTATCATGTTGACATTCC; dG = -34.9
1862 >s_d_s_a_s_b_s_c_s_d_s_c_s_a_s_a_s
GTGACGGTGTCTGTGGCTGTGTGGGTGACGGTGTGGGTGTCTGTGTCTGTG; >clamp_s_c_s_d_s_c_s_a_s_e_s:compl:rev
CACCAGCACAGACACCCACACCGTCACCCACACTATCATGTTGACATTCC; dG = -34.9
1863 >s_d_s_a_s_c_s_c_s_d_s_c_s_a_s_b_s
GTGACGGTGTCTGTGTGGGTGTGGGTGACGGTGTGGGTGTCTGTGGCTGTG; >clamp_s_c_s_d_s_c_s_a_s_e_s:compl:rev
CACCAGCACAGACACCCACACCGTCACCCACACTATCATGTTGACATTCC; dG = -34.9
1864 >s_d_s_a_s_c_s_c_s_d_s_c_s_a_s_a_s
GTGACGGTGTCTGTGTGGGTGTGGGTGACGGTGTGGGTGTCTGTGTCTGTG; >clamp_s_c_s_d_s_c_s_a_s_e_s:compl:rev
CACCAGCACAGACACCCACACCGTCACCCACACTATCATGTTGACATTCC; dG = -34.9
1865 >s_d_s_a_s_d_s_c_s_d_s_c_s_a_s_b_s
GTGACGGTGTCTGTGACGGTGTGGGTGACGGTGTGGGTGTCTGTGGCTGTG; >clamp_s_c_s_d_s_c_s_a_s_e_s:compl:rev
CACCAGCACAGACACCCACACCGTCACCCACACTATCATGTTGACATTCC; dG = -34.9
1866 >s_d_s_a_s_d_s_c_s_d_s_c_s_a_s_a_s
GTGACGGTGTCTGTGACGGTGTGGGTGACGGTGTGGGTGTCTGTGTCTGTG; >clamp_s_c_s_d_s_c_s_a_s_e_s:compl:rev
CACCAGCACAGACACCCACACCGTCACCCACACTATCATGTTGACATTCC; dG = -34.9
1867 >s_d_s_a_s_e_s_c_s_d_s_c_s_a_s_b_s
GTGACGGTGTCTGTGCTGGTGTGGGTGACGGTGTGGGTGTCTGTGGCTGTG; >clamp_s_c_s_d_s_c_s_a_s_e_s:compl:rev
CACCAGCACAGACACCCACACCGTCACCCACACTATCATGTTGACATTCC; dG = -34.9
1868 >s_d_s_a_s_e_s_c_s_d_s_c_s_a_s_a_s
GTGACGGTGTCTGTGCTGGTGTGGGTGACGGTGTGGGTGTCTGTGTCTGTG; >clamp_s_c_s_d_s_c_s_a_s_e_s:compl:rev
CACCAGCACAGACACCCACACCGTCACCCACACTATCATGTTGACATTCC; dG = -34.9
1869 >s_d_s_b_s_a_s_c_s_d_s_c_s_a_s_a_s
GTGACGGTGGCTGTGTCTGTGTGGGTGACGGTGTGGGTGTCTGTGTCTGTG; >clamp_s_c_s_d_s_c_s_a_s_e_s:compl:rev
CACCAGCACAGACACCCACACCGTCACCCACACTATCATGTTGACATTCC; dG = -34.9
1870 >s_d_s_b_s_b_s_c_s_d_s_c_s_a_s_a_s
GTGACGGTGGCTGTGGCTGTGTGGGTGACGGTGTGGGTGTCTGTGTCTGTG; >clamp_s_c_s_d_s_c_s_a_s_e_s:compl:rev
CACCAGCACAGACACCCACACCGTCACCCACACTATCATGTTGACATTCC; dG = -34.9
1871 >s_a_s_b_s_d_s_c_s_d_s_c_s_a_s_b_s
GTGTCTGTGGCTGTGACGGTGTGGGTGACGGTGTGGGTGTCTGTGGCTGTG; >clamp_s_c_s_d_s_c_s_a_s_e_s:compl:rev
CACCAGCACAGACACCCACACCGTCACCCACACTATCATGTTGACATTCC; dG = -34.9
1872 >s_a_s_b_s_d_s_c_s_d_s_c_s_a_s_a_s
GTGTCTGTGGCTGTGACGGTGTGGGTGACGGTGTGGGTGTCTGTGTCTGTG; >clamp_s_c_s_d_s_c_s_a_s_e_s:compl:rev
CACCAGCACAGACACCCACACCGTCACCCACACTATCATGTTGACATTCC; dG = -34.9
1873 >s_d_s_b_s_c_s_c_s_d_s_c_s_a_s_b_s
GTGACGGTGGCTGTGTGGGTGTGGGTGACGGTGTGGGTGTCTGTGGCTGTG; >clamp_s_c_s_d_s_c_s_a_s_e_s:compl:rev
CACCAGCACAGACACCCACACCGTCACCCACACTATCATGTTGACATTCC; dG = -34.9
1874 >s_d_s_b_s_c_s_c_s_d_s_c_s_a_s_a_s
GTGACGGTGGCTGTGTGGGTGTGGGTGACGGTGTGGGTGTCTGTGTCTGTG; >clamp_s_c_s_d_s_c_s_a_s_e_s:compl:rev
CACCAGCACAGACACCCACACCGTCACCCACACTATCATGTTGACATTCC; dG = -34.9
1875 >s_d_s_b_s_d_s_c_s_d_s_c_s_a_s_b_s
GTGACGGTGGCTGTGACGGTGTGGGTGACGGTGTGGGTGTCTGTGGCTGTG; >clamp_s_c_s_d_s_c_s_a_s_e_s:compl:rev
CACCAGCACAGACACCCACACCGTCACCCACACTATCATGTTGACATTCC; dG = -34.9
1876 >s_d_s_b_s_d_s_c_s_d_s_c_s_a_s_a_s
GTGACGGTGGCTGTGACGGTGTGGGTGACGGTGTGGGTGTCTGTGTCTGTG; >clamp_s_c_s_d_s_c_s_a_s_e_s:compl:rev
CACCAGCACAGACACCCACACCGTCACCCACACTATCATGTTGACATTCC; dG = -34.9
1877 >s_d_s_b_s_e_s_c_s_d_s_c_s_a_s_b_s
GTGACGGTGGCTGTGCTGGTGTGGGTGACGGTGTGGGTGTCTGTGGCTGTG; >clamp_s_c_s_d_s_c_s_a_s_e_s:compl:rev
CACCAGCACAGACACCCACACCGTCACCCACACTATCATGTTGACATTCC; dG = -34.9
1878 >s_d_s_b_s_e_s_c_s_d_s_c_s_a_s_a_s
GTGACGGTGGCTGTGCTGGTGTGGGTGACGGTGTGGGTGTCTGTGTCTGTG; >clamp_s_c_s_d_s_c_s_a_s_e_s:compl:rev
CACCAGCACAGACACCCACACCGTCACCCACACTATCATGTTGACATTCC; dG = -34.9
1879 >s_d_s_c_s_a_s_c_s_d_s_c_s_a_s_a_s
GTGACGGTGTGGGTGTCTGTGTGGGTGACGGTGTGGGTGTCTGTGTCTGTG; >clamp_s_c_s_d_s_c_s_a_s_e_s:compl:rev
CACCAGCACAGACACCCACACCGTCACCCACACTATCATGTTGACATTCC; dG = -34.9

1880 >s_d_s_c_s_b_s_c_s_d_s_c_s_a_s_a_s
GTGACGGTGTGGGTGGCTGTGTGGGTGACGGTGTGGGTGTCTGTGTCTGTG; >clamp_s_c_s_d_s_c_s_a_s_e_s:compl:rev
CACCAGCACAGACACCCACACCGTCACCCACACTATCATGTTGACATTCC; dG = -34.9
1881 >s_d_s_c_s_c_s_c_s_d_s_c_s_a_s_b_s
GTGACGGTGTGGGTGTGGGTGTGGGTGACGGTGTGGGTGTCTGTGGCTGTG; >clamp_s_c_s_d_s_c_s_a_s_e_s:compl:rev
CACCAGCACAGACACCCACACCGTCACCCACACTATCATGTTGACATTCC; dG = -34.9
1882 >s_d_s_c_s_c_s_c_s_d_s_c_s_a_s_a_s
GTGACGGTGTGGGTGTGGGTGTGGGTGACGGTGTGGGTGTCTGTGTCTGTG; >clamp_s_c_s_d_s_c_s_a_s_e_s:compl:rev
CACCAGCACAGACACCCACACCGTCACCCACACTATCATGTTGACATTCC; dG = -34.9
1883 >s_d_s_c_s_d_s_c_s_d_s_c_s_a_s_b_s
GTGACGGTGTGGGTGACGGTGTGGGTGACGGTGTGGGTGTCTGTGGCTGTG; >clamp_s_c_s_d_s_c_s_a_s_e_s:compl:rev
CACCAGCACAGACACCCACACCGTCACCCACACTATCATGTTGACATTCC; dG = -34.9
1884 >s_d_s_c_s_d_s_c_s_d_s_c_s_a_s_a_s
GTGACGGTGTGGGTGACGGTGTGGGTGACGGTGTGGGTGTCTGTGTCTGTG; >clamp_s_c_s_d_s_c_s_a_s_e_s:compl:rev
CACCAGCACAGACACCCACACCGTCACCCACACTATCATGTTGACATTCC; dG = -34.9
1885 >s_d_s_c_s_e_s_c_s_d_s_c_s_a_s_b_s
GTGACGGTGTGGGTGCTGGTGTGGGTGACGGTGTGGGTGTCTGTGGCTGTG; >clamp_s_c_s_d_s_c_s_a_s_e_s:compl:rev
CACCAGCACAGACACCCACACCGTCACCCACACTATCATGTTGACATTCC; dG = -34.9
1886 >s_d_s_c_s_e_s_c_s_d_s_c_s_a_s_a_s
GTGACGGTGTGGGTGCTGGTGTGGGTGACGGTGTGGGTGTCTGTGTCTGTG; >clamp_s_c_s_d_s_c_s_a_s_e_s:compl:rev
CACCAGCACAGACACCCACACCGTCACCCACACTATCATGTTGACATTCC; dG = -34.9
1887 >s_d_s_d_s_a_s_c_s_d_s_c_s_a_s_a_s
GTGACGGTGACGGTGTCTGTGTGGGTGACGGTGTGGGTGTCTGTGTCTGTG; >clamp_s_c_s_d_s_c_s_a_s_e_s:compl:rev
CACCAGCACAGACACCCACACCGTCACCCACACTATCATGTTGACATTCC; dG = -34.9
1888 >s_d_s_d_s_b_s_c_s_d_s_c_s_a_s_a_s
GTGACGGTGACGGTGGCTGTGTGGGTGACGGTGTGGGTGTCTGTGTCTGTG; >clamp_s_c_s_d_s_c_s_a_s_e_s:compl:rev
CACCAGCACAGACACCCACACCGTCACCCACACTATCATGTTGACATTCC; dG = -34.9
1889 >s_a_s_b_s_e_s_c_s_d_s_c_s_a_s_b_s
GTGTCTGTGGCTGTGCTGGTGTGGGTGACGGTGTGGGTGTCTGTGGCTGTG; >clamp_s_c_s_d_s_c_s_a_s_e_s:compl:rev
CACCAGCACAGACACCCACACCGTCACCCACACTATCATGTTGACATTCC; dG = -34.9
1890 >s_a_s_b_s_e_s_c_s_d_s_c_s_a_s_a_s
GTGTCTGTGGCTGTGCTGGTGTGGGTGACGGTGTGGGTGTCTGTGTCTGTG; >clamp_s_c_s_d_s_c_s_a_s_e_s:compl:rev
CACCAGCACAGACACCCACACCGTCACCCACACTATCATGTTGACATTCC; dG = -34.9
1891 >s_d_s_d_s_c_s_c_s_d_s_c_s_a_s_b_s
GTGACGGTGACGGTGTGGGTGTGGGTGACGGTGTGGGTGTCTGTGGCTGTG; >clamp_s_c_s_d_s_c_s_a_s_e_s:compl:rev
CACCAGCACAGACACCCACACCGTCACCCACACTATCATGTTGACATTCC; dG = -34.9
1892 >s_d_s_d_s_c_s_c_s_d_s_c_s_a_s_a_s
GTGACGGTGACGGTGTGGGTGTGGGTGACGGTGTGGGTGTCTGTGTCTGTG; >clamp_s_c_s_d_s_c_s_a_s_e_s:compl:rev
CACCAGCACAGACACCCACACCGTCACCCACACTATCATGTTGACATTCC; dG = -34.9
1893 >s_d_s_d_s_d_s_c_s_d_s_c_s_a_s_b_s
GTGACGGTGACGGTGACGGTGTGGGTGACGGTGTGGGTGTCTGTGGCTGTG; >clamp_s_c_s_d_s_c_s_a_s_e_s:compl:rev
CACCAGCACAGACACCCACACCGTCACCCACACTATCATGTTGACATTCC; dG = -34.9
1894 >s_d_s_d_s_d_s_c_s_d_s_c_s_a_s_a_s
GTGACGGTGACGGTGACGGTGTGGGTGACGGTGTGGGTGTCTGTGTCTGTG; >clamp_s_c_s_d_s_c_s_a_s_e_s:compl:rev
CACCAGCACAGACACCCACACCGTCACCCACACTATCATGTTGACATTCC; dG = -34.9
1895 >s_d_s_d_s_e_s_c_s_d_s_c_s_a_s_b_s
GTGACGGTGACGGTGCTGGTGTGGGTGACGGTGTGGGTGTCTGTGGCTGTG; >clamp_s_c_s_d_s_c_s_a_s_e_s:compl:rev
CACCAGCACAGACACCCACACCGTCACCCACACTATCATGTTGACATTCC; dG = -34.9
1896 >s_d_s_d_s_e_s_c_s_d_s_c_s_a_s_a_s
GTGACGGTGACGGTGCTGGTGTGGGTGACGGTGTGGGTGTCTGTGTCTGTG; >clamp_s_c_s_d_s_c_s_a_s_e_s:compl:rev
CACCAGCACAGACACCCACACCGTCACCCACACTATCATGTTGACATTCC; dG = -34.9
1897 >s_d_s_e_s_a_s_c_s_d_s_c_s_a_s_a_s
GTGACGGTGCTGGTGTCTGTGTGGGTGACGGTGTGGGTGTCTGTGTCTGTG; >clamp_s_c_s_d_s_c_s_a_s_e_s:compl:rev
CACCAGCACAGACACCCACACCGTCACCCACACTATCATGTTGACATTCC; dG = -34.9
1898 >s_d_s_e_s_b_s_c_s_d_s_c_s_a_s_a_s
GTGACGGTGCTGGTGGCTGTGTGGGTGACGGTGTGGGTGTCTGTGTCTGTG; >clamp_s_c_s_d_s_c_s_a_s_e_s:compl:rev
CACCAGCACAGACACCCACACCGTCACCCACACTATCATGTTGACATTCC; dG = -34.9
1899 >s_d_s_e_s_c_s_c_s_d_s_c_s_a_s_b_s
GTGACGGTGCTGGTGTGGGTGTGGGTGACGGTGTGGGTGTCTGTGGCTGTG; >clamp_s_c_s_d_s_c_s_a_s_e_s:compl:rev
CACCAGCACAGACACCCACACCGTCACCCACACTATCATGTTGACATTCC; dG = -34.9
1900 >s_d_s_e_s_c_s_c_s_d_s_c_s_a_s_a_s
GTGACGGTGCTGGTGTGGGTGTGGGTGACGGTGTGGGTGTCTGTGTCTGTG; >clamp_s_c_s_d_s_c_s_a_s_e_s:compl:rev
CACCAGCACAGACACCCACACCGTCACCCACACTATCATGTTGACATTCC; dG = -34.9
1901 >s_d_s_e_s_d_s_c_s_d_s_c_s_a_s_b_s
GTGACGGTGCTGGTGACGGTGTGGGTGACGGTGTGGGTGTCTGTGGCTGTG; >clamp_s_c_s_d_s_c_s_a_s_e_s:compl:rev
CACCAGCACAGACACCCACACCGTCACCCACACTATCATGTTGACATTCC; dG = -34.9
1902 >s_d_s_e_s_d_s_c_s_d_s_c_s_a_s_a_s
GTGACGGTGCTGGTGACGGTGTGGGTGACGGTGTGGGTGTCTGTGTCTGTG; >clamp_s_c_s_d_s_c_s_a_s_e_s:compl:rev
CACCAGCACAGACACCCACACCGTCACCCACACTATCATGTTGACATTCC; dG = -34.9
1903 >s_d_s_e_s_e_s_c_s_d_s_c_s_a_s_b_s
GTGACGGTGCTGGTGCTGGTGTGGGTGACGGTGTGGGTGTCTGTGGCTGTG; >clamp_s_c_s_d_s_c_s_a_s_e_s:compl:rev
CACCAGCACAGACACCCACACCGTCACCCACACTATCATGTTGACATTCC; dG = -34.9

1904 >s_d_s_e_s_e_s_c_s_d_s_c_s_a_s_a_s
GTGACGGTGCTGGTGCTGGTGTGGGTGACGGTGTGGGTGTCTGTGTCTGTG; >clamp_s_c_s_d_s_c_s_a_s_e_s:compl:rev
CACCAGCACAGACACCCACACCGTCACCCACACTATCATGTTGACATTCC; dG = -34.9
1905 >s_e_s_a_s_a_s_c_s_d_s_c_s_a_s_a_s
GTGCTGGTGTCTGTGTCTGTGTGGGTGACGGTGTGGGTGTCTGTGTCTGTG; >clamp_s_c_s_d_s_c_s_a_s_e_s:compl:rev
CACCAGCACAGACACCCACACCGTCACCCACACTATCATGTTGACATTCC; dG = -34.9
1906 >s_e_s_a_s_b_s_c_s_d_s_c_s_a_s_a_s
GTGCTGGTGTCTGTGGCTGTGTGGGTGACGGTGTGGGTGTCTGTGTCTGTG; >clamp_s_c_s_d_s_c_s_a_s_e_s:compl:rev
CACCAGCACAGACACCCACACCGTCACCCACACTATCATGTTGACATTCC; dG = -34.9
1907 >s_a_s_c_s_a_s_c_s_d_s_c_s_a_s_a_s
GTGTCTGTGTGGGTGTCTGTGTGGGTGACGGTGTGGGTGTCTGTGTCTGTG; >clamp_s_c_s_d_s_c_s_a_s_e_s:compl:rev
CACCAGCACAGACACCCACACCGTCACCCACACTATCATGTTGACATTCC; dG = -34.9
1908 >s_e_s_a_s_c_s_c_s_d_s_c_s_a_s_b_s
GTGCTGGTGTCTGTGTGGGTGTGGGTGACGGTGTGGGTGTCTGTGGCTGTG; >clamp_s_c_s_d_s_c_s_a_s_e_s:compl:rev
CACCAGCACAGACACCCACACCGTCACCCACACTATCATGTTGACATTCC; dG = -34.9
1909 >s_e_s_a_s_c_s_c_s_d_s_c_s_a_s_a_s
GTGCTGGTGTCTGTGTGGGTGTGGGTGACGGTGTGGGTGTCTGTGTCTGTG; >clamp_s_c_s_d_s_c_s_a_s_e_s:compl:rev
CACCAGCACAGACACCCACACCGTCACCCACACTATCATGTTGACATTCC; dG = -34.9
1910 >s_e_s_a_s_d_s_c_s_d_s_c_s_a_s_b_s
GTGCTGGTGTCTGTGACGGTGTGGGTGACGGTGTGGGTGTCTGTGGCTGTG; >clamp_s_c_s_d_s_c_s_a_s_e_s:compl:rev
CACCAGCACAGACACCCACACCGTCACCCACACTATCATGTTGACATTCC; dG = -34.9
1911 >s_e_s_a_s_d_s_c_s_d_s_c_s_a_s_a_s
GTGCTGGTGTCTGTGACGGTGTGGGTGACGGTGTGGGTGTCTGTGTCTGTG; >clamp_s_c_s_d_s_c_s_a_s_e_s:compl:rev
CACCAGCACAGACACCCACACCGTCACCCACACTATCATGTTGACATTCC; dG = -34.9
1912 >s_e_s_a_s_e_s_c_s_d_s_c_s_a_s_b_s
GTGCTGGTGTCTGTGCTGGTGTGGGTGACGGTGTGGGTGTCTGTGGCTGTG; >clamp_s_c_s_d_s_c_s_a_s_e_s:compl:rev
CACCAGCACAGACACCCACACCGTCACCCACACTATCATGTTGACATTCC; dG = -34.9
1913 >s_e_s_a_s_e_s_c_s_d_s_c_s_a_s_a_s
GTGCTGGTGTCTGTGCTGGTGTGGGTGACGGTGTGGGTGTCTGTGTCTGTG; >clamp_s_c_s_d_s_c_s_a_s_e_s:compl:rev
CACCAGCACAGACACCCACACCGTCACCCACACTATCATGTTGACATTCC; dG = -34.9
1914 >s_e_s_b_s_a_s_c_s_d_s_c_s_a_s_a_s
GTGCTGGTGGCTGTGTCTGTGTGGGTGACGGTGTGGGTGTCTGTGTCTGTG; >clamp_s_c_s_d_s_c_s_a_s_e_s:compl:rev
CACCAGCACAGACACCCACACCGTCACCCACACTATCATGTTGACATTCC; dG = -34.9
1915 >s_e_s_b_s_b_s_c_s_d_s_c_s_a_s_a_s
GTGCTGGTGGCTGTGGCTGTGTGGGTGACGGTGTGGGTGTCTGTGTCTGTG; >clamp_s_c_s_d_s_c_s_a_s_e_s:compl:rev
CACCAGCACAGACACCCACACCGTCACCCACACTATCATGTTGACATTCC; dG = -34.9
1916 >s_e_s_b_s_c_s_c_s_d_s_c_s_a_s_b_s
GTGCTGGTGGCTGTGTGGGTGTGGGTGACGGTGTGGGTGTCTGTGGCTGTG; >clamp_s_c_s_d_s_c_s_a_s_e_s:compl:rev
CACCAGCACAGACACCCACACCGTCACCCACACTATCATGTTGACATTCC; dG = -34.9
1917 >s_e_s_b_s_c_s_c_s_d_s_c_s_a_s_a_s
GTGCTGGTGGCTGTGTGGGTGTGGGTGACGGTGTGGGTGTCTGTGTCTGTG; >clamp_s_c_s_d_s_c_s_a_s_e_s:compl:rev
CACCAGCACAGACACCCACACCGTCACCCACACTATCATGTTGACATTCC; dG = -34.9
1918 >s_e_s_b_s_d_s_c_s_d_s_c_s_a_s_b_s
GTGCTGGTGGCTGTGACGGTGTGGGTGACGGTGTGGGTGTCTGTGGCTGTG; >clamp_s_c_s_d_s_c_s_a_s_e_s:compl:rev
CACCAGCACAGACACCCACACCGTCACCCACACTATCATGTTGACATTCC; dG = -34.9
1919 >s_e_s_b_s_d_s_c_s_d_s_c_s_a_s_a_s
GTGCTGGTGGCTGTGACGGTGTGGGTGACGGTGTGGGTGTCTGTGTCTGTG; >clamp_s_c_s_d_s_c_s_a_s_e_s:compl:rev
CACCAGCACAGACACCCACACCGTCACCCACACTATCATGTTGACATTCC; dG = -34.9
1920 >s_e_s_b_s_e_s_c_s_d_s_c_s_a_s_b_s
GTGCTGGTGGCTGTGCTGGTGTGGGTGACGGTGTGGGTGTCTGTGGCTGTG; >clamp_s_c_s_d_s_c_s_a_s_e_s:compl:rev
CACCAGCACAGACACCCACACCGTCACCCACACTATCATGTTGACATTCC; dG = -34.9
1921 >s_e_s_b_s_e_s_c_s_d_s_c_s_a_s_a_s
GTGCTGGTGGCTGTGCTGGTGTGGGTGACGGTGTGGGTGTCTGTGTCTGTG; >clamp_s_c_s_d_s_c_s_a_s_e_s:compl:rev
CACCAGCACAGACACCCACACCGTCACCCACACTATCATGTTGACATTCC; dG = -34.9
1922 >s_e_s_c_s_a_s_c_s_d_s_c_s_a_s_a_s
GTGCTGGTGTGGGTGTCTGTGTGGGTGACGGTGTGGGTGTCTGTGTCTGTG; >clamp_s_c_s_d_s_c_s_a_s_e_s:compl:rev
CACCAGCACAGACACCCACACCGTCACCCACACTATCATGTTGACATTCC; dG = -34.9
1923 >s_e_s_c_s_b_s_c_s_d_s_c_s_a_s_a_s
GTGCTGGTGTGGGTGGCTGTGTGGGTGACGGTGTGGGTGTCTGTGTCTGTG; >clamp_s_c_s_d_s_c_s_a_s_e_s:compl:rev
CACCAGCACAGACACCCACACCGTCACCCACACTATCATGTTGACATTCC; dG = -34.9
1924 >s_a_s_c_s_b_s_c_s_d_s_c_s_a_s_a_s
GTGTCTGTGTGGGTGGCTGTGTGGGTGACGGTGTGGGTGTCTGTGTCTGTG; >clamp_s_c_s_d_s_c_s_a_s_e_s:compl:rev
CACCAGCACAGACACCCACACCGTCACCCACACTATCATGTTGACATTCC; dG = -34.9
1925 >s_e_s_c_s_c_s_c_s_d_s_c_s_a_s_b_s
GTGCTGGTGTGGGTGTGGGTGTGGGTGACGGTGTGGGTGTCTGTGGCTGTG; >clamp_s_c_s_d_s_c_s_a_s_e_s:compl:rev
CACCAGCACAGACACCCACACCGTCACCCACACTATCATGTTGACATTCC; dG = -34.9
1926 >s_e_s_c_s_c_s_c_s_d_s_c_s_a_s_a_s
GTGCTGGTGTGGGTGTGGGTGTGGGTGACGGTGTGGGTGTCTGTGTCTGTG; >clamp_s_c_s_d_s_c_s_a_s_e_s:compl:rev
CACCAGCACAGACACCCACACCGTCACCCACACTATCATGTTGACATTCC; dG = -34.9
1927 >s_e_s_c_s_d_s_c_s_d_s_c_s_a_s_b_s
GTGCTGGTGTGGGTGACGGTGTGGGTGACGGTGTGGGTGTCTGTGGCTGTG; >clamp_s_c_s_d_s_c_s_a_s_e_s:compl:rev
CACCAGCACAGACACCCACACCGTCACCCACACTATCATGTTGACATTCC; dG = -34.9

1928 >s_e_s_c_s_d_s_c_s_d_s_c_s_a_s_a_s
GTGCTGGTGTGGGTGACGGTGTGGGTGACGGTGTGGGTGTCTGTGTCTGTG;   >clamp_s_c_s_d_s_c_s_a_s_e_s:compl:rev
CACCAGCACAGACACCCACACCGTCACCCACACTATCATGTTGACATTCC; dG = -34.9
1929 >s_e_s_c_s_e_s_c_s_d_s_c_s_a_s_b_s
GTGCTGGTGTGGGTGCTGGTGTGGGTGACGGTGTGGGTGTCTGTGGCTGTG;   >clamp_s_c_s_d_s_c_s_a_s_e_s:compl:rev
CACCAGCACAGACACCCACACCGTCACCCACACTATCATGTTGACATTCC; dG = -34.9
1930 >s_e_s_c_s_e_s_c_s_d_s_c_s_a_s_a_s
GTGCTGGTGTGGGTGCTGGTGTGGGTGACGGTGTGGGTGTCTGTGTCTGTG;   >clamp_s_c_s_d_s_c_s_a_s_e_s:compl:rev
CACCAGCACAGACACCCACACCGTCACCCACACTATCATGTTGACATTCC; dG = -34.9
1931 >s_e_s_d_s_a_s_c_s_d_s_c_s_a_s_a_s
GTGCTGGTGACGGTGTCTGTGTGGGTGACGGTGTGGGTGTCTGTGTCTGTG;   >clamp_s_c_s_d_s_c_s_a_s_e_s:compl:rev
CACCAGCACAGACACCCACACCGTCACCCACACTATCATGTTGACATTCC; dG = -34.9
1932 >s_e_s_d_s_b_s_c_s_d_s_c_s_a_s_a_s
GTGCTGGTGACGGTGGCTGTGTGGGTGACGGTGTGGGTGTCTGTGTCTGTG;   >clamp_s_c_s_d_s_c_s_a_s_e_s:compl:rev
CACCAGCACAGACACCCACACCGTCACCCACACTATCATGTTGACATTCC; dG = -34.9
1933 >s_e_s_d_s_c_s_c_s_d_s_c_s_a_s_b_s
GTGCTGGTGACGGTGTGGGTGTGGGTGACGGTGTGGGTGTCTGTGGCTGTG;   >clamp_s_c_s_d_s_c_s_a_s_e_s:compl:rev
CACCAGCACAGACACCCACACCGTCACCCACACTATCATGTTGACATTCC; dG = -34.9
1934 >s_e_s_d_s_c_s_c_s_d_s_c_s_a_s_a_s
GTGCTGGTGACGGTGTGGGTGTGGGTGACGGTGTGGGTGTCTGTGTCTGTG;   >clamp_s_c_s_d_s_c_s_a_s_e_s:compl:rev
CACCAGCACAGACACCCACACCGTCACCCACACTATCATGTTGACATTCC; dG = -34.9
1935 >s_e_s_d_s_d_s_c_s_d_s_c_s_a_s_b_s
GTGCTGGTGACGGTGACGGTGTGGGTGACGGTGTGGGTGTCTGTGGCTGTG;   >clamp_s_c_s_d_s_c_s_a_s_e_s:compl:rev
CACCAGCACAGACACCCACACCGTCACCCACACTATCATGTTGACATTCC; dG = -34.9
1936 >s_e_s_d_s_d_s_c_s_d_s_c_s_a_s_a_s
GTGCTGGTGACGGTGACGGTGTGGGTGACGGTGTGGGTGTCTGTGTCTGTG;   >clamp_s_c_s_d_s_c_s_a_s_e_s:compl:rev
CACCAGCACAGACACCCACACCGTCACCCACACTATCATGTTGACATTCC; dG = -34.9
1937 >s_e_s_d_s_e_s_c_s_d_s_c_s_a_s_b_s
GTGCTGGTGACGGTGCTGGTGTGGGTGACGGTGTGGGTGTCTGTGGCTGTG;   >clamp_s_c_s_d_s_c_s_a_s_e_s:compl:rev
CACCAGCACAGACACCCACACCGTCACCCACACTATCATGTTGACATTCC; dG = -34.9
1938 >s_e_s_d_s_e_s_c_s_d_s_c_s_a_s_a_s
GTGCTGGTGACGGTGCTGGTGTGGGTGACGGTGTGGGTGTCTGTGTCTGTG;   >clamp_s_c_s_d_s_c_s_a_s_e_s:compl:rev
CACCAGCACAGACACCCACACCGTCACCCACACTATCATGTTGACATTCC; dG = -34.9
1939 >s_a_s_a_s_b_s_c_s_d_s_c_s_a_s_a_s
GTGTCTGTGTCTGTGGCTGTGTGGGTGACGGTGTGGGTGTCTGTGTCTGTG;   >clamp_s_c_s_d_s_c_s_a_s_e_s:compl:rev
CACCAGCACAGACACCCACACCGTCACCCACACTATCATGTTGACATTCC; dG = -34.9
1940 >s_e_s_e_s_a_s_c_s_d_s_c_s_a_s_a_s
GTGCTGGTGCTGGTGTCTGTGTGGGTGACGGTGTGGGTGTCTGTGTCTGTG;   >clamp_s_c_s_d_s_c_s_a_s_e_s:compl:rev
CACCAGCACAGACACCCACACCGTCACCCACACTATCATGTTGACATTCC; dG = -34.9
1941 >s_e_s_e_s_b_s_c_s_d_s_c_s_a_s_a_s
GTGCTGGTGCTGGTGGCTGTGTGGGTGACGGTGTGGGTGTCTGTGTCTGTG;   >clamp_s_c_s_d_s_c_s_a_s_e_s:compl:rev
CACCAGCACAGACACCCACACCGTCACCCACACTATCATGTTGACATTCC; dG = -34.9
1942 >s_a_s_c_s_c_s_c_s_d_s_c_s_a_s_b_s
GTGTCTGTGTGGGTGTGGGTGTGGGTGACGGTGTGGGTGTCTGTGGCTGTG;   >clamp_s_c_s_d_s_c_s_a_s_e_s:compl:rev
CACCAGCACAGACACCCACACCGTCACCCACACTATCATGTTGACATTCC; dG = -34.9
1943 >s_a_s_c_s_c_s_c_s_d_s_c_s_a_s_a_s
GTGTCTGTGTGGGTGTGGGTGTGGGTGACGGTGTGGGTGTCTGTGTCTGTG;   >clamp_s_c_s_d_s_c_s_a_s_e_s:compl:rev
CACCAGCACAGACACCCACACCGTCACCCACACTATCATGTTGACATTCC; dG = -34.9
1944 >s_e_s_e_s_c_s_c_s_d_s_c_s_a_s_b_s
GTGCTGGTGCTGGTGTGGGTGTGGGTGACGGTGTGGGTGTCTGTGGCTGTG;   >clamp_s_c_s_d_s_c_s_a_s_e_s:compl:rev
CACCAGCACAGACACCCACACCGTCACCCACACTATCATGTTGACATTCC; dG = -34.9
1945 >s_e_s_e_s_c_s_c_s_d_s_c_s_a_s_a_s
GTGCTGGTGCTGGTGTGGGTGTGGGTGACGGTGTGGGTGTCTGTGTCTGTG;   >clamp_s_c_s_d_s_c_s_a_s_e_s:compl:rev
CACCAGCACAGACACCCACACCGTCACCCACACTATCATGTTGACATTCC; dG = -34.9
1946 >s_e_s_e_s_d_s_c_s_d_s_c_s_a_s_b_s
GTGCTGGTGCTGGTGACGGTGTGGGTGACGGTGTGGGTGTCTGTGGCTGTG;   >clamp_s_c_s_d_s_c_s_a_s_e_s:compl:rev
CACCAGCACAGACACCCACACCGTCACCCACACTATCATGTTGACATTCC; dG = -34.9
1947 >s_e_s_e_s_d_s_c_s_d_s_c_s_a_s_a_s
GTGCTGGTGCTGGTGACGGTGTGGGTGACGGTGTGGGTGTCTGTGTCTGTG;   >clamp_s_c_s_d_s_c_s_a_s_e_s:compl:rev
CACCAGCACAGACACCCACACCGTCACCCACACTATCATGTTGACATTCC; dG = -34.9
1948 >s_e_s_e_s_e_s_c_s_d_s_c_s_a_s_b_s
GTGCTGGTGCTGGTGCTGGTGTGGGTGACGGTGTGGGTGTCTGTGGCTGTG;   >clamp_s_c_s_d_s_c_s_a_s_e_s:compl:rev
CACCAGCACAGACACCCACACCGTCACCCACACTATCATGTTGACATTCC; dG = -34.9
1949 >s_e_s_e_s_e_s_c_s_d_s_c_s_a_s_a_s
GTGCTGGTGCTGGTGCTGGTGTGGGTGACGGTGTGGGTGTCTGTGTCTGTG;   >clamp_s_c_s_d_s_c_s_a_s_e_s:compl:rev
CACCAGCACAGACACCCACACCGTCACCCACACTATCATGTTGACATTCC; dG = -34.9
1950 >s_a_s_c_s_d_s_c_s_d_s_c_s_a_s_b_s
GTGTCTGTGTGGGTGACGGTGTGGGTGACGGTGTGGGTGTCTGTGGCTGTG;   >clamp_s_c_s_d_s_c_s_a_s_e_s:compl:rev
CACCAGCACAGACACCCACACCGTCACCCACACTATCATGTTGACATTCC; dG = -34.9
1951 >s_a_s_c_s_d_s_c_s_d_s_c_s_a_s_a_s
GTGTCTGTGTGGGTGACGGTGTGGGTGACGGTGTGGGTGTCTGTGTCTGTG;   >clamp_s_c_s_d_s_c_s_a_s_e_s:compl:rev
CACCAGCACAGACACCCACACCGTCACCCACACTATCATGTTGACATTCC; dG = -34.9

1952 >s_a_s_c_s_e_s_c_s_d_s_c_s_a_s_b_s
GTGTCTGTGTGGGTGCTGGTGTGGGTGACGGTGTGGGTGTCTGTGGCTGTG; >clamp_s_c_s_d_s_c_s_a_s_e_s:compl:rev
CACCAGCACAGACACCCACACCGTCACCCACACTATCATGTTGACATTCC; dG = -34.9
1953 >s_a_s_c_s_e_s_c_s_d_s_c_s_a_s_a_s
GTGTCTGTGTGGGTGCTGGTGTGGGTGACGGTGTGGGTGTCTGTGTCTGTG; >clamp_s_c_s_d_s_c_s_a_s_e_s:compl:rev
CACCAGCACAGACACCCACACCGTCACCCACACTATCATGTTGACATTCC; dG = -34.9
1954 >s_a_s_d_s_a_s_c_s_d_s_c_s_a_s_a_s
GTGTCTGTGACGGTGTCTGTGTGGGTGACGGTGTGGGTGTCTGTGTCTGTG; >clamp_s_c_s_d_s_c_s_a_s_e_s:compl:rev
CACCAGCACAGACACCCACACCGTCACCCACACTATCATGTTGACATTCC; dG = -34.9
1955 >s_a_s_d_s_b_s_c_s_d_s_c_s_a_s_a_s
GTGTCTGTGACGGTGGCTGTGTGGGTGACGGTGTGGGTGTCTGTGTCTGTG; >clamp_s_c_s_d_s_c_s_a_s_e_s:compl:rev
CACCAGCACAGACACCCACACCGTCACCCACACTATCATGTTGACATTCC; dG = -34.9
1956 >s_a_s_d_s_c_s_c_s_d_s_c_s_a_s_b_s
GTGTCTGTGACGGTGTGGGTGTGGGTGACGGTGTGGGTGTCTGTGGCTGTG; >clamp_s_c_s_d_s_c_s_a_s_e_s:compl:rev
CACCAGCACAGACACCCACACCGTCACCCACACTATCATGTTGACATTCC; dG = -34.9
1957 >s_a_s_d_s_c_s_c_s_d_s_c_s_a_s_a_s
GTGTCTGTGACGGTGTGGGTGTGGGTGACGGTGTGGGTGTCTGTGTCTGTG; >clamp_s_c_s_d_s_c_s_a_s_e_s:compl:rev
CACCAGCACAGACACCCACACCGTCACCCACACTATCATGTTGACATTCC; dG = -34.9
1958 >s_a_s_d_s_d_s_c_s_d_s_c_s_a_s_b_s
GTGTCTGTGACGGTGACGGTGTGGGTGACGGTGTGGGTGTCTGTGGCTGTG; >clamp_s_c_s_d_s_c_s_a_s_e_s:compl:rev
CACCAGCACAGACACCCACACCGTCACCCACACTATCATGTTGACATTCC; dG = -34.9
1959 >s_a_s_d_s_d_s_c_s_d_s_c_s_a_s_a_s
GTGTCTGTGACGGTGACGGTGTGGGTGACGGTGTGGGTGTCTGTGTCTGTG; >clamp_s_c_s_d_s_c_s_a_s_e_s:compl:rev
CACCAGCACAGACACCCACACCGTCACCCACACTATCATGTTGACATTCC; dG = -34.9
1960 >s_a_s_d_s_e_s_c_s_d_s_c_s_a_s_b_s
GTGTCTGTGACGGTGCTGGTGTGGGTGACGGTGTGGGTGTCTGTGGCTGTG; >clamp_s_c_s_d_s_c_s_a_s_e_s:compl:rev
CACCAGCACAGACACCCACACCGTCACCCACACTATCATGTTGACATTCC; dG = -34.9
1961 >s_a_s_d_s_e_s_c_s_d_s_c_s_a_s_a_s
GTGTCTGTGACGGTGCTGGTGTGGGTGACGGTGTGGGTGTCTGTGTCTGTG; >clamp_s_c_s_d_s_c_s_a_s_e_s:compl:rev
CACCAGCACAGACACCCACACCGTCACCCACACTATCATGTTGACATTCC; dG = -34.9
1962 >s_a_s_a_s_a_s_c_s_d_s_c_s_a_s_a_s
GTGTCTGTGTCTGTGTCTGTGTGGGTGACGGTGTGGGTGTCTGTGTCTGTG; >clamp_s_c_s_d_s_c_s_a_s_e_s:compl:rev
CACCAGCACAGACACCCACACCGTCACCCACACTATCATGTTGACATTCC; dG = -34.9
1963 >s_a_s_e_s_a_s_c_s_d_s_c_s_a_s_a_s
GTGTCTGTGCTGGTGTCTGTGTGGGTGACGGTGTGGGTGTCTGTGTCTGTG; >clamp_s_c_s_d_s_c_s_a_s_e_s:compl:rev
CACCAGCACAGACACCCACACCGTCACCCACACTATCATGTTGACATTCC; dG = -34.9
1964 >s_a_s_e_s_b_s_c_s_d_s_c_s_a_s_a_s
GTGTCTGTGCTGGTGGCTGTGTGGGTGACGGTGTGGGTGTCTGTGTCTGTG; >clamp_s_c_s_d_s_c_s_a_s_e_s:compl:rev
CACCAGCACAGACACCCACACCGTCACCCACACTATCATGTTGACATTCC; dG = -34.9
1965 >s_a_s_a_s_c_s_c_s_d_s_c_s_a_s_b_s
GTGTCTGTGTCTGTGTGGGTGTGGGTGACGGTGTGGGTGTCTGTGGCTGTG; >clamp_s_c_s_d_s_c_s_a_s_e_s:compl:rev
CACCAGCACAGACACCCACACCGTCACCCACACTATCATGTTGACATTCC; dG = -34.9
1966 >s_a_s_a_s_c_s_c_s_d_s_c_s_a_s_a_s
GTGTCTGTGTCTGTGTGGGTGTGGGTGACGGTGTGGGTGTCTGTGTCTGTG; >clamp_s_c_s_d_s_c_s_a_s_e_s:compl:rev
CACCAGCACAGACACCCACACCGTCACCCACACTATCATGTTGACATTCC; dG = -34.9
1967 >s_a_s_e_s_c_s_c_s_d_s_c_s_a_s_b_s
GTGTCTGTGCTGGTGTGGGTGTGGGTGACGGTGTGGGTGTCTGTGGCTGTG; >clamp_s_c_s_d_s_c_s_a_s_e_s:compl:rev
CACCAGCACAGACACCCACACCGTCACCCACACTATCATGTTGACATTCC; dG = -34.9
1968 >s_a_s_e_s_c_s_c_s_d_s_c_s_a_s_a_s
GTGTCTGTGCTGGTGTGGGTGTGGGTGACGGTGTGGGTGTCTGTGTCTGTG; >clamp_s_c_s_d_s_c_s_a_s_e_s:compl:rev
CACCAGCACAGACACCCACACCGTCACCCACACTATCATGTTGACATTCC; dG = -34.9
1969 >s_a_s_e_s_d_s_c_s_d_s_c_s_a_s_b_s
GTGTCTGTGCTGGTGACGGTGTGGGTGACGGTGTGGGTGTCTGTGGCTGTG; >clamp_s_c_s_d_s_c_s_a_s_e_s:compl:rev
CACCAGCACAGACACCCACACCGTCACCCACACTATCATGTTGACATTCC; dG = -34.9
1970 >s_a_s_e_s_d_s_c_s_d_s_c_s_a_s_a_s
GTGTCTGTGCTGGTGACGGTGTGGGTGACGGTGTGGGTGTCTGTGTCTGTG; >clamp_s_c_s_d_s_c_s_a_s_e_s:compl:rev
CACCAGCACAGACACCCACACCGTCACCCACACTATCATGTTGACATTCC; dG = -34.9
1971 >s_a_s_e_s_e_s_c_s_d_s_c_s_a_s_b_s
GTGTCTGTGCTGGTGCTGGTGTGGGTGACGGTGTGGGTGTCTGTGGCTGTG; >clamp_s_c_s_d_s_c_s_a_s_e_s:compl:rev
CACCAGCACAGACACCCACACCGTCACCCACACTATCATGTTGACATTCC; dG = -34.9
1972 >s_a_s_e_s_e_s_c_s_d_s_c_s_a_s_a_s
GTGTCTGTGCTGGTGCTGGTGTGGGTGACGGTGTGGGTGTCTGTGTCTGTG; >clamp_s_c_s_d_s_c_s_a_s_e_s:compl:rev
CACCAGCACAGACACCCACACCGTCACCCACACTATCATGTTGACATTCC; dG = -34.9
1973 >s_b_s_a_s_a_s_c_s_d_s_c_s_a_s_a_s
GTGGCTGTGTCTGTGTCTGTGTGGGTGACGGTGTGGGTGTCTGTGTCTGTG; >clamp_s_c_s_d_s_c_s_a_s_e_s:compl:rev
CACCAGCACAGACACCCACACCGTCACCCACACTATCATGTTGACATTCC; dG = -34.9
1974 >s_b_s_a_s_b_s_c_s_d_s_c_s_a_s_a_s
GTGGCTGTGTCTGTGGCTGTGTGGGTGACGGTGTGGGTGTCTGTGTCTGTG; >clamp_s_c_s_d_s_c_s_a_s_e_s:compl:rev
CACCAGCACAGACACCCACACCGTCACCCACACTATCATGTTGACATTCC; dG = -34.9
1975 >s_b_s_a_s_c_s_c_s_d_s_c_s_a_s_b_s
GTGGCTGTGTCTGTGTGGGTGTGGGTGACGGTGTGGGTGTCTGTGGCTGTG; >clamp_s_c_s_d_s_c_s_a_s_e_s:compl:rev
CACCAGCACAGACACCCACACCGTCACCCACACTATCATGTTGACATTCC; dG = -34.9

```
  1976 >s_b_s_a_s_c_s_c_s_d_s_c_s_a_s_a_s
GTGGCTGTGTCTGTGTGGGTGTGGGTGACGGTGTGGGTGTCTGTGTCTGTG;    >clamp_s_c_s_d_s_c_s_a_s_e_s:compl:rev
CACCAGCACAGACACCCACACCGTCACCCACACTATCATGTTGACATTCC; dG = -34.9
  1977 >s_b_s_a_s_d_s_c_s_d_s_c_s_a_s_b_s
GTGGCTGTGTCTGTGACGGTGTGGGTGACGGTGTGGGTGTCTGTGGCTGTG;    >clamp_s_c_s_d_s_c_s_a_s_e_s:compl:rev
CACCAGCACAGACACCCACACCGTCACCCACACTATCATGTTGACATTCC; dG = -34.9
  1978 >s_b_s_a_s_d_s_c_s_d_s_c_s_a_s_a_s
GTGGCTGTGTCTGTGACGGTGTGGGTGACGGTGTGGGTGTCTGTGTCTGTG;    >clamp_s_c_s_d_s_c_s_a_s_e_s:compl:rev
CACCAGCACAGACACCCACACCGTCACCCACACTATCATGTTGACATTCC; dG = -34.9
  1979 >s_b_s_a_s_e_s_c_s_d_s_c_s_a_s_b_s
GTGGCTGTGTCTGTGCTGGTGTGGGTGACGGTGTGGGTGTCTGTGGCTGTG;    >clamp_s_c_s_d_s_c_s_a_s_e_s:compl:rev
CACCAGCACAGACACCCACACCGTCACCCACACTATCATGTTGACATTCC; dG = -34.9
  1980 >s_b_s_a_s_e_s_c_s_d_s_c_s_a_s_a_s
GTGGCTGTGTCTGTGCTGGTGTGGGTGACGGTGTGGGTGTCTGTGTCTGTG;    >clamp_s_c_s_d_s_c_s_a_s_e_s:compl:rev
CACCAGCACAGACACCCACACCGTCACCCACACTATCATGTTGACATTCC; dG = -34.9
  1981 >s_b_s_b_s_a_s_c_s_d_s_c_s_a_s_a_s
GTGGCTGTGGCTGTGTCTGTGTGGGTGACGGTGTGGGTGTCTGTGTCTGTG;    >clamp_s_c_s_d_s_c_s_a_s_e_s:compl:rev
CACCAGCACAGACACCCACACCGTCACCCACACTATCATGTTGACATTCC; dG = -34.9
  1982 >s_b_s_b_s_b_s_c_s_d_s_c_s_a_s_a_s
GTGGCTGTGGCTGTGGCTGTGTGGGTGACGGTGTGGGTGTCTGTGTCTGTG;    >clamp_s_c_s_d_s_c_s_a_s_e_s:compl:rev
CACCAGCACAGACACCCACACCGTCACCCACACTATCATGTTGACATTCC; dG = -34.9
  1983 >s_a_s_a_s_d_s_c_s_d_s_c_s_a_s_b_s
GTGTCTGTGTCTGTGACGGTGTGGGTGACGGTGTGGGTGTCTGTGGCTGTG;    >clamp_s_c_s_d_s_c_s_a_s_e_s:compl:rev
CACCAGCACAGACACCCACACCGTCACCCACACTATCATGTTGACATTCC; dG = -34.9
  1984 >s_a_s_a_s_d_s_c_s_d_s_c_s_a_s_a_s
GTGTCTGTGTCTGTGACGGTGTGGGTGACGGTGTGGGTGTCTGTGTCTGTG;    >clamp_s_c_s_d_s_c_s_a_s_e_s:compl:rev
CACCAGCACAGACACCCACACCGTCACCCACACTATCATGTTGACATTCC; dG = -34.9
  1985 >s_b_s_b_s_c_s_c_s_d_s_c_s_a_s_b_s
GTGGCTGTGGCTGTGTGGGTGTGGGTGACGGTGTGGGTGTCTGTGGCTGTG;    >clamp_s_c_s_d_s_c_s_a_s_e_s:compl:rev
CACCAGCACAGACACCCACACCGTCACCCACACTATCATGTTGACATTCC; dG = -34.9
  1986 >s_b_s_b_s_c_s_c_s_d_s_c_s_a_s_a_s
GTGGCTGTGGCTGTGTGGGTGTGGGTGACGGTGTGGGTGTCTGTGTCTGTG;    >clamp_s_c_s_d_s_c_s_a_s_e_s:compl:rev
CACCAGCACAGACACCCACACCGTCACCCACACTATCATGTTGACATTCC; dG = -34.9
  1987 >s_b_s_b_s_d_s_c_s_d_s_c_s_a_s_b_s
GTGGCTGTGGCTGTGACGGTGTGGGTGACGGTGTGGGTGTCTGTGGCTGTG;    >clamp_s_c_s_d_s_c_s_a_s_e_s:compl:rev
CACCAGCACAGACACCCACACCGTCACCCACACTATCATGTTGACATTCC; dG = -34.9
  1988 >s_b_s_b_s_d_s_c_s_d_s_c_s_a_s_a_s
GTGGCTGTGGCTGTGACGGTGTGGGTGACGGTGTGGGTGTCTGTGTCTGTG;    >clamp_s_c_s_d_s_c_s_a_s_e_s:compl:rev
CACCAGCACAGACACCCACACCGTCACCCACACTATCATGTTGACATTCC; dG = -34.9
  1989 >s_b_s_b_s_e_s_c_s_d_s_c_s_a_s_b_s
GTGGCTGTGGCTGTGCTGGTGTGGGTGACGGTGTGGGTGTCTGTGGCTGTG;    >clamp_s_c_s_d_s_c_s_a_s_e_s:compl:rev
CACCAGCACAGACACCCACACCGTCACCCACACTATCATGTTGACATTCC; dG = -34.9
  1990 >s_b_s_b_s_e_s_c_s_d_s_c_s_a_s_a_s
GTGGCTGTGGCTGTGCTGGTGTGGGTGACGGTGTGGGTGTCTGTGTCTGTG;    >clamp_s_c_s_d_s_c_s_a_s_e_s:compl:rev
CACCAGCACAGACACCCACACCGTCACCCACACTATCATGTTGACATTCC; dG = -34.9
  1991 >s_b_s_c_s_a_s_c_s_d_s_c_s_a_s_a_s
GTGGCTGTGTGGGTGTCTGTGTGGGTGACGGTGTGGGTGTCTGTGTCTGTG;    >clamp_s_c_s_d_s_c_s_a_s_e_s:compl:rev
CACCAGCACAGACACCCACACCGTCACCCACACTATCATGTTGACATTCC; dG = -34.9
  1992 >s_b_s_c_s_b_s_c_s_d_s_c_s_a_s_a_s
GTGGCTGTGTGGGTGGCTGTGTGGGTGACGGTGTGGGTGTCTGTGTCTGTG;    >clamp_s_c_s_d_s_c_s_a_s_e_s:compl:rev
CACCAGCACAGACACCCACACCGTCACCCACACTATCATGTTGACATTCC; dG = -34.9
  1993 >s_b_s_c_s_c_s_c_s_d_s_c_s_a_s_b_s
GTGGCTGTGTGGGTGTGGGTGTGGGTGACGGTGTGGGTGTCTGTGGCTGTG;    >clamp_s_c_s_d_s_c_s_a_s_e_s:compl:rev
CACCAGCACAGACACCCACACCGTCACCCACACTATCATGTTGACATTCC; dG = -34.9
  1994 >s_b_s_c_s_c_s_c_s_d_s_c_s_a_s_a_s
GTGGCTGTGTGGGTGTGGGTGTGGGTGACGGTGTGGGTGTCTGTGTCTGTG;    >clamp_s_c_s_d_s_c_s_a_s_e_s:compl:rev
CACCAGCACAGACACCCACACCGTCACCCACACTATCATGTTGACATTCC; dG = -34.9
  1995 >s_b_s_c_s_d_s_c_s_d_s_c_s_a_s_b_s
GTGGCTGTGTGGGTGACGGTGTGGGTGACGGTGTGGGTGTCTGTGGCTGTG;    >clamp_s_c_s_d_s_c_s_a_s_e_s:compl:rev
CACCAGCACAGACACCCACACCGTCACCCACACTATCATGTTGACATTCC; dG = -34.9
  1996 >s_b_s_c_s_d_s_c_s_d_s_c_s_a_s_a_s
GTGGCTGTGTGGGTGACGGTGTGGGTGACGGTGTGGGTGTCTGTGTCTGTG;    >clamp_s_c_s_d_s_c_s_a_s_e_s:compl:rev
CACCAGCACAGACACCCACACCGTCACCCACACTATCATGTTGACATTCC; dG = -34.9
  1997 >s_b_s_c_s_e_s_c_s_d_s_c_s_a_s_b_s
GTGGCTGTGTGGGTGCTGGTGTGGGTGACGGTGTGGGTGTCTGTGGCTGTG;    >clamp_s_c_s_d_s_c_s_a_s_e_s:compl:rev
CACCAGCACAGACACCCACACCGTCACCCACACTATCATGTTGACATTCC; dG = -34.9
  1998 >s_b_s_c_s_e_s_c_s_d_s_c_s_a_s_a_s
GTGGCTGTGTGGGTGCTGGTGTGGGTGACGGTGTGGGTGTCTGTGTCTGTG;    >clamp_s_c_s_d_s_c_s_a_s_e_s:compl:rev
CACCAGCACAGACACCCACACCGTCACCCACACTATCATGTTGACATTCC; dG = -34.9
  1999 >s_b_s_d_s_a_s_c_s_d_s_c_s_a_s_a_s
GTGGCTGTGACGGTGTCTGTGTGGGTGACGGTGTGGGTGTCTGTGTCTGTG;    >clamp_s_c_s_d_s_c_s_a_s_e_s:compl:rev
CACCAGCACAGACACCCACACCGTCACCCACACTATCATGTTGACATTCC; dG = -34.9
```

2000 >s_b_s_d_s_b_s_c_s_d_s_c_s_a_s_a_s
GTGGCTGTGACGGTGGCTGTGTGGGTGACGGTGTGGGTGTCTGTGTCTGTG; >clamp_s_c_s_d_s_c_s_a_s_e_s:compl:rev
CACCAGCACAGACACCCACACCGTCACCCACACTATCATGTTGACATTCC; dG = -34.9
2001 >s_a_s_a_s_e_s_c_s_d_s_c_s_a_s_b_s
GTGTCTGTGTCTGTGCTGGTGTGGGTGACGGTGTGGGTGTCTGTGGCTGTG; >clamp_s_c_s_d_s_c_s_a_s_e_s:compl:rev
CACCAGCACAGACACCCACACCGTCACCCACACTATCATGTTGACATTCC; dG = -34.9
2002 >s_a_s_a_s_e_s_c_s_d_s_c_s_a_s_a_s
GTGTCTGTGTCTGTGCTGGTGTGGGTGACGGTGTGGGTGTCTGTGTCTGTG; >clamp_s_c_s_d_s_c_s_a_s_e_s:compl:rev
CACCAGCACAGACACCCACACCGTCACCCACACTATCATGTTGACATTCC; dG = -34.9
2003 >s_b_s_d_s_c_s_c_s_d_s_c_s_a_s_b_s
GTGGCTGTGACGGTGTGGGTGTGGGTGACGGTGTGGGTGTCTGTGGCTGTG; >clamp_s_c_s_d_s_c_s_a_s_e_s:compl:rev
CACCAGCACAGACACCCACACCGTCACCCACACTATCATGTTGACATTCC; dG = -34.9
2004 >s_b_s_d_s_c_s_c_s_d_s_c_s_a_s_a_s
GTGGCTGTGACGGTGTGGGTGTGGGTGACGGTGTGGGTGTCTGTGTCTGTG; >clamp_s_c_s_d_s_c_s_a_s_e_s:compl:rev
CACCAGCACAGACACCCACACCGTCACCCACACTATCATGTTGACATTCC; dG = -34.9
2005 >s_b_s_d_s_d_s_c_s_d_s_c_s_a_s_b_s
GTGGCTGTGACGGTGACGGTGTGGGTGACGGTGTGGGTGTCTGTGGCTGTG; >clamp_s_c_s_d_s_c_s_a_s_e_s:compl:rev
CACCAGCACAGACACCCACACCGTCACCCACACTATCATGTTGACATTCC; dG = -34.9
2006 >s_b_s_d_s_d_s_c_s_d_s_c_s_a_s_a_s
GTGGCTGTGACGGTGACGGTGTGGGTGACGGTGTGGGTGTCTGTGTCTGTG; >clamp_s_c_s_d_s_c_s_a_s_e_s:compl:rev
CACCAGCACAGACACCCACACCGTCACCCACACTATCATGTTGACATTCC; dG = -34.9
2007 >s_b_s_d_s_e_s_c_s_d_s_c_s_a_s_b_s
GTGGCTGTGACGGTGCTGGTGTGGGTGACGGTGTGGGTGTCTGTGGCTGTG; >clamp_s_c_s_d_s_c_s_a_s_e_s:compl:rev
CACCAGCACAGACACCCACACCGTCACCCACACTATCATGTTGACATTCC; dG = -34.9
2008 >s_b_s_d_s_e_s_c_s_d_s_c_s_a_s_a_s
GTGGCTGTGACGGTGCTGGTGTGGGTGACGGTGTGGGTGTCTGTGTCTGTG; >clamp_s_c_s_d_s_c_s_a_s_e_s:compl:rev
CACCAGCACAGACACCCACACCGTCACCCACACTATCATGTTGACATTCC; dG = -34.9
2009 >s_b_s_e_s_a_s_c_s_d_s_c_s_a_s_a_s
GTGGCTGTGCTGGTGTCTGTGTGGGTGACGGTGTGGGTGTCTGTGTCTGTG; >clamp_s_c_s_d_s_c_s_a_s_e_s:compl:rev
CACCAGCACAGACACCCACACCGTCACCCACACTATCATGTTGACATTCC; dG = -34.9
2010 >s_b_s_e_s_b_s_c_s_d_s_c_s_a_s_a_s
GTGGCTGTGCTGGTGGCTGTGTGGGTGACGGTGTGGGTGTCTGTGTCTGTG; >clamp_s_c_s_d_s_c_s_a_s_e_s:compl:rev
CACCAGCACAGACACCCACACCGTCACCCACACTATCATGTTGACATTCC; dG = -34.9
2011 >s_b_s_e_s_c_s_c_s_d_s_c_s_a_s_b_s
GTGGCTGTGCTGGTGTGGGTGTGGGTGACGGTGTGGGTGTCTGTGGCTGTG; >clamp_s_c_s_d_s_c_s_a_s_e_s:compl:rev
CACCAGCACAGACACCCACACCGTCACCCACACTATCATGTTGACATTCC; dG = -34.9
2012 >s_b_s_e_s_c_s_c_s_d_s_c_s_a_s_a_s
GTGGCTGTGCTGGTGTGGGTGTGGGTGACGGTGTGGGTGTCTGTGTCTGTG; >clamp_s_c_s_d_s_c_s_a_s_e_s:compl:rev
CACCAGCACAGACACCCACACCGTCACCCACACTATCATGTTGACATTCC; dG = -34.9
2013 >s_b_s_e_s_d_s_c_s_d_s_c_s_a_s_b_s
GTGGCTGTGCTGGTGACGGTGTGGGTGACGGTGTGGGTGTCTGTGGCTGTG; >clamp_s_c_s_d_s_c_s_a_s_e_s:compl:rev
CACCAGCACAGACACCCACACCGTCACCCACACTATCATGTTGACATTCC; dG = -34.9
2014 >s_b_s_e_s_d_s_c_s_d_s_c_s_a_s_a_s
GTGGCTGTGCTGGTGACGGTGTGGGTGACGGTGTGGGTGTCTGTGTCTGTG; >clamp_s_c_s_d_s_c_s_a_s_e_s:compl:rev
CACCAGCACAGACACCCACACCGTCACCCACACTATCATGTTGACATTCC; dG = -34.9
2015 >s_b_s_e_s_e_s_c_s_d_s_c_s_a_s_b_s
GTGGCTGTGCTGGTGCTGGTGTGGGTGACGGTGTGGGTGTCTGTGGCTGTG; >clamp_s_c_s_d_s_c_s_a_s_e_s:compl:rev
CACCAGCACAGACACCCACACCGTCACCCACACTATCATGTTGACATTCC; dG = -34.9
2016 >s_b_s_e_s_e_s_c_s_d_s_c_s_a_s_a_s
GTGGCTGTGCTGGTGCTGGTGTGGGTGACGGTGTGGGTGTCTGTGTCTGTG; >clamp_s_c_s_d_s_c_s_a_s_e_s:compl:rev
CACCAGCACAGACACCCACACCGTCACCCACACTATCATGTTGACATTCC; dG = -34.9
2017 >s_c_s_a_s_a_s_c_s_d_s_c_s_e_s_e_s
GTGTGGGTGTCTGTGTCTGTGTGGGTGACGGTGTGGGTGCTGGTGCTGGTG; >clamp_s_c_s_d_s_c_s_a_s_e_s:compl:rev
CACCAGCACAGACACCCACACCGTCACCCACACTATCATGTTGACATTCC; dG = -34.7
2018 >s_c_s_a_s_a_s_c_s_d_s_c_s_d_s_e_s
GTGTGGGTGTCTGTGTCTGTGTGGGTGACGGTGTGGGTGACGGTGCTGGTG; >clamp_s_c_s_d_s_c_s_a_s_e_s:compl:rev
CACCAGCACAGACACCCACACCGTCACCCACACTATCATGTTGACATTCC; dG = -34.7
2019 >s_c_s_a_s_b_s_c_s_d_s_c_s_e_s_e_s
GTGTGGGTGTCTGTGGCTGTGTGGGTGACGGTGTGGGTGCTGGTGCTGGTG; >clamp_s_c_s_d_s_c_s_a_s_e_s:compl:rev
CACCAGCACAGACACCCACACCGTCACCCACACTATCATGTTGACATTCC; dG = -34.7
2020 >s_c_s_a_s_b_s_c_s_d_s_c_s_d_s_e_s
GTGTGGGTGTCTGTGGCTGTGTGGGTGACGGTGTGGGTGACGGTGCTGGTG; >clamp_s_c_s_d_s_c_s_a_s_e_s:compl:rev
CACCAGCACAGACACCCACACCGTCACCCACACTATCATGTTGACATTCC; dG = -34.7
2021 >s_a_s_b_s_a_s_c_s_d_s_c_s_e_s_e_s
GTGTCTGTGGCTGTGTCTGTGTGGGTGACGGTGTGGGTGCTGGTGCTGGTG; >clamp_s_c_s_d_s_c_s_a_s_e_s:compl:rev
CACCAGCACAGACACCCACACCGTCACCCACACTATCATGTTGACATTCC; dG = -34.7
2022 >s_a_s_b_s_a_s_c_s_d_s_c_s_d_s_e_s
GTGTCTGTGGCTGTGTCTGTGTGGGTGACGGTGTGGGTGACGGTGCTGGTG; >clamp_s_c_s_d_s_c_s_a_s_e_s:compl:rev
CACCAGCACAGACACCCACACCGTCACCCACACTATCATGTTGACATTCC; dG = -34.7
2023 >s_c_s_b_s_a_s_c_s_d_s_c_s_e_s_e_s
GTGTGGGTGGCTGTGTCTGTGTGGGTGACGGTGTGGGTGCTGGTGCTGGTG; >clamp_s_c_s_d_s_c_s_a_s_e_s:compl:rev
CACCAGCACAGACACCCACACCGTCACCCACACTATCATGTTGACATTCC; dG = -34.7

2024 >s_c_s_b_s_a_s_c_s_d_s_c_s_d_s_e_s
GTGTGGGTGGCTGTGTCTGTGTGGGTGACGGTGTGGGTGACGGTGCTGGTG; >clamp_s_c_s_d_s_c_s_a_s_e_s:compl:rev
CACCAGCACAGACACCCACACCGTCACCCACACTATCATGTTGACATTCC; dG = -34.7
2025 >s_c_s_b_s_b_s_c_s_d_s_c_s_e_s_e_s
GTGTGGGTGGCTGTGGCTGTGTGGGTGACGGTGTGGGTGCTGGTGCTGGTG; >clamp_s_c_s_d_s_c_s_a_s_e_s:compl:rev
CACCAGCACAGACACCCACACCGTCACCCACACTATCATGTTGACATTCC; dG = -34.7
2026 >s_c_s_b_s_b_s_c_s_d_s_c_s_d_s_e_s
GTGTGGGTGGCTGTGGCTGTGTGGGTGACGGTGTGGGTGACGGTGCTGGTG; >clamp_s_c_s_d_s_c_s_a_s_e_s:compl:rev
CACCAGCACAGACACCCACACCGTCACCCACACTATCATGTTGACATTCC; dG = -34.7
2027 >s_c_s_c_s_a_s_c_s_d_s_c_s_e_s_e_s
GTGTGGGTGTGGGTGTCTGTGTGGGTGACGGTGTGGGTGCTGGTGCTGGTG; >clamp_s_c_s_d_s_c_s_a_s_e_s:compl:rev
CACCAGCACAGACACCCACACCGTCACCCACACTATCATGTTGACATTCC; dG = -34.7
2028 >s_c_s_c_s_a_s_c_s_d_s_c_s_d_s_e_s
GTGTGGGTGTGGGTGTCTGTGTGGGTGACGGTGTGGGTGACGGTGCTGGTG; >clamp_s_c_s_d_s_c_s_a_s_e_s:compl:rev
CACCAGCACAGACACCCACACCGTCACCCACACTATCATGTTGACATTCC; dG = -34.7
2029 >s_c_s_c_s_b_s_c_s_d_s_c_s_e_s_e_s
GTGTGGGTGTGGGTGGCTGTGTGGGTGACGGTGTGGGTGCTGGTGCTGGTG; >clamp_s_c_s_d_s_c_s_a_s_e_s:compl:rev
CACCAGCACAGACACCCACACCGTCACCCACACTATCATGTTGACATTCC; dG = -34.7
2030 >s_c_s_c_s_b_s_c_s_d_s_c_s_d_s_e_s
GTGTGGGTGTGGGTGGCTGTGTGGGTGACGGTGTGGGTGACGGTGCTGGTG; >clamp_s_c_s_d_s_c_s_a_s_e_s:compl:rev
CACCAGCACAGACACCCACACCGTCACCCACACTATCATGTTGACATTCC; dG = -34.7
2031 >s_a_s_b_s_b_s_c_s_d_s_c_s_e_s_e_s
GTGTCTGTGGCTGTGGCTGTGTGGGTGACGGTGTGGGTGCTGGTGCTGGTG; >clamp_s_c_s_d_s_c_s_a_s_e_s:compl:rev
CACCAGCACAGACACCCACACCGTCACCCACACTATCATGTTGACATTCC; dG = -34.7
2032 >s_a_s_b_s_b_s_c_s_d_s_c_s_d_s_e_s
GTGTCTGTGGCTGTGGCTGTGTGGGTGACGGTGTGGGTGACGGTGCTGGTG; >clamp_s_c_s_d_s_c_s_a_s_e_s:compl:rev
CACCAGCACAGACACCCACACCGTCACCCACACTATCATGTTGACATTCC; dG = -34.7
2033 >s_c_s_d_s_a_s_c_s_d_s_c_s_e_s_e_s
GTGTGGGTGACGGTGTCTGTGTGGGTGACGGTGTGGGTGCTGGTGCTGGTG; >clamp_s_c_s_d_s_c_s_a_s_e_s:compl:rev
CACCAGCACAGACACCCACACCGTCACCCACACTATCATGTTGACATTCC; dG = -34.7
2034 >s_c_s_d_s_a_s_c_s_d_s_c_s_d_s_e_s
GTGTGGGTGACGGTGTCTGTGTGGGTGACGGTGTGGGTGACGGTGCTGGTG; >clamp_s_c_s_d_s_c_s_a_s_e_s:compl:rev
CACCAGCACAGACACCCACACCGTCACCCACACTATCATGTTGACATTCC; dG = -34.7
2035 >s_c_s_d_s_b_s_c_s_d_s_c_s_e_s_e_s
GTGTGGGTGACGGTGGCTGTGTGGGTGACGGTGTGGGTGCTGGTGCTGGTG; >clamp_s_c_s_d_s_c_s_a_s_e_s:compl:rev
CACCAGCACAGACACCCACACCGTCACCCACACTATCATGTTGACATTCC; dG = -34.7
2036 >s_c_s_d_s_b_s_c_s_d_s_c_s_d_s_e_s
GTGTGGGTGACGGTGGCTGTGTGGGTGACGGTGTGGGTGACGGTGCTGGTG; >clamp_s_c_s_d_s_c_s_a_s_e_s:compl:rev
CACCAGCACAGACACCCACACCGTCACCCACACTATCATGTTGACATTCC; dG = -34.7
2037 >s_c_s_e_s_a_s_c_s_d_s_c_s_e_s_e_s
GTGTGGGTGCTGGTGTCTGTGTGGGTGACGGTGTGGGTGCTGGTGCTGGTG; >clamp_s_c_s_d_s_c_s_a_s_e_s:compl:rev
CACCAGCACAGACACCCACACCGTCACCCACACTATCATGTTGACATTCC; dG = -34.7
2038 >s_c_s_e_s_a_s_c_s_d_s_c_s_d_s_e_s
GTGTGGGTGCTGGTGTCTGTGTGGGTGACGGTGTGGGTGACGGTGCTGGTG; >clamp_s_c_s_d_s_c_s_a_s_e_s:compl:rev
CACCAGCACAGACACCCACACCGTCACCCACACTATCATGTTGACATTCC; dG = -34.7
2039 >s_c_s_e_s_b_s_c_s_d_s_c_s_e_s_e_s
GTGTGGGTGCTGGTGGCTGTGTGGGTGACGGTGTGGGTGCTGGTGCTGGTG; >clamp_s_c_s_d_s_c_s_a_s_e_s:compl:rev
CACCAGCACAGACACCCACACCGTCACCCACACTATCATGTTGACATTCC; dG = -34.7
2040 >s_c_s_e_s_b_s_c_s_d_s_c_s_d_s_e_s
GTGTGGGTGCTGGTGGCTGTGTGGGTGACGGTGTGGGTGACGGTGCTGGTG; >clamp_s_c_s_d_s_c_s_a_s_e_s:compl:rev
CACCAGCACAGACACCCACACCGTCACCCACACTATCATGTTGACATTCC; dG = -34.7
2041 >s_d_s_a_s_a_s_c_s_d_s_c_s_e_s_e_s
GTGACGGTGTCTGTGTCTGTGTGGGTGACGGTGTGGGTGCTGGTGCTGGTG; >clamp_s_c_s_d_s_c_s_a_s_e_s:compl:rev
CACCAGCACAGACACCCACACCGTCACCCACACTATCATGTTGACATTCC; dG = -34.7
2042 >s_d_s_a_s_a_s_c_s_d_s_c_s_d_s_e_s
GTGACGGTGTCTGTGTCTGTGTGGGTGACGGTGTGGGTGACGGTGCTGGTG; >clamp_s_c_s_d_s_c_s_a_s_e_s:compl:rev
CACCAGCACAGACACCCACACCGTCACCCACACTATCATGTTGACATTCC; dG = -34.7
2043 >s_d_s_a_s_b_s_c_s_d_s_c_s_e_s_e_s
GTGACGGTGTCTGTGGCTGTGTGGGTGACGGTGTGGGTGCTGGTGCTGGTG; >clamp_s_c_s_d_s_c_s_a_s_e_s:compl:rev
CACCAGCACAGACACCCACACCGTCACCCACACTATCATGTTGACATTCC; dG = -34.7
2044 >s_d_s_a_s_b_s_c_s_d_s_c_s_d_s_e_s
GTGACGGTGTCTGTGGCTGTGTGGGTGACGGTGTGGGTGACGGTGCTGGTG; >clamp_s_c_s_d_s_c_s_a_s_e_s:compl:rev
CACCAGCACAGACACCCACACCGTCACCCACACTATCATGTTGACATTCC; dG = -34.7
2045 >s_d_s_b_s_a_s_c_s_d_s_c_s_e_s_e_s
GTGACGGTGGCTGTGTCTGTGTGGGTGACGGTGTGGGTGCTGGTGCTGGTG; >clamp_s_c_s_d_s_c_s_a_s_e_s:compl:rev
CACCAGCACAGACACCCACACCGTCACCCACACTATCATGTTGACATTCC; dG = -34.7
2046 >s_d_s_b_s_a_s_c_s_d_s_c_s_d_s_e_s
GTGACGGTGGCTGTGTCTGTGTGGGTGACGGTGTGGGTGACGGTGCTGGTG; >clamp_s_c_s_d_s_c_s_a_s_e_s:compl:rev
CACCAGCACAGACACCCACACCGTCACCCACACTATCATGTTGACATTCC; dG = -34.7
2047 >s_d_s_b_s_b_s_c_s_d_s_c_s_e_s_e_s
GTGACGGTGGCTGTGGCTGTGTGGGTGACGGTGTGGGTGCTGGTGCTGGTG; >clamp_s_c_s_d_s_c_s_a_s_e_s:compl:rev
CACCAGCACAGACACCCACACCGTCACCCACACTATCATGTTGACATTCC; dG = -34.7

2048 >s_d_s_b_s_b_s_c_s_d_s_c_s_d_s_e_s
GTGACGGTGGCTGTGGCTGTGTGGGTGACGGTGTGGGTGACGGTGCTGGTG; >clamp_s_c_s_d_s_c_s_a_s_e_s:compl:rev
CACCAGCACAGACACCCACACCGTCACCCACACTATCATGTTGACATTCC; dG = -34.7
2049 >s_d_s_c_s_a_s_c_s_d_s_c_s_e_s_e_s
GTGACGGTGTGGGTGTCTGTGTGGGTGACGGTGTGGGTGCTGGTGCTGGTG; >clamp_s_c_s_d_s_c_s_a_s_e_s:compl:rev
CACCAGCACAGACACCCACACCGTCACCCACACTATCATGTTGACATTCC; dG = -34.7
2050 >s_d_s_c_s_a_s_c_s_d_s_c_s_d_s_e_s
GTGACGGTGTGGGTGTCTGTGTGGGTGACGGTGTGGGTGACGGTGCTGGTG; >clamp_s_c_s_d_s_c_s_a_s_e_s:compl:rev
CACCAGCACAGACACCCACACCGTCACCCACACTATCATGTTGACATTCC; dG = -34.7
2051 >s_d_s_c_s_b_s_c_s_d_s_c_s_e_s_e_s
GTGACGGTGTGGGTGGCTGTGTGGGTGACGGTGTGGGTGCTGGTGCTGGTG; >clamp_s_c_s_d_s_c_s_a_s_e_s:compl:rev
CACCAGCACAGACACCCACACCGTCACCCACACTATCATGTTGACATTCC; dG = -34.7
2052 >s_d_s_c_s_b_s_c_s_d_s_c_s_d_s_e_s
GTGACGGTGTGGGTGGCTGTGTGGGTGACGGTGTGGGTGACGGTGCTGGTG; >clamp_s_c_s_d_s_c_s_a_s_e_s:compl:rev
CACCAGCACAGACACCCACACCGTCACCCACACTATCATGTTGACATTCC; dG = -34.7
2053 >s_d_s_d_s_a_s_c_s_d_s_c_s_e_s_e_s
GTGACGGTGACGGTGTCTGTGTGGGTGACGGTGTGGGTGCTGGTGCTGGTG; >clamp_s_c_s_d_s_c_s_a_s_e_s:compl:rev
CACCAGCACAGACACCCACACCGTCACCCACACTATCATGTTGACATTCC; dG = -34.7
2054 >s_d_s_d_s_a_s_c_s_d_s_c_s_d_s_e_s
GTGACGGTGACGGTGTCTGTGTGGGTGACGGTGTGGGTGACGGTGCTGGTG; >clamp_s_c_s_d_s_c_s_a_s_e_s:compl:rev
CACCAGCACAGACACCCACACCGTCACCCACACTATCATGTTGACATTCC; dG = -34.7
2055 >s_d_s_d_s_b_s_c_s_d_s_c_s_e_s_e_s
GTGACGGTGACGGTGGCTGTGTGGGTGACGGTGTGGGTGCTGGTGCTGGTG; >clamp_s_c_s_d_s_c_s_a_s_e_s:compl:rev
CACCAGCACAGACACCCACACCGTCACCCACACTATCATGTTGACATTCC; dG = -34.7
2056 >s_d_s_d_s_b_s_c_s_d_s_c_s_d_s_e_s
GTGACGGTGACGGTGGCTGTGTGGGTGACGGTGTGGGTGACGGTGCTGGTG; >clamp_s_c_s_d_s_c_s_a_s_e_s:compl:rev
CACCAGCACAGACACCCACACCGTCACCCACACTATCATGTTGACATTCC; dG = -34.7
2057 >s_d_s_e_s_a_s_c_s_d_s_c_s_e_s_e_s
GTGACGGTGCTGGTGTCTGTGTGGGTGACGGTGTGGGTGCTGGTGCTGGTG; >clamp_s_c_s_d_s_c_s_a_s_e_s:compl:rev
CACCAGCACAGACACCCACACCGTCACCCACACTATCATGTTGACATTCC; dG = -34.7
2058 >s_d_s_e_s_a_s_c_s_d_s_c_s_d_s_e_s
GTGACGGTGCTGGTGTCTGTGTGGGTGACGGTGTGGGTGACGGTGCTGGTG; >clamp_s_c_s_d_s_c_s_a_s_e_s:compl:rev
CACCAGCACAGACACCCACACCGTCACCCACACTATCATGTTGACATTCC; dG = -34.7
2059 >s_d_s_e_s_b_s_c_s_d_s_c_s_e_s_e_s
GTGACGGTGCTGGTGGCTGTGTGGGTGACGGTGTGGGTGCTGGTGCTGGTG; >clamp_s_c_s_d_s_c_s_a_s_e_s:compl:rev
CACCAGCACAGACACCCACACCGTCACCCACACTATCATGTTGACATTCC; dG = -34.7
2060 >s_d_s_e_s_b_s_c_s_d_s_c_s_d_s_e_s
GTGACGGTGCTGGTGGCTGTGTGGGTGACGGTGTGGGTGACGGTGCTGGTG; >clamp_s_c_s_d_s_c_s_a_s_e_s:compl:rev
CACCAGCACAGACACCCACACCGTCACCCACACTATCATGTTGACATTCC; dG = -34.7
2061 >s_e_s_a_s_a_s_c_s_d_s_c_s_e_s_e_s
GTGCTGGTGTCTGTGTCTGTGTGGGTGACGGTGTGGGTGCTGGTGCTGGTG; >clamp_s_c_s_d_s_c_s_a_s_e_s:compl:rev
CACCAGCACAGACACCCACACCGTCACCCACACTATCATGTTGACATTCC; dG = -34.7
2062 >s_e_s_a_s_a_s_c_s_d_s_c_s_d_s_e_s
GTGCTGGTGTCTGTGTCTGTGTGGGTGACGGTGTGGGTGACGGTGCTGGTG; >clamp_s_c_s_d_s_c_s_a_s_e_s:compl:rev
CACCAGCACAGACACCCACACCGTCACCCACACTATCATGTTGACATTCC; dG = -34.7
2063 >s_e_s_a_s_b_s_c_s_d_s_c_s_e_s_e_s
GTGCTGGTGTCTGTGGCTGTGTGGGTGACGGTGTGGGTGCTGGTGCTGGTG; >clamp_s_c_s_d_s_c_s_a_s_e_s:compl:rev
CACCAGCACAGACACCCACACCGTCACCCACACTATCATGTTGACATTCC; dG = -34.7
2064 >s_e_s_a_s_b_s_c_s_d_s_c_s_d_s_e_s
GTGCTGGTGTCTGTGGCTGTGTGGGTGACGGTGTGGGTGACGGTGCTGGTG; >clamp_s_c_s_d_s_c_s_a_s_e_s:compl:rev
CACCAGCACAGACACCCACACCGTCACCCACACTATCATGTTGACATTCC; dG = -34.7
2065 >s_a_s_c_s_a_s_c_s_d_s_c_s_e_s_e_s
GTGTCTGTGTGGGTGTCTGTGTGGGTGACGGTGTGGGTGCTGGTGCTGGTG; >clamp_s_c_s_d_s_c_s_a_s_e_s:compl:rev
CACCAGCACAGACACCCACACCGTCACCCACACTATCATGTTGACATTCC; dG = -34.7
2066 >s_a_s_c_s_a_s_c_s_d_s_c_s_d_s_e_s
GTGTCTGTGTGGGTGTCTGTGTGGGTGACGGTGTGGGTGACGGTGCTGGTG; >clamp_s_c_s_d_s_c_s_a_s_e_s:compl:rev
CACCAGCACAGACACCCACACCGTCACCCACACTATCATGTTGACATTCC; dG = -34.7
2067 >s_e_s_b_s_a_s_c_s_d_s_c_s_e_s_e_s
GTGCTGGTGGCTGTGTCTGTGTGGGTGACGGTGTGGGTGCTGGTGCTGGTG; >clamp_s_c_s_d_s_c_s_a_s_e_s:compl:rev
CACCAGCACAGACACCCACACCGTCACCCACACTATCATGTTGACATTCC; dG = -34.7
2068 >s_e_s_b_s_a_s_c_s_d_s_c_s_d_s_e_s
GTGCTGGTGGCTGTGTCTGTGTGGGTGACGGTGTGGGTGACGGTGCTGGTG; >clamp_s_c_s_d_s_c_s_a_s_e_s:compl:rev
CACCAGCACAGACACCCACACCGTCACCCACACTATCATGTTGACATTCC; dG = -34.7
2069 >s_e_s_b_s_b_s_c_s_d_s_c_s_e_s_e_s
GTGCTGGTGGCTGTGGCTGTGTGGGTGACGGTGTGGGTGCTGGTGCTGGTG; >clamp_s_c_s_d_s_c_s_a_s_e_s:compl:rev
CACCAGCACAGACACCCACACCGTCACCCACACTATCATGTTGACATTCC; dG = -34.7
2070 >s_e_s_b_s_b_s_c_s_d_s_c_s_d_s_e_s
GTGCTGGTGGCTGTGGCTGTGTGGGTGACGGTGTGGGTGACGGTGCTGGTG; >clamp_s_c_s_d_s_c_s_a_s_e_s:compl:rev
CACCAGCACAGACACCCACACCGTCACCCACACTATCATGTTGACATTCC; dG = -34.7
2071 >s_e_s_c_s_a_s_c_s_d_s_c_s_e_s_e_s
GTGCTGGTGTGGGTGTCTGTGTGGGTGACGGTGTGGGTGCTGGTGCTGGTG; >clamp_s_c_s_d_s_c_s_a_s_e_s:compl:rev
CACCAGCACAGACACCCACACCGTCACCCACACTATCATGTTGACATTCC; dG = -34.7

2072 >s_e_s_c_s_a_s_c_s_d_s_c_s_d_s_e_s
GTGCTGGTGTGGGTGTCTGTGTGGGTGACGGTGTGGGTGACGGTGCTGGTG; >clamp_s_c_s_d_s_c_s_a_s_e_s:compl:rev
CACCAGCACAGACACCCACACCGTCACCCACACTATCATGTTGACATTCC; dG = -34.7
2073 >s_e_s_c_s_b_s_c_s_d_s_c_s_e_s_e_s
GTGCTGGTGTGGGTGGCTGTGTGGGTGACGGTGTGGGTGCTGGTGCTGGTG; >clamp_s_c_s_d_s_c_s_a_s_e_s:compl:rev
CACCAGCACAGACACCCACACCGTCACCCACACTATCATGTTGACATTCC; dG = -34.7
2074 >s_e_s_c_s_b_s_c_s_d_s_c_s_d_s_e_s
GTGCTGGTGTGGGTGGCTGTGTGGGTGACGGTGTGGGTGACGGTGCTGGTG; >clamp_s_c_s_d_s_c_s_a_s_e_s:compl:rev
CACCAGCACAGACACCCACACCGTCACCCACACTATCATGTTGACATTCC; dG = -34.7
2075 >s_a_s_c_s_b_s_c_s_d_s_c_s_e_s_e_s
GTGTCTGTGTGGGTGGCTGTGTGGGTGACGGTGTGGGTGCTGGTGCTGGTG; >clamp_s_c_s_d_s_c_s_a_s_e_s:compl:rev
CACCAGCACAGACACCCACACCGTCACCCACACTATCATGTTGACATTCC; dG = -34.7
2076 >s_a_s_c_s_b_s_c_s_d_s_c_s_d_s_e_s
GTGTCTGTGTGGGTGGCTGTGTGGGTGACGGTGTGGGTGACGGTGCTGGTG; >clamp_s_c_s_d_s_c_s_a_s_e_s:compl:rev
CACCAGCACAGACACCCACACCGTCACCCACACTATCATGTTGACATTCC; dG = -34.7
2077 >s_e_s_d_s_a_s_c_s_d_s_c_s_e_s_e_s
GTGCTGGTGACGGTGTCTGTGTGGGTGACGGTGTGGGTGCTGGTGCTGGTG; >clamp_s_c_s_d_s_c_s_a_s_e_s:compl:rev
CACCAGCACAGACACCCACACCGTCACCCACACTATCATGTTGACATTCC; dG = -34.7
2078 >s_e_s_d_s_a_s_c_s_d_s_c_s_d_s_e_s
GTGCTGGTGACGGTGTCTGTGTGGGTGACGGTGTGGGTGACGGTGCTGGTG; >clamp_s_c_s_d_s_c_s_a_s_e_s:compl:rev
CACCAGCACAGACACCCACACCGTCACCCACACTATCATGTTGACATTCC; dG = -34.7
2079 >s_e_s_d_s_b_s_c_s_d_s_c_s_e_s_e_s
GTGCTGGTGACGGTGGCTGTGTGGGTGACGGTGTGGGTGCTGGTGCTGGTG; >clamp_s_c_s_d_s_c_s_a_s_e_s:compl:rev
CACCAGCACAGACACCCACACCGTCACCCACACTATCATGTTGACATTCC; dG = -34.7
2080 >s_e_s_d_s_b_s_c_s_d_s_c_s_d_s_e_s
GTGCTGGTGACGGTGGCTGTGTGGGTGACGGTGTGGGTGACGGTGCTGGTG; >clamp_s_c_s_d_s_c_s_a_s_e_s:compl:rev
CACCAGCACAGACACCCACACCGTCACCCACACTATCATGTTGACATTCC; dG = -34.7
2081 >s_e_s_e_s_a_s_c_s_d_s_c_s_e_s_e_s
GTGCTGGTGCTGGTGTCTGTGTGGGTGACGGTGTGGGTGCTGGTGCTGGTG; >clamp_s_c_s_d_s_c_s_a_s_e_s:compl:rev
CACCAGCACAGACACCCACACCGTCACCCACACTATCATGTTGACATTCC; dG = -34.7
2082 >s_e_s_e_s_a_s_c_s_d_s_c_s_d_s_e_s
GTGCTGGTGCTGGTGTCTGTGTGGGTGACGGTGTGGGTGACGGTGCTGGTG; >clamp_s_c_s_d_s_c_s_a_s_e_s:compl:rev
CACCAGCACAGACACCCACACCGTCACCCACACTATCATGTTGACATTCC; dG = -34.7
2083 >s_e_s_e_s_b_s_c_s_d_s_c_s_e_s_e_s
GTGCTGGTGCTGGTGGCTGTGTGGGTGACGGTGTGGGTGCTGGTGCTGGTG; >clamp_s_c_s_d_s_c_s_a_s_e_s:compl:rev
CACCAGCACAGACACCCACACCGTCACCCACACTATCATGTTGACATTCC; dG = -34.7
2084 >s_e_s_e_s_b_s_c_s_d_s_c_s_d_s_e_s
GTGCTGGTGCTGGTGGCTGTGTGGGTGACGGTGTGGGTGACGGTGCTGGTG; >clamp_s_c_s_d_s_c_s_a_s_e_s:compl:rev
CACCAGCACAGACACCCACACCGTCACCCACACTATCATGTTGACATTCC; dG = -34.7
2085 >s_a_s_a_s_b_s_c_s_d_s_c_s_e_s_e_s
GTGTCTGTGTCTGTGGCTGTGTGGGTGACGGTGTGGGTGCTGGTGCTGGTG; >clamp_s_c_s_d_s_c_s_a_s_e_s:compl:rev
CACCAGCACAGACACCCACACCGTCACCCACACTATCATGTTGACATTCC; dG = -34.7
2086 >s_a_s_a_s_b_s_c_s_d_s_c_s_d_s_e_s
GTGTCTGTGTCTGTGGCTGTGTGGGTGACGGTGTGGGTGACGGTGCTGGTG; >clamp_s_c_s_d_s_c_s_a_s_e_s:compl:rev
CACCAGCACAGACACCCACACCGTCACCCACACTATCATGTTGACATTCC; dG = -34.7
2087 >s_a_s_d_s_a_s_c_s_d_s_c_s_e_s_e_s
GTGTCTGTGACGGTGTCTGTGTGGGTGACGGTGTGGGTGCTGGTGCTGGTG; >clamp_s_c_s_d_s_c_s_a_s_e_s:compl:rev
CACCAGCACAGACACCCACACCGTCACCCACACTATCATGTTGACATTCC; dG = -34.7
2088 >s_a_s_d_s_a_s_c_s_d_s_c_s_d_s_e_s
GTGTCTGTGACGGTGTCTGTGTGGGTGACGGTGTGGGTGACGGTGCTGGTG; >clamp_s_c_s_d_s_c_s_a_s_e_s:compl:rev
CACCAGCACAGACACCCACACCGTCACCCACACTATCATGTTGACATTCC; dG = -34.7
2089 >s_a_s_d_s_b_s_c_s_d_s_c_s_e_s_e_s
GTGTCTGTGACGGTGGCTGTGTGGGTGACGGTGTGGGTGCTGGTGCTGGTG; >clamp_s_c_s_d_s_c_s_a_s_e_s:compl:rev
CACCAGCACAGACACCCACACCGTCACCCACACTATCATGTTGACATTCC; dG = -34.7
2090 >s_a_s_d_s_b_s_c_s_d_s_c_s_d_s_e_s
GTGTCTGTGACGGTGGCTGTGTGGGTGACGGTGTGGGTGACGGTGCTGGTG; >clamp_s_c_s_d_s_c_s_a_s_e_s:compl:rev
CACCAGCACAGACACCCACACCGTCACCCACACTATCATGTTGACATTCC; dG = -34.7
2091 >s_a_s_e_s_a_s_c_s_d_s_c_s_e_s_e_s
GTGTCTGTGCTGGTGTCTGTGTGGGTGACGGTGTGGGTGCTGGTGCTGGTG; >clamp_s_c_s_d_s_c_s_a_s_e_s:compl:rev
CACCAGCACAGACACCCACACCGTCACCCACACTATCATGTTGACATTCC; dG = -34.7
2092 >s_a_s_e_s_a_s_c_s_d_s_c_s_d_s_e_s
GTGTCTGTGCTGGTGTCTGTGTGGGTGACGGTGTGGGTGACGGTGCTGGTG; >clamp_s_c_s_d_s_c_s_a_s_e_s:compl:rev
CACCAGCACAGACACCCACACCGTCACCCACACTATCATGTTGACATTCC; dG = -34.7
2093 >s_a_s_e_s_b_s_c_s_d_s_c_s_e_s_e_s
GTGTCTGTGCTGGTGGCTGTGTGGGTGACGGTGTGGGTGCTGGTGCTGGTG; >clamp_s_c_s_d_s_c_s_a_s_e_s:compl:rev
CACCAGCACAGACACCCACACCGTCACCCACACTATCATGTTGACATTCC; dG = -34.7
2094 >s_a_s_e_s_b_s_c_s_d_s_c_s_d_s_e_s
GTGTCTGTGCTGGTGGCTGTGTGGGTGACGGTGTGGGTGACGGTGCTGGTG; >clamp_s_c_s_d_s_c_s_a_s_e_s:compl:rev
CACCAGCACAGACACCCACACCGTCACCCACACTATCATGTTGACATTCC; dG = -34.7
2095 >s_a_s_a_s_a_s_c_s_d_s_c_s_e_s_e_s
GTGTCTGTGTCTGTGTCTGTGTGGGTGACGGTGTGGGTGCTGGTGCTGGTG; >clamp_s_c_s_d_s_c_s_a_s_e_s:compl:rev
CACCAGCACAGACACCCACACCGTCACCCACACTATCATGTTGACATTCC; dG = -34.7

2096 >s_b_s_a_s_a_s_c_s_d_s_c_s_e_s_e_s
GTGGCTGTGTCTGTGTCTGTGTGGGTGACGGTGTGGGTGCTGGTGCTGGTG; >clamp_s_c_s_d_s_c_s_a_s_e_s:compl:rev
CACCAGCACAGACACCCACACCGTCACCCACACTATCATGTTGACATTCC; dG = -34.7
2097 >s_b_s_a_s_a_s_c_s_d_s_c_s_d_s_e_s
GTGGCTGTGTCTGTGTCTGTGTGGGTGACGGTGTGGGTGACGGTGCTGGTG; >clamp_s_c_s_d_s_c_s_a_s_e_s:compl:rev
CACCAGCACAGACACCCACACCGTCACCCACACTATCATGTTGACATTCC; dG = -34.7
2098 >s_b_s_a_s_b_s_c_s_d_s_c_s_e_s_e_s
GTGGCTGTGTCTGTGGCTGTGTGGGTGACGGTGTGGGTGCTGGTGCTGGTG; >clamp_s_c_s_d_s_c_s_a_s_e_s:compl:rev
CACCAGCACAGACACCCACACCGTCACCCACACTATCATGTTGACATTCC; dG = -34.7
2099 >s_b_s_a_s_b_s_c_s_d_s_c_s_d_s_e_s
GTGGCTGTGTCTGTGGCTGTGTGGGTGACGGTGTGGGTGACGGTGCTGGTG; >clamp_s_c_s_d_s_c_s_a_s_e_s:compl:rev
CACCAGCACAGACACCCACACCGTCACCCACACTATCATGTTGACATTCC; dG = -34.7
2100 >s_a_s_a_s_a_s_c_s_d_s_c_s_d_s_e_s
GTGTCTGTGTCTGTGTCTGTGTGGGTGACGGTGTGGGTGACGGTGCTGGTG; >clamp_s_c_s_d_s_c_s_a_s_e_s:compl:rev
CACCAGCACAGACACCCACACCGTCACCCACACTATCATGTTGACATTCC; dG = -34.7
2101 >s_b_s_b_s_a_s_c_s_d_s_c_s_e_s_e_s
GTGGCTGTGGCTGTGTCTGTGTGGGTGACGGTGTGGGTGCTGGTGCTGGTG; >clamp_s_c_s_d_s_c_s_a_s_e_s:compl:rev
CACCAGCACAGACACCCACACCGTCACCCACACTATCATGTTGACATTCC; dG = -34.7
2102 >s_b_s_b_s_a_s_c_s_d_s_c_s_d_s_e_s
GTGGCTGTGGCTGTGTCTGTGTGGGTGACGGTGTGGGTGACGGTGCTGGTG; >clamp_s_c_s_d_s_c_s_a_s_e_s:compl:rev
CACCAGCACAGACACCCACACCGTCACCCACACTATCATGTTGACATTCC; dG = -34.7
2103 >s_b_s_b_s_b_s_c_s_d_s_c_s_e_s_e_s
GTGGCTGTGGCTGTGGCTGTGTGGGTGACGGTGTGGGTGCTGGTGCTGGTG; >clamp_s_c_s_d_s_c_s_a_s_e_s:compl:rev
CACCAGCACAGACACCCACACCGTCACCCACACTATCATGTTGACATTCC; dG = -34.7
2104 >s_b_s_b_s_b_s_c_s_d_s_c_s_d_s_e_s
GTGGCTGTGGCTGTGGCTGTGTGGGTGACGGTGTGGGTGACGGTGCTGGTG; >clamp_s_c_s_d_s_c_s_a_s_e_s:compl:rev
CACCAGCACAGACACCCACACCGTCACCCACACTATCATGTTGACATTCC; dG = -34.7
2105 >s_b_s_c_s_a_s_c_s_d_s_c_s_e_s_e_s
GTGGCTGTGTGGGTGTCTGTGTGGGTGACGGTGTGGGTGCTGGTGCTGGTG; >clamp_s_c_s_d_s_c_s_a_s_e_s:compl:rev
CACCAGCACAGACACCCACACCGTCACCCACACTATCATGTTGACATTCC; dG = -34.7
2106 >s_b_s_c_s_a_s_c_s_d_s_c_s_d_s_e_s
GTGGCTGTGTGGGTGTCTGTGTGGGTGACGGTGTGGGTGACGGTGCTGGTG; >clamp_s_c_s_d_s_c_s_a_s_e_s:compl:rev
CACCAGCACAGACACCCACACCGTCACCCACACTATCATGTTGACATTCC; dG = -34.7
2107 >s_b_s_c_s_b_s_c_s_d_s_c_s_e_s_e_s
GTGGCTGTGTGGGTGGCTGTGTGGGTGACGGTGTGGGTGCTGGTGCTGGTG; >clamp_s_c_s_d_s_c_s_a_s_e_s:compl:rev
CACCAGCACAGACACCCACACCGTCACCCACACTATCATGTTGACATTCC; dG = -34.7
2108 >s_b_s_c_s_b_s_c_s_d_s_c_s_d_s_e_s
GTGGCTGTGTGGGTGGCTGTGTGGGTGACGGTGTGGGTGACGGTGCTGGTG; >clamp_s_c_s_d_s_c_s_a_s_e_s:compl:rev
CACCAGCACAGACACCCACACCGTCACCCACACTATCATGTTGACATTCC; dG = -34.7
2109 >s_b_s_d_s_a_s_c_s_d_s_c_s_e_s_e_s
GTGGCTGTGACGGTGTCTGTGTGGGTGACGGTGTGGGTGCTGGTGCTGGTG; >clamp_s_c_s_d_s_c_s_a_s_e_s:compl:rev
CACCAGCACAGACACCCACACCGTCACCCACACTATCATGTTGACATTCC; dG = -34.7
2110 >s_b_s_d_s_a_s_c_s_d_s_c_s_d_s_e_s
GTGGCTGTGACGGTGTCTGTGTGGGTGACGGTGTGGGTGACGGTGCTGGTG; >clamp_s_c_s_d_s_c_s_a_s_e_s:compl:rev
CACCAGCACAGACACCCACACCGTCACCCACACTATCATGTTGACATTCC; dG = -34.7
2111 >s_b_s_d_s_b_s_c_s_d_s_c_s_e_s_e_s
GTGGCTGTGACGGTGGCTGTGTGGGTGACGGTGTGGGTGCTGGTGCTGGTG; >clamp_s_c_s_d_s_c_s_a_s_e_s:compl:rev
CACCAGCACAGACACCCACACCGTCACCCACACTATCATGTTGACATTCC; dG = -34.7
2112 >s_b_s_d_s_b_s_c_s_d_s_c_s_d_s_e_s
GTGGCTGTGACGGTGGCTGTGTGGGTGACGGTGTGGGTGACGGTGCTGGTG; >clamp_s_c_s_d_s_c_s_a_s_e_s:compl:rev
CACCAGCACAGACACCCACACCGTCACCCACACTATCATGTTGACATTCC; dG = -34.7
2113 >s_b_s_e_s_a_s_c_s_d_s_c_s_e_s_e_s
GTGGCTGTGCTGGTGTCTGTGTGGGTGACGGTGTGGGTGCTGGTGCTGGTG; >clamp_s_c_s_d_s_c_s_a_s_e_s:compl:rev
CACCAGCACAGACACCCACACCGTCACCCACACTATCATGTTGACATTCC; dG = -34.7
2114 >s_b_s_e_s_a_s_c_s_d_s_c_s_d_s_e_s
GTGGCTGTGCTGGTGTCTGTGTGGGTGACGGTGTGGGTGACGGTGCTGGTG; >clamp_s_c_s_d_s_c_s_a_s_e_s:compl:rev
CACCAGCACAGACACCCACACCGTCACCCACACTATCATGTTGACATTCC; dG = -34.7
2115 >s_b_s_e_s_b_s_c_s_d_s_c_s_e_s_e_s
GTGGCTGTGCTGGTGGCTGTGTGGGTGACGGTGTGGGTGCTGGTGCTGGTG; >clamp_s_c_s_d_s_c_s_a_s_e_s:compl:rev
CACCAGCACAGACACCCACACCGTCACCCACACTATCATGTTGACATTCC; dG = -34.7
2116 >s_b_s_e_s_b_s_c_s_d_s_c_s_d_s_e_s
GTGGCTGTGCTGGTGGCTGTGTGGGTGACGGTGTGGGTGACGGTGCTGGTG; >clamp_s_c_s_d_s_c_s_a_s_e_s:compl:rev
CACCAGCACAGACACCCACACCGTCACCCACACTATCATGTTGACATTCC; dG = -34.7
2117 >s_c_s_a_s_c_s_c_s_d_s_c_s_e_s_e_s
GTGTGGGTGTCTGTGTGGGTGTGGGTGACGGTGTGGGTGCTGGTGCTGGTG; >clamp_s_c_s_d_s_c_s_a_s_e_s:compl:rev
CACCAGCACAGACACCCACACCGTCACCCACACTATCATGTTGACATTCC; dG = -34.6
2118 >s_c_s_a_s_c_s_c_s_d_s_c_s_d_s_e_s
GTGTGGGTGTCTGTGTGGGTGTGGGTGACGGTGTGGGTGACGGTGCTGGTG; >clamp_s_c_s_d_s_c_s_a_s_e_s:compl:rev
CACCAGCACAGACACCCACACCGTCACCCACACTATCATGTTGACATTCC; dG = -34.6
2119 >s_c_s_a_s_d_s_c_s_d_s_c_s_e_s_e_s
GTGTGGGTGTCTGTGACGGTGTGGGTGACGGTGTGGGTGCTGGTGCTGGTG; >clamp_s_c_s_d_s_c_s_a_s_e_s:compl:rev
CACCAGCACAGACACCCACACCGTCACCCACACTATCATGTTGACATTCC; dG = -34.6

2120 >s_c_s_a_s_d_s_c_s_d_s_c_s_d_s_e_s
GTGTGGGTGTCTGTGACGGTGTGGGTGACGGTGTGGGTGACGGTGCTGGTG; >clamp_s_c_s_d_s_c_s_a_s_e_s:compl:rev
CACCAGCACAGACACCCACACCGTCACCCACACTATCATGTTGACATTCC; dG = -34.6
2121 >s_c_s_a_s_e_s_c_s_d_s_c_s_e_s_e_s
GTGTGGGTGTCTGTGCTGGTGTGGGTGACGGTGTGGGTGCTGGTGCTGGTG; >clamp_s_c_s_d_s_c_s_a_s_e_s:compl:rev
CACCAGCACAGACACCCACACCGTCACCCACACTATCATGTTGACATTCC; dG = -34.6
2122 >s_c_s_a_s_e_s_c_s_d_s_c_s_d_s_e_s
GTGTGGGTGTCTGTGCTGGTGTGGGTGACGGTGTGGGTGACGGTGCTGGTG; >clamp_s_c_s_d_s_c_s_a_s_e_s:compl:rev
CACCAGCACAGACACCCACACCGTCACCCACACTATCATGTTGACATTCC; dG = -34.6
2123 >s_c_s_b_s_c_s_c_s_d_s_c_s_e_s_e_s
GTGTGGGTGGCTGTGTGGGTGTGGGTGACGGTGTGGGTGCTGGTGCTGGTG; >clamp_s_c_s_d_s_c_s_a_s_e_s:compl:rev
CACCAGCACAGACACCCACACCGTCACCCACACTATCATGTTGACATTCC; dG = -34.6
2124 >s_c_s_b_s_c_s_c_s_d_s_c_s_d_s_e_s
GTGTGGGTGGCTGTGTGGGTGTGGGTGACGGTGTGGGTGACGGTGCTGGTG; >clamp_s_c_s_d_s_c_s_a_s_e_s:compl:rev
CACCAGCACAGACACCCACACCGTCACCCACACTATCATGTTGACATTCC; dG = -34.6
2125 >s_c_s_b_s_d_s_c_s_d_s_c_s_e_s_e_s
GTGTGGGTGGCTGTGACGGTGTGGGTGACGGTGTGGGTGCTGGTGCTGGTG; >clamp_s_c_s_d_s_c_s_a_s_e_s:compl:rev
CACCAGCACAGACACCCACACCGTCACCCACACTATCATGTTGACATTCC; dG = -34.6
2126 >s_c_s_b_s_d_s_c_s_d_s_c_s_d_s_e_s
GTGTGGGTGGCTGTGACGGTGTGGGTGACGGTGTGGGTGACGGTGCTGGTG; >clamp_s_c_s_d_s_c_s_a_s_e_s:compl:rev
CACCAGCACAGACACCCACACCGTCACCCACACTATCATGTTGACATTCC; dG = -34.6
2127 >s_c_s_b_s_e_s_c_s_d_s_c_s_e_s_e_s
GTGTGGGTGGCTGTGCTGGTGTGGGTGACGGTGTGGGTGCTGGTGCTGGTG; >clamp_s_c_s_d_s_c_s_a_s_e_s:compl:rev
CACCAGCACAGACACCCACACCGTCACCCACACTATCATGTTGACATTCC; dG = -34.6
2128 >s_c_s_b_s_e_s_c_s_d_s_c_s_d_s_e_s
GTGTGGGTGGCTGTGCTGGTGTGGGTGACGGTGTGGGTGACGGTGCTGGTG; >clamp_s_c_s_d_s_c_s_a_s_e_s:compl:rev
CACCAGCACAGACACCCACACCGTCACCCACACTATCATGTTGACATTCC; dG = -34.6
2129 >s_c_s_c_s_c_s_c_s_d_s_c_s_e_s_e_s
GTGTGGGTGTGGGTGTGGGTGTGGGTGACGGTGTGGGTGCTGGTGCTGGTG; >clamp_s_c_s_d_s_c_s_a_s_e_s:compl:rev
CACCAGCACAGACACCCACACCGTCACCCACACTATCATGTTGACATTCC; dG = -34.6
2130 >s_c_s_c_s_c_s_c_s_d_s_c_s_d_s_e_s
GTGTGGGTGTGGGTGTGGGTGTGGGTGACGGTGTGGGTGACGGTGCTGGTG; >clamp_s_c_s_d_s_c_s_a_s_e_s:compl:rev
CACCAGCACAGACACCCACACCGTCACCCACACTATCATGTTGACATTCC; dG = -34.6
2131 >s_c_s_c_s_d_s_c_s_d_s_c_s_e_s_e_s
GTGTGGGTGTGGGTGACGGTGTGGGTGACGGTGTGGGTGCTGGTGCTGGTG; >clamp_s_c_s_d_s_c_s_a_s_e_s:compl:rev
CACCAGCACAGACACCCACACCGTCACCCACACTATCATGTTGACATTCC; dG = -34.6
2132 >s_c_s_c_s_d_s_c_s_d_s_c_s_d_s_e_s
GTGTGGGTGTGGGTGACGGTGTGGGTGACGGTGTGGGTGACGGTGCTGGTG; >clamp_s_c_s_d_s_c_s_a_s_e_s:compl:rev
CACCAGCACAGACACCCACACCGTCACCCACACTATCATGTTGACATTCC; dG = -34.6
2133 >s_c_s_c_s_e_s_c_s_d_s_c_s_e_s_e_s
GTGTGGGTGTGGGTGCTGGTGTGGGTGACGGTGTGGGTGCTGGTGCTGGTG; >clamp_s_c_s_d_s_c_s_a_s_e_s:compl:rev
CACCAGCACAGACACCCACACCGTCACCCACACTATCATGTTGACATTCC; dG = -34.6
2134 >s_c_s_c_s_e_s_c_s_d_s_c_s_d_s_e_s
GTGTGGGTGTGGGTGCTGGTGTGGGTGACGGTGTGGGTGACGGTGCTGGTG; >clamp_s_c_s_d_s_c_s_a_s_e_s:compl:rev
CACCAGCACAGACACCCACACCGTCACCCACACTATCATGTTGACATTCC; dG = -34.6
2135 >s_c_s_d_s_c_s_c_s_d_s_c_s_e_s_e_s
GTGTGGGTGACGGTGTGGGTGTGGGTGACGGTGTGGGTGCTGGTGCTGGTG; >clamp_s_c_s_d_s_c_s_a_s_e_s:compl:rev
CACCAGCACAGACACCCACACCGTCACCCACACTATCATGTTGACATTCC; dG = -34.6
2136 >s_c_s_d_s_c_s_c_s_d_s_c_s_d_s_e_s
GTGTGGGTGACGGTGTGGGTGTGGGTGACGGTGTGGGTGACGGTGCTGGTG; >clamp_s_c_s_d_s_c_s_a_s_e_s:compl:rev
CACCAGCACAGACACCCACACCGTCACCCACACTATCATGTTGACATTCC; dG = -34.6
2137 >s_c_s_d_s_d_s_c_s_d_s_c_s_e_s_e_s
GTGTGGGTGACGGTGACGGTGTGGGTGACGGTGTGGGTGCTGGTGCTGGTG; >clamp_s_c_s_d_s_c_s_a_s_e_s:compl:rev
CACCAGCACAGACACCCACACCGTCACCCACACTATCATGTTGACATTCC; dG = -34.6
2138 >s_c_s_d_s_d_s_c_s_d_s_c_s_d_s_e_s
GTGTGGGTGACGGTGACGGTGTGGGTGACGGTGTGGGTGACGGTGCTGGTG; >clamp_s_c_s_d_s_c_s_a_s_e_s:compl:rev
CACCAGCACAGACACCCACACCGTCACCCACACTATCATGTTGACATTCC; dG = -34.6
2139 >s_c_s_d_s_e_s_c_s_d_s_c_s_e_s_e_s
GTGTGGGTGACGGTGCTGGTGTGGGTGACGGTGTGGGTGCTGGTGCTGGTG; >clamp_s_c_s_d_s_c_s_a_s_e_s:compl:rev
CACCAGCACAGACACCCACACCGTCACCCACACTATCATGTTGACATTCC; dG = -34.6
2140 >s_c_s_d_s_e_s_c_s_d_s_c_s_d_s_e_s
GTGTGGGTGACGGTGCTGGTGTGGGTGACGGTGTGGGTGACGGTGCTGGTG; >clamp_s_c_s_d_s_c_s_a_s_e_s:compl:rev
CACCAGCACAGACACCCACACCGTCACCCACACTATCATGTTGACATTCC; dG = -34.6
2141 >s_c_s_e_s_c_s_c_s_d_s_c_s_e_s_e_s
GTGTGGGTGCTGGTGTGGGTGTGGGTGACGGTGTGGGTGCTGGTGCTGGTG; >clamp_s_c_s_d_s_c_s_a_s_e_s:compl:rev
CACCAGCACAGACACCCACACCGTCACCCACACTATCATGTTGACATTCC; dG = -34.6
2142 >s_c_s_e_s_c_s_c_s_d_s_c_s_d_s_e_s
GTGTGGGTGCTGGTGTGGGTGTGGGTGACGGTGTGGGTGACGGTGCTGGTG; >clamp_s_c_s_d_s_c_s_a_s_e_s:compl:rev
CACCAGCACAGACACCCACACCGTCACCCACACTATCATGTTGACATTCC; dG = -34.6
2143 >s_a_s_b_s_c_s_c_s_d_s_c_s_e_s_e_s
GTGTCTGTGGCTGTGTGGGTGTGGGTGACGGTGTGGGTGCTGGTGCTGGTG; >clamp_s_c_s_d_s_c_s_a_s_e_s:compl:rev
CACCAGCACAGACACCCACACCGTCACCCACACTATCATGTTGACATTCC; dG = -34.6

2144 >s_a_s_b_s_c_s_c_s_d_s_c_s_d_s_e_s
GTGTCTGTGGCTGTGTGGGTGTGGGTGACGGTGTGGGTGACGGTGCTGGTG; >clamp_s_c_s_d_s_c_s_a_s_e_s:compl:rev
CACCAGCACAGACACCCACACCGTCACCCACACTATCATGTTGACATTCC; dG = -34.6
2145 >s_c_s_e_s_d_s_c_s_d_s_c_s_e_s_e_s
GTGTGGGTGCTGGTGACGGTGTGGGTGACGGTGTGGGTGCTGGTGCTGGTG; >clamp_s_c_s_d_s_c_s_a_s_e_s:compl:rev
CACCAGCACAGACACCCACACCGTCACCCACACTATCATGTTGACATTCC; dG = -34.6
2146 >s_c_s_e_s_d_s_c_s_d_s_c_s_d_s_e_s
GTGTGGGTGCTGGTGACGGTGTGGGTGACGGTGTGGGTGACGGTGCTGGTG; >clamp_s_c_s_d_s_c_s_a_s_e_s:compl:rev
CACCAGCACAGACACCCACACCGTCACCCACACTATCATGTTGACATTCC; dG = -34.6
2147 >s_c_s_e_s_e_s_c_s_d_s_c_s_e_s_e_s
GTGTGGGTGCTGGTGCTGGTGTGGGTGACGGTGTGGGTGCTGGTGCTGGTG; >clamp_s_c_s_d_s_c_s_a_s_e_s:compl:rev
CACCAGCACAGACACCCACACCGTCACCCACACTATCATGTTGACATTCC; dG = -34.6
2148 >s_c_s_e_s_e_s_c_s_d_s_c_s_d_s_e_s
GTGTGGGTGCTGGTGCTGGTGTGGGTGACGGTGTGGGTGACGGTGCTGGTG; >clamp_s_c_s_d_s_c_s_a_s_e_s:compl:rev
CACCAGCACAGACACCCACACCGTCACCCACACTATCATGTTGACATTCC; dG = -34.6
2149 >s_d_s_a_s_c_s_c_s_d_s_c_s_e_s_e_s
GTGACGGTGTCTGTGTGGGTGTGGGTGACGGTGTGGGTGCTGGTGCTGGTG; >clamp_s_c_s_d_s_c_s_a_s_e_s:compl:rev
CACCAGCACAGACACCCACACCGTCACCCACACTATCATGTTGACATTCC; dG = -34.6
2150 >s_d_s_a_s_c_s_c_s_d_s_c_s_d_s_e_s
GTGACGGTGTCTGTGTGGGTGTGGGTGACGGTGTGGGTGACGGTGCTGGTG; >clamp_s_c_s_d_s_c_s_a_s_e_s:compl:rev
CACCAGCACAGACACCCACACCGTCACCCACACTATCATGTTGACATTCC; dG = -34.6
2151 >s_d_s_a_s_d_s_c_s_d_s_c_s_e_s_e_s
GTGACGGTGTCTGTGACGGTGTGGGTGACGGTGTGGGTGCTGGTGCTGGTG; >clamp_s_c_s_d_s_c_s_a_s_e_s:compl:rev
CACCAGCACAGACACCCACACCGTCACCCACACTATCATGTTGACATTCC; dG = -34.6
2152 >s_d_s_a_s_d_s_c_s_d_s_c_s_d_s_e_s
GTGACGGTGTCTGTGACGGTGTGGGTGACGGTGTGGGTGACGGTGCTGGTG; >clamp_s_c_s_d_s_c_s_a_s_e_s:compl:rev
CACCAGCACAGACACCCACACCGTCACCCACACTATCATGTTGACATTCC; dG = -34.6
2153 >s_d_s_a_s_e_s_c_s_d_s_c_s_e_s_e_s
GTGACGGTGTCTGTGCTGGTGTGGGTGACGGTGTGGGTGCTGGTGCTGGTG; >clamp_s_c_s_d_s_c_s_a_s_e_s:compl:rev
CACCAGCACAGACACCCACACCGTCACCCACACTATCATGTTGACATTCC; dG = -34.6
2154 >s_d_s_a_s_e_s_c_s_d_s_c_s_d_s_e_s
GTGACGGTGTCTGTGCTGGTGTGGGTGACGGTGTGGGTGACGGTGCTGGTG; >clamp_s_c_s_d_s_c_s_a_s_e_s:compl:rev
CACCAGCACAGACACCCACACCGTCACCCACACTATCATGTTGACATTCC; dG = -34.6
2155 >s_d_s_b_s_c_s_c_s_d_s_c_s_e_s_e_s
GTGACGGTGGCTGTGTGGGTGTGGGTGACGGTGTGGGTGCTGGTGCTGGTG; >clamp_s_c_s_d_s_c_s_a_s_e_s:compl:rev
CACCAGCACAGACACCCACACCGTCACCCACACTATCATGTTGACATTCC; dG = -34.6
2156 >s_d_s_b_s_c_s_c_s_d_s_c_s_d_s_e_s
GTGACGGTGGCTGTGTGGGTGTGGGTGACGGTGTGGGTGACGGTGCTGGTG; >clamp_s_c_s_d_s_c_s_a_s_e_s:compl:rev
CACCAGCACAGACACCCACACCGTCACCCACACTATCATGTTGACATTCC; dG = -34.6
2157 >s_a_s_b_s_d_s_c_s_d_s_c_s_e_s_e_s
GTGTCTGTGGCTGTGACGGTGTGGGTGACGGTGTGGGTGCTGGTGCTGGTG; >clamp_s_c_s_d_s_c_s_a_s_e_s:compl:rev
CACCAGCACAGACACCCACACCGTCACCCACACTATCATGTTGACATTCC; dG = -34.6
2158 >s_a_s_b_s_d_s_c_s_d_s_c_s_d_s_e_s
GTGTCTGTGGCTGTGACGGTGTGGGTGACGGTGTGGGTGACGGTGCTGGTG; >clamp_s_c_s_d_s_c_s_a_s_e_s:compl:rev
CACCAGCACAGACACCCACACCGTCACCCACACTATCATGTTGACATTCC; dG = -34.6
2159 >s_d_s_b_s_d_s_c_s_d_s_c_s_e_s_e_s
GTGACGGTGGCTGTGACGGTGTGGGTGACGGTGTGGGTGCTGGTGCTGGTG; >clamp_s_c_s_d_s_c_s_a_s_e_s:compl:rev
CACCAGCACAGACACCCACACCGTCACCCACACTATCATGTTGACATTCC; dG = -34.6
2160 >s_d_s_b_s_d_s_c_s_d_s_c_s_d_s_e_s
GTGACGGTGGCTGTGACGGTGTGGGTGACGGTGTGGGTGACGGTGCTGGTG; >clamp_s_c_s_d_s_c_s_a_s_e_s:compl:rev
CACCAGCACAGACACCCACACCGTCACCCACACTATCATGTTGACATTCC; dG = -34.6
2161 >s_d_s_b_s_e_s_c_s_d_s_c_s_e_s_e_s
GTGACGGTGGCTGTGCTGGTGTGGGTGACGGTGTGGGTGCTGGTGCTGGTG; >clamp_s_c_s_d_s_c_s_a_s_e_s:compl:rev
CACCAGCACAGACACCCACACCGTCACCCACACTATCATGTTGACATTCC; dG = -34.6
2162 >s_d_s_b_s_e_s_c_s_d_s_c_s_d_s_e_s
GTGACGGTGGCTGTGCTGGTGTGGGTGACGGTGTGGGTGACGGTGCTGGTG; >clamp_s_c_s_d_s_c_s_a_s_e_s:compl:rev
CACCAGCACAGACACCCACACCGTCACCCACACTATCATGTTGACATTCC; dG = -34.6
2163 >s_d_s_c_s_c_s_c_s_d_s_c_s_e_s_e_s
GTGACGGTGTGGGTGTGGGTGTGGGTGACGGTGTGGGTGCTGGTGCTGGTG; >clamp_s_c_s_d_s_c_s_a_s_e_s:compl:rev
CACCAGCACAGACACCCACACCGTCACCCACACTATCATGTTGACATTCC; dG = -34.6
2164 >s_d_s_c_s_c_s_c_s_d_s_c_s_d_s_e_s
GTGACGGTGTGGGTGTGGGTGTGGGTGACGGTGTGGGTGACGGTGCTGGTG; >clamp_s_c_s_d_s_c_s_a_s_e_s:compl:rev
CACCAGCACAGACACCCACACCGTCACCCACACTATCATGTTGACATTCC; dG = -34.6
2165 >s_d_s_c_s_d_s_c_s_d_s_c_s_e_s_e_s
GTGACGGTGTGGGTGACGGTGTGGGTGACGGTGTGGGTGCTGGTGCTGGTG; >clamp_s_c_s_d_s_c_s_a_s_e_s:compl:rev
CACCAGCACAGACACCCACACCGTCACCCACACTATCATGTTGACATTCC; dG = -34.6
2166 >s_d_s_c_s_d_s_c_s_d_s_c_s_d_s_e_s
GTGACGGTGTGGGTGACGGTGTGGGTGACGGTGTGGGTGACGGTGCTGGTG; >clamp_s_c_s_d_s_c_s_a_s_e_s:compl:rev
CACCAGCACAGACACCCACACCGTCACCCACACTATCATGTTGACATTCC; dG = -34.6
2167 >s_d_s_c_s_e_s_c_s_d_s_c_s_e_s_e_s
GTGACGGTGTGGGTGCTGGTGTGGGTGACGGTGTGGGTGCTGGTGCTGGTG; >clamp_s_c_s_d_s_c_s_a_s_e_s:compl:rev
CACCAGCACAGACACCCACACCGTCACCCACACTATCATGTTGACATTCC; dG = -34.6

2168 >s_d_s_c_s_e_s_c_s_d_s_c_s_d_s_e_s
GTGACGGTGTGGGTGCTGGTGTGGGTGACGGTGTGGGTGACGGTGCTGGTG; >clamp_s_c_s_d_s_c_s_a_s_e_s:compl:rev
CACCAGCACAGACACCCACACCGTCACCCACACTATCATGTTGACATTCC; dG = -34.6
2169 >s_d_s_d_s_c_s_c_s_d_s_c_s_e_s_e_s
GTGACGGTGACGGTGTGGGTGTGGGTGACGGTGTGGGTGCTGGTGCTGGTG; >clamp_s_c_s_d_s_c_s_a_s_e_s:compl:rev
CACCAGCACAGACACCCACACCGTCACCCACACTATCATGTTGACATTCC; dG = -34.6
2170 >s_d_s_d_s_c_s_c_s_d_s_c_s_d_s_e_s
GTGACGGTGACGGTGTGGGTGTGGGTGACGGTGTGGGTGACGGTGCTGGTG; >clamp_s_c_s_d_s_c_s_a_s_e_s:compl:rev
CACCAGCACAGACACCCACACCGTCACCCACACTATCATGTTGACATTCC; dG = -34.6
2171 >s_a_s_b_s_e_s_c_s_d_s_c_s_e_s_e_s
GTGTCTGTGGCTGTGCTGGTGTGGGTGACGGTGTGGGTGCTGGTGCTGGTG; >clamp_s_c_s_d_s_c_s_a_s_e_s:compl:rev
CACCAGCACAGACACCCACACCGTCACCCACACTATCATGTTGACATTCC; dG = -34.6
2172 >s_a_s_b_s_e_s_c_s_d_s_c_s_d_s_e_s
GTGTCTGTGGCTGTGCTGGTGTGGGTGACGGTGTGGGTGACGGTGCTGGTG; >clamp_s_c_s_d_s_c_s_a_s_e_s:compl:rev
CACCAGCACAGACACCCACACCGTCACCCACACTATCATGTTGACATTCC; dG = -34.6
2173 >s_d_s_d_s_d_s_c_s_d_s_c_s_e_s_e_s
GTGACGGTGACGGTGACGGTGTGGGTGACGGTGTGGGTGCTGGTGCTGGTG; >clamp_s_c_s_d_s_c_s_a_s_e_s:compl:rev
CACCAGCACAGACACCCACACCGTCACCCACACTATCATGTTGACATTCC; dG = -34.6
2174 >s_d_s_d_s_d_s_c_s_d_s_c_s_d_s_e_s
GTGACGGTGACGGTGACGGTGTGGGTGACGGTGTGGGTGACGGTGCTGGTG; >clamp_s_c_s_d_s_c_s_a_s_e_s:compl:rev
CACCAGCACAGACACCCACACCGTCACCCACACTATCATGTTGACATTCC; dG = -34.6
2175 >s_d_s_d_s_e_s_c_s_d_s_c_s_e_s_e_s
GTGACGGTGACGGTGCTGGTGTGGGTGACGGTGTGGGTGCTGGTGCTGGTG; >clamp_s_c_s_d_s_c_s_a_s_e_s:compl:rev
CACCAGCACAGACACCCACACCGTCACCCACACTATCATGTTGACATTCC; dG = -34.6
2176 >s_d_s_d_s_e_s_c_s_d_s_c_s_d_s_e_s
GTGACGGTGACGGTGCTGGTGTGGGTGACGGTGTGGGTGACGGTGCTGGTG; >clamp_s_c_s_d_s_c_s_a_s_e_s:compl:rev
CACCAGCACAGACACCCACACCGTCACCCACACTATCATGTTGACATTCC; dG = -34.6
2177 >s_d_s_e_s_c_s_c_s_d_s_c_s_e_s_e_s
GTGACGGTGCTGGTGTGGGTGTGGGTGACGGTGTGGGTGCTGGTGCTGGTG; >clamp_s_c_s_d_s_c_s_a_s_e_s:compl:rev
CACCAGCACAGACACCCACACCGTCACCCACACTATCATGTTGACATTCC; dG = -34.6
2178 >s_d_s_e_s_c_s_c_s_d_s_c_s_d_s_e_s
GTGACGGTGCTGGTGTGGGTGTGGGTGACGGTGTGGGTGACGGTGCTGGTG; >clamp_s_c_s_d_s_c_s_a_s_e_s:compl:rev
CACCAGCACAGACACCCACACCGTCACCCACACTATCATGTTGACATTCC; dG = -34.6
2179 >s_d_s_e_s_d_s_c_s_d_s_c_s_e_s_e_s
GTGACGGTGCTGGTGACGGTGTGGGTGACGGTGTGGGTGCTGGTGCTGGTG; >clamp_s_c_s_d_s_c_s_a_s_e_s:compl:rev
CACCAGCACAGACACCCACACCGTCACCCACACTATCATGTTGACATTCC; dG = -34.6
2180 >s_d_s_e_s_d_s_c_s_d_s_c_s_d_s_e_s
GTGACGGTGCTGGTGACGGTGTGGGTGACGGTGTGGGTGACGGTGCTGGTG; >clamp_s_c_s_d_s_c_s_a_s_e_s:compl:rev
CACCAGCACAGACACCCACACCGTCACCCACACTATCATGTTGACATTCC; dG = -34.6
2181 >s_d_s_e_s_e_s_c_s_d_s_c_s_e_s_e_s
GTGACGGTGCTGGTGCTGGTGTGGGTGACGGTGTGGGTGCTGGTGCTGGTG; >clamp_s_c_s_d_s_c_s_a_s_e_s:compl:rev
CACCAGCACAGACACCCACACCGTCACCCACACTATCATGTTGACATTCC; dG = -34.6
2182 >s_d_s_e_s_e_s_c_s_d_s_c_s_d_s_e_s
GTGACGGTGCTGGTGCTGGTGTGGGTGACGGTGTGGGTGACGGTGCTGGTG; >clamp_s_c_s_d_s_c_s_a_s_e_s:compl:rev
CACCAGCACAGACACCCACACCGTCACCCACACTATCATGTTGACATTCC; dG = -34.6
2183 >s_e_s_a_s_c_s_c_s_d_s_c_s_e_s_e_s
GTGCTGGTGTCTGTGTGGGTGTGGGTGACGGTGTGGGTGCTGGTGCTGGTG; >clamp_s_c_s_d_s_c_s_a_s_e_s:compl:rev
CACCAGCACAGACACCCACACCGTCACCCACACTATCATGTTGACATTCC; dG = -34.6
2184 >s_e_s_a_s_c_s_c_s_d_s_c_s_d_s_e_s
GTGCTGGTGTCTGTGTGGGTGTGGGTGACGGTGTGGGTGACGGTGCTGGTG; >clamp_s_c_s_d_s_c_s_a_s_e_s:compl:rev
CACCAGCACAGACACCCACACCGTCACCCACACTATCATGTTGACATTCC; dG = -34.6
2185 >s_e_s_a_s_d_s_c_s_d_s_c_s_e_s_e_s
GTGCTGGTGTCTGTGACGGTGTGGGTGACGGTGTGGGTGCTGGTGCTGGTG; >clamp_s_c_s_d_s_c_s_a_s_e_s:compl:rev
CACCAGCACAGACACCCACACCGTCACCCACACTATCATGTTGACATTCC; dG = -34.6
2186 >s_e_s_a_s_d_s_c_s_d_s_c_s_d_s_e_s
GTGCTGGTGTCTGTGACGGTGTGGGTGACGGTGTGGGTGACGGTGCTGGTG; >clamp_s_c_s_d_s_c_s_a_s_e_s:compl:rev
CACCAGCACAGACACCCACACCGTCACCCACACTATCATGTTGACATTCC; dG = -34.6
2187 >s_e_s_a_s_e_s_c_s_d_s_c_s_e_s_e_s
GTGCTGGTGTCTGTGCTGGTGTGGGTGACGGTGTGGGTGCTGGTGCTGGTG; >clamp_s_c_s_d_s_c_s_a_s_e_s:compl:rev
CACCAGCACAGACACCCACACCGTCACCCACACTATCATGTTGACATTCC; dG = -34.6
2188 >s_e_s_a_s_e_s_c_s_d_s_c_s_d_s_e_s
GTGCTGGTGTCTGTGCTGGTGTGGGTGACGGTGTGGGTGACGGTGCTGGTG; >clamp_s_c_s_d_s_c_s_a_s_e_s:compl:rev
CACCAGCACAGACACCCACACCGTCACCCACACTATCATGTTGACATTCC; dG = -34.6
2189 >s_e_s_b_s_c_s_c_s_d_s_c_s_e_s_e_s
GTGCTGGTGGCTGTGTGGGTGTGGGTGACGGTGTGGGTGCTGGTGCTGGTG; >clamp_s_c_s_d_s_c_s_a_s_e_s:compl:rev
CACCAGCACAGACACCCACACCGTCACCCACACTATCATGTTGACATTCC; dG = -34.6
2190 >s_e_s_b_s_c_s_c_s_d_s_c_s_d_s_e_s
GTGCTGGTGGCTGTGTGGGTGTGGGTGACGGTGTGGGTGACGGTGCTGGTG; >clamp_s_c_s_d_s_c_s_a_s_e_s:compl:rev
CACCAGCACAGACACCCACACCGTCACCCACACTATCATGTTGACATTCC; dG = -34.6
2191 >s_e_s_b_s_d_s_c_s_d_s_c_s_e_s_e_s
GTGCTGGTGGCTGTGACGGTGTGGGTGACGGTGTGGGTGCTGGTGCTGGTG; >clamp_s_c_s_d_s_c_s_a_s_e_s:compl:rev
CACCAGCACAGACACCCACACCGTCACCCACACTATCATGTTGACATTCC; dG = -34.6

2192 >s_e_s_b_s_d_s_c_s_d_s_c_s_d_s_e_s
GTGCTGGTGGCTGTGACGGTGTGGGTGACGGTGTGGGTGACGGTGCTGGTG; >clamp_s_c_s_d_s_c_s_a_s_e_s:compl:rev
CACCAGCACAGACACCCACACCGTCACCCACACTATCATGTTGACATTCC; dG = -34.6
2193 >s_e_s_b_s_e_s_c_s_d_s_c_s_e_s_e_s
GTGCTGGTGGCTGTGCTGGTGTGGGTGACGGTGTGGGTGCTGGTGCTGGTG; >clamp_s_c_s_d_s_c_s_a_s_e_s:compl:rev
CACCAGCACAGACACCCACACCGTCACCCACACTATCATGTTGACATTCC; dG = -34.6
2194 >s_e_s_b_s_e_s_c_s_d_s_c_s_d_s_e_s
GTGCTGGTGGCTGTGCTGGTGTGGGTGACGGTGTGGGTGACGGTGCTGGTG; >clamp_s_c_s_d_s_c_s_a_s_e_s:compl:rev
CACCAGCACAGACACCCACACCGTCACCCACACTATCATGTTGACATTCC; dG = -34.6
2195 >s_e_s_c_s_c_s_c_s_d_s_c_s_e_s_e_s
GTGCTGGTGTGGGTGTGGGTGTGGGTGACGGTGTGGGTGCTGGTGCTGGTG; >clamp_s_c_s_d_s_c_s_a_s_e_s:compl:rev
CACCAGCACAGACACCCACACCGTCACCCACACTATCATGTTGACATTCC; dG = -34.6
2196 >s_e_s_c_s_c_s_c_s_d_s_c_s_d_s_e_s
GTGCTGGTGTGGGTGTGGGTGTGGGTGACGGTGTGGGTGACGGTGCTGGTG; >clamp_s_c_s_d_s_c_s_a_s_e_s:compl:rev
CACCAGCACAGACACCCACACCGTCACCCACACTATCATGTTGACATTCC; dG = -34.6
2197 >s_e_s_c_s_d_s_c_s_d_s_c_s_e_s_e_s
GTGCTGGTGTGGGTGACGGTGTGGGTGACGGTGTGGGTGCTGGTGCTGGTG; >clamp_s_c_s_d_s_c_s_a_s_e_s:compl:rev
CACCAGCACAGACACCCACACCGTCACCCACACTATCATGTTGACATTCC; dG = -34.6
2198 >s_e_s_c_s_d_s_c_s_d_s_c_s_d_s_e_s
GTGCTGGTGTGGGTGACGGTGTGGGTGACGGTGTGGGTGACGGTGCTGGTG; >clamp_s_c_s_d_s_c_s_a_s_e_s:compl:rev
CACCAGCACAGACACCCACACCGTCACCCACACTATCATGTTGACATTCC; dG = -34.6
2199 >s_e_s_c_s_e_s_c_s_d_s_c_s_e_s_e_s
GTGCTGGTGTGGGTGCTGGTGTGGGTGACGGTGTGGGTGCTGGTGCTGGTG; >clamp_s_c_s_d_s_c_s_a_s_e_s:compl:rev
CACCAGCACAGACACCCACACCGTCACCCACACTATCATGTTGACATTCC; dG = -34.6
2200 >s_e_s_c_s_e_s_c_s_d_s_c_s_d_s_e_s
GTGCTGGTGTGGGTGCTGGTGTGGGTGACGGTGTGGGTGACGGTGCTGGTG; >clamp_s_c_s_d_s_c_s_a_s_e_s:compl:rev
CACCAGCACAGACACCCACACCGTCACCCACACTATCATGTTGACATTCC; dG = -34.6
2201 >s_e_s_d_s_c_s_c_s_d_s_c_s_e_s_e_s
GTGCTGGTGACGGTGTGGGTGTGGGTGACGGTGTGGGTGCTGGTGCTGGTG; >clamp_s_c_s_d_s_c_s_a_s_e_s:compl:rev
CACCAGCACAGACACCCACACCGTCACCCACACTATCATGTTGACATTCC; dG = -34.6
2202 >s_e_s_d_s_c_s_c_s_d_s_c_s_d_s_e_s
GTGCTGGTGACGGTGTGGGTGTGGGTGACGGTGTGGGTGACGGTGCTGGTG; >clamp_s_c_s_d_s_c_s_a_s_e_s:compl:rev
CACCAGCACAGACACCCACACCGTCACCCACACTATCATGTTGACATTCC; dG = -34.6
2203 >s_e_s_d_s_d_s_c_s_d_s_c_s_e_s_e_s
GTGCTGGTGACGGTGACGGTGTGGGTGACGGTGTGGGTGCTGGTGCTGGTG; >clamp_s_c_s_d_s_c_s_a_s_e_s:compl:rev
CACCAGCACAGACACCCACACCGTCACCCACACTATCATGTTGACATTCC; dG = -34.6
2204 >s_e_s_d_s_d_s_c_s_d_s_c_s_d_s_e_s
GTGCTGGTGACGGTGACGGTGTGGGTGACGGTGTGGGTGACGGTGCTGGTG; >clamp_s_c_s_d_s_c_s_a_s_e_s:compl:rev
CACCAGCACAGACACCCACACCGTCACCCACACTATCATGTTGACATTCC; dG = -34.6
2205 >s_e_s_d_s_e_s_c_s_d_s_c_s_e_s_e_s
GTGCTGGTGACGGTGCTGGTGTGGGTGACGGTGTGGGTGCTGGTGCTGGTG; >clamp_s_c_s_d_s_c_s_a_s_e_s:compl:rev
CACCAGCACAGACACCCACACCGTCACCCACACTATCATGTTGACATTCC; dG = -34.6
2206 >s_e_s_d_s_e_s_c_s_d_s_c_s_d_s_e_s
GTGCTGGTGACGGTGCTGGTGTGGGTGACGGTGTGGGTGACGGTGCTGGTG; >clamp_s_c_s_d_s_c_s_a_s_e_s:compl:rev
CACCAGCACAGACACCCACACCGTCACCCACACTATCATGTTGACATTCC; dG = -34.6
2207 >s_e_s_e_s_c_s_c_s_d_s_c_s_e_s_e_s
GTGCTGGTGCTGGTGTGGGTGTGGGTGACGGTGTGGGTGCTGGTGCTGGTG; >clamp_s_c_s_d_s_c_s_a_s_e_s:compl:rev
CACCAGCACAGACACCCACACCGTCACCCACACTATCATGTTGACATTCC; dG = -34.6
2208 >s_e_s_e_s_c_s_c_s_d_s_c_s_d_s_e_s
GTGCTGGTGCTGGTGTGGGTGTGGGTGACGGTGTGGGTGACGGTGCTGGTG; >clamp_s_c_s_d_s_c_s_a_s_e_s:compl:rev
CACCAGCACAGACACCCACACCGTCACCCACACTATCATGTTGACATTCC; dG = -34.6
2209 >s_a_s_c_s_c_s_c_s_d_s_c_s_e_s_e_s
GTGTCTGTGTGGGTGTGGGTGTGGGTGACGGTGTGGGTGCTGGTGCTGGTG; >clamp_s_c_s_d_s_c_s_a_s_e_s:compl:rev
CACCAGCACAGACACCCACACCGTCACCCACACTATCATGTTGACATTCC; dG = -34.6
2210 >s_a_s_c_s_c_s_c_s_d_s_c_s_d_s_e_s
GTGTCTGTGTGGGTGTGGGTGTGGGTGACGGTGTGGGTGACGGTGCTGGTG; >clamp_s_c_s_d_s_c_s_a_s_e_s:compl:rev
CACCAGCACAGACACCCACACCGTCACCCACACTATCATGTTGACATTCC; dG = -34.6
2211 >s_e_s_e_s_d_s_c_s_d_s_c_s_e_s_e_s
GTGCTGGTGCTGGTGACGGTGTGGGTGACGGTGTGGGTGCTGGTGCTGGTG; >clamp_s_c_s_d_s_c_s_a_s_e_s:compl:rev
CACCAGCACAGACACCCACACCGTCACCCACACTATCATGTTGACATTCC; dG = -34.6
2212 >s_e_s_e_s_d_s_c_s_d_s_c_s_d_s_e_s
GTGCTGGTGCTGGTGACGGTGTGGGTGACGGTGTGGGTGACGGTGCTGGTG; >clamp_s_c_s_d_s_c_s_a_s_e_s:compl:rev
CACCAGCACAGACACCCACACCGTCACCCACACTATCATGTTGACATTCC; dG = -34.6
2213 >s_e_s_e_s_e_s_c_s_d_s_c_s_e_s_e_s
GTGCTGGTGCTGGTGCTGGTGTGGGTGACGGTGTGGGTGCTGGTGCTGGTG; >clamp_s_c_s_d_s_c_s_a_s_e_s:compl:rev
CACCAGCACAGACACCCACACCGTCACCCACACTATCATGTTGACATTCC; dG = -34.6
2214 >s_e_s_e_s_e_s_c_s_d_s_c_s_d_s_e_s
GTGCTGGTGCTGGTGCTGGTGTGGGTGACGGTGTGGGTGACGGTGCTGGTG; >clamp_s_c_s_d_s_c_s_a_s_e_s:compl:rev
CACCAGCACAGACACCCACACCGTCACCCACACTATCATGTTGACATTCC; dG = -34.6
2215 >s_a_s_c_s_d_s_c_s_d_s_c_s_e_s_e_s
GTGTCTGTGTGGGTGACGGTGTGGGTGACGGTGTGGGTGCTGGTGCTGGTG; >clamp_s_c_s_d_s_c_s_a_s_e_s:compl:rev
CACCAGCACAGACACCCACACCGTCACCCACACTATCATGTTGACATTCC; dG = -34.6

2216 >s_a_s_c_s_d_s_c_s_d_s_c_s_d_s_e_s
GTGTCTGTGTGGGTGACGGTGTGGGTGACGGTGTGGGTGACGGTGCTGGTG; >clamp_s_c_s_d_s_c_s_a_s_e_s:compl:rev
CACCAGCACAGACACCCACACCGTCACCCACACTATCATGTTGACATTCC; dG = -34.6
2217 >s_a_s_c_s_e_s_c_s_d_s_c_s_e_s_e_s
GTGTCTGTGTGGGTGCTGGTGTGGGTGACGGTGTGGGTGCTGGTGCTGGTG; >clamp_s_c_s_d_s_c_s_a_s_e_s:compl:rev
CACCAGCACAGACACCCACACCGTCACCCACACTATCATGTTGACATTCC; dG = -34.6
2218 >s_a_s_c_s_e_s_c_s_d_s_c_s_d_s_e_s
GTGTCTGTGTGGGTGCTGGTGTGGGTGACGGTGTGGGTGACGGTGCTGGTG; >clamp_s_c_s_d_s_c_s_a_s_e_s:compl:rev
CACCAGCACAGACACCCACACCGTCACCCACACTATCATGTTGACATTCC; dG = -34.6
2219 >s_a_s_d_s_c_s_c_s_d_s_c_s_e_s_e_s
GTGTCTGTGACGGTGTGGGTGTGGGTGACGGTGTGGGTGCTGGTGCTGGTG; >clamp_s_c_s_d_s_c_s_a_s_e_s:compl:rev
CACCAGCACAGACACCCACACCGTCACCCACACTATCATGTTGACATTCC; dG = -34.6
2220 >s_a_s_d_s_c_s_c_s_d_s_c_s_d_s_e_s
GTGTCTGTGACGGTGTGGGTGTGGGTGACGGTGTGGGTGACGGTGCTGGTG; >clamp_s_c_s_d_s_c_s_a_s_e_s:compl:rev
CACCAGCACAGACACCCACACCGTCACCCACACTATCATGTTGACATTCC; dG = -34.6
2221 >s_a_s_d_s_d_s_c_s_d_s_c_s_e_s_e_s
GTGTCTGTGACGGTGACGGTGTGGGTGACGGTGTGGGTGCTGGTGCTGGTG; >clamp_s_c_s_d_s_c_s_a_s_e_s:compl:rev
CACCAGCACAGACACCCACACCGTCACCCACACTATCATGTTGACATTCC; dG = -34.6
2222 >s_a_s_d_s_d_s_c_s_d_s_c_s_d_s_e_s
GTGTCTGTGACGGTGACGGTGTGGGTGACGGTGTGGGTGACGGTGCTGGTG; >clamp_s_c_s_d_s_c_s_a_s_e_s:compl:rev
CACCAGCACAGACACCCACACCGTCACCCACACTATCATGTTGACATTCC; dG = -34.6
2223 >s_a_s_d_s_e_s_c_s_d_s_c_s_e_s_e_s
GTGTCTGTGACGGTGCTGGTGTGGGTGACGGTGTGGGTGCTGGTGCTGGTG; >clamp_s_c_s_d_s_c_s_a_s_e_s:compl:rev
CACCAGCACAGACACCCACACCGTCACCCACACTATCATGTTGACATTCC; dG = -34.6
2224 >s_a_s_d_s_e_s_c_s_d_s_c_s_d_s_e_s
GTGTCTGTGACGGTGCTGGTGTGGGTGACGGTGTGGGTGACGGTGCTGGTG; >clamp_s_c_s_d_s_c_s_a_s_e_s:compl:rev
CACCAGCACAGACACCCACACCGTCACCCACACTATCATGTTGACATTCC; dG = -34.6
2225 >s_a_s_e_s_c_s_c_s_d_s_c_s_e_s_e_s
GTGTCTGTGCTGGTGTGGGTGTGGGTGACGGTGTGGGTGCTGGTGCTGGTG; >clamp_s_c_s_d_s_c_s_a_s_e_s:compl:rev
CACCAGCACAGACACCCACACCGTCACCCACACTATCATGTTGACATTCC; dG = -34.6
2226 >s_a_s_e_s_c_s_c_s_d_s_c_s_d_s_e_s
GTGTCTGTGCTGGTGTGGGTGTGGGTGACGGTGTGGGTGACGGTGCTGGTG; >clamp_s_c_s_d_s_c_s_a_s_e_s:compl:rev
CACCAGCACAGACACCCACACCGTCACCCACACTATCATGTTGACATTCC; dG = -34.6
2227 >s_a_s_a_s_c_s_c_s_d_s_c_s_e_s_e_s
GTGTCTGTGTCTGTGTGGGTGTGGGTGACGGTGTGGGTGCTGGTGCTGGTG; >clamp_s_c_s_d_s_c_s_a_s_e_s:compl:rev
CACCAGCACAGACACCCACACCGTCACCCACACTATCATGTTGACATTCC; dG = -34.6
2228 >s_a_s_a_s_c_s_c_s_d_s_c_s_d_s_e_s
GTGTCTGTGTCTGTGTGGGTGTGGGTGACGGTGTGGGTGACGGTGCTGGTG; >clamp_s_c_s_d_s_c_s_a_s_e_s:compl:rev
CACCAGCACAGACACCCACACCGTCACCCACACTATCATGTTGACATTCC; dG = -34.6
2229 >s_a_s_e_s_d_s_c_s_d_s_c_s_e_s_e_s
GTGTCTGTGCTGGTGACGGTGTGGGTGACGGTGTGGGTGCTGGTGCTGGTG; >clamp_s_c_s_d_s_c_s_a_s_e_s:compl:rev
CACCAGCACAGACACCCACACCGTCACCCACACTATCATGTTGACATTCC; dG = -34.6
2230 >s_a_s_e_s_d_s_c_s_d_s_c_s_d_s_e_s
GTGTCTGTGCTGGTGACGGTGTGGGTGACGGTGTGGGTGACGGTGCTGGTG; >clamp_s_c_s_d_s_c_s_a_s_e_s:compl:rev
CACCAGCACAGACACCCACACCGTCACCCACACTATCATGTTGACATTCC; dG = -34.6
2231 >s_a_s_e_s_e_s_c_s_d_s_c_s_e_s_e_s
GTGTCTGTGCTGGTGCTGGTGTGGGTGACGGTGTGGGTGCTGGTGCTGGTG; >clamp_s_c_s_d_s_c_s_a_s_e_s:compl:rev
CACCAGCACAGACACCCACACCGTCACCCACACTATCATGTTGACATTCC; dG = -34.6
2232 >s_a_s_e_s_e_s_c_s_d_s_c_s_d_s_e_s
GTGTCTGTGCTGGTGCTGGTGTGGGTGACGGTGTGGGTGACGGTGCTGGTG; >clamp_s_c_s_d_s_c_s_a_s_e_s:compl:rev
CACCAGCACAGACACCCACACCGTCACCCACACTATCATGTTGACATTCC; dG = -34.6
2233 >s_b_s_a_s_c_s_c_s_d_s_c_s_e_s_e_s
GTGGCTGTGTCTGTGTGGGTGTGGGTGACGGTGTGGGTGCTGGTGCTGGTG; >clamp_s_c_s_d_s_c_s_a_s_e_s:compl:rev
CACCAGCACAGACACCCACACCGTCACCCACACTATCATGTTGACATTCC; dG = -34.6
2234 >s_b_s_a_s_c_s_c_s_d_s_c_s_d_s_e_s
GTGGCTGTGTCTGTGTGGGTGTGGGTGACGGTGTGGGTGACGGTGCTGGTG; >clamp_s_c_s_d_s_c_s_a_s_e_s:compl:rev
CACCAGCACAGACACCCACACCGTCACCCACACTATCATGTTGACATTCC; dG = -34.6
2235 >s_b_s_a_s_d_s_c_s_d_s_c_s_e_s_e_s
GTGGCTGTGTCTGTGACGGTGTGGGTGACGGTGTGGGTGCTGGTGCTGGTG; >clamp_s_c_s_d_s_c_s_a_s_e_s:compl:rev
CACCAGCACAGACACCCACACCGTCACCCACACTATCATGTTGACATTCC; dG = -34.6
2236 >s_b_s_a_s_d_s_c_s_d_s_c_s_d_s_e_s
GTGGCTGTGTCTGTGACGGTGTGGGTGACGGTGTGGGTGACGGTGCTGGTG; >clamp_s_c_s_d_s_c_s_a_s_e_s:compl:rev
CACCAGCACAGACACCCACACCGTCACCCACACTATCATGTTGACATTCC; dG = -34.6
2237 >s_b_s_a_s_e_s_c_s_d_s_c_s_e_s_e_s
GTGGCTGTGTCTGTGCTGGTGTGGGTGACGGTGTGGGTGCTGGTGCTGGTG; >clamp_s_c_s_d_s_c_s_a_s_e_s:compl:rev
CACCAGCACAGACACCCACACCGTCACCCACACTATCATGTTGACATTCC; dG = -34.6
2238 >s_b_s_a_s_e_s_c_s_d_s_c_s_d_s_e_s
GTGGCTGTGTCTGTGCTGGTGTGGGTGACGGTGTGGGTGACGGTGCTGGTG; >clamp_s_c_s_d_s_c_s_a_s_e_s:compl:rev
CACCAGCACAGACACCCACACCGTCACCCACACTATCATGTTGACATTCC; dG = -34.6
2239 >s_b_s_b_s_c_s_c_s_d_s_c_s_e_s_e_s
GTGGCTGTGGCTGTGTGGGTGTGGGTGACGGTGTGGGTGCTGGTGCTGGTG; >clamp_s_c_s_d_s_c_s_a_s_e_s:compl:rev
CACCAGCACAGACACCCACACCGTCACCCACACTATCATGTTGACATTCC; dG = -34.6

2240 >s_b_s_b_s_c_s_c_s_d_s_c_s_d_s_e_s
GTGGCTGTGGCTGTGTGGGTGTGGGTGACGGTGTGGGTGACGGTGCTGGTG; >clamp_s_c_s_d_s_c_s_a_s_e_s:compl:rev
CACCAGCACAGACACCCACACCGTCACCCACACTATCATGTTGACATTCC; dG = -34.6
2241 >s_a_s_a_s_d_s_c_s_d_s_c_s_e_s_e_s
GTGTCTGTGTCTGTGACGGTGTGGGTGACGGTGTGGGTGCTGGTGCTGGTG; >clamp_s_c_s_d_s_c_s_a_s_e_s:compl:rev
CACCAGCACAGACACCCACACCGTCACCCACACTATCATGTTGACATTCC; dG = -34.6
2242 >s_a_s_a_s_d_s_c_s_d_s_c_s_d_s_e_s
GTGTCTGTGTCTGTGACGGTGTGGGTGACGGTGTGGGTGACGGTGCTGGTG; >clamp_s_c_s_d_s_c_s_a_s_e_s:compl:rev
CACCAGCACAGACACCCACACCGTCACCCACACTATCATGTTGACATTCC; dG = -34.6
2243 >s_b_s_b_s_d_s_c_s_d_s_c_s_e_s_e_s
GTGGCTGTGGCTGTGACGGTGTGGGTGACGGTGTGGGTGCTGGTGCTGGTG; >clamp_s_c_s_d_s_c_s_a_s_e_s:compl:rev
CACCAGCACAGACACCCACACCGTCACCCACACTATCATGTTGACATTCC; dG = -34.6
2244 >s_b_s_b_s_d_s_c_s_d_s_c_s_d_s_e_s
GTGGCTGTGGCTGTGACGGTGTGGGTGACGGTGTGGGTGACGGTGCTGGTG; >clamp_s_c_s_d_s_c_s_a_s_e_s:compl:rev
CACCAGCACAGACACCCACACCGTCACCCACACTATCATGTTGACATTCC; dG = -34.6
2245 >s_b_s_b_s_e_s_c_s_d_s_c_s_e_s_e_s
GTGGCTGTGGCTGTGCTGGTGTGGGTGACGGTGTGGGTGCTGGTGCTGGTG; >clamp_s_c_s_d_s_c_s_a_s_e_s:compl:rev
CACCAGCACAGACACCCACACCGTCACCCACACTATCATGTTGACATTCC; dG = -34.6
2246 >s_b_s_b_s_e_s_c_s_d_s_c_s_d_s_e_s
GTGGCTGTGGCTGTGCTGGTGTGGGTGACGGTGTGGGTGACGGTGCTGGTG; >clamp_s_c_s_d_s_c_s_a_s_e_s:compl:rev
CACCAGCACAGACACCCACACCGTCACCCACACTATCATGTTGACATTCC; dG = -34.6
2247 >s_b_s_c_s_c_s_c_s_d_s_c_s_e_s_e_s
GTGGCTGTGTGGGTGTGGGTGTGGGTGACGGTGTGGGTGCTGGTGCTGGTG; >clamp_s_c_s_d_s_c_s_a_s_e_s:compl:rev
CACCAGCACAGACACCCACACCGTCACCCACACTATCATGTTGACATTCC; dG = -34.6
2248 >s_b_s_c_s_c_s_c_s_d_s_c_s_d_s_e_s
GTGGCTGTGTGGGTGTGGGTGTGGGTGACGGTGTGGGTGACGGTGCTGGTG; >clamp_s_c_s_d_s_c_s_a_s_e_s:compl:rev
CACCAGCACAGACACCCACACCGTCACCCACACTATCATGTTGACATTCC; dG = -34.6
2249 >s_b_s_c_s_d_s_c_s_d_s_c_s_e_s_e_s
GTGGCTGTGTGGGTGACGGTGTGGGTGACGGTGTGGGTGCTGGTGCTGGTG; >clamp_s_c_s_d_s_c_s_a_s_e_s:compl:rev
CACCAGCACAGACACCCACACCGTCACCCACACTATCATGTTGACATTCC; dG = -34.6
2250 >s_b_s_c_s_d_s_c_s_d_s_c_s_d_s_e_s
GTGGCTGTGTGGGTGACGGTGTGGGTGACGGTGTGGGTGACGGTGCTGGTG; >clamp_s_c_s_d_s_c_s_a_s_e_s:compl:rev
CACCAGCACAGACACCCACACCGTCACCCACACTATCATGTTGACATTCC; dG = -34.6
2251 >s_b_s_c_s_e_s_c_s_d_s_c_s_e_s_e_s
GTGGCTGTGTGGGTGCTGGTGTGGGTGACGGTGTGGGTGCTGGTGCTGGTG; >clamp_s_c_s_d_s_c_s_a_s_e_s:compl:rev
CACCAGCACAGACACCCACACCGTCACCCACACTATCATGTTGACATTCC; dG = -34.6
2252 >s_b_s_c_s_e_s_c_s_d_s_c_s_d_s_e_s
GTGGCTGTGTGGGTGCTGGTGTGGGTGACGGTGTGGGTGACGGTGCTGGTG; >clamp_s_c_s_d_s_c_s_a_s_e_s:compl:rev
CACCAGCACAGACACCCACACCGTCACCCACACTATCATGTTGACATTCC; dG = -34.6
2253 >s_b_s_d_s_c_s_c_s_d_s_c_s_e_s_e_s
GTGGCTGTGACGGTGTGGGTGTGGGTGACGGTGTGGGTGCTGGTGCTGGTG; >clamp_s_c_s_d_s_c_s_a_s_e_s:compl:rev
CACCAGCACAGACACCCACACCGTCACCCACACTATCATGTTGACATTCC; dG = -34.6
2254 >s_b_s_d_s_c_s_c_s_d_s_c_s_d_s_e_s
GTGGCTGTGACGGTGTGGGTGTGGGTGACGGTGTGGGTGACGGTGCTGGTG; >clamp_s_c_s_d_s_c_s_a_s_e_s:compl:rev
CACCAGCACAGACACCCACACCGTCACCCACACTATCATGTTGACATTCC; dG = -34.6
2255 >s_a_s_a_s_e_s_c_s_d_s_c_s_e_s_e_s
GTGTCTGTGTCTGTGCTGGTGTGGGTGACGGTGTGGGTGCTGGTGCTGGTG; >clamp_s_c_s_d_s_c_s_a_s_e_s:compl:rev
CACCAGCACAGACACCCACACCGTCACCCACACTATCATGTTGACATTCC; dG = -34.6
2256 >s_a_s_a_s_e_s_c_s_d_s_c_s_d_s_e_s
GTGTCTGTGTCTGTGCTGGTGTGGGTGACGGTGTGGGTGACGGTGCTGGTG; >clamp_s_c_s_d_s_c_s_a_s_e_s:compl:rev
CACCAGCACAGACACCCACACCGTCACCCACACTATCATGTTGACATTCC; dG = -34.6
2257 >s_b_s_d_s_d_s_c_s_d_s_c_s_e_s_e_s
GTGGCTGTGACGGTGACGGTGTGGGTGACGGTGTGGGTGCTGGTGCTGGTG; >clamp_s_c_s_d_s_c_s_a_s_e_s:compl:rev
CACCAGCACAGACACCCACACCGTCACCCACACTATCATGTTGACATTCC; dG = -34.6
2258 >s_b_s_d_s_d_s_c_s_d_s_c_s_d_s_e_s
GTGGCTGTGACGGTGACGGTGTGGGTGACGGTGTGGGTGACGGTGCTGGTG; >clamp_s_c_s_d_s_c_s_a_s_e_s:compl:rev
CACCAGCACAGACACCCACACCGTCACCCACACTATCATGTTGACATTCC; dG = -34.6
2259 >s_b_s_d_s_e_s_c_s_d_s_c_s_e_s_e_s
GTGGCTGTGACGGTGCTGGTGTGGGTGACGGTGTGGGTGCTGGTGCTGGTG; >clamp_s_c_s_d_s_c_s_a_s_e_s:compl:rev
CACCAGCACAGACACCCACACCGTCACCCACACTATCATGTTGACATTCC; dG = -34.6
2260 >s_b_s_d_s_e_s_c_s_d_s_c_s_d_s_e_s
GTGGCTGTGACGGTGCTGGTGTGGGTGACGGTGTGGGTGACGGTGCTGGTG; >clamp_s_c_s_d_s_c_s_a_s_e_s:compl:rev
CACCAGCACAGACACCCACACCGTCACCCACACTATCATGTTGACATTCC; dG = -34.6
2261 >s_b_s_e_s_c_s_c_s_d_s_c_s_e_s_e_s
GTGGCTGTGCTGGTGTGGGTGTGGGTGACGGTGTGGGTGCTGGTGCTGGTG; >clamp_s_c_s_d_s_c_s_a_s_e_s:compl:rev
CACCAGCACAGACACCCACACCGTCACCCACACTATCATGTTGACATTCC; dG = -34.6
2262 >s_b_s_e_s_c_s_c_s_d_s_c_s_d_s_e_s
GTGGCTGTGCTGGTGTGGGTGTGGGTGACGGTGTGGGTGACGGTGCTGGTG; >clamp_s_c_s_d_s_c_s_a_s_e_s:compl:rev
CACCAGCACAGACACCCACACCGTCACCCACACTATCATGTTGACATTCC; dG = -34.6
2263 >s_b_s_e_s_d_s_c_s_d_s_c_s_e_s_e_s
GTGGCTGTGCTGGTGACGGTGTGGGTGACGGTGTGGGTGCTGGTGCTGGTG; >clamp_s_c_s_d_s_c_s_a_s_e_s:compl:rev
CACCAGCACAGACACCCACACCGTCACCCACACTATCATGTTGACATTCC; dG = -34.6

2264 >s_b_s_e_s_d_s_c_s_d_s_c_s_d_s_e_s
GTGGCTGTGCTGGTGACGGTGTGGGTGACGGTGTGGGTGACGGTGCTGGTG; >clamp_s_c_s_d_s_c_s_a_s_e_s:compl:rev
CACCAGCACAGACACCCACACCGTCACCCACACTATCATGTTGACATTCC; dG = -34.6
2265 >s_b_s_e_s_e_s_c_s_d_s_c_s_e_s_e_s
GTGGCTGTGCTGGTGCTGGTGTGGGTGACGGTGTGGGTGCTGGTGCTGGTG; >clamp_s_c_s_d_s_c_s_a_s_e_s:compl:rev
CACCAGCACAGACACCCACACCGTCACCCACACTATCATGTTGACATTCC; dG = -34.6
2266 >s_b_s_e_s_e_s_c_s_d_s_c_s_d_s_e_s
GTGGCTGTGCTGGTGCTGGTGTGGGTGACGGTGTGGGTGACGGTGCTGGTG; >clamp_s_c_s_d_s_c_s_a_s_e_s:compl:rev
CACCAGCACAGACACCCACACCGTCACCCACACTATCATGTTGACATTCC; dG = -34.6
2267 >s_c_s_a_s_a_s_c_s_d_s_b_s_a_s_e_s
GTGTGGGTGTCTGTGTCTGTGTGGGTGACGGTGGCTGTGTCTGTGCTGGTG; >clamp_s_c_s_d_s_c_s_a_s_e_s:compl:rev
CACCAGCACAGACACCCACACCGTCACCCACACTATCATGTTGACATTCC; dG = -33.4
2268 >s_c_s_a_s_b_s_c_s_d_s_b_s_a_s_e_s
GTGTGGGTGTCTGTGGCTGTGTGGGTGACGGTGGCTGTGTCTGTGCTGGTG; >clamp_s_c_s_d_s_c_s_a_s_e_s:compl:rev
CACCAGCACAGACACCCACACCGTCACCCACACTATCATGTTGACATTCC; dG = -33.4
2269 >s_a_s_a_s_a_s_c_s_d_s_b_s_a_s_e_s
GTGTCTGTGTCTGTGTCTGTGTGGGTGACGGTGGCTGTGTCTGTGCTGGTG; >clamp_s_c_s_d_s_c_s_a_s_e_s:compl:rev
CACCAGCACAGACACCCACACCGTCACCCACACTATCATGTTGACATTCC; dG = -33.4
2270 >s_a_s_b_s_a_s_c_s_d_s_b_s_a_s_e_s
GTGTCTGTGGCTGTGTCTGTGTGGGTGACGGTGGCTGTGTCTGTGCTGGTG; >clamp_s_c_s_d_s_c_s_a_s_e_s:compl:rev
CACCAGCACAGACACCCACACCGTCACCCACACTATCATGTTGACATTCC; dG = -33.4
2271 >s_c_s_b_s_a_s_c_s_d_s_b_s_a_s_e_s
GTGTGGGTGGCTGTGTCTGTGTGGGTGACGGTGGCTGTGTCTGTGCTGGTG; >clamp_s_c_s_d_s_c_s_a_s_e_s:compl:rev
CACCAGCACAGACACCCACACCGTCACCCACACTATCATGTTGACATTCC; dG = -33.4
2272 >s_c_s_b_s_b_s_c_s_d_s_b_s_a_s_e_s
GTGTGGGTGGCTGTGGCTGTGTGGGTGACGGTGGCTGTGTCTGTGCTGGTG; >clamp_s_c_s_d_s_c_s_a_s_e_s:compl:rev
CACCAGCACAGACACCCACACCGTCACCCACACTATCATGTTGACATTCC; dG = -33.4
2273 >s_c_s_c_s_a_s_c_s_d_s_b_s_a_s_e_s
GTGTGGGTGTGGGTGTCTGTGTGGGTGACGGTGGCTGTGTCTGTGCTGGTG; >clamp_s_c_s_d_s_c_s_a_s_e_s:compl:rev
CACCAGCACAGACACCCACACCGTCACCCACACTATCATGTTGACATTCC; dG = -33.4
2274 >s_c_s_c_s_b_s_c_s_d_s_b_s_a_s_e_s
GTGTGGGTGTGGGTGGCTGTGTGGGTGACGGTGGCTGTGTCTGTGCTGGTG; >clamp_s_c_s_d_s_c_s_a_s_e_s:compl:rev
CACCAGCACAGACACCCACACCGTCACCCACACTATCATGTTGACATTCC; dG = -33.4
2275 >s_a_s_b_s_b_s_c_s_d_s_b_s_a_s_e_s
GTGTCTGTGGCTGTGGCTGTGTGGGTGACGGTGGCTGTGTCTGTGCTGGTG; >clamp_s_c_s_d_s_c_s_a_s_e_s:compl:rev
CACCAGCACAGACACCCACACCGTCACCCACACTATCATGTTGACATTCC; dG = -33.4
2276 >s_c_s_d_s_a_s_c_s_d_s_b_s_a_s_e_s
GTGTGGGTGACGGTGTCTGTGTGGGTGACGGTGGCTGTGTCTGTGCTGGTG; >clamp_s_c_s_d_s_c_s_a_s_e_s:compl:rev
CACCAGCACAGACACCCACACCGTCACCCACACTATCATGTTGACATTCC; dG = -33.4
2277 >s_c_s_d_s_b_s_c_s_d_s_b_s_a_s_e_s
GTGTGGGTGACGGTGGCTGTGTGGGTGACGGTGGCTGTGTCTGTGCTGGTG; >clamp_s_c_s_d_s_c_s_a_s_e_s:compl:rev
CACCAGCACAGACACCCACACCGTCACCCACACTATCATGTTGACATTCC; dG = -33.4
2278 >s_c_s_e_s_a_s_c_s_d_s_b_s_a_s_e_s
GTGTGGGTGCTGGTGTCTGTGTGGGTGACGGTGGCTGTGTCTGTGCTGGTG; >clamp_s_c_s_d_s_c_s_a_s_e_s:compl:rev
CACCAGCACAGACACCCACACCGTCACCCACACTATCATGTTGACATTCC; dG = -33.4
2279 >s_c_s_e_s_b_s_c_s_d_s_b_s_a_s_e_s
GTGTGGGTGCTGGTGGCTGTGTGGGTGACGGTGGCTGTGTCTGTGCTGGTG; >clamp_s_c_s_d_s_c_s_a_s_e_s:compl:rev
CACCAGCACAGACACCCACACCGTCACCCACACTATCATGTTGACATTCC; dG = -33.4
2280 >s_d_s_a_s_a_s_c_s_d_s_b_s_a_s_e_s
GTGACGGTGTCTGTGTCTGTGTGGGTGACGGTGGCTGTGTCTGTGCTGGTG; >clamp_s_c_s_d_s_c_s_a_s_e_s:compl:rev
CACCAGCACAGACACCCACACCGTCACCCACACTATCATGTTGACATTCC; dG = -33.4
2281 >s_d_s_a_s_b_s_c_s_d_s_b_s_a_s_e_s
GTGACGGTGTCTGTGGCTGTGTGGGTGACGGTGGCTGTGTCTGTGCTGGTG; >clamp_s_c_s_d_s_c_s_a_s_e_s:compl:rev
CACCAGCACAGACACCCACACCGTCACCCACACTATCATGTTGACATTCC; dG = -33.4
2282 >s_d_s_b_s_a_s_c_s_d_s_b_s_a_s_e_s
GTGACGGTGGCTGTGTCTGTGTGGGTGACGGTGGCTGTGTCTGTGCTGGTG; >clamp_s_c_s_d_s_c_s_a_s_e_s:compl:rev
CACCAGCACAGACACCCACACCGTCACCCACACTATCATGTTGACATTCC; dG = -33.4
2283 >s_d_s_b_s_b_s_c_s_d_s_b_s_a_s_e_s
GTGACGGTGGCTGTGGCTGTGTGGGTGACGGTGGCTGTGTCTGTGCTGGTG; >clamp_s_c_s_d_s_c_s_a_s_e_s:compl:rev
CACCAGCACAGACACCCACACCGTCACCCACACTATCATGTTGACATTCC; dG = -33.4
2284 >s_d_s_c_s_a_s_c_s_d_s_b_s_a_s_e_s
GTGACGGTGTGGGTGTCTGTGTGGGTGACGGTGGCTGTGTCTGTGCTGGTG; >clamp_s_c_s_d_s_c_s_a_s_e_s:compl:rev
CACCAGCACAGACACCCACACCGTCACCCACACTATCATGTTGACATTCC; dG = -33.4
2285 >s_d_s_c_s_b_s_c_s_d_s_b_s_a_s_e_s
GTGACGGTGTGGGTGGCTGTGTGGGTGACGGTGGCTGTGTCTGTGCTGGTG; >clamp_s_c_s_d_s_c_s_a_s_e_s:compl:rev
CACCAGCACAGACACCCACACCGTCACCCACACTATCATGTTGACATTCC; dG = -33.4
2286 >s_d_s_d_s_a_s_c_s_d_s_b_s_a_s_e_s
GTGACGGTGACGGTGTCTGTGTGGGTGACGGTGGCTGTGTCTGTGCTGGTG; >clamp_s_c_s_d_s_c_s_a_s_e_s:compl:rev
CACCAGCACAGACACCCACACCGTCACCCACACTATCATGTTGACATTCC; dG = -33.4
2287 >s_d_s_d_s_b_s_c_s_d_s_b_s_a_s_e_s
GTGACGGTGACGGTGGCTGTGTGGGTGACGGTGGCTGTGTCTGTGCTGGTG; >clamp_s_c_s_d_s_c_s_a_s_e_s:compl:rev
CACCAGCACAGACACCCACACCGTCACCCACACTATCATGTTGACATTCC; dG = -33.4

2288 >s_d_s_e_s_a_s_c_s_d_s_b_s_a_s_e_s
GTGACGGTGCTGGTGTCTGTGTGGGTGACGGTGGCTGTGTCTGTGCTGGTG; >clamp_s_c_s_d_s_c_s_a_s_e_s:compl:rev
CACCAGCACAGACACCCACACCGTCACCCACACTATCATGTTGACATTCC; dG = -33.4
2289 >s_d_s_e_s_b_s_c_s_d_s_b_s_a_s_e_s
GTGACGGTGCTGGTGGCTGTGTGGGTGACGGTGGCTGTGTCTGTGCTGGTG; >clamp_s_c_s_d_s_c_s_a_s_e_s:compl:rev
CACCAGCACAGACACCCACACCGTCACCCACACTATCATGTTGACATTCC; dG = -33.4
2290 >s_e_s_a_s_a_s_c_s_d_s_b_s_a_s_e_s
GTGCTGGTGTCTGTGTCTGTGTGGGTGACGGTGGCTGTGTCTGTGCTGGTG; >clamp_s_c_s_d_s_c_s_a_s_e_s:compl:rev
CACCAGCACAGACACCCACACCGTCACCCACACTATCATGTTGACATTCC; dG = -33.4
2291 >s_e_s_a_s_b_s_c_s_d_s_b_s_a_s_e_s
GTGCTGGTGTCTGTGGCTGTGTGGGTGACGGTGGCTGTGTCTGTGCTGGTG; >clamp_s_c_s_d_s_c_s_a_s_e_s:compl:rev
CACCAGCACAGACACCCACACCGTCACCCACACTATCATGTTGACATTCC; dG = -33.4
2292 >s_a_s_c_s_a_s_c_s_d_s_b_s_a_s_e_s
GTGTCTGTGTGGGTGTCTGTGTGGGTGACGGTGGCTGTGTCTGTGCTGGTG; >clamp_s_c_s_d_s_c_s_a_s_e_s:compl:rev
CACCAGCACAGACACCCACACCGTCACCCACACTATCATGTTGACATTCC; dG = -33.4
2293 >s_e_s_b_s_a_s_c_s_d_s_b_s_a_s_e_s
GTGCTGGTGGCTGTGTCTGTGTGGGTGACGGTGGCTGTGTCTGTGCTGGTG; >clamp_s_c_s_d_s_c_s_a_s_e_s:compl:rev
CACCAGCACAGACACCCACACCGTCACCCACACTATCATGTTGACATTCC; dG = -33.4
2294 >s_e_s_b_s_b_s_c_s_d_s_b_s_a_s_e_s
GTGCTGGTGGCTGTGGCTGTGTGGGTGACGGTGGCTGTGTCTGTGCTGGTG; >clamp_s_c_s_d_s_c_s_a_s_e_s:compl:rev
CACCAGCACAGACACCCACACCGTCACCCACACTATCATGTTGACATTCC; dG = -33.4
2295 >s_e_s_c_s_a_s_c_s_d_s_b_s_a_s_e_s
GTGCTGGTGTGGGTGTCTGTGTGGGTGACGGTGGCTGTGTCTGTGCTGGTG; >clamp_s_c_s_d_s_c_s_a_s_e_s:compl:rev
CACCAGCACAGACACCCACACCGTCACCCACACTATCATGTTGACATTCC; dG = -33.4
2296 >s_e_s_c_s_b_s_c_s_d_s_b_s_a_s_e_s
GTGCTGGTGTGGGTGGCTGTGTGGGTGACGGTGGCTGTGTCTGTGCTGGTG; >clamp_s_c_s_d_s_c_s_a_s_e_s:compl:rev
CACCAGCACAGACACCCACACCGTCACCCACACTATCATGTTGACATTCC; dG = -33.4
2297 >s_a_s_c_s_b_s_c_s_d_s_b_s_a_s_e_s
GTGTCTGTGTGGGTGGCTGTGTGGGTGACGGTGGCTGTGTCTGTGCTGGTG; >clamp_s_c_s_d_s_c_s_a_s_e_s:compl:rev
CACCAGCACAGACACCCACACCGTCACCCACACTATCATGTTGACATTCC; dG = -33.4
2298 >s_e_s_d_s_a_s_c_s_d_s_b_s_a_s_e_s
GTGCTGGTGACGGTGTCTGTGTGGGTGACGGTGGCTGTGTCTGTGCTGGTG; >clamp_s_c_s_d_s_c_s_a_s_e_s:compl:rev
CACCAGCACAGACACCCACACCGTCACCCACACTATCATGTTGACATTCC; dG = -33.4
2299 >s_e_s_d_s_b_s_c_s_d_s_b_s_a_s_e_s
GTGCTGGTGACGGTGGCTGTGTGGGTGACGGTGGCTGTGTCTGTGCTGGTG; >clamp_s_c_s_d_s_c_s_a_s_e_s:compl:rev
CACCAGCACAGACACCCACACCGTCACCCACACTATCATGTTGACATTCC; dG = -33.4
2300 >s_e_s_e_s_a_s_c_s_d_s_b_s_a_s_e_s
GTGCTGGTGCTGGTGTCTGTGTGGGTGACGGTGGCTGTGTCTGTGCTGGTG; >clamp_s_c_s_d_s_c_s_a_s_e_s:compl:rev
CACCAGCACAGACACCCACACCGTCACCCACACTATCATGTTGACATTCC; dG = -33.4
2301 >s_e_s_e_s_b_s_c_s_d_s_b_s_a_s_e_s
GTGCTGGTGCTGGTGGCTGTGTGGGTGACGGTGGCTGTGTCTGTGCTGGTG; >clamp_s_c_s_d_s_c_s_a_s_e_s:compl:rev
CACCAGCACAGACACCCACACCGTCACCCACACTATCATGTTGACATTCC; dG = -33.4
2302 >s_a_s_a_s_b_s_c_s_d_s_b_s_a_s_e_s
GTGTCTGTGTCTGTGGCTGTGTGGGTGACGGTGGCTGTGTCTGTGCTGGTG; >clamp_s_c_s_d_s_c_s_a_s_e_s:compl:rev
CACCAGCACAGACACCCACACCGTCACCCACACTATCATGTTGACATTCC; dG = -33.4
2303 >s_a_s_d_s_a_s_c_s_d_s_b_s_a_s_e_s
GTGTCTGTGACGGTGTCTGTGTGGGTGACGGTGGCTGTGTCTGTGCTGGTG; >clamp_s_c_s_d_s_c_s_a_s_e_s:compl:rev
CACCAGCACAGACACCCACACCGTCACCCACACTATCATGTTGACATTCC; dG = -33.4
2304 >s_a_s_d_s_b_s_c_s_d_s_b_s_a_s_e_s
GTGTCTGTGACGGTGGCTGTGTGGGTGACGGTGGCTGTGTCTGTGCTGGTG; >clamp_s_c_s_d_s_c_s_a_s_e_s:compl:rev
CACCAGCACAGACACCCACACCGTCACCCACACTATCATGTTGACATTCC; dG = -33.4
2305 >s_a_s_e_s_a_s_c_s_d_s_b_s_a_s_e_s
GTGTCTGTGCTGGTGTCTGTGTGGGTGACGGTGGCTGTGTCTGTGCTGGTG; >clamp_s_c_s_d_s_c_s_a_s_e_s:compl:rev
CACCAGCACAGACACCCACACCGTCACCCACACTATCATGTTGACATTCC; dG = -33.4
2306 >s_a_s_e_s_b_s_c_s_d_s_b_s_a_s_e_s
GTGTCTGTGCTGGTGGCTGTGTGGGTGACGGTGGCTGTGTCTGTGCTGGTG; >clamp_s_c_s_d_s_c_s_a_s_e_s:compl:rev
CACCAGCACAGACACCCACACCGTCACCCACACTATCATGTTGACATTCC; dG = -33.4
2307 >s_b_s_a_s_a_s_c_s_d_s_b_s_a_s_e_s
GTGGCTGTGTCTGTGTCTGTGTGGGTGACGGTGGCTGTGTCTGTGCTGGTG; >clamp_s_c_s_d_s_c_s_a_s_e_s:compl:rev
CACCAGCACAGACACCCACACCGTCACCCACACTATCATGTTGACATTCC; dG = -33.4
2308 >s_b_s_a_s_b_s_c_s_d_s_b_s_a_s_e_s
GTGGCTGTGTCTGTGGCTGTGTGGGTGACGGTGGCTGTGTCTGTGCTGGTG; >clamp_s_c_s_d_s_c_s_a_s_e_s:compl:rev
CACCAGCACAGACACCCACACCGTCACCCACACTATCATGTTGACATTCC; dG = -33.4
2309 >s_b_s_b_s_a_s_c_s_d_s_b_s_a_s_e_s
GTGGCTGTGGCTGTGTCTGTGTGGGTGACGGTGGCTGTGTCTGTGCTGGTG; >clamp_s_c_s_d_s_c_s_a_s_e_s:compl:rev
CACCAGCACAGACACCCACACCGTCACCCACACTATCATGTTGACATTCC; dG = -33.4
2310 >s_b_s_b_s_b_s_c_s_d_s_b_s_a_s_e_s
GTGGCTGTGGCTGTGGCTGTGTGGGTGACGGTGGCTGTGTCTGTGCTGGTG; >clamp_s_c_s_d_s_c_s_a_s_e_s:compl:rev
CACCAGCACAGACACCCACACCGTCACCCACACTATCATGTTGACATTCC; dG = -33.4
2311 >s_b_s_c_s_a_s_c_s_d_s_b_s_a_s_e_s
GTGGCTGTGTGGGTGTCTGTGTGGGTGACGGTGGCTGTGTCTGTGCTGGTG; >clamp_s_c_s_d_s_c_s_a_s_e_s:compl:rev
CACCAGCACAGACACCCACACCGTCACCCACACTATCATGTTGACATTCC; dG = -33.4

2312 >s_b_s_c_s_b_s_c_s_d_s_b_s_a_s_e_s
GTGGCTGTGTGGGTGGCTGTGTGGGTGACGGTGGCTGTGTCTGTGCTGGTG; >clamp_s_c_s_d_s_c_s_a_s_e_s:compl:rev
CACCAGCACAGACACCCACACCGTCACCCACACTATCATGTTGACATTCC; dG = -33.4
2313 >s_b_s_d_s_a_s_c_s_d_s_b_s_a_s_e_s
GTGGCTGTGACGGTGTCTGTGTGGGTGACGGTGGCTGTGTCTGTGCTGGTG; >clamp_s_c_s_d_s_c_s_a_s_e_s:compl:rev
CACCAGCACAGACACCCACACCGTCACCCACACTATCATGTTGACATTCC; dG = -33.4
2314 >s_b_s_d_s_b_s_c_s_d_s_b_s_a_s_e_s
GTGGCTGTGACGGTGGCTGTGTGGGTGACGGTGGCTGTGTCTGTGCTGGTG; >clamp_s_c_s_d_s_c_s_a_s_e_s:compl:rev
CACCAGCACAGACACCCACACCGTCACCCACACTATCATGTTGACATTCC; dG = -33.4
2315 >s_b_s_e_s_a_s_c_s_d_s_b_s_a_s_e_s
GTGGCTGTGCTGGTGTCTGTGTGGGTGACGGTGGCTGTGTCTGTGCTGGTG; >clamp_s_c_s_d_s_c_s_a_s_e_s:compl:rev
CACCAGCACAGACACCCACACCGTCACCCACACTATCATGTTGACATTCC; dG = -33.4
2316 >s_b_s_e_s_b_s_c_s_d_s_b_s_a_s_e_s
GTGGCTGTGCTGGTGGCTGTGTGGGTGACGGTGGCTGTGTCTGTGCTGGTG; >clamp_s_c_s_d_s_c_s_a_s_e_s:compl:rev
CACCAGCACAGACACCCACACCGTCACCCACACTATCATGTTGACATTCC; dG = -33.4
2317 >s_c_s_a_s_c_s_c_s_d_s_b_s_a_s_e_s
GTGTGGGTGTCTGTGTGGGTGTGGGTGACGGTGGCTGTGTCTGTGCTGGTG; >clamp_s_c_s_d_s_c_s_a_s_e_s:compl:rev
CACCAGCACAGACACCCACACCGTCACCCACACTATCATGTTGACATTCC; dG = -33.3
2318 >s_c_s_a_s_d_s_c_s_d_s_b_s_a_s_e_s
GTGTGGGTGTCTGTGACGGTGTGGGTGACGGTGGCTGTGTCTGTGCTGGTG; >clamp_s_c_s_d_s_c_s_a_s_e_s:compl:rev
CACCAGCACAGACACCCACACCGTCACCCACACTATCATGTTGACATTCC; dG = -33.3
2319 >s_c_s_a_s_e_s_c_s_d_s_b_s_a_s_e_s
GTGTGGGTGTCTGTGCTGGTGTGGGTGACGGTGGCTGTGTCTGTGCTGGTG; >clamp_s_c_s_d_s_c_s_a_s_e_s:compl:rev
CACCAGCACAGACACCCACACCGTCACCCACACTATCATGTTGACATTCC; dG = -33.3
2320 >s_c_s_b_s_c_s_c_s_d_s_b_s_a_s_e_s
GTGTGGGTGGCTGTGTGGGTGTGGGTGACGGTGGCTGTGTCTGTGCTGGTG; >clamp_s_c_s_d_s_c_s_a_s_e_s:compl:rev
CACCAGCACAGACACCCACACCGTCACCCACACTATCATGTTGACATTCC; dG = -33.3
2321 >s_c_s_b_s_d_s_c_s_d_s_b_s_a_s_e_s
GTGTGGGTGGCTGTGACGGTGTGGGTGACGGTGGCTGTGTCTGTGCTGGTG; >clamp_s_c_s_d_s_c_s_a_s_e_s:compl:rev
CACCAGCACAGACACCCACACCGTCACCCACACTATCATGTTGACATTCC; dG = -33.3
2322 >s_c_s_b_s_e_s_c_s_d_s_b_s_a_s_e_s
GTGTGGGTGGCTGTGCTGGTGTGGGTGACGGTGGCTGTGTCTGTGCTGGTG; >clamp_s_c_s_d_s_c_s_a_s_e_s:compl:rev
CACCAGCACAGACACCCACACCGTCACCCACACTATCATGTTGACATTCC; dG = -33.3
2323 >s_c_s_c_s_c_s_c_s_d_s_b_s_a_s_e_s
GTGTGGGTGTGGGTGTGGGTGTGGGTGACGGTGGCTGTGTCTGTGCTGGTG; >clamp_s_c_s_d_s_c_s_a_s_e_s:compl:rev
CACCAGCACAGACACCCACACCGTCACCCACACTATCATGTTGACATTCC; dG = -33.3
2324 >s_c_s_c_s_e_s_c_s_d_s_b_s_a_s_e_s
GTGTGGGTGTGGGTGCTGGTGTGGGTGACGGTGGCTGTGTCTGTGCTGGTG; >clamp_s_c_s_d_s_c_s_a_s_e_s:compl:rev
CACCAGCACAGACACCCACACCGTCACCCACACTATCATGTTGACATTCC; dG = -33.3
2325 >s_c_s_d_s_d_s_c_s_d_s_b_s_a_s_e_s
GTGTGGGTGACGGTGACGGTGTGGGTGACGGTGGCTGTGTCTGTGCTGGTG; >clamp_s_c_s_d_s_c_s_a_s_e_s:compl:rev
CACCAGCACAGACACCCACACCGTCACCCACACTATCATGTTGACATTCC; dG = -33.3
2326 >s_c_s_d_s_e_s_c_s_d_s_b_s_a_s_e_s
GTGTGGGTGACGGTGCTGGTGTGGGTGACGGTGGCTGTGTCTGTGCTGGTG; >clamp_s_c_s_d_s_c_s_a_s_e_s:compl:rev
CACCAGCACAGACACCCACACCGTCACCCACACTATCATGTTGACATTCC; dG = -33.3
2327 >s_c_s_e_s_c_s_c_s_d_s_b_s_a_s_e_s
GTGTGGGTGCTGGTGTGGGTGTGGGTGACGGTGGCTGTGTCTGTGCTGGTG; >clamp_s_c_s_d_s_c_s_a_s_e_s:compl:rev
CACCAGCACAGACACCCACACCGTCACCCACACTATCATGTTGACATTCC; dG = -33.3
2328 >s_c_s_e_s_d_s_c_s_d_s_b_s_a_s_e_s
GTGTGGGTGCTGGTGACGGTGTGGGTGACGGTGGCTGTGTCTGTGCTGGTG; >clamp_s_c_s_d_s_c_s_a_s_e_s:compl:rev
CACCAGCACAGACACCCACACCGTCACCCACACTATCATGTTGACATTCC; dG = -33.3
2329 >s_c_s_e_s_e_s_c_s_d_s_b_s_a_s_e_s
GTGTGGGTGCTGGTGCTGGTGTGGGTGACGGTGGCTGTGTCTGTGCTGGTG; >clamp_s_c_s_d_s_c_s_a_s_e_s:compl:rev
CACCAGCACAGACACCCACACCGTCACCCACACTATCATGTTGACATTCC; dG = -33.3
2330 >s_a_s_b_s_c_s_c_s_d_s_b_s_a_s_e_s
GTGTCTGTGGCTGTGTGGGTGTGGGTGACGGTGGCTGTGTCTGTGCTGGTG; >clamp_s_c_s_d_s_c_s_a_s_e_s:compl:rev
CACCAGCACAGACACCCACACCGTCACCCACACTATCATGTTGACATTCC; dG = -33.3
2331 >s_d_s_a_s_c_s_c_s_d_s_b_s_a_s_e_s
GTGACGGTGTCTGTGTGGGTGTGGGTGACGGTGGCTGTGTCTGTGCTGGTG; >clamp_s_c_s_d_s_c_s_a_s_e_s:compl:rev
CACCAGCACAGACACCCACACCGTCACCCACACTATCATGTTGACATTCC; dG = -33.3
2332 >s_d_s_a_s_d_s_c_s_d_s_b_s_a_s_e_s
GTGACGGTGTCTGTGACGGTGTGGGTGACGGTGGCTGTGTCTGTGCTGGTG; >clamp_s_c_s_d_s_c_s_a_s_e_s:compl:rev
CACCAGCACAGACACCCACACCGTCACCCACACTATCATGTTGACATTCC; dG = -33.3
2333 >s_d_s_a_s_e_s_c_s_d_s_b_s_a_s_e_s
GTGACGGTGTCTGTGCTGGTGTGGGTGACGGTGGCTGTGTCTGTGCTGGTG; >clamp_s_c_s_d_s_c_s_a_s_e_s:compl:rev
CACCAGCACAGACACCCACACCGTCACCCACACTATCATGTTGACATTCC; dG = -33.3
2334 >s_d_s_b_s_c_s_c_s_d_s_b_s_a_s_e_s
GTGACGGTGGCTGTGTGGGTGTGGGTGACGGTGGCTGTGTCTGTGCTGGTG; >clamp_s_c_s_d_s_c_s_a_s_e_s:compl:rev
CACCAGCACAGACACCCACACCGTCACCCACACTATCATGTTGACATTCC; dG = -33.3
2335 >s_d_s_b_s_d_s_c_s_d_s_b_s_a_s_e_s
GTGACGGTGGCTGTGACGGTGTGGGTGACGGTGGCTGTGTCTGTGCTGGTG; >clamp_s_c_s_d_s_c_s_a_s_e_s:compl:rev
CACCAGCACAGACACCCACACCGTCACCCACACTATCATGTTGACATTCC; dG = -33.3

2336 >s_d_s_b_s_e_s_c_s_d_s_b_s_a_s_e_s
GTGACGGTGGCTGTGCTGGTGTGGGTGACGGTGGCTGTGTCTGTGCTGGTG; >clamp_s_c_s_d_s_c_s_a_s_e_s:compl:rev
CACCAGCACAGACACCCACACCGTCACCCACACTATCATGTTGACATTCC; dG = -33.3
2337 >s_a_s_b_s_d_s_c_s_d_s_b_s_a_s_e_s
GTGTCTGTGGCTGTGACGGTGTGGGTGACGGTGGCTGTGTCTGTGCTGGTG; >clamp_s_c_s_d_s_c_s_a_s_e_s:compl:rev
CACCAGCACAGACACCCACACCGTCACCCACACTATCATGTTGACATTCC; dG = -33.3
2338 >s_d_s_c_s_c_s_c_s_d_s_b_s_a_s_e_s
GTGACGGTGTGGGTGTGGGTGTGGGTGACGGTGGCTGTGTCTGTGCTGGTG; >clamp_s_c_s_d_s_c_s_a_s_e_s:compl:rev
CACCAGCACAGACACCCACACCGTCACCCACACTATCATGTTGACATTCC; dG = -33.3
2339 >s_d_s_c_s_e_s_c_s_d_s_b_s_a_s_e_s
GTGACGGTGTGGGTGCTGGTGTGGGTGACGGTGGCTGTGTCTGTGCTGGTG; >clamp_s_c_s_d_s_c_s_a_s_e_s:compl:rev
CACCAGCACAGACACCCACACCGTCACCCACACTATCATGTTGACATTCC; dG = -33.3
2340 >s_d_s_d_s_c_s_c_s_d_s_b_s_a_s_e_s
GTGACGGTGACGGTGTGGGTGTGGGTGACGGTGGCTGTGTCTGTGCTGGTG; >clamp_s_c_s_d_s_c_s_a_s_e_s:compl:rev
CACCAGCACAGACACCCACACCGTCACCCACACTATCATGTTGACATTCC; dG = -33.3
2341 >s_d_s_d_s_d_s_c_s_d_s_b_s_a_s_e_s
GTGACGGTGACGGTGACGGTGTGGGTGACGGTGGCTGTGTCTGTGCTGGTG; >clamp_s_c_s_d_s_c_s_a_s_e_s:compl:rev
CACCAGCACAGACACCCACACCGTCACCCACACTATCATGTTGACATTCC; dG = -33.3
2342 >s_d_s_d_s_e_s_c_s_d_s_b_s_a_s_e_s
GTGACGGTGACGGTGCTGGTGTGGGTGACGGTGGCTGTGTCTGTGCTGGTG; >clamp_s_c_s_d_s_c_s_a_s_e_s:compl:rev
CACCAGCACAGACACCCACACCGTCACCCACACTATCATGTTGACATTCC; dG = -33.3
2343 >s_a_s_b_s_e_s_c_s_d_s_b_s_a_s_e_s
GTGTCTGTGGCTGTGCTGGTGTGGGTGACGGTGGCTGTGTCTGTGCTGGTG; >clamp_s_c_s_d_s_c_s_a_s_e_s:compl:rev
CACCAGCACAGACACCCACACCGTCACCCACACTATCATGTTGACATTCC; dG = -33.3
2344 >s_d_s_e_s_c_s_c_s_d_s_b_s_a_s_e_s
GTGACGGTGCTGGTGTGGGTGTGGGTGACGGTGGCTGTGTCTGTGCTGGTG; >clamp_s_c_s_d_s_c_s_a_s_e_s:compl:rev
CACCAGCACAGACACCCACACCGTCACCCACACTATCATGTTGACATTCC; dG = -33.3
2345 >s_d_s_e_s_d_s_c_s_d_s_b_s_a_s_e_s
GTGACGGTGCTGGTGACGGTGTGGGTGACGGTGGCTGTGTCTGTGCTGGTG; >clamp_s_c_s_d_s_c_s_a_s_e_s:compl:rev
CACCAGCACAGACACCCACACCGTCACCCACACTATCATGTTGACATTCC; dG = -33.3
2346 >s_d_s_e_s_e_s_c_s_d_s_b_s_a_s_e_s
GTGACGGTGCTGGTGCTGGTGTGGGTGACGGTGGCTGTGTCTGTGCTGGTG; >clamp_s_c_s_d_s_c_s_a_s_e_s:compl:rev
CACCAGCACAGACACCCACACCGTCACCCACACTATCATGTTGACATTCC; dG = -33.3
2347 >s_e_s_a_s_c_s_c_s_d_s_b_s_a_s_e_s
GTGCTGGTGTCTGTGTGGGTGTGGGTGACGGTGGCTGTGTCTGTGCTGGTG; >clamp_s_c_s_d_s_c_s_a_s_e_s:compl:rev
CACCAGCACAGACACCCACACCGTCACCCACACTATCATGTTGACATTCC; dG = -33.3
2348 >s_e_s_a_s_d_s_c_s_d_s_b_s_a_s_e_s
GTGCTGGTGTCTGTGACGGTGTGGGTGACGGTGGCTGTGTCTGTGCTGGTG; >clamp_s_c_s_d_s_c_s_a_s_e_s:compl:rev
CACCAGCACAGACACCCACACCGTCACCCACACTATCATGTTGACATTCC; dG = -33.3
2349 >s_e_s_a_s_e_s_c_s_d_s_b_s_a_s_e_s
GTGCTGGTGTCTGTGCTGGTGTGGGTGACGGTGGCTGTGTCTGTGCTGGTG; >clamp_s_c_s_d_s_c_s_a_s_e_s:compl:rev
CACCAGCACAGACACCCACACCGTCACCCACACTATCATGTTGACATTCC; dG = -33.3
2350 >s_e_s_b_s_c_s_c_s_d_s_b_s_a_s_e_s
GTGCTGGTGGCTGTGTGGGTGTGGGTGACGGTGGCTGTGTCTGTGCTGGTG; >clamp_s_c_s_d_s_c_s_a_s_e_s:compl:rev
CACCAGCACAGACACCCACACCGTCACCCACACTATCATGTTGACATTCC; dG = -33.3
2351 >s_e_s_b_s_d_s_c_s_d_s_b_s_a_s_e_s
GTGCTGGTGGCTGTGACGGTGTGGGTGACGGTGGCTGTGTCTGTGCTGGTG; >clamp_s_c_s_d_s_c_s_a_s_e_s:compl:rev
CACCAGCACAGACACCCACACCGTCACCCACACTATCATGTTGACATTCC; dG = -33.3
2352 >s_e_s_b_s_e_s_c_s_d_s_b_s_a_s_e_s
GTGCTGGTGGCTGTGCTGGTGTGGGTGACGGTGGCTGTGTCTGTGCTGGTG; >clamp_s_c_s_d_s_c_s_a_s_e_s:compl:rev
CACCAGCACAGACACCCACACCGTCACCCACACTATCATGTTGACATTCC; dG = -33.3
2353 >s_e_s_c_s_c_s_c_s_d_s_b_s_a_s_e_s
GTGCTGGTGTGGGTGTGGGTGTGGGTGACGGTGGCTGTGTCTGTGCTGGTG; >clamp_s_c_s_d_s_c_s_a_s_e_s:compl:rev
CACCAGCACAGACACCCACACCGTCACCCACACTATCATGTTGACATTCC; dG = -33.3
2354 >s_e_s_c_s_e_s_c_s_d_s_b_s_a_s_e_s
GTGCTGGTGTGGGTGCTGGTGTGGGTGACGGTGGCTGTGTCTGTGCTGGTG; >clamp_s_c_s_d_s_c_s_a_s_e_s:compl:rev
CACCAGCACAGACACCCACACCGTCACCCACACTATCATGTTGACATTCC; dG = -33.3
2355 >s_e_s_d_s_c_s_c_s_d_s_b_s_a_s_e_s
GTGCTGGTGACGGTGTGGGTGTGGGTGACGGTGGCTGTGTCTGTGCTGGTG; >clamp_s_c_s_d_s_c_s_a_s_e_s:compl:rev
CACCAGCACAGACACCCACACCGTCACCCACACTATCATGTTGACATTCC; dG = -33.3
2356 >s_e_s_d_s_d_s_c_s_d_s_b_s_a_s_e_s
GTGCTGGTGACGGTGACGGTGTGGGTGACGGTGGCTGTGTCTGTGCTGGTG; >clamp_s_c_s_d_s_c_s_a_s_e_s:compl:rev
CACCAGCACAGACACCCACACCGTCACCCACACTATCATGTTGACATTCC; dG = -33.3
2357 >s_e_s_d_s_e_s_c_s_d_s_b_s_a_s_e_s
GTGCTGGTGACGGTGCTGGTGTGGGTGACGGTGGCTGTGTCTGTGCTGGTG; >clamp_s_c_s_d_s_c_s_a_s_e_s:compl:rev
CACCAGCACAGACACCCACACCGTCACCCACACTATCATGTTGACATTCC; dG = -33.3
2358 >s_e_s_e_s_c_s_c_s_d_s_b_s_a_s_e_s
GTGCTGGTGCTGGTGTGGGTGTGGGTGACGGTGGCTGTGTCTGTGCTGGTG; >clamp_s_c_s_d_s_c_s_a_s_e_s:compl:rev
CACCAGCACAGACACCCACACCGTCACCCACACTATCATGTTGACATTCC; dG = -33.3
2359 >s_e_s_e_s_d_s_c_s_d_s_b_s_a_s_e_s
GTGCTGGTGCTGGTGACGGTGTGGGTGACGGTGGCTGTGTCTGTGCTGGTG; >clamp_s_c_s_d_s_c_s_a_s_e_s:compl:rev
CACCAGCACAGACACCCACACCGTCACCCACACTATCATGTTGACATTCC; dG = -33.3

2360 >s_e_s_e_s_e_s_c_s_d_s_b_s_a_s_e_s
GTGCTGGTGCTGGTGCTGGTGTGGGTGACGGTGGCTGTGTCTGTGCTGGTG; >clamp_s_c_s_d_s_c_s_a_s_e_s:compl:rev
CACCAGCACAGACACCCACACCGTCACCCACACTATCATGTTGACATTCC; dG = -33.3
2361 >s_a_s_c_s_c_s_c_s_d_s_b_s_a_s_e_s
GTGTCTGTGTGGGTGTGGGTGTGGGTGACGGTGGCTGTGTCTGTGCTGGTG; >clamp_s_c_s_d_s_c_s_a_s_e_s:compl:rev
CACCAGCACAGACACCCACACCGTCACCCACACTATCATGTTGACATTCC; dG = -33.3
2362 >s_a_s_c_s_e_s_c_s_d_s_b_s_a_s_e_s
GTGTCTGTGTGGGTGCTGGTGTGGGTGACGGTGGCTGTGTCTGTGCTGGTG; >clamp_s_c_s_d_s_c_s_a_s_e_s:compl:rev
CACCAGCACAGACACCCACACCGTCACCCACACTATCATGTTGACATTCC; dG = -33.3
2363 >s_a_s_d_s_c_s_c_s_d_s_b_s_a_s_e_s
GTGTCTGTGACGGTGTGGGTGTGGGTGACGGTGGCTGTGTCTGTGCTGGTG; >clamp_s_c_s_d_s_c_s_a_s_e_s:compl:rev
CACCAGCACAGACACCCACACCGTCACCCACACTATCATGTTGACATTCC; dG = -33.3
2364 >s_a_s_d_s_d_s_c_s_d_s_b_s_a_s_e_s
GTGTCTGTGACGGTGACGGTGTGGGTGACGGTGGCTGTGTCTGTGCTGGTG; >clamp_s_c_s_d_s_c_s_a_s_e_s:compl:rev
CACCAGCACAGACACCCACACCGTCACCCACACTATCATGTTGACATTCC; dG = -33.3
2365 >s_a_s_d_s_e_s_c_s_d_s_b_s_a_s_e_s
GTGTCTGTGACGGTGCTGGTGTGGGTGACGGTGGCTGTGTCTGTGCTGGTG; >clamp_s_c_s_d_s_c_s_a_s_e_s:compl:rev
CACCAGCACAGACACCCACACCGTCACCCACACTATCATGTTGACATTCC; dG = -33.3
2366 >s_a_s_e_s_c_s_c_s_d_s_b_s_a_s_e_s
GTGTCTGTGCTGGTGTGGGTGTGGGTGACGGTGGCTGTGTCTGTGCTGGTG; >clamp_s_c_s_d_s_c_s_a_s_e_s:compl:rev
CACCAGCACAGACACCCACACCGTCACCCACACTATCATGTTGACATTCC; dG = -33.3
2367 >s_a_s_e_s_d_s_c_s_d_s_b_s_a_s_e_s
GTGTCTGTGCTGGTGACGGTGTGGGTGACGGTGGCTGTGTCTGTGCTGGTG; >clamp_s_c_s_d_s_c_s_a_s_e_s:compl:rev
CACCAGCACAGACACCCACACCGTCACCCACACTATCATGTTGACATTCC; dG = -33.3
2368 >s_a_s_e_s_e_s_c_s_d_s_b_s_a_s_e_s
GTGTCTGTGCTGGTGCTGGTGTGGGTGACGGTGGCTGTGTCTGTGCTGGTG; >clamp_s_c_s_d_s_c_s_a_s_e_s:compl:rev
CACCAGCACAGACACCCACACCGTCACCCACACTATCATGTTGACATTCC; dG = -33.3
2369 >s_a_s_a_s_c_s_c_s_d_s_b_s_a_s_e_s
GTGTCTGTGTCTGTGTGGGTGTGGGTGACGGTGGCTGTGTCTGTGCTGGTG; >clamp_s_c_s_d_s_c_s_a_s_e_s:compl:rev
CACCAGCACAGACACCCACACCGTCACCCACACTATCATGTTGACATTCC; dG = -33.3
2370 >s_b_s_a_s_c_s_c_s_d_s_b_s_a_s_e_s
GTGGCTGTGTCTGTGTGGGTGTGGGTGACGGTGGCTGTGTCTGTGCTGGTG; >clamp_s_c_s_d_s_c_s_a_s_e_s:compl:rev
CACCAGCACAGACACCCACACCGTCACCCACACTATCATGTTGACATTCC; dG = -33.3
2371 >s_b_s_a_s_d_s_c_s_d_s_b_s_a_s_e_s
GTGGCTGTGTCTGTGACGGTGTGGGTGACGGTGGCTGTGTCTGTGCTGGTG; >clamp_s_c_s_d_s_c_s_a_s_e_s:compl:rev
CACCAGCACAGACACCCACACCGTCACCCACACTATCATGTTGACATTCC; dG = -33.3
2372 >s_b_s_a_s_e_s_c_s_d_s_b_s_a_s_e_s
GTGGCTGTGTCTGTGCTGGTGTGGGTGACGGTGGCTGTGTCTGTGCTGGTG; >clamp_s_c_s_d_s_c_s_a_s_e_s:compl:rev
CACCAGCACAGACACCCACACCGTCACCCACACTATCATGTTGACATTCC; dG = -33.3
2373 >s_b_s_b_s_c_s_c_s_d_s_b_s_a_s_e_s
GTGGCTGTGGCTGTGTGGGTGTGGGTGACGGTGGCTGTGTCTGTGCTGGTG; >clamp_s_c_s_d_s_c_s_a_s_e_s:compl:rev
CACCAGCACAGACACCCACACCGTCACCCACACTATCATGTTGACATTCC; dG = -33.3
2374 >s_b_s_b_s_d_s_c_s_d_s_b_s_a_s_e_s
GTGGCTGTGGCTGTGACGGTGTGGGTGACGGTGGCTGTGTCTGTGCTGGTG; >clamp_s_c_s_d_s_c_s_a_s_e_s:compl:rev
CACCAGCACAGACACCCACACCGTCACCCACACTATCATGTTGACATTCC; dG = -33.3
2375 >s_b_s_b_s_e_s_c_s_d_s_b_s_a_s_e_s
GTGGCTGTGGCTGTGCTGGTGTGGGTGACGGTGGCTGTGTCTGTGCTGGTG; >clamp_s_c_s_d_s_c_s_a_s_e_s:compl:rev
CACCAGCACAGACACCCACACCGTCACCCACACTATCATGTTGACATTCC; dG = -33.3
2376 >s_a_s_a_s_d_s_c_s_d_s_b_s_a_s_e_s
GTGTCTGTGTCTGTGACGGTGTGGGTGACGGTGGCTGTGTCTGTGCTGGTG; >clamp_s_c_s_d_s_c_s_a_s_e_s:compl:rev
CACCAGCACAGACACCCACACCGTCACCCACACTATCATGTTGACATTCC; dG = -33.3
2377 >s_b_s_c_s_c_s_c_s_d_s_b_s_a_s_e_s
GTGGCTGTGTGGGTGTGGGTGTGGGTGACGGTGGCTGTGTCTGTGCTGGTG; >clamp_s_c_s_d_s_c_s_a_s_e_s:compl:rev
CACCAGCACAGACACCCACACCGTCACCCACACTATCATGTTGACATTCC; dG = -33.3
2378 >s_b_s_c_s_e_s_c_s_d_s_b_s_a_s_e_s
GTGGCTGTGTGGGTGCTGGTGTGGGTGACGGTGGCTGTGTCTGTGCTGGTG; >clamp_s_c_s_d_s_c_s_a_s_e_s:compl:rev
CACCAGCACAGACACCCACACCGTCACCCACACTATCATGTTGACATTCC; dG = -33.3
2379 >s_b_s_d_s_c_s_c_s_d_s_b_s_a_s_e_s
GTGGCTGTGACGGTGTGGGTGTGGGTGACGGTGGCTGTGTCTGTGCTGGTG; >clamp_s_c_s_d_s_c_s_a_s_e_s:compl:rev
CACCAGCACAGACACCCACACCGTCACCCACACTATCATGTTGACATTCC; dG = -33.3
2380 >s_b_s_d_s_d_s_c_s_d_s_b_s_a_s_e_s
GTGGCTGTGACGGTGACGGTGTGGGTGACGGTGGCTGTGTCTGTGCTGGTG; >clamp_s_c_s_d_s_c_s_a_s_e_s:compl:rev
CACCAGCACAGACACCCACACCGTCACCCACACTATCATGTTGACATTCC; dG = -33.3
2381 >s_b_s_d_s_e_s_c_s_d_s_b_s_a_s_e_s
GTGGCTGTGACGGTGCTGGTGTGGGTGACGGTGGCTGTGTCTGTGCTGGTG; >clamp_s_c_s_d_s_c_s_a_s_e_s:compl:rev
CACCAGCACAGACACCCACACCGTCACCCACACTATCATGTTGACATTCC; dG = -33.3
2382 >s_a_s_a_s_e_s_c_s_d_s_b_s_a_s_e_s
GTGTCTGTGTCTGTGCTGGTGTGGGTGACGGTGGCTGTGTCTGTGCTGGTG; >clamp_s_c_s_d_s_c_s_a_s_e_s:compl:rev
CACCAGCACAGACACCCACACCGTCACCCACACTATCATGTTGACATTCC; dG = -33.3
2383 >s_b_s_e_s_c_s_c_s_d_s_b_s_a_s_e_s
GTGGCTGTGCTGGTGTGGGTGTGGGTGACGGTGGCTGTGTCTGTGCTGGTG; >clamp_s_c_s_d_s_c_s_a_s_e_s:compl:rev
CACCAGCACAGACACCCACACCGTCACCCACACTATCATGTTGACATTCC; dG = -33.3

2384 >s_b_s_e_s_d_s_c_s_d_s_b_s_a_s_e_s
GTGGCTGTGCTGGTGACGGTGTGGGTGACGGTGGCTGTGTCTGTGCTGGTG; >clamp_s_c_s_d_s_c_s_a_s_e_s:compl:rev
CACCAGCACAGACACCCACACCGTCACCCACACTATCATGTTGACATTCC; dG = -33.3
2385 >s_b_s_e_s_e_s_c_s_d_s_b_s_a_s_e_s
GTGGCTGTGCTGGTGCTGGTGTGGGTGACGGTGGCTGTGTCTGTGCTGGTG; >clamp_s_c_s_d_s_c_s_a_s_e_s:compl:rev
CACCAGCACAGACACCCACACCGTCACCCACACTATCATGTTGACATTCC; dG = -33.3
2386 >s_c_s_a_s_a_s_c_s_a_s_c_s_a_s_e_s
GTGTGGGTGTCTGTGTCTGTGTGGGTGTCTGTGTGGGTGTCTGTGCTGGTG; >clamp_s_c_s_d_s_c_s_a_s_e_s:compl:rev
CACCAGCACAGACACCCACACCGTCACCCACACTATCATGTTGACATTCC; dG = -33.1
2387 >s_c_s_a_s_b_s_c_s_a_s_c_s_a_s_e_s
GTGTGGGTGTCTGTGGCTGTGTGGGTGTCTGTGTGGGTGTCTGTGCTGGTG; >clamp_s_c_s_d_s_c_s_a_s_e_s:compl:rev
CACCAGCACAGACACCCACACCGTCACCCACACTATCATGTTGACATTCC; dG = -33.1
2388 >s_c_s_a_s_c_s_c_s_a_s_c_s_a_s_e_s
GTGTGGGTGTCTGTGTGGGTGTGGGTGTCTGTGTGGGTGTCTGTGCTGGTG; >clamp_s_c_s_d_s_c_s_a_s_e_s:compl:rev
CACCAGCACAGACACCCACACCGTCACCCACACTATCATGTTGACATTCC; dG = -33.1
2389 >s_c_s_a_s_d_s_c_s_a_s_c_s_a_s_e_s
GTGTGGGTGTCTGTGACGGTGTGGGTGTCTGTGTGGGTGTCTGTGCTGGTG; >clamp_s_c_s_d_s_c_s_a_s_e_s:compl:rev
CACCAGCACAGACACCCACACCGTCACCCACACTATCATGTTGACATTCC; dG = -33.1
2390 >s_c_s_a_s_e_s_c_s_a_s_c_s_a_s_e_s
GTGTGGGTGTCTGTGCTGGTGTGGGTGTCTGTGTGGGTGTCTGTGCTGGTG; >clamp_s_c_s_d_s_c_s_a_s_e_s:compl:rev
CACCAGCACAGACACCCACACCGTCACCCACACTATCATGTTGACATTCC; dG = -33.1
2391 >s_c_s_b_s_a_s_c_s_a_s_c_s_a_s_e_s
GTGTGGGTGGCTGTGTCTGTGTGGGTGTCTGTGTGGGTGTCTGTGCTGGTG; >clamp_s_c_s_d_s_c_s_a_s_e_s:compl:rev
CACCAGCACAGACACCCACACCGTCACCCACACTATCATGTTGACATTCC; dG = -33.1
2392 >s_c_s_b_s_b_s_c_s_a_s_c_s_a_s_e_s
GTGTGGGTGGCTGTGGCTGTGTGGGTGTCTGTGTGGGTGTCTGTGCTGGTG; >clamp_s_c_s_d_s_c_s_a_s_e_s:compl:rev
CACCAGCACAGACACCCACACCGTCACCCACACTATCATGTTGACATTCC; dG = -33.1
2393 >s_c_s_b_s_c_s_c_s_a_s_c_s_a_s_e_s
GTGTGGGTGGCTGTGTGGGTGTGGGTGTCTGTGTGGGTGTCTGTGCTGGTG; >clamp_s_c_s_d_s_c_s_a_s_e_s:compl:rev
CACCAGCACAGACACCCACACCGTCACCCACACTATCATGTTGACATTCC; dG = -33.1
2394 >s_a_s_b_s_a_s_c_s_a_s_c_s_a_s_e_s
GTGTCTGTGGCTGTGTCTGTGTGGGTGTCTGTGTGGGTGTCTGTGCTGGTG; >clamp_s_c_s_d_s_c_s_a_s_e_s:compl:rev
CACCAGCACAGACACCCACACCGTCACCCACACTATCATGTTGACATTCC; dG = -33.1
2395 >s_c_s_b_s_d_s_c_s_a_s_c_s_a_s_e_s
GTGTGGGTGGCTGTGACGGTGTGGGTGTCTGTGTGGGTGTCTGTGCTGGTG; >clamp_s_c_s_d_s_c_s_a_s_e_s:compl:rev
CACCAGCACAGACACCCACACCGTCACCCACACTATCATGTTGACATTCC; dG = -33.1
2396 >s_c_s_b_s_e_s_c_s_a_s_c_s_a_s_e_s
GTGTGGGTGGCTGTGCTGGTGTGGGTGTCTGTGTGGGTGTCTGTGCTGGTG; >clamp_s_c_s_d_s_c_s_a_s_e_s:compl:rev
CACCAGCACAGACACCCACACCGTCACCCACACTATCATGTTGACATTCC; dG = -33.1
2397 >s_c_s_c_s_a_s_c_s_a_s_c_s_a_s_e_s
GTGTGGGTGTGGGTGTCTGTGTGGGTGTCTGTGTGGGTGTCTGTGCTGGTG; >clamp_s_c_s_d_s_c_s_a_s_e_s:compl:rev
CACCAGCACAGACACCCACACCGTCACCCACACTATCATGTTGACATTCC; dG = -33.1
2398 >s_c_s_c_s_b_s_c_s_a_s_c_s_a_s_e_s
GTGTGGGTGTGGGTGGCTGTGTGGGTGTCTGTGTGGGTGTCTGTGCTGGTG; >clamp_s_c_s_d_s_c_s_a_s_e_s:compl:rev
CACCAGCACAGACACCCACACCGTCACCCACACTATCATGTTGACATTCC; dG = -33.1
2399 >s_c_s_c_s_c_s_c_s_a_s_c_s_a_s_e_s
GTGTGGGTGTGGGTGTGGGTGTGGGTGTCTGTGTGGGTGTCTGTGCTGGTG; >clamp_s_c_s_d_s_c_s_a_s_e_s:compl:rev
CACCAGCACAGACACCCACACCGTCACCCACACTATCATGTTGACATTCC; dG = -33.1
2400 >s_c_s_c_s_e_s_c_s_a_s_c_s_a_s_e_s
GTGTGGGTGTGGGTGCTGGTGTGGGTGTCTGTGTGGGTGTCTGTGCTGGTG; >clamp_s_c_s_d_s_c_s_a_s_e_s:compl:rev
CACCAGCACAGACACCCACACCGTCACCCACACTATCATGTTGACATTCC; dG = -33.1
2401 >s_a_s_b_s_b_s_c_s_a_s_c_s_a_s_e_s
GTGTCTGTGGCTGTGGCTGTGTGGGTGTCTGTGTGGGTGTCTGTGCTGGTG; >clamp_s_c_s_d_s_c_s_a_s_e_s:compl:rev
CACCAGCACAGACACCCACACCGTCACCCACACTATCATGTTGACATTCC; dG = -33.1
2402 >s_c_s_e_s_a_s_c_s_a_s_c_s_a_s_e_s
GTGTGGGTGCTGGTGTCTGTGTGGGTGTCTGTGTGGGTGTCTGTGCTGGTG; >clamp_s_c_s_d_s_c_s_a_s_e_s:compl:rev
CACCAGCACAGACACCCACACCGTCACCCACACTATCATGTTGACATTCC; dG = -33.1
2403 >s_c_s_e_s_b_s_c_s_a_s_c_s_a_s_e_s
GTGTGGGTGCTGGTGGCTGTGTGGGTGTCTGTGTGGGTGTCTGTGCTGGTG; >clamp_s_c_s_d_s_c_s_a_s_e_s:compl:rev
CACCAGCACAGACACCCACACCGTCACCCACACTATCATGTTGACATTCC; dG = -33.1
2404 >s_c_s_e_s_c_s_c_s_a_s_c_s_a_s_e_s
GTGTGGGTGCTGGTGTGGGTGTGGGTGTCTGTGTGGGTGTCTGTGCTGGTG; >clamp_s_c_s_d_s_c_s_a_s_e_s:compl:rev
CACCAGCACAGACACCCACACCGTCACCCACACTATCATGTTGACATTCC; dG = -33.1
2405 >s_c_s_e_s_d_s_c_s_a_s_c_s_a_s_e_s
GTGTGGGTGCTGGTGACGGTGTGGGTGTCTGTGTGGGTGTCTGTGCTGGTG; >clamp_s_c_s_d_s_c_s_a_s_e_s:compl:rev
CACCAGCACAGACACCCACACCGTCACCCACACTATCATGTTGACATTCC; dG = -33.1
2406 >s_c_s_e_s_e_s_c_s_a_s_c_s_a_s_e_s
GTGTGGGTGCTGGTGCTGGTGTGGGTGTCTGTGTGGGTGTCTGTGCTGGTG; >clamp_s_c_s_d_s_c_s_a_s_e_s:compl:rev
CACCAGCACAGACACCCACACCGTCACCCACACTATCATGTTGACATTCC; dG = -33.1
2407 >s_d_s_a_s_a_s_c_s_a_s_c_s_a_s_e_s
GTGACGGTGTCTGTGTCTGTGTGGGTGTCTGTGTGGGTGTCTGTGCTGGTG; >clamp_s_c_s_d_s_c_s_a_s_e_s:compl:rev
CACCAGCACAGACACCCACACCGTCACCCACACTATCATGTTGACATTCC; dG = -33.1

2408 >s_d_s_a_s_b_s_c_s_a_s_c_s_a_s_e_s
GTGACGGTGTCTGTGGCTGTGTGGGTGTCTGTGTGGGTGTCTGTGCTGGTG; >clamp_s_c_s_d_s_c_s_a_s_e_s:compl:rev
CACCAGCACAGACACCCACACCGTCACCCACACTATCATGTTGACATTCC; dG = -33.1
2409 >s_d_s_a_s_c_s_c_s_a_s_c_s_a_s_e_s
GTGACGGTGTCTGTGTGGGTGTGGGTGTCTGTGTGGGTGTCTGTGCTGGTG; >clamp_s_c_s_d_s_c_s_a_s_e_s:compl:rev
CACCAGCACAGACACCCACACCGTCACCCACACTATCATGTTGACATTCC; dG = -33.1
2410 >s_a_s_b_s_c_s_c_s_a_s_c_s_a_s_e_s
GTGTCTGTGGCTGTGTGGGTGTGGGTGTCTGTGTGGGTGTCTGTGCTGGTG; >clamp_s_c_s_d_s_c_s_a_s_e_s:compl:rev
CACCAGCACAGACACCCACACCGTCACCCACACTATCATGTTGACATTCC; dG = -33.1
2411 >s_d_s_a_s_d_s_c_s_a_s_c_s_a_s_e_s
GTGACGGTGTCTGTGACGGTGTGGGTGTCTGTGTGGGTGTCTGTGCTGGTG; >clamp_s_c_s_d_s_c_s_a_s_e_s:compl:rev
CACCAGCACAGACACCCACACCGTCACCCACACTATCATGTTGACATTCC; dG = -33.1
2412 >s_d_s_a_s_e_s_c_s_a_s_c_s_a_s_e_s
GTGACGGTGTCTGTGCTGGTGTGGGTGTCTGTGTGGGTGTCTGTGCTGGTG; >clamp_s_c_s_d_s_c_s_a_s_e_s:compl:rev
CACCAGCACAGACACCCACACCGTCACCCACACTATCATGTTGACATTCC; dG = -33.1
2413 >s_a_s_a_s_a_s_c_s_a_s_c_s_a_s_e_s
GTGTCTGTGTCTGTGTCTGTGTGGGTGTCTGTGTGGGTGTCTGTGCTGGTG; >clamp_s_c_s_d_s_c_s_a_s_e_s:compl:rev
CACCAGCACAGACACCCACACCGTCACCCACACTATCATGTTGACATTCC; dG = -33.1
2414 >s_d_s_b_s_a_s_c_s_a_s_c_s_a_s_e_s
GTGACGGTGGCTGTGTCTGTGTGGGTGTCTGTGTGGGTGTCTGTGCTGGTG; >clamp_s_c_s_d_s_c_s_a_s_e_s:compl:rev
CACCAGCACAGACACCCACACCGTCACCCACACTATCATGTTGACATTCC; dG = -33.1
2415 >s_d_s_b_s_b_s_c_s_a_s_c_s_a_s_e_s
GTGACGGTGGCTGTGGCTGTGTGGGTGTCTGTGTGGGTGTCTGTGCTGGTG; >clamp_s_c_s_d_s_c_s_a_s_e_s:compl:rev
CACCAGCACAGACACCCACACCGTCACCCACACTATCATGTTGACATTCC; dG = -33.1
2416 >s_d_s_b_s_c_s_c_s_a_s_c_s_a_s_e_s
GTGACGGTGGCTGTGTGGGTGTGGGTGTCTGTGTGGGTGTCTGTGCTGGTG; >clamp_s_c_s_d_s_c_s_a_s_e_s:compl:rev
CACCAGCACAGACACCCACACCGTCACCCACACTATCATGTTGACATTCC; dG = -33.1
2417 >s_d_s_b_s_d_s_c_s_a_s_c_s_a_s_e_s
GTGACGGTGGCTGTGACGGTGTGGGTGTCTGTGTGGGTGTCTGTGCTGGTG; >clamp_s_c_s_d_s_c_s_a_s_e_s:compl:rev
CACCAGCACAGACACCCACACCGTCACCCACACTATCATGTTGACATTCC; dG = -33.1
2418 >s_d_s_b_s_e_s_c_s_a_s_c_s_a_s_e_s
GTGACGGTGGCTGTGCTGGTGTGGGTGTCTGTGTGGGTGTCTGTGCTGGTG; >clamp_s_c_s_d_s_c_s_a_s_e_s:compl:rev
CACCAGCACAGACACCCACACCGTCACCCACACTATCATGTTGACATTCC; dG = -33.1
2419 >s_d_s_c_s_a_s_c_s_a_s_c_s_a_s_e_s
GTGACGGTGTGGGTGTCTGTGTGGGTGTCTGTGTGGGTGTCTGTGCTGGTG; >clamp_s_c_s_d_s_c_s_a_s_e_s:compl:rev
CACCAGCACAGACACCCACACCGTCACCCACACTATCATGTTGACATTCC; dG = -33.1
2420 >s_d_s_c_s_b_s_c_s_a_s_c_s_a_s_e_s
GTGACGGTGTGGGTGGCTGTGTGGGTGTCTGTGTGGGTGTCTGTGCTGGTG; >clamp_s_c_s_d_s_c_s_a_s_e_s:compl:rev
CACCAGCACAGACACCCACACCGTCACCCACACTATCATGTTGACATTCC; dG = -33.1
2421 >s_d_s_c_s_c_s_c_s_a_s_c_s_a_s_e_s
GTGACGGTGTGGGTGTGGGTGTGGGTGTCTGTGTGGGTGTCTGTGCTGGTG; >clamp_s_c_s_d_s_c_s_a_s_e_s:compl:rev
CACCAGCACAGACACCCACACCGTCACCCACACTATCATGTTGACATTCC; dG = -33.1
2422 >s_a_s_b_s_d_s_c_s_a_s_c_s_a_s_e_s
GTGTCTGTGGCTGTGACGGTGTGGGTGTCTGTGTGGGTGTCTGTGCTGGTG; >clamp_s_c_s_d_s_c_s_a_s_e_s:compl:rev
CACCAGCACAGACACCCACACCGTCACCCACACTATCATGTTGACATTCC; dG = -33.1
2423 >s_d_s_c_s_e_s_c_s_a_s_c_s_a_s_e_s
GTGACGGTGTGGGTGCTGGTGTGGGTGTCTGTGTGGGTGTCTGTGCTGGTG; >clamp_s_c_s_d_s_c_s_a_s_e_s:compl:rev
CACCAGCACAGACACCCACACCGTCACCCACACTATCATGTTGACATTCC; dG = -33.1
2424 >s_d_s_d_s_a_s_c_s_a_s_c_s_a_s_e_s
GTGACGGTGACGGTGTCTGTGTGGGTGTCTGTGTGGGTGTCTGTGCTGGTG; >clamp_s_c_s_d_s_c_s_a_s_e_s:compl:rev
CACCAGCACAGACACCCACACCGTCACCCACACTATCATGTTGACATTCC; dG = -33.1
2425 >s_d_s_d_s_b_s_c_s_a_s_c_s_a_s_e_s
GTGACGGTGACGGTGGCTGTGTGGGTGTCTGTGTGGGTGTCTGTGCTGGTG; >clamp_s_c_s_d_s_c_s_a_s_e_s:compl:rev
CACCAGCACAGACACCCACACCGTCACCCACACTATCATGTTGACATTCC; dG = -33.1
2426 >s_d_s_d_s_c_s_c_s_a_s_c_s_a_s_e_s
GTGACGGTGACGGTGTGGGTGTGGGTGTCTGTGTGGGTGTCTGTGCTGGTG; >clamp_s_c_s_d_s_c_s_a_s_e_s:compl:rev
CACCAGCACAGACACCCACACCGTCACCCACACTATCATGTTGACATTCC; dG = -33.1
2427 >s_d_s_d_s_d_s_c_s_a_s_c_s_a_s_e_s
GTGACGGTGACGGTGACGGTGTGGGTGTCTGTGTGGGTGTCTGTGCTGGTG; >clamp_s_c_s_d_s_c_s_a_s_e_s:compl:rev
CACCAGCACAGACACCCACACCGTCACCCACACTATCATGTTGACATTCC; dG = -33.1
2428 >s_d_s_d_s_e_s_c_s_a_s_c_s_a_s_e_s
GTGACGGTGACGGTGCTGGTGTGGGTGTCTGTGTGGGTGTCTGTGCTGGTG; >clamp_s_c_s_d_s_c_s_a_s_e_s:compl:rev
CACCAGCACAGACACCCACACCGTCACCCACACTATCATGTTGACATTCC; dG = -33.1
2429 >s_d_s_e_s_a_s_c_s_a_s_c_s_a_s_e_s
GTGACGGTGCTGGTGTCTGTGTGGGTGTCTGTGTGGGTGTCTGTGCTGGTG; >clamp_s_c_s_d_s_c_s_a_s_e_s:compl:rev
CACCAGCACAGACACCCACACCGTCACCCACACTATCATGTTGACATTCC; dG = -33.1
2430 >s_d_s_e_s_b_s_c_s_a_s_c_s_a_s_e_s
GTGACGGTGCTGGTGGCTGTGTGGGTGTCTGTGTGGGTGTCTGTGCTGGTG; >clamp_s_c_s_d_s_c_s_a_s_e_s:compl:rev
CACCAGCACAGACACCCACACCGTCACCCACACTATCATGTTGACATTCC; dG = -33.1
2431 >s_d_s_e_s_c_s_c_s_a_s_c_s_a_s_e_s
GTGACGGTGCTGGTGTGGGTGTGGGTGTCTGTGTGGGTGTCTGTGCTGGTG; >clamp_s_c_s_d_s_c_s_a_s_e_s:compl:rev
CACCAGCACAGACACCCACACCGTCACCCACACTATCATGTTGACATTCC; dG = -33.1

2432 >s_a_s_b_s_e_s_c_s_a_s_c_s_a_s_e_s
GTGTCTGTGGCTGTGCTGGTGTGGGTGTCTGTGTGGGTGTCTGTGCTGGTG; >clamp_s_c_s_d_s_c_s_a_s_e_s:compl:rev
CACCAGCACAGACACCCACACCGTCACCCACACTATCATGTTGACATTCC; dG = -33.1
2433 >s_d_s_e_s_d_s_c_s_a_s_c_s_a_s_e_s
GTGACGGTGCTGGTGACGGTGTGGGTGTCTGTGTGGGTGTCTGTGCTGGTG; >clamp_s_c_s_d_s_c_s_a_s_e_s:compl:rev
CACCAGCACAGACACCCACACCGTCACCCACACTATCATGTTGACATTCC; dG = -33.1
2434 >s_d_s_e_s_e_s_c_s_a_s_c_s_a_s_e_s
GTGACGGTGCTGGTGCTGGTGTGGGTGTCTGTGTGGGTGTCTGTGCTGGTG; >clamp_s_c_s_d_s_c_s_a_s_e_s:compl:rev
CACCAGCACAGACACCCACACCGTCACCCACACTATCATGTTGACATTCC; dG = -33.1
2435 >s_e_s_a_s_a_s_c_s_a_s_c_s_a_s_e_s
GTGCTGGTGTCTGTGTCTGTGTGGGTGTCTGTGTGGGTGTCTGTGCTGGTG; >clamp_s_c_s_d_s_c_s_a_s_e_s:compl:rev
CACCAGCACAGACACCCACACCGTCACCCACACTATCATGTTGACATTCC; dG = -33.1
2436 >s_e_s_a_s_b_s_c_s_a_s_c_s_a_s_e_s
GTGCTGGTGTCTGTGGCTGTGTGGGTGTCTGTGTGGGTGTCTGTGCTGGTG; >clamp_s_c_s_d_s_c_s_a_s_e_s:compl:rev
CACCAGCACAGACACCCACACCGTCACCCACACTATCATGTTGACATTCC; dG = -33.1
2437 >s_e_s_a_s_c_s_c_s_a_s_c_s_a_s_e_s
GTGCTGGTGTCTGTGTGGGTGTGGGTGTCTGTGTGGGTGTCTGTGCTGGTG; >clamp_s_c_s_d_s_c_s_a_s_e_s:compl:rev
CACCAGCACAGACACCCACACCGTCACCCACACTATCATGTTGACATTCC; dG = -33.1
2438 >s_e_s_a_s_d_s_c_s_a_s_c_s_a_s_e_s
GTGCTGGTGTCTGTGACGGTGTGGGTGTCTGTGTGGGTGTCTGTGCTGGTG; >clamp_s_c_s_d_s_c_s_a_s_e_s:compl:rev
CACCAGCACAGACACCCACACCGTCACCCACACTATCATGTTGACATTCC; dG = -33.1
2439 >s_e_s_a_s_e_s_c_s_a_s_c_s_a_s_e_s
GTGCTGGTGTCTGTGCTGGTGTGGGTGTCTGTGTGGGTGTCTGTGCTGGTG; >clamp_s_c_s_d_s_c_s_a_s_e_s:compl:rev
CACCAGCACAGACACCCACACCGTCACCCACACTATCATGTTGACATTCC; dG = -33.1
2440 >s_e_s_b_s_a_s_c_s_a_s_c_s_a_s_e_s
GTGCTGGTGGCTGTGTCTGTGTGGGTGTCTGTGTGGGTGTCTGTGCTGGTG; >clamp_s_c_s_d_s_c_s_a_s_e_s:compl:rev
CACCAGCACAGACACCCACACCGTCACCCACACTATCATGTTGACATTCC; dG = -33.1
2441 >s_e_s_b_s_b_s_c_s_a_s_c_s_a_s_e_s
GTGCTGGTGGCTGTGGCTGTGTGGGTGTCTGTGTGGGTGTCTGTGCTGGTG; >clamp_s_c_s_d_s_c_s_a_s_e_s:compl:rev
CACCAGCACAGACACCCACACCGTCACCCACACTATCATGTTGACATTCC; dG = -33.1
2442 >s_e_s_b_s_c_s_c_s_a_s_c_s_a_s_e_s
GTGCTGGTGGCTGTGTGGGTGTGGGTGTCTGTGTGGGTGTCTGTGCTGGTG; >clamp_s_c_s_d_s_c_s_a_s_e_s:compl:rev
CACCAGCACAGACACCCACACCGTCACCCACACTATCATGTTGACATTCC; dG = -33.1
2443 >s_a_s_c_s_a_s_c_s_a_s_c_s_a_s_e_s
GTGTCTGTGTGGGTGTCTGTGTGGGTGTCTGTGTGGGTGTCTGTGCTGGTG; >clamp_s_c_s_d_s_c_s_a_s_e_s:compl:rev
CACCAGCACAGACACCCACACCGTCACCCACACTATCATGTTGACATTCC; dG = -33.1
2444 >s_e_s_b_s_d_s_c_s_a_s_c_s_a_s_e_s
GTGCTGGTGGCTGTGACGGTGTGGGTGTCTGTGTGGGTGTCTGTGCTGGTG; >clamp_s_c_s_d_s_c_s_a_s_e_s:compl:rev
CACCAGCACAGACACCCACACCGTCACCCACACTATCATGTTGACATTCC; dG = -33.1
2445 >s_e_s_b_s_e_s_c_s_a_s_c_s_a_s_e_s
GTGCTGGTGGCTGTGCTGGTGTGGGTGTCTGTGTGGGTGTCTGTGCTGGTG; >clamp_s_c_s_d_s_c_s_a_s_e_s:compl:rev
CACCAGCACAGACACCCACACCGTCACCCACACTATCATGTTGACATTCC; dG = -33.1
2446 >s_e_s_c_s_a_s_c_s_a_s_c_s_a_s_e_s
GTGCTGGTGTGGGTGTCTGTGTGGGTGTCTGTGTGGGTGTCTGTGCTGGTG; >clamp_s_c_s_d_s_c_s_a_s_e_s:compl:rev
CACCAGCACAGACACCCACACCGTCACCCACACTATCATGTTGACATTCC; dG = -33.1
2447 >s_e_s_c_s_b_s_c_s_a_s_c_s_a_s_e_s
GTGCTGGTGTGGGTGGCTGTGTGGGTGTCTGTGTGGGTGTCTGTGCTGGTG; >clamp_s_c_s_d_s_c_s_a_s_e_s:compl:rev
CACCAGCACAGACACCCACACCGTCACCCACACTATCATGTTGACATTCC; dG = -33.1
2448 >s_e_s_c_s_c_s_c_s_a_s_c_s_a_s_e_s
GTGCTGGTGTGGGTGTGGGTGTGGGTGTCTGTGTGGGTGTCTGTGCTGGTG; >clamp_s_c_s_d_s_c_s_a_s_e_s:compl:rev
CACCAGCACAGACACCCACACCGTCACCCACACTATCATGTTGACATTCC; dG = -33.1
2449 >s_e_s_c_s_e_s_c_s_a_s_c_s_a_s_e_s
GTGCTGGTGTGGGTGCTGGTGTGGGTGTCTGTGTGGGTGTCTGTGCTGGTG; >clamp_s_c_s_d_s_c_s_a_s_e_s:compl:rev
CACCAGCACAGACACCCACACCGTCACCCACACTATCATGTTGACATTCC; dG = -33.1
2450 >s_e_s_d_s_a_s_c_s_a_s_c_s_a_s_e_s
GTGCTGGTGACGGTGTCTGTGTGGGTGTCTGTGTGGGTGTCTGTGCTGGTG; >clamp_s_c_s_d_s_c_s_a_s_e_s:compl:rev
CACCAGCACAGACACCCACACCGTCACCCACACTATCATGTTGACATTCC; dG = -33.1
2451 >s_e_s_d_s_b_s_c_s_a_s_c_s_a_s_e_s
GTGCTGGTGACGGTGGCTGTGTGGGTGTCTGTGTGGGTGTCTGTGCTGGTG; >clamp_s_c_s_d_s_c_s_a_s_e_s:compl:rev
CACCAGCACAGACACCCACACCGTCACCCACACTATCATGTTGACATTCC; dG = -33.1
2452 >s_e_s_d_s_c_s_c_s_a_s_c_s_a_s_e_s
GTGCTGGTGACGGTGTGGGTGTGGGTGTCTGTGTGGGTGTCTGTGCTGGTG; >clamp_s_c_s_d_s_c_s_a_s_e_s:compl:rev
CACCAGCACAGACACCCACACCGTCACCCACACTATCATGTTGACATTCC; dG = -33.1
2453 >s_a_s_c_s_b_s_c_s_a_s_c_s_a_s_e_s
GTGTCTGTGTGGGTGGCTGTGTGGGTGTCTGTGTGGGTGTCTGTGCTGGTG; >clamp_s_c_s_d_s_c_s_a_s_e_s:compl:rev
CACCAGCACAGACACCCACACCGTCACCCACACTATCATGTTGACATTCC; dG = -33.1
2454 >s_e_s_d_s_d_s_c_s_a_s_c_s_a_s_e_s
GTGCTGGTGACGGTGACGGTGTGGGTGTCTGTGTGGGTGTCTGTGCTGGTG; >clamp_s_c_s_d_s_c_s_a_s_e_s:compl:rev
CACCAGCACAGACACCCACACCGTCACCCACACTATCATGTTGACATTCC; dG = -33.1
2455 >s_e_s_d_s_e_s_c_s_a_s_c_s_a_s_e_s
GTGCTGGTGACGGTGCTGGTGTGGGTGTCTGTGTGGGTGTCTGTGCTGGTG; >clamp_s_c_s_d_s_c_s_a_s_e_s:compl:rev
CACCAGCACAGACACCCACACCGTCACCCACACTATCATGTTGACATTCC; dG = -33.1

2456 >s_e_s_e_s_a_s_c_s_a_s_c_s_a_s_e_s
GTGCTGGTGCTGGTGTCTGTGTGGGTGTCTGTGTGGGTGTCTGTGCTGGTG; >clamp_s_c_s_d_s_c_s_a_s_e_s:compl:rev
CACCAGCACAGACACCCACACCGTCACCCACACTATCATGTTGACATTCC; dG = -33.1
2457 >s_e_s_e_s_b_s_c_s_a_s_c_s_a_s_e_s
GTGCTGGTGCTGGTGGCTGTGTGGGTGTCTGTGTGGGTGTCTGTGCTGGTG; >clamp_s_c_s_d_s_c_s_a_s_e_s:compl:rev
CACCAGCACAGACACCCACACCGTCACCCACACTATCATGTTGACATTCC; dG = -33.1
2458 >s_e_s_e_s_c_s_c_s_a_s_c_s_a_s_e_s
GTGCTGGTGCTGGTGTGGGTGTGGGTGTCTGTGTGGGTGTCTGTGCTGGTG; >clamp_s_c_s_d_s_c_s_a_s_e_s:compl:rev
CACCAGCACAGACACCCACACCGTCACCCACACTATCATGTTGACATTCC; dG = -33.1
2459 >s_e_s_e_s_d_s_c_s_a_s_c_s_a_s_e_s
GTGCTGGTGCTGGTGACGGTGTGGGTGTCTGTGTGGGTGTCTGTGCTGGTG; >clamp_s_c_s_d_s_c_s_a_s_e_s:compl:rev
CACCAGCACAGACACCCACACCGTCACCCACACTATCATGTTGACATTCC; dG = -33.1
2460 >s_e_s_e_s_e_s_c_s_a_s_c_s_a_s_e_s
GTGCTGGTGCTGGTGCTGGTGTGGGTGTCTGTGTGGGTGTCTGTGCTGGTG; >clamp_s_c_s_d_s_c_s_a_s_e_s:compl:rev
CACCAGCACAGACACCCACACCGTCACCCACACTATCATGTTGACATTCC; dG = -33.1
2461 >s_a_s_c_s_c_s_c_s_a_s_c_s_a_s_e_s
GTGTCTGTGTGGGTGTGGGTGTGGGTGTCTGTGTGGGTGTCTGTGCTGGTG; >clamp_s_c_s_d_s_c_s_a_s_e_s:compl:rev
CACCAGCACAGACACCCACACCGTCACCCACACTATCATGTTGACATTCC; dG = -33.1
2462 >s_a_s_c_s_e_s_c_s_a_s_c_s_a_s_e_s
GTGTCTGTGTGGGTGCTGGTGTGGGTGTCTGTGTGGGTGTCTGTGCTGGTG; >clamp_s_c_s_d_s_c_s_a_s_e_s:compl:rev
CACCAGCACAGACACCCACACCGTCACCCACACTATCATGTTGACATTCC; dG = -33.1
2463 >s_a_s_d_s_a_s_c_s_a_s_c_s_a_s_e_s
GTGTCTGTGACGGTGTCTGTGTGGGTGTCTGTGTGGGTGTCTGTGCTGGTG; >clamp_s_c_s_d_s_c_s_a_s_e_s:compl:rev
CACCAGCACAGACACCCACACCGTCACCCACACTATCATGTTGACATTCC; dG = -33.1
2464 >s_a_s_d_s_b_s_c_s_a_s_c_s_a_s_e_s
GTGTCTGTGACGGTGGCTGTGTGGGTGTCTGTGTGGGTGTCTGTGCTGGTG; >clamp_s_c_s_d_s_c_s_a_s_e_s:compl:rev
CACCAGCACAGACACCCACACCGTCACCCACACTATCATGTTGACATTCC; dG = -33.1
2465 >s_a_s_d_s_c_s_c_s_a_s_c_s_a_s_e_s
GTGTCTGTGACGGTGTGGGTGTGGGTGTCTGTGTGGGTGTCTGTGCTGGTG; >clamp_s_c_s_d_s_c_s_a_s_e_s:compl:rev
CACCAGCACAGACACCCACACCGTCACCCACACTATCATGTTGACATTCC; dG = -33.1
2466 >s_a_s_a_s_b_s_c_s_a_s_c_s_a_s_e_s
GTGTCTGTGTCTGTGGCTGTGTGGGTGTCTGTGTGGGTGTCTGTGCTGGTG; >clamp_s_c_s_d_s_c_s_a_s_e_s:compl:rev
CACCAGCACAGACACCCACACCGTCACCCACACTATCATGTTGACATTCC; dG = -33.1
2467 >s_a_s_d_s_d_s_c_s_a_s_c_s_a_s_e_s
GTGTCTGTGACGGTGACGGTGTGGGTGTCTGTGTGGGTGTCTGTGCTGGTG; >clamp_s_c_s_d_s_c_s_a_s_e_s:compl:rev
CACCAGCACAGACACCCACACCGTCACCCACACTATCATGTTGACATTCC; dG = -33.1
2468 >s_a_s_d_s_e_s_c_s_a_s_c_s_a_s_e_s
GTGTCTGTGACGGTGCTGGTGTGGGTGTCTGTGTGGGTGTCTGTGCTGGTG; >clamp_s_c_s_d_s_c_s_a_s_e_s:compl:rev
CACCAGCACAGACACCCACACCGTCACCCACACTATCATGTTGACATTCC; dG = -33.1
2469 >s_a_s_e_s_a_s_c_s_a_s_c_s_a_s_e_s
GTGTCTGTGCTGGTGTCTGTGTGGGTGTCTGTGTGGGTGTCTGTGCTGGTG; >clamp_s_c_s_d_s_c_s_a_s_e_s:compl:rev
CACCAGCACAGACACCCACACCGTCACCCACACTATCATGTTGACATTCC; dG = -33.1
2470 >s_a_s_e_s_b_s_c_s_a_s_c_s_a_s_e_s
GTGTCTGTGCTGGTGGCTGTGTGGGTGTCTGTGTGGGTGTCTGTGCTGGTG; >clamp_s_c_s_d_s_c_s_a_s_e_s:compl:rev
CACCAGCACAGACACCCACACCGTCACCCACACTATCATGTTGACATTCC; dG = -33.1
2471 >s_a_s_e_s_c_s_c_s_a_s_c_s_a_s_e_s
GTGTCTGTGCTGGTGTGGGTGTGGGTGTCTGTGTGGGTGTCTGTGCTGGTG; >clamp_s_c_s_d_s_c_s_a_s_e_s:compl:rev
CACCAGCACAGACACCCACACCGTCACCCACACTATCATGTTGACATTCC; dG = -33.1
2472 >s_a_s_e_s_d_s_c_s_a_s_c_s_a_s_e_s
GTGTCTGTGCTGGTGACGGTGTGGGTGTCTGTGTGGGTGTCTGTGCTGGTG; >clamp_s_c_s_d_s_c_s_a_s_e_s:compl:rev
CACCAGCACAGACACCCACACCGTCACCCACACTATCATGTTGACATTCC; dG = -33.1
2473 >s_a_s_e_s_e_s_c_s_a_s_c_s_a_s_e_s
GTGTCTGTGCTGGTGCTGGTGTGGGTGTCTGTGTGGGTGTCTGTGCTGGTG; >clamp_s_c_s_d_s_c_s_a_s_e_s:compl:rev
CACCAGCACAGACACCCACACCGTCACCCACACTATCATGTTGACATTCC; dG = -33.1
2474 >s_b_s_a_s_a_s_c_s_a_s_c_s_a_s_e_s
GTGGCTGTGTCTGTGTCTGTGTGGGTGTCTGTGTGGGTGTCTGTGCTGGTG; >clamp_s_c_s_d_s_c_s_a_s_e_s:compl:rev
CACCAGCACAGACACCCACACCGTCACCCACACTATCATGTTGACATTCC; dG = -33.1
2475 >s_b_s_a_s_b_s_c_s_a_s_c_s_a_s_e_s
GTGGCTGTGTCTGTGGCTGTGTGGGTGTCTGTGTGGGTGTCTGTGCTGGTG; >clamp_s_c_s_d_s_c_s_a_s_e_s:compl:rev
CACCAGCACAGACACCCACACCGTCACCCACACTATCATGTTGACATTCC; dG = -33.1
2476 >s_b_s_a_s_c_s_c_s_a_s_c_s_a_s_e_s
GTGGCTGTGTCTGTGTGGGTGTGGGTGTCTGTGTGGGTGTCTGTGCTGGTG; >clamp_s_c_s_d_s_c_s_a_s_e_s:compl:rev
CACCAGCACAGACACCCACACCGTCACCCACACTATCATGTTGACATTCC; dG = -33.1
2477 >s_a_s_a_s_c_s_c_s_a_s_c_s_a_s_e_s
GTGTCTGTGTCTGTGTGGGTGTGGGTGTCTGTGTGGGTGTCTGTGCTGGTG; >clamp_s_c_s_d_s_c_s_a_s_e_s:compl:rev
CACCAGCACAGACACCCACACCGTCACCCACACTATCATGTTGACATTCC; dG = -33.1
2478 >s_b_s_a_s_d_s_c_s_a_s_c_s_a_s_e_s
GTGGCTGTGTCTGTGACGGTGTGGGTGTCTGTGTGGGTGTCTGTGCTGGTG; >clamp_s_c_s_d_s_c_s_a_s_e_s:compl:rev
CACCAGCACAGACACCCACACCGTCACCCACACTATCATGTTGACATTCC; dG = -33.1
2479 >s_b_s_a_s_e_s_c_s_a_s_c_s_a_s_e_s
GTGGCTGTGTCTGTGCTGGTGTGGGTGTCTGTGTGGGTGTCTGTGCTGGTG; >clamp_s_c_s_d_s_c_s_a_s_e_s:compl:rev
CACCAGCACAGACACCCACACCGTCACCCACACTATCATGTTGACATTCC; dG = -33.1

2480 >s_b_s_b_s_a_s_c_s_a_s_c_s_a_s_e_s
GTGGCTGTGGCTGTGTCTGTGTGGGTGTCTGTGTGGGTGTCTGTGCTGGTG; >clamp_s_c_s_d_s_c_s_a_s_e_s:compl:rev
CACCAGCACAGACACCCACACCGTCACCCACACTATCATGTTGACATTCC; dG = -33.1
2481 >s_b_s_b_s_b_s_c_s_a_s_c_s_a_s_e_s
GTGGCTGTGGCTGTGGCTGTGTGGGTGTCTGTGTGGGTGTCTGTGCTGGTG; >clamp_s_c_s_d_s_c_s_a_s_e_s:compl:rev
CACCAGCACAGACACCCACACCGTCACCCACACTATCATGTTGACATTCC; dG = -33.1
2482 >s_b_s_b_s_c_s_c_s_a_s_c_s_a_s_e_s
GTGGCTGTGGCTGTGTGGGTGTGGGTGTCTGTGTGGGTGTCTGTGCTGGTG; >clamp_s_c_s_d_s_c_s_a_s_e_s:compl:rev
CACCAGCACAGACACCCACACCGTCACCCACACTATCATGTTGACATTCC; dG = -33.1
2483 >s_b_s_b_s_d_s_c_s_a_s_c_s_a_s_e_s
GTGGCTGTGGCTGTGACGGTGTGGGTGTCTGTGTGGGTGTCTGTGCTGGTG; >clamp_s_c_s_d_s_c_s_a_s_e_s:compl:rev
CACCAGCACAGACACCCACACCGTCACCCACACTATCATGTTGACATTCC; dG = -33.1
2484 >s_b_s_b_s_e_s_c_s_a_s_c_s_a_s_e_s
GTGGCTGTGGCTGTGCTGGTGTGGGTGTCTGTGTGGGTGTCTGTGCTGGTG; >clamp_s_c_s_d_s_c_s_a_s_e_s:compl:rev
CACCAGCACAGACACCCACACCGTCACCCACACTATCATGTTGACATTCC; dG = -33.1
2485 >s_b_s_c_s_a_s_c_s_a_s_c_s_a_s_e_s
GTGGCTGTGTGGGTGTCTGTGTGGGTGTCTGTGTGGGTGTCTGTGCTGGTG; >clamp_s_c_s_d_s_c_s_a_s_e_s:compl:rev
CACCAGCACAGACACCCACACCGTCACCCACACTATCATGTTGACATTCC; dG = -33.1
2486 >s_b_s_c_s_b_s_c_s_a_s_c_s_a_s_e_s
GTGGCTGTGTGGGTGGCTGTGTGGGTGTCTGTGTGGGTGTCTGTGCTGGTG; >clamp_s_c_s_d_s_c_s_a_s_e_s:compl:rev
CACCAGCACAGACACCCACACCGTCACCCACACTATCATGTTGACATTCC; dG = -33.1
2487 >s_b_s_c_s_c_s_c_s_a_s_c_s_a_s_e_s
GTGGCTGTGTGGGTGTGGGTGTGGGTGTCTGTGTGGGTGTCTGTGCTGGTG; >clamp_s_c_s_d_s_c_s_a_s_e_s:compl:rev
CACCAGCACAGACACCCACACCGTCACCCACACTATCATGTTGACATTCC; dG = -33.1
2488 >s_a_s_a_s_d_s_c_s_a_s_c_s_a_s_e_s
GTGTCTGTGTCTGTGACGGTGTGGGTGTCTGTGTGGGTGTCTGTGCTGGTG; >clamp_s_c_s_d_s_c_s_a_s_e_s:compl:rev
CACCAGCACAGACACCCACACCGTCACCCACACTATCATGTTGACATTCC; dG = -33.1
2489 >s_b_s_c_s_e_s_c_s_a_s_c_s_a_s_e_s
GTGGCTGTGTGGGTGCTGGTGTGGGTGTCTGTGTGGGTGTCTGTGCTGGTG; >clamp_s_c_s_d_s_c_s_a_s_e_s:compl:rev
CACCAGCACAGACACCCACACCGTCACCCACACTATCATGTTGACATTCC; dG = -33.1
2490 >s_b_s_d_s_a_s_c_s_a_s_c_s_a_s_e_s
GTGGCTGTGACGGTGTCTGTGTGGGTGTCTGTGTGGGTGTCTGTGCTGGTG; >clamp_s_c_s_d_s_c_s_a_s_e_s:compl:rev
CACCAGCACAGACACCCACACCGTCACCCACACTATCATGTTGACATTCC; dG = -33.1
2491 >s_b_s_d_s_b_s_c_s_a_s_c_s_a_s_e_s
GTGGCTGTGACGGTGGCTGTGTGGGTGTCTGTGTGGGTGTCTGTGCTGGTG; >clamp_s_c_s_d_s_c_s_a_s_e_s:compl:rev
CACCAGCACAGACACCCACACCGTCACCCACACTATCATGTTGACATTCC; dG = -33.1
2492 >s_b_s_d_s_c_s_c_s_a_s_c_s_a_s_e_s
GTGGCTGTGACGGTGTGGGTGTGGGTGTCTGTGTGGGTGTCTGTGCTGGTG; >clamp_s_c_s_d_s_c_s_a_s_e_s:compl:rev
CACCAGCACAGACACCCACACCGTCACCCACACTATCATGTTGACATTCC; dG = -33.1
2493 >s_b_s_d_s_d_s_c_s_a_s_c_s_a_s_e_s
GTGGCTGTGACGGTGACGGTGTGGGTGTCTGTGTGGGTGTCTGTGCTGGTG; >clamp_s_c_s_d_s_c_s_a_s_e_s:compl:rev
CACCAGCACAGACACCCACACCGTCACCCACACTATCATGTTGACATTCC; dG = -33.1
2494 >s_b_s_d_s_e_s_c_s_a_s_c_s_a_s_e_s
GTGGCTGTGACGGTGCTGGTGTGGGTGTCTGTGTGGGTGTCTGTGCTGGTG; >clamp_s_c_s_d_s_c_s_a_s_e_s:compl:rev
CACCAGCACAGACACCCACACCGTCACCCACACTATCATGTTGACATTCC; dG = -33.1
2495 >s_b_s_e_s_a_s_c_s_a_s_c_s_a_s_e_s
GTGGCTGTGCTGGTGTCTGTGTGGGTGTCTGTGTGGGTGTCTGTGCTGGTG; >clamp_s_c_s_d_s_c_s_a_s_e_s:compl:rev
CACCAGCACAGACACCCACACCGTCACCCACACTATCATGTTGACATTCC; dG = -33.1
2496 >s_b_s_e_s_b_s_c_s_a_s_c_s_a_s_e_s
GTGGCTGTGCTGGTGGCTGTGTGGGTGTCTGTGTGGGTGTCTGTGCTGGTG; >clamp_s_c_s_d_s_c_s_a_s_e_s:compl:rev
CACCAGCACAGACACCCACACCGTCACCCACACTATCATGTTGACATTCC; dG = -33.1
2497 >s_b_s_e_s_c_s_c_s_a_s_c_s_a_s_e_s
GTGGCTGTGCTGGTGTGGGTGTGGGTGTCTGTGTGGGTGTCTGTGCTGGTG; >clamp_s_c_s_d_s_c_s_a_s_e_s:compl:rev
CACCAGCACAGACACCCACACCGTCACCCACACTATCATGTTGACATTCC; dG = -33.1
2498 >s_a_s_a_s_e_s_c_s_a_s_c_s_a_s_e_s
GTGTCTGTGTCTGTGCTGGTGTGGGTGTCTGTGTGGGTGTCTGTGCTGGTG; >clamp_s_c_s_d_s_c_s_a_s_e_s:compl:rev
CACCAGCACAGACACCCACACCGTCACCCACACTATCATGTTGACATTCC; dG = -33.1
2499 >s_b_s_e_s_d_s_c_s_a_s_c_s_a_s_e_s
GTGGCTGTGCTGGTGACGGTGTGGGTGTCTGTGTGGGTGTCTGTGCTGGTG; >clamp_s_c_s_d_s_c_s_a_s_e_s:compl:rev
CACCAGCACAGACACCCACACCGTCACCCACACTATCATGTTGACATTCC; dG = -33.1
2500 >s_b_s_e_s_e_s_c_s_a_s_c_s_a_s_e_s
GTGGCTGTGCTGGTGCTGGTGTGGGTGTCTGTGTGGGTGTCTGTGCTGGTG; >clamp_s_c_s_d_s_c_s_a_s_e_s:compl:rev
CACCAGCACAGACACCCACACCGTCACCCACACTATCATGTTGACATTCC; dG = -33.1

****

CDCA Matches

1 >s_a_s_a_s_a_s_c_s_d_s_c_s_a_s GTGTCTGTGTCTGTGTCTGTGTGGGTGACGGTGTGGGTGTCTGTG;
>clamp_s_c_s_d_s_c_s_a_s:compl:rev CACAGACACCCACACCGTCACCCACACTATCATGTTGACATTCC; dG = -34.0

```
     2 >s_e_s_a_s_a_s_c_s_d_s_c_s_a_s          GTGCTGGTGTCTGTGTCTGTGTGGGTGACGGTGTGGGTGTCTGTG;
>clamp_s_c_s_d_s_c_s_a_s:compl:rev CACAGACACCCACACCGTCACCCACACTATCATGTTGACATTCC; dG = -34.0
     3 >s_e_s_a_s_b_s_c_s_d_s_c_s_a_s          GTGCTGGTGTCTGTGGCTGTGTGGGTGACGGTGTGGGTGTCTGTG;
>clamp_s_c_s_d_s_c_s_a_s:compl:rev CACAGACACCCACACCGTCACCCACACTATCATGTTGACATTCC; dG = -34.0
     4 >s_e_s_b_s_a_s_c_s_d_s_c_s_a_s          GTGCTGGTGGCTGTGTCTGTGTGGGTGACGGTGTGGGTGTCTGTG;
>clamp_s_c_s_d_s_c_s_a_s:compl:rev CACAGACACCCACACCGTCACCCACACTATCATGTTGACATTCC; dG = -34.0
     5 >s_e_s_b_s_b_s_c_s_d_s_c_s_a_s          GTGCTGGTGGCTGTGGCTGTGTGGGTGACGGTGTGGGTGTCTGTG;
>clamp_s_c_s_d_s_c_s_a_s:compl:rev CACAGACACCCACACCGTCACCCACACTATCATGTTGACATTCC; dG = -34.0
     6 >s_a_s_c_s_a_s_c_s_d_s_c_s_a_s          GTGTCTGTGTGGGTGTCTGTGTGGGTGACGGTGTGGGTGTCTGTG;
>clamp_s_c_s_d_s_c_s_a_s:compl:rev CACAGACACCCACACCGTCACCCACACTATCATGTTGACATTCC; dG = -34.0
     7 >s_e_s_c_s_a_s_c_s_d_s_c_s_a_s          GTGCTGGTGTGGGTGTCTGTGTGGGTGACGGTGTGGGTGTCTGTG;
>clamp_s_c_s_d_s_c_s_a_s:compl:rev CACAGACACCCACACCGTCACCCACACTATCATGTTGACATTCC; dG = -34.0
     8 >s_e_s_c_s_b_s_c_s_d_s_c_s_a_s          GTGCTGGTGTGGGTGGCTGTGTGGGTGACGGTGTGGGTGTCTGTG;
>clamp_s_c_s_d_s_c_s_a_s:compl:rev CACAGACACCCACACCGTCACCCACACTATCATGTTGACATTCC; dG = -34.0
     9 >s_e_s_d_s_a_s_c_s_d_s_c_s_a_s          GTGCTGGTGACGGTGTCTGTGTGGGTGACGGTGTGGGTGTCTGTG;
>clamp_s_c_s_d_s_c_s_a_s:compl:rev CACAGACACCCACACCGTCACCCACACTATCATGTTGACATTCC; dG = -34.0
    10 >s_e_s_d_s_b_s_c_s_d_s_c_s_a_s          GTGCTGGTGACGGTGGCTGTGTGGGTGACGGTGTGGGTGTCTGTG;
>clamp_s_c_s_d_s_c_s_a_s:compl:rev CACAGACACCCACACCGTCACCCACACTATCATGTTGACATTCC; dG = -34.0
    11 >s_a_s_c_s_b_s_c_s_d_s_c_s_a_s          GTGTCTGTGTGGGTGGCTGTGTGGGTGACGGTGTGGGTGTCTGTG;
>clamp_s_c_s_d_s_c_s_a_s:compl:rev CACAGACACCCACACCGTCACCCACACTATCATGTTGACATTCC; dG = -34.0
    12 >s_e_s_e_s_a_s_c_s_d_s_c_s_a_s          GTGCTGGTGCTGGTGTCTGTGTGGGTGACGGTGTGGGTGTCTGTG;
>clamp_s_c_s_d_s_c_s_a_s:compl:rev CACAGACACCCACACCGTCACCCACACTATCATGTTGACATTCC; dG = -34.0
    13 >s_e_s_e_s_b_s_c_s_d_s_c_s_a_s          GTGCTGGTGCTGGTGGCTGTGTGGGTGACGGTGTGGGTGTCTGTG;
>clamp_s_c_s_d_s_c_s_a_s:compl:rev CACAGACACCCACACCGTCACCCACACTATCATGTTGACATTCC; dG = -34.0
    14 >s_a_s_d_s_a_s_c_s_d_s_c_s_a_s          GTGTCTGTGACGGTGTCTGTGTGGGTGACGGTGTGGGTGTCTGTG;
>clamp_s_c_s_d_s_c_s_a_s:compl:rev CACAGACACCCACACCGTCACCCACACTATCATGTTGACATTCC; dG = -34.0
    15 >s_a_s_d_s_b_s_c_s_d_s_c_s_a_s          GTGTCTGTGACGGTGGCTGTGTGGGTGACGGTGTGGGTGTCTGTG;
>clamp_s_c_s_d_s_c_s_a_s:compl:rev CACAGACACCCACACCGTCACCCACACTATCATGTTGACATTCC; dG = -34.0
    16 >s_a_s_a_s_b_s_c_s_d_s_c_s_a_s          GTGTCTGTGTCTGTGGCTGTGTGGGTGACGGTGTGGGTGTCTGTG;
>clamp_s_c_s_d_s_c_s_a_s:compl:rev CACAGACACCCACACCGTCACCCACACTATCATGTTGACATTCC; dG = -34.0
    17 >s_a_s_e_s_a_s_c_s_d_s_c_s_a_s          GTGTCTGTGCTGGTGTCTGTGTGGGTGACGGTGTGGGTGTCTGTG;
>clamp_s_c_s_d_s_c_s_a_s:compl:rev CACAGACACCCACACCGTCACCCACACTATCATGTTGACATTCC; dG = -34.0
    18 >s_a_s_e_s_b_s_c_s_d_s_c_s_a_s          GTGTCTGTGCTGGTGGCTGTGTGGGTGACGGTGTGGGTGTCTGTG;
>clamp_s_c_s_d_s_c_s_a_s:compl:rev CACAGACACCCACACCGTCACCCACACTATCATGTTGACATTCC; dG = -34.0
    19 >s_b_s_a_s_a_s_c_s_d_s_c_s_a_s          GTGGCTGTGTCTGTGTCTGTGTGGGTGACGGTGTGGGTGTCTGTG;
>clamp_s_c_s_d_s_c_s_a_s:compl:rev CACAGACACCCACACCGTCACCCACACTATCATGTTGACATTCC; dG = -34.0
    20 >s_b_s_a_s_b_s_c_s_d_s_c_s_a_s          GTGGCTGTGTCTGTGGCTGTGTGGGTGACGGTGTGGGTGTCTGTG;
>clamp_s_c_s_d_s_c_s_a_s:compl:rev CACAGACACCCACACCGTCACCCACACTATCATGTTGACATTCC; dG = -34.0
    21 >s_b_s_b_s_a_s_c_s_d_s_c_s_a_s          GTGGCTGTGGCTGTGTCTGTGTGGGTGACGGTGTGGGTGTCTGTG;
>clamp_s_c_s_d_s_c_s_a_s:compl:rev CACAGACACCCACACCGTCACCCACACTATCATGTTGACATTCC; dG = -34.0
    22 >s_b_s_b_s_b_s_c_s_d_s_c_s_a_s          GTGGCTGTGGCTGTGGCTGTGTGGGTGACGGTGTGGGTGTCTGTG;
>clamp_s_c_s_d_s_c_s_a_s:compl:rev CACAGACACCCACACCGTCACCCACACTATCATGTTGACATTCC; dG = -34.0
    23 >s_b_s_c_s_a_s_c_s_d_s_c_s_a_s          GTGGCTGTGTGGGTGTCTGTGTGGGTGACGGTGTGGGTGTCTGTG;
>clamp_s_c_s_d_s_c_s_a_s:compl:rev CACAGACACCCACACCGTCACCCACACTATCATGTTGACATTCC; dG = -34.0
    24 >s_b_s_c_s_b_s_c_s_d_s_c_s_a_s          GTGGCTGTGTGGGTGGCTGTGTGGGTGACGGTGTGGGTGTCTGTG;
>clamp_s_c_s_d_s_c_s_a_s:compl:rev CACAGACACCCACACCGTCACCCACACTATCATGTTGACATTCC; dG = -34.0
    25 >s_b_s_d_s_a_s_c_s_d_s_c_s_a_s          GTGGCTGTGACGGTGTCTGTGTGGGTGACGGTGTGGGTGTCTGTG;
>clamp_s_c_s_d_s_c_s_a_s:compl:rev CACAGACACCCACACCGTCACCCACACTATCATGTTGACATTCC; dG = -34.0
    26 >s_b_s_d_s_b_s_c_s_d_s_c_s_a_s          GTGGCTGTGACGGTGGCTGTGTGGGTGACGGTGTGGGTGTCTGTG;
>clamp_s_c_s_d_s_c_s_a_s:compl:rev CACAGACACCCACACCGTCACCCACACTATCATGTTGACATTCC; dG = -34.0
    27 >s_b_s_e_s_a_s_c_s_d_s_c_s_a_s          GTGGCTGTGCTGGTGTCTGTGTGGGTGACGGTGTGGGTGTCTGTG;
>clamp_s_c_s_d_s_c_s_a_s:compl:rev CACAGACACCCACACCGTCACCCACACTATCATGTTGACATTCC; dG = -34.0
    28 >s_b_s_e_s_b_s_c_s_d_s_c_s_a_s          GTGGCTGTGCTGGTGGCTGTGTGGGTGACGGTGTGGGTGTCTGTG;
>clamp_s_c_s_d_s_c_s_a_s:compl:rev CACAGACACCCACACCGTCACCCACACTATCATGTTGACATTCC; dG = -34.0
    29 >s_c_s_a_s_a_s_c_s_d_s_c_s_a_s          GTGTGGGTGTCTGTGTCTGTGTGGGTGACGGTGTGGGTGTCTGTG;
>clamp_s_c_s_d_s_c_s_a_s:compl:rev CACAGACACCCACACCGTCACCCACACTATCATGTTGACATTCC; dG = -34.0
    30 >s_c_s_a_s_b_s_c_s_d_s_c_s_a_s          GTGTGGGTGTCTGTGGCTGTGTGGGTGACGGTGTGGGTGTCTGTG;
>clamp_s_c_s_d_s_c_s_a_s:compl:rev CACAGACACCCACACCGTCACCCACACTATCATGTTGACATTCC; dG = -34.0
    31 >s_c_s_b_s_a_s_c_s_d_s_c_s_a_s          GTGTGGGTGGCTGTGTCTGTGTGGGTGACGGTGTGGGTGTCTGTG;
>clamp_s_c_s_d_s_c_s_a_s:compl:rev CACAGACACCCACACCGTCACCCACACTATCATGTTGACATTCC; dG = -34.0
    32 >s_c_s_b_s_b_s_c_s_d_s_c_s_a_s          GTGTGGGTGGCTGTGGCTGTGTGGGTGACGGTGTGGGTGTCTGTG;
>clamp_s_c_s_d_s_c_s_a_s:compl:rev CACAGACACCCACACCGTCACCCACACTATCATGTTGACATTCC; dG = -34.0
    33 >s_a_s_b_s_a_s_c_s_d_s_c_s_a_s          GTGTCTGTGGCTGTGTCTGTGTGGGTGACGGTGTGGGTGTCTGTG;
>clamp_s_c_s_d_s_c_s_a_s:compl:rev CACAGACACCCACACCGTCACCCACACTATCATGTTGACATTCC; dG = -34.0
    34 >s_c_s_c_s_a_s_c_s_d_s_c_s_a_s          GTGTGGGTGTGGGTGTCTGTGTGGGTGACGGTGTGGGTGTCTGTG;
>clamp_s_c_s_d_s_c_s_a_s:compl:rev CACAGACACCCACACCGTCACCCACACTATCATGTTGACATTCC; dG = -34.0
    35 >s_c_s_c_s_b_s_c_s_d_s_c_s_a_s          GTGTGGGTGTGGGTGGCTGTGTGGGTGACGGTGTGGGTGTCTGTG;
>clamp_s_c_s_d_s_c_s_a_s:compl:rev CACAGACACCCACACCGTCACCCACACTATCATGTTGACATTCC; dG = -34.0
    36 >s_c_s_d_s_a_s_c_s_d_s_c_s_a_s          GTGTGGGTGACGGTGTCTGTGTGGGTGACGGTGTGGGTGTCTGTG;
>clamp_s_c_s_d_s_c_s_a_s:compl:rev CACAGACACCCACACCGTCACCCACACTATCATGTTGACATTCC; dG = -34.0
    37 >s_c_s_d_s_b_s_c_s_d_s_c_s_a_s          GTGTGGGTGACGGTGGCTGTGTGGGTGACGGTGTGGGTGTCTGTG;
>clamp_s_c_s_d_s_c_s_a_s:compl:rev CACAGACACCCACACCGTCACCCACACTATCATGTTGACATTCC; dG = -34.0
    38 >s_a_s_b_s_b_s_c_s_d_s_c_s_a_s          GTGTCTGTGGCTGTGGCTGTGTGGGTGACGGTGTGGGTGTCTGTG;
>clamp_s_c_s_d_s_c_s_a_s:compl:rev CACAGACACCCACACCGTCACCCACACTATCATGTTGACATTCC; dG = -34.0
```

```
    39 >s_c_s_e_s_a_s_c_s_d_s_c_s_a_s          GTGTGGGTGCTGGTGTCTGTGTGGGTGACGGTGTGGGTGTCTGTG;
>clamp_s_c_s_d_s_c_s_a_s:compl:rev CACAGACACCCACACCGTCACCCACACTATCATGTTGACATTCC; dG = -34.0
    40 >s_c_s_e_s_b_s_c_s_d_s_c_s_a_s          GTGTGGGTGCTGGTGGCTGTGTGGGTGACGGTGTGGGTGTCTGTG;
>clamp_s_c_s_d_s_c_s_a_s:compl:rev CACAGACACCCACACCGTCACCCACACTATCATGTTGACATTCC; dG = -34.0
    41 >s_d_s_a_s_a_s_c_s_d_s_c_s_a_s          GTGACGGTGTCTGTGTCTGTGTGGGTGACGGTGTGGGTGTCTGTG;
>clamp_s_c_s_d_s_c_s_a_s:compl:rev CACAGACACCCACACCGTCACCCACACTATCATGTTGACATTCC; dG = -34.0
    42 >s_d_s_a_s_b_s_c_s_d_s_c_s_a_s          GTGACGGTGTCTGTGGCTGTGTGGGTGACGGTGTGGGTGTCTGTG;
>clamp_s_c_s_d_s_c_s_a_s:compl:rev CACAGACACCCACACCGTCACCCACACTATCATGTTGACATTCC; dG = -34.0
    43 >s_d_s_b_s_a_s_c_s_d_s_c_s_a_s          GTGACGGTGGCTGTGTCTGTGTGGGTGACGGTGTGGGTGTCTGTG;
>clamp_s_c_s_d_s_c_s_a_s:compl:rev CACAGACACCCACACCGTCACCCACACTATCATGTTGACATTCC; dG = -34.0
    44 >s_d_s_b_s_b_s_c_s_d_s_c_s_a_s          GTGACGGTGGCTGTGGCTGTGTGGGTGACGGTGTGGGTGTCTGTG;
>clamp_s_c_s_d_s_c_s_a_s:compl:rev CACAGACACCCACACCGTCACCCACACTATCATGTTGACATTCC; dG = -34.0
    45 >s_d_s_c_s_a_s_c_s_d_s_c_s_a_s          GTGACGGTGTGGGTGTCTGTGTGGGTGACGGTGTGGGTGTCTGTG;
>clamp_s_c_s_d_s_c_s_a_s:compl:rev CACAGACACCCACACCGTCACCCACACTATCATGTTGACATTCC; dG = -34.0
    46 >s_d_s_c_s_b_s_c_s_d_s_c_s_a_s          GTGACGGTGTGGGTGGCTGTGTGGGTGACGGTGTGGGTGTCTGTG;
>clamp_s_c_s_d_s_c_s_a_s:compl:rev CACAGACACCCACACCGTCACCCACACTATCATGTTGACATTCC; dG = -34.0
    47 >s_d_s_d_s_a_s_c_s_d_s_c_s_a_s          GTGACGGTGACGGTGTCTGTGTGGGTGACGGTGTGGGTGTCTGTG;
>clamp_s_c_s_d_s_c_s_a_s:compl:rev CACAGACACCCACACCGTCACCCACACTATCATGTTGACATTCC; dG = -34.0
    48 >s_d_s_d_s_b_s_c_s_d_s_c_s_a_s          GTGACGGTGACGGTGGCTGTGTGGGTGACGGTGTGGGTGTCTGTG;
>clamp_s_c_s_d_s_c_s_a_s:compl:rev CACAGACACCCACACCGTCACCCACACTATCATGTTGACATTCC; dG = -34.0
    49 >s_d_s_e_s_a_s_c_s_d_s_c_s_a_s          GTGACGGTGCTGGTGTCTGTGTGGGTGACGGTGTGGGTGTCTGTG;
>clamp_s_c_s_d_s_c_s_a_s:compl:rev CACAGACACCCACACCGTCACCCACACTATCATGTTGACATTCC; dG = -34.0
    50 >s_d_s_e_s_b_s_c_s_d_s_c_s_a_s          GTGACGGTGCTGGTGGCTGTGTGGGTGACGGTGTGGGTGTCTGTG;
>clamp_s_c_s_d_s_c_s_a_s:compl:rev CACAGACACCCACACCGTCACCCACACTATCATGTTGACATTCC; dG = -34.0
    51 >s_a_s_b_s_e_s_c_s_d_s_c_s_a_s          GTGTCTGTGGCTGTGCTGGTGTGGGTGACGGTGTGGGTGTCTGTG;
>clamp_s_c_s_d_s_c_s_a_s:compl:rev CACAGACACCCACACCGTCACCCACACTATCATGTTGACATTCC; dG = -33.9
    52 >s_d_s_e_s_e_s_c_s_d_s_c_s_a_s          GTGACGGTGCTGGTGCTGGTGTGGGTGACGGTGTGGGTGTCTGTG;
>clamp_s_c_s_d_s_c_s_a_s:compl:rev CACAGACACCCACACCGTCACCCACACTATCATGTTGACATTCC; dG = -33.9
    53 >s_e_s_a_s_c_s_c_s_d_s_c_s_a_s          GTGCTGGTGTCTGTGTGGGTGTGGGTGACGGTGTGGGTGTCTGTG;
>clamp_s_c_s_d_s_c_s_a_s:compl:rev CACAGACACCCACACCGTCACCCACACTATCATGTTGACATTCC; dG = -33.9
    54 >s_e_s_a_s_d_s_c_s_d_s_c_s_a_s          GTGCTGGTGTCTGTGACGGTGTGGGTGACGGTGTGGGTGTCTGTG;
>clamp_s_c_s_d_s_c_s_a_s:compl:rev CACAGACACCCACACCGTCACCCACACTATCATGTTGACATTCC; dG = -33.9
    55 >s_e_s_a_s_e_s_c_s_d_s_c_s_a_s          GTGCTGGTGTCTGTGCTGGTGTGGGTGACGGTGTGGGTGTCTGTG;
>clamp_s_c_s_d_s_c_s_a_s:compl:rev CACAGACACCCACACCGTCACCCACACTATCATGTTGACATTCC; dG = -33.9
    56 >s_e_s_b_s_c_s_c_s_d_s_c_s_a_s          GTGCTGGTGGCTGTGTGGGTGTGGGTGACGGTGTGGGTGTCTGTG;
>clamp_s_c_s_d_s_c_s_a_s:compl:rev CACAGACACCCACACCGTCACCCACACTATCATGTTGACATTCC; dG = -33.9
    57 >s_e_s_b_s_d_s_c_s_d_s_c_s_a_s          GTGCTGGTGGCTGTGACGGTGTGGGTGACGGTGTGGGTGTCTGTG;
>clamp_s_c_s_d_s_c_s_a_s:compl:rev CACAGACACCCACACCGTCACCCACACTATCATGTTGACATTCC; dG = -33.9
    58 >s_e_s_b_s_e_s_c_s_d_s_c_s_a_s          GTGCTGGTGGCTGTGCTGGTGTGGGTGACGGTGTGGGTGTCTGTG;
>clamp_s_c_s_d_s_c_s_a_s:compl:rev CACAGACACCCACACCGTCACCCACACTATCATGTTGACATTCC; dG = -33.9
    59 >s_e_s_c_s_c_s_c_s_d_s_c_s_a_s          GTGCTGGTGTGGGTGTGGGTGTGGGTGACGGTGTGGGTGTCTGTG;
>clamp_s_c_s_d_s_c_s_a_s:compl:rev CACAGACACCCACACCGTCACCCACACTATCATGTTGACATTCC; dG = -33.9
    60 >s_e_s_c_s_d_s_c_s_d_s_c_s_a_s          GTGCTGGTGTGGGTGACGGTGTGGGTGACGGTGTGGGTGTCTGTG;
>clamp_s_c_s_d_s_c_s_a_s:compl:rev CACAGACACCCACACCGTCACCCACACTATCATGTTGACATTCC; dG = -33.9
    61 >s_e_s_c_s_e_s_c_s_d_s_c_s_a_s          GTGCTGGTGTGGGTGCTGGTGTGGGTGACGGTGTGGGTGTCTGTG;
>clamp_s_c_s_d_s_c_s_a_s:compl:rev CACAGACACCCACACCGTCACCCACACTATCATGTTGACATTCC; dG = -33.9
    62 >s_e_s_d_s_c_s_c_s_d_s_c_s_a_s          GTGCTGGTGACGGTGTGGGTGTGGGTGACGGTGTGGGTGTCTGTG;
>clamp_s_c_s_d_s_c_s_a_s:compl:rev CACAGACACCCACACCGTCACCCACACTATCATGTTGACATTCC; dG = -33.9
    63 >s_e_s_d_s_d_s_c_s_d_s_c_s_a_s          GTGCTGGTGACGGTGACGGTGTGGGTGACGGTGTGGGTGTCTGTG;
>clamp_s_c_s_d_s_c_s_a_s:compl:rev CACAGACACCCACACCGTCACCCACACTATCATGTTGACATTCC; dG = -33.9
    64 >s_e_s_d_s_e_s_c_s_d_s_c_s_a_s          GTGCTGGTGACGGTGCTGGTGTGGGTGACGGTGTGGGTGTCTGTG;
>clamp_s_c_s_d_s_c_s_a_s:compl:rev CACAGACACCCACACCGTCACCCACACTATCATGTTGACATTCC; dG = -33.9
    65 >s_e_s_e_s_c_s_c_s_d_s_c_s_a_s          GTGCTGGTGCTGGTGTGGGTGTGGGTGACGGTGTGGGTGTCTGTG;
>clamp_s_c_s_d_s_c_s_a_s:compl:rev CACAGACACCCACACCGTCACCCACACTATCATGTTGACATTCC; dG = -33.9
    66 >s_e_s_e_s_d_s_c_s_d_s_c_s_a_s          GTGCTGGTGCTGGTGACGGTGTGGGTGACGGTGTGGGTGTCTGTG;
>clamp_s_c_s_d_s_c_s_a_s:compl:rev CACAGACACCCACACCGTCACCCACACTATCATGTTGACATTCC; dG = -33.9
    67 >s_e_s_e_s_e_s_c_s_d_s_c_s_a_s          GTGCTGGTGCTGGTGCTGGTGTGGGTGACGGTGTGGGTGTCTGTG;
>clamp_s_c_s_d_s_c_s_a_s:compl:rev CACAGACACCCACACCGTCACCCACACTATCATGTTGACATTCC; dG = -33.9
    68 >s_a_s_c_s_c_s_c_s_d_s_c_s_a_s          GTGTCTGTGTGGGTGTGGGTGTGGGTGACGGTGTGGGTGTCTGTG;
>clamp_s_c_s_d_s_c_s_a_s:compl:rev CACAGACACCCACACCGTCACCCACACTATCATGTTGACATTCC; dG = -33.9
    69 >s_a_s_c_s_d_s_c_s_d_s_c_s_a_s          GTGTCTGTGTGGGTGACGGTGTGGGTGACGGTGTGGGTGTCTGTG;
>clamp_s_c_s_d_s_c_s_a_s:compl:rev CACAGACACCCACACCGTCACCCACACTATCATGTTGACATTCC; dG = -33.9
    70 >s_a_s_c_s_e_s_c_s_d_s_c_s_a_s          GTGTCTGTGTGGGTGCTGGTGTGGGTGACGGTGTGGGTGTCTGTG;
>clamp_s_c_s_d_s_c_s_a_s:compl:rev CACAGACACCCACACCGTCACCCACACTATCATGTTGACATTCC; dG = -33.9
    71 >s_a_s_d_s_c_s_c_s_d_s_c_s_a_s          GTGTCTGTGACGGTGTGGGTGTGGGTGACGGTGTGGGTGTCTGTG;
>clamp_s_c_s_d_s_c_s_a_s:compl:rev CACAGACACCCACACCGTCACCCACACTATCATGTTGACATTCC; dG = -33.9
    72 >s_a_s_d_s_d_s_c_s_d_s_c_s_a_s          GTGTCTGTGACGGTGACGGTGTGGGTGACGGTGTGGGTGTCTGTG;
>clamp_s_c_s_d_s_c_s_a_s:compl:rev CACAGACACCCACACCGTCACCCACACTATCATGTTGACATTCC; dG = -33.9
    73 >s_a_s_d_s_e_s_c_s_d_s_c_s_a_s          GTGTCTGTGACGGTGCTGGTGTGGGTGACGGTGTGGGTGTCTGTG;
>clamp_s_c_s_d_s_c_s_a_s:compl:rev CACAGACACCCACACCGTCACCCACACTATCATGTTGACATTCC; dG = -33.9
    74 >s_a_s_e_s_c_s_c_s_d_s_c_s_a_s          GTGTCTGTGCTGGTGTGGGTGTGGGTGACGGTGTGGGTGTCTGTG;
>clamp_s_c_s_d_s_c_s_a_s:compl:rev CACAGACACCCACACCGTCACCCACACTATCATGTTGACATTCC; dG = -33.9
    75 >s_a_s_e_s_d_s_c_s_d_s_c_s_a_s          GTGTCTGTGCTGGTGACGGTGTGGGTGACGGTGTGGGTGTCTGTG;
>clamp_s_c_s_d_s_c_s_a_s:compl:rev CACAGACACCCACACCGTCACCCACACTATCATGTTGACATTCC; dG = -33.9
```

```
    76 >s_a_s_e_s_e_s_c_s_d_s_c_s_a_s          GTGTCTGTGCTGGTGCTGGTGTGGGTGACGGTGTGGGTGTCTGTG;
>clamp_s_c_s_d_s_c_s_a_s:compl:rev CACAGACACCCACACCGTCACCCACACTATCATGTTGACATTCC; dG = -33.9
    77 >s_b_s_a_s_c_s_c_s_d_s_c_s_a_s          GTGGCTGTGTCTGTGTGGGTGTGGGTGACGGTGTGGGTGTCTGTG;
>clamp_s_c_s_d_s_c_s_a_s:compl:rev CACAGACACCCACACCGTCACCCACACTATCATGTTGACATTCC; dG = -33.9
    78 >s_b_s_a_s_d_s_c_s_d_s_c_s_a_s          GTGGCTGTGTCTGTGACGGTGTGGGTGACGGTGTGGGTGTCTGTG;
>clamp_s_c_s_d_s_c_s_a_s:compl:rev CACAGACACCCACACCGTCACCCACACTATCATGTTGACATTCC; dG = -33.9
    79 >s_a_s_a_s_c_s_c_s_d_s_c_s_a_s          GTGTCTGTGTCTGTGTGGGTGTGGGTGACGGTGTGGGTGTCTGTG;
>clamp_s_c_s_d_s_c_s_a_s:compl:rev CACAGACACCCACACCGTCACCCACACTATCATGTTGACATTCC; dG = -33.9
    80 >s_b_s_a_s_e_s_c_s_d_s_c_s_a_s          GTGGCTGTGTCTGTGCTGGTGTGGGTGACGGTGTGGGTGTCTGTG;
>clamp_s_c_s_d_s_c_s_a_s:compl:rev CACAGACACCCACACCGTCACCCACACTATCATGTTGACATTCC; dG = -33.9
    81 >s_b_s_b_s_c_s_c_s_d_s_c_s_a_s          GTGGCTGTGGCTGTGTGGGTGTGGGTGACGGTGTGGGTGTCTGTG;
>clamp_s_c_s_d_s_c_s_a_s:compl:rev CACAGACACCCACACCGTCACCCACACTATCATGTTGACATTCC; dG = -33.9
    82 >s_b_s_b_s_d_s_c_s_d_s_c_s_a_s          GTGGCTGTGGCTGTGACGGTGTGGGTGACGGTGTGGGTGTCTGTG;
>clamp_s_c_s_d_s_c_s_a_s:compl:rev CACAGACACCCACACCGTCACCCACACTATCATGTTGACATTCC; dG = -33.9
    83 >s_b_s_b_s_e_s_c_s_d_s_c_s_a_s          GTGGCTGTGGCTGTGCTGGTGTGGGTGACGGTGTGGGTGTCTGTG;
>clamp_s_c_s_d_s_c_s_a_s:compl:rev CACAGACACCCACACCGTCACCCACACTATCATGTTGACATTCC; dG = -33.9
    84 >s_b_s_c_s_c_s_c_s_d_s_c_s_a_s          GTGGCTGTGTGGGTGTGGGTGTGGGTGACGGTGTGGGTGTCTGTG;
>clamp_s_c_s_d_s_c_s_a_s:compl:rev CACAGACACCCACACCGTCACCCACACTATCATGTTGACATTCC; dG = -33.9
    85 >s_b_s_c_s_d_s_c_s_d_s_c_s_a_s          GTGGCTGTGTGGGTGACGGTGTGGGTGACGGTGTGGGTGTCTGTG;
>clamp_s_c_s_d_s_c_s_a_s:compl:rev CACAGACACCCACACCGTCACCCACACTATCATGTTGACATTCC; dG = -33.9
    86 >s_a_s_a_s_d_s_c_s_d_s_c_s_a_s          GTGTCTGTGTCTGTGACGGTGTGGGTGACGGTGTGGGTGTCTGTG;
>clamp_s_c_s_d_s_c_s_a_s:compl:rev CACAGACACCCACACCGTCACCCACACTATCATGTTGACATTCC; dG = -33.9
    87 >s_b_s_c_s_e_s_c_s_d_s_c_s_a_s          GTGGCTGTGTGGGTGCTGGTGTGGGTGACGGTGTGGGTGTCTGTG;
>clamp_s_c_s_d_s_c_s_a_s:compl:rev CACAGACACCCACACCGTCACCCACACTATCATGTTGACATTCC; dG = -33.9
    88 >s_b_s_d_s_c_s_c_s_d_s_c_s_a_s          GTGGCTGTGACGGTGTGGGTGTGGGTGACGGTGTGGGTGTCTGTG;
>clamp_s_c_s_d_s_c_s_a_s:compl:rev CACAGACACCCACACCGTCACCCACACTATCATGTTGACATTCC; dG = -33.9
    89 >s_b_s_d_s_d_s_c_s_d_s_c_s_a_s          GTGGCTGTGACGGTGACGGTGTGGGTGACGGTGTGGGTGTCTGTG;
>clamp_s_c_s_d_s_c_s_a_s:compl:rev CACAGACACCCACACCGTCACCCACACTATCATGTTGACATTCC; dG = -33.9
    90 >s_b_s_d_s_e_s_c_s_d_s_c_s_a_s          GTGGCTGTGACGGTGCTGGTGTGGGTGACGGTGTGGGTGTCTGTG;
>clamp_s_c_s_d_s_c_s_a_s:compl:rev CACAGACACCCACACCGTCACCCACACTATCATGTTGACATTCC; dG = -33.9
    91 >s_b_s_e_s_c_s_c_s_d_s_c_s_a_s          GTGGCTGTGCTGGTGTGGGTGTGGGTGACGGTGTGGGTGTCTGTG;
>clamp_s_c_s_d_s_c_s_a_s:compl:rev CACAGACACCCACACCGTCACCCACACTATCATGTTGACATTCC; dG = -33.9
    92 >s_b_s_e_s_d_s_c_s_d_s_c_s_a_s          GTGGCTGTGCTGGTGACGGTGTGGGTGACGGTGTGGGTGTCTGTG;
>clamp_s_c_s_d_s_c_s_a_s:compl:rev CACAGACACCCACACCGTCACCCACACTATCATGTTGACATTCC; dG = -33.9
    93 >s_a_s_a_s_e_s_c_s_d_s_c_s_a_s          GTGTCTGTGTCTGTGCTGGTGTGGGTGACGGTGTGGGTGTCTGTG;
>clamp_s_c_s_d_s_c_s_a_s:compl:rev CACAGACACCCACACCGTCACCCACACTATCATGTTGACATTCC; dG = -33.9
    94 >s_b_s_e_s_e_s_c_s_d_s_c_s_a_s          GTGGCTGTGCTGGTGCTGGTGTGGGTGACGGTGTGGGTGTCTGTG;
>clamp_s_c_s_d_s_c_s_a_s:compl:rev CACAGACACCCACACCGTCACCCACACTATCATGTTGACATTCC; dG = -33.9
    95 >s_c_s_a_s_c_s_c_s_d_s_c_s_a_s          GTGTGGGTGTCTGTGTGGGTGTGGGTGACGGTGTGGGTGTCTGTG;
>clamp_s_c_s_d_s_c_s_a_s:compl:rev CACAGACACCCACACCGTCACCCACACTATCATGTTGACATTCC; dG = -33.9
    96 >s_c_s_a_s_d_s_c_s_d_s_c_s_a_s          GTGTGGGTGTCTGTGACGGTGTGGGTGACGGTGTGGGTGTCTGTG;
>clamp_s_c_s_d_s_c_s_a_s:compl:rev CACAGACACCCACACCGTCACCCACACTATCATGTTGACATTCC; dG = -33.9
    97 >s_c_s_a_s_e_s_c_s_d_s_c_s_a_s          GTGTGGGTGTCTGTGCTGGTGTGGGTGACGGTGTGGGTGTCTGTG;
>clamp_s_c_s_d_s_c_s_a_s:compl:rev CACAGACACCCACACCGTCACCCACACTATCATGTTGACATTCC; dG = -33.9
    98 >s_c_s_b_s_c_s_c_s_d_s_c_s_a_s          GTGTGGGTGGCTGTGTGGGTGTGGGTGACGGTGTGGGTGTCTGTG;
>clamp_s_c_s_d_s_c_s_a_s:compl:rev CACAGACACCCACACCGTCACCCACACTATCATGTTGACATTCC; dG = -33.9
    99 >s_c_s_b_s_d_s_c_s_d_s_c_s_a_s          GTGTGGGTGGCTGTGACGGTGTGGGTGACGGTGTGGGTGTCTGTG;
>clamp_s_c_s_d_s_c_s_a_s:compl:rev CACAGACACCCACACCGTCACCCACACTATCATGTTGACATTCC; dG = -33.9
   100 >s_c_s_b_s_e_s_c_s_d_s_c_s_a_s          GTGTGGGTGGCTGTGCTGGTGTGGGTGACGGTGTGGGTGTCTGTG;
>clamp_s_c_s_d_s_c_s_a_s:compl:rev CACAGACACCCACACCGTCACCCACACTATCATGTTGACATTCC; dG = -33.9
   101 >s_c_s_c_s_c_s_c_s_d_s_c_s_a_s          GTGTGGGTGTGGGTGTGGGTGTGGGTGACGGTGTGGGTGTCTGTG;
>clamp_s_c_s_d_s_c_s_a_s:compl:rev CACAGACACCCACACCGTCACCCACACTATCATGTTGACATTCC; dG = -33.9
   102 >s_c_s_c_s_d_s_c_s_d_s_c_s_a_s          GTGTGGGTGTGGGTGACGGTGTGGGTGACGGTGTGGGTGTCTGTG;
>clamp_s_c_s_d_s_c_s_a_s:compl:rev CACAGACACCCACACCGTCACCCACACTATCATGTTGACATTCC; dG = -33.9
   103 >s_c_s_c_s_e_s_c_s_d_s_c_s_a_s          GTGTGGGTGTGGGTGCTGGTGTGGGTGACGGTGTGGGTGTCTGTG;
>clamp_s_c_s_d_s_c_s_a_s:compl:rev CACAGACACCCACACCGTCACCCACACTATCATGTTGACATTCC; dG = -33.9
   104 >s_c_s_d_s_c_s_c_s_d_s_c_s_a_s          GTGTGGGTGACGGTGTGGGTGTGGGTGACGGTGTGGGTGTCTGTG;
>clamp_s_c_s_d_s_c_s_a_s:compl:rev CACAGACACCCACACCGTCACCCACACTATCATGTTGACATTCC; dG = -33.9
   105 >s_c_s_d_s_d_s_c_s_d_s_c_s_a_s          GTGTGGGTGACGGTGACGGTGTGGGTGACGGTGTGGGTGTCTGTG;
>clamp_s_c_s_d_s_c_s_a_s:compl:rev CACAGACACCCACACCGTCACCCACACTATCATGTTGACATTCC; dG = -33.9
   106 >s_c_s_d_s_e_s_c_s_d_s_c_s_a_s          GTGTGGGTGACGGTGCTGGTGTGGGTGACGGTGTGGGTGTCTGTG;
>clamp_s_c_s_d_s_c_s_a_s:compl:rev CACAGACACCCACACCGTCACCCACACTATCATGTTGACATTCC; dG = -33.9
   107 >s_c_s_e_s_c_s_c_s_d_s_c_s_a_s          GTGTGGGTGCTGGTGTGGGTGTGGGTGACGGTGTGGGTGTCTGTG;
>clamp_s_c_s_d_s_c_s_a_s:compl:rev CACAGACACCCACACCGTCACCCACACTATCATGTTGACATTCC; dG = -33.9
   108 >s_c_s_e_s_d_s_c_s_d_s_c_s_a_s          GTGTGGGTGCTGGTGACGGTGTGGGTGACGGTGTGGGTGTCTGTG;
>clamp_s_c_s_d_s_c_s_a_s:compl:rev CACAGACACCCACACCGTCACCCACACTATCATGTTGACATTCC; dG = -33.9
   109 >s_c_s_e_s_e_s_c_s_d_s_c_s_a_s          GTGTGGGTGCTGGTGCTGGTGTGGGTGACGGTGTGGGTGTCTGTG;
>clamp_s_c_s_d_s_c_s_a_s:compl:rev CACAGACACCCACACCGTCACCCACACTATCATGTTGACATTCC; dG = -33.9
   110 >s_d_s_a_s_c_s_c_s_d_s_c_s_a_s          GTGACGGTGTCTGTGTGGGTGTGGGTGACGGTGTGGGTGTCTGTG;
>clamp_s_c_s_d_s_c_s_a_s:compl:rev CACAGACACCCACACCGTCACCCACACTATCATGTTGACATTCC; dG = -33.9
   111 >s_d_s_a_s_d_s_c_s_d_s_c_s_a_s          GTGACGGTGTCTGTGACGGTGTGGGTGACGGTGTGGGTGTCTGTG;
>clamp_s_c_s_d_s_c_s_a_s:compl:rev CACAGACACCCACACCGTCACCCACACTATCATGTTGACATTCC; dG = -33.9
   112 >s_a_s_b_s_c_s_c_s_d_s_c_s_a_s          GTGTCTGTGGCTGTGTGGGTGTGGGTGACGGTGTGGGTGTCTGTG;
>clamp_s_c_s_d_s_c_s_a_s:compl:rev CACAGACACCCACACCGTCACCCACACTATCATGTTGACATTCC; dG = -33.9
```

```
   113 >s_d_s_a_s_e_s_c_s_d_s_c_s_a_s          GTGACGGTGTCTGTGCTGGTGTGGGTGACGGTGTGGGTGTCTGTG;
>clamp_s_c_s_d_s_c_s_a_s:compl:rev CACAGACACCCACACCGTCACCCACACTATCATGTTGACATTCC; dG = -33.9
   114 >s_d_s_b_s_c_s_c_s_d_s_c_s_a_s          GTGACGGTGGCTGTGTGGGTGTGGGTGACGGTGTGGGTGTCTGTG;
>clamp_s_c_s_d_s_c_s_a_s:compl:rev CACAGACACCCACACCGTCACCCACACTATCATGTTGACATTCC; dG = -33.9
   115 >s_d_s_b_s_d_s_c_s_d_s_c_s_a_s          GTGACGGTGGCTGTGACGGTGTGGGTGACGGTGTGGGTGTCTGTG;
>clamp_s_c_s_d_s_c_s_a_s:compl:rev CACAGACACCCACACCGTCACCCACACTATCATGTTGACATTCC; dG = -33.9
   116 >s_d_s_b_s_e_s_c_s_d_s_c_s_a_s          GTGACGGTGGCTGTGCTGGTGTGGGTGACGGTGTGGGTGTCTGTG;
>clamp_s_c_s_d_s_c_s_a_s:compl:rev CACAGACACCCACACCGTCACCCACACTATCATGTTGACATTCC; dG = -33.9
   117 >s_d_s_c_s_c_s_c_s_d_s_c_s_a_s          GTGACGGTGTGGGTGTGGGTGTGGGTGACGGTGTGGGTGTCTGTG;
>clamp_s_c_s_d_s_c_s_a_s:compl:rev CACAGACACCCACACCGTCACCCACACTATCATGTTGACATTCC; dG = -33.9
   118 >s_d_s_c_s_d_s_c_s_d_s_c_s_a_s          GTGACGGTGTGGGTGACGGTGTGGGTGACGGTGTGGGTGTCTGTG;
>clamp_s_c_s_d_s_c_s_a_s:compl:rev CACAGACACCCACACCGTCACCCACACTATCATGTTGACATTCC; dG = -33.9
   119 >s_a_s_b_s_d_s_c_s_d_s_c_s_a_s          GTGTCTGTGGCTGTGACGGTGTGGGTGACGGTGTGGGTGTCTGTG;
>clamp_s_c_s_d_s_c_s_a_s:compl:rev CACAGACACCCACACCGTCACCCACACTATCATGTTGACATTCC; dG = -33.9
   120 >s_d_s_c_s_e_s_c_s_d_s_c_s_a_s          GTGACGGTGTGGGTGCTGGTGTGGGTGACGGTGTGGGTGTCTGTG;
>clamp_s_c_s_d_s_c_s_a_s:compl:rev CACAGACACCCACACCGTCACCCACACTATCATGTTGACATTCC; dG = -33.9
   121 >s_d_s_d_s_c_s_c_s_d_s_c_s_a_s          GTGACGGTGACGGTGTGGGTGTGGGTGACGGTGTGGGTGTCTGTG;
>clamp_s_c_s_d_s_c_s_a_s:compl:rev CACAGACACCCACACCGTCACCCACACTATCATGTTGACATTCC; dG = -33.9
   122 >s_d_s_d_s_d_s_c_s_d_s_c_s_a_s          GTGACGGTGACGGTGACGGTGTGGGTGACGGTGTGGGTGTCTGTG;
>clamp_s_c_s_d_s_c_s_a_s:compl:rev CACAGACACCCACACCGTCACCCACACTATCATGTTGACATTCC; dG = -33.9
   123 >s_d_s_d_s_e_s_c_s_d_s_c_s_a_s          GTGACGGTGACGGTGCTGGTGTGGGTGACGGTGTGGGTGTCTGTG;
>clamp_s_c_s_d_s_c_s_a_s:compl:rev CACAGACACCCACACCGTCACCCACACTATCATGTTGACATTCC; dG = -33.9
   124 >s_d_s_e_s_c_s_c_s_d_s_c_s_a_s          GTGACGGTGCTGGTGTGGGTGTGGGTGACGGTGTGGGTGTCTGTG;
>clamp_s_c_s_d_s_c_s_a_s:compl:rev CACAGACACCCACACCGTCACCCACACTATCATGTTGACATTCC; dG = -33.9
   125 >s_d_s_e_s_d_s_c_s_d_s_c_s_a_s          GTGACGGTGCTGGTGACGGTGTGGGTGACGGTGTGGGTGTCTGTG;
>clamp_s_c_s_d_s_c_s_a_s:compl:rev CACAGACACCCACACCGTCACCCACACTATCATGTTGACATTCC; dG = -33.9

*************************************************************************************
```

CDCA Mismatches

```
     1 >s_c_s_d_s_c_s_b_s_d_s_c_s_a_s          GTGTGGGTGACGGTGTGGGTGGCTGTGACGGTGTGGGTGTCTGTG;
>clamp_s_c_s_d_s_c_s_a_s:compl:rev CACAGACACCCACACCGTCACCCACACTATCATGTTGACATTCC; dG = -32.3
     2 >s_a_s_c_s_d_s_c_s_b_s_c_s_a_s          GTGTCTGTGTGGGTGACGGTGTGGGTGGCTGTGTGGGTGTCTGTG;
>clamp_s_c_s_d_s_c_s_a_s:compl:rev CACAGACACCCACACCGTCACCCACACTATCATGTTGACATTCC; dG = -32.3
     3 >s_b_s_c_s_d_s_c_s_b_s_c_s_a_s          GTGGCTGTGTGGGTGACGGTGTGGGTGGCTGTGTGGGTGTCTGTG;
>clamp_s_c_s_d_s_c_s_a_s:compl:rev CACAGACACCCACACCGTCACCCACACTATCATGTTGACATTCC; dG = -32.3
     4 >s_c_s_c_s_d_s_c_s_b_s_c_s_a_s          GTGTGGGTGTGGGTGACGGTGTGGGTGGCTGTGTGGGTGTCTGTG;
>clamp_s_c_s_d_s_c_s_a_s:compl:rev CACAGACACCCACACCGTCACCCACACTATCATGTTGACATTCC; dG = -32.2
     5 >s_d_s_c_s_d_s_c_s_b_s_c_s_a_s          GTGACGGTGTGGGTGACGGTGTGGGTGGCTGTGTGGGTGTCTGTG;
>clamp_s_c_s_d_s_c_s_a_s:compl:rev CACAGACACCCACACCGTCACCCACACTATCATGTTGACATTCC; dG = -32.2
     6 >s_e_s_c_s_d_s_c_s_b_s_c_s_a_s          GTGCTGGTGTGGGTGACGGTGTGGGTGGCTGTGTGGGTGTCTGTG;
>clamp_s_c_s_d_s_c_s_a_s:compl:rev CACAGACACCCACACCGTCACCCACACTATCATGTTGACATTCC; dG = -32.2
     7 >s_a_s_b_s_b_s_c_s_d_s_c_s_b_s          GTGTCTGTGGCTGTGGCTGTGTGGGTGACGGTGTGGGTGGCTGTG;
>clamp_s_c_s_d_s_c_s_a_s:compl:rev CACAGACACCCACACCGTCACCCACACTATCATGTTGACATTCC; dG = -32.0
     8 >s_c_s_c_s_d_s_c_s_d_s_c_s_b_s          GTGTGGGTGTGGGTGACGGTGTGGGTGACGGTGTGGGTGGCTGTG;
>clamp_s_c_s_d_s_c_s_a_s:compl:rev CACAGACACCCACACCGTCACCCACACTATCATGTTGACATTCC; dG = -32.0
     9 >s_c_s_c_s_e_s_c_s_d_s_c_s_b_s          GTGTGGGTGTGGGTGCTGGTGTGGGTGACGGTGTGGGTGGCTGTG;
>clamp_s_c_s_d_s_c_s_a_s:compl:rev CACAGACACCCACACCGTCACCCACACTATCATGTTGACATTCC; dG = -32.0
    10 >s_c_s_d_s_a_s_c_s_d_s_c_s_b_s          GTGTGGGTGACGGTGTCTGTGTGGGTGACGGTGTGGGTGGCTGTG;
>clamp_s_c_s_d_s_c_s_a_s:compl:rev CACAGACACCCACACCGTCACCCACACTATCATGTTGACATTCC; dG = -32.0
    11 >s_c_s_d_s_b_s_c_s_d_s_c_s_b_s          GTGTGGGTGACGGTGGCTGTGTGGGTGACGGTGTGGGTGGCTGTG;
>clamp_s_c_s_d_s_c_s_a_s:compl:rev CACAGACACCCACACCGTCACCCACACTATCATGTTGACATTCC; dG = -32.0
    12 >s_c_s_d_s_c_s_c_s_d_s_c_s_b_s          GTGTGGGTGACGGTGTGGGTGTGGGTGACGGTGTGGGTGGCTGTG;
>clamp_s_c_s_d_s_c_s_a_s:compl:rev CACAGACACCCACACCGTCACCCACACTATCATGTTGACATTCC; dG = -32.0
    13 >s_c_s_d_s_d_s_c_s_d_s_c_s_b_s          GTGTGGGTGACGGTGACGGTGTGGGTGACGGTGTGGGTGGCTGTG;
>clamp_s_c_s_d_s_c_s_a_s:compl:rev CACAGACACCCACACCGTCACCCACACTATCATGTTGACATTCC; dG = -32.0
    14 >s_c_s_d_s_e_s_c_s_d_s_c_s_b_s          GTGTGGGTGACGGTGCTGGTGTGGGTGACGGTGTGGGTGGCTGTG;
>clamp_s_c_s_d_s_c_s_a_s:compl:rev CACAGACACCCACACCGTCACCCACACTATCATGTTGACATTCC; dG = -32.0
    15 >s_c_s_e_s_a_s_c_s_d_s_c_s_b_s          GTGTGGGTGCTGGTGTCTGTGTGGGTGACGGTGTGGGTGGCTGTG;
>clamp_s_c_s_d_s_c_s_a_s:compl:rev CACAGACACCCACACCGTCACCCACACTATCATGTTGACATTCC; dG = -32.0
    16 >s_c_s_e_s_b_s_c_s_d_s_c_s_b_s          GTGTGGGTGCTGGTGGCTGTGTGGGTGACGGTGTGGGTGGCTGTG;
>clamp_s_c_s_d_s_c_s_a_s:compl:rev CACAGACACCCACACCGTCACCCACACTATCATGTTGACATTCC; dG = -32.0
    17 >s_c_s_e_s_c_s_c_s_d_s_c_s_b_s          GTGTGGGTGCTGGTGTGGGTGTGGGTGACGGTGTGGGTGGCTGTG;
>clamp_s_c_s_d_s_c_s_a_s:compl:rev CACAGACACCCACACCGTCACCCACACTATCATGTTGACATTCC; dG = -32.0
    18 >s_a_s_b_s_c_s_c_s_d_s_c_s_b_s          GTGTCTGTGGCTGTGTGGGTGTGGGTGACGGTGTGGGTGGCTGTG;
>clamp_s_c_s_d_s_c_s_a_s:compl:rev CACAGACACCCACACCGTCACCCACACTATCATGTTGACATTCC; dG = -32.0
    19 >s_c_s_e_s_d_s_c_s_d_s_c_s_b_s          GTGTGGGTGCTGGTGACGGTGTGGGTGACGGTGTGGGTGGCTGTG;
>clamp_s_c_s_d_s_c_s_a_s:compl:rev CACAGACACCCACACCGTCACCCACACTATCATGTTGACATTCC; dG = -32.0
    20 >s_c_s_e_s_e_s_c_s_d_s_c_s_b_s          GTGTGGGTGCTGGTGCTGGTGTGGGTGACGGTGTGGGTGGCTGTG;
>clamp_s_c_s_d_s_c_s_a_s:compl:rev CACAGACACCCACACCGTCACCCACACTATCATGTTGACATTCC; dG = -32.0
```

```
    21 >s_d_s_a_s_a_s_c_s_d_s_c_s_b_s           GTGACGGTGTCTGTGTCTGTGTGGGTGACGGTGTGGGTGGCTGTG;
>clamp_s_c_s_d_s_c_s_a_s:compl:rev CACAGACACCCACACCGTCACCCACACTATCATGTTGACATTCC; dG = -32.0
    22 >s_d_s_a_s_b_s_c_s_d_s_c_s_b_s           GTGACGGTGTCTGTGGCTGTGTGGGTGACGGTGTGGGTGGCTGTG;
>clamp_s_c_s_d_s_c_s_a_s:compl:rev CACAGACACCCACACCGTCACCCACACTATCATGTTGACATTCC; dG = -32.0
    23 >s_d_s_a_s_c_s_c_s_d_s_c_s_b_s           GTGACGGTGTCTGTGTGGGTGTGGGTGACGGTGTGGGTGGCTGTG;
>clamp_s_c_s_d_s_c_s_a_s:compl:rev CACAGACACCCACACCGTCACCCACACTATCATGTTGACATTCC; dG = -32.0
    24 >s_d_s_a_s_d_s_c_s_d_s_c_s_b_s           GTGACGGTGTCTGTGACGGTGTGGGTGACGGTGTGGGTGGCTGTG;
>clamp_s_c_s_d_s_c_s_a_s:compl:rev CACAGACACCCACACCGTCACCCACACTATCATGTTGACATTCC; dG = -32.0
    25 >s_d_s_a_s_e_s_c_s_d_s_c_s_b_s           GTGACGGTGTCTGTGCTGGTGTGGGTGACGGTGTGGGTGGCTGTG;
>clamp_s_c_s_d_s_c_s_a_s:compl:rev CACAGACACCCACACCGTCACCCACACTATCATGTTGACATTCC; dG = -32.0
    26 >s_d_s_b_s_a_s_c_s_d_s_c_s_b_s           GTGACGGTGGCTGTGTCTGTGTGGGTGACGGTGTGGGTGGCTGTG;
>clamp_s_c_s_d_s_c_s_a_s:compl:rev CACAGACACCCACACCGTCACCCACACTATCATGTTGACATTCC; dG = -32.0
    27 >s_d_s_b_s_b_s_c_s_d_s_c_s_b_s           GTGACGGTGGCTGTGGCTGTGTGGGTGACGGTGTGGGTGGCTGTG;
>clamp_s_c_s_d_s_c_s_a_s:compl:rev CACAGACACCCACACCGTCACCCACACTATCATGTTGACATTCC; dG = -32.0
    28 >s_d_s_b_s_c_s_c_s_d_s_c_s_b_s           GTGACGGTGGCTGTGTGGGTGTGGGTGACGGTGTGGGTGGCTGTG;
>clamp_s_c_s_d_s_c_s_a_s:compl:rev CACAGACACCCACACCGTCACCCACACTATCATGTTGACATTCC; dG = -32.0
    29 >s_a_s_b_s_d_s_c_s_d_s_c_s_b_s           GTGTCTGTGGCTGTGACGGTGTGGGTGACGGTGTGGGTGGCTGTG;
>clamp_s_c_s_d_s_c_s_a_s:compl:rev CACAGACACCCACACCGTCACCCACACTATCATGTTGACATTCC; dG = -32.0
    30 >s_d_s_b_s_d_s_c_s_d_s_c_s_b_s           GTGACGGTGGCTGTGACGGTGTGGGTGACGGTGTGGGTGGCTGTG;
>clamp_s_c_s_d_s_c_s_a_s:compl:rev CACAGACACCCACACCGTCACCCACACTATCATGTTGACATTCC; dG = -32.0
    31 >s_d_s_b_s_e_s_c_s_d_s_c_s_b_s           GTGACGGTGGCTGTGCTGGTGTGGGTGACGGTGTGGGTGGCTGTG;
>clamp_s_c_s_d_s_c_s_a_s:compl:rev CACAGACACCCACACCGTCACCCACACTATCATGTTGACATTCC; dG = -32.0
    32 >s_d_s_c_s_a_s_c_s_d_s_c_s_b_s           GTGACGGTGTGGGTGTCTGTGTGGGTGACGGTGTGGGTGGCTGTG;
>clamp_s_c_s_d_s_c_s_a_s:compl:rev CACAGACACCCACACCGTCACCCACACTATCATGTTGACATTCC; dG = -32.0
    33 >s_d_s_c_s_b_s_c_s_d_s_c_s_b_s           GTGACGGTGTGGGTGGCTGTGTGGGTGACGGTGTGGGTGGCTGTG;
>clamp_s_c_s_d_s_c_s_a_s:compl:rev CACAGACACCCACACCGTCACCCACACTATCATGTTGACATTCC; dG = -32.0
    34 >s_d_s_c_s_c_s_c_s_d_s_c_s_b_s           GTGACGGTGTGGGTGTGGGTGTGGGTGACGGTGTGGGTGGCTGTG;
>clamp_s_c_s_d_s_c_s_a_s:compl:rev CACAGACACCCACACCGTCACCCACACTATCATGTTGACATTCC; dG = -32.0
    35 >s_d_s_c_s_d_s_c_s_d_s_c_s_b_s           GTGACGGTGTGGGTGACGGTGTGGGTGACGGTGTGGGTGGCTGTG;
>clamp_s_c_s_d_s_c_s_a_s:compl:rev CACAGACACCCACACCGTCACCCACACTATCATGTTGACATTCC; dG = -32.0
    36 >s_d_s_c_s_e_s_c_s_d_s_c_s_b_s           GTGACGGTGTGGGTGCTGGTGTGGGTGACGGTGTGGGTGGCTGTG;
>clamp_s_c_s_d_s_c_s_a_s:compl:rev CACAGACACCCACACCGTCACCCACACTATCATGTTGACATTCC; dG = -32.0
    37 >s_d_s_d_s_a_s_c_s_d_s_c_s_b_s           GTGACGGTGACGGTGTCTGTGTGGGTGACGGTGTGGGTGGCTGTG;
>clamp_s_c_s_d_s_c_s_a_s:compl:rev CACAGACACCCACACCGTCACCCACACTATCATGTTGACATTCC; dG = -32.0
    38 >s_d_s_d_s_b_s_c_s_d_s_c_s_b_s           GTGACGGTGACGGTGGCTGTGTGGGTGACGGTGTGGGTGGCTGTG;
>clamp_s_c_s_d_s_c_s_a_s:compl:rev CACAGACACCCACACCGTCACCCACACTATCATGTTGACATTCC; dG = -32.0
    39 >s_d_s_d_s_c_s_c_s_d_s_c_s_b_s           GTGACGGTGACGGTGTGGGTGTGGGTGACGGTGTGGGTGGCTGTG;
>clamp_s_c_s_d_s_c_s_a_s:compl:rev CACAGACACCCACACCGTCACCCACACTATCATGTTGACATTCC; dG = -32.0
    40 >s_a_s_b_s_e_s_c_s_d_s_c_s_b_s           GTGTCTGTGGCTGTGCTGGTGTGGGTGACGGTGTGGGTGGCTGTG;
>clamp_s_c_s_d_s_c_s_a_s:compl:rev CACAGACACCCACACCGTCACCCACACTATCATGTTGACATTCC; dG = -32.0
    41 >s_d_s_d_s_d_s_c_s_d_s_c_s_b_s           GTGACGGTGACGGTGACGGTGTGGGTGACGGTGTGGGTGGCTGTG;
>clamp_s_c_s_d_s_c_s_a_s:compl:rev CACAGACACCCACACCGTCACCCACACTATCATGTTGACATTCC; dG = -32.0
    42 >s_d_s_d_s_e_s_c_s_d_s_c_s_b_s           GTGACGGTGACGGTGCTGGTGTGGGTGACGGTGTGGGTGGCTGTG;
>clamp_s_c_s_d_s_c_s_a_s:compl:rev CACAGACACCCACACCGTCACCCACACTATCATGTTGACATTCC; dG = -32.0
    43 >s_d_s_e_s_a_s_c_s_d_s_c_s_b_s           GTGACGGTGCTGGTGTCTGTGTGGGTGACGGTGTGGGTGGCTGTG;
>clamp_s_c_s_d_s_c_s_a_s:compl:rev CACAGACACCCACACCGTCACCCACACTATCATGTTGACATTCC; dG = -32.0
    44 >s_d_s_e_s_b_s_c_s_d_s_c_s_b_s           GTGACGGTGCTGGTGGCTGTGTGGGTGACGGTGTGGGTGGCTGTG;
>clamp_s_c_s_d_s_c_s_a_s:compl:rev CACAGACACCCACACCGTCACCCACACTATCATGTTGACATTCC; dG = -32.0
    45 >s_d_s_e_s_c_s_c_s_d_s_c_s_b_s           GTGACGGTGCTGGTGTGGGTGTGGGTGACGGTGTGGGTGGCTGTG;
>clamp_s_c_s_d_s_c_s_a_s:compl:rev CACAGACACCCACACCGTCACCCACACTATCATGTTGACATTCC; dG = -32.0
    46 >s_d_s_e_s_d_s_c_s_d_s_c_s_b_s           GTGACGGTGCTGGTGACGGTGTGGGTGACGGTGTGGGTGGCTGTG;
>clamp_s_c_s_d_s_c_s_a_s:compl:rev CACAGACACCCACACCGTCACCCACACTATCATGTTGACATTCC; dG = -32.0
    47 >s_d_s_e_s_e_s_c_s_d_s_c_s_b_s           GTGACGGTGCTGGTGCTGGTGTGGGTGACGGTGTGGGTGGCTGTG;
>clamp_s_c_s_d_s_c_s_a_s:compl:rev CACAGACACCCACACCGTCACCCACACTATCATGTTGACATTCC; dG = -32.0
    48 >s_e_s_a_s_a_s_c_s_d_s_c_s_b_s           GTGCTGGTGTCTGTGTCTGTGTGGGTGACGGTGTGGGTGGCTGTG;
>clamp_s_c_s_d_s_c_s_a_s:compl:rev CACAGACACCCACACCGTCACCCACACTATCATGTTGACATTCC; dG = -32.0
    49 >s_e_s_a_s_b_s_c_s_d_s_c_s_b_s           GTGCTGGTGTCTGTGGCTGTGTGGGTGACGGTGTGGGTGGCTGTG;
>clamp_s_c_s_d_s_c_s_a_s:compl:rev CACAGACACCCACACCGTCACCCACACTATCATGTTGACATTCC; dG = -32.0
    50 >s_e_s_a_s_c_s_c_s_d_s_c_s_b_s           GTGCTGGTGTCTGTGTGGGTGTGGGTGACGGTGTGGGTGGCTGTG;
>clamp_s_c_s_d_s_c_s_a_s:compl:rev CACAGACACCCACACCGTCACCCACACTATCATGTTGACATTCC; dG = -32.0
    51 >s_a_s_c_s_a_s_c_s_d_s_c_s_b_s           GTGTCTGTGTGGGTGTCTGTGTGGGTGACGGTGTGGGTGGCTGTG;
>clamp_s_c_s_d_s_c_s_a_s:compl:rev CACAGACACCCACACCGTCACCCACACTATCATGTTGACATTCC; dG = -32.0
    52 >s_e_s_a_s_d_s_c_s_d_s_c_s_b_s           GTGCTGGTGTCTGTGACGGTGTGGGTGACGGTGTGGGTGGCTGTG;
>clamp_s_c_s_d_s_c_s_a_s:compl:rev CACAGACACCCACACCGTCACCCACACTATCATGTTGACATTCC; dG = -32.0
    53 >s_e_s_a_s_e_s_c_s_d_s_c_s_b_s           GTGCTGGTGTCTGTGCTGGTGTGGGTGACGGTGTGGGTGGCTGTG;
>clamp_s_c_s_d_s_c_s_a_s:compl:rev CACAGACACCCACACCGTCACCCACACTATCATGTTGACATTCC; dG = -32.0
    54 >s_e_s_b_s_a_s_c_s_d_s_c_s_b_s           GTGCTGGTGGCTGTGTCTGTGTGGGTGACGGTGTGGGTGGCTGTG;
>clamp_s_c_s_d_s_c_s_a_s:compl:rev CACAGACACCCACACCGTCACCCACACTATCATGTTGACATTCC; dG = -32.0
    55 >s_e_s_b_s_b_s_c_s_d_s_c_s_b_s           GTGCTGGTGGCTGTGGCTGTGTGGGTGACGGTGTGGGTGGCTGTG;
>clamp_s_c_s_d_s_c_s_a_s:compl:rev CACAGACACCCACACCGTCACCCACACTATCATGTTGACATTCC; dG = -32.0
    56 >s_e_s_b_s_c_s_c_s_d_s_c_s_b_s           GTGCTGGTGGCTGTGTGGGTGTGGGTGACGGTGTGGGTGGCTGTG;
>clamp_s_c_s_d_s_c_s_a_s:compl:rev CACAGACACCCACACCGTCACCCACACTATCATGTTGACATTCC; dG = -32.0
    57 >s_e_s_b_s_d_s_c_s_d_s_c_s_b_s           GTGCTGGTGGCTGTGACGGTGTGGGTGACGGTGTGGGTGGCTGTG;
>clamp_s_c_s_d_s_c_s_a_s:compl:rev CACAGACACCCACACCGTCACCCACACTATCATGTTGACATTCC; dG = -32.0
```

```
    58 >s_e_s_b_s_e_s_c_s_d_s_c_s_b_s          GTGCTGGTGGCTGTGCTGGTGTGGGTGACGGTGTGGGTGGCTGTG;
>clamp_s_c_s_d_s_c_s_a_s:compl:rev CACAGACACCCACACCGTCACCCACACTATCATGTTGACATTCC; dG = -32.0
    59 >s_e_s_c_s_a_s_c_s_d_s_c_s_b_s          GTGCTGGTGTGGGTGTCTGTGTGGGTGACGGTGTGGGTGGCTGTG;
>clamp_s_c_s_d_s_c_s_a_s:compl:rev CACAGACACCCACACCGTCACCCACACTATCATGTTGACATTCC; dG = -32.0
    60 >s_e_s_c_s_b_s_c_s_d_s_c_s_b_s          GTGCTGGTGTGGGTGGCTGTGTGGGTGACGGTGTGGGTGGCTGTG;
>clamp_s_c_s_d_s_c_s_a_s:compl:rev CACAGACACCCACACCGTCACCCACACTATCATGTTGACATTCC; dG = -32.0
    61 >s_e_s_c_s_c_s_c_s_d_s_c_s_b_s          GTGCTGGTGTGGGTGTGGGTGTGGGTGACGGTGTGGGTGGCTGTG;
>clamp_s_c_s_d_s_c_s_a_s:compl:rev CACAGACACCCACACCGTCACCCACACTATCATGTTGACATTCC; dG = -32.0
    62 >s_a_s_c_s_b_s_c_s_d_s_c_s_b_s          GTGTCTGTGTGGGTGGCTGTGTGGGTGACGGTGTGGGTGGCTGTG;
>clamp_s_c_s_d_s_c_s_a_s:compl:rev CACAGACACCCACACCGTCACCCACACTATCATGTTGACATTCC; dG = -32.0
    63 >s_e_s_c_s_d_s_c_s_d_s_c_s_b_s          GTGCTGGTGTGGGTGACGGTGTGGGTGACGGTGTGGGTGGCTGTG;
>clamp_s_c_s_d_s_c_s_a_s:compl:rev CACAGACACCCACACCGTCACCCACACTATCATGTTGACATTCC; dG = -32.0
    64 >s_e_s_c_s_e_s_c_s_d_s_c_s_b_s          GTGCTGGTGTGGGTGCTGGTGTGGGTGACGGTGTGGGTGGCTGTG;
>clamp_s_c_s_d_s_c_s_a_s:compl:rev CACAGACACCCACACCGTCACCCACACTATCATGTTGACATTCC; dG = -32.0
    65 >s_e_s_d_s_a_s_c_s_d_s_c_s_b_s          GTGCTGGTGACGGTGTCTGTGTGGGTGACGGTGTGGGTGGCTGTG;
>clamp_s_c_s_d_s_c_s_a_s:compl:rev CACAGACACCCACACCGTCACCCACACTATCATGTTGACATTCC; dG = -32.0
    66 >s_e_s_d_s_b_s_c_s_d_s_c_s_b_s          GTGCTGGTGACGGTGGCTGTGTGGGTGACGGTGTGGGTGGCTGTG;
>clamp_s_c_s_d_s_c_s_a_s:compl:rev CACAGACACCCACACCGTCACCCACACTATCATGTTGACATTCC; dG = -32.0
    67 >s_e_s_d_s_c_s_c_s_d_s_c_s_b_s          GTGCTGGTGACGGTGTGGGTGTGGGTGACGGTGTGGGTGGCTGTG;
>clamp_s_c_s_d_s_c_s_a_s:compl:rev CACAGACACCCACACCGTCACCCACACTATCATGTTGACATTCC; dG = -32.0
    68 >s_e_s_d_s_d_s_c_s_d_s_c_s_b_s          GTGCTGGTGACGGTGACGGTGTGGGTGACGGTGTGGGTGGCTGTG;
>clamp_s_c_s_d_s_c_s_a_s:compl:rev CACAGACACCCACACCGTCACCCACACTATCATGTTGACATTCC; dG = -32.0
    69 >s_e_s_d_s_e_s_c_s_d_s_c_s_b_s          GTGCTGGTGACGGTGCTGGTGTGGGTGACGGTGTGGGTGGCTGTG;
>clamp_s_c_s_d_s_c_s_a_s:compl:rev CACAGACACCCACACCGTCACCCACACTATCATGTTGACATTCC; dG = -32.0
    70 >s_e_s_e_s_a_s_c_s_d_s_c_s_b_s          GTGCTGGTGCTGGTGTCTGTGTGGGTGACGGTGTGGGTGGCTGTG;
>clamp_s_c_s_d_s_c_s_a_s:compl:rev CACAGACACCCACACCGTCACCCACACTATCATGTTGACATTCC; dG = -32.0
    71 >s_e_s_e_s_b_s_c_s_d_s_c_s_b_s          GTGCTGGTGCTGGTGGCTGTGTGGGTGACGGTGTGGGTGGCTGTG;
>clamp_s_c_s_d_s_c_s_a_s:compl:rev CACAGACACCCACACCGTCACCCACACTATCATGTTGACATTCC; dG = -32.0
    72 >s_e_s_e_s_c_s_c_s_d_s_c_s_b_s          GTGCTGGTGCTGGTGTGGGTGTGGGTGACGGTGTGGGTGGCTGTG;
>clamp_s_c_s_d_s_c_s_a_s:compl:rev CACAGACACCCACACCGTCACCCACACTATCATGTTGACATTCC; dG = -32.0
    73 >s_a_s_c_s_c_s_c_s_d_s_c_s_b_s          GTGTCTGTGTGGGTGTGGGTGTGGGTGACGGTGTGGGTGGCTGTG;
>clamp_s_c_s_d_s_c_s_a_s:compl:rev CACAGACACCCACACCGTCACCCACACTATCATGTTGACATTCC; dG = -32.0
    74 >s_e_s_e_s_d_s_c_s_d_s_c_s_b_s          GTGCTGGTGCTGGTGACGGTGTGGGTGACGGTGTGGGTGGCTGTG;
>clamp_s_c_s_d_s_c_s_a_s:compl:rev CACAGACACCCACACCGTCACCCACACTATCATGTTGACATTCC; dG = -32.0
    75 >s_e_s_e_s_e_s_c_s_d_s_c_s_b_s          GTGCTGGTGCTGGTGCTGGTGTGGGTGACGGTGTGGGTGGCTGTG;
>clamp_s_c_s_d_s_c_s_a_s:compl:rev CACAGACACCCACACCGTCACCCACACTATCATGTTGACATTCC; dG = -32.0
    76 >s_a_s_a_s_b_s_c_s_d_s_c_s_b_s          GTGTCTGTGTCTGTGGCTGTGTGGGTGACGGTGTGGGTGGCTGTG;
>clamp_s_c_s_d_s_c_s_a_s:compl:rev CACAGACACCCACACCGTCACCCACACTATCATGTTGACATTCC; dG = -32.0
    77 >s_a_s_c_s_d_s_c_s_d_s_c_s_b_s          GTGTCTGTGTGGGTGACGGTGTGGGTGACGGTGTGGGTGGCTGTG;
>clamp_s_c_s_d_s_c_s_a_s:compl:rev CACAGACACCCACACCGTCACCCACACTATCATGTTGACATTCC; dG = -32.0
    78 >s_a_s_c_s_e_s_c_s_d_s_c_s_b_s          GTGTCTGTGTGGGTGCTGGTGTGGGTGACGGTGTGGGTGGCTGTG;
>clamp_s_c_s_d_s_c_s_a_s:compl:rev CACAGACACCCACACCGTCACCCACACTATCATGTTGACATTCC; dG = -32.0
    79 >s_a_s_d_s_a_s_c_s_d_s_c_s_b_s          GTGTCTGTGACGGTGTCTGTGTGGGTGACGGTGTGGGTGGCTGTG;
>clamp_s_c_s_d_s_c_s_a_s:compl:rev CACAGACACCCACACCGTCACCCACACTATCATGTTGACATTCC; dG = -32.0
    80 >s_a_s_d_s_b_s_c_s_d_s_c_s_b_s          GTGTCTGTGACGGTGGCTGTGTGGGTGACGGTGTGGGTGGCTGTG;
>clamp_s_c_s_d_s_c_s_a_s:compl:rev CACAGACACCCACACCGTCACCCACACTATCATGTTGACATTCC; dG = -32.0
    81 >s_a_s_d_s_c_s_c_s_d_s_c_s_b_s          GTGTCTGTGACGGTGTGGGTGTGGGTGACGGTGTGGGTGGCTGTG;
>clamp_s_c_s_d_s_c_s_a_s:compl:rev CACAGACACCCACACCGTCACCCACACTATCATGTTGACATTCC; dG = -32.0
    82 >s_a_s_d_s_d_s_c_s_d_s_c_s_b_s          GTGTCTGTGACGGTGACGGTGTGGGTGACGGTGTGGGTGGCTGTG;
>clamp_s_c_s_d_s_c_s_a_s:compl:rev CACAGACACCCACACCGTCACCCACACTATCATGTTGACATTCC; dG = -32.0
    83 >s_a_s_d_s_e_s_c_s_d_s_c_s_b_s          GTGTCTGTGACGGTGCTGGTGTGGGTGACGGTGTGGGTGGCTGTG;
>clamp_s_c_s_d_s_c_s_a_s:compl:rev CACAGACACCCACACCGTCACCCACACTATCATGTTGACATTCC; dG = -32.0
    84 >s_a_s_e_s_a_s_c_s_d_s_c_s_b_s          GTGTCTGTGCTGGTGTCTGTGTGGGTGACGGTGTGGGTGGCTGTG;
>clamp_s_c_s_d_s_c_s_a_s:compl:rev CACAGACACCCACACCGTCACCCACACTATCATGTTGACATTCC; dG = -32.0
    85 >s_a_s_e_s_b_s_c_s_d_s_c_s_b_s          GTGTCTGTGCTGGTGGCTGTGTGGGTGACGGTGTGGGTGGCTGTG;
>clamp_s_c_s_d_s_c_s_a_s:compl:rev CACAGACACCCACACCGTCACCCACACTATCATGTTGACATTCC; dG = -32.0
    86 >s_a_s_e_s_c_s_c_s_d_s_c_s_b_s          GTGTCTGTGCTGGTGTGGGTGTGGGTGACGGTGTGGGTGGCTGTG;
>clamp_s_c_s_d_s_c_s_a_s:compl:rev CACAGACACCCACACCGTCACCCACACTATCATGTTGACATTCC; dG = -32.0
    87 >s_a_s_a_s_c_s_c_s_d_s_c_s_b_s          GTGTCTGTGTCTGTGTGGGTGTGGGTGACGGTGTGGGTGGCTGTG;
>clamp_s_c_s_d_s_c_s_a_s:compl:rev CACAGACACCCACACCGTCACCCACACTATCATGTTGACATTCC; dG = -32.0
    88 >s_a_s_e_s_d_s_c_s_d_s_c_s_b_s          GTGTCTGTGCTGGTGACGGTGTGGGTGACGGTGTGGGTGGCTGTG;
>clamp_s_c_s_d_s_c_s_a_s:compl:rev CACAGACACCCACACCGTCACCCACACTATCATGTTGACATTCC; dG = -32.0
    89 >s_a_s_e_s_e_s_c_s_d_s_c_s_b_s          GTGTCTGTGCTGGTGCTGGTGTGGGTGACGGTGTGGGTGGCTGTG;
>clamp_s_c_s_d_s_c_s_a_s:compl:rev CACAGACACCCACACCGTCACCCACACTATCATGTTGACATTCC; dG = -32.0
    90 >s_a_s_a_s_a_s_c_s_d_s_c_s_b_s          GTGTCTGTGTCTGTGTCTGTGTGGGTGACGGTGTGGGTGGCTGTG;
>clamp_s_c_s_d_s_c_s_a_s:compl:rev CACAGACACCCACACCGTCACCCACACTATCATGTTGACATTCC; dG = -32.0
    91 >s_b_s_a_s_a_s_c_s_d_s_c_s_b_s          GTGGCTGTGTCTGTGTCTGTGTGGGTGACGGTGTGGGTGGCTGTG;
>clamp_s_c_s_d_s_c_s_a_s:compl:rev CACAGACACCCACACCGTCACCCACACTATCATGTTGACATTCC; dG = -32.0
    92 >s_b_s_a_s_b_s_c_s_d_s_c_s_b_s          GTGGCTGTGTCTGTGGCTGTGTGGGTGACGGTGTGGGTGGCTGTG;
>clamp_s_c_s_d_s_c_s_a_s:compl:rev CACAGACACCCACACCGTCACCCACACTATCATGTTGACATTCC; dG = -32.0
    93 >s_b_s_a_s_c_s_c_s_d_s_c_s_b_s          GTGGCTGTGTCTGTGTGGGTGTGGGTGACGGTGTGGGTGGCTGTG;
>clamp_s_c_s_d_s_c_s_a_s:compl:rev CACAGACACCCACACCGTCACCCACACTATCATGTTGACATTCC; dG = -32.0
    94 >s_b_s_a_s_d_s_c_s_d_s_c_s_b_s          GTGGCTGTGTCTGTGACGGTGTGGGTGACGGTGTGGGTGGCTGTG;
>clamp_s_c_s_d_s_c_s_a_s:compl:rev CACAGACACCCACACCGTCACCCACACTATCATGTTGACATTCC; dG = -32.0
```

```
    95 >s_b_s_a_s_e_s_c_s_d_s_c_s_b_s          GTGGCTGTGTCTGTGCTGGTGTGGGTGACGGTGTGGGTGGCTGTG;
>clamp_s_c_s_d_s_c_s_a_s:compl:rev CACAGACACCCACACCGTCACCCACACTATCATGTTGACATTCC; dG = -32.0
    96 >s_b_s_b_s_a_s_c_s_d_s_c_s_b_s          GTGGCTGTGGCTGTGTCTGTGTGGGTGACGGTGTGGGTGGCTGTG;
>clamp_s_c_s_d_s_c_s_a_s:compl:rev CACAGACACCCACACCGTCACCCACACTATCATGTTGACATTCC; dG = -32.0
    97 >s_b_s_b_s_b_s_c_s_d_s_c_s_b_s          GTGGCTGTGGCTGTGGCTGTGTGGGTGACGGTGTGGGTGGCTGTG;
>clamp_s_c_s_d_s_c_s_a_s:compl:rev CACAGACACCCACACCGTCACCCACACTATCATGTTGACATTCC; dG = -32.0
    98 >s_b_s_b_s_c_s_c_s_d_s_c_s_b_s          GTGGCTGTGGCTGTGTGGGTGTGGGTGACGGTGTGGGTGGCTGTG;
>clamp_s_c_s_d_s_c_s_a_s:compl:rev CACAGACACCCACACCGTCACCCACACTATCATGTTGACATTCC; dG = -32.0
    99 >s_a_s_a_s_d_s_c_s_d_s_c_s_b_s          GTGTCTGTGTCTGTGACGGTGTGGGTGACGGTGTGGGTGGCTGTG;
>clamp_s_c_s_d_s_c_s_a_s:compl:rev CACAGACACCCACACCGTCACCCACACTATCATGTTGACATTCC; dG = -32.0
   100 >s_b_s_b_s_d_s_c_s_d_s_c_s_b_s          GTGGCTGTGGCTGTGACGGTGTGGGTGACGGTGTGGGTGGCTGTG;
>clamp_s_c_s_d_s_c_s_a_s:compl:rev CACAGACACCCACACCGTCACCCACACTATCATGTTGACATTCC; dG = -32.0
   101 >s_b_s_b_s_e_s_c_s_d_s_c_s_b_s          GTGGCTGTGGCTGTGCTGGTGTGGGTGACGGTGTGGGTGGCTGTG;
>clamp_s_c_s_d_s_c_s_a_s:compl:rev CACAGACACCCACACCGTCACCCACACTATCATGTTGACATTCC; dG = -32.0
   102 >s_b_s_c_s_a_s_c_s_d_s_c_s_b_s          GTGGCTGTGTGGGTGTCTGTGTGGGTGACGGTGTGGGTGGCTGTG;
>clamp_s_c_s_d_s_c_s_a_s:compl:rev CACAGACACCCACACCGTCACCCACACTATCATGTTGACATTCC; dG = -32.0
   103 >s_b_s_c_s_b_s_c_s_d_s_c_s_b_s          GTGGCTGTGTGGGTGGCTGTGTGGGTGACGGTGTGGGTGGCTGTG;
>clamp_s_c_s_d_s_c_s_a_s:compl:rev CACAGACACCCACACCGTCACCCACACTATCATGTTGACATTCC; dG = -32.0
   104 >s_b_s_c_s_c_s_c_s_d_s_c_s_b_s          GTGGCTGTGTGGGTGTGGGTGTGGGTGACGGTGTGGGTGGCTGTG;
>clamp_s_c_s_d_s_c_s_a_s:compl:rev CACAGACACCCACACCGTCACCCACACTATCATGTTGACATTCC; dG = -32.0
   105 >s_b_s_c_s_d_s_c_s_d_s_c_s_b_s          GTGGCTGTGTGGGTGACGGTGTGGGTGACGGTGTGGGTGGCTGTG;
>clamp_s_c_s_d_s_c_s_a_s:compl:rev CACAGACACCCACACCGTCACCCACACTATCATGTTGACATTCC; dG = -32.0
   106 >s_b_s_c_s_e_s_c_s_d_s_c_s_b_s          GTGGCTGTGTGGGTGCTGGTGTGGGTGACGGTGTGGGTGGCTGTG;
>clamp_s_c_s_d_s_c_s_a_s:compl:rev CACAGACACCCACACCGTCACCCACACTATCATGTTGACATTCC; dG = -32.0
   107 >s_b_s_d_s_a_s_c_s_d_s_c_s_b_s          GTGGCTGTGACGGTGTCTGTGTGGGTGACGGTGTGGGTGGCTGTG;
>clamp_s_c_s_d_s_c_s_a_s:compl:rev CACAGACACCCACACCGTCACCCACACTATCATGTTGACATTCC; dG = -32.0
   108 >s_b_s_d_s_b_s_c_s_d_s_c_s_b_s          GTGGCTGTGACGGTGGCTGTGTGGGTGACGGTGTGGGTGGCTGTG;
>clamp_s_c_s_d_s_c_s_a_s:compl:rev CACAGACACCCACACCGTCACCCACACTATCATGTTGACATTCC; dG = -32.0
   109 >s_b_s_d_s_c_s_c_s_d_s_c_s_b_s          GTGGCTGTGACGGTGTGGGTGTGGGTGACGGTGTGGGTGGCTGTG;
>clamp_s_c_s_d_s_c_s_a_s:compl:rev CACAGACACCCACACCGTCACCCACACTATCATGTTGACATTCC; dG = -32.0
   110 >s_a_s_a_s_e_s_c_s_d_s_c_s_b_s          GTGTCTGTGTCTGTGCTGGTGTGGGTGACGGTGTGGGTGGCTGTG;
>clamp_s_c_s_d_s_c_s_a_s:compl:rev CACAGACACCCACACCGTCACCCACACTATCATGTTGACATTCC; dG = -32.0
   111 >s_b_s_d_s_d_s_c_s_d_s_c_s_b_s          GTGGCTGTGACGGTGACGGTGTGGGTGACGGTGTGGGTGGCTGTG;
>clamp_s_c_s_d_s_c_s_a_s:compl:rev CACAGACACCCACACCGTCACCCACACTATCATGTTGACATTCC; dG = -32.0
   112 >s_b_s_d_s_e_s_c_s_d_s_c_s_b_s          GTGGCTGTGACGGTGCTGGTGTGGGTGACGGTGTGGGTGGCTGTG;
>clamp_s_c_s_d_s_c_s_a_s:compl:rev CACAGACACCCACACCGTCACCCACACTATCATGTTGACATTCC; dG = -32.0
   113 >s_b_s_e_s_a_s_c_s_d_s_c_s_b_s          GTGGCTGTGCTGGTGTCTGTGTGGGTGACGGTGTGGGTGGCTGTG;
>clamp_s_c_s_d_s_c_s_a_s:compl:rev CACAGACACCCACACCGTCACCCACACTATCATGTTGACATTCC; dG = -32.0
   114 >s_b_s_e_s_b_s_c_s_d_s_c_s_b_s          GTGGCTGTGCTGGTGGCTGTGTGGGTGACGGTGTGGGTGGCTGTG;
>clamp_s_c_s_d_s_c_s_a_s:compl:rev CACAGACACCCACACCGTCACCCACACTATCATGTTGACATTCC; dG = -32.0
   115 >s_b_s_e_s_c_s_c_s_d_s_c_s_b_s          GTGGCTGTGCTGGTGTGGGTGTGGGTGACGGTGTGGGTGGCTGTG;
>clamp_s_c_s_d_s_c_s_a_s:compl:rev CACAGACACCCACACCGTCACCCACACTATCATGTTGACATTCC; dG = -32.0
   116 >s_b_s_e_s_d_s_c_s_d_s_c_s_b_s          GTGGCTGTGCTGGTGACGGTGTGGGTGACGGTGTGGGTGGCTGTG;
>clamp_s_c_s_d_s_c_s_a_s:compl:rev CACAGACACCCACACCGTCACCCACACTATCATGTTGACATTCC; dG = -32.0
   117 >s_b_s_e_s_e_s_c_s_d_s_c_s_b_s          GTGGCTGTGCTGGTGCTGGTGTGGGTGACGGTGTGGGTGGCTGTG;
>clamp_s_c_s_d_s_c_s_a_s:compl:rev CACAGACACCCACACCGTCACCCACACTATCATGTTGACATTCC; dG = -32.0
   118 >s_c_s_a_s_a_s_c_s_d_s_c_s_b_s          GTGTGGGTGTCTGTGTCTGTGTGGGTGACGGTGTGGGTGGCTGTG;
>clamp_s_c_s_d_s_c_s_a_s:compl:rev CACAGACACCCACACCGTCACCCACACTATCATGTTGACATTCC; dG = -32.0
   119 >s_c_s_a_s_b_s_c_s_d_s_c_s_b_s          GTGTGGGTGTCTGTGGCTGTGTGGGTGACGGTGTGGGTGGCTGTG;
>clamp_s_c_s_d_s_c_s_a_s:compl:rev CACAGACACCCACACCGTCACCCACACTATCATGTTGACATTCC; dG = -32.0
   120 >s_c_s_a_s_c_s_c_s_d_s_c_s_b_s          GTGTGGGTGTCTGTGTGGGTGTGGGTGACGGTGTGGGTGGCTGTG;
>clamp_s_c_s_d_s_c_s_a_s:compl:rev CACAGACACCCACACCGTCACCCACACTATCATGTTGACATTCC; dG = -32.0
   121 >s_a_s_b_s_a_s_c_s_d_s_c_s_b_s          GTGTCTGTGGCTGTGTCTGTGTGGGTGACGGTGTGGGTGGCTGTG;
>clamp_s_c_s_d_s_c_s_a_s:compl:rev CACAGACACCCACACCGTCACCCACACTATCATGTTGACATTCC; dG = -32.0
   122 >s_c_s_a_s_d_s_c_s_d_s_c_s_b_s          GTGTGGGTGTCTGTGACGGTGTGGGTGACGGTGTGGGTGGCTGTG;
>clamp_s_c_s_d_s_c_s_a_s:compl:rev CACAGACACCCACACCGTCACCCACACTATCATGTTGACATTCC; dG = -32.0
   123 >s_c_s_a_s_e_s_c_s_d_s_c_s_b_s          GTGTGGGTGTCTGTGCTGGTGTGGGTGACGGTGTGGGTGGCTGTG;
>clamp_s_c_s_d_s_c_s_a_s:compl:rev CACAGACACCCACACCGTCACCCACACTATCATGTTGACATTCC; dG = -32.0
   124 >s_c_s_b_s_a_s_c_s_d_s_c_s_b_s          GTGTGGGTGGCTGTGTCTGTGTGGGTGACGGTGTGGGTGGCTGTG;
>clamp_s_c_s_d_s_c_s_a_s:compl:rev CACAGACACCCACACCGTCACCCACACTATCATGTTGACATTCC; dG = -32.0
   125 >s_c_s_b_s_b_s_c_s_d_s_c_s_b_s          GTGTGGGTGGCTGTGGCTGTGTGGGTGACGGTGTGGGTGGCTGTG;
>clamp_s_c_s_d_s_c_s_a_s:compl:rev CACAGACACCCACACCGTCACCCACACTATCATGTTGACATTCC; dG = -32.0
   126 >s_c_s_b_s_c_s_c_s_d_s_c_s_b_s          GTGTGGGTGGCTGTGTGGGTGTGGGTGACGGTGTGGGTGGCTGTG;
>clamp_s_c_s_d_s_c_s_a_s:compl:rev CACAGACACCCACACCGTCACCCACACTATCATGTTGACATTCC; dG = -32.0
   127 >s_c_s_b_s_d_s_c_s_d_s_c_s_b_s          GTGTGGGTGGCTGTGACGGTGTGGGTGACGGTGTGGGTGGCTGTG;
>clamp_s_c_s_d_s_c_s_a_s:compl:rev CACAGACACCCACACCGTCACCCACACTATCATGTTGACATTCC; dG = -32.0
   128 >s_c_s_b_s_e_s_c_s_d_s_c_s_b_s          GTGTGGGTGGCTGTGCTGGTGTGGGTGACGGTGTGGGTGGCTGTG;
>clamp_s_c_s_d_s_c_s_a_s:compl:rev CACAGACACCCACACCGTCACCCACACTATCATGTTGACATTCC; dG = -32.0
   129 >s_c_s_c_s_a_s_c_s_d_s_c_s_b_s          GTGTGGGTGTGGGTGTCTGTGTGGGTGACGGTGTGGGTGGCTGTG;
>clamp_s_c_s_d_s_c_s_a_s:compl:rev CACAGACACCCACACCGTCACCCACACTATCATGTTGACATTCC; dG = -32.0
   130 >s_c_s_c_s_b_s_c_s_d_s_c_s_b_s          GTGTGGGTGTGGGTGGCTGTGTGGGTGACGGTGTGGGTGGCTGTG;
>clamp_s_c_s_d_s_c_s_a_s:compl:rev CACAGACACCCACACCGTCACCCACACTATCATGTTGACATTCC; dG = -32.0
   131 >s_c_s_c_s_c_s_c_s_d_s_c_s_b_s          GTGTGGGTGTGGGTGTGGGTGTGGGTGACGGTGTGGGTGGCTGTG;
>clamp_s_c_s_d_s_c_s_a_s:compl:rev CACAGACACCCACACCGTCACCCACACTATCATGTTGACATTCC; dG = -32.0
```

```
   132 >s_c_s_d_s_a_s_c_s_d_s_c_s_c_s          GTGTGGGTGACGGTGTCTGTGTGGGTGACGGTGTGGGTGTGGGTG;
>clamp_s_c_s_d_s_c_s_a_s:compl:rev CACAGACACCCACACCGTCACCCACACTATCATGTTGACATTCC; dG = -28.9
   133 >s_c_s_d_s_b_s_c_s_d_s_c_s_c_s          GTGTGGGTGACGGTGGCTGTGTGGGTGACGGTGTGGGTGTGGGTG;
>clamp_s_c_s_d_s_c_s_a_s:compl:rev CACAGACACCCACACCGTCACCCACACTATCATGTTGACATTCC; dG = -28.9
   134 >s_c_s_e_s_a_s_c_s_d_s_c_s_c_s          GTGTGGGTGCTGGTGTCTGTGTGGGTGACGGTGTGGGTGTGGGTG;
>clamp_s_c_s_d_s_c_s_a_s:compl:rev CACAGACACCCACACCGTCACCCACACTATCATGTTGACATTCC; dG = -28.9
   135 >s_c_s_e_s_b_s_c_s_d_s_c_s_c_s          GTGTGGGTGCTGGTGGCTGTGTGGGTGACGGTGTGGGTGTGGGTG;
>clamp_s_c_s_d_s_c_s_a_s:compl:rev CACAGACACCCACACCGTCACCCACACTATCATGTTGACATTCC; dG = -28.9
   136 >s_d_s_a_s_a_s_c_s_d_s_c_s_c_s          GTGACGGTGTCTGTGTCTGTGTGGGTGACGGTGTGGGTGTGGGTG;
>clamp_s_c_s_d_s_c_s_a_s:compl:rev CACAGACACCCACACCGTCACCCACACTATCATGTTGACATTCC; dG = -28.9
   137 >s_d_s_a_s_b_s_c_s_d_s_c_s_c_s          GTGACGGTGTCTGTGGCTGTGTGGGTGACGGTGTGGGTGTGGGTG;
>clamp_s_c_s_d_s_c_s_a_s:compl:rev CACAGACACCCACACCGTCACCCACACTATCATGTTGACATTCC; dG = -28.9
   138 >s_d_s_b_s_a_s_c_s_d_s_c_s_c_s          GTGACGGTGGCTGTGTCTGTGTGGGTGACGGTGTGGGTGTGGGTG;
>clamp_s_c_s_d_s_c_s_a_s:compl:rev CACAGACACCCACACCGTCACCCACACTATCATGTTGACATTCC; dG = -28.9
   139 >s_d_s_b_s_b_s_c_s_d_s_c_s_c_s          GTGACGGTGGCTGTGGCTGTGTGGGTGACGGTGTGGGTGTGGGTG;
>clamp_s_c_s_d_s_c_s_a_s:compl:rev CACAGACACCCACACCGTCACCCACACTATCATGTTGACATTCC; dG = -28.9
   140 >s_d_s_c_s_a_s_c_s_d_s_c_s_c_s          GTGACGGTGTGGGTGTCTGTGTGGGTGACGGTGTGGGTGTGGGTG;
>clamp_s_c_s_d_s_c_s_a_s:compl:rev CACAGACACCCACACCGTCACCCACACTATCATGTTGACATTCC; dG = -28.9
   141 >s_d_s_c_s_b_s_c_s_d_s_c_s_c_s          GTGACGGTGTGGGTGGCTGTGTGGGTGACGGTGTGGGTGTGGGTG;
>clamp_s_c_s_d_s_c_s_a_s:compl:rev CACAGACACCCACACCGTCACCCACACTATCATGTTGACATTCC; dG = -28.9
   142 >s_d_s_d_s_a_s_c_s_d_s_c_s_c_s          GTGACGGTGACGGTGTCTGTGTGGGTGACGGTGTGGGTGTGGGTG;
>clamp_s_c_s_d_s_c_s_a_s:compl:rev CACAGACACCCACACCGTCACCCACACTATCATGTTGACATTCC; dG = -28.9
   143 >s_d_s_d_s_b_s_c_s_d_s_c_s_c_s          GTGACGGTGACGGTGGCTGTGTGGGTGACGGTGTGGGTGTGGGTG;
>clamp_s_c_s_d_s_c_s_a_s:compl:rev CACAGACACCCACACCGTCACCCACACTATCATGTTGACATTCC; dG = -28.9
   144 >s_d_s_e_s_a_s_c_s_d_s_c_s_c_s          GTGACGGTGCTGGTGTCTGTGTGGGTGACGGTGTGGGTGTGGGTG;
>clamp_s_c_s_d_s_c_s_a_s:compl:rev CACAGACACCCACACCGTCACCCACACTATCATGTTGACATTCC; dG = -28.9
   145 >s_d_s_e_s_b_s_c_s_d_s_c_s_c_s          GTGACGGTGCTGGTGGCTGTGTGGGTGACGGTGTGGGTGTGGGTG;
>clamp_s_c_s_d_s_c_s_a_s:compl:rev CACAGACACCCACACCGTCACCCACACTATCATGTTGACATTCC; dG = -28.9
   146 >s_e_s_a_s_a_s_c_s_d_s_c_s_c_s          GTGCTGGTGTCTGTGTCTGTGTGGGTGACGGTGTGGGTGTGGGTG;
>clamp_s_c_s_d_s_c_s_a_s:compl:rev CACAGACACCCACACCGTCACCCACACTATCATGTTGACATTCC; dG = -28.9
   147 >s_e_s_a_s_b_s_c_s_d_s_c_s_c_s          GTGCTGGTGTCTGTGGCTGTGTGGGTGACGGTGTGGGTGTGGGTG;
>clamp_s_c_s_d_s_c_s_a_s:compl:rev CACAGACACCCACACCGTCACCCACACTATCATGTTGACATTCC; dG = -28.9
   148 >s_a_s_c_s_a_s_c_s_d_s_c_s_c_s          GTGTCTGTGTGGGTGTCTGTGTGGGTGACGGTGTGGGTGTGGGTG;
>clamp_s_c_s_d_s_c_s_a_s:compl:rev CACAGACACCCACACCGTCACCCACACTATCATGTTGACATTCC; dG = -28.9
   149 >s_e_s_b_s_a_s_c_s_d_s_c_s_c_s          GTGCTGGTGGCTGTGTCTGTGTGGGTGACGGTGTGGGTGTGGGTG;
>clamp_s_c_s_d_s_c_s_a_s:compl:rev CACAGACACCCACACCGTCACCCACACTATCATGTTGACATTCC; dG = -28.9
   150 >s_e_s_b_s_b_s_c_s_d_s_c_s_c_s          GTGCTGGTGGCTGTGGCTGTGTGGGTGACGGTGTGGGTGTGGGTG;
>clamp_s_c_s_d_s_c_s_a_s:compl:rev CACAGACACCCACACCGTCACCCACACTATCATGTTGACATTCC; dG = -28.9
   151 >s_e_s_c_s_a_s_c_s_d_s_c_s_c_s          GTGCTGGTGTGGGTGTCTGTGTGGGTGACGGTGTGGGTGTGGGTG;
>clamp_s_c_s_d_s_c_s_a_s:compl:rev CACAGACACCCACACCGTCACCCACACTATCATGTTGACATTCC; dG = -28.9
   152 >s_e_s_c_s_b_s_c_s_d_s_c_s_c_s          GTGCTGGTGTGGGTGGCTGTGTGGGTGACGGTGTGGGTGTGGGTG;
>clamp_s_c_s_d_s_c_s_a_s:compl:rev CACAGACACCCACACCGTCACCCACACTATCATGTTGACATTCC; dG = -28.9
   153 >s_a_s_c_s_b_s_c_s_d_s_c_s_c_s          GTGTCTGTGTGGGTGGCTGTGTGGGTGACGGTGTGGGTGTGGGTG;
>clamp_s_c_s_d_s_c_s_a_s:compl:rev CACAGACACCCACACCGTCACCCACACTATCATGTTGACATTCC; dG = -28.9
   154 >s_e_s_d_s_a_s_c_s_d_s_c_s_c_s          GTGCTGGTGACGGTGTCTGTGTGGGTGACGGTGTGGGTGTGGGTG;
>clamp_s_c_s_d_s_c_s_a_s:compl:rev CACAGACACCCACACCGTCACCCACACTATCATGTTGACATTCC; dG = -28.9
   155 >s_e_s_d_s_b_s_c_s_d_s_c_s_c_s          GTGCTGGTGACGGTGGCTGTGTGGGTGACGGTGTGGGTGTGGGTG;
>clamp_s_c_s_d_s_c_s_a_s:compl:rev CACAGACACCCACACCGTCACCCACACTATCATGTTGACATTCC; dG = -28.9
   156 >s_a_s_a_s_b_s_c_s_d_s_c_s_c_s          GTGTCTGTGTCTGTGGCTGTGTGGGTGACGGTGTGGGTGTGGGTG;
>clamp_s_c_s_d_s_c_s_a_s:compl:rev CACAGACACCCACACCGTCACCCACACTATCATGTTGACATTCC; dG = -28.9
   157 >s_e_s_e_s_a_s_c_s_d_s_c_s_c_s          GTGCTGGTGCTGGTGTCTGTGTGGGTGACGGTGTGGGTGTGGGTG;
>clamp_s_c_s_d_s_c_s_a_s:compl:rev CACAGACACCCACACCGTCACCCACACTATCATGTTGACATTCC; dG = -28.9
   158 >s_e_s_e_s_b_s_c_s_d_s_c_s_c_s          GTGCTGGTGCTGGTGGCTGTGTGGGTGACGGTGTGGGTGTGGGTG;
>clamp_s_c_s_d_s_c_s_a_s:compl:rev CACAGACACCCACACCGTCACCCACACTATCATGTTGACATTCC; dG = -28.9
   159 >s_a_s_c_s_d_s_c_s_c_s_c_s_a_s          GTGTCTGTGTGGGTGACGGTGTGGGTGTGGGTGTGGGTGTCTGTG;
>clamp_s_c_s_d_s_c_s_a_s:compl:rev CACAGACACCCACACCGTCACCCACACTATCATGTTGACATTCC; dG = -28.9
   160 >s_a_s_d_s_a_s_c_s_d_s_c_s_c_s          GTGTCTGTGACGGTGTCTGTGTGGGTGACGGTGTGGGTGTGGGTG;
>clamp_s_c_s_d_s_c_s_a_s:compl:rev CACAGACACCCACACCGTCACCCACACTATCATGTTGACATTCC; dG = -28.9
   161 >s_a_s_d_s_b_s_c_s_d_s_c_s_c_s          GTGTCTGTGACGGTGGCTGTGTGGGTGACGGTGTGGGTGTGGGTG;
>clamp_s_c_s_d_s_c_s_a_s:compl:rev CACAGACACCCACACCGTCACCCACACTATCATGTTGACATTCC; dG = -28.9
   162 >s_a_s_a_s_a_s_c_s_d_s_c_s_c_s          GTGTCTGTGTCTGTGTCTGTGTGGGTGACGGTGTGGGTGTGGGTG;
>clamp_s_c_s_d_s_c_s_a_s:compl:rev CACAGACACCCACACCGTCACCCACACTATCATGTTGACATTCC; dG = -28.9
   163 >s_a_s_e_s_a_s_c_s_d_s_c_s_c_s          GTGTCTGTGCTGGTGTCTGTGTGGGTGACGGTGTGGGTGTGGGTG;
>clamp_s_c_s_d_s_c_s_a_s:compl:rev CACAGACACCCACACCGTCACCCACACTATCATGTTGACATTCC; dG = -28.9
   164 >s_a_s_e_s_b_s_c_s_d_s_c_s_c_s          GTGTCTGTGCTGGTGGCTGTGTGGGTGACGGTGTGGGTGTGGGTG;
>clamp_s_c_s_d_s_c_s_a_s:compl:rev CACAGACACCCACACCGTCACCCACACTATCATGTTGACATTCC; dG = -28.9
   165 >s_b_s_a_s_a_s_c_s_d_s_c_s_c_s          GTGGCTGTGTCTGTGTCTGTGTGGGTGACGGTGTGGGTGTGGGTG;
>clamp_s_c_s_d_s_c_s_a_s:compl:rev CACAGACACCCACACCGTCACCCACACTATCATGTTGACATTCC; dG = -28.9
   166 >s_b_s_a_s_b_s_c_s_d_s_c_s_c_s          GTGGCTGTGTCTGTGGCTGTGTGGGTGACGGTGTGGGTGTGGGTG;
>clamp_s_c_s_d_s_c_s_a_s:compl:rev CACAGACACCCACACCGTCACCCACACTATCATGTTGACATTCC; dG = -28.9
   167 >s_b_s_b_s_a_s_c_s_d_s_c_s_c_s          GTGGCTGTGGCTGTGTCTGTGTGGGTGACGGTGTGGGTGTGGGTG;
>clamp_s_c_s_d_s_c_s_a_s:compl:rev CACAGACACCCACACCGTCACCCACACTATCATGTTGACATTCC; dG = -28.9
   168 >s_b_s_b_s_b_s_c_s_d_s_c_s_c_s          GTGGCTGTGGCTGTGGCTGTGTGGGTGACGGTGTGGGTGTGGGTG;
>clamp_s_c_s_d_s_c_s_a_s:compl:rev CACAGACACCCACACCGTCACCCACACTATCATGTTGACATTCC; dG = -28.9
```

```
   169 >s_b_s_c_s_a_s_c_s_d_s_c_s_c_s          GTGGCTGTGTGGGTGTCTGTGTGGGTGACGGTGTGGGTGTGGGTG;
>clamp_s_c_s_d_s_c_s_a_s:compl:rev CACAGACACCCACACCGTCACCCACACTATCATGTTGACATTCC; dG = -28.9
   170 >s_b_s_c_s_b_s_c_s_d_s_c_s_c_s          GTGGCTGTGTGGGTGGCTGTGTGGGTGACGGTGTGGGTGTGGGTG;
>clamp_s_c_s_d_s_c_s_a_s:compl:rev CACAGACACCCACACCGTCACCCACACTATCATGTTGACATTCC; dG = -28.9
   171 >s_b_s_c_s_d_s_c_s_c_s_c_s_a_s          GTGGCTGTGTGGGTGACGGTGTGGGTGTGGGTGTGGGTGTCTGTG;
>clamp_s_c_s_d_s_c_s_a_s:compl:rev CACAGACACCCACACCGTCACCCACACTATCATGTTGACATTCC; dG = -28.9
   172 >s_b_s_d_s_a_s_c_s_d_s_c_s_c_s          GTGGCTGTGACGGTGTCTGTGTGGGTGACGGTGTGGGTGTGGGTG;
>clamp_s_c_s_d_s_c_s_a_s:compl:rev CACAGACACCCACACCGTCACCCACACTATCATGTTGACATTCC; dG = -28.9
   173 >s_b_s_d_s_b_s_c_s_d_s_c_s_c_s          GTGGCTGTGACGGTGGCTGTGTGGGTGACGGTGTGGGTGTGGGTG;
>clamp_s_c_s_d_s_c_s_a_s:compl:rev CACAGACACCCACACCGTCACCCACACTATCATGTTGACATTCC; dG = -28.9
   174 >s_b_s_e_s_a_s_c_s_d_s_c_s_c_s          GTGGCTGTGCTGGTGTCTGTGTGGGTGACGGTGTGGGTGTGGGTG;
>clamp_s_c_s_d_s_c_s_a_s:compl:rev CACAGACACCCACACCGTCACCCACACTATCATGTTGACATTCC; dG = -28.9
   175 >s_b_s_e_s_b_s_c_s_d_s_c_s_c_s          GTGGCTGTGCTGGTGGCTGTGTGGGTGACGGTGTGGGTGTGGGTG;
>clamp_s_c_s_d_s_c_s_a_s:compl:rev CACAGACACCCACACCGTCACCCACACTATCATGTTGACATTCC; dG = -28.9
   176 >s_c_s_a_s_a_s_c_s_d_s_c_s_c_s          GTGTGGGTGTCTGTGTCTGTGTGGGTGACGGTGTGGGTGTGGGTG;
>clamp_s_c_s_d_s_c_s_a_s:compl:rev CACAGACACCCACACCGTCACCCACACTATCATGTTGACATTCC; dG = -28.9
   177 >s_c_s_a_s_b_s_c_s_d_s_c_s_c_s          GTGTGGGTGTCTGTGGCTGTGTGGGTGACGGTGTGGGTGTGGGTG;
>clamp_s_c_s_d_s_c_s_a_s:compl:rev CACAGACACCCACACCGTCACCCACACTATCATGTTGACATTCC; dG = -28.9
   178 >s_a_s_b_s_a_s_c_s_d_s_c_s_c_s          GTGTCTGTGGCTGTGTCTGTGTGGGTGACGGTGTGGGTGTGGGTG;
>clamp_s_c_s_d_s_c_s_a_s:compl:rev CACAGACACCCACACCGTCACCCACACTATCATGTTGACATTCC; dG = -28.9
   179 >s_c_s_b_s_a_s_c_s_d_s_c_s_c_s          GTGTGGGTGGCTGTGTCTGTGTGGGTGACGGTGTGGGTGTGGGTG;
>clamp_s_c_s_d_s_c_s_a_s:compl:rev CACAGACACCCACACCGTCACCCACACTATCATGTTGACATTCC; dG = -28.9
   180 >s_c_s_b_s_b_s_c_s_d_s_c_s_c_s          GTGTGGGTGGCTGTGGCTGTGTGGGTGACGGTGTGGGTGTGGGTG;
>clamp_s_c_s_d_s_c_s_a_s:compl:rev CACAGACACCCACACCGTCACCCACACTATCATGTTGACATTCC; dG = -28.9
   181 >s_c_s_c_s_a_s_c_s_d_s_c_s_c_s          GTGTGGGTGTGGGTGTCTGTGTGGGTGACGGTGTGGGTGTGGGTG;
>clamp_s_c_s_d_s_c_s_a_s:compl:rev CACAGACACCCACACCGTCACCCACACTATCATGTTGACATTCC; dG = -28.9
   182 >s_c_s_c_s_b_s_c_s_d_s_c_s_c_s          GTGTGGGTGTGGGTGGCTGTGTGGGTGACGGTGTGGGTGTGGGTG;
>clamp_s_c_s_d_s_c_s_a_s:compl:rev CACAGACACCCACACCGTCACCCACACTATCATGTTGACATTCC; dG = -28.9
   183 >s_a_s_b_s_b_s_c_s_d_s_c_s_c_s          GTGTCTGTGGCTGTGGCTGTGTGGGTGACGGTGTGGGTGTGGGTG;
>clamp_s_c_s_d_s_c_s_a_s:compl:rev CACAGACACCCACACCGTCACCCACACTATCATGTTGACATTCC; dG = -28.9
   184 >s_c_s_c_s_d_s_c_s_d_s_c_s_c_s          GTGTGGGTGTGGGTGACGGTGTGGGTGACGGTGTGGGTGTGGGTG;
>clamp_s_c_s_d_s_c_s_a_s:compl:rev CACAGACACCCACACCGTCACCCACACTATCATGTTGACATTCC; dG = -28.8
   185 >s_c_s_c_s_d_s_c_s_c_s_c_s_a_s          GTGTGGGTGTGGGTGACGGTGTGGGTGTGGGTGTGGGTGTCTGTG;
>clamp_s_c_s_d_s_c_s_a_s:compl:rev CACAGACACCCACACCGTCACCCACACTATCATGTTGACATTCC; dG = -28.8
   186 >s_c_s_c_s_e_s_c_s_d_s_c_s_c_s          GTGTGGGTGTGGGTGCTGGTGTGGGTGACGGTGTGGGTGTGGGTG;
>clamp_s_c_s_d_s_c_s_a_s:compl:rev CACAGACACCCACACCGTCACCCACACTATCATGTTGACATTCC; dG = -28.8
   187 >s_c_s_d_s_c_s_c_s_d_s_c_s_c_s          GTGTGGGTGACGGTGTGGGTGTGGGTGACGGTGTGGGTGTGGGTG;
>clamp_s_c_s_d_s_c_s_a_s:compl:rev CACAGACACCCACACCGTCACCCACACTATCATGTTGACATTCC; dG = -28.8
   188 >s_c_s_d_s_d_s_c_s_d_s_c_s_c_s          GTGTGGGTGACGGTGACGGTGTGGGTGACGGTGTGGGTGTGGGTG;
>clamp_s_c_s_d_s_c_s_a_s:compl:rev CACAGACACCCACACCGTCACCCACACTATCATGTTGACATTCC; dG = -28.8
   189 >s_c_s_d_s_e_s_c_s_d_s_c_s_c_s          GTGTGGGTGACGGTGCTGGTGTGGGTGACGGTGTGGGTGTGGGTG;
>clamp_s_c_s_d_s_c_s_a_s:compl:rev CACAGACACCCACACCGTCACCCACACTATCATGTTGACATTCC; dG = -28.8
   190 >s_a_s_b_s_c_s_c_s_d_s_c_s_c_s          GTGTCTGTGGCTGTGTGGGTGTGGGTGACGGTGTGGGTGTGGGTG;
>clamp_s_c_s_d_s_c_s_a_s:compl:rev CACAGACACCCACACCGTCACCCACACTATCATGTTGACATTCC; dG = -28.8
   191 >s_c_s_e_s_c_s_c_s_d_s_c_s_c_s          GTGTGGGTGCTGGTGTGGGTGTGGGTGACGGTGTGGGTGTGGGTG;
>clamp_s_c_s_d_s_c_s_a_s:compl:rev CACAGACACCCACACCGTCACCCACACTATCATGTTGACATTCC; dG = -28.8
   192 >s_c_s_e_s_d_s_c_s_d_s_c_s_c_s          GTGTGGGTGCTGGTGACGGTGTGGGTGACGGTGTGGGTGTGGGTG;
>clamp_s_c_s_d_s_c_s_a_s:compl:rev CACAGACACCCACACCGTCACCCACACTATCATGTTGACATTCC; dG = -28.8
   193 >s_c_s_e_s_e_s_c_s_d_s_c_s_c_s          GTGTGGGTGCTGGTGCTGGTGTGGGTGACGGTGTGGGTGTGGGTG;
>clamp_s_c_s_d_s_c_s_a_s:compl:rev CACAGACACCCACACCGTCACCCACACTATCATGTTGACATTCC; dG = -28.8
   194 >s_d_s_a_s_c_s_c_s_d_s_c_s_c_s          GTGACGGTGTCTGTGTGGGTGTGGGTGACGGTGTGGGTGTGGGTG;
>clamp_s_c_s_d_s_c_s_a_s:compl:rev CACAGACACCCACACCGTCACCCACACTATCATGTTGACATTCC; dG = -28.8
   195 >s_d_s_a_s_d_s_c_s_d_s_c_s_c_s          GTGACGGTGTCTGTGACGGTGTGGGTGACGGTGTGGGTGTGGGTG;
>clamp_s_c_s_d_s_c_s_a_s:compl:rev CACAGACACCCACACCGTCACCCACACTATCATGTTGACATTCC; dG = -28.8
   196 >s_d_s_a_s_e_s_c_s_d_s_c_s_c_s          GTGACGGTGTCTGTGCTGGTGTGGGTGACGGTGTGGGTGTGGGTG;
>clamp_s_c_s_d_s_c_s_a_s:compl:rev CACAGACACCCACACCGTCACCCACACTATCATGTTGACATTCC; dG = -28.8
   197 >s_a_s_b_s_d_s_c_s_d_s_c_s_c_s          GTGTCTGTGGCTGTGACGGTGTGGGTGACGGTGTGGGTGTGGGTG;
>clamp_s_c_s_d_s_c_s_a_s:compl:rev CACAGACACCCACACCGTCACCCACACTATCATGTTGACATTCC; dG = -28.8
   198 >s_d_s_b_s_c_s_c_s_d_s_c_s_c_s          GTGACGGTGGCTGTGTGGGTGTGGGTGACGGTGTGGGTGTGGGTG;
>clamp_s_c_s_d_s_c_s_a_s:compl:rev CACAGACACCCACACCGTCACCCACACTATCATGTTGACATTCC; dG = -28.8
   199 >s_d_s_b_s_d_s_c_s_d_s_c_s_c_s          GTGACGGTGGCTGTGACGGTGTGGGTGACGGTGTGGGTGTGGGTG;
>clamp_s_c_s_d_s_c_s_a_s:compl:rev CACAGACACCCACACCGTCACCCACACTATCATGTTGACATTCC; dG = -28.8
   200 >s_d_s_b_s_e_s_c_s_d_s_c_s_c_s          GTGACGGTGGCTGTGCTGGTGTGGGTGACGGTGTGGGTGTGGGTG;
>clamp_s_c_s_d_s_c_s_a_s:compl:rev CACAGACACCCACACCGTCACCCACACTATCATGTTGACATTCC; dG = -28.8
   201 >s_d_s_c_s_c_s_c_s_d_s_c_s_c_s          GTGACGGTGTGGGTGTGGGTGTGGGTGACGGTGTGGGTGTGGGTG;
>clamp_s_c_s_d_s_c_s_a_s:compl:rev CACAGACACCCACACCGTCACCCACACTATCATGTTGACATTCC; dG = -28.8
   202 >s_d_s_c_s_d_s_c_s_d_s_c_s_c_s          GTGACGGTGTGGGTGACGGTGTGGGTGACGGTGTGGGTGTGGGTG;
>clamp_s_c_s_d_s_c_s_a_s:compl:rev CACAGACACCCACACCGTCACCCACACTATCATGTTGACATTCC; dG = -28.8
   203 >s_d_s_c_s_d_s_c_s_c_s_c_s_a_s          GTGACGGTGTGGGTGACGGTGTGGGTGTGGGTGTGGGTGTCTGTG;
>clamp_s_c_s_d_s_c_s_a_s:compl:rev CACAGACACCCACACCGTCACCCACACTATCATGTTGACATTCC; dG = -28.8
   204 >s_d_s_c_s_e_s_c_s_d_s_c_s_c_s          GTGACGGTGTGGGTGCTGGTGTGGGTGACGGTGTGGGTGTGGGTG;
>clamp_s_c_s_d_s_c_s_a_s:compl:rev CACAGACACCCACACCGTCACCCACACTATCATGTTGACATTCC; dG = -28.8
   205 >s_a_s_b_s_e_s_c_s_d_s_c_s_c_s          GTGTCTGTGGCTGTGCTGGTGTGGGTGACGGTGTGGGTGTGGGTG;
>clamp_s_c_s_d_s_c_s_a_s:compl:rev CACAGACACCCACACCGTCACCCACACTATCATGTTGACATTCC; dG = -28.8
```

```
   206 >s_d_s_d_s_c_s_c_s_d_s_c_s_c_s          GTGACGGTGACGGTGTGGGTGTGGGTGACGGTGTGGGTGTGGGTG;
>clamp_s_c_s_d_s_c_s_a_s:compl:rev CACAGACACCCACACCGTCACCCACACTATCATGTTGACATTCC; dG = -28.8
   207 >s_d_s_d_s_d_s_c_s_d_s_c_s_c_s          GTGACGGTGACGGTGACGGTGTGGGTGACGGTGTGGGTGTGGGTG;
>clamp_s_c_s_d_s_c_s_a_s:compl:rev CACAGACACCCACACCGTCACCCACACTATCATGTTGACATTCC; dG = -28.8
   208 >s_d_s_d_s_e_s_c_s_d_s_c_s_c_s          GTGACGGTGACGGTGCTGGTGTGGGTGACGGTGTGGGTGTGGGTG;
>clamp_s_c_s_d_s_c_s_a_s:compl:rev CACAGACACCCACACCGTCACCCACACTATCATGTTGACATTCC; dG = -28.8
   209 >s_d_s_e_s_c_s_c_s_d_s_c_s_c_s          GTGACGGTGCTGGTGTGGGTGTGGGTGACGGTGTGGGTGTGGGTG;
>clamp_s_c_s_d_s_c_s_a_s:compl:rev CACAGACACCCACACCGTCACCCACACTATCATGTTGACATTCC; dG = -28.8
   210 >s_d_s_e_s_d_s_c_s_d_s_c_s_c_s          GTGACGGTGCTGGTGACGGTGTGGGTGACGGTGTGGGTGTGGGTG;
>clamp_s_c_s_d_s_c_s_a_s:compl:rev CACAGACACCCACACCGTCACCCACACTATCATGTTGACATTCC; dG = -28.8
   211 >s_d_s_e_s_e_s_c_s_d_s_c_s_c_s          GTGACGGTGCTGGTGCTGGTGTGGGTGACGGTGTGGGTGTGGGTG;
>clamp_s_c_s_d_s_c_s_a_s:compl:rev CACAGACACCCACACCGTCACCCACACTATCATGTTGACATTCC; dG = -28.8
   212 >s_e_s_a_s_c_s_c_s_d_s_c_s_c_s          GTGCTGGTGTCTGTGTGGGTGTGGGTGACGGTGTGGGTGTGGGTG;
>clamp_s_c_s_d_s_c_s_a_s:compl:rev CACAGACACCCACACCGTCACCCACACTATCATGTTGACATTCC; dG = -28.8
   213 >s_e_s_a_s_d_s_c_s_d_s_c_s_c_s          GTGCTGGTGTCTGTGACGGTGTGGGTGACGGTGTGGGTGTGGGTG;
>clamp_s_c_s_d_s_c_s_a_s:compl:rev CACAGACACCCACACCGTCACCCACACTATCATGTTGACATTCC; dG = -28.8
   214 >s_e_s_a_s_e_s_c_s_d_s_c_s_c_s          GTGCTGGTGTCTGTGCTGGTGTGGGTGACGGTGTGGGTGTGGGTG;
>clamp_s_c_s_d_s_c_s_a_s:compl:rev CACAGACACCCACACCGTCACCCACACTATCATGTTGACATTCC; dG = -28.8
   215 >s_e_s_b_s_c_s_c_s_d_s_c_s_c_s          GTGCTGGTGGCTGTGTGGGTGTGGGTGACGGTGTGGGTGTGGGTG;
>clamp_s_c_s_d_s_c_s_a_s:compl:rev CACAGACACCCACACCGTCACCCACACTATCATGTTGACATTCC; dG = -28.8
   216 >s_e_s_b_s_d_s_c_s_d_s_c_s_c_s          GTGCTGGTGGCTGTGACGGTGTGGGTGACGGTGTGGGTGTGGGTG;
>clamp_s_c_s_d_s_c_s_a_s:compl:rev CACAGACACCCACACCGTCACCCACACTATCATGTTGACATTCC; dG = -28.8
   217 >s_e_s_b_s_e_s_c_s_d_s_c_s_c_s          GTGCTGGTGGCTGTGCTGGTGTGGGTGACGGTGTGGGTGTGGGTG;
>clamp_s_c_s_d_s_c_s_a_s:compl:rev CACAGACACCCACACCGTCACCCACACTATCATGTTGACATTCC; dG = -28.8
   218 >s_e_s_c_s_c_s_c_s_d_s_c_s_c_s          GTGCTGGTGTGGGTGTGGGTGTGGGTGACGGTGTGGGTGTGGGTG;
>clamp_s_c_s_d_s_c_s_a_s:compl:rev CACAGACACCCACACCGTCACCCACACTATCATGTTGACATTCC; dG = -28.8
   219 >s_e_s_c_s_d_s_c_s_d_s_c_s_c_s          GTGCTGGTGTGGGTGACGGTGTGGGTGACGGTGTGGGTGTGGGTG;
>clamp_s_c_s_d_s_c_s_a_s:compl:rev CACAGACACCCACACCGTCACCCACACTATCATGTTGACATTCC; dG = -28.8
   220 >s_e_s_c_s_d_s_c_s_c_s_c_s_a_s          GTGCTGGTGTGGGTGACGGTGTGGGTGTGGGTGTGGGTGTCTGTG;
>clamp_s_c_s_d_s_c_s_a_s:compl:rev CACAGACACCCACACCGTCACCCACACTATCATGTTGACATTCC; dG = -28.8
   221 >s_e_s_c_s_e_s_c_s_d_s_c_s_c_s          GTGCTGGTGTGGGTGCTGGTGTGGGTGACGGTGTGGGTGTGGGTG;
>clamp_s_c_s_d_s_c_s_a_s:compl:rev CACAGACACCCACACCGTCACCCACACTATCATGTTGACATTCC; dG = -28.8
   222 >s_e_s_d_s_c_s_c_s_d_s_c_s_c_s          GTGCTGGTGACGGTGTGGGTGTGGGTGACGGTGTGGGTGTGGGTG;
>clamp_s_c_s_d_s_c_s_a_s:compl:rev CACAGACACCCACACCGTCACCCACACTATCATGTTGACATTCC; dG = -28.8
   223 >s_e_s_d_s_d_s_c_s_d_s_c_s_c_s          GTGCTGGTGACGGTGACGGTGTGGGTGACGGTGTGGGTGTGGGTG;
>clamp_s_c_s_d_s_c_s_a_s:compl:rev CACAGACACCCACACCGTCACCCACACTATCATGTTGACATTCC; dG = -28.8
   224 >s_e_s_d_s_e_s_c_s_d_s_c_s_c_s          GTGCTGGTGACGGTGCTGGTGTGGGTGACGGTGTGGGTGTGGGTG;
>clamp_s_c_s_d_s_c_s_a_s:compl:rev CACAGACACCCACACCGTCACCCACACTATCATGTTGACATTCC; dG = -28.8
   225 >s_e_s_e_s_c_s_c_s_d_s_c_s_c_s          GTGCTGGTGCTGGTGTGGGTGTGGGTGACGGTGTGGGTGTGGGTG;
>clamp_s_c_s_d_s_c_s_a_s:compl:rev CACAGACACCCACACCGTCACCCACACTATCATGTTGACATTCC; dG = -28.8
   226 >s_a_s_c_s_c_s_c_s_d_s_c_s_c_s          GTGTCTGTGTGGGTGTGGGTGTGGGTGACGGTGTGGGTGTGGGTG;
>clamp_s_c_s_d_s_c_s_a_s:compl:rev CACAGACACCCACACCGTCACCCACACTATCATGTTGACATTCC; dG = -28.8
   227 >s_e_s_e_s_d_s_c_s_d_s_c_s_c_s          GTGCTGGTGCTGGTGACGGTGTGGGTGACGGTGTGGGTGTGGGTG;
>clamp_s_c_s_d_s_c_s_a_s:compl:rev CACAGACACCCACACCGTCACCCACACTATCATGTTGACATTCC; dG = -28.8
   228 >s_e_s_e_s_e_s_c_s_d_s_c_s_c_s          GTGCTGGTGCTGGTGCTGGTGTGGGTGACGGTGTGGGTGTGGGTG;
>clamp_s_c_s_d_s_c_s_a_s:compl:rev CACAGACACCCACACCGTCACCCACACTATCATGTTGACATTCC; dG = -28.8
   229 >s_a_s_c_s_d_s_c_s_d_s_c_s_c_s          GTGTCTGTGTGGGTGACGGTGTGGGTGACGGTGTGGGTGTGGGTG;
>clamp_s_c_s_d_s_c_s_a_s:compl:rev CACAGACACCCACACCGTCACCCACACTATCATGTTGACATTCC; dG = -28.8
   230 >s_a_s_c_s_d_s_c_s_d_s_a_s_a_s          GTGTCTGTGTGGGTGACGGTGTGGGTGACGGTGTCTGTGTCTGTG;
>clamp_s_c_s_d_s_c_s_a_s:compl:rev CACAGACACCCACACCGTCACCCACACTATCATGTTGACATTCC; dG = -28.8
   231 >s_a_s_c_s_e_s_c_s_d_s_c_s_c_s          GTGTCTGTGTGGGTGCTGGTGTGGGTGACGGTGTGGGTGTGGGTG;
>clamp_s_c_s_d_s_c_s_a_s:compl:rev CACAGACACCCACACCGTCACCCACACTATCATGTTGACATTCC; dG = -28.8
   232 >s_a_s_d_s_c_s_c_s_d_s_c_s_c_s          GTGTCTGTGACGGTGTGGGTGTGGGTGACGGTGTGGGTGTGGGTG;
>clamp_s_c_s_d_s_c_s_a_s:compl:rev CACAGACACCCACACCGTCACCCACACTATCATGTTGACATTCC; dG = -28.8
   233 >s_a_s_d_s_d_s_c_s_d_s_c_s_c_s          GTGTCTGTGACGGTGACGGTGTGGGTGACGGTGTGGGTGTGGGTG;
>clamp_s_c_s_d_s_c_s_a_s:compl:rev CACAGACACCCACACCGTCACCCACACTATCATGTTGACATTCC; dG = -28.8
   234 >s_a_s_d_s_e_s_c_s_d_s_c_s_c_s          GTGTCTGTGACGGTGCTGGTGTGGGTGACGGTGTGGGTGTGGGTG;
>clamp_s_c_s_d_s_c_s_a_s:compl:rev CACAGACACCCACACCGTCACCCACACTATCATGTTGACATTCC; dG = -28.8
   235 >s_a_s_a_s_c_s_c_s_d_s_c_s_c_s          GTGTCTGTGTCTGTGTGGGTGTGGGTGACGGTGTGGGTGTGGGTG;
>clamp_s_c_s_d_s_c_s_a_s:compl:rev CACAGACACCCACACCGTCACCCACACTATCATGTTGACATTCC; dG = -28.8
   236 >s_a_s_e_s_c_s_c_s_d_s_c_s_c_s          GTGTCTGTGCTGGTGTGGGTGTGGGTGACGGTGTGGGTGTGGGTG;
>clamp_s_c_s_d_s_c_s_a_s:compl:rev CACAGACACCCACACCGTCACCCACACTATCATGTTGACATTCC; dG = -28.8
   237 >s_a_s_e_s_d_s_c_s_d_s_c_s_c_s          GTGTCTGTGCTGGTGACGGTGTGGGTGACGGTGTGGGTGTGGGTG;
>clamp_s_c_s_d_s_c_s_a_s:compl:rev CACAGACACCCACACCGTCACCCACACTATCATGTTGACATTCC; dG = -28.8
   238 >s_a_s_e_s_e_s_c_s_d_s_c_s_c_s          GTGTCTGTGCTGGTGCTGGTGTGGGTGACGGTGTGGGTGTGGGTG;
>clamp_s_c_s_d_s_c_s_a_s:compl:rev CACAGACACCCACACCGTCACCCACACTATCATGTTGACATTCC; dG = -28.8
   239 >s_b_s_a_s_c_s_c_s_d_s_c_s_c_s          GTGGCTGTGTCTGTGTGGGTGTGGGTGACGGTGTGGGTGTGGGTG;
>clamp_s_c_s_d_s_c_s_a_s:compl:rev CACAGACACCCACACCGTCACCCACACTATCATGTTGACATTCC; dG = -28.8
   240 >s_b_s_a_s_d_s_c_s_d_s_c_s_c_s          GTGGCTGTGTCTGTGACGGTGTGGGTGACGGTGTGGGTGTGGGTG;
>clamp_s_c_s_d_s_c_s_a_s:compl:rev CACAGACACCCACACCGTCACCCACACTATCATGTTGACATTCC; dG = -28.8
   241 >s_b_s_a_s_e_s_c_s_d_s_c_s_c_s          GTGGCTGTGTCTGTGCTGGTGTGGGTGACGGTGTGGGTGTGGGTG;
>clamp_s_c_s_d_s_c_s_a_s:compl:rev CACAGACACCCACACCGTCACCCACACTATCATGTTGACATTCC; dG = -28.8
   242 >s_a_s_a_s_d_s_c_s_d_s_c_s_c_s          GTGTCTGTGTCTGTGACGGTGTGGGTGACGGTGTGGGTGTGGGTG;
>clamp_s_c_s_d_s_c_s_a_s:compl:rev CACAGACACCCACACCGTCACCCACACTATCATGTTGACATTCC; dG = -28.8
```

```
   243 >s_b_s_b_s_c_s_c_s_d_s_c_s_c_s          GTGGCTGTGGCTGTGTGGGTGTGGGTGACGGTGTGGGTGTGGGTG;
>clamp_s_c_s_d_s_c_s_a_s:compl:rev CACAGACACCCACACCGTCACCCACACTATCATGTTGACATTCC; dG = -28.8
   244 >s_b_s_b_s_d_s_c_s_d_s_c_s_c_s          GTGGCTGTGGCTGTGACGGTGTGGGTGACGGTGTGGGTGTGGGTG;
>clamp_s_c_s_d_s_c_s_a_s:compl:rev CACAGACACCCACACCGTCACCCACACTATCATGTTGACATTCC; dG = -28.8
   245 >s_b_s_b_s_e_s_c_s_d_s_c_s_c_s          GTGGCTGTGGCTGTGCTGGTGTGGGTGACGGTGTGGGTGTGGGTG;
>clamp_s_c_s_d_s_c_s_a_s:compl:rev CACAGACACCCACACCGTCACCCACACTATCATGTTGACATTCC; dG = -28.8
   246 >s_b_s_c_s_c_s_c_s_d_s_c_s_c_s          GTGGCTGTGTGGGTGTGGGTGTGGGTGACGGTGTGGGTGTGGGTG;
>clamp_s_c_s_d_s_c_s_a_s:compl:rev CACAGACACCCACACCGTCACCCACACTATCATGTTGACATTCC; dG = -28.8
   247 >s_b_s_c_s_d_s_c_s_d_s_c_s_c_s          GTGGCTGTGTGGGTGACGGTGTGGGTGACGGTGTGGGTGTGGGTG;
>clamp_s_c_s_d_s_c_s_a_s:compl:rev CACAGACACCCACACCGTCACCCACACTATCATGTTGACATTCC; dG = -28.8
   248 >s_b_s_c_s_d_s_c_s_d_s_a_s_a_s          GTGGCTGTGTGGGTGACGGTGTGGGTGACGGTGTCTGTGTCTGTG;
>clamp_s_c_s_d_s_c_s_a_s:compl:rev CACAGACACCCACACCGTCACCCACACTATCATGTTGACATTCC; dG = -28.8
   249 >s_b_s_c_s_e_s_c_s_d_s_c_s_c_s          GTGGCTGTGTGGGTGCTGGTGTGGGTGACGGTGTGGGTGTGGGTG;
>clamp_s_c_s_d_s_c_s_a_s:compl:rev CACAGACACCCACACCGTCACCCACACTATCATGTTGACATTCC; dG = -28.8
   250 >s_a_s_a_s_e_s_c_s_d_s_c_s_c_s          GTGTCTGTGTCTGTGCTGGTGTGGGTGACGGTGTGGGTGTGGGTG;
>clamp_s_c_s_d_s_c_s_a_s:compl:rev CACAGACACCCACACCGTCACCCACACTATCATGTTGACATTCC; dG = -28.8
   251 >s_b_s_d_s_c_s_c_s_d_s_c_s_c_s          GTGGCTGTGACGGTGTGGGTGTGGGTGACGGTGTGGGTGTGGGTG;
>clamp_s_c_s_d_s_c_s_a_s:compl:rev CACAGACACCCACACCGTCACCCACACTATCATGTTGACATTCC; dG = -28.8
   252 >s_b_s_d_s_d_s_c_s_d_s_c_s_c_s          GTGGCTGTGACGGTGACGGTGTGGGTGACGGTGTGGGTGTGGGTG;
>clamp_s_c_s_d_s_c_s_a_s:compl:rev CACAGACACCCACACCGTCACCCACACTATCATGTTGACATTCC; dG = -28.8
   253 >s_b_s_d_s_e_s_c_s_d_s_c_s_c_s          GTGGCTGTGACGGTGCTGGTGTGGGTGACGGTGTGGGTGTGGGTG;
>clamp_s_c_s_d_s_c_s_a_s:compl:rev CACAGACACCCACACCGTCACCCACACTATCATGTTGACATTCC; dG = -28.8
   254 >s_b_s_e_s_c_s_c_s_d_s_c_s_c_s          GTGGCTGTGCTGGTGTGGGTGTGGGTGACGGTGTGGGTGTGGGTG;
>clamp_s_c_s_d_s_c_s_a_s:compl:rev CACAGACACCCACACCGTCACCCACACTATCATGTTGACATTCC; dG = -28.8
   255 >s_b_s_e_s_d_s_c_s_d_s_c_s_c_s          GTGGCTGTGCTGGTGACGGTGTGGGTGACGGTGTGGGTGTGGGTG;
>clamp_s_c_s_d_s_c_s_a_s:compl:rev CACAGACACCCACACCGTCACCCACACTATCATGTTGACATTCC; dG = -28.8
   256 >s_b_s_e_s_e_s_c_s_d_s_c_s_c_s          GTGGCTGTGCTGGTGCTGGTGTGGGTGACGGTGTGGGTGTGGGTG;
>clamp_s_c_s_d_s_c_s_a_s:compl:rev CACAGACACCCACACCGTCACCCACACTATCATGTTGACATTCC; dG = -28.8
   257 >s_c_s_a_s_c_s_c_s_d_s_c_s_c_s          GTGTGGGTGTCTGTGTGGGTGTGGGTGACGGTGTGGGTGTGGGTG;
>clamp_s_c_s_d_s_c_s_a_s:compl:rev CACAGACACCCACACCGTCACCCACACTATCATGTTGACATTCC; dG = -28.8
   258 >s_c_s_a_s_d_s_c_s_d_s_c_s_c_s          GTGTGGGTGTCTGTGACGGTGTGGGTGACGGTGTGGGTGTGGGTG;
>clamp_s_c_s_d_s_c_s_a_s:compl:rev CACAGACACCCACACCGTCACCCACACTATCATGTTGACATTCC; dG = -28.8
   259 >s_c_s_a_s_e_s_c_s_d_s_c_s_c_s          GTGTGGGTGTCTGTGCTGGTGTGGGTGACGGTGTGGGTGTGGGTG;
>clamp_s_c_s_d_s_c_s_a_s:compl:rev CACAGACACCCACACCGTCACCCACACTATCATGTTGACATTCC; dG = -28.8
   260 >s_c_s_b_s_c_s_c_s_d_s_c_s_c_s          GTGTGGGTGGCTGTGTGGGTGTGGGTGACGGTGTGGGTGTGGGTG;
>clamp_s_c_s_d_s_c_s_a_s:compl:rev CACAGACACCCACACCGTCACCCACACTATCATGTTGACATTCC; dG = -28.8
   261 >s_c_s_b_s_d_s_c_s_d_s_c_s_c_s          GTGTGGGTGGCTGTGACGGTGTGGGTGACGGTGTGGGTGTGGGTG;
>clamp_s_c_s_d_s_c_s_a_s:compl:rev CACAGACACCCACACCGTCACCCACACTATCATGTTGACATTCC; dG = -28.8
   262 >s_c_s_b_s_e_s_c_s_d_s_c_s_c_s          GTGTGGGTGGCTGTGCTGGTGTGGGTGACGGTGTGGGTGTGGGTG;
>clamp_s_c_s_d_s_c_s_a_s:compl:rev CACAGACACCCACACCGTCACCCACACTATCATGTTGACATTCC; dG = -28.8
   263 >s_c_s_c_s_c_s_c_s_d_s_c_s_c_s          GTGTGGGTGTGGGTGTGGGTGTGGGTGACGGTGTGGGTGTGGGTG;
>clamp_s_c_s_d_s_c_s_a_s:compl:rev CACAGACACCCACACCGTCACCCACACTATCATGTTGACATTCC; dG = -28.8
   264 >s_c_s_c_s_d_s_c_s_d_s_a_s_a_s          GTGTGGGTGTGGGTGACGGTGTGGGTGACGGTGTCTGTGTCTGTG;
>clamp_s_c_s_d_s_c_s_a_s:compl:rev CACAGACACCCACACCGTCACCCACACTATCATGTTGACATTCC; dG = -28.7
   265 >s_c_s_d_s_c_s_c_s_d_s_c_s_e_s          GTGTGGGTGACGGTGTGGGTGTGGGTGACGGTGTGGGTGCTGGTG;
>clamp_s_c_s_d_s_c_s_a_s:compl:rev CACAGACACCCACACCGTCACCCACACTATCATGTTGACATTCC; dG = -28.7
   266 >s_c_s_d_s_c_s_c_s_d_s_c_s_d_s          GTGTGGGTGACGGTGTGGGTGTGGGTGACGGTGTGGGTGACGGTG;
>clamp_s_c_s_d_s_c_s_a_s:compl:rev CACAGACACCCACACCGTCACCCACACTATCATGTTGACATTCC; dG = -28.7
   267 >s_c_s_d_s_c_s_c_s_d_s_e_s_a_s          GTGTGGGTGACGGTGTGGGTGTGGGTGACGGTGCTGGTGTCTGTG;
>clamp_s_c_s_d_s_c_s_a_s:compl:rev CACAGACACCCACACCGTCACCCACACTATCATGTTGACATTCC; dG = -28.7
   268 >s_c_s_d_s_c_s_c_s_d_s_d_s_a_s          GTGTGGGTGACGGTGTGGGTGTGGGTGACGGTGACGGTGTCTGTG;
>clamp_s_c_s_d_s_c_s_a_s:compl:rev CACAGACACCCACACCGTCACCCACACTATCATGTTGACATTCC; dG = -28.7
   269 >s_c_s_d_s_c_s_c_s_d_s_b_s_a_s          GTGTGGGTGACGGTGTGGGTGTGGGTGACGGTGGCTGTGTCTGTG;
>clamp_s_c_s_d_s_c_s_a_s:compl:rev CACAGACACCCACACCGTCACCCACACTATCATGTTGACATTCC; dG = -28.7
   270 >s_c_s_d_s_c_s_c_s_d_s_a_s_a_s          GTGTGGGTGACGGTGTGGGTGTGGGTGACGGTGTCTGTGTCTGTG;
>clamp_s_c_s_d_s_c_s_a_s:compl:rev CACAGACACCCACACCGTCACCCACACTATCATGTTGACATTCC; dG = -28.7
   271 >s_d_s_a_s_c_s_e_s_d_s_c_s_a_s          GTGACGGTGTCTGTGTGGGTGCTGGTGACGGTGTGGGTGTCTGTG;
>clamp_s_c_s_d_s_c_s_a_s:compl:rev CACAGACACCCACACCGTCACCCACACTATCATGTTGACATTCC; dG = -28.7
   272 >s_d_s_a_s_c_s_d_s_d_s_c_s_a_s          GTGACGGTGTCTGTGTGGGTGACGGTGACGGTGTGGGTGTCTGTG;
>clamp_s_c_s_d_s_c_s_a_s:compl:rev CACAGACACCCACACCGTCACCCACACTATCATGTTGACATTCC; dG = -28.7
   273 >s_d_s_a_s_c_s_b_s_d_s_c_s_a_s          GTGACGGTGTCTGTGTGGGTGGCTGTGACGGTGTGGGTGTCTGTG;
>clamp_s_c_s_d_s_c_s_a_s:compl:rev CACAGACACCCACACCGTCACCCACACTATCATGTTGACATTCC; dG = -28.7
   274 >s_d_s_a_s_c_s_a_s_d_s_c_s_a_s          GTGACGGTGTCTGTGTGGGTGTCTGTGACGGTGTGGGTGTCTGTG;
>clamp_s_c_s_d_s_c_s_a_s:compl:rev CACAGACACCCACACCGTCACCCACACTATCATGTTGACATTCC; dG = -28.7
   275 >s_a_s_b_s_c_s_e_s_d_s_c_s_a_s          GTGTCTGTGGCTGTGTGGGTGCTGGTGACGGTGTGGGTGTCTGTG;
>clamp_s_c_s_d_s_c_s_a_s:compl:rev CACAGACACCCACACCGTCACCCACACTATCATGTTGACATTCC; dG = -28.7
   276 >s_a_s_b_s_c_s_d_s_d_s_c_s_a_s          GTGTCTGTGGCTGTGTGGGTGACGGTGACGGTGTGGGTGTCTGTG;
>clamp_s_c_s_d_s_c_s_a_s:compl:rev CACAGACACCCACACCGTCACCCACACTATCATGTTGACATTCC; dG = -28.7
   277 >s_a_s_b_s_c_s_b_s_d_s_c_s_a_s          GTGTCTGTGGCTGTGTGGGTGGCTGTGACGGTGTGGGTGTCTGTG;
>clamp_s_c_s_d_s_c_s_a_s:compl:rev CACAGACACCCACACCGTCACCCACACTATCATGTTGACATTCC; dG = -28.7
   278 >s_a_s_b_s_c_s_a_s_d_s_c_s_a_s          GTGTCTGTGGCTGTGTGGGTGTCTGTGACGGTGTGGGTGTCTGTG;
>clamp_s_c_s_d_s_c_s_a_s:compl:rev CACAGACACCCACACCGTCACCCACACTATCATGTTGACATTCC; dG = -28.7
   279 >s_d_s_b_s_c_s_e_s_d_s_c_s_a_s          GTGACGGTGGCTGTGTGGGTGCTGGTGACGGTGTGGGTGTCTGTG;
>clamp_s_c_s_d_s_c_s_a_s:compl:rev CACAGACACCCACACCGTCACCCACACTATCATGTTGACATTCC; dG = -28.7
```

```
   280 >s_d_s_b_s_c_s_d_s_d_s_c_s_a_s          GTGACGGTGGCTGTGTGGGTGACGGTGACGGTGTGGGTGTCTGTG;
>clamp_s_c_s_d_s_c_s_a_s:compl:rev CACAGACACCCACACCGTCACCCACACTATCATGTTGACATTCC; dG = -28.7
   281 >s_d_s_b_s_c_s_b_s_d_s_c_s_a_s          GTGACGGTGGCTGTGTGGGTGGCTGTGACGGTGTGGGTGTCTGTG;
>clamp_s_c_s_d_s_c_s_a_s:compl:rev CACAGACACCCACACCGTCACCCACACTATCATGTTGACATTCC; dG = -28.7
   282 >s_d_s_b_s_c_s_a_s_d_s_c_s_a_s          GTGACGGTGGCTGTGTGGGTGTCTGTGACGGTGTGGGTGTCTGTG;
>clamp_s_c_s_d_s_c_s_a_s:compl:rev CACAGACACCCACACCGTCACCCACACTATCATGTTGACATTCC; dG = -28.7
   283 >s_d_s_c_s_d_s_c_s_d_s_a_s_a_s          GTGACGGTGTGGGTGACGGTGTGGGTGACGGTGTCTGTGTCTGTG;
>clamp_s_c_s_d_s_c_s_a_s:compl:rev CACAGACACCCACACCGTCACCCACACTATCATGTTGACATTCC; dG = -28.7
   284 >s_e_s_a_s_c_s_e_s_d_s_c_s_a_s          GTGCTGGTGTCTGTGTGGGTGCTGGTGACGGTGTGGGTGTCTGTG;
>clamp_s_c_s_d_s_c_s_a_s:compl:rev CACAGACACCCACACCGTCACCCACACTATCATGTTGACATTCC; dG = -28.7
   285 >s_e_s_a_s_c_s_d_s_d_s_c_s_a_s          GTGCTGGTGTCTGTGTGGGTGACGGTGACGGTGTGGGTGTCTGTG;
>clamp_s_c_s_d_s_c_s_a_s:compl:rev CACAGACACCCACACCGTCACCCACACTATCATGTTGACATTCC; dG = -28.7
   286 >s_e_s_a_s_c_s_b_s_d_s_c_s_a_s          GTGCTGGTGTCTGTGTGGGTGGCTGTGACGGTGTGGGTGTCTGTG;
>clamp_s_c_s_d_s_c_s_a_s:compl:rev CACAGACACCCACACCGTCACCCACACTATCATGTTGACATTCC; dG = -28.7
   287 >s_e_s_a_s_c_s_a_s_d_s_c_s_a_s          GTGCTGGTGTCTGTGTGGGTGTCTGTGACGGTGTGGGTGTCTGTG;
>clamp_s_c_s_d_s_c_s_a_s:compl:rev CACAGACACCCACACCGTCACCCACACTATCATGTTGACATTCC; dG = -28.7
   288 >s_e_s_b_s_c_s_e_s_d_s_c_s_a_s          GTGCTGGTGGCTGTGTGGGTGCTGGTGACGGTGTGGGTGTCTGTG;
>clamp_s_c_s_d_s_c_s_a_s:compl:rev CACAGACACCCACACCGTCACCCACACTATCATGTTGACATTCC; dG = -28.7
   289 >s_e_s_b_s_c_s_d_s_d_s_c_s_a_s          GTGCTGGTGGCTGTGTGGGTGACGGTGACGGTGTGGGTGTCTGTG;
>clamp_s_c_s_d_s_c_s_a_s:compl:rev CACAGACACCCACACCGTCACCCACACTATCATGTTGACATTCC; dG = -28.7
   290 >s_e_s_b_s_c_s_b_s_d_s_c_s_a_s          GTGCTGGTGGCTGTGTGGGTGGCTGTGACGGTGTGGGTGTCTGTG;
>clamp_s_c_s_d_s_c_s_a_s:compl:rev CACAGACACCCACACCGTCACCCACACTATCATGTTGACATTCC; dG = -28.7
   291 >s_e_s_b_s_c_s_a_s_d_s_c_s_a_s          GTGCTGGTGGCTGTGTGGGTGTCTGTGACGGTGTGGGTGTCTGTG;
>clamp_s_c_s_d_s_c_s_a_s:compl:rev CACAGACACCCACACCGTCACCCACACTATCATGTTGACATTCC; dG = -28.7
   292 >s_e_s_c_s_d_s_c_s_d_s_a_s_a_s          GTGCTGGTGTGGGTGACGGTGTGGGTGACGGTGTCTGTGTCTGTG;
>clamp_s_c_s_d_s_c_s_a_s:compl:rev CACAGACACCCACACCGTCACCCACACTATCATGTTGACATTCC; dG = -28.7
   293 >s_b_s_a_s_c_s_e_s_d_s_c_s_a_s          GTGGCTGTGTCTGTGTGGGTGCTGGTGACGGTGTGGGTGTCTGTG;
>clamp_s_c_s_d_s_c_s_a_s:compl:rev CACAGACACCCACACCGTCACCCACACTATCATGTTGACATTCC; dG = -28.7
   294 >s_b_s_a_s_c_s_d_s_d_s_c_s_a_s          GTGGCTGTGTCTGTGTGGGTGACGGTGACGGTGTGGGTGTCTGTG;
>clamp_s_c_s_d_s_c_s_a_s:compl:rev CACAGACACCCACACCGTCACCCACACTATCATGTTGACATTCC; dG = -28.7
   295 >s_b_s_a_s_c_s_b_s_d_s_c_s_a_s          GTGGCTGTGTCTGTGTGGGTGGCTGTGACGGTGTGGGTGTCTGTG;
>clamp_s_c_s_d_s_c_s_a_s:compl:rev CACAGACACCCACACCGTCACCCACACTATCATGTTGACATTCC; dG = -28.7
   296 >s_b_s_a_s_c_s_a_s_d_s_c_s_a_s          GTGGCTGTGTCTGTGTGGGTGTCTGTGACGGTGTGGGTGTCTGTG;
>clamp_s_c_s_d_s_c_s_a_s:compl:rev CACAGACACCCACACCGTCACCCACACTATCATGTTGACATTCC; dG = -28.7
   297 >s_a_s_a_s_c_s_e_s_d_s_c_s_a_s          GTGTCTGTGTCTGTGTGGGTGCTGGTGACGGTGTGGGTGTCTGTG;
>clamp_s_c_s_d_s_c_s_a_s:compl:rev CACAGACACCCACACCGTCACCCACACTATCATGTTGACATTCC; dG = -28.7
   298 >s_a_s_a_s_c_s_d_s_d_s_c_s_a_s          GTGTCTGTGTCTGTGTGGGTGACGGTGACGGTGTGGGTGTCTGTG;
>clamp_s_c_s_d_s_c_s_a_s:compl:rev CACAGACACCCACACCGTCACCCACACTATCATGTTGACATTCC; dG = -28.7
   299 >s_a_s_a_s_c_s_b_s_d_s_c_s_a_s          GTGTCTGTGTCTGTGTGGGTGGCTGTGACGGTGTGGGTGTCTGTG;
>clamp_s_c_s_d_s_c_s_a_s:compl:rev CACAGACACCCACACCGTCACCCACACTATCATGTTGACATTCC; dG = -28.7
   300 >s_a_s_a_s_c_s_a_s_d_s_c_s_a_s          GTGTCTGTGTCTGTGTGGGTGTCTGTGACGGTGTGGGTGTCTGTG;
>clamp_s_c_s_d_s_c_s_a_s:compl:rev CACAGACACCCACACCGTCACCCACACTATCATGTTGACATTCC; dG = -28.7
   301 >s_b_s_b_s_c_s_e_s_d_s_c_s_a_s          GTGGCTGTGGCTGTGTGGGTGCTGGTGACGGTGTGGGTGTCTGTG;
>clamp_s_c_s_d_s_c_s_a_s:compl:rev CACAGACACCCACACCGTCACCCACACTATCATGTTGACATTCC; dG = -28.7
   302 >s_b_s_b_s_c_s_d_s_d_s_c_s_a_s          GTGGCTGTGGCTGTGTGGGTGACGGTGACGGTGTGGGTGTCTGTG;
>clamp_s_c_s_d_s_c_s_a_s:compl:rev CACAGACACCCACACCGTCACCCACACTATCATGTTGACATTCC; dG = -28.7
   303 >s_b_s_b_s_c_s_b_s_d_s_c_s_a_s          GTGGCTGTGGCTGTGTGGGTGGCTGTGACGGTGTGGGTGTCTGTG;
>clamp_s_c_s_d_s_c_s_a_s:compl:rev CACAGACACCCACACCGTCACCCACACTATCATGTTGACATTCC; dG = -28.7
   304 >s_b_s_b_s_c_s_a_s_d_s_c_s_a_s          GTGGCTGTGGCTGTGTGGGTGTCTGTGACGGTGTGGGTGTCTGTG;
>clamp_s_c_s_d_s_c_s_a_s:compl:rev CACAGACACCCACACCGTCACCCACACTATCATGTTGACATTCC; dG = -28.7
   305 >s_c_s_a_s_c_s_e_s_d_s_c_s_a_s          GTGTGGGTGTCTGTGTGGGTGCTGGTGACGGTGTGGGTGTCTGTG;
>clamp_s_c_s_d_s_c_s_a_s:compl:rev CACAGACACCCACACCGTCACCCACACTATCATGTTGACATTCC; dG = -28.7
   306 >s_c_s_a_s_c_s_d_s_d_s_c_s_a_s          GTGTGGGTGTCTGTGTGGGTGACGGTGACGGTGTGGGTGTCTGTG;
>clamp_s_c_s_d_s_c_s_a_s:compl:rev CACAGACACCCACACCGTCACCCACACTATCATGTTGACATTCC; dG = -28.7
   307 >s_c_s_a_s_c_s_b_s_d_s_c_s_a_s          GTGTGGGTGTCTGTGTGGGTGGCTGTGACGGTGTGGGTGTCTGTG;
>clamp_s_c_s_d_s_c_s_a_s:compl:rev CACAGACACCCACACCGTCACCCACACTATCATGTTGACATTCC; dG = -28.7
   308 >s_c_s_a_s_c_s_a_s_d_s_c_s_a_s          GTGTGGGTGTCTGTGTGGGTGTCTGTGACGGTGTGGGTGTCTGTG;
>clamp_s_c_s_d_s_c_s_a_s:compl:rev CACAGACACCCACACCGTCACCCACACTATCATGTTGACATTCC; dG = -28.7
   309 >s_c_s_b_s_c_s_e_s_d_s_c_s_a_s          GTGTGGGTGGCTGTGTGGGTGCTGGTGACGGTGTGGGTGTCTGTG;
>clamp_s_c_s_d_s_c_s_a_s:compl:rev CACAGACACCCACACCGTCACCCACACTATCATGTTGACATTCC; dG = -28.7
   310 >s_c_s_b_s_c_s_d_s_d_s_c_s_a_s          GTGTGGGTGGCTGTGTGGGTGACGGTGACGGTGTGGGTGTCTGTG;
>clamp_s_c_s_d_s_c_s_a_s:compl:rev CACAGACACCCACACCGTCACCCACACTATCATGTTGACATTCC; dG = -28.7
   311 >s_c_s_b_s_c_s_b_s_d_s_c_s_a_s          GTGTGGGTGGCTGTGTGGGTGGCTGTGACGGTGTGGGTGTCTGTG;
>clamp_s_c_s_d_s_c_s_a_s:compl:rev CACAGACACCCACACCGTCACCCACACTATCATGTTGACATTCC; dG = -28.7
   312 >s_c_s_b_s_c_s_a_s_d_s_c_s_a_s          GTGTGGGTGGCTGTGTGGGTGTCTGTGACGGTGTGGGTGTCTGTG;
>clamp_s_c_s_d_s_c_s_a_s:compl:rev CACAGACACCCACACCGTCACCCACACTATCATGTTGACATTCC; dG = -28.7
   313 >s_c_s_c_s_c_s_e_s_d_s_c_s_a_s          GTGTGGGTGTGGGTGTGGGTGCTGGTGACGGTGTGGGTGTCTGTG;
>clamp_s_c_s_d_s_c_s_a_s:compl:rev CACAGACACCCACACCGTCACCCACACTATCATGTTGACATTCC; dG = -28.6
   314 >s_c_s_c_s_c_s_d_s_d_s_c_s_a_s          GTGTGGGTGTGGGTGTGGGTGACGGTGACGGTGTGGGTGTCTGTG;
>clamp_s_c_s_d_s_c_s_a_s:compl:rev CACAGACACCCACACCGTCACCCACACTATCATGTTGACATTCC; dG = -28.6
   315 >s_c_s_c_s_c_s_b_s_d_s_c_s_a_s          GTGTGGGTGTGGGTGTGGGTGGCTGTGACGGTGTGGGTGTCTGTG;
>clamp_s_c_s_d_s_c_s_a_s:compl:rev CACAGACACCCACACCGTCACCCACACTATCATGTTGACATTCC; dG = -28.6
   316 >s_c_s_c_s_c_s_a_s_d_s_c_s_a_s          GTGTGGGTGTGGGTGTGGGTGTCTGTGACGGTGTGGGTGTCTGTG;
>clamp_s_c_s_d_s_c_s_a_s:compl:rev CACAGACACCCACACCGTCACCCACACTATCATGTTGACATTCC; dG = -28.6
```

```
   317 >s_c_s_d_s_c_s_e_s_d_s_c_s_a_s          GTGTGGGTGACGGTGTGGGTGCTGGTGACGGTGTGGGTGTCTGTG;
>clamp_s_c_s_d_s_c_s_a_s:compl:rev CACAGACACCCACACCGTCACCCACACTATCATGTTGACATTCC; dG = -28.6
   318 >s_c_s_d_s_c_s_d_s_d_s_c_s_a_s          GTGTGGGTGACGGTGTGGGTGACGGTGACGGTGTGGGTGTCTGTG;
>clamp_s_c_s_d_s_c_s_a_s:compl:rev CACAGACACCCACACCGTCACCCACACTATCATGTTGACATTCC; dG = -28.6
   319 >s_c_s_e_s_c_s_e_s_d_s_c_s_a_s          GTGTGGGTGCTGGTGTGGGTGCTGGTGACGGTGTGGGTGTCTGTG;
>clamp_s_c_s_d_s_c_s_a_s:compl:rev CACAGACACCCACACCGTCACCCACACTATCATGTTGACATTCC; dG = -28.6
   320 >s_c_s_e_s_c_s_d_s_d_s_c_s_a_s          GTGTGGGTGCTGGTGTGGGTGACGGTGACGGTGTGGGTGTCTGTG;
>clamp_s_c_s_d_s_c_s_a_s:compl:rev CACAGACACCCACACCGTCACCCACACTATCATGTTGACATTCC; dG = -28.6
   321 >s_c_s_e_s_c_s_b_s_d_s_c_s_a_s          GTGTGGGTGCTGGTGTGGGTGGCTGTGACGGTGTGGGTGTCTGTG;
>clamp_s_c_s_d_s_c_s_a_s:compl:rev CACAGACACCCACACCGTCACCCACACTATCATGTTGACATTCC; dG = -28.6
   322 >s_c_s_e_s_c_s_a_s_d_s_c_s_a_s          GTGTGGGTGCTGGTGTGGGTGTCTGTGACGGTGTGGGTGTCTGTG;
>clamp_s_c_s_d_s_c_s_a_s:compl:rev CACAGACACCCACACCGTCACCCACACTATCATGTTGACATTCC; dG = -28.6
   323 >s_d_s_c_s_c_s_e_s_d_s_c_s_a_s          GTGACGGTGTGGGTGTGGGTGCTGGTGACGGTGTGGGTGTCTGTG;
>clamp_s_c_s_d_s_c_s_a_s:compl:rev CACAGACACCCACACCGTCACCCACACTATCATGTTGACATTCC; dG = -28.6
   324 >s_d_s_c_s_c_s_d_s_d_s_c_s_a_s          GTGACGGTGTGGGTGTGGGTGACGGTGACGGTGTGGGTGTCTGTG;
>clamp_s_c_s_d_s_c_s_a_s:compl:rev CACAGACACCCACACCGTCACCCACACTATCATGTTGACATTCC; dG = -28.6
   325 >s_d_s_c_s_c_s_b_s_d_s_c_s_a_s          GTGACGGTGTGGGTGTGGGTGGCTGTGACGGTGTGGGTGTCTGTG;
>clamp_s_c_s_d_s_c_s_a_s:compl:rev CACAGACACCCACACCGTCACCCACACTATCATGTTGACATTCC; dG = -28.6
   326 >s_d_s_c_s_c_s_a_s_d_s_c_s_a_s          GTGACGGTGTGGGTGTGGGTGTCTGTGACGGTGTGGGTGTCTGTG;
>clamp_s_c_s_d_s_c_s_a_s:compl:rev CACAGACACCCACACCGTCACCCACACTATCATGTTGACATTCC; dG = -28.6
   327 >s_d_s_d_s_c_s_e_s_d_s_c_s_a_s          GTGACGGTGACGGTGTGGGTGCTGGTGACGGTGTGGGTGTCTGTG;
>clamp_s_c_s_d_s_c_s_a_s:compl:rev CACAGACACCCACACCGTCACCCACACTATCATGTTGACATTCC; dG = -28.6
   328 >s_d_s_d_s_c_s_d_s_d_s_c_s_a_s          GTGACGGTGACGGTGTGGGTGACGGTGACGGTGTGGGTGTCTGTG;
>clamp_s_c_s_d_s_c_s_a_s:compl:rev CACAGACACCCACACCGTCACCCACACTATCATGTTGACATTCC; dG = -28.6
   329 >s_d_s_d_s_c_s_b_s_d_s_c_s_a_s          GTGACGGTGACGGTGTGGGTGGCTGTGACGGTGTGGGTGTCTGTG;
>clamp_s_c_s_d_s_c_s_a_s:compl:rev CACAGACACCCACACCGTCACCCACACTATCATGTTGACATTCC; dG = -28.6
   330 >s_d_s_d_s_c_s_a_s_d_s_c_s_a_s          GTGACGGTGACGGTGTGGGTGTCTGTGACGGTGTGGGTGTCTGTG;
>clamp_s_c_s_d_s_c_s_a_s:compl:rev CACAGACACCCACACCGTCACCCACACTATCATGTTGACATTCC; dG = -28.6
   331 >s_d_s_e_s_c_s_e_s_d_s_c_s_a_s          GTGACGGTGCTGGTGTGGGTGCTGGTGACGGTGTGGGTGTCTGTG;
>clamp_s_c_s_d_s_c_s_a_s:compl:rev CACAGACACCCACACCGTCACCCACACTATCATGTTGACATTCC; dG = -28.6
   332 >s_d_s_e_s_c_s_d_s_d_s_c_s_a_s          GTGACGGTGCTGGTGTGGGTGACGGTGACGGTGTGGGTGTCTGTG;
>clamp_s_c_s_d_s_c_s_a_s:compl:rev CACAGACACCCACACCGTCACCCACACTATCATGTTGACATTCC; dG = -28.6
   333 >s_d_s_e_s_c_s_b_s_d_s_c_s_a_s          GTGACGGTGCTGGTGTGGGTGGCTGTGACGGTGTGGGTGTCTGTG;
>clamp_s_c_s_d_s_c_s_a_s:compl:rev CACAGACACCCACACCGTCACCCACACTATCATGTTGACATTCC; dG = -28.6
   334 >s_d_s_e_s_c_s_a_s_d_s_c_s_a_s          GTGACGGTGCTGGTGTGGGTGTCTGTGACGGTGTGGGTGTCTGTG;
>clamp_s_c_s_d_s_c_s_a_s:compl:rev CACAGACACCCACACCGTCACCCACACTATCATGTTGACATTCC; dG = -28.6
   335 >s_e_s_c_s_c_s_e_s_d_s_c_s_a_s          GTGCTGGTGTGGGTGTGGGTGCTGGTGACGGTGTGGGTGTCTGTG;
>clamp_s_c_s_d_s_c_s_a_s:compl:rev CACAGACACCCACACCGTCACCCACACTATCATGTTGACATTCC; dG = -28.6
   336 >s_e_s_c_s_c_s_d_s_d_s_c_s_a_s          GTGCTGGTGTGGGTGTGGGTGACGGTGACGGTGTGGGTGTCTGTG;
>clamp_s_c_s_d_s_c_s_a_s:compl:rev CACAGACACCCACACCGTCACCCACACTATCATGTTGACATTCC; dG = -28.6
   337 >s_e_s_c_s_c_s_b_s_d_s_c_s_a_s          GTGCTGGTGTGGGTGTGGGTGGCTGTGACGGTGTGGGTGTCTGTG;
>clamp_s_c_s_d_s_c_s_a_s:compl:rev CACAGACACCCACACCGTCACCCACACTATCATGTTGACATTCC; dG = -28.6
   338 >s_e_s_c_s_c_s_a_s_d_s_c_s_a_s          GTGCTGGTGTGGGTGTGGGTGTCTGTGACGGTGTGGGTGTCTGTG;
>clamp_s_c_s_d_s_c_s_a_s:compl:rev CACAGACACCCACACCGTCACCCACACTATCATGTTGACATTCC; dG = -28.6
   339 >s_e_s_d_s_c_s_e_s_d_s_c_s_a_s          GTGCTGGTGACGGTGTGGGTGCTGGTGACGGTGTGGGTGTCTGTG;
>clamp_s_c_s_d_s_c_s_a_s:compl:rev CACAGACACCCACACCGTCACCCACACTATCATGTTGACATTCC; dG = -28.6
   340 >s_e_s_d_s_c_s_d_s_d_s_c_s_a_s          GTGCTGGTGACGGTGTGGGTGACGGTGACGGTGTGGGTGTCTGTG;
>clamp_s_c_s_d_s_c_s_a_s:compl:rev CACAGACACCCACACCGTCACCCACACTATCATGTTGACATTCC; dG = -28.6
   341 >s_e_s_d_s_c_s_b_s_d_s_c_s_a_s          GTGCTGGTGACGGTGTGGGTGGCTGTGACGGTGTGGGTGTCTGTG;
>clamp_s_c_s_d_s_c_s_a_s:compl:rev CACAGACACCCACACCGTCACCCACACTATCATGTTGACATTCC; dG = -28.6
   342 >s_e_s_d_s_c_s_a_s_d_s_c_s_a_s          GTGCTGGTGACGGTGTGGGTGTCTGTGACGGTGTGGGTGTCTGTG;
>clamp_s_c_s_d_s_c_s_a_s:compl:rev CACAGACACCCACACCGTCACCCACACTATCATGTTGACATTCC; dG = -28.6
   343 >s_e_s_e_s_c_s_e_s_d_s_c_s_a_s          GTGCTGGTGCTGGTGTGGGTGCTGGTGACGGTGTGGGTGTCTGTG;
>clamp_s_c_s_d_s_c_s_a_s:compl:rev CACAGACACCCACACCGTCACCCACACTATCATGTTGACATTCC; dG = -28.6
   344 >s_e_s_e_s_c_s_d_s_d_s_c_s_a_s          GTGCTGGTGCTGGTGTGGGTGACGGTGACGGTGTGGGTGTCTGTG;
>clamp_s_c_s_d_s_c_s_a_s:compl:rev CACAGACACCCACACCGTCACCCACACTATCATGTTGACATTCC; dG = -28.6
   345 >s_e_s_e_s_c_s_b_s_d_s_c_s_a_s          GTGCTGGTGCTGGTGTGGGTGGCTGTGACGGTGTGGGTGTCTGTG;
>clamp_s_c_s_d_s_c_s_a_s:compl:rev CACAGACACCCACACCGTCACCCACACTATCATGTTGACATTCC; dG = -28.6
   346 >s_e_s_e_s_c_s_a_s_d_s_c_s_a_s          GTGCTGGTGCTGGTGTGGGTGTCTGTGACGGTGTGGGTGTCTGTG;
>clamp_s_c_s_d_s_c_s_a_s:compl:rev CACAGACACCCACACCGTCACCCACACTATCATGTTGACATTCC; dG = -28.6
   347 >s_a_s_c_s_c_s_e_s_d_s_c_s_a_s          GTGTCTGTGTGGGTGTGGGTGCTGGTGACGGTGTGGGTGTCTGTG;
>clamp_s_c_s_d_s_c_s_a_s:compl:rev CACAGACACCCACACCGTCACCCACACTATCATGTTGACATTCC; dG = -28.6
   348 >s_a_s_c_s_c_s_d_s_d_s_c_s_a_s          GTGTCTGTGTGGGTGTGGGTGACGGTGACGGTGTGGGTGTCTGTG;
>clamp_s_c_s_d_s_c_s_a_s:compl:rev CACAGACACCCACACCGTCACCCACACTATCATGTTGACATTCC; dG = -28.6
   349 >s_a_s_c_s_c_s_b_s_d_s_c_s_a_s          GTGTCTGTGTGGGTGTGGGTGGCTGTGACGGTGTGGGTGTCTGTG;
>clamp_s_c_s_d_s_c_s_a_s:compl:rev CACAGACACCCACACCGTCACCCACACTATCATGTTGACATTCC; dG = -28.6
   350 >s_a_s_c_s_c_s_a_s_d_s_c_s_a_s          GTGTCTGTGTGGGTGTGGGTGTCTGTGACGGTGTGGGTGTCTGTG;
>clamp_s_c_s_d_s_c_s_a_s:compl:rev CACAGACACCCACACCGTCACCCACACTATCATGTTGACATTCC; dG = -28.6
   351 >s_a_s_d_s_c_s_e_s_d_s_c_s_a_s          GTGTCTGTGACGGTGTGGGTGCTGGTGACGGTGTGGGTGTCTGTG;
>clamp_s_c_s_d_s_c_s_a_s:compl:rev CACAGACACCCACACCGTCACCCACACTATCATGTTGACATTCC; dG = -28.6
   352 >s_a_s_d_s_c_s_d_s_d_s_c_s_a_s          GTGTCTGTGACGGTGTGGGTGACGGTGACGGTGTGGGTGTCTGTG;
>clamp_s_c_s_d_s_c_s_a_s:compl:rev CACAGACACCCACACCGTCACCCACACTATCATGTTGACATTCC; dG = -28.6
   353 >s_a_s_d_s_c_s_b_s_d_s_c_s_a_s          GTGTCTGTGACGGTGTGGGTGGCTGTGACGGTGTGGGTGTCTGTG;
>clamp_s_c_s_d_s_c_s_a_s:compl:rev CACAGACACCCACACCGTCACCCACACTATCATGTTGACATTCC; dG = -28.6
```

```
   354 >s_a_s_d_s_c_s_a_s_d_s_c_s_a_s           GTGTCTGTGACGGTGTGGGTGTCTGTGACGGTGTGGGTGTCTGTG;
>clamp_s_c_s_d_s_c_s_a_s:compl:rev CACAGACACCCACACCGTCACCCACACTATCATGTTGACATTCC; dG = -28.6
   355 >s_a_s_e_s_c_s_e_s_d_s_c_s_a_s           GTGTCTGTGCTGGTGTGGGTGCTGGTGACGGTGTGGGTGTCTGTG;
>clamp_s_c_s_d_s_c_s_a_s:compl:rev CACAGACACCCACACCGTCACCCACACTATCATGTTGACATTCC; dG = -28.6
   356 >s_a_s_e_s_c_s_d_s_d_s_c_s_a_s           GTGTCTGTGCTGGTGTGGGTGACGGTGACGGTGTGGGTGTCTGTG;
>clamp_s_c_s_d_s_c_s_a_s:compl:rev CACAGACACCCACACCGTCACCCACACTATCATGTTGACATTCC; dG = -28.6
   357 >s_a_s_e_s_c_s_b_s_d_s_c_s_a_s           GTGTCTGTGCTGGTGTGGGTGGCTGTGACGGTGTGGGTGTCTGTG;
>clamp_s_c_s_d_s_c_s_a_s:compl:rev CACAGACACCCACACCGTCACCCACACTATCATGTTGACATTCC; dG = -28.6
   358 >s_a_s_e_s_c_s_a_s_d_s_c_s_a_s           GTGTCTGTGCTGGTGTGGGTGTCTGTGACGGTGTGGGTGTCTGTG;
>clamp_s_c_s_d_s_c_s_a_s:compl:rev CACAGACACCCACACCGTCACCCACACTATCATGTTGACATTCC; dG = -28.6
   359 >s_b_s_c_s_c_s_e_s_d_s_c_s_a_s           GTGGCTGTGTGGGTGTGGGTGCTGGTGACGGTGTGGGTGTCTGTG;
>clamp_s_c_s_d_s_c_s_a_s:compl:rev CACAGACACCCACACCGTCACCCACACTATCATGTTGACATTCC; dG = -28.6
   360 >s_b_s_c_s_c_s_d_s_d_s_c_s_a_s           GTGGCTGTGTGGGTGTGGGTGACGGTGACGGTGTGGGTGTCTGTG;
>clamp_s_c_s_d_s_c_s_a_s:compl:rev CACAGACACCCACACCGTCACCCACACTATCATGTTGACATTCC; dG = -28.6
   361 >s_b_s_c_s_c_s_b_s_d_s_c_s_a_s           GTGGCTGTGTGGGTGTGGGTGGCTGTGACGGTGTGGGTGTCTGTG;
>clamp_s_c_s_d_s_c_s_a_s:compl:rev CACAGACACCCACACCGTCACCCACACTATCATGTTGACATTCC; dG = -28.6
   362 >s_b_s_c_s_c_s_a_s_d_s_c_s_a_s           GTGGCTGTGTGGGTGTGGGTGTCTGTGACGGTGTGGGTGTCTGTG;
>clamp_s_c_s_d_s_c_s_a_s:compl:rev CACAGACACCCACACCGTCACCCACACTATCATGTTGACATTCC; dG = -28.6
   363 >s_b_s_d_s_c_s_e_s_d_s_c_s_a_s           GTGGCTGTGACGGTGTGGGTGCTGGTGACGGTGTGGGTGTCTGTG;
>clamp_s_c_s_d_s_c_s_a_s:compl:rev CACAGACACCCACACCGTCACCCACACTATCATGTTGACATTCC; dG = -28.6
   364 >s_b_s_d_s_c_s_d_s_d_s_c_s_a_s           GTGGCTGTGACGGTGTGGGTGACGGTGACGGTGTGGGTGTCTGTG;
>clamp_s_c_s_d_s_c_s_a_s:compl:rev CACAGACACCCACACCGTCACCCACACTATCATGTTGACATTCC; dG = -28.6
   365 >s_b_s_d_s_c_s_b_s_d_s_c_s_a_s           GTGGCTGTGACGGTGTGGGTGGCTGTGACGGTGTGGGTGTCTGTG;
>clamp_s_c_s_d_s_c_s_a_s:compl:rev CACAGACACCCACACCGTCACCCACACTATCATGTTGACATTCC; dG = -28.6
   366 >s_b_s_d_s_c_s_a_s_d_s_c_s_a_s           GTGGCTGTGACGGTGTGGGTGTCTGTGACGGTGTGGGTGTCTGTG;
>clamp_s_c_s_d_s_c_s_a_s:compl:rev CACAGACACCCACACCGTCACCCACACTATCATGTTGACATTCC; dG = -28.6
   367 >s_b_s_e_s_c_s_e_s_d_s_c_s_a_s           GTGGCTGTGCTGGTGTGGGTGCTGGTGACGGTGTGGGTGTCTGTG;
>clamp_s_c_s_d_s_c_s_a_s:compl:rev CACAGACACCCACACCGTCACCCACACTATCATGTTGACATTCC; dG = -28.6
   368 >s_b_s_e_s_c_s_d_s_d_s_c_s_a_s           GTGGCTGTGCTGGTGTGGGTGACGGTGACGGTGTGGGTGTCTGTG;
>clamp_s_c_s_d_s_c_s_a_s:compl:rev CACAGACACCCACACCGTCACCCACACTATCATGTTGACATTCC; dG = -28.6
   369 >s_b_s_e_s_c_s_b_s_d_s_c_s_a_s           GTGGCTGTGCTGGTGTGGGTGGCTGTGACGGTGTGGGTGTCTGTG;
>clamp_s_c_s_d_s_c_s_a_s:compl:rev CACAGACACCCACACCGTCACCCACACTATCATGTTGACATTCC; dG = -28.6
   370 >s_b_s_e_s_c_s_a_s_d_s_c_s_a_s           GTGGCTGTGCTGGTGTGGGTGTCTGTGACGGTGTGGGTGTCTGTG;
>clamp_s_c_s_d_s_c_s_a_s:compl:rev CACAGACACCCACACCGTCACCCACACTATCATGTTGACATTCC; dG = -28.6
   371 >s_c_s_c_s_d_s_c_s_d_s_c_s_e_s           GTGTGGGTGTGGGTGACGGTGTGGGTGACGGTGTGGGTGCTGGTG;
>clamp_s_c_s_d_s_c_s_a_s:compl:rev CACAGACACCCACACCGTCACCCACACTATCATGTTGACATTCC; dG = -28.4
   372 >s_c_s_c_s_d_s_c_s_d_s_c_s_d_s           GTGTGGGTGTGGGTGACGGTGTGGGTGACGGTGTGGGTGACGGTG;
>clamp_s_c_s_d_s_c_s_a_s:compl:rev CACAGACACCCACACCGTCACCCACACTATCATGTTGACATTCC; dG = -28.4
   373 >s_c_s_c_s_d_s_c_s_d_s_e_s_a_s           GTGTGGGTGTGGGTGACGGTGTGGGTGACGGTGCTGGTGTCTGTG;
>clamp_s_c_s_d_s_c_s_a_s:compl:rev CACAGACACCCACACCGTCACCCACACTATCATGTTGACATTCC; dG = -28.4
   374 >s_c_s_c_s_d_s_c_s_d_s_d_s_a_s           GTGTGGGTGTGGGTGACGGTGTGGGTGACGGTGACGGTGTCTGTG;
>clamp_s_c_s_d_s_c_s_a_s:compl:rev CACAGACACCCACACCGTCACCCACACTATCATGTTGACATTCC; dG = -28.4
   375 >s_c_s_c_s_d_s_c_s_d_s_b_s_a_s           GTGTGGGTGTGGGTGACGGTGTGGGTGACGGTGGCTGTGTCTGTG;
>clamp_s_c_s_d_s_c_s_a_s:compl:rev CACAGACACCCACACCGTCACCCACACTATCATGTTGACATTCC; dG = -28.4
   376 >s_c_s_c_s_e_s_c_s_d_s_c_s_d_s           GTGTGGGTGTGGGTGCTGGTGTGGGTGACGGTGTGGGTGACGGTG;
>clamp_s_c_s_d_s_c_s_a_s:compl:rev CACAGACACCCACACCGTCACCCACACTATCATGTTGACATTCC; dG = -28.4
   377 >s_c_s_d_s_a_s_c_s_d_s_c_s_d_s           GTGTGGGTGACGGTGTCTGTGTGGGTGACGGTGTGGGTGACGGTG;
>clamp_s_c_s_d_s_c_s_a_s:compl:rev CACAGACACCCACACCGTCACCCACACTATCATGTTGACATTCC; dG = -28.4
   378 >s_c_s_d_s_b_s_c_s_d_s_c_s_d_s           GTGTGGGTGACGGTGGCTGTGTGGGTGACGGTGTGGGTGACGGTG;
>clamp_s_c_s_d_s_c_s_a_s:compl:rev CACAGACACCCACACCGTCACCCACACTATCATGTTGACATTCC; dG = -28.4
   379 >s_c_s_d_s_c_s_c_s_e_s_c_s_a_s           GTGTGGGTGACGGTGTGGGTGTGGGTGCTGGTGTGGGTGTCTGTG;
>clamp_s_c_s_d_s_c_s_a_s:compl:rev CACAGACACCCACACCGTCACCCACACTATCATGTTGACATTCC; dG = -28.4
   380 >s_c_s_d_s_c_s_c_s_c_s_c_s_a_s           GTGTGGGTGACGGTGTGGGTGTGGGTGTGGGTGTGGGTGTCTGTG;
>clamp_s_c_s_d_s_c_s_a_s:compl:rev CACAGACACCCACACCGTCACCCACACTATCATGTTGACATTCC; dG = -28.4
   381 >s_c_s_d_s_c_s_c_s_b_s_c_s_a_s           GTGTGGGTGACGGTGTGGGTGTGGGTGGCTGTGTGGGTGTCTGTG;
>clamp_s_c_s_d_s_c_s_a_s:compl:rev CACAGACACCCACACCGTCACCCACACTATCATGTTGACATTCC; dG = -28.4
   382 >s_c_s_d_s_c_s_c_s_a_s_c_s_a_s           GTGTGGGTGACGGTGTGGGTGTGGGTGTCTGTGTGGGTGTCTGTG;
>clamp_s_c_s_d_s_c_s_a_s:compl:rev CACAGACACCCACACCGTCACCCACACTATCATGTTGACATTCC; dG = -28.4
   383 >s_c_s_d_s_d_s_c_s_d_s_c_s_d_s           GTGTGGGTGACGGTGACGGTGTGGGTGACGGTGTGGGTGACGGTG;
>clamp_s_c_s_d_s_c_s_a_s:compl:rev CACAGACACCCACACCGTCACCCACACTATCATGTTGACATTCC; dG = -28.4
   384 >s_c_s_d_s_d_s_c_s_a_s_c_s_a_s           GTGTGGGTGACGGTGACGGTGTGGGTGTCTGTGTGGGTGTCTGTG;
>clamp_s_c_s_d_s_c_s_a_s:compl:rev CACAGACACCCACACCGTCACCCACACTATCATGTTGACATTCC; dG = -28.4
   385 >s_c_s_d_s_e_s_c_s_d_s_c_s_d_s           GTGTGGGTGACGGTGCTGGTGTGGGTGACGGTGTGGGTGACGGTG;
>clamp_s_c_s_d_s_c_s_a_s:compl:rev CACAGACACCCACACCGTCACCCACACTATCATGTTGACATTCC; dG = -28.4
   386 >s_c_s_e_s_a_s_c_s_d_s_c_s_d_s           GTGTGGGTGCTGGTGTCTGTGTGGGTGACGGTGTGGGTGACGGTG;
>clamp_s_c_s_d_s_c_s_a_s:compl:rev CACAGACACCCACACCGTCACCCACACTATCATGTTGACATTCC; dG = -28.4
   387 >s_c_s_e_s_b_s_c_s_d_s_c_s_d_s           GTGTGGGTGCTGGTGGCTGTGTGGGTGACGGTGTGGGTGACGGTG;
>clamp_s_c_s_d_s_c_s_a_s:compl:rev CACAGACACCCACACCGTCACCCACACTATCATGTTGACATTCC; dG = -28.4
   388 >s_a_s_b_s_c_s_c_s_d_s_c_s_d_s           GTGTCTGTGGCTGTGTGGGTGTGGGTGACGGTGTGGGTGACGGTG;
>clamp_s_c_s_d_s_c_s_a_s:compl:rev CACAGACACCCACACCGTCACCCACACTATCATGTTGACATTCC; dG = -28.4
   389 >s_c_s_e_s_c_s_c_s_d_s_c_s_d_s           GTGTGGGTGCTGGTGTGGGTGTGGGTGACGGTGTGGGTGACGGTG;
>clamp_s_c_s_d_s_c_s_a_s:compl:rev CACAGACACCCACACCGTCACCCACACTATCATGTTGACATTCC; dG = -28.4
   390 >s_c_s_e_s_d_s_c_s_d_s_c_s_d_s           GTGTGGGTGCTGGTGACGGTGTGGGTGACGGTGTGGGTGACGGTG;
>clamp_s_c_s_d_s_c_s_a_s:compl:rev CACAGACACCCACACCGTCACCCACACTATCATGTTGACATTCC; dG = -28.4
```

```
   391 >s_c_s_e_s_d_s_c_s_a_s_c_s_a_s          GTGTGGGTGCTGGTGACGGTGTGGGTGTCTGTGTGGGTGTCTGTG;
>clamp_s_c_s_d_s_c_s_a_s:compl:rev CACAGACACCCACACCGTCACCCACACTATCATGTTGACATTCC; dG = -28.4
   392 >s_c_s_e_s_e_s_c_s_d_s_c_s_d_s          GTGTGGGTGCTGGTGCTGGTGTGGGTGACGGTGTGGGTGACGGTG;
>clamp_s_c_s_d_s_c_s_a_s:compl:rev CACAGACACCCACACCGTCACCCACACTATCATGTTGACATTCC; dG = -28.4
   393 >s_d_s_a_s_a_s_c_s_d_s_c_s_d_s          GTGACGGTGTCTGTGTCTGTGTGGGTGACGGTGTGGGTGACGGTG;
>clamp_s_c_s_d_s_c_s_a_s:compl:rev CACAGACACCCACACCGTCACCCACACTATCATGTTGACATTCC; dG = -28.4
   394 >s_d_s_a_s_b_s_c_s_d_s_c_s_d_s          GTGACGGTGTCTGTGGCTGTGTGGGTGACGGTGTGGGTGACGGTG;
>clamp_s_c_s_d_s_c_s_a_s:compl:rev CACAGACACCCACACCGTCACCCACACTATCATGTTGACATTCC; dG = -28.4
   395 >s_d_s_a_s_c_s_c_s_d_s_c_s_d_s          GTGACGGTGTCTGTGTGGGTGTGGGTGACGGTGTGGGTGACGGTG;
>clamp_s_c_s_d_s_c_s_a_s:compl:rev CACAGACACCCACACCGTCACCCACACTATCATGTTGACATTCC; dG = -28.4
   396 >s_d_s_a_s_d_s_c_s_d_s_c_s_d_s          GTGACGGTGTCTGTGACGGTGTGGGTGACGGTGTGGGTGACGGTG;
>clamp_s_c_s_d_s_c_s_a_s:compl:rev CACAGACACCCACACCGTCACCCACACTATCATGTTGACATTCC; dG = -28.4
   397 >s_d_s_a_s_e_s_c_s_d_s_c_s_d_s          GTGACGGTGTCTGTGCTGGTGTGGGTGACGGTGTGGGTGACGGTG;
>clamp_s_c_s_d_s_c_s_a_s:compl:rev CACAGACACCCACACCGTCACCCACACTATCATGTTGACATTCC; dG = -28.4
   398 >s_d_s_b_s_a_s_c_s_d_s_c_s_d_s          GTGACGGTGGCTGTGTCTGTGTGGGTGACGGTGTGGGTGACGGTG;
>clamp_s_c_s_d_s_c_s_a_s:compl:rev CACAGACACCCACACCGTCACCCACACTATCATGTTGACATTCC; dG = -28.4
   399 >s_d_s_b_s_b_s_c_s_d_s_c_s_d_s          GTGACGGTGGCTGTGGCTGTGTGGGTGACGGTGTGGGTGACGGTG;
>clamp_s_c_s_d_s_c_s_a_s:compl:rev CACAGACACCCACACCGTCACCCACACTATCATGTTGACATTCC; dG = -28.4
   400 >s_a_s_b_s_d_s_c_s_d_s_c_s_d_s          GTGTCTGTGGCTGTGACGGTGTGGGTGACGGTGTGGGTGACGGTG;
>clamp_s_c_s_d_s_c_s_a_s:compl:rev CACAGACACCCACACCGTCACCCACACTATCATGTTGACATTCC; dG = -28.4
   401 >s_d_s_b_s_c_s_c_s_d_s_c_s_d_s          GTGACGGTGGCTGTGTGGGTGTGGGTGACGGTGTGGGTGACGGTG;
>clamp_s_c_s_d_s_c_s_a_s:compl:rev CACAGACACCCACACCGTCACCCACACTATCATGTTGACATTCC; dG = -28.4
   402 >s_d_s_b_s_d_s_c_s_d_s_c_s_d_s          GTGACGGTGGCTGTGACGGTGTGGGTGACGGTGTGGGTGACGGTG;
>clamp_s_c_s_d_s_c_s_a_s:compl:rev CACAGACACCCACACCGTCACCCACACTATCATGTTGACATTCC; dG = -28.4
   403 >s_d_s_b_s_e_s_c_s_d_s_c_s_d_s          GTGACGGTGGCTGTGCTGGTGTGGGTGACGGTGTGGGTGACGGTG;
>clamp_s_c_s_d_s_c_s_a_s:compl:rev CACAGACACCCACACCGTCACCCACACTATCATGTTGACATTCC; dG = -28.4
   404 >s_d_s_c_s_a_s_c_s_d_s_c_s_d_s          GTGACGGTGTGGGTGTCTGTGTGGGTGACGGTGTGGGTGACGGTG;
>clamp_s_c_s_d_s_c_s_a_s:compl:rev CACAGACACCCACACCGTCACCCACACTATCATGTTGACATTCC; dG = -28.4
   405 >s_d_s_c_s_b_s_c_s_d_s_c_s_d_s          GTGACGGTGTGGGTGGCTGTGTGGGTGACGGTGTGGGTGACGGTG;
>clamp_s_c_s_d_s_c_s_a_s:compl:rev CACAGACACCCACACCGTCACCCACACTATCATGTTGACATTCC; dG = -28.4
   406 >s_d_s_c_s_c_s_c_s_d_s_c_s_d_s          GTGACGGTGTGGGTGTGGGTGTGGGTGACGGTGTGGGTGACGGTG;
>clamp_s_c_s_d_s_c_s_a_s:compl:rev CACAGACACCCACACCGTCACCCACACTATCATGTTGACATTCC; dG = -28.4
   407 >s_d_s_c_s_d_s_c_s_d_s_c_s_e_s          GTGACGGTGTGGGTGACGGTGTGGGTGACGGTGTGGGTGCTGGTG;
>clamp_s_c_s_d_s_c_s_a_s:compl:rev CACAGACACCCACACCGTCACCCACACTATCATGTTGACATTCC; dG = -28.4
   408 >s_d_s_c_s_d_s_c_s_d_s_c_s_d_s          GTGACGGTGTGGGTGACGGTGTGGGTGACGGTGTGGGTGACGGTG;
>clamp_s_c_s_d_s_c_s_a_s:compl:rev CACAGACACCCACACCGTCACCCACACTATCATGTTGACATTCC; dG = -28.4
   409 >s_d_s_c_s_d_s_c_s_d_s_e_s_a_s          GTGACGGTGTGGGTGACGGTGTGGGTGACGGTGCTGGTGTCTGTG;
>clamp_s_c_s_d_s_c_s_a_s:compl:rev CACAGACACCCACACCGTCACCCACACTATCATGTTGACATTCC; dG = -28.4
   410 >s_d_s_c_s_d_s_c_s_d_s_d_s_a_s          GTGACGGTGTGGGTGACGGTGTGGGTGACGGTGACGGTGTCTGTG;
>clamp_s_c_s_d_s_c_s_a_s:compl:rev CACAGACACCCACACCGTCACCCACACTATCATGTTGACATTCC; dG = -28.4
   411 >s_d_s_c_s_d_s_c_s_d_s_b_s_a_s          GTGACGGTGTGGGTGACGGTGTGGGTGACGGTGGCTGTGTCTGTG;
>clamp_s_c_s_d_s_c_s_a_s:compl:rev CACAGACACCCACACCGTCACCCACACTATCATGTTGACATTCC; dG = -28.4
   412 >s_d_s_c_s_e_s_c_s_d_s_c_s_d_s          GTGACGGTGTGGGTGCTGGTGTGGGTGACGGTGTGGGTGACGGTG;
>clamp_s_c_s_d_s_c_s_a_s:compl:rev CACAGACACCCACACCGTCACCCACACTATCATGTTGACATTCC; dG = -28.4
   413 >s_d_s_d_s_a_s_c_s_d_s_c_s_d_s          GTGACGGTGACGGTGTCTGTGTGGGTGACGGTGTGGGTGACGGTG;
>clamp_s_c_s_d_s_c_s_a_s:compl:rev CACAGACACCCACACCGTCACCCACACTATCATGTTGACATTCC; dG = -28.4
   414 >s_d_s_d_s_b_s_c_s_d_s_c_s_d_s          GTGACGGTGACGGTGGCTGTGTGGGTGACGGTGTGGGTGACGGTG;
>clamp_s_c_s_d_s_c_s_a_s:compl:rev CACAGACACCCACACCGTCACCCACACTATCATGTTGACATTCC; dG = -28.4
   415 >s_a_s_b_s_e_s_c_s_d_s_c_s_d_s          GTGTCTGTGGCTGTGCTGGTGTGGGTGACGGTGTGGGTGACGGTG;
>clamp_s_c_s_d_s_c_s_a_s:compl:rev CACAGACACCCACACCGTCACCCACACTATCATGTTGACATTCC; dG = -28.4
   416 >s_d_s_d_s_c_s_c_s_d_s_c_s_d_s          GTGACGGTGACGGTGTGGGTGTGGGTGACGGTGTGGGTGACGGTG;
>clamp_s_c_s_d_s_c_s_a_s:compl:rev CACAGACACCCACACCGTCACCCACACTATCATGTTGACATTCC; dG = -28.4
   417 >s_d_s_d_s_d_s_c_s_d_s_c_s_d_s          GTGACGGTGACGGTGACGGTGTGGGTGACGGTGTGGGTGACGGTG;
>clamp_s_c_s_d_s_c_s_a_s:compl:rev CACAGACACCCACACCGTCACCCACACTATCATGTTGACATTCC; dG = -28.4
   418 >s_d_s_d_s_e_s_c_s_d_s_c_s_d_s          GTGACGGTGACGGTGCTGGTGTGGGTGACGGTGTGGGTGACGGTG;
>clamp_s_c_s_d_s_c_s_a_s:compl:rev CACAGACACCCACACCGTCACCCACACTATCATGTTGACATTCC; dG = -28.4
   419 >s_d_s_e_s_a_s_c_s_d_s_c_s_d_s          GTGACGGTGCTGGTGTCTGTGTGGGTGACGGTGTGGGTGACGGTG;
>clamp_s_c_s_d_s_c_s_a_s:compl:rev CACAGACACCCACACCGTCACCCACACTATCATGTTGACATTCC; dG = -28.4
   420 >s_d_s_e_s_b_s_c_s_d_s_c_s_d_s          GTGACGGTGCTGGTGGCTGTGTGGGTGACGGTGTGGGTGACGGTG;
>clamp_s_c_s_d_s_c_s_a_s:compl:rev CACAGACACCCACACCGTCACCCACACTATCATGTTGACATTCC; dG = -28.4
   421 >s_d_s_e_s_c_s_c_s_d_s_c_s_d_s          GTGACGGTGCTGGTGTGGGTGTGGGTGACGGTGTGGGTGACGGTG;
>clamp_s_c_s_d_s_c_s_a_s:compl:rev CACAGACACCCACACCGTCACCCACACTATCATGTTGACATTCC; dG = -28.4
   422 >s_d_s_e_s_d_s_c_s_d_s_c_s_d_s          GTGACGGTGCTGGTGACGGTGTGGGTGACGGTGTGGGTGACGGTG;
>clamp_s_c_s_d_s_c_s_a_s:compl:rev CACAGACACCCACACCGTCACCCACACTATCATGTTGACATTCC; dG = -28.4
   423 >s_d_s_e_s_e_s_c_s_d_s_c_s_d_s          GTGACGGTGCTGGTGCTGGTGTGGGTGACGGTGTGGGTGACGGTG;
>clamp_s_c_s_d_s_c_s_a_s:compl:rev CACAGACACCCACACCGTCACCCACACTATCATGTTGACATTCC; dG = -28.4
   424 >s_e_s_a_s_a_s_c_s_d_s_c_s_d_s          GTGCTGGTGTCTGTGTCTGTGTGGGTGACGGTGTGGGTGACGGTG;
>clamp_s_c_s_d_s_c_s_a_s:compl:rev CACAGACACCCACACCGTCACCCACACTATCATGTTGACATTCC; dG = -28.4
   425 >s_e_s_a_s_b_s_c_s_d_s_c_s_d_s          GTGCTGGTGTCTGTGGCTGTGTGGGTGACGGTGTGGGTGACGGTG;
>clamp_s_c_s_d_s_c_s_a_s:compl:rev CACAGACACCCACACCGTCACCCACACTATCATGTTGACATTCC; dG = -28.4
   426 >s_a_s_c_s_a_s_c_s_d_s_c_s_d_s          GTGTCTGTGTGGGTGTCTGTGTGGGTGACGGTGTGGGTGACGGTG;
>clamp_s_c_s_d_s_c_s_a_s:compl:rev CACAGACACCCACACCGTCACCCACACTATCATGTTGACATTCC; dG = -28.4
   427 >s_e_s_a_s_c_s_c_s_d_s_c_s_d_s          GTGCTGGTGTCTGTGTGGGTGTGGGTGACGGTGTGGGTGACGGTG;
>clamp_s_c_s_d_s_c_s_a_s:compl:rev CACAGACACCCACACCGTCACCCACACTATCATGTTGACATTCC; dG = -28.4
```

```
   428 >s_e_s_a_s_d_s_c_s_d_s_c_s_d_s          GTGCTGGTGTCTGTGACGGTGTGGGTGACGGTGTGGGTGACGGTG;
>clamp_s_c_s_d_s_c_s_a_s:compl:rev CACAGACACCCACACCGTCACCCACACTATCATGTTGACATTCC; dG = -28.4
   429 >s_e_s_a_s_e_s_c_s_d_s_c_s_d_s          GTGCTGGTGTCTGTGCTGGTGTGGGTGACGGTGTGGGTGACGGTG;
>clamp_s_c_s_d_s_c_s_a_s:compl:rev CACAGACACCCACACCGTCACCCACACTATCATGTTGACATTCC; dG = -28.4
   430 >s_e_s_b_s_a_s_c_s_d_s_c_s_d_s          GTGCTGGTGGCTGTGTCTGTGTGGGTGACGGTGTGGGTGACGGTG;
>clamp_s_c_s_d_s_c_s_a_s:compl:rev CACAGACACCCACACCGTCACCCACACTATCATGTTGACATTCC; dG = -28.4
   431 >s_e_s_b_s_b_s_c_s_d_s_c_s_d_s          GTGCTGGTGGCTGTGGCTGTGTGGGTGACGGTGTGGGTGACGGTG;
>clamp_s_c_s_d_s_c_s_a_s:compl:rev CACAGACACCCACACCGTCACCCACACTATCATGTTGACATTCC; dG = -28.4
   432 >s_e_s_b_s_c_s_c_s_d_s_c_s_d_s          GTGCTGGTGGCTGTGTGGGTGTGGGTGACGGTGTGGGTGACGGTG;
>clamp_s_c_s_d_s_c_s_a_s:compl:rev CACAGACACCCACACCGTCACCCACACTATCATGTTGACATTCC; dG = -28.4
   433 >s_e_s_b_s_d_s_c_s_d_s_c_s_d_s          GTGCTGGTGGCTGTGACGGTGTGGGTGACGGTGTGGGTGACGGTG;
>clamp_s_c_s_d_s_c_s_a_s:compl:rev CACAGACACCCACACCGTCACCCACACTATCATGTTGACATTCC; dG = -28.4
   434 >s_e_s_b_s_e_s_c_s_d_s_c_s_d_s          GTGCTGGTGGCTGTGCTGGTGTGGGTGACGGTGTGGGTGACGGTG;
>clamp_s_c_s_d_s_c_s_a_s:compl:rev CACAGACACCCACACCGTCACCCACACTATCATGTTGACATTCC; dG = -28.4
   435 >s_e_s_c_s_a_s_c_s_d_s_c_s_d_s          GTGCTGGTGTGGGTGTCTGTGTGGGTGACGGTGTGGGTGACGGTG;
>clamp_s_c_s_d_s_c_s_a_s:compl:rev CACAGACACCCACACCGTCACCCACACTATCATGTTGACATTCC; dG = -28.4
   436 >s_e_s_c_s_b_s_c_s_d_s_c_s_d_s          GTGCTGGTGTGGGTGGCTGTGTGGGTGACGGTGTGGGTGACGGTG;
>clamp_s_c_s_d_s_c_s_a_s:compl:rev CACAGACACCCACACCGTCACCCACACTATCATGTTGACATTCC; dG = -28.4
   437 >s_a_s_c_s_b_s_c_s_d_s_c_s_d_s          GTGTCTGTGTGGGTGGCTGTGTGGGTGACGGTGTGGGTGACGGTG;
>clamp_s_c_s_d_s_c_s_a_s:compl:rev CACAGACACCCACACCGTCACCCACACTATCATGTTGACATTCC; dG = -28.4
   438 >s_e_s_c_s_c_s_c_s_d_s_c_s_d_s          GTGCTGGTGTGGGTGTGGGTGTGGGTGACGGTGTGGGTGACGGTG;
>clamp_s_c_s_d_s_c_s_a_s:compl:rev CACAGACACCCACACCGTCACCCACACTATCATGTTGACATTCC; dG = -28.4
   439 >s_a_s_a_s_b_s_c_s_d_s_c_s_d_s          GTGTCTGTGTCTGTGGCTGTGTGGGTGACGGTGTGGGTGACGGTG;
>clamp_s_c_s_d_s_c_s_a_s:compl:rev CACAGACACCCACACCGTCACCCACACTATCATGTTGACATTCC; dG = -28.4
   440 >s_e_s_c_s_d_s_c_s_d_s_c_s_e_s          GTGCTGGTGTGGGTGACGGTGTGGGTGACGGTGTGGGTGCTGGTG;
>clamp_s_c_s_d_s_c_s_a_s:compl:rev CACAGACACCCACACCGTCACCCACACTATCATGTTGACATTCC; dG = -28.4
   441 >s_e_s_c_s_d_s_c_s_d_s_c_s_d_s          GTGCTGGTGTGGGTGACGGTGTGGGTGACGGTGTGGGTGACGGTG;
>clamp_s_c_s_d_s_c_s_a_s:compl:rev CACAGACACCCACACCGTCACCCACACTATCATGTTGACATTCC; dG = -28.4
   442 >s_e_s_c_s_d_s_c_s_d_s_e_s_a_s          GTGCTGGTGTGGGTGACGGTGTGGGTGACGGTGCTGGTGTCTGTG;
>clamp_s_c_s_d_s_c_s_a_s:compl:rev CACAGACACCCACACCGTCACCCACACTATCATGTTGACATTCC; dG = -28.4
   443 >s_e_s_c_s_d_s_c_s_d_s_d_s_a_s          GTGCTGGTGTGGGTGACGGTGTGGGTGACGGTGACGGTGTCTGTG;
>clamp_s_c_s_d_s_c_s_a_s:compl:rev CACAGACACCCACACCGTCACCCACACTATCATGTTGACATTCC; dG = -28.4
   444 >s_e_s_c_s_d_s_c_s_d_s_b_s_a_s          GTGCTGGTGTGGGTGACGGTGTGGGTGACGGTGGCTGTGTCTGTG;
>clamp_s_c_s_d_s_c_s_a_s:compl:rev CACAGACACCCACACCGTCACCCACACTATCATGTTGACATTCC; dG = -28.4
   445 >s_e_s_c_s_e_s_c_s_d_s_c_s_d_s          GTGCTGGTGTGGGTGCTGGTGTGGGTGACGGTGTGGGTGACGGTG;
>clamp_s_c_s_d_s_c_s_a_s:compl:rev CACAGACACCCACACCGTCACCCACACTATCATGTTGACATTCC; dG = -28.4
   446 >s_e_s_d_s_a_s_c_s_d_s_c_s_d_s          GTGCTGGTGACGGTGTCTGTGTGGGTGACGGTGTGGGTGACGGTG;
>clamp_s_c_s_d_s_c_s_a_s:compl:rev CACAGACACCCACACCGTCACCCACACTATCATGTTGACATTCC; dG = -28.4
   447 >s_e_s_d_s_b_s_c_s_d_s_c_s_d_s          GTGCTGGTGACGGTGGCTGTGTGGGTGACGGTGTGGGTGACGGTG;
>clamp_s_c_s_d_s_c_s_a_s:compl:rev CACAGACACCCACACCGTCACCCACACTATCATGTTGACATTCC; dG = -28.4
   448 >s_e_s_d_s_c_s_c_s_d_s_c_s_d_s          GTGCTGGTGACGGTGTGGGTGTGGGTGACGGTGTGGGTGACGGTG;
>clamp_s_c_s_d_s_c_s_a_s:compl:rev CACAGACACCCACACCGTCACCCACACTATCATGTTGACATTCC; dG = -28.4
   449 >s_e_s_d_s_d_s_c_s_d_s_c_s_d_s          GTGCTGGTGACGGTGACGGTGTGGGTGACGGTGTGGGTGACGGTG;
>clamp_s_c_s_d_s_c_s_a_s:compl:rev CACAGACACCCACACCGTCACCCACACTATCATGTTGACATTCC; dG = -28.4
   450 >s_e_s_d_s_e_s_c_s_d_s_c_s_d_s          GTGCTGGTGACGGTGCTGGTGTGGGTGACGGTGTGGGTGACGGTG;
>clamp_s_c_s_d_s_c_s_a_s:compl:rev CACAGACACCCACACCGTCACCCACACTATCATGTTGACATTCC; dG = -28.4
   451 >s_e_s_e_s_a_s_c_s_d_s_c_s_d_s          GTGCTGGTGCTGGTGTCTGTGTGGGTGACGGTGTGGGTGACGGTG;
>clamp_s_c_s_d_s_c_s_a_s:compl:rev CACAGACACCCACACCGTCACCCACACTATCATGTTGACATTCC; dG = -28.4
   452 >s_e_s_e_s_b_s_c_s_d_s_c_s_d_s          GTGCTGGTGCTGGTGGCTGTGTGGGTGACGGTGTGGGTGACGGTG;
>clamp_s_c_s_d_s_c_s_a_s:compl:rev CACAGACACCCACACCGTCACCCACACTATCATGTTGACATTCC; dG = -28.4
   453 >s_a_s_c_s_c_s_c_s_d_s_c_s_d_s          GTGTCTGTGTGGGTGTGGGTGTGGGTGACGGTGTGGGTGACGGTG;
>clamp_s_c_s_d_s_c_s_a_s:compl:rev CACAGACACCCACACCGTCACCCACACTATCATGTTGACATTCC; dG = -28.4
   454 >s_e_s_e_s_c_s_c_s_d_s_c_s_d_s          GTGCTGGTGCTGGTGTGGGTGTGGGTGACGGTGTGGGTGACGGTG;
>clamp_s_c_s_d_s_c_s_a_s:compl:rev CACAGACACCCACACCGTCACCCACACTATCATGTTGACATTCC; dG = -28.4
   455 >s_e_s_e_s_d_s_c_s_d_s_c_s_d_s          GTGCTGGTGCTGGTGACGGTGTGGGTGACGGTGTGGGTGACGGTG;
>clamp_s_c_s_d_s_c_s_a_s:compl:rev CACAGACACCCACACCGTCACCCACACTATCATGTTGACATTCC; dG = -28.4
   456 >s_e_s_e_s_e_s_c_s_d_s_c_s_d_s          GTGCTGGTGCTGGTGCTGGTGTGGGTGACGGTGTGGGTGACGGTG;
>clamp_s_c_s_d_s_c_s_a_s:compl:rev CACAGACACCCACACCGTCACCCACACTATCATGTTGACATTCC; dG = -28.4
   457 >s_a_s_a_s_a_s_c_s_d_s_c_s_d_s          GTGTCTGTGTCTGTGTCTGTGTGGGTGACGGTGTGGGTGACGGTG;
>clamp_s_c_s_d_s_c_s_a_s:compl:rev CACAGACACCCACACCGTCACCCACACTATCATGTTGACATTCC; dG = -28.4
   458 >s_a_s_c_s_d_s_c_s_d_s_c_s_e_s          GTGTCTGTGTGGGTGACGGTGTGGGTGACGGTGTGGGTGCTGGTG;
>clamp_s_c_s_d_s_c_s_a_s:compl:rev CACAGACACCCACACCGTCACCCACACTATCATGTTGACATTCC; dG = -28.4
   459 >s_a_s_c_s_d_s_c_s_d_s_c_s_d_s          GTGTCTGTGTGGGTGACGGTGTGGGTGACGGTGTGGGTGACGGTG;
>clamp_s_c_s_d_s_c_s_a_s:compl:rev CACAGACACCCACACCGTCACCCACACTATCATGTTGACATTCC; dG = -28.4
   460 >s_a_s_c_s_d_s_c_s_d_s_e_s_a_s          GTGTCTGTGTGGGTGACGGTGTGGGTGACGGTGCTGGTGTCTGTG;
>clamp_s_c_s_d_s_c_s_a_s:compl:rev CACAGACACCCACACCGTCACCCACACTATCATGTTGACATTCC; dG = -28.4
   461 >s_a_s_c_s_d_s_c_s_d_s_d_s_a_s          GTGTCTGTGTGGGTGACGGTGTGGGTGACGGTGACGGTGTCTGTG;
>clamp_s_c_s_d_s_c_s_a_s:compl:rev CACAGACACCCACACCGTCACCCACACTATCATGTTGACATTCC; dG = -28.4
   462 >s_a_s_c_s_d_s_c_s_d_s_b_s_a_s          GTGTCTGTGTGGGTGACGGTGTGGGTGACGGTGGCTGTGTCTGTG;
>clamp_s_c_s_d_s_c_s_a_s:compl:rev CACAGACACCCACACCGTCACCCACACTATCATGTTGACATTCC; dG = -28.4
   463 >s_a_s_c_s_e_s_c_s_d_s_c_s_d_s          GTGTCTGTGTGGGTGCTGGTGTGGGTGACGGTGTGGGTGACGGTG;
>clamp_s_c_s_d_s_c_s_a_s:compl:rev CACAGACACCCACACCGTCACCCACACTATCATGTTGACATTCC; dG = -28.4
   464 >s_a_s_d_s_a_s_c_s_d_s_c_s_d_s          GTGTCTGTGACGGTGTCTGTGTGGGTGACGGTGTGGGTGACGGTG;
>clamp_s_c_s_d_s_c_s_a_s:compl:rev CACAGACACCCACACCGTCACCCACACTATCATGTTGACATTCC; dG = -28.4
```

465 >s_a_s_d_s_b_s_c_s_d_s_c_s_d_s GTGTCTGTGACGGTGGCTGTGTGGGTGACGGTGTGGGTGACGGTG;
>clamp_s_c_s_d_s_c_s_a_s:compl:rev CACAGACACCCACACCGTCACCCACACTATCATGTTGACATTCC; dG = -28.4
466 >s_a_s_d_s_c_s_c_s_d_s_c_s_d_s GTGTCTGTGACGGTGTGGGTGTGGGTGACGGTGTGGGTGACGGTG;
>clamp_s_c_s_d_s_c_s_a_s:compl:rev CACAGACACCCACACCGTCACCCACACTATCATGTTGACATTCC; dG = -28.4
467 >s_a_s_d_s_d_s_c_s_d_s_c_s_d_s GTGTCTGTGACGGTGACGGTGTGGGTGACGGTGTGGGTGACGGTG;
>clamp_s_c_s_d_s_c_s_a_s:compl:rev CACAGACACCCACACCGTCACCCACACTATCATGTTGACATTCC; dG = -28.4
468 >s_a_s_d_s_e_s_c_s_d_s_c_s_d_s GTGTCTGTGACGGTGCTGGTGTGGGTGACGGTGTGGGTGACGGTG;
>clamp_s_c_s_d_s_c_s_a_s:compl:rev CACAGACACCCACACCGTCACCCACACTATCATGTTGACATTCC; dG = -28.4
469 >s_a_s_e_s_a_s_c_s_d_s_c_s_d_s GTGTCTGTGCTGGTGTCTGTGTGGGTGACGGTGTGGGTGACGGTG;
>clamp_s_c_s_d_s_c_s_a_s:compl:rev CACAGACACCCACACCGTCACCCACACTATCATGTTGACATTCC; dG = -28.4
470 >s_a_s_e_s_b_s_c_s_d_s_c_s_d_s GTGTCTGTGCTGGTGGCTGTGTGGGTGACGGTGTGGGTGACGGTG;
>clamp_s_c_s_d_s_c_s_a_s:compl:rev CACAGACACCCACACCGTCACCCACACTATCATGTTGACATTCC; dG = -28.4
471 >s_a_s_a_s_c_s_c_s_d_s_c_s_d_s GTGTCTGTGTCTGTGTGGGTGTGGGTGACGGTGTGGGTGACGGTG;
>clamp_s_c_s_d_s_c_s_a_s:compl:rev CACAGACACCCACACCGTCACCCACACTATCATGTTGACATTCC; dG = -28.4
472 >s_a_s_e_s_c_s_c_s_d_s_c_s_d_s GTGTCTGTGCTGGTGTGGGTGTGGGTGACGGTGTGGGTGACGGTG;
>clamp_s_c_s_d_s_c_s_a_s:compl:rev CACAGACACCCACACCGTCACCCACACTATCATGTTGACATTCC; dG = -28.4
473 >s_a_s_e_s_d_s_c_s_d_s_c_s_d_s GTGTCTGTGCTGGTGACGGTGTGGGTGACGGTGTGGGTGACGGTG;
>clamp_s_c_s_d_s_c_s_a_s:compl:rev CACAGACACCCACACCGTCACCCACACTATCATGTTGACATTCC; dG = -28.4
474 >s_a_s_e_s_e_s_c_s_d_s_c_s_d_s GTGTCTGTGCTGGTGCTGGTGTGGGTGACGGTGTGGGTGACGGTG;
>clamp_s_c_s_d_s_c_s_a_s:compl:rev CACAGACACCCACACCGTCACCCACACTATCATGTTGACATTCC; dG = -28.4
475 >s_b_s_a_s_a_s_c_s_d_s_c_s_d_s GTGGCTGTGTCTGTGTCTGTGTGGGTGACGGTGTGGGTGACGGTG;
>clamp_s_c_s_d_s_c_s_a_s:compl:rev CACAGACACCCACACCGTCACCCACACTATCATGTTGACATTCC; dG = -28.4
476 >s_b_s_a_s_b_s_c_s_d_s_c_s_d_s GTGGCTGTGTCTGTGGCTGTGTGGGTGACGGTGTGGGTGACGGTG;
>clamp_s_c_s_d_s_c_s_a_s:compl:rev CACAGACACCCACACCGTCACCCACACTATCATGTTGACATTCC; dG = -28.4
477 >s_b_s_a_s_c_s_c_s_d_s_c_s_d_s GTGGCTGTGTCTGTGTGGGTGTGGGTGACGGTGTGGGTGACGGTG;
>clamp_s_c_s_d_s_c_s_a_s:compl:rev CACAGACACCCACACCGTCACCCACACTATCATGTTGACATTCC; dG = -28.4
478 >s_b_s_a_s_d_s_c_s_d_s_c_s_d_s GTGGCTGTGTCTGTGACGGTGTGGGTGACGGTGTGGGTGACGGTG;
>clamp_s_c_s_d_s_c_s_a_s:compl:rev CACAGACACCCACACCGTCACCCACACTATCATGTTGACATTCC; dG = -28.4
479 >s_b_s_a_s_e_s_c_s_d_s_c_s_d_s GTGGCTGTGTCTGTGCTGGTGTGGGTGACGGTGTGGGTGACGGTG;
>clamp_s_c_s_d_s_c_s_a_s:compl:rev CACAGACACCCACACCGTCACCCACACTATCATGTTGACATTCC; dG = -28.4
480 >s_b_s_b_s_a_s_c_s_d_s_c_s_d_s GTGGCTGTGGCTGTGTCTGTGTGGGTGACGGTGTGGGTGACGGTG;
>clamp_s_c_s_d_s_c_s_a_s:compl:rev CACAGACACCCACACCGTCACCCACACTATCATGTTGACATTCC; dG = -28.4
481 >s_b_s_b_s_b_s_c_s_d_s_c_s_d_s GTGGCTGTGGCTGTGGCTGTGTGGGTGACGGTGTGGGTGACGGTG;
>clamp_s_c_s_d_s_c_s_a_s:compl:rev CACAGACACCCACACCGTCACCCACACTATCATGTTGACATTCC; dG = -28.4
482 >s_a_s_a_s_d_s_c_s_d_s_c_s_d_s GTGTCTGTGTCTGTGACGGTGTGGGTGACGGTGTGGGTGACGGTG;
>clamp_s_c_s_d_s_c_s_a_s:compl:rev CACAGACACCCACACCGTCACCCACACTATCATGTTGACATTCC; dG = -28.4
483 >s_b_s_b_s_c_s_c_s_d_s_c_s_d_s GTGGCTGTGGCTGTGTGGGTGTGGGTGACGGTGTGGGTGACGGTG;
>clamp_s_c_s_d_s_c_s_a_s:compl:rev CACAGACACCCACACCGTCACCCACACTATCATGTTGACATTCC; dG = -28.4
484 >s_b_s_b_s_d_s_c_s_d_s_c_s_d_s GTGGCTGTGGCTGTGACGGTGTGGGTGACGGTGTGGGTGACGGTG;
>clamp_s_c_s_d_s_c_s_a_s:compl:rev CACAGACACCCACACCGTCACCCACACTATCATGTTGACATTCC; dG = -28.4
485 >s_b_s_b_s_e_s_c_s_d_s_c_s_d_s GTGGCTGTGGCTGTGCTGGTGTGGGTGACGGTGTGGGTGACGGTG;
>clamp_s_c_s_d_s_c_s_a_s:compl:rev CACAGACACCCACACCGTCACCCACACTATCATGTTGACATTCC; dG = -28.4
486 >s_b_s_c_s_a_s_c_s_d_s_c_s_d_s GTGGCTGTGTGGGTGTCTGTGTGGGTGACGGTGTGGGTGACGGTG;
>clamp_s_c_s_d_s_c_s_a_s:compl:rev CACAGACACCCACACCGTCACCCACACTATCATGTTGACATTCC; dG = -28.4
487 >s_b_s_c_s_b_s_c_s_d_s_c_s_d_s GTGGCTGTGTGGGTGGCTGTGTGGGTGACGGTGTGGGTGACGGTG;
>clamp_s_c_s_d_s_c_s_a_s:compl:rev CACAGACACCCACACCGTCACCCACACTATCATGTTGACATTCC; dG = -28.4
488 >s_b_s_c_s_c_s_c_s_d_s_c_s_d_s GTGGCTGTGTGGGTGTGGGTGTGGGTGACGGTGTGGGTGACGGTG;
>clamp_s_c_s_d_s_c_s_a_s:compl:rev CACAGACACCCACACCGTCACCCACACTATCATGTTGACATTCC; dG = -28.4
489 >s_b_s_c_s_d_s_c_s_d_s_c_s_e_s GTGGCTGTGTGGGTGACGGTGTGGGTGACGGTGTGGGTGCTGGTG;
>clamp_s_c_s_d_s_c_s_a_s:compl:rev CACAGACACCCACACCGTCACCCACACTATCATGTTGACATTCC; dG = -28.4
490 >s_b_s_c_s_d_s_c_s_d_s_c_s_d_s GTGGCTGTGTGGGTGACGGTGTGGGTGACGGTGTGGGTGACGGTG;
>clamp_s_c_s_d_s_c_s_a_s:compl:rev CACAGACACCCACACCGTCACCCACACTATCATGTTGACATTCC; dG = -28.4
491 >s_b_s_c_s_d_s_c_s_d_s_e_s_a_s GTGGCTGTGTGGGTGACGGTGTGGGTGACGGTGCTGGTGTCTGTG;
>clamp_s_c_s_d_s_c_s_a_s:compl:rev CACAGACACCCACACCGTCACCCACACTATCATGTTGACATTCC; dG = -28.4
492 >s_b_s_c_s_d_s_c_s_d_s_d_s_a_s GTGGCTGTGTGGGTGACGGTGTGGGTGACGGTGACGGTGTCTGTG;
>clamp_s_c_s_d_s_c_s_a_s:compl:rev CACAGACACCCACACCGTCACCCACACTATCATGTTGACATTCC; dG = -28.4
493 >s_b_s_c_s_d_s_c_s_d_s_b_s_a_s GTGGCTGTGTGGGTGACGGTGTGGGTGACGGTGGCTGTGTCTGTG;
>clamp_s_c_s_d_s_c_s_a_s:compl:rev CACAGACACCCACACCGTCACCCACACTATCATGTTGACATTCC; dG = -28.4
494 >s_b_s_c_s_e_s_c_s_d_s_c_s_d_s GTGGCTGTGTGGGTGCTGGTGTGGGTGACGGTGTGGGTGACGGTG;
>clamp_s_c_s_d_s_c_s_a_s:compl:rev CACAGACACCCACACCGTCACCCACACTATCATGTTGACATTCC; dG = -28.4
495 >s_b_s_d_s_a_s_c_s_d_s_c_s_d_s GTGGCTGTGACGGTGTCTGTGTGGGTGACGGTGTGGGTGACGGTG;
>clamp_s_c_s_d_s_c_s_a_s:compl:rev CACAGACACCCACACCGTCACCCACACTATCATGTTGACATTCC; dG = -28.4
496 >s_b_s_d_s_b_s_c_s_d_s_c_s_d_s GTGGCTGTGACGGTGGCTGTGTGGGTGACGGTGTGGGTGACGGTG;
>clamp_s_c_s_d_s_c_s_a_s:compl:rev CACAGACACCCACACCGTCACCCACACTATCATGTTGACATTCC; dG = -28.4
497 >s_a_s_a_s_e_s_c_s_d_s_c_s_d_s GTGTCTGTGTCTGTGCTGGTGTGGGTGACGGTGTGGGTGACGGTG;
>clamp_s_c_s_d_s_c_s_a_s:compl:rev CACAGACACCCACACCGTCACCCACACTATCATGTTGACATTCC; dG = -28.4
498 >s_b_s_d_s_c_s_c_s_d_s_c_s_d_s GTGGCTGTGACGGTGTGGGTGTGGGTGACGGTGTGGGTGACGGTG;
>clamp_s_c_s_d_s_c_s_a_s:compl:rev CACAGACACCCACACCGTCACCCACACTATCATGTTGACATTCC; dG = -28.4
499 >s_b_s_d_s_d_s_c_s_d_s_c_s_d_s GTGGCTGTGACGGTGACGGTGTGGGTGACGGTGTGGGTGACGGTG;
>clamp_s_c_s_d_s_c_s_a_s:compl:rev CACAGACACCCACACCGTCACCCACACTATCATGTTGACATTCC; dG = -28.4
500 >s_b_s_d_s_e_s_c_s_d_s_c_s_d_s GTGGCTGTGACGGTGCTGGTGTGGGTGACGGTGTGGGTGACGGTG;
>clamp_s_c_s_d_s_c_s_a_s:compl:rev CACAGACACCCACACCGTCACCCACACTATCATGTTGACATTCC; dG = -28.4
501 >s_b_s_e_s_a_s_c_s_d_s_c_s_d_s GTGGCTGTGCTGGTGTCTGTGTGGGTGACGGTGTGGGTGACGGTG;
>clamp_s_c_s_d_s_c_s_a_s:compl:rev CACAGACACCCACACCGTCACCCACACTATCATGTTGACATTCC; dG = -28.4

```
   502 >s_b_s_e_s_b_s_c_s_d_s_c_s_d_s          GTGGCTGTGCTGGTGGCTGTGTGGGTGACGGTGTGGGTGACGGTG;
>clamp_s_c_s_d_s_c_s_a_s:compl:rev CACAGACACCCACACCGTCACCCACACTATCATGTTGACATTCC; dG = -28.4
   503 >s_b_s_e_s_c_s_c_s_d_s_c_s_d_s          GTGGCTGTGCTGGTGTGGGTGTGGGTGACGGTGTGGGTGACGGTG;
>clamp_s_c_s_d_s_c_s_a_s:compl:rev CACAGACACCCACACCGTCACCCACACTATCATGTTGACATTCC; dG = -28.4
   504 >s_b_s_e_s_d_s_c_s_d_s_c_s_d_s          GTGGCTGTGCTGGTGACGGTGTGGGTGACGGTGTGGGTGACGGTG;
>clamp_s_c_s_d_s_c_s_a_s:compl:rev CACAGACACCCACACCGTCACCCACACTATCATGTTGACATTCC; dG = -28.4
   505 >s_b_s_e_s_e_s_c_s_d_s_c_s_d_s          GTGGCTGTGCTGGTGCTGGTGTGGGTGACGGTGTGGGTGACGGTG;
>clamp_s_c_s_d_s_c_s_a_s:compl:rev CACAGACACCCACACCGTCACCCACACTATCATGTTGACATTCC; dG = -28.4
   506 >s_c_s_a_s_a_s_c_s_d_s_c_s_d_s          GTGTGGGTGTCTGTGTCTGTGTGGGTGACGGTGTGGGTGACGGTG;
>clamp_s_c_s_d_s_c_s_a_s:compl:rev CACAGACACCCACACCGTCACCCACACTATCATGTTGACATTCC; dG = -28.4
   507 >s_c_s_a_s_b_s_c_s_d_s_c_s_d_s          GTGTGGGTGTCTGTGGCTGTGTGGGTGACGGTGTGGGTGACGGTG;
>clamp_s_c_s_d_s_c_s_a_s:compl:rev CACAGACACCCACACCGTCACCCACACTATCATGTTGACATTCC; dG = -28.4
   508 >s_a_s_b_s_a_s_c_s_d_s_c_s_d_s          GTGTCTGTGGCTGTGTCTGTGTGGGTGACGGTGTGGGTGACGGTG;
>clamp_s_c_s_d_s_c_s_a_s:compl:rev CACAGACACCCACACCGTCACCCACACTATCATGTTGACATTCC; dG = -28.4
   509 >s_c_s_a_s_c_s_c_s_d_s_c_s_d_s          GTGTGGGTGTCTGTGTGGGTGTGGGTGACGGTGTGGGTGACGGTG;
>clamp_s_c_s_d_s_c_s_a_s:compl:rev CACAGACACCCACACCGTCACCCACACTATCATGTTGACATTCC; dG = -28.4
   510 >s_c_s_a_s_d_s_c_s_d_s_c_s_d_s          GTGTGGGTGTCTGTGACGGTGTGGGTGACGGTGTGGGTGACGGTG;
>clamp_s_c_s_d_s_c_s_a_s:compl:rev CACAGACACCCACACCGTCACCCACACTATCATGTTGACATTCC; dG = -28.4
   511 >s_c_s_a_s_d_s_c_s_a_s_c_s_a_s          GTGTGGGTGTCTGTGACGGTGTGGGTGTCTGTGTGGGTGTCTGTG;
>clamp_s_c_s_d_s_c_s_a_s:compl:rev CACAGACACCCACACCGTCACCCACACTATCATGTTGACATTCC; dG = -28.4
   512 >s_c_s_a_s_e_s_c_s_d_s_c_s_d_s          GTGTGGGTGTCTGTGCTGGTGTGGGTGACGGTGTGGGTGACGGTG;
>clamp_s_c_s_d_s_c_s_a_s:compl:rev CACAGACACCCACACCGTCACCCACACTATCATGTTGACATTCC; dG = -28.4
   513 >s_c_s_b_s_a_s_c_s_d_s_c_s_d_s          GTGTGGGTGGCTGTGTCTGTGTGGGTGACGGTGTGGGTGACGGTG;
>clamp_s_c_s_d_s_c_s_a_s:compl:rev CACAGACACCCACACCGTCACCCACACTATCATGTTGACATTCC; dG = -28.4
   514 >s_c_s_b_s_b_s_c_s_d_s_c_s_d_s          GTGTGGGTGGCTGTGGCTGTGTGGGTGACGGTGTGGGTGACGGTG;
>clamp_s_c_s_d_s_c_s_a_s:compl:rev CACAGACACCCACACCGTCACCCACACTATCATGTTGACATTCC; dG = -28.4
   515 >s_c_s_b_s_c_s_c_s_d_s_c_s_d_s          GTGTGGGTGGCTGTGTGGGTGTGGGTGACGGTGTGGGTGACGGTG;
>clamp_s_c_s_d_s_c_s_a_s:compl:rev CACAGACACCCACACCGTCACCCACACTATCATGTTGACATTCC; dG = -28.4
   516 >s_c_s_b_s_d_s_c_s_d_s_c_s_d_s          GTGTGGGTGGCTGTGACGGTGTGGGTGACGGTGTGGGTGACGGTG;
>clamp_s_c_s_d_s_c_s_a_s:compl:rev CACAGACACCCACACCGTCACCCACACTATCATGTTGACATTCC; dG = -28.4
   517 >s_c_s_b_s_d_s_c_s_a_s_c_s_a_s          GTGTGGGTGGCTGTGACGGTGTGGGTGTCTGTGTGGGTGTCTGTG;
>clamp_s_c_s_d_s_c_s_a_s:compl:rev CACAGACACCCACACCGTCACCCACACTATCATGTTGACATTCC; dG = -28.4
   518 >s_c_s_b_s_e_s_c_s_d_s_c_s_d_s          GTGTGGGTGGCTGTGCTGGTGTGGGTGACGGTGTGGGTGACGGTG;
>clamp_s_c_s_d_s_c_s_a_s:compl:rev CACAGACACCCACACCGTCACCCACACTATCATGTTGACATTCC; dG = -28.4
   519 >s_c_s_c_s_a_s_c_s_d_s_c_s_d_s          GTGTGGGTGTGGGTGTCTGTGTGGGTGACGGTGTGGGTGACGGTG;
>clamp_s_c_s_d_s_c_s_a_s:compl:rev CACAGACACCCACACCGTCACCCACACTATCATGTTGACATTCC; dG = -28.4
   520 >s_c_s_c_s_b_s_c_s_d_s_c_s_d_s          GTGTGGGTGTGGGTGGCTGTGTGGGTGACGGTGTGGGTGACGGTG;
>clamp_s_c_s_d_s_c_s_a_s:compl:rev CACAGACACCCACACCGTCACCCACACTATCATGTTGACATTCC; dG = -28.4
   521 >s_a_s_b_s_b_s_c_s_d_s_c_s_d_s          GTGTCTGTGGCTGTGGCTGTGTGGGTGACGGTGTGGGTGACGGTG;
>clamp_s_c_s_d_s_c_s_a_s:compl:rev CACAGACACCCACACCGTCACCCACACTATCATGTTGACATTCC; dG = -28.4
   522 >s_c_s_c_s_c_s_c_s_d_s_c_s_d_s          GTGTGGGTGTGGGTGTGGGTGTGGGTGACGGTGTGGGTGACGGTG;
>clamp_s_c_s_d_s_c_s_a_s:compl:rev CACAGACACCCACACCGTCACCCACACTATCATGTTGACATTCC; dG = -28.4
   523 >s_c_s_d_s_a_s_c_s_a_s_c_s_a_s          GTGTGGGTGACGGTGTCTGTGTGGGTGTCTGTGTGGGTGTCTGTG;
>clamp_s_c_s_d_s_c_s_a_s:compl:rev CACAGACACCCACACCGTCACCCACACTATCATGTTGACATTCC; dG = -28.3
   524 >s_c_s_d_s_b_s_c_s_a_s_c_s_a_s          GTGTGGGTGACGGTGGCTGTGTGGGTGTCTGTGTGGGTGTCTGTG;
>clamp_s_c_s_d_s_c_s_a_s:compl:rev CACAGACACCCACACCGTCACCCACACTATCATGTTGACATTCC; dG = -28.3
   525 >s_c_s_d_s_e_s_c_s_a_s_c_s_a_s          GTGTGGGTGACGGTGCTGGTGTGGGTGTCTGTGTGGGTGTCTGTG;
>clamp_s_c_s_d_s_c_s_a_s:compl:rev CACAGACACCCACACCGTCACCCACACTATCATGTTGACATTCC; dG = -28.3
   526 >s_a_s_a_s_a_s_c_s_d_s_c_s_e_s          GTGTCTGTGTCTGTGTCTGTGTGGGTGACGGTGTGGGTGCTGGTG;
>clamp_s_c_s_d_s_c_s_a_s:compl:rev CACAGACACCCACACCGTCACCCACACTATCATGTTGACATTCC; dG = -28.2
   527 >s_c_s_d_s_a_s_c_s_d_s_c_s_e_s          GTGTGGGTGACGGTGTCTGTGTGGGTGACGGTGTGGGTGCTGGTG;
>clamp_s_c_s_d_s_c_s_a_s:compl:rev CACAGACACCCACACCGTCACCCACACTATCATGTTGACATTCC; dG = -28.2
   528 >s_c_s_d_s_b_s_c_s_d_s_c_s_e_s          GTGTGGGTGACGGTGGCTGTGTGGGTGACGGTGTGGGTGCTGGTG;
>clamp_s_c_s_d_s_c_s_a_s:compl:rev CACAGACACCCACACCGTCACCCACACTATCATGTTGACATTCC; dG = -28.2
   529 >s_c_s_e_s_a_s_c_s_d_s_c_s_e_s          GTGTGGGTGCTGGTGTCTGTGTGGGTGACGGTGTGGGTGCTGGTG;
>clamp_s_c_s_d_s_c_s_a_s:compl:rev CACAGACACCCACACCGTCACCCACACTATCATGTTGACATTCC; dG = -28.2
   530 >s_c_s_e_s_b_s_c_s_d_s_c_s_e_s          GTGTGGGTGCTGGTGGCTGTGTGGGTGACGGTGTGGGTGCTGGTG;
>clamp_s_c_s_d_s_c_s_a_s:compl:rev CACAGACACCCACACCGTCACCCACACTATCATGTTGACATTCC; dG = -28.2
   531 >s_d_s_a_s_a_s_c_s_d_s_c_s_e_s          GTGACGGTGTCTGTGTCTGTGTGGGTGACGGTGTGGGTGCTGGTG;
>clamp_s_c_s_d_s_c_s_a_s:compl:rev CACAGACACCCACACCGTCACCCACACTATCATGTTGACATTCC; dG = -28.2
   532 >s_d_s_a_s_b_s_c_s_d_s_c_s_e_s          GTGACGGTGTCTGTGGCTGTGTGGGTGACGGTGTGGGTGCTGGTG;
>clamp_s_c_s_d_s_c_s_a_s:compl:rev CACAGACACCCACACCGTCACCCACACTATCATGTTGACATTCC; dG = -28.2
   533 >s_d_s_b_s_a_s_c_s_d_s_c_s_e_s          GTGACGGTGGCTGTGTCTGTGTGGGTGACGGTGTGGGTGCTGGTG;
>clamp_s_c_s_d_s_c_s_a_s:compl:rev CACAGACACCCACACCGTCACCCACACTATCATGTTGACATTCC; dG = -28.2
   534 >s_d_s_b_s_b_s_c_s_d_s_c_s_e_s          GTGACGGTGGCTGTGGCTGTGTGGGTGACGGTGTGGGTGCTGGTG;
>clamp_s_c_s_d_s_c_s_a_s:compl:rev CACAGACACCCACACCGTCACCCACACTATCATGTTGACATTCC; dG = -28.2
   535 >s_d_s_c_s_a_s_c_s_d_s_c_s_e_s          GTGACGGTGTGGGTGTCTGTGTGGGTGACGGTGTGGGTGCTGGTG;
>clamp_s_c_s_d_s_c_s_a_s:compl:rev CACAGACACCCACACCGTCACCCACACTATCATGTTGACATTCC; dG = -28.2
   536 >s_d_s_c_s_b_s_c_s_d_s_c_s_e_s          GTGACGGTGTGGGTGGCTGTGTGGGTGACGGTGTGGGTGCTGGTG;
>clamp_s_c_s_d_s_c_s_a_s:compl:rev CACAGACACCCACACCGTCACCCACACTATCATGTTGACATTCC; dG = -28.2
   537 >s_d_s_d_s_a_s_c_s_d_s_c_s_e_s          GTGACGGTGACGGTGTCTGTGTGGGTGACGGTGTGGGTGCTGGTG;
>clamp_s_c_s_d_s_c_s_a_s:compl:rev CACAGACACCCACACCGTCACCCACACTATCATGTTGACATTCC; dG = -28.2
   538 >s_d_s_d_s_b_s_c_s_d_s_c_s_e_s          GTGACGGTGACGGTGGCTGTGTGGGTGACGGTGTGGGTGCTGGTG;
>clamp_s_c_s_d_s_c_s_a_s:compl:rev CACAGACACCCACACCGTCACCCACACTATCATGTTGACATTCC; dG = -28.2
```

```
   539 >s_d_s_e_s_a_s_c_s_d_s_c_s_e_s          GTGACGGTGCTGGTGTCTGTGTGGGTGACGGTGTGGGTGCTGGTG;
>clamp_s_c_s_d_s_c_s_a_s:compl:rev CACAGACACCCACACCGTCACCCACACTATCATGTTGACATTCC; dG = -28.2
   540 >s_d_s_e_s_b_s_c_s_d_s_c_s_e_s          GTGACGGTGCTGGTGGCTGTGTGGGTGACGGTGTGGGTGCTGGTG;
>clamp_s_c_s_d_s_c_s_a_s:compl:rev CACAGACACCCACACCGTCACCCACACTATCATGTTGACATTCC; dG = -28.2
   541 >s_e_s_a_s_a_s_c_s_d_s_c_s_e_s          GTGCTGGTGTCTGTGTCTGTGTGGGTGACGGTGTGGGTGCTGGTG;
>clamp_s_c_s_d_s_c_s_a_s:compl:rev CACAGACACCCACACCGTCACCCACACTATCATGTTGACATTCC; dG = -28.2
   542 >s_a_s_c_s_a_s_c_s_d_s_c_s_e_s          GTGTCTGTGTGGGTGTCTGTGTGGGTGACGGTGTGGGTGCTGGTG;
>clamp_s_c_s_d_s_c_s_a_s:compl:rev CACAGACACCCACACCGTCACCCACACTATCATGTTGACATTCC; dG = -28.2
   543 >s_e_s_a_s_b_s_c_s_d_s_c_s_e_s          GTGCTGGTGTCTGTGGCTGTGTGGGTGACGGTGTGGGTGCTGGTG;
>clamp_s_c_s_d_s_c_s_a_s:compl:rev CACAGACACCCACACCGTCACCCACACTATCATGTTGACATTCC; dG = -28.2
   544 >s_e_s_b_s_a_s_c_s_d_s_c_s_e_s          GTGCTGGTGGCTGTGTCTGTGTGGGTGACGGTGTGGGTGCTGGTG;
>clamp_s_c_s_d_s_c_s_a_s:compl:rev CACAGACACCCACACCGTCACCCACACTATCATGTTGACATTCC; dG = -28.2
   545 >s_e_s_b_s_b_s_c_s_d_s_c_s_e_s          GTGCTGGTGGCTGTGGCTGTGTGGGTGACGGTGTGGGTGCTGGTG;
>clamp_s_c_s_d_s_c_s_a_s:compl:rev CACAGACACCCACACCGTCACCCACACTATCATGTTGACATTCC; dG = -28.2
   546 >s_a_s_a_s_b_s_c_s_d_s_c_s_e_s          GTGTCTGTGTCTGTGGCTGTGTGGGTGACGGTGTGGGTGCTGGTG;
>clamp_s_c_s_d_s_c_s_a_s:compl:rev CACAGACACCCACACCGTCACCCACACTATCATGTTGACATTCC; dG = -28.2
   547 >s_e_s_c_s_a_s_c_s_d_s_c_s_e_s          GTGCTGGTGTGGGTGTCTGTGTGGGTGACGGTGTGGGTGCTGGTG;
>clamp_s_c_s_d_s_c_s_a_s:compl:rev CACAGACACCCACACCGTCACCCACACTATCATGTTGACATTCC; dG = -28.2
   548 >s_a_s_c_s_b_s_c_s_d_s_c_s_e_s          GTGTCTGTGTGGGTGGCTGTGTGGGTGACGGTGTGGGTGCTGGTG;
>clamp_s_c_s_d_s_c_s_a_s:compl:rev CACAGACACCCACACCGTCACCCACACTATCATGTTGACATTCC; dG = -28.2
   549 >s_e_s_c_s_b_s_c_s_d_s_c_s_e_s          GTGCTGGTGTGGGTGGCTGTGTGGGTGACGGTGTGGGTGCTGGTG;
>clamp_s_c_s_d_s_c_s_a_s:compl:rev CACAGACACCCACACCGTCACCCACACTATCATGTTGACATTCC; dG = -28.2
   550 >s_e_s_d_s_a_s_c_s_d_s_c_s_e_s          GTGCTGGTGACGGTGTCTGTGTGGGTGACGGTGTGGGTGCTGGTG;
>clamp_s_c_s_d_s_c_s_a_s:compl:rev CACAGACACCCACACCGTCACCCACACTATCATGTTGACATTCC; dG = -28.2
   551 >s_e_s_d_s_b_s_c_s_d_s_c_s_e_s          GTGCTGGTGACGGTGGCTGTGTGGGTGACGGTGTGGGTGCTGGTG;
>clamp_s_c_s_d_s_c_s_a_s:compl:rev CACAGACACCCACACCGTCACCCACACTATCATGTTGACATTCC; dG = -28.2
   552 >s_e_s_e_s_a_s_c_s_d_s_c_s_e_s          GTGCTGGTGCTGGTGTCTGTGTGGGTGACGGTGTGGGTGCTGGTG;
>clamp_s_c_s_d_s_c_s_a_s:compl:rev CACAGACACCCACACCGTCACCCACACTATCATGTTGACATTCC; dG = -28.2
   553 >s_e_s_e_s_b_s_c_s_d_s_c_s_e_s          GTGCTGGTGCTGGTGGCTGTGTGGGTGACGGTGTGGGTGCTGGTG;
>clamp_s_c_s_d_s_c_s_a_s:compl:rev CACAGACACCCACACCGTCACCCACACTATCATGTTGACATTCC; dG = -28.2
   554 >s_a_s_c_s_d_s_c_s_e_s_c_s_a_s          GTGTCTGTGTGGGTGACGGTGTGGGTGCTGGTGTGGGTGTCTGTG;
>clamp_s_c_s_d_s_c_s_a_s:compl:rev CACAGACACCCACACCGTCACCCACACTATCATGTTGACATTCC; dG = -28.2
   555 >s_a_s_d_s_a_s_c_s_d_s_c_s_e_s          GTGTCTGTGACGGTGTCTGTGTGGGTGACGGTGTGGGTGCTGGTG;
>clamp_s_c_s_d_s_c_s_a_s:compl:rev CACAGACACCCACACCGTCACCCACACTATCATGTTGACATTCC; dG = -28.2
   556 >s_a_s_d_s_b_s_c_s_d_s_c_s_e_s          GTGTCTGTGACGGTGGCTGTGTGGGTGACGGTGTGGGTGCTGGTG;
>clamp_s_c_s_d_s_c_s_a_s:compl:rev CACAGACACCCACACCGTCACCCACACTATCATGTTGACATTCC; dG = -28.2
   557 >s_a_s_e_s_a_s_c_s_d_s_c_s_e_s          GTGTCTGTGCTGGTGTCTGTGTGGGTGACGGTGTGGGTGCTGGTG;
>clamp_s_c_s_d_s_c_s_a_s:compl:rev CACAGACACCCACACCGTCACCCACACTATCATGTTGACATTCC; dG = -28.2
   558 >s_a_s_e_s_b_s_c_s_d_s_c_s_e_s          GTGTCTGTGCTGGTGGCTGTGTGGGTGACGGTGTGGGTGCTGGTG;
>clamp_s_c_s_d_s_c_s_a_s:compl:rev CACAGACACCCACACCGTCACCCACACTATCATGTTGACATTCC; dG = -28.2
   559 >s_b_s_a_s_a_s_c_s_d_s_c_s_e_s          GTGGCTGTGTCTGTGTCTGTGTGGGTGACGGTGTGGGTGCTGGTG;
>clamp_s_c_s_d_s_c_s_a_s:compl:rev CACAGACACCCACACCGTCACCCACACTATCATGTTGACATTCC; dG = -28.2
   560 >s_b_s_a_s_b_s_c_s_d_s_c_s_e_s          GTGGCTGTGTCTGTGGCTGTGTGGGTGACGGTGTGGGTGCTGGTG;
>clamp_s_c_s_d_s_c_s_a_s:compl:rev CACAGACACCCACACCGTCACCCACACTATCATGTTGACATTCC; dG = -28.2
   561 >s_b_s_b_s_a_s_c_s_d_s_c_s_e_s          GTGGCTGTGGCTGTGTCTGTGTGGGTGACGGTGTGGGTGCTGGTG;
>clamp_s_c_s_d_s_c_s_a_s:compl:rev CACAGACACCCACACCGTCACCCACACTATCATGTTGACATTCC; dG = -28.2
   562 >s_b_s_b_s_b_s_c_s_d_s_c_s_e_s          GTGGCTGTGGCTGTGGCTGTGTGGGTGACGGTGTGGGTGCTGGTG;
>clamp_s_c_s_d_s_c_s_a_s:compl:rev CACAGACACCCACACCGTCACCCACACTATCATGTTGACATTCC; dG = -28.2
   563 >s_b_s_c_s_a_s_c_s_d_s_c_s_e_s          GTGGCTGTGTGGGTGTCTGTGTGGGTGACGGTGTGGGTGCTGGTG;
>clamp_s_c_s_d_s_c_s_a_s:compl:rev CACAGACACCCACACCGTCACCCACACTATCATGTTGACATTCC; dG = -28.2
   564 >s_b_s_c_s_b_s_c_s_d_s_c_s_e_s          GTGGCTGTGTGGGTGGCTGTGTGGGTGACGGTGTGGGTGCTGGTG;
>clamp_s_c_s_d_s_c_s_a_s:compl:rev CACAGACACCCACACCGTCACCCACACTATCATGTTGACATTCC; dG = -28.2
   565 >s_b_s_c_s_d_s_c_s_e_s_c_s_a_s          GTGGCTGTGTGGGTGACGGTGTGGGTGCTGGTGTGGGTGTCTGTG;
>clamp_s_c_s_d_s_c_s_a_s:compl:rev CACAGACACCCACACCGTCACCCACACTATCATGTTGACATTCC; dG = -28.2
   566 >s_b_s_d_s_a_s_c_s_d_s_c_s_e_s          GTGGCTGTGACGGTGTCTGTGTGGGTGACGGTGTGGGTGCTGGTG;
>clamp_s_c_s_d_s_c_s_a_s:compl:rev CACAGACACCCACACCGTCACCCACACTATCATGTTGACATTCC; dG = -28.2
   567 >s_b_s_d_s_b_s_c_s_d_s_c_s_e_s          GTGGCTGTGACGGTGGCTGTGTGGGTGACGGTGTGGGTGCTGGTG;
>clamp_s_c_s_d_s_c_s_a_s:compl:rev CACAGACACCCACACCGTCACCCACACTATCATGTTGACATTCC; dG = -28.2
   568 >s_b_s_e_s_a_s_c_s_d_s_c_s_e_s          GTGGCTGTGCTGGTGTCTGTGTGGGTGACGGTGTGGGTGCTGGTG;
>clamp_s_c_s_d_s_c_s_a_s:compl:rev CACAGACACCCACACCGTCACCCACACTATCATGTTGACATTCC; dG = -28.2
   569 >s_b_s_e_s_b_s_c_s_d_s_c_s_e_s          GTGGCTGTGCTGGTGGCTGTGTGGGTGACGGTGTGGGTGCTGGTG;
>clamp_s_c_s_d_s_c_s_a_s:compl:rev CACAGACACCCACACCGTCACCCACACTATCATGTTGACATTCC; dG = -28.2
   570 >s_c_s_a_s_a_s_c_s_d_s_c_s_e_s          GTGTGGGTGTCTGTGTCTGTGTGGGTGACGGTGTGGGTGCTGGTG;
>clamp_s_c_s_d_s_c_s_a_s:compl:rev CACAGACACCCACACCGTCACCCACACTATCATGTTGACATTCC; dG = -28.2
   571 >s_a_s_b_s_a_s_c_s_d_s_c_s_e_s          GTGTCTGTGGCTGTGTCTGTGTGGGTGACGGTGTGGGTGCTGGTG;
>clamp_s_c_s_d_s_c_s_a_s:compl:rev CACAGACACCCACACCGTCACCCACACTATCATGTTGACATTCC; dG = -28.2
   572 >s_c_s_a_s_b_s_c_s_d_s_c_s_e_s          GTGTGGGTGTCTGTGGCTGTGTGGGTGACGGTGTGGGTGCTGGTG;
>clamp_s_c_s_d_s_c_s_a_s:compl:rev CACAGACACCCACACCGTCACCCACACTATCATGTTGACATTCC; dG = -28.2
   573 >s_c_s_b_s_a_s_c_s_d_s_c_s_e_s          GTGTGGGTGGCTGTGTCTGTGTGGGTGACGGTGTGGGTGCTGGTG;
>clamp_s_c_s_d_s_c_s_a_s:compl:rev CACAGACACCCACACCGTCACCCACACTATCATGTTGACATTCC; dG = -28.2
   574 >s_c_s_b_s_b_s_c_s_d_s_c_s_e_s          GTGTGGGTGGCTGTGGCTGTGTGGGTGACGGTGTGGGTGCTGGTG;
>clamp_s_c_s_d_s_c_s_a_s:compl:rev CACAGACACCCACACCGTCACCCACACTATCATGTTGACATTCC; dG = -28.2
   575 >s_c_s_c_s_a_s_c_s_d_s_c_s_e_s          GTGTGGGTGTGGGTGTCTGTGTGGGTGACGGTGTGGGTGCTGGTG;
>clamp_s_c_s_d_s_c_s_a_s:compl:rev CACAGACACCCACACCGTCACCCACACTATCATGTTGACATTCC; dG = -28.2
```

```
   576 >s_a_s_b_s_b_s_c_s_d_s_c_s_e_s          GTGTCTGTGGCTGTGGCTGTGTGGGTGACGGTGTGGGTGCTGGTG;
>clamp_s_c_s_d_s_c_s_a_s:compl:rev CACAGACACCCACACCGTCACCCACACTATCATGTTGACATTCC; dG = -28.2
   577 >s_c_s_c_s_b_s_c_s_d_s_c_s_e_s          GTGTGGGTGTGGGTGGCTGTGTGGGTGACGGTGTGGGTGCTGGTG;
>clamp_s_c_s_d_s_c_s_a_s:compl:rev CACAGACACCCACACCGTCACCCACACTATCATGTTGACATTCC; dG = -28.2
   578 >s_c_s_c_s_d_s_c_s_e_s_c_s_a_s          GTGTGGGTGTGGGTGACGGTGTGGGTGCTGGTGTGGGTGTCTGTG;
>clamp_s_c_s_d_s_c_s_a_s:compl:rev CACAGACACCCACACCGTCACCCACACTATCATGTTGACATTCC; dG = -28.1
   579 >s_c_s_c_s_e_s_c_s_d_s_c_s_e_s          GTGTGGGTGTGGGTGCTGGTGTGGGTGACGGTGTGGGTGCTGGTG;
>clamp_s_c_s_d_s_c_s_a_s:compl:rev CACAGACACCCACACCGTCACCCACACTATCATGTTGACATTCC; dG = -28.1
   580 >s_c_s_d_s_d_s_c_s_d_s_c_s_e_s          GTGTGGGTGACGGTGACGGTGTGGGTGACGGTGTGGGTGCTGGTG;
>clamp_s_c_s_d_s_c_s_a_s:compl:rev CACAGACACCCACACCGTCACCCACACTATCATGTTGACATTCC; dG = -28.1
   581 >s_c_s_d_s_d_s_a_s_d_s_c_s_a_s          GTGTGGGTGACGGTGACGGTGTCTGTGACGGTGTGGGTGTCTGTG;
>clamp_s_c_s_d_s_c_s_a_s:compl:rev CACAGACACCCACACCGTCACCCACACTATCATGTTGACATTCC; dG = -28.1
   582 >s_c_s_d_s_e_s_c_s_d_s_c_s_e_s          GTGTGGGTGACGGTGCTGGTGTGGGTGACGGTGTGGGTGCTGGTG;
>clamp_s_c_s_d_s_c_s_a_s:compl:rev CACAGACACCCACACCGTCACCCACACTATCATGTTGACATTCC; dG = -28.1
   583 >s_a_s_b_s_c_s_c_s_d_s_c_s_e_s          GTGTCTGTGGCTGTGTGGGTGTGGGTGACGGTGTGGGTGCTGGTG;
>clamp_s_c_s_d_s_c_s_a_s:compl:rev CACAGACACCCACACCGTCACCCACACTATCATGTTGACATTCC; dG = -28.1
   584 >s_c_s_e_s_c_s_c_s_d_s_c_s_e_s          GTGTGGGTGCTGGTGTGGGTGTGGGTGACGGTGTGGGTGCTGGTG;
>clamp_s_c_s_d_s_c_s_a_s:compl:rev CACAGACACCCACACCGTCACCCACACTATCATGTTGACATTCC; dG = -28.1
   585 >s_c_s_e_s_d_s_c_s_d_s_c_s_e_s          GTGTGGGTGCTGGTGACGGTGTGGGTGACGGTGTGGGTGCTGGTG;
>clamp_s_c_s_d_s_c_s_a_s:compl:rev CACAGACACCCACACCGTCACCCACACTATCATGTTGACATTCC; dG = -28.1
   586 >s_c_s_e_s_e_s_c_s_d_s_c_s_e_s          GTGTGGGTGCTGGTGCTGGTGTGGGTGACGGTGTGGGTGCTGGTG;
>clamp_s_c_s_d_s_c_s_a_s:compl:rev CACAGACACCCACACCGTCACCCACACTATCATGTTGACATTCC; dG = -28.1
   587 >s_d_s_a_s_c_s_c_s_d_s_c_s_e_s          GTGACGGTGTCTGTGTGGGTGTGGGTGACGGTGTGGGTGCTGGTG;
>clamp_s_c_s_d_s_c_s_a_s:compl:rev CACAGACACCCACACCGTCACCCACACTATCATGTTGACATTCC; dG = -28.1
   588 >s_d_s_a_s_d_s_c_s_d_s_c_s_e_s          GTGACGGTGTCTGTGACGGTGTGGGTGACGGTGTGGGTGCTGGTG;
>clamp_s_c_s_d_s_c_s_a_s:compl:rev CACAGACACCCACACCGTCACCCACACTATCATGTTGACATTCC; dG = -28.1
   589 >s_d_s_a_s_e_s_c_s_d_s_c_s_e_s          GTGACGGTGTCTGTGCTGGTGTGGGTGACGGTGTGGGTGCTGGTG;
>clamp_s_c_s_d_s_c_s_a_s:compl:rev CACAGACACCCACACCGTCACCCACACTATCATGTTGACATTCC; dG = -28.1
   590 >s_a_s_b_s_d_s_c_s_d_s_c_s_e_s          GTGTCTGTGGCTGTGACGGTGTGGGTGACGGTGTGGGTGCTGGTG;
>clamp_s_c_s_d_s_c_s_a_s:compl:rev CACAGACACCCACACCGTCACCCACACTATCATGTTGACATTCC; dG = -28.1
   591 >s_d_s_b_s_c_s_c_s_d_s_c_s_e_s          GTGACGGTGGCTGTGTGGGTGTGGGTGACGGTGTGGGTGCTGGTG;
>clamp_s_c_s_d_s_c_s_a_s:compl:rev CACAGACACCCACACCGTCACCCACACTATCATGTTGACATTCC; dG = -28.1
   592 >s_d_s_b_s_d_s_c_s_d_s_c_s_e_s          GTGACGGTGGCTGTGACGGTGTGGGTGACGGTGTGGGTGCTGGTG;
>clamp_s_c_s_d_s_c_s_a_s:compl:rev CACAGACACCCACACCGTCACCCACACTATCATGTTGACATTCC; dG = -28.1
   593 >s_d_s_b_s_e_s_c_s_d_s_c_s_e_s          GTGACGGTGGCTGTGCTGGTGTGGGTGACGGTGTGGGTGCTGGTG;
>clamp_s_c_s_d_s_c_s_a_s:compl:rev CACAGACACCCACACCGTCACCCACACTATCATGTTGACATTCC; dG = -28.1
   594 >s_d_s_c_s_c_s_c_s_d_s_c_s_e_s          GTGACGGTGTGGGTGTGGGTGTGGGTGACGGTGTGGGTGCTGGTG;
>clamp_s_c_s_d_s_c_s_a_s:compl:rev CACAGACACCCACACCGTCACCCACACTATCATGTTGACATTCC; dG = -28.1
   595 >s_d_s_c_s_d_s_c_s_e_s_c_s_a_s          GTGACGGTGTGGGTGACGGTGTGGGTGCTGGTGTGGGTGTCTGTG;
>clamp_s_c_s_d_s_c_s_a_s:compl:rev CACAGACACCCACACCGTCACCCACACTATCATGTTGACATTCC; dG = -28.1
   596 >s_d_s_c_s_e_s_c_s_d_s_c_s_e_s          GTGACGGTGTGGGTGCTGGTGTGGGTGACGGTGTGGGTGCTGGTG;
>clamp_s_c_s_d_s_c_s_a_s:compl:rev CACAGACACCCACACCGTCACCCACACTATCATGTTGACATTCC; dG = -28.1
   597 >s_a_s_b_s_e_s_c_s_d_s_c_s_e_s          GTGTCTGTGGCTGTGCTGGTGTGGGTGACGGTGTGGGTGCTGGTG;
>clamp_s_c_s_d_s_c_s_a_s:compl:rev CACAGACACCCACACCGTCACCCACACTATCATGTTGACATTCC; dG = -28.1
   598 >s_d_s_d_s_c_s_c_s_d_s_c_s_e_s          GTGACGGTGACGGTGTGGGTGTGGGTGACGGTGTGGGTGCTGGTG;
>clamp_s_c_s_d_s_c_s_a_s:compl:rev CACAGACACCCACACCGTCACCCACACTATCATGTTGACATTCC; dG = -28.1
   599 >s_d_s_d_s_d_s_c_s_d_s_c_s_e_s          GTGACGGTGACGGTGACGGTGTGGGTGACGGTGTGGGTGCTGGTG;
>clamp_s_c_s_d_s_c_s_a_s:compl:rev CACAGACACCCACACCGTCACCCACACTATCATGTTGACATTCC; dG = -28.1
   600 >s_d_s_d_s_e_s_c_s_d_s_c_s_e_s          GTGACGGTGACGGTGCTGGTGTGGGTGACGGTGTGGGTGCTGGTG;
>clamp_s_c_s_d_s_c_s_a_s:compl:rev CACAGACACCCACACCGTCACCCACACTATCATGTTGACATTCC; dG = -28.1
   601 >s_d_s_e_s_c_s_c_s_d_s_c_s_e_s          GTGACGGTGCTGGTGTGGGTGTGGGTGACGGTGTGGGTGCTGGTG;
>clamp_s_c_s_d_s_c_s_a_s:compl:rev CACAGACACCCACACCGTCACCCACACTATCATGTTGACATTCC; dG = -28.1
   602 >s_d_s_e_s_d_s_c_s_d_s_c_s_e_s          GTGACGGTGCTGGTGACGGTGTGGGTGACGGTGTGGGTGCTGGTG;
>clamp_s_c_s_d_s_c_s_a_s:compl:rev CACAGACACCCACACCGTCACCCACACTATCATGTTGACATTCC; dG = -28.1
   603 >s_d_s_e_s_e_s_c_s_d_s_c_s_e_s          GTGACGGTGCTGGTGCTGGTGTGGGTGACGGTGTGGGTGCTGGTG;
>clamp_s_c_s_d_s_c_s_a_s:compl:rev CACAGACACCCACACCGTCACCCACACTATCATGTTGACATTCC; dG = -28.1
   604 >s_e_s_a_s_c_s_c_s_d_s_c_s_e_s          GTGCTGGTGTCTGTGTGGGTGTGGGTGACGGTGTGGGTGCTGGTG;
>clamp_s_c_s_d_s_c_s_a_s:compl:rev CACAGACACCCACACCGTCACCCACACTATCATGTTGACATTCC; dG = -28.1
   605 >s_e_s_a_s_d_s_c_s_d_s_c_s_e_s          GTGCTGGTGTCTGTGACGGTGTGGGTGACGGTGTGGGTGCTGGTG;
>clamp_s_c_s_d_s_c_s_a_s:compl:rev CACAGACACCCACACCGTCACCCACACTATCATGTTGACATTCC; dG = -28.1
   606 >s_e_s_a_s_e_s_c_s_d_s_c_s_e_s          GTGCTGGTGTCTGTGCTGGTGTGGGTGACGGTGTGGGTGCTGGTG;
>clamp_s_c_s_d_s_c_s_a_s:compl:rev CACAGACACCCACACCGTCACCCACACTATCATGTTGACATTCC; dG = -28.1
   607 >s_e_s_b_s_c_s_c_s_d_s_c_s_e_s          GTGCTGGTGGCTGTGTGGGTGTGGGTGACGGTGTGGGTGCTGGTG;
>clamp_s_c_s_d_s_c_s_a_s:compl:rev CACAGACACCCACACCGTCACCCACACTATCATGTTGACATTCC; dG = -28.1
   608 >s_e_s_b_s_d_s_c_s_d_s_c_s_e_s          GTGCTGGTGGCTGTGACGGTGTGGGTGACGGTGTGGGTGCTGGTG;
>clamp_s_c_s_d_s_c_s_a_s:compl:rev CACAGACACCCACACCGTCACCCACACTATCATGTTGACATTCC; dG = -28.1
   609 >s_e_s_b_s_e_s_c_s_d_s_c_s_e_s          GTGCTGGTGGCTGTGCTGGTGTGGGTGACGGTGTGGGTGCTGGTG;
>clamp_s_c_s_d_s_c_s_a_s:compl:rev CACAGACACCCACACCGTCACCCACACTATCATGTTGACATTCC; dG = -28.1
   610 >s_e_s_c_s_c_s_c_s_d_s_c_s_e_s          GTGCTGGTGTGGGTGTGGGTGTGGGTGACGGTGTGGGTGCTGGTG;
>clamp_s_c_s_d_s_c_s_a_s:compl:rev CACAGACACCCACACCGTCACCCACACTATCATGTTGACATTCC; dG = -28.1
   611 >s_e_s_c_s_d_s_c_s_e_s_c_s_a_s          GTGCTGGTGTGGGTGACGGTGTGGGTGCTGGTGTGGGTGTCTGTG;
>clamp_s_c_s_d_s_c_s_a_s:compl:rev CACAGACACCCACACCGTCACCCACACTATCATGTTGACATTCC; dG = -28.1
   612 >s_e_s_c_s_e_s_c_s_d_s_c_s_e_s          GTGCTGGTGTGGGTGCTGGTGTGGGTGACGGTGTGGGTGCTGGTG;
>clamp_s_c_s_d_s_c_s_a_s:compl:rev CACAGACACCCACACCGTCACCCACACTATCATGTTGACATTCC; dG = -28.1
```

```
   613 >s_e_s_d_s_c_s_c_s_d_s_c_s_e_s          GTGCTGGTGACGGTGTGGGTGTGGGTGACGGTGTGGGTGCTGGTG;
>clamp_s_c_s_d_s_c_s_a_s:compl:rev CACAGACACCCACACCGTCACCCACACTATCATGTTGACATTCC; dG = -28.1
   614 >s_e_s_d_s_d_s_c_s_d_s_c_s_e_s          GTGCTGGTGACGGTGACGGTGTGGGTGACGGTGTGGGTGCTGGTG;
>clamp_s_c_s_d_s_c_s_a_s:compl:rev CACAGACACCCACACCGTCACCCACACTATCATGTTGACATTCC; dG = -28.1
   615 >s_e_s_d_s_e_s_c_s_d_s_c_s_e_s          GTGCTGGTGACGGTGCTGGTGTGGGTGACGGTGTGGGTGCTGGTG;
>clamp_s_c_s_d_s_c_s_a_s:compl:rev CACAGACACCCACACCGTCACCCACACTATCATGTTGACATTCC; dG = -28.1
   616 >s_a_s_c_s_c_s_c_s_d_s_c_s_e_s          GTGTCTGTGTGGGTGTGGGTGTGGGTGACGGTGTGGGTGCTGGTG;
>clamp_s_c_s_d_s_c_s_a_s:compl:rev CACAGACACCCACACCGTCACCCACACTATCATGTTGACATTCC; dG = -28.1
   617 >s_e_s_e_s_c_s_c_s_d_s_c_s_e_s          GTGCTGGTGCTGGTGTGGGTGTGGGTGACGGTGTGGGTGCTGGTG;
>clamp_s_c_s_d_s_c_s_a_s:compl:rev CACAGACACCCACACCGTCACCCACACTATCATGTTGACATTCC; dG = -28.1
   618 >s_e_s_e_s_d_s_c_s_d_s_c_s_e_s          GTGCTGGTGCTGGTGACGGTGTGGGTGACGGTGTGGGTGCTGGTG;
>clamp_s_c_s_d_s_c_s_a_s:compl:rev CACAGACACCCACACCGTCACCCACACTATCATGTTGACATTCC; dG = -28.1
   619 >s_e_s_e_s_e_s_c_s_d_s_c_s_e_s          GTGCTGGTGCTGGTGCTGGTGTGGGTGACGGTGTGGGTGCTGGTG;
>clamp_s_c_s_d_s_c_s_a_s:compl:rev CACAGACACCCACACCGTCACCCACACTATCATGTTGACATTCC; dG = -28.1
   620 >s_a_s_c_s_e_s_c_s_d_s_c_s_e_s          GTGTCTGTGTGGGTGCTGGTGTGGGTGACGGTGTGGGTGCTGGTG;
>clamp_s_c_s_d_s_c_s_a_s:compl:rev CACAGACACCCACACCGTCACCCACACTATCATGTTGACATTCC; dG = -28.1
   621 >s_a_s_d_s_c_s_c_s_d_s_c_s_e_s          GTGTCTGTGACGGTGTGGGTGTGGGTGACGGTGTGGGTGCTGGTG;
>clamp_s_c_s_d_s_c_s_a_s:compl:rev CACAGACACCCACACCGTCACCCACACTATCATGTTGACATTCC; dG = -28.1
   622 >s_a_s_d_s_d_s_c_s_d_s_c_s_e_s          GTGTCTGTGACGGTGACGGTGTGGGTGACGGTGTGGGTGCTGGTG;
>clamp_s_c_s_d_s_c_s_a_s:compl:rev CACAGACACCCACACCGTCACCCACACTATCATGTTGACATTCC; dG = -28.1
   623 >s_a_s_d_s_e_s_c_s_d_s_c_s_e_s          GTGTCTGTGACGGTGCTGGTGTGGGTGACGGTGTGGGTGCTGGTG;
>clamp_s_c_s_d_s_c_s_a_s:compl:rev CACAGACACCCACACCGTCACCCACACTATCATGTTGACATTCC; dG = -28.1
   624 >s_a_s_a_s_c_s_c_s_d_s_c_s_e_s          GTGTCTGTGTCTGTGTGGGTGTGGGTGACGGTGTGGGTGCTGGTG;
>clamp_s_c_s_d_s_c_s_a_s:compl:rev CACAGACACCCACACCGTCACCCACACTATCATGTTGACATTCC; dG = -28.1
   625 >s_a_s_e_s_c_s_c_s_d_s_c_s_e_s          GTGTCTGTGCTGGTGTGGGTGTGGGTGACGGTGTGGGTGCTGGTG;
>clamp_s_c_s_d_s_c_s_a_s:compl:rev CACAGACACCCACACCGTCACCCACACTATCATGTTGACATTCC; dG = -28.1
   626 >s_a_s_e_s_d_s_c_s_d_s_c_s_e_s          GTGTCTGTGCTGGTGACGGTGTGGGTGACGGTGTGGGTGCTGGTG;
>clamp_s_c_s_d_s_c_s_a_s:compl:rev CACAGACACCCACACCGTCACCCACACTATCATGTTGACATTCC; dG = -28.1
   627 >s_a_s_e_s_e_s_c_s_d_s_c_s_e_s          GTGTCTGTGCTGGTGCTGGTGTGGGTGACGGTGTGGGTGCTGGTG;
>clamp_s_c_s_d_s_c_s_a_s:compl:rev CACAGACACCCACACCGTCACCCACACTATCATGTTGACATTCC; dG = -28.1
   628 >s_b_s_a_s_c_s_c_s_d_s_c_s_e_s          GTGGCTGTGTCTGTGTGGGTGTGGGTGACGGTGTGGGTGCTGGTG;
>clamp_s_c_s_d_s_c_s_a_s:compl:rev CACAGACACCCACACCGTCACCCACACTATCATGTTGACATTCC; dG = -28.1
   629 >s_b_s_a_s_d_s_c_s_d_s_c_s_e_s          GTGGCTGTGTCTGTGACGGTGTGGGTGACGGTGTGGGTGCTGGTG;
>clamp_s_c_s_d_s_c_s_a_s:compl:rev CACAGACACCCACACCGTCACCCACACTATCATGTTGACATTCC; dG = -28.1
   630 >s_b_s_a_s_e_s_c_s_d_s_c_s_e_s          GTGGCTGTGTCTGTGCTGGTGTGGGTGACGGTGTGGGTGCTGGTG;
>clamp_s_c_s_d_s_c_s_a_s:compl:rev CACAGACACCCACACCGTCACCCACACTATCATGTTGACATTCC; dG = -28.1
   631 >s_a_s_a_s_d_s_c_s_d_s_c_s_e_s          GTGTCTGTGTCTGTGACGGTGTGGGTGACGGTGTGGGTGCTGGTG;
>clamp_s_c_s_d_s_c_s_a_s:compl:rev CACAGACACCCACACCGTCACCCACACTATCATGTTGACATTCC; dG = -28.1
   632 >s_b_s_b_s_c_s_c_s_d_s_c_s_e_s          GTGGCTGTGGCTGTGTGGGTGTGGGTGACGGTGTGGGTGCTGGTG;
>clamp_s_c_s_d_s_c_s_a_s:compl:rev CACAGACACCCACACCGTCACCCACACTATCATGTTGACATTCC; dG = -28.1
   633 >s_b_s_b_s_d_s_c_s_d_s_c_s_e_s          GTGGCTGTGGCTGTGACGGTGTGGGTGACGGTGTGGGTGCTGGTG;
>clamp_s_c_s_d_s_c_s_a_s:compl:rev CACAGACACCCACACCGTCACCCACACTATCATGTTGACATTCC; dG = -28.1
   634 >s_b_s_b_s_e_s_c_s_d_s_c_s_e_s          GTGGCTGTGGCTGTGCTGGTGTGGGTGACGGTGTGGGTGCTGGTG;
>clamp_s_c_s_d_s_c_s_a_s:compl:rev CACAGACACCCACACCGTCACCCACACTATCATGTTGACATTCC; dG = -28.1
   635 >s_b_s_c_s_c_s_c_s_d_s_c_s_e_s          GTGGCTGTGTGGGTGTGGGTGTGGGTGACGGTGTGGGTGCTGGTG;
>clamp_s_c_s_d_s_c_s_a_s:compl:rev CACAGACACCCACACCGTCACCCACACTATCATGTTGACATTCC; dG = -28.1
   636 >s_b_s_c_s_e_s_c_s_d_s_c_s_e_s          GTGGCTGTGTGGGTGCTGGTGTGGGTGACGGTGTGGGTGCTGGTG;
>clamp_s_c_s_d_s_c_s_a_s:compl:rev CACAGACACCCACACCGTCACCCACACTATCATGTTGACATTCC; dG = -28.1
   637 >s_a_s_a_s_e_s_c_s_d_s_c_s_e_s          GTGTCTGTGTCTGTGCTGGTGTGGGTGACGGTGTGGGTGCTGGTG;
>clamp_s_c_s_d_s_c_s_a_s:compl:rev CACAGACACCCACACCGTCACCCACACTATCATGTTGACATTCC; dG = -28.1
   638 >s_b_s_d_s_c_s_c_s_d_s_c_s_e_s          GTGGCTGTGACGGTGTGGGTGTGGGTGACGGTGTGGGTGCTGGTG;
>clamp_s_c_s_d_s_c_s_a_s:compl:rev CACAGACACCCACACCGTCACCCACACTATCATGTTGACATTCC; dG = -28.1
   639 >s_b_s_d_s_d_s_c_s_d_s_c_s_e_s          GTGGCTGTGACGGTGACGGTGTGGGTGACGGTGTGGGTGCTGGTG;
>clamp_s_c_s_d_s_c_s_a_s:compl:rev CACAGACACCCACACCGTCACCCACACTATCATGTTGACATTCC; dG = -28.1
   640 >s_b_s_d_s_e_s_c_s_d_s_c_s_e_s          GTGGCTGTGACGGTGCTGGTGTGGGTGACGGTGTGGGTGCTGGTG;
>clamp_s_c_s_d_s_c_s_a_s:compl:rev CACAGACACCCACACCGTCACCCACACTATCATGTTGACATTCC; dG = -28.1
   641 >s_b_s_e_s_c_s_c_s_d_s_c_s_e_s          GTGGCTGTGCTGGTGTGGGTGTGGGTGACGGTGTGGGTGCTGGTG;
>clamp_s_c_s_d_s_c_s_a_s:compl:rev CACAGACACCCACACCGTCACCCACACTATCATGTTGACATTCC; dG = -28.1
   642 >s_b_s_e_s_d_s_c_s_d_s_c_s_e_s          GTGGCTGTGCTGGTGACGGTGTGGGTGACGGTGTGGGTGCTGGTG;
>clamp_s_c_s_d_s_c_s_a_s:compl:rev CACAGACACCCACACCGTCACCCACACTATCATGTTGACATTCC; dG = -28.1
   643 >s_b_s_e_s_e_s_c_s_d_s_c_s_e_s          GTGGCTGTGCTGGTGCTGGTGTGGGTGACGGTGTGGGTGCTGGTG;
>clamp_s_c_s_d_s_c_s_a_s:compl:rev CACAGACACCCACACCGTCACCCACACTATCATGTTGACATTCC; dG = -28.1
   644 >s_c_s_a_s_c_s_c_s_d_s_c_s_e_s          GTGTGGGTGTCTGTGTGGGTGTGGGTGACGGTGTGGGTGCTGGTG;
>clamp_s_c_s_d_s_c_s_a_s:compl:rev CACAGACACCCACACCGTCACCCACACTATCATGTTGACATTCC; dG = -28.1
   645 >s_c_s_a_s_d_s_c_s_d_s_c_s_e_s          GTGTGGGTGTCTGTGACGGTGTGGGTGACGGTGTGGGTGCTGGTG;
>clamp_s_c_s_d_s_c_s_a_s:compl:rev CACAGACACCCACACCGTCACCCACACTATCATGTTGACATTCC; dG = -28.1
   646 >s_c_s_a_s_e_s_c_s_d_s_c_s_e_s          GTGTGGGTGTCTGTGCTGGTGTGGGTGACGGTGTGGGTGCTGGTG;
>clamp_s_c_s_d_s_c_s_a_s:compl:rev CACAGACACCCACACCGTCACCCACACTATCATGTTGACATTCC; dG = -28.1
   647 >s_c_s_b_s_c_s_c_s_d_s_c_s_e_s          GTGTGGGTGGCTGTGTGGGTGTGGGTGACGGTGTGGGTGCTGGTG;
>clamp_s_c_s_d_s_c_s_a_s:compl:rev CACAGACACCCACACCGTCACCCACACTATCATGTTGACATTCC; dG = -28.1
   648 >s_c_s_b_s_d_s_c_s_d_s_c_s_e_s          GTGTGGGTGGCTGTGACGGTGTGGGTGACGGTGTGGGTGCTGGTG;
>clamp_s_c_s_d_s_c_s_a_s:compl:rev CACAGACACCCACACCGTCACCCACACTATCATGTTGACATTCC; dG = -28.1
   649 >s_c_s_b_s_e_s_c_s_d_s_c_s_e_s          GTGTGGGTGGCTGTGCTGGTGTGGGTGACGGTGTGGGTGCTGGTG;
>clamp_s_c_s_d_s_c_s_a_s:compl:rev CACAGACACCCACACCGTCACCCACACTATCATGTTGACATTCC; dG = -28.1
```

```
   650 >s_c_s_c_s_c_s_c_s_d_s_c_s_e_s          GTGTGGGTGTGGGTGTGGGTGTGGGTGACGGTGTGGGTGCTGGTG;
>clamp_s_c_s_d_s_c_s_a_s:compl:rev CACAGACACCCACACCGTCACCCACACTATCATGTTGACATTCC; dG = -28.1
   651 >s_c_s_c_s_c_s_c_s_a_s_c_s_a_s          GTGTGGGTGTGGGTGTGGGTGTGGGTGTCTGTGTGGGTGTCTGTG;
>clamp_s_c_s_d_s_c_s_a_s:compl:rev CACAGACACCCACACCGTCACCCACACTATCATGTTGACATTCC; dG = -28.0
   652 >s_c_s_d_s_e_s_a_s_d_s_c_s_a_s          GTGTGGGTGACGGTGCTGGTGTCTGTGACGGTGTGGGTGTCTGTG;
>clamp_s_c_s_d_s_c_s_a_s:compl:rev CACAGACACCCACACCGTCACCCACACTATCATGTTGACATTCC; dG = -28.0
   653 >s_d_s_c_s_c_s_c_s_a_s_c_s_a_s          GTGACGGTGTGGGTGTGGGTGTGGGTGTCTGTGTGGGTGTCTGTG;
>clamp_s_c_s_d_s_c_s_a_s:compl:rev CACAGACACCCACACCGTCACCCACACTATCATGTTGACATTCC; dG = -28.0
   654 >s_d_s_d_s_d_s_c_s_a_s_c_s_a_s          GTGACGGTGACGGTGACGGTGTGGGTGTCTGTGTGGGTGTCTGTG;
>clamp_s_c_s_d_s_c_s_a_s:compl:rev CACAGACACCCACACCGTCACCCACACTATCATGTTGACATTCC; dG = -28.0
   655 >s_e_s_c_s_c_s_c_s_a_s_c_s_a_s          GTGCTGGTGTGGGTGTGGGTGTGGGTGTCTGTGTGGGTGTCTGTG;
>clamp_s_c_s_d_s_c_s_a_s:compl:rev CACAGACACCCACACCGTCACCCACACTATCATGTTGACATTCC; dG = -28.0
   656 >s_e_s_d_s_d_s_c_s_a_s_c_s_a_s          GTGCTGGTGACGGTGACGGTGTGGGTGTCTGTGTGGGTGTCTGTG;
>clamp_s_c_s_d_s_c_s_a_s:compl:rev CACAGACACCCACACCGTCACCCACACTATCATGTTGACATTCC; dG = -28.0
   657 >s_a_s_c_s_c_s_c_s_a_s_c_s_a_s          GTGTCTGTGTGGGTGTGGGTGTGGGTGTCTGTGTGGGTGTCTGTG;
>clamp_s_c_s_d_s_c_s_a_s:compl:rev CACAGACACCCACACCGTCACCCACACTATCATGTTGACATTCC; dG = -28.0
   658 >s_a_s_d_s_d_s_c_s_a_s_c_s_a_s          GTGTCTGTGACGGTGACGGTGTGGGTGTCTGTGTGGGTGTCTGTG;
>clamp_s_c_s_d_s_c_s_a_s:compl:rev CACAGACACCCACACCGTCACCCACACTATCATGTTGACATTCC; dG = -28.0
   659 >s_b_s_c_s_c_s_c_s_a_s_c_s_a_s          GTGGCTGTGTGGGTGTGGGTGTGGGTGTCTGTGTGGGTGTCTGTG;
>clamp_s_c_s_d_s_c_s_a_s:compl:rev CACAGACACCCACACCGTCACCCACACTATCATGTTGACATTCC; dG = -28.0
   660 >s_b_s_d_s_d_s_c_s_a_s_c_s_a_s          GTGGCTGTGACGGTGACGGTGTGGGTGTCTGTGTGGGTGTCTGTG;
>clamp_s_c_s_d_s_c_s_a_s:compl:rev CACAGACACCCACACCGTCACCCACACTATCATGTTGACATTCC; dG = -28.0
   661 >s_d_s_e_s_d_s_c_s_a_s_c_s_a_s          GTGACGGTGCTGGTGACGGTGTGGGTGTCTGTGTGGGTGTCTGTG;
>clamp_s_c_s_d_s_c_s_a_s:compl:rev CACAGACACCCACACCGTCACCCACACTATCATGTTGACATTCC; dG = -27.9
   662 >s_e_s_e_s_d_s_c_s_a_s_c_s_a_s          GTGCTGGTGCTGGTGACGGTGTGGGTGTCTGTGTGGGTGTCTGTG;
>clamp_s_c_s_d_s_c_s_a_s:compl:rev CACAGACACCCACACCGTCACCCACACTATCATGTTGACATTCC; dG = -27.9
   663 >s_a_s_e_s_d_s_c_s_a_s_c_s_a_s          GTGTCTGTGCTGGTGACGGTGTGGGTGTCTGTGTGGGTGTCTGTG;
>clamp_s_c_s_d_s_c_s_a_s:compl:rev CACAGACACCCACACCGTCACCCACACTATCATGTTGACATTCC; dG = -27.9
   664 >s_b_s_e_s_d_s_c_s_a_s_c_s_a_s          GTGGCTGTGCTGGTGACGGTGTGGGTGTCTGTGTGGGTGTCTGTG;
>clamp_s_c_s_d_s_c_s_a_s:compl:rev CACAGACACCCACACCGTCACCCACACTATCATGTTGACATTCC; dG = -27.9
   665 >s_a_s_a_s_a_s_c_s_c_s_c_s_a_s          GTGTCTGTGTCTGTGTCTGTGTGGGTGTGGGTGTGGGTGTCTGTG;
>clamp_s_c_s_d_s_c_s_a_s:compl:rev CACAGACACCCACACCGTCACCCACACTATCATGTTGACATTCC; dG = -27.8
   666 >s_a_s_b_s_b_s_c_s_d_s_d_s_a_s          GTGTCTGTGGCTGTGGCTGTGTGGGTGACGGTGACGGTGTCTGTG;
>clamp_s_c_s_d_s_c_s_a_s:compl:rev CACAGACACCCACACCGTCACCCACACTATCATGTTGACATTCC; dG = -27.8
   667 >s_c_s_d_s_a_s_c_s_d_s_d_s_a_s          GTGTGGGTGACGGTGTCTGTGTGGGTGACGGTGACGGTGTCTGTG;
>clamp_s_c_s_d_s_c_s_a_s:compl:rev CACAGACACCCACACCGTCACCCACACTATCATGTTGACATTCC; dG = -27.8
   668 >s_c_s_d_s_a_s_c_s_c_s_c_s_a_s          GTGTGGGTGACGGTGTCTGTGTGGGTGTGGGTGTGGGTGTCTGTG;
>clamp_s_c_s_d_s_c_s_a_s:compl:rev CACAGACACCCACACCGTCACCCACACTATCATGTTGACATTCC; dG = -27.8
   669 >s_c_s_d_s_a_s_d_s_d_s_c_s_a_s          GTGTGGGTGACGGTGTCTGTGACGGTGACGGTGTGGGTGTCTGTG;
>clamp_s_c_s_d_s_c_s_a_s:compl:rev CACAGACACCCACACCGTCACCCACACTATCATGTTGACATTCC; dG = -27.8
   670 >s_c_s_d_s_b_s_c_s_d_s_d_s_a_s          GTGTGGGTGACGGTGGCTGTGTGGGTGACGGTGACGGTGTCTGTG;
>clamp_s_c_s_d_s_c_s_a_s:compl:rev CACAGACACCCACACCGTCACCCACACTATCATGTTGACATTCC; dG = -27.8
   671 >s_a_s_b_s_b_s_c_s_c_s_c_s_a_s          GTGTCTGTGGCTGTGGCTGTGTGGGTGTGGGTGTGGGTGTCTGTG;
>clamp_s_c_s_d_s_c_s_a_s:compl:rev CACAGACACCCACACCGTCACCCACACTATCATGTTGACATTCC; dG = -27.8
   672 >s_c_s_d_s_b_s_c_s_c_s_c_s_a_s          GTGTGGGTGACGGTGGCTGTGTGGGTGTGGGTGTGGGTGTCTGTG;
>clamp_s_c_s_d_s_c_s_a_s:compl:rev CACAGACACCCACACCGTCACCCACACTATCATGTTGACATTCC; dG = -27.8
   673 >s_c_s_d_s_b_s_d_s_d_s_c_s_a_s          GTGTGGGTGACGGTGGCTGTGACGGTGACGGTGTGGGTGTCTGTG;
>clamp_s_c_s_d_s_c_s_a_s:compl:rev CACAGACACCCACACCGTCACCCACACTATCATGTTGACATTCC; dG = -27.8
   674 >s_a_s_b_s_b_s_d_s_d_s_c_s_a_s          GTGTCTGTGGCTGTGGCTGTGACGGTGACGGTGTGGGTGTCTGTG;
>clamp_s_c_s_d_s_c_s_a_s:compl:rev CACAGACACCCACACCGTCACCCACACTATCATGTTGACATTCC; dG = -27.8
   675 >s_c_s_e_s_a_s_c_s_d_s_d_s_a_s          GTGTGGGTGCTGGTGTCTGTGTGGGTGACGGTGACGGTGTCTGTG;
>clamp_s_c_s_d_s_c_s_a_s:compl:rev CACAGACACCCACACCGTCACCCACACTATCATGTTGACATTCC; dG = -27.8
   676 >s_c_s_e_s_a_s_c_s_c_s_c_s_a_s          GTGTGGGTGCTGGTGTCTGTGTGGGTGTGGGTGTGGGTGTCTGTG;
>clamp_s_c_s_d_s_c_s_a_s:compl:rev CACAGACACCCACACCGTCACCCACACTATCATGTTGACATTCC; dG = -27.8
   677 >s_c_s_e_s_a_s_d_s_d_s_c_s_a_s          GTGTGGGTGCTGGTGTCTGTGACGGTGACGGTGTGGGTGTCTGTG;
>clamp_s_c_s_d_s_c_s_a_s:compl:rev CACAGACACCCACACCGTCACCCACACTATCATGTTGACATTCC; dG = -27.8
   678 >s_c_s_e_s_b_s_c_s_d_s_d_s_a_s          GTGTGGGTGCTGGTGGCTGTGTGGGTGACGGTGACGGTGTCTGTG;
>clamp_s_c_s_d_s_c_s_a_s:compl:rev CACAGACACCCACACCGTCACCCACACTATCATGTTGACATTCC; dG = -27.8
   679 >s_c_s_e_s_b_s_c_s_c_s_c_s_a_s          GTGTGGGTGCTGGTGGCTGTGTGGGTGTGGGTGTGGGTGTCTGTG;
>clamp_s_c_s_d_s_c_s_a_s:compl:rev CACAGACACCCACACCGTCACCCACACTATCATGTTGACATTCC; dG = -27.8
   680 >s_c_s_e_s_b_s_d_s_d_s_c_s_a_s          GTGTGGGTGCTGGTGGCTGTGACGGTGACGGTGTGGGTGTCTGTG;
>clamp_s_c_s_d_s_c_s_a_s:compl:rev CACAGACACCCACACCGTCACCCACACTATCATGTTGACATTCC; dG = -27.8
   681 >s_d_s_a_s_a_s_c_s_d_s_d_s_a_s          GTGACGGTGTCTGTGTCTGTGTGGGTGACGGTGACGGTGTCTGTG;
>clamp_s_c_s_d_s_c_s_a_s:compl:rev CACAGACACCCACACCGTCACCCACACTATCATGTTGACATTCC; dG = -27.8
   682 >s_d_s_a_s_a_s_c_s_c_s_c_s_a_s          GTGACGGTGTCTGTGTCTGTGTGGGTGTGGGTGTGGGTGTCTGTG;
>clamp_s_c_s_d_s_c_s_a_s:compl:rev CACAGACACCCACACCGTCACCCACACTATCATGTTGACATTCC; dG = -27.8
   683 >s_d_s_a_s_a_s_d_s_d_s_c_s_a_s          GTGACGGTGTCTGTGTCTGTGACGGTGACGGTGTGGGTGTCTGTG;
>clamp_s_c_s_d_s_c_s_a_s:compl:rev CACAGACACCCACACCGTCACCCACACTATCATGTTGACATTCC; dG = -27.8
   684 >s_d_s_a_s_b_s_c_s_d_s_d_s_a_s          GTGACGGTGTCTGTGGCTGTGTGGGTGACGGTGACGGTGTCTGTG;
>clamp_s_c_s_d_s_c_s_a_s:compl:rev CACAGACACCCACACCGTCACCCACACTATCATGTTGACATTCC; dG = -27.8
   685 >s_d_s_a_s_b_s_c_s_c_s_c_s_a_s          GTGACGGTGTCTGTGGCTGTGTGGGTGTGGGTGTGGGTGTCTGTG;
>clamp_s_c_s_d_s_c_s_a_s:compl:rev CACAGACACCCACACCGTCACCCACACTATCATGTTGACATTCC; dG = -27.8
   686 >s_d_s_a_s_b_s_d_s_d_s_c_s_a_s          GTGACGGTGTCTGTGGCTGTGACGGTGACGGTGTGGGTGTCTGTG;
>clamp_s_c_s_d_s_c_s_a_s:compl:rev CACAGACACCCACACCGTCACCCACACTATCATGTTGACATTCC; dG = -27.8
```

687 >s_d_s_b_s_a_s_c_s_d_s_d_s_a_s GTGACGGTGGCTGTGTCTGTGTGGGTGACGGTGACGGTGTCTGTG;
>clamp_s_c_s_d_s_c_s_a_s:compl:rev CACAGACACCCACACCGTCACCCACACTATCATGTTGACATTCC; dG = -27.8
688 >s_d_s_b_s_a_s_c_s_c_s_c_s_a_s GTGACGGTGGCTGTGTCTGTGTGGGTGTGGGTGTGGGTGTCTGTG;
>clamp_s_c_s_d_s_c_s_a_s:compl:rev CACAGACACCCACACCGTCACCCACACTATCATGTTGACATTCC; dG = -27.8
689 >s_d_s_b_s_a_s_d_s_d_s_c_s_a_s GTGACGGTGGCTGTGTCTGTGACGGTGACGGTGTGGGTGTCTGTG;
>clamp_s_c_s_d_s_c_s_a_s:compl:rev CACAGACACCCACACCGTCACCCACACTATCATGTTGACATTCC; dG = -27.8
690 >s_d_s_b_s_b_s_c_s_d_s_d_s_a_s GTGACGGTGGCTGTGGCTGTGTGGGTGACGGTGACGGTGTCTGTG;
>clamp_s_c_s_d_s_c_s_a_s:compl:rev CACAGACACCCACACCGTCACCCACACTATCATGTTGACATTCC; dG = -27.8
691 >s_d_s_b_s_b_s_c_s_c_s_c_s_a_s GTGACGGTGGCTGTGGCTGTGTGGGTGTGGGTGTGGGTGTCTGTG;
>clamp_s_c_s_d_s_c_s_a_s:compl:rev CACAGACACCCACACCGTCACCCACACTATCATGTTGACATTCC; dG = -27.8
692 >s_d_s_b_s_b_s_d_s_d_s_c_s_a_s GTGACGGTGGCTGTGGCTGTGACGGTGACGGTGTGGGTGTCTGTG;
>clamp_s_c_s_d_s_c_s_a_s:compl:rev CACAGACACCCACACCGTCACCCACACTATCATGTTGACATTCC; dG = -27.8
693 >s_d_s_c_s_a_s_c_s_d_s_d_s_a_s GTGACGGTGTGGGTGTCTGTGTGGGTGACGGTGACGGTGTCTGTG;
>clamp_s_c_s_d_s_c_s_a_s:compl:rev CACAGACACCCACACCGTCACCCACACTATCATGTTGACATTCC; dG = -27.8
694 >s_d_s_c_s_a_s_c_s_c_s_c_s_a_s GTGACGGTGTGGGTGTCTGTGTGGGTGTGGGTGTGGGTGTCTGTG;
>clamp_s_c_s_d_s_c_s_a_s:compl:rev CACAGACACCCACACCGTCACCCACACTATCATGTTGACATTCC; dG = -27.8
695 >s_d_s_c_s_a_s_d_s_d_s_c_s_a_s GTGACGGTGTGGGTGTCTGTGACGGTGACGGTGTGGGTGTCTGTG;
>clamp_s_c_s_d_s_c_s_a_s:compl:rev CACAGACACCCACACCGTCACCCACACTATCATGTTGACATTCC; dG = -27.8
696 >s_d_s_c_s_b_s_c_s_d_s_d_s_a_s GTGACGGTGTGGGTGGCTGTGTGGGTGACGGTGACGGTGTCTGTG;
>clamp_s_c_s_d_s_c_s_a_s:compl:rev CACAGACACCCACACCGTCACCCACACTATCATGTTGACATTCC; dG = -27.8
697 >s_d_s_c_s_b_s_c_s_c_s_c_s_a_s GTGACGGTGTGGGTGGCTGTGTGGGTGTGGGTGTGGGTGTCTGTG;
>clamp_s_c_s_d_s_c_s_a_s:compl:rev CACAGACACCCACACCGTCACCCACACTATCATGTTGACATTCC; dG = -27.8
698 >s_d_s_c_s_b_s_d_s_d_s_c_s_a_s GTGACGGTGTGGGTGGCTGTGACGGTGACGGTGTGGGTGTCTGTG;
>clamp_s_c_s_d_s_c_s_a_s:compl:rev CACAGACACCCACACCGTCACCCACACTATCATGTTGACATTCC; dG = -27.8
699 >s_a_s_a_s_a_s_d_s_d_s_c_s_a_s GTGTCTGTGTCTGTGTCTGTGACGGTGACGGTGTGGGTGTCTGTG;
>clamp_s_c_s_d_s_c_s_a_s:compl:rev CACAGACACCCACACCGTCACCCACACTATCATGTTGACATTCC; dG = -27.8
700 >s_d_s_d_s_a_s_c_s_d_s_d_s_a_s GTGACGGTGACGGTGTCTGTGTGGGTGACGGTGACGGTGTCTGTG;
>clamp_s_c_s_d_s_c_s_a_s:compl:rev CACAGACACCCACACCGTCACCCACACTATCATGTTGACATTCC; dG = -27.8
701 >s_d_s_d_s_a_s_c_s_c_s_c_s_a_s GTGACGGTGACGGTGTCTGTGTGGGTGTGGGTGTGGGTGTCTGTG;
>clamp_s_c_s_d_s_c_s_a_s:compl:rev CACAGACACCCACACCGTCACCCACACTATCATGTTGACATTCC; dG = -27.8
702 >s_d_s_d_s_a_s_d_s_d_s_c_s_a_s GTGACGGTGACGGTGTCTGTGACGGTGACGGTGTGGGTGTCTGTG;
>clamp_s_c_s_d_s_c_s_a_s:compl:rev CACAGACACCCACACCGTCACCCACACTATCATGTTGACATTCC; dG = -27.8
703 >s_d_s_d_s_b_s_c_s_d_s_d_s_a_s GTGACGGTGACGGTGGCTGTGTGGGTGACGGTGACGGTGTCTGTG;
>clamp_s_c_s_d_s_c_s_a_s:compl:rev CACAGACACCCACACCGTCACCCACACTATCATGTTGACATTCC; dG = -27.8
704 >s_d_s_d_s_b_s_c_s_c_s_c_s_a_s GTGACGGTGACGGTGGCTGTGTGGGTGTGGGTGTGGGTGTCTGTG;
>clamp_s_c_s_d_s_c_s_a_s:compl:rev CACAGACACCCACACCGTCACCCACACTATCATGTTGACATTCC; dG = -27.8
705 >s_d_s_d_s_b_s_d_s_d_s_c_s_a_s GTGACGGTGACGGTGGCTGTGACGGTGACGGTGTGGGTGTCTGTG;
>clamp_s_c_s_d_s_c_s_a_s:compl:rev CACAGACACCCACACCGTCACCCACACTATCATGTTGACATTCC; dG = -27.8
706 >s_d_s_e_s_a_s_c_s_d_s_d_s_a_s GTGACGGTGCTGGTGTCTGTGTGGGTGACGGTGACGGTGTCTGTG;
>clamp_s_c_s_d_s_c_s_a_s:compl:rev CACAGACACCCACACCGTCACCCACACTATCATGTTGACATTCC; dG = -27.8
707 >s_d_s_e_s_a_s_c_s_c_s_c_s_a_s GTGACGGTGCTGGTGTCTGTGTGGGTGTGGGTGTGGGTGTCTGTG;
>clamp_s_c_s_d_s_c_s_a_s:compl:rev CACAGACACCCACACCGTCACCCACACTATCATGTTGACATTCC; dG = -27.8
708 >s_d_s_e_s_a_s_d_s_d_s_c_s_a_s GTGACGGTGCTGGTGTCTGTGACGGTGACGGTGTGGGTGTCTGTG;
>clamp_s_c_s_d_s_c_s_a_s:compl:rev CACAGACACCCACACCGTCACCCACACTATCATGTTGACATTCC; dG = -27.8
709 >s_d_s_e_s_b_s_c_s_d_s_d_s_a_s GTGACGGTGCTGGTGGCTGTGTGGGTGACGGTGACGGTGTCTGTG;
>clamp_s_c_s_d_s_c_s_a_s:compl:rev CACAGACACCCACACCGTCACCCACACTATCATGTTGACATTCC; dG = -27.8
710 >s_d_s_e_s_b_s_c_s_c_s_c_s_a_s GTGACGGTGCTGGTGGCTGTGTGGGTGTGGGTGTGGGTGTCTGTG;
>clamp_s_c_s_d_s_c_s_a_s:compl:rev CACAGACACCCACACCGTCACCCACACTATCATGTTGACATTCC; dG = -27.8
711 >s_d_s_e_s_b_s_d_s_d_s_c_s_a_s GTGACGGTGCTGGTGGCTGTGACGGTGACGGTGTGGGTGTCTGTG;
>clamp_s_c_s_d_s_c_s_a_s:compl:rev CACAGACACCCACACCGTCACCCACACTATCATGTTGACATTCC; dG = -27.8
712 >s_e_s_a_s_a_s_c_s_d_s_d_s_a_s GTGCTGGTGTCTGTGTCTGTGTGGGTGACGGTGACGGTGTCTGTG;
>clamp_s_c_s_d_s_c_s_a_s:compl:rev CACAGACACCCACACCGTCACCCACACTATCATGTTGACATTCC; dG = -27.8
713 >s_e_s_a_s_a_s_c_s_c_s_c_s_a_s GTGCTGGTGTCTGTGTCTGTGTGGGTGTGGGTGTGGGTGTCTGTG;
>clamp_s_c_s_d_s_c_s_a_s:compl:rev CACAGACACCCACACCGTCACCCACACTATCATGTTGACATTCC; dG = -27.8
714 >s_e_s_a_s_a_s_d_s_d_s_c_s_a_s GTGCTGGTGTCTGTGTCTGTGACGGTGACGGTGTGGGTGTCTGTG;
>clamp_s_c_s_d_s_c_s_a_s:compl:rev CACAGACACCCACACCGTCACCCACACTATCATGTTGACATTCC; dG = -27.8
715 >s_e_s_a_s_b_s_c_s_d_s_d_s_a_s GTGCTGGTGTCTGTGGCTGTGTGGGTGACGGTGACGGTGTCTGTG;
>clamp_s_c_s_d_s_c_s_a_s:compl:rev CACAGACACCCACACCGTCACCCACACTATCATGTTGACATTCC; dG = -27.8
716 >s_e_s_a_s_b_s_c_s_c_s_c_s_a_s GTGCTGGTGTCTGTGGCTGTGTGGGTGTGGGTGTGGGTGTCTGTG;
>clamp_s_c_s_d_s_c_s_a_s:compl:rev CACAGACACCCACACCGTCACCCACACTATCATGTTGACATTCC; dG = -27.8
717 >s_e_s_a_s_b_s_d_s_d_s_c_s_a_s GTGCTGGTGTCTGTGGCTGTGACGGTGACGGTGTGGGTGTCTGTG;
>clamp_s_c_s_d_s_c_s_a_s:compl:rev CACAGACACCCACACCGTCACCCACACTATCATGTTGACATTCC; dG = -27.8
718 >s_a_s_c_s_a_s_c_s_d_s_d_s_a_s GTGTCTGTGTGGGTGTCTGTGTGGGTGACGGTGACGGTGTCTGTG;
>clamp_s_c_s_d_s_c_s_a_s:compl:rev CACAGACACCCACACCGTCACCCACACTATCATGTTGACATTCC; dG = -27.8
719 >s_e_s_b_s_a_s_c_s_d_s_d_s_a_s GTGCTGGTGGCTGTGTCTGTGTGGGTGACGGTGACGGTGTCTGTG;
>clamp_s_c_s_d_s_c_s_a_s:compl:rev CACAGACACCCACACCGTCACCCACACTATCATGTTGACATTCC; dG = -27.8
720 >s_e_s_b_s_a_s_c_s_c_s_c_s_a_s GTGCTGGTGGCTGTGTCTGTGTGGGTGTGGGTGTGGGTGTCTGTG;
>clamp_s_c_s_d_s_c_s_a_s:compl:rev CACAGACACCCACACCGTCACCCACACTATCATGTTGACATTCC; dG = -27.8
721 >s_e_s_b_s_a_s_d_s_d_s_c_s_a_s GTGCTGGTGGCTGTGTCTGTGACGGTGACGGTGTGGGTGTCTGTG;
>clamp_s_c_s_d_s_c_s_a_s:compl:rev CACAGACACCCACACCGTCACCCACACTATCATGTTGACATTCC; dG = -27.8
722 >s_e_s_b_s_b_s_c_s_d_s_d_s_a_s GTGCTGGTGGCTGTGGCTGTGTGGGTGACGGTGACGGTGTCTGTG;
>clamp_s_c_s_d_s_c_s_a_s:compl:rev CACAGACACCCACACCGTCACCCACACTATCATGTTGACATTCC; dG = -27.8
723 >s_a_s_c_s_a_s_c_s_c_s_c_s_a_s GTGTCTGTGTGGGTGTCTGTGTGGGTGTGGGTGTGGGTGTCTGTG;
>clamp_s_c_s_d_s_c_s_a_s:compl:rev CACAGACACCCACACCGTCACCCACACTATCATGTTGACATTCC; dG = -27.8

```
   724 >s_e_s_b_s_b_s_c_s_c_s_c_s_a_s          GTGCTGGTGGCTGTGGCTGTGTGGGTGTGGGTGTGGGTGTCTGTG;
>clamp_s_c_s_d_s_c_s_a_s:compl:rev CACAGACACCCACACCGTCACCCACACTATCATGTTGACATTCC; dG = -27.8
   725 >s_e_s_b_s_b_s_d_s_d_s_c_s_a_s          GTGCTGGTGGCTGTGGCTGTGACGGTGACGGTGTGGGTGTCTGTG;
>clamp_s_c_s_d_s_c_s_a_s:compl:rev CACAGACACCCACACCGTCACCCACACTATCATGTTGACATTCC; dG = -27.8
   726 >s_a_s_c_s_a_s_d_s_d_s_c_s_a_s          GTGTCTGTGTGGGTGTCTGTGACGGTGACGGTGTGGGTGTCTGTG;
>clamp_s_c_s_d_s_c_s_a_s:compl:rev CACAGACACCCACACCGTCACCCACACTATCATGTTGACATTCC; dG = -27.8
   727 >s_e_s_c_s_a_s_c_s_d_s_d_s_a_s          GTGCTGGTGTGGGTGTCTGTGTGGGTGACGGTGACGGTGTCTGTG;
>clamp_s_c_s_d_s_c_s_a_s:compl:rev CACAGACACCCACACCGTCACCCACACTATCATGTTGACATTCC; dG = -27.8
   728 >s_e_s_c_s_a_s_c_s_c_s_c_s_a_s          GTGCTGGTGTGGGTGTCTGTGTGGGTGTGGGTGTGGGTGTCTGTG;
>clamp_s_c_s_d_s_c_s_a_s:compl:rev CACAGACACCCACACCGTCACCCACACTATCATGTTGACATTCC; dG = -27.8
   729 >s_e_s_c_s_a_s_d_s_d_s_c_s_a_s          GTGCTGGTGTGGGTGTCTGTGACGGTGACGGTGTGGGTGTCTGTG;
>clamp_s_c_s_d_s_c_s_a_s:compl:rev CACAGACACCCACACCGTCACCCACACTATCATGTTGACATTCC; dG = -27.8
   730 >s_e_s_c_s_b_s_c_s_d_s_d_s_a_s          GTGCTGGTGTGGGTGGCTGTGTGGGTGACGGTGACGGTGTCTGTG;
>clamp_s_c_s_d_s_c_s_a_s:compl:rev CACAGACACCCACACCGTCACCCACACTATCATGTTGACATTCC; dG = -27.8
   731 >s_e_s_c_s_b_s_c_s_c_s_c_s_a_s          GTGCTGGTGTGGGTGGCTGTGTGGGTGTGGGTGTGGGTGTCTGTG;
>clamp_s_c_s_d_s_c_s_a_s:compl:rev CACAGACACCCACACCGTCACCCACACTATCATGTTGACATTCC; dG = -27.8
   732 >s_e_s_c_s_b_s_d_s_d_s_c_s_a_s          GTGCTGGTGTGGGTGGCTGTGACGGTGACGGTGTGGGTGTCTGTG;
>clamp_s_c_s_d_s_c_s_a_s:compl:rev CACAGACACCCACACCGTCACCCACACTATCATGTTGACATTCC; dG = -27.8
   733 >s_a_s_c_s_b_s_c_s_d_s_d_s_a_s          GTGTCTGTGTGGGTGGCTGTGTGGGTGACGGTGACGGTGTCTGTG;
>clamp_s_c_s_d_s_c_s_a_s:compl:rev CACAGACACCCACACCGTCACCCACACTATCATGTTGACATTCC; dG = -27.8
   734 >s_e_s_d_s_a_s_c_s_d_s_d_s_a_s          GTGCTGGTGACGGTGTCTGTGTGGGTGACGGTGACGGTGTCTGTG;
>clamp_s_c_s_d_s_c_s_a_s:compl:rev CACAGACACCCACACCGTCACCCACACTATCATGTTGACATTCC; dG = -27.8
   735 >s_e_s_d_s_a_s_c_s_c_s_c_s_a_s          GTGCTGGTGACGGTGTCTGTGTGGGTGTGGGTGTGGGTGTCTGTG;
>clamp_s_c_s_d_s_c_s_a_s:compl:rev CACAGACACCCACACCGTCACCCACACTATCATGTTGACATTCC; dG = -27.8
   736 >s_e_s_d_s_a_s_d_s_d_s_c_s_a_s          GTGCTGGTGACGGTGTCTGTGACGGTGACGGTGTGGGTGTCTGTG;
>clamp_s_c_s_d_s_c_s_a_s:compl:rev CACAGACACCCACACCGTCACCCACACTATCATGTTGACATTCC; dG = -27.8
   737 >s_e_s_d_s_b_s_c_s_d_s_d_s_a_s          GTGCTGGTGACGGTGGCTGTGTGGGTGACGGTGACGGTGTCTGTG;
>clamp_s_c_s_d_s_c_s_a_s:compl:rev CACAGACACCCACACCGTCACCCACACTATCATGTTGACATTCC; dG = -27.8
   738 >s_a_s_c_s_b_s_c_s_c_s_c_s_a_s          GTGTCTGTGTGGGTGGCTGTGTGGGTGTGGGTGTGGGTGTCTGTG;
>clamp_s_c_s_d_s_c_s_a_s:compl:rev CACAGACACCCACACCGTCACCCACACTATCATGTTGACATTCC; dG = -27.8
   739 >s_e_s_d_s_b_s_c_s_c_s_c_s_a_s          GTGCTGGTGACGGTGGCTGTGTGGGTGTGGGTGTGGGTGTCTGTG;
>clamp_s_c_s_d_s_c_s_a_s:compl:rev CACAGACACCCACACCGTCACCCACACTATCATGTTGACATTCC; dG = -27.8
   740 >s_e_s_d_s_b_s_d_s_d_s_c_s_a_s          GTGCTGGTGACGGTGGCTGTGACGGTGACGGTGTGGGTGTCTGTG;
>clamp_s_c_s_d_s_c_s_a_s:compl:rev CACAGACACCCACACCGTCACCCACACTATCATGTTGACATTCC; dG = -27.8
   741 >s_a_s_c_s_b_s_d_s_d_s_c_s_a_s          GTGTCTGTGTGGGTGGCTGTGACGGTGACGGTGTGGGTGTCTGTG;
>clamp_s_c_s_d_s_c_s_a_s:compl:rev CACAGACACCCACACCGTCACCCACACTATCATGTTGACATTCC; dG = -27.8
   742 >s_e_s_e_s_a_s_c_s_d_s_d_s_a_s          GTGCTGGTGCTGGTGTCTGTGTGGGTGACGGTGACGGTGTCTGTG;
>clamp_s_c_s_d_s_c_s_a_s:compl:rev CACAGACACCCACACCGTCACCCACACTATCATGTTGACATTCC; dG = -27.8
   743 >s_e_s_e_s_a_s_c_s_c_s_c_s_a_s          GTGCTGGTGCTGGTGTCTGTGTGGGTGTGGGTGTGGGTGTCTGTG;
>clamp_s_c_s_d_s_c_s_a_s:compl:rev CACAGACACCCACACCGTCACCCACACTATCATGTTGACATTCC; dG = -27.8
   744 >s_e_s_e_s_a_s_d_s_d_s_c_s_a_s          GTGCTGGTGCTGGTGTCTGTGACGGTGACGGTGTGGGTGTCTGTG;
>clamp_s_c_s_d_s_c_s_a_s:compl:rev CACAGACACCCACACCGTCACCCACACTATCATGTTGACATTCC; dG = -27.8
   745 >s_e_s_e_s_b_s_c_s_d_s_d_s_a_s          GTGCTGGTGCTGGTGGCTGTGTGGGTGACGGTGACGGTGTCTGTG;
>clamp_s_c_s_d_s_c_s_a_s:compl:rev CACAGACACCCACACCGTCACCCACACTATCATGTTGACATTCC; dG = -27.8
   746 >s_e_s_e_s_b_s_c_s_c_s_c_s_a_s          GTGCTGGTGCTGGTGGCTGTGTGGGTGTGGGTGTGGGTGTCTGTG;
>clamp_s_c_s_d_s_c_s_a_s:compl:rev CACAGACACCCACACCGTCACCCACACTATCATGTTGACATTCC; dG = -27.8
   747 >s_e_s_e_s_b_s_d_s_d_s_c_s_a_s          GTGCTGGTGCTGGTGGCTGTGACGGTGACGGTGTGGGTGTCTGTG;
>clamp_s_c_s_d_s_c_s_a_s:compl:rev CACAGACACCCACACCGTCACCCACACTATCATGTTGACATTCC; dG = -27.8
   748 >s_a_s_a_s_b_s_c_s_d_s_d_s_a_s          GTGTCTGTGTCTGTGGCTGTGTGGGTGACGGTGACGGTGTCTGTG;
>clamp_s_c_s_d_s_c_s_a_s:compl:rev CACAGACACCCACACCGTCACCCACACTATCATGTTGACATTCC; dG = -27.8
   749 >s_a_s_d_s_a_s_c_s_d_s_d_s_a_s          GTGTCTGTGACGGTGTCTGTGTGGGTGACGGTGACGGTGTCTGTG;
>clamp_s_c_s_d_s_c_s_a_s:compl:rev CACAGACACCCACACCGTCACCCACACTATCATGTTGACATTCC; dG = -27.8
   750 >s_a_s_d_s_a_s_c_s_c_s_c_s_a_s          GTGTCTGTGACGGTGTCTGTGTGGGTGTGGGTGTGGGTGTCTGTG;
>clamp_s_c_s_d_s_c_s_a_s:compl:rev CACAGACACCCACACCGTCACCCACACTATCATGTTGACATTCC; dG = -27.8
   751 >s_a_s_d_s_a_s_d_s_d_s_c_s_a_s          GTGTCTGTGACGGTGTCTGTGACGGTGACGGTGTGGGTGTCTGTG;
>clamp_s_c_s_d_s_c_s_a_s:compl:rev CACAGACACCCACACCGTCACCCACACTATCATGTTGACATTCC; dG = -27.8
   752 >s_a_s_a_s_b_s_c_s_c_s_c_s_a_s          GTGTCTGTGTCTGTGGCTGTGTGGGTGTGGGTGTGGGTGTCTGTG;
>clamp_s_c_s_d_s_c_s_a_s:compl:rev CACAGACACCCACACCGTCACCCACACTATCATGTTGACATTCC; dG = -27.8
   753 >s_a_s_d_s_b_s_c_s_d_s_d_s_a_s          GTGTCTGTGACGGTGGCTGTGTGGGTGACGGTGACGGTGTCTGTG;
>clamp_s_c_s_d_s_c_s_a_s:compl:rev CACAGACACCCACACCGTCACCCACACTATCATGTTGACATTCC; dG = -27.8
   754 >s_a_s_d_s_b_s_c_s_c_s_c_s_a_s          GTGTCTGTGACGGTGGCTGTGTGGGTGTGGGTGTGGGTGTCTGTG;
>clamp_s_c_s_d_s_c_s_a_s:compl:rev CACAGACACCCACACCGTCACCCACACTATCATGTTGACATTCC; dG = -27.8
   755 >s_a_s_d_s_b_s_d_s_d_s_c_s_a_s          GTGTCTGTGACGGTGGCTGTGACGGTGACGGTGTGGGTGTCTGTG;
>clamp_s_c_s_d_s_c_s_a_s:compl:rev CACAGACACCCACACCGTCACCCACACTATCATGTTGACATTCC; dG = -27.8
   756 >s_a_s_a_s_b_s_d_s_d_s_c_s_a_s          GTGTCTGTGTCTGTGGCTGTGACGGTGACGGTGTGGGTGTCTGTG;
>clamp_s_c_s_d_s_c_s_a_s:compl:rev CACAGACACCCACACCGTCACCCACACTATCATGTTGACATTCC; dG = -27.8
   757 >s_a_s_e_s_a_s_c_s_d_s_d_s_a_s          GTGTCTGTGCTGGTGTCTGTGTGGGTGACGGTGACGGTGTCTGTG;
>clamp_s_c_s_d_s_c_s_a_s:compl:rev CACAGACACCCACACCGTCACCCACACTATCATGTTGACATTCC; dG = -27.8
   758 >s_a_s_e_s_a_s_c_s_c_s_c_s_a_s          GTGTCTGTGCTGGTGTCTGTGTGGGTGTGGGTGTGGGTGTCTGTG;
>clamp_s_c_s_d_s_c_s_a_s:compl:rev CACAGACACCCACACCGTCACCCACACTATCATGTTGACATTCC; dG = -27.8
   759 >s_a_s_e_s_a_s_d_s_d_s_c_s_a_s          GTGTCTGTGCTGGTGTCTGTGACGGTGACGGTGTGGGTGTCTGTG;
>clamp_s_c_s_d_s_c_s_a_s:compl:rev CACAGACACCCACACCGTCACCCACACTATCATGTTGACATTCC; dG = -27.8
   760 >s_a_s_e_s_b_s_c_s_d_s_d_s_a_s          GTGTCTGTGCTGGTGGCTGTGTGGGTGACGGTGACGGTGTCTGTG;
>clamp_s_c_s_d_s_c_s_a_s:compl:rev CACAGACACCCACACCGTCACCCACACTATCATGTTGACATTCC; dG = -27.8
```

761 >s_a_s_e_s_b_s_c_s_c_s_c_s_a_s GTGTCTGTGCTGGTGGCTGTGTGGGTGTGGGTGTGGGTGTCTGTG;
>clamp_s_c_s_d_s_c_s_a_s:compl:rev CACAGACACCCACACCGTCACCCACACTATCATGTTGACATTCC; dG = -27.8
762 >s_a_s_e_s_b_s_d_s_d_s_c_s_a_s GTGTCTGTGCTGGTGGCTGTGACGGTGACGGTGTGGGTGTCTGTG;
>clamp_s_c_s_d_s_c_s_a_s:compl:rev CACAGACACCCACACCGTCACCCACACTATCATGTTGACATTCC; dG = -27.8
763 >s_b_s_a_s_a_s_c_s_d_s_d_s_a_s GTGGCTGTGTCTGTGTCTGTGTGGGTGACGGTGACGGTGTCTGTG;
>clamp_s_c_s_d_s_c_s_a_s:compl:rev CACAGACACCCACACCGTCACCCACACTATCATGTTGACATTCC; dG = -27.8
764 >s_b_s_a_s_a_s_c_s_c_s_c_s_a_s GTGGCTGTGTCTGTGTCTGTGTGGGTGTGGGTGTGGGTGTCTGTG;
>clamp_s_c_s_d_s_c_s_a_s:compl:rev CACAGACACCCACACCGTCACCCACACTATCATGTTGACATTCC; dG = -27.8
765 >s_b_s_a_s_a_s_d_s_d_s_c_s_a_s GTGGCTGTGTCTGTGTCTGTGACGGTGACGGTGTGGGTGTCTGTG;
>clamp_s_c_s_d_s_c_s_a_s:compl:rev CACAGACACCCACACCGTCACCCACACTATCATGTTGACATTCC; dG = -27.8
766 >s_b_s_a_s_b_s_c_s_d_s_d_s_a_s GTGGCTGTGTCTGTGGCTGTGTGGGTGACGGTGACGGTGTCTGTG;
>clamp_s_c_s_d_s_c_s_a_s:compl:rev CACAGACACCCACACCGTCACCCACACTATCATGTTGACATTCC; dG = -27.8
767 >s_b_s_a_s_b_s_c_s_c_s_c_s_a_s GTGGCTGTGTCTGTGGCTGTGTGGGTGTGGGTGTGGGTGTCTGTG;
>clamp_s_c_s_d_s_c_s_a_s:compl:rev CACAGACACCCACACCGTCACCCACACTATCATGTTGACATTCC; dG = -27.8
768 >s_b_s_a_s_b_s_d_s_d_s_c_s_a_s GTGGCTGTGTCTGTGGCTGTGACGGTGACGGTGTGGGTGTCTGTG;
>clamp_s_c_s_d_s_c_s_a_s:compl:rev CACAGACACCCACACCGTCACCCACACTATCATGTTGACATTCC; dG = -27.8
769 >s_b_s_b_s_a_s_c_s_d_s_d_s_a_s GTGGCTGTGGCTGTGTCTGTGTGGGTGACGGTGACGGTGTCTGTG;
>clamp_s_c_s_d_s_c_s_a_s:compl:rev CACAGACACCCACACCGTCACCCACACTATCATGTTGACATTCC; dG = -27.8
770 >s_b_s_b_s_a_s_c_s_c_s_c_s_a_s GTGGCTGTGGCTGTGTCTGTGTGGGTGTGGGTGTGGGTGTCTGTG;
>clamp_s_c_s_d_s_c_s_a_s:compl:rev CACAGACACCCACACCGTCACCCACACTATCATGTTGACATTCC; dG = -27.8
771 >s_b_s_b_s_a_s_d_s_d_s_c_s_a_s GTGGCTGTGGCTGTGTCTGTGACGGTGACGGTGTGGGTGTCTGTG;
>clamp_s_c_s_d_s_c_s_a_s:compl:rev CACAGACACCCACACCGTCACCCACACTATCATGTTGACATTCC; dG = -27.8
772 >s_b_s_b_s_b_s_c_s_d_s_d_s_a_s GTGGCTGTGGCTGTGGCTGTGTGGGTGACGGTGACGGTGTCTGTG;
>clamp_s_c_s_d_s_c_s_a_s:compl:rev CACAGACACCCACACCGTCACCCACACTATCATGTTGACATTCC; dG = -27.8
773 >s_b_s_b_s_b_s_c_s_c_s_c_s_a_s GTGGCTGTGGCTGTGGCTGTGTGGGTGTGGGTGTGGGTGTCTGTG;
>clamp_s_c_s_d_s_c_s_a_s:compl:rev CACAGACACCCACACCGTCACCCACACTATCATGTTGACATTCC; dG = -27.8
774 >s_b_s_b_s_b_s_d_s_d_s_c_s_a_s GTGGCTGTGGCTGTGGCTGTGACGGTGACGGTGTGGGTGTCTGTG;
>clamp_s_c_s_d_s_c_s_a_s:compl:rev CACAGACACCCACACCGTCACCCACACTATCATGTTGACATTCC; dG = -27.8
775 >s_b_s_c_s_a_s_c_s_d_s_d_s_a_s GTGGCTGTGTGGGTGTCTGTGTGGGTGACGGTGACGGTGTCTGTG;
>clamp_s_c_s_d_s_c_s_a_s:compl:rev CACAGACACCCACACCGTCACCCACACTATCATGTTGACATTCC; dG = -27.8
776 >s_b_s_c_s_a_s_c_s_c_s_c_s_a_s GTGGCTGTGTGGGTGTCTGTGTGGGTGTGGGTGTGGGTGTCTGTG;
>clamp_s_c_s_d_s_c_s_a_s:compl:rev CACAGACACCCACACCGTCACCCACACTATCATGTTGACATTCC; dG = -27.8
777 >s_b_s_c_s_a_s_d_s_d_s_c_s_a_s GTGGCTGTGTGGGTGTCTGTGACGGTGACGGTGTGGGTGTCTGTG;
>clamp_s_c_s_d_s_c_s_a_s:compl:rev CACAGACACCCACACCGTCACCCACACTATCATGTTGACATTCC; dG = -27.8
778 >s_b_s_c_s_b_s_c_s_d_s_d_s_a_s GTGGCTGTGTGGGTGGCTGTGTGGGTGACGGTGACGGTGTCTGTG;
>clamp_s_c_s_d_s_c_s_a_s:compl:rev CACAGACACCCACACCGTCACCCACACTATCATGTTGACATTCC; dG = -27.8
779 >s_b_s_c_s_b_s_c_s_c_s_c_s_a_s GTGGCTGTGTGGGTGGCTGTGTGGGTGTGGGTGTGGGTGTCTGTG;
>clamp_s_c_s_d_s_c_s_a_s:compl:rev CACAGACACCCACACCGTCACCCACACTATCATGTTGACATTCC; dG = -27.8
780 >s_b_s_c_s_b_s_d_s_d_s_c_s_a_s GTGGCTGTGTGGGTGGCTGTGACGGTGACGGTGTGGGTGTCTGTG;
>clamp_s_c_s_d_s_c_s_a_s:compl:rev CACAGACACCCACACCGTCACCCACACTATCATGTTGACATTCC; dG = -27.8
781 >s_a_s_a_s_a_s_c_s_d_s_d_s_a_s GTGTCTGTGTCTGTGTCTGTGTGGGTGACGGTGACGGTGTCTGTG;
>clamp_s_c_s_d_s_c_s_a_s:compl:rev CACAGACACCCACACCGTCACCCACACTATCATGTTGACATTCC; dG = -27.8
782 >s_b_s_d_s_a_s_c_s_d_s_d_s_a_s GTGGCTGTGACGGTGTCTGTGTGGGTGACGGTGACGGTGTCTGTG;
>clamp_s_c_s_d_s_c_s_a_s:compl:rev CACAGACACCCACACCGTCACCCACACTATCATGTTGACATTCC; dG = -27.8
783 >s_b_s_d_s_a_s_c_s_c_s_c_s_a_s GTGGCTGTGACGGTGTCTGTGTGGGTGTGGGTGTGGGTGTCTGTG;
>clamp_s_c_s_d_s_c_s_a_s:compl:rev CACAGACACCCACACCGTCACCCACACTATCATGTTGACATTCC; dG = -27.8
784 >s_b_s_d_s_a_s_d_s_d_s_c_s_a_s GTGGCTGTGACGGTGTCTGTGACGGTGACGGTGTGGGTGTCTGTG;
>clamp_s_c_s_d_s_c_s_a_s:compl:rev CACAGACACCCACACCGTCACCCACACTATCATGTTGACATTCC; dG = -27.8
785 >s_b_s_d_s_b_s_c_s_d_s_d_s_a_s GTGGCTGTGACGGTGGCTGTGTGGGTGACGGTGACGGTGTCTGTG;
>clamp_s_c_s_d_s_c_s_a_s:compl:rev CACAGACACCCACACCGTCACCCACACTATCATGTTGACATTCC; dG = -27.8
786 >s_b_s_d_s_b_s_c_s_c_s_c_s_a_s GTGGCTGTGACGGTGGCTGTGTGGGTGTGGGTGTGGGTGTCTGTG;
>clamp_s_c_s_d_s_c_s_a_s:compl:rev CACAGACACCCACACCGTCACCCACACTATCATGTTGACATTCC; dG = -27.8
787 >s_b_s_d_s_b_s_d_s_d_s_c_s_a_s GTGGCTGTGACGGTGGCTGTGACGGTGACGGTGTGGGTGTCTGTG;
>clamp_s_c_s_d_s_c_s_a_s:compl:rev CACAGACACCCACACCGTCACCCACACTATCATGTTGACATTCC; dG = -27.8
788 >s_b_s_e_s_a_s_c_s_d_s_d_s_a_s GTGGCTGTGCTGGTGTCTGTGTGGGTGACGGTGACGGTGTCTGTG;
>clamp_s_c_s_d_s_c_s_a_s:compl:rev CACAGACACCCACACCGTCACCCACACTATCATGTTGACATTCC; dG = -27.8
789 >s_b_s_e_s_a_s_c_s_c_s_c_s_a_s GTGGCTGTGCTGGTGTCTGTGTGGGTGTGGGTGTGGGTGTCTGTG;
>clamp_s_c_s_d_s_c_s_a_s:compl:rev CACAGACACCCACACCGTCACCCACACTATCATGTTGACATTCC; dG = -27.8
790 >s_b_s_e_s_a_s_d_s_d_s_c_s_a_s GTGGCTGTGCTGGTGTCTGTGACGGTGACGGTGTGGGTGTCTGTG;
>clamp_s_c_s_d_s_c_s_a_s:compl:rev CACAGACACCCACACCGTCACCCACACTATCATGTTGACATTCC; dG = -27.8
791 >s_b_s_e_s_b_s_c_s_d_s_d_s_a_s GTGGCTGTGCTGGTGGCTGTGTGGGTGACGGTGACGGTGTCTGTG;
>clamp_s_c_s_d_s_c_s_a_s:compl:rev CACAGACACCCACACCGTCACCCACACTATCATGTTGACATTCC; dG = -27.8
792 >s_b_s_e_s_b_s_c_s_c_s_c_s_a_s GTGGCTGTGCTGGTGGCTGTGTGGGTGTGGGTGTGGGTGTCTGTG;
>clamp_s_c_s_d_s_c_s_a_s:compl:rev CACAGACACCCACACCGTCACCCACACTATCATGTTGACATTCC; dG = -27.8
793 >s_b_s_e_s_b_s_d_s_d_s_c_s_a_s GTGGCTGTGCTGGTGGCTGTGACGGTGACGGTGTGGGTGTCTGTG;
>clamp_s_c_s_d_s_c_s_a_s:compl:rev CACAGACACCCACACCGTCACCCACACTATCATGTTGACATTCC; dG = -27.8
794 >s_c_s_a_s_a_s_c_s_d_s_d_s_a_s GTGTGGGTGTCTGTGTCTGTGTGGGTGACGGTGACGGTGTCTGTG;
>clamp_s_c_s_d_s_c_s_a_s:compl:rev CACAGACACCCACACCGTCACCCACACTATCATGTTGACATTCC; dG = -27.8
795 >s_c_s_a_s_a_s_c_s_c_s_c_s_a_s GTGTGGGTGTCTGTGTCTGTGTGGGTGTGGGTGTGGGTGTCTGTG;
>clamp_s_c_s_d_s_c_s_a_s:compl:rev CACAGACACCCACACCGTCACCCACACTATCATGTTGACATTCC; dG = -27.8
796 >s_c_s_a_s_a_s_d_s_d_s_c_s_a_s GTGTGGGTGTCTGTGTCTGTGACGGTGACGGTGTGGGTGTCTGTG;
>clamp_s_c_s_d_s_c_s_a_s:compl:rev CACAGACACCCACACCGTCACCCACACTATCATGTTGACATTCC; dG = -27.8
797 >s_c_s_a_s_b_s_c_s_d_s_d_s_a_s GTGTGGGTGTCTGTGGCTGTGTGGGTGACGGTGACGGTGTCTGTG;
>clamp_s_c_s_d_s_c_s_a_s:compl:rev CACAGACACCCACACCGTCACCCACACTATCATGTTGACATTCC; dG = -27.8

```
   798 >s_c_s_a_s_b_s_c_s_c_s_c_s_a_s          GTGTGGGTGTCTGTGGCTGTGTGGGTGTGGGTGTGGGTGTCTGTG;
>clamp_s_c_s_d_s_c_s_a_s:compl:rev CACAGACACCCACACCGTCACCCACACTATCATGTTGACATTCC; dG = -27.8
   799 >s_c_s_a_s_b_s_d_s_d_s_c_s_a_s          GTGTGGGTGTCTGTGGCTGTGACGGTGACGGTGTGGGTGTCTGTG;
>clamp_s_c_s_d_s_c_s_a_s:compl:rev CACAGACACCCACACCGTCACCCACACTATCATGTTGACATTCC; dG = -27.8
   800 >s_a_s_b_s_a_s_c_s_d_s_d_s_a_s          GTGTCTGTGGCTGTGTCTGTGTGGGTGACGGTGACGGTGTCTGTG;
>clamp_s_c_s_d_s_c_s_a_s:compl:rev CACAGACACCCACACCGTCACCCACACTATCATGTTGACATTCC; dG = -27.8
   801 >s_c_s_b_s_a_s_c_s_d_s_d_s_a_s          GTGTGGGTGGCTGTGTCTGTGTGGGTGACGGTGACGGTGTCTGTG;
>clamp_s_c_s_d_s_c_s_a_s:compl:rev CACAGACACCCACACCGTCACCCACACTATCATGTTGACATTCC; dG = -27.8
   802 >s_c_s_b_s_a_s_c_s_c_s_c_s_a_s          GTGTGGGTGGCTGTGTCTGTGTGGGTGTGGGTGTGGGTGTCTGTG;
>clamp_s_c_s_d_s_c_s_a_s:compl:rev CACAGACACCCACACCGTCACCCACACTATCATGTTGACATTCC; dG = -27.8
   803 >s_c_s_b_s_a_s_d_s_d_s_c_s_a_s          GTGTGGGTGGCTGTGTCTGTGACGGTGACGGTGTGGGTGTCTGTG;
>clamp_s_c_s_d_s_c_s_a_s:compl:rev CACAGACACCCACACCGTCACCCACACTATCATGTTGACATTCC; dG = -27.8
   804 >s_a_s_b_s_a_s_c_s_c_s_c_s_a_s          GTGTCTGTGGCTGTGTCTGTGTGGGTGTGGGTGTGGGTGTCTGTG;
>clamp_s_c_s_d_s_c_s_a_s:compl:rev CACAGACACCCACACCGTCACCCACACTATCATGTTGACATTCC; dG = -27.8
   805 >s_c_s_b_s_b_s_c_s_d_s_d_s_a_s          GTGTGGGTGGCTGTGGCTGTGTGGGTGACGGTGACGGTGTCTGTG;
>clamp_s_c_s_d_s_c_s_a_s:compl:rev CACAGACACCCACACCGTCACCCACACTATCATGTTGACATTCC; dG = -27.8
   806 >s_c_s_b_s_b_s_c_s_c_s_c_s_a_s          GTGTGGGTGGCTGTGGCTGTGTGGGTGTGGGTGTGGGTGTCTGTG;
>clamp_s_c_s_d_s_c_s_a_s:compl:rev CACAGACACCCACACCGTCACCCACACTATCATGTTGACATTCC; dG = -27.8
   807 >s_c_s_b_s_b_s_d_s_d_s_c_s_a_s          GTGTGGGTGGCTGTGGCTGTGACGGTGACGGTGTGGGTGTCTGTG;
>clamp_s_c_s_d_s_c_s_a_s:compl:rev CACAGACACCCACACCGTCACCCACACTATCATGTTGACATTCC; dG = -27.8
   808 >s_a_s_b_s_a_s_d_s_d_s_c_s_a_s          GTGTCTGTGGCTGTGTCTGTGACGGTGACGGTGTGGGTGTCTGTG;
>clamp_s_c_s_d_s_c_s_a_s:compl:rev CACAGACACCCACACCGTCACCCACACTATCATGTTGACATTCC; dG = -27.8
   809 >s_c_s_c_s_a_s_c_s_d_s_d_s_a_s          GTGTGGGTGTGGGTGTCTGTGTGGGTGACGGTGACGGTGTCTGTG;
>clamp_s_c_s_d_s_c_s_a_s:compl:rev CACAGACACCCACACCGTCACCCACACTATCATGTTGACATTCC; dG = -27.8
   810 >s_c_s_c_s_a_s_c_s_c_s_c_s_a_s          GTGTGGGTGTGGGTGTCTGTGTGGGTGTGGGTGTGGGTGTCTGTG;
>clamp_s_c_s_d_s_c_s_a_s:compl:rev CACAGACACCCACACCGTCACCCACACTATCATGTTGACATTCC; dG = -27.8
   811 >s_c_s_c_s_a_s_d_s_d_s_c_s_a_s          GTGTGGGTGTGGGTGTCTGTGACGGTGACGGTGTGGGTGTCTGTG;
>clamp_s_c_s_d_s_c_s_a_s:compl:rev CACAGACACCCACACCGTCACCCACACTATCATGTTGACATTCC; dG = -27.8
   812 >s_c_s_c_s_b_s_c_s_d_s_d_s_a_s          GTGTGGGTGTGGGTGGCTGTGTGGGTGACGGTGACGGTGTCTGTG;
>clamp_s_c_s_d_s_c_s_a_s:compl:rev CACAGACACCCACACCGTCACCCACACTATCATGTTGACATTCC; dG = -27.8
   813 >s_c_s_c_s_b_s_c_s_c_s_c_s_a_s          GTGTGGGTGTGGGTGGCTGTGTGGGTGTGGGTGTGGGTGTCTGTG;
>clamp_s_c_s_d_s_c_s_a_s:compl:rev CACAGACACCCACACCGTCACCCACACTATCATGTTGACATTCC; dG = -27.8
   814 >s_c_s_c_s_b_s_d_s_d_s_c_s_a_s          GTGTGGGTGTGGGTGGCTGTGACGGTGACGGTGTGGGTGTCTGTG;
>clamp_s_c_s_d_s_c_s_a_s:compl:rev CACAGACACCCACACCGTCACCCACACTATCATGTTGACATTCC; dG = -27.8
   815 >s_c_s_c_s_c_s_c_s_c_s_c_s_a_s          GTGTGGGTGTGGGTGTGGGTGTGGGTGTGGGTGTGGGTGTCTGTG;
>clamp_s_c_s_d_s_c_s_a_s:compl:rev CACAGACACCCACACCGTCACCCACACTATCATGTTGACATTCC; dG = -27.7
   816 >s_a_s_b_s_b_s_c_s_d_s_e_s_a_s          GTGTCTGTGGCTGTGGCTGTGTGGGTGACGGTGCTGGTGTCTGTG;
>clamp_s_c_s_d_s_c_s_a_s:compl:rev CACAGACACCCACACCGTCACCCACACTATCATGTTGACATTCC; dG = -27.7
   817 >s_c_s_c_s_d_s_d_s_d_s_c_s_a_s          GTGTGGGTGTGGGTGACGGTGACGGTGACGGTGTGGGTGTCTGTG;
>clamp_s_c_s_d_s_c_s_a_s:compl:rev CACAGACACCCACACCGTCACCCACACTATCATGTTGACATTCC; dG = -27.7
   818 >s_c_s_c_s_e_s_c_s_d_s_d_s_a_s          GTGTGGGTGTGGGTGCTGGTGTGGGTGACGGTGACGGTGTCTGTG;
>clamp_s_c_s_d_s_c_s_a_s:compl:rev CACAGACACCCACACCGTCACCCACACTATCATGTTGACATTCC; dG = -27.7
   819 >s_c_s_c_s_e_s_c_s_c_s_c_s_a_s          GTGTGGGTGTGGGTGCTGGTGTGGGTGTGGGTGTGGGTGTCTGTG;
>clamp_s_c_s_d_s_c_s_a_s:compl:rev CACAGACACCCACACCGTCACCCACACTATCATGTTGACATTCC; dG = -27.7
   820 >s_c_s_c_s_e_s_d_s_d_s_c_s_a_s          GTGTGGGTGTGGGTGCTGGTGACGGTGACGGTGTGGGTGTCTGTG;
>clamp_s_c_s_d_s_c_s_a_s:compl:rev CACAGACACCCACACCGTCACCCACACTATCATGTTGACATTCC; dG = -27.7
   821 >s_c_s_d_s_a_s_c_s_d_s_e_s_a_s          GTGTGGGTGACGGTGTCTGTGTGGGTGACGGTGCTGGTGTCTGTG;
>clamp_s_c_s_d_s_c_s_a_s:compl:rev CACAGACACCCACACCGTCACCCACACTATCATGTTGACATTCC; dG = -27.7
   822 >s_c_s_d_s_a_s_e_s_d_s_c_s_a_s          GTGTGGGTGACGGTGTCTGTGCTGGTGACGGTGTGGGTGTCTGTG;
>clamp_s_c_s_d_s_c_s_a_s:compl:rev CACAGACACCCACACCGTCACCCACACTATCATGTTGACATTCC; dG = -27.7
   823 >s_c_s_d_s_b_s_c_s_d_s_e_s_a_s          GTGTGGGTGACGGTGGCTGTGTGGGTGACGGTGCTGGTGTCTGTG;
>clamp_s_c_s_d_s_c_s_a_s:compl:rev CACAGACACCCACACCGTCACCCACACTATCATGTTGACATTCC; dG = -27.7
   824 >s_c_s_d_s_b_s_e_s_d_s_c_s_a_s          GTGTGGGTGACGGTGGCTGTGCTGGTGACGGTGTGGGTGTCTGTG;
>clamp_s_c_s_d_s_c_s_a_s:compl:rev CACAGACACCCACACCGTCACCCACACTATCATGTTGACATTCC; dG = -27.7
   825 >s_a_s_b_s_b_s_e_s_d_s_c_s_a_s          GTGTCTGTGGCTGTGGCTGTGCTGGTGACGGTGTGGGTGTCTGTG;
>clamp_s_c_s_d_s_c_s_a_s:compl:rev CACAGACACCCACACCGTCACCCACACTATCATGTTGACATTCC; dG = -27.7
   826 >s_c_s_d_s_d_s_c_s_d_s_d_s_a_s          GTGTGGGTGACGGTGACGGTGTGGGTGACGGTGACGGTGTCTGTG;
>clamp_s_c_s_d_s_c_s_a_s:compl:rev CACAGACACCCACACCGTCACCCACACTATCATGTTGACATTCC; dG = -27.7
   827 >s_c_s_d_s_d_s_c_s_c_s_c_s_a_s          GTGTGGGTGACGGTGACGGTGTGGGTGTGGGTGTGGGTGTCTGTG;
>clamp_s_c_s_d_s_c_s_a_s:compl:rev CACAGACACCCACACCGTCACCCACACTATCATGTTGACATTCC; dG = -27.7
   828 >s_c_s_d_s_d_s_d_s_d_s_c_s_a_s          GTGTGGGTGACGGTGACGGTGACGGTGACGGTGTGGGTGTCTGTG;
>clamp_s_c_s_d_s_c_s_a_s:compl:rev CACAGACACCCACACCGTCACCCACACTATCATGTTGACATTCC; dG = -27.7
   829 >s_c_s_d_s_e_s_c_s_d_s_d_s_a_s          GTGTGGGTGACGGTGCTGGTGTGGGTGACGGTGACGGTGTCTGTG;
>clamp_s_c_s_d_s_c_s_a_s:compl:rev CACAGACACCCACACCGTCACCCACACTATCATGTTGACATTCC; dG = -27.7
   830 >s_c_s_d_s_e_s_c_s_c_s_c_s_a_s          GTGTGGGTGACGGTGCTGGTGTGGGTGTGGGTGTGGGTGTCTGTG;
>clamp_s_c_s_d_s_c_s_a_s:compl:rev CACAGACACCCACACCGTCACCCACACTATCATGTTGACATTCC; dG = -27.7
   831 >s_c_s_d_s_e_s_d_s_d_s_c_s_a_s          GTGTGGGTGACGGTGCTGGTGACGGTGACGGTGTGGGTGTCTGTG;
>clamp_s_c_s_d_s_c_s_a_s:compl:rev CACAGACACCCACACCGTCACCCACACTATCATGTTGACATTCC; dG = -27.7
   832 >s_c_s_e_s_a_s_c_s_d_s_e_s_a_s          GTGTGGGTGCTGGTGTCTGTGTGGGTGACGGTGCTGGTGTCTGTG;
>clamp_s_c_s_d_s_c_s_a_s:compl:rev CACAGACACCCACACCGTCACCCACACTATCATGTTGACATTCC; dG = -27.7
   833 >s_c_s_e_s_a_s_e_s_d_s_c_s_a_s          GTGTGGGTGCTGGTGTCTGTGCTGGTGACGGTGTGGGTGTCTGTG;
>clamp_s_c_s_d_s_c_s_a_s:compl:rev CACAGACACCCACACCGTCACCCACACTATCATGTTGACATTCC; dG = -27.7
   834 >s_c_s_e_s_b_s_c_s_d_s_e_s_a_s          GTGTGGGTGCTGGTGGCTGTGTGGGTGACGGTGCTGGTGTCTGTG;
>clamp_s_c_s_d_s_c_s_a_s:compl:rev CACAGACACCCACACCGTCACCCACACTATCATGTTGACATTCC; dG = -27.7
```

```
   835 >s_c_s_e_s_b_s_e_s_d_s_c_s_a_s          GTGTGGGTGCTGGTGGCTGTGCTGGTGACGGTGTGGGTGTCTGTG;
>clamp_s_c_s_d_s_c_s_a_s:compl:rev CACAGACACCCACACCGTCACCCACACTATCATGTTGACATTCC; dG = -27.7
   836 >s_c_s_e_s_c_s_c_s_d_s_d_s_a_s          GTGTGGGTGCTGGTGTGGGTGTGGGTGACGGTGACGGTGTCTGTG;
>clamp_s_c_s_d_s_c_s_a_s:compl:rev CACAGACACCCACACCGTCACCCACACTATCATGTTGACATTCC; dG = -27.7
   837 >s_c_s_e_s_c_s_c_s_c_s_c_s_a_s          GTGTGGGTGCTGGTGTGGGTGTGGGTGTGGGTGTGGGTGTCTGTG;
>clamp_s_c_s_d_s_c_s_a_s:compl:rev CACAGACACCCACACCGTCACCCACACTATCATGTTGACATTCC; dG = -27.7
   838 >s_c_s_e_s_d_s_c_s_d_s_d_s_a_s          GTGTGGGTGCTGGTGACGGTGTGGGTGACGGTGACGGTGTCTGTG;
>clamp_s_c_s_d_s_c_s_a_s:compl:rev CACAGACACCCACACCGTCACCCACACTATCATGTTGACATTCC; dG = -27.7
   839 >s_c_s_e_s_d_s_c_s_c_s_c_s_a_s          GTGTGGGTGCTGGTGACGGTGTGGGTGTGGGTGTGGGTGTCTGTG;
>clamp_s_c_s_d_s_c_s_a_s:compl:rev CACAGACACCCACACCGTCACCCACACTATCATGTTGACATTCC; dG = -27.7
   840 >s_c_s_e_s_d_s_d_s_d_s_c_s_a_s          GTGTGGGTGCTGGTGACGGTGACGGTGACGGTGTGGGTGTCTGTG;
>clamp_s_c_s_d_s_c_s_a_s:compl:rev CACAGACACCCACACCGTCACCCACACTATCATGTTGACATTCC; dG = -27.7
   841 >s_a_s_b_s_c_s_c_s_d_s_d_s_a_s          GTGTCTGTGGCTGTGTGGGTGTGGGTGACGGTGACGGTGTCTGTG;
>clamp_s_c_s_d_s_c_s_a_s:compl:rev CACAGACACCCACACCGTCACCCACACTATCATGTTGACATTCC; dG = -27.7
   842 >s_c_s_e_s_e_s_c_s_d_s_d_s_a_s          GTGTGGGTGCTGGTGCTGGTGTGGGTGACGGTGACGGTGTCTGTG;
>clamp_s_c_s_d_s_c_s_a_s:compl:rev CACAGACACCCACACCGTCACCCACACTATCATGTTGACATTCC; dG = -27.7
   843 >s_c_s_e_s_e_s_c_s_c_s_c_s_a_s          GTGTGGGTGCTGGTGCTGGTGTGGGTGTGGGTGTGGGTGTCTGTG;
>clamp_s_c_s_d_s_c_s_a_s:compl:rev CACAGACACCCACACCGTCACCCACACTATCATGTTGACATTCC; dG = -27.7
   844 >s_c_s_e_s_e_s_d_s_d_s_c_s_a_s          GTGTGGGTGCTGGTGCTGGTGACGGTGACGGTGTGGGTGTCTGTG;
>clamp_s_c_s_d_s_c_s_a_s:compl:rev CACAGACACCCACACCGTCACCCACACTATCATGTTGACATTCC; dG = -27.7
   845 >s_d_s_a_s_a_s_c_s_d_s_e_s_a_s          GTGACGGTGTCTGTGTCTGTGTGGGTGACGGTGCTGGTGTCTGTG;
>clamp_s_c_s_d_s_c_s_a_s:compl:rev CACAGACACCCACACCGTCACCCACACTATCATGTTGACATTCC; dG = -27.7
   846 >s_d_s_a_s_a_s_e_s_d_s_c_s_a_s          GTGACGGTGTCTGTGTCTGTGCTGGTGACGGTGTGGGTGTCTGTG;
>clamp_s_c_s_d_s_c_s_a_s:compl:rev CACAGACACCCACACCGTCACCCACACTATCATGTTGACATTCC; dG = -27.7
   847 >s_d_s_a_s_b_s_c_s_d_s_e_s_a_s          GTGACGGTGTCTGTGGCTGTGTGGGTGACGGTGCTGGTGTCTGTG;
>clamp_s_c_s_d_s_c_s_a_s:compl:rev CACAGACACCCACACCGTCACCCACACTATCATGTTGACATTCC; dG = -27.7
   848 >s_a_s_b_s_c_s_c_s_c_s_c_s_a_s          GTGTCTGTGGCTGTGTGGGTGTGGGTGTGGGTGTGGGTGTCTGTG;
>clamp_s_c_s_d_s_c_s_a_s:compl:rev CACAGACACCCACACCGTCACCCACACTATCATGTTGACATTCC; dG = -27.7
   849 >s_d_s_a_s_b_s_e_s_d_s_c_s_a_s          GTGACGGTGTCTGTGGCTGTGCTGGTGACGGTGTGGGTGTCTGTG;
>clamp_s_c_s_d_s_c_s_a_s:compl:rev CACAGACACCCACACCGTCACCCACACTATCATGTTGACATTCC; dG = -27.7
   850 >s_d_s_a_s_c_s_c_s_d_s_d_s_a_s          GTGACGGTGTCTGTGTGGGTGTGGGTGACGGTGACGGTGTCTGTG;
>clamp_s_c_s_d_s_c_s_a_s:compl:rev CACAGACACCCACACCGTCACCCACACTATCATGTTGACATTCC; dG = -27.7
   851 >s_d_s_a_s_c_s_c_s_c_s_c_s_a_s          GTGACGGTGTCTGTGTGGGTGTGGGTGTGGGTGTGGGTGTCTGTG;
>clamp_s_c_s_d_s_c_s_a_s:compl:rev CACAGACACCCACACCGTCACCCACACTATCATGTTGACATTCC; dG = -27.7
   852 >s_d_s_a_s_d_s_c_s_d_s_d_s_a_s          GTGACGGTGTCTGTGACGGTGTGGGTGACGGTGACGGTGTCTGTG;
>clamp_s_c_s_d_s_c_s_a_s:compl:rev CACAGACACCCACACCGTCACCCACACTATCATGTTGACATTCC; dG = -27.7
   853 >s_d_s_a_s_d_s_c_s_c_s_c_s_a_s          GTGACGGTGTCTGTGACGGTGTGGGTGTGGGTGTGGGTGTCTGTG;
>clamp_s_c_s_d_s_c_s_a_s:compl:rev CACAGACACCCACACCGTCACCCACACTATCATGTTGACATTCC; dG = -27.7
   854 >s_d_s_a_s_d_s_d_s_d_s_c_s_a_s          GTGACGGTGTCTGTGACGGTGACGGTGACGGTGTGGGTGTCTGTG;
>clamp_s_c_s_d_s_c_s_a_s:compl:rev CACAGACACCCACACCGTCACCCACACTATCATGTTGACATTCC; dG = -27.7
   855 >s_d_s_a_s_e_s_c_s_d_s_d_s_a_s          GTGACGGTGTCTGTGCTGGTGTGGGTGACGGTGACGGTGTCTGTG;
>clamp_s_c_s_d_s_c_s_a_s:compl:rev CACAGACACCCACACCGTCACCCACACTATCATGTTGACATTCC; dG = -27.7
   856 >s_d_s_a_s_e_s_c_s_c_s_c_s_a_s          GTGACGGTGTCTGTGCTGGTGTGGGTGTGGGTGTGGGTGTCTGTG;
>clamp_s_c_s_d_s_c_s_a_s:compl:rev CACAGACACCCACACCGTCACCCACACTATCATGTTGACATTCC; dG = -27.7
   857 >s_d_s_a_s_e_s_d_s_d_s_c_s_a_s          GTGACGGTGTCTGTGCTGGTGACGGTGACGGTGTGGGTGTCTGTG;
>clamp_s_c_s_d_s_c_s_a_s:compl:rev CACAGACACCCACACCGTCACCCACACTATCATGTTGACATTCC; dG = -27.7
   858 >s_d_s_b_s_a_s_c_s_d_s_e_s_a_s          GTGACGGTGGCTGTGTCTGTGTGGGTGACGGTGCTGGTGTCTGTG;
>clamp_s_c_s_d_s_c_s_a_s:compl:rev CACAGACACCCACACCGTCACCCACACTATCATGTTGACATTCC; dG = -27.7
   859 >s_d_s_b_s_a_s_e_s_d_s_c_s_a_s          GTGACGGTGGCTGTGTCTGTGCTGGTGACGGTGTGGGTGTCTGTG;
>clamp_s_c_s_d_s_c_s_a_s:compl:rev CACAGACACCCACACCGTCACCCACACTATCATGTTGACATTCC; dG = -27.7
   860 >s_d_s_b_s_b_s_c_s_d_s_e_s_a_s          GTGACGGTGGCTGTGGCTGTGTGGGTGACGGTGCTGGTGTCTGTG;
>clamp_s_c_s_d_s_c_s_a_s:compl:rev CACAGACACCCACACCGTCACCCACACTATCATGTTGACATTCC; dG = -27.7
   861 >s_a_s_a_s_a_s_e_s_d_s_c_s_a_s          GTGTCTGTGTCTGTGTCTGTGCTGGTGACGGTGTGGGTGTCTGTG;
>clamp_s_c_s_d_s_c_s_a_s:compl:rev CACAGACACCCACACCGTCACCCACACTATCATGTTGACATTCC; dG = -27.7
   862 >s_d_s_b_s_b_s_e_s_d_s_c_s_a_s          GTGACGGTGGCTGTGGCTGTGCTGGTGACGGTGTGGGTGTCTGTG;
>clamp_s_c_s_d_s_c_s_a_s:compl:rev CACAGACACCCACACCGTCACCCACACTATCATGTTGACATTCC; dG = -27.7
   863 >s_d_s_b_s_c_s_c_s_d_s_d_s_a_s          GTGACGGTGGCTGTGTGGGTGTGGGTGACGGTGACGGTGTCTGTG;
>clamp_s_c_s_d_s_c_s_a_s:compl:rev CACAGACACCCACACCGTCACCCACACTATCATGTTGACATTCC; dG = -27.7
   864 >s_d_s_b_s_c_s_c_s_c_s_c_s_a_s          GTGACGGTGGCTGTGTGGGTGTGGGTGTGGGTGTGGGTGTCTGTG;
>clamp_s_c_s_d_s_c_s_a_s:compl:rev CACAGACACCCACACCGTCACCCACACTATCATGTTGACATTCC; dG = -27.7
   865 >s_d_s_b_s_d_s_c_s_d_s_d_s_a_s          GTGACGGTGGCTGTGACGGTGTGGGTGACGGTGACGGTGTCTGTG;
>clamp_s_c_s_d_s_c_s_a_s:compl:rev CACAGACACCCACACCGTCACCCACACTATCATGTTGACATTCC; dG = -27.7
   866 >s_d_s_b_s_d_s_c_s_c_s_c_s_a_s          GTGACGGTGGCTGTGACGGTGTGGGTGTGGGTGTGGGTGTCTGTG;
>clamp_s_c_s_d_s_c_s_a_s:compl:rev CACAGACACCCACACCGTCACCCACACTATCATGTTGACATTCC; dG = -27.7
   867 >s_d_s_b_s_d_s_d_s_d_s_c_s_a_s          GTGACGGTGGCTGTGACGGTGACGGTGACGGTGTGGGTGTCTGTG;
>clamp_s_c_s_d_s_c_s_a_s:compl:rev CACAGACACCCACACCGTCACCCACACTATCATGTTGACATTCC; dG = -27.7
   868 >s_a_s_b_s_d_s_c_s_d_s_d_s_a_s          GTGTCTGTGGCTGTGACGGTGTGGGTGACGGTGACGGTGTCTGTG;
>clamp_s_c_s_d_s_c_s_a_s:compl:rev CACAGACACCCACACCGTCACCCACACTATCATGTTGACATTCC; dG = -27.7
   869 >s_d_s_b_s_e_s_c_s_d_s_d_s_a_s          GTGACGGTGGCTGTGCTGGTGTGGGTGACGGTGACGGTGTCTGTG;
>clamp_s_c_s_d_s_c_s_a_s:compl:rev CACAGACACCCACACCGTCACCCACACTATCATGTTGACATTCC; dG = -27.7
   870 >s_d_s_b_s_e_s_c_s_c_s_c_s_a_s          GTGACGGTGGCTGTGCTGGTGTGGGTGTGGGTGTGGGTGTCTGTG;
>clamp_s_c_s_d_s_c_s_a_s:compl:rev CACAGACACCCACACCGTCACCCACACTATCATGTTGACATTCC; dG = -27.7
   871 >s_d_s_b_s_e_s_d_s_d_s_c_s_a_s          GTGACGGTGGCTGTGCTGGTGACGGTGACGGTGTGGGTGTCTGTG;
>clamp_s_c_s_d_s_c_s_a_s:compl:rev CACAGACACCCACACCGTCACCCACACTATCATGTTGACATTCC; dG = -27.7
```

```
   872 >s_d_s_c_s_a_s_c_s_d_s_e_s_a_s          GTGACGGTGTGGGTGTCTGTGTGGGTGACGGTGCTGGTGTCTGTG;
>clamp_s_c_s_d_s_c_s_a_s:compl:rev CACAGACACCCACACCGTCACCCACACTATCATGTTGACATTCC; dG = -27.7
   873 >s_d_s_c_s_a_s_e_s_d_s_c_s_a_s          GTGACGGTGTGGGTGTCTGTGCTGGTGACGGTGTGGGTGTCTGTG;
>clamp_s_c_s_d_s_c_s_a_s:compl:rev CACAGACACCCACACCGTCACCCACACTATCATGTTGACATTCC; dG = -27.7
   874 >s_d_s_c_s_b_s_c_s_d_s_e_s_a_s          GTGACGGTGTGGGTGGCTGTGTGGGTGACGGTGCTGGTGTCTGTG;
>clamp_s_c_s_d_s_c_s_a_s:compl:rev CACAGACACCCACACCGTCACCCACACTATCATGTTGACATTCC; dG = -27.7
   875 >s_a_s_b_s_d_s_c_s_c_s_c_s_a_s          GTGTCTGTGGCTGTGACGGTGTGGGTGTGGGTGTGGGTGTCTGTG;
>clamp_s_c_s_d_s_c_s_a_s:compl:rev CACAGACACCCACACCGTCACCCACACTATCATGTTGACATTCC; dG = -27.7
   876 >s_d_s_c_s_b_s_e_s_d_s_c_s_a_s          GTGACGGTGTGGGTGGCTGTGCTGGTGACGGTGTGGGTGTCTGTG;
>clamp_s_c_s_d_s_c_s_a_s:compl:rev CACAGACACCCACACCGTCACCCACACTATCATGTTGACATTCC; dG = -27.7
   877 >s_d_s_c_s_c_s_c_s_d_s_d_s_a_s          GTGACGGTGTGGGTGTGGGTGTGGGTGACGGTGACGGTGTCTGTG;
>clamp_s_c_s_d_s_c_s_a_s:compl:rev CACAGACACCCACACCGTCACCCACACTATCATGTTGACATTCC; dG = -27.7
   878 >s_d_s_c_s_c_s_c_s_c_s_c_s_a_s          GTGACGGTGTGGGTGTGGGTGTGGGTGTGGGTGTGGGTGTCTGTG;
>clamp_s_c_s_d_s_c_s_a_s:compl:rev CACAGACACCCACACCGTCACCCACACTATCATGTTGACATTCC; dG = -27.7
   879 >s_d_s_c_s_d_s_d_s_d_s_c_s_a_s          GTGACGGTGTGGGTGACGGTGACGGTGACGGTGTGGGTGTCTGTG;
>clamp_s_c_s_d_s_c_s_a_s:compl:rev CACAGACACCCACACCGTCACCCACACTATCATGTTGACATTCC; dG = -27.7
   880 >s_a_s_b_s_d_s_d_s_d_s_c_s_a_s          GTGTCTGTGGCTGTGACGGTGACGGTGACGGTGTGGGTGTCTGTG;
>clamp_s_c_s_d_s_c_s_a_s:compl:rev CACAGACACCCACACCGTCACCCACACTATCATGTTGACATTCC; dG = -27.7
   881 >s_d_s_c_s_e_s_c_s_d_s_d_s_a_s          GTGACGGTGTGGGTGCTGGTGTGGGTGACGGTGACGGTGTCTGTG;
>clamp_s_c_s_d_s_c_s_a_s:compl:rev CACAGACACCCACACCGTCACCCACACTATCATGTTGACATTCC; dG = -27.7
   882 >s_d_s_c_s_e_s_c_s_c_s_c_s_a_s          GTGACGGTGTGGGTGCTGGTGTGGGTGTGGGTGTGGGTGTCTGTG;
>clamp_s_c_s_d_s_c_s_a_s:compl:rev CACAGACACCCACACCGTCACCCACACTATCATGTTGACATTCC; dG = -27.7
   883 >s_d_s_c_s_e_s_d_s_d_s_c_s_a_s          GTGACGGTGTGGGTGCTGGTGACGGTGACGGTGTGGGTGTCTGTG;
>clamp_s_c_s_d_s_c_s_a_s:compl:rev CACAGACACCCACACCGTCACCCACACTATCATGTTGACATTCC; dG = -27.7
   884 >s_d_s_d_s_a_s_c_s_d_s_e_s_a_s          GTGACGGTGACGGTGTCTGTGTGGGTGACGGTGCTGGTGTCTGTG;
>clamp_s_c_s_d_s_c_s_a_s:compl:rev CACAGACACCCACACCGTCACCCACACTATCATGTTGACATTCC; dG = -27.7
   885 >s_d_s_d_s_a_s_e_s_d_s_c_s_a_s          GTGACGGTGACGGTGTCTGTGCTGGTGACGGTGTGGGTGTCTGTG;
>clamp_s_c_s_d_s_c_s_a_s:compl:rev CACAGACACCCACACCGTCACCCACACTATCATGTTGACATTCC; dG = -27.7
   886 >s_d_s_d_s_b_s_c_s_d_s_e_s_a_s          GTGACGGTGACGGTGGCTGTGTGGGTGACGGTGCTGGTGTCTGTG;
>clamp_s_c_s_d_s_c_s_a_s:compl:rev CACAGACACCCACACCGTCACCCACACTATCATGTTGACATTCC; dG = -27.7
   887 >s_d_s_d_s_b_s_e_s_d_s_c_s_a_s          GTGACGGTGACGGTGGCTGTGCTGGTGACGGTGTGGGTGTCTGTG;
>clamp_s_c_s_d_s_c_s_a_s:compl:rev CACAGACACCCACACCGTCACCCACACTATCATGTTGACATTCC; dG = -27.7
   888 >s_d_s_d_s_c_s_c_s_d_s_d_s_a_s          GTGACGGTGACGGTGTGGGTGTGGGTGACGGTGACGGTGTCTGTG;
>clamp_s_c_s_d_s_c_s_a_s:compl:rev CACAGACACCCACACCGTCACCCACACTATCATGTTGACATTCC; dG = -27.7
   889 >s_d_s_d_s_c_s_c_s_c_s_c_s_a_s          GTGACGGTGACGGTGTGGGTGTGGGTGTGGGTGTGGGTGTCTGTG;
>clamp_s_c_s_d_s_c_s_a_s:compl:rev CACAGACACCCACACCGTCACCCACACTATCATGTTGACATTCC; dG = -27.7
   890 >s_d_s_d_s_d_s_c_s_d_s_d_s_a_s          GTGACGGTGACGGTGACGGTGTGGGTGACGGTGACGGTGTCTGTG;
>clamp_s_c_s_d_s_c_s_a_s:compl:rev CACAGACACCCACACCGTCACCCACACTATCATGTTGACATTCC; dG = -27.7
   891 >s_d_s_d_s_d_s_c_s_c_s_c_s_a_s          GTGACGGTGACGGTGACGGTGTGGGTGTGGGTGTGGGTGTCTGTG;
>clamp_s_c_s_d_s_c_s_a_s:compl:rev CACAGACACCCACACCGTCACCCACACTATCATGTTGACATTCC; dG = -27.7
   892 >s_d_s_d_s_d_s_d_s_d_s_c_s_a_s          GTGACGGTGACGGTGACGGTGACGGTGACGGTGTGGGTGTCTGTG;
>clamp_s_c_s_d_s_c_s_a_s:compl:rev CACAGACACCCACACCGTCACCCACACTATCATGTTGACATTCC; dG = -27.7
   893 >s_a_s_b_s_e_s_c_s_d_s_d_s_a_s          GTGTCTGTGGCTGTGCTGGTGTGGGTGACGGTGACGGTGTCTGTG;
>clamp_s_c_s_d_s_c_s_a_s:compl:rev CACAGACACCCACACCGTCACCCACACTATCATGTTGACATTCC; dG = -27.7
   894 >s_d_s_d_s_e_s_c_s_d_s_d_s_a_s          GTGACGGTGACGGTGCTGGTGTGGGTGACGGTGACGGTGTCTGTG;
>clamp_s_c_s_d_s_c_s_a_s:compl:rev CACAGACACCCACACCGTCACCCACACTATCATGTTGACATTCC; dG = -27.7
   895 >s_d_s_d_s_e_s_c_s_c_s_c_s_a_s          GTGACGGTGACGGTGCTGGTGTGGGTGTGGGTGTGGGTGTCTGTG;
>clamp_s_c_s_d_s_c_s_a_s:compl:rev CACAGACACCCACACCGTCACCCACACTATCATGTTGACATTCC; dG = -27.7
   896 >s_d_s_d_s_e_s_d_s_d_s_c_s_a_s          GTGACGGTGACGGTGCTGGTGACGGTGACGGTGTGGGTGTCTGTG;
>clamp_s_c_s_d_s_c_s_a_s:compl:rev CACAGACACCCACACCGTCACCCACACTATCATGTTGACATTCC; dG = -27.7
   897 >s_d_s_e_s_a_s_c_s_d_s_e_s_a_s          GTGACGGTGCTGGTGTCTGTGTGGGTGACGGTGCTGGTGTCTGTG;
>clamp_s_c_s_d_s_c_s_a_s:compl:rev CACAGACACCCACACCGTCACCCACACTATCATGTTGACATTCC; dG = -27.7
   898 >s_d_s_e_s_a_s_e_s_d_s_c_s_a_s          GTGACGGTGCTGGTGTCTGTGCTGGTGACGGTGTGGGTGTCTGTG;
>clamp_s_c_s_d_s_c_s_a_s:compl:rev CACAGACACCCACACCGTCACCCACACTATCATGTTGACATTCC; dG = -27.7
   899 >s_d_s_e_s_b_s_c_s_d_s_e_s_a_s          GTGACGGTGCTGGTGGCTGTGTGGGTGACGGTGCTGGTGTCTGTG;
>clamp_s_c_s_d_s_c_s_a_s:compl:rev CACAGACACCCACACCGTCACCCACACTATCATGTTGACATTCC; dG = -27.7
   900 >s_a_s_b_s_e_s_c_s_c_s_c_s_a_s          GTGTCTGTGGCTGTGCTGGTGTGGGTGTGGGTGTGGGTGTCTGTG;
>clamp_s_c_s_d_s_c_s_a_s:compl:rev CACAGACACCCACACCGTCACCCACACTATCATGTTGACATTCC; dG = -27.7
   901 >s_d_s_e_s_b_s_e_s_d_s_c_s_a_s          GTGACGGTGCTGGTGGCTGTGCTGGTGACGGTGTGGGTGTCTGTG;
>clamp_s_c_s_d_s_c_s_a_s:compl:rev CACAGACACCCACACCGTCACCCACACTATCATGTTGACATTCC; dG = -27.7
   902 >s_d_s_e_s_c_s_c_s_d_s_d_s_a_s          GTGACGGTGCTGGTGTGGGTGTGGGTGACGGTGACGGTGTCTGTG;
>clamp_s_c_s_d_s_c_s_a_s:compl:rev CACAGACACCCACACCGTCACCCACACTATCATGTTGACATTCC; dG = -27.7
   903 >s_d_s_e_s_c_s_c_s_c_s_c_s_a_s          GTGACGGTGCTGGTGTGGGTGTGGGTGTGGGTGTGGGTGTCTGTG;
>clamp_s_c_s_d_s_c_s_a_s:compl:rev CACAGACACCCACACCGTCACCCACACTATCATGTTGACATTCC; dG = -27.7
   904 >s_d_s_e_s_d_s_c_s_d_s_d_s_a_s          GTGACGGTGCTGGTGACGGTGTGGGTGACGGTGACGGTGTCTGTG;
>clamp_s_c_s_d_s_c_s_a_s:compl:rev CACAGACACCCACACCGTCACCCACACTATCATGTTGACATTCC; dG = -27.7
   905 >s_d_s_e_s_d_s_c_s_c_s_c_s_a_s          GTGACGGTGCTGGTGACGGTGTGGGTGTGGGTGTGGGTGTCTGTG;
>clamp_s_c_s_d_s_c_s_a_s:compl:rev CACAGACACCCACACCGTCACCCACACTATCATGTTGACATTCC; dG = -27.7
   906 >s_d_s_e_s_d_s_d_s_d_s_c_s_a_s          GTGACGGTGCTGGTGACGGTGACGGTGACGGTGTGGGTGTCTGTG;
>clamp_s_c_s_d_s_c_s_a_s:compl:rev CACAGACACCCACACCGTCACCCACACTATCATGTTGACATTCC; dG = -27.7
   907 >s_a_s_b_s_e_s_d_s_d_s_c_s_a_s          GTGTCTGTGGCTGTGCTGGTGACGGTGACGGTGTGGGTGTCTGTG;
>clamp_s_c_s_d_s_c_s_a_s:compl:rev CACAGACACCCACACCGTCACCCACACTATCATGTTGACATTCC; dG = -27.7
   908 >s_d_s_e_s_e_s_c_s_d_s_d_s_a_s          GTGACGGTGCTGGTGCTGGTGTGGGTGACGGTGACGGTGTCTGTG;
>clamp_s_c_s_d_s_c_s_a_s:compl:rev CACAGACACCCACACCGTCACCCACACTATCATGTTGACATTCC; dG = -27.7
```

```
   909 >s_d_s_e_s_e_s_c_s_c_s_c_s_a_s          GTGACGGTGCTGGTGCTGGTGTGGGTGTGGGTGTGGGTGTCTGTG;
>clamp_s_c_s_d_s_c_s_a_s:compl:rev CACAGACACCCACACCGTCACCCACACTATCATGTTGACATTCC; dG = -27.7
   910 >s_d_s_e_s_e_s_d_s_d_s_c_s_a_s          GTGACGGTGCTGGTGCTGGTGACGGTGACGGTGTGGGTGTCTGTG;
>clamp_s_c_s_d_s_c_s_a_s:compl:rev CACAGACACCCACACCGTCACCCACACTATCATGTTGACATTCC; dG = -27.7
   911 >s_e_s_a_s_a_s_c_s_d_s_e_s_a_s          GTGCTGGTGTCTGTGTCTGTGTGGGTGACGGTGCTGGTGTCTGTG;
>clamp_s_c_s_d_s_c_s_a_s:compl:rev CACAGACACCCACACCGTCACCCACACTATCATGTTGACATTCC; dG = -27.7
   912 >s_e_s_a_s_a_s_e_s_d_s_c_s_a_s          GTGCTGGTGTCTGTGTCTGTGCTGGTGACGGTGTGGGTGTCTGTG;
>clamp_s_c_s_d_s_c_s_a_s:compl:rev CACAGACACCCACACCGTCACCCACACTATCATGTTGACATTCC; dG = -27.7
   913 >s_e_s_a_s_b_s_c_s_d_s_e_s_a_s          GTGCTGGTGTCTGTGGCTGTGTGGGTGACGGTGCTGGTGTCTGTG;
>clamp_s_c_s_d_s_c_s_a_s:compl:rev CACAGACACCCACACCGTCACCCACACTATCATGTTGACATTCC; dG = -27.7
   914 >s_e_s_a_s_b_s_e_s_d_s_c_s_a_s          GTGCTGGTGTCTGTGGCTGTGCTGGTGACGGTGTGGGTGTCTGTG;
>clamp_s_c_s_d_s_c_s_a_s:compl:rev CACAGACACCCACACCGTCACCCACACTATCATGTTGACATTCC; dG = -27.7
   915 >s_e_s_a_s_c_s_c_s_d_s_d_s_a_s          GTGCTGGTGTCTGTGTGGGTGTGGGTGACGGTGACGGTGTCTGTG;
>clamp_s_c_s_d_s_c_s_a_s:compl:rev CACAGACACCCACACCGTCACCCACACTATCATGTTGACATTCC; dG = -27.7
   916 >s_e_s_a_s_c_s_c_s_c_s_c_s_a_s          GTGCTGGTGTCTGTGTGGGTGTGGGTGTGGGTGTGGGTGTCTGTG;
>clamp_s_c_s_d_s_c_s_a_s:compl:rev CACAGACACCCACACCGTCACCCACACTATCATGTTGACATTCC; dG = -27.7
   917 >s_e_s_a_s_d_s_c_s_d_s_d_s_a_s          GTGCTGGTGTCTGTGACGGTGTGGGTGACGGTGACGGTGTCTGTG;
>clamp_s_c_s_d_s_c_s_a_s:compl:rev CACAGACACCCACACCGTCACCCACACTATCATGTTGACATTCC; dG = -27.7
   918 >s_a_s_c_s_a_s_c_s_d_s_e_s_a_s          GTGTCTGTGTGGGTGTCTGTGTGGGTGACGGTGCTGGTGTCTGTG;
>clamp_s_c_s_d_s_c_s_a_s:compl:rev CACAGACACCCACACCGTCACCCACACTATCATGTTGACATTCC; dG = -27.7
   919 >s_e_s_a_s_d_s_c_s_c_s_c_s_a_s          GTGCTGGTGTCTGTGACGGTGTGGGTGTGGGTGTGGGTGTCTGTG;
>clamp_s_c_s_d_s_c_s_a_s:compl:rev CACAGACACCCACACCGTCACCCACACTATCATGTTGACATTCC; dG = -27.7
   920 >s_e_s_a_s_d_s_d_s_d_s_c_s_a_s          GTGCTGGTGTCTGTGACGGTGACGGTGACGGTGTGGGTGTCTGTG;
>clamp_s_c_s_d_s_c_s_a_s:compl:rev CACAGACACCCACACCGTCACCCACACTATCATGTTGACATTCC; dG = -27.7
   921 >s_e_s_a_s_e_s_c_s_d_s_d_s_a_s          GTGCTGGTGTCTGTGCTGGTGTGGGTGACGGTGACGGTGTCTGTG;
>clamp_s_c_s_d_s_c_s_a_s:compl:rev CACAGACACCCACACCGTCACCCACACTATCATGTTGACATTCC; dG = -27.7
   922 >s_e_s_a_s_e_s_c_s_c_s_c_s_a_s          GTGCTGGTGTCTGTGCTGGTGTGGGTGTGGGTGTGGGTGTCTGTG;
>clamp_s_c_s_d_s_c_s_a_s:compl:rev CACAGACACCCACACCGTCACCCACACTATCATGTTGACATTCC; dG = -27.7
   923 >s_e_s_a_s_e_s_d_s_d_s_c_s_a_s          GTGCTGGTGTCTGTGCTGGTGACGGTGACGGTGTGGGTGTCTGTG;
>clamp_s_c_s_d_s_c_s_a_s:compl:rev CACAGACACCCACACCGTCACCCACACTATCATGTTGACATTCC; dG = -27.7
   924 >s_e_s_b_s_a_s_c_s_d_s_e_s_a_s          GTGCTGGTGGCTGTGTCTGTGTGGGTGACGGTGCTGGTGTCTGTG;
>clamp_s_c_s_d_s_c_s_a_s:compl:rev CACAGACACCCACACCGTCACCCACACTATCATGTTGACATTCC; dG = -27.7
   925 >s_e_s_b_s_a_s_e_s_d_s_c_s_a_s          GTGCTGGTGGCTGTGTCTGTGCTGGTGACGGTGTGGGTGTCTGTG;
>clamp_s_c_s_d_s_c_s_a_s:compl:rev CACAGACACCCACACCGTCACCCACACTATCATGTTGACATTCC; dG = -27.7
   926 >s_e_s_b_s_b_s_c_s_d_s_e_s_a_s          GTGCTGGTGGCTGTGGCTGTGTGGGTGACGGTGCTGGTGTCTGTG;
>clamp_s_c_s_d_s_c_s_a_s:compl:rev CACAGACACCCACACCGTCACCCACACTATCATGTTGACATTCC; dG = -27.7
   927 >s_e_s_b_s_b_s_e_s_d_s_c_s_a_s          GTGCTGGTGGCTGTGGCTGTGCTGGTGACGGTGTGGGTGTCTGTG;
>clamp_s_c_s_d_s_c_s_a_s:compl:rev CACAGACACCCACACCGTCACCCACACTATCATGTTGACATTCC; dG = -27.7
   928 >s_e_s_b_s_c_s_c_s_d_s_d_s_a_s          GTGCTGGTGGCTGTGTGGGTGTGGGTGACGGTGACGGTGTCTGTG;
>clamp_s_c_s_d_s_c_s_a_s:compl:rev CACAGACACCCACACCGTCACCCACACTATCATGTTGACATTCC; dG = -27.7
   929 >s_e_s_b_s_c_s_c_s_c_s_c_s_a_s          GTGCTGGTGGCTGTGTGGGTGTGGGTGTGGGTGTGGGTGTCTGTG;
>clamp_s_c_s_d_s_c_s_a_s:compl:rev CACAGACACCCACACCGTCACCCACACTATCATGTTGACATTCC; dG = -27.7
   930 >s_e_s_b_s_d_s_c_s_d_s_d_s_a_s          GTGCTGGTGGCTGTGACGGTGTGGGTGACGGTGACGGTGTCTGTG;
>clamp_s_c_s_d_s_c_s_a_s:compl:rev CACAGACACCCACACCGTCACCCACACTATCATGTTGACATTCC; dG = -27.7
   931 >s_a_s_c_s_a_s_e_s_d_s_c_s_a_s          GTGTCTGTGTGGGTGTCTGTGCTGGTGACGGTGTGGGTGTCTGTG;
>clamp_s_c_s_d_s_c_s_a_s:compl:rev CACAGACACCCACACCGTCACCCACACTATCATGTTGACATTCC; dG = -27.7
   932 >s_e_s_b_s_d_s_c_s_c_s_c_s_a_s          GTGCTGGTGGCTGTGACGGTGTGGGTGTGGGTGTGGGTGTCTGTG;
>clamp_s_c_s_d_s_c_s_a_s:compl:rev CACAGACACCCACACCGTCACCCACACTATCATGTTGACATTCC; dG = -27.7
   933 >s_e_s_b_s_d_s_d_s_d_s_c_s_a_s          GTGCTGGTGGCTGTGACGGTGACGGTGACGGTGTGGGTGTCTGTG;
>clamp_s_c_s_d_s_c_s_a_s:compl:rev CACAGACACCCACACCGTCACCCACACTATCATGTTGACATTCC; dG = -27.7
   934 >s_e_s_b_s_e_s_c_s_d_s_d_s_a_s          GTGCTGGTGGCTGTGCTGGTGTGGGTGACGGTGACGGTGTCTGTG;
>clamp_s_c_s_d_s_c_s_a_s:compl:rev CACAGACACCCACACCGTCACCCACACTATCATGTTGACATTCC; dG = -27.7
   935 >s_e_s_b_s_e_s_c_s_c_s_c_s_a_s          GTGCTGGTGGCTGTGCTGGTGTGGGTGTGGGTGTGGGTGTCTGTG;
>clamp_s_c_s_d_s_c_s_a_s:compl:rev CACAGACACCCACACCGTCACCCACACTATCATGTTGACATTCC; dG = -27.7
   936 >s_e_s_b_s_e_s_d_s_d_s_c_s_a_s          GTGCTGGTGGCTGTGCTGGTGACGGTGACGGTGTGGGTGTCTGTG;
>clamp_s_c_s_d_s_c_s_a_s:compl:rev CACAGACACCCACACCGTCACCCACACTATCATGTTGACATTCC; dG = -27.7
   937 >s_e_s_c_s_a_s_c_s_d_s_e_s_a_s          GTGCTGGTGTGGGTGTCTGTGTGGGTGACGGTGCTGGTGTCTGTG;
>clamp_s_c_s_d_s_c_s_a_s:compl:rev CACAGACACCCACACCGTCACCCACACTATCATGTTGACATTCC; dG = -27.7
   938 >s_e_s_c_s_a_s_e_s_d_s_c_s_a_s          GTGCTGGTGTGGGTGTCTGTGCTGGTGACGGTGTGGGTGTCTGTG;
>clamp_s_c_s_d_s_c_s_a_s:compl:rev CACAGACACCCACACCGTCACCCACACTATCATGTTGACATTCC; dG = -27.7
   939 >s_e_s_c_s_b_s_c_s_d_s_e_s_a_s          GTGCTGGTGTGGGTGGCTGTGTGGGTGACGGTGCTGGTGTCTGTG;
>clamp_s_c_s_d_s_c_s_a_s:compl:rev CACAGACACCCACACCGTCACCCACACTATCATGTTGACATTCC; dG = -27.7
   940 >s_e_s_c_s_b_s_e_s_d_s_c_s_a_s          GTGCTGGTGTGGGTGGCTGTGCTGGTGACGGTGTGGGTGTCTGTG;
>clamp_s_c_s_d_s_c_s_a_s:compl:rev CACAGACACCCACACCGTCACCCACACTATCATGTTGACATTCC; dG = -27.7
   941 >s_e_s_c_s_c_s_c_s_d_s_d_s_a_s          GTGCTGGTGTGGGTGTGGGTGTGGGTGACGGTGACGGTGTCTGTG;
>clamp_s_c_s_d_s_c_s_a_s:compl:rev CACAGACACCCACACCGTCACCCACACTATCATGTTGACATTCC; dG = -27.7
   942 >s_e_s_c_s_c_s_c_s_c_s_c_s_a_s          GTGCTGGTGTGGGTGTGGGTGTGGGTGTGGGTGTGGGTGTCTGTG;
>clamp_s_c_s_d_s_c_s_a_s:compl:rev CACAGACACCCACACCGTCACCCACACTATCATGTTGACATTCC; dG = -27.7
   943 >s_a_s_c_s_b_s_c_s_d_s_e_s_a_s          GTGTCTGTGTGGGTGGCTGTGTGGGTGACGGTGCTGGTGTCTGTG;
>clamp_s_c_s_d_s_c_s_a_s:compl:rev CACAGACACCCACACCGTCACCCACACTATCATGTTGACATTCC; dG = -27.7
   944 >s_e_s_c_s_d_s_d_s_d_s_c_s_a_s          GTGCTGGTGTGGGTGACGGTGACGGTGACGGTGTGGGTGTCTGTG;
>clamp_s_c_s_d_s_c_s_a_s:compl:rev CACAGACACCCACACCGTCACCCACACTATCATGTTGACATTCC; dG = -27.7
   945 >s_e_s_c_s_e_s_c_s_d_s_d_s_a_s          GTGCTGGTGTGGGTGCTGGTGTGGGTGACGGTGACGGTGTCTGTG;
>clamp_s_c_s_d_s_c_s_a_s:compl:rev CACAGACACCCACACCGTCACCCACACTATCATGTTGACATTCC; dG = -27.7
```

```
   946 >s_e_s_c_s_e_s_c_s_c_s_c_s_a_s          GTGCTGGTGTGGGTGCTGGTGTGGGTGTGGGTGTGGGTGTCTGTG;
>clamp_s_c_s_d_s_c_s_a_s:compl:rev CACAGACACCCACACCGTCACCCACACTATCATGTTGACATTCC; dG = -27.7
   947 >s_e_s_c_s_e_s_d_s_d_s_c_s_a_s          GTGCTGGTGTGGGTGCTGGTGACGGTGACGGTGTGGGTGTCTGTG;
>clamp_s_c_s_d_s_c_s_a_s:compl:rev CACAGACACCCACACCGTCACCCACACTATCATGTTGACATTCC; dG = -27.7
   948 >s_e_s_d_s_a_s_c_s_d_s_e_s_a_s          GTGCTGGTGACGGTGTCTGTGTGGGTGACGGTGCTGGTGTCTGTG;
>clamp_s_c_s_d_s_c_s_a_s:compl:rev CACAGACACCCACACCGTCACCCACACTATCATGTTGACATTCC; dG = -27.7
   949 >s_e_s_d_s_a_s_e_s_d_s_c_s_a_s          GTGCTGGTGACGGTGTCTGTGCTGGTGACGGTGTGGGTGTCTGTG;
>clamp_s_c_s_d_s_c_s_a_s:compl:rev CACAGACACCCACACCGTCACCCACACTATCATGTTGACATTCC; dG = -27.7
   950 >s_e_s_d_s_b_s_c_s_d_s_e_s_a_s          GTGCTGGTGACGGTGGCTGTGTGGGTGACGGTGCTGGTGTCTGTG;
>clamp_s_c_s_d_s_c_s_a_s:compl:rev CACAGACACCCACACCGTCACCCACACTATCATGTTGACATTCC; dG = -27.7
   951 >s_e_s_d_s_b_s_e_s_d_s_c_s_a_s          GTGCTGGTGACGGTGGCTGTGCTGGTGACGGTGTGGGTGTCTGTG;
>clamp_s_c_s_d_s_c_s_a_s:compl:rev CACAGACACCCACACCGTCACCCACACTATCATGTTGACATTCC; dG = -27.7
   952 >s_e_s_d_s_c_s_c_s_d_s_d_s_a_s          GTGCTGGTGACGGTGTGGGTGTGGGTGACGGTGACGGTGTCTGTG;
>clamp_s_c_s_d_s_c_s_a_s:compl:rev CACAGACACCCACACCGTCACCCACACTATCATGTTGACATTCC; dG = -27.7
   953 >s_e_s_d_s_c_s_c_s_c_s_c_s_a_s          GTGCTGGTGACGGTGTGGGTGTGGGTGTGGGTGTGGGTGTCTGTG;
>clamp_s_c_s_d_s_c_s_a_s:compl:rev CACAGACACCCACACCGTCACCCACACTATCATGTTGACATTCC; dG = -27.7
   954 >s_e_s_d_s_d_s_c_s_d_s_d_s_a_s          GTGCTGGTGACGGTGACGGTGTGGGTGACGGTGACGGTGTCTGTG;
>clamp_s_c_s_d_s_c_s_a_s:compl:rev CACAGACACCCACACCGTCACCCACACTATCATGTTGACATTCC; dG = -27.7
   955 >s_a_s_c_s_b_s_e_s_d_s_c_s_a_s          GTGTCTGTGTGGGTGGCTGTGCTGGTGACGGTGTGGGTGTCTGTG;
>clamp_s_c_s_d_s_c_s_a_s:compl:rev CACAGACACCCACACCGTCACCCACACTATCATGTTGACATTCC; dG = -27.7
   956 >s_e_s_d_s_d_s_c_s_c_s_c_s_a_s          GTGCTGGTGACGGTGACGGTGTGGGTGTGGGTGTGGGTGTCTGTG;
>clamp_s_c_s_d_s_c_s_a_s:compl:rev CACAGACACCCACACCGTCACCCACACTATCATGTTGACATTCC; dG = -27.7
   957 >s_e_s_d_s_d_s_d_s_d_s_c_s_a_s          GTGCTGGTGACGGTGACGGTGACGGTGACGGTGTGGGTGTCTGTG;
>clamp_s_c_s_d_s_c_s_a_s:compl:rev CACAGACACCCACACCGTCACCCACACTATCATGTTGACATTCC; dG = -27.7
   958 >s_e_s_d_s_e_s_c_s_d_s_d_s_a_s          GTGCTGGTGACGGTGCTGGTGTGGGTGACGGTGACGGTGTCTGTG;
>clamp_s_c_s_d_s_c_s_a_s:compl:rev CACAGACACCCACACCGTCACCCACACTATCATGTTGACATTCC; dG = -27.7
   959 >s_e_s_d_s_e_s_c_s_c_s_c_s_a_s          GTGCTGGTGACGGTGCTGGTGTGGGTGTGGGTGTGGGTGTCTGTG;
>clamp_s_c_s_d_s_c_s_a_s:compl:rev CACAGACACCCACACCGTCACCCACACTATCATGTTGACATTCC; dG = -27.7
   960 >s_e_s_d_s_e_s_d_s_d_s_c_s_a_s          GTGCTGGTGACGGTGCTGGTGACGGTGACGGTGTGGGTGTCTGTG;
>clamp_s_c_s_d_s_c_s_a_s:compl:rev CACAGACACCCACACCGTCACCCACACTATCATGTTGACATTCC; dG = -27.7
   961 >s_e_s_e_s_a_s_c_s_d_s_e_s_a_s          GTGCTGGTGCTGGTGTCTGTGTGGGTGACGGTGCTGGTGTCTGTG;
>clamp_s_c_s_d_s_c_s_a_s:compl:rev CACAGACACCCACACCGTCACCCACACTATCATGTTGACATTCC; dG = -27.7
   962 >s_e_s_e_s_a_s_e_s_d_s_c_s_a_s          GTGCTGGTGCTGGTGTCTGTGCTGGTGACGGTGTGGGTGTCTGTG;
>clamp_s_c_s_d_s_c_s_a_s:compl:rev CACAGACACCCACACCGTCACCCACACTATCATGTTGACATTCC; dG = -27.7
   963 >s_e_s_e_s_b_s_c_s_d_s_e_s_a_s          GTGCTGGTGCTGGTGGCTGTGTGGGTGACGGTGCTGGTGTCTGTG;
>clamp_s_c_s_d_s_c_s_a_s:compl:rev CACAGACACCCACACCGTCACCCACACTATCATGTTGACATTCC; dG = -27.7
   964 >s_e_s_e_s_b_s_e_s_d_s_c_s_a_s          GTGCTGGTGCTGGTGGCTGTGCTGGTGACGGTGTGGGTGTCTGTG;
>clamp_s_c_s_d_s_c_s_a_s:compl:rev CACAGACACCCACACCGTCACCCACACTATCATGTTGACATTCC; dG = -27.7
   965 >s_e_s_e_s_c_s_c_s_d_s_d_s_a_s          GTGCTGGTGCTGGTGTGGGTGTGGGTGACGGTGACGGTGTCTGTG;
>clamp_s_c_s_d_s_c_s_a_s:compl:rev CACAGACACCCACACCGTCACCCACACTATCATGTTGACATTCC; dG = -27.7
   966 >s_e_s_e_s_c_s_c_s_c_s_c_s_a_s          GTGCTGGTGCTGGTGTGGGTGTGGGTGTGGGTGTGGGTGTCTGTG;
>clamp_s_c_s_d_s_c_s_a_s:compl:rev CACAGACACCCACACCGTCACCCACACTATCATGTTGACATTCC; dG = -27.7
   967 >s_e_s_e_s_d_s_c_s_d_s_d_s_a_s          GTGCTGGTGCTGGTGACGGTGTGGGTGACGGTGACGGTGTCTGTG;
>clamp_s_c_s_d_s_c_s_a_s:compl:rev CACAGACACCCACACCGTCACCCACACTATCATGTTGACATTCC; dG = -27.7
   968 >s_e_s_e_s_d_s_c_s_c_s_c_s_a_s          GTGCTGGTGCTGGTGACGGTGTGGGTGTGGGTGTGGGTGTCTGTG;
>clamp_s_c_s_d_s_c_s_a_s:compl:rev CACAGACACCCACACCGTCACCCACACTATCATGTTGACATTCC; dG = -27.7
   969 >s_e_s_e_s_d_s_d_s_d_s_c_s_a_s          GTGCTGGTGCTGGTGACGGTGACGGTGACGGTGTGGGTGTCTGTG;
>clamp_s_c_s_d_s_c_s_a_s:compl:rev CACAGACACCCACACCGTCACCCACACTATCATGTTGACATTCC; dG = -27.7
   970 >s_a_s_c_s_c_s_c_s_d_s_d_s_a_s          GTGTCTGTGTGGGTGTGGGTGTGGGTGACGGTGACGGTGTCTGTG;
>clamp_s_c_s_d_s_c_s_a_s:compl:rev CACAGACACCCACACCGTCACCCACACTATCATGTTGACATTCC; dG = -27.7
   971 >s_e_s_e_s_e_s_c_s_d_s_d_s_a_s          GTGCTGGTGCTGGTGCTGGTGTGGGTGACGGTGACGGTGTCTGTG;
>clamp_s_c_s_d_s_c_s_a_s:compl:rev CACAGACACCCACACCGTCACCCACACTATCATGTTGACATTCC; dG = -27.7
   972 >s_e_s_e_s_e_s_c_s_c_s_c_s_a_s          GTGCTGGTGCTGGTGCTGGTGTGGGTGTGGGTGTGGGTGTCTGTG;
>clamp_s_c_s_d_s_c_s_a_s:compl:rev CACAGACACCCACACCGTCACCCACACTATCATGTTGACATTCC; dG = -27.7
   973 >s_e_s_e_s_e_s_d_s_d_s_c_s_a_s          GTGCTGGTGCTGGTGCTGGTGACGGTGACGGTGTGGGTGTCTGTG;
>clamp_s_c_s_d_s_c_s_a_s:compl:rev CACAGACACCCACACCGTCACCCACACTATCATGTTGACATTCC; dG = -27.7
   974 >s_a_s_c_s_c_s_c_s_c_s_c_s_a_s          GTGTCTGTGTGGGTGTGGGTGTGGGTGTGGGTGTGGGTGTCTGTG;
>clamp_s_c_s_d_s_c_s_a_s:compl:rev CACAGACACCCACACCGTCACCCACACTATCATGTTGACATTCC; dG = -27.7
   975 >s_a_s_a_s_b_s_c_s_d_s_e_s_a_s          GTGTCTGTGTCTGTGGCTGTGTGGGTGACGGTGCTGGTGTCTGTG;
>clamp_s_c_s_d_s_c_s_a_s:compl:rev CACAGACACCCACACCGTCACCCACACTATCATGTTGACATTCC; dG = -27.7
   976 >s_a_s_c_s_d_s_d_s_d_s_c_s_a_s          GTGTCTGTGTGGGTGACGGTGACGGTGACGGTGTGGGTGTCTGTG;
>clamp_s_c_s_d_s_c_s_a_s:compl:rev CACAGACACCCACACCGTCACCCACACTATCATGTTGACATTCC; dG = -27.7
   977 >s_a_s_c_s_e_s_c_s_d_s_d_s_a_s          GTGTCTGTGTGGGTGCTGGTGTGGGTGACGGTGACGGTGTCTGTG;
>clamp_s_c_s_d_s_c_s_a_s:compl:rev CACAGACACCCACACCGTCACCCACACTATCATGTTGACATTCC; dG = -27.7
   978 >s_a_s_c_s_e_s_c_s_c_s_c_s_a_s          GTGTCTGTGTGGGTGCTGGTGTGGGTGTGGGTGTGGGTGTCTGTG;
>clamp_s_c_s_d_s_c_s_a_s:compl:rev CACAGACACCCACACCGTCACCCACACTATCATGTTGACATTCC; dG = -27.7
   979 >s_a_s_c_s_e_s_d_s_d_s_c_s_a_s          GTGTCTGTGTGGGTGCTGGTGACGGTGACGGTGTGGGTGTCTGTG;
>clamp_s_c_s_d_s_c_s_a_s:compl:rev CACAGACACCCACACCGTCACCCACACTATCATGTTGACATTCC; dG = -27.7
   980 >s_a_s_d_s_a_s_c_s_d_s_e_s_a_s          GTGTCTGTGACGGTGTCTGTGTGGGTGACGGTGCTGGTGTCTGTG;
>clamp_s_c_s_d_s_c_s_a_s:compl:rev CACAGACACCCACACCGTCACCCACACTATCATGTTGACATTCC; dG = -27.7
   981 >s_a_s_d_s_a_s_e_s_d_s_c_s_a_s          GTGTCTGTGACGGTGTCTGTGCTGGTGACGGTGTGGGTGTCTGTG;
>clamp_s_c_s_d_s_c_s_a_s:compl:rev CACAGACACCCACACCGTCACCCACACTATCATGTTGACATTCC; dG = -27.7
   982 >s_a_s_d_s_b_s_c_s_d_s_e_s_a_s          GTGTCTGTGACGGTGGCTGTGTGGGTGACGGTGCTGGTGTCTGTG;
>clamp_s_c_s_d_s_c_s_a_s:compl:rev CACAGACACCCACACCGTCACCCACACTATCATGTTGACATTCC; dG = -27.7
```

```
   983 >s_a_s_d_s_b_s_e_s_d_s_c_s_a_s          GTGTCTGTGACGGTGGCTGTGCTGGTGACGGTGTGGGTGTCTGTG;
>clamp_s_c_s_d_s_c_s_a_s:compl:rev CACAGACACCCACACCGTCACCCACACTATCATGTTGACATTCC; dG = -27.7
   984 >s_a_s_d_s_c_s_c_s_d_s_d_s_a_s          GTGTCTGTGACGGTGTGGGTGTGGGTGACGGTGACGGTGTCTGTG;
>clamp_s_c_s_d_s_c_s_a_s:compl:rev CACAGACACCCACACCGTCACCCACACTATCATGTTGACATTCC; dG = -27.7
   985 >s_a_s_d_s_c_s_c_s_c_s_c_s_a_s          GTGTCTGTGACGGTGTGGGTGTGGGTGTGGGTGTGGGTGTCTGTG;
>clamp_s_c_s_d_s_c_s_a_s:compl:rev CACAGACACCCACACCGTCACCCACACTATCATGTTGACATTCC; dG = -27.7
   986 >s_a_s_a_s_b_s_e_s_d_s_c_s_a_s          GTGTCTGTGTCTGTGGCTGTGCTGGTGACGGTGTGGGTGTCTGTG;
>clamp_s_c_s_d_s_c_s_a_s:compl:rev CACAGACACCCACACCGTCACCCACACTATCATGTTGACATTCC; dG = -27.7
   987 >s_a_s_d_s_d_s_c_s_d_s_d_s_a_s          GTGTCTGTGACGGTGACGGTGTGGGTGACGGTGACGGTGTCTGTG;
>clamp_s_c_s_d_s_c_s_a_s:compl:rev CACAGACACCCACACCGTCACCCACACTATCATGTTGACATTCC; dG = -27.7
   988 >s_a_s_d_s_d_s_c_s_c_s_c_s_a_s          GTGTCTGTGACGGTGACGGTGTGGGTGTGGGTGTGGGTGTCTGTG;
>clamp_s_c_s_d_s_c_s_a_s:compl:rev CACAGACACCCACACCGTCACCCACACTATCATGTTGACATTCC; dG = -27.7
   989 >s_a_s_d_s_d_s_d_s_d_s_c_s_a_s          GTGTCTGTGACGGTGACGGTGACGGTGACGGTGTGGGTGTCTGTG;
>clamp_s_c_s_d_s_c_s_a_s:compl:rev CACAGACACCCACACCGTCACCCACACTATCATGTTGACATTCC; dG = -27.7
   990 >s_a_s_d_s_e_s_c_s_d_s_d_s_a_s          GTGTCTGTGACGGTGCTGGTGTGGGTGACGGTGACGGTGTCTGTG;
>clamp_s_c_s_d_s_c_s_a_s:compl:rev CACAGACACCCACACCGTCACCCACACTATCATGTTGACATTCC; dG = -27.7
   991 >s_a_s_d_s_e_s_c_s_c_s_c_s_a_s          GTGTCTGTGACGGTGCTGGTGTGGGTGTGGGTGTGGGTGTCTGTG;
>clamp_s_c_s_d_s_c_s_a_s:compl:rev CACAGACACCCACACCGTCACCCACACTATCATGTTGACATTCC; dG = -27.7
   992 >s_a_s_d_s_e_s_d_s_d_s_c_s_a_s          GTGTCTGTGACGGTGCTGGTGACGGTGACGGTGTGGGTGTCTGTG;
>clamp_s_c_s_d_s_c_s_a_s:compl:rev CACAGACACCCACACCGTCACCCACACTATCATGTTGACATTCC; dG = -27.7
   993 >s_a_s_e_s_a_s_c_s_d_s_e_s_a_s          GTGTCTGTGCTGGTGTCTGTGTGGGTGACGGTGCTGGTGTCTGTG;
>clamp_s_c_s_d_s_c_s_a_s:compl:rev CACAGACACCCACACCGTCACCCACACTATCATGTTGACATTCC; dG = -27.7
   994 >s_a_s_e_s_a_s_e_s_d_s_c_s_a_s          GTGTCTGTGCTGGTGTCTGTGCTGGTGACGGTGTGGGTGTCTGTG;
>clamp_s_c_s_d_s_c_s_a_s:compl:rev CACAGACACCCACACCGTCACCCACACTATCATGTTGACATTCC; dG = -27.7
   995 >s_a_s_e_s_b_s_c_s_d_s_e_s_a_s          GTGTCTGTGCTGGTGGCTGTGTGGGTGACGGTGCTGGTGTCTGTG;
>clamp_s_c_s_d_s_c_s_a_s:compl:rev CACAGACACCCACACCGTCACCCACACTATCATGTTGACATTCC; dG = -27.7
   996 >s_a_s_e_s_b_s_e_s_d_s_c_s_a_s          GTGTCTGTGCTGGTGGCTGTGCTGGTGACGGTGTGGGTGTCTGTG;
>clamp_s_c_s_d_s_c_s_a_s:compl:rev CACAGACACCCACACCGTCACCCACACTATCATGTTGACATTCC; dG = -27.7
   997 >s_a_s_e_s_c_s_c_s_d_s_d_s_a_s          GTGTCTGTGCTGGTGTGGGTGTGGGTGACGGTGACGGTGTCTGTG;
>clamp_s_c_s_d_s_c_s_a_s:compl:rev CACAGACACCCACACCGTCACCCACACTATCATGTTGACATTCC; dG = -27.7
   998 >s_a_s_e_s_c_s_c_s_c_s_c_s_a_s          GTGTCTGTGCTGGTGTGGGTGTGGGTGTGGGTGTGGGTGTCTGTG;
>clamp_s_c_s_d_s_c_s_a_s:compl:rev CACAGACACCCACACCGTCACCCACACTATCATGTTGACATTCC; dG = -27.7
   999 >s_a_s_e_s_d_s_c_s_d_s_d_s_a_s          GTGTCTGTGCTGGTGACGGTGTGGGTGACGGTGACGGTGTCTGTG;
>clamp_s_c_s_d_s_c_s_a_s:compl:rev CACAGACACCCACACCGTCACCCACACTATCATGTTGACATTCC; dG = -27.7
  1000 >s_a_s_e_s_d_s_c_s_c_s_c_s_a_s          GTGTCTGTGCTGGTGACGGTGTGGGTGTGGGTGTGGGTGTCTGTG;
>clamp_s_c_s_d_s_c_s_a_s:compl:rev CACAGACACCCACACCGTCACCCACACTATCATGTTGACATTCC; dG = -27.7
  1001 >s_a_s_a_s_c_s_c_s_d_s_d_s_a_s          GTGTCTGTGTCTGTGTGGGTGTGGGTGACGGTGACGGTGTCTGTG;
>clamp_s_c_s_d_s_c_s_a_s:compl:rev CACAGACACCCACACCGTCACCCACACTATCATGTTGACATTCC; dG = -27.7
  1002 >s_a_s_e_s_d_s_d_s_d_s_c_s_a_s          GTGTCTGTGCTGGTGACGGTGACGGTGACGGTGTGGGTGTCTGTG;
>clamp_s_c_s_d_s_c_s_a_s:compl:rev CACAGACACCCACACCGTCACCCACACTATCATGTTGACATTCC; dG = -27.7
  1003 >s_a_s_e_s_e_s_c_s_d_s_d_s_a_s          GTGTCTGTGCTGGTGCTGGTGTGGGTGACGGTGACGGTGTCTGTG;
>clamp_s_c_s_d_s_c_s_a_s:compl:rev CACAGACACCCACACCGTCACCCACACTATCATGTTGACATTCC; dG = -27.7
  1004 >s_a_s_e_s_e_s_c_s_c_s_c_s_a_s          GTGTCTGTGCTGGTGCTGGTGTGGGTGTGGGTGTGGGTGTCTGTG;
>clamp_s_c_s_d_s_c_s_a_s:compl:rev CACAGACACCCACACCGTCACCCACACTATCATGTTGACATTCC; dG = -27.7
  1005 >s_a_s_e_s_e_s_d_s_d_s_c_s_a_s          GTGTCTGTGCTGGTGCTGGTGACGGTGACGGTGTGGGTGTCTGTG;
>clamp_s_c_s_d_s_c_s_a_s:compl:rev CACAGACACCCACACCGTCACCCACACTATCATGTTGACATTCC; dG = -27.7
  1006 >s_b_s_a_s_a_s_c_s_d_s_e_s_a_s          GTGGCTGTGTCTGTGTCTGTGTGGGTGACGGTGCTGGTGTCTGTG;
>clamp_s_c_s_d_s_c_s_a_s:compl:rev CACAGACACCCACACCGTCACCCACACTATCATGTTGACATTCC; dG = -27.7
  1007 >s_b_s_a_s_a_s_e_s_d_s_c_s_a_s          GTGGCTGTGTCTGTGTCTGTGCTGGTGACGGTGTGGGTGTCTGTG;
>clamp_s_c_s_d_s_c_s_a_s:compl:rev CACAGACACCCACACCGTCACCCACACTATCATGTTGACATTCC; dG = -27.7
  1008 >s_a_s_a_s_c_s_c_s_c_s_c_s_a_s          GTGTCTGTGTCTGTGTGGGTGTGGGTGTGGGTGTGGGTGTCTGTG;
>clamp_s_c_s_d_s_c_s_a_s:compl:rev CACAGACACCCACACCGTCACCCACACTATCATGTTGACATTCC; dG = -27.7
  1009 >s_b_s_a_s_b_s_c_s_d_s_e_s_a_s          GTGGCTGTGTCTGTGGCTGTGTGGGTGACGGTGCTGGTGTCTGTG;
>clamp_s_c_s_d_s_c_s_a_s:compl:rev CACAGACACCCACACCGTCACCCACACTATCATGTTGACATTCC; dG = -27.7
  1010 >s_b_s_a_s_b_s_e_s_d_s_c_s_a_s          GTGGCTGTGTCTGTGGCTGTGCTGGTGACGGTGTGGGTGTCTGTG;
>clamp_s_c_s_d_s_c_s_a_s:compl:rev CACAGACACCCACACCGTCACCCACACTATCATGTTGACATTCC; dG = -27.7
  1011 >s_b_s_a_s_c_s_c_s_d_s_d_s_a_s          GTGGCTGTGTCTGTGTGGGTGTGGGTGACGGTGACGGTGTCTGTG;
>clamp_s_c_s_d_s_c_s_a_s:compl:rev CACAGACACCCACACCGTCACCCACACTATCATGTTGACATTCC; dG = -27.7
  1012 >s_b_s_a_s_c_s_c_s_c_s_c_s_a_s          GTGGCTGTGTCTGTGTGGGTGTGGGTGTGGGTGTGGGTGTCTGTG;
>clamp_s_c_s_d_s_c_s_a_s:compl:rev CACAGACACCCACACCGTCACCCACACTATCATGTTGACATTCC; dG = -27.7
  1013 >s_b_s_a_s_d_s_c_s_d_s_d_s_a_s          GTGGCTGTGTCTGTGACGGTGTGGGTGACGGTGACGGTGTCTGTG;
>clamp_s_c_s_d_s_c_s_a_s:compl:rev CACAGACACCCACACCGTCACCCACACTATCATGTTGACATTCC; dG = -27.7
  1014 >s_b_s_a_s_d_s_c_s_c_s_c_s_a_s          GTGGCTGTGTCTGTGACGGTGTGGGTGTGGGTGTGGGTGTCTGTG;
>clamp_s_c_s_d_s_c_s_a_s:compl:rev CACAGACACCCACACCGTCACCCACACTATCATGTTGACATTCC; dG = -27.7
  1015 >s_b_s_a_s_d_s_d_s_d_s_c_s_a_s          GTGGCTGTGTCTGTGACGGTGACGGTGACGGTGTGGGTGTCTGTG;
>clamp_s_c_s_d_s_c_s_a_s:compl:rev CACAGACACCCACACCGTCACCCACACTATCATGTTGACATTCC; dG = -27.7
  1016 >s_b_s_a_s_e_s_c_s_d_s_d_s_a_s          GTGGCTGTGTCTGTGCTGGTGTGGGTGACGGTGACGGTGTCTGTG;
>clamp_s_c_s_d_s_c_s_a_s:compl:rev CACAGACACCCACACCGTCACCCACACTATCATGTTGACATTCC; dG = -27.7
  1017 >s_b_s_a_s_e_s_c_s_c_s_c_s_a_s          GTGGCTGTGTCTGTGCTGGTGTGGGTGTGGGTGTGGGTGTCTGTG;
>clamp_s_c_s_d_s_c_s_a_s:compl:rev CACAGACACCCACACCGTCACCCACACTATCATGTTGACATTCC; dG = -27.7
  1018 >s_b_s_a_s_e_s_d_s_d_s_c_s_a_s          GTGGCTGTGTCTGTGCTGGTGACGGTGACGGTGTGGGTGTCTGTG;
>clamp_s_c_s_d_s_c_s_a_s:compl:rev CACAGACACCCACACCGTCACCCACACTATCATGTTGACATTCC; dG = -27.7
  1019 >s_b_s_b_s_a_s_c_s_d_s_e_s_a_s          GTGGCTGTGGCTGTGTCTGTGTGGGTGACGGTGCTGGTGTCTGTG;
>clamp_s_c_s_d_s_c_s_a_s:compl:rev CACAGACACCCACACCGTCACCCACACTATCATGTTGACATTCC; dG = -27.7
```

```
  1020 >s_b_s_b_s_a_s_e_s_d_s_c_s_a_s          GTGGCTGTGGCTGTGTCTGTGCTGGTGACGGTGTGGGTGTCTGTG;
>clamp_s_c_s_d_s_c_s_a_s:compl:rev CACAGACACCCACACCGTCACCCACACTATCATGTTGACATTCC; dG = -27.7
  1021 >s_a_s_a_s_a_s_c_s_d_s_e_s_a_s          GTGTCTGTGTCTGTGTCTGTGTGGGTGACGGTGCTGGTGTCTGTG;
>clamp_s_c_s_d_s_c_s_a_s:compl:rev CACAGACACCCACACCGTCACCCACACTATCATGTTGACATTCC; dG = -27.7
  1022 >s_b_s_b_s_b_s_c_s_d_s_e_s_a_s          GTGGCTGTGGCTGTGGCTGTGTGGGTGACGGTGCTGGTGTCTGTG;
>clamp_s_c_s_d_s_c_s_a_s:compl:rev CACAGACACCCACACCGTCACCCACACTATCATGTTGACATTCC; dG = -27.7
  1023 >s_b_s_b_s_b_s_e_s_d_s_c_s_a_s          GTGGCTGTGGCTGTGGCTGTGCTGGTGACGGTGTGGGTGTCTGTG;
>clamp_s_c_s_d_s_c_s_a_s:compl:rev CACAGACACCCACACCGTCACCCACACTATCATGTTGACATTCC; dG = -27.7
  1024 >s_b_s_b_s_c_s_c_s_d_s_d_s_a_s          GTGGCTGTGGCTGTGTGGGTGTGGGTGACGGTGACGGTGTCTGTG;
>clamp_s_c_s_d_s_c_s_a_s:compl:rev CACAGACACCCACACCGTCACCCACACTATCATGTTGACATTCC; dG = -27.7
  1025 >s_b_s_b_s_c_s_c_s_c_s_c_s_a_s          GTGGCTGTGGCTGTGTGGGTGTGGGTGTGGGTGTGGGTGTCTGTG;
>clamp_s_c_s_d_s_c_s_a_s:compl:rev CACAGACACCCACACCGTCACCCACACTATCATGTTGACATTCC; dG = -27.7
  1026 >s_b_s_b_s_d_s_c_s_d_s_d_s_a_s          GTGGCTGTGGCTGTGACGGTGTGGGTGACGGTGACGGTGTCTGTG;
>clamp_s_c_s_d_s_c_s_a_s:compl:rev CACAGACACCCACACCGTCACCCACACTATCATGTTGACATTCC; dG = -27.7
  1027 >s_b_s_b_s_d_s_c_s_c_s_c_s_a_s          GTGGCTGTGGCTGTGACGGTGTGGGTGTGGGTGTGGGTGTCTGTG;
>clamp_s_c_s_d_s_c_s_a_s:compl:rev CACAGACACCCACACCGTCACCCACACTATCATGTTGACATTCC; dG = -27.7
  1028 >s_a_s_a_s_d_s_c_s_d_s_d_s_a_s          GTGTCTGTGTCTGTGACGGTGTGGGTGACGGTGACGGTGTCTGTG;
>clamp_s_c_s_d_s_c_s_a_s:compl:rev CACAGACACCCACACCGTCACCCACACTATCATGTTGACATTCC; dG = -27.7
  1029 >s_b_s_b_s_d_s_d_s_d_s_c_s_a_s          GTGGCTGTGGCTGTGACGGTGACGGTGACGGTGTGGGTGTCTGTG;
>clamp_s_c_s_d_s_c_s_a_s:compl:rev CACAGACACCCACACCGTCACCCACACTATCATGTTGACATTCC; dG = -27.7
  1030 >s_b_s_b_s_e_s_c_s_d_s_d_s_a_s          GTGGCTGTGGCTGTGCTGGTGTGGGTGACGGTGACGGTGTCTGTG;
>clamp_s_c_s_d_s_c_s_a_s:compl:rev CACAGACACCCACACCGTCACCCACACTATCATGTTGACATTCC; dG = -27.7
  1031 >s_b_s_b_s_e_s_c_s_c_s_c_s_a_s          GTGGCTGTGGCTGTGCTGGTGTGGGTGTGGGTGTGGGTGTCTGTG;
>clamp_s_c_s_d_s_c_s_a_s:compl:rev CACAGACACCCACACCGTCACCCACACTATCATGTTGACATTCC; dG = -27.7
  1032 >s_b_s_b_s_e_s_d_s_d_s_c_s_a_s          GTGGCTGTGGCTGTGCTGGTGACGGTGACGGTGTGGGTGTCTGTG;
>clamp_s_c_s_d_s_c_s_a_s:compl:rev CACAGACACCCACACCGTCACCCACACTATCATGTTGACATTCC; dG = -27.7
  1033 >s_b_s_c_s_a_s_c_s_d_s_e_s_a_s          GTGGCTGTGTGGGTGTCTGTGTGGGTGACGGTGCTGGTGTCTGTG;
>clamp_s_c_s_d_s_c_s_a_s:compl:rev CACAGACACCCACACCGTCACCCACACTATCATGTTGACATTCC; dG = -27.7
  1034 >s_b_s_c_s_a_s_e_s_d_s_c_s_a_s          GTGGCTGTGTGGGTGTCTGTGCTGGTGACGGTGTGGGTGTCTGTG;
>clamp_s_c_s_d_s_c_s_a_s:compl:rev CACAGACACCCACACCGTCACCCACACTATCATGTTGACATTCC; dG = -27.7
  1035 >s_a_s_a_s_d_s_c_s_c_s_c_s_a_s          GTGTCTGTGTCTGTGACGGTGTGGGTGTGGGTGTGGGTGTCTGTG;
>clamp_s_c_s_d_s_c_s_a_s:compl:rev CACAGACACCCACACCGTCACCCACACTATCATGTTGACATTCC; dG = -27.7
  1036 >s_b_s_c_s_b_s_c_s_d_s_e_s_a_s          GTGGCTGTGTGGGTGGCTGTGTGGGTGACGGTGCTGGTGTCTGTG;
>clamp_s_c_s_d_s_c_s_a_s:compl:rev CACAGACACCCACACCGTCACCCACACTATCATGTTGACATTCC; dG = -27.7
  1037 >s_b_s_c_s_b_s_e_s_d_s_c_s_a_s          GTGGCTGTGTGGGTGGCTGTGCTGGTGACGGTGTGGGTGTCTGTG;
>clamp_s_c_s_d_s_c_s_a_s:compl:rev CACAGACACCCACACCGTCACCCACACTATCATGTTGACATTCC; dG = -27.7
  1038 >s_b_s_c_s_c_s_c_s_d_s_d_s_a_s          GTGGCTGTGTGGGTGTGGGTGTGGGTGACGGTGACGGTGTCTGTG;
>clamp_s_c_s_d_s_c_s_a_s:compl:rev CACAGACACCCACACCGTCACCCACACTATCATGTTGACATTCC; dG = -27.7
  1039 >s_b_s_c_s_c_s_c_s_c_s_c_s_a_s          GTGGCTGTGTGGGTGTGGGTGTGGGTGTGGGTGTGGGTGTCTGTG;
>clamp_s_c_s_d_s_c_s_a_s:compl:rev CACAGACACCCACACCGTCACCCACACTATCATGTTGACATTCC; dG = -27.7
  1040 >s_a_s_a_s_d_s_d_s_d_s_c_s_a_s          GTGTCTGTGTCTGTGACGGTGACGGTGACGGTGTGGGTGTCTGTG;
>clamp_s_c_s_d_s_c_s_a_s:compl:rev CACAGACACCCACACCGTCACCCACACTATCATGTTGACATTCC; dG = -27.7
  1041 >s_b_s_c_s_d_s_d_s_d_s_c_s_a_s          GTGGCTGTGTGGGTGACGGTGACGGTGACGGTGTGGGTGTCTGTG;
>clamp_s_c_s_d_s_c_s_a_s:compl:rev CACAGACACCCACACCGTCACCCACACTATCATGTTGACATTCC; dG = -27.7
  1042 >s_b_s_c_s_e_s_c_s_d_s_d_s_a_s          GTGGCTGTGTGGGTGCTGGTGTGGGTGACGGTGACGGTGTCTGTG;
>clamp_s_c_s_d_s_c_s_a_s:compl:rev CACAGACACCCACACCGTCACCCACACTATCATGTTGACATTCC; dG = -27.7
  1043 >s_b_s_c_s_e_s_c_s_c_s_c_s_a_s          GTGGCTGTGTGGGTGCTGGTGTGGGTGTGGGTGTGGGTGTCTGTG;
>clamp_s_c_s_d_s_c_s_a_s:compl:rev CACAGACACCCACACCGTCACCCACACTATCATGTTGACATTCC; dG = -27.7
  1044 >s_b_s_c_s_e_s_d_s_d_s_c_s_a_s          GTGGCTGTGTGGGTGCTGGTGACGGTGACGGTGTGGGTGTCTGTG;
>clamp_s_c_s_d_s_c_s_a_s:compl:rev CACAGACACCCACACCGTCACCCACACTATCATGTTGACATTCC; dG = -27.7
  1045 >s_b_s_d_s_a_s_c_s_d_s_e_s_a_s          GTGGCTGTGACGGTGTCTGTGTGGGTGACGGTGCTGGTGTCTGTG;
>clamp_s_c_s_d_s_c_s_a_s:compl:rev CACAGACACCCACACCGTCACCCACACTATCATGTTGACATTCC; dG = -27.7
  1046 >s_b_s_d_s_a_s_e_s_d_s_c_s_a_s          GTGGCTGTGACGGTGTCTGTGCTGGTGACGGTGTGGGTGTCTGTG;
>clamp_s_c_s_d_s_c_s_a_s:compl:rev CACAGACACCCACACCGTCACCCACACTATCATGTTGACATTCC; dG = -27.7
  1047 >s_b_s_d_s_b_s_c_s_d_s_e_s_a_s          GTGGCTGTGACGGTGGCTGTGTGGGTGACGGTGCTGGTGTCTGTG;
>clamp_s_c_s_d_s_c_s_a_s:compl:rev CACAGACACCCACACCGTCACCCACACTATCATGTTGACATTCC; dG = -27.7
  1048 >s_b_s_d_s_b_s_e_s_d_s_c_s_a_s          GTGGCTGTGACGGTGGCTGTGCTGGTGACGGTGTGGGTGTCTGTG;
>clamp_s_c_s_d_s_c_s_a_s:compl:rev CACAGACACCCACACCGTCACCCACACTATCATGTTGACATTCC; dG = -27.7
  1049 >s_b_s_d_s_c_s_c_s_d_s_d_s_a_s          GTGGCTGTGACGGTGTGGGTGTGGGTGACGGTGACGGTGTCTGTG;
>clamp_s_c_s_d_s_c_s_a_s:compl:rev CACAGACACCCACACCGTCACCCACACTATCATGTTGACATTCC; dG = -27.7
  1050 >s_b_s_d_s_c_s_c_s_c_s_c_s_a_s          GTGGCTGTGACGGTGTGGGTGTGGGTGTGGGTGTGGGTGTCTGTG;
>clamp_s_c_s_d_s_c_s_a_s:compl:rev CACAGACACCCACACCGTCACCCACACTATCATGTTGACATTCC; dG = -27.7
  1051 >s_b_s_d_s_d_s_c_s_d_s_d_s_a_s          GTGGCTGTGACGGTGACGGTGTGGGTGACGGTGACGGTGTCTGTG;
>clamp_s_c_s_d_s_c_s_a_s:compl:rev CACAGACACCCACACCGTCACCCACACTATCATGTTGACATTCC; dG = -27.7
  1052 >s_b_s_d_s_d_s_c_s_c_s_c_s_a_s          GTGGCTGTGACGGTGACGGTGTGGGTGTGGGTGTGGGTGTCTGTG;
>clamp_s_c_s_d_s_c_s_a_s:compl:rev CACAGACACCCACACCGTCACCCACACTATCATGTTGACATTCC; dG = -27.7
  1053 >s_a_s_a_s_e_s_c_s_d_s_d_s_a_s          GTGTCTGTGTCTGTGCTGGTGTGGGTGACGGTGACGGTGTCTGTG;
>clamp_s_c_s_d_s_c_s_a_s:compl:rev CACAGACACCCACACCGTCACCCACACTATCATGTTGACATTCC; dG = -27.7
  1054 >s_b_s_d_s_d_s_d_s_d_s_c_s_a_s          GTGGCTGTGACGGTGACGGTGACGGTGACGGTGTGGGTGTCTGTG;
>clamp_s_c_s_d_s_c_s_a_s:compl:rev CACAGACACCCACACCGTCACCCACACTATCATGTTGACATTCC; dG = -27.7
  1055 >s_b_s_d_s_e_s_c_s_d_s_d_s_a_s          GTGGCTGTGACGGTGCTGGTGTGGGTGACGGTGACGGTGTCTGTG;
>clamp_s_c_s_d_s_c_s_a_s:compl:rev CACAGACACCCACACCGTCACCCACACTATCATGTTGACATTCC; dG = -27.7
  1056 >s_b_s_d_s_e_s_c_s_c_s_c_s_a_s          GTGGCTGTGACGGTGCTGGTGTGGGTGTGGGTGTGGGTGTCTGTG;
>clamp_s_c_s_d_s_c_s_a_s:compl:rev CACAGACACCCACACCGTCACCCACACTATCATGTTGACATTCC; dG = -27.7
```

```
  1057 >s_b_s_d_s_e_s_d_s_d_s_c_s_a_s          GTGGCTGTGACGGTGCTGGTGACGGTGACGGTGTGGGTGTCTGTG;
>clamp_s_c_s_d_s_c_s_a_s:compl:rev CACAGACACCCACACCGTCACCCACACTATCATGTTGACATTCC; dG = -27.7
  1058 >s_b_s_e_s_a_s_c_s_d_s_e_s_a_s          GTGGCTGTGCTGGTGTCTGTGTGGGTGACGGTGCTGGTGTCTGTG;
>clamp_s_c_s_d_s_c_s_a_s:compl:rev CACAGACACCCACACCGTCACCCACACTATCATGTTGACATTCC; dG = -27.7
  1059 >s_b_s_e_s_a_s_e_s_d_s_c_s_a_s          GTGGCTGTGCTGGTGTCTGTGCTGGTGACGGTGTGGGTGTCTGTG;
>clamp_s_c_s_d_s_c_s_a_s:compl:rev CACAGACACCCACACCGTCACCCACACTATCATGTTGACATTCC; dG = -27.7
  1060 >s_b_s_e_s_b_s_c_s_d_s_e_s_a_s          GTGGCTGTGCTGGTGGCTGTGTGGGTGACGGTGCTGGTGTCTGTG;
>clamp_s_c_s_d_s_c_s_a_s:compl:rev CACAGACACCCACACCGTCACCCACACTATCATGTTGACATTCC; dG = -27.7
  1061 >s_a_s_a_s_e_s_c_s_c_s_c_s_a_s          GTGTCTGTGTCTGTGCTGGTGTGGGTGTGGGTGTGGGTGTCTGTG;
>clamp_s_c_s_d_s_c_s_a_s:compl:rev CACAGACACCCACACCGTCACCCACACTATCATGTTGACATTCC; dG = -27.7
  1062 >s_b_s_e_s_b_s_e_s_d_s_c_s_a_s          GTGGCTGTGCTGGTGGCTGTGCTGGTGACGGTGTGGGTGTCTGTG;
>clamp_s_c_s_d_s_c_s_a_s:compl:rev CACAGACACCCACACCGTCACCCACACTATCATGTTGACATTCC; dG = -27.7
  1063 >s_b_s_e_s_c_s_c_s_d_s_d_s_a_s          GTGGCTGTGCTGGTGTGGGTGTGGGTGACGGTGACGGTGTCTGTG;
>clamp_s_c_s_d_s_c_s_a_s:compl:rev CACAGACACCCACACCGTCACCCACACTATCATGTTGACATTCC; dG = -27.7
  1064 >s_b_s_e_s_c_s_c_s_c_s_c_s_a_s          GTGGCTGTGCTGGTGTGGGTGTGGGTGTGGGTGTGGGTGTCTGTG;
>clamp_s_c_s_d_s_c_s_a_s:compl:rev CACAGACACCCACACCGTCACCCACACTATCATGTTGACATTCC; dG = -27.7
  1065 >s_b_s_e_s_d_s_c_s_d_s_d_s_a_s          GTGGCTGTGCTGGTGACGGTGTGGGTGACGGTGACGGTGTCTGTG;
>clamp_s_c_s_d_s_c_s_a_s:compl:rev CACAGACACCCACACCGTCACCCACACTATCATGTTGACATTCC; dG = -27.7
  1066 >s_b_s_e_s_d_s_c_s_c_s_c_s_a_s          GTGGCTGTGCTGGTGACGGTGTGGGTGTGGGTGTGGGTGTCTGTG;
>clamp_s_c_s_d_s_c_s_a_s:compl:rev CACAGACACCCACACCGTCACCCACACTATCATGTTGACATTCC; dG = -27.7
  1067 >s_a_s_a_s_e_s_d_s_d_s_c_s_a_s          GTGTCTGTGTCTGTGCTGGTGACGGTGACGGTGTGGGTGTCTGTG;
>clamp_s_c_s_d_s_c_s_a_s:compl:rev CACAGACACCCACACCGTCACCCACACTATCATGTTGACATTCC; dG = -27.7
  1068 >s_b_s_e_s_d_s_d_s_d_s_c_s_a_s          GTGGCTGTGCTGGTGACGGTGACGGTGACGGTGTGGGTGTCTGTG;
>clamp_s_c_s_d_s_c_s_a_s:compl:rev CACAGACACCCACACCGTCACCCACACTATCATGTTGACATTCC; dG = -27.7
  1069 >s_b_s_e_s_e_s_c_s_d_s_d_s_a_s          GTGGCTGTGCTGGTGCTGGTGTGGGTGACGGTGACGGTGTCTGTG;
>clamp_s_c_s_d_s_c_s_a_s:compl:rev CACAGACACCCACACCGTCACCCACACTATCATGTTGACATTCC; dG = -27.7
  1070 >s_b_s_e_s_e_s_c_s_c_s_c_s_a_s          GTGGCTGTGCTGGTGCTGGTGTGGGTGTGGGTGTGGGTGTCTGTG;
>clamp_s_c_s_d_s_c_s_a_s:compl:rev CACAGACACCCACACCGTCACCCACACTATCATGTTGACATTCC; dG = -27.7
  1071 >s_b_s_e_s_e_s_d_s_d_s_c_s_a_s          GTGGCTGTGCTGGTGCTGGTGACGGTGACGGTGTGGGTGTCTGTG;
>clamp_s_c_s_d_s_c_s_a_s:compl:rev CACAGACACCCACACCGTCACCCACACTATCATGTTGACATTCC; dG = -27.7
  1072 >s_c_s_a_s_a_s_c_s_d_s_e_s_a_s          GTGTGGGTGTCTGTGTCTGTGTGGGTGACGGTGCTGGTGTCTGTG;
>clamp_s_c_s_d_s_c_s_a_s:compl:rev CACAGACACCCACACCGTCACCCACACTATCATGTTGACATTCC; dG = -27.7
  1073 >s_c_s_a_s_a_s_e_s_d_s_c_s_a_s          GTGTGGGTGTCTGTGTCTGTGCTGGTGACGGTGTGGGTGTCTGTG;
>clamp_s_c_s_d_s_c_s_a_s:compl:rev CACAGACACCCACACCGTCACCCACACTATCATGTTGACATTCC; dG = -27.7
  1074 >s_c_s_a_s_b_s_c_s_d_s_e_s_a_s          GTGTGGGTGTCTGTGGCTGTGTGGGTGACGGTGCTGGTGTCTGTG;
>clamp_s_c_s_d_s_c_s_a_s:compl:rev CACAGACACCCACACCGTCACCCACACTATCATGTTGACATTCC; dG = -27.7
  1075 >s_c_s_a_s_b_s_e_s_d_s_c_s_a_s          GTGTGGGTGTCTGTGGCTGTGCTGGTGACGGTGTGGGTGTCTGTG;
>clamp_s_c_s_d_s_c_s_a_s:compl:rev CACAGACACCCACACCGTCACCCACACTATCATGTTGACATTCC; dG = -27.7
  1076 >s_c_s_a_s_c_s_c_s_d_s_d_s_a_s          GTGTGGGTGTCTGTGTGGGTGTGGGTGACGGTGACGGTGTCTGTG;
>clamp_s_c_s_d_s_c_s_a_s:compl:rev CACAGACACCCACACCGTCACCCACACTATCATGTTGACATTCC; dG = -27.7
  1077 >s_c_s_a_s_c_s_c_s_c_s_c_s_a_s          GTGTGGGTGTCTGTGTGGGTGTGGGTGTGGGTGTGGGTGTCTGTG;
>clamp_s_c_s_d_s_c_s_a_s:compl:rev CACAGACACCCACACCGTCACCCACACTATCATGTTGACATTCC; dG = -27.7
  1078 >s_a_s_b_s_a_s_c_s_d_s_e_s_a_s          GTGTCTGTGGCTGTGTCTGTGTGGGTGACGGTGCTGGTGTCTGTG;
>clamp_s_c_s_d_s_c_s_a_s:compl:rev CACAGACACCCACACCGTCACCCACACTATCATGTTGACATTCC; dG = -27.7
  1079 >s_c_s_a_s_d_s_c_s_d_s_d_s_a_s          GTGTGGGTGTCTGTGACGGTGTGGGTGACGGTGACGGTGTCTGTG;
>clamp_s_c_s_d_s_c_s_a_s:compl:rev CACAGACACCCACACCGTCACCCACACTATCATGTTGACATTCC; dG = -27.7
  1080 >s_c_s_a_s_d_s_c_s_c_s_c_s_a_s          GTGTGGGTGTCTGTGACGGTGTGGGTGTGGGTGTGGGTGTCTGTG;
>clamp_s_c_s_d_s_c_s_a_s:compl:rev CACAGACACCCACACCGTCACCCACACTATCATGTTGACATTCC; dG = -27.7
  1081 >s_c_s_a_s_d_s_d_s_d_s_c_s_a_s          GTGTGGGTGTCTGTGACGGTGACGGTGACGGTGTGGGTGTCTGTG;
>clamp_s_c_s_d_s_c_s_a_s:compl:rev CACAGACACCCACACCGTCACCCACACTATCATGTTGACATTCC; dG = -27.7
  1082 >s_c_s_a_s_e_s_c_s_d_s_d_s_a_s          GTGTGGGTGTCTGTGCTGGTGTGGGTGACGGTGACGGTGTCTGTG;
>clamp_s_c_s_d_s_c_s_a_s:compl:rev CACAGACACCCACACCGTCACCCACACTATCATGTTGACATTCC; dG = -27.7
  1083 >s_c_s_a_s_e_s_c_s_c_s_c_s_a_s          GTGTGGGTGTCTGTGCTGGTGTGGGTGTGGGTGTGGGTGTCTGTG;
>clamp_s_c_s_d_s_c_s_a_s:compl:rev CACAGACACCCACACCGTCACCCACACTATCATGTTGACATTCC; dG = -27.7
  1084 >s_c_s_a_s_e_s_d_s_d_s_c_s_a_s          GTGTGGGTGTCTGTGCTGGTGACGGTGACGGTGTGGGTGTCTGTG;
>clamp_s_c_s_d_s_c_s_a_s:compl:rev CACAGACACCCACACCGTCACCCACACTATCATGTTGACATTCC; dG = -27.7
  1085 >s_c_s_b_s_a_s_c_s_d_s_e_s_a_s          GTGTGGGTGGCTGTGTCTGTGTGGGTGACGGTGCTGGTGTCTGTG;
>clamp_s_c_s_d_s_c_s_a_s:compl:rev CACAGACACCCACACCGTCACCCACACTATCATGTTGACATTCC; dG = -27.7
  1086 >s_c_s_b_s_a_s_e_s_d_s_c_s_a_s          GTGTGGGTGGCTGTGTCTGTGCTGGTGACGGTGTGGGTGTCTGTG;
>clamp_s_c_s_d_s_c_s_a_s:compl:rev CACAGACACCCACACCGTCACCCACACTATCATGTTGACATTCC; dG = -27.7
  1087 >s_c_s_b_s_b_s_c_s_d_s_e_s_a_s          GTGTGGGTGGCTGTGGCTGTGTGGGTGACGGTGCTGGTGTCTGTG;
>clamp_s_c_s_d_s_c_s_a_s:compl:rev CACAGACACCCACACCGTCACCCACACTATCATGTTGACATTCC; dG = -27.7
  1088 >s_c_s_b_s_b_s_e_s_d_s_c_s_a_s          GTGTGGGTGGCTGTGGCTGTGCTGGTGACGGTGTGGGTGTCTGTG;
>clamp_s_c_s_d_s_c_s_a_s:compl:rev CACAGACACCCACACCGTCACCCACACTATCATGTTGACATTCC; dG = -27.7
  1089 >s_c_s_b_s_c_s_c_s_d_s_d_s_a_s          GTGTGGGTGGCTGTGTGGGTGTGGGTGACGGTGACGGTGTCTGTG;
>clamp_s_c_s_d_s_c_s_a_s:compl:rev CACAGACACCCACACCGTCACCCACACTATCATGTTGACATTCC; dG = -27.7
  1090 >s_c_s_b_s_c_s_c_s_c_s_c_s_a_s          GTGTGGGTGGCTGTGTGGGTGTGGGTGTGGGTGTGGGTGTCTGTG;
>clamp_s_c_s_d_s_c_s_a_s:compl:rev CACAGACACCCACACCGTCACCCACACTATCATGTTGACATTCC; dG = -27.7
  1091 >s_a_s_b_s_a_s_e_s_d_s_c_s_a_s          GTGTCTGTGGCTGTGTCTGTGCTGGTGACGGTGTGGGTGTCTGTG;
>clamp_s_c_s_d_s_c_s_a_s:compl:rev CACAGACACCCACACCGTCACCCACACTATCATGTTGACATTCC; dG = -27.7
  1092 >s_c_s_b_s_d_s_c_s_d_s_d_s_a_s          GTGTGGGTGGCTGTGACGGTGTGGGTGACGGTGACGGTGTCTGTG;
>clamp_s_c_s_d_s_c_s_a_s:compl:rev CACAGACACCCACACCGTCACCCACACTATCATGTTGACATTCC; dG = -27.7
  1093 >s_c_s_b_s_d_s_c_s_c_s_c_s_a_s          GTGTGGGTGGCTGTGACGGTGTGGGTGTGGGTGTGGGTGTCTGTG;
>clamp_s_c_s_d_s_c_s_a_s:compl:rev CACAGACACCCACACCGTCACCCACACTATCATGTTGACATTCC; dG = -27.7
```

```
  1094 >s_c_s_b_s_d_s_d_s_d_s_c_s_a_s          GTGTGGGTGGCTGTGACGGTGACGGTGACGGTGTGGGTGTCTGTG;
>clamp_s_c_s_d_s_c_s_a_s:compl:rev CACAGACACCCACACCGTCACCCACACTATCATGTTGACATTCC; dG = -27.7
  1095 >s_c_s_b_s_e_s_c_s_d_s_d_s_a_s          GTGTGGGTGGCTGTGCTGGTGTGGGTGACGGTGACGGTGTCTGTG;
>clamp_s_c_s_d_s_c_s_a_s:compl:rev CACAGACACCCACACCGTCACCCACACTATCATGTTGACATTCC; dG = -27.7
  1096 >s_c_s_b_s_e_s_c_s_c_s_c_s_a_s          GTGTGGGTGGCTGTGCTGGTGTGGGTGTGGGTGTGGGTGTCTGTG;
>clamp_s_c_s_d_s_c_s_a_s:compl:rev CACAGACACCCACACCGTCACCCACACTATCATGTTGACATTCC; dG = -27.7
  1097 >s_c_s_b_s_e_s_d_s_d_s_c_s_a_s          GTGTGGGTGGCTGTGCTGGTGACGGTGACGGTGTGGGTGTCTGTG;
>clamp_s_c_s_d_s_c_s_a_s:compl:rev CACAGACACCCACACCGTCACCCACACTATCATGTTGACATTCC; dG = -27.7
  1098 >s_c_s_c_s_a_s_c_s_d_s_e_s_a_s          GTGTGGGTGTGGGTGTCTGTGTGGGTGACGGTGCTGGTGTCTGTG;
>clamp_s_c_s_d_s_c_s_a_s:compl:rev CACAGACACCCACACCGTCACCCACACTATCATGTTGACATTCC; dG = -27.7
  1099 >s_c_s_c_s_a_s_e_s_d_s_c_s_a_s          GTGTGGGTGTGGGTGTCTGTGCTGGTGACGGTGTGGGTGTCTGTG;
>clamp_s_c_s_d_s_c_s_a_s:compl:rev CACAGACACCCACACCGTCACCCACACTATCATGTTGACATTCC; dG = -27.7
  1100 >s_c_s_c_s_b_s_c_s_d_s_e_s_a_s          GTGTGGGTGTGGGTGGCTGTGTGGGTGACGGTGCTGGTGTCTGTG;
>clamp_s_c_s_d_s_c_s_a_s:compl:rev CACAGACACCCACACCGTCACCCACACTATCATGTTGACATTCC; dG = -27.7
  1101 >s_c_s_c_s_b_s_e_s_d_s_c_s_a_s          GTGTGGGTGTGGGTGGCTGTGCTGGTGACGGTGTGGGTGTCTGTG;
>clamp_s_c_s_d_s_c_s_a_s:compl:rev CACAGACACCCACACCGTCACCCACACTATCATGTTGACATTCC; dG = -27.7
  1102 >s_c_s_c_s_c_s_c_s_d_s_d_s_a_s          GTGTGGGTGTGGGTGTGGGTGTGGGTGACGGTGACGGTGTCTGTG;
>clamp_s_c_s_d_s_c_s_a_s:compl:rev CACAGACACCCACACCGTCACCCACACTATCATGTTGACATTCC; dG = -27.7
  1103 >s_c_s_c_s_d_s_e_s_d_s_c_s_a_s          GTGTGGGTGTGGGTGACGGTGCTGGTGACGGTGTGGGTGTCTGTG;
>clamp_s_c_s_d_s_c_s_a_s:compl:rev CACAGACACCCACACCGTCACCCACACTATCATGTTGACATTCC; dG = -27.6
  1104 >s_c_s_c_s_e_s_c_s_d_s_e_s_a_s          GTGTGGGTGTGGGTGCTGGTGTGGGTGACGGTGCTGGTGTCTGTG;
>clamp_s_c_s_d_s_c_s_a_s:compl:rev CACAGACACCCACACCGTCACCCACACTATCATGTTGACATTCC; dG = -27.6
  1105 >s_c_s_c_s_e_s_e_s_d_s_c_s_a_s          GTGTGGGTGTGGGTGCTGGTGCTGGTGACGGTGTGGGTGTCTGTG;
>clamp_s_c_s_d_s_c_s_a_s:compl:rev CACAGACACCCACACCGTCACCCACACTATCATGTTGACATTCC; dG = -27.6
  1106 >s_c_s_d_s_d_s_c_s_d_s_e_s_a_s          GTGTGGGTGACGGTGACGGTGTGGGTGACGGTGCTGGTGTCTGTG;
>clamp_s_c_s_d_s_c_s_a_s:compl:rev CACAGACACCCACACCGTCACCCACACTATCATGTTGACATTCC; dG = -27.6
  1107 >s_c_s_d_s_d_s_e_s_d_s_c_s_a_s          GTGTGGGTGACGGTGACGGTGCTGGTGACGGTGTGGGTGTCTGTG;
>clamp_s_c_s_d_s_c_s_a_s:compl:rev CACAGACACCCACACCGTCACCCACACTATCATGTTGACATTCC; dG = -27.6
  1108 >s_c_s_d_s_e_s_c_s_d_s_e_s_a_s          GTGTGGGTGACGGTGCTGGTGTGGGTGACGGTGCTGGTGTCTGTG;
>clamp_s_c_s_d_s_c_s_a_s:compl:rev CACAGACACCCACACCGTCACCCACACTATCATGTTGACATTCC; dG = -27.6
  1109 >s_c_s_d_s_e_s_e_s_d_s_c_s_a_s          GTGTGGGTGACGGTGCTGGTGCTGGTGACGGTGTGGGTGTCTGTG;
>clamp_s_c_s_d_s_c_s_a_s:compl:rev CACAGACACCCACACCGTCACCCACACTATCATGTTGACATTCC; dG = -27.6
  1110 >s_c_s_e_s_c_s_c_s_d_s_e_s_a_s          GTGTGGGTGCTGGTGTGGGTGTGGGTGACGGTGCTGGTGTCTGTG;
>clamp_s_c_s_d_s_c_s_a_s:compl:rev CACAGACACCCACACCGTCACCCACACTATCATGTTGACATTCC; dG = -27.6
  1111 >s_c_s_e_s_d_s_c_s_d_s_e_s_a_s          GTGTGGGTGCTGGTGACGGTGTGGGTGACGGTGCTGGTGTCTGTG;
>clamp_s_c_s_d_s_c_s_a_s:compl:rev CACAGACACCCACACCGTCACCCACACTATCATGTTGACATTCC; dG = -27.6
  1112 >s_a_s_b_s_c_s_c_s_d_s_e_s_a_s          GTGTCTGTGGCTGTGTGGGTGTGGGTGACGGTGCTGGTGTCTGTG;
>clamp_s_c_s_d_s_c_s_a_s:compl:rev CACAGACACCCACACCGTCACCCACACTATCATGTTGACATTCC; dG = -27.6
  1113 >s_c_s_e_s_d_s_e_s_d_s_c_s_a_s          GTGTGGGTGCTGGTGACGGTGCTGGTGACGGTGTGGGTGTCTGTG;
>clamp_s_c_s_d_s_c_s_a_s:compl:rev CACAGACACCCACACCGTCACCCACACTATCATGTTGACATTCC; dG = -27.6
  1114 >s_c_s_e_s_e_s_c_s_d_s_e_s_a_s          GTGTGGGTGCTGGTGCTGGTGTGGGTGACGGTGCTGGTGTCTGTG;
>clamp_s_c_s_d_s_c_s_a_s:compl:rev CACAGACACCCACACCGTCACCCACACTATCATGTTGACATTCC; dG = -27.6
  1115 >s_c_s_e_s_e_s_e_s_d_s_c_s_a_s          GTGTGGGTGCTGGTGCTGGTGCTGGTGACGGTGTGGGTGTCTGTG;
>clamp_s_c_s_d_s_c_s_a_s:compl:rev CACAGACACCCACACCGTCACCCACACTATCATGTTGACATTCC; dG = -27.6
  1116 >s_d_s_a_s_c_s_c_s_d_s_e_s_a_s          GTGACGGTGTCTGTGTGGGTGTGGGTGACGGTGCTGGTGTCTGTG;
>clamp_s_c_s_d_s_c_s_a_s:compl:rev CACAGACACCCACACCGTCACCCACACTATCATGTTGACATTCC; dG = -27.6
  1117 >s_d_s_a_s_d_s_c_s_d_s_e_s_a_s          GTGACGGTGTCTGTGACGGTGTGGGTGACGGTGCTGGTGTCTGTG;
>clamp_s_c_s_d_s_c_s_a_s:compl:rev CACAGACACCCACACCGTCACCCACACTATCATGTTGACATTCC; dG = -27.6
  1118 >s_d_s_a_s_d_s_e_s_d_s_c_s_a_s          GTGACGGTGTCTGTGACGGTGCTGGTGACGGTGTGGGTGTCTGTG;
>clamp_s_c_s_d_s_c_s_a_s:compl:rev CACAGACACCCACACCGTCACCCACACTATCATGTTGACATTCC; dG = -27.6
  1119 >s_d_s_a_s_e_s_c_s_d_s_e_s_a_s          GTGACGGTGTCTGTGCTGGTGTGGGTGACGGTGCTGGTGTCTGTG;
>clamp_s_c_s_d_s_c_s_a_s:compl:rev CACAGACACCCACACCGTCACCCACACTATCATGTTGACATTCC; dG = -27.6
  1120 >s_d_s_a_s_e_s_e_s_d_s_c_s_a_s          GTGACGGTGTCTGTGCTGGTGCTGGTGACGGTGTGGGTGTCTGTG;
>clamp_s_c_s_d_s_c_s_a_s:compl:rev CACAGACACCCACACCGTCACCCACACTATCATGTTGACATTCC; dG = -27.6
  1121 >s_d_s_b_s_c_s_c_s_d_s_e_s_a_s          GTGACGGTGGCTGTGTGGGTGTGGGTGACGGTGCTGGTGTCTGTG;
>clamp_s_c_s_d_s_c_s_a_s:compl:rev CACAGACACCCACACCGTCACCCACACTATCATGTTGACATTCC; dG = -27.6
  1122 >s_d_s_b_s_d_s_c_s_d_s_e_s_a_s          GTGACGGTGGCTGTGACGGTGTGGGTGACGGTGCTGGTGTCTGTG;
>clamp_s_c_s_d_s_c_s_a_s:compl:rev CACAGACACCCACACCGTCACCCACACTATCATGTTGACATTCC; dG = -27.6
  1123 >s_a_s_b_s_d_s_c_s_d_s_e_s_a_s          GTGTCTGTGGCTGTGACGGTGTGGGTGACGGTGCTGGTGTCTGTG;
>clamp_s_c_s_d_s_c_s_a_s:compl:rev CACAGACACCCACACCGTCACCCACACTATCATGTTGACATTCC; dG = -27.6
  1124 >s_d_s_b_s_d_s_e_s_d_s_c_s_a_s          GTGACGGTGGCTGTGACGGTGCTGGTGACGGTGTGGGTGTCTGTG;
>clamp_s_c_s_d_s_c_s_a_s:compl:rev CACAGACACCCACACCGTCACCCACACTATCATGTTGACATTCC; dG = -27.6
  1125 >s_d_s_b_s_e_s_c_s_d_s_e_s_a_s          GTGACGGTGGCTGTGCTGGTGTGGGTGACGGTGCTGGTGTCTGTG;
>clamp_s_c_s_d_s_c_s_a_s:compl:rev CACAGACACCCACACCGTCACCCACACTATCATGTTGACATTCC; dG = -27.6
  1126 >s_d_s_b_s_e_s_e_s_d_s_c_s_a_s          GTGACGGTGGCTGTGCTGGTGCTGGTGACGGTGTGGGTGTCTGTG;
>clamp_s_c_s_d_s_c_s_a_s:compl:rev CACAGACACCCACACCGTCACCCACACTATCATGTTGACATTCC; dG = -27.6
  1127 >s_d_s_c_s_c_s_c_s_d_s_e_s_a_s          GTGACGGTGTGGGTGTGGGTGTGGGTGACGGTGCTGGTGTCTGTG;
>clamp_s_c_s_d_s_c_s_a_s:compl:rev CACAGACACCCACACCGTCACCCACACTATCATGTTGACATTCC; dG = -27.6
  1128 >s_a_s_b_s_d_s_e_s_d_s_c_s_a_s          GTGTCTGTGGCTGTGACGGTGCTGGTGACGGTGTGGGTGTCTGTG;
>clamp_s_c_s_d_s_c_s_a_s:compl:rev CACAGACACCCACACCGTCACCCACACTATCATGTTGACATTCC; dG = -27.6
  1129 >s_d_s_c_s_d_s_e_s_d_s_c_s_a_s          GTGACGGTGTGGGTGACGGTGCTGGTGACGGTGTGGGTGTCTGTG;
>clamp_s_c_s_d_s_c_s_a_s:compl:rev CACAGACACCCACACCGTCACCCACACTATCATGTTGACATTCC; dG = -27.6
  1130 >s_d_s_c_s_e_s_c_s_d_s_e_s_a_s          GTGACGGTGTGGGTGCTGGTGTGGGTGACGGTGCTGGTGTCTGTG;
>clamp_s_c_s_d_s_c_s_a_s:compl:rev CACAGACACCCACACCGTCACCCACACTATCATGTTGACATTCC; dG = -27.6
```

1131 >s_d_s_c_s_e_s_e_s_d_s_c_s_a_s GTGACGGTGTGGGTGCTGGTGCTGGTGACGGTGTGGGTGTCTGTG;
>clamp_s_c_s_d_s_c_s_a_s:compl:rev CACAGACACCCACACCGTCACCCACACTATCATGTTGACATTCC; dG = -27.6
1132 >s_d_s_d_s_c_s_c_s_d_s_e_s_a_s GTGACGGTGACGGTGTGGGTGTGGGTGACGGTGCTGGTGTCTGTG;
>clamp_s_c_s_d_s_c_s_a_s:compl:rev CACAGACACCCACACCGTCACCCACACTATCATGTTGACATTCC; dG = -27.6
1133 >s_d_s_d_s_d_s_c_s_d_s_e_s_a_s GTGACGGTGACGGTGACGGTGTGGGTGACGGTGCTGGTGTCTGTG;
>clamp_s_c_s_d_s_c_s_a_s:compl:rev CACAGACACCCACACCGTCACCCACACTATCATGTTGACATTCC; dG = -27.6
1134 >s_a_s_b_s_e_s_c_s_d_s_e_s_a_s GTGTCTGTGGCTGTGCTGGTGTGGGTGACGGTGCTGGTGTCTGTG;
>clamp_s_c_s_d_s_c_s_a_s:compl:rev CACAGACACCCACACCGTCACCCACACTATCATGTTGACATTCC; dG = -27.6
1135 >s_d_s_d_s_d_s_e_s_d_s_c_s_a_s GTGACGGTGACGGTGACGGTGCTGGTGACGGTGTGGGTGTCTGTG;
>clamp_s_c_s_d_s_c_s_a_s:compl:rev CACAGACACCCACACCGTCACCCACACTATCATGTTGACATTCC; dG = -27.6
1136 >s_d_s_d_s_e_s_c_s_d_s_e_s_a_s GTGACGGTGACGGTGCTGGTGTGGGTGACGGTGCTGGTGTCTGTG;
>clamp_s_c_s_d_s_c_s_a_s:compl:rev CACAGACACCCACACCGTCACCCACACTATCATGTTGACATTCC; dG = -27.6
1137 >s_d_s_d_s_e_s_e_s_d_s_c_s_a_s GTGACGGTGACGGTGCTGGTGCTGGTGACGGTGTGGGTGTCTGTG;
>clamp_s_c_s_d_s_c_s_a_s:compl:rev CACAGACACCCACACCGTCACCCACACTATCATGTTGACATTCC; dG = -27.6
1138 >s_d_s_e_s_c_s_c_s_d_s_e_s_a_s GTGACGGTGCTGGTGTGGGTGTGGGTGACGGTGCTGGTGTCTGTG;
>clamp_s_c_s_d_s_c_s_a_s:compl:rev CACAGACACCCACACCGTCACCCACACTATCATGTTGACATTCC; dG = -27.6
1139 >s_d_s_e_s_d_s_c_s_d_s_e_s_a_s GTGACGGTGCTGGTGACGGTGTGGGTGACGGTGCTGGTGTCTGTG;
>clamp_s_c_s_d_s_c_s_a_s:compl:rev CACAGACACCCACACCGTCACCCACACTATCATGTTGACATTCC; dG = -27.6
1140 >s_a_s_b_s_e_s_e_s_d_s_c_s_a_s GTGTCTGTGGCTGTGCTGGTGCTGGTGACGGTGTGGGTGTCTGTG;
>clamp_s_c_s_d_s_c_s_a_s:compl:rev CACAGACACCCACACCGTCACCCACACTATCATGTTGACATTCC; dG = -27.6
1141 >s_d_s_e_s_d_s_e_s_d_s_c_s_a_s GTGACGGTGCTGGTGACGGTGCTGGTGACGGTGTGGGTGTCTGTG;
>clamp_s_c_s_d_s_c_s_a_s:compl:rev CACAGACACCCACACCGTCACCCACACTATCATGTTGACATTCC; dG = -27.6
1142 >s_d_s_e_s_e_s_c_s_d_s_e_s_a_s GTGACGGTGCTGGTGCTGGTGTGGGTGACGGTGCTGGTGTCTGTG;
>clamp_s_c_s_d_s_c_s_a_s:compl:rev CACAGACACCCACACCGTCACCCACACTATCATGTTGACATTCC; dG = -27.6
1143 >s_d_s_e_s_e_s_e_s_d_s_c_s_a_s GTGACGGTGCTGGTGCTGGTGCTGGTGACGGTGTGGGTGTCTGTG;
>clamp_s_c_s_d_s_c_s_a_s:compl:rev CACAGACACCCACACCGTCACCCACACTATCATGTTGACATTCC; dG = -27.6
1144 >s_e_s_a_s_c_s_c_s_d_s_e_s_a_s GTGCTGGTGTCTGTGTGGGTGTGGGTGACGGTGCTGGTGTCTGTG;
>clamp_s_c_s_d_s_c_s_a_s:compl:rev CACAGACACCCACACCGTCACCCACACTATCATGTTGACATTCC; dG = -27.6
1145 >s_e_s_a_s_d_s_c_s_d_s_e_s_a_s GTGCTGGTGTCTGTGACGGTGTGGGTGACGGTGCTGGTGTCTGTG;
>clamp_s_c_s_d_s_c_s_a_s:compl:rev CACAGACACCCACACCGTCACCCACACTATCATGTTGACATTCC; dG = -27.6
1146 >s_e_s_a_s_d_s_e_s_d_s_c_s_a_s GTGCTGGTGTCTGTGACGGTGCTGGTGACGGTGTGGGTGTCTGTG;
>clamp_s_c_s_d_s_c_s_a_s:compl:rev CACAGACACCCACACCGTCACCCACACTATCATGTTGACATTCC; dG = -27.6
1147 >s_e_s_a_s_e_s_c_s_d_s_e_s_a_s GTGCTGGTGTCTGTGCTGGTGTGGGTGACGGTGCTGGTGTCTGTG;
>clamp_s_c_s_d_s_c_s_a_s:compl:rev CACAGACACCCACACCGTCACCCACACTATCATGTTGACATTCC; dG = -27.6
1148 >s_e_s_a_s_e_s_e_s_d_s_c_s_a_s GTGCTGGTGTCTGTGCTGGTGCTGGTGACGGTGTGGGTGTCTGTG;
>clamp_s_c_s_d_s_c_s_a_s:compl:rev CACAGACACCCACACCGTCACCCACACTATCATGTTGACATTCC; dG = -27.6
1149 >s_e_s_b_s_c_s_c_s_d_s_e_s_a_s GTGCTGGTGGCTGTGTGGGTGTGGGTGACGGTGCTGGTGTCTGTG;
>clamp_s_c_s_d_s_c_s_a_s:compl:rev CACAGACACCCACACCGTCACCCACACTATCATGTTGACATTCC; dG = -27.6
1150 >s_e_s_b_s_d_s_c_s_d_s_e_s_a_s GTGCTGGTGGCTGTGACGGTGTGGGTGACGGTGCTGGTGTCTGTG;
>clamp_s_c_s_d_s_c_s_a_s:compl:rev CACAGACACCCACACCGTCACCCACACTATCATGTTGACATTCC; dG = -27.6
1151 >s_e_s_b_s_d_s_e_s_d_s_c_s_a_s GTGCTGGTGGCTGTGACGGTGCTGGTGACGGTGTGGGTGTCTGTG;
>clamp_s_c_s_d_s_c_s_a_s:compl:rev CACAGACACCCACACCGTCACCCACACTATCATGTTGACATTCC; dG = -27.6
1152 >s_e_s_b_s_e_s_c_s_d_s_e_s_a_s GTGCTGGTGGCTGTGCTGGTGTGGGTGACGGTGCTGGTGTCTGTG;
>clamp_s_c_s_d_s_c_s_a_s:compl:rev CACAGACACCCACACCGTCACCCACACTATCATGTTGACATTCC; dG = -27.6
1153 >s_e_s_b_s_e_s_e_s_d_s_c_s_a_s GTGCTGGTGGCTGTGCTGGTGCTGGTGACGGTGTGGGTGTCTGTG;
>clamp_s_c_s_d_s_c_s_a_s:compl:rev CACAGACACCCACACCGTCACCCACACTATCATGTTGACATTCC; dG = -27.6
1154 >s_e_s_c_s_c_s_c_s_d_s_e_s_a_s GTGCTGGTGTGGGTGTGGGTGTGGGTGACGGTGCTGGTGTCTGTG;
>clamp_s_c_s_d_s_c_s_a_s:compl:rev CACAGACACCCACACCGTCACCCACACTATCATGTTGACATTCC; dG = -27.6
1155 >s_e_s_c_s_d_s_e_s_d_s_c_s_a_s GTGCTGGTGTGGGTGACGGTGCTGGTGACGGTGTGGGTGTCTGTG;
>clamp_s_c_s_d_s_c_s_a_s:compl:rev CACAGACACCCACACCGTCACCCACACTATCATGTTGACATTCC; dG = -27.6
1156 >s_e_s_c_s_e_s_c_s_d_s_e_s_a_s GTGCTGGTGTGGGTGCTGGTGTGGGTGACGGTGCTGGTGTCTGTG;
>clamp_s_c_s_d_s_c_s_a_s:compl:rev CACAGACACCCACACCGTCACCCACACTATCATGTTGACATTCC; dG = -27.6
1157 >s_e_s_c_s_e_s_e_s_d_s_c_s_a_s GTGCTGGTGTGGGTGCTGGTGCTGGTGACGGTGTGGGTGTCTGTG;
>clamp_s_c_s_d_s_c_s_a_s:compl:rev CACAGACACCCACACCGTCACCCACACTATCATGTTGACATTCC; dG = -27.6
1158 >s_e_s_d_s_c_s_c_s_d_s_e_s_a_s GTGCTGGTGACGGTGTGGGTGTGGGTGACGGTGCTGGTGTCTGTG;
>clamp_s_c_s_d_s_c_s_a_s:compl:rev CACAGACACCCACACCGTCACCCACACTATCATGTTGACATTCC; dG = -27.6
1159 >s_e_s_d_s_d_s_c_s_d_s_e_s_a_s GTGCTGGTGACGGTGACGGTGTGGGTGACGGTGCTGGTGTCTGTG;
>clamp_s_c_s_d_s_c_s_a_s:compl:rev CACAGACACCCACACCGTCACCCACACTATCATGTTGACATTCC; dG = -27.6
1160 >s_e_s_d_s_d_s_e_s_d_s_c_s_a_s GTGCTGGTGACGGTGACGGTGCTGGTGACGGTGTGGGTGTCTGTG;
>clamp_s_c_s_d_s_c_s_a_s:compl:rev CACAGACACCCACACCGTCACCCACACTATCATGTTGACATTCC; dG = -27.6
1161 >s_e_s_d_s_e_s_c_s_d_s_e_s_a_s GTGCTGGTGACGGTGCTGGTGTGGGTGACGGTGCTGGTGTCTGTG;
>clamp_s_c_s_d_s_c_s_a_s:compl:rev CACAGACACCCACACCGTCACCCACACTATCATGTTGACATTCC; dG = -27.6
1162 >s_e_s_d_s_e_s_e_s_d_s_c_s_a_s GTGCTGGTGACGGTGCTGGTGCTGGTGACGGTGTGGGTGTCTGTG;
>clamp_s_c_s_d_s_c_s_a_s:compl:rev CACAGACACCCACACCGTCACCCACACTATCATGTTGACATTCC; dG = -27.6
1163 >s_e_s_e_s_c_s_c_s_d_s_e_s_a_s GTGCTGGTGCTGGTGTGGGTGTGGGTGACGGTGCTGGTGTCTGTG;
>clamp_s_c_s_d_s_c_s_a_s:compl:rev CACAGACACCCACACCGTCACCCACACTATCATGTTGACATTCC; dG = -27.6
1164 >s_e_s_e_s_d_s_c_s_d_s_e_s_a_s GTGCTGGTGCTGGTGACGGTGTGGGTGACGGTGCTGGTGTCTGTG;
>clamp_s_c_s_d_s_c_s_a_s:compl:rev CACAGACACCCACACCGTCACCCACACTATCATGTTGACATTCC; dG = -27.6
1165 >s_a_s_c_s_c_s_c_s_d_s_e_s_a_s GTGTCTGTGTGGGTGTGGGTGTGGGTGACGGTGCTGGTGTCTGTG;
>clamp_s_c_s_d_s_c_s_a_s:compl:rev CACAGACACCCACACCGTCACCCACACTATCATGTTGACATTCC; dG = -27.6
1166 >s_e_s_e_s_d_s_e_s_d_s_c_s_a_s GTGCTGGTGCTGGTGACGGTGCTGGTGACGGTGTGGGTGTCTGTG;
>clamp_s_c_s_d_s_c_s_a_s:compl:rev CACAGACACCCACACCGTCACCCACACTATCATGTTGACATTCC; dG = -27.6
1167 >s_e_s_e_s_e_s_c_s_d_s_e_s_a_s GTGCTGGTGCTGGTGCTGGTGTGGGTGACGGTGCTGGTGTCTGTG;
>clamp_s_c_s_d_s_c_s_a_s:compl:rev CACAGACACCCACACCGTCACCCACACTATCATGTTGACATTCC; dG = -27.6

```
  1168 >s_e_s_e_s_e_s_e_s_d_s_c_s_a_s          GTGCTGGTGCTGGTGCTGGTGCTGGTGACGGTGTGGGTGTCTGTG;
>clamp_s_c_s_d_s_c_s_a_s:compl:rev CACAGACACCCACACCGTCACCCACACTATCATGTTGACATTCC; dG = -27.6
  1169 >s_a_s_c_s_d_s_e_s_d_s_c_s_a_s          GTGTCTGTGTGGGTGACGGTGCTGGTGACGGTGTGGGTGTCTGTG;
>clamp_s_c_s_d_s_c_s_a_s:compl:rev CACAGACACCCACACCGTCACCCACACTATCATGTTGACATTCC; dG = -27.6
  1170 >s_a_s_c_s_e_s_c_s_d_s_e_s_a_s          GTGTCTGTGTGGGTGCTGGTGTGGGTGACGGTGCTGGTGTCTGTG;
>clamp_s_c_s_d_s_c_s_a_s:compl:rev CACAGACACCCACACCGTCACCCACACTATCATGTTGACATTCC; dG = -27.6
  1171 >s_a_s_c_s_e_s_e_s_d_s_c_s_a_s          GTGTCTGTGTGGGTGCTGGTGCTGGTGACGGTGTGGGTGTCTGTG;
>clamp_s_c_s_d_s_c_s_a_s:compl:rev CACAGACACCCACACCGTCACCCACACTATCATGTTGACATTCC; dG = -27.6
  1172 >s_a_s_d_s_c_s_c_s_d_s_e_s_a_s          GTGTCTGTGACGGTGTGGGTGTGGGTGACGGTGCTGGTGTCTGTG;
>clamp_s_c_s_d_s_c_s_a_s:compl:rev CACAGACACCCACACCGTCACCCACACTATCATGTTGACATTCC; dG = -27.6
  1173 >s_a_s_d_s_d_s_c_s_d_s_e_s_a_s          GTGTCTGTGACGGTGACGGTGTGGGTGACGGTGCTGGTGTCTGTG;
>clamp_s_c_s_d_s_c_s_a_s:compl:rev CACAGACACCCACACCGTCACCCACACTATCATGTTGACATTCC; dG = -27.6
  1174 >s_a_s_d_s_d_s_e_s_d_s_c_s_a_s          GTGTCTGTGACGGTGACGGTGCTGGTGACGGTGTGGGTGTCTGTG;
>clamp_s_c_s_d_s_c_s_a_s:compl:rev CACAGACACCCACACCGTCACCCACACTATCATGTTGACATTCC; dG = -27.6
  1175 >s_a_s_d_s_e_s_c_s_d_s_e_s_a_s          GTGTCTGTGACGGTGCTGGTGTGGGTGACGGTGCTGGTGTCTGTG;
>clamp_s_c_s_d_s_c_s_a_s:compl:rev CACAGACACCCACACCGTCACCCACACTATCATGTTGACATTCC; dG = -27.6
  1176 >s_a_s_d_s_e_s_e_s_d_s_c_s_a_s          GTGTCTGTGACGGTGCTGGTGCTGGTGACGGTGTGGGTGTCTGTG;
>clamp_s_c_s_d_s_c_s_a_s:compl:rev CACAGACACCCACACCGTCACCCACACTATCATGTTGACATTCC; dG = -27.6
  1177 >s_a_s_e_s_c_s_c_s_d_s_e_s_a_s          GTGTCTGTGCTGGTGTGGGTGTGGGTGACGGTGCTGGTGTCTGTG;
>clamp_s_c_s_d_s_c_s_a_s:compl:rev CACAGACACCCACACCGTCACCCACACTATCATGTTGACATTCC; dG = -27.6
  1178 >s_a_s_a_s_c_s_c_s_d_s_e_s_a_s          GTGTCTGTGTCTGTGTGGGTGTGGGTGACGGTGCTGGTGTCTGTG;
>clamp_s_c_s_d_s_c_s_a_s:compl:rev CACAGACACCCACACCGTCACCCACACTATCATGTTGACATTCC; dG = -27.6
  1179 >s_a_s_e_s_d_s_c_s_d_s_e_s_a_s          GTGTCTGTGCTGGTGACGGTGTGGGTGACGGTGCTGGTGTCTGTG;
>clamp_s_c_s_d_s_c_s_a_s:compl:rev CACAGACACCCACACCGTCACCCACACTATCATGTTGACATTCC; dG = -27.6
  1180 >s_a_s_e_s_d_s_e_s_d_s_c_s_a_s          GTGTCTGTGCTGGTGACGGTGCTGGTGACGGTGTGGGTGTCTGTG;
>clamp_s_c_s_d_s_c_s_a_s:compl:rev CACAGACACCCACACCGTCACCCACACTATCATGTTGACATTCC; dG = -27.6
  1181 >s_a_s_e_s_e_s_c_s_d_s_e_s_a_s          GTGTCTGTGCTGGTGCTGGTGTGGGTGACGGTGCTGGTGTCTGTG;
>clamp_s_c_s_d_s_c_s_a_s:compl:rev CACAGACACCCACACCGTCACCCACACTATCATGTTGACATTCC; dG = -27.6
  1182 >s_a_s_e_s_e_s_e_s_d_s_c_s_a_s          GTGTCTGTGCTGGTGCTGGTGCTGGTGACGGTGTGGGTGTCTGTG;
>clamp_s_c_s_d_s_c_s_a_s:compl:rev CACAGACACCCACACCGTCACCCACACTATCATGTTGACATTCC; dG = -27.6
  1183 >s_b_s_a_s_c_s_c_s_d_s_e_s_a_s          GTGGCTGTGTCTGTGTGGGTGTGGGTGACGGTGCTGGTGTCTGTG;
>clamp_s_c_s_d_s_c_s_a_s:compl:rev CACAGACACCCACACCGTCACCCACACTATCATGTTGACATTCC; dG = -27.6
  1184 >s_b_s_a_s_d_s_c_s_d_s_e_s_a_s          GTGGCTGTGTCTGTGACGGTGTGGGTGACGGTGCTGGTGTCTGTG;
>clamp_s_c_s_d_s_c_s_a_s:compl:rev CACAGACACCCACACCGTCACCCACACTATCATGTTGACATTCC; dG = -27.6
  1185 >s_b_s_a_s_d_s_e_s_d_s_c_s_a_s          GTGGCTGTGTCTGTGACGGTGCTGGTGACGGTGTGGGTGTCTGTG;
>clamp_s_c_s_d_s_c_s_a_s:compl:rev CACAGACACCCACACCGTCACCCACACTATCATGTTGACATTCC; dG = -27.6
  1186 >s_b_s_a_s_e_s_c_s_d_s_e_s_a_s          GTGGCTGTGTCTGTGCTGGTGTGGGTGACGGTGCTGGTGTCTGTG;
>clamp_s_c_s_d_s_c_s_a_s:compl:rev CACAGACACCCACACCGTCACCCACACTATCATGTTGACATTCC; dG = -27.6
  1187 >s_b_s_a_s_e_s_e_s_d_s_c_s_a_s          GTGGCTGTGTCTGTGCTGGTGCTGGTGACGGTGTGGGTGTCTGTG;
>clamp_s_c_s_d_s_c_s_a_s:compl:rev CACAGACACCCACACCGTCACCCACACTATCATGTTGACATTCC; dG = -27.6
  1188 >s_b_s_b_s_c_s_c_s_d_s_e_s_a_s          GTGGCTGTGGCTGTGTGGGTGTGGGTGACGGTGCTGGTGTCTGTG;
>clamp_s_c_s_d_s_c_s_a_s:compl:rev CACAGACACCCACACCGTCACCCACACTATCATGTTGACATTCC; dG = -27.6
  1189 >s_a_s_a_s_d_s_c_s_d_s_e_s_a_s          GTGTCTGTGTCTGTGACGGTGTGGGTGACGGTGCTGGTGTCTGTG;
>clamp_s_c_s_d_s_c_s_a_s:compl:rev CACAGACACCCACACCGTCACCCACACTATCATGTTGACATTCC; dG = -27.6
  1190 >s_b_s_b_s_d_s_c_s_d_s_e_s_a_s          GTGGCTGTGGCTGTGACGGTGTGGGTGACGGTGCTGGTGTCTGTG;
>clamp_s_c_s_d_s_c_s_a_s:compl:rev CACAGACACCCACACCGTCACCCACACTATCATGTTGACATTCC; dG = -27.6
  1191 >s_b_s_b_s_d_s_e_s_d_s_c_s_a_s          GTGGCTGTGGCTGTGACGGTGCTGGTGACGGTGTGGGTGTCTGTG;
>clamp_s_c_s_d_s_c_s_a_s:compl:rev CACAGACACCCACACCGTCACCCACACTATCATGTTGACATTCC; dG = -27.6
  1192 >s_b_s_b_s_e_s_c_s_d_s_e_s_a_s          GTGGCTGTGGCTGTGCTGGTGTGGGTGACGGTGCTGGTGTCTGTG;
>clamp_s_c_s_d_s_c_s_a_s:compl:rev CACAGACACCCACACCGTCACCCACACTATCATGTTGACATTCC; dG = -27.6
  1193 >s_b_s_b_s_e_s_e_s_d_s_c_s_a_s          GTGGCTGTGGCTGTGCTGGTGCTGGTGACGGTGTGGGTGTCTGTG;
>clamp_s_c_s_d_s_c_s_a_s:compl:rev CACAGACACCCACACCGTCACCCACACTATCATGTTGACATTCC; dG = -27.6
  1194 >s_b_s_c_s_c_s_c_s_d_s_e_s_a_s          GTGGCTGTGTGGGTGTGGGTGTGGGTGACGGTGCTGGTGTCTGTG;
>clamp_s_c_s_d_s_c_s_a_s:compl:rev CACAGACACCCACACCGTCACCCACACTATCATGTTGACATTCC; dG = -27.6
  1195 >s_a_s_a_s_d_s_e_s_d_s_c_s_a_s          GTGTCTGTGTCTGTGACGGTGCTGGTGACGGTGTGGGTGTCTGTG;
>clamp_s_c_s_d_s_c_s_a_s:compl:rev CACAGACACCCACACCGTCACCCACACTATCATGTTGACATTCC; dG = -27.6
  1196 >s_b_s_c_s_d_s_e_s_d_s_c_s_a_s          GTGGCTGTGTGGGTGACGGTGCTGGTGACGGTGTGGGTGTCTGTG;
>clamp_s_c_s_d_s_c_s_a_s:compl:rev CACAGACACCCACACCGTCACCCACACTATCATGTTGACATTCC; dG = -27.6
  1197 >s_b_s_c_s_e_s_c_s_d_s_e_s_a_s          GTGGCTGTGTGGGTGCTGGTGTGGGTGACGGTGCTGGTGTCTGTG;
>clamp_s_c_s_d_s_c_s_a_s:compl:rev CACAGACACCCACACCGTCACCCACACTATCATGTTGACATTCC; dG = -27.6
  1198 >s_b_s_c_s_e_s_e_s_d_s_c_s_a_s          GTGGCTGTGTGGGTGCTGGTGCTGGTGACGGTGTGGGTGTCTGTG;
>clamp_s_c_s_d_s_c_s_a_s:compl:rev CACAGACACCCACACCGTCACCCACACTATCATGTTGACATTCC; dG = -27.6
  1199 >s_b_s_d_s_c_s_c_s_d_s_e_s_a_s          GTGGCTGTGACGGTGTGGGTGTGGGTGACGGTGCTGGTGTCTGTG;
>clamp_s_c_s_d_s_c_s_a_s:compl:rev CACAGACACCCACACCGTCACCCACACTATCATGTTGACATTCC; dG = -27.6
  1200 >s_a_s_a_s_e_s_c_s_d_s_e_s_a_s          GTGTCTGTGTCTGTGCTGGTGTGGGTGACGGTGCTGGTGTCTGTG;
>clamp_s_c_s_d_s_c_s_a_s:compl:rev CACAGACACCCACACCGTCACCCACACTATCATGTTGACATTCC; dG = -27.6
  1201 >s_b_s_d_s_d_s_c_s_d_s_e_s_a_s          GTGGCTGTGACGGTGACGGTGTGGGTGACGGTGCTGGTGTCTGTG;
>clamp_s_c_s_d_s_c_s_a_s:compl:rev CACAGACACCCACACCGTCACCCACACTATCATGTTGACATTCC; dG = -27.6
  1202 >s_b_s_d_s_d_s_e_s_d_s_c_s_a_s          GTGGCTGTGACGGTGACGGTGCTGGTGACGGTGTGGGTGTCTGTG;
>clamp_s_c_s_d_s_c_s_a_s:compl:rev CACAGACACCCACACCGTCACCCACACTATCATGTTGACATTCC; dG = -27.6
  1203 >s_b_s_d_s_e_s_c_s_d_s_e_s_a_s          GTGGCTGTGACGGTGCTGGTGTGGGTGACGGTGCTGGTGTCTGTG;
>clamp_s_c_s_d_s_c_s_a_s:compl:rev CACAGACACCCACACCGTCACCCACACTATCATGTTGACATTCC; dG = -27.6
  1204 >s_b_s_d_s_e_s_e_s_d_s_c_s_a_s          GTGGCTGTGACGGTGCTGGTGCTGGTGACGGTGTGGGTGTCTGTG;
>clamp_s_c_s_d_s_c_s_a_s:compl:rev CACAGACACCCACACCGTCACCCACACTATCATGTTGACATTCC; dG = -27.6
```

1205 >s_b_s_e_s_c_s_c_s_d_s_e_s_a_s GTGGCTGTGCTGGTGTGGGTGTGGGTGACGGTGCTGGTGTCTGTG;
>clamp_s_c_s_d_s_c_s_a_s:compl:rev CACAGACACCCACACCGTCACCCACACTATCATGTTGACATTCC; dG = -27.6
1206 >s_a_s_a_s_e_s_e_s_d_s_c_s_a_s GTGTCTGTGTCTGTGCTGGTGCTGGTGACGGTGTGGGTGTCTGTG;
>clamp_s_c_s_d_s_c_s_a_s:compl:rev CACAGACACCCACACCGTCACCCACACTATCATGTTGACATTCC; dG = -27.6
1207 >s_b_s_e_s_d_s_c_s_d_s_e_s_a_s GTGGCTGTGCTGGTGACGGTGTGGGTGACGGTGCTGGTGTCTGTG;
>clamp_s_c_s_d_s_c_s_a_s:compl:rev CACAGACACCCACACCGTCACCCACACTATCATGTTGACATTCC; dG = -27.6
1208 >s_b_s_e_s_d_s_e_s_d_s_c_s_a_s GTGGCTGTGCTGGTGACGGTGCTGGTGACGGTGTGGGTGTCTGTG;
>clamp_s_c_s_d_s_c_s_a_s:compl:rev CACAGACACCCACACCGTCACCCACACTATCATGTTGACATTCC; dG = -27.6
1209 >s_b_s_e_s_e_s_c_s_d_s_e_s_a_s GTGGCTGTGCTGGTGCTGGTGTGGGTGACGGTGCTGGTGTCTGTG;
>clamp_s_c_s_d_s_c_s_a_s:compl:rev CACAGACACCCACACCGTCACCCACACTATCATGTTGACATTCC; dG = -27.6
1210 >s_b_s_e_s_e_s_e_s_d_s_c_s_a_s GTGGCTGTGCTGGTGCTGGTGCTGGTGACGGTGTGGGTGTCTGTG;
>clamp_s_c_s_d_s_c_s_a_s:compl:rev CACAGACACCCACACCGTCACCCACACTATCATGTTGACATTCC; dG = -27.6
1211 >s_c_s_a_s_c_s_c_s_d_s_e_s_a_s GTGTGGGTGTCTGTGTGGGTGTGGGTGACGGTGCTGGTGTCTGTG;
>clamp_s_c_s_d_s_c_s_a_s:compl:rev CACAGACACCCACACCGTCACCCACACTATCATGTTGACATTCC; dG = -27.6
1212 >s_c_s_a_s_d_s_c_s_d_s_e_s_a_s GTGTGGGTGTCTGTGACGGTGTGGGTGACGGTGCTGGTGTCTGTG;
>clamp_s_c_s_d_s_c_s_a_s:compl:rev CACAGACACCCACACCGTCACCCACACTATCATGTTGACATTCC; dG = -27.6
1213 >s_c_s_a_s_d_s_e_s_d_s_c_s_a_s GTGTGGGTGTCTGTGACGGTGCTGGTGACGGTGTGGGTGTCTGTG;
>clamp_s_c_s_d_s_c_s_a_s:compl:rev CACAGACACCCACACCGTCACCCACACTATCATGTTGACATTCC; dG = -27.6
1214 >s_c_s_a_s_e_s_c_s_d_s_e_s_a_s GTGTGGGTGTCTGTGCTGGTGTGGGTGACGGTGCTGGTGTCTGTG;
>clamp_s_c_s_d_s_c_s_a_s:compl:rev CACAGACACCCACACCGTCACCCACACTATCATGTTGACATTCC; dG = -27.6
1215 >s_c_s_a_s_e_s_e_s_d_s_c_s_a_s GTGTGGGTGTCTGTGCTGGTGCTGGTGACGGTGTGGGTGTCTGTG;
>clamp_s_c_s_d_s_c_s_a_s:compl:rev CACAGACACCCACACCGTCACCCACACTATCATGTTGACATTCC; dG = -27.6
1216 >s_c_s_b_s_c_s_c_s_d_s_e_s_a_s GTGTGGGTGGCTGTGTGGGTGTGGGTGACGGTGCTGGTGTCTGTG;
>clamp_s_c_s_d_s_c_s_a_s:compl:rev CACAGACACCCACACCGTCACCCACACTATCATGTTGACATTCC; dG = -27.6
1217 >s_c_s_b_s_d_s_c_s_d_s_e_s_a_s GTGTGGGTGGCTGTGACGGTGTGGGTGACGGTGCTGGTGTCTGTG;
>clamp_s_c_s_d_s_c_s_a_s:compl:rev CACAGACACCCACACCGTCACCCACACTATCATGTTGACATTCC; dG = -27.6
1218 >s_c_s_b_s_d_s_e_s_d_s_c_s_a_s GTGTGGGTGGCTGTGACGGTGCTGGTGACGGTGTGGGTGTCTGTG;
>clamp_s_c_s_d_s_c_s_a_s:compl:rev CACAGACACCCACACCGTCACCCACACTATCATGTTGACATTCC; dG = -27.6
1219 >s_c_s_b_s_e_s_c_s_d_s_e_s_a_s GTGTGGGTGGCTGTGCTGGTGTGGGTGACGGTGCTGGTGTCTGTG;
>clamp_s_c_s_d_s_c_s_a_s:compl:rev CACAGACACCCACACCGTCACCCACACTATCATGTTGACATTCC; dG = -27.6
1220 >s_c_s_b_s_e_s_e_s_d_s_c_s_a_s GTGTGGGTGGCTGTGCTGGTGCTGGTGACGGTGTGGGTGTCTGTG;
>clamp_s_c_s_d_s_c_s_a_s:compl:rev CACAGACACCCACACCGTCACCCACACTATCATGTTGACATTCC; dG = -27.6
1221 >s_c_s_c_s_c_s_c_s_d_s_e_s_a_s GTGTGGGTGTGGGTGTGGGTGTGGGTGACGGTGCTGGTGTCTGTG;
>clamp_s_c_s_d_s_c_s_a_s:compl:rev CACAGACACCCACACCGTCACCCACACTATCATGTTGACATTCC; dG = -27.6
1222 >s_c_s_c_s_d_s_b_s_d_s_c_s_a_s GTGTGGGTGTGGGTGACGGTGGCTGTGACGGTGTGGGTGTCTGTG;
>clamp_s_c_s_d_s_c_s_a_s:compl:rev CACAGACACCCACACCGTCACCCACACTATCATGTTGACATTCC; dG = -27.5
1223 >s_c_s_c_s_d_s_a_s_d_s_c_s_a_s GTGTGGGTGTGGGTGACGGTGTCTGTGACGGTGTGGGTGTCTGTG;
>clamp_s_c_s_d_s_c_s_a_s:compl:rev CACAGACACCCACACCGTCACCCACACTATCATGTTGACATTCC; dG = -27.5
1224 >s_c_s_c_s_e_s_b_s_d_s_c_s_a_s GTGTGGGTGTGGGTGCTGGTGGCTGTGACGGTGTGGGTGTCTGTG;
>clamp_s_c_s_d_s_c_s_a_s:compl:rev CACAGACACCCACACCGTCACCCACACTATCATGTTGACATTCC; dG = -27.5
1225 >s_c_s_c_s_e_s_a_s_d_s_c_s_a_s GTGTGGGTGTGGGTGCTGGTGTCTGTGACGGTGTGGGTGTCTGTG;
>clamp_s_c_s_d_s_c_s_a_s:compl:rev CACAGACACCCACACCGTCACCCACACTATCATGTTGACATTCC; dG = -27.5
1226 >s_d_s_c_s_a_s_b_s_d_s_c_s_a_s GTGACGGTGTGGGTGTCTGTGGCTGTGACGGTGTGGGTGTCTGTG;
>clamp_s_c_s_d_s_c_s_a_s:compl:rev CACAGACACCCACACCGTCACCCACACTATCATGTTGACATTCC; dG = -27.5
1227 >s_d_s_c_s_a_s_a_s_d_s_c_s_a_s GTGACGGTGTGGGTGTCTGTGTCTGTGACGGTGTGGGTGTCTGTG;
>clamp_s_c_s_d_s_c_s_a_s:compl:rev CACAGACACCCACACCGTCACCCACACTATCATGTTGACATTCC; dG = -27.5
1228 >s_d_s_c_s_b_s_b_s_d_s_c_s_a_s GTGACGGTGTGGGTGGCTGTGGCTGTGACGGTGTGGGTGTCTGTG;
>clamp_s_c_s_d_s_c_s_a_s:compl:rev CACAGACACCCACACCGTCACCCACACTATCATGTTGACATTCC; dG = -27.5
1229 >s_d_s_c_s_b_s_a_s_d_s_c_s_a_s GTGACGGTGTGGGTGGCTGTGTCTGTGACGGTGTGGGTGTCTGTG;
>clamp_s_c_s_d_s_c_s_a_s:compl:rev CACAGACACCCACACCGTCACCCACACTATCATGTTGACATTCC; dG = -27.5
1230 >s_d_s_c_s_d_s_b_s_d_s_c_s_a_s GTGACGGTGTGGGTGACGGTGGCTGTGACGGTGTGGGTGTCTGTG;
>clamp_s_c_s_d_s_c_s_a_s:compl:rev CACAGACACCCACACCGTCACCCACACTATCATGTTGACATTCC; dG = -27.5
1231 >s_d_s_c_s_d_s_a_s_d_s_c_s_a_s GTGACGGTGTGGGTGACGGTGTCTGTGACGGTGTGGGTGTCTGTG;
>clamp_s_c_s_d_s_c_s_a_s:compl:rev CACAGACACCCACACCGTCACCCACACTATCATGTTGACATTCC; dG = -27.5
1232 >s_d_s_c_s_e_s_b_s_d_s_c_s_a_s GTGACGGTGTGGGTGCTGGTGGCTGTGACGGTGTGGGTGTCTGTG;
>clamp_s_c_s_d_s_c_s_a_s:compl:rev CACAGACACCCACACCGTCACCCACACTATCATGTTGACATTCC; dG = -27.5
1233 >s_d_s_c_s_e_s_a_s_d_s_c_s_a_s GTGACGGTGTGGGTGCTGGTGTCTGTGACGGTGTGGGTGTCTGTG;
>clamp_s_c_s_d_s_c_s_a_s:compl:rev CACAGACACCCACACCGTCACCCACACTATCATGTTGACATTCC; dG = -27.5
1234 >s_a_s_c_s_a_s_b_s_d_s_c_s_a_s GTGTCTGTGTGGGTGTCTGTGGCTGTGACGGTGTGGGTGTCTGTG;
>clamp_s_c_s_d_s_c_s_a_s:compl:rev CACAGACACCCACACCGTCACCCACACTATCATGTTGACATTCC; dG = -27.5
1235 >s_a_s_c_s_a_s_a_s_d_s_c_s_a_s GTGTCTGTGTGGGTGTCTGTGTCTGTGACGGTGTGGGTGTCTGTG;
>clamp_s_c_s_d_s_c_s_a_s:compl:rev CACAGACACCCACACCGTCACCCACACTATCATGTTGACATTCC; dG = -27.5
1236 >s_e_s_c_s_a_s_b_s_d_s_c_s_a_s GTGCTGGTGTGGGTGTCTGTGGCTGTGACGGTGTGGGTGTCTGTG;
>clamp_s_c_s_d_s_c_s_a_s:compl:rev CACAGACACCCACACCGTCACCCACACTATCATGTTGACATTCC; dG = -27.5
1237 >s_e_s_c_s_a_s_a_s_d_s_c_s_a_s GTGCTGGTGTGGGTGTCTGTGTCTGTGACGGTGTGGGTGTCTGTG;
>clamp_s_c_s_d_s_c_s_a_s:compl:rev CACAGACACCCACACCGTCACCCACACTATCATGTTGACATTCC; dG = -27.5
1238 >s_e_s_c_s_b_s_b_s_d_s_c_s_a_s GTGCTGGTGTGGGTGGCTGTGGCTGTGACGGTGTGGGTGTCTGTG;
>clamp_s_c_s_d_s_c_s_a_s:compl:rev CACAGACACCCACACCGTCACCCACACTATCATGTTGACATTCC; dG = -27.5
1239 >s_e_s_c_s_b_s_a_s_d_s_c_s_a_s GTGCTGGTGTGGGTGGCTGTGTCTGTGACGGTGTGGGTGTCTGTG;
>clamp_s_c_s_d_s_c_s_a_s:compl:rev CACAGACACCCACACCGTCACCCACACTATCATGTTGACATTCC; dG = -27.5
1240 >s_e_s_c_s_d_s_b_s_d_s_c_s_a_s GTGCTGGTGTGGGTGACGGTGGCTGTGACGGTGTGGGTGTCTGTG;
>clamp_s_c_s_d_s_c_s_a_s:compl:rev CACAGACACCCACACCGTCACCCACACTATCATGTTGACATTCC; dG = -27.5
1241 >s_e_s_c_s_d_s_a_s_d_s_c_s_a_s GTGCTGGTGTGGGTGACGGTGTCTGTGACGGTGTGGGTGTCTGTG;
>clamp_s_c_s_d_s_c_s_a_s:compl:rev CACAGACACCCACACCGTCACCCACACTATCATGTTGACATTCC; dG = -27.5

1242 >s_e_s_c_s_e_s_b_s_d_s_c_s_a_s GTGCTGGTGTGGGTGCTGGTGGCTGTGACGGTGTGGGTGTCTGTG;
>clamp_s_c_s_d_s_c_s_a_s:compl:rev CACAGACACCCACACCGTCACCCACACTATCATGTTGACATTCC; dG = -27.5
1243 >s_e_s_c_s_e_s_a_s_d_s_c_s_a_s GTGCTGGTGTGGGTGCTGGTGTCTGTGACGGTGTGGGTGTCTGTG;
>clamp_s_c_s_d_s_c_s_a_s:compl:rev CACAGACACCCACACCGTCACCCACACTATCATGTTGACATTCC; dG = -27.5
1244 >s_a_s_c_s_b_s_b_s_d_s_c_s_a_s GTGTCTGTGTGGGTGGCTGTGGCTGTGACGGTGTGGGTGTCTGTG;
>clamp_s_c_s_d_s_c_s_a_s:compl:rev CACAGACACCCACACCGTCACCCACACTATCATGTTGACATTCC; dG = -27.5
1245 >s_a_s_c_s_b_s_a_s_d_s_c_s_a_s GTGTCTGTGTGGGTGGCTGTGTCTGTGACGGTGTGGGTGTCTGTG;
>clamp_s_c_s_d_s_c_s_a_s:compl:rev CACAGACACCCACACCGTCACCCACACTATCATGTTGACATTCC; dG = -27.5
1246 >s_a_s_c_s_d_s_b_s_d_s_c_s_a_s GTGTCTGTGTGGGTGACGGTGGCTGTGACGGTGTGGGTGTCTGTG;
>clamp_s_c_s_d_s_c_s_a_s:compl:rev CACAGACACCCACACCGTCACCCACACTATCATGTTGACATTCC; dG = -27.5
1247 >s_a_s_c_s_d_s_a_s_d_s_c_s_a_s GTGTCTGTGTGGGTGACGGTGTCTGTGACGGTGTGGGTGTCTGTG;
>clamp_s_c_s_d_s_c_s_a_s:compl:rev CACAGACACCCACACCGTCACCCACACTATCATGTTGACATTCC; dG = -27.5
1248 >s_a_s_c_s_e_s_b_s_d_s_c_s_a_s GTGTCTGTGTGGGTGCTGGTGGCTGTGACGGTGTGGGTGTCTGTG;
>clamp_s_c_s_d_s_c_s_a_s:compl:rev CACAGACACCCACACCGTCACCCACACTATCATGTTGACATTCC; dG = -27.5
1249 >s_a_s_c_s_e_s_a_s_d_s_c_s_a_s GTGTCTGTGTGGGTGCTGGTGTCTGTGACGGTGTGGGTGTCTGTG;
>clamp_s_c_s_d_s_c_s_a_s:compl:rev CACAGACACCCACACCGTCACCCACACTATCATGTTGACATTCC; dG = -27.5
1250 >s_b_s_c_s_a_s_b_s_d_s_c_s_a_s GTGGCTGTGTGGGTGTCTGTGGCTGTGACGGTGTGGGTGTCTGTG;
>clamp_s_c_s_d_s_c_s_a_s:compl:rev CACAGACACCCACACCGTCACCCACACTATCATGTTGACATTCC; dG = -27.5
1251 >s_b_s_c_s_a_s_a_s_d_s_c_s_a_s GTGGCTGTGTGGGTGTCTGTGTCTGTGACGGTGTGGGTGTCTGTG;
>clamp_s_c_s_d_s_c_s_a_s:compl:rev CACAGACACCCACACCGTCACCCACACTATCATGTTGACATTCC; dG = -27.5
1252 >s_b_s_c_s_b_s_b_s_d_s_c_s_a_s GTGGCTGTGTGGGTGGCTGTGGCTGTGACGGTGTGGGTGTCTGTG;
>clamp_s_c_s_d_s_c_s_a_s:compl:rev CACAGACACCCACACCGTCACCCACACTATCATGTTGACATTCC; dG = -27.5
1253 >s_b_s_c_s_b_s_a_s_d_s_c_s_a_s GTGGCTGTGTGGGTGGCTGTGTCTGTGACGGTGTGGGTGTCTGTG;
>clamp_s_c_s_d_s_c_s_a_s:compl:rev CACAGACACCCACACCGTCACCCACACTATCATGTTGACATTCC; dG = -27.5
1254 >s_b_s_c_s_d_s_b_s_d_s_c_s_a_s GTGGCTGTGTGGGTGACGGTGGCTGTGACGGTGTGGGTGTCTGTG;
>clamp_s_c_s_d_s_c_s_a_s:compl:rev CACAGACACCCACACCGTCACCCACACTATCATGTTGACATTCC; dG = -27.5
1255 >s_b_s_c_s_d_s_a_s_d_s_c_s_a_s GTGGCTGTGTGGGTGACGGTGTCTGTGACGGTGTGGGTGTCTGTG;
>clamp_s_c_s_d_s_c_s_a_s:compl:rev CACAGACACCCACACCGTCACCCACACTATCATGTTGACATTCC; dG = -27.5
1256 >s_b_s_c_s_e_s_b_s_d_s_c_s_a_s GTGGCTGTGTGGGTGCTGGTGGCTGTGACGGTGTGGGTGTCTGTG;
>clamp_s_c_s_d_s_c_s_a_s:compl:rev CACAGACACCCACACCGTCACCCACACTATCATGTTGACATTCC; dG = -27.5
1257 >s_b_s_c_s_e_s_a_s_d_s_c_s_a_s GTGGCTGTGTGGGTGCTGGTGTCTGTGACGGTGTGGGTGTCTGTG;
>clamp_s_c_s_d_s_c_s_a_s:compl:rev CACAGACACCCACACCGTCACCCACACTATCATGTTGACATTCC; dG = -27.5
1258 >s_c_s_c_s_a_s_b_s_d_s_c_s_a_s GTGTGGGTGTGGGTGTCTGTGGCTGTGACGGTGTGGGTGTCTGTG;
>clamp_s_c_s_d_s_c_s_a_s:compl:rev CACAGACACCCACACCGTCACCCACACTATCATGTTGACATTCC; dG = -27.5
1259 >s_c_s_c_s_a_s_a_s_d_s_c_s_a_s GTGTGGGTGTGGGTGTCTGTGTCTGTGACGGTGTGGGTGTCTGTG;
>clamp_s_c_s_d_s_c_s_a_s:compl:rev CACAGACACCCACACCGTCACCCACACTATCATGTTGACATTCC; dG = -27.5
1260 >s_c_s_c_s_b_s_b_s_d_s_c_s_a_s GTGTGGGTGTGGGTGGCTGTGGCTGTGACGGTGTGGGTGTCTGTG;
>clamp_s_c_s_d_s_c_s_a_s:compl:rev CACAGACACCCACACCGTCACCCACACTATCATGTTGACATTCC; dG = -27.5
1261 >s_c_s_c_s_b_s_a_s_d_s_c_s_a_s GTGTGGGTGTGGGTGGCTGTGTCTGTGACGGTGTGGGTGTCTGTG;
>clamp_s_c_s_d_s_c_s_a_s:compl:rev CACAGACACCCACACCGTCACCCACACTATCATGTTGACATTCC; dG = -27.5
1262 >s_c_s_c_s_e_s_c_s_a_s_c_s_a_s GTGTGGGTGTGGGTGCTGGTGTGGGTGTCTGTGTGGGTGTCTGTG;
>clamp_s_c_s_d_s_c_s_a_s:compl:rev CACAGACACCCACACCGTCACCCACACTATCATGTTGACATTCC; dG = -27.2
1263 >s_a_s_b_s_d_s_c_s_a_s_c_s_a_s GTGTCTGTGGCTGTGACGGTGTGGGTGTCTGTGTGGGTGTCTGTG;
>clamp_s_c_s_d_s_c_s_a_s:compl:rev CACAGACACCCACACCGTCACCCACACTATCATGTTGACATTCC; dG = -27.2
1264 >s_d_s_c_s_e_s_c_s_a_s_c_s_a_s GTGACGGTGTGGGTGCTGGTGTGGGTGTCTGTGTGGGTGTCTGTG;
>clamp_s_c_s_d_s_c_s_a_s:compl:rev CACAGACACCCACACCGTCACCCACACTATCATGTTGACATTCC; dG = -27.2
1265 >s_e_s_c_s_e_s_c_s_a_s_c_s_a_s GTGCTGGTGTGGGTGCTGGTGTGGGTGTCTGTGTGGGTGTCTGTG;
>clamp_s_c_s_d_s_c_s_a_s:compl:rev CACAGACACCCACACCGTCACCCACACTATCATGTTGACATTCC; dG = -27.2
1266 >s_a_s_c_s_e_s_c_s_a_s_c_s_a_s GTGTCTGTGTGGGTGCTGGTGTGGGTGTCTGTGTGGGTGTCTGTG;
>clamp_s_c_s_d_s_c_s_a_s:compl:rev CACAGACACCCACACCGTCACCCACACTATCATGTTGACATTCC; dG = -27.2
1267 >s_b_s_b_s_d_s_c_s_a_s_c_s_a_s GTGGCTGTGGCTGTGACGGTGTGGGTGTCTGTGTGGGTGTCTGTG;
>clamp_s_c_s_d_s_c_s_a_s:compl:rev CACAGACACCCACACCGTCACCCACACTATCATGTTGACATTCC; dG = -27.2
1268 >s_b_s_c_s_e_s_c_s_a_s_c_s_a_s GTGGCTGTGTGGGTGCTGGTGTGGGTGTCTGTGTGGGTGTCTGTG;
>clamp_s_c_s_d_s_c_s_a_s:compl:rev CACAGACACCCACACCGTCACCCACACTATCATGTTGACATTCC; dG = -27.2
1269 >s_c_s_d_s_a_s_a_s_d_s_c_s_a_s GTGTGGGTGACGGTGTCTGTGTCTGTGACGGTGTGGGTGTCTGTG;
>clamp_s_c_s_d_s_c_s_a_s:compl:rev CACAGACACCCACACCGTCACCCACACTATCATGTTGACATTCC; dG = -27.1
1270 >s_d_s_b_s_d_s_c_s_a_s_c_s_a_s GTGACGGTGGCTGTGACGGTGTGGGTGTCTGTGTGGGTGTCTGTG;
>clamp_s_c_s_d_s_c_s_a_s:compl:rev CACAGACACCCACACCGTCACCCACACTATCATGTTGACATTCC; dG = -27.1
1271 >s_e_s_b_s_d_s_c_s_a_s_c_s_a_s GTGCTGGTGGCTGTGACGGTGTGGGTGTCTGTGTGGGTGTCTGTG;
>clamp_s_c_s_d_s_c_s_a_s:compl:rev CACAGACACCCACACCGTCACCCACACTATCATGTTGACATTCC; dG = -27.1
1272 >s_d_s_a_s_d_s_c_s_a_s_c_s_a_s GTGACGGTGTCTGTGACGGTGTGGGTGTCTGTGTGGGTGTCTGTG;
>clamp_s_c_s_d_s_c_s_a_s:compl:rev CACAGACACCCACACCGTCACCCACACTATCATGTTGACATTCC; dG = -27.0
1273 >s_e_s_a_s_d_s_c_s_a_s_c_s_a_s GTGCTGGTGTCTGTGACGGTGTGGGTGTCTGTGTGGGTGTCTGTG;
>clamp_s_c_s_d_s_c_s_a_s:compl:rev CACAGACACCCACACCGTCACCCACACTATCATGTTGACATTCC; dG = -27.0
1274 >s_a_s_c_s_b_s_c_s_a_s_c_s_a_s GTGTCTGTGTGGGTGGCTGTGTGGGTGTCTGTGTGGGTGTCTGTG;
>clamp_s_c_s_d_s_c_s_a_s:compl:rev CACAGACACCCACACCGTCACCCACACTATCATGTTGACATTCC; dG = -27.0
1275 >s_b_s_a_s_d_s_c_s_a_s_c_s_a_s GTGGCTGTGTCTGTGACGGTGTGGGTGTCTGTGTGGGTGTCTGTG;
>clamp_s_c_s_d_s_c_s_a_s:compl:rev CACAGACACCCACACCGTCACCCACACTATCATGTTGACATTCC; dG = -27.0
1276 >s_b_s_c_s_b_s_c_s_a_s_c_s_a_s GTGGCTGTGTGGGTGGCTGTGTGGGTGTCTGTGTGGGTGTCTGTG;
>clamp_s_c_s_d_s_c_s_a_s:compl:rev CACAGACACCCACACCGTCACCCACACTATCATGTTGACATTCC; dG = -27.0
1277 >s_a_s_a_s_d_s_c_s_a_s_c_s_a_s GTGTCTGTGTCTGTGACGGTGTGGGTGTCTGTGTGGGTGTCTGTG;
>clamp_s_c_s_d_s_c_s_a_s:compl:rev CACAGACACCCACACCGTCACCCACACTATCATGTTGACATTCC; dG = -27.0
1278 >s_c_s_c_s_e_s_c_s_e_s_c_s_a_s GTGTGGGTGTGGGTGCTGGTGTGGGTGCTGGTGTGGGTGTCTGTG;
>clamp_s_c_s_d_s_c_s_a_s:compl:rev CACAGACACCCACACCGTCACCCACACTATCATGTTGACATTCC; dG = -26.9

1279 >s_c_s_d_s_a_s_c_s_e_s_c_s_a_s GTGTGGGTGACGGTGTCTGTGTGGGTGCTGGTGTGGGTGTCTGTG;
>clamp_s_c_s_d_s_c_s_a_s:compl:rev CACAGACACCCACACCGTCACCCACACTATCATGTTGACATTCC; dG = -26.9
1280 >s_a_s_b_s_b_s_c_s_e_s_c_s_a_s GTGTCTGTGGCTGTGGCTGTGTGGGTGCTGGTGTGGGTGTCTGTG;
>clamp_s_c_s_d_s_c_s_a_s:compl:rev CACAGACACCCACACCGTCACCCACACTATCATGTTGACATTCC; dG = -26.9
1281 >s_c_s_d_s_a_s_b_s_d_s_c_s_a_s GTGTGGGTGACGGTGTCTGTGGCTGTGACGGTGTGGGTGTCTGTG;
>clamp_s_c_s_d_s_c_s_a_s:compl:rev CACAGACACCCACACCGTCACCCACACTATCATGTTGACATTCC; dG = -26.9
1282 >s_c_s_d_s_b_s_c_s_e_s_c_s_a_s GTGTGGGTGACGGTGGCTGTGTGGGTGCTGGTGTGGGTGTCTGTG;
>clamp_s_c_s_d_s_c_s_a_s:compl:rev CACAGACACCCACACCGTCACCCACACTATCATGTTGACATTCC; dG = -26.9
1283 >s_c_s_d_s_b_s_b_s_d_s_c_s_a_s GTGTGGGTGACGGTGGCTGTGGCTGTGACGGTGTGGGTGTCTGTG;
>clamp_s_c_s_d_s_c_s_a_s:compl:rev CACAGACACCCACACCGTCACCCACACTATCATGTTGACATTCC; dG = -26.9
1284 >s_c_s_d_s_d_s_c_s_e_s_c_s_a_s GTGTGGGTGACGGTGACGGTGTGGGTGCTGGTGTGGGTGTCTGTG;
>clamp_s_c_s_d_s_c_s_a_s:compl:rev CACAGACACCCACACCGTCACCCACACTATCATGTTGACATTCC; dG = -26.9
1285 >s_c_s_d_s_d_s_b_s_d_s_c_s_a_s GTGTGGGTGACGGTGACGGTGGCTGTGACGGTGTGGGTGTCTGTG;
>clamp_s_c_s_d_s_c_s_a_s:compl:rev CACAGACACCCACACCGTCACCCACACTATCATGTTGACATTCC; dG = -26.9
1286 >s_c_s_d_s_e_s_c_s_e_s_c_s_a_s GTGTGGGTGACGGTGCTGGTGTGGGTGCTGGTGTGGGTGTCTGTG;
>clamp_s_c_s_d_s_c_s_a_s:compl:rev CACAGACACCCACACCGTCACCCACACTATCATGTTGACATTCC; dG = -26.9
1287 >s_a_s_b_s_b_s_b_s_d_s_c_s_a_s GTGTCTGTGGCTGTGGCTGTGGCTGTGACGGTGTGGGTGTCTGTG;
>clamp_s_c_s_d_s_c_s_a_s:compl:rev CACAGACACCCACACCGTCACCCACACTATCATGTTGACATTCC; dG = -26.9
1288 >s_c_s_d_s_e_s_b_s_d_s_c_s_a_s GTGTGGGTGACGGTGCTGGTGGCTGTGACGGTGTGGGTGTCTGTG;
>clamp_s_c_s_d_s_c_s_a_s:compl:rev CACAGACACCCACACCGTCACCCACACTATCATGTTGACATTCC; dG = -26.9
1289 >s_c_s_e_s_a_s_c_s_e_s_c_s_a_s GTGTGGGTGCTGGTGTCTGTGTGGGTGCTGGTGTGGGTGTCTGTG;
>clamp_s_c_s_d_s_c_s_a_s:compl:rev CACAGACACCCACACCGTCACCCACACTATCATGTTGACATTCC; dG = -26.9
1290 >s_c_s_e_s_a_s_b_s_d_s_c_s_a_s GTGTGGGTGCTGGTGTCTGTGGCTGTGACGGTGTGGGTGTCTGTG;
>clamp_s_c_s_d_s_c_s_a_s:compl:rev CACAGACACCCACACCGTCACCCACACTATCATGTTGACATTCC; dG = -26.9
1291 >s_c_s_e_s_b_s_c_s_e_s_c_s_a_s GTGTGGGTGCTGGTGGCTGTGTGGGTGCTGGTGTGGGTGTCTGTG;
>clamp_s_c_s_d_s_c_s_a_s:compl:rev CACAGACACCCACACCGTCACCCACACTATCATGTTGACATTCC; dG = -26.9
1292 >s_c_s_e_s_b_s_b_s_d_s_c_s_a_s GTGTGGGTGCTGGTGGCTGTGGCTGTGACGGTGTGGGTGTCTGTG;
>clamp_s_c_s_d_s_c_s_a_s:compl:rev CACAGACACCCACACCGTCACCCACACTATCATGTTGACATTCC; dG = -26.9
1293 >s_c_s_e_s_c_s_c_s_e_s_c_s_a_s GTGTGGGTGCTGGTGTGGGTGTGGGTGCTGGTGTGGGTGTCTGTG;
>clamp_s_c_s_d_s_c_s_a_s:compl:rev CACAGACACCCACACCGTCACCCACACTATCATGTTGACATTCC; dG = -26.9
1294 >s_c_s_e_s_d_s_c_s_e_s_c_s_a_s GTGTGGGTGCTGGTGACGGTGTGGGTGCTGGTGTGGGTGTCTGTG;
>clamp_s_c_s_d_s_c_s_a_s:compl:rev CACAGACACCCACACCGTCACCCACACTATCATGTTGACATTCC; dG = -26.9
1295 >s_c_s_e_s_d_s_b_s_d_s_c_s_a_s GTGTGGGTGCTGGTGACGGTGGCTGTGACGGTGTGGGTGTCTGTG;
>clamp_s_c_s_d_s_c_s_a_s:compl:rev CACAGACACCCACACCGTCACCCACACTATCATGTTGACATTCC; dG = -26.9
1296 >s_c_s_e_s_e_s_c_s_e_s_c_s_a_s GTGTGGGTGCTGGTGCTGGTGTGGGTGCTGGTGTGGGTGTCTGTG;
>clamp_s_c_s_d_s_c_s_a_s:compl:rev CACAGACACCCACACCGTCACCCACACTATCATGTTGACATTCC; dG = -26.9
1297 >s_c_s_e_s_e_s_b_s_d_s_c_s_a_s GTGTGGGTGCTGGTGCTGGTGGCTGTGACGGTGTGGGTGTCTGTG;
>clamp_s_c_s_d_s_c_s_a_s:compl:rev CACAGACACCCACACCGTCACCCACACTATCATGTTGACATTCC; dG = -26.9
1298 >s_d_s_a_s_a_s_c_s_e_s_c_s_a_s GTGACGGTGTCTGTGTCTGTGTGGGTGCTGGTGTGGGTGTCTGTG;
>clamp_s_c_s_d_s_c_s_a_s:compl:rev CACAGACACCCACACCGTCACCCACACTATCATGTTGACATTCC; dG = -26.9
1299 >s_a_s_b_s_c_s_c_s_e_s_c_s_a_s GTGTCTGTGGCTGTGTGGGTGTGGGTGCTGGTGTGGGTGTCTGTG;
>clamp_s_c_s_d_s_c_s_a_s:compl:rev CACAGACACCCACACCGTCACCCACACTATCATGTTGACATTCC; dG = -26.9
1300 >s_d_s_a_s_a_s_b_s_d_s_c_s_a_s GTGACGGTGTCTGTGTCTGTGGCTGTGACGGTGTGGGTGTCTGTG;
>clamp_s_c_s_d_s_c_s_a_s:compl:rev CACAGACACCCACACCGTCACCCACACTATCATGTTGACATTCC; dG = -26.9
1301 >s_d_s_a_s_b_s_c_s_e_s_c_s_a_s GTGACGGTGTCTGTGGCTGTGTGGGTGCTGGTGTGGGTGTCTGTG;
>clamp_s_c_s_d_s_c_s_a_s:compl:rev CACAGACACCCACACCGTCACCCACACTATCATGTTGACATTCC; dG = -26.9
1302 >s_d_s_a_s_b_s_b_s_d_s_c_s_a_s GTGACGGTGTCTGTGGCTGTGGCTGTGACGGTGTGGGTGTCTGTG;
>clamp_s_c_s_d_s_c_s_a_s:compl:rev CACAGACACCCACACCGTCACCCACACTATCATGTTGACATTCC; dG = -26.9
1303 >s_d_s_a_s_c_s_c_s_e_s_c_s_a_s GTGACGGTGTCTGTGTGGGTGTGGGTGCTGGTGTGGGTGTCTGTG;
>clamp_s_c_s_d_s_c_s_a_s:compl:rev CACAGACACCCACACCGTCACCCACACTATCATGTTGACATTCC; dG = -26.9
1304 >s_d_s_a_s_d_s_c_s_e_s_c_s_a_s GTGACGGTGTCTGTGACGGTGTGGGTGCTGGTGTGGGTGTCTGTG;
>clamp_s_c_s_d_s_c_s_a_s:compl:rev CACAGACACCCACACCGTCACCCACACTATCATGTTGACATTCC; dG = -26.9
1305 >s_d_s_a_s_d_s_b_s_d_s_c_s_a_s GTGACGGTGTCTGTGACGGTGGCTGTGACGGTGTGGGTGTCTGTG;
>clamp_s_c_s_d_s_c_s_a_s:compl:rev CACAGACACCCACACCGTCACCCACACTATCATGTTGACATTCC; dG = -26.9
1306 >s_d_s_a_s_e_s_c_s_e_s_c_s_a_s GTGACGGTGTCTGTGCTGGTGTGGGTGCTGGTGTGGGTGTCTGTG;
>clamp_s_c_s_d_s_c_s_a_s:compl:rev CACAGACACCCACACCGTCACCCACACTATCATGTTGACATTCC; dG = -26.9
1307 >s_d_s_a_s_e_s_b_s_d_s_c_s_a_s GTGACGGTGTCTGTGCTGGTGGCTGTGACGGTGTGGGTGTCTGTG;
>clamp_s_c_s_d_s_c_s_a_s:compl:rev CACAGACACCCACACCGTCACCCACACTATCATGTTGACATTCC; dG = -26.9
1308 >s_d_s_b_s_a_s_c_s_e_s_c_s_a_s GTGACGGTGGCTGTGTCTGTGTGGGTGCTGGTGTGGGTGTCTGTG;
>clamp_s_c_s_d_s_c_s_a_s:compl:rev CACAGACACCCACACCGTCACCCACACTATCATGTTGACATTCC; dG = -26.9
1309 >s_d_s_b_s_a_s_b_s_d_s_c_s_a_s GTGACGGTGGCTGTGTCTGTGGCTGTGACGGTGTGGGTGTCTGTG;
>clamp_s_c_s_d_s_c_s_a_s:compl:rev CACAGACACCCACACCGTCACCCACACTATCATGTTGACATTCC; dG = -26.9
1310 >s_d_s_b_s_b_s_c_s_e_s_c_s_a_s GTGACGGTGGCTGTGGCTGTGTGGGTGCTGGTGTGGGTGTCTGTG;
>clamp_s_c_s_d_s_c_s_a_s:compl:rev CACAGACACCCACACCGTCACCCACACTATCATGTTGACATTCC; dG = -26.9
1311 >s_d_s_b_s_b_s_b_s_d_s_c_s_a_s GTGACGGTGGCTGTGGCTGTGGCTGTGACGGTGTGGGTGTCTGTG;
>clamp_s_c_s_d_s_c_s_a_s:compl:rev CACAGACACCCACACCGTCACCCACACTATCATGTTGACATTCC; dG = -26.9
1312 >s_d_s_b_s_c_s_c_s_e_s_c_s_a_s GTGACGGTGGCTGTGTGGGTGTGGGTGCTGGTGTGGGTGTCTGTG;
>clamp_s_c_s_d_s_c_s_a_s:compl:rev CACAGACACCCACACCGTCACCCACACTATCATGTTGACATTCC; dG = -26.9
1313 >s_d_s_b_s_d_s_c_s_e_s_c_s_a_s GTGACGGTGGCTGTGACGGTGTGGGTGCTGGTGTGGGTGTCTGTG;
>clamp_s_c_s_d_s_c_s_a_s:compl:rev CACAGACACCCACACCGTCACCCACACTATCATGTTGACATTCC; dG = -26.9
1314 >s_d_s_b_s_d_s_b_s_d_s_c_s_a_s GTGACGGTGGCTGTGACGGTGGCTGTGACGGTGTGGGTGTCTGTG;
>clamp_s_c_s_d_s_c_s_a_s:compl:rev CACAGACACCCACACCGTCACCCACACTATCATGTTGACATTCC; dG = -26.9
1315 >s_d_s_b_s_e_s_c_s_e_s_c_s_a_s GTGACGGTGGCTGTGCTGGTGTGGGTGCTGGTGTGGGTGTCTGTG;
>clamp_s_c_s_d_s_c_s_a_s:compl:rev CACAGACACCCACACCGTCACCCACACTATCATGTTGACATTCC; dG = -26.9

1316 >s_d_s_b_s_e_s_b_s_d_s_c_s_a_s GTGACGGTGGCTGTGCTGGTGGCTGTGACGGTGTGGGTGTCTGTG;
>clamp_s_c_s_d_s_c_s_a_s:compl:rev CACAGACACCCACACCGTCACCCACACTATCATGTTGACATTCC; dG = -26.9
1317 >s_d_s_c_s_a_s_c_s_e_s_c_s_a_s GTGACGGTGTGGGTGTCTGTGTGGGTGCTGGTGTGGGTGTCTGTG;
>clamp_s_c_s_d_s_c_s_a_s:compl:rev CACAGACACCCACACCGTCACCCACACTATCATGTTGACATTCC; dG = -26.9
1318 >s_a_s_b_s_d_s_c_s_e_s_c_s_a_s GTGTCTGTGGCTGTGACGGTGTGGGTGCTGGTGTGGGTGTCTGTG;
>clamp_s_c_s_d_s_c_s_a_s:compl:rev CACAGACACCCACACCGTCACCCACACTATCATGTTGACATTCC; dG = -26.9
1319 >s_d_s_c_s_b_s_c_s_e_s_c_s_a_s GTGACGGTGTGGGTGGCTGTGTGGGTGCTGGTGTGGGTGTCTGTG;
>clamp_s_c_s_d_s_c_s_a_s:compl:rev CACAGACACCCACACCGTCACCCACACTATCATGTTGACATTCC; dG = -26.9
1320 >s_d_s_c_s_b_s_c_s_a_s_c_s_a_s GTGACGGTGTGGGTGGCTGTGTGGGTGTCTGTGTGGGTGTCTGTG;
>clamp_s_c_s_d_s_c_s_a_s:compl:rev CACAGACACCCACACCGTCACCCACACTATCATGTTGACATTCC; dG = -26.9
1321 >s_d_s_c_s_c_s_c_s_e_s_c_s_a_s GTGACGGTGTGGGTGTGGGTGTGGGTGCTGGTGTGGGTGTCTGTG;
>clamp_s_c_s_d_s_c_s_a_s:compl:rev CACAGACACCCACACCGTCACCCACACTATCATGTTGACATTCC; dG = -26.9
1322 >s_d_s_c_s_e_s_c_s_e_s_c_s_a_s GTGACGGTGTGGGTGCTGGTGTGGGTGCTGGTGTGGGTGTCTGTG;
>clamp_s_c_s_d_s_c_s_a_s:compl:rev CACAGACACCCACACCGTCACCCACACTATCATGTTGACATTCC; dG = -26.9
1323 >s_a_s_b_s_d_s_b_s_d_s_c_s_a_s GTGTCTGTGGCTGTGACGGTGGCTGTGACGGTGTGGGTGTCTGTG;
>clamp_s_c_s_d_s_c_s_a_s:compl:rev CACAGACACCCACACCGTCACCCACACTATCATGTTGACATTCC; dG = -26.9
1324 >s_d_s_d_s_a_s_c_s_e_s_c_s_a_s GTGACGGTGACGGTGTCTGTGTGGGTGCTGGTGTGGGTGTCTGTG;
>clamp_s_c_s_d_s_c_s_a_s:compl:rev CACAGACACCCACACCGTCACCCACACTATCATGTTGACATTCC; dG = -26.9
1325 >s_d_s_d_s_a_s_b_s_d_s_c_s_a_s GTGACGGTGACGGTGTCTGTGGCTGTGACGGTGTGGGTGTCTGTG;
>clamp_s_c_s_d_s_c_s_a_s:compl:rev CACAGACACCCACACCGTCACCCACACTATCATGTTGACATTCC; dG = -26.9
1326 >s_d_s_d_s_b_s_c_s_e_s_c_s_a_s GTGACGGTGACGGTGGCTGTGTGGGTGCTGGTGTGGGTGTCTGTG;
>clamp_s_c_s_d_s_c_s_a_s:compl:rev CACAGACACCCACACCGTCACCCACACTATCATGTTGACATTCC; dG = -26.9
1327 >s_d_s_d_s_b_s_b_s_d_s_c_s_a_s GTGACGGTGACGGTGGCTGTGGCTGTGACGGTGTGGGTGTCTGTG;
>clamp_s_c_s_d_s_c_s_a_s:compl:rev CACAGACACCCACACCGTCACCCACACTATCATGTTGACATTCC; dG = -26.9
1328 >s_d_s_d_s_c_s_c_s_e_s_c_s_a_s GTGACGGTGACGGTGTGGGTGTGGGTGCTGGTGTGGGTGTCTGTG;
>clamp_s_c_s_d_s_c_s_a_s:compl:rev CACAGACACCCACACCGTCACCCACACTATCATGTTGACATTCC; dG = -26.9
1329 >s_d_s_d_s_d_s_c_s_e_s_c_s_a_s GTGACGGTGACGGTGACGGTGTGGGTGCTGGTGTGGGTGTCTGTG;
>clamp_s_c_s_d_s_c_s_a_s:compl:rev CACAGACACCCACACCGTCACCCACACTATCATGTTGACATTCC; dG = -26.9
1330 >s_d_s_d_s_d_s_b_s_d_s_c_s_a_s GTGACGGTGACGGTGACGGTGGCTGTGACGGTGTGGGTGTCTGTG;
>clamp_s_c_s_d_s_c_s_a_s:compl:rev CACAGACACCCACACCGTCACCCACACTATCATGTTGACATTCC; dG = -26.9
1331 >s_a_s_a_s_a_s_b_s_d_s_c_s_a_s GTGTCTGTGTCTGTGTCTGTGGCTGTGACGGTGTGGGTGTCTGTG;
>clamp_s_c_s_d_s_c_s_a_s:compl:rev CACAGACACCCACACCGTCACCCACACTATCATGTTGACATTCC; dG = -26.9
1332 >s_d_s_d_s_e_s_c_s_e_s_c_s_a_s GTGACGGTGACGGTGCTGGTGTGGGTGCTGGTGTGGGTGTCTGTG;
>clamp_s_c_s_d_s_c_s_a_s:compl:rev CACAGACACCCACACCGTCACCCACACTATCATGTTGACATTCC; dG = -26.9
1333 >s_d_s_d_s_e_s_b_s_d_s_c_s_a_s GTGACGGTGACGGTGCTGGTGGCTGTGACGGTGTGGGTGTCTGTG;
>clamp_s_c_s_d_s_c_s_a_s:compl:rev CACAGACACCCACACCGTCACCCACACTATCATGTTGACATTCC; dG = -26.9
1334 >s_d_s_e_s_a_s_c_s_e_s_c_s_a_s GTGACGGTGCTGGTGTCTGTGTGGGTGCTGGTGTGGGTGTCTGTG;
>clamp_s_c_s_d_s_c_s_a_s:compl:rev CACAGACACCCACACCGTCACCCACACTATCATGTTGACATTCC; dG = -26.9
1335 >s_a_s_b_s_e_s_c_s_e_s_c_s_a_s GTGTCTGTGGCTGTGCTGGTGTGGGTGCTGGTGTGGGTGTCTGTG;
>clamp_s_c_s_d_s_c_s_a_s:compl:rev CACAGACACCCACACCGTCACCCACACTATCATGTTGACATTCC; dG = -26.9
1336 >s_d_s_e_s_a_s_b_s_d_s_c_s_a_s GTGACGGTGCTGGTGTCTGTGGCTGTGACGGTGTGGGTGTCTGTG;
>clamp_s_c_s_d_s_c_s_a_s:compl:rev CACAGACACCCACACCGTCACCCACACTATCATGTTGACATTCC; dG = -26.9
1337 >s_d_s_e_s_b_s_c_s_e_s_c_s_a_s GTGACGGTGCTGGTGGCTGTGTGGGTGCTGGTGTGGGTGTCTGTG;
>clamp_s_c_s_d_s_c_s_a_s:compl:rev CACAGACACCCACACCGTCACCCACACTATCATGTTGACATTCC; dG = -26.9
1338 >s_d_s_e_s_b_s_b_s_d_s_c_s_a_s GTGACGGTGCTGGTGGCTGTGGCTGTGACGGTGTGGGTGTCTGTG;
>clamp_s_c_s_d_s_c_s_a_s:compl:rev CACAGACACCCACACCGTCACCCACACTATCATGTTGACATTCC; dG = -26.9
1339 >s_d_s_e_s_c_s_c_s_e_s_c_s_a_s GTGACGGTGCTGGTGTGGGTGTGGGTGCTGGTGTGGGTGTCTGTG;
>clamp_s_c_s_d_s_c_s_a_s:compl:rev CACAGACACCCACACCGTCACCCACACTATCATGTTGACATTCC; dG = -26.9
1340 >s_d_s_e_s_d_s_c_s_e_s_c_s_a_s GTGACGGTGCTGGTGACGGTGTGGGTGCTGGTGTGGGTGTCTGTG;
>clamp_s_c_s_d_s_c_s_a_s:compl:rev CACAGACACCCACACCGTCACCCACACTATCATGTTGACATTCC; dG = -26.9
1341 >s_d_s_e_s_d_s_b_s_d_s_c_s_a_s GTGACGGTGCTGGTGACGGTGGCTGTGACGGTGTGGGTGTCTGTG;
>clamp_s_c_s_d_s_c_s_a_s:compl:rev CACAGACACCCACACCGTCACCCACACTATCATGTTGACATTCC; dG = -26.9
1342 >s_d_s_e_s_e_s_c_s_e_s_c_s_a_s GTGACGGTGCTGGTGCTGGTGTGGGTGCTGGTGTGGGTGTCTGTG;
>clamp_s_c_s_d_s_c_s_a_s:compl:rev CACAGACACCCACACCGTCACCCACACTATCATGTTGACATTCC; dG = -26.9
1343 >s_a_s_b_s_e_s_b_s_d_s_c_s_a_s GTGTCTGTGGCTGTGCTGGTGGCTGTGACGGTGTGGGTGTCTGTG;
>clamp_s_c_s_d_s_c_s_a_s:compl:rev CACAGACACCCACACCGTCACCCACACTATCATGTTGACATTCC; dG = -26.9
1344 >s_d_s_e_s_e_s_b_s_d_s_c_s_a_s GTGACGGTGCTGGTGCTGGTGGCTGTGACGGTGTGGGTGTCTGTG;
>clamp_s_c_s_d_s_c_s_a_s:compl:rev CACAGACACCCACACCGTCACCCACACTATCATGTTGACATTCC; dG = -26.9
1345 >s_e_s_a_s_a_s_c_s_e_s_c_s_a_s GTGCTGGTGTCTGTGTCTGTGTGGGTGCTGGTGTGGGTGTCTGTG;
>clamp_s_c_s_d_s_c_s_a_s:compl:rev CACAGACACCCACACCGTCACCCACACTATCATGTTGACATTCC; dG = -26.9
1346 >s_e_s_a_s_a_s_b_s_d_s_c_s_a_s GTGCTGGTGTCTGTGTCTGTGGCTGTGACGGTGTGGGTGTCTGTG;
>clamp_s_c_s_d_s_c_s_a_s:compl:rev CACAGACACCCACACCGTCACCCACACTATCATGTTGACATTCC; dG = -26.9
1347 >s_e_s_a_s_b_s_c_s_e_s_c_s_a_s GTGCTGGTGTCTGTGGCTGTGTGGGTGCTGGTGTGGGTGTCTGTG;
>clamp_s_c_s_d_s_c_s_a_s:compl:rev CACAGACACCCACACCGTCACCCACACTATCATGTTGACATTCC; dG = -26.9
1348 >s_e_s_a_s_b_s_b_s_d_s_c_s_a_s GTGCTGGTGTCTGTGGCTGTGGCTGTGACGGTGTGGGTGTCTGTG;
>clamp_s_c_s_d_s_c_s_a_s:compl:rev CACAGACACCCACACCGTCACCCACACTATCATGTTGACATTCC; dG = -26.9
1349 >s_e_s_a_s_c_s_c_s_e_s_c_s_a_s GTGCTGGTGTCTGTGTGGGTGTGGGTGCTGGTGTGGGTGTCTGTG;
>clamp_s_c_s_d_s_c_s_a_s:compl:rev CACAGACACCCACACCGTCACCCACACTATCATGTTGACATTCC; dG = -26.9
1350 >s_e_s_a_s_d_s_c_s_e_s_c_s_a_s GTGCTGGTGTCTGTGACGGTGTGGGTGCTGGTGTGGGTGTCTGTG;
>clamp_s_c_s_d_s_c_s_a_s:compl:rev CACAGACACCCACACCGTCACCCACACTATCATGTTGACATTCC; dG = -26.9
1351 >s_e_s_a_s_d_s_b_s_d_s_c_s_a_s GTGCTGGTGTCTGTGACGGTGGCTGTGACGGTGTGGGTGTCTGTG;
>clamp_s_c_s_d_s_c_s_a_s:compl:rev CACAGACACCCACACCGTCACCCACACTATCATGTTGACATTCC; dG = -26.9
1352 >s_e_s_a_s_e_s_c_s_e_s_c_s_a_s GTGCTGGTGTCTGTGCTGGTGTGGGTGCTGGTGTGGGTGTCTGTG;
>clamp_s_c_s_d_s_c_s_a_s:compl:rev CACAGACACCCACACCGTCACCCACACTATCATGTTGACATTCC; dG = -26.9

```
  1353 >s_e_s_a_s_e_s_b_s_d_s_c_s_a_s           GTGCTGGTGTCTGTGCTGGTGGCTGTGACGGTGTGGGTGTCTGTG;
>clamp_s_c_s_d_s_c_s_a_s:compl:rev CACAGACACCCACACCGTCACCCACACTATCATGTTGACATTCC; dG = -26.9
  1354 >s_e_s_b_s_a_s_c_s_e_s_c_s_a_s           GTGCTGGTGGCTGTGTCTGTGTGGGTGCTGGTGTGGGTGTCTGTG;
>clamp_s_c_s_d_s_c_s_a_s:compl:rev CACAGACACCCACACCGTCACCCACACTATCATGTTGACATTCC; dG = -26.9
  1355 >s_a_s_c_s_a_s_c_s_e_s_c_s_a_s           GTGTCTGTGTGGGTGTCTGTGTGGGTGCTGGTGTGGGTGTCTGTG;
>clamp_s_c_s_d_s_c_s_a_s:compl:rev CACAGACACCCACACCGTCACCCACACTATCATGTTGACATTCC; dG = -26.9
  1356 >s_e_s_b_s_a_s_b_s_d_s_c_s_a_s           GTGCTGGTGGCTGTGTCTGTGGCTGTGACGGTGTGGGTGTCTGTG;
>clamp_s_c_s_d_s_c_s_a_s:compl:rev CACAGACACCCACACCGTCACCCACACTATCATGTTGACATTCC; dG = -26.9
  1357 >s_e_s_b_s_b_s_c_s_e_s_c_s_a_s           GTGCTGGTGGCTGTGGCTGTGTGGGTGCTGGTGTGGGTGTCTGTG;
>clamp_s_c_s_d_s_c_s_a_s:compl:rev CACAGACACCCACACCGTCACCCACACTATCATGTTGACATTCC; dG = -26.9
  1358 >s_e_s_b_s_b_s_b_s_d_s_c_s_a_s           GTGCTGGTGGCTGTGGCTGTGGCTGTGACGGTGTGGGTGTCTGTG;
>clamp_s_c_s_d_s_c_s_a_s:compl:rev CACAGACACCCACACCGTCACCCACACTATCATGTTGACATTCC; dG = -26.9
  1359 >s_e_s_b_s_c_s_c_s_e_s_c_s_a_s           GTGCTGGTGGCTGTGTGGGTGTGGGTGCTGGTGTGGGTGTCTGTG;
>clamp_s_c_s_d_s_c_s_a_s:compl:rev CACAGACACCCACACCGTCACCCACACTATCATGTTGACATTCC; dG = -26.9
  1360 >s_e_s_b_s_d_s_c_s_e_s_c_s_a_s           GTGCTGGTGGCTGTGACGGTGTGGGTGCTGGTGTGGGTGTCTGTG;
>clamp_s_c_s_d_s_c_s_a_s:compl:rev CACAGACACCCACACCGTCACCCACACTATCATGTTGACATTCC; dG = -26.9
  1361 >s_e_s_b_s_d_s_b_s_d_s_c_s_a_s           GTGCTGGTGGCTGTGACGGTGGCTGTGACGGTGTGGGTGTCTGTG;
>clamp_s_c_s_d_s_c_s_a_s:compl:rev CACAGACACCCACACCGTCACCCACACTATCATGTTGACATTCC; dG = -26.9
  1362 >s_e_s_b_s_e_s_c_s_e_s_c_s_a_s           GTGCTGGTGGCTGTGCTGGTGTGGGTGCTGGTGTGGGTGTCTGTG;
>clamp_s_c_s_d_s_c_s_a_s:compl:rev CACAGACACCCACACCGTCACCCACACTATCATGTTGACATTCC; dG = -26.9
  1363 >s_e_s_b_s_e_s_b_s_d_s_c_s_a_s           GTGCTGGTGGCTGTGCTGGTGGCTGTGACGGTGTGGGTGTCTGTG;
>clamp_s_c_s_d_s_c_s_a_s:compl:rev CACAGACACCCACACCGTCACCCACACTATCATGTTGACATTCC; dG = -26.9
  1364 >s_e_s_c_s_a_s_c_s_e_s_c_s_a_s           GTGCTGGTGTGGGTGTCTGTGTGGGTGCTGGTGTGGGTGTCTGTG;
>clamp_s_c_s_d_s_c_s_a_s:compl:rev CACAGACACCCACACCGTCACCCACACTATCATGTTGACATTCC; dG = -26.9
  1365 >s_e_s_c_s_b_s_c_s_e_s_c_s_a_s           GTGCTGGTGTGGGTGGCTGTGTGGGTGCTGGTGTGGGTGTCTGTG;
>clamp_s_c_s_d_s_c_s_a_s:compl:rev CACAGACACCCACACCGTCACCCACACTATCATGTTGACATTCC; dG = -26.9
  1366 >s_e_s_c_s_b_s_c_s_a_s_c_s_a_s           GTGCTGGTGTGGGTGGCTGTGTGGGTGTCTGTGTGGGTGTCTGTG;
>clamp_s_c_s_d_s_c_s_a_s:compl:rev CACAGACACCCACACCGTCACCCACACTATCATGTTGACATTCC; dG = -26.9
  1367 >s_e_s_c_s_c_s_c_s_e_s_c_s_a_s           GTGCTGGTGTGGGTGTGGGTGTGGGTGCTGGTGTGGGTGTCTGTG;
>clamp_s_c_s_d_s_c_s_a_s:compl:rev CACAGACACCCACACCGTCACCCACACTATCATGTTGACATTCC; dG = -26.9
  1368 >s_e_s_c_s_e_s_c_s_e_s_c_s_a_s           GTGCTGGTGTGGGTGCTGGTGTGGGTGCTGGTGTGGGTGTCTGTG;
>clamp_s_c_s_d_s_c_s_a_s:compl:rev CACAGACACCCACACCGTCACCCACACTATCATGTTGACATTCC; dG = -26.9
  1369 >s_e_s_d_s_a_s_c_s_e_s_c_s_a_s           GTGCTGGTGACGGTGTCTGTGTGGGTGCTGGTGTGGGTGTCTGTG;
>clamp_s_c_s_d_s_c_s_a_s:compl:rev CACAGACACCCACACCGTCACCCACACTATCATGTTGACATTCC; dG = -26.9
  1370 >s_e_s_d_s_a_s_b_s_d_s_c_s_a_s           GTGCTGGTGACGGTGTCTGTGGCTGTGACGGTGTGGGTGTCTGTG;
>clamp_s_c_s_d_s_c_s_a_s:compl:rev CACAGACACCCACACCGTCACCCACACTATCATGTTGACATTCC; dG = -26.9
  1371 >s_a_s_c_s_b_s_c_s_e_s_c_s_a_s           GTGTCTGTGTGGGTGGCTGTGTGGGTGCTGGTGTGGGTGTCTGTG;
>clamp_s_c_s_d_s_c_s_a_s:compl:rev CACAGACACCCACACCGTCACCCACACTATCATGTTGACATTCC; dG = -26.9
  1372 >s_e_s_d_s_b_s_c_s_e_s_c_s_a_s           GTGCTGGTGACGGTGGCTGTGTGGGTGCTGGTGTGGGTGTCTGTG;
>clamp_s_c_s_d_s_c_s_a_s:compl:rev CACAGACACCCACACCGTCACCCACACTATCATGTTGACATTCC; dG = -26.9
  1373 >s_e_s_d_s_b_s_b_s_d_s_c_s_a_s           GTGCTGGTGACGGTGGCTGTGGCTGTGACGGTGTGGGTGTCTGTG;
>clamp_s_c_s_d_s_c_s_a_s:compl:rev CACAGACACCCACACCGTCACCCACACTATCATGTTGACATTCC; dG = -26.9
  1374 >s_e_s_d_s_c_s_c_s_e_s_c_s_a_s           GTGCTGGTGACGGTGTGGGTGTGGGTGCTGGTGTGGGTGTCTGTG;
>clamp_s_c_s_d_s_c_s_a_s:compl:rev CACAGACACCCACACCGTCACCCACACTATCATGTTGACATTCC; dG = -26.9
  1375 >s_e_s_d_s_d_s_c_s_e_s_c_s_a_s           GTGCTGGTGACGGTGACGGTGTGGGTGCTGGTGTGGGTGTCTGTG;
>clamp_s_c_s_d_s_c_s_a_s:compl:rev CACAGACACCCACACCGTCACCCACACTATCATGTTGACATTCC; dG = -26.9
  1376 >s_e_s_d_s_d_s_b_s_d_s_c_s_a_s           GTGCTGGTGACGGTGACGGTGGCTGTGACGGTGTGGGTGTCTGTG;
>clamp_s_c_s_d_s_c_s_a_s:compl:rev CACAGACACCCACACCGTCACCCACACTATCATGTTGACATTCC; dG = -26.9
  1377 >s_e_s_d_s_e_s_c_s_e_s_c_s_a_s           GTGCTGGTGACGGTGCTGGTGTGGGTGCTGGTGTGGGTGTCTGTG;
>clamp_s_c_s_d_s_c_s_a_s:compl:rev CACAGACACCCACACCGTCACCCACACTATCATGTTGACATTCC; dG = -26.9
  1378 >s_e_s_d_s_e_s_b_s_d_s_c_s_a_s           GTGCTGGTGACGGTGCTGGTGGCTGTGACGGTGTGGGTGTCTGTG;
>clamp_s_c_s_d_s_c_s_a_s:compl:rev CACAGACACCCACACCGTCACCCACACTATCATGTTGACATTCC; dG = -26.9
  1379 >s_e_s_e_s_a_s_c_s_e_s_c_s_a_s           GTGCTGGTGCTGGTGTCTGTGTGGGTGCTGGTGTGGGTGTCTGTG;
>clamp_s_c_s_d_s_c_s_a_s:compl:rev CACAGACACCCACACCGTCACCCACACTATCATGTTGACATTCC; dG = -26.9
  1380 >s_e_s_e_s_a_s_b_s_d_s_c_s_a_s           GTGCTGGTGCTGGTGTCTGTGGCTGTGACGGTGTGGGTGTCTGTG;
>clamp_s_c_s_d_s_c_s_a_s:compl:rev CACAGACACCCACACCGTCACCCACACTATCATGTTGACATTCC; dG = -26.9
  1381 >s_e_s_e_s_b_s_c_s_e_s_c_s_a_s           GTGCTGGTGCTGGTGGCTGTGTGGGTGCTGGTGTGGGTGTCTGTG;
>clamp_s_c_s_d_s_c_s_a_s:compl:rev CACAGACACCCACACCGTCACCCACACTATCATGTTGACATTCC; dG = -26.9
  1382 >s_e_s_e_s_b_s_b_s_d_s_c_s_a_s           GTGCTGGTGCTGGTGGCTGTGGCTGTGACGGTGTGGGTGTCTGTG;
>clamp_s_c_s_d_s_c_s_a_s:compl:rev CACAGACACCCACACCGTCACCCACACTATCATGTTGACATTCC; dG = -26.9
  1383 >s_e_s_e_s_c_s_c_s_e_s_c_s_a_s           GTGCTGGTGCTGGTGTGGGTGTGGGTGCTGGTGTGGGTGTCTGTG;
>clamp_s_c_s_d_s_c_s_a_s:compl:rev CACAGACACCCACACCGTCACCCACACTATCATGTTGACATTCC; dG = -26.9
  1384 >s_e_s_e_s_d_s_c_s_e_s_c_s_a_s           GTGCTGGTGCTGGTGACGGTGTGGGTGCTGGTGTGGGTGTCTGTG;
>clamp_s_c_s_d_s_c_s_a_s:compl:rev CACAGACACCCACACCGTCACCCACACTATCATGTTGACATTCC; dG = -26.9
  1385 >s_e_s_e_s_d_s_b_s_d_s_c_s_a_s           GTGCTGGTGCTGGTGACGGTGGCTGTGACGGTGTGGGTGTCTGTG;
>clamp_s_c_s_d_s_c_s_a_s:compl:rev CACAGACACCCACACCGTCACCCACACTATCATGTTGACATTCC; dG = -26.9
  1386 >s_e_s_e_s_e_s_c_s_e_s_c_s_a_s           GTGCTGGTGCTGGTGCTGGTGTGGGTGCTGGTGTGGGTGTCTGTG;
>clamp_s_c_s_d_s_c_s_a_s:compl:rev CACAGACACCCACACCGTCACCCACACTATCATGTTGACATTCC; dG = -26.9
  1387 >s_e_s_e_s_e_s_b_s_d_s_c_s_a_s           GTGCTGGTGCTGGTGCTGGTGGCTGTGACGGTGTGGGTGTCTGTG;
>clamp_s_c_s_d_s_c_s_a_s:compl:rev CACAGACACCCACACCGTCACCCACACTATCATGTTGACATTCC; dG = -26.9
  1388 >s_a_s_c_s_c_s_c_s_e_s_c_s_a_s           GTGTCTGTGTGGGTGTGGGTGTGGGTGCTGGTGTGGGTGTCTGTG;
>clamp_s_c_s_d_s_c_s_a_s:compl:rev CACAGACACCCACACCGTCACCCACACTATCATGTTGACATTCC; dG = -26.9
  1389 >s_a_s_c_s_e_s_c_s_e_s_c_s_a_s           GTGTCTGTGTGGGTGCTGGTGTGGGTGCTGGTGTGGGTGTCTGTG;
>clamp_s_c_s_d_s_c_s_a_s:compl:rev CACAGACACCCACACCGTCACCCACACTATCATGTTGACATTCC; dG = -26.9
```

```
  1390 >s_a_s_d_s_a_s_c_s_e_s_c_s_a_s          GTGTCTGTGACGGTGTCTGTGTGGGTGCTGGTGTGGGTGTCTGTG;
>clamp_s_c_s_d_s_c_s_a_s:compl:rev CACAGACACCCACACCGTCACCCACACTATCATGTTGACATTCC; dG = -26.9
  1391 >s_a_s_a_s_b_s_c_s_e_s_c_s_a_s          GTGTCTGTGTCTGTGGCTGTGTGGGTGCTGGTGTGGGTGTCTGTG;
>clamp_s_c_s_d_s_c_s_a_s:compl:rev CACAGACACCCACACCGTCACCCACACTATCATGTTGACATTCC; dG = -26.9
  1392 >s_a_s_d_s_a_s_b_s_d_s_c_s_a_s          GTGTCTGTGACGGTGTCTGTGGCTGTGACGGTGTGGGTGTCTGTG;
>clamp_s_c_s_d_s_c_s_a_s:compl:rev CACAGACACCCACACCGTCACCCACACTATCATGTTGACATTCC; dG = -26.9
  1393 >s_a_s_d_s_b_s_c_s_e_s_c_s_a_s          GTGTCTGTGACGGTGGCTGTGTGGGTGCTGGTGTGGGTGTCTGTG;
>clamp_s_c_s_d_s_c_s_a_s:compl:rev CACAGACACCCACACCGTCACCCACACTATCATGTTGACATTCC; dG = -26.9
  1394 >s_a_s_d_s_b_s_b_s_d_s_c_s_a_s          GTGTCTGTGACGGTGGCTGTGGCTGTGACGGTGTGGGTGTCTGTG;
>clamp_s_c_s_d_s_c_s_a_s:compl:rev CACAGACACCCACACCGTCACCCACACTATCATGTTGACATTCC; dG = -26.9
  1395 >s_a_s_d_s_c_s_c_s_e_s_c_s_a_s          GTGTCTGTGACGGTGTGGGTGTGGGTGCTGGTGTGGGTGTCTGTG;
>clamp_s_c_s_d_s_c_s_a_s:compl:rev CACAGACACCCACACCGTCACCCACACTATCATGTTGACATTCC; dG = -26.9
  1396 >s_a_s_d_s_d_s_c_s_e_s_c_s_a_s          GTGTCTGTGACGGTGACGGTGTGGGTGCTGGTGTGGGTGTCTGTG;
>clamp_s_c_s_d_s_c_s_a_s:compl:rev CACAGACACCCACACCGTCACCCACACTATCATGTTGACATTCC; dG = -26.9
  1397 >s_a_s_d_s_d_s_b_s_d_s_c_s_a_s          GTGTCTGTGACGGTGACGGTGGCTGTGACGGTGTGGGTGTCTGTG;
>clamp_s_c_s_d_s_c_s_a_s:compl:rev CACAGACACCCACACCGTCACCCACACTATCATGTTGACATTCC; dG = -26.9
  1398 >s_a_s_a_s_b_s_b_s_d_s_c_s_a_s          GTGTCTGTGTCTGTGGCTGTGGCTGTGACGGTGTGGGTGTCTGTG;
>clamp_s_c_s_d_s_c_s_a_s:compl:rev CACAGACACCCACACCGTCACCCACACTATCATGTTGACATTCC; dG = -26.9
  1399 >s_a_s_d_s_e_s_c_s_e_s_c_s_a_s          GTGTCTGTGACGGTGCTGGTGTGGGTGCTGGTGTGGGTGTCTGTG;
>clamp_s_c_s_d_s_c_s_a_s:compl:rev CACAGACACCCACACCGTCACCCACACTATCATGTTGACATTCC; dG = -26.9
  1400 >s_a_s_d_s_e_s_b_s_d_s_c_s_a_s          GTGTCTGTGACGGTGCTGGTGGCTGTGACGGTGTGGGTGTCTGTG;
>clamp_s_c_s_d_s_c_s_a_s:compl:rev CACAGACACCCACACCGTCACCCACACTATCATGTTGACATTCC; dG = -26.9
  1401 >s_a_s_e_s_a_s_c_s_e_s_c_s_a_s          GTGTCTGTGCTGGTGTCTGTGTGGGTGCTGGTGTGGGTGTCTGTG;
>clamp_s_c_s_d_s_c_s_a_s:compl:rev CACAGACACCCACACCGTCACCCACACTATCATGTTGACATTCC; dG = -26.9
  1402 >s_a_s_e_s_a_s_b_s_d_s_c_s_a_s          GTGTCTGTGCTGGTGTCTGTGGCTGTGACGGTGTGGGTGTCTGTG;
>clamp_s_c_s_d_s_c_s_a_s:compl:rev CACAGACACCCACACCGTCACCCACACTATCATGTTGACATTCC; dG = -26.9
  1403 >s_a_s_e_s_b_s_c_s_e_s_c_s_a_s          GTGTCTGTGCTGGTGGCTGTGTGGGTGCTGGTGTGGGTGTCTGTG;
>clamp_s_c_s_d_s_c_s_a_s:compl:rev CACAGACACCCACACCGTCACCCACACTATCATGTTGACATTCC; dG = -26.9
  1404 >s_a_s_e_s_b_s_b_s_d_s_c_s_a_s          GTGTCTGTGCTGGTGGCTGTGGCTGTGACGGTGTGGGTGTCTGTG;
>clamp_s_c_s_d_s_c_s_a_s:compl:rev CACAGACACCCACACCGTCACCCACACTATCATGTTGACATTCC; dG = -26.9
  1405 >s_a_s_e_s_c_s_c_s_e_s_c_s_a_s          GTGTCTGTGCTGGTGTGGGTGTGGGTGCTGGTGTGGGTGTCTGTG;
>clamp_s_c_s_d_s_c_s_a_s:compl:rev CACAGACACCCACACCGTCACCCACACTATCATGTTGACATTCC; dG = -26.9
  1406 >s_a_s_e_s_d_s_c_s_e_s_c_s_a_s          GTGTCTGTGCTGGTGACGGTGTGGGTGCTGGTGTGGGTGTCTGTG;
>clamp_s_c_s_d_s_c_s_a_s:compl:rev CACAGACACCCACACCGTCACCCACACTATCATGTTGACATTCC; dG = -26.9
  1407 >s_a_s_e_s_d_s_b_s_d_s_c_s_a_s          GTGTCTGTGCTGGTGACGGTGGCTGTGACGGTGTGGGTGTCTGTG;
>clamp_s_c_s_d_s_c_s_a_s:compl:rev CACAGACACCCACACCGTCACCCACACTATCATGTTGACATTCC; dG = -26.9
  1408 >s_a_s_e_s_e_s_c_s_e_s_c_s_a_s          GTGTCTGTGCTGGTGCTGGTGTGGGTGCTGGTGTGGGTGTCTGTG;
>clamp_s_c_s_d_s_c_s_a_s:compl:rev CACAGACACCCACACCGTCACCCACACTATCATGTTGACATTCC; dG = -26.9
  1409 >s_a_s_e_s_e_s_b_s_d_s_c_s_a_s          GTGTCTGTGCTGGTGCTGGTGGCTGTGACGGTGTGGGTGTCTGTG;
>clamp_s_c_s_d_s_c_s_a_s:compl:rev CACAGACACCCACACCGTCACCCACACTATCATGTTGACATTCC; dG = -26.9
  1410 >s_b_s_a_s_a_s_c_s_e_s_c_s_a_s          GTGGCTGTGTCTGTGTCTGTGTGGGTGCTGGTGTGGGTGTCTGTG;
>clamp_s_c_s_d_s_c_s_a_s:compl:rev CACAGACACCCACACCGTCACCCACACTATCATGTTGACATTCC; dG = -26.9
  1411 >s_a_s_a_s_c_s_c_s_e_s_c_s_a_s          GTGTCTGTGTCTGTGTGGGTGTGGGTGCTGGTGTGGGTGTCTGTG;
>clamp_s_c_s_d_s_c_s_a_s:compl:rev CACAGACACCCACACCGTCACCCACACTATCATGTTGACATTCC; dG = -26.9
  1412 >s_b_s_a_s_a_s_b_s_d_s_c_s_a_s          GTGGCTGTGTCTGTGTCTGTGGCTGTGACGGTGTGGGTGTCTGTG;
>clamp_s_c_s_d_s_c_s_a_s:compl:rev CACAGACACCCACACCGTCACCCACACTATCATGTTGACATTCC; dG = -26.9
  1413 >s_b_s_a_s_b_s_c_s_e_s_c_s_a_s          GTGGCTGTGTCTGTGGCTGTGTGGGTGCTGGTGTGGGTGTCTGTG;
>clamp_s_c_s_d_s_c_s_a_s:compl:rev CACAGACACCCACACCGTCACCCACACTATCATGTTGACATTCC; dG = -26.9
  1414 >s_b_s_a_s_b_s_b_s_d_s_c_s_a_s          GTGGCTGTGTCTGTGGCTGTGGCTGTGACGGTGTGGGTGTCTGTG;
>clamp_s_c_s_d_s_c_s_a_s:compl:rev CACAGACACCCACACCGTCACCCACACTATCATGTTGACATTCC; dG = -26.9
  1415 >s_b_s_a_s_c_s_c_s_e_s_c_s_a_s          GTGGCTGTGTCTGTGTGGGTGTGGGTGCTGGTGTGGGTGTCTGTG;
>clamp_s_c_s_d_s_c_s_a_s:compl:rev CACAGACACCCACACCGTCACCCACACTATCATGTTGACATTCC; dG = -26.9
  1416 >s_b_s_a_s_d_s_c_s_e_s_c_s_a_s          GTGGCTGTGTCTGTGACGGTGTGGGTGCTGGTGTGGGTGTCTGTG;
>clamp_s_c_s_d_s_c_s_a_s:compl:rev CACAGACACCCACACCGTCACCCACACTATCATGTTGACATTCC; dG = -26.9
  1417 >s_b_s_a_s_d_s_b_s_d_s_c_s_a_s          GTGGCTGTGTCTGTGACGGTGGCTGTGACGGTGTGGGTGTCTGTG;
>clamp_s_c_s_d_s_c_s_a_s:compl:rev CACAGACACCCACACCGTCACCCACACTATCATGTTGACATTCC; dG = -26.9
  1418 >s_b_s_a_s_e_s_c_s_e_s_c_s_a_s          GTGGCTGTGTCTGTGCTGGTGTGGGTGCTGGTGTGGGTGTCTGTG;
>clamp_s_c_s_d_s_c_s_a_s:compl:rev CACAGACACCCACACCGTCACCCACACTATCATGTTGACATTCC; dG = -26.9
  1419 >s_b_s_a_s_e_s_b_s_d_s_c_s_a_s          GTGGCTGTGTCTGTGCTGGTGGCTGTGACGGTGTGGGTGTCTGTG;
>clamp_s_c_s_d_s_c_s_a_s:compl:rev CACAGACACCCACACCGTCACCCACACTATCATGTTGACATTCC; dG = -26.9
  1420 >s_b_s_b_s_a_s_c_s_e_s_c_s_a_s          GTGGCTGTGGCTGTGTCTGTGTGGGTGCTGGTGTGGGTGTCTGTG;
>clamp_s_c_s_d_s_c_s_a_s:compl:rev CACAGACACCCACACCGTCACCCACACTATCATGTTGACATTCC; dG = -26.9
  1421 >s_b_s_b_s_a_s_b_s_d_s_c_s_a_s          GTGGCTGTGGCTGTGTCTGTGGCTGTGACGGTGTGGGTGTCTGTG;
>clamp_s_c_s_d_s_c_s_a_s:compl:rev CACAGACACCCACACCGTCACCCACACTATCATGTTGACATTCC; dG = -26.9
  1422 >s_b_s_b_s_b_s_c_s_e_s_c_s_a_s          GTGGCTGTGGCTGTGGCTGTGTGGGTGCTGGTGTGGGTGTCTGTG;
>clamp_s_c_s_d_s_c_s_a_s:compl:rev CACAGACACCCACACCGTCACCCACACTATCATGTTGACATTCC; dG = -26.9
  1423 >s_b_s_b_s_b_s_b_s_d_s_c_s_a_s          GTGGCTGTGGCTGTGGCTGTGGCTGTGACGGTGTGGGTGTCTGTG;
>clamp_s_c_s_d_s_c_s_a_s:compl:rev CACAGACACCCACACCGTCACCCACACTATCATGTTGACATTCC; dG = -26.9
  1424 >s_b_s_b_s_c_s_c_s_e_s_c_s_a_s          GTGGCTGTGGCTGTGTGGGTGTGGGTGCTGGTGTGGGTGTCTGTG;
>clamp_s_c_s_d_s_c_s_a_s:compl:rev CACAGACACCCACACCGTCACCCACACTATCATGTTGACATTCC; dG = -26.9
  1425 >s_b_s_b_s_d_s_c_s_e_s_c_s_a_s          GTGGCTGTGGCTGTGACGGTGTGGGTGCTGGTGTGGGTGTCTGTG;
>clamp_s_c_s_d_s_c_s_a_s:compl:rev CACAGACACCCACACCGTCACCCACACTATCATGTTGACATTCC; dG = -26.9
  1426 >s_b_s_b_s_d_s_b_s_d_s_c_s_a_s          GTGGCTGTGGCTGTGACGGTGGCTGTGACGGTGTGGGTGTCTGTG;
>clamp_s_c_s_d_s_c_s_a_s:compl:rev CACAGACACCCACACCGTCACCCACACTATCATGTTGACATTCC; dG = -26.9
```

```
  1427 >s_b_s_b_s_e_s_c_s_e_s_c_s_a_s          GTGGCTGTGGCTGTGCTGGTGTGGGTGCTGGTGTGGGTGTCTGTG;
>clamp_s_c_s_d_s_c_s_a_s:compl:rev CACAGACACCCACACCGTCACCCACACTATCATGTTGACATTCC; dG = -26.9
  1428 >s_b_s_b_s_e_s_b_s_d_s_c_s_a_s          GTGGCTGTGGCTGTGCTGGTGGCTGTGACGGTGTGGGTGTCTGTG;
>clamp_s_c_s_d_s_c_s_a_s:compl:rev CACAGACACCCACACCGTCACCCACACTATCATGTTGACATTCC; dG = -26.9
  1429 >s_b_s_c_s_a_s_c_s_e_s_c_s_a_s          GTGGCTGTGTGGGTGTCTGTGTGGGTGCTGGTGTGGGTGTCTGTG;
>clamp_s_c_s_d_s_c_s_a_s:compl:rev CACAGACACCCACACCGTCACCCACACTATCATGTTGACATTCC; dG = -26.9
  1430 >s_a_s_a_s_d_s_c_s_e_s_c_s_a_s          GTGTCTGTGTCTGTGACGGTGTGGGTGCTGGTGTGGGTGTCTGTG;
>clamp_s_c_s_d_s_c_s_a_s:compl:rev CACAGACACCCACACCGTCACCCACACTATCATGTTGACATTCC; dG = -26.9
  1431 >s_b_s_c_s_b_s_c_s_e_s_c_s_a_s          GTGGCTGTGTGGGTGGCTGTGTGGGTGCTGGTGTGGGTGTCTGTG;
>clamp_s_c_s_d_s_c_s_a_s:compl:rev CACAGACACCCACACCGTCACCCACACTATCATGTTGACATTCC; dG = -26.9
  1432 >s_b_s_c_s_c_s_c_s_e_s_c_s_a_s          GTGGCTGTGTGGGTGTGGGTGTGGGTGCTGGTGTGGGTGTCTGTG;
>clamp_s_c_s_d_s_c_s_a_s:compl:rev CACAGACACCCACACCGTCACCCACACTATCATGTTGACATTCC; dG = -26.9
  1433 >s_a_s_a_s_d_s_b_s_d_s_c_s_a_s          GTGTCTGTGTCTGTGACGGTGGCTGTGACGGTGTGGGTGTCTGTG;
>clamp_s_c_s_d_s_c_s_a_s:compl:rev CACAGACACCCACACCGTCACCCACACTATCATGTTGACATTCC; dG = -26.9
  1434 >s_b_s_c_s_e_s_c_s_e_s_c_s_a_s          GTGGCTGTGTGGGTGCTGGTGTGGGTGCTGGTGTGGGTGTCTGTG;
>clamp_s_c_s_d_s_c_s_a_s:compl:rev CACAGACACCCACACCGTCACCCACACTATCATGTTGACATTCC; dG = -26.9
  1435 >s_b_s_d_s_a_s_c_s_e_s_c_s_a_s          GTGGCTGTGACGGTGTCTGTGTGGGTGCTGGTGTGGGTGTCTGTG;
>clamp_s_c_s_d_s_c_s_a_s:compl:rev CACAGACACCCACACCGTCACCCACACTATCATGTTGACATTCC; dG = -26.9
  1436 >s_b_s_d_s_a_s_b_s_d_s_c_s_a_s          GTGGCTGTGACGGTGTCTGTGGCTGTGACGGTGTGGGTGTCTGTG;
>clamp_s_c_s_d_s_c_s_a_s:compl:rev CACAGACACCCACACCGTCACCCACACTATCATGTTGACATTCC; dG = -26.9
  1437 >s_b_s_d_s_b_s_c_s_e_s_c_s_a_s          GTGGCTGTGACGGTGGCTGTGTGGGTGCTGGTGTGGGTGTCTGTG;
>clamp_s_c_s_d_s_c_s_a_s:compl:rev CACAGACACCCACACCGTCACCCACACTATCATGTTGACATTCC; dG = -26.9
  1438 >s_b_s_d_s_b_s_b_s_d_s_c_s_a_s          GTGGCTGTGACGGTGGCTGTGGCTGTGACGGTGTGGGTGTCTGTG;
>clamp_s_c_s_d_s_c_s_a_s:compl:rev CACAGACACCCACACCGTCACCCACACTATCATGTTGACATTCC; dG = -26.9
  1439 >s_b_s_d_s_c_s_c_s_e_s_c_s_a_s          GTGGCTGTGACGGTGTGGGTGTGGGTGCTGGTGTGGGTGTCTGTG;
>clamp_s_c_s_d_s_c_s_a_s:compl:rev CACAGACACCCACACCGTCACCCACACTATCATGTTGACATTCC; dG = -26.9
  1440 >s_b_s_d_s_d_s_c_s_e_s_c_s_a_s          GTGGCTGTGACGGTGACGGTGTGGGTGCTGGTGTGGGTGTCTGTG;
>clamp_s_c_s_d_s_c_s_a_s:compl:rev CACAGACACCCACACCGTCACCCACACTATCATGTTGACATTCC; dG = -26.9
  1441 >s_b_s_d_s_d_s_b_s_d_s_c_s_a_s          GTGGCTGTGACGGTGACGGTGGCTGTGACGGTGTGGGTGTCTGTG;
>clamp_s_c_s_d_s_c_s_a_s:compl:rev CACAGACACCCACACCGTCACCCACACTATCATGTTGACATTCC; dG = -26.9
  1442 >s_b_s_d_s_e_s_c_s_e_s_c_s_a_s          GTGGCTGTGACGGTGCTGGTGTGGGTGCTGGTGTGGGTGTCTGTG;
>clamp_s_c_s_d_s_c_s_a_s:compl:rev CACAGACACCCACACCGTCACCCACACTATCATGTTGACATTCC; dG = -26.9
  1443 >s_b_s_d_s_e_s_b_s_d_s_c_s_a_s          GTGGCTGTGACGGTGCTGGTGGCTGTGACGGTGTGGGTGTCTGTG;
>clamp_s_c_s_d_s_c_s_a_s:compl:rev CACAGACACCCACACCGTCACCCACACTATCATGTTGACATTCC; dG = -26.9
  1444 >s_b_s_e_s_a_s_c_s_e_s_c_s_a_s          GTGGCTGTGCTGGTGTCTGTGTGGGTGCTGGTGTGGGTGTCTGTG;
>clamp_s_c_s_d_s_c_s_a_s:compl:rev CACAGACACCCACACCGTCACCCACACTATCATGTTGACATTCC; dG = -26.9
  1445 >s_a_s_a_s_e_s_c_s_e_s_c_s_a_s          GTGTCTGTGTCTGTGCTGGTGTGGGTGCTGGTGTGGGTGTCTGTG;
>clamp_s_c_s_d_s_c_s_a_s:compl:rev CACAGACACCCACACCGTCACCCACACTATCATGTTGACATTCC; dG = -26.9
  1446 >s_b_s_e_s_a_s_b_s_d_s_c_s_a_s          GTGGCTGTGCTGGTGTCTGTGGCTGTGACGGTGTGGGTGTCTGTG;
>clamp_s_c_s_d_s_c_s_a_s:compl:rev CACAGACACCCACACCGTCACCCACACTATCATGTTGACATTCC; dG = -26.9
  1447 >s_b_s_e_s_b_s_c_s_e_s_c_s_a_s          GTGGCTGTGCTGGTGGCTGTGTGGGTGCTGGTGTGGGTGTCTGTG;
>clamp_s_c_s_d_s_c_s_a_s:compl:rev CACAGACACCCACACCGTCACCCACACTATCATGTTGACATTCC; dG = -26.9
  1448 >s_b_s_e_s_b_s_b_s_d_s_c_s_a_s          GTGGCTGTGCTGGTGGCTGTGGCTGTGACGGTGTGGGTGTCTGTG;
>clamp_s_c_s_d_s_c_s_a_s:compl:rev CACAGACACCCACACCGTCACCCACACTATCATGTTGACATTCC; dG = -26.9
  1449 >s_b_s_e_s_c_s_c_s_e_s_c_s_a_s          GTGGCTGTGCTGGTGTGGGTGTGGGTGCTGGTGTGGGTGTCTGTG;
>clamp_s_c_s_d_s_c_s_a_s:compl:rev CACAGACACCCACACCGTCACCCACACTATCATGTTGACATTCC; dG = -26.9
  1450 >s_b_s_e_s_d_s_c_s_e_s_c_s_a_s          GTGGCTGTGCTGGTGACGGTGTGGGTGCTGGTGTGGGTGTCTGTG;
>clamp_s_c_s_d_s_c_s_a_s:compl:rev CACAGACACCCACACCGTCACCCACACTATCATGTTGACATTCC; dG = -26.9
  1451 >s_b_s_e_s_d_s_b_s_d_s_c_s_a_s          GTGGCTGTGCTGGTGACGGTGGCTGTGACGGTGTGGGTGTCTGTG;
>clamp_s_c_s_d_s_c_s_a_s:compl:rev CACAGACACCCACACCGTCACCCACACTATCATGTTGACATTCC; dG = -26.9
  1452 >s_a_s_a_s_e_s_b_s_d_s_c_s_a_s          GTGTCTGTGTCTGTGCTGGTGGCTGTGACGGTGTGGGTGTCTGTG;
>clamp_s_c_s_d_s_c_s_a_s:compl:rev CACAGACACCCACACCGTCACCCACACTATCATGTTGACATTCC; dG = -26.9
  1453 >s_b_s_e_s_e_s_c_s_e_s_c_s_a_s          GTGGCTGTGCTGGTGCTGGTGTGGGTGCTGGTGTGGGTGTCTGTG;
>clamp_s_c_s_d_s_c_s_a_s:compl:rev CACAGACACCCACACCGTCACCCACACTATCATGTTGACATTCC; dG = -26.9
  1454 >s_b_s_e_s_e_s_b_s_d_s_c_s_a_s          GTGGCTGTGCTGGTGCTGGTGGCTGTGACGGTGTGGGTGTCTGTG;
>clamp_s_c_s_d_s_c_s_a_s:compl:rev CACAGACACCCACACCGTCACCCACACTATCATGTTGACATTCC; dG = -26.9
  1455 >s_c_s_a_s_a_s_c_s_e_s_c_s_a_s          GTGTGGGTGTCTGTGTCTGTGTGGGTGCTGGTGTGGGTGTCTGTG;
>clamp_s_c_s_d_s_c_s_a_s:compl:rev CACAGACACCCACACCGTCACCCACACTATCATGTTGACATTCC; dG = -26.9
  1456 >s_c_s_a_s_a_s_b_s_d_s_c_s_a_s          GTGTGGGTGTCTGTGTCTGTGGCTGTGACGGTGTGGGTGTCTGTG;
>clamp_s_c_s_d_s_c_s_a_s:compl:rev CACAGACACCCACACCGTCACCCACACTATCATGTTGACATTCC; dG = -26.9
  1457 >s_c_s_a_s_b_s_c_s_e_s_c_s_a_s          GTGTGGGTGTCTGTGGCTGTGTGGGTGCTGGTGTGGGTGTCTGTG;
>clamp_s_c_s_d_s_c_s_a_s:compl:rev CACAGACACCCACACCGTCACCCACACTATCATGTTGACATTCC; dG = -26.9
  1458 >s_c_s_a_s_b_s_b_s_d_s_c_s_a_s          GTGTGGGTGTCTGTGGCTGTGGCTGTGACGGTGTGGGTGTCTGTG;
>clamp_s_c_s_d_s_c_s_a_s:compl:rev CACAGACACCCACACCGTCACCCACACTATCATGTTGACATTCC; dG = -26.9
  1459 >s_c_s_a_s_c_s_c_s_e_s_c_s_a_s          GTGTGGGTGTCTGTGTGGGTGTGGGTGCTGGTGTGGGTGTCTGTG;
>clamp_s_c_s_d_s_c_s_a_s:compl:rev CACAGACACCCACACCGTCACCCACACTATCATGTTGACATTCC; dG = -26.9
  1460 >s_c_s_a_s_d_s_c_s_e_s_c_s_a_s          GTGTGGGTGTCTGTGACGGTGTGGGTGCTGGTGTGGGTGTCTGTG;
>clamp_s_c_s_d_s_c_s_a_s:compl:rev CACAGACACCCACACCGTCACCCACACTATCATGTTGACATTCC; dG = -26.9
  1461 >s_c_s_a_s_d_s_b_s_d_s_c_s_a_s          GTGTGGGTGTCTGTGACGGTGGCTGTGACGGTGTGGGTGTCTGTG;
>clamp_s_c_s_d_s_c_s_a_s:compl:rev CACAGACACCCACACCGTCACCCACACTATCATGTTGACATTCC; dG = -26.9
  1462 >s_c_s_a_s_e_s_c_s_e_s_c_s_a_s          GTGTGGGTGTCTGTGCTGGTGTGGGTGCTGGTGTGGGTGTCTGTG;
>clamp_s_c_s_d_s_c_s_a_s:compl:rev CACAGACACCCACACCGTCACCCACACTATCATGTTGACATTCC; dG = -26.9
  1463 >s_c_s_a_s_e_s_b_s_d_s_c_s_a_s          GTGTGGGTGTCTGTGCTGGTGGCTGTGACGGTGTGGGTGTCTGTG;
>clamp_s_c_s_d_s_c_s_a_s:compl:rev CACAGACACCCACACCGTCACCCACACTATCATGTTGACATTCC; dG = -26.9
```

```
  1464 >s_c_s_b_s_a_s_c_s_e_s_c_s_a_s          GTGTGGGTGGCTGTGTCTGTGTGGGTGCTGGTGTGGGTGTCTGTG;
>clamp_s_c_s_d_s_c_s_a_s:compl:rev CACAGACACCCACACCGTCACCCACACTATCATGTTGACATTCC; dG = -26.9
  1465 >s_a_s_b_s_a_s_c_s_e_s_c_s_a_s          GTGTCTGTGGCTGTGTCTGTGTGGGTGCTGGTGTGGGTGTCTGTG;
>clamp_s_c_s_d_s_c_s_a_s:compl:rev CACAGACACCCACACCGTCACCCACACTATCATGTTGACATTCC; dG = -26.9
  1466 >s_c_s_b_s_a_s_b_s_d_s_c_s_a_s          GTGTGGGTGGCTGTGTCTGTGGCTGTGACGGTGTGGGTGTCTGTG;
>clamp_s_c_s_d_s_c_s_a_s:compl:rev CACAGACACCCACACCGTCACCCACACTATCATGTTGACATTCC; dG = -26.9
  1467 >s_a_s_a_s_a_s_c_s_e_s_c_s_a_s          GTGTCTGTGTCTGTGTCTGTGTGGGTGCTGGTGTGGGTGTCTGTG;
>clamp_s_c_s_d_s_c_s_a_s:compl:rev CACAGACACCCACACCGTCACCCACACTATCATGTTGACATTCC; dG = -26.9
  1468 >s_c_s_b_s_b_s_c_s_e_s_c_s_a_s          GTGTGGGTGGCTGTGGCTGTGTGGGTGCTGGTGTGGGTGTCTGTG;
>clamp_s_c_s_d_s_c_s_a_s:compl:rev CACAGACACCCACACCGTCACCCACACTATCATGTTGACATTCC; dG = -26.9
  1469 >s_c_s_b_s_b_s_b_s_d_s_c_s_a_s          GTGTGGGTGGCTGTGGCTGTGGCTGTGACGGTGTGGGTGTCTGTG;
>clamp_s_c_s_d_s_c_s_a_s:compl:rev CACAGACACCCACACCGTCACCCACACTATCATGTTGACATTCC; dG = -26.9
  1470 >s_c_s_b_s_c_s_c_s_e_s_c_s_a_s          GTGTGGGTGGCTGTGTGGGTGTGGGTGCTGGTGTGGGTGTCTGTG;
>clamp_s_c_s_d_s_c_s_a_s:compl:rev CACAGACACCCACACCGTCACCCACACTATCATGTTGACATTCC; dG = -26.9
  1471 >s_c_s_b_s_d_s_c_s_e_s_c_s_a_s          GTGTGGGTGGCTGTGACGGTGTGGGTGCTGGTGTGGGTGTCTGTG;
>clamp_s_c_s_d_s_c_s_a_s:compl:rev CACAGACACCCACACCGTCACCCACACTATCATGTTGACATTCC; dG = -26.9
  1472 >s_c_s_b_s_d_s_b_s_d_s_c_s_a_s          GTGTGGGTGGCTGTGACGGTGGCTGTGACGGTGTGGGTGTCTGTG;
>clamp_s_c_s_d_s_c_s_a_s:compl:rev CACAGACACCCACACCGTCACCCACACTATCATGTTGACATTCC; dG = -26.9
  1473 >s_a_s_b_s_a_s_b_s_d_s_c_s_a_s          GTGTCTGTGGCTGTGTCTGTGGCTGTGACGGTGTGGGTGTCTGTG;
>clamp_s_c_s_d_s_c_s_a_s:compl:rev CACAGACACCCACACCGTCACCCACACTATCATGTTGACATTCC; dG = -26.9
  1474 >s_c_s_b_s_e_s_c_s_e_s_c_s_a_s          GTGTGGGTGGCTGTGCTGGTGTGGGTGCTGGTGTGGGTGTCTGTG;
>clamp_s_c_s_d_s_c_s_a_s:compl:rev CACAGACACCCACACCGTCACCCACACTATCATGTTGACATTCC; dG = -26.9
  1475 >s_c_s_b_s_e_s_b_s_d_s_c_s_a_s          GTGTGGGTGGCTGTGCTGGTGGCTGTGACGGTGTGGGTGTCTGTG;
>clamp_s_c_s_d_s_c_s_a_s:compl:rev CACAGACACCCACACCGTCACCCACACTATCATGTTGACATTCC; dG = -26.9
  1476 >s_c_s_c_s_a_s_c_s_e_s_c_s_a_s          GTGTGGGTGTGGGTGTCTGTGTGGGTGCTGGTGTGGGTGTCTGTG;
>clamp_s_c_s_d_s_c_s_a_s:compl:rev CACAGACACCCACACCGTCACCCACACTATCATGTTGACATTCC; dG = -26.9
  1477 >s_c_s_c_s_b_s_c_s_e_s_c_s_a_s          GTGTGGGTGTGGGTGGCTGTGTGGGTGCTGGTGTGGGTGTCTGTG;
>clamp_s_c_s_d_s_c_s_a_s:compl:rev CACAGACACCCACACCGTCACCCACACTATCATGTTGACATTCC; dG = -26.9
  1478 >s_c_s_c_s_b_s_c_s_a_s_c_s_a_s          GTGTGGGTGTGGGTGGCTGTGTGGGTGTCTGTGTGGGTGTCTGTG;
>clamp_s_c_s_d_s_c_s_a_s:compl:rev CACAGACACCCACACCGTCACCCACACTATCATGTTGACATTCC; dG = -26.9
  1479 >s_c_s_c_s_c_s_c_s_e_s_c_s_a_s          GTGTGGGTGTGGGTGTGGGTGTGGGTGCTGGTGTGGGTGTCTGTG;
>clamp_s_c_s_d_s_c_s_a_s:compl:rev CACAGACACCCACACCGTCACCCACACTATCATGTTGACATTCC; dG = -26.9
  1480 >s_a_s_b_s_b_s_c_s_d_s_a_s_a_s          GTGTCTGTGGCTGTGGCTGTGTGGGTGACGGTGTCTGTGTCTGTG;
>clamp_s_c_s_d_s_c_s_a_s:compl:rev CACAGACACCCACACCGTCACCCACACTATCATGTTGACATTCC; dG = -26.8
  1481 >s_c_s_d_s_a_s_c_s_d_s_a_s_a_s          GTGTGGGTGACGGTGTCTGTGTGGGTGACGGTGTCTGTGTCTGTG;
>clamp_s_c_s_d_s_c_s_a_s:compl:rev CACAGACACCCACACCGTCACCCACACTATCATGTTGACATTCC; dG = -26.8
  1482 >s_c_s_d_s_b_s_c_s_d_s_a_s_a_s          GTGTGGGTGACGGTGGCTGTGTGGGTGACGGTGTCTGTGTCTGTG;
>clamp_s_c_s_d_s_c_s_a_s:compl:rev CACAGACACCCACACCGTCACCCACACTATCATGTTGACATTCC; dG = -26.8
  1483 >s_c_s_d_s_b_s_a_s_d_s_c_s_a_s          GTGTGGGTGACGGTGGCTGTGTCTGTGACGGTGTGGGTGTCTGTG;
>clamp_s_c_s_d_s_c_s_a_s:compl:rev CACAGACACCCACACCGTCACCCACACTATCATGTTGACATTCC; dG = -26.8
  1484 >s_a_s_b_s_b_s_a_s_d_s_c_s_a_s          GTGTCTGTGGCTGTGGCTGTGTCTGTGACGGTGTGGGTGTCTGTG;
>clamp_s_c_s_d_s_c_s_a_s:compl:rev CACAGACACCCACACCGTCACCCACACTATCATGTTGACATTCC; dG = -26.8
  1485 >s_c_s_e_s_a_s_c_s_d_s_a_s_a_s          GTGTGGGTGCTGGTGTCTGTGTGGGTGACGGTGTCTGTGTCTGTG;
>clamp_s_c_s_d_s_c_s_a_s:compl:rev CACAGACACCCACACCGTCACCCACACTATCATGTTGACATTCC; dG = -26.8
  1486 >s_c_s_e_s_a_s_a_s_d_s_c_s_a_s          GTGTGGGTGCTGGTGTCTGTGTCTGTGACGGTGTGGGTGTCTGTG;
>clamp_s_c_s_d_s_c_s_a_s:compl:rev CACAGACACCCACACCGTCACCCACACTATCATGTTGACATTCC; dG = -26.8
  1487 >s_c_s_e_s_b_s_c_s_d_s_a_s_a_s          GTGTGGGTGCTGGTGGCTGTGTGGGTGACGGTGTCTGTGTCTGTG;
>clamp_s_c_s_d_s_c_s_a_s:compl:rev CACAGACACCCACACCGTCACCCACACTATCATGTTGACATTCC; dG = -26.8
  1488 >s_c_s_e_s_b_s_a_s_d_s_c_s_a_s          GTGTGGGTGCTGGTGGCTGTGTCTGTGACGGTGTGGGTGTCTGTG;
>clamp_s_c_s_d_s_c_s_a_s:compl:rev CACAGACACCCACACCGTCACCCACACTATCATGTTGACATTCC; dG = -26.8
  1489 >s_d_s_a_s_a_s_c_s_d_s_a_s_a_s          GTGACGGTGTCTGTGTCTGTGTGGGTGACGGTGTCTGTGTCTGTG;
>clamp_s_c_s_d_s_c_s_a_s:compl:rev CACAGACACCCACACCGTCACCCACACTATCATGTTGACATTCC; dG = -26.8
  1490 >s_d_s_a_s_a_s_a_s_d_s_c_s_a_s          GTGACGGTGTCTGTGTCTGTGTCTGTGACGGTGTGGGTGTCTGTG;
>clamp_s_c_s_d_s_c_s_a_s:compl:rev CACAGACACCCACACCGTCACCCACACTATCATGTTGACATTCC; dG = -26.8
  1491 >s_d_s_a_s_b_s_c_s_d_s_a_s_a_s          GTGACGGTGTCTGTGGCTGTGTGGGTGACGGTGTCTGTGTCTGTG;
>clamp_s_c_s_d_s_c_s_a_s:compl:rev CACAGACACCCACACCGTCACCCACACTATCATGTTGACATTCC; dG = -26.8
  1492 >s_d_s_a_s_b_s_a_s_d_s_c_s_a_s          GTGACGGTGTCTGTGGCTGTGTCTGTGACGGTGTGGGTGTCTGTG;
>clamp_s_c_s_d_s_c_s_a_s:compl:rev CACAGACACCCACACCGTCACCCACACTATCATGTTGACATTCC; dG = -26.8
  1493 >s_d_s_b_s_a_s_c_s_d_s_a_s_a_s          GTGACGGTGGCTGTGTCTGTGTGGGTGACGGTGTCTGTGTCTGTG;
>clamp_s_c_s_d_s_c_s_a_s:compl:rev CACAGACACCCACACCGTCACCCACACTATCATGTTGACATTCC; dG = -26.8
  1494 >s_d_s_b_s_a_s_a_s_d_s_c_s_a_s          GTGACGGTGGCTGTGTCTGTGTCTGTGACGGTGTGGGTGTCTGTG;
>clamp_s_c_s_d_s_c_s_a_s:compl:rev CACAGACACCCACACCGTCACCCACACTATCATGTTGACATTCC; dG = -26.8
  1495 >s_d_s_b_s_b_s_c_s_d_s_a_s_a_s          GTGACGGTGGCTGTGGCTGTGTGGGTGACGGTGTCTGTGTCTGTG;
>clamp_s_c_s_d_s_c_s_a_s:compl:rev CACAGACACCCACACCGTCACCCACACTATCATGTTGACATTCC; dG = -26.8
  1496 >s_d_s_b_s_b_s_a_s_d_s_c_s_a_s          GTGACGGTGGCTGTGGCTGTGTCTGTGACGGTGTGGGTGTCTGTG;
>clamp_s_c_s_d_s_c_s_a_s:compl:rev CACAGACACCCACACCGTCACCCACACTATCATGTTGACATTCC; dG = -26.8
  1497 >s_d_s_c_s_a_s_c_s_d_s_a_s_a_s          GTGACGGTGTGGGTGTCTGTGTGGGTGACGGTGTCTGTGTCTGTG;
>clamp_s_c_s_d_s_c_s_a_s:compl:rev CACAGACACCCACACCGTCACCCACACTATCATGTTGACATTCC; dG = -26.8
  1498 >s_d_s_c_s_b_s_c_s_d_s_a_s_a_s          GTGACGGTGTGGGTGGCTGTGTGGGTGACGGTGTCTGTGTCTGTG;
>clamp_s_c_s_d_s_c_s_a_s:compl:rev CACAGACACCCACACCGTCACCCACACTATCATGTTGACATTCC; dG = -26.8
  1499 >s_d_s_d_s_a_s_c_s_d_s_a_s_a_s          GTGACGGTGACGGTGTCTGTGTGGGTGACGGTGTCTGTGTCTGTG;
>clamp_s_c_s_d_s_c_s_a_s:compl:rev CACAGACACCCACACCGTCACCCACACTATCATGTTGACATTCC; dG = -26.8
  1500 >s_d_s_d_s_a_s_a_s_d_s_c_s_a_s          GTGACGGTGACGGTGTCTGTGTCTGTGACGGTGTGGGTGTCTGTG;
>clamp_s_c_s_d_s_c_s_a_s:compl:rev CACAGACACCCACACCGTCACCCACACTATCATGTTGACATTCC; dG = -26.8
```

1501 >s_d_s_d_s_b_s_c_s_d_s_a_s_a_s GTGACGGTGACGGTGGCTGTGTGGGTGACGGTGTCTGTGTCTGTG;
>clamp_s_c_s_d_s_c_s_a_s:compl:rev CACAGACACCCACACCGTCACCCACACTATCATGTTGACATTCC; dG = -26.8
1502 >s_d_s_d_s_b_s_a_s_d_s_c_s_a_s GTGACGGTGACGGTGGCTGTGTCTGTGACGGTGTGGGTGTCTGTG;
>clamp_s_c_s_d_s_c_s_a_s:compl:rev CACAGACACCCACACCGTCACCCACACTATCATGTTGACATTCC; dG = -26.8
1503 >s_d_s_e_s_a_s_c_s_d_s_a_s_a_s GTGACGGTGCTGGTGTCTGTGTGGGTGACGGTGTCTGTGTCTGTG;
>clamp_s_c_s_d_s_c_s_a_s:compl:rev CACAGACACCCACACCGTCACCCACACTATCATGTTGACATTCC; dG = -26.8
1504 >s_d_s_e_s_a_s_a_s_d_s_c_s_a_s GTGACGGTGCTGGTGTCTGTGTCTGTGACGGTGTGGGTGTCTGTG;
>clamp_s_c_s_d_s_c_s_a_s:compl:rev CACAGACACCCACACCGTCACCCACACTATCATGTTGACATTCC; dG = -26.8
1505 >s_d_s_e_s_b_s_c_s_d_s_a_s_a_s GTGACGGTGCTGGTGGCTGTGTGGGTGACGGTGTCTGTGTCTGTG;
>clamp_s_c_s_d_s_c_s_a_s:compl:rev CACAGACACCCACACCGTCACCCACACTATCATGTTGACATTCC; dG = -26.8
1506 >s_d_s_e_s_b_s_a_s_d_s_c_s_a_s GTGACGGTGCTGGTGGCTGTGTCTGTGACGGTGTGGGTGTCTGTG;
>clamp_s_c_s_d_s_c_s_a_s:compl:rev CACAGACACCCACACCGTCACCCACACTATCATGTTGACATTCC; dG = -26.8
1507 >s_a_s_a_s_a_s_a_s_d_s_c_s_a_s GTGTCTGTGTCTGTGTCTGTGTCTGTGACGGTGTGGGTGTCTGTG;
>clamp_s_c_s_d_s_c_s_a_s:compl:rev CACAGACACCCACACCGTCACCCACACTATCATGTTGACATTCC; dG = -26.8
1508 >s_e_s_a_s_a_s_c_s_d_s_a_s_a_s GTGCTGGTGTCTGTGTCTGTGTGGGTGACGGTGTCTGTGTCTGTG;
>clamp_s_c_s_d_s_c_s_a_s:compl:rev CACAGACACCCACACCGTCACCCACACTATCATGTTGACATTCC; dG = -26.8
1509 >s_e_s_a_s_a_s_a_s_d_s_c_s_a_s GTGCTGGTGTCTGTGTCTGTGTCTGTGACGGTGTGGGTGTCTGTG;
>clamp_s_c_s_d_s_c_s_a_s:compl:rev CACAGACACCCACACCGTCACCCACACTATCATGTTGACATTCC; dG = -26.8
1510 >s_e_s_a_s_b_s_c_s_d_s_a_s_a_s GTGCTGGTGTCTGTGGCTGTGTGGGTGACGGTGTCTGTGTCTGTG;
>clamp_s_c_s_d_s_c_s_a_s:compl:rev CACAGACACCCACACCGTCACCCACACTATCATGTTGACATTCC; dG = -26.8
1511 >s_e_s_a_s_b_s_a_s_d_s_c_s_a_s GTGCTGGTGTCTGTGGCTGTGTCTGTGACGGTGTGGGTGTCTGTG;
>clamp_s_c_s_d_s_c_s_a_s:compl:rev CACAGACACCCACACCGTCACCCACACTATCATGTTGACATTCC; dG = -26.8
1512 >s_a_s_c_s_a_s_c_s_d_s_a_s_a_s GTGTCTGTGTGGGTGTCTGTGTGGGTGACGGTGTCTGTGTCTGTG;
>clamp_s_c_s_d_s_c_s_a_s:compl:rev CACAGACACCCACACCGTCACCCACACTATCATGTTGACATTCC; dG = -26.8
1513 >s_e_s_b_s_a_s_c_s_d_s_a_s_a_s GTGCTGGTGGCTGTGTCTGTGTGGGTGACGGTGTCTGTGTCTGTG;
>clamp_s_c_s_d_s_c_s_a_s:compl:rev CACAGACACCCACACCGTCACCCACACTATCATGTTGACATTCC; dG = -26.8
1514 >s_e_s_b_s_a_s_a_s_d_s_c_s_a_s GTGCTGGTGGCTGTGTCTGTGTCTGTGACGGTGTGGGTGTCTGTG;
>clamp_s_c_s_d_s_c_s_a_s:compl:rev CACAGACACCCACACCGTCACCCACACTATCATGTTGACATTCC; dG = -26.8
1515 >s_e_s_b_s_b_s_c_s_d_s_a_s_a_s GTGCTGGTGGCTGTGGCTGTGTGGGTGACGGTGTCTGTGTCTGTG;
>clamp_s_c_s_d_s_c_s_a_s:compl:rev CACAGACACCCACACCGTCACCCACACTATCATGTTGACATTCC; dG = -26.8
1516 >s_e_s_b_s_b_s_a_s_d_s_c_s_a_s GTGCTGGTGGCTGTGGCTGTGTCTGTGACGGTGTGGGTGTCTGTG;
>clamp_s_c_s_d_s_c_s_a_s:compl:rev CACAGACACCCACACCGTCACCCACACTATCATGTTGACATTCC; dG = -26.8
1517 >s_e_s_c_s_a_s_c_s_d_s_a_s_a_s GTGCTGGTGTGGGTGTCTGTGTGGGTGACGGTGTCTGTGTCTGTG;
>clamp_s_c_s_d_s_c_s_a_s:compl:rev CACAGACACCCACACCGTCACCCACACTATCATGTTGACATTCC; dG = -26.8
1518 >s_e_s_c_s_b_s_c_s_d_s_a_s_a_s GTGCTGGTGTGGGTGGCTGTGTGGGTGACGGTGTCTGTGTCTGTG;
>clamp_s_c_s_d_s_c_s_a_s:compl:rev CACAGACACCCACACCGTCACCCACACTATCATGTTGACATTCC; dG = -26.8
1519 >s_a_s_c_s_b_s_c_s_d_s_a_s_a_s GTGTCTGTGTGGGTGGCTGTGTGGGTGACGGTGTCTGTGTCTGTG;
>clamp_s_c_s_d_s_c_s_a_s:compl:rev CACAGACACCCACACCGTCACCCACACTATCATGTTGACATTCC; dG = -26.8
1520 >s_e_s_d_s_a_s_c_s_d_s_a_s_a_s GTGCTGGTGACGGTGTCTGTGTGGGTGACGGTGTCTGTGTCTGTG;
>clamp_s_c_s_d_s_c_s_a_s:compl:rev CACAGACACCCACACCGTCACCCACACTATCATGTTGACATTCC; dG = -26.8
1521 >s_e_s_d_s_a_s_a_s_d_s_c_s_a_s GTGCTGGTGACGGTGTCTGTGTCTGTGACGGTGTGGGTGTCTGTG;
>clamp_s_c_s_d_s_c_s_a_s:compl:rev CACAGACACCCACACCGTCACCCACACTATCATGTTGACATTCC; dG = -26.8
1522 >s_e_s_d_s_b_s_c_s_d_s_a_s_a_s GTGCTGGTGACGGTGGCTGTGTGGGTGACGGTGTCTGTGTCTGTG;
>clamp_s_c_s_d_s_c_s_a_s:compl:rev CACAGACACCCACACCGTCACCCACACTATCATGTTGACATTCC; dG = -26.8
1523 >s_e_s_d_s_b_s_a_s_d_s_c_s_a_s GTGCTGGTGACGGTGGCTGTGTCTGTGACGGTGTGGGTGTCTGTG;
>clamp_s_c_s_d_s_c_s_a_s:compl:rev CACAGACACCCACACCGTCACCCACACTATCATGTTGACATTCC; dG = -26.8
1524 >s_e_s_e_s_a_s_c_s_d_s_a_s_a_s GTGCTGGTGCTGGTGTCTGTGTGGGTGACGGTGTCTGTGTCTGTG;
>clamp_s_c_s_d_s_c_s_a_s:compl:rev CACAGACACCCACACCGTCACCCACACTATCATGTTGACATTCC; dG = -26.8
1525 >s_e_s_e_s_a_s_a_s_d_s_c_s_a_s GTGCTGGTGCTGGTGTCTGTGTCTGTGACGGTGTGGGTGTCTGTG;
>clamp_s_c_s_d_s_c_s_a_s:compl:rev CACAGACACCCACACCGTCACCCACACTATCATGTTGACATTCC; dG = -26.8
1526 >s_e_s_e_s_b_s_c_s_d_s_a_s_a_s GTGCTGGTGCTGGTGGCTGTGTGGGTGACGGTGTCTGTGTCTGTG;
>clamp_s_c_s_d_s_c_s_a_s:compl:rev CACAGACACCCACACCGTCACCCACACTATCATGTTGACATTCC; dG = -26.8
1527 >s_e_s_e_s_b_s_a_s_d_s_c_s_a_s GTGCTGGTGCTGGTGGCTGTGTCTGTGACGGTGTGGGTGTCTGTG;
>clamp_s_c_s_d_s_c_s_a_s:compl:rev CACAGACACCCACACCGTCACCCACACTATCATGTTGACATTCC; dG = -26.8
1528 >s_a_s_a_s_b_s_c_s_d_s_a_s_a_s GTGTCTGTGTCTGTGGCTGTGTGGGTGACGGTGTCTGTGTCTGTG;
>clamp_s_c_s_d_s_c_s_a_s:compl:rev CACAGACACCCACACCGTCACCCACACTATCATGTTGACATTCC; dG = -26.8
1529 >s_a_s_d_s_a_s_c_s_d_s_a_s_a_s GTGTCTGTGACGGTGTCTGTGTGGGTGACGGTGTCTGTGTCTGTG;
>clamp_s_c_s_d_s_c_s_a_s:compl:rev CACAGACACCCACACCGTCACCCACACTATCATGTTGACATTCC; dG = -26.8
1530 >s_a_s_d_s_a_s_a_s_d_s_c_s_a_s GTGTCTGTGACGGTGTCTGTGTCTGTGACGGTGTGGGTGTCTGTG;
>clamp_s_c_s_d_s_c_s_a_s:compl:rev CACAGACACCCACACCGTCACCCACACTATCATGTTGACATTCC; dG = -26.8
1531 >s_a_s_d_s_b_s_c_s_d_s_a_s_a_s GTGTCTGTGACGGTGGCTGTGTGGGTGACGGTGTCTGTGTCTGTG;
>clamp_s_c_s_d_s_c_s_a_s:compl:rev CACAGACACCCACACCGTCACCCACACTATCATGTTGACATTCC; dG = -26.8
1532 >s_a_s_d_s_b_s_a_s_d_s_c_s_a_s GTGTCTGTGACGGTGGCTGTGTCTGTGACGGTGTGGGTGTCTGTG;
>clamp_s_c_s_d_s_c_s_a_s:compl:rev CACAGACACCCACACCGTCACCCACACTATCATGTTGACATTCC; dG = -26.8
1533 >s_a_s_a_s_b_s_a_s_d_s_c_s_a_s GTGTCTGTGTCTGTGGCTGTGTCTGTGACGGTGTGGGTGTCTGTG;
>clamp_s_c_s_d_s_c_s_a_s:compl:rev CACAGACACCCACACCGTCACCCACACTATCATGTTGACATTCC; dG = -26.8
1534 >s_a_s_e_s_a_s_c_s_d_s_a_s_a_s GTGTCTGTGCTGGTGTCTGTGTGGGTGACGGTGTCTGTGTCTGTG;
>clamp_s_c_s_d_s_c_s_a_s:compl:rev CACAGACACCCACACCGTCACCCACACTATCATGTTGACATTCC; dG = -26.8
1535 >s_a_s_e_s_a_s_a_s_d_s_c_s_a_s GTGTCTGTGCTGGTGTCTGTGTCTGTGACGGTGTGGGTGTCTGTG;
>clamp_s_c_s_d_s_c_s_a_s:compl:rev CACAGACACCCACACCGTCACCCACACTATCATGTTGACATTCC; dG = -26.8
1536 >s_a_s_e_s_b_s_c_s_d_s_a_s_a_s GTGTCTGTGCTGGTGGCTGTGTGGGTGACGGTGTCTGTGTCTGTG;
>clamp_s_c_s_d_s_c_s_a_s:compl:rev CACAGACACCCACACCGTCACCCACACTATCATGTTGACATTCC; dG = -26.8
1537 >s_a_s_e_s_b_s_a_s_d_s_c_s_a_s GTGTCTGTGCTGGTGGCTGTGTCTGTGACGGTGTGGGTGTCTGTG;
>clamp_s_c_s_d_s_c_s_a_s:compl:rev CACAGACACCCACACCGTCACCCACACTATCATGTTGACATTCC; dG = -26.8

1538 >s_b_s_a_s_a_s_c_s_d_s_a_s_a_s GTGGCTGTGTCTGTGTCTGTGTGGGTGACGGTGTCTGTGTCTGTG;
>clamp_s_c_s_d_s_c_s_a_s:compl:rev CACAGACACCCACACCGTCACCCACACTATCATGTTGACATTCC; dG = -26.8
1539 >s_b_s_a_s_a_s_a_s_d_s_c_s_a_s GTGGCTGTGTCTGTGTCTGTGTCTGTGACGGTGTGGGTGTCTGTG;
>clamp_s_c_s_d_s_c_s_a_s:compl:rev CACAGACACCCACACCGTCACCCACACTATCATGTTGACATTCC; dG = -26.8
1540 >s_b_s_a_s_b_s_c_s_d_s_a_s_a_s GTGGCTGTGTCTGTGGCTGTGTGGGTGACGGTGTCTGTGTCTGTG;
>clamp_s_c_s_d_s_c_s_a_s:compl:rev CACAGACACCCACACCGTCACCCACACTATCATGTTGACATTCC; dG = -26.8
1541 >s_b_s_a_s_b_s_a_s_d_s_c_s_a_s GTGGCTGTGTCTGTGGCTGTGTCTGTGACGGTGTGGGTGTCTGTG;
>clamp_s_c_s_d_s_c_s_a_s:compl:rev CACAGACACCCACACCGTCACCCACACTATCATGTTGACATTCC; dG = -26.8
1542 >s_b_s_b_s_a_s_c_s_d_s_a_s_a_s GTGGCTGTGGCTGTGTCTGTGTGGGTGACGGTGTCTGTGTCTGTG;
>clamp_s_c_s_d_s_c_s_a_s:compl:rev CACAGACACCCACACCGTCACCCACACTATCATGTTGACATTCC; dG = -26.8
1543 >s_b_s_b_s_a_s_a_s_d_s_c_s_a_s GTGGCTGTGGCTGTGTCTGTGTCTGTGACGGTGTGGGTGTCTGTG;
>clamp_s_c_s_d_s_c_s_a_s:compl:rev CACAGACACCCACACCGTCACCCACACTATCATGTTGACATTCC; dG = -26.8
1544 >s_b_s_b_s_b_s_c_s_d_s_a_s_a_s GTGGCTGTGGCTGTGGCTGTGTGGGTGACGGTGTCTGTGTCTGTG;
>clamp_s_c_s_d_s_c_s_a_s:compl:rev CACAGACACCCACACCGTCACCCACACTATCATGTTGACATTCC; dG = -26.8
1545 >s_b_s_b_s_b_s_a_s_d_s_c_s_a_s GTGGCTGTGGCTGTGGCTGTGTCTGTGACGGTGTGGGTGTCTGTG;
>clamp_s_c_s_d_s_c_s_a_s:compl:rev CACAGACACCCACACCGTCACCCACACTATCATGTTGACATTCC; dG = -26.8
1546 >s_b_s_c_s_a_s_c_s_d_s_a_s_a_s GTGGCTGTGTGGGTGTCTGTGTGGGTGACGGTGTCTGTGTCTGTG;
>clamp_s_c_s_d_s_c_s_a_s:compl:rev CACAGACACCCACACCGTCACCCACACTATCATGTTGACATTCC; dG = -26.8
1547 >s_b_s_c_s_b_s_c_s_d_s_a_s_a_s GTGGCTGTGTGGGTGGCTGTGTGGGTGACGGTGTCTGTGTCTGTG;
>clamp_s_c_s_d_s_c_s_a_s:compl:rev CACAGACACCCACACCGTCACCCACACTATCATGTTGACATTCC; dG = -26.8
1548 >s_b_s_d_s_a_s_c_s_d_s_a_s_a_s GTGGCTGTGACGGTGTCTGTGTGGGTGACGGTGTCTGTGTCTGTG;
>clamp_s_c_s_d_s_c_s_a_s:compl:rev CACAGACACCCACACCGTCACCCACACTATCATGTTGACATTCC; dG = -26.8
1549 >s_b_s_d_s_a_s_a_s_d_s_c_s_a_s GTGGCTGTGACGGTGTCTGTGTCTGTGACGGTGTGGGTGTCTGTG;
>clamp_s_c_s_d_s_c_s_a_s:compl:rev CACAGACACCCACACCGTCACCCACACTATCATGTTGACATTCC; dG = -26.8
1550 >s_b_s_d_s_b_s_c_s_d_s_a_s_a_s GTGGCTGTGACGGTGGCTGTGTGGGTGACGGTGTCTGTGTCTGTG;
>clamp_s_c_s_d_s_c_s_a_s:compl:rev CACAGACACCCACACCGTCACCCACACTATCATGTTGACATTCC; dG = -26.8
1551 >s_b_s_d_s_b_s_a_s_d_s_c_s_a_s GTGGCTGTGACGGTGGCTGTGTCTGTGACGGTGTGGGTGTCTGTG;
>clamp_s_c_s_d_s_c_s_a_s:compl:rev CACAGACACCCACACCGTCACCCACACTATCATGTTGACATTCC; dG = -26.8
1552 >s_b_s_e_s_a_s_c_s_d_s_a_s_a_s GTGGCTGTGCTGGTGTCTGTGTGGGTGACGGTGTCTGTGTCTGTG;
>clamp_s_c_s_d_s_c_s_a_s:compl:rev CACAGACACCCACACCGTCACCCACACTATCATGTTGACATTCC; dG = -26.8
1553 >s_b_s_e_s_a_s_a_s_d_s_c_s_a_s GTGGCTGTGCTGGTGTCTGTGTCTGTGACGGTGTGGGTGTCTGTG;
>clamp_s_c_s_d_s_c_s_a_s:compl:rev CACAGACACCCACACCGTCACCCACACTATCATGTTGACATTCC; dG = -26.8
1554 >s_b_s_e_s_b_s_c_s_d_s_a_s_a_s GTGGCTGTGCTGGTGGCTGTGTGGGTGACGGTGTCTGTGTCTGTG;
>clamp_s_c_s_d_s_c_s_a_s:compl:rev CACAGACACCCACACCGTCACCCACACTATCATGTTGACATTCC; dG = -26.8
1555 >s_b_s_e_s_b_s_a_s_d_s_c_s_a_s GTGGCTGTGCTGGTGGCTGTGTCTGTGACGGTGTGGGTGTCTGTG;
>clamp_s_c_s_d_s_c_s_a_s:compl:rev CACAGACACCCACACCGTCACCCACACTATCATGTTGACATTCC; dG = -26.8
1556 >s_a_s_a_s_a_s_c_s_d_s_a_s_a_s GTGTCTGTGTCTGTGTCTGTGTGGGTGACGGTGTCTGTGTCTGTG;
>clamp_s_c_s_d_s_c_s_a_s:compl:rev CACAGACACCCACACCGTCACCCACACTATCATGTTGACATTCC; dG = -26.8
1557 >s_c_s_a_s_a_s_c_s_d_s_a_s_a_s GTGTGGGTGTCTGTGTCTGTGTGGGTGACGGTGTCTGTGTCTGTG;
>clamp_s_c_s_d_s_c_s_a_s:compl:rev CACAGACACCCACACCGTCACCCACACTATCATGTTGACATTCC; dG = -26.8
1558 >s_c_s_a_s_a_s_a_s_d_s_c_s_a_s GTGTGGGTGTCTGTGTCTGTGTCTGTGACGGTGTGGGTGTCTGTG;
>clamp_s_c_s_d_s_c_s_a_s:compl:rev CACAGACACCCACACCGTCACCCACACTATCATGTTGACATTCC; dG = -26.8
1559 >s_c_s_a_s_b_s_c_s_d_s_a_s_a_s GTGTGGGTGTCTGTGGCTGTGTGGGTGACGGTGTCTGTGTCTGTG;
>clamp_s_c_s_d_s_c_s_a_s:compl:rev CACAGACACCCACACCGTCACCCACACTATCATGTTGACATTCC; dG = -26.8
1560 >s_c_s_a_s_b_s_a_s_d_s_c_s_a_s GTGTGGGTGTCTGTGGCTGTGTCTGTGACGGTGTGGGTGTCTGTG;
>clamp_s_c_s_d_s_c_s_a_s:compl:rev CACAGACACCCACACCGTCACCCACACTATCATGTTGACATTCC; dG = -26.8
1561 >s_a_s_b_s_a_s_c_s_d_s_a_s_a_s GTGTCTGTGGCTGTGTCTGTGTGGGTGACGGTGTCTGTGTCTGTG;
>clamp_s_c_s_d_s_c_s_a_s:compl:rev CACAGACACCCACACCGTCACCCACACTATCATGTTGACATTCC; dG = -26.8
1562 >s_c_s_b_s_a_s_c_s_d_s_a_s_a_s GTGTGGGTGGCTGTGTCTGTGTGGGTGACGGTGTCTGTGTCTGTG;
>clamp_s_c_s_d_s_c_s_a_s:compl:rev CACAGACACCCACACCGTCACCCACACTATCATGTTGACATTCC; dG = -26.8
1563 >s_c_s_b_s_a_s_a_s_d_s_c_s_a_s GTGTGGGTGGCTGTGTCTGTGTCTGTGACGGTGTGGGTGTCTGTG;
>clamp_s_c_s_d_s_c_s_a_s:compl:rev CACAGACACCCACACCGTCACCCACACTATCATGTTGACATTCC; dG = -26.8
1564 >s_c_s_b_s_b_s_c_s_d_s_a_s_a_s GTGTGGGTGGCTGTGGCTGTGTGGGTGACGGTGTCTGTGTCTGTG;
>clamp_s_c_s_d_s_c_s_a_s:compl:rev CACAGACACCCACACCGTCACCCACACTATCATGTTGACATTCC; dG = -26.8
1565 >s_c_s_b_s_b_s_a_s_d_s_c_s_a_s GTGTGGGTGGCTGTGGCTGTGTCTGTGACGGTGTGGGTGTCTGTG;
>clamp_s_c_s_d_s_c_s_a_s:compl:rev CACAGACACCCACACCGTCACCCACACTATCATGTTGACATTCC; dG = -26.8
1566 >s_a_s_b_s_a_s_a_s_d_s_c_s_a_s GTGTCTGTGGCTGTGTCTGTGTCTGTGACGGTGTGGGTGTCTGTG;
>clamp_s_c_s_d_s_c_s_a_s:compl:rev CACAGACACCCACACCGTCACCCACACTATCATGTTGACATTCC; dG = -26.8
1567 >s_c_s_c_s_a_s_c_s_d_s_a_s_a_s GTGTGGGTGTGGGTGTCTGTGTGGGTGACGGTGTCTGTGTCTGTG;
>clamp_s_c_s_d_s_c_s_a_s:compl:rev CACAGACACCCACACCGTCACCCACACTATCATGTTGACATTCC; dG = -26.8
1568 >s_c_s_c_s_b_s_c_s_d_s_a_s_a_s GTGTGGGTGTGGGTGGCTGTGTGGGTGACGGTGTCTGTGTCTGTG;
>clamp_s_c_s_d_s_c_s_a_s:compl:rev CACAGACACCCACACCGTCACCCACACTATCATGTTGACATTCC; dG = -26.8
1569 >s_c_s_c_s_c_s_c_s_b_s_c_s_a_s GTGTGGGTGTGGGTGTGGGTGTGGGTGGCTGTGTGGGTGTCTGTG;
>clamp_s_c_s_d_s_c_s_a_s:compl:rev CACAGACACCCACACCGTCACCCACACTATCATGTTGACATTCC; dG = -26.7
1570 >s_c_s_c_s_e_s_c_s_d_s_a_s_a_s GTGTGGGTGTGGGTGCTGGTGTGGGTGACGGTGTCTGTGTCTGTG;
>clamp_s_c_s_d_s_c_s_a_s:compl:rev CACAGACACCCACACCGTCACCCACACTATCATGTTGACATTCC; dG = -26.7
1571 >s_c_s_c_s_e_s_c_s_b_s_c_s_a_s GTGTGGGTGTGGGTGCTGGTGTGGGTGGCTGTGTGGGTGTCTGTG;
>clamp_s_c_s_d_s_c_s_a_s:compl:rev CACAGACACCCACACCGTCACCCACACTATCATGTTGACATTCC; dG = -26.7
1572 >s_c_s_d_s_a_s_c_s_b_s_c_s_a_s GTGTGGGTGACGGTGTCTGTGTGGGTGGCTGTGTGGGTGTCTGTG;
>clamp_s_c_s_d_s_c_s_a_s:compl:rev CACAGACACCCACACCGTCACCCACACTATCATGTTGACATTCC; dG = -26.7
1573 >s_c_s_d_s_b_s_c_s_b_s_c_s_a_s GTGTGGGTGACGGTGGCTGTGTGGGTGGCTGTGTGGGTGTCTGTG;
>clamp_s_c_s_d_s_c_s_a_s:compl:rev CACAGACACCCACACCGTCACCCACACTATCATGTTGACATTCC; dG = -26.7
1574 >s_a_s_b_s_b_s_c_s_b_s_c_s_a_s GTGTCTGTGGCTGTGGCTGTGTGGGTGGCTGTGTGGGTGTCTGTG;
>clamp_s_c_s_d_s_c_s_a_s:compl:rev CACAGACACCCACACCGTCACCCACACTATCATGTTGACATTCC; dG = -26.7

1575 >s_c_s_d_s_d_s_c_s_d_s_a_s_a_s GTGTGGGTGACGGTGACGGTGTGGGTGACGGTGTCTGTGTCTGTG;
>clamp_s_c_s_d_s_c_s_a_s:compl:rev CACAGACACCCACACCGTCACCCACACTATCATGTTGACATTCC; dG = -26.7
1576 >s_c_s_d_s_d_s_c_s_b_s_c_s_a_s GTGTGGGTGACGGTGACGGTGTGGGTGGCTGTGTGGGTGTCTGTG;
>clamp_s_c_s_d_s_c_s_a_s:compl:rev CACAGACACCCACACCGTCACCCACACTATCATGTTGACATTCC; dG = -26.7
1577 >s_a_s_a_s_a_s_c_s_b_s_c_s_a_s GTGTCTGTGTCTGTGTCTGTGTGGGTGGCTGTGTGGGTGTCTGTG;
>clamp_s_c_s_d_s_c_s_a_s:compl:rev CACAGACACCCACACCGTCACCCACACTATCATGTTGACATTCC; dG = -26.7
1578 >s_c_s_d_s_e_s_c_s_d_s_a_s_a_s GTGTGGGTGACGGTGCTGGTGTGGGTGACGGTGTCTGTGTCTGTG;
>clamp_s_c_s_d_s_c_s_a_s:compl:rev CACAGACACCCACACCGTCACCCACACTATCATGTTGACATTCC; dG = -26.7
1579 >s_c_s_d_s_e_s_c_s_b_s_c_s_a_s GTGTGGGTGACGGTGCTGGTGTGGGTGGCTGTGTGGGTGTCTGTG;
>clamp_s_c_s_d_s_c_s_a_s:compl:rev CACAGACACCCACACCGTCACCCACACTATCATGTTGACATTCC; dG = -26.7
1580 >s_c_s_e_s_a_s_c_s_b_s_c_s_a_s GTGTGGGTGCTGGTGTCTGTGTGGGTGGCTGTGTGGGTGTCTGTG;
>clamp_s_c_s_d_s_c_s_a_s:compl:rev CACAGACACCCACACCGTCACCCACACTATCATGTTGACATTCC; dG = -26.7
1581 >s_c_s_e_s_b_s_c_s_b_s_c_s_a_s GTGTGGGTGCTGGTGGCTGTGTGGGTGGCTGTGTGGGTGTCTGTG;
>clamp_s_c_s_d_s_c_s_a_s:compl:rev CACAGACACCCACACCGTCACCCACACTATCATGTTGACATTCC; dG = -26.7
1582 >s_c_s_e_s_c_s_c_s_d_s_a_s_a_s GTGTGGGTGCTGGTGTGGGTGTGGGTGACGGTGTCTGTGTCTGTG;
>clamp_s_c_s_d_s_c_s_a_s:compl:rev CACAGACACCCACACCGTCACCCACACTATCATGTTGACATTCC; dG = -26.7
1583 >s_c_s_e_s_c_s_c_s_b_s_c_s_a_s GTGTGGGTGCTGGTGTGGGTGTGGGTGGCTGTGTGGGTGTCTGTG;
>clamp_s_c_s_d_s_c_s_a_s:compl:rev CACAGACACCCACACCGTCACCCACACTATCATGTTGACATTCC; dG = -26.7
1584 >s_c_s_e_s_d_s_c_s_d_s_a_s_a_s GTGTGGGTGCTGGTGACGGTGTGGGTGACGGTGTCTGTGTCTGTG;
>clamp_s_c_s_d_s_c_s_a_s:compl:rev CACAGACACCCACACCGTCACCCACACTATCATGTTGACATTCC; dG = -26.7
1585 >s_c_s_e_s_d_s_c_s_b_s_c_s_a_s GTGTGGGTGCTGGTGACGGTGTGGGTGGCTGTGTGGGTGTCTGTG;
>clamp_s_c_s_d_s_c_s_a_s:compl:rev CACAGACACCCACACCGTCACCCACACTATCATGTTGACATTCC; dG = -26.7
1586 >s_c_s_e_s_d_s_a_s_d_s_c_s_a_s GTGTGGGTGCTGGTGACGGTGTCTGTGACGGTGTGGGTGTCTGTG;
>clamp_s_c_s_d_s_c_s_a_s:compl:rev CACAGACACCCACACCGTCACCCACACTATCATGTTGACATTCC; dG = -26.7
1587 >s_c_s_e_s_e_s_c_s_d_s_a_s_a_s GTGTGGGTGCTGGTGCTGGTGTGGGTGACGGTGTCTGTGTCTGTG;
>clamp_s_c_s_d_s_c_s_a_s:compl:rev CACAGACACCCACACCGTCACCCACACTATCATGTTGACATTCC; dG = -26.7
1588 >s_c_s_e_s_e_s_c_s_b_s_c_s_a_s GTGTGGGTGCTGGTGCTGGTGTGGGTGGCTGTGTGGGTGTCTGTG;
>clamp_s_c_s_d_s_c_s_a_s:compl:rev CACAGACACCCACACCGTCACCCACACTATCATGTTGACATTCC; dG = -26.7
1589 >s_c_s_e_s_e_s_a_s_d_s_c_s_a_s GTGTGGGTGCTGGTGCTGGTGTCTGTGACGGTGTGGGTGTCTGTG;
>clamp_s_c_s_d_s_c_s_a_s:compl:rev CACAGACACCCACACCGTCACCCACACTATCATGTTGACATTCC; dG = -26.7
1590 >s_a_s_b_s_c_s_c_s_d_s_a_s_a_s GTGTCTGTGGCTGTGTGGGTGTGGGTGACGGTGTCTGTGTCTGTG;
>clamp_s_c_s_d_s_c_s_a_s:compl:rev CACAGACACCCACACCGTCACCCACACTATCATGTTGACATTCC; dG = -26.7
1591 >s_d_s_a_s_a_s_c_s_b_s_c_s_a_s GTGACGGTGTCTGTGTCTGTGTGGGTGGCTGTGTGGGTGTCTGTG;
>clamp_s_c_s_d_s_c_s_a_s:compl:rev CACAGACACCCACACCGTCACCCACACTATCATGTTGACATTCC; dG = -26.7
1592 >s_d_s_a_s_b_s_c_s_b_s_c_s_a_s GTGACGGTGTCTGTGGCTGTGTGGGTGGCTGTGTGGGTGTCTGTG;
>clamp_s_c_s_d_s_c_s_a_s:compl:rev CACAGACACCCACACCGTCACCCACACTATCATGTTGACATTCC; dG = -26.7
1593 >s_a_s_b_s_c_s_c_s_b_s_c_s_a_s GTGTCTGTGGCTGTGTGGGTGTGGGTGGCTGTGTGGGTGTCTGTG;
>clamp_s_c_s_d_s_c_s_a_s:compl:rev CACAGACACCCACACCGTCACCCACACTATCATGTTGACATTCC; dG = -26.7
1594 >s_d_s_a_s_c_s_c_s_d_s_a_s_a_s GTGACGGTGTCTGTGTGGGTGTGGGTGACGGTGTCTGTGTCTGTG;
>clamp_s_c_s_d_s_c_s_a_s:compl:rev CACAGACACCCACACCGTCACCCACACTATCATGTTGACATTCC; dG = -26.7
1595 >s_d_s_a_s_c_s_c_s_b_s_c_s_a_s GTGACGGTGTCTGTGTGGGTGTGGGTGGCTGTGTGGGTGTCTGTG;
>clamp_s_c_s_d_s_c_s_a_s:compl:rev CACAGACACCCACACCGTCACCCACACTATCATGTTGACATTCC; dG = -26.7
1596 >s_d_s_a_s_d_s_c_s_d_s_a_s_a_s GTGACGGTGTCTGTGACGGTGTGGGTGACGGTGTCTGTGTCTGTG;
>clamp_s_c_s_d_s_c_s_a_s:compl:rev CACAGACACCCACACCGTCACCCACACTATCATGTTGACATTCC; dG = -26.7
1597 >s_d_s_a_s_d_s_c_s_b_s_c_s_a_s GTGACGGTGTCTGTGACGGTGTGGGTGGCTGTGTGGGTGTCTGTG;
>clamp_s_c_s_d_s_c_s_a_s:compl:rev CACAGACACCCACACCGTCACCCACACTATCATGTTGACATTCC; dG = -26.7
1598 >s_d_s_a_s_d_s_a_s_d_s_c_s_a_s GTGACGGTGTCTGTGACGGTGTCTGTGACGGTGTGGGTGTCTGTG;
>clamp_s_c_s_d_s_c_s_a_s:compl:rev CACAGACACCCACACCGTCACCCACACTATCATGTTGACATTCC; dG = -26.7
1599 >s_d_s_a_s_e_s_c_s_d_s_a_s_a_s GTGACGGTGTCTGTGCTGGTGTGGGTGACGGTGTCTGTGTCTGTG;
>clamp_s_c_s_d_s_c_s_a_s:compl:rev CACAGACACCCACACCGTCACCCACACTATCATGTTGACATTCC; dG = -26.7
1600 >s_d_s_a_s_e_s_c_s_b_s_c_s_a_s GTGACGGTGTCTGTGCTGGTGTGGGTGGCTGTGTGGGTGTCTGTG;
>clamp_s_c_s_d_s_c_s_a_s:compl:rev CACAGACACCCACACCGTCACCCACACTATCATGTTGACATTCC; dG = -26.7
1601 >s_d_s_a_s_e_s_a_s_d_s_c_s_a_s GTGACGGTGTCTGTGCTGGTGTCTGTGACGGTGTGGGTGTCTGTG;
>clamp_s_c_s_d_s_c_s_a_s:compl:rev CACAGACACCCACACCGTCACCCACACTATCATGTTGACATTCC; dG = -26.7
1602 >s_d_s_b_s_a_s_c_s_b_s_c_s_a_s GTGACGGTGGCTGTGTCTGTGTGGGTGGCTGTGTGGGTGTCTGTG;
>clamp_s_c_s_d_s_c_s_a_s:compl:rev CACAGACACCCACACCGTCACCCACACTATCATGTTGACATTCC; dG = -26.7
1603 >s_d_s_b_s_b_s_c_s_b_s_c_s_a_s GTGACGGTGGCTGTGGCTGTGTGGGTGGCTGTGTGGGTGTCTGTG;
>clamp_s_c_s_d_s_c_s_a_s:compl:rev CACAGACACCCACACCGTCACCCACACTATCATGTTGACATTCC; dG = -26.7
1604 >s_d_s_b_s_c_s_c_s_d_s_a_s_a_s GTGACGGTGGCTGTGTGGGTGTGGGTGACGGTGTCTGTGTCTGTG;
>clamp_s_c_s_d_s_c_s_a_s:compl:rev CACAGACACCCACACCGTCACCCACACTATCATGTTGACATTCC; dG = -26.7
1605 >s_d_s_b_s_c_s_c_s_b_s_c_s_a_s GTGACGGTGGCTGTGTGGGTGTGGGTGGCTGTGTGGGTGTCTGTG;
>clamp_s_c_s_d_s_c_s_a_s:compl:rev CACAGACACCCACACCGTCACCCACACTATCATGTTGACATTCC; dG = -26.7
1606 >s_d_s_b_s_d_s_c_s_d_s_a_s_a_s GTGACGGTGGCTGTGACGGTGTGGGTGACGGTGTCTGTGTCTGTG;
>clamp_s_c_s_d_s_c_s_a_s:compl:rev CACAGACACCCACACCGTCACCCACACTATCATGTTGACATTCC; dG = -26.7
1607 >s_d_s_b_s_d_s_c_s_b_s_c_s_a_s GTGACGGTGGCTGTGACGGTGTGGGTGGCTGTGTGGGTGTCTGTG;
>clamp_s_c_s_d_s_c_s_a_s:compl:rev CACAGACACCCACACCGTCACCCACACTATCATGTTGACATTCC; dG = -26.7
1608 >s_d_s_b_s_d_s_a_s_d_s_c_s_a_s GTGACGGTGGCTGTGACGGTGTCTGTGACGGTGTGGGTGTCTGTG;
>clamp_s_c_s_d_s_c_s_a_s:compl:rev CACAGACACCCACACCGTCACCCACACTATCATGTTGACATTCC; dG = -26.7
1609 >s_d_s_b_s_e_s_c_s_d_s_a_s_a_s GTGACGGTGGCTGTGCTGGTGTGGGTGACGGTGTCTGTGTCTGTG;
>clamp_s_c_s_d_s_c_s_a_s:compl:rev CACAGACACCCACACCGTCACCCACACTATCATGTTGACATTCC; dG = -26.7
1610 >s_d_s_b_s_e_s_c_s_b_s_c_s_a_s GTGACGGTGGCTGTGCTGGTGTGGGTGGCTGTGTGGGTGTCTGTG;
>clamp_s_c_s_d_s_c_s_a_s:compl:rev CACAGACACCCACACCGTCACCCACACTATCATGTTGACATTCC; dG = -26.7
1611 >s_d_s_b_s_e_s_a_s_d_s_c_s_a_s GTGACGGTGGCTGTGCTGGTGTCTGTGACGGTGTGGGTGTCTGTG;
>clamp_s_c_s_d_s_c_s_a_s:compl:rev CACAGACACCCACACCGTCACCCACACTATCATGTTGACATTCC; dG = -26.7

1612 >s_a_s_b_s_d_s_c_s_d_s_a_s_a_s GTGTCTGTGGCTGTGACGGTGTGGGTGACGGTGTCTGTGTCTGTG;
>clamp_s_c_s_d_s_c_s_a_s:compl:rev CACAGACACCCACACCGTCACCCACACTATCATGTTGACATTCC; dG = -26.7
1613 >s_d_s_c_s_a_s_c_s_b_s_c_s_a_s GTGACGGTGTGGGTGTCTGTGTGGGTGGCTGTGTGGGTGTCTGTG;
>clamp_s_c_s_d_s_c_s_a_s:compl:rev CACAGACACCCACACCGTCACCCACACTATCATGTTGACATTCC; dG = -26.7
1614 >s_d_s_c_s_b_s_c_s_b_s_c_s_a_s GTGACGGTGTGGGTGGCTGTGTGGGTGGCTGTGTGGGTGTCTGTG;
>clamp_s_c_s_d_s_c_s_a_s:compl:rev CACAGACACCCACACCGTCACCCACACTATCATGTTGACATTCC; dG = -26.7
1615 >s_a_s_b_s_d_s_c_s_b_s_c_s_a_s GTGTCTGTGGCTGTGACGGTGTGGGTGGCTGTGTGGGTGTCTGTG;
>clamp_s_c_s_d_s_c_s_a_s:compl:rev CACAGACACCCACACCGTCACCCACACTATCATGTTGACATTCC; dG = -26.7
1616 >s_d_s_c_s_c_s_c_s_d_s_a_s_a_s GTGACGGTGTGGGTGTGGGTGTGGGTGACGGTGTCTGTGTCTGTG;
>clamp_s_c_s_d_s_c_s_a_s:compl:rev CACAGACACCCACACCGTCACCCACACTATCATGTTGACATTCC; dG = -26.7
1617 >s_d_s_c_s_c_s_c_s_b_s_c_s_a_s GTGACGGTGTGGGTGTGGGTGTGGGTGGCTGTGTGGGTGTCTGTG;
>clamp_s_c_s_d_s_c_s_a_s:compl:rev CACAGACACCCACACCGTCACCCACACTATCATGTTGACATTCC; dG = -26.7
1618 >s_d_s_c_s_e_s_c_s_d_s_a_s_a_s GTGACGGTGTGGGTGCTGGTGTGGGTGACGGTGTCTGTGTCTGTG;
>clamp_s_c_s_d_s_c_s_a_s:compl:rev CACAGACACCCACACCGTCACCCACACTATCATGTTGACATTCC; dG = -26.7
1619 >s_d_s_c_s_e_s_c_s_b_s_c_s_a_s GTGACGGTGTGGGTGCTGGTGTGGGTGGCTGTGTGGGTGTCTGTG;
>clamp_s_c_s_d_s_c_s_a_s:compl:rev CACAGACACCCACACCGTCACCCACACTATCATGTTGACATTCC; dG = -26.7
1620 >s_a_s_b_s_d_s_a_s_d_s_c_s_a_s GTGTCTGTGGCTGTGACGGTGTCTGTGACGGTGTGGGTGTCTGTG;
>clamp_s_c_s_d_s_c_s_a_s:compl:rev CACAGACACCCACACCGTCACCCACACTATCATGTTGACATTCC; dG = -26.7
1621 >s_d_s_d_s_a_s_c_s_b_s_c_s_a_s GTGACGGTGACGGTGTCTGTGTGGGTGGCTGTGTGGGTGTCTGTG;
>clamp_s_c_s_d_s_c_s_a_s:compl:rev CACAGACACCCACACCGTCACCCACACTATCATGTTGACATTCC; dG = -26.7
1622 >s_d_s_d_s_b_s_c_s_b_s_c_s_a_s GTGACGGTGACGGTGGCTGTGTGGGTGGCTGTGTGGGTGTCTGTG;
>clamp_s_c_s_d_s_c_s_a_s:compl:rev CACAGACACCCACACCGTCACCCACACTATCATGTTGACATTCC; dG = -26.7
1623 >s_d_s_d_s_c_s_c_s_d_s_a_s_a_s GTGACGGTGACGGTGTGGGTGTGGGTGACGGTGTCTGTGTCTGTG;
>clamp_s_c_s_d_s_c_s_a_s:compl:rev CACAGACACCCACACCGTCACCCACACTATCATGTTGACATTCC; dG = -26.7
1624 >s_d_s_d_s_c_s_c_s_b_s_c_s_a_s GTGACGGTGACGGTGTGGGTGTGGGTGGCTGTGTGGGTGTCTGTG;
>clamp_s_c_s_d_s_c_s_a_s:compl:rev CACAGACACCCACACCGTCACCCACACTATCATGTTGACATTCC; dG = -26.7
1625 >s_d_s_d_s_d_s_c_s_d_s_a_s_a_s GTGACGGTGACGGTGACGGTGTGGGTGACGGTGTCTGTGTCTGTG;
>clamp_s_c_s_d_s_c_s_a_s:compl:rev CACAGACACCCACACCGTCACCCACACTATCATGTTGACATTCC; dG = -26.7
1626 >s_d_s_d_s_d_s_c_s_b_s_c_s_a_s GTGACGGTGACGGTGACGGTGTGGGTGGCTGTGTGGGTGTCTGTG;
>clamp_s_c_s_d_s_c_s_a_s:compl:rev CACAGACACCCACACCGTCACCCACACTATCATGTTGACATTCC; dG = -26.7
1627 >s_d_s_d_s_d_s_a_s_d_s_c_s_a_s GTGACGGTGACGGTGACGGTGTCTGTGACGGTGTGGGTGTCTGTG;
>clamp_s_c_s_d_s_c_s_a_s:compl:rev CACAGACACCCACACCGTCACCCACACTATCATGTTGACATTCC; dG = -26.7
1628 >s_d_s_d_s_e_s_c_s_d_s_a_s_a_s GTGACGGTGACGGTGCTGGTGTGGGTGACGGTGTCTGTGTCTGTG;
>clamp_s_c_s_d_s_c_s_a_s:compl:rev CACAGACACCCACACCGTCACCCACACTATCATGTTGACATTCC; dG = -26.7
1629 >s_d_s_d_s_e_s_c_s_b_s_c_s_a_s GTGACGGTGACGGTGCTGGTGTGGGTGGCTGTGTGGGTGTCTGTG;
>clamp_s_c_s_d_s_c_s_a_s:compl:rev CACAGACACCCACACCGTCACCCACACTATCATGTTGACATTCC; dG = -26.7
1630 >s_d_s_d_s_e_s_a_s_d_s_c_s_a_s GTGACGGTGACGGTGCTGGTGTCTGTGACGGTGTGGGTGTCTGTG;
>clamp_s_c_s_d_s_c_s_a_s:compl:rev CACAGACACCCACACCGTCACCCACACTATCATGTTGACATTCC; dG = -26.7
1631 >s_a_s_b_s_e_s_c_s_d_s_a_s_a_s GTGTCTGTGGCTGTGCTGGTGTGGGTGACGGTGTCTGTGTCTGTG;
>clamp_s_c_s_d_s_c_s_a_s:compl:rev CACAGACACCCACACCGTCACCCACACTATCATGTTGACATTCC; dG = -26.7
1632 >s_d_s_e_s_a_s_c_s_b_s_c_s_a_s GTGACGGTGCTGGTGTCTGTGTGGGTGGCTGTGTGGGTGTCTGTG;
>clamp_s_c_s_d_s_c_s_a_s:compl:rev CACAGACACCCACACCGTCACCCACACTATCATGTTGACATTCC; dG = -26.7
1633 >s_d_s_e_s_b_s_c_s_b_s_c_s_a_s GTGACGGTGCTGGTGGCTGTGTGGGTGGCTGTGTGGGTGTCTGTG;
>clamp_s_c_s_d_s_c_s_a_s:compl:rev CACAGACACCCACACCGTCACCCACACTATCATGTTGACATTCC; dG = -26.7
1634 >s_a_s_b_s_e_s_c_s_b_s_c_s_a_s GTGTCTGTGGCTGTGCTGGTGTGGGTGGCTGTGTGGGTGTCTGTG;
>clamp_s_c_s_d_s_c_s_a_s:compl:rev CACAGACACCCACACCGTCACCCACACTATCATGTTGACATTCC; dG = -26.7
1635 >s_d_s_e_s_c_s_c_s_d_s_a_s_a_s GTGACGGTGCTGGTGTGGGTGTGGGTGACGGTGTCTGTGTCTGTG;
>clamp_s_c_s_d_s_c_s_a_s:compl:rev CACAGACACCCACACCGTCACCCACACTATCATGTTGACATTCC; dG = -26.7
1636 >s_d_s_e_s_c_s_c_s_b_s_c_s_a_s GTGACGGTGCTGGTGTGGGTGTGGGTGGCTGTGTGGGTGTCTGTG;
>clamp_s_c_s_d_s_c_s_a_s:compl:rev CACAGACACCCACACCGTCACCCACACTATCATGTTGACATTCC; dG = -26.7
1637 >s_d_s_e_s_d_s_c_s_d_s_a_s_a_s GTGACGGTGCTGGTGACGGTGTGGGTGACGGTGTCTGTGTCTGTG;
>clamp_s_c_s_d_s_c_s_a_s:compl:rev CACAGACACCCACACCGTCACCCACACTATCATGTTGACATTCC; dG = -26.7
1638 >s_d_s_e_s_d_s_c_s_b_s_c_s_a_s GTGACGGTGCTGGTGACGGTGTGGGTGGCTGTGTGGGTGTCTGTG;
>clamp_s_c_s_d_s_c_s_a_s:compl:rev CACAGACACCCACACCGTCACCCACACTATCATGTTGACATTCC; dG = -26.7
1639 >s_d_s_e_s_d_s_a_s_d_s_c_s_a_s GTGACGGTGCTGGTGACGGTGTCTGTGACGGTGTGGGTGTCTGTG;
>clamp_s_c_s_d_s_c_s_a_s:compl:rev CACAGACACCCACACCGTCACCCACACTATCATGTTGACATTCC; dG = -26.7
1640 >s_d_s_e_s_e_s_c_s_d_s_a_s_a_s GTGACGGTGCTGGTGCTGGTGTGGGTGACGGTGTCTGTGTCTGTG;
>clamp_s_c_s_d_s_c_s_a_s:compl:rev CACAGACACCCACACCGTCACCCACACTATCATGTTGACATTCC; dG = -26.7
1641 >s_d_s_e_s_e_s_c_s_b_s_c_s_a_s GTGACGGTGCTGGTGCTGGTGTGGGTGGCTGTGTGGGTGTCTGTG;
>clamp_s_c_s_d_s_c_s_a_s:compl:rev CACAGACACCCACACCGTCACCCACACTATCATGTTGACATTCC; dG = -26.7
1642 >s_d_s_e_s_e_s_a_s_d_s_c_s_a_s GTGACGGTGCTGGTGCTGGTGTCTGTGACGGTGTGGGTGTCTGTG;
>clamp_s_c_s_d_s_c_s_a_s:compl:rev CACAGACACCCACACCGTCACCCACACTATCATGTTGACATTCC; dG = -26.7
1643 >s_a_s_b_s_e_s_a_s_d_s_c_s_a_s GTGTCTGTGGCTGTGCTGGTGTCTGTGACGGTGTGGGTGTCTGTG;
>clamp_s_c_s_d_s_c_s_a_s:compl:rev CACAGACACCCACACCGTCACCCACACTATCATGTTGACATTCC; dG = -26.7
1644 >s_e_s_a_s_a_s_c_s_b_s_c_s_a_s GTGCTGGTGTCTGTGTCTGTGTGGGTGGCTGTGTGGGTGTCTGTG;
>clamp_s_c_s_d_s_c_s_a_s:compl:rev CACAGACACCCACACCGTCACCCACACTATCATGTTGACATTCC; dG = -26.7
1645 >s_e_s_a_s_b_s_c_s_b_s_c_s_a_s GTGCTGGTGTCTGTGGCTGTGTGGGTGGCTGTGTGGGTGTCTGTG;
>clamp_s_c_s_d_s_c_s_a_s:compl:rev CACAGACACCCACACCGTCACCCACACTATCATGTTGACATTCC; dG = -26.7
1646 >s_e_s_a_s_c_s_c_s_d_s_a_s_a_s GTGCTGGTGTCTGTGTGGGTGTGGGTGACGGTGTCTGTGTCTGTG;
>clamp_s_c_s_d_s_c_s_a_s:compl:rev CACAGACACCCACACCGTCACCCACACTATCATGTTGACATTCC; dG = -26.7
1647 >s_e_s_a_s_c_s_c_s_b_s_c_s_a_s GTGCTGGTGTCTGTGTGGGTGTGGGTGGCTGTGTGGGTGTCTGTG;
>clamp_s_c_s_d_s_c_s_a_s:compl:rev CACAGACACCCACACCGTCACCCACACTATCATGTTGACATTCC; dG = -26.7
1648 >s_e_s_a_s_d_s_c_s_d_s_a_s_a_s GTGCTGGTGTCTGTGACGGTGTGGGTGACGGTGTCTGTGTCTGTG;
>clamp_s_c_s_d_s_c_s_a_s:compl:rev CACAGACACCCACACCGTCACCCACACTATCATGTTGACATTCC; dG = -26.7

```
  1649 >s_e_s_a_s_d_s_c_s_b_s_c_s_a_s          GTGCTGGTGTCTGTGACGGTGTGGGTGGCTGTGTGGGTGTCTGTG;
>clamp_s_c_s_d_s_c_s_a_s:compl:rev CACAGACACCCACACCGTCACCCACACTATCATGTTGACATTCC; dG = -26.7
  1650 >s_e_s_a_s_d_s_a_s_d_s_c_s_a_s          GTGCTGGTGTCTGTGACGGTGTCTGTGACGGTGTGGGTGTCTGTG;
>clamp_s_c_s_d_s_c_s_a_s:compl:rev CACAGACACCCACACCGTCACCCACACTATCATGTTGACATTCC; dG = -26.7
  1651 >s_e_s_a_s_e_s_c_s_d_s_a_s_a_s          GTGCTGGTGTCTGTGCTGGTGTGGGTGACGGTGTCTGTGTCTGTG;
>clamp_s_c_s_d_s_c_s_a_s:compl:rev CACAGACACCCACACCGTCACCCACACTATCATGTTGACATTCC; dG = -26.7
  1652 >s_e_s_a_s_e_s_c_s_b_s_c_s_a_s          GTGCTGGTGTCTGTGCTGGTGTGGGTGGCTGTGTGGGTGTCTGTG;
>clamp_s_c_s_d_s_c_s_a_s:compl:rev CACAGACACCCACACCGTCACCCACACTATCATGTTGACATTCC; dG = -26.7
  1653 >s_e_s_a_s_e_s_a_s_d_s_c_s_a_s          GTGCTGGTGTCTGTGCTGGTGTCTGTGACGGTGTGGGTGTCTGTG;
>clamp_s_c_s_d_s_c_s_a_s:compl:rev CACAGACACCCACACCGTCACCCACACTATCATGTTGACATTCC; dG = -26.7
  1654 >s_e_s_b_s_a_s_c_s_b_s_c_s_a_s          GTGCTGGTGGCTGTGTCTGTGTGGGTGGCTGTGTGGGTGTCTGTG;
>clamp_s_c_s_d_s_c_s_a_s:compl:rev CACAGACACCCACACCGTCACCCACACTATCATGTTGACATTCC; dG = -26.7
  1655 >s_e_s_b_s_b_s_c_s_b_s_c_s_a_s          GTGCTGGTGGCTGTGGCTGTGTGGGTGGCTGTGTGGGTGTCTGTG;
>clamp_s_c_s_d_s_c_s_a_s:compl:rev CACAGACACCCACACCGTCACCCACACTATCATGTTGACATTCC; dG = -26.7
  1656 >s_a_s_c_s_a_s_c_s_b_s_c_s_a_s          GTGTCTGTGTGGGTGTCTGTGTGGGTGGCTGTGTGGGTGTCTGTG;
>clamp_s_c_s_d_s_c_s_a_s:compl:rev CACAGACACCCACACCGTCACCCACACTATCATGTTGACATTCC; dG = -26.7
  1657 >s_e_s_b_s_c_s_c_s_d_s_a_s_a_s          GTGCTGGTGGCTGTGTGGGTGTGGGTGACGGTGTCTGTGTCTGTG;
>clamp_s_c_s_d_s_c_s_a_s:compl:rev CACAGACACCCACACCGTCACCCACACTATCATGTTGACATTCC; dG = -26.7
  1658 >s_e_s_b_s_c_s_c_s_b_s_c_s_a_s          GTGCTGGTGGCTGTGTGGGTGTGGGTGGCTGTGTGGGTGTCTGTG;
>clamp_s_c_s_d_s_c_s_a_s:compl:rev CACAGACACCCACACCGTCACCCACACTATCATGTTGACATTCC; dG = -26.7
  1659 >s_e_s_b_s_d_s_c_s_d_s_a_s_a_s          GTGCTGGTGGCTGTGACGGTGTGGGTGACGGTGTCTGTGTCTGTG;
>clamp_s_c_s_d_s_c_s_a_s:compl:rev CACAGACACCCACACCGTCACCCACACTATCATGTTGACATTCC; dG = -26.7
  1660 >s_e_s_b_s_d_s_c_s_b_s_c_s_a_s          GTGCTGGTGGCTGTGACGGTGTGGGTGGCTGTGTGGGTGTCTGTG;
>clamp_s_c_s_d_s_c_s_a_s:compl:rev CACAGACACCCACACCGTCACCCACACTATCATGTTGACATTCC; dG = -26.7
  1661 >s_e_s_b_s_d_s_a_s_d_s_c_s_a_s          GTGCTGGTGGCTGTGACGGTGTCTGTGACGGTGTGGGTGTCTGTG;
>clamp_s_c_s_d_s_c_s_a_s:compl:rev CACAGACACCCACACCGTCACCCACACTATCATGTTGACATTCC; dG = -26.7
  1662 >s_e_s_b_s_e_s_c_s_d_s_a_s_a_s          GTGCTGGTGGCTGTGCTGGTGTGGGTGACGGTGTCTGTGTCTGTG;
>clamp_s_c_s_d_s_c_s_a_s:compl:rev CACAGACACCCACACCGTCACCCACACTATCATGTTGACATTCC; dG = -26.7
  1663 >s_e_s_b_s_e_s_c_s_b_s_c_s_a_s          GTGCTGGTGGCTGTGCTGGTGTGGGTGGCTGTGTGGGTGTCTGTG;
>clamp_s_c_s_d_s_c_s_a_s:compl:rev CACAGACACCCACACCGTCACCCACACTATCATGTTGACATTCC; dG = -26.7
  1664 >s_e_s_b_s_e_s_a_s_d_s_c_s_a_s          GTGCTGGTGGCTGTGCTGGTGTCTGTGACGGTGTGGGTGTCTGTG;
>clamp_s_c_s_d_s_c_s_a_s:compl:rev CACAGACACCCACACCGTCACCCACACTATCATGTTGACATTCC; dG = -26.7
  1665 >s_e_s_c_s_a_s_c_s_b_s_c_s_a_s          GTGCTGGTGTGGGTGTCTGTGTGGGTGGCTGTGTGGGTGTCTGTG;
>clamp_s_c_s_d_s_c_s_a_s:compl:rev CACAGACACCCACACCGTCACCCACACTATCATGTTGACATTCC; dG = -26.7
  1666 >s_e_s_c_s_b_s_c_s_b_s_c_s_a_s          GTGCTGGTGTGGGTGGCTGTGTGGGTGGCTGTGTGGGTGTCTGTG;
>clamp_s_c_s_d_s_c_s_a_s:compl:rev CACAGACACCCACACCGTCACCCACACTATCATGTTGACATTCC; dG = -26.7
  1667 >s_e_s_c_s_c_s_c_s_d_s_a_s_a_s          GTGCTGGTGTGGGTGTGGGTGTGGGTGACGGTGTCTGTGTCTGTG;
>clamp_s_c_s_d_s_c_s_a_s:compl:rev CACAGACACCCACACCGTCACCCACACTATCATGTTGACATTCC; dG = -26.7
  1668 >s_e_s_c_s_c_s_c_s_b_s_c_s_a_s          GTGCTGGTGTGGGTGTGGGTGTGGGTGGCTGTGTGGGTGTCTGTG;
>clamp_s_c_s_d_s_c_s_a_s:compl:rev CACAGACACCCACACCGTCACCCACACTATCATGTTGACATTCC; dG = -26.7
  1669 >s_e_s_c_s_e_s_c_s_d_s_a_s_a_s          GTGCTGGTGTGGGTGCTGGTGTGGGTGACGGTGTCTGTGTCTGTG;
>clamp_s_c_s_d_s_c_s_a_s:compl:rev CACAGACACCCACACCGTCACCCACACTATCATGTTGACATTCC; dG = -26.7
  1670 >s_e_s_c_s_e_s_c_s_b_s_c_s_a_s          GTGCTGGTGTGGGTGCTGGTGTGGGTGGCTGTGTGGGTGTCTGTG;
>clamp_s_c_s_d_s_c_s_a_s:compl:rev CACAGACACCCACACCGTCACCCACACTATCATGTTGACATTCC; dG = -26.7
  1671 >s_e_s_d_s_a_s_c_s_b_s_c_s_a_s          GTGCTGGTGACGGTGTCTGTGTGGGTGGCTGTGTGGGTGTCTGTG;
>clamp_s_c_s_d_s_c_s_a_s:compl:rev CACAGACACCCACACCGTCACCCACACTATCATGTTGACATTCC; dG = -26.7
  1672 >s_e_s_d_s_b_s_c_s_b_s_c_s_a_s          GTGCTGGTGACGGTGGCTGTGTGGGTGGCTGTGTGGGTGTCTGTG;
>clamp_s_c_s_d_s_c_s_a_s:compl:rev CACAGACACCCACACCGTCACCCACACTATCATGTTGACATTCC; dG = -26.7
  1673 >s_a_s_c_s_b_s_c_s_b_s_c_s_a_s          GTGTCTGTGTGGGTGGCTGTGTGGGTGGCTGTGTGGGTGTCTGTG;
>clamp_s_c_s_d_s_c_s_a_s:compl:rev CACAGACACCCACACCGTCACCCACACTATCATGTTGACATTCC; dG = -26.7
  1674 >s_e_s_d_s_c_s_c_s_d_s_a_s_a_s          GTGCTGGTGACGGTGTGGGTGTGGGTGACGGTGTCTGTGTCTGTG;
>clamp_s_c_s_d_s_c_s_a_s:compl:rev CACAGACACCCACACCGTCACCCACACTATCATGTTGACATTCC; dG = -26.7
  1675 >s_e_s_d_s_c_s_c_s_b_s_c_s_a_s          GTGCTGGTGACGGTGTGGGTGTGGGTGGCTGTGTGGGTGTCTGTG;
>clamp_s_c_s_d_s_c_s_a_s:compl:rev CACAGACACCCACACCGTCACCCACACTATCATGTTGACATTCC; dG = -26.7
  1676 >s_e_s_d_s_d_s_c_s_d_s_a_s_a_s          GTGCTGGTGACGGTGACGGTGTGGGTGACGGTGTCTGTGTCTGTG;
>clamp_s_c_s_d_s_c_s_a_s:compl:rev CACAGACACCCACACCGTCACCCACACTATCATGTTGACATTCC; dG = -26.7
  1677 >s_e_s_d_s_d_s_c_s_b_s_c_s_a_s          GTGCTGGTGACGGTGACGGTGTGGGTGGCTGTGTGGGTGTCTGTG;
>clamp_s_c_s_d_s_c_s_a_s:compl:rev CACAGACACCCACACCGTCACCCACACTATCATGTTGACATTCC; dG = -26.7
  1678 >s_e_s_d_s_d_s_a_s_d_s_c_s_a_s          GTGCTGGTGACGGTGACGGTGTCTGTGACGGTGTGGGTGTCTGTG;
>clamp_s_c_s_d_s_c_s_a_s:compl:rev CACAGACACCCACACCGTCACCCACACTATCATGTTGACATTCC; dG = -26.7
  1679 >s_e_s_d_s_e_s_c_s_d_s_a_s_a_s          GTGCTGGTGACGGTGCTGGTGTGGGTGACGGTGTCTGTGTCTGTG;
>clamp_s_c_s_d_s_c_s_a_s:compl:rev CACAGACACCCACACCGTCACCCACACTATCATGTTGACATTCC; dG = -26.7
  1680 >s_e_s_d_s_e_s_c_s_b_s_c_s_a_s          GTGCTGGTGACGGTGCTGGTGTGGGTGGCTGTGTGGGTGTCTGTG;
>clamp_s_c_s_d_s_c_s_a_s:compl:rev CACAGACACCCACACCGTCACCCACACTATCATGTTGACATTCC; dG = -26.7
  1681 >s_e_s_d_s_e_s_a_s_d_s_c_s_a_s          GTGCTGGTGACGGTGCTGGTGTCTGTGACGGTGTGGGTGTCTGTG;
>clamp_s_c_s_d_s_c_s_a_s:compl:rev CACAGACACCCACACCGTCACCCACACTATCATGTTGACATTCC; dG = -26.7
  1682 >s_e_s_e_s_a_s_c_s_b_s_c_s_a_s          GTGCTGGTGCTGGTGTCTGTGTGGGTGGCTGTGTGGGTGTCTGTG;
>clamp_s_c_s_d_s_c_s_a_s:compl:rev CACAGACACCCACACCGTCACCCACACTATCATGTTGACATTCC; dG = -26.7
  1683 >s_e_s_e_s_b_s_c_s_b_s_c_s_a_s          GTGCTGGTGCTGGTGGCTGTGTGGGTGGCTGTGTGGGTGTCTGTG;
>clamp_s_c_s_d_s_c_s_a_s:compl:rev CACAGACACCCACACCGTCACCCACACTATCATGTTGACATTCC; dG = -26.7
  1684 >s_e_s_e_s_c_s_c_s_d_s_a_s_a_s          GTGCTGGTGCTGGTGTGGGTGTGGGTGACGGTGTCTGTGTCTGTG;
>clamp_s_c_s_d_s_c_s_a_s:compl:rev CACAGACACCCACACCGTCACCCACACTATCATGTTGACATTCC; dG = -26.7
  1685 >s_e_s_e_s_c_s_c_s_b_s_c_s_a_s          GTGCTGGTGCTGGTGTGGGTGTGGGTGGCTGTGTGGGTGTCTGTG;
>clamp_s_c_s_d_s_c_s_a_s:compl:rev CACAGACACCCACACCGTCACCCACACTATCATGTTGACATTCC; dG = -26.7
```

```
  1686 >s_e_s_e_s_d_s_c_s_d_s_a_s_a_s           GTGCTGGTGCTGGTGACGGTGTGGGTGACGGTGTCTGTGTCTGTG;
>clamp_s_c_s_d_s_c_s_a_s:compl:rev CACAGACACCCACACCGTCACCCACACTATCATGTTGACATTCC; dG = -26.7
  1687 >s_e_s_e_s_d_s_c_s_b_s_c_s_a_s           GTGCTGGTGCTGGTGACGGTGTGGGTGGCTGTGTGGGTGTCTGTG;
>clamp_s_c_s_d_s_c_s_a_s:compl:rev CACAGACACCCACACCGTCACCCACACTATCATGTTGACATTCC; dG = -26.7
  1688 >s_e_s_e_s_d_s_a_s_d_s_c_s_a_s           GTGCTGGTGCTGGTGACGGTGTCTGTGACGGTGTGGGTGTCTGTG;
>clamp_s_c_s_d_s_c_s_a_s:compl:rev CACAGACACCCACACCGTCACCCACACTATCATGTTGACATTCC; dG = -26.7
  1689 >s_e_s_e_s_e_s_c_s_d_s_a_s_a_s           GTGCTGGTGCTGGTGCTGGTGTGGGTGACGGTGTCTGTGTCTGTG;
>clamp_s_c_s_d_s_c_s_a_s:compl:rev CACAGACACCCACACCGTCACCCACACTATCATGTTGACATTCC; dG = -26.7
  1690 >s_e_s_e_s_e_s_c_s_b_s_c_s_a_s           GTGCTGGTGCTGGTGCTGGTGTGGGTGGCTGTGTGGGTGTCTGTG;
>clamp_s_c_s_d_s_c_s_a_s:compl:rev CACAGACACCCACACCGTCACCCACACTATCATGTTGACATTCC; dG = -26.7
  1691 >s_e_s_e_s_e_s_a_s_d_s_c_s_a_s           GTGCTGGTGCTGGTGCTGGTGTCTGTGACGGTGTGGGTGTCTGTG;
>clamp_s_c_s_d_s_c_s_a_s:compl:rev CACAGACACCCACACCGTCACCCACACTATCATGTTGACATTCC; dG = -26.7
  1692 >s_a_s_c_s_c_s_c_s_d_s_a_s_a_s           GTGTCTGTGTGGGTGTGGGTGTGGGTGACGGTGTCTGTGTCTGTG;
>clamp_s_c_s_d_s_c_s_a_s:compl:rev CACAGACACCCACACCGTCACCCACACTATCATGTTGACATTCC; dG = -26.7
  1693 >s_a_s_c_s_c_s_c_s_b_s_c_s_a_s           GTGTCTGTGTGGGTGTGGGTGTGGGTGGCTGTGTGGGTGTCTGTG;
>clamp_s_c_s_d_s_c_s_a_s:compl:rev CACAGACACCCACACCGTCACCCACACTATCATGTTGACATTCC; dG = -26.7
  1694 >s_a_s_c_s_e_s_c_s_d_s_a_s_a_s           GTGTCTGTGTGGGTGCTGGTGTGGGTGACGGTGTCTGTGTCTGTG;
>clamp_s_c_s_d_s_c_s_a_s:compl:rev CACAGACACCCACACCGTCACCCACACTATCATGTTGACATTCC; dG = -26.7
  1695 >s_a_s_c_s_e_s_c_s_b_s_c_s_a_s           GTGTCTGTGTGGGTGCTGGTGTGGGTGGCTGTGTGGGTGTCTGTG;
>clamp_s_c_s_d_s_c_s_a_s:compl:rev CACAGACACCCACACCGTCACCCACACTATCATGTTGACATTCC; dG = -26.7
  1696 >s_a_s_d_s_a_s_c_s_b_s_c_s_a_s           GTGTCTGTGACGGTGTCTGTGTGGGTGGCTGTGTGGGTGTCTGTG;
>clamp_s_c_s_d_s_c_s_a_s:compl:rev CACAGACACCCACACCGTCACCCACACTATCATGTTGACATTCC; dG = -26.7
  1697 >s_a_s_d_s_b_s_c_s_b_s_c_s_a_s           GTGTCTGTGACGGTGGCTGTGTGGGTGGCTGTGTGGGTGTCTGTG;
>clamp_s_c_s_d_s_c_s_a_s:compl:rev CACAGACACCCACACCGTCACCCACACTATCATGTTGACATTCC; dG = -26.7
  1698 >s_a_s_a_s_b_s_c_s_b_s_c_s_a_s           GTGTCTGTGTCTGTGGCTGTGTGGGTGGCTGTGTGGGTGTCTGTG;
>clamp_s_c_s_d_s_c_s_a_s:compl:rev CACAGACACCCACACCGTCACCCACACTATCATGTTGACATTCC; dG = -26.7
  1699 >s_a_s_d_s_c_s_c_s_d_s_a_s_a_s           GTGTCTGTGACGGTGTGGGTGTGGGTGACGGTGTCTGTGTCTGTG;
>clamp_s_c_s_d_s_c_s_a_s:compl:rev CACAGACACCCACACCGTCACCCACACTATCATGTTGACATTCC; dG = -26.7
  1700 >s_a_s_d_s_c_s_c_s_b_s_c_s_a_s           GTGTCTGTGACGGTGTGGGTGTGGGTGGCTGTGTGGGTGTCTGTG;
>clamp_s_c_s_d_s_c_s_a_s:compl:rev CACAGACACCCACACCGTCACCCACACTATCATGTTGACATTCC; dG = -26.7
  1701 >s_a_s_d_s_d_s_c_s_d_s_a_s_a_s           GTGTCTGTGACGGTGACGGTGTGGGTGACGGTGTCTGTGTCTGTG;
>clamp_s_c_s_d_s_c_s_a_s:compl:rev CACAGACACCCACACCGTCACCCACACTATCATGTTGACATTCC; dG = -26.7
  1702 >s_a_s_d_s_d_s_c_s_b_s_c_s_a_s           GTGTCTGTGACGGTGACGGTGTGGGTGGCTGTGTGGGTGTCTGTG;
>clamp_s_c_s_d_s_c_s_a_s:compl:rev CACAGACACCCACACCGTCACCCACACTATCATGTTGACATTCC; dG = -26.7
  1703 >s_a_s_d_s_d_s_a_s_d_s_c_s_a_s           GTGTCTGTGACGGTGACGGTGTCTGTGACGGTGTGGGTGTCTGTG;
>clamp_s_c_s_d_s_c_s_a_s:compl:rev CACAGACACCCACACCGTCACCCACACTATCATGTTGACATTCC; dG = -26.7
  1704 >s_a_s_d_s_e_s_c_s_d_s_a_s_a_s           GTGTCTGTGACGGTGCTGGTGTGGGTGACGGTGTCTGTGTCTGTG;
>clamp_s_c_s_d_s_c_s_a_s:compl:rev CACAGACACCCACACCGTCACCCACACTATCATGTTGACATTCC; dG = -26.7
  1705 >s_a_s_d_s_e_s_c_s_b_s_c_s_a_s           GTGTCTGTGACGGTGCTGGTGTGGGTGGCTGTGTGGGTGTCTGTG;
>clamp_s_c_s_d_s_c_s_a_s:compl:rev CACAGACACCCACACCGTCACCCACACTATCATGTTGACATTCC; dG = -26.7
  1706 >s_a_s_d_s_e_s_a_s_d_s_c_s_a_s           GTGTCTGTGACGGTGCTGGTGTCTGTGACGGTGTGGGTGTCTGTG;
>clamp_s_c_s_d_s_c_s_a_s:compl:rev CACAGACACCCACACCGTCACCCACACTATCATGTTGACATTCC; dG = -26.7
  1707 >s_a_s_e_s_a_s_c_s_b_s_c_s_a_s           GTGTCTGTGCTGGTGTCTGTGTGGGTGGCTGTGTGGGTGTCTGTG;
>clamp_s_c_s_d_s_c_s_a_s:compl:rev CACAGACACCCACACCGTCACCCACACTATCATGTTGACATTCC; dG = -26.7
  1708 >s_a_s_e_s_b_s_c_s_b_s_c_s_a_s           GTGTCTGTGCTGGTGGCTGTGTGGGTGGCTGTGTGGGTGTCTGTG;
>clamp_s_c_s_d_s_c_s_a_s:compl:rev CACAGACACCCACACCGTCACCCACACTATCATGTTGACATTCC; dG = -26.7
  1709 >s_a_s_e_s_c_s_c_s_d_s_a_s_a_s           GTGTCTGTGCTGGTGTGGGTGTGGGTGACGGTGTCTGTGTCTGTG;
>clamp_s_c_s_d_s_c_s_a_s:compl:rev CACAGACACCCACACCGTCACCCACACTATCATGTTGACATTCC; dG = -26.7
  1710 >s_a_s_e_s_c_s_c_s_b_s_c_s_a_s           GTGTCTGTGCTGGTGTGGGTGTGGGTGGCTGTGTGGGTGTCTGTG;
>clamp_s_c_s_d_s_c_s_a_s:compl:rev CACAGACACCCACACCGTCACCCACACTATCATGTTGACATTCC; dG = -26.7
  1711 >s_a_s_e_s_d_s_c_s_d_s_a_s_a_s           GTGTCTGTGCTGGTGACGGTGTGGGTGACGGTGTCTGTGTCTGTG;
>clamp_s_c_s_d_s_c_s_a_s:compl:rev CACAGACACCCACACCGTCACCCACACTATCATGTTGACATTCC; dG = -26.7
  1712 >s_a_s_e_s_d_s_c_s_b_s_c_s_a_s           GTGTCTGTGCTGGTGACGGTGTGGGTGGCTGTGTGGGTGTCTGTG;
>clamp_s_c_s_d_s_c_s_a_s:compl:rev CACAGACACCCACACCGTCACCCACACTATCATGTTGACATTCC; dG = -26.7
  1713 >s_a_s_e_s_d_s_a_s_d_s_c_s_a_s           GTGTCTGTGCTGGTGACGGTGTCTGTGACGGTGTGGGTGTCTGTG;
>clamp_s_c_s_d_s_c_s_a_s:compl:rev CACAGACACCCACACCGTCACCCACACTATCATGTTGACATTCC; dG = -26.7
  1714 >s_a_s_e_s_e_s_c_s_d_s_a_s_a_s           GTGTCTGTGCTGGTGCTGGTGTGGGTGACGGTGTCTGTGTCTGTG;
>clamp_s_c_s_d_s_c_s_a_s:compl:rev CACAGACACCCACACCGTCACCCACACTATCATGTTGACATTCC; dG = -26.7
  1715 >s_a_s_e_s_e_s_c_s_b_s_c_s_a_s           GTGTCTGTGCTGGTGCTGGTGTGGGTGGCTGTGTGGGTGTCTGTG;
>clamp_s_c_s_d_s_c_s_a_s:compl:rev CACAGACACCCACACCGTCACCCACACTATCATGTTGACATTCC; dG = -26.7
  1716 >s_a_s_e_s_e_s_a_s_d_s_c_s_a_s           GTGTCTGTGCTGGTGCTGGTGTCTGTGACGGTGTGGGTGTCTGTG;
>clamp_s_c_s_d_s_c_s_a_s:compl:rev CACAGACACCCACACCGTCACCCACACTATCATGTTGACATTCC; dG = -26.7
  1717 >s_a_s_a_s_c_s_c_s_d_s_a_s_a_s           GTGTCTGTGTCTGTGTGGGTGTGGGTGACGGTGTCTGTGTCTGTG;
>clamp_s_c_s_d_s_c_s_a_s:compl:rev CACAGACACCCACACCGTCACCCACACTATCATGTTGACATTCC; dG = -26.7
  1718 >s_b_s_a_s_a_s_c_s_b_s_c_s_a_s           GTGGCTGTGTCTGTGTCTGTGTGGGTGGCTGTGTGGGTGTCTGTG;
>clamp_s_c_s_d_s_c_s_a_s:compl:rev CACAGACACCCACACCGTCACCCACACTATCATGTTGACATTCC; dG = -26.7
  1719 >s_b_s_a_s_b_s_c_s_b_s_c_s_a_s           GTGGCTGTGTCTGTGGCTGTGTGGGTGGCTGTGTGGGTGTCTGTG;
>clamp_s_c_s_d_s_c_s_a_s:compl:rev CACAGACACCCACACCGTCACCCACACTATCATGTTGACATTCC; dG = -26.7
  1720 >s_a_s_a_s_c_s_c_s_b_s_c_s_a_s           GTGTCTGTGTCTGTGTGGGTGTGGGTGGCTGTGTGGGTGTCTGTG;
>clamp_s_c_s_d_s_c_s_a_s:compl:rev CACAGACACCCACACCGTCACCCACACTATCATGTTGACATTCC; dG = -26.7
  1721 >s_b_s_a_s_c_s_c_s_d_s_a_s_a_s           GTGGCTGTGTCTGTGTGGGTGTGGGTGACGGTGTCTGTGTCTGTG;
>clamp_s_c_s_d_s_c_s_a_s:compl:rev CACAGACACCCACACCGTCACCCACACTATCATGTTGACATTCC; dG = -26.7
  1722 >s_b_s_a_s_c_s_c_s_b_s_c_s_a_s           GTGGCTGTGTCTGTGTGGGTGTGGGTGGCTGTGTGGGTGTCTGTG;
>clamp_s_c_s_d_s_c_s_a_s:compl:rev CACAGACACCCACACCGTCACCCACACTATCATGTTGACATTCC; dG = -26.7
```

```
1723 >s_b_s_a_s_d_s_c_s_d_s_a_s_a_s GTGGCTGTGTCTGTGACGGTGTGGGTGACGGTGTCTGTGTCTGTG;
>clamp_s_c_s_d_s_c_s_a_s:compl:rev CACAGACACCCACACCGTCACCCACACTATCATGTTGACATTCC; dG = -26.7
1724 >s_b_s_a_s_d_s_c_s_b_s_c_s_a_s GTGGCTGTGTCTGTGACGGTGTGGGTGGCTGTGTGGGTGTCTGTG;
>clamp_s_c_s_d_s_c_s_a_s:compl:rev CACAGACACCCACACCGTCACCCACACTATCATGTTGACATTCC; dG = -26.7
1725 >s_b_s_a_s_d_s_a_s_d_s_c_s_a_s GTGGCTGTGTCTGTGACGGTGTCTGTGACGGTGTGGGTGTCTGTG;
>clamp_s_c_s_d_s_c_s_a_s:compl:rev CACAGACACCCACACCGTCACCCACACTATCATGTTGACATTCC; dG = -26.7
1726 >s_b_s_a_s_e_s_c_s_d_s_a_s_a_s GTGGCTGTGTCTGTGCTGGTGTGGGTGACGGTGTCTGTGTCTGTG;
>clamp_s_c_s_d_s_c_s_a_s:compl:rev CACAGACACCCACACCGTCACCCACACTATCATGTTGACATTCC; dG = -26.7
1727 >s_b_s_a_s_e_s_c_s_b_s_c_s_a_s GTGGCTGTGTCTGTGCTGGTGTGGGTGGCTGTGTGGGTGTCTGTG;
>clamp_s_c_s_d_s_c_s_a_s:compl:rev CACAGACACCCACACCGTCACCCACACTATCATGTTGACATTCC; dG = -26.7
1728 >s_b_s_a_s_e_s_a_s_d_s_c_s_a_s GTGGCTGTGTCTGTGCTGGTGTCTGTGACGGTGTGGGTGTCTGTG;
>clamp_s_c_s_d_s_c_s_a_s:compl:rev CACAGACACCCACACCGTCACCCACACTATCATGTTGACATTCC; dG = -26.7
1729 >s_b_s_b_s_a_s_c_s_b_s_c_s_a_s GTGGCTGTGGCTGTGTCTGTGTGGGTGGCTGTGTGGGTGTCTGTG;
>clamp_s_c_s_d_s_c_s_a_s:compl:rev CACAGACACCCACACCGTCACCCACACTATCATGTTGACATTCC; dG = -26.7
1730 >s_b_s_b_s_b_s_c_s_b_s_c_s_a_s GTGGCTGTGGCTGTGGCTGTGTGGGTGGCTGTGTGGGTGTCTGTG;
>clamp_s_c_s_d_s_c_s_a_s:compl:rev CACAGACACCCACACCGTCACCCACACTATCATGTTGACATTCC; dG = -26.7
1731 >s_b_s_b_s_c_s_c_s_d_s_a_s_a_s GTGGCTGTGGCTGTGTGGGTGTGGGTGACGGTGTCTGTGTCTGTG;
>clamp_s_c_s_d_s_c_s_a_s:compl:rev CACAGACACCCACACCGTCACCCACACTATCATGTTGACATTCC; dG = -26.7
1732 >s_b_s_b_s_c_s_c_s_b_s_c_s_a_s GTGGCTGTGGCTGTGTGGGTGTGGGTGGCTGTGTGGGTGTCTGTG;
>clamp_s_c_s_d_s_c_s_a_s:compl:rev CACAGACACCCACACCGTCACCCACACTATCATGTTGACATTCC; dG = -26.7
1733 >s_b_s_b_s_d_s_c_s_d_s_a_s_a_s GTGGCTGTGGCTGTGACGGTGTGGGTGACGGTGTCTGTGTCTGTG;
>clamp_s_c_s_d_s_c_s_a_s:compl:rev CACAGACACCCACACCGTCACCCACACTATCATGTTGACATTCC; dG = -26.7
1734 >s_b_s_b_s_d_s_c_s_b_s_c_s_a_s GTGGCTGTGGCTGTGACGGTGTGGGTGGCTGTGTGGGTGTCTGTG;
>clamp_s_c_s_d_s_c_s_a_s:compl:rev CACAGACACCCACACCGTCACCCACACTATCATGTTGACATTCC; dG = -26.7
1735 >s_b_s_b_s_d_s_a_s_d_s_c_s_a_s GTGGCTGTGGCTGTGACGGTGTCTGTGACGGTGTGGGTGTCTGTG;
>clamp_s_c_s_d_s_c_s_a_s:compl:rev CACAGACACCCACACCGTCACCCACACTATCATGTTGACATTCC; dG = -26.7
1736 >s_b_s_b_s_e_s_c_s_d_s_a_s_a_s GTGGCTGTGGCTGTGCTGGTGTGGGTGACGGTGTCTGTGTCTGTG;
>clamp_s_c_s_d_s_c_s_a_s:compl:rev CACAGACACCCACACCGTCACCCACACTATCATGTTGACATTCC; dG = -26.7
1737 >s_b_s_b_s_e_s_c_s_b_s_c_s_a_s GTGGCTGTGGCTGTGCTGGTGTGGGTGGCTGTGTGGGTGTCTGTG;
>clamp_s_c_s_d_s_c_s_a_s:compl:rev CACAGACACCCACACCGTCACCCACACTATCATGTTGACATTCC; dG = -26.7
1738 >s_b_s_b_s_e_s_a_s_d_s_c_s_a_s GTGGCTGTGGCTGTGCTGGTGTCTGTGACGGTGTGGGTGTCTGTG;
>clamp_s_c_s_d_s_c_s_a_s:compl:rev CACAGACACCCACACCGTCACCCACACTATCATGTTGACATTCC; dG = -26.7
1739 >s_a_s_a_s_d_s_c_s_d_s_a_s_a_s GTGTCTGTGTCTGTGACGGTGTGGGTGACGGTGTCTGTGTCTGTG;
>clamp_s_c_s_d_s_c_s_a_s:compl:rev CACAGACACCCACACCGTCACCCACACTATCATGTTGACATTCC; dG = -26.7
1740 >s_b_s_c_s_a_s_c_s_b_s_c_s_a_s GTGGCTGTGTGGGTGTCTGTGTGGGTGGCTGTGTGGGTGTCTGTG;
>clamp_s_c_s_d_s_c_s_a_s:compl:rev CACAGACACCCACACCGTCACCCACACTATCATGTTGACATTCC; dG = -26.7
1741 >s_b_s_c_s_b_s_c_s_b_s_c_s_a_s GTGGCTGTGTGGGTGGCTGTGTGGGTGGCTGTGTGGGTGTCTGTG;
>clamp_s_c_s_d_s_c_s_a_s:compl:rev CACAGACACCCACACCGTCACCCACACTATCATGTTGACATTCC; dG = -26.7
1742 >s_a_s_a_s_d_s_c_s_b_s_c_s_a_s GTGTCTGTGTCTGTGACGGTGTGGGTGGCTGTGTGGGTGTCTGTG;
>clamp_s_c_s_d_s_c_s_a_s:compl:rev CACAGACACCCACACCGTCACCCACACTATCATGTTGACATTCC; dG = -26.7
1743 >s_b_s_c_s_c_s_c_s_d_s_a_s_a_s GTGGCTGTGTGGGTGTGGGTGTGGGTGACGGTGTCTGTGTCTGTG;
>clamp_s_c_s_d_s_c_s_a_s:compl:rev CACAGACACCCACACCGTCACCCACACTATCATGTTGACATTCC; dG = -26.7
1744 >s_b_s_c_s_c_s_c_s_b_s_c_s_a_s GTGGCTGTGTGGGTGTGGGTGTGGGTGGCTGTGTGGGTGTCTGTG;
>clamp_s_c_s_d_s_c_s_a_s:compl:rev CACAGACACCCACACCGTCACCCACACTATCATGTTGACATTCC; dG = -26.7
1745 >s_b_s_c_s_e_s_c_s_d_s_a_s_a_s GTGGCTGTGTGGGTGCTGGTGTGGGTGACGGTGTCTGTGTCTGTG;
>clamp_s_c_s_d_s_c_s_a_s:compl:rev CACAGACACCCACACCGTCACCCACACTATCATGTTGACATTCC; dG = -26.7
1746 >s_b_s_c_s_e_s_c_s_b_s_c_s_a_s GTGGCTGTGTGGGTGCTGGTGTGGGTGGCTGTGTGGGTGTCTGTG;
>clamp_s_c_s_d_s_c_s_a_s:compl:rev CACAGACACCCACACCGTCACCCACACTATCATGTTGACATTCC; dG = -26.7
1747 >s_a_s_a_s_d_s_a_s_d_s_c_s_a_s GTGTCTGTGTCTGTGACGGTGTCTGTGACGGTGTGGGTGTCTGTG;
>clamp_s_c_s_d_s_c_s_a_s:compl:rev CACAGACACCCACACCGTCACCCACACTATCATGTTGACATTCC; dG = -26.7
1748 >s_b_s_d_s_a_s_c_s_b_s_c_s_a_s GTGGCTGTGACGGTGTCTGTGTGGGTGGCTGTGTGGGTGTCTGTG;
>clamp_s_c_s_d_s_c_s_a_s:compl:rev CACAGACACCCACACCGTCACCCACACTATCATGTTGACATTCC; dG = -26.7
1749 >s_b_s_d_s_b_s_c_s_b_s_c_s_a_s GTGGCTGTGACGGTGGCTGTGTGGGTGGCTGTGTGGGTGTCTGTG;
>clamp_s_c_s_d_s_c_s_a_s:compl:rev CACAGACACCCACACCGTCACCCACACTATCATGTTGACATTCC; dG = -26.7
1750 >s_b_s_d_s_c_s_c_s_d_s_a_s_a_s GTGGCTGTGACGGTGTGGGTGTGGGTGACGGTGTCTGTGTCTGTG;
>clamp_s_c_s_d_s_c_s_a_s:compl:rev CACAGACACCCACACCGTCACCCACACTATCATGTTGACATTCC; dG = -26.7
1751 >s_b_s_d_s_c_s_c_s_b_s_c_s_a_s GTGGCTGTGACGGTGTGGGTGTGGGTGGCTGTGTGGGTGTCTGTG;
>clamp_s_c_s_d_s_c_s_a_s:compl:rev CACAGACACCCACACCGTCACCCACACTATCATGTTGACATTCC; dG = -26.7
1752 >s_b_s_d_s_d_s_c_s_d_s_a_s_a_s GTGGCTGTGACGGTGACGGTGTGGGTGACGGTGTCTGTGTCTGTG;
>clamp_s_c_s_d_s_c_s_a_s:compl:rev CACAGACACCCACACCGTCACCCACACTATCATGTTGACATTCC; dG = -26.7
1753 >s_b_s_d_s_d_s_c_s_b_s_c_s_a_s GTGGCTGTGACGGTGACGGTGTGGGTGGCTGTGTGGGTGTCTGTG;
>clamp_s_c_s_d_s_c_s_a_s:compl:rev CACAGACACCCACACCGTCACCCACACTATCATGTTGACATTCC; dG = -26.7
1754 >s_b_s_d_s_d_s_a_s_d_s_c_s_a_s GTGGCTGTGACGGTGACGGTGTCTGTGACGGTGTGGGTGTCTGTG;
>clamp_s_c_s_d_s_c_s_a_s:compl:rev CACAGACACCCACACCGTCACCCACACTATCATGTTGACATTCC; dG = -26.7
1755 >s_b_s_d_s_e_s_c_s_d_s_a_s_a_s GTGGCTGTGACGGTGCTGGTGTGGGTGACGGTGTCTGTGTCTGTG;
>clamp_s_c_s_d_s_c_s_a_s:compl:rev CACAGACACCCACACCGTCACCCACACTATCATGTTGACATTCC; dG = -26.7
1756 >s_b_s_d_s_e_s_c_s_b_s_c_s_a_s GTGGCTGTGACGGTGCTGGTGTGGGTGGCTGTGTGGGTGTCTGTG;
>clamp_s_c_s_d_s_c_s_a_s:compl:rev CACAGACACCCACACCGTCACCCACACTATCATGTTGACATTCC; dG = -26.7
1757 >s_b_s_d_s_e_s_a_s_d_s_c_s_a_s GTGGCTGTGACGGTGCTGGTGTCTGTGACGGTGTGGGTGTCTGTG;
>clamp_s_c_s_d_s_c_s_a_s:compl:rev CACAGACACCCACACCGTCACCCACACTATCATGTTGACATTCC; dG = -26.7
1758 >s_a_s_a_s_e_s_c_s_d_s_a_s_a_s GTGTCTGTGTCTGTGCTGGTGTGGGTGACGGTGTCTGTGTCTGTG;
>clamp_s_c_s_d_s_c_s_a_s:compl:rev CACAGACACCCACACCGTCACCCACACTATCATGTTGACATTCC; dG = -26.7
1759 >s_b_s_e_s_a_s_c_s_b_s_c_s_a_s GTGGCTGTGCTGGTGTCTGTGTGGGTGGCTGTGTGGGTGTCTGTG;
>clamp_s_c_s_d_s_c_s_a_s:compl:rev CACAGACACCCACACCGTCACCCACACTATCATGTTGACATTCC; dG = -26.7
```

1760 >s_b_s_e_s_b_s_c_s_b_s_c_s_a_s GTGGCTGTGCTGGTGGCTGTGTGGGTGGCTGTGTGGGTGTCTGTG;
>clamp_s_c_s_d_s_c_s_a_s:compl:rev CACAGACACCCACACCGTCACCCACACTATCATGTTGACATTCC; dG = -26.7
1761 >s_a_s_a_s_e_s_c_s_b_s_c_s_a_s GTGTCTGTGTCTGTGCTGGTGTGGGTGGCTGTGTGGGTGTCTGTG;
>clamp_s_c_s_d_s_c_s_a_s:compl:rev CACAGACACCCACACCGTCACCCACACTATCATGTTGACATTCC; dG = -26.7
1762 >s_b_s_e_s_c_s_c_s_d_s_a_s_a_s GTGGCTGTGCTGGTGTGGGTGTGGGTGACGGTGTCTGTGTCTGTG;
>clamp_s_c_s_d_s_c_s_a_s:compl:rev CACAGACACCCACACCGTCACCCACACTATCATGTTGACATTCC; dG = -26.7
1763 >s_b_s_e_s_c_s_c_s_b_s_c_s_a_s GTGGCTGTGCTGGTGTGGGTGTGGGTGGCTGTGTGGGTGTCTGTG;
>clamp_s_c_s_d_s_c_s_a_s:compl:rev CACAGACACCCACACCGTCACCCACACTATCATGTTGACATTCC; dG = -26.7
1764 >s_b_s_e_s_d_s_c_s_d_s_a_s_a_s GTGGCTGTGCTGGTGACGGTGTGGGTGACGGTGTCTGTGTCTGTG;
>clamp_s_c_s_d_s_c_s_a_s:compl:rev CACAGACACCCACACCGTCACCCACACTATCATGTTGACATTCC; dG = -26.7
1765 >s_b_s_e_s_d_s_c_s_b_s_c_s_a_s GTGGCTGTGCTGGTGACGGTGTGGGTGGCTGTGTGGGTGTCTGTG;
>clamp_s_c_s_d_s_c_s_a_s:compl:rev CACAGACACCCACACCGTCACCCACACTATCATGTTGACATTCC; dG = -26.7
1766 >s_b_s_e_s_d_s_a_s_d_s_c_s_a_s GTGGCTGTGCTGGTGACGGTGTCTGTGACGGTGTGGGTGTCTGTG;
>clamp_s_c_s_d_s_c_s_a_s:compl:rev CACAGACACCCACACCGTCACCCACACTATCATGTTGACATTCC; dG = -26.7
1767 >s_b_s_e_s_e_s_c_s_d_s_a_s_a_s GTGGCTGTGCTGGTGCTGGTGTGGGTGACGGTGTCTGTGTCTGTG;
>clamp_s_c_s_d_s_c_s_a_s:compl:rev CACAGACACCCACACCGTCACCCACACTATCATGTTGACATTCC; dG = -26.7
1768 >s_b_s_e_s_e_s_c_s_b_s_c_s_a_s GTGGCTGTGCTGGTGCTGGTGTGGGTGGCTGTGTGGGTGTCTGTG;
>clamp_s_c_s_d_s_c_s_a_s:compl:rev CACAGACACCCACACCGTCACCCACACTATCATGTTGACATTCC; dG = -26.7
1769 >s_b_s_e_s_e_s_a_s_d_s_c_s_a_s GTGGCTGTGCTGGTGCTGGTGTCTGTGACGGTGTGGGTGTCTGTG;
>clamp_s_c_s_d_s_c_s_a_s:compl:rev CACAGACACCCACACCGTCACCCACACTATCATGTTGACATTCC; dG = -26.7
1770 >s_a_s_a_s_e_s_a_s_d_s_c_s_a_s GTGTCTGTGTCTGTGCTGGTGTCTGTGACGGTGTGGGTGTCTGTG;
>clamp_s_c_s_d_s_c_s_a_s:compl:rev CACAGACACCCACACCGTCACCCACACTATCATGTTGACATTCC; dG = -26.7
1771 >s_c_s_a_s_a_s_c_s_b_s_c_s_a_s GTGTGGGTGTCTGTGTCTGTGTGGGTGGCTGTGTGGGTGTCTGTG;
>clamp_s_c_s_d_s_c_s_a_s:compl:rev CACAGACACCCACACCGTCACCCACACTATCATGTTGACATTCC; dG = -26.7
1772 >s_c_s_a_s_b_s_c_s_b_s_c_s_a_s GTGTGGGTGTCTGTGGCTGTGTGGGTGGCTGTGTGGGTGTCTGTG;
>clamp_s_c_s_d_s_c_s_a_s:compl:rev CACAGACACCCACACCGTCACCCACACTATCATGTTGACATTCC; dG = -26.7
1773 >s_c_s_a_s_c_s_c_s_d_s_a_s_a_s GTGTGGGTGTCTGTGTGGGTGTGGGTGACGGTGTCTGTGTCTGTG;
>clamp_s_c_s_d_s_c_s_a_s:compl:rev CACAGACACCCACACCGTCACCCACACTATCATGTTGACATTCC; dG = -26.7
1774 >s_c_s_a_s_c_s_c_s_b_s_c_s_a_s GTGTGGGTGTCTGTGTGGGTGTGGGTGGCTGTGTGGGTGTCTGTG;
>clamp_s_c_s_d_s_c_s_a_s:compl:rev CACAGACACCCACACCGTCACCCACACTATCATGTTGACATTCC; dG = -26.7
1775 >s_c_s_a_s_d_s_c_s_d_s_a_s_a_s GTGTGGGTGTCTGTGACGGTGTGGGTGACGGTGTCTGTGTCTGTG;
>clamp_s_c_s_d_s_c_s_a_s:compl:rev CACAGACACCCACACCGTCACCCACACTATCATGTTGACATTCC; dG = -26.7
1776 >s_c_s_a_s_d_s_c_s_b_s_c_s_a_s GTGTGGGTGTCTGTGACGGTGTGGGTGGCTGTGTGGGTGTCTGTG;
>clamp_s_c_s_d_s_c_s_a_s:compl:rev CACAGACACCCACACCGTCACCCACACTATCATGTTGACATTCC; dG = -26.7
1777 >s_c_s_a_s_d_s_a_s_d_s_c_s_a_s GTGTGGGTGTCTGTGACGGTGTCTGTGACGGTGTGGGTGTCTGTG;
>clamp_s_c_s_d_s_c_s_a_s:compl:rev CACAGACACCCACACCGTCACCCACACTATCATGTTGACATTCC; dG = -26.7
1778 >s_c_s_a_s_e_s_c_s_d_s_a_s_a_s GTGTGGGTGTCTGTGCTGGTGTGGGTGACGGTGTCTGTGTCTGTG;
>clamp_s_c_s_d_s_c_s_a_s:compl:rev CACAGACACCCACACCGTCACCCACACTATCATGTTGACATTCC; dG = -26.7
1779 >s_c_s_a_s_e_s_c_s_b_s_c_s_a_s GTGTGGGTGTCTGTGCTGGTGTGGGTGGCTGTGTGGGTGTCTGTG;
>clamp_s_c_s_d_s_c_s_a_s:compl:rev CACAGACACCCACACCGTCACCCACACTATCATGTTGACATTCC; dG = -26.7
1780 >s_c_s_a_s_e_s_a_s_d_s_c_s_a_s GTGTGGGTGTCTGTGCTGGTGTCTGTGACGGTGTGGGTGTCTGTG;
>clamp_s_c_s_d_s_c_s_a_s:compl:rev CACAGACACCCACACCGTCACCCACACTATCATGTTGACATTCC; dG = -26.7
1781 >s_c_s_b_s_a_s_c_s_b_s_c_s_a_s GTGTGGGTGGCTGTGTCTGTGTGGGTGGCTGTGTGGGTGTCTGTG;
>clamp_s_c_s_d_s_c_s_a_s:compl:rev CACAGACACCCACACCGTCACCCACACTATCATGTTGACATTCC; dG = -26.7
1782 >s_c_s_b_s_b_s_c_s_b_s_c_s_a_s GTGTGGGTGGCTGTGGCTGTGTGGGTGGCTGTGTGGGTGTCTGTG;
>clamp_s_c_s_d_s_c_s_a_s:compl:rev CACAGACACCCACACCGTCACCCACACTATCATGTTGACATTCC; dG = -26.7
1783 >s_a_s_b_s_a_s_c_s_b_s_c_s_a_s GTGTCTGTGGCTGTGTCTGTGTGGGTGGCTGTGTGGGTGTCTGTG;
>clamp_s_c_s_d_s_c_s_a_s:compl:rev CACAGACACCCACACCGTCACCCACACTATCATGTTGACATTCC; dG = -26.7
1784 >s_c_s_b_s_c_s_c_s_d_s_a_s_a_s GTGTGGGTGGCTGTGTGGGTGTGGGTGACGGTGTCTGTGTCTGTG;
>clamp_s_c_s_d_s_c_s_a_s:compl:rev CACAGACACCCACACCGTCACCCACACTATCATGTTGACATTCC; dG = -26.7
1785 >s_c_s_b_s_c_s_c_s_b_s_c_s_a_s GTGTGGGTGGCTGTGTGGGTGTGGGTGGCTGTGTGGGTGTCTGTG;
>clamp_s_c_s_d_s_c_s_a_s:compl:rev CACAGACACCCACACCGTCACCCACACTATCATGTTGACATTCC; dG = -26.7
1786 >s_c_s_b_s_d_s_c_s_d_s_a_s_a_s GTGTGGGTGGCTGTGACGGTGTGGGTGACGGTGTCTGTGTCTGTG;
>clamp_s_c_s_d_s_c_s_a_s:compl:rev CACAGACACCCACACCGTCACCCACACTATCATGTTGACATTCC; dG = -26.7
1787 >s_c_s_b_s_d_s_c_s_b_s_c_s_a_s GTGTGGGTGGCTGTGACGGTGTGGGTGGCTGTGTGGGTGTCTGTG;
>clamp_s_c_s_d_s_c_s_a_s:compl:rev CACAGACACCCACACCGTCACCCACACTATCATGTTGACATTCC; dG = -26.7
1788 >s_c_s_b_s_d_s_a_s_d_s_c_s_a_s GTGTGGGTGGCTGTGACGGTGTCTGTGACGGTGTGGGTGTCTGTG;
>clamp_s_c_s_d_s_c_s_a_s:compl:rev CACAGACACCCACACCGTCACCCACACTATCATGTTGACATTCC; dG = -26.7
1789 >s_c_s_b_s_e_s_c_s_d_s_a_s_a_s GTGTGGGTGGCTGTGCTGGTGTGGGTGACGGTGTCTGTGTCTGTG;
>clamp_s_c_s_d_s_c_s_a_s:compl:rev CACAGACACCCACACCGTCACCCACACTATCATGTTGACATTCC; dG = -26.7
1790 >s_c_s_b_s_e_s_c_s_b_s_c_s_a_s GTGTGGGTGGCTGTGCTGGTGTGGGTGGCTGTGTGGGTGTCTGTG;
>clamp_s_c_s_d_s_c_s_a_s:compl:rev CACAGACACCCACACCGTCACCCACACTATCATGTTGACATTCC; dG = -26.7
1791 >s_c_s_b_s_e_s_a_s_d_s_c_s_a_s GTGTGGGTGGCTGTGCTGGTGTCTGTGACGGTGTGGGTGTCTGTG;
>clamp_s_c_s_d_s_c_s_a_s:compl:rev CACAGACACCCACACCGTCACCCACACTATCATGTTGACATTCC; dG = -26.7
1792 >s_c_s_c_s_a_s_c_s_b_s_c_s_a_s GTGTGGGTGTGGGTGTCTGTGTGGGTGGCTGTGTGGGTGTCTGTG;
>clamp_s_c_s_d_s_c_s_a_s:compl:rev CACAGACACCCACACCGTCACCCACACTATCATGTTGACATTCC; dG = -26.7
1793 >s_c_s_c_s_b_s_c_s_b_s_c_s_a_s GTGTGGGTGTGGGTGGCTGTGTGGGTGGCTGTGTGGGTGTCTGTG;
>clamp_s_c_s_d_s_c_s_a_s:compl:rev CACAGACACCCACACCGTCACCCACACTATCATGTTGACATTCC; dG = -26.7
1794 >s_c_s_c_s_c_s_c_s_d_s_a_s_a_s GTGTGGGTGTGGGTGTGGGTGTGGGTGACGGTGTCTGTGTCTGTG;
>clamp_s_c_s_d_s_c_s_a_s:compl:rev CACAGACACCCACACCGTCACCCACACTATCATGTTGACATTCC; dG = -26.7
1795 >s_c_s_b_s_c_s_c_s_a_s_c_s_a_s GTGTGGGTGGCTGTGTGGGTGTGGGTGTCTGTGTGGGTGTCTGTG;
>clamp_s_c_s_d_s_c_s_a_s:compl:rev CACAGACACCCACACCGTCACCCACACTATCATGTTGACATTCC; dG = -25.4
1796 >s_c_s_b_s_e_s_c_s_a_s_c_s_a_s GTGTGGGTGGCTGTGCTGGTGTGGGTGTCTGTGTGGGTGTCTGTG;
>clamp_s_c_s_d_s_c_s_a_s:compl:rev CACAGACACCCACACCGTCACCCACACTATCATGTTGACATTCC; dG = -25.4

1797 >s_d_s_c_s_a_s_c_s_d_s_b_s_a_s GTGACGGTGTGGGTGTCTGTGTGGGTGACGGTGGCTGTGTCTGTG;
>clamp_s_c_s_d_s_c_s_a_s:compl:rev CACAGACACCCACACCGTCACCCACACTATCATGTTGACATTCC; dG = -25.2
1798 >s_d_s_c_s_a_s_c_s_a_s_c_s_a_s GTGACGGTGTGGGTGTCTGTGTGGGTGTCTGTGTGGGTGTCTGTG;
>clamp_s_c_s_d_s_c_s_a_s:compl:rev CACAGACACCCACACCGTCACCCACACTATCATGTTGACATTCC; dG = -25.2
1799 >s_c_s_c_s_e_s_c_s_d_s_b_s_a_s GTGTGGGTGTGGGTGCTGGTGTGGGTGACGGTGGCTGTGTCTGTG;
>clamp_s_c_s_d_s_c_s_a_s:compl:rev CACAGACACCCACACCGTCACCCACACTATCATGTTGACATTCC; dG = -24.8
1800 >s_a_s_b_s_b_s_c_s_d_s_b_s_a_s GTGTCTGTGGCTGTGGCTGTGTGGGTGACGGTGGCTGTGTCTGTG;
>clamp_s_c_s_d_s_c_s_a_s:compl:rev CACAGACACCCACACCGTCACCCACACTATCATGTTGACATTCC; dG = -24.8
1801 >s_c_s_d_s_a_s_c_s_d_s_b_s_a_s GTGTGGGTGACGGTGTCTGTGTGGGTGACGGTGGCTGTGTCTGTG;
>clamp_s_c_s_d_s_c_s_a_s:compl:rev CACAGACACCCACACCGTCACCCACACTATCATGTTGACATTCC; dG = -24.8
1802 >s_c_s_d_s_b_s_c_s_d_s_b_s_a_s GTGTGGGTGACGGTGGCTGTGTGGGTGACGGTGGCTGTGTCTGTG;
>clamp_s_c_s_d_s_c_s_a_s:compl:rev CACAGACACCCACACCGTCACCCACACTATCATGTTGACATTCC; dG = -24.8
1803 >s_c_s_d_s_d_s_c_s_d_s_b_s_a_s GTGTGGGTGACGGTGACGGTGTGGGTGACGGTGGCTGTGTCTGTG;
>clamp_s_c_s_d_s_c_s_a_s:compl:rev CACAGACACCCACACCGTCACCCACACTATCATGTTGACATTCC; dG = -24.8
1804 >s_c_s_d_s_e_s_c_s_d_s_b_s_a_s GTGTGGGTGACGGTGCTGGTGTGGGTGACGGTGGCTGTGTCTGTG;
>clamp_s_c_s_d_s_c_s_a_s:compl:rev CACAGACACCCACACCGTCACCCACACTATCATGTTGACATTCC; dG = -24.8
1805 >s_c_s_e_s_a_s_c_s_d_s_b_s_a_s GTGTGGGTGCTGGTGTCTGTGTGGGTGACGGTGGCTGTGTCTGTG;
>clamp_s_c_s_d_s_c_s_a_s:compl:rev CACAGACACCCACACCGTCACCCACACTATCATGTTGACATTCC; dG = -24.8
1806 >s_c_s_e_s_b_s_c_s_d_s_b_s_a_s GTGTGGGTGCTGGTGGCTGTGTGGGTGACGGTGGCTGTGTCTGTG;
>clamp_s_c_s_d_s_c_s_a_s:compl:rev CACAGACACCCACACCGTCACCCACACTATCATGTTGACATTCC; dG = -24.8
1807 >s_c_s_e_s_c_s_c_s_d_s_b_s_a_s GTGTGGGTGCTGGTGTGGGTGTGGGTGACGGTGGCTGTGTCTGTG;
>clamp_s_c_s_d_s_c_s_a_s:compl:rev CACAGACACCCACACCGTCACCCACACTATCATGTTGACATTCC; dG = -24.8
1808 >s_c_s_e_s_d_s_c_s_d_s_b_s_a_s GTGTGGGTGCTGGTGACGGTGTGGGTGACGGTGGCTGTGTCTGTG;
>clamp_s_c_s_d_s_c_s_a_s:compl:rev CACAGACACCCACACCGTCACCCACACTATCATGTTGACATTCC; dG = -24.8
1809 >s_c_s_e_s_e_s_c_s_d_s_b_s_a_s GTGTGGGTGCTGGTGCTGGTGTGGGTGACGGTGGCTGTGTCTGTG;
>clamp_s_c_s_d_s_c_s_a_s:compl:rev CACAGACACCCACACCGTCACCCACACTATCATGTTGACATTCC; dG = -24.8
1810 >s_a_s_b_s_c_s_c_s_d_s_b_s_a_s GTGTCTGTGGCTGTGTGGGTGTGGGTGACGGTGGCTGTGTCTGTG;
>clamp_s_c_s_d_s_c_s_a_s:compl:rev CACAGACACCCACACCGTCACCCACACTATCATGTTGACATTCC; dG = -24.8
1811 >s_d_s_a_s_a_s_c_s_d_s_b_s_a_s GTGACGGTGTCTGTGTCTGTGTGGGTGACGGTGGCTGTGTCTGTG;
>clamp_s_c_s_d_s_c_s_a_s:compl:rev CACAGACACCCACACCGTCACCCACACTATCATGTTGACATTCC; dG = -24.8
1812 >s_d_s_a_s_b_s_c_s_d_s_b_s_a_s GTGACGGTGTCTGTGGCTGTGTGGGTGACGGTGGCTGTGTCTGTG;
>clamp_s_c_s_d_s_c_s_a_s:compl:rev CACAGACACCCACACCGTCACCCACACTATCATGTTGACATTCC; dG = -24.8
1813 >s_d_s_a_s_c_s_c_s_d_s_b_s_a_s GTGACGGTGTCTGTGTGGGTGTGGGTGACGGTGGCTGTGTCTGTG;
>clamp_s_c_s_d_s_c_s_a_s:compl:rev CACAGACACCCACACCGTCACCCACACTATCATGTTGACATTCC; dG = -24.8
1814 >s_d_s_a_s_d_s_c_s_d_s_b_s_a_s GTGACGGTGTCTGTGACGGTGTGGGTGACGGTGGCTGTGTCTGTG;
>clamp_s_c_s_d_s_c_s_a_s:compl:rev CACAGACACCCACACCGTCACCCACACTATCATGTTGACATTCC; dG = -24.8
1815 >s_d_s_a_s_e_s_c_s_d_s_b_s_a_s GTGACGGTGTCTGTGCTGGTGTGGGTGACGGTGGCTGTGTCTGTG;
>clamp_s_c_s_d_s_c_s_a_s:compl:rev CACAGACACCCACACCGTCACCCACACTATCATGTTGACATTCC; dG = -24.8
1816 >s_d_s_b_s_a_s_c_s_d_s_b_s_a_s GTGACGGTGGCTGTGTCTGTGTGGGTGACGGTGGCTGTGTCTGTG;
>clamp_s_c_s_d_s_c_s_a_s:compl:rev CACAGACACCCACACCGTCACCCACACTATCATGTTGACATTCC; dG = -24.8
1817 >s_d_s_b_s_b_s_c_s_d_s_b_s_a_s GTGACGGTGGCTGTGGCTGTGTGGGTGACGGTGGCTGTGTCTGTG;
>clamp_s_c_s_d_s_c_s_a_s:compl:rev CACAGACACCCACACCGTCACCCACACTATCATGTTGACATTCC; dG = -24.8
1818 >s_d_s_b_s_c_s_c_s_d_s_b_s_a_s GTGACGGTGGCTGTGTGGGTGTGGGTGACGGTGGCTGTGTCTGTG;
>clamp_s_c_s_d_s_c_s_a_s:compl:rev CACAGACACCCACACCGTCACCCACACTATCATGTTGACATTCC; dG = -24.8
1819 >s_d_s_b_s_d_s_c_s_d_s_b_s_a_s GTGACGGTGGCTGTGACGGTGTGGGTGACGGTGGCTGTGTCTGTG;
>clamp_s_c_s_d_s_c_s_a_s:compl:rev CACAGACACCCACACCGTCACCCACACTATCATGTTGACATTCC; dG = -24.8
1820 >s_d_s_b_s_e_s_c_s_d_s_b_s_a_s GTGACGGTGGCTGTGCTGGTGTGGGTGACGGTGGCTGTGTCTGTG;
>clamp_s_c_s_d_s_c_s_a_s:compl:rev CACAGACACCCACACCGTCACCCACACTATCATGTTGACATTCC; dG = -24.8
1821 >s_a_s_b_s_d_s_c_s_d_s_b_s_a_s GTGTCTGTGGCTGTGACGGTGTGGGTGACGGTGGCTGTGTCTGTG;
>clamp_s_c_s_d_s_c_s_a_s:compl:rev CACAGACACCCACACCGTCACCCACACTATCATGTTGACATTCC; dG = -24.8
1822 >s_d_s_c_s_b_s_c_s_d_s_b_s_a_s GTGACGGTGTGGGTGGCTGTGTGGGTGACGGTGGCTGTGTCTGTG;
>clamp_s_c_s_d_s_c_s_a_s:compl:rev CACAGACACCCACACCGTCACCCACACTATCATGTTGACATTCC; dG = -24.8
1823 >s_d_s_c_s_c_s_c_s_d_s_b_s_a_s GTGACGGTGTGGGTGTGGGTGTGGGTGACGGTGGCTGTGTCTGTG;
>clamp_s_c_s_d_s_c_s_a_s:compl:rev CACAGACACCCACACCGTCACCCACACTATCATGTTGACATTCC; dG = -24.8
1824 >s_d_s_c_s_e_s_c_s_d_s_b_s_a_s GTGACGGTGTGGGTGCTGGTGTGGGTGACGGTGGCTGTGTCTGTG;
>clamp_s_c_s_d_s_c_s_a_s:compl:rev CACAGACACCCACACCGTCACCCACACTATCATGTTGACATTCC; dG = -24.8
1825 >s_d_s_d_s_a_s_c_s_d_s_b_s_a_s GTGACGGTGACGGTGTCTGTGTGGGTGACGGTGGCTGTGTCTGTG;
>clamp_s_c_s_d_s_c_s_a_s:compl:rev CACAGACACCCACACCGTCACCCACACTATCATGTTGACATTCC; dG = -24.8
1826 >s_d_s_d_s_b_s_c_s_d_s_b_s_a_s GTGACGGTGACGGTGGCTGTGTGGGTGACGGTGGCTGTGTCTGTG;
>clamp_s_c_s_d_s_c_s_a_s:compl:rev CACAGACACCCACACCGTCACCCACACTATCATGTTGACATTCC; dG = -24.8
1827 >s_d_s_d_s_c_s_c_s_d_s_b_s_a_s GTGACGGTGACGGTGTGGGTGTGGGTGACGGTGGCTGTGTCTGTG;
>clamp_s_c_s_d_s_c_s_a_s:compl:rev CACAGACACCCACACCGTCACCCACACTATCATGTTGACATTCC; dG = -24.8
1828 >s_d_s_d_s_d_s_c_s_d_s_b_s_a_s GTGACGGTGACGGTGACGGTGTGGGTGACGGTGGCTGTGTCTGTG;
>clamp_s_c_s_d_s_c_s_a_s:compl:rev CACAGACACCCACACCGTCACCCACACTATCATGTTGACATTCC; dG = -24.8
1829 >s_d_s_d_s_e_s_c_s_d_s_b_s_a_s GTGACGGTGACGGTGCTGGTGTGGGTGACGGTGGCTGTGTCTGTG;
>clamp_s_c_s_d_s_c_s_a_s:compl:rev CACAGACACCCACACCGTCACCCACACTATCATGTTGACATTCC; dG = -24.8
1830 >s_a_s_b_s_e_s_c_s_d_s_b_s_a_s GTGTCTGTGGCTGTGCTGGTGTGGGTGACGGTGGCTGTGTCTGTG;
>clamp_s_c_s_d_s_c_s_a_s:compl:rev CACAGACACCCACACCGTCACCCACACTATCATGTTGACATTCC; dG = -24.8
1831 >s_d_s_e_s_a_s_c_s_d_s_b_s_a_s GTGACGGTGCTGGTGTCTGTGTGGGTGACGGTGGCTGTGTCTGTG;
>clamp_s_c_s_d_s_c_s_a_s:compl:rev CACAGACACCCACACCGTCACCCACACTATCATGTTGACATTCC; dG = -24.8
1832 >s_d_s_e_s_b_s_c_s_d_s_b_s_a_s GTGACGGTGCTGGTGGCTGTGTGGGTGACGGTGGCTGTGTCTGTG;
>clamp_s_c_s_d_s_c_s_a_s:compl:rev CACAGACACCCACACCGTCACCCACACTATCATGTTGACATTCC; dG = -24.8
1833 >s_d_s_e_s_c_s_c_s_d_s_b_s_a_s GTGACGGTGCTGGTGTGGGTGTGGGTGACGGTGGCTGTGTCTGTG;
>clamp_s_c_s_d_s_c_s_a_s:compl:rev CACAGACACCCACACCGTCACCCACACTATCATGTTGACATTCC; dG = -24.8

1834 >s_d_s_e_s_d_s_c_s_d_s_b_s_a_s GTGACGGTGCTGGTGACGGTGTGGGTGACGGTGGCTGTGTCTGTG;
>clamp_s_c_s_d_s_c_s_a_s:compl:rev CACAGACACCCACACCGTCACCCACACTATCATGTTGACATTCC; dG = -24.8
1835 >s_d_s_e_s_e_s_c_s_d_s_b_s_a_s GTGACGGTGCTGGTGCTGGTGTGGGTGACGGTGGCTGTGTCTGTG;
>clamp_s_c_s_d_s_c_s_a_s:compl:rev CACAGACACCCACACCGTCACCCACACTATCATGTTGACATTCC; dG = -24.8
1836 >s_e_s_a_s_a_s_c_s_d_s_b_s_a_s GTGCTGGTGTCTGTGTCTGTGTGGGTGACGGTGGCTGTGTCTGTG;
>clamp_s_c_s_d_s_c_s_a_s:compl:rev CACAGACACCCACACCGTCACCCACACTATCATGTTGACATTCC; dG = -24.8
1837 >s_e_s_a_s_b_s_c_s_d_s_b_s_a_s GTGCTGGTGTCTGTGGCTGTGTGGGTGACGGTGGCTGTGTCTGTG;
>clamp_s_c_s_d_s_c_s_a_s:compl:rev CACAGACACCCACACCGTCACCCACACTATCATGTTGACATTCC; dG = -24.8
1838 >s_e_s_a_s_c_s_c_s_d_s_b_s_a_s GTGCTGGTGTCTGTGTGGGTGTGGGTGACGGTGGCTGTGTCTGTG;
>clamp_s_c_s_d_s_c_s_a_s:compl:rev CACAGACACCCACACCGTCACCCACACTATCATGTTGACATTCC; dG = -24.8
1839 >s_e_s_a_s_d_s_c_s_d_s_b_s_a_s GTGCTGGTGTCTGTGACGGTGTGGGTGACGGTGGCTGTGTCTGTG;
>clamp_s_c_s_d_s_c_s_a_s:compl:rev CACAGACACCCACACCGTCACCCACACTATCATGTTGACATTCC; dG = -24.8
1840 >s_e_s_a_s_e_s_c_s_d_s_b_s_a_s GTGCTGGTGTCTGTGCTGGTGTGGGTGACGGTGGCTGTGTCTGTG;
>clamp_s_c_s_d_s_c_s_a_s:compl:rev CACAGACACCCACACCGTCACCCACACTATCATGTTGACATTCC; dG = -24.8
1841 >s_a_s_c_s_a_s_c_s_d_s_b_s_a_s GTGTCTGTGTGGGTGTCTGTGTGGGTGACGGTGGCTGTGTCTGTG;
>clamp_s_c_s_d_s_c_s_a_s:compl:rev CACAGACACCCACACCGTCACCCACACTATCATGTTGACATTCC; dG = -24.8
1842 >s_e_s_b_s_a_s_c_s_d_s_b_s_a_s GTGCTGGTGGCTGTGTCTGTGTGGGTGACGGTGGCTGTGTCTGTG;
>clamp_s_c_s_d_s_c_s_a_s:compl:rev CACAGACACCCACACCGTCACCCACACTATCATGTTGACATTCC; dG = -24.8
1843 >s_e_s_b_s_b_s_c_s_d_s_b_s_a_s GTGCTGGTGGCTGTGGCTGTGTGGGTGACGGTGGCTGTGTCTGTG;
>clamp_s_c_s_d_s_c_s_a_s:compl:rev CACAGACACCCACACCGTCACCCACACTATCATGTTGACATTCC; dG = -24.8
1844 >s_e_s_b_s_c_s_c_s_d_s_b_s_a_s GTGCTGGTGGCTGTGTGGGTGTGGGTGACGGTGGCTGTGTCTGTG;
>clamp_s_c_s_d_s_c_s_a_s:compl:rev CACAGACACCCACACCGTCACCCACACTATCATGTTGACATTCC; dG = -24.8
1845 >s_a_s_c_s_a_s_c_s_a_s_c_s_a_s GTGTCTGTGTGGGTGTCTGTGTGGGTGTCTGTGTGGGTGTCTGTG;
>clamp_s_c_s_d_s_c_s_a_s:compl:rev CACAGACACCCACACCGTCACCCACACTATCATGTTGACATTCC; dG = -24.8
1846 >s_e_s_b_s_d_s_c_s_d_s_b_s_a_s GTGCTGGTGGCTGTGACGGTGTGGGTGACGGTGGCTGTGTCTGTG;
>clamp_s_c_s_d_s_c_s_a_s:compl:rev CACAGACACCCACACCGTCACCCACACTATCATGTTGACATTCC; dG = -24.8
1847 >s_e_s_b_s_e_s_c_s_d_s_b_s_a_s GTGCTGGTGGCTGTGCTGGTGTGGGTGACGGTGGCTGTGTCTGTG;
>clamp_s_c_s_d_s_c_s_a_s:compl:rev CACAGACACCCACACCGTCACCCACACTATCATGTTGACATTCC; dG = -24.8
1848 >s_e_s_c_s_a_s_c_s_d_s_b_s_a_s GTGCTGGTGTGGGTGTCTGTGTGGGTGACGGTGGCTGTGTCTGTG;
>clamp_s_c_s_d_s_c_s_a_s:compl:rev CACAGACACCCACACCGTCACCCACACTATCATGTTGACATTCC; dG = -24.8
1849 >s_e_s_c_s_b_s_c_s_d_s_b_s_a_s GTGCTGGTGTGGGTGGCTGTGTGGGTGACGGTGGCTGTGTCTGTG;
>clamp_s_c_s_d_s_c_s_a_s:compl:rev CACAGACACCCACACCGTCACCCACACTATCATGTTGACATTCC; dG = -24.8
1850 >s_e_s_c_s_c_s_c_s_d_s_b_s_a_s GTGCTGGTGTGGGTGTGGGTGTGGGTGACGGTGGCTGTGTCTGTG;
>clamp_s_c_s_d_s_c_s_a_s:compl:rev CACAGACACCCACACCGTCACCCACACTATCATGTTGACATTCC; dG = -24.8
1851 >s_e_s_c_s_e_s_c_s_d_s_b_s_a_s GTGCTGGTGTGGGTGCTGGTGTGGGTGACGGTGGCTGTGTCTGTG;
>clamp_s_c_s_d_s_c_s_a_s:compl:rev CACAGACACCCACACCGTCACCCACACTATCATGTTGACATTCC; dG = -24.8
1852 >s_a_s_c_s_b_s_c_s_d_s_b_s_a_s GTGTCTGTGTGGGTGGCTGTGTGGGTGACGGTGGCTGTGTCTGTG;
>clamp_s_c_s_d_s_c_s_a_s:compl:rev CACAGACACCCACACCGTCACCCACACTATCATGTTGACATTCC; dG = -24.8
1853 >s_e_s_d_s_a_s_c_s_d_s_b_s_a_s GTGCTGGTGACGGTGTCTGTGTGGGTGACGGTGGCTGTGTCTGTG;
>clamp_s_c_s_d_s_c_s_a_s:compl:rev CACAGACACCCACACCGTCACCCACACTATCATGTTGACATTCC; dG = -24.8
1854 >s_e_s_d_s_b_s_c_s_d_s_b_s_a_s GTGCTGGTGACGGTGGCTGTGTGGGTGACGGTGGCTGTGTCTGTG;
>clamp_s_c_s_d_s_c_s_a_s:compl:rev CACAGACACCCACACCGTCACCCACACTATCATGTTGACATTCC; dG = -24.8
1855 >s_e_s_d_s_c_s_c_s_d_s_b_s_a_s GTGCTGGTGACGGTGTGGGTGTGGGTGACGGTGGCTGTGTCTGTG;
>clamp_s_c_s_d_s_c_s_a_s:compl:rev CACAGACACCCACACCGTCACCCACACTATCATGTTGACATTCC; dG = -24.8
1856 >s_e_s_d_s_d_s_c_s_d_s_b_s_a_s GTGCTGGTGACGGTGACGGTGTGGGTGACGGTGGCTGTGTCTGTG;
>clamp_s_c_s_d_s_c_s_a_s:compl:rev CACAGACACCCACACCGTCACCCACACTATCATGTTGACATTCC; dG = -24.8
1857 >s_e_s_d_s_e_s_c_s_d_s_b_s_a_s GTGCTGGTGACGGTGCTGGTGTGGGTGACGGTGGCTGTGTCTGTG;
>clamp_s_c_s_d_s_c_s_a_s:compl:rev CACAGACACCCACACCGTCACCCACACTATCATGTTGACATTCC; dG = -24.8
1858 >s_e_s_e_s_a_s_c_s_d_s_b_s_a_s GTGCTGGTGCTGGTGTCTGTGTGGGTGACGGTGGCTGTGTCTGTG;
>clamp_s_c_s_d_s_c_s_a_s:compl:rev CACAGACACCCACACCGTCACCCACACTATCATGTTGACATTCC; dG = -24.8
1859 >s_e_s_e_s_b_s_c_s_d_s_b_s_a_s GTGCTGGTGCTGGTGGCTGTGTGGGTGACGGTGGCTGTGTCTGTG;
>clamp_s_c_s_d_s_c_s_a_s:compl:rev CACAGACACCCACACCGTCACCCACACTATCATGTTGACATTCC; dG = -24.8
1860 >s_e_s_e_s_c_s_c_s_d_s_b_s_a_s GTGCTGGTGCTGGTGTGGGTGTGGGTGACGGTGGCTGTGTCTGTG;
>clamp_s_c_s_d_s_c_s_a_s:compl:rev CACAGACACCCACACCGTCACCCACACTATCATGTTGACATTCC; dG = -24.8
1861 >s_e_s_e_s_d_s_c_s_d_s_b_s_a_s GTGCTGGTGCTGGTGACGGTGTGGGTGACGGTGGCTGTGTCTGTG;
>clamp_s_c_s_d_s_c_s_a_s:compl:rev CACAGACACCCACACCGTCACCCACACTATCATGTTGACATTCC; dG = -24.8
1862 >s_e_s_e_s_e_s_c_s_d_s_b_s_a_s GTGCTGGTGCTGGTGCTGGTGTGGGTGACGGTGGCTGTGTCTGTG;
>clamp_s_c_s_d_s_c_s_a_s:compl:rev CACAGACACCCACACCGTCACCCACACTATCATGTTGACATTCC; dG = -24.8
1863 >s_a_s_c_s_c_s_c_s_d_s_b_s_a_s GTGTCTGTGTGGGTGTGGGTGTGGGTGACGGTGGCTGTGTCTGTG;
>clamp_s_c_s_d_s_c_s_a_s:compl:rev CACAGACACCCACACCGTCACCCACACTATCATGTTGACATTCC; dG = -24.8
1864 >s_a_s_a_s_b_s_c_s_d_s_b_s_a_s GTGTCTGTGTCTGTGGCTGTGTGGGTGACGGTGGCTGTGTCTGTG;
>clamp_s_c_s_d_s_c_s_a_s:compl:rev CACAGACACCCACACCGTCACCCACACTATCATGTTGACATTCC; dG = -24.8
1865 >s_a_s_c_s_e_s_c_s_d_s_b_s_a_s GTGTCTGTGTGGGTGCTGGTGTGGGTGACGGTGGCTGTGTCTGTG;
>clamp_s_c_s_d_s_c_s_a_s:compl:rev CACAGACACCCACACCGTCACCCACACTATCATGTTGACATTCC; dG = -24.8
1866 >s_a_s_d_s_a_s_c_s_d_s_b_s_a_s GTGTCTGTGACGGTGTCTGTGTGGGTGACGGTGGCTGTGTCTGTG;
>clamp_s_c_s_d_s_c_s_a_s:compl:rev CACAGACACCCACACCGTCACCCACACTATCATGTTGACATTCC; dG = -24.8
1867 >s_a_s_d_s_b_s_c_s_d_s_b_s_a_s GTGTCTGTGACGGTGGCTGTGTGGGTGACGGTGGCTGTGTCTGTG;
>clamp_s_c_s_d_s_c_s_a_s:compl:rev CACAGACACCCACACCGTCACCCACACTATCATGTTGACATTCC; dG = -24.8
1868 >s_a_s_d_s_c_s_c_s_d_s_b_s_a_s GTGTCTGTGACGGTGTGGGTGTGGGTGACGGTGGCTGTGTCTGTG;
>clamp_s_c_s_d_s_c_s_a_s:compl:rev CACAGACACCCACACCGTCACCCACACTATCATGTTGACATTCC; dG = -24.8
1869 >s_a_s_d_s_d_s_c_s_d_s_b_s_a_s GTGTCTGTGACGGTGACGGTGTGGGTGACGGTGGCTGTGTCTGTG;
>clamp_s_c_s_d_s_c_s_a_s:compl:rev CACAGACACCCACACCGTCACCCACACTATCATGTTGACATTCC; dG = -24.8
1870 >s_a_s_d_s_e_s_c_s_d_s_b_s_a_s GTGTCTGTGACGGTGCTGGTGTGGGTGACGGTGGCTGTGTCTGTG;
>clamp_s_c_s_d_s_c_s_a_s:compl:rev CACAGACACCCACACCGTCACCCACACTATCATGTTGACATTCC; dG = -24.8

```
  1871 >s_a_s_e_s_a_s_c_s_d_s_b_s_a_s          GTGTCTGTGCTGGTGTCTGTGTGGGTGACGGTGGCTGTGTCTGTG;
>clamp_s_c_s_d_s_c_s_a_s:compl:rev CACAGACACCCACACCGTCACCCACACTATCATGTTGACATTCC; dG = -24.8
  1872 >s_a_s_e_s_b_s_c_s_d_s_b_s_a_s          GTGTCTGTGCTGGTGGCTGTGTGGGTGACGGTGGCTGTGTCTGTG;
>clamp_s_c_s_d_s_c_s_a_s:compl:rev CACAGACACCCACACCGTCACCCACACTATCATGTTGACATTCC; dG = -24.8
  1873 >s_a_s_e_s_c_s_c_s_d_s_b_s_a_s          GTGTCTGTGCTGGTGTGGGTGTGGGTGACGGTGGCTGTGTCTGTG;
>clamp_s_c_s_d_s_c_s_a_s:compl:rev CACAGACACCCACACCGTCACCCACACTATCATGTTGACATTCC; dG = -24.8
  1874 >s_a_s_e_s_d_s_c_s_d_s_b_s_a_s          GTGTCTGTGCTGGTGACGGTGTGGGTGACGGTGGCTGTGTCTGTG;
>clamp_s_c_s_d_s_c_s_a_s:compl:rev CACAGACACCCACACCGTCACCCACACTATCATGTTGACATTCC; dG = -24.8
  1875 >s_a_s_a_s_c_s_c_s_d_s_b_s_a_s          GTGTCTGTGTCTGTGTGGGTGTGGGTGACGGTGGCTGTGTCTGTG;
>clamp_s_c_s_d_s_c_s_a_s:compl:rev CACAGACACCCACACCGTCACCCACACTATCATGTTGACATTCC; dG = -24.8
  1876 >s_a_s_e_s_e_s_c_s_d_s_b_s_a_s          GTGTCTGTGCTGGTGCTGGTGTGGGTGACGGTGGCTGTGTCTGTG;
>clamp_s_c_s_d_s_c_s_a_s:compl:rev CACAGACACCCACACCGTCACCCACACTATCATGTTGACATTCC; dG = -24.8
  1877 >s_b_s_a_s_a_s_c_s_d_s_b_s_a_s          GTGGCTGTGTCTGTGTCTGTGTGGGTGACGGTGGCTGTGTCTGTG;
>clamp_s_c_s_d_s_c_s_a_s:compl:rev CACAGACACCCACACCGTCACCCACACTATCATGTTGACATTCC; dG = -24.8
  1878 >s_b_s_a_s_b_s_c_s_d_s_b_s_a_s          GTGGCTGTGTCTGTGGCTGTGTGGGTGACGGTGGCTGTGTCTGTG;
>clamp_s_c_s_d_s_c_s_a_s:compl:rev CACAGACACCCACACCGTCACCCACACTATCATGTTGACATTCC; dG = -24.8
  1879 >s_b_s_a_s_c_s_c_s_d_s_b_s_a_s          GTGGCTGTGTCTGTGTGGGTGTGGGTGACGGTGGCTGTGTCTGTG;
>clamp_s_c_s_d_s_c_s_a_s:compl:rev CACAGACACCCACACCGTCACCCACACTATCATGTTGACATTCC; dG = -24.8
  1880 >s_b_s_a_s_d_s_c_s_d_s_b_s_a_s          GTGGCTGTGTCTGTGACGGTGTGGGTGACGGTGGCTGTGTCTGTG;
>clamp_s_c_s_d_s_c_s_a_s:compl:rev CACAGACACCCACACCGTCACCCACACTATCATGTTGACATTCC; dG = -24.8
  1881 >s_b_s_a_s_e_s_c_s_d_s_b_s_a_s          GTGGCTGTGTCTGTGCTGGTGTGGGTGACGGTGGCTGTGTCTGTG;
>clamp_s_c_s_d_s_c_s_a_s:compl:rev CACAGACACCCACACCGTCACCCACACTATCATGTTGACATTCC; dG = -24.8
  1882 >s_b_s_b_s_a_s_c_s_d_s_b_s_a_s          GTGGCTGTGGCTGTGTCTGTGTGGGTGACGGTGGCTGTGTCTGTG;
>clamp_s_c_s_d_s_c_s_a_s:compl:rev CACAGACACCCACACCGTCACCCACACTATCATGTTGACATTCC; dG = -24.8
  1883 >s_b_s_b_s_b_s_c_s_d_s_b_s_a_s          GTGGCTGTGGCTGTGGCTGTGTGGGTGACGGTGGCTGTGTCTGTG;
>clamp_s_c_s_d_s_c_s_a_s:compl:rev CACAGACACCCACACCGTCACCCACACTATCATGTTGACATTCC; dG = -24.8
  1884 >s_b_s_b_s_c_s_c_s_d_s_b_s_a_s          GTGGCTGTGGCTGTGTGGGTGTGGGTGACGGTGGCTGTGTCTGTG;
>clamp_s_c_s_d_s_c_s_a_s:compl:rev CACAGACACCCACACCGTCACCCACACTATCATGTTGACATTCC; dG = -24.8
  1885 >s_b_s_b_s_d_s_c_s_d_s_b_s_a_s          GTGGCTGTGGCTGTGACGGTGTGGGTGACGGTGGCTGTGTCTGTG;
>clamp_s_c_s_d_s_c_s_a_s:compl:rev CACAGACACCCACACCGTCACCCACACTATCATGTTGACATTCC; dG = -24.8
  1886 >s_a_s_a_s_d_s_c_s_d_s_b_s_a_s          GTGTCTGTGTCTGTGACGGTGTGGGTGACGGTGGCTGTGTCTGTG;
>clamp_s_c_s_d_s_c_s_a_s:compl:rev CACAGACACCCACACCGTCACCCACACTATCATGTTGACATTCC; dG = -24.8
  1887 >s_b_s_b_s_e_s_c_s_d_s_b_s_a_s          GTGGCTGTGGCTGTGCTGGTGTGGGTGACGGTGGCTGTGTCTGTG;
>clamp_s_c_s_d_s_c_s_a_s:compl:rev CACAGACACCCACACCGTCACCCACACTATCATGTTGACATTCC; dG = -24.8
  1888 >s_b_s_c_s_a_s_c_s_d_s_b_s_a_s          GTGGCTGTGTGGGTGTCTGTGTGGGTGACGGTGGCTGTGTCTGTG;
>clamp_s_c_s_d_s_c_s_a_s:compl:rev CACAGACACCCACACCGTCACCCACACTATCATGTTGACATTCC; dG = -24.8
  1889 >s_b_s_c_s_a_s_c_s_a_s_c_s_a_s          GTGGCTGTGTGGGTGTCTGTGTGGGTGTCTGTGTGGGTGTCTGTG;
>clamp_s_c_s_d_s_c_s_a_s:compl:rev CACAGACACCCACACCGTCACCCACACTATCATGTTGACATTCC; dG = -24.8
  1890 >s_b_s_c_s_b_s_c_s_d_s_b_s_a_s          GTGGCTGTGTGGGTGGCTGTGTGGGTGACGGTGGCTGTGTCTGTG;
>clamp_s_c_s_d_s_c_s_a_s:compl:rev CACAGACACCCACACCGTCACCCACACTATCATGTTGACATTCC; dG = -24.8
  1891 >s_b_s_c_s_c_s_c_s_d_s_b_s_a_s          GTGGCTGTGTGGGTGTGGGTGTGGGTGACGGTGGCTGTGTCTGTG;
>clamp_s_c_s_d_s_c_s_a_s:compl:rev CACAGACACCCACACCGTCACCCACACTATCATGTTGACATTCC; dG = -24.8
  1892 >s_b_s_c_s_e_s_c_s_d_s_b_s_a_s          GTGGCTGTGTGGGTGCTGGTGTGGGTGACGGTGGCTGTGTCTGTG;
>clamp_s_c_s_d_s_c_s_a_s:compl:rev CACAGACACCCACACCGTCACCCACACTATCATGTTGACATTCC; dG = -24.8
  1893 >s_b_s_d_s_a_s_c_s_d_s_b_s_a_s          GTGGCTGTGACGGTGTCTGTGTGGGTGACGGTGGCTGTGTCTGTG;
>clamp_s_c_s_d_s_c_s_a_s:compl:rev CACAGACACCCACACCGTCACCCACACTATCATGTTGACATTCC; dG = -24.8
  1894 >s_b_s_d_s_b_s_c_s_d_s_b_s_a_s          GTGGCTGTGACGGTGGCTGTGTGGGTGACGGTGGCTGTGTCTGTG;
>clamp_s_c_s_d_s_c_s_a_s:compl:rev CACAGACACCCACACCGTCACCCACACTATCATGTTGACATTCC; dG = -24.8
  1895 >s_b_s_d_s_c_s_c_s_d_s_b_s_a_s          GTGGCTGTGACGGTGTGGGTGTGGGTGACGGTGGCTGTGTCTGTG;
>clamp_s_c_s_d_s_c_s_a_s:compl:rev CACAGACACCCACACCGTCACCCACACTATCATGTTGACATTCC; dG = -24.8
  1896 >s_b_s_d_s_d_s_c_s_d_s_b_s_a_s          GTGGCTGTGACGGTGACGGTGTGGGTGACGGTGGCTGTGTCTGTG;
>clamp_s_c_s_d_s_c_s_a_s:compl:rev CACAGACACCCACACCGTCACCCACACTATCATGTTGACATTCC; dG = -24.8
  1897 >s_a_s_a_s_a_s_c_s_d_s_b_s_a_s          GTGTCTGTGTCTGTGTCTGTGTGGGTGACGGTGGCTGTGTCTGTG;
>clamp_s_c_s_d_s_c_s_a_s:compl:rev CACAGACACCCACACCGTCACCCACACTATCATGTTGACATTCC; dG = -24.8
  1898 >s_b_s_d_s_e_s_c_s_d_s_b_s_a_s          GTGGCTGTGACGGTGCTGGTGTGGGTGACGGTGGCTGTGTCTGTG;
>clamp_s_c_s_d_s_c_s_a_s:compl:rev CACAGACACCCACACCGTCACCCACACTATCATGTTGACATTCC; dG = -24.8
  1899 >s_a_s_a_s_e_s_c_s_d_s_b_s_a_s          GTGTCTGTGTCTGTGCTGGTGTGGGTGACGGTGGCTGTGTCTGTG;
>clamp_s_c_s_d_s_c_s_a_s:compl:rev CACAGACACCCACACCGTCACCCACACTATCATGTTGACATTCC; dG = -24.8
  1900 >s_b_s_e_s_a_s_c_s_d_s_b_s_a_s          GTGGCTGTGCTGGTGTCTGTGTGGGTGACGGTGGCTGTGTCTGTG;
>clamp_s_c_s_d_s_c_s_a_s:compl:rev CACAGACACCCACACCGTCACCCACACTATCATGTTGACATTCC; dG = -24.8
  1901 >s_b_s_e_s_b_s_c_s_d_s_b_s_a_s          GTGGCTGTGCTGGTGGCTGTGTGGGTGACGGTGGCTGTGTCTGTG;
>clamp_s_c_s_d_s_c_s_a_s:compl:rev CACAGACACCCACACCGTCACCCACACTATCATGTTGACATTCC; dG = -24.8
  1902 >s_b_s_e_s_c_s_c_s_d_s_b_s_a_s          GTGGCTGTGCTGGTGTGGGTGTGGGTGACGGTGGCTGTGTCTGTG;
>clamp_s_c_s_d_s_c_s_a_s:compl:rev CACAGACACCCACACCGTCACCCACACTATCATGTTGACATTCC; dG = -24.8
  1903 >s_b_s_e_s_d_s_c_s_d_s_b_s_a_s          GTGGCTGTGCTGGTGACGGTGTGGGTGACGGTGGCTGTGTCTGTG;
>clamp_s_c_s_d_s_c_s_a_s:compl:rev CACAGACACCCACACCGTCACCCACACTATCATGTTGACATTCC; dG = -24.8
  1904 >s_b_s_e_s_e_s_c_s_d_s_b_s_a_s          GTGGCTGTGCTGGTGCTGGTGTGGGTGACGGTGGCTGTGTCTGTG;
>clamp_s_c_s_d_s_c_s_a_s:compl:rev CACAGACACCCACACCGTCACCCACACTATCATGTTGACATTCC; dG = -24.8
  1905 >s_c_s_a_s_a_s_c_s_d_s_b_s_a_s          GTGTGGGTGTCTGTGTCTGTGTGGGTGACGGTGGCTGTGTCTGTG;
>clamp_s_c_s_d_s_c_s_a_s:compl:rev CACAGACACCCACACCGTCACCCACACTATCATGTTGACATTCC; dG = -24.8
  1906 >s_c_s_a_s_b_s_c_s_d_s_b_s_a_s          GTGTGGGTGTCTGTGGCTGTGTGGGTGACGGTGGCTGTGTCTGTG;
>clamp_s_c_s_d_s_c_s_a_s:compl:rev CACAGACACCCACACCGTCACCCACACTATCATGTTGACATTCC; dG = -24.8
  1907 >s_c_s_a_s_c_s_c_s_d_s_b_s_a_s          GTGTGGGTGTCTGTGTGGGTGTGGGTGACGGTGGCTGTGTCTGTG;
>clamp_s_c_s_d_s_c_s_a_s:compl:rev CACAGACACCCACACCGTCACCCACACTATCATGTTGACATTCC; dG = -24.8
```

```
  1908 >s_c_s_a_s_d_s_c_s_d_s_b_s_a_s          GTGTGGGTGTCTGTGACGGTGTGGGTGACGGTGGCTGTGTCTGTG;
>clamp_s_c_s_d_s_c_s_a_s:compl:rev CACAGACACCCACACCGTCACCCACACTATCATGTTGACATTCC; dG = -24.8
  1909 >s_c_s_a_s_e_s_c_s_d_s_b_s_a_s          GTGTGGGTGTCTGTGCTGGTGTGGGTGACGGTGGCTGTGTCTGTG;
>clamp_s_c_s_d_s_c_s_a_s:compl:rev CACAGACACCCACACCGTCACCCACACTATCATGTTGACATTCC; dG = -24.8
  1910 >s_a_s_b_s_a_s_c_s_d_s_b_s_a_s          GTGTCTGTGGCTGTGTCTGTGTGGGTGACGGTGGCTGTGTCTGTG;
>clamp_s_c_s_d_s_c_s_a_s:compl:rev CACAGACACCCACACCGTCACCCACACTATCATGTTGACATTCC; dG = -24.8
  1911 >s_c_s_b_s_a_s_c_s_d_s_b_s_a_s          GTGTGGGTGGCTGTGTCTGTGTGGGTGACGGTGGCTGTGTCTGTG;
>clamp_s_c_s_d_s_c_s_a_s:compl:rev CACAGACACCCACACCGTCACCCACACTATCATGTTGACATTCC; dG = -24.8
  1912 >s_c_s_b_s_b_s_c_s_d_s_b_s_a_s          GTGTGGGTGGCTGTGGCTGTGTGGGTGACGGTGGCTGTGTCTGTG;
>clamp_s_c_s_d_s_c_s_a_s:compl:rev CACAGACACCCACACCGTCACCCACACTATCATGTTGACATTCC; dG = -24.8
  1913 >s_c_s_b_s_c_s_c_s_d_s_b_s_a_s          GTGTGGGTGGCTGTGTGGGTGTGGGTGACGGTGGCTGTGTCTGTG;
>clamp_s_c_s_d_s_c_s_a_s:compl:rev CACAGACACCCACACCGTCACCCACACTATCATGTTGACATTCC; dG = -24.8
  1914 >s_c_s_b_s_d_s_c_s_d_s_b_s_a_s          GTGTGGGTGGCTGTGACGGTGTGGGTGACGGTGGCTGTGTCTGTG;
>clamp_s_c_s_d_s_c_s_a_s:compl:rev CACAGACACCCACACCGTCACCCACACTATCATGTTGACATTCC; dG = -24.8
  1915 >s_c_s_b_s_e_s_c_s_d_s_b_s_a_s          GTGTGGGTGGCTGTGCTGGTGTGGGTGACGGTGGCTGTGTCTGTG;
>clamp_s_c_s_d_s_c_s_a_s:compl:rev CACAGACACCCACACCGTCACCCACACTATCATGTTGACATTCC; dG = -24.8
  1916 >s_c_s_c_s_a_s_c_s_d_s_b_s_a_s          GTGTGGGTGTGGGTGTCTGTGTGGGTGACGGTGGCTGTGTCTGTG;
>clamp_s_c_s_d_s_c_s_a_s:compl:rev CACAGACACCCACACCGTCACCCACACTATCATGTTGACATTCC; dG = -24.8
  1917 >s_c_s_c_s_b_s_c_s_d_s_b_s_a_s          GTGTGGGTGTGGGTGGCTGTGTGGGTGACGGTGGCTGTGTCTGTG;
>clamp_s_c_s_d_s_c_s_a_s:compl:rev CACAGACACCCACACCGTCACCCACACTATCATGTTGACATTCC; dG = -24.8
  1918 >s_c_s_c_s_c_s_c_s_d_s_b_s_a_s          GTGTGGGTGTGGGTGTGGGTGTGGGTGACGGTGGCTGTGTCTGTG;
>clamp_s_c_s_d_s_c_s_a_s:compl:rev CACAGACACCCACACCGTCACCCACACTATCATGTTGACATTCC; dG = -24.8
  1919 >s_e_s_c_s_a_s_c_s_a_s_c_s_a_s          GTGCTGGTGTGGGTGTCTGTGTGGGTGTCTGTGTGGGTGTCTGTG;
>clamp_s_c_s_d_s_c_s_a_s:compl:rev CACAGACACCCACACCGTCACCCACACTATCATGTTGACATTCC; dG = -24.7
  1920 >s_c_s_c_s_a_s_c_s_a_s_c_s_a_s          GTGTGGGTGTGGGTGTCTGTGTGGGTGTCTGTGTGGGTGTCTGTG;
>clamp_s_c_s_d_s_c_s_a_s:compl:rev CACAGACACCCACACCGTCACCCACACTATCATGTTGACATTCC; dG = -24.7
  1921 >s_a_s_b_s_b_s_c_s_a_s_c_s_a_s          GTGTCTGTGGCTGTGGCTGTGTGGGTGTCTGTGTGGGTGTCTGTG;
>clamp_s_c_s_d_s_c_s_a_s:compl:rev CACAGACACCCACACCGTCACCCACACTATCATGTTGACATTCC; dG = -24.5
  1922 >s_c_s_e_s_a_s_c_s_a_s_c_s_a_s          GTGTGGGTGCTGGTGTCTGTGTGGGTGTCTGTGTGGGTGTCTGTG;
>clamp_s_c_s_d_s_c_s_a_s:compl:rev CACAGACACCCACACCGTCACCCACACTATCATGTTGACATTCC; dG = -24.5
  1923 >s_c_s_e_s_b_s_c_s_a_s_c_s_a_s          GTGTGGGTGCTGGTGGCTGTGTGGGTGTCTGTGTGGGTGTCTGTG;
>clamp_s_c_s_d_s_c_s_a_s:compl:rev CACAGACACCCACACCGTCACCCACACTATCATGTTGACATTCC; dG = -24.5
  1924 >s_c_s_e_s_c_s_c_s_a_s_c_s_a_s          GTGTGGGTGCTGGTGTGGGTGTGGGTGTCTGTGTGGGTGTCTGTG;
>clamp_s_c_s_d_s_c_s_a_s:compl:rev CACAGACACCCACACCGTCACCCACACTATCATGTTGACATTCC; dG = -24.5
  1925 >s_c_s_e_s_e_s_c_s_a_s_c_s_a_s          GTGTGGGTGCTGGTGCTGGTGTGGGTGTCTGTGTGGGTGTCTGTG;
>clamp_s_c_s_d_s_c_s_a_s:compl:rev CACAGACACCCACACCGTCACCCACACTATCATGTTGACATTCC; dG = -24.5
  1926 >s_a_s_a_s_a_s_c_s_a_s_c_s_a_s          GTGTCTGTGTCTGTGTCTGTGTGGGTGTCTGTGTGGGTGTCTGTG;
>clamp_s_c_s_d_s_c_s_a_s:compl:rev CACAGACACCCACACCGTCACCCACACTATCATGTTGACATTCC; dG = -24.5
  1927 >s_d_s_a_s_a_s_c_s_a_s_c_s_a_s          GTGACGGTGTCTGTGTCTGTGTGGGTGTCTGTGTGGGTGTCTGTG;
>clamp_s_c_s_d_s_c_s_a_s:compl:rev CACAGACACCCACACCGTCACCCACACTATCATGTTGACATTCC; dG = -24.5
  1928 >s_d_s_a_s_b_s_c_s_a_s_c_s_a_s          GTGACGGTGTCTGTGGCTGTGTGGGTGTCTGTGTGGGTGTCTGTG;
>clamp_s_c_s_d_s_c_s_a_s:compl:rev CACAGACACCCACACCGTCACCCACACTATCATGTTGACATTCC; dG = -24.5
  1929 >s_a_s_b_s_c_s_c_s_a_s_c_s_a_s          GTGTCTGTGGCTGTGTGGGTGTGGGTGTCTGTGTGGGTGTCTGTG;
>clamp_s_c_s_d_s_c_s_a_s:compl:rev CACAGACACCCACACCGTCACCCACACTATCATGTTGACATTCC; dG = -24.5
  1930 >s_d_s_a_s_c_s_c_s_a_s_c_s_a_s          GTGACGGTGTCTGTGTGGGTGTGGGTGTCTGTGTGGGTGTCTGTG;
>clamp_s_c_s_d_s_c_s_a_s:compl:rev CACAGACACCCACACCGTCACCCACACTATCATGTTGACATTCC; dG = -24.5
  1931 >s_d_s_a_s_e_s_c_s_a_s_c_s_a_s          GTGACGGTGTCTGTGCTGGTGTGGGTGTCTGTGTGGGTGTCTGTG;
>clamp_s_c_s_d_s_c_s_a_s:compl:rev CACAGACACCCACACCGTCACCCACACTATCATGTTGACATTCC; dG = -24.5
  1932 >s_d_s_b_s_a_s_c_s_a_s_c_s_a_s          GTGACGGTGGCTGTGTCTGTGTGGGTGTCTGTGTGGGTGTCTGTG;
>clamp_s_c_s_d_s_c_s_a_s:compl:rev CACAGACACCCACACCGTCACCCACACTATCATGTTGACATTCC; dG = -24.5
  1933 >s_d_s_b_s_b_s_c_s_a_s_c_s_a_s          GTGACGGTGGCTGTGGCTGTGTGGGTGTCTGTGTGGGTGTCTGTG;
>clamp_s_c_s_d_s_c_s_a_s:compl:rev CACAGACACCCACACCGTCACCCACACTATCATGTTGACATTCC; dG = -24.5
  1934 >s_d_s_b_s_c_s_c_s_a_s_c_s_a_s          GTGACGGTGGCTGTGTGGGTGTGGGTGTCTGTGTGGGTGTCTGTG;
>clamp_s_c_s_d_s_c_s_a_s:compl:rev CACAGACACCCACACCGTCACCCACACTATCATGTTGACATTCC; dG = -24.5
  1935 >s_d_s_b_s_e_s_c_s_a_s_c_s_a_s          GTGACGGTGGCTGTGCTGGTGTGGGTGTCTGTGTGGGTGTCTGTG;
>clamp_s_c_s_d_s_c_s_a_s:compl:rev CACAGACACCCACACCGTCACCCACACTATCATGTTGACATTCC; dG = -24.5
  1936 >s_d_s_d_s_a_s_c_s_a_s_c_s_a_s          GTGACGGTGACGGTGTCTGTGTGGGTGTCTGTGTGGGTGTCTGTG;
>clamp_s_c_s_d_s_c_s_a_s:compl:rev CACAGACACCCACACCGTCACCCACACTATCATGTTGACATTCC; dG = -24.5
  1937 >s_d_s_d_s_b_s_c_s_a_s_c_s_a_s          GTGACGGTGACGGTGGCTGTGTGGGTGTCTGTGTGGGTGTCTGTG;
>clamp_s_c_s_d_s_c_s_a_s:compl:rev CACAGACACCCACACCGTCACCCACACTATCATGTTGACATTCC; dG = -24.5
  1938 >s_d_s_d_s_c_s_c_s_a_s_c_s_a_s          GTGACGGTGACGGTGTGGGTGTGGGTGTCTGTGTGGGTGTCTGTG;
>clamp_s_c_s_d_s_c_s_a_s:compl:rev CACAGACACCCACACCGTCACCCACACTATCATGTTGACATTCC; dG = -24.5
  1939 >s_d_s_d_s_e_s_c_s_a_s_c_s_a_s          GTGACGGTGACGGTGCTGGTGTGGGTGTCTGTGTGGGTGTCTGTG;
>clamp_s_c_s_d_s_c_s_a_s:compl:rev CACAGACACCCACACCGTCACCCACACTATCATGTTGACATTCC; dG = -24.5
  1940 >s_d_s_e_s_a_s_c_s_a_s_c_s_a_s          GTGACGGTGCTGGTGTCTGTGTGGGTGTCTGTGTGGGTGTCTGTG;
>clamp_s_c_s_d_s_c_s_a_s:compl:rev CACAGACACCCACACCGTCACCCACACTATCATGTTGACATTCC; dG = -24.5
  1941 >s_d_s_e_s_b_s_c_s_a_s_c_s_a_s          GTGACGGTGCTGGTGGCTGTGTGGGTGTCTGTGTGGGTGTCTGTG;
>clamp_s_c_s_d_s_c_s_a_s:compl:rev CACAGACACCCACACCGTCACCCACACTATCATGTTGACATTCC; dG = -24.5
  1942 >s_d_s_e_s_c_s_c_s_a_s_c_s_a_s          GTGACGGTGCTGGTGTGGGTGTGGGTGTCTGTGTGGGTGTCTGTG;
>clamp_s_c_s_d_s_c_s_a_s:compl:rev CACAGACACCCACACCGTCACCCACACTATCATGTTGACATTCC; dG = -24.5
  1943 >s_a_s_b_s_e_s_c_s_a_s_c_s_a_s          GTGTCTGTGGCTGTGCTGGTGTGGGTGTCTGTGTGGGTGTCTGTG;
>clamp_s_c_s_d_s_c_s_a_s:compl:rev CACAGACACCCACACCGTCACCCACACTATCATGTTGACATTCC; dG = -24.5
  1944 >s_d_s_e_s_e_s_c_s_a_s_c_s_a_s          GTGACGGTGCTGGTGCTGGTGTGGGTGTCTGTGTGGGTGTCTGTG;
>clamp_s_c_s_d_s_c_s_a_s:compl:rev CACAGACACCCACACCGTCACCCACACTATCATGTTGACATTCC; dG = -24.5
```

```
  1945 >s_e_s_a_s_a_s_c_s_a_s_c_s_a_s          GTGCTGGTGTCTGTGTCTGTGTGGGTGTCTGTGTGGGTGTCTGTG;
>clamp_s_c_s_d_s_c_s_a_s:compl:rev CACAGACACCCACACCGTCACCCACACTATCATGTTGACATTCC; dG = -24.5
  1946 >s_e_s_a_s_b_s_c_s_a_s_c_s_a_s          GTGCTGGTGTCTGTGGCTGTGTGGGTGTCTGTGTGGGTGTCTGTG;
>clamp_s_c_s_d_s_c_s_a_s:compl:rev CACAGACACCCACACCGTCACCCACACTATCATGTTGACATTCC; dG = -24.5
  1947 >s_e_s_a_s_c_s_c_s_a_s_c_s_a_s          GTGCTGGTGTCTGTGTGGGTGTGGGTGTCTGTGTGGGTGTCTGTG;
>clamp_s_c_s_d_s_c_s_a_s:compl:rev CACAGACACCCACACCGTCACCCACACTATCATGTTGACATTCC; dG = -24.5
  1948 >s_e_s_a_s_e_s_c_s_a_s_c_s_a_s          GTGCTGGTGTCTGTGCTGGTGTGGGTGTCTGTGTGGGTGTCTGTG;
>clamp_s_c_s_d_s_c_s_a_s:compl:rev CACAGACACCCACACCGTCACCCACACTATCATGTTGACATTCC; dG = -24.5
  1949 >s_e_s_b_s_a_s_c_s_a_s_c_s_a_s          GTGCTGGTGGCTGTGTCTGTGTGGGTGTCTGTGTGGGTGTCTGTG;
>clamp_s_c_s_d_s_c_s_a_s:compl:rev CACAGACACCCACACCGTCACCCACACTATCATGTTGACATTCC; dG = -24.5
  1950 >s_e_s_b_s_b_s_c_s_a_s_c_s_a_s          GTGCTGGTGGCTGTGGCTGTGTGGGTGTCTGTGTGGGTGTCTGTG;
>clamp_s_c_s_d_s_c_s_a_s:compl:rev CACAGACACCCACACCGTCACCCACACTATCATGTTGACATTCC; dG = -24.5
  1951 >s_e_s_b_s_c_s_c_s_a_s_c_s_a_s          GTGCTGGTGGCTGTGTGGGTGTGGGTGTCTGTGTGGGTGTCTGTG;
>clamp_s_c_s_d_s_c_s_a_s:compl:rev CACAGACACCCACACCGTCACCCACACTATCATGTTGACATTCC; dG = -24.5
  1952 >s_e_s_b_s_e_s_c_s_a_s_c_s_a_s          GTGCTGGTGGCTGTGCTGGTGTGGGTGTCTGTGTGGGTGTCTGTG;
>clamp_s_c_s_d_s_c_s_a_s:compl:rev CACAGACACCCACACCGTCACCCACACTATCATGTTGACATTCC; dG = -24.5
  1953 >s_e_s_d_s_a_s_c_s_a_s_c_s_a_s          GTGCTGGTGACGGTGTCTGTGTGGGTGTCTGTGTGGGTGTCTGTG;
>clamp_s_c_s_d_s_c_s_a_s:compl:rev CACAGACACCCACACCGTCACCCACACTATCATGTTGACATTCC; dG = -24.5
  1954 >s_e_s_d_s_b_s_c_s_a_s_c_s_a_s          GTGCTGGTGACGGTGGCTGTGTGGGTGTCTGTGTGGGTGTCTGTG;
>clamp_s_c_s_d_s_c_s_a_s:compl:rev CACAGACACCCACACCGTCACCCACACTATCATGTTGACATTCC; dG = -24.5
  1955 >s_e_s_d_s_c_s_c_s_a_s_c_s_a_s          GTGCTGGTGACGGTGTGGGTGTGGGTGTCTGTGTGGGTGTCTGTG;
>clamp_s_c_s_d_s_c_s_a_s:compl:rev CACAGACACCCACACCGTCACCCACACTATCATGTTGACATTCC; dG = -24.5
  1956 >s_e_s_d_s_e_s_c_s_a_s_c_s_a_s          GTGCTGGTGACGGTGCTGGTGTGGGTGTCTGTGTGGGTGTCTGTG;
>clamp_s_c_s_d_s_c_s_a_s:compl:rev CACAGACACCCACACCGTCACCCACACTATCATGTTGACATTCC; dG = -24.5
  1957 >s_e_s_e_s_a_s_c_s_a_s_c_s_a_s          GTGCTGGTGCTGGTGTCTGTGTGGGTGTCTGTGTGGGTGTCTGTG;
>clamp_s_c_s_d_s_c_s_a_s:compl:rev CACAGACACCCACACCGTCACCCACACTATCATGTTGACATTCC; dG = -24.5
  1958 >s_e_s_e_s_b_s_c_s_a_s_c_s_a_s          GTGCTGGTGCTGGTGGCTGTGTGGGTGTCTGTGTGGGTGTCTGTG;
>clamp_s_c_s_d_s_c_s_a_s:compl:rev CACAGACACCCACACCGTCACCCACACTATCATGTTGACATTCC; dG = -24.5
  1959 >s_e_s_e_s_c_s_c_s_a_s_c_s_a_s          GTGCTGGTGCTGGTGTGGGTGTGGGTGTCTGTGTGGGTGTCTGTG;
>clamp_s_c_s_d_s_c_s_a_s:compl:rev CACAGACACCCACACCGTCACCCACACTATCATGTTGACATTCC; dG = -24.5
  1960 >s_e_s_e_s_e_s_c_s_a_s_c_s_a_s          GTGCTGGTGCTGGTGCTGGTGTGGGTGTCTGTGTGGGTGTCTGTG;
>clamp_s_c_s_d_s_c_s_a_s:compl:rev CACAGACACCCACACCGTCACCCACACTATCATGTTGACATTCC; dG = -24.5
  1961 >s_a_s_d_s_a_s_c_s_a_s_c_s_a_s          GTGTCTGTGACGGTGTCTGTGTGGGTGTCTGTGTGGGTGTCTGTG;
>clamp_s_c_s_d_s_c_s_a_s:compl:rev CACAGACACCCACACCGTCACCCACACTATCATGTTGACATTCC; dG = -24.5
  1962 >s_a_s_d_s_b_s_c_s_a_s_c_s_a_s          GTGTCTGTGACGGTGGCTGTGTGGGTGTCTGTGTGGGTGTCTGTG;
>clamp_s_c_s_d_s_c_s_a_s:compl:rev CACAGACACCCACACCGTCACCCACACTATCATGTTGACATTCC; dG = -24.5
  1963 >s_a_s_a_s_b_s_c_s_a_s_c_s_a_s          GTGTCTGTGTCTGTGGCTGTGTGGGTGTCTGTGTGGGTGTCTGTG;
>clamp_s_c_s_d_s_c_s_a_s:compl:rev CACAGACACCCACACCGTCACCCACACTATCATGTTGACATTCC; dG = -24.5
  1964 >s_a_s_d_s_c_s_c_s_a_s_c_s_a_s          GTGTCTGTGACGGTGTGGGTGTGGGTGTCTGTGTGGGTGTCTGTG;
>clamp_s_c_s_d_s_c_s_a_s:compl:rev CACAGACACCCACACCGTCACCCACACTATCATGTTGACATTCC; dG = -24.5
  1965 >s_a_s_d_s_e_s_c_s_a_s_c_s_a_s          GTGTCTGTGACGGTGCTGGTGTGGGTGTCTGTGTGGGTGTCTGTG;
>clamp_s_c_s_d_s_c_s_a_s:compl:rev CACAGACACCCACACCGTCACCCACACTATCATGTTGACATTCC; dG = -24.5
  1966 >s_a_s_e_s_a_s_c_s_a_s_c_s_a_s          GTGTCTGTGCTGGTGTCTGTGTGGGTGTCTGTGTGGGTGTCTGTG;
>clamp_s_c_s_d_s_c_s_a_s:compl:rev CACAGACACCCACACCGTCACCCACACTATCATGTTGACATTCC; dG = -24.5
  1967 >s_a_s_e_s_b_s_c_s_a_s_c_s_a_s          GTGTCTGTGCTGGTGGCTGTGTGGGTGTCTGTGTGGGTGTCTGTG;
>clamp_s_c_s_d_s_c_s_a_s:compl:rev CACAGACACCCACACCGTCACCCACACTATCATGTTGACATTCC; dG = -24.5
  1968 >s_a_s_e_s_c_s_c_s_a_s_c_s_a_s          GTGTCTGTGCTGGTGTGGGTGTGGGTGTCTGTGTGGGTGTCTGTG;
>clamp_s_c_s_d_s_c_s_a_s:compl:rev CACAGACACCCACACCGTCACCCACACTATCATGTTGACATTCC; dG = -24.5
  1969 >s_a_s_e_s_e_s_c_s_a_s_c_s_a_s          GTGTCTGTGCTGGTGCTGGTGTGGGTGTCTGTGTGGGTGTCTGTG;
>clamp_s_c_s_d_s_c_s_a_s:compl:rev CACAGACACCCACACCGTCACCCACACTATCATGTTGACATTCC; dG = -24.5
  1970 >s_b_s_a_s_a_s_c_s_a_s_c_s_a_s          GTGGCTGTGTCTGTGTCTGTGTGGGTGTCTGTGTGGGTGTCTGTG;
>clamp_s_c_s_d_s_c_s_a_s:compl:rev CACAGACACCCACACCGTCACCCACACTATCATGTTGACATTCC; dG = -24.5
  1971 >s_b_s_a_s_b_s_c_s_a_s_c_s_a_s          GTGGCTGTGTCTGTGGCTGTGTGGGTGTCTGTGTGGGTGTCTGTG;
>clamp_s_c_s_d_s_c_s_a_s:compl:rev CACAGACACCCACACCGTCACCCACACTATCATGTTGACATTCC; dG = -24.5
  1972 >s_a_s_a_s_c_s_c_s_a_s_c_s_a_s          GTGTCTGTGTCTGTGTGGGTGTGGGTGTCTGTGTGGGTGTCTGTG;
>clamp_s_c_s_d_s_c_s_a_s:compl:rev CACAGACACCCACACCGTCACCCACACTATCATGTTGACATTCC; dG = -24.5
  1973 >s_b_s_a_s_c_s_c_s_a_s_c_s_a_s          GTGGCTGTGTCTGTGTGGGTGTGGGTGTCTGTGTGGGTGTCTGTG;
>clamp_s_c_s_d_s_c_s_a_s:compl:rev CACAGACACCCACACCGTCACCCACACTATCATGTTGACATTCC; dG = -24.5
  1974 >s_b_s_a_s_e_s_c_s_a_s_c_s_a_s          GTGGCTGTGTCTGTGCTGGTGTGGGTGTCTGTGTGGGTGTCTGTG;
>clamp_s_c_s_d_s_c_s_a_s:compl:rev CACAGACACCCACACCGTCACCCACACTATCATGTTGACATTCC; dG = -24.5
  1975 >s_b_s_b_s_a_s_c_s_a_s_c_s_a_s          GTGGCTGTGGCTGTGTCTGTGTGGGTGTCTGTGTGGGTGTCTGTG;
>clamp_s_c_s_d_s_c_s_a_s:compl:rev CACAGACACCCACACCGTCACCCACACTATCATGTTGACATTCC; dG = -24.5
  1976 >s_b_s_b_s_b_s_c_s_a_s_c_s_a_s          GTGGCTGTGGCTGTGGCTGTGTGGGTGTCTGTGTGGGTGTCTGTG;
>clamp_s_c_s_d_s_c_s_a_s:compl:rev CACAGACACCCACACCGTCACCCACACTATCATGTTGACATTCC; dG = -24.5
  1977 >s_b_s_b_s_c_s_c_s_a_s_c_s_a_s          GTGGCTGTGGCTGTGTGGGTGTGGGTGTCTGTGTGGGTGTCTGTG;
>clamp_s_c_s_d_s_c_s_a_s:compl:rev CACAGACACCCACACCGTCACCCACACTATCATGTTGACATTCC; dG = -24.5
  1978 >s_b_s_b_s_e_s_c_s_a_s_c_s_a_s          GTGGCTGTGGCTGTGCTGGTGTGGGTGTCTGTGTGGGTGTCTGTG;
>clamp_s_c_s_d_s_c_s_a_s:compl:rev CACAGACACCCACACCGTCACCCACACTATCATGTTGACATTCC; dG = -24.5
  1979 >s_b_s_d_s_a_s_c_s_a_s_c_s_a_s          GTGGCTGTGACGGTGTCTGTGTGGGTGTCTGTGTGGGTGTCTGTG;
>clamp_s_c_s_d_s_c_s_a_s:compl:rev CACAGACACCCACACCGTCACCCACACTATCATGTTGACATTCC; dG = -24.5
  1980 >s_b_s_d_s_b_s_c_s_a_s_c_s_a_s          GTGGCTGTGACGGTGGCTGTGTGGGTGTCTGTGTGGGTGTCTGTG;
>clamp_s_c_s_d_s_c_s_a_s:compl:rev CACAGACACCCACACCGTCACCCACACTATCATGTTGACATTCC; dG = -24.5
  1981 >s_b_s_d_s_c_s_c_s_a_s_c_s_a_s          GTGGCTGTGACGGTGTGGGTGTGGGTGTCTGTGTGGGTGTCTGTG;
>clamp_s_c_s_d_s_c_s_a_s:compl:rev CACAGACACCCACACCGTCACCCACACTATCATGTTGACATTCC; dG = -24.5
```

1982 >s_b_s_d_s_e_s_c_s_a_s_c_s_a_s GTGGCTGTGACGGTGCTGGTGTGGGTGTCTGTGTGGGTGTCTGTG;
>clamp_s_c_s_d_s_c_s_a_s:compl:rev CACAGACACCCACACCGTCACCCACACTATCATGTTGACATTCC; dG = -24.5
1983 >s_b_s_e_s_a_s_c_s_a_s_c_s_a_s GTGGCTGTGCTGGTGTCTGTGTGGGTGTCTGTGTGGGTGTCTGTG;
>clamp_s_c_s_d_s_c_s_a_s:compl:rev CACAGACACCCACACCGTCACCCACACTATCATGTTGACATTCC; dG = -24.5
1984 >s_b_s_e_s_b_s_c_s_a_s_c_s_a_s GTGGCTGTGCTGGTGGCTGTGTGGGTGTCTGTGTGGGTGTCTGTG;
>clamp_s_c_s_d_s_c_s_a_s:compl:rev CACAGACACCCACACCGTCACCCACACTATCATGTTGACATTCC; dG = -24.5
1985 >s_a_s_a_s_e_s_c_s_a_s_c_s_a_s GTGTCTGTGTCTGTGCTGGTGTGGGTGTCTGTGTGGGTGTCTGTG;
>clamp_s_c_s_d_s_c_s_a_s:compl:rev CACAGACACCCACACCGTCACCCACACTATCATGTTGACATTCC; dG = -24.5
1986 >s_b_s_e_s_c_s_c_s_a_s_c_s_a_s GTGGCTGTGCTGGTGTGGGTGTGGGTGTCTGTGTGGGTGTCTGTG;
>clamp_s_c_s_d_s_c_s_a_s:compl:rev CACAGACACCCACACCGTCACCCACACTATCATGTTGACATTCC; dG = -24.5
1987 >s_b_s_e_s_e_s_c_s_a_s_c_s_a_s GTGGCTGTGCTGGTGCTGGTGTGGGTGTCTGTGTGGGTGTCTGTG;
>clamp_s_c_s_d_s_c_s_a_s:compl:rev CACAGACACCCACACCGTCACCCACACTATCATGTTGACATTCC; dG = -24.5
1988 >s_c_s_a_s_a_s_c_s_a_s_c_s_a_s GTGTGGGTGTCTGTGTCTGTGTGGGTGTCTGTGTGGGTGTCTGTG;
>clamp_s_c_s_d_s_c_s_a_s:compl:rev CACAGACACCCACACCGTCACCCACACTATCATGTTGACATTCC; dG = -24.5
1989 >s_c_s_a_s_b_s_c_s_a_s_c_s_a_s GTGTGGGTGTCTGTGGCTGTGTGGGTGTCTGTGTGGGTGTCTGTG;
>clamp_s_c_s_d_s_c_s_a_s:compl:rev CACAGACACCCACACCGTCACCCACACTATCATGTTGACATTCC; dG = -24.5
1990 >s_c_s_a_s_c_s_c_s_a_s_c_s_a_s GTGTGGGTGTCTGTGTGGGTGTGGGTGTCTGTGTGGGTGTCTGTG;
>clamp_s_c_s_d_s_c_s_a_s:compl:rev CACAGACACCCACACCGTCACCCACACTATCATGTTGACATTCC; dG = -24.5
1991 >s_c_s_a_s_e_s_c_s_a_s_c_s_a_s GTGTGGGTGTCTGTGCTGGTGTGGGTGTCTGTGTGGGTGTCTGTG;
>clamp_s_c_s_d_s_c_s_a_s:compl:rev CACAGACACCCACACCGTCACCCACACTATCATGTTGACATTCC; dG = -24.5
1992 >s_c_s_b_s_a_s_c_s_a_s_c_s_a_s GTGTGGGTGGCTGTGTCTGTGTGGGTGTCTGTGTGGGTGTCTGTG;
>clamp_s_c_s_d_s_c_s_a_s:compl:rev CACAGACACCCACACCGTCACCCACACTATCATGTTGACATTCC; dG = -24.5
1993 >s_c_s_b_s_b_s_c_s_a_s_c_s_a_s GTGTGGGTGGCTGTGGCTGTGTGGGTGTCTGTGTGGGTGTCTGTG;
>clamp_s_c_s_d_s_c_s_a_s:compl:rev CACAGACACCCACACCGTCACCCACACTATCATGTTGACATTCC; dG = -24.5
1994 >s_a_s_b_s_a_s_c_s_a_s_c_s_a_s GTGTCTGTGGCTGTGTCTGTGTGGGTGTCTGTGTGGGTGTCTGTG;
>clamp_s_c_s_d_s_c_s_a_s:compl:rev CACAGACACCCACACCGTCACCCACACTATCATGTTGACATTCC; dG = -24.5

****

CE Matches

1 >s_c_s_e_s_d_s_c_s_e_s GTGTGGGTGCTGGTGACGGTGTGGGTGCTGGTG; >clamp_s_c_s_e_s:compl:rev
CACCAGCACCCACACTATCATGTTGACATTCC; dG = -18.8
2 >s_e_s_c_s_e_s_c_s_e_s GTGCTGGTGTGGGTGCTGGTGTGGGTGCTGGTG; >clamp_s_c_s_e_s:compl:rev
CACCAGCACCCACACTATCATGTTGACATTCC; dG = -18.7
3 >s_a_s_c_s_e_s_c_s_e_s GTGTCTGTGTGGGTGCTGGTGTGGGTGCTGGTG; >clamp_s_c_s_e_s:compl:rev
CACCAGCACCCACACTATCATGTTGACATTCC; dG = -18.7
4 >s_b_s_c_s_e_s_c_s_e_s GTGGCTGTGTGGGTGCTGGTGTGGGTGCTGGTG; >clamp_s_c_s_e_s:compl:rev
CACCAGCACCCACACTATCATGTTGACATTCC; dG = -18.7
5 >s_c_s_c_s_e_s_c_s_e_s GTGTGGGTGTGGGTGCTGGTGTGGGTGCTGGTG; >clamp_s_c_s_e_s:compl:rev
CACCAGCACCCACACTATCATGTTGACATTCC; dG = -18.7
6 >s_d_s_c_s_e_s_c_s_e_s GTGACGGTGTGGGTGCTGGTGTGGGTGCTGGTG; >clamp_s_c_s_e_s:compl:rev
CACCAGCACCCACACTATCATGTTGACATTCC; dG = -18.7
7 >s_a_s_a_s_a_s_c_s_e_s GTGTCTGTGTCTGTGTCTGTGTGGGTGCTGGTG; >clamp_s_c_s_e_s:compl:rev
CACCAGCACCCACACTATCATGTTGACATTCC; dG = -18.4
8 >s_a_s_b_s_e_s_c_s_e_s GTGTCTGTGGCTGTGCTGGTGTGGGTGCTGGTG; >clamp_s_c_s_e_s:compl:rev
CACCAGCACCCACACTATCATGTTGACATTCC; dG = -18.4
9 >s_d_s_e_s_e_s_c_s_e_s GTGACGGTGCTGGTGCTGGTGTGGGTGCTGGTG; >clamp_s_c_s_e_s:compl:rev
CACCAGCACCCACACTATCATGTTGACATTCC; dG = -18.4
10 >s_e_s_a_s_a_s_c_s_e_s GTGCTGGTGTCTGTGTCTGTGTGGGTGCTGGTG; >clamp_s_c_s_e_s:compl:rev
CACCAGCACCCACACTATCATGTTGACATTCC; dG = -18.4
11 >s_e_s_a_s_b_s_c_s_e_s GTGCTGGTGTCTGTGGCTGTGTGGGTGCTGGTG; >clamp_s_c_s_e_s:compl:rev
CACCAGCACCCACACTATCATGTTGACATTCC; dG = -18.4
12 >s_e_s_a_s_c_s_c_s_e_s GTGCTGGTGTCTGTGTGGGTGTGGGTGCTGGTG; >clamp_s_c_s_e_s:compl:rev
CACCAGCACCCACACTATCATGTTGACATTCC; dG = -18.4
13 >s_e_s_a_s_d_s_c_s_e_s GTGCTGGTGTCTGTGACGGTGTGGGTGCTGGTG; >clamp_s_c_s_e_s:compl:rev
CACCAGCACCCACACTATCATGTTGACATTCC; dG = -18.4
14 >s_e_s_a_s_e_s_c_s_e_s GTGCTGGTGTCTGTGCTGGTGTGGGTGCTGGTG; >clamp_s_c_s_e_s:compl:rev
CACCAGCACCCACACTATCATGTTGACATTCC; dG = -18.4
15 >s_e_s_b_s_a_s_c_s_e_s GTGCTGGTGGCTGTGTCTGTGTGGGTGCTGGTG; >clamp_s_c_s_e_s:compl:rev
CACCAGCACCCACACTATCATGTTGACATTCC; dG = -18.4
16 >s_e_s_b_s_b_s_c_s_e_s GTGCTGGTGGCTGTGGCTGTGTGGGTGCTGGTG; >clamp_s_c_s_e_s:compl:rev
CACCAGCACCCACACTATCATGTTGACATTCC; dG = -18.4
17 >s_e_s_b_s_c_s_c_s_e_s GTGCTGGTGGCTGTGTGGGTGTGGGTGCTGGTG; >clamp_s_c_s_e_s:compl:rev
CACCAGCACCCACACTATCATGTTGACATTCC; dG = -18.4
18 >s_e_s_b_s_d_s_c_s_e_s GTGCTGGTGGCTGTGACGGTGTGGGTGCTGGTG; >clamp_s_c_s_e_s:compl:rev
CACCAGCACCCACACTATCATGTTGACATTCC; dG = -18.4
19 >s_a_s_c_s_a_s_c_s_e_s GTGTCTGTGTGGGTGTCTGTGTGGGTGCTGGTG; >clamp_s_c_s_e_s:compl:rev
CACCAGCACCCACACTATCATGTTGACATTCC; dG = -18.4
20 >s_e_s_b_s_e_s_c_s_e_s GTGCTGGTGGCTGTGCTGGTGTGGGTGCTGGTG; >clamp_s_c_s_e_s:compl:rev
CACCAGCACCCACACTATCATGTTGACATTCC; dG = -18.4

21 >s_e_s_c_s_a_s_c_s_e_s GTGCTGGTGTGGGTGTCTGTGTGGGTGCTGGTG; >clamp_s_c_s_e_s:compl:rev
CACCAGCACCCACACTATCATGTTGACATTCC; dG = -18.4
22 >s_e_s_c_s_b_s_c_s_e_s GTGCTGGTGTGGGTGGCTGTGTGGGTGCTGGTG; >clamp_s_c_s_e_s:compl:rev
CACCAGCACCCACACTATCATGTTGACATTCC; dG = -18.4
23 >s_e_s_c_s_c_s_c_s_e_s GTGCTGGTGTGGGTGTGGGTGTGGGTGCTGGTG; >clamp_s_c_s_e_s:compl:rev
CACCAGCACCCACACTATCATGTTGACATTCC; dG = -18.4
24 >s_e_s_c_s_d_s_c_s_e_s GTGCTGGTGTGGGTGACGGTGTGGGTGCTGGTG; >clamp_s_c_s_e_s:compl:rev
CACCAGCACCCACACTATCATGTTGACATTCC; dG = -18.4
25 >s_e_s_d_s_a_s_c_s_e_s GTGCTGGTGACGGTGTCTGTGTGGGTGCTGGTG; >clamp_s_c_s_e_s:compl:rev
CACCAGCACCCACACTATCATGTTGACATTCC; dG = -18.4
26 >s_e_s_d_s_b_s_c_s_e_s GTGCTGGTGACGGTGGCTGTGTGGGTGCTGGTG; >clamp_s_c_s_e_s:compl:rev
CACCAGCACCCACACTATCATGTTGACATTCC; dG = -18.4
27 >s_e_s_d_s_c_s_c_s_e_s GTGCTGGTGACGGTGTGGGTGTGGGTGCTGGTG; >clamp_s_c_s_e_s:compl:rev
CACCAGCACCCACACTATCATGTTGACATTCC; dG = -18.4
28 >s_e_s_d_s_d_s_c_s_e_s GTGCTGGTGACGGTGACGGTGTGGGTGCTGGTG; >clamp_s_c_s_e_s:compl:rev
CACCAGCACCCACACTATCATGTTGACATTCC; dG = -18.4
29 >s_a_s_c_s_b_s_c_s_e_s GTGTCTGTGTGGGTGGCTGTGTGGGTGCTGGTG; >clamp_s_c_s_e_s:compl:rev
CACCAGCACCCACACTATCATGTTGACATTCC; dG = -18.4
30 >s_e_s_d_s_e_s_c_s_e_s GTGCTGGTGACGGTGCTGGTGTGGGTGCTGGTG; >clamp_s_c_s_e_s:compl:rev
CACCAGCACCCACACTATCATGTTGACATTCC; dG = -18.4
31 >s_e_s_e_s_a_s_c_s_e_s GTGCTGGTGCTGGTGTCTGTGTGGGTGCTGGTG; >clamp_s_c_s_e_s:compl:rev
CACCAGCACCCACACTATCATGTTGACATTCC; dG = -18.4
32 >s_e_s_e_s_b_s_c_s_e_s GTGCTGGTGCTGGTGGCTGTGTGGGTGCTGGTG; >clamp_s_c_s_e_s:compl:rev
CACCAGCACCCACACTATCATGTTGACATTCC; dG = -18.4
33 >s_e_s_e_s_c_s_c_s_e_s GTGCTGGTGCTGGTGTGGGTGTGGGTGCTGGTG; >clamp_s_c_s_e_s:compl:rev
CACCAGCACCCACACTATCATGTTGACATTCC; dG = -18.4
34 >s_e_s_e_s_d_s_c_s_e_s GTGCTGGTGCTGGTGACGGTGTGGGTGCTGGTG; >clamp_s_c_s_e_s:compl:rev
CACCAGCACCCACACTATCATGTTGACATTCC; dG = -18.4
35 >s_e_s_e_s_e_s_c_s_e_s GTGCTGGTGCTGGTGCTGGTGTGGGTGCTGGTG; >clamp_s_c_s_e_s:compl:rev
CACCAGCACCCACACTATCATGTTGACATTCC; dG = -18.4
36 >s_a_s_c_s_c_s_c_s_e_s GTGTCTGTGTGGGTGTGGGTGTGGGTGCTGGTG; >clamp_s_c_s_e_s:compl:rev
CACCAGCACCCACACTATCATGTTGACATTCC; dG = -18.4
37 >s_a_s_c_s_d_s_c_s_e_s GTGTCTGTGTGGGTGACGGTGTGGGTGCTGGTG; >clamp_s_c_s_e_s:compl:rev
CACCAGCACCCACACTATCATGTTGACATTCC; dG = -18.4
38 >s_a_s_d_s_a_s_c_s_e_s GTGTCTGTGACGGTGTCTGTGTGGGTGCTGGTG; >clamp_s_c_s_e_s:compl:rev
CACCAGCACCCACACTATCATGTTGACATTCC; dG = -18.4
39 >s_a_s_d_s_b_s_c_s_e_s GTGTCTGTGACGGTGGCTGTGTGGGTGCTGGTG; >clamp_s_c_s_e_s:compl:rev
CACCAGCACCCACACTATCATGTTGACATTCC; dG = -18.4
40 >s_a_s_d_s_c_s_c_s_e_s GTGTCTGTGACGGTGTGGGTGTGGGTGCTGGTG; >clamp_s_c_s_e_s:compl:rev
CACCAGCACCCACACTATCATGTTGACATTCC; dG = -18.4
41 >s_a_s_d_s_d_s_c_s_e_s GTGTCTGTGACGGTGACGGTGTGGGTGCTGGTG; >clamp_s_c_s_e_s:compl:rev
CACCAGCACCCACACTATCATGTTGACATTCC; dG = -18.4
42 >s_a_s_a_s_b_s_c_s_e_s GTGTCTGTGTCTGTGGCTGTGTGGGTGCTGGTG; >clamp_s_c_s_e_s:compl:rev
CACCAGCACCCACACTATCATGTTGACATTCC; dG = -18.4
43 >s_a_s_d_s_e_s_c_s_e_s GTGTCTGTGACGGTGCTGGTGTGGGTGCTGGTG; >clamp_s_c_s_e_s:compl:rev
CACCAGCACCCACACTATCATGTTGACATTCC; dG = -18.4
44 >s_a_s_e_s_a_s_c_s_e_s GTGTCTGTGCTGGTGTCTGTGTGGGTGCTGGTG; >clamp_s_c_s_e_s:compl:rev
CACCAGCACCCACACTATCATGTTGACATTCC; dG = -18.4
45 >s_a_s_e_s_b_s_c_s_e_s GTGTCTGTGCTGGTGGCTGTGTGGGTGCTGGTG; >clamp_s_c_s_e_s:compl:rev
CACCAGCACCCACACTATCATGTTGACATTCC; dG = -18.4
46 >s_a_s_e_s_c_s_c_s_e_s GTGTCTGTGCTGGTGTGGGTGTGGGTGCTGGTG; >clamp_s_c_s_e_s:compl:rev
CACCAGCACCCACACTATCATGTTGACATTCC; dG = -18.4
47 >s_a_s_e_s_d_s_c_s_e_s GTGTCTGTGCTGGTGACGGTGTGGGTGCTGGTG; >clamp_s_c_s_e_s:compl:rev
CACCAGCACCCACACTATCATGTTGACATTCC; dG = -18.4
48 >s_a_s_e_s_e_s_c_s_e_s GTGTCTGTGCTGGTGCTGGTGTGGGTGCTGGTG; >clamp_s_c_s_e_s:compl:rev
CACCAGCACCCACACTATCATGTTGACATTCC; dG = -18.4
49 >s_b_s_a_s_a_s_c_s_e_s GTGGCTGTGTCTGTGTCTGTGTGGGTGCTGGTG; >clamp_s_c_s_e_s:compl:rev
CACCAGCACCCACACTATCATGTTGACATTCC; dG = -18.4
50 >s_b_s_a_s_b_s_c_s_e_s GTGGCTGTGTCTGTGGCTGTGTGGGTGCTGGTG; >clamp_s_c_s_e_s:compl:rev
CACCAGCACCCACACTATCATGTTGACATTCC; dG = -18.4
51 >s_b_s_a_s_c_s_c_s_e_s GTGGCTGTGTCTGTGTGGGTGTGGGTGCTGGTG; >clamp_s_c_s_e_s:compl:rev
CACCAGCACCCACACTATCATGTTGACATTCC; dG = -18.4
52 >s_b_s_a_s_d_s_c_s_e_s GTGGCTGTGTCTGTGACGGTGTGGGTGCTGGTG; >clamp_s_c_s_e_s:compl:rev
CACCAGCACCCACACTATCATGTTGACATTCC; dG = -18.4
53 >s_a_s_a_s_c_s_c_s_e_s GTGTCTGTGTCTGTGTGGGTGTGGGTGCTGGTG; >clamp_s_c_s_e_s:compl:rev
CACCAGCACCCACACTATCATGTTGACATTCC; dG = -18.4
54 >s_b_s_a_s_e_s_c_s_e_s GTGGCTGTGTCTGTGCTGGTGTGGGTGCTGGTG; >clamp_s_c_s_e_s:compl:rev
CACCAGCACCCACACTATCATGTTGACATTCC; dG = -18.4
55 >s_b_s_b_s_a_s_c_s_e_s GTGGCTGTGGCTGTGTCTGTGTGGGTGCTGGTG; >clamp_s_c_s_e_s:compl:rev
CACCAGCACCCACACTATCATGTTGACATTCC; dG = -18.4
56 >s_b_s_b_s_b_s_c_s_e_s GTGGCTGTGGCTGTGGCTGTGTGGGTGCTGGTG; >clamp_s_c_s_e_s:compl:rev
CACCAGCACCCACACTATCATGTTGACATTCC; dG = -18.4
57 >s_b_s_b_s_c_s_c_s_e_s GTGGCTGTGGCTGTGTGGGTGTGGGTGCTGGTG; >clamp_s_c_s_e_s:compl:rev
CACCAGCACCCACACTATCATGTTGACATTCC; dG = -18.4

58 >s_b_s_b_s_d_s_c_s_e_s GTGGCTGTGGCTGTGACGGTGTGGGTGCTGGTG; >clamp_s_c_s_e_s:compl:rev CACCAGCACCCACACTATCATGTTGACATTCC; dG = -18.4
59 >s_b_s_b_s_e_s_c_s_e_s GTGGCTGTGGCTGTGCTGGTGTGGGTGCTGGTG; >clamp_s_c_s_e_s:compl:rev CACCAGCACCCACACTATCATGTTGACATTCC; dG = -18.4
60 >s_b_s_c_s_a_s_c_s_e_s GTGGCTGTGTGGGTGTCTGTGTGGGTGCTGGTG; >clamp_s_c_s_e_s:compl:rev CACCAGCACCCACACTATCATGTTGACATTCC; dG = -18.4
61 >s_b_s_c_s_b_s_c_s_e_s GTGGCTGTGTGGGTGGCTGTGTGGGTGCTGGTG; >clamp_s_c_s_e_s:compl:rev CACCAGCACCCACACTATCATGTTGACATTCC; dG = -18.4
62 >s_b_s_c_s_c_s_c_s_e_s GTGGCTGTGTGGGTGTGGGTGTGGGTGCTGGTG; >clamp_s_c_s_e_s:compl:rev CACCAGCACCCACACTATCATGTTGACATTCC; dG = -18.4
63 >s_b_s_c_s_d_s_c_s_e_s GTGGCTGTGTGGGTGACGGTGTGGGTGCTGGTG; >clamp_s_c_s_e_s:compl:rev CACCAGCACCCACACTATCATGTTGACATTCC; dG = -18.4
64 >s_a_s_a_s_d_s_c_s_e_s GTGTCTGTGTCTGTGACGGTGTGGGTGCTGGTG; >clamp_s_c_s_e_s:compl:rev CACCAGCACCCACACTATCATGTTGACATTCC; dG = -18.4
65 >s_b_s_d_s_a_s_c_s_e_s GTGGCTGTGACGGTGTCTGTGTGGGTGCTGGTG; >clamp_s_c_s_e_s:compl:rev CACCAGCACCCACACTATCATGTTGACATTCC; dG = -18.4
66 >s_b_s_d_s_b_s_c_s_e_s GTGGCTGTGACGGTGGCTGTGTGGGTGCTGGTG; >clamp_s_c_s_e_s:compl:rev CACCAGCACCCACACTATCATGTTGACATTCC; dG = -18.4
67 >s_b_s_d_s_c_s_c_s_e_s GTGGCTGTGACGGTGTGGGTGTGGGTGCTGGTG; >clamp_s_c_s_e_s:compl:rev CACCAGCACCCACACTATCATGTTGACATTCC; dG = -18.4
68 >s_b_s_d_s_d_s_c_s_e_s GTGGCTGTGACGGTGACGGTGTGGGTGCTGGTG; >clamp_s_c_s_e_s:compl:rev CACCAGCACCCACACTATCATGTTGACATTCC; dG = -18.4
69 >s_b_s_d_s_e_s_c_s_e_s GTGGCTGTGACGGTGCTGGTGTGGGTGCTGGTG; >clamp_s_c_s_e_s:compl:rev CACCAGCACCCACACTATCATGTTGACATTCC; dG = -18.4
70 >s_b_s_e_s_a_s_c_s_e_s GTGGCTGTGCTGGTGTCTGTGTGGGTGCTGGTG; >clamp_s_c_s_e_s:compl:rev CACCAGCACCCACACTATCATGTTGACATTCC; dG = -18.4
71 >s_b_s_e_s_b_s_c_s_e_s GTGGCTGTGCTGGTGGCTGTGTGGGTGCTGGTG; >clamp_s_c_s_e_s:compl:rev CACCAGCACCCACACTATCATGTTGACATTCC; dG = -18.4
72 >s_b_s_e_s_c_s_c_s_e_s GTGGCTGTGCTGGTGTGGGTGTGGGTGCTGGTG; >clamp_s_c_s_e_s:compl:rev CACCAGCACCCACACTATCATGTTGACATTCC; dG = -18.4
73 >s_b_s_e_s_d_s_c_s_e_s GTGGCTGTGCTGGTGACGGTGTGGGTGCTGGTG; >clamp_s_c_s_e_s:compl:rev CACCAGCACCCACACTATCATGTTGACATTCC; dG = -18.4
74 >s_a_s_a_s_e_s_c_s_e_s GTGTCTGTGTCTGTGCTGGTGTGGGTGCTGGTG; >clamp_s_c_s_e_s:compl:rev CACCAGCACCCACACTATCATGTTGACATTCC; dG = -18.4
75 >s_b_s_e_s_e_s_c_s_e_s GTGGCTGTGCTGGTGCTGGTGTGGGTGCTGGTG; >clamp_s_c_s_e_s:compl:rev CACCAGCACCCACACTATCATGTTGACATTCC; dG = -18.4
76 >s_c_s_a_s_a_s_c_s_e_s GTGTGGGTGTCTGTGTCTGTGTGGGTGCTGGTG; >clamp_s_c_s_e_s:compl:rev CACCAGCACCCACACTATCATGTTGACATTCC; dG = -18.4
77 >s_c_s_a_s_b_s_c_s_e_s GTGTGGGTGTCTGTGGCTGTGTGGGTGCTGGTG; >clamp_s_c_s_e_s:compl:rev CACCAGCACCCACACTATCATGTTGACATTCC; dG = -18.4
78 >s_c_s_a_s_c_s_c_s_e_s GTGTGGGTGTCTGTGTGGGTGTGGGTGCTGGTG; >clamp_s_c_s_e_s:compl:rev CACCAGCACCCACACTATCATGTTGACATTCC; dG = -18.4
79 >s_c_s_a_s_d_s_c_s_e_s GTGTGGGTGTCTGTGACGGTGTGGGTGCTGGTG; >clamp_s_c_s_e_s:compl:rev CACCAGCACCCACACTATCATGTTGACATTCC; dG = -18.4
80 >s_c_s_a_s_e_s_c_s_e_s GTGTGGGTGTCTGTGCTGGTGTGGGTGCTGGTG; >clamp_s_c_s_e_s:compl:rev CACCAGCACCCACACTATCATGTTGACATTCC; dG = -18.4
81 >s_c_s_b_s_a_s_c_s_e_s GTGTGGGTGGCTGTGTCTGTGTGGGTGCTGGTG; >clamp_s_c_s_e_s:compl:rev CACCAGCACCCACACTATCATGTTGACATTCC; dG = -18.4
82 >s_c_s_b_s_b_s_c_s_e_s GTGTGGGTGGCTGTGGCTGTGTGGGTGCTGGTG; >clamp_s_c_s_e_s:compl:rev CACCAGCACCCACACTATCATGTTGACATTCC; dG = -18.4
83 >s_c_s_b_s_c_s_c_s_e_s GTGTGGGTGGCTGTGTGGGTGTGGGTGCTGGTG; >clamp_s_c_s_e_s:compl:rev CACCAGCACCCACACTATCATGTTGACATTCC; dG = -18.4
84 >s_c_s_b_s_d_s_c_s_e_s GTGTGGGTGGCTGTGACGGTGTGGGTGCTGGTG; >clamp_s_c_s_e_s:compl:rev CACCAGCACCCACACTATCATGTTGACATTCC; dG = -18.4
85 >s_a_s_b_s_a_s_c_s_e_s GTGTCTGTGGCTGTGTCTGTGTGGGTGCTGGTG; >clamp_s_c_s_e_s:compl:rev CACCAGCACCCACACTATCATGTTGACATTCC; dG = -18.4
86 >s_c_s_b_s_e_s_c_s_e_s GTGTGGGTGGCTGTGCTGGTGTGGGTGCTGGTG; >clamp_s_c_s_e_s:compl:rev CACCAGCACCCACACTATCATGTTGACATTCC; dG = -18.4
87 >s_c_s_c_s_a_s_c_s_e_s GTGTGGGTGTGGGTGTCTGTGTGGGTGCTGGTG; >clamp_s_c_s_e_s:compl:rev CACCAGCACCCACACTATCATGTTGACATTCC; dG = -18.4
88 >s_c_s_c_s_b_s_c_s_e_s GTGTGGGTGTGGGTGGCTGTGTGGGTGCTGGTG; >clamp_s_c_s_e_s:compl:rev CACCAGCACCCACACTATCATGTTGACATTCC; dG = -18.4
89 >s_c_s_c_s_c_s_c_s_e_s GTGTGGGTGTGGGTGTGGGTGTGGGTGCTGGTG; >clamp_s_c_s_e_s:compl:rev CACCAGCACCCACACTATCATGTTGACATTCC; dG = -18.4
90 >s_c_s_c_s_d_s_c_s_e_s GTGTGGGTGTGGGTGACGGTGTGGGTGCTGGTG; >clamp_s_c_s_e_s:compl:rev CACCAGCACCCACACTATCATGTTGACATTCC; dG = -18.4
91 >s_c_s_d_s_a_s_c_s_e_s GTGTGGGTGACGGTGTCTGTGTGGGTGCTGGTG; >clamp_s_c_s_e_s:compl:rev CACCAGCACCCACACTATCATGTTGACATTCC; dG = -18.4
92 >s_c_s_d_s_b_s_c_s_e_s GTGTGGGTGACGGTGGCTGTGTGGGTGCTGGTG; >clamp_s_c_s_e_s:compl:rev CACCAGCACCCACACTATCATGTTGACATTCC; dG = -18.4
93 >s_c_s_d_s_c_s_c_s_e_s GTGTGGGTGACGGTGTGGGTGTGGGTGCTGGTG; >clamp_s_c_s_e_s:compl:rev CACCAGCACCCACACTATCATGTTGACATTCC; dG = -18.4
94 >s_c_s_d_s_d_s_c_s_e_s GTGTGGGTGACGGTGACGGTGTGGGTGCTGGTG; >clamp_s_c_s_e_s:compl:rev CACCAGCACCCACACTATCATGTTGACATTCC; dG = -18.4

95 >s_a_s_b_s_b_s_c_s_e_s GTGTCTGTGGCTGTGGCTGTGTGGGTGCTGGTG; >clamp_s_c_s_e_s:compl:rev CACCAGCACCCACACTATCATGTTGACATTCC; dG = -18.4
96 >s_c_s_d_s_e_s_c_s_e_s GTGTGGGTGACGGTGCTGGTGTGGGTGCTGGTG; >clamp_s_c_s_e_s:compl:rev CACCAGCACCCACACTATCATGTTGACATTCC; dG = -18.4
97 >s_c_s_e_s_a_s_c_s_e_s GTGTGGGTGCTGGTGTCTGTGTGGGTGCTGGTG; >clamp_s_c_s_e_s:compl:rev CACCAGCACCCACACTATCATGTTGACATTCC; dG = -18.4
98 >s_c_s_e_s_b_s_c_s_e_s GTGTGGGTGCTGGTGGCTGTGTGGGTGCTGGTG; >clamp_s_c_s_e_s:compl:rev CACCAGCACCCACACTATCATGTTGACATTCC; dG = -18.4
99 >s_c_s_e_s_c_s_c_s_e_s GTGTGGGTGCTGGTGTGGGTGTGGGTGCTGGTG; >clamp_s_c_s_e_s:compl:rev CACCAGCACCCACACTATCATGTTGACATTCC; dG = -18.4
100 >s_c_s_e_s_e_s_c_s_e_s GTGTGGGTGCTGGTGCTGGTGTGGGTGCTGGTG; >clamp_s_c_s_e_s:compl:rev CACCAGCACCCACACTATCATGTTGACATTCC; dG = -18.4
101 >s_d_s_a_s_a_s_c_s_e_s GTGACGGTGTCTGTGTCTGTGTGGGTGCTGGTG; >clamp_s_c_s_e_s:compl:rev CACCAGCACCCACACTATCATGTTGACATTCC; dG = -18.4
102 >s_d_s_a_s_b_s_c_s_e_s GTGACGGTGTCTGTGGCTGTGTGGGTGCTGGTG; >clamp_s_c_s_e_s:compl:rev CACCAGCACCCACACTATCATGTTGACATTCC; dG = -18.4
103 >s_d_s_a_s_c_s_c_s_e_s GTGACGGTGTCTGTGTGGGTGTGGGTGCTGGTG; >clamp_s_c_s_e_s:compl:rev CACCAGCACCCACACTATCATGTTGACATTCC; dG = -18.4
104 >s_d_s_a_s_d_s_c_s_e_s GTGACGGTGTCTGTGACGGTGTGGGTGCTGGTG; >clamp_s_c_s_e_s:compl:rev CACCAGCACCCACACTATCATGTTGACATTCC; dG = -18.4
105 >s_a_s_b_s_c_s_c_s_e_s GTGTCTGTGGCTGTGTGGGTGTGGGTGCTGGTG; >clamp_s_c_s_e_s:compl:rev CACCAGCACCCACACTATCATGTTGACATTCC; dG = -18.4
106 >s_d_s_a_s_e_s_c_s_e_s GTGACGGTGTCTGTGCTGGTGTGGGTGCTGGTG; >clamp_s_c_s_e_s:compl:rev CACCAGCACCCACACTATCATGTTGACATTCC; dG = -18.4
107 >s_d_s_b_s_a_s_c_s_e_s GTGACGGTGGCTGTGTCTGTGTGGGTGCTGGTG; >clamp_s_c_s_e_s:compl:rev CACCAGCACCCACACTATCATGTTGACATTCC; dG = -18.4
108 >s_d_s_b_s_b_s_c_s_e_s GTGACGGTGGCTGTGGCTGTGTGGGTGCTGGTG; >clamp_s_c_s_e_s:compl:rev CACCAGCACCCACACTATCATGTTGACATTCC; dG = -18.4
109 >s_d_s_b_s_c_s_c_s_e_s GTGACGGTGGCTGTGTGGGTGTGGGTGCTGGTG; >clamp_s_c_s_e_s:compl:rev CACCAGCACCCACACTATCATGTTGACATTCC; dG = -18.4
110 >s_d_s_b_s_d_s_c_s_e_s GTGACGGTGGCTGTGACGGTGTGGGTGCTGGTG; >clamp_s_c_s_e_s:compl:rev CACCAGCACCCACACTATCATGTTGACATTCC; dG = -18.4
111 >s_d_s_b_s_e_s_c_s_e_s GTGACGGTGGCTGTGCTGGTGTGGGTGCTGGTG; >clamp_s_c_s_e_s:compl:rev CACCAGCACCCACACTATCATGTTGACATTCC; dG = -18.4
112 >s_d_s_c_s_a_s_c_s_e_s GTGACGGTGTGGGTGTCTGTGTGGGTGCTGGTG; >clamp_s_c_s_e_s:compl:rev CACCAGCACCCACACTATCATGTTGACATTCC; dG = -18.4
113 >s_d_s_c_s_b_s_c_s_e_s GTGACGGTGTGGGTGGCTGTGTGGGTGCTGGTG; >clamp_s_c_s_e_s:compl:rev CACCAGCACCCACACTATCATGTTGACATTCC; dG = -18.4
114 >s_d_s_c_s_c_s_c_s_e_s GTGACGGTGTGGGTGTGGGTGTGGGTGCTGGTG; >clamp_s_c_s_e_s:compl:rev CACCAGCACCCACACTATCATGTTGACATTCC; dG = -18.4
115 >s_d_s_c_s_d_s_c_s_e_s GTGACGGTGTGGGTGACGGTGTGGGTGCTGGTG; >clamp_s_c_s_e_s:compl:rev CACCAGCACCCACACTATCATGTTGACATTCC; dG = -18.4
116 >s_a_s_b_s_d_s_c_s_e_s GTGTCTGTGGCTGTGACGGTGTGGGTGCTGGTG; >clamp_s_c_s_e_s:compl:rev CACCAGCACCCACACTATCATGTTGACATTCC; dG = -18.4
117 >s_d_s_d_s_a_s_c_s_e_s GTGACGGTGACGGTGTCTGTGTGGGTGCTGGTG; >clamp_s_c_s_e_s:compl:rev CACCAGCACCCACACTATCATGTTGACATTCC; dG = -18.4
118 >s_d_s_d_s_b_s_c_s_e_s GTGACGGTGACGGTGGCTGTGTGGGTGCTGGTG; >clamp_s_c_s_e_s:compl:rev CACCAGCACCCACACTATCATGTTGACATTCC; dG = -18.4
119 >s_d_s_d_s_c_s_c_s_e_s GTGACGGTGACGGTGTGGGTGTGGGTGCTGGTG; >clamp_s_c_s_e_s:compl:rev CACCAGCACCCACACTATCATGTTGACATTCC; dG = -18.4
120 >s_d_s_d_s_d_s_c_s_e_s GTGACGGTGACGGTGACGGTGTGGGTGCTGGTG; >clamp_s_c_s_e_s:compl:rev CACCAGCACCCACACTATCATGTTGACATTCC; dG = -18.4
121 >s_d_s_d_s_e_s_c_s_e_s GTGACGGTGACGGTGCTGGTGTGGGTGCTGGTG; >clamp_s_c_s_e_s:compl:rev CACCAGCACCCACACTATCATGTTGACATTCC; dG = -18.4
122 >s_d_s_e_s_a_s_c_s_e_s GTGACGGTGCTGGTGTCTGTGTGGGTGCTGGTG; >clamp_s_c_s_e_s:compl:rev CACCAGCACCCACACTATCATGTTGACATTCC; dG = -18.4
123 >s_d_s_e_s_b_s_c_s_e_s GTGACGGTGCTGGTGGCTGTGTGGGTGCTGGTG; >clamp_s_c_s_e_s:compl:rev CACCAGCACCCACACTATCATGTTGACATTCC; dG = -18.4
124 >s_d_s_e_s_c_s_c_s_e_s GTGACGGTGCTGGTGTGGGTGTGGGTGCTGGTG; >clamp_s_c_s_e_s:compl:rev CACCAGCACCCACACTATCATGTTGACATTCC; dG = -18.4
125 >s_d_s_e_s_d_s_c_s_e_s GTGACGGTGCTGGTGACGGTGTGGGTGCTGGTG; >clamp_s_c_s_e_s:compl:rev CACCAGCACCCACACTATCATGTTGACATTCC; dG = -18.4

****

CE Mismatches

1 >s_a_s_a_s_c_s_c_s_d_s GTGTCTGTGTCTGTGTGGGTGTGGGTGACGGTG; >clamp_s_c_s_e_s:compl:rev CACCAGCACCCACACTATCATGTTGACATTCC; dG = -13.8
2 >s_b_s_a_s_c_s_c_s_d_s GTGGCTGTGTCTGTGTGGGTGTGGGTGACGGTG; >clamp_s_c_s_e_s:compl:rev CACCAGCACCCACACTATCATGTTGACATTCC; dG = -13.8

3 >s_b_s_b_s_c_s_c_s_d_s GTGGCTGTGGCTGTGTGGGTGTGGGTGACGGTG; >clamp_s_c_s_e_s:compl:rev
CACCAGCACCCACACTATCATGTTGACATTCC; dG = -13.8
4 >s_b_s_c_s_c_s_d_s_e_s GTGGCTGTGTGGGTGTGGGTGACGGTGCTGGTG; >clamp_s_c_s_e_s:compl:rev
CACCAGCACCCACACTATCATGTTGACATTCC; dG = -13.8
5 >s_c_s_a_s_c_s_c_s_d_s GTGTGGGTGTCTGTGTGGGTGTGGGTGACGGTG; >clamp_s_c_s_e_s:compl:rev
CACCAGCACCCACACTATCATGTTGACATTCC; dG = -13.8
6 >s_c_s_b_s_c_s_c_s_d_s GTGTGGGTGGCTGTGTGGGTGTGGGTGACGGTG; >clamp_s_c_s_e_s:compl:rev
CACCAGCACCCACACTATCATGTTGACATTCC; dG = -13.8
7 >s_d_s_a_s_c_s_c_s_d_s GTGACGGTGTCTGTGTGGGTGTGGGTGACGGTG; >clamp_s_c_s_e_s:compl:rev
CACCAGCACCCACACTATCATGTTGACATTCC; dG = -13.8
8 >s_a_s_b_s_c_s_c_s_d_s GTGTCTGTGGCTGTGTGGGTGTGGGTGACGGTG; >clamp_s_c_s_e_s:compl:rev
CACCAGCACCCACACTATCATGTTGACATTCC; dG = -13.8
9 >s_d_s_b_s_c_s_c_s_d_s GTGACGGTGGCTGTGTGGGTGTGGGTGACGGTG; >clamp_s_c_s_e_s:compl:rev
CACCAGCACCCACACTATCATGTTGACATTCC; dG = -13.8
10 >s_e_s_a_s_c_s_c_s_d_s GTGCTGGTGTCTGTGTGGGTGTGGGTGACGGTG; >clamp_s_c_s_e_s:compl:rev
CACCAGCACCCACACTATCATGTTGACATTCC; dG = -13.8
11 >s_e_s_b_s_c_s_c_s_d_s GTGCTGGTGGCTGTGTGGGTGTGGGTGACGGTG; >clamp_s_c_s_e_s:compl:rev
CACCAGCACCCACACTATCATGTTGACATTCC; dG = -13.8
12 >s_a_s_c_s_c_s_d_s_e_s GTGTCTGTGTGGGTGTGGGTGACGGTGCTGGTG; >clamp_s_c_s_e_s:compl:rev
CACCAGCACCCACACTATCATGTTGACATTCC; dG = -13.8
13 >s_a_s_d_s_c_s_c_s_d_s GTGTCTGTGACGGTGTGGGTGTGGGTGACGGTG; >clamp_s_c_s_e_s:compl:rev
CACCAGCACCCACACTATCATGTTGACATTCC; dG = -13.7
14 >s_a_s_e_s_c_s_c_s_d_s GTGTCTGTGCTGGTGTGGGTGTGGGTGACGGTG; >clamp_s_c_s_e_s:compl:rev
CACCAGCACCCACACTATCATGTTGACATTCC; dG = -13.7
15 >s_b_s_c_s_c_s_c_s_d_s GTGGCTGTGTGGGTGTGGGTGTGGGTGACGGTG; >clamp_s_c_s_e_s:compl:rev
CACCAGCACCCACACTATCATGTTGACATTCC; dG = -13.7
16 >s_b_s_d_s_c_s_c_s_d_s GTGGCTGTGACGGTGTGGGTGTGGGTGACGGTG; >clamp_s_c_s_e_s:compl:rev
CACCAGCACCCACACTATCATGTTGACATTCC; dG = -13.7
17 >s_b_s_e_s_c_s_c_s_d_s GTGGCTGTGCTGGTGTGGGTGTGGGTGACGGTG; >clamp_s_c_s_e_s:compl:rev
CACCAGCACCCACACTATCATGTTGACATTCC; dG = -13.7
18 >s_c_s_c_s_c_s_c_s_d_s GTGTGGGTGTGGGTGTGGGTGTGGGTGACGGTG; >clamp_s_c_s_e_s:compl:rev
CACCAGCACCCACACTATCATGTTGACATTCC; dG = -13.7
19 >s_c_s_c_s_c_s_d_s_e_s GTGTGGGTGTGGGTGTGGGTGACGGTGCTGGTG; >clamp_s_c_s_e_s:compl:rev
CACCAGCACCCACACTATCATGTTGACATTCC; dG = -13.7
20 >s_c_s_d_s_c_s_c_s_d_s GTGTGGGTGACGGTGTGGGTGTGGGTGACGGTG; >clamp_s_c_s_e_s:compl:rev
CACCAGCACCCACACTATCATGTTGACATTCC; dG = -13.7
21 >s_d_s_c_s_c_s_c_s_d_s GTGACGGTGTGGGTGTGGGTGTGGGTGACGGTG; >clamp_s_c_s_e_s:compl:rev
CACCAGCACCCACACTATCATGTTGACATTCC; dG = -13.7
22 >s_d_s_c_s_c_s_d_s_e_s GTGACGGTGTGGGTGTGGGTGACGGTGCTGGTG; >clamp_s_c_s_e_s:compl:rev
CACCAGCACCCACACTATCATGTTGACATTCC; dG = -13.7
23 >s_d_s_d_s_c_s_c_s_d_s GTGACGGTGACGGTGTGGGTGTGGGTGACGGTG; >clamp_s_c_s_e_s:compl:rev
CACCAGCACCCACACTATCATGTTGACATTCC; dG = -13.7
24 >s_d_s_e_s_c_s_c_s_d_s GTGACGGTGCTGGTGTGGGTGTGGGTGACGGTG; >clamp_s_c_s_e_s:compl:rev
CACCAGCACCCACACTATCATGTTGACATTCC; dG = -13.7
25 >s_e_s_c_s_c_s_c_s_d_s GTGCTGGTGTGGGTGTGGGTGTGGGTGACGGTG; >clamp_s_c_s_e_s:compl:rev
CACCAGCACCCACACTATCATGTTGACATTCC; dG = -13.7
26 >s_e_s_c_s_c_s_d_s_e_s GTGCTGGTGTGGGTGTGGGTGACGGTGCTGGTG; >clamp_s_c_s_e_s:compl:rev
CACCAGCACCCACACTATCATGTTGACATTCC; dG = -13.7
27 >s_e_s_d_s_c_s_c_s_d_s GTGCTGGTGACGGTGTGGGTGTGGGTGACGGTG; >clamp_s_c_s_e_s:compl:rev
CACCAGCACCCACACTATCATGTTGACATTCC; dG = -13.7
28 >s_e_s_e_s_c_s_c_s_d_s GTGCTGGTGCTGGTGTGGGTGTGGGTGACGGTG; >clamp_s_c_s_e_s:compl:rev
CACCAGCACCCACACTATCATGTTGACATTCC; dG = -13.7
29 >s_a_s_c_s_c_s_c_s_d_s GTGTCTGTGTGGGTGTGGGTGTGGGTGACGGTG; >clamp_s_c_s_e_s:compl:rev
CACCAGCACCCACACTATCATGTTGACATTCC; dG = -13.7
30 >s_a_s_c_s_c_s_b_s_e_s GTGTCTGTGTGGGTGTGGGTGGCTGTGCTGGTG; >clamp_s_c_s_e_s:compl:rev
CACCAGCACCCACACTATCATGTTGACATTCC; dG = -13.3
31 >s_a_s_a_s_c_s_c_s_b_s GTGTCTGTGTCTGTGTGGGTGTGGGTGGCTGTG; >clamp_s_c_s_e_s:compl:rev
CACCAGCACCCACACTATCATGTTGACATTCC; dG = -13.3
32 >s_b_s_a_s_c_s_c_s_b_s GTGGCTGTGTCTGTGTGGGTGTGGGTGGCTGTG; >clamp_s_c_s_e_s:compl:rev
CACCAGCACCCACACTATCATGTTGACATTCC; dG = -13.3
33 >s_b_s_b_s_c_s_c_s_b_s GTGGCTGTGGCTGTGTGGGTGTGGGTGGCTGTG; >clamp_s_c_s_e_s:compl:rev
CACCAGCACCCACACTATCATGTTGACATTCC; dG = -13.3
34 >s_b_s_c_s_c_s_b_s_e_s GTGGCTGTGTGGGTGTGGGTGGCTGTGCTGGTG; >clamp_s_c_s_e_s:compl:rev
CACCAGCACCCACACTATCATGTTGACATTCC; dG = -13.3
35 >s_c_s_a_s_c_s_c_s_b_s GTGTGGGTGTCTGTGTGGGTGTGGGTGGCTGTG; >clamp_s_c_s_e_s:compl:rev
CACCAGCACCCACACTATCATGTTGACATTCC; dG = -13.3
36 >s_c_s_a_s_e_s_d_s_e_s GTGTGGGTGTCTGTGCTGGTGACGGTGCTGGTG; >clamp_s_c_s_e_s:compl:rev
CACCAGCACCCACACTATCATGTTGACATTCC; dG = -13.3
37 >s_c_s_b_s_c_s_c_s_b_s GTGTGGGTGGCTGTGTGGGTGTGGGTGGCTGTG; >clamp_s_c_s_e_s:compl:rev
CACCAGCACCCACACTATCATGTTGACATTCC; dG = -13.3
38 >s_c_s_b_s_e_s_d_s_e_s GTGTGGGTGGCTGTGCTGGTGACGGTGCTGGTG; >clamp_s_c_s_e_s:compl:rev
CACCAGCACCCACACTATCATGTTGACATTCC; dG = -13.3
39 >s_c_s_d_s_e_s_d_s_e_s GTGTGGGTGACGGTGCTGGTGACGGTGCTGGTG; >clamp_s_c_s_e_s:compl:rev
CACCAGCACCCACACTATCATGTTGACATTCC; dG = -13.3

40 >s_d_s_a_s_c_s_c_s_b_s GTGACGGTGTCTGTGTGGGTGTGGGTGGCTGTG; >clamp_s_c_s_e_s:compl:rev CACCAGCACCCACACTATCATGTTGACATTCC; dG = -13.3
41 >s_a_s_b_s_c_s_c_s_b_s GTGTCTGTGGCTGTGTGGGTGTGGGTGGCTGTG; >clamp_s_c_s_e_s:compl:rev CACCAGCACCCACACTATCATGTTGACATTCC; dG = -13.3
42 >s_d_s_b_s_c_s_c_s_b_s GTGACGGTGGCTGTGTGGGTGTGGGTGGCTGTG; >clamp_s_c_s_e_s:compl:rev CACCAGCACCCACACTATCATGTTGACATTCC; dG = -13.3
43 >s_e_s_a_s_c_s_c_s_b_s GTGCTGGTGTCTGTGTGGGTGTGGGTGGCTGTG; >clamp_s_c_s_e_s:compl:rev CACCAGCACCCACACTATCATGTTGACATTCC; dG = -13.3
44 >s_e_s_b_s_c_s_c_s_b_s GTGCTGGTGGCTGTGTGGGTGTGGGTGGCTGTG; >clamp_s_c_s_e_s:compl:rev CACCAGCACCCACACTATCATGTTGACATTCC; dG = -13.3
45 >s_a_s_c_s_c_s_a_s_e_s GTGTCTGTGTGGGTGTGGGTGTCTGTGCTGGTG; >clamp_s_c_s_e_s:compl:rev CACCAGCACCCACACTATCATGTTGACATTCC; dG = -13.2
46 >s_a_s_d_s_c_s_c_s_b_s GTGTCTGTGACGGTGTGGGTGTGGGTGGCTGTG; >clamp_s_c_s_e_s:compl:rev CACCAGCACCCACACTATCATGTTGACATTCC; dG = -13.2
47 >s_a_s_e_s_c_s_c_s_b_s GTGTCTGTGCTGGTGTGGGTGTGGGTGGCTGTG; >clamp_s_c_s_e_s:compl:rev CACCAGCACCCACACTATCATGTTGACATTCC; dG = -13.2
48 >s_a_s_a_s_c_s_c_s_c_s GTGTCTGTGTCTGTGTGGGTGTGGGTGTGGGTG; >clamp_s_c_s_e_s:compl:rev CACCAGCACCCACACTATCATGTTGACATTCC; dG = -13.2
49 >s_a_s_a_s_c_s_c_s_a_s GTGTCTGTGTCTGTGTGGGTGTGGGTGTCTGTG; >clamp_s_c_s_e_s:compl:rev CACCAGCACCCACACTATCATGTTGACATTCC; dG = -13.2
50 >s_b_s_a_s_c_s_c_s_c_s GTGGCTGTGTCTGTGTGGGTGTGGGTGTGGGTG; >clamp_s_c_s_e_s:compl:rev CACCAGCACCCACACTATCATGTTGACATTCC; dG = -13.2
51 >s_b_s_a_s_c_s_c_s_a_s GTGGCTGTGTCTGTGTGGGTGTGGGTGTCTGTG; >clamp_s_c_s_e_s:compl:rev CACCAGCACCCACACTATCATGTTGACATTCC; dG = -13.2
52 >s_b_s_b_s_c_s_c_s_c_s GTGGCTGTGGCTGTGTGGGTGTGGGTGTGGGTG; >clamp_s_c_s_e_s:compl:rev CACCAGCACCCACACTATCATGTTGACATTCC; dG = -13.2
53 >s_b_s_b_s_c_s_c_s_a_s GTGGCTGTGGCTGTGTGGGTGTGGGTGTCTGTG; >clamp_s_c_s_e_s:compl:rev CACCAGCACCCACACTATCATGTTGACATTCC; dG = -13.2
54 >s_b_s_c_s_c_s_c_s_c_s GTGGCTGTGTGGGTGTGGGTGTGGGTGTGGGTG; >clamp_s_c_s_e_s:compl:rev CACCAGCACCCACACTATCATGTTGACATTCC; dG = -13.2
55 >s_b_s_c_s_c_s_c_s_b_s GTGGCTGTGTGGGTGTGGGTGTGGGTGGCTGTG; >clamp_s_c_s_e_s:compl:rev CACCAGCACCCACACTATCATGTTGACATTCC; dG = -13.2
56 >s_b_s_c_s_c_s_c_s_a_s GTGGCTGTGTGGGTGTGGGTGTGGGTGTCTGTG; >clamp_s_c_s_e_s:compl:rev CACCAGCACCCACACTATCATGTTGACATTCC; dG = -13.2
57 >s_b_s_c_s_c_s_a_s_e_s GTGGCTGTGTGGGTGTGGGTGTCTGTGCTGGTG; >clamp_s_c_s_e_s:compl:rev CACCAGCACCCACACTATCATGTTGACATTCC; dG = -13.2
58 >s_b_s_d_s_c_s_c_s_b_s GTGGCTGTGACGGTGTGGGTGTGGGTGGCTGTG; >clamp_s_c_s_e_s:compl:rev CACCAGCACCCACACTATCATGTTGACATTCC; dG = -13.2
59 >s_b_s_e_s_c_s_c_s_b_s GTGGCTGTGCTGGTGTGGGTGTGGGTGGCTGTG; >clamp_s_c_s_e_s:compl:rev CACCAGCACCCACACTATCATGTTGACATTCC; dG = -13.2
60 >s_c_s_a_s_c_s_c_s_c_s GTGTGGGTGTCTGTGTGGGTGTGGGTGTGGGTG; >clamp_s_c_s_e_s:compl:rev CACCAGCACCCACACTATCATGTTGACATTCC; dG = -13.2
61 >s_c_s_a_s_c_s_c_s_a_s GTGTGGGTGTCTGTGTGGGTGTGGGTGTCTGTG; >clamp_s_c_s_e_s:compl:rev CACCAGCACCCACACTATCATGTTGACATTCC; dG = -13.2
62 >s_c_s_b_s_c_s_c_s_c_s GTGTGGGTGGCTGTGTGGGTGTGGGTGTGGGTG; >clamp_s_c_s_e_s:compl:rev CACCAGCACCCACACTATCATGTTGACATTCC; dG = -13.2
63 >s_c_s_b_s_c_s_c_s_a_s GTGTGGGTGGCTGTGTGGGTGTGGGTGTCTGTG; >clamp_s_c_s_e_s:compl:rev CACCAGCACCCACACTATCATGTTGACATTCC; dG = -13.2
64 >s_c_s_c_s_c_s_c_s_b_s GTGTGGGTGTGGGTGTGGGTGTGGGTGGCTGTG; >clamp_s_c_s_e_s:compl:rev CACCAGCACCCACACTATCATGTTGACATTCC; dG = -13.2
65 >s_c_s_c_s_c_s_b_s_e_s GTGTGGGTGTGGGTGTGGGTGGCTGTGCTGGTG; >clamp_s_c_s_e_s:compl:rev CACCAGCACCCACACTATCATGTTGACATTCC; dG = -13.2
66 >s_c_s_c_s_d_s_c_s_d_s GTGTGGGTGTGGGTGACGGTGTGGGTGACGGTG; >clamp_s_c_s_e_s:compl:rev CACCAGCACCCACACTATCATGTTGACATTCC; dG = -13.2
67 >s_c_s_c_s_d_s_c_s_c_s GTGTGGGTGTGGGTGACGGTGTGGGTGTGGGTG; >clamp_s_c_s_e_s:compl:rev CACCAGCACCCACACTATCATGTTGACATTCC; dG = -13.2
68 >s_c_s_c_s_d_s_c_s_b_s GTGTGGGTGTGGGTGACGGTGTGGGTGGCTGTG; >clamp_s_c_s_e_s:compl:rev CACCAGCACCCACACTATCATGTTGACATTCC; dG = -13.2
69 >s_c_s_c_s_d_s_c_s_a_s GTGTGGGTGTGGGTGACGGTGTGGGTGTCTGTG; >clamp_s_c_s_e_s:compl:rev CACCAGCACCCACACTATCATGTTGACATTCC; dG = -13.2
70 >s_c_s_c_s_d_s_e_s_e_s GTGTGGGTGTGGGTGACGGTGCTGGTGCTGGTG; >clamp_s_c_s_e_s:compl:rev CACCAGCACCCACACTATCATGTTGACATTCC; dG = -13.2
71 >s_c_s_c_s_d_s_d_s_e_s GTGTGGGTGTGGGTGACGGTGACGGTGCTGGTG; >clamp_s_c_s_e_s:compl:rev CACCAGCACCCACACTATCATGTTGACATTCC; dG = -13.2
72 >s_c_s_c_s_d_s_b_s_e_s GTGTGGGTGTGGGTGACGGTGGCTGTGCTGGTG; >clamp_s_c_s_e_s:compl:rev CACCAGCACCCACACTATCATGTTGACATTCC; dG = -13.2
73 >s_c_s_c_s_d_s_a_s_e_s GTGTGGGTGTGGGTGACGGTGTCTGTGCTGGTG; >clamp_s_c_s_e_s:compl:rev CACCAGCACCCACACTATCATGTTGACATTCC; dG = -13.2
74 >s_c_s_d_s_c_s_c_s_b_s GTGTGGGTGACGGTGTGGGTGTGGGTGGCTGTG; >clamp_s_c_s_e_s:compl:rev CACCAGCACCCACACTATCATGTTGACATTCC; dG = -13.2
75 >s_d_s_a_s_c_s_c_s_c_s GTGACGGTGTCTGTGTGGGTGTGGGTGTGGGTG; >clamp_s_c_s_e_s:compl:rev CACCAGCACCCACACTATCATGTTGACATTCC; dG = -13.2
76 >s_d_s_a_s_c_s_c_s_a_s GTGACGGTGTCTGTGTGGGTGTGGGTGTCTGTG; >clamp_s_c_s_e_s:compl:rev CACCAGCACCCACACTATCATGTTGACATTCC; dG = -13.2

77 >s_a_s_b_s_c_s_c_s_c_s GTGTCTGTGGCTGTGTGGGTGTGGGTGTGGGTG; >clamp_s_c_s_e_s:compl:rev CACCAGCACCCACACTATCATGTTGACATTCC; dG = -13.2
78 >s_d_s_b_s_c_s_c_s_c_s GTGACGGTGGCTGTGTGGGTGTGGGTGTGGGTG; >clamp_s_c_s_e_s:compl:rev CACCAGCACCCACACTATCATGTTGACATTCC; dG = -13.2
79 >s_d_s_b_s_c_s_c_s_a_s GTGACGGTGGCTGTGTGGGTGTGGGTGTCTGTG; >clamp_s_c_s_e_s:compl:rev CACCAGCACCCACACTATCATGTTGACATTCC; dG = -13.2
80 >s_a_s_b_s_c_s_c_s_a_s GTGTCTGTGGCTGTGTGGGTGTGGGTGTCTGTG; >clamp_s_c_s_e_s:compl:rev CACCAGCACCCACACTATCATGTTGACATTCC; dG = -13.2
81 >s_d_s_c_s_c_s_c_s_b_s GTGACGGTGTGGGTGTGGGTGTGGGTGGCTGTG; >clamp_s_c_s_e_s:compl:rev CACCAGCACCCACACTATCATGTTGACATTCC; dG = -13.2
82 >s_d_s_c_s_c_s_b_s_e_s GTGACGGTGTGGGTGTGGGTGGCTGTGCTGGTG; >clamp_s_c_s_e_s:compl:rev CACCAGCACCCACACTATCATGTTGACATTCC; dG = -13.2
83 >s_d_s_d_s_c_s_c_s_b_s GTGACGGTGACGGTGTGGGTGTGGGTGGCTGTG; >clamp_s_c_s_e_s:compl:rev CACCAGCACCCACACTATCATGTTGACATTCC; dG = -13.2
84 >s_d_s_e_s_c_s_c_s_b_s GTGACGGTGCTGGTGTGGGTGTGGGTGGCTGTG; >clamp_s_c_s_e_s:compl:rev CACCAGCACCCACACTATCATGTTGACATTCC; dG = -13.2
85 >s_e_s_a_s_c_s_c_s_c_s GTGCTGGTGTCTGTGTGGGTGTGGGTGTGGGTG; >clamp_s_c_s_e_s:compl:rev CACCAGCACCCACACTATCATGTTGACATTCC; dG = -13.2
86 >s_e_s_a_s_c_s_c_s_a_s GTGCTGGTGTCTGTGTGGGTGTGGGTGTCTGTG; >clamp_s_c_s_e_s:compl:rev CACCAGCACCCACACTATCATGTTGACATTCC; dG = -13.2
87 >s_e_s_b_s_c_s_c_s_c_s GTGCTGGTGGCTGTGTGGGTGTGGGTGTGGGTG; >clamp_s_c_s_e_s:compl:rev CACCAGCACCCACACTATCATGTTGACATTCC; dG = -13.2
88 >s_e_s_b_s_c_s_c_s_a_s GTGCTGGTGGCTGTGTGGGTGTGGGTGTCTGTG; >clamp_s_c_s_e_s:compl:rev CACCAGCACCCACACTATCATGTTGACATTCC; dG = -13.2
89 >s_e_s_c_s_c_s_c_s_b_s GTGCTGGTGTGGGTGTGGGTGTGGGTGGCTGTG; >clamp_s_c_s_e_s:compl:rev CACCAGCACCCACACTATCATGTTGACATTCC; dG = -13.2
90 >s_e_s_c_s_c_s_b_s_e_s GTGCTGGTGTGGGTGTGGGTGGCTGTGCTGGTG; >clamp_s_c_s_e_s:compl:rev CACCAGCACCCACACTATCATGTTGACATTCC; dG = -13.2
91 >s_e_s_d_s_c_s_c_s_b_s GTGCTGGTGACGGTGTGGGTGTGGGTGGCTGTG; >clamp_s_c_s_e_s:compl:rev CACCAGCACCCACACTATCATGTTGACATTCC; dG = -13.2
92 >s_e_s_e_s_c_s_c_s_b_s GTGCTGGTGCTGGTGTGGGTGTGGGTGGCTGTG; >clamp_s_c_s_e_s:compl:rev CACCAGCACCCACACTATCATGTTGACATTCC; dG = -13.2
93 >s_a_s_c_s_c_s_c_s_c_s GTGTCTGTGTGGGTGTGGGTGTGGGTGTGGGTG; >clamp_s_c_s_e_s:compl:rev CACCAGCACCCACACTATCATGTTGACATTCC; dG = -13.2
94 >s_a_s_c_s_c_s_c_s_b_s GTGTCTGTGTGGGTGTGGGTGTGGGTGGCTGTG; >clamp_s_c_s_e_s:compl:rev CACCAGCACCCACACTATCATGTTGACATTCC; dG = -13.2
95 >s_a_s_c_s_c_s_c_s_a_s GTGTCTGTGTGGGTGTGGGTGTGGGTGTCTGTG; >clamp_s_c_s_e_s:compl:rev CACCAGCACCCACACTATCATGTTGACATTCC; dG = -13.2
96 >s_a_s_c_s_d_s_e_s_e_s GTGTCTGTGTGGGTGACGGTGCTGGTGCTGGTG; >clamp_s_c_s_e_s:compl:rev CACCAGCACCCACACTATCATGTTGACATTCC; dG = -13.1
97 >s_a_s_d_s_c_s_c_s_c_s GTGTCTGTGACGGTGTGGGTGTGGGTGTGGGTG; >clamp_s_c_s_e_s:compl:rev CACCAGCACCCACACTATCATGTTGACATTCC; dG = -13.1
98 >s_a_s_d_s_c_s_c_s_a_s GTGTCTGTGACGGTGTGGGTGTGGGTGTCTGTG; >clamp_s_c_s_e_s:compl:rev CACCAGCACCCACACTATCATGTTGACATTCC; dG = -13.1
99 >s_a_s_e_s_c_s_c_s_c_s GTGTCTGTGCTGGTGTGGGTGTGGGTGTGGGTG; >clamp_s_c_s_e_s:compl:rev CACCAGCACCCACACTATCATGTTGACATTCC; dG = -13.1
100 >s_a_s_e_s_c_s_c_s_a_s GTGTCTGTGCTGGTGTGGGTGTGGGTGTCTGTG; >clamp_s_c_s_e_s:compl:rev CACCAGCACCCACACTATCATGTTGACATTCC; dG = -13.1
101 >s_b_s_c_s_a_s_e_s_e_s GTGGCTGTGTGGGTGTCTGTGCTGGTGCTGGTG; >clamp_s_c_s_e_s:compl:rev CACCAGCACCCACACTATCATGTTGACATTCC; dG = -13.1
102 >s_b_s_c_s_b_s_e_s_e_s GTGGCTGTGTGGGTGGCTGTGCTGGTGCTGGTG; >clamp_s_c_s_e_s:compl:rev CACCAGCACCCACACTATCATGTTGACATTCC; dG = -13.1
103 >s_b_s_c_s_d_s_e_s_e_s GTGGCTGTGTGGGTGACGGTGCTGGTGCTGGTG; >clamp_s_c_s_e_s:compl:rev CACCAGCACCCACACTATCATGTTGACATTCC; dG = -13.1
104 >s_b_s_d_s_c_s_c_s_c_s GTGGCTGTGACGGTGTGGGTGTGGGTGTGGGTG; >clamp_s_c_s_e_s:compl:rev CACCAGCACCCACACTATCATGTTGACATTCC; dG = -13.1
105 >s_b_s_d_s_c_s_c_s_a_s GTGGCTGTGACGGTGTGGGTGTGGGTGTCTGTG; >clamp_s_c_s_e_s:compl:rev CACCAGCACCCACACTATCATGTTGACATTCC; dG = -13.1
106 >s_b_s_e_s_c_s_c_s_c_s GTGGCTGTGCTGGTGTGGGTGTGGGTGTGGGTG; >clamp_s_c_s_e_s:compl:rev CACCAGCACCCACACTATCATGTTGACATTCC; dG = -13.1
107 >s_b_s_e_s_c_s_c_s_a_s GTGGCTGTGCTGGTGTGGGTGTGGGTGTCTGTG; >clamp_s_c_s_e_s:compl:rev CACCAGCACCCACACTATCATGTTGACATTCC; dG = -13.1
108 >s_c_s_c_s_a_s_e_s_e_s GTGTGGGTGTGGGTGTCTGTGCTGGTGCTGGTG; >clamp_s_c_s_e_s:compl:rev CACCAGCACCCACACTATCATGTTGACATTCC; dG = -13.1
109 >s_c_s_c_s_b_s_e_s_e_s GTGTGGGTGTGGGTGGCTGTGCTGGTGCTGGTG; >clamp_s_c_s_e_s:compl:rev CACCAGCACCCACACTATCATGTTGACATTCC; dG = -13.1
110 >s_c_s_c_s_c_s_c_s_c_s GTGTGGGTGTGGGTGTGGGTGTGGGTGTGGGTG; >clamp_s_c_s_e_s:compl:rev CACCAGCACCCACACTATCATGTTGACATTCC; dG = -13.1
111 >s_c_s_c_s_c_s_c_s_a_s GTGTGGGTGTGGGTGTGGGTGTGGGTGTCTGTG; >clamp_s_c_s_e_s:compl:rev CACCAGCACCCACACTATCATGTTGACATTCC; dG = -13.1
112 >s_c_s_c_s_c_s_a_s_e_s GTGTGGGTGTGGGTGTGGGTGTCTGTGCTGGTG; >clamp_s_c_s_e_s:compl:rev CACCAGCACCCACACTATCATGTTGACATTCC; dG = -13.1
113 >s_c_s_d_s_c_s_c_s_c_s GTGTGGGTGACGGTGTGGGTGTGGGTGTGGGTG; >clamp_s_c_s_e_s:compl:rev CACCAGCACCCACACTATCATGTTGACATTCC; dG = -13.1

114 >s_c_s_d_s_c_s_c_s_a_s GTGTGGGTGACGGTGTGGGTGTGGGTGTCTGTG; >clamp_s_c_s_e_s:compl:rev CACCAGCACCCACACTATCATGTTGACATTCC; dG = -13.1
115 >s_d_s_c_s_a_s_e_s_e_s GTGACGGTGTGGGTGTCTGTGCTGGTGCTGGTG; >clamp_s_c_s_e_s:compl:rev CACCAGCACCCACACTATCATGTTGACATTCC; dG = -13.1
116 >s_d_s_c_s_b_s_e_s_e_s GTGACGGTGTGGGTGGCTGTGCTGGTGCTGGTG; >clamp_s_c_s_e_s:compl:rev CACCAGCACCCACACTATCATGTTGACATTCC; dG = -13.1
117 >s_d_s_c_s_c_s_c_s_c_s GTGACGGTGTGGGTGTGGGTGTGGGTGTGGGTG; >clamp_s_c_s_e_s:compl:rev CACCAGCACCCACACTATCATGTTGACATTCC; dG = -13.1
118 >s_d_s_c_s_c_s_c_s_a_s GTGACGGTGTGGGTGTGGGTGTGGGTGTCTGTG; >clamp_s_c_s_e_s:compl:rev CACCAGCACCCACACTATCATGTTGACATTCC; dG = -13.1
119 >s_d_s_c_s_c_s_a_s_e_s GTGACGGTGTGGGTGTGGGTGTCTGTGCTGGTG; >clamp_s_c_s_e_s:compl:rev CACCAGCACCCACACTATCATGTTGACATTCC; dG = -13.1
120 >s_d_s_c_s_d_s_e_s_e_s GTGACGGTGTGGGTGACGGTGCTGGTGCTGGTG; >clamp_s_c_s_e_s:compl:rev CACCAGCACCCACACTATCATGTTGACATTCC; dG = -13.1
121 >s_d_s_d_s_c_s_c_s_c_s GTGACGGTGACGGTGTGGGTGTGGGTGTGGGTG; >clamp_s_c_s_e_s:compl:rev CACCAGCACCCACACTATCATGTTGACATTCC; dG = -13.1
122 >s_d_s_d_s_c_s_c_s_a_s GTGACGGTGACGGTGTGGGTGTGGGTGTCTGTG; >clamp_s_c_s_e_s:compl:rev CACCAGCACCCACACTATCATGTTGACATTCC; dG = -13.1
123 >s_d_s_e_s_c_s_c_s_c_s GTGACGGTGCTGGTGTGGGTGTGGGTGTGGGTG; >clamp_s_c_s_e_s:compl:rev CACCAGCACCCACACTATCATGTTGACATTCC; dG = -13.1
124 >s_d_s_e_s_c_s_c_s_a_s GTGACGGTGCTGGTGTGGGTGTGGGTGTCTGTG; >clamp_s_c_s_e_s:compl:rev CACCAGCACCCACACTATCATGTTGACATTCC; dG = -13.1
125 >s_e_s_c_s_a_s_e_s_e_s GTGCTGGTGTGGGTGTCTGTGCTGGTGCTGGTG; >clamp_s_c_s_e_s:compl:rev CACCAGCACCCACACTATCATGTTGACATTCC; dG = -13.1
126 >s_e_s_c_s_b_s_e_s_e_s GTGCTGGTGTGGGTGGCTGTGCTGGTGCTGGTG; >clamp_s_c_s_e_s:compl:rev CACCAGCACCCACACTATCATGTTGACATTCC; dG = -13.1
127 >s_e_s_c_s_c_s_c_s_c_s GTGCTGGTGTGGGTGTGGGTGTGGGTGTGGGTG; >clamp_s_c_s_e_s:compl:rev CACCAGCACCCACACTATCATGTTGACATTCC; dG = -13.1
128 >s_e_s_c_s_c_s_c_s_a_s GTGCTGGTGTGGGTGTGGGTGTGGGTGTCTGTG; >clamp_s_c_s_e_s:compl:rev CACCAGCACCCACACTATCATGTTGACATTCC; dG = -13.1
129 >s_e_s_c_s_c_s_a_s_e_s GTGCTGGTGTGGGTGTGGGTGTCTGTGCTGGTG; >clamp_s_c_s_e_s:compl:rev CACCAGCACCCACACTATCATGTTGACATTCC; dG = -13.1
130 >s_e_s_c_s_d_s_e_s_e_s GTGCTGGTGTGGGTGACGGTGCTGGTGCTGGTG; >clamp_s_c_s_e_s:compl:rev CACCAGCACCCACACTATCATGTTGACATTCC; dG = -13.1
131 >s_a_s_c_s_a_s_e_s_e_s GTGTCTGTGTGGGTGTCTGTGCTGGTGCTGGTG; >clamp_s_c_s_e_s:compl:rev CACCAGCACCCACACTATCATGTTGACATTCC; dG = -13.1
132 >s_e_s_d_s_c_s_c_s_c_s GTGCTGGTGACGGTGTGGGTGTGGGTGTGGGTG; >clamp_s_c_s_e_s:compl:rev CACCAGCACCCACACTATCATGTTGACATTCC; dG = -13.1
133 >s_e_s_d_s_c_s_c_s_a_s GTGCTGGTGACGGTGTGGGTGTGGGTGTCTGTG; >clamp_s_c_s_e_s:compl:rev CACCAGCACCCACACTATCATGTTGACATTCC; dG = -13.1
134 >s_e_s_e_s_c_s_c_s_c_s GTGCTGGTGCTGGTGTGGGTGTGGGTGTGGGTG; >clamp_s_c_s_e_s:compl:rev CACCAGCACCCACACTATCATGTTGACATTCC; dG = -13.1
135 >s_e_s_e_s_c_s_c_s_a_s GTGCTGGTGCTGGTGTGGGTGTGGGTGTCTGTG; >clamp_s_c_s_e_s:compl:rev CACCAGCACCCACACTATCATGTTGACATTCC; dG = -13.1
136 >s_a_s_c_s_b_s_e_s_e_s GTGTCTGTGTGGGTGGCTGTGCTGGTGCTGGTG; >clamp_s_c_s_e_s:compl:rev CACCAGCACCCACACTATCATGTTGACATTCC; dG = -13.1
137 >s_a_s_d_s_e_s_d_s_e_s GTGTCTGTGACGGTGCTGGTGACGGTGCTGGTG; >clamp_s_c_s_e_s:compl:rev CACCAGCACCCACACTATCATGTTGACATTCC; dG = -13.0
138 >s_b_s_a_s_c_s_d_s_e_s GTGGCTGTGTCTGTGTGGGTGACGGTGCTGGTG; >clamp_s_c_s_e_s:compl:rev CACCAGCACCCACACTATCATGTTGACATTCC; dG = -13.0
139 >s_b_s_a_s_c_s_b_s_e_s GTGGCTGTGTCTGTGTGGGTGGCTGTGCTGGTG; >clamp_s_c_s_e_s:compl:rev CACCAGCACCCACACTATCATGTTGACATTCC; dG = -13.0
140 >s_a_s_a_s_c_s_d_s_e_s GTGTCTGTGTCTGTGTGGGTGACGGTGCTGGTG; >clamp_s_c_s_e_s:compl:rev CACCAGCACCCACACTATCATGTTGACATTCC; dG = -13.0
141 >s_b_s_a_s_c_s_a_s_e_s GTGGCTGTGTCTGTGTGGGTGTCTGTGCTGGTG; >clamp_s_c_s_e_s:compl:rev CACCAGCACCCACACTATCATGTTGACATTCC; dG = -13.0
142 >s_a_s_a_s_c_s_b_s_e_s GTGTCTGTGTCTGTGTGGGTGGCTGTGCTGGTG; >clamp_s_c_s_e_s:compl:rev CACCAGCACCCACACTATCATGTTGACATTCC; dG = -13.0
143 >s_a_s_a_s_c_s_a_s_e_s GTGTCTGTGTCTGTGTGGGTGTCTGTGCTGGTG; >clamp_s_c_s_e_s:compl:rev CACCAGCACCCACACTATCATGTTGACATTCC; dG = -13.0
144 >s_b_s_b_s_c_s_d_s_e_s GTGGCTGTGGCTGTGTGGGTGACGGTGCTGGTG; >clamp_s_c_s_e_s:compl:rev CACCAGCACCCACACTATCATGTTGACATTCC; dG = -13.0
145 >s_b_s_b_s_c_s_b_s_e_s GTGGCTGTGGCTGTGTGGGTGGCTGTGCTGGTG; >clamp_s_c_s_e_s:compl:rev CACCAGCACCCACACTATCATGTTGACATTCC; dG = -13.0
146 >s_b_s_b_s_c_s_a_s_e_s GTGGCTGTGGCTGTGTGGGTGTCTGTGCTGGTG; >clamp_s_c_s_e_s:compl:rev CACCAGCACCCACACTATCATGTTGACATTCC; dG = -13.0
147 >s_b_s_d_s_e_s_d_s_e_s GTGGCTGTGACGGTGCTGGTGACGGTGCTGGTG; >clamp_s_c_s_e_s:compl:rev CACCAGCACCCACACTATCATGTTGACATTCC; dG = -13.0
148 >s_c_s_a_s_c_s_d_s_e_s GTGTGGGTGTCTGTGTGGGTGACGGTGCTGGTG; >clamp_s_c_s_e_s:compl:rev CACCAGCACCCACACTATCATGTTGACATTCC; dG = -13.0
149 >s_c_s_a_s_c_s_b_s_e_s GTGTGGGTGTCTGTGTGGGTGGCTGTGCTGGTG; >clamp_s_c_s_e_s:compl:rev CACCAGCACCCACACTATCATGTTGACATTCC; dG = -13.0
150 >s_c_s_a_s_c_s_a_s_e_s GTGTGGGTGTCTGTGTGGGTGTCTGTGCTGGTG; >clamp_s_c_s_e_s:compl:rev CACCAGCACCCACACTATCATGTTGACATTCC; dG = -13.0

151 >s_c_s_b_s_c_s_d_s_e_s GTGTGGGTGGCTGTGTGGGTGACGGTGCTGGTG; >clamp_s_c_s_e_s:compl:rev
CACCAGCACCCACACTATCATGTTGACATTCC; dG = -13.0
152 >s_c_s_b_s_c_s_b_s_e_s GTGTGGGTGGCTGTGTGGGTGGCTGTGCTGGTG; >clamp_s_c_s_e_s:compl:rev
CACCAGCACCCACACTATCATGTTGACATTCC; dG = -13.0
153 >s_c_s_b_s_c_s_a_s_e_s GTGTGGGTGGCTGTGTGGGTGTCTGTGCTGGTG; >clamp_s_c_s_e_s:compl:rev
CACCAGCACCCACACTATCATGTTGACATTCC; dG = -13.0
154 >s_d_s_a_s_c_s_d_s_e_s GTGACGGTGTCTGTGTGGGTGACGGTGCTGGTG; >clamp_s_c_s_e_s:compl:rev
CACCAGCACCCACACTATCATGTTGACATTCC; dG = -13.0
155 >s_d_s_a_s_c_s_b_s_e_s GTGACGGTGTCTGTGTGGGTGGCTGTGCTGGTG; >clamp_s_c_s_e_s:compl:rev
CACCAGCACCCACACTATCATGTTGACATTCC; dG = -13.0
156 >s_d_s_a_s_c_s_a_s_e_s GTGACGGTGTCTGTGTGGGTGTCTGTGCTGGTG; >clamp_s_c_s_e_s:compl:rev
CACCAGCACCCACACTATCATGTTGACATTCC; dG = -13.0
157 >s_d_s_b_s_c_s_d_s_e_s GTGACGGTGGCTGTGTGGGTGACGGTGCTGGTG; >clamp_s_c_s_e_s:compl:rev
CACCAGCACCCACACTATCATGTTGACATTCC; dG = -13.0
158 >s_d_s_b_s_c_s_b_s_e_s GTGACGGTGGCTGTGTGGGTGGCTGTGCTGGTG; >clamp_s_c_s_e_s:compl:rev
CACCAGCACCCACACTATCATGTTGACATTCC; dG = -13.0
159 >s_d_s_b_s_c_s_a_s_e_s GTGACGGTGGCTGTGTGGGTGTCTGTGCTGGTG; >clamp_s_c_s_e_s:compl:rev
CACCAGCACCCACACTATCATGTTGACATTCC; dG = -13.0
160 >s_a_s_b_s_c_s_d_s_e_s GTGTCTGTGGCTGTGTGGGTGACGGTGCTGGTG; >clamp_s_c_s_e_s:compl:rev
CACCAGCACCCACACTATCATGTTGACATTCC; dG = -13.0
161 >s_a_s_b_s_c_s_b_s_e_s GTGTCTGTGGCTGTGTGGGTGGCTGTGCTGGTG; >clamp_s_c_s_e_s:compl:rev
CACCAGCACCCACACTATCATGTTGACATTCC; dG = -13.0
162 >s_a_s_b_s_c_s_a_s_e_s GTGTCTGTGGCTGTGTGGGTGTCTGTGCTGGTG; >clamp_s_c_s_e_s:compl:rev
CACCAGCACCCACACTATCATGTTGACATTCC; dG = -13.0
163 >s_d_s_d_s_e_s_d_s_e_s GTGACGGTGACGGTGCTGGTGACGGTGCTGGTG; >clamp_s_c_s_e_s:compl:rev
CACCAGCACCCACACTATCATGTTGACATTCC; dG = -13.0
164 >s_e_s_a_s_c_s_d_s_e_s GTGCTGGTGTCTGTGTGGGTGACGGTGCTGGTG; >clamp_s_c_s_e_s:compl:rev
CACCAGCACCCACACTATCATGTTGACATTCC; dG = -13.0
165 >s_e_s_a_s_c_s_b_s_e_s GTGCTGGTGTCTGTGTGGGTGGCTGTGCTGGTG; >clamp_s_c_s_e_s:compl:rev
CACCAGCACCCACACTATCATGTTGACATTCC; dG = -13.0
166 >s_e_s_a_s_c_s_a_s_e_s GTGCTGGTGTCTGTGTGGGTGTCTGTGCTGGTG; >clamp_s_c_s_e_s:compl:rev
CACCAGCACCCACACTATCATGTTGACATTCC; dG = -13.0
167 >s_e_s_b_s_c_s_d_s_e_s GTGCTGGTGGCTGTGTGGGTGACGGTGCTGGTG; >clamp_s_c_s_e_s:compl:rev
CACCAGCACCCACACTATCATGTTGACATTCC; dG = -13.0
168 >s_e_s_b_s_c_s_b_s_e_s GTGCTGGTGGCTGTGTGGGTGGCTGTGCTGGTG; >clamp_s_c_s_e_s:compl:rev
CACCAGCACCCACACTATCATGTTGACATTCC; dG = -13.0
169 >s_e_s_b_s_c_s_a_s_e_s GTGCTGGTGGCTGTGTGGGTGTCTGTGCTGGTG; >clamp_s_c_s_e_s:compl:rev
CACCAGCACCCACACTATCATGTTGACATTCC; dG = -13.0
170 >s_e_s_d_s_e_s_d_s_e_s GTGCTGGTGACGGTGCTGGTGACGGTGCTGGTG; >clamp_s_c_s_e_s:compl:rev
CACCAGCACCCACACTATCATGTTGACATTCC; dG = -13.0
171 >s_a_s_a_s_b_s_c_s_c_s GTGTCTGTGTCTGTGGCTGTGTGGGTGTGGGTG; >clamp_s_c_s_e_s:compl:rev
CACCAGCACCCACACTATCATGTTGACATTCC; dG = -12.9
172 >s_a_s_c_s_d_s_c_s_c_s GTGTCTGTGTGGGTGACGGTGTGGGTGTGGGTG; >clamp_s_c_s_e_s:compl:rev
CACCAGCACCCACACTATCATGTTGACATTCC; dG = -12.9
173 >s_a_s_c_s_d_s_d_s_e_s GTGTCTGTGTGGGTGACGGTGACGGTGCTGGTG; >clamp_s_c_s_e_s:compl:rev
CACCAGCACCCACACTATCATGTTGACATTCC; dG = -12.9
174 >s_a_s_d_s_a_s_c_s_c_s GTGTCTGTGACGGTGTCTGTGTGGGTGTGGGTG; >clamp_s_c_s_e_s:compl:rev
CACCAGCACCCACACTATCATGTTGACATTCC; dG = -12.9
175 >s_a_s_d_s_b_s_c_s_c_s GTGTCTGTGACGGTGGCTGTGTGGGTGTGGGTG; >clamp_s_c_s_e_s:compl:rev
CACCAGCACCCACACTATCATGTTGACATTCC; dG = -12.9
176 >s_a_s_d_s_c_s_d_s_e_s GTGTCTGTGACGGTGTGGGTGACGGTGCTGGTG; >clamp_s_c_s_e_s:compl:rev
CACCAGCACCCACACTATCATGTTGACATTCC; dG = -12.9
177 >s_a_s_d_s_c_s_b_s_e_s GTGTCTGTGACGGTGTGGGTGGCTGTGCTGGTG; >clamp_s_c_s_e_s:compl:rev
CACCAGCACCCACACTATCATGTTGACATTCC; dG = -12.9
178 >s_a_s_d_s_c_s_a_s_e_s GTGTCTGTGACGGTGTGGGTGTCTGTGCTGGTG; >clamp_s_c_s_e_s:compl:rev
CACCAGCACCCACACTATCATGTTGACATTCC; dG = -12.9
179 >s_a_s_d_s_d_s_c_s_c_s GTGTCTGTGACGGTGACGGTGTGGGTGTGGGTG; >clamp_s_c_s_e_s:compl:rev
CACCAGCACCCACACTATCATGTTGACATTCC; dG = -12.9
180 >s_a_s_d_s_e_s_c_s_c_s GTGTCTGTGACGGTGCTGGTGTGGGTGTGGGTG; >clamp_s_c_s_e_s:compl:rev
CACCAGCACCCACACTATCATGTTGACATTCC; dG = -12.9
181 >s_a_s_e_s_a_s_c_s_c_s GTGTCTGTGCTGGTGTCTGTGTGGGTGTGGGTG; >clamp_s_c_s_e_s:compl:rev
CACCAGCACCCACACTATCATGTTGACATTCC; dG = -12.9
182 >s_a_s_e_s_b_s_c_s_c_s GTGTCTGTGCTGGTGGCTGTGTGGGTGTGGGTG; >clamp_s_c_s_e_s:compl:rev
CACCAGCACCCACACTATCATGTTGACATTCC; dG = -12.9
183 >s_a_s_e_s_c_s_d_s_e_s GTGTCTGTGCTGGTGTGGGTGACGGTGCTGGTG; >clamp_s_c_s_e_s:compl:rev
CACCAGCACCCACACTATCATGTTGACATTCC; dG = -12.9
184 >s_a_s_e_s_c_s_b_s_e_s GTGTCTGTGCTGGTGTGGGTGGCTGTGCTGGTG; >clamp_s_c_s_e_s:compl:rev
CACCAGCACCCACACTATCATGTTGACATTCC; dG = -12.9
185 >s_a_s_e_s_c_s_a_s_e_s GTGTCTGTGCTGGTGTGGGTGTCTGTGCTGGTG; >clamp_s_c_s_e_s:compl:rev
CACCAGCACCCACACTATCATGTTGACATTCC; dG = -12.9
186 >s_a_s_e_s_d_s_c_s_c_s GTGTCTGTGCTGGTGACGGTGTGGGTGTGGGTG; >clamp_s_c_s_e_s:compl:rev
CACCAGCACCCACACTATCATGTTGACATTCC; dG = -12.9
187 >s_a_s_e_s_e_s_c_s_c_s GTGTCTGTGCTGGTGCTGGTGTGGGTGTGGGTG; >clamp_s_c_s_e_s:compl:rev
CACCAGCACCCACACTATCATGTTGACATTCC; dG = -12.9

188 >s_a_s_e_s_e_s_d_s_e_s GTGTCTGTGCTGGTGCTGGTGACGGTGCTGGTG; >clamp_s_c_s_e_s:compl:rev CACCAGCACCCACACTATCATGTTGACATTCC; dG = -12.9
189 >s_b_s_a_s_a_s_c_s_c_s GTGGCTGTGTCTGTGTCTGTGTGGGTGTGGGTG; >clamp_s_c_s_e_s:compl:rev CACCAGCACCCACACTATCATGTTGACATTCC; dG = -12.9
190 >s_b_s_a_s_b_s_c_s_c_s GTGGCTGTGTCTGTGGCTGTGTGGGTGTGGGTG; >clamp_s_c_s_e_s:compl:rev CACCAGCACCCACACTATCATGTTGACATTCC; dG = -12.9
191 >s_a_s_a_s_a_s_c_s_c_s GTGTCTGTGTCTGTGTCTGTGTGGGTGTGGGTG; >clamp_s_c_s_e_s:compl:rev CACCAGCACCCACACTATCATGTTGACATTCC; dG = -12.9
192 >s_b_s_a_s_d_s_c_s_c_s GTGGCTGTGTCTGTGACGGTGTGGGTGTGGGTG; >clamp_s_c_s_e_s:compl:rev CACCAGCACCCACACTATCATGTTGACATTCC; dG = -12.9
193 >s_b_s_a_s_e_s_c_s_c_s GTGGCTGTGTCTGTGCTGGTGTGGGTGTGGGTG; >clamp_s_c_s_e_s:compl:rev CACCAGCACCCACACTATCATGTTGACATTCC; dG = -12.9
194 >s_b_s_b_s_a_s_c_s_c_s GTGGCTGTGGCTGTGTCTGTGTGGGTGTGGGTG; >clamp_s_c_s_e_s:compl:rev CACCAGCACCCACACTATCATGTTGACATTCC; dG = -12.9
195 >s_b_s_b_s_b_s_c_s_c_s GTGGCTGTGGCTGTGGCTGTGTGGGTGTGGGTG; >clamp_s_c_s_e_s:compl:rev CACCAGCACCCACACTATCATGTTGACATTCC; dG = -12.9
196 >s_a_s_a_s_d_s_c_s_c_s GTGTCTGTGTCTGTGACGGTGTGGGTGTGGGTG; >clamp_s_c_s_e_s:compl:rev CACCAGCACCCACACTATCATGTTGACATTCC; dG = -12.9
197 >s_b_s_b_s_d_s_c_s_c_s GTGGCTGTGGCTGTGACGGTGTGGGTGTGGGTG; >clamp_s_c_s_e_s:compl:rev CACCAGCACCCACACTATCATGTTGACATTCC; dG = -12.9
198 >s_b_s_b_s_e_s_c_s_c_s GTGGCTGTGGCTGTGCTGGTGTGGGTGTGGGTG; >clamp_s_c_s_e_s:compl:rev CACCAGCACCCACACTATCATGTTGACATTCC; dG = -12.9
199 >s_b_s_c_s_a_s_c_s_c_s GTGGCTGTGTGGGTGTCTGTGTGGGTGTGGGTG; >clamp_s_c_s_e_s:compl:rev CACCAGCACCCACACTATCATGTTGACATTCC; dG = -12.9
200 >s_b_s_c_s_b_s_c_s_c_s GTGGCTGTGTGGGTGGCTGTGTGGGTGTGGGTG; >clamp_s_c_s_e_s:compl:rev CACCAGCACCCACACTATCATGTTGACATTCC; dG = -12.9
201 >s_b_s_c_s_d_s_c_s_c_s GTGGCTGTGTGGGTGACGGTGTGGGTGTGGGTG; >clamp_s_c_s_e_s:compl:rev CACCAGCACCCACACTATCATGTTGACATTCC; dG = -12.9
202 >s_b_s_c_s_d_s_d_s_e_s GTGGCTGTGTGGGTGACGGTGACGGTGCTGGTG; >clamp_s_c_s_e_s:compl:rev CACCAGCACCCACACTATCATGTTGACATTCC; dG = -12.9
203 >s_b_s_d_s_a_s_c_s_c_s GTGGCTGTGACGGTGTCTGTGTGGGTGTGGGTG; >clamp_s_c_s_e_s:compl:rev CACCAGCACCCACACTATCATGTTGACATTCC; dG = -12.9
204 >s_b_s_d_s_b_s_c_s_c_s GTGGCTGTGACGGTGGCTGTGTGGGTGTGGGTG; >clamp_s_c_s_e_s:compl:rev CACCAGCACCCACACTATCATGTTGACATTCC; dG = -12.9
205 >s_b_s_d_s_c_s_d_s_e_s GTGGCTGTGACGGTGTGGGTGACGGTGCTGGTG; >clamp_s_c_s_e_s:compl:rev CACCAGCACCCACACTATCATGTTGACATTCC; dG = -12.9
206 >s_b_s_d_s_c_s_b_s_e_s GTGGCTGTGACGGTGTGGGTGGCTGTGCTGGTG; >clamp_s_c_s_e_s:compl:rev CACCAGCACCCACACTATCATGTTGACATTCC; dG = -12.9
207 >s_b_s_d_s_c_s_a_s_e_s GTGGCTGTGACGGTGTGGGTGTCTGTGCTGGTG; >clamp_s_c_s_e_s:compl:rev CACCAGCACCCACACTATCATGTTGACATTCC; dG = -12.9
208 >s_b_s_d_s_d_s_c_s_c_s GTGGCTGTGACGGTGACGGTGTGGGTGTGGGTG; >clamp_s_c_s_e_s:compl:rev CACCAGCACCCACACTATCATGTTGACATTCC; dG = -12.9
209 >s_b_s_d_s_e_s_c_s_c_s GTGGCTGTGACGGTGCTGGTGTGGGTGTGGGTG; >clamp_s_c_s_e_s:compl:rev CACCAGCACCCACACTATCATGTTGACATTCC; dG = -12.9
210 >s_a_s_a_s_e_s_c_s_c_s GTGTCTGTGTCTGTGCTGGTGTGGGTGTGGGTG; >clamp_s_c_s_e_s:compl:rev CACCAGCACCCACACTATCATGTTGACATTCC; dG = -12.9
211 >s_b_s_e_s_a_s_c_s_c_s GTGGCTGTGCTGGTGTCTGTGTGGGTGTGGGTG; >clamp_s_c_s_e_s:compl:rev CACCAGCACCCACACTATCATGTTGACATTCC; dG = -12.9
212 >s_b_s_e_s_b_s_c_s_c_s GTGGCTGTGCTGGTGGCTGTGTGGGTGTGGGTG; >clamp_s_c_s_e_s:compl:rev CACCAGCACCCACACTATCATGTTGACATTCC; dG = -12.9
213 >s_b_s_e_s_c_s_d_s_e_s GTGGCTGTGCTGGTGTGGGTGACGGTGCTGGTG; >clamp_s_c_s_e_s:compl:rev CACCAGCACCCACACTATCATGTTGACATTCC; dG = -12.9
214 >s_b_s_e_s_c_s_b_s_e_s GTGGCTGTGCTGGTGTGGGTGGCTGTGCTGGTG; >clamp_s_c_s_e_s:compl:rev CACCAGCACCCACACTATCATGTTGACATTCC; dG = -12.9
215 >s_b_s_e_s_c_s_a_s_e_s GTGGCTGTGCTGGTGTGGGTGTCTGTGCTGGTG; >clamp_s_c_s_e_s:compl:rev CACCAGCACCCACACTATCATGTTGACATTCC; dG = -12.9
216 >s_b_s_e_s_d_s_c_s_c_s GTGGCTGTGCTGGTGACGGTGTGGGTGTGGGTG; >clamp_s_c_s_e_s:compl:rev CACCAGCACCCACACTATCATGTTGACATTCC; dG = -12.9
217 >s_b_s_e_s_e_s_c_s_c_s GTGGCTGTGCTGGTGCTGGTGTGGGTGTGGGTG; >clamp_s_c_s_e_s:compl:rev CACCAGCACCCACACTATCATGTTGACATTCC; dG = -12.9
218 >s_b_s_e_s_e_s_d_s_e_s GTGGCTGTGCTGGTGCTGGTGACGGTGCTGGTG; >clamp_s_c_s_e_s:compl:rev CACCAGCACCCACACTATCATGTTGACATTCC; dG = -12.9
219 >s_c_s_a_s_a_s_c_s_c_s GTGTGGGTGTCTGTGTCTGTGTGGGTGTGGGTG; >clamp_s_c_s_e_s:compl:rev CACCAGCACCCACACTATCATGTTGACATTCC; dG = -12.9
220 >s_c_s_a_s_b_s_c_s_c_s GTGTGGGTGTCTGTGGCTGTGTGGGTGTGGGTG; >clamp_s_c_s_e_s:compl:rev CACCAGCACCCACACTATCATGTTGACATTCC; dG = -12.9
221 >s_c_s_a_s_d_s_c_s_c_s GTGTGGGTGTCTGTGACGGTGTGGGTGTGGGTG; >clamp_s_c_s_e_s:compl:rev CACCAGCACCCACACTATCATGTTGACATTCC; dG = -12.9
222 >s_c_s_a_s_e_s_c_s_c_s GTGTGGGTGTCTGTGCTGGTGTGGGTGTGGGTG; >clamp_s_c_s_e_s:compl:rev CACCAGCACCCACACTATCATGTTGACATTCC; dG = -12.9
223 >s_a_s_b_s_a_s_c_s_c_s GTGTCTGTGGCTGTGTCTGTGTGGGTGTGGGTG; >clamp_s_c_s_e_s:compl:rev CACCAGCACCCACACTATCATGTTGACATTCC; dG = -12.9
224 >s_c_s_b_s_a_s_c_s_c_s GTGTGGGTGGCTGTGTCTGTGTGGGTGTGGGTG; >clamp_s_c_s_e_s:compl:rev CACCAGCACCCACACTATCATGTTGACATTCC; dG = -12.9

225 >s_c_s_b_s_b_s_c_s_c_s GTGTGGGTGGCTGTGGCTGTGTGGGTGTGGGTG; >clamp_s_c_s_e_s:compl:rev CACCAGCACCCACACTATCATGTTGACATTCC; dG = -12.9
226 >s_c_s_b_s_d_s_c_s_c_s GTGTGGGTGGCTGTGACGGTGTGGGTGTGGGTG; >clamp_s_c_s_e_s:compl:rev CACCAGCACCCACACTATCATGTTGACATTCC; dG = -12.9
227 >s_c_s_b_s_e_s_c_s_c_s GTGTGGGTGGCTGTGCTGGTGTGGGTGTGGGTG; >clamp_s_c_s_e_s:compl:rev CACCAGCACCCACACTATCATGTTGACATTCC; dG = -12.9
228 >s_c_s_c_s_a_s_c_s_c_s GTGTGGGTGTGGGTGTCTGTGTGGGTGTGGGTG; >clamp_s_c_s_e_s:compl:rev CACCAGCACCCACACTATCATGTTGACATTCC; dG = -12.9
229 >s_c_s_c_s_b_s_c_s_c_s GTGTGGGTGTGGGTGGCTGTGTGGGTGTGGGTG; >clamp_s_c_s_e_s:compl:rev CACCAGCACCCACACTATCATGTTGACATTCC; dG = -12.9
230 >s_c_s_d_s_a_s_c_s_c_s GTGTGGGTGACGGTGTCTGTGTGGGTGTGGGTG; >clamp_s_c_s_e_s:compl:rev CACCAGCACCCACACTATCATGTTGACATTCC; dG = -12.9
231 >s_a_s_b_s_b_s_c_s_c_s GTGTCTGTGGCTGTGGCTGTGTGGGTGTGGGTG; >clamp_s_c_s_e_s:compl:rev CACCAGCACCCACACTATCATGTTGACATTCC; dG = -12.9
232 >s_c_s_d_s_b_s_c_s_c_s GTGTGGGTGACGGTGGCTGTGTGGGTGTGGGTG; >clamp_s_c_s_e_s:compl:rev CACCAGCACCCACACTATCATGTTGACATTCC; dG = -12.9
233 >s_c_s_d_s_c_s_d_s_e_s GTGTGGGTGACGGTGTGGGTGACGGTGCTGGTG; >clamp_s_c_s_e_s:compl:rev CACCAGCACCCACACTATCATGTTGACATTCC; dG = -12.9
234 >s_c_s_d_s_c_s_b_s_e_s GTGTGGGTGACGGTGTGGGTGGCTGTGCTGGTG; >clamp_s_c_s_e_s:compl:rev CACCAGCACCCACACTATCATGTTGACATTCC; dG = -12.9
235 >s_c_s_d_s_c_s_a_s_e_s GTGTGGGTGACGGTGTGGGTGTCTGTGCTGGTG; >clamp_s_c_s_e_s:compl:rev CACCAGCACCCACACTATCATGTTGACATTCC; dG = -12.9
236 >s_c_s_d_s_d_s_c_s_c_s GTGTGGGTGACGGTGACGGTGTGGGTGTGGGTG; >clamp_s_c_s_e_s:compl:rev CACCAGCACCCACACTATCATGTTGACATTCC; dG = -12.9
237 >s_c_s_d_s_e_s_c_s_c_s GTGTGGGTGACGGTGCTGGTGTGGGTGTGGGTG; >clamp_s_c_s_e_s:compl:rev CACCAGCACCCACACTATCATGTTGACATTCC; dG = -12.9
238 >s_d_s_a_s_a_s_c_s_c_s GTGACGGTGTCTGTGTCTGTGTGGGTGTGGGTG; >clamp_s_c_s_e_s:compl:rev CACCAGCACCCACACTATCATGTTGACATTCC; dG = -12.9
239 >s_d_s_a_s_b_s_c_s_c_s GTGACGGTGTCTGTGGCTGTGTGGGTGTGGGTG; >clamp_s_c_s_e_s:compl:rev CACCAGCACCCACACTATCATGTTGACATTCC; dG = -12.9
240 >s_d_s_a_s_d_s_c_s_c_s GTGACGGTGTCTGTGACGGTGTGGGTGTGGGTG; >clamp_s_c_s_e_s:compl:rev CACCAGCACCCACACTATCATGTTGACATTCC; dG = -12.9
241 >s_d_s_a_s_e_s_c_s_c_s GTGACGGTGTCTGTGCTGGTGTGGGTGTGGGTG; >clamp_s_c_s_e_s:compl:rev CACCAGCACCCACACTATCATGTTGACATTCC; dG = -12.9
242 >s_d_s_b_s_a_s_c_s_c_s GTGACGGTGGCTGTGTCTGTGTGGGTGTGGGTG; >clamp_s_c_s_e_s:compl:rev CACCAGCACCCACACTATCATGTTGACATTCC; dG = -12.9
243 >s_d_s_b_s_b_s_c_s_c_s GTGACGGTGGCTGTGGCTGTGTGGGTGTGGGTG; >clamp_s_c_s_e_s:compl:rev CACCAGCACCCACACTATCATGTTGACATTCC; dG = -12.9
244 >s_d_s_b_s_d_s_c_s_c_s GTGACGGTGGCTGTGACGGTGTGGGTGTGGGTG; >clamp_s_c_s_e_s:compl:rev CACCAGCACCCACACTATCATGTTGACATTCC; dG = -12.9
245 >s_d_s_b_s_e_s_c_s_c_s GTGACGGTGGCTGTGCTGGTGTGGGTGTGGGTG; >clamp_s_c_s_e_s:compl:rev CACCAGCACCCACACTATCATGTTGACATTCC; dG = -12.9
246 >s_d_s_c_s_a_s_c_s_c_s GTGACGGTGTGGGTGTCTGTGTGGGTGTGGGTG; >clamp_s_c_s_e_s:compl:rev CACCAGCACCCACACTATCATGTTGACATTCC; dG = -12.9
247 >s_d_s_c_s_b_s_c_s_c_s GTGACGGTGTGGGTGGCTGTGTGGGTGTGGGTG; >clamp_s_c_s_e_s:compl:rev CACCAGCACCCACACTATCATGTTGACATTCC; dG = -12.9
248 >s_d_s_c_s_d_s_c_s_c_s GTGACGGTGTGGGTGACGGTGTGGGTGTGGGTG; >clamp_s_c_s_e_s:compl:rev CACCAGCACCCACACTATCATGTTGACATTCC; dG = -12.9
249 >s_d_s_c_s_d_s_d_s_e_s GTGACGGTGTGGGTGACGGTGACGGTGCTGGTG; >clamp_s_c_s_e_s:compl:rev CACCAGCACCCACACTATCATGTTGACATTCC; dG = -12.9
250 >s_d_s_d_s_a_s_c_s_c_s GTGACGGTGACGGTGTCTGTGTGGGTGTGGGTG; >clamp_s_c_s_e_s:compl:rev CACCAGCACCCACACTATCATGTTGACATTCC; dG = -12.9
251 >s_a_s_b_s_d_s_c_s_c_s GTGTCTGTGGCTGTGACGGTGTGGGTGTGGGTG; >clamp_s_c_s_e_s:compl:rev CACCAGCACCCACACTATCATGTTGACATTCC; dG = -12.9
252 >s_d_s_d_s_b_s_c_s_c_s GTGACGGTGACGGTGGCTGTGTGGGTGTGGGTG; >clamp_s_c_s_e_s:compl:rev CACCAGCACCCACACTATCATGTTGACATTCC; dG = -12.9
253 >s_d_s_d_s_c_s_d_s_e_s GTGACGGTGACGGTGTGGGTGACGGTGCTGGTG; >clamp_s_c_s_e_s:compl:rev CACCAGCACCCACACTATCATGTTGACATTCC; dG = -12.9
254 >s_d_s_d_s_c_s_b_s_e_s GTGACGGTGACGGTGTGGGTGGCTGTGCTGGTG; >clamp_s_c_s_e_s:compl:rev CACCAGCACCCACACTATCATGTTGACATTCC; dG = -12.9
255 >s_d_s_d_s_c_s_a_s_e_s GTGACGGTGACGGTGTGGGTGTCTGTGCTGGTG; >clamp_s_c_s_e_s:compl:rev CACCAGCACCCACACTATCATGTTGACATTCC; dG = -12.9
256 >s_d_s_d_s_d_s_c_s_c_s GTGACGGTGACGGTGACGGTGTGGGTGTGGGTG; >clamp_s_c_s_e_s:compl:rev CACCAGCACCCACACTATCATGTTGACATTCC; dG = -12.9
257 >s_d_s_d_s_e_s_c_s_c_s GTGACGGTGACGGTGCTGGTGTGGGTGTGGGTG; >clamp_s_c_s_e_s:compl:rev CACCAGCACCCACACTATCATGTTGACATTCC; dG = -12.9
258 >s_d_s_e_s_a_s_c_s_c_s GTGACGGTGCTGGTGTCTGTGTGGGTGTGGGTG; >clamp_s_c_s_e_s:compl:rev CACCAGCACCCACACTATCATGTTGACATTCC; dG = -12.9
259 >s_d_s_e_s_b_s_c_s_c_s GTGACGGTGCTGGTGGCTGTGTGGGTGTGGGTG; >clamp_s_c_s_e_s:compl:rev CACCAGCACCCACACTATCATGTTGACATTCC; dG = -12.9
260 >s_d_s_e_s_c_s_d_s_e_s GTGACGGTGCTGGTGTGGGTGACGGTGCTGGTG; >clamp_s_c_s_e_s:compl:rev CACCAGCACCCACACTATCATGTTGACATTCC; dG = -12.9
261 >s_d_s_e_s_c_s_b_s_e_s GTGACGGTGCTGGTGTGGGTGGCTGTGCTGGTG; >clamp_s_c_s_e_s:compl:rev CACCAGCACCCACACTATCATGTTGACATTCC; dG = -12.9

262 >s_d_s_e_s_c_s_a_s_e_s GTGACGGTGCTGGTGTGGGTGTCTGTGCTGGTG; >clamp_s_c_s_e_s:compl:rev
CACCAGCACCCACACTATCATGTTGACATTCC; dG = -12.9
263 >s_d_s_e_s_d_s_c_s_c_s GTGACGGTGCTGGTGACGGTGTGGGTGTGGGTG; >clamp_s_c_s_e_s:compl:rev
CACCAGCACCCACACTATCATGTTGACATTCC; dG = -12.9
264 >s_d_s_e_s_e_s_c_s_c_s GTGACGGTGCTGGTGCTGGTGTGGGTGTGGGTG; >clamp_s_c_s_e_s:compl:rev
CACCAGCACCCACACTATCATGTTGACATTCC; dG = -12.9
265 >s_d_s_e_s_e_s_d_s_e_s GTGACGGTGCTGGTGCTGGTGACGGTGCTGGTG; >clamp_s_c_s_e_s:compl:rev
CACCAGCACCCACACTATCATGTTGACATTCC; dG = -12.9
266 >s_e_s_a_s_a_s_c_s_c_s GTGCTGGTGTCTGTGTCTGTGTGGGTGTGGGTG; >clamp_s_c_s_e_s:compl:rev
CACCAGCACCCACACTATCATGTTGACATTCC; dG = -12.9
267 >s_e_s_a_s_b_s_c_s_c_s GTGCTGGTGTCTGTGGCTGTGTGGGTGTGGGTG; >clamp_s_c_s_e_s:compl:rev
CACCAGCACCCACACTATCATGTTGACATTCC; dG = -12.9
268 >s_a_s_b_s_e_s_c_s_c_s GTGTCTGTGGCTGTGCTGGTGTGGGTGTGGGTG; >clamp_s_c_s_e_s:compl:rev
CACCAGCACCCACACTATCATGTTGACATTCC; dG = -12.9
269 >s_e_s_a_s_d_s_c_s_c_s GTGCTGGTGTCTGTGACGGTGTGGGTGTGGGTG; >clamp_s_c_s_e_s:compl:rev
CACCAGCACCCACACTATCATGTTGACATTCC; dG = -12.9
270 >s_e_s_a_s_e_s_c_s_c_s GTGCTGGTGTCTGTGCTGGTGTGGGTGTGGGTG; >clamp_s_c_s_e_s:compl:rev
CACCAGCACCCACACTATCATGTTGACATTCC; dG = -12.9
271 >s_e_s_b_s_a_s_c_s_c_s GTGCTGGTGGCTGTGTCTGTGTGGGTGTGGGTG; >clamp_s_c_s_e_s:compl:rev
CACCAGCACCCACACTATCATGTTGACATTCC; dG = -12.9
272 >s_e_s_b_s_b_s_c_s_c_s GTGCTGGTGGCTGTGGCTGTGTGGGTGTGGGTG; >clamp_s_c_s_e_s:compl:rev
CACCAGCACCCACACTATCATGTTGACATTCC; dG = -12.9
273 >s_e_s_b_s_d_s_c_s_c_s GTGCTGGTGGCTGTGACGGTGTGGGTGTGGGTG; >clamp_s_c_s_e_s:compl:rev
CACCAGCACCCACACTATCATGTTGACATTCC; dG = -12.9
274 >s_e_s_b_s_e_s_c_s_c_s GTGCTGGTGGCTGTGCTGGTGTGGGTGTGGGTG; >clamp_s_c_s_e_s:compl:rev
CACCAGCACCCACACTATCATGTTGACATTCC; dG = -12.9
275 >s_e_s_c_s_a_s_c_s_c_s GTGCTGGTGTGGGTGTCTGTGTGGGTGTGGGTG; >clamp_s_c_s_e_s:compl:rev
CACCAGCACCCACACTATCATGTTGACATTCC; dG = -12.9
276 >s_e_s_c_s_b_s_c_s_c_s GTGCTGGTGTGGGTGGCTGTGTGGGTGTGGGTG; >clamp_s_c_s_e_s:compl:rev
CACCAGCACCCACACTATCATGTTGACATTCC; dG = -12.9
277 >s_a_s_c_s_a_s_c_s_c_s GTGTCTGTGTGGGTGTCTGTGTGGGTGTGGGTG; >clamp_s_c_s_e_s:compl:rev
CACCAGCACCCACACTATCATGTTGACATTCC; dG = -12.9
278 >s_e_s_c_s_d_s_c_s_c_s GTGCTGGTGTGGGTGACGGTGTGGGTGTGGGTG; >clamp_s_c_s_e_s:compl:rev
CACCAGCACCCACACTATCATGTTGACATTCC; dG = -12.9
279 >s_e_s_c_s_d_s_d_s_e_s GTGCTGGTGTGGGTGACGGTGACGGTGCTGGTG; >clamp_s_c_s_e_s:compl:rev
CACCAGCACCCACACTATCATGTTGACATTCC; dG = -12.9
280 >s_e_s_d_s_a_s_c_s_c_s GTGCTGGTGACGGTGTCTGTGTGGGTGTGGGTG; >clamp_s_c_s_e_s:compl:rev
CACCAGCACCCACACTATCATGTTGACATTCC; dG = -12.9
281 >s_e_s_d_s_b_s_c_s_c_s GTGCTGGTGACGGTGGCTGTGTGGGTGTGGGTG; >clamp_s_c_s_e_s:compl:rev
CACCAGCACCCACACTATCATGTTGACATTCC; dG = -12.9
282 >s_e_s_d_s_c_s_d_s_e_s GTGCTGGTGACGGTGTGGGTGACGGTGCTGGTG; >clamp_s_c_s_e_s:compl:rev
CACCAGCACCCACACTATCATGTTGACATTCC; dG = -12.9
283 >s_e_s_d_s_c_s_b_s_e_s GTGCTGGTGACGGTGTGGGTGGCTGTGCTGGTG; >clamp_s_c_s_e_s:compl:rev
CACCAGCACCCACACTATCATGTTGACATTCC; dG = -12.9
284 >s_e_s_d_s_c_s_a_s_e_s GTGCTGGTGACGGTGTGGGTGTCTGTGCTGGTG; >clamp_s_c_s_e_s:compl:rev
CACCAGCACCCACACTATCATGTTGACATTCC; dG = -12.9
285 >s_e_s_d_s_d_s_c_s_c_s GTGCTGGTGACGGTGACGGTGTGGGTGTGGGTG; >clamp_s_c_s_e_s:compl:rev
CACCAGCACCCACACTATCATGTTGACATTCC; dG = -12.9
286 >s_e_s_d_s_e_s_c_s_c_s GTGCTGGTGACGGTGCTGGTGTGGGTGTGGGTG; >clamp_s_c_s_e_s:compl:rev
CACCAGCACCCACACTATCATGTTGACATTCC; dG = -12.9
287 >s_e_s_e_s_a_s_c_s_c_s GTGCTGGTGCTGGTGTCTGTGTGGGTGTGGGTG; >clamp_s_c_s_e_s:compl:rev
CACCAGCACCCACACTATCATGTTGACATTCC; dG = -12.9
288 >s_e_s_e_s_b_s_c_s_c_s GTGCTGGTGCTGGTGGCTGTGTGGGTGTGGGTG; >clamp_s_c_s_e_s:compl:rev
CACCAGCACCCACACTATCATGTTGACATTCC; dG = -12.9
289 >s_e_s_e_s_c_s_d_s_e_s GTGCTGGTGCTGGTGTGGGTGACGGTGCTGGTG; >clamp_s_c_s_e_s:compl:rev
CACCAGCACCCACACTATCATGTTGACATTCC; dG = -12.9
290 >s_e_s_e_s_c_s_b_s_e_s GTGCTGGTGCTGGTGTGGGTGGCTGTGCTGGTG; >clamp_s_c_s_e_s:compl:rev
CACCAGCACCCACACTATCATGTTGACATTCC; dG = -12.9
291 >s_a_s_c_s_b_s_c_s_c_s GTGTCTGTGTGGGTGGCTGTGTGGGTGTGGGTG; >clamp_s_c_s_e_s:compl:rev
CACCAGCACCCACACTATCATGTTGACATTCC; dG = -12.9
292 >s_e_s_e_s_c_s_a_s_e_s GTGCTGGTGCTGGTGTGGGTGTCTGTGCTGGTG; >clamp_s_c_s_e_s:compl:rev
CACCAGCACCCACACTATCATGTTGACATTCC; dG = -12.9
293 >s_e_s_e_s_d_s_c_s_c_s GTGCTGGTGCTGGTGACGGTGTGGGTGTGGGTG; >clamp_s_c_s_e_s:compl:rev
CACCAGCACCCACACTATCATGTTGACATTCC; dG = -12.9
294 >s_e_s_e_s_e_s_c_s_c_s GTGCTGGTGCTGGTGCTGGTGTGGGTGTGGGTG; >clamp_s_c_s_e_s:compl:rev
CACCAGCACCCACACTATCATGTTGACATTCC; dG = -12.9
295 >s_e_s_e_s_e_s_d_s_e_s GTGCTGGTGCTGGTGCTGGTGACGGTGCTGGTG; >clamp_s_c_s_e_s:compl:rev
CACCAGCACCCACACTATCATGTTGACATTCC; dG = -12.9
296 >s_c_s_a_s_e_s_b_s_e_s GTGTGGGTGTCTGTGCTGGTGGCTGTGCTGGTG; >clamp_s_c_s_e_s:compl:rev
CACCAGCACCCACACTATCATGTTGACATTCC; dG = -12.8
297 >s_c_s_b_s_e_s_b_s_e_s GTGTGGGTGGCTGTGCTGGTGGCTGTGCTGGTG; >clamp_s_c_s_e_s:compl:rev
CACCAGCACCCACACTATCATGTTGACATTCC; dG = -12.8
298 >s_c_s_c_s_b_s_c_s_d_s GTGTGGGTGTGGGTGGCTGTGTGGGTGACGGTG; >clamp_s_c_s_e_s:compl:rev
CACCAGCACCCACACTATCATGTTGACATTCC; dG = -12.8

299 >s_c_s_c_s_b_s_c_s_b_s GTGTGGGTGTGGGTGGCTGTGTGGGTGGCTGTG; >clamp_s_c_s_e_s:compl:rev
CACCAGCACCCACACTATCATGTTGACATTCC; dG = -12.8
300 >s_c_s_c_s_b_s_c_s_a_s GTGTGGGTGTGGGTGGCTGTGTGGGTGTCTGTG; >clamp_s_c_s_e_s:compl:rev
CACCAGCACCCACACTATCATGTTGACATTCC; dG = -12.8
301 >s_c_s_c_s_b_s_d_s_e_s GTGTGGGTGTGGGTGGCTGTGACGGTGCTGGTG; >clamp_s_c_s_e_s:compl:rev
CACCAGCACCCACACTATCATGTTGACATTCC; dG = -12.8
302 >s_c_s_c_s_b_s_b_s_e_s GTGTGGGTGTGGGTGGCTGTGGCTGTGCTGGTG; >clamp_s_c_s_e_s:compl:rev
CACCAGCACCCACACTATCATGTTGACATTCC; dG = -12.8
303 >s_c_s_c_s_b_s_a_s_e_s GTGTGGGTGTGGGTGGCTGTGTCTGTGCTGGTG; >clamp_s_c_s_e_s:compl:rev
CACCAGCACCCACACTATCATGTTGACATTCC; dG = -12.8
304 >s_c_s_d_s_e_s_b_s_e_s GTGTGGGTGACGGTGCTGGTGGCTGTGCTGGTG; >clamp_s_c_s_e_s:compl:rev
CACCAGCACCCACACTATCATGTTGACATTCC; dG = -12.8
305 >s_c_s_a_s_e_s_c_s_d_s GTGTGGGTGTCTGTGCTGGTGTGGGTGACGGTG; >clamp_s_c_s_e_s:compl:rev
CACCAGCACCCACACTATCATGTTGACATTCC; dG = -12.7
306 >s_c_s_a_s_e_s_c_s_b_s GTGTGGGTGTCTGTGCTGGTGTGGGTGGCTGTG; >clamp_s_c_s_e_s:compl:rev
CACCAGCACCCACACTATCATGTTGACATTCC; dG = -12.7
307 >s_c_s_a_s_e_s_c_s_a_s GTGTGGGTGTCTGTGCTGGTGTGGGTGTCTGTG; >clamp_s_c_s_e_s:compl:rev
CACCAGCACCCACACTATCATGTTGACATTCC; dG = -12.7
308 >s_c_s_a_s_e_s_a_s_e_s GTGTGGGTGTCTGTGCTGGTGTCTGTGCTGGTG; >clamp_s_c_s_e_s:compl:rev
CACCAGCACCCACACTATCATGTTGACATTCC; dG = -12.7
309 >s_c_s_b_s_e_s_c_s_d_s GTGTGGGTGGCTGTGCTGGTGTGGGTGACGGTG; >clamp_s_c_s_e_s:compl:rev
CACCAGCACCCACACTATCATGTTGACATTCC; dG = -12.7
310 >s_c_s_b_s_e_s_c_s_b_s GTGTGGGTGGCTGTGCTGGTGTGGGTGGCTGTG; >clamp_s_c_s_e_s:compl:rev
CACCAGCACCCACACTATCATGTTGACATTCC; dG = -12.7
311 >s_c_s_b_s_e_s_c_s_a_s GTGTGGGTGGCTGTGCTGGTGTGGGTGTCTGTG; >clamp_s_c_s_e_s:compl:rev
CACCAGCACCCACACTATCATGTTGACATTCC; dG = -12.7
312 >s_c_s_b_s_e_s_a_s_e_s GTGTGGGTGGCTGTGCTGGTGTCTGTGCTGGTG; >clamp_s_c_s_e_s:compl:rev
CACCAGCACCCACACTATCATGTTGACATTCC; dG = -12.7
313 >s_c_s_c_s_a_s_c_s_d_s GTGTGGGTGTGGGTGTCTGTGTGGGTGACGGTG; >clamp_s_c_s_e_s:compl:rev
CACCAGCACCCACACTATCATGTTGACATTCC; dG = -12.7
314 >s_c_s_c_s_a_s_c_s_b_s GTGTGGGTGTGGGTGTCTGTGTGGGTGGCTGTG; >clamp_s_c_s_e_s:compl:rev
CACCAGCACCCACACTATCATGTTGACATTCC; dG = -12.7
315 >s_c_s_c_s_a_s_c_s_a_s GTGTGGGTGTGGGTGTCTGTGTGGGTGTCTGTG; >clamp_s_c_s_e_s:compl:rev
CACCAGCACCCACACTATCATGTTGACATTCC; dG = -12.7
316 >s_c_s_c_s_a_s_d_s_e_s GTGTGGGTGTGGGTGTCTGTGACGGTGCTGGTG; >clamp_s_c_s_e_s:compl:rev
CACCAGCACCCACACTATCATGTTGACATTCC; dG = -12.7
317 >s_c_s_c_s_a_s_b_s_e_s GTGTGGGTGTGGGTGTCTGTGGCTGTGCTGGTG; >clamp_s_c_s_e_s:compl:rev
CACCAGCACCCACACTATCATGTTGACATTCC; dG = -12.7
318 >s_c_s_c_s_a_s_a_s_e_s GTGTGGGTGTGGGTGTCTGTGTCTGTGCTGGTG; >clamp_s_c_s_e_s:compl:rev
CACCAGCACCCACACTATCATGTTGACATTCC; dG = -12.7
319 >s_c_s_d_s_e_s_c_s_d_s GTGTGGGTGACGGTGCTGGTGTGGGTGACGGTG; >clamp_s_c_s_e_s:compl:rev
CACCAGCACCCACACTATCATGTTGACATTCC; dG = -12.7
320 >s_c_s_d_s_e_s_c_s_b_s GTGTGGGTGACGGTGCTGGTGTGGGTGGCTGTG; >clamp_s_c_s_e_s:compl:rev
CACCAGCACCCACACTATCATGTTGACATTCC; dG = -12.7
321 >s_c_s_d_s_e_s_c_s_a_s GTGTGGGTGACGGTGCTGGTGTGGGTGTCTGTG; >clamp_s_c_s_e_s:compl:rev
CACCAGCACCCACACTATCATGTTGACATTCC; dG = -12.7
322 >s_c_s_d_s_e_s_a_s_e_s GTGTGGGTGACGGTGCTGGTGTCTGTGCTGGTG; >clamp_s_c_s_e_s:compl:rev
CACCAGCACCCACACTATCATGTTGACATTCC; dG = -12.7
323 >s_a_s_d_s_e_s_b_s_e_s GTGTCTGTGACGGTGCTGGTGGCTGTGCTGGTG; >clamp_s_c_s_e_s:compl:rev
CACCAGCACCCACACTATCATGTTGACATTCC; dG = -12.6
324 >s_b_s_d_s_e_s_b_s_e_s GTGGCTGTGACGGTGCTGGTGGCTGTGCTGGTG; >clamp_s_c_s_e_s:compl:rev
CACCAGCACCCACACTATCATGTTGACATTCC; dG = -12.6
325 >s_c_s_a_s_e_s_e_s_e_s GTGTGGGTGTCTGTGCTGGTGCTGGTGCTGGTG; >clamp_s_c_s_e_s:compl:rev
CACCAGCACCCACACTATCATGTTGACATTCC; dG = -12.6
326 >s_c_s_b_s_e_s_e_s_e_s GTGTGGGTGGCTGTGCTGGTGCTGGTGCTGGTG; >clamp_s_c_s_e_s:compl:rev
CACCAGCACCCACACTATCATGTTGACATTCC; dG = -12.6
327 >s_c_s_d_s_e_s_e_s_e_s GTGTGGGTGACGGTGCTGGTGCTGGTGCTGGTG; >clamp_s_c_s_e_s:compl:rev
CACCAGCACCCACACTATCATGTTGACATTCC; dG = -12.6
328 >s_a_s_d_s_e_s_c_s_d_s GTGTCTGTGACGGTGCTGGTGTGGGTGACGGTG; >clamp_s_c_s_e_s:compl:rev
CACCAGCACCCACACTATCATGTTGACATTCC; dG = -12.5
329 >s_a_s_d_s_e_s_c_s_b_s GTGTCTGTGACGGTGCTGGTGTGGGTGGCTGTG; >clamp_s_c_s_e_s:compl:rev
CACCAGCACCCACACTATCATGTTGACATTCC; dG = -12.5
330 >s_a_s_d_s_e_s_c_s_a_s GTGTCTGTGACGGTGCTGGTGTGGGTGTCTGTG; >clamp_s_c_s_e_s:compl:rev
CACCAGCACCCACACTATCATGTTGACATTCC; dG = -12.5
331 >s_a_s_d_s_e_s_a_s_e_s GTGTCTGTGACGGTGCTGGTGTCTGTGCTGGTG; >clamp_s_c_s_e_s:compl:rev
CACCAGCACCCACACTATCATGTTGACATTCC; dG = -12.5
332 >s_a_s_e_s_e_s_b_s_e_s GTGTCTGTGCTGGTGCTGGTGGCTGTGCTGGTG; >clamp_s_c_s_e_s:compl:rev
CACCAGCACCCACACTATCATGTTGACATTCC; dG = -12.5
333 >s_b_s_d_s_e_s_c_s_d_s GTGGCTGTGACGGTGCTGGTGTGGGTGACGGTG; >clamp_s_c_s_e_s:compl:rev
CACCAGCACCCACACTATCATGTTGACATTCC; dG = -12.5
334 >s_b_s_d_s_e_s_c_s_b_s GTGGCTGTGACGGTGCTGGTGTGGGTGGCTGTG; >clamp_s_c_s_e_s:compl:rev
CACCAGCACCCACACTATCATGTTGACATTCC; dG = -12.5
335 >s_b_s_d_s_e_s_c_s_a_s GTGGCTGTGACGGTGCTGGTGTGGGTGTCTGTG; >clamp_s_c_s_e_s:compl:rev
CACCAGCACCCACACTATCATGTTGACATTCC; dG = -12.5

336 >s_b_s_d_s_e_s_a_s_e_s GTGGCTGTGACGGTGCTGGTGTCTGTGCTGGTG; >clamp_s_c_s_e_s:compl:rev CACCAGCACCCACACTATCATGTTGACATTCC; dG = -12.5
337 >s_b_s_e_s_e_s_b_s_e_s GTGGCTGTGCTGGTGCTGGTGGCTGTGCTGGTG; >clamp_s_c_s_e_s:compl:rev CACCAGCACCCACACTATCATGTTGACATTCC; dG = -12.5
338 >s_d_s_d_s_e_s_b_s_e_s GTGACGGTGACGGTGCTGGTGGCTGTGCTGGTG; >clamp_s_c_s_e_s:compl:rev CACCAGCACCCACACTATCATGTTGACATTCC; dG = -12.5
339 >s_d_s_e_s_d_s_c_s_d_s GTGACGGTGCTGGTGACGGTGTGGGTGACGGTG; >clamp_s_c_s_e_s:compl:rev CACCAGCACCCACACTATCATGTTGACATTCC; dG = -12.5
340 >s_d_s_e_s_d_s_c_s_b_s GTGACGGTGCTGGTGACGGTGTGGGTGGCTGTG; >clamp_s_c_s_e_s:compl:rev CACCAGCACCCACACTATCATGTTGACATTCC; dG = -12.5
341 >s_d_s_e_s_d_s_c_s_a_s GTGACGGTGCTGGTGACGGTGTGGGTGTCTGTG; >clamp_s_c_s_e_s:compl:rev CACCAGCACCCACACTATCATGTTGACATTCC; dG = -12.5
342 >s_d_s_e_s_d_s_e_s_e_s GTGACGGTGCTGGTGACGGTGCTGGTGCTGGTG; >clamp_s_c_s_e_s:compl:rev CACCAGCACCCACACTATCATGTTGACATTCC; dG = -12.5
343 >s_d_s_e_s_d_s_d_s_e_s GTGACGGTGCTGGTGACGGTGACGGTGCTGGTG; >clamp_s_c_s_e_s:compl:rev CACCAGCACCCACACTATCATGTTGACATTCC; dG = -12.5
344 >s_d_s_e_s_d_s_b_s_e_s GTGACGGTGCTGGTGACGGTGGCTGTGCTGGTG; >clamp_s_c_s_e_s:compl:rev CACCAGCACCCACACTATCATGTTGACATTCC; dG = -12.5
345 >s_d_s_e_s_d_s_a_s_e_s GTGACGGTGCTGGTGACGGTGTCTGTGCTGGTG; >clamp_s_c_s_e_s:compl:rev CACCAGCACCCACACTATCATGTTGACATTCC; dG = -12.5
346 >s_e_s_d_s_e_s_b_s_e_s GTGCTGGTGACGGTGCTGGTGGCTGTGCTGGTG; >clamp_s_c_s_e_s:compl:rev CACCAGCACCCACACTATCATGTTGACATTCC; dG = -12.5
347 >s_a_s_c_s_d_s_b_s_e_s GTGTCTGTGTGGGTGACGGTGGCTGTGCTGGTG; >clamp_s_c_s_e_s:compl:rev CACCAGCACCCACACTATCATGTTGACATTCC; dG = -12.4
348 >s_a_s_d_s_e_s_e_s_e_s GTGTCTGTGACGGTGCTGGTGCTGGTGCTGGTG; >clamp_s_c_s_e_s:compl:rev CACCAGCACCCACACTATCATGTTGACATTCC; dG = -12.4
349 >s_a_s_e_s_e_s_c_s_d_s GTGTCTGTGCTGGTGCTGGTGTGGGTGACGGTG; >clamp_s_c_s_e_s:compl:rev CACCAGCACCCACACTATCATGTTGACATTCC; dG = -12.4
350 >s_a_s_e_s_e_s_c_s_b_s GTGTCTGTGCTGGTGCTGGTGTGGGTGGCTGTG; >clamp_s_c_s_e_s:compl:rev CACCAGCACCCACACTATCATGTTGACATTCC; dG = -12.4
351 >s_a_s_e_s_e_s_c_s_a_s GTGTCTGTGCTGGTGCTGGTGTGGGTGTCTGTG; >clamp_s_c_s_e_s:compl:rev CACCAGCACCCACACTATCATGTTGACATTCC; dG = -12.4
352 >s_a_s_e_s_e_s_a_s_e_s GTGTCTGTGCTGGTGCTGGTGTCTGTGCTGGTG; >clamp_s_c_s_e_s:compl:rev CACCAGCACCCACACTATCATGTTGACATTCC; dG = -12.4
353 >s_b_s_a_s_d_s_e_s_e_s GTGGCTGTGTCTGTGACGGTGCTGGTGCTGGTG; >clamp_s_c_s_e_s:compl:rev CACCAGCACCCACACTATCATGTTGACATTCC; dG = -12.4
354 >s_b_s_b_s_d_s_e_s_e_s GTGGCTGTGGCTGTGACGGTGCTGGTGCTGGTG; >clamp_s_c_s_e_s:compl:rev CACCAGCACCCACACTATCATGTTGACATTCC; dG = -12.4
355 >s_a_s_a_s_d_s_e_s_e_s GTGTCTGTGTCTGTGACGGTGCTGGTGCTGGTG; >clamp_s_c_s_e_s:compl:rev CACCAGCACCCACACTATCATGTTGACATTCC; dG = -12.4
356 >s_b_s_c_s_d_s_b_s_e_s GTGGCTGTGTGGGTGACGGTGGCTGTGCTGGTG; >clamp_s_c_s_e_s:compl:rev CACCAGCACCCACACTATCATGTTGACATTCC; dG = -12.4
357 >s_b_s_d_s_e_s_e_s_e_s GTGGCTGTGACGGTGCTGGTGCTGGTGCTGGTG; >clamp_s_c_s_e_s:compl:rev CACCAGCACCCACACTATCATGTTGACATTCC; dG = -12.4
358 >s_b_s_e_s_e_s_c_s_d_s GTGGCTGTGCTGGTGCTGGTGTGGGTGACGGTG; >clamp_s_c_s_e_s:compl:rev CACCAGCACCCACACTATCATGTTGACATTCC; dG = -12.4
359 >s_b_s_e_s_e_s_c_s_b_s GTGGCTGTGCTGGTGCTGGTGTGGGTGGCTGTG; >clamp_s_c_s_e_s:compl:rev CACCAGCACCCACACTATCATGTTGACATTCC; dG = -12.4
360 >s_b_s_e_s_e_s_c_s_a_s GTGGCTGTGCTGGTGCTGGTGTGGGTGTCTGTG; >clamp_s_c_s_e_s:compl:rev CACCAGCACCCACACTATCATGTTGACATTCC; dG = -12.4
361 >s_b_s_e_s_e_s_a_s_e_s GTGGCTGTGCTGGTGCTGGTGTCTGTGCTGGTG; >clamp_s_c_s_e_s:compl:rev CACCAGCACCCACACTATCATGTTGACATTCC; dG = -12.4
362 >s_c_s_a_s_d_s_e_s_e_s GTGTGGGTGTCTGTGACGGTGCTGGTGCTGGTG; >clamp_s_c_s_e_s:compl:rev CACCAGCACCCACACTATCATGTTGACATTCC; dG = -12.4
363 >s_c_s_b_s_d_s_e_s_e_s GTGTGGGTGGCTGTGACGGTGCTGGTGCTGGTG; >clamp_s_c_s_e_s:compl:rev CACCAGCACCCACACTATCATGTTGACATTCC; dG = -12.4
364 >s_c_s_d_s_d_s_c_s_d_s GTGTGGGTGACGGTGACGGTGTGGGTGACGGTG; >clamp_s_c_s_e_s:compl:rev CACCAGCACCCACACTATCATGTTGACATTCC; dG = -12.4
365 >s_c_s_d_s_d_s_c_s_b_s GTGTGGGTGACGGTGACGGTGTGGGTGGCTGTG; >clamp_s_c_s_e_s:compl:rev CACCAGCACCCACACTATCATGTTGACATTCC; dG = -12.4
366 >s_c_s_d_s_d_s_c_s_a_s GTGTGGGTGACGGTGACGGTGTGGGTGTCTGTG; >clamp_s_c_s_e_s:compl:rev CACCAGCACCCACACTATCATGTTGACATTCC; dG = -12.4
367 >s_c_s_d_s_d_s_e_s_e_s GTGTGGGTGACGGTGACGGTGCTGGTGCTGGTG; >clamp_s_c_s_e_s:compl:rev CACCAGCACCCACACTATCATGTTGACATTCC; dG = -12.4
368 >s_c_s_d_s_d_s_d_s_e_s GTGTGGGTGACGGTGACGGTGACGGTGCTGGTG; >clamp_s_c_s_e_s:compl:rev CACCAGCACCCACACTATCATGTTGACATTCC; dG = -12.4
369 >s_c_s_d_s_d_s_b_s_e_s GTGTGGGTGACGGTGACGGTGGCTGTGCTGGTG; >clamp_s_c_s_e_s:compl:rev CACCAGCACCCACACTATCATGTTGACATTCC; dG = -12.4
370 >s_c_s_d_s_d_s_a_s_e_s GTGTGGGTGACGGTGACGGTGTCTGTGCTGGTG; >clamp_s_c_s_e_s:compl:rev CACCAGCACCCACACTATCATGTTGACATTCC; dG = -12.4
371 >s_d_s_a_s_d_s_e_s_e_s GTGACGGTGTCTGTGACGGTGCTGGTGCTGGTG; >clamp_s_c_s_e_s:compl:rev CACCAGCACCCACACTATCATGTTGACATTCC; dG = -12.4
372 >s_d_s_b_s_d_s_e_s_e_s GTGACGGTGGCTGTGACGGTGCTGGTGCTGGTG; >clamp_s_c_s_e_s:compl:rev CACCAGCACCCACACTATCATGTTGACATTCC; dG = -12.4

373 >s_d_s_c_s_d_s_b_s_e_s GTGACGGTGTGGGTGACGGTGGCTGTGCTGGTG; >clamp_s_c_s_e_s:compl:rev
CACCAGCACCCACACTATCATGTTGACATTCC; dG = -12.4
374 >s_d_s_d_s_e_s_c_s_d_s GTGACGGTGACGGTGCTGGTGTGGGTGACGGTG; >clamp_s_c_s_e_s:compl:rev
CACCAGCACCCACACTATCATGTTGACATTCC; dG = -12.4
375 >s_d_s_d_s_e_s_c_s_b_s GTGACGGTGACGGTGCTGGTGTGGGTGGCTGTG; >clamp_s_c_s_e_s:compl:rev
CACCAGCACCCACACTATCATGTTGACATTCC; dG = -12.4
376 >s_d_s_d_s_e_s_c_s_a_s GTGACGGTGACGGTGCTGGTGTGGGTGTCTGTG; >clamp_s_c_s_e_s:compl:rev
CACCAGCACCCACACTATCATGTTGACATTCC; dG = -12.4
377 >s_a_s_b_s_d_s_e_s_e_s GTGTCTGTGGCTGTGACGGTGCTGGTGCTGGTG; >clamp_s_c_s_e_s:compl:rev
CACCAGCACCCACACTATCATGTTGACATTCC; dG = -12.4
378 >s_d_s_d_s_e_s_a_s_e_s GTGACGGTGACGGTGCTGGTGTCTGTGCTGGTG; >clamp_s_c_s_e_s:compl:rev
CACCAGCACCCACACTATCATGTTGACATTCC; dG = -12.4
379 >s_d_s_e_s_e_s_b_s_e_s GTGACGGTGCTGGTGCTGGTGGCTGTGCTGGTG; >clamp_s_c_s_e_s:compl:rev
CACCAGCACCCACACTATCATGTTGACATTCC; dG = -12.4
380 >s_e_s_a_s_d_s_e_s_e_s GTGCTGGTGTCTGTGACGGTGCTGGTGCTGGTG; >clamp_s_c_s_e_s:compl:rev
CACCAGCACCCACACTATCATGTTGACATTCC; dG = -12.4
381 >s_e_s_b_s_d_s_e_s_e_s GTGCTGGTGGCTGTGACGGTGCTGGTGCTGGTG; >clamp_s_c_s_e_s:compl:rev
CACCAGCACCCACACTATCATGTTGACATTCC; dG = -12.4
382 >s_e_s_c_s_d_s_b_s_e_s GTGCTGGTGTGGGTGACGGTGGCTGTGCTGGTG; >clamp_s_c_s_e_s:compl:rev
CACCAGCACCCACACTATCATGTTGACATTCC; dG = -12.4
383 >s_e_s_d_s_e_s_c_s_d_s GTGCTGGTGACGGTGCTGGTGTGGGTGACGGTG; >clamp_s_c_s_e_s:compl:rev
CACCAGCACCCACACTATCATGTTGACATTCC; dG = -12.4
384 >s_e_s_d_s_e_s_c_s_b_s GTGCTGGTGACGGTGCTGGTGTGGGTGGCTGTG; >clamp_s_c_s_e_s:compl:rev
CACCAGCACCCACACTATCATGTTGACATTCC; dG = -12.4
385 >s_e_s_d_s_e_s_c_s_a_s GTGCTGGTGACGGTGCTGGTGTGGGTGTCTGTG; >clamp_s_c_s_e_s:compl:rev
CACCAGCACCCACACTATCATGTTGACATTCC; dG = -12.4
386 >s_e_s_d_s_e_s_a_s_e_s GTGCTGGTGACGGTGCTGGTGTCTGTGCTGGTG; >clamp_s_c_s_e_s:compl:rev
CACCAGCACCCACACTATCATGTTGACATTCC; dG = -12.4
387 >s_e_s_e_s_d_s_c_s_d_s GTGCTGGTGCTGGTGACGGTGTGGGTGACGGTG; >clamp_s_c_s_e_s:compl:rev
CACCAGCACCCACACTATCATGTTGACATTCC; dG = -12.4
388 >s_e_s_e_s_d_s_c_s_b_s GTGCTGGTGCTGGTGACGGTGTGGGTGGCTGTG; >clamp_s_c_s_e_s:compl:rev
CACCAGCACCCACACTATCATGTTGACATTCC; dG = -12.4
389 >s_e_s_e_s_d_s_c_s_a_s GTGCTGGTGCTGGTGACGGTGTGGGTGTCTGTG; >clamp_s_c_s_e_s:compl:rev
CACCAGCACCCACACTATCATGTTGACATTCC; dG = -12.4
390 >s_e_s_e_s_d_s_e_s_e_s GTGCTGGTGCTGGTGACGGTGCTGGTGCTGGTG; >clamp_s_c_s_e_s:compl:rev
CACCAGCACCCACACTATCATGTTGACATTCC; dG = -12.4
391 >s_e_s_e_s_d_s_d_s_e_s GTGCTGGTGCTGGTGACGGTGACGGTGCTGGTG; >clamp_s_c_s_e_s:compl:rev
CACCAGCACCCACACTATCATGTTGACATTCC; dG = -12.4
392 >s_e_s_e_s_d_s_b_s_e_s GTGCTGGTGCTGGTGACGGTGGCTGTGCTGGTG; >clamp_s_c_s_e_s:compl:rev
CACCAGCACCCACACTATCATGTTGACATTCC; dG = -12.4
393 >s_e_s_e_s_d_s_a_s_e_s GTGCTGGTGCTGGTGACGGTGTCTGTGCTGGTG; >clamp_s_c_s_e_s:compl:rev
CACCAGCACCCACACTATCATGTTGACATTCC; dG = -12.4
394 >s_e_s_e_s_e_s_b_s_e_s GTGCTGGTGCTGGTGCTGGTGGCTGTGCTGGTG; >clamp_s_c_s_e_s:compl:rev
CACCAGCACCCACACTATCATGTTGACATTCC; dG = -12.4
395 >s_a_s_c_s_d_s_c_s_d_s GTGTCTGTGTGGGTGACGGTGTGGGTGACGGTG; >clamp_s_c_s_e_s:compl:rev
CACCAGCACCCACACTATCATGTTGACATTCC; dG = -12.3
396 >s_a_s_c_s_d_s_c_s_b_s GTGTCTGTGTGGGTGACGGTGTGGGTGGCTGTG; >clamp_s_c_s_e_s:compl:rev
CACCAGCACCCACACTATCATGTTGACATTCC; dG = -12.3
397 >s_a_s_c_s_d_s_c_s_a_s GTGTCTGTGTGGGTGACGGTGTGGGTGTCTGTG; >clamp_s_c_s_e_s:compl:rev
CACCAGCACCCACACTATCATGTTGACATTCC; dG = -12.3
398 >s_a_s_c_s_d_s_a_s_e_s GTGTCTGTGTGGGTGACGGTGTCTGTGCTGGTG; >clamp_s_c_s_e_s:compl:rev
CACCAGCACCCACACTATCATGTTGACATTCC; dG = -12.3
399 >s_a_s_d_s_d_s_e_s_e_s GTGTCTGTGACGGTGACGGTGCTGGTGCTGGTG; >clamp_s_c_s_e_s:compl:rev
CACCAGCACCCACACTATCATGTTGACATTCC; dG = -12.3
400 >s_a_s_e_s_d_s_e_s_e_s GTGTCTGTGCTGGTGACGGTGCTGGTGCTGGTG; >clamp_s_c_s_e_s:compl:rev
CACCAGCACCCACACTATCATGTTGACATTCC; dG = -12.3
401 >s_a_s_e_s_e_s_e_s_e_s GTGTCTGTGCTGGTGCTGGTGCTGGTGCTGGTG; >clamp_s_c_s_e_s:compl:rev
CACCAGCACCCACACTATCATGTTGACATTCC; dG = -12.3
402 >s_b_s_a_s_e_s_e_s_e_s GTGGCTGTGTCTGTGCTGGTGCTGGTGCTGGTG; >clamp_s_c_s_e_s:compl:rev
CACCAGCACCCACACTATCATGTTGACATTCC; dG = -12.3
403 >s_b_s_b_s_e_s_e_s_e_s GTGGCTGTGGCTGTGCTGGTGCTGGTGCTGGTG; >clamp_s_c_s_e_s:compl:rev
CACCAGCACCCACACTATCATGTTGACATTCC; dG = -12.3
404 >s_b_s_c_s_d_s_c_s_d_s GTGGCTGTGTGGGTGACGGTGTGGGTGACGGTG; >clamp_s_c_s_e_s:compl:rev
CACCAGCACCCACACTATCATGTTGACATTCC; dG = -12.3
405 >s_b_s_c_s_d_s_c_s_b_s GTGGCTGTGTGGGTGACGGTGTGGGTGGCTGTG; >clamp_s_c_s_e_s:compl:rev
CACCAGCACCCACACTATCATGTTGACATTCC; dG = -12.3
406 >s_b_s_c_s_d_s_c_s_a_s GTGGCTGTGTGGGTGACGGTGTGGGTGTCTGTG; >clamp_s_c_s_e_s:compl:rev
CACCAGCACCCACACTATCATGTTGACATTCC; dG = -12.3
407 >s_b_s_c_s_d_s_a_s_e_s GTGGCTGTGTGGGTGACGGTGTCTGTGCTGGTG; >clamp_s_c_s_e_s:compl:rev
CACCAGCACCCACACTATCATGTTGACATTCC; dG = -12.3
408 >s_b_s_d_s_d_s_e_s_e_s GTGGCTGTGACGGTGACGGTGCTGGTGCTGGTG; >clamp_s_c_s_e_s:compl:rev
CACCAGCACCCACACTATCATGTTGACATTCC; dG = -12.3
409 >s_a_s_a_s_e_s_e_s_e_s GTGTCTGTGTCTGTGCTGGTGCTGGTGCTGGTG; >clamp_s_c_s_e_s:compl:rev
CACCAGCACCCACACTATCATGTTGACATTCC; dG = -12.3

410 >s_b_s_e_s_d_s_e_s_e_s GTGGCTGTGCTGGTGACGGTGCTGGTGCTGGTG; >clamp_s_c_s_e_s:compl:rev CACCAGCACCCACACTATCATGTTGACATTCC; dG = -12.3
411 >s_b_s_e_s_e_s_e_s_e_s GTGGCTGTGCTGGTGCTGGTGCTGGTGCTGGTG; >clamp_s_c_s_e_s:compl:rev CACCAGCACCCACACTATCATGTTGACATTCC; dG = -12.3
412 >s_d_s_a_s_e_s_e_s_e_s GTGACGGTGTCTGTGCTGGTGCTGGTGCTGGTG; >clamp_s_c_s_e_s:compl:rev CACCAGCACCCACACTATCATGTTGACATTCC; dG = -12.3
413 >s_d_s_b_s_e_s_e_s_e_s GTGACGGTGGCTGTGCTGGTGCTGGTGCTGGTG; >clamp_s_c_s_e_s:compl:rev CACCAGCACCCACACTATCATGTTGACATTCC; dG = -12.3
414 >s_d_s_c_s_d_s_c_s_d_s GTGACGGTGTGGGTGACGGTGTGGGTGACGGTG; >clamp_s_c_s_e_s:compl:rev CACCAGCACCCACACTATCATGTTGACATTCC; dG = -12.3
415 >s_d_s_c_s_d_s_c_s_b_s GTGACGGTGTGGGTGACGGTGTGGGTGGCTGTG; >clamp_s_c_s_e_s:compl:rev CACCAGCACCCACACTATCATGTTGACATTCC; dG = -12.3
416 >s_d_s_c_s_d_s_c_s_a_s GTGACGGTGTGGGTGACGGTGTGGGTGTCTGTG; >clamp_s_c_s_e_s:compl:rev CACCAGCACCCACACTATCATGTTGACATTCC; dG = -12.3
417 >s_d_s_c_s_d_s_a_s_e_s GTGACGGTGTGGGTGACGGTGTCTGTGCTGGTG; >clamp_s_c_s_e_s:compl:rev CACCAGCACCCACACTATCATGTTGACATTCC; dG = -12.3
418 >s_d_s_d_s_d_s_e_s_e_s GTGACGGTGACGGTGACGGTGCTGGTGCTGGTG; >clamp_s_c_s_e_s:compl:rev CACCAGCACCCACACTATCATGTTGACATTCC; dG = -12.3
419 >s_d_s_d_s_e_s_e_s_e_s GTGACGGTGACGGTGCTGGTGCTGGTGCTGGTG; >clamp_s_c_s_e_s:compl:rev CACCAGCACCCACACTATCATGTTGACATTCC; dG = -12.3
420 >s_d_s_e_s_e_s_c_s_d_s GTGACGGTGCTGGTGCTGGTGTGGGTGACGGTG; >clamp_s_c_s_e_s:compl:rev CACCAGCACCCACACTATCATGTTGACATTCC; dG = -12.3
421 >s_d_s_e_s_e_s_c_s_b_s GTGACGGTGCTGGTGCTGGTGTGGGTGGCTGTG; >clamp_s_c_s_e_s:compl:rev CACCAGCACCCACACTATCATGTTGACATTCC; dG = -12.3
422 >s_d_s_e_s_e_s_c_s_a_s GTGACGGTGCTGGTGCTGGTGTGGGTGTCTGTG; >clamp_s_c_s_e_s:compl:rev CACCAGCACCCACACTATCATGTTGACATTCC; dG = -12.3
423 >s_d_s_e_s_e_s_a_s_e_s GTGACGGTGCTGGTGCTGGTGTCTGTGCTGGTG; >clamp_s_c_s_e_s:compl:rev CACCAGCACCCACACTATCATGTTGACATTCC; dG = -12.3
424 >s_e_s_a_s_e_s_e_s_e_s GTGCTGGTGTCTGTGCTGGTGCTGGTGCTGGTG; >clamp_s_c_s_e_s:compl:rev CACCAGCACCCACACTATCATGTTGACATTCC; dG = -12.3
425 >s_a_s_b_s_e_s_e_s_e_s GTGTCTGTGGCTGTGCTGGTGCTGGTGCTGGTG; >clamp_s_c_s_e_s:compl:rev CACCAGCACCCACACTATCATGTTGACATTCC; dG = -12.3
426 >s_e_s_b_s_e_s_e_s_e_s GTGCTGGTGGCTGTGCTGGTGCTGGTGCTGGTG; >clamp_s_c_s_e_s:compl:rev CACCAGCACCCACACTATCATGTTGACATTCC; dG = -12.3
427 >s_e_s_c_s_d_s_c_s_d_s GTGCTGGTGTGGGTGACGGTGTGGGTGACGGTG; >clamp_s_c_s_e_s:compl:rev CACCAGCACCCACACTATCATGTTGACATTCC; dG = -12.3
428 >s_e_s_c_s_d_s_c_s_b_s GTGCTGGTGTGGGTGACGGTGTGGGTGGCTGTG; >clamp_s_c_s_e_s:compl:rev CACCAGCACCCACACTATCATGTTGACATTCC; dG = -12.3
429 >s_e_s_c_s_d_s_c_s_a_s GTGCTGGTGTGGGTGACGGTGTGGGTGTCTGTG; >clamp_s_c_s_e_s:compl:rev CACCAGCACCCACACTATCATGTTGACATTCC; dG = -12.3
430 >s_e_s_c_s_d_s_a_s_e_s GTGCTGGTGTGGGTGACGGTGTCTGTGCTGGTG; >clamp_s_c_s_e_s:compl:rev CACCAGCACCCACACTATCATGTTGACATTCC; dG = -12.3
431 >s_e_s_d_s_d_s_e_s_e_s GTGCTGGTGACGGTGACGGTGCTGGTGCTGGTG; >clamp_s_c_s_e_s:compl:rev CACCAGCACCCACACTATCATGTTGACATTCC; dG = -12.3
432 >s_e_s_d_s_e_s_e_s_e_s GTGCTGGTGACGGTGCTGGTGCTGGTGCTGGTG; >clamp_s_c_s_e_s:compl:rev CACCAGCACCCACACTATCATGTTGACATTCC; dG = -12.3
433 >s_e_s_e_s_e_s_c_s_d_s GTGCTGGTGCTGGTGCTGGTGTGGGTGACGGTG; >clamp_s_c_s_e_s:compl:rev CACCAGCACCCACACTATCATGTTGACATTCC; dG = -12.3
434 >s_e_s_e_s_e_s_c_s_b_s GTGCTGGTGCTGGTGCTGGTGTGGGTGGCTGTG; >clamp_s_c_s_e_s:compl:rev CACCAGCACCCACACTATCATGTTGACATTCC; dG = -12.3
435 >s_e_s_e_s_e_s_c_s_a_s GTGCTGGTGCTGGTGCTGGTGTGGGTGTCTGTG; >clamp_s_c_s_e_s:compl:rev CACCAGCACCCACACTATCATGTTGACATTCC; dG = -12.3
436 >s_e_s_e_s_e_s_a_s_e_s GTGCTGGTGCTGGTGCTGGTGTCTGTGCTGGTG; >clamp_s_c_s_e_s:compl:rev CACCAGCACCCACACTATCATGTTGACATTCC; dG = -12.3
437 >s_a_s_d_s_a_s_d_s_e_s GTGTCTGTGACGGTGTCTGTGACGGTGCTGGTG; >clamp_s_c_s_e_s:compl:rev CACCAGCACCCACACTATCATGTTGACATTCC; dG = -12.2
438 >s_a_s_d_s_b_s_d_s_e_s GTGTCTGTGACGGTGGCTGTGACGGTGCTGGTG; >clamp_s_c_s_e_s:compl:rev CACCAGCACCCACACTATCATGTTGACATTCC; dG = -12.2
439 >s_a_s_d_s_d_s_d_s_e_s GTGTCTGTGACGGTGACGGTGACGGTGCTGGTG; >clamp_s_c_s_e_s:compl:rev CACCAGCACCCACACTATCATGTTGACATTCC; dG = -12.2
440 >s_a_s_a_s_b_s_d_s_e_s GTGTCTGTGTCTGTGGCTGTGACGGTGCTGGTG; >clamp_s_c_s_e_s:compl:rev CACCAGCACCCACACTATCATGTTGACATTCC; dG = -12.2
441 >s_a_s_e_s_a_s_d_s_e_s GTGTCTGTGCTGGTGTCTGTGACGGTGCTGGTG; >clamp_s_c_s_e_s:compl:rev CACCAGCACCCACACTATCATGTTGACATTCC; dG = -12.2
442 >s_a_s_e_s_b_s_d_s_e_s GTGTCTGTGCTGGTGGCTGTGACGGTGCTGGTG; >clamp_s_c_s_e_s:compl:rev CACCAGCACCCACACTATCATGTTGACATTCC; dG = -12.2
443 >s_a_s_e_s_d_s_d_s_e_s GTGTCTGTGCTGGTGACGGTGACGGTGCTGGTG; >clamp_s_c_s_e_s:compl:rev CACCAGCACCCACACTATCATGTTGACATTCC; dG = -12.2
444 >s_b_s_a_s_a_s_d_s_e_s GTGGCTGTGTCTGTGTCTGTGACGGTGCTGGTG; >clamp_s_c_s_e_s:compl:rev CACCAGCACCCACACTATCATGTTGACATTCC; dG = -12.2
445 >s_b_s_a_s_b_s_d_s_e_s GTGGCTGTGTCTGTGGCTGTGACGGTGCTGGTG; >clamp_s_c_s_e_s:compl:rev CACCAGCACCCACACTATCATGTTGACATTCC; dG = -12.2
446 >s_b_s_a_s_d_s_d_s_e_s GTGGCTGTGTCTGTGACGGTGACGGTGCTGGTG; >clamp_s_c_s_e_s:compl:rev CACCAGCACCCACACTATCATGTTGACATTCC; dG = -12.2

447 >s_b_s_a_s_e_s_d_s_e_s GTGGCTGTGTCTGTGCTGGTGACGGTGCTGGTG; >clamp_s_c_s_e_s:compl:rev CACCAGCACCCACACTATCATGTTGACATTCC; dG = -12.2
448 >s_b_s_b_s_a_s_d_s_e_s GTGGCTGTGGCTGTGTCTGTGACGGTGCTGGTG; >clamp_s_c_s_e_s:compl:rev CACCAGCACCCACACTATCATGTTGACATTCC; dG = -12.2
449 >s_b_s_b_s_b_s_d_s_e_s GTGGCTGTGGCTGTGGCTGTGACGGTGCTGGTG; >clamp_s_c_s_e_s:compl:rev CACCAGCACCCACACTATCATGTTGACATTCC; dG = -12.2
450 >s_b_s_b_s_d_s_d_s_e_s GTGGCTGTGGCTGTGACGGTGACGGTGCTGGTG; >clamp_s_c_s_e_s:compl:rev CACCAGCACCCACACTATCATGTTGACATTCC; dG = -12.2
451 >s_b_s_b_s_e_s_d_s_e_s GTGGCTGTGGCTGTGCTGGTGACGGTGCTGGTG; >clamp_s_c_s_e_s:compl:rev CACCAGCACCCACACTATCATGTTGACATTCC; dG = -12.2
452 >s_b_s_c_s_a_s_d_s_e_s GTGGCTGTGTGGGTGTCTGTGACGGTGCTGGTG; >clamp_s_c_s_e_s:compl:rev CACCAGCACCCACACTATCATGTTGACATTCC; dG = -12.2
453 >s_b_s_c_s_b_s_d_s_e_s GTGGCTGTGTGGGTGGCTGTGACGGTGCTGGTG; >clamp_s_c_s_e_s:compl:rev CACCAGCACCCACACTATCATGTTGACATTCC; dG = -12.2
454 >s_a_s_a_s_d_s_d_s_e_s GTGTCTGTGTCTGTGACGGTGACGGTGCTGGTG; >clamp_s_c_s_e_s:compl:rev CACCAGCACCCACACTATCATGTTGACATTCC; dG = -12.2
455 >s_b_s_d_s_a_s_d_s_e_s GTGGCTGTGACGGTGTCTGTGACGGTGCTGGTG; >clamp_s_c_s_e_s:compl:rev CACCAGCACCCACACTATCATGTTGACATTCC; dG = -12.2
456 >s_b_s_d_s_b_s_d_s_e_s GTGGCTGTGACGGTGGCTGTGACGGTGCTGGTG; >clamp_s_c_s_e_s:compl:rev CACCAGCACCCACACTATCATGTTGACATTCC; dG = -12.2
457 >s_b_s_d_s_d_s_d_s_e_s GTGGCTGTGACGGTGACGGTGACGGTGCTGGTG; >clamp_s_c_s_e_s:compl:rev CACCAGCACCCACACTATCATGTTGACATTCC; dG = -12.2
458 >s_b_s_e_s_a_s_d_s_e_s GTGGCTGTGCTGGTGTCTGTGACGGTGCTGGTG; >clamp_s_c_s_e_s:compl:rev CACCAGCACCCACACTATCATGTTGACATTCC; dG = -12.2
459 >s_b_s_e_s_b_s_d_s_e_s GTGGCTGTGCTGGTGGCTGTGACGGTGCTGGTG; >clamp_s_c_s_e_s:compl:rev CACCAGCACCCACACTATCATGTTGACATTCC; dG = -12.2
460 >s_b_s_e_s_d_s_d_s_e_s GTGGCTGTGCTGGTGACGGTGACGGTGCTGGTG; >clamp_s_c_s_e_s:compl:rev CACCAGCACCCACACTATCATGTTGACATTCC; dG = -12.2
461 >s_a_s_a_s_e_s_d_s_e_s GTGTCTGTGTCTGTGCTGGTGACGGTGCTGGTG; >clamp_s_c_s_e_s:compl:rev CACCAGCACCCACACTATCATGTTGACATTCC; dG = -12.2
462 >s_c_s_a_s_a_s_d_s_e_s GTGTGGGTGTCTGTGTCTGTGACGGTGCTGGTG; >clamp_s_c_s_e_s:compl:rev CACCAGCACCCACACTATCATGTTGACATTCC; dG = -12.2
463 >s_c_s_a_s_b_s_d_s_e_s GTGTGGGTGTCTGTGGCTGTGACGGTGCTGGTG; >clamp_s_c_s_e_s:compl:rev CACCAGCACCCACACTATCATGTTGACATTCC; dG = -12.2
464 >s_c_s_a_s_d_s_d_s_e_s GTGTGGGTGTCTGTGACGGTGACGGTGCTGGTG; >clamp_s_c_s_e_s:compl:rev CACCAGCACCCACACTATCATGTTGACATTCC; dG = -12.2
465 >s_c_s_b_s_a_s_d_s_e_s GTGTGGGTGGCTGTGTCTGTGACGGTGCTGGTG; >clamp_s_c_s_e_s:compl:rev CACCAGCACCCACACTATCATGTTGACATTCC; dG = -12.2
466 >s_c_s_b_s_b_s_d_s_e_s GTGTGGGTGGCTGTGGCTGTGACGGTGCTGGTG; >clamp_s_c_s_e_s:compl:rev CACCAGCACCCACACTATCATGTTGACATTCC; dG = -12.2
467 >s_c_s_b_s_d_s_d_s_e_s GTGTGGGTGGCTGTGACGGTGACGGTGCTGGTG; >clamp_s_c_s_e_s:compl:rev CACCAGCACCCACACTATCATGTTGACATTCC; dG = -12.2
468 >s_a_s_b_s_a_s_d_s_e_s GTGTCTGTGGCTGTGTCTGTGACGGTGCTGGTG; >clamp_s_c_s_e_s:compl:rev CACCAGCACCCACACTATCATGTTGACATTCC; dG = -12.2
469 >s_c_s_d_s_a_s_d_s_e_s GTGTGGGTGACGGTGTCTGTGACGGTGCTGGTG; >clamp_s_c_s_e_s:compl:rev CACCAGCACCCACACTATCATGTTGACATTCC; dG = -12.2
470 >s_c_s_d_s_b_s_d_s_e_s GTGTGGGTGACGGTGGCTGTGACGGTGCTGGTG; >clamp_s_c_s_e_s:compl:rev CACCAGCACCCACACTATCATGTTGACATTCC; dG = -12.2
471 >s_d_s_a_s_a_s_d_s_e_s GTGACGGTGTCTGTGTCTGTGACGGTGCTGGTG; >clamp_s_c_s_e_s:compl:rev CACCAGCACCCACACTATCATGTTGACATTCC; dG = -12.2
472 >s_a_s_b_s_b_s_d_s_e_s GTGTCTGTGGCTGTGGCTGTGACGGTGCTGGTG; >clamp_s_c_s_e_s:compl:rev CACCAGCACCCACACTATCATGTTGACATTCC; dG = -12.2
473 >s_d_s_a_s_b_s_d_s_e_s GTGACGGTGTCTGTGGCTGTGACGGTGCTGGTG; >clamp_s_c_s_e_s:compl:rev CACCAGCACCCACACTATCATGTTGACATTCC; dG = -12.2
474 >s_d_s_a_s_d_s_d_s_e_s GTGACGGTGTCTGTGACGGTGACGGTGCTGGTG; >clamp_s_c_s_e_s:compl:rev CACCAGCACCCACACTATCATGTTGACATTCC; dG = -12.2
475 >s_d_s_a_s_e_s_d_s_e_s GTGACGGTGTCTGTGCTGGTGACGGTGCTGGTG; >clamp_s_c_s_e_s:compl:rev CACCAGCACCCACACTATCATGTTGACATTCC; dG = -12.2
476 >s_d_s_b_s_a_s_d_s_e_s GTGACGGTGGCTGTGTCTGTGACGGTGCTGGTG; >clamp_s_c_s_e_s:compl:rev CACCAGCACCCACACTATCATGTTGACATTCC; dG = -12.2
477 >s_d_s_b_s_b_s_d_s_e_s GTGACGGTGGCTGTGGCTGTGACGGTGCTGGTG; >clamp_s_c_s_e_s:compl:rev CACCAGCACCCACACTATCATGTTGACATTCC; dG = -12.2
478 >s_d_s_b_s_d_s_d_s_e_s GTGACGGTGGCTGTGACGGTGACGGTGCTGGTG; >clamp_s_c_s_e_s:compl:rev CACCAGCACCCACACTATCATGTTGACATTCC; dG = -12.2
479 >s_d_s_b_s_e_s_d_s_e_s GTGACGGTGGCTGTGCTGGTGACGGTGCTGGTG; >clamp_s_c_s_e_s:compl:rev CACCAGCACCCACACTATCATGTTGACATTCC; dG = -12.2
480 >s_a_s_a_s_a_s_d_s_e_s GTGTCTGTGTCTGTGTCTGTGACGGTGCTGGTG; >clamp_s_c_s_e_s:compl:rev CACCAGCACCCACACTATCATGTTGACATTCC; dG = -12.2
481 >s_d_s_c_s_a_s_d_s_e_s GTGACGGTGTGGGTGTCTGTGACGGTGCTGGTG; >clamp_s_c_s_e_s:compl:rev CACCAGCACCCACACTATCATGTTGACATTCC; dG = -12.2
482 >s_d_s_c_s_b_s_d_s_e_s GTGACGGTGTGGGTGGCTGTGACGGTGCTGGTG; >clamp_s_c_s_e_s:compl:rev CACCAGCACCCACACTATCATGTTGACATTCC; dG = -12.2
483 >s_d_s_d_s_a_s_d_s_e_s GTGACGGTGACGGTGTCTGTGACGGTGCTGGTG; >clamp_s_c_s_e_s:compl:rev CACCAGCACCCACACTATCATGTTGACATTCC; dG = -12.2

484 >s_d_s_d_s_b_s_d_s_e_s GTGACGGTGACGGTGGCTGTGACGGTGCTGGTG; >clamp_s_c_s_e_s:compl:rev CACCAGCACCCACACTATCATGTTGACATTCC; dG = -12.2
485 >s_d_s_d_s_d_s_d_s_e_s GTGACGGTGACGGTGACGGTGACGGTGCTGGTG; >clamp_s_c_s_e_s:compl:rev CACCAGCACCCACACTATCATGTTGACATTCC; dG = -12.2
486 >s_d_s_e_s_a_s_d_s_e_s GTGACGGTGCTGGTGTCTGTGACGGTGCTGGTG; >clamp_s_c_s_e_s:compl:rev CACCAGCACCCACACTATCATGTTGACATTCC; dG = -12.2
487 >s_a_s_b_s_d_s_d_s_e_s GTGTCTGTGGCTGTGACGGTGACGGTGCTGGTG; >clamp_s_c_s_e_s:compl:rev CACCAGCACCCACACTATCATGTTGACATTCC; dG = -12.2
488 >s_d_s_e_s_b_s_d_s_e_s GTGACGGTGCTGGTGGCTGTGACGGTGCTGGTG; >clamp_s_c_s_e_s:compl:rev CACCAGCACCCACACTATCATGTTGACATTCC; dG = -12.2
489 >s_d_s_e_s_e_s_e_s_e_s GTGACGGTGCTGGTGCTGGTGCTGGTGCTGGTG; >clamp_s_c_s_e_s:compl:rev CACCAGCACCCACACTATCATGTTGACATTCC; dG = -12.2
490 >s_e_s_a_s_a_s_d_s_e_s GTGCTGGTGTCTGTGTCTGTGACGGTGCTGGTG; >clamp_s_c_s_e_s:compl:rev CACCAGCACCCACACTATCATGTTGACATTCC; dG = -12.2
491 >s_e_s_a_s_b_s_d_s_e_s GTGCTGGTGTCTGTGGCTGTGACGGTGCTGGTG; >clamp_s_c_s_e_s:compl:rev CACCAGCACCCACACTATCATGTTGACATTCC; dG = -12.2
492 >s_e_s_a_s_d_s_d_s_e_s GTGCTGGTGTCTGTGACGGTGACGGTGCTGGTG; >clamp_s_c_s_e_s:compl:rev CACCAGCACCCACACTATCATGTTGACATTCC; dG = -12.2
493 >s_e_s_a_s_e_s_d_s_e_s GTGCTGGTGTCTGTGCTGGTGACGGTGCTGGTG; >clamp_s_c_s_e_s:compl:rev CACCAGCACCCACACTATCATGTTGACATTCC; dG = -12.2
494 >s_e_s_b_s_a_s_d_s_e_s GTGCTGGTGGCTGTGTCTGTGACGGTGCTGGTG; >clamp_s_c_s_e_s:compl:rev CACCAGCACCCACACTATCATGTTGACATTCC; dG = -12.2
495 >s_a_s_b_s_e_s_d_s_e_s GTGTCTGTGGCTGTGCTGGTGACGGTGCTGGTG; >clamp_s_c_s_e_s:compl:rev CACCAGCACCCACACTATCATGTTGACATTCC; dG = -12.2
496 >s_e_s_b_s_b_s_d_s_e_s GTGCTGGTGGCTGTGGCTGTGACGGTGCTGGTG; >clamp_s_c_s_e_s:compl:rev CACCAGCACCCACACTATCATGTTGACATTCC; dG = -12.2
497 >s_e_s_b_s_d_s_d_s_e_s GTGCTGGTGGCTGTGACGGTGACGGTGCTGGTG; >clamp_s_c_s_e_s:compl:rev CACCAGCACCCACACTATCATGTTGACATTCC; dG = -12.2
498 >s_e_s_b_s_e_s_d_s_e_s GTGCTGGTGGCTGTGCTGGTGACGGTGCTGGTG; >clamp_s_c_s_e_s:compl:rev CACCAGCACCCACACTATCATGTTGACATTCC; dG = -12.2
499 >s_e_s_c_s_a_s_d_s_e_s GTGCTGGTGTGGGTGTCTGTGACGGTGCTGGTG; >clamp_s_c_s_e_s:compl:rev CACCAGCACCCACACTATCATGTTGACATTCC; dG = -12.2
500 >s_e_s_c_s_b_s_d_s_e_s GTGCTGGTGTGGGTGGCTGTGACGGTGCTGGTG; >clamp_s_c_s_e_s:compl:rev CACCAGCACCCACACTATCATGTTGACATTCC; dG = -12.2
501 >s_e_s_d_s_a_s_d_s_e_s GTGCTGGTGACGGTGTCTGTGACGGTGCTGGTG; >clamp_s_c_s_e_s:compl:rev CACCAGCACCCACACTATCATGTTGACATTCC; dG = -12.2
502 >s_e_s_d_s_b_s_d_s_e_s GTGCTGGTGACGGTGGCTGTGACGGTGCTGGTG; >clamp_s_c_s_e_s:compl:rev CACCAGCACCCACACTATCATGTTGACATTCC; dG = -12.2
503 >s_a_s_c_s_a_s_d_s_e_s GTGTCTGTGTGGGTGTCTGTGACGGTGCTGGTG; >clamp_s_c_s_e_s:compl:rev CACCAGCACCCACACTATCATGTTGACATTCC; dG = -12.2
504 >s_e_s_d_s_d_s_d_s_e_s GTGCTGGTGACGGTGACGGTGACGGTGCTGGTG; >clamp_s_c_s_e_s:compl:rev CACCAGCACCCACACTATCATGTTGACATTCC; dG = -12.2
505 >s_e_s_e_s_a_s_d_s_e_s GTGCTGGTGCTGGTGTCTGTGACGGTGCTGGTG; >clamp_s_c_s_e_s:compl:rev CACCAGCACCCACACTATCATGTTGACATTCC; dG = -12.2
506 >s_e_s_e_s_b_s_d_s_e_s GTGCTGGTGCTGGTGGCTGTGACGGTGCTGGTG; >clamp_s_c_s_e_s:compl:rev CACCAGCACCCACACTATCATGTTGACATTCC; dG = -12.2
507 >s_e_s_e_s_e_s_e_s_e_s GTGCTGGTGCTGGTGCTGGTGCTGGTGCTGGTG; >clamp_s_c_s_e_s:compl:rev CACCAGCACCCACACTATCATGTTGACATTCC; dG = -12.2
508 >s_a_s_c_s_b_s_d_s_e_s GTGTCTGTGTGGGTGGCTGTGACGGTGCTGGTG; >clamp_s_c_s_e_s:compl:rev CACCAGCACCCACACTATCATGTTGACATTCC; dG = -12.2
509 >s_a_s_a_s_a_s_c_s_d_s GTGTCTGTGTCTGTGTCTGTGTGGGTGACGGTG; >clamp_s_c_s_e_s:compl:rev CACCAGCACCCACACTATCATGTTGACATTCC; dG = -12.1
510 >s_a_s_d_s_a_s_c_s_d_s GTGTCTGTGACGGTGTCTGTGTGGGTGACGGTG; >clamp_s_c_s_e_s:compl:rev CACCAGCACCCACACTATCATGTTGACATTCC; dG = -12.1
511 >s_a_s_d_s_a_s_e_s_e_s GTGTCTGTGACGGTGTCTGTGCTGGTGCTGGTG; >clamp_s_c_s_e_s:compl:rev CACCAGCACCCACACTATCATGTTGACATTCC; dG = -12.1
512 >s_a_s_d_s_b_s_c_s_d_s GTGTCTGTGACGGTGGCTGTGTGGGTGACGGTG; >clamp_s_c_s_e_s:compl:rev CACCAGCACCCACACTATCATGTTGACATTCC; dG = -12.1
513 >s_a_s_d_s_b_s_e_s_e_s GTGTCTGTGACGGTGGCTGTGCTGGTGCTGGTG; >clamp_s_c_s_e_s:compl:rev CACCAGCACCCACACTATCATGTTGACATTCC; dG = -12.1
514 >s_a_s_a_s_b_s_e_s_e_s GTGTCTGTGTCTGTGGCTGTGCTGGTGCTGGTG; >clamp_s_c_s_e_s:compl:rev CACCAGCACCCACACTATCATGTTGACATTCC; dG = -12.1
515 >s_a_s_e_s_a_s_c_s_d_s GTGTCTGTGCTGGTGTCTGTGTGGGTGACGGTG; >clamp_s_c_s_e_s:compl:rev CACCAGCACCCACACTATCATGTTGACATTCC; dG = -12.1
516 >s_a_s_e_s_a_s_e_s_e_s GTGTCTGTGCTGGTGTCTGTGCTGGTGCTGGTG; >clamp_s_c_s_e_s:compl:rev CACCAGCACCCACACTATCATGTTGACATTCC; dG = -12.1
517 >s_a_s_e_s_b_s_c_s_d_s GTGTCTGTGCTGGTGGCTGTGTGGGTGACGGTG; >clamp_s_c_s_e_s:compl:rev CACCAGCACCCACACTATCATGTTGACATTCC; dG = -12.1
518 >s_a_s_e_s_b_s_e_s_e_s GTGTCTGTGCTGGTGGCTGTGCTGGTGCTGGTG; >clamp_s_c_s_e_s:compl:rev CACCAGCACCCACACTATCATGTTGACATTCC; dG = -12.1
519 >s_b_s_a_s_a_s_c_s_d_s GTGGCTGTGTCTGTGTCTGTGTGGGTGACGGTG; >clamp_s_c_s_e_s:compl:rev CACCAGCACCCACACTATCATGTTGACATTCC; dG = -12.1
520 >s_b_s_a_s_a_s_e_s_e_s GTGGCTGTGTCTGTGTCTGTGCTGGTGCTGGTG; >clamp_s_c_s_e_s:compl:rev CACCAGCACCCACACTATCATGTTGACATTCC; dG = -12.1

521 >s_b_s_a_s_b_s_c_s_d_s GTGGCTGTGTCTGTGGCTGTGTGGGTGACGGTG; >clamp_s_c_s_e_s:compl:rev CACCAGCACCCACACTATCATGTTGACATTCC; dG = -12.1
522 >s_b_s_a_s_b_s_e_s_e_s GTGGCTGTGTCTGTGGCTGTGCTGGTGCTGGTG; >clamp_s_c_s_e_s:compl:rev CACCAGCACCCACACTATCATGTTGACATTCC; dG = -12.1
523 >s_b_s_b_s_a_s_c_s_d_s GTGGCTGTGGCTGTGTCTGTGTGGGTGACGGTG; >clamp_s_c_s_e_s:compl:rev CACCAGCACCCACACTATCATGTTGACATTCC; dG = -12.1
524 >s_b_s_b_s_a_s_e_s_e_s GTGGCTGTGGCTGTGTCTGTGCTGGTGCTGGTG; >clamp_s_c_s_e_s:compl:rev CACCAGCACCCACACTATCATGTTGACATTCC; dG = -12.1
525 >s_b_s_b_s_b_s_c_s_d_s GTGGCTGTGGCTGTGGCTGTGTGGGTGACGGTG; >clamp_s_c_s_e_s:compl:rev CACCAGCACCCACACTATCATGTTGACATTCC; dG = -12.1
526 >s_b_s_b_s_b_s_e_s_e_s GTGGCTGTGGCTGTGGCTGTGCTGGTGCTGGTG; >clamp_s_c_s_e_s:compl:rev CACCAGCACCCACACTATCATGTTGACATTCC; dG = -12.1
527 >s_b_s_c_s_a_s_c_s_d_s GTGGCTGTGTGGGTGTCTGTGTGGGTGACGGTG; >clamp_s_c_s_e_s:compl:rev CACCAGCACCCACACTATCATGTTGACATTCC; dG = -12.1
528 >s_b_s_c_s_b_s_c_s_d_s GTGGCTGTGTGGGTGGCTGTGTGGGTGACGGTG; >clamp_s_c_s_e_s:compl:rev CACCAGCACCCACACTATCATGTTGACATTCC; dG = -12.1
529 >s_b_s_d_s_a_s_c_s_d_s GTGGCTGTGACGGTGTCTGTGTGGGTGACGGTG; >clamp_s_c_s_e_s:compl:rev CACCAGCACCCACACTATCATGTTGACATTCC; dG = -12.1
530 >s_b_s_d_s_a_s_e_s_e_s GTGGCTGTGACGGTGTCTGTGCTGGTGCTGGTG; >clamp_s_c_s_e_s:compl:rev CACCAGCACCCACACTATCATGTTGACATTCC; dG = -12.1
531 >s_b_s_d_s_b_s_c_s_d_s GTGGCTGTGACGGTGGCTGTGTGGGTGACGGTG; >clamp_s_c_s_e_s:compl:rev CACCAGCACCCACACTATCATGTTGACATTCC; dG = -12.1
532 >s_b_s_d_s_b_s_e_s_e_s GTGGCTGTGACGGTGGCTGTGCTGGTGCTGGTG; >clamp_s_c_s_e_s:compl:rev CACCAGCACCCACACTATCATGTTGACATTCC; dG = -12.1
533 >s_b_s_e_s_a_s_c_s_d_s GTGGCTGTGCTGGTGTCTGTGTGGGTGACGGTG; >clamp_s_c_s_e_s:compl:rev CACCAGCACCCACACTATCATGTTGACATTCC; dG = -12.1
534 >s_b_s_e_s_a_s_e_s_e_s GTGGCTGTGCTGGTGTCTGTGCTGGTGCTGGTG; >clamp_s_c_s_e_s:compl:rev CACCAGCACCCACACTATCATGTTGACATTCC; dG = -12.1
535 >s_b_s_e_s_b_s_c_s_d_s GTGGCTGTGCTGGTGGCTGTGTGGGTGACGGTG; >clamp_s_c_s_e_s:compl:rev CACCAGCACCCACACTATCATGTTGACATTCC; dG = -12.1
536 >s_b_s_e_s_b_s_e_s_e_s GTGGCTGTGCTGGTGGCTGTGCTGGTGCTGGTG; >clamp_s_c_s_e_s:compl:rev CACCAGCACCCACACTATCATGTTGACATTCC; dG = -12.1
537 >s_c_s_a_s_a_s_c_s_d_s GTGTGGGTGTCTGTGTCTGTGTGGGTGACGGTG; >clamp_s_c_s_e_s:compl:rev CACCAGCACCCACACTATCATGTTGACATTCC; dG = -12.1
538 >s_c_s_a_s_a_s_e_s_e_s GTGTGGGTGTCTGTGTCTGTGCTGGTGCTGGTG; >clamp_s_c_s_e_s:compl:rev CACCAGCACCCACACTATCATGTTGACATTCC; dG = -12.1
539 >s_c_s_a_s_b_s_c_s_d_s GTGTGGGTGTCTGTGGCTGTGTGGGTGACGGTG; >clamp_s_c_s_e_s:compl:rev CACCAGCACCCACACTATCATGTTGACATTCC; dG = -12.1
540 >s_c_s_a_s_b_s_e_s_e_s GTGTGGGTGTCTGTGGCTGTGCTGGTGCTGGTG; >clamp_s_c_s_e_s:compl:rev CACCAGCACCCACACTATCATGTTGACATTCC; dG = -12.1
541 >s_a_s_b_s_a_s_c_s_d_s GTGTCTGTGGCTGTGTCTGTGTGGGTGACGGTG; >clamp_s_c_s_e_s:compl:rev CACCAGCACCCACACTATCATGTTGACATTCC; dG = -12.1
542 >s_c_s_b_s_a_s_c_s_d_s GTGTGGGTGGCTGTGTCTGTGTGGGTGACGGTG; >clamp_s_c_s_e_s:compl:rev CACCAGCACCCACACTATCATGTTGACATTCC; dG = -12.1
543 >s_c_s_b_s_a_s_e_s_e_s GTGTGGGTGGCTGTGTCTGTGCTGGTGCTGGTG; >clamp_s_c_s_e_s:compl:rev CACCAGCACCCACACTATCATGTTGACATTCC; dG = -12.1
544 >s_c_s_b_s_b_s_c_s_d_s GTGTGGGTGGCTGTGGCTGTGTGGGTGACGGTG; >clamp_s_c_s_e_s:compl:rev CACCAGCACCCACACTATCATGTTGACATTCC; dG = -12.1
545 >s_c_s_b_s_b_s_e_s_e_s GTGTGGGTGGCTGTGGCTGTGCTGGTGCTGGTG; >clamp_s_c_s_e_s:compl:rev CACCAGCACCCACACTATCATGTTGACATTCC; dG = -12.1
546 >s_a_s_b_s_a_s_e_s_e_s GTGTCTGTGGCTGTGTCTGTGCTGGTGCTGGTG; >clamp_s_c_s_e_s:compl:rev CACCAGCACCCACACTATCATGTTGACATTCC; dG = -12.1
547 >s_a_s_b_s_b_s_c_s_d_s GTGTCTGTGGCTGTGGCTGTGTGGGTGACGGTG; >clamp_s_c_s_e_s:compl:rev CACCAGCACCCACACTATCATGTTGACATTCC; dG = -12.1
548 >s_c_s_d_s_a_s_c_s_d_s GTGTGGGTGACGGTGTCTGTGTGGGTGACGGTG; >clamp_s_c_s_e_s:compl:rev CACCAGCACCCACACTATCATGTTGACATTCC; dG = -12.1
549 >s_c_s_d_s_a_s_e_s_e_s GTGTGGGTGACGGTGTCTGTGCTGGTGCTGGTG; >clamp_s_c_s_e_s:compl:rev CACCAGCACCCACACTATCATGTTGACATTCC; dG = -12.1
550 >s_c_s_d_s_b_s_c_s_d_s GTGTGGGTGACGGTGGCTGTGTGGGTGACGGTG; >clamp_s_c_s_e_s:compl:rev CACCAGCACCCACACTATCATGTTGACATTCC; dG = -12.1
551 >s_c_s_d_s_b_s_e_s_e_s GTGTGGGTGACGGTGGCTGTGCTGGTGCTGGTG; >clamp_s_c_s_e_s:compl:rev CACCAGCACCCACACTATCATGTTGACATTCC; dG = -12.1
552 >s_a_s_a_s_a_s_e_s_e_s GTGTCTGTGTCTGTGTCTGTGCTGGTGCTGGTG; >clamp_s_c_s_e_s:compl:rev CACCAGCACCCACACTATCATGTTGACATTCC; dG = -12.1
553 >s_a_s_b_s_b_s_e_s_e_s GTGTCTGTGGCTGTGGCTGTGCTGGTGCTGGTG; >clamp_s_c_s_e_s:compl:rev CACCAGCACCCACACTATCATGTTGACATTCC; dG = -12.1
554 >s_d_s_a_s_a_s_c_s_d_s GTGACGGTGTCTGTGTCTGTGTGGGTGACGGTG; >clamp_s_c_s_e_s:compl:rev CACCAGCACCCACACTATCATGTTGACATTCC; dG = -12.1
555 >s_d_s_a_s_a_s_e_s_e_s GTGACGGTGTCTGTGTCTGTGCTGGTGCTGGTG; >clamp_s_c_s_e_s:compl:rev CACCAGCACCCACACTATCATGTTGACATTCC; dG = -12.1
556 >s_d_s_a_s_b_s_c_s_d_s GTGACGGTGTCTGTGGCTGTGTGGGTGACGGTG; >clamp_s_c_s_e_s:compl:rev CACCAGCACCCACACTATCATGTTGACATTCC; dG = -12.1
557 >s_d_s_a_s_b_s_e_s_e_s GTGACGGTGTCTGTGGCTGTGCTGGTGCTGGTG; >clamp_s_c_s_e_s:compl:rev CACCAGCACCCACACTATCATGTTGACATTCC; dG = -12.1

558 >s_d_s_b_s_a_s_c_s_d_s GTGACGGTGGCTGTGTCTGTGTGGGTGACGGTG; >clamp_s_c_s_e_s:compl:rev CACCAGCACCCACACTATCATGTTGACATTCC; dG = -12.1
559 >s_d_s_b_s_a_s_e_s_e_s GTGACGGTGGCTGTGTCTGTGCTGGTGCTGGTG; >clamp_s_c_s_e_s:compl:rev CACCAGCACCCACACTATCATGTTGACATTCC; dG = -12.1
560 >s_d_s_b_s_b_s_c_s_d_s GTGACGGTGGCTGTGGCTGTGTGGGTGACGGTG; >clamp_s_c_s_e_s:compl:rev CACCAGCACCCACACTATCATGTTGACATTCC; dG = -12.1
561 >s_d_s_b_s_b_s_e_s_e_s GTGACGGTGGCTGTGGCTGTGCTGGTGCTGGTG; >clamp_s_c_s_e_s:compl:rev CACCAGCACCCACACTATCATGTTGACATTCC; dG = -12.1
562 >s_d_s_c_s_a_s_c_s_d_s GTGACGGTGTGGGTGTCTGTGTGGGTGACGGTG; >clamp_s_c_s_e_s:compl:rev CACCAGCACCCACACTATCATGTTGACATTCC; dG = -12.1
563 >s_d_s_c_s_b_s_c_s_d_s GTGACGGTGTGGGTGGCTGTGTGGGTGACGGTG; >clamp_s_c_s_e_s:compl:rev CACCAGCACCCACACTATCATGTTGACATTCC; dG = -12.1
564 >s_d_s_d_s_a_s_c_s_d_s GTGACGGTGACGGTGTCTGTGTGGGTGACGGTG; >clamp_s_c_s_e_s:compl:rev CACCAGCACCCACACTATCATGTTGACATTCC; dG = -12.1
565 >s_d_s_d_s_a_s_e_s_e_s GTGACGGTGACGGTGTCTGTGCTGGTGCTGGTG; >clamp_s_c_s_e_s:compl:rev CACCAGCACCCACACTATCATGTTGACATTCC; dG = -12.1
566 >s_d_s_d_s_b_s_c_s_d_s GTGACGGTGACGGTGGCTGTGTGGGTGACGGTG; >clamp_s_c_s_e_s:compl:rev CACCAGCACCCACACTATCATGTTGACATTCC; dG = -12.1
567 >s_d_s_d_s_b_s_e_s_e_s GTGACGGTGACGGTGGCTGTGCTGGTGCTGGTG; >clamp_s_c_s_e_s:compl:rev CACCAGCACCCACACTATCATGTTGACATTCC; dG = -12.1
568 >s_d_s_e_s_a_s_c_s_d_s GTGACGGTGCTGGTGTCTGTGTGGGTGACGGTG; >clamp_s_c_s_e_s:compl:rev CACCAGCACCCACACTATCATGTTGACATTCC; dG = -12.1
569 >s_d_s_e_s_a_s_e_s_e_s GTGACGGTGCTGGTGTCTGTGCTGGTGCTGGTG; >clamp_s_c_s_e_s:compl:rev CACCAGCACCCACACTATCATGTTGACATTCC; dG = -12.1
570 >s_d_s_e_s_b_s_c_s_d_s GTGACGGTGCTGGTGGCTGTGTGGGTGACGGTG; >clamp_s_c_s_e_s:compl:rev CACCAGCACCCACACTATCATGTTGACATTCC; dG = -12.1
571 >s_d_s_e_s_b_s_e_s_e_s GTGACGGTGCTGGTGGCTGTGCTGGTGCTGGTG; >clamp_s_c_s_e_s:compl:rev CACCAGCACCCACACTATCATGTTGACATTCC; dG = -12.1
572 >s_e_s_a_s_a_s_c_s_d_s GTGCTGGTGTCTGTGTCTGTGTGGGTGACGGTG; >clamp_s_c_s_e_s:compl:rev CACCAGCACCCACACTATCATGTTGACATTCC; dG = -12.1
573 >s_e_s_a_s_a_s_e_s_e_s GTGCTGGTGTCTGTGTCTGTGCTGGTGCTGGTG; >clamp_s_c_s_e_s:compl:rev CACCAGCACCCACACTATCATGTTGACATTCC; dG = -12.1
574 >s_e_s_a_s_b_s_c_s_d_s GTGCTGGTGTCTGTGGCTGTGTGGGTGACGGTG; >clamp_s_c_s_e_s:compl:rev CACCAGCACCCACACTATCATGTTGACATTCC; dG = -12.1
575 >s_e_s_a_s_b_s_e_s_e_s GTGCTGGTGTCTGTGGCTGTGCTGGTGCTGGTG; >clamp_s_c_s_e_s:compl:rev CACCAGCACCCACACTATCATGTTGACATTCC; dG = -12.1
576 >s_e_s_b_s_a_s_c_s_d_s GTGCTGGTGGCTGTGTCTGTGTGGGTGACGGTG; >clamp_s_c_s_e_s:compl:rev CACCAGCACCCACACTATCATGTTGACATTCC; dG = -12.1
577 >s_e_s_b_s_a_s_e_s_e_s GTGCTGGTGGCTGTGTCTGTGCTGGTGCTGGTG; >clamp_s_c_s_e_s:compl:rev CACCAGCACCCACACTATCATGTTGACATTCC; dG = -12.1
578 >s_e_s_b_s_b_s_c_s_d_s GTGCTGGTGGCTGTGGCTGTGTGGGTGACGGTG; >clamp_s_c_s_e_s:compl:rev CACCAGCACCCACACTATCATGTTGACATTCC; dG = -12.1
579 >s_e_s_b_s_b_s_e_s_e_s GTGCTGGTGGCTGTGGCTGTGCTGGTGCTGGTG; >clamp_s_c_s_e_s:compl:rev CACCAGCACCCACACTATCATGTTGACATTCC; dG = -12.1
580 >s_e_s_c_s_a_s_c_s_d_s GTGCTGGTGTGGGTGTCTGTGTGGGTGACGGTG; >clamp_s_c_s_e_s:compl:rev CACCAGCACCCACACTATCATGTTGACATTCC; dG = -12.1
581 >s_a_s_c_s_a_s_c_s_d_s GTGTCTGTGTGGGTGTCTGTGTGGGTGACGGTG; >clamp_s_c_s_e_s:compl:rev CACCAGCACCCACACTATCATGTTGACATTCC; dG = -12.1
582 >s_e_s_c_s_b_s_c_s_d_s GTGCTGGTGTGGGTGGCTGTGTGGGTGACGGTG; >clamp_s_c_s_e_s:compl:rev CACCAGCACCCACACTATCATGTTGACATTCC; dG = -12.1
583 >s_e_s_d_s_a_s_c_s_d_s GTGCTGGTGACGGTGTCTGTGTGGGTGACGGTG; >clamp_s_c_s_e_s:compl:rev CACCAGCACCCACACTATCATGTTGACATTCC; dG = -12.1
584 >s_e_s_d_s_a_s_e_s_e_s GTGCTGGTGACGGTGTCTGTGCTGGTGCTGGTG; >clamp_s_c_s_e_s:compl:rev CACCAGCACCCACACTATCATGTTGACATTCC; dG = -12.1
585 >s_e_s_d_s_b_s_c_s_d_s GTGCTGGTGACGGTGGCTGTGTGGGTGACGGTG; >clamp_s_c_s_e_s:compl:rev CACCAGCACCCACACTATCATGTTGACATTCC; dG = -12.1
586 >s_e_s_d_s_b_s_e_s_e_s GTGCTGGTGACGGTGGCTGTGCTGGTGCTGGTG; >clamp_s_c_s_e_s:compl:rev CACCAGCACCCACACTATCATGTTGACATTCC; dG = -12.1
587 >s_e_s_e_s_a_s_c_s_d_s GTGCTGGTGCTGGTGTCTGTGTGGGTGACGGTG; >clamp_s_c_s_e_s:compl:rev CACCAGCACCCACACTATCATGTTGACATTCC; dG = -12.1
588 >s_e_s_e_s_a_s_e_s_e_s GTGCTGGTGCTGGTGTCTGTGCTGGTGCTGGTG; >clamp_s_c_s_e_s:compl:rev CACCAGCACCCACACTATCATGTTGACATTCC; dG = -12.1
589 >s_e_s_e_s_b_s_c_s_d_s GTGCTGGTGCTGGTGGCTGTGTGGGTGACGGTG; >clamp_s_c_s_e_s:compl:rev CACCAGCACCCACACTATCATGTTGACATTCC; dG = -12.1
590 >s_a_s_c_s_b_s_c_s_d_s GTGTCTGTGTGGGTGGCTGTGTGGGTGACGGTG; >clamp_s_c_s_e_s:compl:rev CACCAGCACCCACACTATCATGTTGACATTCC; dG = -12.1
591 >s_e_s_e_s_b_s_e_s_e_s GTGCTGGTGCTGGTGGCTGTGCTGGTGCTGGTG; >clamp_s_c_s_e_s:compl:rev CACCAGCACCCACACTATCATGTTGACATTCC; dG = -12.1
592 >s_a_s_a_s_b_s_c_s_d_s GTGTCTGTGTCTGTGGCTGTGTGGGTGACGGTG; >clamp_s_c_s_e_s:compl:rev CACCAGCACCCACACTATCATGTTGACATTCC; dG = -12.1
593 >s_a_s_d_s_d_s_c_s_d_s GTGTCTGTGACGGTGACGGTGTGGGTGACGGTG; >clamp_s_c_s_e_s:compl:rev CACCAGCACCCACACTATCATGTTGACATTCC; dG = -12.0
594 >s_a_s_e_s_d_s_c_s_d_s GTGTCTGTGCTGGTGACGGTGTGGGTGACGGTG; >clamp_s_c_s_e_s:compl:rev CACCAGCACCCACACTATCATGTTGACATTCC; dG = -12.0

595 >s_b_s_a_s_d_s_c_s_d_s GTGGCTGTGTCTGTGACGGTGTGGGTGACGGTG; >clamp_s_c_s_e_s:compl:rev CACCAGCACCCACACTATCATGTTGACATTCC; dG = -12.0
596 >s_b_s_a_s_e_s_c_s_d_s GTGGCTGTGTCTGTGCTGGTGTGGGTGACGGTG; >clamp_s_c_s_e_s:compl:rev CACCAGCACCCACACTATCATGTTGACATTCC; dG = -12.0
597 >s_a_s_a_s_d_s_c_s_d_s GTGTCTGTGTCTGTGACGGTGTGGGTGACGGTG; >clamp_s_c_s_e_s:compl:rev CACCAGCACCCACACTATCATGTTGACATTCC; dG = -12.0
598 >s_b_s_b_s_d_s_c_s_d_s GTGGCTGTGGCTGTGACGGTGTGGGTGACGGTG; >clamp_s_c_s_e_s:compl:rev CACCAGCACCCACACTATCATGTTGACATTCC; dG = -12.0
599 >s_b_s_b_s_e_s_c_s_d_s GTGGCTGTGGCTGTGCTGGTGTGGGTGACGGTG; >clamp_s_c_s_e_s:compl:rev CACCAGCACCCACACTATCATGTTGACATTCC; dG = -12.0
600 >s_a_s_a_s_e_s_c_s_d_s GTGTCTGTGTCTGTGCTGGTGTGGGTGACGGTG; >clamp_s_c_s_e_s:compl:rev CACCAGCACCCACACTATCATGTTGACATTCC; dG = -12.0
601 >s_b_s_d_s_d_s_c_s_d_s GTGGCTGTGACGGTGACGGTGTGGGTGACGGTG; >clamp_s_c_s_e_s:compl:rev CACCAGCACCCACACTATCATGTTGACATTCC; dG = -12.0
602 >s_b_s_e_s_d_s_c_s_d_s GTGGCTGTGCTGGTGACGGTGTGGGTGACGGTG; >clamp_s_c_s_e_s:compl:rev CACCAGCACCCACACTATCATGTTGACATTCC; dG = -12.0
603 >s_c_s_a_s_d_s_c_s_d_s GTGTGGGTGTCTGTGACGGTGTGGGTGACGGTG; >clamp_s_c_s_e_s:compl:rev CACCAGCACCCACACTATCATGTTGACATTCC; dG = -12.0
604 >s_c_s_b_s_d_s_c_s_d_s GTGTGGGTGGCTGTGACGGTGTGGGTGACGGTG; >clamp_s_c_s_e_s:compl:rev CACCAGCACCCACACTATCATGTTGACATTCC; dG = -12.0
605 >s_d_s_a_s_d_s_c_s_d_s GTGACGGTGTCTGTGACGGTGTGGGTGACGGTG; >clamp_s_c_s_e_s:compl:rev CACCAGCACCCACACTATCATGTTGACATTCC; dG = -12.0
606 >s_d_s_a_s_e_s_c_s_d_s GTGACGGTGTCTGTGCTGGTGTGGGTGACGGTG; >clamp_s_c_s_e_s:compl:rev CACCAGCACCCACACTATCATGTTGACATTCC; dG = -12.0
607 >s_d_s_b_s_d_s_c_s_d_s GTGACGGTGGCTGTGACGGTGTGGGTGACGGTG; >clamp_s_c_s_e_s:compl:rev CACCAGCACCCACACTATCATGTTGACATTCC; dG = -12.0
608 >s_d_s_b_s_e_s_c_s_d_s GTGACGGTGGCTGTGCTGGTGTGGGTGACGGTG; >clamp_s_c_s_e_s:compl:rev CACCAGCACCCACACTATCATGTTGACATTCC; dG = -12.0
609 >s_a_s_b_s_d_s_c_s_d_s GTGTCTGTGGCTGTGACGGTGTGGGTGACGGTG; >clamp_s_c_s_e_s:compl:rev CACCAGCACCCACACTATCATGTTGACATTCC; dG = -12.0
610 >s_d_s_d_s_d_s_c_s_d_s GTGACGGTGACGGTGACGGTGTGGGTGACGGTG; >clamp_s_c_s_e_s:compl:rev CACCAGCACCCACACTATCATGTTGACATTCC; dG = -12.0
611 >s_d_s_e_s_a_s_c_s_b_s GTGACGGTGCTGGTGTCTGTGTGGGTGGCTGTG; >clamp_s_c_s_e_s:compl:rev CACCAGCACCCACACTATCATGTTGACATTCC; dG = -12.0
612 >s_d_s_e_s_a_s_c_s_a_s GTGACGGTGCTGGTGTCTGTGTGGGTGTCTGTG; >clamp_s_c_s_e_s:compl:rev CACCAGCACCCACACTATCATGTTGACATTCC; dG = -12.0
613 >s_d_s_e_s_a_s_b_s_e_s GTGACGGTGCTGGTGTCTGTGGCTGTGCTGGTG; >clamp_s_c_s_e_s:compl:rev CACCAGCACCCACACTATCATGTTGACATTCC; dG = -12.0
614 >s_d_s_e_s_a_s_a_s_e_s GTGACGGTGCTGGTGTCTGTGTCTGTGCTGGTG; >clamp_s_c_s_e_s:compl:rev CACCAGCACCCACACTATCATGTTGACATTCC; dG = -12.0
615 >s_d_s_e_s_b_s_c_s_b_s GTGACGGTGCTGGTGGCTGTGTGGGTGGCTGTG; >clamp_s_c_s_e_s:compl:rev CACCAGCACCCACACTATCATGTTGACATTCC; dG = -12.0
616 >s_d_s_e_s_b_s_c_s_a_s GTGACGGTGCTGGTGGCTGTGTGGGTGTCTGTG; >clamp_s_c_s_e_s:compl:rev CACCAGCACCCACACTATCATGTTGACATTCC; dG = -12.0
617 >s_d_s_e_s_b_s_b_s_e_s GTGACGGTGCTGGTGGCTGTGGCTGTGCTGGTG; >clamp_s_c_s_e_s:compl:rev CACCAGCACCCACACTATCATGTTGACATTCC; dG = -12.0
618 >s_d_s_e_s_b_s_a_s_e_s GTGACGGTGCTGGTGGCTGTGTCTGTGCTGGTG; >clamp_s_c_s_e_s:compl:rev CACCAGCACCCACACTATCATGTTGACATTCC; dG = -12.0
619 >s_a_s_b_s_e_s_c_s_d_s GTGTCTGTGGCTGTGCTGGTGTGGGTGACGGTG; >clamp_s_c_s_e_s:compl:rev CACCAGCACCCACACTATCATGTTGACATTCC; dG = -12.0
620 >s_e_s_a_s_d_s_c_s_d_s GTGCTGGTGTCTGTGACGGTGTGGGTGACGGTG; >clamp_s_c_s_e_s:compl:rev CACCAGCACCCACACTATCATGTTGACATTCC; dG = -12.0
621 >s_e_s_a_s_e_s_c_s_d_s GTGCTGGTGTCTGTGCTGGTGTGGGTGACGGTG; >clamp_s_c_s_e_s:compl:rev CACCAGCACCCACACTATCATGTTGACATTCC; dG = -12.0
622 >s_e_s_b_s_d_s_c_s_d_s GTGCTGGTGGCTGTGACGGTGTGGGTGACGGTG; >clamp_s_c_s_e_s:compl:rev CACCAGCACCCACACTATCATGTTGACATTCC; dG = -12.0
623 >s_e_s_b_s_e_s_c_s_d_s GTGCTGGTGGCTGTGCTGGTGTGGGTGACGGTG; >clamp_s_c_s_e_s:compl:rev CACCAGCACCCACACTATCATGTTGACATTCC; dG = -12.0
624 >s_e_s_d_s_d_s_c_s_d_s GTGCTGGTGACGGTGACGGTGTGGGTGACGGTG; >clamp_s_c_s_e_s:compl:rev CACCAGCACCCACACTATCATGTTGACATTCC; dG = -12.0
625 >s_c_s_d_s_b_s_c_s_b_s GTGTGGGTGACGGTGGCTGTGTGGGTGGCTGTG; >clamp_s_c_s_e_s:compl:rev CACCAGCACCCACACTATCATGTTGACATTCC; dG = -11.9
626 >s_c_s_d_s_b_s_c_s_a_s GTGTGGGTGACGGTGGCTGTGTGGGTGTCTGTG; >clamp_s_c_s_e_s:compl:rev CACCAGCACCCACACTATCATGTTGACATTCC; dG = -11.9
627 >s_c_s_d_s_b_s_b_s_e_s GTGTGGGTGACGGTGGCTGTGGCTGTGCTGGTG; >clamp_s_c_s_e_s:compl:rev CACCAGCACCCACACTATCATGTTGACATTCC; dG = -11.9
628 >s_c_s_d_s_b_s_a_s_e_s GTGTGGGTGACGGTGGCTGTGTCTGTGCTGGTG; >clamp_s_c_s_e_s:compl:rev CACCAGCACCCACACTATCATGTTGACATTCC; dG = -11.9
629 >s_e_s_e_s_a_s_c_s_b_s GTGCTGGTGCTGGTGTCTGTGTGGGTGGCTGTG; >clamp_s_c_s_e_s:compl:rev CACCAGCACCCACACTATCATGTTGACATTCC; dG = -11.9
630 >s_e_s_e_s_a_s_c_s_a_s GTGCTGGTGCTGGTGTCTGTGTGGGTGTCTGTG; >clamp_s_c_s_e_s:compl:rev CACCAGCACCCACACTATCATGTTGACATTCC; dG = -11.9
631 >s_e_s_e_s_a_s_b_s_e_s GTGCTGGTGCTGGTGTCTGTGGCTGTGCTGGTG; >clamp_s_c_s_e_s:compl:rev CACCAGCACCCACACTATCATGTTGACATTCC; dG = -11.9

632 >s_e_s_e_s_a_s_a_s_e_s GTGCTGGTGCTGGTGTCTGTGTCTGTGCTGGTG; >clamp_s_c_s_e_s:compl:rev CACCAGCACCCACACTATCATGTTGACATTCC; dG = -11.9
633 >s_e_s_e_s_b_s_c_s_b_s GTGCTGGTGCTGGTGGCTGTGTGGGTGGCTGTG; >clamp_s_c_s_e_s:compl:rev CACCAGCACCCACACTATCATGTTGACATTCC; dG = -11.9
634 >s_e_s_e_s_b_s_c_s_a_s GTGCTGGTGCTGGTGGCTGTGTGGGTGTCTGTG; >clamp_s_c_s_e_s:compl:rev CACCAGCACCCACACTATCATGTTGACATTCC; dG = -11.9
635 >s_e_s_e_s_b_s_b_s_e_s GTGCTGGTGCTGGTGGCTGTGGCTGTGCTGGTG; >clamp_s_c_s_e_s:compl:rev CACCAGCACCCACACTATCATGTTGACATTCC; dG = -11.9
636 >s_e_s_e_s_b_s_a_s_e_s GTGCTGGTGCTGGTGGCTGTGTCTGTGCTGGTG; >clamp_s_c_s_e_s:compl:rev CACCAGCACCCACACTATCATGTTGACATTCC; dG = -11.9
637 >s_b_s_c_s_a_s_b_s_e_s GTGGCTGTGTGGGTGTCTGTGGCTGTGCTGGTG; >clamp_s_c_s_e_s:compl:rev CACCAGCACCCACACTATCATGTTGACATTCC; dG = -11.8
638 >s_b_s_c_s_a_s_a_s_e_s GTGGCTGTGTGGGTGTCTGTGTCTGTGCTGGTG; >clamp_s_c_s_e_s:compl:rev CACCAGCACCCACACTATCATGTTGACATTCC; dG = -11.8
639 >s_b_s_c_s_b_s_b_s_e_s GTGGCTGTGTGGGTGGCTGTGGCTGTGCTGGTG; >clamp_s_c_s_e_s:compl:rev CACCAGCACCCACACTATCATGTTGACATTCC; dG = -11.8
640 >s_b_s_c_s_b_s_a_s_e_s GTGGCTGTGTGGGTGGCTGTGTCTGTGCTGGTG; >clamp_s_c_s_e_s:compl:rev CACCAGCACCCACACTATCATGTTGACATTCC; dG = -11.8
641 >s_c_s_d_s_a_s_c_s_b_s GTGTGGGTGACGGTGTCTGTGTGGGTGGCTGTG; >clamp_s_c_s_e_s:compl:rev CACCAGCACCCACACTATCATGTTGACATTCC; dG = -11.8
642 >s_c_s_d_s_a_s_c_s_a_s GTGTGGGTGACGGTGTCTGTGTGGGTGTCTGTG; >clamp_s_c_s_e_s:compl:rev CACCAGCACCCACACTATCATGTTGACATTCC; dG = -11.8
643 >s_c_s_d_s_a_s_b_s_e_s GTGTGGGTGACGGTGTCTGTGGCTGTGCTGGTG; >clamp_s_c_s_e_s:compl:rev CACCAGCACCCACACTATCATGTTGACATTCC; dG = -11.8
644 >s_c_s_d_s_a_s_a_s_e_s GTGTGGGTGACGGTGTCTGTGTCTGTGCTGGTG; >clamp_s_c_s_e_s:compl:rev CACCAGCACCCACACTATCATGTTGACATTCC; dG = -11.8
645 >s_d_s_c_s_a_s_b_s_e_s GTGACGGTGTGGGTGTCTGTGGCTGTGCTGGTG; >clamp_s_c_s_e_s:compl:rev CACCAGCACCCACACTATCATGTTGACATTCC; dG = -11.8
646 >s_d_s_c_s_a_s_a_s_e_s GTGACGGTGTGGGTGTCTGTGTCTGTGCTGGTG; >clamp_s_c_s_e_s:compl:rev CACCAGCACCCACACTATCATGTTGACATTCC; dG = -11.8
647 >s_d_s_c_s_b_s_b_s_e_s GTGACGGTGTGGGTGGCTGTGGCTGTGCTGGTG; >clamp_s_c_s_e_s:compl:rev CACCAGCACCCACACTATCATGTTGACATTCC; dG = -11.8
648 >s_d_s_c_s_b_s_a_s_e_s GTGACGGTGTGGGTGGCTGTGTCTGTGCTGGTG; >clamp_s_c_s_e_s:compl:rev CACCAGCACCCACACTATCATGTTGACATTCC; dG = -11.8
649 >s_e_s_c_s_a_s_b_s_e_s GTGCTGGTGTGGGTGTCTGTGGCTGTGCTGGTG; >clamp_s_c_s_e_s:compl:rev CACCAGCACCCACACTATCATGTTGACATTCC; dG = -11.8
650 >s_e_s_c_s_a_s_a_s_e_s GTGCTGGTGTGGGTGTCTGTGTCTGTGCTGGTG; >clamp_s_c_s_e_s:compl:rev CACCAGCACCCACACTATCATGTTGACATTCC; dG = -11.8
651 >s_e_s_c_s_b_s_b_s_e_s GTGCTGGTGTGGGTGGCTGTGGCTGTGCTGGTG; >clamp_s_c_s_e_s:compl:rev CACCAGCACCCACACTATCATGTTGACATTCC; dG = -11.8
652 >s_e_s_c_s_b_s_a_s_e_s GTGCTGGTGTGGGTGGCTGTGTCTGTGCTGGTG; >clamp_s_c_s_e_s:compl:rev CACCAGCACCCACACTATCATGTTGACATTCC; dG = -11.8
653 >s_a_s_c_s_a_s_b_s_e_s GTGTCTGTGTGGGTGTCTGTGGCTGTGCTGGTG; >clamp_s_c_s_e_s:compl:rev CACCAGCACCCACACTATCATGTTGACATTCC; dG = -11.8
654 >s_a_s_c_s_a_s_a_s_e_s GTGTCTGTGTGGGTGTCTGTGTCTGTGCTGGTG; >clamp_s_c_s_e_s:compl:rev CACCAGCACCCACACTATCATGTTGACATTCC; dG = -11.8
655 >s_a_s_c_s_b_s_b_s_e_s GTGTCTGTGTGGGTGGCTGTGGCTGTGCTGGTG; >clamp_s_c_s_e_s:compl:rev CACCAGCACCCACACTATCATGTTGACATTCC; dG = -11.8
656 >s_a_s_c_s_b_s_a_s_e_s GTGTCTGTGTGGGTGGCTGTGTCTGTGCTGGTG; >clamp_s_c_s_e_s:compl:rev CACCAGCACCCACACTATCATGTTGACATTCC; dG = -11.8
657 >s_b_s_b_s_e_s_c_s_b_s GTGGCTGTGGCTGTGCTGGTGTGGGTGGCTGTG; >clamp_s_c_s_e_s:compl:rev CACCAGCACCCACACTATCATGTTGACATTCC; dG = -11.7
658 >s_b_s_b_s_e_s_c_s_a_s GTGGCTGTGGCTGTGCTGGTGTGGGTGTCTGTG; >clamp_s_c_s_e_s:compl:rev CACCAGCACCCACACTATCATGTTGACATTCC; dG = -11.7
659 >s_b_s_b_s_e_s_b_s_e_s GTGGCTGTGGCTGTGCTGGTGGCTGTGCTGGTG; >clamp_s_c_s_e_s:compl:rev CACCAGCACCCACACTATCATGTTGACATTCC; dG = -11.7
660 >s_b_s_b_s_e_s_a_s_e_s GTGGCTGTGGCTGTGCTGGTGTCTGTGCTGGTG; >clamp_s_c_s_e_s:compl:rev CACCAGCACCCACACTATCATGTTGACATTCC; dG = -11.7
661 >s_b_s_e_s_d_s_c_s_b_s GTGGCTGTGCTGGTGACGGTGTGGGTGGCTGTG; >clamp_s_c_s_e_s:compl:rev CACCAGCACCCACACTATCATGTTGACATTCC; dG = -11.7
662 >s_b_s_e_s_d_s_c_s_a_s GTGGCTGTGCTGGTGACGGTGTGGGTGTCTGTG; >clamp_s_c_s_e_s:compl:rev CACCAGCACCCACACTATCATGTTGACATTCC; dG = -11.7
663 >s_b_s_e_s_d_s_b_s_e_s GTGGCTGTGCTGGTGACGGTGGCTGTGCTGGTG; >clamp_s_c_s_e_s:compl:rev CACCAGCACCCACACTATCATGTTGACATTCC; dG = -11.7
664 >s_b_s_e_s_d_s_a_s_e_s GTGGCTGTGCTGGTGACGGTGTCTGTGCTGGTG; >clamp_s_c_s_e_s:compl:rev CACCAGCACCCACACTATCATGTTGACATTCC; dG = -11.7
665 >s_d_s_b_s_e_s_b_s_e_s GTGACGGTGGCTGTGCTGGTGGCTGTGCTGGTG; >clamp_s_c_s_e_s:compl:rev CACCAGCACCCACACTATCATGTTGACATTCC; dG = -11.7
666 >s_a_s_b_s_e_s_c_s_b_s GTGTCTGTGGCTGTGCTGGTGTGGGTGGCTGTG; >clamp_s_c_s_e_s:compl:rev CACCAGCACCCACACTATCATGTTGACATTCC; dG = -11.7
667 >s_a_s_b_s_e_s_c_s_a_s GTGTCTGTGGCTGTGCTGGTGTGGGTGTCTGTG; >clamp_s_c_s_e_s:compl:rev CACCAGCACCCACACTATCATGTTGACATTCC; dG = -11.7
668 >s_a_s_b_s_e_s_b_s_e_s GTGTCTGTGGCTGTGCTGGTGGCTGTGCTGGTG; >clamp_s_c_s_e_s:compl:rev CACCAGCACCCACACTATCATGTTGACATTCC; dG = -11.7

669 >s_a_s_b_s_e_s_a_s_e_s GTGTCTGTGGCTGTGCTGGTGTCTGTGCTGGTG; >clamp_s_c_s_e_s:compl:rev CACCAGCACCCACACTATCATGTTGACATTCC; dG = -11.7
670 >s_e_s_b_s_e_s_b_s_e_s GTGCTGGTGGCTGTGCTGGTGGCTGTGCTGGTG; >clamp_s_c_s_e_s:compl:rev CACCAGCACCCACACTATCATGTTGACATTCC; dG = -11.7
671 >s_b_s_a_s_e_s_b_s_e_s GTGGCTGTGTCTGTGCTGGTGGCTGTGCTGGTG; >clamp_s_c_s_e_s:compl:rev CACCAGCACCCACACTATCATGTTGACATTCC; dG = -11.6
672 >s_a_s_a_s_e_s_b_s_e_s GTGTCTGTGTCTGTGCTGGTGGCTGTGCTGGTG; >clamp_s_c_s_e_s:compl:rev CACCAGCACCCACACTATCATGTTGACATTCC; dG = -11.6
673 >s_d_s_b_s_e_s_c_s_b_s GTGACGGTGGCTGTGCTGGTGTGGGTGGCTGTG; >clamp_s_c_s_e_s:compl:rev CACCAGCACCCACACTATCATGTTGACATTCC; dG = -11.6
674 >s_d_s_b_s_e_s_c_s_a_s GTGACGGTGGCTGTGCTGGTGTGGGTGTCTGTG; >clamp_s_c_s_e_s:compl:rev CACCAGCACCCACACTATCATGTTGACATTCC; dG = -11.6
675 >s_d_s_b_s_e_s_a_s_e_s GTGACGGTGGCTGTGCTGGTGTCTGTGCTGGTG; >clamp_s_c_s_e_s:compl:rev CACCAGCACCCACACTATCATGTTGACATTCC; dG = -11.6
676 >s_e_s_b_s_e_s_c_s_b_s GTGCTGGTGGCTGTGCTGGTGTGGGTGGCTGTG; >clamp_s_c_s_e_s:compl:rev CACCAGCACCCACACTATCATGTTGACATTCC; dG = -11.6
677 >s_e_s_b_s_e_s_c_s_a_s GTGCTGGTGGCTGTGCTGGTGTGGGTGTCTGTG; >clamp_s_c_s_e_s:compl:rev CACCAGCACCCACACTATCATGTTGACATTCC; dG = -11.6
678 >s_e_s_b_s_e_s_a_s_e_s GTGCTGGTGGCTGTGCTGGTGTCTGTGCTGGTG; >clamp_s_c_s_e_s:compl:rev CACCAGCACCCACACTATCATGTTGACATTCC; dG = -11.6
679 >s_a_s_e_s_d_s_c_s_b_s GTGTCTGTGCTGGTGACGGTGTGGGTGGCTGTG; >clamp_s_c_s_e_s:compl:rev CACCAGCACCCACACTATCATGTTGACATTCC; dG = -11.5
680 >s_a_s_e_s_d_s_c_s_a_s GTGTCTGTGCTGGTGACGGTGTGGGTGTCTGTG; >clamp_s_c_s_e_s:compl:rev CACCAGCACCCACACTATCATGTTGACATTCC; dG = -11.5
681 >s_a_s_e_s_d_s_b_s_e_s GTGTCTGTGCTGGTGACGGTGGCTGTGCTGGTG; >clamp_s_c_s_e_s:compl:rev CACCAGCACCCACACTATCATGTTGACATTCC; dG = -11.5
682 >s_a_s_e_s_d_s_a_s_e_s GTGTCTGTGCTGGTGACGGTGTCTGTGCTGGTG; >clamp_s_c_s_e_s:compl:rev CACCAGCACCCACACTATCATGTTGACATTCC; dG = -11.5
683 >s_b_s_a_s_e_s_c_s_b_s GTGGCTGTGTCTGTGCTGGTGTGGGTGGCTGTG; >clamp_s_c_s_e_s:compl:rev CACCAGCACCCACACTATCATGTTGACATTCC; dG = -11.5
684 >s_b_s_a_s_e_s_c_s_a_s GTGGCTGTGTCTGTGCTGGTGTGGGTGTCTGTG; >clamp_s_c_s_e_s:compl:rev CACCAGCACCCACACTATCATGTTGACATTCC; dG = -11.5
685 >s_b_s_a_s_e_s_a_s_e_s GTGGCTGTGTCTGTGCTGGTGTCTGTGCTGGTG; >clamp_s_c_s_e_s:compl:rev CACCAGCACCCACACTATCATGTTGACATTCC; dG = -11.5
686 >s_a_s_a_s_e_s_c_s_b_s GTGTCTGTGTCTGTGCTGGTGTGGGTGGCTGTG; >clamp_s_c_s_e_s:compl:rev CACCAGCACCCACACTATCATGTTGACATTCC; dG = -11.5
687 >s_a_s_a_s_e_s_c_s_a_s GTGTCTGTGTCTGTGCTGGTGTGGGTGTCTGTG; >clamp_s_c_s_e_s:compl:rev CACCAGCACCCACACTATCATGTTGACATTCC; dG = -11.5
688 >s_a_s_a_s_e_s_a_s_e_s GTGTCTGTGTCTGTGCTGGTGTCTGTGCTGGTG; >clamp_s_c_s_e_s:compl:rev CACCAGCACCCACACTATCATGTTGACATTCC; dG = -11.5
689 >s_d_s_a_s_e_s_b_s_e_s GTGACGGTGTCTGTGCTGGTGGCTGTGCTGGTG; >clamp_s_c_s_e_s:compl:rev CACCAGCACCCACACTATCATGTTGACATTCC; dG = -11.5
690 >s_e_s_a_s_e_s_b_s_e_s GTGCTGGTGTCTGTGCTGGTGGCTGTGCTGGTG; >clamp_s_c_s_e_s:compl:rev CACCAGCACCCACACTATCATGTTGACATTCC; dG = -11.5
691 >s_a_s_d_s_a_s_b_s_e_s GTGTCTGTGACGGTGTCTGTGGCTGTGCTGGTG; >clamp_s_c_s_e_s:compl:rev CACCAGCACCCACACTATCATGTTGACATTCC; dG = -11.4
692 >s_a_s_d_s_b_s_b_s_e_s GTGTCTGTGACGGTGGCTGTGGCTGTGCTGGTG; >clamp_s_c_s_e_s:compl:rev CACCAGCACCCACACTATCATGTTGACATTCC; dG = -11.4
693 >s_a_s_a_s_b_s_b_s_e_s GTGTCTGTGTCTGTGGCTGTGGCTGTGCTGGTG; >clamp_s_c_s_e_s:compl:rev CACCAGCACCCACACTATCATGTTGACATTCC; dG = -11.4
694 >s_a_s_e_s_a_s_b_s_e_s GTGTCTGTGCTGGTGTCTGTGGCTGTGCTGGTG; >clamp_s_c_s_e_s:compl:rev CACCAGCACCCACACTATCATGTTGACATTCC; dG = -11.4
695 >s_a_s_e_s_b_s_b_s_e_s GTGTCTGTGCTGGTGGCTGTGGCTGTGCTGGTG; >clamp_s_c_s_e_s:compl:rev CACCAGCACCCACACTATCATGTTGACATTCC; dG = -11.4
696 >s_b_s_a_s_a_s_b_s_e_s GTGGCTGTGTCTGTGTCTGTGGCTGTGCTGGTG; >clamp_s_c_s_e_s:compl:rev CACCAGCACCCACACTATCATGTTGACATTCC; dG = -11.4
697 >s_b_s_a_s_b_s_b_s_e_s GTGGCTGTGTCTGTGGCTGTGGCTGTGCTGGTG; >clamp_s_c_s_e_s:compl:rev CACCAGCACCCACACTATCATGTTGACATTCC; dG = -11.4
698 >s_b_s_b_s_a_s_b_s_e_s GTGGCTGTGGCTGTGTCTGTGGCTGTGCTGGTG; >clamp_s_c_s_e_s:compl:rev CACCAGCACCCACACTATCATGTTGACATTCC; dG = -11.4
699 >s_b_s_b_s_b_s_b_s_e_s GTGGCTGTGGCTGTGGCTGTGGCTGTGCTGGTG; >clamp_s_c_s_e_s:compl:rev CACCAGCACCCACACTATCATGTTGACATTCC; dG = -11.4
700 >s_b_s_d_s_a_s_b_s_e_s GTGGCTGTGACGGTGTCTGTGGCTGTGCTGGTG; >clamp_s_c_s_e_s:compl:rev CACCAGCACCCACACTATCATGTTGACATTCC; dG = -11.4
701 >s_b_s_d_s_b_s_b_s_e_s GTGGCTGTGACGGTGGCTGTGGCTGTGCTGGTG; >clamp_s_c_s_e_s:compl:rev CACCAGCACCCACACTATCATGTTGACATTCC; dG = -11.4
702 >s_b_s_e_s_a_s_b_s_e_s GTGGCTGTGCTGGTGTCTGTGGCTGTGCTGGTG; >clamp_s_c_s_e_s:compl:rev CACCAGCACCCACACTATCATGTTGACATTCC; dG = -11.4
703 >s_b_s_e_s_b_s_b_s_e_s GTGGCTGTGCTGGTGGCTGTGGCTGTGCTGGTG; >clamp_s_c_s_e_s:compl:rev CACCAGCACCCACACTATCATGTTGACATTCC; dG = -11.4
704 >s_c_s_a_s_a_s_b_s_e_s GTGTGGGTGTCTGTGTCTGTGGCTGTGCTGGTG; >clamp_s_c_s_e_s:compl:rev CACCAGCACCCACACTATCATGTTGACATTCC; dG = -11.4
705 >s_c_s_a_s_b_s_b_s_e_s GTGTGGGTGTCTGTGGCTGTGGCTGTGCTGGTG; >clamp_s_c_s_e_s:compl:rev CACCAGCACCCACACTATCATGTTGACATTCC; dG = -11.4

706 >s_c_s_b_s_a_s_b_s_e_s GTGTGGGTGGCTGTGTCTGTGGCTGTGCTGGTG; >clamp_s_c_s_e_s:compl:rev CACCAGCACCCACACTATCATGTTGACATTCC; dG = -11.4
707 >s_c_s_b_s_b_s_b_s_e_s GTGTGGGTGGCTGTGGCTGTGGCTGTGCTGGTG; >clamp_s_c_s_e_s:compl:rev CACCAGCACCCACACTATCATGTTGACATTCC; dG = -11.4
708 >s_a_s_b_s_a_s_b_s_e_s GTGTCTGTGGCTGTGTCTGTGGCTGTGCTGGTG; >clamp_s_c_s_e_s:compl:rev CACCAGCACCCACACTATCATGTTGACATTCC; dG = -11.4
709 >s_d_s_a_s_a_s_b_s_e_s GTGACGGTGTCTGTGTCTGTGGCTGTGCTGGTG; >clamp_s_c_s_e_s:compl:rev CACCAGCACCCACACTATCATGTTGACATTCC; dG = -11.4
710 >s_d_s_a_s_b_s_b_s_e_s GTGACGGTGTCTGTGGCTGTGGCTGTGCTGGTG; >clamp_s_c_s_e_s:compl:rev CACCAGCACCCACACTATCATGTTGACATTCC; dG = -11.4
711 >s_a_s_b_s_b_s_b_s_e_s GTGTCTGTGGCTGTGGCTGTGGCTGTGCTGGTG; >clamp_s_c_s_e_s:compl:rev CACCAGCACCCACACTATCATGTTGACATTCC; dG = -11.4
712 >s_d_s_a_s_e_s_c_s_b_s GTGACGGTGTCTGTGCTGGTGTGGGTGGCTGTG; >clamp_s_c_s_e_s:compl:rev CACCAGCACCCACACTATCATGTTGACATTCC; dG = -11.4
713 >s_d_s_a_s_e_s_c_s_a_s GTGACGGTGTCTGTGCTGGTGTGGGTGTCTGTG; >clamp_s_c_s_e_s:compl:rev CACCAGCACCCACACTATCATGTTGACATTCC; dG = -11.4
714 >s_d_s_a_s_e_s_a_s_e_s GTGACGGTGTCTGTGCTGGTGTCTGTGCTGGTG; >clamp_s_c_s_e_s:compl:rev CACCAGCACCCACACTATCATGTTGACATTCC; dG = -11.4
715 >s_d_s_b_s_a_s_b_s_e_s GTGACGGTGGCTGTGTCTGTGGCTGTGCTGGTG; >clamp_s_c_s_e_s:compl:rev CACCAGCACCCACACTATCATGTTGACATTCC; dG = -11.4
716 >s_d_s_b_s_b_s_b_s_e_s GTGACGGTGGCTGTGGCTGTGGCTGTGCTGGTG; >clamp_s_c_s_e_s:compl:rev CACCAGCACCCACACTATCATGTTGACATTCC; dG = -11.4
717 >s_d_s_d_s_a_s_b_s_e_s GTGACGGTGACGGTGTCTGTGGCTGTGCTGGTG; >clamp_s_c_s_e_s:compl:rev CACCAGCACCCACACTATCATGTTGACATTCC; dG = -11.4
718 >s_d_s_d_s_b_s_b_s_e_s GTGACGGTGACGGTGGCTGTGGCTGTGCTGGTG; >clamp_s_c_s_e_s:compl:rev CACCAGCACCCACACTATCATGTTGACATTCC; dG = -11.4
719 >s_a_s_a_s_a_s_b_s_e_s GTGTCTGTGTCTGTGTCTGTGGCTGTGCTGGTG; >clamp_s_c_s_e_s:compl:rev CACCAGCACCCACACTATCATGTTGACATTCC; dG = -11.4
720 >s_e_s_a_s_a_s_b_s_e_s GTGCTGGTGTCTGTGTCTGTGGCTGTGCTGGTG; >clamp_s_c_s_e_s:compl:rev CACCAGCACCCACACTATCATGTTGACATTCC; dG = -11.4
721 >s_e_s_a_s_b_s_b_s_e_s GTGCTGGTGTCTGTGGCTGTGGCTGTGCTGGTG; >clamp_s_c_s_e_s:compl:rev CACCAGCACCCACACTATCATGTTGACATTCC; dG = -11.4
722 >s_e_s_a_s_e_s_c_s_b_s GTGCTGGTGTCTGTGCTGGTGTGGGTGGCTGTG; >clamp_s_c_s_e_s:compl:rev CACCAGCACCCACACTATCATGTTGACATTCC; dG = -11.4
723 >s_e_s_a_s_e_s_c_s_a_s GTGCTGGTGTCTGTGCTGGTGTGGGTGTCTGTG; >clamp_s_c_s_e_s:compl:rev CACCAGCACCCACACTATCATGTTGACATTCC; dG = -11.4
724 >s_e_s_a_s_e_s_a_s_e_s GTGCTGGTGTCTGTGCTGGTGTCTGTGCTGGTG; >clamp_s_c_s_e_s:compl:rev CACCAGCACCCACACTATCATGTTGACATTCC; dG = -11.4
725 >s_e_s_b_s_a_s_b_s_e_s GTGCTGGTGGCTGTGTCTGTGGCTGTGCTGGTG; >clamp_s_c_s_e_s:compl:rev CACCAGCACCCACACTATCATGTTGACATTCC; dG = -11.4
726 >s_e_s_b_s_b_s_b_s_e_s GTGCTGGTGGCTGTGGCTGTGGCTGTGCTGGTG; >clamp_s_c_s_e_s:compl:rev CACCAGCACCCACACTATCATGTTGACATTCC; dG = -11.4
727 >s_e_s_d_s_a_s_b_s_e_s GTGCTGGTGACGGTGTCTGTGGCTGTGCTGGTG; >clamp_s_c_s_e_s:compl:rev CACCAGCACCCACACTATCATGTTGACATTCC; dG = -11.4
728 >s_e_s_d_s_b_s_b_s_e_s GTGCTGGTGACGGTGGCTGTGGCTGTGCTGGTG; >clamp_s_c_s_e_s:compl:rev CACCAGCACCCACACTATCATGTTGACATTCC; dG = -11.4
729 >s_a_s_d_s_d_s_b_s_e_s GTGTCTGTGACGGTGACGGTGGCTGTGCTGGTG; >clamp_s_c_s_e_s:compl:rev CACCAGCACCCACACTATCATGTTGACATTCC; dG = -11.3
730 >s_b_s_a_s_d_s_b_s_e_s GTGGCTGTGTCTGTGACGGTGGCTGTGCTGGTG; >clamp_s_c_s_e_s:compl:rev CACCAGCACCCACACTATCATGTTGACATTCC; dG = -11.3
731 >s_b_s_b_s_d_s_b_s_e_s GTGGCTGTGGCTGTGACGGTGGCTGTGCTGGTG; >clamp_s_c_s_e_s:compl:rev CACCAGCACCCACACTATCATGTTGACATTCC; dG = -11.3
732 >s_a_s_a_s_d_s_b_s_e_s GTGTCTGTGTCTGTGACGGTGGCTGTGCTGGTG; >clamp_s_c_s_e_s:compl:rev CACCAGCACCCACACTATCATGTTGACATTCC; dG = -11.3
733 >s_b_s_d_s_d_s_b_s_e_s GTGGCTGTGACGGTGACGGTGGCTGTGCTGGTG; >clamp_s_c_s_e_s:compl:rev CACCAGCACCCACACTATCATGTTGACATTCC; dG = -11.3
734 >s_c_s_a_s_d_s_b_s_e_s GTGTGGGTGTCTGTGACGGTGGCTGTGCTGGTG; >clamp_s_c_s_e_s:compl:rev CACCAGCACCCACACTATCATGTTGACATTCC; dG = -11.3
735 >s_c_s_b_s_d_s_b_s_e_s GTGTGGGTGGCTGTGACGGTGGCTGTGCTGGTG; >clamp_s_c_s_e_s:compl:rev CACCAGCACCCACACTATCATGTTGACATTCC; dG = -11.3
736 >s_d_s_a_s_d_s_b_s_e_s GTGACGGTGTCTGTGACGGTGGCTGTGCTGGTG; >clamp_s_c_s_e_s:compl:rev CACCAGCACCCACACTATCATGTTGACATTCC; dG = -11.3
737 >s_d_s_b_s_d_s_b_s_e_s GTGACGGTGGCTGTGACGGTGGCTGTGCTGGTG; >clamp_s_c_s_e_s:compl:rev CACCAGCACCCACACTATCATGTTGACATTCC; dG = -11.3
738 >s_d_s_d_s_d_s_b_s_e_s GTGACGGTGACGGTGACGGTGGCTGTGCTGGTG; >clamp_s_c_s_e_s:compl:rev CACCAGCACCCACACTATCATGTTGACATTCC; dG = -11.3
739 >s_a_s_b_s_d_s_b_s_e_s GTGTCTGTGGCTGTGACGGTGGCTGTGCTGGTG; >clamp_s_c_s_e_s:compl:rev CACCAGCACCCACACTATCATGTTGACATTCC; dG = -11.3
740 >s_e_s_a_s_d_s_b_s_e_s GTGCTGGTGTCTGTGACGGTGGCTGTGCTGGTG; >clamp_s_c_s_e_s:compl:rev CACCAGCACCCACACTATCATGTTGACATTCC; dG = -11.3
741 >s_e_s_b_s_d_s_b_s_e_s GTGCTGGTGGCTGTGACGGTGGCTGTGCTGGTG; >clamp_s_c_s_e_s:compl:rev CACCAGCACCCACACTATCATGTTGACATTCC; dG = -11.3
742 >s_e_s_d_s_d_s_b_s_e_s GTGCTGGTGACGGTGACGGTGGCTGTGCTGGTG; >clamp_s_c_s_e_s:compl:rev CACCAGCACCCACACTATCATGTTGACATTCC; dG = -11.3

743 >s_a_s_d_s_a_s_a_s_e_s GTGTCTGTGACGGTGTCTGTGTCTGTGCTGGTG; >clamp_s_c_s_e_s:compl:rev CACCAGCACCCACACTATCATGTTGACATTCC; dG = -11.2
744 >s_a_s_d_s_b_s_a_s_e_s GTGTCTGTGACGGTGGCTGTGTCTGTGCTGGTG; >clamp_s_c_s_e_s:compl:rev CACCAGCACCCACACTATCATGTTGACATTCC; dG = -11.2
745 >s_a_s_d_s_d_s_a_s_e_s GTGTCTGTGACGGTGACGGTGTCTGTGCTGGTG; >clamp_s_c_s_e_s:compl:rev CACCAGCACCCACACTATCATGTTGACATTCC; dG = -11.2
746 >s_a_s_e_s_a_s_a_s_e_s GTGTCTGTGCTGGTGTCTGTGTCTGTGCTGGTG; >clamp_s_c_s_e_s:compl:rev CACCAGCACCCACACTATCATGTTGACATTCC; dG = -11.2
747 >s_a_s_a_s_b_s_a_s_e_s GTGTCTGTGTCTGTGGCTGTGTCTGTGCTGGTG; >clamp_s_c_s_e_s:compl:rev CACCAGCACCCACACTATCATGTTGACATTCC; dG = -11.2
748 >s_a_s_e_s_b_s_a_s_e_s GTGTCTGTGCTGGTGGCTGTGTCTGTGCTGGTG; >clamp_s_c_s_e_s:compl:rev CACCAGCACCCACACTATCATGTTGACATTCC; dG = -11.2
749 >s_b_s_a_s_a_s_a_s_e_s GTGGCTGTGTCTGTGTCTGTGTCTGTGCTGGTG; >clamp_s_c_s_e_s:compl:rev CACCAGCACCCACACTATCATGTTGACATTCC; dG = -11.2
750 >s_b_s_a_s_b_s_a_s_e_s GTGGCTGTGTCTGTGGCTGTGTCTGTGCTGGTG; >clamp_s_c_s_e_s:compl:rev CACCAGCACCCACACTATCATGTTGACATTCC; dG = -11.2
751 >s_b_s_a_s_d_s_a_s_e_s GTGGCTGTGTCTGTGACGGTGTCTGTGCTGGTG; >clamp_s_c_s_e_s:compl:rev CACCAGCACCCACACTATCATGTTGACATTCC; dG = -11.2
752 >s_b_s_b_s_a_s_a_s_e_s GTGGCTGTGGCTGTGTCTGTGTCTGTGCTGGTG; >clamp_s_c_s_e_s:compl:rev CACCAGCACCCACACTATCATGTTGACATTCC; dG = -11.2
753 >s_b_s_b_s_b_s_a_s_e_s GTGGCTGTGGCTGTGGCTGTGTCTGTGCTGGTG; >clamp_s_c_s_e_s:compl:rev CACCAGCACCCACACTATCATGTTGACATTCC; dG = -11.2
754 >s_b_s_b_s_d_s_a_s_e_s GTGGCTGTGGCTGTGACGGTGTCTGTGCTGGTG; >clamp_s_c_s_e_s:compl:rev CACCAGCACCCACACTATCATGTTGACATTCC; dG = -11.2
755 >s_b_s_d_s_a_s_a_s_e_s GTGGCTGTGACGGTGTCTGTGTCTGTGCTGGTG; >clamp_s_c_s_e_s:compl:rev CACCAGCACCCACACTATCATGTTGACATTCC; dG = -11.2
756 >s_a_s_a_s_d_s_a_s_e_s GTGTCTGTGTCTGTGACGGTGTCTGTGCTGGTG; >clamp_s_c_s_e_s:compl:rev CACCAGCACCCACACTATCATGTTGACATTCC; dG = -11.2
757 >s_b_s_d_s_b_s_a_s_e_s GTGGCTGTGACGGTGGCTGTGTCTGTGCTGGTG; >clamp_s_c_s_e_s:compl:rev CACCAGCACCCACACTATCATGTTGACATTCC; dG = -11.2
758 >s_b_s_d_s_d_s_a_s_e_s GTGGCTGTGACGGTGACGGTGTCTGTGCTGGTG; >clamp_s_c_s_e_s:compl:rev CACCAGCACCCACACTATCATGTTGACATTCC; dG = -11.2
759 >s_b_s_e_s_a_s_a_s_e_s GTGGCTGTGCTGGTGTCTGTGTCTGTGCTGGTG; >clamp_s_c_s_e_s:compl:rev CACCAGCACCCACACTATCATGTTGACATTCC; dG = -11.2
760 >s_b_s_e_s_b_s_c_s_b_s GTGGCTGTGCTGGTGGCTGTGTGGGTGGCTGTG; >clamp_s_c_s_e_s:compl:rev CACCAGCACCCACACTATCATGTTGACATTCC; dG = -11.2
761 >s_b_s_e_s_b_s_c_s_a_s GTGGCTGTGCTGGTGGCTGTGTGGGTGTCTGTG; >clamp_s_c_s_e_s:compl:rev CACCAGCACCCACACTATCATGTTGACATTCC; dG = -11.2
762 >s_b_s_e_s_b_s_a_s_e_s GTGGCTGTGCTGGTGGCTGTGTCTGTGCTGGTG; >clamp_s_c_s_e_s:compl:rev CACCAGCACCCACACTATCATGTTGACATTCC; dG = -11.2
763 >s_c_s_a_s_a_s_a_s_e_s GTGTGGGTGTCTGTGTCTGTGTCTGTGCTGGTG; >clamp_s_c_s_e_s:compl:rev CACCAGCACCCACACTATCATGTTGACATTCC; dG = -11.2
764 >s_c_s_a_s_b_s_a_s_e_s GTGTGGGTGTCTGTGGCTGTGTCTGTGCTGGTG; >clamp_s_c_s_e_s:compl:rev CACCAGCACCCACACTATCATGTTGACATTCC; dG = -11.2
765 >s_c_s_a_s_d_s_a_s_e_s GTGTGGGTGTCTGTGACGGTGTCTGTGCTGGTG; >clamp_s_c_s_e_s:compl:rev CACCAGCACCCACACTATCATGTTGACATTCC; dG = -11.2
766 >s_c_s_b_s_a_s_a_s_e_s GTGTGGGTGGCTGTGTCTGTGTCTGTGCTGGTG; >clamp_s_c_s_e_s:compl:rev CACCAGCACCCACACTATCATGTTGACATTCC; dG = -11.2
767 >s_c_s_b_s_b_s_a_s_e_s GTGTGGGTGGCTGTGGCTGTGTCTGTGCTGGTG; >clamp_s_c_s_e_s:compl:rev CACCAGCACCCACACTATCATGTTGACATTCC; dG = -11.2
768 >s_c_s_b_s_d_s_a_s_e_s GTGTGGGTGGCTGTGACGGTGTCTGTGCTGGTG; >clamp_s_c_s_e_s:compl:rev CACCAGCACCCACACTATCATGTTGACATTCC; dG = -11.2
769 >s_a_s_b_s_a_s_a_s_e_s GTGTCTGTGGCTGTGTCTGTGTCTGTGCTGGTG; >clamp_s_c_s_e_s:compl:rev CACCAGCACCCACACTATCATGTTGACATTCC; dG = -11.2
770 >s_d_s_a_s_a_s_a_s_e_s GTGACGGTGTCTGTGTCTGTGTCTGTGCTGGTG; >clamp_s_c_s_e_s:compl:rev CACCAGCACCCACACTATCATGTTGACATTCC; dG = -11.2
771 >s_d_s_a_s_b_s_a_s_e_s GTGACGGTGTCTGTGGCTGTGTCTGTGCTGGTG; >clamp_s_c_s_e_s:compl:rev CACCAGCACCCACACTATCATGTTGACATTCC; dG = -11.2
772 >s_a_s_b_s_b_s_a_s_e_s GTGTCTGTGGCTGTGGCTGTGTCTGTGCTGGTG; >clamp_s_c_s_e_s:compl:rev CACCAGCACCCACACTATCATGTTGACATTCC; dG = -11.2
773 >s_d_s_a_s_d_s_a_s_e_s GTGACGGTGTCTGTGACGGTGTCTGTGCTGGTG; >clamp_s_c_s_e_s:compl:rev CACCAGCACCCACACTATCATGTTGACATTCC; dG = -11.2
774 >s_d_s_b_s_a_s_a_s_e_s GTGACGGTGGCTGTGTCTGTGTCTGTGCTGGTG; >clamp_s_c_s_e_s:compl:rev CACCAGCACCCACACTATCATGTTGACATTCC; dG = -11.2
775 >s_d_s_b_s_b_s_a_s_e_s GTGACGGTGGCTGTGGCTGTGTCTGTGCTGGTG; >clamp_s_c_s_e_s:compl:rev CACCAGCACCCACACTATCATGTTGACATTCC; dG = -11.2
776 >s_d_s_b_s_d_s_a_s_e_s GTGACGGTGGCTGTGACGGTGTCTGTGCTGGTG; >clamp_s_c_s_e_s:compl:rev CACCAGCACCCACACTATCATGTTGACATTCC; dG = -11.2
777 >s_d_s_d_s_a_s_a_s_e_s GTGACGGTGACGGTGTCTGTGTCTGTGCTGGTG; >clamp_s_c_s_e_s:compl:rev CACCAGCACCCACACTATCATGTTGACATTCC; dG = -11.2
778 >s_d_s_d_s_b_s_a_s_e_s GTGACGGTGACGGTGGCTGTGTCTGTGCTGGTG; >clamp_s_c_s_e_s:compl:rev CACCAGCACCCACACTATCATGTTGACATTCC; dG = -11.2
779 >s_d_s_d_s_d_s_a_s_e_s GTGACGGTGACGGTGACGGTGTCTGTGCTGGTG; >clamp_s_c_s_e_s:compl:rev CACCAGCACCCACACTATCATGTTGACATTCC; dG = -11.2

780 >s_a_s_b_s_d_s_a_s_e_s GTGTCTGTGGCTGTGACGGTGTCTGTGCTGGTG; >clamp_s_c_s_e_s:compl:rev
CACCAGCACCCACACTATCATGTTGACATTCC; dG = -11.2
781 >s_e_s_a_s_a_s_a_s_e_s GTGCTGGTGTCTGTGTCTGTGTCTGTGCTGGTG; >clamp_s_c_s_e_s:compl:rev
CACCAGCACCCACACTATCATGTTGACATTCC; dG = -11.2
782 >s_e_s_a_s_b_s_a_s_e_s GTGCTGGTGTCTGTGGCTGTGTCTGTGCTGGTG; >clamp_s_c_s_e_s:compl:rev
CACCAGCACCCACACTATCATGTTGACATTCC; dG = -11.2
783 >s_e_s_a_s_d_s_a_s_e_s GTGCTGGTGTCTGTGACGGTGTCTGTGCTGGTG; >clamp_s_c_s_e_s:compl:rev
CACCAGCACCCACACTATCATGTTGACATTCC; dG = -11.2
784 >s_e_s_b_s_a_s_a_s_e_s GTGCTGGTGGCTGTGTCTGTGTCTGTGCTGGTG; >clamp_s_c_s_e_s:compl:rev
CACCAGCACCCACACTATCATGTTGACATTCC; dG = -11.2
785 >s_e_s_b_s_b_s_a_s_e_s GTGCTGGTGGCTGTGGCTGTGTCTGTGCTGGTG; >clamp_s_c_s_e_s:compl:rev
CACCAGCACCCACACTATCATGTTGACATTCC; dG = -11.2
786 >s_e_s_b_s_d_s_a_s_e_s GTGCTGGTGGCTGTGACGGTGTCTGTGCTGGTG; >clamp_s_c_s_e_s:compl:rev
CACCAGCACCCACACTATCATGTTGACATTCC; dG = -11.2
787 >s_a_s_a_s_a_s_a_s_e_s GTGTCTGTGTCTGTGTCTGTGTCTGTGCTGGTG; >clamp_s_c_s_e_s:compl:rev
CACCAGCACCCACACTATCATGTTGACATTCC; dG = -11.2
788 >s_e_s_d_s_a_s_a_s_e_s GTGCTGGTGACGGTGTCTGTGTCTGTGCTGGTG; >clamp_s_c_s_e_s:compl:rev
CACCAGCACCCACACTATCATGTTGACATTCC; dG = -11.2
789 >s_e_s_d_s_b_s_a_s_e_s GTGCTGGTGACGGTGGCTGTGTCTGTGCTGGTG; >clamp_s_c_s_e_s:compl:rev
CACCAGCACCCACACTATCATGTTGACATTCC; dG = -11.2
790 >s_e_s_d_s_d_s_a_s_e_s GTGCTGGTGACGGTGACGGTGTCTGTGCTGGTG; >clamp_s_c_s_e_s:compl:rev
CACCAGCACCCACACTATCATGTTGACATTCC; dG = -11.2
791 >s_b_s_e_s_a_s_c_s_b_s GTGGCTGTGCTGGTGTCTGTGTGGGTGGCTGTG; >clamp_s_c_s_e_s:compl:rev
CACCAGCACCCACACTATCATGTTGACATTCC; dG = -11.1
792 >s_b_s_e_s_a_s_c_s_a_s GTGGCTGTGCTGGTGTCTGTGTGGGTGTCTGTG; >clamp_s_c_s_e_s:compl:rev
CACCAGCACCCACACTATCATGTTGACATTCC; dG = -11.1
793 >s_a_s_e_s_a_s_c_s_b_s GTGTCTGTGCTGGTGTCTGTGTGGGTGGCTGTG; >clamp_s_c_s_e_s:compl:rev
CACCAGCACCCACACTATCATGTTGACATTCC; dG = -11.0
794 >s_a_s_e_s_a_s_c_s_a_s GTGTCTGTGCTGGTGTCTGTGTGGGTGTCTGTG; >clamp_s_c_s_e_s:compl:rev
CACCAGCACCCACACTATCATGTTGACATTCC; dG = -11.0
795 >s_a_s_e_s_b_s_c_s_b_s GTGTCTGTGCTGGTGGCTGTGTGGGTGGCTGTG; >clamp_s_c_s_e_s:compl:rev
CACCAGCACCCACACTATCATGTTGACATTCC; dG = -11.0
796 >s_a_s_e_s_b_s_c_s_a_s GTGTCTGTGCTGGTGGCTGTGTGGGTGTCTGTG; >clamp_s_c_s_e_s:compl:rev
CACCAGCACCCACACTATCATGTTGACATTCC; dG = -11.0
797 >s_a_s_a_s_b_s_c_s_b_s GTGTCTGTGTCTGTGGCTGTGTGGGTGGCTGTG; >clamp_s_c_s_e_s:compl:rev
CACCAGCACCCACACTATCATGTTGACATTCC; dG = -10.8
798 >s_a_s_d_s_a_s_c_s_b_s GTGTCTGTGACGGTGTCTGTGTGGGTGGCTGTG; >clamp_s_c_s_e_s:compl:rev
CACCAGCACCCACACTATCATGTTGACATTCC; dG = -10.8
799 >s_a_s_d_s_a_s_c_s_a_s GTGTCTGTGACGGTGTCTGTGTGGGTGTCTGTG; >clamp_s_c_s_e_s:compl:rev
CACCAGCACCCACACTATCATGTTGACATTCC; dG = -10.8
800 >s_a_s_a_s_b_s_c_s_a_s GTGTCTGTGTCTGTGGCTGTGTGGGTGTCTGTG; >clamp_s_c_s_e_s:compl:rev
CACCAGCACCCACACTATCATGTTGACATTCC; dG = -10.8
801 >s_a_s_d_s_b_s_c_s_b_s GTGTCTGTGACGGTGGCTGTGTGGGTGGCTGTG; >clamp_s_c_s_e_s:compl:rev
CACCAGCACCCACACTATCATGTTGACATTCC; dG = -10.8
802 >s_a_s_d_s_b_s_c_s_a_s GTGTCTGTGACGGTGGCTGTGTGGGTGTCTGTG; >clamp_s_c_s_e_s:compl:rev
CACCAGCACCCACACTATCATGTTGACATTCC; dG = -10.8
803 >s_a_s_d_s_d_s_c_s_b_s GTGTCTGTGACGGTGACGGTGTGGGTGGCTGTG; >clamp_s_c_s_e_s:compl:rev
CACCAGCACCCACACTATCATGTTGACATTCC; dG = -10.8
804 >s_b_s_a_s_a_s_c_s_b_s GTGGCTGTGTCTGTGTCTGTGTGGGTGGCTGTG; >clamp_s_c_s_e_s:compl:rev
CACCAGCACCCACACTATCATGTTGACATTCC; dG = -10.8
805 >s_b_s_a_s_a_s_c_s_a_s GTGGCTGTGTCTGTGTCTGTGTGGGTGTCTGTG; >clamp_s_c_s_e_s:compl:rev
CACCAGCACCCACACTATCATGTTGACATTCC; dG = -10.8
806 >s_b_s_a_s_b_s_c_s_b_s GTGGCTGTGTCTGTGGCTGTGTGGGTGGCTGTG; >clamp_s_c_s_e_s:compl:rev
CACCAGCACCCACACTATCATGTTGACATTCC; dG = -10.8
807 >s_b_s_a_s_b_s_c_s_a_s GTGGCTGTGTCTGTGGCTGTGTGGGTGTCTGTG; >clamp_s_c_s_e_s:compl:rev
CACCAGCACCCACACTATCATGTTGACATTCC; dG = -10.8
808 >s_b_s_a_s_d_s_c_s_b_s GTGGCTGTGTCTGTGACGGTGTGGGTGGCTGTG; >clamp_s_c_s_e_s:compl:rev
CACCAGCACCCACACTATCATGTTGACATTCC; dG = -10.8
809 >s_b_s_b_s_a_s_c_s_b_s GTGGCTGTGGCTGTGTCTGTGTGGGTGGCTGTG; >clamp_s_c_s_e_s:compl:rev
CACCAGCACCCACACTATCATGTTGACATTCC; dG = -10.8
810 >s_b_s_b_s_a_s_c_s_a_s GTGGCTGTGGCTGTGTCTGTGTGGGTGTCTGTG; >clamp_s_c_s_e_s:compl:rev
CACCAGCACCCACACTATCATGTTGACATTCC; dG = -10.8
811 >s_b_s_b_s_b_s_c_s_b_s GTGGCTGTGGCTGTGGCTGTGTGGGTGGCTGTG; >clamp_s_c_s_e_s:compl:rev
CACCAGCACCCACACTATCATGTTGACATTCC; dG = -10.8
812 >s_b_s_b_s_b_s_c_s_a_s GTGGCTGTGGCTGTGGCTGTGTGGGTGTCTGTG; >clamp_s_c_s_e_s:compl:rev
CACCAGCACCCACACTATCATGTTGACATTCC; dG = -10.8
813 >s_b_s_b_s_d_s_c_s_b_s GTGGCTGTGGCTGTGACGGTGTGGGTGGCTGTG; >clamp_s_c_s_e_s:compl:rev
CACCAGCACCCACACTATCATGTTGACATTCC; dG = -10.8
814 >s_a_s_a_s_d_s_c_s_b_s GTGTCTGTGTCTGTGACGGTGTGGGTGGCTGTG; >clamp_s_c_s_e_s:compl:rev
CACCAGCACCCACACTATCATGTTGACATTCC; dG = -10.8
815 >s_b_s_c_s_a_s_c_s_b_s GTGGCTGTGTGGGTGTCTGTGTGGGTGGCTGTG; >clamp_s_c_s_e_s:compl:rev
CACCAGCACCCACACTATCATGTTGACATTCC; dG = -10.8
816 >s_b_s_c_s_a_s_c_s_a_s GTGGCTGTGTGGGTGTCTGTGTGGGTGTCTGTG; >clamp_s_c_s_e_s:compl:rev
CACCAGCACCCACACTATCATGTTGACATTCC; dG = -10.8

817 >s_b_s_c_s_b_s_c_s_b_s GTGGCTGTGTGGGTGGCTGTGTGGGTGGCTGTG; >clamp_s_c_s_e_s:compl:rev CACCAGCACCCACACTATCATGTTGACATTCC; dG = -10.8
818 >s_b_s_c_s_b_s_c_s_a_s GTGGCTGTGTGGGTGGCTGTGTGGGTGTCTGTG; >clamp_s_c_s_e_s:compl:rev CACCAGCACCCACACTATCATGTTGACATTCC; dG = -10.8
819 >s_b_s_d_s_a_s_c_s_b_s GTGGCTGTGACGGTGTCTGTGTGGGTGGCTGTG; >clamp_s_c_s_e_s:compl:rev CACCAGCACCCACACTATCATGTTGACATTCC; dG = -10.8
820 >s_a_s_a_s_a_s_c_s_b_s GTGTCTGTGTCTGTGTCTGTGTGGGTGGCTGTG; >clamp_s_c_s_e_s:compl:rev CACCAGCACCCACACTATCATGTTGACATTCC; dG = -10.8
821 >s_b_s_d_s_a_s_c_s_a_s GTGGCTGTGACGGTGTCTGTGTGGGTGTCTGTG; >clamp_s_c_s_e_s:compl:rev CACCAGCACCCACACTATCATGTTGACATTCC; dG = -10.8
822 >s_b_s_d_s_b_s_c_s_b_s GTGGCTGTGACGGTGGCTGTGTGGGTGGCTGTG; >clamp_s_c_s_e_s:compl:rev CACCAGCACCCACACTATCATGTTGACATTCC; dG = -10.8
823 >s_b_s_d_s_b_s_c_s_a_s GTGGCTGTGACGGTGGCTGTGTGGGTGTCTGTG; >clamp_s_c_s_e_s:compl:rev CACCAGCACCCACACTATCATGTTGACATTCC; dG = -10.8
824 >s_b_s_d_s_d_s_c_s_b_s GTGGCTGTGACGGTGACGGTGTGGGTGGCTGTG; >clamp_s_c_s_e_s:compl:rev CACCAGCACCCACACTATCATGTTGACATTCC; dG = -10.8
825 >s_c_s_a_s_a_s_c_s_b_s GTGTGGGTGTCTGTGTCTGTGTGGGTGGCTGTG; >clamp_s_c_s_e_s:compl:rev CACCAGCACCCACACTATCATGTTGACATTCC; dG = -10.8
826 >s_c_s_a_s_a_s_c_s_a_s GTGTGGGTGTCTGTGTCTGTGTGGGTGTCTGTG; >clamp_s_c_s_e_s:compl:rev CACCAGCACCCACACTATCATGTTGACATTCC; dG = -10.8
827 >s_c_s_a_s_b_s_c_s_b_s GTGTGGGTGTCTGTGGCTGTGTGGGTGGCTGTG; >clamp_s_c_s_e_s:compl:rev CACCAGCACCCACACTATCATGTTGACATTCC; dG = -10.8
828 >s_c_s_a_s_b_s_c_s_a_s GTGTGGGTGTCTGTGGCTGTGTGGGTGTCTGTG; >clamp_s_c_s_e_s:compl:rev CACCAGCACCCACACTATCATGTTGACATTCC; dG = -10.8
829 >s_a_s_a_s_a_s_c_s_a_s GTGTCTGTGTCTGTGTCTGTGTGGGTGTCTGTG; >clamp_s_c_s_e_s:compl:rev CACCAGCACCCACACTATCATGTTGACATTCC; dG = -10.8
830 >s_c_s_a_s_d_s_c_s_b_s GTGTGGGTGTCTGTGACGGTGTGGGTGGCTGTG; >clamp_s_c_s_e_s:compl:rev CACCAGCACCCACACTATCATGTTGACATTCC; dG = -10.8
831 >s_c_s_b_s_a_s_c_s_b_s GTGTGGGTGGCTGTGTCTGTGTGGGTGGCTGTG; >clamp_s_c_s_e_s:compl:rev CACCAGCACCCACACTATCATGTTGACATTCC; dG = -10.8
832 >s_c_s_b_s_a_s_c_s_a_s GTGTGGGTGGCTGTGTCTGTGTGGGTGTCTGTG; >clamp_s_c_s_e_s:compl:rev CACCAGCACCCACACTATCATGTTGACATTCC; dG = -10.8
833 >s_a_s_b_s_a_s_c_s_b_s GTGTCTGTGGCTGTGTCTGTGTGGGTGGCTGTG; >clamp_s_c_s_e_s:compl:rev CACCAGCACCCACACTATCATGTTGACATTCC; dG = -10.8
834 >s_c_s_b_s_b_s_c_s_b_s GTGTGGGTGGCTGTGGCTGTGTGGGTGGCTGTG; >clamp_s_c_s_e_s:compl:rev CACCAGCACCCACACTATCATGTTGACATTCC; dG = -10.8
835 >s_c_s_b_s_b_s_c_s_a_s GTGTGGGTGGCTGTGGCTGTGTGGGTGTCTGTG; >clamp_s_c_s_e_s:compl:rev CACCAGCACCCACACTATCATGTTGACATTCC; dG = -10.8
836 >s_a_s_b_s_a_s_c_s_a_s GTGTCTGTGGCTGTGTCTGTGTGGGTGTCTGTG; >clamp_s_c_s_e_s:compl:rev CACCAGCACCCACACTATCATGTTGACATTCC; dG = -10.8
837 >s_c_s_b_s_d_s_c_s_b_s GTGTGGGTGGCTGTGACGGTGTGGGTGGCTGTG; >clamp_s_c_s_e_s:compl:rev CACCAGCACCCACACTATCATGTTGACATTCC; dG = -10.8
838 >s_a_s_b_s_b_s_c_s_b_s GTGTCTGTGGCTGTGGCTGTGTGGGTGGCTGTG; >clamp_s_c_s_e_s:compl:rev CACCAGCACCCACACTATCATGTTGACATTCC; dG = -10.8
839 >s_a_s_b_s_b_s_c_s_a_s GTGTCTGTGGCTGTGGCTGTGTGGGTGTCTGTG; >clamp_s_c_s_e_s:compl:rev CACCAGCACCCACACTATCATGTTGACATTCC; dG = -10.8
840 >s_d_s_a_s_a_s_c_s_b_s GTGACGGTGTCTGTGTCTGTGTGGGTGGCTGTG; >clamp_s_c_s_e_s:compl:rev CACCAGCACCCACACTATCATGTTGACATTCC; dG = -10.8
841 >s_d_s_a_s_a_s_c_s_a_s GTGACGGTGTCTGTGTCTGTGTGGGTGTCTGTG; >clamp_s_c_s_e_s:compl:rev CACCAGCACCCACACTATCATGTTGACATTCC; dG = -10.8
842 >s_d_s_a_s_b_s_c_s_b_s GTGACGGTGTCTGTGGCTGTGTGGGTGGCTGTG; >clamp_s_c_s_e_s:compl:rev CACCAGCACCCACACTATCATGTTGACATTCC; dG = -10.8
843 >s_d_s_a_s_b_s_c_s_a_s GTGACGGTGTCTGTGGCTGTGTGGGTGTCTGTG; >clamp_s_c_s_e_s:compl:rev CACCAGCACCCACACTATCATGTTGACATTCC; dG = -10.8
844 >s_d_s_a_s_d_s_c_s_b_s GTGACGGTGTCTGTGACGGTGTGGGTGGCTGTG; >clamp_s_c_s_e_s:compl:rev CACCAGCACCCACACTATCATGTTGACATTCC; dG = -10.8
845 >s_d_s_b_s_a_s_c_s_b_s GTGACGGTGGCTGTGTCTGTGTGGGTGGCTGTG; >clamp_s_c_s_e_s:compl:rev CACCAGCACCCACACTATCATGTTGACATTCC; dG = -10.8
846 >s_d_s_b_s_a_s_c_s_a_s GTGACGGTGGCTGTGTCTGTGTGGGTGTCTGTG; >clamp_s_c_s_e_s:compl:rev CACCAGCACCCACACTATCATGTTGACATTCC; dG = -10.8
847 >s_d_s_b_s_b_s_c_s_b_s GTGACGGTGGCTGTGGCTGTGTGGGTGGCTGTG; >clamp_s_c_s_e_s:compl:rev CACCAGCACCCACACTATCATGTTGACATTCC; dG = -10.8
848 >s_d_s_b_s_b_s_c_s_a_s GTGACGGTGGCTGTGGCTGTGTGGGTGTCTGTG; >clamp_s_c_s_e_s:compl:rev CACCAGCACCCACACTATCATGTTGACATTCC; dG = -10.8
849 >s_d_s_b_s_d_s_c_s_b_s GTGACGGTGGCTGTGACGGTGTGGGTGGCTGTG; >clamp_s_c_s_e_s:compl:rev CACCAGCACCCACACTATCATGTTGACATTCC; dG = -10.8
850 >s_d_s_c_s_a_s_c_s_b_s GTGACGGTGTGGGTGTCTGTGTGGGTGGCTGTG; >clamp_s_c_s_e_s:compl:rev CACCAGCACCCACACTATCATGTTGACATTCC; dG = -10.8
851 >s_d_s_c_s_a_s_c_s_a_s GTGACGGTGTGGGTGTCTGTGTGGGTGTCTGTG; >clamp_s_c_s_e_s:compl:rev CACCAGCACCCACACTATCATGTTGACATTCC; dG = -10.8
852 >s_d_s_c_s_b_s_c_s_b_s GTGACGGTGTGGGTGGCTGTGTGGGTGGCTGTG; >clamp_s_c_s_e_s:compl:rev CACCAGCACCCACACTATCATGTTGACATTCC; dG = -10.8
853 >s_d_s_c_s_b_s_c_s_a_s GTGACGGTGTGGGTGGCTGTGTGGGTGTCTGTG; >clamp_s_c_s_e_s:compl:rev CACCAGCACCCACACTATCATGTTGACATTCC; dG = -10.8

854 >s_d_s_d_s_a_s_c_s_b_s GTGACGGTGACGGTGTCTGTGTGGGTGGCTGTG; >clamp_s_c_s_e_s:compl:rev CACCAGCACCCACACTATCATGTTGACATTCC; dG = -10.8
855 >s_d_s_d_s_a_s_c_s_a_s GTGACGGTGACGGTGTCTGTGTGGGTGTCTGTG; >clamp_s_c_s_e_s:compl:rev CACCAGCACCCACACTATCATGTTGACATTCC; dG = -10.8
856 >s_d_s_d_s_b_s_c_s_b_s GTGACGGTGACGGTGGCTGTGTGGGTGGCTGTG; >clamp_s_c_s_e_s:compl:rev CACCAGCACCCACACTATCATGTTGACATTCC; dG = -10.8
857 >s_d_s_d_s_b_s_c_s_a_s GTGACGGTGACGGTGGCTGTGTGGGTGTCTGTG; >clamp_s_c_s_e_s:compl:rev CACCAGCACCCACACTATCATGTTGACATTCC; dG = -10.8
858 >s_a_s_b_s_d_s_c_s_b_s GTGTCTGTGGCTGTGACGGTGTGGGTGGCTGTG; >clamp_s_c_s_e_s:compl:rev CACCAGCACCCACACTATCATGTTGACATTCC; dG = -10.8
859 >s_d_s_d_s_d_s_c_s_b_s GTGACGGTGACGGTGACGGTGTGGGTGGCTGTG; >clamp_s_c_s_e_s:compl:rev CACCAGCACCCACACTATCATGTTGACATTCC; dG = -10.8
860 >s_e_s_a_s_a_s_c_s_b_s GTGCTGGTGTCTGTGTCTGTGTGGGTGGCTGTG; >clamp_s_c_s_e_s:compl:rev CACCAGCACCCACACTATCATGTTGACATTCC; dG = -10.8
861 >s_e_s_a_s_a_s_c_s_a_s GTGCTGGTGTCTGTGTCTGTGTGGGTGTCTGTG; >clamp_s_c_s_e_s:compl:rev CACCAGCACCCACACTATCATGTTGACATTCC; dG = -10.8
862 >s_e_s_a_s_b_s_c_s_b_s GTGCTGGTGTCTGTGGCTGTGTGGGTGGCTGTG; >clamp_s_c_s_e_s:compl:rev CACCAGCACCCACACTATCATGTTGACATTCC; dG = -10.8
863 >s_e_s_a_s_b_s_c_s_a_s GTGCTGGTGTCTGTGGCTGTGTGGGTGTCTGTG; >clamp_s_c_s_e_s:compl:rev CACCAGCACCCACACTATCATGTTGACATTCC; dG = -10.8
864 >s_e_s_a_s_d_s_c_s_b_s GTGCTGGTGTCTGTGACGGTGTGGGTGGCTGTG; >clamp_s_c_s_e_s:compl:rev CACCAGCACCCACACTATCATGTTGACATTCC; dG = -10.8
865 >s_e_s_b_s_a_s_c_s_b_s GTGCTGGTGGCTGTGTCTGTGTGGGTGGCTGTG; >clamp_s_c_s_e_s:compl:rev CACCAGCACCCACACTATCATGTTGACATTCC; dG = -10.8
866 >s_e_s_b_s_a_s_c_s_a_s GTGCTGGTGGCTGTGTCTGTGTGGGTGTCTGTG; >clamp_s_c_s_e_s:compl:rev CACCAGCACCCACACTATCATGTTGACATTCC; dG = -10.8
867 >s_e_s_b_s_b_s_c_s_b_s GTGCTGGTGGCTGTGGCTGTGTGGGTGGCTGTG; >clamp_s_c_s_e_s:compl:rev CACCAGCACCCACACTATCATGTTGACATTCC; dG = -10.8
868 >s_e_s_b_s_b_s_c_s_a_s GTGCTGGTGGCTGTGGCTGTGTGGGTGTCTGTG; >clamp_s_c_s_e_s:compl:rev CACCAGCACCCACACTATCATGTTGACATTCC; dG = -10.8
869 >s_e_s_b_s_d_s_c_s_b_s GTGCTGGTGGCTGTGACGGTGTGGGTGGCTGTG; >clamp_s_c_s_e_s:compl:rev CACCAGCACCCACACTATCATGTTGACATTCC; dG = -10.8
870 >s_e_s_c_s_a_s_c_s_b_s GTGCTGGTGTGGGTGTCTGTGTGGGTGGCTGTG; >clamp_s_c_s_e_s:compl:rev CACCAGCACCCACACTATCATGTTGACATTCC; dG = -10.8
871 >s_e_s_c_s_a_s_c_s_a_s GTGCTGGTGTGGGTGTCTGTGTGGGTGTCTGTG; >clamp_s_c_s_e_s:compl:rev CACCAGCACCCACACTATCATGTTGACATTCC; dG = -10.8
872 >s_e_s_c_s_b_s_c_s_b_s GTGCTGGTGTGGGTGGCTGTGTGGGTGGCTGTG; >clamp_s_c_s_e_s:compl:rev CACCAGCACCCACACTATCATGTTGACATTCC; dG = -10.8
873 >s_e_s_c_s_b_s_c_s_a_s GTGCTGGTGTGGGTGGCTGTGTGGGTGTCTGTG; >clamp_s_c_s_e_s:compl:rev CACCAGCACCCACACTATCATGTTGACATTCC; dG = -10.8
874 >s_a_s_c_s_a_s_c_s_b_s GTGTCTGTGTGGGTGTCTGTGTGGGTGGCTGTG; >clamp_s_c_s_e_s:compl:rev CACCAGCACCCACACTATCATGTTGACATTCC; dG = -10.8
875 >s_a_s_c_s_a_s_c_s_a_s GTGTCTGTGTGGGTGTCTGTGTGGGTGTCTGTG; >clamp_s_c_s_e_s:compl:rev CACCAGCACCCACACTATCATGTTGACATTCC; dG = -10.8
876 >s_e_s_d_s_a_s_c_s_b_s GTGCTGGTGACGGTGTCTGTGTGGGTGGCTGTG; >clamp_s_c_s_e_s:compl:rev CACCAGCACCCACACTATCATGTTGACATTCC; dG = -10.8
877 >s_e_s_d_s_a_s_c_s_a_s GTGCTGGTGACGGTGTCTGTGTGGGTGTCTGTG; >clamp_s_c_s_e_s:compl:rev CACCAGCACCCACACTATCATGTTGACATTCC; dG = -10.8
878 >s_e_s_d_s_b_s_c_s_b_s GTGCTGGTGACGGTGGCTGTGTGGGTGGCTGTG; >clamp_s_c_s_e_s:compl:rev CACCAGCACCCACACTATCATGTTGACATTCC; dG = -10.8
879 >s_e_s_d_s_b_s_c_s_a_s GTGCTGGTGACGGTGGCTGTGTGGGTGTCTGTG; >clamp_s_c_s_e_s:compl:rev CACCAGCACCCACACTATCATGTTGACATTCC; dG = -10.8
880 >s_e_s_d_s_d_s_c_s_b_s GTGCTGGTGACGGTGACGGTGTGGGTGGCTGTG; >clamp_s_c_s_e_s:compl:rev CACCAGCACCCACACTATCATGTTGACATTCC; dG = -10.8
881 >s_a_s_c_s_b_s_c_s_b_s GTGTCTGTGTGGGTGGCTGTGTGGGTGGCTGTG; >clamp_s_c_s_e_s:compl:rev CACCAGCACCCACACTATCATGTTGACATTCC; dG = -10.8
882 >s_a_s_c_s_b_s_c_s_a_s GTGTCTGTGTGGGTGGCTGTGTGGGTGTCTGTG; >clamp_s_c_s_e_s:compl:rev CACCAGCACCCACACTATCATGTTGACATTCC; dG = -10.8
883 >s_a_s_d_s_d_s_c_s_a_s GTGTCTGTGACGGTGACGGTGTGGGTGTCTGTG; >clamp_s_c_s_e_s:compl:rev CACCAGCACCCACACTATCATGTTGACATTCC; dG = -10.7
884 >s_b_s_a_s_d_s_c_s_a_s GTGGCTGTGTCTGTGACGGTGTGGGTGTCTGTG; >clamp_s_c_s_e_s:compl:rev CACCAGCACCCACACTATCATGTTGACATTCC; dG = -10.7
885 >s_b_s_b_s_d_s_c_s_a_s GTGGCTGTGGCTGTGACGGTGTGGGTGTCTGTG; >clamp_s_c_s_e_s:compl:rev CACCAGCACCCACACTATCATGTTGACATTCC; dG = -10.7
886 >s_a_s_a_s_d_s_c_s_a_s GTGTCTGTGTCTGTGACGGTGTGGGTGTCTGTG; >clamp_s_c_s_e_s:compl:rev CACCAGCACCCACACTATCATGTTGACATTCC; dG = -10.7
887 >s_b_s_d_s_d_s_c_s_a_s GTGGCTGTGACGGTGACGGTGTGGGTGTCTGTG; >clamp_s_c_s_e_s:compl:rev CACCAGCACCCACACTATCATGTTGACATTCC; dG = -10.7
888 >s_c_s_a_s_d_s_c_s_a_s GTGTGGGTGTCTGTGACGGTGTGGGTGTCTGTG; >clamp_s_c_s_e_s:compl:rev CACCAGCACCCACACTATCATGTTGACATTCC; dG = -10.7
889 >s_c_s_b_s_d_s_c_s_a_s GTGTGGGTGGCTGTGACGGTGTGGGTGTCTGTG; >clamp_s_c_s_e_s:compl:rev CACCAGCACCCACACTATCATGTTGACATTCC; dG = -10.7
890 >s_d_s_a_s_d_s_c_s_a_s GTGACGGTGTCTGTGACGGTGTGGGTGTCTGTG; >clamp_s_c_s_e_s:compl:rev CACCAGCACCCACACTATCATGTTGACATTCC; dG = -10.7

891 >s_d_s_b_s_d_s_c_s_a_s GTGACGGTGGCTGTGACGGTGTGGGTGTCTGTG; >clamp_s_c_s_e_s:compl:rev
CACCAGCACCCACACTATCATGTTGACATTCC; dG = -10.7
892 >s_d_s_d_s_d_s_c_s_a_s GTGACGGTGACGGTGACGGTGTGGGTGTCTGTG; >clamp_s_c_s_e_s:compl:rev
CACCAGCACCCACACTATCATGTTGACATTCC; dG = -10.7
893 >s_a_s_b_s_d_s_c_s_a_s GTGTCTGTGGCTGTGACGGTGTGGGTGTCTGTG; >clamp_s_c_s_e_s:compl:rev
CACCAGCACCCACACTATCATGTTGACATTCC; dG = -10.7
894 >s_e_s_a_s_d_s_c_s_a_s GTGCTGGTGTCTGTGACGGTGTGGGTGTCTGTG; >clamp_s_c_s_e_s:compl:rev
CACCAGCACCCACACTATCATGTTGACATTCC; dG = -10.7
895 >s_e_s_b_s_d_s_c_s_a_s GTGCTGGTGGCTGTGACGGTGTGGGTGTCTGTG; >clamp_s_c_s_e_s:compl:rev
CACCAGCACCCACACTATCATGTTGACATTCC; dG = -10.7
896 >s_e_s_d_s_d_s_c_s_a_s GTGCTGGTGACGGTGACGGTGTGGGTGTCTGTG; >clamp_s_c_s_e_s:compl:rev
CACCAGCACCCACACTATCATGTTGACATTCC; dG = -10.7

****

DA Matches

1 >s_e_s_d_s_a_s_d_s_a_s GTGCTGGTGACGGTGTCTGTGACGGTGTCTGTG; >clamp_s_d_s_a_s:compl:rev
CACAGACACCGTCACTATCATGTTGACATTCC; dG = -18.1
2 >s_a_s_d_s_a_s_d_s_a_s GTGTCTGTGACGGTGTCTGTGACGGTGTCTGTG; >clamp_s_d_s_a_s:compl:rev
CACAGACACCGTCACTATCATGTTGACATTCC; dG = -18.1
3 >s_b_s_d_s_a_s_d_s_a_s GTGGCTGTGACGGTGTCTGTGACGGTGTCTGTG; >clamp_s_d_s_a_s:compl:rev
CACAGACACCGTCACTATCATGTTGACATTCC; dG = -18.1
4 >s_c_s_d_s_a_s_d_s_a_s GTGTGGGTGACGGTGTCTGTGACGGTGTCTGTG; >clamp_s_d_s_a_s:compl:rev
CACAGACACCGTCACTATCATGTTGACATTCC; dG = -18.1
5 >s_d_s_d_s_a_s_d_s_a_s GTGACGGTGACGGTGTCTGTGACGGTGTCTGTG; >clamp_s_d_s_a_s:compl:rev
CACAGACACCGTCACTATCATGTTGACATTCC; dG = -18.1
6 >s_d_s_a_s_d_s_d_s_a_s GTGACGGTGTCTGTGACGGTGACGGTGTCTGTG; >clamp_s_d_s_a_s:compl:rev
CACAGACACCGTCACTATCATGTTGACATTCC; dG = -17.6
7 >s_a_s_a_s_a_s_d_s_a_s GTGTCTGTGTCTGTGTCTGTGACGGTGTCTGTG; >clamp_s_d_s_a_s:compl:rev
CACAGACACCGTCACTATCATGTTGACATTCC; dG = -17.3
8 >s_e_s_a_s_a_s_d_s_a_s GTGCTGGTGTCTGTGTCTGTGACGGTGTCTGTG; >clamp_s_d_s_a_s:compl:rev
CACAGACACCGTCACTATCATGTTGACATTCC; dG = -17.3
9 >s_e_s_a_s_b_s_d_s_a_s GTGCTGGTGTCTGTGGCTGTGACGGTGTCTGTG; >clamp_s_d_s_a_s:compl:rev
CACAGACACCGTCACTATCATGTTGACATTCC; dG = -17.3
10 >s_e_s_b_s_a_s_d_s_a_s GTGCTGGTGGCTGTGTCTGTGACGGTGTCTGTG; >clamp_s_d_s_a_s:compl:rev
CACAGACACCGTCACTATCATGTTGACATTCC; dG = -17.3
11 >s_e_s_b_s_b_s_d_s_a_s GTGCTGGTGGCTGTGGCTGTGACGGTGTCTGTG; >clamp_s_d_s_a_s:compl:rev
CACAGACACCGTCACTATCATGTTGACATTCC; dG = -17.3
12 >s_a_s_c_s_a_s_d_s_a_s GTGTCTGTGTGGGTGTCTGTGACGGTGTCTGTG; >clamp_s_d_s_a_s:compl:rev
CACAGACACCGTCACTATCATGTTGACATTCC; dG = -17.3
13 >s_e_s_c_s_a_s_d_s_a_s GTGCTGGTGTGGGTGTCTGTGACGGTGTCTGTG; >clamp_s_d_s_a_s:compl:rev
CACAGACACCGTCACTATCATGTTGACATTCC; dG = -17.3
14 >s_e_s_c_s_b_s_d_s_a_s GTGCTGGTGTGGGTGGCTGTGACGGTGTCTGTG; >clamp_s_d_s_a_s:compl:rev
CACAGACACCGTCACTATCATGTTGACATTCC; dG = -17.3
15 >s_e_s_d_s_b_s_d_s_a_s GTGCTGGTGACGGTGGCTGTGACGGTGTCTGTG; >clamp_s_d_s_a_s:compl:rev
CACAGACACCGTCACTATCATGTTGACATTCC; dG = -17.3
16 >s_a_s_c_s_b_s_d_s_a_s GTGTCTGTGTGGGTGGCTGTGACGGTGTCTGTG; >clamp_s_d_s_a_s:compl:rev
CACAGACACCGTCACTATCATGTTGACATTCC; dG = -17.3
17 >s_e_s_e_s_a_s_d_s_a_s GTGCTGGTGCTGGTGTCTGTGACGGTGTCTGTG; >clamp_s_d_s_a_s:compl:rev
CACAGACACCGTCACTATCATGTTGACATTCC; dG = -17.3
18 >s_e_s_e_s_b_s_d_s_a_s GTGCTGGTGCTGGTGGCTGTGACGGTGTCTGTG; >clamp_s_d_s_a_s:compl:rev
CACAGACACCGTCACTATCATGTTGACATTCC; dG = -17.3
19 >s_a_s_d_s_b_s_d_s_a_s GTGTCTGTGACGGTGGCTGTGACGGTGTCTGTG; >clamp_s_d_s_a_s:compl:rev
CACAGACACCGTCACTATCATGTTGACATTCC; dG = -17.3
20 >s_a_s_a_s_b_s_d_s_a_s GTGTCTGTGTCTGTGGCTGTGACGGTGTCTGTG; >clamp_s_d_s_a_s:compl:rev
CACAGACACCGTCACTATCATGTTGACATTCC; dG = -17.3
21 >s_a_s_e_s_a_s_d_s_a_s GTGTCTGTGCTGGTGTCTGTGACGGTGTCTGTG; >clamp_s_d_s_a_s:compl:rev
CACAGACACCGTCACTATCATGTTGACATTCC; dG = -17.3
22 >s_a_s_e_s_b_s_d_s_a_s GTGTCTGTGCTGGTGGCTGTGACGGTGTCTGTG; >clamp_s_d_s_a_s:compl:rev
CACAGACACCGTCACTATCATGTTGACATTCC; dG = -17.3
23 >s_b_s_a_s_a_s_d_s_a_s GTGGCTGTGTCTGTGTCTGTGACGGTGTCTGTG; >clamp_s_d_s_a_s:compl:rev
CACAGACACCGTCACTATCATGTTGACATTCC; dG = -17.3
24 >s_b_s_a_s_b_s_d_s_a_s GTGGCTGTGTCTGTGGCTGTGACGGTGTCTGTG; >clamp_s_d_s_a_s:compl:rev
CACAGACACCGTCACTATCATGTTGACATTCC; dG = -17.3
25 >s_b_s_b_s_a_s_d_s_a_s GTGGCTGTGGCTGTGTCTGTGACGGTGTCTGTG; >clamp_s_d_s_a_s:compl:rev
CACAGACACCGTCACTATCATGTTGACATTCC; dG = -17.3
26 >s_b_s_b_s_b_s_d_s_a_s GTGGCTGTGGCTGTGGCTGTGACGGTGTCTGTG; >clamp_s_d_s_a_s:compl:rev
CACAGACACCGTCACTATCATGTTGACATTCC; dG = -17.3
27 >s_b_s_c_s_a_s_d_s_a_s GTGGCTGTGTGGGTGTCTGTGACGGTGTCTGTG; >clamp_s_d_s_a_s:compl:rev
CACAGACACCGTCACTATCATGTTGACATTCC; dG = -17.3

28 >s_b_s_c_s_b_s_d_s_a_s GTGGCTGTGTGGGTGGCTGTGACGGTGTCTGTG; >clamp_s_d_s_a_s:compl:rev CACAGACACCGTCACTATCATGTTGACATTCC; dG = -17.3
29 >s_b_s_d_s_b_s_d_s_a_s GTGGCTGTGACGGTGGCTGTGACGGTGTCTGTG; >clamp_s_d_s_a_s:compl:rev CACAGACACCGTCACTATCATGTTGACATTCC; dG = -17.3
30 >s_b_s_e_s_a_s_d_s_a_s GTGGCTGTGCTGGTGTCTGTGACGGTGTCTGTG; >clamp_s_d_s_a_s:compl:rev CACAGACACCGTCACTATCATGTTGACATTCC; dG = -17.3
31 >s_b_s_e_s_b_s_d_s_a_s GTGGCTGTGCTGGTGGCTGTGACGGTGTCTGTG; >clamp_s_d_s_a_s:compl:rev CACAGACACCGTCACTATCATGTTGACATTCC; dG = -17.3
32 >s_c_s_a_s_a_s_d_s_a_s GTGTGGGTGTCTGTGTCTGTGACGGTGTCTGTG; >clamp_s_d_s_a_s:compl:rev CACAGACACCGTCACTATCATGTTGACATTCC; dG = -17.3
33 >s_c_s_a_s_b_s_d_s_a_s GTGTGGGTGTCTGTGGCTGTGACGGTGTCTGTG; >clamp_s_d_s_a_s:compl:rev CACAGACACCGTCACTATCATGTTGACATTCC; dG = -17.3
34 >s_c_s_b_s_a_s_d_s_a_s GTGTGGGTGGCTGTGTCTGTGACGGTGTCTGTG; >clamp_s_d_s_a_s:compl:rev CACAGACACCGTCACTATCATGTTGACATTCC; dG = -17.3
35 >s_c_s_b_s_b_s_d_s_a_s GTGTGGGTGGCTGTGGCTGTGACGGTGTCTGTG; >clamp_s_d_s_a_s:compl:rev CACAGACACCGTCACTATCATGTTGACATTCC; dG = -17.3
36 >s_a_s_b_s_a_s_d_s_a_s GTGTCTGTGGCTGTGTCTGTGACGGTGTCTGTG; >clamp_s_d_s_a_s:compl:rev CACAGACACCGTCACTATCATGTTGACATTCC; dG = -17.3
37 >s_c_s_c_s_a_s_d_s_a_s GTGTGGGTGTGGGTGTCTGTGACGGTGTCTGTG; >clamp_s_d_s_a_s:compl:rev CACAGACACCGTCACTATCATGTTGACATTCC; dG = -17.3
38 >s_c_s_c_s_b_s_d_s_a_s GTGTGGGTGTGGGTGGCTGTGACGGTGTCTGTG; >clamp_s_d_s_a_s:compl:rev CACAGACACCGTCACTATCATGTTGACATTCC; dG = -17.3
39 >s_c_s_d_s_b_s_d_s_a_s GTGTGGGTGACGGTGGCTGTGACGGTGTCTGTG; >clamp_s_d_s_a_s:compl:rev CACAGACACCGTCACTATCATGTTGACATTCC; dG = -17.3
40 >s_a_s_b_s_b_s_d_s_a_s GTGTCTGTGGCTGTGGCTGTGACGGTGTCTGTG; >clamp_s_d_s_a_s:compl:rev CACAGACACCGTCACTATCATGTTGACATTCC; dG = -17.3
41 >s_c_s_e_s_a_s_d_s_a_s GTGTGGGTGCTGGTGTCTGTGACGGTGTCTGTG; >clamp_s_d_s_a_s:compl:rev CACAGACACCGTCACTATCATGTTGACATTCC; dG = -17.3
42 >s_c_s_e_s_b_s_d_s_a_s GTGTGGGTGCTGGTGGCTGTGACGGTGTCTGTG; >clamp_s_d_s_a_s:compl:rev CACAGACACCGTCACTATCATGTTGACATTCC; dG = -17.3
43 >s_d_s_a_s_a_s_d_s_a_s GTGACGGTGTCTGTGTCTGTGACGGTGTCTGTG; >clamp_s_d_s_a_s:compl:rev CACAGACACCGTCACTATCATGTTGACATTCC; dG = -17.3
44 >s_d_s_a_s_b_s_d_s_a_s GTGACGGTGTCTGTGGCTGTGACGGTGTCTGTG; >clamp_s_d_s_a_s:compl:rev CACAGACACCGTCACTATCATGTTGACATTCC; dG = -17.3
45 >s_d_s_b_s_a_s_d_s_a_s GTGACGGTGGCTGTGTCTGTGACGGTGTCTGTG; >clamp_s_d_s_a_s:compl:rev CACAGACACCGTCACTATCATGTTGACATTCC; dG = -17.3
46 >s_d_s_b_s_b_s_d_s_a_s GTGACGGTGGCTGTGGCTGTGACGGTGTCTGTG; >clamp_s_d_s_a_s:compl:rev CACAGACACCGTCACTATCATGTTGACATTCC; dG = -17.3
47 >s_d_s_c_s_a_s_d_s_a_s GTGACGGTGTGGGTGTCTGTGACGGTGTCTGTG; >clamp_s_d_s_a_s:compl:rev CACAGACACCGTCACTATCATGTTGACATTCC; dG = -17.3
48 >s_d_s_c_s_b_s_d_s_a_s GTGACGGTGTGGGTGGCTGTGACGGTGTCTGTG; >clamp_s_d_s_a_s:compl:rev CACAGACACCGTCACTATCATGTTGACATTCC; dG = -17.3
49 >s_d_s_d_s_b_s_d_s_a_s GTGACGGTGACGGTGGCTGTGACGGTGTCTGTG; >clamp_s_d_s_a_s:compl:rev CACAGACACCGTCACTATCATGTTGACATTCC; dG = -17.3
50 >s_d_s_e_s_a_s_d_s_a_s GTGACGGTGCTGGTGTCTGTGACGGTGTCTGTG; >clamp_s_d_s_a_s:compl:rev CACAGACACCGTCACTATCATGTTGACATTCC; dG = -17.3
51 >s_d_s_e_s_b_s_d_s_a_s GTGACGGTGCTGGTGGCTGTGACGGTGTCTGTG; >clamp_s_d_s_a_s:compl:rev CACAGACACCGTCACTATCATGTTGACATTCC; dG = -17.3
52 >s_a_s_b_s_e_s_d_s_a_s GTGTCTGTGGCTGTGCTGGTGACGGTGTCTGTG; >clamp_s_d_s_a_s:compl:rev CACAGACACCGTCACTATCATGTTGACATTCC; dG = -17.2
53 >s_d_s_e_s_e_s_d_s_a_s GTGACGGTGCTGGTGCTGGTGACGGTGTCTGTG; >clamp_s_d_s_a_s:compl:rev CACAGACACCGTCACTATCATGTTGACATTCC; dG = -17.2
54 >s_e_s_a_s_c_s_d_s_a_s GTGCTGGTGTCTGTGTGGGTGACGGTGTCTGTG; >clamp_s_d_s_a_s:compl:rev CACAGACACCGTCACTATCATGTTGACATTCC; dG = -17.2
55 >s_e_s_a_s_d_s_d_s_a_s GTGCTGGTGTCTGTGACGGTGACGGTGTCTGTG; >clamp_s_d_s_a_s:compl:rev CACAGACACCGTCACTATCATGTTGACATTCC; dG = -17.2
56 >s_e_s_a_s_e_s_d_s_a_s GTGCTGGTGTCTGTGCTGGTGACGGTGTCTGTG; >clamp_s_d_s_a_s:compl:rev CACAGACACCGTCACTATCATGTTGACATTCC; dG = -17.2
57 >s_e_s_b_s_c_s_d_s_a_s GTGCTGGTGGCTGTGTGGGTGACGGTGTCTGTG; >clamp_s_d_s_a_s:compl:rev CACAGACACCGTCACTATCATGTTGACATTCC; dG = -17.2
58 >s_e_s_b_s_d_s_d_s_a_s GTGCTGGTGGCTGTGACGGTGACGGTGTCTGTG; >clamp_s_d_s_a_s:compl:rev CACAGACACCGTCACTATCATGTTGACATTCC; dG = -17.2
59 >s_e_s_b_s_e_s_d_s_a_s GTGCTGGTGGCTGTGCTGGTGACGGTGTCTGTG; >clamp_s_d_s_a_s:compl:rev CACAGACACCGTCACTATCATGTTGACATTCC; dG = -17.2
60 >s_e_s_c_s_c_s_d_s_a_s GTGCTGGTGTGGGTGTGGGTGACGGTGTCTGTG; >clamp_s_d_s_a_s:compl:rev CACAGACACCGTCACTATCATGTTGACATTCC; dG = -17.2
61 >s_e_s_c_s_d_s_d_s_a_s GTGCTGGTGTGGGTGACGGTGACGGTGTCTGTG; >clamp_s_d_s_a_s:compl:rev CACAGACACCGTCACTATCATGTTGACATTCC; dG = -17.2
62 >s_e_s_c_s_e_s_d_s_a_s GTGCTGGTGTGGGTGCTGGTGACGGTGTCTGTG; >clamp_s_d_s_a_s:compl:rev CACAGACACCGTCACTATCATGTTGACATTCC; dG = -17.2
63 >s_e_s_d_s_c_s_d_s_a_s GTGCTGGTGACGGTGTGGGTGACGGTGTCTGTG; >clamp_s_d_s_a_s:compl:rev CACAGACACCGTCACTATCATGTTGACATTCC; dG = -17.2
64 >s_e_s_d_s_d_s_d_s_a_s GTGCTGGTGACGGTGACGGTGACGGTGTCTGTG; >clamp_s_d_s_a_s:compl:rev CACAGACACCGTCACTATCATGTTGACATTCC; dG = -17.2

65 >s_e_s_d_s_e_s_d_s_a_s GTGCTGGTGACGGTGCTGGTGACGGTGTCTGTG; >clamp_s_d_s_a_s:compl:rev
CACAGACACCGTCACTATCATGTTGACATTCC; dG = -17.2
66 >s_e_s_e_s_c_s_d_s_a_s GTGCTGGTGCTGGTGTGGGTGACGGTGTCTGTG; >clamp_s_d_s_a_s:compl:rev
CACAGACACCGTCACTATCATGTTGACATTCC; dG = -17.2
67 >s_e_s_e_s_d_s_d_s_a_s GTGCTGGTGCTGGTGACGGTGACGGTGTCTGTG; >clamp_s_d_s_a_s:compl:rev
CACAGACACCGTCACTATCATGTTGACATTCC; dG = -17.2
68 >s_e_s_e_s_e_s_d_s_a_s GTGCTGGTGCTGGTGCTGGTGACGGTGTCTGTG; >clamp_s_d_s_a_s:compl:rev
CACAGACACCGTCACTATCATGTTGACATTCC; dG = -17.2
69 >s_a_s_c_s_c_s_d_s_a_s GTGTCTGTGTGGGTGTGGGTGACGGTGTCTGTG; >clamp_s_d_s_a_s:compl:rev
CACAGACACCGTCACTATCATGTTGACATTCC; dG = -17.2
70 >s_a_s_c_s_d_s_d_s_a_s GTGTCTGTGTGGGTGACGGTGACGGTGTCTGTG; >clamp_s_d_s_a_s:compl:rev
CACAGACACCGTCACTATCATGTTGACATTCC; dG = -17.2
71 >s_a_s_c_s_e_s_d_s_a_s GTGTCTGTGTGGGTGCTGGTGACGGTGTCTGTG; >clamp_s_d_s_a_s:compl:rev
CACAGACACCGTCACTATCATGTTGACATTCC; dG = -17.2
72 >s_a_s_d_s_c_s_d_s_a_s GTGTCTGTGACGGTGTGGGTGACGGTGTCTGTG; >clamp_s_d_s_a_s:compl:rev
CACAGACACCGTCACTATCATGTTGACATTCC; dG = -17.2
73 >s_a_s_d_s_d_s_d_s_a_s GTGTCTGTGACGGTGACGGTGACGGTGTCTGTG; >clamp_s_d_s_a_s:compl:rev
CACAGACACCGTCACTATCATGTTGACATTCC; dG = -17.2
74 >s_a_s_d_s_e_s_d_s_a_s GTGTCTGTGACGGTGCTGGTGACGGTGTCTGTG; >clamp_s_d_s_a_s:compl:rev
CACAGACACCGTCACTATCATGTTGACATTCC; dG = -17.2
75 >s_a_s_e_s_c_s_d_s_a_s GTGTCTGTGCTGGTGTGGGTGACGGTGTCTGTG; >clamp_s_d_s_a_s:compl:rev
CACAGACACCGTCACTATCATGTTGACATTCC; dG = -17.2
76 >s_a_s_e_s_d_s_d_s_a_s GTGTCTGTGCTGGTGACGGTGACGGTGTCTGTG; >clamp_s_d_s_a_s:compl:rev
CACAGACACCGTCACTATCATGTTGACATTCC; dG = -17.2
77 >s_a_s_e_s_e_s_d_s_a_s GTGTCTGTGCTGGTGCTGGTGACGGTGTCTGTG; >clamp_s_d_s_a_s:compl:rev
CACAGACACCGTCACTATCATGTTGACATTCC; dG = -17.2
78 >s_b_s_a_s_c_s_d_s_a_s GTGGCTGTGTCTGTGTGGGTGACGGTGTCTGTG; >clamp_s_d_s_a_s:compl:rev
CACAGACACCGTCACTATCATGTTGACATTCC; dG = -17.2
79 >s_b_s_a_s_d_s_d_s_a_s GTGGCTGTGTCTGTGACGGTGACGGTGTCTGTG; >clamp_s_d_s_a_s:compl:rev
CACAGACACCGTCACTATCATGTTGACATTCC; dG = -17.2
80 >s_a_s_a_s_c_s_d_s_a_s GTGTCTGTGTCTGTGTGGGTGACGGTGTCTGTG; >clamp_s_d_s_a_s:compl:rev
CACAGACACCGTCACTATCATGTTGACATTCC; dG = -17.2
81 >s_b_s_a_s_e_s_d_s_a_s GTGGCTGTGTCTGTGCTGGTGACGGTGTCTGTG; >clamp_s_d_s_a_s:compl:rev
CACAGACACCGTCACTATCATGTTGACATTCC; dG = -17.2
82 >s_b_s_b_s_c_s_d_s_a_s GTGGCTGTGGCTGTGTGGGTGACGGTGTCTGTG; >clamp_s_d_s_a_s:compl:rev
CACAGACACCGTCACTATCATGTTGACATTCC; dG = -17.2
83 >s_b_s_b_s_d_s_d_s_a_s GTGGCTGTGGCTGTGACGGTGACGGTGTCTGTG; >clamp_s_d_s_a_s:compl:rev
CACAGACACCGTCACTATCATGTTGACATTCC; dG = -17.2
84 >s_b_s_b_s_e_s_d_s_a_s GTGGCTGTGGCTGTGCTGGTGACGGTGTCTGTG; >clamp_s_d_s_a_s:compl:rev
CACAGACACCGTCACTATCATGTTGACATTCC; dG = -17.2
85 >s_b_s_c_s_c_s_d_s_a_s GTGGCTGTGTGGGTGTGGGTGACGGTGTCTGTG; >clamp_s_d_s_a_s:compl:rev
CACAGACACCGTCACTATCATGTTGACATTCC; dG = -17.2
86 >s_b_s_c_s_d_s_d_s_a_s GTGGCTGTGTGGGTGACGGTGACGGTGTCTGTG; >clamp_s_d_s_a_s:compl:rev
CACAGACACCGTCACTATCATGTTGACATTCC; dG = -17.2
87 >s_a_s_a_s_d_s_d_s_a_s GTGTCTGTGTCTGTGACGGTGACGGTGTCTGTG; >clamp_s_d_s_a_s:compl:rev
CACAGACACCGTCACTATCATGTTGACATTCC; dG = -17.2
88 >s_b_s_c_s_e_s_d_s_a_s GTGGCTGTGTGGGTGCTGGTGACGGTGTCTGTG; >clamp_s_d_s_a_s:compl:rev
CACAGACACCGTCACTATCATGTTGACATTCC; dG = -17.2
89 >s_b_s_d_s_c_s_d_s_a_s GTGGCTGTGACGGTGTGGGTGACGGTGTCTGTG; >clamp_s_d_s_a_s:compl:rev
CACAGACACCGTCACTATCATGTTGACATTCC; dG = -17.2
90 >s_b_s_d_s_d_s_d_s_a_s GTGGCTGTGACGGTGACGGTGACGGTGTCTGTG; >clamp_s_d_s_a_s:compl:rev
CACAGACACCGTCACTATCATGTTGACATTCC; dG = -17.2
91 >s_b_s_d_s_e_s_d_s_a_s GTGGCTGTGACGGTGCTGGTGACGGTGTCTGTG; >clamp_s_d_s_a_s:compl:rev
CACAGACACCGTCACTATCATGTTGACATTCC; dG = -17.2
92 >s_b_s_e_s_c_s_d_s_a_s GTGGCTGTGCTGGTGTGGGTGACGGTGTCTGTG; >clamp_s_d_s_a_s:compl:rev
CACAGACACCGTCACTATCATGTTGACATTCC; dG = -17.2
93 >s_b_s_e_s_d_s_d_s_a_s GTGGCTGTGCTGGTGACGGTGACGGTGTCTGTG; >clamp_s_d_s_a_s:compl:rev
CACAGACACCGTCACTATCATGTTGACATTCC; dG = -17.2
94 >s_a_s_a_s_e_s_d_s_a_s GTGTCTGTGTCTGTGCTGGTGACGGTGTCTGTG; >clamp_s_d_s_a_s:compl:rev
CACAGACACCGTCACTATCATGTTGACATTCC; dG = -17.2
95 >s_b_s_e_s_e_s_d_s_a_s GTGGCTGTGCTGGTGCTGGTGACGGTGTCTGTG; >clamp_s_d_s_a_s:compl:rev
CACAGACACCGTCACTATCATGTTGACATTCC; dG = -17.2
96 >s_c_s_a_s_c_s_d_s_a_s GTGTGGGTGTCTGTGTGGGTGACGGTGTCTGTG; >clamp_s_d_s_a_s:compl:rev
CACAGACACCGTCACTATCATGTTGACATTCC; dG = -17.2
97 >s_c_s_a_s_d_s_d_s_a_s GTGTGGGTGTCTGTGACGGTGACGGTGTCTGTG; >clamp_s_d_s_a_s:compl:rev
CACAGACACCGTCACTATCATGTTGACATTCC; dG = -17.2
98 >s_c_s_a_s_e_s_d_s_a_s GTGTGGGTGTCTGTGCTGGTGACGGTGTCTGTG; >clamp_s_d_s_a_s:compl:rev
CACAGACACCGTCACTATCATGTTGACATTCC; dG = -17.2
99 >s_c_s_b_s_c_s_d_s_a_s GTGTGGGTGGCTGTGTGGGTGACGGTGTCTGTG; >clamp_s_d_s_a_s:compl:rev
CACAGACACCGTCACTATCATGTTGACATTCC; dG = -17.2
100 >s_c_s_b_s_d_s_d_s_a_s GTGTGGGTGGCTGTGACGGTGACGGTGTCTGTG; >clamp_s_d_s_a_s:compl:rev
CACAGACACCGTCACTATCATGTTGACATTCC; dG = -17.2
101 >s_c_s_b_s_e_s_d_s_a_s GTGTGGGTGGCTGTGCTGGTGACGGTGTCTGTG; >clamp_s_d_s_a_s:compl:rev
CACAGACACCGTCACTATCATGTTGACATTCC; dG = -17.2

102 >s_c_s_c_s_c_s_d_s_a_s GTGTGGGTGTGGGTGTGGGTGACGGTGTCTGTG; >clamp_s_d_s_a_s:compl:rev
CACAGACACCGTCACTATCATGTTGACATTCC; dG = -17.2
103 >s_c_s_c_s_d_s_d_s_a_s GTGTGGGTGTGGGTGACGGTGACGGTGTCTGTG; >clamp_s_d_s_a_s:compl:rev
CACAGACACCGTCACTATCATGTTGACATTCC; dG = -17.2
104 >s_c_s_c_s_e_s_d_s_a_s GTGTGGGTGTGGGTGCTGGTGACGGTGTCTGTG; >clamp_s_d_s_a_s:compl:rev
CACAGACACCGTCACTATCATGTTGACATTCC; dG = -17.2
105 >s_c_s_d_s_c_s_d_s_a_s GTGTGGGTGACGGTGTGGGTGACGGTGTCTGTG; >clamp_s_d_s_a_s:compl:rev
CACAGACACCGTCACTATCATGTTGACATTCC; dG = -17.2
106 >s_c_s_d_s_d_s_d_s_a_s GTGTGGGTGACGGTGACGGTGACGGTGTCTGTG; >clamp_s_d_s_a_s:compl:rev
CACAGACACCGTCACTATCATGTTGACATTCC; dG = -17.2
107 >s_c_s_d_s_e_s_d_s_a_s GTGTGGGTGACGGTGCTGGTGACGGTGTCTGTG; >clamp_s_d_s_a_s:compl:rev
CACAGACACCGTCACTATCATGTTGACATTCC; dG = -17.2
108 >s_c_s_e_s_c_s_d_s_a_s GTGTGGGTGCTGGTGTGGGTGACGGTGTCTGTG; >clamp_s_d_s_a_s:compl:rev
CACAGACACCGTCACTATCATGTTGACATTCC; dG = -17.2
109 >s_c_s_e_s_d_s_d_s_a_s GTGTGGGTGCTGGTGACGGTGACGGTGTCTGTG; >clamp_s_d_s_a_s:compl:rev
CACAGACACCGTCACTATCATGTTGACATTCC; dG = -17.2
110 >s_c_s_e_s_e_s_d_s_a_s GTGTGGGTGCTGGTGCTGGTGACGGTGTCTGTG; >clamp_s_d_s_a_s:compl:rev
CACAGACACCGTCACTATCATGTTGACATTCC; dG = -17.2
111 >s_d_s_a_s_c_s_d_s_a_s GTGACGGTGTCTGTGTGGGTGACGGTGTCTGTG; >clamp_s_d_s_a_s:compl:rev
CACAGACACCGTCACTATCATGTTGACATTCC; dG = -17.2
112 >s_a_s_b_s_c_s_d_s_a_s GTGTCTGTGGCTGTGTGGGTGACGGTGTCTGTG; >clamp_s_d_s_a_s:compl:rev
CACAGACACCGTCACTATCATGTTGACATTCC; dG = -17.2
113 >s_d_s_a_s_e_s_d_s_a_s GTGACGGTGTCTGTGCTGGTGACGGTGTCTGTG; >clamp_s_d_s_a_s:compl:rev
CACAGACACCGTCACTATCATGTTGACATTCC; dG = -17.2
114 >s_d_s_b_s_c_s_d_s_a_s GTGACGGTGGCTGTGTGGGTGACGGTGTCTGTG; >clamp_s_d_s_a_s:compl:rev
CACAGACACCGTCACTATCATGTTGACATTCC; dG = -17.2
115 >s_d_s_b_s_d_s_d_s_a_s GTGACGGTGGCTGTGACGGTGACGGTGTCTGTG; >clamp_s_d_s_a_s:compl:rev
CACAGACACCGTCACTATCATGTTGACATTCC; dG = -17.2
116 >s_d_s_b_s_e_s_d_s_a_s GTGACGGTGGCTGTGCTGGTGACGGTGTCTGTG; >clamp_s_d_s_a_s:compl:rev
CACAGACACCGTCACTATCATGTTGACATTCC; dG = -17.2
117 >s_d_s_c_s_c_s_d_s_a_s GTGACGGTGTGGGTGTGGGTGACGGTGTCTGTG; >clamp_s_d_s_a_s:compl:rev
CACAGACACCGTCACTATCATGTTGACATTCC; dG = -17.2
118 >s_d_s_c_s_d_s_d_s_a_s GTGACGGTGTGGGTGACGGTGACGGTGTCTGTG; >clamp_s_d_s_a_s:compl:rev
CACAGACACCGTCACTATCATGTTGACATTCC; dG = -17.2
119 >s_a_s_b_s_d_s_d_s_a_s GTGTCTGTGGCTGTGACGGTGACGGTGTCTGTG; >clamp_s_d_s_a_s:compl:rev
CACAGACACCGTCACTATCATGTTGACATTCC; dG = -17.2
120 >s_d_s_c_s_e_s_d_s_a_s GTGACGGTGTGGGTGCTGGTGACGGTGTCTGTG; >clamp_s_d_s_a_s:compl:rev
CACAGACACCGTCACTATCATGTTGACATTCC; dG = -17.2
121 >s_d_s_d_s_c_s_d_s_a_s GTGACGGTGACGGTGTGGGTGACGGTGTCTGTG; >clamp_s_d_s_a_s:compl:rev
CACAGACACCGTCACTATCATGTTGACATTCC; dG = -17.2
122 >s_d_s_d_s_d_s_d_s_a_s GTGACGGTGACGGTGACGGTGACGGTGTCTGTG; >clamp_s_d_s_a_s:compl:rev
CACAGACACCGTCACTATCATGTTGACATTCC; dG = -17.2
123 >s_d_s_d_s_e_s_d_s_a_s GTGACGGTGACGGTGCTGGTGACGGTGTCTGTG; >clamp_s_d_s_a_s:compl:rev
CACAGACACCGTCACTATCATGTTGACATTCC; dG = -17.2
124 >s_d_s_e_s_c_s_d_s_a_s GTGACGGTGCTGGTGTGGGTGACGGTGTCTGTG; >clamp_s_d_s_a_s:compl:rev
CACAGACACCGTCACTATCATGTTGACATTCC; dG = -17.2
125 >s_d_s_e_s_d_s_d_s_a_s GTGACGGTGCTGGTGACGGTGACGGTGTCTGTG; >clamp_s_d_s_a_s:compl:rev
CACAGACACCGTCACTATCATGTTGACATTCC; dG = -17.2

****

DA Mismatches

1 >s_a_s_d_s_b_s_d_s_e_s GTGTCTGTGACGGTGGCTGTGACGGTGCTGGTG; >clamp_s_d_s_a_s:compl:rev
CACAGACACCGTCACTATCATGTTGACATTCC; dG = -16.2
2 >s_a_s_d_s_b_s_d_s_d_s GTGTCTGTGACGGTGGCTGTGACGGTGACGGTG; >clamp_s_d_s_a_s:compl:rev
CACAGACACCGTCACTATCATGTTGACATTCC; dG = -16.2
3 >s_a_s_d_s_b_s_d_s_c_s GTGTCTGTGACGGTGGCTGTGACGGTGTGGGTG; >clamp_s_d_s_a_s:compl:rev
CACAGACACCGTCACTATCATGTTGACATTCC; dG = -16.2
4 >s_a_s_d_s_b_s_d_s_b_s GTGTCTGTGACGGTGGCTGTGACGGTGGCTGTG; >clamp_s_d_s_a_s:compl:rev
CACAGACACCGTCACTATCATGTTGACATTCC; dG = -16.2
5 >s_b_s_d_s_b_s_d_s_e_s GTGGCTGTGACGGTGGCTGTGACGGTGCTGGTG; >clamp_s_d_s_a_s:compl:rev
CACAGACACCGTCACTATCATGTTGACATTCC; dG = -16.2
6 >s_b_s_d_s_b_s_d_s_d_s GTGGCTGTGACGGTGGCTGTGACGGTGACGGTG; >clamp_s_d_s_a_s:compl:rev
CACAGACACCGTCACTATCATGTTGACATTCC; dG = -16.2
7 >s_b_s_d_s_b_s_d_s_c_s GTGGCTGTGACGGTGGCTGTGACGGTGTGGGTG; >clamp_s_d_s_a_s:compl:rev
CACAGACACCGTCACTATCATGTTGACATTCC; dG = -16.2
8 >s_b_s_d_s_b_s_d_s_b_s GTGGCTGTGACGGTGGCTGTGACGGTGGCTGTG; >clamp_s_d_s_a_s:compl:rev
CACAGACACCGTCACTATCATGTTGACATTCC; dG = -16.2
9 >s_c_s_d_s_b_s_d_s_e_s GTGTGGGTGACGGTGGCTGTGACGGTGCTGGTG; >clamp_s_d_s_a_s:compl:rev
CACAGACACCGTCACTATCATGTTGACATTCC; dG = -16.1

10 >s_c_s_d_s_b_s_d_s_d_s GTGTGGGTGACGGTGGCTGTGACGGTGACGGTG; >clamp_s_d_s_a_s:compl:rev CACAGACACCGTCACTATCATGTTGACATTCC; dG = -16.1

11 >s_c_s_d_s_b_s_d_s_c_s GTGTGGGTGACGGTGGCTGTGACGGTGTGGGTG; >clamp_s_d_s_a_s:compl:rev CACAGACACCGTCACTATCATGTTGACATTCC; dG = -16.1

12 >s_c_s_d_s_b_s_d_s_b_s GTGTGGGTGACGGTGGCTGTGACGGTGGCTGTG; >clamp_s_d_s_a_s:compl:rev CACAGACACCGTCACTATCATGTTGACATTCC; dG = -16.1

13 >s_d_s_d_s_b_s_d_s_e_s GTGACGGTGACGGTGGCTGTGACGGTGCTGGTG; >clamp_s_d_s_a_s:compl:rev CACAGACACCGTCACTATCATGTTGACATTCC; dG = -16.1

14 >s_d_s_d_s_b_s_d_s_d_s GTGACGGTGACGGTGGCTGTGACGGTGACGGTG; >clamp_s_d_s_a_s:compl:rev CACAGACACCGTCACTATCATGTTGACATTCC; dG = -16.1

15 >s_d_s_d_s_b_s_d_s_c_s GTGACGGTGACGGTGGCTGTGACGGTGTGGGTG; >clamp_s_d_s_a_s:compl:rev CACAGACACCGTCACTATCATGTTGACATTCC; dG = -16.1

16 >s_d_s_d_s_b_s_d_s_b_s GTGACGGTGACGGTGGCTGTGACGGTGGCTGTG; >clamp_s_d_s_a_s:compl:rev CACAGACACCGTCACTATCATGTTGACATTCC; dG = -16.1

17 >s_e_s_d_s_b_s_d_s_e_s GTGCTGGTGACGGTGGCTGTGACGGTGCTGGTG; >clamp_s_d_s_a_s:compl:rev CACAGACACCGTCACTATCATGTTGACATTCC; dG = -16.1

18 >s_e_s_d_s_b_s_d_s_d_s GTGCTGGTGACGGTGGCTGTGACGGTGACGGTG; >clamp_s_d_s_a_s:compl:rev CACAGACACCGTCACTATCATGTTGACATTCC; dG = -16.1

19 >s_e_s_d_s_b_s_d_s_c_s GTGCTGGTGACGGTGGCTGTGACGGTGTGGGTG; >clamp_s_d_s_a_s:compl:rev CACAGACACCGTCACTATCATGTTGACATTCC; dG = -16.1

20 >s_e_s_d_s_b_s_d_s_b_s GTGCTGGTGACGGTGGCTGTGACGGTGGCTGTG; >clamp_s_d_s_a_s:compl:rev CACAGACACCGTCACTATCATGTTGACATTCC; dG = -16.1

21 >s_a_s_d_s_b_s_b_s_a_s GTGTCTGTGACGGTGGCTGTGGCTGTGTCTGTG; >clamp_s_d_s_a_s:compl:rev CACAGACACCGTCACTATCATGTTGACATTCC; dG = -15.7

22 >s_b_s_d_s_b_s_b_s_a_s GTGGCTGTGACGGTGGCTGTGGCTGTGTCTGTG; >clamp_s_d_s_a_s:compl:rev CACAGACACCGTCACTATCATGTTGACATTCC; dG = -15.7

23 >s_a_s_d_s_b_s_c_s_a_s GTGTCTGTGACGGTGGCTGTGTGGGTGTCTGTG; >clamp_s_d_s_a_s:compl:rev CACAGACACCGTCACTATCATGTTGACATTCC; dG = -15.6

24 >s_a_s_d_s_b_s_a_s_a_s GTGTCTGTGACGGTGGCTGTGTCTGTGTCTGTG; >clamp_s_d_s_a_s:compl:rev CACAGACACCGTCACTATCATGTTGACATTCC; dG = -15.6

25 >s_b_s_a_s_d_s_b_s_a_s GTGGCTGTGTCTGTGACGGTGGCTGTGTCTGTG; >clamp_s_d_s_a_s:compl:rev CACAGACACCGTCACTATCATGTTGACATTCC; dG = -15.6

26 >s_b_s_b_s_d_s_b_s_a_s GTGGCTGTGGCTGTGACGGTGGCTGTGTCTGTG; >clamp_s_d_s_a_s:compl:rev CACAGACACCGTCACTATCATGTTGACATTCC; dG = -15.6

27 >s_a_s_a_s_d_s_b_s_a_s GTGTCTGTGTCTGTGACGGTGGCTGTGTCTGTG; >clamp_s_d_s_a_s:compl:rev CACAGACACCGTCACTATCATGTTGACATTCC; dG = -15.6

28 >s_b_s_d_s_b_s_c_s_a_s GTGGCTGTGACGGTGGCTGTGTGGGTGTCTGTG; >clamp_s_d_s_a_s:compl:rev CACAGACACCGTCACTATCATGTTGACATTCC; dG = -15.6

29 >s_b_s_d_s_b_s_a_s_a_s GTGGCTGTGACGGTGGCTGTGTCTGTGTCTGTG; >clamp_s_d_s_a_s:compl:rev CACAGACACCGTCACTATCATGTTGACATTCC; dG = -15.6

30 >s_c_s_a_s_d_s_b_s_a_s GTGTGGGTGTCTGTGACGGTGGCTGTGTCTGTG; >clamp_s_d_s_a_s:compl:rev CACAGACACCGTCACTATCATGTTGACATTCC; dG = -15.6

31 >s_c_s_b_s_d_s_b_s_a_s GTGTGGGTGGCTGTGACGGTGGCTGTGTCTGTG; >clamp_s_d_s_a_s:compl:rev CACAGACACCGTCACTATCATGTTGACATTCC; dG = -15.6

32 >s_c_s_d_s_b_s_b_s_a_s GTGTGGGTGACGGTGGCTGTGGCTGTGTCTGTG; >clamp_s_d_s_a_s:compl:rev CACAGACACCGTCACTATCATGTTGACATTCC; dG = -15.6

33 >s_d_s_b_s_d_s_d_s_e_s GTGACGGTGGCTGTGACGGTGACGGTGCTGGTG; >clamp_s_d_s_a_s:compl:rev CACAGACACCGTCACTATCATGTTGACATTCC; dG = -15.6

34 >s_d_s_b_s_d_s_d_s_d_s GTGACGGTGGCTGTGACGGTGACGGTGACGGTG; >clamp_s_d_s_a_s:compl:rev CACAGACACCGTCACTATCATGTTGACATTCC; dG = -15.6

35 >s_d_s_b_s_d_s_d_s_c_s GTGACGGTGGCTGTGACGGTGACGGTGTGGGTG; >clamp_s_d_s_a_s:compl:rev CACAGACACCGTCACTATCATGTTGACATTCC; dG = -15.6

36 >s_d_s_b_s_d_s_d_s_b_s GTGACGGTGGCTGTGACGGTGACGGTGGCTGTG; >clamp_s_d_s_a_s:compl:rev CACAGACACCGTCACTATCATGTTGACATTCC; dG = -15.6

37 >s_d_s_b_s_d_s_e_s_a_s GTGACGGTGGCTGTGACGGTGCTGGTGTCTGTG; >clamp_s_d_s_a_s:compl:rev CACAGACACCGTCACTATCATGTTGACATTCC; dG = -15.6

38 >s_d_s_b_s_d_s_c_s_a_s GTGACGGTGGCTGTGACGGTGTGGGTGTCTGTG; >clamp_s_d_s_a_s:compl:rev CACAGACACCGTCACTATCATGTTGACATTCC; dG = -15.6

39 >s_d_s_b_s_d_s_b_s_a_s GTGACGGTGGCTGTGACGGTGGCTGTGTCTGTG; >clamp_s_d_s_a_s:compl:rev CACAGACACCGTCACTATCATGTTGACATTCC; dG = -15.6

40 >s_d_s_d_s_b_s_b_s_a_s GTGACGGTGACGGTGGCTGTGGCTGTGTCTGTG; >clamp_s_d_s_a_s:compl:rev CACAGACACCGTCACTATCATGTTGACATTCC; dG = -15.6

41 >s_a_s_b_s_d_s_b_s_a_s GTGTCTGTGGCTGTGACGGTGGCTGTGTCTGTG; >clamp_s_d_s_a_s:compl:rev CACAGACACCGTCACTATCATGTTGACATTCC; dG = -15.6

42 >s_e_s_a_s_d_s_b_s_a_s GTGCTGGTGTCTGTGACGGTGGCTGTGTCTGTG; >clamp_s_d_s_a_s:compl:rev CACAGACACCGTCACTATCATGTTGACATTCC; dG = -15.6

43 >s_e_s_b_s_d_s_b_s_a_s GTGCTGGTGGCTGTGACGGTGGCTGTGTCTGTG; >clamp_s_d_s_a_s:compl:rev CACAGACACCGTCACTATCATGTTGACATTCC; dG = -15.6

44 >s_e_s_d_s_b_s_b_s_a_s GTGCTGGTGACGGTGGCTGTGGCTGTGTCTGTG; >clamp_s_d_s_a_s:compl:rev CACAGACACCGTCACTATCATGTTGACATTCC; dG = -15.6

45 >s_a_s_c_s_d_s_b_s_a_s GTGTCTGTGTGGGTGACGGTGGCTGTGTCTGTG; >clamp_s_d_s_a_s:compl:rev CACAGACACCGTCACTATCATGTTGACATTCC; dG = -15.5

46 >s_a_s_d_s_b_s_e_s_a_s GTGTCTGTGACGGTGGCTGTGCTGGTGTCTGTG; >clamp_s_d_s_a_s:compl:rev CACAGACACCGTCACTATCATGTTGACATTCC; dG = -15.5

47 >s_a_s_d_s_d_s_b_s_a_s GTGTCTGTGACGGTGACGGTGGCTGTGTCTGTG; >clamp_s_d_s_a_s:compl:rev CACAGACACCGTCACTATCATGTTGACATTCC; dG = -15.5
48 >s_a_s_e_s_d_s_b_s_a_s GTGTCTGTGCTGGTGACGGTGGCTGTGTCTGTG; >clamp_s_d_s_a_s:compl:rev CACAGACACCGTCACTATCATGTTGACATTCC; dG = -15.5
49 >s_b_s_c_s_d_s_b_s_a_s GTGGCTGTGTGGGTGACGGTGGCTGTGTCTGTG; >clamp_s_d_s_a_s:compl:rev CACAGACACCGTCACTATCATGTTGACATTCC; dG = -15.5
50 >s_b_s_d_s_b_s_e_s_a_s GTGGCTGTGACGGTGGCTGTGCTGGTGTCTGTG; >clamp_s_d_s_a_s:compl:rev CACAGACACCGTCACTATCATGTTGACATTCC; dG = -15.5
51 >s_b_s_d_s_d_s_b_s_a_s GTGGCTGTGACGGTGACGGTGGCTGTGTCTGTG; >clamp_s_d_s_a_s:compl:rev CACAGACACCGTCACTATCATGTTGACATTCC; dG = -15.5
52 >s_b_s_e_s_d_s_b_s_a_s GTGGCTGTGCTGGTGACGGTGGCTGTGTCTGTG; >clamp_s_d_s_a_s:compl:rev CACAGACACCGTCACTATCATGTTGACATTCC; dG = -15.5
53 >s_c_s_c_s_d_s_b_s_a_s GTGTGGGTGTGGGTGACGGTGGCTGTGTCTGTG; >clamp_s_d_s_a_s:compl:rev CACAGACACCGTCACTATCATGTTGACATTCC; dG = -15.5
54 >s_c_s_d_s_b_s_c_s_a_s GTGTGGGTGACGGTGGCTGTGTGGGTGTCTGTG; >clamp_s_d_s_a_s:compl:rev CACAGACACCGTCACTATCATGTTGACATTCC; dG = -15.5
55 >s_c_s_d_s_b_s_a_s_a_s GTGTGGGTGACGGTGGCTGTGTCTGTGTCTGTG; >clamp_s_d_s_a_s:compl:rev CACAGACACCGTCACTATCATGTTGACATTCC; dG = -15.5
56 >s_c_s_d_s_d_s_b_s_a_s GTGTGGGTGACGGTGACGGTGGCTGTGTCTGTG; >clamp_s_d_s_a_s:compl:rev CACAGACACCGTCACTATCATGTTGACATTCC; dG = -15.5
57 >s_c_s_e_s_d_s_b_s_a_s GTGTGGGTGCTGGTGACGGTGGCTGTGTCTGTG; >clamp_s_d_s_a_s:compl:rev CACAGACACCGTCACTATCATGTTGACATTCC; dG = -15.5
58 >s_d_s_c_s_d_s_b_s_a_s GTGACGGTGTGGGTGACGGTGGCTGTGTCTGTG; >clamp_s_d_s_a_s:compl:rev CACAGACACCGTCACTATCATGTTGACATTCC; dG = -15.5
59 >s_d_s_d_s_b_s_c_s_a_s GTGACGGTGACGGTGGCTGTGTGGGTGTCTGTG; >clamp_s_d_s_a_s:compl:rev CACAGACACCGTCACTATCATGTTGACATTCC; dG = -15.5
60 >s_d_s_d_s_b_s_a_s_a_s GTGACGGTGACGGTGGCTGTGTCTGTGTCTGTG; >clamp_s_d_s_a_s:compl:rev CACAGACACCGTCACTATCATGTTGACATTCC; dG = -15.5
61 >s_d_s_d_s_d_s_b_s_a_s GTGACGGTGACGGTGACGGTGGCTGTGTCTGTG; >clamp_s_d_s_a_s:compl:rev CACAGACACCGTCACTATCATGTTGACATTCC; dG = -15.5
62 >s_d_s_e_s_d_s_b_s_a_s GTGACGGTGCTGGTGACGGTGGCTGTGTCTGTG; >clamp_s_d_s_a_s:compl:rev CACAGACACCGTCACTATCATGTTGACATTCC; dG = -15.5
63 >s_e_s_c_s_d_s_b_s_a_s GTGCTGGTGTGGGTGACGGTGGCTGTGTCTGTG; >clamp_s_d_s_a_s:compl:rev CACAGACACCGTCACTATCATGTTGACATTCC; dG = -15.5
64 >s_e_s_d_s_b_s_c_s_a_s GTGCTGGTGACGGTGGCTGTGTGGGTGTCTGTG; >clamp_s_d_s_a_s:compl:rev CACAGACACCGTCACTATCATGTTGACATTCC; dG = -15.5
65 >s_e_s_d_s_b_s_a_s_a_s GTGCTGGTGACGGTGGCTGTGTCTGTGTCTGTG; >clamp_s_d_s_a_s:compl:rev CACAGACACCGTCACTATCATGTTGACATTCC; dG = -15.5
66 >s_e_s_d_s_d_s_b_s_a_s GTGCTGGTGACGGTGACGGTGGCTGTGTCTGTG; >clamp_s_d_s_a_s:compl:rev CACAGACACCGTCACTATCATGTTGACATTCC; dG = -15.5
67 >s_e_s_e_s_d_s_b_s_a_s GTGCTGGTGCTGGTGACGGTGGCTGTGTCTGTG; >clamp_s_d_s_a_s:compl:rev CACAGACACCGTCACTATCATGTTGACATTCC; dG = -15.5
68 >s_c_s_d_s_b_s_e_s_a_s GTGTGGGTGACGGTGGCTGTGCTGGTGTCTGTG; >clamp_s_d_s_a_s:compl:rev CACAGACACCGTCACTATCATGTTGACATTCC; dG = -15.4
69 >s_d_s_d_s_b_s_e_s_a_s GTGACGGTGACGGTGGCTGTGCTGGTGTCTGTG; >clamp_s_d_s_a_s:compl:rev CACAGACACCGTCACTATCATGTTGACATTCC; dG = -15.4
70 >s_e_s_d_s_b_s_e_s_a_s GTGCTGGTGACGGTGGCTGTGCTGGTGTCTGTG; >clamp_s_d_s_a_s:compl:rev CACAGACACCGTCACTATCATGTTGACATTCC; dG = -15.4
71 >s_a_s_c_s_d_s_d_s_b_s GTGTCTGTGTGGGTGACGGTGACGGTGGCTGTG; >clamp_s_d_s_a_s:compl:rev CACAGACACCGTCACTATCATGTTGACATTCC; dG = -15.3
72 >s_a_s_c_s_e_s_d_s_b_s GTGTCTGTGTGGGTGCTGGTGACGGTGGCTGTG; >clamp_s_d_s_a_s:compl:rev CACAGACACCGTCACTATCATGTTGACATTCC; dG = -15.3
73 >s_a_s_a_s_b_s_d_s_b_s GTGTCTGTGTCTGTGGCTGTGACGGTGGCTGTG; >clamp_s_d_s_a_s:compl:rev CACAGACACCGTCACTATCATGTTGACATTCC; dG = -15.3
74 >s_a_s_d_s_c_s_d_s_b_s GTGTCTGTGACGGTGTGGGTGACGGTGGCTGTG; >clamp_s_d_s_a_s:compl:rev CACAGACACCGTCACTATCATGTTGACATTCC; dG = -15.3
75 >s_a_s_d_s_d_s_d_s_b_s GTGTCTGTGACGGTGACGGTGACGGTGGCTGTG; >clamp_s_d_s_a_s:compl:rev CACAGACACCGTCACTATCATGTTGACATTCC; dG = -15.3
76 >s_a_s_d_s_e_s_d_s_b_s GTGTCTGTGACGGTGCTGGTGACGGTGGCTGTG; >clamp_s_d_s_a_s:compl:rev CACAGACACCGTCACTATCATGTTGACATTCC; dG = -15.3
77 >s_a_s_e_s_a_s_d_s_b_s GTGTCTGTGCTGGTGTCTGTGACGGTGGCTGTG; >clamp_s_d_s_a_s:compl:rev CACAGACACCGTCACTATCATGTTGACATTCC; dG = -15.3
78 >s_a_s_e_s_b_s_d_s_b_s GTGTCTGTGCTGGTGGCTGTGACGGTGGCTGTG; >clamp_s_d_s_a_s:compl:rev CACAGACACCGTCACTATCATGTTGACATTCC; dG = -15.3
79 >s_a_s_e_s_c_s_d_s_b_s GTGTCTGTGCTGGTGTGGGTGACGGTGGCTGTG; >clamp_s_d_s_a_s:compl:rev CACAGACACCGTCACTATCATGTTGACATTCC; dG = -15.3
80 >s_a_s_e_s_d_s_d_s_b_s GTGTCTGTGCTGGTGACGGTGACGGTGGCTGTG; >clamp_s_d_s_a_s:compl:rev CACAGACACCGTCACTATCATGTTGACATTCC; dG = -15.3
81 >s_a_s_e_s_e_s_d_s_b_s GTGTCTGTGCTGGTGCTGGTGACGGTGGCTGTG; >clamp_s_d_s_a_s:compl:rev CACAGACACCGTCACTATCATGTTGACATTCC; dG = -15.3
82 >s_b_s_a_s_a_s_d_s_b_s GTGGCTGTGTCTGTGTCTGTGACGGTGGCTGTG; >clamp_s_d_s_a_s:compl:rev CACAGACACCGTCACTATCATGTTGACATTCC; dG = -15.3
83 >s_b_s_a_s_b_s_d_s_b_s GTGGCTGTGTCTGTGGCTGTGACGGTGGCTGTG; >clamp_s_d_s_a_s:compl:rev CACAGACACCGTCACTATCATGTTGACATTCC; dG = -15.3

84 >s_a_s_a_s_c_s_d_s_b_s GTGTCTGTGTCTGTGTGGGTGACGGTGGCTGTG; >clamp_s_d_s_a_s:compl:rev CACAGACACCGTCACTATCATGTTGACATTCC; dG = -15.3
85 >s_b_s_a_s_c_s_d_s_b_s GTGGCTGTGTCTGTGTGGGTGACGGTGGCTGTG; >clamp_s_d_s_a_s:compl:rev CACAGACACCGTCACTATCATGTTGACATTCC; dG = -15.3
86 >s_b_s_a_s_d_s_d_s_b_s GTGGCTGTGTCTGTGACGGTGACGGTGGCTGTG; >clamp_s_d_s_a_s:compl:rev CACAGACACCGTCACTATCATGTTGACATTCC; dG = -15.3
87 >s_b_s_a_s_e_s_d_s_b_s GTGGCTGTGTCTGTGCTGGTGACGGTGGCTGTG; >clamp_s_d_s_a_s:compl:rev CACAGACACCGTCACTATCATGTTGACATTCC; dG = -15.3
88 >s_b_s_b_s_a_s_d_s_b_s GTGGCTGTGGCTGTGTCTGTGACGGTGGCTGTG; >clamp_s_d_s_a_s:compl:rev CACAGACACCGTCACTATCATGTTGACATTCC; dG = -15.3
89 >s_b_s_b_s_b_s_d_s_b_s GTGGCTGTGGCTGTGGCTGTGACGGTGGCTGTG; >clamp_s_d_s_a_s:compl:rev CACAGACACCGTCACTATCATGTTGACATTCC; dG = -15.3
90 >s_b_s_b_s_c_s_d_s_b_s GTGGCTGTGGCTGTGTGGGTGACGGTGGCTGTG; >clamp_s_d_s_a_s:compl:rev CACAGACACCGTCACTATCATGTTGACATTCC; dG = -15.3
91 >s_b_s_b_s_d_s_d_s_b_s GTGGCTGTGGCTGTGACGGTGACGGTGGCTGTG; >clamp_s_d_s_a_s:compl:rev CACAGACACCGTCACTATCATGTTGACATTCC; dG = -15.3
92 >s_b_s_b_s_e_s_d_s_b_s GTGGCTGTGGCTGTGCTGGTGACGGTGGCTGTG; >clamp_s_d_s_a_s:compl:rev CACAGACACCGTCACTATCATGTTGACATTCC; dG = -15.3
93 >s_b_s_c_s_a_s_d_s_b_s GTGGCTGTGTGGGTGTCTGTGACGGTGGCTGTG; >clamp_s_d_s_a_s:compl:rev CACAGACACCGTCACTATCATGTTGACATTCC; dG = -15.3
94 >s_b_s_c_s_b_s_d_s_b_s GTGGCTGTGTGGGTGGCTGTGACGGTGGCTGTG; >clamp_s_d_s_a_s:compl:rev CACAGACACCGTCACTATCATGTTGACATTCC; dG = -15.3
95 >s_a_s_a_s_d_s_d_s_b_s GTGTCTGTGTCTGTGACGGTGACGGTGGCTGTG; >clamp_s_d_s_a_s:compl:rev CACAGACACCGTCACTATCATGTTGACATTCC; dG = -15.3
96 >s_b_s_c_s_c_s_d_s_b_s GTGGCTGTGTGGGTGTGGGTGACGGTGGCTGTG; >clamp_s_d_s_a_s:compl:rev CACAGACACCGTCACTATCATGTTGACATTCC; dG = -15.3
97 >s_b_s_c_s_d_s_d_s_b_s GTGGCTGTGTGGGTGACGGTGACGGTGGCTGTG; >clamp_s_d_s_a_s:compl:rev CACAGACACCGTCACTATCATGTTGACATTCC; dG = -15.3
98 >s_b_s_c_s_e_s_d_s_b_s GTGGCTGTGTGGGTGCTGGTGACGGTGGCTGTG; >clamp_s_d_s_a_s:compl:rev CACAGACACCGTCACTATCATGTTGACATTCC; dG = -15.3
99 >s_b_s_d_s_c_s_d_s_b_s GTGGCTGTGACGGTGTGGGTGACGGTGGCTGTG; >clamp_s_d_s_a_s:compl:rev CACAGACACCGTCACTATCATGTTGACATTCC; dG = -15.3
100 >s_b_s_d_s_d_s_d_s_b_s GTGGCTGTGACGGTGACGGTGACGGTGGCTGTG; >clamp_s_d_s_a_s:compl:rev CACAGACACCGTCACTATCATGTTGACATTCC; dG = -15.3
101 >s_b_s_d_s_e_s_d_s_b_s GTGGCTGTGACGGTGCTGGTGACGGTGGCTGTG; >clamp_s_d_s_a_s:compl:rev CACAGACACCGTCACTATCATGTTGACATTCC; dG = -15.3
102 >s_b_s_e_s_a_s_d_s_b_s GTGGCTGTGCTGGTGTCTGTGACGGTGGCTGTG; >clamp_s_d_s_a_s:compl:rev CACAGACACCGTCACTATCATGTTGACATTCC; dG = -15.3
103 >s_b_s_e_s_b_s_d_s_b_s GTGGCTGTGCTGGTGGCTGTGACGGTGGCTGTG; >clamp_s_d_s_a_s:compl:rev CACAGACACCGTCACTATCATGTTGACATTCC; dG = -15.3
104 >s_a_s_a_s_e_s_d_s_b_s GTGTCTGTGTCTGTGCTGGTGACGGTGGCTGTG; >clamp_s_d_s_a_s:compl:rev CACAGACACCGTCACTATCATGTTGACATTCC; dG = -15.3
105 >s_b_s_e_s_c_s_d_s_b_s GTGGCTGTGCTGGTGTGGGTGACGGTGGCTGTG; >clamp_s_d_s_a_s:compl:rev CACAGACACCGTCACTATCATGTTGACATTCC; dG = -15.3
106 >s_b_s_e_s_d_s_d_s_b_s GTGGCTGTGCTGGTGACGGTGACGGTGGCTGTG; >clamp_s_d_s_a_s:compl:rev CACAGACACCGTCACTATCATGTTGACATTCC; dG = -15.3
107 >s_b_s_e_s_e_s_d_s_b_s GTGGCTGTGCTGGTGCTGGTGACGGTGGCTGTG; >clamp_s_d_s_a_s:compl:rev CACAGACACCGTCACTATCATGTTGACATTCC; dG = -15.3
108 >s_c_s_a_s_a_s_d_s_b_s GTGTGGGTGTCTGTGTCTGTGACGGTGGCTGTG; >clamp_s_d_s_a_s:compl:rev CACAGACACCGTCACTATCATGTTGACATTCC; dG = -15.3
109 >s_c_s_a_s_b_s_d_s_b_s GTGTGGGTGTCTGTGGCTGTGACGGTGGCTGTG; >clamp_s_d_s_a_s:compl:rev CACAGACACCGTCACTATCATGTTGACATTCC; dG = -15.3
110 >s_c_s_a_s_c_s_d_s_b_s GTGTGGGTGTCTGTGTGGGTGACGGTGGCTGTG; >clamp_s_d_s_a_s:compl:rev CACAGACACCGTCACTATCATGTTGACATTCC; dG = -15.3
111 >s_a_s_a_s_a_s_d_s_b_s GTGTCTGTGTCTGTGTCTGTGACGGTGGCTGTG; >clamp_s_d_s_a_s:compl:rev CACAGACACCGTCACTATCATGTTGACATTCC; dG = -15.3
112 >s_c_s_a_s_d_s_d_s_b_s GTGTGGGTGTCTGTGACGGTGACGGTGGCTGTG; >clamp_s_d_s_a_s:compl:rev CACAGACACCGTCACTATCATGTTGACATTCC; dG = -15.3
113 >s_c_s_a_s_e_s_d_s_b_s GTGTGGGTGTCTGTGCTGGTGACGGTGGCTGTG; >clamp_s_d_s_a_s:compl:rev CACAGACACCGTCACTATCATGTTGACATTCC; dG = -15.3
114 >s_c_s_b_s_a_s_d_s_b_s GTGTGGGTGGCTGTGTCTGTGACGGTGGCTGTG; >clamp_s_d_s_a_s:compl:rev CACAGACACCGTCACTATCATGTTGACATTCC; dG = -15.3
115 >s_c_s_b_s_b_s_d_s_b_s GTGTGGGTGGCTGTGGCTGTGACGGTGGCTGTG; >clamp_s_d_s_a_s:compl:rev CACAGACACCGTCACTATCATGTTGACATTCC; dG = -15.3
116 >s_a_s_b_s_a_s_d_s_b_s GTGTCTGTGGCTGTGTCTGTGACGGTGGCTGTG; >clamp_s_d_s_a_s:compl:rev CACAGACACCGTCACTATCATGTTGACATTCC; dG = -15.3
117 >s_c_s_b_s_c_s_d_s_b_s GTGTGGGTGGCTGTGTGGGTGACGGTGGCTGTG; >clamp_s_d_s_a_s:compl:rev CACAGACACCGTCACTATCATGTTGACATTCC; dG = -15.3
118 >s_c_s_b_s_d_s_d_s_b_s GTGTGGGTGGCTGTGACGGTGACGGTGGCTGTG; >clamp_s_d_s_a_s:compl:rev CACAGACACCGTCACTATCATGTTGACATTCC; dG = -15.3
119 >s_c_s_b_s_e_s_d_s_b_s GTGTGGGTGGCTGTGCTGGTGACGGTGGCTGTG; >clamp_s_d_s_a_s:compl:rev CACAGACACCGTCACTATCATGTTGACATTCC; dG = -15.3
120 >s_c_s_c_s_a_s_d_s_b_s GTGTGGGTGTGGGTGTCTGTGACGGTGGCTGTG; >clamp_s_d_s_a_s:compl:rev CACAGACACCGTCACTATCATGTTGACATTCC; dG = -15.3

121 >s_c_s_c_s_b_s_d_s_b_s GTGTGGGTGTGGGTGGCTGTGACGGTGGCTGTG; >clamp_s_d_s_a_s:compl:rev CACAGACACCGTCACTATCATGTTGACATTCC; dG = -15.3
122 >s_c_s_c_s_c_s_d_s_b_s GTGTGGGTGTGGGTGTGGGTGACGGTGGCTGTG; >clamp_s_d_s_a_s:compl:rev CACAGACACCGTCACTATCATGTTGACATTCC; dG = -15.3
123 >s_c_s_c_s_d_s_d_s_b_s GTGTGGGTGTGGGTGACGGTGACGGTGGCTGTG; >clamp_s_d_s_a_s:compl:rev CACAGACACCGTCACTATCATGTTGACATTCC; dG = -15.3
124 >s_c_s_c_s_e_s_d_s_b_s GTGTGGGTGTGGGTGCTGGTGACGGTGGCTGTG; >clamp_s_d_s_a_s:compl:rev CACAGACACCGTCACTATCATGTTGACATTCC; dG = -15.3
125 >s_c_s_d_s_c_s_d_s_b_s GTGTGGGTGACGGTGTGGGTGACGGTGGCTGTG; >clamp_s_d_s_a_s:compl:rev CACAGACACCGTCACTATCATGTTGACATTCC; dG = -15.3
126 >s_a_s_b_s_b_s_d_s_b_s GTGTCTGTGGCTGTGGCTGTGACGGTGGCTGTG; >clamp_s_d_s_a_s:compl:rev CACAGACACCGTCACTATCATGTTGACATTCC; dG = -15.3
127 >s_c_s_d_s_d_s_d_s_b_s GTGTGGGTGACGGTGACGGTGACGGTGGCTGTG; >clamp_s_d_s_a_s:compl:rev CACAGACACCGTCACTATCATGTTGACATTCC; dG = -15.3
128 >s_c_s_d_s_e_s_d_s_b_s GTGTGGGTGACGGTGCTGGTGACGGTGGCTGTG; >clamp_s_d_s_a_s:compl:rev CACAGACACCGTCACTATCATGTTGACATTCC; dG = -15.3
129 >s_c_s_e_s_a_s_d_s_b_s GTGTGGGTGCTGGTGTCTGTGACGGTGGCTGTG; >clamp_s_d_s_a_s:compl:rev CACAGACACCGTCACTATCATGTTGACATTCC; dG = -15.3
130 >s_c_s_e_s_b_s_d_s_b_s GTGTGGGTGCTGGTGGCTGTGACGGTGGCTGTG; >clamp_s_d_s_a_s:compl:rev CACAGACACCGTCACTATCATGTTGACATTCC; dG = -15.3
131 >s_c_s_e_s_c_s_d_s_b_s GTGTGGGTGCTGGTGTGGGTGACGGTGGCTGTG; >clamp_s_d_s_a_s:compl:rev CACAGACACCGTCACTATCATGTTGACATTCC; dG = -15.3
132 >s_c_s_e_s_d_s_d_s_b_s GTGTGGGTGCTGGTGACGGTGACGGTGGCTGTG; >clamp_s_d_s_a_s:compl:rev CACAGACACCGTCACTATCATGTTGACATTCC; dG = -15.3
133 >s_c_s_e_s_e_s_d_s_b_s GTGTGGGTGCTGGTGCTGGTGACGGTGGCTGTG; >clamp_s_d_s_a_s:compl:rev CACAGACACCGTCACTATCATGTTGACATTCC; dG = -15.3
134 >s_d_s_b_s_a_s_d_s_b_s GTGACGGTGGCTGTGTCTGTGACGGTGGCTGTG; >clamp_s_d_s_a_s:compl:rev CACAGACACCGTCACTATCATGTTGACATTCC; dG = -15.3
135 >s_d_s_b_s_b_s_d_s_b_s GTGACGGTGGCTGTGGCTGTGACGGTGGCTGTG; >clamp_s_d_s_a_s:compl:rev CACAGACACCGTCACTATCATGTTGACATTCC; dG = -15.3
136 >s_d_s_b_s_c_s_d_s_b_s GTGACGGTGGCTGTGTGGGTGACGGTGGCTGTG; >clamp_s_d_s_a_s:compl:rev CACAGACACCGTCACTATCATGTTGACATTCC; dG = -15.3
137 >s_a_s_b_s_c_s_d_s_b_s GTGTCTGTGGCTGTGTGGGTGACGGTGGCTGTG; >clamp_s_d_s_a_s:compl:rev CACAGACACCGTCACTATCATGTTGACATTCC; dG = -15.3
138 >s_d_s_b_s_e_s_d_s_b_s GTGACGGTGGCTGTGCTGGTGACGGTGGCTGTG; >clamp_s_d_s_a_s:compl:rev CACAGACACCGTCACTATCATGTTGACATTCC; dG = -15.3
139 >s_d_s_c_s_a_s_d_s_b_s GTGACGGTGTGGGTGTCTGTGACGGTGGCTGTG; >clamp_s_d_s_a_s:compl:rev CACAGACACCGTCACTATCATGTTGACATTCC; dG = -15.3
140 >s_d_s_c_s_b_s_d_s_b_s GTGACGGTGTGGGTGGCTGTGACGGTGGCTGTG; >clamp_s_d_s_a_s:compl:rev CACAGACACCGTCACTATCATGTTGACATTCC; dG = -15.3
141 >s_d_s_c_s_c_s_d_s_b_s GTGACGGTGTGGGTGTGGGTGACGGTGGCTGTG; >clamp_s_d_s_a_s:compl:rev CACAGACACCGTCACTATCATGTTGACATTCC; dG = -15.3
142 >s_d_s_c_s_d_s_d_s_b_s GTGACGGTGTGGGTGACGGTGACGGTGGCTGTG; >clamp_s_d_s_a_s:compl:rev CACAGACACCGTCACTATCATGTTGACATTCC; dG = -15.3
143 >s_d_s_c_s_e_s_d_s_b_s GTGACGGTGTGGGTGCTGGTGACGGTGGCTGTG; >clamp_s_d_s_a_s:compl:rev CACAGACACCGTCACTATCATGTTGACATTCC; dG = -15.3
144 >s_d_s_d_s_c_s_d_s_b_s GTGACGGTGACGGTGTGGGTGACGGTGGCTGTG; >clamp_s_d_s_a_s:compl:rev CACAGACACCGTCACTATCATGTTGACATTCC; dG = -15.3
145 >s_d_s_d_s_d_s_d_s_b_s GTGACGGTGACGGTGACGGTGACGGTGGCTGTG; >clamp_s_d_s_a_s:compl:rev CACAGACACCGTCACTATCATGTTGACATTCC; dG = -15.3
146 >s_d_s_d_s_e_s_d_s_b_s GTGACGGTGACGGTGCTGGTGACGGTGGCTGTG; >clamp_s_d_s_a_s:compl:rev CACAGACACCGTCACTATCATGTTGACATTCC; dG = -15.3
147 >s_a_s_b_s_d_s_d_s_b_s GTGTCTGTGGCTGTGACGGTGACGGTGGCTGTG; >clamp_s_d_s_a_s:compl:rev CACAGACACCGTCACTATCATGTTGACATTCC; dG = -15.3
148 >s_d_s_e_s_a_s_d_s_b_s GTGACGGTGCTGGTGTCTGTGACGGTGGCTGTG; >clamp_s_d_s_a_s:compl:rev CACAGACACCGTCACTATCATGTTGACATTCC; dG = -15.3
149 >s_d_s_e_s_b_s_d_s_b_s GTGACGGTGCTGGTGGCTGTGACGGTGGCTGTG; >clamp_s_d_s_a_s:compl:rev CACAGACACCGTCACTATCATGTTGACATTCC; dG = -15.3
150 >s_d_s_e_s_c_s_d_s_b_s GTGACGGTGCTGGTGTGGGTGACGGTGGCTGTG; >clamp_s_d_s_a_s:compl:rev CACAGACACCGTCACTATCATGTTGACATTCC; dG = -15.3
151 >s_d_s_e_s_d_s_d_s_b_s GTGACGGTGCTGGTGACGGTGACGGTGGCTGTG; >clamp_s_d_s_a_s:compl:rev CACAGACACCGTCACTATCATGTTGACATTCC; dG = -15.3
152 >s_d_s_e_s_e_s_d_s_b_s GTGACGGTGCTGGTGCTGGTGACGGTGGCTGTG; >clamp_s_d_s_a_s:compl:rev CACAGACACCGTCACTATCATGTTGACATTCC; dG = -15.3
153 >s_e_s_a_s_a_s_d_s_b_s GTGCTGGTGTCTGTGTCTGTGACGGTGGCTGTG; >clamp_s_d_s_a_s:compl:rev CACAGACACCGTCACTATCATGTTGACATTCC; dG = -15.3
154 >s_e_s_a_s_b_s_d_s_b_s GTGCTGGTGTCTGTGGCTGTGACGGTGGCTGTG; >clamp_s_d_s_a_s:compl:rev CACAGACACCGTCACTATCATGTTGACATTCC; dG = -15.3
155 >s_e_s_a_s_c_s_d_s_b_s GTGCTGGTGTCTGTGTGGGTGACGGTGGCTGTG; >clamp_s_d_s_a_s:compl:rev CACAGACACCGTCACTATCATGTTGACATTCC; dG = -15.3
156 >s_e_s_a_s_d_s_d_s_b_s GTGCTGGTGTCTGTGACGGTGACGGTGGCTGTG; >clamp_s_d_s_a_s:compl:rev CACAGACACCGTCACTATCATGTTGACATTCC; dG = -15.3
157 >s_a_s_b_s_e_s_d_s_b_s GTGTCTGTGGCTGTGCTGGTGACGGTGGCTGTG; >clamp_s_d_s_a_s:compl:rev CACAGACACCGTCACTATCATGTTGACATTCC; dG = -15.3

158 >s_e_s_a_s_e_s_d_s_b_s GTGCTGGTGTCTGTGCTGGTGACGGTGGCTGTG; >clamp_s_d_s_a_s:compl:rev
CACAGACACCGTCACTATCATGTTGACATTCC; dG = -15.3
159 >s_e_s_b_s_a_s_d_s_b_s GTGCTGGTGGCTGTGTCTGTGACGGTGGCTGTG; >clamp_s_d_s_a_s:compl:rev
CACAGACACCGTCACTATCATGTTGACATTCC; dG = -15.3
160 >s_e_s_b_s_b_s_d_s_b_s GTGCTGGTGGCTGTGGCTGTGACGGTGGCTGTG; >clamp_s_d_s_a_s:compl:rev
CACAGACACCGTCACTATCATGTTGACATTCC; dG = -15.3
161 >s_e_s_b_s_c_s_d_s_b_s GTGCTGGTGGCTGTGTGGGTGACGGTGGCTGTG; >clamp_s_d_s_a_s:compl:rev
CACAGACACCGTCACTATCATGTTGACATTCC; dG = -15.3
162 >s_e_s_b_s_d_s_d_s_b_s GTGCTGGTGGCTGTGACGGTGACGGTGGCTGTG; >clamp_s_d_s_a_s:compl:rev
CACAGACACCGTCACTATCATGTTGACATTCC; dG = -15.3
163 >s_e_s_b_s_e_s_d_s_b_s GTGCTGGTGGCTGTGCTGGTGACGGTGGCTGTG; >clamp_s_d_s_a_s:compl:rev
CACAGACACCGTCACTATCATGTTGACATTCC; dG = -15.3
164 >s_e_s_c_s_a_s_d_s_b_s GTGCTGGTGTGGGTGTCTGTGACGGTGGCTGTG; >clamp_s_d_s_a_s:compl:rev
CACAGACACCGTCACTATCATGTTGACATTCC; dG = -15.3
165 >s_e_s_c_s_b_s_d_s_b_s GTGCTGGTGTGGGTGGCTGTGACGGTGGCTGTG; >clamp_s_d_s_a_s:compl:rev
CACAGACACCGTCACTATCATGTTGACATTCC; dG = -15.3
166 >s_e_s_c_s_c_s_d_s_b_s GTGCTGGTGTGGGTGTGGGTGACGGTGGCTGTG; >clamp_s_d_s_a_s:compl:rev
CACAGACACCGTCACTATCATGTTGACATTCC; dG = -15.3
167 >s_e_s_c_s_d_s_d_s_b_s GTGCTGGTGTGGGTGACGGTGACGGTGGCTGTG; >clamp_s_d_s_a_s:compl:rev
CACAGACACCGTCACTATCATGTTGACATTCC; dG = -15.3
168 >s_a_s_c_s_a_s_d_s_b_s GTGTCTGTGTGGGTGTCTGTGACGGTGGCTGTG; >clamp_s_d_s_a_s:compl:rev
CACAGACACCGTCACTATCATGTTGACATTCC; dG = -15.3
169 >s_e_s_c_s_e_s_d_s_b_s GTGCTGGTGTGGGTGCTGGTGACGGTGGCTGTG; >clamp_s_d_s_a_s:compl:rev
CACAGACACCGTCACTATCATGTTGACATTCC; dG = -15.3
170 >s_e_s_d_s_c_s_d_s_b_s GTGCTGGTGACGGTGTGGGTGACGGTGGCTGTG; >clamp_s_d_s_a_s:compl:rev
CACAGACACCGTCACTATCATGTTGACATTCC; dG = -15.3
171 >s_e_s_d_s_d_s_d_s_b_s GTGCTGGTGACGGTGACGGTGACGGTGGCTGTG; >clamp_s_d_s_a_s:compl:rev
CACAGACACCGTCACTATCATGTTGACATTCC; dG = -15.3
172 >s_e_s_d_s_e_s_d_s_b_s GTGCTGGTGACGGTGCTGGTGACGGTGGCTGTG; >clamp_s_d_s_a_s:compl:rev
CACAGACACCGTCACTATCATGTTGACATTCC; dG = -15.3
173 >s_e_s_e_s_a_s_d_s_b_s GTGCTGGTGCTGGTGTCTGTGACGGTGGCTGTG; >clamp_s_d_s_a_s:compl:rev
CACAGACACCGTCACTATCATGTTGACATTCC; dG = -15.3
174 >s_e_s_e_s_b_s_d_s_b_s GTGCTGGTGCTGGTGGCTGTGACGGTGGCTGTG; >clamp_s_d_s_a_s:compl:rev
CACAGACACCGTCACTATCATGTTGACATTCC; dG = -15.3
175 >s_e_s_e_s_c_s_d_s_b_s GTGCTGGTGCTGGTGTGGGTGACGGTGGCTGTG; >clamp_s_d_s_a_s:compl:rev
CACAGACACCGTCACTATCATGTTGACATTCC; dG = -15.3
176 >s_e_s_e_s_d_s_d_s_b_s GTGCTGGTGCTGGTGACGGTGACGGTGGCTGTG; >clamp_s_d_s_a_s:compl:rev
CACAGACACCGTCACTATCATGTTGACATTCC; dG = -15.3
177 >s_e_s_e_s_e_s_d_s_b_s GTGCTGGTGCTGGTGCTGGTGACGGTGGCTGTG; >clamp_s_d_s_a_s:compl:rev
CACAGACACCGTCACTATCATGTTGACATTCC; dG = -15.3
178 >s_a_s_c_s_b_s_d_s_b_s GTGTCTGTGTGGGTGGCTGTGACGGTGGCTGTG; >clamp_s_d_s_a_s:compl:rev
CACAGACACCGTCACTATCATGTTGACATTCC; dG = -15.3
179 >s_a_s_c_s_c_s_d_s_b_s GTGTCTGTGTGGGTGTGGGTGACGGTGGCTGTG; >clamp_s_d_s_a_s:compl:rev
CACAGACACCGTCACTATCATGTTGACATTCC; dG = -15.3
180 >s_d_s_b_s_b_s_d_s_e_s GTGACGGTGGCTGTGGCTGTGACGGTGCTGGTG; >clamp_s_d_s_a_s:compl:rev
CACAGACACCGTCACTATCATGTTGACATTCC; dG = -15.2
181 >s_d_s_b_s_b_s_d_s_d_s GTGACGGTGGCTGTGGCTGTGACGGTGACGGTG; >clamp_s_d_s_a_s:compl:rev
CACAGACACCGTCACTATCATGTTGACATTCC; dG = -15.2
182 >s_d_s_b_s_b_s_d_s_c_s GTGACGGTGGCTGTGGCTGTGACGGTGTGGGTG; >clamp_s_d_s_a_s:compl:rev
CACAGACACCGTCACTATCATGTTGACATTCC; dG = -15.2
183 >s_d_s_b_s_b_s_e_s_a_s GTGACGGTGGCTGTGGCTGTGCTGGTGTCTGTG; >clamp_s_d_s_a_s:compl:rev
CACAGACACCGTCACTATCATGTTGACATTCC; dG = -15.2
184 >s_d_s_b_s_b_s_c_s_a_s GTGACGGTGGCTGTGGCTGTGTGGGTGTCTGTG; >clamp_s_d_s_a_s:compl:rev
CACAGACACCGTCACTATCATGTTGACATTCC; dG = -15.2
185 >s_d_s_b_s_b_s_b_s_a_s GTGACGGTGGCTGTGGCTGTGGCTGTGTCTGTG; >clamp_s_d_s_a_s:compl:rev
CACAGACACCGTCACTATCATGTTGACATTCC; dG = -15.2
186 >s_d_s_b_s_b_s_a_s_a_s GTGACGGTGGCTGTGGCTGTGTCTGTGTCTGTG; >clamp_s_d_s_a_s:compl:rev
CACAGACACCGTCACTATCATGTTGACATTCC; dG = -15.2
187 >s_d_s_b_s_a_s_d_s_e_s GTGACGGTGGCTGTGTCTGTGACGGTGCTGGTG; >clamp_s_d_s_a_s:compl:rev
CACAGACACCGTCACTATCATGTTGACATTCC; dG = -15.1
188 >s_d_s_b_s_a_s_d_s_d_s GTGACGGTGGCTGTGTCTGTGACGGTGACGGTG; >clamp_s_d_s_a_s:compl:rev
CACAGACACCGTCACTATCATGTTGACATTCC; dG = -15.1
189 >s_d_s_b_s_a_s_d_s_c_s GTGACGGTGGCTGTGTCTGTGACGGTGTGGGTG; >clamp_s_d_s_a_s:compl:rev
CACAGACACCGTCACTATCATGTTGACATTCC; dG = -15.1
190 >s_d_s_b_s_a_s_e_s_a_s GTGACGGTGGCTGTGTCTGTGCTGGTGTCTGTG; >clamp_s_d_s_a_s:compl:rev
CACAGACACCGTCACTATCATGTTGACATTCC; dG = -15.1
191 >s_d_s_b_s_a_s_c_s_a_s GTGACGGTGGCTGTGTCTGTGTGGGTGTCTGTG; >clamp_s_d_s_a_s:compl:rev
CACAGACACCGTCACTATCATGTTGACATTCC; dG = -15.1
192 >s_d_s_b_s_a_s_b_s_a_s GTGACGGTGGCTGTGTCTGTGGCTGTGTCTGTG; >clamp_s_d_s_a_s:compl:rev
CACAGACACCGTCACTATCATGTTGACATTCC; dG = -15.1
193 >s_d_s_b_s_a_s_a_s_a_s GTGACGGTGGCTGTGTCTGTGTCTGTGTCTGTG; >clamp_s_d_s_a_s:compl:rev
CACAGACACCGTCACTATCATGTTGACATTCC; dG = -15.1
194 >s_d_s_b_s_c_s_d_s_e_s GTGACGGTGGCTGTGTGGGTGACGGTGCTGGTG; >clamp_s_d_s_a_s:compl:rev
CACAGACACCGTCACTATCATGTTGACATTCC; dG = -15.1

195 >s_d_s_b_s_c_s_d_s_d_s GTGACGGTGGCTGTGTGGGTGACGGTGACGGTG; >clamp_s_d_s_a_s:compl:rev CACAGACACCGTCACTATCATGTTGACATTCC; dG = -15.1
196 >s_d_s_b_s_c_s_d_s_c_s GTGACGGTGGCTGTGTGGGTGACGGTGTGGGTG; >clamp_s_d_s_a_s:compl:rev CACAGACACCGTCACTATCATGTTGACATTCC; dG = -15.1
197 >s_d_s_b_s_c_s_e_s_a_s GTGACGGTGGCTGTGTGGGTGCTGGTGTCTGTG; >clamp_s_d_s_a_s:compl:rev CACAGACACCGTCACTATCATGTTGACATTCC; dG = -15.1
198 >s_d_s_b_s_c_s_c_s_a_s GTGACGGTGGCTGTGTGGGTGTGGGTGTCTGTG; >clamp_s_d_s_a_s:compl:rev CACAGACACCGTCACTATCATGTTGACATTCC; dG = -15.1
199 >s_d_s_b_s_c_s_b_s_a_s GTGACGGTGGCTGTGTGGGTGGCTGTGTCTGTG; >clamp_s_d_s_a_s:compl:rev CACAGACACCGTCACTATCATGTTGACATTCC; dG = -15.1
200 >s_d_s_b_s_c_s_a_s_a_s GTGACGGTGGCTGTGTGGGTGTCTGTGTCTGTG; >clamp_s_d_s_a_s:compl:rev CACAGACACCGTCACTATCATGTTGACATTCC; dG = -15.1
201 >s_d_s_b_s_e_s_d_s_e_s GTGACGGTGGCTGTGCTGGTGACGGTGCTGGTG; >clamp_s_d_s_a_s:compl:rev CACAGACACCGTCACTATCATGTTGACATTCC; dG = -14.9
202 >s_d_s_b_s_e_s_d_s_d_s GTGACGGTGGCTGTGCTGGTGACGGTGACGGTG; >clamp_s_d_s_a_s:compl:rev CACAGACACCGTCACTATCATGTTGACATTCC; dG = -14.9
203 >s_d_s_b_s_e_s_d_s_c_s GTGACGGTGGCTGTGCTGGTGACGGTGTGGGTG; >clamp_s_d_s_a_s:compl:rev CACAGACACCGTCACTATCATGTTGACATTCC; dG = -14.9
204 >s_d_s_b_s_e_s_e_s_a_s GTGACGGTGGCTGTGCTGGTGCTGGTGTCTGTG; >clamp_s_d_s_a_s:compl:rev CACAGACACCGTCACTATCATGTTGACATTCC; dG = -14.9
205 >s_d_s_b_s_e_s_c_s_a_s GTGACGGTGGCTGTGCTGGTGTGGGTGTCTGTG; >clamp_s_d_s_a_s:compl:rev CACAGACACCGTCACTATCATGTTGACATTCC; dG = -14.9
206 >s_d_s_b_s_e_s_b_s_a_s GTGACGGTGGCTGTGCTGGTGGCTGTGTCTGTG; >clamp_s_d_s_a_s:compl:rev CACAGACACCGTCACTATCATGTTGACATTCC; dG = -14.9
207 >s_d_s_b_s_e_s_a_s_a_s GTGACGGTGGCTGTGCTGGTGTCTGTGTCTGTG; >clamp_s_d_s_a_s:compl:rev CACAGACACCGTCACTATCATGTTGACATTCC; dG = -14.9
208 >s_a_s_d_s_c_s_d_s_e_s GTGTCTGTGACGGTGTGGGTGACGGTGCTGGTG; >clamp_s_d_s_a_s:compl:rev CACAGACACCGTCACTATCATGTTGACATTCC; dG = -12.5
209 >s_a_s_d_s_c_s_d_s_d_s GTGTCTGTGACGGTGTGGGTGACGGTGACGGTG; >clamp_s_d_s_a_s:compl:rev CACAGACACCGTCACTATCATGTTGACATTCC; dG = -12.5
210 >s_a_s_d_s_c_s_d_s_c_s GTGTCTGTGACGGTGTGGGTGACGGTGTGGGTG; >clamp_s_d_s_a_s:compl:rev CACAGACACCGTCACTATCATGTTGACATTCC; dG = -12.5
211 >s_b_s_d_s_c_s_d_s_e_s GTGGCTGTGACGGTGTGGGTGACGGTGCTGGTG; >clamp_s_d_s_a_s:compl:rev CACAGACACCGTCACTATCATGTTGACATTCC; dG = -12.5
212 >s_b_s_d_s_c_s_d_s_d_s GTGGCTGTGACGGTGTGGGTGACGGTGACGGTG; >clamp_s_d_s_a_s:compl:rev CACAGACACCGTCACTATCATGTTGACATTCC; dG = -12.5
213 >s_b_s_d_s_c_s_d_s_c_s GTGGCTGTGACGGTGTGGGTGACGGTGTGGGTG; >clamp_s_d_s_a_s:compl:rev CACAGACACCGTCACTATCATGTTGACATTCC; dG = -12.5
214 >s_c_s_d_s_c_s_d_s_e_s GTGTGGGTGACGGTGTGGGTGACGGTGCTGGTG; >clamp_s_d_s_a_s:compl:rev CACAGACACCGTCACTATCATGTTGACATTCC; dG = -12.4
215 >s_c_s_d_s_c_s_d_s_d_s GTGTGGGTGACGGTGTGGGTGACGGTGACGGTG; >clamp_s_d_s_a_s:compl:rev CACAGACACCGTCACTATCATGTTGACATTCC; dG = -12.4
216 >s_c_s_d_s_c_s_d_s_c_s GTGTGGGTGACGGTGTGGGTGACGGTGTGGGTG; >clamp_s_d_s_a_s:compl:rev CACAGACACCGTCACTATCATGTTGACATTCC; dG = -12.4
217 >s_d_s_d_s_c_s_d_s_e_s GTGACGGTGACGGTGTGGGTGACGGTGCTGGTG; >clamp_s_d_s_a_s:compl:rev CACAGACACCGTCACTATCATGTTGACATTCC; dG = -12.4
218 >s_d_s_d_s_c_s_d_s_d_s GTGACGGTGACGGTGTGGGTGACGGTGACGGTG; >clamp_s_d_s_a_s:compl:rev CACAGACACCGTCACTATCATGTTGACATTCC; dG = -12.4
219 >s_d_s_d_s_c_s_d_s_c_s GTGACGGTGACGGTGTGGGTGACGGTGTGGGTG; >clamp_s_d_s_a_s:compl:rev CACAGACACCGTCACTATCATGTTGACATTCC; dG = -12.4
220 >s_e_s_d_s_c_s_d_s_e_s GTGCTGGTGACGGTGTGGGTGACGGTGCTGGTG; >clamp_s_d_s_a_s:compl:rev CACAGACACCGTCACTATCATGTTGACATTCC; dG = -12.4
221 >s_e_s_d_s_c_s_d_s_d_s GTGCTGGTGACGGTGTGGGTGACGGTGACGGTG; >clamp_s_d_s_a_s:compl:rev CACAGACACCGTCACTATCATGTTGACATTCC; dG = -12.4
222 >s_e_s_d_s_c_s_d_s_c_s GTGCTGGTGACGGTGTGGGTGACGGTGTGGGTG; >clamp_s_d_s_a_s:compl:rev CACAGACACCGTCACTATCATGTTGACATTCC; dG = -12.4
223 >s_d_s_c_s_a_s_d_s_e_s GTGACGGTGTGGGTGTCTGTGACGGTGCTGGTG; >clamp_s_d_s_a_s:compl:rev CACAGACACCGTCACTATCATGTTGACATTCC; dG = -12.3
224 >s_d_s_c_s_a_s_d_s_d_s GTGACGGTGTGGGTGTCTGTGACGGTGACGGTG; >clamp_s_d_s_a_s:compl:rev CACAGACACCGTCACTATCATGTTGACATTCC; dG = -12.3
225 >s_d_s_c_s_a_s_d_s_c_s GTGACGGTGTGGGTGTCTGTGACGGTGTGGGTG; >clamp_s_d_s_a_s:compl:rev CACAGACACCGTCACTATCATGTTGACATTCC; dG = -12.3
226 >s_d_s_c_s_b_s_d_s_e_s GTGACGGTGTGGGTGGCTGTGACGGTGCTGGTG; >clamp_s_d_s_a_s:compl:rev CACAGACACCGTCACTATCATGTTGACATTCC; dG = -12.3
227 >s_d_s_c_s_b_s_d_s_d_s GTGACGGTGTGGGTGGCTGTGACGGTGACGGTG; >clamp_s_d_s_a_s:compl:rev CACAGACACCGTCACTATCATGTTGACATTCC; dG = -12.3
228 >s_d_s_c_s_b_s_d_s_c_s GTGACGGTGTGGGTGGCTGTGACGGTGTGGGTG; >clamp_s_d_s_a_s:compl:rev CACAGACACCGTCACTATCATGTTGACATTCC; dG = -12.3
229 >s_a_s_a_s_b_s_d_s_c_s GTGTCTGTGTCTGTGGCTGTGACGGTGTGGGTG; >clamp_s_d_s_a_s:compl:rev CACAGACACCGTCACTATCATGTTGACATTCC; dG = -12.2
230 >s_a_s_d_s_c_s_e_s_a_s GTGTCTGTGACGGTGTGGGTGCTGGTGTCTGTG; >clamp_s_d_s_a_s:compl:rev CACAGACACCGTCACTATCATGTTGACATTCC; dG = -12.2
231 >s_a_s_d_s_c_s_c_s_a_s GTGTCTGTGACGGTGTGGGTGTGGGTGTCTGTG; >clamp_s_d_s_a_s:compl:rev CACAGACACCGTCACTATCATGTTGACATTCC; dG = -12.2

232 >s_a_s_d_s_c_s_b_s_a_s GTGTCTGTGACGGTGTGGGTGGCTGTGTCTGTG; >clamp_s_d_s_a_s:compl:rev CACAGACACCGTCACTATCATGTTGACATTCC; dG = -12.2
233 >s_a_s_d_s_c_s_a_s_a_s GTGTCTGTGACGGTGTGGGTGTCTGTGTCTGTG; >clamp_s_d_s_a_s:compl:rev CACAGACACCGTCACTATCATGTTGACATTCC; dG = -12.2
234 >s_a_s_e_s_a_s_d_s_c_s GTGTCTGTGCTGGTGTCTGTGACGGTGTGGGTG; >clamp_s_d_s_a_s:compl:rev CACAGACACCGTCACTATCATGTTGACATTCC; dG = -12.2
235 >s_a_s_e_s_b_s_d_s_c_s GTGTCTGTGCTGGTGGCTGTGACGGTGTGGGTG; >clamp_s_d_s_a_s:compl:rev CACAGACACCGTCACTATCATGTTGACATTCC; dG = -12.2
236 >s_b_s_a_s_a_s_d_s_c_s GTGGCTGTGTCTGTGTCTGTGACGGTGTGGGTG; >clamp_s_d_s_a_s:compl:rev CACAGACACCGTCACTATCATGTTGACATTCC; dG = -12.2
237 >s_b_s_a_s_b_s_d_s_c_s GTGGCTGTGTCTGTGGCTGTGACGGTGTGGGTG; >clamp_s_d_s_a_s:compl:rev CACAGACACCGTCACTATCATGTTGACATTCC; dG = -12.2
238 >s_b_s_a_s_d_s_c_s_a_s GTGGCTGTGTCTGTGACGGTGTGGGTGTCTGTG; >clamp_s_d_s_a_s:compl:rev CACAGACACCGTCACTATCATGTTGACATTCC; dG = -12.2
239 >s_b_s_b_s_a_s_d_s_c_s GTGGCTGTGGCTGTGTCTGTGACGGTGTGGGTG; >clamp_s_d_s_a_s:compl:rev CACAGACACCGTCACTATCATGTTGACATTCC; dG = -12.2
240 >s_b_s_b_s_b_s_d_s_c_s GTGGCTGTGGCTGTGGCTGTGACGGTGTGGGTG; >clamp_s_d_s_a_s:compl:rev CACAGACACCGTCACTATCATGTTGACATTCC; dG = -12.2
241 >s_b_s_b_s_d_s_c_s_a_s GTGGCTGTGGCTGTGACGGTGTGGGTGTCTGTG; >clamp_s_d_s_a_s:compl:rev CACAGACACCGTCACTATCATGTTGACATTCC; dG = -12.2
242 >s_b_s_c_s_a_s_d_s_c_s GTGGCTGTGTGGGTGTCTGTGACGGTGTGGGTG; >clamp_s_d_s_a_s:compl:rev CACAGACACCGTCACTATCATGTTGACATTCC; dG = -12.2
243 >s_b_s_c_s_b_s_d_s_c_s GTGGCTGTGTGGGTGGCTGTGACGGTGTGGGTG; >clamp_s_d_s_a_s:compl:rev CACAGACACCGTCACTATCATGTTGACATTCC; dG = -12.2
244 >s_a_s_a_s_a_s_d_s_c_s GTGTCTGTGTCTGTGTCTGTGACGGTGTGGGTG; >clamp_s_d_s_a_s:compl:rev CACAGACACCGTCACTATCATGTTGACATTCC; dG = -12.2
245 >s_a_s_a_s_d_s_c_s_a_s GTGTCTGTGTCTGTGACGGTGTGGGTGTCTGTG; >clamp_s_d_s_a_s:compl:rev CACAGACACCGTCACTATCATGTTGACATTCC; dG = -12.2
246 >s_b_s_d_s_c_s_e_s_a_s GTGGCTGTGACGGTGTGGGTGCTGGTGTCTGTG; >clamp_s_d_s_a_s:compl:rev CACAGACACCGTCACTATCATGTTGACATTCC; dG = -12.2
247 >s_b_s_d_s_c_s_c_s_a_s GTGGCTGTGACGGTGTGGGTGTGGGTGTCTGTG; >clamp_s_d_s_a_s:compl:rev CACAGACACCGTCACTATCATGTTGACATTCC; dG = -12.2
248 >s_b_s_d_s_c_s_b_s_a_s GTGGCTGTGACGGTGTGGGTGGCTGTGTCTGTG; >clamp_s_d_s_a_s:compl:rev CACAGACACCGTCACTATCATGTTGACATTCC; dG = -12.2
249 >s_b_s_d_s_c_s_a_s_a_s GTGGCTGTGACGGTGTGGGTGTCTGTGTCTGTG; >clamp_s_d_s_a_s:compl:rev CACAGACACCGTCACTATCATGTTGACATTCC; dG = -12.2
250 >s_b_s_e_s_a_s_d_s_c_s GTGGCTGTGCTGGTGTCTGTGACGGTGTGGGTG; >clamp_s_d_s_a_s:compl:rev CACAGACACCGTCACTATCATGTTGACATTCC; dG = -12.2
251 >s_b_s_e_s_b_s_d_s_c_s GTGGCTGTGCTGGTGGCTGTGACGGTGTGGGTG; >clamp_s_d_s_a_s:compl:rev CACAGACACCGTCACTATCATGTTGACATTCC; dG = -12.2
252 >s_c_s_a_s_a_s_d_s_c_s GTGTGGGTGTCTGTGTCTGTGACGGTGTGGGTG; >clamp_s_d_s_a_s:compl:rev CACAGACACCGTCACTATCATGTTGACATTCC; dG = -12.2
253 >s_c_s_a_s_b_s_d_s_c_s GTGTGGGTGTCTGTGGCTGTGACGGTGTGGGTG; >clamp_s_d_s_a_s:compl:rev CACAGACACCGTCACTATCATGTTGACATTCC; dG = -12.2
254 >s_c_s_a_s_d_s_c_s_a_s GTGTGGGTGTCTGTGACGGTGTGGGTGTCTGTG; >clamp_s_d_s_a_s:compl:rev CACAGACACCGTCACTATCATGTTGACATTCC; dG = -12.2
255 >s_c_s_b_s_a_s_d_s_c_s GTGTGGGTGGCTGTGTCTGTGACGGTGTGGGTG; >clamp_s_d_s_a_s:compl:rev CACAGACACCGTCACTATCATGTTGACATTCC; dG = -12.2
256 >s_a_s_b_s_a_s_d_s_c_s GTGTCTGTGGCTGTGTCTGTGACGGTGTGGGTG; >clamp_s_d_s_a_s:compl:rev CACAGACACCGTCACTATCATGTTGACATTCC; dG = -12.2
257 >s_c_s_b_s_b_s_d_s_c_s GTGTGGGTGGCTGTGGCTGTGACGGTGTGGGTG; >clamp_s_d_s_a_s:compl:rev CACAGACACCGTCACTATCATGTTGACATTCC; dG = -12.2
258 >s_c_s_b_s_d_s_c_s_a_s GTGTGGGTGGCTGTGACGGTGTGGGTGTCTGTG; >clamp_s_d_s_a_s:compl:rev CACAGACACCGTCACTATCATGTTGACATTCC; dG = -12.2
259 >s_c_s_c_s_a_s_d_s_c_s GTGTGGGTGTGGGTGTCTGTGACGGTGTGGGTG; >clamp_s_d_s_a_s:compl:rev CACAGACACCGTCACTATCATGTTGACATTCC; dG = -12.2
260 >s_c_s_c_s_b_s_d_s_c_s GTGTGGGTGTGGGTGGCTGTGACGGTGTGGGTG; >clamp_s_d_s_a_s:compl:rev CACAGACACCGTCACTATCATGTTGACATTCC; dG = -12.2
261 >s_a_s_b_s_b_s_d_s_c_s GTGTCTGTGGCTGTGGCTGTGACGGTGTGGGTG; >clamp_s_d_s_a_s:compl:rev CACAGACACCGTCACTATCATGTTGACATTCC; dG = -12.2
262 >s_c_s_d_s_c_s_b_s_a_s GTGTGGGTGACGGTGTGGGTGGCTGTGTCTGTG; >clamp_s_d_s_a_s:compl:rev CACAGACACCGTCACTATCATGTTGACATTCC; dG = -12.2
263 >s_c_s_d_s_c_s_a_s_a_s GTGTGGGTGACGGTGTGGGTGTCTGTGTCTGTG; >clamp_s_d_s_a_s:compl:rev CACAGACACCGTCACTATCATGTTGACATTCC; dG = -12.2
264 >s_c_s_e_s_a_s_d_s_c_s GTGTGGGTGCTGGTGTCTGTGACGGTGTGGGTG; >clamp_s_d_s_a_s:compl:rev CACAGACACCGTCACTATCATGTTGACATTCC; dG = -12.2
265 >s_c_s_e_s_b_s_d_s_c_s GTGTGGGTGCTGGTGGCTGTGACGGTGTGGGTG; >clamp_s_d_s_a_s:compl:rev CACAGACACCGTCACTATCATGTTGACATTCC; dG = -12.2
266 >s_d_s_d_s_c_s_b_s_a_s GTGACGGTGACGGTGTGGGTGGCTGTGTCTGTG; >clamp_s_d_s_a_s:compl:rev CACAGACACCGTCACTATCATGTTGACATTCC; dG = -12.2
267 >s_d_s_d_s_c_s_a_s_a_s GTGACGGTGACGGTGTGGGTGTCTGTGTCTGTG; >clamp_s_d_s_a_s:compl:rev CACAGACACCGTCACTATCATGTTGACATTCC; dG = -12.2
268 >s_d_s_e_s_a_s_d_s_c_s GTGACGGTGCTGGTGTCTGTGACGGTGTGGGTG; >clamp_s_d_s_a_s:compl:rev CACAGACACCGTCACTATCATGTTGACATTCC; dG = -12.2

269 >s_d_s_e_s_b_s_d_s_c_s GTGACGGTGCTGGTGGCTGTGACGGTGTGGGTG; >clamp_s_d_s_a_s:compl:rev
CACAGACACCGTCACTATCATGTTGACATTCC; dG = -12.2
270 >s_a_s_b_s_d_s_c_s_a_s GTGTCTGTGGCTGTGACGGTGTGGGTGTCTGTG; >clamp_s_d_s_a_s:compl:rev
CACAGACACCGTCACTATCATGTTGACATTCC; dG = -12.2
271 >s_e_s_a_s_a_s_d_s_c_s GTGCTGGTGTCTGTGTCTGTGACGGTGTGGGTG; >clamp_s_d_s_a_s:compl:rev
CACAGACACCGTCACTATCATGTTGACATTCC; dG = -12.2
272 >s_e_s_a_s_b_s_d_s_c_s GTGCTGGTGTCTGTGGCTGTGACGGTGTGGGTG; >clamp_s_d_s_a_s:compl:rev
CACAGACACCGTCACTATCATGTTGACATTCC; dG = -12.2
273 >s_e_s_a_s_d_s_c_s_a_s GTGCTGGTGTCTGTGACGGTGTGGGTGTCTGTG; >clamp_s_d_s_a_s:compl:rev
CACAGACACCGTCACTATCATGTTGACATTCC; dG = -12.2
274 >s_e_s_b_s_a_s_d_s_c_s GTGCTGGTGGCTGTGTCTGTGACGGTGTGGGTG; >clamp_s_d_s_a_s:compl:rev
CACAGACACCGTCACTATCATGTTGACATTCC; dG = -12.2
275 >s_e_s_b_s_b_s_d_s_c_s GTGCTGGTGGCTGTGGCTGTGACGGTGTGGGTG; >clamp_s_d_s_a_s:compl:rev
CACAGACACCGTCACTATCATGTTGACATTCC; dG = -12.2
276 >s_e_s_b_s_d_s_c_s_a_s GTGCTGGTGGCTGTGACGGTGTGGGTGTCTGTG; >clamp_s_d_s_a_s:compl:rev
CACAGACACCGTCACTATCATGTTGACATTCC; dG = -12.2
277 >s_e_s_c_s_a_s_d_s_c_s GTGCTGGTGTGGGTGTCTGTGACGGTGTGGGTG; >clamp_s_d_s_a_s:compl:rev
CACAGACACCGTCACTATCATGTTGACATTCC; dG = -12.2
278 >s_e_s_c_s_b_s_d_s_c_s GTGCTGGTGTGGGTGGCTGTGACGGTGTGGGTG; >clamp_s_d_s_a_s:compl:rev
CACAGACACCGTCACTATCATGTTGACATTCC; dG = -12.2
279 >s_a_s_c_s_a_s_d_s_c_s GTGTCTGTGTGGGTGTCTGTGACGGTGTGGGTG; >clamp_s_d_s_a_s:compl:rev
CACAGACACCGTCACTATCATGTTGACATTCC; dG = -12.2
280 >s_e_s_d_s_c_s_b_s_a_s GTGCTGGTGACGGTGTGGGTGGCTGTGTCTGTG; >clamp_s_d_s_a_s:compl:rev
CACAGACACCGTCACTATCATGTTGACATTCC; dG = -12.2
281 >s_e_s_d_s_c_s_a_s_a_s GTGCTGGTGACGGTGTGGGTGTCTGTGTCTGTG; >clamp_s_d_s_a_s:compl:rev
CACAGACACCGTCACTATCATGTTGACATTCC; dG = -12.2
282 >s_e_s_e_s_a_s_d_s_c_s GTGCTGGTGCTGGTGTCTGTGACGGTGTGGGTG; >clamp_s_d_s_a_s:compl:rev
CACAGACACCGTCACTATCATGTTGACATTCC; dG = -12.2
283 >s_e_s_e_s_b_s_d_s_c_s GTGCTGGTGCTGGTGGCTGTGACGGTGTGGGTG; >clamp_s_d_s_a_s:compl:rev
CACAGACACCGTCACTATCATGTTGACATTCC; dG = -12.2
284 >s_a_s_c_s_b_s_d_s_c_s GTGTCTGTGTGGGTGGCTGTGACGGTGTGGGTG; >clamp_s_d_s_a_s:compl:rev
CACAGACACCGTCACTATCATGTTGACATTCC; dG = -12.2
285 >s_a_s_c_s_d_s_d_s_c_s GTGTCTGTGTGGGTGACGGTGACGGTGTGGGTG; >clamp_s_d_s_a_s:compl:rev
CACAGACACCGTCACTATCATGTTGACATTCC; dG = -12.1
286 >s_a_s_c_s_d_s_c_s_a_s GTGTCTGTGTGGGTGACGGTGTGGGTGTCTGTG; >clamp_s_d_s_a_s:compl:rev
CACAGACACCGTCACTATCATGTTGACATTCC; dG = -12.1
287 >s_a_s_c_s_e_s_d_s_c_s GTGTCTGTGTGGGTGCTGGTGACGGTGTGGGTG; >clamp_s_d_s_a_s:compl:rev
CACAGACACCGTCACTATCATGTTGACATTCC; dG = -12.1
288 >s_a_s_d_s_d_s_d_s_c_s GTGTCTGTGACGGTGACGGTGACGGTGTGGGTG; >clamp_s_d_s_a_s:compl:rev
CACAGACACCGTCACTATCATGTTGACATTCC; dG = -12.1
289 >s_a_s_d_s_d_s_c_s_a_s GTGTCTGTGACGGTGACGGTGTGGGTGTCTGTG; >clamp_s_d_s_a_s:compl:rev
CACAGACACCGTCACTATCATGTTGACATTCC; dG = -12.1
290 >s_a_s_d_s_e_s_d_s_c_s GTGTCTGTGACGGTGCTGGTGACGGTGTGGGTG; >clamp_s_d_s_a_s:compl:rev
CACAGACACCGTCACTATCATGTTGACATTCC; dG = -12.1
291 >s_a_s_d_s_e_s_a_s_a_s GTGTCTGTGACGGTGCTGGTGTCTGTGTCTGTG; >clamp_s_d_s_a_s:compl:rev
CACAGACACCGTCACTATCATGTTGACATTCC; dG = -12.1
292 >s_a_s_e_s_c_s_d_s_c_s GTGTCTGTGCTGGTGTGGGTGACGGTGTGGGTG; >clamp_s_d_s_a_s:compl:rev
CACAGACACCGTCACTATCATGTTGACATTCC; dG = -12.1
293 >s_a_s_e_s_d_s_d_s_c_s GTGTCTGTGCTGGTGACGGTGACGGTGTGGGTG; >clamp_s_d_s_a_s:compl:rev
CACAGACACCGTCACTATCATGTTGACATTCC; dG = -12.1
294 >s_a_s_e_s_d_s_c_s_a_s GTGTCTGTGCTGGTGACGGTGTGGGTGTCTGTG; >clamp_s_d_s_a_s:compl:rev
CACAGACACCGTCACTATCATGTTGACATTCC; dG = -12.1
295 >s_a_s_e_s_e_s_d_s_c_s GTGTCTGTGCTGGTGCTGGTGACGGTGTGGGTG; >clamp_s_d_s_a_s:compl:rev
CACAGACACCGTCACTATCATGTTGACATTCC; dG = -12.1
296 >s_a_s_a_s_c_s_d_s_c_s GTGTCTGTGTCTGTGTGGGTGACGGTGTGGGTG; >clamp_s_d_s_a_s:compl:rev
CACAGACACCGTCACTATCATGTTGACATTCC; dG = -12.1
297 >s_b_s_a_s_c_s_d_s_c_s GTGGCTGTGTCTGTGTGGGTGACGGTGTGGGTG; >clamp_s_d_s_a_s:compl:rev
CACAGACACCGTCACTATCATGTTGACATTCC; dG = -12.1
298 >s_b_s_a_s_d_s_d_s_c_s GTGGCTGTGTCTGTGACGGTGACGGTGTGGGTG; >clamp_s_d_s_a_s:compl:rev
CACAGACACCGTCACTATCATGTTGACATTCC; dG = -12.1
299 >s_b_s_a_s_e_s_d_s_c_s GTGGCTGTGTCTGTGCTGGTGACGGTGTGGGTG; >clamp_s_d_s_a_s:compl:rev
CACAGACACCGTCACTATCATGTTGACATTCC; dG = -12.1
300 >s_b_s_b_s_c_s_d_s_c_s GTGGCTGTGGCTGTGTGGGTGACGGTGTGGGTG; >clamp_s_d_s_a_s:compl:rev
CACAGACACCGTCACTATCATGTTGACATTCC; dG = -12.1
301 >s_b_s_b_s_d_s_d_s_c_s GTGGCTGTGGCTGTGACGGTGACGGTGTGGGTG; >clamp_s_d_s_a_s:compl:rev
CACAGACACCGTCACTATCATGTTGACATTCC; dG = -12.1
302 >s_b_s_b_s_e_s_d_s_c_s GTGGCTGTGGCTGTGCTGGTGACGGTGTGGGTG; >clamp_s_d_s_a_s:compl:rev
CACAGACACCGTCACTATCATGTTGACATTCC; dG = -12.1
303 >s_a_s_a_s_d_s_d_s_c_s GTGTCTGTGTCTGTGACGGTGACGGTGTGGGTG; >clamp_s_d_s_a_s:compl:rev
CACAGACACCGTCACTATCATGTTGACATTCC; dG = -12.1
304 >s_b_s_c_s_c_s_d_s_c_s GTGGCTGTGTGGGTGTGGGTGACGGTGTGGGTG; >clamp_s_d_s_a_s:compl:rev
CACAGACACCGTCACTATCATGTTGACATTCC; dG = -12.1
305 >s_b_s_c_s_d_s_d_s_c_s GTGGCTGTGTGGGTGACGGTGACGGTGTGGGTG; >clamp_s_d_s_a_s:compl:rev
CACAGACACCGTCACTATCATGTTGACATTCC; dG = -12.1

306 >s_b_s_c_s_d_s_c_s_a_s GTGGCTGTGTGGGTGACGGTGTGGGTGTCTGTG; >clamp_s_d_s_a_s:compl:rev
CACAGACACCGTCACTATCATGTTGACATTCC; dG = -12.1
307 >s_b_s_c_s_e_s_d_s_c_s GTGGCTGTGTGGGTGCTGGTGACGGTGTGGGTG; >clamp_s_d_s_a_s:compl:rev
CACAGACACCGTCACTATCATGTTGACATTCC; dG = -12.1
308 >s_b_s_d_s_d_s_d_s_c_s GTGGCTGTGACGGTGACGGTGACGGTGTGGGTG; >clamp_s_d_s_a_s:compl:rev
CACAGACACCGTCACTATCATGTTGACATTCC; dG = -12.1
309 >s_b_s_d_s_d_s_c_s_a_s GTGGCTGTGACGGTGACGGTGTGGGTGTCTGTG; >clamp_s_d_s_a_s:compl:rev
CACAGACACCGTCACTATCATGTTGACATTCC; dG = -12.1
310 >s_b_s_d_s_e_s_d_s_c_s GTGGCTGTGACGGTGCTGGTGACGGTGTGGGTG; >clamp_s_d_s_a_s:compl:rev
CACAGACACCGTCACTATCATGTTGACATTCC; dG = -12.1
311 >s_b_s_d_s_e_s_a_s_a_s GTGGCTGTGACGGTGCTGGTGTCTGTGTCTGTG; >clamp_s_d_s_a_s:compl:rev
CACAGACACCGTCACTATCATGTTGACATTCC; dG = -12.1
312 >s_a_s_a_s_e_s_d_s_c_s GTGTCTGTGTCTGTGCTGGTGACGGTGTGGGTG; >clamp_s_d_s_a_s:compl:rev
CACAGACACCGTCACTATCATGTTGACATTCC; dG = -12.1
313 >s_b_s_e_s_c_s_d_s_c_s GTGGCTGTGCTGGTGTGGGTGACGGTGTGGGTG; >clamp_s_d_s_a_s:compl:rev
CACAGACACCGTCACTATCATGTTGACATTCC; dG = -12.1
314 >s_b_s_e_s_d_s_d_s_c_s GTGGCTGTGCTGGTGACGGTGACGGTGTGGGTG; >clamp_s_d_s_a_s:compl:rev
CACAGACACCGTCACTATCATGTTGACATTCC; dG = -12.1
315 >s_b_s_e_s_d_s_c_s_a_s GTGGCTGTGCTGGTGACGGTGTGGGTGTCTGTG; >clamp_s_d_s_a_s:compl:rev
CACAGACACCGTCACTATCATGTTGACATTCC; dG = -12.1
316 >s_b_s_e_s_e_s_d_s_c_s GTGGCTGTGCTGGTGCTGGTGACGGTGTGGGTG; >clamp_s_d_s_a_s:compl:rev
CACAGACACCGTCACTATCATGTTGACATTCC; dG = -12.1
317 >s_c_s_a_s_c_s_d_s_c_s GTGTGGGTGTCTGTGTGGGTGACGGTGTGGGTG; >clamp_s_d_s_a_s:compl:rev
CACAGACACCGTCACTATCATGTTGACATTCC; dG = -12.1
318 >s_c_s_a_s_d_s_d_s_c_s GTGTGGGTGTCTGTGACGGTGACGGTGTGGGTG; >clamp_s_d_s_a_s:compl:rev
CACAGACACCGTCACTATCATGTTGACATTCC; dG = -12.1
319 >s_c_s_a_s_e_s_d_s_c_s GTGTGGGTGTCTGTGCTGGTGACGGTGTGGGTG; >clamp_s_d_s_a_s:compl:rev
CACAGACACCGTCACTATCATGTTGACATTCC; dG = -12.1
320 >s_c_s_b_s_c_s_d_s_c_s GTGTGGGTGGCTGTGTGGGTGACGGTGTGGGTG; >clamp_s_d_s_a_s:compl:rev
CACAGACACCGTCACTATCATGTTGACATTCC; dG = -12.1
321 >s_c_s_b_s_d_s_d_s_c_s GTGTGGGTGGCTGTGACGGTGACGGTGTGGGTG; >clamp_s_d_s_a_s:compl:rev
CACAGACACCGTCACTATCATGTTGACATTCC; dG = -12.1
322 >s_c_s_b_s_e_s_d_s_c_s GTGTGGGTGGCTGTGCTGGTGACGGTGTGGGTG; >clamp_s_d_s_a_s:compl:rev
CACAGACACCGTCACTATCATGTTGACATTCC; dG = -12.1
323 >s_c_s_c_s_c_s_d_s_c_s GTGTGGGTGTGGGTGTGGGTGACGGTGTGGGTG; >clamp_s_d_s_a_s:compl:rev
CACAGACACCGTCACTATCATGTTGACATTCC; dG = -12.1
324 >s_c_s_c_s_d_s_d_s_c_s GTGTGGGTGTGGGTGACGGTGACGGTGTGGGTG; >clamp_s_d_s_a_s:compl:rev
CACAGACACCGTCACTATCATGTTGACATTCC; dG = -12.1
325 >s_c_s_c_s_d_s_c_s_a_s GTGTGGGTGTGGGTGACGGTGTGGGTGTCTGTG; >clamp_s_d_s_a_s:compl:rev
CACAGACACCGTCACTATCATGTTGACATTCC; dG = -12.1
326 >s_c_s_c_s_e_s_d_s_c_s GTGTGGGTGTGGGTGCTGGTGACGGTGTGGGTG; >clamp_s_d_s_a_s:compl:rev
CACAGACACCGTCACTATCATGTTGACATTCC; dG = -12.1
327 >s_c_s_d_s_c_s_e_s_a_s GTGTGGGTGACGGTGTGGGTGCTGGTGTCTGTG; >clamp_s_d_s_a_s:compl:rev
CACAGACACCGTCACTATCATGTTGACATTCC; dG = -12.1
328 >s_c_s_d_s_c_s_c_s_a_s GTGTGGGTGACGGTGTGGGTGTGGGTGTCTGTG; >clamp_s_d_s_a_s:compl:rev
CACAGACACCGTCACTATCATGTTGACATTCC; dG = -12.1
329 >s_c_s_d_s_d_s_d_s_c_s GTGTGGGTGACGGTGACGGTGACGGTGTGGGTG; >clamp_s_d_s_a_s:compl:rev
CACAGACACCGTCACTATCATGTTGACATTCC; dG = -12.1
330 >s_c_s_d_s_d_s_c_s_a_s GTGTGGGTGACGGTGACGGTGTGGGTGTCTGTG; >clamp_s_d_s_a_s:compl:rev
CACAGACACCGTCACTATCATGTTGACATTCC; dG = -12.1
331 >s_c_s_d_s_e_s_d_s_c_s GTGTGGGTGACGGTGCTGGTGACGGTGTGGGTG; >clamp_s_d_s_a_s:compl:rev
CACAGACACCGTCACTATCATGTTGACATTCC; dG = -12.1
332 >s_c_s_e_s_c_s_d_s_c_s GTGTGGGTGCTGGTGTGGGTGACGGTGTGGGTG; >clamp_s_d_s_a_s:compl:rev
CACAGACACCGTCACTATCATGTTGACATTCC; dG = -12.1
333 >s_c_s_e_s_d_s_d_s_c_s GTGTGGGTGCTGGTGACGGTGACGGTGTGGGTG; >clamp_s_d_s_a_s:compl:rev
CACAGACACCGTCACTATCATGTTGACATTCC; dG = -12.1
334 >s_c_s_e_s_d_s_c_s_a_s GTGTGGGTGCTGGTGACGGTGTGGGTGTCTGTG; >clamp_s_d_s_a_s:compl:rev
CACAGACACCGTCACTATCATGTTGACATTCC; dG = -12.1
335 >s_c_s_e_s_e_s_d_s_c_s GTGTGGGTGCTGGTGCTGGTGACGGTGTGGGTG; >clamp_s_d_s_a_s:compl:rev
CACAGACACCGTCACTATCATGTTGACATTCC; dG = -12.1
336 >s_a_s_b_s_c_s_d_s_c_s GTGTCTGTGGCTGTGTGGGTGACGGTGTGGGTG; >clamp_s_d_s_a_s:compl:rev
CACAGACACCGTCACTATCATGTTGACATTCC; dG = -12.1
337 >s_d_s_c_s_c_s_d_s_c_s GTGACGGTGTGGGTGTGGGTGACGGTGTGGGTG; >clamp_s_d_s_a_s:compl:rev
CACAGACACCGTCACTATCATGTTGACATTCC; dG = -12.1
338 >s_d_s_c_s_d_s_d_s_c_s GTGACGGTGTGGGTGACGGTGACGGTGTGGGTG; >clamp_s_d_s_a_s:compl:rev
CACAGACACCGTCACTATCATGTTGACATTCC; dG = -12.1
339 >s_d_s_c_s_d_s_c_s_a_s GTGACGGTGTGGGTGACGGTGTGGGTGTCTGTG; >clamp_s_d_s_a_s:compl:rev
CACAGACACCGTCACTATCATGTTGACATTCC; dG = -12.1
340 >s_d_s_c_s_e_s_d_s_c_s GTGACGGTGTGGGTGCTGGTGACGGTGTGGGTG; >clamp_s_d_s_a_s:compl:rev
CACAGACACCGTCACTATCATGTTGACATTCC; dG = -12.1
341 >s_d_s_d_s_c_s_e_s_a_s GTGACGGTGACGGTGTGGGTGCTGGTGTCTGTG; >clamp_s_d_s_a_s:compl:rev
CACAGACACCGTCACTATCATGTTGACATTCC; dG = -12.1
342 >s_d_s_d_s_c_s_c_s_a_s GTGACGGTGACGGTGTGGGTGTGGGTGTCTGTG; >clamp_s_d_s_a_s:compl:rev
CACAGACACCGTCACTATCATGTTGACATTCC; dG = -12.1

343 >s_d_s_d_s_d_s_d_s_c_s GTGACGGTGACGGTGACGGTGACGGTGTGGGTG; >clamp_s_d_s_a_s:compl:rev
CACAGACACCGTCACTATCATGTTGACATTCC; dG = -12.1
344 >s_a_s_b_s_d_s_d_s_c_s GTGTCTGTGGCTGTGACGGTGACGGTGTGGGTG; >clamp_s_d_s_a_s:compl:rev
CACAGACACCGTCACTATCATGTTGACATTCC; dG = -12.1
345 >s_d_s_d_s_d_s_c_s_a_s GTGACGGTGACGGTGACGGTGTGGGTGTCTGTG; >clamp_s_d_s_a_s:compl:rev
CACAGACACCGTCACTATCATGTTGACATTCC; dG = -12.1
346 >s_d_s_d_s_e_s_d_s_c_s GTGACGGTGACGGTGCTGGTGACGGTGTGGGTG; >clamp_s_d_s_a_s:compl:rev
CACAGACACCGTCACTATCATGTTGACATTCC; dG = -12.1
347 >s_d_s_e_s_a_s_d_s_e_s GTGACGGTGCTGGTGTCTGTGACGGTGCTGGTG; >clamp_s_d_s_a_s:compl:rev
CACAGACACCGTCACTATCATGTTGACATTCC; dG = -12.1
348 >s_d_s_e_s_a_s_d_s_d_s GTGACGGTGCTGGTGTCTGTGACGGTGACGGTG; >clamp_s_d_s_a_s:compl:rev
CACAGACACCGTCACTATCATGTTGACATTCC; dG = -12.1
349 >s_d_s_e_s_c_s_d_s_c_s GTGACGGTGCTGGTGTGGGTGACGGTGTGGGTG; >clamp_s_d_s_a_s:compl:rev
CACAGACACCGTCACTATCATGTTGACATTCC; dG = -12.1
350 >s_d_s_e_s_d_s_d_s_c_s GTGACGGTGCTGGTGACGGTGACGGTGTGGGTG; >clamp_s_d_s_a_s:compl:rev
CACAGACACCGTCACTATCATGTTGACATTCC; dG = -12.1
351 >s_d_s_e_s_d_s_c_s_a_s GTGACGGTGCTGGTGACGGTGTGGGTGTCTGTG; >clamp_s_d_s_a_s:compl:rev
CACAGACACCGTCACTATCATGTTGACATTCC; dG = -12.1
352 >s_d_s_e_s_e_s_d_s_c_s GTGACGGTGCTGGTGCTGGTGACGGTGTGGGTG; >clamp_s_d_s_a_s:compl:rev
CACAGACACCGTCACTATCATGTTGACATTCC; dG = -12.1
353 >s_e_s_a_s_c_s_d_s_c_s GTGCTGGTGTCTGTGTGGGTGACGGTGTGGGTG; >clamp_s_d_s_a_s:compl:rev
CACAGACACCGTCACTATCATGTTGACATTCC; dG = -12.1
354 >s_a_s_b_s_e_s_d_s_c_s GTGTCTGTGGCTGTGCTGGTGACGGTGTGGGTG; >clamp_s_d_s_a_s:compl:rev
CACAGACACCGTCACTATCATGTTGACATTCC; dG = -12.1
355 >s_e_s_a_s_d_s_d_s_c_s GTGCTGGTGTCTGTGACGGTGACGGTGTGGGTG; >clamp_s_d_s_a_s:compl:rev
CACAGACACCGTCACTATCATGTTGACATTCC; dG = -12.1
356 >s_e_s_a_s_e_s_d_s_c_s GTGCTGGTGTCTGTGCTGGTGACGGTGTGGGTG; >clamp_s_d_s_a_s:compl:rev
CACAGACACCGTCACTATCATGTTGACATTCC; dG = -12.1
357 >s_e_s_b_s_c_s_d_s_c_s GTGCTGGTGGCTGTGTGGGTGACGGTGTGGGTG; >clamp_s_d_s_a_s:compl:rev
CACAGACACCGTCACTATCATGTTGACATTCC; dG = -12.1
358 >s_e_s_b_s_d_s_d_s_c_s GTGCTGGTGGCTGTGACGGTGACGGTGTGGGTG; >clamp_s_d_s_a_s:compl:rev
CACAGACACCGTCACTATCATGTTGACATTCC; dG = -12.1
359 >s_e_s_b_s_e_s_d_s_c_s GTGCTGGTGGCTGTGCTGGTGACGGTGTGGGTG; >clamp_s_d_s_a_s:compl:rev
CACAGACACCGTCACTATCATGTTGACATTCC; dG = -12.1
360 >s_e_s_c_s_c_s_d_s_c_s GTGCTGGTGTGGGTGTGGGTGACGGTGTGGGTG; >clamp_s_d_s_a_s:compl:rev
CACAGACACCGTCACTATCATGTTGACATTCC; dG = -12.1
361 >s_e_s_c_s_d_s_d_s_c_s GTGCTGGTGTGGGTGACGGTGACGGTGTGGGTG; >clamp_s_d_s_a_s:compl:rev
CACAGACACCGTCACTATCATGTTGACATTCC; dG = -12.1
362 >s_e_s_c_s_d_s_c_s_a_s GTGCTGGTGTGGGTGACGGTGTGGGTGTCTGTG; >clamp_s_d_s_a_s:compl:rev
CACAGACACCGTCACTATCATGTTGACATTCC; dG = -12.1
363 >s_e_s_c_s_e_s_d_s_c_s GTGCTGGTGTGGGTGCTGGTGACGGTGTGGGTG; >clamp_s_d_s_a_s:compl:rev
CACAGACACCGTCACTATCATGTTGACATTCC; dG = -12.1
364 >s_e_s_d_s_c_s_e_s_a_s GTGCTGGTGACGGTGTGGGTGCTGGTGTCTGTG; >clamp_s_d_s_a_s:compl:rev
CACAGACACCGTCACTATCATGTTGACATTCC; dG = -12.1
365 >s_e_s_d_s_c_s_c_s_a_s GTGCTGGTGACGGTGTGGGTGTGGGTGTCTGTG; >clamp_s_d_s_a_s:compl:rev
CACAGACACCGTCACTATCATGTTGACATTCC; dG = -12.1
366 >s_e_s_d_s_d_s_d_s_c_s GTGCTGGTGACGGTGACGGTGACGGTGTGGGTG; >clamp_s_d_s_a_s:compl:rev
CACAGACACCGTCACTATCATGTTGACATTCC; dG = -12.1
367 >s_e_s_d_s_d_s_c_s_a_s GTGCTGGTGACGGTGACGGTGTGGGTGTCTGTG; >clamp_s_d_s_a_s:compl:rev
CACAGACACCGTCACTATCATGTTGACATTCC; dG = -12.1
368 >s_e_s_d_s_e_s_d_s_c_s GTGCTGGTGACGGTGCTGGTGACGGTGTGGGTG; >clamp_s_d_s_a_s:compl:rev
CACAGACACCGTCACTATCATGTTGACATTCC; dG = -12.1
369 >s_e_s_e_s_c_s_d_s_c_s GTGCTGGTGCTGGTGTGGGTGACGGTGTGGGTG; >clamp_s_d_s_a_s:compl:rev
CACAGACACCGTCACTATCATGTTGACATTCC; dG = -12.1
370 >s_e_s_e_s_d_s_d_s_c_s GTGCTGGTGCTGGTGACGGTGACGGTGTGGGTG; >clamp_s_d_s_a_s:compl:rev
CACAGACACCGTCACTATCATGTTGACATTCC; dG = -12.1
371 >s_e_s_e_s_d_s_c_s_a_s GTGCTGGTGCTGGTGACGGTGTGGGTGTCTGTG; >clamp_s_d_s_a_s:compl:rev
CACAGACACCGTCACTATCATGTTGACATTCC; dG = -12.1
372 >s_e_s_e_s_e_s_d_s_c_s GTGCTGGTGCTGGTGCTGGTGACGGTGTGGGTG; >clamp_s_d_s_a_s:compl:rev
CACAGACACCGTCACTATCATGTTGACATTCC; dG = -12.1
373 >s_a_s_c_s_c_s_d_s_c_s GTGTCTGTGTGGGTGTGGGTGACGGTGTGGGTG; >clamp_s_d_s_a_s:compl:rev
CACAGACACCGTCACTATCATGTTGACATTCC; dG = -12.1
374 >s_c_s_d_s_e_s_a_s_a_s GTGTGGGTGACGGTGCTGGTGTCTGTGTCTGTG; >clamp_s_d_s_a_s:compl:rev
CACAGACACCGTCACTATCATGTTGACATTCC; dG = -12.0
375 >s_d_s_c_s_d_s_d_s_e_s GTGACGGTGTGGGTGACGGTGACGGTGCTGGTG; >clamp_s_d_s_a_s:compl:rev
CACAGACACCGTCACTATCATGTTGACATTCC; dG = -12.0
376 >s_d_s_c_s_d_s_d_s_d_s GTGACGGTGTGGGTGACGGTGACGGTGACGGTG; >clamp_s_d_s_a_s:compl:rev
CACAGACACCGTCACTATCATGTTGACATTCC; dG = -12.0
377 >s_d_s_c_s_d_s_e_s_a_s GTGACGGTGTGGGTGACGGTGCTGGTGTCTGTG; >clamp_s_d_s_a_s:compl:rev
CACAGACACCGTCACTATCATGTTGACATTCC; dG = -12.0
378 >s_d_s_d_s_e_s_a_s_a_s GTGACGGTGACGGTGCTGGTGTCTGTGTCTGTG; >clamp_s_d_s_a_s:compl:rev
CACAGACACCGTCACTATCATGTTGACATTCC; dG = -12.0
379 >s_e_s_d_s_e_s_a_s_a_s GTGCTGGTGACGGTGCTGGTGTCTGTGTCTGTG; >clamp_s_d_s_a_s:compl:rev
CACAGACACCGTCACTATCATGTTGACATTCC; dG = -12.0

380 >s_b_s_c_s_a_s_d_s_e_s GTGGCTGTGTGGGTGTCTGTGACGGTGCTGGTG; >clamp_s_d_s_a_s:compl:rev CACAGACACCGTCACTATCATGTTGACATTCC; dG = -11.9
381 >s_b_s_c_s_a_s_d_s_d_s GTGGCTGTGTGGGTGTCTGTGACGGTGACGGTG; >clamp_s_d_s_a_s:compl:rev CACAGACACCGTCACTATCATGTTGACATTCC; dG = -11.9
382 >s_c_s_c_s_a_s_d_s_e_s GTGTGGGTGTGGGTGTCTGTGACGGTGCTGGTG; >clamp_s_d_s_a_s:compl:rev CACAGACACCGTCACTATCATGTTGACATTCC; dG = -11.9
383 >s_c_s_c_s_a_s_d_s_d_s GTGTGGGTGTGGGTGTCTGTGACGGTGACGGTG; >clamp_s_d_s_a_s:compl:rev CACAGACACCGTCACTATCATGTTGACATTCC; dG = -11.9
384 >s_e_s_c_s_a_s_d_s_e_s GTGCTGGTGTGGGTGTCTGTGACGGTGCTGGTG; >clamp_s_d_s_a_s:compl:rev CACAGACACCGTCACTATCATGTTGACATTCC; dG = -11.9
385 >s_e_s_c_s_a_s_d_s_d_s GTGCTGGTGTGGGTGTCTGTGACGGTGACGGTG; >clamp_s_d_s_a_s:compl:rev CACAGACACCGTCACTATCATGTTGACATTCC; dG = -11.9
386 >s_a_s_c_s_a_s_d_s_e_s GTGTCTGTGTGGGTGTCTGTGACGGTGCTGGTG; >clamp_s_d_s_a_s:compl:rev CACAGACACCGTCACTATCATGTTGACATTCC; dG = -11.9
387 >s_a_s_c_s_a_s_d_s_d_s GTGTCTGTGTGGGTGTCTGTGACGGTGACGGTG; >clamp_s_d_s_a_s:compl:rev CACAGACACCGTCACTATCATGTTGACATTCC; dG = -11.9
388 >s_a_s_c_s_d_s_e_s_a_s GTGTCTGTGTGGGTGACGGTGCTGGTGTCTGTG; >clamp_s_d_s_a_s:compl:rev CACAGACACCGTCACTATCATGTTGACATTCC; dG = -11.8
389 >s_a_s_d_s_d_s_e_s_a_s GTGTCTGTGACGGTGACGGTGCTGGTGTCTGTG; >clamp_s_d_s_a_s:compl:rev CACAGACACCGTCACTATCATGTTGACATTCC; dG = -11.8
390 >s_a_s_e_s_d_s_e_s_a_s GTGTCTGTGCTGGTGACGGTGCTGGTGTCTGTG; >clamp_s_d_s_a_s:compl:rev CACAGACACCGTCACTATCATGTTGACATTCC; dG = -11.8
391 >s_b_s_a_s_d_s_e_s_a_s GTGGCTGTGTCTGTGACGGTGCTGGTGTCTGTG; >clamp_s_d_s_a_s:compl:rev CACAGACACCGTCACTATCATGTTGACATTCC; dG = -11.8
392 >s_b_s_b_s_d_s_e_s_a_s GTGGCTGTGGCTGTGACGGTGCTGGTGTCTGTG; >clamp_s_d_s_a_s:compl:rev CACAGACACCGTCACTATCATGTTGACATTCC; dG = -11.8
393 >s_a_s_a_s_d_s_e_s_a_s GTGTCTGTGTCTGTGACGGTGCTGGTGTCTGTG; >clamp_s_d_s_a_s:compl:rev CACAGACACCGTCACTATCATGTTGACATTCC; dG = -11.8
394 >s_b_s_c_s_d_s_e_s_a_s GTGGCTGTGTGGGTGACGGTGCTGGTGTCTGTG; >clamp_s_d_s_a_s:compl:rev CACAGACACCGTCACTATCATGTTGACATTCC; dG = -11.8
395 >s_b_s_d_s_d_s_e_s_a_s GTGGCTGTGACGGTGACGGTGCTGGTGTCTGTG; >clamp_s_d_s_a_s:compl:rev CACAGACACCGTCACTATCATGTTGACATTCC; dG = -11.8
396 >s_b_s_e_s_d_s_e_s_a_s GTGGCTGTGCTGGTGACGGTGCTGGTGTCTGTG; >clamp_s_d_s_a_s:compl:rev CACAGACACCGTCACTATCATGTTGACATTCC; dG = -11.8
397 >s_c_s_a_s_d_s_e_s_a_s GTGTGGGTGTCTGTGACGGTGCTGGTGTCTGTG; >clamp_s_d_s_a_s:compl:rev CACAGACACCGTCACTATCATGTTGACATTCC; dG = -11.8
398 >s_c_s_b_s_d_s_e_s_a_s GTGTGGGTGGCTGTGACGGTGCTGGTGTCTGTG; >clamp_s_d_s_a_s:compl:rev CACAGACACCGTCACTATCATGTTGACATTCC; dG = -11.8
399 >s_c_s_c_s_d_s_e_s_a_s GTGTGGGTGTGGGTGACGGTGCTGGTGTCTGTG; >clamp_s_d_s_a_s:compl:rev CACAGACACCGTCACTATCATGTTGACATTCC; dG = -11.8
400 >s_c_s_d_s_d_s_e_s_a_s GTGTGGGTGACGGTGACGGTGCTGGTGTCTGTG; >clamp_s_d_s_a_s:compl:rev CACAGACACCGTCACTATCATGTTGACATTCC; dG = -11.8
401 >s_c_s_e_s_d_s_e_s_a_s GTGTGGGTGCTGGTGACGGTGCTGGTGTCTGTG; >clamp_s_d_s_a_s:compl:rev CACAGACACCGTCACTATCATGTTGACATTCC; dG = -11.8
402 >s_d_s_c_s_a_s_b_s_a_s GTGACGGTGTGGGTGTCTGTGGCTGTGTCTGTG; >clamp_s_d_s_a_s:compl:rev CACAGACACCGTCACTATCATGTTGACATTCC; dG = -11.8
403 >s_d_s_c_s_b_s_b_s_a_s GTGACGGTGTGGGTGGCTGTGGCTGTGTCTGTG; >clamp_s_d_s_a_s:compl:rev CACAGACACCGTCACTATCATGTTGACATTCC; dG = -11.8
404 >s_d_s_d_s_d_s_e_s_a_s GTGACGGTGACGGTGACGGTGCTGGTGTCTGTG; >clamp_s_d_s_a_s:compl:rev CACAGACACCGTCACTATCATGTTGACATTCC; dG = -11.8
405 >s_a_s_b_s_d_s_e_s_a_s GTGTCTGTGGCTGTGACGGTGCTGGTGTCTGTG; >clamp_s_d_s_a_s:compl:rev CACAGACACCGTCACTATCATGTTGACATTCC; dG = -11.8
406 >s_d_s_e_s_d_s_e_s_a_s GTGACGGTGCTGGTGACGGTGCTGGTGTCTGTG; >clamp_s_d_s_a_s:compl:rev CACAGACACCGTCACTATCATGTTGACATTCC; dG = -11.8
407 >s_e_s_a_s_d_s_e_s_a_s GTGCTGGTGTCTGTGACGGTGCTGGTGTCTGTG; >clamp_s_d_s_a_s:compl:rev CACAGACACCGTCACTATCATGTTGACATTCC; dG = -11.8
408 >s_e_s_b_s_d_s_e_s_a_s GTGCTGGTGGCTGTGACGGTGCTGGTGTCTGTG; >clamp_s_d_s_a_s:compl:rev CACAGACACCGTCACTATCATGTTGACATTCC; dG = -11.8
409 >s_e_s_c_s_d_s_e_s_a_s GTGCTGGTGTGGGTGACGGTGCTGGTGTCTGTG; >clamp_s_d_s_a_s:compl:rev CACAGACACCGTCACTATCATGTTGACATTCC; dG = -11.8
410 >s_e_s_d_s_d_s_e_s_a_s GTGCTGGTGACGGTGACGGTGCTGGTGTCTGTG; >clamp_s_d_s_a_s:compl:rev CACAGACACCGTCACTATCATGTTGACATTCC; dG = -11.8
411 >s_e_s_e_s_d_s_e_s_a_s GTGCTGGTGCTGGTGACGGTGCTGGTGTCTGTG; >clamp_s_d_s_a_s:compl:rev CACAGACACCGTCACTATCATGTTGACATTCC; dG = -11.8
412 >s_a_s_a_s_b_s_d_s_d_s GTGTCTGTGTCTGTGGCTGTGACGGTGACGGTG; >clamp_s_d_s_a_s:compl:rev CACAGACACCGTCACTATCATGTTGACATTCC; dG = -11.7
413 >s_a_s_c_s_d_s_d_s_e_s GTGTCTGTGTGGGTGACGGTGACGGTGCTGGTG; >clamp_s_d_s_a_s:compl:rev CACAGACACCGTCACTATCATGTTGACATTCC; dG = -11.7
414 >s_a_s_c_s_d_s_d_s_d_s GTGTCTGTGTGGGTGACGGTGACGGTGACGGTG; >clamp_s_d_s_a_s:compl:rev CACAGACACCGTCACTATCATGTTGACATTCC; dG = -11.7
415 >s_a_s_c_s_e_s_d_s_d_s GTGTCTGTGTGGGTGCTGGTGACGGTGACGGTG; >clamp_s_d_s_a_s:compl:rev CACAGACACCGTCACTATCATGTTGACATTCC; dG = -11.7
416 >s_a_s_d_s_d_s_d_s_e_s GTGTCTGTGACGGTGACGGTGACGGTGCTGGTG; >clamp_s_d_s_a_s:compl:rev CACAGACACCGTCACTATCATGTTGACATTCC; dG = -11.7

417 >s_a_s_d_s_d_s_d_s_d_s GTGTCTGTGACGGTGACGGTGACGGTGACGGTG; >clamp_s_d_s_a_s:compl:rev CACAGACACCGTCACTATCATGTTGACATTCC; dG = -11.7
418 >s_a_s_d_s_e_s_d_s_d_s GTGTCTGTGACGGTGCTGGTGACGGTGACGGTG; >clamp_s_d_s_a_s:compl:rev CACAGACACCGTCACTATCATGTTGACATTCC; dG = -11.7
419 >s_a_s_e_s_a_s_d_s_d_s GTGTCTGTGCTGGTGTCTGTGACGGTGACGGTG; >clamp_s_d_s_a_s:compl:rev CACAGACACCGTCACTATCATGTTGACATTCC; dG = -11.7
420 >s_a_s_e_s_b_s_d_s_d_s GTGTCTGTGCTGGTGGCTGTGACGGTGACGGTG; >clamp_s_d_s_a_s:compl:rev CACAGACACCGTCACTATCATGTTGACATTCC; dG = -11.7
421 >s_a_s_e_s_c_s_d_s_d_s GTGTCTGTGCTGGTGTGGGTGACGGTGACGGTG; >clamp_s_d_s_a_s:compl:rev CACAGACACCGTCACTATCATGTTGACATTCC; dG = -11.7
422 >s_a_s_e_s_d_s_d_s_e_s GTGTCTGTGCTGGTGACGGTGACGGTGCTGGTG; >clamp_s_d_s_a_s:compl:rev CACAGACACCGTCACTATCATGTTGACATTCC; dG = -11.7
423 >s_a_s_e_s_d_s_d_s_d_s GTGTCTGTGCTGGTGACGGTGACGGTGACGGTG; >clamp_s_d_s_a_s:compl:rev CACAGACACCGTCACTATCATGTTGACATTCC; dG = -11.7
424 >s_a_s_a_s_c_s_d_s_d_s GTGTCTGTGTCTGTGTGGGTGACGGTGACGGTG; >clamp_s_d_s_a_s:compl:rev CACAGACACCGTCACTATCATGTTGACATTCC; dG = -11.7
425 >s_a_s_e_s_e_s_d_s_d_s GTGTCTGTGCTGGTGCTGGTGACGGTGACGGTG; >clamp_s_d_s_a_s:compl:rev CACAGACACCGTCACTATCATGTTGACATTCC; dG = -11.7
426 >s_b_s_a_s_a_s_d_s_d_s GTGGCTGTGTCTGTGTCTGTGACGGTGACGGTG; >clamp_s_d_s_a_s:compl:rev CACAGACACCGTCACTATCATGTTGACATTCC; dG = -11.7
427 >s_b_s_a_s_b_s_d_s_d_s GTGGCTGTGTCTGTGGCTGTGACGGTGACGGTG; >clamp_s_d_s_a_s:compl:rev CACAGACACCGTCACTATCATGTTGACATTCC; dG = -11.7
428 >s_a_s_a_s_a_s_d_s_d_s GTGTCTGTGTCTGTGTCTGTGACGGTGACGGTG; >clamp_s_d_s_a_s:compl:rev CACAGACACCGTCACTATCATGTTGACATTCC; dG = -11.7
429 >s_b_s_a_s_c_s_d_s_d_s GTGGCTGTGTCTGTGTGGGTGACGGTGACGGTG; >clamp_s_d_s_a_s:compl:rev CACAGACACCGTCACTATCATGTTGACATTCC; dG = -11.7
430 >s_b_s_a_s_d_s_d_s_e_s GTGGCTGTGTCTGTGACGGTGACGGTGCTGGTG; >clamp_s_d_s_a_s:compl:rev CACAGACACCGTCACTATCATGTTGACATTCC; dG = -11.7
431 >s_b_s_a_s_d_s_d_s_d_s GTGGCTGTGTCTGTGACGGTGACGGTGACGGTG; >clamp_s_d_s_a_s:compl:rev CACAGACACCGTCACTATCATGTTGACATTCC; dG = -11.7
432 >s_b_s_a_s_e_s_d_s_d_s GTGGCTGTGTCTGTGCTGGTGACGGTGACGGTG; >clamp_s_d_s_a_s:compl:rev CACAGACACCGTCACTATCATGTTGACATTCC; dG = -11.7
433 >s_b_s_b_s_a_s_d_s_d_s GTGGCTGTGGCTGTGTCTGTGACGGTGACGGTG; >clamp_s_d_s_a_s:compl:rev CACAGACACCGTCACTATCATGTTGACATTCC; dG = -11.7
434 >s_b_s_b_s_b_s_d_s_d_s GTGGCTGTGGCTGTGGCTGTGACGGTGACGGTG; >clamp_s_d_s_a_s:compl:rev CACAGACACCGTCACTATCATGTTGACATTCC; dG = -11.7
435 >s_b_s_b_s_c_s_d_s_d_s GTGGCTGTGGCTGTGTGGGTGACGGTGACGGTG; >clamp_s_d_s_a_s:compl:rev CACAGACACCGTCACTATCATGTTGACATTCC; dG = -11.7
436 >s_a_s_a_s_d_s_d_s_e_s GTGTCTGTGTCTGTGACGGTGACGGTGCTGGTG; >clamp_s_d_s_a_s:compl:rev CACAGACACCGTCACTATCATGTTGACATTCC; dG = -11.7
437 >s_b_s_b_s_d_s_d_s_e_s GTGGCTGTGGCTGTGACGGTGACGGTGCTGGTG; >clamp_s_d_s_a_s:compl:rev CACAGACACCGTCACTATCATGTTGACATTCC; dG = -11.7
438 >s_b_s_b_s_d_s_d_s_d_s GTGGCTGTGGCTGTGACGGTGACGGTGACGGTG; >clamp_s_d_s_a_s:compl:rev CACAGACACCGTCACTATCATGTTGACATTCC; dG = -11.7
439 >s_b_s_b_s_e_s_d_s_d_s GTGGCTGTGGCTGTGCTGGTGACGGTGACGGTG; >clamp_s_d_s_a_s:compl:rev CACAGACACCGTCACTATCATGTTGACATTCC; dG = -11.7
440 >s_a_s_a_s_d_s_d_s_d_s GTGTCTGTGTCTGTGACGGTGACGGTGACGGTG; >clamp_s_d_s_a_s:compl:rev CACAGACACCGTCACTATCATGTTGACATTCC; dG = -11.7
441 >s_b_s_c_s_b_s_d_s_d_s GTGGCTGTGTGGGTGGCTGTGACGGTGACGGTG; >clamp_s_d_s_a_s:compl:rev CACAGACACCGTCACTATCATGTTGACATTCC; dG = -11.7
442 >s_b_s_c_s_c_s_d_s_d_s GTGGCTGTGTGGGTGTGGGTGACGGTGACGGTG; >clamp_s_d_s_a_s:compl:rev CACAGACACCGTCACTATCATGTTGACATTCC; dG = -11.7
443 >s_b_s_c_s_d_s_d_s_e_s GTGGCTGTGTGGGTGACGGTGACGGTGCTGGTG; >clamp_s_d_s_a_s:compl:rev CACAGACACCGTCACTATCATGTTGACATTCC; dG = -11.7
444 >s_b_s_c_s_d_s_d_s_d_s GTGGCTGTGTGGGTGACGGTGACGGTGACGGTG; >clamp_s_d_s_a_s:compl:rev CACAGACACCGTCACTATCATGTTGACATTCC; dG = -11.7
445 >s_b_s_c_s_e_s_d_s_d_s GTGGCTGTGTGGGTGCTGGTGACGGTGACGGTG; >clamp_s_d_s_a_s:compl:rev CACAGACACCGTCACTATCATGTTGACATTCC; dG = -11.7
446 >s_b_s_d_s_d_s_d_s_e_s GTGGCTGTGACGGTGACGGTGACGGTGCTGGTG; >clamp_s_d_s_a_s:compl:rev CACAGACACCGTCACTATCATGTTGACATTCC; dG = -11.7
447 >s_b_s_d_s_d_s_d_s_d_s GTGGCTGTGACGGTGACGGTGACGGTGACGGTG; >clamp_s_d_s_a_s:compl:rev CACAGACACCGTCACTATCATGTTGACATTCC; dG = -11.7
448 >s_b_s_d_s_e_s_d_s_d_s GTGGCTGTGACGGTGCTGGTGACGGTGACGGTG; >clamp_s_d_s_a_s:compl:rev CACAGACACCGTCACTATCATGTTGACATTCC; dG = -11.7
449 >s_a_s_a_s_e_s_d_s_d_s GTGTCTGTGTCTGTGCTGGTGACGGTGACGGTG; >clamp_s_d_s_a_s:compl:rev CACAGACACCGTCACTATCATGTTGACATTCC; dG = -11.7
450 >s_b_s_e_s_a_s_d_s_d_s GTGGCTGTGCTGGTGTCTGTGACGGTGACGGTG; >clamp_s_d_s_a_s:compl:rev CACAGACACCGTCACTATCATGTTGACATTCC; dG = -11.7
451 >s_b_s_e_s_b_s_d_s_d_s GTGGCTGTGCTGGTGGCTGTGACGGTGACGGTG; >clamp_s_d_s_a_s:compl:rev CACAGACACCGTCACTATCATGTTGACATTCC; dG = -11.7
452 >s_b_s_e_s_c_s_d_s_d_s GTGGCTGTGCTGGTGTGGGTGACGGTGACGGTG; >clamp_s_d_s_a_s:compl:rev CACAGACACCGTCACTATCATGTTGACATTCC; dG = -11.7
453 >s_b_s_e_s_d_s_d_s_e_s GTGGCTGTGCTGGTGACGGTGACGGTGCTGGTG; >clamp_s_d_s_a_s:compl:rev CACAGACACCGTCACTATCATGTTGACATTCC; dG = -11.7

454 >s_b_s_e_s_d_s_d_s_d_s GTGGCTGTGCTGGTGACGGTGACGGTGACGGTG; >clamp_s_d_s_a_s:compl:rev CACAGACACCGTCACTATCATGTTGACATTCC; dG = -11.7
455 >s_b_s_e_s_e_s_d_s_d_s GTGGCTGTGCTGGTGCTGGTGACGGTGACGGTG; >clamp_s_d_s_a_s:compl:rev CACAGACACCGTCACTATCATGTTGACATTCC; dG = -11.7
456 >s_c_s_a_s_a_s_d_s_d_s GTGTGGGTGTCTGTGTCTGTGACGGTGACGGTG; >clamp_s_d_s_a_s:compl:rev CACAGACACCGTCACTATCATGTTGACATTCC; dG = -11.7
457 >s_c_s_a_s_b_s_d_s_d_s GTGTGGGTGTCTGTGGCTGTGACGGTGACGGTG; >clamp_s_d_s_a_s:compl:rev CACAGACACCGTCACTATCATGTTGACATTCC; dG = -11.7
458 >s_c_s_a_s_c_s_d_s_d_s GTGTGGGTGTCTGTGTGGGTGACGGTGACGGTG; >clamp_s_d_s_a_s:compl:rev CACAGACACCGTCACTATCATGTTGACATTCC; dG = -11.7
459 >s_c_s_a_s_d_s_d_s_e_s GTGTGGGTGTCTGTGACGGTGACGGTGCTGGTG; >clamp_s_d_s_a_s:compl:rev CACAGACACCGTCACTATCATGTTGACATTCC; dG = -11.7
460 >s_c_s_a_s_d_s_d_s_d_s GTGTGGGTGTCTGTGACGGTGACGGTGACGGTG; >clamp_s_d_s_a_s:compl:rev CACAGACACCGTCACTATCATGTTGACATTCC; dG = -11.7
461 >s_c_s_a_s_e_s_d_s_d_s GTGTGGGTGTCTGTGCTGGTGACGGTGACGGTG; >clamp_s_d_s_a_s:compl:rev CACAGACACCGTCACTATCATGTTGACATTCC; dG = -11.7
462 >s_a_s_b_s_a_s_d_s_d_s GTGTCTGTGGCTGTGTCTGTGACGGTGACGGTG; >clamp_s_d_s_a_s:compl:rev CACAGACACCGTCACTATCATGTTGACATTCC; dG = -11.7
463 >s_c_s_b_s_a_s_d_s_d_s GTGTGGGTGGCTGTGTCTGTGACGGTGACGGTG; >clamp_s_d_s_a_s:compl:rev CACAGACACCGTCACTATCATGTTGACATTCC; dG = -11.7
464 >s_c_s_b_s_b_s_d_s_d_s GTGTGGGTGGCTGTGGCTGTGACGGTGACGGTG; >clamp_s_d_s_a_s:compl:rev CACAGACACCGTCACTATCATGTTGACATTCC; dG = -11.7
465 >s_c_s_b_s_c_s_d_s_d_s GTGTGGGTGGCTGTGTGGGTGACGGTGACGGTG; >clamp_s_d_s_a_s:compl:rev CACAGACACCGTCACTATCATGTTGACATTCC; dG = -11.7
466 >s_c_s_b_s_d_s_d_s_e_s GTGTGGGTGGCTGTGACGGTGACGGTGCTGGTG; >clamp_s_d_s_a_s:compl:rev CACAGACACCGTCACTATCATGTTGACATTCC; dG = -11.7
467 >s_c_s_b_s_d_s_d_s_d_s GTGTGGGTGGCTGTGACGGTGACGGTGACGGTG; >clamp_s_d_s_a_s:compl:rev CACAGACACCGTCACTATCATGTTGACATTCC; dG = -11.7
468 >s_c_s_b_s_e_s_d_s_d_s GTGTGGGTGGCTGTGCTGGTGACGGTGACGGTG; >clamp_s_d_s_a_s:compl:rev CACAGACACCGTCACTATCATGTTGACATTCC; dG = -11.7
469 >s_c_s_c_s_b_s_d_s_d_s GTGTGGGTGTGGGTGGCTGTGACGGTGACGGTG; >clamp_s_d_s_a_s:compl:rev CACAGACACCGTCACTATCATGTTGACATTCC; dG = -11.7
470 >s_c_s_c_s_c_s_d_s_d_s GTGTGGGTGTGGGTGTGGGTGACGGTGACGGTG; >clamp_s_d_s_a_s:compl:rev CACAGACACCGTCACTATCATGTTGACATTCC; dG = -11.7
471 >s_c_s_c_s_d_s_d_s_e_s GTGTGGGTGTGGGTGACGGTGACGGTGCTGGTG; >clamp_s_d_s_a_s:compl:rev CACAGACACCGTCACTATCATGTTGACATTCC; dG = -11.7
472 >s_c_s_c_s_d_s_d_s_d_s GTGTGGGTGTGGGTGACGGTGACGGTGACGGTG; >clamp_s_d_s_a_s:compl:rev CACAGACACCGTCACTATCATGTTGACATTCC; dG = -11.7
473 >s_c_s_c_s_e_s_d_s_d_s GTGTGGGTGTGGGTGCTGGTGACGGTGACGGTG; >clamp_s_d_s_a_s:compl:rev CACAGACACCGTCACTATCATGTTGACATTCC; dG = -11.7
474 >s_a_s_b_s_b_s_d_s_d_s GTGTCTGTGGCTGTGGCTGTGACGGTGACGGTG; >clamp_s_d_s_a_s:compl:rev CACAGACACCGTCACTATCATGTTGACATTCC; dG = -11.7
475 >s_c_s_d_s_d_s_d_s_e_s GTGTGGGTGACGGTGACGGTGACGGTGCTGGTG; >clamp_s_d_s_a_s:compl:rev CACAGACACCGTCACTATCATGTTGACATTCC; dG = -11.7
476 >s_c_s_d_s_d_s_d_s_d_s GTGTGGGTGACGGTGACGGTGACGGTGACGGTG; >clamp_s_d_s_a_s:compl:rev CACAGACACCGTCACTATCATGTTGACATTCC; dG = -11.7
477 >s_c_s_d_s_e_s_d_s_d_s GTGTGGGTGACGGTGCTGGTGACGGTGACGGTG; >clamp_s_d_s_a_s:compl:rev CACAGACACCGTCACTATCATGTTGACATTCC; dG = -11.7
478 >s_c_s_e_s_a_s_d_s_d_s GTGTGGGTGCTGGTGTCTGTGACGGTGACGGTG; >clamp_s_d_s_a_s:compl:rev CACAGACACCGTCACTATCATGTTGACATTCC; dG = -11.7
479 >s_c_s_e_s_b_s_d_s_d_s GTGTGGGTGCTGGTGGCTGTGACGGTGACGGTG; >clamp_s_d_s_a_s:compl:rev CACAGACACCGTCACTATCATGTTGACATTCC; dG = -11.7
480 >s_c_s_e_s_c_s_d_s_d_s GTGTGGGTGCTGGTGTGGGTGACGGTGACGGTG; >clamp_s_d_s_a_s:compl:rev CACAGACACCGTCACTATCATGTTGACATTCC; dG = -11.7
481 >s_c_s_e_s_d_s_d_s_e_s GTGTGGGTGCTGGTGACGGTGACGGTGCTGGTG; >clamp_s_d_s_a_s:compl:rev CACAGACACCGTCACTATCATGTTGACATTCC; dG = -11.7
482 >s_c_s_e_s_d_s_d_s_d_s GTGTGGGTGCTGGTGACGGTGACGGTGACGGTG; >clamp_s_d_s_a_s:compl:rev CACAGACACCGTCACTATCATGTTGACATTCC; dG = -11.7
483 >s_c_s_e_s_e_s_d_s_d_s GTGTGGGTGCTGGTGCTGGTGACGGTGACGGTG; >clamp_s_d_s_a_s:compl:rev CACAGACACCGTCACTATCATGTTGACATTCC; dG = -11.7
484 >s_a_s_b_s_c_s_d_s_d_s GTGTCTGTGGCTGTGTGGGTGACGGTGACGGTG; >clamp_s_d_s_a_s:compl:rev CACAGACACCGTCACTATCATGTTGACATTCC; dG = -11.7
485 >s_d_s_c_s_a_s_e_s_a_s GTGACGGTGTGGGTGTCTGTGCTGGTGTCTGTG; >clamp_s_d_s_a_s:compl:rev CACAGACACCGTCACTATCATGTTGACATTCC; dG = -11.7
486 >s_d_s_c_s_a_s_c_s_a_s GTGACGGTGTGGGTGTCTGTGTGGGTGTCTGTG; >clamp_s_d_s_a_s:compl:rev CACAGACACCGTCACTATCATGTTGACATTCC; dG = -11.7
487 >s_d_s_c_s_a_s_a_s_a_s GTGACGGTGTGGGTGTCTGTGTCTGTGTCTGTG; >clamp_s_d_s_a_s:compl:rev CACAGACACCGTCACTATCATGTTGACATTCC; dG = -11.7
488 >s_d_s_c_s_b_s_e_s_a_s GTGACGGTGTGGGTGGCTGTGCTGGTGTCTGTG; >clamp_s_d_s_a_s:compl:rev CACAGACACCGTCACTATCATGTTGACATTCC; dG = -11.7
489 >s_d_s_c_s_b_s_c_s_a_s GTGACGGTGTGGGTGGCTGTGTGGGTGTCTGTG; >clamp_s_d_s_a_s:compl:rev CACAGACACCGTCACTATCATGTTGACATTCC; dG = -11.7
490 >s_d_s_c_s_b_s_a_s_a_s GTGACGGTGTGGGTGGCTGTGTCTGTGTCTGTG; >clamp_s_d_s_a_s:compl:rev CACAGACACCGTCACTATCATGTTGACATTCC; dG = -11.7

491 >s_d_s_c_s_c_s_d_s_e_s GTGACGGTGTGGGTGTGGGTGACGGTGCTGGTG; >clamp_s_d_s_a_s:compl:rev CACAGACACCGTCACTATCATGTTGACATTCC; dG = -11.7
492 >s_d_s_c_s_c_s_d_s_d_s GTGACGGTGTGGGTGTGGGTGACGGTGACGGTG; >clamp_s_d_s_a_s:compl:rev CACAGACACCGTCACTATCATGTTGACATTCC; dG = -11.7
493 >s_d_s_c_s_c_s_e_s_a_s GTGACGGTGTGGGTGTGGGTGCTGGTGTCTGTG; >clamp_s_d_s_a_s:compl:rev CACAGACACCGTCACTATCATGTTGACATTCC; dG = -11.7
494 >s_d_s_c_s_c_s_c_s_a_s GTGACGGTGTGGGTGTGGGTGTGGGTGTCTGTG; >clamp_s_d_s_a_s:compl:rev CACAGACACCGTCACTATCATGTTGACATTCC; dG = -11.7
495 >s_d_s_c_s_c_s_b_s_a_s GTGACGGTGTGGGTGTGGGTGGCTGTGTCTGTG; >clamp_s_d_s_a_s:compl:rev CACAGACACCGTCACTATCATGTTGACATTCC; dG = -11.7
496 >s_d_s_c_s_c_s_a_s_a_s GTGACGGTGTGGGTGTGGGTGTCTGTGTCTGTG; >clamp_s_d_s_a_s:compl:rev CACAGACACCGTCACTATCATGTTGACATTCC; dG = -11.7
497 >s_d_s_c_s_e_s_d_s_e_s GTGACGGTGTGGGTGCTGGTGACGGTGCTGGTG; >clamp_s_d_s_a_s:compl:rev CACAGACACCGTCACTATCATGTTGACATTCC; dG = -11.7
498 >s_d_s_c_s_e_s_d_s_d_s GTGACGGTGTGGGTGCTGGTGACGGTGACGGTG; >clamp_s_d_s_a_s:compl:rev CACAGACACCGTCACTATCATGTTGACATTCC; dG = -11.7
499 >s_d_s_c_s_e_s_e_s_a_s GTGACGGTGTGGGTGCTGGTGCTGGTGTCTGTG; >clamp_s_d_s_a_s:compl:rev CACAGACACCGTCACTATCATGTTGACATTCC; dG = -11.7
500 >s_d_s_c_s_e_s_c_s_a_s GTGACGGTGTGGGTGCTGGTGTGGGTGTCTGTG; >clamp_s_d_s_a_s:compl:rev CACAGACACCGTCACTATCATGTTGACATTCC; dG = -11.7
501 >s_d_s_c_s_e_s_b_s_a_s GTGACGGTGTGGGTGCTGGTGGCTGTGTCTGTG; >clamp_s_d_s_a_s:compl:rev CACAGACACCGTCACTATCATGTTGACATTCC; dG = -11.7
502 >s_d_s_c_s_e_s_a_s_a_s GTGACGGTGTGGGTGCTGGTGTCTGTGTCTGTG; >clamp_s_d_s_a_s:compl:rev CACAGACACCGTCACTATCATGTTGACATTCC; dG = -11.7
503 >s_a_s_b_s_d_s_d_s_e_s GTGTCTGTGGCTGTGACGGTGACGGTGCTGGTG; >clamp_s_d_s_a_s:compl:rev CACAGACACCGTCACTATCATGTTGACATTCC; dG = -11.7
504 >s_a_s_b_s_d_s_d_s_d_s GTGTCTGTGGCTGTGACGGTGACGGTGACGGTG; >clamp_s_d_s_a_s:compl:rev CACAGACACCGTCACTATCATGTTGACATTCC; dG = -11.7
505 >s_d_s_d_s_d_s_d_s_e_s GTGACGGTGACGGTGACGGTGACGGTGCTGGTG; >clamp_s_d_s_a_s:compl:rev CACAGACACCGTCACTATCATGTTGACATTCC; dG = -11.7
506 >s_d_s_d_s_d_s_d_s_d_s GTGACGGTGACGGTGACGGTGACGGTGACGGTG; >clamp_s_d_s_a_s:compl:rev CACAGACACCGTCACTATCATGTTGACATTCC; dG = -11.7
507 >s_d_s_d_s_e_s_d_s_d_s GTGACGGTGACGGTGCTGGTGACGGTGACGGTG; >clamp_s_d_s_a_s:compl:rev CACAGACACCGTCACTATCATGTTGACATTCC; dG = -11.7
508 >s_d_s_e_s_a_s_b_s_a_s GTGACGGTGCTGGTGTCTGTGGCTGTGTCTGTG; >clamp_s_d_s_a_s:compl:rev CACAGACACCGTCACTATCATGTTGACATTCC; dG = -11.7
509 >s_d_s_e_s_b_s_d_s_d_s GTGACGGTGCTGGTGGCTGTGACGGTGACGGTG; >clamp_s_d_s_a_s:compl:rev CACAGACACCGTCACTATCATGTTGACATTCC; dG = -11.7
510 >s_d_s_e_s_c_s_d_s_d_s GTGACGGTGCTGGTGTGGGTGACGGTGACGGTG; >clamp_s_d_s_a_s:compl:rev CACAGACACCGTCACTATCATGTTGACATTCC; dG = -11.7
511 >s_d_s_e_s_d_s_d_s_e_s GTGACGGTGCTGGTGACGGTGACGGTGCTGGTG; >clamp_s_d_s_a_s:compl:rev CACAGACACCGTCACTATCATGTTGACATTCC; dG = -11.7
512 >s_d_s_e_s_d_s_d_s_d_s GTGACGGTGCTGGTGACGGTGACGGTGACGGTG; >clamp_s_d_s_a_s:compl:rev CACAGACACCGTCACTATCATGTTGACATTCC; dG = -11.7
513 >s_d_s_e_s_e_s_d_s_d_s GTGACGGTGCTGGTGCTGGTGACGGTGACGGTG; >clamp_s_d_s_a_s:compl:rev CACAGACACCGTCACTATCATGTTGACATTCC; dG = -11.7
514 >s_e_s_a_s_a_s_d_s_d_s GTGCTGGTGTCTGTGTCTGTGACGGTGACGGTG; >clamp_s_d_s_a_s:compl:rev CACAGACACCGTCACTATCATGTTGACATTCC; dG = -11.7
515 >s_e_s_a_s_b_s_d_s_d_s GTGCTGGTGTCTGTGGCTGTGACGGTGACGGTG; >clamp_s_d_s_a_s:compl:rev CACAGACACCGTCACTATCATGTTGACATTCC; dG = -11.7
516 >s_a_s_b_s_e_s_d_s_d_s GTGTCTGTGGCTGTGCTGGTGACGGTGACGGTG; >clamp_s_d_s_a_s:compl:rev CACAGACACCGTCACTATCATGTTGACATTCC; dG = -11.7
517 >s_e_s_a_s_c_s_d_s_d_s GTGCTGGTGTCTGTGTGGGTGACGGTGACGGTG; >clamp_s_d_s_a_s:compl:rev CACAGACACCGTCACTATCATGTTGACATTCC; dG = -11.7
518 >s_e_s_a_s_d_s_d_s_e_s GTGCTGGTGTCTGTGACGGTGACGGTGCTGGTG; >clamp_s_d_s_a_s:compl:rev CACAGACACCGTCACTATCATGTTGACATTCC; dG = -11.7
519 >s_e_s_a_s_d_s_d_s_d_s GTGCTGGTGTCTGTGACGGTGACGGTGACGGTG; >clamp_s_d_s_a_s:compl:rev CACAGACACCGTCACTATCATGTTGACATTCC; dG = -11.7
520 >s_e_s_a_s_e_s_d_s_d_s GTGCTGGTGTCTGTGCTGGTGACGGTGACGGTG; >clamp_s_d_s_a_s:compl:rev CACAGACACCGTCACTATCATGTTGACATTCC; dG = -11.7
521 >s_e_s_b_s_a_s_d_s_d_s GTGCTGGTGGCTGTGTCTGTGACGGTGACGGTG; >clamp_s_d_s_a_s:compl:rev CACAGACACCGTCACTATCATGTTGACATTCC; dG = -11.7
522 >s_e_s_b_s_b_s_d_s_d_s GTGCTGGTGGCTGTGGCTGTGACGGTGACGGTG; >clamp_s_d_s_a_s:compl:rev CACAGACACCGTCACTATCATGTTGACATTCC; dG = -11.7
523 >s_e_s_b_s_c_s_d_s_d_s GTGCTGGTGGCTGTGTGGGTGACGGTGACGGTG; >clamp_s_d_s_a_s:compl:rev CACAGACACCGTCACTATCATGTTGACATTCC; dG = -11.7
524 >s_e_s_b_s_d_s_d_s_e_s GTGCTGGTGGCTGTGACGGTGACGGTGCTGGTG; >clamp_s_d_s_a_s:compl:rev CACAGACACCGTCACTATCATGTTGACATTCC; dG = -11.7
525 >s_e_s_b_s_d_s_d_s_d_s GTGCTGGTGGCTGTGACGGTGACGGTGACGGTG; >clamp_s_d_s_a_s:compl:rev CACAGACACCGTCACTATCATGTTGACATTCC; dG = -11.7
526 >s_e_s_b_s_e_s_d_s_d_s GTGCTGGTGGCTGTGCTGGTGACGGTGACGGTG; >clamp_s_d_s_a_s:compl:rev CACAGACACCGTCACTATCATGTTGACATTCC; dG = -11.7
527 >s_e_s_c_s_b_s_d_s_d_s GTGCTGGTGTGGGTGGCTGTGACGGTGACGGTG; >clamp_s_d_s_a_s:compl:rev CACAGACACCGTCACTATCATGTTGACATTCC; dG = -11.7

528 >s_e_s_c_s_c_s_d_s_d_s GTGCTGGTGTGGGTGTGGGTGACGGTGACGGTG; >clamp_s_d_s_a_s:compl:rev CACAGACACCGTCACTATCATGTTGACATTCC; dG = -11.7
529 >s_e_s_c_s_d_s_d_s_e_s GTGCTGGTGTGGGTGACGGTGACGGTGCTGGTG; >clamp_s_d_s_a_s:compl:rev CACAGACACCGTCACTATCATGTTGACATTCC; dG = -11.7
530 >s_e_s_c_s_d_s_d_s_d_s GTGCTGGTGTGGGTGACGGTGACGGTGACGGTG; >clamp_s_d_s_a_s:compl:rev CACAGACACCGTCACTATCATGTTGACATTCC; dG = -11.7
531 >s_e_s_c_s_e_s_d_s_d_s GTGCTGGTGTGGGTGCTGGTGACGGTGACGGTG; >clamp_s_d_s_a_s:compl:rev CACAGACACCGTCACTATCATGTTGACATTCC; dG = -11.7
532 >s_e_s_d_s_d_s_d_s_e_s GTGCTGGTGACGGTGACGGTGACGGTGCTGGTG; >clamp_s_d_s_a_s:compl:rev CACAGACACCGTCACTATCATGTTGACATTCC; dG = -11.7
533 >s_e_s_d_s_d_s_d_s_d_s GTGCTGGTGACGGTGACGGTGACGGTGACGGTG; >clamp_s_d_s_a_s:compl:rev CACAGACACCGTCACTATCATGTTGACATTCC; dG = -11.7
534 >s_e_s_d_s_e_s_d_s_d_s GTGCTGGTGACGGTGCTGGTGACGGTGACGGTG; >clamp_s_d_s_a_s:compl:rev CACAGACACCGTCACTATCATGTTGACATTCC; dG = -11.7
535 >s_e_s_e_s_a_s_d_s_d_s GTGCTGGTGCTGGTGTCTGTGACGGTGACGGTG; >clamp_s_d_s_a_s:compl:rev CACAGACACCGTCACTATCATGTTGACATTCC; dG = -11.7
536 >s_e_s_e_s_b_s_d_s_d_s GTGCTGGTGCTGGTGGCTGTGACGGTGACGGTG; >clamp_s_d_s_a_s:compl:rev CACAGACACCGTCACTATCATGTTGACATTCC; dG = -11.7
537 >s_e_s_e_s_c_s_d_s_d_s GTGCTGGTGCTGGTGTGGGTGACGGTGACGGTG; >clamp_s_d_s_a_s:compl:rev CACAGACACCGTCACTATCATGTTGACATTCC; dG = -11.7
538 >s_a_s_c_s_b_s_d_s_d_s GTGTCTGTGTGGGTGGCTGTGACGGTGACGGTG; >clamp_s_d_s_a_s:compl:rev CACAGACACCGTCACTATCATGTTGACATTCC; dG = -11.7
539 >s_e_s_e_s_d_s_d_s_e_s GTGCTGGTGCTGGTGACGGTGACGGTGCTGGTG; >clamp_s_d_s_a_s:compl:rev CACAGACACCGTCACTATCATGTTGACATTCC; dG = -11.7
540 >s_e_s_e_s_d_s_d_s_d_s GTGCTGGTGCTGGTGACGGTGACGGTGACGGTG; >clamp_s_d_s_a_s:compl:rev CACAGACACCGTCACTATCATGTTGACATTCC; dG = -11.7
541 >s_e_s_e_s_e_s_d_s_d_s GTGCTGGTGCTGGTGCTGGTGACGGTGACGGTG; >clamp_s_d_s_a_s:compl:rev CACAGACACCGTCACTATCATGTTGACATTCC; dG = -11.7
542 >s_a_s_c_s_c_s_d_s_d_s GTGTCTGTGTGGGTGTGGGTGACGGTGACGGTG; >clamp_s_d_s_a_s:compl:rev CACAGACACCGTCACTATCATGTTGACATTCC; dG = -11.7
543 >s_d_s_e_s_a_s_c_s_a_s GTGACGGTGCTGGTGTCTGTGTGGGTGTCTGTG; >clamp_s_d_s_a_s:compl:rev CACAGACACCGTCACTATCATGTTGACATTCC; dG = -11.6
544 >s_d_s_e_s_a_s_a_s_a_s GTGACGGTGCTGGTGTCTGTGTCTGTGTCTGTG; >clamp_s_d_s_a_s:compl:rev CACAGACACCGTCACTATCATGTTGACATTCC; dG = -11.6
545 >s_a_s_a_s_a_s_d_s_e_s GTGTCTGTGTCTGTGTCTGTGACGGTGCTGGTG; >clamp_s_d_s_a_s:compl:rev CACAGACACCGTCACTATCATGTTGACATTCC; dG = -11.5
546 >s_a_s_d_s_e_s_d_s_e_s GTGTCTGTGACGGTGCTGGTGACGGTGCTGGTG; >clamp_s_d_s_a_s:compl:rev CACAGACACCGTCACTATCATGTTGACATTCC; dG = -11.5
547 >s_a_s_d_s_e_s_e_s_a_s GTGTCTGTGACGGTGCTGGTGCTGGTGTCTGTG; >clamp_s_d_s_a_s:compl:rev CACAGACACCGTCACTATCATGTTGACATTCC; dG = -11.5
548 >s_a_s_d_s_e_s_c_s_a_s GTGTCTGTGACGGTGCTGGTGTGGGTGTCTGTG; >clamp_s_d_s_a_s:compl:rev CACAGACACCGTCACTATCATGTTGACATTCC; dG = -11.5
549 >s_a_s_d_s_e_s_b_s_a_s GTGTCTGTGACGGTGCTGGTGGCTGTGTCTGTG; >clamp_s_d_s_a_s:compl:rev CACAGACACCGTCACTATCATGTTGACATTCC; dG = -11.5
550 >s_a_s_e_s_a_s_d_s_e_s GTGTCTGTGCTGGTGTCTGTGACGGTGCTGGTG; >clamp_s_d_s_a_s:compl:rev CACAGACACCGTCACTATCATGTTGACATTCC; dG = -11.5
551 >s_a_s_e_s_b_s_d_s_e_s GTGTCTGTGCTGGTGGCTGTGACGGTGCTGGTG; >clamp_s_d_s_a_s:compl:rev CACAGACACCGTCACTATCATGTTGACATTCC; dG = -11.5
552 >s_b_s_a_s_a_s_d_s_e_s GTGGCTGTGTCTGTGTCTGTGACGGTGCTGGTG; >clamp_s_d_s_a_s:compl:rev CACAGACACCGTCACTATCATGTTGACATTCC; dG = -11.5
553 >s_b_s_a_s_b_s_d_s_e_s GTGGCTGTGTCTGTGGCTGTGACGGTGCTGGTG; >clamp_s_d_s_a_s:compl:rev CACAGACACCGTCACTATCATGTTGACATTCC; dG = -11.5
554 >s_b_s_b_s_a_s_d_s_e_s GTGGCTGTGGCTGTGTCTGTGACGGTGCTGGTG; >clamp_s_d_s_a_s:compl:rev CACAGACACCGTCACTATCATGTTGACATTCC; dG = -11.5
555 >s_b_s_b_s_b_s_d_s_e_s GTGGCTGTGGCTGTGGCTGTGACGGTGCTGGTG; >clamp_s_d_s_a_s:compl:rev CACAGACACCGTCACTATCATGTTGACATTCC; dG = -11.5
556 >s_b_s_c_s_b_s_d_s_e_s GTGGCTGTGTGGGTGGCTGTGACGGTGCTGGTG; >clamp_s_d_s_a_s:compl:rev CACAGACACCGTCACTATCATGTTGACATTCC; dG = -11.5
557 >s_b_s_d_s_e_s_d_s_e_s GTGGCTGTGACGGTGCTGGTGACGGTGCTGGTG; >clamp_s_d_s_a_s:compl:rev CACAGACACCGTCACTATCATGTTGACATTCC; dG = -11.5
558 >s_b_s_d_s_e_s_e_s_a_s GTGGCTGTGACGGTGCTGGTGCTGGTGTCTGTG; >clamp_s_d_s_a_s:compl:rev CACAGACACCGTCACTATCATGTTGACATTCC; dG = -11.5
559 >s_b_s_d_s_e_s_c_s_a_s GTGGCTGTGACGGTGCTGGTGTGGGTGTCTGTG; >clamp_s_d_s_a_s:compl:rev CACAGACACCGTCACTATCATGTTGACATTCC; dG = -11.5
560 >s_b_s_d_s_e_s_b_s_a_s GTGGCTGTGACGGTGCTGGTGGCTGTGTCTGTG; >clamp_s_d_s_a_s:compl:rev CACAGACACCGTCACTATCATGTTGACATTCC; dG = -11.5
561 >s_b_s_e_s_a_s_d_s_e_s GTGGCTGTGCTGGTGTCTGTGACGGTGCTGGTG; >clamp_s_d_s_a_s:compl:rev CACAGACACCGTCACTATCATGTTGACATTCC; dG = -11.5
562 >s_b_s_e_s_b_s_d_s_e_s GTGGCTGTGCTGGTGGCTGTGACGGTGCTGGTG; >clamp_s_d_s_a_s:compl:rev CACAGACACCGTCACTATCATGTTGACATTCC; dG = -11.5
563 >s_c_s_a_s_a_s_d_s_e_s GTGTGGGTGTCTGTGTCTGTGACGGTGCTGGTG; >clamp_s_d_s_a_s:compl:rev CACAGACACCGTCACTATCATGTTGACATTCC; dG = -11.5
564 >s_c_s_a_s_b_s_d_s_e_s GTGTGGGTGTCTGTGGCTGTGACGGTGCTGGTG; >clamp_s_d_s_a_s:compl:rev CACAGACACCGTCACTATCATGTTGACATTCC; dG = -11.5

565 >s_a_s_b_s_a_s_d_s_e_s GTGTCTGTGGCTGTGTCTGTGACGGTGCTGGTG; >clamp_s_d_s_a_s:compl:rev
CACAGACACCGTCACTATCATGTTGACATTCC; dG = -11.5
566 >s_c_s_b_s_a_s_d_s_e_s GTGTGGGTGGCTGTGTCTGTGACGGTGCTGGTG; >clamp_s_d_s_a_s:compl:rev
CACAGACACCGTCACTATCATGTTGACATTCC; dG = -11.5
567 >s_c_s_b_s_b_s_d_s_e_s GTGTGGGTGGCTGTGGCTGTGACGGTGCTGGTG; >clamp_s_d_s_a_s:compl:rev
CACAGACACCGTCACTATCATGTTGACATTCC; dG = -11.5
568 >s_c_s_c_s_b_s_d_s_e_s GTGTGGGTGTGGGTGGCTGTGACGGTGCTGGTG; >clamp_s_d_s_a_s:compl:rev
CACAGACACCGTCACTATCATGTTGACATTCC; dG = -11.5
569 >s_a_s_b_s_b_s_d_s_e_s GTGTCTGTGGCTGTGGCTGTGACGGTGCTGGTG; >clamp_s_d_s_a_s:compl:rev
CACAGACACCGTCACTATCATGTTGACATTCC; dG = -11.5
570 >s_c_s_e_s_a_s_d_s_e_s GTGTGGGTGCTGGTGTCTGTGACGGTGCTGGTG; >clamp_s_d_s_a_s:compl:rev
CACAGACACCGTCACTATCATGTTGACATTCC; dG = -11.5
571 >s_c_s_e_s_b_s_d_s_e_s GTGTGGGTGCTGGTGGCTGTGACGGTGCTGGTG; >clamp_s_d_s_a_s:compl:rev
CACAGACACCGTCACTATCATGTTGACATTCC; dG = -11.5
572 >s_d_s_e_s_a_s_e_s_a_s GTGACGGTGCTGGTGTCTGTGCTGGTGTCTGTG; >clamp_s_d_s_a_s:compl:rev
CACAGACACCGTCACTATCATGTTGACATTCC; dG = -11.5
573 >s_d_s_e_s_b_s_d_s_e_s GTGACGGTGCTGGTGGCTGTGACGGTGCTGGTG; >clamp_s_d_s_a_s:compl:rev
CACAGACACCGTCACTATCATGTTGACATTCC; dG = -11.5
574 >s_e_s_a_s_a_s_d_s_e_s GTGCTGGTGTCTGTGTCTGTGACGGTGCTGGTG; >clamp_s_d_s_a_s:compl:rev
CACAGACACCGTCACTATCATGTTGACATTCC; dG = -11.5
575 >s_e_s_a_s_b_s_d_s_e_s GTGCTGGTGTCTGTGGCTGTGACGGTGCTGGTG; >clamp_s_d_s_a_s:compl:rev
CACAGACACCGTCACTATCATGTTGACATTCC; dG = -11.5
576 >s_e_s_b_s_a_s_d_s_e_s GTGCTGGTGGCTGTGTCTGTGACGGTGCTGGTG; >clamp_s_d_s_a_s:compl:rev
CACAGACACCGTCACTATCATGTTGACATTCC; dG = -11.5
577 >s_e_s_b_s_b_s_d_s_e_s GTGCTGGTGGCTGTGGCTGTGACGGTGCTGGTG; >clamp_s_d_s_a_s:compl:rev
CACAGACACCGTCACTATCATGTTGACATTCC; dG = -11.5
578 >s_e_s_c_s_b_s_d_s_e_s GTGCTGGTGTGGGTGGCTGTGACGGTGCTGGTG; >clamp_s_d_s_a_s:compl:rev
CACAGACACCGTCACTATCATGTTGACATTCC; dG = -11.5
579 >s_e_s_e_s_a_s_d_s_e_s GTGCTGGTGCTGGTGTCTGTGACGGTGCTGGTG; >clamp_s_d_s_a_s:compl:rev
CACAGACACCGTCACTATCATGTTGACATTCC; dG = -11.5
580 >s_e_s_e_s_b_s_d_s_e_s GTGCTGGTGCTGGTGGCTGTGACGGTGCTGGTG; >clamp_s_d_s_a_s:compl:rev
CACAGACACCGTCACTATCATGTTGACATTCC; dG = -11.5
581 >s_a_s_c_s_b_s_d_s_e_s GTGTCTGTGTGGGTGGCTGTGACGGTGCTGGTG; >clamp_s_d_s_a_s:compl:rev
CACAGACACCGTCACTATCATGTTGACATTCC; dG = -11.5
582 >s_a_s_a_s_b_s_d_s_e_s GTGTCTGTGTCTGTGGCTGTGACGGTGCTGGTG; >clamp_s_d_s_a_s:compl:rev
CACAGACACCGTCACTATCATGTTGACATTCC; dG = -11.5
583 >s_a_s_c_s_e_s_d_s_e_s GTGTCTGTGTGGGTGCTGGTGACGGTGCTGGTG; >clamp_s_d_s_a_s:compl:rev
CACAGACACCGTCACTATCATGTTGACATTCC; dG = -11.4
584 >s_a_s_e_s_c_s_d_s_e_s GTGTCTGTGCTGGTGTGGGTGACGGTGCTGGTG; >clamp_s_d_s_a_s:compl:rev
CACAGACACCGTCACTATCATGTTGACATTCC; dG = -11.4
585 >s_a_s_a_s_c_s_d_s_e_s GTGTCTGTGTCTGTGTGGGTGACGGTGCTGGTG; >clamp_s_d_s_a_s:compl:rev
CACAGACACCGTCACTATCATGTTGACATTCC; dG = -11.4
586 >s_a_s_e_s_e_s_d_s_e_s GTGTCTGTGCTGGTGCTGGTGACGGTGCTGGTG; >clamp_s_d_s_a_s:compl:rev
CACAGACACCGTCACTATCATGTTGACATTCC; dG = -11.4
587 >s_b_s_a_s_c_s_d_s_e_s GTGGCTGTGTCTGTGTGGGTGACGGTGCTGGTG; >clamp_s_d_s_a_s:compl:rev
CACAGACACCGTCACTATCATGTTGACATTCC; dG = -11.4
588 >s_b_s_a_s_e_s_d_s_e_s GTGGCTGTGTCTGTGCTGGTGACGGTGCTGGTG; >clamp_s_d_s_a_s:compl:rev
CACAGACACCGTCACTATCATGTTGACATTCC; dG = -11.4
589 >s_b_s_b_s_c_s_d_s_e_s GTGGCTGTGGCTGTGTGGGTGACGGTGCTGGTG; >clamp_s_d_s_a_s:compl:rev
CACAGACACCGTCACTATCATGTTGACATTCC; dG = -11.4
590 >s_b_s_b_s_e_s_d_s_e_s GTGGCTGTGGCTGTGCTGGTGACGGTGCTGGTG; >clamp_s_d_s_a_s:compl:rev
CACAGACACCGTCACTATCATGTTGACATTCC; dG = -11.4
591 >s_b_s_c_s_a_s_b_s_a_s GTGGCTGTGTGGGTGTCTGTGGCTGTGTCTGTG; >clamp_s_d_s_a_s:compl:rev
CACAGACACCGTCACTATCATGTTGACATTCC; dG = -11.4
592 >s_b_s_c_s_c_s_d_s_e_s GTGGCTGTGTGGGTGTGGGTGACGGTGCTGGTG; >clamp_s_d_s_a_s:compl:rev
CACAGACACCGTCACTATCATGTTGACATTCC; dG = -11.4
593 >s_b_s_c_s_e_s_d_s_e_s GTGGCTGTGTGGGTGCTGGTGACGGTGCTGGTG; >clamp_s_d_s_a_s:compl:rev
CACAGACACCGTCACTATCATGTTGACATTCC; dG = -11.4
594 >s_a_s_a_s_e_s_d_s_e_s GTGTCTGTGTCTGTGCTGGTGACGGTGCTGGTG; >clamp_s_d_s_a_s:compl:rev
CACAGACACCGTCACTATCATGTTGACATTCC; dG = -11.4
595 >s_b_s_e_s_c_s_d_s_e_s GTGGCTGTGCTGGTGTGGGTGACGGTGCTGGTG; >clamp_s_d_s_a_s:compl:rev
CACAGACACCGTCACTATCATGTTGACATTCC; dG = -11.4
596 >s_b_s_e_s_e_s_d_s_e_s GTGGCTGTGCTGGTGCTGGTGACGGTGCTGGTG; >clamp_s_d_s_a_s:compl:rev
CACAGACACCGTCACTATCATGTTGACATTCC; dG = -11.4
597 >s_c_s_a_s_c_s_d_s_e_s GTGTGGGTGTCTGTGTGGGTGACGGTGCTGGTG; >clamp_s_d_s_a_s:compl:rev
CACAGACACCGTCACTATCATGTTGACATTCC; dG = -11.4
598 >s_c_s_a_s_e_s_d_s_e_s GTGTGGGTGTCTGTGCTGGTGACGGTGCTGGTG; >clamp_s_d_s_a_s:compl:rev
CACAGACACCGTCACTATCATGTTGACATTCC; dG = -11.4
599 >s_c_s_b_s_c_s_d_s_e_s GTGTGGGTGGCTGTGTGGGTGACGGTGCTGGTG; >clamp_s_d_s_a_s:compl:rev
CACAGACACCGTCACTATCATGTTGACATTCC; dG = -11.4
600 >s_c_s_b_s_e_s_d_s_e_s GTGTGGGTGGCTGTGCTGGTGACGGTGCTGGTG; >clamp_s_d_s_a_s:compl:rev
CACAGACACCGTCACTATCATGTTGACATTCC; dG = -11.4
601 >s_c_s_c_s_a_s_b_s_a_s GTGTGGGTGTGGGTGTCTGTGGCTGTGTCTGTG; >clamp_s_d_s_a_s:compl:rev
CACAGACACCGTCACTATCATGTTGACATTCC; dG = -11.4

602 >s_c_s_c_s_c_s_d_s_e_s GTGTGGGTGTGGGTGTGGGTGACGGTGCTGGTG; >clamp_s_d_s_a_s:compl:rev CACAGACACCGTCACTATCATGTTGACATTCC; dG = -11.4
603 >s_c_s_c_s_e_s_d_s_e_s GTGTGGGTGTGGGTGCTGGTGACGGTGCTGGTG; >clamp_s_d_s_a_s:compl:rev CACAGACACCGTCACTATCATGTTGACATTCC; dG = -11.4
604 >s_c_s_d_s_e_s_d_s_e_s GTGTGGGTGACGGTGCTGGTGACGGTGCTGGTG; >clamp_s_d_s_a_s:compl:rev CACAGACACCGTCACTATCATGTTGACATTCC; dG = -11.4
605 >s_c_s_d_s_e_s_e_s_a_s GTGTGGGTGACGGTGCTGGTGCTGGTGTCTGTG; >clamp_s_d_s_a_s:compl:rev CACAGACACCGTCACTATCATGTTGACATTCC; dG = -11.4
606 >s_c_s_d_s_e_s_c_s_a_s GTGTGGGTGACGGTGCTGGTGTGGGTGTCTGTG; >clamp_s_d_s_a_s:compl:rev CACAGACACCGTCACTATCATGTTGACATTCC; dG = -11.4
607 >s_c_s_d_s_e_s_b_s_a_s GTGTGGGTGACGGTGCTGGTGGCTGTGTCTGTG; >clamp_s_d_s_a_s:compl:rev CACAGACACCGTCACTATCATGTTGACATTCC; dG = -11.4
608 >s_c_s_e_s_c_s_d_s_e_s GTGTGGGTGCTGGTGTGGGTGACGGTGCTGGTG; >clamp_s_d_s_a_s:compl:rev CACAGACACCGTCACTATCATGTTGACATTCC; dG = -11.4
609 >s_c_s_e_s_e_s_d_s_e_s GTGTGGGTGCTGGTGCTGGTGACGGTGCTGGTG; >clamp_s_d_s_a_s:compl:rev CACAGACACCGTCACTATCATGTTGACATTCC; dG = -11.4
610 >s_a_s_b_s_c_s_d_s_e_s GTGTCTGTGGCTGTGTGGGTGACGGTGCTGGTG; >clamp_s_d_s_a_s:compl:rev CACAGACACCGTCACTATCATGTTGACATTCC; dG = -11.4
611 >s_d_s_d_s_e_s_d_s_e_s GTGACGGTGACGGTGCTGGTGACGGTGCTGGTG; >clamp_s_d_s_a_s:compl:rev CACAGACACCGTCACTATCATGTTGACATTCC; dG = -11.4
612 >s_d_s_d_s_e_s_e_s_a_s GTGACGGTGACGGTGCTGGTGCTGGTGTCTGTG; >clamp_s_d_s_a_s:compl:rev CACAGACACCGTCACTATCATGTTGACATTCC; dG = -11.4
613 >s_d_s_d_s_e_s_c_s_a_s GTGACGGTGACGGTGCTGGTGTGGGTGTCTGTG; >clamp_s_d_s_a_s:compl:rev CACAGACACCGTCACTATCATGTTGACATTCC; dG = -11.4
614 >s_d_s_d_s_e_s_b_s_a_s GTGACGGTGACGGTGCTGGTGGCTGTGTCTGTG; >clamp_s_d_s_a_s:compl:rev CACAGACACCGTCACTATCATGTTGACATTCC; dG = -11.4
615 >s_d_s_e_s_c_s_d_s_e_s GTGACGGTGCTGGTGTGGGTGACGGTGCTGGTG; >clamp_s_d_s_a_s:compl:rev CACAGACACCGTCACTATCATGTTGACATTCC; dG = -11.4
616 >s_d_s_e_s_e_s_d_s_e_s GTGACGGTGCTGGTGCTGGTGACGGTGCTGGTG; >clamp_s_d_s_a_s:compl:rev CACAGACACCGTCACTATCATGTTGACATTCC; dG = -11.4
617 >s_a_s_b_s_e_s_d_s_e_s GTGTCTGTGGCTGTGCTGGTGACGGTGCTGGTG; >clamp_s_d_s_a_s:compl:rev CACAGACACCGTCACTATCATGTTGACATTCC; dG = -11.4
618 >s_e_s_a_s_c_s_d_s_e_s GTGCTGGTGTCTGTGTGGGTGACGGTGCTGGTG; >clamp_s_d_s_a_s:compl:rev CACAGACACCGTCACTATCATGTTGACATTCC; dG = -11.4
619 >s_e_s_a_s_e_s_d_s_e_s GTGCTGGTGTCTGTGCTGGTGACGGTGCTGGTG; >clamp_s_d_s_a_s:compl:rev CACAGACACCGTCACTATCATGTTGACATTCC; dG = -11.4
620 >s_e_s_b_s_c_s_d_s_e_s GTGCTGGTGGCTGTGTGGGTGACGGTGCTGGTG; >clamp_s_d_s_a_s:compl:rev CACAGACACCGTCACTATCATGTTGACATTCC; dG = -11.4
621 >s_e_s_b_s_e_s_d_s_e_s GTGCTGGTGGCTGTGCTGGTGACGGTGCTGGTG; >clamp_s_d_s_a_s:compl:rev CACAGACACCGTCACTATCATGTTGACATTCC; dG = -11.4
622 >s_e_s_c_s_a_s_b_s_a_s GTGCTGGTGTGGGTGTCTGTGGCTGTGTCTGTG; >clamp_s_d_s_a_s:compl:rev CACAGACACCGTCACTATCATGTTGACATTCC; dG = -11.4
623 >s_e_s_c_s_c_s_d_s_e_s GTGCTGGTGTGGGTGTGGGTGACGGTGCTGGTG; >clamp_s_d_s_a_s:compl:rev CACAGACACCGTCACTATCATGTTGACATTCC; dG = -11.4
624 >s_e_s_c_s_e_s_d_s_e_s GTGCTGGTGTGGGTGCTGGTGACGGTGCTGGTG; >clamp_s_d_s_a_s:compl:rev CACAGACACCGTCACTATCATGTTGACATTCC; dG = -11.4
625 >s_a_s_c_s_a_s_b_s_a_s GTGTCTGTGTGGGTGTCTGTGGCTGTGTCTGTG; >clamp_s_d_s_a_s:compl:rev CACAGACACCGTCACTATCATGTTGACATTCC; dG = -11.4
626 >s_e_s_d_s_e_s_d_s_e_s GTGCTGGTGACGGTGCTGGTGACGGTGCTGGTG; >clamp_s_d_s_a_s:compl:rev CACAGACACCGTCACTATCATGTTGACATTCC; dG = -11.4
627 >s_e_s_d_s_e_s_e_s_a_s GTGCTGGTGACGGTGCTGGTGCTGGTGTCTGTG; >clamp_s_d_s_a_s:compl:rev CACAGACACCGTCACTATCATGTTGACATTCC; dG = -11.4
628 >s_e_s_d_s_e_s_c_s_a_s GTGCTGGTGACGGTGCTGGTGTGGGTGTCTGTG; >clamp_s_d_s_a_s:compl:rev CACAGACACCGTCACTATCATGTTGACATTCC; dG = -11.4
629 >s_e_s_d_s_e_s_b_s_a_s GTGCTGGTGACGGTGCTGGTGGCTGTGTCTGTG; >clamp_s_d_s_a_s:compl:rev CACAGACACCGTCACTATCATGTTGACATTCC; dG = -11.4
630 >s_e_s_e_s_c_s_d_s_e_s GTGCTGGTGCTGGTGTGGGTGACGGTGCTGGTG; >clamp_s_d_s_a_s:compl:rev CACAGACACCGTCACTATCATGTTGACATTCC; dG = -11.4
631 >s_e_s_e_s_e_s_d_s_e_s GTGCTGGTGCTGGTGCTGGTGACGGTGCTGGTG; >clamp_s_d_s_a_s:compl:rev CACAGACACCGTCACTATCATGTTGACATTCC; dG = -11.4
632 >s_a_s_c_s_c_s_d_s_e_s GTGTCTGTGTGGGTGTGGGTGACGGTGCTGGTG; >clamp_s_d_s_a_s:compl:rev CACAGACACCGTCACTATCATGTTGACATTCC; dG = -11.4
633 >s_a_s_c_s_c_s_a_s_a_s GTGTCTGTGTGGGTGTGGGTGTCTGTGTCTGTG; >clamp_s_d_s_a_s:compl:rev CACAGACACCGTCACTATCATGTTGACATTCC; dG = -11.3
634 >s_a_s_e_s_c_s_a_s_a_s GTGTCTGTGCTGGTGTGGGTGTCTGTGTCTGTG; >clamp_s_d_s_a_s:compl:rev CACAGACACCGTCACTATCATGTTGACATTCC; dG = -11.3
635 >s_b_s_a_s_c_s_a_s_a_s GTGGCTGTGTCTGTGTGGGTGTCTGTGTCTGTG; >clamp_s_d_s_a_s:compl:rev CACAGACACCGTCACTATCATGTTGACATTCC; dG = -11.3
636 >s_a_s_a_s_c_s_a_s_a_s GTGTCTGTGTCTGTGTGGGTGTCTGTGTCTGTG; >clamp_s_d_s_a_s:compl:rev CACAGACACCGTCACTATCATGTTGACATTCC; dG = -11.3
637 >s_b_s_b_s_c_s_a_s_a_s GTGGCTGTGGCTGTGTGGGTGTCTGTGTCTGTG; >clamp_s_d_s_a_s:compl:rev CACAGACACCGTCACTATCATGTTGACATTCC; dG = -11.3
638 >s_b_s_c_s_a_s_c_s_a_s GTGGCTGTGTGGGTGTCTGTGTGGGTGTCTGTG; >clamp_s_d_s_a_s:compl:rev CACAGACACCGTCACTATCATGTTGACATTCC; dG = -11.3

639 >s_b_s_c_s_a_s_a_s_a_s GTGGCTGTGTGGGTGTCTGTGTCTGTGTCTGTG; >clamp_s_d_s_a_s:compl:rev CACAGACACCGTCACTATCATGTTGACATTCC; dG = -11.3
640 >s_b_s_c_s_c_s_a_s_a_s GTGGCTGTGTGGGTGTGGGTGTCTGTGTCTGTG; >clamp_s_d_s_a_s:compl:rev CACAGACACCGTCACTATCATGTTGACATTCC; dG = -11.3
641 >s_b_s_e_s_c_s_a_s_a_s GTGGCTGTGCTGGTGTGGGTGTCTGTGTCTGTG; >clamp_s_d_s_a_s:compl:rev CACAGACACCGTCACTATCATGTTGACATTCC; dG = -11.3
642 >s_c_s_a_s_c_s_a_s_a_s GTGTGGGTGTCTGTGTGGGTGTCTGTGTCTGTG; >clamp_s_d_s_a_s:compl:rev CACAGACACCGTCACTATCATGTTGACATTCC; dG = -11.3
643 >s_c_s_b_s_c_s_a_s_a_s GTGTGGGTGGCTGTGTGGGTGTCTGTGTCTGTG; >clamp_s_d_s_a_s:compl:rev CACAGACACCGTCACTATCATGTTGACATTCC; dG = -11.3
644 >s_c_s_c_s_a_s_c_s_a_s GTGTGGGTGTGGGTGTCTGTGTGGGTGTCTGTG; >clamp_s_d_s_a_s:compl:rev CACAGACACCGTCACTATCATGTTGACATTCC; dG = -11.3
645 >s_c_s_c_s_a_s_a_s_a_s GTGTGGGTGTGGGTGTCTGTGTCTGTGTCTGTG; >clamp_s_d_s_a_s:compl:rev CACAGACACCGTCACTATCATGTTGACATTCC; dG = -11.3
646 >s_c_s_c_s_c_s_a_s_a_s GTGTGGGTGTGGGTGTGGGTGTCTGTGTCTGTG; >clamp_s_d_s_a_s:compl:rev CACAGACACCGTCACTATCATGTTGACATTCC; dG = -11.3
647 >s_c_s_e_s_c_s_a_s_a_s GTGTGGGTGCTGGTGTGGGTGTCTGTGTCTGTG; >clamp_s_d_s_a_s:compl:rev CACAGACACCGTCACTATCATGTTGACATTCC; dG = -11.3
648 >s_a_s_b_s_c_s_a_s_a_s GTGTCTGTGGCTGTGTGGGTGTCTGTGTCTGTG; >clamp_s_d_s_a_s:compl:rev CACAGACACCGTCACTATCATGTTGACATTCC; dG = -11.3
649 >s_d_s_e_s_c_s_a_s_a_s GTGACGGTGCTGGTGTGGGTGTCTGTGTCTGTG; >clamp_s_d_s_a_s:compl:rev CACAGACACCGTCACTATCATGTTGACATTCC; dG = -11.3
650 >s_e_s_a_s_c_s_a_s_a_s GTGCTGGTGTCTGTGTGGGTGTCTGTGTCTGTG; >clamp_s_d_s_a_s:compl:rev CACAGACACCGTCACTATCATGTTGACATTCC; dG = -11.3
651 >s_e_s_b_s_c_s_a_s_a_s GTGCTGGTGGCTGTGTGGGTGTCTGTGTCTGTG; >clamp_s_d_s_a_s:compl:rev CACAGACACCGTCACTATCATGTTGACATTCC; dG = -11.3
652 >s_e_s_c_s_a_s_c_s_a_s GTGCTGGTGTGGGTGTCTGTGTGGGTGTCTGTG; >clamp_s_d_s_a_s:compl:rev CACAGACACCGTCACTATCATGTTGACATTCC; dG = -11.3
653 >s_e_s_c_s_a_s_a_s_a_s GTGCTGGTGTGGGTGTCTGTGTCTGTGTCTGTG; >clamp_s_d_s_a_s:compl:rev CACAGACACCGTCACTATCATGTTGACATTCC; dG = -11.3
654 >s_e_s_c_s_c_s_a_s_a_s GTGCTGGTGTGGGTGTGGGTGTCTGTGTCTGTG; >clamp_s_d_s_a_s:compl:rev CACAGACACCGTCACTATCATGTTGACATTCC; dG = -11.3
655 >s_a_s_c_s_a_s_c_s_a_s GTGTCTGTGTGGGTGTCTGTGTGGGTGTCTGTG; >clamp_s_d_s_a_s:compl:rev CACAGACACCGTCACTATCATGTTGACATTCC; dG = -11.3
656 >s_a_s_c_s_a_s_a_s_a_s GTGTCTGTGTGGGTGTCTGTGTCTGTGTCTGTG; >clamp_s_d_s_a_s:compl:rev CACAGACACCGTCACTATCATGTTGACATTCC; dG = -11.3
657 >s_e_s_e_s_c_s_a_s_a_s GTGCTGGTGCTGGTGTGGGTGTCTGTGTCTGTG; >clamp_s_d_s_a_s:compl:rev CACAGACACCGTCACTATCATGTTGACATTCC; dG = -11.3
658 >s_b_s_c_s_a_s_e_s_a_s GTGGCTGTGTGGGTGTCTGTGCTGGTGTCTGTG; >clamp_s_d_s_a_s:compl:rev CACAGACACCGTCACTATCATGTTGACATTCC; dG = -11.2
659 >s_c_s_c_s_a_s_e_s_a_s GTGTGGGTGTGGGTGTCTGTGCTGGTGTCTGTG; >clamp_s_d_s_a_s:compl:rev CACAGACACCGTCACTATCATGTTGACATTCC; dG = -11.2
660 >s_e_s_c_s_a_s_e_s_a_s GTGCTGGTGTGGGTGTCTGTGCTGGTGTCTGTG; >clamp_s_d_s_a_s:compl:rev CACAGACACCGTCACTATCATGTTGACATTCC; dG = -11.2
661 >s_a_s_c_s_a_s_e_s_a_s GTGTCTGTGTGGGTGTCTGTGCTGGTGTCTGTG; >clamp_s_d_s_a_s:compl:rev CACAGACACCGTCACTATCATGTTGACATTCC; dG = -11.2
662 >s_a_s_a_s_b_s_c_s_a_s GTGTCTGTGTCTGTGGCTGTGTGGGTGTCTGTG; >clamp_s_d_s_a_s:compl:rev CACAGACACCGTCACTATCATGTTGACATTCC; dG = -11.1
663 >s_a_s_e_s_a_s_c_s_a_s GTGTCTGTGCTGGTGTCTGTGTGGGTGTCTGTG; >clamp_s_d_s_a_s:compl:rev CACAGACACCGTCACTATCATGTTGACATTCC; dG = -11.1
664 >s_a_s_e_s_b_s_c_s_a_s GTGTCTGTGCTGGTGGCTGTGTGGGTGTCTGTG; >clamp_s_d_s_a_s:compl:rev CACAGACACCGTCACTATCATGTTGACATTCC; dG = -11.1
665 >s_b_s_a_s_a_s_c_s_a_s GTGGCTGTGTCTGTGTCTGTGTGGGTGTCTGTG; >clamp_s_d_s_a_s:compl:rev CACAGACACCGTCACTATCATGTTGACATTCC; dG = -11.1
666 >s_b_s_a_s_b_s_c_s_a_s GTGGCTGTGTCTGTGGCTGTGTGGGTGTCTGTG; >clamp_s_d_s_a_s:compl:rev CACAGACACCGTCACTATCATGTTGACATTCC; dG = -11.1
667 >s_b_s_b_s_a_s_c_s_a_s GTGGCTGTGGCTGTGTCTGTGTGGGTGTCTGTG; >clamp_s_d_s_a_s:compl:rev CACAGACACCGTCACTATCATGTTGACATTCC; dG = -11.1
668 >s_b_s_b_s_b_s_c_s_a_s GTGGCTGTGGCTGTGGCTGTGTGGGTGTCTGTG; >clamp_s_d_s_a_s:compl:rev CACAGACACCGTCACTATCATGTTGACATTCC; dG = -11.1
669 >s_b_s_c_s_b_s_c_s_a_s GTGGCTGTGTGGGTGGCTGTGTGGGTGTCTGTG; >clamp_s_d_s_a_s:compl:rev CACAGACACCGTCACTATCATGTTGACATTCC; dG = -11.1
670 >s_b_s_e_s_a_s_c_s_a_s GTGGCTGTGCTGGTGTCTGTGTGGGTGTCTGTG; >clamp_s_d_s_a_s:compl:rev CACAGACACCGTCACTATCATGTTGACATTCC; dG = -11.1
671 >s_b_s_e_s_b_s_c_s_a_s GTGGCTGTGCTGGTGGCTGTGTGGGTGTCTGTG; >clamp_s_d_s_a_s:compl:rev CACAGACACCGTCACTATCATGTTGACATTCC; dG = -11.1
672 >s_c_s_a_s_a_s_c_s_a_s GTGTGGGTGTCTGTGTCTGTGTGGGTGTCTGTG; >clamp_s_d_s_a_s:compl:rev CACAGACACCGTCACTATCATGTTGACATTCC; dG = -11.1
673 >s_c_s_a_s_b_s_c_s_a_s GTGTGGGTGTCTGTGGCTGTGTGGGTGTCTGTG; >clamp_s_d_s_a_s:compl:rev CACAGACACCGTCACTATCATGTTGACATTCC; dG = -11.1
674 >s_c_s_b_s_a_s_c_s_a_s GTGTGGGTGGCTGTGTCTGTGTGGGTGTCTGTG; >clamp_s_d_s_a_s:compl:rev CACAGACACCGTCACTATCATGTTGACATTCC; dG = -11.1
675 >s_c_s_b_s_b_s_c_s_a_s GTGTGGGTGGCTGTGGCTGTGTGGGTGTCTGTG; >clamp_s_d_s_a_s:compl:rev CACAGACACCGTCACTATCATGTTGACATTCC; dG = -11.1

676 >s_a_s_b_s_a_s_c_s_a_s GTGTCTGTGGCTGTGTCTGTGTGGGTGTCTGTG; >clamp_s_d_s_a_s:compl:rev CACAGACACCGTCACTATCATGTTGACATTCC; dG = -11.1
677 >s_c_s_c_s_b_s_c_s_a_s GTGTGGGTGTGGGTGGCTGTGTGGGTGTCTGTG; >clamp_s_d_s_a_s:compl:rev CACAGACACCGTCACTATCATGTTGACATTCC; dG = -11.1
678 >s_c_s_e_s_a_s_c_s_a_s GTGTGGGTGCTGGTGTCTGTGTGGGTGTCTGTG; >clamp_s_d_s_a_s:compl:rev CACAGACACCGTCACTATCATGTTGACATTCC; dG = -11.1
679 >s_a_s_b_s_b_s_c_s_a_s GTGTCTGTGGCTGTGGCTGTGTGGGTGTCTGTG; >clamp_s_d_s_a_s:compl:rev CACAGACACCGTCACTATCATGTTGACATTCC; dG = -11.1
680 >s_c_s_e_s_b_s_c_s_a_s GTGTGGGTGCTGGTGGCTGTGTGGGTGTCTGTG; >clamp_s_d_s_a_s:compl:rev CACAGACACCGTCACTATCATGTTGACATTCC; dG = -11.1
681 >s_a_s_a_s_a_s_c_s_a_s GTGTCTGTGTCTGTGTCTGTGTGGGTGTCTGTG; >clamp_s_d_s_a_s:compl:rev CACAGACACCGTCACTATCATGTTGACATTCC; dG = -11.1
682 >s_d_s_e_s_b_s_c_s_a_s GTGACGGTGCTGGTGGCTGTGTGGGTGTCTGTG; >clamp_s_d_s_a_s:compl:rev CACAGACACCGTCACTATCATGTTGACATTCC; dG = -11.1
683 >s_e_s_a_s_a_s_c_s_a_s GTGCTGGTGTCTGTGTCTGTGTGGGTGTCTGTG; >clamp_s_d_s_a_s:compl:rev CACAGACACCGTCACTATCATGTTGACATTCC; dG = -11.1
684 >s_e_s_a_s_b_s_c_s_a_s GTGCTGGTGTCTGTGGCTGTGTGGGTGTCTGTG; >clamp_s_d_s_a_s:compl:rev CACAGACACCGTCACTATCATGTTGACATTCC; dG = -11.1
[truncated: 825,989 more chars]
